# Multi-Omics Driven Metabolic Network Reconstruction and Analysis of Lignocellulosic Carbon Utilization in *Rhodosporidium toruloides*

**DOI:** 10.3389/fbioe.2020.612832

**Published:** 2021-01-08

**Authors:** Joonhoon Kim, Samuel T. Coradetti, Young-Mo Kim, Yuqian Gao, Junko Yaegashi, Jeremy D. Zucker, Nathalie Munoz, Erika M. Zink, Kristin E. Burnum-Johnson, Scott E. Baker, Blake A. Simmons, Jeffrey M. Skerker, John M. Gladden, Jon K. Magnuson

**Affiliations:** ^1^Department of Energy, Agile BioFoundry, Emeryville, CA, United States; ^2^Department of Energy, Joint BioEnergy Institute, Emeryville, CA, United States; ^3^Pacific Northwest National Laboratory, Richland, WA, United States; ^4^Sandia National Laboratories, Livermore, CA, United States; ^5^Lawrence Berkeley National Laboratory, Berkeley, CA, United States; ^6^Department of Bioengineering, University of California, Berkeley, Berkeley, CA, United States

**Keywords:** *Rhodosporidium toruloides*, multi-omics, metabolic networks, genome-scale models, lignocellulosic biomass

## Abstract

An oleaginous yeast *Rhodosporidium toruloides* is a promising host for converting lignocellulosic biomass to bioproducts and biofuels. In this work, we performed multi-omics analysis of lignocellulosic carbon utilization in *R. toruloides* and reconstructed the genome-scale metabolic network of *R. toruloides*. High-quality metabolic network models for model organisms and orthologous protein mapping were used to build a draft metabolic network reconstruction. The reconstruction was manually curated to build a metabolic model using functional annotation and multi-omics data including transcriptomics, proteomics, metabolomics, and RB-TDNA sequencing. The multi-omics data and metabolic model were used to investigate *R. toruloides* metabolism including lipid accumulation and lignocellulosic carbon utilization. The developed metabolic model was validated against high-throughput growth phenotyping and gene fitness data, and further refined to resolve the inconsistencies between prediction and data. We believe that this is the most complete and accurate metabolic network model available for *R. toruloides* to date.

## Introduction

An oleaginous yeast *Rhodosporidium toruloides* is a non-model basidiomycete fungus known for its ability to produce carotenoids and accumulate lipids. The high flux in lipid and carotenoid biosynthetic pathways makes *R. toruloides* a promising host organism for producing biofuels and value-added bioproducts from carbon sources derived from lignocellulosic biomass (Wiebe et al., 2012; Fei et al., 2016; Yaegashi et al., 2017; Park et al., 2018; Zhuang et al., 2019). It is also known for the tolerance of inhibitory compounds in lignocellulosic biomass hydrolyzate as well as the ability to consume aromatic compounds related to lignin degradation products (Yaegashi et al., 2017; Sundstrom et al., 2018). For example, *R. toruloides* can utilize hexoses, pentoses, and aromatic compounds that are found in lignocellulosic biomass hydrolyzate such as glucose, xylose, and *p*-coumaric acid, and produce bisabolene or amorphadiene (Yaegashi et al., 2017). Genome sequence and annotation are available for several *R. toruloides* strains and efficient transformation methods have been developed (Zhu et al., 2012; Zhang et al., 2016; Liu et al., 2017; Coradetti et al., 2018). More advanced genetic tools and parts to engineer *R. toruloides* have been recently developed including RB-TDNAseq, CRISPR/Cas9 editing, RNA interference, and promoter libraries (Coradetti et al., 2018; Liu et al., 2019; Nora et al., 2019; Otoupal et al., 2019).

Previous studies of *R. toruloides* metabolism primarily focused on the lipid production and carotenoid production and several multi-omics studies have been performed to date (Zhu et al., 2012; Lee et al., 2014; Bommareddy et al., 2017; Coradetti et al., 2018). However, it is still not fully clear how different carbon sources present in lignocellulosic biomass hydrolyzate are utilized. There are multiple reports indicating that *R. toruloides*’s metabolism of glucose, xylose, or glycerol is different from *Saccharomyces cerevisiae*’s. For example, *R. toruloides* is an oleaginous yeast and generates cytosolic acetyl-CoA from citrate using ATP-citrate lyase whereas *S. cerevisiae* does not have ATP-citrate lyase and uses the pyruvate dehydrogenase bypass (Rodriguez et al., 2016). When grown on D-xylose, *R. toruloides* transiently accumulates D-arabinitol (or D-arabitol) while *S. cerevisiae* accumulates xylitol (Jagtap and Rao, 2018). The regulation of genes involved in glycerol metabolism including glycerol kinase and glycerol 3–phosphate dehydrogenase was also found to be different between *R. toruloides* and *S. cerevisiae* (Bommareddy et al., 2017). Fatty acids are degraded by peroxisomal β-oxidation in *S. cerevisiae*, but both mitochondrial and peroxisomal β-oxidation pathways are shown to be present and necessary for efficient fatty acid degradation in *R. toruloides*. The growth of *S. cerevisiae* is known to be inhibited by some phenolic compounds that are found in lignocellulosic biomass hydrolyzate including *p*-coumaric acid, ferulic acid, and coniferyl aldehyde, and *S. cerevisiae* can convert them to less inhibitory products, but it is unable to grow on them as sole carbon sources (Adeboye et al., 2015). On the other hand, *R. toruloides* grows on *p*-coumaric acid, ferulic acid, vanillic acid, *p*-hydroxybenzoic acid, and benzoic acid (Yaegashi et al., 2017), but the catabolism of aromatic compounds related to lignin is not well studied in fungi yet. Therefore, there is a need for a metabolic network model to systematically investigate the metabolism of non-model oleaginous basidiomycete yeast, *R. toruloides*. In this work, we reconstruct the genome-scale metabolic network of *R. toruloides* using high-quality published models and perform manual curation using functional annotation and multi-omics data in a fully reproducible manner. Every step of the reconstruction and curation was written in electronic notebooks starting from the reconstruction of a draft metabolic network to the evaluation of the resulting metabolic model. The developed metabolic model and multi-omics data were used to study the utilization of carbon sources that are present in lignocellulosic biomass hydrolyzate in *R. toruloides*.

## Materials and Methods

### Metabolic Network Reconstruction

The *R. toruloides* IFO0880 genome sequence, gene models, and gene annotation from a previous study (Coradetti et al., 2018) was used for the metabolic network reconstruction. The same study identified *R. toruloides* proteins that have orthologous proteins in several different eukaryotic organisms using OrthoMCL (Li et al., 2003). To this list we further added orthologous proteins in *Escherichia coli* K-12 MG1655 and *Pseudomonas putida* KT2440, identified with a separate OrthoMCL analysis including proteins from *R. toruloides* NP11, *Saccharomyces cerevisiae*, *Lipomyces starkeyi*, and *Yarrowia lipolytica*. The list of orthologous proteins was used to gather metabolic reactions from BiGG Models (King et al., 2016), a repository containing high-quality manually curated genome-scale metabolic models. Among the models available in BiGG Models, genome-scale metabolic models of *S. cerevisiae* (Mo et al., 2009), *Chlamydomonas reinhardtii* (Chang et al., 2011), human (Duarte et al., 2007), mouse (Sigurdsson et al., 2010), *E*. *coli* (Monk et al., 2017), and *P*. *putida* (Nogales et al., 2008) were used for reconstruction since these models covered most of the orthologous proteins found in *R*. *toruloides*. In addition, a genome-scale metabolic model of another oleaginous yeast *Y*. *lipolytica* CLIB122 (Wei et al., 2017) was included. For each protein in *R*. *toruloides*, metabolic reactions from other metabolic models were added to the *R*. *toruloides* metabolic network if orthologous proteins were associated with the reactions, and their gene association was updated with the *R*. *toruloides* protein identifiers. The function and localization of proteins was determined by Joint Genome Institute’s annotation on MycoCosm (Grigoriev et al., 2014), WoLF PSORT (Horton et al., 2007) prediction, and the presence of peroxisomal targeting sequences PTS1 and PTS2 predicted by FIMO (Grant et al., 2011) using MEME (Bailey and Elkan, 1994) motifs from known peroxisomal protein sequences.

### Metabolic Network Modeling

COBRApy (Ebrahim et al., 2013) was used for curation, evaluation, and modeling of the reconstructed metabolic network. Metabolic models were imported from either the BiGG Models directly or a JSON file constructed from supplemental files from publications. Lieven et al. (2014) was also used to track the development and evaluate the quality of the metabolic model. Escher (King et al., 2015) was used to build metabolic pathway maps and visualize omics data. BOFdat (Lachance et al., 2019) was used to update the biomass composition from experimental data.

### Phenotype Microarrays

Phenotype microarray plates and standard components for yeast phenotypic analysis were obtained from Biolog Inc. (Hayward, CA). Wild type *R. toruloides* IFO0880 was precultured to log phase in LB broth at 30°C, 200 RPM in 10 mL culture tubes. Cells were centrifuged 5 min at 3000 RCF, 22°C, washed twice in sterile water, then resuspended OD 600 of 0.005 in Biolog inoculation fluid IFY-0 with 1 μM nicotinic acid (Sigma, N4126), 1 μM myo-inositol (Sigma, I5125), 1 μM thiamine HCl (Sigma, T1270), 1 μM *p*-aminobenzoic acid (Sigma, A9878), and 1 μM calcium pantothenate (Sigma, 21210) plus Biolog dye mix E (a proprietary, tetrazolium-based dye) and 1 μM menadione sodium bisulfite (Sigma, M5750). For nitrogen, phosphorous, and sulfur sources 100 mM glucose was added to the inoculation fluid. Hundred microliters of the cell suspension was added to each well in plates PM1, PM2, PM3, and PM4. Plates were sealed with clear sealing film (Axygen, CTP-103) and incubated for 120 h at 30°C in the dark. Respiration in each condition was detected by measuring reduction of the dye by comparing absorbance at 590 nm to absorbance at 750 nm.

### Fitness Analysis With RB-TDNAseq

Fitness analysis was performed as described in a previous study (Coradetti et al., 2018). Briefly, the three aliquots of the random insertion mutant pool were thawed on ice and recovered in 100 mL YPD (BD Difco, 242820) for two generations (OD 0.2 to OD 0.8). A 10 mL of each starter culture was pelleted and frozen as an initial “time 0” sample. The remaining cells were pelleted 5 min at 4000 RCF, 22°C, washed twice with sterile water and inoculated at OD 0.1 in 50 mL SD media plus 76 mM KH_2_PO_4_ (Sigma, P9791), 24 mM K_2_HPO_4_ (Sigma, P3786), and 100 nM FeSO_4_ (Sigma, 215422) with 1% w/v carbon source. Cultures were grown to OD 600 = 5–10 (20–50 h depending on carbon source) at 30°C, 200 RPM in baffled flasks (25630-250, DWK Life Sciences). Ten milliliters mL samples were pelleted and frozen for DNA extraction. DNA extraction, barcode amplification, and sequencing was performed as described in Coradetti et al. (2018), except that we used primers including dual indexes to prevent “index swapping” on the HiSeq 4000 instrument (Costello et al., 2018) and different lengths of random bases for improved phasing (Price et al., 2019). Fitness analysis was performed with the RBseq software package version 1.0.6, an updated implementation of the algorithms (available at https://github.com/stcoradetti/RBseq). Raw barcode sequencing data are available at the NCBI Sequence Read Archive (PRJNA595384). Fitness scores are available at the fungal fitness browser.^1^

### Lipid Content by Fatty Acid Methyl-Ester Analysis

Twenty milligrams of lyophilized cell mass was suspended in 750 μl 3N methanolic HCl (Sigma, 40104-U) and 50 μl chloroform (Sigma, CX1050-1). A total of 100 μl of 10 mg/mL tridecanoic acid methyl ester (Sigma, T0627) in methanol (Sigma, 34860) was added as an internal standard and all the resuspended cell mass was transferred to bead-bug screw-top tubes with glass beads. Tubes were shaken vigorously in a Retsch Tissue Lyser at 30 cycles/second for 5 min to break up cell aggregates and disrupt cell walls, then incubated in an 80°C water bath for 2 h with occasional vortexing. fatty acid methyl-esters (FAMEs) were extracted with 500 μl n-hexane (Sigma, 650552), and diluted 1:10 in hexane. Methyl esters of palmitic, palmitoleic, heptadecanoic, stearic, oleic, linoleic, alpha linoleic, arachidic, behenic, and lignoceric acid were separated in a DB-wax column (Agilent, 123-7012) on a Thermo Scientific Focus gas chromatograph (AS 3000 II) with a flame ionization detector. Standard curves of ratios of peak areas from standards of those FAMEs (Sigma) to tridecanoic acid methyl ester (internal standard) were established. FAME concentrations were determined by comparing ratios of peak areas of FAMEs to the internal standard in the samples.

### Media and Growth Conditions for Multi-Omics Experiment

Wild type *R*. *toruloides* IFO0880 was grown in synthetic defined (SD) medium supplemented with different carbon sources (1% glucose, 1% glucose + 1% D-xylose, 1% D-xylose, 1% L-arabinose, or 1% *p*-coumarate). The SD medium was made with Difco yeast nitrogen base without amino acids (Becton, Dickinson & Co., Sparks, MD) and complete supplemental mixture (Sunrise Science Products, San Diego, CA). Cells were pre-cultured in LB media, and pelleted and washed once with sterile ddH2O before inoculation. Initial pH was adjusted to 7.4 with NaOH, and cells were inoculated to 30 mL of medium with a starting optical density at 600 nm of 0.1. Cultures were grown at 30°C and shaken at 200 rpm. Samples were taken in triplicates at 24 and 48 h in SD glucose, at 24, 66, and 90 h in SD glucose + D-xylose, at 66 and 90 h in SD D-xylose, at 40 and 66 h in SD L-arabinose, and at 40 and 90 h in SD *p*-coumarate. RNA extraction was performed on Promega’s Maxwell RSC machine using Plant RNA extraction kit (Promega Corporation, Madison, WI). For proteomics and metabolomics, cells were pelleted and washed twice with 100 mM NH_4_HCO_3_ at pH 7.8, and spent media was filtered through a 0.45 μm filter.

### RNA Sequencing and Analysis

RNA samples were sequenced and processed at Joint Genome Institute (SRP143805, SRP143806, SRP143807, SRP143808, SRP143809, SRP143810, SRP143811, SRP143812, SRP143813, SRP143814, SRP143815, SRP143816, SRP143817, SRP143818, SRP143819, SRP143820, SRP143821, SRP143822, SRP143823, SRP143824, SRP143825, SRP143826, SRP143827, SRP143828, SRP143829, SRP143830, SRP143831, SRP143832, SRP143833, SRP143834, SRP143835, SRP143836, and SRP143838). Raw read counts were used to perform differential gene expression analysis using DESeq2 (Love et al., 2014).

### Metabolite Extraction

Metabolites were extracted from the cell pellets using MPLEx method (Nakayasu et al., 2016; Kim and Heyman, 2018). Briefly, the cell pellets were extracted with a solvent mixture of four volumes of a chloroform and methanol mix (2:1) with a volume of nanopure water. Strong vortexing and ice-cold temperature were also used in the protocol to aid the disruption of the cells. After centrifugation, the aqueous layer and half of the organic layer (containing polar and non-polar metabolites, respectively) were combined for GC-MS analysis. The remaining volume of the organic layer was kept for lipidomics analysis. Collected liquid fractions were transferred to new clean vials and subsequently dried in a speed-vacuum concentrator. The denatured protein disk, located between the aqueous and organic layers during the MPLEx protocol, were separately stored for proteomics analysis. All the samples were dried completely and stored in the −80°C freezer until the instrumental analysis.

### Metabolomics Analysis

The stored metabolite extracts were completely dried under speed-vacuum to remove moisture and chemically derivatized as previously reported (Kim et al., 2015). Briefly, the extracted metabolites were derivatized by methoxyamination and trimethylsilyation (TMS), then the samples were analyzed by GC-MS. GC-MS raw data files were processed using the Metabolite Detector software, version 2.5 beta (Hiller et al., 2009). Retention indices (RI) of detected metabolites were calculated based on the analysis of a FAMEs mixture, followed by their chromatographic alignment across all analyses after deconvolution. Metabolites were initially identified by matching experimental spectra to a PNNL augmented version of Agilent GC-MS metabolomics Library, containing spectra and validated RI for over 850 metabolites. Then, the unknown peaks were additionally matched with the NIST17/Wiley11 GC-MS library. All metabolite identifications and quantification ions were validated and confirmed to reduce deconvolution errors during automated data-processing and to eliminate false identifications. All metabolomics raw data files are available at OSF data depository https://osf.io/tnqwx/.

### Lipidomics

The lipid samples were analyzed using liquid chromatography tandem mass spectrometry (LC-MS/MS) as outlined before (Kyle et al., 2017). Briefly, lipid fractions were re-dried *in vacuo* to remove moisture and reconstituted in 50 μl methanol, 10 μl of which was injected onto a reversed phase Waters CSH column (3.0 mm × 150 mm × 1.7 μm particle size) connected to a Waters Acquity UPLC H class system interfaced with a Velos-ETD Orbitrap mass spectrometer. Lipid molecular species were separated over a 34 min gradient [mobile phase A: acetonitrile/water (40:60) containing 10 mM ammonium acetate; mobile phase B: acetonitrile/isopropanol (10:90) containing 10 mM ammonium acetate] at a flow rate of 250 μl/min. Samples were analyzed in both positive and negative ionization using higher-energy collision dissociation and collision-induced dissociation to obtain high coverage of the lipidome. Confident lipid identifications were made using in-house developed identification software LIQUID (Kyle et al., 2017) where the tandem mass spectrum was examined for diagnostic ion fragments along with associated hydrocarbon chain fragment information. To facilitate quantification of lipids, a reference database for lipids identified from the MS/MS data was created and features from each analysis were then aligned to the reference database based on their identification, m/z and retention time using MZmine 2 (Pluskal et al., 2010). Aligned features were manually curated and peak apex intensity values were generated for subsequent statistical analysis.

### Proteomics

The protein disks were dissolved in 100 mM NH_4_HCO_3_ containing 8 M urea and the protein concentration was measured by BCA assay. Disulfide bonds were reduced by adding dithiothreitol to a final concentration of 5 mM and incubating at 60°C for 30 min. Samples were alkylated with a final concentration of 40 mM iodoacetamide for 1 h at 37°C. The reaction was then diluted 10-fold with 100 mM NH_4_HCO_3_ followed by the addition of CaCl_2_ to 1 mM final concentration. Digestion was carried out for 3 h at 37°C with 1:50 (weight:weight) trypsin-to-protein ratio. Salts and reagents were removed by solid-phase extraction using C18 cartridges according to the manufacturer instructions and the resulting peptides were dried in a vacuum centrifuge. The peptides were then resuspended in milliQ water and 500 ng of material was loaded onto in-house packed reversed-phase capillary columns (70-cm × 75 μm i.d.) with 3-μm Jupiter C18. The separation was carried out using a nanoAcquity HPLC system (Waters Corporation) at room temperature. The mobile phase A is 0.1% formic acid in water while mobile phase B is 0.1% formic acid in acetonitrile. The elution was carried out at 300 nL/min with the following gradient: 0–2 min 1% B; 2–20 min 8% B; 20–75 min 12% B; 75–97 min 30% B; 97–100 min 45% B; 100–105 min 95% B; 105–110 min 95% B; 110–140 min 1% B. MS analysis was carried out using a Q Exactive HF (Thermo Fisher Scientific) in data dependent mode. Mass spectrometer settings were as following: full MS (AGC, 1 × 10^6^; resolution, 30,000; m/z range, 350–2000; maximum ion time, 20 ms); MS/MS (AGC, 1 × 10^5^; resolution, 15,000; m/z range, 200–2000; maximum ion time, 200 ms; minimum signal threshold, 2.5 × 10^4^; isolation width, 2 Da; dynamic exclusion time setting, 45 s; collision energy, NCE 30).

All mass spectrometry data were searched using MS-GF+ (Kim and Pevzner, 2014) and MASIC (Monroe et al., 2008) software. MS-GF+ software was used to identify peptides by scoring MS/MS spectra against peptides derived from the whole protein sequence database. MASIC software was used to generate the selected ion chromatographs (SICs) of all the precursors in MSMS datasets and calculate their peak areas as abundance. MASICResultsMerger^2^ was used to append the relevant MASIC stats for each peptide hit result in MS-GF+. The MS-GF+ data were then filtered based on 1% false discovery rate (FDR) and less than 5-ppm mass accuracy to generate a list of qualified peptide hit results. The abundances of peptides were determined as the highest peak area identified for the peptide within a sample. RRollup algorithm in InfernoRDN software (Polpitiya et al., 2008) was used to calculate the final protein abundance based on peptide abundance.

## Results

### Metabolic Network Reconstruction Workflow

**FIGURE 1:**
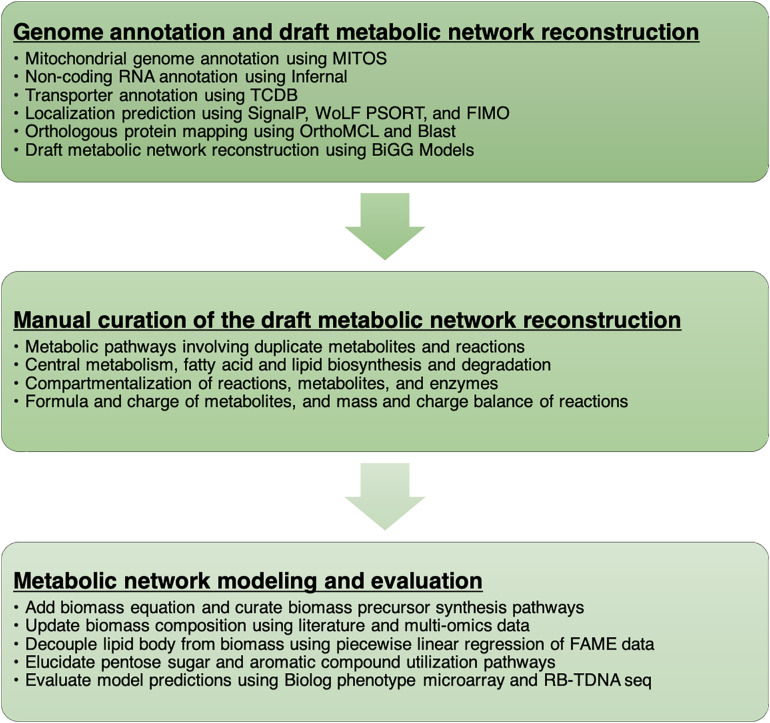
A workflow to develop the metabolic network model of *R. toruloides*.

We documented every step of the metabolic network reconstruction process (Figure 1) using Jupyter Notebooks^3^ to keep records of changes in metabolic network content and the rationale behind them. We divided the process in multiple notebooks for each stage of reconstruction process (Supplementary File 1).

### Genome Annotation and Draft Metabolic Network Reconstruction

First, we used orthologous protein groups from OrthoMCL and published metabolic models to build a draft metabolic network reconstruction. Metabolic reactions were taken from published models when any orthologous protein was found in gene-reaction association information. OrthoMCL orthologous groups consist of orthologs and recent paralogs (i.e., gene duplication after speciation and likely to retain similar function) from at least two species (Li et al., 2003). It is generally thought that gene function is more conserved among orthologous genes than between-species paralogs. Recent studies of gene function versus evolutionary history have demonstrated that paralogs can provide more information that previously thought (Stamboulian et al., 2020) but that this added value comes mostly from within species paralogs in taxa with a large corpus of extant biochemical data. Thus, to build our initial metabolic network we transferred functions from orthologous protein groups only. However, during our manual refinement, in rare cases where we had functional data from *R*. *toruloides* or for filling gaps in pathways for which we have high confidence to exist in *R*. *toruloides*, we transferred functional prediction from paralogs in other species.

The initial draft reconstruction of *R*. *toruloides* metabolic network consisted of 1596 proteins, 3804 reactions, and 3589 metabolites. Among the 1596 proteins, 1137 proteins had orthologous *R*. *toruloides* proteins, but 459 were not yet mapped to *R*. *toruloides* proteins. Manual investigation of functional annotation and BLAST search were used to determine whether the unmapped proteins were present or absent in *R*. *toruloides*. For the reactions associated with a protein absent in *R*. *toruloides*, the absent protein was removed from the gene association if there were isozymes and at least one mapped isozyme was present. A reaction was removed from the metabolic network if the absent protein was a subunit of an enzyme complex and no other isozyme was present. One of the reasons why the number of reactions and metabolites in the initial reconstruction was very large was that protein localization was not yet considered, and same reactions in multiple compartments were present including compartments that are present in other organisms but absent in *R*. *toruloides*. These reactions were examined and either removed or assigned to appropriate compartments based on the localization prediction and the presence of a signal peptide sequence. It was also found that, although reconstructed from published metabolic models, the initial reconstruction contained many erroneous reactions and metabolites including duplicate reactions or metabolites with different names as well as mass or charge imbalanced reactions.

### Manual Curation of the Draft Metabolic Network Reconstruction

Manual curation of the metabolic network reconstruction was performed to first identify and remove duplicate metabolites and reactions. As duplicate or incorrect metabolites and reactions were found, thorough inspection of the metabolic pathway involving them was performed. Functional annotation and localization of each gene in the pathway was confirmed, duplicate or erroneous metabolites and reactions were removed or corrected, and missing metabolites and reactions were either created based on literature and metabolic pathway database or added from other metabolic models in BiGG database. For example, metabolic pathways involving ferricytochrome c were first investigated since duplicate metabolite identifiers exist in BiGG database (ficytc/focytc versus ficytC/focytC). Lactate dehydrogenase and related enzymes involving cytochrome c had many duplicated reactions and incorrect compartment assignments. Cytochrome c oxidase, peroxidase, reductase, NADH:ubiquinone reductase and other mitochondrial electron transport chain reactions were curated. *R*. *toruloides* is known to have coenzyme Q9 (ubiquinone-9) with nine isoprenyl units (Yamada and Kondo, 1973), and our lipidomics analysis also detected coenzyme Q9. The ubiquinone synthesis reactions from other existing models were modified to include 9 isoprenyl units. Heme biosynthesis reactions were also manually curated since heme O and heme A synthesis reactions in BiGG database had incorrect stoichiometries.

There were many incorrect reactions involved in fatty acid biosynthesis and beta-oxidation, especially for unsaturated fatty acids. Impossible lumped reactions were removed considering the cis and trans configuration of fatty acids, and irrelevant fatty acid reactions (bacteria or plant specific unsaturated fatty acids) were removed. Long-chain fatty acid biosynthesis and fatty acid desaturase reactions were moved from cytosol to endoplasmic reticulum, and short-chain peroxisomal fatty acid beta-oxidation reactions were moved to mitochondria since *R*. *toruloides* possesses both mitochondrial and peroxisomal beta-oxidation enzymes. Sphingolipid metabolism was extended to include sphingolipid desaturase, methyltransferase, and fungal ceramide biosynthesis and the reactions were placed in endoplasmic reticulum. Localization of reactions involved in phospholipid biosynthesis and remodeling, and triacylglycerol were also updated.

*Rhodosporidium toruloides* naturally produces and accumulates carotenoids that are derived from mevalonate pathway products. Metabolic reactions and genes in mevalonate pathway and sterol biosynthesis were inspected, and it was found that an ERG27 ortholog is missing in *R*. *toruloides*. A previous phylogenomics study of sterol synthesis found that a 3-ketosteroid reductase exists in vertebrates and fungi (HSD17B7 in vertebrates and ERG27 in *S. cerevisiae*) but is missing in land plants and other eukaryotic phyla (Desmond and Gribaldo, 2009). More recent studies have suggested that enzymes that oxidizes the C-3 hydroxyl group of sterols to a ketone also reduces the C-3 ketone in tomato (Lee et al., 2019) or sterol-producing bacteria (Lee et al., 2018). On the other hand, in human aldo-keto reductases of the 1C subfamily are involved in 3-ketosteroid reduction and known to be promiscuous (Penning et al., 2015). We found three aldo-keto reductase family proteins in *R*. *toruloides* (protein ID 13153, 14209, and 14213) that are homologous to human aldo-keto reductases 1C. One of the aldo-keto reductases (14213) had a predicted signal peptide by SignalP and predicted localization in endoplasmic reticulum by WoLF PSORT, and was assigned to reactions catalyzed by the 3-ketosteroid reductase. Carotenoid biosynthesis in endoplasmic reticulum and accumulation in lipid droplets were added based on previous studies (Sun et al., 2017; Ma et al., 2019; Rabeharindranto et al., 2019).

The reactions and genes in the central metabolic pathways were manually checked for their co-factor usage and localization. Reactions in the compartments that are present in other organisms but irrelevant in *R. toruloides* were either moved to appropriate compartments or removed from the model if redundant. Reactions with incomplete gene-to-protein-to-reaction association (e.g., missing subunits) were either removed from the model or updated with corresponding genes if found. Reactions were checked for mass and charge balance, and chemical formula and charge information was updated for all metabolites. When needed, chemical equations were modified based on metabolic databases or new evidence in literature. The curated metabolic reconstruction consisted of 1106 genes, 1934 reactions, and 2010 metabolites (1246 unique metabolites) in nine compartments.

### Metabolic Network Modeling and Growth Simulation

A biomass reaction from the *S. cerevisiae* metabolic model and exchange reactions for external metabolites were added to the metabolic network reconstruction to develop a draft metabolic model that can be used to make growth and flux predictions. In addition, transport reactions for water, carbon dioxide, and oxygen in different compartments were added. We first examined whether each biomass precursor in the biomass reaction can be synthesized in an aerobic glucose minimal medium. A flux balance analysis (FBA) problem maximizing the biomass production was solved, and the shadow prices of metabolites were examined to identify biomass components that could not be synthesized. The cytosolic components in *S. cerevisiae* biomass reaction whose biosynthesis reactions were moved to endoplasmic reticulum were replaced with the respective metabolites in endoplasmic reticulum, and transport reactions for fatty acids, phospholipids, and sterols were added to allow lipid production. In addition, mitochondrial transport reactions for several amino acids and their precursors were added and incorrect reactions in lysine biosynthesis were manually curated to allow synthesis of all components in *S. cerevisiae* biomass reaction. Several reactions generating a free proton gradient across mitochondrial membrane via a loop were identified by mixed-integer programming and removed to prevent unrealistic ATP production.

**FIGURE 2:**
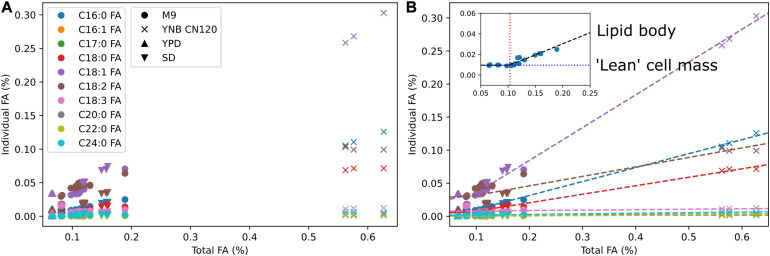
Fatty acid composition of *R. toruloides* in different media conditions and segmented linear regression. **(A)** Fatty acid composition by fatty acid methyl ester analysis in M9, YNB with C to N ratio of 120, YPD, and SD media. Colors indicate different fatty acid species and shapes indicate different media. **(B)** Estimation of fatty acid content in “lean” cell mass and fatty acid content in lipid body using segmented linear regression. Dashed lines are segmented linear regression for each fatty acid *k* using the equation *y_k_* = *m_*k*_(x–x_0_)* + *b*_*k*_. The inset shows the slope *m*_*k*_ in a black dashed line, the *x*-intercept *x*_0_ in a red dotted line, and the *y*-intercept *b*_*k*_ in a blue dotted line for fatty acid *k*.

We used multi-omics and other experimental measurement to update the biomass reaction. The DNA composition was updated using the genome sequence, RNA composition was updated using transcriptomics data, amino acid composition was updated using proteomics data, and lipid composition was updated using fatty acid methyl ester analysis. For lipid composition, we measured fatty acid profiles in multiple media conditions to cover from low lipid to high lipid production states (Figure 2A). We observed that the weight percentages of a subset of individual fatty acids linearly increased from a low lipid condition (e.g., YPD) to a high lipid condition (e.g., YNB CN120) whereas the weight percentages of the other fatty acids remained relatively constant. We assumed those fatty acids that were linearly increasing to be the major components in the lipid body, and used segmented linear regression to estimate the fatty acid composition in “lean” cell mass where the majority of lipids are phospholipids, and the fatty acid composition in lipid body where the majority of lipids are triacylglycerols and sterol esters (Figure 2B). The fatty acid composition in “lean” cell mass was estimated from the y-intercepts and used in the biomass equation, and the fatty acid composition in lipid body was estimated from the slopes and used in demand reactions for triacylglycerols and sterol esters accumulation in lipid droplet. This allows for the simulation of cell growth and lipid accumulation in lipid body separately, and also enables the simulation of lipid mobilization using sink reactions for triacylglycerol and sterol esters in lipid droplet. Next, we added commonly known trace elements including cofactors and vitamins to the biomass equation and examined using FBA whether they could be synthesized. The reactions involved in folate, thiamine pyrophosphate, quinone, and biotin biosynthesis were manually curated to enable their biosynthesis. It was necessary to add demand reactions for 8-amino-7-oxononanoate and lipoate since we were not able to find all the required enzymes for their synthesis.

We tested the model’s capability to predict growth on carbon sources that can be often found in lignocellulosic biomass hydrolyzate – glucose, D-xylose, L-arabinose, and *p*-coumarate. The initial metabolic model was able to predict growth on glucose, but not on D-xylose, L-arabinose, and *p*-coumarate. We examined the existing reactions in the model to identify the missing links within known catabolic pathways. In order to predict growth on D-xylose, the xylose reductase reaction was needed. Two potential xylose reductase encoding genes were found in *R. toruloides* with sequences that are similar to larA and xyrA in *Aspergillus niger*. For growth on L-arabinose, the L-arabinose transporter, L-arabinitol 4-dehydrogenase, and L-xylulose reductase reactions were needed but BLAST of known genes for these reactions resulted in multiple hits with moderate scores. For *p*-coumarate utilization, four reactions in the known *p*-coumarate degradation pathway in bacteria were present in the model. We identified genes that could potentially catalyze the missing reactions in the known *p*-coumarate degradation pathway using functional annotation and BLAST searches. However, additional experimental data was still needed to identify which of these candidates are actually responsible for the missing or added metabolic functions in the D-xylose, L-arabinose, and *p*-coumarate utilization pathways. We therefore performed multi-omics experiments for *R. toruloides* grown in these carbon sources to elucidate the genes and reactions necessary for their utilization.

### Multi-Omics Analysis of Lignocellulosic Carbon Utilization in *R. toruloides*

We performed transcriptomics, proteomics, and metabolomics analysis to investigate genes involved in carbon utilization pathways in *R*. *toruloides*. Cells were grown with glucose, glucose + D-xylose, D-xylose, L-arabinose, or *p*-coumarate as carbon source, and samples were taken during exponential growth phase and stationary phase. An additional sample was taken between exponential and stationary phase for cells grown with glucose + D-xylose to study the co-utilization pattern. Gene expression profiles in cells grown on D-xylose, L-arabinose, or *p*-coumarate was compared to glucose in order to identify genes specifically upregulated or downregulated by each carbon source. RB-TDNA sequencing was also performed in glucose, D-xylose, L-arabinose, *p*-coumarate and other related metabolites. RB-TDNA sequencing uses sequence barcoded random insertions throughout the genome to identify genes required for growth in a given condition. A mixed population of hundreds of thousands of different mutant strains, each bearing an insertion at a different genomic location, is cultured in the condition of interest. The relative abundances of all barcoded strains in the population are simultaneously measured from a single sample by high throughput sequencing. For the more than 6000 genes with three or more independently tracked insertions within their coding sequence, those abundances are aggregated into a single “fitness score” for mutations in each gene in the tested condition. Genes with an essential function in a given condition (e.g., an enzymatic reaction in catabolic pathway) will have negative fitness scores in that condition. Transcriptomics, proteomics, and fitness scores were used to assign genes to reactions when annotations were ambiguous or multiple isozymes with substrate promiscuity were present. Metabolomics data was used to identify intermediates in utilization pathways to provide additional support for the proposed pathways.

**FIGURE 3:**
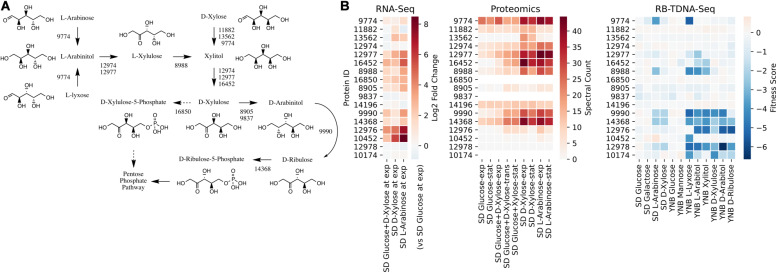
Pentose utilization pathway in *R. toruloides*. **(A)** Pentose sugars and alcohols are converted to D-ribulose-5-phosphate via D-arabinitol dehydrogenases before entering the pentose phosphate pathway. **(B)** Gene expression, protein expression, and fitness scores for pentose utilization pathway genes (exp, exponential phase; trans, transition phase; stat, stationary phase).

We first investigated the metabolic pathways and associated genes involved in D-xylose and L-arabinose utilization using the multi-omics data. The functional annotation and multi-omics data suggested that *R. toruloides* uses an alternative pathway involving D-arabinitol and D-ribulose forming ribulose-5-phosphate instead of the known fungal xylose pathway forming D-xylulose-5-phosphate (Figure 3 and Table 1). Two genes in *R. toruloides* NP11 were annotated as D-arabinitol dehydrogenase, RHTO_07702 and RHTO_07844, and used to identify potential arabinitol dehydrogenases in strain IFO0880. Protein ID 9990 was identified as an ortholog of RHTO_07844 by OrthoMCL, and a BLAST search found matches to D-arabinitol 2-dehydrogenases (converting D-arabinitol to D-ribulose) with relatively high identity (over 50%). Consistent with this annotation, Protein ID 9990 had significant fitness defects in many pentose sugars and alcohols including D-xylose, xylitol, D-xylulose, D-arabinitol, L-arabinose, L-lyxose, and L-arabinitol, but not in D-ribulose. Protein ID 9837 was identified as an ortholog of RHTO_07702 by OrthoMCL, and a BLAST search found matches to D-arabinitol dehydrogenase (NADP^+^), D-arabinitol 2-dehydrogenases, and D-arabinitol 4-dehydrogenase (converting D-xylulose to D-arabinitol) with lower identity (less than 40%). BLAST analysis of *Aspergillus niger* D-arabinitol 4-dehydrogenase (An04g09410) against the *R. toruloides* genome found several hits including protein ID 9837 suggesting its role as D-arabinitol 4-dehydrogenase. However, protein ID 9837 had a weaker fitness defect suggesting that other enzymes participate in the conversion of D-xylulose to D-arabinitol. Among other BLAST hits, protein ID 8905 was upregulated in D-xylose and L-arabinose and had some fitness defect during growth on pentose sugars and alcohols. Therefore, the weak fitness defects for either protein ID 9837 and protein ID 8905 are consistent with genetic redundancy at this step in the xylose utilization pathway. The proposed alternative pathway is supported by our observation that the D-xylulose kinase (protein ID 16850) had very low RNA abundance and no detectable peptides in every condition we tested, and that mutants for protein ID 16850 had no significant fitness defect in any condition tested. Another supporting observation is that the D-ribulose kinase (protein ID 14368) had significant fitness defects in all pentose sugar and alcohol media conditions tested. This pathway is also consistent with recent observations that *R. toruloides* grown on D-xylose transiently accumulates D-arabinitol in the culture media (Jagtap and Rao, 2018). In summary, our omics and genetic data supports an alternative D-xylose and L-arabinose utilization pathway involving a D-ribulose-5-phosphate intermediate rather than a D-xylulose-5-phosphate intermediate (Figure 3).

**TABLE 1:** Genes involved in pentose sugar and alcohol utilization.

**Protein ID**	**Annotation**	***S. cerevisiae* best hit**	**Human best hit**
9774	Alcohol dehydrogenase (NADP+)	YPR1	AKR1A
11882	Glycerol 2-dehydrogenase (NADP+)	YPR1	AKR1A
13562	Alcohol dehydrogenase (NADP+)	ADH7	
12974	Zinc-binding alcohol dehydrogenases	SOR1	SORD
12977	L-iditol 2-dehydrogenase	XYL2	SORD
16452	D-xylulose reductase	SOR1	SORD
8988	Sorbose reductase		DHRS4
16850	Xylulokinase	XKS1	XYLB
8905	Reductases with broad range of substrate specificities	IRC24	DHRS4
9837	D-arabinitol dehydrogenase		
9990	D-arabinitol 2-dehydrogenase	SPS19	CBR4
14368	Ribulose kinase and related carbohydrate kinases	YDR109C	FGGY
12976	Predicted transporter (major facilitator superfamily)	STL1	SLC2A
10452	Predicted transporter (major facilitator superfamily)	RGT2	SLC2A
12978	Fungal specific transcription factor Zn(2)-Cys(6) binuclear cluster domain		
10174	Related to C2H2 zinc finger protein	SDD4	

**FIGURE 4:**
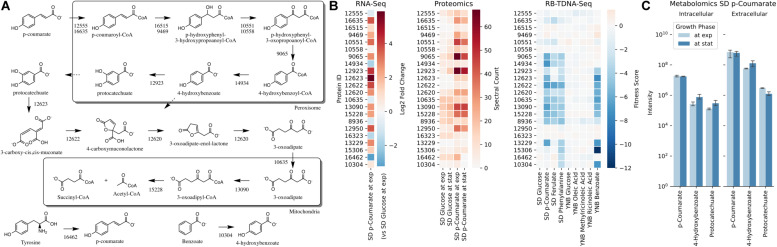
*p*-Coumarate utilization pathway in *R. toruloides*. **(A)** *p*-Coumarate degradation to protocatechuate by a beta-oxidation like pathway in peroxisome, protocatechuate degradation to 3-oxoadipate by the ortho-cleavage pathway in cytosol, and 3-oxoadipate degradation in mitochondria. **(B)** Gene expression, protein expression, and fitness scores for *p*-coumarate utilization pathway genes (exp, exponential phase; stat, stationary phase). **(C)** Intracellular and extracellular measurement of *p*-coumarate and intermediates in *p*-coumarate condition (not detected in glucose, glucose + D-xylose, D-xylose, and L-arabinose conditions).

**FIGURE 5:**
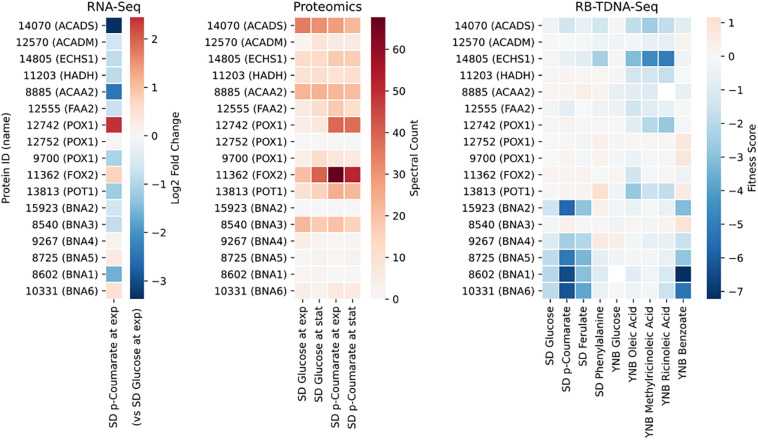
Gene expression, protein expression, and fitness scores for fatty acid beta-oxidation and NAD biosynthesis pathway genes (exp, exponential phase; stat, stationary phase).

Next, we propose that *R. toruloides* metabolizes *p*-coumarate to protocatechuate by a beta-oxidation-like pathway in the peroxisome (Figure 4 and Table 2). Previously known *p*-coumarate utilization pathway in bacteria such as *P. putida* contains *p*-coumaroyl-CoA hydratase/aldolase or feruloyl-CoA hydratase/lyase that hydrolyzes *p*-coumaroyl-CoA to 3S-(4-hydroxyphenyl)-3-hydroxy-propanoyl-CoA and subsequently produces 4-hydroxybenzoyl-CoA and acetyl-CoA. We found that, in *R. toruloides* grown in *p*-coumarate media, enzymes that are similar to peroxisomal fatty acid beta-oxidation enzymes ACSL (long-chain acyl-CoA synthetase), FOX2 (multifunctional enzyme, 3-hydroxyacyl-CoA dehydrogenase, and enoyl-CoA dehydratase), and POT1 (3-ketoacyl-CoA thiolase) were upregulated. Our fitness data from RB-TDNAseq on *p*-coumarate and previously published RB-TDNAseq data on oleic and ricinoleic acid (Coradetti et al., 2018) shows that these enzymes are distinct from the mitochondrial or peroxisomal fatty acid beta-oxidation enzymes (Figure 5 and Table 3) since they did not have a fitness defect in oleic or ricinoleic acid media. Mitochondrial or peroxisomal fatty acid beta-oxidation genes did not have significant fitness defect in *p*-coumarate or ferulate media (Figure 5 and Table 3). The predicted localization of the enzymes involved in *p*-coumarate indicates that *p*-coumarate is first degraded to protocatechuate in the peroxisome, and protocatechuate is transported to the cytosol for further degradation via the 3-oxoadipate pathway. Degradation of *p*-coumarate and ferulate via a beta-oxidation like pathway would result in 4-hydroxybenzoate and vanillate, respectively. Protocatechuate and 4-hydroxybenzoate were detected in the intracellular and extracellular metabolomics of cells grown on *p*-coumarate (Figure 4C). An enzyme similar to kynurenine 3-monooxygenase BNA4 showed fitness defect in *p*-coumarate, but not in ferulate, which indicates it is likely to be a 3-hydroxybenzoate 4-monooxygenase producing protocatechuate from 4-hydroxybenzoate. 4-hydroxybenzoate may also be transported to mitochondria for quinone biosynthesis. A fitness defect in mitochondrial oxoadipate carrier ODC2 suggests that 3-oxoadipate is transported from cytosol to mitochondria for the final steps in the beta-ketoadipate pathway generating succinyl-CoA and acetyl-CoA which can feed into the TCA cycle. Interestingly, several genes involved in aromatic amino acid metabolism showed a significant fitness defect in *p*-coumarate, although they showed some degree of fitness defect in other media conditions. For example, four genes in the tryptophan degradation pathway to 2-amino-3-carboxymuconate semialdehyde via kynurenine (BNA1, BNA2, BNA4, and BNA5) as well as cytosolic aspartate aminotransferase AAT2 had pronounced fitness defects in *p*-coumarate and ferulate (Figure 5 and Table 3). Since *p*-coumarate and 4-hydroxybenzoate are known to be ubiquinone precursors and they are synthesized from aromatic amino acids, it is possible that high concentration of these compounds affects the regulation of aromatic amino acid pathway genes and fitness defect becomes more pronounced. Taken together, the omics and genetic data support a proposed *p*-coumarate utilization pathway that involves formation of protocatechuate in the peroxisome, followed by ortho-cleavage in the cytosol, and then 3-oxoadipate degradation in the mitochondria (Figure 4). Multi-omics analysis and manual curation improved the metabolic model, but their scope was still limited to lignocellulosic carbon utilization pathways. In the next section, we performed a genome-scale evaluation and iteratively improved the model using high-throughput growth phenotyping and functional genomics.

**TABLE 2:** Genes involved in *p*-coumarate utilization.

**Protein ID**	**Annotation**	***S. cerevisiae* best hit**	***P. putida* best hit**	**PTS2 (N-terminal)**	**PTS1 (C-terminal)**	**Pathway**
12555	Long-chain acyl-CoA synthetase	FAA2			AKL*	Peroxisomal *p*-coumarate degradation to protocatechuate
16635	Long-chain acyl-CoA synthetase	PCS60			AKL*	
16515	Enoyl-CoA hydratase/isomerase family				ARL*	
9469	Peroxisomal dehydratase	FOX2			SKL*	
10551	3-oxoacyl-(acyl-carrier protein) reductase	FOX2		6-RLQQVQGQL-14		
10558	3-oxoacyl-(acyl-carrier protein) reductase	FOX2		7-RLSAVSGQL-15		
9065	3-oxoacyl CoA thiolase	POT1				
14934	Alpha/beta hydrolase family				ARL*	
12923	Monooxygenase involved in coenzyme Q (ubiquinone) biosynthesis		pobA		ASL*	
12623	Dioxygenase		pcaH			Protocatechuate degradation via 3-oxoadipate
12622	Lactonase		pcaB			
12620	Carboxymuconolactone decarboxylase family		pcaCD			
13090	3-oxoacid CoA-transferase		pcaIJ			
15228	Acetyl-CoA acyltransferase 1		pcaF			
10635	Mitochondrial 2-oxodicarboxylate transporter	ODC2				
8936	Aspartate aminotransferase, cytoplasmic	AAT2				
12950	Vanillin dehydrogenase	UGA2	ALDH9			
16323	Aldehyde dehydrogenase (NAD+)	HFD1	ALDH3			
13229	Unknown transmembrane protein					
15306	long-chain acyl-CoA synthetase					
16462	Phenylalanine/tyrosine ammonia-lyase					
10304	Cytochrome P450, family 3, subfamily A					

*PTS, peroxisomal targeting sequence.*

**TABLE 3:** Genes involved in fatty acid beta-oxidation and NAD biosynthesis.

**Protein ID**	**Annotation**	***S. cerevisiae* best hit**	**Human best hit**	**Pathway**
14070	Short/branched chain acyl-CoA dehydrogenase		ACADS	Fatty acid beta-oxidation
12570	Acyl-CoA dehydrogenase		ACADM	
14805	Enoyl-CoA hydratase	EHD3	ECHS1	
11203	3-hydroxyacyl-CoA dehydrogenase		HADH	
8885	Acetyl-CoA acyltransferase 2	ERG10	ACAA2	
12555	Long-chain acyl-CoA synthetase	FAA2	ACSL1	
12742	Acyl-CoA oxidase	POX1	ACOX1	
12752	Acyl-CoA oxidase	POX1	ACOX1	
9700	Acyl-CoA oxidase	POX1	ACOX1	
11362	Multifunctional beta-oxidation protein	FOX2	HSD17	
13813	Acetyl-CoA acyltransferase 1	POT1	ACAA1	
15923	Indoleamine 2,3-dioxygenase	BNA2	IDO1	NAD biosynthesis
8540	Kynurenine aminotransferase	BNA3	KYAT3	
9267	Kynurenine 3-monooxygenase	BNA4	KMO	
8725	Kynureninase	BNA5	KYNU	
8602	3-hydroxyanthranilate 3,4-dioxygenase	BNA1	HAAO	
10331	Nicotinate-nucleotide pyrophosphorylase (carboxylating)	BNA6	QPRT	

### Validation and Reconciliation of Growth Phenotype and Gene Essentiality Predictions

**FIGURE 6:**
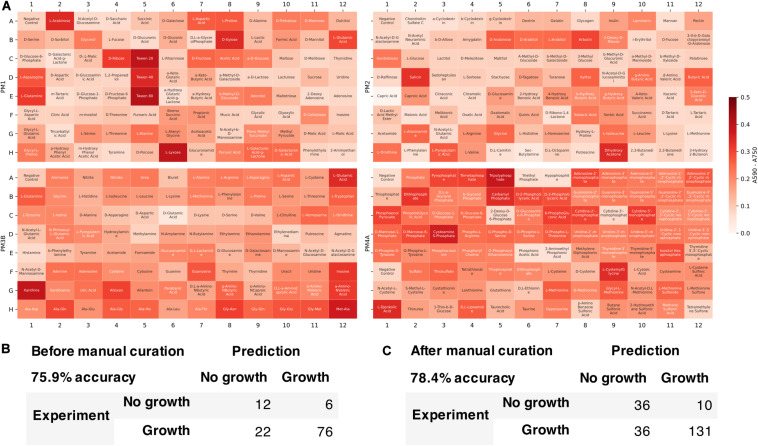
Model evaluation using high-throughput growth phenotype data. **(A)** Biolog phenotype microarray data (white indicates low growth and red indicates high growth). Comparison of model predicted growth and experimental data **(B)** before and **(C)** after manual curation to include more metabolites.

We tested the developed metabolic model’s capability to predict growth on different carbon, nitrogen, sulfur, and phosphate sources. Growth phenotype data from Biolog Phenotype MicroArrays were used to evaluate the model predictions (Figure 6). Among 384 conditions in Biolog plates (PM1, PM2, PM3B, and PM4A), 116 conditions could be simulated with the model since not all the metabolites were present in the metabolic network. Of these 116 conditions, the model correctly predicted 76 positive and 12 negative growth phenotypes, and incorrectly predicted 6 false positive and 22 false negative growth phenotypes. The overall accuracy was 75.9%, comparable to previously published metabolic models of other organisms [e.g., *E. coli i*AF1260 model (Feist et al., 2007) with 75.9% accuracy in 170 conditions]. We then manually refined the model to include more metabolites found in Biolog plates and reconcile the inconsistencies. The updated model was able to simulate 213 conditions in Biolog plates with 78.4% accuracy and Matthew’s correlation coefficient of 0.493.

**FIGURE 7:**
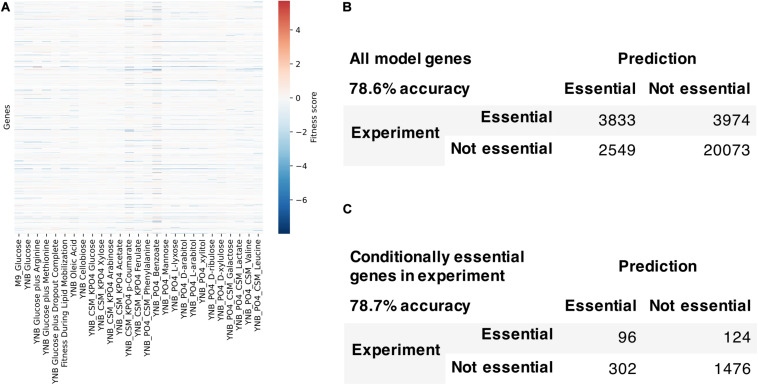
Model evaluation using high-throughput gene essentiality data. **(A)** RB-TDNA sequencing fitness score data for all genes in the model. Comparison of model predicted gene essentiality and experimental data for **(B)** all model genes and **(C)** conditionally essential genes in experiment.

We used the fitness scores from RB-TDNAseq to evaluate the model’s capability to predict conditionally essential genes in different growth conditions (Figure 7). Genes were considered essential if they were classified as essential in our previous RB-TDNAseq study (Coradetti et al., 2018) or fitness score was less than a cut-off value. There were 1147 genes in the model, but 15 genes were mitochondrial and excluded from this analysis since their essentiality was not available from the RB-TDNAseq data. The model predicted gene essentiality for 1132 genes in 27 different growth conditions with 72.7% accuracy and Matthew’s correlation coefficient of 0.388. The model was further refined to resolve the inconsistencies and several genes with erroneous ortholog mapping were removed from the model. The refined model had 1142 genes, 2398 reactions, and 2051 metabolites (1205 unique metabolites), and predicted gene essentiality for 1127 non-mitochondrial genes in 27 conditions with 78.6% accuracy and Matthew’s correlation coefficient of 0.406 [see Supplementary File 5 for a comparison with a previously published model (Dinh et al., 2019)]. Among these 1127 genes, 281 genes were essential across all conditions, 772 genes were not essential under any conditions, and 74 genes were essential under only certain conditions. For these 74 conditionally essential genes, the refined model predicted gene essentiality with 78.7% accuracy.

## Discussion

In this work, we have developed a genome-scale metabolic network model of *R. toruloides* and utilized the model to study the metabolic pathways for utilizing carbon sources derived from lignocellulosic biomass. The initial metabolic network was reconstructed from high-quality published metabolic network models of other organisms using orthologous protein mapping. There is some risk of incorrect reaction identification from the false positives in ortholog identification due to horizontal gene transfer (HGT), and there are many examples that highlight the importance of HGT in ascomycete yeasts (Goncalves et al., 2018; Shen et al., 2018; Kominek et al., 2019; Devia et al., 2020). However, ortholog identification continues to be a standard practice for the initial reconstruction of genome-scale metabolic networks for non-model organisms and currently we do not have evidence of large scale HGT in *R*. *toruloides*. As with any model, our model will need to be improved and re-evaluated over time in cases of HGT and as new metabolic pathways are characterized. The developed model contains 1141 genes, 2398 reactions, and 2051 metabolites (1205 unique metabolites) in nine compartments. The lipid body was separated from the biomass equation allowing the independent simulation of lipid accumulation or mobilization in oleaginous yeasts. The separation of the lipid body from biomass eliminated the need for more than one biomass equation depending on the growth condition or lipid content. Multi-omics analysis and metabolic network reconstruction identified unique reactions and enzymes as well as their localization for unique pentose and aromatic compound utilization pathways in *R. toruloides*. The pentose and aromatic compound utilization pathways proposed in this study have not been suggested in previously published multi-omics studies or genome-scale metabolic models of *R. toruloides* (Bommareddy et al., 2015; Dinh et al., 2019; Tiukova et al., 2019a,b; Lopes et al., 2020; Pinheiro et al., 2020). The first genome-scale metabolic model for *R. toruloides* was recently built for strain NP11 (Tiukova et al., 2019b), and a proteomics study of xylose metabolism was conducted by the same research group (Tiukova et al., 2019a). Another genome-scale metabolic model was shortly after published for strain IFO0880 utilizing the functional genomics data (Coradetti et al., 2018). More recent studies utilized these models to study the utilization of different carbon sources, but their focus was primarily on lipid production (Lopes et al., 2020; Pinheiro et al., 2020). The metabolic network model developed in this study was reconstructed and manually curated reproducibly using multi-omics data and electronic notebooks, and validated against high-throughput growth phenotypes in 213 growth conditions and conditional gene essentiality in 27 growth conditions with high prediction accuracies, significantly expanding the breadth and depth of metabolic coverage from previously published models (Dinh et al., 2019; Tiukova et al., 2019b). We believe that the developed metabolic network for *R. toruloides* is most complete and accurate to date, and the multi-omics data and metabolic model presented in this study will be useful for studying and engineering *R. toruloides* for lignocellulosic biomass conversion.

## Data Availability Statement

The RNA sequencing data (SRP143805–SRP143836 and SRP143838) and RB-TDNA sequencing data (PRJNA595384) are available at the NCBI Sequence Read Archive. Fitness scores are available at the fungal fitness browser (http://fungalfit.genomics.lbl.gov). The mass spectrometry proteomics data are deposited to the ProteomeXchange Consortium via the PRIDE (Perez-Riverol et al., 2019) partner repository with the dataset identifier PXD022377 and 10.6019/PXD02237. The metabolomics data are deposited to the Open Science Framework (OSF) and available at https://osf.io/tnqwx/. Processed multi-omics dataset is available as a Supplementary File. The Jupyter notebooks, processed multi-omics dataset, and genome-scale metabolic network model files will also be made available on a GitHub repository (https://github.com/AgileBioFoundry/Rt_IFO0880).

## Author Contributions

JK, SC, JS, JG, and JM conceived and designed the experiments. JK, SC, JZ, JY, NM, Y-MK, YG, and KB-J performed the experiments and analyzed the data. JK, SC, Y-MK, and YG wrote the initial manuscript. All authors read, revised, and approved the final manuscript.

## Conflict of Interest

The authors declare that the research was conducted in the absence of any commercial or financial relationships that could be construed as a potential conflict of interest.

## Footnotes

1 http://fungalfit.genomics.lbl.gov

2 https://omics.pnl.gov/software/masic-results-merger

3 https://jupyter.org

## Supplementary Material

Supplementary File 1 Jupyter notebooks for metabolic network model development and multi-omics data analysis.

Supplementary File 2 Genome-scale metabolic network model of *R. toruloides* IFO0880 in JSON, SBML, and MAT format and the Memote HTML report.

Supplementary File 3 Genome annotation of *R. toruloides* IFO0880.

Supplementary File 4 Multi-omics dataset for *R. toruloides* IFO0880.

Supplementary File 5 Comparison of gene essentiality predictions by the developed model and a previously published model.
