# Supplementary material for: Multi-Omics Driven Metabolic Network Reconstruction and Analysis of Lignocellulosic Carbon Utilization in Rhodosporidium toruloides
Source: Front Bioeng Biotechnol. 2021 Jan 8;8:612832. doi: 10.3389/fbioe.2020.612832 (PMC7873862; doi:10.3389/fbioe.2020.612832)
Supplement: Supplementary File 4 — Multi-omics dataset for R. toruloides IFO0880. [file Data_Sheet_1.zip › Supplementary File S1/2.Metabolic_modeling/Refinement_2d_Coumarate_Pentose_Lipid_Degradation.html]

Refinement\_2d\_Coumarate\_Pentose\_Lipid\_Degradation


In [1]:

```
%matplotlib inline
from matplotlib import pyplot as plt
from matplotlib import colors
import csv
import numpy as np
import pandas as pd
import cobra
```

In [2]:

```
Annotation = pd.read_excel('../../Data/R_toruloides_Data_for_Reconstruction.xlsx',
                          sheet_name='Annotation', index_col=0)
Annotation.index = Annotation.index.map(str)
Annotation = Annotation.fillna('')
Transcriptomics = pd.read_excel('../../Data/R_toruloides_Data_for_Reconstruction.xlsx',
                          sheet_name='Transcriptomics', header=[0,1,2,3], index_col=0)
Transcriptomics.index = Transcriptomics.index.map(str)
Proteomics = pd.read_excel('../../Data/R_toruloides_Data_for_Reconstruction.xlsx',
                          sheet_name='Proteomics', header=[0,1,2], index_col=0)
Proteomics.index = Proteomics.index.map(str)
Fitness = pd.read_excel('../../Data/R_toruloides_Data_for_Reconstruction.xlsx',
                          sheet_name='Fitness', index_col=0)
Fitness.index = Fitness.index.map(str)
```

In [3]:

```
def background_gradient(s, cmap='seismic', text_color_threshold=0.408):
    lim = max(abs(s.min().min()),abs(s.max().max()))
    rng = 2.0*lim
    norm = colors.Normalize(-lim - (rng * 0.2), lim + (rng * 0.2))
    rgbas = plt.cm.get_cmap(cmap)(norm(s.values))
    def relative_luminance(rgba):
        r, g, b = (x / 12.92 if x <= 0.03928 else ((x + 0.055) / 1.055 ** 2.4) for x in rgba[:3])
        return 0.2126 * r + 0.7152 * g + 0.0722 * b
    def css(rgba):
        dark = relative_luminance(rgba) < text_color_threshold
        text_color = '#f1f1f1' if dark else '#000000'
        return 'background-color: {b};color: {c};'.format(b=colors.rgb2hex(rgba), c=text_color)

    if s.ndim == 1:
        return [css(rgba) for rgba in rgbas]
    else:
        return pd.DataFrame([[css(rgba) for rgba in row] for row in rgbas], index=s.index, columns=s.columns)

def Show_Data(x):
    display(Transcriptomics.loc[x].style.background_gradient(cmap='Reds', low=0.2, high=0.2, axis=None))
    temp = [y for y in x if y in Proteomics.index]
    display(Proteomics.loc[temp].style.background_gradient(cmap='Reds', low=0.2, high=0.2, axis=None))
    temp = [y for y in x if y in Fitness.index]
    display(Fitness.loc[temp].style.apply(background_gradient, cmap='seismic', axis=None))
    return;
```

In [4]:

```
model = cobra.io.load_json_model("IFO0880_GPR_2c.json")
```

In [5]:

```
eco = cobra.io.load_json_model('../../Data/BiGG_Models/iML1515.json')
sce = cobra.io.load_json_model('../../Data/BiGG_Models/iMM904.json')
hsa = cobra.io.load_json_model('../../Data/BiGG_Models/RECON1.json')
hsa2 = cobra.io.load_json_model('../../Data/BiGG_Models/Recon3D.json')
ptri = cobra.io.load_json_model('../../Data/BiGG_Models/iLB1027_lipid.json')
ppu = cobra.io.load_json_model('../../Data/BiGG_Models/iJN746.json')
```

### Coumarate degradation pathway¶

In [6]:

```
for r in sorted(model.metabolites.get_by_id('coucoa_c').reactions, key=lambda x: x.id):
    print(r, r.gene_reaction_rule)
print()
for r in sorted(model.metabolites.get_by_id('coucoa_m').reactions, key=lambda x: x.id):
    print(r, r.gene_reaction_rule)
print()
for r in sorted(model.metabolites.get_by_id('4hbz_c').reactions, key=lambda x: x.id):
    print(r, r.gene_reaction_rule)
print()
for r in sorted(model.metabolites.get_by_id('4hbz_m').reactions, key=lambda x: x.id):
    print(r, r.gene_reaction_rule)
```

```
4CMCOAS: T4hcinnm_c + atp_c + coa_c --> amp_c + coucoa_c + ppi_c 11833 or 13700 or 14802 or 16129

COUCOAFm: T4hcinnm_m + atp_m + coa_m --> amp_m + coucoa_m + ppi_m 16635

4HBHYOX: 4hbz_c + h_c + nadph_c + o2_c --> 34dhbz_c + h2o_c + nadp_c 9267
VNDH_2: 4hbald_c + h2o_c + nad_c --> 4hbz_c + 2.0 h_c + nadh_c 12950

4HBALDDm: 4hbald_m + h2o_m + nad_m --> 4hbz_m + 2.0 h_m + nadh_m 16323
HBZNPT_m: 4hbz_m + npdp_m --> 3nphb_m + ppi_m 15237
```

In [7]:

```
temp = ['11833','13700','14802','16129','16635','9267','12950','16323']
display(Annotation.loc[temp])
Show_Data(temp)
```

|  | Combined Annotations | Signal P | Sc288c Orthologs | Human Orthologs | Sc288 Best Hit | Human Blast | Essential | WolfPSort | C Terminal |
| --- | --- | --- | --- | --- | --- | --- | --- | --- | --- |
| RTO4\_ID |  |  |  |  |  |  |  |  |  |
| 11833 | KOG1176: Acyl-CoA synthetase |  |  |  | PCS60 | ACSF2 | Not Essential | cyto 16, cyto\_nucl 11.5, nucl 5, pero 5 | AKL\* |
| 13700 | K01904: 4CL; 4-coumarate--CoA ligase |  |  |  | PCS60 | ACSM3 | Not Essential | cyto 16.5, cyto\_nucl 12.5, nucl 5.5, mito 3 | LPS\* |
| 14802 | K01904: 4CL; 4-coumarate--CoA ligase |  |  |  | PCS60 | ACSF2 | Not Essential | cyto 17.5, cyto\_nucl 9.5, plas 5, pero 2 | SKL\* |
| 16129 | KOG1176: Acyl-CoA synthetase |  |  |  | PCS60 | ACSF2 | Not Essential | nucl 6.5, cyto\_nucl 6.5, plas 6, cyto 5.5, mit... | AKL\* |
| 16635 | K01897: ACSL, fadD; long-chain acyl-CoA synthe... |  |  |  | PCS60 | ACSF2 | Not Essential | mito 18, cyto 5.5, cyto\_nucl 3.5, pero 3 | AKL\* |
| 9267 | K00486: KMO; kynurenine 3-monooxygenase | S | BNA4 | KMO | BNA4 | KMO | Not Essential | cyto 9, extr 6, E.R. 5, mito 2, plas 2, pero 2 | RVD\* |
| 12950 | KOG2450: Aldehyde dehydrogenase |  |  |  | UGA2 | ALDH9 | Not Essential | cyto 21.5, cyto\_nucl 13, mito 3 | PSA\* |
| 16323 | K00128: ALDH; aldehyde dehydrogenase (NAD+) |  | HFD1 | ALDH3A1,ALDH3A2,ALDH3B1,ALDH3B2 | HFD1 | ALDH3 | Not Essential | cyto 10.5, plas 7, cyto\_nucl 7, mito 3, nucl 2... | GQA\* |

| strain | WT | | | | | | | | | | | | | | | | |
| --- | --- | --- | --- | --- | --- | --- | --- | --- | --- | --- | --- | --- | --- | --- | --- | --- | --- |
| condition | G\_MM | C\_MM | G\_SD | | GX\_SD | | | X\_SD | | A\_SD | | C\_SD | | MM\_CN120 | | MM\_CN5 | Diversity\_Sample |
| phase | exp | exp | exp | stat | exp | trans | stat | exp | stat | exp | stat | exp | stat | exp | stat | exp | exp |
| proteinId | Set1 | Set1 | Set2 | Set2 | Set2 | Set2 | Set2 | Set2 | Set2 | Set2 | Set2 | Set2 | Set2 | Set3 | Set3 | Set3 | Set3 |
| 11833 | 2.462226 | 4.207369 | 3.195762 | 4.475198 | 3.208968 | 3.711798 | 3.109949 | 3.176368 | 4.554590 | 3.825781 | 4.687664 | 3.848133 | 3.231933 | 4.231241 | 3.296411 | 4.262610 | 3.401036 |
| 13700 | 5.747211 | 5.328729 | 7.420104 | 7.100011 | 7.264505 | 6.372336 | 6.236862 | 6.782212 | 5.657107 | 6.704056 | 6.027379 | 5.342598 | 5.030441 | 5.262467 | 5.068991 | 7.325319 | 6.074078 |
| 14802 | 6.193150 | 5.984620 | 6.746985 | 5.399262 | 6.625147 | 5.874359 | 5.806040 | 5.906733 | 6.075098 | 6.203444 | 6.047200 | 5.277981 | 5.425416 | 6.350482 | 5.710859 | 5.527883 | 6.211514 |
| 16129 | 2.392363 | 4.222824 | 3.552490 | 4.796803 | 3.396427 | 3.754885 | 3.956839 | 2.918396 | 4.913993 | 3.579133 | 5.226964 | 1.348858 | 1.271953 | 3.000626 | 2.099691 | 3.781023 | 3.012403 |
| 16635 | 4.274643 | 9.223314 | 3.952433 | 5.180428 | 4.380874 | 5.238679 | 5.008569 | 4.035667 | 4.424341 | 5.107619 | 4.807358 | 7.591964 | 6.556446 | 4.192554 | 3.549286 | 4.917594 | 3.736052 |
| 9267 | 5.805794 | 4.834441 | 5.368139 | 5.400411 | 5.345808 | 5.643300 | 5.797522 | 5.282517 | 5.673452 | 5.219721 | 5.527434 | 5.513204 | 5.148040 | 5.523957 | 5.813412 | 4.820023 | 5.508240 |
| 12950 | 4.923510 | 9.111523 | 5.051287 | 5.980611 | 4.994633 | 4.906592 | 4.935974 | 5.040282 | 5.328005 | 5.319617 | 5.336866 | 8.699229 | 7.154067 | 5.505516 | 5.164858 | 6.161514 | 6.591077 |
| 16323 | 5.541944 | 5.505299 | 6.372264 | 5.686436 | 6.285069 | 6.139204 | 5.868948 | 6.061038 | 5.825760 | 5.509846 | 6.172787 | 4.764325 | 4.749436 | 5.591074 | 6.404342 | 5.999526 | 6.385022 |

| strain | WT | | | | | | | | | | |
| --- | --- | --- | --- | --- | --- | --- | --- | --- | --- | --- | --- |
| condition | G\_SD | | GX\_SD | | | X\_SD | | A\_SD | | C\_SD | |
| proteinId | exp | stat | exp | trans | stat | exp | stat | exp | stat | exp | stat |
| 11833 | 0.000000 | 0.883033 | 0.000000 | 0.000000 | 0.000000 | 0.000000 | 0.000000 | 0.000000 | 0.000000 | 0.220910 | 0.000000 |
| 13700 | 10.763347 | 12.179929 | 12.598575 | 15.469758 | 15.519320 | 17.232456 | 17.711471 | 14.293587 | 13.977507 | 13.459443 | 11.345356 |
| 14802 | 11.901366 | 16.528810 | 11.607841 | 11.890648 | 13.608379 | 12.325402 | 14.650704 | 11.200095 | 11.679182 | 14.535119 | 14.143110 |
| 16129 | 0.000000 | 0.000000 | 0.000000 | 0.000000 | 0.000000 | 0.000000 | 0.000000 | 0.000000 | 0.000000 | 0.431248 | 0.000000 |
| 16635 | 0.738459 | 4.501624 | 2.430184 | 6.308786 | 8.590841 | 4.914107 | 4.728599 | 3.667807 | 6.730556 | 29.904117 | 22.861660 |
| 9267 | 4.803580 | 1.970966 | 5.702218 | 2.974494 | 3.815436 | 1.180981 | 0.198706 | 0.770643 | 0.764589 | 1.285303 | 1.082486 |
| 12950 | 5.168892 | 7.216470 | 6.103060 | 7.460887 | 8.759734 | 11.183392 | 12.600785 | 9.860301 | 10.707912 | 37.599808 | 29.842009 |
| 16323 | 10.990173 | 12.568266 | 13.635080 | 14.134248 | 18.225233 | 11.320916 | 13.924717 | 9.278174 | 10.167519 | 6.607959 | 3.933982 |

|  | Glucose | Xylose | Arabinose | Acetate | Coumarate | Ferulate | YNB Oleic Acid | YNB Ricinoleic Acid | YNB Glucose | YNB Gluc DOC | YPD |
| --- | --- | --- | --- | --- | --- | --- | --- | --- | --- | --- | --- |
| proteinId |  |  |  |  |  |  |  |  |  |  |  |
| 11833 | 0.218588 | -0.139988 | 0.124940 | -0.131598 | -0.032305 | 0.039347 | 0.176377 | 0.128813 | 0.120682 | -0.082954 | -0.133672 |
| 13700 | -0.107884 | -0.155441 | -0.254230 | -0.038800 | 0.149475 | -0.054494 | -0.066590 | -0.169330 | -0.253860 | -0.328580 | -0.324718 |
| 14802 | 0.318433 | 0.250797 | 0.241559 | 0.171794 | -0.011931 | 0.176682 | -0.117700 | -0.091717 | 0.131314 | -0.033473 | -0.125359 |
| 16129 | -0.223651 | -0.013342 | 0.159221 | -0.114595 | -0.029694 | -0.117031 | 0.055164 | 0.063325 | 0.415751 | 0.445395 | -0.195331 |
| 16635 | -0.386094 | 0.074868 | -0.246490 | -0.872329 | -1.765683 | -3.600674 | -0.198188 | -0.037413 | 0.311272 | 0.402185 | 0.872770 |
| 9267 | -1.031030 | 0.119221 | 0.044067 | 0.267081 | -3.396104 | -2.788659 | -0.168550 | -0.540039 | 0.365575 | -0.699194 | -0.124923 |
| 12950 | 0.005965 | -0.171781 | 0.205573 | 0.056949 | 0.122395 | 0.160439 | 0.000605 | 0.089254 | 0.233256 | 0.310560 | 0.190096 |
| 16323 | 0.000210 | -0.017432 | 0.014059 | -0.110443 | 0.004408 | 0.089277 | 0.155043 | 0.103889 | -0.177274 | -0.243731 | -0.140368 |

In [8]:

```
for x in temp:
    if x in model.genes:
        for r in sorted(model.genes.get_by_id(x).reactions, key=lambda x: x.id):
            print(r, r.gene_reaction_rule)
    else:
        print(x, 'no reactions')
    print()
```

```
4CMCOAS: T4hcinnm_c + atp_c + coa_c --> amp_c + coucoa_c + ppi_c 11833 or 13700 or 14802 or 16129

4CMCOAS: T4hcinnm_c + atp_c + coa_c --> amp_c + coucoa_c + ppi_c 11833 or 13700 or 14802 or 16129

4CMCOAS: T4hcinnm_c + atp_c + coa_c --> amp_c + coucoa_c + ppi_c 11833 or 13700 or 14802 or 16129

4CMCOAS: T4hcinnm_c + atp_c + coa_c --> amp_c + coucoa_c + ppi_c 11833 or 13700 or 14802 or 16129

COUCOAFm: T4hcinnm_m + atp_m + coa_m --> amp_m + coucoa_m + ppi_m 16635

4HBHYOX: 4hbz_c + h_c + nadph_c + o2_c --> 34dhbz_c + h2o_c + nadp_c 9267
KYN3OX: Lkynr_c + h_c + nadph_c + o2_c --> h2o_c + hLkynr_c + nadp_c 9267

COALDDH: conialdh_c + h2o_c + nad_c --> fer_c + 2.0 h_c + nadh_c 12950
VNDH: h2o_c + nad_c + vanln_c --> 2.0 h_c + nadh_c + vanlt_c 12950
VNDH_2: 4hbald_c + h2o_c + nad_c --> 4hbz_c + 2.0 h_c + nadh_c 12950
VNDH_3: 34dhbald_c + h2o_c + nad_c --> 34dhbz_c + 2.0 h_c + nadh_c 12950

4HBALDDm: 4hbald_m + h2o_m + nad_m --> 4hbz_m + 2.0 h_m + nadh_m 16323
ALDD16er: h2o_r + hxdcal_r + nad_r --> 2.0 h_r + hdca_r + nadh_r 16323
ALDD21er: h2o_r + nad_r + pristanal_r --> 2.0 h_r + nadh_r + prist_r 16323
ALDD3C161er: h2o_r + hxdceal_r + nad_r --> 2.0 h_r + hdc2ea_r + nadh_r 16323
```

4CMCOAS: T4hcinnm\_c + atp\_c + coa\_c --> amp\_c + coucoa\_c + ppi\_c  
16635 / pero long-chain acyl-CoA synthetase, upregulated and defect in both -> ok, but peroxisomal (AKL\*)

In p. putida, coucoa is converted to 4-hydroxybenzaldehyde and accoa  
COCOAHA: coucoa\_c + h2o\_c --> 4hbald\_c + accoa\_c

If peroxisomal beta-oxidation like pathway, the following reaction needs to happen  
http://www.plantcell.org/content/26/5/1938  
(E)-4-coumaroyl-CoA + H2O -> 3S-(4-hydroxyphenyl)-3-hydroxy-propanoyl-CoA  
3S-(4-hydroxyphenyl)-3-hydroxy-propanoyl-CoA + NAD+ -> 3-(4-hydroxyphenyl)-3-oxo-propanoyl-CoA + NADH + H+  
3-(4-hydroxyphenyl)-3-oxo-propanoyl-CoA + coenzyme A -> 4-hydroxybenzoyl-CoA + acetyl-CoA

VNDH\_2: 4hbald\_c + h2o\_c + nad\_c --> 4hbz\_c + 2.0 h\_c + nadh\_c  
12950 / cyto vanillin dehydrogenase / up / up / no defect in both -> may not be important in beta-oxidation  
4HBALDDm: 4hbald\_m + h2o\_m + nad\_m --> 4hbz\_m + 2.0 h\_m + nadh\_m  
16323 HFD1 known to catalyze this reaction in S. cer / down / down / no defect in both -> more evidence that p-coumarate is directly converted to 4hbz instead of 4hbald

1. T4hcinnm\_x + atp\_x + coa\_x --> amp\_x + coucoa\_x + ppi\_x

In [9]:

```
temp = Annotation.index[Annotation['Combined Annotations'].str.contains('cyl-CoA synthetase') | 
                        Annotation['Combined Annotations'].str.contains('4-coumarate--CoA ligase')]
display(Annotation.loc[temp])
```

|  | Combined Annotations | Signal P | Sc288c Orthologs | Human Orthologs | Sc288 Best Hit | Human Blast | Essential | WolfPSort | C Terminal |
| --- | --- | --- | --- | --- | --- | --- | --- | --- | --- |
| RTO4\_ID |  |  |  |  |  |  |  |  |  |
| 11167 | K01897: ACSL, fadD; long-chain acyl-CoA synthe... |  | FAA3,FAA4,FAA1 | ACSL3,ACSL4 | FAA1 | ACSL4 | Not Essential | cyto 11, cyto\_nucl 8, mito 7, nucl 3, plas 3 | VYP\* |
| 11833 | KOG1176: Acyl-CoA synthetase |  |  |  | PCS60 | ACSF2 | Not Essential | cyto 16, cyto\_nucl 11.5, nucl 5, pero 5 | AKL\* |
| 12272 | KOG1176: Acyl-CoA synthetase |  |  |  |  |  | Not Essential | mito 27 | MSQ\* |
| 12538 | KOG1256: Long-chain acyl-CoA synthetases (AMP-... |  | FAA2 | ACSL1,ACSL5,ACSL6,CTB-127M13.1 | FAA2 | ACSL5 | Not Essential | mito 18.5, cyto\_mito 12.5, cyto 5.5 | GEQ\* |
| 12555 | K01897: ACSL, fadD; long-chain acyl-CoA synthe... |  | FAA2 | ACSL1,ACSL5,ACSL6,CTB-127M13.1 | FAA2 | ACSL1 | Not Essential | cyto 15.5, cyto\_nucl 11.5, pero 6, nucl 4.5 | AKL\* |
| 13700 | K01904: 4CL; 4-coumarate--CoA ligase |  |  |  | PCS60 | ACSM3 | Not Essential | cyto 16.5, cyto\_nucl 12.5, nucl 5.5, mito 3 | LPS\* |
| 14802 | K01904: 4CL; 4-coumarate--CoA ligase |  |  |  | PCS60 | ACSF2 | Not Essential | cyto 17.5, cyto\_nucl 9.5, plas 5, pero 2 | SKL\* |
| 15745 | KOG1256: Long-chain acyl-CoA synthetases (AMP-... |  |  |  |  |  | Not Essential | cyto\_nucl 11.333, nucl 11, cyto 7.5, cyto\_mito... | VDS\* |
| 15746 | KOG1180: Acyl-CoA synthetase |  |  |  | FAA1 | ACSL1 | Not Essential | cyto 13, mito 5, cysk 4, mito\_nucl 4 | EPY\* |
| 15748 | K01897: ACSL, fadD; long-chain acyl-CoA synthe... |  | FAA3,FAA4,FAA1 | ACSL3,ACSL4 | FAA1 | ACSL4 | Not Essential | cyto 23, mito 3 | VYP\* |
| 15900 | K01896: ACSM; medium-chain acyl-CoA synthetase |  |  | ACSM1,ACSM2A,ACSM2B,ACSM3,ACSM4,ACSM5 | ACS2 | ACSM3 | Not Essential | cyto 13, pero 11, cyto\_nucl 8.5 | AKL\* |
| 16129 | KOG1176: Acyl-CoA synthetase |  |  |  | PCS60 | ACSF2 | Not Essential | nucl 6.5, cyto\_nucl 6.5, plas 6, cyto 5.5, mit... | AKL\* |
| 16635 | K01897: ACSL, fadD; long-chain acyl-CoA synthe... |  |  |  | PCS60 | ACSF2 | Not Essential | mito 18, cyto 5.5, cyto\_nucl 3.5, pero 3 | AKL\* |

In [10]:

```
Show_Data(temp)
```

| strain | WT | | | | | | | | | | | | | | | | |
| --- | --- | --- | --- | --- | --- | --- | --- | --- | --- | --- | --- | --- | --- | --- | --- | --- | --- |
| condition | G\_MM | C\_MM | G\_SD | | GX\_SD | | | X\_SD | | A\_SD | | C\_SD | | MM\_CN120 | | MM\_CN5 | Diversity\_Sample |
| phase | exp | exp | exp | stat | exp | trans | stat | exp | stat | exp | stat | exp | stat | exp | stat | exp | exp |
| proteinId | Set1 | Set1 | Set2 | Set2 | Set2 | Set2 | Set2 | Set2 | Set2 | Set2 | Set2 | Set2 | Set2 | Set3 | Set3 | Set3 | Set3 |
| RTO4\_ID |  |  |  |  |  |  |  |  |  |  |  |  |  |  |  |  |  |
| 11167 | 5.962100 | 7.099869 | 4.849938 | 6.051179 | 4.800787 | 5.320248 | 5.450063 | 5.138514 | 5.545723 | 6.104974 | 6.084653 | 7.846552 | 6.746449 | 5.278418 | 5.517599 | 6.016110 | 5.958044 |
| 11833 | 2.462226 | 4.207369 | 3.195762 | 4.475198 | 3.208968 | 3.711798 | 3.109949 | 3.176368 | 4.554590 | 3.825781 | 4.687664 | 3.848133 | 3.231933 | 4.231241 | 3.296411 | 4.262610 | 3.401036 |
| 12272 | 7.163965 | 6.067678 | 9.303781 | 7.718959 | 9.276542 | 9.154729 | 8.824815 | 8.048876 | 7.041213 | 7.273689 | 6.715856 | 5.312591 | 3.331953 | 7.048996 | 7.872964 | 6.576304 | 7.198535 |
| 12538 | 5.193203 | 5.553215 | 4.160336 | 3.684146 | 4.677966 | 4.372388 | 4.526573 | 3.910037 | 4.362894 | 4.968690 | 3.659945 | 6.546178 | 6.535830 | 5.102117 | 4.962309 | 5.508203 | 5.457478 |
| 12555 | 6.453199 | 6.511665 | 5.433267 | 5.782332 | 5.426938 | 5.525507 | 5.263275 | 5.257378 | 6.122703 | 6.156341 | 6.452491 | 4.764078 | 5.102459 | 6.787262 | 5.690798 | 6.007580 | 6.185189 |
| 13700 | 5.747211 | 5.328729 | 7.420104 | 7.100011 | 7.264505 | 6.372336 | 6.236862 | 6.782212 | 5.657107 | 6.704056 | 6.027379 | 5.342598 | 5.030441 | 5.262467 | 5.068991 | 7.325319 | 6.074078 |
| 14802 | 6.193150 | 5.984620 | 6.746985 | 5.399262 | 6.625147 | 5.874359 | 5.806040 | 5.906733 | 6.075098 | 6.203444 | 6.047200 | 5.277981 | 5.425416 | 6.350482 | 5.710859 | 5.527883 | 6.211514 |
| 15745 | 6.956400 | 6.062864 | 5.634024 | 3.392809 | 5.800437 | 3.444051 | 3.852203 | 5.469548 | 4.143451 | 6.260851 | 4.350922 | 5.622096 | 5.248790 | 7.506001 | 6.535418 | 6.971814 | 5.859271 |
| 15746 | 6.175129 | 5.008712 | 5.357361 | 7.018932 | 5.227077 | 6.167502 | 6.494956 | 5.492796 | 6.057375 | 5.800541 | 6.302842 | 4.907049 | 4.687159 | 5.616707 | 6.113755 | 4.261973 | 4.280489 |
| 15748 | 7.663960 | 6.192385 | 6.485672 | 8.825018 | 6.430268 | 7.532425 | 8.157089 | 6.409416 | 7.662987 | 6.287433 | 8.069599 | 7.018318 | 7.075659 | 7.782435 | 7.128693 | 7.435879 | 6.183987 |
| 15900 | 4.895893 | 6.000990 | 6.468589 | 5.988348 | 6.372042 | 5.191828 | 7.416429 | 6.182437 | 4.598664 | 4.897131 | 4.910779 | 4.425038 | 3.574367 | 3.853144 | 3.965801 | 4.415173 | 3.665578 |
| 16129 | 2.392363 | 4.222824 | 3.552490 | 4.796803 | 3.396427 | 3.754885 | 3.956839 | 2.918396 | 4.913993 | 3.579133 | 5.226964 | 1.348858 | 1.271953 | 3.000626 | 2.099691 | 3.781023 | 3.012403 |
| 16635 | 4.274643 | 9.223314 | 3.952433 | 5.180428 | 4.380874 | 5.238679 | 5.008569 | 4.035667 | 4.424341 | 5.107619 | 4.807358 | 7.591964 | 6.556446 | 4.192554 | 3.549286 | 4.917594 | 3.736052 |

| strain | WT | | | | | | | | | | |
| --- | --- | --- | --- | --- | --- | --- | --- | --- | --- | --- | --- |
| condition | G\_SD | | GX\_SD | | | X\_SD | | A\_SD | | C\_SD | |
| proteinId | exp | stat | exp | trans | stat | exp | stat | exp | stat | exp | stat |
| 11167 | 3.853073 | 9.083894 | 3.246704 | 6.152096 | 6.184541 | 6.855645 | 7.095154 | 9.279108 | 10.139292 | 24.000210 | 18.934554 |
| 11833 | 0.000000 | 0.883033 | 0.000000 | 0.000000 | 0.000000 | 0.000000 | 0.000000 | 0.000000 | 0.000000 | 0.220910 | 0.000000 |
| 12272 | 9.635190 | 8.573860 | 8.571738 | 10.417171 | 9.603604 | 10.179092 | 7.892229 | 2.514253 | 3.243563 | 3.194083 | 1.541104 |
| 12538 | 0.000000 | 0.335899 | 0.000000 | 0.189313 | 0.000000 | 0.000000 | 0.393854 | 0.576179 | 1.338001 | 0.439202 | 0.221651 |
| 12555 | 5.371083 | 12.343538 | 4.251359 | 12.086285 | 10.940364 | 7.120259 | 11.219722 | 12.178599 | 13.010334 | 17.546362 | 11.129470 |
| 13700 | 10.763347 | 12.179929 | 12.598575 | 15.469758 | 15.519320 | 17.232456 | 17.711471 | 14.293587 | 13.977507 | 13.459443 | 11.345356 |
| 14802 | 11.901366 | 16.528810 | 11.607841 | 11.890648 | 13.608379 | 12.325402 | 14.650704 | 11.200095 | 11.679182 | 14.535119 | 14.143110 |
| 15746 | 1.361211 | 2.673466 | 1.427725 | 3.344680 | 4.916203 | 1.581813 | 3.365045 | 1.930978 | 3.238722 | 4.050971 | 4.573851 |
| 15748 | 11.135102 | 19.199961 | 10.384382 | 22.137908 | 22.453601 | 9.625781 | 17.154512 | 9.471018 | 17.024149 | 22.199925 | 22.650430 |
| 15900 | 3.255234 | 1.451387 | 3.244096 | 2.980971 | 4.389886 | 4.307486 | 5.078974 | 2.121754 | 1.723179 | 2.544533 | 1.750549 |
| 16129 | 0.000000 | 0.000000 | 0.000000 | 0.000000 | 0.000000 | 0.000000 | 0.000000 | 0.000000 | 0.000000 | 0.431248 | 0.000000 |
| 16635 | 0.738459 | 4.501624 | 2.430184 | 6.308786 | 8.590841 | 4.914107 | 4.728599 | 3.667807 | 6.730556 | 29.904117 | 22.861660 |

|  | Glucose | Xylose | Arabinose | Acetate | Coumarate | Ferulate | YNB Oleic Acid | YNB Ricinoleic Acid | YNB Glucose | YNB Gluc DOC | YPD |
| --- | --- | --- | --- | --- | --- | --- | --- | --- | --- | --- | --- |
| proteinId |  |  |  |  |  |  |  |  |  |  |  |
| 11167 | 0.094600 | -0.123058 | -0.258405 | -0.048598 | -0.094972 | 0.124140 | 0.437731 | 0.018182 | -0.147964 | -0.027676 | -0.200199 |
| 11833 | 0.218588 | -0.139988 | 0.124940 | -0.131598 | -0.032305 | 0.039347 | 0.176377 | 0.128813 | 0.120682 | -0.082954 | -0.133672 |
| 12272 | -0.197686 | -0.196943 | -0.002083 | 0.074668 | 0.025135 | 0.098450 | -0.335589 | -0.358104 | -0.059318 | -0.065698 | 0.074066 |
| 12538 | -0.022874 | -0.002845 | 0.044401 | 0.024594 | 0.047679 | -0.024970 | 0.098187 | 0.240673 | 0.057344 | 0.043576 | 0.066803 |
| 12555 | -0.311448 | -0.162485 | -0.131826 | -0.274268 | -0.854248 | -0.027038 | -1.528607 | -0.659443 | -0.300839 | -0.371389 | -0.434224 |
| 13700 | -0.107884 | -0.155441 | -0.254230 | -0.038800 | 0.149475 | -0.054494 | -0.066590 | -0.169330 | -0.253860 | -0.328580 | -0.324718 |
| 14802 | 0.318433 | 0.250797 | 0.241559 | 0.171794 | -0.011931 | 0.176682 | -0.117700 | -0.091717 | 0.131314 | -0.033473 | -0.125359 |
| 15745 | -0.104500 | -0.034164 | 0.065697 | -0.046831 | 0.013168 | -0.155592 | -0.093087 | -0.055365 | -0.322587 | -0.129447 | 0.125032 |
| 15746 | -0.292855 | -0.044816 | -0.192011 | -0.333648 | -0.131629 | 0.049669 | 0.183731 | -1.144899 | 0.233525 | 0.530271 | 1.095297 |
| 15748 | -0.455473 | 0.172527 | -0.163183 | -0.136613 | -0.855098 | -0.410175 | -0.256403 | -0.113934 | -1.029316 | -0.810487 | 0.034650 |
| 15900 | -0.402251 | 0.038116 | -0.120976 | -0.323788 | -0.290967 | -0.292245 | 0.077503 | 0.423076 | 0.367482 | -0.304034 | -0.394707 |
| 16129 | -0.223651 | -0.013342 | 0.159221 | -0.114595 | -0.029694 | -0.117031 | 0.055164 | 0.063325 | 0.415751 | 0.445395 | -0.195331 |
| 16635 | -0.386094 | 0.074868 | -0.246490 | -0.872329 | -1.765683 | -3.600674 | -0.198188 | -0.037413 | 0.311272 | 0.402185 | 0.872770 |

In [11]:

```
for x in temp:
    if x in model.genes:
        for r in sorted(model.genes.get_by_id(x).reactions, key=lambda x: x.id):
            print(r, r.gene_reaction_rule)
    else:
        print(x, 'no reactions')
    print()
```

```
FACOAL100: atp_c + coa_c + dca_c --> amp_c + dcacoa_c + ppi_c 11167 or 15746 or 15748
FACOAL120: atp_c + coa_c + ddca_c --> amp_c + ddcacoa_c + ppi_c 11167 or 15746 or 15748
FACOAL140: atp_c + coa_c + ttdca_c --> amp_c + ppi_c + tdcoa_c 11167 or 15746 or 15748
FACOAL141: atp_c + coa_c + ttdcea_c --> amp_c + ppi_c + tdecoa_c 11167 or 15746 or 15748
FACOAL150: atp_c + coa_c + ptdca_c --> amp_c + ppi_c + ptdcacoa_c 11167 or 15746 or 15748
FACOAL160: atp_c + coa_c + hdca_c --> amp_c + pmtcoa_c + ppi_c 11167 or 15746 or 15748
FACOAL161: atp_c + coa_c + hdcea_c --> amp_c + hdcoa_c + ppi_c 11167 or 15746 or 15748
FACOAL170: atp_c + coa_c + hpdca_c --> amp_c + hpdcacoa_c + ppi_c 11167 or 15746 or 15748
FACOAL180: atp_c + coa_c + ocdca_c --> amp_c + ppi_c + stcoa_c 11167 or 15746 or 15748
FACOAL181: atp_c + coa_c + ocdcea_c --> amp_c + odecoa_c + ppi_c 11167 or 15746 or 15748
FACOAL1813: atp_c + coa_c + elaid_c --> amp_c + od2coa_c + ppi_c 11167 or 15746 or 15748
FACOAL182: atp_c + coa_c + ocdcya_c --> amp_c + ocdycacoa_c + ppi_c 11167 or 15746 or 15748
FACOAL1831: atp_c + coa_c + lnlncg_c --> amp_c + lnlncgcoa_c + ppi_c 11167 or 15746 or 15748
FACOAL191: atp_c + coa_c + prist_c --> amp_c + ppi_c + pristcoa_c 11167 or 15746 or 15748
FACOAL200: arach_c + atp_c + coa_c --> amp_c + arachcoa_c + ppi_c 11167 or 15746 or 15748
FACOAL206: atp_c + coa_c + phyt_c --> amp_c + phytcoa_c + ppi_c 11167 or 15746 or 15748
FACOAL2251: atp_c + coa_c + dcsptn1_c --> amp_c + dcsptn1coa_c + ppi_c 11167 or 15746 or 15748
FACOAL2252: atp_c + clpnd_c + coa_c --> amp_c + clpndcoa_c + ppi_c 11167 or 15746 or 15748
FACOAL240: atp_c + coa_c + ttc_c --> amp_c + ppi_c + ttccoa_c 11167 or 15746 or 15748
FACOAL241: atp_c + coa_c + nrvnc_c --> amp_c + nrvnccoa_c + ppi_c 11167 or 15746 or 15748
FACOAL244_1: atp_c + coa_c + tettet6_c --> amp_c + ppi_c + tettet6coa_c 11167 or 15746 or 15748
FACOAL260: atp_c + coa_c + hexc_c --> amp_c + hexccoa_c + ppi_c 11167 or 15746 or 15748
FACOAL80: atp_c + coa_c + octa_c --> amp_c + occoa_c + ppi_c 11167 or 15746 or 15748

4CMCOAS: T4hcinnm_c + atp_c + coa_c --> amp_c + coucoa_c + ppi_c 11833 or 13700 or 14802 or 16129

ACSm: ac_m + atp_m + coa_m --> accoa_m + amp_m + ppi_m 12272 or 14597
FACOAL40im: atp_m + but_m + coa_m --> amp_m + btcoa_m + ppi_m 12272

12538 no reactions

FACOAL120p: atp_x + coa_x + ddca_x --> amp_x + ddcacoa_x + ppi_x 12555
FACOAL140p: atp_x + coa_x + ttdca_x --> amp_x + ppi_x + tdcoa_x 12555
FACOAL141p: atp_x + coa_x + ttdcea_x --> amp_x + ppi_x + tdecoa_x 12555
FACOAL160p: atp_x + coa_x + hdca_x --> amp_x + pmtcoa_x + ppi_x 12555
FACOAL161p: atp_x + coa_x + hdcea_x --> amp_x + hdcoa_x + ppi_x 12555
FACOAL180p: atp_x + coa_x + ocdca_x --> amp_x + ppi_x + stcoa_x 12555
FACOAL181p: atp_x + coa_x + ocdcea_x --> amp_x + odecoa_x + ppi_x 12555
FACOAL182p: atp_x + coa_x + ocdcya_x --> amp_x + ocdycacoa_x + ppi_x 12555
FACOAL1831p: atp_x + coa_x + lnlncg_x --> amp_x + lnlncgcoa_x + ppi_x 12555
FACOAL200p: arach_x + atp_x + coa_x --> amp_x + arachcoa_x + ppi_x 12555 or 9912
FACOAL220p: atp_x + coa_x + docosac_x --> amp_x + docoscoa_x + ppi_x 12555 or 9912
FACOAL240p: atp_x + coa_x + ttc_x --> amp_x + ppi_x + ttccoa_x 12555 or 9912
FACOAL260p: atp_x + coa_x + hexc_x --> amp_x + hexccoa_x + ppi_x 12555 or 9912

4CMCOAS: T4hcinnm_c + atp_c + coa_c --> amp_c + coucoa_c + ppi_c 11833 or 13700 or 14802 or 16129

4CMCOAS: T4hcinnm_c + atp_c + coa_c --> amp_c + coucoa_c + ppi_c 11833 or 13700 or 14802 or 16129

15745 no reactions

FACOAL100: atp_c + coa_c + dca_c --> amp_c + dcacoa_c + ppi_c 11167 or 15746 or 15748
FACOAL120: atp_c + coa_c + ddca_c --> amp_c + ddcacoa_c + ppi_c 11167 or 15746 or 15748
FACOAL140: atp_c + coa_c + ttdca_c --> amp_c + ppi_c + tdcoa_c 11167 or 15746 or 15748
FACOAL141: atp_c + coa_c + ttdcea_c --> amp_c + ppi_c + tdecoa_c 11167 or 15746 or 15748
FACOAL150: atp_c + coa_c + ptdca_c --> amp_c + ppi_c + ptdcacoa_c 11167 or 15746 or 15748
FACOAL160: atp_c + coa_c + hdca_c --> amp_c + pmtcoa_c + ppi_c 11167 or 15746 or 15748
FACOAL161: atp_c + coa_c + hdcea_c --> amp_c + hdcoa_c + ppi_c 11167 or 15746 or 15748
FACOAL170: atp_c + coa_c + hpdca_c --> amp_c + hpdcacoa_c + ppi_c 11167 or 15746 or 15748
FACOAL180: atp_c + coa_c + ocdca_c --> amp_c + ppi_c + stcoa_c 11167 or 15746 or 15748
FACOAL181: atp_c + coa_c + ocdcea_c --> amp_c + odecoa_c + ppi_c 11167 or 15746 or 15748
FACOAL1813: atp_c + coa_c + elaid_c --> amp_c + od2coa_c + ppi_c 11167 or 15746 or 15748
FACOAL182: atp_c + coa_c + ocdcya_c --> amp_c + ocdycacoa_c + ppi_c 11167 or 15746 or 15748
FACOAL1831: atp_c + coa_c + lnlncg_c --> amp_c + lnlncgcoa_c + ppi_c 11167 or 15746 or 15748
FACOAL191: atp_c + coa_c + prist_c --> amp_c + ppi_c + pristcoa_c 11167 or 15746 or 15748
FACOAL200: arach_c + atp_c + coa_c --> amp_c + arachcoa_c + ppi_c 11167 or 15746 or 15748
FACOAL206: atp_c + coa_c + phyt_c --> amp_c + phytcoa_c + ppi_c 11167 or 15746 or 15748
FACOAL2251: atp_c + coa_c + dcsptn1_c --> amp_c + dcsptn1coa_c + ppi_c 11167 or 15746 or 15748
FACOAL2252: atp_c + clpnd_c + coa_c --> amp_c + clpndcoa_c + ppi_c 11167 or 15746 or 15748
FACOAL240: atp_c + coa_c + ttc_c --> amp_c + ppi_c + ttccoa_c 11167 or 15746 or 15748
FACOAL241: atp_c + coa_c + nrvnc_c --> amp_c + nrvnccoa_c + ppi_c 11167 or 15746 or 15748
FACOAL244_1: atp_c + coa_c + tettet6_c --> amp_c + ppi_c + tettet6coa_c 11167 or 15746 or 15748
FACOAL260: atp_c + coa_c + hexc_c --> amp_c + hexccoa_c + ppi_c 11167 or 15746 or 15748
FACOAL80: atp_c + coa_c + octa_c --> amp_c + occoa_c + ppi_c 11167 or 15746 or 15748

FACOAL100: atp_c + coa_c + dca_c --> amp_c + dcacoa_c + ppi_c 11167 or 15746 or 15748
FACOAL120: atp_c + coa_c + ddca_c --> amp_c + ddcacoa_c + ppi_c 11167 or 15746 or 15748
FACOAL140: atp_c + coa_c + ttdca_c --> amp_c + ppi_c + tdcoa_c 11167 or 15746 or 15748
FACOAL141: atp_c + coa_c + ttdcea_c --> amp_c + ppi_c + tdecoa_c 11167 or 15746 or 15748
FACOAL150: atp_c + coa_c + ptdca_c --> amp_c + ppi_c + ptdcacoa_c 11167 or 15746 or 15748
FACOAL160: atp_c + coa_c + hdca_c --> amp_c + pmtcoa_c + ppi_c 11167 or 15746 or 15748
FACOAL161: atp_c + coa_c + hdcea_c --> amp_c + hdcoa_c + ppi_c 11167 or 15746 or 15748
FACOAL170: atp_c + coa_c + hpdca_c --> amp_c + hpdcacoa_c + ppi_c 11167 or 15746 or 15748
FACOAL180: atp_c + coa_c + ocdca_c --> amp_c + ppi_c + stcoa_c 11167 or 15746 or 15748
FACOAL181: atp_c + coa_c + ocdcea_c --> amp_c + odecoa_c + ppi_c 11167 or 15746 or 15748
FACOAL1813: atp_c + coa_c + elaid_c --> amp_c + od2coa_c + ppi_c 11167 or 15746 or 15748
FACOAL182: atp_c + coa_c + ocdcya_c --> amp_c + ocdycacoa_c + ppi_c 11167 or 15746 or 15748
FACOAL1831: atp_c + coa_c + lnlncg_c --> amp_c + lnlncgcoa_c + ppi_c 11167 or 15746 or 15748
FACOAL191: atp_c + coa_c + prist_c --> amp_c + ppi_c + pristcoa_c 11167 or 15746 or 15748
FACOAL200: arach_c + atp_c + coa_c --> amp_c + arachcoa_c + ppi_c 11167 or 15746 or 15748
FACOAL206: atp_c + coa_c + phyt_c --> amp_c + phytcoa_c + ppi_c 11167 or 15746 or 15748
FACOAL2251: atp_c + coa_c + dcsptn1_c --> amp_c + dcsptn1coa_c + ppi_c 11167 or 15746 or 15748
FACOAL2252: atp_c + clpnd_c + coa_c --> amp_c + clpndcoa_c + ppi_c 11167 or 15746 or 15748
FACOAL240: atp_c + coa_c + ttc_c --> amp_c + ppi_c + ttccoa_c 11167 or 15746 or 15748
FACOAL241: atp_c + coa_c + nrvnc_c --> amp_c + nrvnccoa_c + ppi_c 11167 or 15746 or 15748
FACOAL244_1: atp_c + coa_c + tettet6_c --> amp_c + ppi_c + tettet6coa_c 11167 or 15746 or 15748
FACOAL260: atp_c + coa_c + hexc_c --> amp_c + hexccoa_c + ppi_c 11167 or 15746 or 15748
FACOAL80: atp_c + coa_c + octa_c --> amp_c + occoa_c + ppi_c 11167 or 15746 or 15748

FACOAL100p: atp_x + coa_x + dca_x --> amp_x + dcacoa_x + ppi_x 15900
FACOAL80p: atp_x + coa_x + octa_x --> amp_x + occoa_x + ppi_x 15900

4CMCOAS: T4hcinnm_c + atp_c + coa_c --> amp_c + coucoa_c + ppi_c 11833 or 13700 or 14802 or 16129

COUCOAFm: T4hcinnm_m + atp_m + coa_m --> amp_m + coucoa_m + ppi_m 16635
```

In [12]:

```
model.metabolites.hxcoa_x
```

Out[12]:

|  |  |
| --- | --- |
| **Metabolite identifier** | hxcoa\_x |
| **Name** | Hexanoyl-CoA (n-C6:0CoA) |
| **Memory address** | 0x07fc99a4f3470 |
| **Formula** | C27H42N7O17P3S |
| **Compartment** | x |
| **In 2 reaction(s)** | ACOAD2p, ACACT3p |

11833 not upregulated, no fitness defect, SKL *-> pero Phenylacetate-CoA ligase by paperblast  
13700 not upregulated, no fitness defect or sigP or PTS -> cyto Phenylacetate-CoA ligase by paperblast  
14802 not upregulated, no fitness defect, SKL* -> pero Phenylacetate-CoA ligase by paperblast  
16129 not upregulated, no fitness defect, AKL\* -> pero long-chain fatty acid CoA ligase? by paperblast

12555 has fitness defect in coumarate and oleic acid, AKL\*, ok  
15475 and 15476 gene models are incorrect  
15476 has fitness defect in Ricinoleic Acid, hydroxy fatty acid specific?  
16635 has most fitness defect in coumarate and ferulate, ferulate more severe, ok

11167 is also upregulated in cou, but no fitness defect or sigP or PTS  
15748 looks okay and has fitness defect in coumarate, VYP\* cyto?  
Change cytosolic 4CMCOAS genes to 11167 or 15748

In [13]:

```
r1 = hsa2.reactions.get_by_id('PACCOAL').copy()
r1.gene_reaction_rule = '13700'
r2 = hsa2.reactions.get_by_id('PACCOALm').copy()
r2.id = 'PACCOALp'
r2.name = 'Phenylacetate Coenzyme A Ligase, Peroxisomal'
r2.gene_reaction_rule = '11833 or 14802'
model.add_reactions([r1,r2])
for m in r2.metabolites:
    if not m.id.replace('_m','_x') in model.metabolites:
        m2 = m.copy()
        m2.id = m.id.replace('_m','_x')
        m2.compartment = 'x'
        model.add_metabolites([m2])
    r2.add_metabolites({m.id: -r2.get_coefficient(m.id), m.id.replace('_m','_x'): r2.get_coefficient(m.id)})
```

In [14]:

```
for r in sorted(model.reactions, key=lambda x: x.id):
    if r.id.startswith('FACOAL') and r.id.endswith('p'):
        print(r, r.gene_reaction_rule)
```

```
FACOAL100p: atp_x + coa_x + dca_x --> amp_x + dcacoa_x + ppi_x 15900
FACOAL120p: atp_x + coa_x + ddca_x --> amp_x + ddcacoa_x + ppi_x 12555
FACOAL140p: atp_x + coa_x + ttdca_x --> amp_x + ppi_x + tdcoa_x 12555
FACOAL141p: atp_x + coa_x + ttdcea_x --> amp_x + ppi_x + tdecoa_x 12555
FACOAL160p: atp_x + coa_x + hdca_x --> amp_x + pmtcoa_x + ppi_x 12555
FACOAL161p: atp_x + coa_x + hdcea_x --> amp_x + hdcoa_x + ppi_x 12555
FACOAL180p: atp_x + coa_x + ocdca_x --> amp_x + ppi_x + stcoa_x 12555
FACOAL181p: atp_x + coa_x + ocdcea_x --> amp_x + odecoa_x + ppi_x 12555
FACOAL182p: atp_x + coa_x + ocdcya_x --> amp_x + ocdycacoa_x + ppi_x 12555
FACOAL1831p: atp_x + coa_x + lnlncg_x --> amp_x + lnlncgcoa_x + ppi_x 12555
FACOAL200p: arach_x + atp_x + coa_x --> amp_x + arachcoa_x + ppi_x 12555 or 9912
FACOAL220p: atp_x + coa_x + docosac_x --> amp_x + docoscoa_x + ppi_x 12555 or 9912
FACOAL240p: atp_x + coa_x + ttc_x --> amp_x + ppi_x + ttccoa_x 12555 or 9912
FACOAL260p: atp_x + coa_x + hexc_x --> amp_x + hexccoa_x + ppi_x 12555 or 9912
FACOAL80p: atp_x + coa_x + octa_x --> amp_x + occoa_x + ppi_x 15900
```

In [15]:

```
r = model.reactions.get_by_id('COUCOAFm')
r.id = 'COUCOALp'
r.gene_reaction_rule = '12555 or 16635'
r.name = '4-Coumarate:Coa Ligase (AMP-Forming), peroxisomal'
for m in r.metabolites:
    if not m.id.replace('_m','_x') in model.metabolites:
        m2 = m.copy()
        m2.id = m.id.replace('_m','_x')
        m2.compartment = 'x'
        model.add_metabolites([m2])
    r.add_metabolites({m.id: -r.get_coefficient(m.id), m.id.replace('_m','_x'): r.get_coefficient(m.id)})
    
model.reactions.get_by_id('4CMCOAS').gene_reaction_rule = '11167 or 15748'
```

1. (E)-4-coumaroyl-CoA + H2O -> 3S-(4-hydroxyphenyl)-3-hydroxy-propanoyl-CoA
2. 3S-(4-hydroxyphenyl)-3-hydroxy-propanoyl-CoA + NAD+ -> 3-(4-hydroxyphenyl)-3-oxo-propanoyl-CoA + NADH + H+

In [16]:

```
temp = Annotation.index[Annotation['Combined Annotations'].str.contains('enoyl-CoA hydratase') |
                        Annotation['Human Blast'].str.contains('ECH')]
display(Annotation.loc[temp])
Show_Data(temp)
```

|  | Combined Annotations | Signal P | Sc288c Orthologs | Human Orthologs | Sc288 Best Hit | Human Blast | Essential | WolfPSort | C Terminal |
| --- | --- | --- | --- | --- | --- | --- | --- | --- | --- |
| RTO4\_ID |  |  |  |  |  |  |  |  |  |
| 8782 | KOG4170: 2-enoyl-CoA hydratase/3-hydroxyacyl-C... |  |  | SCP2D1 |  | HSD17 | Not Essential | cyto 10.5, cyto\_pero 8.5, nucl 7, pero 5.5, mi... | SKL\* |
| 11237 | KOG4170: 2-enoyl-CoA hydratase/3-hydroxyacyl-C... |  |  |  |  |  | Not Essential | mito\_nucl 10.166, nucl 10, mito 10, cyto\_nucl 8 | AKL\* |
| 11907 | K12663: ECH1; Delta(3,5)-Delta(2,4)-dienoyl-Co... |  |  | ECH1 |  | ECH1 | Not Essential | extr 9, mito 8, cyto 5.5, cyto\_nucl 4, pero 3 | AKL\* |
| 14805 | K07511: ECHS1; enoyl-CoA hydratase |  |  | ECHS1 | EHD3 | ECHS1 | Not Essential | mito 26.5, cyto\_mito 14 | TNS\* |
| 16515 | HMMPfam:Enoyl-CoA hydratase/isomerase family:P... |  |  |  |  | ECHS1 | Not Essential | mito 10.5, cyto\_mito 9.833, cyto 8, cyto\_nucl ... | ARL\* |

| strain | WT | | | | | | | | | | | | | | | | |
| --- | --- | --- | --- | --- | --- | --- | --- | --- | --- | --- | --- | --- | --- | --- | --- | --- | --- |
| condition | G\_MM | C\_MM | G\_SD | | GX\_SD | | | X\_SD | | A\_SD | | C\_SD | | MM\_CN120 | | MM\_CN5 | Diversity\_Sample |
| phase | exp | exp | exp | stat | exp | trans | stat | exp | stat | exp | stat | exp | stat | exp | stat | exp | exp |
| proteinId | Set1 | Set1 | Set2 | Set2 | Set2 | Set2 | Set2 | Set2 | Set2 | Set2 | Set2 | Set2 | Set2 | Set3 | Set3 | Set3 | Set3 |
| RTO4\_ID |  |  |  |  |  |  |  |  |  |  |  |  |  |  |  |  |  |
| 8782 | 8.300162 | 8.462562 | 9.007264 | 9.600527 | 8.956773 | 8.369212 | 8.840516 | 7.827337 | 9.211276 | 8.702320 | 9.957014 | 7.255736 | 8.347477 | 7.415559 | 7.872593 | 8.439886 | 7.974047 |
| 11237 | 6.860082 | 6.542515 | 6.034462 | 6.901833 | 5.957620 | 6.078630 | 6.083661 | 5.989742 | 6.613846 | 6.506268 | 6.953158 | 6.056770 | 6.114389 | 6.161046 | 6.409736 | 6.131124 | 5.530005 |
| 11907 | 4.746783 | 5.707813 | 4.463475 | 4.963930 | 4.308148 | 5.018395 | 4.929475 | 4.820192 | 5.305915 | 5.579512 | 5.389401 | 4.737035 | 4.906274 | 4.678545 | 4.750835 | 4.793210 | 4.290084 |
| 14805 | 6.663359 | 7.468676 | 7.289829 | 7.013911 | 7.237435 | 6.957713 | 6.906752 | 7.207778 | 6.454338 | 6.805258 | 6.961815 | 6.468700 | 5.289299 | 6.961100 | 6.884284 | 7.380745 | 7.076806 |
| 16515 | 4.603413 | 7.724820 | 4.508720 | 4.611162 | 4.533973 | 4.195225 | 4.023729 | 4.133973 | 4.837419 | 4.789414 | 5.230861 | 5.639214 | 4.562164 | 4.162955 | 4.147468 | 4.491763 | 4.838867 |

| strain | WT | | | | | | | | | | |
| --- | --- | --- | --- | --- | --- | --- | --- | --- | --- | --- | --- |
| condition | G\_SD | | GX\_SD | | | X\_SD | | A\_SD | | C\_SD | |
| proteinId | exp | stat | exp | trans | stat | exp | stat | exp | stat | exp | stat |
| 8782 | 6.389066 | 8.728867 | 8.344404 | 11.169981 | 10.923474 | 10.162712 | 10.819347 | 11.586292 | 12.829952 | 10.029745 | 9.128371 |
| 11237 | 2.093514 | 5.812726 | 2.836879 | 3.908544 | 4.596553 | 4.705091 | 6.870204 | 5.403808 | 6.520068 | 4.724388 | 3.940110 |
| 11907 | 0.564696 | 3.612806 | 0.615297 | 3.151184 | 4.548558 | 4.342781 | 4.342939 | 4.440655 | 4.797965 | 3.635000 | 2.838800 |
| 14805 | 11.701105 | 12.769114 | 12.825170 | 14.703589 | 16.038353 | 15.836963 | 14.954094 | 14.873176 | 14.940566 | 17.973282 | 16.774977 |
| 16515 | 0.000000 | 0.333333 | 0.000000 | 0.183791 | 1.080446 | 0.000000 | 0.403372 | 0.000000 | 0.000000 | 6.195276 | 3.453420 |

|  | Glucose | Xylose | Arabinose | Acetate | Coumarate | Ferulate | YNB Oleic Acid | YNB Ricinoleic Acid | YNB Glucose | YNB Gluc DOC | YPD |
| --- | --- | --- | --- | --- | --- | --- | --- | --- | --- | --- | --- |
| proteinId |  |  |  |  |  |  |  |  |  |  |  |
| 8782 | 0.055869 | -0.230385 | -0.029543 | 0.284492 | 0.068076 | 0.505709 | -0.161214 | -0.258673 | -0.195698 | -0.284325 | 0.171192 |
| 11237 | -0.057929 | -0.355731 | 0.068947 | 0.085267 | 0.215514 | -0.159126 | -0.115729 | -0.076442 | 0.094597 | 0.206729 | -0.335975 |
| 11907 | 0.114424 | -0.055586 | 0.084374 | 0.095727 | 0.024873 | 0.016917 | -1.153164 | 0.038023 | -0.189194 | -0.250849 | -0.147514 |
| 14805 | 0.195211 | 0.271089 | -0.627815 | -1.435462 | -0.649913 | -0.937919 | -2.906878 | -4.214105 | 0.090276 | -0.076519 | 0.461105 |
| 16515 | -0.001114 | 0.314535 | 0.231734 | 0.369911 | 0.142545 | 0.523174 | -0.065787 | 0.443237 | -0.013139 | -0.094917 | 0.317089 |

In [17]:

```
for x in temp:
    if x in model.genes:
        for r in sorted(model.genes.get_by_id(x).reactions, key=lambda x: x.id):
            print(r, r.gene_reaction_rule)
    else:
        print(x, 'no reactions')
    print()
```

```
8782 no reactions

11237 no reactions

FAO141p_even: 6.0 coa_x + 6.0 h2o_x + 6.0 nad_x + nadph_x + 6.0 o2_x + tdecoa_x --> 7.0 accoa_x + 6.0 h2o2_x + 5.0 h_x + 6.0 nadh_x + nadp_x (10293 and 11362 and 11907 and 12742 and 13228 and 13813) or (10293 and 11362 and 11907 and 12752 and 13228 and 13813) or (10293 and 11362 and 11907 and 13228 and 13813 and 9700)
FAO161p_even: 7.0 coa_x + 7.0 h2o_x + hdcoa_x + 7.0 nad_x + nadph_x + 7.0 o2_x --> 8.0 accoa_x + 7.0 h2o2_x + 6.0 h_x + 7.0 nadh_x + nadp_x (10293 and 11362 and 11907 and 12742 and 13228 and 13813) or (10293 and 11362 and 11907 and 12752 and 13228 and 13813) or (10293 and 11362 and 11907 and 13228 and 13813 and 9700)
FAO181p_even: 8.0 coa_x + 8.0 h2o_x + 8.0 nad_x + nadph_x + 8.0 o2_x + odecoa_x --> 9.0 accoa_x + 8.0 h2o2_x + 7.0 h_x + 8.0 nadh_x + nadp_x (10293 and 11362 and 11907 and 12742 and 13228 and 13813) or (10293 and 11362 and 11907 and 12752 and 13228 and 13813) or (10293 and 11362 and 11907 and 13228 and 13813 and 9700)
FAO182p_even: 8.0 coa_x + 8.0 h2o_x + 8.0 nad_x + 2.0 nadph_x + 8.0 o2_x + ocdycacoa_x --> 9.0 accoa_x + 8.0 h2o2_x + 6.0 h_x + 8.0 nadh_x + 2.0 nadp_x (10293 and 11362 and 11907 and 12742 and 13228 and 13813) or (10293 and 11362 and 11907 and 12752 and 13228 and 13813) or (10293 and 11362 and 11907 and 13228 and 13813 and 9700)
FAO183p_even: 8.0 coa_x + 8.0 h2o_x + lnlncgcoa_x + 8.0 nad_x + 3.0 nadph_x + 8.0 o2_x --> 9.0 accoa_x + 8.0 h2o2_x + 5.0 h_x + 8.0 nadh_x + 3.0 nadp_x (10293 and 11362 and 11907 and 12742 and 13228 and 13813) or (10293 and 11362 and 11907 and 12752 and 13228 and 13813) or (10293 and 11362 and 11907 and 13228 and 13813 and 9700)

ECOAH12m: 2mp2coa_m + h2o_m <=> 3hibutcoa_m 14805
ECOAH1m: 3hbcoa_m <=> b2coa_m + h2o_m 14805
ECOAH2m: 3hhcoa_m <=> h2o_m + hx2coa_m 14805
ECOAH3m: 3hocoa_m <=> h2o_m + oc2coa_m 14805
ECOAH4m: 3hdcoa_m <=> dc2coa_m + h2o_m 14805
ECOAH5m: 3hddcoa_m <=> dd2coa_m + h2o_m 14805
ECOAH6m: 3htdcoa_m <=> h2o_m + td2coa_m 14805
ECOAH7m: 3hhdcoa_m <=> h2o_m + hdd2coa_m 14805
ECOAH9m: 2mb2coa_m + h2o_m <=> 3hmbcoa_m 14805
FAOXC11C9m: coa_m + fad_m + h2o_m + nad_m + undcoa_m --> accoa_m + fadh2_m + h_m + nadh_m + noncoa_m 12570 and 14805 and 11203 and 8885
FAOXC13C11m: coa_m + fad_m + h2o_m + nad_m + tridcoa_m --> accoa_m + fadh2_m + h_m + nadh_m + undcoa_m 12570 and 14805 and 11203 and 8885
FAOXC15C13m: coa_m + fad_m + h2o_m + nad_m + ptdcacoa_m --> accoa_m + fadh2_m + h_m + nadh_m + tridcoa_m 12570 and 14805 and 11203 and 8885
FAOXC170150m: coa_m + fad_m + h2o_m + hpdcacoa_m + nad_m --> accoa_m + fadh2_m + h_m + nadh_m + ptdcacoa_m 12570 and 14805 and 11203 and 8885
FAOXC5C3m: coa_m + fad_m + h2o_m + nad_m + pentcoa_m --> accoa_m + fadh2_m + h_m + nadh_m + ppcoa_m (12570 and 14805 and 11203 and 8885) or (14070 and 14805 and 11203 and 8885)
FAOXC7C5m: coa_m + fad_m + h2o_m + hepcoa_m + nad_m --> accoa_m + fadh2_m + h_m + nadh_m + pentcoa_m 12570 and 14805 and 11203 and 8885
FAOXC9C7m: coa_m + fad_m + h2o_m + nad_m + noncoa_m --> accoa_m + fadh2_m + h_m + hepcoa_m + nadh_m 12570 and 14805 and 11203 and 8885
PRPNCOAHYDm: h2o_m + prpncoa_m --> 3hpcoa_m 14805

16515 no reactions
```

8782 and 11237 are SCP sterol carrier proteins  
14805 mito ECHS1 enoyl-CoA hydratase has some fitness defect, but mito  
make PRPNCOAHYDm reversible  
Add r0365: nad\_m + 3hpp\_m → h\_m + nadh\_m + msa\_m from recon3d and name it 3HPDm  
13694 3-hydroxyisobutyrate dehydrogenase works also on 3HP  
https://www.ncbi.nlm.nih.gov/pubmed/8988636  
16515 ARL\* enoyl-CoA hydratase (2-enoyl + h2o -> 3hydroxy), upregulated but no fitness defect

In [18]:

```
model.reactions.get_by_id('PRPNCOAHYDm').lower_bound = -1000.0
r = hsa2.reactions.get_by_id('r0365').copy()
r.id = '3HPDm'
r.gene_reaction_rule = '13694'
model.add_reactions([r])
```

In [19]:

```
temp = Annotation.index[Annotation['Combined Annotations'].str.contains('multifunctional beta-oxidation') |
                        Annotation['Sc288 Best Hit'].str.contains('FOX2')]
display(Annotation.loc[temp])
Show_Data(temp)
```

|  | Combined Annotations | Signal P | Sc288c Orthologs | Human Orthologs | Sc288 Best Hit | Human Blast | Essential | WolfPSort | C Terminal |
| --- | --- | --- | --- | --- | --- | --- | --- | --- | --- |
| RTO4\_ID |  |  |  |  |  |  |  |  |  |
| 9082 | KOG1201: Hydroxysteroid 17-beta dehydrogenase 11 |  |  |  | FOX2 | DHRS4 | Not Essential | cyto 24, mito 2 | PLQ\* |
| 9469 | KOG1206: Peroxisomal multifunctional beta-oxid... |  |  |  | FOX2 | HSD17 | Not Essential | cyto 25 | SKL\* |
| 9731 | K11147: DHRS4; dehydrogenase/reductase SDR fam... |  |  |  | FOX2 | HSD17B | Not Essential | mito 11, cyto 10, cyto\_nucl 6.5, pero 5 | QFM\* |
| 10551 | K00059: fabG; 3-oxoacyl-[acyl-carrier protein]... |  |  |  | FOX2 | HSD17B | Not Essential | cyto 16, mito 7, pero 3 | MGI\* |
| 10558 | K00059: fabG; 3-oxoacyl-[acyl-carrier protein]... |  |  |  | FOX2 | HSD17B | Not Essential | cyto 20, mito 4, pero 3 | RGI\* |
| 11362 | K14729: FOX2; multifunctional beta-oxidation p... |  | FOX2 | HSD17B4 | FOX2 | HSD17 | Not Essential | cyto 15, mito 6, pero 4, mito\_nucl 4 | LAQ\* |
| 14196 | KOG0725: Reductases with broad range of substr... | S |  | BDH2 | FOX2 | BDH2 | Not Essential | cyto 21.5, cyto\_nucl 12, mito 3 | PKA\* |

| strain | WT | | | | | | | | | | | | | | | | |
| --- | --- | --- | --- | --- | --- | --- | --- | --- | --- | --- | --- | --- | --- | --- | --- | --- | --- |
| condition | G\_MM | C\_MM | G\_SD | | GX\_SD | | | X\_SD | | A\_SD | | C\_SD | | MM\_CN120 | | MM\_CN5 | Diversity\_Sample |
| phase | exp | exp | exp | stat | exp | trans | stat | exp | stat | exp | stat | exp | stat | exp | stat | exp | exp |
| proteinId | Set1 | Set1 | Set2 | Set2 | Set2 | Set2 | Set2 | Set2 | Set2 | Set2 | Set2 | Set2 | Set2 | Set3 | Set3 | Set3 | Set3 |
| RTO4\_ID |  |  |  |  |  |  |  |  |  |  |  |  |  |  |  |  |  |
| 9082 | 6.312844 | 7.771952 | 6.889235 | 3.302427 | 7.179529 | 4.723406 | 4.594308 | 6.347612 | 4.212905 | 5.695956 | 4.322179 | 4.204139 | 3.684672 | 6.704742 | 5.598677 | 5.947749 | 5.323434 |
| 9469 | 5.820048 | 10.594827 | 4.686706 | 5.151856 | 4.837108 | 4.537271 | 4.408129 | 4.972974 | 5.622214 | 6.126097 | 6.424863 | 6.936857 | 6.624843 | 3.889505 | 4.311656 | 5.628766 | 4.859068 |
| 9731 | 3.932942 | 7.167870 | 4.798452 | 5.067240 | 4.781275 | 4.905844 | 4.870672 | 5.084100 | 5.351590 | 5.250732 | 5.588353 | 4.675988 | 4.018411 | 3.476865 | 4.057495 | 4.077026 | 3.868919 |
| 10551 | 5.461825 | 10.365869 | 4.837672 | 5.576077 | 4.888397 | 5.683736 | 5.617345 | 5.184640 | 6.286944 | 6.218729 | 6.674326 | 8.177066 | 7.201104 | 6.285718 | 6.760832 | 6.198634 | 7.423812 |
| 10558 | 4.315543 | 5.737221 | 4.431308 | 5.325381 | 4.278669 | 5.442213 | 5.251694 | 4.735290 | 5.277822 | 5.028327 | 5.384735 | 4.683001 | 3.950123 | 3.767906 | 3.998167 | 4.545969 | 3.389397 |
| 11362 | 6.471399 | 7.867904 | 6.212838 | 7.416520 | 6.150131 | 6.772497 | 7.061987 | 6.332156 | 6.886911 | 6.872667 | 7.257986 | 7.027727 | 6.878984 | 6.721271 | 7.067592 | 5.963518 | 6.446374 |
| 14196 | 7.811461 | 6.830961 | 7.210552 | 6.246278 | 7.322240 | 6.907628 | 6.563977 | 7.540436 | 5.724773 | 6.837532 | 5.945336 | 5.924835 | 6.041888 | 8.812277 | 8.117206 | 8.197228 | 7.950934 |

| strain | WT | | | | | | | | | | |
| --- | --- | --- | --- | --- | --- | --- | --- | --- | --- | --- | --- |
| condition | G\_SD | | GX\_SD | | | X\_SD | | A\_SD | | C\_SD | |
| proteinId | exp | stat | exp | trans | stat | exp | stat | exp | stat | exp | stat |
| 9082 | 15.053159 | 8.692651 | 12.388911 | 6.694969 | 6.037496 | 7.441961 | 7.074928 | 6.961367 | 5.566582 | 0.630494 | 0.432791 |
| 9469 | 2.114280 | 1.995177 | 1.015936 | 0.735718 | 1.807305 | 2.533150 | 2.741914 | 5.027932 | 5.188114 | 26.743015 | 14.384510 |
| 9731 | 1.909239 | 4.293848 | 1.220783 | 3.162756 | 3.630075 | 2.722064 | 3.129075 | 3.474313 | 3.067745 | 6.411952 | 3.049979 |
| 10551 | 2.719587 | 6.542034 | 3.454070 | 8.916621 | 9.495566 | 7.622570 | 8.277752 | 8.508091 | 9.948272 | 46.435645 | 36.834371 |
| 10558 | 1.926841 | 8.336310 | 4.267122 | 11.529846 | 11.851741 | 6.466787 | 6.702920 | 6.565380 | 6.312816 | 17.109420 | 11.081312 |
| 11362 | 20.483068 | 39.756212 | 20.573955 | 30.856187 | 31.455133 | 28.133440 | 34.070261 | 34.587907 | 34.462931 | 67.719650 | 51.512217 |
| 14196 | 10.177624 | 10.438475 | 10.581538 | 10.797013 | 12.139338 | 11.978211 | 12.552946 | 10.422821 | 9.954909 | 13.672393 | 10.023815 |

|  | Glucose | Xylose | Arabinose | Acetate | Coumarate | Ferulate | YNB Oleic Acid | YNB Ricinoleic Acid | YNB Glucose | YNB Gluc DOC | YPD |
| --- | --- | --- | --- | --- | --- | --- | --- | --- | --- | --- | --- |
| proteinId |  |  |  |  |  |  |  |  |  |  |  |
| 9082 | -0.063212 | 0.032908 | -0.166544 | -0.031697 | -0.087610 | 0.010872 | 0.292471 | -0.379542 | -0.062205 | -0.393980 | 0.242774 |
| 9469 | -0.132763 | 0.096170 | -0.120022 | -0.074862 | 0.240087 | -0.313024 | 0.022284 | 0.072853 | 0.013658 | -0.095798 | 0.098296 |
| 9731 | 0.009639 | -0.356818 | -0.433929 | -0.104025 | -0.109207 | -0.251474 | 0.070244 | -0.303031 | 0.410073 | 0.255280 | -0.067573 |
| 10551 | -0.351777 | -0.190585 | -0.003885 | 0.049769 | -0.891608 | -2.059696 | -0.055568 | -0.278141 | 0.377108 | 0.104370 | 0.103662 |
| 10558 | 0.096597 | -0.378480 | 0.037810 | 0.010911 | -0.265278 | -0.378501 | 0.062701 | 0.475345 | -0.019450 | -0.030880 | 0.298025 |
| 11362 | 0.075121 | 0.052146 | 0.008106 | -0.090764 | -0.150565 | 0.023855 | -1.494067 | 0.055192 | 0.104864 | -0.187183 | -0.064219 |
| 14196 | 0.102045 | -0.167104 | -0.051571 | -0.050119 | -0.141501 | -0.055851 | 0.151621 | 0.023838 | -0.049238 | -0.184868 | -0.134232 |

In [20]:

```
for x in temp:
    if x in model.genes:
        for r in sorted(model.genes.get_by_id(x).reactions, key=lambda x: x.id):
            print(r, r.gene_reaction_rule)
    else:
        print(x, 'no reactions')
    print()
```

```
9082 no reactions

9469 no reactions

9731 no reactions

10551 no reactions

3OACOAR: 3ohodcoa_c + h_c + nadph_c --> 3hodcoa_c + nadp_c 10558
3OAR100: 3odecACP_c + h_c + nadph_c <=> 3hdecACP_c + nadp_c 10558
3OAR120: 3oddecACP_c + h_c + nadph_c <=> 3hddecACP_c + nadp_c 10558
3OAR121: 3ocddec5eACP_c + h_c + nadph_c --> 3hcddec5eACP_c + nadp_c 10558
3OAR140: 3omrsACP_c + h_c + nadph_c <=> 3hmrsACP_c + nadp_c 10558
3OAR141: 3ocmrs7eACP_c + h_c + nadph_c --> 3hcmrs7eACP_c + nadp_c 10558
3OAR160: 3opalmACP_c + h_c + nadph_c <=> 3hpalmACP_c + nadp_c 10558
3OAR161: 3ocpalm9eACP_c + h_c + nadph_c --> 3hcpalm9eACP_c + nadp_c 10558
3OAR180: 3ooctdACP_c + h_c + nadph_c <=> 3hoctaACP_c + nadp_c 10558
3OAR181: 3ocvac11eACP_c + h_c + nadph_c --> 3hcvac11eACP_c + nadp_c 10558
3OAR40: actACP_c + h_c + nadph_c <=> 3haACP_c + nadp_c 10558
3OAR60: 3ohexACP_c + h_c + nadph_c <=> 3hhexACP_c + nadp_c 10558
3OAR80: 3ooctACP_c + h_c + nadph_c <=> 3hoctACP_c + nadp_c 10558
OGMEACPR: h_c + nadph_c + ogmeACP_c --> hgmeACP_c + nadp_c 10558
OPMEACPR: h_c + nadph_c + opmeACP_c --> hpmeACP_c + nadp_c 10558
RHACOAR100: 3odcoa_c + h_c + nadph_c <=> R_3hdcoa_c + nadp_c 10558
RHACOAR120: 3oddcoa_c + h_c + nadph_c <=> R_3hddcoa_c + nadp_c 10558
RHACOAR140: 3otdcoa_c + h_c + nadph_c <=> R_3hmrscoa_c + nadp_c 10558
RHACOAR60: 3ohcoa_c + h_c + nadph_c <=> R_3hhcoa_c + nadp_c 10558
RHACOAR80: 3oocoa_c + h_c + nadph_c <=> R_3hocoa_c + nadp_c 10558

ECOAH11p: h2o_x + hxc2coa_x <=> 3hxccoa_x 11362
ECOAH1p: 3hbcoa_x <=> b2coa_x + h2o_x 11362
ECOAH2p: h2o_x + hx2coa_x <=> 3hhcoa_x 11362
ECOAH3p: h2o_x + oc2coa_x <=> 3hocoa_x 11362
ECOAH4p: 3hdcoa_x <=> dc2coa_x + h2o_x 11362
ECOAH5p: 3hddcoa_x <=> dd2coa_x + h2o_x 11362
ECOAH6p: 3htdcoa_x <=> h2o_x + td2coa_x 11362
ECOAH7p: 3hhdcoa_x <=> h2o_x + hdd2coa_x 11362
ECOAH8p: 3hodcoa_x <=> h2o_x + od2coa_x 11362
FAO141p_even: 6.0 coa_x + 6.0 h2o_x + 6.0 nad_x + nadph_x + 6.0 o2_x + tdecoa_x --> 7.0 accoa_x + 6.0 h2o2_x + 5.0 h_x + 6.0 nadh_x + nadp_x (10293 and 11362 and 11907 and 12742 and 13228 and 13813) or (10293 and 11362 and 11907 and 12752 and 13228 and 13813) or (10293 and 11362 and 11907 and 13228 and 13813 and 9700)
FAO141p_odd: 6.0 coa_x + 6.0 h2o_x + 6.0 nad_x + 5.0 o2_x + tdecoa_x --> 7.0 accoa_x + 5.0 h2o2_x + 6.0 h_x + 6.0 nadh_x (10293 and 11362 and 12742 and 13813) or (10293 and 11362 and 12752 and 13813) or (10293 and 11362 and 13813 and 9700)
FAO161p_even: 7.0 coa_x + 7.0 h2o_x + hdcoa_x + 7.0 nad_x + nadph_x + 7.0 o2_x --> 8.0 accoa_x + 7.0 h2o2_x + 6.0 h_x + 7.0 nadh_x + nadp_x (10293 and 11362 and 11907 and 12742 and 13228 and 13813) or (10293 and 11362 and 11907 and 12752 and 13228 and 13813) or (10293 and 11362 and 11907 and 13228 and 13813 and 9700)
FAO161p_odd: 7.0 coa_x + 7.0 h2o_x + hdcoa_x + 7.0 nad_x + 6.0 o2_x --> 8.0 accoa_x + 6.0 h2o2_x + 7.0 h_x + 7.0 nadh_x (10293 and 11362 and 12742 and 13813) or (10293 and 11362 and 12752 and 13813) or (10293 and 11362 and 13813 and 9700)
FAO181p_even: 8.0 coa_x + 8.0 h2o_x + 8.0 nad_x + nadph_x + 8.0 o2_x + odecoa_x --> 9.0 accoa_x + 8.0 h2o2_x + 7.0 h_x + 8.0 nadh_x + nadp_x (10293 and 11362 and 11907 and 12742 and 13228 and 13813) or (10293 and 11362 and 11907 and 12752 and 13228 and 13813) or (10293 and 11362 and 11907 and 13228 and 13813 and 9700)
FAO181p_odd: 8.0 coa_x + 8.0 h2o_x + 8.0 nad_x + 7.0 o2_x + odecoa_x --> 9.0 accoa_x + 7.0 h2o2_x + 8.0 h_x + 8.0 nadh_x (10293 and 11362 and 12742 and 13813) or (10293 and 11362 and 12752 and 13813) or (10293 and 11362 and 13813 and 9700)
FAO182p_even: 8.0 coa_x + 8.0 h2o_x + 8.0 nad_x + 2.0 nadph_x + 8.0 o2_x + ocdycacoa_x --> 9.0 accoa_x + 8.0 h2o2_x + 6.0 h_x + 8.0 nadh_x + 2.0 nadp_x (10293 and 11362 and 11907 and 12742 and 13228 and 13813) or (10293 and 11362 and 11907 and 12752 and 13228 and 13813) or (10293 and 11362 and 11907 and 13228 and 13813 and 9700)
FAO182p_odd: 8.0 coa_x + 8.0 h2o_x + 8.0 nad_x + nadph_x + 7.0 o2_x + ocdycacoa_x --> 9.0 accoa_x + 7.0 h2o2_x + 7.0 h_x + 8.0 nadh_x + nadp_x (10293 and 11362 and 12742 and 13228 and 13813) or (10293 and 11362 and 12752 and 13228 and 13813) or (10293 and 11362 and 13228 and 13813 and 9700)
FAO183p_even: 8.0 coa_x + 8.0 h2o_x + lnlncgcoa_x + 8.0 nad_x + 3.0 nadph_x + 8.0 o2_x --> 9.0 accoa_x + 8.0 h2o2_x + 5.0 h_x + 8.0 nadh_x + 3.0 nadp_x (10293 and 11362 and 11907 and 12742 and 13228 and 13813) or (10293 and 11362 and 11907 and 12752 and 13228 and 13813) or (10293 and 11362 and 11907 and 13228 and 13813 and 9700)
FAO183p_odd: 8.0 coa_x + 8.0 h2o_x + lnlncgcoa_x + 8.0 nad_x + 2.0 nadph_x + 7.0 o2_x --> 9.0 accoa_x + 7.0 h2o2_x + 6.0 h_x + 8.0 nadh_x + 2.0 nadp_x (10293 and 11362 and 12742 and 13228 and 13813) or (10293 and 11362 and 12752 and 13228 and 13813) or (10293 and 11362 and 13228 and 13813 and 9700)
FAO80p: 3.0 coa_x + 3.0 h2o_x + 3.0 nad_x + 3.0 o2_x + occoa_x --> 4.0 accoa_x + 3.0 h2o2_x + 3.0 h_x + 3.0 nadh_x (11362 and 12742 and 13813) or (11362 and 12742 and 9065) or (11362 and 12752 and 13813) or (11362 and 12752 and 9065) or (11362 and 13813 and 9700) or (11362 and 9065 and 9700)
FAOXC200180x: arachcoa_x + coa_x + h2o_x + nad_x + o2_x --> accoa_x + h2o2_x + h_x + nadh_x + stcoa_x (12742 and 11362 and 13813) or (12752 and 11362 and 13813) or (9700 and 11362 and 13813)
FAOXC22C20x: coa_x + docoscoa_x + h2o_x + nad_x + o2_x --> accoa_x + arachcoa_x + h2o2_x + h_x + nadh_x (12742 and 11362 and 13813) or (12752 and 11362 and 13813) or (9700 and 11362 and 13813)
FAOXC241181x: 3.0 coa_x + 3.0 h2o_x + 3.0 nad_x + nrvnccoa_x + 3.0 o2_x --> 3.0 accoa_x + 3.0 h2o2_x + 3.0 h_x + 3.0 nadh_x + odecoa_x (12742 and 11362 and 13813) or (12752 and 11362 and 13813) or (9700 and 11362 and 13813)
FAOXC2442246x: coa_x + h2o_x + nad_x + o2_x + tettet6coa_x --> accoa_x + adrncoa_x + h2o2_x + h_x + nadh_x (12742 and 11362 and 13813) or (12752 and 11362 and 13813) or (9700 and 11362 and 13813)
FAOXC2452253x: coa_x + h2o_x + nad_x + o2_x + tetpent3coa_x --> accoa_x + clpndcoa_x + h2o2_x + h_x + nadh_x (12742 and 11362 and 13813) or (12752 and 11362 and 13813) or (9700 and 11362 and 13813)
FAOXC2452256x: coa_x + h2o_x + nad_x + o2_x + tetpent6coa_x --> accoa_x + dcsptn1coa_x + h2o2_x + h_x + nadh_x (12742 and 11362 and 13813) or (12752 and 11362 and 13813) or (9700 and 11362 and 13813)
FAOXC24C22x: coa_x + h2o_x + nad_x + o2_x + ttccoa_x --> accoa_x + docoscoa_x + h2o2_x + h_x + nadh_x (12742 and 11362 and 13813) or (12752 and 11362 and 13813) or (9700 and 11362 and 13813)
HACD10p: 3hxccoa_x + nad_x <=> 3ohxccoa_x + h_x + nadh_x 11362
HACD1p: aacoa_x + h_x + nadh_x <=> 3hbcoa_x + nad_x 11362
HACD2p: 3hhcoa_x + nad_x <=> 3ohcoa_x + h_x + nadh_x 11362
HACD3p: 3hocoa_x + nad_x <=> 3oocoa_x + h_x + nadh_x 11362
HACD4p: 3odcoa_x + h_x + nadh_x <=> 3hdcoa_x + nad_x 11362
HACD5p: 3oddcoa_x + h_x + nadh_x <=> 3hddcoa_x + nad_x 11362
HACD6p: 3otdcoa_x + h_x + nadh_x <=> 3htdcoa_x + nad_x 11362
HACD7p: 3ohdcoa_x + h_x + nadh_x <=> 3hhdcoa_x + nad_x 11362
HACD8p: 3ohodcoa_x + h_x + nadh_x <=> 3hodcoa_x + nad_x 11362
HSD17B42x: h_x + nadph_x + o2_x + thcholoylcoa_x --> dhocholoylcoa_x + 2.0 h2o_x + nadp_x 11362
HSD17B4x: cholcoads_x + h_x + nadph_x + o2_x --> cholcoaone_x + h2o_x + nadp_x 11362

CHOLID: cholate_c + nad_c --> dhcholn_c + h_c + nadh_c 14196
```

In [21]:

```
temp = Annotation.index[(Annotation['Combined Annotations'].str.contains('3-oxoacyl') &
                         Annotation['Combined Annotations'].str.contains('reductase')) |
                        (Annotation['Combined Annotations'].str.contains('3-hydroxy') &
                         Annotation['Combined Annotations'].str.contains('CoA dehydrogenase')) |
                        Annotation['Human Blast'].str.contains('HADH')]
display(Annotation.loc[temp])
Show_Data(temp)
```

|  | Combined Annotations | Signal P | Sc288c Orthologs | Human Orthologs | Sc288 Best Hit | Human Blast | Essential | WolfPSort | C Terminal |
| --- | --- | --- | --- | --- | --- | --- | --- | --- | --- |
| RTO4\_ID |  |  |  |  |  |  |  |  |  |
| 8782 | KOG4170: 2-enoyl-CoA hydratase/3-hydroxyacyl-C... |  |  | SCP2D1 |  | HSD17 | Not Essential | cyto 10.5, cyto\_pero 8.5, nucl 7, pero 5.5, mi... | SKL\* |
| 10551 | K00059: fabG; 3-oxoacyl-[acyl-carrier protein]... |  |  |  | FOX2 | HSD17B | Not Essential | cyto 16, mito 7, pero 3 | MGI\* |
| 10558 | K00059: fabG; 3-oxoacyl-[acyl-carrier protein]... |  |  |  | FOX2 | HSD17B | Not Essential | cyto 20, mito 4, pero 3 | RGI\* |
| 11203 | K00022: HADH; 3-hydroxyacyl-CoA dehydrogenase |  |  | HADH |  | HADH | Not Essential | mito 20, cyto\_mito 13.333, cyto 4.5, cyto\_nucl... | YSS\* |
| 11237 | KOG4170: 2-enoyl-CoA hydratase/3-hydroxyacyl-C... |  |  |  |  |  | Not Essential | mito\_nucl 10.166, nucl 10, mito 10, cyto\_nucl 8 | AKL\* |
| 13606 | KOG1199: Short-chain alcohol dehydrogenase/3-h... | S |  |  |  | HSD17 | Not Essential | cyto 11.5, cyto\_mito 10, mito 7.5, extr 7 | ASF\* |
| 15180 | K00074: paaH, hbd, fadB, mmgB; 3-hydroxybutyry... |  |  | HADH |  | HADH | Not Essential | cyto 13, cyto\_nucl 8, cysk 8, mito 4 | YTAR |
| 16284 | K08683: HSD17B10; 3-hydroxyacyl-CoA dehydrogen... | S |  | HSD17B10 |  | HSD17 | Not Essential | extr 11, mito 5, plas 5, E.R. 4 | AKM\* |
| 16695 | K10251: HSD17B12, KAR, IFA38; 17beta-estradiol... |  | IFA38 | HSD17B12 | IFA38 | HSD17 | Essential | cyto 12, cyto\_nucl 8.5, mito 7, pero 4, nucl 3 | KGE\* |

| strain | WT | | | | | | | | | | | | | | | | |
| --- | --- | --- | --- | --- | --- | --- | --- | --- | --- | --- | --- | --- | --- | --- | --- | --- | --- |
| condition | G\_MM | C\_MM | G\_SD | | GX\_SD | | | X\_SD | | A\_SD | | C\_SD | | MM\_CN120 | | MM\_CN5 | Diversity\_Sample |
| phase | exp | exp | exp | stat | exp | trans | stat | exp | stat | exp | stat | exp | stat | exp | stat | exp | exp |
| proteinId | Set1 | Set1 | Set2 | Set2 | Set2 | Set2 | Set2 | Set2 | Set2 | Set2 | Set2 | Set2 | Set2 | Set3 | Set3 | Set3 | Set3 |
| RTO4\_ID |  |  |  |  |  |  |  |  |  |  |  |  |  |  |  |  |  |
| 8782 | 8.300162 | 8.462562 | 9.007264 | 9.600527 | 8.956773 | 8.369212 | 8.840516 | 7.827337 | 9.211276 | 8.702320 | 9.957014 | 7.255736 | 8.347477 | 7.415559 | 7.872593 | 8.439886 | 7.974047 |
| 10551 | 5.461825 | 10.365869 | 4.837672 | 5.576077 | 4.888397 | 5.683736 | 5.617345 | 5.184640 | 6.286944 | 6.218729 | 6.674326 | 8.177066 | 7.201104 | 6.285718 | 6.760832 | 6.198634 | 7.423812 |
| 10558 | 4.315543 | 5.737221 | 4.431308 | 5.325381 | 4.278669 | 5.442213 | 5.251694 | 4.735290 | 5.277822 | 5.028327 | 5.384735 | 4.683001 | 3.950123 | 3.767906 | 3.998167 | 4.545969 | 3.389397 |
| 11203 | 6.041437 | 6.525394 | 6.877017 | 5.383443 | 6.849312 | 6.032129 | 5.781838 | 6.320666 | 5.588554 | 5.706189 | 5.514583 | 6.045383 | 5.473750 | 5.184916 | 5.625797 | 5.693375 | 5.761411 |
| 11237 | 6.860082 | 6.542515 | 6.034462 | 6.901833 | 5.957620 | 6.078630 | 6.083661 | 5.989742 | 6.613846 | 6.506268 | 6.953158 | 6.056770 | 6.114389 | 6.161046 | 6.409736 | 6.131124 | 5.530005 |
| 13606 | 4.564166 | 3.980185 | 4.307840 | 4.240558 | 4.154532 | 4.499674 | 4.817458 | 4.133078 | 3.963449 | 4.575894 | 4.571663 | 2.849955 | 2.829355 | 3.001832 | 3.698199 | 2.691355 | 2.920165 |
| 15180 | 6.391310 | 6.939044 | 8.454810 | 7.906982 | 8.287859 | 7.857584 | 7.921010 | 7.726358 | 6.732458 | 6.589121 | 7.204508 | 5.118681 | 2.991233 | 6.784673 | 7.326092 | 6.310684 | 5.765893 |
| 16284 | 3.354767 | 5.767583 | 3.274257 | 3.201308 | 3.259016 | 3.619397 | 3.477925 | 2.828461 | 4.364170 | 4.260539 | 4.476590 | 3.272863 | 3.574331 | 1.866281 | 2.286147 | 1.633156 | 1.931572 |
| 16695 | 7.513154 | 7.869106 | 6.918517 | 6.578633 | 6.859663 | 6.836589 | 6.613833 | 7.622057 | 5.961036 | 6.729662 | 6.211310 | 5.850354 | 5.111494 | 7.924228 | 7.718008 | 8.636205 | 8.918220 |

| strain | WT | | | | | | | | | | |
| --- | --- | --- | --- | --- | --- | --- | --- | --- | --- | --- | --- |
| condition | G\_SD | | GX\_SD | | | X\_SD | | A\_SD | | C\_SD | |
| proteinId | exp | stat | exp | trans | stat | exp | stat | exp | stat | exp | stat |
| 8782 | 6.389066 | 8.728867 | 8.344404 | 11.169981 | 10.923474 | 10.162712 | 10.819347 | 11.586292 | 12.829952 | 10.029745 | 9.128371 |
| 10551 | 2.719587 | 6.542034 | 3.454070 | 8.916621 | 9.495566 | 7.622570 | 8.277752 | 8.508091 | 9.948272 | 46.435645 | 36.834371 |
| 10558 | 1.926841 | 8.336310 | 4.267122 | 11.529846 | 11.851741 | 6.466787 | 6.702920 | 6.565380 | 6.312816 | 17.109420 | 11.081312 |
| 11203 | 9.796469 | 11.404277 | 8.128879 | 8.929695 | 11.316945 | 9.573398 | 13.361649 | 9.658010 | 9.380260 | 9.401637 | 11.145408 |
| 11237 | 2.093514 | 5.812726 | 2.836879 | 3.908544 | 4.596553 | 4.705091 | 6.870204 | 5.403808 | 6.520068 | 4.724388 | 3.940110 |
| 15180 | 12.632877 | 11.128220 | 14.669089 | 13.948857 | 14.761624 | 14.466278 | 16.325710 | 12.943637 | 12.449885 | 11.986097 | 13.081572 |
| 16284 | 0.791247 | 1.029343 | 0.410450 | 1.296446 | 1.281008 | 0.789529 | 0.576330 | 1.542836 | 1.345414 | 4.900659 | 3.497067 |
| 16695 | 6.588829 | 3.616804 | 6.319083 | 6.337211 | 4.198542 | 6.263776 | 5.516612 | 5.211455 | 4.588213 | 5.953288 | 5.420912 |

|  | Glucose | Xylose | Arabinose | Acetate | Coumarate | Ferulate | YNB Oleic Acid | YNB Ricinoleic Acid | YNB Glucose | YNB Gluc DOC | YPD |
| --- | --- | --- | --- | --- | --- | --- | --- | --- | --- | --- | --- |
| proteinId |  |  |  |  |  |  |  |  |  |  |  |
| 8782 | 0.055869 | -0.230385 | -0.029543 | 0.284492 | 0.068076 | 0.505709 | -0.161214 | -0.258673 | -0.195698 | -0.284325 | 0.171192 |
| 10551 | -0.351777 | -0.190585 | -0.003885 | 0.049769 | -0.891608 | -2.059696 | -0.055568 | -0.278141 | 0.377108 | 0.104370 | 0.103662 |
| 10558 | 0.096597 | -0.378480 | 0.037810 | 0.010911 | -0.265278 | -0.378501 | 0.062701 | 0.475345 | -0.019450 | -0.030880 | 0.298025 |
| 11203 | -0.020307 | -0.031901 | 0.142411 | 0.066995 | 0.031557 | 0.080758 | -1.348264 | -1.897919 | 0.232648 | -0.109088 | 0.193759 |
| 11237 | -0.057929 | -0.355731 | 0.068947 | 0.085267 | 0.215514 | -0.159126 | -0.115729 | -0.076442 | 0.094597 | 0.206729 | -0.335975 |
| 13606 | 0.279153 | -0.045962 | 0.327194 | 0.354471 | 0.719525 | 0.273068 | -0.217612 | 0.031970 | 0.373060 | 0.295145 | 0.092554 |
| 15180 | -0.029703 | 0.104901 | -0.197956 | -0.248617 | -0.285895 | -0.000633 | -0.205114 | -0.216211 | -0.184384 | 0.068575 | -0.035094 |
| 16284 | 0.266270 | 0.405879 | 0.100980 | 0.288009 | 0.532942 | 0.271564 | 0.124402 | 0.270842 | -0.788518 | -0.311085 | -0.828540 |

In [22]:

```
for x in temp:
    if x in model.genes:
        for r in sorted(model.genes.get_by_id(x).reactions, key=lambda x: x.id):
            print(r, r.gene_reaction_rule)
    else:
        print(x, 'no reactions')
    print()
```

```
8782 no reactions

10551 no reactions

3OACOAR: 3ohodcoa_c + h_c + nadph_c --> 3hodcoa_c + nadp_c 10558
3OAR100: 3odecACP_c + h_c + nadph_c <=> 3hdecACP_c + nadp_c 10558
3OAR120: 3oddecACP_c + h_c + nadph_c <=> 3hddecACP_c + nadp_c 10558
3OAR121: 3ocddec5eACP_c + h_c + nadph_c --> 3hcddec5eACP_c + nadp_c 10558
3OAR140: 3omrsACP_c + h_c + nadph_c <=> 3hmrsACP_c + nadp_c 10558
3OAR141: 3ocmrs7eACP_c + h_c + nadph_c --> 3hcmrs7eACP_c + nadp_c 10558
3OAR160: 3opalmACP_c + h_c + nadph_c <=> 3hpalmACP_c + nadp_c 10558
3OAR161: 3ocpalm9eACP_c + h_c + nadph_c --> 3hcpalm9eACP_c + nadp_c 10558
3OAR180: 3ooctdACP_c + h_c + nadph_c <=> 3hoctaACP_c + nadp_c 10558
3OAR181: 3ocvac11eACP_c + h_c + nadph_c --> 3hcvac11eACP_c + nadp_c 10558
3OAR40: actACP_c + h_c + nadph_c <=> 3haACP_c + nadp_c 10558
3OAR60: 3ohexACP_c + h_c + nadph_c <=> 3hhexACP_c + nadp_c 10558
3OAR80: 3ooctACP_c + h_c + nadph_c <=> 3hoctACP_c + nadp_c 10558
OGMEACPR: h_c + nadph_c + ogmeACP_c --> hgmeACP_c + nadp_c 10558
OPMEACPR: h_c + nadph_c + opmeACP_c --> hpmeACP_c + nadp_c 10558
RHACOAR100: 3odcoa_c + h_c + nadph_c <=> R_3hdcoa_c + nadp_c 10558
RHACOAR120: 3oddcoa_c + h_c + nadph_c <=> R_3hddcoa_c + nadp_c 10558
RHACOAR140: 3otdcoa_c + h_c + nadph_c <=> R_3hmrscoa_c + nadp_c 10558
RHACOAR60: 3ohcoa_c + h_c + nadph_c <=> R_3hhcoa_c + nadp_c 10558
RHACOAR80: 3oocoa_c + h_c + nadph_c <=> R_3hocoa_c + nadp_c 10558

FAOXC11C9m: coa_m + fad_m + h2o_m + nad_m + undcoa_m --> accoa_m + fadh2_m + h_m + nadh_m + noncoa_m 12570 and 14805 and 11203 and 8885
FAOXC13C11m: coa_m + fad_m + h2o_m + nad_m + tridcoa_m --> accoa_m + fadh2_m + h_m + nadh_m + undcoa_m 12570 and 14805 and 11203 and 8885
FAOXC15C13m: coa_m + fad_m + h2o_m + nad_m + ptdcacoa_m --> accoa_m + fadh2_m + h_m + nadh_m + tridcoa_m 12570 and 14805 and 11203 and 8885
FAOXC170150m: coa_m + fad_m + h2o_m + hpdcacoa_m + nad_m --> accoa_m + fadh2_m + h_m + nadh_m + ptdcacoa_m 12570 and 14805 and 11203 and 8885
FAOXC5C3m: coa_m + fad_m + h2o_m + nad_m + pentcoa_m --> accoa_m + fadh2_m + h_m + nadh_m + ppcoa_m (12570 and 14805 and 11203 and 8885) or (14070 and 14805 and 11203 and 8885)
FAOXC7C5m: coa_m + fad_m + h2o_m + hepcoa_m + nad_m --> accoa_m + fadh2_m + h_m + nadh_m + pentcoa_m 12570 and 14805 and 11203 and 8885
FAOXC9C7m: coa_m + fad_m + h2o_m + nad_m + noncoa_m --> accoa_m + fadh2_m + h_m + hepcoa_m + nadh_m 12570 and 14805 and 11203 and 8885
HACD1m: aacoa_m + h_m + nadh_m <=> 3hbcoa_m + nad_m 11203
HACD2m: 3ohcoa_m + h_m + nadh_m <=> 3hhcoa_m + nad_m 11203
HACD3m: 3oocoa_m + h_m + nadh_m <=> 3hocoa_m + nad_m 11203
HACD4m: 3odcoa_m + h_m + nadh_m <=> 3hdcoa_m + nad_m 11203
HACD5m: 3oddcoa_m + h_m + nadh_m <=> 3hddcoa_m + nad_m 11203
HACD6m: 3otdcoa_m + h_m + nadh_m <=> 3htdcoa_m + nad_m 11203
HACD7m: 3ohdcoa_m + h_m + nadh_m <=> 3hhdcoa_m + nad_m 11203

11237 no reactions

HACD9m: 3hmbcoa_m + nad_m <=> 2maacoa_m + h_m + nadh_m 13606 or 16284

HADPCOADH3: 3hadpcoa_c + nad_c <=> h_c + nadh_c + oxadpcoa_c 15180
HBCO_nadp: aacoa_c + h_c + nadph_c <=> 3hbcoa_c + nadp_c 15180

HACD9m: 3hmbcoa_m + nad_m <=> 2maacoa_m + h_m + nadh_m 13606 or 16284

FAS200COAer: 3.0 h_r + malcoa_r + 2.0 nadph_r + stcoa_r --> arachcoa_r + co2_r + coa_r + h2o_r + 2.0 nadp_r (10677 and 11343 and 16241 and 16695) or (10677 and 16241 and 16655 and 16695)
FAS220COAer: arachcoa_r + 3.0 h_r + malcoa_r + 2.0 nadph_r --> co2_r + coa_r + docoscoa_r + h2o_r + 2.0 nadp_r (10677 and 11343 and 16241 and 16695) or (10677 and 16241 and 16655 and 16695)
FAS240COAer: docoscoa_r + 3.0 h_r + malcoa_r + 2.0 nadph_r --> co2_r + coa_r + h2o_r + 2.0 nadp_r + ttccoa_r (10677 and 11343 and 16241 and 16695) or (10677 and 16241 and 16655 and 16695)
FAS260COAer: 3.0 h_r + malcoa_r + 2.0 nadph_r + ttccoa_r --> co2_r + coa_r + h2o_r + hexccoa_r + 2.0 nadp_r 10677 and 11343 and 16241 and 16695
```

9469 SKL\* has only dehydratase domain, upregulated but no fitness defect  
9731 3-oxoacyl-ACP reductase, but no PTS signal or sigP  
10551 PTS2 3-oxoacyl-ACP reductase, upregulated and fitness defect ok  
10558 PTS2 3-oxoacyl-ACP reductase, upregulated but small fitness defect ok  
11362 pero FOX2, upregulated but no fitness defect

Set 16515 and 9469 for hydratase for coumaroyl-CoA  
Set 10551 and 10558 for 3-hydroxyacyl-CoA dehydrogenase / 3-oxoacyl-ACP reductase  
Remove cyto ACP reactions by 10558

In [23]:

```
print(sce.reactions.get_by_id('4HBZCOAFm'))
```

```
4HBZCOAFm: coa_m + coucoa_m + h2o_m + nad_m --> 4hbzcoa_m + accoa_m + h_m + nadh_m
```

In [24]:

```
r = sce.reactions.get_by_id('4HBZCOAFm').copy()
r.id = 'COUCOAHp'
r.name = '4-Coumarate-CoA hydratase'
r.gene_reaction_rule = '16515 or 9469'
model.add_reactions([r])
for m in r.metabolites:
    if not m.id.replace('_m','_x') in model.metabolites:
        m2 = m.copy()
        m2.id = m.id.replace('_m','_x')
        m2.compartment = 'x'
        model.add_metabolites([m2])
    r.add_metabolites({m.id: -r.get_coefficient(m.id), m.id.replace('_m','_x'): r.get_coefficient(m.id)})
m = model.metabolites.get_by_id('4hbzcoa_x').copy()
m.id = '34hp3hpcoa_x'
m.name = '3S-(4-hydroxyphenyl)-3-hydroxy-propanoyl-CoA'
m.formula = 'C30H40N7O19P3S'
m.charge = -4
model.add_metabolites([m])
r.add_metabolites({'coa_x': 1.0, 'nad_x': 1.0, '4hbzcoa_x': -1.0, 'accoa_x': -1.0, 'h_x': -1.0, 'nadh_x': -1.0,
                   '34hp3hpcoa_x': 1.0})

r = sce.reactions.get_by_id('4HBZCOAFm').copy()
r.id = '4HBZACCOARp'
r.name = '4-hydroxybenzoyl-acetyl-CoA reductase'
r.gene_reaction_rule = '10551 or 10558'
model.add_reactions([r])
for m in r.metabolites:
    if not m.id.replace('_m','_x') in model.metabolites:
        m2 = m.copy()
        m2.id = m.id.replace('_m','_x')
        m2.compartment = 'x'
        model.add_metabolites([m2])
    r.add_metabolites({m.id: -r.get_coefficient(m.id), m.id.replace('_m','_x'): r.get_coefficient(m.id)})
m = model.metabolites.get_by_id('4hbzcoa_x').copy()
m.id = '4hbzaccoa_x'
m.name = '4-hydroxybenzoyl-acetyl-CoA'
m.formula = 'C30H38N7O19P3S'
m.charge = -4
model.add_metabolites([m])
r.add_metabolites({'coa_x': 1.0, 'coucoa_x': 1.0, 'h2o_x': 1.0, '4hbzcoa_x': -1.0, 'accoa_x': -1.0, 
                   '34hp3hpcoa_x': -1.0, '4hbzaccoa_x': 1.0})

temp = ['3OACOAR','3OAR100','3OAR120','3OAR121','3OAR140','3OAR141','3OAR160','3OAR161','3OAR180','3OAR181','3OAR40',
        '3OAR60','3OAR80','OGMEACPR','OPMEACPR','RHACOAR100','RHACOAR120','RHACOAR140','RHACOAR60','RHACOAR80']
model.remove_reactions(temp, remove_orphans=True)
```

1. 3-(4-hydroxyphenyl)-3-oxo-propanoyl-CoA + coenzyme A -> 4-hydroxybenzoyl-CoA + acetyl-CoA

In [25]:

```
temp = Annotation.index[Annotation['Combined Annotations'].str.contains('thiolase') |
                        Annotation['Combined Annotations'].str.contains('CoA acyltransferase')]
display(Annotation.loc[temp])
Show_Data(temp)
```

|  | Combined Annotations | Signal P | Sc288c Orthologs | Human Orthologs | Sc288 Best Hit | Human Blast | Essential | WolfPSort | C Terminal |
| --- | --- | --- | --- | --- | --- | --- | --- | --- | --- |
| RTO4\_ID |  |  |  |  |  |  |  |  |  |
| 8782 | KOG4170: 2-enoyl-CoA hydratase/3-hydroxyacyl-C... |  |  | SCP2D1 |  | HSD17 | Not Essential | cyto 10.5, cyto\_pero 8.5, nucl 7, pero 5.5, mi... | SKL\* |
| 8885 | K07508: ACAA2; acetyl-CoA acyltransferase 2 |  |  | ACAA2 | ERG10 | ACAA2 | Not Essential | mito 12, cyto 11, pero 3 | ERV\* |
| 9065 | KOG1389: 3-oxoacyl CoA thiolase |  | POT1 | ACAA1 | POT1 | ACAA1 | Not Essential | mito 13, cyto 10, nucl 2 | VAE\* |
| 11237 | KOG4170: 2-enoyl-CoA hydratase/3-hydroxyacyl-C... |  |  |  |  |  | Not Essential | mito\_nucl 10.166, nucl 10, mito 10, cyto\_nucl 8 | AKL\* |
| 13813 | K07513: ACAA1; acetyl-CoA acyltransferase 1 |  | POT1 | ACAA1 | POT1 | ACAA1 | Not Essential | mito 18.5, cyto\_mito 13, cyto 6.5 | AEN\* |
| 15228 | K07513: ACAA1; acetyl-CoA acyltransferase 1 |  |  |  | POT1 | ACAA1 | Not Essential | cyto 14, mito 13 | VRD\* |

| strain | WT | | | | | | | | | | | | | | | | |
| --- | --- | --- | --- | --- | --- | --- | --- | --- | --- | --- | --- | --- | --- | --- | --- | --- | --- |
| condition | G\_MM | C\_MM | G\_SD | | GX\_SD | | | X\_SD | | A\_SD | | C\_SD | | MM\_CN120 | | MM\_CN5 | Diversity\_Sample |
| phase | exp | exp | exp | stat | exp | trans | stat | exp | stat | exp | stat | exp | stat | exp | stat | exp | exp |
| proteinId | Set1 | Set1 | Set2 | Set2 | Set2 | Set2 | Set2 | Set2 | Set2 | Set2 | Set2 | Set2 | Set2 | Set3 | Set3 | Set3 | Set3 |
| RTO4\_ID |  |  |  |  |  |  |  |  |  |  |  |  |  |  |  |  |  |
| 8782 | 8.300162 | 8.462562 | 9.007264 | 9.600527 | 8.956773 | 8.369212 | 8.840516 | 7.827337 | 9.211276 | 8.702320 | 9.957014 | 7.255736 | 8.347477 | 7.415559 | 7.872593 | 8.439886 | 7.974047 |
| 8885 | 5.504559 | 6.565118 | 8.065941 | 6.664514 | 8.049050 | 7.554300 | 7.738851 | 7.499308 | 6.910442 | 5.930749 | 6.448773 | 5.706823 | 5.016816 | 5.694920 | 7.324982 | 7.222957 | 6.632954 |
| 9065 | 6.879441 | 10.197874 | 5.144820 | 6.875500 | 5.595462 | 5.112078 | 5.343626 | 5.548889 | 6.853216 | 6.158897 | 6.983728 | 7.896757 | 7.215140 | 6.434061 | 5.640637 | 6.480870 | 5.474475 |
| 11237 | 6.860082 | 6.542515 | 6.034462 | 6.901833 | 5.957620 | 6.078630 | 6.083661 | 5.989742 | 6.613846 | 6.506268 | 6.953158 | 6.056770 | 6.114389 | 6.161046 | 6.409736 | 6.131124 | 5.530005 |
| 13813 | 5.117796 | 7.387603 | 6.033178 | 5.967237 | 5.985038 | 6.213650 | 6.144831 | 6.105746 | 6.681395 | 6.459578 | 7.178775 | 4.891526 | 5.567307 | 6.091379 | 6.739068 | 6.069412 | 5.885319 |
| 15228 | 5.083515 | 8.829758 | 6.510843 | 6.274466 | 6.578483 | 6.366137 | 6.385558 | 5.346932 | 6.750726 | 5.036384 | 6.142249 | 8.950815 | 8.026473 | 5.470105 | 4.793416 | 5.221585 | 5.518555 |

| strain | WT | | | | | | | | | | |
| --- | --- | --- | --- | --- | --- | --- | --- | --- | --- | --- | --- |
| condition | G\_SD | | GX\_SD | | | X\_SD | | A\_SD | | C\_SD | |
| proteinId | exp | stat | exp | trans | stat | exp | stat | exp | stat | exp | stat |
| 8782 | 6.389066 | 8.728867 | 8.344404 | 11.169981 | 10.923474 | 10.162712 | 10.819347 | 11.586292 | 12.829952 | 10.029745 | 9.128371 |
| 8885 | 23.196906 | 23.619082 | 20.995031 | 28.250724 | 31.203200 | 18.993934 | 22.091054 | 13.524671 | 12.638348 | 22.635363 | 20.650153 |
| 9065 | 8.794038 | 15.441107 | 8.726886 | 10.228263 | 9.645741 | 8.994289 | 13.976110 | 15.268987 | 19.731779 | 67.278251 | 45.319038 |
| 11237 | 2.093514 | 5.812726 | 2.836879 | 3.908544 | 4.596553 | 4.705091 | 6.870204 | 5.403808 | 6.520068 | 4.724388 | 3.940110 |
| 13813 | 9.639569 | 15.639286 | 7.316554 | 15.620445 | 17.204833 | 12.553261 | 15.954940 | 15.054102 | 17.617282 | 24.762959 | 22.190499 |
| 15228 | 12.511431 | 17.737287 | 14.842154 | 18.981900 | 18.445357 | 14.681180 | 15.122097 | 11.207129 | 10.704845 | 37.823122 | 38.561263 |

|  | Glucose | Xylose | Arabinose | Acetate | Coumarate | Ferulate | YNB Oleic Acid | YNB Ricinoleic Acid | YNB Glucose | YNB Gluc DOC | YPD |
| --- | --- | --- | --- | --- | --- | --- | --- | --- | --- | --- | --- |
| proteinId |  |  |  |  |  |  |  |  |  |  |  |
| 8782 | 0.055869 | -0.230385 | -0.029543 | 0.284492 | 0.068076 | 0.505709 | -0.161214 | -0.258673 | -0.195698 | -0.284325 | 0.171192 |
| 8885 | -0.216781 | 0.086870 | 0.107291 | -0.144912 | -0.087878 | 0.256151 | -0.409361 | -0.996266 | -0.417173 | -0.594114 | 0.200075 |
| 9065 | -0.350988 | -0.271906 | -0.134182 | -0.284826 | -5.608804 | -3.426361 | -0.290069 | 0.247955 | 0.258871 | 0.400966 | -0.203170 |
| 11237 | -0.057929 | -0.355731 | 0.068947 | 0.085267 | 0.215514 | -0.159126 | -0.115729 | -0.076442 | 0.094597 | 0.206729 | -0.335975 |
| 13813 | 0.126410 | 0.107964 | 0.192915 | 0.252853 | 0.192276 | 0.251335 | -2.491273 | -1.689101 | -0.065264 | -0.276600 | 0.225342 |
| 15228 | -0.223091 | -0.118190 | -0.335936 | -0.277735 | -4.995066 | -4.679965 | 0.138344 | 0.163114 | 0.296363 | 0.541750 | 0.402816 |

In [26]:

```
for x in temp:
    if x in model.genes:
        for r in sorted(model.genes.get_by_id(x).reactions, key=lambda x: x.id):
            print(r, r.gene_reaction_rule)
    else:
        print(x, 'no reactions')
    print()
```

```
8782 no reactions

ACACT2m: accoa_m + btcoa_m <-- 3ohcoa_m + coa_m 8885
ACACT3m: accoa_m + hxcoa_m <-- 3oocoa_m + coa_m 8885
ACACT4m: accoa_m + occoa_m <-- 3odcoa_m + coa_m 8885
ACACT5m: accoa_m + dcacoa_m <-- 3oddcoa_m + coa_m 8885
ACACT6m: accoa_m + ddcacoa_m <-- 3otdcoa_m + coa_m 8885
ACACT7m: accoa_m + tdcoa_m <-- 3ohdcoa_m + coa_m 8885
FAOXC11C9m: coa_m + fad_m + h2o_m + nad_m + undcoa_m --> accoa_m + fadh2_m + h_m + nadh_m + noncoa_m 12570 and 14805 and 11203 and 8885
FAOXC13C11m: coa_m + fad_m + h2o_m + nad_m + tridcoa_m --> accoa_m + fadh2_m + h_m + nadh_m + undcoa_m 12570 and 14805 and 11203 and 8885
FAOXC15C13m: coa_m + fad_m + h2o_m + nad_m + ptdcacoa_m --> accoa_m + fadh2_m + h_m + nadh_m + tridcoa_m 12570 and 14805 and 11203 and 8885
FAOXC170150m: coa_m + fad_m + h2o_m + hpdcacoa_m + nad_m --> accoa_m + fadh2_m + h_m + nadh_m + ptdcacoa_m 12570 and 14805 and 11203 and 8885
FAOXC5C3m: coa_m + fad_m + h2o_m + nad_m + pentcoa_m --> accoa_m + fadh2_m + h_m + nadh_m + ppcoa_m (12570 and 14805 and 11203 and 8885) or (14070 and 14805 and 11203 and 8885)
FAOXC7C5m: coa_m + fad_m + h2o_m + hepcoa_m + nad_m --> accoa_m + fadh2_m + h_m + nadh_m + pentcoa_m 12570 and 14805 and 11203 and 8885
FAOXC9C7m: coa_m + fad_m + h2o_m + nad_m + noncoa_m --> accoa_m + fadh2_m + h_m + hepcoa_m + nadh_m 12570 and 14805 and 11203 and 8885

FAO80p: 3.0 coa_x + 3.0 h2o_x + 3.0 nad_x + 3.0 o2_x + occoa_x --> 4.0 accoa_x + 3.0 h2o2_x + 3.0 h_x + 3.0 nadh_x (11362 and 12742 and 13813) or (11362 and 12742 and 9065) or (11362 and 12752 and 13813) or (11362 and 12752 and 9065) or (11362 and 13813 and 9700) or (11362 and 9065 and 9700)

11237 no reactions

ACACT1p: aacoa_x + coa_x --> 2.0 accoa_x 13813
ACACT2p: 3ohcoa_x + coa_x --> accoa_x + btcoa_x 13813
ACACT3p: 3oocoa_x + coa_x --> accoa_x + hxcoa_x 13813
ACACT4p: 3odcoa_x + coa_x --> accoa_x + occoa_x 13813
ACACT5p: 3oddcoa_x + coa_x --> accoa_x + dcacoa_x 13813
ACACT6p: 3otdcoa_x + coa_x --> accoa_x + ddcacoa_x 13813
ACACT7p: 3ohdcoa_x + coa_x --> accoa_x + tdcoa_x 13813
ACACT8p: 3ohodcoa_x + coa_x --> accoa_x + pmtcoa_x 13813
ACACT9p: 3ohxccoa_x + coa_x --> accoa_x + ttccoa_x 13813
FAO141p_even: 6.0 coa_x + 6.0 h2o_x + 6.0 nad_x + nadph_x + 6.0 o2_x + tdecoa_x --> 7.0 accoa_x + 6.0 h2o2_x + 5.0 h_x + 6.0 nadh_x + nadp_x (10293 and 11362 and 11907 and 12742 and 13228 and 13813) or (10293 and 11362 and 11907 and 12752 and 13228 and 13813) or (10293 and 11362 and 11907 and 13228 and 13813 and 9700)
FAO141p_odd: 6.0 coa_x + 6.0 h2o_x + 6.0 nad_x + 5.0 o2_x + tdecoa_x --> 7.0 accoa_x + 5.0 h2o2_x + 6.0 h_x + 6.0 nadh_x (10293 and 11362 and 12742 and 13813) or (10293 and 11362 and 12752 and 13813) or (10293 and 11362 and 13813 and 9700)
FAO161p_even: 7.0 coa_x + 7.0 h2o_x + hdcoa_x + 7.0 nad_x + nadph_x + 7.0 o2_x --> 8.0 accoa_x + 7.0 h2o2_x + 6.0 h_x + 7.0 nadh_x + nadp_x (10293 and 11362 and 11907 and 12742 and 13228 and 13813) or (10293 and 11362 and 11907 and 12752 and 13228 and 13813) or (10293 and 11362 and 11907 and 13228 and 13813 and 9700)
FAO161p_odd: 7.0 coa_x + 7.0 h2o_x + hdcoa_x + 7.0 nad_x + 6.0 o2_x --> 8.0 accoa_x + 6.0 h2o2_x + 7.0 h_x + 7.0 nadh_x (10293 and 11362 and 12742 and 13813) or (10293 and 11362 and 12752 and 13813) or (10293 and 11362 and 13813 and 9700)
FAO181p_even: 8.0 coa_x + 8.0 h2o_x + 8.0 nad_x + nadph_x + 8.0 o2_x + odecoa_x --> 9.0 accoa_x + 8.0 h2o2_x + 7.0 h_x + 8.0 nadh_x + nadp_x (10293 and 11362 and 11907 and 12742 and 13228 and 13813) or (10293 and 11362 and 11907 and 12752 and 13228 and 13813) or (10293 and 11362 and 11907 and 13228 and 13813 and 9700)
FAO181p_odd: 8.0 coa_x + 8.0 h2o_x + 8.0 nad_x + 7.0 o2_x + odecoa_x --> 9.0 accoa_x + 7.0 h2o2_x + 8.0 h_x + 8.0 nadh_x (10293 and 11362 and 12742 and 13813) or (10293 and 11362 and 12752 and 13813) or (10293 and 11362 and 13813 and 9700)
FAO182p_even: 8.0 coa_x + 8.0 h2o_x + 8.0 nad_x + 2.0 nadph_x + 8.0 o2_x + ocdycacoa_x --> 9.0 accoa_x + 8.0 h2o2_x + 6.0 h_x + 8.0 nadh_x + 2.0 nadp_x (10293 and 11362 and 11907 and 12742 and 13228 and 13813) or (10293 and 11362 and 11907 and 12752 and 13228 and 13813) or (10293 and 11362 and 11907 and 13228 and 13813 and 9700)
FAO182p_odd: 8.0 coa_x + 8.0 h2o_x + 8.0 nad_x + nadph_x + 7.0 o2_x + ocdycacoa_x --> 9.0 accoa_x + 7.0 h2o2_x + 7.0 h_x + 8.0 nadh_x + nadp_x (10293 and 11362 and 12742 and 13228 and 13813) or (10293 and 11362 and 12752 and 13228 and 13813) or (10293 and 11362 and 13228 and 13813 and 9700)
FAO183p_even: 8.0 coa_x + 8.0 h2o_x + lnlncgcoa_x + 8.0 nad_x + 3.0 nadph_x + 8.0 o2_x --> 9.0 accoa_x + 8.0 h2o2_x + 5.0 h_x + 8.0 nadh_x + 3.0 nadp_x (10293 and 11362 and 11907 and 12742 and 13228 and 13813) or (10293 and 11362 and 11907 and 12752 and 13228 and 13813) or (10293 and 11362 and 11907 and 13228 and 13813 and 9700)
FAO183p_odd: 8.0 coa_x + 8.0 h2o_x + lnlncgcoa_x + 8.0 nad_x + 2.0 nadph_x + 7.0 o2_x --> 9.0 accoa_x + 7.0 h2o2_x + 6.0 h_x + 8.0 nadh_x + 2.0 nadp_x (10293 and 11362 and 12742 and 13228 and 13813) or (10293 and 11362 and 12752 and 13228 and 13813) or (10293 and 11362 and 13228 and 13813 and 9700)
FAO80p: 3.0 coa_x + 3.0 h2o_x + 3.0 nad_x + 3.0 o2_x + occoa_x --> 4.0 accoa_x + 3.0 h2o2_x + 3.0 h_x + 3.0 nadh_x (11362 and 12742 and 13813) or (11362 and 12742 and 9065) or (11362 and 12752 and 13813) or (11362 and 12752 and 9065) or (11362 and 13813 and 9700) or (11362 and 9065 and 9700)
FAOXC200180x: arachcoa_x + coa_x + h2o_x + nad_x + o2_x --> accoa_x + h2o2_x + h_x + nadh_x + stcoa_x (12742 and 11362 and 13813) or (12752 and 11362 and 13813) or (9700 and 11362 and 13813)
FAOXC22C20x: coa_x + docoscoa_x + h2o_x + nad_x + o2_x --> accoa_x + arachcoa_x + h2o2_x + h_x + nadh_x (12742 and 11362 and 13813) or (12752 and 11362 and 13813) or (9700 and 11362 and 13813)
FAOXC241181x: 3.0 coa_x + 3.0 h2o_x + 3.0 nad_x + nrvnccoa_x + 3.0 o2_x --> 3.0 accoa_x + 3.0 h2o2_x + 3.0 h_x + 3.0 nadh_x + odecoa_x (12742 and 11362 and 13813) or (12752 and 11362 and 13813) or (9700 and 11362 and 13813)
FAOXC2442246x: coa_x + h2o_x + nad_x + o2_x + tettet6coa_x --> accoa_x + adrncoa_x + h2o2_x + h_x + nadh_x (12742 and 11362 and 13813) or (12752 and 11362 and 13813) or (9700 and 11362 and 13813)
FAOXC2452253x: coa_x + h2o_x + nad_x + o2_x + tetpent3coa_x --> accoa_x + clpndcoa_x + h2o2_x + h_x + nadh_x (12742 and 11362 and 13813) or (12752 and 11362 and 13813) or (9700 and 11362 and 13813)
FAOXC2452256x: coa_x + h2o_x + nad_x + o2_x + tetpent6coa_x --> accoa_x + dcsptn1coa_x + h2o2_x + h_x + nadh_x (12742 and 11362 and 13813) or (12752 and 11362 and 13813) or (9700 and 11362 and 13813)
FAOXC24C22x: coa_x + h2o_x + nad_x + o2_x + ttccoa_x --> accoa_x + docoscoa_x + h2o2_x + h_x + nadh_x (12742 and 11362 and 13813) or (12752 and 11362 and 13813) or (9700 and 11362 and 13813)

15228 no reactions
```

In [27]:

```
temp = Annotation.index[Annotation['Combined Annotations'].str.contains('thioesterase') |
                        Annotation['Combined Annotations'].str.contains('CoA hydrolase')]
display(Annotation.loc[temp])
Show_Data(temp)
```

|  | Combined Annotations | Signal P | Sc288c Orthologs | Human Orthologs | Sc288 Best Hit | Human Blast | Essential | WolfPSort | C Terminal |
| --- | --- | --- | --- | --- | --- | --- | --- | --- | --- |
| RTO4\_ID |  |  |  |  |  |  |  |  |  |
| 8456 | K17361: ACOT9; acyl-coenzyme A thioesterase 9 |  |  | ACOT9 |  | ACOT9 | Not Essential | nucl 13.5, cyto\_nucl 11.5, cyto 8.5, mito 3 | QAP\* |
| 9961 | KOG4366: Predicted thioesterase | S |  |  | YBR096W |  | Not Essential | mito 16, extr 7, cyto 3 | VDL\* |
| 10555 | K09602: OTUB1; ubiquitin thioesterase protein ... |  |  | OTUB2,OTUB1 |  | OTUB1 | Essential | cyto\_nucl 10.5, nucl 9.5, cyto 8.5, cysk 4, mi... | LYR\* |
| 12708 | K01074: PPT; palmitoyl-protein thioesterase | S |  | PPT1 |  | PPT1 | Not Essential | extr 17, mito 5, cyto 4 | ALA\* |
| 13048 | K01067: E3.1.2.1, ACH1; acetyl-CoA hydrolase |  |  |  |  | ACOT12 | Not Essential | cyto 20.5, cyto\_mito 11, nucl 5 | VYA\* |
| 13797 | K01067: E3.1.2.1, ACH1; acetyl-CoA hydrolase |  | ACH1 |  | ACH1 |  | Not Essential | mito 23, cyto 3 | GWS\* |
| 13926 | KOG3328: HGG motif-containing thioesterase |  |  |  |  |  | Not Essential | cyto 13, cyto\_nucl 8, extr 7, mito 3 | AKL\* |
| 14585 | K17362: ACOT13; acyl-coenzyme A thioesterase 13 |  |  | ACOT13 |  | ACOT1 | Not Essential | cysk 9, mito 8, cyto 5.5, cyto\_nucl 5, nucl 3.5 | PSE\* |
| 15218 | K05605: HIBCH; 3-hydroxyisobutyryl-CoA hydrolase |  | EHD3 | HIBCH | EHD3 | HIBCH | Not Essential | mito 22.5, cyto\_mito 14, cyto 4.5 | WVS\* |
| 15321 | K13719: OTU1, YOD1; ubiquitin thioesterase OTU1 |  | OTU1 | YOD1 | OTU1 | YOD1 | Not Essential | cyto\_nucl 10.5, mito 10, nucl 8.5, cyto 7.5 | YDG\* |
| 16048 | K11992: ACOT8, PTE; acyl-CoA thioesterase 8 |  | TES1 | ACOT8 |  | ACOT8 | Not Essential | nucl 8.5, cyto\_nucl 7, pero 7, mito 5, cyto 4.5 | AAL\* |
| 16237 | KOG4366: Predicted thioesterase | S | YBR096W |  |  |  | Not Essential | mito 20, cyto 2, plas 2, extr 2 | QVA\* |

| strain | WT | | | | | | | | | | | | | | | | |
| --- | --- | --- | --- | --- | --- | --- | --- | --- | --- | --- | --- | --- | --- | --- | --- | --- | --- |
| condition | G\_MM | C\_MM | G\_SD | | GX\_SD | | | X\_SD | | A\_SD | | C\_SD | | MM\_CN120 | | MM\_CN5 | Diversity\_Sample |
| phase | exp | exp | exp | stat | exp | trans | stat | exp | stat | exp | stat | exp | stat | exp | stat | exp | exp |
| proteinId | Set1 | Set1 | Set2 | Set2 | Set2 | Set2 | Set2 | Set2 | Set2 | Set2 | Set2 | Set2 | Set2 | Set3 | Set3 | Set3 | Set3 |
| RTO4\_ID |  |  |  |  |  |  |  |  |  |  |  |  |  |  |  |  |  |
| 8456 | 6.433353 | 5.591254 | 6.496609 | 5.414881 | 6.735539 | 5.852896 | 5.853383 | 6.212590 | 6.030268 | 5.997487 | 5.682528 | 6.238931 | 6.983038 | 6.455223 | 5.941573 | 7.756674 | 5.572588 |
| 9961 | 4.495850 | 3.608010 | 4.674156 | 4.218598 | 4.762479 | 4.300008 | 4.208903 | 4.183248 | 3.740842 | 4.153170 | 3.171719 | 2.194673 | 2.855709 | 4.708039 | 3.914848 | 5.450059 | 5.204285 |
| 10555 | 5.319806 | 5.262290 | 4.797780 | 5.534269 | 4.854749 | 5.245510 | 5.046666 | 5.203880 | 4.699953 | 5.115200 | 5.236902 | 5.293648 | 4.448060 | 4.562112 | 4.705076 | 4.662431 | 4.215376 |
| 12708 | 5.424577 | 5.050650 | 4.543740 | 5.407413 | 4.535109 | 5.009180 | 4.804813 | 5.011269 | 4.697303 | 5.067623 | 5.472371 | 4.480345 | 3.763471 | 4.407704 | 4.642598 | 4.425492 | 4.362363 |
| 13048 | 5.101678 | 4.964951 | 4.539066 | 4.468134 | 4.359620 | 4.068779 | 4.084544 | 4.169017 | 4.393575 | 4.457102 | 4.863661 | 4.469643 | 4.178202 | 5.457276 | 5.236640 | 5.285082 | 4.735815 |
| 13797 | 5.628486 | 4.815028 | 8.171250 | 5.050958 | 8.087060 | 6.647683 | 6.378718 | 7.053845 | 5.340168 | 5.190033 | 4.776262 | 5.672364 | 5.477917 | 6.757577 | 7.347255 | 8.418573 | 7.000140 |
| 13926 | 3.932689 | 4.973963 | 3.969918 | 3.400350 | 3.864864 | 3.682200 | 3.775064 | 4.180851 | 4.969104 | 4.482525 | 4.650219 | 3.181818 | 1.998801 | 3.352091 | 2.552751 | 3.572372 | 2.881159 |
| 14585 | 6.515616 | 6.999164 | 6.165002 | 5.663557 | 6.058274 | 5.692616 | 5.832953 | 6.362673 | 6.160310 | 6.679602 | 6.867328 | 6.274651 | 5.640809 | 3.793091 | 3.924944 | 4.871485 | 2.619503 |
| 15218 | 6.864670 | 7.093075 | 6.602717 | 6.705486 | 6.552150 | 6.340783 | 6.390061 | 6.668685 | 6.499981 | 6.384683 | 6.708475 | 7.227468 | 7.167939 | 5.921263 | 5.825528 | 6.532056 | 6.514194 |
| 15321 | 3.741959 | 4.069107 | 4.690566 | 2.596242 | 4.880916 | 3.481731 | 3.268104 | 4.329297 | 3.777246 | 4.202674 | 3.605232 | 3.755475 | 3.590734 | 4.266357 | 3.757620 | 5.211799 | 4.629282 |
| 16048 | 4.849880 | 6.198672 | 4.632170 | 4.181456 | 4.589360 | 4.422483 | 4.282515 | 4.513114 | 4.767136 | 4.236341 | 5.043629 | 4.883313 | 4.607162 | 6.281024 | 6.330490 | 5.407905 | 5.351227 |
| 16237 | 6.175568 | 5.410401 | 5.635182 | 4.524670 | 5.753005 | 4.986377 | 4.988176 | 5.293894 | 5.135045 | 5.085243 | 4.620253 | 5.532479 | 5.741863 | 5.826890 | 5.080445 | 5.722591 | 5.774317 |

| strain | WT | | | | | | | | | | |
| --- | --- | --- | --- | --- | --- | --- | --- | --- | --- | --- | --- |
| condition | G\_SD | | GX\_SD | | | X\_SD | | A\_SD | | C\_SD | |
| proteinId | exp | stat | exp | trans | stat | exp | stat | exp | stat | exp | stat |
| 8456 | 5.382147 | 6.423754 | 5.100165 | 4.645961 | 3.416195 | 4.328608 | 2.539480 | 4.244641 | 2.102469 | 1.067948 | 1.961084 |
| 9961 | 1.154251 | 0.000000 | 1.016462 | 0.000000 | 0.000000 | 0.000000 | 0.000000 | 0.000000 | 0.000000 | 0.000000 | 0.000000 |
| 10555 | 0.180601 | 1.284835 | 0.413883 | 2.042314 | 2.540147 | 2.747058 | 1.975359 | 2.323189 | 2.671203 | 0.433188 | 1.512018 |
| 12708 | 0.762362 | 1.092524 | 0.197656 | 2.592383 | 2.732897 | 1.177419 | 0.189187 | 1.355208 | 0.776074 | 0.637818 | 0.220621 |
| 13048 | 0.000000 | 0.166667 | 0.000000 | 0.000000 | 0.000000 | 0.000000 | 0.188437 | 0.193992 | 0.196097 | 0.000000 | 0.000000 |
| 13797 | 38.793858 | 30.901665 | 34.022717 | 34.830109 | 33.289366 | 18.194751 | 23.443325 | 8.116480 | 10.151072 | 13.250719 | 13.261287 |
| 13926 | 0.190197 | 0.000000 | 0.000000 | 0.000000 | 0.000000 | 0.199906 | 0.000000 | 0.000000 | 0.000000 | 0.632921 | 0.220621 |
| 14585 | 1.122320 | 1.269328 | 1.837318 | 0.751858 | 0.357685 | 0.956129 | 1.171866 | 1.352532 | 1.527934 | 1.711249 | 1.955561 |
| 15218 | 21.177279 | 18.798839 | 21.939978 | 18.443109 | 20.202576 | 20.919152 | 22.503149 | 20.677336 | 20.499395 | 26.939309 | 28.082673 |
| 15321 | 0.190197 | 0.000000 | 0.203508 | 0.000000 | 0.000000 | 0.596794 | 0.404429 | 0.000000 | 0.189478 | 0.211647 | 0.000000 |
| 16048 | 3.244628 | 3.788602 | 3.852815 | 6.513302 | 5.631431 | 4.105917 | 3.895050 | 3.285715 | 2.688339 | 9.425335 | 5.012086 |
| 16237 | 4.994639 | 4.865722 | 3.662350 | 2.043188 | 1.467512 | 3.706536 | 3.137131 | 3.085202 | 2.853273 | 5.097079 | 4.995168 |

|  | Glucose | Xylose | Arabinose | Acetate | Coumarate | Ferulate | YNB Oleic Acid | YNB Ricinoleic Acid | YNB Glucose | YNB Gluc DOC | YPD |
| --- | --- | --- | --- | --- | --- | --- | --- | --- | --- | --- | --- |
| proteinId |  |  |  |  |  |  |  |  |  |  |  |
| 8456 | -0.093474 | -0.207925 | -0.306311 | -0.212615 | -0.296622 | -0.031530 | 0.061205 | 0.055315 | -0.326957 | -0.394361 | -0.012428 |
| 9961 | -0.190319 | 0.156096 | -0.182096 | -0.027216 | -0.122849 | 0.081674 | -0.423321 | -0.904613 | 0.049848 | 0.113205 | 0.222120 |
| 10555 | 0.054346 | 0.050681 | 0.178883 | -0.027631 | -0.165820 | 0.171167 | -0.218187 | 0.285970 | -0.061846 | -0.046716 | -0.268365 |
| 12708 | 0.083158 | -0.299612 | -0.265726 | 0.001657 | -0.558586 | 0.252300 | -0.256762 | -0.383150 | -0.327999 | -0.440711 | -0.316626 |
| 13048 | 0.108406 | 0.006342 | -0.139075 | 0.082046 | 0.153588 | 0.483577 | -0.203929 | -0.246815 | 0.042048 | 0.132070 | 0.511066 |
| 13797 | -0.017241 | -0.175817 | -0.123985 | -0.429914 | -0.168931 | -0.077230 | -0.011927 | 0.333993 | -0.062243 | -0.043898 | -0.158202 |
| 13926 | -0.456230 | 0.041994 | 0.257273 | -0.145215 | -0.050948 | -0.111750 | 0.073652 | -0.140547 | -0.429113 | -0.150206 | -0.282135 |
| 14585 | -0.518157 | 0.276727 | -0.224457 | 0.045163 | -0.339184 | 0.298084 | -0.011245 | -0.068001 | -0.110504 | -0.149529 | 0.172852 |
| 15218 | 0.074529 | -0.021972 | -0.274042 | -1.225263 | -0.919181 | -0.378660 | -0.549135 | 0.000359 | -0.184537 | -0.260827 | 0.215170 |
| 15321 | -0.151307 | -0.176040 | -0.258885 | -0.241727 | -0.164938 | -0.167326 | 0.021240 | -0.196732 | 0.314399 | -0.301713 | -0.071651 |
| 16048 | -0.094170 | -0.037546 | 0.012164 | -0.147875 | -0.012601 | -0.040689 | 0.172548 | 0.036226 | -0.245912 | -0.040811 | 0.228448 |
| 16237 | -0.258299 | 0.087374 | 0.119359 | 0.242647 | 0.215384 | -0.329676 | -0.305929 | -0.188084 | 0.987420 | 0.880214 | -0.112857 |

In [28]:

```
for x in temp:
    if x in model.genes:
        for r in sorted(model.genes.get_by_id(x).reactions, key=lambda x: x.id):
            print(r, r.gene_reaction_rule)
    else:
        print(x, 'no reactions')
    print()
```

```
FACOAE100: dcacoa_c + h2o_c --> coa_c + dca_c + h_c 8456
FACOAE120: ddcacoa_c + h2o_c --> coa_c + ddca_c + h_c 8456
FACOAE140: h2o_c + tdcoa_c --> coa_c + h_c + ttdca_c 8456
FACOAE141: h2o_c + tdecoa_c --> coa_c + h_c + ttdcea_c 8456
FACOAE160: h2o_c + pmtcoa_c --> coa_c + h_c + hdca_c 8456
FACOAE161: h2o_c + hdcoa_c --> coa_c + h_c + hdcea_c 8456
FACOAE180: h2o_c + stcoa_c --> coa_c + h_c + ocdca_c 8456
FACOAE181: h2o_c + odecoa_c --> coa_c + h_c + ocdcea_c 8456
FACOAE1829Z12Z: h2o_c + ocdycacoa_c --> coa_c + h_c + ocdcya_c 8456
FACOAE1836Z9Z12Z: h2o_c + lnlncgcoa_c --> coa_c + h_c + lnlncg_c 8456
FACOAE60: h2o_c + hxcoa_c --> coa_c + h_c + hxa_c 8456
FACOAE80: h2o_c + occoa_c --> coa_c + h_c + octa_c 8456

9961 no reactions

10555 no reactions

12708 no reactions

ACOAH: ac_c + coa_c + h_c <-- accoa_c + h2o_c 13048

ACOAHim: accoa_m + h2o_m --> ac_m + coa_m + h_m 13797

13926 no reactions

PTE10m: dcacoa_m + h2o_m --> coa_m + dca_m + h_m 14585
PTE11m: ddcacoa_m + h2o_m --> coa_m + ddca_m + h_m 14585
PTE2m: h2o_m + pmtcoa_m --> coa_m + h_m + hdca_m 14585
PTE7m: h2o_m + tdcoa_m --> coa_m + h_m + ttdca_m 14585
PTE9m: h2o_m + occoa_m --> coa_m + h_m + octa_m 14585

3HBCOAHLm: 3hibutcoa_m + h2o_m --> 3hmp_m + coa_m + h_m 15218
3HPCOAHYD: 3hpcoa_c + h2o_c --> 3hpp_c + coa_c + h_c 15218

15321 no reactions

PTE10x: dcacoa_x + h2o_x --> coa_x + dca_x + h_x 16048
PTE11x: ddcacoa_x + h2o_x --> coa_x + ddca_x + h_x 16048
PTE12x: h2o_x + ttccoa_x --> coa_x + h_x + ttc_x 16048
PTE2x: h2o_x + pmtcoa_x --> coa_x + h_x + hdca_x 16048
PTE7x: h2o_x + tdcoa_x --> coa_x + h_x + ttdca_x 16048
PTE8x: h2o_x + stcoa_x --> coa_x + h_x + ocdca_x 16048
PTE9x: h2o_x + occoa_x --> coa_x + h_x + octa_x 16048

16237 no reactions
```

9065 pero POT1 3-oxoacyl CoA thiolase, upregulated and defect in both, ok  
15228 also upregulated and defect in both, but no PTS -> pcaF

15218 3-hydroxyisobutyryl-CoA hydrolase upregulated, but mito no PTS  
Change 3HPCOAHYD to 3HPCOAHYDm  
16048 PTE; acyl-CoA thioesterase 8 somewhat upregulated, AAL*, but no fitness defect  
14934 HMMPfam:Alpha/beta hydrolase family:PF12697, fitness defect and ARL*

In [29]:

```
print(sce.reactions.get_by_id('4HBZCOAFm'))
print(sce.reactions.get_by_id('4HBZFm'))
```

```
4HBZCOAFm: coa_m + coucoa_m + h2o_m + nad_m --> 4hbzcoa_m + accoa_m + h_m + nadh_m
4HBZFm: 4hbzcoa_m + h2o_m --> 4hbz_m + coa_m + h_m
```

In [30]:

```
r = sce.reactions.get_by_id('4HBZCOAFm').copy()
r.id = '4HBZACCOATp'
r.name = '4-hydroxybenzoyl-acetyl-CoA thiolase'
r.gene_reaction_rule = '9065'
model.add_reactions([r])
for m in r.metabolites:
    if not m.id.replace('_m','_x') in model.metabolites:
        m2 = m.copy()
        m2.id = m.id.replace('_m','_x')
        m2.compartment = 'x'
        model.add_metabolites([m2])
    r.add_metabolites({m.id: -r.get_coefficient(m.id), m.id.replace('_m','_x'): r.get_coefficient(m.id)})
r.add_metabolites({'coucoa_x': 1.0, 'h2o_x': 1.0, 'nad_x': 1.0, 'h_x': -1.0, 'nadh_x': -1.0,
                   '4hbzaccoa_x': -1.0})

r = model.reactions.get_by_id('3HPCOAHYD')
r.id = '3HPCOAHYDm'
for m in r.metabolites:
    if not m.id.replace('_c','_m') in model.metabolites:
        m2 = m.copy()
        m2.id = m.id.replace('_c','_m')
        m2.compartment = 'm'
        model.add_metabolites([m2])
    r.add_metabolites({m.id: -r.get_coefficient(m.id), m.id.replace('_c','_m'): r.get_coefficient(m.id)})
    
r = sce.reactions.get_by_id('4HBZFm').copy()
r.id = '4HBZCOAEp'
r.name = '4-hydroxybenzoyl-CoA thioesterase'
r.gene_reaction_rule = '14934'
model.add_reactions([r])
for m in r.metabolites:
    if not m.id.replace('_m','_x') in model.metabolites:
        m2 = m.copy()
        m2.id = m.id.replace('_m','_x')
        m2.compartment = 'x'
        model.add_metabolites([m2])
    r.add_metabolites({m.id: -r.get_coefficient(m.id), m.id.replace('_m','_x'): r.get_coefficient(m.id)})

r = sce.reactions.get_by_id('4HBZtm').copy()
r.id = '4HBZtp'
r.name = '4-hydroxybenzoate peroxisomal transport'
r.gene_reaction_rule = '' # find transporter gene?
model.add_reactions([r])
r.add_metabolites({'4hbz_m': -1.0, '4hbz_x': 1.0})

r = sce.reactions.get_by_id('4HBZtm').copy()
model.add_reactions([r]) # find transporter gene?
```

How is coumarate / coumaroyl-CoA transported to peroxisome?

In [31]:

```
# Check fatty acid peroxisomal transport
for r in sorted(model.metabolites.get_by_id('ocdca_x').reactions, key=lambda x: x.id):
    print(r, r.gene_reaction_rule)
```

```
FA180COAabcp: atp_c + 2.0 h2o_c + stcoa_c --> adp_c + coa_c + 2.0 h_c + ocdca_x + pi_c 13167 and 9637
FACOAL180p: atp_x + coa_x + ocdca_x --> amp_x + ppi_x + stcoa_x 12555
PTE8x: h2o_x + stcoa_x --> coa_x + h_x + ocdca_x 16048
```

In [32]:

```
temp = ['9912','13167','9637','12555']
display(Annotation.loc[temp])
Show_Data(temp)
```

|  | Combined Annotations | Signal P | Sc288c Orthologs | Human Orthologs | Sc288 Best Hit | Human Blast | Essential | WolfPSort | C Terminal |
| --- | --- | --- | --- | --- | --- | --- | --- | --- | --- |
| RTO4\_ID |  |  |  |  |  |  |  |  |  |
| 9912 | K08745: SLC27A1\_4, FATP1, FATP4; solute carrie... | S | FAT1 | SLC27A1,SLC27A4,SLC27A2,SLC27A5,SLC27A6 | FAT1 | SLC27 | Not Essential | extr 12, plas 8, pero 4, mito 1, cyto 1, E.R. ... | VRL\* |
| 13167 | K05676: ABCD2, ALDL1; ATP-binding cassette, su... |  | PXA1,PXA2 | ABCD1,ABCD2,ABCD3 | PXA2 | ABCD2 | Not Essential | plas 10, mito 8, cyto 4, pero 2, nucl 1, extr ... | ERI\* |
| 9637 | K15628: PXA; ATP-binding cassette, subfamily D... | S | PXA1,PXA2 | ABCD1,ABCD2,ABCD3 | PXA1 | ABCD2 | Not Essential | extr 14, mito 6, plas 4, cyto\_mito 4 | KET\* |
| 12555 | K01897: ACSL, fadD; long-chain acyl-CoA synthe... |  | FAA2 | ACSL1,ACSL5,ACSL6,CTB-127M13.1 | FAA2 | ACSL1 | Not Essential | cyto 15.5, cyto\_nucl 11.5, pero 6, nucl 4.5 | AKL\* |

| strain | WT | | | | | | | | | | | | | | | | |
| --- | --- | --- | --- | --- | --- | --- | --- | --- | --- | --- | --- | --- | --- | --- | --- | --- | --- |
| condition | G\_MM | C\_MM | G\_SD | | GX\_SD | | | X\_SD | | A\_SD | | C\_SD | | MM\_CN120 | | MM\_CN5 | Diversity\_Sample |
| phase | exp | exp | exp | stat | exp | trans | stat | exp | stat | exp | stat | exp | stat | exp | stat | exp | exp |
| proteinId | Set1 | Set1 | Set2 | Set2 | Set2 | Set2 | Set2 | Set2 | Set2 | Set2 | Set2 | Set2 | Set2 | Set3 | Set3 | Set3 | Set3 |
| 9912 | 5.930735 | 5.408450 | 5.896409 | 6.224796 | 5.878952 | 5.800676 | 5.931129 | 5.504309 | 5.916014 | 5.556604 | 6.229153 | 5.264760 | 4.972355 | 5.889038 | 5.914059 | 5.563289 | 5.572101 |
| 13167 | 6.152975 | 5.743564 | 6.894880 | 7.454562 | 6.811796 | 6.755678 | 7.226369 | 6.442483 | 6.925176 | 6.391643 | 7.334114 | 7.054764 | 8.219373 | 5.566820 | 6.644638 | 7.049615 | 6.207616 |
| 9637 | 4.571137 | 4.276618 | 5.519868 | 5.698537 | 5.534625 | 5.541388 | 5.892257 | 4.925441 | 5.542306 | 4.671716 | 5.292226 | 5.808178 | 6.031330 | 4.282160 | 5.510333 | 4.670796 | 4.495849 |
| 12555 | 6.453199 | 6.511665 | 5.433267 | 5.782332 | 5.426938 | 5.525507 | 5.263275 | 5.257378 | 6.122703 | 6.156341 | 6.452491 | 4.764078 | 5.102459 | 6.787262 | 5.690798 | 6.007580 | 6.185189 |

| strain | WT | | | | | | | | | | |
| --- | --- | --- | --- | --- | --- | --- | --- | --- | --- | --- | --- |
| condition | G\_SD | | GX\_SD | | | X\_SD | | A\_SD | | C\_SD | |
| proteinId | exp | stat | exp | trans | stat | exp | stat | exp | stat | exp | stat |
| 9912 | 4.401893 | 9.062814 | 3.662740 | 8.915689 | 10.377152 | 5.869244 | 6.498924 | 5.023172 | 5.939950 | 4.960270 | 4.999237 |
| 13167 | 2.110223 | 4.532753 | 2.240152 | 2.231675 | 2.904780 | 2.732835 | 2.574824 | 2.124970 | 3.830886 | 3.418130 | 4.128099 |
| 9637 | 3.998634 | 8.815953 | 3.660779 | 8.747234 | 8.604897 | 2.931985 | 4.574291 | 1.739034 | 2.291095 | 5.549274 | 7.849465 |
| 12555 | 5.371083 | 12.343538 | 4.251359 | 12.086285 | 10.940364 | 7.120259 | 11.219722 | 12.178599 | 13.010334 | 17.546362 | 11.129470 |

|  | Glucose | Xylose | Arabinose | Acetate | Coumarate | Ferulate | YNB Oleic Acid | YNB Ricinoleic Acid | YNB Glucose | YNB Gluc DOC | YPD |
| --- | --- | --- | --- | --- | --- | --- | --- | --- | --- | --- | --- |
| proteinId |  |  |  |  |  |  |  |  |  |  |  |
| 9912 | 0.067742 | -0.270103 | -0.302936 | -0.051684 | -0.404229 | 0.068270 | -0.063069 | -0.272301 | -0.340334 | -0.074323 | -0.028894 |
| 13167 | -0.000429 | 0.137701 | -0.047659 | 0.249415 | 0.117662 | 0.180606 | -0.731184 | -0.096239 | -0.064679 | -0.146305 | -0.069131 |
| 9637 | 0.038735 | 0.118884 | -0.169197 | -0.160041 | -0.191542 | -0.008820 | -0.986535 | -0.039732 | -0.079149 | 0.151542 | -0.032181 |
| 12555 | -0.311448 | -0.162485 | -0.131826 | -0.274268 | -0.854248 | -0.027038 | -1.528607 | -0.659443 | -0.300839 | -0.371389 | -0.434224 |

In [33]:

```
for x in temp:
    if x in model.genes:
        for r in sorted(model.genes.get_by_id(x).reactions, key=lambda x: x.id):
            print(r, r.gene_reaction_rule)
    else:
        print(x, 'no reactions')
    print()
```

```
FA200tp: arach_c --> arach_x 9912
FA220tp: docosac_c --> docosac_x 9912
FA240tp: ttc_c --> ttc_x 9912
FA260tp: hexc_c --> hexc_x 9912
FACOAL200p: arach_x + atp_x + coa_x --> amp_x + arachcoa_x + ppi_x 12555 or 9912
FACOAL220p: atp_x + coa_x + docosac_x --> amp_x + docoscoa_x + ppi_x 12555 or 9912
FACOAL240p: atp_x + coa_x + ttc_x --> amp_x + ppi_x + ttccoa_x 12555 or 9912
FACOAL260p: atp_x + coa_x + hexc_x --> amp_x + hexccoa_x + ppi_x 12555 or 9912
VLCS2p: atp_x + coa_x + dhcholestanate_x --> amp_x + dhcholestancoa_x + ppi_x 9912
VLCS2r: atp_r + coa_r + dhcholestanate_r --> amp_r + dhcholestancoa_r + ppi_r 9912
VLCSp: atp_x + coa_x + thcholstoic_x --> amp_x + cholcoar_x + ppi_x 9912
VLCSr: atp_r + coa_r + thcholstoic_r --> amp_r + cholcoar_r + ppi_r 9912

FA140COAabcp: atp_c + 2.0 h2o_c + tdcoa_c --> adp_c + coa_c + 2.0 h_c + pi_c + ttdca_x 13167 and 9637
FA141COAabcp: atp_c + 2.0 h2o_c + tdecoa_c --> adp_c + coa_c + 2.0 h_c + pi_c + ttdcea_x 13167 and 9637
FA160COAabcp: atp_c + 2.0 h2o_c + pmtcoa_c --> adp_c + coa_c + 2.0 h_c + hdca_x + pi_c 13167 and 9637
FA161COAabcp: atp_c + 2.0 h2o_c + hdcoa_c --> adp_c + coa_c + 2.0 h_c + hdcea_x + pi_c 13167 and 9637
FA180COAabcp: atp_c + 2.0 h2o_c + stcoa_c --> adp_c + coa_c + 2.0 h_c + ocdca_x + pi_c 13167 and 9637
FA181COAabcp: atp_c + 2.0 h2o_c + odecoa_c --> adp_c + coa_c + 2.0 h_c + ocdcea_x + pi_c 13167 and 9637
FA182COAabcp: atp_c + 2.0 h2o_c + ocdycacoa_c --> adp_c + coa_c + 2.0 h_c + ocdcya_x + pi_c 13167 and 9637
FA183COAabcp: atp_c + 2.0 h2o_c + lnlncgcoa_c --> adp_c + coa_c + 2.0 h_c + lnlncg_x + pi_c 13167 and 9637

FA140COAabcp: atp_c + 2.0 h2o_c + tdcoa_c --> adp_c + coa_c + 2.0 h_c + pi_c + ttdca_x 13167 and 9637
FA141COAabcp: atp_c + 2.0 h2o_c + tdecoa_c --> adp_c + coa_c + 2.0 h_c + pi_c + ttdcea_x 13167 and 9637
FA160COAabcp: atp_c + 2.0 h2o_c + pmtcoa_c --> adp_c + coa_c + 2.0 h_c + hdca_x + pi_c 13167 and 9637
FA161COAabcp: atp_c + 2.0 h2o_c + hdcoa_c --> adp_c + coa_c + 2.0 h_c + hdcea_x + pi_c 13167 and 9637
FA180COAabcp: atp_c + 2.0 h2o_c + stcoa_c --> adp_c + coa_c + 2.0 h_c + ocdca_x + pi_c 13167 and 9637
FA181COAabcp: atp_c + 2.0 h2o_c + odecoa_c --> adp_c + coa_c + 2.0 h_c + ocdcea_x + pi_c 13167 and 9637
FA182COAabcp: atp_c + 2.0 h2o_c + ocdycacoa_c --> adp_c + coa_c + 2.0 h_c + ocdcya_x + pi_c 13167 and 9637
FA183COAabcp: atp_c + 2.0 h2o_c + lnlncgcoa_c --> adp_c + coa_c + 2.0 h_c + lnlncg_x + pi_c 13167 and 9637

COUCOALp: T4hcinnm_x + atp_x + coa_x --> amp_x + coucoa_x + ppi_x 12555 or 16635
FACOAL120p: atp_x + coa_x + ddca_x --> amp_x + ddcacoa_x + ppi_x 12555
FACOAL140p: atp_x + coa_x + ttdca_x --> amp_x + ppi_x + tdcoa_x 12555
FACOAL141p: atp_x + coa_x + ttdcea_x --> amp_x + ppi_x + tdecoa_x 12555
FACOAL160p: atp_x + coa_x + hdca_x --> amp_x + pmtcoa_x + ppi_x 12555
FACOAL161p: atp_x + coa_x + hdcea_x --> amp_x + hdcoa_x + ppi_x 12555
FACOAL180p: atp_x + coa_x + ocdca_x --> amp_x + ppi_x + stcoa_x 12555
FACOAL181p: atp_x + coa_x + ocdcea_x --> amp_x + odecoa_x + ppi_x 12555
FACOAL182p: atp_x + coa_x + ocdcya_x --> amp_x + ocdycacoa_x + ppi_x 12555
FACOAL1831p: atp_x + coa_x + lnlncg_x --> amp_x + lnlncgcoa_x + ppi_x 12555
FACOAL200p: arach_x + atp_x + coa_x --> amp_x + arachcoa_x + ppi_x 12555 or 9912
FACOAL220p: atp_x + coa_x + docosac_x --> amp_x + docoscoa_x + ppi_x 12555 or 9912
FACOAL240p: atp_x + coa_x + ttc_x --> amp_x + ppi_x + ttccoa_x 12555 or 9912
FACOAL260p: atp_x + coa_x + hexc_x --> amp_x + hexccoa_x + ppi_x 12555 or 9912
```

In [34]:

```
# Fatty acid ATP transporter PXA1 PXA2 do not have fitness defect
# Add a peroxisomal transport reaction for T4hcinnm by 9912 FAT1
r = model.reactions.get_by_id('FA240tp').copy()
r.id = 'T4HCINNMtp'
r.name = '4-Coumarate peroxisomal transport'
r.gene_reaction_rule = '9912'
model.add_reactions([r])
r.add_metabolites({'ttc_c': 1.0, 'ttc_x': -1.0, 'T4hcinnm_c': -1.0, 'T4hcinnm_x': 1.0})
```

In [35]:

```
# Check other possible transporters
temp = Annotation.index[Annotation['Combined Annotations'].str.contains('transporter') |
                        Annotation['Combined Annotations'].str.contains('ABC') |
                        Annotation['Combined Annotations'].str.contains('import')]
temp = [x for x in temp if x in Fitness.index[Fitness['Coumarate'] < -1.0]]
display(Annotation.loc[temp])
```

|  | Combined Annotations | Signal P | Sc288c Orthologs | Human Orthologs | Sc288 Best Hit | Human Blast | Essential | WolfPSort | C Terminal |
| --- | --- | --- | --- | --- | --- | --- | --- | --- | --- |
| RTO4\_ID |  |  |  |  |  |  |  |  |  |
| 8791 | KOG4580: Component of vacuolar transporter cha... |  |  |  | VTC1 |  | Not Essential | mito 14, plas 4, E.R. 3, cyto 2, extr 2 | SSP\* |
| 9588 | KOG0254: Predicted transporter (major facilita... | S | RGT2,SNF3 |  | SNF3 | SLC2A | Not Essential | plas 17, E.R. 4, vacu 4 | NEA\* |
| 9817 | K03325: TC.ACR3; arsenite transporter, ACR3 fa... | A | ARR3 |  | ARR3 |  | Not Essential | plas 26 | EQV\* |
| 10635 | K15110: SLC25A21, ODC; solute carrier family 2... |  | ODC2,ODC1 | SLC25A21 | ODC2 | SLC25 | Not Essential | mito 13, cyto 10, extr 3 | PYI\* |
| 10669 | HMMPfam:Transmembrane amino acid transporter p... |  |  |  |  |  | Not Essential | plas 27 | CAA\* |
| 10704 | KOG0254: Predicted transporter (major facilita... | S | STL1 |  | STL1 | SLC2A | Not Essential | plas 20, E.R. 3, mito 2, vacu 2 | AQV\* |
| 10705 | BLAST: general substrate transporter [Wallemia... |  |  |  |  |  | Not Essential | plas 17, mito 5, E.R. 3 | RHG\* |
| 10922 | BLAST: Amino acid/polyamine transporter I [Spo... |  |  |  |  |  | Not Essential | cysk 24, cyto 3 | LYE\* |
| 12292 | KOG1993: Nuclear transport receptor KAP120 (im... |  | KAP120 | IPO11 | KAP120 | IPO11 | Not Essential | cyto 6, mito 5, plas 5, cyto\_nucl 5, nucl 4, E... | LAG\* |
| 12325 | KOG0254: Predicted transporter (major facilita... |  |  |  | SIT1 |  | Not Essential | plas 27 | AVK\* |
| 12382 | KOG0065: Pleiotropic drug resistance proteins ... |  | SNQ2,PDR15,PDR11,PDR18,AUS1,PDR5,PDR10,PDR12 |  | PDR18 | ABCG2 | Not Essential | plas 22, E.R. 3 | PFS\* |
| 12605 | K15100: SLC25A1, CTP; solute carrier family 25... | S | SFC1 |  | SFC1 | SLC25A | Not Essential | mito 11, cyto 9.5, cyto\_nucl 5.5, plas 3 | YSE\* |
| 14063 | K08869: ADCK, ABC1; aarF domain-containing kinase | S | YPL109C | ADCK2 | YPL109C | ADCK2 | Not Essential | E.R. 6, mito 5, extr 5, cyto 4, plas 4, nucl 1... | YDT\* |
| 14911 | K08712: ABCG2.SNQ2; ATP-binding cassette, subf... |  | SNQ2,PDR15,PDR11,PDR18,AUS1,PDR5,PDR10,PDR12 |  | SNQ2 | ABCG2 | Not Essential | plas 22, E.R. 4 | ELS\* |
| 16428 | KOG0255: Synaptic vesicle transporter SVOP and... |  |  |  | TPO3 |  | Not Essential | plas 25 | VLD\* |

In [36]:

```
Show_Data(temp)
```

| strain | WT | | | | | | | | | | | | | | | | |
| --- | --- | --- | --- | --- | --- | --- | --- | --- | --- | --- | --- | --- | --- | --- | --- | --- | --- |
| condition | G\_MM | C\_MM | G\_SD | | GX\_SD | | | X\_SD | | A\_SD | | C\_SD | | MM\_CN120 | | MM\_CN5 | Diversity\_Sample |
| phase | exp | exp | exp | stat | exp | trans | stat | exp | stat | exp | stat | exp | stat | exp | stat | exp | exp |
| proteinId | Set1 | Set1 | Set2 | Set2 | Set2 | Set2 | Set2 | Set2 | Set2 | Set2 | Set2 | Set2 | Set2 | Set3 | Set3 | Set3 | Set3 |
| 8791 | 6.132815 | 5.965660 | 5.313167 | 5.460195 | 5.283905 | 4.748604 | 4.807544 | 5.339445 | 5.037218 | 5.555025 | 5.155384 | 6.730838 | 6.403707 | 5.652694 | 5.555377 | 5.939521 | 5.721352 |
| 9588 | 4.780557 | 4.210098 | 3.917419 | 4.325914 | 3.954190 | 4.551189 | 4.785825 | 4.309738 | 4.886040 | 4.562954 | 4.715699 | 4.298644 | 3.828880 | 5.657795 | 5.749202 | 4.643719 | 5.046075 |
| 9817 | 2.487074 | 2.999158 | 1.348599 | 1.415753 | 1.225475 | 1.893488 | 2.983447 | 1.782966 | 3.484074 | 2.711775 | 3.749199 | 3.895704 | 4.477763 | 3.400985 | 3.508397 | 3.320303 | 4.120173 |
| 10635 | 7.331124 | 7.612884 | 7.759526 | 6.232002 | 7.912588 | 7.000564 | 7.074152 | 7.612000 | 6.830146 | 6.701988 | 6.345431 | 6.971345 | 6.590608 | 8.449630 | 7.551539 | 8.432385 | 7.967643 |
| 10669 | 2.871422 | 2.309401 | 4.181908 | 4.638191 | 4.157239 | 3.130235 | 3.849301 | 2.255403 | 3.961517 | 1.472285 | 3.582988 | 5.669762 | 5.528743 | 5.334408 | 4.577030 | 4.426247 | 7.152930 |
| 10704 | 6.149007 | 7.368048 | 4.385791 | 3.578195 | 4.666958 | 4.118575 | 3.146945 | 5.937326 | 4.823217 | 4.547818 | 2.595275 | 7.319993 | 5.169154 | 6.944202 | 7.370470 | 7.918796 | 9.250421 |
| 10705 | 6.116982 | 7.407062 | 3.697511 | 3.142039 | 4.184811 | 3.494786 | 2.438119 | 5.381733 | 4.243175 | 3.936176 | 1.773300 | 6.746336 | 4.407741 | 4.147733 | 4.715591 | 5.043871 | 6.438395 |
| 10922 | 9.725501 | 0.111547 | 0.058577 | 0.057951 | 0.000000 | 0.027929 | 0.000000 | 0.027089 | 0.000000 | 0.000000 | 0.073498 | 0.176878 | 0.035313 | 8.555734 | 7.893278 | 1.293748 | 5.576863 |
| 12292 | 3.561035 | 4.859423 | 4.260561 | 3.318377 | 4.322080 | 3.988246 | 3.771992 | 4.161367 | 3.751640 | 3.795807 | 3.370285 | 4.111851 | 3.791877 | 4.851509 | 4.686877 | 5.234027 | 5.581241 |
| 12325 | 3.432416 | 5.498223 | 7.885255 | 7.271954 | 7.771479 | 8.759585 | 9.070192 | 8.157070 | 8.948867 | 6.987649 | 6.670491 | 8.711111 | 8.885285 | 9.873861 | 9.785967 | 8.807354 | 8.805390 |
| 12382 | 4.126399 | 4.436179 | 4.887598 | 4.972062 | 4.870189 | 4.832141 | 4.711787 | 4.649528 | 4.484916 | 4.443539 | 4.155473 | 5.318281 | 5.347534 | 5.399787 | 5.307468 | 5.423672 | 5.417385 |
| 12605 | 3.681421 | 7.627145 | 6.931591 | 5.795483 | 7.108998 | 6.203548 | 6.259582 | 5.075519 | 7.141392 | 5.116748 | 6.202725 | 7.178804 | 6.930891 | 4.276844 | 4.240066 | 5.168802 | 5.356117 |
| 14063 | 5.907892 | 5.107103 | 6.236144 | 6.405614 | 6.204784 | 6.103636 | 6.284790 | 5.948261 | 6.515693 | 6.155545 | 6.262926 | 6.371353 | 7.000409 | 6.377746 | 6.697248 | 6.039125 | 5.777904 |
| 14911 | 6.505891 | 5.309332 | 6.450965 | 6.151209 | 6.283239 | 6.158217 | 5.955974 | 6.446997 | 5.785431 | 6.550844 | 6.079606 | 6.906714 | 6.937283 | 7.767104 | 7.626999 | 6.879699 | 6.971035 |
| 16428 | 3.580104 | 3.911604 | 4.477148 | 5.542588 | 4.615994 | 4.418516 | 5.024662 | 3.081957 | 4.588315 | 4.912990 | 4.777049 | 4.035078 | 6.414670 | 5.254614 | 4.458237 | 4.938106 | 5.285619 |

| strain | WT | | | | | | | | | | |
| --- | --- | --- | --- | --- | --- | --- | --- | --- | --- | --- | --- |
| condition | G\_SD | | GX\_SD | | | X\_SD | | A\_SD | | C\_SD | |
| proteinId | exp | stat | exp | trans | stat | exp | stat | exp | stat | exp | stat |
| 10635 | 17.728464 | 17.363889 | 12.618284 | 14.339407 | 12.921562 | 11.952029 | 10.997038 | 10.433043 | 7.448285 | 14.994109 | 14.572604 |
| 10704 | 0.000000 | 0.000000 | 0.000000 | 0.000000 | 0.000000 | 0.190315 | 0.000000 | 0.000000 | 0.000000 | 0.208996 | 0.000000 |
| 12292 | 2.709359 | 1.744109 | 1.620711 | 1.864116 | 1.288801 | 1.185944 | 1.576008 | 1.545486 | 1.149316 | 0.630494 | 0.000000 |
| 12325 | 4.978764 | 0.000000 | 3.854801 | 3.156707 | 1.831002 | 5.283222 | 0.000000 | 0.000000 | 0.000000 | 3.426683 | 1.518388 |
| 12382 | 0.197667 | 0.000000 | 0.197656 | 0.370143 | 0.180258 | 0.197909 | 0.000000 | 0.000000 | 0.000000 | 0.208996 | 0.000000 |
| 12605 | 7.943939 | 7.960080 | 6.705546 | 6.696635 | 6.410543 | 6.063282 | 6.212271 | 2.319345 | 4.387250 | 16.656289 | 18.100491 |
| 14063 | 2.284201 | 4.083735 | 1.224873 | 3.709708 | 3.798441 | 2.340961 | 3.329589 | 2.903029 | 2.102319 | 0.220910 | 0.211140 |
| 14911 | 12.671319 | 14.853056 | 10.395642 | 21.189762 | 18.416136 | 12.166502 | 11.413459 | 12.183175 | 13.805665 | 9.415389 | 7.386179 |
| 16428 | 0.201691 | 0.000000 | 0.000000 | 0.189769 | 0.000000 | 0.189769 | 0.189187 | 0.190305 | 0.189808 | 0.000000 | 0.000000 |

|  | Glucose | Xylose | Arabinose | Acetate | Coumarate | Ferulate | YNB Oleic Acid | YNB Ricinoleic Acid | YNB Glucose | YNB Gluc DOC | YPD |
| --- | --- | --- | --- | --- | --- | --- | --- | --- | --- | --- | --- |
| proteinId |  |  |  |  |  |  |  |  |  |  |  |
| 8791 | -0.512799 | 0.055060 | 0.352831 | -0.557319 | -1.915137 | -0.486034 | -1.105870 | 0.023703 | -1.087037 | -1.195646 | 0.674743 |
| 9588 | -0.169406 | -0.287542 | -0.513374 | -0.419090 | -2.715290 | -0.791090 | 0.022079 | -1.237434 | -0.196914 | -0.093720 | 0.269282 |
| 9817 | -0.070621 | -0.229735 | 0.034262 | 0.170480 | -1.218102 | 0.051970 | -0.281786 | -0.308958 | 0.015614 | 0.078437 | -0.123666 |
| 10635 | 0.205165 | 0.096462 | 0.203563 | 0.261393 | -3.438625 | -4.081366 | -0.480008 | -0.819799 | 0.253622 | 0.413434 | -0.093817 |
| 10669 | -0.481501 | -1.219224 | -0.816654 | -0.478422 | -1.286889 | -0.425725 | 0.546023 | 0.400691 | 0.417396 | 0.180457 | 0.146982 |
| 10704 | -0.190141 | 0.061148 | 0.142964 | -0.019631 | -2.003941 | -0.111781 | 0.027153 | 0.139080 | -0.218968 | -0.429049 | 0.044097 |
| 10705 | -0.012817 | 0.177850 | 0.125373 | 0.211189 | -1.975652 | -0.002089 | -0.319149 | 0.174455 | -0.078333 | -0.137430 | -0.596689 |
| 10922 | -0.713958 | -0.185319 | -0.567806 | -0.554658 | -1.274269 | -0.736631 | -0.075827 | 0.042362 | -0.290064 | -0.706348 | 0.804357 |
| 12292 | -0.992753 | 0.694427 | 1.270307 | 0.195801 | -3.718814 | -1.683798 | -1.602772 | -0.503818 | -2.506344 | -2.201265 | 1.068949 |
| 12325 | -0.607646 | -0.182508 | -0.331201 | -1.429086 | -1.834039 | -0.514818 | 0.136093 | -0.927292 | -0.153574 | -0.333747 | -0.139890 |
| 12382 | 0.030066 | 0.094737 | 0.007168 | -0.565320 | -1.900454 | -0.076648 | -0.171844 | 0.187256 | 0.118247 | 0.231383 | 0.237073 |
| 12605 | -0.250531 | -1.121243 | -1.276143 | -1.681837 | -1.691549 | -1.568832 | 0.318931 | 0.287611 | 1.114286 | 0.164320 | 2.236104 |
| 14063 | -0.126570 | -0.046877 | -0.259027 | -0.169780 | -3.371237 | -0.465357 | -1.001580 | -1.695241 | -1.034943 | -0.234992 | 0.060567 |
| 14911 | -0.000275 | 0.189047 | -0.087591 | -0.164065 | -4.137395 | -0.204933 | -0.093916 | -0.089997 | 0.043215 | 0.007073 | 0.154653 |
| 16428 | 0.016984 | -0.020539 | 0.227223 | 0.270040 | -1.084491 | -0.294698 | -0.094320 | -1.160762 | -0.089141 | -0.062183 | -0.153238 |

In [37]:

```
for x in temp:
    if x in model.genes:
        for r in sorted(model.genes.get_by_id(x).reactions, key=lambda x: x.id):
            print(r, r.gene_reaction_rule)
    else:
        print(x, 'no reactions')
    print()
```

```
8791 no reactions

9588 no reactions

9817 no reactions

2OXOADPTm: 2oxoadp_c + akg_m <=> 2oxoadp_m + akg_c 10635
AKGMALtm: akg_m + mal__L_c --> akg_c + mal__L_m 10635

10669 no reactions

GLYCt2: glyc_e + h_e --> glyc_c + h_c 10704 and 10705

GLYCt2: glyc_e + h_e --> glyc_c + h_c 10704 and 10705

10922 no reactions

12292 no reactions

12325 no reactions

EPISTt: epist_e <=> epist_c 12382 or 13670 or 14911 or 16585
ERGSTt: ergst_e <=> ergst_c 12382 or 13670 or 14911 or 16585
FECOSTt: fecost_e <=> fecost_c 12382 or 13670 or 14911 or 16585
LANOSTt: lanost_e <=> lanost_c 12382 or 13670 or 14911 or 16585
ZYMSTt: zymst_e <=> zymst_c 12382 or 13670 or 14911 or 16585

SUCFUMtm: fum_m + succ_c --> fum_c + succ_m 12605

14063 no reactions

EPISTt: epist_e <=> epist_c 12382 or 13670 or 14911 or 16585
ERGSTt: ergst_e <=> ergst_c 12382 or 13670 or 14911 or 16585
FECOSTt: fecost_e <=> fecost_c 12382 or 13670 or 14911 or 16585
LANOSTt: lanost_e <=> lanost_c 12382 or 13670 or 14911 or 16585
ZYMSTt: zymst_e <=> zymst_c 12382 or 13670 or 14911 or 16585

16428 no reactions
```

9588 glucose sensor or transporter?  
9817 Bile acid:sodium symporter/arsenical resistance protein Acr3  
10635 ODC -> 3oxoadp mito transport?  
10704 and 10705 gene models wrong, one gene, similar to sugar or quinate transporter -> coumarate transport?

In [38]:

```
# Add coumarate exchange and transport
r1 = ppu.reactions.get_by_id('EX_T4hcinnm_e').copy()
r2 = ppu.reactions.get_by_id('T4HCINNMtex').copy()
r2.id = 'T4HCINNMt'
r2.name = 'Trans-4-hydroxycinnamate transport'
r2.gene_reaction_rule = '10704 and 10705'
model.add_reactions([r1,r2])
r2.add_metabolites({'T4hcinnm_p': -1.0, 'T4hcinnm_c': 1.0})
```

In [39]:

```
# Check genes that have fitness defect and are upregulated
temp = list(Fitness.index[Fitness['Coumarate'] - Fitness['Glucose'] < -1.0])
print(len(temp))
temp = [x for x in temp if x in Transcriptomics.index[Transcriptomics[('WT','C_SD','exp','Set2')] - 
                                                      Transcriptomics[('WT','G_SD','exp','Set2')] > 1]]
print(len(temp))
temp = [x for x in temp if x in Proteomics.index[Proteomics[('WT','C_SD','exp')] - 
                                                 Proteomics[('WT','G_SD','exp')] > 1]]
print(len(temp))
display(Annotation.loc[temp])
```

```
312
46
17
```

|  | Combined Annotations | Signal P | Sc288c Orthologs | Human Orthologs | Sc288 Best Hit | Human Blast | Essential | WolfPSort | C Terminal |
| --- | --- | --- | --- | --- | --- | --- | --- | --- | --- |
| RTO4\_ID |  |  |  |  |  |  |  |  |  |
| 8631 | K01537: E3.6.3.8; Ca2+-transporting ATPase |  | PMC1 | ATP2B1,ATP2B2,ATP2B3,ATP2B4 | PMC1 | ATP2B | Not Essential | plas 19, E.R. 4, cyto 2.5, cyto\_nucl 2 | VKE\* |
| 9065 | KOG1389: 3-oxoacyl CoA thiolase |  | POT1 | ACAA1 | POT1 | ACAA1 | Not Essential | mito 13, cyto 10, nucl 2 | VAE\* |
| 11777 | K05658: ABCB1, CD243; ATP-binding cassette, su... | S | STE6 | ABCB1,ABCB11,ABCB4,ABCB5 | STE6 | ABCB1 | Not Essential | plas 11, E.R. 7, extr 5, mito 3 | KAH\* |
| 11813 | HMMPfam:BRO1-like domain:PF03097,ProSiteProfil... | S |  |  |  |  | Not Essential | cyto 14, extr 7, cyto\_pero 7 | SYF\* |
| 12588 | K19932: NCS1; neuronal calcium sensor 1 |  | FRQ1 | NCS1,HPCAL1 | FRQ1 | NCS1 | Not Essential | cyto 11.5, nucl 10, cyto\_pero 7, mito 2, pero 1.5 | GLV\* |
| 12620 | HMMPfam:Carboxymuconolactone decarboxylase fam... |  |  |  |  |  | Not Essential | mito 20, cyto 4, pero 3 | FLA\* |
| 12622 | HMMPfam:Lactonase, 7-bladed beta-propeller:PF1... |  |  |  |  |  | Not Essential | cyto 12.5, cyto\_mito 11.666, mito 9.5, cyto\_nu... | WLS\* |
| 12623 | HMMPfam:Dioxygenase:PF00775,SUPERFAMILY::SSF49482 | S |  |  |  |  | Not Essential | extr 14, mito 5.5, cyto\_mito 5.5, cyto 4.5 | PKK\* |
| 12923 | KOG3855: Monooxygenase involved in coenzyme Q ... | A |  |  |  |  | Not Essential | mito 14, plas 4, nucl 2, cyto 2, cyto\_nucl 2, ... | ASL\* |
| 13090 | K01027: OXCT; 3-oxoacid CoA-transferase |  |  | OXCT1,OXCT2 |  | OXCT1 | Not Essential | mito 27 | MDA\* |
| 13229 | BLAST: NADH-ubiquinone oxidoreductase [Sanghua... |  |  |  |  |  | Not Essential | plas 20, E.R. 3, mito 2, vacu 2 | KNQ\* |
| 13847 | KOG3070: Predicted RNA-binding protein contain... | S |  |  |  |  | Not Essential | extr 15, mito 6, nucl 5 | LCA\* |
| 14105 | HMMPfam:Arsenite-resistance protein 2:PF04959,... |  |  |  |  |  | Not Essential | nucl 10.5, mito 10, cyto\_nucl 8.5, cyto 5.5 | YADL |
| 15228 | K07513: ACAA1; acetyl-CoA acyltransferase 1 |  |  |  | POT1 | ACAA1 | Not Essential | cyto 14, mito 13 | VRD\* |
| 15833 | K08518: STXBP5, SRO7\_77; syntaxin-binding prot... |  |  |  | SRO7 | STXBP | Not Essential | mito 11.5, cyto\_mito 8.333, nucl 5.5, cyto\_nuc... | RRR\* |
| 16616 | KOG2945: Predicted RNA-binding protein |  |  |  |  |  | Not Essential | nucl 12.5, cyto\_nucl 12, cyto 8.5, mito 3 | ALA\* |
| 16635 | K01897: ACSL, fadD; long-chain acyl-CoA synthe... |  |  |  | PCS60 | ACSF2 | Not Essential | mito 18, cyto 5.5, cyto\_nucl 3.5, pero 3 | AKL\* |

In [40]:

```
Show_Data(temp)
```

| strain | WT | | | | | | | | | | | | | | | | |
| --- | --- | --- | --- | --- | --- | --- | --- | --- | --- | --- | --- | --- | --- | --- | --- | --- | --- |
| condition | G\_MM | C\_MM | G\_SD | | GX\_SD | | | X\_SD | | A\_SD | | C\_SD | | MM\_CN120 | | MM\_CN5 | Diversity\_Sample |
| phase | exp | exp | exp | stat | exp | trans | stat | exp | stat | exp | stat | exp | stat | exp | stat | exp | exp |
| proteinId | Set1 | Set1 | Set2 | Set2 | Set2 | Set2 | Set2 | Set2 | Set2 | Set2 | Set2 | Set2 | Set2 | Set3 | Set3 | Set3 | Set3 |
| 8631 | 6.226570 | 7.219786 | 4.892135 | 4.777245 | 4.954142 | 5.394586 | 5.292039 | 5.028880 | 5.736728 | 4.903980 | 5.172267 | 8.339685 | 8.832668 | 7.500178 | 7.664891 | 5.838210 | 7.192685 |
| 9065 | 6.879441 | 10.197874 | 5.144820 | 6.875500 | 5.595462 | 5.112078 | 5.343626 | 5.548889 | 6.853216 | 6.158897 | 6.983728 | 7.896757 | 7.215140 | 6.434061 | 5.640637 | 6.480870 | 5.474475 |
| 11777 | 6.457995 | 7.256050 | 6.458449 | 6.883010 | 6.375994 | 6.273359 | 6.629518 | 6.329415 | 7.013255 | 6.108229 | 7.155046 | 8.930895 | 9.855801 | 7.231558 | 6.912250 | 6.977503 | 7.562863 |
| 11813 | 4.971221 | 5.124422 | 4.476585 | 4.578494 | 4.640123 | 4.317522 | 4.306302 | 4.161628 | 3.949898 | 4.350433 | 4.113766 | 5.537807 | 5.451472 | 3.876799 | 4.403065 | 3.684084 | 3.901835 |
| 12588 | 7.146184 | 7.816598 | 6.801797 | 6.931421 | 6.778076 | 6.746030 | 6.672289 | 6.584789 | 6.690105 | 6.843305 | 6.745372 | 8.530299 | 7.983152 | 7.516864 | 7.560850 | 6.925153 | 7.651963 |
| 12620 | 4.413272 | 7.771094 | 5.585544 | 5.425382 | 5.530824 | 5.231374 | 5.056305 | 4.950907 | 5.502513 | 4.585628 | 5.126261 | 8.289918 | 7.814177 | 5.124781 | 4.886660 | 5.364061 | 5.658966 |
| 12622 | 5.534603 | 8.903371 | 4.819703 | 5.529464 | 4.834667 | 5.234887 | 5.282115 | 4.699492 | 5.742311 | 4.945875 | 5.590323 | 8.728597 | 8.012084 | 4.281224 | 4.675156 | 4.051080 | 4.144291 |
| 12623 | 4.608380 | 10.415728 | 4.335254 | 5.158892 | 4.231914 | 4.035183 | 4.340782 | 3.187632 | 5.527699 | 3.691363 | 5.515474 | 10.227920 | 9.048972 | 3.669248 | 3.273088 | 3.664992 | 3.600660 |
| 12923 | 3.362166 | 9.354197 | 5.260356 | 6.782160 | 5.221130 | 5.501616 | 5.584604 | 3.983859 | 5.612789 | 4.182859 | 5.415393 | 9.519178 | 7.434039 | 5.299686 | 5.574637 | 6.674650 | 4.559302 |
| 13090 | 5.464366 | 8.899597 | 6.844099 | 7.150481 | 6.967168 | 6.734402 | 6.889335 | 5.741502 | 7.582680 | 5.852255 | 6.979332 | 9.457024 | 8.787576 | 6.683651 | 6.254025 | 6.988210 | 6.813242 |
| 13229 | 3.516976 | 7.126850 | 4.854719 | 5.680387 | 4.761785 | 4.514984 | 4.761143 | 4.338991 | 5.167424 | 4.317091 | 4.889625 | 7.707079 | 6.188071 | 3.319806 | 3.560388 | 4.063428 | 4.321580 |
| 13847 | 3.964453 | 6.543972 | 4.648563 | 4.631487 | 4.825261 | 5.410361 | 5.329121 | 5.142248 | 4.894685 | 4.895182 | 4.725541 | 5.846473 | 6.363216 | 5.074667 | 5.551346 | 5.107200 | 7.259617 |
| 14105 | 4.425629 | 4.905091 | 5.324412 | 4.451432 | 5.490471 | 5.474905 | 5.381377 | 5.669801 | 5.293822 | 5.478555 | 5.387223 | 7.240085 | 7.845309 | 5.293225 | 5.262913 | 5.504009 | 5.918289 |
| 15228 | 5.083515 | 8.829758 | 6.510843 | 6.274466 | 6.578483 | 6.366137 | 6.385558 | 5.346932 | 6.750726 | 5.036384 | 6.142249 | 8.950815 | 8.026473 | 5.470105 | 4.793416 | 5.221585 | 5.518555 |
| 15833 | 4.296553 | 5.519887 | 4.923131 | 4.575786 | 4.787898 | 4.915823 | 4.749071 | 4.447013 | 4.768615 | 4.112793 | 4.502423 | 6.431198 | 6.469101 | 5.233967 | 5.356119 | 5.162554 | 5.672962 |
| 16616 | 5.102604 | 7.171817 | 5.911246 | 5.639201 | 5.964427 | 6.099174 | 5.720832 | 6.657022 | 4.910618 | 6.123293 | 4.782851 | 6.964445 | 6.729841 | 7.743027 | 8.031617 | 9.266731 | 9.919935 |
| 16635 | 4.274643 | 9.223314 | 3.952433 | 5.180428 | 4.380874 | 5.238679 | 5.008569 | 4.035667 | 4.424341 | 5.107619 | 4.807358 | 7.591964 | 6.556446 | 4.192554 | 3.549286 | 4.917594 | 3.736052 |

| strain | WT | | | | | | | | | | |
| --- | --- | --- | --- | --- | --- | --- | --- | --- | --- | --- | --- |
| condition | G\_SD | | GX\_SD | | | X\_SD | | A\_SD | | C\_SD | |
| proteinId | exp | stat | exp | trans | stat | exp | stat | exp | stat | exp | stat |
| 8631 | 0.000000 | 0.000000 | 0.000000 | 0.000000 | 0.000000 | 0.195962 | 0.000000 | 0.388996 | 0.000000 | 1.706577 | 1.525182 |
| 9065 | 8.794038 | 15.441107 | 8.726886 | 10.228263 | 9.645741 | 8.994289 | 13.976110 | 15.268987 | 19.731779 | 67.278251 | 45.319038 |
| 11777 | 2.306535 | 4.199772 | 3.654828 | 8.008216 | 7.133723 | 6.863060 | 7.620974 | 7.528882 | 7.662306 | 15.598264 | 22.216765 |
| 11813 | 0.201691 | 0.354979 | 0.000000 | 0.000000 | 0.000000 | 0.000000 | 0.000000 | 0.386974 | 0.000000 | 1.282652 | 1.312659 |
| 12588 | 2.527259 | 3.587587 | 3.045061 | 4.833923 | 4.392998 | 3.529398 | 3.777915 | 4.440766 | 4.783889 | 4.481376 | 5.017465 |
| 12620 | 6.353038 | 9.469368 | 6.313104 | 7.056756 | 5.991653 | 5.645164 | 6.491256 | 6.196277 | 5.157092 | 26.503242 | 27.236445 |
| 12622 | 0.574292 | 0.735446 | 0.197656 | 0.189313 | 0.889102 | 0.780149 | 0.583328 | 0.193364 | 1.345017 | 7.277259 | 4.783256 |
| 12623 | 0.782821 | 0.895622 | 1.224640 | 0.932688 | 1.076751 | 1.173386 | 0.590346 | 0.579484 | 1.143298 | 14.976311 | 15.099412 |
| 12923 | 4.240959 | 17.188398 | 3.456553 | 15.436691 | 16.198207 | 4.507815 | 15.295459 | 5.407162 | 13.776727 | 66.246276 | 55.355591 |
| 13090 | 17.117931 | 23.037085 | 17.310864 | 22.671171 | 23.207886 | 14.897467 | 16.539875 | 14.296238 | 14.158581 | 45.311824 | 45.747221 |
| 13229 | 0.201691 | 0.338465 | 0.611440 | 0.187227 | 0.173732 | 0.397816 | 1.148351 | 1.548791 | 1.142751 | 4.482679 | 2.620904 |
| 13847 | 0.771959 | 0.000000 | 1.016038 | 0.000000 | 0.000000 | 0.197909 | 0.000000 | 0.000000 | 0.000000 | 4.273191 | 4.364697 |
| 14105 | 0.000000 | 0.552733 | 0.609343 | 1.116479 | 0.726860 | 0.590601 | 0.394160 | 0.773293 | 0.582002 | 1.063051 | 1.085211 |
| 15228 | 12.511431 | 17.737287 | 14.842154 | 18.981900 | 18.445357 | 14.681180 | 15.122097 | 11.207129 | 10.704845 | 37.823122 | 38.561263 |
| 15833 | 2.095970 | 3.060810 | 1.829113 | 3.699188 | 3.498513 | 3.934190 | 2.542457 | 3.676166 | 2.284726 | 7.708458 | 7.642074 |
| 16616 | 17.894048 | 16.739579 | 22.575836 | 11.902944 | 13.137128 | 16.639376 | 15.199378 | 15.648474 | 14.325446 | 22.229306 | 18.984665 |
| 16635 | 0.738459 | 4.501624 | 2.430184 | 6.308786 | 8.590841 | 4.914107 | 4.728599 | 3.667807 | 6.730556 | 29.904117 | 22.861660 |

|  | Glucose | Xylose | Arabinose | Acetate | Coumarate | Ferulate | YNB Oleic Acid | YNB Ricinoleic Acid | YNB Glucose | YNB Gluc DOC | YPD |
| --- | --- | --- | --- | --- | --- | --- | --- | --- | --- | --- | --- |
| proteinId |  |  |  |  |  |  |  |  |  |  |  |
| 8631 | 0.193819 | -0.483699 | 0.096401 | -0.034710 | -1.127991 | -0.201352 | 0.504130 | 0.226385 | 1.130908 | 0.648147 | -0.022761 |
| 9065 | -0.350988 | -0.271906 | -0.134182 | -0.284826 | -5.608804 | -3.426361 | -0.290069 | 0.247955 | 0.258871 | 0.400966 | -0.203170 |
| 11777 | 0.193592 | 0.013685 | 0.098670 | 0.154662 | -0.928078 | 0.036743 | 0.157960 | 0.177089 | -0.064766 | -0.041075 | 0.187794 |
| 11813 | 0.024773 | -0.943552 | -1.180576 | -0.271550 | -3.697693 | -0.334791 | 0.321082 | -0.265125 | 0.778628 | 0.749372 | 0.115452 |
| 12588 | 0.166909 | 0.915210 | 0.384190 | -0.266136 | -5.829157 | -3.146647 | 0.156251 | -3.497062 | 0.252847 | 0.710073 | -0.199311 |
| 12620 | 0.353389 | 0.448131 | -0.451003 | -0.262247 | -4.042890 | -2.583701 | -0.157263 | 0.044296 | 0.092261 | 0.373448 | 0.125082 |
| 12622 | -0.297771 | 0.425898 | -3.410507 | -1.576515 | -4.307831 | -5.744022 | -0.470362 | 0.402838 | -0.812481 | -1.126946 | 1.143412 |
| 12623 | 0.040591 | -0.796384 | -0.693215 | 0.192902 | -5.363135 | -0.719008 | -0.011893 | 0.508726 | -0.030801 | 0.133649 | -0.388432 |
| 12923 | 0.027344 | 0.432376 | -0.079662 | -0.166375 | -3.085008 | -0.006518 | 0.160919 | -0.560441 | -0.142851 | -0.122137 | 0.288931 |
| 13090 | 0.208346 | 0.103291 | 0.189629 | 0.170817 | -5.612075 | -5.268479 | 0.025858 | 0.048927 | -0.017233 | 0.060300 | 0.064795 |
| 13229 | -0.709564 | -0.423434 | -0.754244 | -0.408968 | -3.856215 | -1.466346 | 0.349502 | -0.321974 | -0.326653 | -0.627404 | -0.173793 |
| 13847 | -0.080120 | 0.188129 | 0.359409 | 0.119285 | -1.927753 | -0.556861 | 0.176215 | -0.524063 | -0.830861 | -1.440111 | -0.688506 |
| 14105 | -0.670963 | -0.533398 | 0.264540 | -0.113789 | -1.934841 | -0.787919 | -1.663901 | -1.101238 | -0.823123 | -0.889743 | 2.127998 |
| 15228 | -0.223091 | -0.118190 | -0.335936 | -0.277735 | -4.995066 | -4.679965 | 0.138344 | 0.163114 | 0.296363 | 0.541750 | 0.402816 |
| 15833 | -0.045735 | 0.184423 | 0.352233 | 0.031337 | -1.532957 | 0.019897 | -0.006391 | -0.040208 | 0.047318 | -0.177006 | -0.216892 |
| 16616 | -0.139651 | -0.251655 | -0.549259 | 0.144159 | -2.224111 | -0.830899 | 0.086680 | 0.234170 | 0.164402 | 0.018432 | 0.025799 |
| 16635 | -0.386094 | 0.074868 | -0.246490 | -0.872329 | -1.765683 | -3.600674 | -0.198188 | -0.037413 | 0.311272 | 0.402185 | 0.872770 |

In [41]:

```
for x in temp:
    if x in model.genes:
        for r in sorted(model.genes.get_by_id(x).reactions, key=lambda x: x.id):
            print(r, r.gene_reaction_rule)
    else:
        print(x, 'no reactions')
    print()
```

```
PMCA: atp_c + ca2_c + h2o_c + h_e --> adp_c + ca2_e + 2.0 h_c + pi_c 8631 or 12428

4HBZACCOATp: 4hbzaccoa_x + coa_x --> 4hbzcoa_x + accoa_x 9065
FAO80p: 3.0 coa_x + 3.0 h2o_x + 3.0 nad_x + 3.0 o2_x + occoa_x --> 4.0 accoa_x + 3.0 h2o2_x + 3.0 h_x + 3.0 nadh_x (11362 and 12742 and 13813) or (11362 and 12742 and 9065) or (11362 and 12752 and 13813) or (11362 and 12752 and 9065) or (11362 and 13813 and 9700) or (11362 and 9065 and 9700)

CHOLATEt3: atp_c + cholate_c + h2o_c --> adp_c + cholate_e + h_c + pi_c 11266 or 11777 or 13264 or 14111 or 14463 or 14844 or 8693 or 9145
GCHOLAt3: atp_c + gchola_c + h2o_c --> adp_c + gchola_e + h_c + pi_c 11266 or 11777 or 13264 or 14111 or 14463 or 14844 or 8693 or 9145
TCHOLAt3: atp_c + h2o_c + tchola_c --> adp_c + h_c + pi_c + tchola_e 11266 or 11777 or 13264 or 14111 or 14463 or 14844 or 8693 or 9145

11813 no reactions

12588 no reactions

OXOAEL: 5odhf2a_c + h2o_c --> 3oxoadp_c + h_c 12620

12622 no reactions

12623 no reactions

12923 no reactions

OCOAT1m: acac_m + succoa_m <=> aacoa_m + succ_m 13090

13229 no reactions

13847 no reactions

14105 no reactions

15228 no reactions

15833 no reactions

16616 no reactions

COUCOALp: T4hcinnm_x + atp_x + coa_x --> amp_x + coucoa_x + ppi_x 12555 or 16635
```

4-hydroxybenzoate to protocatechuate

4HBHYOX: 4hbz\_c + h\_c + nadph\_c + o2\_c --> 34dhbz\_c + h2o\_c + nadp\_c  
9267 / mito BNA4 kynurenine 3-monooxygenase / no change / no change / defect in both -> looks okay, but kynurenine  
other BNA genes also have fitness defect in both -> NAD biosynthesis?  
12923 / Anchored, ASL\* 3-hydroxybenzoate 4-monooxygenase / up / up / defect in cou, but not fer -> ok

Protocatechuate degradation (ortho-cleavage, beta-ketoadipate pathway)

PCADYOX: 34dhbz\_c + o2\_c --> CCbuttc\_c + 2.0 h\_c by pcaH/pcaG  
12623 / sigP protocatechuate 3,4-dioxygenase beta chain (3,4-pcd) / up / up / defect in cou, but not in fer -> ok

MUCCY\_kt: CCbuttc\_c + h\_c --> 4cml\_c by pcaB  
12622 / cyto or mito pcaB carboxy-cis,cis-muconate cyclase /  
14052 is also carboxy-cis,cis-muconate cyclase -> no fitness defect and no change in expression

4CMLCL\_kt: 4cml\_c + h\_c --> 5odhf2a\_c + co2\_c by pcaC  
12620 / mito pcaC carboxymuconolactone decarboxylase, and pcaD 3-oxoadipate enol-lactonase 1 /  
12621 unknown, uniprot blast matches to 3-oxoadipate enol-lactonase 2 partially

OXOAEL: 5odhf2a\_c + h2o\_c --> 3oxoadp\_c + h\_c by pcaD  
12620 / mito pcaC carboxymuconolactone decarboxylase, and pcaD 3-oxoadipate enol-lactonase 1 /

3OADPCOAT: 3oxoadp\_c + succoa\_c --> oxadpcoa\_c + succ\_c by pcaI and pcaJ  
13090 / mito 3-oxoacid CoA-transferase, pcaJ and pcaI in one / up / up / defect in both -> ok

10635 ODC2 oxoadipate mito transporter fitness defect -> add 3oxoadp transport

3OXCOAT: coa\_c + oxadpcoa\_c --> accoa\_c + succoa\_c by pcaF  
15228 / mito acetyl-CoA acyltransferase 1 / up / up / defect in both -> ok  
15228 gene model incorrect missing sigP exon at the beginning

In [42]:

```
temp = ['12923','12623','12622','14052','12621','12620','13090','10635','15228']
display(Annotation.loc[temp])
Show_Data(temp)
```

|  | Combined Annotations | Signal P | Sc288c Orthologs | Human Orthologs | Sc288 Best Hit | Human Blast | Essential | WolfPSort | C Terminal |
| --- | --- | --- | --- | --- | --- | --- | --- | --- | --- |
| RTO4\_ID |  |  |  |  |  |  |  |  |  |
| 12923 | KOG3855: Monooxygenase involved in coenzyme Q ... | A |  |  |  |  | Not Essential | mito 14, plas 4, nucl 2, cyto 2, cyto\_nucl 2, ... | ASL\* |
| 12623 | HMMPfam:Dioxygenase:PF00775,SUPERFAMILY::SSF49482 | S |  |  |  |  | Not Essential | extr 14, mito 5.5, cyto\_mito 5.5, cyto 4.5 | PKK\* |
| 12622 | HMMPfam:Lactonase, 7-bladed beta-propeller:PF1... |  |  |  |  |  | Not Essential | cyto 12.5, cyto\_mito 11.666, mito 9.5, cyto\_nu... | WLS\* |
| 14052 | K07404: pgl; 6-phosphogluconolactonase |  |  |  |  |  | Not Essential | cyto 17.5, cyto\_mito 12.166, cyto\_nucl 10.833,... | VWL\* |
| 12621 | BLAST: Dimeric alpha-beta barrel [Ceraceosorus... |  |  |  |  |  | Not Essential | cyto\_nucl 11.5, cyto 11, nucl 10, mito 6 | KDA\* |
| 12620 | HMMPfam:Carboxymuconolactone decarboxylase fam... |  |  |  |  |  | Not Essential | mito 20, cyto 4, pero 3 | FLA\* |
| 13090 | K01027: OXCT; 3-oxoacid CoA-transferase |  |  | OXCT1,OXCT2 |  | OXCT1 | Not Essential | mito 27 | MDA\* |
| 10635 | K15110: SLC25A21, ODC; solute carrier family 2... |  | ODC2,ODC1 | SLC25A21 | ODC2 | SLC25 | Not Essential | mito 13, cyto 10, extr 3 | PYI\* |
| 15228 | K07513: ACAA1; acetyl-CoA acyltransferase 1 |  |  |  | POT1 | ACAA1 | Not Essential | cyto 14, mito 13 | VRD\* |

| strain | WT | | | | | | | | | | | | | | | | |
| --- | --- | --- | --- | --- | --- | --- | --- | --- | --- | --- | --- | --- | --- | --- | --- | --- | --- |
| condition | G\_MM | C\_MM | G\_SD | | GX\_SD | | | X\_SD | | A\_SD | | C\_SD | | MM\_CN120 | | MM\_CN5 | Diversity\_Sample |
| phase | exp | exp | exp | stat | exp | trans | stat | exp | stat | exp | stat | exp | stat | exp | stat | exp | exp |
| proteinId | Set1 | Set1 | Set2 | Set2 | Set2 | Set2 | Set2 | Set2 | Set2 | Set2 | Set2 | Set2 | Set2 | Set3 | Set3 | Set3 | Set3 |
| 12923 | 3.362166 | 9.354197 | 5.260356 | 6.782160 | 5.221130 | 5.501616 | 5.584604 | 3.983859 | 5.612789 | 4.182859 | 5.415393 | 9.519178 | 7.434039 | 5.299686 | 5.574637 | 6.674650 | 4.559302 |
| 12623 | 4.608380 | 10.415728 | 4.335254 | 5.158892 | 4.231914 | 4.035183 | 4.340782 | 3.187632 | 5.527699 | 3.691363 | 5.515474 | 10.227920 | 9.048972 | 3.669248 | 3.273088 | 3.664992 | 3.600660 |
| 12622 | 5.534603 | 8.903371 | 4.819703 | 5.529464 | 4.834667 | 5.234887 | 5.282115 | 4.699492 | 5.742311 | 4.945875 | 5.590323 | 8.728597 | 8.012084 | 4.281224 | 4.675156 | 4.051080 | 4.144291 |
| 14052 | 2.914249 | 4.771960 | 5.298828 | 4.801871 | 5.207516 | 5.129479 | 5.214973 | 5.748074 | 6.160497 | 5.467516 | 6.148511 | 4.084219 | 3.831293 | 3.816111 | 3.389861 | 5.573431 | 4.806654 |
| 12621 | 5.180720 | 8.085616 | 6.035037 | 5.734881 | 6.033639 | 5.547500 | 5.530374 | 5.471405 | 6.127870 | 5.096964 | 6.043611 | 8.038540 | 7.559980 | 3.264468 | 3.329010 | 4.149932 | 3.645859 |
| 12620 | 4.413272 | 7.771094 | 5.585544 | 5.425382 | 5.530824 | 5.231374 | 5.056305 | 4.950907 | 5.502513 | 4.585628 | 5.126261 | 8.289918 | 7.814177 | 5.124781 | 4.886660 | 5.364061 | 5.658966 |
| 13090 | 5.464366 | 8.899597 | 6.844099 | 7.150481 | 6.967168 | 6.734402 | 6.889335 | 5.741502 | 7.582680 | 5.852255 | 6.979332 | 9.457024 | 8.787576 | 6.683651 | 6.254025 | 6.988210 | 6.813242 |
| 10635 | 7.331124 | 7.612884 | 7.759526 | 6.232002 | 7.912588 | 7.000564 | 7.074152 | 7.612000 | 6.830146 | 6.701988 | 6.345431 | 6.971345 | 6.590608 | 8.449630 | 7.551539 | 8.432385 | 7.967643 |
| 15228 | 5.083515 | 8.829758 | 6.510843 | 6.274466 | 6.578483 | 6.366137 | 6.385558 | 5.346932 | 6.750726 | 5.036384 | 6.142249 | 8.950815 | 8.026473 | 5.470105 | 4.793416 | 5.221585 | 5.518555 |

| strain | WT | | | | | | | | | | |
| --- | --- | --- | --- | --- | --- | --- | --- | --- | --- | --- | --- |
| condition | G\_SD | | GX\_SD | | | X\_SD | | A\_SD | | C\_SD | |
| proteinId | exp | stat | exp | trans | stat | exp | stat | exp | stat | exp | stat |
| 12923 | 4.240959 | 17.188398 | 3.456553 | 15.436691 | 16.198207 | 4.507815 | 15.295459 | 5.407162 | 13.776727 | 66.246276 | 55.355591 |
| 12623 | 0.782821 | 0.895622 | 1.224640 | 0.932688 | 1.076751 | 1.173386 | 0.590346 | 0.579484 | 1.143298 | 14.976311 | 15.099412 |
| 12622 | 0.574292 | 0.735446 | 0.197656 | 0.189313 | 0.889102 | 0.780149 | 0.583328 | 0.193364 | 1.345017 | 7.277259 | 4.783256 |
| 14052 | 0.000000 | 0.345922 | 0.000000 | 0.556148 | 1.281008 | 0.398835 | 1.381147 | 0.770260 | 1.526030 | 0.211647 | 0.000000 |
| 12621 | 0.390932 | 0.693444 | 0.598396 | 0.000000 | 0.192891 | 2.548503 | 1.977879 | 2.319686 | 2.670235 | 11.540587 | 10.869417 |
| 12620 | 6.353038 | 9.469368 | 6.313104 | 7.056756 | 5.991653 | 5.645164 | 6.491256 | 6.196277 | 5.157092 | 26.503242 | 27.236445 |
| 13090 | 17.117931 | 23.037085 | 17.310864 | 22.671171 | 23.207886 | 14.897467 | 16.539875 | 14.296238 | 14.158581 | 45.311824 | 45.747221 |
| 10635 | 17.728464 | 17.363889 | 12.618284 | 14.339407 | 12.921562 | 11.952029 | 10.997038 | 10.433043 | 7.448285 | 14.994109 | 14.572604 |
| 15228 | 12.511431 | 17.737287 | 14.842154 | 18.981900 | 18.445357 | 14.681180 | 15.122097 | 11.207129 | 10.704845 | 37.823122 | 38.561263 |

|  | Glucose | Xylose | Arabinose | Acetate | Coumarate | Ferulate | YNB Oleic Acid | YNB Ricinoleic Acid | YNB Glucose | YNB Gluc DOC | YPD |
| --- | --- | --- | --- | --- | --- | --- | --- | --- | --- | --- | --- |
| proteinId |  |  |  |  |  |  |  |  |  |  |  |
| 12923 | 0.027344 | 0.432376 | -0.079662 | -0.166375 | -3.085008 | -0.006518 | 0.160919 | -0.560441 | -0.142851 | -0.122137 | 0.288931 |
| 12623 | 0.040591 | -0.796384 | -0.693215 | 0.192902 | -5.363135 | -0.719008 | -0.011893 | 0.508726 | -0.030801 | 0.133649 | -0.388432 |
| 12622 | -0.297771 | 0.425898 | -3.410507 | -1.576515 | -4.307831 | -5.744022 | -0.470362 | 0.402838 | -0.812481 | -1.126946 | 1.143412 |
| 14052 | 0.101963 | -0.017395 | 0.060814 | 0.178120 | 0.019043 | 0.020340 | 0.115445 | -0.065000 | 0.167203 | 0.104391 | -0.175005 |
| 12620 | 0.353389 | 0.448131 | -0.451003 | -0.262247 | -4.042890 | -2.583701 | -0.157263 | 0.044296 | 0.092261 | 0.373448 | 0.125082 |
| 13090 | 0.208346 | 0.103291 | 0.189629 | 0.170817 | -5.612075 | -5.268479 | 0.025858 | 0.048927 | -0.017233 | 0.060300 | 0.064795 |
| 10635 | 0.205165 | 0.096462 | 0.203563 | 0.261393 | -3.438625 | -4.081366 | -0.480008 | -0.819799 | 0.253622 | 0.413434 | -0.093817 |
| 15228 | -0.223091 | -0.118190 | -0.335936 | -0.277735 | -4.995066 | -4.679965 | 0.138344 | 0.163114 | 0.296363 | 0.541750 | 0.402816 |

In [43]:

```
# 4-hydroxybenzoate to protocatechuate in peroxisome
r = model.reactions.get_by_id('4HBHYOX')
r.id = '4HBHYOXp'
r.name = '4-hydroxybenzoate 3-monooxygenase'
r.gene_reaction_rule = '12923'
for m in r.metabolites:
    if not m.id.replace('_c','_x') in model.metabolites:
        m2 = m.copy()
        m2.id = m.id.replace('_c','_x')
        m2.compartment = 'x'
        model.add_metabolites([m2])
    r.add_metabolites({m.id: -r.get_coefficient(m.id), m.id.replace('_c','_x'): r.get_coefficient(m.id)})
```

In [44]:

```
# protocatechuate transport from peroxisomal to cytosol
r = model.reactions.get_by_id('4HBZtp').copy()
r.id = '34DHBZtp'
r.name = '3,4-dihydroxybenzoate peroxisomal transport'
r.gene_reaction_rule = '' # find gene?
model.add_reactions([r])
r.add_metabolites({'4hbz_c': 1.0, '4hbz_x': -1.0, '34dhbz_c': -1.0, '34dhbz_x': 1.0})
```

In [45]:

```
for x in ['PCADYOX','4CMLCL_kt','3OADPCOAT','3OXCOAT']:
    r = ppu.reactions.get_by_id(x).copy()
    r.gene_reaction_rule = ''
    model.add_reactions([r])
    
model.reactions.get_by_id('PCADYOX').gene_reaction_rule = '12623'
model.reactions.get_by_id('MUCCY_kt').gene_reaction_rule = '12622'
model.reactions.get_by_id('4CMLCL_kt').gene_reaction_rule = '12620'
model.reactions.get_by_id('OXOAEL').gene_reaction_rule = '12620'
model.reactions.get_by_id('3OADPCOAT').gene_reaction_rule = '13090'
model.reactions.get_by_id('3OXCOAT').gene_reaction_rule = '15228'

r = model.reactions.get_by_id('3OADPCOAT')
r.id = '3OADPCOATm'
for m in r.metabolites:
    if not m.id.replace('_c','_m') in model.metabolites:
        m2 = m.copy()
        m2.id = m.id.replace('_c','_m')
        m2.compartment = 'm'
        model.add_metabolites([m2])
    r.add_metabolites({m.id: -r.get_coefficient(m.id), m.id.replace('_c','_m'): r.get_coefficient(m.id)})

r = model.reactions.get_by_id('3OXCOAT')
r.id = '3OXCOATm'
for m in r.metabolites:
    if not m.id.replace('_c','_m') in model.metabolites:
        m2 = m.copy()
        m2.id = m.id.replace('_c','_m')
        m2.compartment = 'm'
        model.add_metabolites([m2])
    r.add_metabolites({m.id: -r.get_coefficient(m.id), m.id.replace('_c','_m'): r.get_coefficient(m.id)})
    
r = model.reactions.get_by_id('2OXOADPTm').copy()
r.id = '3OXOADPTm'
r.name = '3-oxoadipate shuttle (cytosol/mitochondria)'
model.add_reactions([r])
r.gene_reaction_rule = '10635'
r.add_metabolites({'2oxoadp_c': 1.0, '2oxoadp_m': -1.0, '3oxoadp_c': -1.0, '3oxoadp_m': 1.0})
```

In [46]:

```
for r in sorted(model.reactions, key=lambda x: x.id):
    if not r.boundary and sum(abs(x) for x in r.check_mass_balance().values()) > 1e-12:
        print(r, r.gene_reaction_rule, r.check_mass_balance())
```

```
BIOMASS_RT: 0.957502 13BDglcn_c + 0.177315 16BDglcn_c + 0.001283 5mthf_c + 0.577574 alatrna_c + 0.146675 argtrna_c + 0.10797 asntrna_c + 0.197296 asptrna_c + 110.681641 atp_c + 0.002418 btn_m + 0.000832 ca2_c + 0.001792 camp_c + 0.031005 chitin_c + 1.8e-05 clpn_RT_m + 0.00077 coa_c + 0.044881 ctp_c + 0.000525 cu2_c + 0.034269 cystrna_c + 0.002451 datp_c + 0.004285 dctp_c + 0.003763 dgtp_c + 0.002523 dttp_c + 0.004031 ergst_r + 1.6e-05 ergstest_RT_r + 0.000751 fad_c + 0.000597 fe2_c + 0.000597 fe3_c + 0.117898 glntrna_c + 0.208673 glutrna_c + 0.518069 glycogen_c + 0.51322 glytrna_c + 0.00192 gthrd_c + 0.051085 gtp_c + 106.71258 h2o_c + 0.000691 hemeA_m + 0.061167 histrna_c + 0.158569 iletrna_c + 0.585703 k_c + 0.320205 leutrna_c + 0.003107 lipopb_m + 0.172334 lystrna_c + 0.807941 mannan_r + 0.05705 mettrna_c + 0.061716 mg2_c + 0.001292 mlthf_c + 0.000607 mn2_c + 0.026099 na1_c + 0.000888 nad_c + 0.000795 nadp_c + 1.4e-05 pa_RT_r + 0.000172 pc_RT_r + 0.00013 pe_RT_r + 0.095164 phetrna_c + 0.235897 protrna_c + 2e-05 ps_RT_r + 0.004006 psphings_r + 4.7e-05 ptd1ino_RT_r + 0.006524 ptrc_c + 0.0024 pydx5p_c + 0.00074 q9_m + 0.00099 ribflv_c + 0.33687 sertrna_c + 0.003967 spmd_c + 0.001327 thf_c + 0.001393 thmpp_c + 0.21813 thrtrna_c + 0.023372 tre_c + 0.000432 triglyc_RT_r + 0.023319 trptrna_c + 0.062363 tyrtrna_c + 0.051129 utp_c + 0.275168 valtrna_c + 0.00051 zn2_c + 0.004156 zymst_r + 1.7e-05 zymstest_RT_d --> 110.632394 adp_c + 110.632394 h_c + 110.632394 pi_c + 0.209364 ppi_c  {'charge': -4.356142000000063, 'C': -40.73034299999994, 'H': -62.36094499999999, 'O': -17.698876999999808, 'N': -5.868878999999981, 'P': -0.37418499999998533, 'R': -4.027781, 'S': -0.104034, 'Ca': -0.000832, 'Cu': -0.000525, 'Fe': -0.001885, 'K': -0.585703, 'Mg': -0.061716, 'Mn': -0.000607, 'Na': -0.026099, 'Zn': -0.00051}
T4HCINNMt: T4hcinnm_e <=> T4hcinnm_c 10704 and 10705 {'charge': -1.0}
```

In [47]:

```
model.metabolites.get_by_id('CCbuttc_c').charge = -3
model.metabolites.get_by_id('4cml_c').charge = -2
model.metabolites.get_by_id('T4hcinnm_e').charge = -1
```

In [48]:

```
for r in sorted(model.reactions, key=lambda x: x.id):
    if not r.boundary and sum(abs(x) for x in r.check_mass_balance().values()) > 1e-12:
        print(r, r.gene_reaction_rule, r.check_mass_balance())
```

```
BIOMASS_RT: 0.957502 13BDglcn_c + 0.177315 16BDglcn_c + 0.001283 5mthf_c + 0.577574 alatrna_c + 0.146675 argtrna_c + 0.10797 asntrna_c + 0.197296 asptrna_c + 110.681641 atp_c + 0.002418 btn_m + 0.000832 ca2_c + 0.001792 camp_c + 0.031005 chitin_c + 1.8e-05 clpn_RT_m + 0.00077 coa_c + 0.044881 ctp_c + 0.000525 cu2_c + 0.034269 cystrna_c + 0.002451 datp_c + 0.004285 dctp_c + 0.003763 dgtp_c + 0.002523 dttp_c + 0.004031 ergst_r + 1.6e-05 ergstest_RT_r + 0.000751 fad_c + 0.000597 fe2_c + 0.000597 fe3_c + 0.117898 glntrna_c + 0.208673 glutrna_c + 0.518069 glycogen_c + 0.51322 glytrna_c + 0.00192 gthrd_c + 0.051085 gtp_c + 106.71258 h2o_c + 0.000691 hemeA_m + 0.061167 histrna_c + 0.158569 iletrna_c + 0.585703 k_c + 0.320205 leutrna_c + 0.003107 lipopb_m + 0.172334 lystrna_c + 0.807941 mannan_r + 0.05705 mettrna_c + 0.061716 mg2_c + 0.001292 mlthf_c + 0.000607 mn2_c + 0.026099 na1_c + 0.000888 nad_c + 0.000795 nadp_c + 1.4e-05 pa_RT_r + 0.000172 pc_RT_r + 0.00013 pe_RT_r + 0.095164 phetrna_c + 0.235897 protrna_c + 2e-05 ps_RT_r + 0.004006 psphings_r + 4.7e-05 ptd1ino_RT_r + 0.006524 ptrc_c + 0.0024 pydx5p_c + 0.00074 q9_m + 0.00099 ribflv_c + 0.33687 sertrna_c + 0.003967 spmd_c + 0.001327 thf_c + 0.001393 thmpp_c + 0.21813 thrtrna_c + 0.023372 tre_c + 0.000432 triglyc_RT_r + 0.023319 trptrna_c + 0.062363 tyrtrna_c + 0.051129 utp_c + 0.275168 valtrna_c + 0.00051 zn2_c + 0.004156 zymst_r + 1.7e-05 zymstest_RT_d --> 110.632394 adp_c + 110.632394 h_c + 110.632394 pi_c + 0.209364 ppi_c  {'charge': -4.356142000000063, 'C': -40.73034299999994, 'H': -62.36094499999999, 'O': -17.698876999999808, 'N': -5.868878999999981, 'P': -0.37418499999998533, 'R': -4.027781, 'S': -0.104034, 'Ca': -0.000832, 'Cu': -0.000525, 'Fe': -0.001885, 'K': -0.585703, 'Mg': -0.061716, 'Mn': -0.000607, 'Na': -0.026099, 'Zn': -0.00051}
```

In [49]:

```
for x in ['EX_T4hcinnm_e','T4HCINNMt','T4HCINNMtp','COUCOALp','COUCOAHp','4HBZACCOARp','4HBZACCOATp','4HBZCOAEp',
          '4HBHYOXp','34DHBZtp','PCADYOX','MUCCY_kt','4CMLCL_kt','OXOAEL','3OXOADPTm','3OADPCOATm','3OXCOATm']:
    r = model.reactions.get_by_id(x)
    print(r, r.gene_reaction_rule)
```

```
EX_T4hcinnm_e: T4hcinnm_e -->  
T4HCINNMt: T4hcinnm_e <=> T4hcinnm_c 10704 and 10705
T4HCINNMtp: T4hcinnm_c --> T4hcinnm_x 9912
COUCOALp: T4hcinnm_x + atp_x + coa_x --> amp_x + coucoa_x + ppi_x 12555 or 16635
COUCOAHp: coucoa_x + h2o_x --> 34hp3hpcoa_x 16515 or 9469
4HBZACCOARp: 34hp3hpcoa_x + nad_x --> 4hbzaccoa_x + h_x + nadh_x 10551 or 10558
4HBZACCOATp: 4hbzaccoa_x + coa_x --> 4hbzcoa_x + accoa_x 9065
4HBZCOAEp: 4hbzcoa_x + h2o_x --> 4hbz_x + coa_x + h_x 14934
4HBHYOXp: 4hbz_x + h_x + nadph_x + o2_x --> 34dhbz_x + h2o_x + nadp_x 12923
34DHBZtp: 34dhbz_c <=> 34dhbz_x 
PCADYOX: 34dhbz_c + o2_c --> CCbuttc_c + 2.0 h_c 12623
MUCCY_kt: CCbuttc_c + h_c --> 4cml_c 12622
4CMLCL_kt: 4cml_c + h_c --> 5odhf2a_c + co2_c 12620
OXOAEL: 5odhf2a_c + h2o_c --> 3oxoadp_c + h_c 12620
3OXOADPTm: 3oxoadp_c + akg_m <=> 3oxoadp_m + akg_c 10635
3OADPCOATm: 3oxoadp_m + succoa_m --> oxadpcoa_m + succ_m 13090
3OXCOATm: coa_m + oxadpcoa_m --> accoa_m + succoa_m 15228
```

In [50]:

```
# Set AACOATx irreversible
model.reactions.get_by_id('AACOATx').lower_bound = 0.0
# Add ppi_x and h_x transport to allow ppi_x and h_x to leave peroxisome
# ANT1 is known to be proton-coupled, but ppi-coupled would solve this problem
# https://www.frontiersin.org/articles/10.3389/fpls.2012.00003/full
# Hypothesized, but no evidence yet,
# No known inorganic pyrophosphatase targeted to peroxisome
# Simply add ppi_x and h_x transport for now
r = hsa2.reactions.get_by_id('PPItx').copy()
model.add_reactions([r])
r = hsa2.reactions.get_by_id('Htx').copy()
model.add_reactions([r])
# Add coa_x and crn_x transport to allow coa_c and crn_c to enter peroxisome
r = hsa2.reactions.get_by_id('COAtp').copy()
model.add_reactions([r])
r = hsa2.reactions.get_by_id('CRNtp').copy()
model.add_reactions([r])
# Add akg_x transport to allow nadph production
# Add mal__L_x and oaa_x transport to allow nad regeneration
# https://www.sciencedirect.com/science/article/pii/S0167488906002400
r = hsa2.reactions.get_by_id('AKGtp').copy()
model.add_reactions([r])
r = hsa2.reactions.get_by_id('MAL_Ltx').copy()
model.add_reactions([r])
r = hsa2.reactions.get_by_id('HMR_9680').copy()
r.id = 'OAAtp'
model.add_reactions([r])
```

In [51]:

```
with model:
    model.reactions.get_by_id('EX_o2_e').lower_bound = -1000.0
    model.reactions.get_by_id('EX_glc__D_e').lower_bound = 0.0
    model.reactions.get_by_id('EX_T4hcinnm_e').lower_bound = -1.0
    sol = model.optimize()
    print(sol.objective_value)
```

```
0.08000655065055765
```

Other aromatic compounds R. toruloides is known to utilize  
Ferulate - ferulate to vanillate in the same steps as p-coumarate to 4-hydroxybenzoate,  
need vanillate monooxygenase or vanillate O-methyltransferase to convert vanillate to protocatechuate  
but neither is found in R. toruloides  
If feruloyl-CoA is converted to caffeoyl-CoA first, caffeoyl-CoA beta-oxidation to protocatechuate  
13444, 13445 are Caffeoyl-CoA O-methyltransferases, but weak fitness defect in ferulate. redundant?  
Feruloyl-CoA + ahcys <-> Caffeoyl-CoA + amet in E.R. (13444) or cyto (13445)? is it reversible?  
9578 Delta8-fatty-acid desaturase has fitness defect in aromatic compounds, why? DesA?  
https://www.nature.com/articles/s41467-018-04878-2

Benzoate - fitness data suggests benzoate is converted to 4-hydroxybenzoate  
10304 benzoate 4-monooxygenase CYP450, E.R.?  
benzoate + [reduced NADPH---hemoprotein reductase] + O2 = 4-hydroxybenzoate + [oxidized NADPH---hemoprotein reductase] + H2O

In [52]:

```
temp = ['13444','13445','9578','10304']
display(Annotation.loc[temp])
Show_Data(temp)
```

|  | Combined Annotations | Signal P | Sc288c Orthologs | Human Orthologs | Sc288 Best Hit | Human Blast | Essential | WolfPSort | C Terminal |
| --- | --- | --- | --- | --- | --- | --- | --- | --- | --- |
| RTO4\_ID |  |  |  |  |  |  |  |  |  |
| 13444 | K00588: E2.1.1.104; caffeoyl-CoA O-methyltrans... | S |  | COMTD1 |  | COMTD | Not Essential | plas 9, mito 7, E.R. 5, cyto 2, vacu 2 | RRM\* |
| 13445 | KOG1663: O-methyltransferase |  |  | COMTD1 |  | COMTD | Not Essential | cyto 9.5, mito 9, cyto\_nucl 6, extr 2, pero 2,... | RRV\* |
| 9578 | K13076: SLD; Delta8-fatty-acid desaturase |  |  | FADS1,FADS2,FADS3 |  |  | Not Essential | plas 22, mito 3 | LHH\* |
| 10304 | K07424: CYP3A; cytochrome P450, family 3, subf... | A |  |  |  | CYP3A | Not Essential | cyto 10.5, plas 6, cyto\_nucl 6, E.R. 4, mito 2... | FNV\* |

| strain | WT | | | | | | | | | | | | | | | | |
| --- | --- | --- | --- | --- | --- | --- | --- | --- | --- | --- | --- | --- | --- | --- | --- | --- | --- |
| condition | G\_MM | C\_MM | G\_SD | | GX\_SD | | | X\_SD | | A\_SD | | C\_SD | | MM\_CN120 | | MM\_CN5 | Diversity\_Sample |
| phase | exp | exp | exp | stat | exp | trans | stat | exp | stat | exp | stat | exp | stat | exp | stat | exp | exp |
| proteinId | Set1 | Set1 | Set2 | Set2 | Set2 | Set2 | Set2 | Set2 | Set2 | Set2 | Set2 | Set2 | Set2 | Set3 | Set3 | Set3 | Set3 |
| 13444 | 3.998559 | 5.739614 | 2.481996 | 3.407914 | 2.563598 | 1.884579 | 2.173976 | 2.139773 | 2.650392 | 2.111205 | 2.752208 | 2.437136 | 3.270256 | 4.713352 | 3.650538 | 3.372894 | 3.242892 |
| 13445 | 4.844068 | 5.453084 | 7.046451 | 7.243291 | 7.091308 | 6.802879 | 6.680650 | 6.140352 | 6.558608 | 5.804230 | 6.434290 | 5.778070 | 6.617361 | 6.408730 | 6.571836 | 8.422381 | 6.912798 |
| 9578 | 4.590781 | 4.678751 | 4.779547 | 4.371859 | 4.679735 | 5.142119 | 5.200515 | 5.067369 | 5.438483 | 5.120087 | 4.955813 | 5.109503 | 4.902697 | 5.472609 | 5.130125 | 4.734194 | 4.914118 |
| 10304 | 5.813532 | 7.223290 | 6.566576 | 7.869526 | 6.480333 | 6.851217 | 7.166794 | 6.279750 | 7.005338 | 6.820213 | 7.175870 | 7.278576 | 6.335774 | 6.388311 | 6.527305 | 7.219334 | 6.214110 |

| strain | WT | | | | | | | | | | |
| --- | --- | --- | --- | --- | --- | --- | --- | --- | --- | --- | --- |
| condition | G\_SD | | GX\_SD | | | X\_SD | | A\_SD | | C\_SD | |
| proteinId | exp | stat | exp | trans | stat | exp | stat | exp | stat | exp | stat |
| 13445 | 2.866717 | 4.278112 | 2.842629 | 2.594697 | 4.009346 | 1.772055 | 3.324224 | 1.547220 | 2.493360 | 4.044326 | 5.644572 |
| 9578 | 0.000000 | 0.000000 | 0.204847 | 0.183791 | 0.000000 | 0.000000 | 0.189187 | 0.000000 | 0.000000 | 0.000000 | 0.000000 |
| 10304 | 1.526293 | 2.807646 | 1.220359 | 0.745880 | 1.281008 | 0.389675 | 0.782034 | 0.766956 | 3.069048 | 5.327461 | 0.438555 |

|  | Glucose | Xylose | Arabinose | Acetate | Coumarate | Ferulate | YNB Oleic Acid | YNB Ricinoleic Acid | YNB Glucose | YNB Gluc DOC | YPD |
| --- | --- | --- | --- | --- | --- | --- | --- | --- | --- | --- | --- |
| proteinId |  |  |  |  |  |  |  |  |  |  |  |
| 13444 | -0.110618 | 0.074913 | -0.132922 | -0.184650 | -0.456785 | -0.156824 | -0.533952 | 0.014784 | 0.009505 | -0.163047 | -0.297493 |
| 13445 | -0.635958 | -0.527207 | -0.536003 | -0.106179 | -0.605675 | -0.557250 | -1.168182 | -1.548816 | -0.575936 | -0.764138 | -0.712307 |
| 10304 | -0.266672 | -0.167355 | -0.198551 | -0.200802 | 0.409827 | 0.035824 | -0.020535 | 0.192346 | 0.127195 | -0.353236 | -0.334459 |

In [53]:

```
# Ferulate degradation
# Ferulate exchange and transport
r1 = ppu.reactions.get_by_id('EX_fer_e').copy()
r2 = ppu.reactions.get_by_id('FERtex').copy()
r2.id = 'FERt'
r2.name = 'Ferulate transport'
r2.gene_reaction_rule = '' # not clear, no transporter with specific fitness defect in ferulate
model.add_reactions([r1,r2])
r2.add_metabolites({'fer_p': -1.0, 'fer_c': 1.0})
model.metabolites.get_by_id('fer_e').charge = -1
# Assume cytosolic conversion from ferulcoa to caffcoa
# could be E.R. or both, but not likely peroxisomal since no PTS signals in 13444 or 13445
r = ppu.reactions.get_by_id('FERULCOAS').copy()
r.gene_reaction_rule = '' 
model.add_reactions([r])
model.metabolites.get_by_id('ferulcoa_c').charge = -4
model.metabolites.get_by_id('caffcoa_c').charge = -4
model.reactions.get_by_id('CCOAOMT').lower_bound = -1000.0
# caffcoa to 34dhcinm by cysotolic fatty acid thioesterase?
m = ppu.metabolites.get_by_id('34dhcinm_c').copy()
model.add_metabolites([m])
r = model.reactions.get_by_id('FACOAE100').copy()
r.id = 'CAFFCOAE'
r.name = 'Caffeoyl-CoA thioesterase'
r.gene_reaction_rule = '8456'
model.add_reactions([r])
r.add_metabolites({'dcacoa_c': 1.0, 'dca_c': -1.0, 'caffcoa_c': -1.0, '34dhcinm_c': 1.0})
model.metabolites.get_by_id('34dhcinm_c').charge = -1
# Peroxisomal transport reaction for caffcoa by 9912 FAT1?
m = ppu.metabolites.get_by_id('34dhcinm_c').copy()
m.id = '34dhcinm_x'
m.compartment = 'x'
model.add_metabolites([m])
r = model.reactions.get_by_id('FA240tp').copy()
r.id = '34DHCINMtp'
r.name = '3,4-Dihydroxy-trans-cinnamate peroxisomal transport'
r.gene_reaction_rule = '9912'
model.add_reactions([r])
r.add_metabolites({'ttc_c': 1.0, 'ttc_x': -1.0, '34dhcinm_c': -1.0, '34dhcinm_x': 1.0})
model.metabolites.get_by_id('34dhcinm_x').charge = -1
# Peroxisomal caffeoyl-coa ligase
m = model.metabolites.get_by_id('caffcoa_c').copy()
m.id = 'caffcoa_x'
m.compartment = 'x'
model.add_metabolites([m])
r = model.reactions.get_by_id('COUCOALp').copy()
r.id = 'CAFFCOALp'
r.name = 'Caffeate:CoA ligase'
r.gene_reaction_rule = '12555 or 16635'
model.add_reactions([r])
r.add_metabolites({'T4hcinnm_x': 1.0, 'coucoa_x': -1.0, '34dhcinm_x': -1.0, 'caffcoa_x': 1.0})
model.metabolites.get_by_id('caffcoa_x').charge = -4
# Beta-oxidation of caffcoa to protocatechuate
# Hydratase
m = model.metabolites.get_by_id('34hp3hpcoa_x').copy()
m.id = '3dhp3hpcoa_x'
m.name = '3-(3,4-dihydroxyphenyl)-3-hydroxy-propanoyl-CoA'
m.formula = 'C30H40N7O20P3S'
m.charge = -4
model.add_metabolites([m])
r = model.reactions.get_by_id('COUCOAHp').copy()
r.id = 'CAFFCOAHp'
r.name = 'Caffeoyl-CoA hydratase'
r.gene_reaction_rule = '16515 or 9469'
model.add_reactions([r])
r.add_metabolites({'coucoa_x': 1.0, '34hp3hpcoa_x': -1.0, 'caffcoa_x': -1.0, '3dhp3hpcoa_x': 1.0})
# Reductase
m = model.metabolites.get_by_id('4hbzcoa_x').copy()
m.id = '34dhbzaccoa_x'
m.name = '3,4-dihydroxybenzoyl-acetyl-CoA'
m.formula = 'C30H38N7O20P3S'
m.charge = -4
model.add_metabolites([m])
r = model.reactions.get_by_id('4HBZACCOARp').copy()
r.id = '34DHBZACCOARp'
r.name = '3,4-dihydroxybenzoyl-acetyl-CoA reductase'
r.gene_reaction_rule = '10551 or 10558'
model.add_reactions([r])
r.add_metabolites({'34hp3hpcoa_x': 1.0, '4hbzaccoa_x': -1.0, '3dhp3hpcoa_x': -1.0, '34dhbzaccoa_x': 1.0})
# Thiolase
m = model.metabolites.get_by_id('4hbzcoa_x').copy()
m.id = '34dhbzcoa_x'
m.name = '3,4-dihydroxybenzoyl-CoA'
m.formula = 'C28H36N7O19P3S'
m.charge = -4
model.add_metabolites([m])
r = model.reactions.get_by_id('4HBZACCOATp').copy()
r.id = '34DHBZACCOATp'
r.name = '3,4-dihydroxybenzoyl-acetyl-CoA thiolase'
r.gene_reaction_rule = '9065'
model.add_reactions([r])
r.add_metabolites({'4hbzaccoa_x': 1.0, '4hbzcoa_x': -1.0, '34dhbzaccoa_x': -1.0, '34dhbzcoa_x': 1.0})
# Thioesterase
r = model.reactions.get_by_id('4HBZCOAEp').copy()
r.id = '34DHBZCOAEp'
r.name = '3,4-dihydroxybenzoyl-CoA thioesterase'
r.gene_reaction_rule = '14934'
model.add_reactions([r])
r.add_metabolites({'4hbzcoa_x': 1.0, '4hbz_x': -1.0, '34dhbzcoa_x': -1.0, '34dhbz_x': 1.0})
```

In [54]:

```
for x in ['EX_fer_e','FERt','FERULCOAS','CCOAOMT','CAFFCOAE','34DHCINMtp','CAFFCOALp','CAFFCOAHp','34DHBZACCOARp',
          '34DHBZACCOATp','34DHBZCOAEp','34DHBZtp']:
    r = model.reactions.get_by_id(x)
    print(r, r.check_mass_balance())
```

```
EX_fer_e: fer_e -->  {'charge': 1.0, 'C': -10.0, 'H': -9.0, 'O': -4.0}
FERt: fer_e <=> fer_c {}
FERULCOAS: atp_c + coa_c + fer_c --> amp_c + ferulcoa_c + ppi_c {}
CCOAOMT: amet_c + caffcoa_c <=> ahcys_c + ferulcoa_c + h_c {}
CAFFCOAE: caffcoa_c + h2o_c --> 34dhcinm_c + coa_c + h_c {}
34DHCINMtp: 34dhcinm_c --> 34dhcinm_x {}
CAFFCOALp: 34dhcinm_x + atp_x + coa_x --> amp_x + caffcoa_x + ppi_x {}
CAFFCOAHp: caffcoa_x + h2o_x --> 3dhp3hpcoa_x {}
34DHBZACCOARp: 3dhp3hpcoa_x + nad_x --> 34dhbzaccoa_x + h_x + nadh_x {}
34DHBZACCOATp: 34dhbzaccoa_x + coa_x --> 34dhbzcoa_x + accoa_x {}
34DHBZCOAEp: 34dhbzcoa_x + h2o_x --> 34dhbz_x + coa_x + h_x {}
34DHBZtp: 34dhbz_c <=> 34dhbz_x {}
```

In [55]:

```
# Benzoate degradation
# Benzoate exchange and transport
r1 = ppu.reactions.get_by_id('EX_bz_e').copy()
r2 = ppu.reactions.get_by_id('BZtex').copy()
r2.id = 'BZt'
r2.name = 'Benzoate transport'
r2.gene_reaction_rule = '' # not clear
model.add_reactions([r1,r2])
m = model.metabolites.get_by_id('bz_p')
m.id = 'bz_c'
m.compartment = 'c'
model.metabolites.get_by_id('bz_e').charge = -1
model.metabolites.get_by_id('bz_c').charge = -1
# Benzoate 4-monooxygenase (E.R.?, put it in cyto for now)
r = ppu.reactions.get_by_id('BZ12DOX').copy()
r.id = 'BZ4OX'
r.name = 'Benzoate 4-monooxygenase'
r.gene_reaction_rule = '10304'
model.add_reactions([r])
r.add_metabolites({'nadh_c': 1.0, 'nad_c': -1.0, 'nadph_c': -1.0, 'nadp_c': 1.0,
                   'bz12diol_c': -1.0, '4hbz_c': 1.0, 'h2o_c': 1.0})
```

In [56]:

```
for x in ['EX_bz_e','BZt','BZ4OX']:
    r = model.reactions.get_by_id(x)
    print(r, r.check_mass_balance())
```

```
EX_bz_e: bz_e -->  {'charge': 1.0, 'C': -7.0, 'H': -5.0, 'O': -2.0}
BZt: bz_e <=> bz_c {}
BZ4OX: bz_c + h_c + nadph_c + o2_c --> 4hbz_c + h2o_c + nadp_c {}
```

In [57]:

```
with model:
    model.reactions.get_by_id('EX_o2_e').lower_bound = -1000.0
    model.reactions.get_by_id('EX_glc__D_e').lower_bound = 0.0
    model.reactions.get_by_id('EX_fer_e').lower_bound = -2.0
    sol = model.optimize()
    print(sol.status, sol.objective_value)
    model.reactions.get_by_id('EX_fer_e').lower_bound = 0.0
    model.reactions.get_by_id('EX_bz_e').lower_bound = -2.0
    sol = model.optimize()
    print(sol.status, sol.objective_value)
```

```
optimal 0.0009524894124774558
optimal 0.09553995768683396
```

### Xylose and arabinose degradation pathway¶

In [58]:

```
for r in sorted(model.metabolites.get_by_id('xyl__D_c').reactions, key=lambda x: x.id):
    print(r, r.gene_reaction_rule)
print()
for r in sorted(model.metabolites.get_by_id('xylu__D_c').reactions, key=lambda x: x.id):
    print(r, r.gene_reaction_rule)
```

```
XYHDL: h2o_c + iprimv_c --> glc__D_c + xyl__D_c 10170
XYLt: xyl__D_e <=> xyl__D_c 15762

XYLK: atp_c + xylu__D_c --> adp_c + h_c + xu5p__D_c 14368 or 16850
XYLTD_D: nad_c + xylt_c --> h_c + nadh_c + xylu__D_c 16452
```

In [59]:

```
temp = ['10170','15762','14368','16850','16452','12974','12977']
Annotation.loc[temp]
```

Out[59]:

|  | Combined Annotations | Signal P | Sc288c Orthologs | Human Orthologs | Sc288 Best Hit | Human Blast | Essential | WolfPSort | C Terminal |
| --- | --- | --- | --- | --- | --- | --- | --- | --- | --- |
| RTO4\_ID |  |  |  |  |  |  |  |  |  |
| 10170 | K05546: GANAB; alpha 1,3-glucosidase | S | ROT2 | GANAB,GANC | ROT2 | GANAB | Not Essential | mito 17, extr 5, cyto 3 | DEL\* |
| 15762 | KOG0569: Permease of the major facilitator sup... | S | YBR241C | SLC2A1,SLC2A14,SLC2A2,SLC2A3,SLC2A4,SLC2A8 | YBR241C | SLC2A | Not Essential | plas 20, mito 3, E.R. 2 | LMR\* |
| 14368 | KOG2517: Ribulose kinase and related carbohydr... |  | YDR109C | FGGY | YDR109C | FGGY | Not Essential | cyto 14, cysk 6, mito 5 | LGE\* |
| 16850 | K00854: xylB, XYLB; xylulokinase |  |  |  | XKS1 | XYLB | Not Essential | cyto 10.5, cyto\_mito 8, extr 7, mito 4.5, nucl... | GLV\* |
| 16452 | K05351: E1.1.1.9; D-xylulose reductase |  | YDL246C,SOR1,XYL2 | SORD | SOR1 | SORD | Not Essential | cyto 16, cyto\_nucl 10, mito 4, pero 3, nucl 2 | DKF\* |
| 12974 | HMMPfam:Zinc-binding dehydrogenase:PF00107,HMM... |  |  |  | SOR1 | SORD | Not Essential | cyto 12.5, mito 9, cyto\_nucl 7, extr 2, pero 2 | EAL\* |
| 12977 | K00008: SORD, gutB; L-iditol 2-dehydrogenase |  |  |  | XYL2 | SORD | Not Essential | cyto 19.5, cyto\_nucl 12, mito 3 | HSP\* |

In [60]:

```
Show_Data(temp)
```

| strain | WT | | | | | | | | | | | | | | | | |
| --- | --- | --- | --- | --- | --- | --- | --- | --- | --- | --- | --- | --- | --- | --- | --- | --- | --- |
| condition | G\_MM | C\_MM | G\_SD | | GX\_SD | | | X\_SD | | A\_SD | | C\_SD | | MM\_CN120 | | MM\_CN5 | Diversity\_Sample |
| phase | exp | exp | exp | stat | exp | trans | stat | exp | stat | exp | stat | exp | stat | exp | stat | exp | exp |
| proteinId | Set1 | Set1 | Set2 | Set2 | Set2 | Set2 | Set2 | Set2 | Set2 | Set2 | Set2 | Set2 | Set2 | Set3 | Set3 | Set3 | Set3 |
| 10170 | 5.666491 | 5.614234 | 5.866479 | 5.769623 | 5.896109 | 5.537082 | 5.450749 | 5.730279 | 5.784951 | 5.596031 | 5.618704 | 6.193443 | 6.006379 | 5.656993 | 5.540948 | 5.897851 | 5.708526 |
| 15762 | 4.882270 | 4.458294 | 4.521989 | 3.631506 | 4.677238 | 4.425973 | 4.318599 | 4.763567 | 3.992634 | 4.666745 | 3.896071 | 4.506429 | 5.482028 | 4.132238 | 4.052266 | 4.688833 | 3.598391 |
| 14368 | 5.776084 | 6.227531 | 5.811947 | 5.807621 | 7.255347 | 7.811263 | 7.351152 | 6.480310 | 5.229182 | 8.068085 | 4.320579 | 5.204209 | 5.328031 | 6.096076 | 5.921803 | 6.124689 | 6.907272 |
| 16850 | 0.048888 | 4.253379 | 0.000000 | 0.000000 | 0.016744 | 0.000000 | 0.000000 | 0.023802 | 0.006679 | 0.039269 | 0.006831 | 4.367685 | 3.796810 | 0.070525 | 0.019495 | 0.081034 | 0.074838 |
| 16452 | 4.493003 | 5.784615 | 3.483151 | 3.877014 | 6.365064 | 8.412860 | 8.085506 | 5.876326 | 2.054602 | 8.570307 | 1.596919 | 4.774311 | 4.803771 | 4.407766 | 4.129490 | 3.024692 | 7.503153 |
| 12974 | 3.114580 | 3.222719 | 3.662429 | 5.666127 | 3.500368 | 3.942801 | 4.107039 | 4.015275 | 3.717787 | 3.723632 | 3.636170 | 2.884500 | 2.387856 | 4.317949 | 4.775079 | 4.572200 | 4.227138 |
| 12977 | 4.145403 | 6.863003 | 5.821475 | 7.245684 | 7.434302 | 8.322392 | 7.746119 | 8.831253 | 4.493704 | 10.039224 | 2.923550 | 3.904197 | 4.292592 | 4.172015 | 3.619581 | 4.473334 | 7.101817 |

| strain | WT | | | | | | | | | | |
| --- | --- | --- | --- | --- | --- | --- | --- | --- | --- | --- | --- |
| condition | G\_SD | | GX\_SD | | | X\_SD | | A\_SD | | C\_SD | |
| proteinId | exp | stat | exp | trans | stat | exp | stat | exp | stat | exp | stat |
| 10170 | 2.317773 | 5.155588 | 2.647373 | 4.079530 | 2.194652 | 1.747817 | 3.568828 | 3.687671 | 4.973492 | 7.072032 | 8.079749 |
| 14368 | 10.340991 | 11.803987 | 13.224373 | 23.626670 | 22.968584 | 41.126832 | 32.257663 | 39.598535 | 34.104908 | 13.244333 | 10.459359 |
| 16452 | 0.000000 | 0.335899 | 4.659059 | 14.708290 | 18.041717 | 44.504599 | 34.693021 | 37.870811 | 31.535933 | 2.779380 | 2.844356 |
| 12974 | 5.184155 | 5.947413 | 8.330927 | 9.857949 | 10.176895 | 19.593425 | 17.977918 | 24.699510 | 23.352362 | 2.121106 | 2.403355 |
| 12977 | 6.149778 | 9.023107 | 13.626283 | 17.490719 | 19.851293 | 35.252067 | 32.159850 | 44.396025 | 41.922094 | 2.121106 | 2.403355 |

|  | Glucose | Xylose | Arabinose | Acetate | Coumarate | Ferulate | YNB Oleic Acid | YNB Ricinoleic Acid | YNB Glucose | YNB Gluc DOC | YPD |
| --- | --- | --- | --- | --- | --- | --- | --- | --- | --- | --- | --- |
| proteinId |  |  |  |  |  |  |  |  |  |  |  |
| 10170 | -0.348111 | -0.093986 | -0.524100 | -0.453835 | -1.629997 | -0.570911 | -0.277692 | -1.073728 | -0.818833 | -0.702664 | 0.070068 |
| 15762 | -0.402418 | -0.295630 | -0.276055 | -0.280450 | 0.339173 | -0.160004 | 0.086122 | 0.001426 | -0.238339 | -0.097129 | -0.504427 |
| 14368 | -0.063355 | -2.897249 | -3.097320 | -0.236475 | -0.180356 | -0.182745 | -0.133641 | -0.115522 | 0.064443 | 0.127896 | 0.410221 |
| 16850 | 0.093595 | 0.034546 | 0.073679 | 0.073594 | 0.195984 | 0.175782 | 0.039364 | 0.057851 | -0.077381 | 0.018073 | -0.002070 |
| 16452 | -0.300320 | -0.278447 | -0.209842 | 0.129556 | -0.122194 | 0.300891 | -0.304882 | -0.426796 | -0.419360 | -0.138067 | -0.295863 |
| 12974 | 0.078309 | -0.181519 | 0.287525 | -0.291256 | 0.363434 | -0.113870 | 0.129352 | -0.043842 | 0.310271 | 0.186506 | -0.477986 |
| 12977 | -0.301178 | -0.257299 | -0.745618 | 0.039816 | 0.041993 | 0.332437 | 0.104844 | 0.010498 | -0.378356 | -0.719372 | -0.219867 |

In [61]:

```
for x in temp:
    if x in model.genes:
        for r in sorted(model.genes.get_by_id(x).reactions, key=lambda x: x.id):
            print(r, r.gene_reaction_rule)
    else:
        print(x, 'no reactions')
    print()
```

```
MALT: h2o_c + malt_c --> 2.0 glc__D_c 10170 or 9135
MG2A: asnglcnacglcnacman_man_manman_manman_manmanmanglcglc_c + h2o_c --> Glc_aD_c + asnglcnacglcnacman_man_manman_manman_manmanmanglc_c 10170
MG2B: asnglcnacglcnacman_man_manman_manman_manmanmanglcglc_c + h2o_c --> asnglcnacglcnacman_man_manman_manman_manmanmanglc_c + glc__D_c 10170
MG3A: asnglcnacglcnacman_man_manman_manman_manmanmanglc_c + h2o_c --> Glc_aD_c + asnglcnacglcnacman_man_manman_manman_manmanman_c 10170
MG3B: asnglcnacglcnacman_man_manman_manman_manmanmanglc_c + h2o_c --> asnglcnacglcnacman_man_manman_manman_manmanman_c + glc__D_c 10170
MLTG1: h2o_c + malttr_c --> glc__D_c + malt_c 10170
SQGH: h2o_c + sqg_c --> glyc_c + sq_c 10170
XYHDL: h2o_c + iprimv_c --> glc__D_c + xyl__D_c 10170

DHAAt1r: dhdascb_e <=> dhdascb_c 15762
FRUt1r: fru_e <=> fru_c 15762
GALt1r: gal_e <=> gal_c 15762
GAMt1r: gam_e <=> gam_c 15762
GLCt1: glc__D_e --> glc__D_c 10452 or 11075 or 11893 or 15762 or 9102 or 9841
GLCtg: glc__D_c <=> glc__D_g 15762
MALTt1r: malt_e <=> malt_c 15762
MANt1r: man_e <=> man_c 15762
XYLt: xyl__D_e <=> xyl__D_c 15762

RBK_L1: atp_c + rbl__L_c --> adp_c + h_c + ru5p__L_c 14368
XYLK: atp_c + xylu__D_c --> adp_c + h_c + xu5p__D_c 14368 or 16850
XYLK2: atp_c + xylu__L_c --> adp_c + h_c + xu5p__L_c 14368

XYLK: atp_c + xylu__D_c --> adp_c + h_c + xu5p__D_c 14368 or 16850

SBTD_D2: nad_c + sbt__D_c --> fru_c + h_c + nadh_c 16452
SBTD_L: nad_c + sbt__L_c --> h_c + nadh_c + srb__L_c 16452
XYLTD_D: nad_c + xylt_c --> h_c + nadh_c + xylu__D_c 16452

12974 no reactions

12977 no reactions
```

14368 is YDR109C/FGGY D-ribulokinase (D-ribulose -> D-ribulose 5-phosphate)  
16850 is XKS1/XYLB D-xylulokinase (D-xylulose -> D-xylulose 5-phosphate)  
Add RBK\_Dr from Recon3D, but it has a wrong gene (ribokinase)  
Remove RBK\_L1, XYLK2

16452 is D-xylulose reductase EC:1.1.1.9, xylitol + NAD+ = D-xylulose + NADH + H+  
12974 and 12977 is L-iditol 2-dehydrogenase EC:1.1.1.14, L-iditol + NAD+ = L-sorbose + NADH + H+  
L-iditol 2-dehydrogenase also works on xylitol  
Change XYLTD\_D genes to '12974 or 12977 or 16452'  
Change SBTD\_D2 and SBTD\_L genes to '12974 or 12977'

In [62]:

```
r = hsa2.reactions.get_by_id('RBK_Dr').copy()
r.gene_reaction_rule = '14368'
model.add_reactions([r])
model.reactions.get_by_id('XYLK').gene_reaction_rule = '16850'
model.remove_reactions(['RBK_L1','XYLK2'], remove_orphans=True)
model.reactions.get_by_id('XYLTD_D').gene_reaction_rule = '12974 or 12977 or 16452'
model.reactions.get_by_id('SBTD_D2').gene_reaction_rule = '12974 or 12977'
model.reactions.get_by_id('SBTD_L').gene_reaction_rule = '12974 or 12977'
```

In [63]:

```
for r in sorted(model.metabolites.get_by_id('arab__L_c').reactions, key=lambda x: x.id):
    print(r, r.gene_reaction_rule)
```

```
ARABR: arab__L_c + h_c + nadph_c --> abt_c + nadp_c 11882 or 12784 or 9774
```

In [64]:

```
# Add exchange and transport for L-arabinose
for x in ['EX_arab__L_e','ARAB_Lt']:
    r = sce.reactions.get_by_id(x).copy()
    print(r, r.gene_reaction_rule)
    model.add_reactions([r])
```

```
EX_arab__L_e: arab__L_e -->  
ARAB_Lt: arab__L_e <=> arab__L_c
```

In [65]:

```
# Identify minimum set of reactions to support growth on xylose 
Reaction_xylose = ['XYLR'] # S. cerevisiae
# Identify minimum set of reactions to support growth on arabinose
Reaction_arabinose = ['ABTD','XYLUR'] # Human

for x in Reaction_xylose:
    r = sce.reactions.get_by_id(x).copy()
    print(r, r.gene_reaction_rule)
print()
for x in Reaction_arabinose:
    r = hsa2.reactions.get_by_id(x).copy()
    print(r, r.gene_reaction_rule)
```

```
XYLR: h_c + nadph_c + xyl__D_c --> nadp_c + xylt_c YHR104W

ABTD: abt_c + nad_c <=> h_c + nadh_c + xylu__L_c 
XYLUR: h_c + nadph_c + xylu__L_c <=> nadp_c + xylt_c 51181_AT1
```

XYLR - D-xylose reductase (D-xylose to xylitol) -> 11882 or 9774 (larA or xyrA in A. niger, respectively)  
https://www.sciencedirect.com/science/article/pii/S0014579310005326  
blast of xylose reductase (XYLR) genes results in hits with many (top 4 are 9774, 12784, 11756, 11882)  
12784 peroxisomal (AKL\*) no upregulation, 11756 no upregulation

ARABR - L-arabinose reductase (L-arabinose to L-arabinitol, 11882 or 12784 or 9774) -> 9774 (larA in A. niger)  
9774 has fitness defect only in arabinose, not xylose, xyrA works on D-xylose only

In [66]:

```
r = sce.reactions.get_by_id('XYLR').copy()
r.gene_reaction_rule = '11882 or 13562 or 9774'
model.add_reactions([r])
model.reactions.get_by_id('ARABR').gene_reaction_rule = '9774'
```

ABTD - L-arabinitol 4-dehydrogenase (L-arabinitol to L-xylulose) -> 12974 or 12977 (ladA in A. niger)  
blast of L-arabinitol 4-dehydrogenase (ABTD) genes hits 12977 (909) and 12974 (809), 16452 is also high (483)

XYLUR - L-xylulose reductase (L-xylulose to xylitol) -> 8988 (lxrA in A. niger)

In [67]:

```
r = hsa2.reactions.get_by_id('ABTD').copy()
r.gene_reaction_rule = '12974 or 12977'
model.add_reactions([r])
r = hsa2.reactions.get_by_id('XYLUR').copy()
r.gene_reaction_rule = '8988'
model.add_reactions([r])
```

In [68]:

```
temp = ['9774','11882','12784','11756','12977','12974','8988']
Annotation.loc[temp]
```

Out[68]:

|  | Combined Annotations | Signal P | Sc288c Orthologs | Human Orthologs | Sc288 Best Hit | Human Blast | Essential | WolfPSort | C Terminal |
| --- | --- | --- | --- | --- | --- | --- | --- | --- | --- |
| RTO4\_ID |  |  |  |  |  |  |  |  |  |
| 9774 | K00002: AKR1A1, adh; alcohol dehydrogenase (NA... |  | YPR1,GCY1 | AKR1A1 | YPR1 | AKR1A | Not Essential | cyto 17.5, cyto\_mito 13.666, cyto\_nucl 10.333,... | KIK\* |
| 11882 | K18097: GCY1; glycerol 2-dehydrogenase (NADP+) |  | YPR1,GCY1 | AKR1A1 | YPR1 | AKR1A | Not Essential | cyto 24.5, cyto\_nucl 13.5 | SHW\* |
| 12784 | K00002: AKR1A1, adh; alcohol dehydrogenase (NA... |  | YPR1,GCY1 | AKR1A1 | YPR1 | AKR1A | Not Essential | mito 13.5, cyto\_mito 12, cyto 9.5, pero 4 | AKL\* |
| 11756 | KOG1577: Aldo/keto reductase family proteins | S |  |  | GCY1 | AKR1A | Not Essential | cyto 14, cyto\_mito 11.833, mito 8.5, cyto\_nucl... | WMK\* |
| 12977 | K00008: SORD, gutB; L-iditol 2-dehydrogenase |  |  |  | XYL2 | SORD | Not Essential | cyto 19.5, cyto\_nucl 12, mito 3 | HSP\* |
| 12974 | HMMPfam:Zinc-binding dehydrogenase:PF00107,HMM... |  |  |  | SOR1 | SORD | Not Essential | cyto 12.5, mito 9, cyto\_nucl 7, extr 2, pero 2 | EAL\* |
| 8988 | K17742: SOU1; sorbose reductase |  |  |  |  | DHRS4 | Not Essential | cyto 17, cyto\_nucl 10, mito 8 | TLS\* |

In [69]:

```
Show_Data(temp)
```

| strain | WT | | | | | | | | | | | | | | | | |
| --- | --- | --- | --- | --- | --- | --- | --- | --- | --- | --- | --- | --- | --- | --- | --- | --- | --- |
| condition | G\_MM | C\_MM | G\_SD | | GX\_SD | | | X\_SD | | A\_SD | | C\_SD | | MM\_CN120 | | MM\_CN5 | Diversity\_Sample |
| phase | exp | exp | exp | stat | exp | trans | stat | exp | stat | exp | stat | exp | stat | exp | stat | exp | exp |
| proteinId | Set1 | Set1 | Set2 | Set2 | Set2 | Set2 | Set2 | Set2 | Set2 | Set2 | Set2 | Set2 | Set2 | Set3 | Set3 | Set3 | Set3 |
| 9774 | 9.271928 | 8.073898 | 9.068550 | 8.595213 | 9.814367 | 9.928081 | 9.238037 | 9.637172 | 7.833486 | 10.562301 | 7.022552 | 5.690787 | 5.819589 | 10.063638 | 9.334496 | 10.845508 | 10.376461 |
| 11882 | 4.078183 | 5.718606 | 5.836152 | 5.625440 | 5.773365 | 5.296293 | 5.157027 | 7.027547 | 5.003437 | 4.978163 | 5.281302 | 4.361090 | 4.347224 | 6.191333 | 6.372034 | 7.210440 | 7.400484 |
| 12784 | 2.086711 | 1.966555 | 1.059743 | 1.286182 | 1.019274 | 0.689282 | 0.833234 | 1.394698 | 1.871130 | 2.537376 | 1.889525 | 1.630865 | 3.239895 | 0.662714 | 0.423146 | 2.003013 | 1.061628 |
| 11756 | 1.628643 | 3.185600 | 4.554772 | 3.230545 | 4.840388 | 3.760077 | 3.640982 | 4.279339 | 2.741240 | 3.655708 | 2.435367 | 2.565367 | 2.815192 | 3.722068 | 4.259992 | 6.660597 | 6.479482 |
| 12977 | 4.145403 | 6.863003 | 5.821475 | 7.245684 | 7.434302 | 8.322392 | 7.746119 | 8.831253 | 4.493704 | 10.039224 | 2.923550 | 3.904197 | 4.292592 | 4.172015 | 3.619581 | 4.473334 | 7.101817 |
| 12974 | 3.114580 | 3.222719 | 3.662429 | 5.666127 | 3.500368 | 3.942801 | 4.107039 | 4.015275 | 3.717787 | 3.723632 | 3.636170 | 2.884500 | 2.387856 | 4.317949 | 4.775079 | 4.572200 | 4.227138 |
| 8988 | 6.921572 | 6.927083 | 6.704075 | 7.113596 | 8.461796 | 9.022018 | 8.656900 | 7.820671 | 6.173585 | 10.074108 | 4.987534 | 5.788663 | 6.255932 | 7.337214 | 7.159274 | 6.993421 | 8.030642 |

| strain | WT | | | | | | | | | | |
| --- | --- | --- | --- | --- | --- | --- | --- | --- | --- | --- | --- |
| condition | G\_SD | | GX\_SD | | | X\_SD | | A\_SD | | C\_SD | |
| proteinId | exp | stat | exp | trans | stat | exp | stat | exp | stat | exp | stat |
| 9774 | 24.036105 | 20.651004 | 27.252847 | 17.324598 | 17.678054 | 36.939606 | 32.903871 | 40.948526 | 37.872681 | 18.573235 | 14.841079 |
| 11882 | 7.287878 | 9.645819 | 6.913391 | 7.611188 | 9.643068 | 14.690469 | 14.909681 | 6.375137 | 6.336300 | 10.682764 | 9.168715 |
| 12784 | 0.384095 | 0.000000 | 0.204746 | 0.000000 | 0.000000 | 0.000000 | 0.000000 | 0.000000 | 0.189081 | 0.000000 | 0.211140 |
| 11756 | 0.000000 | 0.559723 | 0.000000 | 0.364621 | 0.369051 | 0.000000 | 0.198706 | 0.000000 | 0.189478 | 0.000000 | 0.000000 |
| 12977 | 6.149778 | 9.023107 | 13.626283 | 17.490719 | 19.851293 | 35.252067 | 32.159850 | 44.396025 | 41.922094 | 2.121106 | 2.403355 |
| 12974 | 5.184155 | 5.947413 | 8.330927 | 9.857949 | 10.176895 | 19.593425 | 17.977918 | 24.699510 | 23.352362 | 2.121106 | 2.403355 |
| 8988 | 2.704090 | 3.242278 | 6.707344 | 9.871449 | 8.545995 | 26.373292 | 17.154925 | 29.346260 | 25.139935 | 4.055205 | 2.832854 |

|  | Glucose | Xylose | Arabinose | Acetate | Coumarate | Ferulate | YNB Oleic Acid | YNB Ricinoleic Acid | YNB Glucose | YNB Gluc DOC | YPD |
| --- | --- | --- | --- | --- | --- | --- | --- | --- | --- | --- | --- |
| proteinId |  |  |  |  |  |  |  |  |  |  |  |
| 9774 | 0.023569 | -0.715210 | -3.895191 | -0.087274 | -0.071053 | -0.088020 | -0.002179 | -0.262330 | 0.073917 | -0.149324 | -0.009339 |
| 11882 | 0.155027 | 0.002411 | 0.151468 | -0.006733 | 0.603870 | -0.030919 | 0.675184 | 0.843000 | 0.320601 | 0.279120 | 0.086355 |
| 12784 | -0.069491 | -0.243761 | 0.093312 | 0.089711 | 0.117505 | -0.125559 | 0.078475 | -0.493313 | -0.483602 | -0.361370 | -0.010317 |
| 11756 | -0.317425 | -0.146674 | -0.346224 | -0.090543 | -0.064812 | 0.017614 | 0.238264 | -0.082764 | -0.147349 | -0.323798 | -0.403139 |
| 12977 | -0.301178 | -0.257299 | -0.745618 | 0.039816 | 0.041993 | 0.332437 | 0.104844 | 0.010498 | -0.378356 | -0.719372 | -0.219867 |
| 12974 | 0.078309 | -0.181519 | 0.287525 | -0.291256 | 0.363434 | -0.113870 | 0.129352 | -0.043842 | 0.310271 | 0.186506 | -0.477986 |
| 8988 | 0.022076 | -0.786524 | -3.448062 | -0.079372 | 0.384441 | -0.256518 | 0.013411 | 0.634480 | 0.320769 | 0.290291 | 0.553256 |

In [70]:

```
for x in temp:
    if x in model.genes:
        for r in sorted(model.genes.get_by_id(x).reactions, key=lambda x: x.id):
            print(r, r.gene_reaction_rule)
    else:
        print(x, 'no reactions')
    print()
```

```
ALCD19y: glyald_c + h_c + nadph_c --> glyc_c + nadp_c 11882 or 12784 or 9774
ALCD22yi: 2mbald_c + h_c + nadph_c --> 2mbtoh_c + nadp_c 10029 or 11882 or 12784 or 13554 or 13562 or 13947 or 9774
ALCD2y: etoh_c + nadp_c --> acald_c + h_c + nadph_c 11882 or 12784 or 9774
ALR2: h_c + mthgxl_c + nadph_c --> acetol_c + nadp_c 11882 or 12784 or 9774
ALR3: acetol_c + h_c + nadph_c --> 12ppd__S_c + nadp_c 11882 or 12784 or 9774
ARABR: arab__L_c + h_c + nadph_c --> abt_c + nadp_c 9774
GALOR: gal_c + h_c + nadph_c <=> galt_c + nadp_c 11882 or 12784 or 9774
GLYCDy: glyc_c + nadp_c --> dha_c + h_c + nadph_c 11882 or 12784 or 9774
LALDO2: h_c + mthgxl_c + nadph_c --> lald__D_c + nadp_c 11882 or 12784 or 9774
PPDOy: h_c + lald__D_c + nadph_c --> 12ppd__R_c + nadp_c 10029 or 11882 or 12784 or 13554 or 13562 or 13947 or 9774
SBTR: glc__D_c + h_c + nadph_c --> nadp_c + sbt__D_c 11882 or 12784 or 9774
XYLR: h_c + nadph_c + xyl__D_c --> nadp_c + xylt_c 11882 or 13562 or 9774

ALCD19y: glyald_c + h_c + nadph_c --> glyc_c + nadp_c 11882 or 12784 or 9774
ALCD22yi: 2mbald_c + h_c + nadph_c --> 2mbtoh_c + nadp_c 10029 or 11882 or 12784 or 13554 or 13562 or 13947 or 9774
ALCD2y: etoh_c + nadp_c --> acald_c + h_c + nadph_c 11882 or 12784 or 9774
ALR2: h_c + mthgxl_c + nadph_c --> acetol_c + nadp_c 11882 or 12784 or 9774
ALR3: acetol_c + h_c + nadph_c --> 12ppd__S_c + nadp_c 11882 or 12784 or 9774
DKGLCNR1: 25dkglcn_c + h_c + nadph_c --> 2dhguln_c + nadp_c 11882
GALOR: gal_c + h_c + nadph_c <=> galt_c + nadp_c 11882 or 12784 or 9774
GLYCDy: glyc_c + nadp_c --> dha_c + h_c + nadph_c 11882 or 12784 or 9774
LALDO2: h_c + mthgxl_c + nadph_c --> lald__D_c + nadp_c 11882 or 12784 or 9774
PPDOy: h_c + lald__D_c + nadph_c --> 12ppd__R_c + nadp_c 10029 or 11882 or 12784 or 13554 or 13562 or 13947 or 9774
SBTR: glc__D_c + h_c + nadph_c --> nadp_c + sbt__D_c 11882 or 12784 or 9774
XYLR: h_c + nadph_c + xyl__D_c --> nadp_c + xylt_c 11882 or 13562 or 9774

ALCD19y: glyald_c + h_c + nadph_c --> glyc_c + nadp_c 11882 or 12784 or 9774
ALCD22yi: 2mbald_c + h_c + nadph_c --> 2mbtoh_c + nadp_c 10029 or 11882 or 12784 or 13554 or 13562 or 13947 or 9774
ALCD2y: etoh_c + nadp_c --> acald_c + h_c + nadph_c 11882 or 12784 or 9774
ALR2: h_c + mthgxl_c + nadph_c --> acetol_c + nadp_c 11882 or 12784 or 9774
ALR3: acetol_c + h_c + nadph_c --> 12ppd__S_c + nadp_c 11882 or 12784 or 9774
GALOR: gal_c + h_c + nadph_c <=> galt_c + nadp_c 11882 or 12784 or 9774
GLYCDy: glyc_c + nadp_c --> dha_c + h_c + nadph_c 11882 or 12784 or 9774
LALDO2: h_c + mthgxl_c + nadph_c --> lald__D_c + nadp_c 11882 or 12784 or 9774
PPDOy: h_c + lald__D_c + nadph_c --> 12ppd__R_c + nadp_c 10029 or 11882 or 12784 or 13554 or 13562 or 13947 or 9774
SBTR: glc__D_c + h_c + nadph_c --> nadp_c + sbt__D_c 11882 or 12784 or 9774

11756 no reactions

ABTD: abt_c + nad_c <=> h_c + nadh_c + xylu__L_c 12974 or 12977
SBTD_D2: nad_c + sbt__D_c --> fru_c + h_c + nadh_c 12974 or 12977
SBTD_L: nad_c + sbt__L_c --> h_c + nadh_c + srb__L_c 12974 or 12977
XYLTD_D: nad_c + xylt_c --> h_c + nadh_c + xylu__D_c 12974 or 12977 or 16452

ABTD: abt_c + nad_c <=> h_c + nadh_c + xylu__L_c 12974 or 12977
SBTD_D2: nad_c + sbt__D_c --> fru_c + h_c + nadh_c 12974 or 12977
SBTD_L: nad_c + sbt__L_c --> h_c + nadh_c + srb__L_c 12974 or 12977
XYLTD_D: nad_c + xylt_c --> h_c + nadh_c + xylu__D_c 12974 or 12977 or 16452

DABT2D: abt__D_c + nad_c <=> h_c + nadh_c + rbl__D_c 8988 or 9990
XYLUR: h_c + nadph_c + xylu__L_c <=> nadp_c + xylt_c 8988
```

In [71]:

```
temp = Annotation.index[Annotation['Combined Annotations'].str.contains('D-arab')]
display(Annotation.loc[temp])
Show_Data(temp)
```

|  | Combined Annotations | Signal P | Sc288c Orthologs | Human Orthologs | Sc288 Best Hit | Human Blast | Essential | WolfPSort | C Terminal |
| --- | --- | --- | --- | --- | --- | --- | --- | --- | --- |
| RTO4\_ID |  |  |  |  |  |  |  |  |  |
| 9990 | K17738: ARD; D-arabinitol 2-dehydrogenase | S |  |  | SPS19 | CBR4 | Not Essential | mito 21, cyto 5 | TLT\* |
| 13733 | K00107: ALO; D-arabinono-1,4-lactone oxidase |  |  |  | ALO1 |  | Not Essential | nucl 10, cyto\_nucl 10, cyto 8, mito 6 | EEG\* |
| 16713 | K00062: E1.1.1.116; D-arabinose 1-dehydrogenase |  | ARA2 |  | ARA2 |  | Not Essential | cyto 14, cyto\_nucl 9.5, mito 9, nucl 3 | VGV\* |

| strain | WT | | | | | | | | | | | | | | | | |
| --- | --- | --- | --- | --- | --- | --- | --- | --- | --- | --- | --- | --- | --- | --- | --- | --- | --- |
| condition | G\_MM | C\_MM | G\_SD | | GX\_SD | | | X\_SD | | A\_SD | | C\_SD | | MM\_CN120 | | MM\_CN5 | Diversity\_Sample |
| phase | exp | exp | exp | stat | exp | trans | stat | exp | stat | exp | stat | exp | stat | exp | stat | exp | exp |
| proteinId | Set1 | Set1 | Set2 | Set2 | Set2 | Set2 | Set2 | Set2 | Set2 | Set2 | Set2 | Set2 | Set2 | Set3 | Set3 | Set3 | Set3 |
| RTO4\_ID |  |  |  |  |  |  |  |  |  |  |  |  |  |  |  |  |  |
| 9990 | 5.140148 | 6.270216 | 5.551300 | 6.138047 | 7.867579 | 8.519055 | 7.743922 | 6.670194 | 5.112570 | 9.121079 | 4.531483 | 4.838953 | 5.820834 | 3.984943 | 3.966935 | 4.257002 | 5.559251 |
| 13733 | 4.580478 | 3.300484 | 5.499458 | 7.106518 | 5.694549 | 5.199855 | 5.723329 | 4.299556 | 6.525311 | 4.912754 | 6.230778 | 4.882364 | 6.905751 | 3.365627 | 3.910977 | 3.483697 | 3.174103 |
| 16713 | 4.672241 | 5.657333 | 4.434305 | 5.213714 | 4.574513 | 4.625441 | 4.489629 | 4.880408 | 4.408220 | 5.407260 | 5.515916 | 4.021728 | 5.295094 | 4.185870 | 4.526142 | 4.330894 | 5.508075 |

| strain | WT | | | | | | | | | | |
| --- | --- | --- | --- | --- | --- | --- | --- | --- | --- | --- | --- |
| condition | G\_SD | | GX\_SD | | | X\_SD | | A\_SD | | C\_SD | |
| proteinId | exp | stat | exp | trans | stat | exp | stat | exp | stat | exp | stat |
| 9990 | 2.458974 | 3.619681 | 7.315781 | 17.528749 | 16.692717 | 28.530481 | 23.529465 | 30.329072 | 27.392354 | 4.051601 | 3.506934 |
| 16713 | 3.671515 | 3.254368 | 4.492715 | 1.292670 | 3.708136 | 12.959579 | 11.040823 | 13.908368 | 10.327922 | 8.572039 | 8.254277 |

|  | Glucose | Xylose | Arabinose | Acetate | Coumarate | Ferulate | YNB Oleic Acid | YNB Ricinoleic Acid | YNB Glucose | YNB Gluc DOC | YPD |
| --- | --- | --- | --- | --- | --- | --- | --- | --- | --- | --- | --- |
| proteinId |  |  |  |  |  |  |  |  |  |  |  |
| 9990 | 0.029755 | -2.655320 | -2.324146 | 0.034732 | -0.215495 | 0.067559 | -0.190665 | -0.133194 | 0.095922 | -0.076464 | -0.211712 |
| 13733 | 0.140211 | -0.071017 | 0.107363 | 0.062523 | -0.227245 | 0.259340 | -0.101010 | -0.225138 | 0.118246 | 0.269142 | 0.136270 |
| 16713 | -0.063045 | 0.149404 | -0.075379 | -0.295168 | -0.010354 | -0.080829 | -0.050925 | -0.165690 | -0.258045 | 0.409666 | 0.046358 |

In [72]:

```
for x in temp:
    if x in model.genes:
        for r in sorted(model.genes.get_by_id(x).reactions, key=lambda x: x.id):
            print(r, r.gene_reaction_rule)
    else:
        print(x, 'no reactions')
    print()
```

```
DABT2D: abt__D_c + nad_c <=> h_c + nadh_c + rbl__D_c 8988 or 9990

ARAB14LO: Dara14lac_c + o2_c <=> ertascb__D_c + h2o2_c 10490 or 13733

UDPGALOR: h2o_c + 2.0 nad_c + udpgal_c --> 3.0 h_c + 2.0 nadh_c + udpgalur_c 16713
```

In [73]:

```
# D-arabinitol 2-dehydrogenase (D-arabinitol to D-ribulose), ARD1 9990
model.reactions.get_by_id('DABT2D').gene_reaction_rule = '9990'
r = sce.reactions.get_by_id('ARAB1D2').copy()
r.id = 'ARAB1D'
r.name = 'D-Arabinose 1-dehydrogenase NAD'
r.gene_reaction_rule = '16713'
model.add_reactions([r])
r.add_metabolites({'nadp_c': 1.0, 'nadph_c': -1.0, 'nad_c': -1.0, 'nadh_c': 1.0})
```

In [74]:

```
temp = (Fitness[['Xylose','Arabinose']].mean(axis=1) - Fitness.mean(axis=1)).sort_values().index[0:30]
display(Annotation.loc[temp])
```

|  | Combined Annotations | Signal P | Sc288c Orthologs | Human Orthologs | Sc288 Best Hit | Human Blast | Essential | WolfPSort | C Terminal |
| --- | --- | --- | --- | --- | --- | --- | --- | --- | --- |
| proteinId |  |  |  |  |  |  |  |  |  |
| 14368 | KOG2517: Ribulose kinase and related carbohydr... |  | YDR109C | FGGY | YDR109C | FGGY | Not Essential | cyto 14, cysk 6, mito 5 | LGE\* |
| 9990 | K17738: ARD; D-arabinitol 2-dehydrogenase | S |  |  | SPS19 | CBR4 | Not Essential | mito 21, cyto 5 | TLT\* |
| 8988 | K17742: SOU1; sorbose reductase |  |  |  |  | DHRS4 | Not Essential | cyto 17, cyto\_nucl 10, mito 8 | TLS\* |
| 9774 | K00002: AKR1A1, adh; alcohol dehydrogenase (NA... |  | YPR1,GCY1 | AKR1A1 | YPR1 | AKR1A | Not Essential | cyto 17.5, cyto\_mito 13.666, cyto\_nucl 10.333,... | KIK\* |
| 16825 | K02728: PSMA4; 20S proteasome subunit alpha 3 |  | PRE9 | PSMA4 | PRE9 | PSMA4 | Not Essential | mito 16, cyto\_nucl 6.5, cyto 5.5, nucl 4.5 | AIP\* |
| 12978 | HMMPfam:Fungal Zn(2)-Cys(6) binuclear cluster ... |  |  |  |  |  | Not Essential | mito 12, nucl 10, cyto 3 | DGL\* |
| 14231 | K09281: K09281; copper-fist transcription factor |  | CUP2 |  | CUP2 |  | Not Essential | nucl 11, cyto\_nucl 9.5, mito 7, cyto 6 | AFG\* |
| 14907 | K19800: SCH9; serine/threonine protein kinase ... |  | SCH9 |  | SCH9 | AKT2 | Not Essential | nucl 13.5, cyto\_nucl 10, mito 6, cyto 5.5 | DER\* |
| 10638 | K19833: CLA4; serine/threonine-protein kinase ... |  | CLA4,SKM1 |  | CLA4 | PAK1 | Not Essential | mito 26 | TAR\* |
| 11830 | K17978: NUM1; nuclear migration protein NUM1 |  |  |  | NUM1 |  | Essential | nucl 19, cyto\_nucl 14, cyto 5 | AIA\* |
| 11908 | K00784: rnz; ribonuclease Z |  |  | ELAC1 |  | ELAC1 | Not Essential | cyto 14.5, cyto\_nucl 9.5, mito 8, nucl 3.5 | KRT\* |
| 15310 | K15029: EIF3L; translation initiation factor 3... |  |  | EIF3L |  | EIF3L | Not Essential | cyto 12, cyto\_nucl 11.5, nucl 9, pero 4 | PSSP |
| 12898 | K00605: gcvT, AMT; aminomethyltransferase |  | GCV1 | AMT,RP11-949J7.8 | GCV1 | AMT | Not Essential | mito 23, cyto 4 | RGE\* |
| 16027 | BLAST: acetylornithine transaminase [Moesziomy... |  |  |  |  |  | Not Essential | mito 17, nucl 8 | FSA\* |
| 16630 | K01958: PC, pyc; pyruvate carboxylase |  | PYC2,PYC1 | PC | PYC2 | PCe | Not Essential | mito 19, cyto 7 | VHS\* |
| 11675 | K06268: PPP3R, CNB; serine/threonine-protein p... |  | CNB1 | PPP3R1 | CNB1 | PPP3R | Not Essential | cyto 13.5, cyto\_nucl 12, nucl 9.5, pero 3 | DMW\* |
| 13246 | K04348: PPP3C, CNA; serine/threonine-protein p... |  | CNA1,CMP2 | PPP3CA,PPP3CB,PPP3CC | CMP2 | PPP3C | Not Essential | nucl 16, mito 5, cyto 4 | PAR\* |
| 14687 | KOG1729: FYVE finger containing protein |  |  |  |  |  | Not Essential | nucl 14, mito 11, cyto\_nucl 9 | ATF\* |
| 12960 | K11655: BAZ1A, ACF1; bromodomain adjacent to z... | S | ITC1 |  | ITC1 |  | Not Essential | mito 13, nucl 7, cyto 7, cyto\_nucl 7 | ARF\* |
| 10259 | K03083: GSK3B; glycogen synthase kinase 3 beta |  | MRK1,RIM11 | GSK3A,GSK3B | RIM11 | GSK3B | Not Essential | cyto\_nucl 10.833, nucl 10, cyto 9.5, cyto\_pero... | HLD\* |
| 14088 | K04392: RAC1; Ras-related C3 botulinum toxin s... |  |  | RAC2,RAC3,RAC1 | CDC42 | RAC1 | Not Essential | cyto 22, nucl 3 | VVC\* |
| 11242 | K00555: TRMT1, trm1; tRNA (guanine26-N2/guanin... |  | TRM1 | TRMT1 | TRM1 | TRMT1 | Not Essential | cyto 20.5, cyto\_nucl 12.5, nucl 3.5 | PQK\* |
| 11383 | KOG3252: Uncharacterized conserved protein |  |  | EIF3K |  |  | Not Essential | nucl 10, mito 5, cyto 5, cyto\_mito 5 | SQA\* |
| 12605 | K15100: SLC25A1, CTP; solute carrier family 25... | S | SFC1 |  | SFC1 | SLC25A | Not Essential | mito 11, cyto 9.5, cyto\_nucl 5.5, plas 3 | YSE\* |
| 11667 | K19704: PTC1; protein phosphatase PTC1 |  | PTC1 | PPM1L | PTC1 | PPM1B | Not Essential | nucl 16, cyto\_nucl 15.5, cyto 9 | ASA\* |
| 12007 | K11204: GCLC; glutamate--cysteine ligase catal... | S | GSH1 | GCLC | GSH1 | GCLC | Essential | plas 8, cyto 5, pero 5, cyto\_pero 5, nucl 2, m... | KEA\* |
| 11884 | K01466: allB; allantoinase |  | DAL1 |  | DAL1 |  | Not Essential | mito 17, cyto 6, extr 2, pero 2 | LLL\* |
| 11622 | K12161: URM1; ubiquitin related modifier 1 |  | URM1 | URM1 | URM1 | URM1 | Essential | cysk 24, nucl 1, mito 1, cyto 1, cyto\_nucl 1, ... | HGG\* |
| 10123 | K00326: E1.6.2.2; cytochrome-b5 reductase |  | IRC21 | CYB5R4 | IRC21 | CYB5R | Not Essential | cyto 14.5, cyto\_nucl 9.5, mito 5, nucl 3.5, ex... | VPE\* |
| 14857 | KOG0327: Translation initiation factor 4F, hel... |  |  |  | FAL1 | EIF4A3 | Not Essential | mito 17, nucl 7, cyto 2 | ASV\* |

In [75]:

```
Show_Data(temp)
```

| strain | WT | | | | | | | | | | | | | | | | |
| --- | --- | --- | --- | --- | --- | --- | --- | --- | --- | --- | --- | --- | --- | --- | --- | --- | --- |
| condition | G\_MM | C\_MM | G\_SD | | GX\_SD | | | X\_SD | | A\_SD | | C\_SD | | MM\_CN120 | | MM\_CN5 | Diversity\_Sample |
| phase | exp | exp | exp | stat | exp | trans | stat | exp | stat | exp | stat | exp | stat | exp | stat | exp | exp |
| proteinId | Set1 | Set1 | Set2 | Set2 | Set2 | Set2 | Set2 | Set2 | Set2 | Set2 | Set2 | Set2 | Set2 | Set3 | Set3 | Set3 | Set3 |
| proteinId |  |  |  |  |  |  |  |  |  |  |  |  |  |  |  |  |  |
| 14368 | 5.776084 | 6.227531 | 5.811947 | 5.807621 | 7.255347 | 7.811263 | 7.351152 | 6.480310 | 5.229182 | 8.068085 | 4.320579 | 5.204209 | 5.328031 | 6.096076 | 5.921803 | 6.124689 | 6.907272 |
| 9990 | 5.140148 | 6.270216 | 5.551300 | 6.138047 | 7.867579 | 8.519055 | 7.743922 | 6.670194 | 5.112570 | 9.121079 | 4.531483 | 4.838953 | 5.820834 | 3.984943 | 3.966935 | 4.257002 | 5.559251 |
| 8988 | 6.921572 | 6.927083 | 6.704075 | 7.113596 | 8.461796 | 9.022018 | 8.656900 | 7.820671 | 6.173585 | 10.074108 | 4.987534 | 5.788663 | 6.255932 | 7.337214 | 7.159274 | 6.993421 | 8.030642 |
| 9774 | 9.271928 | 8.073898 | 9.068550 | 8.595213 | 9.814367 | 9.928081 | 9.238037 | 9.637172 | 7.833486 | 10.562301 | 7.022552 | 5.690787 | 5.819589 | 10.063638 | 9.334496 | 10.845508 | 10.376461 |
| 16825 | 7.657076 | 7.295940 | 7.783574 | 7.061624 | 7.843723 | 7.004277 | 7.001619 | 7.411057 | 6.942895 | 7.343842 | 7.016305 | 7.378978 | 7.188635 | 5.148649 | 5.191857 | 6.328836 | 5.262811 |
| 12978 | 4.393423 | 5.194438 | 4.885704 | 3.588745 | 4.824495 | 4.491962 | 4.112972 | 4.695589 | 3.937322 | 4.343928 | 3.496735 | 4.926042 | 4.734740 | 4.609521 | 4.404837 | 4.752908 | 4.446094 |
| 14231 | 3.767794 | 3.275734 | 4.230421 | 4.601045 | 4.061288 | 4.569642 | 4.508152 | 4.305712 | 4.503221 | 4.397954 | 4.873487 | 3.512105 | 3.874199 | 4.182534 | 4.471764 | 3.599620 | 3.488654 |
| 14907 | 5.434381 | 5.923431 | 6.590289 | 6.345614 | 6.524679 | 6.415555 | 6.455967 | 6.295468 | 6.262432 | 6.019095 | 6.055488 | 6.323935 | 7.258583 | 6.452799 | 6.426358 | 6.814517 | 6.286002 |
| 10638 | 5.010081 | 5.200528 | 5.270558 | 4.492927 | 5.264826 | 5.241179 | 5.071123 | 5.567130 | 5.160392 | 5.446604 | 5.117007 | 4.750307 | 4.756007 | 5.825697 | 5.674705 | 6.107995 | 5.963032 |
| 11830 | 5.241348 | 5.117693 | 5.744378 | 3.121037 | 5.711036 | 4.207936 | 3.935266 | 5.427505 | 3.476500 | 5.678690 | 3.678348 | 5.690816 | 6.438362 | 5.595714 | 4.880833 | 5.522870 | 5.341345 |
| 11908 | 4.629421 | 4.505432 | 5.076302 | 5.790723 | 4.838402 | 5.505165 | 5.570286 | 5.073087 | 5.511350 | 4.905311 | 5.315808 | 5.547394 | 5.962090 | 5.191139 | 5.447135 | 5.010241 | 5.229623 |
| 15310 | 4.758671 | 6.623252 | 5.291949 | 5.000084 | 5.278941 | 5.507569 | 5.518476 | 5.954043 | 5.034647 | 5.528235 | 4.916162 | 6.452825 | 6.439472 | 6.108606 | 6.244067 | 7.262856 | 7.931291 |
| 12898 | 5.307788 | 6.161769 | 7.473573 | 6.710837 | 7.643771 | 6.045502 | 6.113622 | 6.043729 | 6.201039 | 5.735288 | 6.243914 | 6.829441 | 6.504322 | 7.567333 | 5.703011 | 8.519396 | 6.162556 |
| 16027 | 6.445118 | 6.194509 | 5.840884 | 7.179176 | 5.869491 | 6.709972 | 6.793854 | 6.150317 | 6.809733 | 6.299903 | 7.314410 | 5.475688 | 5.595994 | 1.821197 | 2.206319 | 1.653705 | 1.616254 |
| 16630 | 8.600103 | 6.017670 | 9.144380 | 6.227308 | 9.344694 | 7.833242 | 7.585591 | 8.482202 | 5.895634 | 6.964134 | 5.591453 | 5.698963 | 4.664978 | 10.595575 | 10.248537 | 10.332566 | 9.338825 |
| 11675 | 6.538519 | 6.410405 | 5.983165 | 6.213769 | 5.970772 | 5.969567 | 5.882642 | 6.125166 | 5.840003 | 6.388899 | 5.969493 | 5.990429 | 5.560666 | 5.977265 | 6.159184 | 6.248522 | 5.760920 |
| 13246 | 6.167613 | 5.621701 | 6.217587 | 5.884295 | 6.257224 | 6.112772 | 6.014357 | 6.082779 | 6.179484 | 5.899378 | 6.051576 | 5.545245 | 5.834088 | 6.586915 | 6.308768 | 6.512005 | 6.305700 |
| 14687 | 6.796413 | 5.931134 | 6.298900 | 6.762061 | 6.254959 | 6.644822 | 6.587171 | 6.653109 | 7.278577 | 6.634430 | 6.760998 | 5.572418 | 6.306180 | 7.768629 | 7.525835 | 7.535958 | 7.777560 |
| 12960 | 4.821116 | 5.244905 | 5.682222 | 4.899644 | 5.780161 | 5.082039 | 5.241646 | 5.227766 | 5.284498 | 5.027882 | 5.400520 | 5.299087 | 5.498660 | 4.973487 | 4.911453 | 5.452867 | 5.592306 |
| 10259 | 8.180610 | 7.858502 | 7.962906 | 8.895652 | 8.049826 | 7.329053 | 7.970500 | 7.743626 | 8.193206 | 7.972524 | 8.403013 | 8.306628 | 9.294979 | 9.243696 | 8.507480 | 9.961993 | 9.200218 |
| 14088 | 7.124818 | 6.545084 | 6.399346 | 6.124604 | 6.477427 | 7.216650 | 6.817667 | 7.151096 | 6.389254 | 7.023833 | 6.251058 | 6.140775 | 6.032613 | 7.992150 | 7.729545 | 7.419086 | 7.312643 |
| 11242 | 4.744573 | 5.670114 | 4.262338 | 3.931542 | 4.341434 | 5.013880 | 5.068279 | 5.137791 | 4.392416 | 4.950080 | 4.506974 | 5.371403 | 5.144817 | 4.083339 | 4.711707 | 4.305553 | 4.952082 |
| 11383 | 5.528824 | 6.476328 | 5.290811 | 5.037567 | 5.538741 | 5.444289 | 5.270147 | 6.029413 | 4.994987 | 5.509489 | 4.896593 | 6.704247 | 6.268307 | 5.120482 | 5.128856 | 6.249786 | 6.034535 |
| 12605 | 3.681421 | 7.627145 | 6.931591 | 5.795483 | 7.108998 | 6.203548 | 6.259582 | 5.075519 | 7.141392 | 5.116748 | 6.202725 | 7.178804 | 6.930891 | 4.276844 | 4.240066 | 5.168802 | 5.356117 |
| 11667 | 6.114910 | 5.939114 | 6.094351 | 6.579407 | 6.063361 | 5.934709 | 5.999736 | 5.848002 | 6.429730 | 6.215963 | 6.160035 | 6.575295 | 6.825235 | 6.194482 | 6.337127 | 5.825841 | 5.931981 |
| 12007 | 6.097840 | 5.754361 | 6.179686 | 5.702429 | 6.255533 | 5.908247 | 5.865802 | 6.368461 | 5.971235 | 6.348352 | 5.852696 | 5.635243 | 6.136678 | 5.868239 | 5.649197 | 6.857549 | 6.412990 |
| 11884 | 5.857438 | 5.132178 | 3.691243 | 3.237350 | 3.589302 | 3.651091 | 4.154785 | 3.899773 | 4.447212 | 4.128709 | 4.533818 | 5.890882 | 7.070183 | 5.167511 | 4.773825 | 3.496817 | 5.398494 |
| 11622 | 7.657299 | 6.569605 | 6.126756 | 6.246573 | 6.052266 | 6.101014 | 5.931865 | 5.849365 | 6.121766 | 5.936027 | 6.520933 | 5.823150 | 5.371949 | 4.552555 | 4.984336 | 4.940352 | 4.448353 |
| 10123 | 4.996453 | 5.145310 | 4.895604 | 5.132273 | 4.953597 | 5.216479 | 5.178914 | 5.182072 | 5.108739 | 5.009333 | 5.266435 | 5.490169 | 5.695681 | 4.628906 | 4.859112 | 4.891222 | 4.616334 |
| 14857 | 3.605963 | 4.620171 | 3.947006 | 2.945722 | 3.998795 | 4.517789 | 4.209226 | 4.413918 | 4.118755 | 4.129911 | 3.414241 | 4.794485 | 4.903466 | 5.409916 | 5.164701 | 4.771891 | 5.194882 |

| strain | WT | | | | | | | | | | |
| --- | --- | --- | --- | --- | --- | --- | --- | --- | --- | --- | --- |
| condition | G\_SD | | GX\_SD | | | X\_SD | | A\_SD | | C\_SD | |
| proteinId | exp | stat | exp | trans | stat | exp | stat | exp | stat | exp | stat |
| 14368 | 10.340991 | 11.803987 | 13.224373 | 23.626670 | 22.968584 | 41.126832 | 32.257663 | 39.598535 | 34.104908 | 13.244333 | 10.459359 |
| 9990 | 2.458974 | 3.619681 | 7.315781 | 17.528749 | 16.692717 | 28.530481 | 23.529465 | 30.329072 | 27.392354 | 4.051601 | 3.506934 |
| 8988 | 2.704090 | 3.242278 | 6.707344 | 9.871449 | 8.545995 | 26.373292 | 17.154925 | 29.346260 | 25.139935 | 4.055205 | 2.832854 |
| 9774 | 24.036105 | 20.651004 | 27.252847 | 17.324598 | 17.678054 | 36.939606 | 32.903871 | 40.948526 | 37.872681 | 18.573235 | 14.841079 |
| 16825 | 5.575624 | 5.921073 | 4.863848 | 5.190346 | 3.630075 | 5.253878 | 3.921091 | 5.408820 | 7.094937 | 5.109249 | 5.012433 |
| 12978 | 0.000000 | 0.000000 | 0.000000 | 0.187227 | 0.191624 | 0.000000 | 0.000000 | 0.000000 | 0.000000 | 0.000000 | 0.000000 |
| 14231 | 0.000000 | 0.000000 | 0.197656 | 0.189313 | 0.000000 | 0.000000 | 0.000000 | 0.193364 | 0.000000 | 0.000000 | 0.000000 |
| 14907 | 1.148424 | 1.221965 | 1.220783 | 0.566765 | 0.179978 | 0.961093 | 0.377624 | 0.000000 | 0.000000 | 0.219601 | 0.657552 |
| 10638 | 4.419640 | 3.207234 | 3.860863 | 3.166471 | 3.831919 | 4.877761 | 3.298743 | 5.026319 | 4.002013 | 1.510662 | 1.516087 |
| 11830 | 0.190197 | 0.000000 | 0.000000 | 0.000000 | 0.173732 | 0.000000 | 0.000000 | 0.000000 | 0.000000 | 0.000000 | 0.217934 |
| 11908 | 0.791247 | 0.726275 | 0.203508 | 1.107620 | 1.113802 | 0.200926 | 0.589595 | 0.577661 | 0.385304 | 0.418217 | 0.867663 |
| 15310 | 13.267448 | 6.964978 | 9.750040 | 6.135045 | 5.799322 | 7.026452 | 6.116496 | 6.568684 | 5.005076 | 11.569232 | 8.905338 |
| 12898 | 16.929126 | 17.166807 | 13.430814 | 12.083050 | 13.134370 | 8.189113 | 11.832851 | 9.288999 | 8.258173 | 12.178332 | 18.309495 |
| 16027 | 1.395364 | 0.905146 | 1.016038 | 0.376996 | 1.632167 | 0.793685 | 0.986719 | 0.965175 | 0.384727 | 1.073656 | 1.072362 |
| 16630 | 88.987656 | 79.271116 | 88.285999 | 80.725819 | 73.994782 | 68.868398 | 69.716810 | 63.943916 | 57.831263 | 53.569103 | 45.691702 |
| 11675 | 0.395333 | 0.585564 | 1.227975 | 1.112259 | 1.635137 | 2.156629 | 1.363573 | 1.156579 | 1.332108 | 1.700869 | 1.740039 |
| 13246 | 7.233820 | 7.981219 | 8.160070 | 8.740758 | 8.593549 | 8.666791 | 7.116156 | 8.491345 | 6.136276 | 5.953106 | 6.979853 |
| 12960 | 4.232749 | 4.686197 | 4.891986 | 6.680994 | 5.130539 | 5.064540 | 5.719182 | 5.224266 | 6.691931 | 5.104761 | 5.669606 |
| 10259 | 11.866510 | 14.255316 | 10.981010 | 11.318092 | 10.770467 | 13.335531 | 12.242184 | 16.611618 | 12.838990 | 16.219809 | 15.005675 |
| 14088 | 3.870824 | 3.942753 | 2.842307 | 5.199966 | 3.635195 | 4.297389 | 4.733541 | 4.826502 | 4.791259 | 2.142690 | 2.838871 |
| 11242 | 2.685686 | 3.183095 | 1.824277 | 2.060647 | 2.000196 | 2.363202 | 2.931427 | 2.704838 | 2.481875 | 1.916021 | 1.515443 |
| 11383 | 3.638877 | 2.899856 | 5.706610 | 2.598034 | 2.887751 | 5.085376 | 3.142035 | 5.020583 | 4.204003 | 4.482635 | 5.439069 |
| 12605 | 7.943939 | 7.960080 | 6.705546 | 6.696635 | 6.410543 | 6.063282 | 6.212271 | 2.319345 | 4.387250 | 16.656289 | 18.100491 |
| 11667 | 5.385591 | 5.370355 | 4.074139 | 5.401948 | 5.799905 | 5.472479 | 5.507094 | 5.027415 | 4.966601 | 5.334302 | 3.925426 |
| 12007 | 6.901959 | 5.290467 | 4.266234 | 3.718712 | 2.878656 | 7.663209 | 5.696262 | 6.573644 | 5.162723 | 0.428597 | 0.000000 |
| 11622 | 0.589555 | 0.886066 | 0.604249 | 0.560368 | 0.192891 | 0.777899 | 0.605825 | 0.776980 | 0.196097 | 0.861785 | 0.874457 |
| 14857 | 1.165091 | 0.000000 | 1.843984 | 0.000000 | 0.192891 | 2.347578 | 0.000000 | 0.963153 | 0.000000 | 0.220910 | 0.000000 |

|  | Glucose | Xylose | Arabinose | Acetate | Coumarate | Ferulate | YNB Oleic Acid | YNB Ricinoleic Acid | YNB Glucose | YNB Gluc DOC | YPD |
| --- | --- | --- | --- | --- | --- | --- | --- | --- | --- | --- | --- |
| proteinId |  |  |  |  |  |  |  |  |  |  |  |
| 14368 | -0.063355 | -2.897249 | -3.097320 | -0.236475 | -0.180356 | -0.182745 | -0.133641 | -0.115522 | 0.064443 | 0.127896 | 0.410221 |
| 9990 | 0.029755 | -2.655320 | -2.324146 | 0.034732 | -0.215495 | 0.067559 | -0.190665 | -0.133194 | 0.095922 | -0.076464 | -0.211712 |
| 8988 | 0.022076 | -0.786524 | -3.448062 | -0.079372 | 0.384441 | -0.256518 | 0.013411 | 0.634480 | 0.320769 | 0.290291 | 0.553256 |
| 9774 | 0.023569 | -0.715210 | -3.895191 | -0.087274 | -0.071053 | -0.088020 | -0.002179 | -0.262330 | 0.073917 | -0.149324 | -0.009339 |
| 16825 | -1.190554 | -2.781091 | -2.456259 | -1.328365 | 0.518200 | -1.247380 | -0.684144 | -2.337145 | 0.316859 | 0.971549 | -0.032245 |
| 12978 | 0.144797 | -2.166005 | -1.714752 | 0.004084 | 0.073762 | 0.106282 | 0.082745 | 0.372322 | 0.001249 | 0.031167 | 0.237146 |
| 14231 | -0.475849 | -1.407831 | -1.093003 | -0.041419 | 2.109775 | -0.143555 | 0.512844 | -0.531368 | 0.754655 | 0.942348 | 1.651097 |
| 14907 | -0.025541 | -0.813168 | -1.090047 | 0.028434 | 2.148325 | 0.191626 | 0.601384 | 1.862324 | 0.860291 | 0.935602 | -0.126388 |
| 10638 | -0.267514 | -0.160118 | -0.156394 | -0.557828 | 3.742110 | -0.128743 | 1.828019 | 3.321855 | 1.519815 | 1.555898 | 0.506878 |
| 11830 | -0.950361 | -1.689199 | -1.283553 | -0.639935 | -0.630597 | -0.412000 | 0.361179 | 0.393314 | 0.357537 | -0.743900 | 1.419607 |
| 11908 | 0.000274 | -1.915485 | -0.953106 | 0.077580 | -0.068595 | -0.163133 | -0.560409 | -0.411017 | 0.138741 | -0.263021 | 0.148978 |
| 15310 | -0.472431 | -0.993298 | -1.492902 | -0.354204 | -0.665789 | -0.026652 | 1.259664 | 0.521623 | -0.399857 | -0.503422 | 0.023608 |
| 12898 | -0.542249 | -1.881551 | -1.449178 | -0.407967 | -1.065138 | -0.406853 | -0.495496 | -1.372018 | 0.206331 | -0.653699 | 0.171222 |
| 16027 | -1.148735 | -2.146438 | -2.458712 | -1.366891 | -0.018297 | -2.008653 | -0.833220 | -3.988051 | -0.523987 | -0.554986 | 0.070170 |
| 16630 | -1.816582 | -1.351364 | -2.020599 | 0.241922 | 0.338143 | 0.009054 | -0.137500 | 0.217258 | -3.248045 | -0.067210 | -0.368913 |
| 11675 | 0.343271 | 0.451518 | 0.303402 | 0.134154 | 2.626488 | 0.586446 | 2.417653 | 3.385683 | 1.921475 | 1.935669 | 0.249082 |
| 13246 | 0.432142 | 0.464680 | 0.497252 | 0.179205 | 3.250962 | 0.554002 | 2.403642 | 3.404119 | 2.080189 | 2.084402 | 0.091696 |
| 14687 | -0.092741 | -1.035040 | -0.528016 | -0.153592 | 2.132973 | -0.177259 | 0.964575 | -0.261815 | 0.328885 | 0.304779 | -0.085737 |
| 12960 | -2.434875 | -2.059322 | -1.607262 | -0.128753 | 2.301998 | -2.454945 | 1.267180 | -0.214968 | -2.058243 | -2.436081 | -0.416200 |
| 10259 | -1.574993 | -2.562452 | -3.456225 | -5.513556 | -3.115012 | -3.269113 | -2.597398 | -2.224493 | 0.105425 | 0.399633 | 0.626319 |
| 14088 | 0.398580 | 0.363226 | 0.348795 | 0.114067 | 4.517712 | 0.267570 | 2.023168 | 3.163266 | 1.173203 | 1.012444 | 0.407168 |
| 11242 | -0.395120 | -1.266916 | -1.004139 | 0.075248 | -1.478580 | -0.366638 | -1.102536 | -0.031490 | 0.315877 | 0.873956 | 1.747957 |
| 11383 | 0.535277 | -0.671012 | -0.728524 | -0.240596 | -0.308197 | -0.203357 | -0.422809 | -0.027091 | 0.648023 | 1.295597 | 2.253522 |
| 12605 | -0.250531 | -1.121243 | -1.276143 | -1.681837 | -1.691549 | -1.568832 | 0.318931 | 0.287611 | 1.114286 | 0.164320 | 2.236104 |
| 11667 | -0.251929 | -0.959013 | -0.680577 | -0.074107 | 0.044549 | -0.102539 | 0.528601 | 0.559751 | 0.360250 | 0.547672 | 0.544112 |
| 12007 | -0.168424 | -0.748662 | -1.271822 | 0.092471 | -0.284501 | -0.368200 | 1.410755 | 0.913490 | -0.266877 | 0.979791 | -2.410653 |
| 11884 | -0.548788 | -0.845153 | -0.394338 | -0.198931 | -0.142190 | -0.293763 | 0.780776 | 0.561183 | 1.451791 | 1.247830 | 0.531065 |
| 11622 | -1.697296 | -0.675386 | -1.461013 | -0.760873 | -0.533253 | -1.707474 | 1.339853 | 1.572138 | 0.573111 | 0.859727 | -0.471581 |
| 10123 | 0.408984 | 0.636526 | 0.447398 | 0.299736 | 2.175350 | 0.260516 | 2.198690 | 4.424063 | 1.771196 | 1.781014 | 0.252354 |
| 14857 | 0.129709 | -0.466578 | -0.236669 | 0.155032 | 0.367799 | 0.098074 | 1.038497 | 1.566958 | 0.888191 | 0.995606 | 0.212929 |

In [76]:

```
for x in temp:
    if x in model.genes:
        for r in sorted(model.genes.get_by_id(x).reactions, key=lambda x: x.id):
            print(r, r.gene_reaction_rule)
    else:
        print(x, 'no reactions')
    print()
```

```
RBK_Dr: atp_c + rbl__D_c --> adp_c + h_c + ru5p__D_c 14368

DABT2D: abt__D_c + nad_c <=> h_c + nadh_c + rbl__D_c 9990

XYLUR: h_c + nadph_c + xylu__L_c <=> nadp_c + xylt_c 8988

ALCD19y: glyald_c + h_c + nadph_c --> glyc_c + nadp_c 11882 or 12784 or 9774
ALCD22yi: 2mbald_c + h_c + nadph_c --> 2mbtoh_c + nadp_c 10029 or 11882 or 12784 or 13554 or 13562 or 13947 or 9774
ALCD2y: etoh_c + nadp_c --> acald_c + h_c + nadph_c 11882 or 12784 or 9774
ALR2: h_c + mthgxl_c + nadph_c --> acetol_c + nadp_c 11882 or 12784 or 9774
ALR3: acetol_c + h_c + nadph_c --> 12ppd__S_c + nadp_c 11882 or 12784 or 9774
ARABR: arab__L_c + h_c + nadph_c --> abt_c + nadp_c 9774
GALOR: gal_c + h_c + nadph_c <=> galt_c + nadp_c 11882 or 12784 or 9774
GLYCDy: glyc_c + nadp_c --> dha_c + h_c + nadph_c 11882 or 12784 or 9774
LALDO2: h_c + mthgxl_c + nadph_c --> lald__D_c + nadp_c 11882 or 12784 or 9774
PPDOy: h_c + lald__D_c + nadph_c --> 12ppd__R_c + nadp_c 10029 or 11882 or 12784 or 13554 or 13562 or 13947 or 9774
SBTR: glc__D_c + h_c + nadph_c --> nadp_c + sbt__D_c 11882 or 12784 or 9774
XYLR: h_c + nadph_c + xyl__D_c --> nadp_c + xylt_c 11882 or 13562 or 9774

16825 no reactions

12978 no reactions

14231 no reactions

14907 no reactions

10638 no reactions

11830 no reactions

11908 no reactions

15310 no reactions

GLYCLm: gly_m + nad_m + thf_m --> co2_m + mlthf_m + nadh_m + nh4_m 10040 and 10205 and 12898 and 15184

16027 no reactions

PC: atp_c + hco3_c + pyr_c --> adp_c + h_c + oaa_c + pi_c 16630

11675 no reactions

13246 no reactions

14687 no reactions

12960 no reactions

10259 no reactions

14088 no reactions

11242 no reactions

11383 no reactions

SUCFUMtm: fum_m + succ_c --> fum_c + succ_m 12605

11667 no reactions

GLUCYS: atp_c + cys__L_c + glu__L_c --> adp_c + glucys_c + h_c + pi_c 12007

ALLTN: alltn_c + h2o_c --> alltt_c + h_c 11884

11622 no reactions

10123 no reactions

14857 no reactions
```

In [77]:

```
temp = (Fitness[['Xylose','Arabinose']].mean(axis=1) - Fitness.mean(axis=1)).sort_values(ascending=False).index[0:30]
display(Annotation.loc[temp])
```

|  | Combined Annotations | Signal P | Sc288c Orthologs | Human Orthologs | Sc288 Best Hit | Human Blast | Essential | WolfPSort | C Terminal |
| --- | --- | --- | --- | --- | --- | --- | --- | --- | --- |
| proteinId |  |  |  |  |  |  |  |  |  |
| 11990 | KOG0254: Predicted transporter (major facilita... |  | RGT2,SNF3 |  | SNF3 | SLC2A | Not Essential | plas 24, mito 1, E.R. 1, golg 1 | LEV\* |
| 12292 | KOG1993: Nuclear transport receptor KAP120 (im... |  | KAP120 | IPO11 | KAP120 | IPO11 | Not Essential | cyto 6, mito 5, plas 5, cyto\_nucl 5, nucl 4, E... | LAG\* |
| 15999 | KOG0226: RNA-binding proteins |  |  | RBM42 | PUB1 | RBM42 | Not Essential | nucl 21.5, cyto\_nucl 12, mito 3 | IRR\* |
| 16280 | K11434: PRMT1; type I protein arginine methylt... |  | HMT1 | PRMT1,PRMT8 | HMT1 | PRMT1 | Not Essential | cyto 20.5, cyto\_nucl 11.5, pero 4 | RMA\* |
| 9331 | K15109: SLC25A20\_29, CACT, CACL, CRC1; solute ... |  | CRC1 | SLC25A20 | CRC1 | SLC25A | Not Essential | mito 13.5, cyto\_mito 12.5, cyto 10.5 | ALF\* |
| 12588 | K19932: NCS1; neuronal calcium sensor 1 |  | FRQ1 | NCS1,HPCAL1 | FRQ1 | NCS1 | Not Essential | cyto 11.5, nucl 10, cyto\_pero 7, mito 2, pero 1.5 | GLV\* |
| 9364 | K19791: FET3\_5; iron transport multicopper oxi... | S | FET5,FET3 |  | FET3 |  | Not Essential | extr 21, plas 3, E.R. 2 | AAQ\* |
| 10955 | K04368: MAP2K1, MEK1; mitogen-activated protei... |  | STE7 | MAP2K1,MAP2K2,RP11-286H14.4 | PBS2 | MAP2K1 | Not Essential | nucl 14, cyto\_nucl 14, cyto 12 | SIP\* |
| 10025 | K08286: E2.7.11.-; protein-serine/threonine ki... |  | SAT4 |  | SAT4 | CHEK1 | Not Essential | nucl 14, cyto\_nucl 12, cyto 8, mito 4 | NAP\* |
| 11412 | K11771: SWI1, ADR6; SWI/SNF chromatin-remodeli... |  |  |  |  |  | Not Essential | nucl 23, cyto\_nucl 14 | SVF\* |
| 11732 | K15423: PPP4C; serine/threonine-protein phosph... |  | PPH3 | PPP4C | PPH3 | PPP4C | Not Essential | cyto\_nucl 9.333, cyto 9, cyto\_pero 8.833, nucl... | YFL\* |
| 15484 | K04393: CDC42; cell division control protein 42 |  | CDC42 | CDC42 | CDC42 | CDC42 | Not Essential | cyto 17, cyto\_nucl 13.833, nucl 8.5, mito\_nucl... | SIL\* |
| 14061 | KOG4186: Peroxisomal biogenesis protein (peroxin) |  | PEX11 |  | PEX11 | PEX11A | Not Essential | mito 17, cyto 5, extr 3 | AAK\* |
| 9193 | HMMPfam:Modifier of rudimentary (Mod(r)) prote... |  |  |  |  |  | Not Essential | nucl 11.5, cyto\_nucl 10, cyto 7.5, mito 4, pero 3 | WMA\* |
| 8667 | ProSiteProfiles:SET domain profile.:PS50280,SU... |  |  |  |  |  | Not Essential | nucl 17.5, cyto\_nucl 13.833, cyto 7, cyto\_mito... | QRS\* |
| 11722 | KOG2395: Protein involved in vacuole import an... |  | VID27 |  | VID27 |  | Not Essential | cyto 12.5, nucl 9, cyto\_mito 8, mito 2.5 | DGL\* |
| 13276 | K15163: SRB8, MED12; mediator of RNA polymeras... |  |  |  | SRB8 |  | Not Essential | mito 10, plas 8, nucl 5, E.R. 2 | RKK\* |
| 10331 | K00767: nadC, QPRT; nicotinate-nucleotide pyro... |  | BNA6 | QPRT | BNA6 | QPRT | Not Essential | mito 14, cyto 12 | PRR\* |
| 14022 | K01637: E4.1.3.1, aceA; isocitrate lyase |  | ICL1,ICL2 |  | ICL1 |  | Not Essential | cyto\_nucl 9.5, cyto 8.5, nucl 7.5, pero 7, mito 2 | QFK\* |
| 15784 |  |  |  |  |  |  | Not Essential | mito 15, nucl 10, cyto\_nucl 7 | IFA\* |
| 15471 |  |  |  |  |  |  | Not Essential | cyto\_nucl 9, nucl 8.5, cyto 6.5, pero 6, mito 5 | LLQ\* |
| 13278 | K14555: UTP13, TBL3; U3 small nucleolar RNA-as... |  | UTP13 | TBL3 | UTP13 | TBL3 | Essential | nucl 11, mito 8, cyto 6 | EEE\* |
| 9226 | K10599: PRPF19, PRP19; pre-mRNA-processing fac... |  | PRP19 | PRPF19 | PRP19 | PRPF19 | Not Essential | cyto 13.5, cyto\_mito 9.5, nucl 8, mito 4.5 | KPE\* |
| 8602 | K00452: HAAO; 3-hydroxyanthranilate 3,4-dioxyg... |  | BNA1 | HAAO | BNA1 | HAAO | Not Essential | cyto\_nucl 12.833, cyto 12.5, nucl 11, cyto\_per... | EAK\* |
| 8664 | K20177: VPS3, TGFBRAP1; vacuolar protein sorti... | S |  |  |  |  | Not Essential | cyto 14.5, nucl 9, cyto\_mito 9 | DLR\* |
| 10274 | KOG0627: Heat shock transcription factor |  |  |  | HSF1 | HSF2 | Not Essential | nucl 13, mito 6, extr 4, cyto\_mito 4 | TDG\* |
| 10310 | HMMPfam:PLD-like domain:PF13091,ProSiteProfile... |  |  |  |  |  | Not Essential | cyto 9, cyto\_nucl 8, pero 8, nucl 5, mito 4 | AVI\* |
| 9964 | K14557: UTP6; U3 small nucleolar RNA-associate... |  | UTP6 | UTP6 | UTP6 |  | Not Essential | nucl 13, cyto 11, mito 2 | FEQ\* |
| 8442 | KOG3600: Thyroid hormone receptor-associated p... |  |  |  |  |  | Not Essential | mito 14, nucl 10, cyto 2 | IIE\* |
| 13851 | KOG1339: Aspartyl protease | S |  |  | PEP4 | CTSD | Not Essential | extr 18, plas 6, E.R. 2 | VAV\* |

In [78]:

```
Show_Data(temp)
```

| strain | WT | | | | | | | | | | | | | | | | |
| --- | --- | --- | --- | --- | --- | --- | --- | --- | --- | --- | --- | --- | --- | --- | --- | --- | --- |
| condition | G\_MM | C\_MM | G\_SD | | GX\_SD | | | X\_SD | | A\_SD | | C\_SD | | MM\_CN120 | | MM\_CN5 | Diversity\_Sample |
| phase | exp | exp | exp | stat | exp | trans | stat | exp | stat | exp | stat | exp | stat | exp | stat | exp | exp |
| proteinId | Set1 | Set1 | Set2 | Set2 | Set2 | Set2 | Set2 | Set2 | Set2 | Set2 | Set2 | Set2 | Set2 | Set3 | Set3 | Set3 | Set3 |
| proteinId |  |  |  |  |  |  |  |  |  |  |  |  |  |  |  |  |  |
| 11990 | 9.833589 | 8.397206 | 8.681860 | 10.416920 | 8.524474 | 8.664173 | 9.167037 | 11.155269 | 11.174203 | 11.704613 | 11.561817 | 6.344171 | 5.306026 | 8.768625 | 8.097153 | 10.502800 | 10.356089 |
| 12292 | 3.561035 | 4.859423 | 4.260561 | 3.318377 | 4.322080 | 3.988246 | 3.771992 | 4.161367 | 3.751640 | 3.795807 | 3.370285 | 4.111851 | 3.791877 | 4.851509 | 4.686877 | 5.234027 | 5.581241 |
| 15999 | 5.876157 | 5.254818 | 5.294241 | 5.549831 | 5.102789 | 5.109696 | 5.325721 | 5.123848 | 5.606917 | 5.019980 | 5.870566 | 5.963301 | 6.148683 | 4.231060 | 4.354456 | 4.445912 | 5.018300 |
| 16280 | 4.111127 | 5.749530 | 4.704382 | 4.062063 | 4.765152 | 4.536371 | 4.688761 | 5.071968 | 4.415042 | 4.913489 | 4.425541 | 5.404074 | 4.856744 | 4.230674 | 4.070631 | 5.177015 | 5.728998 |
| 9331 | 5.750343 | 6.907189 | 6.472043 | 5.740825 | 6.552778 | 5.960345 | 6.050610 | 6.262856 | 6.443843 | 5.921660 | 6.100736 | 6.209495 | 5.938400 | 5.718082 | 5.530642 | 6.083025 | 5.743328 |
| 12588 | 7.146184 | 7.816598 | 6.801797 | 6.931421 | 6.778076 | 6.746030 | 6.672289 | 6.584789 | 6.690105 | 6.843305 | 6.745372 | 8.530299 | 7.983152 | 7.516864 | 7.560850 | 6.925153 | 7.651963 |
| 9364 | 4.650389 | 5.675300 | 9.045215 | 7.183050 | 8.964571 | 9.309306 | 9.313517 | 8.305323 | 8.847896 | 7.970590 | 7.241493 | 7.994688 | 7.694124 | 9.878397 | 9.560561 | 9.381434 | 9.364310 |
| 10955 | 5.312556 | 4.955522 | 5.301127 | 4.429921 | 5.334025 | 4.500051 | 4.386168 | 4.951260 | 4.860752 | 4.828974 | 4.690495 | 4.899960 | 4.753100 | 5.314831 | 4.993568 | 5.464043 | 5.412637 |
| 10025 | 6.445573 | 5.962730 | 5.088660 | 6.554263 | 5.283106 | 5.257568 | 6.051853 | 4.582914 | 6.193997 | 5.720011 | 6.535565 | 5.565489 | 6.425793 | 4.794493 | 4.514718 | 3.221680 | 5.568799 |
| 11412 | 4.259405 | 4.240676 | 5.036322 | 4.054380 | 5.036667 | 5.444166 | 5.190735 | 5.172398 | 5.525014 | 5.073564 | 5.002930 | 4.401927 | 4.928821 | 4.989444 | 4.929466 | 4.690187 | 4.896977 |
| 11732 | 6.389390 | 5.773275 | 6.083267 | 6.022220 | 6.102774 | 5.827775 | 5.943994 | 5.863857 | 6.138251 | 5.976634 | 6.124162 | 5.644302 | 5.523332 | 5.015565 | 4.981186 | 4.965358 | 4.825104 |
| 15484 | 8.940001 | 8.112364 | 8.430384 | 8.676720 | 8.369056 | 8.594319 | 8.299371 | 9.052138 | 8.260951 | 8.940863 | 8.192139 | 7.600335 | 7.648971 | 8.746789 | 8.637409 | 8.502231 | 7.996595 |
| 14061 | 6.840731 | 7.664261 | 7.388000 | 7.001931 | 7.394590 | 7.553228 | 7.521999 | 7.242440 | 7.500803 | 7.577186 | 7.689671 | 7.374080 | 6.896395 | 7.652868 | 7.867076 | 7.548875 | 7.185870 |
| 9193 | 7.527787 | 6.684080 | 6.832502 | 7.111588 | 6.771982 | 6.952441 | 6.640464 | 7.010345 | 6.600346 | 7.144034 | 6.857902 | 6.172903 | 4.292651 | 6.848629 | 6.774856 | 6.732002 | 5.604986 |
| 8667 | 4.487338 | 7.840200 | 6.460510 | 6.343518 | 6.451642 | 6.722551 | 6.451009 | 5.863530 | 6.617526 | 6.118229 | 6.455007 | 6.167024 | 6.076798 | 4.980071 | 5.603579 | 4.854063 | 6.636354 |
| 11722 | 5.652502 | 5.184506 | 5.930322 | 6.563606 | 5.987739 | 5.826364 | 6.051896 | 5.720722 | 5.939486 | 5.916517 | 5.958305 | 5.665362 | 5.639851 | 5.331550 | 5.509983 | 5.277294 | 5.123202 |
| 13276 | 3.667790 | 3.552244 | 3.589897 | 3.572815 | 3.634004 | 3.725230 | 3.520558 | 3.456652 | 3.876966 | 3.305630 | 3.615258 | 3.831866 | 3.531193 | 4.401012 | 4.208472 | 3.673961 | 3.794763 |
| 10331 | 5.794646 | 6.648844 | 6.110708 | 6.131695 | 6.113045 | 6.075187 | 6.123415 | 5.916743 | 6.401240 | 5.711548 | 6.215015 | 6.633974 | 6.400497 | 5.160631 | 4.834324 | 4.753836 | 5.092392 |
| 14022 | 4.686496 | 7.310591 | 8.986223 | 7.363142 | 9.165627 | 7.828568 | 8.008932 | 7.728553 | 8.213910 | 6.124188 | 7.445671 | 7.050714 | 8.078619 | 6.229350 | 7.545095 | 10.114059 | 6.866686 |
| 15784 | 5.394727 | 4.731117 | 5.888345 | 1.824066 | 5.932047 | 3.525213 | 3.368560 | 5.589693 | 1.134390 | 5.932720 | 1.202130 | 4.787871 | 2.801922 | 4.001756 | 3.275728 | 4.483519 | 3.958069 |
| 15471 | 0.801308 | 1.781927 | 0.964576 | 3.485653 | 0.805994 | 1.566187 | 1.419202 | 1.434786 | 2.613560 | 1.289613 | 2.304491 | 1.844323 | 2.564419 | 1.164435 | 1.220662 | 1.432630 | 0.859429 |
| 13278 | 3.358585 | 4.389008 | 3.222548 | 2.991704 | 3.183543 | 4.042715 | 3.967118 | 3.859986 | 3.663943 | 3.911212 | 3.619176 | 4.799471 | 4.748598 | 4.164602 | 4.479046 | 4.216940 | 4.947873 |
| 9226 | 6.201708 | 6.648245 | 6.291009 | 5.769138 | 6.316685 | 6.180294 | 5.910214 | 6.598315 | 5.713844 | 6.357178 | 5.897459 | 6.711183 | 6.747396 | 7.188595 | 7.223271 | 7.768086 | 7.816127 |
| 8602 | 6.113841 | 4.626142 | 6.492428 | 5.976145 | 6.541515 | 5.964060 | 5.893444 | 5.467905 | 5.836117 | 5.200428 | 5.866820 | 4.957258 | 4.348653 | 4.978913 | 4.859631 | 4.860770 | 3.881262 |
| 8664 | 4.529571 | 4.735030 | 4.452975 | 4.378845 | 4.434741 | 4.751615 | 4.435784 | 4.707922 | 4.260515 | 4.687157 | 4.412900 | 4.989577 | 4.755906 | 4.804472 | 4.898025 | 4.771134 | 4.653490 |
| 10274 | 3.811581 | 4.596416 | 5.325504 | 4.628324 | 5.332782 | 5.416318 | 5.196442 | 5.767458 | 5.779932 | 5.868963 | 5.640028 | 4.618445 | 4.725049 | 3.671472 | 4.373551 | 4.565764 | 4.247231 |
| 10310 | 4.689347 | 5.014569 | 5.465456 | 5.280665 | 5.444274 | 5.389589 | 5.292658 | 5.490676 | 5.263380 | 5.025697 | 4.879298 | 6.002791 | 6.924681 | 6.123869 | 6.241373 | 6.235101 | 6.366909 |
| 9964 | 3.776384 | 4.506710 | 4.167369 | 4.908611 | 4.284125 | 4.984287 | 5.006455 | 4.660746 | 4.388074 | 4.465795 | 4.574894 | 5.396713 | 5.218700 | 4.539396 | 5.001421 | 5.169922 | 5.265049 |
| 8442 | 3.672082 | 3.811497 | 4.258336 | 5.054750 | 4.322702 | 4.178402 | 4.363527 | 3.956450 | 4.716279 | 3.879366 | 4.432626 | 4.804286 | 4.464532 | 4.569965 | 4.934220 | 4.596774 | 4.156089 |
| 13851 | 4.833662 | 6.221055 | 5.015399 | 5.917327 | 4.894956 | 5.684087 | 5.734848 | 5.265529 | 5.847822 | 5.376219 | 5.870140 | 7.397095 | 6.793686 | 5.132181 | 5.546418 | 4.728219 | 5.535909 |

| strain | WT | | | | | | | | | | |
| --- | --- | --- | --- | --- | --- | --- | --- | --- | --- | --- | --- |
| condition | G\_SD | | GX\_SD | | | X\_SD | | A\_SD | | C\_SD | |
| proteinId | exp | stat | exp | trans | stat | exp | stat | exp | stat | exp | stat |
| 11990 | 0.197667 | 8.209709 | 0.000000 | 2.603519 | 1.843354 | 15.490644 | 13.214614 | 21.039087 | 13.787164 | 0.000000 | 0.000000 |
| 12292 | 2.709359 | 1.744109 | 1.620711 | 1.864116 | 1.288801 | 1.185944 | 1.576008 | 1.545486 | 1.149316 | 0.630494 | 0.000000 |
| 15999 | 0.000000 | 0.000000 | 0.616535 | 0.000000 | 0.000000 | 0.000000 | 0.000000 | 0.196014 | 0.189207 | 0.211647 | 0.000000 |
| 16280 | 7.859734 | 5.546151 | 7.524834 | 3.134090 | 3.068009 | 5.661995 | 3.337756 | 5.205133 | 2.873072 | 3.196429 | 2.392845 |
| 9331 | 6.348306 | 6.320039 | 6.299912 | 7.465020 | 7.478604 | 8.039252 | 6.468046 | 5.792401 | 6.712627 | 7.272844 | 8.500653 |
| 12588 | 2.527259 | 3.587587 | 3.045061 | 4.833923 | 4.392998 | 3.529398 | 3.777915 | 4.440766 | 4.783889 | 4.481376 | 5.017465 |
| 9364 | 4.270762 | 0.552733 | 5.489146 | 1.844937 | 1.807585 | 2.543067 | 0.778783 | 2.899369 | 0.959442 | 7.058096 | 4.348126 |
| 10955 | 4.014764 | 2.318602 | 2.436138 | 2.598054 | 2.392522 | 2.555625 | 1.771750 | 1.549147 | 0.971900 | 2.795254 | 2.379610 |
| 10025 | 0.197667 | 0.179255 | 0.000000 | 0.000000 | 0.000000 | 0.000000 | 0.000000 | 0.192982 | 0.189081 | 0.000000 | 0.000000 |
| 11412 | 0.197667 | 0.179255 | 0.197656 | 0.000000 | 0.192891 | 0.000000 | 0.195435 | 0.576624 | 0.000000 | 0.000000 | 0.000000 |
| 11732 | 0.935494 | 1.617701 | 2.244335 | 1.298987 | 1.441086 | 2.157606 | 1.000736 | 1.732158 | 1.325669 | 1.056845 | 0.432147 |
| 15484 | 5.895315 | 7.440700 | 6.498413 | 7.820932 | 8.760014 | 5.099053 | 6.498905 | 6.176610 | 7.303697 | 6.190107 | 7.192761 |
| 14061 | 7.917812 | 8.657607 | 6.305921 | 10.066032 | 10.259474 | 8.370130 | 7.669833 | 9.464114 | 7.662060 | 11.764112 | 11.300137 |
| 9193 | 0.000000 | 0.166667 | 0.000000 | 0.000000 | 0.000000 | 0.000000 | 0.188437 | 0.000000 | 0.000000 | 0.000000 | 0.000000 |
| 8667 | 0.943288 | 0.000000 | 1.219444 | 0.000000 | 0.000000 | 0.000000 | 0.000000 | 0.000000 | 0.000000 | 0.000000 | 0.000000 |
| 11722 | 4.045524 | 6.650454 | 2.236821 | 2.794511 | 3.107192 | 2.168805 | 3.521022 | 4.250733 | 4.417753 | 4.086895 | 4.123017 |
| 13276 | 0.000000 | 0.000000 | 0.000000 | 0.000000 | 0.000000 | 0.000000 | 0.000000 | 0.000000 | 0.189081 | 0.000000 | 0.000000 |
| 10331 | 4.416626 | 2.141446 | 2.025689 | 4.476156 | 3.464294 | 3.520166 | 2.159336 | 2.517538 | 0.947506 | 6.843168 | 5.869206 |
| 14022 | 28.964940 | 36.055286 | 32.596178 | 34.252522 | 32.196255 | 21.691514 | 34.507469 | 14.313262 | 21.286186 | 39.899413 | 40.764941 |
| 15784 | 0.000000 | 0.000000 | 0.000000 | 0.000000 | 0.000000 | 0.000000 | 0.000000 | 0.000000 | 0.000000 | 0.000000 | 0.224376 |
| 13278 | 1.744977 | 1.809046 | 2.238482 | 4.840788 | 3.101365 | 2.171395 | 2.732702 | 4.059767 | 2.670085 | 1.073656 | 1.749519 |
| 9226 | 11.579687 | 11.098224 | 13.420024 | 11.543552 | 13.683342 | 13.865857 | 13.980021 | 15.066795 | 14.909205 | 14.766581 | 17.236495 |
| 8602 | 0.755525 | 1.620309 | 0.197656 | 1.847093 | 3.092590 | 2.745535 | 1.786922 | 1.737996 | 1.148920 | 0.000000 | 0.435902 |
| 8664 | 0.190197 | 0.732414 | 0.206941 | 0.937407 | 0.912238 | 1.184925 | 0.566061 | 0.963242 | 1.345719 | 0.211647 | 0.000000 |
| 10274 | 0.761118 | 1.083934 | 1.017275 | 1.119915 | 0.365356 | 1.174787 | 0.787244 | 1.737996 | 1.339371 | 0.633145 | 1.307248 |
| 10310 | 0.948790 | 1.419833 | 1.434916 | 2.230416 | 2.357881 | 0.779896 | 1.379072 | 0.963153 | 1.337796 | 0.420867 | 1.079833 |
| 9964 | 1.344448 | 0.907359 | 0.814191 | 0.000000 | 1.089664 | 0.393872 | 0.394911 | 0.193364 | 0.189808 | 0.000000 | 0.217934 |
| 13851 | 0.000000 | 0.000000 | 0.000000 | 0.000000 | 0.000000 | 0.000000 | 0.000000 | 0.000000 | 0.000000 | 0.000000 | 0.211140 |

|  | Glucose | Xylose | Arabinose | Acetate | Coumarate | Ferulate | YNB Oleic Acid | YNB Ricinoleic Acid | YNB Glucose | YNB Gluc DOC | YPD |
| --- | --- | --- | --- | --- | --- | --- | --- | --- | --- | --- | --- |
| proteinId |  |  |  |  |  |  |  |  |  |  |  |
| 11990 | 0.051506 | 7.429010 | 0.525842 | -0.037608 | 1.068146 | 0.148676 | -1.082252 | -0.854073 | 0.029327 | 0.030189 | -0.052012 |
| 12292 | -0.992753 | 0.694427 | 1.270307 | 0.195801 | -3.718814 | -1.683798 | -1.602772 | -0.503818 | -2.506344 | -2.201265 | 1.068949 |
| 15999 | 0.446315 | 0.805266 | 1.016685 | 0.184550 | -6.200467 | -3.370798 | 0.210868 | -2.955110 | -0.325622 | 0.229329 | 0.181168 |
| 16280 | 0.186450 | 2.107460 | 1.552210 | 0.167253 | -0.058341 | 0.577218 | -0.603866 | -1.270181 | -1.802367 | -1.250431 | 0.933914 |
| 9331 | 0.317304 | 0.660965 | -0.231468 | -1.776454 | 0.262022 | -1.830893 | -3.797246 | -6.149645 | -0.943653 | -0.572548 | -1.193923 |
| 12588 | 0.166909 | 0.915210 | 0.384190 | -0.266136 | -5.829157 | -3.146647 | 0.156251 | -3.497062 | 0.252847 | 0.710073 | -0.199311 |
| 9364 | -0.085059 | -0.217883 | 0.687159 | 0.309115 | 0.098032 | 0.221939 | -1.388785 | -3.417107 | -4.882882 | -3.768761 | -1.123494 |
| 10955 | -1.019127 | -0.487648 | -1.204983 | -0.294673 | -4.683991 | -2.596131 | -1.958550 | -4.952654 | -3.610940 | -1.925370 | -2.597290 |
| 10025 | -0.219634 | 0.444370 | 0.228314 | -0.369603 | -3.624166 | -0.808128 | -1.879124 | -4.811676 | -1.006437 | -0.054364 | 0.167582 |
| 11412 | 0.047278 | 1.396219 | 0.883604 | -0.202440 | -0.515719 | -0.692885 | -1.501443 | -1.178927 | -0.735826 | -0.365748 | 0.142528 |
| 11732 | -0.081075 | 2.120261 | 0.922344 | -0.135453 | -0.580201 | -0.809090 | -0.388174 | -0.202002 | 0.477069 | 0.463531 | -0.253389 |
| 15484 | 0.080904 | 1.531851 | 0.641623 | -0.668669 | -1.104296 | -1.085869 | -0.997969 | -3.320023 | 0.586455 | 1.009565 | 0.130345 |
| 14061 | -0.690796 | -0.415707 | -0.207850 | -1.506291 | -3.907112 | -1.796966 | -3.719036 | -3.912868 | -0.205123 | -0.015974 | -1.340000 |
| 9193 | 0.049079 | 1.289792 | -0.412851 | -0.056239 | -2.037000 | -1.106344 | -0.342138 | -3.991366 | -2.023169 | -0.720471 | 0.006654 |
| 8667 | 0.142409 | -0.043344 | -0.221673 | -2.063714 | -3.075015 | -2.219730 | -2.315443 | -4.827446 | -0.379550 | -0.024526 | 0.138901 |
| 11722 | -0.835464 | 0.468176 | 0.866953 | -0.933932 | 0.629274 | 0.769345 | 0.246433 | -1.044619 | -1.941617 | -2.515287 | -1.477983 |
| 13276 | -0.040690 | 1.704746 | 0.824878 | -0.180223 | -0.166198 | -0.191490 | 0.070605 | -1.283812 | 0.039863 | 0.054341 | -0.000551 |
| 10331 | -1.796683 | 0.010661 | -0.182608 | -0.020225 | -5.740166 | -3.896808 | -0.312468 | -1.404019 | 0.062982 | -0.381056 | -0.356469 |
| 14022 | -0.068436 | -0.040623 | 0.005663 | -3.538774 | -0.672274 | -0.646294 | -3.493515 | -4.315415 | -0.143782 | -0.190331 | 0.037619 |
| 15784 | 0.286015 | 0.891535 | 0.430216 | 0.395019 | -1.262514 | 0.194804 | -1.470394 | -2.573743 | -0.249726 | -1.400371 | -0.590641 |
| 15471 | 0.228247 | 1.790098 | -0.696726 | -0.388581 | -2.592343 | -0.907383 | 1.000376 | -3.492338 | 0.013659 | 0.017784 | -1.574256 |
| 13278 | -0.348674 | 0.451965 | -0.352935 | -0.533417 | -3.755495 | -1.894932 | 0.211774 | -3.111913 | -2.340603 | -1.184131 | 0.834305 |
| 9226 | -0.418199 | 1.026356 | 0.670451 | -1.422232 | -3.245200 | -1.267465 | 0.103730 | -1.944587 | 0.623208 | 0.874015 | 1.954532 |
| 8602 | -1.856955 | -0.183267 | 0.140202 | 0.031840 | -4.537394 | -2.887376 | -0.566587 | -1.120457 | -0.465372 | -1.185903 | 0.238405 |
| 8664 | -0.194781 | 0.839219 | -0.194860 | -0.061245 | -3.764062 | -0.961045 | -1.012643 | -2.021091 | -1.106816 | -0.569772 | 0.474188 |
| 10274 | -0.673477 | 0.557454 | 0.106621 | 0.211326 | -0.706933 | 0.053397 | -2.018768 | -2.578687 | -1.250070 | -1.622092 | -0.474758 |
| 10310 | -0.207733 | 0.741945 | 0.520239 | 0.116020 | -4.135039 | -0.426955 | -0.534712 | -0.623790 | 0.085546 | -0.083434 | -0.532184 |
| 9964 | 0.670378 | 0.410271 | 1.113366 | -1.062492 | -0.330462 | 1.106374 | -0.545423 | -1.829644 | -1.949800 | -1.167399 | -0.036661 |
| 8442 | 0.130288 | 1.315342 | 1.034233 | -0.055871 | -0.356197 | -0.054374 | 0.115307 | -1.009326 | -0.039905 | -0.018325 | -0.045050 |
| 13851 | 0.304502 | 0.422248 | 0.130038 | -0.378786 | -2.515981 | 0.122594 | -0.699164 | -3.733331 | -1.318149 | -1.360415 | 0.298320 |

12978 Fungal specific transcription factor with Zn(2)-Cys(6) binuclear cluster domain  
D-abt -> D-rbl and D-rbl -> D-ru5p both genes have significant fitness defect in both xylose and arabinose  
It seems that D-xylulose is converted to D-arabinitol, but there is no D-arabinitol 4-dehydrogenase  
Production of D-arabitol from D-xylose by the oleaginous yeast Rhodosporidium toruloides IFO0880  
https://www.ncbi.nlm.nih.gov/pubmed/29127468  
Two D-arabinitol dehydrogenase genes from the paper RHTO\_07702 and RHTO\_07844  
9990 RHTO\_07844 blast hits to sorbose reductase NADP and D-arabinitol 2-dehydrogenase NAD, fitness defect, ok  
9837 RHTO\_07702 is annotated as mitochondrial/plastidial beta-ketoacyl-ACP reductase, no fitness defect  
ARD1p in Uromyces fabae works as both D-Arabitol 4-dehydrogenases and D-Arabitol 2-dehydrogenases with NADP,  
but no homologous protein is found
https://www.ncbi.nlm.nih.gov/pubmed/15796718  
Blast hits of A2QK54 A. niger D-arabinitol 4-dehydrogenase An04g09410 are  
['9837','14196','12795','9104','11765','8666','9731','9990','14267','8905','10551']
8905 is upregulated and has weak fitness defect  
Create D-arabinitol 4-dehydrogenase reaction and assign 8905 or 9837

In [79]:

```
temp = ['9837','14196','12795','9104','11765','8666','9731','9990','14267','8905','10551']
display(Annotation.loc[temp])
```

|  | Combined Annotations | Signal P | Sc288c Orthologs | Human Orthologs | Sc288 Best Hit | Human Blast | Essential | WolfPSort | C Terminal |
| --- | --- | --- | --- | --- | --- | --- | --- | --- | --- |
| RTO4\_ID |  |  |  |  |  |  |  |  |  |
| 9837 | KOG1200: Mitochondrial/plastidial beta-ketoacy... |  |  |  |  |  | Not Essential | extr 18, cyto\_mito 5, mito 4.5, cyto 4.5 | SLV\* |
| 14196 | KOG0725: Reductases with broad range of substr... | S |  | BDH2 | FOX2 | BDH2 | Not Essential | cyto 21.5, cyto\_nucl 12, mito 3 | PKA\* |
| 12795 | K11539: CBR4; carbonyl reductase 4 |  |  |  | YMR226C | CBR4 | Not Essential | cyto 16, mito 9, cyto\_nucl 9 | WTA\* |
| 9104 | K00034: gdh; glucose 1-dehydrogenase |  |  |  | YIR035C | DCXR | Not Essential | cyto 19, cyto\_nucl 11, mito 5 | IQA\* |
| 11765 | K13370: HSD17B8; 17beta-estradiol 17-dehydroge... |  |  | BDH2 | SPS19 | DHRS4 | Not Essential | mito 19.5, cyto\_mito 12, cyto 3.5, extr 2 | TPT\* |
| 8666 | KOG0725: Reductases with broad range of substr... |  |  | HSD17B14 | SPS19 | HSD17B | Not Essential | cyto 18.5, cyto\_nucl 12, nucl 2.5, mito 2, ext... | YVH\* |
| 9731 | K11147: DHRS4; dehydrogenase/reductase SDR fam... |  |  |  | FOX2 | HSD17B | Not Essential | mito 11, cyto 10, cyto\_nucl 6.5, pero 5 | QFM\* |
| 9990 | K17738: ARD; D-arabinitol 2-dehydrogenase | S |  |  | SPS19 | CBR4 | Not Essential | mito 21, cyto 5 | TLT\* |
| 14267 | K00038: E1.1.1.53; 3alpha(or 20beta)-hydroxyst... |  |  |  | SPS19 | DHRS4 | Not Essential | cyto 16.5, cyto\_nucl 9, extr 6, mito 4 | KWC\* |
| 8905 | KOG0725: Reductases with broad range of substr... | S |  |  | IRC24 | DHRS4 | Not Essential | extr 13, mito 8, cyto 3.5, cyto\_nucl 3 | SAY\* |
| 10551 | K00059: fabG; 3-oxoacyl-[acyl-carrier protein]... |  |  |  | FOX2 | HSD17B | Not Essential | cyto 16, mito 7, pero 3 | MGI\* |

In [80]:

```
Show_Data(temp)
```

| strain | WT | | | | | | | | | | | | | | | | |
| --- | --- | --- | --- | --- | --- | --- | --- | --- | --- | --- | --- | --- | --- | --- | --- | --- | --- |
| condition | G\_MM | C\_MM | G\_SD | | GX\_SD | | | X\_SD | | A\_SD | | C\_SD | | MM\_CN120 | | MM\_CN5 | Diversity\_Sample |
| phase | exp | exp | exp | stat | exp | trans | stat | exp | stat | exp | stat | exp | stat | exp | stat | exp | exp |
| proteinId | Set1 | Set1 | Set2 | Set2 | Set2 | Set2 | Set2 | Set2 | Set2 | Set2 | Set2 | Set2 | Set2 | Set3 | Set3 | Set3 | Set3 |
| 9837 | 0.865829 | 0.972257 | 1.139759 | 3.177221 | 0.958784 | 1.618141 | 2.096768 | 0.959742 | 2.653801 | 1.287833 | 2.288463 | 2.099555 | 2.747612 | 1.928710 | 2.378149 | 1.253281 | 1.772644 |
| 14196 | 7.811461 | 6.830961 | 7.210552 | 6.246278 | 7.322240 | 6.907628 | 6.563977 | 7.540436 | 5.724773 | 6.837532 | 5.945336 | 5.924835 | 6.041888 | 8.812277 | 8.117206 | 8.197228 | 7.950934 |
| 12795 | 6.009231 | 5.777368 | 5.109421 | 5.369559 | 5.027839 | 4.820215 | 4.772699 | 4.883633 | 5.328962 | 4.886726 | 5.578210 | 4.710982 | 4.055995 | 4.462697 | 4.488295 | 4.147355 | 3.909088 |
| 9104 | 0.519373 | 0.767463 | 0.474285 | 0.141273 | 0.560521 | 0.040141 | 0.101672 | 0.021721 | 0.059857 | 0.000000 | 0.017562 | 0.110029 | 0.259941 | 0.131564 | 0.058696 | 3.833227 | 0.051526 |
| 11765 | 2.911924 | 4.076209 | 3.352397 | 5.319521 | 3.130822 | 3.120180 | 3.737194 | 2.954094 | 4.150098 | 3.046896 | 4.753303 | 4.541225 | 4.059358 | 2.317925 | 2.820377 | 2.998126 | 3.281742 |
| 8666 | 0.116626 | 1.554635 | 0.234326 | 0.253070 | 0.288409 | 0.196665 | 0.048971 | 0.109595 | 1.378694 | 0.101187 | 0.403123 | 3.314838 | 3.554217 | 0.000000 | 0.091063 | 1.152737 | 0.196585 |
| 9731 | 3.932942 | 7.167870 | 4.798452 | 5.067240 | 4.781275 | 4.905844 | 4.870672 | 5.084100 | 5.351590 | 5.250732 | 5.588353 | 4.675988 | 4.018411 | 3.476865 | 4.057495 | 4.077026 | 3.868919 |
| 9990 | 5.140148 | 6.270216 | 5.551300 | 6.138047 | 7.867579 | 8.519055 | 7.743922 | 6.670194 | 5.112570 | 9.121079 | 4.531483 | 4.838953 | 5.820834 | 3.984943 | 3.966935 | 4.257002 | 5.559251 |
| 14267 | 5.829835 | 5.760128 | 4.630589 | 4.668842 | 4.816618 | 3.541195 | 3.452299 | 5.621897 | 5.509234 | 5.350774 | 5.610543 | 4.899809 | 3.907121 | 3.329857 | 2.617179 | 5.458142 | 3.874563 |
| 8905 | 2.095962 | 2.463929 | 4.819721 | 6.295878 | 5.892019 | 8.247780 | 7.089842 | 6.227324 | 6.894446 | 7.823003 | 5.739129 | 3.274304 | 2.630171 | 1.545841 | 0.392913 | 2.960081 | 0.729005 |
| 10551 | 5.461825 | 10.365869 | 4.837672 | 5.576077 | 4.888397 | 5.683736 | 5.617345 | 5.184640 | 6.286944 | 6.218729 | 6.674326 | 8.177066 | 7.201104 | 6.285718 | 6.760832 | 6.198634 | 7.423812 |

| strain | WT | | | | | | | | | | |
| --- | --- | --- | --- | --- | --- | --- | --- | --- | --- | --- | --- |
| condition | G\_SD | | GX\_SD | | | X\_SD | | A\_SD | | C\_SD | |
| proteinId | exp | stat | exp | trans | stat | exp | stat | exp | stat | exp | stat |
| 14196 | 10.177624 | 10.438475 | 10.581538 | 10.797013 | 12.139338 | 11.978211 | 12.552946 | 10.422821 | 9.954909 | 13.672393 | 10.023815 |
| 12795 | 1.732312 | 0.905530 | 1.828033 | 0.366379 | 0.000000 | 0.985019 | 0.957682 | 1.159167 | 1.338127 | 0.212277 | 0.000000 |
| 11765 | 0.000000 | 0.000000 | 0.000000 | 0.000000 | 0.000000 | 0.000000 | 0.000000 | 0.000000 | 0.000000 | 0.854055 | 0.431761 |
| 9731 | 1.909239 | 4.293848 | 1.220783 | 3.162756 | 3.630075 | 2.722064 | 3.129075 | 3.474313 | 3.067745 | 6.411952 | 3.049979 |
| 9990 | 2.458974 | 3.619681 | 7.315781 | 17.528749 | 16.692717 | 28.530481 | 23.529465 | 30.329072 | 27.392354 | 4.051601 | 3.506934 |
| 14267 | 1.725172 | 1.257165 | 2.029880 | 1.113518 | 0.722761 | 2.742812 | 2.377288 | 2.701421 | 2.865480 | 3.431131 | 3.060869 |
| 8905 | 0.000000 | 0.888632 | 0.000000 | 6.716769 | 5.276863 | 1.945092 | 2.560514 | 5.599194 | 5.352923 | 0.000000 | 0.220621 |
| 10551 | 2.719587 | 6.542034 | 3.454070 | 8.916621 | 9.495566 | 7.622570 | 8.277752 | 8.508091 | 9.948272 | 46.435645 | 36.834371 |

|  | Glucose | Xylose | Arabinose | Acetate | Coumarate | Ferulate | YNB Oleic Acid | YNB Ricinoleic Acid | YNB Glucose | YNB Gluc DOC | YPD |
| --- | --- | --- | --- | --- | --- | --- | --- | --- | --- | --- | --- |
| proteinId |  |  |  |  |  |  |  |  |  |  |  |
| 14196 | 0.102045 | -0.167104 | -0.051571 | -0.050119 | -0.141501 | -0.055851 | 0.151621 | 0.023838 | -0.049238 | -0.184868 | -0.134232 |
| 12795 | 0.067355 | 0.953909 | 0.137586 | 0.306703 | -0.069481 | 0.291715 | -0.227028 | -0.390367 | -0.260799 | -0.155835 | -0.199948 |
| 9104 | 0.134223 | 0.087421 | 0.081133 | 0.042388 | -0.269268 | -0.121035 | 0.741374 | 0.529799 | 0.416270 | 0.295256 | 0.049692 |
| 11765 | 0.107044 | 0.135627 | 0.101293 | 0.045089 | 0.014979 | -0.031674 | 0.011288 | 0.070107 | -0.118633 | -0.029956 | 0.387225 |
| 8666 | 0.246518 | -0.053170 | 0.304517 | -0.024053 | -0.100515 | -0.173468 | -0.024925 | -0.343657 | 0.409518 | 0.349750 | -0.016146 |
| 9731 | 0.009639 | -0.356818 | -0.433929 | -0.104025 | -0.109207 | -0.251474 | 0.070244 | -0.303031 | 0.410073 | 0.255280 | -0.067573 |
| 9990 | 0.029755 | -2.655320 | -2.324146 | 0.034732 | -0.215495 | 0.067559 | -0.190665 | -0.133194 | 0.095922 | -0.076464 | -0.211712 |
| 14267 | 0.207207 | -0.248473 | 0.215494 | 0.063958 | -0.173733 | 0.246239 | 0.064674 | 0.027836 | -0.138097 | -0.164489 | 0.459204 |
| 8905 | -0.403369 | -0.325130 | -0.371376 | -0.438335 | -0.426506 | -0.247058 | 0.219238 | 0.223042 | 0.092216 | -0.058310 | 0.417242 |
| 10551 | -0.351777 | -0.190585 | -0.003885 | 0.049769 | -0.891608 | -2.059696 | -0.055568 | -0.278141 | 0.377108 | 0.104370 | 0.103662 |

In [81]:

```
r = model.reactions.get_by_id('DABT2D').copy()
r.id = 'DABT4D'
r.name = 'D-Arabinitol 4-dehydrogenase'
r.gene_reaction_rule = '9837 or 8905'
model.add_reactions([r])
r.add_metabolites({'rbl__D_c': -1.0, 'xylu__D_c': 1.0})
```

In [82]:

```
for x in ['EX_xyl__D_e','XYLt','XYLR','XYLTD_D','XYLK']:
    r = model.reactions.get_by_id(x)
    print(r, r.gene_reaction_rule)
print()
for x in ['EX_arab__L_e','ARAB_Lt','ARABR','ABTD','XYLUR']:
    r = model.reactions.get_by_id(x)
    print(r, r.gene_reaction_rule)
print()
for x in ['DABT4D','DABT2D','RBK_Dr']:
    r = model.reactions.get_by_id(x)
    print(r, r.gene_reaction_rule)
```

```
EX_xyl__D_e: xyl__D_e -->  
XYLt: xyl__D_e <=> xyl__D_c 15762
XYLR: h_c + nadph_c + xyl__D_c --> nadp_c + xylt_c 11882 or 13562 or 9774
XYLTD_D: nad_c + xylt_c --> h_c + nadh_c + xylu__D_c 12974 or 12977 or 16452
XYLK: atp_c + xylu__D_c --> adp_c + h_c + xu5p__D_c 16850

EX_arab__L_e: arab__L_e -->  
ARAB_Lt: arab__L_e <=> arab__L_c 
ARABR: arab__L_c + h_c + nadph_c --> abt_c + nadp_c 9774
ABTD: abt_c + nad_c <=> h_c + nadh_c + xylu__L_c 12974 or 12977
XYLUR: h_c + nadph_c + xylu__L_c <=> nadp_c + xylt_c 8988

DABT4D: abt__D_c + nad_c <=> h_c + nadh_c + xylu__D_c 9837 or 8905
DABT2D: abt__D_c + nad_c <=> h_c + nadh_c + rbl__D_c 9990
RBK_Dr: atp_c + rbl__D_c --> adp_c + h_c + ru5p__D_c 14368
```

In [83]:

```
with model:
    model.reactions.get_by_id('EX_o2_e').lower_bound = -1000.0
    model.reactions.get_by_id('EX_glc__D_e').lower_bound = 0.0
    model.reactions.get_by_id('EX_xyl__D_e').lower_bound = -1.0
    sol = model.optimize()
    print(sol.objective_value)
    print()
    model.reactions.get_by_id('EX_xyl__D_e').lower_bound = 0.0
    model.reactions.get_by_id('EX_arab__L_e').lower_bound = -1.0
    sol = model.optimize()
    print(sol.objective_value)
```

```
0.061030057235400614

0.06103005723540208
```

Other pentose sugars that can be utilized by R. toruloides  
L-lyxose, L-arabitol, xylitol, D-xylulose, D-arabitol, D-ribulose

In [84]:

```
# L-lyxose
r1 = eco.reactions.get_by_id('EX_lyx__L_e').copy()
r2 = eco.reactions.get_by_id('LYXtex').copy()
r2.id = 'LYXt'
r2.name = 'L-Lyxose transport'
r2.gene_reaction_rule = '10452'
model.add_reactions([r1,r2])
m = model.metabolites.get_by_id('lyx__L_p')
m.id = 'lyx__L_c'
m.compartment = 'c'
r = model.reactions.get_by_id('ARABR').copy()
r.id = 'LYXR'
r.name = 'L-lyxose reductase'
model.add_reactions([r])
r.add_metabolites({'arab__L_c': 1.0, 'lyx__L_c': -1.0})
# L-arabitol
r1 = hsa2.reactions.get_by_id('EX_abt_e').copy()
r1.lower_bound = 0.0
r2 = hsa2.reactions.get_by_id('ABTt').copy()
r2.gene_reaction_rule = '12976'
r2.upper_bound = 1000.0
model.add_reactions([r1,r2])
# xylitol
r1 = hsa2.reactions.get_by_id('EX_xylt_e').copy()
r1.lower_bound = 0.0
r2 = hsa2.reactions.get_by_id('XYLTt').copy()
r2.gene_reaction_rule = '12976'
model.add_reactions([r1,r2])
# D-xylulose
r1 = hsa2.reactions.get_by_id('EX_xylu__D_e').copy()
r1.lower_bound = 0.0
r2 = hsa2.reactions.get_by_id('XYLUDte').copy()
r2.gene_reaction_rule = '12976'
model.add_reactions([r1,r2])
# D-arabitol
r1 = hsa2.reactions.get_by_id('EX_abt__D_e').copy()
r1.lower_bound = 0.0
r2 = hsa2.reactions.get_by_id('ABT_Dt').copy()
r2.gene_reaction_rule = '12976'
model.add_reactions([r1,r2])
# D-ribulose
r1 = hsa2.reactions.get_by_id('EX_rbl__D_e').copy()
r1.lower_bound = 0.0
r2 = hsa2.reactions.get_by_id('HMR_9185').copy()
r2.id = 'RBL_Dt'
r2.name = 'D-Ribulose Transport via Diffusion'
r2.gene_reaction_rule = '12976'
model.add_reactions([r1,r2])
```

In [85]:

```
for x in ['EX_lyx__L_e','EX_abt_e','EX_xylt_e','EX_xylu__D_e','EX_abt__D_e','EX_rbl__D_e']:
    with model:
        model.reactions.get_by_id('EX_o2_e').lower_bound = -1000.0
        model.reactions.get_by_id('EX_glc__D_e').lower_bound = 0.0
        model.reactions.get_by_id(x).lower_bound = -2.0
        sol = model.optimize()
        print(x, sol.status, sol.objective_value)
```

```
EX_lyx__L_e optimal 0.1243786738384878
EX_abt_e optimal 0.13371608409296598
EX_xylt_e optimal 0.13371608409296598
EX_xylu__D_e optimal 0.12437867383848569
EX_abt__D_e optimal 0.13371608409296598
EX_rbl__D_e optimal 0.12437867383848569
```

In [86]:

```
# Phosphoketolase is present in R. toruloides
display(Annotation.loc[['13382']])
Show_Data(['13382'])
```

|  | Combined Annotations | Signal P | Sc288c Orthologs | Human Orthologs | Sc288 Best Hit | Human Blast | Essential | WolfPSort | C Terminal |
| --- | --- | --- | --- | --- | --- | --- | --- | --- | --- |
| RTO4\_ID |  |  |  |  |  |  |  |  |  |
| 13382 | K01621: xfp, xpk; xylulose-5-phosphate/fructos... |  |  |  |  |  | Not Essential | cyto 12, cysk 6, cyto\_mito 6, pero 5 | AEQ\* |

| strain | WT | | | | | | | | | | | | | | | | |
| --- | --- | --- | --- | --- | --- | --- | --- | --- | --- | --- | --- | --- | --- | --- | --- | --- | --- |
| condition | G\_MM | C\_MM | G\_SD | | GX\_SD | | | X\_SD | | A\_SD | | C\_SD | | MM\_CN120 | | MM\_CN5 | Diversity\_Sample |
| phase | exp | exp | exp | stat | exp | trans | stat | exp | stat | exp | stat | exp | stat | exp | stat | exp | exp |
| proteinId | Set1 | Set1 | Set2 | Set2 | Set2 | Set2 | Set2 | Set2 | Set2 | Set2 | Set2 | Set2 | Set2 | Set3 | Set3 | Set3 | Set3 |
| 13382 | 8.976534 | 6.867487 | 8.071529 | 7.579503 | 8.036615 | 7.441585 | 7.531568 | 7.132743 | 7.051156 | 6.795327 | 6.404528 | 7.943428 | 7.085854 | 8.988087 | 8.835574 | 8.575917 | 9.595443 |

| strain | WT | | | | | | | | | | |
| --- | --- | --- | --- | --- | --- | --- | --- | --- | --- | --- | --- |
| condition | G\_SD | | GX\_SD | | | X\_SD | | A\_SD | | C\_SD | |
| proteinId | exp | stat | exp | trans | stat | exp | stat | exp | stat | exp | stat |
| 13382 | 37.454848 | 49.146137 | 36.657525 | 45.567608 | 50.296360 | 32.286992 | 40.526080 | 33.812760 | 36.358192 | 49.648174 | 46.565431 |

|  | Glucose | Xylose | Arabinose | Acetate | Coumarate | Ferulate | YNB Oleic Acid | YNB Ricinoleic Acid | YNB Glucose | YNB Gluc DOC | YPD |
| --- | --- | --- | --- | --- | --- | --- | --- | --- | --- | --- | --- |
| proteinId |  |  |  |  |  |  |  |  |  |  |  |
| 13382 | -0.014869 | 0.036227 | -0.027365 | -0.075881 | 0.169243 | -0.030654 | -0.186563 | -0.286777 | -0.356607 | -0.408099 | 0.090867 |

In [87]:

```
for x in ['13382']:
    if x in model.genes:
        for r in sorted(model.genes.get_by_id(x).reactions, key=lambda x: x.id):
            print(r, r.gene_reaction_rule)
    else:
        print(x, 'no reactions')
    print()
```

```
13382 no reactions
```

In [88]:

```
FPK = cobra.Reaction('FPK')
FPK.name = 'Fructose-6-phosphate phosphoketolase'
FPK.lower_bound = 0
FPK.upper_bound = 1000
FPK.add_metabolites({model.metabolites.f6p_c: -1.0, model.metabolites.pi_c: -1.0,
                     model.metabolites.e4p_c: 1.0, model.metabolites.actp_c: 1.0, model.metabolites.h2o_c: 1.0})
FPK.gene_reaction_rule = '13382'

XPK = cobra.Reaction('XPK')
XPK.name = 'Xylulose-5-phosphate phosphoketolase'
XPK.lower_bound = 0
XPK.upper_bound = 1000
XPK.add_metabolites({model.metabolites.xu5p__D_c: -1.0, model.metabolites.pi_c: -1.0,
                     model.metabolites.g3p_c: 1.0, model.metabolites.actp_c: 1.0, model.metabolites.h2o_c: 1.0})
XPK.gene_reaction_rule = '13382'

model.add_reactions([FPK, XPK])
print(model.reactions.FPK)
print(model.reactions.XPK)
```

```
FPK: f6p_c + pi_c --> actp_c + e4p_c + h2o_c
XPK: pi_c + xu5p__D_c --> actp_c + g3p_c + h2o_c
```

In [89]:

```
with model:
    model.reactions.get_by_id('EX_o2_e').lower_bound = -1000.0
    model.reactions.get_by_id('EX_glc__D_e').lower_bound = -1.0
    sol = model.optimize()
    print('Growth on glucose')
    print(sol.status, sol.objective_value)
    print()
    model.reactions.get_by_id('EX_glc__D_e').lower_bound = 0.0
    model.reactions.get_by_id('EX_T4hcinnm_e').lower_bound = -1.0
    sol = model.optimize()
    print('Growth on p-coumarate')
    print(sol.status, sol.objective_value)
    print()
    model.reactions.get_by_id('EX_T4hcinnm_e').lower_bound = 0.0
    model.reactions.get_by_id('EX_xyl__D_e').lower_bound = -1.0
    sol = model.optimize()
    print('Growth on D-xylose')
    print(sol.status, sol.objective_value)
    print()
    model.reactions.get_by_id('EX_xyl__D_e').lower_bound = 0.0
    model.reactions.get_by_id('EX_arab__L_e').lower_bound = -1.0
    sol = model.optimize()
    print('Growth on L-arabinose')
    print(sol.status, sol.objective_value)
```

```
Growth on glucose
optimal 0.07441621983033019

Growth on p-coumarate
optimal 0.08000655065055255

Growth on D-xylose
optimal 0.06163879393904248

Growth on L-arabinose
optimal 0.06163879393904248
```

### Lipid mobilization¶

In [90]:

```
temp =  ['SK_triglyc_RT_d','SK_epistest_RT_d','SK_ergstest_RT_d','SK_fecostest_RT_d','SK_lanostest_RT_d',
         'SK_zymstest_RT_d']
with model:
    model.reactions.get_by_id('EX_o2_e').lower_bound = -1000.0
    model.reactions.get_by_id('EX_glc__D_e').lower_bound = 0.0
    for x in temp:
        model.reactions.get_by_id(x).lower_bound = -0.001
        sol = model.optimize()
        model.reactions.get_by_id(x).lower_bound = 0.0
        print('Growth on '+x.replace('SK_',''))
        print(sol.status, sol.objective_value)
        print()
```

```
Growth on triglyc_RT_d
optimal 0.0829953306710842

Growth on epistest_RT_d
optimal 0.024516652718945087

Growth on ergstest_RT_d
optimal 0.024519922898961523

Growth on fecostest_RT_d
optimal 0.024516652718945087

Growth on lanostest_RT_d
optimal 0.026826463860177157

Growth on zymstest_RT_d
optimal 0.024566313515313215
```

### Add carotenoid production pathway¶

In [91]:

```
Annotation.loc[['8585','8589']]
```

Out[91]:

|  | Combined Annotations | Signal P | Sc288c Orthologs | Human Orthologs | Sc288 Best Hit | Human Blast | Essential | WolfPSort | C Terminal |
| --- | --- | --- | --- | --- | --- | --- | --- | --- | --- |
| RTO4\_ID |  |  |  |  |  |  |  |  |  |
| 8585 | K02292: crtO; beta-carotene ketolase (CrtO type) | S |  |  |  |  | Not Essential | mito 7, plas 5, cyto 4, extr 4, E.R. 4, golg 3 | LRG\* |
| 8589 | HMMPfam:Squalene/phytoene synthase:PF00494,Pro... | A |  |  |  |  | Not Essential | plas 18, extr 3, mito 2, E.R. 2 | QAL\* |

In [92]:

```
# Lycopene biosynthesis in R. toruloides
# https://link.springer.com/article/10.1007%2Fs10529-017-2324-3

m = model.metabolites.get_by_id('ggdp_c').copy()
m.id = 'ggdp_r'
m.compartment = 'r'
model.add_metabolites([m])

r = cobra.Reaction('GGDPter')
r.name = 'Geranylgeranyl diphosphate transport, endoplasmic reticulum'
model.add_reactions([r])
r.add_metabolites({'ggdp_c': -1.0, 'ggdp_r': 1.0})

m1 = cobra.Metabolite('phyto_r', formula='C40H64', name='All-trans-Phytoene', compartment='r')
m2 = cobra.Metabolite('lycop_r', formula='C40H56', name='All-trans-Lycopene', compartment='r')
model.add_metabolites([m1,m2])

r = cobra.Reaction('PSYer')
r.name = 'Phytoene synthase'
r.gene_reaction_rule = '8589'
model.add_reactions([r])
r.add_metabolites({'ggdp_r': -2.0, 'phyto_r': 1.0, 'ppi_r': 2.0})

r = cobra.Reaction('PDSer')
r.name = 'Phytoene dehydrogenase'
r.gene_reaction_rule = '8585'
model.add_reactions([r])
r.add_metabolites({'phyto_r': -1.0, 'nadp_r': -4.0,
                   'lycop_r': 1.0, 'nadph_r': 4.0, 'h_r': 4.0})

m1 = cobra.Metabolite('gcaro_r', formula='C40H56', name='Gamme-Carotene', compartment='r')
m2 = cobra.Metabolite('bcaro_r', formula='C40H56', name='Beta-Carotene', compartment='r')
model.add_metabolites([m1,m2])

r = cobra.Reaction('LYCOPCer')
r.name = 'Lycopene beta-cyclase'
r.gene_reaction_rule = '8589'
model.add_reactions([r])
r.add_metabolites({'lycop_r': -1.0, 'gcaro_r': 1.0})

r = cobra.Reaction('GCAROCer')
r.name = 'Gamma-carotene beta-cyclase'
r.gene_reaction_rule = '8589'
model.add_reactions([r])
r.add_metabolites({'gcaro_r': -1.0, 'bcaro_r': 1.0})

m = model.metabolites.get_by_id('bcaro_r').copy()
m.id = 'bcaro_d'
m.compartment = 'd'
model.add_metabolites([m])

r = cobra.Reaction('BCAROtrd')
r.name = 'Transport of Beta-Carotene from E.R. to lipid droplet'
model.add_reactions([r])
r.add_metabolites({'bcaro_r': -1.0, 'bcaro_d': 1.0})

r = cobra.Reaction('DM_bcaro_d')
r.name = 'Demand needed to allow Beta-Carotene to accumulate in lipid droplet'
model.add_reactions([r])
r.add_metabolites({'bcaro_d': -1.0})
```

In [93]:

```
with model:
    model.reactions.get_by_id('EX_o2_e').lower_bound = -1000.0
    model.reactions.get_by_id('EX_glc__D_e').lower_bound = -1.0
    model.objective = 'DM_bcaro_d'
    sol = model.optimize()
    print('Maximum beta-carotene production')
    print(sol.objective_value)
    model.objective = 'SK_triglyc_RT_d'
    sol = model.optimize()
    print('Maximum triacylglycerol production')
    print(sol.objective_value)
    model.objective = 'SK_zymstest_RT_d'
    sol = model.optimize()
    print('Maximum zymosterol ester production')
    print(sol.objective_value)
```

```
Maximum beta-carotene production
0.0891476274165205
Maximum triacylglycerol production
0.0003847829981437416
Maximum zymosterol ester production
0.0007203185242092204
```

In [94]:

```
# Run pFBA and check what is consumed and produced 
sol = cobra.flux_analysis.pfba(model)
display(model.summary(sol))
```

|  | IN\_FLUXES | | OUT\_FLUXES | | OBJECTIVES | |
| --- | --- | --- | --- | --- | --- | --- |
|  | ID | FLUX | ID | FLUX | ID | FLUX |
| 0 | o2\_e | 2.023367 | h2o\_e | 4.090590 | BIOMASS\_RT | 0.074416 |
| 1 | glc\_\_D\_e | 1.000000 | co2\_e | 2.405858 | NaN | NaN |
| 2 | nh4\_e | 0.596581 | h\_e | 0.117545 | NaN | NaN |
| 3 | k\_e | 0.043586 | spmd\_e | 0.052706 | NaN | NaN |

In [95]:

```
# Model is predicting spermidine production, where is it coming from?
for r in sorted(model.metabolites.get_by_id('spmd_c').reactions, key=lambda x: x.id):
    print(r, r.gene_reaction_rule, sol[r.id])
```

```
BIOMASS_RT: 0.957502 13BDglcn_c + 0.177315 16BDglcn_c + 0.001283 5mthf_c + 0.577574 alatrna_c + 0.146675 argtrna_c + 0.10797 asntrna_c + 0.197296 asptrna_c + 110.681641 atp_c + 0.002418 btn_m + 0.000832 ca2_c + 0.001792 camp_c + 0.031005 chitin_c + 1.8e-05 clpn_RT_m + 0.00077 coa_c + 0.044881 ctp_c + 0.000525 cu2_c + 0.034269 cystrna_c + 0.002451 datp_c + 0.004285 dctp_c + 0.003763 dgtp_c + 0.002523 dttp_c + 0.004031 ergst_r + 1.6e-05 ergstest_RT_r + 0.000751 fad_c + 0.000597 fe2_c + 0.000597 fe3_c + 0.117898 glntrna_c + 0.208673 glutrna_c + 0.518069 glycogen_c + 0.51322 glytrna_c + 0.00192 gthrd_c + 0.051085 gtp_c + 106.71258 h2o_c + 0.000691 hemeA_m + 0.061167 histrna_c + 0.158569 iletrna_c + 0.585703 k_c + 0.320205 leutrna_c + 0.003107 lipopb_m + 0.172334 lystrna_c + 0.807941 mannan_r + 0.05705 mettrna_c + 0.061716 mg2_c + 0.001292 mlthf_c + 0.000607 mn2_c + 0.026099 na1_c + 0.000888 nad_c + 0.000795 nadp_c + 1.4e-05 pa_RT_r + 0.000172 pc_RT_r + 0.00013 pe_RT_r + 0.095164 phetrna_c + 0.235897 protrna_c + 2e-05 ps_RT_r + 0.004006 psphings_r + 4.7e-05 ptd1ino_RT_r + 0.006524 ptrc_c + 0.0024 pydx5p_c + 0.00074 q9_m + 0.00099 ribflv_c + 0.33687 sertrna_c + 0.003967 spmd_c + 0.001327 thf_c + 0.001393 thmpp_c + 0.21813 thrtrna_c + 0.023372 tre_c + 0.000432 triglyc_RT_r + 0.023319 trptrna_c + 0.062363 tyrtrna_c + 0.051129 utp_c + 0.275168 valtrna_c + 0.00051 zn2_c + 0.004156 zymst_r + 1.7e-05 zymstest_RT_d --> 110.632394 adp_c + 110.632394 h_c + 110.632394 pi_c + 0.209364 ppi_c  0.07441621983033947
POLYAO3: h2o_c + o2_c + sprm_c --> bamppald_c + h2o2_c + spmd_c 15939 5.730048926941289e-05
SPMDAT1: accoa_c + spmd_c --> N1aspmd_c + coa_c + h_c 9636 0.0
SPMDAT2: accoa_c + spmd_c --> coa_c + h_c + n8aspmd_c 9636 0.0
SPMDt3i: h_c + spmd_e --> h_e + spmd_c 11017 or 11649 or 13128 or 14774 0.0
SPMDtex2: spmd_c --> spmd_e 13423 0.0527060318570277
SPMDtmr: h_m + spmd_c <=> h_c + spmd_m 11649 or 14774 0.0
SPMS: ametam_c + ptrc_c --> 5mta_c + h_c + spmd_c 16833 0.05300124100109466
SPRMS: ametam_c + spmd_c --> 5mta_c + h_c + sprm_c 16833 5.730048926941289e-05
```

In [96]:

```
# Spermidine is coming from putrescine, do we have all the reactions needed for putrescine degradation?
for r in sorted(model.metabolites.get_by_id('ptrc_c').reactions, key=lambda x: x.id):
    print(r, r.gene_reaction_rule, sol[r.id])
```

```
BIOMASS_RT: 0.957502 13BDglcn_c + 0.177315 16BDglcn_c + 0.001283 5mthf_c + 0.577574 alatrna_c + 0.146675 argtrna_c + 0.10797 asntrna_c + 0.197296 asptrna_c + 110.681641 atp_c + 0.002418 btn_m + 0.000832 ca2_c + 0.001792 camp_c + 0.031005 chitin_c + 1.8e-05 clpn_RT_m + 0.00077 coa_c + 0.044881 ctp_c + 0.000525 cu2_c + 0.034269 cystrna_c + 0.002451 datp_c + 0.004285 dctp_c + 0.003763 dgtp_c + 0.002523 dttp_c + 0.004031 ergst_r + 1.6e-05 ergstest_RT_r + 0.000751 fad_c + 0.000597 fe2_c + 0.000597 fe3_c + 0.117898 glntrna_c + 0.208673 glutrna_c + 0.518069 glycogen_c + 0.51322 glytrna_c + 0.00192 gthrd_c + 0.051085 gtp_c + 106.71258 h2o_c + 0.000691 hemeA_m + 0.061167 histrna_c + 0.158569 iletrna_c + 0.585703 k_c + 0.320205 leutrna_c + 0.003107 lipopb_m + 0.172334 lystrna_c + 0.807941 mannan_r + 0.05705 mettrna_c + 0.061716 mg2_c + 0.001292 mlthf_c + 0.000607 mn2_c + 0.026099 na1_c + 0.000888 nad_c + 0.000795 nadp_c + 1.4e-05 pa_RT_r + 0.000172 pc_RT_r + 0.00013 pe_RT_r + 0.095164 phetrna_c + 0.235897 protrna_c + 2e-05 ps_RT_r + 0.004006 psphings_r + 4.7e-05 ptd1ino_RT_r + 0.006524 ptrc_c + 0.0024 pydx5p_c + 0.00074 q9_m + 0.00099 ribflv_c + 0.33687 sertrna_c + 0.003967 spmd_c + 0.001327 thf_c + 0.001393 thmpp_c + 0.21813 thrtrna_c + 0.023372 tre_c + 0.000432 triglyc_RT_r + 0.023319 trptrna_c + 0.062363 tyrtrna_c + 0.051129 utp_c + 0.275168 valtrna_c + 0.00051 zn2_c + 0.004156 zymst_r + 1.7e-05 zymstest_RT_d --> 110.632394 adp_c + 110.632394 h_c + 110.632394 pi_c + 0.209364 ppi_c  0.07441621983033947
GGPTRCS: atp_c + glu__L_c + ptrc_c --> adp_c + ggptrc_c + h_c + pi_c 8959 0.0
ORNDC: h_c + orn_c --> co2_c + ptrc_c 12635 0.05348673241926779
PTRCAT1: accoa_c + ptrc_c --> aprut_c + coa_c + h_c 16046 0.0
PTRCOX1: h2o_c + o2_c + ptrc_c --> 4abutn_c + h2o2_c + nh4_c 13959 0.0
PTRCt3i: h_c + ptrc_e --> h_e + ptrc_c 11017 or 11649 or 13128 or 14774 0.0
PTRCtex2: ptrc_c --> ptrc_e 13423 0.0
SPMS: ametam_c + ptrc_c --> 5mta_c + h_c + spmd_c 16833 0.05300124100109466
```

In [97]:

```
Annotation.loc[['15939','9636','16833','16046','13959']]
```

Out[97]:

|  | Combined Annotations | Signal P | Sc288c Orthologs | Human Orthologs | Sc288 Best Hit | Human Blast | Essential | WolfPSort | C Terminal |
| --- | --- | --- | --- | --- | --- | --- | --- | --- | --- |
| RTO4\_ID |  |  |  |  |  |  |  |  |  |
| 15939 | K11450: KDM1A, AOF2, LSD1; lysine-specific his... | S | FMS1 |  | FMS1 | KDM1B | Not Essential | cyto 19, mito 4, extr 4 | ALL\* |
| 9636 | K03790: rimJ; ribosomal-protein-alanine N-acet... |  |  |  |  |  | Not Essential | mito 14, cyto 8.5, cyto\_nucl 6, nucl 2.5 | DEV\* |
| 16833 | K00293: LYS9; saccharopine dehydrogenase (NADP... |  | SPE3,LYS9 | SRM,AASS | LYS9 | SRMe | Essential | cyto 19, cysk 6 | KVQ\* |
| 16046 | K00657: speG; diamine N-acetyltransferase |  |  | SAT2,SAT1 |  | SAT2 | Not Essential | cysk 11, mito 7, cyto 4, pero 4, mito\_nucl 4, ... | LAQ\* |
| 13959 | K11182: AOC1, ABP1; diamine oxidase | S |  | AOC2,AOC3 |  | AOC1 | Not Essential | extr 23, E.R. 2 | SIV\* |

In [98]:

```
for x in ['15939','9636','16833','16046','13959']:
    print(x)
    for r in model.genes.get_by_id(x).reactions:
        print(r, sol[r.id])
    print()
```

```
15939
POLYAO: N1aspmd_c + h2o_c + o2_c --> aprut_c + bamppald_c + h2o2_c 0.0
POLYAO2: N1sprm_c + h2o_c + o2_c --> N1aspmd_c + bamppald_c + h2o2_c 0.0
POLYAO3: h2o_c + o2_c + sprm_c --> bamppald_c + h2o2_c + spmd_c 5.730048926941289e-05

9636
SPMDAT1: accoa_c + spmd_c --> N1aspmd_c + coa_c + h_c 0.0
SPMDAT2: accoa_c + spmd_c --> coa_c + h_c + n8aspmd_c 0.0

16833
SPRMS: ametam_c + spmd_c --> 5mta_c + h_c + sprm_c 5.730048926941289e-05
SACCD1: L2aadp6sa_c + glu__L_c + h_c + nadph_c <=> h2o_c + nadp_c + saccrp__L_c 0.01282444482824172
SPMS: ametam_c + ptrc_c --> 5mta_c + h_c + spmd_c 0.05300124100109466

16046
PTRCAT1: accoa_c + ptrc_c --> aprut_c + coa_c + h_c 0.0

13959
AACTOOR: aact_c + h2o_c + o2_c --> h2o2_c + mthgxl_c + nh4_c 0.0
MHISOR: h2o_c + mhista_c + o2_c --> 3mldz_c + h2o2_c + nh4_c 0.0
NMPTRCOX: nmptrc_c + o2_c --> 1mpyr_c + h2o2_c + nh4_c 0.0
42A12BOOX: dopa_c + h2o_c + o2_c --> 34dhpac_c + h2o2_c + nh4_c 0.0
MAOX: h2o_c + mma_c + o2_c --> fald_c + h2o2_c + nh4_c 0.0
PTRCOX1: h2o_c + o2_c + ptrc_c --> 4abutn_c + h2o2_c + nh4_c 0.0
PEAMNO: h2o_c + o2_c + peamn_c --> h2o2_c + nh4_c + pacald_c 0.0
13DAMPPOX: 13dampp_c + h2o_c + o2_c --> bamppald_c + h2o2_c + nh4_c 0.0
TRYPTAOX: h2o_c + o2_c + trypta_c --> h2o2_c + id3acald_c + nh4_c 0.0
HISTASE: h2o_c + hista_c + o2_c --> h2o2_c + im4act_c + nh4_c 0.0
TYROXDAc: h2o_c + o2_c + tym_c --> 4hoxpacd_c + h2o2_c + nh4_c 0.0
```

In [99]:

```
# 15939 FMS1 reactions are incorrect
# POLYAO: N1aspmd_c + h2o_c + o2_c --> ptrc_c + 3aap_c + h2o2_c
# POLYAO2: N1sprm_c + h2o_c + o2_c --> spmd_c + 3aap_c + h2o2_c
# The metabolic fate of 3-acetamidopropanal is unclear, although it may be metabolized within peroxisomes by 
# aldehyde and alcohol dehydrogenases and may give rise to β-alanine via EC 3.5.1.21 (reviewed in [Seiler04])
# Add a demand reaction for 3aap_c for now
m = cobra.Metabolite(id='3aap_c', name='3-acetamidopropanal', formula='C5H9NO2', charge=0, compartment='c')
model.add_metabolites([m])
model.reactions.get_by_id('POLYAO').add_metabolites({'aprut_c': -1.0, 'bamppald_c': -1.0,
                                                     'ptrc_c': 1.0, '3aap_c': 1.0})
model.reactions.get_by_id('POLYAO2').add_metabolites({'N1aspmd_c': -1.0, 'bamppald_c': -1.0,
                                                      'spmd_c': 1.0, '3aap_c': 1.0})
r = cobra.Reaction(id='DM_3aap_c', name='3-acetamidopropanal demand')
r.add_metabolites({m: -1.0})
model.add_reactions([r])
```

In [100]:

```
# PTRCAT1 by 16046 speG, which can also work on spermine and spermidine
# SPMDAT1 and SPMDAT2 have 9636, GNAT family acetyltransferase with 30% identity to spermidine N-acetyltransferase
# iMM904 has DIAT: accoa_c + sprm_c → coa_c + h_c + N1sprm_c without GPR
model.reactions.get_by_id('SPMDAT1').gene_reaction_rule = '16046 or 9636'
model.reactions.get_by_id('SPMDAT2').gene_reaction_rule = '16046 or 9636'
r = sce.reactions.get_by_id('DIAT').copy()
r.gene_reaction_rule = '16046'
model.add_reactions([r])
```

### Check genes¶

In [101]:

```
for g in model.genes:
    if not g.id[0].isdigit():
        print(g)
```

```
RTO3_946004
RTO3_946002
RTO3_979594
RTO3_900622
RTO3_900641
RTO3_879341
RTO3_945994
RTO3_879512
RTO3_900624
RTO3_946005
RTO3_945995
RTO3_874609
RTO3_900623
RTO3_900663
RTO3_879280
COPII
ERG27
```

COPII vesicle comprised of SAR1 (14970), SEC23 (12082), SEC24 (11898 and 16400), SEC13 (16526), SEC31 (12671)  
SEC12 (10521) guanine nucleotide exchange factor converts SAR1-GDP to SAR-GTP  
https://www.sciencedirect.com/science/article/pii/S0014579307001536  
COPII a coatomer that transports proteins from the rough endoplasmic reticulum to the golgi apparatus  
These are essential genes, but only COPII-mediated vesicular transport of ceramide is present in the model

In [102]:

```
for r in model.reactions:
    if 'COPII' in r.gene_reaction_rule:
        print(r.id, r.gene_reaction_rule)
```

```
CER618trg 14324 or COPII
CER124trg 14324 or COPII
CER126trg 14324 or COPII
CER224trg 14324 or COPII
CER226trg 14324 or COPII
CER324trg 14324 or COPII
CER326trg 14324 or COPII
```

ERG27 not in the genome  
Replace with 14213, 3-ketosteroid reductase HSD17B7  
https://pubmed.ncbi.nlm.nih.gov/11165030/, https://pubmed.ncbi.nlm.nih.gov/12829805/

In [103]:

```
for r in model.reactions:
    if 'ERG27' in r.gene_reaction_rule:
        print(r.id, r.gene_reaction_rule)
```

```
C3STKR1er ERG27
C3STKR2er ERG27
```

In [104]:

```
# Replace with 3-ketosteroid reductase 
model.reactions.get_by_id('C3STKR1er').gene_reaction_rule = '14213'
model.reactions.get_by_id('C3STKR2er').gene_reaction_rule = '14213'
```

RTO3\_genes are from the previous annotation that included the mitochondrial genome  
Mitochondrial\_genes  
RTO3\_946004 and RTO3\_946002 : COX1 and COX2 and COX3  
RTO3\_979594 : COB  
RTO3\_874609 and RTO3\_879341 and RTO3\_879512 and RTO3\_900622 and RTO3\_900624 and RTO3\_900641 and RTO3\_945994 and RTO3\_945995 and RTO3\_946005 : NAD1 and NAD2 and NAD3 and NAD4 and NAD4L and NAD5 and NAD6  
RTO3\_879280 and RTO3\_900623 and RTO3\_900663 : ATP6 and ATP8 and ATP9

In [105]:

```
for r in model.reactions:
    if 'RTO3' in r.gene_reaction_rule:
        print(r.id, r.gene_reaction_rule)
```

```
CYOR_u9m RTO3_979594 and 9705 and 9198 and 11618 and 15758 and 15681 and 12966 and 15231 and 13608 and 13614 and 8802
ATPS3m RTO3_879280 and RTO3_900623 and RTO3_900663 and 9080 and 9619 and 9940 and 10674 and 11958 and 11967 and 13053 and 13252 and 13424 and 13759 and 13842 and 14786 and 14912 and 15287 and 15589 and 15880 and 16359
CYOO6m RTO3_946004 and RTO3_946002 and 9165 and 13628 and 10685 and 12506 and 11352 and 13275 and 11769 and 11317 and 12210 and 13007 and 8802
NADH2_u9m2 10010 and 10017 and 10318 and 10541 and 10607 and 11151 and 11411 and 11702 and 11857 and 12482 and 12497 and 12543 and 12653 and 13017 and 13214 and 13268 and 13411 and 13656 and 13925 and 14078 and 14261 and 14418 and 14717 and 14900 and 15179 and 15864 and 15947 and 15973 and 15998 and 8444 and 8446 and 8530 and 8699 and 8817 and 8909 and 9144 and 9348 and RTO3_874609 and RTO3_879341 and RTO3_879512 and RTO3_900622 and RTO3_900624 and RTO3_900641 and RTO3_945994 and RTO3_945995 and RTO3_946005
```

In [106]:

```
model.reactions.get_by_id('CYOR_u9m').gene_reaction_rule = 'COB and 9705 and 9198 and 11618 and 15758 and 15681 and 12966 and 15231 and 13608 and 13614 and 8802'
model.reactions.get_by_id('ATPS3m').gene_reaction_rule = 'ATP6 and ATP8 and ATP9 and 9080 and 9619 and 9940 and 10674 and 11958 and 11967 and 13053 and 13252 and 13424 and 13759 and 13842 and 14786 and 14912 and 15287 and 15589 and 15880 and 16359'
model.reactions.get_by_id('CYOO6m').gene_reaction_rule = 'COX1 and COX2 and COX3 and 9165 and 13628 and 10685 and 12506 and 11352 and 13275 and 11769 and 11317 and 12210 and 13007 and 8802'
model.reactions.get_by_id('NADH2_u9m2').gene_reaction_rule = 'NAD1 and NAD2 and NAD3 and NAD4 and NAD4L and NAD5 and NAD6 and 10010 and 10017 and 10318 and 10541 and 10607 and 11151 and 11411 and 11702 and 11857 and 12482 and 12497 and 12543 and 12653 and 13017 and 13214 and 13268 and 13411 and 13656 and 13925 and 14078 and 14261 and 14418 and 14717 and 14900 and 15179 and 15864 and 15947 and 15973 and 15998 and 8444 and 8446 and 8530 and 8699 and 8817 and 8909 and 9144 and 9348'
```

In [107]:

```
model.summary()
```

Out[107]:

|  | IN\_FLUXES | | OUT\_FLUXES | | OBJECTIVES | |
| --- | --- | --- | --- | --- | --- | --- |
|  | ID | FLUX | ID | FLUX | ID | FLUX |
| 0 | o2\_e | 2.071233 | h2o\_e | 4.119883 | BIOMASS\_RT | 0.077636 |
| 1 | glc\_\_D\_e | 1.000000 | co2\_e | 2.362247 | NaN | NaN |
| 2 | nh4\_e | 0.512423 | h\_e | 0.175673 | NaN | NaN |
| 3 | k\_e | 0.045472 | NaN | NaN | NaN | NaN |

In [108]:

```
# Remove GAM and NGAM to re-estimate
model.reactions.get_by_id('ATPM').lower_bound = 0.0
GAM_old = model.reactions.get_by_id('BIOMASS_RT').get_coefficient('adp_c')
model.reactions.get_by_id('BIOMASS_RT').add_metabolites({'atp_c': GAM_old, 'h2o_c': GAM_old,
                                                         'adp_c': -GAM_old, 'h_c': -GAM_old, 'pi_c': -GAM_old})
```

In [109]:

```
with model:
    model.reactions.get_by_id('EX_o2_e').lower_bound = -1000.0
    model.reactions.get_by_id('EX_glc__D_e').lower_bound = -0.0316392670613915
    model.objective = 'ATPM'
    sol = model.optimize()
    print(sol.objective_value)
    display(model.summary())
NGAM = round(sol.objective_value,2)
```

```
1.2233849930404712
```

|  | IN\_FLUXES | | OUT\_FLUXES | | OBJECTIVES | |
| --- | --- | --- | --- | --- | --- | --- |
|  | ID | FLUX | ID | FLUX | ID | FLUX |
| 0 | o2\_e | 0.189836 | co2\_e | 0.189836 | ATPM | 1.223385 |
| 1 | glc\_\_D\_e | 0.031639 | h2o\_e | 0.189836 | NaN | NaN |

In [110]:

```
temp = np.arange(0.2, 2.5, 0.2)
GAM = []
for x in temp:
    with model:
        model.reactions.get_by_id('EX_o2_e').lower_bound = -1000.0
        model.reactions.get_by_id('EX_glc__D_e').lower_bound = -x
        model.reactions.get_by_id('BIOMASS_RT').lower_bound = 0.0756654696*x
        model.objective = 'ATPM'
        sol = model.optimize()
        GAM.append(sol.objective_value/0.0756654696/x)
plt.scatter(temp,GAM)
print(np.mean(GAM), np.std(GAM))
```

```
140.24938592311105 4.643420789074446e-13
```

In [111]:

```
temp = np.arange(0.2, 2.5, 0.2)
y = []
GAM = round(np.mean(GAM), 6)
for x in temp:
    with model:
        model.reactions.get_by_id('EX_o2_e').lower_bound = -1000.0
        model.reactions.get_by_id('EX_glc__D_e').lower_bound = -x
        model.reactions.get_by_id('BIOMASS_RT').add_metabolites({'atp_c': -GAM, 'h2o_c': -GAM,
                                                                 'adp_c': GAM, 'h_c': GAM, 'pi_c': GAM})
        sol = model.optimize()
        y.append(sol.objective_value)
fit = np.polyfit(temp,y,1)
print(fit)
plt.plot(temp, y, 'o', temp, np.poly1d(fit)(temp), '--k')
```

```
[7.56654696e-02 1.28197512e-16]
```

Out[111]:

```
[<matplotlib.lines.Line2D at 0x7fc9a202e518>,
 <matplotlib.lines.Line2D at 0x7fc9a202eef0>]
```

In [112]:

```
# Set NGAM and GAM to new values
model.reactions.get_by_id('ATPM').lower_bound = NGAM
model.reactions.get_by_id('BIOMASS_RT').add_metabolites({'atp_c': -GAM, 'h2o_c': -GAM,
                                                         'adp_c': GAM, 'h_c': GAM, 'pi_c': GAM})
```

In [113]:

```
sol = cobra.flux_analysis.pfba(model)
display(model.summary(sol))
```

|  | IN\_FLUXES | | OUT\_FLUXES | | OBJECTIVES | |
| --- | --- | --- | --- | --- | --- | --- |
|  | ID | FLUX | ID | FLUX | ID | FLUX |
| 0 | o2\_e | 2.286682 | h2o\_e | 4.221148 | BIOMASS\_RT | 0.073379 |
| 1 | glc\_\_D\_e | 1.000000 | co2\_e | 2.559898 | NaN | NaN |
| 2 | nh4\_e | 0.484323 | h\_e | 0.167878 | NaN | NaN |
| 3 | k\_e | 0.042978 | NaN | NaN | NaN | NaN |

In [114]:

```
for r in sorted(model.reactions, key=lambda x: x.id):
    if not r.boundary and sum(abs(x) for x in r.check_mass_balance().values()) > 1e-12:
        print(r, r.gene_reaction_rule, r.check_mass_balance())
```

```
BIOMASS_RT: 0.957502 13BDglcn_c + 0.177315 16BDglcn_c + 0.001283 5mthf_c + 0.577574 alatrna_c + 0.146675 argtrna_c + 0.10797 asntrna_c + 0.197296 asptrna_c + 140.298633 atp_c + 0.002418 btn_m + 0.000832 ca2_c + 0.001792 camp_c + 0.031005 chitin_c + 1.8e-05 clpn_RT_m + 0.00077 coa_c + 0.044881 ctp_c + 0.000525 cu2_c + 0.034269 cystrna_c + 0.002451 datp_c + 0.004285 dctp_c + 0.003763 dgtp_c + 0.002523 dttp_c + 0.004031 ergst_r + 1.6e-05 ergstest_RT_r + 0.000751 fad_c + 0.000597 fe2_c + 0.000597 fe3_c + 0.117898 glntrna_c + 0.208673 glutrna_c + 0.518069 glycogen_c + 0.51322 glytrna_c + 0.00192 gthrd_c + 0.051085 gtp_c + 136.32957199999998 h2o_c + 0.000691 hemeA_m + 0.061167 histrna_c + 0.158569 iletrna_c + 0.585703 k_c + 0.320205 leutrna_c + 0.003107 lipopb_m + 0.172334 lystrna_c + 0.807941 mannan_r + 0.05705 mettrna_c + 0.061716 mg2_c + 0.001292 mlthf_c + 0.000607 mn2_c + 0.026099 na1_c + 0.000888 nad_c + 0.000795 nadp_c + 1.4e-05 pa_RT_r + 0.000172 pc_RT_r + 0.00013 pe_RT_r + 0.095164 phetrna_c + 0.235897 protrna_c + 2e-05 ps_RT_r + 0.004006 psphings_r + 4.7e-05 ptd1ino_RT_r + 0.006524 ptrc_c + 0.0024 pydx5p_c + 0.00074 q9_m + 0.00099 ribflv_c + 0.33687 sertrna_c + 0.003967 spmd_c + 0.001327 thf_c + 0.001393 thmpp_c + 0.21813 thrtrna_c + 0.023372 tre_c + 0.000432 triglyc_RT_r + 0.023319 trptrna_c + 0.062363 tyrtrna_c + 0.051129 utp_c + 0.275168 valtrna_c + 0.00051 zn2_c + 0.004156 zymst_r + 1.7e-05 zymstest_RT_d --> 140.249386 adp_c + 140.249386 h_c + 140.249386 pi_c + 0.209364 ppi_c  {'charge': -4.356142000000034, 'C': -40.730343000000175, 'H': -62.36094499999962, 'O': -17.698877000000152, 'N': -5.868878999999993, 'R': -4.027781, 'P': -0.3741850000001534, 'S': -0.104034, 'Ca': -0.000832, 'Cu': -0.000525, 'Fe': -0.001885, 'K': -0.585703, 'Mg': -0.061716, 'Mn': -0.000607, 'Na': -0.026099, 'Zn': -0.00051}
```

In [115]:

```
print(len(model.genes))
print(len(model.reactions))
print(len(model.metabolites))
model
```

```
1152
2216
1968
```

Out[115]:

|  |  |
| --- | --- |
| **Name** | R. toruloides |
| **Memory address** | 0x07fc99a3f8908 |
| **Number of metabolites** | 1968 |
| **Number of reactions** | 2216 |
| **Number of groups** | 0 |
| **Objective expression** | 1.0\*BIOMASS\_RT - 1.0\*BIOMASS\_RT\_reverse\_2b3e0 |
| **Compartments** | c, x, m, e, r, v, n, g, d, p |

In [116]:

```
for x in sorted(model.genes, key=lambda x: x.id):
    if not x.reactions:
        print(x)
print()
for x in sorted(model.metabolites, key=lambda x: x.id):
    if not x.reactions:
        print(x)
```

```
16129
ERG27
RTO3_874609
RTO3_879280
RTO3_879341
RTO3_879512
RTO3_900622
RTO3_900623
RTO3_900624
RTO3_900641
RTO3_900663
RTO3_945994
RTO3_945995
RTO3_946002
RTO3_946004
RTO3_946005
RTO3_979594

3hpcoa_c
4hbzcoa_m
T4hcinnm_m
T4hcinnm_p
bz12diol_c
coucoa_m
fer_p
pac_m
phaccoa_m
succoa_c
```

In [117]:

```
cobra.manipulation.remove_genes(model, [x for x in model.genes if not x.reactions])
model.remove_metabolites([x for x in model.metabolites if not x.reactions])
```

In [118]:

```
print(len(model.genes))
print(len(model.reactions))
print(len(model.metabolites))
print(len(set([m.id.rsplit('_',1)[0] for m in model.metabolites])))
print(len(model.compartments))
model
```

```
1135
2216
1958
1211
9
```

Out[118]:

|  |  |
| --- | --- |
| **Name** | R. toruloides |
| **Memory address** | 0x07fc99a3f8908 |
| **Number of metabolites** | 1958 |
| **Number of reactions** | 2216 |
| **Number of groups** | 0 |
| **Objective expression** | 1.0\*BIOMASS\_RT - 1.0\*BIOMASS\_RT\_reverse\_2b3e0 |
| **Compartments** | c, x, m, e, r, v, n, g, d |

In [119]:

```
cobra.io.save_json_model(model, "IFO0880_GPR_2d.json")
```

In [120]:

```
model_old = cobra.io.load_json_model("IFO0880_GPR_2c.json")
model_new = cobra.io.load_json_model("IFO0880_GPR_2d.json")
```

In [121]:

```
print('Removed reactions\n')
for r in sorted(model_old.reactions, key=lambda x: x.id):
    if r not in model_new.reactions:
        print(r)
```

```
Removed reactions

3HPCOAHYD: 3hpcoa_c + h2o_c --> 3hpp_c + coa_c + h_c
3OACOAR: 3ohodcoa_c + h_c + nadph_c --> 3hodcoa_c + nadp_c
3OAR100: 3odecACP_c + h_c + nadph_c <=> 3hdecACP_c + nadp_c
3OAR120: 3oddecACP_c + h_c + nadph_c <=> 3hddecACP_c + nadp_c
3OAR121: 3ocddec5eACP_c + h_c + nadph_c --> 3hcddec5eACP_c + nadp_c
3OAR140: 3omrsACP_c + h_c + nadph_c <=> 3hmrsACP_c + nadp_c
3OAR141: 3ocmrs7eACP_c + h_c + nadph_c --> 3hcmrs7eACP_c + nadp_c
3OAR160: 3opalmACP_c + h_c + nadph_c <=> 3hpalmACP_c + nadp_c
3OAR161: 3ocpalm9eACP_c + h_c + nadph_c --> 3hcpalm9eACP_c + nadp_c
3OAR180: 3ooctdACP_c + h_c + nadph_c <=> 3hoctaACP_c + nadp_c
3OAR181: 3ocvac11eACP_c + h_c + nadph_c --> 3hcvac11eACP_c + nadp_c
3OAR40: actACP_c + h_c + nadph_c <=> 3haACP_c + nadp_c
3OAR60: 3ohexACP_c + h_c + nadph_c <=> 3hhexACP_c + nadp_c
3OAR80: 3ooctACP_c + h_c + nadph_c <=> 3hoctACP_c + nadp_c
4HBHYOX: 4hbz_c + h_c + nadph_c + o2_c --> 34dhbz_c + h2o_c + nadp_c
COUCOAFm: T4hcinnm_m + atp_m + coa_m --> amp_m + coucoa_m + ppi_m
OGMEACPR: h_c + nadph_c + ogmeACP_c --> hgmeACP_c + nadp_c
OPMEACPR: h_c + nadph_c + opmeACP_c --> hpmeACP_c + nadp_c
RBK_L1: atp_c + rbl__L_c --> adp_c + h_c + ru5p__L_c
RHACOAR100: 3odcoa_c + h_c + nadph_c <=> R_3hdcoa_c + nadp_c
RHACOAR120: 3oddcoa_c + h_c + nadph_c <=> R_3hddcoa_c + nadp_c
RHACOAR140: 3otdcoa_c + h_c + nadph_c <=> R_3hmrscoa_c + nadp_c
RHACOAR60: 3ohcoa_c + h_c + nadph_c <=> R_3hhcoa_c + nadp_c
RHACOAR80: 3oocoa_c + h_c + nadph_c <=> R_3hocoa_c + nadp_c
XYLK2: atp_c + xylu__L_c --> adp_c + h_c + xu5p__L_c
```

In [122]:

```
print('Updated reactions\n')
for r in sorted(model_old.reactions, key=lambda x: x.id):
    if r in model_new.reactions:
        r2 = model_new.reactions.get_by_id(r.id)
        if (r.name == r2.name and r.reaction == r2.reaction and r.gene_reaction_rule == r2.gene_reaction_rule and
            r.lower_bound == r2.lower_bound and r.upper_bound == r2.upper_bound):
            pass
        else:
            print('Old', r, r.gene_reaction_rule)
            print('New', r2, r2.gene_reaction_rule)
            print()
```

```
Updated reactions

Old 4CMCOAS: T4hcinnm_c + atp_c + coa_c --> amp_c + coucoa_c + ppi_c 11833 or 13700 or 14802 or 16129
New 4CMCOAS: T4hcinnm_c + atp_c + coa_c --> amp_c + coucoa_c + ppi_c 11167 or 15748

Old AACOATx: acac_x + atp_x + coa_x <=> aacoa_x + amp_x + ppi_x 15276
New AACOATx: acac_x + atp_x + coa_x --> aacoa_x + amp_x + ppi_x 15276

Old ARABR: arab__L_c + h_c + nadph_c --> abt_c + nadp_c 11882 or 12784 or 9774
New ARABR: arab__L_c + h_c + nadph_c --> abt_c + nadp_c 9774

Old ATPS3m: adp_m + 3.0 h_c + pi_m --> atp_m + h2o_m + 2.0 h_m RTO3_879280 and RTO3_900623 and RTO3_900663 and 9080 and 9619 and 9940 and 10674 and 11958 and 11967 and 13053 and 13252 and 13424 and 13759 and 13842 and 14786 and 14912 and 15287 and 15589 and 15880 and 16359
New ATPS3m: adp_m + 3.0 h_c + pi_m --> atp_m + h2o_m + 2.0 h_m ATP6 and ATP8 and ATP9 and 9080 and 9619 and 9940 and 10674 and 11958 and 11967 and 13053 and 13252 and 13424 and 13759 and 13842 and 14786 and 14912 and 15287 and 15589 and 15880 and 16359

Old BIOMASS_RT: 0.957502 13BDglcn_c + 0.177315 16BDglcn_c + 0.001283 5mthf_c + 0.577574 alatrna_c + 0.146675 argtrna_c + 0.10797 asntrna_c + 0.197296 asptrna_c + 110.681641 atp_c + 0.002418 btn_m + 0.000832 ca2_c + 0.001792 camp_c + 0.031005 chitin_c + 1.8e-05 clpn_RT_m + 0.00077 coa_c + 0.044881 ctp_c + 0.000525 cu2_c + 0.034269 cystrna_c + 0.002451 datp_c + 0.004285 dctp_c + 0.003763 dgtp_c + 0.002523 dttp_c + 0.004031 ergst_r + 1.6e-05 ergstest_RT_r + 0.000751 fad_c + 0.000597 fe2_c + 0.000597 fe3_c + 0.117898 glntrna_c + 0.208673 glutrna_c + 0.518069 glycogen_c + 0.51322 glytrna_c + 0.00192 gthrd_c + 0.051085 gtp_c + 106.71258 h2o_c + 0.000691 hemeA_m + 0.061167 histrna_c + 0.158569 iletrna_c + 0.585703 k_c + 0.320205 leutrna_c + 0.003107 lipopb_m + 0.172334 lystrna_c + 0.807941 mannan_r + 0.05705 mettrna_c + 0.061716 mg2_c + 0.001292 mlthf_c + 0.000607 mn2_c + 0.026099 na1_c + 0.000888 nad_c + 0.000795 nadp_c + 1.4e-05 pa_RT_r + 0.000172 pc_RT_r + 0.00013 pe_RT_r + 0.095164 phetrna_c + 0.235897 protrna_c + 2e-05 ps_RT_r + 0.004006 psphings_r + 4.7e-05 ptd1ino_RT_r + 0.006524 ptrc_c + 0.0024 pydx5p_c + 0.00074 q9_m + 0.00099 ribflv_c + 0.33687 sertrna_c + 0.003967 spmd_c + 0.001327 thf_c + 0.001393 thmpp_c + 0.21813 thrtrna_c + 0.023372 tre_c + 0.000432 triglyc_RT_r + 0.023319 trptrna_c + 0.062363 tyrtrna_c + 0.051129 utp_c + 0.275168 valtrna_c + 0.00051 zn2_c + 0.004156 zymst_r + 1.7e-05 zymstest_RT_d --> 110.632394 adp_c + 110.632394 h_c + 110.632394 pi_c + 0.209364 ppi_c 
New BIOMASS_RT: 0.957502 13BDglcn_c + 0.177315 16BDglcn_c + 0.001283 5mthf_c + 0.577574 alatrna_c + 0.146675 argtrna_c + 0.10797 asntrna_c + 0.197296 asptrna_c + 140.298633 atp_c + 0.002418 btn_m + 0.000832 ca2_c + 0.001792 camp_c + 0.031005 chitin_c + 1.8e-05 clpn_RT_m + 0.00077 coa_c + 0.044881 ctp_c + 0.000525 cu2_c + 0.034269 cystrna_c + 0.002451 datp_c + 0.004285 dctp_c + 0.003763 dgtp_c + 0.002523 dttp_c + 0.004031 ergst_r + 1.6e-05 ergstest_RT_r + 0.000751 fad_c + 0.000597 fe2_c + 0.000597 fe3_c + 0.117898 glntrna_c + 0.208673 glutrna_c + 0.518069 glycogen_c + 0.51322 glytrna_c + 0.00192 gthrd_c + 0.051085 gtp_c + 136.32957199999998 h2o_c + 0.000691 hemeA_m + 0.061167 histrna_c + 0.158569 iletrna_c + 0.585703 k_c + 0.320205 leutrna_c + 0.003107 lipopb_m + 0.172334 lystrna_c + 0.807941 mannan_r + 0.05705 mettrna_c + 0.061716 mg2_c + 0.001292 mlthf_c + 0.000607 mn2_c + 0.026099 na1_c + 0.000888 nad_c + 0.000795 nadp_c + 1.4e-05 pa_RT_r + 0.000172 pc_RT_r + 0.00013 pe_RT_r + 0.095164 phetrna_c + 0.235897 protrna_c + 2e-05 ps_RT_r + 0.004006 psphings_r + 4.7e-05 ptd1ino_RT_r + 0.006524 ptrc_c + 0.0024 pydx5p_c + 0.00074 q9_m + 0.00099 ribflv_c + 0.33687 sertrna_c + 0.003967 spmd_c + 0.001327 thf_c + 0.001393 thmpp_c + 0.21813 thrtrna_c + 0.023372 tre_c + 0.000432 triglyc_RT_r + 0.023319 trptrna_c + 0.062363 tyrtrna_c + 0.051129 utp_c + 0.275168 valtrna_c + 0.00051 zn2_c + 0.004156 zymst_r + 1.7e-05 zymstest_RT_d --> 140.249386 adp_c + 140.249386 h_c + 140.249386 pi_c + 0.209364 ppi_c 

Old C3STKR1er: 4mzym_int2_r + h_r + nadph_r --> 4mzym_r + nadp_r ERG27
New C3STKR1er: 4mzym_int2_r + h_r + nadph_r --> 4mzym_r + nadp_r 14213

Old C3STKR2er: h_r + nadph_r + zym_int2_r --> nadp_r + zymst_r ERG27
New C3STKR2er: h_r + nadph_r + zym_int2_r --> nadp_r + zymst_r 14213

Old CCOAOMT: amet_c + caffcoa_c --> ahcys_c + ferulcoa_c + h_c 13444 or 13445
New CCOAOMT: amet_c + caffcoa_c <=> ahcys_c + ferulcoa_c + h_c 13444 or 13445

Old CYOO6m: 4.0 focytc_m + 8.0 h_m + o2_m --> 4.0 ficytc_m + 2.0 h2o_m + 4.0 h_c RTO3_946004 and RTO3_946002 and 9165 and 13628 and 10685 and 12506 and 11352 and 13275 and 11769 and 11317 and 12210 and 13007 and 8802
New CYOO6m: 4.0 focytc_m + 8.0 h_m + o2_m --> 4.0 ficytc_m + 2.0 h2o_m + 4.0 h_c COX1 and COX2 and COX3 and 9165 and 13628 and 10685 and 12506 and 11352 and 13275 and 11769 and 11317 and 12210 and 13007 and 8802

Old CYOR_u9m: 2.0 ficytc_m + 2.0 h_m + q9h2_m --> 2.0 focytc_m + 4.0 h_c + q9_m RTO3_979594 and 9705 and 9198 and 11618 and 15758 and 15681 and 12966 and 15231 and 13608 and 13614 and 8802
New CYOR_u9m: 2.0 ficytc_m + 2.0 h_m + q9h2_m --> 2.0 focytc_m + 4.0 h_c + q9_m COB and 9705 and 9198 and 11618 and 15758 and 15681 and 12966 and 15231 and 13608 and 13614 and 8802

Old DABT2D: abt__D_c + nad_c <=> h_c + nadh_c + rbl__D_c 8988 or 9990
New DABT2D: abt__D_c + nad_c <=> h_c + nadh_c + rbl__D_c 9990

Old MUCCY_kt: CCbuttc_c + h_c --> 4cml_c 15805
New MUCCY_kt: CCbuttc_c + h_c --> 4cml_c 12622

Old NADH2_u9m2: 5.0 h_m + nadh_m + q9_m --> 4.0 h_c + nad_m + q9h2_m 10010 and 10017 and 10318 and 10541 and 10607 and 11151 and 11411 and 11702 and 11857 and 12482 and 12497 and 12543 and 12653 and 13017 and 13214 and 13268 and 13411 and 13656 and 13925 and 14078 and 14261 and 14418 and 14717 and 14900 and 15179 and 15864 and 15947 and 15973 and 15998 and 8444 and 8446 and 8530 and 8699 and 8817 and 8909 and 9144 and 9348 and RTO3_874609 and RTO3_879341 and RTO3_879512 and RTO3_900622 and RTO3_900624 and RTO3_900641 and RTO3_945994 and RTO3_945995 and RTO3_946005
New NADH2_u9m2: 5.0 h_m + nadh_m + q9_m --> 4.0 h_c + nad_m + q9h2_m NAD1 and NAD2 and NAD3 and NAD4 and NAD4L and NAD5 and NAD6 and 10010 and 10017 and 10318 and 10541 and 10607 and 11151 and 11411 and 11702 and 11857 and 12482 and 12497 and 12543 and 12653 and 13017 and 13214 and 13268 and 13411 and 13656 and 13925 and 14078 and 14261 and 14418 and 14717 and 14900 and 15179 and 15864 and 15947 and 15973 and 15998 and 8444 and 8446 and 8530 and 8699 and 8817 and 8909 and 9144 and 9348

Old POLYAO: N1aspmd_c + h2o_c + o2_c --> aprut_c + bamppald_c + h2o2_c 15939
New POLYAO: N1aspmd_c + h2o_c + o2_c --> 3aap_c + h2o2_c + ptrc_c 15939

Old POLYAO2: N1sprm_c + h2o_c + o2_c --> N1aspmd_c + bamppald_c + h2o2_c 15939
New POLYAO2: N1sprm_c + h2o_c + o2_c --> 3aap_c + h2o2_c + spmd_c 15939

Old PRPNCOAHYDm: h2o_m + prpncoa_m --> 3hpcoa_m 14805
New PRPNCOAHYDm: h2o_m + prpncoa_m <=> 3hpcoa_m 14805

Old SBTD_D2: nad_c + sbt__D_c --> fru_c + h_c + nadh_c 16452
New SBTD_D2: nad_c + sbt__D_c --> fru_c + h_c + nadh_c 12974 or 12977

Old SBTD_L: nad_c + sbt__L_c --> h_c + nadh_c + srb__L_c 16452
New SBTD_L: nad_c + sbt__L_c --> h_c + nadh_c + srb__L_c 12974 or 12977

Old SPMDAT1: accoa_c + spmd_c --> N1aspmd_c + coa_c + h_c 9636
New SPMDAT1: accoa_c + spmd_c --> N1aspmd_c + coa_c + h_c 16046 or 9636

Old SPMDAT2: accoa_c + spmd_c --> coa_c + h_c + n8aspmd_c 9636
New SPMDAT2: accoa_c + spmd_c --> coa_c + h_c + n8aspmd_c 16046 or 9636

Old XYLK: atp_c + xylu__D_c --> adp_c + h_c + xu5p__D_c 14368 or 16850
New XYLK: atp_c + xylu__D_c --> adp_c + h_c + xu5p__D_c 16850

Old XYLTD_D: nad_c + xylt_c --> h_c + nadh_c + xylu__D_c 16452
New XYLTD_D: nad_c + xylt_c --> h_c + nadh_c + xylu__D_c 12974 or 12977 or 16452
```

In [123]:

```
print('Added reactions\n')
for r in sorted(model_new.reactions, key=lambda x: x.id):
    if r not in model_old.reactions:
        print(r)
```

```
Added reactions

34DHBZACCOARp: 3dhp3hpcoa_x + nad_x --> 34dhbzaccoa_x + h_x + nadh_x
34DHBZACCOATp: 34dhbzaccoa_x + coa_x --> 34dhbzcoa_x + accoa_x
34DHBZCOAEp: 34dhbzcoa_x + h2o_x --> 34dhbz_x + coa_x + h_x
34DHBZtp: 34dhbz_c <=> 34dhbz_x
34DHCINMtp: 34dhcinm_c --> 34dhcinm_x
3HPCOAHYDm: 3hpcoa_m + h2o_m --> 3hpp_m + coa_m + h_m
3HPDm: 3hpp_m + nad_m --> h_m + msa_m + nadh_m
3OADPCOATm: 3oxoadp_m + succoa_m --> oxadpcoa_m + succ_m
3OXCOATm: coa_m + oxadpcoa_m --> accoa_m + succoa_m
3OXOADPTm: 3oxoadp_c + akg_m <=> 3oxoadp_m + akg_c
4CMLCL_kt: 4cml_c + h_c --> 5odhf2a_c + co2_c
4HBHYOXp: 4hbz_x + h_x + nadph_x + o2_x --> 34dhbz_x + h2o_x + nadp_x
4HBZACCOARp: 34hp3hpcoa_x + nad_x --> 4hbzaccoa_x + h_x + nadh_x
4HBZACCOATp: 4hbzaccoa_x + coa_x --> 4hbzcoa_x + accoa_x
4HBZCOAEp: 4hbzcoa_x + h2o_x --> 4hbz_x + coa_x + h_x
4HBZtm: 4hbz_c <=> 4hbz_m
4HBZtp: 4hbz_c <=> 4hbz_x
ABTD: abt_c + nad_c <=> h_c + nadh_c + xylu__L_c
ABT_Dt: abt__D_e <=> abt__D_c
ABTt: abt_e <=> abt_c
AKGtp: akg_c <=> akg_x
ARAB1D: arab__D_c + nad_c --> Dara14lac_c + h_c + nadh_c
ARAB_Lt: arab__L_e <=> arab__L_c
BCAROtrd: bcaro_r --> bcaro_d
BZ4OX: bz_c + h_c + nadph_c + o2_c --> 4hbz_c + h2o_c + nadp_c
BZt: bz_e <=> bz_c
CAFFCOAE: caffcoa_c + h2o_c --> 34dhcinm_c + coa_c + h_c
CAFFCOAHp: caffcoa_x + h2o_x --> 3dhp3hpcoa_x
CAFFCOALp: 34dhcinm_x + atp_x + coa_x --> amp_x + caffcoa_x + ppi_x
COAtp: coa_c <=> coa_x
COUCOAHp: coucoa_x + h2o_x --> 34hp3hpcoa_x
COUCOALp: T4hcinnm_x + atp_x + coa_x --> amp_x + coucoa_x + ppi_x
CRNtp: crn_c <=> crn_x
DABT4D: abt__D_c + nad_c <=> h_c + nadh_c + xylu__D_c
DIAT: accoa_c + sprm_c --> N1sprm_c + coa_c + h_c
DM_3aap_c: 3aap_c --> 
DM_bcaro_d: bcaro_d --> 
EX_T4hcinnm_e: T4hcinnm_e --> 
EX_abt__D_e: abt__D_e --> 
EX_abt_e: abt_e --> 
EX_arab__L_e: arab__L_e --> 
EX_bz_e: bz_e --> 
EX_fer_e: fer_e --> 
EX_lyx__L_e: lyx__L_e --> 
EX_rbl__D_e: rbl__D_e --> 
EX_xylt_e: xylt_e --> 
EX_xylu__D_e: xylu__D_e --> 
FERULCOAS: atp_c + coa_c + fer_c --> amp_c + ferulcoa_c + ppi_c
FERt: fer_e <=> fer_c
FPK: f6p_c + pi_c --> actp_c + e4p_c + h2o_c
GCAROCer: gcaro_r --> bcaro_r
GGDPter: ggdp_c --> ggdp_r
Htx: h_c <=> h_x
LYCOPCer: lycop_r --> gcaro_r
LYXR: h_c + lyx__L_c + nadph_c --> abt_c + nadp_c
LYXt: lyx__L_e <=> lyx__L_c
MAL_Ltx: mal__L_x <=> mal__L_c
OAAtp: oaa_x <=> oaa_c
PACCOAL: atp_c + coa_c + pac_c --> amp_c + phaccoa_c + ppi_c
PACCOALp: atp_x + coa_x + pac_x --> amp_x + phaccoa_x + ppi_x
PCADYOX: 34dhbz_c + o2_c --> CCbuttc_c + 2.0 h_c
PDSer: 4.0 nadp_r + phyto_r --> 4.0 h_r + lycop_r + 4.0 nadph_r
PPItx: ppi_c <=> ppi_x
PSYer: 2.0 ggdp_r --> phyto_r + 2.0 ppi_r
RBK_Dr: atp_c + rbl__D_c --> adp_c + h_c + ru5p__D_c
RBL_Dt: rbl__D_c <=> rbl__D_e
T4HCINNMt: T4hcinnm_e <=> T4hcinnm_c
T4HCINNMtp: T4hcinnm_c --> T4hcinnm_x
XPK: pi_c + xu5p__D_c --> actp_c + g3p_c + h2o_c
XYLR: h_c + nadph_c + xyl__D_c --> nadp_c + xylt_c
XYLTt: xylt_e <=> xylt_c
XYLUDte: xylu__D_c <=> xylu__D_e
XYLUR: h_c + nadph_c + xylu__L_c <=> nadp_c + xylt_c
```

In [ ]:

```

```
