# Supplementary material for: Multi-Omics Driven Metabolic Network Reconstruction and Analysis of Lignocellulosic Carbon Utilization in Rhodosporidium toruloides
Source: Front Bioeng Biotechnol. 2021 Jan 8;8:612832. doi: 10.3389/fbioe.2020.612832 (PMC7873862; doi:10.3389/fbioe.2020.612832)
Supplement: Supplementary File 4 — Multi-omics dataset for R. toruloides IFO0880. [file Data_Sheet_1.zip › Supplementary File S1/2.Metabolic_modeling/Refinement_2e_Growth_Phenotype_Gene_Essentiality_Evaluation.html]

Refinement\_2e\_Growth\_Phenotype\_Gene\_Essentiality\_Evaluation


In [1]:

```
%matplotlib inline
from matplotlib import pyplot as plt
from matplotlib import colors
import numpy as np
import pandas as pd
import cobra
import seaborn as sns
```

In [2]:

```
Annotation = pd.read_excel('../../Data/R_toruloides_Data_for_Reconstruction.xlsx',
                          sheet_name='Annotation', index_col=0)
Annotation.index = Annotation.index.map(str)
Annotation = Annotation.fillna('')
```

In [3]:

```
def background_gradient(s, cmap='seismic', text_color_threshold=0.408):
    lim = max(abs(s.min().min()),abs(s.max().max()))
    rng = 2.0*lim
    norm = colors.Normalize(-lim - (rng * 0.2), lim + (rng * 0.2))
    rgbas = plt.cm.get_cmap(cmap)(norm(s.values))
    def relative_luminance(rgba):
        r, g, b = (x / 12.92 if x <= 0.03928 else ((x + 0.055) / 1.055 ** 2.4) for x in rgba[:3])
        return 0.2126 * r + 0.7152 * g + 0.0722 * b
    def css(rgba):
        dark = relative_luminance(rgba) < text_color_threshold
        text_color = '#f1f1f1' if dark else '#000000'
        return 'background-color: {b};color: {c};'.format(b=colors.rgb2hex(rgba), c=text_color)

    if s.ndim == 1:
        return [css(rgba) for rgba in rgbas]
    else:
        return pd.DataFrame([[css(rgba) for rgba in row] for row in rgbas], index=s.index, columns=s.columns)
    
def Show_Data(x):
    display(Fitness.loc[temp].style.apply(background_gradient, cmap='seismic', axis=None).highlight_null('lightgrey'))
    return;
```

In [4]:

```
model = cobra.io.load_json_model("IFO0880_GPR_2d.json")
```

In [5]:

```
eco = cobra.io.load_json_model('../../Data/BiGG_Models/iML1515.json')
sce = cobra.io.load_json_model('../../Data/BiGG_Models/iMM904.json')
hsa2 = cobra.io.load_json_model('../../Data/BiGG_Models/Recon3D.json')
ptri = cobra.io.load_json_model('../../Data/BiGG_Models/iLB1027_lipid.json')
```

In [6]:

```
Biolog = pd.read_excel('../../Data/Biolog_Media.xlsx',
                        usecols=list(range(0,11)))
Biolog['Row'] = Biolog['Row'].replace(list(range(1,9)), list('ABCDEFGH'))
Biolog = Biolog.sort_values(by=['PlateType','Row','Column'])
Biolog.index = Biolog['PlateType']+'_'+Biolog['Well']
with pd.option_context('display.max_rows', None, 'display.max_columns', None):
    display(Biolog)
```

|  | PlateType | Experiment | Well | Row | Column | Compound | Average | All > NC | Average > 0.1 | Pass | Ttest |
| --- | --- | --- | --- | --- | --- | --- | --- | --- | --- | --- | --- |
| PM1\_A1 | PM1 | Carbon | A1 | A | 1 | Negative Control | 0.086400 | False | False | False | 5.000000e-01 |
| PM1\_A2 | PM1 | Carbon | A2 | A | 2 | L-Arabinose | 0.344767 | True | True | True | 8.193098e-05 |
| PM1\_A3 | PM1 | Carbon | A3 | A | 3 | N-Acetyl-D-Glucosamine | 0.068867 | False | False | False | 3.061006e-02 |
| PM1\_A4 | PM1 | Carbon | A4 | A | 4 | D-Saccharic Acid | 0.071200 | False | False | False | 1.513148e-02 |
| PM1\_A5 | PM1 | Carbon | A5 | A | 5 | Succinic Acid | 0.111200 | True | True | True | 8.159577e-02 |
| PM1\_A6 | PM1 | Carbon | A6 | A | 6 | D-Galactose | 0.143300 | True | True | True | 1.097891e-05 |
| PM1\_A7 | PM1 | Carbon | A7 | A | 7 | L-Aspartic Acid | 0.267867 | True | True | True | 8.807619e-06 |
| PM1\_A8 | PM1 | Carbon | A8 | A | 8 | L-Proline | 0.272400 | True | True | True | 5.782247e-06 |
| PM1\_A9 | PM1 | Carbon | A9 | A | 9 | D-Alanine | 0.121500 | True | True | True | 4.121000e-03 |
| PM1\_A10 | PM1 | Carbon | A10 | A | 10 | D-Trehalose | 0.221300 | True | True | True | 7.633336e-06 |
| PM1\_A11 | PM1 | Carbon | A11 | A | 11 | D-Mannose | 0.212667 | True | True | True | 8.873841e-04 |
| PM1\_A12 | PM1 | Carbon | A12 | A | 12 | Dulcitol | 0.132067 | True | True | True | 2.355727e-04 |
| PM1\_B1 | PM1 | Carbon | B1 | B | 1 | D-Serine | 0.150600 | True | True | True | 7.067355e-03 |
| PM1\_B2 | PM1 | Carbon | B2 | B | 2 | D-Sorbitol | 0.124767 | True | True | True | 1.433546e-03 |
| PM1\_B3 | PM1 | Carbon | B3 | B | 3 | Glycerol | 0.224300 | True | True | True | 8.813718e-04 |
| PM1\_B4 | PM1 | Carbon | B4 | B | 4 | L-Fucose | 0.099667 | False | False | False | 8.722623e-02 |
| PM1\_B5 | PM1 | Carbon | B5 | B | 5 | D-Glucuronic Acid | 0.071833 | False | False | False | 2.200902e-02 |
| PM1\_B6 | PM1 | Carbon | B6 | B | 6 | D-Gluconic Acid | 0.143467 | True | True | True | 2.630279e-03 |
| PM1\_B7 | PM1 | Carbon | B7 | B | 7 | D,L-a-GlycerolPhosphate | 0.072200 | False | False | False | 3.713293e-03 |
| PM1\_B8 | PM1 | Carbon | B8 | B | 8 | D-Xylose | 0.342267 | True | True | True | 1.162623e-05 |
| PM1\_B9 | PM1 | Carbon | B9 | B | 9 | L-Lactic Acid | 0.122067 | True | True | True | 2.821432e-04 |
| PM1\_B10 | PM1 | Carbon | B10 | B | 10 | Formic Acid | 0.177800 | True | True | True | 1.459995e-04 |
| PM1\_B11 | PM1 | Carbon | B11 | B | 11 | D-Mannitol | 0.120767 | True | True | True | 1.252326e-03 |
| PM1\_B12 | PM1 | Carbon | B12 | B | 12 | L-Glutamic Acid | 0.297367 | True | True | True | 6.618403e-04 |
| PM1\_C1 | PM1 | Carbon | C1 | C | 1 | D-Glucose-6-Phosphate | 0.087033 | False | False | False | 4.804190e-01 |
| PM1\_C2 | PM1 | Carbon | C2 | C | 2 | D-Galactonic Acid-g-Lactone | 0.055900 | False | False | False | 1.732839e-03 |
| PM1\_C3 | PM1 | Carbon | C3 | C | 3 | D-,L-Malic Acid | 0.114800 | True | True | True | 2.508493e-04 |
| PM1\_C4 | PM1 | Carbon | C4 | C | 4 | D-Ribose | 0.312900 | True | True | True | 4.262355e-05 |
| PM1\_C5 | PM1 | Carbon | C5 | C | 5 | Tween 20 | 0.397733 | True | True | True | 3.562568e-05 |
| PM1\_C6 | PM1 | Carbon | C6 | C | 6 | L-Rhamnose | 0.093733 | False | False | False | 8.019996e-02 |
| PM1\_C7 | PM1 | Carbon | C7 | C | 7 | D-Fructose | 0.257533 | True | True | True | 1.024063e-05 |
| PM1\_C8 | PM1 | Carbon | C8 | C | 8 | Acetic Acid | 0.243067 | True | True | True | 6.450955e-05 |
| PM1\_C9 | PM1 | Carbon | C9 | C | 9 | a-D-Glucose | 0.215400 | True | True | True | 1.236420e-05 |
| PM1\_C10 | PM1 | Carbon | C10 | C | 10 | Maltose | 0.105733 | False | True | False | 1.022524e-01 |
| PM1\_C11 | PM1 | Carbon | C11 | C | 11 | D-Melibiose | 0.076300 | False | False | False | 6.700306e-02 |
| PM1\_C12 | PM1 | Carbon | C12 | C | 12 | Thymidine | 0.089500 | False | False | False | 3.290534e-01 |
| PM1\_D1 | PM1 | Carbon | D1 | D | 1 | L-Asparagine | 0.279933 | True | True | True | 4.461910e-04 |
| PM1\_D2 | PM1 | Carbon | D2 | D | 2 | D-Aspartic Acid | 0.095967 | False | False | False | 5.278631e-02 |
| PM1\_D3 | PM1 | Carbon | D3 | D | 3 | D-Glucosaminic Acid | 0.083900 | False | False | False | 2.956786e-01 |
| PM1\_D4 | PM1 | Carbon | D4 | D | 4 | 1,2-Propanediol | 0.081500 | False | False | False | 3.418909e-01 |
| PM1\_D5 | PM1 | Carbon | D5 | D | 5 | Tween 40 | 0.347467 | True | True | True | 3.498285e-06 |
| PM1\_D6 | PM1 | Carbon | D6 | D | 6 | a-Keto-Glutaric Acid | 0.103400 | True | True | True | 3.181628e-03 |
| PM1\_D7 | PM1 | Carbon | D7 | D | 7 | a-Keto-Butyric Acid | 0.154233 | True | True | True | 1.671211e-04 |
| PM1\_D8 | PM1 | Carbon | D8 | D | 8 | a-Methyl-D-Galactoside | 0.081267 | False | False | False | 2.263187e-01 |
| PM1\_D9 | PM1 | Carbon | D9 | D | 9 | a-D-Lactose | 0.081033 | False | False | False | 2.823806e-01 |
| PM1\_D10 | PM1 | Carbon | D10 | D | 10 | Lactulose | 0.083933 | False | False | False | 4.201821e-01 |
| PM1\_D11 | PM1 | Carbon | D11 | D | 11 | Sucrose | 0.125267 | True | True | True | 4.238808e-02 |
| PM1\_D12 | PM1 | Carbon | D12 | D | 12 | Uridine | 0.090700 | True | False | False | 5.349142e-02 |
| PM1\_E1 | PM1 | Carbon | E1 | E | 1 | L-Glutamine | 0.315700 | True | True | True | 2.516058e-03 |
| PM1\_E2 | PM1 | Carbon | E2 | E | 2 | m-Tartaric Acid | 0.067167 | False | False | False | 1.348157e-02 |
| PM1\_E3 | PM1 | Carbon | E3 | E | 3 | D-Glucose-1-Phosphate | 0.078633 | False | False | False | 2.621608e-01 |
| PM1\_E4 | PM1 | Carbon | E4 | E | 4 | D-Fructose-6-Phosphate | 0.097133 | False | False | False | 5.156235e-02 |
| PM1\_E5 | PM1 | Carbon | E5 | E | 5 | Tween 80 | 0.408233 | True | True | True | 8.547917e-04 |
| PM1\_E6 | PM1 | Carbon | E6 | E | 6 | a-Hydroxy Glutaric Acid-g-Lactone | 0.064367 | False | False | False | 3.419270e-02 |
| PM1\_E7 | PM1 | Carbon | E7 | E | 7 | a-Hydroxy Butyric Acid | 0.099200 | True | False | False | 2.900810e-02 |
| PM1\_E8 | PM1 | Carbon | E8 | E | 8 | b-Methyl-D-Glucoside | 0.212600 | True | True | True | 1.180755e-05 |
| PM1\_E9 | PM1 | Carbon | E9 | E | 9 | Adonitol | 0.225933 | True | True | True | 2.780261e-04 |
| PM1\_E10 | PM1 | Carbon | E10 | E | 10 | Maltotriose | 0.124433 | True | True | True | 2.869355e-02 |
| PM1\_E11 | PM1 | Carbon | E11 | E | 11 | 2-Deoxy Adenosine | 0.068633 | False | False | False | 2.661486e-02 |
| PM1\_E12 | PM1 | Carbon | E12 | E | 12 | Adenosine | 0.077000 | False | False | False | 9.859285e-02 |
| PM1\_F1 | PM1 | Carbon | F1 | F | 1 | Glycyl-L-Aspartic Acid | 0.086133 | False | False | False | 4.892884e-01 |
| PM1\_F2 | PM1 | Carbon | F2 | F | 2 | Citric Acid | 0.087633 | False | False | False | 4.243018e-01 |
| PM1\_F3 | PM1 | Carbon | F3 | F | 3 | m-Inositol | 0.100567 | False | True | False | 2.799850e-01 |
| PM1\_F4 | PM1 | Carbon | F4 | F | 4 | D-Threonine | 0.112333 | False | True | False | 8.638349e-02 |
| PM1\_F5 | PM1 | Carbon | F5 | F | 5 | Fumaric Acid | 0.093367 | False | False | False | 2.834587e-01 |
| PM1\_F6 | PM1 | Carbon | F6 | F | 6 | Bromo Succinic Acid | 0.173133 | True | True | True | 1.310181e-05 |
| PM1\_F7 | PM1 | Carbon | F7 | F | 7 | Propionic Acid | 0.182700 | True | True | True | 3.044667e-02 |
| PM1\_F8 | PM1 | Carbon | F8 | F | 8 | Mucic Acid | 0.087533 | False | False | False | 4.622348e-01 |
| PM1\_F9 | PM1 | Carbon | F9 | F | 9 | Glycolic Acid | 0.082433 | False | False | False | 3.446353e-01 |
| PM1\_F10 | PM1 | Carbon | F10 | F | 10 | Glyoxylic Acid | 0.078933 | False | False | False | 1.097401e-01 |
| PM1\_F11 | PM1 | Carbon | F11 | F | 11 | D-Cellobiose | 0.198867 | True | True | True | 2.978958e-05 |
| PM1\_F12 | PM1 | Carbon | F12 | F | 12 | Inosine | 0.093200 | False | False | False | 2.054701e-01 |
| PM1\_G1 | PM1 | Carbon | G1 | G | 1 | Glycyl-L-Glutamic Acid | 0.153067 | True | True | True | 7.060863e-02 |
| PM1\_G2 | PM1 | Carbon | G2 | G | 2 | Tricarballylic Acid | 0.063433 | False | False | False | 3.115519e-03 |
| PM1\_G3 | PM1 | Carbon | G3 | G | 3 | L-Serine | 0.189367 | True | True | True | 1.104250e-02 |
| PM1\_G4 | PM1 | Carbon | G4 | G | 4 | L-Threonine | 0.160133 | True | True | True | 2.374893e-05 |
| PM1\_G5 | PM1 | Carbon | G5 | G | 5 | L-Alanine | 0.241200 | True | True | True | 6.271767e-07 |
| PM1\_G6 | PM1 | Carbon | G6 | G | 6 | L-Alanyl-Glycine | 0.186600 | True | True | True | 5.390148e-05 |
| PM1\_G7 | PM1 | Carbon | G7 | G | 7 | Acetoacetic Acid | 0.161467 | True | True | True | 6.929044e-03 |
| PM1\_G8 | PM1 | Carbon | G8 | G | 8 | N-Acetyl-b-D-Mannosamine | 0.084433 | False | False | False | 3.883473e-01 |
| PM1\_G9 | PM1 | Carbon | G9 | G | 9 | Mono Methyl Succinate | 0.200767 | True | True | True | 3.537269e-03 |
| PM1\_G10 | PM1 | Carbon | G10 | G | 10 | Methyl Pyruvate | 0.170767 | True | True | True | 4.498965e-03 |
| PM1\_G11 | PM1 | Carbon | G11 | G | 11 | D-Malic Acid | 0.121733 | True | True | True | 1.988904e-02 |
| PM1\_G12 | PM1 | Carbon | G12 | G | 12 | L-Malic Acid | 0.107933 | False | True | False | 1.918242e-01 |
| PM1\_H1 | PM1 | Carbon | H1 | H | 1 | Glycyl-L-Proline | 0.225233 | True | True | True | 8.064704e-03 |
| PM1\_H2 | PM1 | Carbon | H2 | H | 2 | p-Hydroxy Phenyl Acetic Acid | 0.139733 | True | True | True | 1.469080e-02 |
| PM1\_H3 | PM1 | Carbon | H3 | H | 3 | m-Hydroxy Phenyl Acetic Acid | 0.082633 | False | False | False | 3.493750e-01 |
| PM1\_H4 | PM1 | Carbon | H4 | H | 4 | Tyramine | 0.070267 | False | False | False | 8.877945e-02 |
| PM1\_H5 | PM1 | Carbon | H5 | H | 5 | D-Psicose | 0.122867 | True | True | True | 3.424499e-04 |
| PM1\_H6 | PM1 | Carbon | H6 | H | 6 | L-Lyxose | 0.393067 | True | True | True | 4.629262e-07 |
| PM1\_H7 | PM1 | Carbon | H7 | H | 7 | Glucuronamide | 0.112100 | True | True | True | 3.057506e-03 |
| PM1\_H8 | PM1 | Carbon | H8 | H | 8 | Pyruvic Acid | 0.215867 | True | True | True | 2.793350e-03 |
| PM1\_H9 | PM1 | Carbon | H9 | H | 9 | L-Galactonic Acid-g-Lactone | 0.252700 | True | True | True | 1.810548e-02 |
| PM1\_H10 | PM1 | Carbon | H10 | H | 10 | D-Galacturonic Acid | 0.248833 | True | True | True | 1.185145e-05 |
| PM1\_H11 | PM1 | Carbon | H11 | H | 11 | Phenylethylamine | 0.082833 | False | False | False | 3.853989e-01 |
| PM1\_H12 | PM1 | Carbon | H12 | H | 12 | 2-Aminoethanol | 0.075267 | False | False | False | 1.452555e-02 |
| PM2\_A1 | PM2 | Carbon | A1 | A | 1 | Negative Control | 0.082233 | False | False | False | 1.270398e-01 |
| PM2\_A2 | PM2 | Carbon | A2 | A | 2 | Chondroitin Sulfate C | 0.057667 | False | False | False | 1.231307e-02 |
| PM2\_A3 | PM2 | Carbon | A3 | A | 3 | a-Cyclodextrin | 0.059600 | False | False | False | 1.856112e-02 |
| PM2\_A4 | PM2 | Carbon | A4 | A | 4 | b-Cyclodextrin | 0.079833 | False | False | False | 2.570013e-01 |
| PM2\_A5 | PM2 | Carbon | A5 | A | 5 | g-Cyclodextrin | 0.102967 | False | True | False | 8.502013e-02 |
| PM2\_A6 | PM2 | Carbon | A6 | A | 6 | Dextrin | 0.129000 | True | True | True | 5.008316e-03 |
| PM2\_A7 | PM2 | Carbon | A7 | A | 7 | Gelatin | 0.133233 | True | True | True | 1.962280e-05 |
| PM2\_A8 | PM2 | Carbon | A8 | A | 8 | Glycogen | 0.074300 | False | False | False | 1.237706e-01 |
| PM2\_A9 | PM2 | Carbon | A9 | A | 9 | Inulin | 0.165867 | True | True | True | 6.155844e-04 |
| PM2\_A10 | PM2 | Carbon | A10 | A | 10 | Laminarin | 0.203733 | True | True | True | 6.241022e-05 |
| PM2\_A11 | PM2 | Carbon | A11 | A | 11 | Mannan | 0.066133 | False | False | False | 2.261493e-02 |
| PM2\_A12 | PM2 | Carbon | A12 | A | 12 | Pectin | 0.159867 | True | True | True | 2.488824e-02 |
| PM2\_B1 | PM2 | Carbon | B1 | B | 1 | N-Acetyl-D-Galactosamine | 0.077967 | False | False | False | 1.156505e-01 |
| PM2\_B2 | PM2 | Carbon | B2 | B | 2 | N-Acetyl Neuraminic Acid | 0.072467 | False | False | False | 8.815494e-02 |
| PM2\_B3 | PM2 | Carbon | B3 | B | 3 | b-D-Allose | 0.107000 | False | True | False | 9.224988e-02 |
| PM2\_B4 | PM2 | Carbon | B4 | B | 4 | Amygdalin | 0.105433 | True | True | True | 5.890414e-02 |
| PM2\_B5 | PM2 | Carbon | B5 | B | 5 | D-Arabinose | 0.269833 | True | True | True | 3.588621e-05 |
| PM2\_B6 | PM2 | Carbon | B6 | B | 6 | D-Arabitol | 0.244600 | True | True | True | 2.646561e-04 |
| PM2\_B7 | PM2 | Carbon | B7 | B | 7 | L-Arabitol | 0.249133 | True | True | True | 2.740648e-04 |
| PM2\_B8 | PM2 | Carbon | B8 | B | 8 | Arbutin | 0.300467 | True | True | True | 3.748186e-04 |
| PM2\_B9 | PM2 | Carbon | B9 | B | 9 | 2-Deoxy-D-Ribose | 0.209900 | True | True | True | 6.514437e-03 |
| PM2\_B10 | PM2 | Carbon | B10 | B | 10 | i-Erythritol | 0.076100 | False | False | False | 1.513530e-01 |
| PM2\_B11 | PM2 | Carbon | B11 | B | 11 | D-Fucose | 0.110233 | True | True | True | 8.409343e-02 |
| PM2\_B12 | PM2 | Carbon | B12 | B | 12 | 3-0-b-D-Galactopyranosyl-D-Arabinose | 0.114800 | True | True | True | 1.631917e-02 |
| PM2\_C1 | PM2 | Carbon | C1 | C | 1 | Gentiobiose | 0.230400 | True | True | True | 2.071380e-03 |
| PM2\_C2 | PM2 | Carbon | C2 | C | 2 | L-Glucose | 0.115167 | True | True | True | 3.659123e-02 |
| PM2\_C3 | PM2 | Carbon | C3 | C | 3 | Lactitol | 0.086033 | False | False | False | 4.874053e-01 |
| PM2\_C4 | PM2 | Carbon | C4 | C | 4 | D-Melezitose | 0.086133 | False | False | False | 4.904291e-01 |
| PM2\_C5 | PM2 | Carbon | C5 | C | 5 | Maltitol | 0.089767 | False | False | False | 4.086976e-01 |
| PM2\_C6 | PM2 | Carbon | C6 | C | 6 | A-Methyl-D-Glucoside | 0.086867 | False | False | False | 4.844072e-01 |
| PM2\_C7 | PM2 | Carbon | C7 | C | 7 | b-Methyl-D-Galactoside | 0.101900 | False | True | False | 1.717256e-01 |
| PM2\_C8 | PM2 | Carbon | C8 | C | 8 | 3-Methyl Glucose | 0.092833 | False | False | False | 2.970214e-01 |
| PM2\_C9 | PM2 | Carbon | C9 | C | 9 | b-Methyl-D-Glucuronic Acid | 0.080167 | False | False | False | 2.713970e-01 |
| PM2\_C10 | PM2 | Carbon | C10 | C | 10 | a-Methyl-D-Mannoside | 0.092733 | False | False | False | 2.002651e-01 |
| PM2\_C11 | PM2 | Carbon | C11 | C | 11 | b-Methyl-D-Xyloside | 0.078433 | False | False | False | 2.175206e-01 |
| PM2\_C12 | PM2 | Carbon | C12 | C | 12 | Palatinose | 0.088200 | False | False | False | 3.936345e-01 |
| PM2\_D1 | PM2 | Carbon | D1 | D | 1 | D-Raffinose | 0.075867 | False | False | False | 1.162669e-01 |
| PM2\_D2 | PM2 | Carbon | D2 | D | 2 | Salicin | 0.328200 | True | True | True | 4.908107e-05 |
| PM2\_D3 | PM2 | Carbon | D3 | D | 3 | Sedoheptulosan | 0.090200 | False | False | False | 4.071660e-01 |
| PM2\_D4 | PM2 | Carbon | D4 | D | 4 | L-Sorbose | 0.094133 | False | False | False | 3.205580e-01 |
| PM2\_D5 | PM2 | Carbon | D5 | D | 5 | Stachyose | 0.084233 | False | False | False | 4.452672e-01 |
| PM2\_D6 | PM2 | Carbon | D6 | D | 6 | D-Tagatose | 0.163200 | True | True | True | 1.845691e-04 |
| PM2\_D7 | PM2 | Carbon | D7 | D | 7 | Turanose | 0.122033 | True | True | True | 2.933866e-02 |
| PM2\_D8 | PM2 | Carbon | D8 | D | 8 | Xylitol | 0.260667 | True | True | True | 8.445740e-05 |
| PM2\_D9 | PM2 | Carbon | D9 | D | 9 | N-Acetyl-D-Glucosaminitol | 0.078800 | False | False | False | 2.986646e-01 |
| PM2\_D10 | PM2 | Carbon | D10 | D | 10 | g-Amino Butyric Acid | 0.240867 | True | True | True | 6.580347e-04 |
| PM2\_D11 | PM2 | Carbon | D11 | D | 11 | d-Amino Valeric Acid | 0.097467 | False | False | False | 1.141363e-01 |
| PM2\_D12 | PM2 | Carbon | D12 | D | 12 | Butyric Acid | 0.265533 | True | True | True | 6.380314e-05 |
| PM2\_E1 | PM2 | Carbon | E1 | E | 1 | Capric Acid | 0.006800 | False | False | False | 5.089092e-05 |
| PM2\_E2 | PM2 | Carbon | E2 | E | 2 | Caproic Acid | 0.182000 | True | True | True | 9.302030e-03 |
| PM2\_E3 | PM2 | Carbon | E3 | E | 3 | Citraconic Acid | 0.071400 | False | False | False | 1.990501e-01 |
| PM2\_E4 | PM2 | Carbon | E4 | E | 4 | Citramalic Acid | 0.073900 | False | False | False | 2.331099e-01 |
| PM2\_E5 | PM2 | Carbon | E5 | E | 5 | D-Glucosamine | 0.171867 | True | True | True | 2.187429e-02 |
| PM2\_E6 | PM2 | Carbon | E6 | E | 6 | 2-Hydroxy Benzoic Acid | 0.100167 | False | True | False | 1.260118e-01 |
| PM2\_E7 | PM2 | Carbon | E7 | E | 7 | 4-Hydroxy Benzoic Acid | 0.186267 | True | True | True | 1.136073e-02 |
| PM2\_E8 | PM2 | Carbon | E8 | E | 8 | b-Hydroxy Butyric Acid | 0.196300 | True | True | True | 4.789428e-03 |
| PM2\_E9 | PM2 | Carbon | E9 | E | 9 | g-Hydroxy Butyric Acid | 0.212067 | True | True | True | 3.849800e-04 |
| PM2\_E10 | PM2 | Carbon | E10 | E | 10 | A-Keto-Valeric Acid | 0.156967 | True | True | True | 2.352901e-02 |
| PM2\_E11 | PM2 | Carbon | E11 | E | 11 | Itaconic Acid | 0.071800 | False | False | False | 1.355016e-01 |
| PM2\_E12 | PM2 | Carbon | E12 | E | 12 | 5-Keto-D-Gluconic Acid | 0.219967 | True | True | True | 1.231725e-03 |
| PM2\_F1 | PM2 | Carbon | F1 | F | 1 | D-Lactic Acid Methyl Ester | 0.111333 | True | True | True | 6.542987e-02 |
| PM2\_F2 | PM2 | Carbon | F2 | F | 2 | Malonic Acid | 0.058900 | False | False | False | 4.172815e-02 |
| PM2\_F3 | PM2 | Carbon | F3 | F | 3 | Melibionic Acid | 0.118367 | True | True | True | 5.318862e-02 |
| PM2\_F4 | PM2 | Carbon | F4 | F | 4 | Oxalic Acid | 0.075933 | False | False | False | 2.556046e-01 |
| PM2\_F5 | PM2 | Carbon | F5 | F | 5 | Oxalomalic Acid | 0.146133 | True | True | True | 2.907240e-03 |
| PM2\_F6 | PM2 | Carbon | F6 | F | 6 | Quinic Acid | 0.149700 | True | True | True | 2.455034e-03 |
| PM2\_F7 | PM2 | Carbon | F7 | F | 7 | D-Ribono-1,4-Lactone | 0.057833 | False | False | False | 3.572564e-02 |
| PM2\_F8 | PM2 | Carbon | F8 | F | 8 | Sebacic Acid | 0.230433 | True | True | True | 9.563650e-04 |
| PM2\_F9 | PM2 | Carbon | F9 | F | 9 | Sorbic Acid | 0.184000 | True | True | True | 8.510466e-03 |
| PM2\_F10 | PM2 | Carbon | F10 | F | 10 | Succinamic Acid | 0.128233 | True | True | True | 2.613341e-02 |
| PM2\_F11 | PM2 | Carbon | F11 | F | 11 | D-Tartaric Acid | 0.070400 | False | False | False | 1.166823e-01 |
| PM2\_F12 | PM2 | Carbon | F12 | F | 12 | L-Tartaric Acid | 0.075800 | False | False | False | 1.772723e-01 |
| PM2\_G1 | PM2 | Carbon | G1 | G | 1 | Acetamide | 0.088133 | False | False | False | 4.441336e-01 |
| PM2\_G2 | PM2 | Carbon | G2 | G | 2 | L-Alaninamide | 0.254367 | True | True | True | 1.792628e-03 |
| PM2\_G3 | PM2 | Carbon | G3 | G | 3 | N-Acetyl-L-Glutamic Acid | 0.071800 | False | False | False | 2.438114e-01 |
| PM2\_G4 | PM2 | Carbon | G4 | G | 4 | L-Arginine | 0.188467 | True | True | True | 1.647968e-02 |
| PM2\_G5 | PM2 | Carbon | G5 | G | 5 | Glycine | 0.254033 | True | True | True | 1.333848e-04 |
| PM2\_G6 | PM2 | Carbon | G6 | G | 6 | L-Histidine | 0.114700 | False | True | False | 1.304654e-01 |
| PM2\_G7 | PM2 | Carbon | G7 | G | 7 | L-Homoserine | 0.164767 | True | True | True | 3.741022e-03 |
| PM2\_G8 | PM2 | Carbon | G8 | G | 8 | Hydroxy-L-Proline | 0.093567 | False | False | False | 3.738221e-01 |
| PM2\_G9 | PM2 | Carbon | G9 | G | 9 | L-Isoleucine | 0.241867 | True | True | True | 5.080988e-05 |
| PM2\_G10 | PM2 | Carbon | G10 | G | 10 | L-Leucine | 0.173533 | True | True | True | 6.836260e-03 |
| PM2\_G11 | PM2 | Carbon | G11 | G | 11 | L-Lysine | 0.091567 | False | False | False | 3.015676e-01 |
| PM2\_G12 | PM2 | Carbon | G12 | G | 12 | L-Methionine | 0.062633 | False | False | False | 3.395955e-02 |
| PM2\_H1 | PM2 | Carbon | H1 | H | 1 | L-Ornithine | 0.238633 | True | True | True | 1.775401e-04 |
| PM2\_H2 | PM2 | Carbon | H2 | H | 2 | L-Phenylalanine | 0.061000 | False | False | False | 2.519256e-02 |
| PM2\_H3 | PM2 | Carbon | H3 | H | 3 | L-Pyroglutamic Acid | 0.272200 | True | True | True | 3.183625e-03 |
| PM2\_H4 | PM2 | Carbon | H4 | H | 4 | L-Valine | 0.179467 | True | True | True | 6.748991e-03 |
| PM2\_H5 | PM2 | Carbon | H5 | H | 5 | D,L-Carnitine | 0.064333 | False | False | False | 1.062199e-01 |
| PM2\_H6 | PM2 | Carbon | H6 | H | 6 | Sec-Butylamine | 0.038367 | False | False | False | 3.474074e-03 |
| PM2\_H7 | PM2 | Carbon | H7 | H | 7 | D,L-Octopamine | 0.091033 | False | False | False | 3.924378e-01 |
| PM2\_H8 | PM2 | Carbon | H8 | H | 8 | Putrescine | 0.066933 | False | False | False | 1.451333e-01 |
| PM2\_H9 | PM2 | Carbon | H9 | H | 9 | Dihydroxy Acetone | 0.340200 | True | True | True | 2.433408e-03 |
| PM2\_H10 | PM2 | Carbon | H10 | H | 10 | 2,3-Butanediol | 0.072550 | False | False | False | 2.131868e-01 |
| PM2\_H11 | PM2 | Carbon | H11 | H | 11 | 2,3-Butanedione | 0.086967 | False | False | False | 4.768224e-01 |
| PM2\_H12 | PM2 | Carbon | H12 | H | 12 | 3-Hydroxy 2-Butanon | 0.060133 | False | False | False | 7.483330e-02 |
| PM3B\_A1 | PM3B | Nitrogen | A1 | A | 1 | Negative Control | 0.100600 | False | True | False | 5.000000e-01 |
| PM3B\_A2 | PM3B | Nitrogen | A2 | A | 2 | Ammonia | 0.215700 | True | True | True | 8.714590e-03 |
| PM3B\_A3 | PM3B | Nitrogen | A3 | A | 3 | Nitrite | 0.157167 | True | True | True | 7.583231e-04 |
| PM3B\_A4 | PM3B | Nitrogen | A4 | A | 4 | Nitrate | 0.214200 | True | True | True | 2.441368e-04 |
| PM3B\_A5 | PM3B | Nitrogen | A5 | A | 5 | Urea | 0.228100 | True | True | True | 1.453080e-02 |
| PM3B\_A6 | PM3B | Nitrogen | A6 | A | 6 | Biuret | 0.112633 | False | True | False | 1.172464e-01 |
| PM3B\_A7 | PM3B | Nitrogen | A7 | A | 7 | L-Alanine | 0.216500 | True | True | True | 1.082364e-02 |
| PM3B\_A8 | PM3B | Nitrogen | A8 | A | 8 | L-Arginine | 0.228267 | True | True | True | 6.174921e-04 |
| PM3B\_A9 | PM3B | Nitrogen | A9 | A | 9 | L-Asparagine | 0.211933 | True | True | True | 3.106471e-03 |
| PM3B\_A10 | PM3B | Nitrogen | A10 | A | 10 | L-Aspartic Acid | 0.236567 | True | True | True | 4.961819e-04 |
| PM3B\_A11 | PM3B | Nitrogen | A11 | A | 11 | L-Cysteine | 0.162167 | True | True | True | 7.715002e-03 |
| PM3B\_A12 | PM3B | Nitrogen | A12 | A | 12 | L-Glutamic Acid | 0.343500 | True | True | True | 4.482125e-03 |
| PM3B\_B1 | PM3B | Nitrogen | B1 | B | 1 | L-Glutamine | 0.269500 | True | True | True | 5.119435e-02 |
| PM3B\_B2 | PM3B | Nitrogen | B2 | B | 2 | Glycine | 0.200800 | True | True | True | 9.850523e-03 |
| PM3B\_B3 | PM3B | Nitrogen | B3 | B | 3 | L-Histidine | 0.140533 | True | True | True | 4.905889e-02 |
| PM3B\_B4 | PM3B | Nitrogen | B4 | B | 4 | L-Isoleucine | 0.183400 | True | True | True | 1.143585e-04 |
| PM3B\_B5 | PM3B | Nitrogen | B5 | B | 5 | L-Leucine | 0.175600 | True | True | True | 1.474676e-03 |
| PM3B\_B6 | PM3B | Nitrogen | B6 | B | 6 | L-Lysine | 0.159900 | True | True | True | 3.055213e-03 |
| PM3B\_B7 | PM3B | Nitrogen | B7 | B | 7 | L-Methionine | 0.218600 | True | True | True | 1.130262e-03 |
| PM3B\_B8 | PM3B | Nitrogen | B8 | B | 8 | L-Phenylalanine | 0.173667 | True | True | True | 6.966396e-03 |
| PM3B\_B9 | PM3B | Nitrogen | B9 | B | 9 | L-Proline | 0.228133 | True | True | True | 6.912024e-03 |
| PM3B\_B10 | PM3B | Nitrogen | B10 | B | 10 | L-Serine | 0.174900 | True | True | True | 4.967680e-03 |
| PM3B\_B11 | PM3B | Nitrogen | B11 | B | 11 | L-Threonine | 0.162267 | True | True | True | 4.021312e-03 |
| PM3B\_B12 | PM3B | Nitrogen | B12 | B | 12 | L-Tryptophan | 0.265533 | True | True | True | 1.556645e-04 |
| PM3B\_C1 | PM3B | Nitrogen | C1 | C | 1 | L-Tyrosine | 0.197500 | True | True | True | 1.702870e-02 |
| PM3B\_C2 | PM3B | Nitrogen | C2 | C | 2 | L-Valine | 0.203267 | True | True | True | 2.117481e-02 |
| PM3B\_C3 | PM3B | Nitrogen | C3 | C | 3 | D-Alanine | 0.146133 | True | True | True | 3.364843e-02 |
| PM3B\_C4 | PM3B | Nitrogen | C4 | C | 4 | D-Asparagine | 0.111333 | True | True | True | 2.873781e-02 |
| PM3B\_C5 | PM3B | Nitrogen | C5 | C | 5 | D-Aspartic Acid | 0.100800 | False | True | False | 4.881286e-01 |
| PM3B\_C6 | PM3B | Nitrogen | C6 | C | 6 | D-Glutamic Acid | 0.046600 | False | False | False | 1.008160e-04 |
| PM3B\_C7 | PM3B | Nitrogen | C7 | C | 7 | D-Lysine | 0.110933 | False | True | False | 2.718926e-01 |
| PM3B\_C8 | PM3B | Nitrogen | C8 | C | 8 | D-Serine | 0.108500 | False | True | False | 3.342331e-01 |
| PM3B\_C9 | PM3B | Nitrogen | C9 | C | 9 | D-Valine | 0.121267 | True | True | True | 2.694548e-02 |
| PM3B\_C10 | PM3B | Nitrogen | C10 | C | 10 | L-Citrulline | 0.190100 | True | True | True | 2.708958e-03 |
| PM3B\_C11 | PM3B | Nitrogen | C11 | C | 11 | L-Homoserine | 0.252767 | True | True | True | 2.503726e-03 |
| PM3B\_C12 | PM3B | Nitrogen | C12 | C | 12 | L-Ornithine | 0.223733 | True | True | True | 3.565946e-02 |
| PM3B\_D1 | PM3B | Nitrogen | D1 | D | 1 | N-Acetyl-L-Glutamic Acid | 0.112500 | False | True | False | 1.245772e-01 |
| PM3B\_D2 | PM3B | Nitrogen | D2 | D | 2 | N-Phthaloyl-L-Glutamic Acid | 0.217400 | True | True | True | 1.587860e-03 |
| PM3B\_D3 | PM3B | Nitrogen | D3 | D | 3 | L-Pyroglutamic Acid | 0.202267 | True | True | True | 1.195223e-03 |
| PM3B\_D4 | PM3B | Nitrogen | D4 | D | 4 | Hydroxylamine | 0.150133 | True | True | True | 1.341796e-02 |
| PM3B\_D5 | PM3B | Nitrogen | D5 | D | 5 | Methylamine | 0.087867 | False | False | False | 1.085812e-01 |
| PM3B\_D6 | PM3B | Nitrogen | D6 | D | 6 | N-Amylamine | 0.163700 | True | True | True | 6.949713e-03 |
| PM3B\_D7 | PM3B | Nitrogen | D7 | D | 7 | N-Butylamine | 0.151933 | True | True | True | 1.113801e-02 |
| PM3B\_D8 | PM3B | Nitrogen | D8 | D | 8 | Ethylamine | 0.159533 | True | True | True | 1.669074e-02 |
| PM3B\_D9 | PM3B | Nitrogen | D9 | D | 9 | Ethanolamine | 0.182600 | True | True | True | 3.882741e-03 |
| PM3B\_D10 | PM3B | Nitrogen | D10 | D | 10 | Ethylenediamine | 0.079633 | False | False | False | 1.199674e-02 |
| PM3B\_D11 | PM3B | Nitrogen | D11 | D | 11 | Putrescine | 0.122467 | True | True | True | 9.223327e-02 |
| PM3B\_D12 | PM3B | Nitrogen | D12 | D | 12 | Agmatine | 0.103200 | False | True | False | 3.430667e-01 |
| PM3B\_E1 | PM3B | Nitrogen | E1 | E | 1 | Histamine | 0.073933 | False | False | False | 1.162502e-02 |
| PM3B\_E2 | PM3B | Nitrogen | E2 | E | 2 | b-Phenylethylamine | 0.118233 | False | True | False | 1.679197e-01 |
| PM3B\_E3 | PM3B | Nitrogen | E3 | E | 3 | Tyramine | 0.145667 | True | True | True | 4.030644e-02 |
| PM3B\_E4 | PM3B | Nitrogen | E4 | E | 4 | Acetamide | 0.149867 | True | True | True | 3.123878e-03 |
| PM3B\_E5 | PM3B | Nitrogen | E5 | E | 5 | Formamide | 0.161000 | True | True | True | 8.973497e-04 |
| PM3B\_E6 | PM3B | Nitrogen | E6 | E | 6 | Glucuronamide | 0.203700 | True | True | True | 1.693989e-04 |
| PM3B\_E7 | PM3B | Nitrogen | E7 | E | 7 | D,L-Lactamide | 0.215433 | True | True | True | 4.106176e-05 |
| PM3B\_E8 | PM3B | Nitrogen | E8 | E | 8 | D-Glucosamine | 0.129100 | True | True | True | 6.595409e-02 |
| PM3B\_E9 | PM3B | Nitrogen | E9 | E | 9 | D-Galactosamine | 0.181900 | True | True | True | 1.433956e-03 |
| PM3B\_E10 | PM3B | Nitrogen | E10 | E | 10 | D-Mannosamine | 0.130233 | True | True | True | 2.913399e-02 |
| PM3B\_E11 | PM3B | Nitrogen | E11 | E | 11 | N-Acetyl-D-Glucosamine | 0.101067 | False | True | False | 4.752756e-01 |
| PM3B\_E12 | PM3B | Nitrogen | E12 | E | 12 | N-Acetyl-D-Galactosamine | 0.090833 | False | False | False | 9.226927e-02 |
| PM3B\_F1 | PM3B | Nitrogen | F1 | F | 1 | N-Acetyl-D-Mannosamine | 0.096367 | False | False | False | 1.965995e-01 |
| PM3B\_F2 | PM3B | Nitrogen | F2 | F | 2 | Adenine | 0.239667 | True | True | True | 2.159428e-04 |
| PM3B\_F3 | PM3B | Nitrogen | F3 | F | 3 | Adenosine | 0.238100 | True | True | True | 1.283275e-03 |
| PM3B\_F4 | PM3B | Nitrogen | F4 | F | 4 | Cytidine | 0.196300 | True | True | True | 7.562988e-04 |
| PM3B\_F5 | PM3B | Nitrogen | F5 | F | 5 | Cytosine | 0.178733 | True | True | True | 1.172453e-03 |
| PM3B\_F6 | PM3B | Nitrogen | F6 | F | 6 | Guanine | 0.127233 | False | True | False | 1.606233e-01 |
| PM3B\_F7 | PM3B | Nitrogen | F7 | F | 7 | Guanosine | 0.275767 | True | True | True | 1.620693e-06 |
| PM3B\_F8 | PM3B | Nitrogen | F8 | F | 8 | Thymine | 0.153600 | True | True | True | 3.230379e-02 |
| PM3B\_F9 | PM3B | Nitrogen | F9 | F | 9 | Thymidine | 0.119500 | True | True | True | 4.397306e-03 |
| PM3B\_F10 | PM3B | Nitrogen | F10 | F | 10 | Uracil | 0.193733 | True | True | True | 6.049486e-04 |
| PM3B\_F11 | PM3B | Nitrogen | F11 | F | 11 | Uridine | 0.189400 | True | True | True | 3.280129e-04 |
| PM3B\_F12 | PM3B | Nitrogen | F12 | F | 12 | Inosine | 0.324900 | True | True | True | 9.910421e-03 |
| PM3B\_G1 | PM3B | Nitrogen | G1 | G | 1 | Xanthine | 0.387333 | True | True | True | 8.866356e-04 |
| PM3B\_G2 | PM3B | Nitrogen | G2 | G | 2 | Xanthosine | 0.200100 | True | True | True | 1.147614e-03 |
| PM3B\_G3 | PM3B | Nitrogen | G3 | G | 3 | Uric Acid | 0.239433 | True | True | True | 1.655418e-02 |
| PM3B\_G4 | PM3B | Nitrogen | G4 | G | 4 | Alloxan | 0.306567 | True | True | True | 2.486531e-04 |
| PM3B\_G5 | PM3B | Nitrogen | G5 | G | 5 | Allantoin | 0.194900 | True | True | True | 6.702298e-04 |
| PM3B\_G6 | PM3B | Nitrogen | G6 | G | 6 | Parabanic Acid | 0.215033 | True | True | True | 2.236734e-04 |
| PM3B\_G7 | PM3B | Nitrogen | G7 | G | 7 | D,L-a-Amino-NButyric Acid | 0.157433 | True | True | True | 2.738293e-03 |
| PM3B\_G8 | PM3B | Nitrogen | G8 | G | 8 | g-Amino-NButyric Acid | 0.258267 | True | True | True | 4.858764e-04 |
| PM3B\_G9 | PM3B | Nitrogen | G9 | G | 9 | e-Amino-NCaproic Acid | 0.138967 | True | True | True | 1.193646e-02 |
| PM3B\_G10 | PM3B | Nitrogen | G10 | G | 10 | D,L-a-AminoCaprylic Acid | 0.228933 | True | True | True | 2.814331e-03 |
| PM3B\_G11 | PM3B | Nitrogen | G11 | G | 11 | d-Amino-NValeric Acid | 0.200433 | True | True | True | 1.199652e-02 |
| PM3B\_G12 | PM3B | Nitrogen | G12 | G | 12 | a-Amino-NValeric Acid | 0.314867 | True | True | True | 3.285660e-06 |
| PM3B\_H1 | PM3B | Nitrogen | H1 | H | 1 | Ala-Asp | 0.196167 | True | True | True | 2.372107e-03 |
| PM3B\_H2 | PM3B | Nitrogen | H2 | H | 2 | Ala-Gln | 0.294933 | True | True | True | 2.293602e-02 |
| PM3B\_H3 | PM3B | Nitrogen | H3 | H | 3 | Ala-Glu | 0.187667 | True | True | True | 1.707807e-03 |
| PM3B\_H4 | PM3B | Nitrogen | H4 | H | 4 | Ala-Gly | 0.268000 | True | True | True | 1.143225e-04 |
| PM3B\_H5 | PM3B | Nitrogen | H5 | H | 5 | Ala-His | 0.258600 | True | True | True | 1.010602e-03 |
| PM3B\_H6 | PM3B | Nitrogen | H6 | H | 6 | Ala-Leu | 0.162200 | True | True | True | 3.614322e-03 |
| PM3B\_H7 | PM3B | Nitrogen | H7 | H | 7 | Ala-Thr | 0.206067 | True | True | True | 9.619428e-04 |
| PM3B\_H8 | PM3B | Nitrogen | H8 | H | 8 | Gly-Asn | 0.308767 | True | True | True | 6.690069e-06 |
| PM3B\_H9 | PM3B | Nitrogen | H9 | H | 9 | Gly-Gln | 0.258733 | True | True | True | 3.041417e-03 |
| PM3B\_H10 | PM3B | Nitrogen | H10 | H | 10 | Gly-Glu | 0.280533 | True | True | True | 1.963066e-03 |
| PM3B\_H11 | PM3B | Nitrogen | H11 | H | 11 | Gly-Met | 0.283833 | True | True | True | 9.115125e-04 |
| PM3B\_H12 | PM3B | Nitrogen | H12 | H | 12 | Met-Ala | 0.370500 | True | True | True | 1.290200e-06 |
| PM4A\_A1 | PM4A | Phosphorus | A1 | A | 1 | Negative Control | 0.067500 | False | False | False | 5.000000e-01 |
| PM4A\_A2 | PM4A | Phosphorus | A2 | A | 2 | Phosphate | 0.217267 | True | True | True | 1.585756e-04 |
| PM4A\_A3 | PM4A | Phosphorus | A3 | A | 3 | Pyrophosphate | 0.299700 | True | True | True | 4.010296e-03 |
| PM4A\_A4 | PM4A | Phosphorus | A4 | A | 4 | Trimetaphosphate | 0.305667 | True | True | True | 3.375760e-06 |
| PM4A\_A5 | PM4A | Phosphorus | A5 | A | 5 | Tripolyphosphate | 0.383400 | True | True | True | 5.212308e-05 |
| PM4A\_A6 | PM4A | Phosphorus | A6 | A | 6 | Triethyl Phosphate | 0.065967 | False | False | False | 4.594924e-01 |
| PM4A\_A7 | PM4A | Phosphorus | A7 | A | 7 | Hypophosphite | 0.061300 | False | False | False | 2.813323e-01 |
| PM4A\_A8 | PM4A | Phosphorus | A8 | A | 8 | Adenosine-2’-monophosphate | 0.298300 | True | True | True | 5.019726e-06 |
| PM4A\_A9 | PM4A | Phosphorus | A9 | A | 9 | Adenosine-3’-monophosphate | 0.237633 | True | True | True | 3.409667e-05 |
| PM4A\_A10 | PM4A | Phosphorus | A10 | A | 10 | Adenosine-5’-monophosphate | 0.220933 | True | True | True | 4.235822e-05 |
| PM4A\_A11 | PM4A | Phosphorus | A11 | A | 11 | Adenosine-2’,3’-Cyclic monophosphate | 0.209733 | True | True | True | 1.333630e-04 |
| PM4A\_A12 | PM4A | Phosphorus | A12 | A | 12 | Adenosine-3’,5’-Cyclic monophosphate | 0.272933 | True | True | True | 5.038275e-03 |
| PM4A\_B1 | PM4A | Phosphorus | B1 | B | 1 | Thiophosphate | 0.124300 | True | True | True | 4.549269e-02 |
| PM4A\_B2 | PM4A | Phosphorus | B2 | B | 2 | Dithiophosphate | 0.324100 | True | True | True | 1.682775e-04 |
| PM4A\_B3 | PM4A | Phosphorus | B3 | B | 3 | D,L-a-Glycerol Phosphate | 0.212700 | True | True | True | 3.261109e-04 |
| PM4A\_B4 | PM4A | Phosphorus | B4 | B | 4 | b-Glycerol Phosphate | 0.203100 | True | True | True | 1.637802e-04 |
| PM4A\_B5 | PM4A | Phosphorus | B5 | B | 5 | Carbamyl Phosphate | 0.337933 | True | True | True | 3.379950e-03 |
| PM4A\_B6 | PM4A | Phosphorus | B6 | B | 6 | D-2-PhosphoGlyceric Acid | 0.295633 | True | True | True | 2.394438e-05 |
| PM4A\_B7 | PM4A | Phosphorus | B7 | B | 7 | D-3-PhosphoGlyceric Acid | 0.286367 | True | True | True | 1.612982e-04 |
| PM4A\_B8 | PM4A | Phosphorus | B8 | B | 8 | Guanosine-2’-monophosphate | 0.201267 | True | True | True | 5.019368e-04 |
| PM4A\_B9 | PM4A | Phosphorus | B9 | B | 9 | Guanosine-3’-monophosphate | 0.224833 | True | True | True | 5.198822e-04 |
| PM4A\_B10 | PM4A | Phosphorus | B10 | B | 10 | Guanosine-5’-monophosphate | 0.222300 | True | True | True | 3.780977e-04 |
| PM4A\_B11 | PM4A | Phosphorus | B11 | B | 11 | Guanosine-2’,3’-Cyclic monophosphate | 0.233500 | True | True | True | 3.055379e-04 |
| PM4A\_B12 | PM4A | Phosphorus | B12 | B | 12 | Guanosine-3’,5’-Cyclic monophosphate | 0.218567 | True | True | True | 2.440755e-03 |
| PM4A\_C1 | PM4A | Phosphorus | C1 | C | 1 | Phosphoenol Pyruvate | 0.304033 | True | True | True | 6.867371e-03 |
| PM4A\_C2 | PM4A | Phosphorus | C2 | C | 2 | PhosphoGlycolic Acid | 0.238667 | True | True | True | 1.280709e-03 |
| PM4A\_C3 | PM4A | Phosphorus | C3 | C | 3 | D-Glucose-1-Phosphate | 0.217267 | True | True | True | 3.173362e-04 |
| PM4A\_C4 | PM4A | Phosphorus | C4 | C | 4 | D-Glucose-6-Phosphate | 0.248967 | True | True | True | 6.147327e-04 |
| PM4A\_C5 | PM4A | Phosphorus | C5 | C | 5 | 2-Deoxy-D-Glucose 6-Phosphate | 0.074400 | False | False | False | 3.175716e-01 |
| PM4A\_C6 | PM4A | Phosphorus | C6 | C | 6 | D-Glucosamine-6-Phosphate | 0.219933 | True | True | True | 5.541472e-04 |
| PM4A\_C7 | PM4A | Phosphorus | C7 | C | 7 | 6-PhosphoGluconic Acid | 0.308633 | True | True | True | 4.517791e-04 |
| PM4A\_C8 | PM4A | Phosphorus | C8 | C | 8 | Cytidine-2’-monophosphate | 0.302167 | True | True | True | 2.416267e-02 |
| PM4A\_C9 | PM4A | Phosphorus | C9 | C | 9 | Cytidine-3’-monophosphate | 0.075300 | False | False | False | 2.159127e-01 |
| PM4A\_C10 | PM4A | Phosphorus | C10 | C | 10 | Cytidine-5’-monophosphate | 0.313933 | True | True | True | 1.391660e-05 |
| PM4A\_C11 | PM4A | Phosphorus | C11 | C | 11 | Cytidine-2’,3’-Cyclic monophosphate | 0.197300 | True | True | True | 1.095209e-04 |
| PM4A\_C12 | PM4A | Phosphorus | C12 | C | 12 | Cytidine-3’,5’-Cyclic monophosphate | 0.196100 | True | True | True | 9.250467e-05 |
| PM4A\_D1 | PM4A | Phosphorus | D1 | D | 1 | D-Mannose-1-Phosphate | 0.252833 | True | True | True | 2.062550e-02 |
| PM4A\_D2 | PM4A | Phosphorus | D2 | D | 2 | D-Mannose-6-Phosphate | 0.294767 | True | True | True | 2.941949e-03 |
| PM4A\_D3 | PM4A | Phosphorus | D3 | D | 3 | Cysteamine S-Phosphate | 0.366467 | True | True | True | 3.695288e-04 |
| PM4A\_D4 | PM4A | Phosphorus | D4 | D | 4 | Phospho-L-Arginine | 0.242233 | True | True | True | 4.000312e-03 |
| PM4A\_D5 | PM4A | Phosphorus | D5 | D | 5 | O-Phospho-D-Serine | 0.251967 | True | True | True | 2.123975e-02 |
| PM4A\_D6 | PM4A | Phosphorus | D6 | D | 6 | O-Phospho-L-Serine | 0.236900 | True | True | True | 1.400548e-03 |
| PM4A\_D7 | PM4A | Phosphorus | D7 | D | 7 | O-Phospho-L-Threonine | 0.270133 | True | True | True | 1.482491e-02 |
| PM4A\_D8 | PM4A | Phosphorus | D8 | D | 8 | Uridine-2’-monophosphate | 0.230733 | True | True | True | 1.467996e-03 |
| PM4A\_D9 | PM4A | Phosphorus | D9 | D | 9 | Uridine-3’-monophosphate | 0.212100 | True | True | True | 3.079172e-04 |
| PM4A\_D10 | PM4A | Phosphorus | D10 | D | 10 | Uridine-5’-monophosphate | 0.231200 | True | True | True | 3.357194e-03 |
| PM4A\_D11 | PM4A | Phosphorus | D11 | D | 11 | Uridine-2’,3’-Cyclic monophosphate | 0.225300 | True | True | True | 1.081703e-03 |
| PM4A\_D12 | PM4A | Phosphorus | D12 | D | 12 | Uridine-3’,5’-Cyclic monophosphate | 0.218200 | True | True | True | 1.001221e-03 |
| PM4A\_E1 | PM4A | Phosphorus | E1 | E | 1 | O-Phospho-D-Tyrosine | 0.235467 | True | True | True | 1.478754e-02 |
| PM4A\_E2 | PM4A | Phosphorus | E2 | E | 2 | O-Phospho-L-Tyrosine | 0.192400 | True | True | True | 2.108053e-03 |
| PM4A\_E3 | PM4A | Phosphorus | E3 | E | 3 | Phosphocreatine | 0.218667 | True | True | True | 4.066979e-03 |
| PM4A\_E4 | PM4A | Phosphorus | E4 | E | 4 | Phosphoryl Choline | 0.216200 | True | True | True | 3.415909e-03 |
| PM4A\_E5 | PM4A | Phosphorus | E5 | E | 5 | O-PhosphorylEthanolamine | 0.211700 | True | True | True | 3.846899e-04 |
| PM4A\_E6 | PM4A | Phosphorus | E6 | E | 6 | Phosphono Acetic Acid | 0.062000 | False | False | False | 3.426570e-01 |
| PM4A\_E7 | PM4A | Phosphorus | E7 | E | 7 | 2-Aminoethyl Phosphonic Acid | 0.060400 | False | False | False | 2.340544e-01 |
| PM4A\_E8 | PM4A | Phosphorus | E8 | E | 8 | Methylene Diphosphonic Acid | 0.175633 | True | True | True | 8.483050e-03 |
| PM4A\_E9 | PM4A | Phosphorus | E9 | E | 9 | Thymidine-3’-monophosphate | 0.198100 | True | True | True | 1.259104e-04 |
| PM4A\_E10 | PM4A | Phosphorus | E10 | E | 10 | Thymidine-5’-monophosphate | 0.190300 | True | True | True | 1.393921e-04 |
| PM4A\_E11 | PM4A | Phosphorus | E11 | E | 11 | Inositol Hexaphosphate | 0.288667 | True | True | True | 3.242554e-03 |
| PM4A\_E12 | PM4A | Phosphorus | E12 | E | 12 | Thymidine 3’,5’-Cyclic monophosphate | 0.124133 | False | True | False | 8.604169e-02 |
| PM4A\_F1 | PM4A | Sulfur | F1 | F | 1 | Negative Control | 0.078800 | False | False | False | 5.000000e-01 |
| PM4A\_F2 | PM4A | Sulfur | F2 | F | 2 | Sulfate | 0.233500 | True | True | True | 8.887386e-03 |
| PM4A\_F3 | PM4A | Sulfur | F3 | F | 3 | Thiosulfate | 0.256500 | True | True | True | 6.445097e-04 |
| PM4A\_F4 | PM4A | Sulfur | F4 | F | 4 | Tetrathionate | 0.194367 | True | True | True | 1.837971e-03 |
| PM4A\_F5 | PM4A | Sulfur | F5 | F | 5 | Thiophosphate | 0.216967 | True | True | True | 1.118004e-05 |
| PM4A\_F6 | PM4A | Sulfur | F6 | F | 6 | Dithiophosphate | 0.200267 | True | True | True | 4.704902e-04 |
| PM4A\_F7 | PM4A | Sulfur | F7 | F | 7 | L-Cysteine | 0.180100 | True | True | True | 3.284224e-04 |
| PM4A\_F8 | PM4A | Sulfur | F8 | F | 8 | D-Cysteine | 0.121600 | True | True | True | 1.278542e-02 |
| PM4A\_F9 | PM4A | Sulfur | F9 | F | 9 | L-CysteinylGlycine | 0.339133 | True | True | True | 2.062261e-03 |
| PM4A\_F10 | PM4A | Sulfur | F10 | F | 10 | L-Cysteic Acid | 0.141700 | True | True | True | 1.010716e-05 |
| PM4A\_F11 | PM4A | Sulfur | F11 | F | 11 | Cysteamine | 0.186700 | True | True | True | 4.672723e-04 |
| PM4A\_F12 | PM4A | Sulfur | F12 | F | 12 | L-Cysteine Sulfinic Acid | 0.193200 | True | True | True | 2.658437e-03 |
| PM4A\_G1 | PM4A | Sulfur | G1 | G | 1 | N-Acetyl-L-Cysteine | 0.121333 | True | True | True | 9.561798e-04 |
| PM4A\_G2 | PM4A | Sulfur | G2 | G | 2 | S-Methyl-L-Cysteine | 0.155867 | True | True | True | 4.744089e-04 |
| PM4A\_G3 | PM4A | Sulfur | G3 | G | 3 | Cystathionine | 0.183667 | True | True | True | 3.791719e-05 |
| PM4A\_G4 | PM4A | Sulfur | G4 | G | 4 | Lanthionine | 0.182200 | True | True | True | 2.997179e-05 |
| PM4A\_G5 | PM4A | Sulfur | G5 | G | 5 | Glutathione | 0.132700 | True | True | True | 1.199680e-04 |
| PM4A\_G6 | PM4A | Sulfur | G6 | G | 6 | D,L-Ethionine | 0.106300 | True | True | True | 1.750069e-04 |
| PM4A\_G7 | PM4A | Sulfur | G7 | G | 7 | L-Methionine | 0.268700 | True | True | True | 8.399395e-05 |
| PM4A\_G8 | PM4A | Sulfur | G8 | G | 8 | D-Methionine | 0.206700 | True | True | True | 5.975166e-05 |
| PM4A\_G9 | PM4A | Sulfur | G9 | G | 9 | Glycyl-L-Methionine | 0.268467 | True | True | True | 6.250130e-05 |
| PM4A\_G10 | PM4A | Sulfur | G10 | G | 10 | N-Acetyl-D,L-Methionine | 0.174567 | True | True | True | 2.345897e-04 |
| PM4A\_G11 | PM4A | Sulfur | G11 | G | 11 | L-Methionine Sulfoxide | 0.259267 | True | True | True | 5.559775e-04 |
| PM4A\_G12 | PM4A | Sulfur | G12 | G | 12 | L-Methionine Sulfone | 0.174233 | True | True | True | 1.221453e-04 |
| PM4A\_H1 | PM4A | Sulfur | H1 | H | 1 | L-Djenkolic Acid | 0.343900 | True | True | True | 9.628870e-04 |
| PM4A\_H2 | PM4A | Sulfur | H2 | H | 2 | Thiourea | 0.167867 | True | True | True | 1.579425e-03 |
| PM4A\_H3 | PM4A | Sulfur | H3 | H | 3 | 1-Thio-b-D-Glucose | 0.144700 | True | True | True | 5.054776e-05 |
| PM4A\_H4 | PM4A | Sulfur | H4 | H | 4 | D,L-Lipoamide | 0.269967 | True | True | True | 4.905622e-07 |
| PM4A\_H5 | PM4A | Sulfur | H5 | H | 5 | Taurocholic Acid | 0.131533 | True | True | True | 1.336562e-05 |
| PM4A\_H6 | PM4A | Sulfur | H6 | H | 6 | Taurine | 0.182767 | True | True | True | 5.280316e-04 |
| PM4A\_H7 | PM4A | Sulfur | H7 | H | 7 | Hypotaurine | 0.219867 | True | True | True | 8.591333e-04 |
| PM4A\_H8 | PM4A | Sulfur | H8 | H | 8 | p-Amino Benzene Sulfonic Acid | 0.086933 | False | False | False | 2.063828e-01 |
| PM4A\_H9 | PM4A | Sulfur | H9 | H | 9 | Butane Sulfonic Acid | 0.188767 | True | True | True | 1.278648e-03 |
| PM4A\_H10 | PM4A | Sulfur | H10 | H | 10 | 2-Hydroxyethane Sulfonic Acid | 0.177900 | True | True | True | 3.339269e-05 |
| PM4A\_H11 | PM4A | Sulfur | H11 | H | 11 | Methane Sulfonic Acid | 0.206367 | True | True | True | 3.593406e-03 |
| PM4A\_H12 | PM4A | Sulfur | H12 | H | 12 | Tetramethylene Sulfone | 0.079033 | False | False | False | 4.767091e-01 |

In [7]:

```
Biolog_Media = pd.read_excel('../../Data/Biolog_Media.xlsx',
                             usecols=list(range(11,17)), index_col=0)
Biolog_Media = Biolog_Media.loc[Biolog_Media[['Carbon','Nitrogen','Phosphorus','Sulfur']].dropna(axis=0, how='all').index]
Biolog_Media
```

Out[7]:

|  | Source | Carbon | Nitrogen | Phosphorus | Sulfur |
| --- | --- | --- | --- | --- | --- |
| Biolog |  |  |  |  |  |
| PM1\_A2 | Carbon | EX\_arab\_\_L\_e | EX\_nh4\_e | EX\_pi\_e | EX\_so4\_e |
| PM1\_A3 | Carbon | EX\_acgam\_e | EX\_nh4\_e | EX\_pi\_e | EX\_so4\_e |
| PM1\_A4 | Carbon | EX\_glcr\_e | EX\_nh4\_e | EX\_pi\_e | EX\_so4\_e |
| PM1\_A5 | Carbon | EX\_succ\_e | EX\_nh4\_e | EX\_pi\_e | EX\_so4\_e |
| PM1\_A6 | Carbon | EX\_gal\_e | EX\_nh4\_e | EX\_pi\_e | EX\_so4\_e |
| ... | ... | ... | ... | ... | ... |
| PM4A\_H6 | Sulfur | EX\_pyr\_e | EX\_nh4\_e | EX\_pi\_e | EX\_taur\_e |
| PM4A\_H7 | Sulfur | EX\_pyr\_e | EX\_nh4\_e | EX\_pi\_e | EX\_hyptaur\_e |
| PM4A\_H9 | Sulfur | EX\_pyr\_e | EX\_nh4\_e | EX\_pi\_e | EX\_butso3\_e |
| PM4A\_H10 | Sulfur | EX\_pyr\_e | EX\_nh4\_e | EX\_pi\_e | EX\_isetac\_e |
| PM4A\_H11 | Sulfur | EX\_pyr\_e | EX\_nh4\_e | EX\_pi\_e | EX\_mso3\_e |

285 rows × 5 columns

In [8]:

```
print(Biolog.shape)
print(Biolog_Media.shape)
```

```
(384, 11)
(285, 5)
```

In [9]:

```
with pd.option_context('display.max_rows', None, 'display.max_columns', None):
    display(Biolog[~Biolog.index.isin(Biolog_Media.index)])
```

|  | PlateType | Experiment | Well | Row | Column | Compound | Average | All > NC | Average > 0.1 | Pass | Ttest |
| --- | --- | --- | --- | --- | --- | --- | --- | --- | --- | --- | --- |
| PM1\_A1 | PM1 | Carbon | A1 | A | 1 | Negative Control | 0.086400 | False | False | False | 0.500000 |
| PM1\_C5 | PM1 | Carbon | C5 | C | 5 | Tween 20 | 0.397733 | True | True | True | 0.000036 |
| PM1\_D3 | PM1 | Carbon | D3 | D | 3 | D-Glucosaminic Acid | 0.083900 | False | False | False | 0.295679 |
| PM1\_D5 | PM1 | Carbon | D5 | D | 5 | Tween 40 | 0.347467 | True | True | True | 0.000003 |
| PM1\_D8 | PM1 | Carbon | D8 | D | 8 | a-Methyl-D-Galactoside | 0.081267 | False | False | False | 0.226319 |
| PM1\_D10 | PM1 | Carbon | D10 | D | 10 | Lactulose | 0.083933 | False | False | False | 0.420182 |
| PM1\_E5 | PM1 | Carbon | E5 | E | 5 | Tween 80 | 0.408233 | True | True | True | 0.000855 |
| PM1\_E6 | PM1 | Carbon | E6 | E | 6 | a-Hydroxy Glutaric Acid-g-Lactone | 0.064367 | False | False | False | 0.034193 |
| PM1\_F4 | PM1 | Carbon | F4 | F | 4 | D-Threonine | 0.112333 | False | True | False | 0.086383 |
| PM1\_F6 | PM1 | Carbon | F6 | F | 6 | Bromo Succinic Acid | 0.173133 | True | True | True | 0.000013 |
| PM1\_G10 | PM1 | Carbon | G10 | G | 10 | Methyl Pyruvate | 0.170767 | True | True | True | 0.004499 |
| PM1\_H5 | PM1 | Carbon | H5 | H | 5 | D-Psicose | 0.122867 | True | True | True | 0.000342 |
| PM1\_H7 | PM1 | Carbon | H7 | H | 7 | Glucuronamide | 0.112100 | True | True | True | 0.003058 |
| PM2\_A1 | PM2 | Carbon | A1 | A | 1 | Negative Control | 0.082233 | False | False | False | 0.127040 |
| PM2\_A2 | PM2 | Carbon | A2 | A | 2 | Chondroitin Sulfate C | 0.057667 | False | False | False | 0.012313 |
| PM2\_A3 | PM2 | Carbon | A3 | A | 3 | a-Cyclodextrin | 0.059600 | False | False | False | 0.018561 |
| PM2\_A4 | PM2 | Carbon | A4 | A | 4 | b-Cyclodextrin | 0.079833 | False | False | False | 0.257001 |
| PM2\_A5 | PM2 | Carbon | A5 | A | 5 | g-Cyclodextrin | 0.102967 | False | True | False | 0.085020 |
| PM2\_A7 | PM2 | Carbon | A7 | A | 7 | Gelatin | 0.133233 | True | True | True | 0.000020 |
| PM2\_A9 | PM2 | Carbon | A9 | A | 9 | Inulin | 0.165867 | True | True | True | 0.000616 |
| PM2\_A10 | PM2 | Carbon | A10 | A | 10 | Laminarin | 0.203733 | True | True | True | 0.000062 |
| PM2\_B4 | PM2 | Carbon | B4 | B | 4 | Amygdalin | 0.105433 | True | True | True | 0.058904 |
| PM2\_B10 | PM2 | Carbon | B10 | B | 10 | i-Erythritol | 0.076100 | False | False | False | 0.151353 |
| PM2\_B11 | PM2 | Carbon | B11 | B | 11 | D-Fucose | 0.110233 | True | True | True | 0.084093 |
| PM2\_B12 | PM2 | Carbon | B12 | B | 12 | 3-0-b-D-Galactopyranosyl-D-Arabinose | 0.114800 | True | True | True | 0.016319 |
| PM2\_C1 | PM2 | Carbon | C1 | C | 1 | Gentiobiose | 0.230400 | True | True | True | 0.002071 |
| PM2\_C2 | PM2 | Carbon | C2 | C | 2 | L-Glucose | 0.115167 | True | True | True | 0.036591 |
| PM2\_C3 | PM2 | Carbon | C3 | C | 3 | Lactitol | 0.086033 | False | False | False | 0.487405 |
| PM2\_C4 | PM2 | Carbon | C4 | C | 4 | D-Melezitose | 0.086133 | False | False | False | 0.490429 |
| PM2\_C5 | PM2 | Carbon | C5 | C | 5 | Maltitol | 0.089767 | False | False | False | 0.408698 |
| PM2\_C6 | PM2 | Carbon | C6 | C | 6 | A-Methyl-D-Glucoside | 0.086867 | False | False | False | 0.484407 |
| PM2\_C7 | PM2 | Carbon | C7 | C | 7 | b-Methyl-D-Galactoside | 0.101900 | False | True | False | 0.171726 |
| PM2\_C8 | PM2 | Carbon | C8 | C | 8 | 3-Methyl Glucose | 0.092833 | False | False | False | 0.297021 |
| PM2\_C9 | PM2 | Carbon | C9 | C | 9 | b-Methyl-D-Glucuronic Acid | 0.080167 | False | False | False | 0.271397 |
| PM2\_C10 | PM2 | Carbon | C10 | C | 10 | a-Methyl-D-Mannoside | 0.092733 | False | False | False | 0.200265 |
| PM2\_C11 | PM2 | Carbon | C11 | C | 11 | b-Methyl-D-Xyloside | 0.078433 | False | False | False | 0.217521 |
| PM2\_C12 | PM2 | Carbon | C12 | C | 12 | Palatinose | 0.088200 | False | False | False | 0.393634 |
| PM2\_D1 | PM2 | Carbon | D1 | D | 1 | D-Raffinose | 0.075867 | False | False | False | 0.116267 |
| PM2\_D3 | PM2 | Carbon | D3 | D | 3 | Sedoheptulosan | 0.090200 | False | False | False | 0.407166 |
| PM2\_D5 | PM2 | Carbon | D5 | D | 5 | Stachyose | 0.084233 | False | False | False | 0.445267 |
| PM2\_D7 | PM2 | Carbon | D7 | D | 7 | Turanose | 0.122033 | True | True | True | 0.029339 |
| PM2\_D9 | PM2 | Carbon | D9 | D | 9 | N-Acetyl-D-Glucosaminitol | 0.078800 | False | False | False | 0.298665 |
| PM2\_D11 | PM2 | Carbon | D11 | D | 11 | d-Amino Valeric Acid | 0.097467 | False | False | False | 0.114136 |
| PM2\_E6 | PM2 | Carbon | E6 | E | 6 | 2-Hydroxy Benzoic Acid | 0.100167 | False | True | False | 0.126012 |
| PM2\_E10 | PM2 | Carbon | E10 | E | 10 | A-Keto-Valeric Acid | 0.156967 | True | True | True | 0.023529 |
| PM2\_F1 | PM2 | Carbon | F1 | F | 1 | D-Lactic Acid Methyl Ester | 0.111333 | True | True | True | 0.065430 |
| PM2\_F3 | PM2 | Carbon | F3 | F | 3 | Melibionic Acid | 0.118367 | True | True | True | 0.053189 |
| PM2\_F5 | PM2 | Carbon | F5 | F | 5 | Oxalomalic Acid | 0.146133 | True | True | True | 0.002907 |
| PM2\_F7 | PM2 | Carbon | F7 | F | 7 | D-Ribono-1,4-Lactone | 0.057833 | False | False | False | 0.035726 |
| PM2\_F8 | PM2 | Carbon | F8 | F | 8 | Sebacic Acid | 0.230433 | True | True | True | 0.000956 |
| PM2\_F9 | PM2 | Carbon | F9 | F | 9 | Sorbic Acid | 0.184000 | True | True | True | 0.008510 |
| PM2\_F10 | PM2 | Carbon | F10 | F | 10 | Succinamic Acid | 0.128233 | True | True | True | 0.026133 |
| PM2\_G2 | PM2 | Carbon | G2 | G | 2 | L-Alaninamide | 0.254367 | True | True | True | 0.001793 |
| PM2\_G8 | PM2 | Carbon | G8 | G | 8 | Hydroxy-L-Proline | 0.093567 | False | False | False | 0.373822 |
| PM2\_H3 | PM2 | Carbon | H3 | H | 3 | L-Pyroglutamic Acid | 0.272200 | True | True | True | 0.003184 |
| PM2\_H6 | PM2 | Carbon | H6 | H | 6 | Sec-Butylamine | 0.038367 | False | False | False | 0.003474 |
| PM2\_H7 | PM2 | Carbon | H7 | H | 7 | D,L-Octopamine | 0.091033 | False | False | False | 0.392438 |
| PM2\_H11 | PM2 | Carbon | H11 | H | 11 | 2,3-Butanedione | 0.086967 | False | False | False | 0.476822 |
| PM2\_H12 | PM2 | Carbon | H12 | H | 12 | 3-Hydroxy 2-Butanon | 0.060133 | False | False | False | 0.074833 |
| PM3B\_A1 | PM3B | Nitrogen | A1 | A | 1 | Negative Control | 0.100600 | False | True | False | 0.500000 |
| PM3B\_A6 | PM3B | Nitrogen | A6 | A | 6 | Biuret | 0.112633 | False | True | False | 0.117246 |
| PM3B\_D2 | PM3B | Nitrogen | D2 | D | 2 | N-Phthaloyl-L-Glutamic Acid | 0.217400 | True | True | True | 0.001588 |
| PM3B\_D3 | PM3B | Nitrogen | D3 | D | 3 | L-Pyroglutamic Acid | 0.202267 | True | True | True | 0.001195 |
| PM3B\_D6 | PM3B | Nitrogen | D6 | D | 6 | N-Amylamine | 0.163700 | True | True | True | 0.006950 |
| PM3B\_D7 | PM3B | Nitrogen | D7 | D | 7 | N-Butylamine | 0.151933 | True | True | True | 0.011138 |
| PM3B\_D8 | PM3B | Nitrogen | D8 | D | 8 | Ethylamine | 0.159533 | True | True | True | 0.016691 |
| PM3B\_D10 | PM3B | Nitrogen | D10 | D | 10 | Ethylenediamine | 0.079633 | False | False | False | 0.011997 |
| PM3B\_E6 | PM3B | Nitrogen | E6 | E | 6 | Glucuronamide | 0.203700 | True | True | True | 0.000169 |
| PM3B\_E7 | PM3B | Nitrogen | E7 | E | 7 | D,L-Lactamide | 0.215433 | True | True | True | 0.000041 |
| PM3B\_E10 | PM3B | Nitrogen | E10 | E | 10 | D-Mannosamine | 0.130233 | True | True | True | 0.029134 |
| PM3B\_G6 | PM3B | Nitrogen | G6 | G | 6 | Parabanic Acid | 0.215033 | True | True | True | 0.000224 |
| PM3B\_G9 | PM3B | Nitrogen | G9 | G | 9 | e-Amino-NCaproic Acid | 0.138967 | True | True | True | 0.011936 |
| PM3B\_G10 | PM3B | Nitrogen | G10 | G | 10 | D,L-a-AminoCaprylic Acid | 0.228933 | True | True | True | 0.002814 |
| PM3B\_G11 | PM3B | Nitrogen | G11 | G | 11 | d-Amino-NValeric Acid | 0.200433 | True | True | True | 0.011997 |
| PM3B\_G12 | PM3B | Nitrogen | G12 | G | 12 | a-Amino-NValeric Acid | 0.314867 | True | True | True | 0.000003 |
| PM4A\_A1 | PM4A | Phosphorus | A1 | A | 1 | Negative Control | 0.067500 | False | False | False | 0.500000 |
| PM4A\_A6 | PM4A | Phosphorus | A6 | A | 6 | Triethyl Phosphate | 0.065967 | False | False | False | 0.459492 |
| PM4A\_A7 | PM4A | Phosphorus | A7 | A | 7 | Hypophosphite | 0.061300 | False | False | False | 0.281332 |
| PM4A\_B1 | PM4A | Phosphorus | B1 | B | 1 | Thiophosphate | 0.124300 | True | True | True | 0.045493 |
| PM4A\_B2 | PM4A | Phosphorus | B2 | B | 2 | Dithiophosphate | 0.324100 | True | True | True | 0.000168 |
| PM4A\_B8 | PM4A | Phosphorus | B8 | B | 8 | Guanosine-2’-monophosphate | 0.201267 | True | True | True | 0.000502 |
| PM4A\_C8 | PM4A | Phosphorus | C8 | C | 8 | Cytidine-2’-monophosphate | 0.302167 | True | True | True | 0.024163 |
| PM4A\_D3 | PM4A | Phosphorus | D3 | D | 3 | Cysteamine S-Phosphate | 0.366467 | True | True | True | 0.000370 |
| PM4A\_D8 | PM4A | Phosphorus | D8 | D | 8 | Uridine-2’-monophosphate | 0.230733 | True | True | True | 0.001468 |
| PM4A\_D12 | PM4A | Phosphorus | D12 | D | 12 | Uridine-3’,5’-Cyclic monophosphate | 0.218200 | True | True | True | 0.001001 |
| PM4A\_E1 | PM4A | Phosphorus | E1 | E | 1 | O-Phospho-D-Tyrosine | 0.235467 | True | True | True | 0.014788 |
| PM4A\_E8 | PM4A | Phosphorus | E8 | E | 8 | Methylene Diphosphonic Acid | 0.175633 | True | True | True | 0.008483 |
| PM4A\_E9 | PM4A | Phosphorus | E9 | E | 9 | Thymidine-3’-monophosphate | 0.198100 | True | True | True | 0.000126 |
| PM4A\_E12 | PM4A | Phosphorus | E12 | E | 12 | Thymidine 3’,5’-Cyclic monophosphate | 0.124133 | False | True | False | 0.086042 |
| PM4A\_F1 | PM4A | Sulfur | F1 | F | 1 | Negative Control | 0.078800 | False | False | False | 0.500000 |
| PM4A\_F5 | PM4A | Sulfur | F5 | F | 5 | Thiophosphate | 0.216967 | True | True | True | 0.000011 |
| PM4A\_F6 | PM4A | Sulfur | F6 | F | 6 | Dithiophosphate | 0.200267 | True | True | True | 0.000470 |
| PM4A\_G2 | PM4A | Sulfur | G2 | G | 2 | S-Methyl-L-Cysteine | 0.155867 | True | True | True | 0.000474 |
| PM4A\_G6 | PM4A | Sulfur | G6 | G | 6 | D,L-Ethionine | 0.106300 | True | True | True | 0.000175 |
| PM4A\_G12 | PM4A | Sulfur | G12 | G | 12 | L-Methionine Sulfone | 0.174233 | True | True | True | 0.000122 |
| PM4A\_H2 | PM4A | Sulfur | H2 | H | 2 | Thiourea | 0.167867 | True | True | True | 0.001579 |
| PM4A\_H3 | PM4A | Sulfur | H3 | H | 3 | 1-Thio-b-D-Glucose | 0.144700 | True | True | True | 0.000051 |
| PM4A\_H8 | PM4A | Sulfur | H8 | H | 8 | p-Amino Benzene Sulfonic Acid | 0.086933 | False | False | False | 0.206383 |
| PM4A\_H12 | PM4A | Sulfur | H12 | H | 12 | Tetramethylene Sulfone | 0.079033 | False | False | False | 0.476709 |

In [10]:

```
Biolog_in_model = pd.DataFrame(columns=['Biolog','Model','Exchange','Metabolite',
                                        'Internal','External','Average','Pass'])
for i, row in Biolog.iterrows():
    if i in Biolog_Media.index:
        x = Biolog_Media.loc[i]
        Biolog_in_model.loc[i,'Exchange'] = x[x['Source']]
        Biolog_in_model.loc[i,'Metabolite'] = x[x['Source']].replace('EX_','')
        if ',' in Biolog_in_model.loc[i,'Metabolite']:
            Biolog_in_model.loc[i,'Model'] = [model.metabolites.get_by_id(x).name if x in model.metabolites \
                else next((model.metabolites.get_by_id(x.rsplit('_',1)[0]+'_'+c).name for c in model.compartments \
                    if x.rsplit('_',1)[0]+'_'+c in model.metabolites), None) \
                for x in Biolog_in_model.loc[i,'Metabolite'].split(',')]
            Biolog_in_model.loc[i,'Internal'] = all(any(x.rsplit('_',1)[0]+'_'+c in model.metabolites
                                                        for c in model.compartments) \
                                                     for x in Biolog_in_model.loc[i,'Metabolite'].split(','))
            Biolog_in_model.loc[i,'External'] = all(x in model.metabolites \
                                                    for x in Biolog_in_model.loc[i,'Metabolite'].split(','))
        else:
            Biolog_in_model.loc[i,'Model'] = model.metabolites.get_by_id(Biolog_in_model.loc[i,'Metabolite']).name \
                if Biolog_in_model.loc[i,'Metabolite'] in model.metabolites \
                else next((model.metabolites.get_by_id(Biolog_in_model.loc[i,'Metabolite'].rsplit('_',1)[0]+'_'+c).name \
                    for c in model.compartments 
                          if Biolog_in_model.loc[i,'Metabolite'].rsplit('_',1)[0]+c in model.metabolites), None)
            Biolog_in_model.loc[i,'Internal'] = any(Biolog_in_model.loc[i,'Metabolite'].rsplit('_',1)[0]+'_'+c
                                                    in model.metabolites for c in model.compartments)
            Biolog_in_model.loc[i,'External'] = Biolog_in_model.loc[i,'Metabolite'] in model.metabolites
    else:
        Biolog_in_model.loc[i] = None
    Biolog_in_model.loc[i,'Biolog'] = row['Compound']
    Biolog_in_model.loc[i,'Average'] = row['Average']
    Biolog_in_model.loc[i,'Pass'] = row['Pass']
```

In [11]:

```
model.medium
```

Out[11]:

```
{'EX_h_e': 1000.0,
 'EX_h2o_e': 1000.0,
 'EX_nh4_e': 1000.0,
 'EX_o2_e': 1000.0,
 'EX_pi_e': 1000.0,
 'EX_so4_e': 1000.0,
 'EX_glc__D_e': 1.0,
 'EX_ca2_e': 1000.0,
 'EX_fe2_e': 1000.0,
 'EX_fe3_e': 1000.0,
 'EX_k_e': 1000.0,
 'EX_na1_e': 1000.0,
 'EX_mg2_e': 1000.0,
 'EX_mn2_e': 1000.0,
 'EX_cu2_e': 1000.0,
 'EX_zn2_e': 1000.0}
```

In [12]:

```
Biolog_Prediction = pd.DataFrame(columns=['PlateType','Experiment','Row','Column',
                                          'Data','Data_TF','Prediction','Prediction_TF'])
with model:
    model.reactions.get_by_id('ATPM').lower_bound = 0.0
    model.reactions.get_by_id('EX_glc__D_e').lower_bound = 0.0
    model.reactions.get_by_id('EX_nh4_e').lower_bound = 0.0
    model.reactions.get_by_id('EX_pi_e').lower_bound = 0.0
    model.reactions.get_by_id('EX_so4_e').lower_bound = 0.0
    for i, row in Biolog.iterrows():
        Biolog_Prediction.loc[i,'PlateType'] = row['PlateType']
        Biolog_Prediction.loc[i,'Experiment'] = row['Experiment']
        Biolog_Prediction.loc[i,'Row'] = row['Row']
        Biolog_Prediction.loc[i,'Column'] = row['Column']
        Biolog_Prediction.loc[i,'Data'] = row['Average']
        Biolog_Prediction.loc[i,'Data_TF'] = row['Pass']
        if i in Biolog_Media.index and Biolog_in_model.loc[i,'External']:
            for x in Biolog_Media.loc[i][1:]:
                if ',' in x:
                    for y in x.split(','):
                        model.reactions.get_by_id(y).lower_bound = -10.0
                else:
                    model.reactions.get_by_id(x).lower_bound = -10.0
            sol = model.optimize()
            for x in Biolog_Media.loc[i][1:]:
                if ',' in x:
                    for y in x.split(','):
                        model.reactions.get_by_id(y).lower_bound = 0.0
                else:
                    model.reactions.get_by_id(x).lower_bound = 0.0
            if sol.status == 'optimal':
                Biolog_Prediction.loc[i,'Prediction'] = sol.objective_value
                Biolog_Prediction.loc[i,'Prediction_TF'] = sol.objective_value > 1e-3
            else:
                print(i, 'non-optimal')
                Biolog_Prediction.loc[i,'Prediction'] = np.nan
                Biolog_Prediction.loc[i,'Prediction_TF'] = np.nan
        elif i in Biolog_Media.index and Biolog_in_model.loc[i,'Internal']:
            continue
            for x in Biolog_Media.loc[i,Biolog_Media.loc[i,'Source']].split(','):
                if not x in model.reactions:
                    r = model.reactions.get_by_id('EX_h_e').copy()
                    r.id = x
                    model.add_reactions([r])
                    r.add_metabolites({'h_e': 1.0, x.replace('EX_','').rsplit('_',1)[0]+'_c': -1.0})
            for x in Biolog_Media.loc[i][1:]:
                if ',' in x:
                    for y in x.split(','):
                        model.reactions.get_by_id(y).lower_bound = -10.0
                else:
                    model.reactions.get_by_id(x).lower_bound = -10.0
            sol = model.optimize()
            for x in Biolog_Media.loc[i][1:]:
                if ',' in x:
                    for y in x.split(','):
                        model.reactions.get_by_id(y).lower_bound = 0.0
                else:
                    model.reactions.get_by_id(x).lower_bound = 0.0
            if sol.status == 'optimal':
                Biolog_Prediction.loc[i,'Prediction'] = sol.objective_value
                Biolog_Prediction.loc[i,'Prediction_TF'] = sol.objective_value > 1e-3
            else:
                print(i, 'non-optimal')
                Biolog_Prediction.loc[i,'Prediction'] = np.nan
                Biolog_Prediction.loc[i,'Prediction_TF'] = np.nan
        else:
            Biolog_Prediction.loc[i,'Prediction'] = np.nan
            Biolog_Prediction.loc[i,'Prediction_TF'] =np.nan
            #Biolog_Prediction.loc[i,'Prediction'] = 0.0
            #Biolog_Prediction.loc[i,'Prediction_TF'] = False
```

In [13]:

```
Biolog_Prediction['Data'] = Biolog_Prediction['Data'].astype(float)
Biolog_Prediction['Prediction'] = Biolog_Prediction['Prediction'].astype(float)
Biolog_Prediction.loc[abs(Biolog_Prediction.Prediction) < 1e-6, 'Prediction'] = 0
```

In [14]:

```
with pd.option_context('display.max_rows', None, 'display.max_columns', None):
    display(Biolog_Prediction)
```

|  | PlateType | Experiment | Row | Column | Data | Data\_TF | Prediction | Prediction\_TF |
| --- | --- | --- | --- | --- | --- | --- | --- | --- |
| PM1\_A1 | PM1 | Carbon | A | 1 | 0.086400 | False | NaN | NaN |
| PM1\_A2 | PM1 | Carbon | A | 2 | 0.344767 | True | 0.630546 | True |
| PM1\_A3 | PM1 | Carbon | A | 3 | 0.068867 | False | 0.000000 | False |
| PM1\_A4 | PM1 | Carbon | A | 4 | 0.071200 | False | NaN | NaN |
| PM1\_A5 | PM1 | Carbon | A | 5 | 0.111200 | True | NaN | NaN |
| PM1\_A6 | PM1 | Carbon | A | 6 | 0.143300 | True | 0.756655 | True |
| PM1\_A7 | PM1 | Carbon | A | 7 | 0.267867 | True | 0.356350 | True |
| PM1\_A8 | PM1 | Carbon | A | 8 | 0.272400 | True | 0.000000 | False |
| PM1\_A9 | PM1 | Carbon | A | 9 | 0.121500 | True | 0.076596 | True |
| PM1\_A10 | PM1 | Carbon | A | 10 | 0.221300 | True | NaN | NaN |
| PM1\_A11 | PM1 | Carbon | A | 11 | 0.212667 | True | 0.756655 | True |
| PM1\_A12 | PM1 | Carbon | A | 12 | 0.132067 | True | NaN | NaN |
| PM1\_B1 | PM1 | Carbon | B | 1 | 0.150600 | True | NaN | NaN |
| PM1\_B2 | PM1 | Carbon | B | 2 | 0.124767 | True | NaN | NaN |
| PM1\_B3 | PM1 | Carbon | B | 3 | 0.224300 | True | 0.414484 | True |
| PM1\_B4 | PM1 | Carbon | B | 4 | 0.099667 | False | NaN | NaN |
| PM1\_B5 | PM1 | Carbon | B | 5 | 0.071833 | False | NaN | NaN |
| PM1\_B6 | PM1 | Carbon | B | 6 | 0.143467 | True | NaN | NaN |
| PM1\_B7 | PM1 | Carbon | B | 7 | 0.072200 | False | NaN | NaN |
| PM1\_B8 | PM1 | Carbon | B | 8 | 0.342267 | True | 0.630546 | True |
| PM1\_B9 | PM1 | Carbon | B | 9 | 0.122067 | True | 0.310909 | True |
| PM1\_B10 | PM1 | Carbon | B | 10 | 0.177800 | True | 0.000000 | False |
| PM1\_B11 | PM1 | Carbon | B | 11 | 0.120767 | True | NaN | NaN |
| PM1\_B12 | PM1 | Carbon | B | 12 | 0.297367 | True | 0.541195 | True |
| PM1\_C1 | PM1 | Carbon | C | 1 | 0.087033 | False | NaN | NaN |
| PM1\_C2 | PM1 | Carbon | C | 2 | 0.055900 | False | NaN | NaN |
| PM1\_C3 | PM1 | Carbon | C | 3 | 0.114800 | True | NaN | NaN |
| PM1\_C4 | PM1 | Carbon | C | 4 | 0.312900 | True | NaN | NaN |
| PM1\_C5 | PM1 | Carbon | C | 5 | 0.397733 | True | NaN | NaN |
| PM1\_C6 | PM1 | Carbon | C | 6 | 0.093733 | False | NaN | NaN |
| PM1\_C7 | PM1 | Carbon | C | 7 | 0.257533 | True | 0.756655 | True |
| PM1\_C8 | PM1 | Carbon | C | 8 | 0.243067 | True | 0.132660 | True |
| PM1\_C9 | PM1 | Carbon | C | 9 | 0.215400 | True | 0.756655 | True |
| PM1\_C10 | PM1 | Carbon | C | 10 | 0.105733 | False | 1.513309 | True |
| PM1\_C11 | PM1 | Carbon | C | 11 | 0.076300 | False | NaN | NaN |
| PM1\_C12 | PM1 | Carbon | C | 12 | 0.089500 | False | NaN | NaN |
| PM1\_D1 | PM1 | Carbon | D | 1 | 0.279933 | True | 0.356491 | True |
| PM1\_D2 | PM1 | Carbon | D | 2 | 0.095967 | False | NaN | NaN |
| PM1\_D3 | PM1 | Carbon | D | 3 | 0.083900 | False | NaN | NaN |
| PM1\_D4 | PM1 | Carbon | D | 4 | 0.081500 | False | NaN | NaN |
| PM1\_D5 | PM1 | Carbon | D | 5 | 0.347467 | True | NaN | NaN |
| PM1\_D6 | PM1 | Carbon | D | 6 | 0.103400 | True | NaN | NaN |
| PM1\_D7 | PM1 | Carbon | D | 7 | 0.154233 | True | 0.445621 | True |
| PM1\_D8 | PM1 | Carbon | D | 8 | 0.081267 | False | NaN | NaN |
| PM1\_D9 | PM1 | Carbon | D | 9 | 0.081033 | False | NaN | NaN |
| PM1\_D10 | PM1 | Carbon | D | 10 | 0.083933 | False | NaN | NaN |
| PM1\_D11 | PM1 | Carbon | D | 11 | 0.125267 | True | 1.513309 | True |
| PM1\_D12 | PM1 | Carbon | D | 12 | 0.090700 | False | 0.000000 | False |
| PM1\_E1 | PM1 | Carbon | E | 1 | 0.315700 | True | 0.541853 | True |
| PM1\_E2 | PM1 | Carbon | E | 2 | 0.067167 | False | NaN | NaN |
| PM1\_E3 | PM1 | Carbon | E | 3 | 0.078633 | False | NaN | NaN |
| PM1\_E4 | PM1 | Carbon | E | 4 | 0.097133 | False | NaN | NaN |
| PM1\_E5 | PM1 | Carbon | E | 5 | 0.408233 | True | NaN | NaN |
| PM1\_E6 | PM1 | Carbon | E | 6 | 0.064367 | False | NaN | NaN |
| PM1\_E7 | PM1 | Carbon | E | 7 | 0.099200 | False | NaN | NaN |
| PM1\_E8 | PM1 | Carbon | E | 8 | 0.212600 | True | NaN | NaN |
| PM1\_E9 | PM1 | Carbon | E | 9 | 0.225933 | True | NaN | NaN |
| PM1\_E10 | PM1 | Carbon | E | 10 | 0.124433 | True | NaN | NaN |
| PM1\_E11 | PM1 | Carbon | E | 11 | 0.068633 | False | NaN | NaN |
| PM1\_E12 | PM1 | Carbon | E | 12 | 0.077000 | False | 0.752495 | True |
| PM1\_F1 | PM1 | Carbon | F | 1 | 0.086133 | False | 0.368529 | True |
| PM1\_F2 | PM1 | Carbon | F | 2 | 0.087633 | False | NaN | NaN |
| PM1\_F3 | PM1 | Carbon | F | 3 | 0.100567 | False | 0.000000 | False |
| PM1\_F4 | PM1 | Carbon | F | 4 | 0.112333 | False | NaN | NaN |
| PM1\_F5 | PM1 | Carbon | F | 5 | 0.093367 | False | NaN | NaN |
| PM1\_F6 | PM1 | Carbon | F | 6 | 0.173133 | True | NaN | NaN |
| PM1\_F7 | PM1 | Carbon | F | 7 | 0.182700 | True | NaN | NaN |
| PM1\_F8 | PM1 | Carbon | F | 8 | 0.087533 | False | NaN | NaN |
| PM1\_F9 | PM1 | Carbon | F | 9 | 0.082433 | False | 0.010806 | True |
| PM1\_F10 | PM1 | Carbon | F | 10 | 0.078933 | False | 0.000000 | False |
| PM1\_F11 | PM1 | Carbon | F | 11 | 0.198867 | True | NaN | NaN |
| PM1\_F12 | PM1 | Carbon | F | 12 | 0.093200 | False | 0.000000 | False |
| PM1\_G1 | PM1 | Carbon | G | 1 | 0.153067 | True | 0.562131 | True |
| PM1\_G2 | PM1 | Carbon | G | 2 | 0.063433 | False | NaN | NaN |
| PM1\_G3 | PM1 | Carbon | G | 3 | 0.189367 | True | 0.311824 | True |
| PM1\_G4 | PM1 | Carbon | G | 4 | 0.160133 | True | 0.452060 | True |
| PM1\_G5 | PM1 | Carbon | G | 5 | 0.241200 | True | 0.076596 | True |
| PM1\_G6 | PM1 | Carbon | G | 6 | 0.186600 | True | 0.076596 | True |
| PM1\_G7 | PM1 | Carbon | G | 7 | 0.161467 | True | NaN | NaN |
| PM1\_G8 | PM1 | Carbon | G | 8 | 0.084433 | False | NaN | NaN |
| PM1\_G9 | PM1 | Carbon | G | 9 | 0.200767 | True | NaN | NaN |
| PM1\_G10 | PM1 | Carbon | G | 10 | 0.170767 | True | NaN | NaN |
| PM1\_G11 | PM1 | Carbon | G | 11 | 0.121733 | True | NaN | NaN |
| PM1\_G12 | PM1 | Carbon | G | 12 | 0.107933 | False | NaN | NaN |
| PM1\_H1 | PM1 | Carbon | H | 1 | 0.225233 | True | 0.000000 | False |
| PM1\_H2 | PM1 | Carbon | H | 2 | 0.139733 | True | NaN | NaN |
| PM1\_H3 | PM1 | Carbon | H | 3 | 0.082633 | False | NaN | NaN |
| PM1\_H4 | PM1 | Carbon | H | 4 | 0.070267 | False | NaN | NaN |
| PM1\_H5 | PM1 | Carbon | H | 5 | 0.122867 | True | NaN | NaN |
| PM1\_H6 | PM1 | Carbon | H | 6 | 0.393067 | True | 0.630546 | True |
| PM1\_H7 | PM1 | Carbon | H | 7 | 0.112100 | True | NaN | NaN |
| PM1\_H8 | PM1 | Carbon | H | 8 | 0.215867 | True | 0.287444 | True |
| PM1\_H9 | PM1 | Carbon | H | 9 | 0.252700 | True | NaN | NaN |
| PM1\_H10 | PM1 | Carbon | H | 10 | 0.248833 | True | 0.000000 | False |
| PM1\_H11 | PM1 | Carbon | H | 11 | 0.082833 | False | NaN | NaN |
| PM1\_H12 | PM1 | Carbon | H | 12 | 0.075267 | False | NaN | NaN |
| PM2\_A1 | PM2 | Carbon | A | 1 | 0.082233 | False | NaN | NaN |
| PM2\_A2 | PM2 | Carbon | A | 2 | 0.057667 | False | NaN | NaN |
| PM2\_A3 | PM2 | Carbon | A | 3 | 0.059600 | False | NaN | NaN |
| PM2\_A4 | PM2 | Carbon | A | 4 | 0.079833 | False | NaN | NaN |
| PM2\_A5 | PM2 | Carbon | A | 5 | 0.102967 | False | NaN | NaN |
| PM2\_A6 | PM2 | Carbon | A | 6 | 0.129000 | True | NaN | NaN |
| PM2\_A7 | PM2 | Carbon | A | 7 | 0.133233 | True | NaN | NaN |
| PM2\_A8 | PM2 | Carbon | A | 8 | 0.074300 | False | NaN | NaN |
| PM2\_A9 | PM2 | Carbon | A | 9 | 0.165867 | True | NaN | NaN |
| PM2\_A10 | PM2 | Carbon | A | 10 | 0.203733 | True | NaN | NaN |
| PM2\_A11 | PM2 | Carbon | A | 11 | 0.066133 | False | NaN | NaN |
| PM2\_A12 | PM2 | Carbon | A | 12 | 0.159867 | True | NaN | NaN |
| PM2\_B1 | PM2 | Carbon | B | 1 | 0.077967 | False | NaN | NaN |
| PM2\_B2 | PM2 | Carbon | B | 2 | 0.072467 | False | NaN | NaN |
| PM2\_B3 | PM2 | Carbon | B | 3 | 0.107000 | False | NaN | NaN |
| PM2\_B4 | PM2 | Carbon | B | 4 | 0.105433 | True | NaN | NaN |
| PM2\_B5 | PM2 | Carbon | B | 5 | 0.269833 | True | NaN | NaN |
| PM2\_B6 | PM2 | Carbon | B | 6 | 0.244600 | True | 0.672832 | True |
| PM2\_B7 | PM2 | Carbon | B | 7 | 0.249133 | True | 0.672832 | True |
| PM2\_B8 | PM2 | Carbon | B | 8 | 0.300467 | True | NaN | NaN |
| PM2\_B9 | PM2 | Carbon | B | 9 | 0.209900 | True | NaN | NaN |
| PM2\_B10 | PM2 | Carbon | B | 10 | 0.076100 | False | NaN | NaN |
| PM2\_B11 | PM2 | Carbon | B | 11 | 0.110233 | True | NaN | NaN |
| PM2\_B12 | PM2 | Carbon | B | 12 | 0.114800 | True | NaN | NaN |
| PM2\_C1 | PM2 | Carbon | C | 1 | 0.230400 | True | NaN | NaN |
| PM2\_C2 | PM2 | Carbon | C | 2 | 0.115167 | True | NaN | NaN |
| PM2\_C3 | PM2 | Carbon | C | 3 | 0.086033 | False | NaN | NaN |
| PM2\_C4 | PM2 | Carbon | C | 4 | 0.086133 | False | NaN | NaN |
| PM2\_C5 | PM2 | Carbon | C | 5 | 0.089767 | False | NaN | NaN |
| PM2\_C6 | PM2 | Carbon | C | 6 | 0.086867 | False | NaN | NaN |
| PM2\_C7 | PM2 | Carbon | C | 7 | 0.101900 | False | NaN | NaN |
| PM2\_C8 | PM2 | Carbon | C | 8 | 0.092833 | False | NaN | NaN |
| PM2\_C9 | PM2 | Carbon | C | 9 | 0.080167 | False | NaN | NaN |
| PM2\_C10 | PM2 | Carbon | C | 10 | 0.092733 | False | NaN | NaN |
| PM2\_C11 | PM2 | Carbon | C | 11 | 0.078433 | False | NaN | NaN |
| PM2\_C12 | PM2 | Carbon | C | 12 | 0.088200 | False | NaN | NaN |
| PM2\_D1 | PM2 | Carbon | D | 1 | 0.075867 | False | NaN | NaN |
| PM2\_D2 | PM2 | Carbon | D | 2 | 0.328200 | True | NaN | NaN |
| PM2\_D3 | PM2 | Carbon | D | 3 | 0.090200 | False | NaN | NaN |
| PM2\_D4 | PM2 | Carbon | D | 4 | 0.094133 | False | NaN | NaN |
| PM2\_D5 | PM2 | Carbon | D | 5 | 0.084233 | False | NaN | NaN |
| PM2\_D6 | PM2 | Carbon | D | 6 | 0.163200 | True | NaN | NaN |
| PM2\_D7 | PM2 | Carbon | D | 7 | 0.122033 | True | NaN | NaN |
| PM2\_D8 | PM2 | Carbon | D | 8 | 0.260667 | True | 0.672832 | True |
| PM2\_D9 | PM2 | Carbon | D | 9 | 0.078800 | False | NaN | NaN |
| PM2\_D10 | PM2 | Carbon | D | 10 | 0.240867 | True | 0.502522 | True |
| PM2\_D11 | PM2 | Carbon | D | 11 | 0.097467 | False | NaN | NaN |
| PM2\_D12 | PM2 | Carbon | D | 12 | 0.265533 | True | NaN | NaN |
| PM2\_E1 | PM2 | Carbon | E | 1 | 0.006800 | False | 0.000000 | False |
| PM2\_E2 | PM2 | Carbon | E | 2 | 0.182000 | True | NaN | NaN |
| PM2\_E3 | PM2 | Carbon | E | 3 | 0.071400 | False | NaN | NaN |
| PM2\_E4 | PM2 | Carbon | E | 4 | 0.073900 | False | NaN | NaN |
| PM2\_E5 | PM2 | Carbon | E | 5 | 0.171867 | True | 0.000000 | False |
| PM2\_E6 | PM2 | Carbon | E | 6 | 0.100167 | False | NaN | NaN |
| PM2\_E7 | PM2 | Carbon | E | 7 | 0.186267 | True | NaN | NaN |
| PM2\_E8 | PM2 | Carbon | E | 8 | 0.196300 | True | NaN | NaN |
| PM2\_E9 | PM2 | Carbon | E | 9 | 0.212067 | True | NaN | NaN |
| PM2\_E10 | PM2 | Carbon | E | 10 | 0.156967 | True | NaN | NaN |
| PM2\_E11 | PM2 | Carbon | E | 11 | 0.071800 | False | NaN | NaN |
| PM2\_E12 | PM2 | Carbon | E | 12 | 0.219967 | True | NaN | NaN |
| PM2\_F1 | PM2 | Carbon | F | 1 | 0.111333 | True | NaN | NaN |
| PM2\_F2 | PM2 | Carbon | F | 2 | 0.058900 | False | NaN | NaN |
| PM2\_F3 | PM2 | Carbon | F | 3 | 0.118367 | True | NaN | NaN |
| PM2\_F4 | PM2 | Carbon | F | 4 | 0.075933 | False | 0.000000 | False |
| PM2\_F5 | PM2 | Carbon | F | 5 | 0.146133 | True | NaN | NaN |
| PM2\_F6 | PM2 | Carbon | F | 6 | 0.149700 | True | NaN | NaN |
| PM2\_F7 | PM2 | Carbon | F | 7 | 0.057833 | False | NaN | NaN |
| PM2\_F8 | PM2 | Carbon | F | 8 | 0.230433 | True | NaN | NaN |
| PM2\_F9 | PM2 | Carbon | F | 9 | 0.184000 | True | NaN | NaN |
| PM2\_F10 | PM2 | Carbon | F | 10 | 0.128233 | True | NaN | NaN |
| PM2\_F11 | PM2 | Carbon | F | 11 | 0.070400 | False | NaN | NaN |
| PM2\_F12 | PM2 | Carbon | F | 12 | 0.075800 | False | NaN | NaN |
| PM2\_G1 | PM2 | Carbon | G | 1 | 0.088133 | False | NaN | NaN |
| PM2\_G2 | PM2 | Carbon | G | 2 | 0.254367 | True | NaN | NaN |
| PM2\_G3 | PM2 | Carbon | G | 3 | 0.071800 | False | NaN | NaN |
| PM2\_G4 | PM2 | Carbon | G | 4 | 0.188467 | True | 0.581976 | True |
| PM2\_G5 | PM2 | Carbon | G | 5 | 0.254033 | True | 0.000000 | False |
| PM2\_G6 | PM2 | Carbon | G | 6 | 0.114700 | False | 0.000000 | False |
| PM2\_G7 | PM2 | Carbon | G | 7 | 0.164767 | True | NaN | NaN |
| PM2\_G8 | PM2 | Carbon | G | 8 | 0.093567 | False | NaN | NaN |
| PM2\_G9 | PM2 | Carbon | G | 9 | 0.241867 | True | 0.796474 | True |
| PM2\_G10 | PM2 | Carbon | G | 10 | 0.173533 | True | 0.696876 | True |
| PM2\_G11 | PM2 | Carbon | G | 11 | 0.091567 | False | 0.000000 | False |
| PM2\_G12 | PM2 | Carbon | G | 12 | 0.062633 | False | 0.000000 | False |
| PM2\_H1 | PM2 | Carbon | H | 1 | 0.238633 | True | 0.581375 | True |
| PM2\_H2 | PM2 | Carbon | H | 2 | 0.061000 | False | 0.000000 | False |
| PM2\_H3 | PM2 | Carbon | H | 3 | 0.272200 | True | NaN | NaN |
| PM2\_H4 | PM2 | Carbon | H | 4 | 0.179467 | True | 0.656339 | True |
| PM2\_H5 | PM2 | Carbon | H | 5 | 0.064333 | False | NaN | NaN |
| PM2\_H6 | PM2 | Carbon | H | 6 | 0.038367 | False | NaN | NaN |
| PM2\_H7 | PM2 | Carbon | H | 7 | 0.091033 | False | NaN | NaN |
| PM2\_H8 | PM2 | Carbon | H | 8 | 0.066933 | False | 0.578705 | True |
| PM2\_H9 | PM2 | Carbon | H | 9 | 0.340200 | True | NaN | NaN |
| PM2\_H10 | PM2 | Carbon | H | 10 | 0.072550 | False | NaN | NaN |
| PM2\_H11 | PM2 | Carbon | H | 11 | 0.086967 | False | NaN | NaN |
| PM2\_H12 | PM2 | Carbon | H | 12 | 0.060133 | False | NaN | NaN |
| PM3B\_A1 | PM3B | Nitrogen | A | 1 | 0.100600 | False | NaN | NaN |
| PM3B\_A2 | PM3B | Nitrogen | A | 2 | 0.215700 | True | 0.287444 | True |
| PM3B\_A3 | PM3B | Nitrogen | A | 3 | 0.157167 | True | 0.253313 | True |
| PM3B\_A4 | PM3B | Nitrogen | A | 4 | 0.214200 | True | 0.243668 | True |
| PM3B\_A5 | PM3B | Nitrogen | A | 5 | 0.228100 | True | 0.285784 | True |
| PM3B\_A6 | PM3B | Nitrogen | A | 6 | 0.112633 | False | NaN | NaN |
| PM3B\_A7 | PM3B | Nitrogen | A | 7 | 0.216500 | True | 0.100837 | True |
| PM3B\_A8 | PM3B | Nitrogen | A | 8 | 0.228267 | True | 0.897019 | True |
| PM3B\_A9 | PM3B | Nitrogen | A | 9 | 0.211933 | True | 0.654757 | True |
| PM3B\_A10 | PM3B | Nitrogen | A | 10 | 0.236567 | True | 0.654502 | True |
| PM3B\_A11 | PM3B | Nitrogen | A | 11 | 0.162167 | True | 0.000000 | False |
| PM3B\_A12 | PM3B | Nitrogen | A | 12 | 0.343500 | True | 0.832591 | True |
| PM3B\_B1 | PM3B | Nitrogen | B | 1 | 0.269500 | True | 0.833676 | True |
| PM3B\_B2 | PM3B | Nitrogen | B | 2 | 0.200800 | True | 0.071201 | True |
| PM3B\_B3 | PM3B | Nitrogen | B | 3 | 0.140533 | True | 0.000000 | False |
| PM3B\_B4 | PM3B | Nitrogen | B | 4 | 0.183400 | True | 1.123017 | True |
| PM3B\_B5 | PM3B | Nitrogen | B | 5 | 0.175600 | True | 1.056977 | True |
| PM3B\_B6 | PM3B | Nitrogen | B | 6 | 0.159900 | True | 0.000000 | False |
| PM3B\_B7 | PM3B | Nitrogen | B | 7 | 0.218600 | True | 0.000000 | False |
| PM3B\_B8 | PM3B | Nitrogen | B | 8 | 0.173667 | True | 0.000000 | False |
| PM3B\_B9 | PM3B | Nitrogen | B | 9 | 0.228133 | True | 0.000000 | False |
| PM3B\_B10 | PM3B | Nitrogen | B | 10 | 0.174900 | True | 0.623649 | True |
| PM3B\_B11 | PM3B | Nitrogen | B | 11 | 0.162267 | True | 0.743519 | True |
| PM3B\_B12 | PM3B | Nitrogen | B | 12 | 0.265533 | True | 1.310924 | True |
| PM3B\_C1 | PM3B | Nitrogen | C | 1 | 0.197500 | True | 1.114540 | True |
| PM3B\_C2 | PM3B | Nitrogen | C | 2 | 0.203267 | True | 0.964357 | True |
| PM3B\_C3 | PM3B | Nitrogen | C | 3 | 0.146133 | True | 0.100837 | True |
| PM3B\_C4 | PM3B | Nitrogen | C | 4 | 0.111333 | True | NaN | NaN |
| PM3B\_C5 | PM3B | Nitrogen | C | 5 | 0.100800 | False | NaN | NaN |
| PM3B\_C6 | PM3B | Nitrogen | C | 6 | 0.046600 | False | NaN | NaN |
| PM3B\_C7 | PM3B | Nitrogen | C | 7 | 0.110933 | False | NaN | NaN |
| PM3B\_C8 | PM3B | Nitrogen | C | 8 | 0.108500 | False | NaN | NaN |
| PM3B\_C9 | PM3B | Nitrogen | C | 9 | 0.121267 | True | NaN | NaN |
| PM3B\_C10 | PM3B | Nitrogen | C | 10 | 0.190100 | True | NaN | NaN |
| PM3B\_C11 | PM3B | Nitrogen | C | 11 | 0.252767 | True | NaN | NaN |
| PM3B\_C12 | PM3B | Nitrogen | C | 12 | 0.223733 | True | 0.896009 | True |
| PM3B\_D1 | PM3B | Nitrogen | D | 1 | 0.112500 | False | NaN | NaN |
| PM3B\_D2 | PM3B | Nitrogen | D | 2 | 0.217400 | True | NaN | NaN |
| PM3B\_D3 | PM3B | Nitrogen | D | 3 | 0.202267 | True | NaN | NaN |
| PM3B\_D4 | PM3B | Nitrogen | D | 4 | 0.150133 | True | NaN | NaN |
| PM3B\_D5 | PM3B | Nitrogen | D | 5 | 0.087867 | False | NaN | NaN |
| PM3B\_D6 | PM3B | Nitrogen | D | 6 | 0.163700 | True | NaN | NaN |
| PM3B\_D7 | PM3B | Nitrogen | D | 7 | 0.151933 | True | NaN | NaN |
| PM3B\_D8 | PM3B | Nitrogen | D | 8 | 0.159533 | True | NaN | NaN |
| PM3B\_D9 | PM3B | Nitrogen | D | 9 | 0.182600 | True | NaN | NaN |
| PM3B\_D10 | PM3B | Nitrogen | D | 10 | 0.079633 | False | NaN | NaN |
| PM3B\_D11 | PM3B | Nitrogen | D | 11 | 0.122467 | True | 0.893100 | True |
| PM3B\_D12 | PM3B | Nitrogen | D | 12 | 0.103200 | False | NaN | NaN |
| PM3B\_E1 | PM3B | Nitrogen | E | 1 | 0.073933 | False | NaN | NaN |
| PM3B\_E2 | PM3B | Nitrogen | E | 2 | 0.118233 | False | NaN | NaN |
| PM3B\_E3 | PM3B | Nitrogen | E | 3 | 0.145667 | True | NaN | NaN |
| PM3B\_E4 | PM3B | Nitrogen | E | 4 | 0.149867 | True | NaN | NaN |
| PM3B\_E5 | PM3B | Nitrogen | E | 5 | 0.161000 | True | NaN | NaN |
| PM3B\_E6 | PM3B | Nitrogen | E | 6 | 0.203700 | True | NaN | NaN |
| PM3B\_E7 | PM3B | Nitrogen | E | 7 | 0.215433 | True | NaN | NaN |
| PM3B\_E8 | PM3B | Nitrogen | E | 8 | 0.129100 | True | 0.000000 | False |
| PM3B\_E9 | PM3B | Nitrogen | E | 9 | 0.181900 | True | NaN | NaN |
| PM3B\_E10 | PM3B | Nitrogen | E | 10 | 0.130233 | True | NaN | NaN |
| PM3B\_E11 | PM3B | Nitrogen | E | 11 | 0.101067 | False | 0.000000 | False |
| PM3B\_E12 | PM3B | Nitrogen | E | 12 | 0.090833 | False | NaN | NaN |
| PM3B\_F1 | PM3B | Nitrogen | F | 1 | 0.096367 | False | NaN | NaN |
| PM3B\_F2 | PM3B | Nitrogen | F | 2 | 0.239667 | True | 0.400319 | True |
| PM3B\_F3 | PM3B | Nitrogen | F | 3 | 0.238100 | True | 1.081567 | True |
| PM3B\_F4 | PM3B | Nitrogen | F | 4 | 0.196300 | True | 0.000000 | False |
| PM3B\_F5 | PM3B | Nitrogen | F | 5 | 0.178733 | True | 0.000000 | False |
| PM3B\_F6 | PM3B | Nitrogen | F | 6 | 0.127233 | False | 0.352032 | True |
| PM3B\_F7 | PM3B | Nitrogen | F | 7 | 0.275767 | True | NaN | NaN |
| PM3B\_F8 | PM3B | Nitrogen | F | 8 | 0.153600 | True | NaN | NaN |
| PM3B\_F9 | PM3B | Nitrogen | F | 9 | 0.119500 | True | NaN | NaN |
| PM3B\_F10 | PM3B | Nitrogen | F | 10 | 0.193733 | True | 0.000000 | False |
| PM3B\_F11 | PM3B | Nitrogen | F | 11 | 0.189400 | True | 0.000000 | False |
| PM3B\_F12 | PM3B | Nitrogen | F | 12 | 0.324900 | True | 0.000000 | False |
| PM3B\_G1 | PM3B | Nitrogen | G | 1 | 0.387333 | True | NaN | NaN |
| PM3B\_G2 | PM3B | Nitrogen | G | 2 | 0.200100 | True | NaN | NaN |
| PM3B\_G3 | PM3B | Nitrogen | G | 3 | 0.239433 | True | 0.000000 | False |
| PM3B\_G4 | PM3B | Nitrogen | G | 4 | 0.306567 | True | NaN | NaN |
| PM3B\_G5 | PM3B | Nitrogen | G | 5 | 0.194900 | True | 0.315487 | True |
| PM3B\_G6 | PM3B | Nitrogen | G | 6 | 0.215033 | True | NaN | NaN |
| PM3B\_G7 | PM3B | Nitrogen | G | 7 | 0.157433 | True | NaN | NaN |
| PM3B\_G8 | PM3B | Nitrogen | G | 8 | 0.258267 | True | 0.814883 | True |
| PM3B\_G9 | PM3B | Nitrogen | G | 9 | 0.138967 | True | NaN | NaN |
| PM3B\_G10 | PM3B | Nitrogen | G | 10 | 0.228933 | True | NaN | NaN |
| PM3B\_G11 | PM3B | Nitrogen | G | 11 | 0.200433 | True | NaN | NaN |
| PM3B\_G12 | PM3B | Nitrogen | G | 12 | 0.314867 | True | NaN | NaN |
| PM3B\_H1 | PM3B | Nitrogen | H | 1 | 0.196167 | True | 0.840371 | True |
| PM3B\_H2 | PM3B | Nitrogen | H | 2 | 0.294933 | True | 1.026642 | True |
| PM3B\_H3 | PM3B | Nitrogen | H | 3 | 0.187667 | True | 1.025235 | True |
| PM3B\_H4 | PM3B | Nitrogen | H | 4 | 0.268000 | True | 0.100837 | True |
| PM3B\_H5 | PM3B | Nitrogen | H | 5 | 0.258600 | True | 0.105272 | True |
| PM3B\_H6 | PM3B | Nitrogen | H | 6 | 0.162200 | True | 1.314530 | True |
| PM3B\_H7 | PM3B | Nitrogen | H | 7 | 0.206067 | True | 0.924552 | True |
| PM3B\_H8 | PM3B | Nitrogen | H | 8 | 0.308767 | True | 0.679091 | True |
| PM3B\_H9 | PM3B | Nitrogen | H | 9 | 0.258733 | True | 0.864521 | True |
| PM3B\_H10 | PM3B | Nitrogen | H | 10 | 0.280533 | True | 0.863355 | True |
| PM3B\_H11 | PM3B | Nitrogen | H | 11 | 0.283833 | True | 0.074864 | True |
| PM3B\_H12 | PM3B | Nitrogen | H | 12 | 0.370500 | True | 0.105594 | True |
| PM4A\_A1 | PM4A | Phosphorus | A | 1 | 0.067500 | False | NaN | NaN |
| PM4A\_A2 | PM4A | Phosphorus | A | 2 | 0.217267 | True | 0.287444 | True |
| PM4A\_A3 | PM4A | Phosphorus | A | 3 | 0.299700 | True | NaN | NaN |
| PM4A\_A4 | PM4A | Phosphorus | A | 4 | 0.305667 | True | NaN | NaN |
| PM4A\_A5 | PM4A | Phosphorus | A | 5 | 0.383400 | True | NaN | NaN |
| PM4A\_A6 | PM4A | Phosphorus | A | 6 | 0.065967 | False | NaN | NaN |
| PM4A\_A7 | PM4A | Phosphorus | A | 7 | 0.061300 | False | NaN | NaN |
| PM4A\_A8 | PM4A | Phosphorus | A | 8 | 0.298300 | True | NaN | NaN |
| PM4A\_A9 | PM4A | Phosphorus | A | 9 | 0.237633 | True | NaN | NaN |
| PM4A\_A10 | PM4A | Phosphorus | A | 10 | 0.220933 | True | NaN | NaN |
| PM4A\_A11 | PM4A | Phosphorus | A | 11 | 0.209733 | True | NaN | NaN |
| PM4A\_A12 | PM4A | Phosphorus | A | 12 | 0.272933 | True | 0.000000 | False |
| PM4A\_B1 | PM4A | Phosphorus | B | 1 | 0.124300 | True | NaN | NaN |
| PM4A\_B2 | PM4A | Phosphorus | B | 2 | 0.324100 | True | NaN | NaN |
| PM4A\_B3 | PM4A | Phosphorus | B | 3 | 0.212700 | True | NaN | NaN |
| PM4A\_B4 | PM4A | Phosphorus | B | 4 | 0.203100 | True | NaN | NaN |
| PM4A\_B5 | PM4A | Phosphorus | B | 5 | 0.337933 | True | NaN | NaN |
| PM4A\_B6 | PM4A | Phosphorus | B | 6 | 0.295633 | True | NaN | NaN |
| PM4A\_B7 | PM4A | Phosphorus | B | 7 | 0.286367 | True | NaN | NaN |
| PM4A\_B8 | PM4A | Phosphorus | B | 8 | 0.201267 | True | NaN | NaN |
| PM4A\_B9 | PM4A | Phosphorus | B | 9 | 0.224833 | True | NaN | NaN |
| PM4A\_B10 | PM4A | Phosphorus | B | 10 | 0.222300 | True | NaN | NaN |
| PM4A\_B11 | PM4A | Phosphorus | B | 11 | 0.233500 | True | NaN | NaN |
| PM4A\_B12 | PM4A | Phosphorus | B | 12 | 0.218567 | True | 0.000000 | False |
| PM4A\_C1 | PM4A | Phosphorus | C | 1 | 0.304033 | True | NaN | NaN |
| PM4A\_C2 | PM4A | Phosphorus | C | 2 | 0.238667 | True | NaN | NaN |
| PM4A\_C3 | PM4A | Phosphorus | C | 3 | 0.217267 | True | NaN | NaN |
| PM4A\_C4 | PM4A | Phosphorus | C | 4 | 0.248967 | True | NaN | NaN |
| PM4A\_C5 | PM4A | Phosphorus | C | 5 | 0.074400 | False | NaN | NaN |
| PM4A\_C6 | PM4A | Phosphorus | C | 6 | 0.219933 | True | NaN | NaN |
| PM4A\_C7 | PM4A | Phosphorus | C | 7 | 0.308633 | True | NaN | NaN |
| PM4A\_C8 | PM4A | Phosphorus | C | 8 | 0.302167 | True | NaN | NaN |
| PM4A\_C9 | PM4A | Phosphorus | C | 9 | 0.075300 | False | NaN | NaN |
| PM4A\_C10 | PM4A | Phosphorus | C | 10 | 0.313933 | True | NaN | NaN |
| PM4A\_C11 | PM4A | Phosphorus | C | 11 | 0.197300 | True | NaN | NaN |
| PM4A\_C12 | PM4A | Phosphorus | C | 12 | 0.196100 | True | NaN | NaN |
| PM4A\_D1 | PM4A | Phosphorus | D | 1 | 0.252833 | True | NaN | NaN |
| PM4A\_D2 | PM4A | Phosphorus | D | 2 | 0.294767 | True | NaN | NaN |
| PM4A\_D3 | PM4A | Phosphorus | D | 3 | 0.366467 | True | NaN | NaN |
| PM4A\_D4 | PM4A | Phosphorus | D | 4 | 0.242233 | True | NaN | NaN |
| PM4A\_D5 | PM4A | Phosphorus | D | 5 | 0.251967 | True | NaN | NaN |
| PM4A\_D6 | PM4A | Phosphorus | D | 6 | 0.236900 | True | NaN | NaN |
| PM4A\_D7 | PM4A | Phosphorus | D | 7 | 0.270133 | True | NaN | NaN |
| PM4A\_D8 | PM4A | Phosphorus | D | 8 | 0.230733 | True | NaN | NaN |
| PM4A\_D9 | PM4A | Phosphorus | D | 9 | 0.212100 | True | NaN | NaN |
| PM4A\_D10 | PM4A | Phosphorus | D | 10 | 0.231200 | True | NaN | NaN |
| PM4A\_D11 | PM4A | Phosphorus | D | 11 | 0.225300 | True | NaN | NaN |
| PM4A\_D12 | PM4A | Phosphorus | D | 12 | 0.218200 | True | NaN | NaN |
| PM4A\_E1 | PM4A | Phosphorus | E | 1 | 0.235467 | True | NaN | NaN |
| PM4A\_E2 | PM4A | Phosphorus | E | 2 | 0.192400 | True | NaN | NaN |
| PM4A\_E3 | PM4A | Phosphorus | E | 3 | 0.218667 | True | NaN | NaN |
| PM4A\_E4 | PM4A | Phosphorus | E | 4 | 0.216200 | True | NaN | NaN |
| PM4A\_E5 | PM4A | Phosphorus | E | 5 | 0.211700 | True | NaN | NaN |
| PM4A\_E6 | PM4A | Phosphorus | E | 6 | 0.062000 | False | NaN | NaN |
| PM4A\_E7 | PM4A | Phosphorus | E | 7 | 0.060400 | False | NaN | NaN |
| PM4A\_E8 | PM4A | Phosphorus | E | 8 | 0.175633 | True | NaN | NaN |
| PM4A\_E9 | PM4A | Phosphorus | E | 9 | 0.198100 | True | NaN | NaN |
| PM4A\_E10 | PM4A | Phosphorus | E | 10 | 0.190300 | True | NaN | NaN |
| PM4A\_E11 | PM4A | Phosphorus | E | 11 | 0.288667 | True | NaN | NaN |
| PM4A\_E12 | PM4A | Phosphorus | E | 12 | 0.124133 | False | NaN | NaN |
| PM4A\_F1 | PM4A | Sulfur | F | 1 | 0.078800 | False | NaN | NaN |
| PM4A\_F2 | PM4A | Sulfur | F | 2 | 0.233500 | True | 0.287444 | True |
| PM4A\_F3 | PM4A | Sulfur | F | 3 | 0.256500 | True | NaN | NaN |
| PM4A\_F4 | PM4A | Sulfur | F | 4 | 0.194367 | True | NaN | NaN |
| PM4A\_F5 | PM4A | Sulfur | F | 5 | 0.216967 | True | NaN | NaN |
| PM4A\_F6 | PM4A | Sulfur | F | 6 | 0.200267 | True | NaN | NaN |
| PM4A\_F7 | PM4A | Sulfur | F | 7 | 0.180100 | True | 0.290329 | True |
| PM4A\_F8 | PM4A | Sulfur | F | 8 | 0.121600 | True | NaN | NaN |
| PM4A\_F9 | PM4A | Sulfur | F | 9 | 0.339133 | True | 0.300877 | True |
| PM4A\_F10 | PM4A | Sulfur | F | 10 | 0.141700 | True | NaN | NaN |
| PM4A\_F11 | PM4A | Sulfur | F | 11 | 0.186700 | True | NaN | NaN |
| PM4A\_F12 | PM4A | Sulfur | F | 12 | 0.193200 | True | NaN | NaN |
| PM4A\_G1 | PM4A | Sulfur | G | 1 | 0.121333 | True | NaN | NaN |
| PM4A\_G2 | PM4A | Sulfur | G | 2 | 0.155867 | True | NaN | NaN |
| PM4A\_G3 | PM4A | Sulfur | G | 3 | 0.183667 | True | NaN | NaN |
| PM4A\_G4 | PM4A | Sulfur | G | 4 | 0.182200 | True | NaN | NaN |
| PM4A\_G5 | PM4A | Sulfur | G | 5 | 0.132700 | True | 0.871562 | True |
| PM4A\_G6 | PM4A | Sulfur | G | 6 | 0.106300 | True | NaN | NaN |
| PM4A\_G7 | PM4A | Sulfur | G | 7 | 0.268700 | True | 0.293527 | True |
| PM4A\_G8 | PM4A | Sulfur | G | 8 | 0.206700 | True | NaN | NaN |
| PM4A\_G9 | PM4A | Sulfur | G | 9 | 0.268467 | True | 0.304724 | True |
| PM4A\_G10 | PM4A | Sulfur | G | 10 | 0.174567 | True | NaN | NaN |
| PM4A\_G11 | PM4A | Sulfur | G | 11 | 0.259267 | True | NaN | NaN |
| PM4A\_G12 | PM4A | Sulfur | G | 12 | 0.174233 | True | NaN | NaN |
| PM4A\_H1 | PM4A | Sulfur | H | 1 | 0.343900 | True | NaN | NaN |
| PM4A\_H2 | PM4A | Sulfur | H | 2 | 0.167867 | True | NaN | NaN |
| PM4A\_H3 | PM4A | Sulfur | H | 3 | 0.144700 | True | NaN | NaN |
| PM4A\_H4 | PM4A | Sulfur | H | 4 | 0.269967 | True | NaN | NaN |
| PM4A\_H5 | PM4A | Sulfur | H | 5 | 0.131533 | True | 0.000000 | False |
| PM4A\_H6 | PM4A | Sulfur | H | 6 | 0.182767 | True | NaN | NaN |
| PM4A\_H7 | PM4A | Sulfur | H | 7 | 0.219867 | True | NaN | NaN |
| PM4A\_H8 | PM4A | Sulfur | H | 8 | 0.086933 | False | NaN | NaN |
| PM4A\_H9 | PM4A | Sulfur | H | 9 | 0.188767 | True | NaN | NaN |
| PM4A\_H10 | PM4A | Sulfur | H | 10 | 0.177900 | True | NaN | NaN |
| PM4A\_H11 | PM4A | Sulfur | H | 11 | 0.206367 | True | NaN | NaN |
| PM4A\_H12 | PM4A | Sulfur | H | 12 | 0.079033 | False | NaN | NaN |

In [15]:

```
from sklearn.metrics import confusion_matrix, matthews_corrcoef
```

In [16]:

```
temp = Biolog_Prediction.index[~np.isnan(Biolog_Prediction.Prediction)]
y_data = Biolog_Prediction.Data_TF[temp].astype(int)
y_pred = Biolog_Prediction.Prediction_TF[temp].astype(int)
TN, FP, FN, TP = confusion_matrix(y_data, y_pred).ravel()
print(TN, FP, FN, TP, sum([TN, FP, FN, TP]))
```

```
12 6 22 76 116
```

In [17]:

```
df_confusion = pd.DataFrame(confusion_matrix(y_data, y_pred),
                            index = pd.MultiIndex.from_product([['Experiment'],['No growth', 'Growth']]),
                            columns = pd.MultiIndex.from_product([['Prediction'],['No growth', 'Growth']]))
df_confusion
```

Out[17]:

|  |  | Prediction | |
| --- | --- | --- | --- |
|  |  | No growth | Growth |
| Experiment | No growth | 12 | 6 |
|  | Growth | 22 | 76 |

In [18]:

```
# Sensitivity, hit rate, recall, or true positive rate
TPR = TP/(TP+FN)
# Specificity or true negative rate
TNR = TN/(TN+FP) 
# Precision or positive predictive value
PPV = TP/(TP+FP)
# Negative predictive value
NPV = TN/(TN+FN)
# Fall out or false positive rate
FPR = FP/(FP+TN)
# False negative rate
FNR = FN/(TP+FN)
# False discovery rate
FDR = FP/(TP+FP)
# Overall accuracy
ACC = (TP+TN)/(TP+FP+FN+TN)
# Matthew's
MCC = matthews_corrcoef(y_data, y_pred)
print('Recall:', TPR.round(3))
print('Precision:', PPV.round(3))
print('Accuracy:', ACC.round(3))
print('Matthew\'s correlation:', MCC.round(3))
```

```
Recall: 0.776
Precision: 0.927
Accuracy: 0.759
Matthew's correlation: 0.352
```

In [19]:

```
print(sum(~Biolog_in_model['Exchange'].isna()))
print(len(Biolog_in_model.query('External == True and Internal == False')))
print(len(Biolog_in_model.query('External == False and Internal == True')))
print(len(Biolog_in_model.query('External == True and Internal == True')))
print(len(Biolog_in_model.query('External == False and Internal == False')))
```

```
285
0
93
116
76
```

In [20]:

```
with pd.option_context('display.max_rows', None, 'display.max_columns', None):
    display(Biolog_in_model)
```

|  | Biolog | Model | Exchange | Metabolite | Internal | External | Average | Pass |
| --- | --- | --- | --- | --- | --- | --- | --- | --- |
| PM1\_A1 | Negative Control | NaN | NaN | NaN | NaN | NaN | 0.0864 | False |
| PM1\_A2 | L-Arabinose | L-Arabinose | EX\_arab\_\_L\_e | arab\_\_L\_e | True | True | 0.344767 | True |
| PM1\_A3 | N-Acetyl-D-Glucosamine | N-Acetyl-D-glucosamine | EX\_acgam\_e | acgam\_e | True | True | 0.0688667 | False |
| PM1\_A4 | D-Saccharic Acid | None | EX\_glcr\_e | glcr\_e | False | False | 0.0712 | False |
| PM1\_A5 | Succinic Acid | None | EX\_succ\_e | succ\_e | True | False | 0.1112 | True |
| PM1\_A6 | D-Galactose | D-Galactose | EX\_gal\_e | gal\_e | True | True | 0.1433 | True |
| PM1\_A7 | L-Aspartic Acid | L-Aspartate | EX\_asp\_\_L\_e | asp\_\_L\_e | True | True | 0.267867 | True |
| PM1\_A8 | L-Proline | L-Proline | EX\_pro\_\_L\_e | pro\_\_L\_e | True | True | 0.2724 | True |
| PM1\_A9 | D-Alanine | D-Alanine | EX\_ala\_\_D\_e | ala\_\_D\_e | True | True | 0.1215 | True |
| PM1\_A10 | D-Trehalose | None | EX\_tre\_e | tre\_e | True | False | 0.2213 | True |
| PM1\_A11 | D-Mannose | D-Mannose | EX\_man\_e | man\_e | True | True | 0.212667 | True |
| PM1\_A12 | Dulcitol | None | EX\_galt\_e | galt\_e | True | False | 0.132067 | True |
| PM1\_B1 | D-Serine | None | EX\_ser\_\_D\_e | ser\_\_D\_e | True | False | 0.1506 | True |
| PM1\_B2 | D-Sorbitol | None | EX\_sbt\_\_D\_e | sbt\_\_D\_e | True | False | 0.124767 | True |
| PM1\_B3 | Glycerol | Glycerol | EX\_glyc\_e | glyc\_e | True | True | 0.2243 | True |
| PM1\_B4 | L-Fucose | None | EX\_fuc\_\_L\_e | fuc\_\_L\_e | False | False | 0.0996667 | False |
| PM1\_B5 | D-Glucuronic Acid | None | EX\_glcur\_e | glcur\_e | False | False | 0.0718333 | False |
| PM1\_B6 | D-Gluconic Acid | None | EX\_glcn\_e | glcn\_e | True | False | 0.143467 | True |
| PM1\_B7 | D,L-a-GlycerolPhosphate | None | EX\_glyc3p\_e | glyc3p\_e | True | False | 0.0722 | False |
| PM1\_B8 | D-Xylose | D-Xylose | EX\_xyl\_\_D\_e | xyl\_\_D\_e | True | True | 0.342267 | True |
| PM1\_B9 | L-Lactic Acid | L-Lactate | EX\_lac\_\_L\_e | lac\_\_L\_e | True | True | 0.122067 | True |
| PM1\_B10 | Formic Acid | Formate | EX\_for\_e | for\_e | True | True | 0.1778 | True |
| PM1\_B11 | D-Mannitol | None | EX\_mnl\_e | mnl\_e | False | False | 0.120767 | True |
| PM1\_B12 | L-Glutamic Acid | L-Glutamate | EX\_glu\_\_L\_e | glu\_\_L\_e | True | True | 0.297367 | True |
| PM1\_C1 | D-Glucose-6-Phosphate | None | EX\_g6p\_e | g6p\_e | True | False | 0.0870333 | False |
| PM1\_C2 | D-Galactonic Acid-g-Lactone | None | EX\_galctn\_\_D\_e | galctn\_\_D\_e | True | False | 0.0559 | False |
| PM1\_C3 | D-,L-Malic Acid | [None, L-Malate] | EX\_mal\_\_D\_e,EX\_mal\_\_L\_e | mal\_\_D\_e,mal\_\_L\_e | False | False | 0.1148 | True |
| PM1\_C4 | D-Ribose | None | EX\_rib\_\_D\_e | rib\_\_D\_e | True | False | 0.3129 | True |
| PM1\_C5 | Tween 20 | NaN | NaN | NaN | NaN | NaN | 0.397733 | True |
| PM1\_C6 | L-Rhamnose | None | EX\_rmn\_e | rmn\_e | False | False | 0.0937333 | False |
| PM1\_C7 | D-Fructose | D-Fructose | EX\_fru\_e | fru\_e | True | True | 0.257533 | True |
| PM1\_C8 | Acetic Acid | Acetate | EX\_ac\_e | ac\_e | True | True | 0.243067 | True |
| PM1\_C9 | a-D-Glucose | D-Glucose | EX\_glc\_\_D\_e | glc\_\_D\_e | True | True | 0.2154 | True |
| PM1\_C10 | Maltose | Maltose C12H22O11 | EX\_malt\_e | malt\_e | True | True | 0.105733 | False |
| PM1\_C11 | D-Melibiose | None | EX\_melib\_e | melib\_e | False | False | 0.0763 | False |
| PM1\_C12 | Thymidine | None | EX\_thymd\_e | thymd\_e | True | False | 0.0895 | False |
| PM1\_D1 | L-Asparagine | L-Asparagine | EX\_asn\_\_L\_e | asn\_\_L\_e | True | True | 0.279933 | True |
| PM1\_D2 | D-Aspartic Acid | None | EX\_asp\_\_D\_e | asp\_\_D\_e | True | False | 0.0959667 | False |
| PM1\_D3 | D-Glucosaminic Acid | NaN | NaN | NaN | NaN | NaN | 0.0839 | False |
| PM1\_D4 | 1,2-Propanediol | None | EX\_12ppd\_\_S\_e | 12ppd\_\_S\_e | True | False | 0.0815 | False |
| PM1\_D5 | Tween 40 | NaN | NaN | NaN | NaN | NaN | 0.347467 | True |
| PM1\_D6 | a-Keto-Glutaric Acid | None | EX\_akg\_e | akg\_e | True | False | 0.1034 | True |
| PM1\_D7 | a-Keto-Butyric Acid | 2-Oxobutanoate | EX\_2obut\_e | 2obut\_e | True | True | 0.154233 | True |
| PM1\_D8 | a-Methyl-D-Galactoside | NaN | NaN | NaN | NaN | NaN | 0.0812667 | False |
| PM1\_D9 | a-D-Lactose | None | EX\_lcts\_e | lcts\_e | False | False | 0.0810333 | False |
| PM1\_D10 | Lactulose | NaN | NaN | NaN | NaN | NaN | 0.0839333 | False |
| PM1\_D11 | Sucrose | Sucrose C12H22O11 | EX\_sucr\_e | sucr\_e | True | True | 0.125267 | True |
| PM1\_D12 | Uridine | Uridine | EX\_uri\_e | uri\_e | True | True | 0.0907 | False |
| PM1\_E1 | L-Glutamine | L-Glutamine | EX\_gln\_\_L\_e | gln\_\_L\_e | True | True | 0.3157 | True |
| PM1\_E2 | m-Tartaric Acid | None | EX\_tartr\_\_M\_e | tartr\_\_M\_e | False | False | 0.0671667 | False |
| PM1\_E3 | D-Glucose-1-Phosphate | None | EX\_g1p\_e | g1p\_e | True | False | 0.0786333 | False |
| PM1\_E4 | D-Fructose-6-Phosphate | None | EX\_f6p\_e | f6p\_e | True | False | 0.0971333 | False |
| PM1\_E5 | Tween 80 | NaN | NaN | NaN | NaN | NaN | 0.408233 | True |
| PM1\_E6 | a-Hydroxy Glutaric Acid-g-Lactone | NaN | NaN | NaN | NaN | NaN | 0.0643667 | False |
| PM1\_E7 | a-Hydroxy Butyric Acid | None | EX\_2hb\_e | 2hb\_e | False | False | 0.0992 | False |
| PM1\_E8 | b-Methyl-D-Glucoside | None | EX\_mbdg\_e | mbdg\_e | False | False | 0.2126 | True |
| PM1\_E9 | Adonitol | None | EX\_rbt\_e | rbt\_e | False | False | 0.225933 | True |
| PM1\_E10 | Maltotriose | None | EX\_malttr\_e | malttr\_e | True | False | 0.124433 | True |
| PM1\_E11 | 2-Deoxy Adenosine | None | EX\_dad\_\_2\_e | dad\_\_2\_e | False | False | 0.0686333 | False |
| PM1\_E12 | Adenosine | Adenosine | EX\_adn\_e | adn\_e | True | True | 0.077 | False |
| PM1\_F1 | Glycyl-L-Aspartic Acid | [Glycine, L-Aspartate] | EX\_gly\_e,EX\_asp\_\_L\_e | gly\_e,asp\_\_L\_e | True | True | 0.0861333 | False |
| PM1\_F2 | Citric Acid | None | EX\_cit\_e | cit\_e | True | False | 0.0876333 | False |
| PM1\_F3 | m-Inositol | Myo-Inositol | EX\_inost\_e | inost\_e | True | True | 0.100567 | False |
| PM1\_F4 | D-Threonine | NaN | NaN | NaN | NaN | NaN | 0.112333 | False |
| PM1\_F5 | Fumaric Acid | None | EX\_fum\_e | fum\_e | True | False | 0.0933667 | False |
| PM1\_F6 | Bromo Succinic Acid | NaN | NaN | NaN | NaN | NaN | 0.173133 | True |
| PM1\_F7 | Propionic Acid | None | EX\_ppa\_e | ppa\_e | True | False | 0.1827 | True |
| PM1\_F8 | Mucic Acid | None | EX\_galct\_\_D\_e | galct\_\_D\_e | False | False | 0.0875333 | False |
| PM1\_F9 | Glycolic Acid | Glycolate C2H3O3 | EX\_glyclt\_e | glyclt\_e | True | True | 0.0824333 | False |
| PM1\_F10 | Glyoxylic Acid | Glyoxylate | EX\_glx\_e | glx\_e | True | True | 0.0789333 | False |
| PM1\_F11 | D-Cellobiose | None | EX\_cellb\_e | cellb\_e | True | False | 0.198867 | True |
| PM1\_F12 | Inosine | Inosine | EX\_ins\_e | ins\_e | True | True | 0.0932 | False |
| PM1\_G1 | Glycyl-L-Glutamic Acid | [Glycine, L-Glutamate] | EX\_gly\_e,EX\_glu\_\_L\_e | gly\_e,glu\_\_L\_e | True | True | 0.153067 | True |
| PM1\_G2 | Tricarballylic Acid | None | EX\_tcb\_e | tcb\_e | False | False | 0.0634333 | False |
| PM1\_G3 | L-Serine | L-Serine | EX\_ser\_\_L\_e | ser\_\_L\_e | True | True | 0.189367 | True |
| PM1\_G4 | L-Threonine | L-Threonine | EX\_thr\_\_L\_e | thr\_\_L\_e | True | True | 0.160133 | True |
| PM1\_G5 | L-Alanine | L-Alanine | EX\_ala\_\_L\_e | ala\_\_L\_e | True | True | 0.2412 | True |
| PM1\_G6 | L-Alanyl-Glycine | [L-Alanine, Glycine] | EX\_ala\_\_L\_e,EX\_gly\_e | ala\_\_L\_e,gly\_e | True | True | 0.1866 | True |
| PM1\_G7 | Acetoacetic Acid | None | EX\_acac\_e | acac\_e | True | False | 0.161467 | True |
| PM1\_G8 | N-Acetyl-b-D-Mannosamine | None | EX\_acmana\_e | acmana\_e | False | False | 0.0844333 | False |
| PM1\_G9 | Mono Methyl Succinate | None | EX\_methsucc\_e | methsucc\_e | False | False | 0.200767 | True |
| PM1\_G10 | Methyl Pyruvate | NaN | NaN | NaN | NaN | NaN | 0.170767 | True |
| PM1\_G11 | D-Malic Acid | None | EX\_mal\_\_D\_e | mal\_\_D\_e | False | False | 0.121733 | True |
| PM1\_G12 | L-Malic Acid | None | EX\_mal\_\_L\_e | mal\_\_L\_e | True | False | 0.107933 | False |
| PM1\_H1 | Glycyl-L-Proline | [Glycine, L-Proline] | EX\_gly\_e,EX\_pro\_\_L\_e | gly\_e,pro\_\_L\_e | True | True | 0.225233 | True |
| PM1\_H2 | p-Hydroxy Phenyl Acetic Acid | None | EX\_4hoxpac\_e | 4hoxpac\_e | False | False | 0.139733 | True |
| PM1\_H3 | m-Hydroxy Phenyl Acetic Acid | None | EX\_3hoxpac\_e | 3hoxpac\_e | False | False | 0.0826333 | False |
| PM1\_H4 | Tyramine | None | EX\_tym\_e | tym\_e | True | False | 0.0702667 | False |
| PM1\_H5 | D-Psicose | NaN | NaN | NaN | NaN | NaN | 0.122867 | True |
| PM1\_H6 | L-Lyxose | L-Lyxose | EX\_lyx\_\_L\_e | lyx\_\_L\_e | True | True | 0.393067 | True |
| PM1\_H7 | Glucuronamide | NaN | NaN | NaN | NaN | NaN | 0.1121 | True |
| PM1\_H8 | Pyruvic Acid | Pyruvate | EX\_pyr\_e | pyr\_e | True | True | 0.215867 | True |
| PM1\_H9 | L-Galactonic Acid-g-Lactone | None | EX\_galctn\_\_L\_e | galctn\_\_L\_e | False | False | 0.2527 | True |
| PM1\_H10 | D-Galacturonic Acid | D-Galacturonate | EX\_galur\_e | galur\_e | True | True | 0.248833 | True |
| PM1\_H11 | Phenylethylamine | None | EX\_peamn\_e | peamn\_e | True | False | 0.0828333 | False |
| PM1\_H12 | 2-Aminoethanol | None | EX\_etha\_e | etha\_e | True | False | 0.0752667 | False |
| PM2\_A1 | Negative Control | NaN | NaN | NaN | NaN | NaN | 0.0822333 | False |
| PM2\_A2 | Chondroitin Sulfate C | NaN | NaN | NaN | NaN | NaN | 0.0576667 | False |
| PM2\_A3 | a-Cyclodextrin | NaN | NaN | NaN | NaN | NaN | 0.0596 | False |
| PM2\_A4 | b-Cyclodextrin | NaN | NaN | NaN | NaN | NaN | 0.0798333 | False |
| PM2\_A5 | g-Cyclodextrin | NaN | NaN | NaN | NaN | NaN | 0.102967 | False |
| PM2\_A6 | Dextrin | None | EX\_dextrin\_e | dextrin\_e | False | False | 0.129 | True |
| PM2\_A7 | Gelatin | NaN | NaN | NaN | NaN | NaN | 0.133233 | True |
| PM2\_A8 | Glycogen | None | EX\_glycogen\_e | glycogen\_e | True | False | 0.0743 | False |
| PM2\_A9 | Inulin | NaN | NaN | NaN | NaN | NaN | 0.165867 | True |
| PM2\_A10 | Laminarin | NaN | NaN | NaN | NaN | NaN | 0.203733 | True |
| PM2\_A11 | Mannan | None | EX\_mannan\_e | mannan\_e | True | False | 0.0661333 | False |
| PM2\_A12 | Pectin | None | EX\_pect\_e | pect\_e | False | False | 0.159867 | True |
| PM2\_B1 | N-Acetyl-D-Galactosamine | None | EX\_acgal\_e | acgal\_e | False | False | 0.0779667 | False |
| PM2\_B2 | N-Acetyl Neuraminic Acid | None | EX\_acnam\_e | acnam\_e | False | False | 0.0724667 | False |
| PM2\_B3 | b-D-Allose | None | EX\_all\_\_D\_e | all\_\_D\_e | False | False | 0.107 | False |
| PM2\_B4 | Amygdalin | NaN | NaN | NaN | NaN | NaN | 0.105433 | True |
| PM2\_B5 | D-Arabinose | None | EX\_arab\_\_D\_e | arab\_\_D\_e | True | False | 0.269833 | True |
| PM2\_B6 | D-Arabitol | D-Arabitol | EX\_abt\_\_D\_e | abt\_\_D\_e | True | True | 0.2446 | True |
| PM2\_B7 | L-Arabitol | L Arabinitol C5H12O5 | EX\_abt\_e | abt\_e | True | True | 0.249133 | True |
| PM2\_B8 | Arbutin | None | EX\_arbt\_e | arbt\_e | False | False | 0.300467 | True |
| PM2\_B9 | 2-Deoxy-D-Ribose | None | EX\_drib\_e | drib\_e | True | False | 0.2099 | True |
| PM2\_B10 | i-Erythritol | NaN | NaN | NaN | NaN | NaN | 0.0761 | False |
| PM2\_B11 | D-Fucose | NaN | NaN | NaN | NaN | NaN | 0.110233 | True |
| PM2\_B12 | 3-0-b-D-Galactopyranosyl-D-Arabinose | NaN | NaN | NaN | NaN | NaN | 0.1148 | True |
| PM2\_C1 | Gentiobiose | NaN | NaN | NaN | NaN | NaN | 0.2304 | True |
| PM2\_C2 | L-Glucose | NaN | NaN | NaN | NaN | NaN | 0.115167 | True |
| PM2\_C3 | Lactitol | NaN | NaN | NaN | NaN | NaN | 0.0860333 | False |
| PM2\_C4 | D-Melezitose | NaN | NaN | NaN | NaN | NaN | 0.0861333 | False |
| PM2\_C5 | Maltitol | NaN | NaN | NaN | NaN | NaN | 0.0897667 | False |
| PM2\_C6 | A-Methyl-D-Glucoside | NaN | NaN | NaN | NaN | NaN | 0.0868667 | False |
| PM2\_C7 | b-Methyl-D-Galactoside | NaN | NaN | NaN | NaN | NaN | 0.1019 | False |
| PM2\_C8 | 3-Methyl Glucose | NaN | NaN | NaN | NaN | NaN | 0.0928333 | False |
| PM2\_C9 | b-Methyl-D-Glucuronic Acid | NaN | NaN | NaN | NaN | NaN | 0.0801667 | False |
| PM2\_C10 | a-Methyl-D-Mannoside | NaN | NaN | NaN | NaN | NaN | 0.0927333 | False |
| PM2\_C11 | b-Methyl-D-Xyloside | NaN | NaN | NaN | NaN | NaN | 0.0784333 | False |
| PM2\_C12 | Palatinose | NaN | NaN | NaN | NaN | NaN | 0.0882 | False |
| PM2\_D1 | D-Raffinose | NaN | NaN | NaN | NaN | NaN | 0.0758667 | False |
| PM2\_D2 | Salicin | None | EX\_salcn\_e | salcn\_e | False | False | 0.3282 | True |
| PM2\_D3 | Sedoheptulosan | NaN | NaN | NaN | NaN | NaN | 0.0902 | False |
| PM2\_D4 | L-Sorbose | None | EX\_srb\_\_L\_e | srb\_\_L\_e | True | False | 0.0941333 | False |
| PM2\_D5 | Stachyose | NaN | NaN | NaN | NaN | NaN | 0.0842333 | False |
| PM2\_D6 | D-Tagatose | None | EX\_tag\_\_D\_e | tag\_\_D\_e | False | False | 0.1632 | True |
| PM2\_D7 | Turanose | NaN | NaN | NaN | NaN | NaN | 0.122033 | True |
| PM2\_D8 | Xylitol | Xylitol C5H12O5 | EX\_xylt\_e | xylt\_e | True | True | 0.260667 | True |
| PM2\_D9 | N-Acetyl-D-Glucosaminitol | NaN | NaN | NaN | NaN | NaN | 0.0788 | False |
| PM2\_D10 | g-Amino Butyric Acid | 4-Aminobutanoate | EX\_4abut\_e | 4abut\_e | True | True | 0.240867 | True |
| PM2\_D11 | d-Amino Valeric Acid | NaN | NaN | NaN | NaN | NaN | 0.0974667 | False |
| PM2\_D12 | Butyric Acid | None | EX\_but\_e | but\_e | True | False | 0.265533 | True |
| PM2\_E1 | Capric Acid | Decanoate (n-C10:0) | EX\_dca\_e | dca\_e | True | True | 0.0068 | False |
| PM2\_E2 | Caproic Acid | None | EX\_hxa\_e | hxa\_e | True | False | 0.182 | True |
| PM2\_E3 | Citraconic Acid | None | EX\_citac\_e | citac\_e | False | False | 0.0714 | False |
| PM2\_E4 | Citramalic Acid | None | EX\_citm\_e | citm\_e | False | False | 0.0739 | False |
| PM2\_E5 | D-Glucosamine | D-Glucosamine | EX\_gam\_e | gam\_e | True | True | 0.171867 | True |
| PM2\_E6 | 2-Hydroxy Benzoic Acid | NaN | NaN | NaN | NaN | NaN | 0.100167 | False |
| PM2\_E7 | 4-Hydroxy Benzoic Acid | None | EX\_4hbz\_e | 4hbz\_e | True | False | 0.186267 | True |
| PM2\_E8 | b-Hydroxy Butyric Acid | None | EX\_bhb\_e | bhb\_e | True | False | 0.1963 | True |
| PM2\_E9 | g-Hydroxy Butyric Acid | None | EX\_ghb\_e | ghb\_e | True | False | 0.212067 | True |
| PM2\_E10 | A-Keto-Valeric Acid | NaN | NaN | NaN | NaN | NaN | 0.156967 | True |
| PM2\_E11 | Itaconic Acid | None | EX\_itacon\_e | itacon\_e | False | False | 0.0718 | False |
| PM2\_E12 | 5-Keto-D-Gluconic Acid | None | EX\_5dglcn\_e | 5dglcn\_e | True | False | 0.219967 | True |
| PM2\_F1 | D-Lactic Acid Methyl Ester | NaN | NaN | NaN | NaN | NaN | 0.111333 | True |
| PM2\_F2 | Malonic Acid | None | EX\_malon\_e | malon\_e | False | False | 0.0589 | False |
| PM2\_F3 | Melibionic Acid | NaN | NaN | NaN | NaN | NaN | 0.118367 | True |
| PM2\_F4 | Oxalic Acid | Oxalate | EX\_oxa\_e | oxa\_e | True | True | 0.0759333 | False |
| PM2\_F5 | Oxalomalic Acid | NaN | NaN | NaN | NaN | NaN | 0.146133 | True |
| PM2\_F6 | Quinic Acid | None | EX\_quin\_e | quin\_e | True | False | 0.1497 | True |
| PM2\_F7 | D-Ribono-1,4-Lactone | NaN | NaN | NaN | NaN | NaN | 0.0578333 | False |
| PM2\_F8 | Sebacic Acid | NaN | NaN | NaN | NaN | NaN | 0.230433 | True |
| PM2\_F9 | Sorbic Acid | NaN | NaN | NaN | NaN | NaN | 0.184 | True |
| PM2\_F10 | Succinamic Acid | NaN | NaN | NaN | NaN | NaN | 0.128233 | True |
| PM2\_F11 | D-Tartaric Acid | None | EX\_tartr\_\_D\_e | tartr\_\_D\_e | False | False | 0.0704 | False |
| PM2\_F12 | L-Tartaric Acid | None | EX\_tartr\_\_L\_e | tartr\_\_L\_e | False | False | 0.0758 | False |
| PM2\_G1 | Acetamide | None | EX\_ad\_e | ad\_e | True | False | 0.0881333 | False |
| PM2\_G2 | L-Alaninamide | NaN | NaN | NaN | NaN | NaN | 0.254367 | True |
| PM2\_G3 | N-Acetyl-L-Glutamic Acid | None | EX\_acglu\_e | acglu\_e | True | False | 0.0718 | False |
| PM2\_G4 | L-Arginine | L-Arginine | EX\_arg\_\_L\_e | arg\_\_L\_e | True | True | 0.188467 | True |
| PM2\_G5 | Glycine | Glycine | EX\_gly\_e | gly\_e | True | True | 0.254033 | True |
| PM2\_G6 | L-Histidine | L-Histidine | EX\_his\_\_L\_e | his\_\_L\_e | True | True | 0.1147 | False |
| PM2\_G7 | L-Homoserine | None | EX\_hom\_\_L\_e | hom\_\_L\_e | True | False | 0.164767 | True |
| PM2\_G8 | Hydroxy-L-Proline | NaN | NaN | NaN | NaN | NaN | 0.0935667 | False |
| PM2\_G9 | L-Isoleucine | L-Isoleucine | EX\_ile\_\_L\_e | ile\_\_L\_e | True | True | 0.241867 | True |
| PM2\_G10 | L-Leucine | L-Leucine | EX\_leu\_\_L\_e | leu\_\_L\_e | True | True | 0.173533 | True |
| PM2\_G11 | L-Lysine | L-Lysine | EX\_lys\_\_L\_e | lys\_\_L\_e | True | True | 0.0915667 | False |
| PM2\_G12 | L-Methionine | L-Methionine | EX\_met\_\_L\_e | met\_\_L\_e | True | True | 0.0626333 | False |
| PM2\_H1 | L-Ornithine | Ornithine | EX\_orn\_e | orn\_e | True | True | 0.238633 | True |
| PM2\_H2 | L-Phenylalanine | L-Phenylalanine | EX\_phe\_\_L\_e | phe\_\_L\_e | True | True | 0.061 | False |
| PM2\_H3 | L-Pyroglutamic Acid | NaN | NaN | NaN | NaN | NaN | 0.2722 | True |
| PM2\_H4 | L-Valine | L-Valine | EX\_val\_\_L\_e | val\_\_L\_e | True | True | 0.179467 | True |
| PM2\_H5 | D,L-Carnitine | [None, L-Carnitine] | EX\_crn\_\_D\_e,EX\_crn\_e | crn\_\_D\_e,crn\_e | False | False | 0.0643333 | False |
| PM2\_H6 | Sec-Butylamine | NaN | NaN | NaN | NaN | NaN | 0.0383667 | False |
| PM2\_H7 | D,L-Octopamine | NaN | NaN | NaN | NaN | NaN | 0.0910333 | False |
| PM2\_H8 | Putrescine | Putrescine | EX\_ptrc\_e | ptrc\_e | True | True | 0.0669333 | False |
| PM2\_H9 | Dihydroxy Acetone | None | EX\_dha\_e | dha\_e | True | False | 0.3402 | True |
| PM2\_H10 | 2,3-Butanediol | None | EX\_btd\_RR\_e | btd\_RR\_e | True | False | 0.07255 | False |
| PM2\_H11 | 2,3-Butanedione | NaN | NaN | NaN | NaN | NaN | 0.0869667 | False |
| PM2\_H12 | 3-Hydroxy 2-Butanon | NaN | NaN | NaN | NaN | NaN | 0.0601333 | False |
| PM3B\_A1 | Negative Control | NaN | NaN | NaN | NaN | NaN | 0.1006 | False |
| PM3B\_A2 | Ammonia | Ammonium | EX\_nh4\_e | nh4\_e | True | True | 0.2157 | True |
| PM3B\_A3 | Nitrite | Nitrite | EX\_no2\_e | no2\_e | True | True | 0.157167 | True |
| PM3B\_A4 | Nitrate | Nitrate | EX\_no3\_e | no3\_e | True | True | 0.2142 | True |
| PM3B\_A5 | Urea | Urea CH4N2O | EX\_urea\_e | urea\_e | True | True | 0.2281 | True |
| PM3B\_A6 | Biuret | NaN | NaN | NaN | NaN | NaN | 0.112633 | False |
| PM3B\_A7 | L-Alanine | L-Alanine | EX\_ala\_\_L\_e | ala\_\_L\_e | True | True | 0.2165 | True |
| PM3B\_A8 | L-Arginine | L-Arginine | EX\_arg\_\_L\_e | arg\_\_L\_e | True | True | 0.228267 | True |
| PM3B\_A9 | L-Asparagine | L-Asparagine | EX\_asn\_\_L\_e | asn\_\_L\_e | True | True | 0.211933 | True |
| PM3B\_A10 | L-Aspartic Acid | L-Aspartate | EX\_asp\_\_L\_e | asp\_\_L\_e | True | True | 0.236567 | True |
| PM3B\_A11 | L-Cysteine | L-Cysteine | EX\_cys\_\_L\_e | cys\_\_L\_e | True | True | 0.162167 | True |
| PM3B\_A12 | L-Glutamic Acid | L-Glutamate | EX\_glu\_\_L\_e | glu\_\_L\_e | True | True | 0.3435 | True |
| PM3B\_B1 | L-Glutamine | L-Glutamine | EX\_gln\_\_L\_e | gln\_\_L\_e | True | True | 0.2695 | True |
| PM3B\_B2 | Glycine | Glycine | EX\_gly\_e | gly\_e | True | True | 0.2008 | True |
| PM3B\_B3 | L-Histidine | L-Histidine | EX\_his\_\_L\_e | his\_\_L\_e | True | True | 0.140533 | True |
| PM3B\_B4 | L-Isoleucine | L-Isoleucine | EX\_ile\_\_L\_e | ile\_\_L\_e | True | True | 0.1834 | True |
| PM3B\_B5 | L-Leucine | L-Leucine | EX\_leu\_\_L\_e | leu\_\_L\_e | True | True | 0.1756 | True |
| PM3B\_B6 | L-Lysine | L-Lysine | EX\_lys\_\_L\_e | lys\_\_L\_e | True | True | 0.1599 | True |
| PM3B\_B7 | L-Methionine | L-Methionine | EX\_met\_\_L\_e | met\_\_L\_e | True | True | 0.2186 | True |
| PM3B\_B8 | L-Phenylalanine | L-Phenylalanine | EX\_phe\_\_L\_e | phe\_\_L\_e | True | True | 0.173667 | True |
| PM3B\_B9 | L-Proline | L-Proline | EX\_pro\_\_L\_e | pro\_\_L\_e | True | True | 0.228133 | True |
| PM3B\_B10 | L-Serine | L-Serine | EX\_ser\_\_L\_e | ser\_\_L\_e | True | True | 0.1749 | True |
| PM3B\_B11 | L-Threonine | L-Threonine | EX\_thr\_\_L\_e | thr\_\_L\_e | True | True | 0.162267 | True |
| PM3B\_B12 | L-Tryptophan | L-Tryptophan | EX\_trp\_\_L\_e | trp\_\_L\_e | True | True | 0.265533 | True |
| PM3B\_C1 | L-Tyrosine | L-Tyrosine | EX\_tyr\_\_L\_e | tyr\_\_L\_e | True | True | 0.1975 | True |
| PM3B\_C2 | L-Valine | L-Valine | EX\_val\_\_L\_e | val\_\_L\_e | True | True | 0.203267 | True |
| PM3B\_C3 | D-Alanine | D-Alanine | EX\_ala\_\_D\_e | ala\_\_D\_e | True | True | 0.146133 | True |
| PM3B\_C4 | D-Asparagine | None | EX\_asn\_\_D\_e | asn\_\_D\_e | False | False | 0.111333 | True |
| PM3B\_C5 | D-Aspartic Acid | None | EX\_asp\_\_D\_e | asp\_\_D\_e | True | False | 0.1008 | False |
| PM3B\_C6 | D-Glutamic Acid | None | EX\_glu\_\_D\_e | glu\_\_D\_e | False | False | 0.0466 | False |
| PM3B\_C7 | D-Lysine | None | EX\_lys\_\_D\_e | lys\_\_D\_e | False | False | 0.110933 | False |
| PM3B\_C8 | D-Serine | None | EX\_ser\_\_D\_e | ser\_\_D\_e | True | False | 0.1085 | False |
| PM3B\_C9 | D-Valine | None | EX\_val\_\_D\_e | val\_\_D\_e | False | False | 0.121267 | True |
| PM3B\_C10 | L-Citrulline | None | EX\_citr\_\_L\_e | citr\_\_L\_e | True | False | 0.1901 | True |
| PM3B\_C11 | L-Homoserine | None | EX\_hom\_\_L\_e | hom\_\_L\_e | True | False | 0.252767 | True |
| PM3B\_C12 | L-Ornithine | Ornithine | EX\_orn\_e | orn\_e | True | True | 0.223733 | True |
| PM3B\_D1 | N-Acetyl-L-Glutamic Acid | None | EX\_acglu\_e | acglu\_e | True | False | 0.1125 | False |
| PM3B\_D2 | N-Phthaloyl-L-Glutamic Acid | NaN | NaN | NaN | NaN | NaN | 0.2174 | True |
| PM3B\_D3 | L-Pyroglutamic Acid | NaN | NaN | NaN | NaN | NaN | 0.202267 | True |
| PM3B\_D4 | Hydroxylamine | None | EX\_ham\_e | ham\_e | False | False | 0.150133 | True |
| PM3B\_D5 | Methylamine | None | EX\_mma\_e | mma\_e | True | False | 0.0878667 | False |
| PM3B\_D6 | N-Amylamine | NaN | NaN | NaN | NaN | NaN | 0.1637 | True |
| PM3B\_D7 | N-Butylamine | NaN | NaN | NaN | NaN | NaN | 0.151933 | True |
| PM3B\_D8 | Ethylamine | NaN | NaN | NaN | NaN | NaN | 0.159533 | True |
| PM3B\_D9 | Ethanolamine | None | EX\_etha\_e | etha\_e | True | False | 0.1826 | True |
| PM3B\_D10 | Ethylenediamine | NaN | NaN | NaN | NaN | NaN | 0.0796333 | False |
| PM3B\_D11 | Putrescine | Putrescine | EX\_ptrc\_e | ptrc\_e | True | True | 0.122467 | True |
| PM3B\_D12 | Agmatine | None | EX\_agm\_e | agm\_e | True | False | 0.1032 | False |
| PM3B\_E1 | Histamine | None | EX\_hista\_e | hista\_e | True | False | 0.0739333 | False |
| PM3B\_E2 | b-Phenylethylamine | None | EX\_peamn\_e | peamn\_e | True | False | 0.118233 | False |
| PM3B\_E3 | Tyramine | None | EX\_tym\_e | tym\_e | True | False | 0.145667 | True |
| PM3B\_E4 | Acetamide | None | EX\_ad\_e | ad\_e | True | False | 0.149867 | True |
| PM3B\_E5 | Formamide | None | EX\_frmd\_e | frmd\_e | True | False | 0.161 | True |
| PM3B\_E6 | Glucuronamide | NaN | NaN | NaN | NaN | NaN | 0.2037 | True |
| PM3B\_E7 | D,L-Lactamide | NaN | NaN | NaN | NaN | NaN | 0.215433 | True |
| PM3B\_E8 | D-Glucosamine | D-Glucosamine | EX\_gam\_e | gam\_e | True | True | 0.1291 | True |
| PM3B\_E9 | D-Galactosamine | None | EX\_galam\_e | galam\_e | False | False | 0.1819 | True |
| PM3B\_E10 | D-Mannosamine | NaN | NaN | NaN | NaN | NaN | 0.130233 | True |
| PM3B\_E11 | N-Acetyl-D-Glucosamine | N-Acetyl-D-glucosamine | EX\_acgam\_e | acgam\_e | True | True | 0.101067 | False |
| PM3B\_E12 | N-Acetyl-D-Galactosamine | None | EX\_acgal\_e | acgal\_e | False | False | 0.0908333 | False |
| PM3B\_F1 | N-Acetyl-D-Mannosamine | None | EX\_acmana\_e | acmana\_e | False | False | 0.0963667 | False |
| PM3B\_F2 | Adenine | Adenine | EX\_ade\_e | ade\_e | True | True | 0.239667 | True |
| PM3B\_F3 | Adenosine | Adenosine | EX\_adn\_e | adn\_e | True | True | 0.2381 | True |
| PM3B\_F4 | Cytidine | Cytidine | EX\_cytd\_e | cytd\_e | True | True | 0.1963 | True |
| PM3B\_F5 | Cytosine | Cytosine | EX\_csn\_e | csn\_e | True | True | 0.178733 | True |
| PM3B\_F6 | Guanine | Guanine | EX\_gua\_e | gua\_e | True | True | 0.127233 | False |
| PM3B\_F7 | Guanosine | None | EX\_gsn\_e | gsn\_e | True | False | 0.275767 | True |
| PM3B\_F8 | Thymine | None | EX\_thym\_e | thym\_e | True | False | 0.1536 | True |
| PM3B\_F9 | Thymidine | None | EX\_thymd\_e | thymd\_e | True | False | 0.1195 | True |
| PM3B\_F10 | Uracil | Uracil | EX\_ura\_e | ura\_e | True | True | 0.193733 | True |
| PM3B\_F11 | Uridine | Uridine | EX\_uri\_e | uri\_e | True | True | 0.1894 | True |
| PM3B\_F12 | Inosine | Inosine | EX\_ins\_e | ins\_e | True | True | 0.3249 | True |
| PM3B\_G1 | Xanthine | None | EX\_xan\_e | xan\_e | True | False | 0.387333 | True |
| PM3B\_G2 | Xanthosine | None | EX\_xtsn\_e | xtsn\_e | True | False | 0.2001 | True |
| PM3B\_G3 | Uric Acid | Urate C5H4N4O3 | EX\_urate\_e | urate\_e | True | True | 0.239433 | True |
| PM3B\_G4 | Alloxan | None | EX\_CE0074\_e | CE0074\_e | False | False | 0.306567 | True |
| PM3B\_G5 | Allantoin | Allantoin | EX\_alltn\_e | alltn\_e | True | True | 0.1949 | True |
| PM3B\_G6 | Parabanic Acid | NaN | NaN | NaN | NaN | NaN | 0.215033 | True |
| PM3B\_G7 | D,L-a-Amino-NButyric Acid | None | EX\_C02356\_e | C02356\_e | False | False | 0.157433 | True |
| PM3B\_G8 | g-Amino-NButyric Acid | 4-Aminobutanoate | EX\_4abut\_e | 4abut\_e | True | True | 0.258267 | True |
| PM3B\_G9 | e-Amino-NCaproic Acid | NaN | NaN | NaN | NaN | NaN | 0.138967 | True |
| PM3B\_G10 | D,L-a-AminoCaprylic Acid | NaN | NaN | NaN | NaN | NaN | 0.228933 | True |
| PM3B\_G11 | d-Amino-NValeric Acid | NaN | NaN | NaN | NaN | NaN | 0.200433 | True |
| PM3B\_G12 | a-Amino-NValeric Acid | NaN | NaN | NaN | NaN | NaN | 0.314867 | True |
| PM3B\_H1 | Ala-Asp | [L-Alanine, L-Aspartate] | EX\_ala\_\_L\_e,EX\_asp\_\_L\_e | ala\_\_L\_e,asp\_\_L\_e | True | True | 0.196167 | True |
| PM3B\_H2 | Ala-Gln | [L-Alanine, L-Glutamine] | EX\_ala\_\_L\_e,EX\_gln\_\_L\_e | ala\_\_L\_e,gln\_\_L\_e | True | True | 0.294933 | True |
| PM3B\_H3 | Ala-Glu | [L-Alanine, L-Glutamate] | EX\_ala\_\_L\_e,EX\_glu\_\_L\_e | ala\_\_L\_e,glu\_\_L\_e | True | True | 0.187667 | True |
| PM3B\_H4 | Ala-Gly | [L-Alanine, Glycine] | EX\_ala\_\_L\_e,EX\_gly\_e | ala\_\_L\_e,gly\_e | True | True | 0.268 | True |
| PM3B\_H5 | Ala-His | [L-Alanine, L-Histidine] | EX\_ala\_\_L\_e,EX\_his\_\_L\_e | ala\_\_L\_e,his\_\_L\_e | True | True | 0.2586 | True |
| PM3B\_H6 | Ala-Leu | [L-Alanine, L-Leucine] | EX\_ala\_\_L\_e,EX\_leu\_\_L\_e | ala\_\_L\_e,leu\_\_L\_e | True | True | 0.1622 | True |
| PM3B\_H7 | Ala-Thr | [L-Alanine, L-Threonine] | EX\_ala\_\_L\_e,EX\_thr\_\_L\_e | ala\_\_L\_e,thr\_\_L\_e | True | True | 0.206067 | True |
| PM3B\_H8 | Gly-Asn | [Glycine, L-Asparagine] | EX\_gly\_e,EX\_asn\_\_L\_e | gly\_e,asn\_\_L\_e | True | True | 0.308767 | True |
| PM3B\_H9 | Gly-Gln | [Glycine, L-Glutamine] | EX\_gly\_e,EX\_gln\_\_L\_e | gly\_e,gln\_\_L\_e | True | True | 0.258733 | True |
| PM3B\_H10 | Gly-Glu | [Glycine, L-Glutamate] | EX\_gly\_e,EX\_glu\_\_L\_e | gly\_e,glu\_\_L\_e | True | True | 0.280533 | True |
| PM3B\_H11 | Gly-Met | [Glycine, L-Methionine] | EX\_gly\_e,EX\_met\_\_L\_e | gly\_e,met\_\_L\_e | True | True | 0.283833 | True |
| PM3B\_H12 | Met-Ala | [L-Methionine, L-Alanine] | EX\_met\_\_L\_e,EX\_ala\_\_L\_e | met\_\_L\_e,ala\_\_L\_e | True | True | 0.3705 | True |
| PM4A\_A1 | Negative Control | NaN | NaN | NaN | NaN | NaN | 0.0675 | False |
| PM4A\_A2 | Phosphate | Phosphate | EX\_pi\_e | pi\_e | True | True | 0.217267 | True |
| PM4A\_A3 | Pyrophosphate | None | EX\_ppi\_e | ppi\_e | True | False | 0.2997 | True |
| PM4A\_A4 | Trimetaphosphate | None | EX\_tmp\_e | tmp\_e | False | False | 0.305667 | True |
| PM4A\_A5 | Tripolyphosphate | None | EX\_pppi\_e | pppi\_e | True | False | 0.3834 | True |
| PM4A\_A6 | Triethyl Phosphate | NaN | NaN | NaN | NaN | NaN | 0.0659667 | False |
| PM4A\_A7 | Hypophosphite | NaN | NaN | NaN | NaN | NaN | 0.0613 | False |
| PM4A\_A8 | Adenosine-2’-monophosphate | None | EX\_amp2p\_e | amp2p\_e | False | False | 0.2983 | True |
| PM4A\_A9 | Adenosine-3’-monophosphate | None | EX\_3amp\_e | 3amp\_e | False | False | 0.237633 | True |
| PM4A\_A10 | Adenosine-5’-monophosphate | None | EX\_amp\_e | amp\_e | True | False | 0.220933 | True |
| PM4A\_A11 | Adenosine-2’,3’-Cyclic monophosphate | None | EX\_23camp\_e | 23camp\_e | False | False | 0.209733 | True |
| PM4A\_A12 | Adenosine-3’,5’-Cyclic monophosphate | CAMP C10H11N5O6P | EX\_camp\_e | camp\_e | True | True | 0.272933 | True |
| PM4A\_B1 | Thiophosphate | NaN | NaN | NaN | NaN | NaN | 0.1243 | True |
| PM4A\_B2 | Dithiophosphate | NaN | NaN | NaN | NaN | NaN | 0.3241 | True |
| PM4A\_B3 | D,L-a-Glycerol Phosphate | None | EX\_glyc3p\_e | glyc3p\_e | True | False | 0.2127 | True |
| PM4A\_B4 | b-Glycerol Phosphate | None | EX\_glyc2p\_e | glyc2p\_e | True | False | 0.2031 | True |
| PM4A\_B5 | Carbamyl Phosphate | None | EX\_cbp\_e | cbp\_e | True | False | 0.337933 | True |
| PM4A\_B6 | D-2-PhosphoGlyceric Acid | None | EX\_2pg\_e | 2pg\_e | True | False | 0.295633 | True |
| PM4A\_B7 | D-3-PhosphoGlyceric Acid | None | EX\_3pg\_e | 3pg\_e | True | False | 0.286367 | True |
| PM4A\_B8 | Guanosine-2’-monophosphate | NaN | NaN | NaN | NaN | NaN | 0.201267 | True |
| PM4A\_B9 | Guanosine-3’-monophosphate | None | EX\_3gmp\_e | 3gmp\_e | False | False | 0.224833 | True |
| PM4A\_B10 | Guanosine-5’-monophosphate | None | EX\_gmp\_e | gmp\_e | True | False | 0.2223 | True |
| PM4A\_B11 | Guanosine-2’,3’-Cyclic monophosphate | None | EX\_23cgmp\_e | 23cgmp\_e | False | False | 0.2335 | True |
| PM4A\_B12 | Guanosine-3’,5’-Cyclic monophosphate | 3',5'-Cyclic GMP | EX\_35cgmp\_e | 35cgmp\_e | True | True | 0.218567 | True |
| PM4A\_C1 | Phosphoenol Pyruvate | None | EX\_pep\_e | pep\_e | True | False | 0.304033 | True |
| PM4A\_C2 | PhosphoGlycolic Acid | None | EX\_2pglyc\_e | 2pglyc\_e | False | False | 0.238667 | True |
| PM4A\_C3 | D-Glucose-1-Phosphate | None | EX\_g1p\_e | g1p\_e | True | False | 0.217267 | True |
| PM4A\_C4 | D-Glucose-6-Phosphate | None | EX\_g6p\_e | g6p\_e | True | False | 0.248967 | True |
| PM4A\_C5 | 2-Deoxy-D-Glucose 6-Phosphate | None | EX\_2doxg6p\_e | 2doxg6p\_e | True | False | 0.0744 | False |
| PM4A\_C6 | D-Glucosamine-6-Phosphate | None | EX\_gam6p\_e | gam6p\_e | True | False | 0.219933 | True |
| PM4A\_C7 | 6-PhosphoGluconic Acid | None | EX\_6pgc\_e | 6pgc\_e | True | False | 0.308633 | True |
| PM4A\_C8 | Cytidine-2’-monophosphate | NaN | NaN | NaN | NaN | NaN | 0.302167 | True |
| PM4A\_C9 | Cytidine-3’-monophosphate | None | EX\_3cmp\_e | 3cmp\_e | False | False | 0.0753 | False |
| PM4A\_C10 | Cytidine-5’-monophosphate | None | EX\_cmp\_e | cmp\_e | True | False | 0.313933 | True |
| PM4A\_C11 | Cytidine-2’,3’-Cyclic monophosphate | None | EX\_23ccmp\_e | 23ccmp\_e | False | False | 0.1973 | True |
| PM4A\_C12 | Cytidine-3’,5’-Cyclic monophosphate | None | EX\_35ccmp\_e | 35ccmp\_e | True | False | 0.1961 | True |
| PM4A\_D1 | D-Mannose-1-Phosphate | None | EX\_man1p\_e | man1p\_e | True | False | 0.252833 | True |
| PM4A\_D2 | D-Mannose-6-Phosphate | None | EX\_man6p\_e | man6p\_e | True | False | 0.294767 | True |
| PM4A\_D3 | Cysteamine S-Phosphate | NaN | NaN | NaN | NaN | NaN | 0.366467 | True |
| PM4A\_D4 | Phospho-L-Arginine | None | EX\_argp\_e | argp\_e | False | False | 0.242233 | True |
| PM4A\_D5 | O-Phospho-D-Serine | None | EX\_pser\_\_D\_e | pser\_\_D\_e | False | False | 0.251967 | True |
| PM4A\_D6 | O-Phospho-L-Serine | None | EX\_pser\_\_L\_e | pser\_\_L\_e | True | False | 0.2369 | True |
| PM4A\_D7 | O-Phospho-L-Threonine | None | EX\_thrp\_e | thrp\_e | False | False | 0.270133 | True |
| PM4A\_D8 | Uridine-2’-monophosphate | NaN | NaN | NaN | NaN | NaN | 0.230733 | True |
| PM4A\_D9 | Uridine-3’-monophosphate | None | EX\_3ump\_e | 3ump\_e | False | False | 0.2121 | True |
| PM4A\_D10 | Uridine-5’-monophosphate | None | EX\_ump\_e | ump\_e | True | False | 0.2312 | True |
| PM4A\_D11 | Uridine-2’,3’-Cyclic monophosphate | None | EX\_23cump\_e | 23cump\_e | False | False | 0.2253 | True |
| PM4A\_D12 | Uridine-3’,5’-Cyclic monophosphate | NaN | NaN | NaN | NaN | NaN | 0.2182 | True |
| PM4A\_E1 | O-Phospho-D-Tyrosine | NaN | NaN | NaN | NaN | NaN | 0.235467 | True |
| PM4A\_E2 | O-Phospho-L-Tyrosine | None | EX\_tyrp\_e | tyrp\_e | False | False | 0.1924 | True |
| PM4A\_E3 | Phosphocreatine | None | EX\_pcreat\_e | pcreat\_e | False | False | 0.218667 | True |
| PM4A\_E4 | Phosphoryl Choline | None | EX\_cholp\_e | cholp\_e | True | False | 0.2162 | True |
| PM4A\_E5 | O-PhosphorylEthanolamine | None | EX\_ethamp\_e | ethamp\_e | True | False | 0.2117 | True |
| PM4A\_E6 | Phosphono Acetic Acid | None | EX\_phnac\_e | phnac\_e | False | False | 0.062 | False |
| PM4A\_E7 | 2-Aminoethyl Phosphonic Acid | None | EX\_2ameph\_e | 2ameph\_e | False | False | 0.0604 | False |
| PM4A\_E8 | Methylene Diphosphonic Acid | NaN | NaN | NaN | NaN | NaN | 0.175633 | True |
| PM4A\_E9 | Thymidine-3’-monophosphate | NaN | NaN | NaN | NaN | NaN | 0.1981 | True |
| PM4A\_E10 | Thymidine-5’-monophosphate | None | EX\_dtmp\_e | dtmp\_e | True | False | 0.1903 | True |
| PM4A\_E11 | Inositol Hexaphosphate | None | EX\_minohp\_e | minohp\_e | True | False | 0.288667 | True |
| PM4A\_E12 | Thymidine 3’,5’-Cyclic monophosphate | NaN | NaN | NaN | NaN | NaN | 0.124133 | False |
| PM4A\_F1 | Negative Control | NaN | NaN | NaN | NaN | NaN | 0.0788 | False |
| PM4A\_F2 | Sulfate | Sulfate | EX\_so4\_e | so4\_e | True | True | 0.2335 | True |
| PM4A\_F3 | Thiosulfate | None | EX\_tsul\_e | tsul\_e | True | False | 0.2565 | True |
| PM4A\_F4 | Tetrathionate | None | EX\_tet\_e | tet\_e | False | False | 0.194367 | True |
| PM4A\_F5 | Thiophosphate | NaN | NaN | NaN | NaN | NaN | 0.216967 | True |
| PM4A\_F6 | Dithiophosphate | NaN | NaN | NaN | NaN | NaN | 0.200267 | True |
| PM4A\_F7 | L-Cysteine | L-Cysteine | EX\_cys\_\_L\_e | cys\_\_L\_e | True | True | 0.1801 | True |
| PM4A\_F8 | D-Cysteine | None | EX\_cys\_\_D\_e | cys\_\_D\_e | False | False | 0.1216 | True |
| PM4A\_F9 | L-CysteinylGlycine | Cys Gly C5H10N2O3S | EX\_cgly\_e | cgly\_e | True | True | 0.339133 | True |
| PM4A\_F10 | L-Cysteic Acid | None | EX\_Lcyst\_e | Lcyst\_e | True | False | 0.1417 | True |
| PM4A\_F11 | Cysteamine | None | EX\_cysam\_e | cysam\_e | False | False | 0.1867 | True |
| PM4A\_F12 | L-Cysteine Sulfinic Acid | None | EX\_3sala\_e | 3sala\_e | True | False | 0.1932 | True |
| PM4A\_G1 | N-Acetyl-L-Cysteine | None | EX\_CE1310\_e | CE1310\_e | False | False | 0.121333 | True |
| PM4A\_G2 | S-Methyl-L-Cysteine | NaN | NaN | NaN | NaN | NaN | 0.155867 | True |
| PM4A\_G3 | Cystathionine | None | EX\_cyst\_\_L\_e | cyst\_\_L\_e | True | False | 0.183667 | True |
| PM4A\_G4 | Lanthionine | None | EX\_lanth\_e | lanth\_e | False | False | 0.1822 | True |
| PM4A\_G5 | Glutathione | Reduced glutathione | EX\_gthrd\_e | gthrd\_e | True | True | 0.1327 | True |
| PM4A\_G6 | D,L-Ethionine | NaN | NaN | NaN | NaN | NaN | 0.1063 | True |
| PM4A\_G7 | L-Methionine | L-Methionine | EX\_met\_\_L\_e | met\_\_L\_e | True | True | 0.2687 | True |
| PM4A\_G8 | D-Methionine | None | EX\_met\_\_D\_e | met\_\_D\_e | False | False | 0.2067 | True |
| PM4A\_G9 | Glycyl-L-Methionine | [Glycine, L-Methionine] | EX\_gly\_e,EX\_met\_\_L\_e | gly\_e,met\_\_L\_e | True | True | 0.268467 | True |
| PM4A\_G10 | N-Acetyl-D,L-Methionine | None | EX\_acmet\_e | acmet\_e | False | False | 0.174567 | True |
| PM4A\_G11 | L-Methionine Sulfoxide | [L-methionine-R-sulfoxide, L-Methionine Sulfox... | EX\_metsox\_R\_\_L\_e,EX\_metsox\_S\_\_L\_e | metsox\_R\_\_L\_e,metsox\_S\_\_L\_e | True | False | 0.259267 | True |
| PM4A\_G12 | L-Methionine Sulfone | NaN | NaN | NaN | NaN | NaN | 0.174233 | True |
| PM4A\_H1 | L-Djenkolic Acid | None | EX\_djenk\_e | djenk\_e | False | False | 0.3439 | True |
| PM4A\_H2 | Thiourea | NaN | NaN | NaN | NaN | NaN | 0.167867 | True |
| PM4A\_H3 | 1-Thio-b-D-Glucose | NaN | NaN | NaN | NaN | NaN | 0.1447 | True |
| PM4A\_H4 | D,L-Lipoamide | None | EX\_lpam\_e | lpam\_e | False | False | 0.269967 | True |
| PM4A\_H5 | Taurocholic Acid | Taurocholic acid C26H45NO7S | EX\_tchola\_e | tchola\_e | True | True | 0.131533 | True |
| PM4A\_H6 | Taurine | None | EX\_taur\_e | taur\_e | True | False | 0.182767 | True |
| PM4A\_H7 | Hypotaurine | None | EX\_hyptaur\_e | hyptaur\_e | True | False | 0.219867 | True |
| PM4A\_H8 | p-Amino Benzene Sulfonic Acid | NaN | NaN | NaN | NaN | NaN | 0.0869333 | False |
| PM4A\_H9 | Butane Sulfonic Acid | None | EX\_butso3\_e | butso3\_e | False | False | 0.188767 | True |
| PM4A\_H10 | 2-Hydroxyethane Sulfonic Acid | None | EX\_isetac\_e | isetac\_e | False | False | 0.1779 | True |
| PM4A\_H11 | Methane Sulfonic Acid | None | EX\_mso3\_e | mso3\_e | False | False | 0.206367 | True |
| PM4A\_H12 | Tetramethylene Sulfone | NaN | NaN | NaN | NaN | NaN | 0.0790333 | False |

In [21]:

```
with pd.option_context('display.max_rows', None, 'display.max_columns', None):
    display(Biolog_in_model.loc[Biolog_in_model['Biolog'].duplicated(keep=False)].sort_values('Biolog'))
```

|  | Biolog | Model | Exchange | Metabolite | Internal | External | Average | Pass |
| --- | --- | --- | --- | --- | --- | --- | --- | --- |
| PM2\_G1 | Acetamide | None | EX\_ad\_e | ad\_e | True | False | 0.0881333 | False |
| PM3B\_E4 | Acetamide | None | EX\_ad\_e | ad\_e | True | False | 0.149867 | True |
| PM3B\_F3 | Adenosine | Adenosine | EX\_adn\_e | adn\_e | True | True | 0.2381 | True |
| PM1\_E12 | Adenosine | Adenosine | EX\_adn\_e | adn\_e | True | True | 0.077 | False |
| PM1\_A9 | D-Alanine | D-Alanine | EX\_ala\_\_D\_e | ala\_\_D\_e | True | True | 0.1215 | True |
| PM3B\_C3 | D-Alanine | D-Alanine | EX\_ala\_\_D\_e | ala\_\_D\_e | True | True | 0.146133 | True |
| PM3B\_C5 | D-Aspartic Acid | None | EX\_asp\_\_D\_e | asp\_\_D\_e | True | False | 0.1008 | False |
| PM1\_D2 | D-Aspartic Acid | None | EX\_asp\_\_D\_e | asp\_\_D\_e | True | False | 0.0959667 | False |
| PM2\_E5 | D-Glucosamine | D-Glucosamine | EX\_gam\_e | gam\_e | True | True | 0.171867 | True |
| PM3B\_E8 | D-Glucosamine | D-Glucosamine | EX\_gam\_e | gam\_e | True | True | 0.1291 | True |
| PM1\_E3 | D-Glucose-1-Phosphate | None | EX\_g1p\_e | g1p\_e | True | False | 0.0786333 | False |
| PM4A\_C3 | D-Glucose-1-Phosphate | None | EX\_g1p\_e | g1p\_e | True | False | 0.217267 | True |
| PM4A\_C4 | D-Glucose-6-Phosphate | None | EX\_g6p\_e | g6p\_e | True | False | 0.248967 | True |
| PM1\_C1 | D-Glucose-6-Phosphate | None | EX\_g6p\_e | g6p\_e | True | False | 0.0870333 | False |
| PM3B\_C8 | D-Serine | None | EX\_ser\_\_D\_e | ser\_\_D\_e | True | False | 0.1085 | False |
| PM1\_B1 | D-Serine | None | EX\_ser\_\_D\_e | ser\_\_D\_e | True | False | 0.1506 | True |
| PM4A\_F6 | Dithiophosphate | NaN | NaN | NaN | NaN | NaN | 0.200267 | True |
| PM4A\_B2 | Dithiophosphate | NaN | NaN | NaN | NaN | NaN | 0.3241 | True |
| PM1\_H7 | Glucuronamide | NaN | NaN | NaN | NaN | NaN | 0.1121 | True |
| PM3B\_E6 | Glucuronamide | NaN | NaN | NaN | NaN | NaN | 0.2037 | True |
| PM3B\_B2 | Glycine | Glycine | EX\_gly\_e | gly\_e | True | True | 0.2008 | True |
| PM2\_G5 | Glycine | Glycine | EX\_gly\_e | gly\_e | True | True | 0.254033 | True |
| PM1\_F12 | Inosine | Inosine | EX\_ins\_e | ins\_e | True | True | 0.0932 | False |
| PM3B\_F12 | Inosine | Inosine | EX\_ins\_e | ins\_e | True | True | 0.3249 | True |
| PM1\_G5 | L-Alanine | L-Alanine | EX\_ala\_\_L\_e | ala\_\_L\_e | True | True | 0.2412 | True |
| PM3B\_A7 | L-Alanine | L-Alanine | EX\_ala\_\_L\_e | ala\_\_L\_e | True | True | 0.2165 | True |
| PM3B\_A8 | L-Arginine | L-Arginine | EX\_arg\_\_L\_e | arg\_\_L\_e | True | True | 0.228267 | True |
| PM2\_G4 | L-Arginine | L-Arginine | EX\_arg\_\_L\_e | arg\_\_L\_e | True | True | 0.188467 | True |
| PM3B\_A9 | L-Asparagine | L-Asparagine | EX\_asn\_\_L\_e | asn\_\_L\_e | True | True | 0.211933 | True |
| PM1\_D1 | L-Asparagine | L-Asparagine | EX\_asn\_\_L\_e | asn\_\_L\_e | True | True | 0.279933 | True |
| PM3B\_A10 | L-Aspartic Acid | L-Aspartate | EX\_asp\_\_L\_e | asp\_\_L\_e | True | True | 0.236567 | True |
| PM1\_A7 | L-Aspartic Acid | L-Aspartate | EX\_asp\_\_L\_e | asp\_\_L\_e | True | True | 0.267867 | True |
| PM3B\_A11 | L-Cysteine | L-Cysteine | EX\_cys\_\_L\_e | cys\_\_L\_e | True | True | 0.162167 | True |
| PM4A\_F7 | L-Cysteine | L-Cysteine | EX\_cys\_\_L\_e | cys\_\_L\_e | True | True | 0.1801 | True |
| PM3B\_A12 | L-Glutamic Acid | L-Glutamate | EX\_glu\_\_L\_e | glu\_\_L\_e | True | True | 0.3435 | True |
| PM1\_B12 | L-Glutamic Acid | L-Glutamate | EX\_glu\_\_L\_e | glu\_\_L\_e | True | True | 0.297367 | True |
| PM3B\_B1 | L-Glutamine | L-Glutamine | EX\_gln\_\_L\_e | gln\_\_L\_e | True | True | 0.2695 | True |
| PM1\_E1 | L-Glutamine | L-Glutamine | EX\_gln\_\_L\_e | gln\_\_L\_e | True | True | 0.3157 | True |
| PM3B\_B3 | L-Histidine | L-Histidine | EX\_his\_\_L\_e | his\_\_L\_e | True | True | 0.140533 | True |
| PM2\_G6 | L-Histidine | L-Histidine | EX\_his\_\_L\_e | his\_\_L\_e | True | True | 0.1147 | False |
| PM2\_G7 | L-Homoserine | None | EX\_hom\_\_L\_e | hom\_\_L\_e | True | False | 0.164767 | True |
| PM3B\_C11 | L-Homoserine | None | EX\_hom\_\_L\_e | hom\_\_L\_e | True | False | 0.252767 | True |
| PM2\_G9 | L-Isoleucine | L-Isoleucine | EX\_ile\_\_L\_e | ile\_\_L\_e | True | True | 0.241867 | True |
| PM3B\_B4 | L-Isoleucine | L-Isoleucine | EX\_ile\_\_L\_e | ile\_\_L\_e | True | True | 0.1834 | True |
| PM2\_G10 | L-Leucine | L-Leucine | EX\_leu\_\_L\_e | leu\_\_L\_e | True | True | 0.173533 | True |
| PM3B\_B5 | L-Leucine | L-Leucine | EX\_leu\_\_L\_e | leu\_\_L\_e | True | True | 0.1756 | True |
| PM3B\_B6 | L-Lysine | L-Lysine | EX\_lys\_\_L\_e | lys\_\_L\_e | True | True | 0.1599 | True |
| PM2\_G11 | L-Lysine | L-Lysine | EX\_lys\_\_L\_e | lys\_\_L\_e | True | True | 0.0915667 | False |
| PM3B\_B7 | L-Methionine | L-Methionine | EX\_met\_\_L\_e | met\_\_L\_e | True | True | 0.2186 | True |
| PM4A\_G7 | L-Methionine | L-Methionine | EX\_met\_\_L\_e | met\_\_L\_e | True | True | 0.2687 | True |
| PM2\_G12 | L-Methionine | L-Methionine | EX\_met\_\_L\_e | met\_\_L\_e | True | True | 0.0626333 | False |
| PM2\_H1 | L-Ornithine | Ornithine | EX\_orn\_e | orn\_e | True | True | 0.238633 | True |
| PM3B\_C12 | L-Ornithine | Ornithine | EX\_orn\_e | orn\_e | True | True | 0.223733 | True |
| PM3B\_B8 | L-Phenylalanine | L-Phenylalanine | EX\_phe\_\_L\_e | phe\_\_L\_e | True | True | 0.173667 | True |
| PM2\_H2 | L-Phenylalanine | L-Phenylalanine | EX\_phe\_\_L\_e | phe\_\_L\_e | True | True | 0.061 | False |
| PM3B\_B9 | L-Proline | L-Proline | EX\_pro\_\_L\_e | pro\_\_L\_e | True | True | 0.228133 | True |
| PM1\_A8 | L-Proline | L-Proline | EX\_pro\_\_L\_e | pro\_\_L\_e | True | True | 0.2724 | True |
| PM2\_H3 | L-Pyroglutamic Acid | NaN | NaN | NaN | NaN | NaN | 0.2722 | True |
| PM3B\_D3 | L-Pyroglutamic Acid | NaN | NaN | NaN | NaN | NaN | 0.202267 | True |
| PM3B\_B10 | L-Serine | L-Serine | EX\_ser\_\_L\_e | ser\_\_L\_e | True | True | 0.1749 | True |
| PM1\_G3 | L-Serine | L-Serine | EX\_ser\_\_L\_e | ser\_\_L\_e | True | True | 0.189367 | True |
| PM1\_G4 | L-Threonine | L-Threonine | EX\_thr\_\_L\_e | thr\_\_L\_e | True | True | 0.160133 | True |
| PM3B\_B11 | L-Threonine | L-Threonine | EX\_thr\_\_L\_e | thr\_\_L\_e | True | True | 0.162267 | True |
| PM2\_H4 | L-Valine | L-Valine | EX\_val\_\_L\_e | val\_\_L\_e | True | True | 0.179467 | True |
| PM3B\_C2 | L-Valine | L-Valine | EX\_val\_\_L\_e | val\_\_L\_e | True | True | 0.203267 | True |
| PM2\_B1 | N-Acetyl-D-Galactosamine | None | EX\_acgal\_e | acgal\_e | False | False | 0.0779667 | False |
| PM3B\_E12 | N-Acetyl-D-Galactosamine | None | EX\_acgal\_e | acgal\_e | False | False | 0.0908333 | False |
| PM3B\_E11 | N-Acetyl-D-Glucosamine | N-Acetyl-D-glucosamine | EX\_acgam\_e | acgam\_e | True | True | 0.101067 | False |
| PM1\_A3 | N-Acetyl-D-Glucosamine | N-Acetyl-D-glucosamine | EX\_acgam\_e | acgam\_e | True | True | 0.0688667 | False |
| PM3B\_D1 | N-Acetyl-L-Glutamic Acid | None | EX\_acglu\_e | acglu\_e | True | False | 0.1125 | False |
| PM2\_G3 | N-Acetyl-L-Glutamic Acid | None | EX\_acglu\_e | acglu\_e | True | False | 0.0718 | False |
| PM4A\_F1 | Negative Control | NaN | NaN | NaN | NaN | NaN | 0.0788 | False |
| PM4A\_A1 | Negative Control | NaN | NaN | NaN | NaN | NaN | 0.0675 | False |
| PM1\_A1 | Negative Control | NaN | NaN | NaN | NaN | NaN | 0.0864 | False |
| PM2\_A1 | Negative Control | NaN | NaN | NaN | NaN | NaN | 0.0822333 | False |
| PM3B\_A1 | Negative Control | NaN | NaN | NaN | NaN | NaN | 0.1006 | False |
| PM3B\_D11 | Putrescine | Putrescine | EX\_ptrc\_e | ptrc\_e | True | True | 0.122467 | True |
| PM2\_H8 | Putrescine | Putrescine | EX\_ptrc\_e | ptrc\_e | True | True | 0.0669333 | False |
| PM4A\_B1 | Thiophosphate | NaN | NaN | NaN | NaN | NaN | 0.1243 | True |
| PM4A\_F5 | Thiophosphate | NaN | NaN | NaN | NaN | NaN | 0.216967 | True |
| PM1\_C12 | Thymidine | None | EX\_thymd\_e | thymd\_e | True | False | 0.0895 | False |
| PM3B\_F9 | Thymidine | None | EX\_thymd\_e | thymd\_e | True | False | 0.1195 | True |
| PM1\_H4 | Tyramine | None | EX\_tym\_e | tym\_e | True | False | 0.0702667 | False |
| PM3B\_E3 | Tyramine | None | EX\_tym\_e | tym\_e | True | False | 0.145667 | True |
| PM3B\_F11 | Uridine | Uridine | EX\_uri\_e | uri\_e | True | True | 0.1894 | True |
| PM1\_D12 | Uridine | Uridine | EX\_uri\_e | uri\_e | True | True | 0.0907 | False |

In [22]:

```
with pd.option_context('display.max_rows', None, 'display.max_columns', None):
    display(Biolog_in_model[Biolog_in_model['Exchange'].isna()])
```

|  | Biolog | Model | Exchange | Metabolite | Internal | External | Average | Pass |
| --- | --- | --- | --- | --- | --- | --- | --- | --- |
| PM1\_A1 | Negative Control | NaN | NaN | NaN | NaN | NaN | 0.0864 | False |
| PM1\_C5 | Tween 20 | NaN | NaN | NaN | NaN | NaN | 0.397733 | True |
| PM1\_D3 | D-Glucosaminic Acid | NaN | NaN | NaN | NaN | NaN | 0.0839 | False |
| PM1\_D5 | Tween 40 | NaN | NaN | NaN | NaN | NaN | 0.347467 | True |
| PM1\_D8 | a-Methyl-D-Galactoside | NaN | NaN | NaN | NaN | NaN | 0.0812667 | False |
| PM1\_D10 | Lactulose | NaN | NaN | NaN | NaN | NaN | 0.0839333 | False |
| PM1\_E5 | Tween 80 | NaN | NaN | NaN | NaN | NaN | 0.408233 | True |
| PM1\_E6 | a-Hydroxy Glutaric Acid-g-Lactone | NaN | NaN | NaN | NaN | NaN | 0.0643667 | False |
| PM1\_F4 | D-Threonine | NaN | NaN | NaN | NaN | NaN | 0.112333 | False |
| PM1\_F6 | Bromo Succinic Acid | NaN | NaN | NaN | NaN | NaN | 0.173133 | True |
| PM1\_G10 | Methyl Pyruvate | NaN | NaN | NaN | NaN | NaN | 0.170767 | True |
| PM1\_H5 | D-Psicose | NaN | NaN | NaN | NaN | NaN | 0.122867 | True |
| PM1\_H7 | Glucuronamide | NaN | NaN | NaN | NaN | NaN | 0.1121 | True |
| PM2\_A1 | Negative Control | NaN | NaN | NaN | NaN | NaN | 0.0822333 | False |
| PM2\_A2 | Chondroitin Sulfate C | NaN | NaN | NaN | NaN | NaN | 0.0576667 | False |
| PM2\_A3 | a-Cyclodextrin | NaN | NaN | NaN | NaN | NaN | 0.0596 | False |
| PM2\_A4 | b-Cyclodextrin | NaN | NaN | NaN | NaN | NaN | 0.0798333 | False |
| PM2\_A5 | g-Cyclodextrin | NaN | NaN | NaN | NaN | NaN | 0.102967 | False |
| PM2\_A7 | Gelatin | NaN | NaN | NaN | NaN | NaN | 0.133233 | True |
| PM2\_A9 | Inulin | NaN | NaN | NaN | NaN | NaN | 0.165867 | True |
| PM2\_A10 | Laminarin | NaN | NaN | NaN | NaN | NaN | 0.203733 | True |
| PM2\_B4 | Amygdalin | NaN | NaN | NaN | NaN | NaN | 0.105433 | True |
| PM2\_B10 | i-Erythritol | NaN | NaN | NaN | NaN | NaN | 0.0761 | False |
| PM2\_B11 | D-Fucose | NaN | NaN | NaN | NaN | NaN | 0.110233 | True |
| PM2\_B12 | 3-0-b-D-Galactopyranosyl-D-Arabinose | NaN | NaN | NaN | NaN | NaN | 0.1148 | True |
| PM2\_C1 | Gentiobiose | NaN | NaN | NaN | NaN | NaN | 0.2304 | True |
| PM2\_C2 | L-Glucose | NaN | NaN | NaN | NaN | NaN | 0.115167 | True |
| PM2\_C3 | Lactitol | NaN | NaN | NaN | NaN | NaN | 0.0860333 | False |
| PM2\_C4 | D-Melezitose | NaN | NaN | NaN | NaN | NaN | 0.0861333 | False |
| PM2\_C5 | Maltitol | NaN | NaN | NaN | NaN | NaN | 0.0897667 | False |
| PM2\_C6 | A-Methyl-D-Glucoside | NaN | NaN | NaN | NaN | NaN | 0.0868667 | False |
| PM2\_C7 | b-Methyl-D-Galactoside | NaN | NaN | NaN | NaN | NaN | 0.1019 | False |
| PM2\_C8 | 3-Methyl Glucose | NaN | NaN | NaN | NaN | NaN | 0.0928333 | False |
| PM2\_C9 | b-Methyl-D-Glucuronic Acid | NaN | NaN | NaN | NaN | NaN | 0.0801667 | False |
| PM2\_C10 | a-Methyl-D-Mannoside | NaN | NaN | NaN | NaN | NaN | 0.0927333 | False |
| PM2\_C11 | b-Methyl-D-Xyloside | NaN | NaN | NaN | NaN | NaN | 0.0784333 | False |
| PM2\_C12 | Palatinose | NaN | NaN | NaN | NaN | NaN | 0.0882 | False |
| PM2\_D1 | D-Raffinose | NaN | NaN | NaN | NaN | NaN | 0.0758667 | False |
| PM2\_D3 | Sedoheptulosan | NaN | NaN | NaN | NaN | NaN | 0.0902 | False |
| PM2\_D5 | Stachyose | NaN | NaN | NaN | NaN | NaN | 0.0842333 | False |
| PM2\_D7 | Turanose | NaN | NaN | NaN | NaN | NaN | 0.122033 | True |
| PM2\_D9 | N-Acetyl-D-Glucosaminitol | NaN | NaN | NaN | NaN | NaN | 0.0788 | False |
| PM2\_D11 | d-Amino Valeric Acid | NaN | NaN | NaN | NaN | NaN | 0.0974667 | False |
| PM2\_E6 | 2-Hydroxy Benzoic Acid | NaN | NaN | NaN | NaN | NaN | 0.100167 | False |
| PM2\_E10 | A-Keto-Valeric Acid | NaN | NaN | NaN | NaN | NaN | 0.156967 | True |
| PM2\_F1 | D-Lactic Acid Methyl Ester | NaN | NaN | NaN | NaN | NaN | 0.111333 | True |
| PM2\_F3 | Melibionic Acid | NaN | NaN | NaN | NaN | NaN | 0.118367 | True |
| PM2\_F5 | Oxalomalic Acid | NaN | NaN | NaN | NaN | NaN | 0.146133 | True |
| PM2\_F7 | D-Ribono-1,4-Lactone | NaN | NaN | NaN | NaN | NaN | 0.0578333 | False |
| PM2\_F8 | Sebacic Acid | NaN | NaN | NaN | NaN | NaN | 0.230433 | True |
| PM2\_F9 | Sorbic Acid | NaN | NaN | NaN | NaN | NaN | 0.184 | True |
| PM2\_F10 | Succinamic Acid | NaN | NaN | NaN | NaN | NaN | 0.128233 | True |
| PM2\_G2 | L-Alaninamide | NaN | NaN | NaN | NaN | NaN | 0.254367 | True |
| PM2\_G8 | Hydroxy-L-Proline | NaN | NaN | NaN | NaN | NaN | 0.0935667 | False |
| PM2\_H3 | L-Pyroglutamic Acid | NaN | NaN | NaN | NaN | NaN | 0.2722 | True |
| PM2\_H6 | Sec-Butylamine | NaN | NaN | NaN | NaN | NaN | 0.0383667 | False |
| PM2\_H7 | D,L-Octopamine | NaN | NaN | NaN | NaN | NaN | 0.0910333 | False |
| PM2\_H11 | 2,3-Butanedione | NaN | NaN | NaN | NaN | NaN | 0.0869667 | False |
| PM2\_H12 | 3-Hydroxy 2-Butanon | NaN | NaN | NaN | NaN | NaN | 0.0601333 | False |
| PM3B\_A1 | Negative Control | NaN | NaN | NaN | NaN | NaN | 0.1006 | False |
| PM3B\_A6 | Biuret | NaN | NaN | NaN | NaN | NaN | 0.112633 | False |
| PM3B\_D2 | N-Phthaloyl-L-Glutamic Acid | NaN | NaN | NaN | NaN | NaN | 0.2174 | True |
| PM3B\_D3 | L-Pyroglutamic Acid | NaN | NaN | NaN | NaN | NaN | 0.202267 | True |
| PM3B\_D6 | N-Amylamine | NaN | NaN | NaN | NaN | NaN | 0.1637 | True |
| PM3B\_D7 | N-Butylamine | NaN | NaN | NaN | NaN | NaN | 0.151933 | True |
| PM3B\_D8 | Ethylamine | NaN | NaN | NaN | NaN | NaN | 0.159533 | True |
| PM3B\_D10 | Ethylenediamine | NaN | NaN | NaN | NaN | NaN | 0.0796333 | False |
| PM3B\_E6 | Glucuronamide | NaN | NaN | NaN | NaN | NaN | 0.2037 | True |
| PM3B\_E7 | D,L-Lactamide | NaN | NaN | NaN | NaN | NaN | 0.215433 | True |
| PM3B\_E10 | D-Mannosamine | NaN | NaN | NaN | NaN | NaN | 0.130233 | True |
| PM3B\_G6 | Parabanic Acid | NaN | NaN | NaN | NaN | NaN | 0.215033 | True |
| PM3B\_G9 | e-Amino-NCaproic Acid | NaN | NaN | NaN | NaN | NaN | 0.138967 | True |
| PM3B\_G10 | D,L-a-AminoCaprylic Acid | NaN | NaN | NaN | NaN | NaN | 0.228933 | True |
| PM3B\_G11 | d-Amino-NValeric Acid | NaN | NaN | NaN | NaN | NaN | 0.200433 | True |
| PM3B\_G12 | a-Amino-NValeric Acid | NaN | NaN | NaN | NaN | NaN | 0.314867 | True |
| PM4A\_A1 | Negative Control | NaN | NaN | NaN | NaN | NaN | 0.0675 | False |
| PM4A\_A6 | Triethyl Phosphate | NaN | NaN | NaN | NaN | NaN | 0.0659667 | False |
| PM4A\_A7 | Hypophosphite | NaN | NaN | NaN | NaN | NaN | 0.0613 | False |
| PM4A\_B1 | Thiophosphate | NaN | NaN | NaN | NaN | NaN | 0.1243 | True |
| PM4A\_B2 | Dithiophosphate | NaN | NaN | NaN | NaN | NaN | 0.3241 | True |
| PM4A\_B8 | Guanosine-2’-monophosphate | NaN | NaN | NaN | NaN | NaN | 0.201267 | True |
| PM4A\_C8 | Cytidine-2’-monophosphate | NaN | NaN | NaN | NaN | NaN | 0.302167 | True |
| PM4A\_D3 | Cysteamine S-Phosphate | NaN | NaN | NaN | NaN | NaN | 0.366467 | True |
| PM4A\_D8 | Uridine-2’-monophosphate | NaN | NaN | NaN | NaN | NaN | 0.230733 | True |
| PM4A\_D12 | Uridine-3’,5’-Cyclic monophosphate | NaN | NaN | NaN | NaN | NaN | 0.2182 | True |
| PM4A\_E1 | O-Phospho-D-Tyrosine | NaN | NaN | NaN | NaN | NaN | 0.235467 | True |
| PM4A\_E8 | Methylene Diphosphonic Acid | NaN | NaN | NaN | NaN | NaN | 0.175633 | True |
| PM4A\_E9 | Thymidine-3’-monophosphate | NaN | NaN | NaN | NaN | NaN | 0.1981 | True |
| PM4A\_E12 | Thymidine 3’,5’-Cyclic monophosphate | NaN | NaN | NaN | NaN | NaN | 0.124133 | False |
| PM4A\_F1 | Negative Control | NaN | NaN | NaN | NaN | NaN | 0.0788 | False |
| PM4A\_F5 | Thiophosphate | NaN | NaN | NaN | NaN | NaN | 0.216967 | True |
| PM4A\_F6 | Dithiophosphate | NaN | NaN | NaN | NaN | NaN | 0.200267 | True |
| PM4A\_G2 | S-Methyl-L-Cysteine | NaN | NaN | NaN | NaN | NaN | 0.155867 | True |
| PM4A\_G6 | D,L-Ethionine | NaN | NaN | NaN | NaN | NaN | 0.1063 | True |
| PM4A\_G12 | L-Methionine Sulfone | NaN | NaN | NaN | NaN | NaN | 0.174233 | True |
| PM4A\_H2 | Thiourea | NaN | NaN | NaN | NaN | NaN | 0.167867 | True |
| PM4A\_H3 | 1-Thio-b-D-Glucose | NaN | NaN | NaN | NaN | NaN | 0.1447 | True |
| PM4A\_H8 | p-Amino Benzene Sulfonic Acid | NaN | NaN | NaN | NaN | NaN | 0.0869333 | False |
| PM4A\_H12 | Tetramethylene Sulfone | NaN | NaN | NaN | NaN | NaN | 0.0790333 | False |

In [23]:

```
with pd.option_context('display.max_rows', None, 'display.max_columns', None):
    display(Biolog_in_model.query('External == False and Internal == False'))
```

|  | Biolog | Model | Exchange | Metabolite | Internal | External | Average | Pass |
| --- | --- | --- | --- | --- | --- | --- | --- | --- |
| PM1\_A4 | D-Saccharic Acid | None | EX\_glcr\_e | glcr\_e | False | False | 0.0712 | False |
| PM1\_B4 | L-Fucose | None | EX\_fuc\_\_L\_e | fuc\_\_L\_e | False | False | 0.0996667 | False |
| PM1\_B5 | D-Glucuronic Acid | None | EX\_glcur\_e | glcur\_e | False | False | 0.0718333 | False |
| PM1\_B11 | D-Mannitol | None | EX\_mnl\_e | mnl\_e | False | False | 0.120767 | True |
| PM1\_C3 | D-,L-Malic Acid | [None, L-Malate] | EX\_mal\_\_D\_e,EX\_mal\_\_L\_e | mal\_\_D\_e,mal\_\_L\_e | False | False | 0.1148 | True |
| PM1\_C6 | L-Rhamnose | None | EX\_rmn\_e | rmn\_e | False | False | 0.0937333 | False |
| PM1\_C11 | D-Melibiose | None | EX\_melib\_e | melib\_e | False | False | 0.0763 | False |
| PM1\_D9 | a-D-Lactose | None | EX\_lcts\_e | lcts\_e | False | False | 0.0810333 | False |
| PM1\_E2 | m-Tartaric Acid | None | EX\_tartr\_\_M\_e | tartr\_\_M\_e | False | False | 0.0671667 | False |
| PM1\_E7 | a-Hydroxy Butyric Acid | None | EX\_2hb\_e | 2hb\_e | False | False | 0.0992 | False |
| PM1\_E8 | b-Methyl-D-Glucoside | None | EX\_mbdg\_e | mbdg\_e | False | False | 0.2126 | True |
| PM1\_E9 | Adonitol | None | EX\_rbt\_e | rbt\_e | False | False | 0.225933 | True |
| PM1\_E11 | 2-Deoxy Adenosine | None | EX\_dad\_\_2\_e | dad\_\_2\_e | False | False | 0.0686333 | False |
| PM1\_F8 | Mucic Acid | None | EX\_galct\_\_D\_e | galct\_\_D\_e | False | False | 0.0875333 | False |
| PM1\_G2 | Tricarballylic Acid | None | EX\_tcb\_e | tcb\_e | False | False | 0.0634333 | False |
| PM1\_G8 | N-Acetyl-b-D-Mannosamine | None | EX\_acmana\_e | acmana\_e | False | False | 0.0844333 | False |
| PM1\_G9 | Mono Methyl Succinate | None | EX\_methsucc\_e | methsucc\_e | False | False | 0.200767 | True |
| PM1\_G11 | D-Malic Acid | None | EX\_mal\_\_D\_e | mal\_\_D\_e | False | False | 0.121733 | True |
| PM1\_H2 | p-Hydroxy Phenyl Acetic Acid | None | EX\_4hoxpac\_e | 4hoxpac\_e | False | False | 0.139733 | True |
| PM1\_H3 | m-Hydroxy Phenyl Acetic Acid | None | EX\_3hoxpac\_e | 3hoxpac\_e | False | False | 0.0826333 | False |
| PM1\_H9 | L-Galactonic Acid-g-Lactone | None | EX\_galctn\_\_L\_e | galctn\_\_L\_e | False | False | 0.2527 | True |
| PM2\_A6 | Dextrin | None | EX\_dextrin\_e | dextrin\_e | False | False | 0.129 | True |
| PM2\_A12 | Pectin | None | EX\_pect\_e | pect\_e | False | False | 0.159867 | True |
| PM2\_B1 | N-Acetyl-D-Galactosamine | None | EX\_acgal\_e | acgal\_e | False | False | 0.0779667 | False |
| PM2\_B2 | N-Acetyl Neuraminic Acid | None | EX\_acnam\_e | acnam\_e | False | False | 0.0724667 | False |
| PM2\_B3 | b-D-Allose | None | EX\_all\_\_D\_e | all\_\_D\_e | False | False | 0.107 | False |
| PM2\_B8 | Arbutin | None | EX\_arbt\_e | arbt\_e | False | False | 0.300467 | True |
| PM2\_D2 | Salicin | None | EX\_salcn\_e | salcn\_e | False | False | 0.3282 | True |
| PM2\_D6 | D-Tagatose | None | EX\_tag\_\_D\_e | tag\_\_D\_e | False | False | 0.1632 | True |
| PM2\_E3 | Citraconic Acid | None | EX\_citac\_e | citac\_e | False | False | 0.0714 | False |
| PM2\_E4 | Citramalic Acid | None | EX\_citm\_e | citm\_e | False | False | 0.0739 | False |
| PM2\_E11 | Itaconic Acid | None | EX\_itacon\_e | itacon\_e | False | False | 0.0718 | False |
| PM2\_F2 | Malonic Acid | None | EX\_malon\_e | malon\_e | False | False | 0.0589 | False |
| PM2\_F11 | D-Tartaric Acid | None | EX\_tartr\_\_D\_e | tartr\_\_D\_e | False | False | 0.0704 | False |
| PM2\_F12 | L-Tartaric Acid | None | EX\_tartr\_\_L\_e | tartr\_\_L\_e | False | False | 0.0758 | False |
| PM2\_H5 | D,L-Carnitine | [None, L-Carnitine] | EX\_crn\_\_D\_e,EX\_crn\_e | crn\_\_D\_e,crn\_e | False | False | 0.0643333 | False |
| PM3B\_C4 | D-Asparagine | None | EX\_asn\_\_D\_e | asn\_\_D\_e | False | False | 0.111333 | True |
| PM3B\_C6 | D-Glutamic Acid | None | EX\_glu\_\_D\_e | glu\_\_D\_e | False | False | 0.0466 | False |
| PM3B\_C7 | D-Lysine | None | EX\_lys\_\_D\_e | lys\_\_D\_e | False | False | 0.110933 | False |
| PM3B\_C9 | D-Valine | None | EX\_val\_\_D\_e | val\_\_D\_e | False | False | 0.121267 | True |
| PM3B\_D4 | Hydroxylamine | None | EX\_ham\_e | ham\_e | False | False | 0.150133 | True |
| PM3B\_E9 | D-Galactosamine | None | EX\_galam\_e | galam\_e | False | False | 0.1819 | True |
| PM3B\_E12 | N-Acetyl-D-Galactosamine | None | EX\_acgal\_e | acgal\_e | False | False | 0.0908333 | False |
| PM3B\_F1 | N-Acetyl-D-Mannosamine | None | EX\_acmana\_e | acmana\_e | False | False | 0.0963667 | False |
| PM3B\_G4 | Alloxan | None | EX\_CE0074\_e | CE0074\_e | False | False | 0.306567 | True |
| PM3B\_G7 | D,L-a-Amino-NButyric Acid | None | EX\_C02356\_e | C02356\_e | False | False | 0.157433 | True |
| PM4A\_A4 | Trimetaphosphate | None | EX\_tmp\_e | tmp\_e | False | False | 0.305667 | True |
| PM4A\_A8 | Adenosine-2’-monophosphate | None | EX\_amp2p\_e | amp2p\_e | False | False | 0.2983 | True |
| PM4A\_A9 | Adenosine-3’-monophosphate | None | EX\_3amp\_e | 3amp\_e | False | False | 0.237633 | True |
| PM4A\_A11 | Adenosine-2’,3’-Cyclic monophosphate | None | EX\_23camp\_e | 23camp\_e | False | False | 0.209733 | True |
| PM4A\_B9 | Guanosine-3’-monophosphate | None | EX\_3gmp\_e | 3gmp\_e | False | False | 0.224833 | True |
| PM4A\_B11 | Guanosine-2’,3’-Cyclic monophosphate | None | EX\_23cgmp\_e | 23cgmp\_e | False | False | 0.2335 | True |
| PM4A\_C2 | PhosphoGlycolic Acid | None | EX\_2pglyc\_e | 2pglyc\_e | False | False | 0.238667 | True |
| PM4A\_C9 | Cytidine-3’-monophosphate | None | EX\_3cmp\_e | 3cmp\_e | False | False | 0.0753 | False |
| PM4A\_C11 | Cytidine-2’,3’-Cyclic monophosphate | None | EX\_23ccmp\_e | 23ccmp\_e | False | False | 0.1973 | True |
| PM4A\_D4 | Phospho-L-Arginine | None | EX\_argp\_e | argp\_e | False | False | 0.242233 | True |
| PM4A\_D5 | O-Phospho-D-Serine | None | EX\_pser\_\_D\_e | pser\_\_D\_e | False | False | 0.251967 | True |
| PM4A\_D7 | O-Phospho-L-Threonine | None | EX\_thrp\_e | thrp\_e | False | False | 0.270133 | True |
| PM4A\_D9 | Uridine-3’-monophosphate | None | EX\_3ump\_e | 3ump\_e | False | False | 0.2121 | True |
| PM4A\_D11 | Uridine-2’,3’-Cyclic monophosphate | None | EX\_23cump\_e | 23cump\_e | False | False | 0.2253 | True |
| PM4A\_E2 | O-Phospho-L-Tyrosine | None | EX\_tyrp\_e | tyrp\_e | False | False | 0.1924 | True |
| PM4A\_E3 | Phosphocreatine | None | EX\_pcreat\_e | pcreat\_e | False | False | 0.218667 | True |
| PM4A\_E6 | Phosphono Acetic Acid | None | EX\_phnac\_e | phnac\_e | False | False | 0.062 | False |
| PM4A\_E7 | 2-Aminoethyl Phosphonic Acid | None | EX\_2ameph\_e | 2ameph\_e | False | False | 0.0604 | False |
| PM4A\_F4 | Tetrathionate | None | EX\_tet\_e | tet\_e | False | False | 0.194367 | True |
| PM4A\_F8 | D-Cysteine | None | EX\_cys\_\_D\_e | cys\_\_D\_e | False | False | 0.1216 | True |
| PM4A\_F11 | Cysteamine | None | EX\_cysam\_e | cysam\_e | False | False | 0.1867 | True |
| PM4A\_G1 | N-Acetyl-L-Cysteine | None | EX\_CE1310\_e | CE1310\_e | False | False | 0.121333 | True |
| PM4A\_G4 | Lanthionine | None | EX\_lanth\_e | lanth\_e | False | False | 0.1822 | True |
| PM4A\_G8 | D-Methionine | None | EX\_met\_\_D\_e | met\_\_D\_e | False | False | 0.2067 | True |
| PM4A\_G10 | N-Acetyl-D,L-Methionine | None | EX\_acmet\_e | acmet\_e | False | False | 0.174567 | True |
| PM4A\_H1 | L-Djenkolic Acid | None | EX\_djenk\_e | djenk\_e | False | False | 0.3439 | True |
| PM4A\_H4 | D,L-Lipoamide | None | EX\_lpam\_e | lpam\_e | False | False | 0.269967 | True |
| PM4A\_H9 | Butane Sulfonic Acid | None | EX\_butso3\_e | butso3\_e | False | False | 0.188767 | True |
| PM4A\_H10 | 2-Hydroxyethane Sulfonic Acid | None | EX\_isetac\_e | isetac\_e | False | False | 0.1779 | True |
| PM4A\_H11 | Methane Sulfonic Acid | None | EX\_mso3\_e | mso3\_e | False | False | 0.206367 | True |

In [24]:

```
with pd.option_context('display.max_rows', None, 'display.max_columns', None):
    display(Biolog_in_model.query('External == False and Internal == True'))
```

|  | Biolog | Model | Exchange | Metabolite | Internal | External | Average | Pass |
| --- | --- | --- | --- | --- | --- | --- | --- | --- |
| PM1\_A5 | Succinic Acid | None | EX\_succ\_e | succ\_e | True | False | 0.1112 | True |
| PM1\_A10 | D-Trehalose | None | EX\_tre\_e | tre\_e | True | False | 0.2213 | True |
| PM1\_A12 | Dulcitol | None | EX\_galt\_e | galt\_e | True | False | 0.132067 | True |
| PM1\_B1 | D-Serine | None | EX\_ser\_\_D\_e | ser\_\_D\_e | True | False | 0.1506 | True |
| PM1\_B2 | D-Sorbitol | None | EX\_sbt\_\_D\_e | sbt\_\_D\_e | True | False | 0.124767 | True |
| PM1\_B6 | D-Gluconic Acid | None | EX\_glcn\_e | glcn\_e | True | False | 0.143467 | True |
| PM1\_B7 | D,L-a-GlycerolPhosphate | None | EX\_glyc3p\_e | glyc3p\_e | True | False | 0.0722 | False |
| PM1\_C1 | D-Glucose-6-Phosphate | None | EX\_g6p\_e | g6p\_e | True | False | 0.0870333 | False |
| PM1\_C2 | D-Galactonic Acid-g-Lactone | None | EX\_galctn\_\_D\_e | galctn\_\_D\_e | True | False | 0.0559 | False |
| PM1\_C4 | D-Ribose | None | EX\_rib\_\_D\_e | rib\_\_D\_e | True | False | 0.3129 | True |
| PM1\_C12 | Thymidine | None | EX\_thymd\_e | thymd\_e | True | False | 0.0895 | False |
| PM1\_D2 | D-Aspartic Acid | None | EX\_asp\_\_D\_e | asp\_\_D\_e | True | False | 0.0959667 | False |
| PM1\_D4 | 1,2-Propanediol | None | EX\_12ppd\_\_S\_e | 12ppd\_\_S\_e | True | False | 0.0815 | False |
| PM1\_D6 | a-Keto-Glutaric Acid | None | EX\_akg\_e | akg\_e | True | False | 0.1034 | True |
| PM1\_E3 | D-Glucose-1-Phosphate | None | EX\_g1p\_e | g1p\_e | True | False | 0.0786333 | False |
| PM1\_E4 | D-Fructose-6-Phosphate | None | EX\_f6p\_e | f6p\_e | True | False | 0.0971333 | False |
| PM1\_E10 | Maltotriose | None | EX\_malttr\_e | malttr\_e | True | False | 0.124433 | True |
| PM1\_F2 | Citric Acid | None | EX\_cit\_e | cit\_e | True | False | 0.0876333 | False |
| PM1\_F5 | Fumaric Acid | None | EX\_fum\_e | fum\_e | True | False | 0.0933667 | False |
| PM1\_F7 | Propionic Acid | None | EX\_ppa\_e | ppa\_e | True | False | 0.1827 | True |
| PM1\_F11 | D-Cellobiose | None | EX\_cellb\_e | cellb\_e | True | False | 0.198867 | True |
| PM1\_G7 | Acetoacetic Acid | None | EX\_acac\_e | acac\_e | True | False | 0.161467 | True |
| PM1\_G12 | L-Malic Acid | None | EX\_mal\_\_L\_e | mal\_\_L\_e | True | False | 0.107933 | False |
| PM1\_H4 | Tyramine | None | EX\_tym\_e | tym\_e | True | False | 0.0702667 | False |
| PM1\_H11 | Phenylethylamine | None | EX\_peamn\_e | peamn\_e | True | False | 0.0828333 | False |
| PM1\_H12 | 2-Aminoethanol | None | EX\_etha\_e | etha\_e | True | False | 0.0752667 | False |
| PM2\_A8 | Glycogen | None | EX\_glycogen\_e | glycogen\_e | True | False | 0.0743 | False |
| PM2\_A11 | Mannan | None | EX\_mannan\_e | mannan\_e | True | False | 0.0661333 | False |
| PM2\_B5 | D-Arabinose | None | EX\_arab\_\_D\_e | arab\_\_D\_e | True | False | 0.269833 | True |
| PM2\_B9 | 2-Deoxy-D-Ribose | None | EX\_drib\_e | drib\_e | True | False | 0.2099 | True |
| PM2\_D4 | L-Sorbose | None | EX\_srb\_\_L\_e | srb\_\_L\_e | True | False | 0.0941333 | False |
| PM2\_D12 | Butyric Acid | None | EX\_but\_e | but\_e | True | False | 0.265533 | True |
| PM2\_E2 | Caproic Acid | None | EX\_hxa\_e | hxa\_e | True | False | 0.182 | True |
| PM2\_E7 | 4-Hydroxy Benzoic Acid | None | EX\_4hbz\_e | 4hbz\_e | True | False | 0.186267 | True |
| PM2\_E8 | b-Hydroxy Butyric Acid | None | EX\_bhb\_e | bhb\_e | True | False | 0.1963 | True |
| PM2\_E9 | g-Hydroxy Butyric Acid | None | EX\_ghb\_e | ghb\_e | True | False | 0.212067 | True |
| PM2\_E12 | 5-Keto-D-Gluconic Acid | None | EX\_5dglcn\_e | 5dglcn\_e | True | False | 0.219967 | True |
| PM2\_F6 | Quinic Acid | None | EX\_quin\_e | quin\_e | True | False | 0.1497 | True |
| PM2\_G1 | Acetamide | None | EX\_ad\_e | ad\_e | True | False | 0.0881333 | False |
| PM2\_G3 | N-Acetyl-L-Glutamic Acid | None | EX\_acglu\_e | acglu\_e | True | False | 0.0718 | False |
| PM2\_G7 | L-Homoserine | None | EX\_hom\_\_L\_e | hom\_\_L\_e | True | False | 0.164767 | True |
| PM2\_H9 | Dihydroxy Acetone | None | EX\_dha\_e | dha\_e | True | False | 0.3402 | True |
| PM2\_H10 | 2,3-Butanediol | None | EX\_btd\_RR\_e | btd\_RR\_e | True | False | 0.07255 | False |
| PM3B\_C5 | D-Aspartic Acid | None | EX\_asp\_\_D\_e | asp\_\_D\_e | True | False | 0.1008 | False |
| PM3B\_C8 | D-Serine | None | EX\_ser\_\_D\_e | ser\_\_D\_e | True | False | 0.1085 | False |
| PM3B\_C10 | L-Citrulline | None | EX\_citr\_\_L\_e | citr\_\_L\_e | True | False | 0.1901 | True |
| PM3B\_C11 | L-Homoserine | None | EX\_hom\_\_L\_e | hom\_\_L\_e | True | False | 0.252767 | True |
| PM3B\_D1 | N-Acetyl-L-Glutamic Acid | None | EX\_acglu\_e | acglu\_e | True | False | 0.1125 | False |
| PM3B\_D5 | Methylamine | None | EX\_mma\_e | mma\_e | True | False | 0.0878667 | False |
| PM3B\_D9 | Ethanolamine | None | EX\_etha\_e | etha\_e | True | False | 0.1826 | True |
| PM3B\_D12 | Agmatine | None | EX\_agm\_e | agm\_e | True | False | 0.1032 | False |
| PM3B\_E1 | Histamine | None | EX\_hista\_e | hista\_e | True | False | 0.0739333 | False |
| PM3B\_E2 | b-Phenylethylamine | None | EX\_peamn\_e | peamn\_e | True | False | 0.118233 | False |
| PM3B\_E3 | Tyramine | None | EX\_tym\_e | tym\_e | True | False | 0.145667 | True |
| PM3B\_E4 | Acetamide | None | EX\_ad\_e | ad\_e | True | False | 0.149867 | True |
| PM3B\_E5 | Formamide | None | EX\_frmd\_e | frmd\_e | True | False | 0.161 | True |
| PM3B\_F7 | Guanosine | None | EX\_gsn\_e | gsn\_e | True | False | 0.275767 | True |
| PM3B\_F8 | Thymine | None | EX\_thym\_e | thym\_e | True | False | 0.1536 | True |
| PM3B\_F9 | Thymidine | None | EX\_thymd\_e | thymd\_e | True | False | 0.1195 | True |
| PM3B\_G1 | Xanthine | None | EX\_xan\_e | xan\_e | True | False | 0.387333 | True |
| PM3B\_G2 | Xanthosine | None | EX\_xtsn\_e | xtsn\_e | True | False | 0.2001 | True |
| PM4A\_A3 | Pyrophosphate | None | EX\_ppi\_e | ppi\_e | True | False | 0.2997 | True |
| PM4A\_A5 | Tripolyphosphate | None | EX\_pppi\_e | pppi\_e | True | False | 0.3834 | True |
| PM4A\_A10 | Adenosine-5’-monophosphate | None | EX\_amp\_e | amp\_e | True | False | 0.220933 | True |
| PM4A\_B3 | D,L-a-Glycerol Phosphate | None | EX\_glyc3p\_e | glyc3p\_e | True | False | 0.2127 | True |
| PM4A\_B4 | b-Glycerol Phosphate | None | EX\_glyc2p\_e | glyc2p\_e | True | False | 0.2031 | True |
| PM4A\_B5 | Carbamyl Phosphate | None | EX\_cbp\_e | cbp\_e | True | False | 0.337933 | True |
| PM4A\_B6 | D-2-PhosphoGlyceric Acid | None | EX\_2pg\_e | 2pg\_e | True | False | 0.295633 | True |
| PM4A\_B7 | D-3-PhosphoGlyceric Acid | None | EX\_3pg\_e | 3pg\_e | True | False | 0.286367 | True |
| PM4A\_B10 | Guanosine-5’-monophosphate | None | EX\_gmp\_e | gmp\_e | True | False | 0.2223 | True |
| PM4A\_C1 | Phosphoenol Pyruvate | None | EX\_pep\_e | pep\_e | True | False | 0.304033 | True |
| PM4A\_C3 | D-Glucose-1-Phosphate | None | EX\_g1p\_e | g1p\_e | True | False | 0.217267 | True |
| PM4A\_C4 | D-Glucose-6-Phosphate | None | EX\_g6p\_e | g6p\_e | True | False | 0.248967 | True |
| PM4A\_C5 | 2-Deoxy-D-Glucose 6-Phosphate | None | EX\_2doxg6p\_e | 2doxg6p\_e | True | False | 0.0744 | False |
| PM4A\_C6 | D-Glucosamine-6-Phosphate | None | EX\_gam6p\_e | gam6p\_e | True | False | 0.219933 | True |
| PM4A\_C7 | 6-PhosphoGluconic Acid | None | EX\_6pgc\_e | 6pgc\_e | True | False | 0.308633 | True |
| PM4A\_C10 | Cytidine-5’-monophosphate | None | EX\_cmp\_e | cmp\_e | True | False | 0.313933 | True |
| PM4A\_C12 | Cytidine-3’,5’-Cyclic monophosphate | None | EX\_35ccmp\_e | 35ccmp\_e | True | False | 0.1961 | True |
| PM4A\_D1 | D-Mannose-1-Phosphate | None | EX\_man1p\_e | man1p\_e | True | False | 0.252833 | True |
| PM4A\_D2 | D-Mannose-6-Phosphate | None | EX\_man6p\_e | man6p\_e | True | False | 0.294767 | True |
| PM4A\_D6 | O-Phospho-L-Serine | None | EX\_pser\_\_L\_e | pser\_\_L\_e | True | False | 0.2369 | True |
| PM4A\_D10 | Uridine-5’-monophosphate | None | EX\_ump\_e | ump\_e | True | False | 0.2312 | True |
| PM4A\_E4 | Phosphoryl Choline | None | EX\_cholp\_e | cholp\_e | True | False | 0.2162 | True |
| PM4A\_E5 | O-PhosphorylEthanolamine | None | EX\_ethamp\_e | ethamp\_e | True | False | 0.2117 | True |
| PM4A\_E10 | Thymidine-5’-monophosphate | None | EX\_dtmp\_e | dtmp\_e | True | False | 0.1903 | True |
| PM4A\_E11 | Inositol Hexaphosphate | None | EX\_minohp\_e | minohp\_e | True | False | 0.288667 | True |
| PM4A\_F3 | Thiosulfate | None | EX\_tsul\_e | tsul\_e | True | False | 0.2565 | True |
| PM4A\_F10 | L-Cysteic Acid | None | EX\_Lcyst\_e | Lcyst\_e | True | False | 0.1417 | True |
| PM4A\_F12 | L-Cysteine Sulfinic Acid | None | EX\_3sala\_e | 3sala\_e | True | False | 0.1932 | True |
| PM4A\_G3 | Cystathionine | None | EX\_cyst\_\_L\_e | cyst\_\_L\_e | True | False | 0.183667 | True |
| PM4A\_G11 | L-Methionine Sulfoxide | [L-methionine-R-sulfoxide, L-Methionine Sulfox... | EX\_metsox\_R\_\_L\_e,EX\_metsox\_S\_\_L\_e | metsox\_R\_\_L\_e,metsox\_S\_\_L\_e | True | False | 0.259267 | True |
| PM4A\_H6 | Taurine | None | EX\_taur\_e | taur\_e | True | False | 0.182767 | True |
| PM4A\_H7 | Hypotaurine | None | EX\_hyptaur\_e | hyptaur\_e | True | False | 0.219867 | True |

Carbon sources

S. cer JEN1 does not transport succinate or malate, but K. lactis's does  
https://onlinelibrary.wiley.com/doi/full/10.1111/j.1574-6976.2008.00128.x  
Growth on succinate or malate is weak, do not add transport reactions  
Trehalase 8580 (cyto) and 8376 (extr), remove TREHv by 8376 and add TREHe  
Trehalose proton symporter 9137 and 13485 MALTt2 (also maltose MALTt2)  
R. toruloides did not consume much akg in IL hydrolysate and growth is weak, do not add transport  
For metabolites with unknown transporters, add diffusion if available

In [25]:

```
# Succinate
r = sce.reactions.get_by_id('EX_succ_e').copy()
r.lower_bound = 0.0
model.add_reactions([r])
# Trehalose
r1 = hsa2.reactions.get_by_id('EX_tre_e').copy()
r1.lower_bound = 0.0
r2 = hsa2.reactions.get_by_id('TREHe').copy()
r2.gene_reaction_rule = '8376'
r3 = sce.reactions.get_by_id('TREt2').copy()
r3.gene_reaction_rule = '9137 or 13485'
model.add_reactions([r1, r2, r3])
model.remove_reactions(['TREHv'], remove_orphans=True)
model.reactions.get_by_id('MALTt2').gene_reaction_rule = '9137 or 13485'
# Galactitol
r1 = hsa2.reactions.get_by_id('EX_galt_e').copy()
r1.lower_bound = 0.0
r2 = hsa2.reactions.get_by_id('GALTt').copy()
r2.gene_reaction_rule = ''
model.add_reactions([r1, r2])
# D-Serine
r1 = eco.reactions.get_by_id('EX_ser__D_e').copy()
r1.lower_bound = 0.0
r2 = hsa2.reactions.get_by_id('HMR_9191').copy()
r2.id = 'SER_Dt'
r2.name = 'D-serine transport'
r2.gene_reaction_rule = ''
model.add_reactions([r1, r2])
# D-Sorbitol
r1 = sce.reactions.get_by_id('EX_sbt__D_e').copy()
r1.lower_bound = 0.0
r2 = sce.reactions.get_by_id('SBT_Dt').copy()
r2.gene_reaction_rule = ''
model.add_reactions([r1, r2])
# D-Gluconate
r1 = hsa2.reactions.get_by_id('EX_glcn_e').copy()
r1.lower_bound = 0.0
r2 = hsa2.reactions.get_by_id('GLCNte').copy()
r2.gene_reaction_rule = ''
model.add_reactions([r1, r2])
# Glycerol 3-phosphate
r = hsa2.reactions.get_by_id('EX_glyc3p_e').copy()
r.lower_bound = 0.0
model.add_reactions([r])
# D-Glucose 6-phosphate
r = eco.reactions.get_by_id('EX_g6p_e').copy()
r.lower_bound = 0.0
model.add_reactions([r])
```

In [26]:

```
for r in model.metabolites.get_by_id('galctn__D_n').reactions:
    print(r, r.gene_reaction_rule)
```

```
GALO: gal_n + h2o_n + o2_n --> galctn__D_n + h2o2_n + h_n 16104
```

In [27]:

```
Annotation.loc[['16104','8542']]
```

Out[27]:

|  | Combined Annotations | Signal P | Sc288c Orthologs | Human Orthologs | Sc288 Best Hit | Human Blast | Essential | WolfPSort | C Terminal |
| --- | --- | --- | --- | --- | --- | --- | --- | --- | --- |
| RTO4\_ID |  |  |  |  |  |  |  |  |  |
| 16104 | HMMPfam:Glyoxal oxidase N-terminus:PF07250,HMM... | S |  |  |  |  | Essential | extr 27 | WAL\* |
| 8542 | HMMPfam:Glyoxal oxidase N-terminus:PF07250,HMM... | S |  |  |  |  | Not Essential | extr 27 | SAS\* |

In [28]:

```
# 16104 is glyoxal oxidase, not Galactose oxidase. Substrates include, methylglyoxal, glycolaldehyde,
## acetaldehyde, formaldehyde and glyoxal.
gxl_e = cobra.Metabolite(id='gxl_e', name='Glyoxal', formula='C2H2O2', charge=0, compartment='e')
m = model.metabolites.get_by_id('mthgxl_c').copy()
m.id = 'mthgxl_e'
m.compartment = 'e'
model.add_metabolites([gxl_e, m])
# Add exchange for h2o2
r1 = hsa2.reactions.get_by_id('EX_h2o2_e').copy()
r1.lower_bound = 0.0
r2 = hsa2.reactions.get_by_id('H2O2t').copy()
model.add_reactions([r1, r2])
# Add glyoxal and methylglyoxal oxidase
r1 = cobra.Reaction(id='GXLOe', name='Glyoxal oxidase, extracellular')
r1.gene_reaction_rule = '16104 or 8542'
r2 = cobra.Reaction(id='MTHGXLe', name='Methylglyoxal oxidase, extracellular')
r2.gene_reaction_rule = '16104 or 8542'
model.add_reactions([r1, r2])
r1.add_metabolites({'gxl_e': -1.0, 'o2_e': -1.0, 'h2o_e': -1.0, 'glx_e': 1.0, 'h2o2_e': 1.0, 'h_e': 1.0})
r2.add_metabolites({'mthgxl_e': -1.0, 'o2_e': -1.0, 'h2o_e': -1.0, 'pyr_e': 1.0, 'h2o2_e': 1.0, 'h_e': 1.0})
# Remove wrong reaction by 16104
model.remove_reactions(['GALO'], remove_orphans=True)
```

In [29]:

```
# D-Ribose
r1 = hsa2.reactions.get_by_id('EX_rib__D_e').copy()
r1.lower_bound = 0.0
r2 = hsa2.reactions.get_by_id('RIBt').copy()
r2.gene_reaction_rule = ''
model.add_reactions([r1, r2])
# Thymidine
r1 = hsa2.reactions.get_by_id('EX_thymd_e').copy()
r1.lower_bound = 0.0
r2 = hsa2.reactions.get_by_id('THYMDt1').copy()
r2.gene_reaction_rule = '13969'
model.add_reactions([r1, r2])
# D-Aspartic Acid
r1 = hsa2.reactions.get_by_id('EX_asp__D_e').copy()
r1.lower_bound = 0.0
r2 = hsa2.reactions.get_by_id('ASPDTDe').copy()
r2.gene_reaction_rule = ''
model.add_reactions([r1, r2])
# 1,2-Propanediol
r1 = eco.reactions.get_by_id('EX_12ppd__S_e').copy()
r1.lower_bound = 0.0
r2 = eco.reactions.get_by_id('12PPDStpp').copy()
r2.id = '12PPDSt'
r2.name = '(R)-Propane-1,2-diol facilitated transport'
r2.gene_reaction_rule = ''
model.add_reactions([r1, r2])
r2.add_metabolites({'12ppd__S_p': 1.0, '12ppd__S_e': -1.0})
# R-form needs to be secreted out
r1 = hsa2.reactions.get_by_id('EX_12ppd__R_e').copy()
r1.lower_bound = 0.0
r2 = hsa2.reactions.get_by_id('12PPDRte').copy()
r2.gene_reaction_rule = ''
model.add_reactions([r1, r2])
# 2-Oxoglutarate
r = sce.reactions.get_by_id('EX_akg_e').copy()
r.lower_bound = 0.0
model.add_reactions([r])
# D-Glucose 1-phosphate
r = hsa2.reactions.get_by_id('EX_g1p_e').copy()
r.lower_bound = 0.0
model.add_reactions([r])
# D-Fructose-6-Phosphate
r = eco.reactions.get_by_id('EX_f6p_e').copy()
r.lower_bound = 0.0
model.add_reactions([r])
# Maltotriose
r1 = hsa2.reactions.get_by_id('EX_malttr_e').copy()
r1.lower_bound = 0.0
r2 = hsa2.reactions.get_by_id('MLTG1e').copy()
r2.gene_reaction_rule = '14743'
r3 = hsa2.reactions.get_by_id('MALTe').copy()
r3.gene_reaction_rule = '14743'
model.add_reactions([r1, r2, r3])
```

10170 is mannosyl-oligosaccharide alpha-1,3-glucosidase [EC:3.2.1.207]  
cleaves off sequentially the 2 innermost α-1,3-linked glucose residues  
GlcMan9GlcNAc2-[protein] + H2O → Man9GlcNAc2-[protein] + α-D-glucopyranose
Glc2Man9GlcNAc2-[protein] + H2O → GlcMan9GlcNAc2-[protein] + α-D-glucopyranose

9135 is oligo-1,6-glucosidase [EC:3.2.1.10]  
hdrolysis of (1→6)-α-D-glucosidic linkages in some oligosaccharides  
Sucrose + H2O <=> D-Fructose + D-Glucose  
Isomaltose + H2O <=> alpha-D-Glucose + D-Glucose  
Starch + H2O <=> D-Glucose + Starch  
Dextrin + H2O <=> D-Glucose + Dextrin

12639 is glycogen debranching enzyme [EC:2.4.1.25 3.2.1.33]  
2.4.1.25 transfers a segment of a (1→4)-α-D-glucan to a new position
Amylose + n D-Glucose <=> n Maltose  
1,4-alpha-D-Glucan + n D-Glucose <=> n Maltose  
3.2.1.33 hydrolyses an unsubstituted glucose unit linked by an α(1→6) bond to an α(1→4) glucose chain
Starch + H2O <=> Amylose + alpha-D-Glucose  
Starch + H2O <=> 1,4-alpha-D-Glucan + alpha-D-Glucose

14743 is glucoamylase (glucan 1,4-α-glucosidase) [EC:3.2.1.3]  
hydrolysis of terminal (1→4)-linked α-D-glucose residues  
Starch + H2O <=> D-Glucose + Starch  
Dextrin + H2O <=> D-Glucose + Dextrin

11993 is alpha-amylase (1,4-α-D-glucan glucanohydrolase) [EC:3.2.1.1]  
catalyzes the endohydrolysis of (1→4)-α-D-glucosidic linkages in polysaccharides  
Starch + H2O <=> Dextrin + Starch  
Starch <=> Dextrin + Maltose  
Starch(n+m) + H2O <=> Starch(n) + Starch(m)  
Maltodextrin + H2O <=> Maltose

12594 is 1,4-alpha-glucan branching enzyme [EC:2.4.1.18]  
transfers a segment of a 1,4-α-D-glucan chain to a primary hydroxyl group in a similar glucan chain, forming a 1,6 branch point.
Amylose <=> Starch  
1,4-alpha-D-Glucan <=> Starch

13553 is glucan 1,3-beta-glucosidase [EC:3.2.1.58]  
1,3-beta-D-Glucan(n+1) + H2O <=> D-Glucose + 1,3-beta-D-Glucan(n)

In [30]:

```
temp = ['10170','9135','14743','12639','11993','12594','13553','14256']
Annotation.loc[temp]
```

Out[30]:

|  | Combined Annotations | Signal P | Sc288c Orthologs | Human Orthologs | Sc288 Best Hit | Human Blast | Essential | WolfPSort | C Terminal |
| --- | --- | --- | --- | --- | --- | --- | --- | --- | --- |
| RTO4\_ID |  |  |  |  |  |  |  |  |  |
| 10170 | K05546: GANAB; alpha 1,3-glucosidase | S | ROT2 | GANAB,GANC | ROT2 | GANAB | Not Essential | mito 17, extr 5, cyto 3 | DEL\* |
| 9135 | K01182: IMA, malL; oligo-1,6-glucosidase |  | MAL32,IMA1,MAL12,YIL172C,IMA5,IMA4,IMA2 | SLC3A1 | IMA1 | SLC3A | Not Essential | cyto 11.5, cyto\_nucl 10, pero 8, nucl 5.5 | FVF\* |
| 14743 | K01178: E3.2.1.3; glucoamylase |  | SGA1 |  | SGA1 |  | Not Essential | mito 22, cyto 4 | GRE\* |
| 12639 | K01196: AGL; glycogen debranching enzyme | S | GDB1 | AC096949.1,AGL | GDB1 | AGL | Not Essential | cyto\_mito 9.666, cyto 9.5, mito 8.5, cyto\_nucl... | ARN\* |
| 11993 | K01176: AMY, amyA, malS; alpha-amylase |  |  |  |  |  | Not Essential | cyto 10.5, nucl 9, cyto\_mito 8, mito 4.5 | GKK\* |
| 12594 | K00700: GBE1, glgB; 1,4-alpha-glucan branching... |  | GLC3 | GBE1 | GLC3 | GBE1 | Not Essential | cyto 13, cyto\_nucl 10.333, cyto\_pero 9.333, nu... | LAH\* |
| 13553 | K01210: E3.2.1.58; glucan 1,3-beta-glucosidase | A |  |  | EXG1 |  | Essential | extr 19, plas 4, mito 1, E.R. 1, golg 1, vacu 1 | GRQ\* |
| 14256 | K00688: PYG, glgP; glycogen phosphorylase |  | GPH1 | PYGB,PYGL,PYGM | GPH1 | PYGL | Not Essential | cyto 12.5, cyto\_nucl 10.5, nucl 7.5, pero 6 | QDA\* |

In [31]:

```
for x in temp:
    for r in model.genes.get_by_id(x).reactions:
        print(r.id, r.reaction, r.gene_reaction_rule)
    print()
```

```
XYHDL h2o_c + iprimv_c --> glc__D_c + xyl__D_c 10170
MG3A asnglcnacglcnacman_man_manman_manman_manmanmanglc_c + h2o_c --> Glc_aD_c + asnglcnacglcnacman_man_manman_manman_manmanman_c 10170
MG2A asnglcnacglcnacman_man_manman_manman_manmanmanglcglc_c + h2o_c --> Glc_aD_c + asnglcnacglcnacman_man_manman_manman_manmanmanglc_c 10170
SQGH h2o_c + sqg_c --> glyc_c + sq_c 10170
MG3B asnglcnacglcnacman_man_manman_manman_manmanmanglc_c + h2o_c --> asnglcnacglcnacman_man_manman_manman_manmanman_c + glc__D_c 10170
MLTG1 h2o_c + malttr_c --> glc__D_c + malt_c 10170
MG2B asnglcnacglcnacman_man_manman_manman_manmanmanglcglc_c + h2o_c --> asnglcnacglcnacman_man_manman_manman_manmanmanglc_c + glc__D_c 10170
MALT h2o_c + malt_c --> 2.0 glc__D_c 10170 or 9135

AMY1e 8.0 h2o_e + strch1_e --> 8.0 glc__D_e + strch2_e 9135
AMY2e glygn2_e + 8.0 h2o_e --> 8.0 glc__D_e + glygn4_e 9135
DGGH 6dg_c + h2o_c --> gal_c + glc__D_c 9135
TRE6PH h2o_c + tre6p_c --> g6p_c + glc__D_c 9135
MALT h2o_c + malt_c --> 2.0 glc__D_c 10170 or 9135

MALTe h2o_e + malt_e --> 2.0 glc__D_e 14743
MLTG1e h2o_e + malttr_e --> glc__D_e + malt_e 14743
GLCGSDv glycogen_v + h2o_v --> glc__D_v 14743

GLDBRAN dxtrn_c + h2o_c --> glc__D_c + glygn3_c 12639
GLCGSD glycogen_c + h2o_c --> glc__D_c 12639

AAMYL 14glucan_c --> malthx_c 11993

GLBRAN2 glycogen_c --> bglycogen_c 12594
GBEZ 14glun_c --> glycogen_c + h2o_c 12594
GLBRAN glygn1_c --> glygn2_c 12594

13BGHe 13BDglcn_e + h2o_e --> glc__D_e 13553

GLCP2 bglycogen_c + pi_c --> g1p_c 14256
MLTP1 maltpt_c + pi_c <=> g1p_c + maltttr_c 14256
MLTP2 malthx_c + pi_c <=> g1p_c + maltpt_c 14256
GLPASE2 glygn3_c + 7.0 h2o_c --> Tyr_ggn_c + 7.0 glc__D_c 14256
GLCP glycogen_c + pi_c --> g1p_c 14256
MLTP3 malthp_c + pi_c <=> g1p_c + malthx_c 14256
GLPASE1 glygn2_c + 3.0 pi_c --> dxtrn_c + 3.0 g1p_c 14256
```

In [32]:

```
# Not found in R. toruloides, only in iML1515
model.remove_reactions(['XYHDL','SQGH'], remove_orphans=True)
# Maltose breakdown by α-1,4-glucosidase
model.reactions.get_by_id('MLTG1').gene_reaction_rule = '14743'
model.reactions.get_by_id('MALT').gene_reaction_rule = '14743'
model.remove_reactions(['GLCGSDv'], remove_orphans=True)
# 6dg_c is melibiose replace DGGH with GALS3 by 9135
r1 = sce.reactions.get_by_id('EX_melib_e').copy()
r1.lower_bound = 0.0
r2 = sce.reactions.get_by_id('MELIBt2').copy()
r2.gene_reaction_rule = ''
r3 = sce.reactions.get_by_id('GALS3').copy()
r3.gene_reaction_rule = '9135'
model.add_reactions([r1, r2, r3])
model.remove_reactions(['DGGH'], remove_orphans=True)
# Glycogen breakdown by 1,4 or 1,6
model.reactions.get_by_id('GLCGSD').gene_reaction_rule = '12639 or 14743'
```

Glycogen metabolism  
In S. cerevisiae  
synthesis GLYGS h2o\_c + udpg\_c --> 14glun\_c + h\_c + udp\_c 14002 and 9596  
synthesis GLCS2 udpg\_c → glycogen\_c + h\_c + udp\_c 14002 and 9596  
branching GBEZ 14glun\_c → glycogen\_c + h2o\_c 12594  
phosphorylase GLCP glycogen\_c + pi\_c → g1p\_c 14256  
breakdown GLCGSD glycogen\_c + h2o\_c → glc**D\_c 12639  
In E. coli  
synthesis GLCS1 adpglc\_c → adp\_c + glycogen\_c + h\_c #absent  
branching GLBRAN2 glycogen\_c → bglycogen\_c 12594  
debranching GLDBRAN2 bglycogen\_c → glycogen\_c #absent  
phosphorylase GLCP glycogen\_c + pi\_c → g1p\_c 14256  
phosphorylase GLCP2 bglycogen\_c + pi\_c → g1p\_c 14256  
In Human  
synthesis GGNG Tyr\_ggn\_c + 8.0 udpg\_c --> ggn\_c + 8.0 h\_c + 8.0 udp\_c 14002 and 9596  
synthesis GLGNS1 ggn\_c + 3.0 udpg\_c --> glygn1\_c + 3.0 h\_c + 3.0 udp\_c 14002 and 9596  
branching GLBRAN glygn1\_c --> glygn2\_c 12594  
debranching GLDBRAN dxtrn\_c + h2o\_c --> glc**D\_c + glygn3\_c 12639  
breakdown GLPASE1 glygn2\_c + 3.0 pi\_c --> dxtrn\_c + 3.0 g1p\_c 14256  
breakdown GLPASE2 glygn3\_c + 7.0 h2o\_c --> Tyr\_ggn\_c + 7.0 glc\_\_D\_c 14256  
Discrepancies between models
14glun\_c is unbranched glycogen in S. cer (glycogen\_c in E. coli, glygn1\_c in Human)  
glycogen\_c is branched glycogen in S. cer (bglycogen\_c in E. coli, glygn2\_c in Human)  
14glucan\_c is 1,4 glucan of 6 glucose units in E. coli, replace with 14glun

In [33]:

```
model.remove_reactions(['GLBRAN2','GLCP2','GGNG','GLGNS1','GLBRAN','GLDBRAN','GLPASE1','GLPASE2'], remove_orphans=True)
# update AAMYL replacing 14glucan_c with 14glun_c
r = model.reactions.get_by_id('AAMYL')
r.id = 'AAMYLASE'
r.add_metabolites({'14glucan_c': 1.0, '14glun_c': -6.0, 'h2o_c': 5.0})
# 14glun is break down to malthx_c, starting from 6 unit add maltase reactions, remove 7 unit reactions
model.remove_reactions(['MLTP3'], remove_orphans=True)
for x in ['MLTG4','MLTG3','MLTG2']:
    r = eco.reactions.get_by_id(x).copy()
    r.gene_reaction_rule = '14743'
    model.add_reactions([r])
```

In [34]:

```
# Citrate
r = sce.reactions.get_by_id('EX_cit_e').copy()
r.lower_bound = 0.0
model.add_reactions([r])
# Fumarate
r = sce.reactions.get_by_id('EX_fum_e').copy()
r.lower_bound = 0.0
model.add_reactions([r])
# Propionate
r1 = hsa2.reactions.get_by_id('EX_ppa_e').copy()
r1.lower_bound = 0.0
r2 = hsa2.reactions.get_by_id('PPAtr').copy()
r2.gene_reaction_rule = ''
model.add_reactions([r1, r2])
# Cellobiose
# Add extracellular beta-glucosidase reaction for cellobiose, BG_CELLB exists in iYO844
# 16716 and 16717 are annotated as extracellular beta-glucosidase
r1 = eco.reactions.get_by_id('EX_cellb_e').copy()
r1.lower_bound = 0.0
r2 = model.reactions.get_by_id('BGLA').copy()
r2.id = 'BG_CELLB'
r2.name = 'Beta glucosidase cellobiose'
r2.gene_reaction_rule = '16716 or 16717'
model.add_reactions([r1, r2])
for m, v in r2.metabolites.items():
    if m.id.replace('_c','_e') not in model.metabolites:
        m2 = m.copy()
        m2.id = m.id.replace('_c','_e')
        m2.compartment = 'e'
        model.add_metabolites([m2])
    r2.add_metabolites({m.id: -v, m.id.replace('_c','_e'): v})
# Acetoacetate
r1 = hsa2.reactions.get_by_id('EX_acac_e').copy()
r1.lower_bound = 0.0
r2 = hsa2.reactions.get_by_id('ACACt2').copy()
r2.gene_reaction_rule = '11257' #SLC16A7
model.add_reactions([r1, r2])
r1 = hsa2.reactions.get_by_id('ACACt2m').copy()
r1.gene_reaction_rule = '15773 and 16581' #MPC
r2 = hsa2.reactions.get_by_id('ACACtx').copy()
r2.gene_reaction_rule = '' #diffusion
model.add_reactions([r1, r2])
# L-Malate
r = sce.reactions.get_by_id('EX_mal__L_e').copy()
r.lower_bound = 0.0
model.add_reactions([r])
# Tyramine
r1 = hsa2.reactions.get_by_id('EX_tym_e').copy()
r1.lower_bound = 0.0
r2 = hsa2.reactions.get_by_id('TYMte').copy()
r2.gene_reaction_rule = ''
model.add_reactions([r1, r2])
# Phenethylamine
r = eco.reactions.get_by_id('EX_peamn_e').copy()
r.lower_bound = 0.0
model.add_reactions([r])
# Ethanolamine
r1 = hsa2.reactions.get_by_id('EX_etha_e').copy()
r1.lower_bound = 0.0
r2 = hsa2.reactions.get_by_id('ETHAt').copy()
r2.gene_reaction_rule = ''
model.add_reactions([r1, r2])
```

In [35]:

```
# Glycogen
m = model.metabolites.get_by_id('glycogen_c').copy()
m.id = 'glycogen_e'
m.compartment = 'e'
model.add_metabolites([m])
r = model.reactions.get_by_id('EX_h_e').copy()
r.id = 'EX_glycogen_e'
r.name = 'Glycogen exchange'
r.gene_reaction_rule = ''
r.lower_bound = 0.0
model.add_reactions([r])
r.add_metabolites({'h_e': 1.0, 'glycogen_e': -1.0})
# Mannan
m = model.metabolites.get_by_id('mannan_r').copy()
m.id = 'mannan_e'
m.compartment = 'e'
model.add_metabolites([m])
r = model.reactions.get_by_id('EX_h_e').copy()
r.id = 'EX_mannan_e'
r.name = 'Mannan exchange'
r.gene_reaction_rule = ''
r.lower_bound = 0.0
model.add_reactions([r])
r.add_metabolites({'h_e': 1.0, 'mannan_e': -1.0})
# D-arabinose
r1 = sce.reactions.get_by_id('EX_arab__D_e').copy()
r1.lower_bound = 0.0
r2 = sce.reactions.get_by_id('ARAB_Dt').copy()
r2.gene_reaction_rule = ''
model.add_reactions([r1, r2])
# D-arabinose can also be converted to D-arabitol by XYL1
# https://www.tandfonline.com/doi/full/10.1080/09168451.2016.1204221
r = model.reactions.get_by_id('ARABR').copy()
r.id = 'DARABR'
r.name = 'D-Arabinose reductase'
r.gene_reaction_rule = '9774'
model.add_reactions([r])
r.add_metabolites({'arab__L_c': 1.0, 'abt_c': -1.0, 'arab__D_c': -1.0, 'abt__D_c': 1.0})
# Deoxyribose
r1 = hsa2.reactions.get_by_id('EX_drib_e').copy()
r1.lower_bound = 0.0
r2 = hsa2.reactions.get_by_id('DRIBt').copy()
r2.gene_reaction_rule = ''
model.add_reactions([r1, r2])
# L-Sorbose
r1 = sce.reactions.get_by_id('EX_srb__L_e').copy()
r1.lower_bound = 0.0
r2 = sce.reactions.get_by_id('SRB_Lt').copy()
r2.gene_reaction_rule = ''
model.add_reactions([r1, r2])
# Butyrate
r1 = hsa2.reactions.get_by_id('EX_but_e').copy()
r1.lower_bound = 0.0
r2 = hsa2.reactions.get_by_id('BUTt').copy()
r2.gene_reaction_rule = ''
model.add_reactions([r1, r2])
# Hexanoate
r1 = hsa2.reactions.get_by_id('EX_hxa_e').copy()
r1.lower_bound = 0.0
r2 = hsa2.reactions.get_by_id('HXAt3').copy()
r2.gene_reaction_rule = ''
model.add_reactions([r1, r2])
```

In [36]:

```
# Add degradation pathways for C4 and C6 fatty acids
# Fatty acid CoA ligase
r1 = hsa2.reactions.get_by_id('HMR_0156').copy()
r1.id = 'FACOAL40'
r1.name = 'Fatty-acid--CoA ligase (butanoate)'
r1.gene_reaction_rule = '11167 or 15746 or 15748'
r2 = model.reactions.get_by_id('FACOAL80').copy()
r2.id = 'FACOAL60'
r2.name = 'Fatty-acid--CoA ligase (hexanoate)'
r2.gene_reaction_rule = '11167 or 15746 or 15748'
model.add_reactions([r1, r2])
r2.add_metabolites({'octa_c': 1.0, 'occoa_c': -1.0, 'hxa_c': -1.0, 'hxcoa_c': 1.0})
# CRN O-acetyltransferase in cyto
r1 = hsa2.reactions.get_by_id('C40CPT1').copy()
r1.lower_bound = 0.0
r1.gene_reaction_rule = '9315'
r2 = hsa2.reactions.get_by_id('C60CPT1').copy()
r2.lower_bound = 0.0
r2.gene_reaction_rule = '9315'
model.add_reactions([r1, r2])
# CRN transport mitochondrial
r1 = model.reactions.get_by_id('C160CRNt').copy()
r1.id = 'C40CRNt'
r1.name = 'C40 transport into the mitochondria'
r1.gene_reaction_rule = '9331'
r2 = model.reactions.get_by_id('C160CRNt').copy()
r2.id = 'C60CRNt'
r2.name = 'C60 transport into the mitochondria'
r2.gene_reaction_rule = '9331'
model.add_reactions([r1, r2])
m1 = hsa2.metabolites.get_by_id('c4crn_m').copy()
m2 = hsa2.metabolites.get_by_id('c6crn_c').copy()
m2.id = 'c6crn_m'
m2.compartment = 'm'
model.add_metabolites([m1, m2])
r1.add_metabolites({'pmtcrn_c': 1.0, 'pmtcrn_m': -1.0, 'c4crn_c': -1.0, 'c4crn_m': 1.0})
r2.add_metabolites({'pmtcrn_c': 1.0, 'pmtcrn_m': -1.0, 'c6crn_c': -1.0, 'c6crn_m': 1.0})
# CRN O-acetyltransferase in mito
r1 = model.reactions.get_by_id('C160CPT2').copy()
r1.id = 'C40CPT2'
r1.name = 'C40 transport into the mitochondria'
r1.gene_reaction_rule = '14245'
r2 = model.reactions.get_by_id('C160CPT2').copy()
r2.id = 'C60CPT2'
r2.name = 'C60 transport into the mitochondria'
r2.gene_reaction_rule = '14245'
model.add_reactions([r1, r2])
r1.add_metabolites({'pmtcrn_m': 1.0, 'pmtcoa_m': -1.0, 'c4crn_m': -1.0, 'btcoa_m': 1.0})
r2.add_metabolites({'pmtcrn_m': 1.0, 'pmtcoa_m': -1.0, 'c6crn_m': -1.0, 'hxcoa_m': 1.0})
# CRN transport peroxisomal
model.reactions.get_by_id('CRNtp').gene_reaction_rule = '10149'
```

In [37]:

```
# 4-hydroxybenzoate
r1 = hsa2.reactions.get_by_id('EX_4hbz_e').copy()
r1.lower_bound = 0.0
r2 = hsa2.reactions.get_by_id('4HBZte').copy()
r2.lower_bound = -1000.0
r2.gene_reaction_rule = ''
model.add_reactions([r1, r2])
# beta-hydroxybutyrate
r1 = hsa2.reactions.get_by_id('EX_bhb_e').copy()
r1.name = '(R)-3-Hydroxybutanoate exchange'
r1.lower_bound = 0.0
r2 = hsa2.reactions.get_by_id('BHBt').copy()
r2.gene_reaction_rule = ''
r3 = hsa2.reactions.get_by_id('BHBtm').copy()
r3.gene_reaction_rule = ''
model.add_reactions([r1, r2, r3])
# gamma-hydroxybutyrate
r1 = hsa2.reactions.get_by_id('EX_bhb_e').copy()
r1.id = 'EX_ghb_e'
r1.name = 'Gamma-hydroxybutyrate exchange'
r1.lower_bound = 0.0
r2 = hsa2.reactions.get_by_id('BHBt').copy()
r2.id = 'GHBt'
r2.name = 'Gamma-hydroxybutyrate transport via H+ symport'
r2.gene_reaction_rule = ''
r3 = hsa2.reactions.get_by_id('BHBtm').copy()
r3.id = 'GHBtm'
r3.name = 'Gamma-hydroxybutyrate mitochondrial transport via H+ symport'
r3.gene_reaction_rule = ''
r4 = hsa2.reactions.get_by_id('4ABUTtm').copy()
r4.gene_reaction_rule = ''
model.add_reactions([r1, r2, r3, r4])
m1 = model.metabolites.get_by_id('ghb_m').copy()
m1.id = 'ghb_e'
m1.compartment = 'e'
m2 = model.metabolites.get_by_id('ghb_m').copy()
m2.id = 'ghb_c'
m2.compartment = 'c'
model.add_metabolites([m1, m2])
r1.add_metabolites({'bhb_e': 1.0, 'ghb_e': -1.0})
r2.add_metabolites({'bhb_e': 1.0, 'bhb_c': -1.0, 'ghb_e': -1.0, 'ghb_c': 1.0})
r3.add_metabolites({'bhb_c': 1.0, 'bhb_m': -1.0, 'ghb_c': -1.0, 'ghb_m': 1.0})
# 5-ketogluconate
r1 = eco.reactions.get_by_id('EX_5dglcn_e').copy()
r1.lower_bound = 0.0
r2 = model.reactions.get_by_id('CO2t').copy()
r2.id = '5DGLCNt'
r2.name = '5-ketogluconate transport via diffusion'
r2.gene_reaction_rule = ''
model.add_reactions([r1, r2])
r2.add_metabolites({'co2_e': 1.0, 'co2_c': -1.0, '5dglcn_e': -1.0, '5dglcn_c': 1.0})
# Quinic Acid
r1 = eco.reactions.get_by_id('EX_quin_e').copy()
r1.lower_bound = 0.0
r2 = eco.reactions.get_by_id('QUIN2tex').copy()
r2.id = 'QUINt'
r2.name = 'Quinate transport via diffusion'
r2.gene_reaction_rule = ''
model.add_reactions([r1, r2])
r2.add_metabolites({'quin_p': -1.0, 'quin_c': 1.0})
```

In [38]:

```
# Acetamide
m = model.metabolites.get_by_id('ad_c').copy()
m.id = 'ad_e'
m.compartment = 'e'
model.add_metabolites([m])
r = model.reactions.get_by_id('EX_h_e').copy()
r.id = 'EX_ad_e'
r.name = 'Acetamide exchange'
r.gene_reaction_rule = ''
r.lower_bound = 0.0
model.add_reactions([r])
r.add_metabolites({'h_e': 1.0, 'ad_e': -1.0})
r = model.reactions.get_by_id('CO2t').copy()
r.id = 'ADtr'
r.name = 'Acetamide transport, extracellular'
r.gene_reaction_rule = ''
model.add_reactions([r])
r.add_metabolites({'co2_e': 1.0, 'co2_c': -1.0, 'ad_e': -1.0, 'ad_c': 1.0})
# N-Acetyl-L-Glutamic Acid
r1 = hsa2.reactions.get_by_id('EX_acglu_e').copy()
r1.lower_bound = 0.0
r2 = hsa2.reactions.get_by_id('ACGLUtd').copy()
r2.lower_bound = -1000.0
r2.gene_reaction_rule = ''
model.add_reactions([r1, r2])
# L-Homoserine
r1 = hsa2.reactions.get_by_id('EX_hom__L_e').copy()
r1.lower_bound = 0.0
r2 = hsa2.reactions.get_by_id('r2535').copy()
r2.id = 'HOMt'
r2.name = 'L-Homoserine transport via diffusion'
r2.gene_reaction_rule = ''
model.add_reactions([r1, r2])
# Dihydroxyacetone
r1 = eco.reactions.get_by_id('EX_dha_e').copy()
r1.lower_bound = 0.0
r2 = eco.reactions.get_by_id('DHAtex').copy()
r2.id = 'DHAt'
r2.name = 'Dihydroxyacetone transport via diffusion'
r2.gene_reaction_rule = ''
model.add_reactions([r1, r2])
r2.add_metabolites({'dha_p': -1.0, 'dha_c': 1.0})
# 2,3-Butanediol
r1 = sce.reactions.get_by_id('EX_btd_RR_e').copy()
r1.lower_bound = 0.0
r2 = sce.reactions.get_by_id('BTDt_RR').copy()
r2.lower_bound = -1000.0
r2.gene_reaction_rule = ''
model.add_reactions([r1, r2])
```

Nitrogen, Phosphate, and Sulfur sources

In [39]:

```
# L-Citrulline
r1 = hsa2.reactions.get_by_id('EX_citr__L_e').copy()
r1.lower_bound = 0.0
r2 = hsa2.reactions.get_by_id('r0817').copy()
r2.id = 'CITRte'
r2.name = 'L-Citrulline transport via diffusion'
r2.gene_reaction_rule = ''
model.add_reactions([r1, r2])
# Methylamine
r1 = hsa2.reactions.get_by_id('EX_mma_e').copy()
r1.lower_bound = 0.0
r2 = hsa2.reactions.get_by_id('MMAt2e').copy()
r2.gene_reaction_rule = ''
model.add_reactions([r1, r2])
# Agmatine
r1 = hsa2.reactions.get_by_id('EX_agm_e').copy()
r1.lower_bound = 0.0
r2 = hsa2.reactions.get_by_id('AGRMte').copy()
r2.gene_reaction_rule = ''
r3 = hsa2.reactions.get_by_id('AGMt_m').copy()
r3.gene_reaction_rule = ''
model.add_reactions([r1, r2, r3])
# Histamine
r1 = hsa2.reactions.get_by_id('EX_hista_e').copy()
r1.lower_bound = 0.0
r2 = hsa2.reactions.get_by_id('HISTAtu').copy()
r2.gene_reaction_rule = ''
model.add_reactions([r1, r2])
# Formamide
m = model.metabolites.get_by_id('frmd_c').copy()
m.id = 'frmd_e'
m.compartment = 'e'
model.add_metabolites([m])
r = model.reactions.get_by_id('EX_h_e').copy()
r.id = 'EX_frmd_e'
r.name = 'Formamide exchange'
r.gene_reaction_rule = ''
r.lower_bound = 0.0
model.add_reactions([r])
r.add_metabolites({'h_e': 1.0, 'frmd_e': -1.0})
r = model.reactions.get_by_id('CO2t').copy()
r.id = 'FRMDt'
r.name = 'Formamide transport via diffusion'
r.gene_reaction_rule = ''
model.add_reactions([r])
r.add_metabolites({'co2_e': 1.0, 'co2_c': -1.0, 'frmd_e': -1.0, 'frmd_c': 1.0})
```

In [40]:

```
# L-Guanosine
r1 = hsa2.reactions.get_by_id('EX_gsn_e').copy()
r1.lower_bound = 0.0
r2 = hsa2.reactions.get_by_id('GSNt').copy()
r2.gene_reaction_rule = ''
model.add_reactions([r1, r2])
# Thymine
r1 = hsa2.reactions.get_by_id('EX_thym_e').copy()
r1.lower_bound = 0.0
r2 = hsa2.reactions.get_by_id('THYMt').copy()
r2.gene_reaction_rule = ''
model.add_reactions([r1, r2])
# Xanthine
r1 = hsa2.reactions.get_by_id('EX_xan_e').copy()
r1.lower_bound = 0.0
r2 = hsa2.reactions.get_by_id('XANt').copy()
r2.gene_reaction_rule = ''
model.add_reactions([r1, r2])
# Xanthosine
r1 = hsa2.reactions.get_by_id('EX_xtsn_e').copy()
r1.lower_bound = 0.0
r2 = hsa2.reactions.get_by_id('XTSNtr').copy()
r2.gene_reaction_rule = ''
model.add_reactions([r1, r2])
# Pyrophosphate
r1 = hsa2.reactions.get_by_id('EX_ppi_e').copy()
r1.lower_bound = 0.0
r2 = model.reactions.get_by_id('PIt2r').copy()
r2.id = 'PPIt2r'
r2.name = 'Diphosphate reversible transport via symport'
r2.gene_reaction_rule = ''
model.add_reactions([r1, r2])
r2.add_metabolites({'pi_e': 1.0, 'pi_c': -1.0, 'ppi_e': -1.0, 'ppi_c': 1.0})
# Tripolyphosphate
m = model.metabolites.get_by_id('pppi_c').copy()
m.id = 'pppi_e'
m.compartment = 'e'
model.add_metabolites([m])
r1 = hsa2.reactions.get_by_id('EX_ppi_e').copy()
r1.id = 'EX_pppi_e'
r1.name = 'Triphosphate exchange'
r1.lower_bound = 0.0
r2 = model.reactions.get_by_id('PIt2r').copy()
r2.id = 'PPPIt2r'
r2.name = 'Triphosphate reversible transport via symport'
r2.gene_reaction_rule = ''
model.add_reactions([r1, r2])
r1.add_metabolites({'ppi_e': 1.0, 'pppi_e': -1.0})
r2.add_metabolites({'pi_e': 1.0, 'pi_c': -1.0, 'pppi_e': -1.0, 'pppi_c': 1.0})
```

Multiple alkaline phosphatases in cyto (13409, 11597) and extr (15429)  
Two purple acid phosphatase in cyto (9345) and extr (9531)
PAP12 functions as a phosphate scavenger for plants during instances of nutritional phosphate deprivation.  
PAP12 is able to act on various substrates, with the highest level of activity attained using PEP (treated as 100% activity). Lower levels of activity were seen using phosphotyrosine (60%), D-glucose-6-phosphate (40%), D-ribose 5-phosphate (9%), phosphothreonine (29%),(19%), ATP (81%), ADP (68%), GTP (45%), dAMP (12%), and AMP (9%). It did not show activity against phytic acid or phosphocholine, and β-D-fructofuranose 6-phosphate.

In [41]:

```
temp = ['13409','11597','14013','15429','9345','9531','16648','9995']
Annotation.loc[temp]
```

Out[41]:

|  | Combined Annotations | Signal P | Sc288c Orthologs | Human Orthologs | Sc288 Best Hit | Human Blast | Essential | WolfPSort | C Terminal |
| --- | --- | --- | --- | --- | --- | --- | --- | --- | --- |
| RTO4\_ID |  |  |  |  |  |  |  |  |  |
| 13409 | K01077: E3.1.3.1, phoA, phoB; alkaline phospha... |  | PHO8 | ALPI,ALPL,ALPP,ALPPL2 | PHO8 | ALPL | Not Essential | plas 14, nucl 4.5, cyto\_nucl 4.5, cyto 3.5, mi... | GDF\* |
| 11597 | K01113: phoD; alkaline phosphatase D |  |  |  |  |  | Not Essential | cyto 12, cyto\_mito 8.833, cyto\_nucl 7.333, ext... | WFS\* |
| 14013 | BLAST: putative alkaline phosphatase [Phaeomon... | S |  |  |  |  | Not Essential | mito 9.5, nucl 9, cyto\_mito 9, cyto 7.5 | DYL\* |
| 15429 | K01077: E3.1.3.1, phoA, phoB; alkaline phospha... | S |  |  | PHO8 | ALPPL2 | Not Essential | extr 17, mito 6, cyto 2, vacu 2 | PRH\* |
| 9345 | KOG1378: Purple acid phosphatase |  |  | ACP7 |  | ACP7 | Not Essential | cyto\_nucl 11, nucl 9.5, cyto 9.5, extr 3, pero 3 | LHF\* |
| 9531 | KOG1378: Purple acid phosphatase | S |  |  |  |  | Not Essential | extr 27 | SQN\* |
| 16648 | KOG2157: Predicted tubulin-tyrosine ligase |  |  |  | PBY1 |  | Not Essential | mito 17, cyto 5, nucl 4 | WKI\* |
| 9995 | K18551: SDT1; pyrimidine and pyridine-specific... |  | SDT1,PHM8 |  | SDT1 |  | Not Essential | mito 17, nucl 5, pero 3 | GSS\* |

In [42]:

```
for x in temp:
    if x in model.genes:
        for r in sorted(model.genes.get_by_id(x).reactions, key=lambda x: x.id):
            print(r, r.gene_reaction_rule)
    else:
        print(x, 'no reactions')
    print()
```

```
ALKP: dhap_c + h2o_c --> dha_c + pi_c 13409
NMNHYD: h2o_c + nmn_c --> pi_c + rnam_c 13409

11597 no reactions

14013 no reactions

15429 no reactions

9345 no reactions

9531 no reactions

NTD1: dump_c + h2o_c --> duri_c + pi_c 16648
NTD10: h2o_c + xmp_c --> pi_c + xtsn_c 16648
NTD11: h2o_c + imp_c --> ins_c + pi_c 16648
NTD12: dimp_c + h2o_c --> din_c + pi_c 16648
NTD2: h2o_c + ump_c --> pi_c + uri_c 16648 or 9995
NTD3: dcmp_c + h2o_c --> dcyt_c + pi_c 16648
NTD4: cmp_c + h2o_c --> cytd_c + pi_c 16648 or 9995
NTD5: dtmp_c + h2o_c --> pi_c + thymd_c 16648
NTD6: damp_c + h2o_c --> dad_2_c + pi_c 16648
NTD7: amp_c + h2o_c --> adn_c + pi_c 16648
NTD8: dgmp_c + h2o_c --> dgsn_c + pi_c 16648
NTD9: gmp_c + h2o_c --> gsn_c + pi_c 16648
PPA: h2o_c + ppi_c --> h_c + 2.0 pi_c 15879 or 16648
PPA2: h2o_c + pppi_c --> h_c + pi_c + ppi_c 16648

NTD2: h2o_c + ump_c --> pi_c + uri_c 16648 or 9995
NTD4: cmp_c + h2o_c --> cytd_c + pi_c 16648 or 9995
```

Acid phosphatases  
12870 hypothetical protein or putative histidine acid phosphatase, extr  
13856 acid phosphatase, extr  
14049 Dolichyl pyrophosphate phosphatase  
15091 Lysophosphatidic acid phosphatase type 6; Acid phosphatase 6

In [43]:

```
temp = ['10885','13419','12870','13856','14049','15091']
Annotation.loc[temp]
```

Out[43]:

|  | Combined Annotations | Signal P | Sc288c Orthologs | Human Orthologs | Sc288 Best Hit | Human Blast | Essential | WolfPSort | C Terminal |
| --- | --- | --- | --- | --- | --- | --- | --- | --- | --- |
| RTO4\_ID |  |  |  |  |  |  |  |  |  |
| 10885 | K01078: E3.1.3.2; acid phosphatase | A |  |  | DIA3 |  | Not Essential | mito 9, plas 6, cyto 4.5, cyto\_nucl 3.5, pero ... | PHS\* |
| 13419 | KOG1382: Multiple inositol polyphosphate phosp... | S |  |  |  |  | Not Essential | cyto 9.5, extr 6, cyto\_nucl 6, E.R. 5, pero 2,... | KDE\* |
| 12870 | KOG3720: Lysosomal & prostatic acid phosphatases |  |  |  |  |  | Not Essential | extr 25 | RQY\* |
| 13856 | K01078: E3.1.3.2; acid phosphatase | S |  |  |  |  | Not Essential | extr 27 | ALL\* |
| 14049 | KOG3146: Dolichyl pyrophosphate phosphatase an... |  |  |  |  |  | Not Essential | mito 13, extr 12 | GEL\* |
| 15091 | K01078: E3.1.3.2; acid phosphatase |  |  | ACP6 |  | ACP6 | Not Essential | mito 16.5, cyto\_mito 11.5, cyto 5.5, nucl 4 | TVE\* |

In [44]:

```
for x in temp:
    if x in model.genes:
        for r in sorted(model.genes.get_by_id(x).reactions, key=lambda x: x.id):
            print(r, r.gene_reaction_rule)
    else:
        print(x, 'no reactions')
    print()
```

```
10885 no reactions

13419 no reactions

12870 no reactions

ACP1e: fmn_e + h2o_e --> pi_e + ribflv_e 13856
THMPe: h2o_e + thmmp_e --> pi_e + thm_e 13856

14049 no reactions

15091 no reactions
```

In [45]:

```
for r in model.metabolites.get_by_id('1ag3p_RT_r').reactions:
    print(r, r.gene_reaction_rule)
```

```
GAT1er_RT: 0.01 arachcoa_r + glyc3p_r + 0.02 hpdcacoa_r + 0.1 lnlncgcoa_r + 0.37 ocdycacoa_r + 0.32 odecoa_r + 0.09 pmtcoa_r + 0.07 stcoa_r + 0.02 ttccoa_r --> 0.01 1ag3p_RT_r + coa_r 15435
AGATer_RT: 0.01 1ag3p_RT_r + 0.01 arachcoa_r + 0.02 hpdcacoa_r + 0.1 lnlncgcoa_r + 0.37 ocdycacoa_r + 0.32 odecoa_r + 0.09 pmtcoa_r + 0.07 stcoa_r + 0.02 ttccoa_r --> coa_r + 0.01 pa_RT_r 10427 or 16030 or 16779 or 9746
ADHAPRer_RT: 0.01 1agly3p_RT_r + h_r + nadph_r --> 0.01 1ag3p_RT_r + nadp_r 15575
```

In [46]:

```
model.metabolites.get_by_id('1ag3p_RT_r')
```

Out[46]:

|  |  |
| --- | --- |
| **Metabolite identifier** | 1ag3p\_RT\_r |
| **Name** | 1-Acyl-sn-glycerol 3-phosphate |
| **Memory address** | 0x07fea9786ad30 |
| **Formula** | C2094H3816O700P100 |
| **Compartment** | r |
| **In 3 reaction(s)** | GAT1er\_RT, AGATer\_RT, ADHAPRer\_RT |

In [47]:

```
# nucleoside-5’-monophosphate (amp, gmp, cmp, ump)
for x in ['EX_amp_e','EX_cmp_e','EX_gmp_e','EX_ump_e']:
    r = hsa2.reactions.get_by_id(x).copy()
    r.lower_bound = 0.0
    model.add_reactions([r])    
for x in ['NTD2e','NTD4e','NTD7e','NTD9e']:
    r = hsa2.reactions.get_by_id(x).copy()
    r.gene_reaction_rule = '15429 or 9531'
    model.add_reactions([r])
# b-Glycerol Phosphate
r1 = hsa2.reactions.get_by_id('EX_glyc2p_e').copy()
r1.lower_bound = 0.0
r2 = model.reactions.get_by_id('G2PP').copy()
r2.id = 'G2PPe'
r2.name = 'Glycerol-2-phosphate phosphatase, extracellular'
r2.gene_reaction_rule = '15429'
model.add_reactions([r1, r2])
for m, v in r2.metabolites.items():
    if m.id.replace('_c','_e') not in model.metabolites:
        m2 = m.copy()
        m2.id = m.id.replace('_c','_e')
        m2.compartment = 'e'
        model.add_metabolites([m2])
    r2.add_metabolites({m.id: -v, m.id.replace('_c','_e'): v})
# Carbamyl Phosphate
m = model.metabolites.get_by_id('cbp_c').copy()
m.id = 'cbp_e'
m.compartment = 'e'
model.add_metabolites([m])
r1 = model.reactions.get_by_id('EX_h_e').copy()
r1.id = 'EX_cbp_e'
r1.name = 'Carbamoyl phosphate exchange'
r1.lower_bound = 0.0
r2 = model.reactions.get_by_id('CO2t').copy()
r2.id = 'CBPt'
r2.name = 'Carbamoyl phosphate transport via diffusion'
r2.gene_reaction_rule = ''
model.add_reactions([r1, r2])
r1.add_metabolites({'h_e': 1.0, 'cbp_e': -1.0})
r2.add_metabolites({'co2_e': 1.0, 'co2_c': -1.0, 'cbp_e': -1.0, 'cbp_c': 1.0})
# D-2-PhosphoGlyceric Acid
r1 = hsa2.reactions.get_by_id('EX_2pg_e').copy()
r1.lower_bound = 0.0
r2 = hsa2.reactions.get_by_id('EX_glyc__R_e').copy()
r2.lower_bound = 0.0
model.add_reactions([r1, r2])
r1 = model.reactions.get_by_id('G2PPe').copy()
r1.id = '2PGPe'
r1.name = 'D-Glycerate 2-phosphate phosphatase, extracellular'
r1.gene_reaction_rule = '15429'
r2 = hsa2.reactions.get_by_id('GLYC_Rt').copy()
r2.gene_reaction_rule = ''
model.add_reactions([r1, r2])
r1.add_metabolites({'glyc2p_e': 1.0, 'glyc_e': -1.0, '2pg_e': -1.0, 'glyc__R_e': 1.0})
# D-3-PhosphoGlyceric Acid
r1 = hsa2.reactions.get_by_id('EX_3pg_e').copy()
r1.lower_bound = 0.0
r2 = model.reactions.get_by_id('G2PPe').copy()
r2.id = '3PGPe'
r2.name = '3-Phospho-D-glycerate phosphatase, extracellular'
r2.gene_reaction_rule = '15429'
model.add_reactions([r1, r2])
r2.add_metabolites({'glyc2p_e': 1.0, 'glyc_e': -1.0, '3pg_e': -1.0, 'glyc__R_e': 1.0})
# Phosphoenol Pyruvate
r1 = hsa2.reactions.get_by_id('EX_pep_e').copy()
r1.lower_bound = 0.0
r2 = model.reactions.get_by_id('G2PPe').copy()
r2.id = 'PEPPe'
r2.name = 'Phosphoenolpyruvate phosphatase, extracellular'
r2.gene_reaction_rule = '15429 or 9531'
model.add_reactions([r1, r2])
r2.add_metabolites({'glyc2p_e': 1.0, 'glyc_e': -1.0, 'pep_e': -1.0, 'pyr_e': 1.0})
```

In [48]:

```
# 2-Deoxy-D-Glucose 6-Phosphate
m = model.metabolites.get_by_id('2doxg6p_c').copy()
m.id = '2doxg6p_e'
m.compartment = 'e'
model.add_metabolites([m])
r = model.reactions.get_by_id('EX_h_e').copy()
r.id = 'EX_2doxg6p_e'
r.name = '2-Deoxy-D-Glucose 6-Phosphate exchange'
r.lower_bound = 0.0
model.add_reactions([r])
r.add_metabolites({'h_e': 1.0, '2doxg6p_e': -1.0})
# D-Glucosamine-6-Phosphate
r1 = sce.reactions.get_by_id('EX_gam6p_e').copy()
r1.lower_bound = 0.0
r2 = model.reactions.get_by_id('G2PPe').copy()
r2.id = 'GAM6PPe'
r2.name = 'D-Glucosamine-6-Phosphate phosphatase, extracellular'
r2.gene_reaction_rule = '15429'
model.add_reactions([r1, r2])
r2.add_metabolites({'glyc2p_e': 1.0, 'glyc_e': -1.0, 'gam6p_e': -1.0, 'gam_e': 1.0})
# 6-PhosphoGluconic Acid
m = model.metabolites.get_by_id('6pgc_c').copy()
m.id = '6pgc_e'
m.compartment = 'e'
model.add_metabolites([m])
r1 = model.reactions.get_by_id('EX_h_e').copy()
r1.id = 'EX_6pgc_e'
r1.name = '6-Phospho-D-gluconate exchange'
r1.lower_bound = 0.0
r2 = model.reactions.get_by_id('GNP').copy()
r2.id = 'GNPe'
r2.name = 'Phosphogluconate phosphatase, extracellular'
r2.gene_reaction_rule = '15429'
model.add_reactions([r1, r2])
r1.add_metabolites({'h_e': 1.0, '6pgc_e': -1.0})
for m, v in r2.metabolites.items():
    if m.id.replace('_c','_e') not in model.metabolites:
        m2 = m.copy()
        m2.id = m.id.replace('_c','_e')
        m2.compartment = 'e'
        model.add_metabolites([m2])
    r2.add_metabolites({m.id: -v, m.id.replace('_c','_e'): v})
# Cytidine-3’,5’-Cyclic monophosphate
# cyclic monophosphates - are these transported into cytoplasm or hydrolyzed extracellularly?
# No reaction exists for either case in BiGG, but cAMP transmembrane transport exists in BioCyc
# https://biocyc.org/META/NEW-IMAGE?type=REACTION&object=TRANS-RXN0-564
m = model.metabolites.get_by_id('35ccmp_c').copy()
m.id = '35ccmp_e'
m.compartment = 'e'
model.add_metabolites([m])
r1 = model.reactions.get_by_id('EX_h_e').copy()
r1.id = 'EX_35ccmp_e'
r1.name = "3',5'-Cyclic CMP exchange"
r1.lower_bound = 0.0
r2 = model.reactions.get_by_id('CO2t').copy()
r2.id = 'CCMPt2'
r2.name = "3',5'-Cyclic CMP transport via diffusion"
r2.gene_reaction_rule = ''
model.add_reactions([r1, r2])
r1.add_metabolites({'h_e': 1.0, '35ccmp_e': -1.0})
r2.add_metabolites({'co2_e': 1.0, 'co2_c': -1.0, '35ccmp_e': -1.0, '35ccmp_c': 1.0})
# cAMP and 35ccmp
r1 = model.reactions.get_by_id('CO2t').copy()
r1.id = 'CAMPt2'
r1.name = "3',5'-Cyclic AMP transport via diffusion"
r1.gene_reaction_rule = ''
r2 = model.reactions.get_by_id('CO2t').copy()
r2.id = 'CGMPt2'
r2.name = "3',5'-Cyclic GMP transport via diffusion"
r2.gene_reaction_rule = ''
model.add_reactions([r1, r2])
r1.add_metabolites({'co2_e': 1.0, 'co2_c': -1.0, 'camp_e': -1.0, 'camp_c': 1.0})
r2.add_metabolites({'co2_e': 1.0, 'co2_c': -1.0, '35cgmp_e': -1.0, '35cgmp_c': 1.0})
```

In [49]:

```
# D-Mannose-1-Phosphate
m = model.metabolites.get_by_id('man1p_c').copy()
m.id = 'man1p_e'
m.compartment = 'e'
model.add_metabolites([m])
r1 = model.reactions.get_by_id('EX_h_e').copy()
r1.id = 'EX_man1p_e'
r1.name = 'D-Mannose-1-Phosphate exchange'
r1.lower_bound = 0.0
r2 = model.reactions.get_by_id('G2PPe').copy()
r2.id = 'MAN1PPe'
r2.name = 'D-Mannose-1-Phosphate phosphatase, extracellular'
r2.gene_reaction_rule = '15429'
model.add_reactions([r1, r2])
r1.add_metabolites({'h_e': 1.0, 'man1p_e': -1.0})
r2.add_metabolites({'glyc2p_e': 1.0, 'glyc_e': -1.0, 'man1p_e': -1.0, 'man_e': 1.0})
# D-Mannose-6-Phosphate
r1 = eco.reactions.get_by_id('EX_man6p_e').copy()
r1.lower_bound = 0.0
r2 = model.reactions.get_by_id('G2PPe').copy()
r2.id = 'MAN6PPe'
r2.name = 'D-Mannose-6-Phosphate phosphatase, extracellular'
r2.gene_reaction_rule = '15429'
model.add_reactions([r1, r2])
r2.add_metabolites({'glyc2p_e': 1.0, 'glyc_e': -1.0, 'man6p_e': -1.0, 'man_e': 1.0})
# O-Phospho-L-Serine
r1 = hsa2.reactions.get_by_id('EX_pser__L_e').copy()
r1.lower_bound = 0.0
r2 = hsa2.reactions.get_by_id('PSERtr').copy()
r2.gene_reaction_rule = ''
model.add_reactions([r1, r2])
# Phosphoryl Choline
r1 = hsa2.reactions.get_by_id('EX_cholp_e').copy()
r1.lower_bound = 0.0
r2 = hsa2.reactions.get_by_id('CHOLPtr').copy()
r2.gene_reaction_rule = ''
model.add_reactions([r1, r2])
# O-PhosphorylEthanolamine
r1 = hsa2.reactions.get_by_id('EX_ethamp_e').copy()
r1.lower_bound = 0.0
r2 = hsa2.reactions.get_by_id('ETHAMPtr').copy()
r2.gene_reaction_rule = ''
model.add_reactions([r1, r2])
# Thymidine-5’-monophosphate
r1 = hsa2.reactions.get_by_id('EX_dtmp_e').copy()
r1.lower_bound = 0.0
r2 = model.reactions.get_by_id('NTD5').copy()
r2.id = 'NTD5e'
r2.name = "5'-nucleotidase (dTMP), extracellular"
r2.gene_reaction_rule = '13856'
model.add_reactions([r1, r2])
for m, v in r2.metabolites.items():
    if m.id.replace('_c','_e') not in model.metabolites:
        m2 = m.copy()
        m2.id = m.id.replace('_c','_e')
        m2.compartment = 'e'
        model.add_metabolites([m2])
    r2.add_metabolites({m.id: -v, m.id.replace('_c','_e'): v})
```

In [50]:

```
# Inositol Hexaphosphate
# 10885 myo-inositol-hexakisphosphate 3-phosphohydrolase (phytase/3-phytase), secreted in Aspergillus  
r1 = eco.reactions.get_by_id('EX_minohp_e').copy()
r1.lower_bound = 0.0
r2 = hsa2.reactions.get_by_id('MINOHPtn').copy()
r2.gene_reaction_rule = ''
model.add_reactions([r1, r2])
r = eco.reactions.get_by_id('PHYTSpp').copy()
r.id = 'PHYTSe'
r.name = 'Phytase, extracellular'
r.gene_reaction_rule = '10885'
model.add_reactions([r])
for m, v in r.metabolites.items():
    if m.id.replace('_p','_e') not in model.metabolites:
        m2 = m.copy()
        m2.id = m.id.replace('_p','_e')
        m2.compartment = 'e'
        model.add_metabolites([m2])
    r.add_metabolites({m.id: -v, m.id.replace('_p','_e'): v})
# 13419 Multiple inositol polyphosphate phosphatase, E.R. in human  
m1 = model.metabolites.get_by_id('minohp_n').copy()
m1.id = 'minohp_r'
m1.compartment = 'r'
m2 = model.metabolites.get_by_id('mi13456p_n').copy()
m2.id = 'mi13456p_r'
m2.compartment = 'r'
m3 = model.metabolites.get_by_id('mi1345p_n').copy()
m3.id = 'mi1345p_r'
m3.compartment = 'r'
m4 = model.metabolites.get_by_id('mi1456p_n').copy()
m4.id = 'mi1456p_r'
m4.compartment = 'r'
m5 = model.metabolites.get_by_id('mi145p_n').copy()
m5.id = 'mi145p_r'
m5.compartment = 'r'
model.add_metabolites([m1,m2,m3,m4,m5])
r1 = cobra.Reaction(id='MINPP1er', name='Multiple inositol polyphosphate phosphatase')
r1.gene_reaction_rule = '13419'
r2 = cobra.Reaction(id='MINPP2er', name='Multiple inositol polyphosphate phosphatase')
r2.gene_reaction_rule = '13419'
r3 = cobra.Reaction(id='MINPP3er', name='Multiple inositol polyphosphate phosphatase')
r3.gene_reaction_rule = '13419'
r4 = cobra.Reaction(id='MINPP4er', name='Multiple inositol polyphosphate phosphatase')
r4.gene_reaction_rule = '13419'
model.add_reactions([r1,r2,r3,r4])
r1.add_metabolites({'minohp_r': -1.0, 'h2o_r': -1.0, 'mi13456p_r': 1.0, 'pi_r': 1.0})
r2.add_metabolites({'mi13456p_r': -1.0, 'h2o_r': -1.0, 'mi1456p_r': 1.0, 'pi_r': 1.0})
r3.add_metabolites({'mi1456p_r': -1.0, 'h2o_r': -1.0, 'mi145p_r': 1.0, 'pi_r': 1.0})
r4.add_metabolites({'mi1345p_r': -1.0, 'h2o_r': -1.0, 'mi145p_r': 1.0, 'pi_r': 1.0})
```

In [51]:

```
for r in model.reactions:
    if r.id.startswith('MI') and r.id.endswith('PPn'):
        print(r, r.gene_reaction_rule)
print()
for r in model.reactions:
    if r.id.startswith('MI') and r.id.endswith('PP'):
        print(r, r.gene_reaction_rule)
```

```
MI145PPn: h2o_n + mi145p_n --> mi14p_n + pi_n 13609 or 15340
MI1345PPn: h2o_n + mi1345p_n --> mi134p_n + pi_n 13609 or 15340

MI1PP: h2o_c + mi1p__D_c --> inost_c + pi_c 13708 or 16369
MI4PP: h2o_c + mi4p__D_c --> inost_c + pi_c 13708 or 16369
MI3PP: h2o_c + mi3p__D_c --> inost_c + pi_c 13708 or 16369
MI14PP: h2o_c + mi14p_c --> mi4p__D_c + pi_c 10673
```

In [52]:

```
temp = ['13609','15340','15545','11381','16619','13708','16369','10673']
display(Annotation.loc[temp])
for x in temp:
    if x in model.genes:
        for r in sorted(model.genes.get_by_id(x).reactions, key=lambda x: x.id):
            print(r, r.gene_reaction_rule)
    else:
        print(x, 'no reactions')
    print()
```

|  | Combined Annotations | Signal P | Sc288c Orthologs | Human Orthologs | Sc288 Best Hit | Human Blast | Essential | WolfPSort | C Terminal |
| --- | --- | --- | --- | --- | --- | --- | --- | --- | --- |
| RTO4\_ID |  |  |  |  |  |  |  |  |  |
| 13609 | K01099: INPP5B\_F; inositol polyphosphate 5-pho... |  |  | INPP5B,OCRL | INP51 | OCRL | Not Essential | nucl 16.5, cyto\_nucl 13.333, cyto 9, cyto\_mito... | LEE\* |
| 15340 | K20279: SYNJ; synaptojanin |  | INP52,INP53,INP51 | SYNJ1,SYNJ2 | INP53 | SYNJ1 | Not Essential | extr 10, cyto 4, mito 3, plas 3, cyto\_nucl 3, ... | LLS\* |
| 15545 | KOG0565: Inositol polyphosphate 5-phosphatase ... |  |  |  | INP51 | INPP5 | Not Essential | mito 8.5, cyto\_mito 8, cyto 6.5, plas 5, nucl 4 | ALL\* |
| 11381 | KOG1889: Putative phosphoinositide phosphatase |  | SAC1 | SACM1L | SAC1 | SACM1 | Not Essential | plas 18, E.R. 5, vacu 2 | KQE\* |
| 16619 | HMMPfam:SacI homology domain:PF02383,ProSitePr... |  | FIG4 | FIG4 | FIG4 | FIG4 | Not Essential | mito 6, plas 6, nucl 5.5, cyto\_nucl 5.5, cyto ... | AEG\* |
| 13708 | K01092: E3.1.3.25, IMPA, suhB; myo-inositol-1(... |  | INM2,INM1 | IMPA1 | INM1 | IMPA1 | Not Essential | extr 12, cyto 10, mito 2, plas 2 | RPT\* |
| 16369 | K01092: E3.1.3.25, IMPA, suhB; myo-inositol-1(... |  | INM2,INM1 | IMPA1 | INM1 | IMPA1 | Not Essential | cyto 20.5, cyto\_nucl 11.5, pero 3 | WDV\* |
| 10673 | K01082: cysQ, MET22, BPNT1; 3'(2'), 5'-bisphos... |  | MET22 |  | MET22 |  | Essential | mito 16.5, cyto\_mito 10.5, extr 7 | EKK\* |

```
MI1345PPn: h2o_n + mi1345p_n --> mi134p_n + pi_n 13609 or 15340
MI145PPn: h2o_n + mi145p_n --> mi14p_n + pi_n 13609 or 15340
PI45BP5Pn_RT: h2o_n + 0.01 ptd145bp_RT_n --> pi_n + 0.01 ptd4ino_RT_n 13609 or 15545

MI1345PPn: h2o_n + mi1345p_n --> mi134p_n + pi_n 13609 or 15340
MI145PPn: h2o_n + mi145p_n --> mi14p_n + pi_n 13609 or 15340
PI35BP5Per_RT: h2o_r + 0.01 ptd135bp_RT_r --> pi_r + 0.01 ptd3ino_RT_r 11381 or 15340 or 16619
PI45BP5Per_RT: h2o_r + 0.01 ptd145bp_RT_r --> pi_r + 0.01 ptd4ino_RT_r 15340

PI45BP5Pn_RT: h2o_n + 0.01 ptd145bp_RT_n --> pi_n + 0.01 ptd4ino_RT_n 13609 or 15545

PI35BP5Per_RT: h2o_r + 0.01 ptd135bp_RT_r --> pi_r + 0.01 ptd3ino_RT_r 11381 or 15340 or 16619
PIN3Per_RT: h2o_r + 0.01 ptd3ino_RT_r --> pi_r + 0.01 ptd1ino_RT_r 11381
PIN4Per_RT: h2o_r + 0.01 ptd4ino_RT_r --> pi_r + 0.01 ptd1ino_RT_r 11381

PI35BP5Per_RT: h2o_r + 0.01 ptd135bp_RT_r --> pi_r + 0.01 ptd3ino_RT_r 11381 or 15340 or 16619

MI1PP: h2o_c + mi1p__D_c --> inost_c + pi_c 13708 or 16369
MI3PP: h2o_c + mi3p__D_c --> inost_c + pi_c 13708 or 16369
MI4PP: h2o_c + mi4p__D_c --> inost_c + pi_c 13708 or 16369

G2PP: glyc2p_c + h2o_c --> glyc_c + pi_c 16369
MI1PP: h2o_c + mi1p__D_c --> inost_c + pi_c 13708 or 16369
MI3PP: h2o_c + mi3p__D_c --> inost_c + pi_c 13708 or 16369
MI4PP: h2o_c + mi4p__D_c --> inost_c + pi_c 13708 or 16369

BPNT: h2o_c + pap_c --> amp_c + pi_c 10673
BPNT2: h2o_c + paps_c --> aps_c + pi_c 10673
MI14PP: h2o_c + mi14p_c --> mi4p__D_c + pi_c 10673
```

In [53]:

```
model.remove_reactions(['MI14PP'], remove_orphans=True)
```

In [54]:

```
# Thiosulfate
r1 = hsa2.reactions.get_by_id('EX_tsul_e').copy()
r1.lower_bound = 0.0
r2 = hsa2.reactions.get_by_id('TSULt4_3').copy()
r2.gene_reaction_rule = ''
model.add_reactions([r1, r2])
# L-Cysteic Acid
r1 = hsa2.reactions.get_by_id('EX_Lcyst_e').copy()
r1.lower_bound = 0.0
r2 = hsa2.reactions.get_by_id('LCYSTt').copy()
r2.gene_reaction_rule = ''
model.add_reactions([r1, r2])
# L-Cysteine Sulfinic Acid
m = model.metabolites.get_by_id('3sala_c').copy()
m.id = '3sala_e'
m.compartment = 'e'
model.add_metabolites([m])
r = model.reactions.get_by_id('EX_h_e').copy()
r.id = 'EX_3sala_e'
r.name = '3-Sulfino-L-alanine exchange'
r.gene_reaction_rule = ''
r.lower_bound = 0.0
model.add_reactions([r])
r.add_metabolites({'h_e': 1.0, '3sala_e': -1.0})
r = model.reactions.get_by_id('CO2t').copy()
r.id = '3SALAt'
r.name = '3-Sulfino-L-alanine transport via diffusion'
r.gene_reaction_rule = ''
model.add_reactions([r])
r.add_metabolites({'co2_e': 1.0, 'co2_c': -1.0, '3sala_e': -1.0, '3sala_c': 1.0})
# Cystathionine
r1 = hsa2.reactions.get_by_id('EX_cyst__L_e').copy()
r1.lower_bound = 0.0
r2 = hsa2.reactions.get_by_id('CYST_Ltr').copy()
r2.gene_reaction_rule = ''
model.add_reactions([r1, r2])
# L-Methionine Sulfoxide
r1 = eco.reactions.get_by_id('EX_metsox_S__L_e').copy()
r1.lower_bound = 0.0
r2 = eco.reactions.get_by_id('EX_metsox_R__L_e').copy()
r2.lower_bound = 0.0
model.add_reactions([r1, r2])
r1 = model.reactions.get_by_id('CO2t').copy()
r1.id = 'METSOX1t'
r1.name = 'L-Methionine Sulfoxide transport via diffusion'
r1.gene_reaction_rule = ''
r2 = model.reactions.get_by_id('CO2t').copy()
r2.id = 'METSOX2t'
r2.name = 'L-methionine-R-sulfoxide transport via diffusion'
r2.gene_reaction_rule = ''
model.add_reactions([r1, r2])
r1.add_metabolites({'co2_e': 1.0, 'co2_c': -1.0, 'metsox_S__L_e': -1.0, 'metsox_S__L_c': 1.0})
r2.add_metabolites({'co2_e': 1.0, 'co2_c': -1.0, 'metsox_R__L_e': -1.0, 'metsox_R__L_c': 1.0})
# Taurine
r1 = hsa2.reactions.get_by_id('EX_taur_e').copy()
r1.lower_bound = 0.0
r2 = hsa2.reactions.get_by_id('TAURt').copy()
r2.lower_bound = -1000.0
r2.gene_reaction_rule = ''
model.add_reactions([r1, r2])
# Hypotaurine
r = hsa2.reactions.get_by_id('EX_hyptaur_e').copy()
r.lower_bound = 0.0
model.add_reactions([r])
```

In [55]:

```
for r in sorted(model.reactions, key=lambda x: x.id):
    if not r.boundary and sum(abs(x) for x in r.check_mass_balance().values()) > 1e-12:
        print(r, r.gene_reaction_rule, r.check_mass_balance())
```

```
BIOMASS_RT: 0.957502 13BDglcn_c + 0.177315 16BDglcn_c + 0.001283 5mthf_c + 0.577574 alatrna_c + 0.146675 argtrna_c + 0.10797 asntrna_c + 0.197296 asptrna_c + 140.298633 atp_c + 0.002418 btn_m + 0.000832 ca2_c + 0.001792 camp_c + 0.031005 chitin_c + 1.8e-05 clpn_RT_m + 0.00077 coa_c + 0.044881 ctp_c + 0.000525 cu2_c + 0.034269 cystrna_c + 0.002451 datp_c + 0.004285 dctp_c + 0.003763 dgtp_c + 0.002523 dttp_c + 0.004031 ergst_r + 1.6e-05 ergstest_RT_r + 0.000751 fad_c + 0.000597 fe2_c + 0.000597 fe3_c + 0.117898 glntrna_c + 0.208673 glutrna_c + 0.518069 glycogen_c + 0.51322 glytrna_c + 0.00192 gthrd_c + 0.051085 gtp_c + 136.32957199999998 h2o_c + 0.000691 hemeA_m + 0.061167 histrna_c + 0.158569 iletrna_c + 0.585703 k_c + 0.320205 leutrna_c + 0.003107 lipopb_m + 0.172334 lystrna_c + 0.807941 mannan_r + 0.05705 mettrna_c + 0.061716 mg2_c + 0.001292 mlthf_c + 0.000607 mn2_c + 0.026099 na1_c + 0.000888 nad_c + 0.000795 nadp_c + 1.4e-05 pa_RT_r + 0.000172 pc_RT_r + 0.00013 pe_RT_r + 0.095164 phetrna_c + 0.235897 protrna_c + 2e-05 ps_RT_r + 0.004006 psphings_r + 4.7e-05 ptd1ino_RT_r + 0.006524 ptrc_c + 0.0024 pydx5p_c + 0.00074 q9_m + 0.00099 ribflv_c + 0.33687 sertrna_c + 0.003967 spmd_c + 0.001327 thf_c + 0.001393 thmpp_c + 0.21813 thrtrna_c + 0.023372 tre_c + 0.000432 triglyc_RT_r + 0.023319 trptrna_c + 0.062363 tyrtrna_c + 0.051129 utp_c + 0.275168 valtrna_c + 0.00051 zn2_c + 0.004156 zymst_r + 1.7e-05 zymstest_RT_d --> 140.249386 adp_c + 140.249386 h_c + 140.249386 pi_c + 0.209364 ppi_c  {'charge': -4.356142000000063, 'C': -40.73034300000017, 'H': -62.360945000000214, 'O': -17.69887699999992, 'N': -5.868879000000095, 'P': -0.3741850000000422, 'R': -4.027781, 'S': -0.104034, 'Ca': -0.000832, 'Cu': -0.000525, 'Fe': -0.001885, 'K': -0.585703, 'Mg': -0.061716, 'Mn': -0.000607, 'Na': -0.026099, 'Zn': -0.00051}
```

In [56]:

```
with pd.option_context('display.max_rows', None, 'display.max_columns', None):
    display(Biolog_in_model.query('External == False and Internal == False'))
```

|  | Biolog | Model | Exchange | Metabolite | Internal | External | Average | Pass |
| --- | --- | --- | --- | --- | --- | --- | --- | --- |
| PM1\_A4 | D-Saccharic Acid | None | EX\_glcr\_e | glcr\_e | False | False | 0.0712 | False |
| PM1\_B4 | L-Fucose | None | EX\_fuc\_\_L\_e | fuc\_\_L\_e | False | False | 0.0996667 | False |
| PM1\_B5 | D-Glucuronic Acid | None | EX\_glcur\_e | glcur\_e | False | False | 0.0718333 | False |
| PM1\_B11 | D-Mannitol | None | EX\_mnl\_e | mnl\_e | False | False | 0.120767 | True |
| PM1\_C3 | D-,L-Malic Acid | [None, L-Malate] | EX\_mal\_\_D\_e,EX\_mal\_\_L\_e | mal\_\_D\_e,mal\_\_L\_e | False | False | 0.1148 | True |
| PM1\_C6 | L-Rhamnose | None | EX\_rmn\_e | rmn\_e | False | False | 0.0937333 | False |
| PM1\_C11 | D-Melibiose | None | EX\_melib\_e | melib\_e | False | False | 0.0763 | False |
| PM1\_D9 | a-D-Lactose | None | EX\_lcts\_e | lcts\_e | False | False | 0.0810333 | False |
| PM1\_E2 | m-Tartaric Acid | None | EX\_tartr\_\_M\_e | tartr\_\_M\_e | False | False | 0.0671667 | False |
| PM1\_E7 | a-Hydroxy Butyric Acid | None | EX\_2hb\_e | 2hb\_e | False | False | 0.0992 | False |
| PM1\_E8 | b-Methyl-D-Glucoside | None | EX\_mbdg\_e | mbdg\_e | False | False | 0.2126 | True |
| PM1\_E9 | Adonitol | None | EX\_rbt\_e | rbt\_e | False | False | 0.225933 | True |
| PM1\_E11 | 2-Deoxy Adenosine | None | EX\_dad\_\_2\_e | dad\_\_2\_e | False | False | 0.0686333 | False |
| PM1\_F8 | Mucic Acid | None | EX\_galct\_\_D\_e | galct\_\_D\_e | False | False | 0.0875333 | False |
| PM1\_G2 | Tricarballylic Acid | None | EX\_tcb\_e | tcb\_e | False | False | 0.0634333 | False |
| PM1\_G8 | N-Acetyl-b-D-Mannosamine | None | EX\_acmana\_e | acmana\_e | False | False | 0.0844333 | False |
| PM1\_G9 | Mono Methyl Succinate | None | EX\_methsucc\_e | methsucc\_e | False | False | 0.200767 | True |
| PM1\_G11 | D-Malic Acid | None | EX\_mal\_\_D\_e | mal\_\_D\_e | False | False | 0.121733 | True |
| PM1\_H2 | p-Hydroxy Phenyl Acetic Acid | None | EX\_4hoxpac\_e | 4hoxpac\_e | False | False | 0.139733 | True |
| PM1\_H3 | m-Hydroxy Phenyl Acetic Acid | None | EX\_3hoxpac\_e | 3hoxpac\_e | False | False | 0.0826333 | False |
| PM1\_H9 | L-Galactonic Acid-g-Lactone | None | EX\_galctn\_\_L\_e | galctn\_\_L\_e | False | False | 0.2527 | True |
| PM2\_A6 | Dextrin | None | EX\_dextrin\_e | dextrin\_e | False | False | 0.129 | True |
| PM2\_A12 | Pectin | None | EX\_pect\_e | pect\_e | False | False | 0.159867 | True |
| PM2\_B1 | N-Acetyl-D-Galactosamine | None | EX\_acgal\_e | acgal\_e | False | False | 0.0779667 | False |
| PM2\_B2 | N-Acetyl Neuraminic Acid | None | EX\_acnam\_e | acnam\_e | False | False | 0.0724667 | False |
| PM2\_B3 | b-D-Allose | None | EX\_all\_\_D\_e | all\_\_D\_e | False | False | 0.107 | False |
| PM2\_B8 | Arbutin | None | EX\_arbt\_e | arbt\_e | False | False | 0.300467 | True |
| PM2\_D2 | Salicin | None | EX\_salcn\_e | salcn\_e | False | False | 0.3282 | True |
| PM2\_D6 | D-Tagatose | None | EX\_tag\_\_D\_e | tag\_\_D\_e | False | False | 0.1632 | True |
| PM2\_E3 | Citraconic Acid | None | EX\_citac\_e | citac\_e | False | False | 0.0714 | False |
| PM2\_E4 | Citramalic Acid | None | EX\_citm\_e | citm\_e | False | False | 0.0739 | False |
| PM2\_E11 | Itaconic Acid | None | EX\_itacon\_e | itacon\_e | False | False | 0.0718 | False |
| PM2\_F2 | Malonic Acid | None | EX\_malon\_e | malon\_e | False | False | 0.0589 | False |
| PM2\_F11 | D-Tartaric Acid | None | EX\_tartr\_\_D\_e | tartr\_\_D\_e | False | False | 0.0704 | False |
| PM2\_F12 | L-Tartaric Acid | None | EX\_tartr\_\_L\_e | tartr\_\_L\_e | False | False | 0.0758 | False |
| PM2\_H5 | D,L-Carnitine | [None, L-Carnitine] | EX\_crn\_\_D\_e,EX\_crn\_e | crn\_\_D\_e,crn\_e | False | False | 0.0643333 | False |
| PM3B\_C4 | D-Asparagine | None | EX\_asn\_\_D\_e | asn\_\_D\_e | False | False | 0.111333 | True |
| PM3B\_C6 | D-Glutamic Acid | None | EX\_glu\_\_D\_e | glu\_\_D\_e | False | False | 0.0466 | False |
| PM3B\_C7 | D-Lysine | None | EX\_lys\_\_D\_e | lys\_\_D\_e | False | False | 0.110933 | False |
| PM3B\_C9 | D-Valine | None | EX\_val\_\_D\_e | val\_\_D\_e | False | False | 0.121267 | True |
| PM3B\_D4 | Hydroxylamine | None | EX\_ham\_e | ham\_e | False | False | 0.150133 | True |
| PM3B\_E9 | D-Galactosamine | None | EX\_galam\_e | galam\_e | False | False | 0.1819 | True |
| PM3B\_E12 | N-Acetyl-D-Galactosamine | None | EX\_acgal\_e | acgal\_e | False | False | 0.0908333 | False |
| PM3B\_F1 | N-Acetyl-D-Mannosamine | None | EX\_acmana\_e | acmana\_e | False | False | 0.0963667 | False |
| PM3B\_G4 | Alloxan | None | EX\_CE0074\_e | CE0074\_e | False | False | 0.306567 | True |
| PM3B\_G7 | D,L-a-Amino-NButyric Acid | None | EX\_C02356\_e | C02356\_e | False | False | 0.157433 | True |
| PM4A\_A4 | Trimetaphosphate | None | EX\_tmp\_e | tmp\_e | False | False | 0.305667 | True |
| PM4A\_A8 | Adenosine-2’-monophosphate | None | EX\_amp2p\_e | amp2p\_e | False | False | 0.2983 | True |
| PM4A\_A9 | Adenosine-3’-monophosphate | None | EX\_3amp\_e | 3amp\_e | False | False | 0.237633 | True |
| PM4A\_A11 | Adenosine-2’,3’-Cyclic monophosphate | None | EX\_23camp\_e | 23camp\_e | False | False | 0.209733 | True |
| PM4A\_B9 | Guanosine-3’-monophosphate | None | EX\_3gmp\_e | 3gmp\_e | False | False | 0.224833 | True |
| PM4A\_B11 | Guanosine-2’,3’-Cyclic monophosphate | None | EX\_23cgmp\_e | 23cgmp\_e | False | False | 0.2335 | True |
| PM4A\_C2 | PhosphoGlycolic Acid | None | EX\_2pglyc\_e | 2pglyc\_e | False | False | 0.238667 | True |
| PM4A\_C9 | Cytidine-3’-monophosphate | None | EX\_3cmp\_e | 3cmp\_e | False | False | 0.0753 | False |
| PM4A\_C11 | Cytidine-2’,3’-Cyclic monophosphate | None | EX\_23ccmp\_e | 23ccmp\_e | False | False | 0.1973 | True |
| PM4A\_D4 | Phospho-L-Arginine | None | EX\_argp\_e | argp\_e | False | False | 0.242233 | True |
| PM4A\_D5 | O-Phospho-D-Serine | None | EX\_pser\_\_D\_e | pser\_\_D\_e | False | False | 0.251967 | True |
| PM4A\_D7 | O-Phospho-L-Threonine | None | EX\_thrp\_e | thrp\_e | False | False | 0.270133 | True |
| PM4A\_D9 | Uridine-3’-monophosphate | None | EX\_3ump\_e | 3ump\_e | False | False | 0.2121 | True |
| PM4A\_D11 | Uridine-2’,3’-Cyclic monophosphate | None | EX\_23cump\_e | 23cump\_e | False | False | 0.2253 | True |
| PM4A\_E2 | O-Phospho-L-Tyrosine | None | EX\_tyrp\_e | tyrp\_e | False | False | 0.1924 | True |
| PM4A\_E3 | Phosphocreatine | None | EX\_pcreat\_e | pcreat\_e | False | False | 0.218667 | True |
| PM4A\_E6 | Phosphono Acetic Acid | None | EX\_phnac\_e | phnac\_e | False | False | 0.062 | False |
| PM4A\_E7 | 2-Aminoethyl Phosphonic Acid | None | EX\_2ameph\_e | 2ameph\_e | False | False | 0.0604 | False |
| PM4A\_F4 | Tetrathionate | None | EX\_tet\_e | tet\_e | False | False | 0.194367 | True |
| PM4A\_F8 | D-Cysteine | None | EX\_cys\_\_D\_e | cys\_\_D\_e | False | False | 0.1216 | True |
| PM4A\_F11 | Cysteamine | None | EX\_cysam\_e | cysam\_e | False | False | 0.1867 | True |
| PM4A\_G1 | N-Acetyl-L-Cysteine | None | EX\_CE1310\_e | CE1310\_e | False | False | 0.121333 | True |
| PM4A\_G4 | Lanthionine | None | EX\_lanth\_e | lanth\_e | False | False | 0.1822 | True |
| PM4A\_G8 | D-Methionine | None | EX\_met\_\_D\_e | met\_\_D\_e | False | False | 0.2067 | True |
| PM4A\_G10 | N-Acetyl-D,L-Methionine | None | EX\_acmet\_e | acmet\_e | False | False | 0.174567 | True |
| PM4A\_H1 | L-Djenkolic Acid | None | EX\_djenk\_e | djenk\_e | False | False | 0.3439 | True |
| PM4A\_H4 | D,L-Lipoamide | None | EX\_lpam\_e | lpam\_e | False | False | 0.269967 | True |
| PM4A\_H9 | Butane Sulfonic Acid | None | EX\_butso3\_e | butso3\_e | False | False | 0.188767 | True |
| PM4A\_H10 | 2-Hydroxyethane Sulfonic Acid | None | EX\_isetac\_e | isetac\_e | False | False | 0.1779 | True |
| PM4A\_H11 | Methane Sulfonic Acid | None | EX\_mso3\_e | mso3\_e | False | False | 0.206367 | True |

In [57]:

```
import urllib
bsub = cobra.io.load_json_model(urllib.request.urlopen('http://bigg.ucsd.edu/static/models/iYO844.json'))
ecoO = cobra.io.load_json_model(urllib.request.urlopen('http://bigg.ucsd.edu/static/models/iE2348C_1286.json'))
```

In [58]:

```
# Methyl-DGlucoside  
# 16716 and 16717 K05349: bglX; beta-glucosidase, extracellular
r1 = bsub.reactions.get_by_id('BG_MBDG').copy()
r1.gene_reaction_rule = '16716 or 16717'
r2 = bsub.reactions.get_by_id('EX_mbdg_e').copy()
r2.lower_bound = 0.0
model.add_reactions([r1,r2])
# Add meoh transport (diffusion) and exchange
r1 = hsa2.reactions.get_by_id('MEOHt2').copy()
r1.gene_reaction_rule = ''
r2 = hsa2.reactions.get_by_id('EX_meoh_e').copy()
r2.lower_bound = 0.0
model.add_reactions([r1,r2])
# Adonitol (D-ribitol)  
# Add D-ribitol transport (diffusion) and exchange
r1 = hsa2.reactions.get_by_id('RBTt').copy()
r1.gene_reaction_rule = ''
r2 = hsa2.reactions.get_by_id('EX_rbt_e').copy()
r2.lower_bound = 0.0
model.add_reactions([r1,r2])
# D-ribitol dehydrogenase (to D-ribulose)
# There are many short chain dehydrogenase, Glucose/ribitol dehydrogenase family genes
# Blast of known ribitol 2-dehydrogenase -> 1st hit is 11362 perox beta-ox, 2nd hit is 8666 with homolog hits
# 8666 Reductases with broad range of substrate specificities
r = ecoO.reactions.get_by_id('RBTDG').copy()
r.gene_reaction_rule = '8666'
model.add_reactions([r])
# Arbutin
# betaglucosidase releases a glucose and hydroquinone (hqn)
r1 = bsub.reactions.get_by_id('EX_arbt_e').copy()
r1.lower_bound = 0.0
r2 = bsub.reactions.get_by_id('EX_hqn_e').copy()
r2.lower_bound = 0.0
model.add_reactions([r1,r2])
r = model.reactions.get_by_id('BG_MBDG').copy()
r.id = 'ARBTHe'
r.name = 'Beta-glucosidase (arbutin), extracellular'
model.add_reactions([r])
r.add_metabolites({'mbdg_e': 1.0, 'meoh_e': -1.0, 'arbt_e': -1.0, 'hqn_e': 1.0})
# Salicin
# betaglucosidase releases a glucose and salicyl alcohol (2-hydroxybenzyl alcohol or 2-hydroxymethyl phenol)
# BiGG has two versions (2hxmp or 2hymeph), just take 2hxmp for now
r1 = ecoO.reactions.get_by_id('EX_salcn_e').copy()
r1.lower_bound = 0.0
r2 = bsub.reactions.get_by_id('EX_2hxmp_e').copy()
r2.lower_bound = 0.0
model.add_reactions([r1,r2])
r = model.reactions.get_by_id('BG_MBDG').copy()
r.id = 'SALCNHe'
r.name = 'Beta-glucosidase (salicin), extracellular'
model.add_reactions([r])
r.add_metabolites({'mbdg_e': 1.0, 'meoh_e': -1.0, 'salcn_e': -1.0, '2hxmp_e': 1.0})
```

In [59]:

```
Biolog_in_model = pd.DataFrame(columns=['Biolog','Model','Exchange','Metabolite',
                                        'Internal','External','Average','Pass'])
for i, row in Biolog.iterrows():
    if i in Biolog_Media.index:
        x = Biolog_Media.loc[i]
        Biolog_in_model.loc[i,'Exchange'] = x[x['Source']]
        Biolog_in_model.loc[i,'Metabolite'] = x[x['Source']].replace('EX_','')
        if ',' in Biolog_in_model.loc[i,'Metabolite']:
            Biolog_in_model.loc[i,'Model'] = [model.metabolites.get_by_id(x).name if x in model.metabolites \
                else next((model.metabolites.get_by_id(x.rsplit('_',1)[0]+'_'+c).name for c in model.compartments \
                    if x.rsplit('_',1)[0]+'_'+c in model.metabolites), None) \
                for x in Biolog_in_model.loc[i,'Metabolite'].split(',')]
            Biolog_in_model.loc[i,'Internal'] = all(any(x.rsplit('_',1)[0]+'_'+c in model.metabolites
                                                        for c in model.compartments) \
                                                     for x in Biolog_in_model.loc[i,'Metabolite'].split(','))
            Biolog_in_model.loc[i,'External'] = all(x in model.metabolites \
                                                    for x in Biolog_in_model.loc[i,'Metabolite'].split(','))
        else:
            Biolog_in_model.loc[i,'Model'] = model.metabolites.get_by_id(Biolog_in_model.loc[i,'Metabolite']).name \
                if Biolog_in_model.loc[i,'Metabolite'] in model.metabolites \
                else next((model.metabolites.get_by_id(Biolog_in_model.loc[i,'Metabolite'].rsplit('_',1)[0]+'_'+c).name \
                    for c in model.compartments 
                          if Biolog_in_model.loc[i,'Metabolite'].rsplit('_',1)[0]+c in model.metabolites), None)
            Biolog_in_model.loc[i,'Internal'] = any(Biolog_in_model.loc[i,'Metabolite'].rsplit('_',1)[0]+'_'+c
                                                    in model.metabolites for c in model.compartments)
            Biolog_in_model.loc[i,'External'] = Biolog_in_model.loc[i,'Metabolite'] in model.metabolites
    else:
        Biolog_in_model.loc[i] = None
    Biolog_in_model.loc[i,'Biolog'] = row['Compound']
    Biolog_in_model.loc[i,'Average'] = row['Average']
    Biolog_in_model.loc[i,'Pass'] = row['Pass']
```

In [60]:

```
print(Biolog_in_model.Internal.sum())
print(Biolog_in_model.External.sum())
```

```
213
213
```

In [61]:

```
model.medium
```

Out[61]:

```
{'EX_h_e': 1000.0,
 'EX_h2o_e': 1000.0,
 'EX_nh4_e': 1000.0,
 'EX_o2_e': 1000.0,
 'EX_pi_e': 1000.0,
 'EX_so4_e': 1000.0,
 'EX_glc__D_e': 1.0,
 'EX_ca2_e': 1000.0,
 'EX_fe2_e': 1000.0,
 'EX_fe3_e': 1000.0,
 'EX_k_e': 1000.0,
 'EX_na1_e': 1000.0,
 'EX_mg2_e': 1000.0,
 'EX_mn2_e': 1000.0,
 'EX_cu2_e': 1000.0,
 'EX_zn2_e': 1000.0}
```

In [62]:

```
Biolog_Prediction = pd.DataFrame(columns=['PlateType','Experiment','Row','Column',
                                          'Data','Data_TF','Prediction','Prediction_TF'])
with model:
    model.reactions.get_by_id('ATPM').lower_bound = 0.0
    model.reactions.get_by_id('EX_glc__D_e').lower_bound = 0.0
    model.reactions.get_by_id('EX_nh4_e').lower_bound = 0.0
    model.reactions.get_by_id('EX_pi_e').lower_bound = 0.0
    model.reactions.get_by_id('EX_so4_e').lower_bound = 0.0
    for i, row in Biolog.iterrows():
        Biolog_Prediction.loc[i,'PlateType'] = row['PlateType']
        Biolog_Prediction.loc[i,'Experiment'] = row['Experiment']
        Biolog_Prediction.loc[i,'Row'] = row['Row']
        Biolog_Prediction.loc[i,'Column'] = row['Column']
        Biolog_Prediction.loc[i,'Data'] = row['Average']
        Biolog_Prediction.loc[i,'Data_TF'] = row['Pass']
        if i in Biolog_Media.index and Biolog_in_model.loc[i,'External']:
            for x in Biolog_Media.loc[i][1:]:
                if ',' in x:
                    for y in x.split(','):
                        model.reactions.get_by_id(y).lower_bound = -10.0
                else:
                    model.reactions.get_by_id(x).lower_bound = -10.0
            sol = model.optimize()
            for x in Biolog_Media.loc[i][1:]:
                if ',' in x:
                    for y in x.split(','):
                        model.reactions.get_by_id(y).lower_bound = 0.0
                else:
                    model.reactions.get_by_id(x).lower_bound = 0.0
            if sol.status == 'optimal':
                Biolog_Prediction.loc[i,'Prediction'] = sol.objective_value
                Biolog_Prediction.loc[i,'Prediction_TF'] = sol.objective_value > 1e-3
            else:
                print(i, 'non-optimal')
                Biolog_Prediction.loc[i,'Prediction'] = np.nan
                Biolog_Prediction.loc[i,'Prediction_TF'] = np.nan
        elif i in Biolog_Media.index and Biolog_in_model.loc[i,'Internal']:
            continue
            for x in Biolog_Media.loc[i,Biolog_Media.loc[i,'Source']].split(','):
                if not x in model.reactions:
                    r = model.reactions.get_by_id('EX_h_e').copy()
                    r.id = x
                    model.add_reactions([r])
                    r.add_metabolites({'h_e': 1.0, x.replace('EX_','').rsplit('_',1)[0]+'_c': -1.0})
            for x in Biolog_Media.loc[i][1:]:
                if ',' in x:
                    for y in x.split(','):
                        model.reactions.get_by_id(y).lower_bound = -10.0
                else:
                    model.reactions.get_by_id(x).lower_bound = -10.0
            sol = model.optimize()
            for x in Biolog_Media.loc[i][1:]:
                if ',' in x:
                    for y in x.split(','):
                        model.reactions.get_by_id(y).lower_bound = 0.0
                else:
                    model.reactions.get_by_id(x).lower_bound = 0.0
            if sol.status == 'optimal':
                Biolog_Prediction.loc[i,'Prediction'] = sol.objective_value
                Biolog_Prediction.loc[i,'Prediction_TF'] = sol.objective_value > 1e-3
            else:
                print(i, 'non-optimal')
                Biolog_Prediction.loc[i,'Prediction'] = np.nan
                Biolog_Prediction.loc[i,'Prediction_TF'] = np.nan
        else:
            Biolog_Prediction.loc[i,'Prediction'] = np.nan
            Biolog_Prediction.loc[i,'Prediction_TF'] =np.nan
            #Biolog_Prediction.loc[i,'Prediction'] = 0.0
            #Biolog_Prediction.loc[i,'Prediction_TF'] = False
```

In [63]:

```
Biolog_Prediction['Data'] = Biolog_Prediction['Data'].astype(float)
Biolog_Prediction['Prediction'] = Biolog_Prediction['Prediction'].astype(float)
Biolog_Prediction.loc[abs(Biolog_Prediction.Prediction) < 1e-6, 'Prediction'] = 0
```

In [64]:

```
with pd.option_context('display.max_rows', None, 'display.max_columns', None):
    display(Biolog_Prediction)
```

|  | PlateType | Experiment | Row | Column | Data | Data\_TF | Prediction | Prediction\_TF |
| --- | --- | --- | --- | --- | --- | --- | --- | --- |
| PM1\_A1 | PM1 | Carbon | A | 1 | 0.086400 | False | NaN | NaN |
| PM1\_A2 | PM1 | Carbon | A | 2 | 0.344767 | True | 0.631354 | True |
| PM1\_A3 | PM1 | Carbon | A | 3 | 0.068867 | False | 0.000000 | False |
| PM1\_A4 | PM1 | Carbon | A | 4 | 0.071200 | False | NaN | NaN |
| PM1\_A5 | PM1 | Carbon | A | 5 | 0.111200 | True | 0.000000 | False |
| PM1\_A6 | PM1 | Carbon | A | 6 | 0.143300 | True | 0.757625 | True |
| PM1\_A7 | PM1 | Carbon | A | 7 | 0.267867 | True | 0.362006 | True |
| PM1\_A8 | PM1 | Carbon | A | 8 | 0.272400 | True | 0.000000 | False |
| PM1\_A9 | PM1 | Carbon | A | 9 | 0.121500 | True | 0.094657 | True |
| PM1\_A10 | PM1 | Carbon | A | 10 | 0.221300 | True | 1.515162 | True |
| PM1\_A11 | PM1 | Carbon | A | 11 | 0.212667 | True | 0.757625 | True |
| PM1\_A12 | PM1 | Carbon | A | 12 | 0.132067 | True | 0.799547 | True |
| PM1\_B1 | PM1 | Carbon | B | 1 | 0.150600 | True | 0.312158 | True |
| PM1\_B2 | PM1 | Carbon | B | 2 | 0.124767 | True | 0.799547 | True |
| PM1\_B3 | PM1 | Carbon | B | 3 | 0.224300 | True | 0.414492 | True |
| PM1\_B4 | PM1 | Carbon | B | 4 | 0.099667 | False | NaN | NaN |
| PM1\_B5 | PM1 | Carbon | B | 5 | 0.071833 | False | NaN | NaN |
| PM1\_B6 | PM1 | Carbon | B | 6 | 0.143467 | True | 0.672843 | True |
| PM1\_B7 | PM1 | Carbon | B | 7 | 0.072200 | False | 0.000000 | False |
| PM1\_B8 | PM1 | Carbon | B | 8 | 0.342267 | True | 0.631354 | True |
| PM1\_B9 | PM1 | Carbon | B | 9 | 0.122067 | True | 0.311209 | True |
| PM1\_B10 | PM1 | Carbon | B | 10 | 0.177800 | True | 0.000000 | False |
| PM1\_B11 | PM1 | Carbon | B | 11 | 0.120767 | True | NaN | NaN |
| PM1\_B12 | PM1 | Carbon | B | 12 | 0.297367 | True | 0.541215 | True |
| PM1\_C1 | PM1 | Carbon | C | 1 | 0.087033 | False | 0.000000 | False |
| PM1\_C2 | PM1 | Carbon | C | 2 | 0.055900 | False | NaN | NaN |
| PM1\_C3 | PM1 | Carbon | C | 3 | 0.114800 | True | NaN | NaN |
| PM1\_C4 | PM1 | Carbon | C | 4 | 0.312900 | True | 0.631354 | True |
| PM1\_C5 | PM1 | Carbon | C | 5 | 0.397733 | True | NaN | NaN |
| PM1\_C6 | PM1 | Carbon | C | 6 | 0.093733 | False | NaN | NaN |
| PM1\_C7 | PM1 | Carbon | C | 7 | 0.257533 | True | 0.757625 | True |
| PM1\_C8 | PM1 | Carbon | C | 8 | 0.243067 | True | 0.147001 | True |
| PM1\_C9 | PM1 | Carbon | C | 9 | 0.215400 | True | 0.757625 | True |
| PM1\_C10 | PM1 | Carbon | C | 10 | 0.105733 | False | 1.515120 | True |
| PM1\_C11 | PM1 | Carbon | C | 11 | 0.076300 | False | 1.515120 | True |
| PM1\_C12 | PM1 | Carbon | C | 12 | 0.089500 | False | 0.000000 | False |
| PM1\_D1 | PM1 | Carbon | D | 1 | 0.279933 | True | 0.362153 | True |
| PM1\_D2 | PM1 | Carbon | D | 2 | 0.095967 | False | 0.000000 | False |
| PM1\_D3 | PM1 | Carbon | D | 3 | 0.083900 | False | NaN | NaN |
| PM1\_D4 | PM1 | Carbon | D | 4 | 0.081500 | False | 0.000000 | False |
| PM1\_D5 | PM1 | Carbon | D | 5 | 0.347467 | True | NaN | NaN |
| PM1\_D6 | PM1 | Carbon | D | 6 | 0.103400 | True | 0.000000 | False |
| PM1\_D7 | PM1 | Carbon | D | 7 | 0.154233 | True | 0.446171 | True |
| PM1\_D8 | PM1 | Carbon | D | 8 | 0.081267 | False | NaN | NaN |
| PM1\_D9 | PM1 | Carbon | D | 9 | 0.081033 | False | NaN | NaN |
| PM1\_D10 | PM1 | Carbon | D | 10 | 0.083933 | False | NaN | NaN |
| PM1\_D11 | PM1 | Carbon | D | 11 | 0.125267 | True | 1.515120 | True |
| PM1\_D12 | PM1 | Carbon | D | 12 | 0.090700 | False | 0.000000 | False |
| PM1\_E1 | PM1 | Carbon | E | 1 | 0.315700 | True | 0.541872 | True |
| PM1\_E2 | PM1 | Carbon | E | 2 | 0.067167 | False | NaN | NaN |
| PM1\_E3 | PM1 | Carbon | E | 3 | 0.078633 | False | 0.000000 | False |
| PM1\_E4 | PM1 | Carbon | E | 4 | 0.097133 | False | 0.000000 | False |
| PM1\_E5 | PM1 | Carbon | E | 5 | 0.408233 | True | NaN | NaN |
| PM1\_E6 | PM1 | Carbon | E | 6 | 0.064367 | False | NaN | NaN |
| PM1\_E7 | PM1 | Carbon | E | 7 | 0.099200 | False | NaN | NaN |
| PM1\_E8 | PM1 | Carbon | E | 8 | 0.212600 | True | 0.860927 | True |
| PM1\_E9 | PM1 | Carbon | E | 9 | 0.225933 | True | 0.000000 | False |
| PM1\_E10 | PM1 | Carbon | E | 10 | 0.124433 | True | 1.677069 | True |
| PM1\_E11 | PM1 | Carbon | E | 11 | 0.068633 | False | NaN | NaN |
| PM1\_E12 | PM1 | Carbon | E | 12 | 0.077000 | False | 0.752506 | True |
| PM1\_F1 | PM1 | Carbon | F | 1 | 0.086133 | False | 0.373519 | True |
| PM1\_F2 | PM1 | Carbon | F | 2 | 0.087633 | False | 0.000000 | False |
| PM1\_F3 | PM1 | Carbon | F | 3 | 0.100567 | False | 0.000000 | False |
| PM1\_F4 | PM1 | Carbon | F | 4 | 0.112333 | False | NaN | NaN |
| PM1\_F5 | PM1 | Carbon | F | 5 | 0.093367 | False | 0.000000 | False |
| PM1\_F6 | PM1 | Carbon | F | 6 | 0.173133 | True | NaN | NaN |
| PM1\_F7 | PM1 | Carbon | F | 7 | 0.182700 | True | 0.346714 | True |
| PM1\_F8 | PM1 | Carbon | F | 8 | 0.087533 | False | NaN | NaN |
| PM1\_F9 | PM1 | Carbon | F | 9 | 0.082433 | False | 0.081819 | True |
| PM1\_F10 | PM1 | Carbon | F | 10 | 0.078933 | False | 0.000000 | False |
| PM1\_F11 | PM1 | Carbon | F | 11 | 0.198867 | True | 1.515120 | True |
| PM1\_F12 | PM1 | Carbon | F | 12 | 0.093200 | False | 0.000000 | False |
| PM1\_G1 | PM1 | Carbon | G | 1 | 0.153067 | True | 0.562184 | True |
| PM1\_G2 | PM1 | Carbon | G | 2 | 0.063433 | False | NaN | NaN |
| PM1\_G3 | PM1 | Carbon | G | 3 | 0.189367 | True | 0.312158 | True |
| PM1\_G4 | PM1 | Carbon | G | 4 | 0.160133 | True | 0.452342 | True |
| PM1\_G5 | PM1 | Carbon | G | 5 | 0.241200 | True | 0.094657 | True |
| PM1\_G6 | PM1 | Carbon | G | 6 | 0.186600 | True | 0.094657 | True |
| PM1\_G7 | PM1 | Carbon | G | 7 | 0.161467 | True | 0.425940 | True |
| PM1\_G8 | PM1 | Carbon | G | 8 | 0.084433 | False | NaN | NaN |
| PM1\_G9 | PM1 | Carbon | G | 9 | 0.200767 | True | NaN | NaN |
| PM1\_G10 | PM1 | Carbon | G | 10 | 0.170767 | True | NaN | NaN |
| PM1\_G11 | PM1 | Carbon | G | 11 | 0.121733 | True | NaN | NaN |
| PM1\_G12 | PM1 | Carbon | G | 12 | 0.107933 | False | 0.000000 | False |
| PM1\_H1 | PM1 | Carbon | H | 1 | 0.225233 | True | 0.000000 | False |
| PM1\_H2 | PM1 | Carbon | H | 2 | 0.139733 | True | NaN | NaN |
| PM1\_H3 | PM1 | Carbon | H | 3 | 0.082633 | False | NaN | NaN |
| PM1\_H4 | PM1 | Carbon | H | 4 | 0.070267 | False | 0.000000 | False |
| PM1\_H5 | PM1 | Carbon | H | 5 | 0.122867 | True | NaN | NaN |
| PM1\_H6 | PM1 | Carbon | H | 6 | 0.393067 | True | 0.631354 | True |
| PM1\_H7 | PM1 | Carbon | H | 7 | 0.112100 | True | NaN | NaN |
| PM1\_H8 | PM1 | Carbon | H | 8 | 0.215867 | True | 0.287721 | True |
| PM1\_H9 | PM1 | Carbon | H | 9 | 0.252700 | True | NaN | NaN |
| PM1\_H10 | PM1 | Carbon | H | 10 | 0.248833 | True | 0.000000 | False |
| PM1\_H11 | PM1 | Carbon | H | 11 | 0.082833 | False | 0.000000 | False |
| PM1\_H12 | PM1 | Carbon | H | 12 | 0.075267 | False | 0.000000 | False |
| PM2\_A1 | PM2 | Carbon | A | 1 | 0.082233 | False | NaN | NaN |
| PM2\_A2 | PM2 | Carbon | A | 2 | 0.057667 | False | NaN | NaN |
| PM2\_A3 | PM2 | Carbon | A | 3 | 0.059600 | False | NaN | NaN |
| PM2\_A4 | PM2 | Carbon | A | 4 | 0.079833 | False | NaN | NaN |
| PM2\_A5 | PM2 | Carbon | A | 5 | 0.102967 | False | NaN | NaN |
| PM2\_A6 | PM2 | Carbon | A | 6 | 0.129000 | True | NaN | NaN |
| PM2\_A7 | PM2 | Carbon | A | 7 | 0.133233 | True | NaN | NaN |
| PM2\_A8 | PM2 | Carbon | A | 8 | 0.074300 | False | 0.000000 | False |
| PM2\_A9 | PM2 | Carbon | A | 9 | 0.165867 | True | NaN | NaN |
| PM2\_A10 | PM2 | Carbon | A | 10 | 0.203733 | True | NaN | NaN |
| PM2\_A11 | PM2 | Carbon | A | 11 | 0.066133 | False | 0.000000 | False |
| PM2\_A12 | PM2 | Carbon | A | 12 | 0.159867 | True | NaN | NaN |
| PM2\_B1 | PM2 | Carbon | B | 1 | 0.077967 | False | NaN | NaN |
| PM2\_B2 | PM2 | Carbon | B | 2 | 0.072467 | False | NaN | NaN |
| PM2\_B3 | PM2 | Carbon | B | 3 | 0.107000 | False | NaN | NaN |
| PM2\_B4 | PM2 | Carbon | B | 4 | 0.105433 | True | NaN | NaN |
| PM2\_B5 | PM2 | Carbon | B | 5 | 0.269833 | True | 0.631354 | True |
| PM2\_B6 | PM2 | Carbon | B | 6 | 0.244600 | True | 0.672843 | True |
| PM2\_B7 | PM2 | Carbon | B | 7 | 0.249133 | True | 0.672843 | True |
| PM2\_B8 | PM2 | Carbon | B | 8 | 0.300467 | True | 0.757625 | True |
| PM2\_B9 | PM2 | Carbon | B | 9 | 0.209900 | True | 0.000000 | False |
| PM2\_B10 | PM2 | Carbon | B | 10 | 0.076100 | False | NaN | NaN |
| PM2\_B11 | PM2 | Carbon | B | 11 | 0.110233 | True | NaN | NaN |
| PM2\_B12 | PM2 | Carbon | B | 12 | 0.114800 | True | NaN | NaN |
| PM2\_C1 | PM2 | Carbon | C | 1 | 0.230400 | True | NaN | NaN |
| PM2\_C2 | PM2 | Carbon | C | 2 | 0.115167 | True | NaN | NaN |
| PM2\_C3 | PM2 | Carbon | C | 3 | 0.086033 | False | NaN | NaN |
| PM2\_C4 | PM2 | Carbon | C | 4 | 0.086133 | False | NaN | NaN |
| PM2\_C5 | PM2 | Carbon | C | 5 | 0.089767 | False | NaN | NaN |
| PM2\_C6 | PM2 | Carbon | C | 6 | 0.086867 | False | NaN | NaN |
| PM2\_C7 | PM2 | Carbon | C | 7 | 0.101900 | False | NaN | NaN |
| PM2\_C8 | PM2 | Carbon | C | 8 | 0.092833 | False | NaN | NaN |
| PM2\_C9 | PM2 | Carbon | C | 9 | 0.080167 | False | NaN | NaN |
| PM2\_C10 | PM2 | Carbon | C | 10 | 0.092733 | False | NaN | NaN |
| PM2\_C11 | PM2 | Carbon | C | 11 | 0.078433 | False | NaN | NaN |
| PM2\_C12 | PM2 | Carbon | C | 12 | 0.088200 | False | NaN | NaN |
| PM2\_D1 | PM2 | Carbon | D | 1 | 0.075867 | False | NaN | NaN |
| PM2\_D2 | PM2 | Carbon | D | 2 | 0.328200 | True | 0.757625 | True |
| PM2\_D3 | PM2 | Carbon | D | 3 | 0.090200 | False | NaN | NaN |
| PM2\_D4 | PM2 | Carbon | D | 4 | 0.094133 | False | 0.000000 | False |
| PM2\_D5 | PM2 | Carbon | D | 5 | 0.084233 | False | NaN | NaN |
| PM2\_D6 | PM2 | Carbon | D | 6 | 0.163200 | True | NaN | NaN |
| PM2\_D7 | PM2 | Carbon | D | 7 | 0.122033 | True | NaN | NaN |
| PM2\_D8 | PM2 | Carbon | D | 8 | 0.260667 | True | 0.672843 | True |
| PM2\_D9 | PM2 | Carbon | D | 9 | 0.078800 | False | NaN | NaN |
| PM2\_D10 | PM2 | Carbon | D | 10 | 0.240867 | True | 0.502531 | True |
| PM2\_D11 | PM2 | Carbon | D | 11 | 0.097467 | False | NaN | NaN |
| PM2\_D12 | PM2 | Carbon | D | 12 | 0.265533 | True | 0.500384 | True |
| PM2\_E1 | PM2 | Carbon | E | 1 | 0.006800 | False | 0.000000 | False |
| PM2\_E2 | PM2 | Carbon | E | 2 | 0.182000 | True | 0.811095 | True |
| PM2\_E3 | PM2 | Carbon | E | 3 | 0.071400 | False | NaN | NaN |
| PM2\_E4 | PM2 | Carbon | E | 4 | 0.073900 | False | NaN | NaN |
| PM2\_E5 | PM2 | Carbon | E | 5 | 0.171867 | True | 0.000000 | False |
| PM2\_E6 | PM2 | Carbon | E | 6 | 0.100167 | False | NaN | NaN |
| PM2\_E7 | PM2 | Carbon | E | 7 | 0.186267 | True | 0.554280 | True |
| PM2\_E8 | PM2 | Carbon | E | 8 | 0.196300 | True | 0.482732 | True |
| PM2\_E9 | PM2 | Carbon | E | 9 | 0.212067 | True | 0.488933 | True |
| PM2\_E10 | PM2 | Carbon | E | 10 | 0.156967 | True | NaN | NaN |
| PM2\_E11 | PM2 | Carbon | E | 11 | 0.071800 | False | NaN | NaN |
| PM2\_E12 | PM2 | Carbon | E | 12 | 0.219967 | True | 0.631354 | True |
| PM2\_F1 | PM2 | Carbon | F | 1 | 0.111333 | True | NaN | NaN |
| PM2\_F2 | PM2 | Carbon | F | 2 | 0.058900 | False | NaN | NaN |
| PM2\_F3 | PM2 | Carbon | F | 3 | 0.118367 | True | NaN | NaN |
| PM2\_F4 | PM2 | Carbon | F | 4 | 0.075933 | False | 0.000000 | False |
| PM2\_F5 | PM2 | Carbon | F | 5 | 0.146133 | True | NaN | NaN |
| PM2\_F6 | PM2 | Carbon | F | 6 | 0.149700 | True | 0.699300 | True |
| PM2\_F7 | PM2 | Carbon | F | 7 | 0.057833 | False | NaN | NaN |
| PM2\_F8 | PM2 | Carbon | F | 8 | 0.230433 | True | NaN | NaN |
| PM2\_F9 | PM2 | Carbon | F | 9 | 0.184000 | True | NaN | NaN |
| PM2\_F10 | PM2 | Carbon | F | 10 | 0.128233 | True | NaN | NaN |
| PM2\_F11 | PM2 | Carbon | F | 11 | 0.070400 | False | NaN | NaN |
| PM2\_F12 | PM2 | Carbon | F | 12 | 0.075800 | False | NaN | NaN |
| PM2\_G1 | PM2 | Carbon | G | 1 | 0.088133 | False | 0.147001 | True |
| PM2\_G2 | PM2 | Carbon | G | 2 | 0.254367 | True | NaN | NaN |
| PM2\_G3 | PM2 | Carbon | G | 3 | 0.071800 | False | 0.000000 | False |
| PM2\_G4 | PM2 | Carbon | G | 4 | 0.188467 | True | 0.581986 | True |
| PM2\_G5 | PM2 | Carbon | G | 5 | 0.254033 | True | 0.000000 | False |
| PM2\_G6 | PM2 | Carbon | G | 6 | 0.114700 | False | 0.000000 | False |
| PM2\_G7 | PM2 | Carbon | G | 7 | 0.164767 | True | 0.465643 | True |
| PM2\_G8 | PM2 | Carbon | G | 8 | 0.093567 | False | NaN | NaN |
| PM2\_G9 | PM2 | Carbon | G | 9 | 0.241867 | True | 0.836939 | True |
| PM2\_G10 | PM2 | Carbon | G | 10 | 0.173533 | True | 0.818448 | True |
| PM2\_G11 | PM2 | Carbon | G | 11 | 0.091567 | False | 0.000000 | False |
| PM2\_G12 | PM2 | Carbon | G | 12 | 0.062633 | False | 0.000000 | False |
| PM2\_H1 | PM2 | Carbon | H | 1 | 0.238633 | True | 0.581384 | True |
| PM2\_H2 | PM2 | Carbon | H | 2 | 0.061000 | False | 0.000000 | False |
| PM2\_H3 | PM2 | Carbon | H | 3 | 0.272200 | True | NaN | NaN |
| PM2\_H4 | PM2 | Carbon | H | 4 | 0.179467 | True | 0.669992 | True |
| PM2\_H5 | PM2 | Carbon | H | 5 | 0.064333 | False | NaN | NaN |
| PM2\_H6 | PM2 | Carbon | H | 6 | 0.038367 | False | NaN | NaN |
| PM2\_H7 | PM2 | Carbon | H | 7 | 0.091033 | False | NaN | NaN |
| PM2\_H8 | PM2 | Carbon | H | 8 | 0.066933 | False | 0.578715 | True |
| PM2\_H9 | PM2 | Carbon | H | 9 | 0.340200 | True | 0.374990 | True |
| PM2\_H10 | PM2 | Carbon | H | 10 | 0.072550 | False | 0.000000 | False |
| PM2\_H11 | PM2 | Carbon | H | 11 | 0.086967 | False | NaN | NaN |
| PM2\_H12 | PM2 | Carbon | H | 12 | 0.060133 | False | NaN | NaN |
| PM3B\_A1 | PM3B | Nitrogen | A | 1 | 0.100600 | False | NaN | NaN |
| PM3B\_A2 | PM3B | Nitrogen | A | 2 | 0.215700 | True | 0.287721 | True |
| PM3B\_A3 | PM3B | Nitrogen | A | 3 | 0.157167 | True | 0.253528 | True |
| PM3B\_A4 | PM3B | Nitrogen | A | 4 | 0.214200 | True | 0.243867 | True |
| PM3B\_A5 | PM3B | Nitrogen | A | 5 | 0.228100 | True | 0.286058 | True |
| PM3B\_A6 | PM3B | Nitrogen | A | 6 | 0.112633 | False | NaN | NaN |
| PM3B\_A7 | PM3B | Nitrogen | A | 7 | 0.216500 | True | 0.103576 | True |
| PM3B\_A8 | PM3B | Nitrogen | A | 8 | 0.228267 | True | 0.897035 | True |
| PM3B\_A9 | PM3B | Nitrogen | A | 9 | 0.211933 | True | 0.659058 | True |
| PM3B\_A10 | PM3B | Nitrogen | A | 10 | 0.236567 | True | 0.658804 | True |
| PM3B\_A11 | PM3B | Nitrogen | A | 11 | 0.162167 | True | 0.561895 | True |
| PM3B\_A12 | PM3B | Nitrogen | A | 12 | 0.343500 | True | 0.832622 | True |
| PM3B\_B1 | PM3B | Nitrogen | B | 1 | 0.269500 | True | 0.833707 | True |
| PM3B\_B2 | PM3B | Nitrogen | B | 2 | 0.200800 | True | 0.071218 | True |
| PM3B\_B3 | PM3B | Nitrogen | B | 3 | 0.140533 | True | 0.000000 | False |
| PM3B\_B4 | PM3B | Nitrogen | B | 4 | 0.183400 | True | 1.128228 | True |
| PM3B\_B5 | PM3B | Nitrogen | B | 5 | 0.175600 | True | 1.118530 | True |
| PM3B\_B6 | PM3B | Nitrogen | B | 6 | 0.159900 | True | 0.000000 | False |
| PM3B\_B7 | PM3B | Nitrogen | B | 7 | 0.218600 | True | 0.058960 | True |
| PM3B\_B8 | PM3B | Nitrogen | B | 8 | 0.173667 | True | 0.000000 | False |
| PM3B\_B9 | PM3B | Nitrogen | B | 9 | 0.228133 | True | 0.000000 | False |
| PM3B\_B10 | PM3B | Nitrogen | B | 10 | 0.174900 | True | 0.624317 | True |
| PM3B\_B11 | PM3B | Nitrogen | B | 11 | 0.162267 | True | 0.743984 | True |
| PM3B\_B12 | PM3B | Nitrogen | B | 12 | 0.265533 | True | 1.311039 | True |
| PM3B\_C1 | PM3B | Nitrogen | C | 1 | 0.197500 | True | 1.121971 | True |
| PM3B\_C2 | PM3B | Nitrogen | C | 2 | 0.203267 | True | 0.964393 | True |
| PM3B\_C3 | PM3B | Nitrogen | C | 3 | 0.146133 | True | 0.103576 | True |
| PM3B\_C4 | PM3B | Nitrogen | C | 4 | 0.111333 | True | NaN | NaN |
| PM3B\_C5 | PM3B | Nitrogen | C | 5 | 0.100800 | False | 0.000000 | False |
| PM3B\_C6 | PM3B | Nitrogen | C | 6 | 0.046600 | False | NaN | NaN |
| PM3B\_C7 | PM3B | Nitrogen | C | 7 | 0.110933 | False | NaN | NaN |
| PM3B\_C8 | PM3B | Nitrogen | C | 8 | 0.108500 | False | 0.624317 | True |
| PM3B\_C9 | PM3B | Nitrogen | C | 9 | 0.121267 | True | NaN | NaN |
| PM3B\_C10 | PM3B | Nitrogen | C | 10 | 0.190100 | True | 0.897285 | True |
| PM3B\_C11 | PM3B | Nitrogen | C | 11 | 0.252767 | True | 0.777625 | True |
| PM3B\_C12 | PM3B | Nitrogen | C | 12 | 0.223733 | True | 0.896025 | True |
| PM3B\_D1 | PM3B | Nitrogen | D | 1 | 0.112500 | False | 0.000000 | False |
| PM3B\_D2 | PM3B | Nitrogen | D | 2 | 0.217400 | True | NaN | NaN |
| PM3B\_D3 | PM3B | Nitrogen | D | 3 | 0.202267 | True | NaN | NaN |
| PM3B\_D4 | PM3B | Nitrogen | D | 4 | 0.150133 | True | NaN | NaN |
| PM3B\_D5 | PM3B | Nitrogen | D | 5 | 0.087867 | False | 0.470716 | True |
| PM3B\_D6 | PM3B | Nitrogen | D | 6 | 0.163700 | True | NaN | NaN |
| PM3B\_D7 | PM3B | Nitrogen | D | 7 | 0.151933 | True | NaN | NaN |
| PM3B\_D8 | PM3B | Nitrogen | D | 8 | 0.159533 | True | NaN | NaN |
| PM3B\_D9 | PM3B | Nitrogen | D | 9 | 0.182600 | True | 0.000000 | False |
| PM3B\_D10 | PM3B | Nitrogen | D | 10 | 0.079633 | False | NaN | NaN |
| PM3B\_D11 | PM3B | Nitrogen | D | 11 | 0.122467 | True | 0.893116 | True |
| PM3B\_D12 | PM3B | Nitrogen | D | 12 | 0.103200 | False | 0.000000 | False |
| PM3B\_E1 | PM3B | Nitrogen | E | 1 | 0.073933 | False | 0.000000 | False |
| PM3B\_E2 | PM3B | Nitrogen | E | 2 | 0.118233 | False | 0.000000 | False |
| PM3B\_E3 | PM3B | Nitrogen | E | 3 | 0.145667 | True | 0.000000 | False |
| PM3B\_E4 | PM3B | Nitrogen | E | 4 | 0.149867 | True | 0.457976 | True |
| PM3B\_E5 | PM3B | Nitrogen | E | 5 | 0.161000 | True | 0.376572 | True |
| PM3B\_E6 | PM3B | Nitrogen | E | 6 | 0.203700 | True | NaN | NaN |
| PM3B\_E7 | PM3B | Nitrogen | E | 7 | 0.215433 | True | NaN | NaN |
| PM3B\_E8 | PM3B | Nitrogen | E | 8 | 0.129100 | True | 0.000000 | False |
| PM3B\_E9 | PM3B | Nitrogen | E | 9 | 0.181900 | True | NaN | NaN |
| PM3B\_E10 | PM3B | Nitrogen | E | 10 | 0.130233 | True | NaN | NaN |
| PM3B\_E11 | PM3B | Nitrogen | E | 11 | 0.101067 | False | 0.000000 | False |
| PM3B\_E12 | PM3B | Nitrogen | E | 12 | 0.090833 | False | NaN | NaN |
| PM3B\_F1 | PM3B | Nitrogen | F | 1 | 0.096367 | False | NaN | NaN |
| PM3B\_F2 | PM3B | Nitrogen | F | 2 | 0.239667 | True | 0.400327 | True |
| PM3B\_F3 | PM3B | Nitrogen | F | 3 | 0.238100 | True | 1.081587 | True |
| PM3B\_F4 | PM3B | Nitrogen | F | 4 | 0.196300 | True | 0.000000 | False |
| PM3B\_F5 | PM3B | Nitrogen | F | 5 | 0.178733 | True | 0.000000 | False |
| PM3B\_F6 | PM3B | Nitrogen | F | 6 | 0.127233 | False | 0.355268 | True |
| PM3B\_F7 | PM3B | Nitrogen | F | 7 | 0.275767 | True | 1.023991 | True |
| PM3B\_F8 | PM3B | Nitrogen | F | 8 | 0.153600 | True | 0.000000 | False |
| PM3B\_F9 | PM3B | Nitrogen | F | 9 | 0.119500 | True | 0.000000 | False |
| PM3B\_F10 | PM3B | Nitrogen | F | 10 | 0.193733 | True | 0.000000 | False |
| PM3B\_F11 | PM3B | Nitrogen | F | 11 | 0.189400 | True | 0.000000 | False |
| PM3B\_F12 | PM3B | Nitrogen | F | 12 | 0.324900 | True | 0.000000 | False |
| PM3B\_G1 | PM3B | Nitrogen | G | 1 | 0.387333 | True | 0.000000 | False |
| PM3B\_G2 | PM3B | Nitrogen | G | 2 | 0.200100 | True | 0.000000 | False |
| PM3B\_G3 | PM3B | Nitrogen | G | 3 | 0.239433 | True | 0.000000 | False |
| PM3B\_G4 | PM3B | Nitrogen | G | 4 | 0.306567 | True | NaN | NaN |
| PM3B\_G5 | PM3B | Nitrogen | G | 5 | 0.194900 | True | 0.414006 | True |
| PM3B\_G6 | PM3B | Nitrogen | G | 6 | 0.215033 | True | NaN | NaN |
| PM3B\_G7 | PM3B | Nitrogen | G | 7 | 0.157433 | True | NaN | NaN |
| PM3B\_G8 | PM3B | Nitrogen | G | 8 | 0.258267 | True | 0.814905 | True |
| PM3B\_G9 | PM3B | Nitrogen | G | 9 | 0.138967 | True | NaN | NaN |
| PM3B\_G10 | PM3B | Nitrogen | G | 10 | 0.228933 | True | NaN | NaN |
| PM3B\_G11 | PM3B | Nitrogen | G | 11 | 0.200433 | True | NaN | NaN |
| PM3B\_G12 | PM3B | Nitrogen | G | 12 | 0.314867 | True | NaN | NaN |
| PM3B\_H1 | PM3B | Nitrogen | H | 1 | 0.196167 | True | 0.843702 | True |
| PM3B\_H2 | PM3B | Nitrogen | H | 2 | 0.294933 | True | 1.026682 | True |
| PM3B\_H3 | PM3B | Nitrogen | H | 3 | 0.187667 | True | 1.025275 | True |
| PM3B\_H4 | PM3B | Nitrogen | H | 4 | 0.268000 | True | 0.103576 | True |
| PM3B\_H5 | PM3B | Nitrogen | H | 5 | 0.258600 | True | 0.108096 | True |
| PM3B\_H6 | PM3B | Nitrogen | H | 6 | 0.162200 | True | 1.336765 | True |
| PM3B\_H7 | PM3B | Nitrogen | H | 7 | 0.206067 | True | 0.925134 | True |
| PM3B\_H8 | PM3B | Nitrogen | H | 8 | 0.308767 | True | 0.683531 | True |
| PM3B\_H9 | PM3B | Nitrogen | H | 9 | 0.258733 | True | 0.864603 | True |
| PM3B\_H10 | PM3B | Nitrogen | H | 10 | 0.280533 | True | 0.863437 | True |
| PM3B\_H11 | PM3B | Nitrogen | H | 11 | 0.283833 | True | 0.191168 | True |
| PM3B\_H12 | PM3B | Nitrogen | H | 12 | 0.370500 | True | 0.287581 | True |
| PM4A\_A1 | PM4A | Phosphorus | A | 1 | 0.067500 | False | NaN | NaN |
| PM4A\_A2 | PM4A | Phosphorus | A | 2 | 0.217267 | True | 0.287721 | True |
| PM4A\_A3 | PM4A | Phosphorus | A | 3 | 0.299700 | True | 0.287721 | True |
| PM4A\_A4 | PM4A | Phosphorus | A | 4 | 0.305667 | True | NaN | NaN |
| PM4A\_A5 | PM4A | Phosphorus | A | 5 | 0.383400 | True | 0.287721 | True |
| PM4A\_A6 | PM4A | Phosphorus | A | 6 | 0.065967 | False | NaN | NaN |
| PM4A\_A7 | PM4A | Phosphorus | A | 7 | 0.061300 | False | NaN | NaN |
| PM4A\_A8 | PM4A | Phosphorus | A | 8 | 0.298300 | True | NaN | NaN |
| PM4A\_A9 | PM4A | Phosphorus | A | 9 | 0.237633 | True | NaN | NaN |
| PM4A\_A10 | PM4A | Phosphorus | A | 10 | 0.220933 | True | 1.081587 | True |
| PM4A\_A11 | PM4A | Phosphorus | A | 11 | 0.209733 | True | NaN | NaN |
| PM4A\_A12 | PM4A | Phosphorus | A | 12 | 0.272933 | True | 1.083006 | True |
| PM4A\_B1 | PM4A | Phosphorus | B | 1 | 0.124300 | True | NaN | NaN |
| PM4A\_B2 | PM4A | Phosphorus | B | 2 | 0.324100 | True | NaN | NaN |
| PM4A\_B3 | PM4A | Phosphorus | B | 3 | 0.212700 | True | 0.000000 | False |
| PM4A\_B4 | PM4A | Phosphorus | B | 4 | 0.203100 | True | 0.730628 | True |
| PM4A\_B5 | PM4A | Phosphorus | B | 5 | 0.337933 | True | 0.288065 | True |
| PM4A\_B6 | PM4A | Phosphorus | B | 6 | 0.295633 | True | 0.588167 | True |
| PM4A\_B7 | PM4A | Phosphorus | B | 7 | 0.286367 | True | 0.588167 | True |
| PM4A\_B8 | PM4A | Phosphorus | B | 8 | 0.201267 | True | NaN | NaN |
| PM4A\_B9 | PM4A | Phosphorus | B | 9 | 0.224833 | True | NaN | NaN |
| PM4A\_B10 | PM4A | Phosphorus | B | 10 | 0.222300 | True | 1.023991 | True |
| PM4A\_B11 | PM4A | Phosphorus | B | 11 | 0.233500 | True | NaN | NaN |
| PM4A\_B12 | PM4A | Phosphorus | B | 12 | 0.218567 | True | 1.024229 | True |
| PM4A\_C1 | PM4A | Phosphorus | C | 1 | 0.304033 | True | 0.575442 | True |
| PM4A\_C2 | PM4A | Phosphorus | C | 2 | 0.238667 | True | NaN | NaN |
| PM4A\_C3 | PM4A | Phosphorus | C | 3 | 0.217267 | True | 0.000000 | False |
| PM4A\_C4 | PM4A | Phosphorus | C | 4 | 0.248967 | True | 0.000000 | False |
| PM4A\_C5 | PM4A | Phosphorus | C | 5 | 0.074400 | False | 0.000000 | False |
| PM4A\_C6 | PM4A | Phosphorus | C | 6 | 0.219933 | True | 0.288452 | True |
| PM4A\_C7 | PM4A | Phosphorus | C | 7 | 0.308633 | True | 0.991006 | True |
| PM4A\_C8 | PM4A | Phosphorus | C | 8 | 0.302167 | True | NaN | NaN |
| PM4A\_C9 | PM4A | Phosphorus | C | 9 | 0.075300 | False | NaN | NaN |
| PM4A\_C10 | PM4A | Phosphorus | C | 10 | 0.313933 | True | 0.290925 | True |
| PM4A\_C11 | PM4A | Phosphorus | C | 11 | 0.197300 | True | NaN | NaN |
| PM4A\_C12 | PM4A | Phosphorus | C | 12 | 0.196100 | True | 0.000000 | False |
| PM4A\_D1 | PM4A | Phosphorus | D | 1 | 0.252833 | True | 1.058832 | True |
| PM4A\_D2 | PM4A | Phosphorus | D | 2 | 0.294767 | True | 1.058832 | True |
| PM4A\_D3 | PM4A | Phosphorus | D | 3 | 0.366467 | True | NaN | NaN |
| PM4A\_D4 | PM4A | Phosphorus | D | 4 | 0.242233 | True | NaN | NaN |
| PM4A\_D5 | PM4A | Phosphorus | D | 5 | 0.251967 | True | NaN | NaN |
| PM4A\_D6 | PM4A | Phosphorus | D | 6 | 0.236900 | True | 0.000000 | False |
| PM4A\_D7 | PM4A | Phosphorus | D | 7 | 0.270133 | True | NaN | NaN |
| PM4A\_D8 | PM4A | Phosphorus | D | 8 | 0.230733 | True | NaN | NaN |
| PM4A\_D9 | PM4A | Phosphorus | D | 9 | 0.212100 | True | NaN | NaN |
| PM4A\_D10 | PM4A | Phosphorus | D | 10 | 0.231200 | True | 0.290902 | True |
| PM4A\_D11 | PM4A | Phosphorus | D | 11 | 0.225300 | True | NaN | NaN |
| PM4A\_D12 | PM4A | Phosphorus | D | 12 | 0.218200 | True | NaN | NaN |
| PM4A\_E1 | PM4A | Phosphorus | E | 1 | 0.235467 | True | NaN | NaN |
| PM4A\_E2 | PM4A | Phosphorus | E | 2 | 0.192400 | True | NaN | NaN |
| PM4A\_E3 | PM4A | Phosphorus | E | 3 | 0.218667 | True | NaN | NaN |
| PM4A\_E4 | PM4A | Phosphorus | E | 4 | 0.216200 | True | 0.000000 | False |
| PM4A\_E5 | PM4A | Phosphorus | E | 5 | 0.211700 | True | 0.287721 | True |
| PM4A\_E6 | PM4A | Phosphorus | E | 6 | 0.062000 | False | NaN | NaN |
| PM4A\_E7 | PM4A | Phosphorus | E | 7 | 0.060400 | False | NaN | NaN |
| PM4A\_E8 | PM4A | Phosphorus | E | 8 | 0.175633 | True | NaN | NaN |
| PM4A\_E9 | PM4A | Phosphorus | E | 9 | 0.198100 | True | NaN | NaN |
| PM4A\_E10 | PM4A | Phosphorus | E | 10 | 0.190300 | True | 0.287721 | True |
| PM4A\_E11 | PM4A | Phosphorus | E | 11 | 0.288667 | True | 0.287832 | True |
| PM4A\_E12 | PM4A | Phosphorus | E | 12 | 0.124133 | False | NaN | NaN |
| PM4A\_F1 | PM4A | Sulfur | F | 1 | 0.078800 | False | NaN | NaN |
| PM4A\_F2 | PM4A | Sulfur | F | 2 | 0.233500 | True | 0.287721 | True |
| PM4A\_F3 | PM4A | Sulfur | F | 3 | 0.256500 | True | 0.000000 | False |
| PM4A\_F4 | PM4A | Sulfur | F | 4 | 0.194367 | True | NaN | NaN |
| PM4A\_F5 | PM4A | Sulfur | F | 5 | 0.216967 | True | NaN | NaN |
| PM4A\_F6 | PM4A | Sulfur | F | 6 | 0.200267 | True | NaN | NaN |
| PM4A\_F7 | PM4A | Sulfur | F | 7 | 0.180100 | True | 0.290612 | True |
| PM4A\_F8 | PM4A | Sulfur | F | 8 | 0.121600 | True | NaN | NaN |
| PM4A\_F9 | PM4A | Sulfur | F | 9 | 0.339133 | True | 0.301188 | True |
| PM4A\_F10 | PM4A | Sulfur | F | 10 | 0.141700 | True | 0.287731 | True |
| PM4A\_F11 | PM4A | Sulfur | F | 11 | 0.186700 | True | NaN | NaN |
| PM4A\_F12 | PM4A | Sulfur | F | 12 | 0.193200 | True | 0.700712 | True |
| PM4A\_G1 | PM4A | Sulfur | G | 1 | 0.121333 | True | NaN | NaN |
| PM4A\_G2 | PM4A | Sulfur | G | 2 | 0.155867 | True | NaN | NaN |
| PM4A\_G3 | PM4A | Sulfur | G | 3 | 0.183667 | True | 0.743643 | True |
| PM4A\_G4 | PM4A | Sulfur | G | 4 | 0.182200 | True | NaN | NaN |
| PM4A\_G5 | PM4A | Sulfur | G | 5 | 0.132700 | True | 0.871645 | True |
| PM4A\_G6 | PM4A | Sulfur | G | 6 | 0.106300 | True | NaN | NaN |
| PM4A\_G7 | PM4A | Sulfur | G | 7 | 0.268700 | True | 0.293816 | True |
| PM4A\_G8 | PM4A | Sulfur | G | 8 | 0.206700 | True | NaN | NaN |
| PM4A\_G9 | PM4A | Sulfur | G | 9 | 0.268467 | True | 0.305043 | True |
| PM4A\_G10 | PM4A | Sulfur | G | 10 | 0.174567 | True | NaN | NaN |
| PM4A\_G11 | PM4A | Sulfur | G | 11 | 0.259267 | True | 0.293573 | True |
| PM4A\_G12 | PM4A | Sulfur | G | 12 | 0.174233 | True | NaN | NaN |
| PM4A\_H1 | PM4A | Sulfur | H | 1 | 0.343900 | True | NaN | NaN |
| PM4A\_H2 | PM4A | Sulfur | H | 2 | 0.167867 | True | NaN | NaN |
| PM4A\_H3 | PM4A | Sulfur | H | 3 | 0.144700 | True | NaN | NaN |
| PM4A\_H4 | PM4A | Sulfur | H | 4 | 0.269967 | True | NaN | NaN |
| PM4A\_H5 | PM4A | Sulfur | H | 5 | 0.131533 | True | 0.000000 | False |
| PM4A\_H6 | PM4A | Sulfur | H | 6 | 0.182767 | True | 0.287731 | True |
| PM4A\_H7 | PM4A | Sulfur | H | 7 | 0.219867 | True | 0.000000 | False |
| PM4A\_H8 | PM4A | Sulfur | H | 8 | 0.086933 | False | NaN | NaN |
| PM4A\_H9 | PM4A | Sulfur | H | 9 | 0.188767 | True | NaN | NaN |
| PM4A\_H10 | PM4A | Sulfur | H | 10 | 0.177900 | True | NaN | NaN |
| PM4A\_H11 | PM4A | Sulfur | H | 11 | 0.206367 | True | NaN | NaN |
| PM4A\_H12 | PM4A | Sulfur | H | 12 | 0.079033 | False | NaN | NaN |

In [65]:

```
temp = Biolog_Prediction.index[~np.isnan(Biolog_Prediction.Prediction)]
y_data = Biolog_Prediction.Data_TF[temp].astype(int)
y_pred = Biolog_Prediction.Prediction_TF[temp].astype(int)
TN, FP, FN, TP = confusion_matrix(y_data, y_pred).ravel()
print(TN, FP, FN, TP, sum([TN, FP, FN, TP]))
```

```
36 10 36 131 213
```

In [66]:

```
df_confusion = pd.DataFrame(confusion_matrix(y_data, y_pred),
                            index = pd.MultiIndex.from_product([['Experiment'],['No growth', 'Growth']]),
                            columns = pd.MultiIndex.from_product([['Prediction'],['No growth', 'Growth']]))
df_confusion
```

Out[66]:

|  |  | Prediction | |
| --- | --- | --- | --- |
|  |  | No growth | Growth |
| Experiment | No growth | 36 | 10 |
|  | Growth | 36 | 131 |

In [67]:

```
# Sensitivity, hit rate, recall, or true positive rate
TPR = TP/(TP+FN)
# Specificity or true negative rate
TNR = TN/(TN+FP) 
# Precision or positive predictive value
PPV = TP/(TP+FP)
# Negative predictive value
NPV = TN/(TN+FN)
# Fall out or false positive rate
FPR = FP/(FP+TN)
# False negative rate
FNR = FN/(TP+FN)
# False discovery rate
FDR = FP/(TP+FP)
# Overall accuracy
ACC = (TP+TN)/(TP+FP+FN+TN)
# Matthew's
MCC = matthews_corrcoef(y_data, y_pred)
print('Recall:', TPR.round(3))
print('Precision:', PPV.round(3))
print('Accuracy:', ACC.round(3))
print('Matthew\'s correlation:', MCC.round(3))
```

```
Recall: 0.784
Precision: 0.929
Accuracy: 0.784
Matthew's correlation: 0.493
```

In [68]:

```
Biolog_Prediction_Normalized = Biolog_Prediction.copy()
Biolog_Prediction_Normalized.loc['Data'] = Biolog_Prediction_Normalized['Data'].astype(float)
Biolog_Prediction_Normalized.loc[0:96,'Data'] = Biolog_Prediction_Normalized['Data'][0:96] - Biolog_Prediction_Normalized['Data'][0]
Biolog_Prediction_Normalized.loc[96:192,'Data'] = Biolog_Prediction_Normalized['Data'][96:192] - Biolog_Prediction_Normalized['Data'][96]
Biolog_Prediction_Normalized.loc[192:288,'Data'] = Biolog_Prediction_Normalized['Data'][192:288] - Biolog_Prediction_Normalized['Data'][192]
Biolog_Prediction_Normalized.loc[288:384,'Data'] = Biolog_Prediction_Normalized['Data'][288:384] - Biolog_Prediction_Normalized['Data'][288]

temp = Biolog_Prediction[['Data_TF','Prediction_TF']].copy()
#temp = Biolog_Prediction_Normalized[['Data','Prediction_TF']]
temp.columns = ['Data','Model']
temp['Data'] = temp['Data'].astype(float)
temp['Model'] = temp['Model'].astype(float)*temp['Data'].max()
temp.index = temp.index+': '+Biolog.loc[temp.index,'Compound']

len(temp)
```

```
/Users/kimj972/anaconda3/envs/python3_cobrapy0.17.1/lib/python3.6/site-packages/ipykernel_launcher.py:3 FutureWarning: Slicing a positional slice with .loc is not supported, and will raise TypeError in a future version.  Use .loc with labels or .iloc with positions instead.
/Users/kimj972/anaconda3/envs/python3_cobrapy0.17.1/lib/python3.6/site-packages/ipykernel_launcher.py:4 FutureWarning: Slicing a positional slice with .loc is not supported, and will raise TypeError in a future version.  Use .loc with labels or .iloc with positions instead.
/Users/kimj972/anaconda3/envs/python3_cobrapy0.17.1/lib/python3.6/site-packages/ipykernel_launcher.py:5 FutureWarning: Slicing a positional slice with .loc is not supported, and will raise TypeError in a future version.  Use .loc with labels or .iloc with positions instead.
/Users/kimj972/anaconda3/envs/python3_cobrapy0.17.1/lib/python3.6/site-packages/ipykernel_launcher.py:6 FutureWarning: Slicing a positional slice with .loc is not supported, and will raise TypeError in a future version.  Use .loc with labels or .iloc with positions instead.
```

Out[68]:

```
384
```

In [69]:

```
fig, ax = plt.subplots(figsize=(20,20), ncols=4)
sns.heatmap(temp[0:96], linewidths=1, linecolor='black', ax=ax[0], cmap='Reds', cbar=False, mask=temp[0:96].isnull())
sns.heatmap(temp[96:192], linewidths=1, linecolor='black', ax=ax[1], cmap='Reds', cbar=False, mask=temp[96:192].isnull())
sns.heatmap(temp[192:288], linewidths=1, linecolor='black', ax=ax[2], cmap='Reds', cbar=False, mask=temp[192:288].isnull())
sns.heatmap(temp[288:384], linewidths=1, linecolor='black', ax=ax[3], cmap='Reds', cbar=False, mask=temp[288:384].isnull())
ax[0].xaxis.tick_top()
ax[1].xaxis.tick_top()
ax[2].xaxis.tick_top()
ax[3].xaxis.tick_top()
ax[0].set_facecolor('xkcd:grey')
ax[1].set_facecolor('xkcd:grey')
ax[2].set_facecolor('xkcd:grey')
ax[3].set_facecolor('xkcd:grey')
plt.tight_layout()
```

In [70]:

```
temp = Biolog_Prediction.pivot_table(index=['PlateType','Row'], columns='Column', values='Data')
temp.style.background_gradient(cmap='Reds', low=0, high=0.5, axis=None).set_precision(2)
```

Out[70]:

|  | Column | 1 | 2 | 3 | 4 | 5 | 6 | 7 | 8 | 9 | 10 | 11 | 12 |
| --- | --- | --- | --- | --- | --- | --- | --- | --- | --- | --- | --- | --- | --- |
| PlateType | Row |  |  |  |  |  |  |  |  |  |  |  |  |
| PM1 | A | 0.09 | 0.34 | 0.07 | 0.07 | 0.11 | 0.14 | 0.27 | 0.27 | 0.12 | 0.22 | 0.21 | 0.13 |
|  | B | 0.15 | 0.12 | 0.22 | 0.10 | 0.07 | 0.14 | 0.07 | 0.34 | 0.12 | 0.18 | 0.12 | 0.30 |
|  | C | 0.09 | 0.06 | 0.11 | 0.31 | 0.40 | 0.09 | 0.26 | 0.24 | 0.22 | 0.11 | 0.08 | 0.09 |
|  | D | 0.28 | 0.10 | 0.08 | 0.08 | 0.35 | 0.10 | 0.15 | 0.08 | 0.08 | 0.08 | 0.13 | 0.09 |
|  | E | 0.32 | 0.07 | 0.08 | 0.10 | 0.41 | 0.06 | 0.10 | 0.21 | 0.23 | 0.12 | 0.07 | 0.08 |
|  | F | 0.09 | 0.09 | 0.10 | 0.11 | 0.09 | 0.17 | 0.18 | 0.09 | 0.08 | 0.08 | 0.20 | 0.09 |
|  | G | 0.15 | 0.06 | 0.19 | 0.16 | 0.24 | 0.19 | 0.16 | 0.08 | 0.20 | 0.17 | 0.12 | 0.11 |
|  | H | 0.23 | 0.14 | 0.08 | 0.07 | 0.12 | 0.39 | 0.11 | 0.22 | 0.25 | 0.25 | 0.08 | 0.08 |
| PM2 | A | 0.08 | 0.06 | 0.06 | 0.08 | 0.10 | 0.13 | 0.13 | 0.07 | 0.17 | 0.20 | 0.07 | 0.16 |
|  | B | 0.08 | 0.07 | 0.11 | 0.11 | 0.27 | 0.24 | 0.25 | 0.30 | 0.21 | 0.08 | 0.11 | 0.11 |
|  | C | 0.23 | 0.12 | 0.09 | 0.09 | 0.09 | 0.09 | 0.10 | 0.09 | 0.08 | 0.09 | 0.08 | 0.09 |
|  | D | 0.08 | 0.33 | 0.09 | 0.09 | 0.08 | 0.16 | 0.12 | 0.26 | 0.08 | 0.24 | 0.10 | 0.27 |
|  | E | 0.01 | 0.18 | 0.07 | 0.07 | 0.17 | 0.10 | 0.19 | 0.20 | 0.21 | 0.16 | 0.07 | 0.22 |
|  | F | 0.11 | 0.06 | 0.12 | 0.08 | 0.15 | 0.15 | 0.06 | 0.23 | 0.18 | 0.13 | 0.07 | 0.08 |
|  | G | 0.09 | 0.25 | 0.07 | 0.19 | 0.25 | 0.11 | 0.16 | 0.09 | 0.24 | 0.17 | 0.09 | 0.06 |
|  | H | 0.24 | 0.06 | 0.27 | 0.18 | 0.06 | 0.04 | 0.09 | 0.07 | 0.34 | 0.07 | 0.09 | 0.06 |
| PM3B | A | 0.10 | 0.22 | 0.16 | 0.21 | 0.23 | 0.11 | 0.22 | 0.23 | 0.21 | 0.24 | 0.16 | 0.34 |
|  | B | 0.27 | 0.20 | 0.14 | 0.18 | 0.18 | 0.16 | 0.22 | 0.17 | 0.23 | 0.17 | 0.16 | 0.27 |
|  | C | 0.20 | 0.20 | 0.15 | 0.11 | 0.10 | 0.05 | 0.11 | 0.11 | 0.12 | 0.19 | 0.25 | 0.22 |
|  | D | 0.11 | 0.22 | 0.20 | 0.15 | 0.09 | 0.16 | 0.15 | 0.16 | 0.18 | 0.08 | 0.12 | 0.10 |
|  | E | 0.07 | 0.12 | 0.15 | 0.15 | 0.16 | 0.20 | 0.22 | 0.13 | 0.18 | 0.13 | 0.10 | 0.09 |
|  | F | 0.10 | 0.24 | 0.24 | 0.20 | 0.18 | 0.13 | 0.28 | 0.15 | 0.12 | 0.19 | 0.19 | 0.32 |
|  | G | 0.39 | 0.20 | 0.24 | 0.31 | 0.19 | 0.22 | 0.16 | 0.26 | 0.14 | 0.23 | 0.20 | 0.31 |
|  | H | 0.20 | 0.29 | 0.19 | 0.27 | 0.26 | 0.16 | 0.21 | 0.31 | 0.26 | 0.28 | 0.28 | 0.37 |
| PM4A | A | 0.07 | 0.22 | 0.30 | 0.31 | 0.38 | 0.07 | 0.06 | 0.30 | 0.24 | 0.22 | 0.21 | 0.27 |
|  | B | 0.12 | 0.32 | 0.21 | 0.20 | 0.34 | 0.30 | 0.29 | 0.20 | 0.22 | 0.22 | 0.23 | 0.22 |
|  | C | 0.30 | 0.24 | 0.22 | 0.25 | 0.07 | 0.22 | 0.31 | 0.30 | 0.08 | 0.31 | 0.20 | 0.20 |
|  | D | 0.25 | 0.29 | 0.37 | 0.24 | 0.25 | 0.24 | 0.27 | 0.23 | 0.21 | 0.23 | 0.23 | 0.22 |
|  | E | 0.24 | 0.19 | 0.22 | 0.22 | 0.21 | 0.06 | 0.06 | 0.18 | 0.20 | 0.19 | 0.29 | 0.12 |
|  | F | 0.08 | 0.23 | 0.26 | 0.19 | 0.22 | 0.20 | 0.18 | 0.12 | 0.34 | 0.14 | 0.19 | 0.19 |
|  | G | 0.12 | 0.16 | 0.18 | 0.18 | 0.13 | 0.11 | 0.27 | 0.21 | 0.27 | 0.17 | 0.26 | 0.17 |
|  | H | 0.34 | 0.17 | 0.14 | 0.27 | 0.13 | 0.18 | 0.22 | 0.09 | 0.19 | 0.18 | 0.21 | 0.08 |

In [71]:

```
temp = Biolog_Prediction.pivot_table(index=['PlateType','Row'], columns='Column', values='Prediction', dropna=False)
temp.style.apply(background_gradient, cmap='RdBu_r', axis=None).highlight_null('lightgrey').set_precision(2)
```

Out[71]:

|  | Column | 1 | 2 | 3 | 4 | 5 | 6 | 7 | 8 | 9 | 10 | 11 | 12 |
| --- | --- | --- | --- | --- | --- | --- | --- | --- | --- | --- | --- | --- | --- |
| PlateType | Row |  |  |  |  |  |  |  |  |  |  |  |  |
| PM1 | A | nan | 0.63 | 0.00 | nan | 0.00 | 0.76 | 0.36 | 0.00 | 0.09 | 1.52 | 0.76 | 0.80 |
|  | B | 0.31 | 0.80 | 0.41 | nan | nan | 0.67 | 0.00 | 0.63 | 0.31 | 0.00 | nan | 0.54 |
|  | C | 0.00 | nan | nan | 0.63 | nan | nan | 0.76 | 0.15 | 0.76 | 1.52 | 1.52 | 0.00 |
|  | D | 0.36 | 0.00 | nan | 0.00 | nan | 0.00 | 0.45 | nan | nan | nan | 1.52 | 0.00 |
|  | E | 0.54 | nan | 0.00 | 0.00 | nan | nan | nan | 0.86 | 0.00 | 1.68 | nan | 0.75 |
|  | F | 0.37 | 0.00 | 0.00 | nan | 0.00 | nan | 0.35 | nan | 0.08 | 0.00 | 1.52 | 0.00 |
|  | G | 0.56 | nan | 0.31 | 0.45 | 0.09 | 0.09 | 0.43 | nan | nan | nan | nan | 0.00 |
|  | H | 0.00 | nan | nan | 0.00 | nan | 0.63 | nan | 0.29 | nan | 0.00 | 0.00 | 0.00 |
| PM2 | A | nan | nan | nan | nan | nan | nan | nan | 0.00 | nan | nan | 0.00 | nan |
|  | B | nan | nan | nan | nan | 0.63 | 0.67 | 0.67 | 0.76 | 0.00 | nan | nan | nan |
|  | C | nan | nan | nan | nan | nan | nan | nan | nan | nan | nan | nan | nan |
|  | D | nan | 0.76 | nan | 0.00 | nan | nan | nan | 0.67 | nan | 0.50 | nan | 0.50 |
|  | E | 0.00 | 0.81 | nan | nan | 0.00 | nan | 0.55 | 0.48 | 0.49 | nan | nan | 0.63 |
|  | F | nan | nan | nan | 0.00 | nan | 0.70 | nan | nan | nan | nan | nan | nan |
|  | G | 0.15 | nan | 0.00 | 0.58 | 0.00 | 0.00 | 0.47 | nan | 0.84 | 0.82 | 0.00 | 0.00 |
|  | H | 0.58 | 0.00 | nan | 0.67 | nan | nan | nan | 0.58 | 0.37 | 0.00 | nan | nan |
| PM3B | A | nan | 0.29 | 0.25 | 0.24 | 0.29 | nan | 0.10 | 0.90 | 0.66 | 0.66 | 0.56 | 0.83 |
|  | B | 0.83 | 0.07 | 0.00 | 1.13 | 1.12 | 0.00 | 0.06 | 0.00 | 0.00 | 0.62 | 0.74 | 1.31 |
|  | C | 1.12 | 0.96 | 0.10 | nan | 0.00 | nan | nan | 0.62 | nan | 0.90 | 0.78 | 0.90 |
|  | D | 0.00 | nan | nan | nan | 0.47 | nan | nan | nan | 0.00 | nan | 0.89 | 0.00 |
|  | E | 0.00 | 0.00 | 0.00 | 0.46 | 0.38 | nan | nan | 0.00 | nan | nan | 0.00 | nan |
|  | F | nan | 0.40 | 1.08 | 0.00 | 0.00 | 0.36 | 1.02 | 0.00 | 0.00 | 0.00 | 0.00 | 0.00 |
|  | G | 0.00 | 0.00 | 0.00 | nan | 0.41 | nan | nan | 0.81 | nan | nan | nan | nan |
|  | H | 0.84 | 1.03 | 1.03 | 0.10 | 0.11 | 1.34 | 0.93 | 0.68 | 0.86 | 0.86 | 0.19 | 0.29 |
| PM4A | A | nan | 0.29 | 0.29 | nan | 0.29 | nan | nan | nan | nan | 1.08 | nan | 1.08 |
|  | B | nan | nan | 0.00 | 0.73 | 0.29 | 0.59 | 0.59 | nan | nan | 1.02 | nan | 1.02 |
|  | C | 0.58 | nan | 0.00 | 0.00 | 0.00 | 0.29 | 0.99 | nan | nan | 0.29 | nan | 0.00 |
|  | D | 1.06 | 1.06 | nan | nan | nan | 0.00 | nan | nan | nan | 0.29 | nan | nan |
|  | E | nan | nan | nan | 0.00 | 0.29 | nan | nan | nan | nan | 0.29 | 0.29 | nan |
|  | F | nan | 0.29 | 0.00 | nan | nan | nan | 0.29 | nan | 0.30 | 0.29 | nan | 0.70 |
|  | G | nan | nan | 0.74 | nan | 0.87 | nan | 0.29 | nan | 0.31 | nan | 0.29 | nan |
|  | H | nan | nan | nan | nan | 0.00 | 0.29 | 0.00 | nan | nan | nan | nan | nan |

In [72]:

```
temp = Biolog_Prediction.pivot_table(index=['PlateType','Row'], columns='Column', values='Data')
temp_annot = Biolog.pivot_table(index=['PlateType','Row'], columns='Column', values='Compound',
                                aggfunc=lambda x: ', '.join(x.unique())).apply(lambda x: x.str.wrap(12))

fig, axes = plt.subplots(figsize=(18,8), nrows=2, ncols=2, sharex=True, sharey=True)
cbar_ax = fig.add_axes([.96, .3, .01, .4])
i = 0
j = 0
for k, df in temp.groupby(level=0):
    sns.heatmap(df.droplevel(level=0), annot=temp_annot.loc[k], fmt="", linewidth=0.1, cmap='Reds',
                annot_kws={"fontsize": 6}, vmin=0, vmax=0.5, ax=axes[i,j],
                cbar=(i == 0), cbar_ax=None if i else cbar_ax, cbar_kws={'label': 'A590 - A750'})
    axes[i,j].set_ylabel(k)
    axes[i,j].set_xlabel('')
    if j % 2 == 1:
        i = 1
        j = 0
    else:
        j = j+1
axes[0,0].xaxis.set_ticks_position('top')
axes[0,1].xaxis.set_ticks_position('top')
axes[1,0].xaxis.set_ticks_position('none')
axes[1,1].xaxis.set_ticks_position('none')
axes[0,0].set_yticklabels(axes[0,0].get_yticklabels(), rotation=0)
axes[1,0].set_yticklabels(axes[1,0].get_yticklabels(), rotation=0)
plt.tight_layout(rect=[0, 0, .95, 1])
plt.savefig('Biolog.svg', dpi=300)
```

```
/Users/kimj972/anaconda3/envs/python3_cobrapy0.17.1/lib/python3.6/site-packages/ipykernel_launcher.py:26 UserWarning: This figure includes Axes that are not compatible with tight_layout, so results might be incorrect.
```

In [73]:

```
temp = Biolog_Prediction.pivot_table(index=['PlateType','Row'], columns='Column', values='Data')
fig, axes = plt.subplots(figsize=(10,20), nrows=4)
i = 0
for k, df in temp.groupby(level=0):
    sns.heatmap(df.droplevel(level=0), annot=True, fmt="0.2f", linewidth=0.1, cmap='Reds', 
                vmin=0, vmax=temp.max().max(), ax=axes[i])
    axes[i].set_ylabel(k)
    axes[i].xaxis.set_ticks_position('top')
    i = i+1
```

In [74]:

```
temp = Biolog_Prediction_Normalized.pivot_table(index=['PlateType','Row'], columns='Column', values='Data')
temp.style.background_gradient(cmap='Reds', low=-0.2, high=0.5, axis=None).set_precision(2)
```

Out[74]:

|  | Column | 1 | 2 | 3 | 4 | 5 | 6 | 7 | 8 | 9 | 10 | 11 | 12 |
| --- | --- | --- | --- | --- | --- | --- | --- | --- | --- | --- | --- | --- | --- |
| PlateType | Row |  |  |  |  |  |  |  |  |  |  |  |  |
| PM1 | A | 0.00 | 0.26 | -0.02 | -0.02 | 0.02 | 0.06 | 0.18 | 0.19 | 0.04 | 0.13 | 0.13 | 0.05 |
|  | B | 0.06 | 0.04 | 0.14 | 0.01 | -0.01 | 0.06 | -0.01 | 0.26 | 0.04 | 0.09 | 0.03 | 0.21 |
|  | C | 0.00 | -0.03 | 0.03 | 0.23 | 0.31 | 0.01 | 0.17 | 0.16 | 0.13 | 0.02 | -0.01 | 0.00 |
|  | D | 0.19 | 0.01 | -0.00 | -0.00 | 0.26 | 0.02 | 0.07 | -0.01 | -0.01 | -0.00 | 0.04 | 0.00 |
|  | E | 0.23 | -0.02 | -0.01 | 0.01 | 0.32 | -0.02 | 0.01 | 0.13 | 0.14 | 0.04 | -0.02 | -0.01 |
|  | F | -0.00 | 0.00 | 0.01 | 0.03 | 0.01 | 0.09 | 0.10 | 0.00 | -0.00 | -0.01 | 0.11 | 0.01 |
|  | G | 0.07 | -0.02 | 0.10 | 0.07 | 0.15 | 0.10 | 0.08 | -0.00 | 0.11 | 0.08 | 0.04 | 0.02 |
|  | H | 0.14 | 0.05 | -0.00 | -0.02 | 0.04 | 0.31 | 0.03 | 0.13 | 0.17 | 0.16 | -0.00 | -0.01 |
| PM2 | A | 0.00 | -0.02 | -0.02 | -0.00 | 0.02 | 0.05 | 0.05 | -0.01 | 0.08 | 0.12 | -0.02 | 0.08 |
|  | B | -0.00 | -0.01 | 0.02 | 0.02 | 0.19 | 0.16 | 0.17 | 0.22 | 0.13 | -0.01 | 0.03 | 0.03 |
|  | C | 0.15 | 0.03 | 0.00 | 0.00 | 0.01 | 0.00 | 0.02 | 0.01 | -0.00 | 0.01 | -0.00 | 0.01 |
|  | D | -0.01 | 0.25 | 0.01 | 0.01 | 0.00 | 0.08 | 0.04 | 0.18 | -0.00 | 0.16 | 0.02 | 0.18 |
|  | E | -0.08 | 0.10 | -0.01 | -0.01 | 0.09 | 0.02 | 0.10 | 0.11 | 0.13 | 0.07 | -0.01 | 0.14 |
|  | F | 0.03 | -0.02 | 0.04 | -0.01 | 0.06 | 0.07 | -0.02 | 0.15 | 0.10 | 0.05 | -0.01 | -0.01 |
|  | G | 0.01 | 0.17 | -0.01 | 0.11 | 0.17 | 0.03 | 0.08 | 0.01 | 0.16 | 0.09 | 0.01 | -0.02 |
|  | H | 0.16 | -0.02 | 0.19 | 0.10 | -0.02 | -0.04 | 0.01 | -0.02 | 0.26 | -0.01 | 0.00 | -0.02 |
| PM3B | A | 0.00 | 0.12 | 0.06 | 0.11 | 0.13 | 0.01 | 0.12 | 0.13 | 0.11 | 0.14 | 0.06 | 0.24 |
|  | B | 0.17 | 0.10 | 0.04 | 0.08 | 0.08 | 0.06 | 0.12 | 0.07 | 0.13 | 0.07 | 0.06 | 0.16 |
|  | C | 0.10 | 0.10 | 0.05 | 0.01 | 0.00 | -0.05 | 0.01 | 0.01 | 0.02 | 0.09 | 0.15 | 0.12 |
|  | D | 0.01 | 0.12 | 0.10 | 0.05 | -0.01 | 0.06 | 0.05 | 0.06 | 0.08 | -0.02 | 0.02 | 0.00 |
|  | E | -0.03 | 0.02 | 0.05 | 0.05 | 0.06 | 0.10 | 0.11 | 0.03 | 0.08 | 0.03 | 0.00 | -0.01 |
|  | F | -0.00 | 0.14 | 0.14 | 0.10 | 0.08 | 0.03 | 0.18 | 0.05 | 0.02 | 0.09 | 0.09 | 0.22 |
|  | G | 0.29 | 0.10 | 0.14 | 0.21 | 0.09 | 0.11 | 0.06 | 0.16 | 0.04 | 0.13 | 0.10 | 0.21 |
|  | H | 0.10 | 0.19 | 0.09 | 0.17 | 0.16 | 0.06 | 0.11 | 0.21 | 0.16 | 0.18 | 0.18 | 0.27 |
| PM4A | A | 0.00 | 0.15 | 0.23 | 0.24 | 0.32 | -0.00 | -0.01 | 0.23 | 0.17 | 0.15 | 0.14 | 0.21 |
|  | B | 0.06 | 0.26 | 0.15 | 0.14 | 0.27 | 0.23 | 0.22 | 0.13 | 0.16 | 0.15 | 0.17 | 0.15 |
|  | C | 0.24 | 0.17 | 0.15 | 0.18 | 0.01 | 0.15 | 0.24 | 0.23 | 0.01 | 0.25 | 0.13 | 0.13 |
|  | D | 0.19 | 0.23 | 0.30 | 0.17 | 0.18 | 0.17 | 0.20 | 0.16 | 0.14 | 0.16 | 0.16 | 0.15 |
|  | E | 0.17 | 0.12 | 0.15 | 0.15 | 0.14 | -0.01 | -0.01 | 0.11 | 0.13 | 0.12 | 0.22 | 0.06 |
|  | F | 0.01 | 0.17 | 0.19 | 0.13 | 0.15 | 0.13 | 0.11 | 0.05 | 0.27 | 0.07 | 0.12 | 0.13 |
|  | G | 0.05 | 0.09 | 0.12 | 0.11 | 0.07 | 0.04 | 0.20 | 0.14 | 0.20 | 0.11 | 0.19 | 0.11 |
|  | H | 0.28 | 0.10 | 0.08 | 0.20 | 0.06 | 0.12 | 0.15 | 0.02 | 0.12 | 0.11 | 0.14 | 0.01 |

### Fitness¶

In [75]:

```
Fitness_metadata = pd.read_csv('../../Data/Fitness_Extended_Metadata.txt',
                               sep='\t', index_col=[0])
Fitness_metadata
```

Out[75]:

|  | Media | Format | Temperature | Starting pH | Shaking | StartOD | EndOD | Hours |
| --- | --- | --- | --- | --- | --- | --- | --- | --- |
| Condition |  |  |  |  |  |  |  |  |
| M9\_Glucose | M9 salts, Trace Elements, 1% Glucose | 10 mL in glass test tubes | 30.0 | 7 | 200 | 0.1 | 5.4 | 72.0 |
| YNB Glucose | YNB no Amino Acids 6.7 g/L, 2% Glucose | 100 mL in 250 mL baffled flask | 30.0 | approx 7 | 200 RPM | 0.1 | ~5 | 12.0 |
| YNB Glucose plus Arginine | YNB no Amino Acids 6.7 g/L, 2% Glucose, 75 mM ... | 100 mL in 250 mL baffled flask | 30.0 | approx 7 | 200 RPM | 0.1 | ~5 | 12.0 |
| YNB Glucose plus Methionine | YNB no Amino Acids 6.7 g/L, 2% Glucose, 75 mM ... | 100 mL in 250 mL baffled flask | 30.0 | approx 7 | 200 RPM | 0.1 | ~5 | 12.0 |
| YNB Glucose plus Dropout Complete | YNB no Amino Acids 6.7 g/L, 2% Glucose, 0.2% D... | 100 mL in 250 mL baffled flask | 30.0 | approx 7 | 200 RPM | 0.1 | ~5 | 12.0 |
| Fitness During Lipid Mobilization | YNB no Amino Acids or Ammonium Sulfate, 25 mM ... | 100 mL in 250 mL baffled flask | 30.0 | approx 7 | 200 RPM | 0.1 | ~10 | 88.0 |
| YNB Oleic Acid | YNB no Amino Acids 6.7 g/L, 1% Oleic Acid | 100 mL in 250 mL baffled flask | 30.0 | approx 7 | 200 RPM | 0.1 | ~3 | 72.0 |
| YNB Cellobiose | YNB no Amino Acids 6.7 g/L, 2% Cellobiose | 50 mL in 250 mL baffled flask | 30.0 | approx 7 | 200 RPM | 0.1 | 0.8 | 108.0 |
| YNB\_CSM\_KPO4 Glucose | YNB no Amino Acids 6.7 g/L, Complete Supplemen... | 50 mL in 250 mL baffled flask | 30.0 | 6 | 200 RPM | 0.1 | 11 | 20.0 |
| YNB\_CSM\_KPO4 Xylose | YNB no Amino Acids 6.7 g/L, Complete Supplemen... | 50 mL in 250 mL baffled flask | 30.0 | 6 | 200 RPM | 0.1 | 6 | 50.0 |
| YNB\_CSM\_KPO4 Arabinose | YNB no Amino Acids 6.7 g/L, Complete Supplemen... | 50 mL in 250 mL baffled flask | 30.0 | 6 | 200 RPM | 0.1 | 9.5 | 40.0 |
| YNB\_CSM\_KPO4 Acetate | YNB no Amino Acids 6.7 g/L, Complete Supplemen... | 50 mL in 250 mL baffled flask | 30.0 | 6 | 200 RPM | 0.1 | 2 | 20.0 |
| YNB\_CSM\_KPO4 p-Coumarate | YNB no Amino Acids 6.7 g/L, Complete Supplemen... | 50 mL in 250 mL baffled flask | 30.0 | 6 | 200 RPM | 0.1 | 5 | 50.0 |
| YNB\_CSM\_KPO4 Ferulate | YNB no Amino Acids 6.7 g/L, Complete Supplemen... | 50 mL in 250 mL baffled flask | 30.0 | 6 | 200 RPM | 0.1 | 5 | 50.0 |
| YNB\_PO4\_Benzoate | YNB no Amino Acids 6.7 g/L, 100mM PO4, 0.25% B... | 10 mL in glass test tubes | 30.0 | 7 | 200 | 0.1 | 1.9 | 72.0 |
| YNB\_PO4\_Mannose | YNB no Amino Acids 6.7 g/L, 100mM PO4, 1% Mannose | 10 mL in glass test tubes | 30.0 | 6 | 200 | 0.1 | 6 | 24.0 |
| YNB\_PO4\_L-lyxose | YNB no Amino Acids 6.7 g/L, 100mM PO4, 1% L-ly... | 10 mL in glass test tubes | 30.0 | 5 | 200 | 0.1 | 4.2826 | 96.0 |
| YNB\_PO4\_D-arabitol | YNB no Amino Acids 6.7 g/L, 100mM PO4, 1% D-ar... | 10 mL in glass test tubes | 30.0 | 5 | 200 | 0.1 | 7.3209 | 48.0 |
| YNB\_PO4\_L-arabitol | YNB no Amino Acids 6.7 g/L, 100mM PO4, 1% L-ar... | 10 mL in glass test tubes | 30.0 | 5 | 200 | 0.1 | 5.4947 | 48.0 |
| YNB\_PO4\_xylitol | YNB no Amino Acids 6.7 g/L, 100mM PO4, 1% xylitol | 10 mL in glass test tubes | 30.0 | 5 | 200 | 0.1 | 4.3677 | 48.0 |
| YNB\_PO4\_D-ribulose | YNB no Amino Acids 6.7 g/L, 100mM PO4, 0.25% D... | 10 mL in glass test tubes | 30.0 | 5 | 200 | 0.1 | 0.9085 | 96.0 |
| YNB\_PO4\_D-xylulose | YNB no Amino Acids 6.7 g/L, 100mM PO4, 0.25% D... | 10 mL in glass test tubes | 30.0 | 5 | 200 | 0.1 | 1.2144 | 96.0 |
| YNB\_PO4\_CSM\_Galactose | YNB no Amino Acids 6.7 g/L, 100mM PO4, 0.79 g/... | 10 mL in glass test tubes | 30.0 | 7 | 200 | 0.1 | 6.8 | 120.0 |
| YNB\_PO4\_CSM\_Lactate | YNB no Amino Acids 6.7 g/L, 100mM PO4, 0.79 g/... | 10 mL in glass test tubes | 30.0 | 7 | 200 | 0.1 | 1.1 | 72.0 |
| YNB\_PO4\_CSM\_Valine | YNB no Amino Acids 6.7 g/L, 100mM PO4, 0.79 g/... | 10 mL in glass test tubes | 30.0 | 6 | 200 | 0.1 | 3.5 | 72.0 |
| YNB\_PO4\_CSM\_Leucine | YNB no Amino Acids 6.7 g/L, 100mM PO4, 0.79 g/... | 10 mL in glass test tubes | 30.0 | 6 | 200 | 0.1 | 4 | 72.0 |
| YNB\_PO4\_CSM\_Phenylalanine | YNB no Amino Acids 6.7 g/L, 100mM PO4, 0.79 g/... | 10 mL in glass test tubes | 30.0 | 7 | 200 | 0.1 | 4.2 | 120.0 |

In [76]:

```
# Biotin supplement in YNB
# S. cer VHT1 biotin transporter blasts to 12353 and 12740 H+-pantothenate symporter FEN2
# 12353 essential, 12740 not essential
# VHT1 does not transport pantothenate, FEN2 is not known to transport biotin
# https://www.longdom.org/open-access/mechanisms-of-biotin-transport-2161-1009-1000210.pdf
r1 = sce.reactions.get_by_id('EX_btn_e').copy()
r1.lower_bound = 0.0
r2 = sce.reactions.get_by_id('BTNt2i').copy()
r2.gene_reaction_rule = ''
model.add_reactions([r1,r2])
# Folate supplement in YNB
r1 = hsa2.reactions.get_by_id('EX_fol_e').copy()
r1.lower_bound = 0.0
r2 = hsa2.reactions.get_by_id('FOLt').copy()
r2.gene_reaction_rule = ''
model.add_reactions([r1,r2])
model.metabolites.get_by_id('fol_e').formula = 'C19H17N7O6'
model.metabolites.get_by_id('fol_e').charge = -2
# folate mitochondrial transporter 
# 14159 SLC25A32 - FLX1 yeast mitochondrial FAD transporter, ok assigned to FADFMNtm
# 10961 SLC25A32 - YEA6, YIA6 yeast mitochondrial NAD transporter, missing reaction
# https://www.ncbi.nlm.nih.gov/pubmed/16291748
r = hsa2.reactions.get_by_id('NADtm').copy()
r.gene_reaction_rule = '10961'
r.lower_bound = 0.0
model.add_reactions([r])
# Pyridoxine supplement in YNB
# Diffusion in Recon3D
r1 = hsa2.reactions.get_by_id('EX_pydxn_e').copy()
r1.lower_bound = 0.0
r2 = hsa2.reactions.get_by_id('PYDXNtr').copy()
r2.gene_reaction_rule = ''
model.add_reactions([r1,r2])
# Riboflavin supplement in YNB
# Transport by MCH5 in yeast, by SLC52A1-3 in human
# SLC52A1-3 not found, but many MCH5 homologs - MCH5 blast best hits are 9772 and 9771
r = sce.reactions.get_by_id('RIBFLVt2').copy()
r.gene_reaction_rule = '9771 or 9772'
model.add_reactions([r])
# 11257 Slc16a7 catalyzes the rapid transport across the plasma membrane of many monocarboxylates such as
# lactate, pyruvate, branched-chain oxo acids derived from leucine, valine and isoleucine, and 
# the ketone bodies acetoacetate, beta-hydroxybutyrate and acetate. Functions as high-affinity pyruvate transporter
```

In [77]:

```
temp = Annotation.index[Annotation['Sc288 Best Hit'].str.contains('MCH5')]
display(Annotation.loc[temp])
```

|  | Combined Annotations | Signal P | Sc288c Orthologs | Human Orthologs | Sc288 Best Hit | Human Blast | Essential | WolfPSort | C Terminal |
| --- | --- | --- | --- | --- | --- | --- | --- | --- | --- |
| RTO4\_ID |  |  |  |  |  |  |  |  |  |
| 9254 | KOG2504: Monocarboxylate transporter |  |  |  | MCH5 |  | Not Essential | plas 25 | QWV\* |
| 9771 | K08190: SLC16A14; MFS transporter, MCP family,... |  |  |  | MCH5 | SLC16 | Not Essential | plas 24, E.R. 2 | PWV\* |
| 9772 | KOG2504: Monocarboxylate transporter |  |  |  | MCH5 | SLC16 | Not Essential | plas 27 | RWV\* |
| 10001 | HMMPfam:Major Facilitator Superfamily:PF07690,... |  |  |  | MCH5 | SLC16 | Not Essential | plas 23, mito 3 | SVV\* |
| 11257 | K08184: SLC16A7; MFS transporter, MCP family, ... | A |  |  | MCH5 | SLC16A | Not Essential | plas 23, E.R. 3 | KKM\* |
| 16137 | KOG2504: Monocarboxylate transporter |  |  |  | MCH5 |  | Not Essential | plas 21, mito 4 | ARY\* |

In [78]:

```
for x in temp:
    if x in model.genes:
        for r in sorted(model.genes.get_by_id(x).reactions, key=lambda x: x.id):
            print(r, r.gene_reaction_rule)
    else:
        print(x, 'no reactions')
    print()
```

```
9254 no reactions

RIBFLVt2: h_e + ribflv_e --> h_c + ribflv_c 9771 or 9772

RIBFLVt2: h_e + ribflv_e --> h_c + ribflv_c 9771 or 9772

10001 no reactions

2OBUTt: 2obut_e + h_e <=> 2obut_c + h_c 11257
ACACt2: acac_e + h_e <=> acac_c + h_c 11257

16137 no reactions
```

In [79]:

```
Fitness = pd.read_csv('../../Data/Fitness_Extended.txt',
                      sep='\t', index_col=[0])
Fitness.head()
```

Out[79]:

|  | M9\_Glucose | YNB Glucose | YNB Glucose plus Arginine | YNB Glucose plus Methionine | YNB Glucose plus Dropout Complete | Fitness During Lipid Mobilization | YNB Oleic Acid | YNB Cellobiose | YNB\_CSM\_KPO4 Glucose | YNB\_CSM\_KPO4 Xylose | ... | YNB\_PO4\_D-arabitol | YNB\_PO4\_L-arabitol | YNB\_PO4\_xylitol | YNB\_PO4\_D-ribulose | YNB\_PO4\_D-xylulose | YNB\_PO4\_CSM\_Galactose | YNB\_PO4\_CSM\_Lactate | YNB\_PO4\_CSM\_Valine | YNB\_PO4\_CSM\_Leucine | YNB\_PO4\_CSM\_Phenylalanine |
| --- | --- | --- | --- | --- | --- | --- | --- | --- | --- | --- | --- | --- | --- | --- | --- | --- | --- | --- | --- | --- | --- |
| NearestGene |  |  |  |  |  |  |  |  |  |  |  |  |  |  |  |  |  |  |  |  |  |
| 10000 | NaN | NaN | NaN | NaN | NaN | NaN | NaN | NaN | NaN | NaN | ... | NaN | NaN | NaN | NaN | NaN | NaN | NaN | NaN | NaN | NaN |
| 10001 | 0.470687 | NaN | NaN | NaN | NaN | NaN | 0.355042 | -1.380790 | 0.232644 | -0.611164 | ... | -0.058498 | NaN | -0.173179 | NaN | NaN | 0.805186 | -0.305508 | -1.859958 | 0.478936 | NaN |
| 10002 | 0.283788 | 0.038678 | 0.172165 | 0.063986 | 0.265484 | 0.090129 | 0.037532 | -0.219215 | -0.610762 | -0.284108 | ... | 0.158657 | -0.020557 | -0.202506 | 0.005331 | 0.172014 | 0.420393 | -0.879143 | 0.209051 | 0.048033 | 0.510014 |
| 10003 | -0.030815 | -0.411329 | -0.366240 | -0.373962 | -0.535817 | -0.213208 | -0.241435 | 0.042549 | -0.069536 | -0.283891 | ... | -0.299174 | -0.374318 | -0.240221 | -0.231199 | 0.032861 | -0.268949 | -0.249481 | -0.722290 | -0.262344 | -0.712309 |
| 10004 | -0.071440 | -0.088858 | -0.191260 | -0.096023 | -0.159386 | -0.203134 | -0.045141 | -0.009610 | 0.068061 | -0.033638 | ... | 0.149967 | 0.053322 | 0.003410 | 0.189365 | 0.237623 | -0.058611 | 0.193184 | 0.089955 | 0.166444 | 0.246537 |

5 rows × 27 columns

In [80]:

```
YNB = ['EX_nh4_e','EX_btn_e','EX_pnto__R_e','EX_fol_e','EX_nac_e','EX_4abz_e','EX_pydxn_e','EX_thm_e']
DOC = ['EX_ala__L_e','EX_arg__L_e','EX_asn__L_e','EX_asp__L_e','EX_cys__L_e','EX_gln__L_e','EX_glu__L_e','EX_gly_e',
      'EX_his__L_e','EX_ile__L_e','EX_leu__L_e','EX_lys__L_e','EX_met__L_e','EX_phe__L_e','EX_pro__L_e','EX_ser__L_e',
      'EX_thr__L_e','EX_trp__L_e','EX_tyr__L_e','EX_val__L_e','EX_ade_e','EX_ura_e','EX_4abz_e','EX_inost_e']
CSM = ['EX_ade_e','EX_arg__L_e','EX_asp__L_e','EX_his__L_e','EX_ile__L_e','EX_leu__L_e','EX_lys__L_e','EX_met__L_e',
       'EX_phe__L_e','EX_thr__L_e','EX_trp__L_e','EX_tyr__L_e','EX_val__L_e','EX_ura_e']
Fitness_Media = {'M9_Glucose': ['EX_glc__D_e','EX_nh4_e'],
                 'YNB Glucose': ['EX_glc__D_e']+YNB,
                 'YNB Glucose plus Arginine': ['EX_glc__D_e','EX_arg__L_e']+YNB,
                 'YNB Glucose plus Methionine': ['EX_glc__D_e','EX_met__L_e']+YNB,
                 'YNB Glucose plus Dropout Complete': ['EX_glc__D_e']+YNB+DOC,
                 'Fitness During Lipid Mobilization': ['SK_triglyc_RT_d']+YNB,
                 'YNB Oleic Acid': ['EX_ocdcea_e']+YNB,
                 'YNB Cellobiose': ['EX_cellb_e']+YNB,
                 'YNB_CSM_KPO4 Glucose': ['EX_glc__D_e']+YNB+CSM,
                 'YNB_CSM_KPO4 Xylose': ['EX_xyl__D_e']+YNB+CSM,
                 'YNB_CSM_KPO4 Arabinose': ['EX_arab__L_e']+YNB+CSM,
                 'YNB_CSM_KPO4 Acetate': ['EX_ac_e']+YNB+CSM,
                 'YNB_CSM_KPO4 p-Coumarate': ['EX_T4hcinnm_e']+YNB+CSM,
                 'YNB_CSM_KPO4 Ferulate': ['EX_fer_e']+YNB+CSM,
                 'YNB_PO4_CSM_Phenylalanine': ['EX_phe__L_e']+YNB+CSM,
                 'YNB_PO4_Benzoate': ['EX_bz_e']+YNB,
                 'YNB_PO4_Mannose': ['EX_man_e']+YNB,
                 'YNB_PO4_L-lyxose': ['EX_lyx__L_e']+YNB,
                 'YNB_PO4_D-arabitol': ['EX_abt__D_e']+YNB,
                 'YNB_PO4_L-arabitol': ['EX_abt_e']+YNB,
                 'YNB_PO4_xylitol': ['EX_xylt_e']+YNB,
                 'YNB_PO4_D-ribulose': ['EX_rbl__D_e']+YNB,
                 'YNB_PO4_D-xylulose': ['EX_xylu__D_e']+YNB,
                 'YNB_PO4_CSM_Galactose': ['EX_gal_e']+YNB+CSM,
                 'YNB_PO4_CSM_Lactate': ['EX_lac__D_e','EX_lac__L_e']+YNB+CSM,
                 'YNB_PO4_CSM_Valine': ['EX_val__L_e']+YNB+CSM,
                 'YNB_PO4_CSM_Leucine': ['EX_leu__L_e']+YNB+CSM}
```

In [81]:

```
model.medium
```

Out[81]:

```
{'EX_h_e': 1000.0,
 'EX_h2o_e': 1000.0,
 'EX_nh4_e': 1000.0,
 'EX_o2_e': 1000.0,
 'EX_pi_e': 1000.0,
 'EX_so4_e': 1000.0,
 'EX_glc__D_e': 1.0,
 'EX_ca2_e': 1000.0,
 'EX_fe2_e': 1000.0,
 'EX_fe3_e': 1000.0,
 'EX_k_e': 1000.0,
 'EX_na1_e': 1000.0,
 'EX_mg2_e': 1000.0,
 'EX_mn2_e': 1000.0,
 'EX_cu2_e': 1000.0,
 'EX_zn2_e': 1000.0}
```

Use uptake rate of -1000.0 for metabolites in YNB, they are not growth limiting  
Use uptake rate of -1.0 for main carbon sources, and -0.01 for supplements  
Use uptake rate of -0.01 for metabolites in DOC or CSM, they are supplements in small amount  
Use lipid mobilization rate of -0.001 to account for the high molecular weight

In [82]:

```
Growth_Prediction_All = pd.DataFrame()
with model:
    model.reactions.get_by_id('ATPM').lower_bound = 0.0
    model.reactions.get_by_id('EX_glc__D_e').lower_bound = 0.0
    for k, v in Fitness_Media.items():
        for x in v:
            if x in YNB:
                model.reactions.get_by_id(x).lower_bound = -1000.0
            elif x in DOC or x in CSM:
                if v.index(x) == 0:
                    model.reactions.get_by_id(x).lower_bound = -1.0
                else:
                    model.reactions.get_by_id(x).lower_bound = -0.01
            elif x == 'SK_triglyc_RT_d':
                model.reactions.get_by_id(x).lower_bound = -0.001
            else:
                model.reactions.get_by_id(x).lower_bound = -1.0
        temp = cobra.flux_analysis.single_gene_deletion(model)
        for x in v:
            model.reactions.get_by_id(x).lower_bound = 0.0
        Growth_Prediction_All[k] = temp['growth']
```

In [83]:

```
Growth_Prediction_All.index = [list(x)[0] for x in Growth_Prediction_All.index]
```

In [84]:

```
print('Genes with fitness score:', len(Fitness))
print('Model genes:', len(Growth_Prediction_All))
print('Model genes excluding mitochondrial and unknown:', len([x for x in Growth_Prediction_All.index if x[0].isdigit()]))
```

```
Genes with fitness score: 8345
Model genes: 1147
Model genes excluding mitochondrial and unknown: 1132
```

In [85]:

```
[x for x in Growth_Prediction_All.index if not x[0].isdigit()]
```

Out[85]:

```
['COX1',
 'NAD4L',
 'ATP9',
 'NAD4',
 'NAD1',
 'NAD2',
 'NAD6',
 'COPII',
 'COB',
 'COX3',
 'NAD3',
 'ATP8',
 'NAD5',
 'ATP6',
 'COX2']
```

In [86]:

```
genes_with_fitness = set(Growth_Prediction_All.index).intersection(Fitness.index)
print('Model genes with fitness score:', len(genes_with_fitness))
genes_without_fitness = set(x.id for x in model.genes if x.id not in genes_with_fitness and x.id[0].isdigit())
print('Model genes without fitness score:', len(genes_without_fitness))
temp = sorted(list(genes_with_fitness.union(genes_without_fitness)))
print('Model genes that can be simulated:', len(temp))
```

```
Model genes with fitness score: 1116
Model genes without fitness score: 16
Model genes that can be simulated: 1132
```

In [87]:

```
Growth_Data = Fitness.reindex(index=temp, columns=Growth_Prediction_All.columns)
Growth_Prediction = Growth_Prediction_All.loc[temp]
display(Growth_Data.head().style.background_gradient(cmap='RdBu_r', axis=None).highlight_null('lightgrey'))
display(Growth_Prediction.head().style.background_gradient(cmap='RdBu_r', axis=None))
```

|  | M9\_Glucose | YNB Glucose | YNB Glucose plus Arginine | YNB Glucose plus Methionine | YNB Glucose plus Dropout Complete | Fitness During Lipid Mobilization | YNB Oleic Acid | YNB Cellobiose | YNB\_CSM\_KPO4 Glucose | YNB\_CSM\_KPO4 Xylose | YNB\_CSM\_KPO4 Arabinose | YNB\_CSM\_KPO4 Acetate | YNB\_CSM\_KPO4 p-Coumarate | YNB\_CSM\_KPO4 Ferulate | YNB\_PO4\_CSM\_Phenylalanine | YNB\_PO4\_Benzoate | YNB\_PO4\_Mannose | YNB\_PO4\_L-lyxose | YNB\_PO4\_D-arabitol | YNB\_PO4\_L-arabitol | YNB\_PO4\_xylitol | YNB\_PO4\_D-ribulose | YNB\_PO4\_D-xylulose | YNB\_PO4\_CSM\_Galactose | YNB\_PO4\_CSM\_Lactate | YNB\_PO4\_CSM\_Valine | YNB\_PO4\_CSM\_Leucine |
| --- | --- | --- | --- | --- | --- | --- | --- | --- | --- | --- | --- | --- | --- | --- | --- | --- | --- | --- | --- | --- | --- | --- | --- | --- | --- | --- | --- |
| NearestGene |  |  |  |  |  |  |  |  |  |  |  |  |  |  |  |  |  |  |  |  |  |  |  |  |  |  |  |
| 10000 | nan | nan | nan | nan | nan | nan | nan | nan | nan | nan | nan | nan | nan | nan | nan | nan | nan | nan | nan | nan | nan | nan | nan | nan | nan | nan | nan |
| 10007 | nan | nan | nan | nan | nan | nan | nan | nan | nan | nan | nan | nan | nan | nan | nan | nan | nan | nan | nan | nan | nan | nan | nan | nan | nan | nan | nan |
| 10010 | -0.130360 | -0.558565 | -0.332740 | -0.260252 | -0.383313 | -0.030872 | -0.611898 | -0.097438 | -0.035705 | -0.405832 | -0.077257 | -0.114424 | -2.825808 | -0.313043 | -0.376989 | -1.005593 | -0.068720 | -0.902581 | -0.966503 | -0.732452 | -0.836823 | -0.490688 | -0.534801 | -0.035402 | -0.427705 | -0.143231 | -0.240726 |
| 10012 | 0.120928 | -0.056685 | -0.143451 | -0.205516 | -0.240177 | -0.282986 | -0.039371 | 0.021825 | -0.185444 | 0.247584 | -0.357236 | -0.279080 | -0.284744 | -0.191232 | -0.586259 | 0.419109 | 0.055791 | -0.243860 | 0.006924 | -0.185901 | -0.347117 | -0.043478 | 0.049640 | -0.330369 | -0.571357 | -0.390520 | -4.921371 |
| 10017 | -0.117902 | 0.001326 | 0.216430 | 0.169223 | -0.072431 | 0.382748 | 0.455964 | 0.161420 | 0.012681 | 0.187815 | 0.037770 | 0.028149 | -0.167706 | -0.147833 | -0.086100 | 0.326169 | -0.004083 | -0.188072 | -0.253541 | 0.073421 | 0.076093 | -0.266397 | 0.038548 | 0.024525 | 0.112220 | -0.159979 | -0.229817 |

|  | M9\_Glucose | YNB Glucose | YNB Glucose plus Arginine | YNB Glucose plus Methionine | YNB Glucose plus Dropout Complete | Fitness During Lipid Mobilization | YNB Oleic Acid | YNB Cellobiose | YNB\_CSM\_KPO4 Glucose | YNB\_CSM\_KPO4 Xylose | YNB\_CSM\_KPO4 Arabinose | YNB\_CSM\_KPO4 Acetate | YNB\_CSM\_KPO4 p-Coumarate | YNB\_CSM\_KPO4 Ferulate | YNB\_PO4\_CSM\_Phenylalanine | YNB\_PO4\_Benzoate | YNB\_PO4\_Mannose | YNB\_PO4\_L-lyxose | YNB\_PO4\_D-arabitol | YNB\_PO4\_L-arabitol | YNB\_PO4\_xylitol | YNB\_PO4\_D-ribulose | YNB\_PO4\_D-xylulose | YNB\_PO4\_CSM\_Galactose | YNB\_PO4\_CSM\_Lactate | YNB\_PO4\_CSM\_Valine | YNB\_PO4\_CSM\_Leucine |
| --- | --- | --- | --- | --- | --- | --- | --- | --- | --- | --- | --- | --- | --- | --- | --- | --- | --- | --- | --- | --- | --- | --- | --- | --- | --- | --- | --- |
| 10000 | 0.075763 | 0.075961 | 0.076815 | 0.077491 | 0.096124 | 0.085168 | 0.257937 | 0.151923 | 0.091300 | 0.077833 | 0.077833 | 0.023544 | 0.096940 | 0.048618 | 0.007028 | 0.047933 | 0.075961 | 0.063301 | 0.067460 | 0.067460 | 0.067460 | 0.063301 | 0.063301 | 0.091300 | 0.075805 | 0.081519 | 0.096762 |
| 10007 | 0.074169 | 0.074360 | 0.075155 | 0.075789 | 0.093805 | 0.085585 | 0.259253 | 0.148719 | 0.089184 | 0.075961 | 0.075961 | 0.024133 | 0.096026 | 0.049045 | 0.006890 | 0.048049 | 0.074360 | 0.061966 | 0.066132 | 0.066132 | 0.066132 | 0.061966 | 0.061966 | 0.089184 | 0.075731 | 0.081503 | 0.096491 |
| 10010 | 0.066110 | 0.066270 | 0.067092 | 0.067496 | 0.084007 | 0.072205 | 0.216104 | 0.132540 | 0.079559 | 0.067648 | 0.067648 | 0.017800 | 0.084590 | 0.036073 | 0.005985 | 0.043659 | 0.066270 | 0.055225 | 0.059959 | 0.059959 | 0.059959 | 0.055225 | 0.055225 | 0.079559 | 0.067189 | 0.070366 | 0.084595 |
| 10012 | 0.075763 | 0.075961 | 0.076815 | 0.077491 | 0.096124 | 0.085585 | 0.259253 | 0.151923 | 0.091300 | 0.077833 | 0.077833 | 0.024259 | 0.097926 | 0.049847 | 0.006229 | 0.048517 | 0.075961 | 0.063301 | 0.067460 | 0.067460 | 0.067460 | 0.063301 | 0.063301 | 0.091300 | 0.076995 | 0.075973 | 0.006229 |
| 10017 | 0.066110 | 0.066270 | 0.067092 | 0.067496 | 0.084007 | 0.072205 | 0.216104 | 0.132540 | 0.079559 | 0.067648 | 0.067648 | 0.017800 | 0.084590 | 0.036073 | 0.005985 | 0.043659 | 0.066270 | 0.055225 | 0.059959 | 0.059959 | 0.059959 | 0.055225 | 0.055225 | 0.079559 | 0.067189 | 0.070366 | 0.084595 |

In [88]:

```
print(Growth_Data.shape)
print(Growth_Prediction.shape)
```

```
(1132, 27)
(1132, 27)
```

In [89]:

```
fig, ax = plt.subplots(figsize=(12,7), ncols=2)
sns.heatmap(Growth_Data, ax=ax[0], cmap='RdBu_r', xticklabels=True, yticklabels=False, mask=Growth_Data.isnull())
sns.heatmap(Growth_Prediction, ax=ax[1], cmap='RdBu_r', xticklabels=True, yticklabels=False)
ax[0].set_ylabel('Genes')
ax[1].set_ylabel('Genes')
plt.tight_layout()
```

In [90]:

```
fig, ax = plt.subplots(figsize=(6,7), ncols=1)
sns.heatmap(Growth_Data, ax=ax, cmap='RdBu_r', xticklabels=True, yticklabels=False, mask=Growth_Data.isnull(),
            cbar_kws={'label': 'Fitness score'}, center=0)
ax.set_ylabel('Genes')
plt.tight_layout()
plt.savefig("Fitness.svg", dpi=300)
```

In [91]:

```
Essential_genes = pd.read_csv('../../Data/eLife_essential_genes.txt',
                              sep='\t', index_col=0)
Essential_genes.index = Essential_genes.index.map(str)
Essential_genes.head()
```

Out[91]:

|  | Essential |
| --- | --- |
| Protein ID |  |
| 15174 | Yes |
| 15278 | Yes |
| 16786 | Yes |
| 14470 | Yes |
| 16628 | Yes |

In [92]:

```
y_data = ~(Growth_Data < -4)
y_data.loc[Growth_Data.index[Essential_genes.loc[Growth_Data.index,'Essential'] == 'Yes']] = False
y_pred = Growth_Prediction > 0.01*Growth_Prediction.max()
```

In [93]:

```
fig, ax = plt.subplots(figsize=(12,7), ncols=2)
sns.heatmap(y_data, ax=ax[0], cmap='Reds', xticklabels=True, yticklabels=False, mask=y_data.isnull())
sns.heatmap(y_pred, ax=ax[1], cmap='Reds', xticklabels=True, yticklabels=False)
ax[0].set_ylabel('Genes')
ax[1].set_ylabel('Genes')
plt.tight_layout()
```

In [94]:

```
TN, FP, FN, TP = confusion_matrix(y_data.values.ravel(), y_pred.values.ravel()).ravel()
print(TN, FP, FN, TP, sum([TN, FP, FN, TP]))
# Sensitivity, hit rate, recall, or true positive rate
TPR = TP/(TP+FN)
# Specificity or true negative rate
TNR = TN/(TN+FP) 
# Precision or positive predictive value
PPV = TP/(TP+FP)
# Negative predictive value
NPV = TN/(TN+FN)
# Fall out or false positive rate
FPR = FP/(FP+TN)
# False negative rate
FNR = FN/(TP+FN)
# False discovery rate
FDR = FP/(TP+FP)
# Overall accuracy
ACC = (TP+TN)/(TP+FP+FN+TN)
# Matthew's
MCC = matthews_corrcoef(y_data.values.ravel(), y_pred.values.ravel())
print('Recall:', TPR.round(3))
print('Precision:', PPV.round(3))
print('Accuracy:', ACC.round(3))
print('Matthew\'s correlation:', MCC.round(3))
```

```
3430 4350 2549 20235 30564
Recall: 0.888
Precision: 0.823
Accuracy: 0.774
Matthew's correlation: 0.361
```

In [95]:

```
df_confusion = pd.DataFrame(confusion_matrix(y_data.values.ravel(), y_pred.values.ravel()),
                            index = pd.MultiIndex.from_product([['Experiment'],['No growth', 'Growth']]),
                            columns = pd.MultiIndex.from_product([['Prediction'],['No growth', 'Growth']]))
df_confusion
```

Out[95]:

|  |  | Prediction | |
| --- | --- | --- | --- |
|  |  | No growth | Growth |
| Experiment | No growth | 3430 | 4350 |
|  | Growth | 2549 | 20235 |

In [96]:

```
TN, FP, FN, TP = (confusion_matrix(y_data.values.ravel(), y_pred.values.ravel()).ravel()/y_data.shape[1]).round()
print(TN, FP, FN, TP, sum([TN, FP, FN, TP]))
# Sensitivity, hit rate, recall, or true positive rate
TPR = TP/(TP+FN)
# Specificity or true negative rate
TNR = TN/(TN+FP) 
# Precision or positive predictive value
PPV = TP/(TP+FP)
# Negative predictive value
NPV = TN/(TN+FN)
# Fall out or false positive rate
FPR = FP/(FP+TN)
# False negative rate
FNR = FN/(TP+FN)
# False discovery rate
FDR = FP/(TP+FP)
# Overall accuracy
ACC = (TP+TN)/(TP+FP+FN+TN)
# Matthew's
MCC = matthews_corrcoef(y_data.values.ravel(), y_pred.values.ravel())
print('Recall:', TPR.round(3))
print('Precision:', PPV.round(3))
print('Accuracy:', ACC.round(3))
print('Matthew\'s correlation:', MCC.round(3))
```

```
127.0 161.0 94.0 749.0 1131.0
Recall: 0.888
Precision: 0.823
Accuracy: 0.775
Matthew's correlation: 0.361
```

In [97]:

```
df_confusion = pd.DataFrame((confusion_matrix(y_data.values.ravel(), y_pred.values.ravel())/y_data.shape[1]).round(),
                            index = pd.MultiIndex.from_product([['Experiment'],['No growth', 'Growth']]),
                            columns = pd.MultiIndex.from_product([['Prediction'],['No growth', 'Growth']]))
df_confusion
```

Out[97]:

|  |  | Prediction | |
| --- | --- | --- | --- |
|  |  | No growth | Growth |
| Experiment | No growth | 127.0 | 161.0 |
|  | Growth | 94.0 | 749.0 |

In [98]:

```
thresholds = np.arange(-7, 0, 0.1)
TPR = {}
FPR = {}
ACC = {}
MCC = {}
for x in thresholds:
    
    y_data = ~(Growth_Data < x)
    y_data.loc[Growth_Data.index[Essential_genes.loc[Growth_Data.index,'Essential'] == 'Yes']] = False
    y_pred = Growth_Prediction > 0.01*Growth_Prediction.max()
    TN, FP, FN, TP = confusion_matrix(y_data.values.ravel(), y_pred.values.ravel()).ravel()
    # Sensitivity, hit rate, recall, or true positive rate
    TPR[x] = TP/(TP+FN)
    # Fall out or false positive rate
    FPR[x] = FP/(FP+TN)
    # Overall accuracy
    ACC[x] = (TP+TN)/(TP+FP+FN+TN)
    # Matthew's
    MCC[x] = matthews_corrcoef(y_data.values.ravel(), y_pred.values.ravel())
```

In [99]:

```
fig, axes = plt.subplots(1, 2, figsize=(8,4))

axes[0].plot(list(FPR.values()),list(TPR.values()))
axes[0].set_xlabel('FPR')
axes[0].set_ylabel('TPR')

axes[1].plot(thresholds,list(TPR.values()))
axes[1].plot(thresholds,list(FPR.values()))
axes[1].plot(thresholds,list(ACC.values()))
axes[1].plot(thresholds,list(MCC.values()))
axes[1].set_xlabel('Cutoff')
axes[1].legend(['TPR','FPR','ACC','MCC'])

plt.show()
```

In [100]:

```
print(thresholds[np.argmax(list(TPR.values()))])
print(thresholds[np.argmin(list(FPR.values()))])
print(thresholds[np.argmax(list(ACC.values()))])
print(thresholds[np.argmax(list(MCC.values()))])
```

```
-0.6000000000000227
-4.9000000000000075
-6.800000000000001
-3.2000000000000135
```

In [101]:

```
for x in Growth_Prediction.index:
    if Growth_Prediction.loc[x,'YNB Glucose'] > 1e-6 and not Growth_Data.loc[x,'YNB Glucose'] > -3:
        print(x, Annotation.loc[x,'Combined Annotations'])
        print(Growth_Prediction.loc[x,'YNB Glucose'], Growth_Data.loc[x,'YNB Glucose'])
        print(' Model')
        for r in sorted(model.genes.get_by_id(x).reactions, key=lambda x: x.id):
            print(' ', r, r.gene_reaction_rule)
        print()
```

```
10000 K00036: G6PD, zwf; glucose-6-phosphate 1-dehydrogenase
0.07596130136997142 nan
 Model
  G6PDH2r: g6p_c + nadp_c <=> 6pgl_c + h_c + nadph_c 10000
  G6PDH2rp: g6p_x + nadp_x <=> 6pgl_x + h_x + nadph_x 10000

10007 K00164: OGDH, sucA; 2-oxoglutarate dehydrogenase E1 component
0.07435968324826536 nan
 Model
  AKGDm: akg_m + coa_m + nad_m --> co2_m + nadh_m + succoa_m 10007 and 10040 and 12116

10095 K03843: ALG2; alpha-1,3/alpha-1,6-mannosyltransferase
0.07596130137114704 nan
 Model
  G13MT_L: gdpmann_c + 0.1 mpdol__L_c --> gdp_c + h_c + 0.1 m1mpdol__L_c 10095
  G13MT_U: gdpmann_c + 0.1 mpdol_U_c --> gdp_c + h_c + 0.1 m1mpdol_U_c 10095

10173 K00616: E2.2.1.2, talA, talB; transaldolase
0.07571403578180025 nan
 Model
  TALA: g3p_c + s7p_c <=> e4p_c + f6p_c 10173

10197 K20238: E2.1.1.317; sphingolipid C9-methyltransferase
0.07596130136997237 nan
 Model
  CERMT618er: amet_r + cer5_18_r --> ahcys_r + cer6_18_r + h_r 10197

10293 K13239: PECI; peroxisomal 3,2-trans-enoyl-CoA isomerase
0.07596130136997425 nan
 Model
  FAO141p_even: 6.0 coa_x + 6.0 h2o_x + 6.0 nad_x + nadph_x + 6.0 o2_x + tdecoa_x --> 7.0 accoa_x + 6.0 h2o2_x + 5.0 h_x + 6.0 nadh_x + nadp_x (10293 and 11362 and 11907 and 12742 and 13228 and 13813) or (10293 and 11362 and 11907 and 12752 and 13228 and 13813) or (10293 and 11362 and 11907 and 13228 and 13813 and 9700)
  FAO141p_odd: 6.0 coa_x + 6.0 h2o_x + 6.0 nad_x + 5.0 o2_x + tdecoa_x --> 7.0 accoa_x + 5.0 h2o2_x + 6.0 h_x + 6.0 nadh_x (10293 and 11362 and 12742 and 13813) or (10293 and 11362 and 12752 and 13813) or (10293 and 11362 and 13813 and 9700)
  FAO161p_even: 7.0 coa_x + 7.0 h2o_x + hdcoa_x + 7.0 nad_x + nadph_x + 7.0 o2_x --> 8.0 accoa_x + 7.0 h2o2_x + 6.0 h_x + 7.0 nadh_x + nadp_x (10293 and 11362 and 11907 and 12742 and 13228 and 13813) or (10293 and 11362 and 11907 and 12752 and 13228 and 13813) or (10293 and 11362 and 11907 and 13228 and 13813 and 9700)
  FAO161p_odd: 7.0 coa_x + 7.0 h2o_x + hdcoa_x + 7.0 nad_x + 6.0 o2_x --> 8.0 accoa_x + 6.0 h2o2_x + 7.0 h_x + 7.0 nadh_x (10293 and 11362 and 12742 and 13813) or (10293 and 11362 and 12752 and 13813) or (10293 and 11362 and 13813 and 9700)
  FAO181p_even: 8.0 coa_x + 8.0 h2o_x + 8.0 nad_x + nadph_x + 8.0 o2_x + odecoa_x --> 9.0 accoa_x + 8.0 h2o2_x + 7.0 h_x + 8.0 nadh_x + nadp_x (10293 and 11362 and 11907 and 12742 and 13228 and 13813) or (10293 and 11362 and 11907 and 12752 and 13228 and 13813) or (10293 and 11362 and 11907 and 13228 and 13813 and 9700)
  FAO181p_odd: 8.0 coa_x + 8.0 h2o_x + 8.0 nad_x + 7.0 o2_x + odecoa_x --> 9.0 accoa_x + 7.0 h2o2_x + 8.0 h_x + 8.0 nadh_x (10293 and 11362 and 12742 and 13813) or (10293 and 11362 and 12752 and 13813) or (10293 and 11362 and 13813 and 9700)
  FAO182p_even: 8.0 coa_x + 8.0 h2o_x + 8.0 nad_x + 2.0 nadph_x + 8.0 o2_x + ocdycacoa_x --> 9.0 accoa_x + 8.0 h2o2_x + 6.0 h_x + 8.0 nadh_x + 2.0 nadp_x (10293 and 11362 and 11907 and 12742 and 13228 and 13813) or (10293 and 11362 and 11907 and 12752 and 13228 and 13813) or (10293 and 11362 and 11907 and 13228 and 13813 and 9700)
  FAO182p_odd: 8.0 coa_x + 8.0 h2o_x + 8.0 nad_x + nadph_x + 7.0 o2_x + ocdycacoa_x --> 9.0 accoa_x + 7.0 h2o2_x + 7.0 h_x + 8.0 nadh_x + nadp_x (10293 and 11362 and 12742 and 13228 and 13813) or (10293 and 11362 and 12752 and 13228 and 13813) or (10293 and 11362 and 13228 and 13813 and 9700)
  FAO183p_even: 8.0 coa_x + 8.0 h2o_x + lnlncgcoa_x + 8.0 nad_x + 3.0 nadph_x + 8.0 o2_x --> 9.0 accoa_x + 8.0 h2o2_x + 5.0 h_x + 8.0 nadh_x + 3.0 nadp_x (10293 and 11362 and 11907 and 12742 and 13228 and 13813) or (10293 and 11362 and 11907 and 12752 and 13228 and 13813) or (10293 and 11362 and 11907 and 13228 and 13813 and 9700)
  FAO183p_odd: 8.0 coa_x + 8.0 h2o_x + lnlncgcoa_x + 8.0 nad_x + 2.0 nadph_x + 7.0 o2_x --> 9.0 accoa_x + 7.0 h2o2_x + 6.0 h_x + 8.0 nadh_x + 2.0 nadp_x (10293 and 11362 and 12742 and 13228 and 13813) or (10293 and 11362 and 12752 and 13228 and 13813) or (10293 and 11362 and 13228 and 13813 and 9700)

10318 K03965: NDUFB9; NADH dehydrogenase (ubiquinone) 1 beta subcomplex subunit 9
0.06627011871621578 nan
 Model
  NADH2_u9m2: 5.0 h_m + nadh_m + q9_m --> 4.0 h_c + nad_m + q9h2_m NAD1 and NAD2 and NAD3 and NAD4 and NAD4L and NAD5 and NAD6 and 10010 and 10017 and 10318 and 10541 and 10607 and 11151 and 11411 and 11702 and 11857 and 12482 and 12497 and 12543 and 12653 and 13017 and 13214 and 13268 and 13411 and 13656 and 13925 and 14078 and 14261 and 14418 and 14717 and 14900 and 15179 and 15864 and 15947 and 15973 and 15998 and 8444 and 8446 and 8530 and 8699 and 8817 and 8909 and 9144 and 9348

10332 K01495: GCH1, folE; GTP cyclohydrolase I
0.07596130137115692 nan
 Model
  GTPCI: gtp_c + h2o_c --> ahdt_c + for_c + h_c 10332
  GTPCIn: gtp_n + h2o_n --> ahdt_n + for_n + h_n 10332

10427 K13509: AGPAT1_2; lysophosphatidate acyltransferase
0.07596130136793124 nan
 Model
  AGATer_RT: 0.01 1ag3p_RT_r + 0.01 arachcoa_r + 0.02 hpdcacoa_r + 0.1 lnlncgcoa_r + 0.37 ocdycacoa_r + 0.32 odecoa_r + 0.09 pmtcoa_r + 0.07 stcoa_r + 0.02 ttccoa_r --> coa_r + 0.01 pa_RT_r 10427 or 16030 or 16779 or 9746

10428 K05824: LYS12; homoisocitrate dehydrogenase
0.07551171266248174 -3.2079395578822925
 Model
  HICITDm: hicit_m + nad_m <=> 2oxoadp_m + co2_m + nadh_m 10428

10460 K20457: DHFS; dihydrofolate synthase
0.07596130136997607 nan
 Model
  DHFS: atp_c + dhpt_c + glu__L_c --> adp_c + dhf_c + h_c + pi_c 10460

10540 K00968: PCYT1; choline-phosphate cytidylyltransferase
0.07596130136988427 nan
 Model
  CHLPCTD: cholp_c + ctp_c + h_c --> cdpchol_c + ppi_c 10540
  CPCTDTX: ctp_c + h_c + ntm2amep_c --> cmpntm2amep_c + ppi_c 10540

10613 K00134: GAPDH, gapA; glyceraldehyde 3-phosphate dehydrogenase
0.06559327664045211 nan
 Model
  E4PD: e4p_c + h2o_c + nad_c <=> 4per_c + 2.0 h_c + nadh_c 10613
  GAPD: g3p_c + nad_c + pi_c <=> 13dpg_c + h_c + nadh_c 10613
  NADHHR: h2o_c + nadh_c --> nadhx__R_c 10613
  NADHHS: h2o_c + nadh_c --> nadhx__S_c 10613
  NADPHHR: h2o_c + nadph_c --> nadphx__R_c 10613
  NADPHHS: h2o_c + nadph_c --> nadphx__S_c 10613

10629 K12670: WBP1; oligosaccharyltransferase complex subunit beta
0.0759613013701044 nan
 Model
  DOLASNT: Asn_X_Ser_Thr_c + doldpglcnacglcnacman_man_manman_manman_manmanmanglcglcglc_c --> asnglcnacglcnacman_man_manman_manman_manmanmanglcglcglc_c + doldp_c + 3.0 h_c 10629 and 11804 and 12158 and 12815

10673 K01082: cysQ, MET22, BPNT1; 3'(2'), 5'-bisphosphate nucleotidase
0.07596130136755619 nan
 Model
  BPNT: h2o_c + pap_c --> amp_c + pi_c 10673
  BPNT2: h2o_c + paps_c --> aps_c + pi_c 10673

10674 HMMPfam:ATP synthase complex subunit h:PF10775
0.019318790828581754 nan
 Model
  ATPS3m: adp_m + 3.0 h_c + pi_m --> atp_m + h2o_m + 2.0 h_m ATP6 and ATP8 and ATP9 and 9080 and 9619 and 9940 and 10674 and 11958 and 11967 and 13053 and 13252 and 13424 and 13759 and 13842 and 14786 and 14912 and 15287 and 15589 and 15880 and 16359

10680 K00275: pdxH, PNPO; pyridoxamine 5'-phosphate oxidase
0.07567088224219555 nan
 Model
  PDX5POi: o2_c + pdx5p_c --> h2o2_c + pydx5p_c 10680
  PYAM5PO: h2o_c + o2_c + pyam5p_c --> h2o2_c + nh4_c + pydx5p_c 10680
  PYDXNO: o2_c + pydxn_c <=> h2o2_c + pydx_c 10680
  PYDXO_1: h2o_c + o2_c + pydam_c <=> h2o2_c + nh4_c + pydx_c 10680

10802 HMMPfam:ATP synthase subunit H:PF05493
0.07596130137185197 nan
 Model
  ATPS2v: atp_c + h2o_c + h_c --> adp_c + 2.0 h_v + pi_c 10802 and 11025 and 11052 and 11064 and 11117 and 11403 and 14971 and 15309 and 15351 and 15963 and 16397 and 9210 and 9622 and 9748

10848 KOG0907: Thioredoxin
0.07596130136995456 nan
 Model
  METSOXR1: metsox_S__L_c + trdrd_c --> h2o_c + met__L_c + trdox_c (10848 and 15902) or (12730 and 15902) or (12737 and 15902) or (15339 and 15902)
  METSOXR2: metsox_R__L_c + trdrd_c --> h2o_c + met__L_c + trdox_c (10848 and 15469) or (12730 and 15469) or (12737 and 15469) or (15339 and 15469)
  PAPSR: paps_c + trdrd_c --> 2.0 h_c + pap_c + so3_c + trdox_c (10848 and 11741) or (11741 and 12730) or (11741 and 12737) or (11741 and 15339)
  RNDR1: adp_c + trdrd_c --> dadp_c + h2o_c + trdox_c (10848 and 11172 and 11290 and 14237) or (11172 and 11290 and 12730 and 14237) or (11172 and 11290 and 12737 and 14237) or (11172 and 11290 and 14237 and 15339)
  RNDR1n: adp_n + trdrd_n --> dadp_n + h2o_n + trdox_n (10848 and 11172 and 11290 and 14237) or (11172 and 11290 and 12730 and 14237) or (11172 and 11290 and 12737 and 14237)
  RNDR2: gdp_c + trdrd_c --> dgdp_c + h2o_c + trdox_c (10848 and 11172 and 11290 and 14237) or (11172 and 11290 and 12730 and 14237) or (11172 and 11290 and 12737 and 14237) or (11172 and 11290 and 14237 and 15339)
  RNDR2n: gdp_n + trdrd_n --> dgdp_n + h2o_n + trdox_n (10848 and 11172 and 11290 and 14237) or (11172 and 11290 and 12730 and 14237) or (11172 and 11290 and 12737 and 14237)
  RNDR3: cdp_c + trdrd_c --> dcdp_c + h2o_c + trdox_c (10848 and 11172 and 11290 and 14237) or (11172 and 11290 and 12730 and 14237) or (11172 and 11290 and 12737 and 14237) or (11172 and 11290 and 14237 and 15339)
  RNDR3n: cdp_n + trdrd_n --> dcdp_n + h2o_n + trdox_n (10848 and 11172 and 11290 and 14237) or (11172 and 11290 and 12730 and 14237) or (11172 and 11290 and 12737 and 14237)
  RNDR4: trdrd_c + udp_c --> dudp_c + h2o_c + trdox_c (10848 and 11172 and 11290 and 14237) or (11172 and 11290 and 12730 and 14237) or (11172 and 11290 and 12737 and 14237) or (11172 and 11290 and 14237 and 15339)
  RNDR4n: trdrd_n + udp_n --> dudp_n + h2o_n + trdox_n (10848 and 11172 and 11290 and 14237) or (11172 and 11290 and 12730 and 14237) or (11172 and 11290 and 12737 and 14237)
  THIORDXi: h2o2_c + trdrd_c --> 2.0 h2o_c + trdox_c (10848 and 12715) or (12715 and 12730) or (12715 and 12737)
  THIORDXni: h2o2_n + trdrd_n --> 2.0 h2o_n + trdox_n (10848 and 15037) or (12730 and 15037) or (12737 and 15037)
  TRDR: h_c + nadph_c + trdox_c --> nadp_c + trdrd_c (10848 and 9688) or (12730 and 9688) or (12737 and 9688)

10961 K15115: SLC25A32, MFT; solute carrier family 25 (mitochondrial folate transporter), member 32
0.07596130136968925 nan
 Model
  NADtm: nad_c --> nad_m 10961

11025 K02155: ATPeV0C, ATP6L; V-type H+-transporting ATPase 16kDa proteolipid subunit
0.0759613013714965 nan
 Model
  ATPS2v: atp_c + h2o_c + h_c --> adp_c + 2.0 h_v + pi_c 10802 and 11025 and 11052 and 11064 and 11117 and 11403 and 14971 and 15309 and 15351 and 15963 and 16397 and 9210 and 9622 and 9748

11050 K00589: MET1; uroporphyrin-III C-methyltransferase
0.0759613013707557 -3.6345639193821184
 Model
  UPP3MT: 2.0 amet_c + uppg3_c --> 2.0 ahcys_c + dscl_c + h_c 11050

11052 K02144: ATPeV1H; V-type H+-transporting ATPase subunit H
0.07596130136899605 nan
 Model
  ATPS2v: atp_c + h2o_c + h_c --> adp_c + 2.0 h_v + pi_c 10802 and 11025 and 11052 and 11064 and 11117 and 11403 and 14971 and 15309 and 15351 and 15963 and 16397 and 9210 and 9622 and 9748

11064 K02147: ATPeV1B, ATP6B; V-type H+-transporting ATPase subunit B
0.07596130137021107 nan
 Model
  ATPS2v: atp_c + h2o_c + h_c --> adp_c + 2.0 h_v + pi_c 10802 and 11025 and 11052 and 11064 and 11117 and 11403 and 14971 and 15309 and 15351 and 15963 and 16397 and 9210 and 9622 and 9748

11094 K01725: cynS; cyanate lyase
0.07596130137966384 nan
 Model
  CYNTAH: cynt_c + 3.0 h_c + hco3_c --> 2.0 co2_c + nh4_c 11094

11117 K02146: ATPeV0D, ATP6D; V-type H+-transporting ATPase subunit d
0.07596130136899605 nan
 Model
  ATPS2v: atp_c + h2o_c + h_c --> adp_c + 2.0 h_v + pi_c 10802 and 11025 and 11052 and 11064 and 11117 and 11403 and 14971 and 15309 and 15351 and 15963 and 16397 and 9210 and 9622 and 9748

11145 K00948: PRPS, prsA; ribose-phosphate pyrophosphokinase
0.0759613013699694 nan
 Model
  PRPPS: atp_c + r5p_c <=> amp_c + h_c + prpp_c 11145 or 16592

11151 BLAST: NADH dehydrogenase (ubiquinone) Fe-S protein 5 [C...
0.06627011871460528 nan
 Model
  NADH2_u9m2: 5.0 h_m + nadh_m + q9_m --> 4.0 h_c + nad_m + q9h2_m NAD1 and NAD2 and NAD3 and NAD4 and NAD4L and NAD5 and NAD6 and 10010 and 10017 and 10318 and 10541 and 10607 and 11151 and 11411 and 11702 and 11857 and 12482 and 12497 and 12543 and 12653 and 13017 and 13214 and 13268 and 13411 and 13656 and 13925 and 14078 and 14261 and 14418 and 14717 and 14900 and 15179 and 15864 and 15947 and 15973 and 15998 and 8444 and 8446 and 8530 and 8699 and 8817 and 8909 and 9144 and 9348

11178 K00026: MDH2; malate dehydrogenase
0.07519026044695952 nan
 Model
  MDHm: mal__L_m + nad_m <=> h_m + nadh_m + oaa_m 11178

11183 K09699: DBT, bkdB; 2-oxoisovalerate dehydrogenase E2 component (dihydrolipoyl transacylase)
0.07596130137450857 nan
 Model
  OBDHm: 2obut_m + coa_m + nad_m --> co2_m + nadh_m + ppcoa_m (10040 and 11183 and 12566 and 15436) or (10040 and 11188 and 12566 and 15436)
  OIVD1m: 4mop_m + coa_m + nad_m --> co2_m + ivcoa_m + nadh_m (10040 and 11183 and 12566 and 15436) or (10040 and 11188 and 12566 and 15436)
  OIVD2m: 3mob_m + coa_m + nad_m --> co2_m + ibcoa_m + nadh_m (10040 and 11183 and 12566 and 15436) or (10040 and 11188 and 12566 and 15436)
  OIVD3m: 3mop_m + coa_m + nad_m --> 2mbcoa_m + co2_m + nadh_m (10040 and 11183 and 12566 and 15436) or (10040 and 11188 and 12566 and 15436)

11188 HMMPfam:2-oxoacid dehydrogenases acyltransferase (catalytic domain):PF00198,HMMPfam:Biotin-requiring enzyme:PF00364,HMMPfam:e3 binding domain:PF02817,SUPERFAMILY::SSF47005,SUPERFAMILY::SSF51230,SUPERFAMILY::SSF52777
0.075961301369976 nan
 Model
  OBDHm: 2obut_m + coa_m + nad_m --> co2_m + nadh_m + ppcoa_m (10040 and 11183 and 12566 and 15436) or (10040 and 11188 and 12566 and 15436)
  OIVD1m: 4mop_m + coa_m + nad_m --> co2_m + ivcoa_m + nadh_m (10040 and 11183 and 12566 and 15436) or (10040 and 11188 and 12566 and 15436)
  OIVD2m: 3mob_m + coa_m + nad_m --> co2_m + ibcoa_m + nadh_m (10040 and 11183 and 12566 and 15436) or (10040 and 11188 and 12566 and 15436)
  OIVD3m: 3mop_m + coa_m + nad_m --> 2mbcoa_m + co2_m + nadh_m (10040 and 11183 and 12566 and 15436) or (10040 and 11188 and 12566 and 15436)

11202 K01001: ALG7; UDP-N-acetylglucosamine--dolichyl-phosphate N-acetylglucosaminephosphotransferase
0.07596130136745266 nan
 Model
  GLCNACPT: dolp_c + 2.0 h_c + uacgam_c --> doldpglcnac_c + ump_c 11202
  GLCNACPT_L: 0.1 dolp__L_c + uacgam_c --> 0.1 naglc2p__L_c + ump_c 11202
  GLCNACPT_U: 0.1 dolp_U_c + uacgam_c --> 0.1 naglc2p_U_c + ump_c 11202

11229 KOG4754: Predicted phosphoglycerate mutase
0.07596130136914532 nan
 Model
  PGM: 2pg_c <=> 3pg_c 11229 or 12393 or 15425 or 9910

11328 KOG2617: Citrate synthase
0.07596130137270207 nan
 Model
  MCITSm: h2o_m + oaa_m + ppcoa_m --> 2mcit_m + coa_m + h_m 11328

11331 K01647: CS, gltA; citrate synthase
0.0757006846632597 nan
 Model
  CSm: accoa_m + h2o_m + oaa_m --> cit_m + coa_m + h_m 11331

11375 K15119: SLC25A39_40; solute carrier family 25, member 39/40
0.07596130136745266 nan
 Model
  PYDX5Ptm: pydx5p_c <=> pydx5p_m 11375

11381 KOG1889: Putative phosphoinositide phosphatase
0.07596130136760809 nan
 Model
  PI35BP5Per_RT: h2o_r + 0.01 ptd135bp_RT_r --> pi_r + 0.01 ptd3ino_RT_r 11381 or 15340 or 16619
  PIN3Per_RT: h2o_r + 0.01 ptd3ino_RT_r --> pi_r + 0.01 ptd1ino_RT_r 11381
  PIN4Per_RT: h2o_r + 0.01 ptd4ino_RT_r --> pi_r + 0.01 ptd1ino_RT_r 11381

11391 K04709: LAG1; Acyl-CoA-dependent ceramide synthase
0.0759613013714965 nan
 Model
  CERS118er: sphgn_r + stcoa_r --> cer1_18_r + coa_r + h_r 11391 or 15168
  CERS124er: sphgn_r + ttccoa_r --> cer1_24_r + coa_r + h_r 11391 or 15168
  CERS126er: hexccoa_r + sphgn_r --> cer1_26_r + coa_r + h_r 11391 or 15168
  CERS224er: psphings_r + ttccoa_r --> cer2_24_r + coa_r + h_r 11391 or 15168
  CERS226er: hexccoa_r + psphings_r --> cer2_26_r + coa_r + h_r 11391 or 15168

11403 K03661: ATPeV0B, ATP6F; V-type H+-transporting ATPase 21kDa proteolipid subunit
0.07596130136977523 nan
 Model
  ATPS2v: atp_c + h2o_c + h_c --> adp_c + 2.0 h_v + pi_c 10802 and 11025 and 11052 and 11064 and 11117 and 11403 and 14971 and 15309 and 15351 and 15963 and 16397 and 9210 and 9622 and 9748

11411 K18159: NDUFAF1, CIA30; NADH dehydrogenase [ubiquinone] 1 alpha subcomplex assembly factor 1
0.0662701187159571 nan
 Model
  NADH2_u9m2: 5.0 h_m + nadh_m + q9_m --> 4.0 h_c + nad_m + q9h2_m NAD1 and NAD2 and NAD3 and NAD4 and NAD4L and NAD5 and NAD6 and 10010 and 10017 and 10318 and 10541 and 10607 and 11151 and 11411 and 11702 and 11857 and 12482 and 12497 and 12543 and 12653 and 13017 and 13214 and 13268 and 13411 and 13656 and 13925 and 14078 and 14261 and 14418 and 14717 and 14900 and 15179 and 15864 and 15947 and 15973 and 15998 and 8444 and 8446 and 8530 and 8699 and 8817 and 8909 and 9144 and 9348

11418 K14430: PHO87_91; phosphate transporter
0.0759613013699707 nan
 Model
  PIt2r: h_e + pi_e <=> h_c + pi_c 10316 or 11409 or 11410 or 11418 or 11534

11534 K14430: PHO87_91; phosphate transporter
0.07596130137431857 nan
 Model
  PIt2r: h_e + pi_e <=> h_c + pi_c 10316 or 11409 or 11410 or 11418 or 11534

11536 KOG2404: Fumarate reductase, flavoprotein subunit
0.0759613013705777 nan
 Model
  FRDcm: fadh2_m + fum_c --> fad_m + succ_c 11420 or 11536

11610 K01814: hisA; phosphoribosylformimino-5-aminoimidazole carboxamide ribotide isomerase
0.07596130136914532 nan
 Model
  PRMICI: prfp_c <=> prlp_c 11610 or 9084

11681 K00030: IDH3; isocitrate dehydrogenase (NAD+)
0.07360869865042646 nan
 Model
  ICDHxm: icit_m + nad_m --> akg_m + co2_m + nadh_m 11681 and 11682

11682 K00030: IDH3; isocitrate dehydrogenase (NAD+)
0.07360869865212294 nan
 Model
  ICDHxm: icit_m + nad_m --> akg_m + co2_m + nadh_m 11681 and 11682

11702 K03936: NDUFS3; NADH dehydrogenase (ubiquinone) Fe-S protein 3
0.06627011871507331 nan
 Model
  NADH2_u9m2: 5.0 h_m + nadh_m + q9_m --> 4.0 h_c + nad_m + q9h2_m NAD1 and NAD2 and NAD3 and NAD4 and NAD4L and NAD5 and NAD6 and 10010 and 10017 and 10318 and 10541 and 10607 and 11151 and 11411 and 11702 and 11857 and 12482 and 12497 and 12543 and 12653 and 13017 and 13214 and 13268 and 13411 and 13656 and 13925 and 14078 and 14261 and 14418 and 14717 and 14900 and 15179 and 15864 and 15947 and 15973 and 15998 and 8444 and 8446 and 8530 and 8699 and 8817 and 8909 and 9144 and 9348

11752 K00963: UGP2, galU, galF; UTP--glucose-1-phosphate uridylyltransferase
0.07596130137115692 nan
 Model
  GALUi: g1p_c + h_c + utp_c --> ppi_c + udpg_c 11752

11844 K01915: glnA, GLUL; glutamine synthetase
0.07596130137014753 nan
 Model
  GLNS: atp_c + glu__L_c + nh4_c --> adp_c + gln__L_c + h_c + pi_c 11844 or 8959

11878 K00889: PIP5K; 1-phosphatidylinositol-4-phosphate 5-kinase
0.0759613013699707 nan
 Model
  PI4P5Kn_RT: atp_n + 0.01 ptd4ino_RT_n --> adp_n + h_n + 0.01 ptd145bp_RT_n 11878

11899 K00604: MTFMT, fmt; methionyl-tRNA formyltransferase
0.07596130136997425 nan
 Model
  FMETTRSm: 10fthf_m + mettrna_m --> fmettrna_m + h_m + thf_m 11899
  FTHFD: 10fthf_c + h2o_c --> for_c + h_c + thf_c 11899

11952 K00122: FDH; formate dehydrogenase
0.07596130137139687 nan
 Model
  FDH: for_c + nad_c --> co2_c + nadh_c 11952

11958 K02136: ATPeF1G, ATP5C1, ATP3; F-type H+-transporting ATPase subunit gamma
0.019318790827511267 nan
 Model
  ATPS3m: adp_m + 3.0 h_c + pi_m --> atp_m + h2o_m + 2.0 h_m ATP6 and ATP8 and ATP9 and 9080 and 9619 and 9940 and 10674 and 11958 and 11967 and 13053 and 13252 and 13424 and 13759 and 13842 and 14786 and 14912 and 15287 and 15589 and 15880 and 16359

11967 K02134: ATPeF1D, ATP5D, ATP16; F-type H+-transporting ATPase subunit delta
0.01931879082688633 nan
 Model
  ATPS3m: adp_m + 3.0 h_c + pi_m --> atp_m + h2o_m + 2.0 h_m ATP6 and ATP8 and ATP9 and 9080 and 9619 and 9940 and 10674 and 11958 and 11967 and 13053 and 13252 and 13424 and 13759 and 13842 and 14786 and 14912 and 15287 and 15589 and 15880 and 16359

12031 K01738: cysK; cysteine synthase A
0.07596130137127213 -3.1094068564790103
 Model
  CYSS: acser_c + h2s_c --> ac_c + cys__L_c + h_c 12031 or 13106

12080 K00003: E1.1.1.3; homoserine dehydrogenase
0.07596130137019827 nan
 Model
  HSDxi: aspsa_c + h_c + nadh_c --> hom__L_c + nad_c 12080 or 16738
  HSDy: hom__L_c + nadp_c <=> aspsa_c + h_c + nadph_c 12080 or 16738

12116 K00658: DLST, sucB; 2-oxoglutarate dehydrogenase E2 component (dihydrolipoamide succinyltransferase)
0.07410532596555526 nan
 Model
  2OXOADOXm: 2oxoadp_m + coa_m + nad_m --> co2_m + glutcoa_m + nadh_m 10040 and 12116 and 9274
  AKGDm: akg_m + coa_m + nad_m --> co2_m + nadh_m + succoa_m 10007 and 10040 and 12116

12123 K01893: NARS, asnS; asparaginyl-tRNA synthetase
0.0759613013699694 nan
 Model
  ASNTRSm: asn__L_m + atp_m + trnaasn_m --> amp_m + asntrna_m + ppi_m 12123

12158 K07151: STT3; dolichyl-diphosphooligosaccharide--protein glycosyltransferase
0.07596130137270207 nan
 Model
  DOLASNT: Asn_X_Ser_Thr_c + doldpglcnacglcnacman_man_manman_manman_manmanmanglcglcglc_c --> asnglcnacglcnacman_man_manman_manman_manmanmanglcglcglc_c + doldp_c + 3.0 h_c 10629 and 11804 and 12158 and 12815

12248 K00262: E1.4.1.4, gdhA; glutamate dehydrogenase (NADP+)
0.0751378768077882 nan
 Model
  GLUDy: glu__L_c + h2o_c + nadp_c <=> akg_c + h_c + nadph_c + nh4_c 12248

12302 K01955: carB, CPA2; carbamoyl-phosphate synthase large subunit
0.0759613013699707 -4.924379789084965
 Model
  CBPSm: 2.0 atp_m + gln__L_m + h2o_m + hco3_m --> 2.0 adp_m + cbp_m + glu__L_m + 2.0 h_m + pi_m 12302 and 13297

12353 K03448: FEN2, LIZ1; MFS transporter, ACS family, pantothenate transporter
0.07596130137114704 nan
 Model
  PNTOt2: h_e + pnto__R_e <=> h_c + pnto__R_c 12353 or 12740

12446 K15113: SLC25A28_37, MFRN; solute carrier family 25 (mitochondrial iron transporter), member 28/37
0.07596130137450857 nan
 Model
  FE2tm: fe2_c + h_c --> fe2_m + h_m 12446

12471 K00326: E1.6.2.2; cytochrome-b5 reductase
0.07596130137325818 nan
 Model
  C22STDSrx: ergtrol_r + h_r + nadh_r + o2_r --> ergtetrol_r + 2.0 h2o_r + nad_r (11716 and 12471 and 16097) or (11716 and 13206 and 16097)
  CERD418er: cer2p_18_r + h_r + nadph_r + o2_r --> cer4_18_r + 2.0 h2o_r + nadp_r (12471 and 16097 and 9629) or (13206 and 16097 and 9629)
  DESAT1829Z12Zer: h_r + nadh_r + o2_r + odecoa_r --> 2.0 h2o_r + nad_r + ocdycacoa_r (12471 and 16097 and 8845) or (13206 and 16097 and 8845)
  LNS14DMrx: 2.0 h_r + lanost_r + 3.0 nadh_r + 3.0 o2_r --> 44mctr_r + for_r + 4.0 h2o_r + 3.0 nad_r (12843 and 12471 and 16097) or (12843 and 13206 and 16097)
  SQLErx: h_r + nadh_r + o2_r + sql_r --> Ssq23epx_r + h2o_r + nad_r (13729 and 12471 and 16097) or (13729 and 13206 and 16097)

12485 K15728: LPIN; phosphatidate phosphatase LPIN
0.07596130136755619 nan
 Model
  DAGPYPer_RT: h2o_r + 0.01 pa_RT_r --> 0.01 12dgr_RT_r + pi_r 12485 or 13087

12497 K03949: NDUFA5; NADH dehydrogenase (ubiquinone) 1 alpha subcomplex subunit 5
0.06627011871558734 nan
 Model
  NADH2_u9m2: 5.0 h_m + nadh_m + q9_m --> 4.0 h_c + nad_m + q9h2_m NAD1 and NAD2 and NAD3 and NAD4 and NAD4L and NAD5 and NAD6 and 10010 and 10017 and 10318 and 10541 and 10607 and 11151 and 11411 and 11702 and 11857 and 12482 and 12497 and 12543 and 12653 and 13017 and 13214 and 13268 and 13411 and 13656 and 13925 and 14078 and 14261 and 14418 and 14717 and 14900 and 15179 and 15864 and 15947 and 15973 and 15998 and 8444 and 8446 and 8530 and 8699 and 8817 and 8909 and 9144 and 9348

12513 K00641: metX; homoserine O-acetyltransferase
0.07596130136997425 -4.825283815793256
 Model
  HSERTA: accoa_c + hom__L_c <=> achms_c + coa_c 12513 or 15248

12540 KOG1212: Amidases
0.07596130137014993 nan
 Model
  AMID: 4gudbd_c + h2o_c --> 4gudbutn_c + nh4_c 10276 or 10277 or 12540 or 12553 or 12842 or 13791
  AMID2: h2o_c + pad_c --> nh4_c + pac_c 10276 or 10277 or 12540 or 12553 or 12842 or 13791
  AMID3: h2o_c + iad_c --> ind3ac_c + nh4_c 10276 or 10277 or 12540 or 12553 or 12842 or 13791
  AMID_1: ad_c + h2o_c --> ac_c + nh4_c 10276 or 10277 or 12540 or 12553 or 12842 or 13791

12551 K01497: ribA, RIB1; GTP cyclohydrolase II
0.0759613013714965 nan
 Model
  GTPCII: gtp_c + 3.0 h2o_c --> 25dhpp_c + for_c + 2.0 h_c + ppi_c 12551 or 16666
  GTPCII2: gtp_c + 3.0 h2o_c --> 25drapp_c + for_c + 2.0 h_c + ppi_c 12551

12649 K11778: DHDDS, RER2, SRT1; ditrans,polycis-polyprenyl diphosphate synthase
0.0759613013695713 nan
 Model
  FT: frdp_c + ipdp_c --> ppi_c + ttc_ggdp_c 12649

12653 K18162: NDUFAF5; NADH dehydrogenase [ubiquinone] 1 alpha subcomplex assembly factor 5
0.0662701187150738 nan
 Model
  MALCOAMT: amet_c + malcoa_c --> ahcys_c + malcoame_c 12653
  NADH2_u9m2: 5.0 h_m + nadh_m + q9_m --> 4.0 h_c + nad_m + q9h2_m NAD1 and NAD2 and NAD3 and NAD4 and NAD4L and NAD5 and NAD6 and 10010 and 10017 and 10318 and 10541 and 10607 and 11151 and 11411 and 11702 and 11857 and 12482 and 12497 and 12543 and 12653 and 13017 and 13214 and 13268 and 13411 and 13656 and 13925 and 14078 and 14261 and 14418 and 14717 and 14900 and 15179 and 15864 and 15947 and 15973 and 15998 and 8444 and 8446 and 8530 and 8699 and 8817 and 8909 and 9144 and 9348

12704 K05863: SLC25A4S, ANT; solute carrier family 25 (mitochondrial adenine nucleotide translocator), member 4/5/6/31
0.01351887875457856 nan
 Model
  ATPtm: adp_c + atp_m --> adp_m + atp_c 12704

12715 K03386: PRDX2_4, ahpC; peroxiredoxin (alkyl hydroperoxide reductase subunit C)
0.07596130137824275 nan
 Model
  THIORDXi: h2o2_c + trdrd_c --> 2.0 h2o_c + trdox_c (10848 and 12715) or (12715 and 12730) or (12715 and 12737)

12736 K00652: bioF; 8-amino-7-oxononanoate synthase
0.07596130137013613 nan
 Model
  AOXSp: ala__L_x + h_x + pimcoa_x --> 8aonn_x + co2_x + coa_x 12736

12737 K03671: trxA; thioredoxin 1
0.07596130136899605 nan
 Model
  METSOXR1: metsox_S__L_c + trdrd_c --> h2o_c + met__L_c + trdox_c (10848 and 15902) or (12730 and 15902) or (12737 and 15902) or (15339 and 15902)
  METSOXR2: metsox_R__L_c + trdrd_c --> h2o_c + met__L_c + trdox_c (10848 and 15469) or (12730 and 15469) or (12737 and 15469) or (15339 and 15469)
  PAPSR: paps_c + trdrd_c --> 2.0 h_c + pap_c + so3_c + trdox_c (10848 and 11741) or (11741 and 12730) or (11741 and 12737) or (11741 and 15339)
  RNDR1: adp_c + trdrd_c --> dadp_c + h2o_c + trdox_c (10848 and 11172 and 11290 and 14237) or (11172 and 11290 and 12730 and 14237) or (11172 and 11290 and 12737 and 14237) or (11172 and 11290 and 14237 and 15339)
  RNDR1n: adp_n + trdrd_n --> dadp_n + h2o_n + trdox_n (10848 and 11172 and 11290 and 14237) or (11172 and 11290 and 12730 and 14237) or (11172 and 11290 and 12737 and 14237)
  RNDR2: gdp_c + trdrd_c --> dgdp_c + h2o_c + trdox_c (10848 and 11172 and 11290 and 14237) or (11172 and 11290 and 12730 and 14237) or (11172 and 11290 and 12737 and 14237) or (11172 and 11290 and 14237 and 15339)
  RNDR2n: gdp_n + trdrd_n --> dgdp_n + h2o_n + trdox_n (10848 and 11172 and 11290 and 14237) or (11172 and 11290 and 12730 and 14237) or (11172 and 11290 and 12737 and 14237)
  RNDR3: cdp_c + trdrd_c --> dcdp_c + h2o_c + trdox_c (10848 and 11172 and 11290 and 14237) or (11172 and 11290 and 12730 and 14237) or (11172 and 11290 and 12737 and 14237) or (11172 and 11290 and 14237 and 15339)
  RNDR3n: cdp_n + trdrd_n --> dcdp_n + h2o_n + trdox_n (10848 and 11172 and 11290 and 14237) or (11172 and 11290 and 12730 and 14237) or (11172 and 11290 and 12737 and 14237)
  RNDR4: trdrd_c + udp_c --> dudp_c + h2o_c + trdox_c (10848 and 11172 and 11290 and 14237) or (11172 and 11290 and 12730 and 14237) or (11172 and 11290 and 12737 and 14237) or (11172 and 11290 and 14237 and 15339)
  RNDR4n: trdrd_n + udp_n --> dudp_n + h2o_n + trdox_n (10848 and 11172 and 11290 and 14237) or (11172 and 11290 and 12730 and 14237) or (11172 and 11290 and 12737 and 14237)
  THIORDXi: h2o2_c + trdrd_c --> 2.0 h2o_c + trdox_c (10848 and 12715) or (12715 and 12730) or (12715 and 12737)
  THIORDXni: h2o2_n + trdrd_n --> 2.0 h2o_n + trdox_n (10848 and 15037) or (12730 and 15037) or (12737 and 15037)
  TRDR: h_c + nadph_c + trdox_c --> nadp_c + trdrd_c (10848 and 9688) or (12730 and 9688) or (12737 and 9688)

12815 K12666: OST1, RPN1; oligosaccharyltransferase complex subunit alpha (ribophorin I)
0.0759613013699906 nan
 Model
  DOLASNT: Asn_X_Ser_Thr_c + doldpglcnacglcnacman_man_manman_manman_manmanmanglcglcglc_c --> asnglcnacglcnacman_man_manman_manman_manmanmanglcglcglc_c + doldp_c + 3.0 h_c 10629 and 11804 and 12158 and 12815

12855 K05857: PLCD; phosphatidylinositol phospholipase C, delta
0.075961301370527 nan
 Model
  PI45BPPn_RT: h2o_n + 0.01 ptd145bp_RT_n --> 0.01 12dgr_RT_n + h_n + mi145p_n 12855

12927 HMMPfam:PAP2 superfamily:PF01569,SMART:Acid phosphatase homologues:SM00014,SUPERFAMILY::SSF48317
0.07596130137014859 nan
 Model
  IPCS124g_RT: cer1_24_g + 0.01 ptd1ino_RT_g --> 0.01 12dgr_RT_g + 0.01 ipc124_RT_g 12927 and 8747
  IPCS126g_RT: cer1_26_g + 0.01 ptd1ino_RT_g --> 0.01 12dgr_RT_g + 0.01 ipc126_RT_g 12927 and 8747
  IPCS224g_RT: cer2_24_g + 0.01 ptd1ino_RT_g --> 0.01 12dgr_RT_g + 0.01 ipc224_RT_g 12927 and 8747
  IPCS226g_RT: cer2_26_g + 0.01 ptd1ino_RT_g --> 0.01 12dgr_RT_g + 0.01 ipc226_RT_g 12927 and 8747
  IPCS324g_RT: cer3_24_g + 0.01 ptd1ino_RT_g --> 0.01 12dgr_RT_g + 0.01 ipc324_RT_g 12927 and 8747
  IPCS326g_RT: cer3_26_g + 0.01 ptd1ino_RT_g --> 0.01 12dgr_RT_g + 0.01 ipc326_RT_g 12927 and 8747

13002 K00873: PK, pyk; pyruvate kinase
0.06699222198176358 nan
 Model
  PYK: adp_c + h_c + pep_c --> atp_c + pyr_c 13002

13017 K03934: NDUFS1; NADH dehydrogenase (ubiquinone) Fe-S protein 1
0.0662701187150738 nan
 Model
  NADH2_u9m2: 5.0 h_m + nadh_m + q9_m --> 4.0 h_c + nad_m + q9h2_m NAD1 and NAD2 and NAD3 and NAD4 and NAD4L and NAD5 and NAD6 and 10010 and 10017 and 10318 and 10541 and 10607 and 11151 and 11411 and 11702 and 11857 and 12482 and 12497 and 12543 and 12653 and 13017 and 13214 and 13268 and 13411 and 13656 and 13925 and 14078 and 14261 and 14418 and 14717 and 14900 and 15179 and 15864 and 15947 and 15973 and 15998 and 8444 and 8446 and 8530 and 8699 and 8817 and 8909 and 9144 and 9348

13044 K01101: E3.1.3.41; 4-nitrophenyl phosphatase
0.07596130136996843 nan
 Model
  HYPOE: h2o_c + pyam5p_c --> pi_c + pydam_c 13044
  PDXPP: h2o_c + pdx5p_c --> pi_c + pydxn_c 13044
  PYDXPP: h2o_c + pydx5p_c --> pi_c + pydx_c 13044

13053 K18192: ATP10; mitochondrial ATPase complex subunit ATP10
0.019318790827387695 nan
 Model
  ATPS3m: adp_m + 3.0 h_c + pi_m --> atp_m + h2o_m + 2.0 h_m ATP6 and ATP8 and ATP9 and 9080 and 9619 and 9940 and 10674 and 11958 and 11967 and 13053 and 13252 and 13424 and 13759 and 13842 and 14786 and 14912 and 15287 and 15589 and 15880 and 16359

13137 K01866: YARS, tyrS; tyrosyl-tRNA synthetase
0.07596130136996895 nan
 Model
  TYRTRSm: atp_m + trnatyr_m + tyr__L_m --> amp_m + ppi_m + tyrtrna_m 13137

13175 K03119: tauD; taurine dioxygenase
0.07596130137072579 nan
 Model
  TAUDO: akg_c + o2_c + taur_c --> aacald_c + co2_c + h_c + so3_c + succ_c 10226 or 13175 or 14698 or 8618

13206 K00326: E1.6.2.2; cytochrome-b5 reductase
0.07596130136961309 nan
 Model
  C22STDSrx: ergtrol_r + h_r + nadh_r + o2_r --> ergtetrol_r + 2.0 h2o_r + nad_r (11716 and 12471 and 16097) or (11716 and 13206 and 16097)
  CERD418er: cer2p_18_r + h_r + nadph_r + o2_r --> cer4_18_r + 2.0 h2o_r + nadp_r (12471 and 16097 and 9629) or (13206 and 16097 and 9629)
  DESAT1829Z12Zer: h_r + nadh_r + o2_r + odecoa_r --> 2.0 h2o_r + nad_r + ocdycacoa_r (12471 and 16097 and 8845) or (13206 and 16097 and 8845)
  LNS14DMrx: 2.0 h_r + lanost_r + 3.0 nadh_r + 3.0 o2_r --> 44mctr_r + for_r + 4.0 h2o_r + 3.0 nad_r (12843 and 12471 and 16097) or (12843 and 13206 and 16097)
  SQLErx: h_r + nadh_r + o2_r + sql_r --> Ssq23epx_r + h2o_r + nad_r (13729 and 12471 and 16097) or (13729 and 13206 and 16097)

13208 KOG4526: Predicted membrane protein
0.07596130136911045 nan
 Model
  PEPAT: accoa_c + pepd_c --> apep_c + coa_c + h_c 13208 or 9438

13214 K03943: NDUFV2; NADH dehydrogenase (ubiquinone) flavoprotein 2
0.06627011871507285 nan
 Model
  NADH2_u9m2: 5.0 h_m + nadh_m + q9_m --> 4.0 h_c + nad_m + q9h2_m NAD1 and NAD2 and NAD3 and NAD4 and NAD4L and NAD5 and NAD6 and 10010 and 10017 and 10318 and 10541 and 10607 and 11151 and 11411 and 11702 and 11857 and 12482 and 12497 and 12543 and 12653 and 13017 and 13214 and 13268 and 13411 and 13656 and 13925 and 14078 and 14261 and 14418 and 14717 and 14900 and 15179 and 15864 and 15947 and 15973 and 15998 and 8444 and 8446 and 8530 and 8699 and 8817 and 8909 and 9144 and 9348

13252 K07555: ATPeAF1, ATPAF1, ATP11; ATP synthase mitochondrial F1 complex assembly factor 1
0.019318790827504782 nan
 Model
  ATPS3m: adp_m + 3.0 h_c + pi_m --> atp_m + h2o_m + 2.0 h_m ATP6 and ATP8 and ATP9 and 9080 and 9619 and 9940 and 10674 and 11958 and 11967 and 13053 and 13252 and 13424 and 13759 and 13842 and 14786 and 14912 and 15287 and 15589 and 15880 and 16359

13260 K00915: IPMK, IPK2; inositol-polyphosphate multikinase
0.07596130137014753 nan
 Model
  MI1345PKn: atp_n + mi1345p_n --> adp_n + h_n + mi13456p_n 13260
  MI1456PKn: atp_n + mi1456p_n --> adp_n + h_n + mi13456p_n 13260
  MI145P6Kn: atp_n + mi145p_n --> adp_n + h_n + mi1456p_n 13260
  MI145PKn: atp_n + mi145p_n --> adp_n + h_n + mi1345p_n 13260

13265 K01759: GLO1, gloA; lactoylglutathione lyase
0.07596130136995781 nan
 Model
  LGTHL: gthrd_c + mthgxl_c --> lgt__S_c 13265 or 16520

13268 HMMPfam:NADH-ubiquinone oxidoreductase B12 subunit family:PF08122
0.0662701187150717 nan
 Model
  NADH2_u9m2: 5.0 h_m + nadh_m + q9_m --> 4.0 h_c + nad_m + q9h2_m NAD1 and NAD2 and NAD3 and NAD4 and NAD4L and NAD5 and NAD6 and 10010 and 10017 and 10318 and 10541 and 10607 and 11151 and 11411 and 11702 and 11857 and 12482 and 12497 and 12543 and 12653 and 13017 and 13214 and 13268 and 13411 and 13656 and 13925 and 14078 and 14261 and 14418 and 14717 and 14900 and 15179 and 15864 and 15947 and 15973 and 15998 and 8444 and 8446 and 8530 and 8699 and 8817 and 8909 and 9144 and 9348

13288 K01807: rpiA; ribose 5-phosphate isomerase A
0.07591539273812817 nan
 Model
  RPI: r5p_c <=> ru5p__D_c 13288

13297 K01956: carA, CPA1; carbamoyl-phosphate synthase small subunit
0.07596130136743372 -3.8118928917474233
 Model
  CBPSm: 2.0 atp_m + gln__L_m + h2o_m + hco3_m --> 2.0 adp_m + cbp_m + glu__L_m + 2.0 h_m + pi_m 12302 and 13297

13348 K07432: ALG13; beta-1,4-N-acetylglucosaminyltransferase
0.075961301370527 nan
 Model
  GLCNACT: doldpglcnac_c + uacgam_c --> doldpglcnacglcnac_c + h_c + udp_c 13348 or 14170

13398 K01889: FARSA, pheS; phenylalanyl-tRNA synthetase alpha chain
0.07596130137289103 nan
 Model
  PHETRSm: atp_m + phe__L_m + trnaphe_m --> amp_m + phetrna_m + ppi_m 13398

13424 K02137: ATPeF0O, ATP5O, ATP5; F-type H+-transporting ATPase subunit O
0.019318790825105528 nan
 Model
  ATPS3m: adp_m + 3.0 h_c + pi_m --> atp_m + h2o_m + 2.0 h_m ATP6 and ATP8 and ATP9 and 9080 and 9619 and 9940 and 10674 and 11958 and 11967 and 13053 and 13252 and 13424 and 13759 and 13842 and 14786 and 14912 and 15287 and 15589 and 15880 and 16359

13432 K00286: proC; pyrroline-5-carboxylate reductase
0.07587294327415464 nan
 Model
  HPROa: 1p3h5c_c + 2.0 h_c + nadh_c --> 4hpro_LT_c + nad_c 13432
  HPROb: 1p3h5c_c + 2.0 h_c + nadph_c --> 4hpro_LT_c + nadp_c 13432
  P5CR: 1pyr5c_c + 2.0 h_c + nadph_c --> nadp_c + pro__L_c 13432
  P5CRx: 1pyr5c_c + 2.0 h_c + nadh_c --> nad_c + pro__L_c 13432

13492 K13248: PHOSPHO2; pyridoxal phosphate phosphatase PHOSPHO2
0.07596130137027578 nan
 Model
  CHLP: cholp_c + h2o_c --> chol_c + pi_c 13492
  ETHP: ethamp_c + h2o_c --> etha_c + pi_c 13492

13553 K01210: E3.2.1.58; glucan 1,3-beta-glucosidase
0.07596130137334717 nan
 Model
  13BGHe: 13BDglcn_e + h2o_e --> glc__D_e 13553

13555 KOG2533: Permease of the major facilitator superfamily
0.07596130136997191 nan
 Model
  NACt: nac_e <=> nac_c 12221 or 13555 or 9534 or 9938

13569 K02304: MET8; precorrin-2 dehydrogenase / sirohydrochlorin ferrochelatase
0.07596130137010333 nan
 Model
  SHCHD2: dscl_c + nad_c --> h_c + nadh_c + scl_c 13569
  SHCHF: fe2_c + scl_c --> 3.0 h_c + sheme_c 13569

13617 K01535: PMA1, PMA2; H+-transporting ATPase
0.07596130136998831 nan
 Model
  ATPS: atp_c + h2o_c --> adp_c + h_e + pi_c 13617 or 15584

13630 K00161: PDHA, pdhA; pyruvate dehydrogenase E1 component alpha subunit
0.0736473636230317 nan
 Model
  PDHm: coa_m + nad_m + pyr_m --> accoa_m + co2_m + nadh_m 10040 and 13630 and 13722 and 13948 and 14126

13759 HMMPfam:ATP synthase j chain:PF04911
0.019318790827458916 nan
 Model
  ATPS3m: adp_m + 3.0 h_c + pi_m --> atp_m + h2o_m + 2.0 h_m ATP6 and ATP8 and ATP9 and 9080 and 9619 and 9940 and 10674 and 11958 and 11967 and 13053 and 13252 and 13424 and 13759 and 13842 and 14786 and 14912 and 15287 and 15589 and 15880 and 16359

13842 HMMPfam:Mitochondrial ATP synthase epsilon chain:PF04627,SUPERFAMILY::SSF48690
0.019318790828151577 nan
 Model
  ATPS3m: adp_m + 3.0 h_c + pi_m --> atp_m + h2o_m + 2.0 h_m ATP6 and ATP8 and ATP9 and 9080 and 9619 and 9940 and 10674 and 11958 and 11967 and 13053 and 13252 and 13424 and 13759 and 13842 and 14786 and 14912 and 15287 and 15589 and 15880 and 16359

13935 K14394: ACP1; low molecular weight phosphotyrosine protein phosphatase
0.07596130136897777 nan
 Model
  ACP1_FMN: fmn_c + h2o_c --> pi_c + ribflv_c 13935
  THMP: h2o_c + thmmp_c --> pi_c + thm_c 13935

13948 K00162: PDHB, pdhB; pyruvate dehydrogenase E1 component beta subunit
0.07364736362303066 nan
 Model
  PDHm: coa_m + nad_m + pyr_m --> accoa_m + co2_m + nadh_m 10040 and 13630 and 13722 and 13948 and 14126

13987 K09885: AQPF; aquaporin rerated protein, other eukaryote
0.07596130136745266 nan
 Model
  H2Ot: h2o_e <=> h2o_c 13986 or 13987 or 9014 or 9015

14031 K15441: TAD2, ADAT2; tRNA-specific adenosine deaminase 2
0.0759613013698403 nan
 Model
  CSND: csn_c + h2o_c + h_c --> nh4_c + ura_c 14031 or 14559

14078 K03963: NDUFB7; NADH dehydrogenase (ubiquinone) 1 beta subcomplex subunit 7
0.06627011871267646 nan
 Model
  NADH2_u9m2: 5.0 h_m + nadh_m + q9_m --> 4.0 h_c + nad_m + q9h2_m NAD1 and NAD2 and NAD3 and NAD4 and NAD4L and NAD5 and NAD6 and 10010 and 10017 and 10318 and 10541 and 10607 and 11151 and 11411 and 11702 and 11857 and 12482 and 12497 and 12543 and 12653 and 13017 and 13214 and 13268 and 13411 and 13656 and 13925 and 14078 and 14261 and 14418 and 14717 and 14900 and 15179 and 15864 and 15947 and 15973 and 15998 and 8444 and 8446 and 8530 and 8699 and 8817 and 8909 and 9144 and 9348

14096 K03644: lipA; lipoyl synthase
0.07596130136979587 nan
 Model
  LIPOSm: 4fe4s_m + 2.0 amet_m + h_m + nad_m + octapb_m --> 2fe2s_m + 2.0 dad_5_m + 2.0 fe2_m + lipopb_m + 2.0 met__L_m + nadh_m 14096

14109 K00121: frmA, ADH5, adhC; S-(hydroxymethyl)glutathione dehydrogenase / alcohol dehydrogenase
0.07596130136597552 nan
 Model
  ALCD22xi: 2mbald_c + h_c + nadh_c --> 2mbtoh_c + nad_c 14108 or 14109 or 15438
  ALCD23xi: 2mppal_c + h_c + nadh_c --> ibutoh_c + nad_c 14108 or 14109 or 15438
  ALCD24xi: 3mbald_c + h_c + nadh_c --> iamoh_c + nad_c 14108 or 14109 or 15438
  ALCD25xi: h_c + nadh_c + pacald_c --> 2phetoh_c + nad_c 14108 or 14109 or 15438
  ALCD26xi: h_c + id3acald_c + nadh_c --> ind3eth_c + nad_c 14108 or 14109 or 15438
  FALDH2: hmgth_c + nad_c <=> Sfglutth_c + h_c + nadh_c 14108 or 14109

14126 K00627: DLAT, aceF, pdhC; pyruvate dehydrogenase E2 component (dihydrolipoamide acetyltransferase)
0.07364736362524127 nan
 Model
  PDHm: coa_m + nad_m + pyr_m --> accoa_m + co2_m + nadh_m 10040 and 13630 and 13722 and 13948 and 14126

14159 K15115: SLC25A32, MFT; solute carrier family 25 (mitochondrial folate transporter), member 32
0.07596130136737093 nan
 Model
  FADFMNtm: fad_c + fmn_m --> fad_m + fmn_c 14159

14225 K01875: SARS, serS; seryl-tRNA synthetase
0.0759613013699687 nan
 Model
  SERTRSm: atp_m + ser__L_m + trnaser_m --> amp_m + ppi_m + sertrna_m 14225

14261 KOG1748: Acyl carrier protein/NADH-ubiquinone oxidoreductase, NDUFAB1/SDAP subunit
0.06627011871484001 nan
 Model
  MCOATAm: ACP_m + malcoa_m <=> coa_m + malACP_m 14261 and 16542
  NADH2_u9m2: 5.0 h_m + nadh_m + q9_m --> 4.0 h_c + nad_m + q9h2_m NAD1 and NAD2 and NAD3 and NAD4 and NAD4L and NAD5 and NAD6 and 10010 and 10017 and 10318 and 10541 and 10607 and 11151 and 11411 and 11702 and 11857 and 12482 and 12497 and 12543 and 12653 and 13017 and 13214 and 13268 and 13411 and 13656 and 13925 and 14078 and 14261 and 14418 and 14717 and 14900 and 15179 and 15864 and 15947 and 15973 and 15998 and 8444 and 8446 and 8530 and 8699 and 8817 and 8909 and 9144 and 9348

14277 K00077: panE, apbA; 2-dehydropantoate 2-reductase
0.07596130136997384 nan
 Model
  DPR: 2dhp_c + h_c + nadph_c --> nadp_c + pant__R_c 14277 or 16522

14377 K13939: FOL1; dihydroneopterin aldolase / 2-amino-4-hydroxy-6-hydroxymethyldihydropteridine diphosphokinase / dihydropteroate synthase
0.07596130136997117 nan
 Model
  DHNPA2r: dhnpt_c <=> 6hmhpt_c + gcald_c 14377
  DHPS2: 4abz_c + 6hmhptpp_c --> dhpt_c + ppi_c 14377
  HPPK2: 6hmhpt_c + atp_c --> 6hmhptpp_c + amp_c + h_c 14377

14418 K03942: NDUFV1; NADH dehydrogenase (ubiquinone) flavoprotein 1
0.06627011871507611 nan
 Model
  NADH2_u9m2: 5.0 h_m + nadh_m + q9_m --> 4.0 h_c + nad_m + q9h2_m NAD1 and NAD2 and NAD3 and NAD4 and NAD4L and NAD5 and NAD6 and 10010 and 10017 and 10318 and 10541 and 10607 and 11151 and 11411 and 11702 and 11857 and 12482 and 12497 and 12543 and 12653 and 13017 and 13214 and 13268 and 13411 and 13656 and 13925 and 14078 and 14261 and 14418 and 14717 and 14900 and 15179 and 15864 and 15947 and 15973 and 15998 and 8444 and 8446 and 8530 and 8699 and 8817 and 8909 and 9144 and 9348

14499 K01057: PGLS, pgl, devB; 6-phosphogluconolactonase
0.07596130137289103 nan
 Model
  PGLp: 6pgl_x + h2o_x --> 6pgc_x + h_x 14499

14565 K01885: EARS, gltX; glutamyl-tRNA synthetase
0.0759613013714965 nan
 Model
  GLUTRSm: atp_m + glu__L_m + trnaglu_m --> amp_m + glutrna_m + ppi_m 14565

14574 K10524: NRK1_2; nicotinamide/nicotinate riboside kinase
0.07596106159095774 nan
 Model
  NICRNS: atp_c + nicrns_c --> adp_c + h_c + nicrnt_c 14574
  RNMK: atp_c + rnam_c --> adp_c + h_c + nmn_c 14574

14666 K03842: ALG1; beta-1,4-mannosyltransferase
0.07596130136606769 nan
 Model
  BDMT: doldpglcnacglcnac_c + gdpmann_c --> doldpglcnacglcnacman_c + gdp_c + h_c 14666
  BDMT_L: 0.1 chito2pdol__L_c + gdpmann_c --> gdp_c + h_c + 0.1 mpdol__L_c 14666
  BDMT_U: 0.1 chito2pdol_U_c + gdpmann_c --> gdp_c + h_c + 0.1 mpdol_U_c 14666

14717 BLAST: NADH dehydrogenase [ubiquinone] 1 beta subcomplex...
0.06627011871506874 nan
 Model
  NADH2_u9m2: 5.0 h_m + nadh_m + q9_m --> 4.0 h_c + nad_m + q9h2_m NAD1 and NAD2 and NAD3 and NAD4 and NAD4L and NAD5 and NAD6 and 10010 and 10017 and 10318 and 10541 and 10607 and 11151 and 11411 and 11702 and 11857 and 12482 and 12497 and 12543 and 12653 and 13017 and 13214 and 13268 and 13411 and 13656 and 13925 and 14078 and 14261 and 14418 and 14717 and 14900 and 15179 and 15864 and 15947 and 15973 and 15998 and 8444 and 8446 and 8530 and 8699 and 8817 and 8909 and 9144 and 9348

14786 K02127: ATPeF0B, ATP5F1, ATP4; F-type H+-transporting ATPase subunit b
0.019318790827008617 nan
 Model
  ATPS3m: adp_m + 3.0 h_c + pi_m --> atp_m + h2o_m + 2.0 h_m ATP6 and ATP8 and ATP9 and 9080 and 9619 and 9940 and 10674 and 11958 and 11967 and 13053 and 13252 and 13424 and 13759 and 13842 and 14786 and 14912 and 15287 and 15589 and 15880 and 16359

14803 K01930: FPGS; folylpolyglutamate synthase
0.07596130136786104 nan
 Model
  10FTHFGLULLm: 10fthf_m + atp_m + glu__L_m --> 10fthfglu__L_m + adp_m + pi_m 14803
  FPGS: 4.0 atp_c + 4.0 glu__L_c + thf_c --> 5thf_c + 4.0 adp_c + 4.0 h_c + 4.0 pi_c 14803
  FPGS2: 5thf_c + atp_c + glu__L_c --> 6thf_c + adp_c + h_c + pi_c 14803
  FPGS2m: 5thf_m + atp_m + glu__L_m --> 6thf_m + adp_m + h_m + pi_m 14803
  FPGS3: 6thf_c + atp_c + glu__L_c --> 7thf_c + adp_c + h_c + pi_c 14803
  FPGS3m: 6thf_m + atp_m + glu__L_m --> 7thf_m + adp_m + h_m + pi_m 14803
  FPGS4: 4.0 atp_c + dhf_c + 4.0 glu__L_c --> 5dhf_c + 4.0 adp_c + 4.0 h_c + 4.0 pi_c 14803
  FPGS4m: 4.0 atp_m + dhf_m + 4.0 glu__L_m --> 5dhf_m + 4.0 adp_m + 4.0 h_m + 4.0 pi_m 14803
  FPGS5: 5dhf_c + atp_c + glu__L_c --> 6dhf_c + adp_c + h_c + pi_c 14803
  FPGS5m: 5dhf_m + atp_m + glu__L_m --> 6dhf_m + adp_m + h_m + pi_m 14803
  FPGS6: 6dhf_c + atp_c + glu__L_c --> 7dhf_c + adp_c + h_c + pi_c 14803
  FPGS6m: 6dhf_m + atp_m + glu__L_m --> 7dhf_m + adp_m + h_m + pi_m 14803
  FPGS7: 10fthf_c + 4.0 atp_c + 4.0 glu__L_c --> 10fthf5glu_c + 4.0 adp_c + 4.0 h_c + 4.0 pi_c 14803
  FPGS7m: 10fthf_m + 4.0 atp_m + 4.0 glu__L_m --> 10fthf5glu_m + 4.0 adp_m + 4.0 h_m + 4.0 pi_m 14803
  FPGS8: 10fthf5glu_c + atp_c + glu__L_c --> 10fthf6glu_c + adp_c + h_c + pi_c 14803
  FPGS8m: 10fthf5glu_m + atp_m + glu__L_m --> 10fthf6glu_m + adp_m + h_m + pi_m 14803
  FPGS9: 10fthf6glu_c + atp_c + glu__L_c --> 10fthf7glu_c + adp_c + h_c + pi_c 14803
  FPGS9m: 10fthf6glu_m + atp_m + glu__L_m --> 10fthf7glu_m + adp_m + h_m + pi_m 14803
  FPGSm: 4.0 atp_m + 4.0 glu__L_m + thf_m --> 5thf_m + 4.0 adp_m + 4.0 h_m + 4.0 pi_m 14803
  THFGLUS: atp_c + glu__L_c + thf_c <=> adp_c + h_c + pi_c + thfglu_c 14803

14821 K03801: lipB; lipoyl(octanoyl) transferase
0.07596130136784464 nan
 Model
  LIPOCTm: h_m + ocACP_m --> ACP_m + octapb_m 14821

14828 K10427: DCTN5; dynactin 5
0.07596130136996941 nan
 Model
  HCO3Em: co2_m + h2o_m <=> h_m + hco3_m 14828 or 14831

14831 K10427: DCTN5; dynactin 5
0.07596130136864415 nan
 Model
  HCO3Em: co2_m + h2o_m <=> h_m + hco3_m 14828 or 14831

14856 K01655: LYS21, LYS20; homocitrate synthase
0.07551171266036474 -6.013768844028507
 Model
  HCITSm: accoa_m + akg_m + h2o_m --> coa_m + h_m + hcit_m 14856

14912 K02138: ATPeF0D, ATP5H, ATP7; F-type H+-transporting ATPase subunit d
0.01931879082759984 nan
 Model
  ATPS3m: adp_m + 3.0 h_c + pi_m --> atp_m + h2o_m + 2.0 h_m ATP6 and ATP8 and ATP9 and 9080 and 9619 and 9940 and 10674 and 11958 and 11967 and 13053 and 13252 and 13424 and 13759 and 13842 and 14786 and 14912 and 15287 and 15589 and 15880 and 16359

14956 K00966: GMPP; mannose-1-phosphate guanylyltransferase
0.07596130137114704 nan
 Model
  G1PTT: dttp_c + g1p_c + h_c --> dtdpglu_c + ppi_c 14956
  MAN1PT: gtp_c + h_c + man1p_c --> gdpmann_c + ppi_c 10964 or 14956
  MAN1PT2: gdp_c + h_c + man1p_c --> gdpmann_c + pi_c 10964 or 14956

14971 K02149: ATPeV1D, ATP6M; V-type H+-transporting ATPase subunit D
0.07596130136914532 nan
 Model
  ATPS2v: atp_c + h2o_c + h_c --> adp_c + 2.0 h_v + pi_c 10802 and 11025 and 11052 and 11064 and 11117 and 11403 and 14971 and 15309 and 15351 and 15963 and 16397 and 9210 and 9622 and 9748

15038 K03676: grxC, GLRX, GLRX2; glutaredoxin 3
0.07596130136743372 nan
 Model
  DHAOX_c: dhdascb_c + 2.0 gthrd_c --> ascb__L_c + gthox_c + h_c 15038
  GRXR: grxox_c + 2.0 gthrd_c --> grxrd_c + gthox_c 15038 or 8790

15060 KOG3888: Gamma-butyrobetaine,2-oxoglutarate dioxygenase
0.07596130136792231 nan
 Model
  GBBOX_m: akg_m + gbbtn_m + o2_m --> co2_m + crn_m + succ_m 15060

15137 K01953: asnB, ASNS; asparagine synthase (glutamine-hydrolysing)
0.07594592177382317 nan
 Model
  ASNS1: asp__L_c + atp_c + gln__L_c + h2o_c --> amp_c + asn__L_c + glu__L_c + h_c + ppi_c 14762 or 15137
  ASNS2: asp__L_c + atp_c + nh4_c --> amp_c + asn__L_c + h_c + ppi_c 15137

15140 KOG0202: Ca2+ transporting ATPase
0.075961301370527 nan
 Model
  SERCA: atp_c + 2.0 ca2_c + h2o_c + 2.0 h_r --> adp_c + 2.0 ca2_r + 3.0 h_c + pi_c (15140 and 15141) or 15156

15141 HMMPfam:Cation transporter/ATPase, N-terminus:PF00690,SUPERFAMILY::SSF81665
0.075961301369876 nan
 Model
  SERCA: atp_c + 2.0 ca2_c + h2o_c + 2.0 h_r --> adp_c + 2.0 ca2_r + 3.0 h_c + pi_c (15140 and 15141) or 15156

15158 K00671: E2.3.1.97, NMT; glycylpeptide N-tetradecanoyltransferase
0.07596130136755619 nan
 Model
  GLPT: glp_c + tdcoa_c --> coa_c + h_c + tglp_c 15158

15167 K00033: PGD, gnd, gntZ; 6-phosphogluconate dehydrogenase
0.07596130137114704 nan
 Model
  GND: 6pgc_c + nadp_c --> co2_c + nadph_c + ru5p__D_c 15167

15179 K03953: NDUFA9; NADH dehydrogenase (ubiquinone) 1 alpha subcomplex subunit 9
0.06627011871507324 nan
 Model
  NADH2_u9m2: 5.0 h_m + nadh_m + q9_m --> 4.0 h_c + nad_m + q9h2_m NAD1 and NAD2 and NAD3 and NAD4 and NAD4L and NAD5 and NAD6 and 10010 and 10017 and 10318 and 10541 and 10607 and 11151 and 11411 and 11702 and 11857 and 12482 and 12497 and 12543 and 12653 and 13017 and 13214 and 13268 and 13411 and 13656 and 13925 and 14078 and 14261 and 14418 and 14717 and 14900 and 15179 and 15864 and 15947 and 15973 and 15998 and 8444 and 8446 and 8530 and 8699 and 8817 and 8909 and 9144 and 9348

15184 K02437: gcvH, GCSH; glycine cleavage system H protein
0.07596130137026993 nan
 Model
  GLYCLm: gly_m + nad_m + thf_m --> co2_m + mlthf_m + nadh_m + nh4_m 10040 and 10205 and 12898 and 15184

15188 K00931: proB; glutamate 5-kinase
0.07587294325881833 nan
 Model
  GLU5K: atp_c + glu__L_c --> adp_c + glu5p_c 15188

15248 K00641: metX; homoserine O-acetyltransferase
0.07596130136996944 -3.084081837506256
 Model
  HSERTA: accoa_c + hom__L_c <=> achms_c + coa_c 12513 or 15248

15287 K07556: ATPeAF2, ATPAF2, ATP12; ATP synthase mitochondrial F1 complex assembly factor 2
0.01931879082802085 nan
 Model
  ATPS3m: adp_m + 3.0 h_c + pi_m --> atp_m + h2o_m + 2.0 h_m ATP6 and ATP8 and ATP9 and 9080 and 9619 and 9940 and 10674 and 11958 and 11967 and 13053 and 13252 and 13424 and 13759 and 13842 and 14786 and 14912 and 15287 and 15589 and 15880 and 16359

15309 K02154: ATPeV0A, ATP6N; V-type H+-transporting ATPase subunit a
0.07596130137289103 nan
 Model
  ATPS2v: atp_c + h2o_c + h_c --> adp_c + 2.0 h_v + pi_c 10802 and 11025 and 11052 and 11064 and 11117 and 11403 and 14971 and 15309 and 15351 and 15963 and 16397 and 9210 and 9622 and 9748

15339 K03671: trxA; thioredoxin 1
0.07596130136745266 nan
 Model
  METSOXR1: metsox_S__L_c + trdrd_c --> h2o_c + met__L_c + trdox_c (10848 and 15902) or (12730 and 15902) or (12737 and 15902) or (15339 and 15902)
  METSOXR2: metsox_R__L_c + trdrd_c --> h2o_c + met__L_c + trdox_c (10848 and 15469) or (12730 and 15469) or (12737 and 15469) or (15339 and 15469)
  PAPSR: paps_c + trdrd_c --> 2.0 h_c + pap_c + so3_c + trdox_c (10848 and 11741) or (11741 and 12730) or (11741 and 12737) or (11741 and 15339)
  RNDR1: adp_c + trdrd_c --> dadp_c + h2o_c + trdox_c (10848 and 11172 and 11290 and 14237) or (11172 and 11290 and 12730 and 14237) or (11172 and 11290 and 12737 and 14237) or (11172 and 11290 and 14237 and 15339)
  RNDR2: gdp_c + trdrd_c --> dgdp_c + h2o_c + trdox_c (10848 and 11172 and 11290 and 14237) or (11172 and 11290 and 12730 and 14237) or (11172 and 11290 and 12737 and 14237) or (11172 and 11290 and 14237 and 15339)
  RNDR3: cdp_c + trdrd_c --> dcdp_c + h2o_c + trdox_c (10848 and 11172 and 11290 and 14237) or (11172 and 11290 and 12730 and 14237) or (11172 and 11290 and 12737 and 14237) or (11172 and 11290 and 14237 and 15339)
  RNDR4: trdrd_c + udp_c --> dudp_c + h2o_c + trdox_c (10848 and 11172 and 11290 and 14237) or (11172 and 11290 and 12730 and 14237) or (11172 and 11290 and 12737 and 14237) or (11172 and 11290 and 14237 and 15339)
  THIORDXp: h2o2_x + trdrd_x <=> 2.0 h2o_x + trdox_x 13262 and 15339

15342 K17686: copA, ATP7; Cu+-exporting ATPase
0.07596130136997607 nan
 Model
  Cu1ATPase: atp_c + cu_c + h2o_c --> adp_c + cu_e + h_c + pi_c 15342

15351 K02145: ATPeV1A, ATP6A; V-type H+-transporting ATPase subunit A
0.07596130136996015 nan
 Model
  ATPS2v: atp_c + h2o_c + h_c --> adp_c + 2.0 h_v + pi_c 10802 and 11025 and 11052 and 11064 and 11117 and 11403 and 14971 and 15309 and 15351 and 15963 and 16397 and 9210 and 9622 and 9748

15404 K00831: serC, PSAT1; phosphoserine aminotransferase
0.07596130137010333 nan
 Model
  PSERT: 3php_c + glu__L_c --> akg_c + pser__L_c 15404
  SDPTA: akg_c + sl26da_c <=> glu__L_c + sl2a6o_c 15404

15420 K01624: FBA, fbaA; fructose-bisphosphate aldolase, class II
0.07308391673471581 nan
 Model
  FBA: fdp_c <=> dhap_c + g3p_c 15420
  FBA2: f1p_c <=> dhap_c + glyald_c 15420
  FBA3: s17bp_c <=> dhap_c + e4p_c 15420

15482 K00383: GSR, gor; glutathione reductase (NADPH)
0.07596130137370447 nan
 Model
  GTHOm: gthox_m + h_m + nadph_m --> 2.0 gthrd_m + nadp_m 15482
  GTHOr: gthox_c + h_c + nadph_c <=> 2.0 gthrd_c + nadp_c 15482

15483 K00297: metF, MTHFR; methylenetetrahydrofolate reductase (NADPH)
0.0759613013712417 -3.8947759470510768
 Model
  MTHFR2: 2.0 h_c + mlthf_c + nadh_c --> 5mthf_c + nad_c 15483 or 9244
  MTHFR3: 2.0 h_c + mlthf_c + nadph_c --> 5mthf_c + nadp_c 15483 or 9244

15518 K01874: MARS, metG; methionyl-tRNA synthetase
0.07596130136737093 nan
 Model
  METTRSm: atp_m + met__L_m + trnamet_m --> amp_m + mettrna_m + ppi_m 15518

15533 K01867: WARS, trpS; tryptophanyl-tRNA synthetase
0.07596130137060628 nan
 Model
  TRPTRSm: atp_m + trnatrp_m + trp__L_m --> amp_m + ppi_m + trptrna_m 15533

15555 K00799: GST, gst; glutathione S-transferase
0.07596130137824275 nan
 Model
  LTC4Sr: gthrd_r + leuktrA4_r --> leuktrC4_r 15555

15556 K01870: IARS, ileS; isoleucyl-tRNA synthetase
0.07596130137494583 nan
 Model
  ILETRSm: atp_m + ile__L_m + trnaile_m --> amp_m + iletrna_m + ppi_m 15556

15589 K02133: ATPeF1B, ATP5B, ATP2; F-type H+-transporting ATPase subunit beta
0.019318790827534488 nan
 Model
  ATPS3m: adp_m + 3.0 h_c + pi_m --> atp_m + h2o_m + 2.0 h_m ATP6 and ATP8 and ATP9 and 9080 and 9619 and 9940 and 10674 and 11958 and 11967 and 13053 and 13252 and 13424 and 13759 and 13842 and 14786 and 14912 and 15287 and 15589 and 15880 and 16359

15600 K10572: IPPK; inositol-pentakisphosphate 2-kinase
0.07596130137011117 nan
 Model
  MI13456PKn: atp_n + mi13456p_n --> adp_n + h_n + minohp_n 15600

15631 K00615: E2.2.1.1, tktA, tktB; transketolase
0.07519231006285435 nan
 Model
  TKT1: r5p_c + xu5p__D_c <=> g3p_c + s7p_c 15631
  TKT2: e4p_c + xu5p__D_c <=> f6p_c + g3p_c 15631

15706 K00451: HGD, hmgA; homogentisate 1,2-dioxygenase
0.07596130137014859 nan
 Model
  HGNTOR: hgentis_c + o2_c --> 4mlacac_c + h_c 15706

15721 K00927: PGK, pgk; phosphoglycerate kinase
0.0737116024847904 nan
 Model
  PGK: 3pg_c + atp_c <=> 13dpg_c + adp_c 15721

15773 KOG1589: Uncharacterized conserved protein
0.07220717705475185 nan
 Model
  ACACt2m: acac_c + h_c <=> acac_m + h_m 15773 and 16581
  PYRt2m: h_c + pyr_c <=> h_m + pyr_m 15773 and 16581

15864 K03935: NDUFS2; NADH dehydrogenase (ubiquinone) Fe-S protein 2
0.06627011871507321 nan
 Model
  NADH2_u9m2: 5.0 h_m + nadh_m + q9_m --> 4.0 h_c + nad_m + q9h2_m NAD1 and NAD2 and NAD3 and NAD4 and NAD4L and NAD5 and NAD6 and 10010 and 10017 and 10318 and 10541 and 10607 and 11151 and 11411 and 11702 and 11857 and 12482 and 12497 and 12543 and 12653 and 13017 and 13214 and 13268 and 13411 and 13656 and 13925 and 14078 and 14261 and 14418 and 14717 and 14900 and 15179 and 15864 and 15947 and 15973 and 15998 and 8444 and 8446 and 8530 and 8699 and 8817 and 8909 and 9144 and 9348

15880 HMMPfam:ATP synthase E chain:PF05680
0.01931879082856625 nan
 Model
  ATPS3m: adp_m + 3.0 h_c + pi_m --> atp_m + h2o_m + 2.0 h_m ATP6 and ATP8 and ATP9 and 9080 and 9619 and 9940 and 10674 and 11958 and 11967 and 13053 and 13252 and 13424 and 13759 and 13842 and 14786 and 14912 and 15287 and 15589 and 15880 and 16359

15939 K11450: KDM1A, AOF2, LSD1; lysine-specific histone demethylase 1A
0.07328528129286956 nan
 Model
  POLYAO: N1aspmd_c + h2o_c + o2_c --> 3aap_c + h2o2_c + ptrc_c 15939
  POLYAO2: N1sprm_c + h2o_c + o2_c --> 3aap_c + h2o2_c + spmd_c 15939
  POLYAO3: h2o_c + o2_c + sprm_c --> bamppald_c + h2o2_c + spmd_c 15939

15942 K15275: SLC35B1; solute carrier family 35 (UDP-galactose transporter), member B1
0.07596130136914532 nan
 Model
  UDPGALt2g: udpgal_c --> udpgal_g 15942

15947 K03952: NDUFA8; NADH dehydrogenase (ubiquinone) 1 alpha subcomplex subunit 8
0.06627011871507639 nan
 Model
  NADH2_u9m2: 5.0 h_m + nadh_m + q9_m --> 4.0 h_c + nad_m + q9h2_m NAD1 and NAD2 and NAD3 and NAD4 and NAD4L and NAD5 and NAD6 and 10010 and 10017 and 10318 and 10541 and 10607 and 11151 and 11411 and 11702 and 11857 and 12482 and 12497 and 12543 and 12653 and 13017 and 13214 and 13268 and 13411 and 13656 and 13925 and 14078 and 14261 and 14418 and 14717 and 14900 and 15179 and 15864 and 15947 and 15973 and 15998 and 8444 and 8446 and 8530 and 8699 and 8817 and 8909 and 9144 and 9348

15963 K02151: ATPeV1F, ATP6S14; V-type H+-transporting ATPase subunit F
0.0759613013699687 nan
 Model
  ATPS2v: atp_c + h2o_c + h_c --> adp_c + 2.0 h_v + pi_c 10802 and 11025 and 11052 and 11064 and 11117 and 11403 and 14971 and 15309 and 15351 and 15963 and 16397 and 9210 and 9622 and 9748

15967 K01900: LSC2; succinyl-CoA synthetase beta subunit
0.07463628837051073 nan
 Model
  SUCOASm: atp_m + coa_m + succ_m <=> adp_m + pi_m + succoa_m 15967 and 16144

15973 K03941: NDUFS8; NADH dehydrogenase (ubiquinone) Fe-S protein 8
0.06627011871611854 nan
 Model
  NADH2_u9m2: 5.0 h_m + nadh_m + q9_m --> 4.0 h_c + nad_m + q9h2_m NAD1 and NAD2 and NAD3 and NAD4 and NAD4L and NAD5 and NAD6 and 10010 and 10017 and 10318 and 10541 and 10607 and 11151 and 11411 and 11702 and 11857 and 12482 and 12497 and 12543 and 12653 and 13017 and 13214 and 13268 and 13411 and 13656 and 13925 and 14078 and 14261 and 14418 and 14717 and 14900 and 15179 and 15864 and 15947 and 15973 and 15998 and 8444 and 8446 and 8530 and 8699 and 8817 and 8909 and 9144 and 9348

15998 K11353: NDUFA13; NADH dehydrogenase (ubiquinone) 1 alpha subcomplex subunit 13
0.06627011871507323 nan
 Model
  NADH2_u9m2: 5.0 h_m + nadh_m + q9_m --> 4.0 h_c + nad_m + q9h2_m NAD1 and NAD2 and NAD3 and NAD4 and NAD4L and NAD5 and NAD6 and 10010 and 10017 and 10318 and 10541 and 10607 and 11151 and 11411 and 11702 and 11857 and 12482 and 12497 and 12543 and 12653 and 13017 and 13214 and 13268 and 13411 and 13656 and 13925 and 14078 and 14261 and 14418 and 14717 and 14900 and 15179 and 15864 and 15947 and 15973 and 15998 and 8444 and 8446 and 8530 and 8699 and 8817 and 8909 and 9144 and 9348

16016 K01705: LYS4; homoaconitate hydratase
0.07551171266034903 -4.84389788794991
 Model
  HACONTbm: h2o_m + hacon_C_m <=> hicit_m 16016

16104 HMMPfam:Glyoxal oxidase N-terminus:PF07250,HMMPfam:Domain of unknown function (DUF1929):PF09118,SUPERFAMILY::SSF50965,SUPERFAMILY::SSF81296
0.07596130136996895 nan
 Model
  GXLOe: gxl_e + h2o_e + o2_e --> glx_e + h2o2_e + h_e 16104 or 8542
  MTHGXLe: h2o_e + mthgxl_e + o2_e --> h2o2_e + h_e + pyr_e 16104 or 8542

16144 K01899: LSC1; succinyl-CoA synthetase alpha subunit
0.07463628837041848 nan
 Model
  SUCOASm: atp_m + coa_m + succ_m <=> adp_m + pi_m + succoa_m 15967 and 16144

16145 KOG0769: Predicted mitochondrial carrier protein
0.07585473177578719 nan
 Model
  ATP2tp_H: amp_x + atp_c + 2.0 h_c --> amp_c + atp_x + 2.0 h_x 16145

16181 K01476: E3.5.3.1, rocF, arg; arginase
0.07596130136989368 nan
 Model
  ARGN: arg__L_c + h2o_c --> orn_c + urea_c 16181
  GUDBUTNAH: 4gudbutn_c + h2o_c --> 4abut_c + urea_c 16181

16264 K01783: rpe, RPE; ribulose-phosphate 3-epimerase
0.07591564358196497 nan
 Model
  RPE: ru5p__D_c <=> xu5p__D_c 16264

16273 K00147: proA; glutamate-5-semialdehyde dehydrogenase
0.07587294327280357 nan
 Model
  G5SD: glu5p_c + h_c + nadph_c --> glu5sa_c + nadp_c + pi_c 16273

16359 K02132: ATPeF1A, ATP5A1, ATP1; F-type H+-transporting ATPase subunit alpha
0.01931879082755995 nan
 Model
  ATPS3m: adp_m + 3.0 h_c + pi_m --> atp_m + h2o_m + 2.0 h_m ATP6 and ATP8 and ATP9 and 9080 and 9619 and 9940 and 10674 and 11958 and 11967 and 13053 and 13252 and 13424 and 13759 and 13842 and 14786 and 14912 and 15287 and 15589 and 15880 and 16359

16397 K02152: ATPeV1G, ATP6G; V-type H+-transporting ATPase subunit G
0.07596130136895185 nan
 Model
  ATPS2v: atp_c + h2o_c + h_c --> adp_c + 2.0 h_v + pi_c 10802 and 11025 and 11052 and 11064 and 11117 and 11403 and 14971 and 15309 and 15351 and 15963 and 16397 and 9210 and 9622 and 9748

16404 K01942: HLCS; biotin--protein ligase
0.07596130136997908 nan
 Model
  BACCL: atp_c + btn_c + h_c --> btamp_c + ppi_c 16404
  BACCLm: atp_m + btn_m + h_m --> btamp_m + ppi_m 16404
  BTNPL: apoC_Lys_c + btamp_c --> amp_c + apoC_Lys_btn_c + h_c 16404
  BTNPLm: apoC_Lys_m + btamp_m --> amp_m + apoC_Lys_btn_m + h_m 16404

16423 K06173: truA, PUS1; tRNA pseudouridine38-40 synthase
0.07596130136778377 nan
 Model
  YUMPS: r5p_c + ura_c <=> h2o_c + psd5p_c 14436 or 16088 or 16423 or 16646

16507 K01139: spoT, HDDC3; guanosine-3',5'-bis(diphosphate) 3'-pyrophosphohydrolase
0.07596130136999202 nan
 Model
  GDPDPK: atp_c + gdp_c --> amp_c + h_c + ppgpp_c 16507
  GDPTPDP: gdptp_c + h2o_c --> gtp_c + ppi_c 16507
  PPGPPDP: h2o_c + ppgpp_c --> gdp_c + ppi_c 16507

16511 K01515: nudF; ADP-ribose pyrophosphatase
0.07596130137292237 nan
 Model
  ADPGLC: adpglc_c + h2o_c --> amp_c + g1p_c + 2.0 h_c 16511
  ADPMAN: adpman_c + h2o_c --> amp_c + 2.0 h_c + man1p_c 16511
  ADPRDP: adprib_c + h2o_c --> amp_c + 2.0 h_c + r5p_c 16511

16592 K00948: PRPS, prsA; ribose-phosphate pyrophosphokinase
0.07596130137271784 nan
 Model
  PRPPS: atp_c + r5p_c <=> amp_c + h_c + prpp_c 11145 or 16592

16746 KOG0756: Mitochondrial tricarboxylate/dicarboxylate carrier proteins
0.07596130137125853 nan
 Model
  AKGCITtm: akg_c + cit_m --> akg_m + cit_c 16746

8385 K00856: E2.7.1.20, ADK; adenosine kinase
0.07585246780698304 nan
 Model
  ADNK1: adn_c + atp_c --> adp_c + amp_c + h_c 8385

8395 K00088: IMPDH, guaB; IMP dehydrogenase
0.07594518008676232 nan
 Model
  6TINS5MPOR: 6tins5mp_c + h2o_c + nad_c --> 6txan5mp_c + h_c + nadh_c 8395
  IMPD: h2o_c + imp_c + nad_c --> h_c + nadh_c + xmp_c 8395
  IMPDm: h2o_m + imp_m + nad_m --> h_m + nadh_m + xmp_m 8395

8431 K01800: maiA, GSTZ1; maleylacetoacetate isomerase
0.07596130136997749 nan
 Model
  MACACI: 4mlacac_c --> 4fumacac_c 8431

8444 K18164: NDUFAF7; NADH dehydrogenase [ubiquinone] 1 alpha subcomplex assembly factor 7
0.06627011871375998 nan
 Model
  NADH2_u9m2: 5.0 h_m + nadh_m + q9_m --> 4.0 h_c + nad_m + q9h2_m NAD1 and NAD2 and NAD3 and NAD4 and NAD4L and NAD5 and NAD6 and 10010 and 10017 and 10318 and 10541 and 10607 and 11151 and 11411 and 11702 and 11857 and 12482 and 12497 and 12543 and 12653 and 13017 and 13214 and 13268 and 13411 and 13656 and 13925 and 14078 and 14261 and 14418 and 14717 and 14900 and 15179 and 15864 and 15947 and 15973 and 15998 and 8444 and 8446 and 8530 and 8699 and 8817 and 8909 and 9144 and 9348

8446 HMMPfam:NADH-ubiquinone oxidoreductase B15 subunit (NDUFB4):PF07225
0.06627011871507334 nan
 Model
  NADH2_u9m2: 5.0 h_m + nadh_m + q9_m --> 4.0 h_c + nad_m + q9h2_m NAD1 and NAD2 and NAD3 and NAD4 and NAD4L and NAD5 and NAD6 and 10010 and 10017 and 10318 and 10541 and 10607 and 11151 and 11411 and 11702 and 11857 and 12482 and 12497 and 12543 and 12653 and 13017 and 13214 and 13268 and 13411 and 13656 and 13925 and 14078 and 14261 and 14418 and 14717 and 14900 and 15179 and 15864 and 15947 and 15973 and 15998 and 8444 and 8446 and 8530 and 8699 and 8817 and 8909 and 9144 and 9348

8452 K01522: FHIT; bis(5'-adenosyl)-triphosphatase
0.07596130137276705 nan
 Model
  AP4AH: ap4a_c + h2o_c --> 2.0 adp_c + 2.0 h_c 8452

8460 KOG2914: Predicted haloacid-halidohydrolase and related hydrolases
0.07595976270265925 nan
 Model
  G3PT: glyc3p_c + h2o_c --> glyc_c + pi_c 8460
  GAPP: g3p_c + h2o_c --> glyald_c + pi_c 8460
  MN6PP: h2o_c + man6p_c --> man_c + pi_c 8460

8579 K00432: gpx; glutathione peroxidase
0.07596130136997106 nan
 Model
  GTHPi: 2.0 gthrd_c + h2o2_c --> gthox_c + 2.0 h2o_c 8579

8602 K00452: HAAO; 3-hydroxyanthranilate 3,4-dioxygenase
0.0759613013709219 nan
 Model
  3HAO: 3hanthrn_c + o2_c --> cmusa_c + h_c 8602

8635 K03381: catA; catechol 1,2-dioxygenase
0.07596130136988427 nan
 Model
  CATDOX: catechol_c + o2_c --> ccmuac_c + 2.0 h_c 8635

8699 K03940: NDUFS7; NADH dehydrogenase (ubiquinone) Fe-S protein 7
0.06627011871639743 nan
 Model
  NADH2_u9m2: 5.0 h_m + nadh_m + q9_m --> 4.0 h_c + nad_m + q9h2_m NAD1 and NAD2 and NAD3 and NAD4 and NAD4L and NAD5 and NAD6 and 10010 and 10017 and 10318 and 10541 and 10607 and 11151 and 11411 and 11702 and 11857 and 12482 and 12497 and 12543 and 12653 and 13017 and 13214 and 13268 and 13411 and 13656 and 13925 and 14078 and 14261 and 14418 and 14717 and 14900 and 15179 and 15864 and 15947 and 15973 and 15998 and 8444 and 8446 and 8530 and 8699 and 8817 and 8909 and 9144 and 9348

8712 K00914: PIK3C3, VPS34; phosphatidylinositol 3-kinase
0.07596130136838075 nan
 Model
  PIN3Kn_RT: atp_n + 0.01 ptd1ino_RT_n --> adp_n + h_n + 0.01 ptd3ino_RT_n 8712

8759 K01760: metC; cystathionine beta-lyase
0.07596130136997006 nan
 Model
  CYSTL: cyst__L_c + h2o_c --> hcys__L_c + nh4_c + pyr_c 8759

8790 KOG0911: Glutaredoxin-related protein
0.07596130136995671 nan
 Model
  GRXR: grxox_c + 2.0 gthrd_c --> grxrd_c + gthox_c 15038 or 8790

8814 K00254: DHODH, pyrD; dihydroorotate dehydrogenase
0.07596130137115692 nan
 Model
  DHORD_u9m: dhor__S_c + q9_m --> orot_c + q9h2_m 8814

8817 HMMPfam:ESSS subunit of NADH:ubiquinone oxidoreductase (complex I):PF10183
0.06627011871393051 nan
 Model
  NADH2_u9m2: 5.0 h_m + nadh_m + q9_m --> 4.0 h_c + nad_m + q9h2_m NAD1 and NAD2 and NAD3 and NAD4 and NAD4L and NAD5 and NAD6 and 10010 and 10017 and 10318 and 10541 and 10607 and 11151 and 11411 and 11702 and 11857 and 12482 and 12497 and 12543 and 12653 and 13017 and 13214 and 13268 and 13411 and 13656 and 13925 and 14078 and 14261 and 14418 and 14717 and 14900 and 15179 and 15864 and 15947 and 15973 and 15998 and 8444 and 8446 and 8530 and 8699 and 8817 and 8909 and 9144 and 9348

8835 K07748: E1.1.1.170, NSDHL, ERG26; sterol-4alpha-carboxylate 3-dehydrogenase (decarboxylating)
0.07596130137174742 nan
 Model
  C3STDH1Pr: 4mzym_int1_r + nadp_r --> 4mzym_int2_r + co2_r + h_r + nadph_r 13724 or 8835
  C3STDH1r: 4mzym_int1_r + nad_r --> 4mzym_int2_r + co2_r + h_r + nadh_r 13724 or 8835
  C3STDH2er: nad_r + zym_int1_r --> co2_r + h_r + nadh_r + zym_int2_r 13724 or 8835

8859 K00850: pfkA, PFK; 6-phosphofructokinase 1
0.07596130136780077 nan
 Model
  PFK: atp_c + f6p_c --> adp_c + fdp_c + h_c 8859 or 8863 or 8867
  PFK_2: atp_c + tag6p__D_c --> adp_c + h_c + tagdp__D_c 8859 or 8863 or 8867
  PFK_3: atp_c + s7p_c --> adp_c + h_c + s17bp_c 8859 or 8863 or 8867

8863 K00850: pfkA, PFK; 6-phosphofructokinase 1
0.07596130136846956 nan
 Model
  PFK: atp_c + f6p_c --> adp_c + fdp_c + h_c 8859 or 8863 or 8867
  PFK_2: atp_c + tag6p__D_c --> adp_c + h_c + tagdp__D_c 8859 or 8863 or 8867
  PFK_3: atp_c + s7p_c --> adp_c + h_c + s17bp_c 8859 or 8863 or 8867

8867 K00850: pfkA, PFK; 6-phosphofructokinase 1
0.07596130136755619 nan
 Model
  PFK: atp_c + f6p_c --> adp_c + fdp_c + h_c 8859 or 8863 or 8867
  PFK_2: atp_c + tag6p__D_c --> adp_c + h_c + tagdp__D_c 8859 or 8863 or 8867
  PFK_3: atp_c + s7p_c --> adp_c + h_c + s17bp_c 8859 or 8863 or 8867

8878 K01922: PPCS, coaB; phosphopantothenate-cysteine ligase
0.07596130136745266 nan
 Model
  PPNCL2: 4ppan_c + ctp_c + cys__L_c --> 4ppcys_c + cmp_c + h_c + ppi_c 8536 or 8878
  PPNCL3: 4ppan_c + atp_c + cys__L_c --> 4ppcys_c + amp_c + h_c + ppi_c 8878

8889 K15102: SLC25A3, PHC, PIC; solute carrier family 25 (mitochondrial phosphate transporter), member 3
0.07596130137148778 nan
 Model
  PIt2m: h_c + pi_c --> h_m + pi_m 15874 or 8889

8909 HMMPfam:NADH-ubiquinone oxidoreductase ASHI subunit (CI-ASHI or NDUFB8):PF05821
0.0662701187150685 nan
 Model
  NADH2_u9m2: 5.0 h_m + nadh_m + q9_m --> 4.0 h_c + nad_m + q9h2_m NAD1 and NAD2 and NAD3 and NAD4 and NAD4L and NAD5 and NAD6 and 10010 and 10017 and 10318 and 10541 and 10607 and 11151 and 11411 and 11702 and 11857 and 12482 and 12497 and 12543 and 12653 and 13017 and 13214 and 13268 and 13411 and 13656 and 13925 and 14078 and 14261 and 14418 and 14717 and 14900 and 15179 and 15864 and 15947 and 15973 and 15998 and 8444 and 8446 and 8530 and 8699 and 8817 and 8909 and 9144 and 9348

8916 K15084: SLC25A16, GDA, LEU5; solute carrier family 25 (mitochondrial carrier protein), member 16
0.0759613013699707 nan
 Model
  COAtim: coa_c --> coa_m 8916

8969 K01784: galE, GALE; UDP-glucose 4-epimerase
0.07596130136955835 nan
 Model
  UA4E: udpxyl_c <=> udparab_c 8969
  UAG4Ei: uacgam_c <=> udpacgal_c 8969
  UDPG4E: udpg_c <=> udpgal_c 8969

9011 K15117: SLC25A34_35, OAC1; solute carrier family 25, member 34/35
0.07590052300521756 nan
 Model
  OAAIPMtm: 3c3hmp_m + oaa_c --> 3c3hmp_c + oaa_m 9011

9015 K09885: AQPF; aquaporin rerated protein, other eukaryote
0.07596130136913248 nan
 Model
  H2Ot: h2o_e <=> h2o_c 13986 or 13987 or 9014 or 9015

9080 KOG4103: Mitochondrial F1F0-ATP synthase, subunit g/ATP20
0.019318790827515347 nan
 Model
  ATPS3m: adp_m + 3.0 h_c + pi_m --> atp_m + h2o_m + 2.0 h_m ATP6 and ATP8 and ATP9 and 9080 and 9619 and 9940 and 10674 and 11958 and 11967 and 13053 and 13252 and 13424 and 13759 and 13842 and 14786 and 14912 and 15287 and 15589 and 15880 and 16359

9085 K00058: serA, PHGDH; D-3-phosphoglycerate dehydrogenase
0.07596130137009396 nan
 Model
  ARHGDx: nad_c + r2hglut_c <=> akg_c + h_c + nadh_c 9085
  PGCD: 3pg_c + nad_c --> 3php_c + h_c + nadh_c 9085

9093 KOG2702: Predicted panthothenate kinase/uridine kinase-related protein
0.07596130137025799 nan
 Model
  CYTDK1: atp_c + cytd_c --> adp_c + cmp_c + h_c 8633 or 9093
  CYTDK2: cytd_c + gtp_c --> cmp_c + gdp_c + h_c 8633 or 9093
  DATCY: cytd_c + datp_c --> cmp_c + dadp_c + h_c 8633 or 9093
  DATUP: datp_c + uri_c --> dadp_c + h_c + ump_c 8633 or 9093
  DCTCP: cytd_c + dctp_c --> cmp_c + dcdp_c + h_c 8633 or 9093
  DCTUP: dctp_c + uri_c --> dcdp_c + h_c + ump_c 8633 or 9093
  DGTCY: cytd_c + dgtp_c --> cmp_c + dgdp_c + h_c 8633 or 9093
  DGTUP: dgtp_c + uri_c --> dgdp_c + h_c + ump_c 8633 or 9093
  DTTGY: cytd_c + dttp_c --> cmp_c + dtdp_c + h_c 8633 or 9093
  DTTUP: dttp_c + uri_c --> dtdp_c + h_c + ump_c 8633 or 9093
  DUTCP: cytd_c + dutp_c --> cmp_c + dudp_c + h_c 8633 or 9093
  DUTUP: dutp_c + uri_c --> dudp_c + h_c + ump_c 8633 or 9093
  ITCY: cytd_c + itp_c --> cmp_c + h_c + idp_c 8633 or 9093
  URIK1: atp_c + uri_c --> adp_c + h_c + ump_c 8633 or 9093
  URIK2: gtp_c + uri_c --> gdp_c + h_c + ump_c 8633 or 9093
  URIK3: itp_c + uri_c --> h_c + idp_c + ump_c 8633 or 9093
  UTCY: cytd_c + utp_c --> cmp_c + h_c + udp_c 8633 or 9093
  UTUP: uri_c + utp_c --> h_c + udp_c + ump_c 8633 or 9093

9144 BLAST: NADH:ubiquinone oxidoreductase kD subunit [Pseudo...
0.06627011871507277 nan
 Model
  NADH2_u9m2: 5.0 h_m + nadh_m + q9_m --> 4.0 h_c + nad_m + q9h2_m NAD1 and NAD2 and NAD3 and NAD4 and NAD4L and NAD5 and NAD6 and 10010 and 10017 and 10318 and 10541 and 10607 and 11151 and 11411 and 11702 and 11857 and 12482 and 12497 and 12543 and 12653 and 13017 and 13214 and 13268 and 13411 and 13656 and 13925 and 14078 and 14261 and 14418 and 14717 and 14900 and 15179 and 15864 and 15947 and 15973 and 15998 and 8444 and 8446 and 8530 and 8699 and 8817 and 8909 and 9144 and 9348

9180 K00474: TMLHE; trimethyllysine dioxygenase
0.07596130136964689 nan
 Model
  TMLOX_m: akg_m + o2_m + tmlys_m --> 3htmelys_m + co2_m + succ_m 9180

9210 K02148: ATPeV1C, ATP6C; V-type H+-transporting ATPase subunit C
0.0759613013740823 nan
 Model
  ATPS2v: atp_c + h2o_c + h_c --> adp_c + 2.0 h_v + pi_c 10802 and 11025 and 11052 and 11064 and 11117 and 11403 and 14971 and 15309 and 15351 and 15963 and 16397 and 9210 and 9622 and 9748

9250 K07390: grxD, GLRX5; monothiol glutaredoxin
0.07596130136997749 nan
 Model
  GRXRm: grxox_m + 2.0 gthrd_m --> grxrd_m + gthox_m 13734 or 16549 or 9250

9300 K01803: TPI, tpiA; triosephosphate isomerase (TIM)
0.07426313812352472 nan
 Model
  TPI: dhap_c <=> g3p_c 9300

9348 K03946: NDUFA2; NADH dehydrogenase (ubiquinone) 1 alpha subcomplex subunit 2
0.06627011871387176 nan
 Model
  NADH2_u9m2: 5.0 h_m + nadh_m + q9_m --> 4.0 h_c + nad_m + q9h2_m NAD1 and NAD2 and NAD3 and NAD4 and NAD4L and NAD5 and NAD6 and 10010 and 10017 and 10318 and 10541 and 10607 and 11151 and 11411 and 11702 and 11857 and 12482 and 12497 and 12543 and 12653 and 13017 and 13214 and 13268 and 13411 and 13656 and 13925 and 14078 and 14261 and 14418 and 14717 and 14900 and 15179 and 15864 and 15947 and 15973 and 15998 and 8444 and 8446 and 8530 and 8699 and 8817 and 8909 and 9144 and 9348

9589 K01810: GPI, pgi; glucose-6-phosphate isomerase
0.07596130136913248 nan
 Model
  PGI: g6p_c <=> f6p_c 9589

9604 K02377: TSTA3, fcl; GDP-L-fucose synthase
0.0759613013703494 nan
 Model
  GDMANE: gdpddman_c --> gdpofuc_c 9604
  GFUCS: gdpddman_c + h_c + nadph_c --> gdpfuc_c + nadp_c 9604
  GOFUCR: gdpofuc_c + h_c + nadph_c --> gdpfuc_c + nadp_c 9604

9622 K02150: ATPeV1E, ATP6E; V-type H+-transporting ATPase subunit E
0.07596130136997266 nan
 Model
  ATPS2v: atp_c + h2o_c + h_c --> adp_c + 2.0 h_v + pi_c 10802 and 11025 and 11052 and 11064 and 11117 and 11403 and 14971 and 15309 and 15351 and 15963 and 16397 and 9210 and 9622 and 9748

9629 K04712: DEGS; sphingolipid Delta-4 desaturase
0.07596130136968925 nan
 Model
  CERD418er: cer2p_18_r + h_r + nadph_r + o2_r --> cer4_18_r + 2.0 h2o_r + nadp_r (12471 and 16097 and 9629) or (13206 and 16097 and 9629)

9664 K19703: FA2H, SCS7; 4-hydroxysphinganine ceramide fatty acyl 2-hydroxylase
0.07596130137375212 nan
 Model
  CERS2p18er: cer1_18_r + h_r + nadph_r + o2_r --> cer2p_18_r + h2o_r + nadp_r 9664
  CERS2p24er: cer1_24_r + h_r + nadph_r + o2_r --> cer2p_24_r + h2o_r + nadp_r 9664
  CERS2p26er: cer1_26_r + h_r + nadph_r + o2_r --> cer2p_26_r + h2o_r + nadp_r 9664
  CERS324er: cer2_24_r + h_r + nadph_r + o2_r --> cer3_24_r + h2o_r + nadp_r 9664
  CERS326er: cer2_26_r + h_r + nadph_r + o2_r --> cer3_26_r + h2o_r + nadp_r 9664

9689 K01687: ilvD; dihydroxy-acid dehydratase
0.0759613013697442 nan
 Model
  DHAD1m: 23dhmb_m --> 3mob_m + h2o_m 14626 or 9689
  DHAD2m: 23dhmp_m --> 3mop_m + h2o_m 14626 or 9689

9726 K01648: ACLY; ATP citrate (pro-S)-lyase
0.07595740045643908 nan
 Model
  ACITL: atp_c + cit_c + coa_c --> accoa_c + adp_c + oaa_c + pi_c 9726

9748 K02155: ATPeV0C, ATP6L; V-type H+-transporting ATPase 16kDa proteolipid subunit
0.07596130137846592 nan
 Model
  ATPS2v: atp_c + h2o_c + h_c --> adp_c + 2.0 h_v + pi_c 10802 and 11025 and 11052 and 11064 and 11117 and 11403 and 14971 and 15309 and 15351 and 15963 and 16397 and 9210 and 9622 and 9748

9782 K19801: PI4KB; phosphatidylinositol 4-kinase B
0.07596130137148778 nan
 Model
  PIN4Kn_RT: atp_n + 0.01 ptd1ino_RT_n --> adp_n + h_n + 0.01 ptd4ino_RT_n 15086 or 9782

9805 K01526: E3.6.1.42; guanosine-diphosphatase
0.07596130136996941 nan
 Model
  NDP3g: gdp_g + h2o_g --> gmp_g + h_g + pi_g 9805

9825 K00549: metE; 5-methyltetrahydropteroyltriglutamate--homocysteine methyltransferase
0.07596130136997749 -3.8444193177584105
 Model
  METS: 5mthf_c + hcys__L_c --> h_c + met__L_c + thf_c 12876 or 12920 or 9825

9830 K01764: E4.4.1.17; cytochrome c heme-lyase
0.07596130136994475 nan
 Model
  HEMELm: apocytc_m + hemeC_m <=> cytc_m 9830

9857 K09022: ridA, tdcF; 2-iminobutanoate/2-iminopropanoate deaminase
0.07596130137270207 nan
 Model
  AMCOXO: amuco_c + h2o_c + h_c + nadph_c --> 2oxoadp_c + nadp_c + nh4_c 9857

9905 K05284: PIGM; phosphatidylinositol glycan, class M
0.07596130136794996 nan
 Model
  G12MT1: doldpglcnacglcnacman_man_man_c + gdpmann_c --> doldpglcnacglcnacman_man_manman_c + gdp_c + h_c 9905
  G12MT2: doldpglcnacglcnacman_man_manman_c + gdpmann_c --> doldpglcnacglcnacman_man_manmanman_c + gdp_c + h_c 9905
  G12MT3: doldpglcnacglcnacman_manman_manmanman_c + dolmanp_c --> doldpglcnacglcnacman_manmanman_manmanman_c + dolp_c + h_c 9905
  G12MT4: doldpglcnacglcnacman_man_man_manman_manmanman_c + dolmanp_c --> doldpglcnacglcnacman_man_manman_manman_manmanman_c + dolp_c + h_c 9905
  G13MT: doldpglcnacglcnacman_c + gdpmann_c --> doldpglcnacglcnacmanman_c + gdp_c + h_c 9905
  G16MT: doldpglcnacglcnacmanman_c + gdpmann_c --> doldpglcnacglcnacman_man_man_c + gdp_c + h_c 9905

9910 K01834: PGAM, gpmA; 2,3-bisphosphoglycerate-dependent phosphoglycerate mutase
0.07596130137099522 nan
 Model
  DPGM: 13dpg_c <=> 23dpg_c + h_c 9910
  DPGase: 23dpg_c + h2o_c --> 3pg_c + pi_c 9910
  PGM: 2pg_c <=> 3pg_c 11229 or 12393 or 15425 or 9910

9940 K02139: ATPeFF, ATP17; F-type H+-transporting ATPase subunit f
0.01931879082718314 nan
 Model
  ATPS3m: adp_m + 3.0 h_c + pi_m --> atp_m + h2o_m + 2.0 h_m ATP6 and ATP8 and ATP9 and 9080 and 9619 and 9940 and 10674 and 11958 and 11967 and 13053 and 13252 and 13424 and 13759 and 13842 and 14786 and 14912 and 15287 and 15589 and 15880 and 16359
```

In [102]:

```
# 8959 is Lengsin, Glutamate-ammonia ligase domain-containing protein 1 or Lens glutamine synthase-like
model.reactions.get_by_id('GLNS').gene_reaction_rule = '11844'
# 14762 is Asparagine synthetase domain-containing protein 1
model.reactions.get_by_id('ASNS1').gene_reaction_rule = '15137'
# PRPS https://bmcbiotechnol.biomedcentral.com/articles/10.1186/1472-6750-8-67
# Thus, in S. cerevisiae PRPP synthetase is organized in two interacting complexes or functional entities: 
# a heterodimer comprising Prs1p-Prs3p; and a heterotrimer consisting of Prs2p-Prs4p-Prs5p [15].
# 11145 and 16592 are both essential, they could be forming a heterodimer
# 11145	K00948: PRPS, prsA; ribose-phosphate pyrophosphokinase		PRS4,PRS2,PRS3	PRPS1,PRPS1L1,PRPS2	PRS3	PRPS2	Essential	mito 20, cyto 5	VVR*
# 16592	K00948: PRPS, prsA; ribose-phosphate pyrophosphokinase		PRS4,PRS2,PRS3	PRPS1,PRPS1L1,PRPS2	PRS2	PRPS1	Essential	cyto 13, cyto_nucl 10.333, cyto_pero 7.333, mito 7, nucl 6.5	LFK*
model.reactions.get_by_id('PRPPS').gene_reaction_rule = '11145 and 16592'
# ERG26 8835 is right size, 13724 is much longer at the C-terminus, RNA data suggests two transcripts for 13724
model.reactions.get_by_id('C3STDH1Pr').gene_reaction_rule = '8835'
model.reactions.get_by_id('C3STDH1r').gene_reaction_rule = '8835'
model.reactions.get_by_id('C3STDH2er').gene_reaction_rule = '8835'
# 8878 is PPCS/CAB2 (ATP eukaryotic and CTP prokaryotic), 8536 is PPCDC/CAB3
# PPNCL is incorrect
model.remove_reactions(['PPNCL','PPNCL2'], remove_orphans=True)
# CSm is essential without CITtp
# Remove CITtp (added in Refinement_1a_Duplicate_Metabolites)
# ICITtp is needed for growth on fatty acids
model.remove_reactions(['CITtp'], remove_orphans=True)
# AKGDm can be made essential if SUCOASm is irreversible in the reverse direction (normal TCA cycle direction)
# and OCOAT1m is irreversible in the forward direction (consistent with metacyc description for its function)
# but both SUCOASm and OCOAT1m are reversible in metacyc
model.reactions.get_by_id('OCOAT1m').lower_bound = 0.0
model.reactions.get_by_id('SUCOASm').upper_bound = 0.0
# GALUi is essential if UGLT is irreversible in the forward direction
model.reactions.get_by_id('UGLT').lower_bound = 0.0
# 11610 is HIS6 (PRMICI) and 9084 is HIS7 (IG3PS)
model.reactions.get_by_id('PRMICI').gene_reaction_rule = '11610'
# 14626 and 9689 are both ILV3, but only 9689 is essential
# In A. fumigatus, there are two ILV3, Ilv3A (essential) and Ilv3B (not essential)
# https://journals.plos.org/plosone/article?id=10.1371/journal.pone.0043559
# It is not clear what Ilv3B's role is, remove it for now
model.reactions.get_by_id('DHAD1m').gene_reaction_rule = '9689'
model.reactions.get_by_id('DHAD2m').gene_reaction_rule = '9689'
# 16404 BPL1 acts on many biotin-dependent carboxylases including acetyl-CoA-carboxylase, pyruvate carboxylase,
# propionyl CoA carboxylase, and 3-methylcrotonyl CoA carboxylase.
model.reactions.get_by_id('ACCOAC').gene_reaction_rule = '8639 and 16404'
model.reactions.get_by_id('PC').gene_reaction_rule = '16630 and 16404'
model.reactions.get_by_id('MCCCrm').gene_reaction_rule = '12867 and 15694 and 16404'
model.reactions.get_by_id('UREASE').gene_reaction_rule = '9326 and 16404'
# 12551 RIB1 is GTP cyclohydrolase, but 16666 RIB3 is 3,4-dihydroxy 2-butanone 4-phosphate synthase
# A main difference between the fungal and bacterial/plant pathways (see flavin biosynthesis I (bacteria and plants)) 
# is in the order in which two of the enzymes act. Two enzymatic steps are required to convert 
# 2,5-diamino-6-(5-phospho-D-ribosylamino)pyrimidin-4(3H)-one to 5-amino-6-(5-phospho-D-ribitylamino)uracil -
# a demaination and a reduction. In bacteria and plants, the deaminase acts first, followed by the reductase 
# (although both steps are catalyzed by a single bifunctional enzyme in some organisms), 
# while in fungi the reductase acts first, followed by the deaminase.
# 25dhpp_c (GTPCII) and 25drapp_c (GTPCII2) are the same
# 25dhpp_c is converted to 25dthpp_c by 16837 RIB7 reductase, and converted to 5apru_c by RIB2 deaminase
# metacyc reaction is using NADH, but model has DROPPRy (NADPH), add DROPPRx from BiGG
# 12663 is K15454: PUS9; tRNA pseudouridine synthase 9, not RIB2
# 15506 is K14655: RIB2, PUS8; tRNA pseudouridine synthase 8 / 2,5-diamino-6-(5-phospho-D-ribitylamino)-pyrimidin-4(3H)-one deaminase
# GTPCII2, DHPPDA/DHPPDA2, APRAUR is bacterial
r = model.reactions.get_by_id('DROPPRy')
r.id = 'DROPPRx'
r.name = '2 5 diamino 6 ribosylamino 4 3H pyrimidinone 5 phosphate reductase nadh'
r.add_metabolites({'nadph_c': 1.0, 'nadp_c': -1.0, 'nadh_c': -1.0, 'nad_c': 1.0})
model.reactions.get_by_id('GTPCII').gene_reaction_rule = '12551'
model.reactions.get_by_id('DRTPPD').gene_reaction_rule = '15506'
model.remove_reactions(['GTPCII2','DHPPDA','DHPPDA2','APRAUR'], remove_orphans=True)
# 10964 is GMPPA and 14956 is GMPPB/PSA1, UniProt states GMPPA may serve as a regulatory subunit and 
# allow allosteric feedback inhibition of GMPPB by GDP-mannose
# remove G1PTT and MAN1PT2
model.reactions.get_by_id('MAN1PT').gene_reaction_rule = '14956'
model.remove_reactions(['G1PTT','MAN1PT2'], remove_orphans=True)
# GLCNACT is by 13348 ALG13 and 14170 ALG14
model.reactions.get_by_id('GLCNACT').gene_reaction_rule = '13348 and 14170'
# Three carbonic anhydrases ['16143','12581','10985']
# Add HCO3En with 16143, change HCO3Em to 10985
model.reactions.get_by_id('HCO3Em').gene_reaction_rule = '10985'
# PYDXS is incorrect, replace ru5p__D_c with r5p_c
# https://metacyc.org/META/NEW-IMAGE?type=REACTION&object=RXN-11322
model.reactions.get_by_id('PYDXS').add_metabolites({'ru5p__D_c': 1.0, 'r5p_c': -1.0})
# 9830 is Cytochrome c1 heme lyase; involved in maturation of cytochrome c1, which is a subunit of 
# the mitochondrial ubiquinol-cytochrome-c reductase, CYOR_u9m
model.reactions.get_by_id('CYOR_u9m').gene_reaction_rule = 'COB and 9705 and 9198 and 11618 and 15758 and 15681 and 12966 and 15231 and 13608 and 13614 and 8802 and 9830'
# 14856 is essential for making 2oxoadp_m, HCITSm is essential if AMCOXO is removed
# check if AM6SAD and AMCOXO are reasonable (Refinement_2a_Add_Biomass_Reaction)
# https://metacyc.org/META/NEW-IMAGE?type=PATHWAY&object=PWY-5652&&detail-level=2&ENZORG=NIL
# In any case, HICITDm is irreversible
model.reactions.get_by_id('HICITDm').lower_bound = 0.0

print('Removed genes', [g.id for g in model.genes if not g.reactions])
cobra.manipulation.remove_genes(model, [g.id for g in model.genes if not g.reactions])
```

```
Removed genes ['13724', '14626', '12663', '14762', '14831', '14828']
```

In [103]:

```
Growth_Prediction_All = pd.DataFrame()
with model:
    model.reactions.get_by_id('ATPM').lower_bound = 0.0
    model.reactions.get_by_id('EX_glc__D_e').lower_bound = 0.0
    for k, v in Fitness_Media.items():
        for x in v:
            if x in YNB:
                model.reactions.get_by_id(x).lower_bound = -1000.0
            elif x in DOC or x in CSM:
                if v.index(x) == 0:
                    model.reactions.get_by_id(x).lower_bound = -1.0
                else:
                    model.reactions.get_by_id(x).lower_bound = -0.01
            elif x == 'SK_triglyc_RT_d':
                model.reactions.get_by_id(x).lower_bound = -0.001
            else:
                model.reactions.get_by_id(x).lower_bound = -1.0
        temp = cobra.flux_analysis.single_gene_deletion(model)
        for x in v:
            model.reactions.get_by_id(x).lower_bound = 0.0
        Growth_Prediction_All[k] = temp['growth']
```

In [104]:

```
Growth_Prediction_All.index = [list(x)[0] for x in Growth_Prediction_All.index]
```

In [105]:

```
print('Genes with fitness score:', len(Fitness))
print('Model genes:', len(Growth_Prediction_All))
print('Model genes excluding mitochondrial and unknown:', len([x for x in Growth_Prediction_All.index if x[0].isdigit()]))
```

```
Genes with fitness score: 8345
Model genes: 1142
Model genes excluding mitochondrial and unknown: 1127
```

In [106]:

```
genes_with_fitness = set(Growth_Prediction_All.index).intersection(Fitness.index)
print('Model genes with fitness score:', len(genes_with_fitness))
genes_without_fitness = set(x.id for x in model.genes if x.id not in genes_with_fitness and x.id[0].isdigit())
print('Model genes without fitness score:', len(genes_without_fitness))
temp = sorted(list(genes_with_fitness.union(genes_without_fitness)))
print('Model genes that can be simulated:', len(temp))
```

```
Model genes with fitness score: 1111
Model genes without fitness score: 16
Model genes that can be simulated: 1127
```

In [107]:

```
Growth_Data = Fitness.reindex(index=temp, columns=Growth_Prediction_All.columns)
Growth_Prediction = Growth_Prediction_All.loc[temp]
```

In [108]:

```
print(Growth_Data.shape)
print(Growth_Prediction.shape)
```

```
(1127, 27)
(1127, 27)
```

In [109]:

```
fig, ax = plt.subplots(figsize=(6,7), ncols=1)
sns.heatmap(Growth_Data, ax=ax, cmap='RdBu_r', xticklabels=True, yticklabels=False, mask=Growth_Data.isnull())
ax.set_ylabel('Genes')
plt.tight_layout()
```

In [110]:

```
y_data = ~(Growth_Data < -4)
y_data.loc[Growth_Data.index[Essential_genes.loc[Growth_Data.index,'Essential'] == 'Yes']] = False
y_pred = Growth_Prediction > 0.01*Growth_Prediction.max()
```

In [111]:

```
TN, FP, FN, TP = confusion_matrix(y_data.values.ravel(), y_pred.values.ravel()).ravel()
print(TN, FP, FN, TP, sum([TN, FP, FN, TP]))
# Sensitivity, hit rate, recall, or true positive rate
TPR = TP/(TP+FN)
# Specificity or true negative rate
TNR = TN/(TN+FP) 
# Precision or positive predictive value
PPV = TP/(TP+FP)
# Negative predictive value
NPV = TN/(TN+FN)
# Fall out or false positive rate
FPR = FP/(FP+TN)
# False negative rate
FNR = FN/(TP+FN)
# False discovery rate
FDR = FP/(TP+FP)
# Overall accuracy
ACC = (TP+TN)/(TP+FP+FN+TN)
# Matthew's
MCC = matthews_corrcoef(y_data.values.ravel(), y_pred.values.ravel())
print('Recall:', TPR.round(3))
print('Precision:', PPV.round(3))
print('Accuracy:', ACC.round(3))
print('Matthew\'s correlation:', MCC.round(3))
```

```
3833 3974 2549 20073 30429
Recall: 0.887
Precision: 0.835
Accuracy: 0.786
Matthew's correlation: 0.406
```

In [112]:

```
df_confusion = pd.DataFrame(confusion_matrix(y_data.values.ravel(), y_pred.values.ravel()),
                            index = pd.MultiIndex.from_product([['Experiment'],['No growth', 'Growth']]),
                            columns = pd.MultiIndex.from_product([['Prediction'],['No growth', 'Growth']]))
df_confusion
```

Out[112]:

|  |  | Prediction | |
| --- | --- | --- | --- |
|  |  | No growth | Growth |
| Experiment | No growth | 3833 | 3974 |
|  | Growth | 2549 | 20073 |

In [113]:

```
genes_essential = Growth_Data.index[Essential_genes.loc[Growth_Data.index,'Essential'] == 'Yes']
print('Experimentally essential in all conditions:', len(genes_essential))
genes_nonessential = Growth_Data.index[(Essential_genes.loc[Growth_Data.index,'Essential'] != 'Yes') & 
                                       ~((Growth_Data < -4).any(axis=1))]
print('Experimentally non-essential in all conditions:', len(genes_nonessential))
genes_conditionally_essential = Growth_Data.index[(Essential_genes.loc[Growth_Data.index,'Essential'] != 'Yes') & 
                                                  (Growth_Data < -4).any(axis=1)]
print('Experimentally essential in only certain conditions:', len(genes_conditionally_essential))
```

```
Experimentally essential in all conditions: 281
Experimentally non-essential in all conditions: 772
Experimentally essential in only certain conditions: 74
```

In [114]:

```
y_data = ~(Growth_Data.loc[genes_conditionally_essential] < -4)
y_pred = Growth_Prediction.loc[genes_conditionally_essential] > 0.01*Growth_Prediction.max()
```

In [115]:

```
TN, FP, FN, TP = confusion_matrix(y_data.values.ravel(), y_pred.values.ravel()).ravel()
print(TN, FP, FN, TP, sum([TN, FP, FN, TP]))
# Sensitivity, hit rate, recall, or true positive rate
TPR = TP/(TP+FN)
# Specificity or true negative rate
TNR = TN/(TN+FP) 
# Precision or positive predictive value
PPV = TP/(TP+FP)
# Negative predictive value
NPV = TN/(TN+FN)
# Fall out or false positive rate
FPR = FP/(FP+TN)
# False negative rate
FNR = FN/(TP+FN)
# False discovery rate
FDR = FP/(TP+FP)
# Overall accuracy
ACC = (TP+TN)/(TP+FP+FN+TN)
# Matthew's
MCC = matthews_corrcoef(y_data.values.ravel(), y_pred.values.ravel())
print('Recall:', TPR.round(3))
print('Precision:', PPV.round(3))
print('Accuracy:', ACC.round(3))
print('Matthew\'s correlation:', MCC.round(3))
```

```
96 124 302 1476 1998
Recall: 0.83
Precision: 0.922
Accuracy: 0.787
Matthew's correlation: 0.209
```

In [116]:

```
df_confusion = pd.DataFrame(confusion_matrix(y_data.values.ravel(), y_pred.values.ravel()),
                            index = pd.MultiIndex.from_product([['Experiment'],['No growth', 'Growth']]),
                            columns = pd.MultiIndex.from_product([['Prediction'],['No growth', 'Growth']]))
df_confusion
```

Out[116]:

|  |  | Prediction | |
| --- | --- | --- | --- |
|  |  | No growth | Growth |
| Experiment | No growth | 96 | 124 |
|  | Growth | 302 | 1476 |

In [117]:

```
model.medium
```

Out[117]:

```
{'EX_h_e': 1000.0,
 'EX_h2o_e': 1000.0,
 'EX_nh4_e': 1000.0,
 'EX_o2_e': 1000.0,
 'EX_pi_e': 1000.0,
 'EX_so4_e': 1000.0,
 'EX_glc__D_e': 1.0,
 'EX_ca2_e': 1000.0,
 'EX_fe2_e': 1000.0,
 'EX_fe3_e': 1000.0,
 'EX_k_e': 1000.0,
 'EX_na1_e': 1000.0,
 'EX_mg2_e': 1000.0,
 'EX_mn2_e': 1000.0,
 'EX_cu2_e': 1000.0,
 'EX_zn2_e': 1000.0}
```

In [118]:

```
sol = cobra.flux_analysis.pfba(model)
display(model.summary(sol))
```

|  | IN\_FLUXES | | OUT\_FLUXES | | OBJECTIVES | |
| --- | --- | --- | --- | --- | --- | --- |
|  | ID | FLUX | ID | FLUX | ID | FLUX |
| 0 | o2\_e | 2.291609 | h2o\_e | 4.223508 | BIOMASS\_RT | 0.073282 |
| 1 | glc\_\_D\_e | 1.000000 | co2\_e | 2.564463 | NaN | NaN |
| 2 | nh4\_e | 0.483680 | h\_e | 0.167655 | NaN | NaN |
| 3 | k\_e | 0.042921 | NaN | NaN | NaN | NaN |

In [119]:

```
# Remove GAM and NGAM to re-estimate
model.reactions.get_by_id('ATPM').lower_bound = 0.0
GAM_old = model.reactions.get_by_id('BIOMASS_RT').get_coefficient('adp_c')
model.reactions.get_by_id('BIOMASS_RT').add_metabolites({'atp_c': GAM_old, 'h2o_c': GAM_old,
                                                         'adp_c': -GAM_old, 'h_c': -GAM_old, 'pi_c': -GAM_old})
```

In [120]:

```
with model:
    model.reactions.get_by_id('EX_o2_e').lower_bound = -1000.0
    model.reactions.get_by_id('EX_glc__D_e').lower_bound = -0.0316392670613915
    model.objective = 'ATPM'
    sol = model.optimize()
    print(sol.objective_value)
    display(model.summary())
NGAM = round(sol.objective_value,2)
```

```
1.2233849930404712
```

|  | IN\_FLUXES | | OUT\_FLUXES | | OBJECTIVES | |
| --- | --- | --- | --- | --- | --- | --- |
|  | ID | FLUX | ID | FLUX | ID | FLUX |
| 0 | o2\_e | 0.189836 | co2\_e | 0.189836 | ATPM | 1.223385 |
| 1 | glc\_\_D\_e | 0.031639 | h2o\_e | 0.189836 | NaN | NaN |

In [121]:

```
temp = np.arange(0.2, 2.5, 0.2)
GAM = []
for x in temp:
    with model:
        model.reactions.get_by_id('EX_o2_e').lower_bound = -1000.0
        model.reactions.get_by_id('EX_glc__D_e').lower_bound = -x
        model.reactions.get_by_id('BIOMASS_RT').lower_bound = 0.0756654696*x
        model.objective = 'ATPM'
        sol = model.optimize()
        GAM.append(sol.objective_value/0.0756654696/x)
plt.scatter(temp,GAM)
print(np.mean(GAM), np.std(GAM))
```

```
139.63945341448806 1.200039158308405e-12
```

In [122]:

```
temp = np.arange(0.2, 2.5, 0.2)
y = []
GAM = round(np.mean(GAM), 6)
for x in temp:
    with model:
        model.reactions.get_by_id('EX_o2_e').lower_bound = -1000.0
        model.reactions.get_by_id('EX_glc__D_e').lower_bound = -x
        model.reactions.get_by_id('BIOMASS_RT').add_metabolites({'atp_c': -GAM, 'h2o_c': -GAM,
                                                                 'adp_c': GAM, 'h_c': GAM, 'pi_c': GAM})
        sol = model.optimize()
        y.append(sol.objective_value)
fit = np.polyfit(temp,y,1)
print(fit)
plt.plot(temp, y, 'o', temp, np.poly1d(fit)(temp), '--k')
```

```
[ 7.56654697e-02 -1.92296269e-16]
```

Out[122]:

```
[<matplotlib.lines.Line2D at 0x7fea86f33c50>,
 <matplotlib.lines.Line2D at 0x7fea86f33d30>]
```

In [123]:

```
# Set NGAM and GAM to new values
model.reactions.get_by_id('ATPM').lower_bound = NGAM
model.reactions.get_by_id('BIOMASS_RT').add_metabolites({'atp_c': -GAM, 'h2o_c': -GAM,
                                                         'adp_c': GAM, 'h_c': GAM, 'pi_c': GAM})
```

In [124]:

```
sol = cobra.flux_analysis.pfba(model)
display(model.summary(sol))
```

|  | IN\_FLUXES | | OUT\_FLUXES | | OBJECTIVES | |
| --- | --- | --- | --- | --- | --- | --- |
|  | ID | FLUX | ID | FLUX | ID | FLUX |
| 0 | o2\_e | 2.287342 | h2o\_e | 4.221464 | BIOMASS\_RT | 0.073366 |
| 1 | glc\_\_D\_e | 1.000000 | co2\_e | 2.560510 | NaN | NaN |
| 2 | nh4\_e | 0.484237 | h\_e | 0.167848 | NaN | NaN |
| 3 | k\_e | 0.042971 | NaN | NaN | NaN | NaN |

In [125]:

```
for r in sorted(model.reactions, key=lambda x: x.id):
    if not r.boundary and sum(abs(x) for x in r.check_mass_balance().values()) > 1e-12:
        print(r, r.gene_reaction_rule, r.check_mass_balance())
```

```
BIOMASS_RT: 0.957502 13BDglcn_c + 0.177315 16BDglcn_c + 0.001283 5mthf_c + 0.577574 alatrna_c + 0.146675 argtrna_c + 0.10797 asntrna_c + 0.197296 asptrna_c + 139.6887 atp_c + 0.002418 btn_m + 0.000832 ca2_c + 0.001792 camp_c + 0.031005 chitin_c + 1.8e-05 clpn_RT_m + 0.00077 coa_c + 0.044881 ctp_c + 0.000525 cu2_c + 0.034269 cystrna_c + 0.002451 datp_c + 0.004285 dctp_c + 0.003763 dgtp_c + 0.002523 dttp_c + 0.004031 ergst_r + 1.6e-05 ergstest_RT_r + 0.000751 fad_c + 0.000597 fe2_c + 0.000597 fe3_c + 0.117898 glntrna_c + 0.208673 glutrna_c + 0.518069 glycogen_c + 0.51322 glytrna_c + 0.00192 gthrd_c + 0.051085 gtp_c + 135.719639 h2o_c + 0.000691 hemeA_m + 0.061167 histrna_c + 0.158569 iletrna_c + 0.585703 k_c + 0.320205 leutrna_c + 0.003107 lipopb_m + 0.172334 lystrna_c + 0.807941 mannan_r + 0.05705 mettrna_c + 0.061716 mg2_c + 0.001292 mlthf_c + 0.000607 mn2_c + 0.026099 na1_c + 0.000888 nad_c + 0.000795 nadp_c + 1.4e-05 pa_RT_r + 0.000172 pc_RT_r + 0.00013 pe_RT_r + 0.095164 phetrna_c + 0.235897 protrna_c + 2e-05 ps_RT_r + 0.004006 psphings_r + 4.7e-05 ptd1ino_RT_r + 0.006524 ptrc_c + 0.0024 pydx5p_c + 0.00074 q9_m + 0.00099 ribflv_c + 0.33687 sertrna_c + 0.003967 spmd_c + 0.001327 thf_c + 0.001393 thmpp_c + 0.21813 thrtrna_c + 0.023372 tre_c + 0.000432 triglyc_RT_r + 0.023319 trptrna_c + 0.062363 tyrtrna_c + 0.051129 utp_c + 0.275168 valtrna_c + 0.00051 zn2_c + 0.004156 zymst_r + 1.7e-05 zymstest_RT_d --> 139.639453 adp_c + 139.639453 h_c + 139.639453 pi_c + 0.209364 ppi_c  {'charge': -4.356141999999977, 'C': -40.730343000000175, 'H': -62.360944999999674, 'O': -17.698877000000266, 'N': -5.868878999999993, 'R': -4.027781, 'P': -0.374185000000125, 'S': -0.104034, 'Ca': -0.000832, 'Cu': -0.000525, 'Fe': -0.001885, 'K': -0.585703, 'Mg': -0.061716, 'Mn': -0.000607, 'Na': -0.026099, 'Zn': -0.00051}
```

In [126]:

```
print(len(model.genes))
print(len(model.reactions))
print(len(model.metabolites))
model
```

```
1142
2398
2059
```

Out[126]:

|  |  |
| --- | --- |
| **Name** | R. toruloides |
| **Memory address** | 0x07fea977e5f60 |
| **Number of metabolites** | 2059 |
| **Number of reactions** | 2398 |
| **Number of groups** | 0 |
| **Objective expression** | 1.0\*BIOMASS\_RT - 1.0\*BIOMASS\_RT\_reverse\_2b3e0 |
| **Compartments** | c, x, m, e, r, v, n, g, d, p |

In [127]:

```
for x in sorted(model.genes, key=lambda x: x.id):
    if not x.reactions:
        print(x)
print()
for x in sorted(model.metabolites, key=lambda x: x.id):
    if not x.reactions:
        print(x)
```

```
12ppd__S_p
14glucan_c
dha_p
h2o_p
inost_p
minohp_p
pi_p
quin_p
```

In [128]:

```
cobra.manipulation.remove_genes(model, [x for x in model.genes if not x.reactions])
model.remove_metabolites([x for x in model.metabolites if not x.reactions])
```

In [129]:

```
print(len(model.genes))
print(len(model.reactions))
print(len(model.metabolites))
[truncated: 16,641 more chars]
