# Supplementary material for: Multi-Omics Driven Metabolic Network Reconstruction and Analysis of Lignocellulosic Carbon Utilization in Rhodosporidium toruloides
Source: Front Bioeng Biotechnol. 2021 Jan 8;8:612832. doi: 10.3389/fbioe.2020.612832 (PMC7873862; doi:10.3389/fbioe.2020.612832)
Supplement: Supplementary File 4 — Multi-omics dataset for R. toruloides IFO0880. [file Data_Sheet_1.zip › Supplementary File S1/2.Metabolic_modeling/Refinement_2f_Model_Annotation_and_Memote.html]

Refinement\_2f\_Model\_Annotation\_and\_Memote


In [1]:

```
%matplotlib inline
from matplotlib import pyplot as plt
import numpy as np
import pandas as pd
import json
import urllib
import cobra
```

json.load(urllib.request.urlopen('http://bigg.ucsd.edu/api/v2/database\_version'))  
{'api\_version': 'v2',  
'last\_updated': '2019-10-31 10:05:54.157598',  
'bigg\_models\_version': '1.6.0'}

In [2]:

```
bigg_universal = cobra.io.load_json_model(urllib.request.urlopen('http://bigg.ucsd.edu/static/namespace/universal_model.json'))
cobra.io.save_json_model(bigg_universal, "../../Data/BiGG_Models/BiGG_universal.json")
```

In [3]:

```
bigg_universal = cobra.io.load_json_model("../../Data/BiGG_Models/BiGG_universal.json")
```

In [4]:

```
model = cobra.io.load_json_model("IFO0880_GPR_2e.json")
```

In [5]:

```
for x in sorted(model.genes, key=lambda x: x.id):
    if not x.reactions:
        print(x)
print()
for x in sorted(model.metabolites, key=lambda x: x.id):
    if not x.reactions:
        print(x)
```

```

```

#### Metabolite annotation¶

In [6]:

```
for m in model.metabolites:
    if m.notes:
        print(m, m.notes)
```

```
adp_c {'original_bigg_ids': ['adp_c']}
atp_c {'original_bigg_ids': ['atp_c']}
atp_x {'original_bigg_ids': ['atp_x']}
h_c {'original_bigg_ids': ['h_c']}
h_x {'original_bigg_ids': ['h_x']}
2phetoh_c {'original_bigg_ids': ['2phetoh_c']}
nadp_c {'original_bigg_ids': ['nadp_c']}
nadph_c {'original_bigg_ids': ['nadph_c']}
pacald_c {'original_bigg_ids': ['pacald_c']}
10fthf_m {'original_bigg_ids': ['10fthf_m']}
h2o_m {'original_bigg_ids': ['h2o_m']}
h_m {'original_bigg_ids': ['h_m']}
methf_m {'original_bigg_ids': ['methf_m']}
ade_c {'original_bigg_ids': ['ade_c']}
amp_c {'original_bigg_ids': ['amp_c']}
h2o_c {'original_bigg_ids': ['h2o_c']}
r5p_c {'original_bigg_ids': ['r5p_c']}
cdpchol_c {'original_bigg_ids': ['cdpchol_c']}
cmp_c {'original_bigg_ids': ['cmp_c']}
nad_m {'original_bigg_ids': ['nad_m']}
nadh_m {'original_bigg_ids': ['nadh_m']}
succ_m {'original_bigg_ids': ['succ_m']}
sucsal_m {'original_bigg_ids': ['sucsal_m']}
h_e {'original_bigg_ids': ['h_e']}
pyr_c {'original_bigg_ids': ['pyr_c']}
pyr_e {'original_bigg_ids': ['pyr_e']}
co2_m {'original_bigg_ids': ['co2_m']}
nicrnt_m {'original_bigg_ids': ['nicrnt_m']}
ppi_m {'original_bigg_ids': ['ppi_m']}
prpp_m {'original_bigg_ids': ['prpp_m']}
quln_m {'original_bigg_ids': ['quln_m']}
aacoa_m {'original_bigg_ids': ['aacoa_m']}
accoa_m {'original_bigg_ids': ['accoa_m']}
coa_m {'original_bigg_ids': ['coa_m']}
hmgcoa_m {'original_bigg_ids': ['hmgcoa_m']}
35cgmp_c {'original_bigg_ids': ['35cgmp_c']}
gmp_c {'original_bigg_ids': ['gmp_c']}
pap_c {'original_bigg_ids': ['pap_c']}
paps_c {'original_bigg_ids': ['paps_c']}
so3_c {'original_bigg_ids': ['so3_c']}
trdox_c {'original_bigg_ids': ['trdox_c']}
trdrd_c {'original_bigg_ids': ['trdrd_c']}
amp_x {'original_bigg_ids': ['amp_x']}
coa_x {'original_bigg_ids': ['coa_x']}
occoa_x {'original_bigg_ids': ['occoa_x']}
octa_x {'original_bigg_ids': ['octa_x']}
ppi_x {'original_bigg_ids': ['ppi_x']}
rib__D_c {'original_bigg_ids': ['rib__D_c']}
ura_c {'original_bigg_ids': ['ura_c']}
uri_c {'original_bigg_ids': ['uri_c']}
acald_m {'original_bigg_ids': ['acald_m']}
dc2coa_x {'original_bigg_ids': ['dc2coa_x']}
dcacoa_x {'original_bigg_ids': ['dcacoa_x']}
h2o2_x {'original_bigg_ids': ['h2o2_x']}
o2_x {'original_bigg_ids': ['o2_x']}
3c2hmp_c {'original_bigg_ids': ['3c2hmp_c']}
3ipmmest_c {'original_bigg_ids': ['3ipmmest_c']}
ahcys_c {'original_bigg_ids': ['ahcys_c']}
amet_c {'original_bigg_ids': ['amet_c']}
ACP_m {'original_bigg_ids': ['ACP_m']}
malACP_m {'original_bigg_ids': ['malACP_m']}
nadp_m {'original_bigg_ids': ['nadp_m']}
nadph_m {'original_bigg_ids': ['nadph_m']}
nad_c {'original_bigg_ids': ['nad_c']}
nadh_c {'original_bigg_ids': ['nadh_c']}
udpg_c {'original_bigg_ids': ['udpg_c']}
udpglcur_c {'original_bigg_ids': ['udpglcur_c']}
cytd_c {'original_bigg_ids': ['cytd_c']}
gdp_c {'original_bigg_ids': ['gdp_c']}
gtp_c {'original_bigg_ids': ['gtp_c']}
ac_c {'original_bigg_ids': ['ac_c']}
chitin_c {'original_bigg_ids': ['chitin_c']}
chitos_c {'original_bigg_ids': ['chitos_c']}
coa_c {'original_bigg_ids': ['coa_c']}
tdecoa_c {'original_bigg_ids': ['tdecoa_c']}
ttdcea_c {'original_bigg_ids': ['ttdcea_c']}
accoa_c {'original_bigg_ids': ['accoa_c']}
co2_c {'original_bigg_ids': ['co2_c']}
malcoa_c {'original_bigg_ids': ['malcoa_c']}
octa_c {'original_bigg_ids': ['octa_c']}
pi_c {'original_bigg_ids': ['pi_c']}
3ohdcoa_x {'original_bigg_ids': ['3ohdcoa_x']}
accoa_x {'original_bigg_ids': ['accoa_x']}
tdcoa_x {'original_bigg_ids': ['tdcoa_x']}
datp_c {'original_bigg_ids': ['datp_c']}
N1aspmd_c {'original_bigg_ids': ['N1aspmd_c']}
aprut_c {'original_bigg_ids': ['aprut_c']}
h2o2_c {'original_bigg_ids': ['h2o2_c']}
5pmev_c {'original_bigg_ids': ['5pmev_c']}
mev__R_c {'original_bigg_ids': ['mev_R_c']}
ala__L_c {'original_bigg_ids': ['ala_DASH_L_c']}
dadp_c {'original_bigg_ids': ['dadp_c']}
ump_c {'original_bigg_ids': ['ump_c']}
h2o_e {'original_bigg_ids': ['h2o_e']}
hdca_e {'original_bigg_ids': ['hdca_e']}
hdcea_e {'original_bigg_ids': ['hdcea_e']}
ocdca_e {'original_bigg_ids': ['ocdca_e']}
ocdcea_e {'original_bigg_ids': ['ocdcea_e']}
ttdca_e {'original_bigg_ids': ['ttdca_e']}
zymst_e {'original_bigg_ids': ['zymst_e']}
aacald_c {'original_bigg_ids': ['aacald_c']}
succ_c {'original_bigg_ids': ['succ_c']}
taur_c {'original_bigg_ids': ['taur_c']}
cit_x {'original_bigg_ids': ['cit_x']}
h2o_x {'original_bigg_ids': ['h2o_x']}
oaa_x {'original_bigg_ids': ['oaa_x']}
3php_c {'original_bigg_ids': ['3php_c']}
pser__L_c {'original_bigg_ids': ['pser__L_c']}
cys__L_e {'original_bigg_ids': ['cys_L_e']}
1pyr5c_m {'original_bigg_ids': ['1pyr5c_m']}
pro__L_m {'original_bigg_ids': ['pro_DASH_L_m']}
adp_v {'original_bigg_ids': ['adp_v']}
atp_v {'original_bigg_ids': ['atp_v']}
h2o_v {'original_bigg_ids': ['h2o_v']}
h_v {'original_bigg_ids': ['h_v']}
pi_v {'original_bigg_ids': ['pi_v']}
ethamp_c {'original_bigg_ids': ['ethamp_c']}
dca_e {'original_bigg_ids': ['dca_e']}
ddca_e {'original_bigg_ids': ['ddca_e']}
g3pc_e {'original_bigg_ids': ['g3pc_e']}
ocdcya_e {'original_bigg_ids': ['ocdcya_e']}
pc_RT_e {'original_bigg_ids': ['pc_SC_e']}
pac_c {'original_bigg_ids': ['pac_c']}
ahdt_c {'original_bigg_ids': ['ahdt_c']}
dhnpt_c {'original_bigg_ids': ['dhnpt_c']}
hexc_c {'original_bigg_ids': ['hexc_c']}
ttc_c {'original_bigg_ids': ['ttc_c']}
dcdp_c {'original_bigg_ids': ['dcdp_c']}
dctp_c {'original_bigg_ids': ['dctp_c']}
12ppd__R_c {'original_bigg_ids': ['12ppd__R_c']}
lald__D_c {'original_bigg_ids': ['lald__D_c']}
glyclt_c {'original_bigg_ids': ['glyclt_c']}
dgdp_c {'original_bigg_ids': ['dgdp_c']}
dgmp_c {'original_bigg_ids': ['dgmp_c']}
cer1_26_r {'original_bigg_ids': ['cer1_26_r']}
coa_r {'original_bigg_ids': ['coa_r']}
hexccoa_r {'original_bigg_ids': ['hexccoa_r']}
sphgn_r {'original_bigg_ids': ['sphgn_r']}
cit_m {'original_bigg_ids': ['cit_m']}
oaa_m {'original_bigg_ids': ['oaa_m']}
adp_n {'original_bigg_ids': ['adp_n']}
atp_n {'original_bigg_ids': ['atp_n']}
h_n {'original_bigg_ids': ['h_n']}
mi1456p_n {'original_bigg_ids': ['mi1456p_n']}
mi145p_n {'original_bigg_ids': ['mi145p_n']}
amp_m {'original_bigg_ids': ['amp_m']}
gdp_m {'original_bigg_ids': ['gdp_m']}
gtp_m {'original_bigg_ids': ['gtp_m']}
dmpp_c {'original_bigg_ids': ['dmpp_c']}
grdp_c {'original_bigg_ids': ['grdp_c']}
ipdp_c {'original_bigg_ids': ['ipdp_c']}
2mahmp_c {'original_bigg_ids': ['2mahmp_c']}
4ampm_c {'original_bigg_ids': ['4ampm_c']}
lgt__S_c {'original_bigg_ids': ['lgt__S_c']}
mthgxl_c {'original_bigg_ids': ['mthgxl_c']}
gsn_m {'original_bigg_ids': ['gsn_m']}
gua_m {'original_bigg_ids': ['gua_m']}
r1p_m {'original_bigg_ids': ['r1p_m']}
ocdca_c {'original_bigg_ids': ['ocdca_c']}
phpyr_c {'original_bigg_ids': ['phpyr_c']}
pphn_c {'original_bigg_ids': ['pphn_c']}
glytrna_c {'original_bigg_ids': ['glytrna_c']}
trnagly_c {'original_bigg_ids': ['trnagly_c']}
3hdcoa_x {'original_bigg_ids': ['3hdcoa_x']}
3odcoa_x {'original_bigg_ids': ['3odcoa_x']}
dcacoa_c {'original_bigg_ids': ['dcacoa_c']}
ddcacoa_c {'original_bigg_ids': ['ddcacoa_c']}
dhap_c {'original_bigg_ids': ['dhap_c']}
hdcoa_c {'original_bigg_ids': ['hdcoa_c']}
ocdycacoa_c {'original_bigg_ids': ['ocdycacoa_c']}
odecoa_c {'original_bigg_ids': ['odecoa_c']}
pmtcoa_c {'original_bigg_ids': ['pmtcoa_c']}
stcoa_c {'original_bigg_ids': ['stcoa_c']}
ctp_c {'original_bigg_ids': ['ctp_c']}
3mb2coa_m {'original_bigg_ids': ['3mb2coa_m']}
fad_m {'original_bigg_ids': ['fad_m']}
fadh2_m {'original_bigg_ids': ['fadh2_m']}
ivcoa_m {'original_bigg_ids': ['ivcoa_m']}
3hbcoa_x {'original_bigg_ids': ['3hbcoa_x']}
b2coa_x {'original_bigg_ids': ['b2coa_x']}
Lkynr_c {'original_bigg_ids': ['Lkynr_c']}
anth_c {'original_bigg_ids': ['anth_c']}
hisp_c {'original_bigg_ids': ['hisp_c']}
imacp_c {'original_bigg_ids': ['imacp_c']}
tdecoa_x {'original_bigg_ids': ['tdecoa_x']}
ttdcea_x {'original_bigg_ids': ['ttdcea_x']}
fe2_c {'original_bigg_ids': ['fe2_c']}
fe2_e {'original_bigg_ids': ['fe2_e']}
2mbald_c {'original_bigg_ids': ['2mbald_c']}
3mop_c {'original_bigg_ids': ['3mop_c']}
ddca_x {'original_bigg_ids': ['ddca_x']}
ddcacoa_x {'original_bigg_ids': ['ddcacoa_x']}
glutrna_c {'original_bigg_ids': ['glutrna_c']}
trnaglu_c {'original_bigg_ids': ['trnaglu_c']}
10fthf_c {'original_bigg_ids': ['10fthf_c']}
aicar_c {'original_bigg_ids': ['aicar_c']}
fprica_c {'original_bigg_ids': ['fprica_c']}
thf_c {'original_bigg_ids': ['thf_c']}
nh4_c {'original_bigg_ids': ['nh4_c']}
fad_c {'original_bigg_ids': ['fad_c']}
13dpg_c {'original_bigg_ids': ['13dpg_c']}
23dpg_c {'original_bigg_ids': ['23dpg_c']}
g3p_c {'original_bigg_ids': ['g3p_c']}
dnad_c {'original_bigg_ids': ['dnad_c']}
13dampp_c {'original_bigg_ids': ['13dampp_c']}
4abutn_c {'original_bigg_ids': ['4abutn_c']}
spmd_c {'original_bigg_ids': ['spmd_c']}
his__L_c {'original_bigg_ids': ['his_L_c']}
histrna_c {'original_bigg_ids': ['histrna_c']}
trnahis_c {'original_bigg_ids': ['trnahis_c']}
phe__L_m {'original_bigg_ids': ['phe_L_m']}
phetrna_m {'original_bigg_ids': ['phetrna_m']}
trnaphe_m {'original_bigg_ids': ['trnaphe_m']}
ddca_c {'original_bigg_ids': ['ddca_c']}
cit_c {'original_bigg_ids': ['cit_c']}
pep_c {'original_bigg_ids': ['pep_c']}
pep_m {'original_bigg_ids': ['pep_m']}
3c4mop_c {'original_bigg_ids': ['3c4mop_c']}
4mop_c {'original_bigg_ids': ['4mop_c']}
udp_c {'original_bigg_ids': ['udp_c']}
utp_c {'original_bigg_ids': ['utp_c']}
leu__L_c {'original_bigg_ids': ['leu_L_c']}
dtdp_c {'original_bigg_ids': ['dtdp_c']}
dtmp_c {'original_bigg_ids': ['dtmp_c']}
2mppal_c {'original_bigg_ids': ['2mppal_c']}
ibutoh_c {'original_bigg_ids': ['ibutoh_c']}
pi_x {'original_bigg_ids': ['pi_x']}
hdca_x {'original_bigg_ids': ['hdca_x']}
pmtcoa_x {'original_bigg_ids': ['pmtcoa_x']}
6pgc_c {'original_bigg_ids': ['6pgc_c']}
6pgl_c {'original_bigg_ids': ['6pgl_c']}
2obut_m {'original_bigg_ids': ['2obut_m']}
nh4_m {'original_bigg_ids': ['nh4_m']}
thr__L_m {'original_bigg_ids': ['thr_L_m']}
glu5sa_m {'original_bigg_ids': ['glu5sa_m']}
trnatrp_c {'original_bigg_ids': ['trnatrp_c']}
trp__L_c {'original_bigg_ids': ['trp_L_c']}
trptrna_c {'original_bigg_ids': ['trptrna_c']}
cyst__L_c {'original_bigg_ids': ['cyst__L_c']}
suchms_c {'original_bigg_ids': ['suchms_c']}
achms_c {'original_bigg_ids': ['achms_c']}
ch4s_c {'original_bigg_ids': ['ch4s_c']}
met__L_c {'original_bigg_ids': ['met_L_c']}
ac_e {'original_bigg_ids': ['ac_e']}
dkmpp_c {'original_bigg_ids': ['dkmpp_c']}
lanost_c {'original_bigg_ids': ['lanost_c']}
2mb2coa_m {'original_bigg_ids': ['2mb2coa_m']}
2mbcoa_m {'original_bigg_ids': ['2mbcoa_m']}
2dr1p_c {'original_bigg_ids': ['2dr1p_c']}
thym_c {'original_bigg_ids': ['thym_c']}
thymd_c {'original_bigg_ids': ['thymd_c']}
inost_c {'original_bigg_ids': ['inost_c']}
inost_e {'original_bigg_ids': ['inost_e']}
mi1p__D_c {'original_bigg_ids': ['mi1p_D_c']}
trnatrp_m {'original_bigg_ids': ['trnatrp_m']}
trp__L_m {'original_bigg_ids': ['trp_L_m']}
trptrna_m {'original_bigg_ids': ['trptrna_m']}
dutp_c {'original_bigg_ids': ['dutp_c']}
icit_c {'original_bigg_ids': ['icit_c']}
fru_c {'original_bigg_ids': ['fru_c']}
hicit_m {'original_bigg_ids': ['hicit_m']}
mal__L_c {'original_bigg_ids': ['mal_L_c']}
mal__L_m {'original_bigg_ids': ['mal_L_m']}
4gudbd_c {'original_bigg_ids': ['4gudbd_c']}
4gudbutn_c {'original_bigg_ids': ['4gudbutn_c']}
2mcit_m {'original_bigg_ids': ['2mcit_m']}
ppcoa_m {'original_bigg_ids': ['ppcoa_m']}
gsn_c {'original_bigg_ids': ['gsn_c']}
gua_c {'original_bigg_ids': ['gua_c']}
4ppan_c {'original_bigg_ids': ['4ppan_c']}
pnto__R_c {'original_bigg_ids': ['pnto_R_c']}
adn_c {'original_bigg_ids': ['adn_c']}
ins_c {'original_bigg_ids': ['ins_c']}
ficytc_m {'original_bigg_ids': ['ficytc_m']}
focytc_m {'original_bigg_ids': ['focytc_m']}
lac__L_c {'original_bigg_ids': ['lac_L_c']}
ura_e {'original_bigg_ids': ['ura_e']}
stcoa_x {'original_bigg_ids': ['stcoa_x']}
pad_c {'original_bigg_ids': ['pad_c']}
ergst_c {'original_bigg_ids': ['ergst_c']}
ergst_e {'original_bigg_ids': ['ergst_e']}
acrn_x {'original_bigg_ids': ['acrn_x']}
crn_x {'original_bigg_ids': ['crn_x']}
udpgal_c {'original_bigg_ids': ['udpgal_c']}
3dhsk_c {'original_bigg_ids': ['3dhsk_c']}
skm_c {'original_bigg_ids': ['skm_c']}
sucsal_c {'original_bigg_ids': ['sucsal_c']}
h2s_c {'original_bigg_ids': ['h2s_c']}
succoa_m {'original_bigg_ids': ['succoa_m']}
2dhp_c {'original_bigg_ids': ['2dhp_c']}
3mob_c {'original_bigg_ids': ['3mob_c']}
mlthf_c {'original_bigg_ids': ['mlthf_c']}
fgam_c {'original_bigg_ids': ['fgam_c']}
gar_c {'original_bigg_ids': ['gar_c']}
pyam5p_c {'original_bigg_ids': ['pyam5p_c']}
pydam_c {'original_bigg_ids': ['pydam_c']}
3hanthrn_c {'original_bigg_ids': ['3hanthrn_c']}
hLkynr_c {'original_bigg_ids': ['hLkynr_c']}
dnad_n {'original_bigg_ids': ['dnad_n']}
nicrnt_n {'original_bigg_ids': ['nicrnt_n']}
ppi_n {'original_bigg_ids': ['ppi_n']}
met__L_m {'original_bigg_ids': ['met_L_m']}
mettrna_m {'original_bigg_ids': ['mettrna_m']}
trnamet_m {'original_bigg_ids': ['trnamet_m']}
Sfglutth_c {'original_bigg_ids': ['Sfglutth_c']}
fmn_e {'original_bigg_ids': ['fmn_e']}
pi_e {'original_bigg_ids': ['pi_e']}
ribflv_e {'original_bigg_ids': ['ribflv_e']}
nadp_x {'original_bigg_ids': ['nadp_x']}
nadph_x {'original_bigg_ids': ['nadph_x']}
4pasp_c {'original_bigg_ids': ['4pasp_c']}
aspsa_c {'original_bigg_ids': ['aspsa_c']}
3hhdcoa_x {'original_bigg_ids': ['3hhdcoa_x']}
hdd2coa_x {'original_bigg_ids': ['hdd2coa_x']}
ttdca_x {'original_bigg_ids': ['ttdca_x']}
3hmbcoa_m {'original_bigg_ids': ['3hmbcoa_m']}
glyc3p_m {'original_bigg_ids': ['glyc3p_m']}
ocdcya_c {'original_bigg_ids': ['ocdcya_c']}
hxan_c {'original_bigg_ids': ['hxan_c']}
imp_c {'original_bigg_ids': ['imp_c']}
prpp_c {'original_bigg_ids': ['prpp_c']}
aprop_c {'original_bigg_ids': ['aprop_c']}
23dhmp_m {'original_bigg_ids': ['23dhmp_m']}
3mop_m {'original_bigg_ids': ['3mop_m']}
ergst3glc_c {'original_bigg_ids': ['ergst3glc_c']}
1pyr5c_c {'original_bigg_ids': ['1pyr5c_c']}
pro__L_c {'original_bigg_ids': ['pro__L_c']}
na1_c {'original_bigg_ids': ['na1_c']}
na1_e {'original_bigg_ids': ['na1_e']}
aps_c {'original_bigg_ids': ['aps_c']}
4hbz_m {'original_bigg_ids': ['4hbz_m']}
cdp_n {'original_bigg_ids': ['cdp_n']}
dcdp_n {'original_bigg_ids': ['dcdp_n']}
h2o_n {'original_bigg_ids': ['h2o_n']}
trdox_n {'original_bigg_ids': ['trdox_n']}
trdrd_n {'original_bigg_ids': ['trdrd_n']}
cpppg3_c {'original_bigg_ids': ['cpppg3_c']}
pppg9_c {'original_bigg_ids': ['pppg9_c']}
ptrc_c {'original_bigg_ids': ['ptrc_c']}
ptrc_e {'original_bigg_ids': ['ptrc_e']}
nac_c {'original_bigg_ids': ['nac_c']}
nicrnt_c {'original_bigg_ids': ['nicrnt_c']}
etoh_c {'original_bigg_ids': ['etoh_c']}
cdp_c {'original_bigg_ids': ['cdp_c']}
hdcoa_x {'original_bigg_ids': ['hdcoa_x']}
lanost_e {'original_bigg_ids': ['lanost_e']}
3hddcoa_m {'original_bigg_ids': ['3hddcoa_m']}
dd2coa_m {'original_bigg_ids': ['dd2coa_m']}
dudp_c {'original_bigg_ids': ['dudp_c']}
dump_c {'original_bigg_ids': ['dump_c']}
allphn_c {'original_bigg_ids': ['allphn_c']}
duri_c {'original_bigg_ids': ['duri_c']}
hom__L_c {'original_bigg_ids': ['hom_L_c']}
phe__L_c {'original_bigg_ids': ['phe_L_c']}
phetrna_c {'original_bigg_ids': ['phetrna_c']}
trnaphe_c {'original_bigg_ids': ['trnaphe_c']}
ttdca_c {'original_bigg_ids': ['ttdca_c']}
fmn_c {'original_bigg_ids': ['fmn_c']}
trnatyr_c {'original_bigg_ids': ['trnatyr_c']}
tyr__L_c {'original_bigg_ids': ['tyr_L_c']}
tyrtrna_c {'original_bigg_ids': ['tyrtrna_c']}
trnatyr_m {'original_bigg_ids': ['trnatyr_m']}
tyr__L_m {'original_bigg_ids': ['tyr_L_m']}
tyrtrna_m {'original_bigg_ids': ['tyrtrna_m']}
acybut_c {'original_bigg_ids': ['acybut_c']}
ile__L_m {'original_bigg_ids': ['ile_L_m']}
iletrna_m {'original_bigg_ids': ['iletrna_m']}
trnaile_m {'original_bigg_ids': ['trnaile_m']}
asp__L_c {'original_bigg_ids': ['asp__L_c']}
caphis_c {'original_bigg_ids': ['caphis_c']}
cmaphis_c {'original_bigg_ids': ['cmaphis_c']}
udpgal_g {'original_bigg_ids': ['udpgal_g']}
lald__L_c {'original_bigg_ids': ['lald_L_c']}
phom_c {'original_bigg_ids': ['phom_c']}
4ppcys_c {'original_bigg_ids': ['4ppcys_c']}
pan4p_c {'original_bigg_ids': ['pan4p_c']}
ap4a_c {'original_bigg_ids': ['ap4a_c']}
prbamp_c {'original_bigg_ids': ['prbamp_c']}
prbatp_c {'original_bigg_ids': ['prbatp_c']}
asptrna_c {'original_bigg_ids': ['asptrna_c']}
trnaasp_c {'original_bigg_ids': ['trnaasp_c']}
tyr__L_v {'original_bigg_ids': ['tyr_L_v']}
co2_v {'original_bigg_ids': ['co2_v']}
pe_RT_v {'original_bigg_ids': ['pe_SC_v']}
ps_RT_v {'original_bigg_ids': ['ps_SC_v']}
dgtp_c {'original_bigg_ids': ['dgtp_c']}
35ccmp_c {'original_bigg_ids': ['35ccmp_c']}
dudp_n {'original_bigg_ids': ['dudp_n']}
udp_n {'original_bigg_ids': ['udp_n']}
fru_e {'original_bigg_ids': ['fru_e']}
glc__D_e {'original_bigg_ids': ['glc_D_e']}
sucr_e {'original_bigg_ids': ['sucr_e']}
ru5p__D_c {'original_bigg_ids': ['ru5p__D_c']}
xu5p__D_c {'original_bigg_ids': ['xu5p__D_c']}
hco3_m {'original_bigg_ids': ['hco3_m']}
malcoa_m {'original_bigg_ids': ['malcoa_m']}
man6p_c {'original_bigg_ids': ['man6p_c']}
man_c {'original_bigg_ids': ['man_c']}
ile__L_c {'original_bigg_ids': ['ile_L_c']}
ile__L_v {'original_bigg_ids': ['ile_L_v']}
12ppd__S_c {'original_bigg_ids': ['12ppd__S_c']}
3dhq_c {'original_bigg_ids': ['3dhq_c']}
orn_c {'original_bigg_ids': ['orn_c']}
orn_m {'original_bigg_ids': ['orn_m']}
4mop_m {'original_bigg_ids': ['4mop_m']}
leu__L_m {'original_bigg_ids': ['leu_L_m']}
3htdcoa_m {'original_bigg_ids': ['3htdcoa_m']}
td2coa_m {'original_bigg_ids': ['td2coa_m']}
3c4mop_m {'original_bigg_ids': ['3c4mop_m']}
ocdycacoa_x {'original_bigg_ids': ['ocdycacoa_x']}
cholp_c {'original_bigg_ids': ['cholp_c']}
trdox_m {'original_bigg_ids': ['trdox_m']}
trdrd_m {'original_bigg_ids': ['trdrd_m']}
prpncoa_m {'original_bigg_ids': ['prpncoa_m']}
3oddcoa_x {'original_bigg_ids': ['3oddcoa_x']}
alltt_c {'original_bigg_ids': ['alltt_c']}
urdglyc_c {'original_bigg_ids': ['urdglyc_c']}
urea_c {'original_bigg_ids': ['urea_c']}
gam1p_c {'original_bigg_ids': ['gam1p_c']}
gam6p_c {'original_bigg_ids': ['gam6p_c']}
5dpmev_c {'original_bigg_ids': ['5dpmev_c']}
occoa_c {'original_bigg_ids': ['occoa_c']}
dhf_c {'original_bigg_ids': ['dhf_c']}
dhpt_c {'original_bigg_ids': ['dhpt_c']}
acg5p_m {'original_bigg_ids': ['acg5p_m']}
acglu_m {'original_bigg_ids': ['acglu_m']}
3pg_c {'original_bigg_ids': ['3pg_c']}
5aop_c {'original_bigg_ids': ['5aop_c']}
ppbng_c {'original_bigg_ids': ['ppbng_c']}
prfp_c {'original_bigg_ids': ['prfp_c']}
prlp_c {'original_bigg_ids': ['prlp_c']}
oaa_c {'original_bigg_ids': ['oaa_c']}
nac_m {'original_bigg_ids': ['nac_m']}
ncam_m {'original_bigg_ids': ['ncam_m']}
actn__R_c {'original_bigg_ids': ['actn_R_c']}
2pg_c {'original_bigg_ids': ['2pg_c']}
gluala_c {'original_bigg_ids': ['gluala_c']}
g1p_c {'original_bigg_ids': ['g1p_c']}
g6p_c {'original_bigg_ids': ['g6p_c']}
gthrd_v {'original_bigg_ids': ['gthrd_v']}
dolp_r {'original_bigg_ids': ['dolp_r']}
mannan_r {'original_bigg_ids': ['mannan_r']}
damp_c {'original_bigg_ids': ['damp_c']}
dttp_c {'original_bigg_ids': ['dttp_c']}
zymst_c {'original_bigg_ids': ['zymst_c']}
camp_c {'original_bigg_ids': ['camp_c']}
uri_e {'original_bigg_ids': ['uri_e']}
cbasp_c {'original_bigg_ids': ['cbasp_c']}
cbp_c {'original_bigg_ids': ['cbp_c']}
cystrna_c {'original_bigg_ids': ['cystrna_c']}
trnacys_c {'original_bigg_ids': ['trnacys_c']}
arg__L_c {'original_bigg_ids': ['arg_L_c']}
arg__L_e {'original_bigg_ids': ['arg_L_e']}
gdp_g {'original_bigg_ids': ['gdp_g']}
gdpmann_g {'original_bigg_ids': ['gdpmann_g']}
h_g {'original_bigg_ids': ['h_g']}
m3macchitppdol_g {'original_bigg_ids': ['m3macchitppdol_g']}
m4macchitppdol_g {'original_bigg_ids': ['m4macchitppdol_g']}
tag6p__D_c {'original_bigg_ids': ['tag6p__D_c']}
tagdp__D_c {'original_bigg_ids': ['tagdp__D_c']}
peamn_c {'original_bigg_ids': ['peamn_c']}
dha_c {'original_bigg_ids': ['dha_c']}
3hpp_c {'original_bigg_ids': ['3hpp_c']}
gal_c {'original_bigg_ids': ['gal_c']}
asn__L_c {'original_bigg_ids': ['asn_L_c']}
asn__L_v {'original_bigg_ids': ['asn_L_v']}
acgam1p_c {'original_bigg_ids': ['acgam1p_c']}
acgam6p_c {'original_bigg_ids': ['acgam6p_c']}
gam_c {'original_bigg_ids': ['gam_c']}
16BDglcn_c {'original_bigg_ids': ['16BDglcn_c']}
amp_n {'original_bigg_ids': ['amp_n']}
nad_n {'original_bigg_ids': ['nad_n']}
nh4_n {'original_bigg_ids': ['nh4_n']}
thfglu_c {'original_bigg_ids': ['thfglu_c']}
aacoa_c {'original_bigg_ids': ['aacoa_c']}
acac_c {'original_bigg_ids': ['acac_c']}
btd_RR_c {'original_bigg_ids': ['btd_RR_c']}
hmgcoa_c {'original_bigg_ids': ['hmgcoa_c']}
gdpddman_c {'original_bigg_ids': ['gdpddman_c']}
mlthf_m {'original_bigg_ids': ['mlthf_m']}
acrn_c {'original_bigg_ids': ['acrn_c']}
crn_c {'original_bigg_ids': ['crn_c']}
leutrna_c {'original_bigg_ids': ['leutrna_c']}
trnaleu_c {'original_bigg_ids': ['trnaleu_c']}
glyc3p_c {'original_bigg_ids': ['glyc3p_c']}
ncam_c {'original_bigg_ids': ['ncam_c']}
glp_c {'original_bigg_ids': ['glp_c']}
tglp_c {'original_bigg_ids': ['tglp_c']}
2dr5p_c {'original_bigg_ids': ['2dr5p_c']}
drib_c {'original_bigg_ids': ['drib_c']}
adn_e {'original_bigg_ids': ['adn_e']}
urea_e {'original_bigg_ids': ['urea_e']}
dcamp_c {'original_bigg_ids': ['dcamp_c']}
fum_c {'original_bigg_ids': ['fum_c']}
akg_x {'original_bigg_ids': ['akg_x']}
icit_x {'original_bigg_ids': ['icit_x']}
e4p_c {'original_bigg_ids': ['e4p_c']}
fecost_c {'original_bigg_ids': ['fecost_c']}
gthox_c {'original_bigg_ids': ['gthox_c']}
methf_c {'original_bigg_ids': ['methf_c']}
met__L_e {'original_bigg_ids': ['met_L_e']}
3hbcoa_c {'original_bigg_ids': ['3hbcoa_c']}
3mob_m {'original_bigg_ids': ['3mob_m']}
val__L_m {'original_bigg_ids': ['val_L_m']}
gthrd_e {'original_bigg_ids': ['gthrd_e']}
metsox_S__L_c {'original_bigg_ids': ['metsox_S__L_c']}
hexc_x {'original_bigg_ids': ['hexc_x']}
hexccoa_x {'original_bigg_ids': ['hexccoa_x']}
pdx5p_c {'original_bigg_ids': ['pdx5p_c']}
pydxn_c {'original_bigg_ids': ['pydxn_c']}
eig3p_c {'original_bigg_ids': ['eig3p_c']}
cytd_e {'original_bigg_ids': ['cytd_e']}
2mp2coa_m {'original_bigg_ids': ['2mp2coa_m']}
3hibutcoa_m {'original_bigg_ids': ['3hibutcoa_m']}
frdp_c {'original_bigg_ids': ['frdp_c']}
hexccoa_c {'original_bigg_ids': ['hexccoa_c']}
iletrna_c {'original_bigg_ids': ['iletrna_c']}
trnaile_c {'original_bigg_ids': ['trnaile_c']}
pydx_c {'original_bigg_ids': ['pydx_c']}
hco3_c {'original_bigg_ids': ['hco3_c']}
34hpp_c {'original_bigg_ids': ['34hpp_c']}
glycogen_c {'original_bigg_ids': ['glycogen_c']}
quln_c {'original_bigg_ids': ['quln_c']}
glu__L_x {'original_bigg_ids': ['glu_L_x']}
4r5au_c {'original_bigg_ids': ['4r5au_c']}
db4p_c {'original_bigg_ids': ['db4p_c']}
dmlz_c {'original_bigg_ids': ['dmlz_c']}
man1p_c {'original_bigg_ids': ['man1p_c']}
dca_c {'original_bigg_ids': ['dca_c']}
mettrna_c {'original_bigg_ids': ['mettrna_c']}
trnamet_c {'original_bigg_ids': ['trnamet_c']}
co2_g {'original_bigg_ids': ['co2_g']}
pe_RT_g {'original_bigg_ids': ['pe_SC_g']}
ps_RT_g {'original_bigg_ids': ['ps_SC_g']}
lac__D_c {'original_bigg_ids': ['lac__D_c']}
dd2coa_x {'original_bigg_ids': ['dd2coa_x']}
25dthpp_c {'original_bigg_ids': ['25dthpp_c']}
5aprbu_c {'original_bigg_ids': ['5aprbu_c']}
5aop_m {'original_bigg_ids': ['5aop_m']}
gly_m {'original_bigg_ids': ['gly_m']}
3mbald_c {'original_bigg_ids': ['3mbald_c']}
iamoh_c {'original_bigg_ids': ['iamoh_c']}
gmp_g {'original_bigg_ids': ['gmp_g']}
h2o_g {'original_bigg_ids': ['h2o_g']}
pi_g {'original_bigg_ids': ['pi_g']}
spmd_e {'original_bigg_ids': ['spmd_e']}
dca_x {'original_bigg_ids': ['dca_x']}
sertrna_c {'original_bigg_ids': ['sertrna_c']}
trnaser_c {'original_bigg_ids': ['trnaser_c']}
s7p_c {'original_bigg_ids': ['s7p_c']}
ptcys_c {'original_bigg_ids': ['4pcys_c']}
epist_c {'original_bigg_ids': ['epist_c']}
icit_m {'original_bigg_ids': ['icit_m']}
13BDglcn_c {'original_bigg_ids': ['13BDglcn_c']}
dscl_c {'original_bigg_ids': ['dscl_c']}
scl_c {'original_bigg_ids': ['scl_c']}
gal1p_c {'original_bigg_ids': ['gal1p_c']}
thm_c {'original_bigg_ids': ['thm_c']}
glyclt_x {'original_bigg_ids': ['glyclt_x']}
hdcea_c {'original_bigg_ids': ['hdcea_c']}
ocdcea_c {'original_bigg_ids': ['ocdcea_c']}
thr__L_c {'original_bigg_ids': ['thr_L_c']}
thr__L_e {'original_bigg_ids': ['thr_L_e']}
etfox_m {'original_bigg_ids': ['etfox_m']}
etfrd_m {'original_bigg_ids': ['etfrd_m']}
nicrns_c {'original_bigg_ids': ['nicrns_c']}
dpcoa_c {'original_bigg_ids': ['dpcoa_c']}
2cpr5p_c {'original_bigg_ids': ['2cpr5p_c']}
3ig3p_c {'original_bigg_ids': ['3ig3p_c']}
lys__L_c {'original_bigg_ids': ['lys_L_c']}
lys__L_v {'original_bigg_ids': ['lys_L_v']}
5mthf_c {'original_bigg_ids': ['5mthf_c']}
hcys__L_c {'original_bigg_ids': ['hcys__L_c']}
cdpdag_RT_m {'original_bigg_ids': ['cdpdag_SC_m']}
pgp_RT_m {'original_bigg_ids': ['pgp_SC_m']}
histd_c {'original_bigg_ids': ['histd_c']}
hdcea_x {'original_bigg_ids': ['hdcea_x']}
ibutac_c {'original_bigg_ids': ['ibutac_c']}
3otdcoa_x {'original_bigg_ids': ['3otdcoa_x']}
xmp_c {'original_bigg_ids': ['xmp_c']}
4adcho_c {'original_bigg_ids': ['4adcho_c']}
chor_c {'original_bigg_ids': ['chor_c']}
indpyr_c {'original_bigg_ids': ['indpyr_c']}
pan4p_x {'original_bigg_ids': ['pan4p_x']}
pap_x {'original_bigg_ids': ['pap_x']}
acorn_m {'original_bigg_ids': ['acorn_m']}
val__L_c {'original_bigg_ids': ['val_L_c']}
val__L_e {'original_bigg_ids': ['val_L_e']}
ergst_r {'original_bigg_ids': ['ergst_r']}
psd5p_c {'original_bigg_ids': ['psd5p_c']}
alac__S_m {'original_bigg_ids': ['alac_S_m']}
pyr_m {'original_bigg_ids': ['pyr_m']}
nh4_e {'original_bigg_ids': ['nh4_e']}
1p3h5c_m {'original_bigg_ids': ['1p3h5c_m']}
4hpro_LT_m {'original_bigg_ids': ['4hpro_LT_m']}
glucys_c {'original_bigg_ids': ['glucys_c']}
alatrna_c {'original_bigg_ids': ['alatrna_c']}
trnaala_c {'original_bigg_ids': ['trnaala_c']}
glntrna_c {'original_bigg_ids': ['glntrna_c']}
trnagln_c {'original_bigg_ids': ['trnagln_c']}
L2aadp6sa_c {'original_bigg_ids': ['L2aadp6sa_c']}
citr__L_c {'original_bigg_ids': ['citr__L_c']}
ade_e {'original_bigg_ids': ['ade_e']}
fecost_e {'original_bigg_ids': ['fecost_e']}
tre_c {'original_bigg_ids': ['tre_c']}
glu__L_e {'original_bigg_ids': ['glu_L_e']}
2dda7p_m {'original_bigg_ids': ['2dda7p_m']}
e4p_m {'original_bigg_ids': ['e4p_m']}
14glun_c {'original_bigg_ids': ['14glun_c']}
1p3h5c_c {'original_bigg_ids': ['1p3h5c_c']}
4hpro_LT_c {'original_bigg_ids': ['4hpro_LT_c']}
4mpetz_c {'original_bigg_ids': ['4mpetz_c']}
thmmp_c {'original_bigg_ids': ['thmmp_c']}
fdp_c {'original_bigg_ids': ['fdp_c']}
argsuc_c {'original_bigg_ids': ['argsuc_c']}
2dda7p_c {'original_bigg_ids': ['2dda7p_c']}
leu__L_e {'original_bigg_ids': ['leu_L_e']}
3psme_c {'original_bigg_ids': ['3psme_c']}
skm3p_c {'original_bigg_ids': ['skm5p_c']}
4abut_e {'original_bigg_ids': ['4abut_e']}
3htdcoa_x {'original_bigg_ids': ['3htdcoa_x']}
td2coa_x {'original_bigg_ids': ['td2coa_x']}
3hddcoa_x {'original_bigg_ids': ['3hddcoa_x']}
25aics_c {'original_bigg_ids': ['25aics_c']}
5aizc_c {'original_bigg_ids': ['5aizc_c']}
pram_c {'original_bigg_ids': ['pram_c']}
glu5p_c {'original_bigg_ids': ['glu5p_c']}
glu5sa_c {'original_bigg_ids': ['glu5sa_c']}
5mdr1p_c {'original_bigg_ids': ['5mdr1p_c']}
5mdru1p_c {'original_bigg_ids': ['5mdru1p_c']}
his__L_e {'original_bigg_ids': ['his_L_e']}
2aobut_c {'original_bigg_ids': ['2aobut_c']}
athr__L_c {'original_bigg_ids': ['athr__L_c']}
dolp_c {'original_bigg_ids': ['dolp_c']}
apep_c {'original_bigg_ids': ['apep_c']}
pepd_c {'original_bigg_ids': ['pepd_c']}
mercppyr_c {'original_bigg_ids': ['mercppyr_c']}
fald_c {'original_bigg_ids': ['fald_c']}
pro__L_e {'original_bigg_ids': ['pro_L_e']}
ibcoa_m {'original_bigg_ids': ['ibcoa_m']}
25dhpp_c {'original_bigg_ids': ['25dhpp_c']}
m2macchitppdol_g {'original_bigg_ids': ['m2macchitppdol_g']}
air_c {'original_bigg_ids': ['air_c']}
glyc_c {'original_bigg_ids': ['glyc_c']}
gthox_m {'original_bigg_ids': ['gthox_m']}
gthrd_m {'original_bigg_ids': ['gthrd_m']}
dimp_c {'original_bigg_ids': ['dimp_c']}
dad_2_c {'original_bigg_ids': ['dad_2_c']}
frdp_m {'original_bigg_ids': ['frdp_m']}
hemeO_m {'original_bigg_ids': ['hemeO_m']}
pheme_m {'original_bigg_ids': ['pheme_m']}
ptd1ino_RT_n {'original_bigg_ids': ['ptd1ino_SC_n']}
ptd4ino_RT_n {'original_bigg_ids': ['ptd4ino_SC_n']}
ala__L_e {'original_bigg_ids': ['ala_L_e']}
odecoa_x {'original_bigg_ids': ['odecoa_x']}
csn_c {'original_bigg_ids': ['csn_c']}
csn_e {'original_bigg_ids': ['csn_e']}
gln__L_v {'original_bigg_ids': ['gln_L_v']}
23dhmb_m {'original_bigg_ids': ['23dhmb_m']}
ump_n {'original_bigg_ids': ['ump_n']}
mi13456p_n {'original_bigg_ids': ['mi13456p_n']}
mi1345p_n {'original_bigg_ids': ['mi1345p_n']}
argtrna_c {'original_bigg_ids': ['argtrna_c']}
trnaarg_c {'original_bigg_ids': ['trnaarg_c']}
34hpp_x {'original_bigg_ids': ['34hpp_x']}
tyr__L_x {'original_bigg_ids': ['tyr_L_x']}
f1p_c {'original_bigg_ids': ['f1p_c']}
glyald_c {'original_bigg_ids': ['glyald_c']}
asn__L_m {'original_bigg_ids': ['asn_L_m']}
asntrna_m {'original_bigg_ids': ['asntrna_m']}
trnaasn_m {'original_bigg_ids': ['trnaasn_m']}
asp__L_v {'original_bigg_ids': ['asp_L_v']}
adprib_c {'original_bigg_ids': ['adprib_c']}
fpram_c {'original_bigg_ids': ['fpram_c']}
ind3acnl_c {'original_bigg_ids': ['ind3acnl_c']}
phe__L_e {'original_bigg_ids': ['phe_L_e']}
3ohxccoa_x {'original_bigg_ids': ['3ohxccoa_x']}
ttccoa_x {'original_bigg_ids': ['ttccoa_x']}
fum_m {'original_bigg_ids': ['fum_m']}
ggdp_c {'original_bigg_ids': ['ggdp_c']}
pnto__R_e {'original_bigg_ids': ['pnto_R_e']}
pant__R_c {'original_bigg_ids': ['pant__R_c']}
hmgth_c {'original_bigg_ids': ['hmgth_c']}
lys__L_e {'original_bigg_ids': ['lys_L_e']}
glyc_e {'original_bigg_ids': ['glyc_e']}
ametam_c {'original_bigg_ids': ['ametam_c']}
tyr__L_e {'original_bigg_ids': ['tyr_L_e']}
2mbtoh_c {'original_bigg_ids': ['2mbtoh_c']}
Dara14lac_c {'original_bigg_ids': ['Dara14lac_c']}
ertascb__D_c {'original_bigg_ids': ['ertascb_D_c']}
uppg3_c {'original_bigg_ids': ['uppg3_c']}
2amsa_c {'original_bigg_ids': ['2amsa_c']}
4mhetz_c {'original_bigg_ids': ['4mhetz_c']}
hmbil_c {'original_bigg_ids': ['hmbil_c']}
xylt_c {'original_bigg_ids': ['xylt_c']}
xylu__D_c {'original_bigg_ids': ['xylu_D_c']}
trnaval_c {'original_bigg_ids': ['trnaval_c']}
valtrna_c {'original_bigg_ids': ['valtrna_c']}
pydx5p_c {'original_bigg_ids': ['pydx5p_c']}
udpacgal_c {'original_bigg_ids': ['udpacgal_c']}
epist_e {'original_bigg_ids': ['epist_e']}
man_e {'original_bigg_ids': ['man_e']}
2maacoa_m {'original_bigg_ids': ['2maacoa_m']}
clpn_RT_m {'original_bigg_ids': ['clpn_SC_m']}
pg_RT_m {'original_bigg_ids': ['pg_SC_m']}
2dglc_c {'original_bigg_ids': ['2dglc_c']}
2doxg6p_c {'original_bigg_ids': ['2doxg6p_c']}
hacon_C_m {'original_bigg_ids': ['b124tc_m']}
ser__L_m {'original_bigg_ids': ['ser_L_m']}
4abz_c {'original_bigg_ids': ['4abz_c']}
msa_m {'original_bigg_ids': ['msa_m']}
glcn_c {'original_bigg_ids': ['glcn_c']}
Ssq23epx_r {'original_bigg_ids': ['Ssq23epx_r']}
ac_m {'original_bigg_ids': ['ac_m']}
chol_e {'original_bigg_ids': ['chol_e']}
ade_m {'original_bigg_ids': ['ade_m']}
adn_m {'original_bigg_ids': ['adn_m']}
iad_c {'original_bigg_ids': ['iad_c']}
fmettrna_m {'original_bigg_ids': ['fmettrna_m']}
leu__L_v {'original_bigg_ids': ['leu_L_v']}
mi4p__D_c {'original_bigg_ids': ['mi4p_DASH_D_c']}
fmn_m {'original_bigg_ids': ['fmn_m']}
aces_c {'original_bigg_ids': ['aces_c']}
35cdamp_c {'original_bigg_ids': ['35cdamp_c']}
gua_e {'original_bigg_ids': ['gua_e']}
glu__L_v {'original_bigg_ids': ['glu_L_v']}
saccrp__L_c {'original_bigg_ids': ['saccrp_L_c']}
ipdp_m {'original_bigg_ids': ['ipdp_m']}
2ippm_c {'original_bigg_ids': ['2ippm_c']}
3c3hmp_c {'original_bigg_ids': ['3c3hmp_c']}
g3pi_e {'original_bigg_ids': ['g3pi_e']}
ptd1ino_RT_e {'original_bigg_ids': ['ptd1ino_SC_e']}
ptth_c {'original_bigg_ids': ['ptth_c']}
ctp_m {'original_bigg_ids': ['ctp_m']}
xan_c {'original_bigg_ids': ['xan_c']}
xtsn_c {'original_bigg_ids': ['xtsn_c']}
arg__L_m {'original_bigg_ids': ['arg_L_m']}
argtrna_m {'original_bigg_ids': ['argtrna_m']}
trnaarg_m {'original_bigg_ids': ['trnaarg_m']}
ppa_c {'original_bigg_ids': ['ppa_c']}
orn_e {'original_bigg_ids': ['orn_e']}
2obut_c {'original_bigg_ids': ['2obut_c']}
3hdcoa_m {'original_bigg_ids': ['3hdcoa_m']}
his__L_m {'original_bigg_ids': ['his_L_m']}
histrna_m {'original_bigg_ids': ['histrna_m']}
trnahis_m {'original_bigg_ids': ['trnahis_m']}
g3pc_c {'original_bigg_ids': ['g3pc_c']}
asp__L_x {'original_bigg_ids': ['asp_L_x']}
s17bp_c {'original_bigg_ids': ['s17bp_c']}
protrna_c {'original_bigg_ids': ['protrna_c']}
trnapro_c {'original_bigg_ids': ['trnapro_c']}
od2coa_x {'original_bigg_ids': ['od2coa_x']}
nmn_c {'original_bigg_ids': ['nmn_c']}
ind3eth_c {'original_bigg_ids': ['ind3eth_c']}
sheme_c {'original_bigg_ids': ['sheme_c']}
dgsn_c {'original_bigg_ids': ['dgsn_c']}
ap4g_c {'original_bigg_ids': ['ap4g_c']}
dump_n {'original_bigg_ids': ['dump_n']}
gal_e {'original_bigg_ids': ['gal_e']}
trnaval_m {'original_bigg_ids': ['trnaval_m']}
valtrna_m {'original_bigg_ids': ['valtrna_m']}
alltn_c {'original_bigg_ids': ['alltn_c']}
h2o2_n {'original_bigg_ids': ['h2o2_n']}
xtp_c {'original_bigg_ids': ['xtp_c']}
cysi__L_c {'original_bigg_ids': ['Lcystin_c']}
cysi__L_v {'original_bigg_ids': ['Lcystin_v']}
g3pi_c {'original_bigg_ids': ['g3pi_c']}
acetol_c {'original_bigg_ids': ['acetol_c']}
lac__D_e {'original_bigg_ids': ['lac_D_e']}
gly_e {'original_bigg_ids': ['gly_e']}
tchola_c {'original_bigg_ids': ['tchola_c']}
tchola_v {'original_bigg_ids': ['tchola_v']}
lystrna_c {'original_bigg_ids': ['lystrna_c']}
trnalys_c {'original_bigg_ids': ['trnalys_c']}
3mgcoa_m {'original_bigg_ids': ['3mgcoa_m']}
4hglusa_m {'original_bigg_ids': ['4hglusa_m']}
e4hglu_m {'original_bigg_ids': ['e4hglu_m']}
ttc_x {'original_bigg_ids': ['ttc_x']}
mi3p__D_c {'original_bigg_ids': ['mi3p_DASH_D_c']}
cys__L_m {'original_bigg_ids': ['cys_DASH_L_m']}
mercppyr_m {'original_bigg_ids': ['mercppyr_m']}
ile__L_e {'original_bigg_ids': ['ile_L_e']}
gln__L_e {'original_bigg_ids': ['gln_L_e']}
pa_RT_m {'original_bigg_ids': ['pa_SC_m']}
din_c {'original_bigg_ids': ['din_c']}
cer2_24_r {'original_bigg_ids': ['cer2_24_r']}
ttccoa_r {'original_bigg_ids': ['ttccoa_r']}
acon5m_c {'original_bigg_ids': ['acon5m_c']}
acon_T_c {'original_bigg_ids': ['acon_T_c']}
3hodcoa_x {'original_bigg_ids': ['3hodcoa_x']}
3ohodcoa_x {'original_bigg_ids': ['3ohodcoa_x']}
cyan_c {'original_bigg_ids': ['cyan_c']}
tcynt_c {'original_bigg_ids': ['tcynt_c']}
asn__L_e {'original_bigg_ids': ['asn_L_e']}
C04051_c {'original_bigg_ids': ['C04051_c']}
acg5sa_m {'original_bigg_ids': ['acg5sa_m']}
1mncam_c {'original_bigg_ids': ['1mncam_c']}
3hxccoa_x {'original_bigg_ids': ['3hxccoa_x']}
dadp_n {'original_bigg_ids': ['dadp_n']}
sbt__L_c {'original_bigg_ids': ['sbt_L_c']}
srb__L_c {'original_bigg_ids': ['srb_L_c']}
psph1p_r {'original_bigg_ids': ['psph1p_r']}
dhpmp_c {'original_bigg_ids': ['dhpmp_c']}
orot5p_c {'original_bigg_ids': ['orot5p_c']}
ribflv_m {'original_bigg_ids': ['ribflv_m']}
nac_e {'original_bigg_ids': ['nac_e']}
im4ac_c {'original_bigg_ids': ['im4ac_c']}
im4act_c {'original_bigg_ids': ['im4act_c']}
3hbcoa_m {'original_bigg_ids': ['3hbcoa_m']}
b2coa_m {'original_bigg_ids': ['b2coa_m']}
thrtrna_c {'original_bigg_ids': ['thrtrna_c']}
trnathr_c {'original_bigg_ids': ['trnathr_c']}
cmusa_c {'original_bigg_ids': ['cmusa_c']}
ser__L_e {'original_bigg_ids': ['ser_L_e']}
malt_e {'original_bigg_ids': ['malt_e']}
sprm_c {'original_bigg_ids': ['sprm_c']}
ala__L_m {'original_bigg_ids': ['ala_L_m']}
pe_RT_m {'original_bigg_ids': ['pe_SC_m']}
ps_RT_m {'original_bigg_ids': ['ps_SC_m']}
sprm_e {'original_bigg_ids': ['sprm_e']}
galur_e {'original_bigg_ids': ['galur_e']}
pectin_e {'original_bigg_ids': ['pectin_e']}
4ahmmp_c {'original_bigg_ids': ['4ahmmp_c']}
hxc2coa_x {'original_bigg_ids': ['hxc2coa_x']}
glutrna_m {'original_bigg_ids': ['glutrna_m']}
trnaglu_m {'original_bigg_ids': ['trnaglu_m']}
so3_e {'original_bigg_ids': ['so3_e']}
mma_c {'original_bigg_ids': ['mma_c']}
nmn_m {'original_bigg_ids': ['nmn_m']}
5mta_c {'original_bigg_ids': ['5mta_c']}
asp__L_m {'original_bigg_ids': ['asp_DASH_L_m']}
gp4g_c {'original_bigg_ids': ['gp4g_c']}
cer2_26_r {'original_bigg_ids': ['cer2_26_r']}
alltn_e {'original_bigg_ids': ['alltn_e']}
dcyt_c {'original_bigg_ids': ['dcyt_c']}
oh1_c {'original_bigg_ids': ['oh1_c']}
asp__L_e {'original_bigg_ids': ['asp_L_e']}
4abut_m {'original_bigg_ids': ['4abut_m']}
pran_c {'original_bigg_ids': ['pran_c']}
iamac_c {'original_bigg_ids': ['iamac_c']}
hemeA_m {'original_bigg_ids': ['hemeA_m']}
ocdca_x {'original_bigg_ids': ['ocdca_x']}
dgdp_n {'original_bigg_ids': ['dgdp_n']}
gdp_n {'original_bigg_ids': ['gdp_n']}
micit_m {'original_bigg_ids': ['micit_m']}
alltt_e {'original_bigg_ids': ['alltt_e']}
35cimp_c {'original_bigg_ids': ['35cimp_c']}
N1sprm_c {'original_bigg_ids': ['N1sprm_c']}
NPmehis_c {'original_bigg_ids': ['NPmehis_c']}
3hpcoa_m {'original_bigg_ids': ['3hpcoa_m']}
ribflv_c {'original_bigg_ids': ['ribflv_c']}
lnlncg_c {'original_bigg_ids': ['lnlncg_c']}
lnlncgcoa_c {'original_bigg_ids': ['lnlncgcoa_c']}
lac__L_e {'original_bigg_ids': ['lac_L_e']}
3spyr_m {'original_bigg_ids': ['3spyr_m']}
Lcyst_m {'original_bigg_ids': ['Lcyst_m']}
cer1_24_r {'original_bigg_ids': ['cer1_24_r']}
ump_m {'original_bigg_ids': ['ump_m']}
utp_m {'original_bigg_ids': ['utp_m']}
4mlacac_c {'original_bigg_ids': ['4mlacac_c']}
hgentis_c {'original_bigg_ids': ['hgentis_c']}
dnad_m {'original_bigg_ids': ['dnad_m']}
3hhdcoa_m {'original_bigg_ids': ['3hhdcoa_m']}
asntrna_c {'original_bigg_ids': ['asntrna_c']}
trnaasn_c {'original_bigg_ids': ['trnaasn_c']}
idp_m {'original_bigg_ids': ['idp_m']}
itp_m {'original_bigg_ids': ['itp_m']}
trp__L_e {'original_bigg_ids': ['trp_L_e']}
trdox_x {'original_bigg_ids': ['trdox_x']}
trdrd_x {'original_bigg_ids': ['trdrd_x']}
pepd_e {'original_bigg_ids': ['pepd_e']}
so4_e {'original_bigg_ids': ['so4_e']}
for_m {'original_bigg_ids': ['for_m']}
his__L_v {'original_bigg_ids': ['his_L_v']}
4fumacac_c {'original_bigg_ids': ['4fumacac_c']}
asnglcnacglcnacman_man_manman_man_manmanman_c {'original_bigg_ids': ['asnglcnacglcnacman_DASH_LPAREN_DASH_man_DASH_LPAREN_DASH_manman_DASH_RPAREN_DASH_man_DASH_RPAREN_DASH_manmanman_c']}
asnglcnacglcnacman_man_manman_manman_manmanman_c {'original_bigg_ids': ['asnglcnacglcnacman_DASH_LPAREN_DASH_man_DASH_LPAREN_DASH_manman_DASH_RPAREN_DASH_manman_DASH_RPAREN_DASH_manmanman_c']}
metsox_R__L_c {'original_bigg_ids': ['metsox_R__L_c']}
pe1801829Z12Z_c {'original_bigg_ids': ['pe1801829Z12Z_c']}
chols_c {'original_bigg_ids': ['chols_c']}
g3pe_c {'original_bigg_ids': ['g3pe_c']}
no3_c {'original_bigg_ids': ['no3_c']}
no3_e {'original_bigg_ids': ['no3_e']}
uacmam_c {'original_bigg_ids': ['uacmam_c']}
uacmamu_c {'original_bigg_ids': ['uacmamu_c']}
sl26da_c {'original_bigg_ids': ['sl26da_c']}
sl2a6o_c {'original_bigg_ids': ['sl2a6o_c']}
dadp_m {'original_bigg_ids': ['dadp_m']}
dolpglc_c {'original_bigg_ids': ['dolpglc_c']}
gdptp_c {'original_bigg_ids': ['gdptp_c']}
vanln_c {'original_bigg_ids': ['vanln_c']}
vanlt_c {'original_bigg_ids': ['vanlt_c']}
malthx_c {'original_bigg_ids': ['malthx_c']}
camp_n {'original_bigg_ids': ['camp_n']}
grxox_c {'original_bigg_ids': ['grxox_c']}
grxrd_c {'original_bigg_ids': ['grxrd_c']}
Glc_aD_c {'original_bigg_ids': ['glc_DASH_A_c']}
pe1801819Z_c {'original_bigg_ids': ['pe1801819Z_c']}
udpLa4o_c {'original_bigg_ids': ['udpLa4o_c']}
12dgr1819Z1819Z_c {'original_bigg_ids': ['12dgr1819Z1819Z_c']}
tgua_c {'original_bigg_ids': ['tgua_c']}
tgua_e {'original_bigg_ids': ['tgua_e']}
hcys__L_m {'original_bigg_ids': ['hcys_DASH_L_m']}
3hocoa_m {'original_bigg_ids': ['3hocoa_m']}
3oocoa_m {'original_bigg_ids': ['3oocoa_m']}
k_c {'original_bigg_ids': ['k_c']}
fol_c {'original_bigg_ids': ['fol_c']}
2dhguln_c {'original_bigg_ids': ['2dhguln_c']}
doldpglcnacglcnacman_manman_manmanman_c {'original_bigg_ids': ['doldpglcnacglcnacman_DASH_LPAREN_DASH_manman_DASH_RPAREN_DASH_manmanman_c']}
doldpglcnacglcnacman_manmanman_manmanman_c {'original_bigg_ids': ['doldpglcnacglcnacman_DASH_LPAREN_DASH_manmanman_DASH_RPAREN_DASH_manmanman_c']}
12dgr1601819Z_c {'original_bigg_ids': ['12dgr1601819Z_c']}
nadhx__S_c {'original_bigg_ids': ['nadhx__S_c']}
na1_m {'original_bigg_ids': ['na1_m']}
man6pglyc_c {'original_bigg_ids': ['man6pglyc_c']}
udpLa4fn_c {'original_bigg_ids': ['udpLa4fn_c']}
udpLa4n_c {'original_bigg_ids': ['udpLa4n_c']}
doldpglcnac_c {'original_bigg_ids': ['doldpglcnac_c']}
doldpglcnacglcnac_c {'original_bigg_ids': ['doldpglcnacglcnac_c']}
lystrna_m {'original_bigg_ids': ['lystrna_m']}
trnalys_m {'original_bigg_ids': ['trnalys_m']}
dghs1819Z1819Z_c {'original_bigg_ids': ['dghs1819Z1819Z_c']}
pi_n {'original_bigg_ids': ['pi_n']}
datp_m {'original_bigg_ids': ['datp_m']}
1btol_c {'original_bigg_ids': ['1btol_c']}
btal_c {'original_bigg_ids': ['btal_c']}
no2_c {'original_bigg_ids': ['no2_c']}
no2_e {'original_bigg_ids': ['no2_e']}
dtgcl_c {'original_bigg_ids': ['dtgcl_c']}
mercpeth_c {'original_bigg_ids': ['mercpeth_c']}
3amac_c {'original_bigg_ids': ['3amac_c']}
pe1819Z1829Z12Z_c {'original_bigg_ids': ['pe1819Z1829Z12Z_c']}
hista_c {'original_bigg_ids': ['hista_c']}
malcoame_c {'original_bigg_ids': ['malcoame_c']}
acanth_c {'original_bigg_ids': ['acanth_c']}
ppal_c {'original_bigg_ids': ['ppal_c']}
maltpt_c {'original_bigg_ids': ['maltpt_c']}
acac_x {'original_bigg_ids': ['acac_x']}
spmd_m {'original_bigg_ids': ['spmd_m']}
actp_m {'original_bigg_ids': ['actp_m']}
ggptrc_c {'original_bigg_ids': ['ggptrc_c']}
apppa_n {'original_bigg_ids': ['apppa_n']}
ans_c {'original_bigg_ids': ['ans_c']}
dna5mtc_c {'original_bigg_ids': ['dna5mtc_c']}
dna_c {'original_bigg_ids': ['dna_c']}
cholate_c {'original_bigg_ids': ['cholate_c']}
dhcholn_c {'original_bigg_ids': ['dhcholn_c']}
pppi_c {'original_bigg_ids': ['pppi_c']}
56dh5flura_c {'original_bigg_ids': ['56dh5flura_c']}
aflburppa_c {'original_bigg_ids': ['aflburppa_c']}
asptrna_m {'original_bigg_ids': ['asptrna_m']}
trnaasp_m {'original_bigg_ids': ['trnaasp_m']}
25dkglcn_c {'original_bigg_ids': ['25dkglcn_c']}
pgp1819Z160_c {'original_bigg_ids': ['pgp1819Z160_c']}
pgp1819Z160_e {'original_bigg_ids': ['pgp1819Z160_e']}
13BDglcn_e {'original_bigg_ids': ['13BDglcn_e']}
34dhbald_c {'original_bigg_ids': ['34dhbald_c']}
34dhbz_c {'original_bigg_ids': ['34dhbz_c']}
3hcinnm_c {'original_bigg_ids': ['3hcinnm_c']}
dhcinnm_c {'original_bigg_ids': ['dhcinnm_c']}
imp_m {'original_bigg_ids': ['imp_m']}
xmp_m {'original_bigg_ids': ['xmp_m']}
6hmhptpp_c {'original_bigg_ids': ['6hmhptpp_c']}
ala__D_c {'original_bigg_ids': ['ala_DASH_D_c']}
cpppg1_c {'original_bigg_ids': ['cpppg1_c']}
uppg1_c {'original_bigg_ids': ['uppg1_c']}
dhcur_c {'original_bigg_ids': ['dhcur_c']}
thcur_c {'original_bigg_ids': ['thcur_c']}
apocytc_m {'original_bigg_ids': ['apocytc_m']}
cytc_m {'original_bigg_ids': ['cytc_m']}
frmd_c {'original_bigg_ids': ['frmd_c']}
dcacoa_m {'original_bigg_ids': ['dcacoa_m']}
urate_m {'original_bigg_ids': ['urate_m']}
lkdr_c {'original_bigg_ids': ['lkdr_c']}
thmmp_e {'original_bigg_ids': ['thmmp_e']}
fad_x {'original_bigg_ids': ['fad_x']}
fadh2_x {'original_bigg_ids': ['fadh2_x']}
cu_c {'original_bigg_ids': ['cu_c']}
dtdp4d6dm_c {'original_bigg_ids': ['dtdp4d6dm_c']}
dtdprmn_c {'original_bigg_ids': ['dtdprmn_c']}
asnglcnacglcnacman_man_manman_manman_manmanmanglc_c {'original_bigg_ids': ['asnglcnacglcnacman_DASH_LPAREN_DASH_man_DASH_LPAREN_DASH_manman_DASH_RPAREN_DASH_manman_DASH_RPAREN_DASH_manmanmanglc_c']}
ppgpp_c {'original_bigg_ids': ['ppgpp_c']}
gdpfuc_c {'original_bigg_ids': ['gdpfuc_c']}
gdpofuc_c {'original_bigg_ids': ['gdpofuc_c']}
oxa_c {'original_bigg_ids': ['oxa_c']}
oxalcoa_c {'original_bigg_ids': ['oxalcoa_c']}
thrtrna_m {'original_bigg_ids': ['thrtrna_m']}
trnathr_m {'original_bigg_ids': ['trnathr_m']}
pe1819Z1819Z_c {'original_bigg_ids': ['pe1819Z1819Z_c']}
pe1819Z1819Z_e {'original_bigg_ids': ['pe1819Z1819Z_e']}
pail1819Z160_c {'original_bigg_ids': ['pail1819Z160_c']}
pail1819Z160_e {'original_bigg_ids': ['pail1819Z160_e']}
5phdt_c {'original_bigg_ids': ['5phdt_c']}
5phua_c {'original_bigg_ids': ['5phua_c']}
dghs1601819Z_c {'original_bigg_ids': ['dghs1601819Z_c']}
dgts1601819Z_c {'original_bigg_ids': ['dgts1601819Z_c']}
gmplys_c {'original_bigg_ids': ['gmplys_c']}
nalme_c {'original_bigg_ids': ['nalme_c']}
ad_c {'original_bigg_ids': ['ad_c']}
cu2_c {'original_bigg_ids': ['cu2_c']}
mobd_c {'original_bigg_ids': ['mobd_c']}
moco_c {'original_bigg_ids': ['moco_c']}
mptamp_c {'original_bigg_ids': ['mptamp_c']}
6mpur_c {'original_bigg_ids': ['6mpur_c']}
6tins5mp_c {'original_bigg_ids': ['6tins5mp_c']}
cl_c {'original_bigg_ids': ['cl_c']}
hdd2coa_c {'original_bigg_ids': ['hdd2coa_c']}
xyl__D_c {'original_bigg_ids': ['xyl__D_c']}
Ncbmpts_m {'original_bigg_ids': ['cbmp_m']}
ptrc_m {'original_bigg_ids': ['ptrc_m']}
10fthf_x {'original_bigg_ids': ['10fthf_x']}
methf_x {'original_bigg_ids': ['methf_x']}
acg5sa_c {'original_bigg_ids': ['acg5sa_c']}
acorn_c {'original_bigg_ids': ['acorn_c']}
doldpglcnacglcnacman_c {'original_bigg_ids': ['doldpglcnacglcnacman_c']}
5dglcn_c {'original_bigg_ids': ['5dglcn_c']}
hxcoa_c {'original_bigg_ids': ['hxcoa_c']}
asnglcnacglcnacman_man_man_man_man_c {'original_bigg_ids': ['asnglcnacglcnacman_DASH_LPAREN_DASH_man_DASH_LPAREN_DASH_man_DASH_RPAREN_DASH_man_DASH_RPAREN_DASH_man_c']}
oc2coa_x {'original_bigg_ids': ['oc2coa_x']}
4cml_c {'original_bigg_ids': ['4cml_c']}
CCbuttc_c {'original_bigg_ids': ['CCbuttc_c']}
indole_c {'original_bigg_ids': ['indole_c']}
26dap_LL_c {'original_bigg_ids': ['26dap_LL_c']}
dgdp_m {'original_bigg_ids': ['dgdp_m']}
dgtp_m {'original_bigg_ids': ['dgtp_m']}
acglc__D_c {'original_bigg_ids': ['acglc__D_c']}
actp_c {'original_bigg_ids': ['actp_c']}
3hhcoa_m {'original_bigg_ids': ['3hhcoa_m']}
hx2coa_m {'original_bigg_ids': ['hx2coa_m']}
mag160_c {'original_bigg_ids': ['mag160_c']}
pg1819Z160_c {'original_bigg_ids': ['pg1819Z160_c']}
pg1819Z160_e {'original_bigg_ids': ['pg1819Z160_e']}
5aop_e {'original_bigg_ids': ['5aop_e']}
6txan5mp_c {'original_bigg_ids': ['6txan5mp_c']}
bhb_m {'original_bigg_ids': ['bhb_m']}
btcoa_x {'original_bigg_ids': ['btcoa_x']}
cynt_c {'original_bigg_ids': ['cynt_c']}
ara5p_c {'original_bigg_ids': ['ara5p_c']}
conialdh_c {'original_bigg_ids': ['conialdh_c']}
fer_c {'original_bigg_ids': ['fer_c']}
5flura_c {'original_bigg_ids': ['5flura_c']}
5flura_e {'original_bigg_ids': ['5flura_e']}
od2coa_c {'original_bigg_ids': ['od2coa_c']}
g3ps_c {'original_bigg_ids': ['g3ps_c']}
ppa_m {'original_bigg_ids': ['ppa_m']}
progly_c {'original_bigg_ids': ['progly_c']}
3hocoa_x {'original_bigg_ids': ['3hocoa_x']}
3oocoa_x {'original_bigg_ids': ['3oocoa_x']}
bwco_c {'original_bigg_ids': ['bwco_c']}
wco_c {'original_bigg_ids': ['wco_c']}
thmpp_e {'original_bigg_ids': ['thmpp_e']}
h2co3_c {'original_bigg_ids': ['h2co3_c']}
acglu_c {'original_bigg_ids': ['acglu_c']}
udpgalur_c {'original_bigg_ids': ['udpgalur_c']}
ser__D_c {'original_bigg_ids': ['ser__D_c']}
fc1p_c {'original_bigg_ids': ['fc1p_c']}
ttc_ggdp_c {'original_bigg_ids': ['ttc_DASH_ggdp_c']}
gln__L_m {'original_bigg_ids': ['gln_DASH_L_m']}
1acpc_c {'original_bigg_ids': ['1acpc_c']}
4hbz_c {'original_bigg_ids': ['4hbz_c']}
gcald_c {'original_bigg_ids': ['gcald_c']}
apoACP_c {'original_bigg_ids': ['apoACP_c']}
hxcoa_m {'original_bigg_ids': ['hxcoa_m']}
3hadpcoa_c {'original_bigg_ids': ['3hadpcoa_c']}
acmum6p_c {'original_bigg_ids': ['acmum6p_c']}
anhm_c {'original_bigg_ids': ['anhm_c']}
nadhx__R_c {'original_bigg_ids': ['nadhx__R_c']}
for_x {'original_bigg_ids': ['for_x']}
frmd_x {'original_bigg_ids': ['frmd_x']}
asnglcnacglcnacman_man_man_c {'original_bigg_ids': ['asnglcnacglcnacman_DASH_LPAREN_DASH_man_DASH_RPAREN_DASH_man_c']}
asnglcnacglcnacman_man_manman_manman_manmanmanglcglc_c {'original_bigg_ids': ['asnglcnacglcnacman_DASH_LPAREN_DASH_man_DASH_LPAREN_DASH_manman_DASH_RPAREN_DASH_manman_DASH_RPAREN_DASH_manmanmanglcglc_c']}
asnglcnacglcnacman_man_manman_manman_manmanmanglcglcglc_c {'original_bigg_ids': ['asnglcnacglcnacman_DASH_LPAREN_DASH_man_DASH_LPAREN_DASH_manman_DASH_RPAREN_DASH_manman_DASH_RPAREN_DASH_manmanmanglcglcglc_c']}
dgts1819Z1819Z_c {'original_bigg_ids': ['dgts1819Z1819Z_c']}
cur_c {'original_bigg_ids': ['cur_c']}
56dura_c {'original_bigg_ids': ['56dura_c']}
cala_c {'original_bigg_ids': ['cala_c']}
acmalt_c {'original_bigg_ids': ['acmalt_c']}
erthrs_c {'original_bigg_ids': ['erthrs_c']}
urate_e {'original_bigg_ids': ['urate_e']}
mag180_c {'original_bigg_ids': ['mag180_c']}
6mpur_e {'original_bigg_ids': ['6mpur_e']}
rbl__D_c {'original_bigg_ids': ['rbl__D_c']}
r2hglut_c {'original_bigg_ids': ['r2hglut_c']}
3sala_m {'original_bigg_ids': ['3sala_m']}
3snpyr_m {'original_bigg_ids': ['3snpyr_m']}
minohp_n {'original_bigg_ids': ['minohp_n']}
hxa_c {'original_bigg_ids': ['hxa_c']}
3oxoadp_c {'original_bigg_ids': ['3oxoadp_c']}
5odhf2a_c {'original_bigg_ids': ['5odhf2a_c']}
nmn_n {'original_bigg_ids': ['nmn_n']}
g3pg_c {'original_bigg_ids': ['g3pg_c']}
dutp_m {'original_bigg_ids': ['dutp_m']}
3hhcoa_x {'original_bigg_ids': ['3hhcoa_x']}
hx2coa_x {'original_bigg_ids': ['hx2coa_x']}
maltttr_c {'original_bigg_ids': ['maltttr_c']}
5hiu_m {'original_bigg_ids': ['5hiu_m']}
Asn_X_Ser_Thr_c {'original_bigg_ids': ['Asn_DASH_X_DASH_Ser_DASH_FSLASH_DASH_Thr_c']}
doldp_c {'original_bigg_ids': ['doldp_c']}
doldpglcnacglcnacman_man_manman_manman_manmanmanglcglcglc_c {'original_bigg_ids': ['doldpglcnacglcnacman_DASH_LPAREN_DASH_man_DASH_LPAREN_DASH_manman_DASH_RPAREN_DASH_manman_DASH_RPAREN_DASH_manmanmanglcglcglc_c']}
10fthfglu__L_m {'original_bigg_ids': ['10fthfglu_DASH_L_m']}
fe3_c {'original_bigg_ids': ['fe3_c']}
fe3_e {'original_bigg_ids': ['fe3_e']}
udparab_c {'original_bigg_ids': ['udparab_c']}
so3_m {'original_bigg_ids': ['hso3_m']}
so4_m {'original_bigg_ids': ['so4_m']}
mag1819Z_c {'original_bigg_ids': ['mag1819Z_c']}
urea_m {'original_bigg_ids': ['urea_m']}
pe1801829Z12Z_e {'original_bigg_ids': ['pe1801829Z12Z_e']}
nadphx__S_c {'original_bigg_ids': ['nadphx__S_c']}
moadamp_c {'original_bigg_ids': ['moadamp_c']}
ala_B_m {'original_bigg_ids': ['ala_DASH_B_m']}
doldpglcnacglcnacman_man_man_manman_manmanman_c {'original_bigg_ids': ['doldpglcnacglcnacman_DASH_LPAREN_DASH_man_DASH_LPAREN_DASH_man_DASH_RPAREN_DASH_manman_DASH_RPAREN_DASH_manmanman_c']}
doldpglcnacglcnacman_man_manman_manman_manmanman_c {'original_bigg_ids': ['doldpglcnacglcnacman_DASH_LPAREN_DASH_man_DASH_LPAREN_DASH_manman_DASH_RPAREN_DASH_manman_DASH_RPAREN_DASH_manmanman_c']}
6tgsnmp_c {'original_bigg_ids': ['6tgsnmp_c']}
arso4_c {'original_bigg_ids': ['arso4_c']}
phenol_c {'original_bigg_ids': ['phen_c']}
acg5p_c {'original_bigg_ids': ['acg5p_c']}
doldpglcnacglcnacman_man_man_c {'original_bigg_ids': ['doldpglcnacglcnacman_DASH_LPAREN_DASH_man_DASH_RPAREN_DASH_man_c']}
doldpglcnacglcnacmanman_c {'original_bigg_ids': ['doldpglcnacglcnacmanman_c']}
3ohcoa_x {'original_bigg_ids': ['3ohcoa_x']}
fgam_m {'original_bigg_ids': ['fgam_m']}
gar_m {'original_bigg_ids': ['gar_m']}
aflbala_c {'original_bigg_ids': ['aflbala_c']}
moadcoo_c {'original_bigg_ids': ['moadcoo_c']}
o2_n {'original_bigg_ids': ['o2_n']}
oxadpcoa_c {'original_bigg_ids': ['oxadpcoa_c']}
mmet_c {'original_bigg_ids': ['mmet_c']}
glyclt_m {'original_bigg_ids': ['glyclt_m']}
ca2_c {'original_bigg_ids': ['ca2_c']}
betald_c {'original_bigg_ids': ['betald_c']}
pe1819Z1829Z12Z_e {'original_bigg_ids': ['pe1819Z1829Z12Z_e']}
oc2coa_m {'original_bigg_ids': ['oc2coa_m']}
glytrna_m {'original_bigg_ids': ['glytrna_m']}
trnagly_m {'original_bigg_ids': ['trnagly_m']}
bmoco_c {'original_bigg_ids': ['bmoco_c']}
seramp_c {'original_bigg_ids': ['seramp_c']}
n8aspmd_c {'original_bigg_ids': ['n8aspmd_c']}
pe1801819Z_e {'original_bigg_ids': ['pe1801819Z_e']}
tega_c {'original_bigg_ids': ['tega_c']}
tega_e {'original_bigg_ids': ['tega_e']}
hxcoa_x {'original_bigg_ids': ['hxcoa_x']}
doldpglcnacglcnacman_man_manman_c {'original_bigg_ids': ['doldpglcnacglcnacman_DASH_LPAREN_DASH_man_DASH_RPAREN_DASH_manman_c']}
doldpglcnacglcnacman_man_manmanman_c {'original_bigg_ids': ['doldpglcnacglcnacman_DASH_LPAREN_DASH_man_DASH_RPAREN_DASH_manmanman_c']}
3ohcoa_m {'original_bigg_ids': ['3ohcoa_m']}
btcoa_m {'original_bigg_ids': ['btcoa_m']}
nadphx__R_c {'original_bigg_ids': ['nadphx__R_c']}
glyc2p_c {'original_bigg_ids': ['glyc2p_c']}
catechol_c {'original_bigg_ids': ['catechol_c']}
ccmuac_c {'original_bigg_ids': ['ccmuac_c']}
ppap_m {'original_bigg_ids': ['ppap_m']}
arab__L_c {'original_bigg_ids': ['arab__L_c']}
2ddara_c {'original_bigg_ids': ['2ddara_c']}
4per_c {'original_bigg_ids': ['4per_c']}
3hpppn_c {'original_bigg_ids': ['3hpppn_c']}
dhpppn_c {'original_bigg_ids': ['dhpppn_c']}
5mthf_m {'original_bigg_ids': ['5mthf_m']}
uLa4fn_c {'original_bigg_ids': ['uLa4fn_c']}
udcpp_c {'original_bigg_ids': ['udcpp_c']}
4hbald_c {'original_bigg_ids': ['4hbald_c']}
35cgmp_n {'original_bigg_ids': ['35cgmp_n']}
gmp_n {'original_bigg_ids': ['gmp_n']}
tungs_c {'original_bigg_ids': ['tungs_c']}
dolmanp_U_r {'original_bigg_ids': ['dolmanp_U_r']}
dolp_U_r {'original_bigg_ids': ['dolp_U_r']}
clpndcrn_c {'original_bigg_ids': ['clpndcrn_c']}
clpndcrn_m {'original_bigg_ids': ['clpndcrn_m']}
nrvnc_c {'original_bigg_ids': ['nrvnc_c']}
nrvnc_e {'original_bigg_ids': ['nrvnc_e']}
dhocholoylcoa_x {'original_bigg_ids': ['dhocholoylcoa_x']}
thcholoylcoa_x {'original_bigg_ids': ['thcholoylcoa_x']}
pcrn_m {'original_bigg_ids': ['pcrn_m']}
m7masnB_r {'original_bigg_ids': ['m7masnB_r']}
m8masn_r {'original_bigg_ids': ['m8masn_r']}
man_r {'original_bigg_ids': ['man_r']}
btamp_m {'original_bigg_ids': ['btamp_m']}
btn_m {'original_bigg_ids': ['btn_m']}
dolmanp__L_r {'original_bigg_ids': ['dolmanp_L_r']}
dolp__L_r {'original_bigg_ids': ['dolp_L_r']}
carn_c {'original_bigg_ids': ['carn_c']}
gdpfuc_g {'original_bigg_ids': ['gdpfuc_g']}
3hxkynam_c {'original_bigg_ids': ['3hxkynam_c']}
hmgcoa_r {'original_bigg_ids': ['hmgcoa_r']}
mev__R_r {'original_bigg_ids': ['mev_DASH_R_r']}
dctp_n {'original_bigg_ids': ['dctp_n']}
fna5moxam_c {'original_bigg_ids': ['fna5moxam_c']}
melatn_c {'original_bigg_ids': ['melatn_c']}
accoa_g {'original_bigg_ids': ['accoa_g']}
lnlncgcoa_x {'original_bigg_ids': ['lnlncgcoa_x']}
clpndcoa_c {'original_bigg_ids': ['clpndcoa_c']}
estroneglc_c {'original_bigg_ids': ['estroneglc_c']}
estroneglc_e {'original_bigg_ids': ['estroneglc_e']}
dolglcp_U_r {'original_bigg_ids': ['dolglcp_U_r']}
g2m8mpdol_U_r {'original_bigg_ids': ['g2m8mpdol_U_r']}
g3m8mpdol_U_r {'original_bigg_ids': ['g3m8mpdol_U_r']}
5thf_c {'original_bigg_ids': ['5thf_c']}
dhcholestanate_x {'original_bigg_ids': ['dhcholestanate_x']}
dhcholestancoa_x {'original_bigg_ids': ['dhcholestancoa_x']}
datp_n {'original_bigg_ids': ['datp_n']}
k_e {'original_bigg_ids': ['k_e']}
o2s_m {'original_bigg_ids': ['o2s_m']}
f5hoxkyn_c {'original_bigg_ids': ['f5hoxkyn_c']}
srtn_c {'original_bigg_ids': ['srtn_c']}
ump_g {'original_bigg_ids': ['ump_g']}
hpdcacoa_c {'original_bigg_ids': ['hpdcacoa_c']}
hpdcacrn_c {'original_bigg_ids': ['hpdcacrn_c']}
1mpyr_c {'original_bigg_ids': ['1mpyr_c']}
nmptrc_c {'original_bigg_ids': ['nmptrc_c']}
odecoa_m {'original_bigg_ids': ['odecoa_m']}
odecrn_m {'original_bigg_ids': ['odecrn_m']}
6thf_c {'original_bigg_ids': ['6thf_c']}
dhcholoylcoa_x {'original_bigg_ids': ['dhcholoylcoa_x']}
dolglcp__L_r {'original_bigg_ids': ['dolglcp_L_r']}
g1m8mpdol__L_r {'original_bigg_ids': ['g1m8mpdol_L_r']}
m8mpdol__L_r {'original_bigg_ids': ['m8mpdol_L_r']}
5htrp_c {'original_bigg_ids': ['5htrp_c']}
agm_m {'original_bigg_ids': ['agm_m']}
urate_x {'original_bigg_ids': ['urate_x']}
xan_x {'original_bigg_ids': ['xan_x']}
aact_c {'original_bigg_ids': ['aact_c']}
cholcoar_r {'original_bigg_ids': ['cholcoar_r']}
cholcoas_r {'original_bigg_ids': ['cholcoas_r']}
dgtp_n {'original_bigg_ids': ['dgtp_n']}
betald_m {'original_bigg_ids': ['betald_m']}
chol_m {'original_bigg_ids': ['chol_m']}
abt_c {'original_bigg_ids': ['abt_c']}
2mop_m {'original_bigg_ids': ['2mop_m']}
3hmp_m {'original_bigg_ids': ['3hmp_m']}
clpndcoa_x {'original_bigg_ids': ['clpndcoa_x']}
tetpent3coa_x {'original_bigg_ids': ['tetpent3coa_x']}
k_g {'original_bigg_ids': ['k_g']}
lnlncgcrn_c {'original_bigg_ids': ['lnlncgcrn_c']}
lnlncgcrn_m {'original_bigg_ids': ['lnlncgcrn_m']}
ttdcrn_m {'original_bigg_ids': ['ttdcrn_m']}
lnlncgcoa_m {'original_bigg_ids': ['lnlncgcoa_m']}
tettet6_c {'original_bigg_ids': ['tettet6_c']}
tettet6coa_c {'original_bigg_ids': ['tettet6coa_c']}
cmp_n {'original_bigg_ids': ['cmp_n']}
malttr_c {'original_bigg_ids': ['malttr_c']}
3snpyr_c {'original_bigg_ids': ['3snpyr_c']}
hpdcacoa_m {'original_bigg_ids': ['hpdcacoa_m']}
dcsptn1coa_c {'original_bigg_ids': ['dcsptn1coa_c']}
dcsptn1crn_c {'original_bigg_ids': ['dcsptn1crn_c']}
dmnoncoa_x {'original_bigg_ids': ['dmnoncoa_x']}
ppcoa_x {'original_bigg_ids': ['ppcoa_x']}
4mzym_int2_r {'original_bigg_ids': ['4mzym_int2_r']}
cl_e {'original_bigg_ids': ['cl_e']}
oxa_e {'original_bigg_ids': ['oxa_e']}
10fthf5glu_m {'original_bigg_ids': ['10fthf5glu_m']}
10fthf6glu_m {'original_bigg_ids': ['10fthf6glu_m']}
estradiolglc_c {'original_bigg_ids': ['estradiolglc_c']}
estradiolglc_e {'original_bigg_ids': ['estradiolglc_e']}
gtp_n {'original_bigg_ids': ['gtp_n']}
dcsptn1coa_m {'original_bigg_ids': ['dcsptn1coa_m']}
dcsptn1crn_m {'original_bigg_ids': ['dcsptn1crn_m']}
estriolglc_c {'original_bigg_ids': ['estriolglc_c']}
estriolglc_e {'original_bigg_ids': ['estriolglc_e']}
cgly_e {'original_bigg_ids': ['cgly_e']}
gluala_e {'original_bigg_ids': ['gluala_e']}
ach_e {'original_bigg_ids': ['ach_e']}
cholcoa_x {'original_bigg_ids': ['cholcoa_x']}
cholcoaone_x {'original_bigg_ids': ['cholcoaone_x']}
hexccrn_c {'original_bigg_ids': ['hexccrn_c']}
dolglcp__L_c {'original_bigg_ids': ['dolglcp_L_c']}
dolp__L_c {'original_bigg_ids': ['dolp_L_c']}
gchola_c {'original_bigg_ids': ['gchola_c']}
gchola_e {'original_bigg_ids': ['gchola_e']}
nrvnccrn_c {'original_bigg_ids': ['nrvnccrn_c']}
nrvnccrn_m {'original_bigg_ids': ['nrvnccrn_m']}
clpnd_c {'original_bigg_ids': ['clpnd_c']}
4h2oglt_m {'original_bigg_ids': ['4h2oglt_m']}
tettet6coa_m {'original_bigg_ids': ['tettet6coa_m']}
tettet6crn_m {'original_bigg_ids': ['tettet6crn_m']}
hpdcacrn_m {'original_bigg_ids': ['hpdcacrn_m']}
s2l2n2m2m_c {'original_bigg_ids': ['s2l2n2m2m_c']}
s2l2n2m2mn_c {'original_bigg_ids': ['s2l2n2m2mn_c']}
dcmp_n {'original_bigg_ids': ['dcmp_n']}
dcsptn1coa_x {'original_bigg_ids': ['dcsptn1coa_x']}
tetpent6coa_x {'original_bigg_ids': ['tetpent6coa_x']}
g2m8masn_r {'original_bigg_ids': ['g2m8masn_r']}
g3m8masn_r {'original_bigg_ids': ['g3m8masn_r']}
glc__D_r {'original_bigg_ids': ['glc_DASH_D_r']}
andrstndn_r {'original_bigg_ids': ['andrstndn_r']}
tststerone_r {'original_bigg_ids': ['tststerone_r']}
dmnoncoa_c {'original_bigg_ids': ['dmnoncoa_c']}
cholcoads_x {'original_bigg_ids': ['cholcoads_x']}
cholcoas_x {'original_bigg_ids': ['cholcoas_x']}
hdcecrn_m {'original_bigg_ids': ['hdcecrn_m']}
hdcoa_m {'original_bigg_ids': ['hdcoa_m']}
xyl__D_e {'original_bigg_ids': ['xyl_DASH_D_e']}
m2mn_c {'original_bigg_ids': ['m2mn_c']}
mn_c {'original_bigg_ids': ['mn_c']}
6pthp_c {'original_bigg_ids': ['6pthp_c']}
pmtcrn_m {'original_bigg_ids': ['pmtcrn_m']}
g2m8mpdol__L_r {'original_bigg_ids': ['g2m8mpdol_L_r']}
5dhf_m {'original_bigg_ids': ['5dhf_m']}
dhbpt_c {'original_bigg_ids': ['dhbpt_c']}
thbpt4acam_c {'original_bigg_ids': ['thbpt4acam_c']}
g3m8mpdol__L_r {'original_bigg_ids': ['g3m8mpdol_L_r']}
10fthf6glu_c {'original_bigg_ids': ['10fthf6glu_c']}
10fthf7glu_c {'original_bigg_ids': ['10fthf7glu_c']}
udp_g {'original_bigg_ids': ['udp_g']}
udpglcur_g {'original_bigg_ids': ['udpglcur_g']}
tym_c {'original_bigg_ids': ['tym_c']}
amp_r {'original_bigg_ids': ['amp_r']}
thcholstoic_r {'original_bigg_ids': ['thcholstoic_r']}
dump_m {'original_bigg_ids': ['dump_m']}
44mctr_r {'original_bigg_ids': ['44mctr_r']}
44mzym_r {'original_bigg_ids': ['44mzym_r']}
utp_n {'original_bigg_ids': ['utp_n']}
dolp_U_c {'original_bigg_ids': ['dolp_U_c']}
tststeroneglc_c {'original_bigg_ids': ['tststeroneglc_c']}
tststeroneglc_e {'original_bigg_ids': ['tststeroneglc_e']}
udpg_g {'original_bigg_ids': ['udpg_g']}
ala_B_e {'original_bigg_ids': ['ala_DASH_B_e']}
tettet6crn_c {'original_bigg_ids': ['tettet6crn_c']}
ptdca_c {'original_bigg_ids': ['ptdca_c']}
ptdcacoa_c {'original_bigg_ids': ['ptdcacoa_c']}
oh1_e {'original_bigg_ids': ['oh1_e']}
hco3_e {'original_bigg_ids': ['hco3_e']}
adrnl_c {'original_bigg_ids': ['adrnl_c']}
mepi_c {'original_bigg_ids': ['mepi_c']}
bilglcur_c {'original_bigg_ids': ['bilglcur_c']}
bilglcur_e {'original_bigg_ids': ['bilglcur_e']}
prist_c {'original_bigg_ids': ['prist_c']}
pristcoa_c {'original_bigg_ids': ['pristcoa_c']}
m4mpdol__L_r {'original_bigg_ids': ['m4mpdol_L_r']}
m5mpdol__L_r {'original_bigg_ids': ['m5mpdol_L_r']}
dcholcoa_x {'original_bigg_ids': ['dcholcoa_x']}
ppmi12346p_n {'original_bigg_ids': ['ppmi12346p_n']}
g1m6masnB1_r {'original_bigg_ids': ['g1m6masnB1_r']}
g1m7masnC_r {'original_bigg_ids': ['g1m7masnC_r']}
ttdcrn_c {'original_bigg_ids': ['ttdcrn_c']}
ptdcacrn_c {'original_bigg_ids': ['ptdcacrn_c']}
ptdcacrn_m {'original_bigg_ids': ['ptdcacrn_m']}
5adtststerone_r {'original_bigg_ids': ['5adtststerone_r']}
arach_c {'original_bigg_ids': ['arach_c']}
arachcoa_c {'original_bigg_ids': ['arachcoa_c']}
nrvnccoa_m {'original_bigg_ids': ['nrvnccoa_m']}
nrvnccoa_c {'original_bigg_ids': ['nrvnccoa_c']}
adrncoa_x {'original_bigg_ids': ['adrncoa_x']}
adpman_c {'original_bigg_ids': ['adpman_c']}
6dhf_m {'original_bigg_ids': ['6dhf_m']}
7dhf_m {'original_bigg_ids': ['7dhf_m']}
3spyr_c {'original_bigg_ids': ['3spyr_c']}
cholate_e {'original_bigg_ids': ['cholate_e']}
dutp_n {'original_bigg_ids': ['dutp_n']}
gam_e {'original_bigg_ids': ['gam_e']}
cmpntm2amep_c {'original_bigg_ids': ['cmpntm2amep_c']}
ntm2amep_c {'original_bigg_ids': ['ntm2amep_c']}
hdd2crn_c {'original_bigg_ids': ['hdd2crn_c']}
6dhf_c {'original_bigg_ids': ['6dhf_c']}
7dhf_c {'original_bigg_ids': ['7dhf_c']}
hdd2crn_m {'original_bigg_ids': ['hdd2crn_m']}
bamppald_c {'original_bigg_ids': ['bamppald_c']}
ptdcacoa_m {'original_bigg_ids': ['ptdcacoa_m']}
camp_e {'original_bigg_ids': ['camp_e']}
4aphdob_c {'original_bigg_ids': ['4aphdob_c']}
paps_g {'original_bigg_ids': ['paps_g']}
2aobut_m {'original_bigg_ids': ['2aobut_m']}
glygn2_e {'original_bigg_ids': ['glygn2_e']}
glygn4_e {'original_bigg_ids': ['glygn4_e']}
phyt_c {'original_bigg_ids': ['phyt_c']}
phytcoa_c {'original_bigg_ids': ['phytcoa_c']}
prostge2_c {'original_bigg_ids': ['prostge2_c']}
prostge2_e {'original_bigg_ids': ['prostge2_e']}
pro__D_c {'original_bigg_ids': ['pro_DASH_D_c']}
pro__D_e {'original_bigg_ids': ['pro_DASH_D_e']}
3aib_m {'original_bigg_ids': ['3aib_m']}
arachcoa_x {'original_bigg_ids': ['arachcoa_x']}
udpacgal_g {'original_bigg_ids': ['udpacgal_g']}
17ahprgnlone_r {'original_bigg_ids': ['17ahprgnlone_r']}
prgnlone_r {'original_bigg_ids': ['prgnlone_r']}
6pthp_n {'original_bigg_ids': ['6pthp_n']}
ahdt_n {'original_bigg_ids': ['ahdt_n']}
pppi_n {'original_bigg_ids': ['pppi_n']}
elaidcrn_c {'original_bigg_ids': ['elaidcrn_c']}
elaidcrn_m {'original_bigg_ids': ['elaidcrn_m']}
oxa_x {'original_bigg_ids': ['oxa_x']}
but_m {'original_bigg_ids': ['but_m']}
gthrd_r {'original_bigg_ids': ['gthrd_r']}
leuktrA4_r {'original_bigg_ids': ['leuktrA4_r']}
leuktrC4_r {'original_bigg_ids': ['leuktrC4_r']}
ca2_e {'original_bigg_ids': ['ca2_e']}
hxan_x {'original_bigg_ids': ['hxan_x']}
hexccrn_m {'original_bigg_ids': ['hexccrn_m']}
dad_2_e {'original_bigg_ids': ['dad_DASH_2_e']}
din_e {'original_bigg_ids': ['din_e']}
dhcholestancoa_r {'original_bigg_ids': ['dhcholestancoa_r']}
dhcholoylcoa_r {'original_bigg_ids': ['dhcholoylcoa_r']}
leuktrA4_c {'original_bigg_ids': ['leuktrA4_c']}
leuktrB4_c {'original_bigg_ids': ['leuktrB4_c']}
m1mpdol__L_c {'original_bigg_ids': ['m1mpdol_L_c']}
mpdol__L_c {'original_bigg_ids': ['mpdol_L_c']}
naglc2p__L_c {'original_bigg_ids': ['naglc2p_L_c']}
nrvnccoa_x {'original_bigg_ids': ['nrvnccoa_x']}
5thf_m {'original_bigg_ids': ['5thf_m']}
6thf_m {'original_bigg_ids': ['6thf_m']}
35cgmp_e {'original_bigg_ids': ['35cgmp_e']}
for_e {'original_bigg_ids': ['for_e']}
10fthf5glu_c {'original_bigg_ids': ['10fthf5glu_c']}
hdcecrn_c {'original_bigg_ids': ['hdcecrn_c']}
adpglc_c {'original_bigg_ids': ['adpglc_c']}
accoa_r {'original_bigg_ids': ['accoa_r']}
acrn_r {'original_bigg_ids': ['acrn_r']}
crn_r {'original_bigg_ids': ['crn_r']}
odecrn_c {'original_bigg_ids': ['odecrn_c']}
elaid_c {'original_bigg_ids': ['elaid_c']}
glc__D_g {'original_bigg_ids': ['glc_DASH_D_g']}
4mzym_int1_r {'original_bigg_ids': ['4mzym_int1_r']}
ascb__L_c {'original_bigg_ids': ['ascb_DASH_L_c']}
dhdascb_c {'original_bigg_ids': ['dhdascb_c']}
n2m2masn_g {'original_bigg_ids': ['n2m2masn_g']}
n2m2nmasn_g {'original_bigg_ids': ['n2m2nmasn_g']}
uacgam_g {'original_bigg_ids': ['uacgam_g']}
5dhf_c {'original_bigg_ids': ['5dhf_c']}
acald_x {'original_bigg_ids': ['acald_x']}
etoh_x {'original_bigg_ids': ['etoh_x']}
g1m8mpdol_U_r {'original_bigg_ids': ['g1m8mpdol_U_r']}
cholcoar_x {'original_bigg_ids': ['cholcoar_x']}
thcholstoic_x {'original_bigg_ids': ['thcholstoic_x']}
hexccoa_m {'original_bigg_ids': ['hexccoa_m']}
adsel_c {'original_bigg_ids': ['adsel_c']}
sel_c {'original_bigg_ids': ['sel_c']}
trypta_c {'original_bigg_ids': ['trypta_c']}
17ahprgstrn_r {'original_bigg_ids': ['17ahprgstrn_r']}
prgstrn_r {'original_bigg_ids': ['prgstrn_r']}
10fthf7glu_m {'original_bigg_ids': ['10fthf7glu_m']}
tettet6coa_x {'original_bigg_ids': ['tettet6coa_x']}
hmgcoa_x {'original_bigg_ids': ['hmgcoa_x']}
m6mpdol_U_r {'original_bigg_ids': ['m6mpdol_U_r']}
m7mpdol_U_r {'original_bigg_ids': ['m7mpdol_U_r']}
na1_g {'original_bigg_ids': ['na1_g']}
ala__D_e {'original_bigg_ids': ['ala_DASH_D_e']}
chito2pdol_U_c {'original_bigg_ids': ['chito2pdol_U_c']}
mpdol_U_c {'original_bigg_ids': ['mpdol_U_c']}
7thf_c {'original_bigg_ids': ['7thf_c']}
ctp_n {'original_bigg_ids': ['ctp_n']}
udpxyl_g {'original_bigg_ids': ['udpxyl_g']}
5adtststeroneglc_c {'original_bigg_ids': ['5adtststeroneglc_c']}
5adtststeroneglc_e {'original_bigg_ids': ['5adtststeroneglc_e']}
phytcoa_x {'original_bigg_ids': ['phytcoa_x']}
pmtcrn_c {'original_bigg_ids': ['pmtcrn_c']}
n2m2nm_c {'original_bigg_ids': ['n2m2nm_c']}
n2m2nmn_c {'original_bigg_ids': ['n2m2nmn_c']}
chito2pdol__L_c {'original_bigg_ids': ['chito2pdol_L_c']}
g1m7masnB_r {'original_bigg_ids': ['g1m7masnB_r']}
g1m8masn_r {'original_bigg_ids': ['g1m8masn_r']}
dhcholestanate_r {'original_bigg_ids': ['dhcholestanate_r']}
bildglcur_c {'original_bigg_ids': ['bildglcur_c']}
bildglcur_e {'original_bigg_ids': ['bildglcur_e']}
dmnoncrn_x {'original_bigg_ids': ['dmnoncrn_x']}
dcsptn1_c {'original_bigg_ids': ['dcsptn1_c']}
naglc2p_U_c {'original_bigg_ids': ['naglc2p_U_c']}
acgam_e {'original_bigg_ids': ['acgam_e']}
chtn_e {'original_bigg_ids': ['chtn_e']}
m4mpdol_U_r {'original_bigg_ids': ['m4mpdol_U_r']}
m5mpdol_U_r {'original_bigg_ids': ['m5mpdol_U_r']}
3mlda_c {'original_bigg_ids': ['3mlda_c']}
3mldz_c {'original_bigg_ids': ['3mldz_c']}
clpndcoa_m {'original_bigg_ids': ['clpndcoa_m']}
apoC_Lys_btn_m {'original_bigg_ids': ['apoC_DASH_Lys_btn_m']}
apoC_Lys_m {'original_bigg_ids': ['apoC_DASH_Lys_m']}
dolglcp_U_c {'original_bigg_ids': ['dolglcp_U_c']}
5hoxnfkyn_c {'original_bigg_ids': ['5hoxnfkyn_c']}
for_n {'original_bigg_ids': ['for_n']}
hxdcal_r {'original_bigg_ids': ['hxdcal_r']}
dgcholcoa_x {'original_bigg_ids': ['dgcholcoa_x']}
apoC_Lys_btn_c {'original_bigg_ids': ['apoC_DASH_Lys_btn_c']}
apoC_Lys_c {'original_bigg_ids': ['apoC_DASH_Lys_c']}
5hxkyn_c {'original_bigg_ids': ['5hxkyn_c']}
5hxkynam_c {'original_bigg_ids': ['5hxkynam_c']}
m1mpdol_U_c {'original_bigg_ids': ['m1mpdol_U_c']}
chlstol_r {'original_bigg_ids': ['chlstol_r']}
ddsmsterol_r {'original_bigg_ids': ['ddsmsterol_r']}
hpdca_c {'original_bigg_ids': ['hpdca_c']}
acald_r {'original_bigg_ids': ['acald_r']}
3padsel_c {'original_bigg_ids': ['3padsel_c']}
pcrn_x {'original_bigg_ids': ['pcrn_x']}
7thf_m {'original_bigg_ids': ['7thf_m']}
ahandrostanglc_c {'original_bigg_ids': ['ahandrostanglc_c']}
ahandrostanglc_e {'original_bigg_ids': ['ahandrostanglc_e']}
glyc_m {'original_bigg_ids': ['glyc_m']}
arach_e {'original_bigg_ids': ['arach_e']}
ins_e {'original_bigg_ids': ['ins_e']}
asp__D_x {'original_bigg_ids': ['asp_DASH_D_x']}
mhista_c {'original_bigg_ids': ['mhista_c']}
tchola_e {'original_bigg_ids': ['tchola_e']}
am6sa_c {'original_bigg_ids': ['am6sa_c']}
m6mpdol__L_r {'original_bigg_ids': ['m6mpdol_L_r']}
m7mpdol__L_r {'original_bigg_ids': ['m7mpdol_L_r']}
cysi__L_e {'original_bigg_ids': ['Lcystin_e']}
34dhphe_c {'original_bigg_ids': ['34dhphe_c']}
nformanth_c {'original_bigg_ids': ['nformanth_c']}
galt_c {'original_bigg_ids': ['galt_c']}
andrstrnglc_c {'original_bigg_ids': ['andrstrnglc_c']}
andrstrnglc_e {'original_bigg_ids': ['andrstrnglc_e']}
andrstandn_r {'original_bigg_ids': ['andrstandn_r']}
normete__L_c {'original_bigg_ids': ['normete_DASH_L_c']}
nrpphr_c {'original_bigg_ids': ['nrpphr_c']}
4hoxpacd_c {'original_bigg_ids': ['4hoxpacd_c']}
4hphac_c {'original_bigg_ids': ['4hphac_c']}
prostge1_c {'original_bigg_ids': ['prostge1_c']}
prostge1_e {'original_bigg_ids': ['prostge1_e']}
42A3HP24DB_c {'original_bigg_ids': ['42A3HP24DB_c']}
strch1_e {'original_bigg_ids': ['strch1_e']}
strch2_e {'original_bigg_ids': ['strch2_e']}
m8mpdol_U_r {'original_bigg_ids': ['m8mpdol_U_r']}
dhdascb_e {'original_bigg_ids': ['dhdascb_e']}
dhea_r {'original_bigg_ids': ['dhea_r']}
r2hglut_m {'original_bigg_ids': ['r2hglut_c']}
s2hglut_m {'original_bigg_ids': ['S2hglut_c']}
s3hb_m {'original_bigg_ids': ['bhb_m']}
ghb_m {'original_bigg_ids': ['ghb_c']}
hemeC_m {'original_bigg_ids': ['pheme_m']}
grdp_m {'original_bigg_ids': ['grdp_m']}
npdp_m {'original_bigg_ids': ['npdp_m']}
3nphb_m {'original_bigg_ids': ['3nphb_m']}
3npdhb_m {'original_bigg_ids': ['3npdhb_m']}
me3dhnpdh_m {'original_bigg_ids': ['me3dhnpdh_m']}
2np6mep_m {'original_bigg_ids': ['2np6mep_m']}
2np6mobq_m {'original_bigg_ids': ['2np6mobq_m']}
me2np6mobq_m {'original_bigg_ids': ['me2np6mobq_m']}
2npmhmobq_m {'original_bigg_ids': ['2npmhmobq_m']}
q9_m {'original_bigg_ids': ['q9_m']}
q9h2_m {'original_bigg_ids': ['q9h2_m']}
ca2_r {'original_bigg_ids': ['ca2_c']}
cu_e {'original_bigg_ids': ['cu_p']}
hkmpp_c {'original_bigg_ids': ['2h3kmtp_c']}
ala__L_x {'original_bigg_ids': ['ala_DASH_L_c']}
pimcoa_x {'original_bigg_ids': ['pimACP_c']}
dtbt_m {'original_bigg_ids': ['dtbt_c']}
2fe1s_m {'original_bigg_ids': ['2fe1s_c']}
2fe2s_m {'original_bigg_ids': ['2fe2s_c']}
dad_5_m {'original_bigg_ids': ['dad_5_c']}
5fthf_m {'original_bigg_ids': ['5fthf_m']}
3htmelys_m {'original_bigg_ids': ['3htmelys_m']}
4tmeabut_m {'original_bigg_ids': ['4tmeabut_m']}
tmlys_m {'original_bigg_ids': ['tmlys_m']}
gbbtn_m {'original_bigg_ids': ['gbbtn_m']}
cer2p_24_r {'original_bigg_ids': ['cer2_24_c', 'cer2__24_c']}
cer2p_26_r {'original_bigg_ids': ['cer2_26_c', 'cer2__26_c']}
cer3_24_r {'original_bigg_ids': ['cer3_24_c']}
cer3_26_r {'original_bigg_ids': ['cer3_26_c']}
2hhxdal_r {'original_bigg_ids': ['2hhxdal_c']}
grxox_m {'original_bigg_ids': ['grxox_c']}
grxrd_m {'original_bigg_ids': ['grxrd_c']}
didp_m {'original_bigg_ids': ['didp_m']}
ditp_m {'original_bigg_ids': ['ditp_m']}
udp_m {'original_bigg_ids': ['udp_m']}
cdp_m {'original_bigg_ids': ['cdp_m']}
dtdp_m {'original_bigg_ids': ['dtdp_m']}
dttp_m {'original_bigg_ids': ['dttp_m']}
dudp_m {'original_bigg_ids': ['dudp_m']}
dcdp_m {'original_bigg_ids': ['dcdp_m']}
dctp_m {'original_bigg_ids': ['dctp_m']}
hyptaur_c {'original_bigg_ids': ['hyptaur_c']}
pentcoa_m {'original_bigg_ids': ['pentcoa[m]']}
hepcoa_m {'original_bigg_ids': ['hepcoa[m]']}
noncoa_m {'original_bigg_ids': ['noncoa[m]']}
undcoa_m {'original_bigg_ids': ['undcoa[m]']}
tridcoa_m {'original_bigg_ids': ['tridcoa[m]']}
ttc_e {'original_bigg_ids': ['ttc_c']}
ttccoa_c {'original_bigg_ids': ['ttccoa_x']}
T4hcinnm_c {'original_bigg_ids': ['T4hcinnm[c]']}
coucoa_c {'original_bigg_ids': ['coucoa[c]']}
hdcoa_r {'original_bigg_ids': ['hdcoa_c']}
odecoa_r {'original_bigg_ids': ['odecoa_c']}
ocdycacoa_r {'original_bigg_ids': ['ocdycacoa_c']}
lnlncgcoa_r {'original_bigg_ids': ['lnlncgcoa_c']}
cer1_18_r {'original_bigg_ids': ['cer1_24_r']}
cer2p_18_r {'original_bigg_ids': ['cer2_24_c', 'cer2__24_c']}
cer4_18_r {'original_bigg_ids': ['cer2_24_c', 'cer2__24_c']}
cer5_18_r {'original_bigg_ids': ['cer2_24_c', 'cer2__24_c']}
cer6_18_r {'original_bigg_ids': ['cer2_24_c', 'cer2__24_c']}
cer6_18_g {'original_bigg_ids': ['cer2_24_c', 'cer2__24_c']}
glccer_18_g {'original_bigg_ids': ['cer2_24_c', 'cer2__24_c']}
12dgr_RT_g {'original_bigg_ids': ['12dgr_SC_c']}
cer2_24_g {'original_bigg_ids': ['cer2_24_c', 'cer2__24_c']}
ipc224_RT_g {'original_bigg_ids': ['ipc224_SC_c']}
ptd1ino_RT_g {'original_bigg_ids': ['ptd1ino_SC_c']}
cer2_26_g {'original_bigg_ids': ['cer2_26_c', 'cer2__26_c']}
ipc226_RT_g {'original_bigg_ids': ['ipc226_SC_c']}
cer3_24_g {'original_bigg_ids': ['cer3_24_c']}
ipc324_RT_g {'original_bigg_ids': ['ipc324_SC_c']}
cer1_26_g {'original_bigg_ids': ['cer1_26_c']}
ipc126_RT_g {'original_bigg_ids': ['ipc126_SC_c']}
cer3_26_g {'original_bigg_ids': ['cer3_26_c']}
ipc326_RT_g {'original_bigg_ids': ['ipc326_SC_c']}
cer1_24_g {'original_bigg_ids': ['cer1_24_c']}
ipc124_RT_g {'original_bigg_ids': ['ipc124_SC_c']}
mipc226_RT_g {'original_bigg_ids': ['mipc226_SC_c']}
mipc126_RT_g {'original_bigg_ids': ['mipc126_SC_c']}
mipc224_RT_g {'original_bigg_ids': ['mipc224_SC_c']}
mipc324_RT_g {'original_bigg_ids': ['mipc324_SC_c']}
mipc326_RT_g {'original_bigg_ids': ['mipc326_SC_c']}
mipc124_RT_g {'original_bigg_ids': ['mipc124_SC_c']}
mip2c224_RT_g {'original_bigg_ids': ['mip2c224_SC_c']}
mip2c324_RT_g {'original_bigg_ids': ['mip2c324_SC_c']}
mip2c226_RT_g {'original_bigg_ids': ['mip2c226_SC_c']}
mip2c126_RT_g {'original_bigg_ids': ['mip2c126_SC_c']}
mip2c124_RT_g {'original_bigg_ids': ['mip2c124_SC_c']}
mip2c326_RT_g {'original_bigg_ids': ['mip2c326_SC_c']}
manmi1p__D_g {'original_bigg_ids': ['manmi1p_D_c']}
man2mi1p__D_g {'original_bigg_ids': ['man2mi1p_D_c']}
mi1p__D_g {'original_bigg_ids': ['mi1p_D_c']}
ergtrol_r {'original_bigg_ids': ['ergtrol_c']}
4mzym_r {'original_bigg_ids': ['4mzym_c']}
zym_int1_r {'original_bigg_ids': ['zym_int1_c']}
zym_int2_r {'original_bigg_ids': ['zym_int2_c']}
zymst_r {'original_bigg_ids': ['zymst_c']}
fecost_r {'original_bigg_ids': ['fecost_c']}
epist_r {'original_bigg_ids': ['epist_c']}
malcoa_r {'original_bigg_ids': ['malcoa_c']}
lipoamp_m {'original_bigg_ids': ['lipoamp_c']}
lipopb_m {'original_bigg_ids': ['lipopb_c']}
lipoate_m {'original_bigg_ids': ['lipoate_c']}
octapb_m {'original_bigg_ids': ['octapb_c']}
ocACP_m {'original_bigg_ids': ['ocACP_c']}
4fe4s_m {'original_bigg_ids': ['4fe4s_c']}
fe2_m {'original_bigg_ids': ['fe2_c']}
dcacoa_r {'original_bigg_ids': ['dcacoa_c']}
1agly3p_RT_r {'original_bigg_ids': ['1agly3p_SC_c']}
pa_RT_r {'original_bigg_ids': ['pa_SC_c']}
12dgr_RT_r {'original_bigg_ids': ['12dgr_SC_c']}
dagpy_RT_r {'original_bigg_ids': ['dagpy_SC_c']}
triglyc_RT_r {'original_bigg_ids': ['triglyc_SC_c']}
epistest_RT_r {'original_bigg_ids': ['epistest_SC_c']}
ergstest_RT_r {'original_bigg_ids': ['ergstest_SC_c']}
fecostest_RT_r {'original_bigg_ids': ['fecostest_SC_c']}
lanostest_RT_r {'original_bigg_ids': ['lanostest_SC_c']}
zymstest_RT_r {'original_bigg_ids': ['zymstest_SC_c']}
1agpc_RT_r {'original_bigg_ids': ['1agpc_SC_c']}
pc_RT_r {'original_bigg_ids': ['pc_SC_c']}
pe_RT_e {'original_bigg_ids': ['pe_SC_c']}
g3pe_e {'original_bigg_ids': ['g3pe_c']}
triglyc_RT_d {'original_bigg_ids': ['triglyc_SC_c']}
12dgr_RT_d {'original_bigg_ids': ['12dgr_SC_c']}
epistest_RT_d {'original_bigg_ids': ['epistest_SC_c']}
ergstest_RT_d {'original_bigg_ids': ['ergstest_SC_c']}
fecostest_RT_d {'original_bigg_ids': ['fecostest_SC_c']}
lanostest_RT_d {'original_bigg_ids': ['lanostest_SC_c']}
zymstest_RT_d {'original_bigg_ids': ['zymstest_SC_c']}
mag_RT_d {'original_bigg_ids': ['12dgr_SC_c']}
cdpdag_RT_r {'original_bigg_ids': ['cdpdag_SC_c']}
ptd1ino_RT_r {'original_bigg_ids': ['ptd1ino_SC_c']}
ptd4ino_RT_r {'original_bigg_ids': ['ptd4ino_SC_c']}
ptd135bp_RT_r {'original_bigg_ids': ['ptd135bp_SC_c']}
ptd3ino_RT_r {'original_bigg_ids': ['ptd3ino_SC_c']}
ptd145bp_RT_r {'original_bigg_ids': ['ptd145bp_SC_c']}
ps_RT_r {'original_bigg_ids': ['ps_SC_c']}
ptdmeeta_RT_r {'original_bigg_ids': ['ptdmeeta_SC_c']}
ptd2meeta_RT_r {'original_bigg_ids': ['ptd2meeta_SC_c']}
pa_RT_n {'original_bigg_ids': ['pa_SC_c']}
pc_RT_n {'original_bigg_ids': ['pc_SC_c']}
ptd3ino_RT_n {'original_bigg_ids': ['ptd3ino_SC_c']}
ptd135bp_RT_n {'original_bigg_ids': ['ptd135bp_SC_c']}
ptd145bp_RT_n {'original_bigg_ids': ['ptd145bp_SC_c']}
12dgr_RT_n {'original_bigg_ids': ['12dgr_SC_c']}
mi134p_n {'original_bigg_ids': ['mi134p_c']}
mi14p_n {'original_bigg_ids': ['mi14p_c']}
iscs_m {'original_bigg_ids': ['iscs_c']}
iscssh_m {'original_bigg_ids': ['iscssh_c']}
iscu_2fe2s_m {'original_bigg_ids': ['iscu_2fe2s_c']}
iscu_m {'original_bigg_ids': ['iscu_c']}
iscu_2fe2s2_m {'original_bigg_ids': ['iscu_2fe2s2_c']}
iscu_4fe4s_m {'original_bigg_ids': ['iscu_4fe4s_c']}
6pgl_x {'original_bigg_ids': ['6pgl_c']}
g6p_x {'original_bigg_ids': ['g6p_c']}
6pgc_x {'original_bigg_ids': ['6pgc_c']}
ru5p__D_x {'original_bigg_ids': ['ru5p__D_c']}
hcit_m {'original_bigg_ids': ['hcit_m']}
co2_e {'original_bigg_ids': ['co2_e']}
o2_e {'original_bigg_ids': ['o2_e']}
co2_n {'original_bigg_ids': ['co2_n']}
pcrn_c {'original_bigg_ids': ['pcrn[c]']}
ocdcea_x {'original_bigg_ids': ['ocdcea_c']}
ocdcya_x {'original_bigg_ids': ['ocdcya_c']}
lnlncg_x {'original_bigg_ids': ['lnlncg_c']}
kynate_c {'original_bigg_ids': ['kynate[c]']}
C02470_c {'original_bigg_ids': ['C02470[c]']}
ind3eth_e {'original_bigg_ids': ['ind3eth_e']}
amuco_c {'original_bigg_ids': ['amuco_c']}
2obut_e {'original_bigg_ids': ['2obut[e]']}
pydx5p_m {'original_bigg_ids': ['pydx5p_m']}
psuri_c {'original_bigg_ids': ['psuri_c']}
6hmhpt_c {'original_bigg_ids': ['6hmhpt_c']}
ocdcead_c {'original_bigg_ids': ['ocdcea_c']}
prist_r {'original_bigg_ids': ['prist_c']}
pristanal_r {'original_bigg_ids': ['pristanal_c']}
hdc2ea_r {'original_bigg_ids': ['hdc2ea_c']}
hxdceal_r {'original_bigg_ids': ['hxdceal_c']}
4hbald_m {'original_bigg_ids': ['4hbald_c']}
glyclt_e {'original_bigg_ids': ['glyclt[e]']}
glx_e {'original_bigg_ids': ['glx[e]']}
quin_c {'original_bigg_ids': ['quin_c']}
cinnm_c {'original_bigg_ids': ['cinnm_c']}
caffcoa_c {'original_bigg_ids': ['caffcoa_c']}
ferulcoa_c {'original_bigg_ids': ['ferulcoa_c']}
4abz_e {'original_bigg_ids': ['4abz_e']}
4abz_m {'original_bigg_ids': ['4abz_m']}
3npab_m {'original_bigg_ids': ['3nphb_m']}
3npahb_m {'original_bigg_ids': ['3npdhb_m']}
sertrna_m {'original_bigg_ids': ['sertrna_c']}
trnaser_m {'original_bigg_ids': ['trnaser_c']}
hpdcacoa_r {'original_bigg_ids': ['hpdcacoa_c']}
arachcoa_r {'original_bigg_ids': ['arachcoa_c']}
docoscoa_r {'original_bigg_ids': ['docoscoa[r]']}
docoscoa_x {'original_bigg_ids': ['docoscoa[x]']}
arach_x {'original_bigg_ids': ['arach_c']}
docosac_c {'original_bigg_ids': ['docosac[c]']}
docosac_x {'original_bigg_ids': ['docosac[c]']}
lnlncg_e {'original_bigg_ids': ['lnlncg[e]']}
hpdca_e {'original_bigg_ids': ['hpdca[e]']}
docosac_e {'original_bigg_ids': ['docosac[e]']}
mg2_c {'original_bigg_ids': ['mg2_c']}
mg2_e {'original_bigg_ids': ['mg2_e']}
mn2_c {'original_bigg_ids': ['mn2_c']}
mn2_e {'original_bigg_ids': ['mn2_e']}
cu2_e {'original_bigg_ids': ['cu2_e']}
zn2_c {'original_bigg_ids': ['zn2_c']}
zn2_e {'original_bigg_ids': ['zn2_e']}
phaccoa_c {'original_bigg_ids': ['phaccoa[c]']}
pac_x {'original_bigg_ids': ['pac[m]']}
phaccoa_x {'original_bigg_ids': ['phaccoa[m]']}
T4hcinnm_x {'original_bigg_ids': ['T4hcinnm[m]']}
coucoa_x {'original_bigg_ids': ['coucoa[m]']}
3hpp_m {'original_bigg_ids': ['3hpp[m]']}
4hbzcoa_x {'original_bigg_ids': ['4hbzcoa_m']}
34hp3hpcoa_x {'original_bigg_ids': ['4hbzcoa_m']}
4hbzaccoa_x {'original_bigg_ids': ['4hbzcoa_m']}
4hbz_x {'original_bigg_ids': ['4hbz_m']}
T4hcinnm_e {'original_bigg_ids': ['T4hcinnm_e']}
34dhbz_x {'original_bigg_ids': ['34dhbz_c']}
3oxoadp_m {'original_bigg_ids': ['3oxoadp_c']}
oxadpcoa_m {'original_bigg_ids': ['oxadpcoa_c']}
fer_e {'original_bigg_ids': ['fer_e']}
34dhcinm_c {'original_bigg_ids': ['34dhcinm_c']}
34dhcinm_x {'original_bigg_ids': ['34dhcinm_c']}
caffcoa_x {'original_bigg_ids': ['caffcoa_c']}
3dhp3hpcoa_x {'original_bigg_ids': ['4hbzcoa_m']}
34dhbzaccoa_x {'original_bigg_ids': ['4hbzcoa_m']}
34dhbzcoa_x {'original_bigg_ids': ['4hbzcoa_m']}
bz_e {'original_bigg_ids': ['bz_e']}
bz_c {'original_bigg_ids': ['bz_p']}
arab__L_e {'original_bigg_ids': ['arab_L_e']}
xylu__L_c {'original_bigg_ids': ['xylu_L[c]']}
arab__D_c {'original_bigg_ids': ['arab_D_c']}
lyx__L_e {'original_bigg_ids': ['lyx__L_e']}
lyx__L_c {'original_bigg_ids': ['lyx__L_p']}
abt_e {'original_bigg_ids': ['abt[e]']}
xylt_e {'original_bigg_ids': ['xylt[e]']}
xylu__D_e {'original_bigg_ids': ['xylu_D[e]']}
abt__D_e {'original_bigg_ids': ['abt_D[e]']}
rbl__D_e {'original_bigg_ids': ['rbl_D[e]']}
ggdp_r {'original_bigg_ids': ['ggdp_c']}
succ_e {'original_bigg_ids': ['succ_e']}
tre_e {'original_bigg_ids': ['tre[e]']}
galt_e {'original_bigg_ids': ['galt[e]']}
ser__D_e {'original_bigg_ids': ['ser__D_e']}
sbt__D_e {'original_bigg_ids': ['sbt_D_e']}
glcn_e {'original_bigg_ids': ['glcn[e]']}
glyc3p_e {'original_bigg_ids': ['glyc3p[e]']}
g6p_e {'original_bigg_ids': ['g6p_e']}
mthgxl_e {'original_bigg_ids': ['mthgxl_c']}
h2o2_e {'original_bigg_ids': ['h2o2[e]']}
rib__D_e {'original_bigg_ids': ['rib_D[e]']}
thymd_e {'original_bigg_ids': ['thymd[e]']}
asp__D_e {'original_bigg_ids': ['asp_D[e]']}
asp__D_c {'original_bigg_ids': ['asp_D[c]']}
12ppd__S_e {'original_bigg_ids': ['12ppd__S_e']}
12ppd__R_e {'original_bigg_ids': ['12ppd_R[e]']}
akg_e {'original_bigg_ids': ['akg_e']}
g1p_e {'original_bigg_ids': ['g1p[e]']}
f6p_e {'original_bigg_ids': ['f6p_e']}
malttr_e {'original_bigg_ids': ['malttr[e]']}
melib_e {'original_bigg_ids': ['melib_e']}
melib_c {'original_bigg_ids': ['melib_c']}
cit_e {'original_bigg_ids': ['cit_e']}
fum_e {'original_bigg_ids': ['fum_e']}
ppa_e {'original_bigg_ids': ['ppa[e]']}
cellb_e {'original_bigg_ids': ['cellb_e']}
acac_e {'original_bigg_ids': ['acac[e]']}
mal__L_e {'original_bigg_ids': ['mal_L_e']}
tym_e {'original_bigg_ids': ['tym[e]']}
peamn_e {'original_bigg_ids': ['peamn_e']}
etha_e {'original_bigg_ids': ['etha[e]']}
glycogen_e {'original_bigg_ids': ['glycogen_c']}
mannan_e {'original_bigg_ids': ['mannan_r']}
arab__D_e {'original_bigg_ids': ['arab_D_e']}
drib_e {'original_bigg_ids': ['drib[e]']}
srb__L_e {'original_bigg_ids': ['srb_L_e']}
but_e {'original_bigg_ids': ['but[e]']}
but_c {'original_bigg_ids': ['but[c]']}
hxa_e {'original_bigg_ids': ['hxa[e]']}
btcoa_c {'original_bigg_ids': ['btcoa[c]']}
c4crn_c {'original_bigg_ids': ['c4crn[c]']}
c6crn_c {'original_bigg_ids': ['c6crn[c]']}
c4crn_m {'original_bigg_ids': ['c4crn[m]']}
c6crn_m {'original_bigg_ids': ['c6crn[c]']}
4hbz_e {'original_bigg_ids': ['4hbz[e]']}
bhb_e {'original_bigg_ids': ['bhb[e]']}
bhb_c {'original_bigg_ids': ['bhb[c]']}
ghb_e {'original_bigg_ids': ['ghb_c']}
ghb_c {'original_bigg_ids': ['ghb_c']}
5dglcn_e {'original_bigg_ids': ['5dglcn_e']}
quin_e {'original_bigg_ids': ['quin_e']}
ad_e {'original_bigg_ids': ['ad_c']}
acglu_e {'original_bigg_ids': ['acglu[e]']}
hom__L_e {'original_bigg_ids': ['hom_L[e]']}
dha_e {'original_bigg_ids': ['dha_e']}
btd_RR_e {'original_bigg_ids': ['btd_RR_e']}
citr__L_e {'original_bigg_ids': ['citr_L[e]']}
mma_e {'original_bigg_ids': ['mma[e]']}
agm_e {'original_bigg_ids': ['agm[e]']}
agm_c {'original_bigg_ids': ['agm[c]']}
hista_e {'original_bigg_ids': ['hista[e]']}
frmd_e {'original_bigg_ids': ['frmd_c']}
gsn_e {'original_bigg_ids': ['gsn[e]']}
thym_e {'original_bigg_ids': ['thym[e]']}
xan_e {'original_bigg_ids': ['xan[e]']}
xtsn_e {'original_bigg_ids': ['xtsn[e]']}
ppi_e {'original_bigg_ids': ['ppi[e]']}
pppi_e {'original_bigg_ids': ['pppi_c']}
amp_e {'original_bigg_ids': ['amp[e]']}
cmp_e {'original_bigg_ids': ['cmp[e]']}
gmp_e {'original_bigg_ids': ['gmp[e]']}
ump_e {'original_bigg_ids': ['ump[e]']}
glyc2p_e {'original_bigg_ids': ['glyc2p[e]']}
cbp_e {'original_bigg_ids': ['cbp_c']}
2pg_e {'original_bigg_ids': ['2pg[e]']}
glyc__R_e {'original_bigg_ids': ['glyc_R[e]']}
3pg_e {'original_bigg_ids': ['3pg[e]']}
pep_e {'original_bigg_ids': ['pep[e]']}
2doxg6p_e {'original_bigg_ids': ['2doxg6p_c']}
gam6p_e {'original_bigg_ids': ['gam6p_e']}
6pgc_e {'original_bigg_ids': ['6pgc_c']}
35ccmp_e {'original_bigg_ids': ['35ccmp_c']}
man1p_e {'original_bigg_ids': ['man1p_c']}
man6p_e {'original_bigg_ids': ['man6p_e']}
pser__L_e {'original_bigg_ids': ['pser_L[e]']}
cholp_e {'original_bigg_ids': ['cholp[e]']}
ethamp_e {'original_bigg_ids': ['ethamp[e]']}
dtmp_e {'original_bigg_ids': ['dtmp[e]']}
minohp_e {'original_bigg_ids': ['minohp_e']}
minohp_c {'original_bigg_ids': ['minohp[c]']}
minohp_r {'original_bigg_ids': ['minohp_n']}
mi13456p_r {'original_bigg_ids': ['mi13456p_n']}
mi1345p_r {'original_bigg_ids': ['mi1345p_n']}
mi1456p_r {'original_bigg_ids': ['mi1456p_n']}
mi145p_r {'original_bigg_ids': ['mi145p_n']}
tsul_e {'original_bigg_ids': ['tsul[e]']}
Lcyst_e {'original_bigg_ids': ['Lcyst[e]']}
cyst__L_e {'original_bigg_ids': ['cyst_L[e]']}
metsox_S__L_e {'original_bigg_ids': ['metsox_S__L_e']}
metsox_R__L_e {'original_bigg_ids': ['metsox_R__L_e']}
taur_e {'original_bigg_ids': ['taur[e]']}
hyptaur_e {'original_bigg_ids': ['hyptaur[e]']}
mbdg_e {'original_bigg_ids': ['mbdg_e']}
meoh_e {'original_bigg_ids': ['meoh_e']}
rbt_c {'original_bigg_ids': ['rbt[c]']}
rbt_e {'original_bigg_ids': ['rbt[e]']}
arbt_e {'original_bigg_ids': ['arbt_e']}
hqn_e {'original_bigg_ids': ['hqn_e']}
salcn_e {'original_bigg_ids': ['salcn_e']}
2hxmp_e {'original_bigg_ids': ['2hxmp_e']}
btn_e {'original_bigg_ids': ['btn_e']}
fol_e {'original_bigg_ids': ['fol[e]']}
pydxn_e {'original_bigg_ids': ['pydxn[e]']}
```

In [7]:

```
# check original_bigg_ids for met and rxn
```

In [8]:

```
for m in model.metabolites:
    if m.annotation:
        print(m, m.annotation)
```

```
mbdg_e {'bigg.metabolite': ['mbdg'], 'metanetx.chemical': ['MNXM10816'], 'sbo': 'SBO:0000247', 'seed.compound': ['cpd15585']}
meoh_e {'bigg.metabolite': ['meoh'], 'biocyc': ['META:ALCOHOL-GROUP', 'META:METOH'], 'chebi': ['CHEBI:52090', 'CHEBI:25227', 'CHEBI:44080', 'CHEBI:17790', 'CHEBI:44553', 'CHEBI:14588', 'CHEBI:6816'], 'envipath': ['650babc9-9d68-4b73-9332-11972ca26f7b/compound/2c247069-759d-4108-ba1b-b1c81678bab7', '32de3cf4-e3e6-4168-956e-32fa5ddb0ce1/compound/b5522308-bb5c-41bb-a11a-e9b9ae61da85'], 'hmdb': ['HMDB01875'], 'inchi_key': ['OKKJLVBELUTLKV-UHFFFAOYSA-N'], 'kegg.compound': ['C00132'], 'kegg.drug': ['D02309'], 'metanetx.chemical': ['MNXM157'], 'reactome.compound': ['5693711', '5359061'], 'sabiork': ['55'], 'sbo': 'SBO:0000247', 'seed.compound': ['cpd00116']}
arbt_e {'bigg.metabolite': ['arbt'], 'biocyc': ['META:HYDROQUINONE-O-BETA-D-GLUCOPYRANOSIDE'], 'chebi': ['CHEBI:2806', 'CHEBI:18305', 'CHEBI:14417'], 'hmdb': ['HMDB29943'], 'inchi_key': ['BJRNKVDFDLYUGJ-RMPHRYRLSA-N'], 'kegg.compound': ['C06186'], 'metanetx.chemical': ['MNXM2683'], 'sabiork': ['3779'], 'sbo': 'SBO:0000247', 'seed.compound': ['cpd03696']}
hqn_e {'bigg.metabolite': ['hqn'], 'biocyc': ['META:HYDROQUINONE'], 'chebi': ['CHEBI:17594', 'CHEBI:24645', 'CHEBI:14416', 'CHEBI:5793'], 'envipath': ['32de3cf4-e3e6-4168-956e-32fa5ddb0ce1/compound/792b6781-d85c-4c1d-bd82-a082fef85353', '650babc9-9d68-4b73-9332-11972ca26f7b/compound/019d5653-a5fc-4ab4-8c6f-47577e8ef560', '4fd7f3e0-dd25-43ac-9453-dda3e52396e4/compound/44858aec-2eb4-41d1-a154-388fd87dccac', '5882df9c-dae1-4d80-a40e-db4724271456/compound/33365a7e-24d4-4212-ad80-7b9a92572df5'], 'hmdb': ['HMDB02434'], 'inchi_key': ['QIGBRXMKCJKVMJ-UHFFFAOYSA-N'], 'kegg.compound': ['C00530'], 'kegg.drug': ['D00073'], 'metanetx.chemical': ['MNXM376'], 'sabiork': ['2076'], 'sbo': 'SBO:0000247', 'seed.compound': ['cpd00415']}
salcn_e {'bigg.metabolite': ['salcn'], 'biocyc': ['META:CPD-1142'], 'chebi': ['CHEBI:15058', 'CHEBI:26591', 'CHEBI:9002', 'CHEBI:17814'], 'hmdb': ['HMDB03546'], 'inchi_key': ['NGFMICBWJRZIBI-UJPOAAIJSA-N'], 'kegg.compound': ['C01451'], 'metanetx.chemical': ['MNXM2561'], 'sabiork': ['3778'], 'sbo': 'SBO:0000247', 'seed.compound': ['cpd01030']}
2hxmp_e {'bigg.metabolite': ['2hxmp'], 'biocyc': ['META:CPD-173'], 'chebi': ['CHEBI:974', 'CHEBI:15059', 'CHEBI:16464', 'CHEBI:9004', 'CHEBI:26592'], 'envipath': ['650babc9-9d68-4b73-9332-11972ca26f7b/compound/3935c8e4-2be9-4f36-8bb2-8e2b2180cfa7'], 'hmdb': ['HMDB59709'], 'inchi_key': ['CQRYARSYNCAZFO-UHFFFAOYSA-N'], 'kegg.compound': ['C02323'], 'kegg.drug': ['D05790'], 'metanetx.chemical': ['MNXM1855'], 'sabiork': ['6141'], 'sbo': 'SBO:0000247', 'seed.compound': ['cpd01553']}
```

In [9]:

```
set(sum([list(m.annotation.keys()) for m in model.metabolites],[]))
```

Out[9]:

```
{'bigg.metabolite',
 'biocyc',
 'chebi',
 'envipath',
 'hmdb',
 'inchi_key',
 'kegg.compound',
 'kegg.drug',
 'metanetx.chemical',
 'reactome.compound',
 'sabiork',
 'sbo',
 'seed.compound'}
```

In [10]:

```
bigg_universal.metabolites.get_by_id('glc__D_c').annotation
```

Out[10]:

```
[['KEGG Compound', 'http://identifiers.org/kegg.compound/C00031'],
 ['CHEBI', 'http://identifiers.org/chebi/CHEBI:12965'],
 ['CHEBI', 'http://identifiers.org/chebi/CHEBI:17634'],
 ['CHEBI', 'http://identifiers.org/chebi/CHEBI:20999'],
 ['CHEBI', 'http://identifiers.org/chebi/CHEBI:4167'],
 ['KEGG Drug', 'http://identifiers.org/kegg.drug/D00009'],
 ['Human Metabolome Database', 'http://identifiers.org/hmdb/HMDB00122'],
 ['Human Metabolome Database', 'http://identifiers.org/hmdb/HMDB06564'],
 ['BioCyc', 'http://identifiers.org/biocyc/META:Glucopyranose'],
 ['MetaNetX (MNX) Chemical',
  'http://identifiers.org/metanetx.chemical/MNXM41'],
 ['InChI Key', 'https://identifiers.org/inchikey/WQZGKKKJIJFFOK-GASJEMHNSA-N'],
 ['SEED Compound', 'http://identifiers.org/seed.compound/cpd00027'],
 ['SEED Compound', 'http://identifiers.org/seed.compound/cpd26821']]
```

In [11]:

```
# How to deal with multiple annotations for the same field?
dict(bigg_universal.metabolites.get_by_id('glc__D_c').annotation)
```

Out[11]:

```
{'KEGG Compound': 'http://identifiers.org/kegg.compound/C00031',
 'CHEBI': 'http://identifiers.org/chebi/CHEBI:4167',
 'KEGG Drug': 'http://identifiers.org/kegg.drug/D00009',
 'Human Metabolome Database': 'http://identifiers.org/hmdb/HMDB06564',
 'BioCyc': 'http://identifiers.org/biocyc/META:Glucopyranose',
 'MetaNetX (MNX) Chemical': 'http://identifiers.org/metanetx.chemical/MNXM41',
 'InChI Key': 'https://identifiers.org/inchikey/WQZGKKKJIJFFOK-GASJEMHNSA-N',
 'SEED Compound': 'http://identifiers.org/seed.compound/cpd26821'}
```

In [12]:

```
set(sum([[x[0] for x in m.annotation] for m in bigg_universal.metabolites],[]))
```

Out[12]:

```
{'BioCyc',
 'CHEBI',
 'Human Metabolome Database',
 'InChI Key',
 'KEGG Compound',
 'KEGG Drug',
 'KEGG Glycan',
 'LipidMaps',
 'MetaNetX (MNX) Chemical',
 'Reactome Compound',
 'SEED Compound'}
```

In [13]:

```
for m in model.metabolites:
    if m.id in bigg_universal.metabolites:
        #m.notes.update(dict(bigg_universal.metabolites.get_by_id(m.id).notes))
        #m.annotation.update(dict(bigg_universal.metabolites.get_by_id(m.id).annotation))
        m.annotation = dict(bigg_universal.metabolites.get_by_id(m.id).annotation)
```

In [14]:

```
# Reformat to memote style
m_annotation = dict({'BioCyc': 'biocyc',
                     'CHEBI': 'chebi',
                     'Human Metabolome Database': 'hmdb',
                     'InChI Key': 'inchikey',
                     'KEGG Compound': 'kegg.compound',
                     'KEGG Drug': 'kegg.drug',
                     'KEGG Glycan': 'kegg.glycan',
                     'LipidMaps': 'lipidmaps',
                     'MetaNetX (MNX) Chemical': 'metanetx.chemical',
                     'Reactome Compound': 'reactome',
                     'SEED Compound': 'seed.compound'})
```

In [15]:

```
for m in model.metabolites:
    if m.annotation:
        m.annotation = dict((m_annotation[k], v.rsplit('/',1)[-1]) for k, v in m.annotation.items())
    m.annotation['bigg.metabolite'] = m.id
```

In [16]:

```
for m in model.metabolites:
    m.annotation['sbo'] = 'SBO:0000247'
```

In [17]:

```
model.metabolites.get_by_id('glc__D_c').annotation
```

Out[17]:

```
{'kegg.compound': 'C00031',
 'chebi': 'CHEBI:4167',
 'kegg.drug': 'D00009',
 'hmdb': 'HMDB06564',
 'biocyc': 'META:Glucopyranose',
 'metanetx.chemical': 'MNXM41',
 'inchikey': 'WQZGKKKJIJFFOK-GASJEMHNSA-N',
 'seed.compound': 'cpd26821',
 'bigg.metabolite': 'glc__D_c',
 'sbo': 'SBO:0000247'}
```

#### Reaction annotation¶

In [18]:

```
for r in sorted(model.reactions, key=lambda x: x.id):
    if r.notes:
        print(r, r.notes)
```

```
10FTHFGLULLm: 10fthf_m + atp_m + glu__L_m --> 10fthfglu__L_m + adp_m + pi_m {'original_bigg_ids': ['10FTHFGLULLm']}
12PPDRte: 12ppd__R_c <=> 12ppd__R_e {'original_bigg_ids': ['12PPDRte']}
12PPDSt: 12ppd__S_e <=> 12ppd__S_c {'original_bigg_ids': ['12PPDStpp']}
13BGHe: 13BDglcn_e + h2o_e --> glc__D_e {'original_bigg_ids': ['13BGHe']}
13DAMPPOX: 13dampp_c + h2o_c + o2_c --> bamppald_c + h2o2_c + nh4_c {'original_bigg_ids': ['13DAMPPOX']}
13GS: udpg_c --> 13BDglcn_c + h_c + udp_c {'original_bigg_ids': ['13GS']}
16GS: udpg_c --> 16BDglcn_c + h_c + udp_c {'original_bigg_ids': ['16GS']}
2AMACHYD: 2amac_c + h2o_c --> nh4_c + pyr_c {'original_bigg_ids': ['2AMACHYD']}
2DDARAA: 2ddara_c <=> gcald_c + pyr_c {'original_bigg_ids': ['2DDARAA']}
2DOXG6PP: 2doxg6p_c + h2o_c --> 2dglc_c + pi_c {'original_bigg_ids': ['2DOXG6PP']}
2HESR: dtgcl_c + 2.0 gthrd_c --> gthox_c + 2.0 mercpeth_c {'original_bigg_ids': ['2HESR']}
2OBUTt: 2obut_e + h_e <=> 2obut_c + h_c {'original_bigg_ids': ['2OBUTt']}
2OBUTtm: 2obut_c <=> 2obut_m {'original_bigg_ids': ['2OBUTtm']}
2OH3K5MPPISO: h2o_c + hkmpp_c --> dhmtp_c + pi_c {'original_bigg_ids': ['ACRSP']}
2OXOADOXm: 2oxoadp_m + coa_m + nad_m --> co2_m + glutcoa_m + nadh_m {'original_bigg_ids': ['2OXOADOXm']}
2OXOADPTm: 2oxoadp_c + akg_m <=> 2oxoadp_m + akg_c {'original_bigg_ids': ['2OXOADPTm']}
2PGPe: 2pg_e + h2o_e --> glyc__R_e + pi_e {'original_bigg_ids': ['G2PP']}
34DHALDD: 34dhpac_c + h2o_c + nad_c --> 34dhpha_c + 2.0 h_c + nadh_c {'original_bigg_ids': ['34DHPLACOX']}
34DHBZACCOARp: 3dhp3hpcoa_x + nad_x --> 34dhbzaccoa_x + h_x + nadh_x {'original_bigg_ids': ['4HBZCOAFm']}
34DHBZACCOATp: 34dhbzaccoa_x + coa_x --> 34dhbzcoa_x + accoa_x {'original_bigg_ids': ['4HBZCOAFm']}
34DHBZCOAEp: 34dhbzcoa_x + h2o_x --> 34dhbz_x + coa_x + h_x {'original_bigg_ids': ['4HBZFm']}
34DHBZtp: 34dhbz_c <=> 34dhbz_x {'original_bigg_ids': ['4HBZtm']}
34DHCINMtp: 34dhcinm_c --> 34dhcinm_x {'original_bigg_ids': ['FA240tp']}
34DHPLACOX_NADP: 34dhpac_c + h2o_c + nadp_c <=> 34dhpha_c + 2.0 h_c + nadph_c {'original_bigg_ids': ['34DHPLACOX_LPAREN_NADP_RPAREN_']}
34HPPOR: 34hpp_c + o2_c --> co2_c + hgentis_c {'original_bigg_ids': ['34HPPOR']}
34HPPYRDC: 34hpp_c + h_c --> 4hoxpacd_c + co2_c {'original_bigg_ids': ['PPYRDC']}
3AIBTm: 2mop_m + glu__L_m <=> 3aib_m + akg_m {'original_bigg_ids': ['3AIBTm']}
3AMACHYD: 3amac_c + h2o_c + h_c --> msa_c + nh4_c {'original_bigg_ids': ['3AMACHYD']}
3HAO: 3hanthrn_c + o2_c --> cmusa_c + h_c {'original_bigg_ids': ['3HAO']}
3HBCOAHLm: 3hibutcoa_m + h2o_m --> 3hmp_m + coa_m + h_m {'original_bigg_ids': ['3HBCOAHLm']}
3HCINNMH: 3hcinnm_c + h_c + nadh_c + o2_c --> dhcinnm_c + h2o_c + nad_c {'original_bigg_ids': ['3HCINNMH']}
3HKYNAKGAT: akg_c + hLkynr_c --> 42A3HP24DB_c + glu__L_c {'original_bigg_ids': ['3HKYNAKGAT']}
3HLYTCL: 34dhphe_c + h_c --> co2_c + dopa_c {'original_bigg_ids': ['3HLYTCL']}
3HPCOAHYDm: 3hpcoa_m + h2o_m --> 3hpp_m + coa_m + h_m {'original_bigg_ids': ['HICH']}
3HPDm: 3hpp_m + nad_m --> h_m + msa_m + nadh_m {'original_bigg_ids': ['r0365']}
3HPPPNH: 3hpppn_c + h_c + nadh_c + o2_c --> dhpppn_c + h2o_c + nad_c {'original_bigg_ids': ['3HPPPNH']}
3HXKYNDCL: hLkynr_c + h_c --> 3hxkynam_c + co2_c {'original_bigg_ids': ['3HXKYNDCL']}
3IPM3MT: 3c2hmp_c + amet_c --> 3ipmmest_c + ahcys_c {'original_bigg_ids': ['3IPM3MT']}
3MOBDC: 3mob_c + h_c --> 2mppal_c + co2_c {'original_bigg_ids': ['3MOBDC']}
3MOBtm: 3mob_c <=> 3mob_m {'original_bigg_ids': ['3MOBtm']}
3MOPDC: 3mop_c + h_c --> 2mbald_c + co2_c {'original_bigg_ids': ['3MOPDC']}
3MOPtm: 3mop_c <=> 3mop_m {'original_bigg_ids': ['3MOPtm']}
3NPABH_m: 3npab_m + h_m + nadph_m + o2_m --> 3npahb_m + h2o_m + nadp_m {'original_bigg_ids': ['3NPHBH2_m']}
3NPAHBDH_m: 3npahb_m + 3.0 h_m + 2.0 nadph_m + o2_m --> 3npdhb_m + h2o_m + 2.0 nadp_m + nh4_m {'original_bigg_ids': ['3NPHBH2_m']}
3NPHBH2_m: 3nphb_m + h_m + nadph_m + o2_m --> 3npdhb_m + h2o_m + nadp_m {'original_bigg_ids': ['3NPHBH2_m']}
3OADPCOATm: 3oxoadp_m + succoa_m --> oxadpcoa_m + succ_m {'original_bigg_ids': ['3OADPCOAT']}
3OXCOATm: coa_m + oxadpcoa_m --> accoa_m + succoa_m {'original_bigg_ids': ['3OXCOAT']}
3OXOADPTm: 3oxoadp_c + akg_m <=> 3oxoadp_m + akg_c {'original_bigg_ids': ['2OXOADPTm']}
3PGPe: 3pg_e + h2o_e --> glyc__R_e + pi_e {'original_bigg_ids': ['G2PP']}
3SALAASPm: 3sala_m + asp__L_c <=> 3sala_c + asp__L_m {'original_bigg_ids': ['3SALAASPm']}
3SALACBOXL: 3sala_c + h_c --> co2_c + hyptaur_c {'original_bigg_ids': ['3SALACBOXL']}
3SALAOX: 2.0 3sala_c + 2.0 h_c + o2_c --> 2.0 Lcyst_c {'original_bigg_ids': ['3SALAOX']}
3SALATAi: 3sala_c + akg_c + h_c --> 3snpyr_c + glu__L_c {'original_bigg_ids': ['3SALATAi']}
3SALATAim: 3sala_m + akg_m + h_m --> 3snpyr_m + glu__L_m {'original_bigg_ids': ['3SALATAim']}
3SALAt: 3sala_e <=> 3sala_c {'original_bigg_ids': ['CO2t']}
3SPYRSP: 3snpyr_c + h2o_c --> h_c + pyr_c + so3_c {'original_bigg_ids': ['3SPYRSP']}
3SPYRSPm: 3snpyr_m + h2o_m --> h_m + pyr_m + so3_m {'original_bigg_ids': ['3SPYRSPm']}
4ABUTtm: 4abut_c <=> 4abut_m {'original_bigg_ids': ['4ABUTtm']}
4ABZt: 4abz_c <=> 4abz_e {'original_bigg_ids': ['4ABZt']}
4ABZtm: 4abz_c <=> 4abz_m {'original_bigg_ids': ['4ABZtm']}
4CMCOAS: T4hcinnm_c + atp_c + coa_c --> amp_c + coucoa_c + ppi_c {'original_bigg_ids': ['HMR_6784']}
4CMLCL_kt: 4cml_c + h_c --> 5odhf2a_c + co2_c {'original_bigg_ids': ['4CMLCL_kt']}
4HBALDDm: 4hbald_m + h2o_m + nad_m --> 4hbz_m + 2.0 h_m + nadh_m {'original_bigg_ids': ['VNDH_2']}
4HBHYOXp: 4hbz_x + h_x + nadph_x + o2_x --> 34dhbz_x + h2o_x + nadp_x {'original_bigg_ids': ['4HBHYOX']}
4HBZACCOARp: 34hp3hpcoa_x + nad_x --> 4hbzaccoa_x + h_x + nadh_x {'original_bigg_ids': ['4HBZCOAFm']}
4HBZACCOATp: 4hbzaccoa_x + coa_x --> 4hbzcoa_x + accoa_x {'original_bigg_ids': ['4HBZCOAFm']}
4HBZCOAEp: 4hbzcoa_x + h2o_x --> 4hbz_x + coa_x + h_x {'original_bigg_ids': ['4HBZFm']}
4HBZte: 4hbz_c <=> 4hbz_e {'original_bigg_ids': ['4HBZte']}
4HBZtm: 4hbz_c <=> 4hbz_m {'original_bigg_ids': ['4HBZtm']}
4HBZtp: 4hbz_c <=> 4hbz_x {'original_bigg_ids': ['4HBZtm']}
4HGLSDm: 4hglusa_m + h2o_m + nad_m --> e4hglu_m + 2.0 h_m + nadh_m {'original_bigg_ids': ['4HGLSDm']}
4HOXPACDOX_NADP: 4hoxpacd_c + h2o_c + nadp_c <=> 4hphac_c + 2.0 h_c + nadph_c {'original_bigg_ids': ['4HOXPACDOX_LPAREN_NADP_RPAREN_']}
4MOPDC: 4mop_c + h_c --> 3mbald_c + co2_c {'original_bigg_ids': ['4MOPDC']}
4MOPtm: 4mop_c <=> 4mop_m {'original_bigg_ids': ['3MOPtm']}
56DH5FLURAAMH: 56dh5flura_c + h2o_c --> aflburppa_c {'original_bigg_ids': ['56DH5FLURAAMH']}
5ADTSTSTERONEGLCte: 5adtststeroneglc_c + atp_c + h2o_c --> 5adtststeroneglc_e + adp_c + h_c + pi_c {'original_bigg_ids': ['5ADTSTSTERONEGLCte']}
5AOPt2: 5aop_e + h_e --> 5aop_c + h_c {'original_bigg_ids': ['5AOPt2']}
5AOPtm: 5aop_c <=> 5aop_m {'original_bigg_ids': ['5AOPtm']}
5DGLCNR: 5dglcn_c + h_c + nadph_c --> glcn_c + nadp_c {'original_bigg_ids': ['5DGLCNR']}
5DGLCNt: 5dglcn_e <=> 5dglcn_c {'original_bigg_ids': ['CO2t']}
5DKGR: glcn_c + nad_c <=> 5dglcn_c + h_c + nadh_c {'original_bigg_ids': ['5DKGR']}
5FLURAt: 5flura_e <=> 5flura_c {'original_bigg_ids': ['5FLURAt']}
5HLTDL: 5htrp_c + h_c --> co2_c + srtn_c {'original_bigg_ids': ['5HLTDL']}
5HTRPDOX: 5htrp_c + o2_c --> 5hoxnfkyn_c {'original_bigg_ids': ['5HTRPDOX']}
5HXKYNDCL: 5hxkyn_c + h_c --> 5hxkynam_c + co2_c {'original_bigg_ids': ['5HXKYNDCL']}
6MPURPRT: 6mpur_c + prpp_c --> 6tins5mp_c + ppi_c {'original_bigg_ids': ['6MPURPRT']}
6MPURt: 6mpur_e <=> 6mpur_c {'original_bigg_ids': ['6MPURt']}
6TINS5MPOR: 6tins5mp_c + h2o_c + nad_c --> 6txan5mp_c + h_c + nadh_c {'original_bigg_ids': ['6TINS5MPOR']}
6TXAN5MPAML: 6txan5mp_c + atp_c + gln__L_c + h2o_c --> 6tgsnmp_c + amp_c + glu__L_c + 2.0 h_c + ppi_c {'original_bigg_ids': ['6TXAN5MPAML']}
A1E: Glc_aD_c <=> glc__D_c {'original_bigg_ids': ['A1E']}
A5PISO: ru5p__D_c <=> ara5p_c {'original_bigg_ids': ['A5PISO']}
AACOATx: acac_x + atp_x + coa_x --> aacoa_x + amp_x + ppi_x {'original_bigg_ids': ['AACOAT']}
AACTOOR: aact_c + h2o_c + o2_c --> h2o2_c + mthgxl_c + nh4_c {'original_bigg_ids': ['AACTOOR']}
AAMYLASE: 6.0 14glun_c --> 5.0 h2o_c + malthx_c {'original_bigg_ids': ['AAMYL']}
AASAD1: L2aadp_c + atp_c + h_c + nadph_c --> L2aadp6sa_c + amp_c + nadp_c + ppi_c {'original_bigg_ids': ['AASAD1']}
AASAD3m: L2aadp6sa_m + h2o_m + nad_m --> L2aadp_m + 2.0 h_m + nadh_m {'original_bigg_ids': ['AASAD3m']}
AATA: 2oxoadp_c + glu__L_c <=> L2aadp_c + akg_c {'original_bigg_ids': ['AATA']}
AATHA: h2o_c + tre_c --> 2.0 Glc_aD_c {'original_bigg_ids': ['AATHA']}
ABTA: 4abut_c + akg_c --> glu__L_c + sucsal_c {'original_bigg_ids': ['ABTA']}
ABTArm: 4abut_m + akg_m <=> glu__L_m + sucsal_m {'original_bigg_ids': ['ABTArm']}
ABTD: abt_c + nad_c <=> h_c + nadh_c + xylu__L_c {'original_bigg_ids': ['ABTD']}
ABT_Dt: abt__D_e <=> abt__D_c {'original_bigg_ids': ['ABT_Dt']}
ABTt: abt_e <=> abt_c {'original_bigg_ids': ['ABTti']}
ABUTt2r: 4abut_e + h_e <=> 4abut_c + h_c {'original_bigg_ids': ['ABUTt2r']}
ABZNPT_m: 4abz_m + npdp_m --> 3npab_m + ppi_m {'original_bigg_ids': ['HBZNPT_m']}
ACACT10m: 2maacoa_m + coa_m <=> accoa_m + ppcoa_m {'original_bigg_ids': ['ACACT10m']}
ACACT1m: 2.0 accoa_m <=> aacoa_m + coa_m {'original_bigg_ids': ['ACACT1m']}
ACACT1p: aacoa_x + coa_x --> 2.0 accoa_x {'original_bigg_ids': ['ACACT1']}
ACACT1r: 2.0 accoa_c <=> aacoa_c + coa_c {'original_bigg_ids': ['ACACT1r']}
ACACT2m: accoa_m + btcoa_m <-- 3ohcoa_m + coa_m {'original_bigg_ids': ['ACACT2m']}
ACACT2p: 3ohcoa_x + coa_x --> accoa_x + btcoa_x {'original_bigg_ids': ['ACACT2']}
ACACT3m: accoa_m + hxcoa_m <-- 3oocoa_m + coa_m {'original_bigg_ids': ['ACACT3m']}
ACACT3p: 3oocoa_x + coa_x --> accoa_x + hxcoa_x {'original_bigg_ids': ['ACACT3']}
ACACT4p: 3odcoa_x + coa_x --> accoa_x + occoa_x {'original_bigg_ids': ['ACACT4p']}
ACACT5m: accoa_m + dcacoa_m <-- 3oddcoa_m + coa_m {'original_bigg_ids': ['ACACT5m']}
ACACT5p: 3oddcoa_x + coa_x --> accoa_x + dcacoa_x {'original_bigg_ids': ['ACACT5p']}
ACACT6p: 3otdcoa_x + coa_x --> accoa_x + ddcacoa_x {'original_bigg_ids': ['ACACT6p']}
ACACT7p: 3ohdcoa_x + coa_x --> accoa_x + tdcoa_x {'original_bigg_ids': ['ACACT7p']}
ACACT8p: 3ohodcoa_x + coa_x --> accoa_x + pmtcoa_x {'original_bigg_ids': ['ACACT8p']}
ACACT9p: 3ohxccoa_x + coa_x --> accoa_x + ttccoa_x {'original_bigg_ids': ['ACACT9p']}
ACACt2: acac_e + h_e <=> acac_c + h_c {'original_bigg_ids': ['ACACt2']}
ACACt2m: acac_c + h_c <=> acac_m + h_m {'original_bigg_ids': ['ACACt2m']}
ACACtx: acac_c <=> acac_x {'original_bigg_ids': ['ACACtx']}
ACALDCD: 2.0 acald_c --> actn__R_c {'original_bigg_ids': ['ACALDCD']}
ACALDtm: acald_m <=> acald_c {'original_bigg_ids': ['ACALDtm']}
ACANTHAT: accoa_c + anth_c --> acanth_c + coa_c {'original_bigg_ids': ['ACANTHAT']}
ACCOAC: accoa_c + atp_c + hco3_c --> adp_c + h_c + malcoa_c + pi_c {'original_bigg_ids': ['ACCOAC']}
ACCOAgt: accoa_c <=> accoa_g {'original_bigg_ids': ['ACCOAgt']}
ACCOAtr: accoa_c <=> accoa_r {'original_bigg_ids': ['ACCOAtr']}
ACDO: dhmtp_c + o2_c --> 2kmb_c + for_c + h_c {'original_bigg_ids': ['ACDO']}
ACGAM6PS: accoa_c + gam6p_c <=> acgam6p_c + coa_c + h_c {'original_bigg_ids': ['ACGAM6PS']}
ACGAMPM: acgam6p_c <=> acgam1p_c {'original_bigg_ids': ['ACGAMPM']}
ACGKm: acglu_m + atp_m --> acg5p_m + adp_m {'original_bigg_ids': ['ACGKm']}
ACGLUtd: acglu_c <=> acglu_e {'original_bigg_ids': ['ACGLUtd']}
ACGS: accoa_c + glu__L_c --> acglu_c + coa_c + h_c {'original_bigg_ids': ['ACGS']}
ACGSm: accoa_m + glu__L_m --> acglu_m + coa_m + h_m {'original_bigg_ids': ['ACGSm']}
ACHBSm: 2obut_m + h_m + pyr_m --> 2ahbut_m + co2_m {'original_bigg_ids': ['ACHBSm']}
ACHEe: ach_e + h2o_e --> ac_e + chol_e + h_e {'original_bigg_ids': ['ACHEe']}
ACHLE1: h2o_c + iamac_c --> ac_c + h_c + iamoh_c {'original_bigg_ids': ['ACHLE1']}
ACHLE2: h2o_c + ibutac_c --> ac_c + h_c + ibutoh_c {'original_bigg_ids': ['ACHLE2']}
ACHLE3: aces_c + h2o_c --> ac_c + etoh_c + h_c {'original_bigg_ids': ['ACHLE3']}
ACITL: atp_c + cit_c + coa_c --> accoa_c + adp_c + oaa_c + pi_c {'original_bigg_ids': ['ATPCS']}
ACKr: ac_c + atp_c <=> actp_c + adp_c {'original_bigg_ids': ['ACKr']}
ACKrm: ac_m + atp_m <=> actp_m + adp_m {'original_bigg_ids': ['ACKrm']}
ACLSm: h_m + 2.0 pyr_m --> alac__S_m + co2_m {'original_bigg_ids': ['ACLSm']}
ACOAD10m: 2mbcoa_m + fad_m --> 2mb2coa_m + fadh2_m {'original_bigg_ids': ['MCDH_LPAREN_2mb2coa_RPAREN_']}
ACOAD1m: btcoa_m + fad_m --> b2coa_m + fadh2_m {'original_bigg_ids': ['ACOAD1fm']}
ACOAD1p: btcoa_x + fad_x --> b2coa_x + fadh2_x {'original_bigg_ids': ['ACOA40OR']}
ACOAD2m: fad_m + hxcoa_m --> fadh2_m + hx2coa_m {'original_bigg_ids': ['ACOAD2f']}
ACOAD2p: fad_x + hxcoa_x --> fadh2_x + hx2coa_x {'original_bigg_ids': ['ACOA60OR']}
ACOAD3m: fad_m + occoa_m --> fadh2_m + oc2coa_m {'original_bigg_ids': ['ACOAD2f']}
ACOAD3p: fad_x + occoa_x --> fadh2_x + oc2coa_x {'original_bigg_ids': ['ACOA80OR']}
ACOAD4m: dcacoa_m + fad_m --> dc2coa_m + fadh2_m {'original_bigg_ids': ['ACOAD2f']}
ACOAD4p: dcacoa_x + fad_x --> dc2coa_x + fadh2_x {'original_bigg_ids': ['ACOA100OR']}
ACOAD5m: ddcacoa_m + fad_m --> dd2coa_m + fadh2_m {'original_bigg_ids': ['ACOAD2f']}
ACOAD5p: ddcacoa_x + fad_x --> dd2coa_x + fadh2_x {'original_bigg_ids': ['ACOA120OR']}
ACOAD6m: fad_m + tdcoa_m --> fadh2_m + td2coa_m {'original_bigg_ids': ['ACOAD2f']}
ACOAD6p: fad_x + tdcoa_x --> fadh2_x + td2coa_x {'original_bigg_ids': ['ACOA140OR']}
ACOAD7m: fad_m + pmtcoa_m --> fadh2_m + hdd2coa_m {'original_bigg_ids': ['ACOAD2f']}
ACOAD7p: fad_x + pmtcoa_x --> fadh2_x + hdd2coa_x {'original_bigg_ids': ['ACOA160OR']}
ACOAD8m: fad_m + ivcoa_m --> 3mb2coa_m + fadh2_m {'original_bigg_ids': ['IVCDH']}
ACOAD8p: fad_x + stcoa_x --> fadh2_x + od2coa_x {'original_bigg_ids': ['ACOA160OR']}
ACOAD9m: fad_m + ibcoa_m --> 2mp2coa_m + fadh2_m {'original_bigg_ids': ['MCDH']}
ACOAHim: accoa_m + h2o_m --> ac_m + coa_m + h_m {'original_bigg_ids': ['ACOAHim']}
ACOAO4p: dcacoa_x + o2_x --> dc2coa_x + h2o2_x {'original_bigg_ids': ['ACOAO4p']}
ACOAO5p: ddcacoa_x + o2_x --> dd2coa_x + h2o2_x {'original_bigg_ids': ['ACOAO5p']}
ACOAO6p: o2_x + tdcoa_x --> h2o2_x + td2coa_x {'original_bigg_ids': ['ACOAO6p']}
ACOAO7p: o2_x + pmtcoa_x --> h2o2_x + hdd2coa_x {'original_bigg_ids': ['ACOAO7p']}
ACOAO8p: o2_x + stcoa_x --> h2o2_x + od2coa_x {'original_bigg_ids': ['ACOAO8p']}
ACOAO9p: hexccoa_x + o2_x --> h2o2_x + hxc2coa_x {'original_bigg_ids': ['ACOAO9p']}
ACOAR2m: h_m + hx2coa_m + nadph_m --> hxcoa_m + nadp_m {'original_bigg_ids': ['ACOAR2m']}
ACOAR3m: h_m + nadph_m + oc2coa_m --> nadp_m + occoa_m {'original_bigg_ids': ['ACOAR3m']}
ACOAR4m: dc2coa_m + h_m + nadph_m --> dcacoa_m + nadp_m {'original_bigg_ids': ['ACOAR4m']}
ACOAR5m: dd2coa_m + h_m + nadph_m --> ddcacoa_m + nadp_m {'original_bigg_ids': ['ACOAR5m']}
ACOAR6m: h_m + nadph_m + td2coa_m --> nadp_m + tdcoa_m {'original_bigg_ids': ['ACOAR6m']}
ACOAR7m: h_m + hdd2coa_m + nadph_m --> nadp_m + pmtcoa_m {'original_bigg_ids': ['ACOAR7m']}
ACON3MT: acon_T_c + amet_c --> acon5m_c + ahcys_c {'original_bigg_ids': ['ACON3MT']}
ACONT3m: 2mcit_m <=> micit_m {'original_bigg_ids': ['ACONT3m']}
ACOTAim: acg5sa_m + glu__L_m --> acorn_m + akg_m {'original_bigg_ids': ['ACOTAim']}
ACOX22x: dhcholoylcoa_x + fadh2_x + 0.5 o2_x --> fad_x + thcholoylcoa_x {'original_bigg_ids': ['ACOX22x']}
ACOX2x: cholcoas_x + fadh2_x + o2_x --> cholcoads_x + fad_x + 2.0 h2o_x {'original_bigg_ids': ['ACOX2x']}
ACOXT: accoa_c + oxa_c <=> ac_c + oxalcoa_c {'original_bigg_ids': ['ACOXT']}
ACP1_FMN: fmn_c + h2o_c --> pi_c + ribflv_c {'original_bigg_ids': ['ACP1']}
ACP1e: fmn_e + h2o_e --> pi_e + ribflv_e {'original_bigg_ids': ['ACP1e']}
ACPCS: amet_c --> 1acpc_c + 5mta_c + h_c {'original_bigg_ids': ['ACPCS']}
ACPS1: apoACP_c + coa_c --> ACP_c + h_c + pap_c {'original_bigg_ids': ['ACPS1']}
ACPT1601819Z: 12dgr1601819Z_c + amet_c --> 5mta_c + dghs1601819Z_c + h_c {'original_bigg_ids': ['ACPT1601819Z']}
ACPT1819Z1819Z: 12dgr1819Z1819Z_c + amet_c --> 5mta_c + dghs1819Z1819Z_c + h_c {'original_bigg_ids': ['ACPT1819Z1819Z']}
ACRNtm: acrn_c --> acrn_m {'original_bigg_ids': ['ACRNtm']}
ACRNtp: acrn_x --> acrn_c {'original_bigg_ids': ['ACRNtp']}
ACRS: dkmpp_c --> h_c + hkmpp_c {'original_bigg_ids': ['ACRS']}
ACS: ac_c + atp_c + coa_c --> accoa_c + amp_c + ppi_c {'original_bigg_ids': ['ACS']}
ACS2: atp_c + coa_c + ppa_c --> amp_c + ppcoa_c + ppi_c {'original_bigg_ids': ['PPACOAL']}
ACSm: ac_m + atp_m + coa_m --> accoa_m + amp_m + ppi_m {'original_bigg_ids': ['ACSm']}
ACtr: ac_e <=> ac_c {'original_bigg_ids': ['ACtr']}
ADA: adn_c + h2o_c + h_c --> ins_c + nh4_c {'original_bigg_ids': ['ADA']}
ADAe: adn_e + h2o_e + h_e --> ins_e + nh4_e {'original_bigg_ids': ['ADAe']}
ADCL: 4adcho_c --> 4abz_c + h_c + pyr_c {'original_bigg_ids': ['ADCL']}
ADCS: chor_c + gln__L_c --> 4adcho_c + glu__L_c {'original_bigg_ids': ['ADCS']}
ADD: ade_c + h2o_c + h_c --> hxan_c + nh4_c {'original_bigg_ids': ['ADD']}
ADEt2: ade_e + h_e --> ade_c + h_c {'original_bigg_ids': ['ADEt2']}
ADHAPRer_RT: 0.01 1agly3p_RT_r + h_r + nadph_r --> 0.01 1ag3p_RT_r + nadp_r {'original_bigg_ids': ['ADHAPR_SC']}
ADK1: amp_c + atp_c <=> 2.0 adp_c {'original_bigg_ids': ['ADK1']}
ADK3m: amp_m + gtp_m <=> adp_m + gdp_m {'original_bigg_ids': ['ADK3m']}
ADK4m: amp_m + itp_m <=> adp_m + idp_m {'original_bigg_ids': ['ADK4m']}
ADMDC: amet_c + h_c --> ametam_c + co2_c {'original_bigg_ids': ['ADMDC']}
ADNCYC: atp_c --> camp_c + ppi_c {'original_bigg_ids': ['ADNCYC']}
ADNK1: adn_c + atp_c --> adp_c + amp_c + h_c {'original_bigg_ids': ['ADNK1']}
ADNUC: adn_c + h2o_c --> ade_c + rib__D_c {'original_bigg_ids': ['ADNUC']}
ADNt2: adn_e + h_e --> adn_c + h_c {'original_bigg_ids': ['ADNt2']}
ADNtm: adn_c <=> adn_m {'original_bigg_ids': ['ADNtm']}
ADPGLC: adpglc_c + h2o_c --> amp_c + g1p_c + 2.0 h_c {'original_bigg_ids': ['ADPGLC']}
ADPMAN: adpman_c + h2o_c --> amp_c + 2.0 h_c + man1p_c {'original_bigg_ids': ['ADPMAN']}
ADPRDP: adprib_c + h2o_c --> amp_c + 2.0 h_c + r5p_c {'original_bigg_ids': ['ADPRDP']}
ADPT: ade_c + prpp_c --> amp_c + ppi_c {'original_bigg_ids': ['ADPT']}
ADPT2: aicar_c + ppi_c <=> C04051_c + prpp_c {'original_bigg_ids': ['ADPT2']}
ADPter: adp_r <=> adp_c {'original_bigg_ids': ['Htr']}
ADSELK: adsel_c + atp_c --> 3padsel_c + adp_c + h_c {'original_bigg_ids': ['ADSELK']}
ADSHm: ahcys_m + h2o_m <=> adn_m + hcys__L_m {'original_bigg_ids': ['ADSHm']}
ADSK: aps_c + atp_c --> adp_c + h_c + paps_c {'original_bigg_ids': ['ADSK']}
ADSL1r: dcamp_c <=> amp_c + fum_c {'original_bigg_ids': ['ADSL1r']}
ADSL2r: 25aics_c <=> aicar_c + fum_c {'original_bigg_ids': ['ADSL2r']}
ADSS: asp__L_c + gtp_c + imp_c --> dcamp_c + gdp_c + 2.0 h_c + pi_c {'original_bigg_ids': ['ADSS']}
ADtr: ad_e <=> ad_c {'original_bigg_ids': ['CO2t']}
AFLBURPPAAMH: aflburppa_c + h2o_c + h_c --> aflbala_c + co2_c + nh4_c {'original_bigg_ids': ['AFLBURPPAAMH']}
AGATer_RT: 0.01 1ag3p_RT_r + 0.01 arachcoa_r + 0.02 hpdcacoa_r + 0.1 lnlncgcoa_r + 0.37 ocdycacoa_r + 0.32 odecoa_r + 0.09 pmtcoa_r + 0.07 stcoa_r + 0.02 ttccoa_r --> coa_r + 0.01 pa_RT_r {'original_bigg_ids': ['AGAT_SC']}
AGMTm: agm_m + h2o_m --> ptrc_m + urea_m {'original_bigg_ids': ['AGMTm']}
AGMt_m: agm_c <=> agm_m {'original_bigg_ids': ['AGRMtm']}
AGPR: acg5sa_c + nadp_c + pi_c <=> acg5p_c + h_c + nadph_c {'original_bigg_ids': ['AGPR']}
AGPRim: acg5p_m + h_m + nadph_m --> acg5sa_m + nadp_m + pi_m {'original_bigg_ids': ['AGPRim']}
AGRMte: agm_c <=> agm_e {'original_bigg_ids': ['AGRMte']}
AGTi: ala__L_c + glx_c --> gly_c + pyr_c {'original_bigg_ids': ['AGTi']}
AGTim: ala__L_m + glx_m --> gly_m + pyr_m {'original_bigg_ids': ['AGTim']}
AHANDROSTANGLCte: ahandrostanglc_c + atp_c + h2o_c --> adp_c + ahandrostanglc_e + h_c + pi_c {'original_bigg_ids': ['AHANDROSTANGLCte']}
AHCYStr: ahcys_c <=> ahcys_r {'original_bigg_ids': ['AHCYStr']}
AHCi: ahcys_c + h2o_c --> adn_c + hcys__L_c {'original_bigg_ids': ['AHCi']}
AHGDxm: fad_m + s2hglut_m <=> akg_m + fadh2_m {'original_bigg_ids': ['AHGDx_1']}
AHSERL: achms_c + ch4s_c --> ac_c + h_c + met__L_c {'original_bigg_ids': ['AHSERL']}
AHSERL2: achms_c + h2s_c --> ac_c + h_c + hcys__L_c {'original_bigg_ids': ['AHSERL2']}
AICART: 10fthf_c + aicar_c <=> fprica_c + thf_c {'original_bigg_ids': ['AICART']}
AIRCr: air_c + co2_c <=> 5aizc_c + h_c {'original_bigg_ids': ['AIRCr']}
AKGCITtm: akg_c + cit_m --> akg_m + cit_c {'original_bigg_ids': ['AKGCITtm']}
AKGDm: akg_m + coa_m + nad_m --> co2_m + nadh_m + succoa_m {'original_bigg_ids': ['AKGDm']}
AKGMALtm: akg_m + mal__L_c --> akg_c + mal__L_m {'original_bigg_ids': ['MALAKGtm']}
AKGtp: akg_c <=> akg_x {'original_bigg_ids': ['AKGtp']}
ALAR: ala__L_c <=> ala__D_c {'original_bigg_ids': ['ALAR']}
ALASm: gly_m + h_m + succoa_m --> 5aop_m + co2_m + coa_m {'original_bigg_ids': ['ALASm']}
ALATA_Lm: akg_m + ala__L_m <=> glu__L_m + pyr_m {'original_bigg_ids': ['ALATA_Lm']}
ALATRS: ala__L_c + atp_c + trnaala_c --> alatrna_c + amp_c + ppi_c {'original_bigg_ids': ['ALATRS']}
ALAt2r: ala__L_e + h_e <=> ala__L_c + h_c {'original_bigg_ids': ['ALAt2r']}
ALAtN1: ala__L_e + h_c + 2.0 na1_e <=> ala__L_c + h_e + 2.0 na1_c {'original_bigg_ids': ['ALAtN1']}
ALCD19: glyald_c + h_c + nadh_c <=> glyc_c + nad_c {'original_bigg_ids': ['ALCD19']}
ALCD19y: glyald_c + h_c + nadph_c --> glyc_c + nadp_c {'original_bigg_ids': ['ALCD19y']}
ALCD22xi: 2mbald_c + h_c + nadh_c --> 2mbtoh_c + nad_c {'original_bigg_ids': ['ALCD22xi']}
ALCD22yi: 2mbald_c + h_c + nadph_c --> 2mbtoh_c + nadp_c {'original_bigg_ids': ['ALCD22yi']}
ALCD23xi: 2mppal_c + h_c + nadh_c --> ibutoh_c + nad_c {'original_bigg_ids': ['ALCD23xi']}
ALCD23yi: 2mppal_c + h_c + nadph_c --> ibutoh_c + nadp_c {'original_bigg_ids': ['ALCD23yi']}
ALCD24xi: 3mbald_c + h_c + nadh_c --> iamoh_c + nad_c {'original_bigg_ids': ['ALCD24xi']}
ALCD24yi: 3mbald_c + h_c + nadph_c --> iamoh_c + nadp_c {'original_bigg_ids': ['ALCD24yi']}
ALCD25xi: h_c + nadh_c + pacald_c --> 2phetoh_c + nad_c {'original_bigg_ids': ['ALCD25xi']}
ALCD25yi: h_c + nadph_c + pacald_c --> 2phetoh_c + nadp_c {'original_bigg_ids': ['ALCD25yi']}
ALCD26xi: h_c + id3acald_c + nadh_c --> ind3eth_c + nad_c {'original_bigg_ids': ['ALCD26xi']}
ALCD2x: etoh_c + nad_c <=> acald_c + h_c + nadh_c {'original_bigg_ids': ['ALCD2x']}
ALCD2y: etoh_c + nadp_c --> acald_c + h_c + nadph_c {'original_bigg_ids': ['ALCD2yf']}
ALDD16er: h2o_r + hxdcal_r + nad_r --> 2.0 h_r + hdca_r + nadh_r {'original_bigg_ids': ['ALDD3C16_c']}
ALDD19x_P: h2o_c + nadp_c + pacald_c --> 2.0 h_c + nadph_c + pac_c {'original_bigg_ids': ['ALDD19x_LPAREN_P_RPAREN_']}
ALDD19xr: h2o_c + nad_c + pacald_c <=> 2.0 h_c + nadh_c + pac_c {'original_bigg_ids': ['ALDD19xr']}
ALDD20y: h2o_c + id3acald_c + nadp_c --> 2.0 h_c + ind3ac_c + nadph_c {'original_bigg_ids': ['ALDD20y']}
ALDD21er: h2o_r + nad_r + pristanal_r --> 2.0 h_r + nadh_r + prist_r {'original_bigg_ids': ['ALDD21']}
ALDD2x: acald_c + h2o_c + nad_c --> ac_c + 2.0 h_c + nadh_c {'original_bigg_ids': ['ALDDH']}
ALDD2xm: acald_m + h2o_m + nad_m --> ac_m + 2.0 h_m + nadh_m {'original_bigg_ids': ['ALDD2xm']}
ALDD2y: acald_c + h2o_c + nadp_c --> ac_c + 2.0 h_c + nadph_c {'original_bigg_ids': ['ALDD2y']}
ALDD2ym: acald_m + h2o_m + nadp_m --> ac_m + 2.0 h_m + nadph_m {'original_bigg_ids': ['ALDD2ym']}
ALDD3C161er: h2o_r + hxdceal_r + nad_r --> 2.0 h_r + hdc2ea_r + nadh_r {'original_bigg_ids': ['ALDD3C161_c']}
ALDD3y: h2o_c + nadp_c + ppal_c --> 2.0 h_c + nadph_c + ppa_c {'original_bigg_ids': ['ALDD3y']}
ALKP: dhap_c + h2o_c --> dha_c + pi_c {'original_bigg_ids': ['ALKP']}
ALLTAHr: alltt_c + h2o_c <=> urdglyc_c + urea_c {'original_bigg_ids': ['ALLTAHr']}
ALLTN: alltn_c + h2o_c --> alltt_c + h_c {'original_bigg_ids': ['ALLTN']}
ALLTNti: alltn_e --> alltn_c {'original_bigg_ids': ['ALLTNti']}
ALLTTti: alltt_e --> alltt_c {'original_bigg_ids': ['ALLTTti']}
ALPHNH: allphn_c + h2o_c + 3.0 h_c --> 2.0 co2_c + 2.0 nh4_c {'original_bigg_ids': ['ALPHNH']}
ALR2: h_c + mthgxl_c + nadph_c --> acetol_c + nadp_c {'original_bigg_ids': ['ALR2']}
ALR3: acetol_c + h_c + nadph_c --> 12ppd__S_c + nadp_c {'original_bigg_ids': ['ALR3']}
AM6SAD: am6sa_c + h2o_c + nad_c --> amuco_c + 2.0 h_c + nadh_c {'original_bigg_ids': ['AM6SAD']}
AMACR2p: dhcholestancoa_x + 0.5 o2_x --> dhcholoylcoa_x + h2o_x {'original_bigg_ids': ['AMACR2p']}
AMACR2r: dhcholestancoa_r + 0.5 o2_r --> dhcholoylcoa_r + h2o_r {'original_bigg_ids': ['AMACR2r']}
AMACRp: cholcoar_x --> cholcoas_x {'original_bigg_ids': ['AMACRp']}
AMACRr: cholcoar_r --> cholcoas_r {'original_bigg_ids': ['AMACRr']}
AMCOXO: amuco_c + h2o_c + h_c + nadph_c --> 2oxoadp_c + nadp_c + nh4_c {'original_bigg_ids': ['AMCOXO']}
AMETr: amet_c <=> amet_r {'original_bigg_ids': ['AMETr']}
AMETt2m: ahcys_m + amet_c <=> ahcys_c + amet_m {'original_bigg_ids': ['AMETt2m']}
AMETtm: amet_c <=> amet_m {'original_bigg_ids': ['AMETtm']}
AMID: 4gudbd_c + h2o_c --> 4gudbutn_c + nh4_c {'original_bigg_ids': ['AMID']}
AMID2: h2o_c + pad_c --> nh4_c + pac_c {'original_bigg_ids': ['AMID2']}
AMID3: h2o_c + iad_c --> ind3ac_c + nh4_c {'original_bigg_ids': ['AMID3']}
AMID_1: ad_c + h2o_c --> ac_c + nh4_c {'original_bigg_ids': ['AMID']}
AMPDA: amp_c + h2o_c + h_c --> imp_c + nh4_c {'original_bigg_ids': ['AMPDA']}
AMPN: amp_c + h2o_c --> ade_c + r5p_c {'original_bigg_ids': ['AMPN']}
AMPTASECG: cgly_c + h2o_c --> cys__L_c + gly_c {'original_bigg_ids': ['AMPTASECG']}
AMPTASEPG: h2o_c + progly_c --> gly_c + pro__L_c {'original_bigg_ids': ['AMPTASEPG']}
AMPter: amp_c <=> amp_r {'original_bigg_ids': ['Htr']}
AMY1e: 8.0 h2o_e + strch1_e --> 8.0 glc__D_e + strch2_e {'original_bigg_ids': ['AMY1e']}
AMY2e: glygn2_e + 8.0 h2o_e --> 8.0 glc__D_e + glygn4_e {'original_bigg_ids': ['AMY2e']}
ANDRSTRNGLCte: andrstrnglc_c + atp_c + h2o_c --> adp_c + andrstrnglc_e + h_c + pi_c {'original_bigg_ids': ['ANDRSTRNGLCte']}
ANHMK: anhm_c + atp_c + h2o_c --> acmum6p_c + adp_c + h_c {'original_bigg_ids': ['ANHMK']}
ANPRT: anth_c + prpp_c --> ppi_c + pran_c {'original_bigg_ids': ['ANPRT']}
ANS: chor_c + gln__L_c --> anth_c + glu__L_c + h_c + pyr_c {'original_bigg_ids': ['ANS']}
AOXSp: ala__L_x + h_x + pimcoa_x --> 8aonn_x + co2_x + coa_x {'original_bigg_ids': ['AOXSr2']}
AP4AH: ap4a_c + h2o_c --> 2.0 adp_c + 2.0 h_c {'original_bigg_ids': ['AP4AH']}
APAT2rm: akg_m + ala_B_m <=> glu__L_m + msa_m {'original_bigg_ids': ['APAT2rm']}
APCPT: atp_c + ptcys_c --> 4ppcys_c + adp_c + h_c {'original_bigg_ids': ['APCPT']}
APNPT: atp_c + ptth_c --> adp_c + h_c + pan4p_c {'original_bigg_ids': ['APNPT']}
APNn: camp_n + h2o_n --> amp_n + h_n {'original_bigg_ids': ['APNn']}
APPTm: atp_m + ppa_m <=> adp_m + ppap_m {'original_bigg_ids': ['APPTm']}
APSR: aps_c + trdrd_c --> amp_c + 2.0 h_c + so3_c + trdox_c {'original_bigg_ids': ['APSR']}
ARAB14LO: Dara14lac_c + o2_c <=> ertascb__D_c + h2o2_c {'original_bigg_ids': ['ARAB14LO']}
ARAB1D: arab__D_c + nad_c --> Dara14lac_c + h_c + nadh_c {'original_bigg_ids': ['ARAB1D2']}
ARABR: arab__L_c + h_c + nadph_c --> abt_c + nadp_c {'original_bigg_ids': ['ARABR']}
ARAB_Dt: arab__D_e <=> arab__D_c {'original_bigg_ids': ['ARAB_Dt']}
ARAB_Lt: arab__L_e <=> arab__L_c {'original_bigg_ids': ['ARAB_Lt']}
ARACHt: arach_e --> arach_c {'original_bigg_ids': ['ARACHt']}
ARBTHe: arbt_e + h2o_e --> glc__D_e + hqn_e {'original_bigg_ids': ['BG_MBDG_']}
ARGN: arg__L_c + h2o_c --> orn_c + urea_c {'original_bigg_ids': ['ARGN']}
ARGSL: argsuc_c <=> arg__L_c + fum_c {'original_bigg_ids': ['ARGSL']}
ARGSS: asp__L_c + atp_c + citr__L_c --> amp_c + argsuc_c + h_c + ppi_c {'original_bigg_ids': ['ARGSS']}
ARGTRS: arg__L_c + atp_c + trnaarg_c --> amp_c + argtrna_c + ppi_c {'original_bigg_ids': ['ARGTRS']}
ARGTRSm: arg__L_m + atp_m + trnaarg_m --> amp_m + argtrna_m + ppi_m {'original_bigg_ids': ['ARGTRSm']}
ARGt2r: arg__L_e + h_e <=> arg__L_c + h_c {'original_bigg_ids': ['ARGt2r']}
ARGt7m: arg__L_c --> arg__L_m {'original_bigg_ids': ['GLUt7m']}
ARHGDx: nad_c + r2hglut_c <=> akg_c + h_c + nadh_c {'original_bigg_ids': ['ARHGDx']}
ARHGDxm: fad_m + r2hglut_m <=> akg_m + fadh2_m {'original_bigg_ids': ['ARHGDx']}
ARMT: amet_c + his__L_c --> NPmehis_c + ahcys_c + h_c {'original_bigg_ids': ['ARMT']}
ARSO4SH: arso4_c + h2o_c --> 2.0 h_c + phenol_c + so4_c {'original_bigg_ids': ['ARSO4SH']}
ASAD: aspsa_c + nadp_c + pi_c <=> 4pasp_c + h_c + nadph_c {'original_bigg_ids': ['ASAD']}
ASNN: asn__L_c + h2o_c --> asp__L_c + nh4_c {'original_bigg_ids': ['ASNN']}
ASNNe: asn__L_e + h2o_e --> asp__L_e + nh4_e {'original_bigg_ids': ['ASNNe']}
ASNS1: asp__L_c + atp_c + gln__L_c + h2o_c --> amp_c + asn__L_c + glu__L_c + h_c + ppi_c {'original_bigg_ids': ['ASNS1']}
ASNS2: asp__L_c + atp_c + nh4_c --> amp_c + asn__L_c + h_c + ppi_c {'original_bigg_ids': ['ASNS2']}
ASNTRS: asn__L_c + atp_c + trnaasn_c --> amp_c + asntrna_c + ppi_c {'original_bigg_ids': ['ASNTRS']}
ASNTRSm: asn__L_m + atp_m + trnaasn_m --> amp_m + asntrna_m + ppi_m {'original_bigg_ids': ['ASNTRSm']}
ASNt2r: asn__L_e + h_e <=> asn__L_c + h_c {'original_bigg_ids': ['ASNt2r']}
ASNt6: asn__L_c + h_v --> asn__L_v + h_c {'original_bigg_ids': ['ASNt6']}
ASNt7: asn__L_v + h_v --> asn__L_c + h_c {'original_bigg_ids': ['ASNt7']}
ASNtN1: asn__L_e + h_c + 2.0 na1_e <=> asn__L_c + h_e + 2.0 na1_c {'original_bigg_ids': ['ASNtN1']}
ASPCT: asp__L_c + cbp_c --> cbasp_c + h_c + pi_c {'original_bigg_ids': ['ASPCT']}
ASPDTDe: asp__D_c <=> asp__D_e {'original_bigg_ids': ['ASPDTDe']}
ASPGLUm: asp__L_m + glu__L_c + h_c --> asp__L_c + glu__L_m + h_m {'original_bigg_ids': ['ASPGLUm']}
ASPK: asp__L_c + atp_c <=> 4pasp_c + adp_c {'original_bigg_ids': ['ASPK']}
ASPTA: akg_c + asp__L_c <=> glu__L_c + oaa_c {'original_bigg_ids': ['ASPTA']}
ASPTAm: akg_m + asp__L_m <=> glu__L_m + oaa_m {'original_bigg_ids': ['ASPATm']}
ASPTAp: akg_x + asp__L_x <=> glu__L_x + oaa_x {'original_bigg_ids': ['ASPTAp']}
ASPTRS: asp__L_c + atp_c + trnaasp_c --> amp_c + asptrna_c + ppi_c {'original_bigg_ids': ['ASPTRS']}
ASPTRSm: asp__L_m + atp_m + trnaasp_m --> amp_m + asptrna_m + ppi_m {'original_bigg_ids': ['ASPTL']}
ASPt2r: asp__L_e + h_e <=> asp__L_c + h_c {'original_bigg_ids': ['ASPt2r']}
ASPt7: asp__L_v + h_v --> asp__L_c + h_c {'original_bigg_ids': ['ASPt7']}
ATHRDHr: athr__L_c + nadp_c <=> 2aobut_c + h_c + nadph_c {'original_bigg_ids': ['ATHRDHr']}
ATP2tp_H: amp_x + atp_c + 2.0 h_c --> amp_c + atp_x + 2.0 h_x {'original_bigg_ids': ['ATP2tp_H']}
ATPATF1: adp_c + atp_c + h_c --> ap4a_c + pi_c {'original_bigg_ids': ['ATPATF1']}
ATPATF2: adp_c + gtp_c + h_c --> ap4g_c + pi_c {'original_bigg_ids': ['ATPATF2']}
ATPATF3: gdp_c + gtp_c + h_c --> gp4g_c + pi_c {'original_bigg_ids': ['ATPATF3']}
ATPM: atp_c + h2o_c --> adp_c + h_c + pi_c {'original_bigg_ids': ['ATPM']}
ATPPRT: atp_c + prpp_c --> ppi_c + prbatp_c {'original_bigg_ids': ['ATPPRT']}
ATPS: atp_c + h2o_c --> adp_c + h_e + pi_c {'original_bigg_ids': ['ATPS']}
ATPS2v: atp_c + h2o_c + h_c --> adp_c + 2.0 h_v + pi_c {'original_bigg_ids': ['ATPS3v']}
ATPS3m: adp_m + 3.0 h_c + pi_m --> atp_m + h2o_m + 2.0 h_m {'original_bigg_ids': ['ATPS3m']}
ATPter: atp_c <=> atp_r {'original_bigg_ids': ['Htr']}
ATPtm: adp_c + atp_m --> adp_m + atp_c {'original_bigg_ids': ['ATPtm']}
A_MANASE: 2.0 h2o_c + m2mn_c --> 2.0 man_c + mn_c {'original_bigg_ids': ['A_MANASE']}
BACCL: atp_c + btn_c + h_c --> btamp_c + ppi_c {'original_bigg_ids': ['BACCL']}
BACCLm: atp_m + btn_m + h_m --> btamp_m + ppi_m {'original_bigg_ids': ['BACCLm']}
BADP: ans_c + h2o_c --> NPmehis_c + ala_B_c {'original_bigg_ids': ['BADP']}
BAMPPALDOX: bamppald_c + h2o_c + nad_c --> ala_B_c + 2.0 h_c + nadh_c {'original_bigg_ids': ['BAMPPALDOX']}
BDHm: bhb_m + nad_m <=> acac_m + h_m + nadh_m {'original_bigg_ids': ['HBNOm']}
BDMT: doldpglcnacglcnac_c + gdpmann_c --> doldpglcnacglcnacman_c + gdp_c + h_c {'original_bigg_ids': ['BDMT']}
BDMT_L: 0.1 chito2pdol__L_c + gdpmann_c --> gdp_c + h_c + 0.1 mpdol__L_c {'original_bigg_ids': ['BDMT_L']}
BDMT_U: 0.1 chito2pdol_U_c + gdpmann_c --> gdp_c + h_c + 0.1 mpdol_U_c {'original_bigg_ids': ['BDMT_U']}
BG_MBDG: h2o_e + mbdg_e --> glc__D_e + meoh_e {'original_bigg_ids': ['BG_MBDG_']}
BHBt: bhb_e + h_e <=> bhb_c + h_c {'original_bigg_ids': ['BHBt']}
BHBtm: bhb_c + h_c <=> bhb_m + h_m {'original_bigg_ids': ['BHBtm']}
BILDGLCURte: atp_c + bildglcur_c + h2o_c <=> adp_c + bildglcur_e + h_c + pi_c {'original_bigg_ids': ['BILDGLCURte']}
BILGLCURte: atp_c + bilglcur_c + h2o_c <=> adp_c + bilglcur_e + h_c + pi_c {'original_bigg_ids': ['BILGLCURte']}
BIOMASS_RT: 0.957502 13BDglcn_c + 0.177315 16BDglcn_c + 0.001283 5mthf_c + 0.577574 alatrna_c + 0.146675 argtrna_c + 0.10797 asntrna_c + 0.197296 asptrna_c + 139.6887 atp_c + 0.002418 btn_m + 0.000832 ca2_c + 0.001792 camp_c + 0.031005 chitin_c + 1.8e-05 clpn_RT_m + 0.00077 coa_c + 0.044881 ctp_c + 0.000525 cu2_c + 0.034269 cystrna_c + 0.002451 datp_c + 0.004285 dctp_c + 0.003763 dgtp_c + 0.002523 dttp_c + 0.004031 ergst_r + 1.6e-05 ergstest_RT_r + 0.000751 fad_c + 0.000597 fe2_c + 0.000597 fe3_c + 0.117898 glntrna_c + 0.208673 glutrna_c + 0.518069 glycogen_c + 0.51322 glytrna_c + 0.00192 gthrd_c + 0.051085 gtp_c + 135.719639 h2o_c + 0.000691 hemeA_m + 0.061167 histrna_c + 0.158569 iletrna_c + 0.585703 k_c + 0.320205 leutrna_c + 0.003107 lipopb_m + 0.172334 lystrna_c + 0.807941 mannan_r + 0.05705 mettrna_c + 0.061716 mg2_c + 0.001292 mlthf_c + 0.000607 mn2_c + 0.026099 na1_c + 0.000888 nad_c + 0.000795 nadp_c + 1.4e-05 pa_RT_r + 0.000172 pc_RT_r + 0.00013 pe_RT_r + 0.095164 phetrna_c + 0.235897 protrna_c + 2e-05 ps_RT_r + 0.004006 psphings_r + 4.7e-05 ptd1ino_RT_r + 0.006524 ptrc_c + 0.0024 pydx5p_c + 0.00074 q9_m + 0.00099 ribflv_c + 0.33687 sertrna_c + 0.003967 spmd_c + 0.001327 thf_c + 0.001393 thmpp_c + 0.21813 thrtrna_c + 0.023372 tre_c + 0.000432 triglyc_RT_r + 0.023319 trptrna_c + 0.062363 tyrtrna_c + 0.051129 utp_c + 0.275168 valtrna_c + 0.00051 zn2_c + 0.004156 zymst_r + 1.7e-05 zymstest_RT_d --> 139.639453 adp_c + 139.639453 h_c + 139.639453 pi_c + 0.209364 ppi_c {'original_bigg_ids': ['biomass_SC5_notrace']}
BMOCOS: moco_c + mptamp_c --> amp_c + bmoco_c + cu2_c {'original_bigg_ids': ['BMOCOS']}
BPNT: h2o_c + pap_c --> amp_c + pi_c {'original_bigg_ids': ['BPNT']}
BPNT2: h2o_c + paps_c --> aps_c + pi_c {'original_bigg_ids': ['BPNT2']}
BTDD_RR: btd_RR_c + nad_c <=> actn__R_c + h_c + nadh_c {'original_bigg_ids': ['BTDD_RR']}
BTDt_RR: btd_RR_c <=> btd_RR_e {'original_bigg_ids': ['BTDt_RR']}
BTNPL: apoC_Lys_c + btamp_c --> amp_c + apoC_Lys_btn_c + h_c {'original_bigg_ids': ['BTNPL']}
BTNPLm: apoC_Lys_m + btamp_m --> amp_m + apoC_Lys_btn_m + h_m {'original_bigg_ids': ['BTNPLm']}
BTNt2i: btn_e + h_e --> btn_c + h_c {'original_bigg_ids': ['BTNt2i']}
BTP: apppa_n + h2o_n --> adp_n + amp_n + 2.0 h_n {'original_bigg_ids': ['BTP']}
BTS: btal_c + h_c + nadh_c --> 1btol_c + nad_c {'original_bigg_ids': ['BTS']}
BTS5m: 2fe2s_m + amet_m + dtbt_m --> 2fe1s_m + btn_m + dad_5_m + h_m + met__L_m {'original_bigg_ids': ['BTS5']}
BTS_nadph: btal_c + h_c + nadph_c --> 1btol_c + nadp_c {'original_bigg_ids': ['BTS_LPAREN_nadph_RPAREN_']}
BUPN: cala_c + h2o_c + 2.0 h_c --> ala_B_c + co2_c + nh4_c {'original_bigg_ids': ['BUPN']}
BUTt: but_e <=> but_c {'original_bigg_ids': ['HMR_0155']}
BWCOS: mptamp_c + wco_c --> amp_c + bwco_c + cu2_c {'original_bigg_ids': ['BWCOS']}
BZ4OX: bz_c + h_c + nadph_c + o2_c --> 4hbz_c + h2o_c + nadp_c {'original_bigg_ids': ['BZ12DOX']}
BZt: bz_e <=> bz_c {'original_bigg_ids': ['BZtex']}
C14STRr: 44mctr_r + h_r + nadph_r --> 44mzym_r + nadp_r {'original_bigg_ids': ['C14STRr']}
C160CPT1: crn_c + pmtcoa_c --> coa_c + pmtcrn_c {'original_bigg_ids': ['C160CPT1']}
C160CPT2: coa_m + pmtcrn_m --> crn_m + pmtcoa_m {'original_bigg_ids': ['C160CPT2']}
C160CRNt: pmtcrn_c --> pmtcrn_m {'original_bigg_ids': ['C160CRNt']}
C161CPT1: crn_c + hdcoa_c --> coa_c + hdcecrn_c {'original_bigg_ids': ['C161CPT1']}
C161CPT12: crn_c + hdd2coa_c --> coa_c + hdd2crn_c {'original_bigg_ids': ['C161CPT12']}
C161CPT2: coa_m + hdcecrn_m --> crn_m + hdcoa_m {'original_bigg_ids': ['C161CPT2']}
C161CPT22: coa_m + hdd2crn_m --> crn_m + hdd2coa_m {'original_bigg_ids': ['C161CPT22']}
C161CRN2t: hdd2crn_c --> hdd2crn_m {'original_bigg_ids': ['C161CRN2t']}
C161CRNt: hdcecrn_c --> hdcecrn_m {'original_bigg_ids': ['C161CRNt']}
C181CPT1: crn_c + odecoa_c --> coa_c + odecrn_c {'original_bigg_ids': ['C181CPT1']}
C181CPT2: coa_m + odecrn_m --> crn_m + odecoa_m {'original_bigg_ids': ['C181CPT2']}
C181CRNt: odecrn_c --> odecrn_m {'original_bigg_ids': ['C181CRNt']}
C22STDSer: ergtrol_r + h_r + nadph_r + o2_r --> ergtetrol_r + 2.0 h2o_r + nadp_r {'original_bigg_ids': ['C22STDS']}
C22STDSrx: ergtrol_r + h_r + nadh_r + o2_r --> ergtetrol_r + 2.0 h2o_r + nad_r {'original_bigg_ids': ['C22STDSx']}
C24STRer: ergtetrol_r + h_r + nadph_r --> ergst_r + nadp_r {'original_bigg_ids': ['C24STRer']}
C30CPT1: crn_c + ppcoa_c <=> coa_c + pcrn_c {'original_bigg_ids': ['C30CPT1']}
C3STDH1Pr: 4mzym_int1_r + nadp_r --> 4mzym_int2_r + co2_r + h_r + nadph_r {'original_bigg_ids': ['C3STDH1Pr']}
C3STDH1r: 4mzym_int1_r + nad_r --> 4mzym_int2_r + co2_r + h_r + nadh_r {'original_bigg_ids': ['C3STDH1r']}
C3STDH2er: nad_r + zym_int1_r --> co2_r + h_r + nadh_r + zym_int2_r {'original_bigg_ids': ['C3STDH2']}
C3STKR1er: 4mzym_int2_r + h_r + nadph_r --> 4mzym_r + nadp_r {'original_bigg_ids': ['C3STKR1']}
C3STKR2er: h_r + nadph_r + zym_int2_r --> nadp_r + zymst_r {'original_bigg_ids': ['C3STKR2']}
C40CPT1: btcoa_c + crn_c --> c4crn_c + coa_c {'original_bigg_ids': ['C40CPT1']}
C40CPT2: c4crn_m + coa_m --> btcoa_m + crn_m {'original_bigg_ids': ['C160CPT2']}
C40CRNt: c4crn_c --> c4crn_m {'original_bigg_ids': ['C160CRNt']}
C4STMO1r: 44mzym_r + 3.0 h_r + 3.0 nadph_r + 3.0 o2_r --> 4mzym_int1_r + 4.0 h2o_r + 3.0 nadp_r {'original_bigg_ids': ['C4STMO1r']}
C4STMO2er: 4mzym_r + 3.0 h_r + 3.0 nadph_r + 3.0 o2_r --> 4.0 h2o_r + 3.0 nadp_r + zym_int1_r {'original_bigg_ids': ['C4STMO2']}
C5STDSer: epist_r + h_r + nadph_r + o2_r --> ergtrol_r + 2.0 h2o_r + nadp_r {'original_bigg_ids': ['C5STDS']}
C60CPT1: crn_c + hxcoa_c --> c6crn_c + coa_c {'original_bigg_ids': ['C60CPT1']}
C60CPT2: c6crn_m + coa_m --> crn_m + hxcoa_m {'original_bigg_ids': ['C160CPT2']}
C60CRNt: c6crn_c --> c6crn_m {'original_bigg_ids': ['C160CRNt']}
C8STIer: fecost_r --> epist_r {'original_bigg_ids': ['C8STI']}
CA2t: ca2_e <=> ca2_c {'original_bigg_ids': ['CA2t']}
CAFFCOAE: caffcoa_c + h2o_c --> 34dhcinm_c + coa_c + h_c {'original_bigg_ids': ['FACOAE100']}
CAFFCOAHp: caffcoa_x + h2o_x --> 3dhp3hpcoa_x {'original_bigg_ids': ['4HBZCOAFm']}
CAFFCOALp: 34dhcinm_x + atp_x + coa_x --> amp_x + caffcoa_x + ppi_x {'original_bigg_ids': ['COUCOAFm']}
CAMPt: atp_c + camp_c + h2o_c --> adp_c + camp_e + h_c + pi_c {'original_bigg_ids': ['CAMPt']}
CAMPt2: camp_e <=> camp_c {'original_bigg_ids': ['CO2t']}
CAT: 2.0 h2o2_c --> 2.0 h2o_c + o2_c {'original_bigg_ids': ['CAT']}
CAT2p: etoh_x + h2o2_x --> acald_x + 2.0 h2o_x {'original_bigg_ids': ['CAT2p']}
CATDOX: catechol_c + o2_c --> ccmuac_c + 2.0 h_c {'original_bigg_ids': ['CATDOX']}
CATm: 2.0 h2o2_m --> 2.0 h2o_m + o2_m {'original_bigg_ids': ['CATm']}
CATp: 2.0 h2o2_x --> 2.0 h2o_x + o2_x {'original_bigg_ids': ['CATp']}
CBPS: 2.0 atp_c + gln__L_c + h2o_c + hco3_c --> 2.0 adp_c + cbp_c + glu__L_c + 2.0 h_c + pi_c {'original_bigg_ids': ['CBPS']}
CBPSm: 2.0 atp_m + gln__L_m + h2o_m + hco3_m --> 2.0 adp_m + cbp_m + glu__L_m + 2.0 h_m + pi_m {'original_bigg_ids': ['HCGALm']}
CBPt: cbp_e <=> cbp_c {'original_bigg_ids': ['CO2t']}
CCMPt2: 35ccmp_e <=> 35ccmp_c {'original_bigg_ids': ['CO2t']}
CCP2_m: 2.0 focytc_m + h2o2_m + 2.0 h_m --> 2.0 ficytc_m + 2.0 h2o_m {'original_bigg_ids': ['CCP2_m']}
CDPCHOLter: cdpchol_c <=> cdpchol_r {'original_bigg_ids': ['r1159']}
CDPDAGterm_RT: cdpdag_RT_r <=> cdpdag_RT_m {'original_bigg_ids': ['GLYCtm']}
CDPDGPm_RT: 0.01 cdpdag_RT_m + glyc3p_m --> cmp_m + h_m + 0.01 pgp_RT_m {'original_bigg_ids': ['CDPDGPm_SC']}
CDPter: cdp_r <=> cdp_c {'original_bigg_ids': ['Htr']}
CERD418er: cer2p_18_r + h_r + nadph_r + o2_r --> cer4_18_r + 2.0 h2o_r + nadp_r {'original_bigg_ids': ['CERH124', 'CERS2_24']}
CERD518er: cer4_18_r + h_r + nadph_r + o2_r --> cer5_18_r + 2.0 h2o_r + nadp_r {'original_bigg_ids': ['CERH124', 'CERS2_24']}
CERH124er: cer1_24_r + h_r + nadph_r + o2_r --> cer2_24_r + h2o_r + nadp_r {'original_bigg_ids': ['CERH124', 'CERS2_24']}
CERH126er: cer1_26_r + h_r + nadph_r + o2_r --> cer2_26_r + h2o_r + nadp_r {'original_bigg_ids': ['CERH126', 'CERS2_26']}
CERS118er: sphgn_r + stcoa_r --> cer1_18_r + coa_r + h_r {'original_bigg_ids': ['CERS124er']}
CERS124er: sphgn_r + ttccoa_r --> cer1_24_r + coa_r + h_r {'original_bigg_ids': ['CERS124er']}
CERS126er: hexccoa_r + sphgn_r --> cer1_26_r + coa_r + h_r {'original_bigg_ids': ['CERS126er']}
CERS224er: psphings_r + ttccoa_r --> cer2_24_r + coa_r + h_r {'original_bigg_ids': ['CERS224er']}
CERS226er: hexccoa_r + psphings_r --> cer2_26_r + coa_r + h_r {'original_bigg_ids': ['CERS226er']}
CERS2p18er: cer1_18_r + h_r + nadph_r + o2_r --> cer2p_18_r + h2o_r + nadp_r {'original_bigg_ids': ['CERH124', 'CERS2_24']}
CERS2p24er: cer1_24_r + h_r + nadph_r + o2_r --> cer2p_24_r + h2o_r + nadp_r {'original_bigg_ids': ['CERH124', 'CERS2_24']}
CERS2p26er: cer1_26_r + h_r + nadph_r + o2_r --> cer2p_26_r + h2o_r + nadp_r {'original_bigg_ids': ['CERH126', 'CERS2_26']}
CERS324er: cer2_24_r + h_r + nadph_r + o2_r --> cer3_24_r + h2o_r + nadp_r {'original_bigg_ids': ['CERS324']}
CERS326er: cer2_26_r + h_r + nadph_r + o2_r --> cer3_26_r + h2o_r + nadp_r {'original_bigg_ids': ['CERS326']}
CGLYt3_2: cgly_e + 2.0 h_e --> cgly_c + 2.0 h_c {'original_bigg_ids': ['CGLYt3_LPAREN_2_RPAREN_']}
CGMPt: 35cgmp_c + atp_c + h2o_c --> 35cgmp_e + adp_c + h_c + pi_c {'original_bigg_ids': ['CGMPt']}
CGMPt2: 35cgmp_e <=> 35cgmp_c {'original_bigg_ids': ['CO2t']}
CHLP: cholp_c + h2o_c --> chol_c + pi_c {'original_bigg_ids': ['CHLP']}
CHLPCTD: cholp_c + ctp_c + h_c --> cdpchol_c + ppi_c {'original_bigg_ids': ['CHLPCTD']}
CHLSTI_1er: zymst_r --> chlstol_r {'original_bigg_ids': ['CHLSTI']}
CHLt2: chol_e + h_e --> chol_c + h_c {'original_bigg_ids': ['CHLt2']}
CHOLATEt3: atp_c + cholate_c + h2o_c --> adp_c + cholate_e + h_c + pi_c {'original_bigg_ids': ['CHOLATEt3']}
CHOLD: chol_c + nad_c --> betald_c + h_c + nadh_c {'original_bigg_ids': ['CHOLD']}
CHOLD2m: chol_m + fad_m --> betald_m + fadh2_m {'original_bigg_ids': ['CHOLD2m']}
CHOLID: cholate_c + nad_c --> dhcholn_c + h_c + nadh_c {'original_bigg_ids': ['CHOLID']}
CHOLK: atp_c + chol_c --> adp_c + cholp_c + h_c {'original_bigg_ids': ['CHOLK']}
CHOLPtr: cholp_e <=> cholp_c {'original_bigg_ids': ['CHOLPtr']}
CHOLSH: chols_c + h2o_c --> chol_c + h_c + so4_c {'original_bigg_ids': ['CHOLSH']}
CHORM: chor_c --> pphn_c {'original_bigg_ids': ['CHORM']}
CHORS: 3psme_c --> chor_c + pi_c {'original_bigg_ids': ['CHORS']}
CHTNASE: chtn_c + 2.0 h2o_c --> 3.0 acgam_c {'original_bigg_ids': ['CHTNASE']}
CHTNASEe: chtn_e + 2.0 h2o_e --> 3.0 acgam_e {'original_bigg_ids': ['CHTNASEe']}
CHTNDA: chitin_c + h2o_c --> ac_c + chitos_c + h_c {'original_bigg_ids': ['CHTNDA']}
CHTNS: udpacgal_c --> chitin_c + h_c + udp_c {'original_bigg_ids': ['CHTNS']}
CITRte: citr__L_c <=> citr__L_e {'original_bigg_ids': ['r0817']}
CITtam: cit_c + mal__L_m <=> cit_m + mal__L_c {'original_bigg_ids': ['CITtam']}
CITtcm: cit_c + icit_m <=> cit_m + icit_c {'original_bigg_ids': ['CITtcm']}
CLFORtex2: 2.0 cl_e + for_c --> 2.0 cl_c + for_e {'original_bigg_ids': ['CLFORtex2']}
CLHCO3tex2: 2.0 cl_e + hco3_c --> 2.0 cl_c + hco3_e {'original_bigg_ids': ['CLHCO3tex2']}
CLHCOtex: cl_e + 2.0 hco3_c --> cl_c + 2.0 hco3_e {'original_bigg_ids': ['CLHCOtex']}
CLOHtex2: 2.0 cl_e + oh1_c --> 2.0 cl_c + oh1_e {'original_bigg_ids': ['CLOHtex2']}
CLOXAtex2: 2.0 cl_e + oxa_c --> 2.0 cl_c + oxa_e {'original_bigg_ids': ['CLOXAtex2']}
CLPNDCPT1: clpndcoa_c + crn_c --> clpndcrn_c + coa_c {'original_bigg_ids': ['CLPNDCPT1']}
CLPNDCPT2: clpndcrn_m + coa_m --> clpndcoa_m + crn_m {'original_bigg_ids': ['CLPNDCPT2']}
CLPNDCRNt: clpndcrn_c --> clpndcrn_m {'original_bigg_ids': ['CLPNDCRNt']}
CLPNSm_RT: 0.01 cdpdag_RT_m + 0.01 pg_RT_m --> 0.01 clpn_RT_m + cmp_m + h_m {'original_bigg_ids': ['CLPNSm_SC']}
CMPA: Ncbmpts_m + h2o_m + 2.0 h_m --> co2_m + nh4_m + ptrc_m {'original_bigg_ids': ['CMPA']}
CMPter: cmp_c <=> cmp_r {'original_bigg_ids': ['Htr']}
CO2t: co2_e <=> co2_c {'original_bigg_ids': ['CO2t']}
CO2ter: co2_c <=> co2_r {'original_bigg_ids': ['CO2ter']}
CO2tg: co2_c <=> co2_g {'original_bigg_ids': ['CO2tg']}
CO2tm: co2_c <=> co2_m {'original_bigg_ids': ['CO2tm']}
CO2tn: co2_n <=> co2_c {'original_bigg_ids': ['CO2tn']}
CO2tp: co2_c <=> co2_x {'original_bigg_ids': ['CO2tp']}
CO2tv: co2_c <=> co2_v {'original_bigg_ids': ['CO2tv']}
COAHLp: coa_x + h2o_x --> 2.0 h_x + pan4p_x + pap_x {'original_bigg_ids': ['COAHLp']}
COALDDH: conialdh_c + h2o_c + nad_c --> fer_c + 2.0 h_c + nadh_c {'original_bigg_ids': ['COALDDH']}
COAtim: coa_c --> coa_m {'original_bigg_ids': ['COAtim']}
COAtp: coa_c <=> coa_x {'original_bigg_ids': ['COAtp']}
COAtr: coa_c <=> coa_r {'original_bigg_ids': ['COAtr']}
COQ3_m: 2npmhmobq_m + amet_m --> ahcys_m + h_m + q9_m {'original_bigg_ids': ['COQ3_m']}
COQ5_m: 2np6mobq_m + amet_m --> ahcys_m + h_m + me2np6mobq_m {'original_bigg_ids': ['COQ5_m']}
COQ6_m: 2np6mep_m + o2_m --> 2np6mobq_m + h2o_m {'original_bigg_ids': ['COQ6_m']}
COQ7_m: h_m + me2np6mobq_m + nadph_m + o2_m --> 2npmhmobq_m + h2o_m + nadp_m {'original_bigg_ids': ['COQ7_m']}
COUCOAHp: coucoa_x + h2o_x --> 34hp3hpcoa_x {'original_bigg_ids': ['4HBZCOAFm']}
COUCOALp: T4hcinnm_x + atp_x + coa_x --> amp_x + coucoa_x + ppi_x {'original_bigg_ids': ['COUCOAFm']}
CPCTDTX: ctp_c + h_c + ntm2amep_c --> cmpntm2amep_c + ppi_c {'original_bigg_ids': ['CPCTDTX']}
CPK1: cmp_c + ctp_c <=> 2.0 cdp_c {'original_bigg_ids': ['CYTK6']}
CPPPGO: cpppg3_c + 2.0 h_c + o2_c --> 2.0 co2_c + 2.0 h2o_c + pppg9_c {'original_bigg_ids': ['CPPPGO']}
CRNtim: crn_m --> crn_c {'original_bigg_ids': ['CRNtim']}
CRNtp: crn_c <=> crn_x {'original_bigg_ids': ['CRNrtx']}
CSNAT2m: coa_m + pcrn_m <=> crn_m + ppcoa_m {'original_bigg_ids': ['CSNAT2m']}
CSNAT2x: crn_x + dmnoncoa_x <=> coa_x + dmnoncrn_x {'original_bigg_ids': ['CSNAT2x']}
CSNAT3x: crn_x + ppcoa_x <=> coa_x + pcrn_x {'original_bigg_ids': ['CSNAT3x']}
CSNATer: accoa_r + crn_r <=> acrn_r + coa_r {'original_bigg_ids': ['CSNATer']}
CSNATm: acrn_m + coa_m <=> accoa_m + crn_m {'original_bigg_ids': ['CSNATm']}
CSNATp: accoa_x + crn_x <=> acrn_x + coa_x {'original_bigg_ids': ['CSNATp']}
CSND: csn_c + h2o_c + h_c --> nh4_c + ura_c {'original_bigg_ids': ['CSND']}
CSNt2: csn_e + h_e --> csn_c + h_c {'original_bigg_ids': ['CSNt2']}
CSm: accoa_m + h2o_m + oaa_m --> cit_m + coa_m + h_m {'original_bigg_ids': ['CSm']}
CSp: accoa_x + h2o_x + oaa_x --> cit_x + coa_x + h_x {'original_bigg_ids': ['CSp']}
CTPS1: atp_c + nh4_c + utp_c --> adp_c + ctp_c + 2.0 h_c + pi_c {'original_bigg_ids': ['CTPS1']}
CTPS2: atp_c + gln__L_c + h2o_c + utp_c --> adp_c + ctp_c + glu__L_c + 2.0 h_c + pi_c {'original_bigg_ids': ['CTPS2']}
CTPter: ctp_c <=> ctp_r {'original_bigg_ids': ['Htr']}
CTPtm: cmp_m + ctp_c + 2.0 h_c --> cmp_c + ctp_m + 2.0 h_m {'original_bigg_ids': ['CTPtm']}
CTPtm2: cmp_m + ctp_c + fe2_c --> cmp_c + ctp_m + fe2_m {'original_bigg_ids': ['CTPtm']}
CU2t: cu2_e <=> cu2_c {'original_bigg_ids': ['CU2tex']}
CURR: cur_c + h_c + nadph_c --> dhcur_c + nadp_c {'original_bigg_ids': ['CURR']}
CYANST: cyan_c + tsul_c --> h_c + so3_c + tcynt_c {'original_bigg_ids': ['CYANST']}
CYNTAH: cynt_c + 3.0 h_c + hco3_c --> 2.0 co2_c + nh4_c {'original_bigg_ids': ['CYNTAH']}
CYOO6m: 4.0 focytc_m + 8.0 h_m + o2_m --> 4.0 ficytc_m + 2.0 h2o_m + 4.0 h_c {'original_bigg_ids': ['CYOO6m']}
CYOR_u9m: 2.0 ficytc_m + 2.0 h_m + q9h2_m --> 2.0 focytc_m + 4.0 h_c + q9_m {'original_bigg_ids': ['CYOR_u6m']}
CYSDS: cys__L_c + h2o_c --> h2s_c + nh4_c + pyr_c {'original_bigg_ids': ['CYSDS']}
CYSS: acser_c + h2s_c --> ac_c + cys__L_c + h_c {'original_bigg_ids': ['CYSS']}
CYSTA: akg_c + cys__L_c <=> glu__L_c + mercppyr_c {'original_bigg_ids': ['CYSTA']}
CYSTAm: akg_m + cys__L_m <=> glu__L_m + mercppyr_m {'original_bigg_ids': ['CYSTAm']}
CYSTGL: cyst__L_c + h2o_c --> 2obut_c + cys__L_c + nh4_c {'original_bigg_ids': ['CYSTGL']}
CYSTRS: atp_c + cys__L_c + trnacys_c --> amp_c + cystrna_c + ppi_c {'original_bigg_ids': ['CYSTRS']}
CYSTS: hcys__L_c + ser__L_c --> cyst__L_c + h2o_c {'original_bigg_ids': ['CYSTS']}
CYSTSERex: cysi__L_e + ser__L_c --> cysi__L_c + ser__L_e {'original_bigg_ids': ['CYSTSERex']}
CYST_Ltr: cyst__L_e <=> cyst__L_c {'original_bigg_ids': ['CYST_Ltr']}
CYSt2r: cys__L_e + h_e <=> cys__L_c + h_c {'original_bigg_ids': ['CYSt2r']}
CYStm: cys__L_c <=> cys__L_m {'original_bigg_ids': ['r1437']}
CYTD: cytd_c + h2o_c + h_c --> nh4_c + uri_c {'original_bigg_ids': ['CYTD']}
CYTDH: cytd_c + h2o_c --> csn_c + rib__D_c {'original_bigg_ids': ['CYTDH']}
CYTDK1: atp_c + cytd_c --> adp_c + cmp_c + h_c {'original_bigg_ids': ['CYTDK1']}
CYTDK2: cytd_c + gtp_c --> cmp_c + gdp_c + h_c {'original_bigg_ids': ['CYTDK2']}
CYTDt2: cytd_e + h_e --> cytd_c + h_c {'original_bigg_ids': ['CYTDt2']}
CYTK10: cmp_c + dgtp_c <=> cdp_c + dgdp_c {'original_bigg_ids': ['CYTK10']}
CYTK10n: cmp_n + dgtp_n <=> cdp_n + dgdp_n {'original_bigg_ids': ['CYTK10n']}
CYTK11: dcmp_c + dgtp_c <=> dcdp_c + dgdp_c {'original_bigg_ids': ['CYTK11']}
CYTK11n: dcmp_n + dgtp_n <=> dcdp_n + dgdp_n {'original_bigg_ids': ['CYTK11n']}
CYTK12: dcmp_c + dctp_c <=> 2.0 dcdp_c {'original_bigg_ids': ['CYTK12']}
CYTK12n: dcmp_n + dctp_n <=> 2.0 dcdp_n {'original_bigg_ids': ['CYTK12n']}
CYTK13: datp_c + dcmp_c <=> dadp_c + dcdp_c {'original_bigg_ids': ['CYTK13']}
CYTK13n: datp_n + dcmp_n <=> dadp_n + dcdp_n {'original_bigg_ids': ['CYTK13n']}
CYTK14: dcmp_c + utp_c <=> dcdp_c + udp_c {'original_bigg_ids': ['CYTK14']}
CYTK14n: dcmp_n + utp_n <=> dcdp_n + udp_n {'original_bigg_ids': ['CYTK14n']}
CYTK1n: atp_n + cmp_n <=> adp_n + cdp_n {'original_bigg_ids': ['CYTK1n']}
CYTK2_1: ctp_c + dcmp_c <=> cdp_c + dcdp_c {'original_bigg_ids': ['CYTK5']}
CYTK2n: atp_n + dcmp_n <=> adp_n + dcdp_n {'original_bigg_ids': ['CYTK2n']}
CYTK3n: ctp_n + dcmp_n <=> cdp_n + dcdp_n {'original_bigg_ids': ['CYTK3n']}
CYTK4n: dcmp_n + gtp_n <=> dcdp_n + gdp_n {'original_bigg_ids': ['CYTK4n']}
CYTK5n: cmp_n + gtp_n <=> cdp_n + gdp_n {'original_bigg_ids': ['CYTK5n']}
CYTK6n: cmp_n + ctp_n <=> 2.0 cdp_n {'original_bigg_ids': ['CYTK6n']}
CYTK7: cmp_c + utp_c <=> cdp_c + udp_c {'original_bigg_ids': ['CYTK7']}
CYTK7n: cmp_n + utp_n <=> cdp_n + udp_n {'original_bigg_ids': ['CYTK7n']}
CYTK8: cmp_c + datp_c <=> cdp_c + dadp_c {'original_bigg_ids': ['CYTK8']}
CYTK8n: cmp_n + datp_n <=> cdp_n + dadp_n {'original_bigg_ids': ['CYTK8n']}
CYTK9: cmp_c + dctp_c <=> cdp_c + dcdp_c {'original_bigg_ids': ['CYTK9']}
CYTK9n: cmp_n + dctp_n <=> cdp_n + dcdp_n {'original_bigg_ids': ['CYTK9n']}
Cu1ATPase: atp_c + cu_c + h2o_c --> adp_c + cu_e + h_c + pi_c {'original_bigg_ids': ['CU1abcpp']}
DADA: dad_2_c + h2o_c + h_c --> din_c + nh4_c {'original_bigg_ids': ['DADA']}
DADAe: dad_2_e + h2o_e + h_e --> din_e + nh4_e {'original_bigg_ids': ['DADAe']}
DADK: atp_c + damp_c <=> adp_c + dadp_c {'original_bigg_ids': ['DADK']}
DAGCPTer_RT: 0.01 12dgr_RT_r + cdpchol_r --> cmp_r + h_r + 0.01 pc_RT_r {'original_bigg_ids': ['DAGCPT_SC']}
DAGL_RT: 0.01 12dgr_RT_d + h2o_c --> h_c + 0.23 hdca_c + 0.01 hdcea_c + 0.01 lnlncg_c + 0.01 mag_RT_d + 0.12 ocdca_c + 0.48 ocdcea_c + 0.14 ocdcya_c + 0.01 ttc_c {'original_bigg_ids': ['TAGL_SC']}
DAGPYPer_RT: h2o_r + 0.01 pa_RT_r --> 0.01 12dgr_RT_r + pi_r {'original_bigg_ids': ['DAGPYP_SC']}
DALAt2r: ala__D_e + h_e <=> ala__D_c + h_c {'original_bigg_ids': ['DALAt2r']}
DARABR: arab__D_c + h_c + nadph_c --> abt__D_c + nadp_c {'original_bigg_ids': ['ARABR']}
DASPO1p: asp__D_x + h2o_x + o2_x --> h2o2_x + nh4_x + oaa_x {'original_bigg_ids': ['DASPO1p']}
DASYNer_RT: ctp_r + h_r + 0.01 pa_RT_r --> 0.01 cdpdag_RT_r + ppi_r {'original_bigg_ids': ['DASYN_SC']}
DASYNm_RT: ctp_m + h_m + 0.01 pa_RT_m --> 0.01 cdpdag_RT_m + ppi_m {'original_bigg_ids': ['DASYNm_SC']}
DATCY: cytd_c + datp_c --> cmp_c + dadp_c + h_c {'original_bigg_ids': ['DATCY']}
DATUP: datp_c + uri_c --> dadp_c + h_c + ump_c {'original_bigg_ids': ['DATUP']}
DB4PS: ru5p__D_c --> db4p_c + for_c + h_c {'original_bigg_ids': ['DB4PS']}
DBTSm: atp_m + co2_m + dann_m <=> adp_m + dtbt_m + 3.0 h_m + pi_m {'original_bigg_ids': ['DBTSr']}
DCACOAtr: dcacoa_c <=> dcacoa_r {'original_bigg_ids': ['ACCOAtr']}
DCMPDA: dcmp_c + h2o_c + h_c <=> dump_c + nh4_c {'original_bigg_ids': ['DCMPDA']}
DCSPTN1CPT1: crn_c + dcsptn1coa_c --> coa_c + dcsptn1crn_c {'original_bigg_ids': ['DCSPTN1CPT1']}
DCSPTN1CPT2: coa_m + dcsptn1crn_m --> crn_m + dcsptn1coa_m {'original_bigg_ids': ['DCSPTN1CPT2']}
DCSPTN1CRNt: dcsptn1crn_c --> dcsptn1crn_m {'original_bigg_ids': ['DCSPTN1CRNt']}
DCTCP: cytd_c + dctp_c --> cmp_c + dcdp_c + h_c {'original_bigg_ids': ['DCTCP']}
DCTPD: dctp_c + h2o_c + h_c --> dutp_c + nh4_c {'original_bigg_ids': ['DCTPD']}
DCTUP: dctp_c + uri_c --> dcdp_c + h_c + ump_c {'original_bigg_ids': ['DCTUP']}
DCYTD: dcyt_c + h2o_c + h_c --> duri_c + nh4_c {'original_bigg_ids': ['DCYTD']}
DDCACOAtr: ddcacoa_c <=> ddcacoa_r {'original_bigg_ids': ['ACCOAtr']}
DDPA: e4p_c + h2o_c + pep_c --> 2dda7p_c + pi_c {'original_bigg_ids': ['DDPA']}
DDPAm: e4p_m + h2o_m + pep_m --> 2dda7p_m + pi_m {'original_bigg_ids': ['DDPAm']}
DDPGAm: 4h2oglt_m <=> glx_m + pyr_m {'original_bigg_ids': ['DDPGAm']}
DESAT1619Zer: h_r + nadh_r + o2_r + pmtcoa_r --> 2.0 h2o_r + hdcoa_r + nad_r {'original_bigg_ids': ['DESAT16_2']}
DESAT1819Zer: h_r + nadh_r + o2_r + stcoa_r --> 2.0 h2o_r + nad_r + odecoa_r {'original_bigg_ids': ['DESAT18_3']}
DESAT1829Z12Zer: h_r + nadh_r + o2_r + odecoa_r --> 2.0 h2o_r + nad_r + ocdycacoa_r {'original_bigg_ids': ['DESAT18_2']}
DESAT1836Z9Z12Zer: h_r + nadh_r + o2_r + ocdycacoa_r --> 2.0 h2o_r + lnlncgcoa_r + nad_r {'original_bigg_ids': ['DESAT18_9']}
DGK1: atp_c + dgmp_c <=> adp_c + dgdp_c {'original_bigg_ids': ['DGK1']}
DGTCY: cytd_c + dgtp_c --> cmp_c + dgdp_c + h_c {'original_bigg_ids': ['DGTCY']}
DGTUP: dgtp_c + uri_c --> dgdp_c + h_c + ump_c {'original_bigg_ids': ['DGTUP']}
DHAAt1r: dhdascb_e <=> dhdascb_c {'original_bigg_ids': ['DHAAt1r']}
DHAD1m: 23dhmb_m --> 3mob_m + h2o_m {'original_bigg_ids': ['DHAD1m']}
DHAD2m: 23dhmp_m --> 3mop_m + h2o_m {'original_bigg_ids': ['DHAD2m']}
DHAK: atp_c + dha_c --> adp_c + dhap_c + h_c {'original_bigg_ids': ['DHAK']}
DHAOX_c: dhdascb_c + 2.0 gthrd_c --> ascb__L_c + gthox_c + h_c {'original_bigg_ids': ['DHAOX_c']}
DHAt: dha_e <=> dha_c {'original_bigg_ids': ['DHAtex']}
DHCURR: dhcur_c + h_c + nadph_c --> nadp_c + thcur_c {'original_bigg_ids': ['DHCURR']}
DHFR: dhf_c + h_c + nadph_c <=> nadp_c + thf_c {'original_bigg_ids': ['DHFR']}
DHFRim: dhf_m + h_m + nadph_m --> nadp_m + thf_m {'original_bigg_ids': ['DHFRim']}
DHFS: atp_c + dhpt_c + glu__L_c --> adp_c + dhf_c + h_c + pi_c {'original_bigg_ids': ['DHFS']}
DHNPA2r: dhnpt_c <=> 6hmhpt_c + gcald_c {'original_bigg_ids': ['DHNPA2r']}
DHNPBMT_m: 3npdhb_m + amet_m --> ahcys_m + h_m + me3dhnpdh_m {'original_bigg_ids': ['DHNPBMT_m']}
DHORD_u9m: dhor__S_c + q9_m --> orot_c + q9h2_m {'original_bigg_ids': ['DHORD4i']}
DHORDfum: dhor__S_c + fum_c --> orot_c + succ_c {'original_bigg_ids': ['DHORDfum']}
DHORTS: dhor__S_c + h2o_c <=> cbasp_c + h_c {'original_bigg_ids': ['DHORTS']}
DHPM1: 56dura_c + h2o_c --> cala_c + h_c {'original_bigg_ids': ['DHPM1']}
DHPS2: 4abz_c + 6hmhptpp_c --> dhpt_c + ppi_c {'original_bigg_ids': ['DHPS2']}
DHQS: 2dda7p_c --> 3dhq_c + pi_c {'original_bigg_ids': ['DHQS']}
DHQTi: 3dhq_c --> 3dhsk_c + h2o_c {'original_bigg_ids': ['DHQTi']}
DHSKDH: 3dhsk_c --> 34dhbz_c + h2o_c {'original_bigg_ids': ['DHSKDH']}
DIAT: accoa_c + sprm_c --> N1sprm_c + coa_c + h_c {'original_bigg_ids': ['DIAT']}
DIPS: amet_c + caphis_c --> ahcys_c + cmaphis_c + h_c {'original_bigg_ids': ['DIPS']}
DKGLCNR1: 25dkglcn_c + h_c + nadph_c --> 2dhguln_c + nadp_c {'original_bigg_ids': ['DKGLCNR1']}
DMATT: dmpp_c + ipdp_c --> grdp_c + ppi_c {'original_bigg_ids': ['DMATT']}
DM_aacald_c: aacald_c -->  {'original_bigg_ids': ['DM_aacald_c']}
DM_amob_m: amob_m -->  {'original_bigg_ids': ['DM_aacald_c']}
DM_dad_5_m: dad_5_m -->  {'original_bigg_ids': ['DM_aacald_c']}
DNADDP: dnad_c + h2o_c --> amp_c + 2.0 h_c + nicrnt_c {'original_bigg_ids': ['DNNH']}
DNAMTn2r: amet_c + dna_c <=> ahcys_c + dna5mtc_c + h_c {'original_bigg_ids': ['DNAMTn2r']}
DNMPPA: dhpmp_c + h2o_c --> dhnpt_c + pi_c {'original_bigg_ids': ['DNMPPA']}
DNTPPA: ahdt_c + h2o_c --> dhpmp_c + h_c + ppi_c {'original_bigg_ids': ['DNTPPA']}
DOCOSACt: docosac_e --> docosac_c {'original_bigg_ids': ['DOCOSACTDe']}
DOLASNT: Asn_X_Ser_Thr_c + doldpglcnacglcnacman_man_manman_manman_manmanmanglcglcglc_c --> asnglcnacglcnacman_man_manman_manman_manmanmanglcglcglc_c + doldp_c + 3.0 h_c {'original_bigg_ids': ['DOLASNT']}
DOLPGT1_Ler: 0.1 dolglcp__L_r + 0.1 m8mpdol__L_r --> 0.1 dolp__L_r + 0.1 g1m8mpdol__L_r + h_r {'original_bigg_ids': ['DOLPGT1_Ler']}
DOLPGT1_Uer: 0.1 dolglcp_U_r + 0.1 m8mpdol_U_r --> 0.1 dolp_U_r + 0.1 g1m8mpdol_U_r + h_r {'original_bigg_ids': ['DOLPGT1_Uer']}
DOLPGT2_Ler: 0.1 dolglcp__L_r + 0.1 g1m8mpdol__L_r --> 0.1 dolp__L_r + 0.1 g2m8mpdol__L_r + h_r {'original_bigg_ids': ['DOLPGT2_Ler']}
DOLPGT2_Uer: 0.1 dolglcp_U_r + 0.1 g1m8mpdol_U_r --> 0.1 dolp_U_r + 0.1 g2m8mpdol_U_r + h_r {'original_bigg_ids': ['DOLPGT2_Uer']}
DOLPGT3_Ler: 0.1 dolglcp__L_r + 0.1 g2m8mpdol__L_r --> 0.1 dolp__L_r + 0.1 g3m8mpdol__L_r + h_r {'original_bigg_ids': ['DOLPGT3_Ler']}
DOLPGT3_Uer: 0.1 dolglcp_U_r + 0.1 g2m8mpdol_U_r --> 0.1 dolp_U_r + 0.1 g3m8mpdol_U_r + h_r {'original_bigg_ids': ['DOLPGT3_Uer']}
DOLPMMer: dolmanp_r --> dolp_r + h_r + mannan_r {'original_bigg_ids': ['DOLPMMer']}
DOLPMT: dolp_c + gdpmann_c --> dolmanp_c + gdp_c {'original_bigg_ids': ['DOLPMT']}
DOLPMT1_Ler: 0.1 dolmanp__L_r + 0.1 m4mpdol__L_r --> 0.1 dolp__L_r + h_r + 0.1 m5mpdol__L_r {'original_bigg_ids': ['DOLPMT1_Ler']}
DOLPMT1_Uer: 0.1 dolmanp_U_r + 0.1 m4mpdol_U_r --> 0.1 dolp_U_r + h_r + 0.1 m5mpdol_U_r {'original_bigg_ids': ['DOLPMT1_Uer']}
DOLPMT3_Ler: 0.1 dolmanp__L_r + 0.1 m6mpdol__L_r --> 0.1 dolp__L_r + h_r + 0.1 m7mpdol__L_r {'original_bigg_ids': ['DOLPMT3_Ler']}
DOLPMT3_Uer: 0.1 dolmanp_U_r + 0.1 m6mpdol_U_r --> 0.1 dolp_U_r + h_r + 0.1 m7mpdol_U_r {'original_bigg_ids': ['DOLPMT3_Uer']}
DOLPMTcer: dolp_c + gdpmann_c --> dolmanp_r + gdp_c {'original_bigg_ids': ['DOLPMTcer']}
DPCOAK: atp_c + dpcoa_c --> adp_c + coa_c + h_c {'original_bigg_ids': ['DPCOAK']}
DPGM: 13dpg_c <=> 23dpg_c + h_c {'original_bigg_ids': ['DPGM']}
DPGase: 23dpg_c + h2o_c --> 3pg_c + pi_c {'original_bigg_ids': ['DPGase']}
DPMVD: 5dpmev_c + atp_c --> adp_c + co2_c + ipdp_c + pi_c {'original_bigg_ids': ['DPMVD']}
DPR: 2dhp_c + h_c + nadph_c --> nadp_c + pant__R_c {'original_bigg_ids': ['DPR']}
DRBK: atp_c + drib_c --> 2dr5p_c + adp_c + h_c {'original_bigg_ids': ['DRBK']}
DRIBt: drib_e <=> drib_c {'original_bigg_ids': ['DRIBt']}
DROPPRx: 25dhpp_c + h_c + nadh_c --> 25dthpp_c + nad_c {'original_bigg_ids': ['DROPPRy']}
DRTPPD: 25dthpp_c + h2o_c + h_c --> 5aprbu_c + nh4_c {'original_bigg_ids': ['DRTPPD']}
DSERDHr: nadp_c + ser__D_c <=> 2amsa_c + h_c + nadph_c {'original_bigg_ids': ['DSERDHr']}
DTMPK: atp_c + dtmp_c <=> adp_c + dtdp_c {'original_bigg_ids': ['DTMPK']}
DTTGY: cytd_c + dttp_c --> cmp_c + dtdp_c + h_c {'original_bigg_ids': ['DTTGY']}
DTTUP: dttp_c + uri_c --> dtdp_c + h_c + ump_c {'original_bigg_ids': ['DTTUP']}
DURIPP: duri_c + pi_c <=> 2dr1p_c + ura_c {'original_bigg_ids': ['DURIPP']}
DUTCP: cytd_c + dutp_c --> cmp_c + dudp_c + h_c {'original_bigg_ids': ['DUTCP']}
DUTPDP: dutp_c + h2o_c --> dump_c + h_c + ppi_c {'original_bigg_ids': ['DUTPDP']}
DUTPDPm: dutp_m + h2o_m --> dump_m + h_m + ppi_m {'original_bigg_ids': ['DUTPDPm']}
DUTPDPn: dutp_n + h2o_n --> dump_n + h_n + ppi_n {'original_bigg_ids': ['DUTPDPn']}
DUTUP: dutp_c + uri_c --> dudp_c + h_c + ump_c {'original_bigg_ids': ['DUTUP']}
D_LACDcm: 2.0 ficytc_m + lac__D_c --> 2.0 focytc_m + 2.0 h_c + pyr_c {'original_bigg_ids': ['D_LACDcm']}
D_LACt2: h_e + lac__D_e <=> h_c + lac__D_c {'original_bigg_ids': ['D_LACt2']}
E4PD: e4p_c + h2o_c + nad_c <=> 4per_c + 2.0 h_c + nadh_c {'original_bigg_ids': ['E4PD']}
E4PP: e4p_c + h2o_c --> erthrs_c + pi_c {'original_bigg_ids': ['E4PP']}
ECOAH11p: h2o_x + hxc2coa_x <=> 3hxccoa_x {'original_bigg_ids': ['ECOAH11p']}
ECOAH12m: 2mp2coa_m + h2o_m <=> 3hibutcoa_m {'original_bigg_ids': ['ECOAH12m']}
ECOAH1m: 3hbcoa_m <=> b2coa_m + h2o_m {'original_bigg_ids': ['ECOAH1m']}
ECOAH1p: 3hbcoa_x <=> b2coa_x + h2o_x {'original_bigg_ids': ['ECOAH1']}
ECOAH2m: 3hhcoa_m <=> h2o_m + hx2coa_m {'original_bigg_ids': ['ECOAH2m']}
ECOAH2p: h2o_x + hx2coa_x <=> 3hhcoa_x {'original_bigg_ids': ['ECOAH2']}
ECOAH3m: 3hocoa_m <=> h2o_m + oc2coa_m {'original_bigg_ids': ['ECOAH3m']}
ECOAH3p: h2o_x + oc2coa_x <=> 3hocoa_x {'original_bigg_ids': ['ECOAH3']}
ECOAH4m: 3hdcoa_m <=> dc2coa_m + h2o_m {'original_bigg_ids': ['ECOAH4m']}
ECOAH4p: 3hdcoa_x <=> dc2coa_x + h2o_x {'original_bigg_ids': ['ECOAH4p']}
ECOAH5m: 3hddcoa_m <=> dd2coa_m + h2o_m {'original_bigg_ids': ['ECOAH5m']}
ECOAH5p: 3hddcoa_x <=> dd2coa_x + h2o_x {'original_bigg_ids': ['ECOAH5p']}
ECOAH6m: 3htdcoa_m <=> h2o_m + td2coa_m {'original_bigg_ids': ['ECOAH6m']}
ECOAH6p: 3htdcoa_x <=> h2o_x + td2coa_x {'original_bigg_ids': ['ECOAH6p']}
ECOAH7m: 3hhdcoa_m <=> h2o_m + hdd2coa_m {'original_bigg_ids': ['ECOAH7m']}
ECOAH7p: 3hhdcoa_x <=> h2o_x + hdd2coa_x {'original_bigg_ids': ['ECOAH7p']}
ECOAH8p: 3hodcoa_x <=> h2o_x + od2coa_x {'original_bigg_ids': ['ECOAH8p']}
ECOAH9m: 2mb2coa_m + h2o_m <=> 3hmbcoa_m {'original_bigg_ids': ['ECOAH9m']}
EHGLAT2m: e4hglu_m + oaa_m --> 4h2oglt_m + asp__L_m {'original_bigg_ids': ['EHGLAT2m']}
EHGLATm: akg_m + e4hglu_m --> 4h2oglt_m + glu__L_m {'original_bigg_ids': ['EHGLATm']}
ELAIDCPT1: crn_c + od2coa_c --> coa_c + elaidcrn_c {'original_bigg_ids': ['ELAIDCPT1']}
ELAIDCPT2: coa_m + elaidcrn_m --> crn_m + od2coa_m {'original_bigg_ids': ['ELAIDCPT2']}
ELAIDCRNt: elaidcrn_c --> elaidcrn_m {'original_bigg_ids': ['ELAIDCRNt']}
ENGASE: h2o_c + s2l2n2m2m_c --> acgam_c + s2l2n2m2mn_c {'original_bigg_ids': ['ENGASE']}
ENGASE2: h2o_c + n2m2nm_c --> acgam_c + n2m2nmn_c {'original_bigg_ids': ['ENGASE2']}
ENO: 2pg_c <=> h2o_c + pep_c {'original_bigg_ids': ['ENO']}
EPGALURSe: h2o_e + pectin_e --> galur_e {'original_bigg_ids': ['EPGALURSe']}
EPISTATer_RT: epist_r + 0.01 hdcoa_r + 0.01 lnlncgcoa_r + 0.14 ocdycacoa_r + 0.48 odecoa_r + 0.23 pmtcoa_r + 0.12 stcoa_r + 0.01 ttccoa_r --> coa_r + 0.01 epistest_RT_r {'original_bigg_ids': ['EPISTAT_SC']}
EPISTESTH_RT: 0.01 epistest_RT_d + h2o_c --> epist_c + h_c + 0.23 hdca_c + 0.01 hdcea_c + 0.01 lnlncg_c + 0.12 ocdca_c + 0.48 ocdcea_c + 0.14 ocdcya_c + 0.01 ttc_c {'original_bigg_ids': ['EPISTESTH_SC']}
EPISTESTtrd: epistest_RT_r --> epistest_RT_d {'original_bigg_ids': ['FRDPtcr']}
EPISTt: epist_e <=> epist_c {'original_bigg_ids': ['EPISTt']}
EPISTtr: epist_c <=> epist_r {'original_bigg_ids': ['EPISTt']}
ERGSTATer_RT: ergst_r + 0.01 hdcoa_r + 0.01 lnlncgcoa_r + 0.14 ocdycacoa_r + 0.48 odecoa_r + 0.23 pmtcoa_r + 0.12 stcoa_r + 0.01 ttccoa_r --> coa_r + 0.01 ergstest_RT_r {'original_bigg_ids': ['ERGSTAT_SC']}
ERGSTESTH_RT: 0.01 ergstest_RT_d + h2o_c --> ergst_c + h_c + 0.23 hdca_c + 0.01 hdcea_c + 0.01 lnlncg_c + 0.12 ocdca_c + 0.48 ocdcea_c + 0.14 ocdcya_c + 0.01 ttc_c {'original_bigg_ids': ['ERGSTESTH_SC']}
ERGSTESTtrd: ergstest_RT_r --> ergstest_RT_d {'original_bigg_ids': ['FRDPtcr']}
ERGSTGLCT: ergst_c + udpg_c --> ergst3glc_c + h_c + udp_c {'original_bigg_ids': ['ERGSTGLCT']}
ERGSTt: ergst_e <=> ergst_c {'original_bigg_ids': ['ERGSTt']}
ERGSTtr: ergst_c <=> ergst_r {'original_bigg_ids': ['ERGSTt']}
ESTRADIOLGLCt2: atp_c + estradiolglc_c + h2o_c --> adp_c + estradiolglc_e + h_c + pi_c {'original_bigg_ids': ['ESTRADIOLGLCt2']}
ESTRIOLGLCte: atp_c + estriolglc_c + h2o_c --> adp_c + estriolglc_e + h_c + pi_c {'original_bigg_ids': ['ESTRIOLGLCte']}
ESTRONEGLCt: atp_c + estroneglc_c + h2o_c --> adp_c + estroneglc_e + h_c + pi_c {'original_bigg_ids': ['ESTRONEGLCt']}
ETF: etfox_m + fadh2_m --> etfrd_m + fad_m {'original_bigg_ids': ['ETF']}
ETFQO_m: etfrd_m + q9_m --> etfox_m + q9h2_m {'original_bigg_ids': ['ETFQO_m']}
ETHAK: atp_c + etha_c --> adp_c + ethamp_c + h_c {'original_bigg_ids': ['ETHAK']}
ETHAMPtr: ethamp_e <=> ethamp_c {'original_bigg_ids': ['ETHAMPtr']}
ETHAPTer_RT: 0.01 12dgr_RT_r + cdpea_r --> cmp_r + h_r + 0.01 pe_RT_r {'original_bigg_ids': ['ETHAPT_SC']}
ETHAt: etha_e <=> etha_c {'original_bigg_ids': ['ETHAt']}
ETHP: ethamp_c + h2o_c --> etha_c + pi_c {'original_bigg_ids': ['ETHP']}
EX_12ppd__R_e: 12ppd__R_e -->  {'original_bigg_ids': ['EX_12ppd_R[e]']}
EX_12ppd__S_e: 12ppd__S_e -->  {'original_bigg_ids': ['EX_12ppd__S_e']}
EX_13BDglcn_e: 13BDglcn_e -->  {'original_bigg_ids': ['EX_h_e_']}
EX_2doxg6p_e: 2doxg6p_e -->  {'original_bigg_ids': ['EX_h_e_']}
EX_2hxmp_e: 2hxmp_e -->  {'original_bigg_ids': ['EX_2hxmp_e_']}
EX_2obut_e: 2obut_e -->  {'original_bigg_ids': ['EX_2obut[e]']}
EX_2pg_e: 2pg_e -->  {'original_bigg_ids': ['EX_2pg[e]']}
EX_35ccmp_e: 35ccmp_e -->  {'original_bigg_ids': ['EX_h_e_']}
EX_35cgmp_e: 35cgmp_e -->  {'original_bigg_ids': ['EX_h_e_']}
EX_3pg_e: 3pg_e -->  {'original_bigg_ids': ['EX_3pg[e]']}
EX_3sala_e: 3sala_e -->  {'original_bigg_ids': ['EX_h_e_']}
EX_4abut_e: 4abut_e -->  {'original_bigg_ids': ['EX_h_e_']}
EX_4abz_e: 4abz_e -->  {'original_bigg_ids': ['EX_4abz_e_']}
EX_4hbz_e: 4hbz_e -->  {'original_bigg_ids': ['EX_4hbz[e]']}
EX_5adtststeroneglc_e: 5adtststeroneglc_e -->  {'original_bigg_ids': ['EX_h_e_']}
EX_5aop_e: 5aop_e -->  {'original_bigg_ids': ['EX_h_e_']}
EX_5dglcn_e: 5dglcn_e -->  {'original_bigg_ids': ['EX_5dglcn_e']}
EX_5flura_e: 5flura_e -->  {'original_bigg_ids': ['EX_h_e_']}
EX_6mpur_e: 6mpur_e -->  {'original_bigg_ids': ['EX_h_e_']}
EX_6pgc_e: 6pgc_e -->  {'original_bigg_ids': ['EX_h_e_']}
EX_Lcyst_e: Lcyst_e -->  {'original_bigg_ids': ['EX_Lcyst[e]']}
EX_T4hcinnm_e: T4hcinnm_e -->  {'original_bigg_ids': ['EX_T4hcinnm_LPAREN_e_RPAREN_']}
EX_abt__D_e: abt__D_e -->  {'original_bigg_ids': ['EX_abt_D[e]']}
EX_abt_e: abt_e -->  {'original_bigg_ids': ['EX_abt[e]']}
EX_ac_e: ac_e -->  {'original_bigg_ids': ['EX_h_e_']}
EX_acac_e: acac_e -->  {'original_bigg_ids': ['EX_acac[e]']}
EX_acgam_e: acgam_e -->  {'original_bigg_ids': ['EX_h_e_']}
EX_acglu_e: acglu_e -->  {'original_bigg_ids': ['EX_acglu[e]']}
EX_ach_e: ach_e -->  {'original_bigg_ids': ['EX_h_e_']}
EX_ad_e: ad_e -->  {'original_bigg_ids': ['EX_h_e_']}
EX_ade_e: ade_e -->  {'original_bigg_ids': ['EX_h_e_']}
EX_adn_e: adn_e -->  {'original_bigg_ids': ['EX_h_e_']}
EX_agm_e: agm_e -->  {'original_bigg_ids': ['EX_agm[e]']}
EX_ahandrostanglc_e: ahandrostanglc_e -->  {'original_bigg_ids': ['EX_h_e_']}
EX_akg_e: akg_e -->  {'original_bigg_ids': ['EX_akg_e_']}
EX_ala_B_e: ala_B_e -->  {'original_bigg_ids': ['EX_h_e_']}
EX_ala__D_e: ala__D_e -->  {'original_bigg_ids': ['EX_h_e_']}
EX_ala__L_e: ala__L_e -->  {'original_bigg_ids': ['EX_h_e_']}
EX_alltn_e: alltn_e -->  {'original_bigg_ids': ['EX_h_e_']}
EX_alltt_e: alltt_e -->  {'original_bigg_ids': ['EX_h_e_']}
EX_amet_e: amet_e -->  {'original_bigg_ids': ['EX_h_e_']}
EX_amp_e: amp_e -->  {'original_bigg_ids': ['EX_amp[e]']}
EX_andrstrnglc_e: andrstrnglc_e -->  {'original_bigg_ids': ['EX_h_e_']}
EX_arab__D_e: arab__D_e -->  {'original_bigg_ids': ['EX_arab_D_e_']}
EX_arab__L_e: arab__L_e -->  {'original_bigg_ids': ['EX_arab_L_e_']}
EX_arach_e: arach_e -->  {'original_bigg_ids': ['EX_h_e_']}
EX_arbt_e: arbt_e -->  {'original_bigg_ids': ['EX_arbt_e_']}
EX_arg__L_e: arg__L_e -->  {'original_bigg_ids': ['EX_h_e_']}
EX_asn__L_e: asn__L_e -->  {'original_bigg_ids': ['EX_h_e_']}
EX_asp__D_e: asp__D_e -->  {'original_bigg_ids': ['EX_asp_D[e]']}
EX_asp__L_e: asp__L_e -->  {'original_bigg_ids': ['EX_h_e_']}
EX_bhb_e: bhb_e -->  {'original_bigg_ids': ['EX_bhb[e]']}
EX_bildglcur_e: bildglcur_e -->  {'original_bigg_ids': ['EX_h_e_']}
EX_bilglcur_e: bilglcur_e -->  {'original_bigg_ids': ['EX_h_e_']}
EX_btd_RR_e: btd_RR_e -->  {'original_bigg_ids': ['EX_btd_RR_e_']}
EX_btn_e: btn_e -->  {'original_bigg_ids': ['EX_btn_e_']}
EX_but_e: but_e -->  {'original_bigg_ids': ['EX_but[e]']}
EX_bz_e: bz_e -->  {'original_bigg_ids': ['EX_bz_LPAREN_e_RPAREN_']}
EX_ca2_e: ca2_e <=>  {'original_bigg_ids': ['EX_h_e_']}
EX_camp_e: camp_e -->  {'original_bigg_ids': ['EX_h_e_']}
EX_cbp_e: cbp_e -->  {'original_bigg_ids': ['EX_h_e_']}
EX_cellb_e: cellb_e -->  {'original_bigg_ids': ['EX_cellb_e']}
EX_cgly_e: cgly_e -->  {'original_bigg_ids': ['EX_h_e_']}
EX_chol_e: chol_e -->  {'original_bigg_ids': ['EX_h_e_']}
EX_cholate_e: cholate_e -->  {'original_bigg_ids': ['EX_h_e_']}
EX_cholp_e: cholp_e -->  {'original_bigg_ids': ['EX_cholp[e]']}
EX_chtn_e: chtn_e -->  {'original_bigg_ids': ['EX_h_e_']}
EX_cit_e: cit_e -->  {'original_bigg_ids': ['EX_cit_e_']}
EX_citr__L_e: citr__L_e -->  {'original_bigg_ids': ['EX_citr_L[e]']}
EX_cl_e: cl_e -->  {'original_bigg_ids': ['EX_h_e_']}
EX_cmp_e: cmp_e -->  {'original_bigg_ids': ['EX_cmp[e]']}
EX_co2_e: co2_e -->  {'original_bigg_ids': ['EX_co2_e_']}
EX_csn_e: csn_e -->  {'original_bigg_ids': ['EX_h_e_']}
EX_cu2_e: cu2_e <=>  {'original_bigg_ids': ['EX_cu2_e']}
EX_cu_e: cu_e -->  {'original_bigg_ids': ['EX_h_e_']}
EX_cys__L_e: cys__L_e -->  {'original_bigg_ids': ['EX_h_e_']}
EX_cysi__L_e: cysi__L_e -->  {'original_bigg_ids': ['EX_h_e_']}
EX_cyst__L_e: cyst__L_e -->  {'original_bigg_ids': ['EX_cyst_L[e]']}
EX_cytd_e: cytd_e -->  {'original_bigg_ids': ['EX_h_e_']}
EX_dad_2_e: dad_2_e -->  {'original_bigg_ids': ['EX_h_e_']}
EX_dca_e: dca_e -->  {'original_bigg_ids': ['EX_h_e_']}
EX_ddca_e: ddca_e -->  {'original_bigg_ids': ['EX_h_e_']}
EX_dha_e: dha_e -->  {'original_bigg_ids': ['EX_dha_e']}
EX_dhdascb_e: dhdascb_e -->  {'original_bigg_ids': ['EX_h_e_']}
EX_din_e: din_e -->  {'original_bigg_ids': ['EX_h_e_']}
EX_drib_e: drib_e -->  {'original_bigg_ids': ['EX_drib[e]']}
EX_dtmp_e: dtmp_e -->  {'original_bigg_ids': ['EX_dtmp[e]']}
EX_epist_e: epist_e -->  {'original_bigg_ids': ['EX_h_e_']}
EX_ergst_e: ergst_e -->  {'original_bigg_ids': ['EX_h_e_']}
EX_estradiolglc_e: estradiolglc_e -->  {'original_bigg_ids': ['EX_h_e_']}
EX_estriolglc_e: estriolglc_e -->  {'original_bigg_ids': ['EX_h_e_']}
EX_estroneglc_e: estroneglc_e -->  {'original_bigg_ids': ['EX_h_e_']}
EX_etha_e: etha_e -->  {'original_bigg_ids': ['EX_etha[e]']}
EX_ethamp_e: ethamp_e -->  {'original_bigg_ids': ['EX_ethamp[e]']}
EX_f6p_e: f6p_e -->  {'original_bigg_ids': ['EX_f6p_e']}
EX_fe2_e: fe2_e <=>  {'original_bigg_ids': ['EX_h_e_']}
EX_fe3_e: fe3_e <=>  {'original_bigg_ids': ['EX_h_e_']}
EX_fecost_e: fecost_e -->  {'original_bigg_ids': ['EX_h_e_']}
EX_fer_e: fer_e -->  {'original_bigg_ids': ['EX_fer_LPAREN_e_RPAREN_']}
EX_fmn_e: fmn_e -->  {'original_bigg_ids': ['EX_h_e_']}
EX_fol_e: fol_e -->  {'original_bigg_ids': ['EX_fol[e]']}
EX_for_e: for_e -->  {'original_bigg_ids': ['EX_h_e_']}
EX_frmd_e: frmd_e -->  {'original_bigg_ids': ['EX_h_e_']}
EX_fru_e: fru_e -->  {'original_bigg_ids': ['EX_h_e_']}
EX_fum_e: fum_e -->  {'original_bigg_ids': ['EX_fum_e_']}
EX_g1p_e: g1p_e -->  {'original_bigg_ids': ['EX_g1p[e]']}
EX_g3pc_e: g3pc_e -->  {'original_bigg_ids': ['EX_h_e_']}
EX_g3pe_e: g3pe_e -->  {'original_bigg_ids': ['EX_h_e_']}
EX_g3pi_e: g3pi_e -->  {'original_bigg_ids': ['EX_h_e_']}
EX_g6p_e: g6p_e -->  {'original_bigg_ids': ['EX_g6p_e']}
EX_gal_e: gal_e -->  {'original_bigg_ids': ['EX_h_e_']}
EX_galt_e: galt_e -->  {'original_bigg_ids': ['EX_galt[e]']}
EX_galur_e: galur_e -->  {'original_bigg_ids': ['EX_h_e_']}
EX_gam6p_e: gam6p_e -->  {'original_bigg_ids': ['EX_gam6p_e_']}
EX_gam_e: gam_e -->  {'original_bigg_ids': ['EX_h_e_']}
EX_gchola_e: gchola_e -->  {'original_bigg_ids': ['EX_h_e_']}
EX_ghb_e: ghb_e -->  {'original_bigg_ids': ['EX_bhb[e]']}
EX_glc__D_e: glc__D_e <=>  {'original_bigg_ids': ['EX_glc_e_']}
EX_glcn_e: glcn_e -->  {'original_bigg_ids': ['EX_glcn[e]']}
EX_gln__L_e: gln__L_e -->  {'original_bigg_ids': ['EX_h_e_']}
EX_glu__L_e: glu__L_e -->  {'original_bigg_ids': ['EX_h_e_']}
EX_gluala_e: gluala_e -->  {'original_bigg_ids': ['EX_h_e_']}
EX_glx_e: glx_e -->  {'original_bigg_ids': ['EX_glx[e]']}
EX_gly_e: gly_e -->  {'original_bigg_ids': ['EX_h_e_']}
EX_glyc2p_e: glyc2p_e -->  {'original_bigg_ids': ['EX_glyc2p[e]']}
EX_glyc3p_e: glyc3p_e -->  {'original_bigg_ids': ['EX_glyc3p[e]']}
EX_glyc__R_e: glyc__R_e -->  {'original_bigg_ids': ['EX_glyc_R[e]']}
EX_glyc_e: glyc_e -->  {'original_bigg_ids': ['EX_h_e_']}
EX_glyclt_e: glyclt_e -->  {'original_bigg_ids': ['EX_glyclt[e]']}
EX_glycogen_e: glycogen_e -->  {'original_bigg_ids': ['EX_h_e_']}
EX_glygn2_e: glygn2_e -->  {'original_bigg_ids': ['EX_h_e_']}
EX_glygn4_e: glygn4_e -->  {'original_bigg_ids': ['EX_h_e_']}
EX_gmp_e: gmp_e -->  {'original_bigg_ids': ['EX_gmp[e]']}
EX_gsn_e: gsn_e -->  {'original_bigg_ids': ['EX_gsn[e]']}
EX_gthrd_e: gthrd_e -->  {'original_bigg_ids': ['EX_h_e_']}
EX_gua_e: gua_e -->  {'original_bigg_ids': ['EX_h_e_']}
EX_h2o2_e: h2o2_e -->  {'original_bigg_ids': ['EX_h2o2[e]']}
EX_h2o_e: h2o_e <=>  {'original_bigg_ids': ['EX_h2o_e_']}
EX_h_e: h_e <=>  {'original_bigg_ids': ['EX_h_e_']}
EX_hco3_e: hco3_e -->  {'original_bigg_ids': ['EX_h_e_']}
EX_hdca_e: hdca_e -->  {'original_bigg_ids': ['EX_h_e_']}
EX_hdcea_e: hdcea_e -->  {'original_bigg_ids': ['EX_h_e_']}
EX_his__L_e: his__L_e -->  {'original_bigg_ids': ['EX_h_e_']}
EX_hista_e: hista_e -->  {'original_bigg_ids': ['EX_hista[e]']}
EX_hom__L_e: hom__L_e -->  {'original_bigg_ids': ['EX_hom_L[e]']}
EX_hqn_e: hqn_e -->  {'original_bigg_ids': ['EX_hqn_e_']}
EX_hxa_e: hxa_e -->  {'original_bigg_ids': ['EX_hxa[e]']}
EX_hyptaur_e: hyptaur_e -->  {'original_bigg_ids': ['EX_hyptaur[e]']}
EX_ile__L_e: ile__L_e -->  {'original_bigg_ids': ['EX_h_e_']}
EX_ind3eth_e: ind3eth_e -->  {'original_bigg_ids': ['EX_ind3eth_e_']}
EX_inost_e: inost_e -->  {'original_bigg_ids': ['EX_h_e_']}
EX_ins_e: ins_e -->  {'original_bigg_ids': ['EX_h_e_']}
EX_k_e: k_e <=>  {'original_bigg_ids': ['EX_h_e_']}
EX_lac__D_e: lac__D_e -->  {'original_bigg_ids': ['EX_h_e_']}
EX_lac__L_e: lac__L_e -->  {'original_bigg_ids': ['EX_h_e_']}
EX_lanost_e: lanost_e -->  {'original_bigg_ids': ['EX_h_e_']}
EX_leu__L_e: leu__L_e -->  {'original_bigg_ids': ['EX_h_e_']}
EX_lys__L_e: lys__L_e -->  {'original_bigg_ids': ['EX_h_e_']}
EX_lyx__L_e: lyx__L_e -->  {'original_bigg_ids': ['EX_lyx__L_e']}
EX_mal__L_e: mal__L_e -->  {'original_bigg_ids': ['EX_mal_L_e_']}
EX_malt_e: malt_e -->  {'original_bigg_ids': ['EX_h_e_']}
EX_malttr_e: malttr_e -->  {'original_bigg_ids': ['EX_malttr[e]']}
EX_man1p_e: man1p_e -->  {'original_bigg_ids': ['EX_h_e_']}
EX_man6p_e: man6p_e -->  {'original_bigg_ids': ['EX_man6p_e']}
EX_man_e: man_e -->  {'original_bigg_ids': ['EX_h_e_']}
EX_mannan_e: mannan_e -->  {'original_bigg_ids': ['EX_h_e_']}
EX_mbdg_e: mbdg_e -->  {'original_bigg_ids': ['EX_mbdg_e_']}
EX_melib_e: melib_e -->  {'original_bigg_ids': ['EX_melib_e_']}
EX_meoh_e: meoh_e -->  {'original_bigg_ids': ['EX_meoh[e]']}
EX_met__L_e: met__L_e -->  {'original_bigg_ids': ['EX_h_e_']}
EX_metsox_R__L_e: metsox_R__L_e -->  {'original_bigg_ids': ['EX_metsox_R__L_e']}
EX_metsox_S__L_e: metsox_S__L_e -->  {'original_bigg_ids': ['EX_metsox_S__L_e']}
EX_mg2_e: mg2_e <=>  {'original_bigg_ids': ['EX_mg2_e']}
EX_minohp_e: minohp_e -->  {'original_bigg_ids': ['EX_minohp_e']}
EX_mma_e: mma_e -->  {'original_bigg_ids': ['EX_mma[e]']}
EX_mn2_e: mn2_e <=>  {'original_bigg_ids': ['EX_mn2_e']}
EX_na1_e: na1_e <=>  {'original_bigg_ids': ['EX_h_e_']}
EX_nac_e: nac_e -->  {'original_bigg_ids': ['EX_h_e_']}
EX_nh4_e: nh4_e <=>  {'original_bigg_ids': ['EX_nh4_e_']}
EX_no2_e: no2_e -->  {'original_bigg_ids': ['EX_h_e_']}
EX_no3_e: no3_e -->  {'original_bigg_ids': ['EX_h_e_']}
EX_nrvnc_e: nrvnc_e -->  {'original_bigg_ids': ['EX_h_e_']}
EX_o2_e: o2_e <=>  {'original_bigg_ids': ['EX_o2_e_']}
EX_ocdca_e: ocdca_e -->  {'original_bigg_ids': ['EX_h_e_']}
EX_ocdcea_e: ocdcea_e -->  {'original_bigg_ids': ['EX_h_e_']}
EX_ocdcya_e: ocdcya_e -->  {'original_bigg_ids': ['EX_h_e_']}
EX_oh1_e: oh1_e -->  {'original_bigg_ids': ['EX_h_e_']}
EX_orn_e: orn_e -->  {'original_bigg_ids': ['EX_h_e_']}
EX_oxa_e: oxa_e -->  {'original_bigg_ids': ['EX_h_e_']}
EX_pail1819Z160_e: pail1819Z160_e -->  {'original_bigg_ids': ['EX_h_e_']}
EX_pc_RT_e: pc_RT_e -->  {'original_bigg_ids': ['EX_h_e_']}
EX_pe1801819Z_e: pe1801819Z_e -->  {'original_bigg_ids': ['EX_h_e_']}
EX_pe1801829Z12Z_e: pe1801829Z12Z_e -->  {'original_bigg_ids': ['EX_h_e_']}
EX_pe1819Z1819Z_e: pe1819Z1819Z_e -->  {'original_bigg_ids': ['EX_h_e_']}
EX_pe1819Z1829Z12Z_e: pe1819Z1829Z12Z_e -->  {'original_bigg_ids': ['EX_h_e_']}
EX_pe_RT_e: pe_RT_e -->  {'original_bigg_ids': ['EX_h_e_']}
EX_peamn_e: peamn_e -->  {'original_bigg_ids': ['EX_peamn_e']}
EX_pectin_e: pectin_e -->  {'original_bigg_ids': ['EX_h_e_']}
EX_pep_e: pep_e -->  {'original_bigg_ids': ['EX_pep[e]']}
EX_pepd_e: pepd_e -->  {'original_bigg_ids': ['EX_h_e_']}
EX_pg1819Z160_e: pg1819Z160_e -->  {'original_bigg_ids': ['EX_h_e_']}
EX_pgp1819Z160_e: pgp1819Z160_e -->  {'original_bigg_ids': ['EX_h_e_']}
EX_phe__L_e: phe__L_e -->  {'original_bigg_ids': ['EX_h_e_']}
EX_pi_e: pi_e <=>  {'original_bigg_ids': ['EX_pi_e_']}
EX_pnto__R_e: pnto__R_e -->  {'original_bigg_ids': ['EX_h_e_']}
EX_ppa_e: ppa_e -->  {'original_bigg_ids': ['EX_ppa[e]']}
EX_ppi_e: ppi_e -->  {'original_bigg_ids': ['EX_ppi[e]']}
EX_pppi_e: pppi_e -->  {'original_bigg_ids': ['EX_ppi[e]']}
EX_pro__D_e: pro__D_e -->  {'original_bigg_ids': ['EX_h_e_']}
EX_pro__L_e: pro__L_e -->  {'original_bigg_ids': ['EX_h_e_']}
EX_prostge1_e: prostge1_e -->  {'original_bigg_ids': ['EX_h_e_']}
EX_prostge2_e: prostge2_e -->  {'original_bigg_ids': ['EX_h_e_']}
EX_pser__L_e: pser__L_e -->  {'original_bigg_ids': ['EX_pser_L[e]']}
EX_ptd1ino_RT_e: ptd1ino_RT_e -->  {'original_bigg_ids': ['EX_h_e_']}
EX_ptrc_e: ptrc_e -->  {'original_bigg_ids': ['EX_h_e_']}
EX_pydxn_e: pydxn_e -->  {'original_bigg_ids': ['EX_pydxn[e]']}
EX_pyr_e: pyr_e -->  {'original_bigg_ids': ['EX_h_e_']}
EX_quin_e: quin_e -->  {'original_bigg_ids': ['EX_quin_e']}
EX_rbl__D_e: rbl__D_e -->  {'original_bigg_ids': ['EX_rbl_D[e]']}
EX_rbt_e: rbt_e -->  {'original_bigg_ids': ['EX_rbt[e]']}
EX_rib__D_e: rib__D_e -->  {'original_bigg_ids': ['EX_rib_D[e]']}
EX_ribflv_e: ribflv_e -->  {'original_bigg_ids': ['EX_h_e_']}
EX_salcn_e: salcn_e -->  {'original_bigg_ids': ['EX_salcn_LPAREN_e_RPAREN_']}
EX_sbt__D_e: sbt__D_e -->  {'original_bigg_ids': ['EX_sbt_D_e_']}
EX_ser__D_e: ser__D_e -->  {'original_bigg_ids': ['EX_ser__D_e']}
EX_ser__L_e: ser__L_e -->  {'original_bigg_ids': ['EX_h_e_']}
EX_so3_e: so3_e -->  {'original_bigg_ids': ['EX_h_e_']}
EX_so4_e: so4_e <=>  {'original_bigg_ids': ['EX_so4_e_']}
EX_spmd_e: spmd_e -->  {'original_bigg_ids': ['EX_h_e_']}
EX_sprm_e: sprm_e -->  {'original_bigg_ids': ['EX_h_e_']}
EX_srb__L_e: srb__L_e -->  {'original_bigg_ids': ['EX_srb_L_e_']}
EX_strch1_e: strch1_e -->  {'original_bigg_ids': ['EX_h_e_']}
EX_strch2_e: strch2_e -->  {'original_bigg_ids': ['EX_h_e_']}
EX_succ_e: succ_e -->  {'original_bigg_ids': ['EX_succ_e_']}
EX_sucr_e: sucr_e -->  {'original_bigg_ids': ['EX_h_e_']}
EX_taur_e: taur_e -->  {'original_bigg_ids': ['EX_taur[e]']}
EX_tchola_e: tchola_e -->  {'original_bigg_ids': ['EX_h_e_']}
EX_tega_e: tega_e -->  {'original_bigg_ids': ['EX_h_e_']}
EX_tgua_e: tgua_e -->  {'original_bigg_ids': ['EX_h_e_']}
EX_thm_e: thm_e -->  {'original_bigg_ids': ['EX_h_e_']}
EX_thmmp_e: thmmp_e -->  {'original_bigg_ids': ['EX_h_e_']}
EX_thmpp_e: thmpp_e -->  {'original_bigg_ids': ['EX_h_e_']}
EX_thr__L_e: thr__L_e -->  {'original_bigg_ids': ['EX_h_e_']}
EX_thym_e: thym_e -->  {'original_bigg_ids': ['EX_thym[e]']}
EX_thymd_e: thymd_e -->  {'original_bigg_ids': ['EX_thymd[e]']}
EX_tre_e: tre_e -->  {'original_bigg_ids': ['EX_tre[e]']}
EX_trp__L_e: trp__L_e -->  {'original_bigg_ids': ['EX_h_e_']}
EX_tststeroneglc_e: tststeroneglc_e -->  {'original_bigg_ids': ['EX_h_e_']}
EX_tsul_e: tsul_e -->  {'original_bigg_ids': ['EX_tsul[e]']}
EX_ttc_e: ttc_e -->  {'original_bigg_ids': ['EX_h_e_']}
EX_ttdca_e: ttdca_e -->  {'original_bigg_ids': ['EX_h_e_']}
EX_tym_e: tym_e -->  {'original_bigg_ids': ['EX_tym[e]']}
EX_tyr__L_e: tyr__L_e -->  {'original_bigg_ids': ['EX_h_e_']}
EX_ump_e: ump_e -->  {'original_bigg_ids': ['EX_ump[e]']}
EX_ura_e: ura_e -->  {'original_bigg_ids': ['EX_h_e_']}
EX_urate_e: urate_e -->  {'original_bigg_ids': ['EX_h_e_']}
EX_urea_e: urea_e -->  {'original_bigg_ids': ['EX_h_e_']}
EX_uri_e: uri_e -->  {'original_bigg_ids': ['EX_h_e_']}
EX_val__L_e: val__L_e -->  {'original_bigg_ids': ['EX_h_e_']}
EX_xan_e: xan_e -->  {'original_bigg_ids': ['EX_xan[e]']}
EX_xtsn_e: xtsn_e -->  {'original_bigg_ids': ['EX_xtsn[e]']}
EX_xyl__D_e: xyl__D_e -->  {'original_bigg_ids': ['EX_h_e_']}
EX_xylt_e: xylt_e -->  {'original_bigg_ids': ['EX_xylt[e]']}
EX_xylu__D_e: xylu__D_e -->  {'original_bigg_ids': ['EX_xylu_D[e]']}
EX_zn2_e: zn2_e <=>  {'original_bigg_ids': ['EX_zn2_e']}
EX_zymst_e: zymst_e -->  {'original_bigg_ids': ['EX_h_e_']}
F1PP: f1p_c + h2o_c --> fru_c + pi_c {'original_bigg_ids': ['F1PP']}
F6PP: f6p_c + h2o_c --> fru_c + pi_c {'original_bigg_ids': ['F6PP']}
FA140COAabcp: atp_c + 2.0 h2o_c + tdcoa_c --> adp_c + coa_c + 2.0 h_c + pi_c + ttdca_x {'original_bigg_ids': ['FA140COAabcp']}
FA141COAabcp: atp_c + 2.0 h2o_c + tdecoa_c --> adp_c + coa_c + 2.0 h_c + pi_c + ttdcea_x {'original_bigg_ids': ['FA141COAabcp']}
FA160COAabcp: atp_c + 2.0 h2o_c + pmtcoa_c --> adp_c + coa_c + 2.0 h_c + hdca_x + pi_c {'original_bigg_ids': ['FA160COAabcp']}
FA161COAabcp: atp_c + 2.0 h2o_c + hdcoa_c --> adp_c + coa_c + 2.0 h_c + hdcea_x + pi_c {'original_bigg_ids': ['FA161COAabcp']}
FA180COAabcp: atp_c + 2.0 h2o_c + stcoa_c --> adp_c + coa_c + 2.0 h_c + ocdca_x + pi_c {'original_bigg_ids': ['FA180COAabcp']}
FA181COAabcp: atp_c + 2.0 h2o_c + odecoa_c --> adp_c + coa_c + 2.0 h_c + ocdcea_x + pi_c {'original_bigg_ids': ['FA181COAabcp']}
FA182COAabcp: atp_c + 2.0 h2o_c + ocdycacoa_c --> adp_c + coa_c + 2.0 h_c + ocdcya_x + pi_c {'original_bigg_ids': ['FA182COAabcp']}
FA183COAabcp: atp_c + 2.0 h2o_c + lnlncgcoa_c --> adp_c + coa_c + 2.0 h_c + lnlncg_x + pi_c {'original_bigg_ids': ['FA140COAabcp']}
FA200tp: arach_c --> arach_x {'original_bigg_ids': ['FA240tp']}
FA220tp: docosac_c --> docosac_x {'original_bigg_ids': ['FA240tp']}
FA240tp: ttc_c --> ttc_x {'original_bigg_ids': ['FA240tp']}
FA260tp: hexc_c --> hexc_x {'original_bigg_ids': ['FA260tp']}
FAAH: h2o_c + ocdcead_c --> nh4_c + ocdcea_c {'original_bigg_ids': ['AMID']}
FACOAE100: dcacoa_c + h2o_c --> coa_c + dca_c + h_c {'original_bigg_ids': ['FACOAE100']}
FACOAE120: ddcacoa_c + h2o_c --> coa_c + ddca_c + h_c {'original_bigg_ids': ['FACOAE120']}
FACOAE140: h2o_c + tdcoa_c --> coa_c + h_c + ttdca_c {'original_bigg_ids': ['FACOAE140']}
FACOAE141: h2o_c + tdecoa_c --> coa_c + h_c + ttdcea_c {'original_bigg_ids': ['FACOAE141']}
FACOAE160: h2o_c + pmtcoa_c --> coa_c + h_c + hdca_c {'original_bigg_ids': ['FACOAE160']}
FACOAE161: h2o_c + hdcoa_c --> coa_c + h_c + hdcea_c {'original_bigg_ids': ['FACOAE161']}
FACOAE180: h2o_c + stcoa_c --> coa_c + h_c + ocdca_c {'original_bigg_ids': ['FACOAE180']}
FACOAE181: h2o_c + odecoa_c --> coa_c + h_c + ocdcea_c {'original_bigg_ids': ['FACOAE181']}
FACOAE1829Z12Z: h2o_c + ocdycacoa_c --> coa_c + h_c + ocdcya_c {'original_bigg_ids': ['FACOAE1829Z12Z']}
FACOAE1836Z9Z12Z: h2o_c + lnlncgcoa_c --> coa_c + h_c + lnlncg_c {'original_bigg_ids': ['FACOAE1836Z9Z12Z']}
FACOAE60: h2o_c + hxcoa_c --> coa_c + h_c + hxa_c {'original_bigg_ids': ['FACOAE60']}
FACOAE80: h2o_c + occoa_c --> coa_c + h_c + octa_c {'original_bigg_ids': ['FACOAE80']}
FACOAL100p: atp_x + coa_x + dca_x --> amp_x + dcacoa_x + ppi_x {'original_bigg_ids': ['FACOAL100p']}
FACOAL120p: atp_x + coa_x + ddca_x --> amp_x + ddcacoa_x + ppi_x {'original_bigg_ids': ['FACOAL120p']}
FACOAL140: atp_c + coa_c + ttdca_c --> amp_c + ppi_c + tdcoa_c {'original_bigg_ids': ['FACOAL140']}
FACOAL140p: atp_x + coa_x + ttdca_x --> amp_x + ppi_x + tdcoa_x {'original_bigg_ids': ['FACOAL140p']}
FACOAL141: atp_c + coa_c + ttdcea_c --> amp_c + ppi_c + tdecoa_c {'original_bigg_ids': ['FACOAL141']}
FACOAL141p: atp_x + coa_x + ttdcea_x --> amp_x + ppi_x + tdecoa_x {'original_bigg_ids': ['FACOAL141p']}
FACOAL150: atp_c + coa_c + ptdca_c --> amp_c + ppi_c + ptdcacoa_c {'original_bigg_ids': ['FACOAL150']}
FACOAL160: atp_c + coa_c + hdca_c --> amp_c + pmtcoa_c + ppi_c {'original_bigg_ids': ['FACOAL160']}
FACOAL160p: atp_x + coa_x + hdca_x --> amp_x + pmtcoa_x + ppi_x {'original_bigg_ids': ['FACOAL160p']}
FACOAL161: atp_c + coa_c + hdcea_c --> amp_c + hdcoa_c + ppi_c {'original_bigg_ids': ['FACOAL161']}
FACOAL161p: atp_x + coa_x + hdcea_x --> amp_x + hdcoa_x + ppi_x {'original_bigg_ids': ['FACOAL161p']}
FACOAL170: atp_c + coa_c + hpdca_c --> amp_c + hpdcacoa_c + ppi_c {'original_bigg_ids': ['FACOAL170']}
FACOAL180: atp_c + coa_c + ocdca_c --> amp_c + ppi_c + stcoa_c {'original_bigg_ids': ['FACOAL180']}
FACOAL180p: atp_x + coa_x + ocdca_x --> amp_x + ppi_x + stcoa_x {'original_bigg_ids': ['FACOAL180']}
FACOAL181: atp_c + coa_c + ocdcea_c --> amp_c + odecoa_c + ppi_c {'original_bigg_ids': ['FACOAL181']}
FACOAL1813: atp_c + coa_c + elaid_c --> amp_c + od2coa_c + ppi_c {'original_bigg_ids': ['FACOAL1813']}
FACOAL181p: atp_x + coa_x + ocdcea_x --> amp_x + odecoa_x + ppi_x {'original_bigg_ids': ['FACOAL181']}
FACOAL182: atp_c + coa_c + ocdcya_c --> amp_c + ocdycacoa_c + ppi_c {'original_bigg_ids': ['FACOAL182']}
FACOAL182p: atp_x + coa_x + ocdcya_x --> amp_x + ocdycacoa_x + ppi_x {'original_bigg_ids': ['FACOAL182']}
FACOAL1831: atp_c + coa_c + lnlncg_c --> amp_c + lnlncgcoa_c + ppi_c {'original_bigg_ids': ['FACOAL1831']}
FACOAL1831p: atp_x + coa_x + lnlncg_x --> amp_x + lnlncgcoa_x + ppi_x {'original_bigg_ids': ['FACOAL1831']}
FACOAL191: atp_c + coa_c + prist_c --> amp_c + ppi_c + pristcoa_c {'original_bigg_ids': ['FACOAL191']}
FACOAL200: arach_c + atp_c + coa_c --> amp_c + arachcoa_c + ppi_c {'original_bigg_ids': ['FACOAL200']}
FACOAL200p: arach_x + atp_x + coa_x --> amp_x + arachcoa_x + ppi_x {'original_bigg_ids': ['FACOAL240p']}
FACOAL206: atp_c + coa_c + phyt_c --> amp_c + phytcoa_c + ppi_c {'original_bigg_ids': ['FACOAL206']}
FACOAL220p: atp_x + coa_x + docosac_x --> amp_x + docoscoa_x + ppi_x {'original_bigg_ids': ['FACOAL240p']}
FACOAL2251: atp_c + coa_c + dcsptn1_c --> amp_c + dcsptn1coa_c + ppi_c {'original_bigg_ids': ['FACOAL2251']}
FACOAL2252: atp_c + clpnd_c + coa_c --> amp_c + clpndcoa_c + ppi_c {'original_bigg_ids': ['FACOAL2252']}
FACOAL240: atp_c + coa_c + ttc_c --> amp_c + ppi_c + ttccoa_c {'original_bigg_ids': ['FACOAL240']}
FACOAL240p: atp_x + coa_x + ttc_x --> amp_x + ppi_x + ttccoa_x {'original_bigg_ids': ['FACOAL240p']}
FACOAL241: atp_c + coa_c + nrvnc_c --> amp_c + nrvnccoa_c + ppi_c {'original_bigg_ids': ['FACOAL241']}
FACOAL244_1: atp_c + coa_c + tettet6_c --> amp_c + ppi_c + tettet6coa_c {'original_bigg_ids': ['FACOAL244_1']}
FACOAL260: atp_c + coa_c + hexc_c --> amp_c + hexccoa_c + ppi_c {'original_bigg_ids': ['FACOAL260']}
FACOAL260p: atp_x + coa_x + hexc_x --> amp_x + hexccoa_x + ppi_x {'original_bigg_ids': ['FACOAL260p']}
FACOAL40: atp_c + but_c + coa_c --> amp_c + btcoa_c + ppi_c {'original_bigg_ids': ['HMR_0156']}
FACOAL40im: atp_m + but_m + coa_m --> amp_m + btcoa_m + ppi_m {'original_bigg_ids': ['FACOAL40im']}
FACOAL60: atp_c + coa_c + hxa_c --> amp_c + hxcoa_c + ppi_c {'original_bigg_ids': ['FACOAL80i']}
FACOAL80: atp_c + coa_c + octa_c --> amp_c + occoa_c + ppi_c {'original_bigg_ids': ['FACOAL80i']}
FACOAL80p: atp_x + coa_x + octa_x --> amp_x + occoa_x + ppi_x {'original_bigg_ids': ['FACOAL80p']}
FADDP: fad_c + h2o_c --> amp_c + fmn_c + 2.0 h_c {'original_bigg_ids': ['FADDP']}
FADFMNtm: fad_c + fmn_m --> fad_m + fmn_c {'original_bigg_ids': ['FADFMNtm']}
FALDH2: hmgth_c + nad_c <=> Sfglutth_c + h_c + nadh_c {'original_bigg_ids': ['FALDH2']}
FALGTHLs: fald_c + gthrd_c <=> hmgth_c {'original_bigg_ids': ['FALGTHLs']}
FAO141p_even: 6.0 coa_x + 6.0 h2o_x + 6.0 nad_x + nadph_x + 6.0 o2_x + tdecoa_x --> 7.0 accoa_x + 6.0 h2o2_x + 5.0 h_x + 6.0 nadh_x + nadp_x {'original_bigg_ids': ['FAO141p_even']}
FAO141p_odd: 6.0 coa_x + 6.0 h2o_x + 6.0 nad_x + 5.0 o2_x + tdecoa_x --> 7.0 accoa_x + 5.0 h2o2_x + 6.0 h_x + 6.0 nadh_x {'original_bigg_ids': ['FAO141p_odd']}
FAO161p_even: 7.0 coa_x + 7.0 h2o_x + hdcoa_x + 7.0 nad_x + nadph_x + 7.0 o2_x --> 8.0 accoa_x + 7.0 h2o2_x + 6.0 h_x + 7.0 nadh_x + nadp_x {'original_bigg_ids': ['FAO161p_even']}
FAO161p_odd: 7.0 coa_x + 7.0 h2o_x + hdcoa_x + 7.0 nad_x + 6.0 o2_x --> 8.0 accoa_x + 6.0 h2o2_x + 7.0 h_x + 7.0 nadh_x {'original_bigg_ids': ['FAO161p_odd']}
FAO181p_even: 8.0 coa_x + 8.0 h2o_x + 8.0 nad_x + nadph_x + 8.0 o2_x + odecoa_x --> 9.0 accoa_x + 8.0 h2o2_x + 7.0 h_x + 8.0 nadh_x + nadp_x {'original_bigg_ids': ['FAO181p_even']}
FAO181p_odd: 8.0 coa_x + 8.0 h2o_x + 8.0 nad_x + 7.0 o2_x + odecoa_x --> 9.0 accoa_x + 7.0 h2o2_x + 8.0 h_x + 8.0 nadh_x {'original_bigg_ids': ['FAO181p_odd']}
FAO182p_even: 8.0 coa_x + 8.0 h2o_x + 8.0 nad_x + 2.0 nadph_x + 8.0 o2_x + ocdycacoa_x --> 9.0 accoa_x + 8.0 h2o2_x + 6.0 h_x + 8.0 nadh_x + 2.0 nadp_x {'original_bigg_ids': ['FAO182p_eveneven']}
FAO182p_odd: 8.0 coa_x + 8.0 h2o_x + 8.0 nad_x + nadph_x + 7.0 o2_x + ocdycacoa_x --> 9.0 accoa_x + 7.0 h2o2_x + 7.0 h_x + 8.0 nadh_x + nadp_x {'original_bigg_ids': ['FAO182p_evenodd']}
FAO183p_even: 8.0 coa_x + 8.0 h2o_x + lnlncgcoa_x + 8.0 nad_x + 3.0 nadph_x + 8.0 o2_x --> 9.0 accoa_x + 8.0 h2o2_x + 5.0 h_x + 8.0 nadh_x + 3.0 nadp_x {'original_bigg_ids': ['FAO182p_eveneven']}
FAO183p_odd: 8.0 coa_x + 8.0 h2o_x + lnlncgcoa_x + 8.0 nad_x + 2.0 nadph_x + 7.0 o2_x --> 9.0 accoa_x + 7.0 h2o2_x + 6.0 h_x + 8.0 nadh_x + 2.0 nadp_x {'original_bigg_ids': ['FAO182p_evenodd']}
FAO80p: 3.0 coa_x + 3.0 h2o_x + 3.0 nad_x + 3.0 o2_x + occoa_x --> 4.0 accoa_x + 3.0 h2o2_x + 3.0 h_x + 3.0 nadh_x {'original_bigg_ids': ['FAO80p']}
FAOXC11C9m: coa_m + fad_m + h2o_m + nad_m + undcoa_m --> accoa_m + fadh2_m + h_m + nadh_m + noncoa_m {'original_bigg_ids': ['FAOXC11C9m']}
FAOXC13C11m: coa_m + fad_m + h2o_m + nad_m + tridcoa_m --> accoa_m + fadh2_m + h_m + nadh_m + undcoa_m {'original_bigg_ids': ['FAOXC13C11m']}
FAOXC15C13m: coa_m + fad_m + h2o_m + nad_m + ptdcacoa_m --> accoa_m + fadh2_m + h_m + nadh_m + tridcoa_m {'original_bigg_ids': ['FAOXC15C13m']}
FAOXC170150m: coa_m + fad_m + h2o_m + hpdcacoa_m + nad_m --> accoa_m + fadh2_m + h_m + nadh_m + ptdcacoa_m {'original_bigg_ids': ['FAOXC17C15m']}
FAOXC200180x: arachcoa_x + coa_x + h2o_x + nad_x + o2_x --> accoa_x + h2o2_x + h_x + nadh_x + stcoa_x {'original_bigg_ids': ['FAOXC200180x']}
FAOXC22C20x: coa_x + docoscoa_x + h2o_x + nad_x + o2_x --> accoa_x + arachcoa_x + h2o2_x + h_x + nadh_x {'original_bigg_ids': ['FAOXC22C20x']}
FAOXC241181x: 3.0 coa_x + 3.0 h2o_x + 3.0 nad_x + nrvnccoa_x + 3.0 o2_x --> 3.0 accoa_x + 3.0 h2o2_x + 3.0 h_x + 3.0 nadh_x + odecoa_x {'original_bigg_ids': ['FAOXC241181x']}
FAOXC2442246x: coa_x + h2o_x + nad_x + o2_x + tettet6coa_x --> accoa_x + adrncoa_x + h2o2_x + h_x + nadh_x {'original_bigg_ids': ['FAOXC2442246x']}
FAOXC2452253x: coa_x + h2o_x + nad_x + o2_x + tetpent3coa_x --> accoa_x + clpndcoa_x + h2o2_x + h_x + nadh_x {'original_bigg_ids': ['FAOXC2452253x']}
FAOXC2452256x: coa_x + h2o_x + nad_x + o2_x + tetpent6coa_x --> accoa_x + dcsptn1coa_x + h2o2_x + h_x + nadh_x {'original_bigg_ids': ['FAOXC2452256x']}
FAOXC24C22x: coa_x + h2o_x + nad_x + o2_x + ttccoa_x --> accoa_x + docoscoa_x + h2o2_x + h_x + nadh_x {'original_bigg_ids': ['FAOXC24C22x']}
FAOXC5C3m: coa_m + fad_m + h2o_m + nad_m + pentcoa_m --> accoa_m + fadh2_m + h_m + nadh_m + ppcoa_m {'original_bigg_ids': ['FAOXC5C3x']}
FAOXC7C5m: coa_m + fad_m + h2o_m + hepcoa_m + nad_m --> accoa_m + fadh2_m + h_m + nadh_m + pentcoa_m {'original_bigg_ids': ['FAOXC7C5m']}
FAOXC9C7m: coa_m + fad_m + h2o_m + nad_m + noncoa_m --> accoa_m + fadh2_m + h_m + hepcoa_m + nadh_m {'original_bigg_ids': ['FAOXC9C7m']}
FAS100COA: 3.0 h_c + malcoa_c + 2.0 nadph_c + occoa_c --> co2_c + coa_c + dcacoa_c + h2o_c + 2.0 nadp_c {'original_bigg_ids': ['FAS100COA']}
FAS120COA: dcacoa_c + 3.0 h_c + malcoa_c + 2.0 nadph_c --> co2_c + coa_c + ddcacoa_c + h2o_c + 2.0 nadp_c {'original_bigg_ids': ['FAS120COA']}
FAS140COA: ddcacoa_c + 3.0 h_c + malcoa_c + 2.0 nadph_c --> co2_c + coa_c + h2o_c + 2.0 nadp_c + tdcoa_c {'original_bigg_ids': ['FAS140COA']}
FAS160COA: 3.0 h_c + malcoa_c + 2.0 nadph_c + tdcoa_c --> co2_c + coa_c + h2o_c + 2.0 nadp_c + pmtcoa_c {'original_bigg_ids': ['FAS160COA']}
FAS170COA_L: 21.0 h_c + 7.0 malcoa_c + 14.0 nadph_c + ppcoa_c --> 7.0 co2_c + 7.0 coa_c + 7.0 h2o_c + hpdcacoa_c + 14.0 nadp_c {'original_bigg_ids': ['FAS80COA_L']}
FAS180COA: 3.0 h_c + malcoa_c + 2.0 nadph_c + pmtcoa_c --> co2_c + coa_c + h2o_c + 2.0 nadp_c + stcoa_c {'original_bigg_ids': ['FAS180COA']}
FAS200COAer: 3.0 h_r + malcoa_r + 2.0 nadph_r + stcoa_r --> arachcoa_r + co2_r + coa_r + h2o_r + 2.0 nadp_r {'original_bigg_ids': ['FAS260']}
FAS220COAer: arachcoa_r + 3.0 h_r + malcoa_r + 2.0 nadph_r --> co2_r + coa_r + docoscoa_r + h2o_r + 2.0 nadp_r {'original_bigg_ids': ['FAS260']}
FAS240COAer: docoscoa_r + 3.0 h_r + malcoa_r + 2.0 nadph_r --> co2_r + coa_r + h2o_r + 2.0 nadp_r + ttccoa_r {'original_bigg_ids': ['FAS240_L']}
FAS260COAer: 3.0 h_r + malcoa_r + 2.0 nadph_r + ttccoa_r --> co2_r + coa_r + h2o_r + hexccoa_r + 2.0 nadp_r {'original_bigg_ids': ['FAS260']}
FAS80COA_L: accoa_c + 9.0 h_c + 3.0 malcoa_c + 6.0 nadph_c --> 3.0 co2_c + 3.0 coa_c + 3.0 h2o_c + 6.0 nadp_c + occoa_c {'original_bigg_ids': ['FAS80COA_L']}
FBA: fdp_c <=> dhap_c + g3p_c {'original_bigg_ids': ['FBA']}
FBA2: f1p_c <=> dhap_c + glyald_c {'original_bigg_ids': ['FBA2']}
FBA3: s17bp_c <=> dhap_c + e4p_c {'original_bigg_ids': ['FBA3']}
FBP: fdp_c + h2o_c --> f6p_c + pi_c {'original_bigg_ids': ['FBP']}
FBP26: f26bp_c + h2o_c --> f6p_c + pi_c {'original_bigg_ids': ['FBP26']}
FCLPA: fc1p_c <=> dhap_c + lald__L_c {'original_bigg_ids': ['FCLPA']}
FCLTm: fe2_m + ppp9_m --> 2.0 h_m + pheme_m {'original_bigg_ids': ['FCLTm']}
FDH: for_c + nad_c --> co2_c + nadh_c {'original_bigg_ids': ['FDH']}
FE2GTPabc: fe2_e + gtp_c + h2o_c --> fe2_c + gdp_c + h_c + pi_c {'original_bigg_ids': ['FE2GTPabc']}
FE2t: fe2_e <=> fe2_c {'original_bigg_ids': ['FE2t']}
FE2tm: fe2_c + h_c --> fe2_m + h_m {'original_bigg_ids': ['FE2tm']}
FE3t: fe3_e <=> fe3_c {'original_bigg_ids': ['FE3t']}
FECOSTATer_RT: fecost_r + 0.01 hdcoa_r + 0.01 lnlncgcoa_r + 0.14 ocdycacoa_r + 0.48 odecoa_r + 0.23 pmtcoa_r + 0.12 stcoa_r + 0.01 ttccoa_r --> coa_r + 0.01 fecostest_RT_r {'original_bigg_ids': ['FECOSTAT_SC']}
FECOSTESTH_RT: 0.01 fecostest_RT_d + h2o_c --> fecost_c + h_c + 0.23 hdca_c + 0.01 hdcea_c + 0.01 lnlncg_c + 0.12 ocdca_c + 0.48 ocdcea_c + 0.14 ocdcya_c + 0.01 ttc_c {'original_bigg_ids': ['FECOSTESTH_SC']}
FECOSTESTtrd: fecostest_RT_r --> fecostest_RT_d {'original_bigg_ids': ['FRDPtcr']}
FECOSTt: fecost_e <=> fecost_c {'original_bigg_ids': ['FECOSTt']}
FECOSTtr: fecost_c <=> fecost_r {'original_bigg_ids': ['FECOSTt']}
FERULCOAS: atp_c + coa_c + fer_c --> amp_c + ferulcoa_c + ppi_c {'original_bigg_ids': ['FERULCOAS']}
FERt: fer_e <=> fer_c {'original_bigg_ids': ['FERtex']}
FKYNH: Lfmkynr_c + h2o_c --> Lkynr_c + for_c + h_c {'original_bigg_ids': ['FKYNH']}
FMETTRSm: 10fthf_m + mettrna_m --> fmettrna_m + h_m + thf_m {'original_bigg_ids': ['FMETTRSm']}
FMNAT: atp_c + fmn_c + h_c --> fad_c + ppi_c {'original_bigg_ids': ['FMNAT']}
FOLR2_1: fol_c + h_c + nadph_c --> dhf_c + nadp_c {'original_bigg_ids': ['FOLR2']}
FOLt: fol_e + h_e --> fol_c + h_c {'original_bigg_ids': ['FOLTle']}
FORA: frmd_c + h2o_c --> for_c + nh4_c {'original_bigg_ids': ['FORA']}
FORAMDx: frmd_x + h2o_x --> for_x + nh4_x {'original_bigg_ids': ['FORAMDx']}
FORtr: for_c <=> for_r {'original_bigg_ids': ['FORtr']}
FPGFTm: 10fthf_m + gar_m <=> fgam_m + h_m + thf_m {'original_bigg_ids': ['FPGFTm']}
FPGS: 4.0 atp_c + 4.0 glu__L_c + thf_c --> 5thf_c + 4.0 adp_c + 4.0 h_c + 4.0 pi_c {'original_bigg_ids': ['FPGS']}
FPGS2: 5thf_c + atp_c + glu__L_c --> 6thf_c + adp_c + h_c + pi_c {'original_bigg_ids': ['FPGS2']}
FPGS2m: 5thf_m + atp_m + glu__L_m --> 6thf_m + adp_m + h_m + pi_m {'original_bigg_ids': ['FPGS2m']}
FPGS3: 6thf_c + atp_c + glu__L_c --> 7thf_c + adp_c + h_c + pi_c {'original_bigg_ids': ['FPGS3']}
FPGS3m: 6thf_m + atp_m + glu__L_m --> 7thf_m + adp_m + h_m + pi_m {'original_bigg_ids': ['FPGS3m']}
FPGS4: 4.0 atp_c + dhf_c + 4.0 glu__L_c --> 5dhf_c + 4.0 adp_c + 4.0 h_c + 4.0 pi_c {'original_bigg_ids': ['FPGS4']}
FPGS4m: 4.0 atp_m + dhf_m + 4.0 glu__L_m --> 5dhf_m + 4.0 adp_m + 4.0 h_m + 4.0 pi_m {'original_bigg_ids': ['FPGS4m']}
FPGS5: 5dhf_c + atp_c + glu__L_c --> 6dhf_c + adp_c + h_c + pi_c {'original_bigg_ids': ['FPGS5']}
FPGS5m: 5dhf_m + atp_m + glu__L_m --> 6dhf_m + adp_m + h_m + pi_m {'original_bigg_ids': ['FPGS5m']}
FPGS6: 6dhf_c + atp_c + glu__L_c --> 7dhf_c + adp_c + h_c + pi_c {'original_bigg_ids': ['FPGS6']}
FPGS6m: 6dhf_m + atp_m + glu__L_m --> 7dhf_m + adp_m + h_m + pi_m {'original_bigg_ids': ['FPGS6m']}
FPGS7: 10fthf_c + 4.0 atp_c + 4.0 glu__L_c --> 10fthf5glu_c + 4.0 adp_c + 4.0 h_c + 4.0 pi_c {'original_bigg_ids': ['FPGS7']}
FPGS7m: 10fthf_m + 4.0 atp_m + 4.0 glu__L_m --> 10fthf5glu_m + 4.0 adp_m + 4.0 h_m + 4.0 pi_m {'original_bigg_ids': ['FPGS7m']}
FPGS8: 10fthf5glu_c + atp_c + glu__L_c --> 10fthf6glu_c + adp_c + h_c + pi_c {'original_bigg_ids': ['FPGS8']}
FPGS8m: 10fthf5glu_m + atp_m + glu__L_m --> 10fthf6glu_m + adp_m + h_m + pi_m {'original_bigg_ids': ['FPGS8m']}
FPGS9: 10fthf6glu_c + atp_c + glu__L_c --> 10fthf7glu_c + adp_c + h_c + pi_c {'original_bigg_ids': ['FPGS9']}
FPGS9m: 10fthf6glu_m + atp_m + glu__L_m --> 10fthf7glu_m + adp_m + h_m + pi_m {'original_bigg_ids': ['FPGS9m']}
FPGSm: 4.0 atp_m + 4.0 glu__L_m + thf_m --> 5thf_m + 4.0 adp_m + 4.0 h_m + 4.0 pi_m {'original_bigg_ids': ['FPGSm']}
FRDPtcr: frdp_c <=> frdp_r {'original_bigg_ids': ['FRDPtcr']}
FRDPtm: frdp_c <=> frdp_m {'original_bigg_ids': ['FRDPtm']}
FRDcm: fadh2_m + fum_c --> fad_m + succ_c {'original_bigg_ids': ['FRDcm']}
FRMDt: frmd_e <=> frmd_c {'original_bigg_ids': ['CO2t']}
FRTT: frdp_c + ipdp_c --> ggdp_c + ppi_c {'original_bigg_ids': ['FRTT']}
FRUt1r: fru_e <=> fru_c {'original_bigg_ids': ['FRUt1r']}
FRUt2: fru_e + h_e --> fru_c + h_c {'original_bigg_ids': ['FRUt2']}
FT: frdp_c + ipdp_c --> ppi_c + ttc_ggdp_c {'original_bigg_ids': ['TTCGGDPS']}
FTHFCLm: 5fthf_m + atp_m --> adp_m + methf_m + pi_m {'original_bigg_ids': ['FTHFCLm']}
FTHFD: 10fthf_c + h2o_c --> for_c + h_c + thf_c {'original_bigg_ids': ['FTHDF']}
FTHFDH: 10fthf_c + h2o_c + nadp_c --> co2_c + h_c + nadph_c + thf_c {'original_bigg_ids': ['FTHFO']}
FTHFLi: atp_c + for_c + thf_c --> 10fthf_c + adp_c + pi_c {'original_bigg_ids': ['FTHFLi']}
FTHFLmi: atp_m + for_m + thf_m --> 10fthf_m + adp_m + pi_m {'original_bigg_ids': ['FTHFLmi']}
FUMAC: 4fumacac_c + h2o_c --> acac_c + fum_c + h_c {'original_bigg_ids': ['FUMAC']}
FUMm: fum_m + h2o_m <=> mal__L_m {'original_bigg_ids': ['FUMm']}
G12MT1: doldpglcnacglcnacman_man_man_c + gdpmann_c --> doldpglcnacglcnacman_man_manman_c + gdp_c + h_c {'original_bigg_ids': ['G12MT1']}
G12MT1g: gdpmann_g + m2macchitppdol_g --> gdp_g + h_g + m3macchitppdol_g {'original_bigg_ids': ['G12MT1g']}
G12MT2: doldpglcnacglcnacman_man_manman_c + gdpmann_c --> doldpglcnacglcnacman_man_manmanman_c + gdp_c + h_c {'original_bigg_ids': ['G12MT2']}
G12MT2g: gdpmann_g + m3macchitppdol_g --> gdp_g + h_g + m4macchitppdol_g {'original_bigg_ids': ['G12MT2g']}
G12MT3: doldpglcnacglcnacman_manman_manmanman_c + dolmanp_c --> doldpglcnacglcnacman_manmanman_manmanman_c + dolp_c + h_c {'original_bigg_ids': ['G12MT3']}
G12MT4: doldpglcnacglcnacman_man_man_manman_manmanman_c + dolmanp_c --> doldpglcnacglcnacman_man_manman_manman_manmanman_c + dolp_c + h_c {'original_bigg_ids': ['G12MT4']}
G13MT: doldpglcnacglcnacman_c + gdpmann_c --> doldpglcnacglcnacmanman_c + gdp_c + h_c {'original_bigg_ids': ['G13MT']}
G13MT_L: gdpmann_c + 0.1 mpdol__L_c --> gdp_c + h_c + 0.1 m1mpdol__L_c {'original_bigg_ids': ['G13MT_L']}
G13MT_U: gdpmann_c + 0.1 mpdol_U_c --> gdp_c + h_c + 0.1 m1mpdol_U_c {'original_bigg_ids': ['G13MT_U']}
G16MT: doldpglcnacglcnacmanman_c + gdpmann_c --> doldpglcnacglcnacman_man_man_c + gdp_c + h_c {'original_bigg_ids': ['G16MT']}
G1PP: g1p_c + h2o_c --> glc__D_c + pi_c {'original_bigg_ids': ['G1PP']}
G2PP: glyc2p_c + h2o_c --> glyc_c + pi_c {'original_bigg_ids': ['G2PP']}
G2PPe: glyc2p_e + h2o_e --> glyc_e + pi_e {'original_bigg_ids': ['G2PP']}
G3PCt: g3pc_e <=> g3pc_c {'original_bigg_ids': ['G3PCt']}
G3PD1ir: dhap_c + h_c + nadh_c --> glyc3p_c + nad_c {'original_bigg_ids': ['G3PD1ir']}
G3PD_u9cm: glyc3p_c + q9_m --> dhap_c + q9h2_m {'original_bigg_ids': ['G3PD']}
G3PIt: g3pi_e <=> g3pi_c {'original_bigg_ids': ['G3PIt']}
G3PT: glyc3p_c + h2o_c --> glyc_c + pi_c {'original_bigg_ids': ['G3PT']}
G5SADrm: glu5sa_m <=> 1pyr5c_m + h2o_m + h_m {'original_bigg_ids': ['G5SADrm']}
G5SADs: glu5sa_c <=> 1pyr5c_c + h2o_c + h_c {'original_bigg_ids': ['G5SADr']}
G5SD: glu5p_c + h_c + nadph_c --> glu5sa_c + nadp_c + pi_c {'original_bigg_ids': ['G5SD']}
G6PDH2r: g6p_c + nadp_c <=> 6pgl_c + h_c + nadph_c {'original_bigg_ids': ['G6PDH2r']}
G6PDH2rp: g6p_x + nadp_x <=> 6pgl_x + h_x + nadph_x {'original_bigg_ids': ['G6PDH2r']}
G6PP: g6p_c + h2o_c --> glc__D_c + pi_c {'original_bigg_ids': ['G6PP']}
GALKr: atp_c + gal_c <=> adp_c + gal1p_c + h_c {'original_bigg_ids': ['GALKr']}
GALOR: gal_c + h_c + nadph_c <=> galt_c + nadp_c {'original_bigg_ids': ['GALOR']}
GALS3: h2o_c + melib_c --> gal_c + glc__D_c {'original_bigg_ids': ['GALS3']}
GALT: gal1p_c + h_c + utp_c <=> ppi_c + udpgal_c {'original_bigg_ids': ['GALT']}
GALTt: galt_c <=> galt_e {'original_bigg_ids': ['GALTt']}
GALUi: g1p_c + h_c + utp_c --> ppi_c + udpg_c {'original_bigg_ids': ['GALUi']}
GALt1r: gal_e <=> gal_c {'original_bigg_ids': ['GALt1r']}
GALt2: gal_e + h_e --> gal_c + h_c {'original_bigg_ids': ['GALt2']}
GAM6PPe: gam6p_e + h2o_e --> gam_e + pi_e {'original_bigg_ids': ['G2PP']}
GAMt1r: gam_e <=> gam_c {'original_bigg_ids': ['GAMt1r']}
GAPD: g3p_c + nad_c + pi_c <=> 13dpg_c + h_c + nadh_c {'original_bigg_ids': ['GAPD']}
GAPP: g3p_c + h2o_c --> glyald_c + pi_c {'original_bigg_ids': ['GAPP']}
GARFT: 10fthf_c + gar_c <=> fgam_c + h_c + thf_c {'original_bigg_ids': ['GARFT']}
GAT1er_RT: 0.01 arachcoa_r + glyc3p_r + 0.02 hpdcacoa_r + 0.1 lnlncgcoa_r + 0.37 ocdycacoa_r + 0.32 odecoa_r + 0.09 pmtcoa_r + 0.07 stcoa_r + 0.02 ttccoa_r --> 0.01 1ag3p_RT_r + coa_r {'original_bigg_ids': ['GAT1_SC']}
GAT2er_RT: 0.01 arachcoa_r + dhap_r + 0.02 hpdcacoa_r + 0.1 lnlncgcoa_r + 0.37 ocdycacoa_r + 0.32 odecoa_r + 0.09 pmtcoa_r + 0.07 stcoa_r + 0.02 ttccoa_r --> 0.01 1agly3p_RT_r + coa_r {'original_bigg_ids': ['GAT2_SC']}
GBBOX_m: akg_m + gbbtn_m + o2_m --> co2_m + crn_m + succ_m {'original_bigg_ids': ['GBBOX_m']}
GBEZ: 14glun_c --> glycogen_c + h2o_c {'original_bigg_ids': ['GBEZ']}
GCALDD: gcald_c + h2o_c + nad_c --> glyclt_c + 2.0 h_c + nadh_c {'original_bigg_ids': ['GCALDD']}
GCHOLAt3: atp_c + gchola_c + h2o_c --> adp_c + gchola_e + h_c + pi_c {'original_bigg_ids': ['GCHOLAt3']}
GCPNn: 35cgmp_n + h2o_n --> gmp_n + h_n {'original_bigg_ids': ['GCPNn']}
GDMANE: gdpddman_c --> gdpofuc_c {'original_bigg_ids': ['GDMANE']}
GDPDPK: atp_c + gdp_c --> amp_c + h_c + ppgpp_c {'original_bigg_ids': ['GDPDPK']}
GDPFUCtg: gdpfuc_c + gmp_g <=> gdpfuc_g + gmp_c {'original_bigg_ids': ['GDPFUCtg']}
GDPMANNtg: gdpmann_c + gmp_g --> gdpmann_g + gmp_c {'original_bigg_ids': ['GDPMANNtg']}
GDPTPDP: gdptp_c + h2o_c --> gtp_c + ppi_c {'original_bigg_ids': ['GDPTPDP']}
GF6PTA: f6p_c + gln__L_c --> gam6p_c + glu__L_c {'original_bigg_ids': ['GF6PTA']}
GFUCS: gdpddman_c + h_c + nadph_c --> gdpfuc_c + nadp_c {'original_bigg_ids': ['GFUCS']}
GGPTRCS: atp_c + glu__L_c + ptrc_c --> adp_c + ggptrc_c + h_c + pi_c {'original_bigg_ids': ['GGPTRCS']}
GHBTRHDm: akg_m + ghb_m <=> r2hglut_m + sucsal_m {'original_bigg_ids': ['ARHGDx']}
GHBt: ghb_e + h_e <=> ghb_c + h_c {'original_bigg_ids': ['BHBt']}
GHBtm: ghb_c + h_c <=> ghb_m + h_m {'original_bigg_ids': ['BHBtm']}
GHMT2r: ser__L_c + thf_c <=> gly_c + h2o_c + mlthf_c {'original_bigg_ids': ['GHMT2r']}
GK1: atp_c + gmp_c <=> adp_c + gdp_c {'original_bigg_ids': ['GK1']}
GK2: datp_c + gmp_c <=> dadp_c + gdp_c {'original_bigg_ids': ['GK2']}
GLCATr: accoa_c + glc__D_c <=> acglc__D_c + coa_c {'original_bigg_ids': ['GLCATr']}
GLCGSD: glycogen_c + h2o_c --> glc__D_c {'original_bigg_ids': ['GLCGSD']}
GLCNACPT: dolp_c + 2.0 h_c + uacgam_c --> doldpglcnac_c + ump_c {'original_bigg_ids': ['GLCNACPT']}
GLCNACPT_L: 0.1 dolp__L_c + uacgam_c --> 0.1 naglc2p__L_c + ump_c {'original_bigg_ids': ['GLCNACPT_L']}
GLCNACPT_U: 0.1 dolp_U_c + uacgam_c --> 0.1 naglc2p_U_c + ump_c {'original_bigg_ids': ['GLCNACPT_U']}
GLCNACT: doldpglcnac_c + uacgam_c --> doldpglcnacglcnac_c + h_c + udp_c {'original_bigg_ids': ['GLCNACT']}
GLCNte: glcn_c <=> glcn_e {'original_bigg_ids': ['GLCNte']}
GLCP: glycogen_c + pi_c --> g1p_c {'original_bigg_ids': ['GLCP']}
GLCS2: udpg_c --> glycogen_c + h_c + udp_c {'original_bigg_ids': ['GLCS2']}
GLCt1: glc__D_e --> glc__D_c {'original_bigg_ids': ['GLCt1']}
GLCtg: glc__D_c <=> glc__D_g {'original_bigg_ids': ['GLCtg']}
GLNS: atp_c + glu__L_c + nh4_c --> adp_c + gln__L_c + h_c + pi_c {'original_bigg_ids': ['GLNS']}
GLNTRS: atp_c + gln__L_c + trnagln_c --> amp_c + glntrna_c + ppi_c {'original_bigg_ids': ['GLNTRS']}
GLNt2r: gln__L_e + h_e <=> gln__L_c + h_c {'original_bigg_ids': ['GLNt2r']}
GLNt6: gln__L_c + h_v --> gln__L_v + h_c {'original_bigg_ids': ['GLNt6']}
GLNt7: gln__L_v + h_v --> gln__L_c + h_c {'original_bigg_ids': ['GLNt7']}
GLNtN1: gln__L_e + h_c + 2.0 na1_e <=> gln__L_c + h_e + 2.0 na1_c {'original_bigg_ids': ['GLNtN1']}
GLPT: glp_c + tdcoa_c --> coa_c + h_c + tglp_c {'original_bigg_ids': ['GLPT']}
GLU5K: atp_c + glu__L_c --> adp_c + glu5p_c {'original_bigg_ids': ['GLU5K']}
GLUCYS: atp_c + cys__L_c + glu__L_c --> adp_c + glucys_c + h_c + pi_c {'original_bigg_ids': ['GLUCYS']}
GLUDC: glu__L_c + h_c --> 4abut_c + co2_c {'original_bigg_ids': ['GLUDC']}
GLUDxi: glu__L_c + h2o_c + nad_c --> akg_c + h_c + nadh_c + nh4_c {'original_bigg_ids': ['GLUDxi']}
GLUDy: glu__L_c + h2o_c + nadp_c <=> akg_c + h_c + nadph_c + nh4_c {'original_bigg_ids': ['GLUDy']}
GLUN: gln__L_c + h2o_c --> glu__L_c + nh4_c {'original_bigg_ids': ['GLUN']}
GLUPRT: gln__L_c + h2o_c + prpp_c --> glu__L_c + ppi_c + pram_c {'original_bigg_ids': ['GLUPRT']}
GLUSx: akg_c + gln__L_c + h_c + nadh_c --> 2.0 glu__L_c + nad_c {'original_bigg_ids': ['GLUSx']}
GLUTRS: atp_c + glu__L_c + trnaglu_c --> amp_c + glutrna_c + ppi_c {'original_bigg_ids': ['GLUTRS']}
GLUTRSm: atp_m + glu__L_m + trnaglu_m --> amp_m + glutrna_m + ppi_m {'original_bigg_ids': ['GLUTRSm']}
GLUt2r: glu__L_e + h_e <=> glu__L_c + h_c {'original_bigg_ids': ['GLUt2r']}
GLUt7: glu__L_v + h_v --> glu__L_c + h_c {'original_bigg_ids': ['GLUt7']}
GLUt7m: glu__L_c --> glu__L_m {'original_bigg_ids': ['GLUt7m']}
GLXO2p: glx_x + h2o_x + o2_x --> h2o2_x + h_x + oxa_x {'original_bigg_ids': ['GLXO2p']}
GLXt: glx_c --> glx_e {'original_bigg_ids': ['GLXte']}
GLXtm: glx_c <=> glx_m {'original_bigg_ids': ['GLXtm']}
GLXtp: glx_c <=> glx_x {'original_bigg_ids': ['GLXtp']}
GLYAT: accoa_c + gly_c <=> 2aobut_c + coa_c {'original_bigg_ids': ['GLYAT']}
GLYATm: accoa_m + gly_m <=> 2aobut_m + coa_m {'original_bigg_ids': ['GLYATm']}
GLYCDy: glyc_c + nadp_c --> dha_c + h_c + nadph_c {'original_bigg_ids': ['GLYCDy']}
GLYCK: atp_c + glyc__R_c --> 3pg_c + adp_c + h_c {'original_bigg_ids': ['GLYCK']}
GLYCLTDxm: glx_m + h_m + nadh_m --> glyclt_m + nad_m {'original_bigg_ids': ['GLYCLTDx']}
GLYCLTDy: glx_c + h_c + nadph_c --> glyclt_c + nadp_c {'original_bigg_ids': ['GLYCLTDy']}
GLYCLTDyp: glx_x + h_x + nadph_x --> glyclt_x + nadp_x {'original_bigg_ids': ['GLYCLTDy']}
GLYCLTt: glyclt_e <=> glyclt_c {'original_bigg_ids': ['GLYCLTtd']}
GLYCLTtm: glyclt_c <=> glyclt_m {'original_bigg_ids': ['GLYCLTtm']}
GLYCLTtp: glyclt_c --> glyclt_x {'original_bigg_ids': ['GLYCLTtp']}
GLYCLm: gly_m + nad_m + thf_m --> co2_m + mlthf_m + nadh_m + nh4_m {'original_bigg_ids': ['GLYCLm']}
GLYCTO1p: glyclt_x + o2_x --> glx_x + h2o2_x {'original_bigg_ids': ['HDAO10x']}
GLYC_Rt: glyc__R_c <=> glyc__R_e {'original_bigg_ids': ['GLYC_Rt']}
GLYCt2: glyc_e + h_e --> glyc_c + h_c {'original_bigg_ids': ['GLYCt2']}
GLYCtm: glyc_c <=> glyc_m {'original_bigg_ids': ['GLYCtm']}
GLYGS: h2o_c + udpg_c --> 14glun_c + h_c + udp_c {'original_bigg_ids': ['GLYGS']}
GLYK: atp_c + glyc_c --> adp_c + glyc3p_c + h_c {'original_bigg_ids': ['GLYK']}
GLYKm: atp_m + glyc_m --> adp_m + glyc3p_m + h_m {'original_bigg_ids': ['GLYKm']}
GLYOX: h2o_c + lgt__S_c --> gthrd_c + h_c + lac__D_c {'original_bigg_ids': ['GLYOX']}
GLYOp: gly_x + h2o_x + o2_x --> glx_x + h2o2_x + nh4_x {'original_bigg_ids': ['GLYOp']}
GLYTLm: atp_m + gly_m + trnagly_m --> amp_m + glytrna_m + ppi_m {'original_bigg_ids': ['GLYTLm']}
GLYTRS: atp_c + gly_c + trnagly_c --> amp_c + glytrna_c + ppi_c {'original_bigg_ids': ['GLYTRS']}
GLYt2r: gly_e + h_e <=> gly_c + h_c {'original_bigg_ids': ['GLYt2r']}
GLYtm: gly_c <=> gly_m {'original_bigg_ids': ['GLYtm']}
GMAND: gdpmann_c --> gdpddman_c + h2o_c {'original_bigg_ids': ['GMAND']}
GMPS: atp_c + nh4_c + xmp_c --> amp_c + gmp_c + 2.0 h_c + ppi_c {'original_bigg_ids': ['GMPS']}
GMPS2: atp_c + gln__L_c + h2o_c + xmp_c --> amp_c + glu__L_c + gmp_c + 2.0 h_c + ppi_c {'original_bigg_ids': ['GMPS2']}
GND: 6pgc_c + nadp_c --> co2_c + nadph_c + ru5p__D_c {'original_bigg_ids': ['GND']}
GNDp: 6pgc_x + nadp_x --> co2_x + nadph_x + ru5p__D_x {'original_bigg_ids': ['GND']}
GNK: atp_c + glcn_c --> 6pgc_c + adp_c + h_c {'original_bigg_ids': ['GNK']}
GNNUC: gsn_c + h2o_c --> gua_c + rib__D_c {'original_bigg_ids': ['GNNUC']}
GNP: 6pgc_c + h2o_c --> glcn_c + pi_c {'original_bigg_ids': ['GNP']}
GNPe: 6pgc_e + h2o_e --> glcn_e + pi_e {'original_bigg_ids': ['GNP']}
GOFUCR: gdpofuc_c + h_c + nadph_c --> gdpfuc_c + nadp_c {'original_bigg_ids': ['GOFUCR']}
GPDDA1: g3pc_c + h2o_c --> chol_c + glyc3p_c + h_c {'original_bigg_ids': ['GPDDA1']}
GPDDA2: g3pe_c + h2o_c --> etha_c + glyc3p_c + h_c {'original_bigg_ids': ['GPDDA2']}
GPDDA3: g3ps_c + h2o_c --> glyc3p_c + h_c + ser__L_c {'original_bigg_ids': ['GPDDA3']}
GPDDA4: g3pg_c + h2o_c --> glyc3p_c + glyc_c + h_c {'original_bigg_ids': ['GPDDA4']}
GPDDA5: g3pi_c + h2o_c --> glyc3p_c + h_c + inost_c {'original_bigg_ids': ['GPDDA5']}
GRDPtm: grdp_c <=> grdp_m {'original_bigg_ids': ['GRDPt_c']}
GRTT: grdp_c + ipdp_c --> frdp_c + ppi_c {'original_bigg_ids': ['GRTT']}
GRXR: grxox_c + 2.0 gthrd_c --> grxrd_c + gthox_c {'original_bigg_ids': ['GRXR']}
GRXRm: grxox_m + 2.0 gthrd_m --> grxrd_m + gthox_m {'original_bigg_ids': ['GRXR']}
GSNt: gsn_e <=> gsn_c {'original_bigg_ids': ['GSNt']}
GTHOm: gthox_m + h_m + nadph_m --> 2.0 gthrd_m + nadp_m {'original_bigg_ids': ['GTHOm']}
GTHOr: gthox_c + h_c + nadph_c <=> 2.0 gthrd_c + nadp_c {'original_bigg_ids': ['GTHOr']}
GTHPi: 2.0 gthrd_c + h2o2_c --> gthox_c + 2.0 h2o_c {'original_bigg_ids': ['GTHPi']}
GTHRDabcv: atp_v + gthrd_c + h2o_v --> adp_v + gthrd_v + h_v + pi_v {'original_bigg_ids': ['GTHRDabcv']}
GTHRDt2: gthrd_e --> gthrd_c {'original_bigg_ids': ['GTHRDt2']}
GTHS: atp_c + glucys_c + gly_c --> adp_c + gthrd_c + h_c + pi_c {'original_bigg_ids': ['GTHS']}
GTMLT: ala__L_c + gthrd_c --> cgly_c + gluala_c {'original_bigg_ids': ['GTMLT']}
GTMLTe: ala__L_c + gthrd_c --> cgly_e + gluala_e {'original_bigg_ids': ['GTMLTe']}
GTPCI: gtp_c + h2o_c --> ahdt_c + for_c + h_c {'original_bigg_ids': ['GTPCI']}
GTPCII: gtp_c + 3.0 h2o_c --> 25dhpp_c + for_c + 2.0 h_c + ppi_c {'original_bigg_ids': ['GTPCII']}
GTPCIn: gtp_n + h2o_n --> ahdt_n + for_n + h_n {'original_bigg_ids': ['GTPCIn']}
GTPt2m: gdp_m + gtp_c + h_c --> gdp_c + gtp_m + h_m {'original_bigg_ids': ['GTPt2m']}
GUAD: gua_c + h2o_c + h_c --> nh4_c + xan_c {'original_bigg_ids': ['GUAD']}
GUAt2r: gua_e + h_e <=> gua_c + h_c {'original_bigg_ids': ['GUAt2r']}
GUDBUTNAH: 4gudbutn_c + h2o_c --> 4abut_c + urea_c {'original_bigg_ids': ['GUDBUTNAH']}
H2CO3D: co2_c + h2o_c <=> h2co3_c {'original_bigg_ids': ['H2CO3D']}
H2O2syn: h_c + nadph_c + o2_c --> h2o2_c + nadp_c {'original_bigg_ids': ['H2O2syn']}
H2O2t: h2o2_e <=> h2o2_c {'original_bigg_ids': ['H2O2t']}
H2Ot: h2o_e <=> h2o_c {'original_bigg_ids': ['H2Ot']}
H2Oter: h2o_c <=> h2o_r {'original_bigg_ids': ['H2Oter']}
H2Otg: h2o_c <=> h2o_g {'original_bigg_ids': ['H2Otg']}
H2Otm: h2o_c <=> h2o_m {'original_bigg_ids': ['H2Otm']}
H2Otn: h2o_n <=> h2o_c {'original_bigg_ids': ['H2Otn']}
H2Otp: h2o_c <=> h2o_x {'original_bigg_ids': ['H2Otx']}
H2Otv: h2o_c <=> h2o_v {'original_bigg_ids': ['H2Otv']}
HACD10p: 3hxccoa_x + nad_x <=> 3ohxccoa_x + h_x + nadh_x {'original_bigg_ids': ['HACD10p']}
HACD1m: aacoa_m + h_m + nadh_m <=> 3hbcoa_m + nad_m {'original_bigg_ids': ['HACD1m']}
HACD1p: aacoa_x + h_x + nadh_x <=> 3hbcoa_x + nad_x {'original_bigg_ids': ['HACD1']}
HACD2m: 3ohcoa_m + h_m + nadh_m <=> 3hhcoa_m + nad_m {'original_bigg_ids': ['HACD2m']}
HACD2p: 3hhcoa_x + nad_x <=> 3ohcoa_x + h_x + nadh_x {'original_bigg_ids': ['HACD2']}
HACD3m: 3oocoa_m + h_m + nadh_m <=> 3hocoa_m + nad_m {'original_bigg_ids': ['HACD3m']}
HACD3p: 3hocoa_x + nad_x <=> 3oocoa_x + h_x + nadh_x {'original_bigg_ids': ['HACD3']}
HACD4m: 3odcoa_m + h_m + nadh_m <=> 3hdcoa_m + nad_m {'original_bigg_ids': ['HACD4m']}
HACD4p: 3odcoa_x + h_x + nadh_x <=> 3hdcoa_x + nad_x {'original_bigg_ids': ['HACD4p']}
HACD5m: 3oddcoa_m + h_m + nadh_m <=> 3hddcoa_m + nad_m {'original_bigg_ids': ['HACD5m']}
HACD5p: 3oddcoa_x + h_x + nadh_x <=> 3hddcoa_x + nad_x {'original_bigg_ids': ['HACD5p']}
HACD6m: 3otdcoa_m + h_m + nadh_m <=> 3htdcoa_m + nad_m {'original_bigg_ids': ['HACD6m']}
HACD6p: 3otdcoa_x + h_x + nadh_x <=> 3htdcoa_x + nad_x {'original_bigg_ids': ['HACD6p']}
HACD7m: 3ohdcoa_m + h_m + nadh_m <=> 3hhdcoa_m + nad_m {'original_bigg_ids': ['HACD7m']}
HACD7p: 3ohdcoa_x + h_x + nadh_x <=> 3hhdcoa_x + nad_x {'original_bigg_ids': ['HACD7p']}
HACD8p: 3ohodcoa_x + h_x + nadh_x <=> 3hodcoa_x + nad_x {'original_bigg_ids': ['HACD8p']}
HACD9m: 3hmbcoa_m + nad_m <=> 2maacoa_m + h_m + nadh_m {'original_bigg_ids': ['HMNOS']}
HACONTam: hcit_m <=> h2o_m + hacon_C_m {'original_bigg_ids': ['MCITDm']}
HACONTbm: h2o_m + hacon_C_m <=> hicit_m {'original_bigg_ids': ['HACNHm']}
HADPCOADH3: 3hadpcoa_c + nad_c <=> h_c + nadh_c + oxadpcoa_c {'original_bigg_ids': ['HADPCOADH3']}
HBCO_nadp: aacoa_c + h_c + nadph_c <=> 3hbcoa_c + nadp_c {'original_bigg_ids': ['HBCO_LPAREN_nadp_RPAREN_']}
HBZNPT_m: 4hbz_m + npdp_m --> 3nphb_m + ppi_m {'original_bigg_ids': ['HBZNPT_m']}
HCITSm: accoa_m + akg_m + h2o_m --> coa_m + h_m + hcit_m {'original_bigg_ids': ['HCITSm']}
HCO3E: co2_c + h2o_c <=> h_c + hco3_c {'original_bigg_ids': ['HCO3E']}
HCO3Em: co2_m + h2o_m <=> h_m + hco3_m {'original_bigg_ids': ['HCO3Em']}
HCYSMT: amet_c + hcys__L_c --> ahcys_c + h_c + met__L_c {'original_bigg_ids': ['HCYSMT']}
HCYSMT2: hcys__L_c + mmet_c --> h_c + 2.0 met__L_c {'original_bigg_ids': ['HCYSMT2']}
HDCAt: hdca_e --> hdca_c {'original_bigg_ids': ['HDCAt']}
HDCEAt: hdcea_e --> hdcea_c {'original_bigg_ids': ['HDCEAt']}
HDCOAtr: hdcoa_c <=> hdcoa_r {'original_bigg_ids': ['ACCOAtr']}
HEMEASm: 2.0 h_m + hemeO_m + 2.0 nadph_m + 2.0 o2_m --> 3.0 h2o_m + hemeA_m + 2.0 nadp_m {'original_bigg_ids': ['HEMEOMOm']}
HEMELm: apocytc_m + hemeC_m <=> cytc_m {'original_bigg_ids': ['HEMELm']}
HEMEOSm: frdp_m + h2o_m + pheme_m --> hemeO_m + ppi_m {'original_bigg_ids': ['HEMEOSm']}
HETZK: 4mhetz_c + atp_c --> 4mpetz_c + adp_c + h_c {'original_bigg_ids': ['HETZK']}
HEX1: atp_c + glc__D_c --> adp_c + g6p_c + h_c {'original_bigg_ids': ['HEX1']}
HEX10: atp_c + gam_c --> adp_c + gam6p_c + h_c {'original_bigg_ids': ['HEX10']}
HEX4: atp_c + man_c --> adp_c + h_c + man6p_c {'original_bigg_ids': ['HEX4']}
HEX7: atp_c + fru_c --> adp_c + f6p_c + h_c {'original_bigg_ids': ['HEX7']}
HEXCCOAtr: hexccoa_c <=> hexccoa_r {'original_bigg_ids': ['ACCOAtr']}
HEXCCPT1: crn_c + hexccoa_c --> coa_c + hexccrn_c {'original_bigg_ids': ['HEXCCPT1']}
HEXCCPT2: coa_m + hexccrn_m --> crn_m + hexccoa_m {'original_bigg_ids': ['HEXCCPT2']}
HEXCCRNt: hexccrn_c --> hexccrn_m {'original_bigg_ids': ['HEXCCRNt']}
HGNTOR: hgentis_c + o2_c --> 4mlacac_c + h_c {'original_bigg_ids': ['HGNTOR']}
HIBDm: 3hmp_m + nad_m <=> 2mop_m + h_m + nadh_m {'original_bigg_ids': ['HIBDm']}
HICITDm: hicit_m + nad_m --> 2oxoadp_m + co2_m + nadh_m {'original_bigg_ids': ['HICITDm']}
HISDC: h_c + his__L_c --> co2_c + hista_c {'original_bigg_ids': ['HISDC']}
HISTASE: h2o_c + hista_c + o2_c --> h2o2_c + im4act_c + nh4_c {'original_bigg_ids': ['HISTASE']}
HISTAtu: hista_e <=> hista_c {'original_bigg_ids': ['HISTAtu']}
HISTD: h2o_c + histd_c + 2.0 nad_c --> 3.0 h_c + his__L_c + 2.0 nadh_c {'original_bigg_ids': ['HISTD']}
HISTP: h2o_c + hisp_c --> histd_c + pi_c {'original_bigg_ids': ['HISTP']}
HISTRS: atp_c + his__L_c + trnahis_c --> amp_c + histrna_c + ppi_c {'original_bigg_ids': ['HISTRS']}
HISTRSm: atp_m + his__L_m + trnahis_m --> amp_m + histrna_m + ppi_m {'original_bigg_ids': ['HISTRSm']}
HISt2r: h_e + his__L_e <=> h_c + his__L_c {'original_bigg_ids': ['HISt2r']}
HISt6: h_v + his__L_c --> h_c + his__L_v {'original_bigg_ids': ['HISt6']}
HIStN1: h_c + his__L_e + 2.0 na1_e <=> h_e + his__L_c + 2.0 na1_c {'original_bigg_ids': ['HIStN1']}
HKYNH: h2o_c + hLkynr_c --> 3hanthrn_c + ala__L_c {'original_bigg_ids': ['HKYNH']}
HMBS: h2o_c + 4.0 ppbng_c --> hmbil_c + 4.0 nh4_c {'original_bigg_ids': ['HMBS']}
HMGCOAR: coa_c + mev__R_c + 2.0 nadp_c <=> 2.0 h_c + hmgcoa_c + 2.0 nadph_c {'original_bigg_ids': ['HMGCOAR']}
HMGCOARr: 2.0 h_r + hmgcoa_r + 2.0 nadph_r --> coa_r + mev__R_r + 2.0 nadp_r {'original_bigg_ids': ['HMGCOARr']}
HMGCOAS: coa_c + h_c + hmgcoa_c <=> aacoa_c + accoa_c + h2o_c {'original_bigg_ids': ['HMGCOAS']}
HMGCOASm: coa_m + h_m + hmgcoa_m <=> aacoa_m + accoa_m + h2o_m {'original_bigg_ids': ['HMGCOASm']}
HMGCOAtr: hmgcoa_c <=> hmgcoa_r {'original_bigg_ids': ['ACCOAtr']}
HMGLm: hmgcoa_m --> acac_m + accoa_m {'original_bigg_ids': ['HMGLm']}
HMGLx: hmgcoa_x --> acac_x + accoa_x {'original_bigg_ids': ['HMGLx']}
HMPK1: 4ahmmp_c + atp_c --> 4ampm_c + adp_c + h_c {'original_bigg_ids': ['HMPK1']}
HOMt: hom__L_e <=> hom__L_c {'original_bigg_ids': ['r2535']}
HPDCACOAtr: hpdcacoa_c <=> hpdcacoa_r {'original_bigg_ids': ['ACCOAtr']}
HPDCACRNCPT1: crn_c + hpdcacoa_c --> coa_c + hpdcacrn_c {'original_bigg_ids': ['HPDCACRNCPT1']}
HPDCACRNCPT2: coa_m + hpdcacrn_m --> crn_m + hpdcacoa_m {'original_bigg_ids': ['HPDCACRNCPT2']}
HPDCACRNt: hpdcacrn_c --> hpdcacrn_m {'original_bigg_ids': ['HPDCACRNt']}
HPDCAt: hpdca_e --> hpdca_c {'original_bigg_ids': ['HPDCAt']}
HPPK2: 6hmhpt_c + atp_c --> 6hmhptpp_c + amp_c + h_c {'original_bigg_ids': ['HPPK2']}
HPRO_u9m: 4hpro_LT_m + q9_m --> 1p3h5c_m + h_m + q9h2_m {'original_bigg_ids': ['HPROxm']}
HPROa: 1p3h5c_c + 2.0 h_c + nadh_c --> 4hpro_LT_c + nad_c {'original_bigg_ids': ['HPROa']}
HPROb: 1p3h5c_c + 2.0 h_c + nadph_c --> 4hpro_LT_c + nadp_c {'original_bigg_ids': ['HPROb']}
HPYRP: 3php_c + h2o_c --> hpyr_c + pi_c {'original_bigg_ids': ['HPYRP']}
HPYRRx: h_c + hpyr_c + nadh_c --> glyc__R_c + nad_c {'original_bigg_ids': ['HPYRRx']}
HPYRRy: h_c + hpyr_c + nadph_c --> glyc__R_c + nadp_c {'original_bigg_ids': ['HPYRRy']}
HSD17B42x: h_x + nadph_x + o2_x + thcholoylcoa_x --> dhocholoylcoa_x + 2.0 h2o_x + nadp_x {'original_bigg_ids': ['HSD17B42x']}
HSD17B4x: cholcoads_x + h_x + nadph_x + o2_x --> cholcoaone_x + h2o_x + nadp_x {'original_bigg_ids': ['HSD17B4x']}
HSDxi: aspsa_c + h_c + nadh_c --> hom__L_c + nad_c {'original_bigg_ids': ['HSDxi']}
HSDy: hom__L_c + nadp_c <=> aspsa_c + h_c + nadph_c {'original_bigg_ids': ['HSDy']}
HSERTA: accoa_c + hom__L_c <=> achms_c + coa_c {'original_bigg_ids': ['HSERTA']}
HSK: atp_c + hom__L_c --> adp_c + h_c + phom_c {'original_bigg_ids': ['HSK']}
HSTPT: glu__L_c + imacp_c --> akg_c + hisp_c {'original_bigg_ids': ['HSTPT']}
HTMLA_m: 3htmelys_m --> 4tmeabut_m + gly_m {'original_bigg_ids': ['HTMLA_m']}
HXAND: h2o_c + hxan_c + nad_c --> h_c + nadh_c + xan_c {'original_bigg_ids': ['HXAND']}
HXAt3: hxa_c <=> hxa_e {'original_bigg_ids': ['HXAt3']}
HXPRT: hxan_c + prpp_c --> imp_c + ppi_c {'original_bigg_ids': ['HXPRT']}
HYPOE: h2o_c + pyam5p_c --> pi_c + pydam_c {'original_bigg_ids': ['PYAMPP']}
Htr: h_c <=> h_r {'original_bigg_ids': ['Htr']}
Htx: h_c <=> h_x {'original_bigg_ids': ['Htx']}
I2FE2SRm: 2fe1s_m + iscssh_m + iscu_m --> 4.0 h_m + iscs_m + iscu_2fe2s_m {'original_bigg_ids': ['I2FE2SR']}
I2FE2SS2m: fadh2_m + 2.0 fe2_m + 2.0 iscssh_m + iscu_2fe2s_m --> fad_m + 6.0 h_m + 2.0 iscs_m + iscu_2fe2s2_m {'original_bigg_ids': ['I2FE2SS2']}
I2FE2SSm: fadh2_m + 2.0 fe2_m + 2.0 iscssh_m + iscu_m --> fad_m + 6.0 h_m + 2.0 iscs_m + iscu_2fe2s_m {'original_bigg_ids': ['I2FE2SS']}
I2FE2STm: 4.0 h_m + iscu_2fe2s_m --> 2fe2s_m + iscu_m {'original_bigg_ids': ['I2FE2ST']}
I4FE4SRm: fadh2_m + 2.0 h_m + iscu_2fe2s2_m --> fad_m + iscu_4fe4s_m {'original_bigg_ids': ['I4FE4SR']}
I4FE4STm: 4.0 h_m + iscu_4fe4s_m --> 4fe4s_m + iscu_m {'original_bigg_ids': ['I4FE4ST']}
ICDHxm: icit_m + nad_m --> akg_m + co2_m + nadh_m {'original_bigg_ids': ['ICDHxm']}
ICDHyp: icit_x + nadp_x --> akg_x + co2_x + nadph_x {'original_bigg_ids': ['ICDHyp']}
ICITtp: icit_c <=> icit_x {'original_bigg_ids': ['SUCCtp']}
ICLp: icit_x --> glx_x + succ_x {'original_bigg_ids': ['ICL']}
ICYSDSm: cys__L_m + iscs_m --> ala__L_m + iscssh_m {'original_bigg_ids': ['ICYSDS']}
IG3PS: gln__L_c + prlp_c --> aicar_c + eig3p_c + glu__L_c + h_c {'original_bigg_ids': ['IG3PS']}
IGPDH: eig3p_c --> h2o_c + imacp_c {'original_bigg_ids': ['IGPDH']}
IGPS: 2cpr5p_c + h_c --> 3ig3p_c + co2_c + h2o_c {'original_bigg_ids': ['IGPS']}
ILETA: akg_c + ile__L_c <=> 3mop_c + glu__L_c {'original_bigg_ids': ['ILETA']}
ILETAm: akg_m + ile__L_m <=> 3mop_m + glu__L_m {'original_bigg_ids': ['ILETAm']}
ILETRS: atp_c + ile__L_c + trnaile_c --> amp_c + iletrna_c + ppi_c {'original_bigg_ids': ['ILETRS']}
ILETRSm: atp_m + ile__L_m + trnaile_m --> amp_m + iletrna_m + ppi_m {'original_bigg_ids': ['ILETRSm']}
ILEt2r: h_e + ile__L_e <=> h_c + ile__L_c {'original_bigg_ids': ['ILEt2r']}
ILEt6: h_v + ile__L_c --> h_c + ile__L_v {'original_bigg_ids': ['ILEt6']}
ILEt7: h_v + ile__L_v --> h_c + ile__L_c {'original_bigg_ids': ['ILEt7']}
ILEtmi: ile__L_m <=> ile__L_c {'original_bigg_ids': ['ILEt5m']}
IMACTD: h2o_c + im4act_c + nad_c --> 2.0 h_c + im4ac_c + nadh_c {'original_bigg_ids': ['ALDH_LPAREN_im4ac_RPAREN_']}
IMPC: h2o_c + imp_c <=> fprica_c {'original_bigg_ids': ['IMPC']}
IMPD: h2o_c + imp_c + nad_c --> h_c + nadh_c + xmp_c {'original_bigg_ids': ['IMPD']}
IMPDm: h2o_m + imp_m + nad_m --> h_m + nadh_m + xmp_m {'original_bigg_ids': ['IMPDm']}
IND3ETHt: ind3eth_c <=> ind3eth_e {'original_bigg_ids': ['IND3ETHt']}
INDPYRD: h_c + indpyr_c <=> co2_c + id3acald_c {'original_bigg_ids': ['INDPYRD']}
INOSTter: inost_c --> inost_r {'original_bigg_ids': ['r0826']}
INSH: h2o_c + ins_c --> hxan_c + rib__D_c {'original_bigg_ids': ['INSH']}
INSTt2: h_e + inost_e --> h_c + inost_c {'original_bigg_ids': ['INSTt2']}
IPC124PLCg_RT: h2o_g + 0.01 ipc124_RT_g --> cer1_24_g + h_g + mi1p__D_g {'original_bigg_ids': ['IPC124PLC_SC']}
IPC126PLCg_RT: h2o_g + 0.01 ipc126_RT_g --> cer1_26_g + h_g + mi1p__D_g {'original_bigg_ids': ['IPC126PLC_SC']}
IPC224PLCg_RT: h2o_g + 0.01 ipc224_RT_g --> cer2_24_g + h_g + mi1p__D_g {'original_bigg_ids': ['IPC224PLC_SC']}
IPC226PLCg_RT: h2o_g + 0.01 ipc226_RT_g --> cer2_26_g + h_g + mi1p__D_g {'original_bigg_ids': ['IPC226PLC_SC']}
IPC324PLCg_RT: h2o_g + 0.01 ipc324_RT_g --> cer3_24_g + h_g + mi1p__D_g {'original_bigg_ids': ['IPC324PLC_SC']}
IPC326PLCg_RT: h2o_g + 0.01 ipc326_RT_g --> cer3_26_g + h_g + mi1p__D_g {'original_bigg_ids': ['IPC326PLC_SC']}
IPCS124g_RT: cer1_24_g + 0.01 ptd1ino_RT_g --> 0.01 12dgr_RT_g + 0.01 ipc124_RT_g {'original_bigg_ids': ['IPCS124_SC']}
IPCS126g_RT: cer1_26_g + 0.01 ptd1ino_RT_g --> 0.01 12dgr_RT_g + 0.01 ipc126_RT_g {'original_bigg_ids': ['IPCS126_SC']}
IPCS224g_RT: cer2_24_g + 0.01 ptd1ino_RT_g --> 0.01 12dgr_RT_g + 0.01 ipc224_RT_g {'original_bigg_ids': ['IPCS224_SC']}
IPCS226g_RT: cer2_26_g + 0.01 ptd1ino_RT_g --> 0.01 12dgr_RT_g + 0.01 ipc226_RT_g {'original_bigg_ids': ['IPCS226_SC']}
IPCS324g_RT: cer3_24_g + 0.01 ptd1ino_RT_g --> 0.01 12dgr_RT_g + 0.01 ipc324_RT_g {'original_bigg_ids': ['IPCS324_SC']}
IPCS326g_RT: cer3_26_g + 0.01 ptd1ino_RT_g --> 0.01 12dgr_RT_g + 0.01 ipc326_RT_g {'original_bigg_ids': ['IPCS326_SC']}
IPDDI: ipdp_c <=> dmpp_c {'original_bigg_ids': ['IPDDI']}
IPDPtm: ipdp_c <=> ipdp_m {'original_bigg_ids': ['IPDPt_m']}
IPMD: 3c2hmp_c + nad_c --> 3c4mop_c + h_c + nadh_c {'original_bigg_ids': ['IPMD']}
IPPMIa: 3c2hmp_c <=> 2ippm_c + h2o_c {'original_bigg_ids': ['IPPMIa']}
IPPMIb: 2ippm_c + h2o_c <=> 3c3hmp_c {'original_bigg_ids': ['IPPMIb']}
IPPS: 3mob_c + accoa_c + h2o_c --> 3c3hmp_c + coa_c + h_c {'original_bigg_ids': ['IPPS']}
IPPSm: 3mob_m + accoa_m + h2o_m --> 3c3hmp_m + coa_m + h_m {'original_bigg_ids': ['IPPSm']}
ITCY: cytd_c + itp_c --> cmp_c + h_c + idp_c {'original_bigg_ids': ['ITCY']}
KARA1im: alac__S_m + h_m + nadph_m --> 23dhmb_m + nadp_m {'original_bigg_ids': ['KARA1im']}
KARA2im: 2ahbut_m + h_m + nadph_m --> 23dhmp_m + nadp_m {'original_bigg_ids': ['KARA2im']}
KATPase: atp_c + h2o_c + h_e + k_c --> adp_c + 2.0 h_c + k_e + pi_c {'original_bigg_ids': ['HKt']}
KYN: Lkynr_c + h2o_c --> ala__L_c + anth_c + h_c {'original_bigg_ids': ['KYN']}
KYN3OX: Lkynr_c + h_c + nadph_c + o2_c --> h2o_c + hLkynr_c + nadp_c {'original_bigg_ids': ['KYN3OX']}
KYNAKGAT: Lkynr_c + akg_c --> 4aphdob_c + glu__L_c {'original_bigg_ids': ['KYNAKGAT']}
KYNATESYN: 4aphdob_c --> h2o_c + kynate_c {'original_bigg_ids': ['KYNATESYN']}
Kt2r: h_e + k_e <=> h_c + k_c {'original_bigg_ids': ['Kt2r']}
Kt3g: h_g + k_c <=> h_c + k_g {'original_bigg_ids': ['Kt3g']}
LALDO2: h_c + mthgxl_c + nadph_c --> lald__D_c + nadp_c {'original_bigg_ids': ['LALDO2']}
LALDO3: h_c + mthgxl_c + nadph_c --> lald__L_c + nadp_c {'original_bigg_ids': ['LALDO3']}
LANOSTATer_RT: 0.01 hdcoa_r + lanost_r + 0.01 lnlncgcoa_r + 0.14 ocdycacoa_r + 0.48 odecoa_r + 0.23 pmtcoa_r + 0.12 stcoa_r + 0.01 ttccoa_r --> coa_r + 0.01 lanostest_RT_r {'original_bigg_ids': ['LANOSTAT_SC']}
LANOSTESTH_RT: h2o_c + 0.01 lanostest_RT_d --> h_c + 0.23 hdca_c + 0.01 hdcea_c + lanost_c + 0.01 lnlncg_c + 0.12 ocdca_c + 0.48 ocdcea_c + 0.14 ocdcya_c + 0.01 ttc_c {'original_bigg_ids': ['LANOSTESTH_SC']}
LANOSTESTtrd: lanostest_RT_r --> lanostest_RT_d {'original_bigg_ids': ['FRDPtcr']}
LANOSTt: lanost_e <=> lanost_c {'original_bigg_ids': ['LANOSTt']}
LANOSTtr: lanost_c <=> lanost_r {'original_bigg_ids': ['LANOSTt']}
LCADi: h2o_c + lald__L_c + nad_c --> 2.0 h_c + lac__L_c + nadh_c {'original_bigg_ids': ['LCADi']}
LCADi_D: h2o_c + lald__D_c + nad_c --> 2.0 h_c + lac__D_c + nadh_c {'original_bigg_ids': ['LCADi_D']}
LCARSyi: h_c + lald__L_c + nadph_c --> 12ppd__S_c + nadp_c {'original_bigg_ids': ['LCARSyi']}
LCYSTAT: Lcyst_c + akg_c <=> 3spyr_c + glu__L_c {'original_bigg_ids': ['LCYSTAT']}
LCYSTATm: Lcyst_m + akg_m <=> 3spyr_m + glu__L_m {'original_bigg_ids': ['AATCm']}
LCYSTintv: cysi__L_v + h_v --> cysi__L_c + h_c {'original_bigg_ids': ['LCYSTintv']}
LCYSTt: Lcyst_e + cl_e + 2.0 na1_e <=> Lcyst_c + cl_c + 2.0 na1_c {'original_bigg_ids': ['LCYSTt']}
LEUTA: akg_c + leu__L_c <=> 4mop_c + glu__L_c {'original_bigg_ids': ['LEUTA']}
LEUTAm: akg_m + leu__L_m <=> 4mop_m + glu__L_m {'original_bigg_ids': ['LEUTAm']}
LEUTRS: atp_c + leu__L_c + trnaleu_c --> amp_c + leutrna_c + ppi_c {'original_bigg_ids': ['LEUTRS']}
LEUt2r: h_e + leu__L_e <=> h_c + leu__L_c {'original_bigg_ids': ['LEUt2r']}
LEUt5m: leu__L_c <=> leu__L_m {'original_bigg_ids': ['LEUt5m']}
LEUt6: h_v + leu__L_c --> h_c + leu__L_v {'original_bigg_ids': ['LEUt6']}
LEUt7: h_v + leu__L_v --> h_c + leu__L_c {'original_bigg_ids': ['LEUt7']}
LFORKYNHYD: Lfmkynr_c + h2o_c --> ala__L_c + h_c + nformanth_c {'original_bigg_ids': ['LFORKYNHYD']}
LGTHL: gthrd_c + mthgxl_c --> lgt__S_c {'original_bigg_ids': ['LGTHL']}
LIPAMPLm: lipoamp_m --> amp_m + lipopb_m {'original_bigg_ids': ['LIPAMPL']}
LIPATPTm: atp_m + lipoate_m --> lipoamp_m + ppi_m {'original_bigg_ids': ['LIPATPT']}
LIPOCTm: h_m + ocACP_m --> ACP_m + octapb_m {'original_bigg_ids': ['LIPOCT']}
LIPOSm: 4fe4s_m + 2.0 amet_m + h_m + nad_m + octapb_m --> 2fe2s_m + 2.0 dad_5_m + 2.0 fe2_m + lipopb_m + 2.0 met__L_m + nadh_m {'original_bigg_ids': ['LIPOS']}
LKDRA: lkdr_c <=> lald__L_c + pyr_c {'original_bigg_ids': ['LKDRA']}
LNLNCGCOAtr: lnlncgcoa_c <=> lnlncgcoa_r {'original_bigg_ids': ['ACCOAtr']}
LNLNCGCPT1: crn_c + lnlncgcoa_c --> coa_c + lnlncgcrn_c {'original_bigg_ids': ['LNLNCGCPT1']}
LNLNCGCPT2: coa_m + lnlncgcrn_m --> crn_m + lnlncgcoa_m {'original_bigg_ids': ['LNLNCGCPT2']}
LNLNCGCRNt: lnlncgcrn_c --> lnlncgcrn_m {'original_bigg_ids': ['LNLNCGCRNt']}
LNLNCGt: lnlncg_e --> lnlncg_c {'original_bigg_ids': ['LNLNCGt']}
LNS14DMr: 2.0 h_r + lanost_r + 3.0 nadph_r + 3.0 o2_r --> 44mctr_r + for_r + 4.0 h2o_r + 3.0 nadp_r {'original_bigg_ids': ['LNS14DMr']}
LNS14DMrx: 2.0 h_r + lanost_r + 3.0 nadh_r + 3.0 o2_r --> 44mctr_r + for_r + 4.0 h2o_r + 3.0 nad_r {'original_bigg_ids': ['LNS14DMx']}
LPCATer_RT: 0.01 1agpc_RT_r + 0.01 arachcoa_r + 0.02 hpdcacoa_r + 0.1 lnlncgcoa_r + 0.37 ocdycacoa_r + 0.32 odecoa_r + 0.09 pmtcoa_r + 0.07 stcoa_r + 0.02 ttccoa_r --> coa_r + 0.01 pc_RT_r {'original_bigg_ids': ['LPCAT_SC']}
LPPer_RT: 0.01 dagpy_RT_r + h2o_r --> h_r + 0.01 pa_RT_r + pi_r {'original_bigg_ids': ['LPP_SC']}
LSERDHr: nadp_c + ser__L_c <=> 2amsa_c + h_c + nadph_c {'original_bigg_ids': ['LSERDHr']}
LSTO1r: chlstol_r + h_r + nadph_r + o2_r --> ddsmsterol_r + 2.0 h2o_r + nadp_r {'original_bigg_ids': ['LSTO1r']}
LTA4H: h2o_c + leuktrA4_c --> leuktrB4_c {'original_bigg_ids': ['LTA4H']}
LTC4Sr: gthrd_r + leuktrA4_r --> leuktrC4_r {'original_bigg_ids': ['LTC4Sr']}
LTDCL: h_c + trp__L_c --> co2_c + trypta_c {'original_bigg_ids': ['LTDCL']}
LYSTRS: atp_c + lys__L_c + trnalys_c --> amp_c + lystrna_c + ppi_c {'original_bigg_ids': ['LYSTRS']}
LYSTRSm: atp_m + lys__L_m + trnalys_m --> amp_m + lystrna_m + ppi_m {'original_bigg_ids': ['LYSTL']}
LYSt2r: h_e + lys__L_e <=> h_c + lys__L_c {'original_bigg_ids': ['LYSt2r']}
LYSt6: h_v + lys__L_c --> h_c + lys__L_v {'original_bigg_ids': ['LYSt6']}
LYSt7m: lys__L_c --> lys__L_m {'original_bigg_ids': ['GLUt7m']}
LYXR: h_c + lyx__L_c + nadph_c --> abt_c + nadp_c {'original_bigg_ids': ['ARABR']}
LYXt: lyx__L_e <=> lyx__L_c {'original_bigg_ids': ['LYXtex']}
L_LACDcm: 2.0 ficytc_m + lac__L_c --> 2.0 focytc_m + 2.0 h_c + pyr_c {'original_bigg_ids': ['L_LACDcm']}
L_LACt2r: h_e + lac__L_e <=> h_c + lac__L_c {'original_bigg_ids': ['L_LACt2r']}
M14NTg: n2m2masn_g + uacgam_g --> h_g + n2m2nmasn_g + udp_g {'original_bigg_ids': ['M14NTg']}
MACACI: 4mlacac_c --> 4fumacac_c {'original_bigg_ids': ['MACACI']}
MACOXO: 3mldz_c + h2o_c + nad_c --> 3mlda_c + 2.0 h_c + nadh_c {'original_bigg_ids': ['MACOXO']}
MAGAH160: h2o_c + mag160_c --> glyc_c + h_c + hdca_c {'original_bigg_ids': ['MAGAH160']}
MAGAH180: h2o_c + mag180_c --> glyc_c + h_c + ocdca_c {'original_bigg_ids': ['MAGAH180']}
MAGAH1819Z: h2o_c + mag1819Z_c --> glyc_c + h_c + ocdcea_c {'original_bigg_ids': ['MAGAH1819Z']}
MAGL_RT: h2o_c + 0.01 mag_RT_d --> glyc_c + h_c + 0.23 hdca_c + 0.01 hdcea_c + 0.01 lnlncg_c + 0.12 ocdca_c + 0.48 ocdcea_c + 0.14 ocdcya_c + 0.01 ttc_c {'original_bigg_ids': ['TAGL_SC']}
MALCOAMT: amet_c + malcoa_c --> ahcys_c + malcoame_c {'original_bigg_ids': ['MALCOAMT']}
MALCOAtr: malcoa_c <=> malcoa_r {'original_bigg_ids': ['ACCOAtr']}
MALSp: accoa_x + glx_x + h2o_x --> coa_x + h_x + mal__L_x {'original_bigg_ids': ['MALSp']}
MALT: h2o_c + malt_c --> 2.0 glc__D_c {'original_bigg_ids': ['MALT']}
MALTATr: accoa_c + malt_c <=> acmalt_c + coa_c {'original_bigg_ids': ['MALTATr']}
MALTe: h2o_e + malt_e --> 2.0 glc__D_e {'original_bigg_ids': ['MALTe']}
MALTt1r: malt_e <=> malt_c {'original_bigg_ids': ['MALTt1r']}
MALTt2: h_e + malt_e --> h_c + malt_c {'original_bigg_ids': ['MALTt2']}
MAL_Ltx: mal__L_x <=> mal__L_c {'original_bigg_ids': ['MAL_Ltx']}
MALtm: mal__L_c + pi_m --> mal__L_m + pi_c {'original_bigg_ids': ['MALtm']}
MAN1PPe: h2o_e + man1p_e --> man_e + pi_e {'original_bigg_ids': ['G2PP']}
MAN1PT: gtp_c + h_c + man1p_c --> gdpmann_c + ppi_c {'original_bigg_ids': ['MAN1PT']}
MAN1_6B1er: g1m7masnC_r + h2o_r --> g1m6masnB1_r + man_r {'original_bigg_ids': ['MAN1_6B1er']}
MAN1_7Ber: g1m8masn_r + h2o_r --> g1m7masnB_r + man_r {'original_bigg_ids': ['MAN1_7Ber']}
MAN6PI: man6p_c <=> f6p_c {'original_bigg_ids': ['MAN6PI']}
MAN6PPe: h2o_e + man6p_e --> man_e + pi_e {'original_bigg_ids': ['G2PP']}
MANPGH: h2o_c + man6pglyc_c --> glyc__R_c + man6p_c {'original_bigg_ids': ['MANPGH']}
MANt1r: man_e <=> man_c {'original_bigg_ids': ['MANt1r']}
MANt2: h_e + man_e --> h_c + man_c {'original_bigg_ids': ['MANt2']}
MAOX: h2o_c + mma_c + o2_c --> fald_c + h2o2_c + nh4_c {'original_bigg_ids': ['MAOX']}
MCCCrm: 3mb2coa_m + atp_m + hco3_m <=> 3mgcoa_m + adp_m + h_m + pi_m {'original_bigg_ids': ['MCCCrm']}
MCITL2m: micit_m --> pyr_m + succ_m {'original_bigg_ids': ['MCITL2m']}
MCITSm: h2o_m + oaa_m + ppcoa_m --> 2mcit_m + coa_m + h_m {'original_bigg_ids': ['MCITSm']}
MCOATAm: ACP_m + malcoa_m <=> coa_m + malACP_m {'original_bigg_ids': ['MCOATAm']}
MCPST: cyan_c + mercppyr_c --> h_c + pyr_c + tcynt_c {'original_bigg_ids': ['MCPST']}
MDHm: mal__L_m + nad_m <=> h_m + nadh_m + oaa_m {'original_bigg_ids': ['MDHm']}
MDHp: mal__L_x + nad_x <=> h_x + nadh_x + oaa_x {'original_bigg_ids': ['MDHx']}
ME1m: mal__L_m + nad_m --> co2_m + nadh_m + pyr_m {'original_bigg_ids': ['ME1m']}
ME2: mal__L_c + nadp_c --> co2_c + nadph_c + pyr_c {'original_bigg_ids': ['ME2']}
MELATN23DOX: melatn_c + o2_c --> fna5moxam_c {'original_bigg_ids': ['MELATN23DOX']}
MELIBt2: h_e + melib_e --> h_c + melib_c {'original_bigg_ids': ['MELIBt2']}
MEOHt2: meoh_e <=> meoh_c {'original_bigg_ids': ['MEOHt2']}
METAT: atp_c + h2o_c + met__L_c --> amet_c + pi_c + ppi_c {'original_bigg_ids': ['METAT']}
METS: 5mthf_c + hcys__L_c --> h_c + met__L_c + thf_c {'original_bigg_ids': ['METS']}
METSOX1t: metsox_S__L_e <=> metsox_S__L_c {'original_bigg_ids': ['CO2t']}
METSOX2t: metsox_R__L_e <=> metsox_R__L_c {'original_bigg_ids': ['CO2t']}
METSOXR1: metsox_S__L_c + trdrd_c --> h2o_c + met__L_c + trdox_c {'original_bigg_ids': ['METSOXR1']}
METSOXR2: metsox_R__L_c + trdrd_c --> h2o_c + met__L_c + trdox_c {'original_bigg_ids': ['METSOXR2']}
METTRS: atp_c + met__L_c + trnamet_c --> amp_c + mettrna_c + ppi_c {'original_bigg_ids': ['METTRS']}
METTRSm: atp_m + met__L_m + trnamet_m --> amp_m + mettrna_m + ppi_m {'original_bigg_ids': ['METTRSm']}
METt2r: h_e + met__L_e <=> h_c + met__L_c {'original_bigg_ids': ['METt2r']}
METtm: met__L_c <=> met__L_m {'original_bigg_ids': ['r1436']}
MEVK1: atp_c + mev__R_c --> 5pmev_c + adp_c + h_c {'original_bigg_ids': ['MEVK1']}
MFAPSer_RT: amet_r + 0.01 ptdmeeta_RT_r --> ahcys_r + h_r + 0.01 ptd2meeta_RT_r {'original_bigg_ids': ['MFAPS_SC']}
MG1A: asnglcnacglcnacman_man_manman_manman_manmanmanglcglcglc_c + h2o_c --> Glc_aD_c + asnglcnacglcnacman_man_manman_manman_manmanmanglcglc_c {'original_bigg_ids': ['MG1A']}
MG1B: asnglcnacglcnacman_man_manman_manman_manmanmanglcglcglc_c + h2o_c --> asnglcnacglcnacman_man_manman_manman_manmanmanglcglc_c + glc__D_c {'original_bigg_ids': ['MG1B']}
MG1er: g3m8masn_r + h2o_r --> g2m8masn_r + glc__D_r {'original_bigg_ids': ['MG1er']}
MG2A: asnglcnacglcnacman_man_manman_manman_manmanmanglcglc_c + h2o_c --> Glc_aD_c + asnglcnacglcnacman_man_manman_manman_manmanmanglc_c {'original_bigg_ids': ['MG2A']}
MG2B: asnglcnacglcnacman_man_manman_manman_manmanmanglcglc_c + h2o_c --> asnglcnacglcnacman_man_manman_manman_manmanmanglc_c + glc__D_c {'original_bigg_ids': ['MG2B']}
MG2t: mg2_e <=> mg2_c {'original_bigg_ids': ['MG2tex']}
MG3A: asnglcnacglcnacman_man_manman_manman_manmanmanglc_c + h2o_c --> Glc_aD_c + asnglcnacglcnacman_man_manman_manman_manmanman_c {'original_bigg_ids': ['MG3A']}
MG3B: asnglcnacglcnacman_man_manman_manman_manmanmanglc_c + h2o_c --> asnglcnacglcnacman_man_manman_manman_manmanman_c + glc__D_c {'original_bigg_ids': ['MG3B']}
MGCHrm: 3mgcoa_m + h2o_m <=> hmgcoa_m {'original_bigg_ids': ['MGC']}
MHISOR: h2o_c + mhista_c + o2_c --> 3mldz_c + h2o2_c + nh4_c {'original_bigg_ids': ['MHISOR']}
MI13456PKn: atp_n + mi13456p_n --> adp_n + h_n + minohp_n {'original_bigg_ids': ['MI13456PKn']}
MI1345PKn: atp_n + mi1345p_n --> adp_n + h_n + mi13456p_n {'original_bigg_ids': ['MI1345PKn']}
MI1345PPn: h2o_n + mi1345p_n --> mi134p_n + pi_n {'original_bigg_ids': ['MI1345PP']}
MI1456PKn: atp_n + mi1456p_n --> adp_n + h_n + mi13456p_n {'original_bigg_ids': ['MI1456PKn']}
MI145P6Kn: atp_n + mi145p_n --> adp_n + h_n + mi1456p_n {'original_bigg_ids': ['MI145P6Kn']}
MI145PKn: atp_n + mi145p_n --> adp_n + h_n + mi1345p_n {'original_bigg_ids': ['MI145PKn']}
MI145PPn: h2o_n + mi145p_n --> mi14p_n + pi_n {'original_bigg_ids': ['MI145PP']}
MI1PP: h2o_c + mi1p__D_c --> inost_c + pi_c {'original_bigg_ids': ['MI1PP']}
MI3PP: h2o_c + mi3p__D_c --> inost_c + pi_c {'original_bigg_ids': ['MI3PP']}
MI3PS: g6p_c --> mi3p__D_c {'original_bigg_ids': ['HMR_6572']}
MI4PP: h2o_c + mi4p__D_c --> inost_c + pi_c {'original_bigg_ids': ['MI4PP']}
MINOHPtn: minohp_c <=> minohp_n {'original_bigg_ids': ['MINOHPtn']}
MIP2C124PLCg_RT: h2o_g + 0.01 mip2c124_RT_g --> cer1_24_g + h_g + man2mi1p__D_g {'original_bigg_ids': ['MIP2C124PLC_SC']}
MIP2C126PLCg_RT: h2o_g + 0.01 mip2c126_RT_g --> cer1_26_g + h_g + man2mi1p__D_g {'original_bigg_ids': ['MIP2C126PLC_SC']}
MIP2C224PLCg_RT: h2o_g + 0.01 mip2c224_RT_g --> cer2_24_g + h_g + man2mi1p__D_g {'original_bigg_ids': ['MIP2C224PLC_SC']}
MIP2C226PLCg_RT: h2o_g + 0.01 mip2c226_RT_g --> cer2_26_g + h_g + man2mi1p__D_g {'original_bigg_ids': ['MIP2C226PLC_SC']}
MIP2C324PLCg_RT: h2o_g + 0.01 mip2c324_RT_g --> cer3_24_g + h_g + man2mi1p__D_g {'original_bigg_ids': ['MIP2C324PLC_SC']}
MIP2C326PLCg_RT: h2o_g + 0.01 mip2c326_RT_g --> cer3_26_g + h_g + man2mi1p__D_g {'original_bigg_ids': ['MIP2C326PLC_SC']}
MIP2CS124g_RT: 0.01 mipc124_RT_g + 0.01 ptd1ino_RT_g --> 0.01 12dgr_RT_g + 0.01 mip2c124_RT_g {'original_bigg_ids': ['MIP2CS124_SC']}
MIP2CS126g_RT: 0.01 mipc126_RT_g + 0.01 ptd1ino_RT_g --> 0.01 12dgr_RT_g + 0.01 mip2c126_RT_g {'original_bigg_ids': ['MIP2CS126_SC']}
MIP2CS224g_RT: 0.01 mipc224_RT_g + 0.01 ptd1ino_RT_g --> 0.01 12dgr_RT_g + 0.01 mip2c224_RT_g {'original_bigg_ids': ['MIP2CS224_SC']}
MIP2CS226g_RT: 0.01 mipc226_RT_g + 0.01 ptd1ino_RT_g --> 0.01 12dgr_RT_g + 0.01 mip2c226_RT_g {'original_bigg_ids': ['MIP2CS226_SC']}
MIP2CS324g_RT: 0.01 mipc324_RT_g + 0.01 ptd1ino_RT_g --> 0.01 12dgr_RT_g + 0.01 mip2c324_RT_g {'original_bigg_ids': ['MIP2CS324_SC']}
MIP2CS326g_RT: 0.01 mipc326_RT_g + 0.01 ptd1ino_RT_g --> 0.01 12dgr_RT_g + 0.01 mip2c326_RT_g {'original_bigg_ids': ['MIP2CS326_SC']}
MIPC124PLCg_RT: h2o_g + 0.01 mipc124_RT_g --> cer1_24_g + h_g + manmi1p__D_g {'original_bigg_ids': ['MIPC124PLC_SC']}
MIPC126PLCg_RT: h2o_g + 0.01 mipc126_RT_g --> cer1_26_g + h_g + manmi1p__D_g {'original_bigg_ids': ['MIPC126PLC_SC']}
MIPC224PLCg_RT: h2o_g + 0.01 mipc224_RT_g --> cer2_24_g + h_g + manmi1p__D_g {'original_bigg_ids': ['MIPC224PLC_SC']}
MIPC226PLCg_RT: h2o_g + 0.01 mipc226_RT_g --> cer2_26_g + h_g + manmi1p__D_g {'original_bigg_ids': ['MIPC226PLC_SC']}
MIPC324PLCg_RT: h2o_g + 0.01 mipc324_RT_g --> cer3_24_g + h_g + manmi1p__D_g {'original_bigg_ids': ['MIPC324PLC_SC']}
MIPC326PLCg_RT: h2o_g + 0.01 mipc326_RT_g --> cer3_26_g + h_g + manmi1p__D_g {'original_bigg_ids': ['MIPC326PLC_SC']}
MIPCS124g_RT: gdpmann_g + 0.01 ipc124_RT_g --> gdp_g + h_g + 0.01 mipc124_RT_g {'original_bigg_ids': ['MIPCS124_SC']}
MIPCS126g_RT: gdpmann_g + 0.01 ipc126_RT_g --> gdp_g + h_g + 0.01 mipc126_RT_g {'original_bigg_ids': ['MIPCS126_SC']}
MIPCS224g_RT: gdpmann_g + 0.01 ipc224_RT_g --> gdp_g + h_g + 0.01 mipc224_RT_g {'original_bigg_ids': ['MIPCS224_SC']}
MIPCS226g_RT: gdpmann_g + 0.01 ipc226_RT_g --> gdp_g + h_g + 0.01 mipc226_RT_g {'original_bigg_ids': ['MIPCS226_SC']}
MIPCS324g_RT: gdpmann_g + 0.01 ipc324_RT_g --> gdp_g + h_g + 0.01 mipc324_RT_g {'original_bigg_ids': ['MIPCS324_SC']}
MIPCS326g_RT: gdpmann_g + 0.01 ipc326_RT_g --> gdp_g + h_g + 0.01 mipc326_RT_g {'original_bigg_ids': ['MIPCS326_SC']}
MLTG1: h2o_c + malttr_c --> glc__D_c + malt_c {'original_bigg_ids': ['MLTG1']}
MLTG1e: h2o_e + malttr_e --> glc__D_e + malt_e {'original_bigg_ids': ['MLTG1e']}
MLTG2: h2o_c + maltttr_c --> glc__D_c + malttr_c {'original_bigg_ids': ['MLTG2']}
MLTG3: h2o_c + maltpt_c --> glc__D_c + maltttr_c {'original_bigg_ids': ['MLTG3']}
MLTG4: h2o_c + malthx_c --> glc__D_c + maltpt_c {'original_bigg_ids': ['MLTG4']}
MLTP1: maltpt_c + pi_c <=> g1p_c + maltttr_c {'original_bigg_ids': ['MLTP1']}
MLTP2: malthx_c + pi_c <=> g1p_c + maltpt_c {'original_bigg_ids': ['MLTP2']}
MM1: asnglcnacglcnacman_man_manman_manman_manmanman_c + 4.0 h2o_c --> asnglcnacglcnacman_man_man_man_man_c + 4.0 man_c {'original_bigg_ids': ['MM1']}
MM2: asnglcnacglcnacman_man_man_man_man_c + 2.0 h2o_c --> asnglcnacglcnacman_man_man_c + 2.0 man_c {'original_bigg_ids': ['MM2']}
MM3: asnglcnacglcnacman_man_manman_manman_manmanman_c + h2o_c --> asnglcnacglcnacman_man_manman_man_manmanman_c + man_c {'original_bigg_ids': ['MM3']}
MM8Ber: h2o_r + m8masn_r --> m7masnB_r + man_r {'original_bigg_ids': ['MM8Ber']}
MMAt2e: mma_c <=> mma_e {'original_bigg_ids': ['MMAt2e']}
MMSAD1m: 2mop_m + coa_m + nad_m --> co2_m + nadh_m + ppcoa_m {'original_bigg_ids': ['MMSAD1m']}
MMSAD3m: coa_m + msa_m + nad_m --> accoa_m + co2_m + nadh_m {'original_bigg_ids': ['MMSD']}
MN6PP: h2o_c + man6p_c --> man_c + pi_c {'original_bigg_ids': ['MN6PP']}
MNt: mn2_e <=> mn2_c {'original_bigg_ids': ['MNtex']}
MOCOS: 2.0 h_c + mobd_c + mptamp_c --> amp_c + cu2_c + h2o_c + moco_c {'original_bigg_ids': ['MOCOS']}
MOHMT: 3mob_c + h2o_c + mlthf_c --> 2dhp_c + thf_c {'original_bigg_ids': ['MOHMT']}
MPTSS: atp_c + h_c + moadcoo_c --> moadamp_c + ppi_c {'original_bigg_ids': ['MPTSS']}
MSAR: h_c + msa_c + nadph_c --> 3hpp_c + nadp_c {'original_bigg_ids': ['MSAR']}
MTAP: 5mta_c + pi_c --> 5mdr1p_c + ade_c {'original_bigg_ids': ['MTAP']}
MTHFC: h2o_c + methf_c <=> 10fthf_c + h_c {'original_bigg_ids': ['MTHFC']}
MTHFCm: h2o_m + methf_m <=> 10fthf_m + h_m {'original_bigg_ids': ['MTHFCm']}
MTHFCx: 10fthf_x + h_x --> h2o_x + methf_x {'original_bigg_ids': ['MTHFCx']}
MTHFD: mlthf_c + nadp_c <=> methf_c + nadph_c {'original_bigg_ids': ['MTHFD']}
MTHFD2i: mlthf_c + nad_c --> methf_c + nadh_c {'original_bigg_ids': ['MTHFD2i']}
MTHFDm: mlthf_m + nadp_m <=> methf_m + nadph_m {'original_bigg_ids': ['MTHFDm']}
MTHFR2: 2.0 h_c + mlthf_c + nadh_c --> 5mthf_c + nad_c {'original_bigg_ids': ['MTHFR2']}
MTHFR2m: 2.0 h_m + mlthf_m + nadh_m --> 5mthf_m + nad_m {'original_bigg_ids': ['MTHFO']}
MTHFR3: 2.0 h_c + mlthf_c + nadph_c --> 5mthf_c + nadp_c {'original_bigg_ids': ['MTHFR3']}
MTHFR3m: 2.0 h_m + mlthf_m + nadph_m --> 5mthf_m + nadp_m {'original_bigg_ids': ['MTHFO_LPAREN_nadp_RPAREN_']}
MTRI: 5mdr1p_c <=> 5mdru1p_c {'original_bigg_ids': ['MTRI']}
MUCCY_kt: CCbuttc_c + h_c --> 4cml_c {'original_bigg_ids': ['MUCCY_kt']}
NA1tm: na1_c <=> na1_m {'original_bigg_ids': ['NA1tm']}
NACt: nac_e <=> nac_c {'original_bigg_ids': ['NACt']}
NADDP: h2o_c + nad_c --> amp_c + 2.0 h_c + nmn_c {'original_bigg_ids': ['NADDP']}
NADH2_u9cm: h_c + nadh_c + q9_m --> nad_c + q9h2_m {'original_bigg_ids': ['NADH2_u6cm']}
NADH2_u9m1: h_m + nadh_m + q9_m --> nad_m + q9h2_m {'original_bigg_ids': ['NADH2_u6m']}
NADH2_u9m2: 5.0 h_m + nadh_m + q9_m --> 4.0 h_c + nad_m + q9h2_m {'original_bigg_ids': ['NADH2_u10m']}
NADHHR: h2o_c + nadh_c --> nadhx__R_c {'original_bigg_ids': ['NADHHR']}
NADHHS: h2o_c + nadh_c --> nadhx__S_c {'original_bigg_ids': ['NADHHS']}
NADHXD2: atp_c + nadhx__S_c --> adp_c + h_c + nadh_c + pi_c {'original_bigg_ids': ['NADHXD']}
NADHXE: nadhx__S_c <=> nadhx__R_c {'original_bigg_ids': ['NADHXE']}
NADK: atp_c + nad_c --> adp_c + h_c + nadp_c {'original_bigg_ids': ['NADK']}
NADPHHR: h2o_c + nadph_c --> nadphx__R_c {'original_bigg_ids': ['NADPHHR']}
NADPHHS: h2o_c + nadph_c --> nadphx__S_c {'original_bigg_ids': ['NADPHHS']}
NADPHXD2: atp_c + nadphx__S_c --> adp_c + h_c + nadph_c + pi_c {'original_bigg_ids': ['NADPHXD']}
NADPHXE: nadphx__R_c <=> nadphx__S_c {'original_bigg_ids': ['NADPHXE']}
NADPHtru: nadph_c --> nadph_r {'original_bigg_ids': ['NADPHtru']}
NADPtru: nadp_r --> nadp_c {'original_bigg_ids': ['NADPtru']}
NADS1: atp_c + dnad_c + nh4_c --> amp_c + h_c + nad_c + ppi_c {'original_bigg_ids': ['NADS1']}
NADS1n: atp_n + dnad_n + nh4_n --> amp_n + h_n + nad_n + ppi_n {'original_bigg_ids': ['NADS1n']}
NADS2: atp_c + dnad_c + gln__L_c + h2o_c --> amp_c + glu__L_c + h_c + nad_c + ppi_c {'original_bigg_ids': ['DNGAL']}
NADtm: nad_c --> nad_m {'original_bigg_ids': ['NADtm']}
NAMNPP: atp_c + h2o_c + nac_c + prpp_c --> adp_c + nicrnt_c + pi_c + ppi_c {'original_bigg_ids': ['NAMNPP']}
NAPRT: h_c + nac_c + prpp_c --> nicrnt_c + ppi_c {'original_bigg_ids': ['NAPRT']}
NAPRTm: h_m + nac_m + prpp_m --> nicrnt_m + ppi_m {'original_bigg_ids': ['NAPRTm']}
NARK: no2_c <=> no2_e {'original_bigg_ids': ['NO2t']}
NAt3_1: h_e + na1_c <=> h_c + na1_e {'original_bigg_ids': ['NAt3_1']}
NAt3_1g: h_g + na1_c <=> h_c + na1_g {'original_bigg_ids': ['NAt3_1g']}
NBAHH_ir: carn_c + h2o_c --> ala_B_c + his__L_c {'original_bigg_ids': ['NBAHH_ir']}
NDP3g: gdp_g + h2o_g --> gmp_g + h_g + pi_g {'original_bigg_ids': ['NDP3g']}
NDP7g: h2o_g + udp_g --> h_g + pi_g + ump_g {'original_bigg_ids': ['NDP7g']}
NDP8: dudp_c + h2o_c --> dump_c + h_c + pi_c {'original_bigg_ids': ['NDP8']}
NDPK1: atp_c + gdp_c <=> adp_c + gtp_c {'original_bigg_ids': ['NDPK1']}
NDPK10m: atp_m + didp_m <=> adp_m + ditp_m {'original_bigg_ids': ['NDPK10m']}
NDPK1m: atp_m + gdp_m <=> adp_m + gtp_m {'original_bigg_ids': ['NDPK1m']}
NDPK2: atp_c + udp_c <=> adp_c + utp_c {'original_bigg_ids': ['NDPK2']}
NDPK2m: atp_m + udp_m <=> adp_m + utp_m {'original_bigg_ids': ['NDPK2m']}
NDPK3: atp_c + cdp_c <=> adp_c + ctp_c {'original_bigg_ids': ['NDPK3']}
NDPK3m: atp_m + cdp_m <=> adp_m + ctp_m {'original_bigg_ids': ['NDPK3m']}
NDPK4: atp_c + dtdp_c <=> adp_c + dttp_c {'original_bigg_ids': ['NDPK4']}
NDPK4m: atp_m + dtdp_m <=> adp_m + dttp_m {'original_bigg_ids': ['NDPK4m']}
NDPK5: atp_c + dgdp_c <=> adp_c + dgtp_c {'original_bigg_ids': ['NDPK5']}
NDPK5m: atp_m + dgdp_m <=> adp_m + dgtp_m {'original_bigg_ids': ['NDPK5m']}
NDPK6: atp_c + dudp_c <=> adp_c + dutp_c {'original_bigg_ids': ['NDPK6']}
NDPK6m: atp_m + dudp_m <=> adp_m + dutp_m {'original_bigg_ids': ['NDPK6m']}
NDPK7: atp_c + dcdp_c <=> adp_c + dctp_c {'original_bigg_ids': ['NDPK7']}
NDPK7m: atp_m + dcdp_m <=> adp_m + dctp_m {'original_bigg_ids': ['NDPK7m']}
NDPK8: atp_c + dadp_c <=> adp_c + datp_c {'original_bigg_ids': ['NDPK8']}
NDPK8m: atp_m + dadp_m <=> adp_m + datp_m {'original_bigg_ids': ['NDPK8m']}
NDPK9: atp_c + idp_c <=> adp_c + itp_c {'original_bigg_ids': ['NDPK9']}
NDPK9m: atp_m + idp_m <=> adp_m + itp_m {'original_bigg_ids': ['NDPK9m']}
NH4t: nh4_e <=> nh4_c {'original_bigg_ids': ['NH4t']}
NH4tm: nh4_c <=> nh4_m {'original_bigg_ids': ['NH4tm']}
NH4tp: nh4_c <=> nh4_x {'original_bigg_ids': ['NH4tx']}
NITR: h_c + nadh_c + no3_c --> h2o_c + nad_c + no2_c {'original_bigg_ids': ['NITR']}
NMNAT: atp_c + h_c + nmn_c --> nad_c + ppi_c {'original_bigg_ids': ['NMNAT']}
NMNATm: atp_m + h_m + nmn_m --> nad_m + ppi_m {'original_bigg_ids': ['NMNATm']}
NMNATn: atp_n + h_n + nmn_n --> nad_n + ppi_n {'original_bigg_ids': ['NMNATn']}
NMNHYD: h2o_c + nmn_c --> pi_c + rnam_c {'original_bigg_ids': ['NTD5_a']}
NMPTRCOX: nmptrc_c + o2_c --> 1mpyr_c + h2o2_c + nh4_c {'original_bigg_ids': ['NMPTRCOX']}
NNAM: h2o_c + ncam_c --> nac_c + nh4_c {'original_bigg_ids': ['NNAM']}
NNAMrm: h2o_m + ncam_m <=> nac_m + nh4_m {'original_bigg_ids': ['NNAMrm']}
NNAT: atp_c + h_c + nicrnt_c --> dnad_c + ppi_c {'original_bigg_ids': ['NNATr']}
NNATm: atp_m + h_m + nicrnt_m --> dnad_m + ppi_m {'original_bigg_ids': ['NNATm']}
NNATn: atp_n + h_n + nicrnt_n --> dnad_n + ppi_n {'original_bigg_ids': ['NNATn']}
NNDPR: 2.0 h_c + prpp_c + quln_c --> co2_c + nicrnt_c + ppi_c {'original_bigg_ids': ['NNDPR']}
NNDPRm: 2.0 h_m + prpp_m + quln_m --> co2_m + nicrnt_m + ppi_m {'original_bigg_ids': ['NNDPRm']}
NNMT: amet_c + ncam_c --> 1mncam_c + ahcys_c {'original_bigg_ids': ['NNMT']}
NO3t: no3_e <=> no3_c {'original_bigg_ids': ['NO3t']}
NP1: h_c + nac_c + r1p_c --> nicrns_c + pi_c {'original_bigg_ids': ['NP1']}
NPDPS_m: grdp_m + 7.0 ipdp_m --> npdp_m + 7.0 ppi_m {'original_bigg_ids': ['NPDPS_m']}
NPHMBDC_m: h_m + me3dhnpdh_m --> 2np6mep_m + co2_m {'original_bigg_ids': ['NPHMBDC_m']}
NRVNCCPT1: crn_c + nrvnccoa_c --> coa_c + nrvnccrn_c {'original_bigg_ids': ['NRVNCCPT1']}
NRVNCCPT2: coa_m + nrvnccrn_m --> crn_m + nrvnccoa_m {'original_bigg_ids': ['NRVNCCPT2']}
NRVNCCRNt: nrvnccrn_c --> nrvnccrn_m {'original_bigg_ids': ['NRVNCCRNt']}
NTD1: dump_c + h2o_c --> duri_c + pi_c {'original_bigg_ids': ['NTD1']}
NTD10: h2o_c + xmp_c --> pi_c + xtsn_c {'original_bigg_ids': ['NTD10']}
NTD11: h2o_c + imp_c --> ins_c + pi_c {'original_bigg_ids': ['NTD11']}
NTD12: dimp_c + h2o_c --> din_c + pi_c {'original_bigg_ids': ['NTD12']}
NTD2: h2o_c + ump_c --> pi_c + uri_c {'original_bigg_ids': ['NTD2']}
NTD2e: h2o_e + ump_e --> pi_e + uri_e {'original_bigg_ids': ['NTD2e']}
NTD3: dcmp_c + h2o_c --> dcyt_c + pi_c {'original_bigg_ids': ['NTD3']}
NTD4: cmp_c + h2o_c --> cytd_c + pi_c {'original_bigg_ids': ['NTD4']}
NTD4e: cmp_e + h2o_e --> cytd_e + pi_e {'original_bigg_ids': ['NTD4e']}
NTD5: dtmp_c + h2o_c --> pi_c + thymd_c {'original_bigg_ids': ['NTD5']}
NTD5e: dtmp_e + h2o_e --> pi_e + thymd_e {'original_bigg_ids': ['NTD5']}
NTD6: damp_c + h2o_c --> dad_2_c + pi_c {'original_bigg_ids': ['NTD6']}
NTD7: amp_c + h2o_c --> adn_c + pi_c {'original_bigg_ids': ['NTD7']}
NTD7e: amp_e + h2o_e --> adn_e + pi_e {'original_bigg_ids': ['NTD7e']}
NTD8: dgmp_c + h2o_c --> dgsn_c + pi_c {'original_bigg_ids': ['NTD8']}
NTD9: gmp_c + h2o_c --> gsn_c + pi_c {'original_bigg_ids': ['NTD9']}
NTD9e: gmp_e + h2o_e --> gsn_e + pi_e {'original_bigg_ids': ['NTD9e']}
NTPP1: dgtp_c + h2o_c --> dgmp_c + h_c + ppi_c {'original_bigg_ids': ['DGTD']}
NTPP10: ditp_c + h2o_c --> dimp_c + h_c + ppi_c {'original_bigg_ids': ['NTPP10']}
NTPP11: h2o_c + xtp_c --> h_c + ppi_c + xmp_c {'original_bigg_ids': ['NTPP11']}
NTPP2: gtp_c + h2o_c --> gmp_c + h_c + ppi_c {'original_bigg_ids': ['NTDP']}
NTPP7: dttp_c + h2o_c --> dtmp_c + h_c + ppi_c {'original_bigg_ids': ['NTPP7']}
NTPP8: h2o_c + utp_c --> h_c + ppi_c + ump_c {'original_bigg_ids': ['NTPP8']}
NTPP9: h2o_c + itp_c --> h_c + imp_c + ppi_c {'original_bigg_ids': ['NTPP9']}
NTRIR2x: 5.0 h_c + 3.0 nadh_c + no2_c --> 2.0 h2o_c + 3.0 nad_c + nh4_c {'original_bigg_ids': ['NTRIR2x']}
NTRLASE: 2.0 h2o_c + ind3acnl_c --> ind3ac_c + nh4_c {'original_bigg_ids': ['NTRLASE']}
NTRLASE2: aprop_c + 2.0 h2o_c --> ala__L_c + nh4_c {'original_bigg_ids': ['NTRLASE2']}
NTRLASE3: acybut_c + 2.0 h2o_c --> glu__L_c + nh4_c {'original_bigg_ids': ['NTRLASE3']}
Na1ATPase: atp_c + h2o_c + h_e + na1_c --> adp_c + 2.0 h_c + na1_e + pi_c {'original_bigg_ids': ['NaKt']}
O2t: o2_e <=> o2_c {'original_bigg_ids': ['O2t']}
O2ter: o2_c <=> o2_r {'original_bigg_ids': ['O2ter']}
O2tm: o2_c <=> o2_m {'original_bigg_ids': ['O2tm']}
O2tn: o2_c <=> o2_n {'original_bigg_ids': ['O2tn']}
O2tp: o2_c <=> o2_x {'original_bigg_ids': ['O2tp']}
OAADC: h_c + oaa_c --> co2_c + pyr_c {'original_bigg_ids': ['OAADC']}
OAADCm: h_m + oaa_m --> co2_m + pyr_m {'original_bigg_ids': ['OAADC']}
OAAIPMtm: 3c3hmp_m + oaa_c --> 3c3hmp_c + oaa_m {'original_bigg_ids': ['OAAAKGtm']}
OAAtp: oaa_x <=> oaa_c {'original_bigg_ids': ['HMR_9680']}
OBDHm: 2obut_m + coa_m + nad_m --> co2_m + nadh_m + ppcoa_m {'original_bigg_ids': ['OBDHm']}
OCBT: cbp_c + orn_c <=> citr__L_c + h_c + pi_c {'original_bigg_ids': ['OCBT']}
OCDCAt: ocdca_e --> ocdca_c {'original_bigg_ids': ['OCDCAt']}
OCDCEAt: ocdcea_e --> ocdcea_c {'original_bigg_ids': ['OCDCEAt']}
OCDCYAt: ocdcya_e --> ocdcya_c {'original_bigg_ids': ['OCDCYAt']}
OCDYCACOAtr: ocdycacoa_c <=> ocdycacoa_r {'original_bigg_ids': ['ACCOAtr']}
OCTNLLm: atp_m + h_m + octa_m --> amp_m + octapb_m + ppi_m {'original_bigg_ids': ['OCTNLL']}
ODECOAtr: odecoa_c <=> odecoa_r {'original_bigg_ids': ['ACCOAtr']}
OIVD1m: 4mop_m + coa_m + nad_m --> co2_m + ivcoa_m + nadh_m {'original_bigg_ids': ['OIVD1m']}
OIVD2m: 3mob_m + coa_m + nad_m --> co2_m + ibcoa_m + nadh_m {'original_bigg_ids': ['OIVD2m']}
OIVD3m: 3mop_m + coa_m + nad_m --> 2mbcoa_m + co2_m + nadh_m {'original_bigg_ids': ['OIVD3m']}
OMCDC: 3c4mop_c + h_c --> 4mop_c + co2_c {'original_bigg_ids': ['OMCDC']}
OMCDCm: 3c4mop_m + h_m --> 4mop_m + co2_m {'original_bigg_ids': ['OMCDCm']}
OMPDC: h_c + orot5p_c --> co2_c + ump_c {'original_bigg_ids': ['OMPDC']}
ORNARGtm: arg__L_c + orn_m --> arg__L_m + orn_c {'original_bigg_ids': ['ORNt3m']}
ORNCD: orn_c --> nh4_c + pro__L_c {'original_bigg_ids': ['ORNCD']}
ORNDC: h_c + orn_c --> co2_c + ptrc_c {'original_bigg_ids': ['ORNDC']}
ORNLYStm: lys__L_c + orn_m --> lys__L_m + orn_c {'original_bigg_ids': ['ORNt3m']}
ORNTA: akg_c + orn_c --> glu5sa_c + glu__L_c {'original_bigg_ids': ['ORNTA']}
ORNTAC: acorn_c + glu__L_c <=> acglu_c + orn_c {'original_bigg_ids': ['ORNTAC']}
ORNTACim: acorn_m + glu__L_m --> acglu_m + orn_m {'original_bigg_ids': ['ORNTACim']}
ORNTArm: akg_m + orn_m <=> glu5sa_m + glu__L_m {'original_bigg_ids': ['ORNTArm']}
ORNt2r: h_e + orn_e <=> h_c + orn_c {'original_bigg_ids': ['ORNt2r']}
ORNt3m: h_c + orn_m --> h_m + orn_c {'original_bigg_ids': ['ORNt3m']}
ORPT: orot5p_c + ppi_c <=> orot_c + prpp_c {'original_bigg_ids': ['ORPT']}
OXAHCOtex: 2.0 hco3_c + oxa_e --> 2.0 hco3_e + oxa_c {'original_bigg_ids': ['OXAHCOtex']}
OXOAEL: 5odhf2a_c + h2o_c --> 3oxoadp_c + h_c {'original_bigg_ids': ['OXOAEL']}
P45017A1r: h_r + nadph_r + o2_r + prgnlone_r --> 17ahprgnlone_r + h2o_r + nadp_r {'original_bigg_ids': ['P45017A1r']}
P45017A2r: 17ahprgnlone_r + h_r + nadph_r + 0.5 o2_r --> acald_r + dhea_r + h2o_r + nadp_r {'original_bigg_ids': ['P45017A2r']}
P45017A3r: h_r + nadph_r + o2_r + prgstrn_r --> 17ahprgstrn_r + h2o_r + nadp_r {'original_bigg_ids': ['P45017A3r']}
P45017A4r: 17ahprgstrn_r + h_r + nadph_r + 0.5 o2_r --> acald_r + andrstndn_r + h2o_r + nadp_r {'original_bigg_ids': ['P45017A4r']}
P5CR: 1pyr5c_c + 2.0 h_c + nadph_c --> nadp_c + pro__L_c {'original_bigg_ids': ['P5CR']}
P5CRx: 1pyr5c_c + 2.0 h_c + nadh_c --> nad_c + pro__L_c {'original_bigg_ids': ['P5CRx']}
PACCOAL: atp_c + coa_c + pac_c --> amp_c + phaccoa_c + ppi_c {'original_bigg_ids': ['PACCOAL']}
PACCOALp: atp_x + coa_x + pac_x --> amp_x + phaccoa_x + ppi_x {'original_bigg_ids': ['PACCOALm']}
PAIL1819Z160t: atp_c + h2o_c + pail1819Z160_c --> adp_c + h_c + pail1819Z160_e + pi_c {'original_bigg_ids': ['PAIL1819Z160t']}
PAL: phe__L_c --> cinnm_c + nh4_c {'original_bigg_ids': ['HISD']}
PANTS: ala_B_c + atp_c + pant__R_c --> amp_c + h_c + pnto__R_c + ppi_c {'original_bigg_ids': ['PANTS']}
PAPSR: paps_c + trdrd_c --> 2.0 h_c + pap_c + so3_c + trdox_c {'original_bigg_ids': ['PAPSR']}
PAPStg: paps_c <=> paps_g {'original_bigg_ids': ['PAPStg']}
PAterm_RT: pa_RT_r <=> pa_RT_m {'original_bigg_ids': ['GLYCtm']}
PC: atp_c + hco3_c + pyr_c --> adp_c + h_c + oaa_c + pi_c {'original_bigg_ids': ['PC']}
PCADYOX: 34dhbz_c + o2_c --> CCbuttc_c + 2.0 h_c {'original_bigg_ids': ['PCADYOX']}
PCDAGATer_RT: 12dgr_RT_r + pc_RT_r --> 1agpc_RT_r + triglyc_RT_r {'original_bigg_ids': ['PCDAGAT']}
PCLAD: cmusa_c + h_c --> am6sa_c + co2_c {'original_bigg_ids': ['PCLAD']}
PCRNtc: pcrn_x --> pcrn_c {'original_bigg_ids': ['PCRNtc']}
PCRNtm: pcrn_c <=> pcrn_m {'original_bigg_ids': ['PCRNtm']}
PDE1: camp_c + h2o_c --> amp_c + h_c {'original_bigg_ids': ['PDE1']}
PDE2: 35cdamp_c + h2o_c --> damp_c + h_c {'original_bigg_ids': ['PDE2']}
PDE3: 35cimp_c + h2o_c --> h_c + imp_c {'original_bigg_ids': ['PDE3']}
PDE4: 35cgmp_c + h2o_c --> gmp_c + h_c {'original_bigg_ids': ['PDE4']}
PDE5: 35ccmp_c + h2o_c --> cmp_c + h_c {'original_bigg_ids': ['PDE5']}
PDHm: coa_m + nad_m + pyr_m --> accoa_m + co2_m + nadh_m {'original_bigg_ids': ['PDHm']}
PDX5POi: o2_c + pdx5p_c --> h2o2_c + pydx5p_c {'original_bigg_ids': ['PDX5POi']}
PDXPP: h2o_c + pdx5p_c --> pi_c + pydxn_c {'original_bigg_ids': ['PDXPP']}
PE1801819Zt: atp_c + h2o_c + pe1801819Z_c --> adp_c + h_c + pe1801819Z_e + pi_c {'original_bigg_ids': ['PE1801819Zt']}
PE1801829Z12Zt: atp_c + h2o_c + pe1801829Z12Z_c --> adp_c + h_c + pe1801829Z12Z_e + pi_c {'original_bigg_ids': ['PE1801829Z12Zt']}
PE1819Z1819Zt: atp_c + h2o_c + pe1819Z1819Z_c --> adp_c + h_c + pe1819Z1819Z_e + pi_c {'original_bigg_ids': ['PE1819Z1819Zt']}
PE1819Z1829Z12Zt: atp_c + h2o_c + pe1819Z1829Z12Z_c --> adp_c + h_c + pe1819Z1829Z12Z_e + pi_c {'original_bigg_ids': ['PE1819Z1829Z12Zt']}
PEAMNO: h2o_c + o2_c + peamn_c --> h2o2_c + nh4_c + pacald_c {'original_bigg_ids': ['PEAMNO']}
PEPAT: accoa_c + pepd_c --> apep_c + coa_c + h_c {'original_bigg_ids': ['PEPAT']}
PEPDt3: h_e + pepd_e --> h_c + pepd_c {'original_bigg_ids': ['PEPDt3']}
PEPPe: h2o_e + pep_e --> pi_e + pyr_e {'original_bigg_ids': ['G2PP']}
PETOHMer_RT: amet_r + 0.01 pe_RT_r --> ahcys_r + h_r + 0.01 ptdmeeta_RT_r {'original_bigg_ids': ['PETOHM_SC']}
PEterm_RT: pe_RT_r <=> pe_RT_m {'original_bigg_ids': ['GLYCtm']}
PFK: atp_c + f6p_c --> adp_c + fdp_c + h_c {'original_bigg_ids': ['PFK']}
PFK_2: atp_c + tag6p__D_c --> adp_c + h_c + tagdp__D_c {'original_bigg_ids': ['PFK_2']}
PFK_3: atp_c + s7p_c --> adp_c + h_c + s17bp_c {'original_bigg_ids': ['PFK_3']}
PG1819Z160t: atp_c + h2o_c + pg1819Z160_c --> adp_c + h_c + pg1819Z160_e + pi_c {'original_bigg_ids': ['PG1819Z160t']}
PGAMT: gam1p_c <=> gam6p_c {'original_bigg_ids': ['PGAMT']}
PGCD: 3pg_c + nad_c --> 3php_c + h_c + nadh_c {'original_bigg_ids': ['PGCD']}
PGI: g6p_c <=> f6p_c {'original_bigg_ids': ['PGI']}
PGK: 3pg_c + atp_c <=> 13dpg_c + adp_c {'original_bigg_ids': ['PGK']}
PGL: 6pgl_c + h2o_c --> 6pgc_c + h_c {'original_bigg_ids': ['PGL']}
PGLp: 6pgl_x + h2o_x --> 6pgc_x + h_x {'original_bigg_ids': ['PGL']}
PGM: 2pg_c <=> 3pg_c {'original_bigg_ids': ['PGM']}
PGMT: g1p_c <=> g6p_c {'original_bigg_ids': ['PGMT']}
PGP1819Z160t: atp_c + h2o_c + pgp1819Z160_c --> adp_c + h_c + pgp1819Z160_e + pi_c {'original_bigg_ids': ['PGP1819Z160t']}
PGPPAm_RT: h2o_m + 0.01 pgp_RT_m --> 0.01 pg_RT_m + pi_m {'original_bigg_ids': ['PGPPAm_SC']}
PHCHGSm: 1p3h5c_m + h2o_m + h_m <=> 4hglusa_m {'original_bigg_ids': ['PHCHGSm']}
PHDA: 5phdt_c + h2o_c --> 5phua_c {'original_bigg_ids': ['PHDA']}
PHETA1: akg_c + phe__L_c <=> glu__L_c + phpyr_c {'original_bigg_ids': ['PHETA1']}
PHETRS: atp_c + phe__L_c + trnaphe_c --> amp_c + phetrna_c + ppi_c {'original_bigg_ids': ['PHETRS']}
PHETRSm: atp_m + phe__L_m + trnaphe_m --> amp_m + phetrna_m + ppi_m {'original_bigg_ids': ['PHETRSm']}
PHEt2r: h_e + phe__L_e <=> h_c + phe__L_c {'original_bigg_ids': ['PHEt2r']}
PHYCBOXL: h_c + phe__L_c --> co2_c + peamn_c {'original_bigg_ids': ['PHYCBOXL']}
PHYTSe: 6.0 h2o_e + minohp_e --> inost_e + 6.0 pi_e {'original_bigg_ids': ['PHYTSpp']}
PI35BP5Per_RT: h2o_r + 0.01 ptd135bp_RT_r --> pi_r + 0.01 ptd3ino_RT_r {'original_bigg_ids': ['PI35BP5P_SC']}
PI3P5Kn_RT: atp_n + 0.01 ptd3ino_RT_n --> adp_n + h_n + 0.01 ptd135bp_RT_n {'original_bigg_ids': ['PI3P5K_SC']}
PI45BP5Per_RT: h2o_r + 0.01 ptd145bp_RT_r --> pi_r + 0.01 ptd4ino_RT_r {'original_bigg_ids': ['PI45BP5P_SC']}
PI45BP5Pn_RT: h2o_n + 0.01 ptd145bp_RT_n --> pi_n + 0.01 ptd4ino_RT_n {'original_bigg_ids': ['PI45BP5P_SC']}
PI45BPPn_RT: h2o_n + 0.01 ptd145bp_RT_n --> 0.01 12dgr_RT_n + h_n + mi145p_n {'original_bigg_ids': ['PI45BPP_SC']}
PI4P5Kn_RT: atp_n + 0.01 ptd4ino_RT_n --> adp_n + h_n + 0.01 ptd145bp_RT_n {'original_bigg_ids': ['PI4P5K_SC']}
PIN3Kn_RT: atp_n + 0.01 ptd1ino_RT_n --> adp_n + h_n + 0.01 ptd3ino_RT_n {'original_bigg_ids': ['PIN3K_SC']}
PIN3Per_RT: h2o_r + 0.01 ptd3ino_RT_r --> pi_r + 0.01 ptd1ino_RT_r {'original_bigg_ids': ['PI35BP5P_SC']}
PIN3Pn_RT: h2o_n + 0.01 ptd3ino_RT_n --> pi_n + 0.01 ptd1ino_RT_n {'original_bigg_ids': ['PI45BP5P_SC']}
PIN4Ker_RT: atp_r + 0.01 ptd1ino_RT_r --> adp_r + h_r + 0.01 ptd4ino_RT_r {'original_bigg_ids': ['PIN4K_SC']}
PIN4Kn_RT: atp_n + 0.01 ptd1ino_RT_n --> adp_n + h_n + 0.01 ptd4ino_RT_n {'original_bigg_ids': ['PIN4Kn_SC']}
PIN4Per_RT: h2o_r + 0.01 ptd4ino_RT_r --> pi_r + 0.01 ptd1ino_RT_r {'original_bigg_ids': ['PI45BP5P_SC']}
PINOSer_RT: 0.01 cdpdag_RT_r + inost_r --> cmp_r + h_r + 0.01 ptd1ino_RT_r {'original_bigg_ids': ['PINOS_SC']}
PIt2m: h_c + pi_c --> h_m + pi_m {'original_bigg_ids': ['PIt2m']}
PIt2r: h_e + pi_e <=> h_c + pi_c {'original_bigg_ids': ['PIt2r']}
PIt9: 2.0 na1_e + pi_e <=> 2.0 na1_c + pi_c {'original_bigg_ids': ['PIt9']}
PIter: pi_r <=> pi_c {'original_bigg_ids': ['PIter']}
PItn: pi_n <=> pi_c {'original_bigg_ids': ['PItn']}
PItx: pi_c <=> pi_x {'original_bigg_ids': ['PItx']}
PLBP1Ie_RT: h2o_e + 0.005 ptd1ino_RT_e --> 0.01 arach_e + 0.5 g3pi_e + h_e + 0.09 hdca_e + 0.02 hpdca_e + 0.1 lnlncg_e + 0.07 ocdca_e + 0.32 ocdcea_e + 0.37 ocdcya_e + 0.02 ttc_e {'original_bigg_ids': ['PLBP1I_SCe']}
PLBPC_RT: h2o_c + 0.005 pc_RT_r --> 0.01 arach_c + 0.5 g3pc_c + h_c + 0.09 hdca_c + 0.02 hpdca_c + 0.1 lnlncg_c + 0.07 ocdca_c + 0.32 ocdcea_c + 0.37 ocdcya_c + 0.02 ttc_c {'original_bigg_ids': ['PLBPC_SC']}
PLBPCe_RT: h2o_e + 0.005 pc_RT_e --> 0.01 arach_e + 0.5 g3pc_e + h_e + 0.09 hdca_e + 0.02 hpdca_e + 0.1 lnlncg_e + 0.07 ocdca_e + 0.32 ocdcea_e + 0.37 ocdcya_e + 0.02 ttc_e {'original_bigg_ids': ['PLBPC_SCe']}
PLBPEe_RT: h2o_e + 0.005 pe_RT_e --> 0.01 arach_e + 0.5 g3pe_e + h_e + 0.09 hdca_e + 0.02 hpdca_e + 0.1 lnlncg_e + 0.07 ocdca_e + 0.32 ocdcea_e + 0.37 ocdcya_e + 0.02 ttc_e {'original_bigg_ids': ['PLBPC_SCe']}
PLDn_RT: h2o_n + 0.01 pc_RT_n --> chol_n + h_n + 0.01 pa_RT_n {'original_bigg_ids': ['PLD_SC']}
PMANM: man1p_c <=> man6p_c {'original_bigg_ids': ['PMANM']}
PMCA: atp_c + ca2_c + h2o_c + h_e --> adp_c + ca2_e + 2.0 h_c + pi_c {'original_bigg_ids': ['CAATPS']}
PMDPHT: 5aprbu_c + h2o_c --> 4r5au_c + pi_c {'original_bigg_ids': ['PMDPHT']}
PMETMer_RT: amet_r + 0.01 ptd2meeta_RT_r --> ahcys_r + h_r + 0.01 pc_RT_r {'original_bigg_ids': ['PMETM_SC']}
PMEVK: 5pmev_c + atp_c --> 5dpmev_c + adp_c {'original_bigg_ids': ['PMEVK']}
PMI12346PSn: atp_n + minohp_n --> adp_n + ppmi12346p_n {'original_bigg_ids': ['PMI12346PS']}
PMPK: 4ampm_c + atp_c --> 2mahmp_c + adp_c {'original_bigg_ids': ['PMPK']}
PMTCOAtr: pmtcoa_c <=> pmtcoa_r {'original_bigg_ids': ['ACCOAtr']}
PNSPA: gmplys_c + h2o_c --> gmp_c + nalme_c {'original_bigg_ids': ['PNSPA']}
PNTK: atp_c + pnto__R_c --> 4ppan_c + adp_c + h_c {'original_bigg_ids': ['PNTK']}
PNTOt2: h_e + pnto__R_e <=> h_c + pnto__R_c {'original_bigg_ids': ['PNTOt2']}
POLYAO: N1aspmd_c + h2o_c + o2_c --> 3aap_c + h2o2_c + ptrc_c {'original_bigg_ids': ['POLYAO']}
POLYAO2: N1sprm_c + h2o_c + o2_c --> 3aap_c + h2o2_c + spmd_c {'original_bigg_ids': ['POLYAO2']}
POLYAO3: h2o_c + o2_c + sprm_c --> bamppald_c + h2o2_c + spmd_c {'original_bigg_ids': ['POLYAO3']}
PPA: h2o_c + ppi_c --> h_c + 2.0 pi_c {'original_bigg_ids': ['PPA']}
PPA2: h2o_c + pppi_c --> h_c + pi_c + ppi_c {'original_bigg_ids': ['PPA2']}
PPAtr: ppa_e --> ppa_c {'original_bigg_ids': ['PPAt']}
PPBNGS: 2.0 5aop_c --> 2.0 h2o_c + h_c + ppbng_c {'original_bigg_ids': ['PPBNGS']}
PPCDC: 4ppcys_c + h_c --> co2_c + pan4p_c {'original_bigg_ids': ['PPCDC']}
PPCK: atp_c + oaa_c --> adp_c + co2_c + pep_c {'original_bigg_ids': ['PPCK']}
PPCOAOm: fad_m + ppcoa_m --> fadh2_m + prpncoa_m {'original_bigg_ids': ['PPCOAOm']}
PPDOy: h_c + lald__D_c + nadph_c --> 12ppd__R_c + nadp_c {'original_bigg_ids': ['PPDOy']}
PPGPPDP: h2o_c + ppgpp_c --> gdp_c + ppi_c {'original_bigg_ids': ['PPGPPDP']}
PPIt2r: h_e + ppi_e <=> h_c + ppi_c {'original_bigg_ids': ['PIt2r']}
PPItr: ppi_c <=> ppi_r {'original_bigg_ids': ['PPItr']}
PPItx: ppi_c <=> ppi_x {'original_bigg_ids': ['PPItx']}
PPM: r1p_c <=> r5p_c {'original_bigg_ids': ['PPM']}
PPNCL3: 4ppan_c + atp_c + cys__L_c --> 4ppcys_c + amp_c + h_c + ppi_c {'original_bigg_ids': ['PPCL']}
PPND2: nadp_c + pphn_c --> 34hpp_c + co2_c + nadph_c {'original_bigg_ids': ['PPND2']}
PPNDH: h_c + pphn_c --> co2_c + h2o_c + phpyr_c {'original_bigg_ids': ['PPNDH']}
PPPG9tm: pppg9_c <=> pppg9_m {'original_bigg_ids': ['PPPG9tm']}
PPPGOm: 3.0 o2_m + 2.0 pppg9_m --> 6.0 h2o_m + 2.0 ppp9_m {'original_bigg_ids': ['PPPGOm']}
PPPIt2r: h_e + pppi_e <=> h_c + pppi_c {'original_bigg_ids': ['PIt2r']}
PPYRDC: h_c + phpyr_c --> co2_c + pacald_c {'original_bigg_ids': ['PPYRDC']}
PRAGSr: atp_c + gly_c + pram_c <=> adp_c + gar_c + h_c + pi_c {'original_bigg_ids': ['PRAGSr']}
PRAIS: atp_c + fpram_c --> adp_c + air_c + 2.0 h_c + pi_c {'original_bigg_ids': ['PRAIS']}
PRAIi: pran_c --> 2cpr5p_c {'original_bigg_ids': ['PRAIi']}
PRAMPC: h2o_c + prbamp_c --> prfp_c {'original_bigg_ids': ['PRAMPC']}
PRASCSi: 5aizc_c + asp__L_c + atp_c --> 25aics_c + adp_c + h_c + pi_c {'original_bigg_ids': ['PRASCSi']}
PRATPP: h2o_c + prbatp_c --> h_c + ppi_c + prbamp_c {'original_bigg_ids': ['PRATPP']}
PRFGS: atp_c + fgam_c + gln__L_c + h2o_c --> adp_c + fpram_c + glu__L_c + h_c + pi_c {'original_bigg_ids': ['PRFGS']}
PRMICI: prfp_c <=> prlp_c {'original_bigg_ids': ['PRMICI']}
PROD_u9m: pro__L_m + q9_m --> 1pyr5c_m + h_m + q9h2_m {'original_bigg_ids': ['PROD2m']}
PRODt2r: h_e + pro__D_e <=> h_c + pro__D_c {'original_bigg_ids': ['PRODt2r']}
PROSTGE1t3: atp_c + h2o_c + prostge1_c --> adp_c + h_c + pi_c + prostge1_e {'original_bigg_ids': ['PROSTGE1t3']}
PROSTGE2t3: atp_c + h2o_c + prostge2_c --> adp_c + h_c + pi_c + prostge2_e {'original_bigg_ids': ['PROSTGE2t3']}
PROTRS: atp_c + pro__L_c + trnapro_c --> amp_c + ppi_c + protrna_c {'original_bigg_ids': ['PROTRS']}
PROt2r: h_e + pro__L_e <=> h_c + pro__L_c {'original_bigg_ids': ['PROt2r']}
PRPNCOAHYDm: h2o_m + prpncoa_m <=> 3hpcoa_m {'original_bigg_ids': ['PRPNCOAHYDm']}
PRPPS: atp_c + r5p_c <=> amp_c + h_c + prpp_c {'original_bigg_ids': ['PRPPS']}
PSCVT: pep_c + skm3p_c <=> 3psme_c + pi_c {'original_bigg_ids': ['PSCVT']}
PSERDer_RT: h_r + 0.01 ps_RT_r --> co2_r + 0.01 pe_RT_r {'original_bigg_ids': ['PSERDm_SC']}
PSERDg_RT: h_g + 0.01 ps_RT_g --> co2_g + 0.01 pe_RT_g {'original_bigg_ids': ['PSERDg_SC']}
PSERDm_RT: h_m + 0.01 ps_RT_m --> co2_m + 0.01 pe_RT_m {'original_bigg_ids': ['PSERDm_SC']}
PSERDv_RT: h_v + 0.01 ps_RT_v --> co2_v + 0.01 pe_RT_v {'original_bigg_ids': ['PSERDv_SC']}
PSERSer_RT: 0.01 cdpdag_RT_r + ser__L_r --> cmp_r + h_r + 0.01 ps_RT_r {'original_bigg_ids': ['PSERS_SC']}
PSERSm_RT: 0.01 cdpdag_RT_m + ser__L_m --> cmp_m + h_m + 0.01 ps_RT_m {'original_bigg_ids': ['PSERSm_SC']}
PSERT: 3php_c + glu__L_c --> akg_c + pser__L_c {'original_bigg_ids': ['PSERT']}
PSERtr: pser__L_c <=> pser__L_e {'original_bigg_ids': ['PSERtr']}
PSPHPLer: psph1p_r --> 2hhxdal_r + ethamp_r {'original_bigg_ids': ['PSPHPL']}
PSURIK: atp_c + psuri_c --> adp_c + h_c + psd5p_c {'original_bigg_ids': ['PSURIK']}
PSURIP: h2o_c + psd5p_c --> pi_c + psuri_c {'original_bigg_ids': ['ALKP']}
PSterm_RT: ps_RT_r <=> ps_RT_m {'original_bigg_ids': ['GLYCtm']}
PTDCACRNCPT1: crn_c + ptdcacoa_c --> coa_c + ptdcacrn_c {'original_bigg_ids': ['PTDCACRNCPT1']}
PTDCACRNCPT2: coa_m + ptdcacrn_m --> crn_m + ptdcacoa_m {'original_bigg_ids': ['PTDCACRNCPT2']}
PTDCACRNt: ptdcacrn_c --> ptdcacrn_m {'original_bigg_ids': ['PTDCACRNt']}
PTE10x: dcacoa_x + h2o_x --> coa_x + dca_x + h_x {'original_bigg_ids': ['PTE10x']}
PTE11x: ddcacoa_x + h2o_x --> coa_x + ddca_x + h_x {'original_bigg_ids': ['PTE11x']}
PTE12x: h2o_x + ttccoa_x --> coa_x + h_x + ttc_x {'original_bigg_ids': ['PTE12x']}
PTE2x: h2o_x + pmtcoa_x --> coa_x + h_x + hdca_x {'original_bigg_ids': ['PTE2x']}
PTE7x: h2o_x + tdcoa_x --> coa_x + h_x + ttdca_x {'original_bigg_ids': ['PTE7x']}
PTE8x: h2o_x + stcoa_x --> coa_x + h_x + ocdca_x {'original_bigg_ids': ['PTE8x']}
PTE9x: h2o_x + occoa_x --> coa_x + h_x + octa_x {'original_bigg_ids': ['PTE9x']}
PTHPS: ahdt_c --> 6pthp_c + pppi_c {'original_bigg_ids': ['PTHPS']}
PTHPSn: ahdt_n --> 6pthp_n + pppi_n {'original_bigg_ids': ['PTHPSn']}
PTPATi: atp_c + h_c + pan4p_c --> dpcoa_c + ppi_c {'original_bigg_ids': ['PTPATi']}
PTRCAT1: accoa_c + ptrc_c --> aprut_c + coa_c + h_c {'original_bigg_ids': ['PTRCAT1']}
PTRCOX1: h2o_c + o2_c + ptrc_c --> 4abutn_c + h2o2_c + nh4_c {'original_bigg_ids': ['PTRCOX1']}
PTRCt3i: h_c + ptrc_e --> h_e + ptrc_c {'original_bigg_ids': ['PTRCt3i']}
PUNP1: adn_c + pi_c <=> ade_c + r1p_c {'original_bigg_ids': ['PUNP1']}
PUNP1m: adn_m + pi_m <=> ade_m + r1p_m {'original_bigg_ids': ['PUNP1m']}
PUNP2: dad_2_c + pi_c <=> 2dr1p_c + ade_c {'original_bigg_ids': ['PUNP2']}
PUNP3: gsn_c + pi_c <=> gua_c + r1p_c {'original_bigg_ids': ['PUNP3']}
PUNP3m: gsn_m + pi_m <=> gua_m + r1p_m {'original_bigg_ids': ['PUNP3m']}
PUNP4: dgsn_c + pi_c <=> 2dr1p_c + gua_c {'original_bigg_ids': ['PUNP4']}
PUNP5: ins_c + pi_c <=> hxan_c + r1p_c {'original_bigg_ids': ['PUNP5']}
PUNP6: din_c + pi_c <=> 2dr1p_c + hxan_c {'original_bigg_ids': ['PUNP6']}
PUNP7: pi_c + xtsn_c <=> r1p_c + xan_c {'original_bigg_ids': ['PUNP7']}
PYAM5PO: h2o_c + o2_c + pyam5p_c --> h2o2_c + nh4_c + pydx5p_c {'original_bigg_ids': ['PYAM5PO']}
PYDAMK: atp_c + pydam_c --> adp_c + h_c + pyam5p_c {'original_bigg_ids': ['PYDAMK']}
PYDX5Ptm: pydx5p_c <=> pydx5p_m {'original_bigg_ids': ['PYDX5Ptm']}
PYDXK: atp_c + pydx_c --> adp_c + h_c + pydx5p_c {'original_bigg_ids': ['PYDXK']}
PYDXNK: atp_c + pydxn_c --> adp_c + h_c + pdx5p_c {'original_bigg_ids': ['PYDXNK']}
PYDXNO: o2_c + pydxn_c <=> h2o2_c + pydx_c {'original_bigg_ids': ['PYDXNO']}
PYDXNtr: pydxn_e <=> pydxn_c {'original_bigg_ids': ['PYDXNtr']}
PYDXOR: h_c + nadph_c + pydx_c <=> nadp_c + pydxn_c {'original_bigg_ids': ['PYDXOR']}
PYDXPP: h2o_c + pydx5p_c --> pi_c + pydx_c {'original_bigg_ids': ['PYDXPP']}
PYDXS: g3p_c + gln__L_c + r5p_c --> glu__L_c + 3.0 h2o_c + h_c + pi_c + pydx5p_c {'original_bigg_ids': ['PYDXS']}
PYK: adp_c + h_c + pep_c --> atp_c + pyr_c {'original_bigg_ids': ['PYK']}
PYNP2r: pi_c + uri_c <=> r1p_c + ura_c {'original_bigg_ids': ['PYNP2r']}
PYRDC: h_c + pyr_c --> acald_c + co2_c {'original_bigg_ids': ['PYRDC']}
PYRt2: h_e + pyr_e --> h_c + pyr_c {'original_bigg_ids': ['PYRt2']}
PYRt2m: h_c + pyr_c <=> h_m + pyr_m {'original_bigg_ids': ['PYRt2m']}
QUILSYN: cmusa_c --> h2o_c + h_c + quln_c {'original_bigg_ids': ['QUILSYN']}
QUINDH: nad_c + quin_c --> 3dhq_c + 2.0 h_c + nadh_c {'original_bigg_ids': ['QUINDH']}
QUINt: quin_e <=> quin_c {'original_bigg_ids': ['QUIN2tex']}
R5PP: h2o_c + r5p_c --> pi_c + rib__D_c {'original_bigg_ids': ['R5PP']}
RBFK: atp_c + ribflv_c --> adp_c + fmn_c + h_c {'original_bigg_ids': ['RBFK']}
RBFKm: atp_m + ribflv_m --> adp_m + fmn_m + h_m {'original_bigg_ids': ['RBFKm']}
RBFSa: 4r5au_c + db4p_c --> dmlz_c + 2.0 h2o_c + pi_c {'original_bigg_ids': ['RBFSa']}
RBFSb: 2.0 dmlz_c --> 4r5au_c + ribflv_c {'original_bigg_ids': ['RBFSb']}
RBK: atp_c + rib__D_c --> adp_c + h_c + r5p_c {'original_bigg_ids': ['RBK']}
RBK_Dr: atp_c + rbl__D_c --> adp_c + h_c + ru5p__D_c {'original_bigg_ids': ['RBK_D']}
RBL_Dt: rbl__D_c <=> rbl__D_e {'original_bigg_ids': ['HMR_9185']}
RBTDG: nad_c + rbt_c <=> h_c + nadh_c + rbl__D_c {'original_bigg_ids': ['RBTDG']}
RBTt: rbt_c --> rbt_e {'original_bigg_ids': ['RBTt']}
RIBFLVt2: h_e + ribflv_e --> h_c + ribflv_c {'original_bigg_ids': ['RIBFLVt2']}
RIBt: rib__D_e <=> rib__D_c {'original_bigg_ids': ['RIBt']}
RNDR1: adp_c + trdrd_c --> dadp_c + h2o_c + trdox_c {'original_bigg_ids': ['RNDR1']}
RNDR1n: adp_n + trdrd_n --> dadp_n + h2o_n + trdox_n {'original_bigg_ids': ['RNDR1n']}
RNDR2: gdp_c + trdrd_c --> dgdp_c + h2o_c + trdox_c {'original_bigg_ids': ['RNDR2']}
RNDR2n: gdp_n + trdrd_n --> dgdp_n + h2o_n + trdox_n {'original_bigg_ids': ['RNDR2n']}
RNDR3: cdp_c + trdrd_c --> dcdp_c + h2o_c + trdox_c {'original_bigg_ids': ['RNDR3']}
RNDR3n: cdp_n + trdrd_n --> dcdp_n + h2o_n + trdox_n {'original_bigg_ids': ['RNDR3n']}
RNDR4: trdrd_c + udp_c --> dudp_c + h2o_c + trdox_c {'original_bigg_ids': ['RNDR4']}
RNDR4n: trdrd_n + udp_n --> dudp_n + h2o_n + trdox_n {'original_bigg_ids': ['RNDR4n']}
RNMK: atp_c + rnam_c --> adp_c + h_c + nmn_c {'original_bigg_ids': ['RNMK']}
RPE: ru5p__D_c <=> xu5p__D_c {'original_bigg_ids': ['RPE']}
RPI: r5p_c <=> ru5p__D_c {'original_bigg_ids': ['RPI']}
RU5PP: h2o_c + ru5p__D_c --> pi_c + rbl__D_c {'original_bigg_ids': ['RU5PP']}
S3HBTRHDm: akg_m + s3hb_m <=> acac_m + r2hglut_m {'original_bigg_ids': ['ARHGDx']}
SACCD1: L2aadp6sa_c + glu__L_c + h_c + nadph_c <=> h2o_c + nadp_c + saccrp__L_c {'original_bigg_ids': ['SACCD1']}
SACCD2: h2o_c + nad_c + saccrp__L_c <=> akg_c + h_c + lys__L_c + nadh_c {'original_bigg_ids': ['SACCD2']}
SACCD3m: akg_m + h_m + lys__L_m + nadph_m --> h2o_m + nadp_m + saccrp__L_m {'original_bigg_ids': ['SACCD3m']}
SADT: atp_c + h_c + so4_c --> aps_c + ppi_c {'original_bigg_ids': ['SADT']}
SALCNHe: h2o_e + salcn_e --> 2hxmp_e + glc__D_e {'original_bigg_ids': ['BG_MBDG_']}
SALMCOM: amet_c + nrpphr_c --> ahcys_c + h_c + normete__L_c {'original_bigg_ids': ['SALMCOM']}
SALMCOM2: adrnl_c + amet_c --> ahcys_c + h_c + mepi_c {'original_bigg_ids': ['SALMCOM2']}
SAM24MTer: amet_r + zymst_r --> ahcys_r + fecost_r + h_r {'original_bigg_ids': ['SAM24MT']}
SBP: h2o_c + s17bp_c --> pi_c + s7p_c {'original_bigg_ids': ['FBP26']}
SBPP1er: h2o_r + sph1p_r --> pi_r + sphgn_r {'original_bigg_ids': ['SBPP1er']}
SBPP2er: h2o_r + psph1p_r --> pi_r + psphings_r {'original_bigg_ids': ['SBPP2er']}
SBTD_D2: nad_c + sbt__D_c --> fru_c + h_c + nadh_c {'original_bigg_ids': ['SBTD_D2']}
SBTD_L: nad_c + sbt__L_c --> h_c + nadh_c + srb__L_c {'original_bigg_ids': ['SBTD_L']}
SBTR: glc__D_c + h_c + nadph_c --> nadp_c + sbt__D_c {'original_bigg_ids': ['SBTR']}
SBT_Dt: sbt__D_e <=> sbt__D_c {'original_bigg_ids': ['SBT_Dt']}
SCP21x: phytcoa_c <=> phytcoa_x {'original_bigg_ids': ['SCP21x']}
SCP22x: dmnoncoa_c <=> dmnoncoa_x {'original_bigg_ids': ['SCP22x']}
SCP2x: coa_x + dhocholoylcoa_x --> dgcholcoa_x + ppcoa_x {'original_bigg_ids': ['SCP2x']}
SCP3x: coa_x + dhcholestancoa_x + o2_x --> dcholcoa_x + h2o_x + ppcoa_x {'original_bigg_ids': ['SCP3x']}
SCPx: cholcoaone_x + coa_x --> cholcoa_x + ppcoa_x {'original_bigg_ids': ['SCPx']}
SDPDS: h2o_c + sl26da_c --> 26dap_LL_c + succ_c {'original_bigg_ids': ['SDPDS']}
SDPTA: akg_c + sl26da_c <=> glu__L_c + sl2a6o_c {'original_bigg_ids': ['SDPTA']}
SELADT: atp_c + h_c + sel_c --> adsel_c + ppi_c {'original_bigg_ids': ['SELADT']}
SERASr: atp_c + h_c + ser__L_c <=> ppi_c + seramp_c {'original_bigg_ids': ['SERASr']}
SERCA: atp_c + 2.0 ca2_c + h2o_c + 2.0 h_r --> adp_c + 2.0 ca2_r + 3.0 h_c + pi_c {'original_bigg_ids': ['CAATPS']}
SERDC: h_c + ser__L_c --> co2_c + etha_c {'original_bigg_ids': ['SERDC']}
SERD_L: ser__L_c --> nh4_c + pyr_c {'original_bigg_ids': ['SERD_L']}
SERHL: ser__L_c --> 2amac_c + h2o_c {'original_bigg_ids': ['SERHL']}
SERLYSNaex: lys__L_c + na1_e + ser__L_e --> lys__L_e + na1_c + ser__L_c {'original_bigg_ids': ['SERLYSNaex']}
SERTRS: atp_c + ser__L_c + trnaser_c --> amp_c + ppi_c + sertrna_c {'original_bigg_ids': ['SERTRS']}
SERTRSm: atp_m + ser__L_m + trnaser_m --> amp_m + ppi_m + sertrna_m {'original_bigg_ids': ['SERTRS']}
SER_Dt: ser__D_e --> ser__D_c {'original_bigg_ids': ['HMR_9191']}
SERt2r: h_e + ser__L_e <=> h_c + ser__L_c {'original_bigg_ids': ['SERt2r']}
SERter: ser__L_c <=> ser__L_r {'original_bigg_ids': ['Htr']}
SFGTHi: Sfglutth_c + h2o_c --> for_c + gthrd_c + h_c {'original_bigg_ids': ['SFGTHi']}
SHCHD2: dscl_c + nad_c --> h_c + nadh_c + scl_c {'original_bigg_ids': ['SHCHD2']}
SHCHF: fe2_c + scl_c --> 3.0 h_c + sheme_c {'original_bigg_ids': ['SHCHF']}
SHK3Dr: 3dhsk_c + h_c + nadph_c <=> nadp_c + skm_c {'original_bigg_ids': ['SHK3Dr']}
SHKK: atp_c + skm_c --> adp_c + h_c + skm3p_c {'original_bigg_ids': ['SHKK']}
SHSL1: cys__L_c + suchms_c --> cyst__L_c + h_c + succ_c {'original_bigg_ids': ['SHSL1']}
SLCBK1er: atp_r + sphgn_r --> adp_r + h_r + sph1p_r {'original_bigg_ids': ['SLCBK1']}
SLCBK2er: atp_r + psphings_r --> adp_r + h_r + psph1p_r {'original_bigg_ids': ['SLCBK2']}
SLFAT: adp_c + h_c + so4_c <=> aps_c + pi_c {'original_bigg_ids': ['SLFAT']}
SO3ti: so3_c --> so3_e {'original_bigg_ids': ['SO3ti']}
SO4CLtex2: cl_c + 2.0 so4_e --> cl_e + 2.0 so4_c {'original_bigg_ids': ['SO4CLtex2']}
SO4HCOtex: 2.0 hco3_c + so4_e --> 2.0 hco3_e + so4_c {'original_bigg_ids': ['SO4HCOtex']}
SO4OXAtex2: oxa_c + 2.0 so4_e --> oxa_e + 2.0 so4_c {'original_bigg_ids': ['SO4OXAtex2']}
SO4t2: h_e + so4_e <=> h_c + so4_c {'original_bigg_ids': ['SO4t']}
SO4ti: so4_e --> so4_c {'original_bigg_ids': ['SO4ti']}
SPHPLer: sph1p_r --> ethamp_r + hxdcal_r {'original_bigg_ids': ['SPHPL']}
SPMDAT1: accoa_c + spmd_c --> N1aspmd_c + coa_c + h_c {'original_bigg_ids': ['SPMDAT1']}
SPMDAT2: accoa_c + spmd_c --> coa_c + h_c + n8aspmd_c {'original_bigg_ids': ['SPMDAT2']}
SPMDt3i: h_c + spmd_e --> h_e + spmd_c {'original_bigg_ids': ['SPMDt3i']}
SPMDtmr: h_m + spmd_c <=> h_c + spmd_m {'original_bigg_ids': ['SPMDtmr']}
SPMS: ametam_c + ptrc_c --> 5mta_c + h_c + spmd_c {'original_bigg_ids': ['SPMS']}
SPODM: 2.0 h_c + 2.0 o2s_c --> h2o2_c + o2_c {'original_bigg_ids': ['SPODM']}
SPODMm: 2.0 h_m + 2.0 o2s_m --> h2o2_m + o2_m {'original_bigg_ids': ['SPODMm']}
SPRMS: ametam_c + spmd_c --> 5mta_c + h_c + sprm_c {'original_bigg_ids': ['SPRMS']}
SPRMt2i: h_c + sprm_e --> h_e + sprm_c {'original_bigg_ids': ['SPRMt2i']}
SQLEr: h_r + nadph_r + o2_r + sql_r --> Ssq23epx_r + h2o_r + nadp_r {'original_bigg_ids': ['SQLEr']}
SQLErx: h_r + nadh_r + o2_r + sql_r --> Ssq23epx_r + h2o_r + nad_r {'original_bigg_ids': ['SQLErx']}
SQLSr: 2.0 frdp_r + h_r + nadph_r --> nadp_r + 2.0 ppi_r + sql_r {'original_bigg_ids': ['SQLSr']}
SR5AR2r: andrstndn_r + h_r + nadph_r --> andrstandn_r + nadp_r {'original_bigg_ids': ['SR5AR2r']}
SR5ARr: h_r + nadph_r + tststerone_r --> 5adtststerone_r + nadp_r {'original_bigg_ids': ['SR5ARr']}
SRB_Lt: srb__L_e <=> srb__L_c {'original_bigg_ids': ['SRB_Lt']}
SRC_8aonn_m:  --> 8aonn_m {'original_bigg_ids': ['DM_aacald_c']}
SRC_lipoate_m:  --> lipoate_m {'original_bigg_ids': ['DM_aacald_c']}
SRC_trnaala_c:  --> trnaala_c {'original_bigg_ids': ['DM_aacald_c']}
SRC_trnaarg_c:  --> trnaarg_c {'original_bigg_ids': ['DM_aacald_c']}
SRC_trnaasn_c:  --> trnaasn_c {'original_bigg_ids': ['DM_aacald_c']}
SRC_trnaasp_c:  --> trnaasp_c {'original_bigg_ids': ['DM_aacald_c']}
SRC_trnacys_c:  --> trnacys_c {'original_bigg_ids': ['DM_aacald_c']}
SRC_trnagln_c:  --> trnagln_c {'original_bigg_ids': ['DM_aacald_c']}
SRC_trnaglu_c:  --> trnaglu_c {'original_bigg_ids': ['DM_aacald_c']}
SRC_trnagly_c:  --> trnagly_c {'original_bigg_ids': ['DM_aacald_c']}
SRC_trnahis_c:  --> trnahis_c {'original_bigg_ids': ['DM_aacald_c']}
SRC_trnaile_c:  --> trnaile_c {'original_bigg_ids': ['DM_aacald_c']}
SRC_trnaleu_c:  --> trnaleu_c {'original_bigg_ids': ['DM_aacald_c']}
SRC_trnalys_c:  --> trnalys_c {'original_bigg_ids': ['DM_aacald_c']}
SRC_trnamet_c:  --> trnamet_c {'original_bigg_ids': ['DM_aacald_c']}
SRC_trnaphe_c:  --> trnaphe_c {'original_bigg_ids': ['DM_aacald_c']}
SRC_trnapro_c:  --> trnapro_c {'original_bigg_ids': ['DM_aacald_c']}
SRC_trnaser_c:  --> trnaser_c {'original_bigg_ids': ['DM_aacald_c']}
SRC_trnathr_c:  --> trnathr_c {'original_bigg_ids': ['DM_aacald_c']}
SRC_trnatrp_c:  --> trnatrp_c {'original_bigg_ids': ['DM_aacald_c']}
SRC_trnatyr_c:  --> trnatyr_c {'original_bigg_ids': ['DM_aacald_c']}
SRC_trnaval_c:  --> trnaval_c {'original_bigg_ids': ['DM_aacald_c']}
SRTN23OX: o2_c + srtn_c --> f5hoxkyn_c {'original_bigg_ids': ['SRTN23OX']}
SSALy: h2o_c + nadp_c + sucsal_c --> 2.0 h_c + nadph_c + succ_c {'original_bigg_ids': ['SSALy']}
STCOAtr: stcoa_c <=> stcoa_r {'original_bigg_ids': ['ACCOAtr']}
SUCCtm: pi_m + succ_c --> pi_c + succ_m {'original_bigg_ids': ['SUCCtm']}
SUCCtp: succ_c <=> succ_x {'original_bigg_ids': ['SUCCtp']}
SUCD2_u9m: q9_m + succ_m --> fum_m + q9h2_m {'original_bigg_ids': ['SUCD2_u6m']}
SUCFUMtm: fum_m + succ_c --> fum_c + succ_m {'original_bigg_ids': ['SUCFUMtm']}
SUCOASm: atp_m + coa_m + succ_m <-- adp_m + pi_m + succoa_m {'original_bigg_ids': ['SUCOASm']}
SUCRe: h2o_e + sucr_e --> fru_e + glc__D_e {'original_bigg_ids': ['SUCRe']}
SULO: h2o_c + o2_c + so3_c --> h2o2_c + so4_c {'original_bigg_ids': ['SULO']}
SULOm: h2o_m + o2_m + so3_m --> h2o2_m + so4_m {'original_bigg_ids': ['SULOm']}
SULR: 5.0 h_c + 3.0 nadph_c + so3_c --> 3.0 h2o_c + h2s_c + 3.0 nadp_c {'original_bigg_ids': ['SULR']}
T4HCINNMt: T4hcinnm_e <=> T4hcinnm_c {'original_bigg_ids': ['T4HCINNMtex']}
T4HCINNMtp: T4hcinnm_c --> T4hcinnm_x {'original_bigg_ids': ['FA240tp']}
TAGL_RT: h2o_c + 0.01 triglyc_RT_d --> 0.01 12dgr_RT_d + h_c + 0.23 hdca_c + 0.01 hdcea_c + 0.01 lnlncg_c + 0.12 ocdca_c + 0.48 ocdcea_c + 0.14 ocdcya_c + 0.01 ttc_c {'original_bigg_ids': ['TAGL_SC']}
TAGtrd: 0.03 hdcoa_r + 0.48 odecoa_r + 0.42 pmtcoa_r + 0.15 stcoa_r + 0.01 triglyc_RT_r --> 0.03 arachcoa_r + 0.06 hpdcacoa_r + 0.27 lnlncgcoa_r + 0.69 ocdycacoa_r + 0.01 triglyc_RT_d + 0.03 ttccoa_r {'original_bigg_ids': ['FRDPtcr']}
TAL: tyr__L_c --> T4hcinnm_c + nh4_c {'original_bigg_ids': ['HISD']}
TALA: g3p_c + s7p_c <=> e4p_c + f6p_c {'original_bigg_ids': ['TALA']}
TAUDO: akg_c + o2_c + taur_c --> aacald_c + co2_c + h_c + so3_c + succ_c {'original_bigg_ids': ['TAUDO']}
TAURt: taur_c <=> taur_e {'original_bigg_ids': ['TAURCHAe']}
TCHOLAabcv: atp_v + h2o_v + tchola_c --> adp_v + h_v + pi_v + tchola_v {'original_bigg_ids': ['TCHOLAabcv']}
TCHOLAt3: atp_c + h2o_c + tchola_c --> adp_c + h_c + pi_c + tchola_e {'original_bigg_ids': ['TCHOLAt3']}
TDCOAtr: tdcoa_c <=> tdcoa_r {'original_bigg_ids': ['ACCOAtr']}
TDP: h2o_c + thmpp_c --> h_c + pi_c + thmmp_c {'original_bigg_ids': ['TDP']}
TDPDRR: dtdp4d6dm_c + h_c + nadph_c --> dtdprmn_c + nadp_c {'original_bigg_ids': ['TDPDRR']}
TEGAt: tega_e <=> tega_c {'original_bigg_ids': ['TEGAt']}
TETTET6CPT1: crn_c + tettet6coa_c --> coa_c + tettet6crn_c {'original_bigg_ids': ['TETTET6CPT1']}
TETTET6CPT2: coa_m + tettet6crn_m --> crn_m + tettet6coa_m {'original_bigg_ids': ['TETTET6CPT2']}
TETTET6CRNt: tettet6crn_c --> tettet6crn_m {'original_bigg_ids': ['TETTET6CRNt']}
TGUAPRT: prpp_c + tgua_c --> 6tgsnmp_c + ppi_c {'original_bigg_ids': ['TGUAPRT']}
TGUAt: tgua_e <=> tgua_c {'original_bigg_ids': ['TGUAt']}
THBPT4ACAMDASE: thbpt4acam_c --> dhbpt_c + h2o_c {'original_bigg_ids': ['THBPT4ACAMDASE']}
THFGLUS: atp_c + glu__L_c + thf_c <=> adp_c + h_c + pi_c + thfglu_c {'original_bigg_ids': ['THFGLUS']}
THIORDXi: h2o2_c + trdrd_c --> 2.0 h2o_c + trdox_c {'original_bigg_ids': ['THIORDXi']}
THIORDXm: h2o2_m + trdrd_m <=> 2.0 h2o_m + trdox_m {'original_bigg_ids': ['THIORDXm']}
THIORDXni: h2o2_n + trdrd_n --> 2.0 h2o_n + trdox_n {'original_bigg_ids': ['THIORDXni']}
THIORDXp: h2o2_x + trdrd_x <=> 2.0 h2o_x + trdox_x {'original_bigg_ids': ['THIORDXp']}
THMP: h2o_c + thmmp_c --> pi_c + thm_c {'original_bigg_ids': ['THMP']}
THMPe: h2o_e + thmmp_e --> pi_e + thm_e {'original_bigg_ids': ['THMPe']}
THRA: thr__L_c --> acald_c + gly_c {'original_bigg_ids': ['THRAi']}
THRA2: athr__L_c --> acald_c + gly_c {'original_bigg_ids': ['THRA2i']}
THRD_L: thr__L_c --> 2obut_c + nh4_c {'original_bigg_ids': ['THRD_L']}
THRD_Lm: thr__L_m --> 2obut_m + nh4_m {'original_bigg_ids': ['THRD_Lm']}
THRS: h2o_c + phom_c --> pi_c + thr__L_c {'original_bigg_ids': ['THRS']}
THRTRS: atp_c + thr__L_c + trnathr_c --> amp_c + ppi_c + thrtrna_c {'original_bigg_ids': ['THRTRS']}
THRTRSm: atp_m + thr__L_m + trnathr_m --> amp_m + ppi_m + thrtrna_m {'original_bigg_ids': ['THRTLm']}
THRt2r: h_e + thr__L_e <=> h_c + thr__L_c {'original_bigg_ids': ['THRt2r']}
THRtm: thr__L_c <=> thr__L_m {'original_bigg_ids': ['r1440']}
THYMDt1: thymd_e --> thymd_c {'original_bigg_ids': ['THYMDt1']}
THYMt: thym_e <=> thym_c {'original_bigg_ids': ['THYMt']}
THZPSN4: cys__L_c + gly_c + nad_c --> 2amac_c + 4mpetz_c + amp_c + co2_c + 2.0 h2o_c + 3.0 h_c + ncam_c {'original_bigg_ids': ['THZPSN2_SC']}
TKT1: r5p_c + xu5p__D_c <=> g3p_c + s7p_c {'original_bigg_ids': ['TKT1']}
TKT2: e4p_c + xu5p__D_c <=> f6p_c + g3p_c {'original_bigg_ids': ['TKT2']}
TM1601819Z: 3.0 amet_c + dghs1601819Z_c --> 3.0 ahcys_c + dgts1601819Z_c + 3.0 h_c {'original_bigg_ids': ['TM1601819Z']}
TM1819Z1819Z: 3.0 amet_c + dghs1819Z1819Z_c --> 3.0 ahcys_c + dgts1819Z1819Z_c + 3.0 h_c {'original_bigg_ids': ['TM1819Z1819Z']}
TMABDH1_m: 4tmeabut_m + h2o_m + nad_m --> gbbtn_m + 2.0 h_m + nadh_m {'original_bigg_ids': ['TMABDH1_m']}
TMDPK: atp_c + thm_c --> amp_c + h_c + thmpp_c {'original_bigg_ids': ['TMDPK']}
TMDPP: pi_c + thymd_c <=> 2dr1p_c + thym_c {'original_bigg_ids': ['TMDPP']}
TMDS: dump_c + mlthf_c --> dhf_c + dtmp_c {'original_bigg_ids': ['TMDS']}
TMLOX_m: akg_m + o2_m + tmlys_m --> 3htmelys_m + co2_m + succ_m {'original_bigg_ids': ['TMLOX_m']}
TMN: h2o_c + thm_c --> 4ahmmp_c + 4mhetz_c + h_c {'original_bigg_ids': ['TMN']}
TMPPP: 2mahmp_c + 4mpetz_c + h_c --> ppi_c + thmmp_c {'original_bigg_ids': ['TMPPP']}
TPI: dhap_c <=> g3p_c {'original_bigg_ids': ['TPI']}
TRDR: h_c + nadph_c + trdox_c --> nadp_c + trdrd_c {'original_bigg_ids': ['TRDR']}
TRDRm: h_m + nadph_m + trdox_m --> nadp_m + trdrd_m {'original_bigg_ids': ['TRDRm']}
TRE6PH: h2o_c + tre6p_c --> g6p_c + glc__D_c {'original_bigg_ids': ['TRE6PH']}
TRE6PP: h2o_c + tre6p_c --> pi_c + tre_c {'original_bigg_ids': ['TRE6PP']}
TRE6PS: g6p_c + udpg_c --> h_c + tre6p_c + udp_c {'original_bigg_ids': ['TRE6PS']}
TREH: h2o_c + tre_c --> 2.0 glc__D_c {'original_bigg_ids': ['TREH']}
TREHe: h2o_e + tre_e --> 2.0 glc__D_e {'original_bigg_ids': ['TREHe']}
TREt2: h_e + tre_e --> h_c + tre_c {'original_bigg_ids': ['TREt2']}
TRIGSer_RT: 0.01 12dgr_RT_r + 0.01 arachcoa_r + 0.02 hpdcacoa_r + 0.1 lnlncgcoa_r + 0.37 ocdycacoa_r + 0.32 odecoa_r + 0.09 pmtcoa_r + 0.07 stcoa_r + 0.02 ttccoa_r --> coa_r + 0.01 triglyc_RT_r {'original_bigg_ids': ['TRIGS_SC']}
TRPO2: o2_c + trp__L_c --> Lfmkynr_c {'original_bigg_ids': ['TRPO2']}
TRPS1: 3ig3p_c + ser__L_c --> g3p_c + h2o_c + trp__L_c {'original_bigg_ids': ['TRPS1']}
TRPS2: indole_c + ser__L_c --> h2o_c + trp__L_c {'original_bigg_ids': ['TRPS2']}
TRPTA: akg_c + trp__L_c <=> glu__L_c + indpyr_c {'original_bigg_ids': ['TRPTA']}
TRPTRS: atp_c + trnatrp_c + trp__L_c --> amp_c + ppi_c + trptrna_c {'original_bigg_ids': ['TRPTRS']}
TRPTRSm: atp_m + trnatrp_m + trp__L_m --> amp_m + ppi_m + trptrna_m {'original_bigg_ids': ['TRPTRSm']}
TRPt2r: h_e + trp__L_e <=> h_c + trp__L_c {'original_bigg_ids': ['TRPt2r']}
TRYPTAOX: h2o_c + o2_c + trypta_c --> h2o2_c + id3acald_c + nh4_c {'original_bigg_ids': ['TRYPTAOX']}
TSTSTERONEGLCte: atp_c + h2o_c + tststeroneglc_c --> adp_c + h_c + pi_c + tststeroneglc_e {'original_bigg_ids': ['TSTSTERONEGLCte']}
TSULt4_3: 3.0 na1_e + tsul_e <=> 3.0 na1_c + tsul_c {'original_bigg_ids': ['TSULt4_3']}
TTCCOAtr: ttccoa_c <=> ttccoa_r {'original_bigg_ids': ['ACCOAtr']}
TTCt: ttc_e --> ttc_c {'original_bigg_ids': ['DOCOSACTDe']}
TTDCPT1: crn_c + tdcoa_c --> coa_c + ttdcrn_c {'original_bigg_ids': ['TTDCPT1']}
TTDCPT2: coa_m + ttdcrn_m --> crn_m + tdcoa_m {'original_bigg_ids': ['TTDCPT2']}
TTDCRNt: ttdcrn_c --> ttdcrn_m {'original_bigg_ids': ['TTDCRNt']}
TYMte: tym_c <=> tym_e {'original_bigg_ids': ['TYMte']}
TYRCBOX: h_c + tyr__L_c --> co2_c + tym_c {'original_bigg_ids': ['TYRCBOX']}
TYROXDAc: h2o_c + o2_c + tym_c --> 4hoxpacd_c + h2o2_c + nh4_c {'original_bigg_ids': ['TYROXDAc']}
TYRTA: akg_c + tyr__L_c <=> 34hpp_c + glu__L_c {'original_bigg_ids': ['TYRTA']}
TYRTAip: 34hpp_x + glu__L_x --> akg_x + tyr__L_x {'original_bigg_ids': ['TYRTAip']}
TYRTRS: atp_c + trnatyr_c + tyr__L_c --> amp_c + ppi_c + tyrtrna_c {'original_bigg_ids': ['TYRTRS']}
TYRTRSm: atp_m + trnatyr_m + tyr__L_m --> amp_m + ppi_m + tyrtrna_m {'original_bigg_ids': ['TYRTRSm']}
TYRt2r: h_e + tyr__L_e <=> h_c + tyr__L_c {'original_bigg_ids': ['TYRt2r']}
TYRt6: h_v + tyr__L_c --> h_c + tyr__L_v {'original_bigg_ids': ['TYRt6']}
TYRt7: h_v + tyr__L_v --> h_c + tyr__L_c {'original_bigg_ids': ['TYRt7']}
UA4E: udpxyl_c <=> udparab_c {'original_bigg_ids': ['UA4E']}
UACMAMO: h2o_c + 2.0 nad_c + uacmam_c --> 3.0 h_c + 2.0 nadh_c + uacmamu_c {'original_bigg_ids': ['UACMAMO']}
UAG4Ei: uacgam_c <=> udpacgal_c {'original_bigg_ids': ['UAG4E']}
UAGDP: acgam1p_c + h_c + utp_c --> ppi_c + uacgam_c {'original_bigg_ids': ['UAGDP']}
UCP2ASPtm: asp__L_m + h_c + pi_c --> asp__L_c + h_m + pi_m {'original_bigg_ids': ['OAAt2m']}
UCP2MALtm: h_c + mal__L_m + pi_c --> h_m + mal__L_c + pi_m {'original_bigg_ids': ['OAAt2m']}
UCP2OAAtm: h_c + oaa_m + pi_c --> h_m + oaa_c + pi_m {'original_bigg_ids': ['OAAt2m']}
UDPACGLP: acgam1p_c + h_c + utp_c <=> ppi_c + udpacgal_c {'original_bigg_ids': ['UDPACGLP']}
UDPDOLPT: dolp_c + udpg_c --> dolpglc_c + udp_c {'original_bigg_ids': ['UDPDOLPT']}
UDPDOLPT_L: 0.1 dolp__L_c + udpg_c --> 0.1 dolglcp__L_c + udp_c {'original_bigg_ids': ['UDPDOLPT_L']}
UDPDOLPT_U: 0.1 dolp_U_c + udpg_c --> 0.1 dolglcp_U_c + udp_c {'original_bigg_ids': ['UDPDOLPT_U']}
UDPG4E: udpg_c <=> udpgal_c {'original_bigg_ids': ['UDPG4E']}
UDPGALOR: h2o_c + 2.0 nad_c + udpgal_c --> 3.0 h_c + 2.0 nadh_c + udpgalur_c {'original_bigg_ids': ['UDPGALOR']}
UDPGALt2g: udpgal_c --> udpgal_g {'original_bigg_ids': ['UDPGALt2g']}
UDPGALtg: udpgal_c + ump_g <=> udpgal_g + ump_c {'original_bigg_ids': ['UDPGALtg']}
UDPGD: h2o_c + 2.0 nad_c + udpg_c --> 3.0 h_c + 2.0 nadh_c + udpglcur_c {'original_bigg_ids': ['UDPGD']}
UDPGDC: nad_c + udpglcur_c --> co2_c + nadh_c + udpLa4o_c {'original_bigg_ids': ['UDPGDC']}
UDPGLCtg: udpg_c + ump_g <=> udpg_g + ump_c {'original_bigg_ids': ['UDPGLCtg']}
UDPGLDC: h_c + udpglcur_c --> co2_c + udpxyl_c {'original_bigg_ids': ['UGDC']}
UDPGLDCg: h_g + udpglcur_g --> co2_g + udpxyl_g {'original_bigg_ids': ['UDPGLDCg']}
UDPGP: h2o_c + udpg_c --> g1p_c + 2.0 h_c + ump_c {'original_bigg_ids': ['UDPGP']}
UGALNACtg: udpacgal_c + ump_g <=> udpacgal_g + ump_c {'original_bigg_ids': ['UGALNACtg']}
UGLT: gal1p_c + udpg_c --> g1p_c + udpgal_c {'original_bigg_ids': ['UGLT']}
UGLYCH: h2o_c + 2.0 h_c + urdglyc_c --> co2_c + glx_c + 2.0 nh4_c {'original_bigg_ids': ['UGLYCH']}
ULA4NFT: 10fthf_c + udpLa4n_c --> h_c + thf_c + udpLa4fn_c {'original_bigg_ids': ['ULA4NFT']}
UMPK: atp_c + ump_c <=> adp_c + udp_c {'original_bigg_ids': ['UMPK']}
UMPK2: ctp_c + ump_c <=> cdp_c + udp_c {'original_bigg_ids': ['UMPK2']}
UMPK2n: ctp_n + ump_n <=> cdp_n + udp_n {'original_bigg_ids': ['UMPK2n']}
UMPK3: ump_c + utp_c <=> 2.0 udp_c {'original_bigg_ids': ['UMPK3']}
UMPK3n: ump_n + utp_n <=> 2.0 udp_n {'original_bigg_ids': ['UMPK3n']}
UMPK4: gtp_c + ump_c <=> gdp_c + udp_c {'original_bigg_ids': ['UMPK4']}
UMPK4n: gtp_n + ump_n <=> gdp_n + udp_n {'original_bigg_ids': ['UMPK4n']}
UMPK5: datp_c + ump_c <=> dadp_c + udp_c {'original_bigg_ids': ['UMPK5']}
UMPK5n: datp_n + ump_n <=> dadp_n + udp_n {'original_bigg_ids': ['UMPK5n']}
UMPK6: dctp_c + ump_c <=> dcdp_c + udp_c {'original_bigg_ids': ['UMPK6']}
UMPK6n: dctp_n + ump_n <=> dcdp_n + udp_n {'original_bigg_ids': ['UMPK6n']}
UMPK7: dgtp_c + ump_c <=> dgdp_c + udp_c {'original_bigg_ids': ['UMPK7']}
UMPK7n: dgtp_n + ump_n <=> dgdp_n + udp_n {'original_bigg_ids': ['UMPK7n']}
UMPKn: atp_n + ump_n <=> adp_n + udp_n {'original_bigg_ids': ['UMPKn']}
UNK3: 2kmb_c + glu__L_c --> akg_c + met__L_c {'original_bigg_ids': ['UNK3']}
UPLA4FNT: udcpp_c + udpLa4fn_c --> uLa4fn_c + udp_c {'original_bigg_ids': ['UPLA4FNT']}
UPP3MT: 2.0 amet_c + uppg3_c --> 2.0 ahcys_c + dscl_c + h_c {'original_bigg_ids': ['UPP3MT']}
UPP3S: hmbil_c --> h2o_c + uppg3_c {'original_bigg_ids': ['UPP3S']}
UPPDC1: 4.0 h_c + uppg3_c --> 4.0 co2_c + cpppg3_c {'original_bigg_ids': ['UPPDC1']}
UPPDC2: 4.0 h_c + uppg1_c --> 4.0 co2_c + cpppg1_c {'original_bigg_ids': ['UPPDC2']}
UPPRT: prpp_c + ura_c --> ppi_c + ump_c {'original_bigg_ids': ['UPPRT']}
URATEt_1: h_e + urate_e <=> h_c + urate_c {'original_bigg_ids': ['URATEt']}
URATEtm: h_c + urate_c <=> h_m + urate_m {'original_bigg_ids': ['URATEtm']}
URAt2: h_e + ura_e --> h_c + ura_c {'original_bigg_ids': ['URAt2']}
UREA2t2: 2.0 h_e + urea_e <=> 2.0 h_c + urea_c {'original_bigg_ids': ['UREA2t2']}
UREASE: atp_c + hco3_c + urea_c <=> adp_c + allphn_c + h_c + pi_c {'original_bigg_ids': ['UREASE']}
UREAt: urea_e <=> urea_c {'original_bigg_ids': ['UREAt']}
UREAtm: urea_c <=> urea_m {'original_bigg_ids': ['UREAtm']}
URIDK2r: atp_c + dump_c <=> adp_c + dudp_c {'original_bigg_ids': ['URIDK2r']}
URIDK2rn: atp_n + dump_n <=> adp_n + dudp_n {'original_bigg_ids': ['URIDK2rn']}
URIH: h2o_c + uri_c --> rib__D_c + ura_c {'original_bigg_ids': ['URIH']}
URIK1: atp_c + uri_c --> adp_c + h_c + ump_c {'original_bigg_ids': ['URIK1']}
URIK2: gtp_c + uri_c --> gdp_c + h_c + ump_c {'original_bigg_ids': ['URIK2']}
URIK3: itp_c + uri_c --> h_c + idp_c + ump_c {'original_bigg_ids': ['ITUP']}
URIt2: h_e + uri_e --> h_c + uri_c {'original_bigg_ids': ['URIt2']}
URO: h2o_m + o2_m + urate_m --> 5hiu_m + h2o2_m + h_m {'original_bigg_ids': ['URO']}
UTCY: cytd_c + utp_c --> cmp_c + h_c + udp_c {'original_bigg_ids': ['UTCY']}
UTPtm: 2.0 h_c + ump_m + utp_c --> 2.0 h_m + ump_c + utp_m {'original_bigg_ids': ['UTPtm']}
UTPtm2: fe2_c + ump_m + utp_c --> fe2_m + ump_c + utp_m {'original_bigg_ids': ['UTPtm']}
UTUP: uri_c + utp_c --> h_c + udp_c + ump_c {'original_bigg_ids': ['UTUP']}
VALTA: akg_c + val__L_c <=> 3mob_c + glu__L_c {'original_bigg_ids': ['VALTA']}
VALTAm: akg_m + val__L_m <=> 3mob_m + glu__L_m {'original_bigg_ids': ['VALTAim']}
VALTRS: atp_c + trnaval_c + val__L_c --> amp_c + ppi_c + valtrna_c {'original_bigg_ids': ['VALTRS']}
VALTRSm: atp_m + trnaval_m + val__L_m --> amp_m + ppi_m + valtrna_m {'original_bigg_ids': ['VALTRSm']}
VALt2r: h_e + val__L_e <=> h_c + val__L_c {'original_bigg_ids': ['VALt2r']}
VALt5m: val__L_c <=> val__L_m {'original_bigg_ids': ['VALt5m']}
VLCS2p: atp_x + coa_x + dhcholestanate_x --> amp_x + dhcholestancoa_x + ppi_x {'original_bigg_ids': ['VLCS2p']}
VLCS2r: atp_r + coa_r + dhcholestanate_r --> amp_r + dhcholestancoa_r + ppi_r {'original_bigg_ids': ['VLCS2r']}
VLCSp: atp_x + coa_x + thcholstoic_x --> amp_x + cholcoar_x + ppi_x {'original_bigg_ids': ['VLCSp']}
VLCSr: atp_r + coa_r + thcholstoic_r --> amp_r + cholcoar_r + ppi_r {'original_bigg_ids': ['VLCSr']}
VNDH: h2o_c + nad_c + vanln_c --> 2.0 h_c + nadh_c + vanlt_c {'original_bigg_ids': ['VNDH']}
VNDH_2: 4hbald_c + h2o_c + nad_c --> 4hbz_c + 2.0 h_c + nadh_c {'original_bigg_ids': ['VNDH_2']}
VNDH_3: 34dhbald_c + h2o_c + nad_c --> 34dhbz_c + 2.0 h_c + nadh_c {'original_bigg_ids': ['VNDH_3']}
WCOS: 2.0 h_c + mptamp_c + tungs_c --> amp_c + cu2_c + h2o_c + wco_c {'original_bigg_ids': ['WCOS']}
XAND: h2o_c + nad_c + xan_c --> h_c + nadh_c + urate_c {'original_bigg_ids': ['XAND']}
XANDp: h2o_x + nad_x + xan_x --> h_x + nadh_x + urate_x {'original_bigg_ids': ['XANDp']}
XANt: xan_e <=> xan_c {'original_bigg_ids': ['XANtr']}
XAO2x: h2o_x + hxan_x + o2_x --> h2o2_x + xan_x {'original_bigg_ids': ['XAO2x']}
XAOx: h2o_x + o2_x + xan_x --> h2o2_x + urate_x {'original_bigg_ids': ['XAOx']}
XPPT: prpp_c + xan_c --> ppi_c + xmp_c {'original_bigg_ids': ['XPPT']}
XTSNH: h2o_c + xtsn_c --> rib__D_c + xan_c {'original_bigg_ids': ['XTSNH']}
XTSNtr: xtsn_e <=> xtsn_c {'original_bigg_ids': ['XTSNtr']}
XYLK: atp_c + xylu__D_c --> adp_c + h_c + xu5p__D_c {'original_bigg_ids': ['XYLK']}
XYLR: h_c + nadph_c + xyl__D_c --> nadp_c + xylt_c {'original_bigg_ids': ['XYLR']}
XYLTD_D: nad_c + xylt_c --> h_c + nadh_c + xylu__D_c {'original_bigg_ids': ['XYLTD_D']}
XYLTt: xylt_e <=> xylt_c {'original_bigg_ids': ['XYLTt']}
XYLUDte: xylu__D_c <=> xylu__D_e {'original_bigg_ids': ['XYLUDte']}
XYLUR: h_c + nadph_c + xylu__L_c <=> nadp_c + xylt_c {'original_bigg_ids': ['XYLUR']}
XYLt: xyl__D_e <=> xyl__D_c {'original_bigg_ids': ['XYLt']}
YUMPS: r5p_c + ura_c <=> h2o_c + psd5p_c {'original_bigg_ids': ['YUMPS']}
ZYMSTATer_RT: 0.01 hdcoa_r + 0.01 lnlncgcoa_r + 0.14 ocdycacoa_r + 0.48 odecoa_r + 0.23 pmtcoa_r + 0.12 stcoa_r + 0.01 ttccoa_r + zymst_r --> coa_r + 0.01 zymstest_RT_r {'original_bigg_ids': ['ZYMSTAT_SC']}
ZYMSTESTH_RT: h2o_c + 0.01 zymstest_RT_d --> h_c + 0.23 hdca_c + 0.01 hdcea_c + 0.01 lnlncg_c + 0.12 ocdca_c + 0.48 ocdcea_c + 0.14 ocdcya_c + 0.01 ttc_c + zymst_c {'original_bigg_ids': ['ZYMSTESTH_SC']}
ZYMSTESTtrd: zymstest_RT_r --> zymstest_RT_d {'original_bigg_ids': ['FRDPtcr']}
ZYMSTt: zymst_e <=> zymst_c {'original_bigg_ids': ['ZYMSTt']}
ZYMSTtr: zymst_c <=> zymst_r {'original_bigg_ids': ['ZYMSTt']}
Zn2t: zn2_e <=> zn2_c {'original_bigg_ids': ['Zn2tex']}
r0647: 42A3HP24DB_c <=> C02470_c + h2o_c {'original_bigg_ids': ['r0647']}
```

In [19]:

```
for r in sorted(model.reactions, key=lambda x: x.id):
    if r.annotation:
        print(r, r.annotation)
```

```
ARBTHe: arbt_e + h2o_e --> glc__D_e + hqn_e {'bigg.reaction': ['BG_MBDG'], 'ec-code': ['3.2.1.21'], 'metanetx.reaction': ['MNXR96248'], 'sbo': 'SBO:0000176', 'seed.reaction': ['rxn09979']}
BG_MBDG: h2o_e + mbdg_e --> glc__D_e + meoh_e {'bigg.reaction': ['BG_MBDG'], 'ec-code': ['3.2.1.21'], 'metanetx.reaction': ['MNXR96248'], 'sbo': 'SBO:0000176', 'seed.reaction': ['rxn09979']}
DM_aacald_c: aacald_c -->  {'SBO': 'SBO:0000628'}
DM_amob_m: amob_m -->  {'SBO': 'SBO:0000628'}
DM_dad_5_m: dad_5_m -->  {'SBO': 'SBO:0000628'}
EX_12ppd__R_e: 12ppd__R_e -->  {'SBO': 'SBO:0000627'}
EX_12ppd__S_e: 12ppd__S_e -->  {'SBO': 'SBO:0000627'}
EX_13BDglcn_e: 13BDglcn_e -->  {'SBO': 'SBO:0000627'}
EX_2doxg6p_e: 2doxg6p_e -->  {'SBO': 'SBO:0000627'}
EX_2hxmp_e: 2hxmp_e -->  {'bigg.reaction': ['EX_2hxmp_e'], 'biocyc': ['META:TRANS-RXN0-528'], 'metanetx.reaction': ['MNXR124044'], 'sbo': 'SBO:0000627'}
EX_2obut_e: 2obut_e -->  {'SBO': 'SBO:0000627'}
EX_2pg_e: 2pg_e -->  {'SBO': 'SBO:0000627'}
EX_35ccmp_e: 35ccmp_e -->  {'SBO': 'SBO:0000627'}
EX_35cgmp_e: 35cgmp_e -->  {'SBO': 'SBO:0000627'}
EX_3pg_e: 3pg_e -->  {'SBO': 'SBO:0000627'}
EX_3sala_e: 3sala_e -->  {'SBO': 'SBO:0000627'}
EX_4abut_e: 4abut_e -->  {'SBO': 'SBO:0000627'}
EX_4abz_e: 4abz_e -->  {'SBO': 'SBO:0000627'}
EX_4hbz_e: 4hbz_e -->  {'SBO': 'SBO:0000627'}
EX_5adtststeroneglc_e: 5adtststeroneglc_e -->  {'SBO': 'SBO:0000627'}
EX_5aop_e: 5aop_e -->  {'SBO': 'SBO:0000627'}
EX_5dglcn_e: 5dglcn_e -->  {'SBO': 'SBO:0000627'}
EX_5flura_e: 5flura_e -->  {'SBO': 'SBO:0000627'}
EX_6mpur_e: 6mpur_e -->  {'SBO': 'SBO:0000627'}
EX_6pgc_e: 6pgc_e -->  {'SBO': 'SBO:0000627'}
EX_Lcyst_e: Lcyst_e -->  {'SBO': 'SBO:0000627'}
EX_T4hcinnm_e: T4hcinnm_e -->  {'SBO': 'SBO:0000627'}
EX_abt__D_e: abt__D_e -->  {'SBO': 'SBO:0000627'}
EX_abt_e: abt_e -->  {'SBO': 'SBO:0000627'}
EX_ac_e: ac_e -->  {'SBO': 'SBO:0000627'}
EX_acac_e: acac_e -->  {'SBO': 'SBO:0000627'}
EX_acgam_e: acgam_e -->  {'SBO': 'SBO:0000627'}
EX_acglu_e: acglu_e -->  {'SBO': 'SBO:0000627'}
EX_ach_e: ach_e -->  {'SBO': 'SBO:0000627'}
EX_ad_e: ad_e -->  {'SBO': 'SBO:0000627'}
EX_ade_e: ade_e -->  {'SBO': 'SBO:0000627'}
EX_adn_e: adn_e -->  {'SBO': 'SBO:0000627'}
EX_agm_e: agm_e -->  {'SBO': 'SBO:0000627'}
EX_ahandrostanglc_e: ahandrostanglc_e -->  {'SBO': 'SBO:0000627'}
EX_akg_e: akg_e -->  {'SBO': 'SBO:0000627'}
EX_ala_B_e: ala_B_e -->  {'SBO': 'SBO:0000627'}
EX_ala__D_e: ala__D_e -->  {'SBO': 'SBO:0000627'}
EX_ala__L_e: ala__L_e -->  {'SBO': 'SBO:0000627'}
EX_alltn_e: alltn_e -->  {'SBO': 'SBO:0000627'}
EX_alltt_e: alltt_e -->  {'SBO': 'SBO:0000627'}
EX_amet_e: amet_e -->  {'SBO': 'SBO:0000627'}
EX_amp_e: amp_e -->  {'SBO': 'SBO:0000627'}
EX_andrstrnglc_e: andrstrnglc_e -->  {'SBO': 'SBO:0000627'}
EX_arab__D_e: arab__D_e -->  {'SBO': 'SBO:0000627'}
EX_arab__L_e: arab__L_e -->  {'SBO': 'SBO:0000627'}
EX_arach_e: arach_e -->  {'SBO': 'SBO:0000627'}
EX_arbt_e: arbt_e -->  {'bigg.reaction': ['EX_arbt_e'], 'metanetx.reaction': ['MNXR95933'], 'rhea': ['35024', '35025', '35023', '35026'], 'sbo': 'SBO:0000627'}
EX_arg__L_e: arg__L_e -->  {'SBO': 'SBO:0000627'}
EX_asn__L_e: asn__L_e -->  {'SBO': 'SBO:0000627'}
EX_asp__D_e: asp__D_e -->  {'SBO': 'SBO:0000627'}
EX_asp__L_e: asp__L_e -->  {'SBO': 'SBO:0000627'}
EX_bhb_e: bhb_e -->  {'SBO': 'SBO:0000627'}
EX_bildglcur_e: bildglcur_e -->  {'SBO': 'SBO:0000627'}
EX_bilglcur_e: bilglcur_e -->  {'SBO': 'SBO:0000627'}
EX_btd_RR_e: btd_RR_e -->  {'SBO': 'SBO:0000627'}
EX_btn_e: btn_e -->  {'SBO': 'SBO:0000627'}
EX_but_e: but_e -->  {'SBO': 'SBO:0000627'}
EX_bz_e: bz_e -->  {'SBO': 'SBO:0000627'}
EX_ca2_e: ca2_e <=>  {'SBO': 'SBO:0000627'}
EX_camp_e: camp_e -->  {'SBO': 'SBO:0000627'}
EX_cbp_e: cbp_e -->  {'SBO': 'SBO:0000627'}
EX_cellb_e: cellb_e -->  {'SBO': 'SBO:0000627'}
EX_cgly_e: cgly_e -->  {'SBO': 'SBO:0000627'}
EX_chol_e: chol_e -->  {'SBO': 'SBO:0000627'}
EX_cholate_e: cholate_e -->  {'SBO': 'SBO:0000627'}
EX_cholp_e: cholp_e -->  {'SBO': 'SBO:0000627'}
EX_chtn_e: chtn_e -->  {'SBO': 'SBO:0000627'}
EX_cit_e: cit_e -->  {'SBO': 'SBO:0000627'}
EX_citr__L_e: citr__L_e -->  {'SBO': 'SBO:0000627'}
EX_cl_e: cl_e -->  {'SBO': 'SBO:0000627'}
EX_cmp_e: cmp_e -->  {'SBO': 'SBO:0000627'}
EX_co2_e: co2_e -->  {'SBO': 'SBO:0000627'}
EX_csn_e: csn_e -->  {'SBO': 'SBO:0000627'}
EX_cu2_e: cu2_e <=>  {'SBO': 'SBO:0000627'}
EX_cu_e: cu_e -->  {'SBO': 'SBO:0000627'}
EX_cys__L_e: cys__L_e -->  {'SBO': 'SBO:0000627'}
EX_cysi__L_e: cysi__L_e -->  {'SBO': 'SBO:0000627'}
EX_cyst__L_e: cyst__L_e -->  {'SBO': 'SBO:0000627'}
EX_cytd_e: cytd_e -->  {'SBO': 'SBO:0000627'}
EX_dad_2_e: dad_2_e -->  {'SBO': 'SBO:0000627'}
EX_dca_e: dca_e -->  {'SBO': 'SBO:0000627'}
EX_ddca_e: ddca_e -->  {'SBO': 'SBO:0000627'}
EX_dha_e: dha_e -->  {'SBO': 'SBO:0000627'}
EX_dhdascb_e: dhdascb_e -->  {'SBO': 'SBO:0000627'}
EX_din_e: din_e -->  {'SBO': 'SBO:0000627'}
EX_drib_e: drib_e -->  {'SBO': 'SBO:0000627'}
EX_dtmp_e: dtmp_e -->  {'SBO': 'SBO:0000627'}
EX_epist_e: epist_e -->  {'SBO': 'SBO:0000627'}
EX_ergst_e: ergst_e -->  {'SBO': 'SBO:0000627'}
EX_estradiolglc_e: estradiolglc_e -->  {'SBO': 'SBO:0000627'}
EX_estriolglc_e: estriolglc_e -->  {'SBO': 'SBO:0000627'}
EX_estroneglc_e: estroneglc_e -->  {'SBO': 'SBO:0000627'}
EX_etha_e: etha_e -->  {'SBO': 'SBO:0000627'}
EX_ethamp_e: ethamp_e -->  {'SBO': 'SBO:0000627'}
EX_f6p_e: f6p_e -->  {'SBO': 'SBO:0000627'}
EX_fe2_e: fe2_e <=>  {'SBO': 'SBO:0000627'}
EX_fe3_e: fe3_e <=>  {'SBO': 'SBO:0000627'}
EX_fecost_e: fecost_e -->  {'SBO': 'SBO:0000627'}
EX_fer_e: fer_e -->  {'SBO': 'SBO:0000627'}
EX_fmn_e: fmn_e -->  {'SBO': 'SBO:0000627'}
EX_fol_e: fol_e -->  {'SBO': 'SBO:0000627'}
EX_for_e: for_e -->  {'SBO': 'SBO:0000627'}
EX_frmd_e: frmd_e -->  {'SBO': 'SBO:0000627'}
EX_fru_e: fru_e -->  {'SBO': 'SBO:0000627'}
EX_fum_e: fum_e -->  {'SBO': 'SBO:0000627'}
EX_g1p_e: g1p_e -->  {'SBO': 'SBO:0000627'}
EX_g3pc_e: g3pc_e -->  {'SBO': 'SBO:0000627'}
EX_g3pe_e: g3pe_e -->  {'SBO': 'SBO:0000627'}
EX_g3pi_e: g3pi_e -->  {'SBO': 'SBO:0000627'}
EX_g6p_e: g6p_e -->  {'SBO': 'SBO:0000627'}
EX_gal_e: gal_e -->  {'SBO': 'SBO:0000627'}
EX_galt_e: galt_e -->  {'SBO': 'SBO:0000627'}
EX_galur_e: galur_e -->  {'SBO': 'SBO:0000627'}
EX_gam6p_e: gam6p_e -->  {'SBO': 'SBO:0000627'}
EX_gam_e: gam_e -->  {'SBO': 'SBO:0000627'}
EX_gchola_e: gchola_e -->  {'SBO': 'SBO:0000627'}
EX_ghb_e: ghb_e -->  {'SBO': 'SBO:0000627'}
EX_glc__D_e: glc__D_e <=>  {'SBO': 'SBO:0000627'}
EX_glcn_e: glcn_e -->  {'SBO': 'SBO:0000627'}
EX_gln__L_e: gln__L_e -->  {'SBO': 'SBO:0000627'}
EX_glu__L_e: glu__L_e -->  {'SBO': 'SBO:0000627'}
EX_gluala_e: gluala_e -->  {'SBO': 'SBO:0000627'}
EX_glx_e: glx_e -->  {'SBO': 'SBO:0000627'}
EX_gly_e: gly_e -->  {'SBO': 'SBO:0000627'}
EX_glyc2p_e: glyc2p_e -->  {'SBO': 'SBO:0000627'}
EX_glyc3p_e: glyc3p_e -->  {'SBO': 'SBO:0000627'}
EX_glyc__R_e: glyc__R_e -->  {'SBO': 'SBO:0000627'}
EX_glyc_e: glyc_e -->  {'SBO': 'SBO:0000627'}
EX_glyclt_e: glyclt_e -->  {'SBO': 'SBO:0000627'}
EX_glycogen_e: glycogen_e -->  {'SBO': 'SBO:0000627'}
EX_glygn2_e: glygn2_e -->  {'SBO': 'SBO:0000627'}
EX_glygn4_e: glygn4_e -->  {'SBO': 'SBO:0000627'}
EX_gmp_e: gmp_e -->  {'SBO': 'SBO:0000627'}
EX_gsn_e: gsn_e -->  {'SBO': 'SBO:0000627'}
EX_gthrd_e: gthrd_e -->  {'SBO': 'SBO:0000627'}
EX_gua_e: gua_e -->  {'SBO': 'SBO:0000627'}
EX_h2o2_e: h2o2_e -->  {'SBO': 'SBO:0000627'}
EX_h2o_e: h2o_e <=>  {'SBO': 'SBO:0000627'}
EX_h_e: h_e <=>  {'SBO': 'SBO:0000627'}
EX_hco3_e: hco3_e -->  {'SBO': 'SBO:0000627'}
EX_hdca_e: hdca_e -->  {'SBO': 'SBO:0000627'}
EX_hdcea_e: hdcea_e -->  {'SBO': 'SBO:0000627'}
EX_his__L_e: his__L_e -->  {'SBO': 'SBO:0000627'}
EX_hista_e: hista_e -->  {'SBO': 'SBO:0000627'}
EX_hom__L_e: hom__L_e -->  {'SBO': 'SBO:0000627'}
EX_hqn_e: hqn_e -->  {'bigg.reaction': ['EX_hqn_e'], 'biocyc': ['META:TRANS-RXN0-529'], 'metanetx.reaction': ['MNXR124045'], 'sbo': 'SBO:0000627'}
EX_hxa_e: hxa_e -->  {'SBO': 'SBO:0000627'}
EX_hyptaur_e: hyptaur_e -->  {'SBO': 'SBO:0000627'}
EX_ile__L_e: ile__L_e -->  {'SBO': 'SBO:0000627'}
EX_ind3eth_e: ind3eth_e -->  {'SBO': 'SBO:0000627'}
EX_inost_e: inost_e -->  {'SBO': 'SBO:0000627'}
EX_ins_e: ins_e -->  {'SBO': 'SBO:0000627'}
EX_k_e: k_e <=>  {'SBO': 'SBO:0000627'}
EX_lac__D_e: lac__D_e -->  {'SBO': 'SBO:0000627'}
EX_lac__L_e: lac__L_e -->  {'SBO': 'SBO:0000627'}
EX_lanost_e: lanost_e -->  {'SBO': 'SBO:0000627'}
EX_leu__L_e: leu__L_e -->  {'SBO': 'SBO:0000627'}
EX_lys__L_e: lys__L_e -->  {'SBO': 'SBO:0000627'}
EX_lyx__L_e: lyx__L_e -->  {'SBO': 'SBO:0000627'}
EX_mal__L_e: mal__L_e -->  {'SBO': 'SBO:0000627'}
EX_malt_e: malt_e -->  {'SBO': 'SBO:0000627'}
EX_malttr_e: malttr_e -->  {'SBO': 'SBO:0000627'}
EX_man1p_e: man1p_e -->  {'SBO': 'SBO:0000627'}
EX_man6p_e: man6p_e -->  {'SBO': 'SBO:0000627'}
EX_man_e: man_e -->  {'SBO': 'SBO:0000627'}
EX_mannan_e: mannan_e -->  {'SBO': 'SBO:0000627'}
EX_mbdg_e: mbdg_e -->  {'bigg.reaction': ['EX_mbdg_e'], 'metanetx.reaction': ['MNXR143029'], 'sbo': 'SBO:0000627'}
EX_melib_e: melib_e -->  {'SBO': 'SBO:0000627'}
EX_meoh_e: meoh_e -->  {'SBO': 'SBO:0000627'}
EX_met__L_e: met__L_e -->  {'SBO': 'SBO:0000627'}
EX_metsox_R__L_e: metsox_R__L_e -->  {'SBO': 'SBO:0000627'}
EX_metsox_S__L_e: metsox_S__L_e -->  {'SBO': 'SBO:0000627'}
EX_mg2_e: mg2_e <=>  {'SBO': 'SBO:0000627'}
EX_minohp_e: minohp_e -->  {'SBO': 'SBO:0000627'}
EX_mma_e: mma_e -->  {'SBO': 'SBO:0000627'}
EX_mn2_e: mn2_e <=>  {'SBO': 'SBO:0000627'}
EX_na1_e: na1_e <=>  {'SBO': 'SBO:0000627'}
EX_nac_e: nac_e -->  {'SBO': 'SBO:0000627'}
EX_nh4_e: nh4_e <=>  {'SBO': 'SBO:0000627'}
EX_no2_e: no2_e -->  {'SBO': 'SBO:0000627'}
EX_no3_e: no3_e -->  {'SBO': 'SBO:0000627'}
EX_nrvnc_e: nrvnc_e -->  {'SBO': 'SBO:0000627'}
EX_o2_e: o2_e <=>  {'SBO': 'SBO:0000627'}
EX_ocdca_e: ocdca_e -->  {'SBO': 'SBO:0000627'}
EX_ocdcea_e: ocdcea_e -->  {'SBO': 'SBO:0000627'}
EX_ocdcya_e: ocdcya_e -->  {'SBO': 'SBO:0000627'}
EX_oh1_e: oh1_e -->  {'SBO': 'SBO:0000627'}
EX_orn_e: orn_e -->  {'SBO': 'SBO:0000627'}
EX_oxa_e: oxa_e -->  {'SBO': 'SBO:0000627'}
EX_pail1819Z160_e: pail1819Z160_e -->  {'SBO': 'SBO:0000627'}
EX_pc_RT_e: pc_RT_e -->  {'SBO': 'SBO:0000627'}
EX_pe1801819Z_e: pe1801819Z_e -->  {'SBO': 'SBO:0000627'}
EX_pe1801829Z12Z_e: pe1801829Z12Z_e -->  {'SBO': 'SBO:0000627'}
EX_pe1819Z1819Z_e: pe1819Z1819Z_e -->  {'SBO': 'SBO:0000627'}
EX_pe1819Z1829Z12Z_e: pe1819Z1829Z12Z_e -->  {'SBO': 'SBO:0000627'}
EX_pe_RT_e: pe_RT_e -->  {'SBO': 'SBO:0000627'}
EX_peamn_e: peamn_e -->  {'SBO': 'SBO:0000627'}
EX_pectin_e: pectin_e -->  {'SBO': 'SBO:0000627'}
EX_pep_e: pep_e -->  {'SBO': 'SBO:0000627'}
EX_pepd_e: pepd_e -->  {'SBO': 'SBO:0000627'}
EX_pg1819Z160_e: pg1819Z160_e -->  {'SBO': 'SBO:0000627'}
EX_pgp1819Z160_e: pgp1819Z160_e -->  {'SBO': 'SBO:0000627'}
EX_phe__L_e: phe__L_e -->  {'SBO': 'SBO:0000627'}
EX_pi_e: pi_e <=>  {'SBO': 'SBO:0000627'}
EX_pnto__R_e: pnto__R_e -->  {'SBO': 'SBO:0000627'}
EX_ppa_e: ppa_e -->  {'SBO': 'SBO:0000627'}
EX_ppi_e: ppi_e -->  {'SBO': 'SBO:0000627'}
EX_pppi_e: pppi_e -->  {'SBO': 'SBO:0000627'}
EX_pro__D_e: pro__D_e -->  {'SBO': 'SBO:0000627'}
EX_pro__L_e: pro__L_e -->  {'SBO': 'SBO:0000627'}
EX_prostge1_e: prostge1_e -->  {'SBO': 'SBO:0000627'}
EX_prostge2_e: prostge2_e -->  {'SBO': 'SBO:0000627'}
EX_pser__L_e: pser__L_e -->  {'SBO': 'SBO:0000627'}
EX_ptd1ino_RT_e: ptd1ino_RT_e -->  {'SBO': 'SBO:0000627'}
EX_ptrc_e: ptrc_e -->  {'SBO': 'SBO:0000627'}
EX_pydxn_e: pydxn_e -->  {'SBO': 'SBO:0000627'}
EX_pyr_e: pyr_e -->  {'SBO': 'SBO:0000627'}
EX_quin_e: quin_e -->  {'SBO': 'SBO:0000627'}
EX_rbl__D_e: rbl__D_e -->  {'SBO': 'SBO:0000627'}
EX_rbt_e: rbt_e -->  {'SBO': 'SBO:0000627'}
EX_rib__D_e: rib__D_e -->  {'SBO': 'SBO:0000627'}
EX_ribflv_e: ribflv_e -->  {'SBO': 'SBO:0000627'}
EX_salcn_e: salcn_e -->  {'bigg.reaction': ['EX_salcn_e'], 'metanetx.reaction': ['MNXR104264'], 'sbo': 'SBO:0000627'}
EX_sbt__D_e: sbt__D_e -->  {'SBO': 'SBO:0000627'}
EX_ser__D_e: ser__D_e -->  {'SBO': 'SBO:0000627'}
EX_ser__L_e: ser__L_e -->  {'SBO': 'SBO:0000627'}
EX_so3_e: so3_e -->  {'SBO': 'SBO:0000627'}
EX_so4_e: so4_e <=>  {'SBO': 'SBO:0000627'}
EX_spmd_e: spmd_e -->  {'SBO': 'SBO:0000627'}
EX_sprm_e: sprm_e -->  {'SBO': 'SBO:0000627'}
EX_srb__L_e: srb__L_e -->  {'SBO': 'SBO:0000627'}
EX_strch1_e: strch1_e -->  {'SBO': 'SBO:0000627'}
EX_strch2_e: strch2_e -->  {'SBO': 'SBO:0000627'}
EX_succ_e: succ_e -->  {'SBO': 'SBO:0000627'}
EX_sucr_e: sucr_e -->  {'SBO': 'SBO:0000627'}
EX_taur_e: taur_e -->  {'SBO': 'SBO:0000627'}
EX_tchola_e: tchola_e -->  {'SBO': 'SBO:0000627'}
EX_tega_e: tega_e -->  {'SBO': 'SBO:0000627'}
EX_tgua_e: tgua_e -->  {'SBO': 'SBO:0000627'}
EX_thm_e: thm_e -->  {'SBO': 'SBO:0000627'}
EX_thmmp_e: thmmp_e -->  {'SBO': 'SBO:0000627'}
EX_thmpp_e: thmpp_e -->  {'SBO': 'SBO:0000627'}
EX_thr__L_e: thr__L_e -->  {'SBO': 'SBO:0000627'}
EX_thym_e: thym_e -->  {'SBO': 'SBO:0000627'}
EX_thymd_e: thymd_e -->  {'SBO': 'SBO:0000627'}
EX_tre_e: tre_e -->  {'SBO': 'SBO:0000627'}
EX_trp__L_e: trp__L_e -->  {'SBO': 'SBO:0000627'}
EX_tststeroneglc_e: tststeroneglc_e -->  {'SBO': 'SBO:0000627'}
EX_tsul_e: tsul_e -->  {'SBO': 'SBO:0000627'}
EX_ttc_e: ttc_e -->  {'SBO': 'SBO:0000627'}
EX_ttdca_e: ttdca_e -->  {'SBO': 'SBO:0000627'}
EX_tym_e: tym_e -->  {'SBO': 'SBO:0000627'}
EX_tyr__L_e: tyr__L_e -->  {'SBO': 'SBO:0000627'}
EX_ump_e: ump_e -->  {'SBO': 'SBO:0000627'}
EX_ura_e: ura_e -->  {'SBO': 'SBO:0000627'}
EX_urate_e: urate_e -->  {'SBO': 'SBO:0000627'}
EX_urea_e: urea_e -->  {'SBO': 'SBO:0000627'}
EX_uri_e: uri_e -->  {'SBO': 'SBO:0000627'}
EX_val__L_e: val__L_e -->  {'SBO': 'SBO:0000627'}
EX_xan_e: xan_e -->  {'SBO': 'SBO:0000627'}
EX_xtsn_e: xtsn_e -->  {'SBO': 'SBO:0000627'}
EX_xyl__D_e: xyl__D_e -->  {'SBO': 'SBO:0000627'}
EX_xylt_e: xylt_e -->  {'SBO': 'SBO:0000627'}
EX_xylu__D_e: xylu__D_e -->  {'SBO': 'SBO:0000627'}
EX_zn2_e: zn2_e <=>  {'SBO': 'SBO:0000627'}
EX_zymst_e: zymst_e -->  {'SBO': 'SBO:0000627'}
RBTDG: nad_c + rbt_c <=> h_c + nadh_c + rbl__D_c {'bigg.reaction': ['RBTDG'], 'biocyc': ['META:RIBITOL-2-DEHYDROGENASE-RXN'], 'ec-code': ['1.1.1.56'], 'kegg.reaction': ['R01895'], 'metanetx.reaction': ['MNXR103437'], 'rhea': ['20056', '20053', '20055', '20054'], 'sabiork': ['1952', '1953'], 'sbo': 'SBO:0000176', 'seed.reaction': ['rxn01383']}
SALCNHe: h2o_e + salcn_e --> 2hxmp_e + glc__D_e {'bigg.reaction': ['BG_MBDG'], 'ec-code': ['3.2.1.21'], 'metanetx.reaction': ['MNXR96248'], 'sbo': 'SBO:0000176', 'seed.reaction': ['rxn09979']}
SRC_8aonn_m:  --> 8aonn_m {'SBO': 'SBO:0000628'}
SRC_lipoate_m:  --> lipoate_m {'SBO': 'SBO:0000628'}
SRC_trnaala_c:  --> trnaala_c {'SBO': 'SBO:0000628'}
SRC_trnaarg_c:  --> trnaarg_c {'SBO': 'SBO:0000628'}
SRC_trnaasn_c:  --> trnaasn_c {'SBO': 'SBO:0000628'}
SRC_trnaasp_c:  --> trnaasp_c {'SBO': 'SBO:0000628'}
SRC_trnacys_c:  --> trnacys_c {'SBO': 'SBO:0000628'}
SRC_trnagln_c:  --> trnagln_c {'SBO': 'SBO:0000628'}
SRC_trnaglu_c:  --> trnaglu_c {'SBO': 'SBO:0000628'}
SRC_trnagly_c:  --> trnagly_c {'SBO': 'SBO:0000628'}
SRC_trnahis_c:  --> trnahis_c {'SBO': 'SBO:0000628'}
SRC_trnaile_c:  --> trnaile_c {'SBO': 'SBO:0000628'}
SRC_trnaleu_c:  --> trnaleu_c {'SBO': 'SBO:0000628'}
SRC_trnalys_c:  --> trnalys_c {'SBO': 'SBO:0000628'}
SRC_trnamet_c:  --> trnamet_c {'SBO': 'SBO:0000628'}
SRC_trnaphe_c:  --> trnaphe_c {'SBO': 'SBO:0000628'}
SRC_trnapro_c:  --> trnapro_c {'SBO': 'SBO:0000628'}
SRC_trnaser_c:  --> trnaser_c {'SBO': 'SBO:0000628'}
SRC_trnathr_c:  --> trnathr_c {'SBO': 'SBO:0000628'}
SRC_trnatrp_c:  --> trnatrp_c {'SBO': 'SBO:0000628'}
SRC_trnatyr_c:  --> trnatyr_c {'SBO': 'SBO:0000628'}
SRC_trnaval_c:  --> trnaval_c {'SBO': 'SBO:0000628'}
```

In [20]:

```
for r in sorted(model.reactions, key=lambda x: x.id):
    r.annotation = {}
```

In [21]:

```
for r in model.reactions:
    if r.boundary:
        if r.id.startswith('DM_') or r.id.startswith('SRC_'):
            r.annotation = {'SBO': 'SBO:0000628'}
        elif r.id.startswith('EX_'):
            r.annotation = {'SBO': 'SBO:0000627'}
        elif r.id.startswith('SK_'):
            r.annotation = {'SBO': 'SBO:0000632'}
```

In [22]:

```
for r in model.reactions:
    if len(r.compartments) > 1 and 't' in r.id:
        print(r)
```

```
PYRt2: h_e + pyr_e --> h_c + pyr_c
CYSt2r: cys__L_e + h_e <=> cys__L_c + h_c
FE2t: fe2_e <=> fe2_c
COAtim: coa_c --> coa_m
AMETt2: amet_e + h_e --> amet_c + h_c
ACtr: ac_e <=> ac_c
INSTt2: h_e + inost_e --> h_c + inost_c
MALtm: mal__L_c + pi_m --> mal__L_m + pi_c
URAt2: h_e + ura_e --> h_c + ura_c
ERGSTt: ergst_e <=> ergst_c
H2Otm: h2o_c <=> h2o_m
NAt3_1: h_e + na1_c <=> h_c + na1_e
PTRCt3i: h_c + ptrc_e --> h_e + ptrc_c
LANOSTt: lanost_e <=> lanost_c
CITtam: cit_c + mal__L_m <=> cit_m + mal__L_c
UDPGALt2g: udpgal_c --> udpgal_g
GTPt2m: gdp_m + gtp_c + h_c --> gdp_c + gtp_m + h_m
TYRt6: h_v + tyr__L_c --> h_c + tyr__L_v
TYRt7: h_v + tyr__L_v --> h_c + tyr__L_c
ILEt7: h_v + ile__L_v --> h_c + ile__L_c
ORNt3m: h_c + orn_m --> h_m + orn_c
ZYMSTt: zymst_e <=> zymst_c
URIt2: h_e + uri_e --> h_c + uri_c
ARGt2r: arg__L_e + h_e <=> arg__L_c + h_c
ASNt6: asn__L_c + h_v --> asn__L_v + h_c
ADNt2: adn_e + h_e --> adn_c + h_c
UREA2t2: 2.0 h_e + urea_e <=> 2.0 h_c + urea_c
ATP2tp_H: amp_x + atp_c + 2.0 h_c --> amp_c + atp_x + 2.0 h_x
METt2r: h_e + met__L_e <=> h_c + met__L_c
GTHRDt2: gthrd_e --> gthrd_c
CYTDt2: cytd_e + h_e --> cytd_c + h_c
PIt2m: h_c + pi_c --> h_m + pi_m
SPMDt3i: h_c + spmd_e --> h_e + spmd_c
CITtcm: cit_c + icit_m <=> cit_m + icit_c
THRt2r: h_e + thr__L_e <=> h_c + thr__L_c
LYSt6: h_v + lys__L_c --> h_c + lys__L_v
VALt2r: h_e + val__L_e <=> h_c + val__L_c
ASNt7: asn__L_v + h_v --> asn__L_c + h_c
NH4t: nh4_e <=> nh4_c
ADEt2: ade_e + h_e --> ade_c + h_c
GLUt2r: glu__L_e + h_e <=> glu__L_c + h_c
FECOSTt: fecost_e <=> fecost_c
LEUt2r: h_e + leu__L_e <=> h_c + leu__L_c
ABUTt2r: 4abut_e + h_e <=> 4abut_c + h_c
GLUt7m: glu__L_c --> glu__L_m
HISt2r: h_e + his__L_e <=> h_c + his__L_c
PROt2r: h_e + pro__L_e <=> h_c + pro__L_c
ALAt2r: ala__L_e + h_e <=> ala__L_c + h_c
CSNt2: csn_e + h_e --> csn_c + h_c
GLNt6: gln__L_c + h_v --> gln__L_v + h_c
H2Ot: h2o_e <=> h2o_c
ASPt7: asp__L_v + h_v --> asp__L_c + h_c
PHEt2r: h_e + phe__L_e <=> h_c + phe__L_c
PNTOt2: h_e + pnto__R_e <=> h_c + pnto__R_c
LYSt2r: h_e + lys__L_e <=> h_c + lys__L_c
GLYCt2: glyc_e + h_e --> glyc_c + h_c
TYRt2r: h_e + tyr__L_e <=> h_c + tyr__L_c
FRUt2: fru_e + h_e --> fru_c + h_c
THMt2: h_e + thm_e --> h_c + thm_c
MANt2: h_e + man_e --> h_c + man_c
CHLt2: chol_e + h_e --> chol_c + h_c
LEUt6: h_v + leu__L_c --> h_c + leu__L_v
FADFMNtm: fad_c + fmn_m --> fad_m + fmn_c
GUAt2r: gua_e + h_e <=> gua_c + h_c
GLUt7: glu__L_v + h_v --> glu__L_c + h_c
EPISTt: epist_e <=> epist_c
CTPtm: cmp_m + ctp_c + 2.0 h_c --> cmp_c + ctp_m + 2.0 h_m
ORNt2r: h_e + orn_e <=> h_c + orn_c
LEUt7: h_v + leu__L_v --> h_c + leu__L_c
ILEt6: h_v + ile__L_c --> h_c + ile__L_v
GALt2: gal_e + h_e --> gal_c + h_c
SUCFUMtm: fum_m + succ_c --> fum_c + succ_m
LCYSTintv: cysi__L_v + h_v --> cysi__L_c + h_c
G3PIt: g3pi_e <=> g3pi_c
D_LACt2: h_e + lac__D_e <=> h_c + lac__D_c
GLYCt: glyc_c <=> glyc_e
GLYt2r: gly_e + h_e <=> gly_c + h_c
FA240tp: ttc_c --> ttc_x
ILEt2r: h_e + ile__L_e <=> h_c + ile__L_c
GLNt2r: gln__L_e + h_e <=> gln__L_c + h_c
FA260tp: hexc_c --> hexc_x
ASNt2r: asn__L_e + h_e <=> asn__L_c + h_c
AMETtm: amet_c <=> amet_m
NACt: nac_e <=> nac_c
SERt2r: h_e + ser__L_e <=> h_c + ser__L_c
MALTt2: h_e + malt_e --> h_c + malt_c
AMETt2m: ahcys_m + amet_c <=> ahcys_c + amet_m
GDPMANNtg: gdpmann_c + gmp_g --> gdpmann_g + gmp_c
SPRMt2i: h_c + sprm_e --> h_e + sprm_c
SPMDtex2: spmd_c --> spmd_e
SO3ti: so3_c --> so3_e
SUCCtm: pi_m + succ_c --> pi_c + succ_m
ALLTNti: alltn_e --> alltn_c
G3PCt: g3pc_e <=> g3pc_c
GLNt7: gln__L_v + h_v --> gln__L_c + h_c
ASPt2r: asp__L_e + h_e <=> asp__L_c + h_c
PIt2r: h_e + pi_e <=> h_c + pi_c
PTRCtex2: ptrc_c --> ptrc_e
ALLTTti: alltt_e --> alltt_c
L_LACt2r: h_e + lac__L_e <=> h_c + lac__L_c
UTPtm: 2.0 h_c + ump_m + utp_c --> 2.0 h_m + ump_c + utp_m
GLCt1: glc__D_e --> glc__D_c
TRPt2r: h_e + trp__L_e <=> h_c + trp__L_c
PEPDt3: h_e + pepd_e --> h_c + pepd_c
SO4ti: so4_e --> so4_c
HISt6: h_v + his__L_c --> h_c + his__L_v
NO3t: no3_e <=> no3_c
TGUAt: tgua_e <=> tgua_c
NA1tm: na1_c <=> na1_m
ADNtm: adn_c <=> adn_m
SPMDtmr: h_m + spmd_c <=> h_c + spmd_m
PGP1819Z160t: atp_c + h2o_c + pgp1819Z160_c --> adp_c + h_c + pgp1819Z160_e + pi_c
URATEtm: h_c + urate_c <=> h_m + urate_m
PE1819Z1819Zt: atp_c + h2o_c + pe1819Z1819Z_c --> adp_c + h_c + pe1819Z1819Z_e + pi_c
PAIL1819Z160t: atp_c + h2o_c + pail1819Z160_c --> adp_c + h_c + pail1819Z160_e + pi_c
PG1819Z160t: atp_c + h2o_c + pg1819Z160_c --> adp_c + h_c + pg1819Z160_e + pi_c
5AOPt2: 5aop_e + h_e --> 5aop_c + h_c
5FLURAt: 5flura_e <=> 5flura_c
SO4t2: h_e + so4_e <=> h_c + so4_c
URATEt_1: h_e + urate_e <=> h_c + urate_c
6MPURt: 6mpur_e <=> 6mpur_c
PItn: pi_n <=> pi_c
FE3t: fe3_e <=> fe3_c
PE1801829Z12Zt: atp_c + h2o_c + pe1801829Z12Z_c --> adp_c + h_c + pe1801829Z12Z_e + pi_c
H2Otp: h2o_c <=> h2o_x
NH4tp: nh4_c <=> nh4_x
PE1819Z1829Z12Zt: atp_c + h2o_c + pe1819Z1829Z12Z_c --> adp_c + h_c + pe1819Z1829Z12Z_e + pi_c
PE1801819Zt: atp_c + h2o_c + pe1801819Z_c --> adp_c + h_c + pe1801819Z_e + pi_c
TEGAt: tega_e <=> tega_c
CLPNDCRNt: clpndcrn_c --> clpndcrn_m
GALt1r: gal_e <=> gal_c
MANt1r: man_e <=> man_c
GDPFUCtg: gdpfuc_c + gmp_g <=> gdpfuc_g + gmp_c
ESTRONEGLCt: atp_c + estroneglc_c + h2o_c --> adp_c + estroneglc_e + h_c + pi_c
CRNtim: crn_m --> crn_c
UDPGALtg: udpgal_c + ump_g <=> udpgal_g + ump_c
Kt3g: h_g + k_c <=> h_c + k_g
LNLNCGCRNt: lnlncgcrn_c --> lnlncgcrn_m
UREAtm: urea_c <=> urea_m
CLOXAtex2: 2.0 cl_e + oxa_c --> 2.0 cl_c + oxa_e
ESTRADIOLGLCt2: atp_c + estradiolglc_c + h2o_c --> adp_c + estradiolglc_e + h_c + pi_c
ESTRIOLGLCte: atp_c + estriolglc_c + h2o_c --> adp_c + estriolglc_e + h_c + pi_c
GCHOLAt3: atp_c + gchola_c + h2o_c --> adp_c + gchola_e + h_c + pi_c
NRVNCCRNt: nrvnccrn_c --> nrvnccrn_m
DCSPTN1CRNt: dcsptn1crn_c --> dcsptn1crn_m
XYLt: xyl__D_e <=> xyl__D_c
MALTt1r: malt_e <=> malt_c
SO4OXAtex2: oxa_c + 2.0 so4_e --> oxa_e + 2.0 so4_c
FRUt1r: fru_e <=> fru_c
TSTSTERONEGLCte: atp_c + h2o_c + tststeroneglc_c --> adp_c + h_c + pi_c + tststeroneglc_e
UDPGLCtg: udpg_c + ump_g <=> udpg_g + ump_c
TETTET6CRNt: tettet6crn_c --> tettet6crn_m
CLOHtex2: 2.0 cl_e + oh1_c --> 2.0 cl_c + oh1_e
CLHCOtex: cl_e + 2.0 hco3_c --> cl_c + 2.0 hco3_e
BILGLCURte: atp_c + bilglcur_c + h2o_c <=> adp_c + bilglcur_e + h_c + pi_c
SO4CLtex2: cl_c + 2.0 so4_e --> cl_e + 2.0 so4_c
TTDCRNt: ttdcrn_c --> ttdcrn_m
PTDCACRNt: ptdcacrn_c --> ptdcacrn_m
ASNtN1: asn__L_e + h_c + 2.0 na1_e <=> asn__L_c + h_e + 2.0 na1_c
CHOLATEt3: atp_c + cholate_c + h2o_c --> adp_c + cholate_e + h_c + pi_c
GAMt1r: gam_e <=> gam_c
C161CRN2t: hdd2crn_c --> hdd2crn_m
CAMPt: atp_c + camp_c + h2o_c --> adp_c + camp_e + h_c + pi_c
PAPStg: paps_c <=> paps_g
PROSTGE2t3: atp_c + h2o_c + prostge2_c --> adp_c + h_c + pi_c + prostge2_e
PRODt2r: h_e + pro__D_e <=> h_c + pro__D_c
UGALNACtg: udpacgal_c + ump_g <=> udpacgal_g + ump_c
ELAIDCRNt: elaidcrn_c --> elaidcrn_m
HEXCCRNt: hexccrn_c --> hexccrn_m
CGMPt: 35cgmp_c + atp_c + h2o_c --> 35cgmp_e + adp_c + h_c + pi_c
CLFORtex2: 2.0 cl_e + for_c --> 2.0 cl_c + for_e
GLCtg: glc__D_c <=> glc__D_g
NAt3_1g: h_g + na1_c <=> h_c + na1_g
DALAt2r: ala__D_e + h_e <=> ala__D_c + h_c
ALAtN1: ala__L_e + h_c + 2.0 na1_e <=> ala__L_c + h_e + 2.0 na1_c
HPDCACRNt: hpdcacrn_c --> hpdcacrn_m
5ADTSTSTERONEGLCte: 5adtststeroneglc_c + atp_c + h2o_c --> 5adtststeroneglc_e + adp_c + h_c + pi_c
C160CRNt: pmtcrn_c --> pmtcrn_m
C181CRNt: odecrn_c --> odecrn_m
BILDGLCURte: atp_c + bildglcur_c + h2o_c <=> adp_c + bildglcur_e + h_c + pi_c
CGLYt3_2: cgly_e + 2.0 h_e --> cgly_c + 2.0 h_c
ACRNtm: acrn_c --> acrn_m
OXAHCOtex: 2.0 hco3_c + oxa_e --> 2.0 hco3_e + oxa_c
C161CRNt: hdcecrn_c --> hdcecrn_m
GLNtN1: gln__L_e + h_c + 2.0 na1_e <=> gln__L_c + h_e + 2.0 na1_c
SO4HCOtex: 2.0 hco3_c + so4_e --> 2.0 hco3_e + so4_c
AHANDROSTANGLCte: ahandrostanglc_c + atp_c + h2o_c --> adp_c + ahandrostanglc_e + h_c + pi_c
ATPtm: adp_c + atp_m --> adp_m + atp_c
ACCOAtr: accoa_c <=> accoa_r
HIStN1: h_c + his__L_e + 2.0 na1_e <=> h_e + his__L_c + 2.0 na1_c
TCHOLAt3: atp_c + h2o_c + tchola_c --> adp_c + h_c + pi_c + tchola_e
ACCOAgt: accoa_c <=> accoa_g
ANDRSTRNGLCte: andrstrnglc_c + atp_c + h2o_c --> adp_c + andrstrnglc_e + h_c + pi_c
PROSTGE1t3: atp_c + h2o_c + prostge1_c --> adp_c + h_c + pi_c + prostge1_e
CLHCO3tex2: 2.0 cl_e + hco3_c --> 2.0 cl_c + hco3_e
DHAAt1r: dhdascb_e <=> dhdascb_c
SUCCtp: succ_c <=> succ_x
ICITtp: icit_c <=> icit_x
FRDPtm: frdp_c <=> frdp_m
IPDPtm: ipdp_c <=> ipdp_m
GRDPtm: grdp_c <=> grdp_m
PItx: pi_c <=> pi_x
PIt9: 2.0 na1_e + pi_e <=> 2.0 na1_c + pi_c
CER618trg: cer6_18_r --> cer6_18_g
CER124trg: cer1_24_r --> cer1_24_g
CER126trg: cer1_26_r --> cer1_26_g
CER224trg: cer2_24_r --> cer2_24_g
CER226trg: cer2_26_r --> cer2_26_g
CER324trg: cer3_24_r --> cer3_24_g
CER326trg: cer3_26_r --> cer3_26_g
FRDPtcr: frdp_c <=> frdp_r
TAGtrd: 0.03 hdcoa_r + 0.48 odecoa_r + 0.42 pmtcoa_r + 0.15 stcoa_r + 0.01 triglyc_RT_r --> 0.03 arachcoa_r + 0.06 hpdcacoa_r + 0.27 lnlncgcoa_r + 0.69 ocdycacoa_r + 0.01 triglyc_RT_d + 0.03 ttccoa_r
EPISTESTtrd: epistest_RT_r --> epistest_RT_d
ERGSTESTtrd: ergstest_RT_r --> ergstest_RT_d
FECOSTESTtrd: fecostest_RT_r --> fecostest_RT_d
LANOSTESTtrd: lanostest_RT_r --> lanostest_RT_d
ZYMSTESTtrd: zymstest_RT_r --> zymstest_RT_d
GLYCtm: glyc_c <=> glyc_m
PAterm_RT: pa_RT_r <=> pa_RT_m
CDPDAGterm_RT: cdpdag_RT_r <=> cdpdag_RT_m
PSterm_RT: ps_RT_r <=> ps_RT_m
PEterm_RT: pe_RT_r <=> pe_RT_m
UREAt: urea_e <=> urea_c
H2Oter: h2o_c <=> h2o_r
H2Otg: h2o_c <=> h2o_g
H2Otn: h2o_n <=> h2o_c
H2Otv: h2o_c <=> h2o_v
CO2t: co2_e <=> co2_c
CO2ter: co2_c <=> co2_r
CO2tg: co2_c <=> co2_g
CO2tm: co2_c <=> co2_m
CO2tn: co2_n <=> co2_c
CO2tp: co2_c <=> co2_x
CO2tv: co2_c <=> co2_v
O2t: o2_e <=> o2_c
O2ter: o2_c <=> o2_r
O2tm: o2_c <=> o2_m
O2tn: o2_c <=> o2_n
O2tp: o2_c <=> o2_x
PCRNtm: pcrn_c <=> pcrn_m
PCRNtc: pcrn_x --> pcrn_c
ACRNtp: acrn_x --> acrn_c
DCACOAtr: dcacoa_c <=> dcacoa_r
DDCACOAtr: ddcacoa_c <=> ddcacoa_r
HDCOAtr: hdcoa_c <=> hdcoa_r
HEXCCOAtr: hexccoa_c <=> hexccoa_r
HMGCOAtr: hmgcoa_c <=> hmgcoa_r
LNLNCGCOAtr: lnlncgcoa_c <=> lnlncgcoa_r
MALCOAtr: malcoa_c <=> malcoa_r
OCDYCACOAtr: ocdycacoa_c <=> ocdycacoa_r
ODECOAtr: odecoa_c <=> odecoa_r
PMTCOAtr: pmtcoa_c <=> pmtcoa_r
STCOAtr: stcoa_c <=> stcoa_r
TDCOAtr: tdcoa_c <=> tdcoa_r
TTCCOAtr: ttccoa_c <=> ttccoa_r
GLYC3Pter: glyc3p_c <=> glyc3p_r
DHAPter: dhap_c <=> dhap_r
DOLPter: dolp_r --> dolp_c
IND3ETHt: ind3eth_c <=> ind3eth_e
PYRt2m: h_c + pyr_c <=> h_m + pyr_m
LEUt5m: leu__L_c <=> leu__L_m
VALt5m: val__L_c <=> val__L_m
ILEtmi: ile__L_m <=> ile__L_c
3MOPtm: 3mop_c <=> 3mop_m
3MOBtm: 3mob_c <=> 3mob_m
4MOPtm: 4mop_c <=> 4mop_m
THRtm: thr__L_c <=> thr__L_m
2OBUTtm: 2obut_c <=> 2obut_m
2OBUTt: 2obut_e + h_e <=> 2obut_c + h_c
NH4tm: nh4_c <=> nh4_m
ACALDtm: acald_m <=> acald_c
Htr: h_c <=> h_r
PIter: pi_r <=> pi_c
PPItr: ppi_c <=> ppi_r
COAtr: coa_c <=> coa_r
NADPHtru: nadph_c --> nadph_r
NADPtru: nadp_r --> nadp_c
INOSTter: inost_c --> inost_r
CDPCHOLter: cdpchol_c <=> cdpchol_r
FORtr: for_c <=> for_r
AHCYStr: ahcys_c <=> ahcys_r
ATPter: atp_c <=> atp_r
ADPter: adp_r <=> adp_c
AMPter: amp_c <=> amp_r
CTPter: ctp_c <=> ctp_r
CDPter: cdp_r <=> cdp_c
CMPter: cmp_c <=> cmp_r
SERter: ser__L_c <=> ser__L_r
AKGMALtm: akg_m + mal__L_c --> akg_c + mal__L_m
AKGCITtm: akg_c + cit_m --> akg_m + cit_c
UCP2ASPtm: asp__L_m + h_c + pi_c --> asp__L_c + h_m + pi_m
UCP2MALtm: h_c + mal__L_m + pi_c --> h_m + mal__L_c + pi_m
UCP2OAAtm: h_c + oaa_m + pi_c --> h_m + oaa_c + pi_m
OAAIPMtm: 3c3hmp_m + oaa_c --> 3c3hmp_c + oaa_m
ORNARGtm: arg__L_c + orn_m --> arg__L_m + orn_c
ORNLYStm: lys__L_c + orn_m --> lys__L_m + orn_c
ARGt7m: arg__L_c --> arg__L_m
LYSt7m: lys__L_c --> lys__L_m
FE2tm: fe2_c + h_c --> fe2_m + h_m
CTPtm2: cmp_m + ctp_c + fe2_c --> cmp_c + ctp_m + fe2_m
UTPtm2: fe2_c + ump_m + utp_c --> fe2_m + ump_c + utp_m
GLYtm: gly_c <=> gly_m
PYDX5Ptm: pydx5p_c <=> pydx5p_m
5AOPtm: 5aop_c <=> 5aop_m
PPPG9tm: pppg9_c <=> pppg9_m
GLYCLTt: glyclt_e <=> glyclt_c
GLYCLTtm: glyclt_c <=> glyclt_m
GLYCLTtp: glyclt_c --> glyclt_x
GLXt: glx_c --> glx_e
GLXtm: glx_c <=> glx_m
GLXtp: glx_c <=> glx_x
4ABZt: 4abz_c <=> 4abz_e
4ABZtm: 4abz_c <=> 4abz_m
METtm: met__L_c <=> met__L_m
CYStm: cys__L_c <=> cys__L_m
HPDCACOAtr: hpdcacoa_c <=> hpdcacoa_r
FA200tp: arach_c --> arach_x
FA220tp: docosac_c --> docosac_x
HDCAt: hdca_e --> hdca_c
HDCEAt: hdcea_e --> hdcea_c
OCDCAt: ocdca_e --> ocdca_c
OCDCEAt: ocdcea_e --> ocdcea_c
OCDCYAt: ocdcya_e --> ocdcya_c
LNLNCGt: lnlncg_e --> lnlncg_c
HPDCAt: hpdca_e --> hpdca_c
ARACHt: arach_e --> arach_c
DOCOSACt: docosac_e --> docosac_c
TTCt: ttc_e --> ttc_c
EPISTtr: epist_c <=> epist_r
ERGSTtr: ergst_c <=> ergst_r
FECOSTtr: fecost_c <=> fecost_r
LANOSTtr: lanost_c <=> lanost_r
ZYMSTtr: zymst_c <=> zymst_r
MG2t: mg2_e <=> mg2_c
MNt: mn2_e <=> mn2_c
CU2t: cu2_e <=> cu2_c
Zn2t: zn2_e <=> zn2_c
Kt2r: h_e + k_e <=> h_c + k_c
CA2t: ca2_e <=> ca2_c
4HBZtp: 4hbz_c <=> 4hbz_x
4HBZtm: 4hbz_c <=> 4hbz_m
T4HCINNMtp: T4hcinnm_c --> T4hcinnm_x
T4HCINNMt: T4hcinnm_e <=> T4hcinnm_c
34DHBZtp: 34dhbz_c <=> 34dhbz_x
PPItx: ppi_c <=> ppi_x
Htx: h_c <=> h_x
COAtp: coa_c <=> coa_x
CRNtp: crn_c <=> crn_x
AKGtp: akg_c <=> akg_x
MAL_Ltx: mal__L_x <=> mal__L_c
OAAtp: oaa_x <=> oaa_c
FERt: fer_e <=> fer_c
34DHCINMtp: 34dhcinm_c --> 34dhcinm_x
BZt: bz_e <=> bz_c
ARAB_Lt: arab__L_e <=> arab__L_c
LYXt: lyx__L_e <=> lyx__L_c
ABTt: abt_e <=> abt_c
XYLTt: xylt_e <=> xylt_c
XYLUDte: xylu__D_c <=> xylu__D_e
ABT_Dt: abt__D_e <=> abt__D_c
RBL_Dt: rbl__D_c <=> rbl__D_e
GGDPter: ggdp_c --> ggdp_r
BCAROtrd: bcaro_r --> bcaro_d
TREt2: h_e + tre_e --> h_c + tre_c
GALTt: galt_c <=> galt_e
SER_Dt: ser__D_e --> ser__D_c
SBT_Dt: sbt__D_e <=> sbt__D_c
GLCNte: glcn_c <=> glcn_e
H2O2t: h2o2_e <=> h2o2_c
RIBt: rib__D_e <=> rib__D_c
THYMDt1: thymd_e --> thymd_c
12PPDSt: 12ppd__S_e <=> 12ppd__S_c
12PPDRte: 12ppd__R_c <=> 12ppd__R_e
MELIBt2: h_e + melib_e --> h_c + melib_c
PPAtr: ppa_e --> ppa_c
ACACt2: acac_e + h_e <=> acac_c + h_c
ACACt2m: acac_c + h_c <=> acac_m + h_m
ACACtx: acac_c <=> acac_x
TYMte: tym_c <=> tym_e
ETHAt: etha_e <=> etha_c
ARAB_Dt: arab__D_e <=> arab__D_c
DRIBt: drib_e <=> drib_c
SRB_Lt: srb__L_e <=> srb__L_c
BUTt: but_e <=> but_c
HXAt3: hxa_c <=> hxa_e
C40CRNt: c4crn_c --> c4crn_m
C60CRNt: c6crn_c --> c6crn_m
4HBZte: 4hbz_c <=> 4hbz_e
BHBt: bhb_e + h_e <=> bhb_c + h_c
BHBtm: bhb_c + h_c <=> bhb_m + h_m
GHBt: ghb_e + h_e <=> ghb_c + h_c
GHBtm: ghb_c + h_c <=> ghb_m + h_m
4ABUTtm: 4abut_c <=> 4abut_m
5DGLCNt: 5dglcn_e <=> 5dglcn_c
QUINt: quin_e <=> quin_c
ADtr: ad_e <=> ad_c
ACGLUtd: acglu_c <=> acglu_e
HOMt: hom__L_e <=> hom__L_c
DHAt: dha_e <=> dha_c
BTDt_RR: btd_RR_c <=> btd_RR_e
CITRte: citr__L_c <=> citr__L_e
MMAt2e: mma_c <=> mma_e
AGRMte: agm_c <=> agm_e
AGMt_m: agm_c <=> agm_m
HISTAtu: hista_e <=> hista_c
FRMDt: frmd_e <=> frmd_c
GSNt: gsn_e <=> gsn_c
THYMt: thym_e <=> thym_c
XANt: xan_e <=> xan_c
XTSNtr: xtsn_e <=> xtsn_c
PPIt2r: h_e + ppi_e <=> h_c + ppi_c
PPPIt2r: h_e + pppi_e <=> h_c + pppi_c
CBPt: cbp_e <=> cbp_c
GLYC_Rt: glyc__R_c <=> glyc__R_e
CCMPt2: 35ccmp_e <=> 35ccmp_c
CAMPt2: camp_e <=> camp_c
CGMPt2: 35cgmp_e <=> 35cgmp_c
PSERtr: pser__L_c <=> pser__L_e
CHOLPtr: cholp_e <=> cholp_c
ETHAMPtr: ethamp_e <=> ethamp_c
MINOHPtn: minohp_c <=> minohp_n
TSULt4_3: 3.0 na1_e + tsul_e <=> 3.0 na1_c + tsul_c
LCYSTt: Lcyst_e + cl_e + 2.0 na1_e <=> Lcyst_c + cl_c + 2.0 na1_c
3SALAt: 3sala_e <=> 3sala_c
CYST_Ltr: cyst__L_e <=> cyst__L_c
METSOX1t: metsox_S__L_e <=> metsox_S__L_c
METSOX2t: metsox_R__L_e <=> metsox_R__L_c
TAURt: taur_c <=> taur_e
MEOHt2: meoh_e <=> meoh_c
RBTt: rbt_c --> rbt_e
BTNt2i: btn_e + h_e --> btn_c + h_c
FOLt: fol_e + h_e --> fol_c + h_c
NADtm: nad_c --> nad_m
PYDXNtr: pydxn_e <=> pydxn_c
RIBFLVt2: h_e + ribflv_e --> h_c + ribflv_c
```

In [23]:

```
for r in sorted(model.reactions, key=lambda x: x.id):
    if (len(r.compartments) > 1 and not 't' in r.id and not 'd' in r.compartments and
        not any(x in [m.id for m in r.metabolites] for x in ['focytc_m','q9_m','fadh2_m','dolmanp_r','pc_RT_r'])):
        print(r)
```

```
2OXOADPTm: 2oxoadp_c + akg_m <=> 2oxoadp_m + akg_c
3OXOADPTm: 3oxoadp_c + akg_m <=> 3oxoadp_m + akg_c
3SALAASPm: 3sala_m + asp__L_c <=> 3sala_c + asp__L_m
AMETr: amet_c <=> amet_r
ASPDTDe: asp__D_c <=> asp__D_e
ASPGLUm: asp__L_m + glu__L_c + h_c --> asp__L_c + glu__L_m + h_m
ATPS: atp_c + h2o_c --> adp_c + h_e + pi_c
ATPS2v: atp_c + h2o_c + h_c --> adp_c + 2.0 h_v + pi_c
ATPS3m: adp_m + 3.0 h_c + pi_m --> atp_m + h2o_m + 2.0 h_m
CYSTSERex: cysi__L_e + ser__L_c --> cysi__L_c + ser__L_e
Cu1ATPase: atp_c + cu_c + h2o_c --> adp_c + cu_e + h_c + pi_c
FA140COAabcp: atp_c + 2.0 h2o_c + tdcoa_c --> adp_c + coa_c + 2.0 h_c + pi_c + ttdca_x
FA141COAabcp: atp_c + 2.0 h2o_c + tdecoa_c --> adp_c + coa_c + 2.0 h_c + pi_c + ttdcea_x
FA160COAabcp: atp_c + 2.0 h2o_c + pmtcoa_c --> adp_c + coa_c + 2.0 h_c + hdca_x + pi_c
FA161COAabcp: atp_c + 2.0 h2o_c + hdcoa_c --> adp_c + coa_c + 2.0 h_c + hdcea_x + pi_c
FA180COAabcp: atp_c + 2.0 h2o_c + stcoa_c --> adp_c + coa_c + 2.0 h_c + ocdca_x + pi_c
FA181COAabcp: atp_c + 2.0 h2o_c + odecoa_c --> adp_c + coa_c + 2.0 h_c + ocdcea_x + pi_c
FA182COAabcp: atp_c + 2.0 h2o_c + ocdycacoa_c --> adp_c + coa_c + 2.0 h_c + ocdcya_x + pi_c
FA183COAabcp: atp_c + 2.0 h2o_c + lnlncgcoa_c --> adp_c + coa_c + 2.0 h_c + lnlncg_x + pi_c
FE2GTPabc: fe2_e + gtp_c + h2o_c --> fe2_c + gdp_c + h_c + pi_c
GTHRDabcv: atp_v + gthrd_c + h2o_v --> adp_v + gthrd_v + h_v + pi_v
GTMLTe: ala__L_c + gthrd_c --> cgly_e + gluala_e
KATPase: atp_c + h2o_c + h_e + k_c --> adp_c + 2.0 h_c + k_e + pi_c
NARK: no2_c <=> no2_e
Na1ATPase: atp_c + h2o_c + h_e + na1_c --> adp_c + 2.0 h_c + na1_e + pi_c
PMCA: atp_c + ca2_c + h2o_c + h_e --> adp_c + ca2_e + 2.0 h_c + pi_c
SCP21x: phytcoa_c <=> phytcoa_x
SCP22x: dmnoncoa_c <=> dmnoncoa_x
SERCA: atp_c + 2.0 ca2_c + h2o_c + 2.0 h_r --> adp_c + 2.0 ca2_r + 3.0 h_c + pi_c
SERLYSNaex: lys__L_c + na1_e + ser__L_e --> lys__L_e + na1_c + ser__L_c
TCHOLAabcv: atp_v + h2o_v + tchola_c --> adp_v + h_v + pi_v + tchola_v
```

In [24]:

```
for r in model.reactions:
    if len(r.compartments) > 1 and 't' in r.id:
        r.annotation['SBO'] = 'SBO:0000655'
    if (len(r.compartments) > 1 and not 't' in r.id and not ('d' in r.compartments or
        any(x in [m.id for m in r.metabolites] for x in ['focytc_m','q9_m','fadh2_m','dolmanp_r','pc_RT_r']))):
        r.annotation['SBO'] = 'SBO:0000655'
```

In [25]:

```
for r in model.reactions:
    if len(r.compartments) == 1 and not r.boundary:
        r.annotation['SBO'] = 'SBO:0000176'
    if (len(r.compartments) > 1 and not 't' in r.id and ('d' in r.compartments or
        any(x in [m.id for m in r.metabolites] for x in ['focytc_m','q9_m','fadh2_m','dolmanp_r','pc_RT_r']))):
        r.annotation['SBO'] = 'SBO:0000176'
```

In [26]:

```
model.reactions.get_by_id('BIOMASS_RT').annotation['SBO'] = 'SBO:0000629'
```

In [27]:

```
set(sum([list(r.annotation.keys()) for r in model.reactions],[]))
```

Out[27]:

```
{'SBO'}
```

In [28]:

```
for r in model.reactions:
    if r.id in bigg_universal.reactions:
        #r.notes.update(dict(bigg_universal.reactions.get_by_id(r.id).notes))
        r.annotation.update(dict(bigg_universal.reactions.get_by_id(r.id).annotation))
```

In [29]:

```
set(sum([list(r.annotation.keys()) for r in model.reactions],[]))
```

Out[29]:

```
{'BioCyc',
 'EC Number',
 'KEGG Reaction',
 'MetaNetX (MNX) Equation',
 'RHEA',
 'Reactome Reaction',
 'SBO',
 'SEED Reaction'}
```

In [30]:

```
model.reactions.get_by_id('PGI').annotation
```

Out[30]:

```
{'SBO': 'SBO:0000176',
 'EC Number': 'http://identifiers.org/ec-code/5.3.1.9',
 'BioCyc': 'http://identifiers.org/biocyc/META:PGLUCISOM-RXN',
 'MetaNetX (MNX) Equation': 'http://identifiers.org/metanetx.reaction/MNXR102535'}
```

In [31]:

```
# Reformat to memote style
r_annotation = dict({'BioCyc': 'biocyc',
                     'EC Number': 'ec-code',
                     'KEGG Reaction': 'kegg.reaction',
                     'MetaNetX (MNX) Equation': 'metanetx.reaction',
                     'RHEA': 'rhea',
                     'Reactome Reaction': 'reactome',
                     'SBO': 'sbo',
                     'SEED Reaction': 'seed.reaction'})
```

In [32]:

```
for r in model.reactions:
    if r.annotation:
        r.annotation = dict((r_annotation[k], v.rsplit('/',1)[-1]) for k, v in r.annotation.items())
    r.annotation['bigg.reaction'] = r.id
```

In [33]:

```
model.reactions.get_by_id('PGI').annotation
```

Out[33]:

```
{'sbo': 'SBO:0000176',
 'ec-code': '5.3.1.9',
 'biocyc': 'META:PGLUCISOM-RXN',
 'metanetx.reaction': 'MNXR102535',
 'bigg.reaction': 'PGI'}
```

In [34]:

```
set(r.subsystem for r in model.reactions)
```

Out[34]:

```
{'',
 'Alanine and Aspartate Metabolism',
 'Alanine and aspartate metabolism',
 'Aldehyde degradation',
 'Alkaloid biosynthesis II',
 'Alternate Carbon Metabolism',
 'Amino sugar and nucleotide sugar metabolism',
 'Aminosugar Metabolism',
 'Anaplerotic Reactions',
 'Arginine and Proline Metabolism',
 'Arginine and proline metabolism',
 'Ascorbate metabolism',
 'B-Ketoadipate pathway',
 'Bile Acid Biosynthesis',
 'Biomass and maintenance functions',
 'Biosynthesis of steroids',
 'Biosynthesis of steroids: Terpenoid backbone synthesis',
 'Biosynthesis of steroids: Ubiquinone/Terpenoid-quinone biosynthesis',
 'Biosynthesis of unsaturated fatty acids',
 'Biotin Metabolism',
 'Butanoate Metabolism',
 'Butanoate metabolism',
 'Calvin cycle/Pentose phosphate pathway',
 'Carbon fixation',
 'Carbon fixation;Glutamate metabolism;Alanine and aspartate metabolism',
 'Carnitine shuttle',
 'Cell Envelope Biosynthesis',
 'Cholesterol Metabolism',
 'Citric Acid Cycle',
 'Cofactor and Prosthetic Group Biosynthesis',
 'Cofactor biosynthesis: Carnitine biosynthesis',
 'Cofactor recycling',
 'Cysteine Metabolism',
 'Cysteine and methionine metabolism',
 'Cysteine metabolism',
 'Drug metabolism - other enzymes',
 'Eicosanoid Metabolism',
 'Extracellular exchange',
 'Fatty Acid Metabolism',
 'Fatty acid activation',
 'Fatty acid elongation',
 'Fatty acid elongation in mitochondria',
 'Fatty acid metabolism',
 'Fatty acid oxidation',
 'Fatty acid oxidation, peroxisome',
 'Fatty acid synthesis',
 'Folate Metabolism',
 'Folate biosynthesis',
 'Fructose and Mannose Metabolism',
 'Galactose metabolism',
 'Glutamate Metabolism',
 'Glutamate metabolism',
 'Glutathione Metabolism',
 'Glycerolipid metabolism',
 'Glycerophospholipid Metabolism',
 'Glycerophospholipid metabolism',
 'Glycine and Serine Metabolism',
 'Glycine, Serine and threonine metabolism',
 'Glycine, Serine, and Threonine Metabolism',
 'Glycine, serine and threonine metabolism',
 'Glycine, serine, alanine, and threonine metabolism',
 'Glycolysis',
 'Glycolysis / Gluconeogenesis',
 'Glycolysis/Gluconeogenesis',
 'Glyoxylate Metabolism',
 'Glyoxylate and Dicarboxylate Metabolism',
 'Glyoxylate metabolism',
 'High-mannose type N-glycan biosynthesis',
 'Histidine Metabolism',
 'Histidine metabolism',
 'Homogentisate Pathway',
 'Inorganic Ion Transport and Metabolism',
 'Inositol Phosphate Metabolism',
 'Inositol phosphate metabolism',
 'Intracellular demand',
 'Lipopolysaccharide Biosynthesis / Recycling',
 'Lysine Metabolism',
 'Lysine biosynthesis',
 'Membrane Lipid Metabolism',
 'Metabolite Repair',
 'Methionine Metabolism',
 'Methionine and cysteine metabolism',
 'Methionine metabolism',
 'Methylglyoxal Metabolism',
 'Murein Recycling',
 'N-Glycan Biosynthesis',
 'N-Glycan Degradation',
 'N-Glycan biosynthesis',
 'NAD Metabolism',
 'Nicotinate and nicotinamide metabolism',
 'Nitrogen Metabolism',
 'Nitrogen metabolism',
 'Nucleotide Salvage Pathway',
 'Nucleotide Sugar Metabolism',
 'Nucleotide interconversion',
 'Nucleotides',
 'One carbon pool by folate',
 'Oxidative Phosphorylation',
 'Oxidative phosphorylation',
 'Pantothenate and CoA biosynthesis',
 'Pentose Phosphate Pathway',
 'Pentose and Glucuronate Interconversions',
 'Pentose phosphate pathway',
 'Phenylalanine metabolism',
 'Polyamine metabolism',
 'Porphyrin and Chlorophyll Metabolism',
 'Porphyrin and chlorophyll metabolism',
 'Propanoate Metabolism',
 'Propanoate metabolism',
 'Purine Catabolism',
 'Purine Metabolism',
 'Purine and Pyrimidine Biosynthesis',
 'Purine metabolism',
 'Pyrimidine Catabolism',
 'Pyrimidine metabolism',
 'Pyruvate Metabolism',
 'Pyruvate metabolism',
 'ROS Detoxification',
 'Riboflavin Metabolism',
 'S_Alanine_and_Aspartate_Metabolism',
 'S_Alternate_Carbon_Metabolism',
 'S_Anaplerotic_reactions',
 'S_Arabinose_Metabolism',
 'S_Arginine_and_Proline_Metabolism',
 'S_Asparagine_metabolism',
 'S_Carbohydrates_and_related_molecules',
 'S_Citric_Acid_Cycle',
 'S_Complex_Alcohol_Metabolism',
 'S_Cysteine_Metabolism',
 'S_Fatty_Acid_Degradation',
 'S_Fatty_Acid_Metabolism',
 'S_Fatty_Acid__Biosynthesis',
 'S_Folate_Metabolism',
 'S_Fructose_and_Mannose_Metabolism',
 'S_Galactose_metabolism',
 'S_Glutamate_metabolism',
 'S_Glutamine_Metabolism',
 'S_Glycerolipid_Metabolism',
 'S_Glycine_and_Serine_Metabolism',
 'S_GlycolysisGluconeogenesis',
 'S_Glycoprotein_Metabolism',
 'S_Histidine_Metabolism',
 'S_Methane_Metabolism',
 'S_Methionine_Metabolism',
 'S_NAD_Biosynthesis',
 'S_Nitrogen_Metabolism',
 'S_Nucleotide_Salvage_Pathway',
 'S_Other',
 'S_Other_Amino_Acid_Metabolism',
 'S_Oxidative_Phosphorylation',
 'S_Pantothenate_and_CoA_Biosynthesis',
 'S_Pentose_Phosphate_Pathway',
 'S_Phospholipid_Biosynthesis',
 'S_Phospholipid_Metabolism',
 'S_Porphyrin_and_Chlorophyll_Metabolism',
 'S_Purine_and_Pyrimidine_Biosynthesis',
 'S_Pyridoxine_Metabolism',
 'S_Pyruvate_Metabolism',
 'S_Quinone_Biosynthesis',
 'S_Riboflavin_Metabolism',
 'S_Sphingolipid_Metabolism',
 'S_Starch_and_Sucrose_Metabolism',
 'S_Sterol_Metabolism',
 'S_Thiamine_Metabolism',
 'S_Threonine_and_Lysine_Metabolism',
 'S_Transport__Endoplasmic_Reticular',
 'S_Transport__Extracellular',
 'S_Transport__Golgi_Apparatus',
 'S_Transport__Mitochondrial',
 'S_Transport__Nuclear',
 'S_Transport__Peroxisomal',
 'S_Transport__Vacuolar',
 'S_Tyrosine__Tryptophan__and_Phenylalanine_Metabolism',
 'S_Valine__Leucine__and_Isoleucine_Metabolism',
 'S_Xylose_Metabolism',
 'S_tRNA_charging',
 'Selenoamino acid metabolism',
 'Starch and Sucrose Metabolism',
 'Starch and sucrose metabolism',
 'Steroid Metabolism',
 'Sulfur Metabolism',
 'Sulfur metabolism',
 'TCA cycle;Carbon fixation;CO2 fixation',
 'Taurine and hypotaurine metabolism',
 'Terpenoid backbone biosynthesis',
 'Tetrahydrobiopterin',
 'Threonine and Lysine Metabolism',
 'Transport',
 'Transport, Endoplasmic Reticular',
 'Transport, Extracellular',
 'Transport, Golgi Apparatus',
 'Transport, Inner Membrane',
 'Transport, Mitochondrial',
 'Transport, Nuclear',
 'Transport, Outer Membrane Porin',
 'Transport, Peroxisomal',
 'Transport, endoplasmic reticular',
 'Transport, extracellular',
 'Transport, glyoxysome',
 'Transport, mitochondria',
 'Transport, mitochondrial',
 'Transport, nuclear',
 'Transport, nucleus',
 'Transport, peroxisomal',
 'Tryptophan metabolism',
 'Tyrosine metabolism',
 'Tyrosine, Tryptophan, and Phenylalanine Metabolism',
 'Ubiquinone synthesis',
 'Unassigned',
 'Urea cycle/amino group metabolism',
 'Valine, Leucine, and Isoleucine Metabolism',
 'Valine, leucine and isoleucine degradation',
 'Valine, leucine, and isoleucine metabolism',
 'Vitamin B2 metabolism',
 'Vitamin B6 Metabolism',
 'Vitamin B6 metabolism',
 'beta-Alanine metabolism',
 'beta-Alanine metabolism;Pyrimidine metabolism',
 'tRNA Charging'}
```

In [35]:

```
for r in model.reactions:
    if r.subsystem.startswith('S_'):
        r.subsystem = r.subsystem.replace('S_','').replace('__',', ').replace('_',' ')
    r.subsystem = r.subsystem.replace('Glycolysis / Gluconeogenesis','Glycolysis/Gluconeogenesis')
    r.subsystem = r.subsystem.replace('GlycolysisGluconeogenesis','Glycolysis/Gluconeogenesis')
    r.subsystem = r.subsystem.replace('metabolism','Metabolism')
    r.subsystem = r.subsystem.replace('aspartate','Aspartate')
    r.subsystem = r.subsystem.replace('reactions','Reactions')
    r.subsystem = r.subsystem.replace('proline','Proline')
    r.subsystem = r.subsystem.replace('serine','Serine')
    r.subsystem = r.subsystem.replace('Serine and threonine','Serine, and Threonine')
    r.subsystem = r.subsystem.replace(', alanine',', Alanine')
    r.subsystem = r.subsystem.replace('phosphate','Phosphate')
    r.subsystem = r.subsystem.replace('pathway','Pathway')
    r.subsystem = r.subsystem.replace('biosynthesis','Biosynthesis')
    r.subsystem = r.subsystem.replace('chlorophyll','Chlorophyll')
    r.subsystem = r.subsystem.replace('sucrose','Sucrose')
    r.subsystem = r.subsystem.replace('endoplasmic reticular','Endoplasmic Reticular')
    r.subsystem = r.subsystem.replace('extracellular','Extracellular')
    r.subsystem = r.subsystem.replace('Transport, mitochondria','Transport, Mitochondrial')
    r.subsystem = r.subsystem.replace('Transport, mitochondrial','Transport, Mitochondrial')
    r.subsystem = r.subsystem.replace('peroxisomal','Peroxisomal')
    r.subsystem = r.subsystem.replace('leucine and isoleucine','Leucine, and Isoleucine')
    r.subsystem = r.subsystem.replace('leucine, and isoleucine','Leucine, and Isoleucine')
    r.subsystem = r.subsystem.replace('charging','Charging')
    r.subsystem = r.subsystem.replace('phosphorylation','Phosphorylation')
    r.subsystem = r.subsystem.replace('degradation','Degradation')
```

In [36]:

```
temp = [r.subsystem for r in model.reactions]
for k in sorted(set(temp)):
    print(k, temp.count(k))
```

```
 86
Alanine and Aspartate Metabolism 10
Aldehyde Degradation 2
Alkaloid Biosynthesis II 1
Alternate Carbon Metabolism 88
Amino sugar and nucleotide sugar Metabolism 2
Aminosugar Metabolism 4
Anaplerotic Reactions 11
Arabinose Metabolism 2
Arginine and Proline Metabolism 42
Ascorbate Metabolism 1
Asparagine Metabolism 1
B-Ketoadipate Pathway 17
Bile Acid Biosynthesis 15
Biomass and maintenance functions 2
Biosynthesis of steroids 1
Biosynthesis of steroids: Terpenoid backbone synthesis 1
Biosynthesis of steroids: Ubiquinone/Terpenoid-quinone Biosynthesis 11
Biosynthesis of unsaturated fatty acids 2
Biotin Metabolism 3
Butanoate Metabolism 5
Calvin cycle/Pentose Phosphate Pathway 1
Carbohydrates and related molecules 3
Carbon fixation 1
Carbon fixation;Glutamate Metabolism;Alanine and Aspartate Metabolism 1
Carnitine shuttle 48
Cell Envelope Biosynthesis 21
Cholesterol Metabolism 10
Citric Acid Cycle 7
Cofactor Biosynthesis: Carnitine Biosynthesis 4
Cofactor and Prosthetic Group Biosynthesis 68
Cofactor recycling 1
Complex Alcohol Metabolism 18
Cysteine Metabolism 15
Cysteine and methionine Metabolism 1
Drug Metabolism - other enzymes 6
Eicosanoid Metabolism 2
Extracellular exchange 266
Fatty Acid Degradation 42
Fatty Acid Metabolism 11
Fatty Acid, Biosynthesis 35
Fatty acid Metabolism 17
Fatty acid activation 17
Fatty acid elongation 3
Fatty acid elongation in mitochondria 21
Fatty acid oxidation 16
Fatty acid oxidation, peroxisome 5
Fatty acid synthesis 1
Folate Biosynthesis 1
Folate Metabolism 33
Fructose and Mannose Metabolism 5
Galactose Metabolism 4
Glutamate Metabolism 13
Glutamine Metabolism 1
Glutathione Metabolism 1
Glycerolipid Metabolism 16
Glycerophospholipid Metabolism 12
Glycine and Serine Metabolism 16
Glycine, Serine, Alanine, and threonine Metabolism 1
Glycine, Serine, and Threonine Metabolism 8
Glycolysis 2
Glycolysis/Gluconeogenesis 23
Glycoprotein Metabolism 4
Glyoxylate Metabolism 9
Glyoxylate and Dicarboxylate Metabolism 2
High-mannose type N-glycan Biosynthesis 1
Histidine Metabolism 19
Homogentisate Pathway 2
Inorganic Ion Transport and Metabolism 1
Inositol Phosphate Metabolism 6
Intracellular demand 25
Lipopolysaccharide Biosynthesis / Recycling 3
Lysine Biosynthesis 1
Lysine Metabolism 3
Membrane Lipid Metabolism 3
Metabolite Repair 8
Methane Metabolism 1
Methionine Metabolism 23
Methionine and cysteine Metabolism 2
Methylglyoxal Metabolism 3
Murein Recycling 1
N-Glycan Biosynthesis 43
N-Glycan Degradation 3
NAD Biosynthesis 13
NAD Metabolism 3
Nicotinate and nicotinamide Metabolism 2
Nitrogen Metabolism 10
Nucleotide Salvage Pathway 85
Nucleotide Sugar Metabolism 1
Nucleotide interconversion 4
Nucleotides 54
One carbon pool by folate 5
Other 4
Other Amino Acid Metabolism 7
Oxidative Phosphorylation 13
Pantothenate and CoA Biosynthesis 10
Pentose Phosphate Pathway 18
Pentose and Glucuronate Interconversions 4
Phenylalanine Metabolism 6
Phospholipid Biosynthesis 44
Phospholipid Metabolism 6
Polyamine Metabolism 1
Porphyrin and Chlorophyll Metabolism 8
Propanoate Metabolism 6
Purine Catabolism 3
Purine Metabolism 8
Purine and Pyrimidine Biosynthesis 41
Pyridoxine Metabolism 1
Pyrimidine Catabolism 1
Pyrimidine Metabolism 15
Pyruvate Metabolism 16
Quinone Biosynthesis 9
ROS Detoxification 2
Riboflavin Metabolism 10
Selenoamino acid Metabolism 2
Sphingolipid Metabolism 65
Starch and Sucrose Metabolism 10
Steroid Metabolism 6
Sterol Metabolism 33
Sulfur Metabolism 5
TCA cycle;Carbon fixation;CO2 fixation 1
Taurine and hypotaurine Metabolism 2
Terpenoid backbone Biosynthesis 1
Tetrahydrobiopterin 4
Thiamine Metabolism 5
Threonine and Lysine Metabolism 19
Transport 2
Transport, Endoplasmic Reticular 43
Transport, Extracellular 220
Transport, Golgi Apparatus 12
Transport, Inner Membrane 1
Transport, Mitochondrial 67
Transport, Mitochondriall 11
Transport, Nuclear 3
Transport, Outer Membrane Porin 7
Transport, Peroxisomal 33
Transport, Vacuolar 19
Transport, glyoxysome 2
Transport, nuclear 1
Transport, nucleus 1
Tryptophan Metabolism 20
Tyrosine Metabolism 10
Tyrosine, Tryptophan, and Phenylalanine Metabolism 37
Ubiquinone synthesis 2
Unassigned 9
Urea cycle/amino group Metabolism 4
Valine, Leucine, and Isoleucine Degradation 6
Valine, Leucine, and Isoleucine Metabolism 30
Vitamin B2 Metabolism 1
Vitamin B6 Metabolism 6
Xylose Metabolism 2
beta-Alanine Metabolism 6
beta-Alanine Metabolism;Pyrimidine Metabolism 1
tRNA Charging 32
```

In [37]:

```
for r in sorted(model.reactions, key=lambda x: x.id):
    if not r.subsystem:
        print(r)
        for m in r.metabolites:
            if len(m.reactions) < 7:
                print([(r2.id, r2.subsystem) for r2 in m.reactions if r2 is not r])
```

```
34DHPHAMT: 34dhpha_c + amet_c --> ahcys_c + h_c + homoval_c
[('34DHPLACOX_NADP', 'Tyrosine Metabolism'), ('34DHALDD', 'Tyrosine Metabolism')]
[]
3DSPHRer: 3dsphgn_r + h_r + nadph_r --> nadp_r + sphgn_r
[('SERPTer', '')]
42A12BOOX: dopa_c + h2o_c + o2_c --> 34dhpac_c + h2o2_c + nh4_c
[('34DHALDD', 'Tyrosine Metabolism'), ('34DHPLACOX_NADP', 'Tyrosine Metabolism')]
[('3HLYTCL', 'Tyrosine Metabolism')]
ABUTD: 4abutn_c + h2o_c + nad_c --> 4abut_c + 2.0 h_c + nadh_c
[('ABUTt2r', 'Transport, Extracellular'), ('ABTA', 'Arginine and Proline Metabolism'), ('GLUDC', 'Glutamate Metabolism'), ('4ABUTtm', 'Transport, Mitochondriall'), ('GUDBUTNAH', 'Arginine and Proline Metabolism')]
[('PTRCOX1', 'Arginine and Proline Metabolism')]
ACACT4m: accoa_m + occoa_m <-- 3odcoa_m + coa_m
[('HACD4m', 'Fatty acid elongation in mitochondria')]
[('ACOAD3m', 'Fatty Acid Metabolism'), ('ACOAR3m', 'Fatty acid elongation in mitochondria'), ('PTE9m', '')]
ACACT6m: accoa_m + ddcacoa_m <-- 3otdcoa_m + coa_m
[('HACD6m', 'Fatty acid elongation in mitochondria')]
[('PTE11m', ''), ('ACOAD5m', 'Fatty Acid Metabolism'), ('ACOAR5m', 'Fatty acid elongation in mitochondria')]
ACACT7m: accoa_m + tdcoa_m <-- 3ohdcoa_m + coa_m
[('HACD7m', 'Fatty acid elongation in mitochondria')]
[('TTDCPT2', 'Carnitine shuttle'), ('ACOAD6m', 'Fatty Acid Metabolism'), ('ACOAR6m', 'Fatty acid elongation in mitochondria'), ('PTE7m', '')]
ACOAH: ac_c + coa_c + h_c <-- accoa_c + h2o_c
ACONTam: acon_C_m + h2o_m <=> cit_m
[('ACONTbm', '')]
[('CSm', 'Citric Acid Cycle'), ('CITtcm', 'Transport, Mitochondrial'), ('AKGCITtm', 'Transport, Mitochondrial'), ('CITtam', 'Transport, Mitochondrial')]
ACONTbm: acon_C_m + h2o_m <=> icit_m
[('ACONTam', '')]
[('ICDHxm', 'Citric Acid Cycle'), ('CITtcm', 'Transport, Mitochondrial')]
ALDD20x: h2o_c + id3acald_c + nad_c --> 2.0 h_c + ind3ac_c + nadh_c
[('INDPYRD', 'Tyrosine, Tryptophan, and Phenylalanine Metabolism'), ('TRYPTAOX', 'Tryptophan Metabolism'), ('ALDD20y', 'Tyrosine, Tryptophan, and Phenylalanine Metabolism'), ('ALCD26xi', 'Complex Alcohol Metabolism')]
[('AMID3', 'Tyrosine, Tryptophan, and Phenylalanine Metabolism'), ('ALDD20y', 'Tyrosine, Tryptophan, and Phenylalanine Metabolism'), ('NTRLASE', 'Nitrogen Metabolism')]
AMAOTrm: 8aonn_m + amet_m <=> amob_m + dann_m
[('SRC_8aonn_m', 'Intracellular demand')]
[('DM_amob_m', 'Intracellular demand')]
[('DBTSm', 'Pantothenate and CoA Biosynthesis')]
AMETt2: amet_e + h_e --> amet_c + h_c
[('EX_amet_e', 'Extracellular exchange')]
ASP1DC: asp__L_c + h_c --> ala_B_c + co2_c
[('BADP', 'beta-Alanine Metabolism'), ('PANTS', 'Cofactor and Prosthetic Group Biosynthesis'), ('BUPN', 'beta-Alanine Metabolism;Pyrimidine Metabolism'), ('NBAHH_ir', 'beta-Alanine Metabolism'), ('BAMPPALDOX', 'beta-Alanine Metabolism')]
BCAROtrd: bcaro_r --> bcaro_d
[('DM_bcaro_d', '')]
[('GCAROCer', '')]
BFFS: h2o_c + suc6p_c <=> fru_c + g6p_c
[]
BGLA: cellb_c + h2o_c --> 2.0 glc__D_c
[]
BG_CELLB: cellb_e + h2o_e --> 2.0 glc__D_e
[('EX_cellb_e', 'Extracellular exchange')]
CCOAOMT: amet_c + caffcoa_c <=> ahcys_c + ferulcoa_c + h_c
[('CAFFCOAE', 'Cell Envelope Biosynthesis')]
[('FERULCOAS', 'B-Ketoadipate Pathway')]
CYSTL: cyst__L_c + h2o_c --> hcys__L_c + nh4_c + pyr_c
[('CYST_Ltr', 'Transport, Extracellular'), ('CYSTS', 'Glycine and Serine Metabolism'), ('CYSTGL', 'Methionine Metabolism'), ('SHSL1', 'Methionine Metabolism')]
CYTK1: atp_c + cmp_c <=> adp_c + cdp_c
CYTK2: atp_c + dcmp_c <=> adp_c + dcdp_c
DABT2D: abt__D_c + nad_c <=> h_c + nadh_c + rbl__D_c
[('ABT_Dt', 'Transport, Extracellular'), ('DARABR', 'Pentose and Glucuronate Interconversions'), ('DABT4D', '')]
[('RBTDG', 'Pentose and Glucuronate Interconversions'), ('RBK_Dr', 'Pentose Phosphate Pathway'), ('RU5PP', 'Alternate Carbon Metabolism'), ('RBL_Dt', 'Transport, Extracellular')]
DABT4D: abt__D_c + nad_c <=> h_c + nadh_c + xylu__D_c
[('ABT_Dt', 'Transport, Extracellular'), ('DARABR', 'Pentose and Glucuronate Interconversions'), ('DABT2D', '')]
[('XYLK', 'Alternate Carbon Metabolism'), ('XYLTD_D', 'Xylose Metabolism'), ('XYLUDte', 'Transport, Extracellular')]
DAGKer_RT: 0.01 12dgr_RT_r + ctp_r --> cdp_r + h_r + 0.01 pa_RT_r
[('ETHAPTer_RT', 'Phospholipid Biosynthesis'), ('PCDAGATer_RT', 'Glycerolipid Metabolism'), ('DAGPYPer_RT', 'Phospholipid Biosynthesis'), ('TRIGSer_RT', 'Glycerolipid Metabolism'), ('DAGCPTer_RT', 'Phospholipid Biosynthesis')]
[('CDPter', 'Transport, Endoplasmic Reticular')]
[('CTPter', 'Transport, Endoplasmic Reticular'), ('DASYNer_RT', 'Phospholipid Biosynthesis')]
DHAPter: dhap_c <=> dhap_r
[('GAT2er_RT', 'Phospholipid Biosynthesis')]
DM_3aap_c: 3aap_c --> 
[('POLYAO2', 'Pantothenate and CoA Biosynthesis'), ('POLYAO', 'Pantothenate and CoA Biosynthesis')]
DM_4oglu_c: 4oglu_c --> 
[('HMPPS', '')]
DM_bcaro_d: bcaro_d --> 
[('BCAROtrd', '')]
DOLPter: dolp_r --> dolp_c
[('DOLPMMer', 'Glycoprotein Metabolism')]
FACOAL100: atp_c + coa_c + dca_c --> amp_c + dcacoa_c + ppi_c
[('FACOAE100', 'Cell Envelope Biosynthesis')]
[('FACOAE100', 'Cell Envelope Biosynthesis'), ('DCACOAtr', 'Transport, Endoplasmic Reticular'), ('FAS120COA', 'Fatty Acid, Biosynthesis'), ('FAS100COA', 'Fatty Acid, Biosynthesis')]
FACOAL120: atp_c + coa_c + ddca_c --> amp_c + ddcacoa_c + ppi_c
[('FACOAE120', 'Cell Envelope Biosynthesis')]
[('DDCACOAtr', 'Transport, Endoplasmic Reticular'), ('FAS120COA', 'Fatty Acid, Biosynthesis'), ('FACOAE120', 'Cell Envelope Biosynthesis'), ('FAS140COA', 'Fatty Acid, Biosynthesis')]
FMNATm: atp_m + fmn_m + h_m --> fad_m + ppi_m
[('FADFMNtm', 'Transport, Mitochondrial'), ('RBFKm', 'Riboflavin Metabolism')]
FPK: f6p_c + pi_c --> actp_c + e4p_c + h2o_c
[('ACKr', 'Pyruvate Metabolism'), ('XPK', '')]
G5SADm: glu5sa_m + h2o_m + nad_m --> glu__L_m + 2.0 h_m + nadh_m
[('ORNTArm', 'Arginine and Proline Metabolism'), ('G5SADrm', 'Arginine and Proline Metabolism')]
GCAROCer: gcaro_r --> bcaro_r
[('BCAROtrd', '')]
[('LYCOPCer', '')]
GGDPter: ggdp_c --> ggdp_r
[('FRTT', 'Sterol Metabolism')]
[('PSYer', '')]
GLUTCOADHm: fad_m + glutcoa_m + h_m --> b2coa_m + co2_m + fadh2_m
[('ECOAH1m', 'Tryptophan Metabolism'), ('ACOAD1m', 'Butanoate Metabolism')]
[('2OXOADOXm', 'Lysine Metabolism')]
GLYC3Pter: glyc3p_c <=> glyc3p_r
[('GAT1er_RT', 'Phospholipid Biosynthesis')]
GLYCt: glyc_c <=> glyc_e
[('G2PPe', 'Alternate Carbon Metabolism'), ('GLYCt2', 'Transport, Extracellular'), ('EX_glyc_e', 'Extracellular exchange')]
GTHRDH_syn: gthrd_c + h2o_c --> cgly_c + glu__L_c
[('AMPTASECG', 'Cysteine Metabolism'), ('GTMLT', 'Other Amino Acid Metabolism'), ('CGLYt3_2', 'Transport, Extracellular')]
GUACYC: gtp_c --> 35cgmp_c + ppi_c
[('PDE4', 'Purine and Pyrimidine Biosynthesis'), ('CGMPt', 'Transport, Extracellular'), ('CGMPt2', 'Transport, Extracellular')]
GUAPRT: gua_c + prpp_c --> gmp_c + ppi_c
[('GUAt2r', 'Transport, Extracellular'), ('GUAD', 'Nucleotide Salvage Pathway'), ('GNNUC', 'Nucleotide Salvage Pathway'), ('PUNP4', 'Nucleotide Salvage Pathway'), ('PUNP3', 'Nucleotide Salvage Pathway')]
GXLOe: gxl_e + h2o_e + o2_e --> glx_e + h2o2_e + h_e
[('GLXt', 'Transport, Extracellular'), ('EX_glx_e', 'Extracellular exchange')]
[]
[('EX_h2o2_e', 'Extracellular exchange'), ('MTHGXLe', ''), ('H2O2t', 'Transport, Extracellular')]
[('MTHGXLe', ''), ('O2t', 'Transport, Extracellular'), ('EX_o2_e', 'Extracellular exchange')]
HMPPS: 4.0 fe3_c + h2o2_c + his__L_c + 2.0 o2_c + pdx5p_c --> 4ampm_c + 4oglu_c + co2_c + 4.0 fe2_c + glx_c + 6.0 h_c
[('PMPK', 'Cofactor and Prosthetic Group Biosynthesis'), ('HMPK1', 'Cofactor and Prosthetic Group Biosynthesis')]
[('DM_4oglu_c', '')]
[('BIOMASS_RT', 'Biomass and maintenance functions'), ('FE3t', 'Transport, Extracellular')]
[('PDX5POi', 'Cofactor and Prosthetic Group Biosynthesis'), ('PYDXNK', 'Vitamin B6 Metabolism'), ('PDXPP', 'Vitamin B6 Metabolism')]
HMR_6515: mercppyr_c + so3_c --> pyr_c + tsul_c
[('CYSTA', 'Cysteine Metabolism'), ('MCPST', 'Cysteine Metabolism')]
[('TSULt4_3', 'Transport, Extracellular'), ('CYANST', 'Unassigned')]
LCYSTCBOXL: Lcyst_c + h_c --> co2_c + taur_c
[('3SALAOX', 'Taurine and hypotaurine Metabolism'), ('LCYSTAT', 'Cysteine Metabolism'), ('LCYSTt', 'Transport, Extracellular')]
[('TAUDO', 'Alternate Carbon Metabolism'), ('TAURt', 'Transport, Extracellular')]
LEUTRSm: atp_m + leu__L_m + trnaleu_m --> amp_m + leutrna_m + ppi_m
[('LEUt5m', 'Transport, Mitochondrial'), ('LEUTAm', 'Valine, Leucine, and Isoleucine Metabolism')]
[]
[]
LNSTLSr: Ssq23epx_r --> lanost_r
[('SQLEr', 'Sterol Metabolism'), ('SQLErx', 'Sterol Metabolism')]
[('LANOSTtr', 'Transport, Extracellular'), ('LANOSTATer_RT', 'Sterol Metabolism'), ('LNS14DMr', 'Cholesterol Metabolism'), ('LNS14DMrx', 'Sterol Metabolism')]
LYCOPCer: lycop_r --> gcaro_r
[('GCAROCer', '')]
[('PDSer', '')]
MDRPD: 5mdru1p_c --> dkmpp_c + h2o_c
[('MTRI', 'Methionine Metabolism')]
[('ACRS', 'Methionine Metabolism')]
MINPP1er: h2o_r + minohp_r --> mi13456p_r + pi_r
[('MINPP2er', '')]
[]
MINPP2er: h2o_r + mi13456p_r --> mi1456p_r + pi_r
[('MINPP1er', '')]
[('MINPP3er', '')]
MINPP3er: h2o_r + mi1456p_r --> mi145p_r + pi_r
[('MINPP2er', '')]
[('MINPP4er', '')]
MINPP4er: h2o_r + mi1345p_r --> mi145p_r + pi_r
[]
[('MINPP3er', '')]
MTHGXLe: h2o_e + mthgxl_e + o2_e --> h2o2_e + h_e + pyr_e
[('EX_h2o2_e', 'Extracellular exchange'), ('GXLOe', ''), ('H2O2t', 'Transport, Extracellular')]
[]
[('GXLOe', ''), ('O2t', 'Transport, Extracellular'), ('EX_o2_e', 'Extracellular exchange')]
[('PEPPe', 'Alternate Carbon Metabolism'), ('EX_pyr_e', 'Extracellular exchange'), ('PYRt2', 'Transport, Extracellular')]
NDPK10: atp_c + didp_c <=> adp_c + ditp_c
[]
[('NTPP10', 'Nucleotide Salvage Pathway')]
NICRNS: atp_c + nicrns_c --> adp_c + h_c + nicrnt_c
[('NP1', 'NAD Metabolism')]
[('NAPRT', 'NAD Biosynthesis'), ('NAMNPP', 'Cofactor and Prosthetic Group Biosynthesis'), ('NNAT', 'Nucleotide Salvage Pathway'), ('DNADDP', 'Nicotinate and nicotinamide Metabolism'), ('NNDPR', 'Cofactor and Prosthetic Group Biosynthesis')]
OCBTm: cbp_m + orn_m --> citr__L_m + h_m + pi_m
[('CBPSm', 'Pyrimidine Metabolism')]
[]
[('ORNt3m', 'Transport, Mitochondrial'), ('ORNTArm', 'Arginine and Proline Metabolism'), ('ORNTACim', 'Arginine and Proline Metabolism'), ('ORNLYStm', 'Transport, Mitochondrial'), ('ORNARGtm', 'Transport, Mitochondrial')]
OCOAT1m: acac_m + succoa_m --> aacoa_m + succ_m
[('HACD1m', 'Tryptophan Metabolism'), ('HMGCOASm', 'Sterol Metabolism'), ('ACACT1m', 'Fatty Acid, Biosynthesis')]
[('BDHm', 'Butanoate Metabolism'), ('S3HBTRHDm', 'Glycine and Serine Metabolism'), ('ACACt2m', 'Transport, Mitochondriall'), ('HMGLm', 'Cholesterol Metabolism')]
[('3OXCOATm', 'B-Ketoadipate Pathway'), ('SUCOASm', 'Citric Acid Cycle'), ('AKGDm', 'Citric Acid Cycle'), ('3OADPCOATm', 'B-Ketoadipate Pathway'), ('ALASm', 'Porphyrin and Chlorophyll Metabolism')]
OPAH: 5oxpro_c + atp_c + 2.0 h2o_c --> adp_c + glu__L_c + h_c + pi_c
[]
PDSer: 4.0 nadp_r + phyto_r --> 4.0 h_r + lycop_r + 4.0 nadph_r
[('LYCOPCer', '')]
[('PSYer', '')]
PFK26: atp_c + f6p_c --> adp_c + f26bp_c + h_c
[('FBP26', 'Fructose and Mannose Metabolism')]
PNP: pi_c + rnam_c <=> h_c + ncam_c + r1p_c
[('NNAM', 'Cofactor and Prosthetic Group Biosynthesis'), ('NNMT', 'NAD Biosynthesis'), ('THZPSN4', 'Thiamine Metabolism')]
[('NMNHYD', 'Vitamin B2 Metabolism'), ('RNMK', 'NAD Biosynthesis')]
PPAm: h2o_m + ppi_m --> h_m + 2.0 pi_m
PRDX: h2o2_c + meoh_c --> fald_c + 2.0 h2o_c
[('MAOX', 'Tyrosine Metabolism'), ('FALGTHLs', 'Cofactor and Prosthetic Group Biosynthesis')]
[('MEOHt2', 'Transport, Extracellular')]
PSPHSer: h_r + nadph_r + o2_r + sphgn_r --> h2o_r + nadp_r + psphings_r
[('BIOMASS_RT', 'Biomass and maintenance functions'), ('SBPP2er', 'Sphingolipid Metabolism'), ('CERS226er', 'Sphingolipid Metabolism'), ('CERS224er', 'Sphingolipid Metabolism'), ('SLCBK2er', 'Sphingolipid Metabolism')]
PSYer: 2.0 ggdp_r --> phyto_r + 2.0 ppi_r
[('GGDPter', '')]
[('PDSer', '')]
[('SQLSr', 'Cholesterol Metabolism'), ('VLCSr', 'Bile Acid Biosynthesis'), ('PPItr', 'Transport, Endoplasmic Reticular'), ('DASYNer_RT', 'Phospholipid Biosynthesis'), ('VLCS2r', 'Bile Acid Biosynthesis')]
PTE10m: dcacoa_m + h2o_m --> coa_m + dca_m + h_m
[]
[('ACOAR4m', 'Fatty acid elongation in mitochondria'), ('ACACT5m', 'Fatty acid elongation in mitochondria'), ('ACOAD4m', 'Fatty Acid Metabolism')]
PTE11m: ddcacoa_m + h2o_m --> coa_m + ddca_m + h_m
[]
[('ACACT6m', ''), ('ACOAD5m', 'Fatty Acid Metabolism'), ('ACOAR5m', 'Fatty acid elongation in mitochondria')]
PTE2m: h2o_m + pmtcoa_m --> coa_m + h_m + hdca_m
[]
[('ACOAD7m', 'Fatty Acid Metabolism'), ('C160CPT2', 'Carnitine shuttle'), ('ACOAR7m', 'Fatty acid elongation in mitochondria')]
PTE7m: h2o_m + tdcoa_m --> coa_m + h_m + ttdca_m
[('TTDCPT2', 'Carnitine shuttle'), ('ACACT7m', ''), ('ACOAD6m', 'Fatty Acid Metabolism'), ('ACOAR6m', 'Fatty acid elongation in mitochondria')]
[]
PTE9m: h2o_m + occoa_m --> coa_m + h_m + octa_m
[('ACOAD3m', 'Fatty Acid Metabolism'), ('ACACT4m', ''), ('ACOAR3m', 'Fatty acid elongation in mitochondria')]
[('OCTNLLm', 'Cofactor and Prosthetic Group Biosynthesis')]
PTRCtex2: ptrc_c --> ptrc_e
[('EX_ptrc_e', 'Extracellular exchange'), ('PTRCt3i', 'Transport, Extracellular')]
PYDXO_1: h2o_c + o2_c + pydam_c <=> h2o2_c + nh4_c + pydx_c
[('HYPOE', 'Vitamin B6 Metabolism'), ('PYDAMK', 'Vitamin B6 Metabolism')]
[('PYDXNO', 'Pyridoxine Metabolism'), ('PYDXPP', 'Cofactor and Prosthetic Group Biosynthesis'), ('PYDXOR', 'Vitamin B6 Metabolism'), ('PYDXK', 'Cofactor and Prosthetic Group Biosynthesis')]
SACCD4m: h2o_m + nadp_m + saccrp__L_m --> L2aadp6sa_m + glu__L_m + h_m + nadph_m
[('AASAD3m', 'Lysine Metabolism')]
[('SACCD3m', 'Lysine Metabolism')]
SERPTer: h_r + pmtcoa_r + ser__L_r --> 3dsphgn_r + co2_r + coa_r
[('3DSPHRer', '')]
[('SERter', 'Transport, Endoplasmic Reticular'), ('PSERSer_RT', 'Phospholipid Biosynthesis')]
SK_epistest_RT_d: epistest_RT_d --> 
[('EPISTESTtrd', 'Transport, Endoplasmic Reticular'), ('EPISTESTH_RT', 'Sterol Metabolism')]
SK_ergstest_RT_d: ergstest_RT_d --> 
[('ERGSTESTH_RT', 'Sterol Metabolism'), ('ERGSTESTtrd', 'Transport, Endoplasmic Reticular')]
SK_fecostest_RT_d: fecostest_RT_d --> 
[('FECOSTESTH_RT', 'Sterol Metabolism'), ('FECOSTESTtrd', 'Transport, Endoplasmic Reticular')]
SK_lanostest_RT_d: lanostest_RT_d --> 
[('LANOSTESTH_RT', 'Sterol Metabolism'), ('LANOSTESTtrd', 'Transport, Endoplasmic Reticular')]
SK_triglyc_RT_d: triglyc_RT_d --> 
[('TAGL_RT', 'Glycerolipid Metabolism'), ('TAGtrd', 'Transport, Endoplasmic Reticular')]
SK_zymstest_RT_d: zymstest_RT_d --> 
[('BIOMASS_RT', 'Biomass and maintenance functions'), ('ZYMSTESTH_RT', 'Sterol Metabolism'), ('ZYMSTESTtrd', 'Transport, Endoplasmic Reticular')]
SPMDtex2: spmd_c --> spmd_e
[('SPMDt3i', 'Transport, Extracellular'), ('EX_spmd_e', 'Extracellular exchange')]
THMt2: h_e + thm_e --> h_c + thm_c
[('TMDPK', 'Thiamine Metabolism'), ('TMN', 'Thiamine Metabolism'), ('THMP', 'Thiamine Metabolism')]
[('EX_thm_e', 'Extracellular exchange'), ('THMPe', 'Thiamine Metabolism')]
XPK: pi_c + xu5p__D_c --> actp_c + g3p_c + h2o_c
[('ACKr', 'Pyruvate Metabolism'), ('FPK', '')]
[('XYLK', 'Alternate Carbon Metabolism'), ('TKT2', 'Pentose Phosphate Pathway'), ('RPE', 'Pentose Phosphate Pathway'), ('TKT1', 'Pentose Phosphate Pathway')]
```

In [38]:

```
model.reactions.get_by_id('34DHPHAMT').subsystem = 'Tyrosine Metabolism'
model.reactions.get_by_id('3DSPHRer').subsystem = 'Sphingolipid Metabolism'
model.reactions.get_by_id('42A12BOOX').subsystem = 'Tyrosine Metabolism'
model.reactions.get_by_id('ABUTD').subsystem = 'Arginine and Proline Metabolism'
model.reactions.get_by_id('ACACT4m').subsystem = 'Fatty acid elongation in mitochondria'
model.reactions.get_by_id('ACACT6m').subsystem = 'Fatty acid elongation in mitochondria'
model.reactions.get_by_id('ACACT7m').subsystem = 'Fatty acid elongation in mitochondria'
model.reactions.get_by_id('ACOAH').subsystem = 'Pyruvate metabolism'
model.reactions.get_by_id('ACONTam').subsystem = 'Citric Acid Cycle'
model.reactions.get_by_id('ACONTbm').subsystem = 'Citric Acid Cycle'
model.reactions.get_by_id('ALDD20x').subsystem = 'Tyrosine, Tryptophan, and Phenylalanine Metabolism'
model.reactions.get_by_id('AMAOTrm').subsystem = 'Pantothenate and CoA Biosynthesis'
model.reactions.get_by_id('AMETt2').subsystem = 'Transport, Extracellular'
model.reactions.get_by_id('ASP1DC').subsystem = 'beta-Alanine Metabolism'
model.reactions.get_by_id('LEUTRSm').subsystem = 'Valine, Leucine, and Isoleucine Metabolism'
model.reactions.get_by_id('PYDXO_1').subsystem = 'Vitamin B6 Metabolism'
```

#### Gene annotation¶

In [39]:

```
for g in sorted(model.genes, key=lambda x: x.id):
    if g.annotation:
        print(g)
```

In [40]:

```
for g in sorted(model.genes, key=lambda x: x.id):
    if g.name:
        print(g, g.name, g.reactions)
```

In [41]:

```
Uniprot = pd.read_csv('../../Data/IFO0880_4_Uniprot.txt',
                      sep='\t', index_col=0)
Uniprot.index = Uniprot.index.map(str)
Uniprot
```

Out[41]:

|  | UniProt |
| --- | --- |
| RTO4\_ID |  |
| 10632 | B3TQJ2 |
| 10637 | B3TQJ2 |
| 10640 | B3TQJ2 |
| 15931 | A0A2T0A585 |
| 15937 | A0A2T0A585 |
| ... | ... |
| 15267 | A0A0K3CG50 |
| 11828 | A0A0K3CS30 |
| 9774 | A0A0K3CLY8 |
| 11638 | A0A0K3CVN2 |
| 15831 | A0A0K3CHJ3 |

8490 rows × 1 columns

In [42]:

```
for g in model.genes:
    if g.id not in Uniprot.index:
        print(g)
```

```
COPII
COB
ATP6
ATP9
ATP8
COX1
COX3
COX2
NAD3
NAD2
NAD4
NAD5
NAD1
NAD4L
NAD6
```

In [43]:

```
for g in model.genes:
    if g.id in Uniprot.index:
        g.annotation['uniprot'] = Uniprot.loc[g.id,'UniProt']
        g.annotation['jgi'] = 'Rhoto_IFO0880_4_'+g.id
        g.annotation['ncbiprotein'] = 'AAT19DRAFT_'+g.id
        g.name = 'jgi|Rhoto_IFO0880_4|'+g.id
```

In [44]:

```
for g in model.genes:
    g.annotation['sbo'] = 'SBO:0000243'
```

In [45]:

```
for x in sorted(model.genes, key=lambda x: x.id):
    if not x.reactions:
        print(x)
print()
for x in sorted(model.metabolites, key=lambda x: x.id):
    if not x.reactions:
        print(x)
```

```

```

In [46]:

```
cobra.manipulation.remove_genes(model, [x for x in model.genes if not x.reactions])
model.remove_metabolites([x for x in model.metabolites if not x.reactions])
```

In [47]:

```
for r in sorted(model.reactions, key=lambda x: x.id):
    if not r.boundary and sum(abs(x) for x in r.check_mass_balance().values()) > 1e-12:
        print(r, r.gene_reaction_rule, r.check_mass_balance())
```

```
BIOMASS_RT: 0.957502 13BDglcn_c + 0.177315 16BDglcn_c + 0.001283 5mthf_c + 0.577574 alatrna_c + 0.146675 argtrna_c + 0.10797 asntrna_c + 0.197296 asptrna_c + 139.6887 atp_c + 0.002418 btn_m + 0.000832 ca2_c + 0.001792 camp_c + 0.031005 chitin_c + 1.8e-05 clpn_RT_m + 0.00077 coa_c + 0.044881 ctp_c + 0.000525 cu2_c + 0.034269 cystrna_c + 0.002451 datp_c + 0.004285 dctp_c + 0.003763 dgtp_c + 0.002523 dttp_c + 0.004031 ergst_r + 1.6e-05 ergstest_RT_r + 0.000751 fad_c + 0.000597 fe2_c + 0.000597 fe3_c + 0.117898 glntrna_c + 0.208673 glutrna_c + 0.518069 glycogen_c + 0.51322 glytrna_c + 0.00192 gthrd_c + 0.051085 gtp_c + 135.719639 h2o_c + 0.000691 hemeA_m + 0.061167 histrna_c + 0.158569 iletrna_c + 0.585703 k_c + 0.320205 leutrna_c + 0.003107 lipopb_m + 0.172334 lystrna_c + 0.807941 mannan_r + 0.05705 mettrna_c + 0.061716 mg2_c + 0.001292 mlthf_c + 0.000607 mn2_c + 0.026099 na1_c + 0.000888 nad_c + 0.000795 nadp_c + 1.4e-05 pa_RT_r + 0.000172 pc_RT_r + 0.00013 pe_RT_r + 0.095164 phetrna_c + 0.235897 protrna_c + 2e-05 ps_RT_r + 0.004006 psphings_r + 4.7e-05 ptd1ino_RT_r + 0.006524 ptrc_c + 0.0024 pydx5p_c + 0.00074 q9_m + 0.00099 ribflv_c + 0.33687 sertrna_c + 0.003967 spmd_c + 0.001327 thf_c + 0.001393 thmpp_c + 0.21813 thrtrna_c + 0.023372 tre_c + 0.000432 triglyc_RT_r + 0.023319 trptrna_c + 0.062363 tyrtrna_c + 0.051129 utp_c + 0.275168 valtrna_c + 0.00051 zn2_c + 0.004156 zymst_r + 1.7e-05 zymstest_RT_d --> 139.639453 adp_c + 139.639453 h_c + 139.639453 pi_c + 0.209364 ppi_c  {'charge': -4.356142000000063, 'C': -40.73034300000017, 'H': -62.360945000000214, 'O': -17.69887699999992, 'N': -5.868879000000095, 'P': -0.37418500000001376, 'R': -4.027781, 'S': -0.104034, 'Ca': -0.000832, 'Cu': -0.000525, 'Fe': -0.001885, 'K': -0.585703, 'Mg': -0.061716, 'Mn': -0.000607, 'Na': -0.026099, 'Zn': -0.00051}
```

In [48]:

```
print(len(model.genes))
print(len(model.reactions))
print(len(model.metabolites))
print(len(set([m.id.rsplit('_',1)[0] for m in model.metabolites])))
print(len(model.compartments))
model
```

```
1142
2398
2051
1205
9
```

Out[48]:

|  |  |
| --- | --- |
| **Name** | R. toruloides |
| **Memory address** | 0x07fd26f46c4a8 |
| **Number of metabolites** | 2051 |
| **Number of reactions** | 2398 |
| **Number of groups** | 0 |
| **Objective expression** | 1.0\*BIOMASS\_RT - 1.0\*BIOMASS\_RT\_reverse\_2b3e0 |
| **Compartments** | c, x, m, e, r, v, n, g, d |

In [49]:

```
model.id = 'Rt_IFO0880'
model.name = 'Rt_IFO0880'
model.description = 'R. toruloides IFO0880'
```

In [50]:

```
cobra.io.save_json_model(model, "../../Model/Rt_IFO0880.json")
```

In [51]:

```
cobra.io.write_sbml_model(model, "../../Model/Rt_IFO0880.xml")
```

In [52]:

```
cobra.io.save_matlab_model(model, "../../Model/Rt_IFO0880.mat")
```

In [53]:

```
%%bash
source activate python3_cobrapy
cd ../../Model
memote report snapshot --solver cplex Rt_IFO0880.xml
mv index.html Rt_IFO0880.html
```

```
============================= test session starts ==============================
platform darwin -- Python 3.6.12, pytest-4.6.11, py-1.9.0, pluggy-0.13.1
rootdir: /Users/kimj972
collected 145 items

../../../../anaconda3/envs/python3_cobrapy/lib/python3.6/site-packages/memote/suite/tests/test_annotation.py . [  0%]
..FFFFFFFFF.FFFFF.FFFFFFFFFFFFFFF...F......FF....FF.F.FFFF.FFF..         [ 44%]
../../../../anaconda3/envs/python3_cobrapy/lib/python3.6/site-packages/memote/suite/tests/test_basic.py . [ 45%]
.....FF.......F..FF.FF                                                   [ 60%]
../../../../anaconda3/envs/python3_cobrapy/lib/python3.6/site-packages/memote/suite/tests/test_biomass.py . [ 61%]
F....FF.F                                                                [ 67%]
../../../../anaconda3/envs/python3_cobrapy/lib/python3.6/site-packages/memote/suite/tests/test_consistency.py . [ 68%]
ssssssssssssssssFFFFFF.FF.                                               [ 86%]
../../../../anaconda3/envs/python3_cobrapy/lib/python3.6/site-packages/memote/suite/tests/test_essentiality.py s [ 86%]
                                                                         [ 86%]
../../../../anaconda3/envs/python3_cobrapy/lib/python3.6/site-packages/memote/suite/tests/test_growth.py s [ 87%]
                                                                         [ 87%]
../../../../anaconda3/envs/python3_cobrapy/lib/python3.6/site-packages/memote/suite/tests/test_matrix.py . [ 88%]
...                                                                      [ 90%]
../../../../anaconda3/envs/python3_cobrapy/lib/python3.6/site-packages/memote/suite/tests/test_sbml.py . [ 91%]
.                                                                        [ 91%]
../../../../anaconda3/envs/python3_cobrapy/lib/python3.6/site-packages/memote/suite/tests/test_sbo.py F [ 92%]
FFFFFFFFF.                                                               [ 99%]
../../../../anaconda3/envs/python3_cobrapy/lib/python3.6/site-packages/memote/suite/tests/test_thermodynamics.py . [100%]

=============================== warnings summary ===============================
anaconda3/envs/python3_cobrapy/lib/python3.6/site-packages/memote/suite/tests/test_biomass.py::test_biomass_consistency[BIOMASS_RT]
  /Users/kimj972/anaconda3/envs/python3_cobrapy/lib/python3.6/site-packages/cobra/core/metabolite.py:136: UserWarning: The element 'R' does not appear in the periodic table
    warn("The element %s does not appear in the periodic table" % e)

-- Docs: https://docs.pytest.org/en/latest/warnings.html
======== 71 failed, 56 passed, 18 skipped, 1 warnings in 47.54 seconds =========
```

```
Writing snapshot report to 'index.html'.
```

In [ ]:

```

```
