# Supplementary material for: Multi-Omics Driven Metabolic Network Reconstruction and Analysis of Lignocellulosic Carbon Utilization in Rhodosporidium toruloides
Source: Front Bioeng Biotechnol. 2021 Jan 8;8:612832. doi: 10.3389/fbioe.2020.612832 (PMC7873862; doi:10.3389/fbioe.2020.612832)
Supplement: Supplementary File 4 — Multi-omics dataset for R. toruloides IFO0880. [file Data_Sheet_1.zip › Supplementary File S1/2.Metabolic_modeling/Refinement_2b_Add_Trace_Elements_to_Biomass.html]

Refinement\_2b\_Add\_Trace\_Elements\_to\_Biomass


In [1]:

```
%matplotlib inline
from matplotlib import pyplot as plt
from matplotlib import colors
import csv
import numpy as np
import pandas as pd
import cobra
```

In [2]:

```
Annotation = pd.read_excel('../../Data/R_toruloides_Data_for_Reconstruction.xlsx',
                          sheet_name='Annotation', index_col=0)
Annotation.index = Annotation.index.map(str)
Annotation = Annotation.fillna('')
Transcriptomics = pd.read_excel('../../Data/R_toruloides_Data_for_Reconstruction.xlsx',
                          sheet_name='Transcriptomics', header=[0,1,2,3], index_col=0)
Transcriptomics.index = Transcriptomics.index.map(str)
Proteomics = pd.read_excel('../../Data/R_toruloides_Data_for_Reconstruction.xlsx',
                          sheet_name='Proteomics', header=[0,1,2], index_col=0)
Proteomics.index = Proteomics.index.map(str)
Fitness = pd.read_excel('../../Data/R_toruloides_Data_for_Reconstruction.xlsx',
                          sheet_name='Fitness', index_col=0)
Fitness.index = Fitness.index.map(str)
```

In [3]:

```
def background_gradient(s, cmap='seismic', text_color_threshold=0.408):
    lim = max(abs(s.min().min()),abs(s.max().max()))
    rng = 2.0*lim
    norm = colors.Normalize(-lim - (rng * 0.2), lim + (rng * 0.2))
    rgbas = plt.cm.get_cmap(cmap)(norm(s.values))
    def relative_luminance(rgba):
        r, g, b = (x / 12.92 if x <= 0.03928 else ((x + 0.055) / 1.055 ** 2.4) for x in rgba[:3])
        return 0.2126 * r + 0.7152 * g + 0.0722 * b
    def css(rgba):
        dark = relative_luminance(rgba) < text_color_threshold
        text_color = '#f1f1f1' if dark else '#000000'
        return 'background-color: {b};color: {c};'.format(b=colors.rgb2hex(rgba), c=text_color)

    if s.ndim == 1:
        return [css(rgba) for rgba in rgbas]
    else:
        return pd.DataFrame([[css(rgba) for rgba in row] for row in rgbas], index=s.index, columns=s.columns)

def Show_Data(x):
    display(Transcriptomics.loc[x].style.background_gradient(cmap='Reds', low=0.2, high=0.2, axis=None))
    temp = [y for y in x if y in Proteomics.index]
    display(Proteomics.loc[temp].style.background_gradient(cmap='Reds', low=0.2, high=0.2, axis=None))
    temp = [y for y in x if y in Fitness.index]
    display(Fitness.loc[temp].style.apply(background_gradient, cmap='seismic', axis=None))
    return;
```

In [4]:

```
model = cobra.io.load_json_model("IFO0880_GPR_2a.json")
```

In [5]:

```
eco = cobra.io.load_json_model('../../Data/BiGG_Models/iML1515.json')
sce = cobra.io.load_json_model('../../Data/BiGG_Models/iMM904.json')
hsa = cobra.io.load_json_model('../../Data/BiGG_Models/RECON1.json')
hsa2 = cobra.io.load_json_model('../../Data/BiGG_Models/Recon3D.json')
ptri = cobra.io.load_json_model('../../Data/BiGG_Models/iLB1027_lipid.json')
ppu = cobra.io.load_json_model('../../Data/BiGG_Models/iJN746.json')
```

In [6]:

```
with model:
    sol = model.optimize()
    print(sol)
    print()
    for k, v in sol.shadow_prices.items():
        if v and k in [m.id for m in model.reactions.get_by_id('BIOMASS_RT').metabolites]:
            print(k, v)
```

```
<Solution 0.137 at 0x1033dbbd68>

adp_c -0.4595342375266865
atp_c -0.4708528148056687
amp_c -0.44821566024770426
cmp_c -0.05885660185070177
glu__L_c -0.028673729106749368
gmp_c -0.47462567389865584
ser__L_c -0.15996922554294643
cys__L_c -0.1373320709849902
ala__L_c -0.09658519278065125
ump_c -0.047538024571719545
gln__L_c -0.03999230638573159
dgmp_c -0.4633070966196757
pe_RT_r -264.0141153222689
his__L_c -0.25806356196080116
dtmp_c -0.18487009555670572
trp__L_c -0.22788068921684132
met__L_c -0.12601349370600967
pro__L_c -0.0173551518277677
phe__L_c -0.022637154557965222
tyr__L_c -0.033955731836945335
asp__L_c 1.1102230246251565e-15
ile__L_c -0.012073149097580949
dcmp_c -0.036219447292739376
mannan_r -0.011318577278982224
damp_c -0.4368970829687241
arg__L_c -0.10488548278523885
asn__L_c -0.022637154557963335
glycogen_c -0.022637154557964445
13BDglcn_c -0.022637154557964445
thr__L_c 4.440892098500626e-16
lys__L_c -0.027164585469555635
val__L_c -0.015846008190575023
ergst_r 1.9671687310871127
tre_c -0.03395573183694667
ribflv_c -0.028673729106771573
zymst_r 2.2637154557964543
pa_RT_r -246.1307632214772
triglyc_RT_r -369.1961448322157
pc_RT_r -318.79602935254275
ptd1ino_RT_r -249.52633640517186
ps_RT_r -264.3914012315683
```

In [7]:

```
for m in model.reactions.get_by_id('BIOMASS_RT').reactants:
    print(m.id, model.reactions.get_by_id('BIOMASS_RT').get_coefficient(m.id))
```

```
13BDglcn_c -1.1348
ala__L_c -0.4588
amp_c -0.046
arg__L_c -0.1607
asn__L_c -0.1017
asp__L_c -0.2975
atp_c -59.276
cmp_c -0.0447
cys__L_c -0.0066
damp_c -0.0036
dcmp_c -0.0024
dgmp_c -0.0024
dtmp_c -0.0036
ergst_r -0.0007
gln__L_c -0.1054
glu__L_c -0.3018
gly_c -0.2904
glycogen_c -0.5185
gmp_c -0.046
h2o_c -59.276
his__L_c -0.0663
ile__L_c -0.1927
leu__L_c -0.2964
lys__L_c -0.2862
mannan_r -0.8079
met__L_c -0.0507
pa_RT_r -6e-06
pc_RT_r -6e-05
pe_RT_r -4.5e-05
phe__L_c -0.1339
pro__L_c -0.1647
ps_RT_r -1.7e-05
ptd1ino_RT_r -5.3e-05
ribflv_c -0.00099
ser__L_c -0.1854
so4_c -0.02
thr__L_c -0.1914
tre_c -0.0234
triglyc_RT_r -6.6e-05
trp__L_c -0.0284
tyr__L_c -0.102
ump_c -0.0599
val__L_c -0.2646
zymst_r -0.0015
```

In [8]:

```
model.medium
```

Out[8]:

```
{'EX_h_e': 1000.0,
 'EX_h2o_e': 1000.0,
 'EX_nh4_e': 1000.0,
 'EX_o2_e': 2.0,
 'EX_pi_e': 1000.0,
 'EX_so4_e': 1000.0,
 'EX_glc__D_e': 10.0}
```

In [9]:

```
# Check if model can produce chitin and other cofactors
# chitin and cofactors is missing in iMM904 biomass
# nmp and dnmp in iMM904, check ntp and dntp biosynthesis
cofactors = ['camp_c','coa_c','fad_c','gthrd_c','hemeA_m','nad_c','nadp_c','q9_m','thf_c','thmpp_c',
             '5mthf_c','btn_m','lipopb_m','mlthf_c','ptrc_c','pydx5p_c','spmd_c',
             'chitin_c','ctp_c','gtp_c','utp_c','datp_c','dctp_c','dgtp_c','dttp_c']
with model:
    for x in cofactors:
        model.reactions.get_by_id('BIOMASS_RT').add_metabolites({x: -1e-2})
        sol = model.optimize()
        print(x, sol.objective_value)
        if abs(sol.objective_value) < 1e-6:
            for k, v in sol.shadow_prices.items():
                if v and k in [m.id for m in model.reactions.get_by_id('BIOMASS_RT').metabolites]:
                    print('\t',k, v)
        model.reactions.get_by_id('BIOMASS_RT').add_metabolites({x: 1e-2})
```

```
camp_c 0.13593743393234042
coa_c 0.13547854502629142
fad_c 0.13588334572423133
gthrd_c 0.13632034044837388
hemeA_m 0.0
	 hemeA_m -100.0
nad_c 0.13561558122628062
nadp_c 0.13560034129332552
q9_m 0.0
	 q9_m -100.0
thf_c 0.0
	 thf_c -100.0
thmpp_c 0.0
	 thmpp_c -100.0
5mthf_c 0.0
	 5mthf_c -99.99999999999991
btn_m 0.0
	 btn_m -100.0
lipopb_m 0.0
	 lipopb_m -100.0
mlthf_c 0.0
	 mlthf_c -99.99999999999991
ptrc_c 0.136506426473676
pydx5p_c 0.1365311390189468
spmd_c 0.1365774991663941
chitin_c 0.1364848103339987
ctp_c 0.13646628766234234
gtp_c 0.13590171047694038
utp_c 0.13648172287288304
datp_c 0.13595274973450394
dctp_c 0.136497161575454
dgtp_c 0.13591701822992183
dttp_c 0.13629467782836888
```

Folate biosynthesis (from chorismate via 4-aminobenzoate)  
GTPCI: gtp\_c + h2o\_c --> ahdt\_c + for\_c + h\_c 10332  
AKP1: ahdt\_c + 3.0 h2o\_c --> dhnpt\_c + 2.0 h\_c + 3.0 pi\_c 13409  
PTHPS: ahdt\_c --> 6pthp\_c + pppi\_c 10583  
DNTPPA: ahdt\_c + h2o\_c --> dhpmp\_c + h\_c + ppi\_c 15385  
DNMPPA: dhpmp\_c + h2o\_c --> dhnpt\_c + pi\_c 14615 or 14875 or 15385

DHNPAm: dhnpt\_m --> 2ahhmp\_m + gcald\_m + h\_m 14377  
HPPKm: 2ahhmp\_m + atp\_m --> 2ahhmd\_m + amp\_m + h\_m 14377  
DHPSm: 2ahhmp\_m + 4abz\_m --> dhpt\_m + h2o\_m 14377  
FOLD3m: 2ahhmd\_m + 4abz\_m --> dhpt\_m + ppi\_m 14377

DHPS: 2ahhmp\_c + 4abz\_c --> dhpt\_c + h2o\_c 14377  
FOLD3: 2ahhmd\_c + 4abz\_c --> dhpt\_c + ppi\_c 14377  
FOLD3\_1: 2ahhmd\_c + 4abz\_c --> dhpt\_c + h\_c + ppi\_c 14377

HPPK\_1: 2ahhmp\_c + atp\_c --> 2ahhmd\_c + amp\_c 10332  
HPPK2: 6hmhpt\_c + atp\_c → 6hmhptpp\_c + amp\_c + h\_c  
DHPS2: 4abz\_c + 6hmhptpp\_c --> dhpt\_c + ppi\_c 14377  
6hmhpt (E. coli) is same as 2ahhmp (S. cer)  
6hmhptpp (E. coli) is same as 2ahhmd (S. cer)

DHFS: atp\_c + dhpt\_c + glu\_\_L\_c --> adp\_c + dhf\_c + h\_c + pi\_c 10460 or 14803  
DHFR: dhf\_c + h\_c + nadph\_c <=> nadp\_c + thf\_c 10845  
DHFR2i: dhf\_c + h\_c + nadh\_c --> nad\_c + thf\_c 10845

DHFOR: fol\_c + h\_c + nadh\_c --> dhf\_c + nad\_c 10845  
FOLR2\_1: fol\_c + h\_c + nadph\_c --> dhf\_c + nadp\_c 10845  
FPGS4: 4.0 atp\_c + dhf\_c + 4.0 glu\_\_L\_c --> 5dhf\_c + 4.0 adp\_c + 4.0 h\_c + 4.0 pi\_c 14803  
TMDS: dump\_c + mlthf\_c --> dhf\_c + dtmp\_c 10845

FPGS4m: 4.0 atp\_m + dhf\_m + 4.0 glu\_\_L\_m --> 5dhf\_m + 4.0 adp\_m + 4.0 h\_m + 4.0 pi\_m 14803

In [10]:

```
temp = ['10332','13409','15429','14546','8460','8576','13711','9959','11513','13413','9241',
        '10583','15385','14638','12434','14615','14875','14377','10460','14803','10845','12159','15483','9244']
display(Annotation.loc[temp])
Show_Data(temp)
```

|  | Combined Annotations | Signal P | Sc288c Orthologs | Human Orthologs | Sc288 Best Hit | Human Blast | Essential | WolfPSort | C Terminal |
| --- | --- | --- | --- | --- | --- | --- | --- | --- | --- |
| RTO4\_ID |  |  |  |  |  |  |  |  |  |
| 10332 | K01495: GCH1, folE; GTP cyclohydrolase I |  | FOL2 | GCH1 | FOL2 | GCH1 | Essential | cysk 13, nucl 4, mito 4, cyto 4, cyto\_nucl 4, ... | PQR\* |
| 13409 | K01077: E3.1.3.1, phoA, phoB; alkaline phospha... |  | PHO8 | ALPI,ALPL,ALPP,ALPPL2 | PHO8 | ALPL | Not Essential | plas 14, nucl 4.5, cyto\_nucl 4.5, cyto 3.5, mi... | GDF\* |
| 15429 | K01077: E3.1.3.1, phoA, phoB; alkaline phospha... | S |  |  | PHO8 | ALPPL2 | Not Essential | extr 17, mito 6, cyto 2, vacu 2 | PRH\* |
| 14546 | K17623: HDHD1; pseudouridine 5'-phosphatase |  | YKL033W-A | PUDP | YKL033W-A | PUDP | Not Essential | cyto 10.5, cysk 9, cyto\_nucl 8, nucl 4.5 | YDQ\* |
| 8460 | KOG2914: Predicted haloacid-halidohydrolase an... |  |  |  | DOG2 |  | Not Essential | cyto 13.5, cyto\_nucl 12.5, nucl 10.5 | TIDA |
| 8576 | K19270: yfbT, yniC; sugar-phosphatase |  |  |  | GPP2 |  | Not Essential | cyto 18, cysk 8 | FVV\* |
| 13711 | K01835: pgm; phosphoglucomutase |  | PRM15 | PGM2,PGM2L1 | PRM15 | PGM2 | Not Essential | cyto 15.5, cyto\_nucl 9, mito 6, pero 2, cysk 2 | KPE\* |
| 9959 | K01835: pgm; phosphoglucomutase |  | PGM1,PGM2 | PGM1,PGM5 | PGM2 | PGM1 | Not Essential | cyto 13.5, cyto\_nucl 10, pero 5, nucl 3.5, mito 3 | VIT\* |
| 11513 | HMMPfam:haloacid dehalogenase-like hydrolase:P... |  |  |  |  |  | Not Essential | mito 10, cyto 7.5, cyto\_nucl 6.5, pero 5, nucl... | AYP\* |
| 13413 | K01111: E3.1.3.68; 2-deoxyglucose-6-phosphatase |  | GPP2,GPP1,DOG2,DOG1 |  | DOG1 |  | Not Essential | cysk 17, cyto 10 | GTA\* |
| 9241 | K17497: PMM; phosphomannomutase |  | SEC53 | PMM2,PMM1 | SEC53 | PMM1 | Essential | cyto 17, cyto\_nucl 14.5, nucl 8 | FKL\* |
| 10583 | K01737: queD, ptpS, PTS; 6-pyruvoyltetrahydrop... |  |  | PTS |  | PTSe | Not Essential | cyto\_nucl 12.5, cyto 11.5, nucl 10.5, mito 4 | LPA\* |
| 15385 | K03574: mutT, NUDT15, MTH2; 8-oxo-dGTP diphosp... |  |  | NUDT15 |  | NUDT15 | Not Essential | cyto\_mito 7, mito 6.5, cyto 6.5, pero 5, nucl ... | LVL\* |
| 14638 | KOG2645: Type I phosphodiesterase/nucleotide p... |  | NPP1,NPP2 | ENPP1,ENPP2,ENPP3 | NPP1 | ENPP5 | Not Essential | cyto 8.5, extr 7, mito 6, cyto\_nucl 5, plas 2 | EGV\* |
| 12434 | K03426: E3.6.1.22, NUDT12, nudC; NAD+ diphosph... |  | NPY1 | NUDT12 | NPY1 | NUDT12 | Not Essential | mito 13.5, cyto\_mito 10.833, cyto 7, cyto\_nucl... | SKM\* |
| 14615 | K01551: arsA, ASNA1; arsenite-transporting ATPase |  | YDJ1,GET3 | DNAJA1,DNAJA4,ASNA1,DNAJA2 | GET3 | ASNA1 | Not Essential | nucl 10.5, cyto\_nucl 10.5, cyto 9.5, cysk 5 | VLS\* |
| 14875 | K09503: DNAJA2; DnaJ homolog subfamily A member 2 |  | YDJ1,GET3 | DNAJA1,DNAJA4,ASNA1,DNAJA2 | YDJ1 | DNAJA | Essential | cyto 13.5, cyto\_nucl 13.333, nucl 12, mito\_nuc... | ANS\* |
| 14377 | K13939: FOL1; dihydroneopterin aldolase / 2-am... |  | FOL1 |  | FOL1 |  | Essential | cyto 19, nucl 6 | GEA\* |
| 10460 | K20457: DHFS; dihydrofolate synthase |  | FOL3 |  | FOL3 | FPGS | Essential | cyto 17.5, cyto\_mito 12, mito 5.5, nucl 3 | EER\* |
| 14803 | K01930: FPGS; folylpolyglutamate synthase |  | MET7 | FPGS | MET7 | FPGS | Essential | mito 24, cyto 2 | LPL\* |
| 10845 | K00560: thyA, TYMS; thymidylate synthase |  | CDC21 | TYMS | CDC21 | TYMS | Essential | cyto\_nucl 11, nucl 10.5, cyto 8.5, pero 7 | MSV\* |
| 12159 | K00287: folA; dihydrofolate reductase |  |  |  | DFR1 | DHFR | Essential | mito 12, nucl 7, cyto 3, plas 2, cysk 2, cyto\_... | GAG\* |
| 15483 | K00297: metF, MTHFR; methylenetetrahydrofolate... |  | MET12 |  | MET13 | MTHFR | Not Essential | mito 10, cyto 8.5, cyto\_nucl 7.833, cyto\_pero ... | EGR\* |
| 9244 | K00297: metF, MTHFR; methylenetetrahydrofolate... |  | MET13 | MTHFR | MET13 | MTHFR | Not Essential | cyto 10, mito 9, pero 7 | NGH\* |

| strain | WT | | | | | | | | | | | | | | | | |
| --- | --- | --- | --- | --- | --- | --- | --- | --- | --- | --- | --- | --- | --- | --- | --- | --- | --- |
| condition | G\_MM | C\_MM | G\_SD | | GX\_SD | | | X\_SD | | A\_SD | | C\_SD | | MM\_CN120 | | MM\_CN5 | Diversity\_Sample |
| phase | exp | exp | exp | stat | exp | trans | stat | exp | stat | exp | stat | exp | stat | exp | stat | exp | exp |
| proteinId | Set1 | Set1 | Set2 | Set2 | Set2 | Set2 | Set2 | Set2 | Set2 | Set2 | Set2 | Set2 | Set2 | Set3 | Set3 | Set3 | Set3 |
| 10332 | 7.06609 | 6.36037 | 6.44329 | 5.62387 | 6.8928 | 5.78003 | 5.74998 | 6.19453 | 5.73599 | 6.03366 | 5.6755 | 6.17638 | 6.61001 | 6.43256 | 6.14908 | 7.69653 | 6.5479 |
| 13409 | 5.77406 | 5.75485 | 5.08031 | 5.84881 | 5.01275 | 5.64916 | 5.6641 | 5.35795 | 5.43869 | 5.71891 | 5.48581 | 5.4486 | 5.29291 | 5.63199 | 5.79844 | 5.51138 | 5.14926 |
| 15429 | 1.74095 | 2.85469 | 2.22407 | 2.04805 | 2.3777 | 1.58903 | 1.73116 | 1.459 | 1.44493 | 1.29614 | 1.36645 | 4.76133 | 4.62134 | 2.43615 | 1.84085 | 2.95883 | 6.22924 |
| 14546 | 6.70306 | 6.64705 | 6.55577 | 6.15421 | 6.48538 | 6.55323 | 6.32456 | 6.58575 | 5.78236 | 6.22821 | 6.08386 | 6.23016 | 5.42504 | 5.40667 | 5.47056 | 5.79605 | 5.61121 |
| 8460 | 5.80692 | 6.20037 | 6.49302 | 5.62712 | 6.52974 | 5.7109 | 5.41669 | 5.81656 | 7.02602 | 5.76869 | 5.59903 | 5.54068 | 4.17386 | 5.96325 | 6.34736 | 6.33874 | 6.54362 |
| 8576 | 1.0794 | 0.991857 | 0.705599 | 2.30449 | 0.641345 | 0.640812 | 1.05624 | 0.535925 | 1.06389 | 0.7932 | 1.83137 | 0.86341 | 1.07793 | 0.529077 | 0.652997 | 0.461709 | 0.63206 |
| 13711 | 5.4756 | 4.99893 | 5.59893 | 5.45759 | 5.63608 | 5.5111 | 5.36929 | 5.30838 | 5.05155 | 5.32124 | 5.33168 | 5.04332 | 3.83139 | 5.33363 | 5.28411 | 5.89922 | 5.26643 |
| 9959 | 7.59496 | 7.91456 | 7.8573 | 8.41921 | 7.89378 | 7.29494 | 7.42608 | 7.64315 | 7.18423 | 7.65505 | 7.29434 | 7.85281 | 7.92367 | 7.32321 | 7.07996 | 7.91676 | 7.83097 |
| 11513 | 7.63976 | 6.34911 | 8.82403 | 8.57657 | 8.90305 | 8.55555 | 8.46384 | 7.2824 | 8.63429 | 6.87232 | 7.27209 | 8.07761 | 9.22487 | 8.65123 | 8.62297 | 9.0862 | 8.26419 |
| 13413 | 5.60139 | 6.34458 | 5.56352 | 5.20157 | 5.68008 | 5.59498 | 5.59586 | 5.83845 | 5.32538 | 5.79563 | 5.33654 | 5.97306 | 5.86648 | 5.23733 | 5.21087 | 5.93192 | 5.5741 |
| 9241 | 7.82136 | 7.9438 | 8.09824 | 5.57212 | 8.2003 | 7.49193 | 6.97899 | 8.20321 | 6.39014 | 7.95272 | 6.51467 | 7.12766 | 5.95166 | 8.95421 | 8.36603 | 9.1758 | 9.02435 |
| 10583 | 6.53364 | 5.97007 | 6.59258 | 6.58938 | 6.39192 | 7.37787 | 7.21915 | 5.97587 | 6.5588 | 6.21376 | 6.65525 | 5.97022 | 5.80215 | 5.35725 | 5.90763 | 4.16865 | 5.11483 |
| 15385 | 4.58125 | 5.79123 | 5.3584 | 3.80447 | 5.35956 | 4.13412 | 3.91884 | 5.05498 | 4.35897 | 4.93059 | 4.45503 | 3.97967 | 3.02766 | 3.67539 | 3.43641 | 5.47745 | 4.36837 |
| 14638 | 5.15844 | 5.21161 | 5.35331 | 5.58536 | 5.32859 | 5.72897 | 5.88648 | 5.67892 | 6.22445 | 6.03511 | 6.36047 | 4.30281 | 4.59106 | 3.84878 | 4.13769 | 3.50472 | 3.45622 |
| 12434 | 6.28869 | 6.1311 | 5.5469 | 4.9578 | 5.58953 | 5.08688 | 4.89047 | 5.48647 | 4.68415 | 5.4815 | 4.67004 | 4.49138 | 3.68946 | 5.54848 | 5.08118 | 4.84835 | 4.45017 |
| 14615 | 7.14504 | 6.9622 | 6.91378 | 6.84301 | 7.17327 | 5.99007 | 7.2352 | 6.42542 | 6.22747 | 6.29883 | 6.2649 | 6.53152 | 5.96692 | 6.82485 | 6.40134 | 7.38119 | 6.95379 |
| 14875 | 6.5373 | 7.54361 | 7.87672 | 7.19907 | 8.19053 | 7.2348 | 7.46078 | 7.34744 | 6.95584 | 7.0422 | 5.95737 | 7.42084 | 7.57197 | 6.781 | 6.78673 | 7.04605 | 7.17177 |
| 14377 | 5.12747 | 5.04855 | 5.32372 | 5.3983 | 5.3609 | 5.18591 | 5.27585 | 5.25818 | 5.06955 | 5.29864 | 4.9039 | 5.3784 | 5.0016 | 5.87658 | 6.15521 | 6.39064 | 6.37909 |
| 10460 | 4.50304 | 5.13608 | 4.6477 | 4.86495 | 4.60092 | 4.9379 | 4.79914 | 4.8652 | 4.394 | 4.608 | 4.37076 | 5.27325 | 4.40392 | 4.18791 | 4.24482 | 4.04906 | 4.21169 |
| 14803 | 5.94759 | 5.18542 | 5.92156 | 6.2209 | 5.98205 | 5.95006 | 6.04518 | 5.46967 | 6.60713 | 5.68099 | 6.42864 | 5.60703 | 5.45604 | 5.83519 | 5.82525 | 5.52874 | 5.60252 |
| 10845 | 6.30809 | 5.78895 | 6.91411 | 4.65532 | 7.26598 | 5.95169 | 5.63916 | 6.30129 | 5.04668 | 5.91707 | 4.83108 | 5.08404 | 4.41255 | 6.7228 | 5.98244 | 6.89292 | 5.9381 |
| 12159 | 5.0043 | 5.34171 | 4.58722 | 3.19025 | 4.77926 | 4.38627 | 4.06675 | 4.56901 | 3.80087 | 4.20101 | 3.25109 | 4.14056 | 3.38487 | 3.90128 | 3.59928 | 4.66567 | 4.60867 |
| 15483 | 5.17651 | 5.31455 | 5.44125 | 4.47879 | 5.5053 | 5.20672 | 5.24453 | 5.47806 | 5.24311 | 5.17248 | 5.17106 | 5.76289 | 5.74671 | 4.94549 | 4.83827 | 5.37441 | 5.59654 |
| 9244 | 5.11237 | 6.4343 | 6.34447 | 4.44966 | 6.69229 | 5.63542 | 5.72295 | 6.78737 | 5.50549 | 6.32221 | 5.24142 | 5.62205 | 5.72199 | 5.33791 | 5.60354 | 7.78719 | 7.49567 |

| strain | WT | | | | | | | | | | |
| --- | --- | --- | --- | --- | --- | --- | --- | --- | --- | --- | --- |
| condition | G\_SD | | GX\_SD | | | X\_SD | | A\_SD | | C\_SD | |
| proteinId | exp | stat | exp | trans | stat | exp | stat | exp | stat | exp | stat |
| 10332 | 6.1363 | 3.49125 | 4.87667 | 4.66801 | 4.36318 | 3.3401 | 2.74895 | 3.66828 | 3.44132 | 3.20812 | 4.30892 |
| 13409 | 0 | 0 | 0 | 0 | 0 | 0 | 0 | 0 | 0 | 0.420867 | 0.21114 |
| 15429 | 0.188629 | 0 | 0.204847 | 0 | 0 | 0 | 0 | 0 | 0 | 0.432557 | 0.663961 |
| 14546 | 5.7393 | 6.66996 | 5.30115 | 7.26182 | 7.70842 | 8.0393 | 8.65673 | 6.96264 | 7.85284 | 5.34428 | 7.41173 |
| 8460 | 3.80752 | 3.24578 | 2.84273 | 3.34472 | 1.81485 | 2.54896 | 1.77875 | 1.54611 | 1.35191 | 7.26893 | 0.660206 |
| 13711 | 4.81902 | 6.62224 | 3.67416 | 5.77438 | 7.14804 | 6.6619 | 6.73257 | 5.21338 | 5.74632 | 5.08845 | 7.15348 |
| 9959 | 24.6548 | 22.9486 | 19.9315 | 19.7296 | 22.5406 | 27.4887 | 27.2109 | 26.0918 | 27.7944 | 31.4035 | 25.4687 |
| 11513 | 16.1014 | 20.8312 | 16.2718 | 17.1194 | 17.8821 | 7.0392 | 12.9913 | 5.60657 | 9.95543 | 20.0688 | 19.362 |
| 13413 | 3.98763 | 1.28818 | 4.88072 | 0.737724 | 1.48285 | 3.73392 | 1.79096 | 1.93779 | 2.09957 | 4.26527 | 3.7106 |
| 9241 | 21.1019 | 17.0658 | 19.3706 | 20.3072 | 17.0843 | 24.8765 | 24.1496 | 22.6009 | 23.3337 | 20.7038 | 15.6842 |
| 10583 | 0.559427 | 1.26319 | 0.40783 | 0.941627 | 0.903019 | 0.974881 | 0.976431 | 0.775498 | 0.763742 | 0.649507 | 0.42946 |
| 15385 | 1.11453 | 1.97353 | 0.822239 | 1.86194 | 1.44297 | 1.17673 | 1.77146 | 1.54614 | 0.959442 | 0.428597 | 0.211526 |
| 14638 | 0.39032 | 0.373592 | 0.203084 | 0.55439 | 0.179978 | 0.390221 | 0.384622 | 0.579683 | 0.384331 | 0 | 0 |
| 12434 | 5.35949 | 7.91423 | 6.71517 | 7.06516 | 6.42319 | 5.69736 | 5.1247 | 5.20413 | 6.522 | 5.12621 | 3.94029 |
| 14615 | 7.13591 | 5.39733 | 5.8877 | 6.1228 | 5.81565 | 6.06371 | 6.50989 | 5.01328 | 7.2781 | 8.99448 | 5.40251 |
| 14875 | 9.78597 | 7.44295 | 8.96989 | 5.95977 | 7.06358 | 8.20408 | 7.83817 | 6.36685 | 5.75114 | 6.62248 | 5.65495 |
| 14377 | 2.70786 | 4.11241 | 2.84649 | 5.03446 | 4.96375 | 2.93058 | 4.49743 | 3.47914 | 4.77984 | 1.71988 | 3.03448 |
| 10460 | 0 | 0.166667 | 0 | 0 | 0.173732 | 0 | 0 | 0.193364 | 0 | 0.22091 | 0.448751 |
| 14803 | 14.9116 | 15.7727 | 12.8494 | 21.3646 | 18.9026 | 15.6539 | 17.1504 | 13.7229 | 14.7659 | 15.4291 | 17.1757 |
| 10845 | 7.409 | 0.867101 | 7.52225 | 0.748421 | 0.540356 | 5.28361 | 0.378375 | 3.67156 | 0.189081 | 2.56639 | 1.74209 |
| 12159 | 0 | 0 | 0.197656 | 0.371018 | 0 | 0.400832 | 0.195435 | 0.190305 | 0.189478 | 0.211647 | 0.422666 |
| 15483 | 11.5369 | 7.32878 | 10.5995 | 4.83355 | 4.37217 | 9.0158 | 5.71207 | 7.34113 | 4.78377 | 5.32019 | 4.5925 |
| 9244 | 19.5188 | 8.12481 | 19.7383 | 5.56662 | 3.99885 | 11.9421 | 8.0981 | 8.49945 | 7.84562 | 6.61308 | 2.80296 |

|  | Glucose | Xylose | Arabinose | Acetate | Coumarate | Ferulate | YNB Oleic Acid | YNB Ricinoleic Acid | YNB Glucose | YNB Gluc DOC | YPD |
| --- | --- | --- | --- | --- | --- | --- | --- | --- | --- | --- | --- |
| proteinId |  |  |  |  |  |  |  |  |  |  |  |
| 13409 | 0.0829495 | 0.0345418 | 0.0179658 | 0.0812198 | 0.411433 | -0.0471804 | 0.110333 | 0.114602 | 0.275288 | 0.104641 | 0.279144 |
| 15429 | 0.25048 | 0.082651 | 0.0362311 | 0.0420706 | 0.202925 | 0.132586 | 0.246786 | 0.268539 | 0.135507 | 0.150893 | -0.180486 |
| 14546 | 0.441895 | 0.052392 | 0.302713 | 0.220549 | -0.147649 | 0.167133 | -0.101398 | -0.225625 | -0.193782 | -0.238061 | 0.105055 |
| 8460 | -0.0791731 | -0.223637 | 0.153426 | -0.225143 | 0.229381 | -0.210056 | -0.184662 | 0.201914 | -0.0619675 | -0.0264 | -0.0905085 |
| 8576 | 0.0928334 | 0.0403074 | 0.342139 | 0.0272539 | 1.10922 | 0.219086 | -0.299253 | 0.0333091 | 0.136365 | -0.0567877 | 0.190766 |
| 13711 | -0.0790543 | -0.0552581 | 0.00927533 | -0.070228 | -0.0211338 | -0.295143 | 0.100453 | 0.339881 | 0.282623 | 0.339392 | -0.0223127 |
| 9959 | -0.127159 | -0.335271 | 0.0959883 | -0.361527 | -0.571583 | -0.0651405 | 0.117287 | -0.424015 | 0.209902 | -0.174774 | -0.293563 |
| 11513 | 0.0450204 | 0.0302811 | -0.04327 | 0.0507433 | -0.535173 | 0.0529926 | -0.0692324 | -0.128559 | -0.175321 | -0.272272 | -0.0418747 |
| 13413 | 0.0653619 | -0.121519 | 0.17951 | 0.0909367 | 0.399522 | 0.263557 | -0.530393 | 0.667075 | 0.651914 | 0.610799 | 0.728724 |
| 10583 | 0.163529 | -0.157861 | 0.0348188 | -0.195808 | -0.11856 | -0.468373 | 0.641369 | -0.150546 | 0.237656 | -0.246356 | -0.0819575 |
| 15385 | 0.143052 | 0.199756 | 0.117154 | -0.6569 | 0.223566 | 0.0301745 | -0.131316 | 0.0372969 | 0.79271 | 0.52207 | -0.949059 |
| 14638 | 0.114456 | 0.0848906 | 0.0696892 | 0.259241 | 0.125445 | 0.160165 | 0.295706 | 0.252442 | -0.580114 | -0.284104 | 0.149297 |
| 12434 | -0.0170235 | 0.261878 | -0.0985772 | -0.063162 | 0.234502 | -0.0581464 | 0.139611 | -0.43109 | -0.359522 | -0.510862 | -0.0743243 |
| 14615 | 0.17739 | 0.510393 | 0.607001 | 0.642465 | 1.28267 | 0.218209 | -0.141567 | -1.09105 | -1.47497 | -1.19205 | -0.168373 |
| 15483 | -0.603864 | -0.13241 | -0.837909 | 0.229329 | -0.188361 | -0.366465 | -3.472 | -4.68714 | -4.18645 | -0.165798 | -0.00174497 |
| 9244 | -1.9293 | -1.86496 | -2.41047 | -1.2578 | -4.55257 | -0.106164 | -2.43985 | -3.67819 | -1.8172 | 0.647837 | 0.00767542 |

In [11]:

```
for x in temp:
    if x in model.genes:
        for r in sorted(model.genes.get_by_id(x).reactions, key=lambda x: x.id):
            print(r, r.gene_reaction_rule)
    else:
        print(x, 'no reactions')
    print()
```

```
GTPCI: gtp_c + h2o_c --> ahdt_c + for_c + h_c 10332
GTPCIn: gtp_n + h2o_n --> ahdt_n + for_n + h_n 10332

AKP1: ahdt_c + 3.0 h2o_c --> dhnpt_c + 2.0 h_c + 3.0 pi_c 13409
ALKP: dhap_c + h2o_c --> dha_c + pi_c 13409 or 14546

15429 no reactions

ALKP: dhap_c + h2o_c --> dha_c + pi_c 13409 or 14546
E4PP: e4p_c + h2o_c --> erthrs_c + pi_c 14546 or 8460 or 8576
F1PP: f1p_c + h2o_c --> fru_c + pi_c 14546 or 8576
F6PP: f6p_c + h2o_c --> fru_c + pi_c 14546 or 8576
GNP: 6pgc_c + h2o_c --> glcn_c + pi_c 14546
PGMT: g1p_c <=> g6p_c 13711 or 14546 or 8460 or 9959
R5PP: h2o_c + r5p_c --> pi_c + rib__D_c 14546 or 8576

2DOXG6PP: 2doxg6p_c + h2o_c --> 2dglc_c + pi_c 13413 or 8460 or 8576
E4PP: e4p_c + h2o_c --> erthrs_c + pi_c 14546 or 8460 or 8576
GAPP: g3p_c + h2o_c --> glyald_c + pi_c 8460
MN6PP: h2o_c + man6p_c --> man_c + pi_c 8460
PGMT: g1p_c <=> g6p_c 13711 or 14546 or 8460 or 9959

2DOXG6PP: 2doxg6p_c + h2o_c --> 2dglc_c + pi_c 13413 or 8460 or 8576
E4PP: e4p_c + h2o_c --> erthrs_c + pi_c 14546 or 8460 or 8576
F1PP: f1p_c + h2o_c --> fru_c + pi_c 14546 or 8576
F6PP: f6p_c + h2o_c --> fru_c + pi_c 14546 or 8576
G1PP: g1p_c + h2o_c --> glc__D_c + pi_c 8576
G6PP: g6p_c + h2o_c --> glc__D_c + pi_c 8576
R5PP: h2o_c + r5p_c --> pi_c + rib__D_c 14546 or 8576
RU5PP: h2o_c + ru5p__D_c --> pi_c + rbl__D_c 8576

PGMT: g1p_c <=> g6p_c 13711 or 14546 or 8460 or 9959
PMANM: man1p_c <=> man6p_c 13711 or 9241
PPM: r1p_c <=> r5p_c 13711 or 9959

PGMT: g1p_c <=> g6p_c 13711 or 14546 or 8460 or 9959
PPM: r1p_c <=> r5p_c 13711 or 9959

PMDPHT: 5aprbu_c + h2o_c --> 4r5au_c + pi_c 11513

2DOXG6PP: 2doxg6p_c + h2o_c --> 2dglc_c + pi_c 13413 or 8460 or 8576
G3PT: glyc3p_c + h2o_c --> glyc_c + pi_c 13413

PMANM: man1p_c <=> man6p_c 13711 or 9241

PTHPS: ahdt_c --> 6pthp_c + pppi_c 10583
PTHPSn: ahdt_n --> 6pthp_n + pppi_n 10583

DNADDP: dnad_c + h2o_c --> amp_c + 2.0 h_c + nicrnt_c 14638 or 15385
DNMPPA: dhpmp_c + h2o_c --> dhnpt_c + pi_c 14615 or 14875 or 15385
DNTPPA: ahdt_c + h2o_c --> dhpmp_c + h_c + ppi_c 15385

DNADDP: dnad_c + h2o_c --> amp_c + 2.0 h_c + nicrnt_c 14638 or 15385
FADDP: fad_c + h2o_c --> amp_c + fmn_c + 2.0 h_c 14638
UDPGP: h2o_c + udpg_c --> g1p_c + 2.0 h_c + ump_c 14638

NADDPp: h2o_x + nad_x --> amp_x + 2.0 h_x + nmn_x 12434

DNMPPA: dhpmp_c + h2o_c --> dhnpt_c + pi_c 14615 or 14875 or 15385

DNMPPA: dhpmp_c + h2o_c --> dhnpt_c + pi_c 14615 or 14875 or 15385

DHNPAm: dhnpt_m --> 2ahhmp_m + gcald_m 14377
DHPS: 2ahhmp_c + 4abz_c --> dhpt_c + h2o_c 14377
DHPS2: 4abz_c + 6hmhptpp_c --> dhpt_c + ppi_c 14377
DHPSm: 2ahhmp_m + 4abz_m --> dhpt_m + h2o_m 14377
FOLD3: 2ahhmd_c + 4abz_c --> dhpt_c + ppi_c 14377
FOLD3m: 2ahhmd_m + 4abz_m --> dhpt_m + ppi_m 14377
HPPKm: 2ahhmp_m + atp_m --> 2ahhmd_m + amp_m + h_m 14377

DHFS: atp_c + dhpt_c + glu__L_c --> adp_c + dhf_c + h_c + pi_c 10460 or 14803
THFGLUS: atp_c + glu__L_c + thf_c <=> adp_c + h_c + pi_c + thfglu_c 10460 or 14803

10FTHFGLULLm: 10fthf_m + atp_m + glu__L_m --> 10fthfglu__L_m + adp_m + pi_m 14803
DHFS: atp_c + dhpt_c + glu__L_c --> adp_c + dhf_c + h_c + pi_c 10460 or 14803
FPGS: 4.0 atp_c + 4.0 glu__L_c + thf_c --> 5thf_c + 4.0 adp_c + 4.0 h_c + 4.0 pi_c 14803
FPGS2: 5thf_c + atp_c + glu__L_c --> 6thf_c + adp_c + h_c + pi_c 14803
FPGS2m: 5thf_m + atp_m + glu__L_m --> 6thf_m + adp_m + h_m + pi_m 14803
FPGS3: 6thf_c + atp_c + glu__L_c --> 7thf_c + adp_c + h_c + pi_c 14803
FPGS3m: 6thf_m + atp_m + glu__L_m --> 7thf_m + adp_m + h_m + pi_m 14803
FPGS4: 4.0 atp_c + dhf_c + 4.0 glu__L_c --> 5dhf_c + 4.0 adp_c + 4.0 h_c + 4.0 pi_c 14803
FPGS4m: 4.0 atp_m + dhf_m + 4.0 glu__L_m --> 5dhf_m + 4.0 adp_m + 4.0 h_m + 4.0 pi_m 14803
FPGS5: 5dhf_c + atp_c + glu__L_c --> 6dhf_c + adp_c + h_c + pi_c 14803
FPGS5m: 5dhf_m + atp_m + glu__L_m --> 6dhf_m + adp_m + h_m + pi_m 14803
FPGS6: 6dhf_c + atp_c + glu__L_c --> 7dhf_c + adp_c + h_c + pi_c 14803
FPGS6m: 6dhf_m + atp_m + glu__L_m --> 7dhf_m + adp_m + h_m + pi_m 14803
FPGS7: 10fthf_c + 4.0 atp_c + 4.0 glu__L_c --> 10fthf5glu_c + 4.0 adp_c + 4.0 h_c + 4.0 pi_c 14803
FPGS7m: 10fthf_m + 4.0 atp_m + 4.0 glu__L_m --> 10fthf5glu_m + 4.0 adp_m + 4.0 h_m + 4.0 pi_m 14803
FPGS8: 10fthf5glu_c + atp_c + glu__L_c --> 10fthf6glu_c + adp_c + h_c + pi_c 14803
FPGS8m: 10fthf5glu_m + atp_m + glu__L_m --> 10fthf6glu_m + adp_m + h_m + pi_m 14803
FPGS9: 10fthf6glu_c + atp_c + glu__L_c --> 10fthf7glu_c + adp_c + h_c + pi_c 14803
FPGS9m: 10fthf6glu_m + atp_m + glu__L_m --> 10fthf7glu_m + adp_m + h_m + pi_m 14803
FPGSm: 4.0 atp_m + 4.0 glu__L_m + thf_m --> 5thf_m + 4.0 adp_m + 4.0 h_m + 4.0 pi_m 14803
THFGLUS: atp_c + glu__L_c + thf_c <=> adp_c + h_c + pi_c + thfglu_c 10460 or 14803

DHFOR: fol_c + h_c + nadh_c --> dhf_c + nad_c 10845
DHFR: dhf_c + h_c + nadph_c <=> nadp_c + thf_c 10845
DHFR2i: dhf_c + h_c + nadh_c --> nad_c + thf_c 10845
FOLR2_1: fol_c + h_c + nadph_c --> dhf_c + nadp_c 10845
THFOR1: fol_c + 2.0 h_c + 2.0 nadh_c --> 2.0 nad_c + thf_c 10845
THFOR2: fol_c + 2.0 h_c + 2.0 nadph_c --> 2.0 nadp_c + thf_c 10845
TMDS: dump_c + mlthf_c --> dhf_c + dtmp_c 10845

12159 no reactions

MTHFR2: 2.0 h_c + mlthf_c + nadh_c --> 5mthf_c + nad_c 15483 or 9244
MTHFR3: 2.0 h_c + mlthf_c + nadph_c --> 5mthf_c + nadp_c 15483 or 9244

MTHFR2: 2.0 h_c + mlthf_c + nadh_c --> 5mthf_c + nad_c 15483 or 9244
MTHFR2m: 2.0 h_m + mlthf_m + nadh_m --> 5mthf_m + nad_m 9244
MTHFR3: 2.0 h_c + mlthf_c + nadph_c --> 5mthf_c + nadp_c 15483 or 9244
MTHFR3m: 2.0 h_m + mlthf_m + nadph_m --> 5mthf_m + nadp_m 9244
```

In [12]:

```
# 13409 is PHO8 -> make nicotinamide riboside from NMN, add NMNHYD from Recon3D
# Also farnesyl diphosphatase, frdp + h2o -> farnesol + ppi
r = hsa2.reactions.get_by_id('NMNHYD').copy()
r.gene_reaction_rule = '13409'
model.add_reactions([r])
# ALKP is by phoA in E. coli, AKP1 is wrong, remove
model.reactions.get_by_id('ALKP').gene_reaction_rule = '13409'
model.remove_reactions(['AKP1'], remove_orphans=True)
# 14546 is pseudouridine 5'-phosphatase, 16646 pseudouridine kinase
# add PSURIK from eco, set gene to 16646
# modify ALKP to PSURIP and set gene to 14546
r = eco.reactions.get_by_id('PSURIK').copy()
r.gene_reaction_rule = '16646'
model.add_reactions([r])
r = model.reactions.get_by_id('ALKP').copy()
r.id = 'PSURIP'
r.name = 'Pseudouridine 5-phosphatase'
r.gene_reaction_rule = '14546'
model.add_reactions([r])
r.add_metabolites({'dhap_c': 1.0, 'dha_c': -1.0, 'psd5p_c': -1.0, 'psuri_c': 1.0})
# 8460 is glycerol-1-phosphatase, also glyc3p
model.reactions.get_by_id('G3PT').gene_reaction_rule = '8460'
# 8576 is sugar phosphatase
model.reactions.get_by_id('E4PP').gene_reaction_rule = '8576'
model.reactions.get_by_id('F1PP').gene_reaction_rule = '8576'
model.reactions.get_by_id('F6PP').gene_reaction_rule = '8576'
model.reactions.get_by_id('GNP').gene_reaction_rule = '8576'
model.reactions.get_by_id('R5PP').gene_reaction_rule = '8576'
# 13413 is 2-deoxyglucose-6-phosphatase
model.reactions.get_by_id('2DOXG6PP').gene_reaction_rule = '13413'
# 13711 and 9959 PGM2 and PGM1
model.reactions.get_by_id('PGMT').gene_reaction_rule = '13711 or 9959'
# 9241 is phosphomannomutase
model.reactions.get_by_id('PMANM').gene_reaction_rule = '9241'
# DNADDP is by 12434 NUDT12 or 14638 NPP/ENPP
# replace NADDPp with NADDP from eco, gene is 12434
model.reactions.get_by_id('DNADDP').gene_reaction_rule = '12434 or 14638'
r = eco.reactions.get_by_id('NADDP').copy()
r.gene_reaction_rule = '12434'
model.add_reactions([r])
model.remove_reactions(['NADDPp'], remove_orphans=True)
# FADDP and UDPGP is okay, by ENPP
# DNTPPA is correct, by 15385 8-oxo-dGTP diphosphatase
# DNMPPA is monophosphate, no gene is known in metacyc, iMM904 gene GET3 is wrong
model.reactions.get_by_id('DNMPPA').gene_reaction_rule = ''
# 14377 FOL1 cytosolic, old reactions are wrong, add DHNPA2r and HPPK2 from eco, keep DHPS2
# remove DHNPAm,DHPS,DHPSm,FOLD3,FOLD3m,HPPKm
r1 = eco.reactions.get_by_id('DHNPA2r').copy()
r1.gene_reaction_rule = '14377'
r2 = eco.reactions.get_by_id('HPPK2').copy()
r2.gene_reaction_rule = '14377'
model.add_reactions([r1,r2])
remove = ['DHNPAm','DHPS','DHPSm','FOLD3','FOLD3m','HPPKm']
model.remove_reactions(remove, remove_orphans=True)
# 10460 is DHFS, 14803 is FPGS
model.reactions.get_by_id('DHFS').gene_reaction_rule = '10460'
model.reactions.get_by_id('THFGLUS').gene_reaction_rule = '14803'
# 10845 is thymidylate synthase, TMDS is correct
# dihydrofolate reductase is 12159 (gene model is wrong, 947542 correct with sigP)
# Add DHFRim from sce, and set gene to 12159 (NADP)
# mlthf reductase is 15483 or 9244
# remove wrong folate reductase reactions
model.reactions.get_by_id('DHFR').gene_reaction_rule = '12159'
model.reactions.get_by_id('FOLR2_1').gene_reaction_rule = '12159'
r = sce.reactions.get_by_id('DHFRim').copy()
r.gene_reaction_rule = '12159'
model.add_reactions([r])
remove = ['DHFOR','DHFR2i','THFOR1','THFOR2']
model.remove_reactions(remove, remove_orphans=True)
```

In [13]:

```
# Still need to make thf, check where glycald is going
for r in sorted(model.metabolites.get_by_id('gcald_c').reactions, key=lambda x: x.id):
    print(r, r.gene_reaction_rule)
print()
for r in sorted(model.metabolites.get_by_id('gcald_m').reactions, key=lambda x: x.id):
    print(r, r.gene_reaction_rule)
```

```
2DDARAA: 2ddara_c <=> gcald_c + pyr_c 12061
DHNPA2r: dhnpt_c <=> 6hmhpt_c + gcald_c 14377
GCALDD: gcald_c + h2o_c + nad_c --> glyclt_c + 2.0 h_c + nadh_c 12042 or 13426 or 16323

GCALDDm: gcald_m + h2o_m + nad_m --> glyclt_m + 2.0 h_m + nadh_m 12042 or 13426
```

In [14]:

```
temp = Annotation.index[Annotation['Combined Annotations'].str.contains('Aldehyde dehydrogenase') |
                        Annotation['Combined Annotations'].str.contains(' aldehyde dehydrogenase') |
                        Annotation['Combined Annotations'].str.contains(' retinal dehydrogenase') ]
display(Annotation.loc[temp])
Show_Data(temp)
```

|  | Combined Annotations | Signal P | Sc288c Orthologs | Human Orthologs | Sc288 Best Hit | Human Blast | Essential | WolfPSort | C Terminal |
| --- | --- | --- | --- | --- | --- | --- | --- | --- | --- |
| RTO4\_ID |  |  |  |  |  |  |  |  |  |
| 10421 | KOG2450: Aldehyde dehydrogenase |  |  |  | ALD5 | ALDH1 | Not Essential | cyto 19.5, cyto\_nucl 14.5, nucl 6.5 | RYG\* |
| 10657 | KOG2454: Betaine aldehyde dehydrogenase | A | MSC7 |  | MSC7 | ALDH1 | Not Essential | plas 15, mito 5, cyto 3, E.R. 2, cyto\_pero 2 | SAK\* |
| 11124 | KOG2450: Aldehyde dehydrogenase |  |  |  | ALD4 | ALDH1 | Not Essential | cyto 22.5, cyto\_nucl 12, mito 2 | GAI\* |
| 12002 | KOG2456: Aldehyde dehydrogenase |  |  |  |  | ALDH3 | Not Essential | mito 8, extr 7, cyto 5, mito\_nucl 5 | FFV\* |
| 12042 | K00128: ALDH; aldehyde dehydrogenase (NAD+) |  | ALD5,ALD4,ALD6,ALD3,ALD2 | ALDH1A1,ALDH1A2,ALDH1A3,ALDH1B1,ALDH2 | ALD5 | ALDH2 | Not Essential | cyto 18.5, cyto\_nucl 10, mito 4, pero 4 | NPL\* |
| 12950 | KOG2450: Aldehyde dehydrogenase |  |  |  | UGA2 | ALDH9 | Not Essential | cyto 21.5, cyto\_nucl 13, mito 3 | PSA\* |
| 13426 | K07249: E1.2.1.36; retinal dehydrogenase |  | ALD5,ALD4,ALD6,ALD3,ALD2 | ALDH1A1,ALDH1A2,ALDH1A3,ALDH1B1,ALDH2 | ALD5 | ALDH1 | Not Essential | mito 25.5, cyto\_mito 14 | WPL\* |
| 15814 | K14085: ALDH7A1; aldehyde dehydrogenase family... | S |  | ALDH7A1 | ALD4 | ALDH7 | Not Essential | mito 26 | TFD\* |
| 16323 | K00128: ALDH; aldehyde dehydrogenase (NAD+) |  | HFD1 | ALDH3A1,ALDH3A2,ALDH3B1,ALDH3B2 | HFD1 | ALDH3 | Not Essential | cyto 10.5, plas 7, cyto\_nucl 7, mito 3, nucl 2... | GQA\* |

| strain | WT | | | | | | | | | | | | | | | | |
| --- | --- | --- | --- | --- | --- | --- | --- | --- | --- | --- | --- | --- | --- | --- | --- | --- | --- |
| condition | G\_MM | C\_MM | G\_SD | | GX\_SD | | | X\_SD | | A\_SD | | C\_SD | | MM\_CN120 | | MM\_CN5 | Diversity\_Sample |
| phase | exp | exp | exp | stat | exp | trans | stat | exp | stat | exp | stat | exp | stat | exp | stat | exp | exp |
| proteinId | Set1 | Set1 | Set2 | Set2 | Set2 | Set2 | Set2 | Set2 | Set2 | Set2 | Set2 | Set2 | Set2 | Set3 | Set3 | Set3 | Set3 |
| RTO4\_ID |  |  |  |  |  |  |  |  |  |  |  |  |  |  |  |  |  |
| 10421 | 5.03396 | 3.8538 | 4.4605 | 3.96951 | 4.65717 | 4.54788 | 4.45432 | 3.91481 | 3.97507 | 4.65033 | 3.9581 | 3.95946 | 2.90532 | 5.73493 | 4.37571 | 3.09717 | 4.21234 |
| 10657 | 4.43268 | 4.93535 | 4.28278 | 3.7805 | 4.26482 | 3.93578 | 3.69934 | 4.37594 | 3.84255 | 3.99426 | 3.44949 | 4.95043 | 4.21765 | 4.05869 | 3.65482 | 4.05272 | 4.00843 |
| 11124 | 6.09909 | 8.08878 | 4.97345 | 6.02468 | 4.90969 | 6.53754 | 6.28127 | 7.16586 | 5.72809 | 7.55822 | 5.7529 | 5.76947 | 6.03021 | 7.04008 | 6.42934 | 6.38792 | 6.47123 |
| 12002 | 1.42351 | 3.00645 | 1.46495 | 1.73158 | 1.81397 | 1.48549 | 1.32265 | 1.2798 | 1.31252 | 1.03704 | 1.95806 | 2.41684 | 3.02324 | 1.32884 | 1.65213 | 2.70295 | 2.05964 |
| 12042 | 8.00849 | 8.563 | 8.41698 | 9.34145 | 8.34137 | 8.32176 | 8.06734 | 8.44974 | 8.36047 | 8.84866 | 8.59447 | 7.17248 | 5.67475 | 7.81326 | 7.51984 | 8.29774 | 8.31731 |
| 12950 | 4.92351 | 9.11152 | 5.05129 | 5.98061 | 4.99463 | 4.90659 | 4.93597 | 5.04028 | 5.328 | 5.31962 | 5.33687 | 8.69923 | 7.15407 | 5.50552 | 5.16486 | 6.16151 | 6.59108 |
| 13426 | 7.30781 | 4.89844 | 3.84807 | 2.79984 | 3.78882 | 3.98344 | 3.73207 | 4.58197 | 3.36082 | 4.5625 | 3.35098 | 4.25235 | 3.1633 | 6.93547 | 6.81825 | 3.29168 | 5.38811 |
| 15814 | 6.79043 | 6.58728 | 6.74196 | 7.35713 | 6.45038 | 5.89362 | 6.3322 | 4.9806 | 6.79946 | 5.56262 | 7.47028 | 5.93923 | 5.07561 | 6.72499 | 6.05561 | 6.66991 | 6.07386 |
| 16323 | 5.54194 | 5.5053 | 6.37226 | 5.68644 | 6.28507 | 6.1392 | 5.86895 | 6.06104 | 5.82576 | 5.50985 | 6.17279 | 4.76433 | 4.74944 | 5.59107 | 6.40434 | 5.99953 | 6.38502 |

| strain | WT | | | | | | | | | | |
| --- | --- | --- | --- | --- | --- | --- | --- | --- | --- | --- | --- |
| condition | G\_SD | | GX\_SD | | | X\_SD | | A\_SD | | C\_SD | |
| proteinId | exp | stat | exp | trans | stat | exp | stat | exp | stat | exp | stat |
| 10421 | 0.775983 | 1.26319 | 1.22078 | 2.23128 | 3.11072 | 4.88892 | 4.91813 | 6.38233 | 5.35902 | 1.29303 | 0.872376 |
| 10657 | 0.387864 | 0 | 0 | 0.189769 | 0.177427 | 0.197909 | 0 | 0 | 0 | 0 | 0 |
| 11124 | 10.2577 | 20.6079 | 11.5958 | 18.6341 | 22.4319 | 24.648 | 25.2443 | 26.8531 | 23.5393 | 20.5504 | 13.0738 |
| 12042 | 33.9947 | 44.5614 | 32.5616 | 41.5121 | 40.2993 | 56.5808 | 63.4798 | 61.9973 | 72.5595 | 57.7357 | 40.7488 |
| 12950 | 5.16889 | 7.21647 | 6.10306 | 7.46089 | 8.75973 | 11.1834 | 12.6008 | 9.8603 | 10.7079 | 37.5998 | 29.842 |
| 13426 | 0.757094 | 0.533653 | 1.41886 | 0.929251 | 0.72686 | 2.14514 | 0.960201 | 3.46858 | 3.64334 | 1.06907 | 0.864976 |
| 15814 | 3.29054 | 5.82245 | 5.71037 | 5.95711 | 7.26142 | 4.32025 | 6.69429 | 6.37318 | 8.43457 | 13.4882 | 9.81823 |
| 16323 | 10.9902 | 12.5683 | 13.6351 | 14.1342 | 18.2252 | 11.3209 | 13.9247 | 9.27817 | 10.1675 | 6.60796 | 3.93398 |

|  | Glucose | Xylose | Arabinose | Acetate | Coumarate | Ferulate | YNB Oleic Acid | YNB Ricinoleic Acid | YNB Glucose | YNB Gluc DOC | YPD |
| --- | --- | --- | --- | --- | --- | --- | --- | --- | --- | --- | --- |
| proteinId |  |  |  |  |  |  |  |  |  |  |  |
| 10421 | 0.0359244 | 0.158257 | -0.042663 | 0.144297 | 0.101933 | 0.159197 | -0.0958431 | -0.19259 | 0.219806 | 0.0679839 | -0.0525745 |
| 10657 | -0.469803 | -0.464668 | -0.230016 | -0.51407 | -0.156593 | -0.504589 | -0.23201 | -0.350682 | 0.27828 | 0.223645 | -0.472739 |
| 11124 | -0.225491 | 0.0952583 | -0.0901236 | -0.197801 | -0.387562 | -0.0191805 | 0.037875 | -0.0790279 | -0.0604181 | -0.154212 | 0.18216 |
| 12002 | -0.514533 | -0.440842 | -0.333472 | -0.175652 | -0.520258 | -0.329939 | -0.0362967 | 0.48597 | 0.281434 | 0.230279 | 0.0668551 |
| 12042 | 0.170666 | -0.143043 | 0.000953096 | -0.0230376 | -0.521499 | 0.0146534 | 0.144875 | -0.295284 | 0.392859 | 0.454577 | -0.242457 |
| 12950 | 0.00596512 | -0.171781 | 0.205573 | 0.0569485 | 0.122395 | 0.160439 | 0.000604604 | 0.0892542 | 0.233256 | 0.31056 | 0.190096 |
| 13426 | -0.226122 | -0.181863 | -0.169891 | -0.0897023 | -0.0133507 | -0.00809092 | -1.1502 | -2.84044 | -0.85113 | -0.458514 | -0.0243377 |
| 15814 | -0.464573 | -0.6107 | -0.102958 | -0.543758 | -0.425179 | -0.212745 | -0.360015 | -1.03866 | 0.403738 | -0.219486 | -0.625104 |
| 16323 | 0.000210473 | -0.0174318 | 0.0140589 | -0.110443 | 0.00440814 | 0.0892768 | 0.155043 | 0.103889 | -0.177274 | -0.243731 | -0.140368 |

In [15]:

```
for x in temp:
    if x in model.genes:
        for r in sorted(model.genes.get_by_id(x).reactions, key=lambda x: x.id):
            print(r, r.gene_reaction_rule)
    else:
        print(x, 'no reactions')
    print()
```

```
10421 no reactions

10657 no reactions

11124 no reactions

12002 no reactions

34DHALDD: 34dhpac_c + h2o_c + nad_c --> 34dhpha_c + 2.0 h_c + nadh_c 12042 or 13426 or 16323
34DHPLACOX_NADP: 34dhpac_c + h2o_c + nadp_c <=> 34dhpha_c + 2.0 h_c + nadph_c 12042 or 13426 or 16323
3HPADHi: 3hppnl_c + h2o_c + nad_c --> 3hpp_c + 2.0 h_c + nadh_c 12042 or 13426
3M4HDXPAC: 3mox4hpac_c + h2o_c + nad_c <=> 2.0 h_c + homoval_c + nadh_c 12042 or 13426 or 16323
3MOX4HOXPGALDOX: 3m4hpga_c + h2o_c + nad_c --> 3mox4hoxm_c + 2.0 h_c + nadh_c 12042 or 13426 or 16323
3MOX4HOXPGALDOX_NADP: 3m4hpga_c + h2o_c + nadp_c <=> 3mox4hoxm_c + 2.0 h_c + nadph_c 12042 or 13426 or 16323
4HOXPACDOX_NADP: 4hoxpacd_c + h2o_c + nadp_c <=> 4hphac_c + 2.0 h_c + nadph_c 12042 or 13426 or 16323
5HOXINDACTOX: 5hoxindact_c + h2o_c + nad_c --> 5hoxindoa_c + 2.0 h_c + nadh_c 12042 or 13426 or 16323
5HOXINDACTOXm: 5hoxindact_m + h2o_m + nad_m --> 5hoxindoa_m + 2.0 h_m + nadh_m 12042 or 13426
ABOR: 4abutn_m + h2o_m + nadp_m --> 4abut_m + 2.0 h_m + nadph_m 12042 or 13426
ABUTD: 4abutn_c + h2o_c + nad_c --> 4abut_c + 2.0 h_c + nadh_c 12042 or 13426 or 16323
ABUTDm: 4abutn_m + h2o_m + nad_m --> 4abut_m + 2.0 h_m + nadh_m 12042 or 13426
ALDD19x_P: h2o_c + nadp_c + pacald_c --> 2.0 h_c + nadph_c + pac_c 12042 or 13426 or 16323
ALDD19xr: h2o_c + nad_c + pacald_c <=> 2.0 h_c + nadh_c + pac_c 12042 or 13426 or 16323
ALDD20x: h2o_c + id3acald_c + nad_c --> 2.0 h_c + ind3ac_c + nadh_c 12042 or 13426 or 16323
ALDD20xm: h2o_m + id3acald_m + nad_m --> 2.0 h_m + ind3ac_m + nadh_m 12042 or 13426
ALDD20y: h2o_c + id3acald_c + nadp_c --> 2.0 h_c + ind3ac_c + nadph_c 12042 or 13426
ALDD20ym: h2o_m + id3acald_m + nadp_m --> 2.0 h_m + ind3ac_m + nadph_m 12042 or 13426
ALDD2x: acald_c + h2o_c + nad_c --> ac_c + 2.0 h_c + nadh_c 12042 or 13426 or 16323
ALDD2xm: acald_m + h2o_m + nad_m --> ac_m + 2.0 h_m + nadh_m 12042 or 13426 or 16323
ALDD2y: acald_c + h2o_c + nadp_c --> ac_c + 2.0 h_c + nadph_c 11650 or 12042 or 13426 or 14700 or 16323 or 8666
ALDD2ym: acald_m + h2o_m + nadp_m --> ac_m + 2.0 h_m + nadph_m 12042 or 13426
AM6SAD: am6sa_c + h2o_c + nad_c --> amuco_c + 2.0 h_c + nadh_c 12042 or 13426
BAMPPALDOX: bamppald_c + h2o_c + nad_c --> ala_B_c + 2.0 h_c + nadh_c 12042 or 13426 or 16323
BAMPPALDOXm: bamppald_m + h2o_m + nad_m --> ala_B_m + 2.0 h_m + nadh_m 12042 or 13426
FTHFDH: 10fthf_c + h2o_c + nadp_c --> co2_c + h_c + nadph_c + thf_c 12042 or 13426
GCALDD: gcald_c + h2o_c + nad_c --> glyclt_c + 2.0 h_c + nadh_c 12042 or 13426 or 16323
GCALDDm: gcald_m + h2o_m + nad_m --> glyclt_m + 2.0 h_m + nadh_m 12042 or 13426
GDBTALDH: gdbtal_c + h2o_c + nad_c --> 4gudbutn_c + 2.0 h_c + nadh_c 12042 or 13426
GGGABADr: ggbutal_c + h2o_c + nadp_c <=> gg4abut_c + 2.0 h_c + nadph_c 12042 or 13426
GLACO: glac_c + 2.0 h2o_c + nad_c --> glcr_c + 3.0 h_c + nadh_c 12042 or 13426 or 16323
GLACOm: glac_m + 2.0 h2o_m + nad_m --> glcr_m + 3.0 h_m + nadh_m 12042 or 13426
IMACTD: h2o_c + im4act_c + nad_c --> 2.0 h_c + im4ac_c + nadh_c 12042 or 13426 or 16323
IMACTD_m: h2o_m + im4act_m + nad_m --> 2.0 h_m + im4ac_m + nadh_m 12042 or 13426
LCADi: h2o_c + lald__L_c + nad_c --> 2.0 h_c + lac__L_c + nadh_c 12042 or 13426 or 16323
LCADi_D: h2o_c + lald__D_c + nad_c --> 2.0 h_c + lac__D_c + nadh_c 12042 or 13426 or 16323
MACOXO: 3mldz_c + h2o_c + nad_c --> 3mlda_c + 2.0 h_c + nadh_c 12042 or 13426 or 16323
NABTNO: h2o_c + n4abutn_c + nad_c --> 4aabutn_c + 2.0 h_c + nadh_c 12042 or 13426 or 16323
NABTNOm: h2o_m + n4abutn_m + nad_m --> 4aabutn_m + 2.0 h_m + nadh_m 12042 or 13426
PYLALDOX: h2o_c + nad_c + pylald_c --> 2.0 h_c + nadh_c + peracd_c 12042 or 13426 or 16323
PYLALDOXm: h2o_m + nad_m + pylald_m --> 2.0 h_m + nadh_m + peracd_m 12042 or 13426

VNDH: h2o_c + nad_c + vanln_c --> 2.0 h_c + nadh_c + vanlt_c 12950
VNDH_2: 4hbald_c + h2o_c + nad_c --> 4hbz_c + 2.0 h_c + nadh_c 12950
VNDH_3: 34dhbald_c + h2o_c + nad_c --> 34dhbz_c + 2.0 h_c + nadh_c 12950

34DHALDD: 34dhpac_c + h2o_c + nad_c --> 34dhpha_c + 2.0 h_c + nadh_c 12042 or 13426 or 16323
34DHPLACOX_NADP: 34dhpac_c + h2o_c + nadp_c <=> 34dhpha_c + 2.0 h_c + nadph_c 12042 or 13426 or 16323
3HPADHi: 3hppnl_c + h2o_c + nad_c --> 3hpp_c + 2.0 h_c + nadh_c 12042 or 13426
3M4HDXPAC: 3mox4hpac_c + h2o_c + nad_c <=> 2.0 h_c + homoval_c + nadh_c 12042 or 13426 or 16323
3MOX4HOXPGALDOX: 3m4hpga_c + h2o_c + nad_c --> 3mox4hoxm_c + 2.0 h_c + nadh_c 12042 or 13426 or 16323
3MOX4HOXPGALDOX_NADP: 3m4hpga_c + h2o_c + nadp_c <=> 3mox4hoxm_c + 2.0 h_c + nadph_c 12042 or 13426 or 16323
4HOXPACDOX_NADP: 4hoxpacd_c + h2o_c + nadp_c <=> 4hphac_c + 2.0 h_c + nadph_c 12042 or 13426 or 16323
5HOXINDACTOX: 5hoxindact_c + h2o_c + nad_c --> 5hoxindoa_c + 2.0 h_c + nadh_c 12042 or 13426 or 16323
5HOXINDACTOXm: 5hoxindact_m + h2o_m + nad_m --> 5hoxindoa_m + 2.0 h_m + nadh_m 12042 or 13426
ABOR: 4abutn_m + h2o_m + nadp_m --> 4abut_m + 2.0 h_m + nadph_m 12042 or 13426
ABUTD: 4abutn_c + h2o_c + nad_c --> 4abut_c + 2.0 h_c + nadh_c 12042 or 13426 or 16323
ABUTDm: 4abutn_m + h2o_m + nad_m --> 4abut_m + 2.0 h_m + nadh_m 12042 or 13426
ALDD19x_P: h2o_c + nadp_c + pacald_c --> 2.0 h_c + nadph_c + pac_c 12042 or 13426 or 16323
ALDD19xr: h2o_c + nad_c + pacald_c <=> 2.0 h_c + nadh_c + pac_c 12042 or 13426 or 16323
ALDD20x: h2o_c + id3acald_c + nad_c --> 2.0 h_c + ind3ac_c + nadh_c 12042 or 13426 or 16323
ALDD20xm: h2o_m + id3acald_m + nad_m --> 2.0 h_m + ind3ac_m + nadh_m 12042 or 13426
ALDD20y: h2o_c + id3acald_c + nadp_c --> 2.0 h_c + ind3ac_c + nadph_c 12042 or 13426
ALDD20ym: h2o_m + id3acald_m + nadp_m --> 2.0 h_m + ind3ac_m + nadph_m 12042 or 13426
ALDD2x: acald_c + h2o_c + nad_c --> ac_c + 2.0 h_c + nadh_c 12042 or 13426 or 16323
ALDD2xm: acald_m + h2o_m + nad_m --> ac_m + 2.0 h_m + nadh_m 12042 or 13426 or 16323
ALDD2y: acald_c + h2o_c + nadp_c --> ac_c + 2.0 h_c + nadph_c 11650 or 12042 or 13426 or 14700 or 16323 or 8666
ALDD2ym: acald_m + h2o_m + nadp_m --> ac_m + 2.0 h_m + nadph_m 12042 or 13426
AM6SAD: am6sa_c + h2o_c + nad_c --> amuco_c + 2.0 h_c + nadh_c 12042 or 13426
BAMPPALDOX: bamppald_c + h2o_c + nad_c --> ala_B_c + 2.0 h_c + nadh_c 12042 or 13426 or 16323
BAMPPALDOXm: bamppald_m + h2o_m + nad_m --> ala_B_m + 2.0 h_m + nadh_m 12042 or 13426
FTHFDH: 10fthf_c + h2o_c + nadp_c --> co2_c + h_c + nadph_c + thf_c 12042 or 13426
GCALDD: gcald_c + h2o_c + nad_c --> glyclt_c + 2.0 h_c + nadh_c 12042 or 13426 or 16323
GCALDDm: gcald_m + h2o_m + nad_m --> glyclt_m + 2.0 h_m + nadh_m 12042 or 13426
GDBTALDH: gdbtal_c + h2o_c + nad_c --> 4gudbutn_c + 2.0 h_c + nadh_c 12042 or 13426
GGGABADr: ggbutal_c + h2o_c + nadp_c <=> gg4abut_c + 2.0 h_c + nadph_c 12042 or 13426
GLACO: glac_c + 2.0 h2o_c + nad_c --> glcr_c + 3.0 h_c + nadh_c 12042 or 13426 or 16323
GLACOm: glac_m + 2.0 h2o_m + nad_m --> glcr_m + 3.0 h_m + nadh_m 12042 or 13426
IMACTD: h2o_c + im4act_c + nad_c --> 2.0 h_c + im4ac_c + nadh_c 12042 or 13426 or 16323
IMACTD_m: h2o_m + im4act_m + nad_m --> 2.0 h_m + im4ac_m + nadh_m 12042 or 13426
LCADi: h2o_c + lald__L_c + nad_c --> 2.0 h_c + lac__L_c + nadh_c 12042 or 13426 or 16323
LCADi_D: h2o_c + lald__D_c + nad_c --> 2.0 h_c + lac__D_c + nadh_c 12042 or 13426 or 16323
MACOXO: 3mldz_c + h2o_c + nad_c --> 3mlda_c + 2.0 h_c + nadh_c 12042 or 13426 or 16323
NABTNO: h2o_c + n4abutn_c + nad_c --> 4aabutn_c + 2.0 h_c + nadh_c 12042 or 13426 or 16323
NABTNOm: h2o_m + n4abutn_m + nad_m --> 4aabutn_m + 2.0 h_m + nadh_m 12042 or 13426
PYLALDOX: h2o_c + nad_c + pylald_c --> 2.0 h_c + nadh_c + peracd_c 12042 or 13426 or 16323
PYLALDOXm: h2o_m + nad_m + pylald_m --> 2.0 h_m + nadh_m + peracd_m 12042 or 13426
TMABDH1_m: 4tmeabut_m + h2o_m + nad_m --> gbbtn_m + 2.0 h_m + nadh_m 13426

AASAD3m: L2aadp6sa_m + h2o_m + nad_m --> L2aadp_m + 2.0 h_m + nadh_m 15814

34DHALDD: 34dhpac_c + h2o_c + nad_c --> 34dhpha_c + 2.0 h_c + nadh_c 12042 or 13426 or 16323
34DHPLACOX_NADP: 34dhpac_c + h2o_c + nadp_c <=> 34dhpha_c + 2.0 h_c + nadph_c 12042 or 13426 or 16323
3M4HDXPAC: 3mox4hpac_c + h2o_c + nad_c <=> 2.0 h_c + homoval_c + nadh_c 12042 or 13426 or 16323
3MOX4HOXPGALDOX: 3m4hpga_c + h2o_c + nad_c --> 3mox4hoxm_c + 2.0 h_c + nadh_c 12042 or 13426 or 16323
3MOX4HOXPGALDOX_NADP: 3m4hpga_c + h2o_c + nadp_c <=> 3mox4hoxm_c + 2.0 h_c + nadph_c 12042 or 13426 or 16323
4HOXPACDOX_NADP: 4hoxpacd_c + h2o_c + nadp_c <=> 4hphac_c + 2.0 h_c + nadph_c 12042 or 13426 or 16323
5HOXINDACTOX: 5hoxindact_c + h2o_c + nad_c --> 5hoxindoa_c + 2.0 h_c + nadh_c 12042 or 13426 or 16323
ABUTD: 4abutn_c + h2o_c + nad_c --> 4abut_c + 2.0 h_c + nadh_c 12042 or 13426 or 16323
ALDD19x_P: h2o_c + nadp_c + pacald_c --> 2.0 h_c + nadph_c + pac_c 12042 or 13426 or 16323
ALDD19xr: h2o_c + nad_c + pacald_c <=> 2.0 h_c + nadh_c + pac_c 12042 or 13426 or 16323
ALDD20x: h2o_c + id3acald_c + nad_c --> 2.0 h_c + ind3ac_c + nadh_c 12042 or 13426 or 16323
ALDD21: h2o_c + nad_c + pristanal_c --> 2.0 h_c + nadh_c + prist_c 16323
ALDD2x: acald_c + h2o_c + nad_c --> ac_c + 2.0 h_c + nadh_c 12042 or 13426 or 16323
ALDD2xm: acald_m + h2o_m + nad_m --> ac_m + 2.0 h_m + nadh_m 12042 or 13426 or 16323
ALDD2y: acald_c + h2o_c + nadp_c --> ac_c + 2.0 h_c + nadph_c 11650 or 12042 or 13426 or 14700 or 16323 or 8666
BAMPPALDOX: bamppald_c + h2o_c + nad_c --> ala_B_c + 2.0 h_c + nadh_c 12042 or 13426 or 16323
COALDDH: conialdh_c + h2o_c + nad_c --> fer_c + 2.0 h_c + nadh_c 16323
GCALDD: gcald_c + h2o_c + nad_c --> glyclt_c + 2.0 h_c + nadh_c 12042 or 13426 or 16323
GLACO: glac_c + 2.0 h2o_c + nad_c --> glcr_c + 3.0 h_c + nadh_c 12042 or 13426 or 16323
IMACTD: h2o_c + im4act_c + nad_c --> 2.0 h_c + im4ac_c + nadh_c 12042 or 13426 or 16323
LCADi: h2o_c + lald__L_c + nad_c --> 2.0 h_c + lac__L_c + nadh_c 12042 or 13426 or 16323
LCADi_D: h2o_c + lald__D_c + nad_c --> 2.0 h_c + lac__D_c + nadh_c 12042 or 13426 or 16323
MACOXO: 3mldz_c + h2o_c + nad_c --> 3mlda_c + 2.0 h_c + nadh_c 12042 or 13426 or 16323
NABTNO: h2o_c + n4abutn_c + nad_c --> 4aabutn_c + 2.0 h_c + nadh_c 12042 or 13426 or 16323
PYLALDOX: h2o_c + nad_c + pylald_c --> 2.0 h_c + nadh_c + peracd_c 12042 or 13426 or 16323
```

12042 cyto ALD  
12950 cyto vdh  
13426 mito ALD  
15814 mito 2-aminoadipate semialdehyde  
16323 mito/ER HFD1 hexadecenal and 4-hydroxybenzaldehyde

In [16]:

```
temp = ['11650','14700','8666']
display(Annotation.loc[temp])
Show_Data(temp)
```

|  | Combined Annotations | Signal P | Sc288c Orthologs | Human Orthologs | Sc288 Best Hit | Human Blast | Essential | WolfPSort | C Terminal |
| --- | --- | --- | --- | --- | --- | --- | --- | --- | --- |
| RTO4\_ID |  |  |  |  |  |  |  |  |  |
| 11650 | KOG0023: Alcohol dehydrogenase, class V |  |  |  | ADH2 | ADH1B | Not Essential | cyto 9.5, extr 8, mito 6, cyto\_nucl 6, nucl 1.5 | VIP\* |
| 14700 | KOG0725: Reductases with broad range of substr... |  |  | HSD17B14 | YMR226C | DECR2 | Not Essential | cyto 14.5, cyto\_nucl 11.5, nucl 5.5, pero 3, c... | YLV\* |
| 8666 | KOG0725: Reductases with broad range of substr... |  |  | HSD17B14 | SPS19 | HSD17B | Not Essential | cyto 18.5, cyto\_nucl 12, nucl 2.5, mito 2, ext... | YVH\* |

| strain | WT | | | | | | | | | | | | | | | | |
| --- | --- | --- | --- | --- | --- | --- | --- | --- | --- | --- | --- | --- | --- | --- | --- | --- | --- |
| condition | G\_MM | C\_MM | G\_SD | | GX\_SD | | | X\_SD | | A\_SD | | C\_SD | | MM\_CN120 | | MM\_CN5 | Diversity\_Sample |
| phase | exp | exp | exp | stat | exp | trans | stat | exp | stat | exp | stat | exp | stat | exp | stat | exp | exp |
| proteinId | Set1 | Set1 | Set2 | Set2 | Set2 | Set2 | Set2 | Set2 | Set2 | Set2 | Set2 | Set2 | Set2 | Set3 | Set3 | Set3 | Set3 |
| 11650 | 1.37514 | 2.41086 | 1.41178 | 3.1204 | 1.56558 | 2.07352 | 2.58627 | 1.40096 | 2.16051 | 1.2817 | 2.88878 | 2.57003 | 1.84991 | 2.31936 | 1.83456 | 1.11962 | 2.89719 |
| 14700 | 3.68256 | 4.08361 | 4.44055 | 3.4422 | 4.80507 | 1.56428 | 1.06717 | 2.03973 | 4.6801 | 2.95861 | 4.34493 | 5.79966 | 5.57785 | 1.47714 | 0.854975 | 4.55847 | 3.8255 |
| 8666 | 0.116626 | 1.55463 | 0.234326 | 0.25307 | 0.288409 | 0.196665 | 0.0489713 | 0.109595 | 1.37869 | 0.101187 | 0.403123 | 3.31484 | 3.55422 | 0 | 0.0910628 | 1.15274 | 0.196585 |

| strain | WT | | | | | | | | | | |
| --- | --- | --- | --- | --- | --- | --- | --- | --- | --- | --- | --- |
| condition | G\_SD | | GX\_SD | | | X\_SD | | A\_SD | | C\_SD | |
| proteinId | exp | stat | exp | trans | stat | exp | stat | exp | stat | exp | stat |
| 14700 | 0.391889 | 0.540259 | 1.01019 | 0 | 0 | 0 | 1.16487 | 1.15964 | 1.34502 | 13.0174 | 12.8551 |

|  | Glucose | Xylose | Arabinose | Acetate | Coumarate | Ferulate | YNB Oleic Acid | YNB Ricinoleic Acid | YNB Glucose | YNB Gluc DOC | YPD |
| --- | --- | --- | --- | --- | --- | --- | --- | --- | --- | --- | --- |
| proteinId |  |  |  |  |  |  |  |  |  |  |  |
| 11650 | 0.162296 | -0.125414 | 0.239373 | 0.0824604 | 0.133479 | 0.152742 | 0.151596 | -0.00688522 | -0.0872696 | -0.226133 | 0.148191 |
| 14700 | -0.136366 | 0.186102 | -0.178815 | -0.232998 | -0.0364499 | -0.0311361 | 0.24188 | -0.184481 | -0.138316 | 0.201217 | 0.511467 |
| 8666 | 0.246518 | -0.0531704 | 0.304517 | -0.024053 | -0.100515 | -0.173468 | -0.0249248 | -0.343657 | 0.409518 | 0.34975 | -0.0161461 |

In [17]:

```
for x in temp:
    if x in model.genes:
        for r in sorted(model.genes.get_by_id(x).reactions, key=lambda x: x.id):
            print(r, r.gene_reaction_rule)
    else:
        print(x, 'no reactions')
    print()
```

```
ALDD2y: acald_c + h2o_c + nadp_c --> ac_c + 2.0 h_c + nadph_c 11650 or 12042 or 13426 or 14700 or 16323 or 8666
ALDD3y: h2o_c + nadp_c + ppal_c --> 2.0 h_c + nadph_c + ppa_c 11650

ALDD2y: acald_c + h2o_c + nadp_c --> ac_c + 2.0 h_c + nadph_c 11650 or 12042 or 13426 or 14700 or 16323 or 8666

ALDD2y: acald_c + h2o_c + nadp_c --> ac_c + 2.0 h_c + nadph_c 11650 or 12042 or 13426 or 14700 or 16323 or 8666
```

11650 Alcohol dehydrogenase patD; Patulin biosynthesis cluster protein D?  
14700 oxidoreductase, short chain dehydrogenase/reductase family, upregulated in p-coumarate  
8666 oxidoreductase

In [18]:

```
for r in sorted(model.reactions, key=lambda x: x.id):
    if any(x in r.gene_reaction_rule for x in ['12042','13426']):
        if 'c' in r.compartments:
            print('model.reactions.get_by_id(\''+r.id+'\').gene_reaction_rule = \'12042\'')
print()
for r in sorted(model.reactions, key=lambda x: x.id):
    if any(x in r.gene_reaction_rule for x in ['12042','13426']):
        if 'm' in r.compartments:
            print('model.reactions.get_by_id(\''+r.id+'\').gene_reaction_rule = \'13426\'')
```

```
model.reactions.get_by_id('34DHALDD').gene_reaction_rule = '12042'
model.reactions.get_by_id('34DHPLACOX_NADP').gene_reaction_rule = '12042'
model.reactions.get_by_id('3HPADHi').gene_reaction_rule = '12042'
model.reactions.get_by_id('3M4HDXPAC').gene_reaction_rule = '12042'
model.reactions.get_by_id('3MOX4HOXPGALDOX').gene_reaction_rule = '12042'
model.reactions.get_by_id('3MOX4HOXPGALDOX_NADP').gene_reaction_rule = '12042'
model.reactions.get_by_id('4HOXPACDOX_NADP').gene_reaction_rule = '12042'
model.reactions.get_by_id('5HOXINDACTOX').gene_reaction_rule = '12042'
model.reactions.get_by_id('ABUTD').gene_reaction_rule = '12042'
model.reactions.get_by_id('ALDD19x_P').gene_reaction_rule = '12042'
model.reactions.get_by_id('ALDD19xr').gene_reaction_rule = '12042'
model.reactions.get_by_id('ALDD20x').gene_reaction_rule = '12042'
model.reactions.get_by_id('ALDD20y').gene_reaction_rule = '12042'
model.reactions.get_by_id('ALDD2x').gene_reaction_rule = '12042'
model.reactions.get_by_id('ALDD2y').gene_reaction_rule = '12042'
model.reactions.get_by_id('AM6SAD').gene_reaction_rule = '12042'
model.reactions.get_by_id('BAMPPALDOX').gene_reaction_rule = '12042'
model.reactions.get_by_id('FTHFDH').gene_reaction_rule = '12042'
model.reactions.get_by_id('GCALDD').gene_reaction_rule = '12042'
model.reactions.get_by_id('GDBTALDH').gene_reaction_rule = '12042'
model.reactions.get_by_id('GGGABADr').gene_reaction_rule = '12042'
model.reactions.get_by_id('GLACO').gene_reaction_rule = '12042'
model.reactions.get_by_id('IMACTD').gene_reaction_rule = '12042'
model.reactions.get_by_id('LCADi').gene_reaction_rule = '12042'
model.reactions.get_by_id('LCADi_D').gene_reaction_rule = '12042'
model.reactions.get_by_id('MACOXO').gene_reaction_rule = '12042'
model.reactions.get_by_id('NABTNO').gene_reaction_rule = '12042'
model.reactions.get_by_id('PYLALDOX').gene_reaction_rule = '12042'

model.reactions.get_by_id('5HOXINDACTOXm').gene_reaction_rule = '13426'
model.reactions.get_by_id('ABOR').gene_reaction_rule = '13426'
model.reactions.get_by_id('ABUTDm').gene_reaction_rule = '13426'
model.reactions.get_by_id('ALDD20xm').gene_reaction_rule = '13426'
model.reactions.get_by_id('ALDD20ym').gene_reaction_rule = '13426'
model.reactions.get_by_id('ALDD2xm').gene_reaction_rule = '13426'
model.reactions.get_by_id('ALDD2ym').gene_reaction_rule = '13426'
model.reactions.get_by_id('BAMPPALDOXm').gene_reaction_rule = '13426'
model.reactions.get_by_id('GCALDDm').gene_reaction_rule = '13426'
model.reactions.get_by_id('GLACOm').gene_reaction_rule = '13426'
model.reactions.get_by_id('IMACTD_m').gene_reaction_rule = '13426'
model.reactions.get_by_id('NABTNOm').gene_reaction_rule = '13426'
model.reactions.get_by_id('PYLALDOXm').gene_reaction_rule = '13426'
model.reactions.get_by_id('TMABDH1_m').gene_reaction_rule = '13426'
```

In [19]:

```
model.reactions.get_by_id('34DHALDD').gene_reaction_rule = '12042'
model.reactions.get_by_id('34DHPLACOX_NADP').gene_reaction_rule = '12042'
model.reactions.get_by_id('3HPADHi').gene_reaction_rule = '12042'
model.reactions.get_by_id('3M4HDXPAC').gene_reaction_rule = '12042'
model.reactions.get_by_id('3MOX4HOXPGALDOX').gene_reaction_rule = '12042'
model.reactions.get_by_id('3MOX4HOXPGALDOX_NADP').gene_reaction_rule = '12042'
model.reactions.get_by_id('4HOXPACDOX_NADP').gene_reaction_rule = '12042'
model.reactions.get_by_id('5HOXINDACTOX').gene_reaction_rule = '12042'
model.reactions.get_by_id('ABUTD').gene_reaction_rule = '12042'
model.reactions.get_by_id('ALDD19x_P').gene_reaction_rule = '12042'
model.reactions.get_by_id('ALDD19xr').gene_reaction_rule = '12042'
model.reactions.get_by_id('ALDD20x').gene_reaction_rule = '12042'
model.reactions.get_by_id('ALDD20y').gene_reaction_rule = '12042'
model.reactions.get_by_id('ALDD2x').gene_reaction_rule = '12042'
model.reactions.get_by_id('ALDD2y').gene_reaction_rule = '12042'
model.reactions.get_by_id('AM6SAD').gene_reaction_rule = '12042'
model.reactions.get_by_id('BAMPPALDOX').gene_reaction_rule = '12042'
model.reactions.get_by_id('FTHFDH').gene_reaction_rule = '12042'
model.reactions.get_by_id('GCALDD').gene_reaction_rule = '12042'
model.reactions.get_by_id('GDBTALDH').gene_reaction_rule = '12042'
model.reactions.get_by_id('GGGABADr').gene_reaction_rule = '12042'
model.reactions.get_by_id('GLACO').gene_reaction_rule = '12042'
model.reactions.get_by_id('IMACTD').gene_reaction_rule = '12042'
model.reactions.get_by_id('LCADi').gene_reaction_rule = '12042'
model.reactions.get_by_id('LCADi_D').gene_reaction_rule = '12042'
model.reactions.get_by_id('MACOXO').gene_reaction_rule = '12042'
model.reactions.get_by_id('NABTNO').gene_reaction_rule = '12042'
model.reactions.get_by_id('PYLALDOX').gene_reaction_rule = '12042'

model.reactions.get_by_id('5HOXINDACTOXm').gene_reaction_rule = '13426'
model.reactions.get_by_id('ABOR').gene_reaction_rule = '13426'
model.reactions.get_by_id('ABUTDm').gene_reaction_rule = '13426'
model.reactions.get_by_id('ALDD20xm').gene_reaction_rule = '13426'
model.reactions.get_by_id('ALDD20ym').gene_reaction_rule = '13426'
model.reactions.get_by_id('ALDD2xm').gene_reaction_rule = '13426'
model.reactions.get_by_id('ALDD2ym').gene_reaction_rule = '13426'
model.reactions.get_by_id('BAMPPALDOXm').gene_reaction_rule = '13426'
model.reactions.get_by_id('GCALDDm').gene_reaction_rule = '13426'
model.reactions.get_by_id('GLACOm').gene_reaction_rule = '13426'
model.reactions.get_by_id('IMACTD_m').gene_reaction_rule = '13426'
model.reactions.get_by_id('NABTNOm').gene_reaction_rule = '13426'
model.reactions.get_by_id('PYLALDOXm').gene_reaction_rule = '13426'
model.reactions.get_by_id('TMABDH1_m').gene_reaction_rule = '13426'

model.reactions.get_by_id('ALDD21').gene_reaction_rule = '16323'
model.reactions.get_by_id('COALDDH').gene_reaction_rule = '16323'
```

In [20]:

```
for r in sorted(model.genes.get_by_id('12042').reactions, key=lambda x: x.id):
    for m in r.reactants:
        if not any(x == m.id for x in ['h2o_c','nad_c','nadp_c','h2o_m','nad_m','nadp_m']):
            print(m.id)
            for r2 in m.reactions:
                print(r2, r2.gene_reaction_rule)
            print()
```

```
34dhpac_c
34DHALDD: 34dhpac_c + h2o_c + nad_c --> 34dhpha_c + 2.0 h_c + nadh_c 12042
42A12BOOX: dopa_c + h2o_c + o2_c --> 34dhpac_c + h2o2_c + nh4_c 10308 or 13959
34DHPLACOX_NADP: 34dhpac_c + h2o_c + nadp_c <=> 34dhpha_c + 2.0 h_c + nadph_c 12042

34dhpac_c
34DHALDD: 34dhpac_c + h2o_c + nad_c --> 34dhpha_c + 2.0 h_c + nadh_c 12042
42A12BOOX: dopa_c + h2o_c + o2_c --> 34dhpac_c + h2o2_c + nh4_c 10308 or 13959
34DHPLACOX_NADP: 34dhpac_c + h2o_c + nadp_c <=> 34dhpha_c + 2.0 h_c + nadph_c 12042

3hppnl_c
3HPADHi: 3hppnl_c + h2o_c + nad_c --> 3hpp_c + 2.0 h_c + nadh_c 12042

3mox4hpac_c
3M4HDXPAC: 3mox4hpac_c + h2o_c + nad_c <=> 2.0 h_c + homoval_c + nadh_c 12042

3m4hpga_c
3MOX4HOXPGALDOX: 3m4hpga_c + h2o_c + nad_c --> 3mox4hoxm_c + 2.0 h_c + nadh_c 12042
3MOX4HOXPGALDOX_NADP: 3m4hpga_c + h2o_c + nadp_c <=> 3mox4hoxm_c + 2.0 h_c + nadph_c 12042

3m4hpga_c
3MOX4HOXPGALDOX: 3m4hpga_c + h2o_c + nad_c --> 3mox4hoxm_c + 2.0 h_c + nadh_c 12042
3MOX4HOXPGALDOX_NADP: 3m4hpga_c + h2o_c + nadp_c <=> 3mox4hoxm_c + 2.0 h_c + nadph_c 12042

4hoxpacd_c
TYROXDAc: h2o_c + o2_c + tym_c --> 4hoxpacd_c + h2o2_c + nh4_c 10308 or 13959
4HOXPACDOX_NADP: 4hoxpacd_c + h2o_c + nadp_c <=> 4hphac_c + 2.0 h_c + nadph_c 12042
34HPPYRDC: 34hpp_c + h_c --> 4hoxpacd_c + co2_c 15791

5hoxindact_c
5HOXINDACTOX: 5hoxindact_c + h2o_c + nad_c --> 5hoxindoa_c + 2.0 h_c + nadh_c 12042

4abutn_c
PTRCOX1: h2o_c + o2_c + ptrc_c --> 4abutn_c + h2o2_c + nh4_c 13959
ABUTD: 4abutn_c + h2o_c + nad_c --> 4abut_c + 2.0 h_c + nadh_c 12042

pacald_c
ALCD25yi: h_c + nadph_c + pacald_c --> 2phetoh_c + nadp_c 10029 or 13554 or 13562 or 13947
PEAMNO: h2o_c + o2_c + peamn_c --> h2o2_c + nh4_c + pacald_c 10308 or 13959
PPYRDC: h_c + phpyr_c --> co2_c + pacald_c 15791
ALDD19xr: h2o_c + nad_c + pacald_c <=> 2.0 h_c + nadh_c + pac_c 12042
ALCD25xi: h_c + nadh_c + pacald_c --> 2phetoh_c + nad_c 14108 or 14109 or 15438
ALDD19x_P: h2o_c + nadp_c + pacald_c --> 2.0 h_c + nadph_c + pac_c 12042

pacald_c
ALCD25yi: h_c + nadph_c + pacald_c --> 2phetoh_c + nadp_c 10029 or 13554 or 13562 or 13947
PEAMNO: h2o_c + o2_c + peamn_c --> h2o2_c + nh4_c + pacald_c 10308 or 13959
PPYRDC: h_c + phpyr_c --> co2_c + pacald_c 15791
ALDD19xr: h2o_c + nad_c + pacald_c <=> 2.0 h_c + nadh_c + pac_c 12042
ALCD25xi: h_c + nadh_c + pacald_c --> 2phetoh_c + nad_c 14108 or 14109 or 15438
ALDD19x_P: h2o_c + nadp_c + pacald_c --> 2.0 h_c + nadph_c + pac_c 12042

id3acald_c
ALDD20x: h2o_c + id3acald_c + nad_c --> 2.0 h_c + ind3ac_c + nadh_c 12042
ALCD26xi: h_c + id3acald_c + nadh_c --> ind3eth_c + nad_c 14108 or 14109 or 15438
TRYPTAOX: h2o_c + o2_c + trypta_c --> h2o2_c + id3acald_c + nh4_c 13959
ALDD20y: h2o_c + id3acald_c + nadp_c --> 2.0 h_c + ind3ac_c + nadph_c 12042
INDPYRD: h_c + indpyr_c <=> co2_c + id3acald_c 15791

id3acald_c
ALDD20x: h2o_c + id3acald_c + nad_c --> 2.0 h_c + ind3ac_c + nadh_c 12042
ALCD26xi: h_c + id3acald_c + nadh_c --> ind3eth_c + nad_c 14108 or 14109 or 15438
TRYPTAOX: h2o_c + o2_c + trypta_c --> h2o2_c + id3acald_c + nh4_c 13959
ALDD20y: h2o_c + id3acald_c + nadp_c --> 2.0 h_c + ind3ac_c + nadph_c 12042
INDPYRD: h_c + indpyr_c <=> co2_c + id3acald_c 15791

acald_c
ACALDtm: acald_m <=> acald_c 
ALCD2y: etoh_c + nadp_c --> acald_c + h_c + nadph_c 11882 or 12784 or 9774
ACALDCD: 2.0 acald_c --> actn__R_c 15791
ALDD2y: acald_c + h2o_c + nadp_c --> ac_c + 2.0 h_c + nadph_c 12042
ALCD2x: etoh_c + nad_c <=> acald_c + h_c + nadh_c 15438
ALDD2x: acald_c + h2o_c + nad_c --> ac_c + 2.0 h_c + nadh_c 12042
THRA: thr__L_c --> acald_c + gly_c 16182
THRA2: athr__L_c --> acald_c + gly_c 16182
PYRDC: h_c + pyr_c --> acald_c + co2_c 15791

acald_c
ACALDtm: acald_m <=> acald_c 
ALCD2y: etoh_c + nadp_c --> acald_c + h_c + nadph_c 11882 or 12784 or 9774
ACALDCD: 2.0 acald_c --> actn__R_c 15791
ALDD2y: acald_c + h2o_c + nadp_c --> ac_c + 2.0 h_c + nadph_c 12042
ALCD2x: etoh_c + nad_c <=> acald_c + h_c + nadh_c 15438
ALDD2x: acald_c + h2o_c + nad_c --> ac_c + 2.0 h_c + nadh_c 12042
THRA: thr__L_c --> acald_c + gly_c 16182
THRA2: athr__L_c --> acald_c + gly_c 16182
PYRDC: h_c + pyr_c --> acald_c + co2_c 15791

am6sa_c
PCLAD: cmusa_c + h_c --> am6sa_c + co2_c 12916
AM6SAD: am6sa_c + h2o_c + nad_c --> amuco_c + 2.0 h_c + nadh_c 12042

bamppald_c
POLYAO3: h2o_c + o2_c + sprm_c --> bamppald_c + h2o2_c + spmd_c 15939
POLYAO: N1aspmd_c + h2o_c + o2_c --> aprut_c + bamppald_c + h2o2_c 15939
BAMPPALDOX: bamppald_c + h2o_c + nad_c --> ala_B_c + 2.0 h_c + nadh_c 12042
13DAMPPOX: 13dampp_c + h2o_c + o2_c --> bamppald_c + h2o2_c + nh4_c 13959
POLYAO2: N1sprm_c + h2o_c + o2_c --> N1aspmd_c + bamppald_c + h2o2_c 15939

10fthf_c
FPGS7: 10fthf_c + 4.0 atp_c + 4.0 glu__L_c --> 10fthf5glu_c + 4.0 adp_c + 4.0 h_c + 4.0 pi_c 14803
ULA4NFT: 10fthf_c + udpLa4n_c --> h_c + thf_c + udpLa4fn_c 15418
MTHFC: h2o_c + methf_c <=> 10fthf_c + h_c 15366
AICART: 10fthf_c + aicar_c <=> fprica_c + thf_c 12421
FTHFDH: 10fthf_c + h2o_c + nadp_c --> co2_c + h_c + nadph_c + thf_c 12042
FMETTRS: 10fthf_c + mettrna_c --> fmettrna_c + h_c + thf_c 11899
FTHFD: 10fthf_c + h2o_c --> for_c + h_c + thf_c 11899
GARFT: 10fthf_c + gar_c <=> fgam_c + h_c + thf_c 13595
FTHFLi: atp_c + for_c + thf_c --> 10fthf_c + adp_c + pi_c 15366

gcald_c
2DDARAA: 2ddara_c <=> gcald_c + pyr_c 12061
DHNPA2r: dhnpt_c <=> 6hmhpt_c + gcald_c 14377
GCALDD: gcald_c + h2o_c + nad_c --> glyclt_c + 2.0 h_c + nadh_c 12042

gdbtal_c
GDBTALDH: gdbtal_c + h2o_c + nad_c --> 4gudbutn_c + 2.0 h_c + nadh_c 12042

ggbutal_c
GGGABADr: ggbutal_c + h2o_c + nadp_c <=> gg4abut_c + 2.0 h_c + nadph_c 12042

glac_c
GLACO: glac_c + 2.0 h2o_c + nad_c --> glcr_c + 3.0 h_c + nadh_c 12042

im4act_c
IMACTD: h2o_c + im4act_c + nad_c --> 2.0 h_c + im4ac_c + nadh_c 12042
HISTASE: h2o_c + hista_c + o2_c --> h2o2_c + im4act_c + nh4_c 13959

lald__L_c
LALDO3: h_c + mthgxl_c + nadph_c --> lald__L_c + nadp_c 10497 or 11160 or 12151 or 12883
LCADi: h2o_c + lald__L_c + nad_c --> 2.0 h_c + lac__L_c + nadh_c 12042
FCLPA: fc1p_c <=> dhap_c + lald__L_c 11873
LCARSyi: h_c + lald__L_c + nadph_c --> 12ppd__S_c + nadp_c 10029 or 13554 or 13562 or 13947
LKDRA: lkdr_c <=> lald__L_c + pyr_c 16266

lald__D_c
PPDOy: h_c + lald__D_c + nadph_c --> 12ppd__R_c + nadp_c 10029 or 11882 or 12784 or 13554 or 13562 or 13947 or 9774
LALDO2: h_c + mthgxl_c + nadph_c --> lald__D_c + nadp_c 11882 or 12784 or 9774
LCADi_D: h2o_c + lald__D_c + nad_c --> 2.0 h_c + lac__D_c + nadh_c 12042

3mldz_c
MHISOR: h2o_c + mhista_c + o2_c --> 3mldz_c + h2o2_c + nh4_c 13959
MACOXO: 3mldz_c + h2o_c + nad_c --> 3mlda_c + 2.0 h_c + nadh_c 12042

n4abutn_c
NABTNO: h2o_c + n4abutn_c + nad_c --> 4aabutn_c + 2.0 h_c + nadh_c 12042

pylald_c
PYLALDOX: h2o_c + nad_c + pylald_c --> 2.0 h_c + nadh_c + peracd_c 12042
```

In [21]:

```
# 3MOX4HOXPGALDOX metanephrine degradation in human
# 5HOXINDACTOX serotonin degradation in human
# GGGABADr/GDBTALDH bacterial putrescine/arginine degradation
# remove blocked reactions 3HPADHi, 3M4HDXPAC, GLACO, NABTNO
remove = ['3MOX4HOXPGALDOX','3MOX4HOXPGALDOX_NADP','5HOXINDACTOX','GGGABADr','GDBTALDH','3HPADHi','3M4HDXPAC','GLACO',
          'NABTNO','PYLALDOX']
model.remove_reactions(remove, remove_orphans=True)
```

In [22]:

```
# GDBTALDH is connected to amidase reaction
for r in sorted(model.genes.get_by_id('10276').reactions, key=lambda x: x.id):
    print(r, r.gene_reaction_rule)
```

```
AMID: 4gudbd_c + h2o_c --> 4gudbutn_c + nh4_c 10276 or 10277 or 12540 or 12553 or 12640 or 12842
AMID2: h2o_c + pad_c --> nh4_c + pac_c 10276 or 10277 or 12161 or 12540 or 12553 or 12842 or 13791
AMID3: h2o_c + iad_c --> ind3ac_c + nh4_c 10276 or 10277 or 12161 or 12540 or 12553 or 12842 or 13791
```

In [23]:

```
temp = ['10276','10277','12540','12553','12842','13791','12161','12640','11436','13380','9167','12123']
display(Annotation.loc[temp])
Show_Data(temp)
```

|  | Combined Annotations | Signal P | Sc288c Orthologs | Human Orthologs | Sc288 Best Hit | Human Blast | Essential | WolfPSort | C Terminal |
| --- | --- | --- | --- | --- | --- | --- | --- | --- | --- |
| RTO4\_ID |  |  |  |  |  |  |  |  |  |
| 10276 | K01426: E3.5.1.4, amiE; amidase |  | AMD2 |  | AMD2 | FAAH | Not Essential | cyto 13.5, cyto\_nucl 10.5, nucl 4.5, mito 3, p... | WAE\* |
| 10277 | K01426: E3.5.1.4, amiE; amidase |  | AMD2 |  | AMD2 | FAAH | Not Essential | mito 14, cyto 9, nucl 2, pero 2 | WAA\* |
| 12540 | KOG1212: Amidases |  | AMD2 |  | AMD2 | FAAH | Not Essential | mito 10, cysk 6, cyto 5, pero 3, extr 2 | QSA\* |
| 12553 | KOG1212: Amidases | S | AMD2 |  | AMD2 | FAAH | Not Essential | mito 27 | QSA\* |
| 12842 | K01426: E3.5.1.4, amiE; amidase |  | AMD2 |  | AMD2 | FAAH | Not Essential | cyto 13.5, cyto\_nucl 10, nucl 5.5, pero 5 | KKA\* |
| 13791 | K01426: E3.5.1.4, amiE; amidase |  |  |  | AMD2 | FAAH | Not Essential | cyto 13.5, cyto\_mito 11.5, mito 8.5, nucl 2, p... | EEQ\* |
| 12161 | K15528: FAAH; fatty acid amide hydrolase |  |  | FAAH | AMD2 | FAAH | Not Essential | cyto 11, mito 10, nucl 3, pero 2 | RVY\* |
| 12640 | K02433: gatA, QRSL1; aspartyl-tRNA(Asn)/glutam... |  | HER2 | QRSL1 | HER2 | QRSL1 | Essential | mito 25 | KAE\* |
| 11436 | K02434: gatB, PET112; aspartyl-tRNA(Asn)/gluta... | S | PET112 | GATB | PET112 | GATB | Not Essential | mito 24, cyto 3 | LGV\* |
| 13380 | K01886: QARS, glnS; glutaminyl-tRNA synthetase |  | GLN4 | QARS | GLN4 | QARS | Essential | cyto 16.5, cyto\_nucl 11.5, nucl 5.5, pero 5 | KGR\* |
| 9167 | K01893: NARS, asnS; asparaginyl-tRNA synthetase |  | DED81 | NARS | DED81 | NARS | Essential | cyto 7.5, cysk 7, cyto\_nucl 5.5, mito 5, pero ... | CTP\* |
| 12123 | K01893: NARS, asnS; asparaginyl-tRNA synthetase | S | SLM5 | NARS2 | SLM5 | NARS2 | Essential | mito 24, nucl 1, cyto 1, cyto\_nucl 1, pero 1, ... | SRF\* |

| strain | WT | | | | | | | | | | | | | | | | |
| --- | --- | --- | --- | --- | --- | --- | --- | --- | --- | --- | --- | --- | --- | --- | --- | --- | --- |
| condition | G\_MM | C\_MM | G\_SD | | GX\_SD | | | X\_SD | | A\_SD | | C\_SD | | MM\_CN120 | | MM\_CN5 | Diversity\_Sample |
| phase | exp | exp | exp | stat | exp | trans | stat | exp | stat | exp | stat | exp | stat | exp | stat | exp | exp |
| proteinId | Set1 | Set1 | Set2 | Set2 | Set2 | Set2 | Set2 | Set2 | Set2 | Set2 | Set2 | Set2 | Set2 | Set3 | Set3 | Set3 | Set3 |
| 10276 | 3.15429 | 3.36948 | 2.6701 | 2.30977 | 3.0374 | 1.57321 | 1.48973 | 2.32877 | 1.63896 | 2.49123 | 2.07225 | 3.34966 | 4.47407 | 3.14403 | 3.69471 | 4.02683 | 1.97517 |
| 10277 | 5.34588 | 5.58006 | 4.1053 | 3.88726 | 4.61931 | 3.52581 | 3.47827 | 3.5902 | 3.67328 | 4.2043 | 4.01799 | 5.98716 | 7.0036 | 4.94884 | 5.13663 | 6.0111 | 3.90538 |
| 12540 | 1.55994 | 0.308965 | 1.11492 | 1.97622 | 1.18187 | 1.25621 | 1.19815 | 1.48809 | 1.39891 | 1.70189 | 1.77721 | 1.68702 | 1.40805 | 4.17143 | 4.58674 | 1.78701 | 3.47272 |
| 12553 | 7.24969 | 4.15575 | 3.94645 | 5.16328 | 3.8075 | 4.10641 | 4.03111 | 4.36158 | 4.0417 | 4.59273 | 4.78232 | 4.66484 | 4.41267 | 7.25157 | 7.52111 | 3.93988 | 6.2874 |
| 12842 | 7.71865 | 6.30078 | 3.96539 | 6.55085 | 3.97341 | 5.03384 | 4.29265 | 3.62493 | 4.40198 | 3.7654 | 4.68818 | 7.57077 | 6.69475 | 8.02319 | 8.18109 | 5.11062 | 7.67516 |
| 13791 | 2.50432 | 2.91952 | 2.99014 | 4.62504 | 2.75351 | 2.99432 | 3.41392 | 2.63787 | 3.46481 | 2.43439 | 3.84491 | 3.36642 | 3.40616 | 2.29347 | 2.85922 | 2.93671 | 2.40948 |
| 12161 | 4.57775 | 4.90366 | 4.034 | 4.68712 | 3.8866 | 4.06412 | 4.26078 | 3.87598 | 4.63356 | 4.57754 | 5.48447 | 2.718 | 2.40096 | 4.81874 | 4.26639 | 4.32178 | 4.77091 |
| 12640 | 4.72337 | 4.67068 | 4.54807 | 4.16471 | 4.60462 | 4.66302 | 4.71802 | 4.78206 | 4.88462 | 4.78575 | 4.83495 | 5.01524 | 4.50581 | 4.45699 | 4.42594 | 4.1347 | 4.82639 |
| 11436 | 5.97298 | 6.02637 | 6.30339 | 6.1655 | 6.30505 | 6.40165 | 6.43813 | 6.1688 | 6.33189 | 6.22155 | 6.27676 | 7.13991 | 8.10268 | 5.07018 | 5.30716 | 5.6739 | 5.84654 |
| 13380 | 6.44596 | 6.22254 | 6.42365 | 5.39934 | 6.44485 | 5.65898 | 5.82887 | 6.02897 | 5.66281 | 5.73484 | 5.5412 | 7.03683 | 8.0008 | 7.60585 | 7.3392 | 8.0525 | 7.76654 |
| 9167 | 7.31786 | 6.92274 | 7.36809 | 5.72268 | 7.70801 | 6.31477 | 6.33639 | 6.54584 | 5.84019 | 6.0349 | 5.75525 | 7.29172 | 7.90533 | 6.05743 | 5.80145 | 7.96328 | 6.70836 |
| 12123 | 4.83165 | 5.11938 | 4.28754 | 4.29641 | 4.28059 | 4.56924 | 4.60539 | 4.60529 | 4.87018 | 4.36486 | 4.68175 | 5.3067 | 5.40623 | 2.91112 | 3.11052 | 3.2877 | 3.41716 |

| strain | WT | | | | | | | | | | |
| --- | --- | --- | --- | --- | --- | --- | --- | --- | --- | --- | --- |
| condition | G\_SD | | GX\_SD | | | X\_SD | | A\_SD | | C\_SD | |
| proteinId | exp | stat | exp | trans | stat | exp | stat | exp | stat | exp | stat |
| 10276 | 0.190197 | 0 | 0 | 0 | 0 | 0 | 0.189187 | 0 | 0.378559 | 0.211647 | 0 |
| 12540 | 0 | 0 | 0 | 0 | 0.180258 | 0.200926 | 0 | 0 | 0.189808 | 0 | 0 |
| 12553 | 0 | 0 | 0 | 0 | 0.180258 | 0.200926 | 0 | 0 | 0.379285 | 0 | 0 |
| 12842 | 4.22787 | 9.52695 | 5.07788 | 7.62377 | 9.47954 | 8.05696 | 10.8031 | 11.0265 | 14.1575 | 32.6833 | 32.0136 |
| 12161 | 0.581761 | 5.0617 | 0.816288 | 9.66316 | 11.521 | 3.33454 | 4.73458 | 5.40794 | 7.4729 | 1.48832 | 2.38474 |
| 12640 | 2.89122 | 1.99226 | 2.4349 | 2.23254 | 1.2786 | 1.97251 | 1.1741 | 1.35206 | 0.575052 | 0.22091 | 0.211526 |
| 11436 | 1.73538 | 1.96009 | 2.02425 | 1.31001 | 2.01439 | 1.97051 | 1.37761 | 0.575797 | 0.385057 | 2.55492 | 1.07378 |
| 13380 | 22.1379 | 20.4416 | 24.0083 | 18.4137 | 20.3454 | 24.2712 | 21.2518 | 20.8526 | 19.1839 | 21.9887 | 30.3015 |
| 9167 | 18.029 | 16.2329 | 17.4771 | 12.6469 | 13.8957 | 11.3458 | 11.2215 | 12.3556 | 9.57064 | 11.1383 | 16.3228 |
| 12123 | 1.12412 | 1.30423 | 0 | 1.11902 | 1.44978 | 0.787109 | 0.97047 | 0.382815 | 0.764134 | 0 | 0 |

|  | Glucose | Xylose | Arabinose | Acetate | Coumarate | Ferulate | YNB Oleic Acid | YNB Ricinoleic Acid | YNB Glucose | YNB Gluc DOC | YPD |
| --- | --- | --- | --- | --- | --- | --- | --- | --- | --- | --- | --- |
| proteinId |  |  |  |  |  |  |  |  |  |  |  |
| 10276 | 0.0590597 | -0.183785 | 0.0217332 | 0.217737 | 0.0962688 | 0.400174 | -0.602794 | 0.20458 | -0.0993253 | -0.246385 | 0.0940215 |
| 10277 | -0.0317559 | -0.00631653 | -0.182822 | -0.0983566 | -0.30335 | 0.0500607 | -0.0843293 | -0.292154 | -0.291562 | -0.141471 | 0.55294 |
| 12553 | -0.0449658 | -0.0975784 | -0.208307 | -0.150953 | 0.248676 | -0.226523 | 0.18321 | 0.155096 | 0.137944 | -0.184634 | 0.0300694 |
| 12842 | -0.0326962 | 0.0985683 | 0.114314 | 0.138411 | 0.448384 | 0.230723 | 0.456779 | -0.0532847 | 0.146205 | 0.0458719 | -0.0169437 |
| 13791 | 0.20527 | 0.0745708 | 0.0792896 | 0.103773 | 0.000193625 | 0.194923 | 0.216039 | 0.139521 | -0.0856743 | -0.17181 | 0.102706 |
| 12161 | -0.266226 | -0.209809 | -0.279097 | -0.156056 | -0.336039 | -0.0991283 | -0.226048 | 0.175646 | -0.254703 | 0.0193068 | 0.345903 |
| 11436 | -0.125742 | -0.00340533 | -0.101468 | 0.00454544 | -0.101642 | -0.139944 | 0.119935 | 0.000208259 | 0.0940378 | 0.207925 | -0.528785 |
| 13380 | 0.212952 | 0.48865 | 0.134225 | 0.480758 | -0.595638 | 0.105697 | -0.289997 | -0.915948 | 0.122397 | -0.08248 | -0.750243 |

In [24]:

```
for x in temp:
    if x in model.genes:
        for r in sorted(model.genes.get_by_id(x).reactions, key=lambda x: x.id):
            print(r, r.gene_reaction_rule)
    else:
        print(x, 'no reactions')
    print()
```

```
AMID: 4gudbd_c + h2o_c --> 4gudbutn_c + nh4_c 10276 or 10277 or 12540 or 12553 or 12640 or 12842
AMID2: h2o_c + pad_c --> nh4_c + pac_c 10276 or 10277 or 12161 or 12540 or 12553 or 12842 or 13791
AMID3: h2o_c + iad_c --> ind3ac_c + nh4_c 10276 or 10277 or 12161 or 12540 or 12553 or 12842 or 13791

AMID: 4gudbd_c + h2o_c --> 4gudbutn_c + nh4_c 10276 or 10277 or 12540 or 12553 or 12640 or 12842
AMID2: h2o_c + pad_c --> nh4_c + pac_c 10276 or 10277 or 12161 or 12540 or 12553 or 12842 or 13791
AMID3: h2o_c + iad_c --> ind3ac_c + nh4_c 10276 or 10277 or 12161 or 12540 or 12553 or 12842 or 13791

AMID: 4gudbd_c + h2o_c --> 4gudbutn_c + nh4_c 10276 or 10277 or 12540 or 12553 or 12640 or 12842
AMID2: h2o_c + pad_c --> nh4_c + pac_c 10276 or 10277 or 12161 or 12540 or 12553 or 12842 or 13791
AMID3: h2o_c + iad_c --> ind3ac_c + nh4_c 10276 or 10277 or 12161 or 12540 or 12553 or 12842 or 13791

AMID: 4gudbd_c + h2o_c --> 4gudbutn_c + nh4_c 10276 or 10277 or 12540 or 12553 or 12640 or 12842
AMID2: h2o_c + pad_c --> nh4_c + pac_c 10276 or 10277 or 12161 or 12540 or 12553 or 12842 or 13791
AMID3: h2o_c + iad_c --> ind3ac_c + nh4_c 10276 or 10277 or 12161 or 12540 or 12553 or 12842 or 13791

AMID: 4gudbd_c + h2o_c --> 4gudbutn_c + nh4_c 10276 or 10277 or 12540 or 12553 or 12640 or 12842
AMID2: h2o_c + pad_c --> nh4_c + pac_c 10276 or 10277 or 12161 or 12540 or 12553 or 12842 or 13791
AMID3: h2o_c + iad_c --> ind3ac_c + nh4_c 10276 or 10277 or 12161 or 12540 or 12553 or 12842 or 13791

AMID2: h2o_c + pad_c --> nh4_c + pac_c 10276 or 10277 or 12161 or 12540 or 12553 or 12842 or 13791
AMID3: h2o_c + iad_c --> ind3ac_c + nh4_c 10276 or 10277 or 12161 or 12540 or 12553 or 12842 or 13791
AMID_1: ad_c + h2o_c --> ac_c + nh4_c 13791

AMID2: h2o_c + pad_c --> nh4_c + pac_c 10276 or 10277 or 12161 or 12540 or 12553 or 12842 or 13791
AMID3: h2o_c + iad_c --> ind3ac_c + nh4_c 10276 or 10277 or 12161 or 12540 or 12553 or 12842 or 13791

AMID: 4gudbd_c + h2o_c --> 4gudbutn_c + nh4_c 10276 or 10277 or 12540 or 12553 or 12640 or 12842

11436 no reactions

GLNTRS: atp_c + gln__L_c + trnagln_c --> amp_c + glntrna_c + ppi_c 13380

ASNTRS: asn__L_c + atp_c + trnaasn_c --> amp_c + asntrna_c + ppi_c 9167

ASNTRSm: asn__L_m + atp_m + trnaasn_m --> amp_m + asntrna_m + ppi_m 12123
```

In [25]:

```
# 12161 is fatty acid amide hydrolase
m = model.metabolites.get_by_id('ocdcea_c').copy()
m.id = 'ocdcead_c'
m.name = 'Oleamide'
m.formula = 'C18H35NO'
m.charge = 0
model.add_metabolites([m])
r = model.reactions.get_by_id('AMID').copy()
r.id = 'FAAH'
r.name = 'Fatty acid amide hydrolase'
r.gene_reaction_rule = '12161'
model.add_reactions([r])
r.add_metabolites({'4gudbd_c': 1.0, '4gudbutn_c': -1.0, 'ocdcead_c': -1.0, 'ocdcea_c': 1.0})
# 12640 and 11436 are aspartyl-tRNA(Asn)/glutamyl-tRNA(Gln) amidotransferase subunits gatA and gatB
# gatC is missing -> check tRNA synthesis later 
# https://www.ncbi.nlm.nih.gov/pmc/articles/PMC4301749/
model.reactions.get_by_id('AMID').gene_reaction_rule = '10276 or 10277 or 12540 or 12553 or 12842 or 13791'
model.reactions.get_by_id('AMID2').gene_reaction_rule = '10276 or 10277 or 12540 or 12553 or 12842 or 13791'
model.reactions.get_by_id('AMID3').gene_reaction_rule = '10276 or 10277 or 12540 or 12553 or 12842 or 13791'
model.reactions.get_by_id('AMID_1').gene_reaction_rule = '10276 or 10277 or 12540 or 12553 or 12842 or 13791'
```

In [26]:

```
for r in sorted(model.genes.get_by_id('13426').reactions, key=lambda x: x.id):
    for m in r.reactants:
        if not any(x == m.id for x in ['h2o_c','nad_c','nadp_c','h2o_m','nad_m','nadp_m']):
            print(m.id)
            for r2 in m.reactions:
                print(r2, r2.gene_reaction_rule)
            print()
```

```
5hoxindact_m
5HOXINDACTOXm: 5hoxindact_m + h2o_m + nad_m --> 5hoxindoa_m + 2.0 h_m + nadh_m 13426

4abutn_m
ABUTDm: 4abutn_m + h2o_m + nad_m --> 4abut_m + 2.0 h_m + nadh_m 13426
ABOR: 4abutn_m + h2o_m + nadp_m --> 4abut_m + 2.0 h_m + nadph_m 13426

4abutn_m
ABUTDm: 4abutn_m + h2o_m + nad_m --> 4abut_m + 2.0 h_m + nadh_m 13426
ABOR: 4abutn_m + h2o_m + nadp_m --> 4abut_m + 2.0 h_m + nadph_m 13426

id3acald_m
ALDD20xm: h2o_m + id3acald_m + nad_m --> 2.0 h_m + ind3ac_m + nadh_m 13426
ALDD20ym: h2o_m + id3acald_m + nadp_m --> 2.0 h_m + ind3ac_m + nadph_m 13426

id3acald_m
ALDD20xm: h2o_m + id3acald_m + nad_m --> 2.0 h_m + ind3ac_m + nadh_m 13426
ALDD20ym: h2o_m + id3acald_m + nadp_m --> 2.0 h_m + ind3ac_m + nadph_m 13426

acald_m
ALDD2xm: acald_m + h2o_m + nad_m --> ac_m + 2.0 h_m + nadh_m 13426
ALDD2ym: acald_m + h2o_m + nadp_m --> ac_m + 2.0 h_m + nadph_m 13426
ACALDtm: acald_m <=> acald_c 

acald_m
ALDD2xm: acald_m + h2o_m + nad_m --> ac_m + 2.0 h_m + nadh_m 13426
ALDD2ym: acald_m + h2o_m + nadp_m --> ac_m + 2.0 h_m + nadph_m 13426
ACALDtm: acald_m <=> acald_c 

bamppald_m
BAMPPALDOXm: bamppald_m + h2o_m + nad_m --> ala_B_m + 2.0 h_m + nadh_m 13426

gcald_m
GCALDDm: gcald_m + h2o_m + nad_m --> glyclt_m + 2.0 h_m + nadh_m 13426

glac_m
GLACOm: glac_m + 2.0 h2o_m + nad_m --> glcr_m + 3.0 h_m + nadh_m 13426

im4act_m
IMACTD_m: h2o_m + im4act_m + nad_m --> 2.0 h_m + im4ac_m + nadh_m 13426

n4abutn_m
NABTNOm: h2o_m + n4abutn_m + nad_m --> 4aabutn_m + 2.0 h_m + nadh_m 13426

pylald_m
PYLALDOXm: h2o_m + nad_m + pylald_m --> 2.0 h_m + nadh_m + peracd_m 13426

4tmeabut_m
HTMLA_m: 3htmelys_m --> 4tmeabut_m + gly_m 9222
TMABDH1_m: 4tmeabut_m + h2o_m + nad_m --> gbbtn_m + 2.0 h_m + nadh_m 13426
```

In [27]:

```
# 5HOXINDACTOXm serotonin degradation in human
# 4abutn is produced only in cyto, remove ABOR and ABUTDm
# id3acald is produced only in cyto, remove ALDD20ym and ALDD20xm
# bamppald is produced only in cyto, remove BAMPPALDOXm
# similar reasons, remove GCALDDm, GLACOm, IMACTD_m, NABTNOm, PYLALDOXm
remove = ['5HOXINDACTOXm','ABOR','ABUTDm','ALDD20xm','ALDD20ym','BAMPPALDOXm','GCALDDm','GLACOm','IMACTD_m','NABTNOm',
          'PYLALDOXm']
model.remove_reactions(remove, remove_orphans=True)
```

In [28]:

```
for r in model.reactions:
    if r.gene_reaction_rule == '16323':
        print(r, r.gene_reaction_rule)
```

```
COALDDH: conialdh_c + h2o_c + nad_c --> fer_c + 2.0 h_c + nadh_c 16323
ALDD21: h2o_c + nad_c + pristanal_c --> 2.0 h_c + nadh_c + prist_c 16323
```

In [29]:

```
temp = ['16323','12950']
display(Annotation.loc[temp])
Show_Data(temp)
```

|  | Combined Annotations | Signal P | Sc288c Orthologs | Human Orthologs | Sc288 Best Hit | Human Blast | Essential | WolfPSort | C Terminal |
| --- | --- | --- | --- | --- | --- | --- | --- | --- | --- |
| RTO4\_ID |  |  |  |  |  |  |  |  |  |
| 16323 | K00128: ALDH; aldehyde dehydrogenase (NAD+) |  | HFD1 | ALDH3A1,ALDH3A2,ALDH3B1,ALDH3B2 | HFD1 | ALDH3 | Not Essential | cyto 10.5, plas 7, cyto\_nucl 7, mito 3, nucl 2... | GQA\* |
| 12950 | KOG2450: Aldehyde dehydrogenase |  |  |  | UGA2 | ALDH9 | Not Essential | cyto 21.5, cyto\_nucl 13, mito 3 | PSA\* |

| strain | WT | | | | | | | | | | | | | | | | |
| --- | --- | --- | --- | --- | --- | --- | --- | --- | --- | --- | --- | --- | --- | --- | --- | --- | --- |
| condition | G\_MM | C\_MM | G\_SD | | GX\_SD | | | X\_SD | | A\_SD | | C\_SD | | MM\_CN120 | | MM\_CN5 | Diversity\_Sample |
| phase | exp | exp | exp | stat | exp | trans | stat | exp | stat | exp | stat | exp | stat | exp | stat | exp | exp |
| proteinId | Set1 | Set1 | Set2 | Set2 | Set2 | Set2 | Set2 | Set2 | Set2 | Set2 | Set2 | Set2 | Set2 | Set3 | Set3 | Set3 | Set3 |
| 16323 | 5.54194 | 5.5053 | 6.37226 | 5.68644 | 6.28507 | 6.1392 | 5.86895 | 6.06104 | 5.82576 | 5.50985 | 6.17279 | 4.76433 | 4.74944 | 5.59107 | 6.40434 | 5.99953 | 6.38502 |
| 12950 | 4.92351 | 9.11152 | 5.05129 | 5.98061 | 4.99463 | 4.90659 | 4.93597 | 5.04028 | 5.328 | 5.31962 | 5.33687 | 8.69923 | 7.15407 | 5.50552 | 5.16486 | 6.16151 | 6.59108 |

| strain | WT | | | | | | | | | | |
| --- | --- | --- | --- | --- | --- | --- | --- | --- | --- | --- | --- |
| condition | G\_SD | | GX\_SD | | | X\_SD | | A\_SD | | C\_SD | |
| proteinId | exp | stat | exp | trans | stat | exp | stat | exp | stat | exp | stat |
| 16323 | 10.9902 | 12.5683 | 13.6351 | 14.1342 | 18.2252 | 11.3209 | 13.9247 | 9.27817 | 10.1675 | 6.60796 | 3.93398 |
| 12950 | 5.16889 | 7.21647 | 6.10306 | 7.46089 | 8.75973 | 11.1834 | 12.6008 | 9.8603 | 10.7079 | 37.5998 | 29.842 |

|  | Glucose | Xylose | Arabinose | Acetate | Coumarate | Ferulate | YNB Oleic Acid | YNB Ricinoleic Acid | YNB Glucose | YNB Gluc DOC | YPD |
| --- | --- | --- | --- | --- | --- | --- | --- | --- | --- | --- | --- |
| proteinId |  |  |  |  |  |  |  |  |  |  |  |
| 16323 | 0.000210473 | -0.0174318 | 0.0140589 | -0.110443 | 0.00440814 | 0.0892768 | 0.155043 | 0.103889 | -0.177274 | -0.243731 | -0.140368 |
| 12950 | 0.00596512 | -0.171781 | 0.205573 | 0.0569485 | 0.122395 | 0.160439 | 0.000604604 | 0.0892542 | 0.233256 | 0.31056 | 0.190096 |

In [30]:

```
Annotation.loc['12950','WolfPSort']
```

Out[30]:

```
'cyto 21.5, cyto_nucl 13, mito 3'
```

16323 HFD1 is ER hexadecenal and mito 4-hydroxybenzaldehyde dehydrogenase with nadh  
https://www.sciencedirect.com/science/article/pii/S1097276512003954  
https://www.sciencedirect.com/science/article/pii/S2451945616302902

hexadecenal (hxdceal) is generated from sphingosine phosphate lyase  
if coming from cer4\_18\_r, Sphingosine:n-C18:0OH, Cer(d18:1(4E)/18:0(2OH))  
we get a sphingosine (sphings) and a 2-hydroxyhexadecanoate  
if coming from desaturated version of cer1\_18\_r, Sphingosine:n-C18:0, Cer(d18:1(4E)/18:0)  
we get a sphingosine (sphings) and a normal hydroxy fatty acid  
sphingosine 1-phosphate is degraded to ehamp and hexadecenal  
2-hydroxy fatty acid needs alpha-oxidation  
hexadecenal is oxidized to hexadecenoate, and activated to hdd2coa, enters beta-oxidation

phytosphingosine degradation results in a 2-hydroxy fatty aldehyde,  
which can be oxidized to 2-hydroxy fatty acid by HFD1/ALDH3A2  
fatty acid alpha-oxydation happens in E.R. in human, by HACL2 (not found in Rhodo) and PECR (10912?)  
https://www.pnas.org/content/114/13/E2616

12950 bacterial vdh can also oxidize 4-hydroxybenzaldehyde, weak sigP, alternative splicing?  
Assign to cyto reactions for now

In [31]:

```
r = ptri.reactions.get_by_id('ALDD16').copy()
r.gene_reaction_rule = '16323'
model.add_reactions([r])

r = ptri.reactions.get_by_id('ALDD3C161_c').copy()
r.id = 'ALDD3C161'
r.gene_reaction_rule = '16323'
model.add_reactions([r])

for x in ['ALDD21','ALDD16','ALDD3C161']:
    r = model.reactions.get_by_id(x)
    r.id = r.id + 'er'
    for m in r.metabolites:
        if not m.id.replace('_c','_r') in model.metabolites:
            m2 = m.copy()
            m2.id = m.id.replace('_c','_r')
            m2.compartment = 'r'
            model.add_metabolites([m2])
        r.add_metabolites({m.id: -r.get_coefficient(m.id), m.id.replace('_c','_r'): r.get_coefficient(m.id)})

r = model.reactions.get_by_id('VNDH_2').copy()
r.id = '4HBALDDm'
r.gene_reaction_rule = '16323'
model.add_reactions([r])
for m in r.metabolites:
    if not m.id.replace('_c','_m') in model.metabolites:
        m2 = m.copy()
        m2.id = m.id.replace('_c','_m')
        m2.compartment = 'm'
        model.add_metabolites([m2])
    r.add_metabolites({m.id: -r.get_coefficient(m.id), m.id.replace('_c','_m'): r.get_coefficient(m.id)})

for x in ['COALDDH','VNDH','VNDH_2','VNDH_3']:
    r = model.reactions.get_by_id(x)
    r.gene_reaction_rule = '12950'
```

In [32]:

```
for r in sorted(model.genes.get_by_id('16323').reactions, key=lambda x: x.id):
    print(r, r.gene_reaction_rule)
print()
for r in sorted(model.genes.get_by_id('12950').reactions, key=lambda x: x.id):
    print(r, r.gene_reaction_rule)
```

```
4HBALDDm: 4hbald_m + h2o_m + nad_m --> 4hbz_m + 2.0 h_m + nadh_m 16323
ALDD16er: h2o_r + hxdcal_r + nad_r --> 2.0 h_r + hdca_r + nadh_r 16323
ALDD21er: h2o_r + nad_r + pristanal_r --> 2.0 h_r + nadh_r + prist_r 16323
ALDD3C161er: h2o_r + hxdceal_r + nad_r --> 2.0 h_r + hdc2ea_r + nadh_r 16323

COALDDH: conialdh_c + h2o_c + nad_c --> fer_c + 2.0 h_c + nadh_c 12950
VNDH: h2o_c + nad_c + vanln_c --> 2.0 h_c + nadh_c + vanlt_c 12950
VNDH_2: 4hbald_c + h2o_c + nad_c --> 4hbz_c + 2.0 h_c + nadh_c 12950
VNDH_3: 34dhbald_c + h2o_c + nad_c --> 34dhbz_c + 2.0 h_c + nadh_c 12950
```

glycolate is oxidized to glyoxylate in peroxisome by 2-hydroxy acid oxidase

In [33]:

```
for r in sorted(model.metabolites.get_by_id('glyclt_c').reactions, key=lambda x: x.id):
    print(r, r.gene_reaction_rule)
print()
for r in sorted(model.metabolites.get_by_id('glyclt_m').reactions, key=lambda x: x.id):
    print(r, r.gene_reaction_rule)
print()
for r in sorted(model.metabolites.get_by_id('glyclt_x').reactions, key=lambda x: x.id):
    print(r, r.gene_reaction_rule)
```

```
GCALDD: gcald_c + h2o_c + nad_c --> glyclt_c + 2.0 h_c + nadh_c 12042
GLYCLTDy: glx_c + h_c + nadph_c --> glyclt_c + nadp_c 11036 or 9515 or 9520

GLYCLTDxm: glx_m + h_m + nadh_m --> glyclt_m + nad_m 12051

GLYCLTDyp: glx_x + h_x + nadph_x --> glyclt_x + nadp_x 11036
GLYCTO1p: glyclt_x + o2_x --> glx_x + h2o2_x 14950 or 16607
```

In [34]:

```
temp = ['EX_glyclt_e','GLYCLTt','GLYCLTtm','GLYCLTtp']
for x in temp:
    r = hsa2.reactions.get_by_id(x).copy()
    r.gene_reaction_rule = ''
    model.add_reactions([r])
model.reactions.get_by_id('EX_glyclt_e').lower_bound = 0.0
```

In [35]:

```
for r in sorted(model.metabolites.get_by_id('glx_c').reactions, key=lambda x: x.id):
    print(r, r.gene_reaction_rule)
print()
for r in sorted(model.metabolites.get_by_id('glx_m').reactions, key=lambda x: x.id):
    print(r, r.gene_reaction_rule)
print()
for r in sorted(model.metabolites.get_by_id('glx_x').reactions, key=lambda x: x.id):
    print(r, r.gene_reaction_rule)
```

```
AGTi: ala__L_c + glx_c --> gly_c + pyr_c 8819
GLYCLTDy: glx_c + h_c + nadph_c --> glyclt_c + nadp_c 11036 or 9515 or 9520
UGLYCH: h2o_c + 2.0 h_c + urdglyc_c --> co2_c + glx_c + 2.0 nh4_c 14724

AGTim: ala__L_m + glx_m --> gly_m + pyr_m 8819
DDPGAm: 4h2oglt_m <=> glx_m + pyr_m 12061
GLYCLTDxm: glx_m + h_m + nadh_m --> glyclt_m + nad_m 12051

GLXO2p: glx_x + h2o_x + o2_x --> h2o2_x + h_x + oxa_x 14950 or 16607
GLYCLTDyp: glx_x + h_x + nadph_x --> glyclt_x + nadp_x 11036
GLYCTO1p: glyclt_x + o2_x --> glx_x + h2o2_x 14950 or 16607
GLYOp: gly_x + h2o_x + o2_x --> glx_x + h2o2_x + nh4_x 15994
ICLp: icit_x --> glx_x + succ_x 14022
MALSp: accoa_x + glx_x + h2o_x --> coa_x + h_x + mal__L_x 9457
```

In [36]:

```
temp = ['EX_glx_e','GLXt','GLXtm','GLXtp']
for x in temp:
    r = hsa2.reactions.get_by_id(x).copy()
    r.gene_reaction_rule = ''
    model.add_reactions([r])
model.reactions.get_by_id('EX_glx_e').lower_bound = 0.0
```

In [37]:

```
# check if model can produce cofactors
cofactors = ['camp_c','coa_c','fad_c','gthrd_c','hemeA_m','nad_c','nadp_c','q9_m','thf_c','thmpp_c',
             '5mthf_c','btn_m','lipopb_m','mlthf_c','ptrc_c','pydx5p_c','spmd_c']
with model:
    for x in cofactors:
        model.reactions.get_by_id('BIOMASS_RT').add_metabolites({x: -1e-2})
        sol = model.optimize()
        print(x, sol.objective_value)
        if abs(sol.objective_value) < 1e-6:
            for k, v in sol.shadow_prices.items():
                if v and k in [m.id for m in model.reactions.get_by_id('BIOMASS_RT').metabolites]:
                    print('\t',k, v)
        model.reactions.get_by_id('BIOMASS_RT').add_metabolites({x: 1e-2})
```

```
camp_c 0.13593743393234803
coa_c 0.1354785450262918
fad_c 0.1358833457242384
gthrd_c 0.13632034044837113
hemeA_m 0.0
	 hemeA_m -100.0
nad_c 0.1356155812262751
nadp_c 0.13560034129332388
q9_m 0.0
	 q9_m -100.0
thf_c 0.13597215470566093
thmpp_c 0.0
	 thmpp_c -100.0
5mthf_c 0.13577122443666576
btn_m 0.0
	 btn_m -100.0
lipopb_m 0.0
	 lipopb_m -100.0
mlthf_c 0.1357559495044811
ptrc_c 0.13650642647367617
pydx5p_c 0.13653113901894953
spmd_c 0.13657749916639828
```

Thiamine biosynthesis  
HETZK: 4mhetz\_c + atp\_c --> 4mpetz\_c + adp\_c + h\_c 16213  
TMPPP: 2mahmp\_c + 4mpetz\_c + h\_c --> ppi\_c + thmmp\_c 16213  
TMDPK: atp\_c + thm\_c --> amp\_c + h\_c + thmpp\_c 16484  
TMDPPK: atp\_c + thmpp\_c --> adp\_c + thmtp\_c 16484

Need to add TDP by 14465  
TDP: h2o\_c + thmpp\_c --> h\_c + pi\_c + thmmp\_c  
Need to add THMP by thiamine phosphate phosphatase (?)  
THMP: h2o\_c + thmmp\_c --> pi\_c + thm\_c

2mahmp\_c  
Need to add TMN by THI20 12778  
TMN: h2o\_c + thm\_c --> 4ahmmp\_c + 4mhetz\_c + h\_c missing  
HMPK1: 4ahmmp\_c + atp\_c --> 4ampm\_c + adp\_c + h\_c 12778  
PMPK: 4ampm\_c + atp\_c --> 2mahmp\_c + adp\_c 12778  
There are several more reactions by 12778 THI20 that can synthesis precursors

4mpetz\_c
synthesis reactions are hypothetical by 16324 THI4  
gly + [protein]-cys**L + nad -> ADP-5-ethyl-4-methylthiazole-2-carboxylate + [protein]-2amac + 2 h + 3 h2o + ncam  
ADP-5-ethyl-4-methylthiazole-2-carboxylate + h2o -> 4mpetz + h + co2 + AMP  
make a new overall reaction THZPSN4  
gly + cys**L + nad -> 4mpetz + 2amac + co2 + amp + ncam + 3 h + 2 h2o

2ahmmp\_c (from 4ahmmp\_c or 4ampm\_c)  
there are bacterial synthesis reactions (from air) in BiGG  
AHMMPS: air\_c + 2.0 h\_c → 4ahmmp\_c + gcald\_c + pi\_c (iMM904)  
AMPMS2: air\_c + h2o\_c + nad\_c → 4ampm\_c + 2.0 for\_c + 3.0 h\_c + nadh\_c (iML1515)  
AMPMS3: air\_c + amet\_c ⇌ 4ampm\_c + dad\_5\_c + for\_c + 2.0 h\_c + met\_\_L\_c + co\_c (iJB785)  
Fungal synthesis by HMP-P synthase THI5 is very recent discovery  
https://www.ncbi.nlm.nih.gov/pubmed/22568620  
https://oaktrust.library.tamu.edu/handle/1969.1/155385  
Create a new reaction HMPPS  
pdx5p + his-L + 2 o2 + h2o2 + 2 fe3 -> 4ampm + 4oglu + glx + co2 + 2 fe2 + 4 h

In [38]:

```
temp = ['16213','16484','12778','16324','11147','14465']
display(Annotation.loc[temp])
Show_Data(temp)
```

|  | Combined Annotations | Signal P | Sc288c Orthologs | Human Orthologs | Sc288 Best Hit | Human Blast | Essential | WolfPSort | C Terminal |
| --- | --- | --- | --- | --- | --- | --- | --- | --- | --- |
| RTO4\_ID |  |  |  |  |  |  |  |  |  |
| 16213 | K14154: THI6; thiamine-phosphate diphosphoryla... |  | THI6 |  | THI6 |  | Not Essential | mito 11, cyto 7, extr 4, cyto\_nucl 4 | QRV\* |
| 16484 | K00949: thiN, TPK1, THI80; thiamine pyrophosph... |  | THI80 | TPK1 | THI80 | TPK1 | Essential | nucl 10.5, cyto\_nucl 10, cyto 8.5, mito 5 | GAE\* |
| 12778 | K00877: THI20; hydroxymethylpyrimidine/phospho... |  | THI20,THI21,THI22 |  | THI20 |  | Not Essential | cyto 15.5, cyto\_nucl 8.5, mito 8 | MPQ\* |
| 16324 | K03146: THI4, THI1; thiamine thiazole synthase |  | THI4 |  | THI4 |  | Not Essential | cyto 20, mito 7 | ISA\* |
| 11147 | K18278: THI5; pyrimidine precursor biosynthesi... |  | THI13,YFL058W,YJR156C,THI12 |  | THI5 |  | Not Essential | cyto 14, E.R. 5, mito 3, extr 3 | VAA\* |
| 14465 | KOG4313: Thiamine pyrophosphokinase |  | YJR142W |  | YJR142W |  | Not Essential | cyto 8, mito 7, cyto\_nucl 6.333, cyto\_pero 6.3... | GPA\* |

| strain | WT | | | | | | | | | | | | | | | | |
| --- | --- | --- | --- | --- | --- | --- | --- | --- | --- | --- | --- | --- | --- | --- | --- | --- | --- |
| condition | G\_MM | C\_MM | G\_SD | | GX\_SD | | | X\_SD | | A\_SD | | C\_SD | | MM\_CN120 | | MM\_CN5 | Diversity\_Sample |
| phase | exp | exp | exp | stat | exp | trans | stat | exp | stat | exp | stat | exp | stat | exp | stat | exp | exp |
| proteinId | Set1 | Set1 | Set2 | Set2 | Set2 | Set2 | Set2 | Set2 | Set2 | Set2 | Set2 | Set2 | Set2 | Set3 | Set3 | Set3 | Set3 |
| 16213 | 4.81309 | 4.89148 | 4.83198 | 4.05331 | 4.748 | 4.93295 | 4.81588 | 4.82558 | 4.71979 | 4.59339 | 4.44003 | 5.25192 | 4.77541 | 4.57317 | 4.61025 | 4.75554 | 4.62232 |
| 16484 | 4.38788 | 3.99024 | 4.38679 | 4.65291 | 4.53257 | 4.62049 | 4.62611 | 4.38352 | 5.2266 | 4.8717 | 5.7625 | 4.43056 | 4.82527 | 3.43244 | 3.73435 | 3.44117 | 3.04767 |
| 12778 | 5.90323 | 5.62428 | 5.55726 | 5.22359 | 5.54935 | 6.03522 | 5.88355 | 5.998 | 5.35261 | 5.91296 | 5.84426 | 5.47944 | 4.11869 | 6.04004 | 6.01528 | 5.80453 | 5.62412 |
| 16324 | 8.77404 | 9.56479 | 7.61304 | 5.43187 | 7.58099 | 6.77067 | 6.47918 | 7.88397 | 4.42388 | 7.00318 | 3.38646 | 7.75762 | 5.79927 | 10.0994 | 10.0325 | 9.40644 | 9.19961 |
| 11147 | 9.25676 | 7.75752 | 9.30126 | 6.71582 | 9.24485 | 8.48123 | 8.41708 | 8.90518 | 4.30593 | 8.48646 | 4.63163 | 3.34688 | 1.39671 | 10.3197 | 10.3502 | 10.5869 | 8.9695 |
| 14465 | 5.58739 | 5.69374 | 5.83264 | 5.3976 | 5.87591 | 5.61624 | 5.44709 | 5.78252 | 5.19988 | 5.50733 | 5.51123 | 7.14826 | 6.37856 | 6.47374 | 6.16327 | 6.58145 | 6.8356 |

| strain | WT | | | | | | | | | | |
| --- | --- | --- | --- | --- | --- | --- | --- | --- | --- | --- | --- |
| condition | G\_SD | | GX\_SD | | | X\_SD | | A\_SD | | C\_SD | |
| proteinId | exp | stat | exp | trans | stat | exp | stat | exp | stat | exp | stat |
| 16213 | 1.32179 | 1.84973 | 2.25609 | 2.2419 | 1.81526 | 2.16368 | 1.01025 | 1.74067 | 1.51427 | 3.44744 | 3.25672 |
| 16484 | 0 | 0 | 0 | 0.370599 | 0.180258 | 0 | 0 | 0 | 0 | 0 | 0.21114 |
| 12778 | 3.40224 | 3.41101 | 2.84316 | 5.0203 | 4.20165 | 5.30399 | 2.96316 | 5.21404 | 2.68105 | 1.91252 | 1.09883 |
| 16324 | 3.22913 | 1.46009 | 4.27277 | 1.12159 | 1.09591 | 3.50409 | 0.755248 | 3.28647 | 0.196097 | 2.77412 | 0 |
| 11147 | 3.49934 | 0 | 2.43314 | 0.559108 | 0.720334 | 2.74841 | 0.204666 | 1.54385 | 0 | 0 | 0 |
| 14465 | 2.12701 | 3.07235 | 1.41501 | 2.4107 | 3.30705 | 2.73136 | 2.94093 | 2.312 | 2.67553 | 5.76974 | 5.88654 |

|  | Glucose | Xylose | Arabinose | Acetate | Coumarate | Ferulate | YNB Oleic Acid | YNB Ricinoleic Acid | YNB Glucose | YNB Gluc DOC | YPD |
| --- | --- | --- | --- | --- | --- | --- | --- | --- | --- | --- | --- |
| proteinId |  |  |  |  |  |  |  |  |  |  |  |
| 16213 | -0.296936 | -0.341591 | -0.0041986 | -0.184173 | 0.0422894 | -0.124728 | -0.00794017 | -0.0866272 | 0.142589 | 0.226911 | 0.0872267 |
| 16484 | -0.833702 | -2.52844 | -1.67896 | -0.683333 | -4.02708 | -2.29738 | -0.947317 | -5.03069 | -2.83414 | -2.67902 | 0.361126 |
| 12778 | 0.0606635 | 0.189572 | 0.155593 | 0.30207 | 0.344105 | 0.0254225 | 0.372177 | -0.114837 | -0.202211 | -0.113041 | 0.0202546 |
| 16324 | 0.186695 | -0.0225794 | 0.00910147 | 0.144733 | 0.221646 | -0.0879462 | 0.23013 | -0.296677 | 0.210096 | 0.205255 | 0.164019 |
| 11147 | -0.106836 | -0.173124 | -0.204399 | -0.0157074 | -0.273489 | -0.327768 | 0.176538 | 0.136414 | -0.530394 | -0.165471 | -0.378923 |
| 14465 | 0.350408 | 0.211929 | 0.157372 | 0.0222189 | 0.572482 | -0.0365795 | 0.16677 | 0.293391 | 0.178531 | 0.416711 | -0.0397786 |

In [39]:

```
for x in temp:
    if x in model.genes:
        for r in sorted(model.genes.get_by_id(x).reactions, key=lambda x: x.id):
            print(r, r.gene_reaction_rule)
    else:
        print(x, 'no reactions')
    print()
```

```
HETZK: 4mhetz_c + atp_c --> 4mpetz_c + adp_c + h_c 16213
TMPPP: 2mahmp_c + 4mpetz_c + h_c --> ppi_c + thmmp_c 16213

TMDPK: atp_c + thm_c --> amp_c + h_c + thmpp_c 16484
TMDPPK: atp_c + thmpp_c --> adp_c + thmtp_c 16484

HMPK1: 4ahmmp_c + atp_c --> 4ampm_c + adp_c + h_c 12778
PMPK: 4ampm_c + atp_c --> 2mahmp_c + adp_c 12778

16324 no reactions

11147 no reactions

14465 no reactions
```

In [40]:

```
# 16484 is pyrophosphokinase, remove triphosphate
model.remove_reactions(['TMDPPK'], remove_orphans=True)
# 14465 is Thiamin pyrophosphatase, not kinase - has Nudix hydrolase domain
r = eco.reactions.get_by_id('TDP').copy()
r.gene_reaction_rule = '14465'
model.add_reactions([r])
# Add thiaminase reaction by 12778
r = sce.reactions.get_by_id('TMN').copy()
r.gene_reaction_rule = '12778'
model.add_reactions([r])
# Add thiazole phosphate synthesis reaction by 16324 THI4
r = sce.reactions.get_by_id('THZPSN2_SC').copy()
r.id = 'THZPSN4'
r.name = 'Thiamine thiazole synthase'
r.gene_reaction_rule = '16324'
model.add_reactions([r])
r.add_metabolites({'achms_c': 1.0, 'r5p_c': 1.0, '4abut_c': -1.0, 'ac_c': -1.0, 'nh4_c': -1.0, 'pyr_c': -1.0,
                   'nad_c': -1.0, 'h_c': 4.0, '2amac_c': 1.0, 'amp_c': 1.0, 'ncam_c': 1.0, 'h2o_c': -1.0})
# Add HMP-P synthase reaction by 11147 THI5 and 2-oxo-L-glutamate demand
m = cobra.Metabolite('4oglu_c', name='4-oxo-L-glutamate', formula='C5H6NO5', charge=-1, compartment='c')
model.add_metabolites([m])
r = cobra.Reaction('HMPPS', name='4-amino-5-hydroxymethyl-2-methylpyrimidine phosphate synthase')
r.gene_reaction_rule = '11147'
model.add_reactions([r])
r.add_metabolites({'pdx5p_c': -1.0, 'his__L_c': -1.0, 'o2_c': -2.0, 'h2o2_c': -1.0, 'fe3_c': -4.0,
                   '4ampm_c': 1.0, '4oglu_c': 1.0, 'glx_c': 1.0, 'co2_c': 1.0, 'fe2_c': 4.0, 'h_c': 6.0})
r = cobra.Reaction('DM_4oglu_c', name='4-oxo-L-glutamate demand')
model.add_reactions([r])
r.add_metabolites({'4oglu_c': -1.0})
```

In [41]:

```
temp = ['10885','13856','13935','9324','10359','10921','11871','12902','13969']
display(Annotation.loc[temp])
Show_Data(temp)
```

|  | Combined Annotations | Signal P | Sc288c Orthologs | Human Orthologs | Sc288 Best Hit | Human Blast | Essential | WolfPSort | C Terminal |
| --- | --- | --- | --- | --- | --- | --- | --- | --- | --- |
| RTO4\_ID |  |  |  |  |  |  |  |  |  |
| 10885 | K01078: E3.1.3.2; acid phosphatase | A |  |  | DIA3 |  | Not Essential | mito 9, plas 6, cyto 4.5, cyto\_nucl 3.5, pero ... | PHS\* |
| 13856 | K01078: E3.1.3.2; acid phosphatase | S |  |  |  |  | Not Essential | extr 27 | ALL\* |
| 13935 | K14394: ACP1; low molecular weight phosphotyro... | S | LTP1 | ACP1 | LTP1 | ACP1 | Not Essential | mito 10, cyto 9.5, cyto\_nucl 7.5, pero 3, nucl... | GKL\* |
| 9324 | KOG2466: Uridine permease/thiamine transporter... |  |  |  | DAL4 |  | Not Essential | plas 27 | VAV\* |
| 10359 | K03457: TC.NCS1; nucleobase:cation symporter-1... |  |  |  | FUI1 |  | Not Essential | plas 26 | VHA\* |
| 10921 | KOG2466: Uridine permease/thiamine transporter... | A |  |  | FUR4 |  | Not Essential | plas 26 | EHA\* |
| 11871 | K03457: TC.NCS1; nucleobase:cation symporter-1... |  |  |  | DAL4 |  | Not Essential | plas 27 | VAV\* |
| 12902 | K03457: TC.NCS1; nucleobase:cation symporter-1... | A | FUI1,FUR4,DAL4 |  | DAL4 |  | Not Essential | plas 27 | ELV\* |
| 13969 | K15014: SLC29A1\_2\_3, ENT1\_2\_3; solute carrier ... |  | FUN26 | SLC29A3 |  | SLC29 | Not Essential | plas 17, E.R. 8 | PFV\* |

| strain | WT | | | | | | | | | | | | | | | | |
| --- | --- | --- | --- | --- | --- | --- | --- | --- | --- | --- | --- | --- | --- | --- | --- | --- | --- |
| condition | G\_MM | C\_MM | G\_SD | | GX\_SD | | | X\_SD | | A\_SD | | C\_SD | | MM\_CN120 | | MM\_CN5 | Diversity\_Sample |
| phase | exp | exp | exp | stat | exp | trans | stat | exp | stat | exp | stat | exp | stat | exp | stat | exp | exp |
| proteinId | Set1 | Set1 | Set2 | Set2 | Set2 | Set2 | Set2 | Set2 | Set2 | Set2 | Set2 | Set2 | Set2 | Set3 | Set3 | Set3 | Set3 |
| 10885 | 5.27185 | 4.82499 | 5.2096 | 5.99336 | 5.13688 | 6.08261 | 6.1692 | 5.61282 | 6.03404 | 5.7127 | 5.89019 | 5.34397 | 5.58436 | 5.62315 | 6.02913 | 5.15284 | 5.1842 |
| 13856 | 2.8427 | 2.68561 | 3.39498 | 4.13674 | 3.26146 | 3.50804 | 3.63072 | 3.63612 | 3.74065 | 3.47702 | 3.84538 | 4.29174 | 3.51592 | 3.55472 | 3.06683 | 3.54583 | 5.71469 |
| 13935 | 6.432 | 6.23831 | 6.72828 | 7.04166 | 6.65742 | 6.75824 | 6.66222 | 6.95567 | 6.23222 | 6.65139 | 6.50462 | 5.85581 | 5.24993 | 3.3872 | 3.90333 | 4.0671 | 3.41575 |
| 9324 | 6.18668 | 3.41832 | 1.72273 | 3.92615 | 1.48347 | 2.14285 | 3.04163 | 2.71787 | 2.70306 | 2.39242 | 3.62013 | 3.63183 | 4.12953 | 7.70839 | 7.27459 | 2.55925 | 5.9587 |
| 10359 | 6.00893 | 5.18488 | 5.68414 | 4.06275 | 5.69971 | 6.39187 | 6.21034 | 5.9226 | 4.29561 | 3.38493 | 1.90812 | 4.21495 | 2.7907 | 8.28382 | 8.42949 | 8.34369 | 7.79966 |
| 10921 | 9.11615 | 3.74959 | 3.25081 | 5.51642 | 2.9931 | 3.28825 | 3.37009 | 3.4852 | 4.63672 | 3.5884 | 5.30777 | 2.9804 | 2.72336 | 9.08856 | 8.31255 | 3.40673 | 6.70464 |
| 11871 | 7.34732 | 2.7515 | 1.10275 | 0.591233 | 0.921368 | 1.07688 | 1.34994 | 1.34029 | 1.27659 | 0.986886 | 0.749476 | 4.14901 | 4.95234 | 9.26656 | 9.30171 | 2.43521 | 6.96783 |
| 12902 | 7.72974 | 3.38774 | 1.93551 | 5.04464 | 1.77171 | 3.1743 | 3.7558 | 3.49217 | 2.75304 | 4.30574 | 4.32905 | 2.14502 | 0.298019 | 8.89377 | 8.64075 | 2.58406 | 7.48828 |
| 13969 | 4.77836 | 4.71399 | 5.33796 | 6.7602 | 5.26059 | 5.86708 | 6.03702 | 5.51241 | 5.96645 | 5.317 | 5.65621 | 5.61667 | 5.27335 | 5.90366 | 6.17908 | 5.58594 | 5.6646 |

| strain | WT | | | | | | | | | | |
| --- | --- | --- | --- | --- | --- | --- | --- | --- | --- | --- | --- |
| condition | G\_SD | | GX\_SD | | | X\_SD | | A\_SD | | C\_SD | |
| proteinId | exp | stat | exp | trans | stat | exp | stat | exp | stat | exp | stat |
| 10885 | 0.579959 | 1.58001 | 0.605486 | 2.42699 | 0.743204 | 2.343 | 1.78202 | 0.585393 | 0.574264 | 0 | 0 |
| 13935 | 1.70062 | 2.1545 | 2.0325 | 1.85563 | 1.84124 | 2.91589 | 3.14788 | 2.1261 | 2.1083 | 0.854055 | 0.442271 |
| 11871 | 0 | 0 | 0 | 0 | 0 | 0 | 0 | 0 | 0.189808 | 0 | 0 |
| 13969 | 0 | 0.351946 | 0.203084 | 0.564631 | 1.09591 | 0.974881 | 1.18215 | 0.775498 | 0.96029 | 0.632921 | 0.224376 |

|  | Glucose | Xylose | Arabinose | Acetate | Coumarate | Ferulate | YNB Oleic Acid | YNB Ricinoleic Acid | YNB Glucose | YNB Gluc DOC | YPD |
| --- | --- | --- | --- | --- | --- | --- | --- | --- | --- | --- | --- |
| proteinId |  |  |  |  |  |  |  |  |  |  |  |
| 10885 | -0.067265 | -0.106314 | 0.0205561 | -0.0456077 | -0.324245 | 0.0817338 | -0.0342517 | 0.178912 | -0.00788415 | -0.039932 | -0.030814 |
| 13856 | 0.0352738 | 0.140121 | 0.0520424 | -0.219534 | -0.195112 | -0.308844 | 0.222728 | -0.16456 | 0.125953 | -0.0656023 | 0.542956 |
| 13935 | -0.0733152 | -0.0910469 | 0.177923 | 0.0527015 | 0.639309 | -0.0737553 | -0.126422 | -0.201468 | -0.305069 | -0.392733 | -0.1807 |
| 9324 | 0.143381 | 0.177886 | 0.137487 | 0.276363 | 0.211538 | -0.0416871 | -0.0968904 | 0.137922 | 0.239205 | -0.0403884 | 0.140725 |
| 10359 | -0.0718355 | 0.205283 | -0.276995 | 0.00506491 | 0.481236 | -0.246722 | 0.455706 | 0.582832 | -0.121553 | -0.101068 | 0.118733 |
| 10921 | 0.0884597 | 0.127497 | 0.0667718 | 0.118477 | 0.227692 | 0.434115 | -0.34908 | -0.0758182 | -0.140076 | -0.193443 | 0.172072 |
| 11871 | 0.00466906 | -0.159493 | -0.121645 | -0.124357 | -0.127033 | -0.129729 | 0.26642 | 0.155626 | 0.289326 | 0.181374 | -0.510964 |
| 12902 | 0.157584 | 0.274742 | -0.026447 | 0.145726 | 0.0343194 | -0.0428231 | 0.00108723 | 0.310765 | 0.356296 | 0.0756319 | -0.146704 |
| 13969 | -0.0391677 | -0.338096 | -0.0310784 | -0.20435 | 0.209895 | -0.0194941 | 0.0241269 | 0.533762 | 0.189846 | 0.301947 | 0.461926 |

In [42]:

```
for x in temp:
    if x in model.genes:
        for r in sorted(model.genes.get_by_id(x).reactions, key=lambda x: x.id):
            print(r, r.gene_reaction_rule)
    else:
        print(x, 'no reactions')
    print()
```

```
ACP1e: fmn_e + h2o_e --> pi_e + ribflv_e 10885 or 13856 or 13935
THMDPe: 2.0 h2o_e + thmpp_e --> h_e + 2.0 pi_e + thm_e 10885
THMPe: h2o_e + thmmp_e --> pi_e + thm_e 10885

ACP1e: fmn_e + h2o_e --> pi_e + ribflv_e 10885 or 13856 or 13935

ACP1_FMN: fmn_c + h2o_c --> pi_c + ribflv_c 13935
ACP1e: fmn_e + h2o_e --> pi_e + ribflv_e 10885 or 13856 or 13935

9324 no reactions

10359 no reactions

ALLTNti: alltn_e --> alltn_c 10921 or 11871 or 12902
THMt2: h_e + thm_e --> h_c + thm_c 10921 or 11871 or 12902
URAt2: h_e + ura_e --> h_c + ura_c 10921 or 11871 or 12902
URIt2: h_e + uri_e --> h_c + uri_c 10921 or 11871 or 12902 or 13969 or (10921 and 13969) or (11871 and 13969) or (12902 and 13969)

ALLTNti: alltn_e --> alltn_c 10921 or 11871 or 12902
THMt2: h_e + thm_e --> h_c + thm_c 10921 or 11871 or 12902
URAt2: h_e + ura_e --> h_c + ura_c 10921 or 11871 or 12902
URIt2: h_e + uri_e --> h_c + uri_c 10921 or 11871 or 12902 or 13969 or (10921 and 13969) or (11871 and 13969) or (12902 and 13969)

ALLTNti: alltn_e --> alltn_c 10921 or 11871 or 12902
THMt2: h_e + thm_e --> h_c + thm_c 10921 or 11871 or 12902
URAt2: h_e + ura_e --> h_c + ura_c 10921 or 11871 or 12902
URIt2: h_e + uri_e --> h_c + uri_c 10921 or 11871 or 12902 or 13969 or (10921 and 13969) or (11871 and 13969) or (12902 and 13969)

ADNt2: adn_e + h_e --> adn_c + h_c 13969
ADNtm: adn_c <=> adn_m 13969
CYTDt2: cytd_e + h_e --> cytd_c + h_c 13969
URIt2: h_e + uri_e --> h_c + uri_c 10921 or 11871 or 12902 or 13969 or (10921 and 13969) or (11871 and 13969) or (12902 and 13969)
```

In [43]:

```
# 10885 myo-inositol-hexakisphosphate 3-phosphohydrolase
# Phytic acid + H2O <=> D-myo-Inositol 1,2,4,5,6-pentakisphosphate + Orthophosphate
# No reaction in BiGG, remove 10885 from other reactions
model.remove_reactions(['THMDPe'], remove_orphans=True)
# 13856 acid phosphatase (extra), and 13935 low molecular weight phosphotyrosine protein phosphatase (cyto?)
# FMN + H2O <=> Riboflavin + Orthophosphate
# Thiamin monophosphate + H2O <=> Thiamine + Orthophosphate
# 4-Nitrophenyl phosphate + H2O <=> 4-Nitrophenol + Orthophosphate
# 1-Acyl-sn-glycerol 3-phosphate + H2O <=> 1-Acylglycerol + Orthophosphate
r = sce.reactions.get_by_id('THMP').copy()
r.gene_reaction_rule = '13935'
model.add_reactions([r])
model.reactions.get_by_id('ACP1e').gene_reaction_rule = '13856'
model.reactions.get_by_id('THMPe').gene_reaction_rule = '13856'
# 9324 is similar to 10921 -> add, 13969 is SLC29A3 -> remove
# SLC29A3 mediates both influx and efflux of nucleosides across the membrane (equilibrative transporter).
# Mediates transport of adenine, adenosine and uridine, as well as several nucleoside analog drugs,
# such as anticancer and antiviral agents, including cladribine, cordycepin, tubercidin and AZT. 
# Does not transport hypoxanthine.
model.reactions.get_by_id('ALLTNti').gene_reaction_rule = '9324 or 10921 or 11871 or 12902'
model.reactions.get_by_id('THMt2').gene_reaction_rule = '9324 or 10921 or 11871 or 12902'
model.reactions.get_by_id('URAt2').gene_reaction_rule = '9324 or 10921 or 11871 or 12902'
model.reactions.get_by_id('URIt2').gene_reaction_rule = '9324 or 10921 or 11871 or 12902'
```

In [81]:

```
Annotation.loc['10885','WolfPSort']
```

Out[81]:

```
'mito 9, plas 6, cyto 4.5, cyto_nucl 3.5, pero 2, nucl 1.5, E.R. 1, golg 1, cysk 1, vacu 1'
```

In [79]:

```
for r in model.metabolites.minohp_n.reactions:
    print(r, r.gene_reaction_rule)
```

```
PMI12346PSn: atp_n + minohp_n --> adp_n + ppmi12346p_n 9763
MI13456PKn: atp_n + mi13456p_n --> adp_n + h_n + minohp_n 15600
```

In [82]:

```
for r in model.genes.get_by_id('13260').reactions:
    print(r, r.gene_reaction_rule)
```

```
MI1345PKn: atp_n + mi1345p_n --> adp_n + h_n + mi13456p_n 13260
MI145P6Kn: atp_n + mi145p_n --> adp_n + h_n + mi1456p_n 13260
MI145PKn: atp_n + mi145p_n --> adp_n + h_n + mi1345p_n 13260
MI1456PKn: atp_n + mi1456p_n --> adp_n + h_n + mi13456p_n 13260
```

In [44]:

```
# check if model can produce cofactors
cofactors = ['camp_c','coa_c','fad_c','gthrd_c','hemeA_m','nad_c','nadp_c','q9_m','thf_c','thmpp_c',
             '5mthf_c','btn_m','lipopb_m','mlthf_c','ptrc_c','pydx5p_c','spmd_c']
with model:
    model.reactions.get_by_id('FE2t').lower_bound = -1000.0
    model.reactions.get_by_id('FE3t').lower_bound = -1000.0
    model.reactions.get_by_id('EX_fe2_e').lower_bound = -1000.0
    model.reactions.get_by_id('EX_fe3_e').lower_bound = -1000.0
    for x in cofactors:
        model.reactions.get_by_id('BIOMASS_RT').add_metabolites({x: -1e-2})
        sol = model.optimize()
        print(x, sol.objective_value)
        if abs(sol.objective_value) < 1e-6:
            for k, v in sol.shadow_prices.items():
                #if v and k in [m.id for m in model.reactions.get_by_id('BIOMASS_RT').metabolites]:
                if abs(v) > 1:
                    print('\t',k, v)
        model.reactions.get_by_id('BIOMASS_RT').add_metabolites({x: 1e-2})
```

```
camp_c 0.13593743393234387
coa_c 0.13547854502629672
fad_c 0.13588334572423844
gthrd_c 0.13632034044837513
hemeA_m 0.13525177641251737
nad_c 0.135615581226282
nadp_c 0.13560034129332244
q9_m 0.0
	 4hbz_m -100.0
	 3nphb_m -100.0
	 3npdhb_m -100.0
	 me3dhnpdh_m -100.0
	 2np6mep_m -100.0
	 2np6mobq_m -100.0
	 me2np6mobq_m -100.0
	 2npmhmobq_m -100.0
	 q9_m -100.0
	 q9h2_m -100.0
	 4hbald_m -100.0
thf_c 0.13597215470564794
thmpp_c 0.13561761347615886
5mthf_c 0.13577122443666242
btn_m 0.0
	 btamp_m -100.0
	 btn_m -100.0
	 apoC_Lys_btn_m -100.0
	 dad_5_m 100.0
lipopb_m 0.0
	 dad_5_m 50.0
	 lipoamp_m -100.0
	 lipopb_m -100.0
	 lipoate_m -100.0
mlthf_c 0.13575594950448788
ptrc_c 0.1365064264736814
pydx5p_c 0.13653113901894684
spmd_c 0.13657749916638803
```

In [45]:

```
model.reactions.get_by_id('FE2t').lower_bound = -1000.0
model.reactions.get_by_id('FE3t').lower_bound = -1000.0
model.reactions.get_by_id('EX_fe2_e').lower_bound = -1000.0
model.reactions.get_by_id('EX_fe3_e').lower_bound = -1000.0
```

q9 biosynthesis  
HBZNPT\_m: 4hbz\_m + npdp\_m --> 3nphb\_m + ppi\_m 9681

If mito, need to add  
TYRTAim: 34hpp\_m + glu**L\_m <=> akg\_m + tyr**L\_m GOT2 or TAT  
34HPLFM: 34hpp\_m + h\_m + nadh\_m --> 34hpl\_m + nad\_m ?  
T4HCINNMFM: 34hpl\_m --> T4hcinnm\_m + h2o\_m ?  
COUCOAFm: T4hcinnm\_m + atp\_m + coa\_m --> amp\_m + coucoa\_m + ppi\_m 16635  
4HBZCOAFm: coa\_m + coucoa\_m + h2o\_m + nad\_m --> 4hbzcoa\_m + accoa\_m + h\_m + nadh\_m ?  
4HBZFm: 4hbzcoa\_m + h2o\_m --> 4hbz\_m + coa\_m + h\_m ?

4CMCOAS: T4hcinnm\_c + atp\_c + coa\_c --> amp\_c + coucoa\_c + ppi\_c 11833 or 13700 or 14802 or 16129

Where is 34hpp coming from?  
(e4p + pyr -> skm -> chor -> pphn -> 34hpp)

Chorismate biosynthesis (via shikimate)  
DDPA: e4p\_c + h2o\_c + pep\_c --> 2dda7p\_c + pi\_c 10602 or 12155 or 15534  
DHQS: 2dda7p\_c --> 3dhq\_c + pi\_c 14252  
DHQTi: 3dhq\_c --> 3dhsk\_c + h2o\_c 10572 or 14252  
SHK3Dr: 3dhsk\_c + h\_c + nadph\_c <=> nadp\_c + skm\_c 14252  
SHKK: atp\_c + skm\_c --> adp\_c + h\_c + skm5p\_c 13706 or 14252  
PSCVT: pep\_c + skm5p\_c <=> 3psme\_c + pi\_c 14252  
CHORS: 3psme\_c --> chor\_c + pi\_c 14037

dehydroshikimate dehydratase (3-dehydroshikimate -> protocatechuate + h2o) is missing  
9106 paperblast to 3-dehydroshikimate dehydratase  
DHSKDH: 3dhsk\_c → h2o\_c + 34dhbz\_c 9106  
protocatechuate is 34dhbz\_c

Phenylalanine biosynthesis (from chorismate via prephenate)  
CHORM: chor\_c --> pphn\_c 14195 or 16225 or 9704  
ADCS: chor\_c + gln**L\_c --> 4adcho\_c + glu**L\_c 13669  
ADCL: 4adcho\_c --> 4abz\_c + h\_c + pyr\_c 12476  
PPNDH: h\_c + pphn\_c --> co2\_c + h2o\_c + phpyr\_c 14195  
PHETA1: akg\_c + phe**L\_c <=> glu**L\_c + phpyr\_c 14908 or 15839  
PPYRDC: h\_c + phpyr\_c --> co2\_c + pacald\_c 15791  
ALCD25xi: h\_c + nadh\_c + pacald\_c --> 2phetoh\_c + nad\_c 14108 or 14109 or 15438  
ALCD25yi: h\_c + nadph\_c + pacald\_c --> 2phetoh\_c + nadp\_c 10029 or 13554 or 13562 or 13947  
ALDD19x\_P: h2o\_c + nadp\_c + pacald\_c --> 2.0 h\_c + nadph\_c + pac\_c 12042 or 13426 or 16323  
ALDD19xr: h2o\_c + nad\_c + pacald\_c <=> 2.0 h\_c + nadh\_c + pac\_c 12042 or 13426 or 16323

16462 phenylalanine ammonia-lyase

Tyrosine biosynthesis (from prephenate)  
PPND: nad\_c + pphn\_c --> 34hpp\_c + co2\_c + nadh\_c 14252 or 16225  
PPND2: nadp\_c + pphn\_c --> 34hpp\_c + co2\_c + nadph\_c 16225  
TYRTA: akg\_c + tyr**L\_c <=> 34hpp\_c + glu**L\_c 14908 or 15839 or 16065

In [46]:

```
# 10602 ARO4 cyto, 12155 ARO3 mito, 15534 mito
model.reactions.get_by_id('DDPA').gene_reaction_rule = '10602'
model.reactions.get_by_id('DDPAm').gene_reaction_rule = '12155 or 15534'
# 14252 is ARO1 pentafunctional protein, but separate enzymes exist in rhodo
# Separate enzymes look like they are in mito, create mito reactions? keep cyto for now
# PPND is not catalyzed by 14252 ARO1, PPND2 is correct by 16225 TYR1 prephenate dehydrogenase (NADP+)
model.remove_reactions(['PPND'], remove_orphans=True)
# 13706 mito shikimate kinase / 3-dehydroquinate dehydratase type I (two functions), fitness defect in cou and fer
# SHKK okay, add 13706 to DHQTi
# 10572 looks like a mito aroQ 3-dehydroquinate dehydratase
model.reactions.get_by_id('DHQTi').gene_reaction_rule = '10572 or 13706 or 14252'
# 13709 shikimate / quinate 5-dehydrogenase (5' alternate splicing?), KEGG matches are quinate dehydrogenase
# Add QUINDH from eco, and set genes to 13709
r = eco.reactions.get_by_id('QUINDH').copy()
r.gene_reaction_rule = '13709'
model.add_reactions([r])
# 13710 (sigP) shikimate / quinate 5-dehydrogenase, but coverage is < 50%, leave it out for now
# shikimate kinase makes shikimate 3-phosphate, replace skm5p with skm3p
model.metabolites.get_by_id('skm5p_c').id = 'skm3p_c'
model.metabolites.get_by_id('skm3p_c').name = 'Shikimate 3-phosphate'
# Add DHSKDH from ppu, and set genes to 9106
r = ppu.reactions.get_by_id('DHSKDH').copy()
r.gene_reaction_rule = '9106'
model.add_reactions([r])
# 14195 prephenate dehydratase only (not bifunctional chorismate mutase/prephenate dehydratase), PPNDH correct
# 16225 prephenate dehydrogenase (NADP+), PPND2 correct
# 9704 chorismate mutase, CHORM
model.reactions.get_by_id('CHORM').gene_reaction_rule = '9704'
# 13669 Para-aminobenzoate (PABA) synthase ABZ1, ADCS correct
# 12476 ABZ2, ADCL is correct
# 4-aminobenzoate can be a precursor to ubiquinone biosynthesis -> add reactions
```

In [47]:

```
temp = ['10602','12155','15534','14252','10572','13706','13709','13710','14037','9106',
        '14195','16225','9704','13669','12476','14908','15839','15791',
        '14108','14109','15438','10029','13554','13562','13947','12042','13426','16323',
        '15434','16462','13444','13445']
display(Annotation.loc[temp])
Show_Data(temp)
```

|  | Combined Annotations | Signal P | Sc288c Orthologs | Human Orthologs | Sc288 Best Hit | Human Blast | Essential | WolfPSort | C Terminal |
| --- | --- | --- | --- | --- | --- | --- | --- | --- | --- |
| RTO4\_ID |  |  |  |  |  |  |  |  |  |
| 10602 | K01626: E2.5.1.54, aroF, aroG, aroH; 3-deoxy-7... |  | ARO3,ARO4 |  | ARO4 |  | Essential | cyto 22.5, cyto\_mito 12, nucl 2 | EKA\* |
| 12155 | K01626: E2.5.1.54, aroF, aroG, aroH; 3-deoxy-7... |  | ARO3,ARO4 |  | ARO3 |  | Not Essential | mito 20, cyto 6.5, cyto\_nucl 4 | PAA\* |
| 15534 | K01626: E2.5.1.54, aroF, aroG, aroH; 3-deoxy-7... |  |  |  |  |  | Not Essential | mito 10, cyto\_nucl 8, nucl 7.5, cyto 7.5 | VKQ\* |
| 14252 | K13830: ARO1; pentafunctional AROM polypeptide |  | ARO1 |  | ARO1 |  | Essential | cyto 12.5, cyto\_nucl 7.5, plas 3, extr 3, E.R.... | RDN\* |
| 10572 | K03786: aroQ, qutE; 3-dehydroquinate dehydrata... |  |  |  |  |  | Not Essential | mito 16, cyto 10.5, cyto\_nucl 6 | KQK\* |
| 13706 | K00891: E2.7.1.71, aroK, aroL; shikimate kinase |  |  |  | ARO1 |  | Not Essential | mito 15, cyto 5.5, cyto\_nucl 5.5, nucl 4.5 | RMG\* |
| 13709 | KOG0692: Pentafunctional AROM protein |  |  |  | ARO1 |  | Not Essential | cyto 15, cyto\_nucl 10.5, mito 5, nucl 4 | PFE\* |
| 13710 | KOG0692: Pentafunctional AROM protein | S |  |  | ARO1 |  | Not Essential | mito 10, cyto 6, extr 5, nucl 3, plas 3, cyto\_... | IGS\* |
| 14037 | K01736: aroC; chorismate synthase |  | ARO2 |  | ARO2 |  | Essential | cyto 15.5, cyto\_nucl 9, mito 7, pero 2 | CSV\* |
| 9106 | K06606: iolI; 2-keto-myo-inositol isomerase |  |  |  |  |  | Not Essential | cyto 10, mito 8, pero 3, extr 2, vacu 2 | PFW\* |
| 14195 | K04518: pheA2; prephenate dehydratase |  | PHA2 |  |  |  | Not Essential | mito 18.5, cyto\_mito 12.333, cyto 5, cyto\_nucl... | SEQ\* |
| 16225 | K00211: TYR1; prephenate dehydrogenase (NADP+) |  | TYR1 |  | TYR1 |  | Essential | cyto 10, cyto\_nucl 7.5, mito 5, nucl 3, extr 3... | RAL\* |
| 9704 | K01850: E5.4.99.5; chorismate mutase |  | ARO7 |  | ARO7 |  | Essential | nucl 13.5, cyto\_nucl 12.5, cyto 8.5, mito 2 | KSG\* |
| 13669 | K13950: pabAB; para-aminobenzoate synthetase |  | ABZ1 |  | ABZ1 |  | Not Essential | extr 9, cyto 8, cyto\_mito 7.333, cyto\_nucl 6.8... | KAS\* |
| 12476 | HMMPfam:Aminotransferase class IV:PF01063,SUPE... |  | ABZ2 |  | ABZ2 |  | Not Essential | mito 11, cyto\_nucl 9, cyto 8.5, nucl 6.5 | DLQ\* |
| 14908 | K00838: ARO8; aromatic amino acid aminotransfe... |  | ARO8 | AADAT | ARO8 | AADAT | Not Essential | cyto 7, cysk 7, mito 5, cyto\_nucl 5, pero 4, m... | WAY\* |
| 15839 | K00838: ARO8; aromatic amino acid aminotransfe... |  | ARO8 | AADAT | ARO8 | AADAT | Not Essential | cysk 9, cyto 6, nucl 4, mito 4, pero 4, mito\_n... | FKD\* |
| 15791 | K01568: PDC, pdc; pyruvate decarboxylase |  | THI3,PDC6,PDC1,PDC5 |  | PDC1 |  | Not Essential | cyto 21.5, cyto\_mito 11.5, nucl 3 | NAA\* |
| 14108 | K00121: frmA, ADH5, adhC; S-(hydroxymethyl)glu... |  | SFA1 | ADH5 | SFA1 | ADH5 | Unclear (ambiguous TDNA mapping) | cyto 19, extr 6 | MWA\* |
| 14109 | K00121: frmA, ADH5, adhC; S-(hydroxymethyl)glu... |  | SFA1 | ADH5 | SFA1 | ADH5 | Unclear (ambiguous TDNA mapping) | cyto 19, extr 6 | MWA\* |
| 15438 | K13953: adhP; alcohol dehydrogenase, propanol-... |  | ADH5,ADH3,ADH2,ADH1 |  | ADH3 | ZADH2 | Not Essential | cyto 25.5, cyto\_nucl 13.5 | KNF\* |
| 10029 | K13979: yahK; uncharacterized zinc-type alcoho... |  | ADH7,ADH6 |  | ADH6 | ADH1C | Not Essential | cyto 24, cyto\_nucl 13.5 | KQV\* |
| 13554 | KOG0023: Alcohol dehydrogenase, class V |  | ADH7,ADH6 |  | ADH6 |  | Not Essential | cyto 27 | SSR\* |
| 13562 | K00002: AKR1A1, adh; alcohol dehydrogenase (NA... |  | ADH7,ADH6 |  | ADH7 |  | Not Essential | cyto 23, mito 2, pero 2 | GKL\* |
| 13947 | HMMPfam:Zinc-binding dehydrogenase:PF00107,HMM... |  | ADH7,ADH6 |  | ADH6 |  | Not Essential | cyto 12, cysk 8, mito 6, cyto\_nucl 6 | LKN\* |
| 12042 | K00128: ALDH; aldehyde dehydrogenase (NAD+) |  | ALD5,ALD4,ALD6,ALD3,ALD2 | ALDH1A1,ALDH1A2,ALDH1A3,ALDH1B1,ALDH2 | ALD5 | ALDH2 | Not Essential | cyto 18.5, cyto\_nucl 10, mito 4, pero 4 | NPL\* |
| 13426 | K07249: E1.2.1.36; retinal dehydrogenase |  | ALD5,ALD4,ALD6,ALD3,ALD2 | ALDH1A1,ALDH1A2,ALDH1A3,ALDH1B1,ALDH2 | ALD5 | ALDH1 | Not Essential | mito 25.5, cyto\_mito 14 | WPL\* |
| 16323 | K00128: ALDH; aldehyde dehydrogenase (NAD+) |  | HFD1 | ALDH3A1,ALDH3A2,ALDH3B1,ALDH3B2 | HFD1 | ALDH3 | Not Essential | cyto 10.5, plas 7, cyto\_nucl 7, mito 3, nucl 2... | GQA\* |
| 15434 | K00799: GST, gst; glutathione S-transferase |  |  |  |  |  | Not Essential | cyto 19, cyto\_nucl 14.5, nucl 8 | GEE\* |
| 16462 | K10775: PAL; phenylalanine ammonia-lyase |  |  | HAL |  | HAL | Not Essential | cyto 9, mito 8, cyto\_nucl 7, extr 5, nucl 3 | MLA\* |
| 13444 | K00588: E2.1.1.104; caffeoyl-CoA O-methyltrans... | S |  | COMTD1 |  | COMTD | Not Essential | plas 9, mito 7, E.R. 5, cyto 2, vacu 2 | RRM\* |
| 13445 | KOG1663: O-methyltransferase |  |  | COMTD1 |  | COMTD | Not Essential | cyto 9.5, mito 9, cyto\_nucl 6, extr 2, pero 2,... | RRV\* |

| strain | WT | | | | | | | | | | | | | | | | |
| --- | --- | --- | --- | --- | --- | --- | --- | --- | --- | --- | --- | --- | --- | --- | --- | --- | --- |
| condition | G\_MM | C\_MM | G\_SD | | GX\_SD | | | X\_SD | | A\_SD | | C\_SD | | MM\_CN120 | | MM\_CN5 | Diversity\_Sample |
| phase | exp | exp | exp | stat | exp | trans | stat | exp | stat | exp | stat | exp | stat | exp | stat | exp | exp |
| proteinId | Set1 | Set1 | Set2 | Set2 | Set2 | Set2 | Set2 | Set2 | Set2 | Set2 | Set2 | Set2 | Set2 | Set3 | Set3 | Set3 | Set3 |
| 10602 | 3.98679 | 6.38918 | 5.08137 | 3.44606 | 5.76821 | 4.3107 | 4.6397 | 4.84295 | 4.42766 | 4.02116 | 4.07531 | 6.56661 | 7.15696 | 4.31981 | 5.17107 | 7.94952 | 6.30001 |
| 12155 | 7.16288 | 7.32845 | 7.83452 | 6.32751 | 8.27745 | 7.04311 | 7.2524 | 7.02109 | 6.42256 | 6.33931 | 6.42862 | 8.2368 | 9.38795 | 6.13139 | 6.07861 | 8.94615 | 7.1063 |
| 15534 | 5.10412 | 5.92078 | 5.72848 | 5.38788 | 5.89942 | 5.26317 | 5.30391 | 5.54401 | 5.09664 | 4.98359 | 5.26134 | 5.45861 | 4.61196 | 4.81831 | 4.90519 | 6.68599 | 5.32389 |
| 14252 | 4.3579 | 5.37763 | 5.81766 | 3.75466 | 6.0588 | 6.07259 | 5.95803 | 5.87346 | 4.47816 | 4.70146 | 3.87656 | 6.3327 | 7.04239 | 6.59833 | 6.3641 | 7.83028 | 7.39287 |
| 10572 | 4.5071 | 5.90804 | 4.93043 | 4.75075 | 4.83152 | 4.18322 | 4.35291 | 4.47909 | 5.49514 | 4.43917 | 4.89364 | 5.25072 | 4.54698 | 3.25956 | 2.97179 | 3.46119 | 4.30885 |
| 13706 | 4.09458 | 4.00909 | 3.86033 | 4.20533 | 3.8023 | 4.22179 | 3.94129 | 4.20055 | 4.26681 | 4.3585 | 4.7482 | 4.3399 | 4.56569 | 4.0823 | 3.91478 | 3.96903 | 3.7488 |
| 13709 | 4.34639 | 4.88345 | 4.67997 | 4.89132 | 4.60904 | 4.38206 | 4.3332 | 4.53881 | 4.47233 | 4.25719 | 4.44202 | 4.35909 | 3.39849 | 4.46046 | 4.28413 | 4.42993 | 4.43512 |
| 13710 | 6.14093 | 6.29232 | 6.27351 | 8.71031 | 6.00268 | 6.25877 | 7.03931 | 5.93598 | 7.53159 | 6.37648 | 7.68426 | 5.23142 | 4.40282 | 5.13727 | 5.32825 | 5.35098 | 4.86762 |
| 14037 | 6.5182 | 6.54923 | 6.82456 | 5.54941 | 7.01828 | 6.21798 | 6.26565 | 6.33919 | 5.6532 | 5.56498 | 5.36878 | 6.76885 | 7.1861 | 6.44868 | 6.45185 | 7.76119 | 7.37878 |
| 9106 | 1.40824 | 3.97918 | 1.99162 | 3.39814 | 1.88494 | 2.22254 | 2.3709 | 1.7515 | 2.6332 | 1.60284 | 3.06702 | 2.77249 | 2.5707 | 2.00176 | 2.22485 | 1.53442 | 1.94948 |
| 14195 | 6.10071 | 5.51242 | 5.47145 | 5.81413 | 5.55005 | 5.93499 | 5.75172 | 6.07805 | 5.16291 | 5.7594 | 5.4537 | 5.11936 | 4.66111 | 6.97861 | 6.76951 | 6.68949 | 6.12953 |
| 16225 | 7.02463 | 7.10042 | 6.7291 | 4.18853 | 7.24131 | 4.6127 | 4.70133 | 5.02002 | 4.75579 | 4.56894 | 4.38512 | 7.23066 | 8.16255 | 4.70602 | 4.53012 | 7.90915 | 6.15688 |
| 9704 | 5.52994 | 5.53119 | 4.55295 | 3.94229 | 4.57396 | 4.70832 | 4.65477 | 4.92544 | 4.45244 | 4.67973 | 4.74249 | 5.26298 | 5.69957 | 5.39717 | 5.01189 | 5.02707 | 5.49674 |
| 13669 | 4.31736 | 3.91914 | 4.44627 | 4.5636 | 4.5273 | 4.68787 | 4.73919 | 4.20105 | 4.80266 | 4.03218 | 4.51465 | 3.91098 | 3.2547 | 5.72401 | 5.78354 | 5.14876 | 5.73864 |
| 12476 | 4.74343 | 4.6576 | 5.1642 | 4.77906 | 5.10108 | 4.99438 | 4.96114 | 4.49367 | 4.45783 | 4.35516 | 4.62875 | 4.83815 | 4.48134 | 5.51202 | 5.344 | 4.81587 | 5.29143 |
| 14908 | 4.96188 | 5.39382 | 6.32914 | 4.57254 | 6.87228 | 3.93282 | 4.6105 | 2.86689 | 4.55744 | 3.57167 | 4.54445 | 5.45156 | 8.85544 | 4.48318 | 3.71453 | 9.27804 | 5.94028 |
| 15839 | 8.11963 | 6.83965 | 6.43567 | 6.23959 | 6.4065 | 6.29378 | 6.4164 | 6.59727 | 6.49423 | 6.81951 | 6.69291 | 6.85344 | 6.82691 | 7.91155 | 7.40486 | 6.5373 | 6.73561 |
| 15791 | 7.84603 | 5.59 | 8.00875 | 7.69016 | 7.99933 | 7.37604 | 7.53144 | 7.37936 | 7.66072 | 7.3849 | 7.52336 | 6.39243 | 7.14277 | 7.57892 | 7.20347 | 7.69795 | 7.22035 |
| 14108 | 7.23326 | 7.01358 | 5.64402 | 6.69668 | 5.53004 | 5.99657 | 6.08719 | 5.36359 | 6.35115 | 5.52325 | 6.40565 | 6.79692 | 6.55453 | 6.97915 | 7.14294 | 5.17651 | 7.49575 |
| 14109 | 8.1968 | 7.85284 | 6.09397 | 7.21435 | 5.98603 | 6.42928 | 6.60693 | 5.78854 | 6.82888 | 5.9483 | 6.79863 | 7.20147 | 6.86586 | 7.25272 | 7.30374 | 5.27859 | 7.86155 |
| 15438 | 10.1609 | 8.53977 | 5.43727 | 5.78674 | 5.14966 | 5.82878 | 5.74105 | 5.70476 | 6.17528 | 5.33619 | 5.18161 | 5.6251 | 3.78025 | 8.62195 | 9.06836 | 5.22118 | 8.47463 |
| 10029 | 0.842122 | 0.725473 | 0.654647 | 0.486899 | 0.788714 | 0.350695 | 0.212935 | 0.337656 | 2.74871 | 0.49429 | 1.05144 | 0.700215 | 0.890117 | 1.33775 | 0.314616 | 0.841001 | 0.885068 |
| 13554 | 6.22436 | 5.15024 | 2.60124 | 6.33352 | 2.3 | 3.16147 | 3.37554 | 5.13312 | 4.16605 | 4.86668 | 3.67605 | 4.94462 | 2.88809 | 7.49317 | 6.72913 | 1.90543 | 6.80605 |
| 13562 | 3.25924 | 1.79687 | 3.21586 | 6.41048 | 2.88199 | 3.72041 | 4.2892 | 5.59293 | 6.51501 | 5.15441 | 5.88998 | 4.34403 | 4.22994 | 4.08001 | 4.46089 | 2.00505 | 6.3307 |
| 13947 | 5.22842 | 5.55835 | 4.23722 | 4.69839 | 4.95399 | 4.63487 | 4.63343 | 3.94145 | 5.15667 | 3.82245 | 4.37562 | 4.61502 | 3.97329 | 4.97202 | 5.56916 | 4.24401 | 6.12808 |
| 12042 | 8.00849 | 8.563 | 8.41698 | 9.34145 | 8.34137 | 8.32176 | 8.06734 | 8.44974 | 8.36047 | 8.84866 | 8.59447 | 7.17248 | 5.67475 | 7.81326 | 7.51984 | 8.29774 | 8.31731 |
| 13426 | 7.30781 | 4.89844 | 3.84807 | 2.79984 | 3.78882 | 3.98344 | 3.73207 | 4.58197 | 3.36082 | 4.5625 | 3.35098 | 4.25235 | 3.1633 | 6.93547 | 6.81825 | 3.29168 | 5.38811 |
| 16323 | 5.54194 | 5.5053 | 6.37226 | 5.68644 | 6.28507 | 6.1392 | 5.86895 | 6.06104 | 5.82576 | 5.50985 | 6.17279 | 4.76433 | 4.74944 | 5.59107 | 6.40434 | 5.99953 | 6.38502 |
| 15434 | 5.80565 | 6.08142 | 6.52684 | 6.31524 | 6.37302 | 6.1649 | 5.978 | 6.37637 | 4.97335 | 6.20587 | 5.3655 | 6.05685 | 5.52107 | 6.04486 | 6.6216 | 6.3371 | 6.98472 |
| 16462 | 3.90264 | 4.62789 | 6.18747 | 7.55717 | 5.38365 | 5.68582 | 6.61188 | 2.28084 | 7.3195 | 3.52111 | 8.01509 | 3.01366 | 0.419363 | 7.37249 | 6.26534 | 6.48645 | 4.86941 |
| 13444 | 3.99856 | 5.73961 | 2.482 | 3.40791 | 2.5636 | 1.88458 | 2.17398 | 2.13977 | 2.65039 | 2.1112 | 2.75221 | 2.43714 | 3.27026 | 4.71335 | 3.65054 | 3.37289 | 3.24289 |
| 13445 | 4.84407 | 5.45308 | 7.04645 | 7.24329 | 7.09131 | 6.80288 | 6.68065 | 6.14035 | 6.55861 | 5.80423 | 6.43429 | 5.77807 | 6.61736 | 6.40873 | 6.57184 | 8.42238 | 6.9128 |

| strain | WT | | | | | | | | | | |
| --- | --- | --- | --- | --- | --- | --- | --- | --- | --- | --- | --- |
| condition | G\_SD | | GX\_SD | | | X\_SD | | A\_SD | | C\_SD | |
| proteinId | exp | stat | exp | trans | stat | exp | stat | exp | stat | exp | stat |
| 10602 | 11.8307 | 7.93172 | 13.2212 | 7.27551 | 6.21975 | 9.39519 | 7.33007 | 9.09225 | 8.04921 | 12.7972 | 15.6311 |
| 12155 | 15.9461 | 16.0935 | 16.644 | 14.8764 | 15.2957 | 16.0935 | 17.049 | 18.3503 | 15.6904 | 12.1972 | 21.5122 |
| 15534 | 9.53687 | 7.42471 | 8.96953 | 6.14533 | 5.68819 | 7.06292 | 4.898 | 4.25483 | 4.59431 | 7.47955 | 5.90987 |
| 14252 | 36.5349 | 29.8852 | 36.8114 | 26.7819 | 28.0485 | 26.2271 | 20.3172 | 21.2641 | 15.5215 | 25.6195 | 34.0537 |
| 10572 | 1.56445 | 1.07103 | 1.42387 | 1.12032 | 0.745032 | 0.99461 | 0.80453 | 1.54816 | 0.769635 | 2.99413 | 2.61615 |
| 13706 | 0 | 0.376624 | 0.409593 | 1.31563 | 1.45359 | 0.979371 | 0.993718 | 1.73932 | 2.68387 | 1.06174 | 0.647042 |
| 13709 | 0 | 0.166667 | 0 | 0 | 0 | 0.197909 | 0 | 0 | 0.189808 | 0.212277 | 0 |
| 13710 | 1.37985 | 1.99864 | 1.22611 | 0.744201 | 0.545614 | 1.17597 | 0.994024 | 1.92278 | 2.11478 | 1.26768 | 0.448751 |
| 14037 | 11.4613 | 7.99233 | 12.7892 | 8.73599 | 8.23685 | 10.321 | 9.44507 | 7.13976 | 7.83382 | 9.80669 | 12.4264 |
| 14195 | 0.574292 | 0.559608 | 0.197656 | 1.1148 | 0.903019 | 1.58113 | 0.999965 | 0.58337 | 0.580553 | 0 | 0 |
| 16225 | 5.36263 | 5.40374 | 6.1182 | 3.52178 | 2.52125 | 3.51477 | 2.93737 | 5.41473 | 4.40031 | 4.91604 | 11.1009 |
| 9704 | 4.43083 | 3.41416 | 4.67328 | 4.08091 | 3.98537 | 2.94145 | 2.78529 | 2.31348 | 2.86049 | 5.57471 | 7.62463 |
| 13669 | 0 | 0.179255 | 0.204746 | 0.562089 | 0.179978 | 0 | 0.589289 | 0 | 0 | 0 | 0 |
| 12476 | 0.180601 | 0 | 0.206941 | 0 | 0.173732 | 0 | 0 | 0 | 0 | 0 | 0.21114 |
| 14908 | 9.31562 | 0 | 11.3891 | 0 | 0 | 3.31911 | 0 | 1.54769 | 0 | 2.57326 | 8.71334 |
| 15839 | 4.43453 | 11.9185 | 6.09339 | 17.1048 | 18.0361 | 9.79935 | 13.8093 | 7.91426 | 12.8202 | 11.5438 | 12.6204 |
| 15791 | 18.6789 | 22.6393 | 20.5475 | 21.5711 | 21.3974 | 18.4088 | 20.9127 | 17.6003 | 20.8646 | 9.62964 | 10.8778 |
| 14108 | 18.0857 | 18.8911 | 18.7317 | 20.2976 | 20.2797 | 27.8013 | 24.3021 | 25.503 | 21.4289 | 38.0378 | 35.2758 |
| 15438 | 1.92087 | 3.42103 | 2.02435 | 4.84423 | 5.98016 | 5.47116 | 7.05095 | 7.14776 | 7.4443 | 7.46885 | 9.36764 |
| 13554 | 0 | 0 | 0 | 0 | 0 | 6.64594 | 5.10549 | 0.966657 | 0 | 0.440511 | 1.52514 |
| 13562 | 0 | 0 | 0 | 0 | 0 | 19.9627 | 13.2261 | 2.51583 | 1.14892 | 1.08098 | 2.83239 |
| 13947 | 4.00856 | 1.4515 | 3.66111 | 0.374862 | 0 | 1.95935 | 1.17292 | 0.386502 | 0 | 3.01683 | 1.96377 |
| 12042 | 33.9947 | 44.5614 | 32.5616 | 41.5121 | 40.2993 | 56.5808 | 63.4798 | 61.9973 | 72.5595 | 57.7357 | 40.7488 |
| 13426 | 0.757094 | 0.533653 | 1.41886 | 0.929251 | 0.72686 | 2.14514 | 0.960201 | 3.46858 | 3.64334 | 1.06907 | 0.864976 |
| 16323 | 10.9902 | 12.5683 | 13.6351 | 14.1342 | 18.2252 | 11.3209 | 13.9247 | 9.27817 | 10.1675 | 6.60796 | 3.93398 |
| 15434 | 8.06945 | 8.61257 | 7.33533 | 8.40182 | 10.7788 | 10.3689 | 11.0012 | 10.8043 | 10.3272 | 10.9133 | 10.6735 |
| 16462 | 12.004 | 13.131 | 10.1528 | 7.7987 | 4.60551 | 10.0032 | 11.394 | 3.0952 | 8.80573 | 22.8165 | 0.220621 |
| 13445 | 2.86672 | 4.27811 | 2.84263 | 2.5947 | 4.00935 | 1.77205 | 3.32422 | 1.54722 | 2.49336 | 4.04433 | 5.64457 |

|  | Glucose | Xylose | Arabinose | Acetate | Coumarate | Ferulate | YNB Oleic Acid | YNB Ricinoleic Acid | YNB Glucose | YNB Gluc DOC | YPD |
| --- | --- | --- | --- | --- | --- | --- | --- | --- | --- | --- | --- |
| proteinId |  |  |  |  |  |  |  |  |  |  |  |
| 12155 | -0.310447 | 0.288119 | 0.166192 | 0.156131 | -1.09158 | 0.22266 | 0.616564 | -0.0241481 | -0.33732 | -0.126719 | -0.268415 |
| 15534 | -0.124421 | -0.387767 | -0.320253 | -0.0883859 | 0.059986 | 0.319192 | -0.181263 | -0.91462 | 0.160326 | -0.138136 | -0.137485 |
| 10572 | -0.119734 | -0.168895 | -0.116319 | 0.0274772 | -0.107331 | 0.105408 | -0.00101536 | 0.251161 | 0.256098 | 0.183985 | -0.411911 |
| 13706 | 0.00469469 | 0.241651 | 0.155219 | -0.0508848 | -1.80215 | -0.994622 | -0.320292 | -1.71794 | -0.409246 | -0.139143 | -0.046574 |
| 13709 | 0.128287 | -0.0819031 | 0.241922 | 0.225473 | 0.355881 | 0.0161442 | 0.147499 | -0.0217116 | -0.0521156 | 0.00706958 | -0.0412034 |
| 13710 | 0.0339886 | -0.0967401 | 0.149373 | 0.0257554 | 0.117254 | -0.00539465 | -0.0998042 | -0.246703 | 0.168151 | -0.0269269 | 0.00424615 |
| 9106 | -0.0566865 | -0.376082 | -0.0190414 | -0.191385 | 0.00924402 | -0.172002 | -0.0708188 | -0.207445 | 0.0876317 | 0.27866 | 0.0112956 |
| 14195 | -0.355315 | -0.506645 | -0.987004 | 0.022568 | -3.17046 | -0.42575 | -3.15518 | -2.42223 | -4.24174 | -3.07117 | -0.325324 |
| 13669 | 0.0145212 | -0.149855 | -0.0347267 | 0.0314421 | 0.0858744 | 0.127857 | 0.079359 | -0.172931 | 0.00296095 | 0.0207803 | -0.20695 |
| 12476 | 0.100719 | -0.523567 | 0.0361221 | 0.0739901 | 0.0207199 | -0.198743 | 0.184802 | -0.0292378 | 0.139254 | -0.101526 | -0.77324 |
| 14908 | 0.0451271 | -0.280956 | 0.220759 | -0.0377905 | 0.21851 | -0.259388 | -0.292657 | 0.206026 | 0.339453 | 0.398586 | -0.0659921 |
| 15839 | -0.0847761 | -0.12423 | 0.269542 | 0.0647239 | 0.154571 | 0.146919 | -0.346466 | -0.214054 | -0.177142 | -0.229588 | -0.0156292 |
| 15791 | -0.154956 | -0.0421572 | -0.0907286 | -0.0167367 | -0.120817 | -0.129008 | -0.170373 | -0.0587459 | -0.473236 | -0.697073 | -0.423493 |
| 14108 | -0.211659 | -0.531723 | -0.079527 | 0.0229318 | -0.277901 | -0.0256706 | 0.156921 | 0.219306 | -0.220427 | -0.0578182 | 0.207023 |
| 15438 | 0.0161124 | -0.202524 | -0.327434 | -0.164034 | -0.309855 | 0.00473032 | -0.0374177 | -0.0303498 | 0.195573 | 0.369537 | -0.187797 |
| 10029 | 0.0595242 | 0.392557 | 0.0821196 | 0.0512548 | 0.0185552 | 0.16491 | -0.18167 | -0.21429 | 0.238796 | -0.00558567 | -0.579144 |
| 13554 | -0.196593 | 0.10526 | -0.0874651 | -0.335435 | -0.481545 | 0.0486948 | 0.125232 | 0.334176 | 0.0365657 | 0.0984233 | 0.116142 |
| 13562 | -0.446997 | -0.151925 | -0.143913 | -0.311324 | -0.0296998 | -0.213521 | 0.338785 | 0.669978 | 0.49722 | 0.468333 | 0.253026 |
| 13947 | 0.344057 | 0.282048 | -0.084196 | -0.0429453 | 0.431049 | 0.386405 | 0.300993 | 0.272082 | -0.679036 | -0.677594 | 0.545753 |
| 12042 | 0.170666 | -0.143043 | 0.000953096 | -0.0230376 | -0.521499 | 0.0146534 | 0.144875 | -0.295284 | 0.392859 | 0.454577 | -0.242457 |
| 13426 | -0.226122 | -0.181863 | -0.169891 | -0.0897023 | -0.0133507 | -0.00809092 | -1.1502 | -2.84044 | -0.85113 | -0.458514 | -0.0243377 |
| 16323 | 0.000210473 | -0.0174318 | 0.0140589 | -0.110443 | 0.00440814 | 0.0892768 | 0.155043 | 0.103889 | -0.177274 | -0.243731 | -0.140368 |
| 15434 | -0.229413 | 0.369782 | -0.275873 | -0.187233 | -0.529117 | -0.384708 | 0.654654 | -0.566257 | 0.27908 | 0.452524 | 0.107615 |
| 16462 | -0.312151 | -0.304914 | -0.265122 | -0.170161 | -0.412883 | 0.239843 | -0.224816 | -0.294722 | -0.101513 | -0.0397254 | -0.389717 |
| 13444 | -0.110618 | 0.074913 | -0.132922 | -0.18465 | -0.456785 | -0.156824 | -0.533952 | 0.0147839 | 0.00950462 | -0.163047 | -0.297493 |
| 13445 | -0.635958 | -0.527207 | -0.536003 | -0.106179 | -0.605675 | -0.55725 | -1.16818 | -1.54882 | -0.575936 | -0.764138 | -0.712307 |

In [48]:

```
for x in temp:
    if x in model.genes:
        for r in sorted(model.genes.get_by_id(x).reactions, key=lambda x: x.id):
            print(r, r.gene_reaction_rule)
    else:
        print(x, 'no reactions')
    print()
```

```
DDPA: e4p_c + h2o_c + pep_c --> 2dda7p_c + pi_c 10602

DDPAm: e4p_m + h2o_m + pep_m --> 2dda7p_m + pi_m 12155 or 15534

DDPAm: e4p_m + h2o_m + pep_m --> 2dda7p_m + pi_m 12155 or 15534

DHQS: 2dda7p_c --> 3dhq_c + pi_c 14252
DHQTi: 3dhq_c --> 3dhsk_c + h2o_c 10572 or 13706 or 14252
PSCVT: pep_c + skm3p_c <=> 3psme_c + pi_c 14252
SHK3Dr: 3dhsk_c + h_c + nadph_c <=> nadp_c + skm_c 14252
SHKK: atp_c + skm_c --> adp_c + h_c + skm3p_c 13706 or 14252

DHQTi: 3dhq_c --> 3dhsk_c + h2o_c 10572 or 13706 or 14252

DHQTi: 3dhq_c --> 3dhsk_c + h2o_c 10572 or 13706 or 14252
SHKK: atp_c + skm_c --> adp_c + h_c + skm3p_c 13706 or 14252

QUINDH: nad_c + quin_c --> 3dhq_c + 2.0 h_c + nadh_c 13709

13710 no reactions

CHORS: 3psme_c --> chor_c + pi_c 14037

DHSKDH: 3dhsk_c --> 34dhbz_c + h2o_c 9106

PPNDH: h_c + pphn_c --> co2_c + h2o_c + phpyr_c 14195

PPND2: nadp_c + pphn_c --> 34hpp_c + co2_c + nadph_c 16225

CHORM: chor_c --> pphn_c 9704

ADCS: chor_c + gln__L_c --> 4adcho_c + glu__L_c 13669

ADCL: 4adcho_c --> 4abz_c + h_c + pyr_c 12476

3HKYNAKGAT: akg_c + hLkynr_c --> 42A3HP24DB_c + glu__L_c 14908 or 15839 or 8540
AATA: 2oxoadp_c + glu__L_c <=> L2aadp_c + akg_c 14908 or 15839
KYNAKGAT: Lkynr_c + akg_c --> 4aphdob_c + glu__L_c 14908 or 15839 or 8540
PHETA1: akg_c + phe__L_c <=> glu__L_c + phpyr_c 14908 or 15839
TRPTA: akg_c + trp__L_c <=> glu__L_c + indpyr_c 12407 or 14908 or 15839
TYRTA: akg_c + tyr__L_c <=> 34hpp_c + glu__L_c 14908 or 15839 or 16065

3HKYNAKGAT: akg_c + hLkynr_c --> 42A3HP24DB_c + glu__L_c 14908 or 15839 or 8540
AATA: 2oxoadp_c + glu__L_c <=> L2aadp_c + akg_c 14908 or 15839
KYNAKGAT: Lkynr_c + akg_c --> 4aphdob_c + glu__L_c 14908 or 15839 or 8540
PHETA1: akg_c + phe__L_c <=> glu__L_c + phpyr_c 14908 or 15839
TRPTA: akg_c + trp__L_c <=> glu__L_c + indpyr_c 12407 or 14908 or 15839
TYRTA: akg_c + tyr__L_c <=> 34hpp_c + glu__L_c 14908 or 15839 or 16065

34HPPYRDC: 34hpp_c + h_c --> 4hoxpacd_c + co2_c 15791
3MOBDC: 3mob_c + h_c --> 2mppal_c + co2_c 15791
3MOPDC: 3mop_c + h_c --> 2mbald_c + co2_c 15791
4MOPDC: 4mop_c + h_c --> 3mbald_c + co2_c 15791
ACALDCD: 2.0 acald_c --> actn__R_c 15791
INDPYRD: h_c + indpyr_c <=> co2_c + id3acald_c 15791
PPYRDC: h_c + phpyr_c --> co2_c + pacald_c 15791
PYRDC: h_c + pyr_c --> acald_c + co2_c 15791

ALCD22xi: 2mbald_c + h_c + nadh_c --> 2mbtoh_c + nad_c 14108 or 14109 or 15438
ALCD23xi: 2mppal_c + h_c + nadh_c --> ibutoh_c + nad_c 14108 or 14109 or 15438
ALCD24xi: 3mbald_c + h_c + nadh_c --> iamoh_c + nad_c 14108 or 14109 or 15438
ALCD25xi: h_c + nadh_c + pacald_c --> 2phetoh_c + nad_c 14108 or 14109 or 15438
ALCD26xi: h_c + id3acald_c + nadh_c --> ind3eth_c + nad_c 14108 or 14109 or 15438
FALDH2: hmgth_c + nad_c <=> Sfglutth_c + h_c + nadh_c 14108 or 14109

ALCD22xi: 2mbald_c + h_c + nadh_c --> 2mbtoh_c + nad_c 14108 or 14109 or 15438
ALCD23xi: 2mppal_c + h_c + nadh_c --> ibutoh_c + nad_c 14108 or 14109 or 15438
ALCD24xi: 3mbald_c + h_c + nadh_c --> iamoh_c + nad_c 14108 or 14109 or 15438
ALCD25xi: h_c + nadh_c + pacald_c --> 2phetoh_c + nad_c 14108 or 14109 or 15438
ALCD26xi: h_c + id3acald_c + nadh_c --> ind3eth_c + nad_c 14108 or 14109 or 15438
FALDH2: hmgth_c + nad_c <=> Sfglutth_c + h_c + nadh_c 14108 or 14109

ALCD19: glyald_c + h_c + nadh_c <=> glyc_c + nad_c 15438
ALCD22xi: 2mbald_c + h_c + nadh_c --> 2mbtoh_c + nad_c 14108 or 14109 or 15438
ALCD23xi: 2mppal_c + h_c + nadh_c --> ibutoh_c + nad_c 14108 or 14109 or 15438
ALCD24xi: 3mbald_c + h_c + nadh_c --> iamoh_c + nad_c 14108 or 14109 or 15438
ALCD25xi: h_c + nadh_c + pacald_c --> 2phetoh_c + nad_c 14108 or 14109 or 15438
ALCD26xi: h_c + id3acald_c + nadh_c --> ind3eth_c + nad_c 14108 or 14109 or 15438
ALCD2x: etoh_c + nad_c <=> acald_c + h_c + nadh_c 15438

ALCD22yi: 2mbald_c + h_c + nadph_c --> 2mbtoh_c + nadp_c 10029 or 11882 or 12784 or 13554 or 13562 or 13947 or 9774
ALCD23yi: 2mppal_c + h_c + nadph_c --> ibutoh_c + nadp_c 10029 or 13554 or 13562 or 13947
ALCD24yi: 3mbald_c + h_c + nadph_c --> iamoh_c + nadp_c 10029 or 13554 or 13562 or 13947
ALCD25yi: h_c + nadph_c + pacald_c --> 2phetoh_c + nadp_c 10029 or 13554 or 13562 or 13947
LCARSyi: h_c + lald__L_c + nadph_c --> 12ppd__S_c + nadp_c 10029 or 13554 or 13562 or 13947
PPDOy: h_c + lald__D_c + nadph_c --> 12ppd__R_c + nadp_c 10029 or 11882 or 12784 or 13554 or 13562 or 13947 or 9774

ALCD22yi: 2mbald_c + h_c + nadph_c --> 2mbtoh_c + nadp_c 10029 or 11882 or 12784 or 13554 or 13562 or 13947 or 9774
ALCD23yi: 2mppal_c + h_c + nadph_c --> ibutoh_c + nadp_c 10029 or 13554 or 13562 or 13947
ALCD24yi: 3mbald_c + h_c + nadph_c --> iamoh_c + nadp_c 10029 or 13554 or 13562 or 13947
ALCD25yi: h_c + nadph_c + pacald_c --> 2phetoh_c + nadp_c 10029 or 13554 or 13562 or 13947
LCARSyi: h_c + lald__L_c + nadph_c --> 12ppd__S_c + nadp_c 10029 or 13554 or 13562 or 13947
PPDOy: h_c + lald__D_c + nadph_c --> 12ppd__R_c + nadp_c 10029 or 11882 or 12784 or 13554 or 13562 or 13947 or 9774

ALCD22yi: 2mbald_c + h_c + nadph_c --> 2mbtoh_c + nadp_c 10029 or 11882 or 12784 or 13554 or 13562 or 13947 or 9774
ALCD23yi: 2mppal_c + h_c + nadph_c --> ibutoh_c + nadp_c 10029 or 13554 or 13562 or 13947
ALCD24yi: 3mbald_c + h_c + nadph_c --> iamoh_c + nadp_c 10029 or 13554 or 13562 or 13947
ALCD25yi: h_c + nadph_c + pacald_c --> 2phetoh_c + nadp_c 10029 or 13554 or 13562 or 13947
LCARSyi: h_c + lald__L_c + nadph_c --> 12ppd__S_c + nadp_c 10029 or 13554 or 13562 or 13947
PPDOy: h_c + lald__D_c + nadph_c --> 12ppd__R_c + nadp_c 10029 or 11882 or 12784 or 13554 or 13562 or 13947 or 9774

ALCD22yi: 2mbald_c + h_c + nadph_c --> 2mbtoh_c + nadp_c 10029 or 11882 or 12784 or 13554 or 13562 or 13947 or 9774
ALCD23yi: 2mppal_c + h_c + nadph_c --> ibutoh_c + nadp_c 10029 or 13554 or 13562 or 13947
ALCD24yi: 3mbald_c + h_c + nadph_c --> iamoh_c + nadp_c 10029 or 13554 or 13562 or 13947
ALCD25yi: h_c + nadph_c + pacald_c --> 2phetoh_c + nadp_c 10029 or 13554 or 13562 or 13947
LCARSyi: h_c + lald__L_c + nadph_c --> 12ppd__S_c + nadp_c 10029 or 13554 or 13562 or 13947
PPDOy: h_c + lald__D_c + nadph_c --> 12ppd__R_c + nadp_c 10029 or 11882 or 12784 or 13554 or 13562 or 13947 or 9774

34DHALDD: 34dhpac_c + h2o_c + nad_c --> 34dhpha_c + 2.0 h_c + nadh_c 12042
34DHPLACOX_NADP: 34dhpac_c + h2o_c + nadp_c <=> 34dhpha_c + 2.0 h_c + nadph_c 12042
4HOXPACDOX_NADP: 4hoxpacd_c + h2o_c + nadp_c <=> 4hphac_c + 2.0 h_c + nadph_c 12042
ABUTD: 4abutn_c + h2o_c + nad_c --> 4abut_c + 2.0 h_c + nadh_c 12042
ALDD19x_P: h2o_c + nadp_c + pacald_c --> 2.0 h_c + nadph_c + pac_c 12042
ALDD19xr: h2o_c + nad_c + pacald_c <=> 2.0 h_c + nadh_c + pac_c 12042
ALDD20x: h2o_c + id3acald_c + nad_c --> 2.0 h_c + ind3ac_c + nadh_c 12042
ALDD20y: h2o_c + id3acald_c + nadp_c --> 2.0 h_c + ind3ac_c + nadph_c 12042
ALDD2x: acald_c + h2o_c + nad_c --> ac_c + 2.0 h_c + nadh_c 12042
ALDD2y: acald_c + h2o_c + nadp_c --> ac_c + 2.0 h_c + nadph_c 12042
AM6SAD: am6sa_c + h2o_c + nad_c --> amuco_c + 2.0 h_c + nadh_c 12042
BAMPPALDOX: bamppald_c + h2o_c + nad_c --> ala_B_c + 2.0 h_c + nadh_c 12042
FTHFDH: 10fthf_c + h2o_c + nadp_c --> co2_c + h_c + nadph_c + thf_c 12042
GCALDD: gcald_c + h2o_c + nad_c --> glyclt_c + 2.0 h_c + nadh_c 12042
IMACTD: h2o_c + im4act_c + nad_c --> 2.0 h_c + im4ac_c + nadh_c 12042
LCADi: h2o_c + lald__L_c + nad_c --> 2.0 h_c + lac__L_c + nadh_c 12042
LCADi_D: h2o_c + lald__D_c + nad_c --> 2.0 h_c + lac__D_c + nadh_c 12042
MACOXO: 3mldz_c + h2o_c + nad_c --> 3mlda_c + 2.0 h_c + nadh_c 12042

ALDD2xm: acald_m + h2o_m + nad_m --> ac_m + 2.0 h_m + nadh_m 13426
ALDD2ym: acald_m + h2o_m + nadp_m --> ac_m + 2.0 h_m + nadph_m 13426
TMABDH1_m: 4tmeabut_m + h2o_m + nad_m --> gbbtn_m + 2.0 h_m + nadh_m 13426

4HBALDDm: 4hbald_m + h2o_m + nad_m --> 4hbz_m + 2.0 h_m + nadh_m 16323
ALDD16er: h2o_r + hxdcal_r + nad_r --> 2.0 h_r + hdca_r + nadh_r 16323
ALDD21er: h2o_r + nad_r + pristanal_r --> 2.0 h_r + nadh_r + prist_r 16323
ALDD3C161er: h2o_r + hxdceal_r + nad_r --> 2.0 h_r + hdc2ea_r + nadh_r 16323

HISDr: his__L_c --> nh4_c + urcan_c 15434 or 16462

HISDr: his__L_c --> nh4_c + urcan_c 15434 or 16462

SALMCOM: amet_c + nrpphr_c --> ahcys_c + h_c + normete__L_c 13444 or 13445
SALMCOM2: adrnl_c + amet_c --> ahcys_c + h_c + mepi_c 13444 or 13445

34DHPHAMT: 34dhpha_c + amet_c --> ahcys_c + h_c + homoval_c 13445
SALMCOM: amet_c + nrpphr_c --> ahcys_c + h_c + normete__L_c 13444 or 13445
SALMCOM2: adrnl_c + amet_c --> ahcys_c + h_c + mepi_c 13444 or 13445
```

15434 is glutathione S-transferase  
16462 Phenylalanine/tyrosine amino-lyase  
https://www.ncbi.nlm.nih.gov/pubmed/25911487  
Add PAL and TAL reactions  
13444, 13445 caffeoyl-CoA O-methyltransferase?

In [49]:

```
r = model.reactions.get_by_id('HISDr')
r.id = 'PAL'
r.name = 'Phenylalanine ammonia-lyase'
r.gene_reaction_rule = '16462'
model.add_metabolites([eco.metabolites.get_by_id('cinnm_c').copy()])
r.add_metabolites({'his__L_c': 1.0, 'urcan_c': -1.0, 'phe__L_c': -1.0, 'cinnm_c': 1.0})

r = model.reactions.get_by_id('PAL').copy()
r.id = 'TAL'
r.name = 'Tyrosine ammonia-lyase'
r.gene_reaction_rule = '16462'
model.add_reactions([r])
r.add_metabolites({'phe__L_c': 1.0, 'cinnm_c': -1.0, 'tyr__L_c': -1.0, 'T4hcinnm_c': 1.0})
```

In [50]:

```
m1 = ppu.metabolites.get_by_id('caffcoa_c').copy()
m2 = ppu.metabolites.get_by_id('ferulcoa_c').copy()
model.add_metabolites([m1,m2])
r = model.reactions.get_by_id('34DHPHAMT').copy()
r.id = 'CCOAOMT'
r.name = 'Caffeoyl-CoA O-methyltransferase'
r.gene_reaction_rule = '13444 or 13445'
model.add_reactions([r])
r.add_metabolites({'34dhpha_c': 1.0, 'homoval_c': -1.0, 'caffcoa_c': -1.0, 'ferulcoa_c': 1.0})
```

In [51]:

```
for r in sorted(model.metabolites.get_by_id('T4hcinnm_c').reactions, key=lambda x: x.id):
    print(r, r.gene_reaction_rule)
print()
for r in sorted(model.metabolites.get_by_id('T4hcinnm_m').reactions, key=lambda x: x.id):
    print(r, r.gene_reaction_rule)
print()
for r in sorted(model.metabolites.get_by_id('ferulcoa_c').reactions, key=lambda x: x.id):
    print(r, r.gene_reaction_rule)
```

```
4CMCOAS: T4hcinnm_c + atp_c + coa_c --> amp_c + coucoa_c + ppi_c 11833 or 13700 or 14802 or 16129
TAL: tyr__L_c --> T4hcinnm_c + nh4_c 16462

COUCOAFm: T4hcinnm_m + atp_m + coa_m --> amp_m + coucoa_m + ppi_m 16635

CCOAOMT: amet_c + caffcoa_c --> ahcys_c + ferulcoa_c + h_c 13444 or 13445
```

q9 biosynthesis

If coumarate is coming from 34hpp instead of TAL,  
TYRTAim: 34hpp\_m + glu**L\_m <=> akg\_m + tyr**L\_m GOT2 or TAT (this step is by mito aminotransferase)  
34HPLFM: 34hpp\_m + h\_m + nadh\_m --> 34hpl\_m + nad\_m ?  
T4HCINNMFM: 34hpl\_m --> T4hcinnm\_m + h2o\_m ?

S.cer https://www.nature.com/articles/nbt.3683.pdf  
tyr\_\_L -> 34hpp by Aro9 (no ortholog, ARO8 could do it in cyto)  
34hpp -> 34hpacald by Aro10 (no ortholog, PDC could do it in cyto)  
34hpacald -> T4hcinnm by Aim18? (16521)

C. sporogenes https://www.nature.com/articles/nature24661.pdf  
tyr\_\_L -> 34hpp by aat (many)  
34hpp -> 34hpac by porA pyruvate:ferredoxin dehydrogenase (no ortholog)  
34hpp -> 34hpl by fldH aromatic 2-oxoacid reductase (blast to 9085 D-3-phosphoglycerate dehydrogenase)  
34hpl -> 34hpl-CoA by fldA (9923 succinate---hydroxymethylglutarate CoA-transferase)  
34hpl-CoA -> T4hcinnm-CoA by fldBC (no ortholog)  
34hpl -> 3-hydroxyphenylpropanoate by acdA (14070 ACADSB / 12570 ACADM / 10012 IVDe)

no complete pathway from 34hpp to coumarate except 34hpp -> tyr -> T4hcinnm

In [52]:

```
Annotation.loc[['16521','9085','9923','14070','12570','10012']]
```

Out[52]:

|  | Combined Annotations | Signal P | Sc288c Orthologs | Human Orthologs | Sc288 Best Hit | Human Blast | Essential | WolfPSort | C Terminal |
| --- | --- | --- | --- | --- | --- | --- | --- | --- | --- |
| RTO4\_ID |  |  |  |  |  |  |  |  |  |
| 16521 | HMMPfam:Chalcone-flavanone isomerase:PF02431,S... | A | AIM18 |  |  |  | Not Essential | mito 26 | GAA\* |
| 9085 | K00058: serA, PHGDH; D-3-phosphoglycerate dehy... |  | SER3,SER33 | PHGDH | SER33 | PHGDH | Not Essential | extr 12, mito 10, pero 2, nucl 1, cyto 1, cyto... | MLF\* |
| 9923 | K18703: SUGCT; succinate---hydroxymethylglutar... | S |  | SUGCT |  | SUGCT | Not Essential | mito 26.5, cyto\_mito 14 | GVV\* |
| 14070 | K09478: ACADSB; short/branched chain acyl-CoA ... |  |  | ACADSB |  | ACADS | Not Essential | mito 26, cyto\_mito 14.5 | EYS\* |
| 12570 | K00249: ACADM, acd; acyl-CoA dehydrogenase |  |  | ACADM |  | ACADM | Not Essential | mito 26 | DYA\* |
| 10012 | K00253: IVD, ivd; isovaleryl-CoA dehydrogenase | S |  | IVD |  | IVDe | Not Essential | mito 25.5, cyto\_mito 14 | GAL\* |

In [53]:

```
# 4HB can be made from coumarate, or para-aminobenzoate can substitute 4HB for ubiquinone synthesis
r1 = sce.reactions.get_by_id('EX_4abz_e').copy()
r2 = sce.reactions.get_by_id('4ABZt').copy()
r3 = sce.reactions.get_by_id('4ABZtm').copy()
model.add_reactions([r1,r2,r3])

m1 = model.metabolites.get_by_id('3nphb_m').copy()
m1.id = '3npab_m'
m1.name = '3-Nonaprenyl-4-aminobenzoate'
m1.formula = 'C52H78NO2'
m2 = model.metabolites.get_by_id('3npdhb_m').copy()
m2.id = '3npahb_m'
m2.name = '3-Nonaprenyl-4-amino-5-hydroxybenzoate'
m2.formula = 'C52H78NO3'
model.add_metabolites([m1,m2])

r = model.reactions.get_by_id('HBZNPT_m').copy()
r.id = 'ABZNPT_m'
r.name = 'Aminobenzoate Nonaprenyltransferase'
model.add_reactions([r])
r.add_metabolites({'4hbz_m': 1.0, '3nphb_m': -1.0, '4abz_m': -1.0, '3npab_m': 1.0})

r = model.reactions.get_by_id('3NPHBH2_m').copy()
r.id = '3NPABH_m'
r.name = 'Hydroxylation of 3-nonaaprenyl-4-aminobenzoate (NADP)'
model.add_reactions([r])
r.add_metabolites({'3nphb_m': 1.0, '3npdhb_m': -1.0, '3npab_m': -1.0, '3npahb_m': 1.0})

r = model.reactions.get_by_id('3NPHBH2_m').copy()
r.id = '3NPAHBDH_m'
r.name = 'Deamination and hydroxylation of 3-nonaaprenyl-4-amino-5-hydroxybenzoate (NADP)'
model.add_reactions([r])
r.add_metabolites({'3nphb_m': 1.0, '3npahb_m': -1.0, 'nadph_m': -1.0, 'h_m': -2.0, 'nadp_m': 1.0, 'nh4_m': 1.0})
```

In [54]:

```
# check if model can produce cofactors
cofactors = ['camp_c','coa_c','fad_c','gthrd_c','hemeA_m','nad_c','nadp_c','q9_m','thf_c','thmpp_c',
             '5mthf_c','btn_m','lipopb_m','mlthf_c','ptrc_c','pydx5p_c','spmd_c']
with model:
    for x in cofactors:
        model.reactions.get_by_id('BIOMASS_RT').add_metabolites({x: -1e-2})
        sol = model.optimize()
        print(x, sol.objective_value)
        if abs(sol.objective_value) < 1e-6:
            for k, v in sol.shadow_prices.items():
                #if v and k in [m.id for m in model.reactions.get_by_id('BIOMASS_RT').metabolites]:
                if abs(v) > 1:
                    print('\t',k, v)
        model.reactions.get_by_id('BIOMASS_RT').add_metabolites({x: 1e-2})
```

```
camp_c 0.13593743393234278
coa_c 0.13547854502629483
fad_c 0.1358833457242419
gthrd_c 0.13632034044837316
hemeA_m 0.1352517764125231
nad_c 0.13561558122627718
nadp_c 0.1356003412933258
q9_m 0.13393271057829534
thf_c 0.1359721547056612
thmpp_c 0.13561761347616483
5mthf_c 0.13577122443665932
btn_m 0.0
	 btn_m -100.0
	 dad_5_m 100.0
lipopb_m 0.0
	 met__L_m 33.33333333333333
	 mettrna_m 33.33333333333333
	 fmettrna_m 33.33333333333333
	 2fe2s_m 33.33333333333333
	 lipoamp_m -100.0
	 lipopb_m -100.0
	 lipoate_m -100.0
mlthf_c 0.135755949504487
ptrc_c 0.13650642647367803
pydx5p_c 0.13653113901894637
spmd_c 0.1365774991663852
```

Replace amino acids with aminoacyl-tRNA for protein?  
Check aminoacyl-tRNA synthetase

In [55]:

```
temp = Annotation.index[Annotation['Combined Annotations'].str.contains('tRNA synthetase')]
display(Annotation.loc[temp])
Show_Data(temp)
```

|  | Combined Annotations | Signal P | Sc288c Orthologs | Human Orthologs | Sc288 Best Hit | Human Blast | Essential | WolfPSort | C Terminal |
| --- | --- | --- | --- | --- | --- | --- | --- | --- | --- |
| RTO4\_ID |  |  |  |  |  |  |  |  |  |
| 8575 | K01892: HARS, hisS; histidyl-tRNA synthetase |  | HTS1 | HARS,HARS2 | HTS1 | HARS | Essential | mito 25.5, cyto\_mito 14 | GKK\* |
| 9167 | K01893: NARS, asnS; asparaginyl-tRNA synthetase |  | DED81 | NARS | DED81 | NARS | Essential | cyto 7.5, cysk 7, cyto\_nucl 5.5, mito 5, pero ... | CTP\* |
| 9334 | BLAST: probable GUS1-Glutamyl-tRNA synthetase ... |  |  |  |  |  | Not Essential | nucl 11, cyto\_nucl 9, mito 7, cyto 5, extr 3 | ALV\* |
| 9855 | K01883: CARS, cysS; cysteinyl-tRNA synthetase |  | YNL247W | CARS,CARS2 | YNL247W | CARS | Essential | cyto 13.5, cyto\_mito 9.5, nucl 5, mito 4.5, pe... | QSA\* |
| 10079 | K01887: RARS, argS; arginyl-tRNA synthetase |  | YDR341C,MSR1 | RARS2,RARS | YDR341C | RARS2 | Essential | cyto 20, cyto\_nucl 12, mito 5 | ERM\* |
| 10144 | K01869: LARS, leuS; leucyl-tRNA synthetase |  | CDC60 | LARS | CDC60 | LARS | Essential | cyto 13.5, cyto\_mito 11, mito 7.5, nucl 4 | YNI\* |
| 10246 | K01875: SARS, serS; seryl-tRNA synthetase |  | SES1 | SARS | SES1 | SARS | Essential | cyto 14.5, cyto\_nucl 12, nucl 8.5, pero 2 | QKH\* |
| 10753 | K01885: EARS, gltX; glutamyl-tRNA synthetase |  | GUS1 |  | GUS1 | EPRS | Essential | cyto\_mito 10.5, mito 10, cyto 9, nucl 5 | INE\* |
| 11486 | K01881: PARS, proS; prolyl-tRNA synthetase |  | YHR020W | EPRS | YHR020W | EPRS | Not Essential | cyto 17, cyto\_mito 11.833, cyto\_nucl 9.833, mi... | RSY\* |
| 11945 | K04567: KARS, lysS; lysyl-tRNA synthetase, cla... |  | KRS1 | KARS | KRS1 | KARS | Not Essential | mito 23, mito\_nucl 13.833, cyto\_mito 12.333 | SRR\* |
| 11973 | K01872: AARS, alaS; alanyl-tRNA synthetase |  | ALA1 | AARS,AARS2 | ALA1 | AARS | Essential | cyto 21, cyto\_nucl 12.5, pero 3 | ESR\* |
| 12123 | K01893: NARS, asnS; asparaginyl-tRNA synthetase | S | SLM5 | NARS2 | SLM5 | NARS2 | Essential | mito 24, nucl 1, cyto 1, cyto\_nucl 1, pero 1, ... | SRF\* |
| 12470 | K01889: FARSA, pheS; phenylalanyl-tRNA synthet... |  | FRS2 | FARSA | FRS2 | FARSA | Essential | cysk 10, cyto 8, nucl 4, mito 4, mito\_nucl 4 | VRF\* |
| 13137 | K01866: YARS, tyrS; tyrosyl-tRNA synthetase | S | MSY1 | YARS2 | MSY1 | YARS2 | Essential | mito 21.5, cyto\_mito 12.5, cyto 2.5 | LLE\* |
| 13140 | K01874: MARS, metG; methionyl-tRNA synthetase |  | MES1 | MARS | MES1 | MARS | Essential | cyto 8.5, mito 8, cyto\_nucl 7, nucl 4.5, pero ... | VQL\* |
| 13249 | K01890: FARSB, pheT; phenylalanyl-tRNA synthet... |  | FRS1 | FARSB | FRS1 | FARSB | Essential | cyto 12.5, cyto\_nucl 11.5, mito 8, nucl 5.5 | PFL\* |
| 13380 | K01886: QARS, glnS; glutaminyl-tRNA synthetase |  | GLN4 | QARS | GLN4 | QARS | Essential | cyto 16.5, cyto\_nucl 11.5, nucl 5.5, pero 5 | KGR\* |
| 13398 | K01889: FARSA, pheS; phenylalanyl-tRNA synthet... |  | MSF1 | FARS2 | MSF1 | FARS2 | Essential | mito 22, cyto 4 | EVR\* |
| 13446 | K01870: IARS, ileS; isoleucyl-tRNA synthetase |  | ILS1 | IARS | ILS1 | IARS | Essential | cyto\_nucl 11, nucl 10.5, cyto 10.5, mito 3 | VWA\* |
| 14225 | K01875: SARS, serS; seryl-tRNA synthetase | S | DIA4 | CTC-360G5.8,SARS2 | DIA4 | SARS2 | Essential | mito 27 | VQQ\* |
| 14258 | K01876: DARS, aspS; aspartyl-tRNA synthetase |  | DPS1 | DARS | DPS1 | DARS | Essential | mito 24.5, cyto\_mito 14 | TSP\* |
| 14565 | K01885: EARS, gltX; glutamyl-tRNA synthetase |  | MSE1 | EARS2 | MSE1 | EARS2 | Essential | mito 25.5, cyto\_mito 14 | EHA\* |
| 14709 | K01867: WARS, trpS; tryptophanyl-tRNA synthetase |  | WRS1 | WARS | WRS1 | WARS | Essential | cyto 12.5, cyto\_nucl 11.5, nucl 9.5, pero 3 | STA\* |
| 15504 | K01880: GARS, glyS1; glycyl-tRNA synthetase | S | GRS1,YPR081C | GARS | GRS1 | GARS | Essential | mito 22, cyto 4 | QDA\* |
| 15518 | K01874: MARS, metG; methionyl-tRNA synthetase |  | MSM1 | MARS2 | MSM1 | MARS2 | Essential | mito 23, cyto\_mito 14 | KGK\* |
| 15533 | K01867: WARS, trpS; tryptophanyl-tRNA synthetase |  | MSW1 | WARS2 | MSW1 | WARS2 | Essential | mito 26 | GLD\* |
| 15556 | K01870: IARS, ileS; isoleucyl-tRNA synthetase |  | ISM1 | IARS2 | ISM1 | IARS2 | Essential | mito 20, cyto 4, nucl 3 | VSA\* |
| 15848 | K01868: TARS, thrS; threonyl-tRNA synthetase | S | THS1 | TARS,TARS2,TARSL2 | THS1 | TARS | Essential | mito 21, cyto\_mito 12.833, mito\_nucl 11.833, c... | ELR\* |
| 16528 | K01873: VARS, valS; valyl-tRNA synthetase | S | VAS1 | VARS,VARS2 | VAS1 | VARS | Essential | mito 15.5, cyto\_mito 11.833, cyto 7, cyto\_nucl... | LRV\* |
| 16641 | K01866: YARS, tyrS; tyrosyl-tRNA synthetase |  | TYS1 | YARS | TYS1 | YARS | Essential | cyto 20.5, cyto\_nucl 12.833, nucl 4, mito\_nucl... | LSA\* |

| strain | WT | | | | | | | | | | | | | | | | |
| --- | --- | --- | --- | --- | --- | --- | --- | --- | --- | --- | --- | --- | --- | --- | --- | --- | --- |
| condition | G\_MM | C\_MM | G\_SD | | GX\_SD | | | X\_SD | | A\_SD | | C\_SD | | MM\_CN120 | | MM\_CN5 | Diversity\_Sample |
| phase | exp | exp | exp | stat | exp | trans | stat | exp | stat | exp | stat | exp | stat | exp | stat | exp | exp |
| proteinId | Set1 | Set1 | Set2 | Set2 | Set2 | Set2 | Set2 | Set2 | Set2 | Set2 | Set2 | Set2 | Set2 | Set3 | Set3 | Set3 | Set3 |
| RTO4\_ID |  |  |  |  |  |  |  |  |  |  |  |  |  |  |  |  |  |
| 8575 | 6.05881 | 6.40004 | 5.36813 | 4.7866 | 5.50675 | 5.55138 | 5.48989 | 6.01434 | 5.40234 | 5.66144 | 5.37761 | 6.19163 | 6.0076 | 5.02952 | 5.17475 | 5.90318 | 5.63833 |
| 9167 | 7.31786 | 6.92274 | 7.36809 | 5.72268 | 7.70801 | 6.31477 | 6.33639 | 6.54584 | 5.84019 | 6.0349 | 5.75525 | 7.29172 | 7.90533 | 6.05743 | 5.80145 | 7.96328 | 6.70836 |
| 9334 | 5.49526 | 5.08708 | 4.54032 | 4.93814 | 4.51925 | 4.85948 | 4.44842 | 4.73151 | 4.53714 | 4.76604 | 4.97204 | 5.06523 | 5.05958 | 4.47232 | 4.14952 | 3.49519 | 3.65786 |
| 9855 | 4.80542 | 5.45591 | 5.30644 | 3.57824 | 5.73652 | 4.52058 | 4.51006 | 4.97264 | 4.32004 | 4.48613 | 4.11283 | 5.88492 | 6.29967 | 4.71888 | 4.77876 | 6.95199 | 6.25162 |
| 10079 | 6.40796 | 6.64322 | 7.06016 | 5.90628 | 7.40764 | 6.22291 | 6.26246 | 6.51973 | 5.66779 | 6.14351 | 5.60319 | 7.10212 | 7.71407 | 6.29707 | 6.23331 | 8.30928 | 6.80331 |
| 10144 | 5.28024 | 6.29388 | 6.10584 | 4.64947 | 6.39427 | 5.42995 | 5.51394 | 5.66942 | 4.8861 | 5.16299 | 4.41526 | 7.05069 | 7.83129 | 6.95207 | 6.8628 | 8.36295 | 8.00918 |
| 10246 | 5.27896 | 6.88364 | 5.67446 | 5.02349 | 5.8517 | 5.85649 | 6.01192 | 6.31778 | 5.29938 | 5.89671 | 5.32933 | 7.13197 | 7.90831 | 6.89588 | 6.98522 | 8.19399 | 8.36539 |
| 10753 | 5.90519 | 6.49779 | 6.6231 | 5.16279 | 7.10354 | 5.87061 | 5.73871 | 6.25847 | 5.07628 | 5.72519 | 4.80697 | 6.99094 | 7.61781 | 6.60874 | 6.57683 | 8.6694 | 7.52913 |
| 11486 | 7.77673 | 7.66072 | 7.9391 | 7.42264 | 8.1018 | 7.6099 | 7.51401 | 7.86168 | 7.17692 | 7.4761 | 7.0446 | 7.92266 | 8.20729 | 8.13712 | 7.87847 | 8.69799 | 8.81189 |
| 11945 | 6.25922 | 6.35467 | 7.12177 | 6.29758 | 7.35047 | 6.78881 | 6.66436 | 6.89706 | 5.99613 | 6.21558 | 5.7276 | 7.21095 | 8.49133 | 7.08419 | 7.02055 | 8.2492 | 7.65108 |
| 11973 | 7.03366 | 6.87635 | 6.39792 | 5.03871 | 6.65624 | 5.71571 | 5.71709 | 6.04595 | 5.62836 | 5.9109 | 5.68666 | 6.8667 | 6.96244 | 6.42575 | 6.28584 | 7.3301 | 7.06233 |
| 12123 | 4.83165 | 5.11938 | 4.28754 | 4.29641 | 4.28059 | 4.56924 | 4.60539 | 4.60529 | 4.87018 | 4.36486 | 4.68175 | 5.3067 | 5.40623 | 2.91112 | 3.11052 | 3.2877 | 3.41716 |
| 12470 | 6.42634 | 6.64362 | 6.94296 | 6.08359 | 7.26418 | 6.38454 | 6.38097 | 6.48157 | 6.23637 | 6.12981 | 6.09405 | 7.04272 | 7.30237 | 5.7371 | 5.87262 | 7.31853 | 6.72797 |
| 13137 | 4.6149 | 5.33004 | 5.50746 | 5.1702 | 5.48784 | 5.65296 | 5.46171 | 5.78508 | 4.795 | 5.08064 | 4.70401 | 6.0649 | 7.39659 | 4.4088 | 4.18961 | 4.79112 | 4.57402 |
| 13140 | 6.02947 | 5.79311 | 6.24968 | 6.31578 | 6.28361 | 6.35491 | 6.42526 | 6.60024 | 6.31109 | 6.53924 | 6.48153 | 6.31376 | 6.38062 | 7.07357 | 6.83471 | 7.92136 | 7.41805 |
| 13249 | 5.78137 | 6.10883 | 6.14493 | 5.93719 | 6.27621 | 6.08087 | 6.02481 | 6.25418 | 5.54188 | 5.92668 | 5.55026 | 6.796 | 7.04988 | 7.15479 | 7.10609 | 8.08867 | 8.06445 |
| 13380 | 6.44596 | 6.22254 | 6.42365 | 5.39934 | 6.44485 | 5.65898 | 5.82887 | 6.02897 | 5.66281 | 5.73484 | 5.5412 | 7.03683 | 8.0008 | 7.60585 | 7.3392 | 8.0525 | 7.76654 |
| 13398 | 6.6167 | 6.66118 | 7.65404 | 6.38937 | 7.6774 | 6.51358 | 6.7289 | 7.00813 | 7.40112 | 7.07803 | 7.39992 | 6.67221 | 6.35053 | 6.92189 | 6.35342 | 7.52977 | 8.00568 |
| 13446 | 6.29045 | 6.44689 | 6.85996 | 5.20898 | 7.28578 | 5.94924 | 5.90092 | 6.27526 | 5.4352 | 5.66776 | 5.08721 | 6.86215 | 8.77516 | 7.1975 | 7.01547 | 9.90927 | 8.60887 |
| 14225 | 5.34376 | 5.33535 | 5.09191 | 3.99261 | 5.10577 | 4.71025 | 4.72532 | 4.89119 | 4.67486 | 4.77683 | 4.50383 | 4.7837 | 4.80266 | 4.49752 | 4.14504 | 5.14546 | 5.08923 |
| 14258 | 7.34753 | 7.04298 | 7.65447 | 7.23351 | 7.6869 | 7.34888 | 7.30124 | 7.36809 | 7.08829 | 7.11225 | 6.67594 | 6.72858 | 6.26603 | 7.98163 | 7.84666 | 8.11256 | 8.13374 |
| 14565 | 4.92229 | 4.26284 | 5.53308 | 5.39289 | 5.52954 | 5.41592 | 5.46257 | 5.06213 | 5.25611 | 4.94004 | 5.07734 | 4.489 | 4.33901 | 4.82619 | 4.88169 | 5.08511 | 5.33634 |
| 14709 | 6.482 | 6.29116 | 6.62375 | 5.51804 | 6.69295 | 6.1073 | 5.98059 | 6.52512 | 5.42229 | 6.22893 | 5.50368 | 5.95575 | 6.24629 | 7.28761 | 7.12288 | 8.39255 | 7.95277 |
| 15504 | 5.38129 | 6.61729 | 6.09784 | 5.79833 | 6.23726 | 6.17174 | 6.10136 | 6.47785 | 5.67068 | 6.05229 | 5.6845 | 6.62478 | 6.39933 | 5.58817 | 5.79317 | 7.00509 | 7.00623 |
| 15518 | 5.38458 | 5.10264 | 5.97062 | 6.3679 | 6.03946 | 6.44423 | 6.33426 | 6.25984 | 5.70963 | 5.71325 | 5.7035 | 5.73513 | 4.57198 | 5.78838 | 5.97832 | 5.8254 | 5.81304 |
| 15533 | 5.6567 | 5.42986 | 6.27533 | 5.81764 | 6.1594 | 6.56376 | 6.45369 | 6.51018 | 5.59409 | 5.31988 | 5.19531 | 6.46253 | 5.83565 | 5.14632 | 5.19554 | 5.02622 | 5.84459 |
| 15556 | 4.3147 | 4.3728 | 5.85209 | 4.96724 | 5.79969 | 5.36095 | 5.08279 | 5.46143 | 4.50372 | 5.42262 | 4.53018 | 5.26314 | 5.21676 | 5.02292 | 5.03868 | 6.35509 | 5.9945 |
| 15848 | 6.00872 | 6.55516 | 5.8614 | 5.24436 | 6.13215 | 5.63305 | 5.66313 | 5.79048 | 5.62595 | 5.58945 | 5.64164 | 6.5921 | 6.94182 | 5.80674 | 6.05274 | 6.93054 | 7.36609 |
| 16528 | 5.83323 | 6.07082 | 6.73082 | 4.87153 | 7.01552 | 5.74748 | 5.61988 | 5.99348 | 5.14614 | 5.34936 | 4.65258 | 6.71624 | 7.67826 | 6.17908 | 5.99888 | 8.24533 | 7.14784 |
| 16641 | 6.58207 | 6.55522 | 6.63809 | 6.31759 | 6.92415 | 6.22947 | 6.28546 | 6.31109 | 6.30821 | 6.23252 | 6.57565 | 6.62251 | 6.66037 | 6.62968 | 6.70263 | 7.9117 | 7.00222 |

| strain | WT | | | | | | | | | | |
| --- | --- | --- | --- | --- | --- | --- | --- | --- | --- | --- | --- |
| condition | G\_SD | | GX\_SD | | | X\_SD | | A\_SD | | C\_SD | |
| proteinId | exp | stat | exp | trans | stat | exp | stat | exp | stat | exp | stat |
| 8575 | 8.64136 | 7.05676 | 10.7738 | 8.37404 | 8.55989 | 10.558 | 7.43273 | 9.27283 | 8.40676 | 11.3115 | 9.59442 |
| 9167 | 18.029 | 16.2329 | 17.4771 | 12.6469 | 13.8957 | 11.3458 | 11.2215 | 12.3556 | 9.57064 | 11.1383 | 16.3228 |
| 9855 | 14.4145 | 6.58832 | 16.2828 | 5.56121 | 5.2757 | 7.25798 | 5.54671 | 7.72142 | 5.76449 | 7.49793 | 9.33989 |
| 10079 | 26.8376 | 20.061 | 26.8432 | 16.5405 | 18.3172 | 17.2023 | 19.6967 | 17.1907 | 17.2524 | 20.5322 | 28.7483 |
| 10144 | 49.2838 | 38.4873 | 42.0542 | 26.0076 | 24.5459 | 32.4489 | 26.2234 | 29.3692 | 23.5277 | 37.5847 | 52.2132 |
| 10246 | 17.6839 | 13.9206 | 18.9122 | 12.8365 | 13.4425 | 17.5971 | 15.0835 | 16.4184 | 15.8996 | 16.901 | 23.2744 |
| 10753 | 36.8113 | 25.8272 | 36.0151 | 20.9941 | 21.9173 | 25.2218 | 20.1148 | 23.5771 | 18.3662 | 27.7605 | 34.881 |
| 11486 | 54.4539 | 46.4512 | 57.3783 | 46.2788 | 47.1616 | 47.7606 | 48.5151 | 47.1198 | 50.7194 | 55.4815 | 54.2207 |
| 11945 | 25.1957 | 23.4441 | 23.8144 | 23.5981 | 22.4235 | 20.3438 | 22.8433 | 19.1263 | 18.9511 | 18.2087 | 23.9155 |
| 11973 | 25.2758 | 21.637 | 24.6097 | 16.5442 | 19.3354 | 24.1027 | 25.7758 | 25.3214 | 22.0349 | 22.4391 | 16.7124 |
| 12123 | 1.12412 | 1.30423 | 0 | 1.11902 | 1.44978 | 0.787109 | 0.97047 | 0.382815 | 0.764134 | 0 | 0 |
| 12470 | 16.8987 | 14.6513 | 17.6721 | 11.6861 | 13.3448 | 15.2694 | 13.381 | 13.8921 | 11.672 | 14.3339 | 19.5951 |
| 13137 | 2.34329 | 1.77738 | 1.82669 | 2.40404 | 2.75249 | 2.74539 | 2.56051 | 2.51084 | 0.965155 | 2.118 | 1.50822 |
| 13140 | 10.9791 | 12.7928 | 13.424 | 10.9896 | 12.6054 | 12.5494 | 11.6 | 13.1205 | 16.8615 | 11.5344 | 11.5453 |
| 13249 | 27.5906 | 19.962 | 23.5807 | 18.7845 | 17.069 | 21.4883 | 20.6888 | 18.3434 | 15.5149 | 16.4567 | 19.1354 |
| 13380 | 22.1379 | 20.4416 | 24.0083 | 18.4137 | 20.3454 | 24.2712 | 21.2518 | 20.8526 | 19.1839 | 21.9887 | 30.3015 |
| 13398 | 10.7927 | 10.4966 | 9.34468 | 9.68347 | 10.9165 | 7.83792 | 9.42228 | 7.13468 | 7.09427 | 9.39764 | 8.69966 |
| 13446 | 35.2241 | 23.5188 | 36.1914 | 19.3302 | 18.9875 | 25.5816 | 23.5865 | 24.3267 | 20.7187 | 25.2334 | 38.3194 |
| 14225 | 0.95256 | 0.380468 | 0.204746 | 0 | 0.711395 | 0.199906 | 0 | 0 | 0 | 0 | 0.211526 |
| 14258 | 23.1999 | 19.6551 | 23.9881 | 19.8977 | 22.6314 | 23.8352 | 19.0557 | 15.843 | 15.1256 | 19.0229 | 16.1094 |
| 14565 | 1.33979 | 1.48616 | 0 | 1.10034 | 2.15989 | 0 | 0 | 0 | 0 | 0 | 0 |
| 14709 | 8.081 | 6.8015 | 7.32598 | 5.76267 | 5.84885 | 8.60478 | 5.12384 | 6.17919 | 4.77872 | 6.61917 | 4.78357 |
| 15504 | 26.553 | 23.5036 | 30.7657 | 19.8736 | 21.7561 | 26.233 | 25.0088 | 22.5954 | 23.7486 | 22.5519 | 21.5311 |
| 15518 | 4.63627 | 5.92002 | 4.25908 | 7.25202 | 6.19705 | 6.06658 | 3.37947 | 4.438 | 2.86037 | 2.98438 | 2.4047 |
| 15533 | 3.8456 | 8.27088 | 2.63161 | 10.9656 | 10.417 | 4.11998 | 6.89345 | 1.54735 | 2.10135 | 4.48937 | 6.11263 |
| 15556 | 4.61118 | 4.92087 | 3.4408 | 3.53365 | 4.2038 | 0.978443 | 1.56304 | 2.31641 | 2.67015 | 2.15681 | 1.97492 |
| 15848 | 17.8176 | 12.1926 | 19.7243 | 9.45844 | 9.80469 | 14.7078 | 11.8443 | 15.0653 | 9.95495 | 20.9066 | 22.0113 |
| 16528 | 32.2982 | 26.5728 | 33.9816 | 20.2776 | 20.1194 | 21.746 | 22.0328 | 25.6918 | 20.3139 | 20.4882 | 26.1486 |
| 16641 | 20.1686 | 16.8781 | 19.5427 | 15.0647 | 16.2303 | 16.0509 | 15.1979 | 18.3294 | 19.5192 | 10.8658 | 14.3797 |

|  | Glucose | Xylose | Arabinose | Acetate | Coumarate | Ferulate | YNB Oleic Acid | YNB Ricinoleic Acid | YNB Glucose | YNB Gluc DOC | YPD |
| --- | --- | --- | --- | --- | --- | --- | --- | --- | --- | --- | --- |
| proteinId |  |  |  |  |  |  |  |  |  |  |  |
| 9334 | -0.054738 | -0.115217 | -0.158594 | 0.0709991 | -0.514091 | -0.15432 | -0.0958847 | 0.106688 | -0.283772 | -0.107636 | 0.0440375 |
| 9855 | -0.127481 | 0.180375 | 0.178513 | 0.249167 | 1.66084 | 0.563794 | 0.0563758 | 0.586144 | 0.594192 | 0.61092 | -0.179258 |
| 11486 | -0.105912 | -0.0575314 | -0.199827 | -0.0594206 | 0.190392 | -0.00969138 | -0.0508443 | -0.138312 | -0.210517 | -0.183923 | -0.0170305 |
| 11945 | -0.475597 | -0.0471994 | -0.467938 | 0.159971 | 0.078547 | -0.217404 | -0.410717 | 0.0768835 | -0.145783 | 0.330613 | 0.626294 |
| 13380 | 0.212952 | 0.48865 | 0.134225 | 0.480758 | -0.595638 | 0.105697 | -0.289997 | -0.915948 | 0.122397 | -0.08248 | -0.750243 |
| 15848 | -1.02181 | -0.967317 | -1.48895 | -0.786704 | -0.959296 | -1.31833 | -0.0313898 | 0.319973 | 0.277672 | 0.15802 | 0.360635 |

In [56]:

```
for x in temp:
    if x in model.genes:
        for r in sorted(model.genes.get_by_id(x).reactions, key=lambda x: x.id):
            print(r, r.gene_reaction_rule)
    else:
        print(x, 'no reactions')
    print()
```

```
HISTRS: atp_c + his__L_c + trnahis_c --> amp_c + histrna_c + ppi_c 8575
HISTRSm: atp_m + his__L_m + trnahis_m --> amp_m + histrna_m + ppi_m 8575

ASNTRS: asn__L_c + atp_c + trnaasn_c --> amp_c + asntrna_c + ppi_c 9167

9334 no reactions

CYSTRS: atp_c + cys__L_c + trnacys_c --> amp_c + cystrna_c + ppi_c 9855

ARGTRS: arg__L_c + atp_c + trnaarg_c --> amp_c + argtrna_c + ppi_c 10079
ARGTRSm: arg__L_m + atp_m + trnaarg_m --> amp_m + argtrna_m + ppi_m 10079

LEUTRS: atp_c + leu__L_c + trnaleu_c --> amp_c + leutrna_c + ppi_c 10144
LEUTRSm: atp_m + leu__L_m + trnaleu_m --> amp_m + leutrna_m + ppi_m 10144

SECYSTL: atp_m + ser__L_m + trnasecys_m --> amp_m + ppi_m + sertrna_sec_m 10246 or 14225
SERTRS: atp_c + ser__L_c + trnaser_c --> amp_c + ppi_c + sertrna_c 10246 or 14225

GLUTRS: atp_c + glu__L_c + trnaglu_c --> amp_c + glutrna_c + ppi_c 10753 or 14565

PROTRS: atp_c + pro__L_c + trnapro_c --> amp_c + ppi_c + protrna_c 11486

AP4AS: 2.0 atp_c + h_c --> ap4a_c + ppi_c 11945
LYSTRS: atp_c + lys__L_c + trnalys_c --> amp_c + lystrna_c + ppi_c 11945
LYSTRSm: atp_m + lys__L_m + trnalys_m --> amp_m + lystrna_m + ppi_m 11945

ALATL: ala__L_m + atp_m + trnaala_m --> alatrna_m + amp_m + ppi_m 11973
ALATRS: ala__L_c + atp_c + trnaala_c --> alatrna_c + amp_c + ppi_c 11973

ASNTRSm: asn__L_m + atp_m + trnaasn_m --> amp_m + asntrna_m + ppi_m 12123

PHETRS: atp_c + phe__L_c + trnaphe_c --> amp_c + phetrna_c + ppi_c 12470 and 13249

TYRTRS: atp_c + trnatyr_c + tyr__L_c --> amp_c + ppi_c + tyrtrna_c 13137 or 16641
TYRTRSm: atp_m + trnatyr_m + tyr__L_m --> amp_m + ppi_m + tyrtrna_m 13137

METTRS: atp_c + met__L_c + trnamet_c --> amp_c + mettrna_c + ppi_c 13140 or 15518

PHETRS: atp_c + phe__L_c + trnaphe_c --> amp_c + phetrna_c + ppi_c 12470 and 13249

GLNTRS: atp_c + gln__L_c + trnagln_c --> amp_c + glntrna_c + ppi_c 13380

PHETRSm: atp_m + phe__L_m + trnaphe_m --> amp_m + phetrna_m + ppi_m 13398

ILETRS: atp_c + ile__L_c + trnaile_c --> amp_c + iletrna_c + ppi_c 13446

SECYSTL: atp_m + ser__L_m + trnasecys_m --> amp_m + ppi_m + sertrna_sec_m 10246 or 14225
SERTRS: atp_c + ser__L_c + trnaser_c --> amp_c + ppi_c + sertrna_c 10246 or 14225

ASPTRS: asp__L_c + atp_c + trnaasp_c --> amp_c + asptrna_c + ppi_c 14258
ASPTRSm: asp__L_m + atp_m + trnaasp_m --> amp_m + asptrna_m + ppi_m 14258

GLUTRS: atp_c + glu__L_c + trnaglu_c --> amp_c + glutrna_c + ppi_c 10753 or 14565
GLUTRSm: atp_m + glu__L_m + trnaglu_m --> amp_m + glutrna_m + ppi_m 14565

TRPTRS: atp_c + trnatrp_c + trp__L_c --> amp_c + ppi_c + trptrna_c 14709 or 15533

GLYTLm: atp_m + gly_m + trnagly_m --> amp_m + glytrna_m + ppi_m 15504
GLYTRS: atp_c + gly_c + trnagly_c --> amp_c + glytrna_c + ppi_c 15504

METTRS: atp_c + met__L_c + trnamet_c --> amp_c + mettrna_c + ppi_c 13140 or 15518
METTRSm: atp_m + met__L_m + trnamet_m --> amp_m + mettrna_m + ppi_m 15518

TRPTRS: atp_c + trnatrp_c + trp__L_c --> amp_c + ppi_c + trptrna_c 14709 or 15533
TRPTRSm: atp_m + trnatrp_m + trp__L_m --> amp_m + ppi_m + trptrna_m 15533

ILETRSm: atp_m + ile__L_m + trnaile_m --> amp_m + iletrna_m + ppi_m 15556

THRTRS: atp_c + thr__L_c + trnathr_c --> amp_c + ppi_c + thrtrna_c 15848
THRTRSm: atp_m + thr__L_m + trnathr_m --> amp_m + ppi_m + thrtrna_m 15848

VALTRS: atp_c + trnaval_c + val__L_c --> amp_c + ppi_c + valtrna_c 16528
VALTRSm: atp_m + trnaval_m + val__L_m --> amp_m + ppi_m + valtrna_m 16528

TYRTRS: atp_c + trnatyr_c + tyr__L_c --> amp_c + ppi_c + tyrtrna_c 13137 or 16641
```

In [57]:

```
for m in sorted(model.metabolites, key=lambda x: x.id):
    if m.id.endswith('trna_c'):
        for r in sorted(m.reactions, key=lambda x: x.id):
            print(r, r.gene_reaction_rule)
        if m.id.replace('_c','_m') in model.metabolites:
            for r in sorted(model.metabolites.get_by_id(m.id.replace('_c','_m')).reactions, key=lambda x: x.id):
                print(r, r.gene_reaction_rule)
        print()
```

```
ALATRS: ala__L_c + atp_c + trnaala_c --> alatrna_c + amp_c + ppi_c 11973
ALATL: ala__L_m + atp_m + trnaala_m --> alatrna_m + amp_m + ppi_m 11973

ARGTRS: arg__L_c + atp_c + trnaarg_c --> amp_c + argtrna_c + ppi_c 10079
ARGTRSm: arg__L_m + atp_m + trnaarg_m --> amp_m + argtrna_m + ppi_m 10079

ASNTRS: asn__L_c + atp_c + trnaasn_c --> amp_c + asntrna_c + ppi_c 9167
ASNTRSm: asn__L_m + atp_m + trnaasn_m --> amp_m + asntrna_m + ppi_m 12123

ASPTRS: asp__L_c + atp_c + trnaasp_c --> amp_c + asptrna_c + ppi_c 14258
ASPTRSm: asp__L_m + atp_m + trnaasp_m --> amp_m + asptrna_m + ppi_m 14258

CYSTRS: atp_c + cys__L_c + trnacys_c --> amp_c + cystrna_c + ppi_c 9855

FMETTRS: 10fthf_c + mettrna_c --> fmettrna_c + h_c + thf_c 11899
FMETTRSm: 10fthf_m + mettrna_m --> fmettrna_m + h_m + thf_m 11899

GLNTRS: atp_c + gln__L_c + trnagln_c --> amp_c + glntrna_c + ppi_c 13380

GLUTRS: atp_c + glu__L_c + trnaglu_c --> amp_c + glutrna_c + ppi_c 10753 or 14565
GLUTRSm: atp_m + glu__L_m + trnaglu_m --> amp_m + glutrna_m + ppi_m 14565

GLYTRS: atp_c + gly_c + trnagly_c --> amp_c + glytrna_c + ppi_c 15504
GLYTLm: atp_m + gly_m + trnagly_m --> amp_m + glytrna_m + ppi_m 15504

HISTRS: atp_c + his__L_c + trnahis_c --> amp_c + histrna_c + ppi_c 8575
HISTRSm: atp_m + his__L_m + trnahis_m --> amp_m + histrna_m + ppi_m 8575

ILETRS: atp_c + ile__L_c + trnaile_c --> amp_c + iletrna_c + ppi_c 13446
ILETRSm: atp_m + ile__L_m + trnaile_m --> amp_m + iletrna_m + ppi_m 15556

LEUTRS: atp_c + leu__L_c + trnaleu_c --> amp_c + leutrna_c + ppi_c 10144
LEUTRSm: atp_m + leu__L_m + trnaleu_m --> amp_m + leutrna_m + ppi_m 10144

LYSTRS: atp_c + lys__L_c + trnalys_c --> amp_c + lystrna_c + ppi_c 11945
LYSTRSm: atp_m + lys__L_m + trnalys_m --> amp_m + lystrna_m + ppi_m 11945

FMETTRS: 10fthf_c + mettrna_c --> fmettrna_c + h_c + thf_c 11899
METTRS: atp_c + met__L_c + trnamet_c --> amp_c + mettrna_c + ppi_c 13140 or 15518
FMETTRSm: 10fthf_m + mettrna_m --> fmettrna_m + h_m + thf_m 11899
METTRSm: atp_m + met__L_m + trnamet_m --> amp_m + mettrna_m + ppi_m 15518

PHETRS: atp_c + phe__L_c + trnaphe_c --> amp_c + phetrna_c + ppi_c 12470 and 13249
PHETRSm: atp_m + phe__L_m + trnaphe_m --> amp_m + phetrna_m + ppi_m 13398

PROTRS: atp_c + pro__L_c + trnapro_c --> amp_c + ppi_c + protrna_c 11486

SERTRS: atp_c + ser__L_c + trnaser_c --> amp_c + ppi_c + sertrna_c 10246 or 14225

THRTRS: atp_c + thr__L_c + trnathr_c --> amp_c + ppi_c + thrtrna_c 15848
THRTRSm: atp_m + thr__L_m + trnathr_m --> amp_m + ppi_m + thrtrna_m 15848

TRPTRS: atp_c + trnatrp_c + trp__L_c --> amp_c + ppi_c + trptrna_c 14709 or 15533
TRPTRSm: atp_m + trnatrp_m + trp__L_m --> amp_m + ppi_m + trptrna_m 15533

TYRTRS: atp_c + trnatyr_c + tyr__L_c --> amp_c + ppi_c + tyrtrna_c 13137 or 16641
TYRTRSm: atp_m + trnatyr_m + tyr__L_m --> amp_m + ppi_m + tyrtrna_m 13137

VALTRS: atp_c + trnaval_c + val__L_c --> amp_c + ppi_c + valtrna_c 16528
VALTRSm: atp_m + trnaval_m + val__L_m --> amp_m + ppi_m + valtrna_m 16528
```

In [58]:

```
for r in sorted(model.metabolites.get_by_id('mettrna_c').reactions, key=lambda x: x.id):
    print(r, r.gene_reaction_rule)
print()
for r in sorted(model.metabolites.get_by_id('mettrna_m').reactions, key=lambda x: x.id):
    print(r, r.gene_reaction_rule)
```

```
FMETTRS: 10fthf_c + mettrna_c --> fmettrna_c + h_c + thf_c 11899
METTRS: atp_c + met__L_c + trnamet_c --> amp_c + mettrna_c + ppi_c 13140 or 15518

FMETTRSm: 10fthf_m + mettrna_m --> fmettrna_m + h_m + thf_m 11899
METTRSm: atp_m + met__L_m + trnamet_m --> amp_m + mettrna_m + ppi_m 15518
```

In [59]:

```
model.reactions.get_by_id('GLUTRS').gene_reaction_rule = '10753'
model.reactions.get_by_id('METTRS').gene_reaction_rule = '13140'
model.reactions.get_by_id('SERTRS').gene_reaction_rule = '10246'
model.reactions.get_by_id('TRPTRS').gene_reaction_rule = '14709'
model.reactions.get_by_id('TYRTRS').gene_reaction_rule = '16641'

# Make mito seryl-tRNA synthetase by 14225
r = model.reactions.get_by_id('SERTRS').copy()
r.id = 'SERTRSm'
r.gene_reaction_rule = '14225'
model.add_reactions([r])
for m in r.metabolites:
    if not m.id.replace('_c','_m') in model.metabolites:
        m2 = m.copy()
        m2.id = m.id.replace('_c','_m')
        m2.compartment = 'm'
        model.add_metabolites([m2])
    r.add_metabolites({m.id: -r.get_coefficient(m.id), m.id.replace('_c','_m'): r.get_coefficient(m.id)})

# AP4AS by E. coli lysU, but not by KRS1 -> remove AP4AS
# 11973 cyto, no ALATL in iMM904 -> remove ALATL
# 11899 mito -> remove FMETTRS
# no selenocysteine synthase in annotation -> remove SECYSTL
model.remove_reactions(['AP4AS','ALATL','FMETTRS','SECYSTL'], remove_orphans=True)
```

In [60]:

```
# check if model can produce cofactors
cofactors = ['camp_c','coa_c','fad_c','gthrd_c','hemeA_m','nad_c','nadp_c','q9_m','thf_c','thmpp_c',
             '5mthf_c','btn_m','lipopb_m','mlthf_c','ptrc_c','pydx5p_c','spmd_c']
with model:
    for x in cofactors:
        model.reactions.get_by_id('BIOMASS_RT').add_metabolites({x: -1e-2})
        sol = model.optimize()
        print(x, sol.objective_value)
        if abs(sol.objective_value) < 1e-6:
            for k, v in sol.shadow_prices.items():
                #if v and k in [m.id for m in model.reactions.get_by_id('BIOMASS_RT').metabolites]:
                if abs(v) > 1:
                    print('\t',k, v)
        model.reactions.get_by_id('BIOMASS_RT').add_metabolites({x: 1e-2})
```

```
camp_c 0.13593743393234173
coa_c 0.13547854502629292
fad_c 0.13588334572424138
gthrd_c 0.13632034044837502
hemeA_m 0.13525177641252495
nad_c 0.13561558122627657
nadp_c 0.13560034129332058
q9_m 0.13393271057829698
thf_c 0.13597215470565893
thmpp_c 0.13561761347616552
5mthf_c 0.13577122443665735
btn_m 0.0
	 adp_m 100.0
	 atp_m 100.0
	 amp_m 100.0
	 fad_m 100.0
	 fadh2_m 100.0
	 met__L_m 100.0
	 mettrna_m 100.0
	 fmettrna_m 100.0
	 nmn_m -100.0
	 dnad_m 100.0
	 btn_m -100.0
	 lipoamp_m -100.0
	 lipopb_m -200.0
	 lipoate_m -200.0
lipopb_m 0.0
	 adp_m 49.99999999999999
	 atp_m 49.99999999999999
	 amp_m 49.99999999999999
	 fad_m 49.99999999999999
	 fadh2_m 49.99999999999999
	 met__L_m 49.99999999999999
	 mettrna_m 49.99999999999999
	 fmettrna_m 49.99999999999999
	 nmn_m -49.99999999999999
	 dnad_m 49.99999999999999
	 btn_m -49.99999999999999
	 lipoamp_m -49.99999999999999
	 lipopb_m -99.99999999999999
	 lipoate_m -99.99999999999999
mlthf_c 0.13575594950448788
ptrc_c 0.13650642647368216
pydx5p_c 0.1365311390189492
spmd_c 0.13657749916637818
```

In [61]:

```
for r in sorted(model.metabolites.get_by_id('dann_m').reactions, key=lambda x: x.id):
    print(r, r.gene_reaction_rule)
for r in sorted(model.metabolites.get_by_id('btn_m').reactions, key=lambda x: x.id):
    print(r, r.gene_reaction_rule)
print()
for r in sorted(model.metabolites.get_by_id('lipoamp_m').reactions, key=lambda x: x.id):
    print(r, r.gene_reaction_rule)
```

```
AMAOTrm: 8aonn_m + amet_m <=> amob_m + dann_m 12731
DBTSm: atp_m + co2_m + dann_m <=> adp_m + dtbt_m + 3.0 h_m + pi_m 12731
BACCLm: atp_m + btn_m + h_m --> btamp_m + ppi_m 16404
BTS5m: 2fe2s_m + amet_m + dtbt_m --> 2fe1s_m + btn_m + dad_5_m + h_m + met__L_m 15908

LIPAMPLm: lipoamp_m --> amp_m + lipopb_m 13899
LIPATPTm: atp_m + lipoate_m --> lipoamp_m + ppi_m 13899
```

In [62]:

```
temp = ['12731','15908','16404','13899']
display(Annotation.loc[temp])
Show_Data(temp)
```

|  | Combined Annotations | Signal P | Sc288c Orthologs | Human Orthologs | Sc288 Best Hit | Human Blast | Essential | WolfPSort | C Terminal |
| --- | --- | --- | --- | --- | --- | --- | --- | --- | --- |
| RTO4\_ID |  |  |  |  |  |  |  |  |  |
| 12731 | K19562: BIO3-BIO1; bifunctional dethiobiotin s... |  |  |  | BIO3 |  | Essential | mito 11, extr 10, cyto 3, pero 2 | GAS\* |
| 15908 | K01012: bioB; biotin synthase |  | BIO2 |  | BIO2 |  | Essential | mito 26.5, cyto\_mito 14 | VAA\* |
| 16404 | K01942: HLCS; biotin--protein ligase |  | BPL1 | HLCS | BPL1 | HLCS | Essential | extr 12, cyto 6, cyto\_nucl 5.5, mito 4, nucl 3 | KSG\* |
| 13899 | K03800: lplA, lplJ; lipoate---protein ligase |  | AIM22 | LIPT1 | AIM22 | LIPT1 | Essential | mito 20, nucl 4, cyto 1, plas 1, pero 1, cyto\_... | GEM\* |

| strain | WT | | | | | | | | | | | | | | | | |
| --- | --- | --- | --- | --- | --- | --- | --- | --- | --- | --- | --- | --- | --- | --- | --- | --- | --- |
| condition | G\_MM | C\_MM | G\_SD | | GX\_SD | | | X\_SD | | A\_SD | | C\_SD | | MM\_CN120 | | MM\_CN5 | Diversity\_Sample |
| phase | exp | exp | exp | stat | exp | trans | stat | exp | stat | exp | stat | exp | stat | exp | stat | exp | exp |
| proteinId | Set1 | Set1 | Set2 | Set2 | Set2 | Set2 | Set2 | Set2 | Set2 | Set2 | Set2 | Set2 | Set2 | Set3 | Set3 | Set3 | Set3 |
| 12731 | 6.02185 | 4.60555 | 6.45171 | 5.35008 | 6.39028 | 6.2194 | 5.81525 | 6.0943 | 4.79923 | 4.92084 | 4.63383 | 4.19327 | 3.7021 | 7.16018 | 6.81399 | 7.2256 | 6.71275 |
| 15908 | 7.52668 | 7.0794 | 6.1455 | 4.45929 | 6.24934 | 4.98317 | 4.78694 | 6.18894 | 4.23801 | 5.67966 | 4.58943 | 5.42106 | 6.46046 | 7.44985 | 6.84422 | 7.47498 | 6.98186 |
| 16404 | 5.14898 | 5.19209 | 5.68806 | 4.45038 | 5.78277 | 5.12319 | 4.79587 | 5.48776 | 4.40562 | 5.33835 | 4.57964 | 4.22056 | 4.62725 | 5.50854 | 5.4644 | 6.08156 | 6.255 |
| 13899 | 6.13776 | 5.38147 | 5.93976 | 7.0956 | 5.87833 | 6.48813 | 6.6631 | 6.19008 | 6.10676 | 6.23258 | 6.50523 | 5.72275 | 4.91908 | 4.86625 | 4.95779 | 4.72677 | 3.95335 |

| strain | WT | | | | | | | | | | |
| --- | --- | --- | --- | --- | --- | --- | --- | --- | --- | --- | --- |
| condition | G\_SD | | GX\_SD | | | X\_SD | | A\_SD | | C\_SD | |
| proteinId | exp | stat | exp | trans | stat | exp | stat | exp | stat | exp | stat |
| 12731 | 10.3494 | 10.6314 | 10.7826 | 13.9404 | 14.2423 | 9.78049 | 10.1996 | 5.98241 | 7.46515 | 8.755 | 7.20604 |
| 15908 | 4.95587 | 0.18528 | 3.66997 | 0.187227 | 0 | 0.795632 | 0 | 0 | 0 | 1.91908 | 0.666647 |
| 16404 | 0.579561 | 0 | 1.42376 | 0.185549 | 0.173732 | 0.200926 | 0.189187 | 0.579683 | 0.19525 | 0 | 0 |
| 13899 | 0.587987 | 1.08393 | 1.22078 | 1.30235 | 1.09591 | 1.17479 | 1.18215 | 0.775498 | 1.14892 | 0.208996 | 0.42946 |

|  | Glucose | Xylose | Arabinose | Acetate | Coumarate | Ferulate | YNB Oleic Acid | YNB Ricinoleic Acid | YNB Glucose | YNB Gluc DOC | YPD |
| --- | --- | --- | --- | --- | --- | --- | --- | --- | --- | --- | --- |
| proteinId |  |  |  |  |  |  |  |  |  |  |  |
| 16404 | 0.224466 | -0.772008 | -0.234875 | -0.5554 | -0.0770478 | 0.701184 | 0.14778 | -0.208173 | -1.19924 | -0.980346 | 0.058461 |

In [63]:

```
cofactors = ['btn_m','lipopb_m']

with model:
    r = model.reactions.get_by_id('DM_aacald_c').copy()
    r.id = 'SRC_8aonn_m'
    r.name = 'Source needed to allow 8-amino-7-oxononanoate to enter system'
    model.add_reactions([r])
    r.add_metabolites({'aacald_c': 1.0, '8aonn_m': 1.0})

    r = model.reactions.get_by_id('DM_aacald_c').copy()
    r.id = 'DM_amob_m'
    r.name = 'Demand needed to allow S-Adenosyl-4-methylthio-2-oxobutanoate to leave system'
    model.add_reactions([r])
    r.add_metabolites({'aacald_c': 1.0, 'amob_m': -1.0})

    r = model.reactions.get_by_id('DM_aacald_c').copy()
    r.id = 'DM_dad_5_m'
    r.name = 'Demand needed to allow 5-Deoxyadenosine to leave system'
    model.add_reactions([r])
    r.add_metabolites({'aacald_c': 1.0, 'dad_5_m': -1.0})
    
    r1 = hsa2.reactions.get_by_id('METtm').copy()
    r1.lower_bound = -1000.0
    r2 = hsa2.reactions.get_by_id('r1437').copy()
    r2.id = 'CYStm'
    r2.lower_bound = -1000.0
    model.add_reactions([r1,r2])

    r = model.reactions.get_by_id('DM_aacald_c').copy()
    r.id = 'SRC_lipoate_m'
    r.name = 'Source needed to allow lipoate to enter system'
    model.add_reactions([r])
    r.add_metabolites({'aacald_c': 1.0, 'lipoate_m': 1.0})

    for x in cofactors:
        model.reactions.get_by_id('BIOMASS_RT').add_metabolites({x: -1e-2})
        sol = model.optimize()
        print(x, sol.objective_value)
        if abs(sol.objective_value) < 1e-6:
            for k, v in sol.shadow_prices.items():
                #if v and k in [m.id for m in model.reactions.get_by_id('BIOMASS_RT').metabolites]:
                if abs(v) > 1:
                    print('\t',k, v)
        model.reactions.get_by_id('BIOMASS_RT').add_metabolites({x: 1e-2})
```

```
btn_m 0.13491103213722255
lipopb_m 0.13655173964275558
```

In [64]:

```
r = model.reactions.get_by_id('DM_aacald_c').copy()
r.id = 'SRC_8aonn_m'
r.name = 'Source needed to allow 8-amino-7-oxononanoate to enter system'
model.add_reactions([r])
r.add_metabolites({'aacald_c': 1.0, '8aonn_m': 1.0})

r = model.reactions.get_by_id('DM_aacald_c').copy()
r.id = 'DM_amob_m'
r.name = 'Demand needed to allow S-Adenosyl-4-methylthio-2-oxobutanoate to leave system'
model.add_reactions([r])
r.add_metabolites({'aacald_c': 1.0, 'amob_m': -1.0})

r = model.reactions.get_by_id('DM_aacald_c').copy()
r.id = 'DM_dad_5_m'
r.name = 'Demand needed to allow 5-Deoxyadenosine to leave system'
model.add_reactions([r])
r.add_metabolites({'aacald_c': 1.0, 'dad_5_m': -1.0})

r1 = hsa2.reactions.get_by_id('METtm').copy()
r1.lower_bound = -1000.0
r2 = hsa2.reactions.get_by_id('r1437').copy()
r2.id = 'CYStm'
r2.lower_bound = -1000.0
model.add_reactions([r1,r2])

r = model.reactions.get_by_id('DM_aacald_c').copy()
r.id = 'SRC_lipoate_m'
r.name = 'Source needed to allow lipoate to enter system'
model.add_reactions([r])
r.add_metabolites({'aacald_c': 1.0, 'lipoate_m': 1.0})
```

In [65]:

```
# check if model can produce cofactors
cofactors = ['camp_c','coa_c','fad_c','gthrd_c','hemeA_m','nad_c','nadp_c','q9_m','thf_c','thmpp_c',
             '5mthf_c','btn_m','lipopb_m','mlthf_c','ptrc_c','pydx5p_c','spmd_c']
with model:
    for x in cofactors:
        model.reactions.get_by_id('BIOMASS_RT').add_metabolites({x: -1e-2})
        sol = model.optimize()
        print(x, sol.objective_value)
        if abs(sol.objective_value) < 1e-6:
            for k, v in sol.shadow_prices.items():
                #if v and k in [m.id for m in model.reactions.get_by_id('BIOMASS_RT').metabolites]:
                if abs(v) > 1:
                    print('\t',k, v)
        model.reactions.get_by_id('BIOMASS_RT').add_metabolites({x: 1e-2})
```

```
camp_c 0.13593743393234295
coa_c 0.13547854502629367
fad_c 0.1358833457242399
gthrd_c 0.1363203404483766
hemeA_m 0.13525177641251918
nad_c 0.13561558122627665
nadp_c 0.13560034129332127
q9_m 0.1339327105783163
thf_c 0.13597215470565932
thmpp_c 0.13561761347616408
5mthf_c 0.13577122443665915
btn_m 0.1349110321372241
lipopb_m 0.13655173964275663
mlthf_c 0.13575594950448663
ptrc_c 0.1365064264736743
pydx5p_c 0.1365311390189447
spmd_c 0.13657749916638584
```

In [66]:

```
cofactors = ['camp_c','coa_c','fad_c','gthrd_c','hemeA_m','nad_c','nadp_c','q9_m','thf_c','thmpp_c',
             '5mthf_c','btn_m','lipopb_m','mlthf_c','ptrc_c','pydx5p_c','spmd_c']
[x for x in cofactors if x in [m.id for m in model.reactions.get_by_id('BIOMASS_RT').metabolites]]
```

Out[66]:

```
[]
```

In [67]:

```
for x in cofactors:
    model.reactions.get_by_id('BIOMASS_RT').add_metabolites({x: -1e-6})
```

In [68]:

```
sol = model.optimize()
print(sol.objective_value)
```

```
0.13657612542330205
```

In [69]:

```
print(len(model.genes))
print(len(model.reactions))
print(len(model.metabolites))
model
```

```
1127
2117
1956
```

Out[69]:

|  |  |
| --- | --- |
| **Name** | R. toruloides |
| **Memory address** | 0x0102adc2e10 |
| **Number of metabolites** | 1956 |
| **Number of reactions** | 2117 |
| **Number of groups** | 0 |
| **Objective expression** | 1.0\*BIOMASS\_RT - 1.0\*BIOMASS\_RT\_reverse\_2b3e0 |
| **Compartments** | c, x, m, e, r, v, n, g, d |

In [70]:

```
for x in sorted(model.genes, key=lambda x: x.id):
    if not x.reactions:
        print(x)
print()
for x in sorted(model.metabolites, key=lambda x: x.id):
    if not x.reactions:
        print(x)
```

```
10885
12640
14615
14700
14875
15434
8666

hdc2ea_c
hxdcal_c
hxdceal_c
pristanal_c
urcan_c
```

In [71]:

```
cobra.manipulation.remove_genes(model, [x for x in model.genes if not x.reactions])
model.remove_metabolites([x for x in model.metabolites if not x.reactions])
```

In [72]:

```
print(len(model.genes))
print(len(model.reactions))
print(len(model.metabolites))
print(len(set([m.id.rsplit('_',1)[0] for m in model.metabolites])))
print(len(model.compartments))
model
```

```
1120
2117
1951
1227
9
```

Out[72]:

|  |  |
| --- | --- |
| **Name** | R. toruloides |
| **Memory address** | 0x0102adc2e10 |
| **Number of metabolites** | 1951 |
| **Number of reactions** | 2117 |
| **Number of groups** | 0 |
| **Objective expression** | 1.0\*BIOMASS\_RT - 1.0\*BIOMASS\_RT\_reverse\_2b3e0 |
| **Compartments** | c, x, m, e, r, v, n, g, d |

In [73]:

```
cobra.io.save_json_model(model, "IFO0880_GPR_2b.json")
```

In [74]:

```
model_old = cobra.io.load_json_model("IFO0880_GPR_2a.json")
model_new = cobra.io.load_json_model("IFO0880_GPR_2b.json")
```

In [75]:

```
print('Removed reactions\n')
for r in sorted(model_old.reactions, key=lambda x: x.id):
    if r not in model_new.reactions:
        print(r)
```

```
Removed reactions

3HPADHi: 3hppnl_c + h2o_c + nad_c --> 3hpp_c + 2.0 h_c + nadh_c
3M4HDXPAC: 3mox4hpac_c + h2o_c + nad_c <=> 2.0 h_c + homoval_c + nadh_c
3MOX4HOXPGALDOX: 3m4hpga_c + h2o_c + nad_c --> 3mox4hoxm_c + 2.0 h_c + nadh_c
3MOX4HOXPGALDOX_NADP: 3m4hpga_c + h2o_c + nadp_c <=> 3mox4hoxm_c + 2.0 h_c + nadph_c
5HOXINDACTOX: 5hoxindact_c + h2o_c + nad_c --> 5hoxindoa_c + 2.0 h_c + nadh_c
5HOXINDACTOXm: 5hoxindact_m + h2o_m + nad_m --> 5hoxindoa_m + 2.0 h_m + nadh_m
ABOR: 4abutn_m + h2o_m + nadp_m --> 4abut_m + 2.0 h_m + nadph_m
ABUTDm: 4abutn_m + h2o_m + nad_m --> 4abut_m + 2.0 h_m + nadh_m
AKP1: ahdt_c + 3.0 h2o_c --> dhnpt_c + 2.0 h_c + 3.0 pi_c
ALATL: ala__L_m + atp_m + trnaala_m --> alatrna_m + amp_m + ppi_m
ALDD20xm: h2o_m + id3acald_m + nad_m --> 2.0 h_m + ind3ac_m + nadh_m
ALDD20ym: h2o_m + id3acald_m + nadp_m --> 2.0 h_m + ind3ac_m + nadph_m
ALDD21: h2o_c + nad_c + pristanal_c --> 2.0 h_c + nadh_c + prist_c
AP4AS: 2.0 atp_c + h_c --> ap4a_c + ppi_c
BAMPPALDOXm: bamppald_m + h2o_m + nad_m --> ala_B_m + 2.0 h_m + nadh_m
DHFOR: fol_c + h_c + nadh_c --> dhf_c + nad_c
DHFR2i: dhf_c + h_c + nadh_c --> nad_c + thf_c
DHNPAm: dhnpt_m --> 2ahhmp_m + gcald_m
DHPS: 2ahhmp_c + 4abz_c --> dhpt_c + h2o_c
DHPSm: 2ahhmp_m + 4abz_m --> dhpt_m + h2o_m
FMETTRS: 10fthf_c + mettrna_c --> fmettrna_c + h_c + thf_c
FOLD3: 2ahhmd_c + 4abz_c --> dhpt_c + ppi_c
FOLD3m: 2ahhmd_m + 4abz_m --> dhpt_m + ppi_m
GCALDDm: gcald_m + h2o_m + nad_m --> glyclt_m + 2.0 h_m + nadh_m
GDBTALDH: gdbtal_c + h2o_c + nad_c --> 4gudbutn_c + 2.0 h_c + nadh_c
GGGABADr: ggbutal_c + h2o_c + nadp_c <=> gg4abut_c + 2.0 h_c + nadph_c
GLACO: glac_c + 2.0 h2o_c + nad_c --> glcr_c + 3.0 h_c + nadh_c
GLACOm: glac_m + 2.0 h2o_m + nad_m --> glcr_m + 3.0 h_m + nadh_m
HISDr: his__L_c --> nh4_c + urcan_c
HPPKm: 2ahhmp_m + atp_m --> 2ahhmd_m + amp_m + h_m
IMACTD_m: h2o_m + im4act_m + nad_m --> 2.0 h_m + im4ac_m + nadh_m
NABTNO: h2o_c + n4abutn_c + nad_c --> 4aabutn_c + 2.0 h_c + nadh_c
NABTNOm: h2o_m + n4abutn_m + nad_m --> 4aabutn_m + 2.0 h_m + nadh_m
NADDPp: h2o_x + nad_x --> amp_x + 2.0 h_x + nmn_x
PPND: nad_c + pphn_c --> 34hpp_c + co2_c + nadh_c
PYLALDOX: h2o_c + nad_c + pylald_c --> 2.0 h_c + nadh_c + peracd_c
PYLALDOXm: h2o_m + nad_m + pylald_m --> 2.0 h_m + nadh_m + peracd_m
SECYSTL: atp_m + ser__L_m + trnasecys_m --> amp_m + ppi_m + sertrna_sec_m
THFOR1: fol_c + 2.0 h_c + 2.0 nadh_c --> 2.0 nad_c + thf_c
THFOR2: fol_c + 2.0 h_c + 2.0 nadph_c --> 2.0 nadp_c + thf_c
THMDPe: 2.0 h2o_e + thmpp_e --> h_e + 2.0 pi_e + thm_e
TMDPPK: atp_c + thmpp_c --> adp_c + thmtp_c
```

In [76]:

```
print('Updated reactions\n')
for r in sorted(model_old.reactions, key=lambda x: x.id):
    if r in model_new.reactions:
        r2 = model_new.reactions.get_by_id(r.id)
        if (r.name == r2.name and r.reaction == r2.reaction and r.gene_reaction_rule == r2.gene_reaction_rule and
            r.lower_bound == r2.lower_bound and r.upper_bound == r2.upper_bound):
            pass
        else:
            print('Old', r, r.gene_reaction_rule)
            print('New', r2, r2.gene_reaction_rule)
            print()
```

```
Updated reactions

Old 2DOXG6PP: 2doxg6p_c + h2o_c --> 2dglc_c + pi_c 13413 or 8460 or 8576
New 2DOXG6PP: 2doxg6p_c + h2o_c --> 2dglc_c + pi_c 13413

Old 34DHALDD: 34dhpac_c + h2o_c + nad_c --> 34dhpha_c + 2.0 h_c + nadh_c 12042 or 13426 or 16323
New 34DHALDD: 34dhpac_c + h2o_c + nad_c --> 34dhpha_c + 2.0 h_c + nadh_c 12042

Old 34DHPLACOX_NADP: 34dhpac_c + h2o_c + nadp_c <=> 34dhpha_c + 2.0 h_c + nadph_c 12042 or 13426 or 16323
New 34DHPLACOX_NADP: 34dhpac_c + h2o_c + nadp_c <=> 34dhpha_c + 2.0 h_c + nadph_c 12042

Old 4HOXPACDOX_NADP: 4hoxpacd_c + h2o_c + nadp_c <=> 4hphac_c + 2.0 h_c + nadph_c 12042 or 13426 or 16323
New 4HOXPACDOX_NADP: 4hoxpacd_c + h2o_c + nadp_c <=> 4hphac_c + 2.0 h_c + nadph_c 12042

Old ABUTD: 4abutn_c + h2o_c + nad_c --> 4abut_c + 2.0 h_c + nadh_c 12042 or 13426 or 16323
New ABUTD: 4abutn_c + h2o_c + nad_c --> 4abut_c + 2.0 h_c + nadh_c 12042

Old ACP1e: fmn_e + h2o_e --> pi_e + ribflv_e 10885 or 13856 or 13935
New ACP1e: fmn_e + h2o_e --> pi_e + ribflv_e 13856

Old ALDD19x_P: h2o_c + nadp_c + pacald_c --> 2.0 h_c + nadph_c + pac_c 12042 or 13426 or 16323
New ALDD19x_P: h2o_c + nadp_c + pacald_c --> 2.0 h_c + nadph_c + pac_c 12042

Old ALDD19xr: h2o_c + nad_c + pacald_c <=> 2.0 h_c + nadh_c + pac_c 12042 or 13426 or 16323
New ALDD19xr: h2o_c + nad_c + pacald_c <=> 2.0 h_c + nadh_c + pac_c 12042

Old ALDD20x: h2o_c + id3acald_c + nad_c --> 2.0 h_c + ind3ac_c + nadh_c 12042 or 13426 or 16323
New ALDD20x: h2o_c + id3acald_c + nad_c --> 2.0 h_c + ind3ac_c + nadh_c 12042

Old ALDD20y: h2o_c + id3acald_c + nadp_c --> 2.0 h_c + ind3ac_c + nadph_c 12042 or 13426
New ALDD20y: h2o_c + id3acald_c + nadp_c --> 2.0 h_c + ind3ac_c + nadph_c 12042

Old ALDD2x: acald_c + h2o_c + nad_c --> ac_c + 2.0 h_c + nadh_c 12042 or 13426 or 16323
New ALDD2x: acald_c + h2o_c + nad_c --> ac_c + 2.0 h_c + nadh_c 12042

Old ALDD2xm: acald_m + h2o_m + nad_m --> ac_m + 2.0 h_m + nadh_m 12042 or 13426 or 16323
New ALDD2xm: acald_m + h2o_m + nad_m --> ac_m + 2.0 h_m + nadh_m 13426

Old ALDD2y: acald_c + h2o_c + nadp_c --> ac_c + 2.0 h_c + nadph_c 11650 or 12042 or 13426 or 14700 or 16323 or 8666
New ALDD2y: acald_c + h2o_c + nadp_c --> ac_c + 2.0 h_c + nadph_c 12042

Old ALDD2ym: acald_m + h2o_m + nadp_m --> ac_m + 2.0 h_m + nadph_m 12042 or 13426
New ALDD2ym: acald_m + h2o_m + nadp_m --> ac_m + 2.0 h_m + nadph_m 13426

Old ALKP: dhap_c + h2o_c --> dha_c + pi_c 13409 or 14546
New ALKP: dhap_c + h2o_c --> dha_c + pi_c 13409

Old ALLTNti: alltn_e --> alltn_c 10921 or 11871 or 12902
New ALLTNti: alltn_e --> alltn_c 9324 or 10921 or 11871 or 12902

Old AM6SAD: am6sa_c + h2o_c + nad_c --> amuco_c + 2.0 h_c + nadh_c 12042 or 13426
New AM6SAD: am6sa_c + h2o_c + nad_c --> amuco_c + 2.0 h_c + nadh_c 12042

Old AMID: 4gudbd_c + h2o_c --> 4gudbutn_c + nh4_c 10276 or 10277 or 12540 or 12553 or 12640 or 12842
New AMID: 4gudbd_c + h2o_c --> 4gudbutn_c + nh4_c 10276 or 10277 or 12540 or 12553 or 12842 or 13791

Old AMID2: h2o_c + pad_c --> nh4_c + pac_c 10276 or 10277 or 12161 or 12540 or 12553 or 12842 or 13791
New AMID2: h2o_c + pad_c --> nh4_c + pac_c 10276 or 10277 or 12540 or 12553 or 12842 or 13791

Old AMID3: h2o_c + iad_c --> ind3ac_c + nh4_c 10276 or 10277 or 12161 or 12540 or 12553 or 12842 or 13791
New AMID3: h2o_c + iad_c --> ind3ac_c + nh4_c 10276 or 10277 or 12540 or 12553 or 12842 or 13791

Old AMID_1: ad_c + h2o_c --> ac_c + nh4_c 13791
New AMID_1: ad_c + h2o_c --> ac_c + nh4_c 10276 or 10277 or 12540 or 12553 or 12842 or 13791

Old BAMPPALDOX: bamppald_c + h2o_c + nad_c --> ala_B_c + 2.0 h_c + nadh_c 12042 or 13426 or 16323
New BAMPPALDOX: bamppald_c + h2o_c + nad_c --> ala_B_c + 2.0 h_c + nadh_c 12042

Old BIOMASS_RT: 1.1348 13BDglcn_c + 0.4588 ala__L_c + 0.046 amp_c + 0.1607 arg__L_c + 0.1017 asn__L_c + 0.2975 asp__L_c + 59.276 atp_c + 0.0447 cmp_c + 0.0066 cys__L_c + 0.0036 damp_c + 0.0024 dcmp_c + 0.0024 dgmp_c + 0.0036 dtmp_c + 0.0007 ergst_r + 0.1054 gln__L_c + 0.3018 glu__L_c + 0.2904 gly_c + 0.5185 glycogen_c + 0.046 gmp_c + 59.276 h2o_c + 0.0663 his__L_c + 0.1927 ile__L_c + 0.2964 leu__L_c + 0.2862 lys__L_c + 0.8079 mannan_r + 0.0507 met__L_c + 6e-06 pa_RT_r + 6e-05 pc_RT_r + 4.5e-05 pe_RT_r + 0.1339 phe__L_c + 0.1647 pro__L_c + 1.7e-05 ps_RT_r + 5.3e-05 ptd1ino_RT_r + 0.00099 ribflv_c + 0.1854 ser__L_c + 0.02 so4_c + 0.1914 thr__L_c + 0.0234 tre_c + 6.6e-05 triglyc_RT_r + 0.0284 trp__L_c + 0.102 tyr__L_c + 0.0599 ump_c + 0.2646 val__L_c + 0.0015 zymst_r --> 59.276 adp_c + 58.70001 h_c + 59.305 pi_c 
New BIOMASS_RT: 1.1348 13BDglcn_c + 1e-06 5mthf_c + 0.4588 ala__L_c + 0.046 amp_c + 0.1607 arg__L_c + 0.1017 asn__L_c + 0.2975 asp__L_c + 59.276 atp_c + 1e-06 btn_m + 1e-06 camp_c + 0.0447 cmp_c + 1e-06 coa_c + 0.0066 cys__L_c + 0.0036 damp_c + 0.0024 dcmp_c + 0.0024 dgmp_c + 0.0036 dtmp_c + 0.0007 ergst_r + 1e-06 fad_c + 0.1054 gln__L_c + 0.3018 glu__L_c + 0.2904 gly_c + 0.5185 glycogen_c + 0.046 gmp_c + 1e-06 gthrd_c + 59.276 h2o_c + 1e-06 hemeA_m + 0.0663 his__L_c + 0.1927 ile__L_c + 0.2964 leu__L_c + 1e-06 lipopb_m + 0.2862 lys__L_c + 0.8079 mannan_r + 0.0507 met__L_c + 1e-06 mlthf_c + 1e-06 nad_c + 1e-06 nadp_c + 6e-06 pa_RT_r + 6e-05 pc_RT_r + 4.5e-05 pe_RT_r + 0.1339 phe__L_c + 0.1647 pro__L_c + 1.7e-05 ps_RT_r + 5.3e-05 ptd1ino_RT_r + 1e-06 ptrc_c + 1e-06 pydx5p_c + 1e-06 q9_m + 0.00099 ribflv_c + 0.1854 ser__L_c + 0.02 so4_c + 1e-06 spmd_c + 1e-06 thf_c + 1e-06 thmpp_c + 0.1914 thr__L_c + 0.0234 tre_c + 6.6e-05 triglyc_RT_r + 0.0284 trp__L_c + 0.102 tyr__L_c + 0.0599 ump_c + 0.2646 val__L_c + 0.0015 zymst_r --> 59.276 adp_c + 58.70001 h_c + 59.305 pi_c 

Old CHORM: chor_c --> pphn_c 14195 or 16225 or 9704
New CHORM: chor_c --> pphn_c 9704

Old COALDDH: conialdh_c + h2o_c + nad_c --> fer_c + 2.0 h_c + nadh_c 16323
New COALDDH: conialdh_c + h2o_c + nad_c --> fer_c + 2.0 h_c + nadh_c 12950

Old DDPA: e4p_c + h2o_c + pep_c --> 2dda7p_c + pi_c 10602 or 12155 or 15534
New DDPA: e4p_c + h2o_c + pep_c --> 2dda7p_c + pi_c 10602

Old DDPAm: e4p_m + h2o_m + pep_m --> 2dda7p_m + pi_m 10602 or 12155
New DDPAm: e4p_m + h2o_m + pep_m --> 2dda7p_m + pi_m 12155 or 15534

Old DHFR: dhf_c + h_c + nadph_c <=> nadp_c + thf_c 10845
New DHFR: dhf_c + h_c + nadph_c <=> nadp_c + thf_c 12159

Old DHFS: atp_c + dhpt_c + glu__L_c --> adp_c + dhf_c + h_c + pi_c 10460 or 14803
New DHFS: atp_c + dhpt_c + glu__L_c --> adp_c + dhf_c + h_c + pi_c 10460

Old DHQTi: 3dhq_c --> 3dhsk_c + h2o_c 10572 or 14252
New DHQTi: 3dhq_c --> 3dhsk_c + h2o_c 10572 or 13706 or 14252

Old DNADDP: dnad_c + h2o_c --> amp_c + 2.0 h_c + nicrnt_c 14638 or 15385
New DNADDP: dnad_c + h2o_c --> amp_c + 2.0 h_c + nicrnt_c 12434 or 14638

Old DNMPPA: dhpmp_c + h2o_c --> dhnpt_c + pi_c 14615 or 14875 or 15385
New DNMPPA: dhpmp_c + h2o_c --> dhnpt_c + pi_c 

Old E4PP: e4p_c + h2o_c --> erthrs_c + pi_c 14546 or 8460 or 8576
New E4PP: e4p_c + h2o_c --> erthrs_c + pi_c 8576

Old EX_fe2_e: fe2_e -->  
New EX_fe2_e: fe2_e <=>  

Old EX_fe3_e: fe3_e -->  
New EX_fe3_e: fe3_e <=>  

Old F1PP: f1p_c + h2o_c --> fru_c + pi_c 14546 or 8576
New F1PP: f1p_c + h2o_c --> fru_c + pi_c 8576

Old F6PP: f6p_c + h2o_c --> fru_c + pi_c 14546 or 8576
New F6PP: f6p_c + h2o_c --> fru_c + pi_c 8576

Old FE2t: fe2_e --> fe2_c 10455
New FE2t: fe2_e <=> fe2_c 10455

Old FE3t: fe3_e --> fe3_c 13857 or 15844 or 15845
New FE3t: fe3_e <=> fe3_c 13857 or 15844 or 15845

Old FOLR2_1: fol_c + h_c + nadph_c --> dhf_c + nadp_c 10845
New FOLR2_1: fol_c + h_c + nadph_c --> dhf_c + nadp_c 12159

Old FTHFDH: 10fthf_c + h2o_c + nadp_c --> co2_c + h_c + nadph_c + thf_c 12042 or 13426
New FTHFDH: 10fthf_c + h2o_c + nadp_c --> co2_c + h_c + nadph_c + thf_c 12042

Old G3PT: glyc3p_c + h2o_c --> glyc_c + pi_c 13413
New G3PT: glyc3p_c + h2o_c --> glyc_c + pi_c 8460

Old GCALDD: gcald_c + h2o_c + nad_c --> glyclt_c + 2.0 h_c + nadh_c 12042 or 13426 or 16323
New GCALDD: gcald_c + h2o_c + nad_c --> glyclt_c + 2.0 h_c + nadh_c 12042

Old GLUTRS: atp_c + glu__L_c + trnaglu_c --> amp_c + glutrna_c + ppi_c 10753 or 14565
New GLUTRS: atp_c + glu__L_c + trnaglu_c --> amp_c + glutrna_c + ppi_c 10753

Old GNP: 6pgc_c + h2o_c --> glcn_c + pi_c 14546
New GNP: 6pgc_c + h2o_c --> glcn_c + pi_c 8576

Old IMACTD: h2o_c + im4act_c + nad_c --> 2.0 h_c + im4ac_c + nadh_c 12042 or 13426 or 16323
New IMACTD: h2o_c + im4act_c + nad_c --> 2.0 h_c + im4ac_c + nadh_c 12042

Old LCADi: h2o_c + lald__L_c + nad_c --> 2.0 h_c + lac__L_c + nadh_c 12042 or 13426 or 16323
New LCADi: h2o_c + lald__L_c + nad_c --> 2.0 h_c + lac__L_c + nadh_c 12042

Old LCADi_D: h2o_c + lald__D_c + nad_c --> 2.0 h_c + lac__D_c + nadh_c 12042 or 13426 or 16323
New LCADi_D: h2o_c + lald__D_c + nad_c --> 2.0 h_c + lac__D_c + nadh_c 12042

Old MACOXO: 3mldz_c + h2o_c + nad_c --> 3mlda_c + 2.0 h_c + nadh_c 12042 or 13426 or 16323
New MACOXO: 3mldz_c + h2o_c + nad_c --> 3mlda_c + 2.0 h_c + nadh_c 12042

Old METTRS: atp_c + met__L_c + trnamet_c --> amp_c + mettrna_c + ppi_c 13140 or 15518
New METTRS: atp_c + met__L_c + trnamet_c --> amp_c + mettrna_c + ppi_c 13140

Old PGMT: g1p_c <=> g6p_c 13711 or 14546 or 8460 or 9959
New PGMT: g1p_c <=> g6p_c 13711 or 9959

Old PMANM: man1p_c <=> man6p_c 13711 or 9241
New PMANM: man1p_c <=> man6p_c 9241

Old PSCVT: pep_c + skm5p_c <=> 3psme_c + pi_c 14252
New PSCVT: pep_c + skm3p_c <=> 3psme_c + pi_c 14252

Old R5PP: h2o_c + r5p_c --> pi_c + rib__D_c 14546 or 8576
New R5PP: h2o_c + r5p_c --> pi_c + rib__D_c 8576

Old SERTRS: atp_c + ser__L_c + trnaser_c --> amp_c + ppi_c + sertrna_c 10246 or 14225
New SERTRS: atp_c + ser__L_c + trnaser_c --> amp_c + ppi_c + sertrna_c 10246

Old SHKK: atp_c + skm_c --> adp_c + h_c + skm5p_c 13706 or 14252
New SHKK: atp_c + skm_c --> adp_c + h_c + skm3p_c 13706 or 14252

Old THFGLUS: atp_c + glu__L_c + thf_c <=> adp_c + h_c + pi_c + thfglu_c 10460 or 14803
New THFGLUS: atp_c + glu__L_c + thf_c <=> adp_c + h_c + pi_c + thfglu_c 14803

Old THMPe: h2o_e + thmmp_e --> pi_e + thm_e 10885
New THMPe: h2o_e + thmmp_e --> pi_e + thm_e 13856

Old THMt2: h_e + thm_e --> h_c + thm_c 10921 or 11871 or 12902
New THMt2: h_e + thm_e --> h_c + thm_c 9324 or 10921 or 11871 or 12902

Old TRPTRS: atp_c + trnatrp_c + trp__L_c --> amp_c + ppi_c + trptrna_c 14709 or 15533
New TRPTRS: atp_c + trnatrp_c + trp__L_c --> amp_c + ppi_c + trptrna_c 14709

Old TYRTRS: atp_c + trnatyr_c + tyr__L_c --> amp_c + ppi_c + tyrtrna_c 13137 or 16641
New TYRTRS: atp_c + trnatyr_c + tyr__L_c --> amp_c + ppi_c + tyrtrna_c 16641

Old URAt2: h_e + ura_e --> h_c + ura_c 10921 or 11871 or 12902
New URAt2: h_e + ura_e --> h_c + ura_c 9324 or 10921 or 11871 or 12902

Old URIt2: h_e + uri_e --> h_c + uri_c 10921 or 11871 or 12902 or 13969 or (10921 and 13969) or (11871 and 13969) or (12902 and 13969)
New URIt2: h_e + uri_e --> h_c + uri_c 9324 or 10921 or 11871 or 12902
```

In [77]:

```
print('Added reactions\n')
for r in sorted(model_new.reactions, key=lambda x: x.id):
    if r not in model_old.reactions:
        print(r)
```

```
Added reactions

3NPABH_m: 3npab_m + h_m + nadph_m + o2_m --> 3npahb_m + h2o_m + nadp_m
3NPAHBDH_m: 3npahb_m + 3.0 h_m + 2.0 nadph_m + o2_m --> 3npdhb_m + h2o_m + 2.0 nadp_m + nh4_m
4ABZt: 4abz_c <=> 4abz_e
4ABZtm: 4abz_c <=> 4abz_m
4HBALDDm: 4hbald_m + h2o_m + nad_m --> 4hbz_m + 2.0 h_m + nadh_m
ABZNPT_m: 4abz_m + npdp_m --> 3npab_m + ppi_m
ALDD16er: h2o_r + hxdcal_r + nad_r --> 2.0 h_r + hdca_r + nadh_r
ALDD21er: h2o_r + nad_r + pristanal_r --> 2.0 h_r + nadh_r + prist_r
ALDD3C161er: h2o_r + hxdceal_r + nad_r --> 2.0 h_r + hdc2ea_r + nadh_r
CCOAOMT: amet_c + caffcoa_c --> ahcys_c + ferulcoa_c + h_c
CYStm: cys__L_c <=> cys__L_m
DHFRim: dhf_m + h_m + nadph_m --> nadp_m + thf_m
DHNPA2r: dhnpt_c <=> 6hmhpt_c + gcald_c
DHSKDH: 3dhsk_c --> 34dhbz_c + h2o_c
DM_4oglu_c: 4oglu_c --> 
DM_amob_m: amob_m --> 
DM_dad_5_m: dad_5_m --> 
EX_4abz_e: 4abz_e --> 
EX_glx_e: glx_e --> 
EX_glyclt_e: glyclt_e --> 
FAAH: h2o_c + ocdcead_c --> nh4_c + ocdcea_c
GLXt: glx_c --> glx_e
GLXtm: glx_c <=> glx_m
GLXtp: glx_c <=> glx_x
GLYCLTt: glyclt_e <=> glyclt_c
GLYCLTtm: glyclt_c <=> glyclt_m
GLYCLTtp: glyclt_c --> glyclt_x
HMPPS: 4.0 fe3_c + h2o2_c + his__L_c + 2.0 o2_c + pdx5p_c --> 4ampm_c + 4oglu_c + co2_c + 4.0 fe2_c + glx_c + 6.0 h_c
HPPK2: 6hmhpt_c + atp_c --> 6hmhptpp_c + amp_c + h_c
METtm: met__L_c <=> met__L_m
NADDP: h2o_c + nad_c --> amp_c + 2.0 h_c + nmn_c
NMNHYD: h2o_c + nmn_c --> pi_c + rnam_c
PAL: phe__L_c --> cinnm_c + nh4_c
PSURIK: atp_c + psuri_c --> adp_c + h_c + psd5p_c
PSURIP: h2o_c + psd5p_c --> pi_c + psuri_c
QUINDH: nad_c + quin_c --> 3dhq_c + 2.0 h_c + nadh_c
SERTRSm: atp_m + ser__L_m + trnaser_m --> amp_m + ppi_m + sertrna_m
SRC_8aonn_m:  --> 8aonn_m
SRC_lipoate_m:  --> lipoate_m
TAL: tyr__L_c --> T4hcinnm_c + nh4_c
TDP: h2o_c + thmpp_c --> h_c + pi_c + thmmp_c
THMP: h2o_c + thmmp_c --> pi_c + thm_c
THZPSN4: cys__L_c + gly_c + nad_c --> 2amac_c + 4mpetz_c + amp_c + co2_c + 2.0 h2o_c + 3.0 h_c + ncam_c
TMN: h2o_c + thm_c --> 4ahmmp_c + 4mhetz_c + h_c
```

In [ ]:

```

```
