# Supplementary material for: Multi-Omics Driven Metabolic Network Reconstruction and Analysis of Lignocellulosic Carbon Utilization in Rhodosporidium toruloides
Source: Front Bioeng Biotechnol. 2021 Jan 8;8:612832. doi: 10.3389/fbioe.2020.612832 (PMC7873862; doi:10.3389/fbioe.2020.612832)
Supplement: Supplementary File 4 — Multi-omics dataset for R. toruloides IFO0880. [file Data_Sheet_1.zip › Supplementary File S1/2.Metabolic_modeling/Refinement_2a_Add_Biomass_Reaction.html]

Refinement\_2a\_Add\_Biomass\_Reaction


In [1]:

```
%matplotlib inline
from matplotlib import pyplot as plt
from matplotlib import colors
import csv
import numpy as np
import pandas as pd
import cobra
```

In [2]:

```
Annotation = pd.read_excel('../../Data/R_toruloides_Data_for_Reconstruction.xlsx',
                          sheet_name='Annotation', index_col=0)
Annotation.index = Annotation.index.map(str)
Annotation = Annotation.fillna('')
Transcriptomics = pd.read_excel('../../Data/R_toruloides_Data_for_Reconstruction.xlsx',
                          sheet_name='Transcriptomics', header=[0,1,2,3], index_col=0)
Transcriptomics.index = Transcriptomics.index.map(str)
Proteomics = pd.read_excel('../../Data/R_toruloides_Data_for_Reconstruction.xlsx',
                          sheet_name='Proteomics', header=[0,1,2], index_col=0)
Proteomics.index = Proteomics.index.map(str)
Fitness = pd.read_excel('../../Data/R_toruloides_Data_for_Reconstruction.xlsx',
                          sheet_name='Fitness', index_col=0)
Fitness.index = Fitness.index.map(str)
```

In [3]:

```
def background_gradient(s, cmap='seismic', text_color_threshold=0.408):
    lim = max(abs(s.min().min()),abs(s.max().max()))
    rng = 2.0*lim
    norm = colors.Normalize(-lim - (rng * 0.2), lim + (rng * 0.2))
    rgbas = plt.cm.get_cmap(cmap)(norm(s.values))
    def relative_luminance(rgba):
        r, g, b = (x / 12.92 if x <= 0.03928 else ((x + 0.055) / 1.055 ** 2.4) for x in rgba[:3])
        return 0.2126 * r + 0.7152 * g + 0.0722 * b
    def css(rgba):
        dark = relative_luminance(rgba) < text_color_threshold
        text_color = '#f1f1f1' if dark else '#000000'
        return 'background-color: {b};color: {c};'.format(b=colors.rgb2hex(rgba), c=text_color)

    if s.ndim == 1:
        return [css(rgba) for rgba in rgbas]
    else:
        return pd.DataFrame([[css(rgba) for rgba in row] for row in rgbas], index=s.index, columns=s.columns)

def Show_Data(x):
    display(Transcriptomics.loc[x].style.background_gradient(cmap='Reds', low=0.2, high=0.2, axis=None))
    temp = [y for y in x if y in Proteomics.index]
    display(Proteomics.loc[temp].style.background_gradient(cmap='Reds', low=0.2, high=0.2, axis=None))
    temp = [y for y in x if y in Fitness.index]
    display(Fitness.loc[temp].style.apply(background_gradient, cmap='seismic', axis=None))
    return;
```

In [4]:

```
model = cobra.io.load_json_model("../1.Manual_curation/IFO0880_GPR_1f.json")
```

In [5]:

```
eco = cobra.io.load_json_model('../../Data/BiGG_Models/iML1515.json')
sce = cobra.io.load_json_model('../../Data/BiGG_Models/iMM904.json')
hsa = cobra.io.load_json_model('../../Data/BiGG_Models/RECON1.json')
hsa2 = cobra.io.load_json_model('../../Data/BiGG_Models/Recon3D.json')
ptri = cobra.io.load_json_model('../../Data/BiGG_Models/iLB1027_lipid.json')
```

In [6]:

```
Reaction_manual = ['EX_co2_e','EX_h_e','EX_h2o_e','EX_nh4_e','EX_o2_e','EX_pi_e','EX_so4_e','EX_glc__D_e',
                   'H2Ot','H2Oter','H2Otg','H2Otm','H2Otn','H2Otp','H2Otv',
                   'CO2t','CO2ter','CO2tg','CO2tm','CO2tn','CO2tp','CO2tv',
                   'O2t','O2ter','O2tm','O2tn','O2tp']
print([x for x in Reaction_manual if x in model.reactions])
print([(r.id, r.gene_reaction_rule)  for r in sce.reactions if r.id in Reaction_manual and r.gene_reaction_rule])
print([(r.id, r.gene_reaction_rule)  for r in hsa.reactions if r.id in Reaction_manual and r.gene_reaction_rule])
```

```
['H2Ot', 'H2Otm', 'H2Otp']
[('H2Ot', 'YPR192W or YLL052C')]
[('H2Ot', '6523_AT1'), ('H2Otm', '343_AT1')]
```

In [7]:

```
for x in ['H2Ot', 'H2Otm', 'H2Otp']:
    print(x,model.reactions.get_by_id(x).gene_reaction_rule)
```

```
H2Ot 13986 or 13987 or 9014 or 9015
H2Otm 13986 or 13987 or 9014 or 9015
H2Otp 13986
```

In [8]:

```
#temp = Annotation.index[Annotation['Combined Annotations'].str.contains('aquaporin')]
temp = Annotation.index[Annotation['Human Blast'].str.contains('AQP', na=False)]
display(Annotation.loc[temp])
Show_Data(temp)
```

|  | Combined Annotations | Signal P | Sc288c Orthologs | Human Orthologs | Sc288 Best Hit | Human Blast | Essential | WolfPSort | C Terminal |
| --- | --- | --- | --- | --- | --- | --- | --- | --- | --- |
| RTO4\_ID |  |  |  |  |  |  |  |  |  |
| 9014 | K09885: AQPF; aquaporin rerated protein, other... |  | AQY1 | AQP4,AQP1,AQP8 | AQY1 | AQP4 | Not Essential | plas 18, mito 4, E.R. 2, cyto\_mito 2 | EKV\* |
| 9015 | K09885: AQPF; aquaporin rerated protein, other... | A | AQY1 | AQP4,AQP1,AQP8 | AQY1 | AQP1 | Not Essential | plas 27 | SAS\* |
| 13100 | HMMPfam:Major intrinsic protein:PF00230,PRINTS... |  |  |  | YFL054C | AQP10 | Not Essential | plas 21, E.R. 4 | LKA\* |
| 13986 | K09866: AQP4; aquaporin-4 |  | AQY1 | AQP4,AQP1,AQP8 | AQY1 | AQP4 | Not Essential | plas 23, E.R. 3 | SPV\* |
| 13987 | K09885: AQPF; aquaporin rerated protein, other... |  | AQY1 | AQP4,AQP1,AQP8 | AQY1 | AQP1 | Not Essential | plas 24, E.R. 2 | RFA\* |
| 15470 | K03441: GLP-F; aquaglyceroporin related protei... |  | YFL054C,FPS1 | AQP3,AQP9 | YFL054C | AQP3 | Not Essential | plas 17, nucl 6, mito 1, cyto 1, E.R. 1, vacu ... | WRQ\* |

| strain | WT | | | | | | | | | | | | | | | | |
| --- | --- | --- | --- | --- | --- | --- | --- | --- | --- | --- | --- | --- | --- | --- | --- | --- | --- |
| condition | G\_MM | C\_MM | G\_SD | | GX\_SD | | | X\_SD | | A\_SD | | C\_SD | | MM\_CN120 | | MM\_CN5 | Diversity\_Sample |
| phase | exp | exp | exp | stat | exp | trans | stat | exp | stat | exp | stat | exp | stat | exp | stat | exp | exp |
| proteinId | Set1 | Set1 | Set2 | Set2 | Set2 | Set2 | Set2 | Set2 | Set2 | Set2 | Set2 | Set2 | Set2 | Set3 | Set3 | Set3 | Set3 |
| RTO4\_ID |  |  |  |  |  |  |  |  |  |  |  |  |  |  |  |  |  |
| 9014 | 4.2994 | 3.8223 | 5.17239 | 3.2933 | 5.6173 | 1.75055 | 2.25894 | 1.90922 | 2.01577 | 1.47985 | 2.40971 | 3.37265 | 8.22473 | 3.62799 | 2.25471 | 6.95379 | 4.30783 |
| 9015 | 1.00476 | 2.38813 | 2.74547 | 2.28042 | 2.74675 | 0.663524 | 1.23034 | 0.712135 | 0.857415 | 0.992198 | 1.5919 | 1.04885 | 4.22185 | 1.01805 | 0.669088 | 3.32354 | 1.77993 |
| 13100 | 3.79856 | 0.554947 | 3.07605 | 7.26083 | 3.02172 | 4.73937 | 4.93495 | 5.27856 | 6.89231 | 6.8639 | 8.15457 | 1.18047 | 0.934462 | 4.43998 | 2.43789 | 3.4705 | 2.73492 |
| 13986 | 0.811067 | 1.2042 | 1.0854 | 2.79969 | 0.770469 | 1.29013 | 1.65201 | 1.17297 | 0.872764 | 0.822605 | 1.83409 | 0.547347 | 1.06074 | 0.743115 | 0.489299 | 0.67146 | 1.08878 |
| 13987 | 0.220805 | 0.542888 | 0.525869 | 1.27328 | 0.374866 | 0.527462 | 0.897064 | 0.553509 | 0.659766 | 0.322068 | 0.501118 | 0.637008 | 0.338122 | 0.278834 | 0.301224 | 0.196509 | 0.830183 |
| 15470 | 2.58482 | 2.6645 | 3.34569 | 4.55699 | 3.28507 | 3.35072 | 3.38165 | 3.06554 | 3.80438 | 2.88507 | 3.38184 | 3.12887 | 3.08643 | 3.45926 | 3.81437 | 3.23958 | 3.79991 |

| strain | WT | | | | | | | | | | |
| --- | --- | --- | --- | --- | --- | --- | --- | --- | --- | --- | --- |
| condition | G\_SD | | GX\_SD | | | X\_SD | | A\_SD | | C\_SD | |
| proteinId | exp | stat | exp | trans | stat | exp | stat | exp | stat | exp | stat |
| 13100 | 0 | 0.195188 | 0 | 0 | 0 | 0 | 0.795012 | 0.962299 | 1.54664 | 0 | 0 |
| 13987 | 0 | 0 | 0 | 0 | 0 | 0 | 0.188437 | 0 | 0 | 0 | 0 |

|  | Glucose | Xylose | Arabinose | Acetate | Coumarate | Ferulate | YNB Oleic Acid | YNB Ricinoleic Acid | YNB Glucose | YNB Gluc DOC | YPD |
| --- | --- | --- | --- | --- | --- | --- | --- | --- | --- | --- | --- |
| proteinId |  |  |  |  |  |  |  |  |  |  |  |
| 9014 | -0.0989641 | -0.0117056 | -0.217921 | -0.106086 | -0.110371 | 0.18789 | -0.442884 | -0.248592 | -0.165871 | -0.342108 | -0.131261 |
| 9015 | -0.0247873 | -0.200422 | -0.0550922 | -0.197392 | 0.177297 | -0.108184 | 0.511344 | -0.225037 | -0.229903 | -0.0266842 | -0.165627 |
| 13100 | 0.110137 | 0.219541 | 0.237074 | 0.153246 | 0.575455 | 0.53134 | -0.0581222 | -0.293301 | -0.353711 | -0.27729 | 0.269053 |
| 13986 | 0.266698 | 0.148965 | 0.291259 | 0.155225 | 0.191408 | 0.108453 | 0.340606 | 0.278426 | 0.062012 | 0.0155407 | 0.383375 |
| 13987 | 0.171695 | 0.299718 | -0.0115636 | 0.288009 | -0.0296966 | 0.106285 | 0.263578 | -0.712035 | -0.210913 | -0.0287829 | 1.6117 |
| 15470 | 0.00318651 | 0.0512761 | -0.167947 | 0.0916783 | 0.0498898 | -0.185868 | 0.158407 | -0.399259 | 0.128998 | -0.140828 | -0.115934 |

In [9]:

```
for x in temp:
    if x in model.genes:
        for r in sorted(model.genes.get_by_id(x).reactions, key=lambda x: x.id):
            print(r.id, r.reaction, r.gene_reaction_rule)
    else:
        print(x, 'no reactions')
    print()
```

```
H2Ot h2o_e <=> h2o_c 13986 or 13987 or 9014 or 9015
H2Otm h2o_c <=> h2o_m 13986 or 13987 or 9014 or 9015

H2Ot h2o_e <=> h2o_c 13986 or 13987 or 9014 or 9015
H2Otm h2o_c <=> h2o_m 13986 or 13987 or 9014 or 9015

13100 no reactions

H2Ot h2o_e <=> h2o_c 13986 or 13987 or 9014 or 9015
H2Otm h2o_c <=> h2o_m 13986 or 13987 or 9014 or 9015
H2Otp h2o_c <=> h2o_x 13986

H2Ot h2o_e <=> h2o_c 13986 or 13987 or 9014 or 9015
H2Otm h2o_c <=> h2o_m 13986 or 13987 or 9014 or 9015

GLYCt glyc_c <=> glyc_e 15470
L_LACtcm lac__L_c --> lac__L_m 15470
UREAtm urea_c <=> urea_m 15470
```

In [10]:

```
# H2Otm in Recon is by AQP8, leave 9014 since it has mito in WolfPSort
model.reactions.get_by_id('H2Otm').gene_reaction_rule = '9014'
model.reactions.get_by_id('H2Otp').gene_reaction_rule = ''
# 15470 is Aquaglyceroporin, remove L_LACtcm
# 13100 is also Aquaglyceroporin
model.reactions.get_by_id('GLYCt').gene_reaction_rule = '13100 or 15470'
r = hsa2.reactions.get_by_id('UREAt').copy()
r.gene_reaction_rule = '13100 or 15470'
model.add_reactions([r])
```

In [11]:

```
for x in Reaction_manual:
    if x not in model.reactions:
        if x in sce.reactions:
            r = sce.reactions.get_by_id(x)
            print(r.id, r.reaction, r.gene_reaction_rule)
        elif x in hsa.reactions:
            r = hsa.reactions.get_by_id(x)
            print(r.id, r.reaction, r.gene_reaction_rule)
        else:
            print(x, 'not found in sce or hsa')
```

```
EX_co2_e co2_e -->  
EX_h_e h_e <=>  
EX_h2o_e h2o_e <=>  
EX_nh4_e nh4_e <=>  
EX_o2_e o2_e <=>  
EX_pi_e pi_e <=>  
EX_so4_e so4_e <=>  
EX_glc__D_e glc__D_e <=>  
H2Oter h2o_c <=> h2o_r 
H2Otg h2o_c <=> h2o_g 
H2Otn h2o_n <=> h2o_c 
H2Otv h2o_c <=> h2o_v 
CO2t co2_e <=> co2_c 
CO2ter co2_c <=> co2_r 
CO2tg co2_c <=> co2_g 
CO2tm co2_c <=> co2_m 
CO2tn co2_n <=> co2_c 
CO2tp co2_c <=> co2_x 
CO2tv co2_c <=> co2_v 
O2t o2_e <=> o2_c 
O2ter o2_c <=> o2_r 
O2tm o2_c <=> o2_m 
O2tn o2_c <=> o2_n 
O2tp o2_c <=> o2_x
```

In [12]:

```
for x in Reaction_manual:
    if x not in model.reactions:
        if x in sce.reactions:
            model.add_reaction(sce.reactions.get_by_id(x).copy())
        elif x in hsa.reactions:
            model.add_reaction(hsa.reactions.get_by_id(x).copy())
        else:
            print(x, 'not found in sce or hsa')
```

In [13]:

```
r = sce.reactions.get_by_id('ATPM').copy()
model.add_reactions([r])
r
```

Out[13]:

|  |  |
| --- | --- |
| **Reaction identifier** | ATPM |
| **Name** | ATP maintenance requirement |
| **Memory address** | 0x0102b9d6b00 |
| **Stoichiometry** | atp\_c + h2o\_c --> adp\_c + h\_c + pi\_c  ATP C10H12N5O13P3 + H2O H2O --> ADP C10H12N5O10P2 + H+ + Phosphate |
| **GPR** |  |
| **Lower bound** | 1.0 |
| **Upper bound** | 1.0 |

In [14]:

```
Biomass = sce.reactions.get_by_id('BIOMASS_SC5_notrace').copy()
Biomass.id = 'BIOMASS_RT'
Biomass.name = 'Biomass'
Biomass
```

Out[14]:

|  |  |
| --- | --- |
| **Reaction identifier** | BIOMASS\_RT |
| **Name** | Biomass |
| **Memory address** | 0x0102ba6abe0 |
| **Stoichiometry** | 1.1348 13BDglcn\_c + 0.4588 ala\_\_L\_c + 0.046 amp\_c + 0.1607 arg\_\_L\_c + 0.1017 asn\_\_L\_c + 0.2975 asp\_\_L\_c + 59.276 atp\_c + 0.0447 cmp\_c + 0.0066 cys\_\_L\_c + 0.0036 damp\_c + 0.0024 dcmp\_c + 0.0024 dgmp...  1.1348 1 3 beta D Glucan C6H10O5 + 0.4588 L-Alanine + 0.046 AMP C10H12N5O7P + 0.1607 L-Arginine + 0.1017 L-Asparagine + 0.2975 L-Aspartate + 59.276 ATP C10H12N5O13P3 + 0.0447 CMP C9H12N3O8P + 0.006... |
| **GPR** |  |
| **Lower bound** | 0.0 |
| **Upper bound** | 999999.0 |

In [15]:

```
for m in Biomass.metabolites:
    if not m.id in model.metabolites:
        if not 'SC' in m.id:
            print(m.id)
        elif m.id.replace('_SC','_RT') in model.metabolites:
            print(m.id, m.id.replace('_SC','_RT'))
        elif m.id.replace('_SC_c','_RT_r') in model.metabolites:
            print(m.id, m.id.replace('_SC_c','_RT_r'))
```

```
mannan_c
pa_SC_c pa_RT_r
pc_SC_c pc_RT_r
pe_SC_c pe_RT_r
ps_SC_c ps_RT_r
ptd1ino_SC_c ptd1ino_RT_r
triglyc_SC_c triglyc_RT_r
```

In [16]:

```
for r in sorted(model.metabolites.get_by_id('mannan_r').reactions, key=lambda x: x.id):
    print(r, r.gene_reaction_rule)
print()
for r in sorted(model.metabolites.get_by_id('dolmanp_r').reactions, key=lambda x: x.id):
    print(r, r.gene_reaction_rule)
```

```
DOLPMMer: dolmanp_r --> dolp_r + h_r + mannan_r 12121 or 13840 or 8768 or (13840 and 8768)

DOLPMMer: dolmanp_r --> dolp_r + h_r + mannan_r 12121 or 13840 or 8768 or (13840 and 8768)
DOLPMTcer: dolp_c + gdpmann_c --> dolmanp_r + gdp_c 13484
```

In [17]:

```
model.reactions.get_by_id('DOLPMMer').gene_reaction_rule = '12121 or 13840 or 8768'
model.add_reactions([Biomass])
Biomass.add_metabolites({'mannan_c': -Biomass.get_coefficient('mannan_c'),
                         'mannan_r': Biomass.get_coefficient('mannan_c')})
Biomass.add_metabolites({'pa_SC_c': -Biomass.get_coefficient('pa_SC_c'),
                         'pa_RT_r': Biomass.get_coefficient('pa_SC_c')})
Biomass.add_metabolites({'pc_SC_c': -Biomass.get_coefficient('pc_SC_c'),
                         'pc_RT_r': Biomass.get_coefficient('pc_SC_c')})
Biomass.add_metabolites({'pe_SC_c': -Biomass.get_coefficient('pe_SC_c'),
                         'pe_RT_r': Biomass.get_coefficient('pe_SC_c')})
Biomass.add_metabolites({'ps_SC_c': -Biomass.get_coefficient('ps_SC_c'),
                         'ps_RT_r': Biomass.get_coefficient('ps_SC_c')})
Biomass.add_metabolites({'ptd1ino_SC_c': -Biomass.get_coefficient('ptd1ino_SC_c'),
                         'ptd1ino_RT_r': Biomass.get_coefficient('ptd1ino_SC_c')})
Biomass.add_metabolites({'triglyc_SC_c': -Biomass.get_coefficient('triglyc_SC_c'),
                         'triglyc_RT_r': Biomass.get_coefficient('triglyc_SC_c')})
```

In [18]:

```
for x in sorted(model.metabolites, key=lambda x: x.id):
    if not x.reactions:
        print(x)
model.remove_metabolites([x for x in model.metabolites if not x.reactions])
```

```
mannan_c
pa_SC_c
pc_SC_c
pe_SC_c
ps_SC_c
ptd1ino_SC_c
triglyc_SC_c
```

In [19]:

```
for r in sorted(model.reactions, key=lambda x: x.id):
    if r.check_mass_balance() and not r.boundary:
        if sum(abs(x) for x in r.check_mass_balance().values()) > 1e-6:
            print(r, r.check_mass_balance())
```

```
BIOMASS_RT: 1.1348 13BDglcn_c + 0.4588 ala__L_c + 0.046 amp_c + 0.1607 arg__L_c + 0.1017 asn__L_c + 0.2975 asp__L_c + 59.276 atp_c + 0.0447 cmp_c + 0.0066 cys__L_c + 0.0036 damp_c + 0.0024 dcmp_c + 0.0024 dgmp_c + 0.0036 dtmp_c + 0.0007 ergst_c + 0.1054 gln__L_c + 0.3018 glu__L_c + 0.2904 gly_c + 0.5185 glycogen_c + 0.046 gmp_c + 59.276 h2o_c + 0.0663 his__L_c + 0.1927 ile__L_c + 0.2964 leu__L_c + 0.2862 lys__L_c + 0.8079 mannan_r + 0.0507 met__L_c + 6e-06 pa_RT_r + 6e-05 pc_RT_r + 4.5e-05 pe_RT_r + 0.1339 phe__L_c + 0.1647 pro__L_c + 1.7e-05 ps_RT_r + 5.3e-05 ptd1ino_RT_r + 0.00099 ribflv_c + 0.1854 ser__L_c + 0.02 so4_c + 0.1914 thr__L_c + 0.0234 tre_c + 6.6e-05 triglyc_RT_r + 0.0284 trp__L_c + 0.102 tyr__L_c + 0.0599 ump_c + 0.2646 val__L_c + 0.0015 zymst_c --> 59.276 adp_c + 58.70001 h_c + 59.305 pi_c {'charge': -0.01618999999994411, 'C': -35.94203000000009, 'H': -65.57310999999986, 'O': -23.6762400000002, 'N': -5.596460000000039, 'P': -0.1977000000000017, 'S': -0.07730000000000001}
```

## Fatty acid transport reactions for lipid biosynthesis and mobilization¶

In [20]:

```
# Mito fatty acid transport
for r in sorted(model.metabolites.get_by_id('crn_c').reactions, key=lambda x: x.id):
    print(r, r.gene_reaction_rule)
print()
for r in sorted(model.metabolites.get_by_id('crn_m').reactions, key=lambda x: x.id):
    print(r, r.gene_reaction_rule)
print()
for r in sorted(model.metabolites.get_by_id('crn_x').reactions, key=lambda x: x.id):
    print(r, r.gene_reaction_rule)
print()
for r in sorted(model.metabolites.get_by_id('crn_r').reactions, key=lambda x: x.id):
    print(r, r.gene_reaction_rule)
```

```
ARACHCPT1: arachcoa_c + crn_c --> arachcrn_c + coa_c 9315
C160CPT1: crn_c + pmtcoa_c --> coa_c + pmtcrn_c 9315
C161CPT1: crn_c + hdcoa_c --> coa_c + hdcecrn_c 9315
C161CPT12: crn_c + hdd2coa_c --> coa_c + hdd2crn_c 9315
C181CPT1: crn_c + odecoa_c --> coa_c + odecrn_c 9315
CLPNDCPT1: clpndcoa_c + crn_c --> clpndcrn_c + coa_c 9315
CRNCARtm: acrn_c + crn_m --> acrn_m + crn_c 9331
CRNtim: crn_m --> crn_c 9331
CSNATr: accoa_c + crn_c <=> acrn_c + coa_c 13580 or 14245
DCSPTN1CPT1: crn_c + dcsptn1coa_c --> coa_c + dcsptn1crn_c 9315
ELAIDCPT1: crn_c + od2coa_c --> coa_c + elaidcrn_c 9315
HEXCCPT1: crn_c + hexccoa_c --> coa_c + hexccrn_c 9315
HPDCACRNCPT1: crn_c + hpdcacoa_c --> coa_c + hpdcacrn_c 9315
LNLNCGCPT1: crn_c + lnlncgcoa_c --> coa_c + lnlncgcrn_c 9315
NRVNCCPT1: crn_c + nrvnccoa_c --> coa_c + nrvnccrn_c 9315
PTDCACRNCPT1: crn_c + ptdcacoa_c --> coa_c + ptdcacrn_c 9315
RTOTAL2CRNCPT1: Rtotal2coa_c + crn_c --> Rtotal2crn_c + coa_c 9315
RTOTAL3CRNCPT1: Rtotal3coa_c + crn_c --> Rtotal3crn_c + coa_c 9315
RTOTALCRNCPT1: Rtotalcoa_c + crn_c --> Rtotalcrn_c + coa_c 9315
TETTET6CPT1: crn_c + tettet6coa_c --> coa_c + tettet6crn_c 9315
TTDCPT1: crn_c + tdcoa_c --> coa_c + ttdcrn_c 9315

ARACHCPT2: arachcrn_m + coa_m --> arachcoa_m + crn_m 13580
C160CPT2: coa_m + pmtcrn_m --> crn_m + pmtcoa_m 13580
C161CPT2: coa_m + hdcecrn_m --> crn_m + hdcoa_m 13580
C161CPT22: coa_m + hdd2crn_m --> crn_m + hdd2coa_m 13580
C181CPT2: coa_m + odecrn_m --> crn_m + odecoa_m 13580
CLPNDCPT2: clpndcrn_m + coa_m --> clpndcoa_m + crn_m 13580
CRNCARtm: acrn_c + crn_m --> acrn_m + crn_c 9331
CRNtim: crn_m --> crn_c 9331
CSNAT2m: coa_m + pcrn_m <=> crn_m + ppcoa_m 14245
CSNATirm: accoa_m + crn_m <=> acrn_m + coa_m 13580 or 14245
CSNATm: acrn_m + coa_m <=> accoa_m + crn_m 14245
DCSPTN1CPT2: coa_m + dcsptn1crn_m --> crn_m + dcsptn1coa_m 13580
ELAIDCPT2: coa_m + elaidcrn_m --> crn_m + od2coa_m 13580
GBBOX_m: akg_m + gbbtn_m + o2_m --> co2_m + crn_m + succ_m 15060
HEXCCPT2: coa_m + hexccrn_m --> crn_m + hexccoa_m 13580
HPDCACRNCPT2: coa_m + hpdcacrn_m --> crn_m + hpdcacoa_m 13580
LNLNCGCPT2: coa_m + lnlncgcrn_m --> crn_m + lnlncgcoa_m 13580
NRVNCCPT2: coa_m + nrvnccrn_m --> crn_m + nrvnccoa_m 13580
PTDCACRNCPT2: coa_m + ptdcacrn_m --> crn_m + ptdcacoa_m 13580
RTOTAL2CRNCPT2: Rtotal2crn_m + coa_m --> Rtotal2coa_m + crn_m 13580
RTOTAL3CRNCPT2: Rtotal3crn_m + coa_m --> Rtotal3coa_m + crn_m 13580
RTOTALCRNCPT2: Rtotalcrn_m + coa_m --> Rtotalcoa_m + crn_m 13580
TETTET6CPT2: coa_m + tettet6crn_m --> crn_m + tettet6coa_m 13580
TTDCPT2: coa_m + ttdcrn_m --> crn_m + tdcoa_m 13580

CSNAT2x: crn_x + dmnoncoa_x <=> coa_x + dmnoncrn_x 14245
CSNAT3x: crn_x + ppcoa_x <=> coa_x + pcrn_x 14245
CSNATp: accoa_x + crn_x <=> acrn_x + coa_x 14245

CSNATer: accoa_r + crn_r <=> acrn_r + coa_r 14245
```

In [21]:

```
temp = Annotation.index[Annotation['Combined Annotations'].str.contains('choline O-acetyltransferase')]
display(Annotation.loc[temp])
Show_Data(temp)
```

|  | Combined Annotations | Signal P | Sc288c Orthologs | Human Orthologs | Sc288 Best Hit | Human Blast | Essential | WolfPSort | C Terminal |
| --- | --- | --- | --- | --- | --- | --- | --- | --- | --- |
| RTO4\_ID |  |  |  |  |  |  |  |  |  |
| 9315 | K00623: CHAT; choline O-acetyltransferase | S |  | CPT1A,CPT1B,CPT1C | CAT2 | CRAT | Not Essential | mito 24.5, cyto\_mito 13.5 | AKA\* |
| 15783 | K00623: CHAT; choline O-acetyltransferase |  |  |  | CAT2 | CRAT | Not Essential | mito 25 | AKL\* |

| strain | WT | | | | | | | | | | | | | | | | |
| --- | --- | --- | --- | --- | --- | --- | --- | --- | --- | --- | --- | --- | --- | --- | --- | --- | --- |
| condition | G\_MM | C\_MM | G\_SD | | GX\_SD | | | X\_SD | | A\_SD | | C\_SD | | MM\_CN120 | | MM\_CN5 | Diversity\_Sample |
| phase | exp | exp | exp | stat | exp | trans | stat | exp | stat | exp | stat | exp | stat | exp | stat | exp | exp |
| proteinId | Set1 | Set1 | Set2 | Set2 | Set2 | Set2 | Set2 | Set2 | Set2 | Set2 | Set2 | Set2 | Set2 | Set3 | Set3 | Set3 | Set3 |
| RTO4\_ID |  |  |  |  |  |  |  |  |  |  |  |  |  |  |  |  |  |
| 9315 | 4.83819 | 4.97685 | 7.01529 | 4.25404 | 6.89698 | 5.8326 | 5.34129 | 6.126 | 5.08243 | 4.4436 | 4.81725 | 4.03008 | 4.23203 | 5.51854 | 5.34193 | 5.47882 | 5.34975 |
| 15783 | 5.39557 | 6.12538 | 5.34963 | 4.72463 | 5.41244 | 4.76387 | 4.84051 | 5.25698 | 5.58384 | 5.52205 | 5.92832 | 6.0202 | 6.26982 | 5.02665 | 4.69026 | 5.2436 | 5.46712 |

| strain | WT | | | | | | | | | | |
| --- | --- | --- | --- | --- | --- | --- | --- | --- | --- | --- | --- |
| condition | G\_SD | | GX\_SD | | | X\_SD | | A\_SD | | C\_SD | |
| proteinId | exp | stat | exp | trans | stat | exp | stat | exp | stat | exp | stat |
| 9315 | 15.3637 | 12.474 | 16.8732 | 14.5076 | 18.9601 | 7.22872 | 9.84082 | 2.12229 | 3.62822 | 5.55516 | 3.2787 |
| 15783 | 3.248 | 3.46634 | 5.08664 | 4.64716 | 5.64037 | 5.29905 | 3.53768 | 4.82477 | 4.7967 | 7.64822 | 5.64045 |

|  | Glucose | Xylose | Arabinose | Acetate | Coumarate | Ferulate | YNB Oleic Acid | YNB Ricinoleic Acid | YNB Glucose | YNB Gluc DOC | YPD |
| --- | --- | --- | --- | --- | --- | --- | --- | --- | --- | --- | --- |
| proteinId |  |  |  |  |  |  |  |  |  |  |  |
| 9315 | 0.100782 | 0.0170754 | 0.0600288 | 0.236768 | 0.0167474 | 0.169648 | 0.0449251 | -0.315115 | 0.690845 | 0.75223 | 0.0392563 |

In [22]:

```
for x in temp:
    if x in model.genes:
        for r in sorted(model.genes.get_by_id(x).reactions, key=lambda x: x.id):
            print(r.id, r.reaction, r.gene_reaction_rule)
    else:
        print(x, 'no reactions')
    print()
```

```
ARACHCPT1 arachcoa_c + crn_c --> arachcrn_c + coa_c 9315
C160CPT1 crn_c + pmtcoa_c --> coa_c + pmtcrn_c 9315
C161CPT1 crn_c + hdcoa_c --> coa_c + hdcecrn_c 9315
C161CPT12 crn_c + hdd2coa_c --> coa_c + hdd2crn_c 9315
C181CPT1 crn_c + odecoa_c --> coa_c + odecrn_c 9315
CLPNDCPT1 clpndcoa_c + crn_c --> clpndcrn_c + coa_c 9315
DCSPTN1CPT1 crn_c + dcsptn1coa_c --> coa_c + dcsptn1crn_c 9315
ELAIDCPT1 crn_c + od2coa_c --> coa_c + elaidcrn_c 9315
HEXCCPT1 crn_c + hexccoa_c --> coa_c + hexccrn_c 9315
HPDCACRNCPT1 crn_c + hpdcacoa_c --> coa_c + hpdcacrn_c 9315
LNLNCGCPT1 crn_c + lnlncgcoa_c --> coa_c + lnlncgcrn_c 9315
NRVNCCPT1 crn_c + nrvnccoa_c --> coa_c + nrvnccrn_c 9315
PTDCACRNCPT1 crn_c + ptdcacoa_c --> coa_c + ptdcacrn_c 9315
RTOTAL2CRNCPT1 Rtotal2coa_c + crn_c --> Rtotal2crn_c + coa_c 9315
RTOTAL3CRNCPT1 Rtotal3coa_c + crn_c --> Rtotal3crn_c + coa_c 9315
RTOTALCRNCPT1 Rtotalcoa_c + crn_c --> Rtotalcrn_c + coa_c 9315
TETTET6CPT1 crn_c + tettet6coa_c --> coa_c + tettet6crn_c 9315
TTDCPT1 crn_c + tdcoa_c --> coa_c + ttdcrn_c 9315

15783 no reactions
```

In [23]:

```
model.remove_reactions(['RTOTAL2CRNCPT1','RTOTAL3CRNCPT1','RTOTALCRNCPT1',
                        'RTOTAL2CRNCPT2','RTOTAL3CRNCPT2','RTOTALCRNCPT2',
                        'RTOTAL2CRNt','RTOTAL3CRNt','RTOTALCRNt'],remove_orphans=True)
```

In [24]:

```
temp = Annotation.index[Annotation['Combined Annotations'].str.contains('carnitine')]
display(Annotation.loc[temp])
Show_Data(temp)
```

|  | Combined Annotations | Signal P | Sc288c Orthologs | Human Orthologs | Sc288 Best Hit | Human Blast | Essential | WolfPSort | C Terminal |
| --- | --- | --- | --- | --- | --- | --- | --- | --- | --- |
| RTO4\_ID |  |  |  |  |  |  |  |  |  |
| 9331 | K15109: SLC25A20\_29, CACT, CACL, CRC1; solute ... |  | CRC1 | SLC25A20 | CRC1 | SLC25A | Not Essential | mito 13.5, cyto\_mito 12.5, cyto 10.5 | ALF\* |
| 10149 | K15109: SLC25A20\_29, CACT, CACL, CRC1; solute ... |  |  |  | YMC2 | SLC25 | Not Essential | mito 11, plas 10, nucl 1, cyto 1, extr 1, cyto... | TSD\* |
| 12591 | KOG3957: Predicted L-carnitine dehydratase/alp... | S |  |  |  | SUGCT | Not Essential | mito 11, extr 5, cyto 4.5, cyto\_nucl 4.5, nucl... | TSE\* |
| 13580 | K00624: E2.3.1.7; carnitine O-acetyltransferase |  | YAT1 | CPT2 | YAT1 | CPT2 | Not Essential | cyto 12, cysk 6, nucl 5, mito 3 | DFV\* |
| 13666 | KOG0758: Mitochondrial carnitine-acylcarnitine... |  |  |  |  | SLC25 | Not Essential | mito 18, cyto 4, extr 4 | GVG\* |
| 14245 | K00624: E2.3.1.7; carnitine O-acetyltransferase |  | CAT2 | CRAT | CAT2 | CRAT | Not Essential | nucl 11.5, cyto\_nucl 8, pero 6, mito 5, cyto 3.5 | AKL\* |
| 15390 | KOG0758: Mitochondrial carnitine-acylcarnitine... |  |  |  | ORT1 | SLC25 | Not Essential | cyto\_mito 10.332, mito 10, cyto 10, cyto\_nucl 9.5 | RIE\* |

| strain | WT | | | | | | | | | | | | | | | | |
| --- | --- | --- | --- | --- | --- | --- | --- | --- | --- | --- | --- | --- | --- | --- | --- | --- | --- |
| condition | G\_MM | C\_MM | G\_SD | | GX\_SD | | | X\_SD | | A\_SD | | C\_SD | | MM\_CN120 | | MM\_CN5 | Diversity\_Sample |
| phase | exp | exp | exp | stat | exp | trans | stat | exp | stat | exp | stat | exp | stat | exp | stat | exp | exp |
| proteinId | Set1 | Set1 | Set2 | Set2 | Set2 | Set2 | Set2 | Set2 | Set2 | Set2 | Set2 | Set2 | Set2 | Set3 | Set3 | Set3 | Set3 |
| RTO4\_ID |  |  |  |  |  |  |  |  |  |  |  |  |  |  |  |  |  |
| 9331 | 5.75034 | 6.90719 | 6.47204 | 5.74082 | 6.55278 | 5.96035 | 6.05061 | 6.26286 | 6.44384 | 5.92166 | 6.10074 | 6.2095 | 5.9384 | 5.71808 | 5.53064 | 6.08302 | 5.74333 |
| 10149 | 4.39904 | 4.09667 | 5.06018 | 4.97188 | 5.16128 | 4.82506 | 4.96388 | 4.68437 | 5.28909 | 4.66677 | 5.26263 | 4.70503 | 3.82369 | 3.34746 | 3.44983 | 3.0779 | 3.20093 |
| 12591 | 3.73466 | 4.87196 | 5.10381 | 3.96578 | 5.16791 | 4.44783 | 4.21332 | 4.68985 | 3.81088 | 4.34143 | 3.67514 | 4.38706 | 5.39394 | 3.41792 | 3.1951 | 4.54651 | 3.26433 |
| 13580 | 5.54131 | 5.35065 | 7.85727 | 7.00135 | 7.9002 | 6.84984 | 7.07869 | 6.23154 | 7.7904 | 5.85422 | 7.08069 | 6.31985 | 7.36375 | 7.61777 | 7.40081 | 8.83529 | 7.75281 |
| 13666 | 5.37408 | 5.11721 | 5.82946 | 4.37274 | 5.79234 | 5.06525 | 4.92656 | 5.11797 | 4.8888 | 4.65015 | 4.4935 | 5.2846 | 5.51131 | 4.08986 | 3.93964 | 4.53848 | 5.0126 |
| 14245 | 6.06317 | 7.70984 | 7.99279 | 7.29384 | 8.05087 | 7.70814 | 7.47044 | 7.51113 | 8.39524 | 7.44235 | 7.25858 | 7.74462 | 7.59851 | 6.14024 | 6.15318 | 7.50772 | 6.58116 |
| 15390 | 4.88318 | 5.34429 | 4.99969 | 4.9015 | 5.4048 | 5.14553 | 5.31671 | 4.84671 | 5.42026 | 4.68338 | 5.13838 | 5.76557 | 6.15043 | 4.01139 | 4.14294 | 5.36492 | 4.27319 |

| strain | WT | | | | | | | | | | |
| --- | --- | --- | --- | --- | --- | --- | --- | --- | --- | --- | --- |
| condition | G\_SD | | GX\_SD | | | X\_SD | | A\_SD | | C\_SD | |
| proteinId | exp | stat | exp | trans | stat | exp | stat | exp | stat | exp | stat |
| 9331 | 6.34831 | 6.32004 | 6.29991 | 7.46502 | 7.4786 | 8.03925 | 6.46805 | 5.7924 | 6.71263 | 7.27284 | 8.50065 |
| 10149 | 0.954128 | 0.351946 | 0.822239 | 1.67041 | 0.564493 | 1.17177 | 1.18291 | 0 | 0.391347 | 0.22091 | 0.44231 |
| 12591 | 1.32958 | 1.08393 | 0.819619 | 0.37654 | 1.08557 | 0.385731 | 0.599864 | 0.580338 | 0.574385 | 0.208996 | 0 |
| 13580 | 14.3748 | 15.9317 | 13.8056 | 15.6473 | 16.712 | 8.23336 | 12.6022 | 8.67938 | 11.1015 | 8.94503 | 10.6636 |
| 13666 | 0.387864 | 0.345922 | 0.606012 | 0 | 0 | 0 | 0 | 0 | 0 | 0 | 0 |
| 14245 | 14.5259 | 27.3807 | 11.8075 | 28.2696 | 30.4572 | 23.1041 | 26.0315 | 16.2216 | 19.336 | 40.8914 | 39.1811 |
| 15390 | 0.180601 | 0.36442 | 0 | 0 | 0 | 0 | 0 | 0 | 0 | 0 | 0.211526 |

|  | Glucose | Xylose | Arabinose | Acetate | Coumarate | Ferulate | YNB Oleic Acid | YNB Ricinoleic Acid | YNB Glucose | YNB Gluc DOC | YPD |
| --- | --- | --- | --- | --- | --- | --- | --- | --- | --- | --- | --- |
| proteinId |  |  |  |  |  |  |  |  |  |  |  |
| 9331 | 0.317304 | 0.660965 | -0.231468 | -1.77645 | 0.262022 | -1.83089 | -3.79725 | -6.14964 | -0.943653 | -0.572548 | -1.19392 |
| 10149 | -0.075474 | -0.4456 | -0.241651 | 0.0656419 | -0.293397 | -0.0232707 | -0.142486 | 0.752866 | -0.199261 | -0.496042 | -0.194102 |
| 12591 | 0.194622 | 0.213333 | 0.153395 | 0.184751 | 0.0930738 | 0.0114473 | 0.11552 | -0.453092 | -0.126999 | -0.166318 | 0.367602 |
| 13580 | -0.239219 | 3.78134e-05 | -0.00584095 | -1.86406 | -0.310162 | -0.0740489 | -0.0123864 | 0.374486 | 0.231935 | 0.164481 | 0.439824 |
| 14245 | 0.0908612 | 0.110605 | 0.0366664 | -0.673789 | -1.23329 | -1.08932 | -2.16438 | -2.74615 | -0.209163 | -0.174774 | 0.0767215 |
| 15390 | 0.0647533 | 0.144211 | -0.0323675 | -0.139986 | -0.405733 | 0.0388373 | -0.199415 | -0.347569 | -0.159976 | 0.0398413 | 0.038154 |

In [25]:

```
print(Annotation.loc['9331','Combined Annotations'])
print(Annotation.loc['10149','WolfPSort'])
print(Annotation.loc['12591','Combined Annotations'])
print(Annotation.loc['12591','WolfPSort'])
print(Annotation.loc['13666','Combined Annotations'])
```

```
K15109: SLC25A20_29, CACT, CACL, CRC1; solute carrier family 25 (mitochondrial carnitine/acylcarnitine transporter), member 20/29
mito 11, plas 10, nucl 1, cyto 1, extr 1, cyto_nucl 1, pero 1, E.R. 1, golg 1, cyto_pero 1
KOG3957: Predicted L-carnitine dehydratase/alpha-methylacyl-CoA racemase
mito 11, extr 5, cyto 4.5, cyto_nucl 4.5, nucl 3.5
KOG0758: Mitochondrial carnitine-acylcarnitine carrier protein
```

In [26]:

```
for x in temp:
    if x in model.genes:
        for r in sorted(model.genes.get_by_id(x).reactions, key=lambda x: x.id):
            print(r.id, r.reaction, r.gene_reaction_rule)
    else:
        print(x, 'no reactions')
    print()
```

```
ACRNtm acrn_c --> acrn_m 9331
ARACHCRNt arachcrn_c --> arachcrn_m 9331
C160CRNt pmtcrn_c --> pmtcrn_m 9331
C161CRN2t hdd2crn_c --> hdd2crn_m 9331
C161CRNt hdcecrn_c --> hdcecrn_m 9331
C181CRNt odecrn_c --> odecrn_m 9331
CLPNDCRNt clpndcrn_c --> clpndcrn_m 9331
CRNCARtm acrn_c + crn_m --> acrn_m + crn_c 9331
CRNtim crn_m --> crn_c 9331
DCSPTN1CRNt dcsptn1crn_c --> dcsptn1crn_m 9331
ELAIDCRNt elaidcrn_c --> elaidcrn_m 9331
HEXCCRNt hexccrn_c --> hexccrn_m 9331
HPDCACRNt hpdcacrn_c --> hpdcacrn_m 9331
LNLNCGCRNt lnlncgcrn_c --> lnlncgcrn_m 9331
NRVNCCRNt nrvnccrn_c --> nrvnccrn_m 9331
PTDCACRNt ptdcacrn_c --> ptdcacrn_m 9331
TETTET6CRNt tettet6crn_c --> tettet6crn_m 9331
TTDCRNt ttdcrn_c --> ttdcrn_m 9331

10149 no reactions

12591 no reactions

ARACHCPT2 arachcrn_m + coa_m --> arachcoa_m + crn_m 13580
C160CPT2 coa_m + pmtcrn_m --> crn_m + pmtcoa_m 13580
C161CPT2 coa_m + hdcecrn_m --> crn_m + hdcoa_m 13580
C161CPT22 coa_m + hdd2crn_m --> crn_m + hdd2coa_m 13580
C181CPT2 coa_m + odecrn_m --> crn_m + odecoa_m 13580
CLPNDCPT2 clpndcrn_m + coa_m --> clpndcoa_m + crn_m 13580
CSNATirm accoa_m + crn_m <=> acrn_m + coa_m 13580 or 14245
CSNATr accoa_c + crn_c <=> acrn_c + coa_c 13580 or 14245
DCSPTN1CPT2 coa_m + dcsptn1crn_m --> crn_m + dcsptn1coa_m 13580
ELAIDCPT2 coa_m + elaidcrn_m --> crn_m + od2coa_m 13580
HEXCCPT2 coa_m + hexccrn_m --> crn_m + hexccoa_m 13580
HPDCACRNCPT2 coa_m + hpdcacrn_m --> crn_m + hpdcacoa_m 13580
LNLNCGCPT2 coa_m + lnlncgcrn_m --> crn_m + lnlncgcoa_m 13580
NRVNCCPT2 coa_m + nrvnccrn_m --> crn_m + nrvnccoa_m 13580
PTDCACRNCPT2 coa_m + ptdcacrn_m --> crn_m + ptdcacoa_m 13580
TETTET6CPT2 coa_m + tettet6crn_m --> crn_m + tettet6coa_m 13580
TTDCPT2 coa_m + ttdcrn_m --> crn_m + tdcoa_m 13580

13666 no reactions

CSNAT2m coa_m + pcrn_m <=> crn_m + ppcoa_m 14245
CSNAT2x crn_x + dmnoncoa_x <=> coa_x + dmnoncrn_x 14245
CSNAT3x crn_x + ppcoa_x <=> coa_x + pcrn_x 14245
CSNATer accoa_r + crn_r <=> acrn_r + coa_r 14245
CSNATirm accoa_m + crn_m <=> acrn_m + coa_m 13580 or 14245
CSNATm acrn_m + coa_m <=> accoa_m + crn_m 14245
CSNATp accoa_x + crn_x <=> acrn_x + coa_x 14245
CSNATr accoa_c + crn_c <=> acrn_c + coa_c 13580 or 14245

15390 no reactions
```

In [27]:

```
# CRNCARtm redundant with ACRNtm and CRNtim
# CSNATirm redundant with CSNATm
# CRAT is not in cytosol remove CSNATr
model.remove_reactions(['CRNCARtm','CSNATirm','CSNATr'],remove_orphans=True)
```

cyto carnitine acyltransferase CPT1 9315 (anchored in mito outermembrane facing cytosol)  
mito carnitine acyltransferase CPT2 13580  
pero carnitine acyltransferase 15783

mitochondrial/peroxisome/ER carnitine acetyltransferase CRAT 14245

mito carnitine transport 9331
pero carnitine transport 10149

In [28]:

```
for m in model.metabolites:
    if m.id.endswith('crn_x'):
        for r in m.reactions:
            print(r, r.gene_reaction_rule)
```

```
CSNATp: accoa_x + crn_x <=> acrn_x + coa_x 14245
CSNATp: accoa_x + crn_x <=> acrn_x + coa_x 14245
CSNAT2x: crn_x + dmnoncoa_x <=> coa_x + dmnoncrn_x 14245
CSNAT3x: crn_x + ppcoa_x <=> coa_x + pcrn_x 14245
CSNAT2x: crn_x + dmnoncoa_x <=> coa_x + dmnoncrn_x 14245
CSNAT3x: crn_x + ppcoa_x <=> coa_x + pcrn_x 14245
```

In [29]:

```
model.reactions.get_by_id('CSNAT2x').gene_reaction_rule = '15783'
# pcrn cyto transferase is missing
# transport of acrn and pcrn from pero and into mito is missing
r1 = hsa2.reactions.get_by_id('C30CPT1').copy()
r1.gene_reaction_rule = '9315'
r2 = hsa2.reactions.get_by_id('PCRNtm').copy()
r2.gene_reaction_rule = '9331'
r3 = hsa2.reactions.get_by_id('PCRNtc').copy()
r3.gene_reaction_rule = '10149'
r4 = sce.reactions.get_by_id('ACRNtp').copy()
r4.gene_reaction_rule = '10149'
model.add_reactions([r1,r2,r3,r4])
# also add medium chain crn transport from pero to cyto? further degradation in mito
```

ER and pero fatty acid transport  
https://www.ncbi.nlm.nih.gov/pmc/articles/PMC2846691/  
ER  
10946 ortholog of SLC33A1 ER acyl-coa transporter  
9912 FATP4 http://jcs.biologists.org/content/119/22/4678  
AtABCA9 https://www.pnas.org/content/110/2/773  
AT-1 https://www.ncbi.nlm.nih.gov/pmc/articles/PMC2939804/  
pero  
9637 PXA1 and 13167 PXA2 https://www.ncbi.nlm.nih.gov/pmc/articles/PMC3370197/

In [30]:

```
temp = ['10946','9912']
display(Annotation.loc[temp])
Show_Data(temp)
```

|  | Combined Annotations | Signal P | Sc288c Orthologs | Human Orthologs | Sc288 Best Hit | Human Blast | Essential | WolfPSort | C Terminal |
| --- | --- | --- | --- | --- | --- | --- | --- | --- | --- |
| RTO4\_ID |  |  |  |  |  |  |  |  |  |
| 10946 | KOG3574: Acetyl-CoA transporter |  | YBR220C | SLC33A1 | YBR220C | SLC33 | Not Essential | plas 26 | NGR\* |
| 9912 | K08745: SLC27A1\_4, FATP1, FATP4; solute carrie... | S | FAT1 | SLC27A1,SLC27A4,SLC27A2,SLC27A5,SLC27A6 | FAT1 | SLC27 | Not Essential | extr 12, plas 8, pero 4, mito 1, cyto 1, E.R. ... | VRL\* |

| strain | WT | | | | | | | | | | | | | | | | |
| --- | --- | --- | --- | --- | --- | --- | --- | --- | --- | --- | --- | --- | --- | --- | --- | --- | --- |
| condition | G\_MM | C\_MM | G\_SD | | GX\_SD | | | X\_SD | | A\_SD | | C\_SD | | MM\_CN120 | | MM\_CN5 | Diversity\_Sample |
| phase | exp | exp | exp | stat | exp | trans | stat | exp | stat | exp | stat | exp | stat | exp | stat | exp | exp |
| proteinId | Set1 | Set1 | Set2 | Set2 | Set2 | Set2 | Set2 | Set2 | Set2 | Set2 | Set2 | Set2 | Set2 | Set3 | Set3 | Set3 | Set3 |
| 10946 | 5.41534 | 5.5384 | 5.23328 | 4.48787 | 5.30221 | 5.27407 | 5.16605 | 5.37864 | 4.99623 | 5.25112 | 4.81155 | 5.38352 | 5.5417 | 6.48125 | 6.29298 | 6.63921 | 6.93083 |
| 9912 | 5.93073 | 5.40845 | 5.89641 | 6.2248 | 5.87895 | 5.80068 | 5.93113 | 5.50431 | 5.91601 | 5.5566 | 6.22915 | 5.26476 | 4.97236 | 5.88904 | 5.91406 | 5.56329 | 5.5721 |

| strain | WT | | | | | | | | | | |
| --- | --- | --- | --- | --- | --- | --- | --- | --- | --- | --- | --- |
| condition | G\_SD | | GX\_SD | | | X\_SD | | A\_SD | | C\_SD | |
| proteinId | exp | stat | exp | trans | stat | exp | stat | exp | stat | exp | stat |
| 10946 | 1.33766 | 0.351946 | 0.819619 | 0.7501 | 0.564773 | 0 | 0.377624 | 0.772848 | 0 | 0.211647 | 0 |
| 9912 | 4.40189 | 9.06281 | 3.66274 | 8.91569 | 10.3772 | 5.86924 | 6.49892 | 5.02317 | 5.93995 | 4.96027 | 4.99924 |

|  | Glucose | Xylose | Arabinose | Acetate | Coumarate | Ferulate | YNB Oleic Acid | YNB Ricinoleic Acid | YNB Glucose | YNB Gluc DOC | YPD |
| --- | --- | --- | --- | --- | --- | --- | --- | --- | --- | --- | --- |
| proteinId |  |  |  |  |  |  |  |  |  |  |  |
| 10946 | 0.0358943 | 0.227035 | 0.149761 | 0.0525809 | -0.825083 | 0.314581 | 0.515223 | 0.992047 | 0.412156 | -0.0177003 | 0.296114 |
| 9912 | 0.0677425 | -0.270103 | -0.302936 | -0.051684 | -0.404229 | 0.0682701 | -0.0630695 | -0.272301 | -0.340334 | -0.0743227 | -0.0288945 |

In [31]:

```
for x in temp:
    if x in model.genes:
        for r in sorted(model.genes.get_by_id(x).reactions, key=lambda x: x.id):
            print(r.id, r.reaction, r.gene_reaction_rule)
    else:
        print(x, 'no reactions')
    print()
```

```
ACCOAgt accoa_c <=> accoa_g 10946
ACCOAtm accoa_c + coa_m <=> accoa_m + coa_c 10946
ACCOAtr accoa_c <=> accoa_r 10946
ACCOAtx accoa_c + coa_x <=> accoa_x + coa_c 10946

FA240tp ttc_c --> ttc_x 9912
FA260tp hexc_c --> hexc_x 9912
FACOAL240p atp_x + coa_x + ttc_x --> amp_x + ppi_x + ttccoa_x 9912
FACOAL260p atp_x + coa_x + hexc_x --> amp_x + hexccoa_x + ppi_x 9912
FATP1t hdca_c + na1_c <=> hdca_e + na1_e 9912
FATP2t na1_c + ocdcea_c <=> na1_e + ocdcea_e 9912
FATP3t na1_c + ocdca_c <=> na1_e + ocdca_e 9912
FATP4t arach_c + na1_c <=> arach_e + na1_e 9912
FATP8t na1_c + ttc_c <=> na1_e + ttc_e 9912
FATP9t na1_c + nrvnc_c <=> na1_e + nrvnc_e 9912
VLCS2p atp_x + coa_x + dhcholestanate_x --> amp_x + dhcholestancoa_x + ppi_x 9912
VLCS2r atp_r + coa_r + dhcholestanate_r --> amp_r + dhcholestancoa_r + ppi_r 9912
VLCSp atp_x + coa_x + thcholstoic_x --> amp_x + cholcoar_x + ppi_x 9912
VLCSr atp_r + coa_r + thcholstoic_r --> amp_r + cholcoar_r + ppi_r 9912
```

In [32]:

```
model.remove_reactions(['ACCOAtm','ACCOAtx'],remove_orphans=True)
```

In [33]:

```
for m in sorted(model.metabolites, key=lambda x: x.id):
    if m.id.endswith('coa_r') and not m.id == 'coa_r' and not m.id == 'accoa_r':
        print(m)
        for r in m.reactions:
            print(r, r.gene_reaction_rule)
        print()
```

```
dcacoa_r
TRIGSer_RT: 0.01 12dgr_RT_r + 0.02 dcacoa_r + 0.06 ddcacoa_r + 0.17 hdcoa_r + 0.09 ocdycacoa_r + 0.24 odecoa_r + 0.27 pmtcoa_r + 0.05 stcoa_r + 0.1 tdcoa_r --> coa_r + 0.01 triglyc_RT_r 16460
AGATer_RT: 0.01 1ag3p_RT_r + 0.02 dcacoa_r + 0.06 ddcacoa_r + 0.17 hdcoa_r + 0.09 ocdycacoa_r + 0.24 odecoa_r + 0.27 pmtcoa_r + 0.05 stcoa_r + 0.1 tdcoa_r --> coa_r + 0.01 pa_RT_r 10427 or 16030 or 16779 or 9746
LPCATer_RT: 0.01 1agpc_RT_r + 0.02 dcacoa_r + 0.06 ddcacoa_r + 0.17 hdcoa_r + 0.09 ocdycacoa_r + 0.24 odecoa_r + 0.27 pmtcoa_r + 0.05 stcoa_r + 0.1 tdcoa_r --> coa_r + 0.01 pc_RT_r 16030
GAT2er_RT: 0.02 dcacoa_r + 0.06 ddcacoa_r + dhap_r + 0.17 hdcoa_r + 0.09 ocdycacoa_r + 0.24 odecoa_r + 0.27 pmtcoa_r + 0.05 stcoa_r + 0.1 tdcoa_r --> 0.01 1agly3p_RT_r + coa_r 13369 or 15435
GAT1er_RT: 0.02 dcacoa_r + 0.06 ddcacoa_r + glyc3p_r + 0.17 hdcoa_r + 0.09 ocdycacoa_r + 0.24 odecoa_r + 0.27 pmtcoa_r + 0.05 stcoa_r + 0.1 tdcoa_r --> 0.01 1ag3p_RT_r + coa_r 15435

ddcacoa_r
TRIGSer_RT: 0.01 12dgr_RT_r + 0.02 dcacoa_r + 0.06 ddcacoa_r + 0.17 hdcoa_r + 0.09 ocdycacoa_r + 0.24 odecoa_r + 0.27 pmtcoa_r + 0.05 stcoa_r + 0.1 tdcoa_r --> coa_r + 0.01 triglyc_RT_r 16460
AGATer_RT: 0.01 1ag3p_RT_r + 0.02 dcacoa_r + 0.06 ddcacoa_r + 0.17 hdcoa_r + 0.09 ocdycacoa_r + 0.24 odecoa_r + 0.27 pmtcoa_r + 0.05 stcoa_r + 0.1 tdcoa_r --> coa_r + 0.01 pa_RT_r 10427 or 16030 or 16779 or 9746
LPCATer_RT: 0.01 1agpc_RT_r + 0.02 dcacoa_r + 0.06 ddcacoa_r + 0.17 hdcoa_r + 0.09 ocdycacoa_r + 0.24 odecoa_r + 0.27 pmtcoa_r + 0.05 stcoa_r + 0.1 tdcoa_r --> coa_r + 0.01 pc_RT_r 16030
GAT2er_RT: 0.02 dcacoa_r + 0.06 ddcacoa_r + dhap_r + 0.17 hdcoa_r + 0.09 ocdycacoa_r + 0.24 odecoa_r + 0.27 pmtcoa_r + 0.05 stcoa_r + 0.1 tdcoa_r --> 0.01 1agly3p_RT_r + coa_r 13369 or 15435
GAT1er_RT: 0.02 dcacoa_r + 0.06 ddcacoa_r + glyc3p_r + 0.17 hdcoa_r + 0.09 ocdycacoa_r + 0.24 odecoa_r + 0.27 pmtcoa_r + 0.05 stcoa_r + 0.1 tdcoa_r --> 0.01 1ag3p_RT_r + coa_r 15435

dhcholestancoa_r
VLCS2r: atp_r + coa_r + dhcholestanate_r --> amp_r + dhcholestancoa_r + ppi_r 9912
AMACR2r: dhcholestancoa_r + 0.5 o2_r --> dhcholoylcoa_r + h2o_r 8616

dhcholoylcoa_r
AMACR2r: dhcholestancoa_r + 0.5 o2_r --> dhcholoylcoa_r + h2o_r 8616

hdcoa_r
DESAT1619Zer: h_r + nadh_r + o2_r + pmtcoa_r --> 2.0 h2o_r + hdcoa_r + nad_r 9730
ERGSTATer_RT: 0.01 ergst_r + 0.655 hdcoa_r + 0.01 hexccoa_r + 0.27 odecoa_r + 0.02 pmtcoa_r + 0.03 stcoa_r + 0.015 tdcoa_r --> coa_r + 0.01 ergstest_RT_r 11799
EPISTATer_RT: 0.01 epist_r + 0.655 hdcoa_r + 0.01 hexccoa_r + 0.27 odecoa_r + 0.02 pmtcoa_r + 0.03 stcoa_r + 0.015 tdcoa_r --> coa_r + 0.01 epistest_RT_r 11799
LPCATer_RT: 0.01 1agpc_RT_r + 0.02 dcacoa_r + 0.06 ddcacoa_r + 0.17 hdcoa_r + 0.09 ocdycacoa_r + 0.24 odecoa_r + 0.27 pmtcoa_r + 0.05 stcoa_r + 0.1 tdcoa_r --> coa_r + 0.01 pc_RT_r 16030
GAT2er_RT: 0.02 dcacoa_r + 0.06 ddcacoa_r + dhap_r + 0.17 hdcoa_r + 0.09 ocdycacoa_r + 0.24 odecoa_r + 0.27 pmtcoa_r + 0.05 stcoa_r + 0.1 tdcoa_r --> 0.01 1agly3p_RT_r + coa_r 13369 or 15435
GAT1er_RT: 0.02 dcacoa_r + 0.06 ddcacoa_r + glyc3p_r + 0.17 hdcoa_r + 0.09 ocdycacoa_r + 0.24 odecoa_r + 0.27 pmtcoa_r + 0.05 stcoa_r + 0.1 tdcoa_r --> 0.01 1ag3p_RT_r + coa_r 15435
TRIGSer_RT: 0.01 12dgr_RT_r + 0.02 dcacoa_r + 0.06 ddcacoa_r + 0.17 hdcoa_r + 0.09 ocdycacoa_r + 0.24 odecoa_r + 0.27 pmtcoa_r + 0.05 stcoa_r + 0.1 tdcoa_r --> coa_r + 0.01 triglyc_RT_r 16460
FECOSTATer_RT: 0.01 fecost_r + 0.655 hdcoa_r + 0.01 hexccoa_r + 0.27 odecoa_r + 0.02 pmtcoa_r + 0.03 stcoa_r + 0.015 tdcoa_r --> coa_r + 0.01 fecostest_RT_r 11799
ZYMSTATer_RT: 0.655 hdcoa_r + 0.01 hexccoa_r + 0.27 odecoa_r + 0.02 pmtcoa_r + 0.03 stcoa_r + 0.015 tdcoa_r + 0.01 zymst_r --> coa_r + 0.01 zymstest_RT_r 11799
LANOSTATer_RT: 0.655 hdcoa_r + 0.01 hexccoa_r + 0.01 lanost_r + 0.27 odecoa_r + 0.02 pmtcoa_r + 0.03 stcoa_r + 0.015 tdcoa_r --> coa_r + 0.01 lanostest_RT_r 11799
AGATer_RT: 0.01 1ag3p_RT_r + 0.02 dcacoa_r + 0.06 ddcacoa_r + 0.17 hdcoa_r + 0.09 ocdycacoa_r + 0.24 odecoa_r + 0.27 pmtcoa_r + 0.05 stcoa_r + 0.1 tdcoa_r --> coa_r + 0.01 pa_RT_r 10427 or 16030 or 16779 or 9746

hexccoa_r
ERGSTATer_RT: 0.01 ergst_r + 0.655 hdcoa_r + 0.01 hexccoa_r + 0.27 odecoa_r + 0.02 pmtcoa_r + 0.03 stcoa_r + 0.015 tdcoa_r --> coa_r + 0.01 ergstest_RT_r 11799
EPISTATer_RT: 0.01 epist_r + 0.655 hdcoa_r + 0.01 hexccoa_r + 0.27 odecoa_r + 0.02 pmtcoa_r + 0.03 stcoa_r + 0.015 tdcoa_r --> coa_r + 0.01 epistest_RT_r 11799
FAS260COAer: 3.0 h_r + malcoa_r + 2.0 nadph_r + ttccoa_r --> co2_r + coa_r + h2o_r + hexccoa_r + 2.0 nadp_r 10677 and 11343 and 16241 and 16695
CERS226er: hexccoa_r + psphings_r --> cer2_26_r + coa_r + h_r 11391 or 15168
FECOSTATer_RT: 0.01 fecost_r + 0.655 hdcoa_r + 0.01 hexccoa_r + 0.27 odecoa_r + 0.02 pmtcoa_r + 0.03 stcoa_r + 0.015 tdcoa_r --> coa_r + 0.01 fecostest_RT_r 11799
ZYMSTATer_RT: 0.655 hdcoa_r + 0.01 hexccoa_r + 0.27 odecoa_r + 0.02 pmtcoa_r + 0.03 stcoa_r + 0.015 tdcoa_r + 0.01 zymst_r --> coa_r + 0.01 zymstest_RT_r 11799
LANOSTATer_RT: 0.655 hdcoa_r + 0.01 hexccoa_r + 0.01 lanost_r + 0.27 odecoa_r + 0.02 pmtcoa_r + 0.03 stcoa_r + 0.015 tdcoa_r --> coa_r + 0.01 lanostest_RT_r 11799
CERS126er: hexccoa_r + sphgn_r --> cer1_26_r + coa_r + h_r 11391 or 15168

hmgcoa_r
HMGCOARr: 2.0 h_r + hmgcoa_r + 2.0 nadph_r --> coa_r + mev__R_r + 2.0 nadp_r 9574

lnlncgcoa_r
DESAT1836Z9Z12Zer: h_r + nadh_r + o2_r + ocdycacoa_r --> 2.0 h2o_r + lnlncgcoa_r + nad_r 9578

malcoa_r
FAS260COAer: 3.0 h_r + malcoa_r + 2.0 nadph_r + ttccoa_r --> co2_r + coa_r + h2o_r + hexccoa_r + 2.0 nadp_r 10677 and 11343 and 16241 and 16695
FAS240COAer: 9.0 h_r + 3.0 malcoa_r + 6.0 nadph_r + stcoa_r --> 3.0 co2_r + 3.0 coa_r + 3.0 h2o_r + 6.0 nadp_r + ttccoa_r (10677 and 11343 and 16241 and 16695) or (10677 and 16241 and 16655 and 16695)

ocdycacoa_r
TRIGSer_RT: 0.01 12dgr_RT_r + 0.02 dcacoa_r + 0.06 ddcacoa_r + 0.17 hdcoa_r + 0.09 ocdycacoa_r + 0.24 odecoa_r + 0.27 pmtcoa_r + 0.05 stcoa_r + 0.1 tdcoa_r --> coa_r + 0.01 triglyc_RT_r 16460
AGATer_RT: 0.01 1ag3p_RT_r + 0.02 dcacoa_r + 0.06 ddcacoa_r + 0.17 hdcoa_r + 0.09 ocdycacoa_r + 0.24 odecoa_r + 0.27 pmtcoa_r + 0.05 stcoa_r + 0.1 tdcoa_r --> coa_r + 0.01 pa_RT_r 10427 or 16030 or 16779 or 9746
LPCATer_RT: 0.01 1agpc_RT_r + 0.02 dcacoa_r + 0.06 ddcacoa_r + 0.17 hdcoa_r + 0.09 ocdycacoa_r + 0.24 odecoa_r + 0.27 pmtcoa_r + 0.05 stcoa_r + 0.1 tdcoa_r --> coa_r + 0.01 pc_RT_r 16030
DESAT1836Z9Z12Zer: h_r + nadh_r + o2_r + ocdycacoa_r --> 2.0 h2o_r + lnlncgcoa_r + nad_r 9578
DESAT1829Z12Zer: h_r + nadh_r + o2_r + odecoa_r --> 2.0 h2o_r + nad_r + ocdycacoa_r (12471 and 16097 and 8845) or (13206 and 16097 and 8845)
GAT2er_RT: 0.02 dcacoa_r + 0.06 ddcacoa_r + dhap_r + 0.17 hdcoa_r + 0.09 ocdycacoa_r + 0.24 odecoa_r + 0.27 pmtcoa_r + 0.05 stcoa_r + 0.1 tdcoa_r --> 0.01 1agly3p_RT_r + coa_r 13369 or 15435
GAT1er_RT: 0.02 dcacoa_r + 0.06 ddcacoa_r + glyc3p_r + 0.17 hdcoa_r + 0.09 ocdycacoa_r + 0.24 odecoa_r + 0.27 pmtcoa_r + 0.05 stcoa_r + 0.1 tdcoa_r --> 0.01 1ag3p_RT_r + coa_r 15435

odecoa_r
ERGSTATer_RT: 0.01 ergst_r + 0.655 hdcoa_r + 0.01 hexccoa_r + 0.27 odecoa_r + 0.02 pmtcoa_r + 0.03 stcoa_r + 0.015 tdcoa_r --> coa_r + 0.01 ergstest_RT_r 11799
EPISTATer_RT: 0.01 epist_r + 0.655 hdcoa_r + 0.01 hexccoa_r + 0.27 odecoa_r + 0.02 pmtcoa_r + 0.03 stcoa_r + 0.015 tdcoa_r --> coa_r + 0.01 epistest_RT_r 11799
LPCATer_RT: 0.01 1agpc_RT_r + 0.02 dcacoa_r + 0.06 ddcacoa_r + 0.17 hdcoa_r + 0.09 ocdycacoa_r + 0.24 odecoa_r + 0.27 pmtcoa_r + 0.05 stcoa_r + 0.1 tdcoa_r --> coa_r + 0.01 pc_RT_r 16030
DESAT1819Zer: h_r + nadh_r + o2_r + stcoa_r --> 2.0 h2o_r + nad_r + odecoa_r 9730
GAT2er_RT: 0.02 dcacoa_r + 0.06 ddcacoa_r + dhap_r + 0.17 hdcoa_r + 0.09 ocdycacoa_r + 0.24 odecoa_r + 0.27 pmtcoa_r + 0.05 stcoa_r + 0.1 tdcoa_r --> 0.01 1agly3p_RT_r + coa_r 13369 or 15435
GAT1er_RT: 0.02 dcacoa_r + 0.06 ddcacoa_r + glyc3p_r + 0.17 hdcoa_r + 0.09 ocdycacoa_r + 0.24 odecoa_r + 0.27 pmtcoa_r + 0.05 stcoa_r + 0.1 tdcoa_r --> 0.01 1ag3p_RT_r + coa_r 15435
TRIGSer_RT: 0.01 12dgr_RT_r + 0.02 dcacoa_r + 0.06 ddcacoa_r + 0.17 hdcoa_r + 0.09 ocdycacoa_r + 0.24 odecoa_r + 0.27 pmtcoa_r + 0.05 stcoa_r + 0.1 tdcoa_r --> coa_r + 0.01 triglyc_RT_r 16460
FECOSTATer_RT: 0.01 fecost_r + 0.655 hdcoa_r + 0.01 hexccoa_r + 0.27 odecoa_r + 0.02 pmtcoa_r + 0.03 stcoa_r + 0.015 tdcoa_r --> coa_r + 0.01 fecostest_RT_r 11799
ZYMSTATer_RT: 0.655 hdcoa_r + 0.01 hexccoa_r + 0.27 odecoa_r + 0.02 pmtcoa_r + 0.03 stcoa_r + 0.015 tdcoa_r + 0.01 zymst_r --> coa_r + 0.01 zymstest_RT_r 11799
LANOSTATer_RT: 0.655 hdcoa_r + 0.01 hexccoa_r + 0.01 lanost_r + 0.27 odecoa_r + 0.02 pmtcoa_r + 0.03 stcoa_r + 0.015 tdcoa_r --> coa_r + 0.01 lanostest_RT_r 11799
DESAT1829Z12Zer: h_r + nadh_r + o2_r + odecoa_r --> 2.0 h2o_r + nad_r + ocdycacoa_r (12471 and 16097 and 8845) or (13206 and 16097 and 8845)
AGATer_RT: 0.01 1ag3p_RT_r + 0.02 dcacoa_r + 0.06 ddcacoa_r + 0.17 hdcoa_r + 0.09 ocdycacoa_r + 0.24 odecoa_r + 0.27 pmtcoa_r + 0.05 stcoa_r + 0.1 tdcoa_r --> coa_r + 0.01 pa_RT_r 10427 or 16030 or 16779 or 9746

pmtcoa_r
DESAT1619Zer: h_r + nadh_r + o2_r + pmtcoa_r --> 2.0 h2o_r + hdcoa_r + nad_r 9730
ERGSTATer_RT: 0.01 ergst_r + 0.655 hdcoa_r + 0.01 hexccoa_r + 0.27 odecoa_r + 0.02 pmtcoa_r + 0.03 stcoa_r + 0.015 tdcoa_r --> coa_r + 0.01 ergstest_RT_r 11799
EPISTATer_RT: 0.01 epist_r + 0.655 hdcoa_r + 0.01 hexccoa_r + 0.27 odecoa_r + 0.02 pmtcoa_r + 0.03 stcoa_r + 0.015 tdcoa_r --> coa_r + 0.01 epistest_RT_r 11799
LPCATer_RT: 0.01 1agpc_RT_r + 0.02 dcacoa_r + 0.06 ddcacoa_r + 0.17 hdcoa_r + 0.09 ocdycacoa_r + 0.24 odecoa_r + 0.27 pmtcoa_r + 0.05 stcoa_r + 0.1 tdcoa_r --> coa_r + 0.01 pc_RT_r 16030
GAT2er_RT: 0.02 dcacoa_r + 0.06 ddcacoa_r + dhap_r + 0.17 hdcoa_r + 0.09 ocdycacoa_r + 0.24 odecoa_r + 0.27 pmtcoa_r + 0.05 stcoa_r + 0.1 tdcoa_r --> 0.01 1agly3p_RT_r + coa_r 13369 or 15435
GAT1er_RT: 0.02 dcacoa_r + 0.06 ddcacoa_r + glyc3p_r + 0.17 hdcoa_r + 0.09 ocdycacoa_r + 0.24 odecoa_r + 0.27 pmtcoa_r + 0.05 stcoa_r + 0.1 tdcoa_r --> 0.01 1ag3p_RT_r + coa_r 15435
TRIGSer_RT: 0.01 12dgr_RT_r + 0.02 dcacoa_r + 0.06 ddcacoa_r + 0.17 hdcoa_r + 0.09 ocdycacoa_r + 0.24 odecoa_r + 0.27 pmtcoa_r + 0.05 stcoa_r + 0.1 tdcoa_r --> coa_r + 0.01 triglyc_RT_r 16460
FECOSTATer_RT: 0.01 fecost_r + 0.655 hdcoa_r + 0.01 hexccoa_r + 0.27 odecoa_r + 0.02 pmtcoa_r + 0.03 stcoa_r + 0.015 tdcoa_r --> coa_r + 0.01 fecostest_RT_r 11799
ZYMSTATer_RT: 0.655 hdcoa_r + 0.01 hexccoa_r + 0.27 odecoa_r + 0.02 pmtcoa_r + 0.03 stcoa_r + 0.015 tdcoa_r + 0.01 zymst_r --> coa_r + 0.01 zymstest_RT_r 11799
SERPTer: h_r + pmtcoa_r + ser__L_r --> 3dsphgn_r + co2_r + coa_r 10303 and 9394 and 9425
LANOSTATer_RT: 0.655 hdcoa_r + 0.01 hexccoa_r + 0.01 lanost_r + 0.27 odecoa_r + 0.02 pmtcoa_r + 0.03 stcoa_r + 0.015 tdcoa_r --> coa_r + 0.01 lanostest_RT_r 11799
AGATer_RT: 0.01 1ag3p_RT_r + 0.02 dcacoa_r + 0.06 ddcacoa_r + 0.17 hdcoa_r + 0.09 ocdycacoa_r + 0.24 odecoa_r + 0.27 pmtcoa_r + 0.05 stcoa_r + 0.1 tdcoa_r --> coa_r + 0.01 pa_RT_r 10427 or 16030 or 16779 or 9746

stcoa_r
ERGSTATer_RT: 0.01 ergst_r + 0.655 hdcoa_r + 0.01 hexccoa_r + 0.27 odecoa_r + 0.02 pmtcoa_r + 0.03 stcoa_r + 0.015 tdcoa_r --> coa_r + 0.01 ergstest_RT_r 11799
EPISTATer_RT: 0.01 epist_r + 0.655 hdcoa_r + 0.01 hexccoa_r + 0.27 odecoa_r + 0.02 pmtcoa_r + 0.03 stcoa_r + 0.015 tdcoa_r --> coa_r + 0.01 epistest_RT_r 11799
LPCATer_RT: 0.01 1agpc_RT_r + 0.02 dcacoa_r + 0.06 ddcacoa_r + 0.17 hdcoa_r + 0.09 ocdycacoa_r + 0.24 odecoa_r + 0.27 pmtcoa_r + 0.05 stcoa_r + 0.1 tdcoa_r --> coa_r + 0.01 pc_RT_r 16030
DESAT1819Zer: h_r + nadh_r + o2_r + stcoa_r --> 2.0 h2o_r + nad_r + odecoa_r 9730
GAT2er_RT: 0.02 dcacoa_r + 0.06 ddcacoa_r + dhap_r + 0.17 hdcoa_r + 0.09 ocdycacoa_r + 0.24 odecoa_r + 0.27 pmtcoa_r + 0.05 stcoa_r + 0.1 tdcoa_r --> 0.01 1agly3p_RT_r + coa_r 13369 or 15435
CERS118er: sphgn_r + stcoa_r --> cer1_18_r + coa_r + h_r 11391 or 15168
GAT1er_RT: 0.02 dcacoa_r + 0.06 ddcacoa_r + glyc3p_r + 0.17 hdcoa_r + 0.09 ocdycacoa_r + 0.24 odecoa_r + 0.27 pmtcoa_r + 0.05 stcoa_r + 0.1 tdcoa_r --> 0.01 1ag3p_RT_r + coa_r 15435
TRIGSer_RT: 0.01 12dgr_RT_r + 0.02 dcacoa_r + 0.06 ddcacoa_r + 0.17 hdcoa_r + 0.09 ocdycacoa_r + 0.24 odecoa_r + 0.27 pmtcoa_r + 0.05 stcoa_r + 0.1 tdcoa_r --> coa_r + 0.01 triglyc_RT_r 16460
FECOSTATer_RT: 0.01 fecost_r + 0.655 hdcoa_r + 0.01 hexccoa_r + 0.27 odecoa_r + 0.02 pmtcoa_r + 0.03 stcoa_r + 0.015 tdcoa_r --> coa_r + 0.01 fecostest_RT_r 11799
ZYMSTATer_RT: 0.655 hdcoa_r + 0.01 hexccoa_r + 0.27 odecoa_r + 0.02 pmtcoa_r + 0.03 stcoa_r + 0.015 tdcoa_r + 0.01 zymst_r --> coa_r + 0.01 zymstest_RT_r 11799
LANOSTATer_RT: 0.655 hdcoa_r + 0.01 hexccoa_r + 0.01 lanost_r + 0.27 odecoa_r + 0.02 pmtcoa_r + 0.03 stcoa_r + 0.015 tdcoa_r --> coa_r + 0.01 lanostest_RT_r 11799
FAS240COAer: 9.0 h_r + 3.0 malcoa_r + 6.0 nadph_r + stcoa_r --> 3.0 co2_r + 3.0 coa_r + 3.0 h2o_r + 6.0 nadp_r + ttccoa_r (10677 and 11343 and 16241 and 16695) or (10677 and 16241 and 16655 and 16695)
AGATer_RT: 0.01 1ag3p_RT_r + 0.02 dcacoa_r + 0.06 ddcacoa_r + 0.17 hdcoa_r + 0.09 ocdycacoa_r + 0.24 odecoa_r + 0.27 pmtcoa_r + 0.05 stcoa_r + 0.1 tdcoa_r --> coa_r + 0.01 pa_RT_r 10427 or 16030 or 16779 or 9746

tdcoa_r
ERGSTATer_RT: 0.01 ergst_r + 0.655 hdcoa_r + 0.01 hexccoa_r + 0.27 odecoa_r + 0.02 pmtcoa_r + 0.03 stcoa_r + 0.015 tdcoa_r --> coa_r + 0.01 ergstest_RT_r 11799
EPISTATer_RT: 0.01 epist_r + 0.655 hdcoa_r + 0.01 hexccoa_r + 0.27 odecoa_r + 0.02 pmtcoa_r + 0.03 stcoa_r + 0.015 tdcoa_r --> coa_r + 0.01 epistest_RT_r 11799
LPCATer_RT: 0.01 1agpc_RT_r + 0.02 dcacoa_r + 0.06 ddcacoa_r + 0.17 hdcoa_r + 0.09 ocdycacoa_r + 0.24 odecoa_r + 0.27 pmtcoa_r + 0.05 stcoa_r + 0.1 tdcoa_r --> coa_r + 0.01 pc_RT_r 16030
GAT2er_RT: 0.02 dcacoa_r + 0.06 ddcacoa_r + dhap_r + 0.17 hdcoa_r + 0.09 ocdycacoa_r + 0.24 odecoa_r + 0.27 pmtcoa_r + 0.05 stcoa_r + 0.1 tdcoa_r --> 0.01 1agly3p_RT_r + coa_r 13369 or 15435
GAT1er_RT: 0.02 dcacoa_r + 0.06 ddcacoa_r + glyc3p_r + 0.17 hdcoa_r + 0.09 ocdycacoa_r + 0.24 odecoa_r + 0.27 pmtcoa_r + 0.05 stcoa_r + 0.1 tdcoa_r --> 0.01 1ag3p_RT_r + coa_r 15435
TRIGSer_RT: 0.01 12dgr_RT_r + 0.02 dcacoa_r + 0.06 ddcacoa_r + 0.17 hdcoa_r + 0.09 ocdycacoa_r + 0.24 odecoa_r + 0.27 pmtcoa_r + 0.05 stcoa_r + 0.1 tdcoa_r --> coa_r + 0.01 triglyc_RT_r 16460
FECOSTATer_RT: 0.01 fecost_r + 0.655 hdcoa_r + 0.01 hexccoa_r + 0.27 odecoa_r + 0.02 pmtcoa_r + 0.03 stcoa_r + 0.015 tdcoa_r --> coa_r + 0.01 fecostest_RT_r 11799
ZYMSTATer_RT: 0.655 hdcoa_r + 0.01 hexccoa_r + 0.27 odecoa_r + 0.02 pmtcoa_r + 0.03 stcoa_r + 0.015 tdcoa_r + 0.01 zymst_r --> coa_r + 0.01 zymstest_RT_r 11799
LANOSTATer_RT: 0.655 hdcoa_r + 0.01 hexccoa_r + 0.01 lanost_r + 0.27 odecoa_r + 0.02 pmtcoa_r + 0.03 stcoa_r + 0.015 tdcoa_r --> coa_r + 0.01 lanostest_RT_r 11799
AGATer_RT: 0.01 1ag3p_RT_r + 0.02 dcacoa_r + 0.06 ddcacoa_r + 0.17 hdcoa_r + 0.09 ocdycacoa_r + 0.24 odecoa_r + 0.27 pmtcoa_r + 0.05 stcoa_r + 0.1 tdcoa_r --> coa_r + 0.01 pa_RT_r 10427 or 16030 or 16779 or 9746

ttccoa_r
CERS224er: psphings_r + ttccoa_r --> cer2_24_r + coa_r + h_r 11391 or 15168
CERS124er: sphgn_r + ttccoa_r --> cer1_24_r + coa_r + h_r 11391 or 15168
FAS260COAer: 3.0 h_r + malcoa_r + 2.0 nadph_r + ttccoa_r --> co2_r + coa_r + h2o_r + hexccoa_r + 2.0 nadp_r 10677 and 11343 and 16241 and 16695
FAS240COAer: 9.0 h_r + 3.0 malcoa_r + 6.0 nadph_r + stcoa_r --> 3.0 co2_r + 3.0 coa_r + 3.0 h2o_r + 6.0 nadp_r + ttccoa_r (10677 and 11343 and 16241 and 16695) or (10677 and 16241 and 16655 and 16695)
```

In [34]:

```
for m in sorted(model.metabolites, key=lambda x: x.id):
    if m.id.endswith('coa_r') and not m.id == 'coa_r' and not m.id == 'accoa_r' and not m.id.startswith('dhchol'):
        print(m, m.name, m.id.upper().replace('_R','tr'), m.id.upper().replace('_R','tr') in model.reactions)
```

```
dcacoa_r Decanoyl-CoA (n-C10:0CoA) DCACOAtr False
ddcacoa_r Lauroyl-CoA DDCACOAtr False
hdcoa_r Hexadecenoyl-CoA (n-C16:1CoA) HDCOAtr False
hexccoa_r Hexacosanoyl CoA  n C260CoA  C47H82N7O17P3S HEXCCOAtr False
hmgcoa_r Hydroxymethylglutaryl CoA C27H39N7O20P3S HMGCOAtr False
lnlncgcoa_r Gamma-linolenoyl-CoA LNLNCGCOAtr False
malcoa_r Malonyl CoA C24H33N7O19P3S MALCOAtr False
ocdycacoa_r Octadecynoyl CoA  n C182CoA  C39H62N7O17P3S OCDYCACOAtr False
odecoa_r Octadecenoyl-CoA (n-C18:1CoA) ODECOAtr False
pmtcoa_r Palmitoyl-CoA PMTCOAtr False
stcoa_r Stearoyl-CoA STCOAtr False
tdcoa_r Tetradecanoyl-CoA TDCOAtr False
ttccoa_r Tetracosanoyl CoA   n C240CoA  C45H78N7O17P3S TTCCOAtr False
```

In [35]:

```
for m in sorted(model.metabolites, key=lambda x: x.id):
    if m.id.endswith('coa_r') and not m.id == 'coa_r' and not m.id == 'accoa_r' and not m.id.startswith('dhchol'):
        print('model.reactions.get_by_id(\''+m.id.upper().replace('_R','tr')+'\').name = \''+m.name+' transport, endoplasmic reticulum\'')
```

```
model.reactions.get_by_id('DCACOAtr').name = 'Decanoyl-CoA (n-C10:0CoA) transport, endoplasmic reticulum'
model.reactions.get_by_id('DDCACOAtr').name = 'Lauroyl-CoA transport, endoplasmic reticulum'
model.reactions.get_by_id('HDCOAtr').name = 'Hexadecenoyl-CoA (n-C16:1CoA) transport, endoplasmic reticulum'
model.reactions.get_by_id('HEXCCOAtr').name = 'Hexacosanoyl CoA  n C260CoA  C47H82N7O17P3S transport, endoplasmic reticulum'
model.reactions.get_by_id('HMGCOAtr').name = 'Hydroxymethylglutaryl CoA C27H39N7O20P3S transport, endoplasmic reticulum'
model.reactions.get_by_id('LNLNCGCOAtr').name = 'Gamma-linolenoyl-CoA transport, endoplasmic reticulum'
model.reactions.get_by_id('MALCOAtr').name = 'Malonyl CoA C24H33N7O19P3S transport, endoplasmic reticulum'
model.reactions.get_by_id('OCDYCACOAtr').name = 'Octadecynoyl CoA  n C182CoA  C39H62N7O17P3S transport, endoplasmic reticulum'
model.reactions.get_by_id('ODECOAtr').name = 'Octadecenoyl-CoA (n-C18:1CoA) transport, endoplasmic reticulum'
model.reactions.get_by_id('PMTCOAtr').name = 'Palmitoyl-CoA transport, endoplasmic reticulum'
model.reactions.get_by_id('STCOAtr').name = 'Stearoyl-CoA transport, endoplasmic reticulum'
model.reactions.get_by_id('TDCOAtr').name = 'Tetradecanoyl-CoA transport, endoplasmic reticulum'
model.reactions.get_by_id('TTCCOAtr').name = 'Tetracosanoyl CoA   n C240CoA  C45H78N7O17P3S transport, endoplasmic reticulum'
```

In [36]:

```
for m in sorted(model.metabolites, key=lambda x: x.id):
    if m.id.endswith('coa_r') and not m.id == 'coa_r' and not m.id == 'accoa_r' and not m.id.startswith('dhchol'):
        r = model.reactions.get_by_id('ACCOAtr').copy()
        r.id = m.id.upper().replace('_R','tr')
        model.add_reactions([r])
        r.add_metabolites({'accoa_c': 1.0, 'accoa_r': -1.0, m.id: 1.0, m.id.replace('_r','_c'): -1.0})

model.reactions.get_by_id('DCACOAtr').name = 'Decanoyl-CoA transport, endoplasmic reticulum'
model.reactions.get_by_id('DDCACOAtr').name = 'Lauroyl-CoA transport, endoplasmic reticulum'
model.reactions.get_by_id('HDCOAtr').name = 'Hexadecenoyl-CoA transport, endoplasmic reticulum'
model.reactions.get_by_id('HEXCCOAtr').name = 'Hexacosanoyl-CoA transport, endoplasmic reticulum'
model.reactions.get_by_id('HMGCOAtr').name = 'Hydroxymethylglutaryl-CoA transport, endoplasmic reticulum'
model.reactions.get_by_id('LNLNCGCOAtr').name = 'Gamma-linolenoyl-CoA transport, endoplasmic reticulum'
model.reactions.get_by_id('MALCOAtr').name = 'Malonyl-CoA transport, endoplasmic reticulum'
model.reactions.get_by_id('OCDYCACOAtr').name = 'Octadecynoyl-CoA transport, endoplasmic reticulum'
model.reactions.get_by_id('ODECOAtr').name = 'Octadecenoyl-CoA transport, endoplasmic reticulum'
model.reactions.get_by_id('PMTCOAtr').name = 'Palmitoyl-CoA transport, endoplasmic reticulum'
model.reactions.get_by_id('STCOAtr').name = 'Stearoyl-CoA transport, endoplasmic reticulum'
model.reactions.get_by_id('TDCOAtr').name = 'Tetradecanoyl-CoA transport, endoplasmic reticulum'
model.reactions.get_by_id('TTCCOAtr').name = 'Tetracosanoyl-CoA transport, endoplasmic reticulum'
```

In [37]:

```
for r in sorted(model.metabolites.pmtcoa_x.reactions, key=lambda x: x.id):
    print(r, r.gene_reaction_rule)
```

```
ACACT8p: 3ohodcoa_x + coa_x --> accoa_x + pmtcoa_x 13813
ACOAD7p: fad_x + pmtcoa_x --> fadh2_x + hdd2coa_x 12989 or 16253
ACOAO7p: o2_x + pmtcoa_x --> h2o2_x + hdd2coa_x 12742 or 12752 or 9700
FA160COAabcp: atp_x + h2o_x + pmtcoa_c --> adp_x + h_x + pi_x + pmtcoa_x 13167 or 9637
FA160COAabcp_1: atp_c + h2o_c + pmtcoa_c --> adp_c + h_c + pi_c + pmtcoa_x 13167 or 9637
FACOAL160p: atp_x + coa_x + hdca_x --> amp_x + pmtcoa_x + ppi_x 12555 or 15900
PTE2x: h2o_x + pmtcoa_x --> coa_x + h_x + hdca_x 16048
```

In [38]:

```
temp = ['9637','13167','12538','12555','15900','9912']
display(Annotation.loc[temp])
Show_Data(temp)
```

|  | Combined Annotations | Signal P | Sc288c Orthologs | Human Orthologs | Sc288 Best Hit | Human Blast | Essential | WolfPSort | C Terminal |
| --- | --- | --- | --- | --- | --- | --- | --- | --- | --- |
| RTO4\_ID |  |  |  |  |  |  |  |  |  |
| 9637 | K15628: PXA; ATP-binding cassette, subfamily D... | S | PXA1,PXA2 | ABCD1,ABCD2,ABCD3 | PXA1 | ABCD2 | Not Essential | extr 14, mito 6, plas 4, cyto\_mito 4 | KET\* |
| 13167 | K05676: ABCD2, ALDL1; ATP-binding cassette, su... |  | PXA1,PXA2 | ABCD1,ABCD2,ABCD3 | PXA2 | ABCD2 | Not Essential | plas 10, mito 8, cyto 4, pero 2, nucl 1, extr ... | ERI\* |
| 12538 | KOG1256: Long-chain acyl-CoA synthetases (AMP-... |  | FAA2 | ACSL1,ACSL5,ACSL6,CTB-127M13.1 | FAA2 | ACSL5 | Not Essential | mito 18.5, cyto\_mito 12.5, cyto 5.5 | GEQ\* |
| 12555 | K01897: ACSL, fadD; long-chain acyl-CoA synthe... |  | FAA2 | ACSL1,ACSL5,ACSL6,CTB-127M13.1 | FAA2 | ACSL1 | Not Essential | cyto 15.5, cyto\_nucl 11.5, pero 6, nucl 4.5 | AKL\* |
| 15900 | K01896: ACSM; medium-chain acyl-CoA synthetase |  |  | ACSM1,ACSM2A,ACSM2B,ACSM3,ACSM4,ACSM5 | ACS2 | ACSM3 | Not Essential | cyto 13, pero 11, cyto\_nucl 8.5 | AKL\* |
| 9912 | K08745: SLC27A1\_4, FATP1, FATP4; solute carrie... | S | FAT1 | SLC27A1,SLC27A4,SLC27A2,SLC27A5,SLC27A6 | FAT1 | SLC27 | Not Essential | extr 12, plas 8, pero 4, mito 1, cyto 1, E.R. ... | VRL\* |

| strain | WT | | | | | | | | | | | | | | | | |
| --- | --- | --- | --- | --- | --- | --- | --- | --- | --- | --- | --- | --- | --- | --- | --- | --- | --- |
| condition | G\_MM | C\_MM | G\_SD | | GX\_SD | | | X\_SD | | A\_SD | | C\_SD | | MM\_CN120 | | MM\_CN5 | Diversity\_Sample |
| phase | exp | exp | exp | stat | exp | trans | stat | exp | stat | exp | stat | exp | stat | exp | stat | exp | exp |
| proteinId | Set1 | Set1 | Set2 | Set2 | Set2 | Set2 | Set2 | Set2 | Set2 | Set2 | Set2 | Set2 | Set2 | Set3 | Set3 | Set3 | Set3 |
| 9637 | 4.57114 | 4.27662 | 5.51987 | 5.69854 | 5.53462 | 5.54139 | 5.89226 | 4.92544 | 5.54231 | 4.67172 | 5.29223 | 5.80818 | 6.03133 | 4.28216 | 5.51033 | 4.6708 | 4.49585 |
| 13167 | 6.15297 | 5.74356 | 6.89488 | 7.45456 | 6.8118 | 6.75568 | 7.22637 | 6.44248 | 6.92518 | 6.39164 | 7.33411 | 7.05476 | 8.21937 | 5.56682 | 6.64464 | 7.04961 | 6.20762 |
| 12538 | 5.1932 | 5.55322 | 4.16034 | 3.68415 | 4.67797 | 4.37239 | 4.52657 | 3.91004 | 4.36289 | 4.96869 | 3.65995 | 6.54618 | 6.53583 | 5.10212 | 4.96231 | 5.5082 | 5.45748 |
| 12555 | 6.4532 | 6.51166 | 5.43327 | 5.78233 | 5.42694 | 5.52551 | 5.26328 | 5.25738 | 6.1227 | 6.15634 | 6.45249 | 4.76408 | 5.10246 | 6.78726 | 5.6908 | 6.00758 | 6.18519 |
| 15900 | 4.89589 | 6.00099 | 6.46859 | 5.98835 | 6.37204 | 5.19183 | 7.41643 | 6.18244 | 4.59866 | 4.89713 | 4.91078 | 4.42504 | 3.57437 | 3.85314 | 3.9658 | 4.41517 | 3.66558 |
| 9912 | 5.93073 | 5.40845 | 5.89641 | 6.2248 | 5.87895 | 5.80068 | 5.93113 | 5.50431 | 5.91601 | 5.5566 | 6.22915 | 5.26476 | 4.97236 | 5.88904 | 5.91406 | 5.56329 | 5.5721 |

| strain | WT | | | | | | | | | | |
| --- | --- | --- | --- | --- | --- | --- | --- | --- | --- | --- | --- |
| condition | G\_SD | | GX\_SD | | | X\_SD | | A\_SD | | C\_SD | |
| proteinId | exp | stat | exp | trans | stat | exp | stat | exp | stat | exp | stat |
| 9637 | 3.99863 | 8.81595 | 3.66078 | 8.74723 | 8.6049 | 2.93199 | 4.57429 | 1.73903 | 2.29109 | 5.54927 | 7.84946 |
| 13167 | 2.11022 | 4.53275 | 2.24015 | 2.23168 | 2.90478 | 2.73284 | 2.57482 | 2.12497 | 3.83089 | 3.41813 | 4.1281 |
| 12538 | 0 | 0.335899 | 0 | 0.189313 | 0 | 0 | 0.393854 | 0.576179 | 1.338 | 0.439202 | 0.221651 |
| 12555 | 5.37108 | 12.3435 | 4.25136 | 12.0863 | 10.9404 | 7.12026 | 11.2197 | 12.1786 | 13.0103 | 17.5464 | 11.1295 |
| 15900 | 3.25523 | 1.45139 | 3.2441 | 2.98097 | 4.38989 | 4.30749 | 5.07897 | 2.12175 | 1.72318 | 2.54453 | 1.75055 |
| 9912 | 4.40189 | 9.06281 | 3.66274 | 8.91569 | 10.3772 | 5.86924 | 6.49892 | 5.02317 | 5.93995 | 4.96027 | 4.99924 |

|  | Glucose | Xylose | Arabinose | Acetate | Coumarate | Ferulate | YNB Oleic Acid | YNB Ricinoleic Acid | YNB Glucose | YNB Gluc DOC | YPD |
| --- | --- | --- | --- | --- | --- | --- | --- | --- | --- | --- | --- |
| proteinId |  |  |  |  |  |  |  |  |  |  |  |
| 9637 | 0.0387351 | 0.118884 | -0.169197 | -0.160041 | -0.191542 | -0.00882013 | -0.986535 | -0.0397322 | -0.0791493 | 0.151542 | -0.0321814 |
| 13167 | -0.000429381 | 0.137701 | -0.0476588 | 0.249415 | 0.117662 | 0.180606 | -0.731184 | -0.0962394 | -0.0646791 | -0.146305 | -0.0691314 |
| 12538 | -0.0228742 | -0.00284459 | 0.0444012 | 0.0245939 | 0.0476792 | -0.0249695 | 0.0981869 | 0.240673 | 0.0573442 | 0.0435764 | 0.0668027 |
| 12555 | -0.311448 | -0.162485 | -0.131826 | -0.274268 | -0.854248 | -0.027038 | -1.52861 | -0.659443 | -0.300839 | -0.371389 | -0.434224 |
| 15900 | -0.402251 | 0.0381164 | -0.120976 | -0.323788 | -0.290967 | -0.292245 | 0.0775032 | 0.423076 | 0.367482 | -0.304034 | -0.394707 |
| 9912 | 0.0677425 | -0.270103 | -0.302936 | -0.051684 | -0.404229 | 0.0682701 | -0.0630695 | -0.272301 | -0.340334 | -0.0743227 | -0.0288945 |

In [39]:

```
for x in temp:
    if x in model.genes:
        for r in sorted(model.genes.get_by_id(x).reactions, key=lambda x: x.id):
            print(r.id, r.reaction, r.gene_reaction_rule)
    else:
        print(x, 'no reactions')
    print()
```

```
FA140COAabcp atp_x + h2o_x + tdcoa_c --> adp_x + h_x + pi_x + tdcoa_x 13167 or 9637
FA141COAabcp atp_x + h2o_x + tdecoa_c --> adp_x + h_x + pi_x + tdecoa_x 13167 or 9637
FA160COAabcp atp_x + h2o_x + pmtcoa_c --> adp_x + h_x + pi_x + pmtcoa_x 13167 or 9637
FA160COAabcp_1 atp_c + h2o_c + pmtcoa_c --> adp_c + h_c + pi_c + pmtcoa_x 13167 or 9637
FA161COAabcp atp_x + h2o_x + hdcoa_c --> adp_x + h_x + hdcoa_x + pi_x 13167 or 9637
FA180COAabcp atp_x + h2o_x + stcoa_c --> adp_x + h_x + pi_x + stcoa_x 13167 or 9637
FA181COAabcp atp_x + h2o_x + odecoa_c --> adp_x + h_x + odecoa_x + pi_x 13167 or 9637
FA182COAabcp atp_x + h2o_x + ocdycacoa_c --> adp_x + h_x + ocdycacoa_x + pi_x 13167 or 9637

FA140COAabcp atp_x + h2o_x + tdcoa_c --> adp_x + h_x + pi_x + tdcoa_x 13167 or 9637
FA141COAabcp atp_x + h2o_x + tdecoa_c --> adp_x + h_x + pi_x + tdecoa_x 13167 or 9637
FA160COAabcp atp_x + h2o_x + pmtcoa_c --> adp_x + h_x + pi_x + pmtcoa_x 13167 or 9637
FA160COAabcp_1 atp_c + h2o_c + pmtcoa_c --> adp_c + h_c + pi_c + pmtcoa_x 13167 or 9637
FA161COAabcp atp_x + h2o_x + hdcoa_c --> adp_x + h_x + hdcoa_x + pi_x 13167 or 9637
FA180COAabcp atp_x + h2o_x + stcoa_c --> adp_x + h_x + pi_x + stcoa_x 13167 or 9637
FA181COAabcp atp_x + h2o_x + odecoa_c --> adp_x + h_x + odecoa_x + pi_x 13167 or 9637
FA182COAabcp atp_x + h2o_x + ocdycacoa_c --> adp_x + h_x + ocdycacoa_x + pi_x 13167 or 9637

12538 no reactions

FACOAL100p atp_x + coa_x + dca_x --> amp_x + dcacoa_x + ppi_x 12555 or 15900
FACOAL120p atp_x + coa_x + ddca_x --> amp_x + ddcacoa_x + ppi_x 12555 or 15900
FACOAL140p atp_x + coa_x + ttdca_x --> amp_x + ppi_x + tdcoa_x 12555 or 15900
FACOAL141p atp_x + coa_x + ttdcea_x --> amp_x + ppi_x + tdecoa_x 12555 or 15900
FACOAL160p atp_x + coa_x + hdca_x --> amp_x + pmtcoa_x + ppi_x 12555 or 15900
FACOAL161p atp_x + coa_x + hdcea_x --> amp_x + hdcoa_x + ppi_x 12555 or 15900
FACOAL80p atp_x + coa_x + octa_x --> amp_x + occoa_x + ppi_x 12555 or 15900

FACOAL100p atp_x + coa_x + dca_x --> amp_x + dcacoa_x + ppi_x 12555 or 15900
FACOAL120p atp_x + coa_x + ddca_x --> amp_x + ddcacoa_x + ppi_x 12555 or 15900
FACOAL140p atp_x + coa_x + ttdca_x --> amp_x + ppi_x + tdcoa_x 12555 or 15900
FACOAL141p atp_x + coa_x + ttdcea_x --> amp_x + ppi_x + tdecoa_x 12555 or 15900
FACOAL160p atp_x + coa_x + hdca_x --> amp_x + pmtcoa_x + ppi_x 12555 or 15900
FACOAL161p atp_x + coa_x + hdcea_x --> amp_x + hdcoa_x + ppi_x 12555 or 15900
FACOAL80p atp_x + coa_x + octa_x --> amp_x + occoa_x + ppi_x 12555 or 15900

FA240tp ttc_c --> ttc_x 9912
FA260tp hexc_c --> hexc_x 9912
FACOAL240p atp_x + coa_x + ttc_x --> amp_x + ppi_x + ttccoa_x 9912
FACOAL260p atp_x + coa_x + hexc_x --> amp_x + hexccoa_x + ppi_x 9912
FATP1t hdca_c + na1_c <=> hdca_e + na1_e 9912
FATP2t na1_c + ocdcea_c <=> na1_e + ocdcea_e 9912
FATP3t na1_c + ocdca_c <=> na1_e + ocdca_e 9912
FATP4t arach_c + na1_c <=> arach_e + na1_e 9912
FATP8t na1_c + ttc_c <=> na1_e + ttc_e 9912
FATP9t na1_c + nrvnc_c <=> na1_e + nrvnc_e 9912
VLCS2p atp_x + coa_x + dhcholestanate_x --> amp_x + dhcholestancoa_x + ppi_x 9912
VLCS2r atp_r + coa_r + dhcholestanate_r --> amp_r + dhcholestancoa_r + ppi_r 9912
VLCSp atp_x + coa_x + thcholstoic_x --> amp_x + cholcoar_x + ppi_x 9912
VLCSr atp_r + coa_r + thcholstoic_r --> amp_r + cholcoar_r + ppi_r 9912
```

In [40]:

```
# fatty acid peroxisomal transport
model.remove_reactions(['FA160COAabcp_1'],remove_orphans=True)
# acyl-coa is hydrolyzed and transported by pxa1 and pxa2
# active domain on cytosolic side
for r in sorted(model.reactions, key=lambda x: x.id):
    if r.id.startswith('FA') and r.id.endswith('abcp'):
        r.gene_reaction_rule = '13167 and 9637'
        r.add_metabolites({'atp_x': 1.0, 'h2o_x': 1.0, 'adp_x': -1.0, 'h_x': -1.0, 'pi_x': -1.0,
                           'atp_c': -1.0, 'h2o_c': -1.0, 'adp_c': 1.0, 'h_c': 1.0, 'pi_c': 1.0})
model.reactions.get_by_id('FA140COAabcp').add_metabolites({'tdcoa_x': -1.0, 'ttdca_x': 1.0,
                                                           'h2o_c': -1.0, 'coa_c': 1.0, 'h_c': 1.0})
model.reactions.get_by_id('FA141COAabcp').add_metabolites({'tdecoa_x': -1.0, 'ttdcea_x': 1.0,
                                                           'h2o_c': -1.0, 'coa_c': 1.0, 'h_c': 1.0})
model.reactions.get_by_id('FA160COAabcp').add_metabolites({'pmtcoa_x': -1.0, 'hdca_x': 1.0,
                                                           'h2o_c': -1.0, 'coa_c': 1.0, 'h_c': 1.0})
model.reactions.get_by_id('FA161COAabcp').add_metabolites({'hdcoa_x': -1.0, 'hdcea_x': 1.0,
                                                           'h2o_c': -1.0, 'coa_c': 1.0, 'h_c': 1.0})
model.reactions.get_by_id('FA180COAabcp').add_metabolites({'stcoa_x': -1.0, 'ocdca_x': 1.0,
                                                           'h2o_c': -1.0, 'coa_c': 1.0, 'h_c': 1.0})
m1 = model.metabolites.get_by_id('ocdcea_c').copy()
m1.id = 'ocdcea_x'
m1.compartment = 'x'
m2 = model.metabolites.get_by_id('ocdcya_c').copy()
m2.id = 'ocdcya_x'
m2.compartment = 'x'
model.add_metabolites([m1,m2])
model.reactions.get_by_id('FA181COAabcp').add_metabolites({'odecoa_x': -1.0, 'ocdcea_x': 1.0,
                                                           'h2o_c': -1.0, 'coa_c': 1.0, 'h_c': 1.0})
model.reactions.get_by_id('FA182COAabcp').add_metabolites({'ocdycacoa_x': -1.0, 'ocdcya_x': 1.0,
                                                           'h2o_c': -1.0, 'coa_c': 1.0, 'h_c': 1.0})
r = model.reactions.get_by_id('FA140COAabcp').copy()
r.id = 'FA183COAabcp'
model.add_reactions([r])
m = model.metabolites.get_by_id('lnlncg_c').copy()
m.id = 'lnlncg_x'
m.compartment = 'x'
model.add_metabolites([m])
r.add_metabolites({'tdcoa_c': 1.0, 'ttdca_x': -1.0, 'lnlncgcoa_c': -1.0, 'lnlncg_x': 1.0})

# free fatty acid is activated again by 12555 pero faa2
# 15900 ACSM is for C4 to C11
model.reactions.get_by_id('FACOAL80p').gene_reaction_rule = '15900'
model.reactions.get_by_id('FACOAL100p').gene_reaction_rule = '15900'
model.reactions.get_by_id('FACOAL120p').gene_reaction_rule = '12555'
model.reactions.get_by_id('FACOAL140p').gene_reaction_rule = '12555'
model.reactions.get_by_id('FACOAL141p').gene_reaction_rule = '12555'
model.reactions.get_by_id('FACOAL160p').gene_reaction_rule = '12555'
model.reactions.get_by_id('FACOAL161p').gene_reaction_rule = '12555'
for x in ['FACOAL180','FACOAL181','FACOAL182','FACOAL1831']:
    r = model.reactions.get_by_id(x).copy()
    r.id = r.id + 'p'
    r.gene_reaction_rule = '12555'
    model.add_reactions([r])
    for m in r.metabolites:
        if not m.id.replace('_c','_x') in model.metabolites:
            m2 = m.copy()
            m2.id = m.id.replace('_c','_x')
            m2.compartment = 'x'
            model.add_metabolites([m2])
        r.add_metabolites({m.id: -r.get_coefficient(m.id), m.id.replace('_c','_x'): r.get_coefficient(m.id)})
# free very long chain fatty acid transported and activated by 9912 fat1
# very long chain fatty acid also activated by faa2
model.reactions.get_by_id('FACOAL240p').gene_reaction_rule = '12555 or 9912'
model.reactions.get_by_id('FACOAL260p').gene_reaction_rule = '12555 or 9912'
```

In [41]:

```
for x in temp:
    if x in model.genes:
        for r in sorted(model.genes.get_by_id(x).reactions, key=lambda x: x.id):
            print(r.id, r.reaction, r.gene_reaction_rule)
    else:
        print(x, 'no reactions')
    print()
```

```
FA140COAabcp atp_c + 2.0 h2o_c + tdcoa_c --> adp_c + coa_c + 2.0 h_c + pi_c + ttdca_x 13167 and 9637
FA141COAabcp atp_c + 2.0 h2o_c + tdecoa_c --> adp_c + coa_c + 2.0 h_c + pi_c + ttdcea_x 13167 and 9637
FA160COAabcp atp_c + 2.0 h2o_c + pmtcoa_c --> adp_c + coa_c + 2.0 h_c + hdca_x + pi_c 13167 and 9637
FA161COAabcp atp_c + 2.0 h2o_c + hdcoa_c --> adp_c + coa_c + 2.0 h_c + hdcea_x + pi_c 13167 and 9637
FA180COAabcp atp_c + 2.0 h2o_c + stcoa_c --> adp_c + coa_c + 2.0 h_c + ocdca_x + pi_c 13167 and 9637
FA181COAabcp atp_c + 2.0 h2o_c + odecoa_c --> adp_c + coa_c + 2.0 h_c + ocdcea_x + pi_c 13167 and 9637
FA182COAabcp atp_c + 2.0 h2o_c + ocdycacoa_c --> adp_c + coa_c + 2.0 h_c + ocdcya_x + pi_c 13167 and 9637
FA183COAabcp atp_c + 2.0 h2o_c + lnlncgcoa_c --> adp_c + coa_c + 2.0 h_c + lnlncg_x + pi_c 13167 and 9637

FA140COAabcp atp_c + 2.0 h2o_c + tdcoa_c --> adp_c + coa_c + 2.0 h_c + pi_c + ttdca_x 13167 and 9637
FA141COAabcp atp_c + 2.0 h2o_c + tdecoa_c --> adp_c + coa_c + 2.0 h_c + pi_c + ttdcea_x 13167 and 9637
FA160COAabcp atp_c + 2.0 h2o_c + pmtcoa_c --> adp_c + coa_c + 2.0 h_c + hdca_x + pi_c 13167 and 9637
FA161COAabcp atp_c + 2.0 h2o_c + hdcoa_c --> adp_c + coa_c + 2.0 h_c + hdcea_x + pi_c 13167 and 9637
FA180COAabcp atp_c + 2.0 h2o_c + stcoa_c --> adp_c + coa_c + 2.0 h_c + ocdca_x + pi_c 13167 and 9637
FA181COAabcp atp_c + 2.0 h2o_c + odecoa_c --> adp_c + coa_c + 2.0 h_c + ocdcea_x + pi_c 13167 and 9637
FA182COAabcp atp_c + 2.0 h2o_c + ocdycacoa_c --> adp_c + coa_c + 2.0 h_c + ocdcya_x + pi_c 13167 and 9637
FA183COAabcp atp_c + 2.0 h2o_c + lnlncgcoa_c --> adp_c + coa_c + 2.0 h_c + lnlncg_x + pi_c 13167 and 9637

12538 no reactions

FACOAL120p atp_x + coa_x + ddca_x --> amp_x + ddcacoa_x + ppi_x 12555
FACOAL140p atp_x + coa_x + ttdca_x --> amp_x + ppi_x + tdcoa_x 12555
FACOAL141p atp_x + coa_x + ttdcea_x --> amp_x + ppi_x + tdecoa_x 12555
FACOAL160p atp_x + coa_x + hdca_x --> amp_x + pmtcoa_x + ppi_x 12555
FACOAL161p atp_x + coa_x + hdcea_x --> amp_x + hdcoa_x + ppi_x 12555
FACOAL180p atp_x + coa_x + ocdca_x --> amp_x + ppi_x + stcoa_x 12555
FACOAL181p atp_x + coa_x + ocdcea_x --> amp_x + odecoa_x + ppi_x 12555
FACOAL182p atp_x + coa_x + ocdcya_x --> amp_x + ocdycacoa_x + ppi_x 12555
FACOAL1831p atp_x + coa_x + lnlncg_x --> amp_x + lnlncgcoa_x + ppi_x 12555
FACOAL240p atp_x + coa_x + ttc_x --> amp_x + ppi_x + ttccoa_x 12555 or 9912
FACOAL260p atp_x + coa_x + hexc_x --> amp_x + hexccoa_x + ppi_x 12555 or 9912

FACOAL100p atp_x + coa_x + dca_x --> amp_x + dcacoa_x + ppi_x 15900
FACOAL80p atp_x + coa_x + octa_x --> amp_x + occoa_x + ppi_x 15900

FA240tp ttc_c --> ttc_x 9912
FA260tp hexc_c --> hexc_x 9912
FACOAL240p atp_x + coa_x + ttc_x --> amp_x + ppi_x + ttccoa_x 12555 or 9912
FACOAL260p atp_x + coa_x + hexc_x --> amp_x + hexccoa_x + ppi_x 12555 or 9912
FATP1t hdca_c + na1_c <=> hdca_e + na1_e 9912
FATP2t na1_c + ocdcea_c <=> na1_e + ocdcea_e 9912
FATP3t na1_c + ocdca_c <=> na1_e + ocdca_e 9912
FATP4t arach_c + na1_c <=> arach_e + na1_e 9912
FATP8t na1_c + ttc_c <=> na1_e + ttc_e 9912
FATP9t na1_c + nrvnc_c <=> na1_e + nrvnc_e 9912
VLCS2p atp_x + coa_x + dhcholestanate_x --> amp_x + dhcholestancoa_x + ppi_x 9912
VLCS2r atp_r + coa_r + dhcholestanate_r --> amp_r + dhcholestancoa_r + ppi_r 9912
VLCSp atp_x + coa_x + thcholstoic_x --> amp_x + cholcoar_x + ppi_x 9912
VLCSr atp_r + coa_r + thcholstoic_r --> amp_r + cholcoar_r + ppi_r 9912
```

In [42]:

```
r = model.reactions.get_by_id('BIOMASS_RT')
for m in sorted(r.metabolites, key=lambda x: x.id):
    print(m, r.get_coefficient(m.id))
```

```
13BDglcn_c -1.1348
adp_c 59.276
ala__L_c -0.4588
amp_c -0.046
arg__L_c -0.1607
asn__L_c -0.1017
asp__L_c -0.2975
atp_c -59.276
cmp_c -0.0447
cys__L_c -0.0066
damp_c -0.0036
dcmp_c -0.0024
dgmp_c -0.0024
dtmp_c -0.0036
ergst_c -0.0007
gln__L_c -0.1054
glu__L_c -0.3018
gly_c -0.2904
glycogen_c -0.5185
gmp_c -0.046
h2o_c -59.276
h_c 58.70001
his__L_c -0.0663
ile__L_c -0.1927
leu__L_c -0.2964
lys__L_c -0.2862
mannan_r -0.8079
met__L_c -0.0507
pa_RT_r -6e-06
pc_RT_r -6e-05
pe_RT_r -4.5e-05
phe__L_c -0.1339
pi_c 59.305
pro__L_c -0.1647
ps_RT_r -1.7e-05
ptd1ino_RT_r -5.3e-05
ribflv_c -0.00099
ser__L_c -0.1854
so4_c -0.02
thr__L_c -0.1914
tre_c -0.0234
triglyc_RT_r -6.6e-05
trp__L_c -0.0284
tyr__L_c -0.102
ump_c -0.0599
val__L_c -0.2646
zymst_c -0.0015
```

In [43]:

```
r = model.reactions.get_by_id('BIOMASS_RT')
# Change sterols in biomass to ER
for x in ['zymst_c','ergst_c']:
    r.add_metabolites({x: -r.get_coefficient(x),
                       x.replace('_c','_r'): r.get_coefficient(x)})
```

In [44]:

```
r = model.reactions.get_by_id('BIOMASS_RT')
for m in r.metabolites:
    if len(m.reactions) < 2:
        print(m.id)
```

In [45]:

```
temp = ['16430','10400','12097','10888','14882','16411','13481','15169']
display(Annotation.loc[temp])
for x in temp:
    if x in model.genes:
        for r in sorted(model.genes.get_by_id(x).reactions, key=lambda x: x.id):
            print(r, r.gene_reaction_rule)
    else:
        print(x, 'no reactions')
    print()
```

|  | Combined Annotations | Signal P | Sc288c Orthologs | Human Orthologs | Sc288 Best Hit | Human Blast | Essential | WolfPSort | C Terminal |
| --- | --- | --- | --- | --- | --- | --- | --- | --- | --- |
| RTO4\_ID |  |  |  |  |  |  |  |  |  |
| 16430 | KOG1441: Glucose-6-phosphate/phosphate and pho... |  |  |  |  | SLC35 | Not Essential | plas 26 | RLG\* |
| 10400 | KOG1441: Glucose-6-phosphate/phosphate and pho... | S |  |  |  | SLC35 | Not Essential | plas 19, E.R. 6 | KQQ\* |
| 12097 | K15280: SLC35C2; solute carrier family 35, mem... |  |  |  |  | SLC35 | Not Essential | plas 21, mito 3, E.R. 2 | KLG\* |
| 10888 | KOG1441: Glucose-6-phosphate/phosphate and pho... | A |  |  |  | SLC35 | Not Essential | plas 25 | VGR\* |
| 14882 | KOG1441: Glucose-6-phosphate/phosphate and pho... | A |  |  |  | SLC35 | Not Essential | plas 17, E.R. 5, mito 4 | TPN\* |
| 16411 | K15283: SLC35E1; solute carrier family 35, mem... |  | SLY41 | SLC35E1 | SLY41 | SLC35 | Not Essential | plas 21, nucl 3, mito 1, extr 1, vacu 1 | QLR\* |
| 13481 | KOG1441: Glucose-6-phosphate/phosphate and pho... |  |  |  |  |  | Not Essential | plas 12, extr 5, mito 4, E.R. 4 | FLD\* |
| 15169 | K15279: SLC35C1, FUCT1; solute carrier family ... |  |  | SLC35C1 |  | SLC35C | Not Essential | plas 26 | ERR\* |

```
16430 no reactions

10400 no reactions

12097 no reactions

10888 no reactions

14882 no reactions

16411 no reactions

13481 no reactions

GDPFUCtg: gdpfuc_c + gmp_g <=> gdpfuc_g + gmp_c 15169
```

In [46]:

```
r = cobra.Reaction('GLYC3Pter')
r.name = 'Glycerol 3-phosphate endoplasmic reticular transport'
#r.gene_reaction_rule = ''
r.lower_bound = -1000.0
r.upper_bound = 1000.0
r.add_metabolites({model.metabolites.glyc3p_c: -1.0, model.metabolites.glyc3p_r: 1.0})
model.add_reactions([r])

r = cobra.Reaction('DHAPter')
r.name = 'Dihydroxyacetone phosphate endoplasmic reticular transport'
#r.gene_reaction_rule = ''
r.lower_bound = -1000.0
r.upper_bound = 1000.0
r.add_metabolites({model.metabolites.dhap_c: -1.0, model.metabolites.dhap_r: 1.0})
model.add_reactions([r])
```

In [47]:

```
model.objective = 'BIOMASS_RT'
```

In [48]:

```
for x in sorted(model.reactions, key=lambda x: x.id):
    if x.boundary:
        print(x.id, x.lower_bound, x.upper_bound)
```

```
EX_co2_e 0.0 999999.0
EX_glc__D_e -10.0 999999.0
EX_h2o_e -999999.0 999999.0
EX_h_e -999999.0 999999.0
EX_nh4_e -999999.0 999999.0
EX_o2_e -2.0 999999.0
EX_pi_e -999999.0 999999.0
EX_so4_e -999999.0 999999.0
```

In [49]:

```
for m in sorted(model.metabolites, key=lambda x: x.id):
    if 'e' in m.compartment:
        if 'EX_'+m.id in model.reactions:
            print(m.id)
```

```
co2_e
glc__D_e
h2o_e
h_e
nh4_e
o2_e
pi_e
so4_e
```

In [50]:

```
for m in sorted(model.metabolites, key=lambda x: x.id):
    if 'e' in m.compartment:
        if not 'EX_'+m.id in model.reactions:
            print(m.id)
```

```
13BDglcn_e
35cgmp_e
4abut_e
5adtststeroneglc_e
5aop_e
5flura_e
6mpur_e
ac_e
acgam_e
ach_e
ade_e
adn_e
ahandrostanglc_e
ala_B_e
ala__D_e
ala__L_e
alltn_e
alltt_e
amet_e
andrstrnglc_e
arach_e
arg__L_e
asn__L_e
asp__L_e
bildglcur_e
bilglcur_e
ca2_e
camp_e
cgly_e
chol_e
cholate_e
chtn_e
cl_e
csn_e
cu_e
cys__L_e
cysi__L_e
cytd_e
dad_2_e
dca_e
ddca_e
dhdascb_e
din_e
epist_e
ergst_e
estradiolglc_e
estriolglc_e
estroneglc_e
fe2_e
fe3_e
fecost_e
fmn_e
for_e
fru_e
g3pc_e
g3pe_e
g3pi_e
gal_e
galur_e
gam_e
gchola_e
gln__L_e
glu__L_e
gluala_e
gly_e
glyc_e
glygn2_e
glygn4_e
gthrd_e
gua_e
hco3_e
hdca_e
hdcea_e
his__L_e
ile__L_e
inost_e
ins_e
k_e
lac__D_e
lac__L_e
lanost_e
leu__L_e
lys__L_e
malt_e
man_e
met__L_e
na1_e
nac_e
no2_e
no3_e
nrvnc_e
ocdca_e
ocdcea_e
ocdcya_e
oh1_e
orn_e
oxa_e
pail1819Z160_e
pc_RT_e
pe1801819Z_e
pe1801829Z12Z_e
pe1819Z1819Z_e
pe1819Z1829Z12Z_e
pe_RT_e
pectin_e
pepd_e
pg1819Z160_e
pgp1819Z160_e
phe__L_e
pnto__R_e
pro__D_e
pro__L_e
prostge1_e
prostge2_e
ptd1ino_RT_e
ptrc_e
pyr_e
ribflv_e
ser__L_e
so3_e
spmd_e
sprm_e
strch1_e
strch2_e
sucr_e
tchola_e
tega_e
tgua_e
thm_e
thmmp_e
thmpp_e
thr__L_e
trp__L_e
tststeroneglc_e
ttc_e
ttdca_e
tyr__L_e
ura_e
urate_e
urea_e
uri_e
val__L_e
xyl__D_e
zymst_e
```

In [51]:

```
for m in sorted(model.metabolites, key=lambda x: x.id):
    if 'e' in m.compartment:
        if not m.id.endswith('_e'):
            print(m.id)
```

In [52]:

```
for m in sorted(model.metabolites, key=lambda x: x.id):
    if 'e' in m.compartment:
        if 'EX_'+m.id not in model.reactions:
            r = model.reactions.get_by_id('EX_h_e').copy()
            r.id = 'EX_'+m.id
            r.name = m.name + ' exchange'
            model.add_reactions([r])
            model.reactions.get_by_id('EX_'+m.id).add_metabolites({'h_e': 1.0, m.id: -1.0})
            model.reactions.get_by_id('EX_'+m.id).lower_bound = 0.0
```

In [53]:

```
for r in model.reactions:
    if r.lower_bound < -1000.0:
        r.lower_bound = -1000.0
    if r.upper_bound > 1000.0:
        r.upper_bound = 1000.0
```

In [54]:

```
model.medium
```

Out[54]:

```
{'EX_h_e': 1000.0,
 'EX_h2o_e': 1000.0,
 'EX_nh4_e': 1000.0,
 'EX_o2_e': 2.0,
 'EX_pi_e': 1000.0,
 'EX_so4_e': 1000.0,
 'EX_glc__D_e': 10.0}
```

In [55]:

```
sol = model.optimize()
print(sol.objective_value)
for k, v in sol.shadow_prices.items():
    if v:
        print(k, v)
```

```
0.0
dolp_r 1.2377769525931428
mannan_r -1.2377769525931428
```

In [56]:

```
for r in sorted(model.metabolites.get_by_id('dolmanp_r').reactions, key=lambda x: x.id):
    print(r, r.gene_reaction_rule)
print()
for r in sorted(model.metabolites.get_by_id('dolp_c').reactions, key=lambda x: x.id):
    print(r, r.gene_reaction_rule)
```

```
DOLPMMer: dolmanp_r --> dolp_r + h_r + mannan_r 12121 or 13840 or 8768
DOLPMTcer: dolp_c + gdpmann_c --> dolmanp_r + gdp_c 13484

DOLPMT: dolp_c + gdpmann_c --> dolmanp_c + gdp_c 13484
DOLPMTcer: dolp_c + gdpmann_c --> dolmanp_r + gdp_c 13484
G12MT3: doldpglcnacglcnacman_manman_manmanman_c + dolmanp_c --> doldpglcnacglcnacman_manmanman_manmanman_c + dolp_c + h_c 9905
G12MT4: doldpglcnacglcnacman_man_man_manman_manmanman_c + dolmanp_c --> doldpglcnacglcnacman_man_manman_manman_manmanman_c + dolp_c + h_c 9905
GLCNACPT: dolp_c + 2.0 h_c + uacgam_c --> doldpglcnac_c + ump_c 11202
UDPDOLPT: dolp_c + udpg_c --> dolpglc_c + udp_c 11809
```

In [57]:

```
r = cobra.Reaction('DOLPter')
r.name = 'Dolichol phosphate flippase'
r.lower_bound = 0.0
r.upper_bound = 1000.0
r.add_metabolites({model.metabolites.dolp_r: -1.0, model.metabolites.dolp_c: 1.0})
model.add_reactions([r])
```

In [58]:

```
sol = model.optimize()
print(sol.objective_value)

for k, v in sol.shadow_prices.items():
    if v and k in [m.id for m in model.reactions.get_by_id('BIOMASS_RT').metabolites]:
        print(k, v)
```

```
0.0
ile__L_c -5.189413596263622
```

### amino acid transporters¶

In [59]:

```
temp = list(Annotation.index[Annotation['Combined Annotations'].str.contains('amino acid transporter')]) + \
       list(Annotation.index[Annotation['Combined Annotations'].str.contains('Amino acid transporter')]) + \
       ['15991','16258','9135','11017','13128','16454','11649','14774']
display(Annotation.loc[temp])
Show_Data(temp)
```

|  | Combined Annotations | Signal P | Sc288c Orthologs | Human Orthologs | Sc288 Best Hit | Human Blast | Essential | WolfPSort | C Terminal |
| --- | --- | --- | --- | --- | --- | --- | --- | --- | --- |
| RTO4\_ID |  |  |  |  |  |  |  |  |  |
| 8962 | K16261: YAT; yeast amino acid transporter |  | DIP5 |  | DIP5 |  | Not Essential | plas 27 | AIM\* |
| 9319 | K16261: YAT; yeast amino acid transporter | A | DIP5 |  | DIP5 |  | Not Essential | plas 21, mito 2, vacu 2 | WLM\* |
| 9322 | K16261: YAT; yeast amino acid transporter | A | DIP5 |  | DIP5 |  | Not Essential | plas 27 | WLM\* |
| 9962 | K16261: YAT; yeast amino acid transporter | S | DIP5 |  | DIP5 |  | Not Essential | plas 21, E.R. 2, vacu 2 | DAA\* |
| 10059 | K16261: YAT; yeast amino acid transporter |  |  |  | DIP5 |  | Not Essential | plas 27 | YVL\* |
| 10669 | HMMPfam:Transmembrane amino acid transporter p... |  |  |  |  |  | Not Essential | plas 27 | CAA\* |
| 11026 | BLAST: transmembrane amino acid transporter pr... |  |  |  |  |  | Not Essential | plas 24, E.R. 2 | VRG\* |
| 11625 | K13869: SLC7A11; solute carrier family 7 (L-ty... |  |  | SLC7A6,SLC7A7,SLC7A9,SLC7A5 | MUP1 | SLC7A | Not Essential | plas 27 | EMT\* |
| 12743 | K14209: SLC36A, PAT; solute carrier family 36 ... |  | AVT3,AVT4 | SLC36A1,SLC36A2,SLC36A3,SLC36A4 | AVT3 | SLC36 | Not Essential | plas 13, E.R. 6, extr 5, vacu 2 | PRA\* |
| 12758 | K16261: YAT; yeast amino acid transporter |  |  |  | PUT4 |  | Not Essential | plas 26 | WLF\* |
| 13601 | HMMPfam:Transmembrane amino acid transporter p... |  |  |  |  |  | Not Essential | plas 20, E.R. 4, vacu 3 | DFR\* |
| 14129 | K14997: SLC38A11; solute carrier family 38 (so... |  | AVT2 | SLC38A11,SLC38A3 | AVT2 | SLC38A | Not Essential | plas 24, E.R. 2 | KQC\* |
| 14229 | K16261: YAT; yeast amino acid transporter |  | CAN1,LYP1,ALP1,HIP1,GAP1,TAT2 |  | GAP1 |  | Not Essential | plas 27 | FTN\* |
| 14391 | K03293: TC.AAT; amino acid transporter, AAT fa... |  |  |  | DIP5 |  | Not Essential | plas 27 | WVA\* |
| 15074 | K16261: YAT; yeast amino acid transporter |  | CAN1,LYP1,ALP1,HIP1,GAP1,TAT2 |  | GAP1 |  | Not Essential | plas 22, E.R. 2, vacu 2 | FTY\* |
| 16187 | K15015: SLC32A, VGAT; solute carrier family 32... |  | AVT1 | SLC32A1 | AVT1 | SLC32A | Not Essential | plas 20, mito 4, E.R. 2, mito\_nucl 2 | GLQ\* |
| 8552 | KOG1286: Amino acid transporters |  |  |  | DIP5 |  | Not Essential | plas 27 | AFL\* |
| 8766 | KOG1305: Amino acid transporter protein |  | AVT6,AVT5 |  | AVT6 | SLC38 | Not Essential | plas 26 | ARR\* |
| 9616 | KOG1289: Amino acid transporters |  |  |  | HNM1 |  | Not Essential | plas 24, E.R. 2 | SIG\* |
| 10057 | KOG1286: Amino acid transporters | A |  |  | CAN1 |  | Not Essential | plas 24, mito 1, E.R. 1, vacu 1 | ALV\* |
| 10143 | KOG1289: Amino acid transporters |  |  |  | HNM1 |  | Not Essential | plas 24, mito 1, pero 1, E.R. 1 | AAV\* |
| 11269 | KOG1289: Amino acid transporters |  |  |  | UGA4 |  | Not Essential | plas 27 | EVK\* |
| 12567 | KOG1303: Amino acid transporters |  |  |  |  |  | Not Essential | plas 27 | DTF\* |
| 13178 | KOG1304: Amino acid transporters | A |  |  |  |  | Not Essential | plas 20, vacu 4, mito 2 | NSS\* |
| 13423 | KOG1289: Amino acid transporters |  | TPO5 |  | HNM1 |  | Not Essential | plas 27 | HHR\* |
| 14071 | KOG1289: Amino acid transporters |  |  |  | UGA4 |  | Not Essential | plas 27 | AAR\* |
| 14469 | KOG1303: Amino acid transporters |  |  |  |  |  | Not Essential | plas 27 | GSV\* |
| 15991 | K19564: CTR, HNM1; choline transport protein |  | HNM1 |  | HNM1 |  | Not Essential | plas 26 | EEE\* |
| 16258 | K19564: CTR, HNM1; choline transport protein |  | HNM1 |  | HNM1 |  | Not Essential | plas 24, mito 1, pero 1, E.R. 1 | TVA\* |
| 9135 | K01182: IMA, malL; oligo-1,6-glucosidase |  | MAL32,IMA1,MAL12,YIL172C,IMA5,IMA4,IMA2 | SLC3A1 | IMA1 | SLC3A | Not Essential | cyto 11.5, cyto\_nucl 10, pero 8, nucl 5.5 | FVF\* |
| 11017 | K08157: TPO1; MFS transporter, DHA1 family, mu... |  | TPO1 |  | TPO1 |  | Not Essential | plas 22, mito 2, E.R. 2 | EGV\* |
| 13128 | K08157: TPO1; MFS transporter, DHA1 family, mu... |  |  |  | TPO1 |  | Not Essential | plas 25 | KAG\* |
| 16454 | KOG0255: Synaptic vesicle transporter SVOP and... | A | YHR048W |  | YHK8 |  | Not Essential | plas 25 | TED\* |
| 11649 | KOG0255: Synaptic vesicle transporter SVOP and... |  |  |  | TPO3 |  | Not Essential | plas 26 | SQA\* |
| 14774 | KOG0255: Synaptic vesicle transporter SVOP and... |  | TPO2,TPO3 |  | TPO2 |  | Not Essential | plas 27 | ATS\* |

| strain | WT | | | | | | | | | | | | | | | | |
| --- | --- | --- | --- | --- | --- | --- | --- | --- | --- | --- | --- | --- | --- | --- | --- | --- | --- |
| condition | G\_MM | C\_MM | G\_SD | | GX\_SD | | | X\_SD | | A\_SD | | C\_SD | | MM\_CN120 | | MM\_CN5 | Diversity\_Sample |
| phase | exp | exp | exp | stat | exp | trans | stat | exp | stat | exp | stat | exp | stat | exp | stat | exp | exp |
| proteinId | Set1 | Set1 | Set2 | Set2 | Set2 | Set2 | Set2 | Set2 | Set2 | Set2 | Set2 | Set2 | Set2 | Set3 | Set3 | Set3 | Set3 |
| 8962 | 9.40669 | 4.21147 | 3.75418 | 5.18817 | 3.45813 | 5.35275 | 5.33798 | 5.92402 | 5.72123 | 6.73371 | 6.31699 | 5.21098 | 4.57923 | 10.7988 | 10.3077 | 5.57621 | 8.4861 |
| 9319 | 4.31343 | 5.61405 | 6.56642 | 4.89862 | 6.86377 | 5.18923 | 5.46303 | 4.20479 | 4.63713 | 2.71395 | 4.82923 | 4.95565 | 4.98932 | 6.27998 | 5.72736 | 8.59793 | 7.30291 |
| 9322 | 4.24776 | 3.49915 | 5.07674 | 3.73918 | 5.3914 | 4.58124 | 4.6639 | 3.06761 | 4.06643 | 2.74115 | 2.97376 | 3.91655 | 5.15789 | 3.64587 | 3.95377 | 5.63037 | 5.37934 |
| 9962 | 8.83094 | 7.86758 | 1.96822 | 4.1115 | 2.08733 | 2.24492 | 2.6758 | 2.74331 | 3.83402 | 4.41682 | 4.38619 | 5.73599 | 3.76163 | 9.49677 | 9.42918 | 3.61019 | 7.47773 |
| 10059 | 9.88164 | 7.73995 | 6.0904 | 9.01983 | 6.09768 | 7.59086 | 8.86184 | 8.02948 | 10.7582 | 9.29069 | 11.1036 | 5.43482 | 5.65156 | 11.1167 | 11.2406 | 6.75045 | 9.19265 |
| 10669 | 2.87142 | 2.3094 | 4.18191 | 4.63819 | 4.15724 | 3.13023 | 3.8493 | 2.2554 | 3.96152 | 1.47228 | 3.58299 | 5.66976 | 5.52874 | 5.33441 | 4.57703 | 4.42625 | 7.15293 |
| 11026 | 5.25222 | 4.75689 | 4.67811 | 5.55299 | 4.55917 | 5.07308 | 4.99382 | 4.85569 | 4.85005 | 5.0938 | 5.1087 | 4.96635 | 4.73275 | 7.29879 | 7.36444 | 6.60863 | 7.24308 |
| 11625 | 4.31131 | 4.28584 | 4.19956 | 3.77024 | 4.1102 | 4.53594 | 4.27176 | 4.42923 | 3.7883 | 4.45479 | 3.95697 | 3.9239 | 3.82572 | 4.83317 | 4.88451 | 4.58114 | 4.54674 |
| 12743 | 4.88961 | 4.62487 | 5.07013 | 5.19661 | 5.0602 | 5.22374 | 5.20224 | 5.04997 | 4.97569 | 5.09596 | 5.00232 | 5.05031 | 5.25329 | 5.7298 | 5.78227 | 5.50328 | 5.42846 |
| 12758 | 1.21233 | 1.64366 | 1.53296 | 3.41733 | 1.24655 | 2.39 | 3.67876 | 2.19561 | 4.35085 | 0.986836 | 3.49317 | 1.61649 | 1.64726 | 3.42146 | 4.23344 | 3.33855 | 5.67115 |
| 13601 | 8.55474 | 6.26767 | 8.14305 | 8.79613 | 7.9683 | 7.42414 | 7.53177 | 8.41874 | 9.20901 | 8.80709 | 9.75598 | 1.4016 | 1.19204 | 9.13146 | 7.81609 | 8.28332 | 8.8821 |
| 14129 | 4.79103 | 4.67934 | 4.56625 | 4.59331 | 4.47219 | 4.81185 | 4.75584 | 4.99885 | 4.80568 | 5.17577 | 4.79144 | 4.31526 | 4.06162 | 6.07475 | 5.63371 | 5.83408 | 5.65605 |
| 14229 | 7.34242 | 7.35983 | 2.50673 | 5.16458 | 2.60551 | 4.71047 | 4.43101 | 3.10506 | 3.17976 | 1.96968 | 3.49584 | 5.40335 | 6.0118 | 6.80212 | 7.25017 | 3.19076 | 5.81833 |
| 14391 | 2.60356 | 3.25213 | 2.1557 | 4.80222 | 2.6098 | 2.33842 | 1.81526 | 4.2286 | 3.97552 | 4.8507 | 5.08444 | 4.15572 | 4.73008 | 2.5055 | 2.48072 | 6.67515 | 4.36564 |
| 15074 | 3.3455 | 5.05748 | 2.4274 | 3.59277 | 2.22147 | 2.77927 | 3.24951 | 2.69885 | 2.98712 | 2.69954 | 2.80385 | 5.52326 | 5.45594 | 3.68592 | 4.11958 | 2.80343 | 5.12872 |
| 16187 | 5.4007 | 4.32309 | 4.48496 | 4.28106 | 4.40388 | 4.64908 | 4.37014 | 4.69955 | 4.11518 | 4.84497 | 4.25246 | 5.04559 | 4.08946 | 6.5704 | 6.45253 | 4.90486 | 5.52041 |
| 8552 | 4.08452 | 3.21257 | 4.95925 | 3.48866 | 5.0902 | 5.19435 | 6.0672 | 4.04669 | 4.70073 | 2.36553 | 3.00349 | 3.23585 | 3.57009 | 6.18718 | 4.85022 | 4.64461 | 4.26554 |
| 8766 | 5.30612 | 5.7717 | 4.68587 | 5.39994 | 4.56915 | 5.18808 | 5.30781 | 5.15294 | 5.79277 | 5.43298 | 5.81497 | 7.14508 | 7.33851 | 5.86785 | 6.10512 | 5.20377 | 5.98726 |
| 9616 | 8.53063 | 5.4158 | 5.53469 | 5.89674 | 5.69978 | 5.55203 | 5.96014 | 5.37888 | 6.20651 | 5.91506 | 6.24991 | 5.89116 | 6.1231 | 8.37535 | 7.36564 | 6.75664 | 6.8151 |
| 10057 | 2.88399 | 3.40973 | 1.75835 | 4.29631 | 1.79211 | 1.65367 | 1.94408 | 1.26529 | 2.20306 | 1.57415 | 3.10867 | 3.92446 | 3.7086 | 4.50454 | 3.41322 | 2.85561 | 3.62671 |
| 10143 | 4.85203 | 4.02518 | 2.85321 | 3.18585 | 2.79596 | 2.55107 | 2.53955 | 3.07649 | 3.14264 | 3.37325 | 3.61355 | 2.69367 | 3.12648 | 6.03368 | 5.82901 | 4.50873 | 4.85485 |
| 11269 | 4.80841 | 7.60637 | 3.94892 | 3.59945 | 4.02136 | 4.28514 | 4.36041 | 5.55072 | 4.39582 | 5.07934 | 4.17402 | 6.61203 | 6.2505 | 4.51437 | 4.44383 | 5.92538 | 6.60381 |
| 12567 | 4.61299 | 4.67382 | 5.46171 | 5.76861 | 5.55588 | 5.74744 | 5.92856 | 5.95208 | 7.18366 | 6.99491 | 8.2339 | 4.10774 | 5.56662 | 6.3865 | 5.69017 | 7.15255 | 5.65068 |
| 13178 | 5.53161 | 3.95831 | 6.41901 | 4.95777 | 6.36087 | 5.08926 | 5.31759 | 3.04429 | 4.57959 | 1.99751 | 2.46513 | 5.84412 | 5.86528 | 8.39699 | 6.97934 | 6.8331 | 7.23337 |
| 13423 | 7.06139 | 3.56389 | 2.18037 | 4.70638 | 2.00664 | 3.50729 | 3.49868 | 3.94701 | 3.77595 | 5.03549 | 4.53948 | 4.51806 | 4.61194 | 8.05732 | 7.81529 | 3.33987 | 6.09669 |
| 14071 | 7.77271 | 7.64957 | 5.12607 | 7.3259 | 5.02588 | 5.8963 | 5.54121 | 5.43334 | 5.65054 | 5.57201 | 5.57369 | 5.85967 | 5.8897 | 6.29154 | 6.28644 | 4.90622 | 5.5518 |
| 14469 | 1.64486 | 2.87639 | 1.77685 | 2.67379 | 1.62135 | 1.70021 | 1.72328 | 1.31951 | 1.66421 | 1.45783 | 2.39095 | 1.91336 | 1.99715 | 2.03129 | 2.11207 | 1.69224 | 2.41999 |
| 15991 | 3.09396 | 2.45267 | 3.37056 | 3.36614 | 3.26373 | 3.11204 | 2.90434 | 3.11694 | 2.84616 | 2.73968 | 3.53606 | 2.74484 | 2.28994 | 3.6629 | 3.55649 | 3.3503 | 2.73951 |
| 16258 | 5.30293 | 2.39891 | 5.29654 | 4.14757 | 5.44465 | 5.1772 | 4.85869 | 5.46291 | 4.7875 | 4.96302 | 5.445 | 2.67762 | 2.45806 | 8.16443 | 7.07669 | 7.58019 | 7.20374 |
| 9135 | 9.49406 | 0.873723 | 6.06576 | 5.15088 | 5.97749 | 6.13543 | 5.10501 | 6.10322 | 4.43986 | 8.13123 | 5.36761 | 0.0470114 | 0.0161395 | 10.3289 | 8.25615 | 9.24043 | 7.64627 |
| 11017 | 3.35254 | 3.25576 | 3.1118 | 3.78046 | 3.62787 | 2.2995 | 2.35772 | 2.88435 | 4.0967 | 3.90694 | 4.01468 | 2.93721 | 5.19391 | 4.26473 | 3.43194 | 4.33086 | 3.54139 |
| 13128 | 1.54206 | 1.49946 | 1.4009 | 4.15745 | 1.26371 | 1.80213 | 2.54512 | 1.37778 | 2.71309 | 1.70487 | 2.9778 | 1.55676 | 1.67873 | 1.58157 | 1.9881 | 1.35839 | 1.83167 |
| 16454 | 2.88784 | 3.98979 | 4.43134 | 4.52037 | 4.92808 | 3.99491 | 4.10789 | 3.52258 | 4.25586 | 3.98244 | 3.52756 | 4.14656 | 3.6833 | 4.19595 | 3.65707 | 5.89973 | 4.30239 |
| 11649 | 3.43775 | 4.62787 | 6.43242 | 7.06456 | 6.67773 | 6.29142 | 6.86093 | 4.14974 | 4.317 | 6.14037 | 4.46694 | 2.93763 | 4.49621 | 5.03848 | 3.70794 | 5.21708 | 5.4498 |
| 14774 | 4.59016 | 1.76869 | 9.14238 | 4.63531 | 9.2258 | 7.64782 | 7.75554 | 5.68199 | 6.43342 | 1.65968 | 2.98774 | 2.62868 | 2.54596 | 6.04054 | 7.04763 | 6.96532 | 7.76192 |

| strain | WT | | | | | | | | | | |
| --- | --- | --- | --- | --- | --- | --- | --- | --- | --- | --- | --- |
| condition | G\_SD | | GX\_SD | | | X\_SD | | A\_SD | | C\_SD | |
| proteinId | exp | stat | exp | trans | stat | exp | stat | exp | stat | exp | stat |
| 9319 | 0.384095 | 0 | 1.01594 | 0 | 0 | 0 | 0 | 0 | 0 | 0 | 0 |
| 9322 | 0.384095 | 0 | 1.01594 | 0 | 0 | 0 | 0 | 0 | 0 | 0 | 0 |
| 9962 | 0 | 0 | 0 | 0 | 0 | 0 | 0 | 0.193364 | 0 | 0 | 0 |
| 10059 | 0 | 3.45394 | 0 | 0.18083 | 0.177427 | 2.91247 | 4.32092 | 3.8676 | 4.97494 | 0 | 0 |
| 11026 | 0 | 0 | 0 | 0 | 0 | 0 | 0.195435 | 0 | 0.189081 | 0 | 0 |
| 12743 | 0.197667 | 0.531087 | 0 | 1.67263 | 1.09846 | 0 | 0 | 0 | 0.965882 | 0 | 0 |
| 13601 | 1.13435 | 1.23455 | 1.2115 | 2.06183 | 0.553127 | 6.4423 | 5.33625 | 4.25338 | 4.40666 | 0 | 0 |
| 16187 | 0.58333 | 1.05541 | 0.605588 | 0.7501 | 0.904286 | 0.984473 | 0.788301 | 1.92965 | 1.33755 | 0.842141 | 0.435516 |
| 16258 | 0.566264 | 0.169232 | 0.408254 | 1.48156 | 0.725592 | 1.9633 | 0 | 1.54466 | 0 | 0 | 0 |
| 9135 | 2.09696 | 0.195188 | 2.43009 | 1.1275 | 0 | 1.17479 | 0 | 14.0854 | 2.48003 | 0 | 0 |
| 13128 | 0.197667 | 0.166667 | 0 | 0 | 0 | 0 | 0 | 0 | 0 | 0 | 0 |
| 14774 | 4.40005 | 2.91544 | 6.30919 | 1.66665 | 1.65785 | 0 | 0 | 0 | 0 | 0 | 0 |

|  | Glucose | Xylose | Arabinose | Acetate | Coumarate | Ferulate | YNB Oleic Acid | YNB Ricinoleic Acid | YNB Glucose | YNB Gluc DOC | YPD |
| --- | --- | --- | --- | --- | --- | --- | --- | --- | --- | --- | --- |
| proteinId |  |  |  |  |  |  |  |  |  |  |  |
| 8962 | 0.141486 | 0.0706017 | -0.390702 | -0.181357 | -0.0403765 | -0.347303 | 0.133173 | 0.00723612 | -0.0278378 | -0.0333285 | 0.404273 |
| 9319 | 0.28395 | -0.120032 | -0.0979726 | 0.212506 | -0.238418 | 0.249639 | -0.131144 | -0.0190663 | -0.498955 | -0.281257 | 0.264164 |
| 9322 | 0.284803 | 0.183181 | 0.0765583 | -0.0782087 | -0.472852 | -0.120008 | -0.373087 | -0.742947 | -0.260077 | -0.47357 | -0.526786 |
| 9962 | 0.00129231 | -0.155615 | -0.265223 | 0.0187776 | -0.279612 | -0.163992 | -0.205995 | -0.156544 | 0.138236 | 0.0485481 | 0.402889 |
| 10059 | 0.147659 | -0.475336 | -0.336757 | -0.0222465 | 0.0354311 | 0.201796 | -0.224187 | -0.162138 | -0.0949714 | -0.0628296 | 0.0267071 |
| 10669 | -0.481501 | -1.21922 | -0.816654 | -0.478422 | -1.28689 | -0.425725 | 0.546023 | 0.400691 | 0.417396 | 0.180457 | 0.146982 |
| 11026 | 0.0181579 | -0.0239085 | -0.118443 | -0.387647 | -0.432678 | -0.32265 | -0.175959 | 0.0511242 | -0.0657003 | 0.0282204 | -0.483335 |
| 11625 | -0.14636 | -0.104615 | -0.242208 | -0.0680301 | 0.268976 | -0.149826 | 0.108663 | -0.199959 | 0.0126483 | 0.160107 | 0.0637124 |
| 12743 | -0.121988 | 0.0384331 | 0.14049 | 0.117804 | 0.432167 | 0.381956 | 0.211245 | 0.007785 | 0.125206 | 0.169535 | -0.0687908 |
| 12758 | -0.19741 | 0.139069 | -0.213048 | -0.00655189 | 0.331348 | 0.282728 | 0.732525 | 1.14909 | 0.267083 | 0.0514948 | 0.812044 |
| 13601 | 0.141954 | -0.264735 | 0.0394291 | 0.0376471 | -0.06481 | 0.105764 | 0.0874236 | 0.303779 | 0.158006 | 0.128562 | -0.0135884 |
| 14129 | 0.148251 | -0.0203626 | 0.144737 | -0.104494 | 0.777341 | -0.0563806 | 0.269715 | 0.274875 | 0.211093 | 0.196924 | -0.0616991 |
| 14229 | 0.00533457 | -0.173265 | -0.154243 | -0.242561 | -0.0740997 | -0.364293 | 0.0654025 | -0.154642 | -0.0162818 | 0.0682648 | 0.22377 |
| 14391 | 0.255832 | -0.2209 | -0.012261 | -0.0885669 | -0.03941 | -0.123938 | -0.0640727 | -0.26578 | -0.270392 | -0.0629986 | -0.0813395 |
| 15074 | -0.337726 | -0.280806 | -0.18878 | -0.132357 | 0.160661 | -0.159963 | -0.167013 | -0.114776 | -0.97439 | -0.660703 | -0.364153 |
| 16187 | -0.199182 | -0.0444003 | 0.229648 | -0.0344641 | -0.316065 | 0.0106524 | -0.281907 | 0.108378 | -0.0667902 | -0.249493 | -0.122693 |
| 8552 | -0.146125 | -0.277436 | -0.132178 | -0.0560574 | -0.231995 | -0.187831 | 0.208797 | 0.0257344 | 0.141455 | 0.029351 | -0.0862794 |
| 8766 | 0.2145 | 0.144916 | -0.00311138 | 0.270145 | 0.315071 | 0.0459327 | 0.18641 | 0.488652 | -0.0720502 | -0.314925 | 0.269857 |
| 9616 | -0.0175774 | 0.0383898 | 0.0510782 | 0.102134 | -0.0448857 | -0.0109879 | -0.0899286 | -0.00487041 | 0.0319209 | 0.148193 | -0.0582659 |
| 10057 | 0.0890031 | 0.0919 | 0.0404123 | 0.140596 | 0.220633 | 0.243692 | 0.0166057 | -0.0678822 | 0.231666 | 0.130517 | 0.121888 |
| 10143 | -0.254464 | -0.0213609 | 0.142103 | 0.0296966 | -0.094582 | 0.145843 | -0.0813105 | -0.134965 | -0.260261 | -0.255154 | 0.140303 |
| 11269 | -0.485226 | -0.248686 | -0.215953 | -0.303509 | 0.154373 | -0.193979 | 0.389631 | 0.15886 | 0.0541594 | -0.059126 | 0.0127811 |
| 12567 | -0.0341913 | 0.135271 | 0.0339091 | -0.10744 | -0.0628957 | -0.302262 | -0.0520553 | -0.077944 | -0.105648 | -0.0954301 | 0.0216707 |
| 13178 | -0.298758 | -0.139066 | 0.0784444 | 0.0604963 | -0.0313833 | -0.321889 | -0.313487 | 0.238667 | 0.0566631 | 0.1038 | -0.40749 |
| 13423 | -0.0679298 | -0.100835 | 0.080249 | 0.0345801 | 0.321922 | -0.17617 | 0.146782 | -0.079178 | 0.834333 | 0.530939 | -0.120765 |
| 14071 | -0.102314 | -0.26938 | -0.0187161 | -0.340502 | -0.436314 | -0.298031 | 0.142043 | 0.257511 | 0.356636 | 0.137425 | -0.0154546 |
| 14469 | 0.0153256 | -0.20857 | 0.0354788 | -0.00468889 | -0.279354 | -0.0589968 | -0.0950973 | 0.170976 | -0.289312 | -0.300754 | 0.0138053 |
| 15991 | -0.288875 | -0.152167 | -0.154714 | -0.192469 | -0.553862 | -0.281901 | 0.204088 | 0.518778 | 0.0234849 | 0.116907 | -0.0470714 |
| 16258 | -0.209806 | -0.165133 | -0.273176 | -0.202161 | 0.0750265 | -0.319361 | 0.0401007 | 0.373834 | 0.160624 | -0.0177312 | 0.0337607 |
| 9135 | -0.096142 | -0.0936169 | -0.207051 | -0.143251 | -0.122962 | -0.0498735 | 0.234996 | 0.109655 | 0.189304 | -0.0106298 | 0.241318 |
| 11017 | -0.300448 | -0.246203 | -0.350006 | -0.26054 | -0.26677 | -0.166425 | 0.284098 | 0.112294 | 0.286884 | 0.0262498 | -0.440491 |
| 13128 | -0.0137505 | 0.0899395 | -0.0590193 | 0.0703256 | 0.166176 | 0.138183 | 0.316875 | -0.0871419 | -0.179964 | -0.122323 | 0.034754 |
| 16454 | 0.075807 | 0.0335049 | 0.129629 | -0.11518 | -0.0182373 | -0.0103804 | -0.0403416 | 0.166222 | -0.250331 | -0.0595196 | 0.0155462 |
| 11649 | 0.010138 | 0.223662 | -0.0770604 | -0.0121978 | 0.226909 | -0.11174 | 0.248165 | -0.343117 | 0.216925 | 0.0728566 | -0.409145 |
| 14774 | -0.156627 | -0.174322 | -0.300691 | -0.167797 | -0.335762 | -0.128901 | 0.0621872 | 0.0473526 | 0.0510012 | -0.112789 | -0.323977 |

In [60]:

```
for x in temp:
    if x in model.genes:
        for r in sorted(model.genes.get_by_id(x).reactions, key=lambda x: x.id):
            print(r, r.gene_reaction_rule)
    else:
        print(x, 'no reactions')
    print()
```

```
ALAt2r: ala__L_e + h_e <=> ala__L_c + h_c 12743 or 14229 or 15074 or 8962 or 9319 or 9322 or 9962
ARGt2r: arg__L_e + h_e <=> arg__L_c + h_c 14229 or 15074 or 8962 or 9319 or 9322 or (14229 and 8962) or (14229 and 9319) or (14229 and 9322) or (15074 and 8962) or (15074 and 9319) or (15074 and 9322)
ASNt2r: asn__L_e + h_e <=> asn__L_c + h_c 14229 or 15074 or 8962 or 9319 or 9322 or 9962 or (14229 and 8962) or (14229 and 9319) or (14229 and 9322) or (14229 and 9962) or (15074 and 8962) or (15074 and 9319) or (15074 and 9322) or (15074 and 9962)
ASPt2r: asp__L_e + h_e <=> asp__L_c + h_c 14229 or 15074 or 8962 or 9319 or 9322 or 9962
GLNt2r: gln__L_e + h_e <=> gln__L_c + h_c 14229 or 15074 or 8962 or 9319 or 9322 or 9962 or (14229 and 8962) or (14229 and 9319) or (14229 and 9322) or (14229 and 9962) or (15074 and 8962) or (15074 and 9319) or (15074 and 9322) or (15074 and 9962)
GLUt2r: glu__L_e + h_e <=> glu__L_c + h_c 14229 or 15074 or 8962 or 9319 or 9322 or 9962
GLYt2r: gly_e + h_e <=> gly_c + h_c 12743 or 14229 or 15074 or 8962 or 9319 or 9322 or 9962
LYSt2r: h_e + lys__L_e <=> h_c + lys__L_c 14229 or 15074 or 8962 or 9319 or 9322 or (14229 and 8962) or (14229 and 9319) or (14229 and 9322) or (15074 and 8962) or (15074 and 9319) or (15074 and 9322)
ORNt2r: h_e + orn_e <=> h_c + orn_c 14229 or 15074 or 8962 or 9319 or 9322 or (14229 and 8962) or (14229 and 9319) or (14229 and 9322) or (15074 and 8962) or (15074 and 9319) or (15074 and 9322)
SERt2r: h_e + ser__L_e <=> h_c + ser__L_c 14229 or 15074 or 8962 or 9319 or 9322 or 9962

ALAt2r: ala__L_e + h_e <=> ala__L_c + h_c 12743 or 14229 or 15074 or 8962 or 9319 or 9322 or 9962
ARGt2r: arg__L_e + h_e <=> arg__L_c + h_c 14229 or 15074 or 8962 or 9319 or 9322 or (14229 and 8962) or (14229 and 9319) or (14229 and 9322) or (15074 and 8962) or (15074 and 9319) or (15074 and 9322)
ASNt2r: asn__L_e + h_e <=> asn__L_c + h_c 14229 or 15074 or 8962 or 9319 or 9322 or 9962 or (14229 and 8962) or (14229 and 9319) or (14229 and 9322) or (14229 and 9962) or (15074 and 8962) or (15074 and 9319) or (15074 and 9322) or (15074 and 9962)
ASPt2r: asp__L_e + h_e <=> asp__L_c + h_c 14229 or 15074 or 8962 or 9319 or 9322 or 9962
GLNt2r: gln__L_e + h_e <=> gln__L_c + h_c 14229 or 15074 or 8962 or 9319 or 9322 or 9962 or (14229 and 8962) or (14229 and 9319) or (14229 and 9322) or (14229 and 9962) or (15074 and 8962) or (15074 and 9319) or (15074 and 9322) or (15074 and 9962)
GLUt2r: glu__L_e + h_e <=> glu__L_c + h_c 14229 or 15074 or 8962 or 9319 or 9322 or 9962
GLYt2r: gly_e + h_e <=> gly_c + h_c 12743 or 14229 or 15074 or 8962 or 9319 or 9322 or 9962
LYSt2r: h_e + lys__L_e <=> h_c + lys__L_c 14229 or 15074 or 8962 or 9319 or 9322 or (14229 and 8962) or (14229 and 9319) or (14229 and 9322) or (15074 and 8962) or (15074 and 9319) or (15074 and 9322)
ORNt2r: h_e + orn_e <=> h_c + orn_c 14229 or 15074 or 8962 or 9319 or 9322 or (14229 and 8962) or (14229 and 9319) or (14229 and 9322) or (15074 and 8962) or (15074 and 9319) or (15074 and 9322)
SERt2r: h_e + ser__L_e <=> h_c + ser__L_c 14229 or 15074 or 8962 or 9319 or 9322 or 9962

ALAt2r: ala__L_e + h_e <=> ala__L_c + h_c 12743 or 14229 or 15074 or 8962 or 9319 or 9322 or 9962
ARGt2r: arg__L_e + h_e <=> arg__L_c + h_c 14229 or 15074 or 8962 or 9319 or 9322 or (14229 and 8962) or (14229 and 9319) or (14229 and 9322) or (15074 and 8962) or (15074 and 9319) or (15074 and 9322)
ASNt2r: asn__L_e + h_e <=> asn__L_c + h_c 14229 or 15074 or 8962 or 9319 or 9322 or 9962 or (14229 and 8962) or (14229 and 9319) or (14229 and 9322) or (14229 and 9962) or (15074 and 8962) or (15074 and 9319) or (15074 and 9322) or (15074 and 9962)
ASPt2r: asp__L_e + h_e <=> asp__L_c + h_c 14229 or 15074 or 8962 or 9319 or 9322 or 9962
GLNt2r: gln__L_e + h_e <=> gln__L_c + h_c 14229 or 15074 or 8962 or 9319 or 9322 or 9962 or (14229 and 8962) or (14229 and 9319) or (14229 and 9322) or (14229 and 9962) or (15074 and 8962) or (15074 and 9319) or (15074 and 9322) or (15074 and 9962)
GLUt2r: glu__L_e + h_e <=> glu__L_c + h_c 14229 or 15074 or 8962 or 9319 or 9322 or 9962
GLYt2r: gly_e + h_e <=> gly_c + h_c 12743 or 14229 or 15074 or 8962 or 9319 or 9322 or 9962
LYSt2r: h_e + lys__L_e <=> h_c + lys__L_c 14229 or 15074 or 8962 or 9319 or 9322 or (14229 and 8962) or (14229 and 9319) or (14229 and 9322) or (15074 and 8962) or (15074 and 9319) or (15074 and 9322)
ORNt2r: h_e + orn_e <=> h_c + orn_c 14229 or 15074 or 8962 or 9319 or 9322 or (14229 and 8962) or (14229 and 9319) or (14229 and 9322) or (15074 and 8962) or (15074 and 9319) or (15074 and 9322)
SERt2r: h_e + ser__L_e <=> h_c + ser__L_c 14229 or 15074 or 8962 or 9319 or 9322 or 9962

ALAt2r: ala__L_e + h_e <=> ala__L_c + h_c 12743 or 14229 or 15074 or 8962 or 9319 or 9322 or 9962
ASNt2r: asn__L_e + h_e <=> asn__L_c + h_c 14229 or 15074 or 8962 or 9319 or 9322 or 9962 or (14229 and 8962) or (14229 and 9319) or (14229 and 9322) or (14229 and 9962) or (15074 and 8962) or (15074 and 9319) or (15074 and 9322) or (15074 and 9962)
ASPt2r: asp__L_e + h_e <=> asp__L_c + h_c 14229 or 15074 or 8962 or 9319 or 9322 or 9962
GLNt2r: gln__L_e + h_e <=> gln__L_c + h_c 14229 or 15074 or 8962 or 9319 or 9322 or 9962 or (14229 and 8962) or (14229 and 9319) or (14229 and 9322) or (14229 and 9962) or (15074 and 8962) or (15074 and 9319) or (15074 and 9322) or (15074 and 9962)
GLUt2r: glu__L_e + h_e <=> glu__L_c + h_c 14229 or 15074 or 8962 or 9319 or 9322 or 9962
GLYt2r: gly_e + h_e <=> gly_c + h_c 12743 or 14229 or 15074 or 8962 or 9319 or 9322 or 9962
SERt2r: h_e + ser__L_e <=> h_c + ser__L_c 14229 or 15074 or 8962 or 9319 or 9322 or 9962

10059 no reactions

10669 no reactions

11026 no reactions

AMY1e: 8.0 h2o_e + strch1_e --> 8.0 glc__D_e + strch2_e 11625 and 9135
AMY2e: glygn2_e + 8.0 h2o_e --> 8.0 glc__D_e + glygn4_e 11625 and 9135
CYSTSERex: cysi__L_e + ser__L_c --> cysi__L_c + ser__L_e 11625 and 9135
SERLYSNaex: lys__L_c + na1_e + ser__L_e --> lys__L_e + na1_c + ser__L_c 11625 and 9135

ABUTt2r: 4abut_e + h_e <=> 4abut_c + h_c 11269 or 12743 or 13423
ALAt2r: ala__L_e + h_e <=> ala__L_c + h_c 12743 or 14229 or 15074 or 8962 or 9319 or 9322 or 9962
ASNt7: asn__L_v + h_v --> asn__L_c + h_c 12743
DALAt2r: ala__D_e + h_e <=> ala__D_c + h_c 12743
GLNt7: gln__L_v + h_v --> gln__L_c + h_c 12743
GLYt2r: gly_e + h_e <=> gly_c + h_c 12743 or 14229 or 15074 or 8962 or 9319 or 9322 or 9962
ILEt7: h_v + ile__L_v --> h_c + ile__L_c 12743
LEUt7: h_v + leu__L_v --> h_c + leu__L_c 12743
PRODt2r: h_e + pro__D_e <=> h_c + pro__D_c 12743
PROt2r: h_e + pro__L_e <=> h_c + pro__L_c 12743 or 14229 or 15074
TYRt7: h_v + tyr__L_v --> h_c + tyr__L_c 12743

12758 no reactions

13601 no reactions

ALAtN1: ala__L_e + h_c + 2.0 na1_e <=> ala__L_c + h_e + 2.0 na1_c 14129
ASNtN1: asn__L_e + h_c + 2.0 na1_e <=> asn__L_c + h_e + 2.0 na1_c 14129
GLNtN1: gln__L_e + h_c + 2.0 na1_e <=> gln__L_c + h_e + 2.0 na1_c 14129
HIStN1: h_c + his__L_e + 2.0 na1_e <=> h_e + his__L_c + 2.0 na1_c 14129

ALAt2r: ala__L_e + h_e <=> ala__L_c + h_c 12743 or 14229 or 15074 or 8962 or 9319 or 9322 or 9962
AMETt2: amet_e + h_e --> amet_c + h_c 14229 or 15074
ARGt2r: arg__L_e + h_e <=> arg__L_c + h_c 14229 or 15074 or 8962 or 9319 or 9322 or (14229 and 8962) or (14229 and 9319) or (14229 and 9322) or (15074 and 8962) or (15074 and 9319) or (15074 and 9322)
ASNt2r: asn__L_e + h_e <=> asn__L_c + h_c 14229 or 15074 or 8962 or 9319 or 9322 or 9962 or (14229 and 8962) or (14229 and 9319) or (14229 and 9322) or (14229 and 9962) or (15074 and 8962) or (15074 and 9319) or (15074 and 9322) or (15074 and 9962)
ASPt2r: asp__L_e + h_e <=> asp__L_c + h_c 14229 or 15074 or 8962 or 9319 or 9322 or 9962
CYSt2r: cys__L_e + h_e <=> cys__L_c + h_c 14229 or 15074
GLNt2r: gln__L_e + h_e <=> gln__L_c + h_c 14229 or 15074 or 8962 or 9319 or 9322 or 9962 or (14229 and 8962) or (14229 and 9319) or (14229 and 9322) or (14229 and 9962) or (15074 and 8962) or (15074 and 9319) or (15074 and 9322) or (15074 and 9962)
GLUt2r: glu__L_e + h_e <=> glu__L_c + h_c 14229 or 15074 or 8962 or 9319 or 9322 or 9962
GLYt2r: gly_e + h_e <=> gly_c + h_c 12743 or 14229 or 15074 or 8962 or 9319 or 9322 or 9962
HISt2r: h_e + his__L_e <=> h_c + his__L_c 14229 or 15074
ILEt2r: h_e + ile__L_e <=> h_c + ile__L_c 14229 or 15074
LEUt2r: h_e + leu__L_e <=> h_c + leu__L_c 14229 or 15074
LYSt2r: h_e + lys__L_e <=> h_c + lys__L_c 14229 or 15074 or 8962 or 9319 or 9322 or (14229 and 8962) or (14229 and 9319) or (14229 and 9322) or (15074 and 8962) or (15074 and 9319) or (15074 and 9322)
METt2r: h_e + met__L_e <=> h_c + met__L_c 14229 or 15074
ORNt2r: h_e + orn_e <=> h_c + orn_c 14229 or 15074 or 8962 or 9319 or 9322 or (14229 and 8962) or (14229 and 9319) or (14229 and 9322) or (15074 and 8962) or (15074 and 9319) or (15074 and 9322)
PHEt2r: h_e + phe__L_e <=> h_c + phe__L_c 14229 or 15074
PROt2r: h_e + pro__L_e <=> h_c + pro__L_c 12743 or 14229 or 15074
SERt2r: h_e + ser__L_e <=> h_c + ser__L_c 14229 or 15074 or 8962 or 9319 or 9322 or 9962
THRt2r: h_e + thr__L_e <=> h_c + thr__L_c 14229 or 15074
TRPt2r: h_e + trp__L_e <=> h_c + trp__L_c 14229 or 15074
TYRt2r: h_e + tyr__L_e <=> h_c + tyr__L_c 14229 or 15074
VALt2r: h_e + val__L_e <=> h_c + val__L_c 14229 or 15074

14391 no reactions

ALAt2r: ala__L_e + h_e <=> ala__L_c + h_c 12743 or 14229 or 15074 or 8962 or 9319 or 9322 or 9962
AMETt2: amet_e + h_e --> amet_c + h_c 14229 or 15074
ARGt2r: arg__L_e + h_e <=> arg__L_c + h_c 14229 or 15074 or 8962 or 9319 or 9322 or (14229 and 8962) or (14229 and 9319) or (14229 and 9322) or (15074 and 8962) or (15074 and 9319) or (15074 and 9322)
ASNt2r: asn__L_e + h_e <=> asn__L_c + h_c 14229 or 15074 or 8962 or 9319 or 9322 or 9962 or (14229 and 8962) or (14229 and 9319) or (14229 and 9322) or (14229 and 9962) or (15074 and 8962) or (15074 and 9319) or (15074 and 9322) or (15074 and 9962)
ASPt2r: asp__L_e + h_e <=> asp__L_c + h_c 14229 or 15074 or 8962 or 9319 or 9322 or 9962
CYSt2r: cys__L_e + h_e <=> cys__L_c + h_c 14229 or 15074
GLNt2r: gln__L_e + h_e <=> gln__L_c + h_c 14229 or 15074 or 8962 or 9319 or 9322 or 9962 or (14229 and 8962) or (14229 and 9319) or (14229 and 9322) or (14229 and 9962) or (15074 and 8962) or (15074 and 9319) or (15074 and 9322) or (15074 and 9962)
GLUt2r: glu__L_e + h_e <=> glu__L_c + h_c 14229 or 15074 or 8962 or 9319 or 9322 or 9962
GLYt2r: gly_e + h_e <=> gly_c + h_c 12743 or 14229 or 15074 or 8962 or 9319 or 9322 or 9962
HISt2r: h_e + his__L_e <=> h_c + his__L_c 14229 or 15074
ILEt2r: h_e + ile__L_e <=> h_c + ile__L_c 14229 or 15074
LEUt2r: h_e + leu__L_e <=> h_c + leu__L_c 14229 or 15074
LYSt2r: h_e + lys__L_e <=> h_c + lys__L_c 14229 or 15074 or 8962 or 9319 or 9322 or (14229 and 8962) or (14229 and 9319) or (14229 and 9322) or (15074 and 8962) or (15074 and 9319) or (15074 and 9322)
METt2r: h_e + met__L_e <=> h_c + met__L_c 14229 or 15074
ORNt2r: h_e + orn_e <=> h_c + orn_c 14229 or 15074 or 8962 or 9319 or 9322 or (14229 and 8962) or (14229 and 9319) or (14229 and 9322) or (15074 and 8962) or (15074 and 9319) or (15074 and 9322)
PHEt2r: h_e + phe__L_e <=> h_c + phe__L_c 14229 or 15074
PROt2r: h_e + pro__L_e <=> h_c + pro__L_c 12743 or 14229 or 15074
SERt2r: h_e + ser__L_e <=> h_c + ser__L_c 14229 or 15074 or 8962 or 9319 or 9322 or 9962
THRt2r: h_e + thr__L_e <=> h_c + thr__L_c 14229 or 15074
TRPt2r: h_e + trp__L_e <=> h_c + trp__L_c 14229 or 15074
TYRt2r: h_e + tyr__L_e <=> h_c + tyr__L_c 14229 or 15074
VALt2r: h_e + val__L_e <=> h_c + val__L_c 14229 or 15074

ASNt6: asn__L_c + h_v --> asn__L_v + h_c 16187
ASNtx: asn__L_c + h_c <=> asn__L_x + h_x 16187
BALAVECSEC: 3.0 ala_B_c + atp_c + h2o_c --> adp_c + 3.0 ala_B_e + h_c + pi_c 16187
GABAVESSEC: 3.0 4abut_c + atp_c + h2o_c --> 3.0 4abut_e + adp_c + h_c + pi_c 16187
GLNt6: gln__L_c + h_v --> gln__L_v + h_c 16187
GLYVESSEC: atp_c + 3.0 gly_c + h2o_c --> adp_c + 3.0 gly_e + h_c + pi_c 16187
ILEt6: h_v + ile__L_c --> h_c + ile__L_v 16187
LEUt6: h_v + leu__L_c --> h_c + leu__L_v 16187
TYRt6: h_v + tyr__L_c --> h_c + tyr__L_v 16187

8552 no reactions

ASPt2m: asp__L_c + h_c <=> asp__L_m + h_m 8766
ASPt7: asp__L_v + h_v --> asp__L_c + h_c 8766
ASPtx: asp__L_x + h_x <=> asp__L_c + h_c 8766
GLUt2m: glu__L_c + h_c <=> glu__L_m + h_m 16799 or 8766
GLUt7: glu__L_v + h_v --> glu__L_c + h_c 8766

9616 no reactions

10057 no reactions

10143 no reactions

4ABUTtmi: 4abut_c + h_c --> 4abut_m + h_m 11269 or 13423
5AOPt2: 5aop_e + h_e --> 5aop_c + h_c 11269 or 13423
ABUTt2r: 4abut_e + h_e <=> 4abut_c + h_c 11269 or 12743 or 13423

12567 no reactions

13178 no reactions

4ABUTtmi: 4abut_c + h_c --> 4abut_m + h_m 11269 or 13423
5AOPt2: 5aop_e + h_e --> 5aop_c + h_c 11269 or 13423
ABUTt2r: 4abut_e + h_e <=> 4abut_c + h_c 11269 or 12743 or 13423
PTRCtex2: ptrc_c --> ptrc_e 13423 or 15991 or 16258
SPMDtex2: spmd_c --> spmd_e 13423 or 15991 or 16258

14071 no reactions

14469 no reactions

CHLt2: chol_e + h_e --> chol_c + h_c 15991 or 16258
PTRCtex2: ptrc_c --> ptrc_e 13423 or 15991 or 16258
SPMDtex2: spmd_c --> spmd_e 13423 or 15991 or 16258

CHLt2: chol_e + h_e --> chol_c + h_c 15991 or 16258
PTRCtex2: ptrc_c --> ptrc_e 13423 or 15991 or 16258
SPMDtex2: spmd_c --> spmd_e 13423 or 15991 or 16258

AMY1e: 8.0 h2o_e + strch1_e --> 8.0 glc__D_e + strch2_e 11625 and 9135
AMY2e: glygn2_e + 8.0 h2o_e --> 8.0 glc__D_e + glygn4_e 11625 and 9135
CYSTSERex: cysi__L_e + ser__L_c --> cysi__L_c + ser__L_e 11625 and 9135
DGGH: 6dg_c + h2o_c --> gal_c + glc__D_c 9135
MALT: h2o_c + malt_c --> 2.0 glc__D_c 10170 or 9135
SERLYSNaex: lys__L_c + na1_e + ser__L_e --> lys__L_e + na1_c + ser__L_c 11625 and 9135
TRE6PH: h2o_c + tre6p_c --> g6p_c + glc__D_c 9135

PTRCt3i: h_c + ptrc_e --> h_e + ptrc_c 11017 or 13128 or (11017 and 16454) or (13128 and 16454)
SPMDt3i: h_c + spmd_e --> h_e + spmd_c 11017 or 13128 or (11017 and 16454) or (13128 and 16454)
SPRMt2i: h_c + sprm_e --> h_e + sprm_c 11017 or 11649 or 13128 or 14774 or (11017 and 11649 and 16454) or (11017 and 14774 and 16454) or (11649 and 13128 and 16454) or (13128 and 14774 and 16454)

PTRCt3i: h_c + ptrc_e --> h_e + ptrc_c 11017 or 13128 or (11017 and 16454) or (13128 and 16454)
SPMDt3i: h_c + spmd_e --> h_e + spmd_c 11017 or 13128 or (11017 and 16454) or (13128 and 16454)
SPRMt2i: h_c + sprm_e --> h_e + sprm_c 11017 or 11649 or 13128 or 14774 or (11017 and 11649 and 16454) or (11017 and 14774 and 16454) or (11649 and 13128 and 16454) or (13128 and 14774 and 16454)

PTRCt3i: h_c + ptrc_e --> h_e + ptrc_c 11017 or 13128 or (11017 and 16454) or (13128 and 16454)
SPMDt3i: h_c + spmd_e --> h_e + spmd_c 11017 or 13128 or (11017 and 16454) or (13128 and 16454)
SPRMt2i: h_c + sprm_e --> h_e + sprm_c 11017 or 11649 or 13128 or 14774 or (11017 and 11649 and 16454) or (11017 and 14774 and 16454) or (11649 and 13128 and 16454) or (13128 and 14774 and 16454)

SPMDtmr: h_m + spmd_c <=> h_c + spmd_m 11649 or 14774
SPRMt2i: h_c + sprm_e --> h_e + sprm_c 11017 or 11649 or 13128 or 14774 or (11017 and 11649 and 16454) or (11017 and 14774 and 16454) or (11649 and 13128 and 16454) or (13128 and 14774 and 16454)

SPMDtmr: h_m + spmd_c <=> h_c + spmd_m 11649 or 14774
SPRMt2i: h_c + sprm_e --> h_e + sprm_c 11017 or 11649 or 13128 or 14774 or (11017 and 11649 and 16454) or (11017 and 14774 and 16454) or (11649 and 13128 and 16454) or (13128 and 14774 and 16454)
```

In [61]:

```
# 14229 and 15074 GAP1 general amino acid permease
# 8962, 9319, 9322, 9962 DIP5 dicarboxylic aa permease glu, asp, gln, asn, ser, ala, gly
# 11625 SLC7A11 Sodium-independent, high-affinity exchange of anionic amino acids with 
#       high specificity for anionic form of cystine and glutamate -> orthoMCL found different SLC7?
# 9135 is oligo-1,6-glucosidase, remove them from transport reactions
# 14129 SLC38A3/SLC38A11 sodium-coupled neutral amino acid transporter, ok
# 11269 UGA4 gamma aminobutyrate permease putrescine, delta-aminolevulinic acid
# 13423 TPO5 secretion of putrescine, spermidine
# 15991 and 16258 HNM1 plasma membrane transporter for choline, ethanolamine, and carnitine, ok
# 16187 vacuolar import large neutral aa
# 12743 vacuolar export large neutral aa
# 8766 vacuolar export asp and glu
# 11017, 13128, 11649, 14774 TPO1-4 polyamine MFS transporter
# 16454 YHK8 presumed MFS drug:H antiporter

model.reactions.get_by_id('AMY1e').gene_reaction_rule = '9135'
model.reactions.get_by_id('AMY2e').gene_reaction_rule = '9135'
model.reactions.get_by_id('CYSTSERex').gene_reaction_rule = '11625'
model.reactions.get_by_id('SERLYSNaex').gene_reaction_rule = '11625'

model.reactions.get_by_id('ABUTt2r').gene_reaction_rule = '11269'
model.reactions.get_by_id('5AOPt2').gene_reaction_rule = '11269'
model.reactions.get_by_id('SPMDtex2').gene_reaction_rule = '13423'
model.reactions.get_by_id('PTRCtex2').gene_reaction_rule = '13423'

model.reactions.get_by_id('PTRCt3i').gene_reaction_rule = '11017 or 11649 or 13128 or 14774'
model.reactions.get_by_id('SPMDt3i').gene_reaction_rule = '11017 or 11649 or 13128 or 14774'
model.reactions.get_by_id('SPRMt2i').gene_reaction_rule = '11017 or 11649 or 13128 or 14774'

model.reactions.get_by_id('ALAt2r').gene_reaction_rule = '14229 or 15074 or 8962 or 9319 or 9322 or 9962'
model.reactions.get_by_id('GLNt2r').gene_reaction_rule = '14229 or 15074 or 8962 or 9319 or 9322 or 9962'
model.reactions.get_by_id('ASNt2r').gene_reaction_rule = '14229 or 15074 or 8962 or 9319 or 9322 or 9962'
model.reactions.get_by_id('ASPt2r').gene_reaction_rule = '14229 or 15074 or 8962 or 9319 or 9322 or 9962'
model.reactions.get_by_id('SERt2r').gene_reaction_rule = '14229 or 15074 or 8962 or 9319 or 9322 or 9962'
model.reactions.get_by_id('GLUt2r').gene_reaction_rule = '14229 or 15074 or 8962 or 9319 or 9322 or 9962'
model.reactions.get_by_id('GLYt2r').gene_reaction_rule = '14229 or 15074 or 8962 or 9319 or 9322 or 9962'

model.reactions.get_by_id('ARGt2r').gene_reaction_rule = '14229 or 15074'
model.reactions.get_by_id('LYSt2r').gene_reaction_rule = '14229 or 15074'
model.reactions.get_by_id('ORNt2r').gene_reaction_rule = '14229 or 15074'
model.reactions.get_by_id('PROt2r').gene_reaction_rule = '14229 or 15074'
model.reactions.get_by_id('DALAt2r').gene_reaction_rule = '14229 or 15074'
model.reactions.get_by_id('PRODt2r').gene_reaction_rule = '14229 or 15074'

remove = ['GLYVESSEC','BALAVECSEC','GABAVESSEC','4ABUTtmi','ASPt2m','GLUt2m','ASNtx','ASPtx']
model.remove_reactions(remove ,remove_orphans=True)
```

In [62]:

```
for r in sorted(model.metabolites.get_by_id('val__L_c').reactions, key=lambda x: x.id):
    print(r, r.gene_reaction_rule)
print()
for r in sorted(model.metabolites.get_by_id('leu__L_c').reactions, key=lambda x: x.id):
    print(r, r.gene_reaction_rule)
print()
for r in sorted(model.metabolites.get_by_id('ile__L_c').reactions, key=lambda x: x.id):
    print(r, r.gene_reaction_rule)
```

```
BIOMASS_RT: 1.1348 13BDglcn_c + 0.4588 ala__L_c + 0.046 amp_c + 0.1607 arg__L_c + 0.1017 asn__L_c + 0.2975 asp__L_c + 59.276 atp_c + 0.0447 cmp_c + 0.0066 cys__L_c + 0.0036 damp_c + 0.0024 dcmp_c + 0.0024 dgmp_c + 0.0036 dtmp_c + 0.0007 ergst_r + 0.1054 gln__L_c + 0.3018 glu__L_c + 0.2904 gly_c + 0.5185 glycogen_c + 0.046 gmp_c + 59.276 h2o_c + 0.0663 his__L_c + 0.1927 ile__L_c + 0.2964 leu__L_c + 0.2862 lys__L_c + 0.8079 mannan_r + 0.0507 met__L_c + 6e-06 pa_RT_r + 6e-05 pc_RT_r + 4.5e-05 pe_RT_r + 0.1339 phe__L_c + 0.1647 pro__L_c + 1.7e-05 ps_RT_r + 5.3e-05 ptd1ino_RT_r + 0.00099 ribflv_c + 0.1854 ser__L_c + 0.02 so4_c + 0.1914 thr__L_c + 0.0234 tre_c + 6.6e-05 triglyc_RT_r + 0.0284 trp__L_c + 0.102 tyr__L_c + 0.0599 ump_c + 0.2646 val__L_c + 0.0015 zymst_r --> 59.276 adp_c + 58.70001 h_c + 59.305 pi_c 
VALTA: akg_c + val__L_c <=> 3mob_c + glu__L_c 14014 or 14610 or 14853
VALTRS: atp_c + trnaval_c + val__L_c --> amp_c + ppi_c + valtrna_c 16528
VALt2r: h_e + val__L_e <=> h_c + val__L_c 14229 or 15074

BIOMASS_RT: 1.1348 13BDglcn_c + 0.4588 ala__L_c + 0.046 amp_c + 0.1607 arg__L_c + 0.1017 asn__L_c + 0.2975 asp__L_c + 59.276 atp_c + 0.0447 cmp_c + 0.0066 cys__L_c + 0.0036 damp_c + 0.0024 dcmp_c + 0.0024 dgmp_c + 0.0036 dtmp_c + 0.0007 ergst_r + 0.1054 gln__L_c + 0.3018 glu__L_c + 0.2904 gly_c + 0.5185 glycogen_c + 0.046 gmp_c + 59.276 h2o_c + 0.0663 his__L_c + 0.1927 ile__L_c + 0.2964 leu__L_c + 0.2862 lys__L_c + 0.8079 mannan_r + 0.0507 met__L_c + 6e-06 pa_RT_r + 6e-05 pc_RT_r + 4.5e-05 pe_RT_r + 0.1339 phe__L_c + 0.1647 pro__L_c + 1.7e-05 ps_RT_r + 5.3e-05 ptd1ino_RT_r + 0.00099 ribflv_c + 0.1854 ser__L_c + 0.02 so4_c + 0.1914 thr__L_c + 0.0234 tre_c + 6.6e-05 triglyc_RT_r + 0.0284 trp__L_c + 0.102 tyr__L_c + 0.0599 ump_c + 0.2646 val__L_c + 0.0015 zymst_r --> 59.276 adp_c + 58.70001 h_c + 59.305 pi_c 
LEUTA: akg_c + leu__L_c <=> 4mop_c + glu__L_c 14014 or 14610 or 14853
LEUTAi: 4mop_c + glu__L_c --> akg_c + leu__L_c 14610 or 14853 or 8936
LEUTRS: atp_c + leu__L_c + trnaleu_c --> amp_c + leutrna_c + ppi_c 10144
LEUt2r: h_e + leu__L_e <=> h_c + leu__L_c 14229 or 15074
LEUt6: h_v + leu__L_c --> h_c + leu__L_v 16187
LEUt7: h_v + leu__L_v --> h_c + leu__L_c 12743

BIOMASS_RT: 1.1348 13BDglcn_c + 0.4588 ala__L_c + 0.046 amp_c + 0.1607 arg__L_c + 0.1017 asn__L_c + 0.2975 asp__L_c + 59.276 atp_c + 0.0447 cmp_c + 0.0066 cys__L_c + 0.0036 damp_c + 0.0024 dcmp_c + 0.0024 dgmp_c + 0.0036 dtmp_c + 0.0007 ergst_r + 0.1054 gln__L_c + 0.3018 glu__L_c + 0.2904 gly_c + 0.5185 glycogen_c + 0.046 gmp_c + 59.276 h2o_c + 0.0663 his__L_c + 0.1927 ile__L_c + 0.2964 leu__L_c + 0.2862 lys__L_c + 0.8079 mannan_r + 0.0507 met__L_c + 6e-06 pa_RT_r + 6e-05 pc_RT_r + 4.5e-05 pe_RT_r + 0.1339 phe__L_c + 0.1647 pro__L_c + 1.7e-05 ps_RT_r + 5.3e-05 ptd1ino_RT_r + 0.00099 ribflv_c + 0.1854 ser__L_c + 0.02 so4_c + 0.1914 thr__L_c + 0.0234 tre_c + 6.6e-05 triglyc_RT_r + 0.0284 trp__L_c + 0.102 tyr__L_c + 0.0599 ump_c + 0.2646 val__L_c + 0.0015 zymst_r --> 59.276 adp_c + 58.70001 h_c + 59.305 pi_c 
ILETA: akg_c + ile__L_c <=> 3mop_c + glu__L_c 14014 or 14610 or 14853
ILETRS: atp_c + ile__L_c + trnaile_c --> amp_c + iletrna_c + ppi_c 13446 or 15556
ILEt2r: h_e + ile__L_e <=> h_c + ile__L_c 14229 or 15074
ILEt6: h_v + ile__L_c --> h_c + ile__L_v 16187
ILEt7: h_v + ile__L_v --> h_c + ile__L_c 12743
```

In [63]:

```
for r in sorted(model.metabolites.get_by_id('val__L_m').reactions, key=lambda x: x.id):
    print(r, r.gene_reaction_rule)
print()
for r in sorted(model.metabolites.get_by_id('leu__L_m').reactions, key=lambda x: x.id):
    print(r, r.gene_reaction_rule)
print()
for r in sorted(model.metabolites.get_by_id('ile__L_m').reactions, key=lambda x: x.id):
    print(r, r.gene_reaction_rule)
```

```
VALTAim: akg_m + val__L_m --> 3mob_m + glu__L_m 14014 or 14610 or 14853
VALTRSm: atp_m + trnaval_m + val__L_m --> amp_m + ppi_m + valtrna_m 16528

LEUTAm: akg_m + leu__L_m <=> 4mop_m + glu__L_m 14014 or 14610 or 14853
LEUTRSm: atp_m + leu__L_m + trnaleu_m --> amp_m + leutrna_m + ppi_m 10144

ILETAm: akg_m + ile__L_m <=> 3mop_m + glu__L_m 14014 or 14610 or 14853
ILETRSm: atp_m + ile__L_m + trnaile_m --> amp_m + iletrna_m + ppi_m 13446 or 15556
```

In [64]:

```
temp = ['16528','10144','13446','15556']
display(Annotation.loc[temp])
Show_Data(temp)
```

|  | Combined Annotations | Signal P | Sc288c Orthologs | Human Orthologs | Sc288 Best Hit | Human Blast | Essential | WolfPSort | C Terminal |
| --- | --- | --- | --- | --- | --- | --- | --- | --- | --- |
| RTO4\_ID |  |  |  |  |  |  |  |  |  |
| 16528 | K01873: VARS, valS; valyl-tRNA synthetase | S | VAS1 | VARS,VARS2 | VAS1 | VARS | Essential | mito 15.5, cyto\_mito 11.833, cyto 7, cyto\_nucl... | LRV\* |
| 10144 | K01869: LARS, leuS; leucyl-tRNA synthetase |  | CDC60 | LARS | CDC60 | LARS | Essential | cyto 13.5, cyto\_mito 11, mito 7.5, nucl 4 | YNI\* |
| 13446 | K01870: IARS, ileS; isoleucyl-tRNA synthetase |  | ILS1 | IARS | ILS1 | IARS | Essential | cyto\_nucl 11, nucl 10.5, cyto 10.5, mito 3 | VWA\* |
| 15556 | K01870: IARS, ileS; isoleucyl-tRNA synthetase |  | ISM1 | IARS2 | ISM1 | IARS2 | Essential | mito 20, cyto 4, nucl 3 | VSA\* |

| strain | WT | | | | | | | | | | | | | | | | |
| --- | --- | --- | --- | --- | --- | --- | --- | --- | --- | --- | --- | --- | --- | --- | --- | --- | --- |
| condition | G\_MM | C\_MM | G\_SD | | GX\_SD | | | X\_SD | | A\_SD | | C\_SD | | MM\_CN120 | | MM\_CN5 | Diversity\_Sample |
| phase | exp | exp | exp | stat | exp | trans | stat | exp | stat | exp | stat | exp | stat | exp | stat | exp | exp |
| proteinId | Set1 | Set1 | Set2 | Set2 | Set2 | Set2 | Set2 | Set2 | Set2 | Set2 | Set2 | Set2 | Set2 | Set3 | Set3 | Set3 | Set3 |
| 16528 | 5.83323 | 6.07082 | 6.73082 | 4.87153 | 7.01552 | 5.74748 | 5.61988 | 5.99348 | 5.14614 | 5.34936 | 4.65258 | 6.71624 | 7.67826 | 6.17908 | 5.99888 | 8.24533 | 7.14784 |
| 10144 | 5.28024 | 6.29388 | 6.10584 | 4.64947 | 6.39427 | 5.42995 | 5.51394 | 5.66942 | 4.8861 | 5.16299 | 4.41526 | 7.05069 | 7.83129 | 6.95207 | 6.8628 | 8.36295 | 8.00918 |
| 13446 | 6.29045 | 6.44689 | 6.85996 | 5.20898 | 7.28578 | 5.94924 | 5.90092 | 6.27526 | 5.4352 | 5.66776 | 5.08721 | 6.86215 | 8.77516 | 7.1975 | 7.01547 | 9.90927 | 8.60887 |
| 15556 | 4.3147 | 4.3728 | 5.85209 | 4.96724 | 5.79969 | 5.36095 | 5.08279 | 5.46143 | 4.50372 | 5.42262 | 4.53018 | 5.26314 | 5.21676 | 5.02292 | 5.03868 | 6.35509 | 5.9945 |

| strain | WT | | | | | | | | | | |
| --- | --- | --- | --- | --- | --- | --- | --- | --- | --- | --- | --- |
| condition | G\_SD | | GX\_SD | | | X\_SD | | A\_SD | | C\_SD | |
| proteinId | exp | stat | exp | trans | stat | exp | stat | exp | stat | exp | stat |
| 16528 | 32.2982 | 26.5728 | 33.9816 | 20.2776 | 20.1194 | 21.746 | 22.0328 | 25.6918 | 20.3139 | 20.4882 | 26.1486 |
| 10144 | 49.2838 | 38.4873 | 42.0542 | 26.0076 | 24.5459 | 32.4489 | 26.2234 | 29.3692 | 23.5277 | 37.5847 | 52.2132 |
| 13446 | 35.2241 | 23.5188 | 36.1914 | 19.3302 | 18.9875 | 25.5816 | 23.5865 | 24.3267 | 20.7187 | 25.2334 | 38.3194 |
| 15556 | 4.61118 | 4.92087 | 3.4408 | 3.53365 | 4.2038 | 0.978443 | 1.56304 | 2.31641 | 2.67015 | 2.15681 | 1.97492 |

|  | Glucose | Xylose | Arabinose | Acetate | Coumarate | Ferulate | YNB Oleic Acid | YNB Ricinoleic Acid | YNB Glucose | YNB Gluc DOC | YPD |
| --- | --- | --- | --- | --- | --- | --- | --- | --- | --- | --- | --- |
| proteinId |  |  |  |  |  |  |  |  |  |  |  |

In [65]:

```
# 13446 cyto 15556 mito
model.reactions.get_by_id('ILETRS').gene_reaction_rule = '13446'
model.reactions.get_by_id('ILETRSm').gene_reaction_rule = '15556'
```

In [66]:

```
temp = Annotation.index[Annotation['Combined Annotations'].str.contains('aminotransferase')]
display(Annotation.loc[temp])
Show_Data(temp)
```

|  | Combined Annotations | Signal P | Sc288c Orthologs | Human Orthologs | Sc288 Best Hit | Human Blast | Essential | WolfPSort | C Terminal |
| --- | --- | --- | --- | --- | --- | --- | --- | --- | --- |
| RTO4\_ID |  |  |  |  |  |  |  |  |  |
| 8540 | K14264: BNA3; kynurenine aminotransferase |  | BNA3 | KYAT3,KYAT1 | BNA3 | KYAT3 | Not Essential | cyto 17, cyto\_nucl 11, pero 4, nucl 3 | RDA\* |
| 8936 | K14454: GOT1; aspartate aminotransferase, cyto... |  | AAT2 | GOT1 | AAT2 | GOT2 | Not Essential | cyto 18.5, cyto\_nucl 12.5, nucl 3.5, pero 3 | GQL\* |
| 10152 | KOG0257: Kynurenine aminotransferase, glutamin... |  |  |  |  | KYAT3 | Not Essential | cyto 11.5, cyto\_nucl 10.833, cyto\_pero 9.166, ... | LKK\* |
| 10937 | K07250: gabT; 4-aminobutyrate aminotransferase... |  |  | AGXT2 | ARG8 | AGXT2 | Not Essential | cyto 15, cyto\_mito 12.333, cyto\_nucl 8.833, mi... | HEG\* |
| 12099 | K00820: glmS, GFPT; glucosamine--fructose-6-ph... |  | GFA1,YMR085W | GFPT1,GFPT2 | GFA1 | GFPT2 | Essential | cysk 12, cyto 6.5, mito 6, cyto\_nucl 5 | TVE\* |
| 12407 | K00825: AADAT, KAT2; kynurenine/2-aminoadipate... |  | ARO8 | AADAT | ARO8 | AADAT | Not Essential | cyto 11.5, cyto\_nucl 11.5, nucl 8.5, pero 6 | EKA\* |
| 12731 | K19562: BIO3-BIO1; bifunctional dethiobiotin s... |  |  |  | BIO3 |  | Essential | mito 11, extr 10, cyto 3, pero 2 | GAS\* |
| 13230 | K00817: hisC; histidinol-phosphate aminotransf... |  | HIS5 |  | HIS5 |  | Essential | mito 13.5, cyto\_mito 12.333, cyto 10, cyto\_nuc... | FLQ\* |
| 13930 | KOG0257: Kynurenine aminotransferase, glutamin... |  |  |  | BNA3 | TAT | Not Essential | cyto 10.5, cyto\_nucl 8, mito 6, nucl 4.5, pero 4 | LQE\* |
| 14014 | K00826: E2.6.1.42, ilvE; branched-chain amino ... |  | BAT1,BAT2 | BCAT1 | BAT2 | BCAT1 | Not Essential | cyto 12, mito 11, cyto\_nucl 6.833, cyto\_pero 6... | VVV\* |
| 14281 | K14455: GOT2; aspartate aminotransferase, mito... |  | AAT1 | GOT2 | AAT2 | GOT2 | Not Essential | mito 26 | NDA\* |
| 14610 | K00826: E2.6.1.42, ilvE; branched-chain amino ... | S | BAT1,BAT2 | BCAT1 | BAT1 | BCAT2 | Not Essential | mito 20, cyto 2, E.R. 2, plas 1, extr 1, cyto\_... | VDS\* |
| 14853 | K00826: E2.6.1.42, ilvE; branched-chain amino ... | S | BAT1,BAT2 | BCAT1 | BAT2 | BCAT2 | Not Essential | mito 8.5, cyto\_mito 7.833, pero 7, cyto 6, ext... | SKA\* |
| 14878 | K00818: E2.6.1.11, argD; acetylornithine amino... | S | ARG8 |  | ARG8 | OAT | Not Essential | mito 26 | EGL\* |
| 14908 | K00838: ARO8; aromatic amino acid aminotransfe... |  | ARO8 | AADAT | ARO8 | AADAT | Not Essential | cyto 7, cysk 7, mito 5, cyto\_nucl 5, pero 4, m... | WAY\* |
| 15404 | K00831: serC, PSAT1; phosphoserine aminotransf... | S | SER1 | PSAT1 | SER1 | PSAT1 | Essential | cyto 15, cyto\_nucl 9, mito 5, extr 4 | QNQ\* |
| 15839 | K00838: ARO8; aromatic amino acid aminotransfe... |  | ARO8 | AADAT | ARO8 | AADAT | Not Essential | cysk 9, cyto 6, nucl 4, mito 4, pero 4, mito\_n... | FKD\* |
| 15905 | K13524: ABAT; 4-aminobutyrate aminotransferase... |  | UGA1 | ABAT | UGA1 | ABAT | Not Essential | mito 26 | NMS\* |
| 16065 | K00815: TAT; tyrosine aminotransferase |  |  | TAT | ALT2 | TAT | Not Essential | cyto 14.5, cyto\_nucl 11, nucl 6.5, mito 3, pero 3 | VDA\* |

| strain | WT | | | | | | | | | | | | | | | | |
| --- | --- | --- | --- | --- | --- | --- | --- | --- | --- | --- | --- | --- | --- | --- | --- | --- | --- |
| condition | G\_MM | C\_MM | G\_SD | | GX\_SD | | | X\_SD | | A\_SD | | C\_SD | | MM\_CN120 | | MM\_CN5 | Diversity\_Sample |
| phase | exp | exp | exp | stat | exp | trans | stat | exp | stat | exp | stat | exp | stat | exp | stat | exp | exp |
| proteinId | Set1 | Set1 | Set2 | Set2 | Set2 | Set2 | Set2 | Set2 | Set2 | Set2 | Set2 | Set2 | Set2 | Set3 | Set3 | Set3 | Set3 |
| RTO4\_ID |  |  |  |  |  |  |  |  |  |  |  |  |  |  |  |  |  |
| 8540 | 6.55727 | 6.83994 | 6.85377 | 5.75216 | 6.97296 | 6.18698 | 5.69389 | 7.05604 | 5.13742 | 6.82076 | 5.11155 | 6.06275 | 6.07168 | 6.93708 | 6.32795 | 8.5621 | 7.8333 |
| 8936 | 7.86637 | 6.64116 | 7.91375 | 7.07647 | 8.09211 | 6.95363 | 6.89341 | 6.93058 | 7.53621 | 6.38309 | 7.15592 | 7.01203 | 7.52195 | 7.45909 | 6.9531 | 7.81801 | 7.00633 |
| 10152 | 4.5867 | 4.36561 | 5.60601 | 6.35034 | 5.44614 | 5.33316 | 5.18555 | 5.22831 | 5.41855 | 5.1139 | 5.12219 | 4.1552 | 4.13047 | 3.39529 | 3.40656 | 4.56702 | 4.35344 |
| 10937 | 5.34598 | 5.55945 | 6.51015 | 6.67274 | 7.00851 | 6.72084 | 6.82701 | 6.38013 | 6.7731 | 6.07713 | 6.8177 | 7.30439 | 7.56095 | 4.77018 | 5.34697 | 7.52402 | 5.67411 |
| 12099 | 6.94216 | 7.79938 | 6.94639 | 5.14515 | 7.00162 | 6.64935 | 6.35393 | 7.00836 | 5.54101 | 6.8942 | 5.48702 | 6.09179 | 5.09134 | 8.48387 | 7.77371 | 7.45166 | 8.05406 |
| 12407 | 7.16156 | 6.96812 | 7.22568 | 5.61243 | 7.56705 | 6.22319 | 6.25552 | 6.21589 | 5.90204 | 5.8889 | 6.07415 | 7.3238 | 8.10277 | 5.28312 | 5.16308 | 7.46782 | 5.58338 |
| 12731 | 6.02185 | 4.60555 | 6.45171 | 5.35008 | 6.39028 | 6.2194 | 5.81525 | 6.0943 | 4.79923 | 4.92084 | 4.63383 | 4.19327 | 3.7021 | 7.16018 | 6.81399 | 7.2256 | 6.71275 |
| 13230 | 6.13722 | 5.54775 | 5.82637 | 4.90553 | 6.41469 | 5.41282 | 5.49459 | 5.22864 | 5.40033 | 4.91983 | 4.98875 | 6.14577 | 7.09142 | 6.88296 | 6.80054 | 8.25489 | 6.52053 |
| 13930 | 3.29171 | 4.15008 | 4.22635 | 1.76161 | 4.10496 | 3.49021 | 3.01426 | 4.08026 | 1.99706 | 3.85205 | 1.41317 | 2.55389 | 1.86367 | 3.46643 | 3.08362 | 4.28526 | 3.75983 |
| 14014 | 2.77869 | 2.40508 | 5.58636 | 5.56856 | 5.84731 | 3.74791 | 4.71441 | 2.22746 | 3.55795 | 2.60791 | 4.6743 | 2.74453 | 2.98602 | 3.74213 | 3.448 | 5.31524 | 3.5165 |
| 14281 | 6.58722 | 7.20393 | 8.20142 | 6.09041 | 8.52252 | 7.21017 | 7.01216 | 7.85368 | 6.20268 | 7.13853 | 6.15274 | 7.58338 | 7.76082 | 9.02 | 8.16336 | 10.7998 | 10.0833 |
| 14610 | 6.35581 | 6.93464 | 7.40831 | 6.37511 | 7.74917 | 6.71342 | 6.67106 | 6.40937 | 6.14813 | 5.59469 | 6.53238 | 6.02404 | 7.27274 | 7.37942 | 6.60182 | 9.56953 | 7.78925 |
| 14853 | 4.7362 | 4.5406 | 4.88838 | 5.4074 | 4.98216 | 5.05629 | 5.10921 | 4.7381 | 5.09388 | 4.7394 | 5.03763 | 5.19336 | 5.50641 | 5.46496 | 5.90049 | 5.73904 | 5.14016 |
| 14878 | 6.87638 | 6.32167 | 6.38109 | 5.99617 | 6.51843 | 6.44142 | 6.32239 | 6.58497 | 6.16439 | 6.29796 | 6.29575 | 6.41516 | 6.40775 | 5.6833 | 5.33991 | 6.21991 | 5.11105 |
| 14908 | 4.96188 | 5.39382 | 6.32914 | 4.57254 | 6.87228 | 3.93282 | 4.6105 | 2.86689 | 4.55744 | 3.57167 | 4.54445 | 5.45156 | 8.85544 | 4.48318 | 3.71453 | 9.27804 | 5.94028 |
| 15404 | 7.37367 | 6.48891 | 7.18959 | 5.31336 | 7.62295 | 5.75183 | 5.7009 | 6.06478 | 5.31379 | 5.63253 | 5.39557 | 7.08458 | 7.81355 | 5.20601 | 5.04839 | 7.68141 | 5.53268 |
| 15839 | 8.11963 | 6.83965 | 6.43567 | 6.23959 | 6.4065 | 6.29378 | 6.4164 | 6.59727 | 6.49423 | 6.81951 | 6.69291 | 6.85344 | 6.82691 | 7.91155 | 7.40486 | 6.5373 | 6.73561 |
| 15905 | 7.00614 | 6.60151 | 5.42188 | 4.85478 | 5.9331 | 3.2202 | 3.52389 | 4.11749 | 4.27075 | 4.56288 | 5.03435 | 5.76963 | 5.55895 | 4.79904 | 4.96018 | 6.42678 | 6.38085 |
| 16065 | 6.21029 | 5.22606 | 6.84916 | 4.50063 | 7.28519 | 5.3224 | 5.87781 | 4.34718 | 4.97603 | 3.1559 | 4.00366 | 6.05556 | 6.25728 | 8.47076 | 7.6981 | 8.80249 | 7.77073 |

| strain | WT | | | | | | | | | | |
| --- | --- | --- | --- | --- | --- | --- | --- | --- | --- | --- | --- |
| condition | G\_SD | | GX\_SD | | | X\_SD | | A\_SD | | C\_SD | |
| proteinId | exp | stat | exp | trans | stat | exp | stat | exp | stat | exp | stat |
| 8540 | 21.8496 | 17.2693 | 18.8959 | 16.1895 | 17.5183 | 24.4495 | 22.2304 | 23.1722 | 18.9557 | 19.8966 | 15.4585 |
| 8936 | 14.1864 | 16.949 | 11.5716 | 18.0127 | 17.684 | 12.1475 | 12.4499 | 13.8959 | 12.0653 | 10.9247 | 14.1547 |
| 10152 | 0.564696 | 1.07488 | 0.605588 | 2.2193 | 2.17393 | 0.58915 | 0.204666 | 0.582516 | 0.763742 | 0.418217 | 0 |
| 10937 | 16.5246 | 15.5808 | 17.6871 | 18.2392 | 18.7639 | 18.3695 | 17.2973 | 20.1003 | 19.5082 | 14.4206 | 20.8634 |
| 12099 | 37.0529 | 37.3537 | 37.4084 | 31.837 | 26.4381 | 28.21 | 29.3437 | 28.1821 | 28.1873 | 33.2662 | 26.1069 |
| 12407 | 7.11912 | 7.07756 | 7.33971 | 5.944 | 5.30084 | 6.87491 | 5.50339 | 7.70989 | 5.56004 | 7.71286 | 9.59344 |
| 12731 | 10.3494 | 10.6314 | 10.7826 | 13.9404 | 14.2423 | 9.78049 | 10.1996 | 5.98241 | 7.46515 | 8.755 | 7.20604 |
| 13230 | 11.6827 | 7.27433 | 13.8235 | 5.38016 | 4.19573 | 8.81754 | 5.49931 | 8.10677 | 7.06313 | 5.32037 | 9.81267 |
| 13930 | 1.52348 | 1.08048 | 1.01604 | 0.741161 | 0.359956 | 1.37028 | 0.789821 | 1.54066 | 2.10863 | 0.22091 | 0.21114 |
| 14014 | 5.16681 | 0 | 7.11355 | 0 | 0 | 0 | 0 | 0 | 0 | 0 | 0 |
| 14281 | 44.8268 | 45.1951 | 51.0269 | 41.2985 | 42.3767 | 45.6448 | 45.2421 | 43.2537 | 42.8765 | 41.581 | 46.6144 |
| 14610 | 19.4505 | 16.9826 | 20.3233 | 15.4321 | 16.7866 | 21.5237 | 17.317 | 18.5377 | 16.843 | 14.9946 | 14.1686 |
| 14878 | 17.3324 | 17.529 | 16.2456 | 20.6565 | 22.0728 | 17.2263 | 19.3814 | 17.0232 | 16.2795 | 17.1024 | 20.4469 |
| 14908 | 9.31562 | 0 | 11.3891 | 0 | 0 | 3.31911 | 0 | 1.54769 | 0 | 2.57326 | 8.71334 |
| 15404 | 8.48871 | 5.5987 | 8.51087 | 5.77136 | 5.67114 | 5.85913 | 5.32308 | 9.47351 | 6.89022 | 2.5796 | 6.95859 |
| 15839 | 4.43453 | 11.9185 | 6.09339 | 17.1048 | 18.0361 | 9.79935 | 13.8093 | 7.91426 | 12.8202 | 11.5438 | 12.6204 |
| 15905 | 11.4696 | 8.46692 | 11.182 | 4.46295 | 4.38324 | 7.67606 | 9.04404 | 17.194 | 13.3959 | 11.9937 | 10.2171 |
| 16065 | 4.41183 | 0 | 7.52777 | 0.189313 | 0 | 0 | 0 | 0 | 0 | 2.14646 | 2.8314 |

|  | Glucose | Xylose | Arabinose | Acetate | Coumarate | Ferulate | YNB Oleic Acid | YNB Ricinoleic Acid | YNB Glucose | YNB Gluc DOC | YPD |
| --- | --- | --- | --- | --- | --- | --- | --- | --- | --- | --- | --- |
| proteinId |  |  |  |  |  |  |  |  |  |  |  |
| 8540 | -0.157945 | -0.017177 | -0.0517655 | -0.107362 | 0.311773 | 0.0292196 | 0.160261 | 0.186308 | -0.0791432 | -0.421139 | 0.0482614 |
| 8936 | -0.354295 | -0.277708 | -0.923495 | -1.45071 | -4.24826 | -4.247 | -0.440484 | -0.917334 | -0.37578 | 0.0703331 | -0.272326 |
| 10152 | -0.028338 | -0.135224 | -0.0818158 | -0.123987 | 0.0632719 | -0.0688348 | -0.0579668 | 0.600573 | -0.00597398 | -0.188567 | 0.396747 |
| 10937 | 0.125487 | 0.362162 | 0.242396 | 0.177756 | 0.0130856 | 0.391006 | -0.254579 | 0.281721 | -0.244334 | -0.480414 | -0.324646 |
| 12407 | -0.120884 | 0.0459414 | -0.0417789 | -0.263358 | -0.162155 | -0.400014 | 0.275532 | 0.206367 | 0.0858757 | 0.335526 | 1.09564 |
| 13930 | -0.35523 | -0.34978 | -0.178866 | -0.644413 | -0.310697 | -0.499722 | 0.290919 | -0.0131707 | -0.21765 | -0.179061 | -0.0730051 |
| 14014 | 0.277808 | 0.132282 | 0.304391 | 0.220584 | 0.430317 | 0.603165 | -0.337377 | -0.942644 | -0.236419 | -0.266986 | -0.316429 |
| 14281 | -0.134443 | 0.0025888 | -0.314789 | -0.228214 | -0.0206698 | -0.461234 | -0.785788 | -2.1105 | -0.415618 | -0.136043 | -0.195287 |
| 14610 | -0.00277608 | -0.0846046 | -0.813033 | -0.269283 | 0.667987 | -0.430488 | -0.572899 | -1.45413 | 0.0684007 | 0.163426 | 0.132251 |
| 14853 | -0.137609 | -0.301241 | -0.25221 | 0.0016244 | 0.0216384 | -0.339282 | -0.120204 | 0.862746 | -0.101846 | 0.237236 | -0.385014 |
| 14878 | -0.211372 | 0.257549 | -0.386031 | -0.166675 | 0.469444 | -0.0262115 | 0.339558 | -0.421871 | -1.53088 | -0.0115705 | -0.29169 |
| 14908 | 0.0451271 | -0.280956 | 0.220759 | -0.0377905 | 0.21851 | -0.259388 | -0.292657 | 0.206026 | 0.339453 | 0.398586 | -0.0659921 |
| 15839 | -0.0847761 | -0.12423 | 0.269542 | 0.0647239 | 0.154571 | 0.146919 | -0.346466 | -0.214054 | -0.177142 | -0.229588 | -0.0156292 |
| 15905 | -0.0550694 | 0.0820966 | 0.104398 | 0.0213273 | -0.363552 | -0.282914 | -0.248534 | 0.46351 | -0.303281 | -0.307461 | -0.0522098 |
| 16065 | -0.100963 | -0.276939 | 0.0184184 | -0.00602183 | 0.244104 | 0.0553499 | -0.0261936 | -0.470801 | -0.0251039 | -0.0482782 | 0.0523716 |

In [67]:

```
for x in temp:
    if x in model.genes:
        for r in sorted(model.genes.get_by_id(x).reactions, key=lambda x: x.id):
            print(r, r.gene_reaction_rule)
    else:
        print(x, 'no reactions')
    print()
```

```
3HKYNAKGAT: akg_c + hLkynr_c --> 42A3HP24DB_c + glu__L_c 12407 or 14908 or 15839 or 8540
FKYNH: Lfmkynr_c + h2o_c --> Lkynr_c + for_c + h_c 8540
KYNAKGAT: Lkynr_c + akg_c --> 4aphdob_c + glu__L_c 12407 or 14908 or 15839 or 8540

3SALATAi: 3sala_c + akg_c + h_c --> 3snpyr_c + glu__L_c 8936
ASPTA: akg_c + asp__L_c <=> glu__L_c + oaa_c 14281 or 8936
ASPTAp: akg_x + asp__L_x <=> glu__L_x + oaa_x 8936
CYSTA: akg_c + cys__L_c --> glu__L_c + mercppyr_c 14281 or 8936
DLYSOXGAT: akg_c + lys__D_c <=> 6a2ohxnt_c + glu__D_c 8936
DLYSPYRAT: lys__D_c + pyr_c <=> 6a2ohxnt_c + ala__D_c 8936
EHGLAT: akg_c + e4hglu_c --> 4h2oglt_c + glu__L_c 8936
EHGLATp: akg_x + e4hglu_x --> 4h2oglt_x + glu__L_x 8936
LCYSTAT: Lcyst_c + akg_c <=> 3spyr_c + glu__L_c 8936
LEUTAi: 4mop_c + glu__L_c --> akg_c + leu__L_c 14610 or 14853 or 8936
PHETA1: akg_c + phe__L_c <=> glu__L_c + phpyr_c 12407 or 13230 or 14281 or 14610 or 14853 or 14908 or 15839 or 8936
TYRTA: akg_c + tyr__L_c <=> 34hpp_c + glu__L_c 12407 or 13230 or 14281 or 14610 or 14853 or 14908 or 15839 or 8936
TYRTAi: 34hpp_c + glu__L_c --> akg_c + tyr__L_c 12407 or 13230 or 14281 or 14908 or 15839 or 8936
TYRTAip: 34hpp_x + glu__L_x --> akg_x + tyr__L_x 8936
UNK3: 2kmb_c + glu__L_c --> akg_c + met__L_c 12407 or 14281 or 14908 or 15839 or 8936

10152 no reactions

ABTA: 4abut_c + akg_c --> glu__L_c + sucsal_c 10937

GF6PTA: f6p_c + gln__L_c --> gam6p_c + glu__L_c 12099

3HKYNAKGAT: akg_c + hLkynr_c --> 42A3HP24DB_c + glu__L_c 12407 or 14908 or 15839 or 8540
AATA: 2oxoadp_c + glu__L_c <=> L2aadp_c + akg_c 12407 or 14908 or 15839
KYNAKGAT: Lkynr_c + akg_c --> 4aphdob_c + glu__L_c 12407 or 14908 or 15839 or 8540
PHETA1: akg_c + phe__L_c <=> glu__L_c + phpyr_c 12407 or 13230 or 14281 or 14610 or 14853 or 14908 or 15839 or 8936
PHETA1m: akg_m + phe__L_m <=> glu__L_m + phpyr_m 12407 or 13230 or 14281 or 14908 or 15839 or 16065
TRPTA: akg_c + trp__L_c <=> glu__L_c + indpyr_c 12407 or 14908 or 15839
TYRTA: akg_c + tyr__L_c <=> 34hpp_c + glu__L_c 12407 or 13230 or 14281 or 14610 or 14853 or 14908 or 15839 or 8936
TYRTAi: 34hpp_c + glu__L_c --> akg_c + tyr__L_c 12407 or 13230 or 14281 or 14908 or 15839 or 8936
TYRTAim: 34hpp_m + glu__L_m --> akg_m + tyr__L_m 12407 or 13230 or 14281 or 14908 or 15839 or 16065
UNK3: 2kmb_c + glu__L_c --> akg_c + met__L_c 12407 or 14281 or 14908 or 15839 or 8936

AMAOTrm: 8aonn_m + amet_m <=> amob_m + dann_m 12731
DBTSm: atp_m + co2_m + dann_m <=> adp_m + dtbt_m + 3.0 h_m + pi_m 12731

HSTPT: glu__L_c + imacp_c --> akg_c + hisp_c 13230
PHETA1: akg_c + phe__L_c <=> glu__L_c + phpyr_c 12407 or 13230 or 14281 or 14610 or 14853 or 14908 or 15839 or 8936
PHETA1m: akg_m + phe__L_m <=> glu__L_m + phpyr_m 12407 or 13230 or 14281 or 14908 or 15839 or 16065
TYRTA: akg_c + tyr__L_c <=> 34hpp_c + glu__L_c 12407 or 13230 or 14281 or 14610 or 14853 or 14908 or 15839 or 8936
TYRTAi: 34hpp_c + glu__L_c --> akg_c + tyr__L_c 12407 or 13230 or 14281 or 14908 or 15839 or 8936
TYRTAim: 34hpp_m + glu__L_m --> akg_m + tyr__L_m 12407 or 13230 or 14281 or 14908 or 15839 or 16065

13930 no reactions

ILETA: akg_c + ile__L_c <=> 3mop_c + glu__L_c 14014 or 14610 or 14853
ILETAm: akg_m + ile__L_m <=> 3mop_m + glu__L_m 14014 or 14610 or 14853
LEUTA: akg_c + leu__L_c <=> 4mop_c + glu__L_c 14014 or 14610 or 14853
LEUTAm: akg_m + leu__L_m <=> 4mop_m + glu__L_m 14014 or 14610 or 14853
OMCDC: 3c4mop_c + h_c --> 4mop_c + co2_c 14014 or 14610 or 14853
OMCDCm: 3c4mop_m + h_m --> 4mop_m + co2_m 14014 or 14610 or 14853
VALTA: akg_c + val__L_c <=> 3mob_c + glu__L_c 14014 or 14610 or 14853
VALTAim: akg_m + val__L_m --> 3mob_m + glu__L_m 14014 or 14610 or 14853

3SALATAim: 3sala_m + akg_m + h_m --> 3snpyr_m + glu__L_m 14281
ASPTA: akg_c + asp__L_c <=> glu__L_c + oaa_c 14281 or 8936
ASPTAm: akg_m + asp__L_m <=> glu__L_m + oaa_m 14281
CYSATm: glu__L_m + mercppyr_m --> akg_m + cys__L_m 14281
CYSTA: akg_c + cys__L_c --> glu__L_c + mercppyr_c 14281 or 8936
CYSTAm: akg_m + cys__L_m <=> glu__L_m + mercppyr_m 14281
EHGLAT2m: e4hglu_m + oaa_m --> 4h2oglt_m + asp__L_m 14281
EHGLATm: akg_m + e4hglu_m --> 4h2oglt_m + glu__L_m 14281
LCYSTATm: Lcyst_m + akg_m <=> 3spyr_m + glu__L_m 14281
PHETA1: akg_c + phe__L_c <=> glu__L_c + phpyr_c 12407 or 13230 or 14281 or 14610 or 14853 or 14908 or 15839 or 8936
PHETA1m: akg_m + phe__L_m <=> glu__L_m + phpyr_m 12407 or 13230 or 14281 or 14908 or 15839 or 16065
TYRTA: akg_c + tyr__L_c <=> 34hpp_c + glu__L_c 12407 or 13230 or 14281 or 14610 or 14853 or 14908 or 15839 or 8936
TYRTAi: 34hpp_c + glu__L_c --> akg_c + tyr__L_c 12407 or 13230 or 14281 or 14908 or 15839 or 8936
TYRTAim: 34hpp_m + glu__L_m --> akg_m + tyr__L_m 12407 or 13230 or 14281 or 14908 or 15839 or 16065
UNK3: 2kmb_c + glu__L_c --> akg_c + met__L_c 12407 or 14281 or 14908 or 15839 or 8936

ILETA: akg_c + ile__L_c <=> 3mop_c + glu__L_c 14014 or 14610 or 14853
ILETAm: akg_m + ile__L_m <=> 3mop_m + glu__L_m 14014 or 14610 or 14853
LEUTA: akg_c + leu__L_c <=> 4mop_c + glu__L_c 14014 or 14610 or 14853
LEUTAi: 4mop_c + glu__L_c --> akg_c + leu__L_c 14610 or 14853 or 8936
LEUTAm: akg_m + leu__L_m <=> 4mop_m + glu__L_m 14014 or 14610 or 14853
OMCDC: 3c4mop_c + h_c --> 4mop_c + co2_c 14014 or 14610 or 14853
OMCDCm: 3c4mop_m + h_m --> 4mop_m + co2_m 14014 or 14610 or 14853
PHETA1: akg_c + phe__L_c <=> glu__L_c + phpyr_c 12407 or 13230 or 14281 or 14610 or 14853 or 14908 or 15839 or 8936
TYRTA: akg_c + tyr__L_c <=> 34hpp_c + glu__L_c 12407 or 13230 or 14281 or 14610 or 14853 or 14908 or 15839 or 8936
VALTA: akg_c + val__L_c <=> 3mob_c + glu__L_c 14014 or 14610 or 14853
VALTAim: akg_m + val__L_m --> 3mob_m + glu__L_m 14014 or 14610 or 14853

ILETA: akg_c + ile__L_c <=> 3mop_c + glu__L_c 14014 or 14610 or 14853
ILETAm: akg_m + ile__L_m <=> 3mop_m + glu__L_m 14014 or 14610 or 14853
LEUTA: akg_c + leu__L_c <=> 4mop_c + glu__L_c 14014 or 14610 or 14853
LEUTAi: 4mop_c + glu__L_c --> akg_c + leu__L_c 14610 or 14853 or 8936
LEUTAm: akg_m + leu__L_m <=> 4mop_m + glu__L_m 14014 or 14610 or 14853
OMCDC: 3c4mop_c + h_c --> 4mop_c + co2_c 14014 or 14610 or 14853
OMCDCm: 3c4mop_m + h_m --> 4mop_m + co2_m 14014 or 14610 or 14853
PHETA1: akg_c + phe__L_c <=> glu__L_c + phpyr_c 12407 or 13230 or 14281 or 14610 or 14853 or 14908 or 15839 or 8936
TYRTA: akg_c + tyr__L_c <=> 34hpp_c + glu__L_c 12407 or 13230 or 14281 or 14610 or 14853 or 14908 or 15839 or 8936
VALTA: akg_c + val__L_c <=> 3mob_c + glu__L_c 14014 or 14610 or 14853
VALTAim: akg_m + val__L_m --> 3mob_m + glu__L_m 14014 or 14610 or 14853

ACOTAim: acg5sa_m + glu__L_m --> acorn_m + akg_m 14878

3HKYNAKGAT: akg_c + hLkynr_c --> 42A3HP24DB_c + glu__L_c 12407 or 14908 or 15839 or 8540
AATA: 2oxoadp_c + glu__L_c <=> L2aadp_c + akg_c 12407 or 14908 or 15839
KYNAKGAT: Lkynr_c + akg_c --> 4aphdob_c + glu__L_c 12407 or 14908 or 15839 or 8540
PHETA1: akg_c + phe__L_c <=> glu__L_c + phpyr_c 12407 or 13230 or 14281 or 14610 or 14853 or 14908 or 15839 or 8936
PHETA1m: akg_m + phe__L_m <=> glu__L_m + phpyr_m 12407 or 13230 or 14281 or 14908 or 15839 or 16065
TRPTA: akg_c + trp__L_c <=> glu__L_c + indpyr_c 12407 or 14908 or 15839
TYRTA: akg_c + tyr__L_c <=> 34hpp_c + glu__L_c 12407 or 13230 or 14281 or 14610 or 14853 or 14908 or 15839 or 8936
TYRTAi: 34hpp_c + glu__L_c --> akg_c + tyr__L_c 12407 or 13230 or 14281 or 14908 or 15839 or 8936
TYRTAim: 34hpp_m + glu__L_m --> akg_m + tyr__L_m 12407 or 13230 or 14281 or 14908 or 15839 or 16065
UNK3: 2kmb_c + glu__L_c --> akg_c + met__L_c 12407 or 14281 or 14908 or 15839 or 8936

PSERT: 3php_c + glu__L_c --> akg_c + pser__L_c 15404
SDPTA: akg_c + sl26da_c <=> glu__L_c + sl2a6o_c 15404

3HKYNAKGAT: akg_c + hLkynr_c --> 42A3HP24DB_c + glu__L_c 12407 or 14908 or 15839 or 8540
AATA: 2oxoadp_c + glu__L_c <=> L2aadp_c + akg_c 12407 or 14908 or 15839
KYNAKGAT: Lkynr_c + akg_c --> 4aphdob_c + glu__L_c 12407 or 14908 or 15839 or 8540
PHETA1: akg_c + phe__L_c <=> glu__L_c + phpyr_c 12407 or 13230 or 14281 or 14610 or 14853 or 14908 or 15839 or 8936
PHETA1m: akg_m + phe__L_m <=> glu__L_m + phpyr_m 12407 or 13230 or 14281 or 14908 or 15839 or 16065
TRPTA: akg_c + trp__L_c <=> glu__L_c + indpyr_c 12407 or 14908 or 15839
TYRTA: akg_c + tyr__L_c <=> 34hpp_c + glu__L_c 12407 or 13230 or 14281 or 14610 or 14853 or 14908 or 15839 or 8936
TYRTAi: 34hpp_c + glu__L_c --> akg_c + tyr__L_c 12407 or 13230 or 14281 or 14908 or 15839 or 8936
TYRTAim: 34hpp_m + glu__L_m --> akg_m + tyr__L_m 12407 or 13230 or 14281 or 14908 or 15839 or 16065
UNK3: 2kmb_c + glu__L_c --> akg_c + met__L_c 12407 or 14281 or 14908 or 15839 or 8936

3AIBTm: 2mop_m + glu__L_m <=> 3aib_m + akg_m 15905
ABTArm: 4abut_m + akg_m <=> glu__L_m + sucsal_m 15905
APAT2rm: akg_m + ala_B_m <=> glu__L_m + msa_m 15905

PHETA1m: akg_m + phe__L_m <=> glu__L_m + phpyr_m 12407 or 13230 or 14281 or 14908 or 15839 or 16065
TYRTAim: 34hpp_m + glu__L_m --> akg_m + tyr__L_m 12407 or 13230 or 14281 or 14908 or 15839 or 16065
```

gene annotation and blast search  
12407 (tryptophan), 13230 (histidinol-phosphate), 14281 (aspartate, mito), 8936 (aspartate, cyto)  
14908, 15839 (aromatic, phenylalanine/tyrosine/3-methylphenylalanine, 2-aminoadipate, tryptophan, tyrosine)  
16065 TAT (tyrosine)

In [68]:

```
# 8540 BNA3 (kynurenine, cyto), also catalyzed by ARO8 and ARO9
# https://www.nature.com/articles/s41598-017-12392-6
# 12407 is for tryptophan by KEGG matches, 14908 and 15839 are ARO8
model.reactions.get_by_id('3HKYNAKGAT').gene_reaction_rule = '14908 or 15839 or 8540'
model.reactions.get_by_id('KYNAKGAT').gene_reaction_rule = '14908 or 15839 or 8540'
# KYNAKGAT and 3HKYNAKGAT needs following spontaneous reactions 'KYNATESYN','r0647'
for x in ['KYNATESYN','r0647']:
    r = hsa2.reactions.get_by_id(x).copy()
    model.add_reactions([r])
# FKYNH is 14788 BNA7, not BNA3 (iMM904 and metacyc wrong)
model.reactions.get_by_id('FKYNH').gene_reaction_rule = '14788'
# 8936 (aspartate, cyto), 14281 (aspartate, mito)
model.reactions.get_by_id('ASPTA').gene_reaction_rule = '8936'
model.reactions.get_by_id('CYSTA').gene_reaction_rule = '8936'
model.reactions.get_by_id('CYSTA').lower_bound = -1000.0
# 3SALATAi is blocked, need to add 3SPYRSP and 3SPYRSPm (spontaneous) to degrade 3snpyr to pyr and so3  
# 3sala is 3-sulfinoalanine / L-cysteine Sulfinic acid in biolog
# In metacyc, 3sala is degraded to Lcyst spontaneously using nad, but 3SALAOX is using O2 
# Lcyst can be converted to taurine, and taurine is degraded to aacald, succ, and so3
# aacald needs to be secreted out, DM_aacald_c is sink in iML1515
for x in ['3SPYRSP','3SPYRSPm','3SALAOX']:
    r = hsa2.reactions.get_by_id(x).copy()
    r.gene_reaction_rule = '12061'
    model.add_reactions([r])
for x in ['DM_aacald_c']:
    r = eco.reactions.get_by_id(x).copy()
    model.add_reactions([r])
# DLYSOXGAT, DLYSPYRAT, EHGLAT, EHGLATp are blocked - remove
# CYSTAm is correct remove CYSATm (wrong)
# EHGLATm/EHGLAT2m leads to 4-hydroxy-2-oxoglutarate, which can be degraded by 12061 HOGA1 (mito in uniprot)
# Add DDPGAm from hsa2 and change gene to 12061
for x in ['DDPGAm']:
    r = hsa2.reactions.get_by_id(x).copy()
    r.gene_reaction_rule = '12061'
    model.add_reactions([r])
# 14610, 14853 (branched, leu/val/ile, mito), 14014 (branched, cyto)
# Remove LEUTAi
model.reactions.get_by_id('VALTA').gene_reaction_rule = '14014'
model.reactions.get_by_id('LEUTA').gene_reaction_rule = '14014'
model.reactions.get_by_id('ILETA').gene_reaction_rule = '14014'
model.reactions.get_by_id('VALTAim').gene_reaction_rule = '14610 or 14853'
model.reactions.get_by_id('LEUTAm').gene_reaction_rule = '14610 or 14853'
model.reactions.get_by_id('ILETAm').gene_reaction_rule = '14610 or 14853'
# VALTAim reversible
model.reactions.get_by_id('VALTAim').id = 'VALTAm'
model.reactions.get_by_id('VALTAm').lower_bound = -1000.0
# OMCDC/OMCDCm spontaneous
model.reactions.get_by_id('OMCDC').gene_reaction_rule = ''
model.reactions.get_by_id('OMCDCm').gene_reaction_rule = ''
# TRPTA is by 12407 and ARO8, correct
# TYRTA is by TAT or ARO8/9, remove TYRTAi and TYRTAim
# Keep? needed for 4hb production in pero TYRTAip: 34hpp_x + glu__L_x --> akg_x + tyr__L_x 8936
model.reactions.get_by_id('TYRTA').gene_reaction_rule = '14908 or 15839 or 16065'
# TYRTA makes 34hpp_c, where is it going?
# PHETA1 is by ARO8, remove PHETA1m
model.reactions.get_by_id('PHETA1').gene_reaction_rule = '14908 or 15839'
# AATA is by AADAT and ARO8
model.reactions.get_by_id('AATA').gene_reaction_rule = '14908 or 15839'
# UNK3 is unknown 2-oxo-4-methylthiobutanoate-glutamine aminotransferase 
# Known e. coli gene ybdL blast to 8540 BNA3 with 37% id, A. thaliana BCAT4 blast to 14014 cyto BCAT with 39.5% id
# BCAT makes sense since it is active on oxo substrates
model.reactions.get_by_id('UNK3').gene_reaction_rule = '8540 or 14014'
# 13930 (unclear, similar to Sulfolobus AspAT, aspartate/aromatic or kynurenine or arginine-pyruvate)  
# 10152 (unclear, similar to Sulfolobus AspAT, and eukaryotic bifunctional aspartate/prephenate)

remove = ['DLYSOXGAT','DLYSPYRAT','EHGLAT','EHGLATp','CYSATm','LEUTAi','TYRTAi','TYRTAim','PHETA1m']
model.remove_reactions(remove, remove_orphans=True)
```

In [69]:

```
temp = Annotation.index[Annotation['Combined Annotations'].str.contains('aminotransferase')]
display(Annotation.loc[temp])
```

|  | Combined Annotations | Signal P | Sc288c Orthologs | Human Orthologs | Sc288 Best Hit | Human Blast | Essential | WolfPSort | C Terminal |
| --- | --- | --- | --- | --- | --- | --- | --- | --- | --- |
| RTO4\_ID |  |  |  |  |  |  |  |  |  |
| 8540 | K14264: BNA3; kynurenine aminotransferase |  | BNA3 | KYAT3,KYAT1 | BNA3 | KYAT3 | Not Essential | cyto 17, cyto\_nucl 11, pero 4, nucl 3 | RDA\* |
| 8936 | K14454: GOT1; aspartate aminotransferase, cyto... |  | AAT2 | GOT1 | AAT2 | GOT2 | Not Essential | cyto 18.5, cyto\_nucl 12.5, nucl 3.5, pero 3 | GQL\* |
| 10152 | KOG0257: Kynurenine aminotransferase, glutamin... |  |  |  |  | KYAT3 | Not Essential | cyto 11.5, cyto\_nucl 10.833, cyto\_pero 9.166, ... | LKK\* |
| 10937 | K07250: gabT; 4-aminobutyrate aminotransferase... |  |  | AGXT2 | ARG8 | AGXT2 | Not Essential | cyto 15, cyto\_mito 12.333, cyto\_nucl 8.833, mi... | HEG\* |
| 12099 | K00820: glmS, GFPT; glucosamine--fructose-6-ph... |  | GFA1,YMR085W | GFPT1,GFPT2 | GFA1 | GFPT2 | Essential | cysk 12, cyto 6.5, mito 6, cyto\_nucl 5 | TVE\* |
| 12407 | K00825: AADAT, KAT2; kynurenine/2-aminoadipate... |  | ARO8 | AADAT | ARO8 | AADAT | Not Essential | cyto 11.5, cyto\_nucl 11.5, nucl 8.5, pero 6 | EKA\* |
| 12731 | K19562: BIO3-BIO1; bifunctional dethiobiotin s... |  |  |  | BIO3 |  | Essential | mito 11, extr 10, cyto 3, pero 2 | GAS\* |
| 13230 | K00817: hisC; histidinol-phosphate aminotransf... |  | HIS5 |  | HIS5 |  | Essential | mito 13.5, cyto\_mito 12.333, cyto 10, cyto\_nuc... | FLQ\* |
| 13930 | KOG0257: Kynurenine aminotransferase, glutamin... |  |  |  | BNA3 | TAT | Not Essential | cyto 10.5, cyto\_nucl 8, mito 6, nucl 4.5, pero 4 | LQE\* |
| 14014 | K00826: E2.6.1.42, ilvE; branched-chain amino ... |  | BAT1,BAT2 | BCAT1 | BAT2 | BCAT1 | Not Essential | cyto 12, mito 11, cyto\_nucl 6.833, cyto\_pero 6... | VVV\* |
| 14281 | K14455: GOT2; aspartate aminotransferase, mito... |  | AAT1 | GOT2 | AAT2 | GOT2 | Not Essential | mito 26 | NDA\* |
| 14610 | K00826: E2.6.1.42, ilvE; branched-chain amino ... | S | BAT1,BAT2 | BCAT1 | BAT1 | BCAT2 | Not Essential | mito 20, cyto 2, E.R. 2, plas 1, extr 1, cyto\_... | VDS\* |
| 14853 | K00826: E2.6.1.42, ilvE; branched-chain amino ... | S | BAT1,BAT2 | BCAT1 | BAT2 | BCAT2 | Not Essential | mito 8.5, cyto\_mito 7.833, pero 7, cyto 6, ext... | SKA\* |
| 14878 | K00818: E2.6.1.11, argD; acetylornithine amino... | S | ARG8 |  | ARG8 | OAT | Not Essential | mito 26 | EGL\* |
| 14908 | K00838: ARO8; aromatic amino acid aminotransfe... |  | ARO8 | AADAT | ARO8 | AADAT | Not Essential | cyto 7, cysk 7, mito 5, cyto\_nucl 5, pero 4, m... | WAY\* |
| 15404 | K00831: serC, PSAT1; phosphoserine aminotransf... | S | SER1 | PSAT1 | SER1 | PSAT1 | Essential | cyto 15, cyto\_nucl 9, mito 5, extr 4 | QNQ\* |
| 15839 | K00838: ARO8; aromatic amino acid aminotransfe... |  | ARO8 | AADAT | ARO8 | AADAT | Not Essential | cysk 9, cyto 6, nucl 4, mito 4, pero 4, mito\_n... | FKD\* |
| 15905 | K13524: ABAT; 4-aminobutyrate aminotransferase... |  | UGA1 | ABAT | UGA1 | ABAT | Not Essential | mito 26 | NMS\* |
| 16065 | K00815: TAT; tyrosine aminotransferase |  |  | TAT | ALT2 | TAT | Not Essential | cyto 14.5, cyto\_nucl 11, nucl 6.5, mito 3, pero 3 | VDA\* |

In [70]:

```
for x in temp:
    if x in model.genes:
        for r in sorted(model.genes.get_by_id(x).reactions, key=lambda x: x.id):
            print(r, r.gene_reaction_rule)
    else:
        print(x, 'no reactions')
    print()
```

```
3HKYNAKGAT: akg_c + hLkynr_c --> 42A3HP24DB_c + glu__L_c 14908 or 15839 or 8540
KYNAKGAT: Lkynr_c + akg_c --> 4aphdob_c + glu__L_c 14908 or 15839 or 8540
UNK3: 2kmb_c + glu__L_c --> akg_c + met__L_c 8540 or 14014

3SALATAi: 3sala_c + akg_c + h_c --> 3snpyr_c + glu__L_c 8936
ASPTA: akg_c + asp__L_c <=> glu__L_c + oaa_c 8936
ASPTAp: akg_x + asp__L_x <=> glu__L_x + oaa_x 8936
CYSTA: akg_c + cys__L_c <=> glu__L_c + mercppyr_c 8936
LCYSTAT: Lcyst_c + akg_c <=> 3spyr_c + glu__L_c 8936
TYRTAip: 34hpp_x + glu__L_x --> akg_x + tyr__L_x 8936

10152 no reactions

ABTA: 4abut_c + akg_c --> glu__L_c + sucsal_c 10937

GF6PTA: f6p_c + gln__L_c --> gam6p_c + glu__L_c 12099

TRPTA: akg_c + trp__L_c <=> glu__L_c + indpyr_c 12407 or 14908 or 15839

AMAOTrm: 8aonn_m + amet_m <=> amob_m + dann_m 12731
DBTSm: atp_m + co2_m + dann_m <=> adp_m + dtbt_m + 3.0 h_m + pi_m 12731

HSTPT: glu__L_c + imacp_c --> akg_c + hisp_c 13230

13930 no reactions

ILETA: akg_c + ile__L_c <=> 3mop_c + glu__L_c 14014
LEUTA: akg_c + leu__L_c <=> 4mop_c + glu__L_c 14014
UNK3: 2kmb_c + glu__L_c --> akg_c + met__L_c 8540 or 14014
VALTA: akg_c + val__L_c <=> 3mob_c + glu__L_c 14014

3SALATAim: 3sala_m + akg_m + h_m --> 3snpyr_m + glu__L_m 14281
ASPTAm: akg_m + asp__L_m <=> glu__L_m + oaa_m 14281
CYSTAm: akg_m + cys__L_m <=> glu__L_m + mercppyr_m 14281
EHGLAT2m: e4hglu_m + oaa_m --> 4h2oglt_m + asp__L_m 14281
EHGLATm: akg_m + e4hglu_m --> 4h2oglt_m + glu__L_m 14281
LCYSTATm: Lcyst_m + akg_m <=> 3spyr_m + glu__L_m 14281

ILETAm: akg_m + ile__L_m <=> 3mop_m + glu__L_m 14610 or 14853
LEUTAm: akg_m + leu__L_m <=> 4mop_m + glu__L_m 14610 or 14853
VALTAm: akg_m + val__L_m <=> 3mob_m + glu__L_m 14610 or 14853

ILETAm: akg_m + ile__L_m <=> 3mop_m + glu__L_m 14610 or 14853
LEUTAm: akg_m + leu__L_m <=> 4mop_m + glu__L_m 14610 or 14853
VALTAm: akg_m + val__L_m <=> 3mob_m + glu__L_m 14610 or 14853

ACOTAim: acg5sa_m + glu__L_m --> acorn_m + akg_m 14878

3HKYNAKGAT: akg_c + hLkynr_c --> 42A3HP24DB_c + glu__L_c 14908 or 15839 or 8540
AATA: 2oxoadp_c + glu__L_c <=> L2aadp_c + akg_c 14908 or 15839
KYNAKGAT: Lkynr_c + akg_c --> 4aphdob_c + glu__L_c 14908 or 15839 or 8540
PHETA1: akg_c + phe__L_c <=> glu__L_c + phpyr_c 14908 or 15839
TRPTA: akg_c + trp__L_c <=> glu__L_c + indpyr_c 12407 or 14908 or 15839
TYRTA: akg_c + tyr__L_c <=> 34hpp_c + glu__L_c 14908 or 15839 or 16065

PSERT: 3php_c + glu__L_c --> akg_c + pser__L_c 15404
SDPTA: akg_c + sl26da_c <=> glu__L_c + sl2a6o_c 15404

3HKYNAKGAT: akg_c + hLkynr_c --> 42A3HP24DB_c + glu__L_c 14908 or 15839 or 8540
AATA: 2oxoadp_c + glu__L_c <=> L2aadp_c + akg_c 14908 or 15839
KYNAKGAT: Lkynr_c + akg_c --> 4aphdob_c + glu__L_c 14908 or 15839 or 8540
PHETA1: akg_c + phe__L_c <=> glu__L_c + phpyr_c 14908 or 15839
TRPTA: akg_c + trp__L_c <=> glu__L_c + indpyr_c 12407 or 14908 or 15839
TYRTA: akg_c + tyr__L_c <=> 34hpp_c + glu__L_c 14908 or 15839 or 16065

3AIBTm: 2mop_m + glu__L_m <=> 3aib_m + akg_m 15905
ABTArm: 4abut_m + akg_m <=> glu__L_m + sucsal_m 15905
APAT2rm: akg_m + ala_B_m <=> glu__L_m + msa_m 15905

TYRTA: akg_c + tyr__L_c <=> 34hpp_c + glu__L_c 14908 or 15839 or 16065
```

TYRTA generates 3-(4-Hydroxyphenyl)pyruvate, but it is blocked

11840 is mito 4-hydroxyphenylpyruvate dioxygenase  
Add 34HPPOR from sce, and change genes to '11840'  
Next step is HGNTOR by 15706 mito homogentisate 1,2-dioxygenase
Next step is MACACI by 8431 maleylacetoacetate isomerase  
Add MACACI from sce, and change genes to '8431'  
Next step is FUMAC by 11962

34HPPOR: 34hpp\_c + o2\_c → co2\_c + hgentis\_c 11840 or 9106  
HGNTOR: hgentis\_c + o2\_c --> 4mlacac\_c + h\_c 15706  
MACACI: 4mlacac\_c --> 4fumacac\_c 8431  
FUMAC: 4fumacac\_c + h2o\_c --> acac\_c + fum\_c + h\_c 11962

Can Rhodo grow on Tyrosine as a sole carbon source?

3-(4-Hydroxyphenyl)pyruvate can also be decarboxylated by PDC  
Make 34HPPYRDC and add gene 15791 PDC  
PPYRDC: h\_c + 34hpp\_c --> co2\_c + 4hoxpacd\_c 15791  
4HOXPACDOX\_NADP: 4hoxpacd\_c + h2o\_c + nadp\_c <=> 4hphac\_c + 2.0 h\_c + nadph\_c 12042 or 13426 or 16323

Many reactions possible from (4-hydroxyphenyl)acetate, check later

In [71]:

```
r1 = sce.reactions.get_by_id('34HPPOR').copy()
r2 = sce.reactions.get_by_id('MACACI').copy()
r1.gene_reaction_rule = '11840'
r2.gene_reaction_rule = '8431'
model.add_reactions([r1,r2])
model.reactions.get_by_id('PHETA1').gene_reaction_rule = '14908 or 15839'

r = model.reactions.get_by_id('PPYRDC').copy()
r.id = '34HPPYRDC'
r.name = '3-(4-Hydroxyphenyl)pyruvate decarboxylase'
r.gene_reaction_rule = '15791'
model.add_reactions([r])
r.add_metabolites({'phpyr_c': 1.0, 'pacald_c': -1.0, '34hpp_c': -1.0, '4hoxpacd_c': 1.0})
```

In [72]:

```
Annotation.loc[['11840','15706','8431','11962']]
```

Out[72]:

|  | Combined Annotations | Signal P | Sc288c Orthologs | Human Orthologs | Sc288 Best Hit | Human Blast | Essential | WolfPSort | C Terminal |
| --- | --- | --- | --- | --- | --- | --- | --- | --- | --- |
| RTO4\_ID |  |  |  |  |  |  |  |  |  |
| 11840 | HMMPfam:Xylose isomerase-like TIM barrel:PF012... | S |  |  |  |  | Not Essential | mito 9.5, extr 9, cyto\_mito 6.5, cyto 2.5, nuc... | ADD\* |
| 15706 | K00451: HGD, hmgA; homogentisate 1,2-dioxygenase |  |  | HGD |  | HGD | Not Essential | mito 13.5, cyto\_mito 11.333, cyto 8, cyto\_nucl... | NGH\* |
| 8431 | K01800: maiA, GSTZ1; maleylacetoacetate isomerase |  |  |  |  | GSTO1 | Not Essential | mito 21.5, cyto\_mito 13, cyto 3.5 | QKL\* |
| 11962 | K01555: FAH, fahA; fumarylacetoacetase |  |  | FAH |  | FAH | Not Essential | cyto 7.5, mito 6, cyto\_nucl 6, cysk 6, extr 3,... | RMA\* |

Tryptophan can be degraded via indolepyruvate  
TRPTA: akg\_c + trp**L\_c <=> glu**L\_c + indpyr\_c 12407 or 14908 or 15839  
INDPYRD: h\_c + indpyr\_c <=> co2\_c + id3acald\_c 15791  
ALDD20y: h2o\_c + id3acald\_c + nadp\_c --> 2.0 h\_c + ind3ac\_c + nadph\_c 12042 or 13426  
ALDD20x: h2o\_c + id3acald\_c + nad\_c --> 2.0 h\_c + ind3ac\_c + nadh\_c 12042 or 13426 or 15814 or 16323  
ALCD26xi: h\_c + id3acald\_c + nadh\_c --> ind3eth\_c + nad\_c 14108 or 14109 or 15438  
ALDD20x: h2o\_c + id3acald\_c + nad\_c --> 2.0 h\_c + ind3ac\_c + nadh\_c 12042 or 13426 or 15814 or 16323  
NTRLASE: 2.0 h2o\_c + ind3acnl\_c --> ind3ac\_c + nh4\_c 12410 or 14925 or 15730  
AMID3: h2o\_c + iad\_c --> ind3ac\_c + nh4\_c 10276 or 10277 or 12161 or 12540 or 12553 or 12842 or 13791  
ALCD26xi: h\_c + id3acald\_c + nadh\_c --> ind3eth\_c + nad\_c 14108 or 14109 or 15438  
Need to get rid of ind3eth  
Add IND3ETHt and EX\_ind3eth\_e from sce

Tryptophan can also be degraded via kynurenine  
TRPO2: o2\_c + trp**L\_c --> Lfmkynr\_c 15923  
FKYNH: Lfmkynr\_c + h2o\_c --> Lkynr\_c + for\_c + h\_c 14788  
KYN3OX: Lkynr\_c + h\_c + nadph\_c + o2\_c --> h2o\_c + hLkynr\_c + nadp\_c 9267  
HKYNH: h2o\_c + hLkynr\_c --> 3hanthrn\_c + ala**L\_c 8725  
3HAO: 3hanthrn\_c + o2\_c --> cmusa\_c + h\_c 8602  
PCLAD: cmusa\_c + h\_c --> am6sa\_c + co2\_c 12916  
Need to add next steps AM6SAD and AMCOXO are missing  
AM6SAD known genes blast to general aldehyde dehydrogenases (12042 or 13426) with high %id  
AMCOXO looks like a lumped reaction including deaminase (9857) and reductase (unknown)

NAD biosynthesis from 2-amino-3-carboxymuconate semialdehyde (cmusa)  
Add QUILSYN (spontaneous) from sce  
QUILSYN: cmusa\_c --> h2o\_c + h\_c + quln\_c  
NNDPR: 2.0 h\_c + prpp\_c + quln\_c --> co2\_c + nicrnt\_c + ppi\_c 10331  
NICRNS: atp\_c + nicrns\_c --> adp\_c + h\_c + nicrnt\_c 14574  
NAPRT: h\_c + nac\_c + prpp\_c --> nicrnt\_c + ppi\_c 10292  
NNAT: atp\_c + h\_c + nicrnt\_c --> dnad\_c + ppi\_c 10430  
NAMNPP: atp\_c + h2o\_c + nac\_c + prpp\_c --> adp\_c + nicrnt\_c + pi\_c + ppi\_c 10292  
NNDPR: 2.0 h\_c + prpp\_c + quln\_c --> co2\_c + nicrnt\_c + ppi\_c 10331  
DNADDP: dnad\_c + h2o\_c --> amp\_c + 2.0 h\_c + nicrnt\_c 14638 or 15385  
NADS1: atp\_c + dnad\_c + nh4\_c --> amp\_c + h\_c + nad\_c + ppi\_c 9283  
NADS2: atp\_c + dnad\_c + gln**L\_c + h2o\_c --> amp\_c + glu**L\_c + h\_c + nad\_c + ppi\_c 9283

Tryptophan can also be degraded via anthranilate  
KYN: Lkynr\_c + h2o\_c --> ala\_\_L\_c + anth\_c + h\_c 8725  
ACANTHAT: accoa\_c + anth\_c --> acanth\_c + coa\_c 15430

Tryptophan biosynthesis  
ANS: chor\_c + gln**L\_c --> anth\_c + glu**L\_c + h\_c + pyr\_c 15109 and 16564  
ANPRT: anth\_c + prpp\_c --> ppi\_c + pran\_c 9900  
PRAIi: pran\_c --> 2cpr5p\_c 16564  
IGPS: 2cpr5p\_c + h\_c --> 3ig3p\_c + co2\_c + h2o\_c 16564  
TRPS1: 3ig3p\_c + ser**L\_c --> g3p\_c + h2o\_c + trp**L\_c 9262  
16564 is anthranilate synthase / indole-3-glycerol phosphate synthase / phosphoribosylanthranilate isomerase  
(three enzyme in one, gene model is wrong at the beginning)

In [73]:

```
r1 = sce.reactions.get_by_id('IND3ETHt').copy()
r2 = sce.reactions.get_by_id('EX_ind3eth_e').copy()
model.add_reactions([r1,r2])

r1 = hsa.reactions.get_by_id('AM6SAD').copy()
r2 = hsa.reactions.get_by_id('AMCOXO').copy()
r1.gene_reaction_rule = '12042 or 13426'
r2.gene_reaction_rule = '9857'
model.add_reactions([r1,r2])

r = sce.reactions.get_by_id('QUILSYN').copy()
model.add_reactions([r])

#Fix GPR
model.reactions.get_by_id('ANS').gene_reaction_rule = '15109 and 16564'
```

In [74]:

```
temp = ['15923','14788','9267','8725','8602','12916','15430','15109','16564','9900','9262','13669']
display(Annotation.loc[temp])
Show_Data(temp)
```

|  | Combined Annotations | Signal P | Sc288c Orthologs | Human Orthologs | Sc288 Best Hit | Human Blast | Essential | WolfPSort | C Terminal |
| --- | --- | --- | --- | --- | --- | --- | --- | --- | --- |
| RTO4\_ID |  |  |  |  |  |  |  |  |  |
| 15923 | K00463: IDO, INDO; indoleamine 2,3-dioxygenase |  | BNA2 | IDO1,IDO2 | BNA2 | IDO1 | Not Essential | mito 21, cyto 3, nucl 2, cyto\_pero 2 | QQE\* |
| 14788 | K14263: BNA7; kynurenine formamidase |  | BNA7 |  | BNA7 |  | Not Essential | cyto 15, cyto\_nucl 11.833, cyto\_mito 9.499, nu... | VRK\* |
| 9267 | K00486: KMO; kynurenine 3-monooxygenase | S | BNA4 | KMO | BNA4 | KMO | Not Essential | cyto 9, extr 6, E.R. 5, mito 2, plas 2, pero 2 | RVD\* |
| 8725 | K01556: KYNU, kynU; kynureninase | S | BNA5 | KYNU | BNA5 | KYNU | Not Essential | mito 12.5, cyto\_mito 10, cyto 6.5, nucl 4, extr 3 | LRQ\* |
| 8602 | K00452: HAAO; 3-hydroxyanthranilate 3,4-dioxyg... |  | BNA1 | HAAO | BNA1 | HAAO | Not Essential | cyto\_nucl 12.833, cyto 12.5, nucl 11, cyto\_per... | EAK\* |
| 12916 | K03392: ACMSD; aminocarboxymuconate-semialdehy... |  |  | ACMSD |  | ACMSD | Not Essential | cyto 13.5, cyto\_nucl 8, cysk 8, mito 3 | LEI\* |
| 15430 | HMMPfam:N-acetyltransferase:PF00797,SUPERFAMIL... |  |  | NAT2 |  |  | Not Essential | extr 13, mito 7, cyto 3.5, pero 3 | QFQ\* |
| 15109 | K01657: trpE; anthranilate synthase component I | S | TRP2 |  | TRP2 |  | Essential | cyto 14, cyto\_mito 12.333, mito 9.5, cyto\_nucl... | RQA\* |
| 16564 | K13501: TRP1; anthranilate synthase / indole-3... |  | TRP3 |  | TRP3 |  | Essential | cyto 14.5, cyto\_nucl 8.5, mito 6, extr 3 | KEA\* |
| 9900 | K00766: trpD; anthranilate phosphoribosyltrans... |  | TRP4 |  | TRP4 |  | Not Essential | cyto 8.5, mito 8, cyto\_nucl 6.5, extr 5, nucl 3.5 | LIG\* |
| 9262 | K01694: TRP; tryptophan synthase |  | TRP5 |  | TRP5 |  | Essential | cyto 12, cysk 11, mito 4 | VQG\* |
| 13669 | K13950: pabAB; para-aminobenzoate synthetase |  | ABZ1 |  | ABZ1 |  | Not Essential | extr 9, cyto 8, cyto\_mito 7.333, cyto\_nucl 6.8... | KAS\* |

| strain | WT | | | | | | | | | | | | | | | | |
| --- | --- | --- | --- | --- | --- | --- | --- | --- | --- | --- | --- | --- | --- | --- | --- | --- | --- |
| condition | G\_MM | C\_MM | G\_SD | | GX\_SD | | | X\_SD | | A\_SD | | C\_SD | | MM\_CN120 | | MM\_CN5 | Diversity\_Sample |
| phase | exp | exp | exp | stat | exp | trans | stat | exp | stat | exp | stat | exp | stat | exp | stat | exp | exp |
| proteinId | Set1 | Set1 | Set2 | Set2 | Set2 | Set2 | Set2 | Set2 | Set2 | Set2 | Set2 | Set2 | Set2 | Set3 | Set3 | Set3 | Set3 |
| 15923 | 5.33721 | 5.27405 | 6.78725 | 5.85274 | 6.92079 | 6.70644 | 6.60769 | 6.15287 | 6.8321 | 6.16545 | 5.95556 | 6.19269 | 6.07501 | 6.9287 | 6.85916 | 6.09835 | 6.8305 |
| 14788 | 6.6301 | 3.17126 | 3.23491 | 2.70728 | 3.08463 | 2.11032 | 2.33245 | 2.03558 | 2.23456 | 2.07861 | 1.26881 | 1.73431 | 1.55017 | 5.37646 | 3.29976 | 2.60208 | 3.76722 |
| 9267 | 5.80579 | 4.83444 | 5.36814 | 5.40041 | 5.34581 | 5.6433 | 5.79752 | 5.28252 | 5.67345 | 5.21972 | 5.52743 | 5.5132 | 5.14804 | 5.52396 | 5.81341 | 4.82002 | 5.50824 |
| 8725 | 5.26695 | 4.74813 | 4.89925 | 5.19964 | 4.87629 | 5.40814 | 5.40369 | 5.15125 | 5.32725 | 5.08769 | 5.24412 | 5.24333 | 5.16677 | 6.08564 | 5.67467 | 5.05532 | 5.75231 |
| 8602 | 6.11384 | 4.62614 | 6.49243 | 5.97615 | 6.54151 | 5.96406 | 5.89344 | 5.4679 | 5.83612 | 5.20043 | 5.86682 | 4.95726 | 4.34865 | 4.97891 | 4.85963 | 4.86077 | 3.88126 |
| 12916 | 6.11378 | 5.22846 | 5.6284 | 4.99706 | 5.63114 | 5.03594 | 5.06049 | 5.73198 | 5.21899 | 5.43441 | 5.01116 | 5.10263 | 4.4664 | 4.96705 | 4.48538 | 3.82088 | 4.2383 |
| 15430 | 6.37194 | 5.44655 | 6.42215 | 6.08485 | 7.03107 | 5.68479 | 6.2328 | 5.01507 | 5.07883 | 5.57224 | 5.17559 | 6.56254 | 7.83445 | 5.1754 | 5.05752 | 8.3594 | 5.62781 |
| 15109 | 5.724 | 5.90008 | 6.26505 | 5.21375 | 6.64499 | 5.48574 | 5.62565 | 5.68005 | 5.49855 | 5.23151 | 5.26422 | 6.37503 | 6.75081 | 4.98576 | 4.8497 | 7.27174 | 6.01453 |
| 16564 | 6.26733 | 6.18235 | 6.48428 | 5.1047 | 6.90093 | 5.78441 | 5.6582 | 5.83949 | 5.63211 | 5.36699 | 5.27117 | 7.27322 | 7.88227 | 6.35111 | 5.94342 | 8.69663 | 7.19855 |
| 9900 | 6.49713 | 6.05646 | 6.28967 | 5.98646 | 6.70774 | 5.75215 | 5.87655 | 5.98361 | 5.98121 | 5.89019 | 5.96411 | 6.52356 | 7.16357 | 5.9321 | 5.97166 | 7.8289 | 6.53705 |
| 9262 | 6.12762 | 6.14506 | 5.80105 | 5.11905 | 5.95766 | 5.4806 | 5.4839 | 5.70657 | 5.07119 | 5.23851 | 5.07851 | 6.02309 | 8.59626 | 5.18814 | 4.97477 | 6.76938 | 6.10403 |
| 13669 | 4.31736 | 3.91914 | 4.44627 | 4.5636 | 4.5273 | 4.68787 | 4.73919 | 4.20105 | 4.80266 | 4.03218 | 4.51465 | 3.91098 | 3.2547 | 5.72401 | 5.78354 | 5.14876 | 5.73864 |

| strain | WT | | | | | | | | | | |
| --- | --- | --- | --- | --- | --- | --- | --- | --- | --- | --- | --- |
| condition | G\_SD | | GX\_SD | | | X\_SD | | A\_SD | | C\_SD | |
| proteinId | exp | stat | exp | trans | stat | exp | stat | exp | stat | exp | stat |
| 15923 | 0 | 0 | 0 | 0 | 0 | 0 | 0 | 0 | 0 | 0 | 0.211526 |
| 9267 | 4.80358 | 1.97097 | 5.70222 | 2.97449 | 3.81544 | 1.18098 | 0.198706 | 0.770643 | 0.764589 | 1.2853 | 1.08249 |
| 8725 | 0.764563 | 2.32362 | 0.203084 | 2.77247 | 1.64649 | 1.75648 | 2.3591 | 1.54299 | 1.34417 | 1.26278 | 2.39285 |
| 8602 | 0.755525 | 1.62031 | 0.197656 | 1.84709 | 3.09259 | 2.74553 | 1.78692 | 1.738 | 1.14892 | 0 | 0.435902 |
| 12916 | 2.30349 | 1.78437 | 1.82461 | 1.48286 | 1.44863 | 1.15955 | 2.3521 | 1.93098 | 1.91278 | 2.13648 | 1.72991 |
| 15430 | 1.70715 | 0 | 3.05477 | 0 | 0 | 0.975427 | 0 | 0.964164 | 0.189808 | 0.219601 | 3.92784 |
| 15109 | 8.09146 | 5.20237 | 8.13768 | 5.58285 | 5.4679 | 7.85191 | 7.08785 | 7.34538 | 6.12237 | 6.65098 | 7.81128 |
| 16564 | 23.8866 | 14.0932 | 22.7706 | 9.66202 | 8.38586 | 15.683 | 14.1552 | 13.72 | 11.8543 | 16.8902 | 24.8764 |
| 9900 | 5.25522 | 4.34444 | 5.50751 | 4.84971 | 4.53793 | 5.0718 | 4.93088 | 3.85609 | 5.53499 | 5.56226 | 5.86552 |
| 9262 | 20.6092 | 11.5145 | 15.8096 | 9.66499 | 8.72314 | 10.1819 | 7.41383 | 12.7586 | 7.0833 | 15.5535 | 17.6502 |
| 13669 | 0 | 0.179255 | 0.204746 | 0.562089 | 0.179978 | 0 | 0.589289 | 0 | 0 | 0 | 0 |

|  | Glucose | Xylose | Arabinose | Acetate | Coumarate | Ferulate | YNB Oleic Acid | YNB Ricinoleic Acid | YNB Glucose | YNB Gluc DOC | YPD |
| --- | --- | --- | --- | --- | --- | --- | --- | --- | --- | --- | --- |
| proteinId |  |  |  |  |  |  |  |  |  |  |  |
| 15923 | -1.58327 | -0.187605 | -0.00698488 | -0.0148311 | -5.22999 | -2.92776 | -0.544326 | -0.413142 | -0.115497 | -0.427772 | -0.127409 |
| 14788 | -0.233399 | -0.305637 | -0.0800108 | 0.195436 | -0.295385 | 0.0592889 | 0.0538321 | -0.0922441 | 0.176823 | 0.0955898 | -0.0658581 |
| 9267 | -1.03103 | 0.119221 | 0.0440669 | 0.267081 | -3.3961 | -2.78866 | -0.16855 | -0.540039 | 0.365575 | -0.699194 | -0.124923 |
| 8725 | -1.59329 | 0.192628 | -0.118265 | -0.0429016 | -5.08803 | -3.28412 | -0.138058 | -0.339698 | -0.0896783 | -0.755181 | -0.0748085 |
| 8602 | -1.85696 | -0.183267 | 0.140202 | 0.0318403 | -4.53739 | -2.88738 | -0.566587 | -1.12046 | -0.465372 | -1.1859 | 0.238405 |
| 12916 | 0.264166 | 0.0723649 | 0.043976 | 0.0056242 | 1.21605 | 0.0696509 | 0.200722 | -0.111672 | 0.480955 | 0.417124 | -0.207886 |
| 15430 | -0.0495182 | 0.0447665 | -0.357126 | -0.188565 | 0.279091 | 0.485558 | -0.995476 | 0.160607 | 0.0361053 | 0.262345 | -0.402873 |
| 13669 | 0.0145212 | -0.149855 | -0.0347267 | 0.0314421 | 0.0858744 | 0.127857 | 0.079359 | -0.172931 | 0.00296095 | 0.0207803 | -0.20695 |

In [75]:

```
for x in temp:
    if x in model.genes:
        for r in sorted(model.genes.get_by_id(x).reactions, key=lambda x: x.id):
            print(r, r.gene_reaction_rule)
    else:
        print(x, 'no reactions')
    print()
```

```
5HTRPDOX: 5htrp_c + o2_c --> 5hoxnfkyn_c 15923
MELATN23DOX: melatn_c + o2_c --> fna5moxam_c 15923
SRTN23OX: o2_c + srtn_c --> f5hoxkyn_c 15923
TRPO2: o2_c + trp__L_c --> Lfmkynr_c 15923

FKYNH: Lfmkynr_c + h2o_c --> Lkynr_c + for_c + h_c 14788

4HBHYOX: 4hbz_c + h_c + nadph_c + o2_c --> 34dhbz_c + h2o_c + nadp_c 9267
KYN3OX: Lkynr_c + h_c + nadph_c + o2_c --> h2o_c + hLkynr_c + nadp_c 9267

HKYNH: h2o_c + hLkynr_c --> 3hanthrn_c + ala__L_c 8725
KYN: Lkynr_c + h2o_c --> ala__L_c + anth_c + h_c 8725
LFORKYNHYD: Lfmkynr_c + h2o_c --> ala__L_c + h_c + nformanth_c 8725

3HAO: 3hanthrn_c + o2_c --> cmusa_c + h_c 8602

PCLAD: cmusa_c + h_c --> am6sa_c + co2_c 12916

ACANTHAT: accoa_c + anth_c --> acanth_c + coa_c 15430

ANS: chor_c + gln__L_c --> anth_c + glu__L_c + h_c + pyr_c 15109 and 16564

ADCS: chor_c + gln__L_c --> 4adcho_c + glu__L_c 13669 or (13669 and 16564)
ANS: chor_c + gln__L_c --> anth_c + glu__L_c + h_c + pyr_c 15109 and 16564
IGPS: 2cpr5p_c + h_c --> 3ig3p_c + co2_c + h2o_c 16564
PRAIi: pran_c --> 2cpr5p_c 16564

ANPRT: anth_c + prpp_c --> ppi_c + pran_c 9900

TRPS1: 3ig3p_c + ser__L_c --> g3p_c + h2o_c + trp__L_c 9262
TRPS2: indole_c + ser__L_c --> h2o_c + trp__L_c 9262

ADCS: chor_c + gln__L_c --> 4adcho_c + glu__L_c 13669 or (13669 and 16564)
PABB: chor_c + nh4_c --> 4adcho_c + h2o_c 13669
```

In [76]:

```
# 13669 ABZ1 para-aminobenzoate synthetase using glutamine
model.reactions.get_by_id('ADCS').gene_reaction_rule = '13669'
model.remove_reactions(['PABB'],remove_orphans=True)
```

Valine biosynthesis  
cyto  
KARA1: 23dhmb\_c + nadp\_c <=> alac**S\_c + h\_c + nadph\_c 9176  
DHAD1: 23dhmb\_c --> 3mob\_c + h2o\_c 14626 or 9689  
VALTA: akg\_c + val**L\_c <=> 3mob\_c + glu**L\_c 14014  
mito  
ACLSm: h\_m + 2.0 pyr\_m --> alac**S\_m + co2\_m 15685 and 9800  
KARA1im: alac**S\_m + h\_m + nadph\_m --> 23dhmb\_m + nadp\_m 9176  
DHAD1m: 23dhmb\_m --> 3mob\_m + h2o\_m 14626 or 9689  
VALTAm: akg\_m + val**L\_m <=> 3mob\_m + glu\_\_L\_m 14610 or 14853

Leucine biosynthesis  
cyto  
IPPS: 3mob\_c + accoa\_c + h2o\_c --> 3c3hmp\_c + coa\_c + h\_c 14856 or 15488  
IPPMIb: 2ippm\_c + h2o\_c <=> 3c3hmp\_c 14914  
IPPMIa: 3c2hmp\_c <=> 2ippm\_c + h2o\_c 14914  
IPMD: 3c2hmp\_c + nad\_c --> 3c4mop\_c + h\_c + nadh\_c 10428 or 13894  
OMCDC: 3c4mop\_c + h\_c --> 4mop\_c + co2\_c 14014  
LEUTA: akg\_c + leu**L\_c <=> 4mop\_c + glu**L\_c 14014  
mito  
IPPSm: 3mob\_m + accoa\_m + h2o\_m --> 3c3hmp\_m + coa\_m + h\_m 15488  
missing 3c3hmp -> 3c2hmp (IPPMIb and IPPMIa)  
missing 3c2hmp -> 3c4mop (IPMD)  
OMCDCm: 3c4mop\_m + h\_m --> 4mop\_m + co2\_m 14610 or 14853  
LEUTAm: akg\_m + leu**L\_m <=> 4mop\_m + glu**L\_m 14610 or 14853

Isoleucine biosynthesis  
cyto  
DHAD2: 23dhmp\_c --> 3mop\_c + h2o\_c 14626 or 9689  
LETA: akg\_c + ile**L\_c <=> 3mop\_c + glu**L\_c 14014  
mito
THRD\_Lm: thr**L\_m --> 2obut\_m + nh4\_m 11909  
ACHBSm: 2obut\_m + h\_m + pyr\_m --> 2ahbut\_m + co2\_m 15685 and 9800  
KARA2im: 2ahbut\_m + h\_m + nadph\_m --> 23dhmp\_m + nadp\_m 9176  
DHAD2m: 23dhmp\_m --> 3mop\_m + h2o\_m 14626 or 9689  
ILETAm: akg\_m + ile**L\_m <=> 3mop\_m + glu\_\_L\_m 14610 or 14853  
ok, need to make threonine

In [77]:

```
temp = ['15685','9800','9176','14626','9689','14014','14610','14853',
        '14856','15488','14914','10428','13894']
display(Annotation.loc[temp])
Show_Data(temp)
```

|  | Combined Annotations | Signal P | Sc288c Orthologs | Human Orthologs | Sc288 Best Hit | Human Blast | Essential | WolfPSort | C Terminal |
| --- | --- | --- | --- | --- | --- | --- | --- | --- | --- |
| RTO4\_ID |  |  |  |  |  |  |  |  |  |
| 15685 | K01652: E2.2.1.6L, ilvB, ilvG, ilvI; acetolact... | S | ILV2 |  | ILV2 | HACL1 | Essential | mito 26.5, cyto\_mito 14 | FAK\* |
| 9800 | K01653: E2.2.1.6S, ilvH, ilvN; acetolactate sy... | S | ILV6 |  | ILV6 |  | Not Essential | mito 26 | PPG\* |
| 9176 | K00053: ilvC; ketol-acid reductoisomerase |  | ILV5 |  | ILV5 |  | Essential | mito 24.5, cyto\_mito 14 | KNN\* |
| 14626 | K01687: ilvD; dihydroxy-acid dehydratase |  | ILV3 |  | ILV3 |  | Not Essential | cyto 21, cyto\_nucl 13.5, nucl 4 | YTD\* |
| 9689 | K01687: ilvD; dihydroxy-acid dehydratase |  | ILV3 |  | ILV3 |  | Essential | cyto 23.5, cyto\_nucl 14 | DGP\* |
| 14014 | K00826: E2.6.1.42, ilvE; branched-chain amino ... |  | BAT1,BAT2 | BCAT1 | BAT2 | BCAT1 | Not Essential | cyto 12, mito 11, cyto\_nucl 6.833, cyto\_pero 6... | VVV\* |
| 14610 | K00826: E2.6.1.42, ilvE; branched-chain amino ... | S | BAT1,BAT2 | BCAT1 | BAT1 | BCAT2 | Not Essential | mito 20, cyto 2, E.R. 2, plas 1, extr 1, cyto\_... | VDS\* |
| 14853 | K00826: E2.6.1.42, ilvE; branched-chain amino ... | S | BAT1,BAT2 | BCAT1 | BAT2 | BCAT2 | Not Essential | mito 8.5, cyto\_mito 7.833, pero 7, cyto 6, ext... | SKA\* |
| 14856 | K01655: LYS21, LYS20; homocitrate synthase |  | LYS21,LYS20 |  | LYS20 |  | Not Essential | cyto 17.5, cyto\_nucl 15.5, nucl 8.5 | TSA\* |
| 15488 | K01649: leuA; 2-isopropylmalate synthase |  | LEU4,LEU9 |  | LEU4 |  | Not Essential | mito 18.5, cyto\_mito 11.833, cyto 4, pero 4 | GRV\* |
| 14914 | K01702: LEU1; 3-isopropylmalate dehydratase |  | LEU1 |  | LEU1 | IREB2 | Not Essential | cyto 13, cyto\_nucl 9.5, pero 5, nucl 4, cysk 4 | TDW\* |
| 10428 | K05824: LYS12; homoisocitrate dehydrogenase |  | LYS12 |  | LYS12 | IDH3A | Not Essential | mito 15, cyto 12 | KQL\* |
| 13894 | K00052: leuB; 3-isopropylmalate dehydrogenase |  | LEU2 |  | LEU2 |  | Not Essential | cyto 19.5, cyto\_nucl 10.5, cysk 5 | SKK\* |

| strain | WT | | | | | | | | | | | | | | | | |
| --- | --- | --- | --- | --- | --- | --- | --- | --- | --- | --- | --- | --- | --- | --- | --- | --- | --- |
| condition | G\_MM | C\_MM | G\_SD | | GX\_SD | | | X\_SD | | A\_SD | | C\_SD | | MM\_CN120 | | MM\_CN5 | Diversity\_Sample |
| phase | exp | exp | exp | stat | exp | trans | stat | exp | stat | exp | stat | exp | stat | exp | stat | exp | exp |
| proteinId | Set1 | Set1 | Set2 | Set2 | Set2 | Set2 | Set2 | Set2 | Set2 | Set2 | Set2 | Set2 | Set2 | Set3 | Set3 | Set3 | Set3 |
| 15685 | 4.77072 | 5.8768 | 5.70414 | 4.73389 | 6.11419 | 5.91613 | 5.78912 | 5.83106 | 3.84626 | 4.48862 | 3.54703 | 7.37206 | 8.39005 | 6.30499 | 5.95482 | 8.41215 | 7.29474 |
| 9800 | 6.32412 | 7.25166 | 7.53837 | 5.65275 | 7.82612 | 6.7912 | 6.97343 | 6.80425 | 5.48086 | 5.61202 | 5.25958 | 8.01455 | 8.89711 | 6.68387 | 6.31772 | 8.57819 | 7.26386 |
| 9176 | 6.19789 | 8.34984 | 7.53869 | 5.93458 | 7.74068 | 7.87645 | 7.837 | 8.33781 | 6.30239 | 6.76229 | 5.66163 | 8.26595 | 8.94042 | 8.04193 | 7.81227 | 9.85569 | 9.73602 |
| 14626 | 5.08767 | 5.09035 | 4.65376 | 6.68103 | 4.49497 | 4.51506 | 4.76778 | 4.38213 | 4.99576 | 5.42286 | 5.66505 | 2.769 | 2.00379 | 4.71399 | 4.88003 | 5.4148 | 6.4164 |
| 9689 | 7.46058 | 7.5321 | 7.95539 | 5.73128 | 8.3509 | 6.60228 | 6.56024 | 6.96945 | 5.57709 | 6.3408 | 5.68655 | 8.02851 | 8.84294 | 6.42819 | 6.28558 | 8.35659 | 7.28829 |
| 14014 | 2.77869 | 2.40508 | 5.58636 | 5.56856 | 5.84731 | 3.74791 | 4.71441 | 2.22746 | 3.55795 | 2.60791 | 4.6743 | 2.74453 | 2.98602 | 3.74213 | 3.448 | 5.31524 | 3.5165 |
| 14610 | 6.35581 | 6.93464 | 7.40831 | 6.37511 | 7.74917 | 6.71342 | 6.67106 | 6.40937 | 6.14813 | 5.59469 | 6.53238 | 6.02404 | 7.27274 | 7.37942 | 6.60182 | 9.56953 | 7.78925 |
| 14853 | 4.7362 | 4.5406 | 4.88838 | 5.4074 | 4.98216 | 5.05629 | 5.10921 | 4.7381 | 5.09388 | 4.7394 | 5.03763 | 5.19336 | 5.50641 | 5.46496 | 5.90049 | 5.73904 | 5.14016 |
| 14856 | 7.06277 | 7.64718 | 7.71952 | 6.84579 | 8.04825 | 7.27894 | 7.24555 | 7.49081 | 6.40468 | 6.66873 | 6.64251 | 8.046 | 8.80539 | 8.713 | 8.69493 | 10.3276 | 9.37678 |
| 15488 | 6.28101 | 7.35254 | 7.44341 | 5.87795 | 7.80891 | 6.73351 | 6.6735 | 7.22184 | 5.25894 | 6.05889 | 5.48361 | 7.75099 | 8.88047 | 6.35347 | 6.13102 | 8.97025 | 8.08185 |
| 14914 | 5.62765 | 6.51879 | 5.07049 | 5.99872 | 5.35954 | 5.95493 | 6.81603 | 6.06848 | 5.43264 | 5.2887 | 5.47478 | 4.62242 | 6.67896 | 4.5949 | 4.32192 | 6.2286 | 6.32912 |
| 10428 | 5.88933 | 7.89489 | 6.82443 | 5.55507 | 6.92434 | 6.38563 | 6.42311 | 6.88477 | 5.7152 | 6.03695 | 5.43536 | 7.38936 | 6.49532 | 7.20884 | 7.15186 | 8.16188 | 8.87302 |
| 13894 | 5.93444 | 7.10758 | 6.99072 | 5.40606 | 7.22745 | 6.85489 | 6.84543 | 7.25474 | 4.87398 | 5.97372 | 4.47006 | 6.54287 | 7.53049 | 6.71483 | 6.0947 | 8.40918 | 7.43845 |

| strain | WT | | | | | | | | | | |
| --- | --- | --- | --- | --- | --- | --- | --- | --- | --- | --- | --- |
| condition | G\_SD | | GX\_SD | | | X\_SD | | A\_SD | | C\_SD | |
| proteinId | exp | stat | exp | trans | stat | exp | stat | exp | stat | exp | stat |
| 15685 | 25.4124 | 24.9145 | 22.9967 | 21.9311 | 28.5391 | 22.5044 | 21.6688 | 20.2808 | 16.6334 | 20.469 | 35.7014 |
| 9800 | 15.8981 | 17.6088 | 18.9464 | 13.9729 | 16.9157 | 14.2408 | 12.7677 | 10.8225 | 11.8564 | 15.1347 | 22.6449 |
| 9176 | 34.9546 | 31.9793 | 36.382 | 29.9508 | 29.0601 | 36.7434 | 32.1382 | 30.5018 | 29.8441 | 37.2473 | 38.309 |
| 14626 | 0.773528 | 1.47315 | 0.411687 | 2.24102 | 2.9377 | 2.94734 | 3.33584 | 3.08437 | 6.71647 | 0 | 0 |
| 9689 | 16.104 | 12.284 | 15.4336 | 10.4055 | 11.1308 | 11.3576 | 10.871 | 11.5971 | 9.37307 | 13.2356 | 17.2174 |
| 14014 | 5.16681 | 0 | 7.11355 | 0 | 0 | 0 | 0 | 0 | 0 | 0 | 0 |
| 14610 | 19.4505 | 16.9826 | 20.3233 | 15.4321 | 16.7866 | 21.5237 | 17.317 | 18.5377 | 16.843 | 14.9946 | 14.1686 |
| 14856 | 27.7406 | 22.5671 | 29.2617 | 25.3093 | 23.2998 | 19.5737 | 20.3033 | 21.4361 | 20.466 | 27.7783 | 31.5448 |
| 15488 | 50.2258 | 36.0327 | 48.4576 | 35.9037 | 36.5373 | 35.4218 | 38.4594 | 39.4312 | 36.9226 | 42.5377 | 51.1645 |
| 14914 | 13.491 | 4.86729 | 11.5805 | 2.42616 | 3.85706 | 9.42596 | 3.9263 | 11.599 | 4.20932 | 9.62272 | 9.20358 |
| 10428 | 17.6535 | 16.8612 | 16.4479 | 14.9055 | 16.103 | 15.0332 | 14.6223 | 11.5922 | 13.8129 | 25.3987 | 26.7699 |
| 13894 | 36.8803 | 31.2576 | 33.7625 | 25.4761 | 28.0464 | 32.114 | 24.9683 | 26.6611 | 20.3026 | 31.6014 | 36.1719 |

|  | Glucose | Xylose | Arabinose | Acetate | Coumarate | Ferulate | YNB Oleic Acid | YNB Ricinoleic Acid | YNB Glucose | YNB Gluc DOC | YPD |
| --- | --- | --- | --- | --- | --- | --- | --- | --- | --- | --- | --- |
| proteinId |  |  |  |  |  |  |  |  |  |  |  |
| 14626 | -0.0945652 | -0.537756 | -0.266318 | -0.0308197 | -0.122558 | -0.179321 | -0.354095 | 0.163809 | -0.20504 | 0.172839 | -0.00261887 |
| 9689 | 0.196183 | -0.0642164 | 0.0647096 | 0.198793 | -0.269961 | -0.509114 | 0.343713 | 0.642502 | -0.236652 | -0.991276 | -0.694307 |
| 14014 | 0.277808 | 0.132282 | 0.304391 | 0.220584 | 0.430317 | 0.603165 | -0.337377 | -0.942644 | -0.236419 | -0.266986 | -0.316429 |
| 14610 | -0.00277608 | -0.0846046 | -0.813033 | -0.269283 | 0.667987 | -0.430488 | -0.572899 | -1.45413 | 0.0684007 | 0.163426 | 0.132251 |
| 14853 | -0.137609 | -0.301241 | -0.25221 | 0.0016244 | 0.0216384 | -0.339282 | -0.120204 | 0.862746 | -0.101846 | 0.237236 | -0.385014 |
| 14856 | -2.41996 | -2.32883 | -3.21568 | -1.90593 | -3.79053 | -1.27159 | -2.91199 | -2.78208 | -3.91013 | -1.46531 | -0.724535 |
| 10428 | -0.412945 | 0.364408 | -1.37317 | -0.878157 | -0.883249 | -0.442528 | -1.48324 | -2.05839 | -2.52439 | -0.284016 | -0.218092 |

In [78]:

```
for x in temp:
    if x in model.genes:
        for r in sorted(model.genes.get_by_id(x).reactions, key=lambda x: x.id):
            print(r, r.gene_reaction_rule)
    else:
        print(x, 'no reactions')
    print()
```

```
ACHBSm: 2obut_m + h_m + pyr_m --> 2ahbut_m + co2_m 15685 and 9800
ACLSm: h_m + 2.0 pyr_m --> alac__S_m + co2_m 15685 and 9800

ACHBSm: 2obut_m + h_m + pyr_m --> 2ahbut_m + co2_m 15685 and 9800
ACLSm: h_m + 2.0 pyr_m --> alac__S_m + co2_m 15685 and 9800

DPR: 2dhp_c + h_c + nadph_c --> nadp_c + pant__R_c 14277 or 16522 or 9176
DPRm: 2dhp_m + h_m + nadph_m --> nadp_m + pant__R_m 14277 or 9176
KARA1: 23dhmb_c + nadp_c <=> alac__S_c + h_c + nadph_c 9176
KARA1im: alac__S_m + h_m + nadph_m --> 23dhmb_m + nadp_m 9176
KARA2: 2ahbut_c + h_c + nadph_c <=> 23dhmp_c + nadp_c 9176
KARA2im: 2ahbut_m + h_m + nadph_m --> 23dhmp_m + nadp_m 9176

DHAD1: 23dhmb_c --> 3mob_c + h2o_c 14626 or 9689
DHAD1m: 23dhmb_m --> 3mob_m + h2o_m 14626 or 9689
DHAD2: 23dhmp_c --> 3mop_c + h2o_c 14626 or 9689
DHAD2m: 23dhmp_m --> 3mop_m + h2o_m 14626 or 9689

DHAD1: 23dhmb_c --> 3mob_c + h2o_c 14626 or 9689
DHAD1m: 23dhmb_m --> 3mob_m + h2o_m 14626 or 9689
DHAD2: 23dhmp_c --> 3mop_c + h2o_c 14626 or 9689
DHAD2m: 23dhmp_m --> 3mop_m + h2o_m 14626 or 9689

ILETA: akg_c + ile__L_c <=> 3mop_c + glu__L_c 14014
LEUTA: akg_c + leu__L_c <=> 4mop_c + glu__L_c 14014
UNK3: 2kmb_c + glu__L_c --> akg_c + met__L_c 8540 or 14014
VALTA: akg_c + val__L_c <=> 3mob_c + glu__L_c 14014

ILETAm: akg_m + ile__L_m <=> 3mop_m + glu__L_m 14610 or 14853
LEUTAm: akg_m + leu__L_m <=> 4mop_m + glu__L_m 14610 or 14853
VALTAm: akg_m + val__L_m <=> 3mob_m + glu__L_m 14610 or 14853

ILETAm: akg_m + ile__L_m <=> 3mop_m + glu__L_m 14610 or 14853
LEUTAm: akg_m + leu__L_m <=> 4mop_m + glu__L_m 14610 or 14853
VALTAm: akg_m + val__L_m <=> 3mob_m + glu__L_m 14610 or 14853

HCITSn: accoa_n + akg_n + h2o_n --> coa_n + h_n + hcit_n 14856
IPPS: 3mob_c + accoa_c + h2o_c --> 3c3hmp_c + coa_c + h_c 14856 or 15488

IPPS: 3mob_c + accoa_c + h2o_c --> 3c3hmp_c + coa_c + h_c 14856 or 15488
IPPSm: 3mob_m + accoa_m + h2o_m --> 3c3hmp_m + coa_m + h_m 15488

IPPMIa: 3c2hmp_c <=> 2ippm_c + h2o_c 14914
IPPMIb: 2ippm_c + h2o_c <=> 3c3hmp_c 14914

HICITDm: hicit_m + nad_m <=> h_m + nadh_m + oxag_m 10428
IPMD: 3c2hmp_c + nad_c --> 3c4mop_c + h_c + nadh_c 10428 or 13894
MALDDH: mal__D_c + nad_c --> co2_c + nadh_c + pyr_c 10428

IPMD: 3c2hmp_c + nad_c --> 3c4mop_c + h_c + nadh_c 10428 or 13894
```

In [79]:

```
# 9176 ILV5 mito, 14626/9689 ILV3 says cyto but ILV3 mito in S. cer
model.remove_reactions(['KARA1','KARA2','DHAD1','DHAD2'],remove_orphans=True)
# 14856 is for lysine and 15488 LEU4 in both cyto and mito in S. cer
model.reactions.get_by_id('IPPS').gene_reaction_rule = '15488'
# 10428 is for lysine, MALDDH in E. coli different gene
model.reactions.get_by_id('IPMD').gene_reaction_rule = '13894'
model.remove_reactions(['MALDDH'],remove_orphans=True)
```

phosphopantothenate biosynthesis  
2DHPFALDL: 2dhp\_c <=> 3mob\_c + fald\_c 8443  
MOHMT: 3mob\_c + h2o\_c + mlthf\_c --> 2dhp\_c + thf\_c 8443  
DPR: 2dhp\_c + h\_c + nadph\_c --> nadp\_c + pant**R\_c 14277 or 16522 or 9176  
PANTS: ala\_B\_c + atp\_c + pant**R\_c --> amp\_c + h\_c + pnto\_\_R\_c + ppi\_c 10475

MTMOHT: 3mob\_m + h2o\_m + mlthf\_m --> 2dhp\_m + thf\_m 8443  
DPRm: 2dhp\_m + h\_m + nadph\_m --> nadp\_m + pant\_\_R\_m 14277 or 9176

In [80]:

```
temp = ['8443','14277','16522','10475']
display(Annotation.loc[temp])
Show_Data(temp)
```

|  | Combined Annotations | Signal P | Sc288c Orthologs | Human Orthologs | Sc288 Best Hit | Human Blast | Essential | WolfPSort | C Terminal |
| --- | --- | --- | --- | --- | --- | --- | --- | --- | --- |
| RTO4\_ID |  |  |  |  |  |  |  |  |  |
| 8443 | K00606: panB; 3-methyl-2-oxobutanoate hydroxym... |  | ECM31 |  | ECM31 |  | Not Essential | cyto 18, mito 6, nucl 2 | REA\* |
| 14277 | K00077: panE, apbA; 2-dehydropantoate 2-reductase |  | YHR063C |  |  |  | Not Essential | cyto\_nucl 14, nucl 13, cyto 11 | SSR\* |
| 16522 | HMMPfam:Ketopantoate reductase PanE/ApbA:PF025... | S | YDL144C |  | YDL144C |  | Not Essential | extr 13, plas 5, cyto 4, E.R. 3 | KSA\* |
| 10475 | K01918: panC; pantoate--beta-alanine ligase |  | PAN6 |  | PAN6 |  | Not Essential | cyto 11.5, cyto\_mito 10.5, mito 8.5, nucl 4 | NPQ\* |

| strain | WT | | | | | | | | | | | | | | | | |
| --- | --- | --- | --- | --- | --- | --- | --- | --- | --- | --- | --- | --- | --- | --- | --- | --- | --- |
| condition | G\_MM | C\_MM | G\_SD | | GX\_SD | | | X\_SD | | A\_SD | | C\_SD | | MM\_CN120 | | MM\_CN5 | Diversity\_Sample |
| phase | exp | exp | exp | stat | exp | trans | stat | exp | stat | exp | stat | exp | stat | exp | stat | exp | exp |
| proteinId | Set1 | Set1 | Set2 | Set2 | Set2 | Set2 | Set2 | Set2 | Set2 | Set2 | Set2 | Set2 | Set2 | Set3 | Set3 | Set3 | Set3 |
| 8443 | 3.45967 | 4.80159 | 3.29678 | 3.08217 | 3.42933 | 3.46913 | 3.21668 | 4.13509 | 3.84075 | 3.77114 | 3.69081 | 3.64389 | 3.7098 | 3.02908 | 3.48832 | 4.58781 | 4.05644 |
| 14277 | 3.89017 | 4.31966 | 4.66867 | 4.79236 | 4.62198 | 5.16278 | 5.25471 | 4.84903 | 5.47629 | 4.84527 | 5.25706 | 5.03354 | 5.01387 | 5.11589 | 5.38363 | 5.3395 | 6.093 |
| 16522 | 6.89197 | 6.03441 | 6.89046 | 6.2812 | 6.94421 | 6.50629 | 6.46171 | 6.48772 | 6.26786 | 6.0824 | 6.0298 | 5.72289 | 5.20306 | 6.55196 | 6.29893 | 6.37711 | 6.50675 |
| 10475 | 3.26999 | 4.95918 | 4.17557 | 3.85013 | 4.24659 | 4.18121 | 4.42698 | 4.24126 | 4.70378 | 4.07398 | 4.28513 | 4.90202 | 4.86675 | 3.96173 | 4.18114 | 4.38108 | 5.26038 |

| strain | WT | | | | | | | | | | |
| --- | --- | --- | --- | --- | --- | --- | --- | --- | --- | --- | --- |
| condition | G\_SD | | GX\_SD | | | X\_SD | | A\_SD | | C\_SD | |
| proteinId | exp | stat | exp | trans | stat | exp | stat | exp | stat | exp | stat |
| 8443 | 0.754893 | 0.719399 | 0.606012 | 0.932688 | 0.922178 | 0.794704 | 0.976431 | 0.578829 | 0.378288 | 0.858728 | 1.30725 |
| 14277 | 0.201691 | 0.166667 | 0 | 0.370599 | 0.191624 | 0 | 0 | 0 | 0.189207 | 0 | 0 |
| 16522 | 8.08378 | 11.2184 | 9.56809 | 14.8792 | 12.8912 | 11.3635 | 9.08891 | 9.27211 | 8.62477 | 9.6385 | 7.41095 |
| 10475 | 4.37133 | 2.5324 | 2.44972 | 2.03257 | 2.56596 | 1.76092 | 1.96942 | 1.93855 | 2.10751 | 2.77763 | 2.37154 |

|  | Glucose | Xylose | Arabinose | Acetate | Coumarate | Ferulate | YNB Oleic Acid | YNB Ricinoleic Acid | YNB Glucose | YNB Gluc DOC | YPD |
| --- | --- | --- | --- | --- | --- | --- | --- | --- | --- | --- | --- |
| proteinId |  |  |  |  |  |  |  |  |  |  |  |
| 8443 | -0.0514645 | -0.0487158 | 0.0612808 | -0.113505 | -0.0411842 | -0.480022 | 0.28359 | 0.201074 | 0.460733 | 0.427401 | 0.0130797 |
| 16522 | -0.110156 | -0.221079 | 0.00235083 | -0.151444 | -0.380114 | -0.212064 | 0.137293 | -0.179819 | 0.0525662 | -0.117143 | 0.0325771 |
| 10475 | 0.480227 | 0.33277 | 0.617152 | 0.59239 | 0.159394 | 0.27627 | 0.026824 | -0.255013 | -0.188392 | -0.201929 | 0.0510207 |

In [81]:

```
for x in temp:
    if x in model.genes:
        for r in sorted(model.genes.get_by_id(x).reactions, key=lambda x: x.id):
            print(r, r.gene_reaction_rule)
    else:
        print(x, 'no reactions')
    print()
```

```
2DHPFALDL: 2dhp_c <=> 3mob_c + fald_c 8443
MOHMT: 3mob_c + h2o_c + mlthf_c --> 2dhp_c + thf_c 8443
MTMOHT: 3mob_m + h2o_m + mlthf_m --> 2dhp_m + thf_m 8443

DPR: 2dhp_c + h_c + nadph_c --> nadp_c + pant__R_c 14277 or 16522 or 9176
DPRm: 2dhp_m + h_m + nadph_m --> nadp_m + pant__R_m 14277 or 9176
PNORn: nadp_n + pant__R_n <=> 2dhp_n + h_n + nadph_n 14277

DPR: 2dhp_c + h_c + nadph_c --> nadp_c + pant__R_c 14277 or 16522 or 9176

PANTS: ala_B_c + atp_c + pant__R_c --> amp_c + h_c + pnto__R_c + ppi_c 10475
```

In [82]:

```
# 8443 cyto
# 14277 cyto and 16522 is ?, it is not known that 9716 ILV5 has DPR activity like ilvC 
model.reactions.get_by_id('DPR').gene_reaction_rule = '14277 or 16522'
model.remove_reactions(['MTMOHT','2DHPFALDL','DPRm','PNORn'],remove_orphans=True)
```

In [83]:

```
temp = ['15773','16581']
display(Annotation.loc[temp])
Show_Data(temp)
```

|  | Combined Annotations | Signal P | Sc288c Orthologs | Human Orthologs | Sc288 Best Hit | Human Blast | Essential | WolfPSort | C Terminal |
| --- | --- | --- | --- | --- | --- | --- | --- | --- | --- |
| RTO4\_ID |  |  |  |  |  |  |  |  |  |
| 15773 | KOG1589: Uncharacterized conserved protein |  | MPC3,MPC2 | MPC2 | MPC3 | MPC2 | Not Essential | mito 13.5, cyto\_mito 11.333, cyto 8, cyto\_nucl... | LAA\* |
| 16581 | KOG1590: Uncharacterized conserved protein |  | MPC1 | MPC1,MPC1L | MPC1 | MPC1 | Not Essential | mito 21, cyto 3, nucl 1, plas 1, pero 1 | ALK\* |

| strain | WT | | | | | | | | | | | | | | | | |
| --- | --- | --- | --- | --- | --- | --- | --- | --- | --- | --- | --- | --- | --- | --- | --- | --- | --- |
| condition | G\_MM | C\_MM | G\_SD | | GX\_SD | | | X\_SD | | A\_SD | | C\_SD | | MM\_CN120 | | MM\_CN5 | Diversity\_Sample |
| phase | exp | exp | exp | stat | exp | trans | stat | exp | stat | exp | stat | exp | stat | exp | stat | exp | exp |
| proteinId | Set1 | Set1 | Set2 | Set2 | Set2 | Set2 | Set2 | Set2 | Set2 | Set2 | Set2 | Set2 | Set2 | Set3 | Set3 | Set3 | Set3 |
| 15773 | 7.96284 | 6.88291 | 7.03641 | 6.02659 | 7.13969 | 6.40684 | 6.24606 | 6.97534 | 5.88906 | 6.69975 | 5.82715 | 6.35278 | 5.75793 | 8.42449 | 7.96252 | 8.59533 | 7.93077 |
| 16581 | 8.37542 | 7.26983 | 7.25141 | 7.01541 | 7.37745 | 6.98988 | 6.91277 | 7.33769 | 6.70705 | 7.25664 | 6.77322 | 6.40658 | 6.09586 | 6.93194 | 6.77741 | 7.30661 | 6.52741 |

| strain | WT | | | | | | | | | | |
| --- | --- | --- | --- | --- | --- | --- | --- | --- | --- | --- | --- |
| condition | G\_SD | | GX\_SD | | | X\_SD | | A\_SD | | C\_SD | |
| proteinId | exp | stat | exp | trans | stat | exp | stat | exp | stat | exp | stat |
| 15773 | 3.64579 | 3.05965 | 2.83822 | 2.77461 | 2.72296 | 2.13511 | 2.19568 | 2.70648 | 2.68834 | 1.93481 | 3.03755 |
| 16581 | 2.71165 | 0.877009 | 0.610101 | 1.4811 | 1.26709 | 0.776426 | 0.198706 | 0.959937 | 0.385057 | 0.441821 | 0.435902 |

|  | Glucose | Xylose | Arabinose | Acetate | Coumarate | Ferulate | YNB Oleic Acid | YNB Ricinoleic Acid | YNB Glucose | YNB Gluc DOC | YPD |
| --- | --- | --- | --- | --- | --- | --- | --- | --- | --- | --- | --- |
| proteinId |  |  |  |  |  |  |  |  |  |  |  |
| 16581 | -1.05737 | 1.06236 | -0.0930612 | -1.09979 | 0.0681052 | -0.245449 | 0.297283 | -2.5247 | 0.690153 | 0.325869 | 0.53652 |

In [84]:

```
r = sce.reactions.get_by_id('PYRt2m').copy()
r.gene_reaction_rule = '15773 and 16581'
model.add_reactions([r])

for x in ['LEUt5m','VALt5m','ILEtmi']:
    r = hsa.reactions.get_by_id(x).copy()
    r.gene_reaction_rule = ''
    model.add_reactions([r])

# Add keto acid transporters
# https://mmbr.asm.org/content/67/1/1
# proton symport reactions in RECON1/3D - generate proton gradient when transporting keto acids out
# makes it better to make amino acids in cyto, but mito BAT1 is for biosynthesis and cyto BAT2 is for degradation
# Mito carriers are usually exchange mechanism, but it is not known -> just add diffusion like transport
for x in ['3MOPtm','3MOBtm']:
    r = sce.reactions.get_by_id(x).copy()
    r.gene_reaction_rule = ''
    model.add_reactions([r])

r = model.reactions.get_by_id('3MOPtm').copy()
r.id = '4MOPtm'
r.name = '4-Methyl-2-oxopentanoate transport diffusion mitochondrial'
model.add_reactions([r])
r.add_metabolites({'3mop_c': 1.0, '3mop_m': -1.0, '4mop_c': -1.0, '4mop_m': 1.0})
```

In [85]:

```
sol = model.optimize()
print(sol.objective_value)

for k, v in sol.shadow_prices.items():
    if v and k in [m.id for m in model.reactions.get_by_id('BIOMASS_RT').metabolites]:
        print(k, v)
```

```
0.0
ser__L_c -0.8858729835316214
leu__L_c -0.8858729835316214
dtmp_c -0.8858729835316214
trp__L_c -0.8858729835316214
ile__L_c -0.8858729835316214
arg__L_c -0.8858729835316214
val__L_c -0.8858729835316214
ribflv_c 2.657618950594864
```

In [86]:

```
with model:
    r = model.reactions.get_by_id('EX_h_e').copy()
    r.id = 'EX_temp_e'
    model.add_reactions([r])
    r.add_metabolites({'h_e': 1.0, 'thr__L_m': -1.0})
    model.objective = 'EX_temp_e'
    sol = model.optimize()
    print(sol.objective_value)
    r.add_metabolites({'thr__L_m': 1.0, 'thr__L_c': -1.0})
    model.objective = 'EX_temp_e'
    sol = model.optimize()
    print(sol.objective_value)
```

```
0.0
14.999999999999996
```

In [87]:

```
# ile_L comes from thr_L
for r in sorted(model.metabolites.get_by_id('thr__L_c').reactions, key=lambda x: x.id):
    print(r, r.gene_reaction_rule)
print()
for r in sorted(model.metabolites.get_by_id('thr__L_m').reactions, key=lambda x: x.id):
    print(r, r.gene_reaction_rule)
```

```
BIOMASS_RT: 1.1348 13BDglcn_c + 0.4588 ala__L_c + 0.046 amp_c + 0.1607 arg__L_c + 0.1017 asn__L_c + 0.2975 asp__L_c + 59.276 atp_c + 0.0447 cmp_c + 0.0066 cys__L_c + 0.0036 damp_c + 0.0024 dcmp_c + 0.0024 dgmp_c + 0.0036 dtmp_c + 0.0007 ergst_r + 0.1054 gln__L_c + 0.3018 glu__L_c + 0.2904 gly_c + 0.5185 glycogen_c + 0.046 gmp_c + 59.276 h2o_c + 0.0663 his__L_c + 0.1927 ile__L_c + 0.2964 leu__L_c + 0.2862 lys__L_c + 0.8079 mannan_r + 0.0507 met__L_c + 6e-06 pa_RT_r + 6e-05 pc_RT_r + 4.5e-05 pe_RT_r + 0.1339 phe__L_c + 0.1647 pro__L_c + 1.7e-05 ps_RT_r + 5.3e-05 ptd1ino_RT_r + 0.00099 ribflv_c + 0.1854 ser__L_c + 0.02 so4_c + 0.1914 thr__L_c + 0.0234 tre_c + 6.6e-05 triglyc_RT_r + 0.0284 trp__L_c + 0.102 tyr__L_c + 0.0599 ump_c + 0.2646 val__L_c + 0.0015 zymst_r --> 59.276 adp_c + 58.70001 h_c + 59.305 pi_c 
THRA: thr__L_c --> acald_c + gly_c 16182
THRD_L: thr__L_c --> 2obut_c + nh4_c 11909 or 9216
THRS: h2o_c + phom_c --> pi_c + thr__L_c 9742
THRTRS: atp_c + thr__L_c + trnathr_c --> amp_c + ppi_c + thrtrna_c 15848
THRt2r: h_e + thr__L_e <=> h_c + thr__L_c 14229 or 15074

THRD_Lm: thr__L_m --> 2obut_m + nh4_m 11909
THRTRSm: atp_m + thr__L_m + trnathr_m --> amp_m + ppi_m + thrtrna_m 15848
```

In [88]:

```
temp = ['9742','11909','9216','11849']
display(Annotation.loc[temp])
Show_Data(temp)
```

|  | Combined Annotations | Signal P | Sc288c Orthologs | Human Orthologs | Sc288 Best Hit | Human Blast | Essential | WolfPSort | C Terminal |
| --- | --- | --- | --- | --- | --- | --- | --- | --- | --- |
| RTO4\_ID |  |  |  |  |  |  |  |  |  |
| 9742 | K01733: thrC; threonine synthase |  | THR4 | THNSL2 | THR4 | THNSL | Essential | cyto 18, cyto\_nucl 12.5, nucl 5, pero 2, cysk 2 | SGL\* |
| 11909 | K01754: E4.3.1.19, ilvA, tdcB; threonine dehyd... |  | ILV1 |  | ILV1 | SRR | Not Essential | cyto 13.5, cyto\_nucl 9.5, mito 7, nucl 4.5 | DAE\* |
| 9216 | K17989: SDS, SDH, CHA1; L-serine/L-threonine a... |  | CHA1,YIL167W | SDS,SDSL | SDL1 | SDS | Not Essential | mito 12.5, cyto\_mito 12.5, cyto 11.5 | VEA\* |
| 11849 | K01752: E4.3.1.17, sdaA, sdaB, tdcG; L-serine ... |  |  |  |  |  | Not Essential | mito 26.5, cyto\_mito 14 | PAC\* |

| strain | WT | | | | | | | | | | | | | | | | |
| --- | --- | --- | --- | --- | --- | --- | --- | --- | --- | --- | --- | --- | --- | --- | --- | --- | --- |
| condition | G\_MM | C\_MM | G\_SD | | GX\_SD | | | X\_SD | | A\_SD | | C\_SD | | MM\_CN120 | | MM\_CN5 | Diversity\_Sample |
| phase | exp | exp | exp | stat | exp | trans | stat | exp | stat | exp | stat | exp | stat | exp | stat | exp | exp |
| proteinId | Set1 | Set1 | Set2 | Set2 | Set2 | Set2 | Set2 | Set2 | Set2 | Set2 | Set2 | Set2 | Set2 | Set3 | Set3 | Set3 | Set3 |
| 9742 | 7.90983 | 7.1252 | 7.96339 | 6.72313 | 8.27653 | 6.75093 | 6.88205 | 7.66045 | 7.23251 | 7.19305 | 7.29636 | 7.39503 | 8.0496 | 9.12655 | 8.75224 | 10.1053 | 8.86808 |
| 11909 | 5.31118 | 5.08388 | 6.17871 | 5.29515 | 6.36711 | 5.61243 | 5.80521 | 5.3497 | 5.16685 | 4.73669 | 5.16398 | 5.89176 | 6.40268 | 5.34339 | 5.00204 | 6.72593 | 6.03529 |
| 9216 | 5.15958 | 5.09801 | 5.24913 | 3.90544 | 6.00156 | 3.95406 | 3.34307 | 4.58521 | 3.68645 | 4.42569 | 3.59233 | 3.88971 | 6.03636 | 3.28899 | 3.26756 | 7.08022 | 5.18274 |
| 11849 | 3.90525 | 4.34293 | 3.29415 | 3.80516 | 3.20581 | 3.82732 | 4.10255 | 3.82424 | 5.09652 | 3.60623 | 4.82892 | 4.81815 | 4.82469 | 5.00494 | 4.53925 | 4.21855 | 4.03297 |

| strain | WT | | | | | | | | | | |
| --- | --- | --- | --- | --- | --- | --- | --- | --- | --- | --- | --- |
| condition | G\_SD | | GX\_SD | | | X\_SD | | A\_SD | | C\_SD | |
| proteinId | exp | stat | exp | trans | stat | exp | stat | exp | stat | exp | stat |
| 9742 | 24.3253 | 19.3143 | 23.9989 | 16.1646 | 17.1198 | 17.2166 | 15.5564 | 18.3476 | 14.1536 | 22.0805 | 23.0151 |
| 11909 | 10.9387 | 7.74686 | 10.1522 | 5.00197 | 6.37603 | 6.8153 | 3.75932 | 3.85933 | 2.86536 | 5.12255 | 6.30634 |
| 9216 | 1.51882 | 0.738863 | 2.85378 | 0.929251 | 1.09591 | 1.56446 | 1.38682 | 1.74011 | 1.91303 | 2.15064 | 3.26462 |
| 11849 | 0.188629 | 0 | 0 | 0 | 0.179978 | 0.773955 | 0.41039 | 0.965803 | 1.338 | 0.854055 | 1.30353 |

|  | Glucose | Xylose | Arabinose | Acetate | Coumarate | Ferulate | YNB Oleic Acid | YNB Ricinoleic Acid | YNB Glucose | YNB Gluc DOC | YPD |
| --- | --- | --- | --- | --- | --- | --- | --- | --- | --- | --- | --- |
| proteinId |  |  |  |  |  |  |  |  |  |  |  |
| 11909 | -0.644152 | 0.0708412 | -0.968321 | 0.291933 | -0.126688 | 0.609645 | -0.56859 | -0.287953 | -0.564922 | -0.208981 | -0.120712 |
| 9216 | -0.0217597 | -0.217502 | 0.0258124 | 0.0953588 | -0.281999 | -0.329755 | 0.728678 | -0.168594 | 0.245015 | 0.086814 | -0.14243 |
| 11849 | 0.0856094 | 0.0176457 | 0.0441559 | -0.0559005 | -0.105301 | 0.0302815 | 0.0638251 | -0.342133 | 0.150496 | -0.00587411 | 0.0607802 |

In [89]:

```
for x in temp:
    if x in model.genes:
        for r in sorted(model.genes.get_by_id(x).reactions, key=lambda x: x.id):
            print(r, r.gene_reaction_rule)
    else:
        print(x, 'no reactions')
    print()
```

```
THRS: h2o_c + phom_c --> pi_c + thr__L_c 9742

SERD_L: ser__L_c --> nh4_c + pyr_c 11849 or 11909 or 9216
SER_AL: ser__L_c <=> h_c + nh3_c + pyr_c 11909
THRD_L: thr__L_c --> 2obut_c + nh4_c 11909 or 9216
THRD_Lm: thr__L_m --> 2obut_m + nh4_m 11909

SERD_L: ser__L_c --> nh4_c + pyr_c 11849 or 11909 or 9216
SERHL: ser__L_c --> 2amac_c + h2o_c 9216
THRD_L: thr__L_c --> 2obut_c + nh4_c 11909 or 9216

SERD_L: ser__L_c --> nh4_c + pyr_c 11849 or 11909 or 9216
```

In [90]:

```
# 11909 mito thr
# 9216 cyto CHA1/SDS? ser and thr deaminase, also SERHL and 2AMACHYD
# 11849 mito? cyto? bacterial SDH
model.reactions.get_by_id('SERD_L').gene_reaction_rule = '11849 or 9216'
model.reactions.get_by_id('THRD_L').gene_reaction_rule = '9216'
r = hsa2.reactions.get_by_id('2AMACHYD').copy()
r.gene_reaction_rule = '9216'
model.add_reactions([r])
model.remove_reactions(['SER_AL'],remove_orphans=True)

# Threonine is synthesized in cyto
r = hsa2.reactions.get_by_id('thr_mt').copy()
r.id = 'THRtm'
r.lower_bound = -1000.0
model.add_reactions([r])

# cyto 2obut needs to be either transported out or to mito (known to happen)
r = sce.reactions.get_by_id('2OBUTtm').copy()
r.gene_reaction_rule = ''
model.add_reactions([r])
r = hsa2.reactions.get_by_id('2OBUTt').copy()
r.gene_reaction_rule = '11257'
model.add_reactions([r])
r = hsa2.reactions.get_by_id('EX_2obut_e').copy()
r.lower_bound = 0.0
model.add_reactions([r])

# Need to get rid of nh4_m from THRD_Lm somehow
r = sce.reactions.get_by_id('NH4tm').copy()
# find transporter gene
#r.gene_reaction_rule = ''
model.add_reactions([r])
```

In [91]:

```
with model:
    r = model.reactions.get_by_id('EX_h_e').copy()
    r.id = 'EX_temp_e'
    model.add_reactions([r])
    r.add_metabolites({'h_e': 1.0, '2ahbut_m': -1.0})
    model.objective = 'EX_temp_e'
    sol = model.optimize()
    print(sol.objective_value)
    r.add_metabolites({'2ahbut_m': 1.0, '23dhmp_m': -1.0})
    model.objective = 'EX_temp_e'
    sol = model.optimize()
    print(sol.objective_value)
```

```
9.999999999999996
0.0
```

Cannot make nadph\_m, need to find a source  
In S. cer, the NADH kinase encoded by the POS5 gene is considered to be the main provider of NADPH in the mitochondrion by phosphorylation of NADH. POS5 also works as NAD kinase, this reaction followed by the dehydrogenase reaction of ALD4/ALD5 is the other source for the synthesis of NADPH. http://www.jbc.org/content/284/12/7553.full  
There is no POS5 in R. toruloides, but cyto NAD+ kinase YEF1/UTR1 10182 exists

In [92]:

```
for r in sorted(model.metabolites.get_by_id('nadp_m').reactions, key=lambda x: x.id):
    if 'nadph_m' in [m.id for m in r.products]:
        print(r, r.gene_reaction_rule)
    if 'nadph_m' in [m.id for m in r.reactants] and r.reversibility:
        print(r, r.gene_reaction_rule)
```

```
ABOR: 4abutn_m + h2o_m + nadp_m --> 4abut_m + 2.0 h_m + nadph_m 12042 or 13426
ALDD20ym: h2o_m + id3acald_m + nadp_m --> 2.0 h_m + ind3ac_m + nadph_m 12042 or 13426
ALDD2ym: acald_m + h2o_m + nadp_m --> ac_m + 2.0 h_m + nadph_m 12042 or 13426
MTHFDm: mlthf_m + nadp_m <=> methf_m + nadph_m 15366
SACCD4m: h2o_m + nadp_m + saccrp__L_m --> L2aadp6sa_m + glu__L_m + h_m + nadph_m 10577 or 16833
```

In [93]:

```
for r in sorted(model.genes.get_by_id('10182').reactions, key=lambda x: x.id):
    print(r, r.gene_reaction_rule)
```

```
NADK: atp_c + nad_c --> adp_c + h_c + nadp_c 10182
NADKm: atp_m + nad_m --> adp_m + h_m + nadp_m 10182
```

In [94]:

```
model.remove_reactions(['NADKm'], remove_orphans=True)
```

In [95]:

```
for r in sorted(model.genes.get_by_id('15814').reactions, key=lambda x: x.id):
    print(r, r.gene_reaction_rule)
```

```
5HOXINDACTOX: 5hoxindact_c + h2o_c + nad_c --> 5hoxindoa_c + 2.0 h_c + nadh_c 12042 or 13426 or 15814 or 16323
AASAD3: L2aadp6sa_c + h2o_c + nad_c --> L2aadp_c + 2.0 h_c + nadh_c 15814
ALDD20x: h2o_c + id3acald_c + nad_c --> 2.0 h_c + ind3ac_c + nadh_c 12042 or 13426 or 15814 or 16323
ALDD2x: acald_c + h2o_c + nad_c --> ac_c + 2.0 h_c + nadh_c 12042 or 13426 or 15814 or 16323
BAMPPALDOX: bamppald_c + h2o_c + nad_c --> ala_B_c + 2.0 h_c + nadh_c 12042 or 13426 or 15814 or 16323
GCALDD: gcald_c + h2o_c + nad_c --> glyclt_c + 2.0 h_c + nadh_c 12042 or 13426 or 15814 or 16323
IMACTD: h2o_c + im4act_c + nad_c --> 2.0 h_c + im4ac_c + nadh_c 12042 or 13426 or 15814 or 16323
LCADi: h2o_c + lald__L_c + nad_c --> 2.0 h_c + lac__L_c + nadh_c 12042 or 13426 or 15814 or 16323
LCADi_D: h2o_c + lald__D_c + nad_c --> 2.0 h_c + lac__D_c + nadh_c 12042 or 13426 or 15814 or 16323
NABTNO: h2o_c + n4abutn_c + nad_c --> 4aabutn_c + 2.0 h_c + nadh_c 12042 or 13426 or 15814 or 16323
PYLALDOX: h2o_c + nad_c + pylald_c --> 2.0 h_c + nadh_c + peracd_c 12042 or 13426 or 15814 or 16323
```

In [96]:

```
for r in sorted(model.metabolites.get_by_id('acald_c').reactions, key=lambda x: x.id):
    print(r, r.gene_reaction_rule)
print()
for r in sorted(model.metabolites.get_by_id('acald_m').reactions, key=lambda x: x.id):
    print(r, r.gene_reaction_rule)
```

```
ACALDCD: 2.0 acald_c --> actn__R_c 15791
ALCD2x: etoh_c + nad_c <=> acald_c + h_c + nadh_c 15438
ALCD2y: etoh_c + nadp_c --> acald_c + h_c + nadph_c 11882 or 12784 or 9774
ALDD2x: acald_c + h2o_c + nad_c --> ac_c + 2.0 h_c + nadh_c 12042 or 13426 or 15814 or 16323
ALDD2y: acald_c + h2o_c + nadp_c --> ac_c + 2.0 h_c + nadph_c 11650 or 12042 or 13426 or 14700 or 16323 or 8666
PYRDC: h_c + pyr_c --> acald_c + co2_c 15791
THRA: thr__L_c --> acald_c + gly_c 16182
THRA2: athr__L_c --> acald_c + gly_c 16182

ALDD2xm: acald_m + h2o_m + nad_m --> ac_m + 2.0 h_m + nadh_m 12042 or 13426 or 16323
ALDD2ym: acald_m + h2o_m + nadp_m --> ac_m + 2.0 h_m + nadph_m 12042 or 13426
```

In [97]:

```
temp = ['12042','13426','16323']
display(Annotation.loc[temp])
Show_Data(temp)
```

|  | Combined Annotations | Signal P | Sc288c Orthologs | Human Orthologs | Sc288 Best Hit | Human Blast | Essential | WolfPSort | C Terminal |
| --- | --- | --- | --- | --- | --- | --- | --- | --- | --- |
| RTO4\_ID |  |  |  |  |  |  |  |  |  |
| 12042 | K00128: ALDH; aldehyde dehydrogenase (NAD+) |  | ALD5,ALD4,ALD6,ALD3,ALD2 | ALDH1A1,ALDH1A2,ALDH1A3,ALDH1B1,ALDH2 | ALD5 | ALDH2 | Not Essential | cyto 18.5, cyto\_nucl 10, mito 4, pero 4 | NPL\* |
| 13426 | K07249: E1.2.1.36; retinal dehydrogenase |  | ALD5,ALD4,ALD6,ALD3,ALD2 | ALDH1A1,ALDH1A2,ALDH1A3,ALDH1B1,ALDH2 | ALD5 | ALDH1 | Not Essential | mito 25.5, cyto\_mito 14 | WPL\* |
| 16323 | K00128: ALDH; aldehyde dehydrogenase (NAD+) |  | HFD1 | ALDH3A1,ALDH3A2,ALDH3B1,ALDH3B2 | HFD1 | ALDH3 | Not Essential | cyto 10.5, plas 7, cyto\_nucl 7, mito 3, nucl 2... | GQA\* |

| strain | WT | | | | | | | | | | | | | | | | |
| --- | --- | --- | --- | --- | --- | --- | --- | --- | --- | --- | --- | --- | --- | --- | --- | --- | --- |
| condition | G\_MM | C\_MM | G\_SD | | GX\_SD | | | X\_SD | | A\_SD | | C\_SD | | MM\_CN120 | | MM\_CN5 | Diversity\_Sample |
| phase | exp | exp | exp | stat | exp | trans | stat | exp | stat | exp | stat | exp | stat | exp | stat | exp | exp |
| proteinId | Set1 | Set1 | Set2 | Set2 | Set2 | Set2 | Set2 | Set2 | Set2 | Set2 | Set2 | Set2 | Set2 | Set3 | Set3 | Set3 | Set3 |
| 12042 | 8.00849 | 8.563 | 8.41698 | 9.34145 | 8.34137 | 8.32176 | 8.06734 | 8.44974 | 8.36047 | 8.84866 | 8.59447 | 7.17248 | 5.67475 | 7.81326 | 7.51984 | 8.29774 | 8.31731 |
| 13426 | 7.30781 | 4.89844 | 3.84807 | 2.79984 | 3.78882 | 3.98344 | 3.73207 | 4.58197 | 3.36082 | 4.5625 | 3.35098 | 4.25235 | 3.1633 | 6.93547 | 6.81825 | 3.29168 | 5.38811 |
| 16323 | 5.54194 | 5.5053 | 6.37226 | 5.68644 | 6.28507 | 6.1392 | 5.86895 | 6.06104 | 5.82576 | 5.50985 | 6.17279 | 4.76433 | 4.74944 | 5.59107 | 6.40434 | 5.99953 | 6.38502 |

| strain | WT | | | | | | | | | | |
| --- | --- | --- | --- | --- | --- | --- | --- | --- | --- | --- | --- |
| condition | G\_SD | | GX\_SD | | | X\_SD | | A\_SD | | C\_SD | |
| proteinId | exp | stat | exp | trans | stat | exp | stat | exp | stat | exp | stat |
| 12042 | 33.9947 | 44.5614 | 32.5616 | 41.5121 | 40.2993 | 56.5808 | 63.4798 | 61.9973 | 72.5595 | 57.7357 | 40.7488 |
| 13426 | 0.757094 | 0.533653 | 1.41886 | 0.929251 | 0.72686 | 2.14514 | 0.960201 | 3.46858 | 3.64334 | 1.06907 | 0.864976 |
| 16323 | 10.9902 | 12.5683 | 13.6351 | 14.1342 | 18.2252 | 11.3209 | 13.9247 | 9.27817 | 10.1675 | 6.60796 | 3.93398 |

|  | Glucose | Xylose | Arabinose | Acetate | Coumarate | Ferulate | YNB Oleic Acid | YNB Ricinoleic Acid | YNB Glucose | YNB Gluc DOC | YPD |
| --- | --- | --- | --- | --- | --- | --- | --- | --- | --- | --- | --- |
| proteinId |  |  |  |  |  |  |  |  |  |  |  |
| 12042 | 0.170666 | -0.143043 | 0.000953096 | -0.0230376 | -0.521499 | 0.0146534 | 0.144875 | -0.295284 | 0.392859 | 0.454577 | -0.242457 |
| 13426 | -0.226122 | -0.181863 | -0.169891 | -0.0897023 | -0.0133507 | -0.00809092 | -1.1502 | -2.84044 | -0.85113 | -0.458514 | -0.0243377 |
| 16323 | 0.000210473 | -0.0174318 | 0.0140589 | -0.110443 | 0.00440814 | 0.0892768 | 0.155043 | 0.103889 | -0.177274 | -0.243731 | -0.140368 |

In [98]:

```
temp = ['11650','14700','8666']
display(Annotation.loc[temp])
Show_Data(temp)
```

|  | Combined Annotations | Signal P | Sc288c Orthologs | Human Orthologs | Sc288 Best Hit | Human Blast | Essential | WolfPSort | C Terminal |
| --- | --- | --- | --- | --- | --- | --- | --- | --- | --- |
| RTO4\_ID |  |  |  |  |  |  |  |  |  |
| 11650 | KOG0023: Alcohol dehydrogenase, class V |  |  |  | ADH2 | ADH1B | Not Essential | cyto 9.5, extr 8, mito 6, cyto\_nucl 6, nucl 1.5 | VIP\* |
| 14700 | KOG0725: Reductases with broad range of substr... |  |  | HSD17B14 | YMR226C | DECR2 | Not Essential | cyto 14.5, cyto\_nucl 11.5, nucl 5.5, pero 3, c... | YLV\* |
| 8666 | KOG0725: Reductases with broad range of substr... |  |  | HSD17B14 | SPS19 | HSD17B | Not Essential | cyto 18.5, cyto\_nucl 12, nucl 2.5, mito 2, ext... | YVH\* |

| strain | WT | | | | | | | | | | | | | | | | |
| --- | --- | --- | --- | --- | --- | --- | --- | --- | --- | --- | --- | --- | --- | --- | --- | --- | --- |
| condition | G\_MM | C\_MM | G\_SD | | GX\_SD | | | X\_SD | | A\_SD | | C\_SD | | MM\_CN120 | | MM\_CN5 | Diversity\_Sample |
| phase | exp | exp | exp | stat | exp | trans | stat | exp | stat | exp | stat | exp | stat | exp | stat | exp | exp |
| proteinId | Set1 | Set1 | Set2 | Set2 | Set2 | Set2 | Set2 | Set2 | Set2 | Set2 | Set2 | Set2 | Set2 | Set3 | Set3 | Set3 | Set3 |
| 11650 | 1.37514 | 2.41086 | 1.41178 | 3.1204 | 1.56558 | 2.07352 | 2.58627 | 1.40096 | 2.16051 | 1.2817 | 2.88878 | 2.57003 | 1.84991 | 2.31936 | 1.83456 | 1.11962 | 2.89719 |
| 14700 | 3.68256 | 4.08361 | 4.44055 | 3.4422 | 4.80507 | 1.56428 | 1.06717 | 2.03973 | 4.6801 | 2.95861 | 4.34493 | 5.79966 | 5.57785 | 1.47714 | 0.854975 | 4.55847 | 3.8255 |
| 8666 | 0.116626 | 1.55463 | 0.234326 | 0.25307 | 0.288409 | 0.196665 | 0.0489713 | 0.109595 | 1.37869 | 0.101187 | 0.403123 | 3.31484 | 3.55422 | 0 | 0.0910628 | 1.15274 | 0.196585 |

| strain | WT | | | | | | | | | | |
| --- | --- | --- | --- | --- | --- | --- | --- | --- | --- | --- | --- |
| condition | G\_SD | | GX\_SD | | | X\_SD | | A\_SD | | C\_SD | |
| proteinId | exp | stat | exp | trans | stat | exp | stat | exp | stat | exp | stat |
| 14700 | 0.391889 | 0.540259 | 1.01019 | 0 | 0 | 0 | 1.16487 | 1.15964 | 1.34502 | 13.0174 | 12.8551 |

|  | Glucose | Xylose | Arabinose | Acetate | Coumarate | Ferulate | YNB Oleic Acid | YNB Ricinoleic Acid | YNB Glucose | YNB Gluc DOC | YPD |
| --- | --- | --- | --- | --- | --- | --- | --- | --- | --- | --- | --- |
| proteinId |  |  |  |  |  |  |  |  |  |  |  |
| 11650 | 0.162296 | -0.125414 | 0.239373 | 0.0824604 | 0.133479 | 0.152742 | 0.151596 | -0.00688522 | -0.0872696 | -0.226133 | 0.148191 |
| 14700 | -0.136366 | 0.186102 | -0.178815 | -0.232998 | -0.0364499 | -0.0311361 | 0.24188 | -0.184481 | -0.138316 | 0.201217 | 0.511467 |
| 8666 | 0.246518 | -0.0531704 | 0.304517 | -0.024053 | -0.100515 | -0.173468 | -0.0249248 | -0.343657 | 0.409518 | 0.34975 | -0.0161461 |

In [99]:

```
with model:
    r = sce.reactions.get_by_id('ACALDtm').copy()
    model.add_reactions([r])
    sol = model.optimize()
    print(sol.objective_value)
    
    for k, v in sol.shadow_prices.items():
        if v and k in [m.id for m in model.reactions.get_by_id('BIOMASS_RT').metabolites]:
            print(k, v)
```

```
0.0
pe_RT_r 124.47099825740602
mannan_r -1.2447099825740602
```

In [100]:

```
r = sce.reactions.get_by_id('ACALDtm').copy()
model.add_reactions([r])
```

In [101]:

```
for r in sorted(model.metabolites.get_by_id('saccrp__L_m').reactions, key=lambda x: x.id):
    print(r, r.gene_reaction_rule)
print()
for r in sorted(model.metabolites.get_by_id('L2aadp6sa_m').reactions, key=lambda x: x.id):
    print(r, r.gene_reaction_rule)
print()
for r in sorted(model.metabolites.get_by_id('L2aadp_m').reactions, key=lambda x: x.id):
    print(r, r.gene_reaction_rule)
```

```
SACCD3m: akg_m + h_m + lys__L_m + nadph_m --> h2o_m + nadp_m + saccrp__L_m 10577 or 16833
SACCD4m: h2o_m + nadp_m + saccrp__L_m --> L2aadp6sa_m + glu__L_m + h_m + nadph_m 10577 or 16833

SACCD4m: h2o_m + nadp_m + saccrp__L_m --> L2aadp6sa_m + glu__L_m + h_m + nadph_m 10577 or 16833

2AMADPTm: L2aadp_c + akg_m <=> L2aadp_m + akg_c 10635
```

In [102]:

```
temp = ['10577','16833','14087','9495','10220']
display(Annotation.loc[temp])
Show_Data(temp)
```

|  | Combined Annotations | Signal P | Sc288c Orthologs | Human Orthologs | Sc288 Best Hit | Human Blast | Essential | WolfPSort | C Terminal |
| --- | --- | --- | --- | --- | --- | --- | --- | --- | --- |
| RTO4\_ID |  |  |  |  |  |  |  |  |  |
| 10577 | K14157: AASS; alpha-aminoadipic semialdehyde s... |  | SPE3,LYS9 | SRM,AASS | LYS9 | AASS | Not Essential | mito 21, cyto 3, pero 2 | ARF\* |
| 16833 | K00293: LYS9; saccharopine dehydrogenase (NADP... |  | SPE3,LYS9 | SRM,AASS | LYS9 | SRMe | Essential | cyto 19, cysk 6 | KVQ\* |
| 14087 | K00290: LYS1; saccharopine dehydrogenase (NAD+... |  | LYS1 |  | LYS1 |  | Not Essential | cyto 16.5, cyto\_nucl 10, pero 5, nucl 2.5 | KKQ\* |
| 9495 | K00143: LYS2; L-aminoadipate-semialdehyde dehy... |  | LYS2 |  | LYS2 | AASDH | Not Essential | mito 9, cyto 8.5, cyto\_nucl 5.5, extr 5, pero 2 | GVV\* |
| 10220 | K06133: LYS5, acpT; 4'-phosphopantetheinyl tra... |  |  | AASDHPPT |  |  | Not Essential | nucl 12, cyto\_nucl 10, mito 6, cyto 6 | AVD\* |

| strain | WT | | | | | | | | | | | | | | | | |
| --- | --- | --- | --- | --- | --- | --- | --- | --- | --- | --- | --- | --- | --- | --- | --- | --- | --- |
| condition | G\_MM | C\_MM | G\_SD | | GX\_SD | | | X\_SD | | A\_SD | | C\_SD | | MM\_CN120 | | MM\_CN5 | Diversity\_Sample |
| phase | exp | exp | exp | stat | exp | trans | stat | exp | stat | exp | stat | exp | stat | exp | stat | exp | exp |
| proteinId | Set1 | Set1 | Set2 | Set2 | Set2 | Set2 | Set2 | Set2 | Set2 | Set2 | Set2 | Set2 | Set2 | Set3 | Set3 | Set3 | Set3 |
| 10577 | 6.93825 | 6.51413 | 4.93966 | 6.72379 | 4.72929 | 4.32338 | 4.9139 | 3.42089 | 5.96694 | 3.35347 | 6.55975 | 5.82237 | 5.1241 | 7.06085 | 6.1541 | 4.83314 | 6.54823 |
| 16833 | 4.23834 | 6.81843 | 5.63912 | 4.87843 | 5.60182 | 6.13682 | 5.77789 | 6.51094 | 5.17185 | 5.79962 | 4.61771 | 6.47684 | 6.18936 | 6.01351 | 6.43293 | 7.32623 | 8.13086 |
| 14087 | 6.49169 | 7.66131 | 7.21483 | 6.34303 | 7.54094 | 6.05278 | 6.32643 | 6.4245 | 6.09519 | 5.91083 | 5.79607 | 8.23445 | 9.00442 | 6.38311 | 6.71187 | 8.39366 | 7.75438 |
| 9495 | 4.99092 | 5.42381 | 6.35839 | 4.54241 | 6.61367 | 5.24037 | 5.18124 | 5.62019 | 5.25199 | 5.13713 | 4.78198 | 6.88201 | 7.4782 | 5.72535 | 5.58315 | 7.96304 | 6.9019 |
| 10220 | 6.08332 | 5.58961 | 5.30673 | 5.38475 | 5.17929 | 5.58642 | 5.41775 | 5.47459 | 4.52614 | 5.73158 | 5.409 | 4.07171 | 3.79137 | 5.48093 | 5.412 | 5.2834 | 5.17945 |

| strain | WT | | | | | | | | | | |
| --- | --- | --- | --- | --- | --- | --- | --- | --- | --- | --- | --- |
| condition | G\_SD | | GX\_SD | | | X\_SD | | A\_SD | | C\_SD | |
| proteinId | exp | stat | exp | trans | stat | exp | stat | exp | stat | exp | stat |
| 10577 | 4.60528 | 14.6285 | 3.6463 | 7.44721 | 8.77835 | 11.1558 | 15.7276 | 16.8009 | 15.8869 | 30.3513 | 25.0464 |
| 16833 | 43.1584 | 35.8344 | 50.7022 | 37.4319 | 37.2344 | 42.2953 | 41.4076 | 38.8457 | 40.0537 | 51.2791 | 53.2254 |
| 14087 | 14.0501 | 10.0588 | 15.8828 | 5.03914 | 9.15155 | 9.39594 | 8.41597 | 9.66871 | 10.748 | 18.1339 | 24.6929 |
| 9495 | 38.2663 | 25.8364 | 44.5155 | 16.7301 | 16.7625 | 30.7141 | 24.8014 | 34.5918 | 29.3337 | 28.8223 | 41.3364 |

|  | Glucose | Xylose | Arabinose | Acetate | Coumarate | Ferulate | YNB Oleic Acid | YNB Ricinoleic Acid | YNB Glucose | YNB Gluc DOC | YPD |
| --- | --- | --- | --- | --- | --- | --- | --- | --- | --- | --- | --- |
| proteinId |  |  |  |  |  |  |  |  |  |  |  |
| 10577 | -0.0823193 | -0.0536589 | -0.172111 | -0.277613 | -0.0621896 | -0.131564 | -0.239781 | -0.0105686 | 0.20184 | 0.345546 | 0.0251315 |
| 9495 | -0.852305 | -0.88796 | -1.33062 | -0.950872 | -1.39312 | -1.26513 | -1.29749 | -1.72025 | -1.59537 | -1.27751 | 0.0278676 |

In [103]:

```
for x in temp:
    if x in model.genes:
        for r in sorted(model.genes.get_by_id(x).reactions, key=lambda x: x.id):
            print(r, r.gene_reaction_rule)
    else:
        print(x, 'no reactions')
    print()
```

```
SACCD1: L2aadp6sa_c + glu__L_c + h_c + nadph_c <=> h2o_c + nadp_c + saccrp__L_c 10577 or 16833
SACCD3m: akg_m + h_m + lys__L_m + nadph_m --> h2o_m + nadp_m + saccrp__L_m 10577 or 16833
SACCD4m: h2o_m + nadp_m + saccrp__L_m --> L2aadp6sa_m + glu__L_m + h_m + nadph_m 10577 or 16833
SPMS: ametam_c + ptrc_c --> 5mta_c + h_c + spmd_c 10577 or 16833
SPRMS: ametam_c + spmd_c --> 5mta_c + h_c + sprm_c 10577 or 16833

SACCD1: L2aadp6sa_c + glu__L_c + h_c + nadph_c <=> h2o_c + nadp_c + saccrp__L_c 10577 or 16833
SACCD3m: akg_m + h_m + lys__L_m + nadph_m --> h2o_m + nadp_m + saccrp__L_m 10577 or 16833
SACCD4m: h2o_m + nadp_m + saccrp__L_m --> L2aadp6sa_m + glu__L_m + h_m + nadph_m 10577 or 16833
SPMS: ametam_c + ptrc_c --> 5mta_c + h_c + spmd_c 10577 or 16833
SPRMS: ametam_c + spmd_c --> 5mta_c + h_c + sprm_c 10577 or 16833

SACCD2: h2o_c + nad_c + saccrp__L_c <=> akg_c + h_c + lys__L_c + nadh_c 14087

AASAD1: L2aadp_c + atp_c + h_c + nadph_c --> L2aadp6sa_c + amp_c + nadp_c + ppi_c 9495 or (10220 and 9495)
AASAD2: L2aadp_c + atp_c + h_c + nadh_c --> L2aadp6sa_c + amp_c + nad_c + ppi_c 9495 or (10220 and 9495)

AASAD1: L2aadp_c + atp_c + h_c + nadph_c --> L2aadp6sa_c + amp_c + nadp_c + ppi_c 9495 or (10220 and 9495)
AASAD2: L2aadp_c + atp_c + h_c + nadh_c --> L2aadp6sa_c + amp_c + nad_c + ppi_c 9495 or (10220 and 9495)
```

In [104]:

```
# Lysine biosynthesis
temp = ['14856','14600','16016','10428','14908','15839','9495','10220','10577','16833','14087','15814']
display(Annotation.loc[temp])
Show_Data(temp)
```

|  | Combined Annotations | Signal P | Sc288c Orthologs | Human Orthologs | Sc288 Best Hit | Human Blast | Essential | WolfPSort | C Terminal |
| --- | --- | --- | --- | --- | --- | --- | --- | --- | --- |
| RTO4\_ID |  |  |  |  |  |  |  |  |  |
| 14856 | K01655: LYS21, LYS20; homocitrate synthase |  | LYS21,LYS20 |  | LYS20 |  | Not Essential | cyto 17.5, cyto\_nucl 15.5, nucl 8.5 | TSA\* |
| 14600 | K17450: ACO2; homoaconitase |  | ACO2,ACO1 | ACO1,IREB2,ACO2 | ACO2 | ACO2 | Not Essential | mito 20, cyto 4.5, cyto\_nucl 3.5 | GQA\* |
| 16016 | K01705: LYS4; homoaconitate hydratase |  | LYS4 |  | LYS4 | ACO2 | Not Essential | mito 23, cyto 4 | ISA\* |
| 10428 | K05824: LYS12; homoisocitrate dehydrogenase |  | LYS12 |  | LYS12 | IDH3A | Not Essential | mito 15, cyto 12 | KQL\* |
| 14908 | K00838: ARO8; aromatic amino acid aminotransfe... |  | ARO8 | AADAT | ARO8 | AADAT | Not Essential | cyto 7, cysk 7, mito 5, cyto\_nucl 5, pero 4, m... | WAY\* |
| 15839 | K00838: ARO8; aromatic amino acid aminotransfe... |  | ARO8 | AADAT | ARO8 | AADAT | Not Essential | cysk 9, cyto 6, nucl 4, mito 4, pero 4, mito\_n... | FKD\* |
| 9495 | K00143: LYS2; L-aminoadipate-semialdehyde dehy... |  | LYS2 |  | LYS2 | AASDH | Not Essential | mito 9, cyto 8.5, cyto\_nucl 5.5, extr 5, pero 2 | GVV\* |
| 10220 | K06133: LYS5, acpT; 4'-phosphopantetheinyl tra... |  |  | AASDHPPT |  |  | Not Essential | nucl 12, cyto\_nucl 10, mito 6, cyto 6 | AVD\* |
| 10577 | K14157: AASS; alpha-aminoadipic semialdehyde s... |  | SPE3,LYS9 | SRM,AASS | LYS9 | AASS | Not Essential | mito 21, cyto 3, pero 2 | ARF\* |
| 16833 | K00293: LYS9; saccharopine dehydrogenase (NADP... |  | SPE3,LYS9 | SRM,AASS | LYS9 | SRMe | Essential | cyto 19, cysk 6 | KVQ\* |
| 14087 | K00290: LYS1; saccharopine dehydrogenase (NAD+... |  | LYS1 |  | LYS1 |  | Not Essential | cyto 16.5, cyto\_nucl 10, pero 5, nucl 2.5 | KKQ\* |
| 15814 | K14085: ALDH7A1; aldehyde dehydrogenase family... | S |  | ALDH7A1 | ALD4 | ALDH7 | Not Essential | mito 26 | TFD\* |

| strain | WT | | | | | | | | | | | | | | | | |
| --- | --- | --- | --- | --- | --- | --- | --- | --- | --- | --- | --- | --- | --- | --- | --- | --- | --- |
| condition | G\_MM | C\_MM | G\_SD | | GX\_SD | | | X\_SD | | A\_SD | | C\_SD | | MM\_CN120 | | MM\_CN5 | Diversity\_Sample |
| phase | exp | exp | exp | stat | exp | trans | stat | exp | stat | exp | stat | exp | stat | exp | stat | exp | exp |
| proteinId | Set1 | Set1 | Set2 | Set2 | Set2 | Set2 | Set2 | Set2 | Set2 | Set2 | Set2 | Set2 | Set2 | Set3 | Set3 | Set3 | Set3 |
| 14856 | 7.06277 | 7.64718 | 7.71952 | 6.84579 | 8.04825 | 7.27894 | 7.24555 | 7.49081 | 6.40468 | 6.66873 | 6.64251 | 8.046 | 8.80539 | 8.713 | 8.69493 | 10.3276 | 9.37678 |
| 14600 | 4.23002 | 6.82861 | 3.04574 | 3.75571 | 2.88192 | 4.01126 | 4.14943 | 3.80416 | 3.69202 | 3.80241 | 4.05072 | 4.9755 | 4.72552 | 4.21007 | 4.34772 | 4.50387 | 6.4354 |
| 16016 | 4.96913 | 5.81973 | 5.19863 | 2.95433 | 5.41004 | 4.81655 | 4.90984 | 4.88237 | 4.38389 | 3.847 | 4.14014 | 5.57342 | 6.38216 | 5.7524 | 5.7551 | 6.34446 | 6.17036 |
| 10428 | 5.88933 | 7.89489 | 6.82443 | 5.55507 | 6.92434 | 6.38563 | 6.42311 | 6.88477 | 5.7152 | 6.03695 | 5.43536 | 7.38936 | 6.49532 | 7.20884 | 7.15186 | 8.16188 | 8.87302 |
| 14908 | 4.96188 | 5.39382 | 6.32914 | 4.57254 | 6.87228 | 3.93282 | 4.6105 | 2.86689 | 4.55744 | 3.57167 | 4.54445 | 5.45156 | 8.85544 | 4.48318 | 3.71453 | 9.27804 | 5.94028 |
| 15839 | 8.11963 | 6.83965 | 6.43567 | 6.23959 | 6.4065 | 6.29378 | 6.4164 | 6.59727 | 6.49423 | 6.81951 | 6.69291 | 6.85344 | 6.82691 | 7.91155 | 7.40486 | 6.5373 | 6.73561 |
| 9495 | 4.99092 | 5.42381 | 6.35839 | 4.54241 | 6.61367 | 5.24037 | 5.18124 | 5.62019 | 5.25199 | 5.13713 | 4.78198 | 6.88201 | 7.4782 | 5.72535 | 5.58315 | 7.96304 | 6.9019 |
| 10220 | 6.08332 | 5.58961 | 5.30673 | 5.38475 | 5.17929 | 5.58642 | 5.41775 | 5.47459 | 4.52614 | 5.73158 | 5.409 | 4.07171 | 3.79137 | 5.48093 | 5.412 | 5.2834 | 5.17945 |
| 10577 | 6.93825 | 6.51413 | 4.93966 | 6.72379 | 4.72929 | 4.32338 | 4.9139 | 3.42089 | 5.96694 | 3.35347 | 6.55975 | 5.82237 | 5.1241 | 7.06085 | 6.1541 | 4.83314 | 6.54823 |
| 16833 | 4.23834 | 6.81843 | 5.63912 | 4.87843 | 5.60182 | 6.13682 | 5.77789 | 6.51094 | 5.17185 | 5.79962 | 4.61771 | 6.47684 | 6.18936 | 6.01351 | 6.43293 | 7.32623 | 8.13086 |
| 14087 | 6.49169 | 7.66131 | 7.21483 | 6.34303 | 7.54094 | 6.05278 | 6.32643 | 6.4245 | 6.09519 | 5.91083 | 5.79607 | 8.23445 | 9.00442 | 6.38311 | 6.71187 | 8.39366 | 7.75438 |
| 15814 | 6.79043 | 6.58728 | 6.74196 | 7.35713 | 6.45038 | 5.89362 | 6.3322 | 4.9806 | 6.79946 | 5.56262 | 7.47028 | 5.93923 | 5.07561 | 6.72499 | 6.05561 | 6.66991 | 6.07386 |

| strain | WT | | | | | | | | | | |
| --- | --- | --- | --- | --- | --- | --- | --- | --- | --- | --- | --- |
| condition | G\_SD | | GX\_SD | | | X\_SD | | A\_SD | | C\_SD | |
| proteinId | exp | stat | exp | trans | stat | exp | stat | exp | stat | exp | stat |
| 14856 | 27.7406 | 22.5671 | 29.2617 | 25.3093 | 23.2998 | 19.5737 | 20.3033 | 21.4361 | 20.466 | 27.7783 | 31.5448 |
| 14600 | 0.382292 | 0 | 0.41045 | 0 | 0 | 0 | 0 | 0.776735 | 0 | 0 | 0 |
| 16016 | 11.9133 | 5.71703 | 14.2237 | 9.49844 | 9.4943 | 9.60274 | 9.21253 | 7.5461 | 3.63413 | 5.98956 | 15.4644 |
| 10428 | 17.6535 | 16.8612 | 16.4479 | 14.9055 | 16.103 | 15.0332 | 14.6223 | 11.5922 | 13.8129 | 25.3987 | 26.7699 |
| 14908 | 9.31562 | 0 | 11.3891 | 0 | 0 | 3.31911 | 0 | 1.54769 | 0 | 2.57326 | 8.71334 |
| 15839 | 4.43453 | 11.9185 | 6.09339 | 17.1048 | 18.0361 | 9.79935 | 13.8093 | 7.91426 | 12.8202 | 11.5438 | 12.6204 |
| 9495 | 38.2663 | 25.8364 | 44.5155 | 16.7301 | 16.7625 | 30.7141 | 24.8014 | 34.5918 | 29.3337 | 28.8223 | 41.3364 |
| 10577 | 4.60528 | 14.6285 | 3.6463 | 7.44721 | 8.77835 | 11.1558 | 15.7276 | 16.8009 | 15.8869 | 30.3513 | 25.0464 |
| 16833 | 43.1584 | 35.8344 | 50.7022 | 37.4319 | 37.2344 | 42.2953 | 41.4076 | 38.8457 | 40.0537 | 51.2791 | 53.2254 |
| 14087 | 14.0501 | 10.0588 | 15.8828 | 5.03914 | 9.15155 | 9.39594 | 8.41597 | 9.66871 | 10.748 | 18.1339 | 24.6929 |
| 15814 | 3.29054 | 5.82245 | 5.71037 | 5.95711 | 7.26142 | 4.32025 | 6.69429 | 6.37318 | 8.43457 | 13.4882 | 9.81823 |

|  | Glucose | Xylose | Arabinose | Acetate | Coumarate | Ferulate | YNB Oleic Acid | YNB Ricinoleic Acid | YNB Glucose | YNB Gluc DOC | YPD |
| --- | --- | --- | --- | --- | --- | --- | --- | --- | --- | --- | --- |
| proteinId |  |  |  |  |  |  |  |  |  |  |  |
| 14856 | -2.41996 | -2.32883 | -3.21568 | -1.90593 | -3.79053 | -1.27159 | -2.91199 | -2.78208 | -3.91013 | -1.46531 | -0.724535 |
| 14600 | -0.10018 | 0.289792 | -0.141329 | 0.0684176 | -0.00584732 | -0.323588 | -0.218815 | -1.45316 | -1.32814 | -0.239199 | 0.545846 |
| 16016 | -2.28259 | -1.40215 | -1.81138 | -3.40624 | -2.91691 | -1.86657 | -3.35685 | -4.04351 | -3.50648 | -0.684523 | 0.0693203 |
| 10428 | -0.412945 | 0.364408 | -1.37317 | -0.878157 | -0.883249 | -0.442528 | -1.48324 | -2.05839 | -2.52439 | -0.284016 | -0.218092 |
| 14908 | 0.0451271 | -0.280956 | 0.220759 | -0.0377905 | 0.21851 | -0.259388 | -0.292657 | 0.206026 | 0.339453 | 0.398586 | -0.0659921 |
| 15839 | -0.0847761 | -0.12423 | 0.269542 | 0.0647239 | 0.154571 | 0.146919 | -0.346466 | -0.214054 | -0.177142 | -0.229588 | -0.0156292 |
| 9495 | -0.852305 | -0.88796 | -1.33062 | -0.950872 | -1.39312 | -1.26513 | -1.29749 | -1.72025 | -1.59537 | -1.27751 | 0.0278676 |
| 10577 | -0.0823193 | -0.0536589 | -0.172111 | -0.277613 | -0.0621896 | -0.131564 | -0.239781 | -0.0105686 | 0.20184 | 0.345546 | 0.0251315 |
| 15814 | -0.464573 | -0.6107 | -0.102958 | -0.543758 | -0.425179 | -0.212745 | -0.360015 | -1.03866 | 0.403738 | -0.219486 | -0.625104 |

In [105]:

```
for x in temp:
    if x in model.genes:
        for r in sorted(model.genes.get_by_id(x).reactions, key=lambda x: x.id):
            print(r, r.gene_reaction_rule)
    else:
        print(x, 'no reactions')
    print()
```

```
HCITSn: accoa_n + akg_n + h2o_n --> coa_n + h_n + hcit_n 14856

HACONTam: hcit_m <=> h2o_m + hacon_C_m 14600

HACONTbm: h2o_m + hacon_C_m <=> hicit_m 16016

HICITDm: hicit_m + nad_m <=> h_m + nadh_m + oxag_m 10428

3HKYNAKGAT: akg_c + hLkynr_c --> 42A3HP24DB_c + glu__L_c 14908 or 15839 or 8540
AATA: 2oxoadp_c + glu__L_c <=> L2aadp_c + akg_c 14908 or 15839
KYNAKGAT: Lkynr_c + akg_c --> 4aphdob_c + glu__L_c 14908 or 15839 or 8540
PHETA1: akg_c + phe__L_c <=> glu__L_c + phpyr_c 14908 or 15839
TRPTA: akg_c + trp__L_c <=> glu__L_c + indpyr_c 12407 or 14908 or 15839
TYRTA: akg_c + tyr__L_c <=> 34hpp_c + glu__L_c 14908 or 15839 or 16065

3HKYNAKGAT: akg_c + hLkynr_c --> 42A3HP24DB_c + glu__L_c 14908 or 15839 or 8540
AATA: 2oxoadp_c + glu__L_c <=> L2aadp_c + akg_c 14908 or 15839
KYNAKGAT: Lkynr_c + akg_c --> 4aphdob_c + glu__L_c 14908 or 15839 or 8540
PHETA1: akg_c + phe__L_c <=> glu__L_c + phpyr_c 14908 or 15839
TRPTA: akg_c + trp__L_c <=> glu__L_c + indpyr_c 12407 or 14908 or 15839
TYRTA: akg_c + tyr__L_c <=> 34hpp_c + glu__L_c 14908 or 15839 or 16065

AASAD1: L2aadp_c + atp_c + h_c + nadph_c --> L2aadp6sa_c + amp_c + nadp_c + ppi_c 9495 or (10220 and 9495)
AASAD2: L2aadp_c + atp_c + h_c + nadh_c --> L2aadp6sa_c + amp_c + nad_c + ppi_c 9495 or (10220 and 9495)

AASAD1: L2aadp_c + atp_c + h_c + nadph_c --> L2aadp6sa_c + amp_c + nadp_c + ppi_c 9495 or (10220 and 9495)
AASAD2: L2aadp_c + atp_c + h_c + nadh_c --> L2aadp6sa_c + amp_c + nad_c + ppi_c 9495 or (10220 and 9495)

SACCD1: L2aadp6sa_c + glu__L_c + h_c + nadph_c <=> h2o_c + nadp_c + saccrp__L_c 10577 or 16833
SACCD3m: akg_m + h_m + lys__L_m + nadph_m --> h2o_m + nadp_m + saccrp__L_m 10577 or 16833
SACCD4m: h2o_m + nadp_m + saccrp__L_m --> L2aadp6sa_m + glu__L_m + h_m + nadph_m 10577 or 16833
SPMS: ametam_c + ptrc_c --> 5mta_c + h_c + spmd_c 10577 or 16833
SPRMS: ametam_c + spmd_c --> 5mta_c + h_c + sprm_c 10577 or 16833

SACCD1: L2aadp6sa_c + glu__L_c + h_c + nadph_c <=> h2o_c + nadp_c + saccrp__L_c 10577 or 16833
SACCD3m: akg_m + h_m + lys__L_m + nadph_m --> h2o_m + nadp_m + saccrp__L_m 10577 or 16833
SACCD4m: h2o_m + nadp_m + saccrp__L_m --> L2aadp6sa_m + glu__L_m + h_m + nadph_m 10577 or 16833
SPMS: ametam_c + ptrc_c --> 5mta_c + h_c + spmd_c 10577 or 16833
SPRMS: ametam_c + spmd_c --> 5mta_c + h_c + sprm_c 10577 or 16833

SACCD2: h2o_c + nad_c + saccrp__L_c <=> akg_c + h_c + lys__L_c + nadh_c 14087

5HOXINDACTOX: 5hoxindact_c + h2o_c + nad_c --> 5hoxindoa_c + 2.0 h_c + nadh_c 12042 or 13426 or 15814 or 16323
AASAD3: L2aadp6sa_c + h2o_c + nad_c --> L2aadp_c + 2.0 h_c + nadh_c 15814
ALDD20x: h2o_c + id3acald_c + nad_c --> 2.0 h_c + ind3ac_c + nadh_c 12042 or 13426 or 15814 or 16323
ALDD2x: acald_c + h2o_c + nad_c --> ac_c + 2.0 h_c + nadh_c 12042 or 13426 or 15814 or 16323
BAMPPALDOX: bamppald_c + h2o_c + nad_c --> ala_B_c + 2.0 h_c + nadh_c 12042 or 13426 or 15814 or 16323
GCALDD: gcald_c + h2o_c + nad_c --> glyclt_c + 2.0 h_c + nadh_c 12042 or 13426 or 15814 or 16323
IMACTD: h2o_c + im4act_c + nad_c --> 2.0 h_c + im4ac_c + nadh_c 12042 or 13426 or 15814 or 16323
LCADi: h2o_c + lald__L_c + nad_c --> 2.0 h_c + lac__L_c + nadh_c 12042 or 13426 or 15814 or 16323
LCADi_D: h2o_c + lald__D_c + nad_c --> 2.0 h_c + lac__D_c + nadh_c 12042 or 13426 or 15814 or 16323
NABTNO: h2o_c + n4abutn_c + nad_c --> 4aabutn_c + 2.0 h_c + nadh_c 12042 or 13426 or 15814 or 16323
PYLALDOX: h2o_c + nad_c + pylald_c --> 2.0 h_c + nadh_c + peracd_c 12042 or 13426 or 15814 or 16323
```

Lysine biosynthesis and aconitase/homoaconitase  
14856 LYS20 is nucl in S. cer, but 14856 has weak sigalP and KEGG matches are mito, add mito reaction  
https://onlinelibrary.wiley.com/doi/pdf/10.1111/mmi.12076  
HICITDm is incorrect, update the reaction to  
hicit\_m + nad\_m <=> 2oxoadp\_m + co2\_m + nadh\_m  
Change AASAD1 genes to 10220 and 9495, remove AASAD2 (NADH)  
15814 is ALDH7A1 mito degradation, change AASAD3 to mito and remove gene from other rxns  
16833 LYS9 cyto lysine biosynthesis, 10577 AASS mito lysine degradation bifunctional  
Change SACCD1 gene to 16833, SACCD3m and SACCD4m to 10577  
16833 has both SPE3 and LYS9 domains  
Change SPMS and SPRMS genes to 16833

In [106]:

```
r = sce.reactions.get_by_id('HCITSm').copy()
r.gene_reaction_rule = '14856'
model.remove_reactions(['HCITSn'],remove_orphans=True)
model.add_reactions([r])
model.reactions.get_by_id('HICITDm').add_metabolites({'h_m': -1.0, 'oxag_m': -1.0,
                                                      'co2_m': 1.0, '2oxoadp_m': 1.0})

model.reactions.get_by_id('AASAD1').gene_reaction_rule = '10220 and 9495'
r = hsa.reactions.get_by_id('AASAD3m').copy()
r.gene_reaction_rule = '15814'
model.add_reactions([r])
model.remove_reactions(['AASAD2','AASAD3'],remove_orphans=True)

model.reactions.get_by_id('5HOXINDACTOX').gene_reaction_rule = '12042 or 13426 or 16323'
model.reactions.get_by_id('ALDD20x').gene_reaction_rule = '12042 or 13426 or 16323'
model.reactions.get_by_id('ALDD2x').gene_reaction_rule = '12042 or 13426 or 16323'
model.reactions.get_by_id('BAMPPALDOX').gene_reaction_rule = '12042 or 13426 or 16323'
model.reactions.get_by_id('GCALDD').gene_reaction_rule = '12042 or 13426 or 16323'
model.reactions.get_by_id('IMACTD').gene_reaction_rule = '12042 or 13426 or 16323'
model.reactions.get_by_id('LCADi').gene_reaction_rule = '12042 or 13426 or 16323'
model.reactions.get_by_id('LCADi_D').gene_reaction_rule = '12042 or 13426 or 16323'
model.reactions.get_by_id('NABTNO').gene_reaction_rule = '12042 or 13426 or 16323'
model.reactions.get_by_id('PYLALDOX').gene_reaction_rule = '12042 or 13426 or 16323'

model.reactions.get_by_id('SACCD1').gene_reaction_rule = '16833'
model.reactions.get_by_id('SACCD3m').gene_reaction_rule = '10577'
model.reactions.get_by_id('SACCD4m').gene_reaction_rule = '10577'
model.reactions.get_by_id('SPMS').gene_reaction_rule = '16833'
model.reactions.get_by_id('SPRMS').gene_reaction_rule = '16833'
```

In [107]:

```
sol = model.optimize()
print(sol.objective_value)

for k, v in sol.shadow_prices.items():
    if v and k in [m.id for m in model.reactions.get_by_id('BIOMASS_RT').metabolites]:
        print(k, v)
```

```
0.0
pe_RT_r 124.47099825740602
mannan_r -1.2447099825740602
```

In [108]:

```
with model:
    model.add_reactions([hsa2.reactions.Htr.copy()])
    model.add_reactions([hsa2.reactions.PIter.copy()])
    model.add_reactions([hsa2.reactions.PPItr.copy()])
    model.add_reactions([hsa2.reactions.COAtr.copy()])
    model.add_reactions([hsa2.reactions.NADPHtru.copy()])
    model.add_reactions([hsa2.reactions.NADPtru.copy()])
    model.add_reactions([hsa2.reactions.r0826.copy()]) # inost_r transport
    model.add_reactions([hsa2.reactions.r1159.copy()]) # cdpchol_r transport
    model.add_reactions([hsa2.reactions.FORtr.copy()]) # for_r transport
    model.add_reactions([hsa2.reactions.AMETr.copy()]) # amet_r transport
    model.add_reactions([hsa2.reactions.AHCYStr.copy()]) # ahcys_r transport

    r = hsa2.reactions.Htr.copy()
    r.id = 'ATPter'
    r.name = 'ATP transporter, endoplasmic reticulum'
    model.add_reactions([r])
    r.add_metabolites({'h_c': 1.0, 'h_r': -1.0, 'atp_c': -1.0, 'atp_r': 1.0})
    r = hsa2.reactions.Htr.copy()
    r.id = 'ADPter'
    r.name = 'ADP transporter, endoplasmic reticulum'
    model.add_reactions([r])
    r.add_metabolites({'h_c': 1.0, 'h_r': -1.0, 'adp_r': -1.0, 'adp_c': 1.0})
    r = hsa2.reactions.Htr.copy()
    r.id = 'AMPter'
    r.name = 'AMP transporter, endoplasmic reticulum'
    model.add_reactions([r])
    r.add_metabolites({'h_c': 1.0, 'h_r': -1.0, 'amp_c': -1.0, 'amp_r': 1.0})
    r = hsa2.reactions.Htr.copy()
    r.id = 'CTPter'
    r.name = 'CTP transporter, endoplasmic reticulum'
    model.add_reactions([r])
    r.add_metabolites({'h_c': 1.0, 'h_r': -1.0, 'ctp_c': -1.0, 'ctp_r': 1.0})
    r = hsa2.reactions.Htr.copy()
    r.id = 'CDPter'
    r.name = 'CDP transporter, endoplasmic reticulum'
    model.add_reactions([r])
    r.add_metabolites({'h_c': 1.0, 'h_r': -1.0, 'cdp_r': -1.0, 'cdp_c': 1.0})
    r = hsa2.reactions.Htr.copy()
    r.id = 'CMPter'
    r.name = 'CMP transporter, endoplasmic reticulum'
    model.add_reactions([r])
    r.add_metabolites({'h_c': 1.0, 'h_r': -1.0, 'cmp_c': -1.0, 'cmp_r': 1.0})
    r = hsa2.reactions.Htr.copy()
    r.id = 'SERter'
    r.name = 'L-serine transporter, endoplasmic reticulum'
    model.add_reactions([r])
    r.add_metabolites({'h_c': 1.0, 'h_r': -1.0, 'ser__L_c': -1.0, 'ser__L_r': 1.0})

    sol = model.optimize()
    print(sol.objective_value)
    for k, v in sol.shadow_prices.items():
        if v and k in [m.id for m in model.reactions.get_by_id('BIOMASS_RT').metabolites]:
            print(k, v)
```

```
1.4134701823753533
adp_c -0.6360615820689088
atp_c -0.6360615820689088
amp_c -0.6360615820689088
cmp_c -0.18846269098338064
glu__L_c -0.14134701823753534
gmp_c -0.6360615820689088
gly_c -1.3877787807814457e-17
ser__L_c -0.259136200102148
cys__L_c -0.2591362001021481
ala__L_c -0.047115672745844966
ump_c -0.18846269098338064
gln__L_c -0.14134701823753534
dgmp_c -0.6360615820689088
pe_RT_r -207.2382865726006
his__L_c -0.37692538196676084
leu__L_c -0.21202052735630303
dtmp_c -0.44759889108552864
trp__L_c -0.47115672745845094
met__L_c -0.3298097092209158
pro__L_c -0.14134701823753534
phe__L_c -0.23557836372922558
tyr__L_c -0.23557836372922558
asp__L_c -0.07067350911876767
ile__L_c -0.14134701823753534
dcmp_c -0.18846269098338064
mannan_r -0.14134701823753537
damp_c -0.6360615820689088
arg__L_c -0.14134701823753534
asn__L_c -0.07067350911876769
glycogen_c -0.14134701823753543
13BDglcn_c -0.14134701823753543
thr__L_c -0.07067350911876769
lys__L_c -0.16490485461045792
val__L_c -0.14134701823753534
ergst_r -0.2591362001021368
tre_c -0.28269403647507085
ribflv_c -0.09423134549169038
zymst_r 1.1102230246251565e-14
pa_RT_r -181.3246665623858
triglyc_RT_r -268.45332438764035
pc_RT_r -284.979146603245
ptd1ino_RT_r -195.45936838613935
ps_RT_r -207.2382865726006
```

In [109]:

```
temp = ['Htr','PIter','PPItr','COAtr','NADPHtru','NADPtru','r0826','r1159','FORtr','AMETr','AHCYStr']
for x in temp:
    model.add_reactions([hsa2.reactions.get_by_id(x).copy()])    

model.reactions.get_by_id('PIter').gene_reaction_rule = ''
model.reactions.get_by_id('r0826').id = 'INOSTter'
model.reactions.get_by_id('r1159').id = 'CDPCHOLter'

r = hsa2.reactions.Htr.copy()
r.id = 'ATPter'
r.name = 'ATP transporter, endoplasmic reticulum'
model.add_reactions([r])
r.add_metabolites({'h_c': 1.0, 'h_r': -1.0, 'atp_c': -1.0, 'atp_r': 1.0})
r = hsa2.reactions.Htr.copy()
r.id = 'ADPter'
r.name = 'ADP transporter, endoplasmic reticulum'
model.add_reactions([r])
r.add_metabolites({'h_c': 1.0, 'h_r': -1.0, 'adp_r': -1.0, 'adp_c': 1.0})
r = hsa2.reactions.Htr.copy()
r.id = 'AMPter'
r.name = 'AMP transporter, endoplasmic reticulum'
model.add_reactions([r])
r.add_metabolites({'h_c': 1.0, 'h_r': -1.0, 'amp_c': -1.0, 'amp_r': 1.0})
r = hsa2.reactions.Htr.copy()
r.id = 'CTPter'
r.name = 'CTP transporter, endoplasmic reticulum'
model.add_reactions([r])
r.add_metabolites({'h_c': 1.0, 'h_r': -1.0, 'ctp_c': -1.0, 'ctp_r': 1.0})
r = hsa2.reactions.Htr.copy()
r.id = 'CDPter'
r.name = 'CDP transporter, endoplasmic reticulum'
model.add_reactions([r])
r.add_metabolites({'h_c': 1.0, 'h_r': -1.0, 'cdp_r': -1.0, 'cdp_c': 1.0})
r = hsa2.reactions.Htr.copy()
r.id = 'CMPter'
r.name = 'CMP transporter, endoplasmic reticulum'
model.add_reactions([r])
r.add_metabolites({'h_c': 1.0, 'h_r': -1.0, 'cmp_c': -1.0, 'cmp_r': 1.0})
r = hsa2.reactions.Htr.copy()
r.id = 'SERter'
r.name = 'L-serine transporter, endoplasmic reticulum'
model.add_reactions([r])
r.add_metabolites({'h_c': 1.0, 'h_r': -1.0, 'ser__L_c': -1.0, 'ser__L_r': 1.0})
```

In [110]:

```
sol = model.optimize()
print(sol.objective_value)

for k, v in sol.shadow_prices.items():
    if v and k in [m.id for m in model.reactions.get_by_id('BIOMASS_RT').metabolites]:
        print(k, v)
```

```
1.4134701823753493
adp_c -0.6360615820689072
atp_c -0.6360615820689072
amp_c -0.6360615820689072
cmp_c -0.18846269098337992
glu__L_c -0.1413470182375349
gmp_c -0.6360615820689072
ser__L_c -0.25913620010214733
cys__L_c -0.2591362001021471
ala__L_c -0.047115672745844994
ump_c -0.18846269098337992
gln__L_c -0.1413470182375349
dgmp_c -0.6360615820689072
pe_RT_r -207.2382865725995
his__L_c -0.37692538196675984
leu__L_c -0.2120205273563024
dtmp_c -0.44759889108552725
trp__L_c -0.4711567274584497
met__L_c -0.32980970922091457
pro__L_c -0.1413470182375349
phe__L_c -0.23557836372922486
tyr__L_c -0.23557836372922486
asp__L_c -0.07067350911876746
ile__L_c -0.14134701823753493
dcmp_c -0.18846269098337992
mannan_r -0.14134701823753482
damp_c -0.6360615820689072
arg__L_c -0.1413470182375349
asn__L_c -0.07067350911876746
glycogen_c -0.14134701823753487
13BDglcn_c -0.14134701823753487
thr__L_c -0.07067350911876746
lys__L_c -0.1649048546104574
val__L_c -0.14134701823753493
ergst_r -0.25913620010213934
tre_c -0.28269403647506974
ribflv_c -0.09423134549168999
zymst_r 7.993605777301127e-15
pa_RT_r -181.32466656238478
triglyc_RT_r -268.45332438763876
pc_RT_r -284.97914660324363
ptd1ino_RT_r -195.45936838613827
ps_RT_r -207.2382865725995
```

In [111]:

```
# Identify the minimum set of reactions to keep the maximum growth under a reasonable value
remove = ['NA1Htm','UREAtm_1','PYDXO','ASPGLUm','AKGCITtm','AKGMALtm','AKGICITtm','OAAAKGtm']

with model:
    model.remove_reactions(remove, remove_orphans=True)
    sol = model.optimize()
    print(sol.objective_value)
```

```
0.12105280269848512
```

In [112]:

```
for x in remove:
    with model:
        model.remove_reactions([y for y in remove if not y == x], remove_orphans=True)
        sol = model.optimize()
        print(x, sol.objective_value)
```

```
NA1Htm 0.6474314774710036
UREAtm_1 0.12105280269848379
PYDXO 1.3848778722367265
ASPGLUm 1.3733526777687943
AKGCITtm 1.3733526777687748
AKGMALtm 1.3733526777687723
AKGICITtm 1.3733526777687624
OAAAKGtm 1.3733526777687675
```

In [113]:

```
for x in remove:
    r = model.reactions.get_by_id(x)
    print(r, r.gene_reaction_rule, ['sce' if r in sce.reactions else 'none'], ['hsa' if r in hsa.reactions else 'none'])
```

```
NA1Htm: h_m + na1_c <=> h_c + na1_m 10469 ['none'] ['none']
UREAtm_1: na1_c + urea_c <=> na1_m + urea_m 15807 ['none'] ['none']
PYDXO: 2.0 h2o_c + nh4_c + 0.5 o2_c + pydx_c <=> 2.0 h2o2_c + pydam_c 10680 ['sce'] ['none']
ASPGLUm: asp__L_m + glu__L_c + h_c <=> asp__L_c + glu__L_m + h_m 16799 ['none'] ['hsa']
AKGCITtm: akg_c + cit_m <=> akg_m + cit_c 11740 or 13510 ['none'] ['none']
AKGMALtm: akg_m + mal__L_c <=> akg_c + mal__L_m 11740 or 13510 ['none'] ['hsa']
AKGICITtm: akg_c + icit_m <=> akg_m + icit_c 11740 or 13510 ['none'] ['none']
OAAAKGtm: akg_m + oaa_c <=> akg_c + oaa_m 11740 or 13510 ['none'] ['none']
```

In [114]:

```
# first half
temp = ['10469','15807','10680']
display(Annotation.loc[temp])
Show_Data(temp)
```

|  | Combined Annotations | Signal P | Sc288c Orthologs | Human Orthologs | Sc288 Best Hit | Human Blast | Essential | WolfPSort | C Terminal |
| --- | --- | --- | --- | --- | --- | --- | --- | --- | --- |
| RTO4\_ID |  |  |  |  |  |  |  |  |  |
| 10469 | K12041: SLC9A6\_7, NHE6\_7; solute carrier famil... | A | NHX1 | SLC9A6,SLC9A7,SLC9A9,SLC9A8 | NHX1 | SLC9A | Not Essential | plas 24, E.R. 2 | GRR\* |
| 15807 | K20989: DUR3; urea-proton symporter | A | DUR3 |  | DUR3 |  | Not Essential | plas 21, vacu 3, mito 2 | GKK\* |
| 10680 | K00275: pdxH, PNPO; pyridoxamine 5'-phosphate ... |  | PDX3 | PNPO | PDX3 | PNPO | Not Essential | cyto\_mito 10.166, cyto\_nucl 9.833, mito 9.5, c... | LSP\* |

| strain | WT | | | | | | | | | | | | | | | | |
| --- | --- | --- | --- | --- | --- | --- | --- | --- | --- | --- | --- | --- | --- | --- | --- | --- | --- |
| condition | G\_MM | C\_MM | G\_SD | | GX\_SD | | | X\_SD | | A\_SD | | C\_SD | | MM\_CN120 | | MM\_CN5 | Diversity\_Sample |
| phase | exp | exp | exp | stat | exp | trans | stat | exp | stat | exp | stat | exp | stat | exp | stat | exp | exp |
| proteinId | Set1 | Set1 | Set2 | Set2 | Set2 | Set2 | Set2 | Set2 | Set2 | Set2 | Set2 | Set2 | Set2 | Set3 | Set3 | Set3 | Set3 |
| 10469 | 6.18807 | 5.79617 | 4.8731 | 4.3291 | 4.84976 | 5.16965 | 5.2313 | 5.3075 | 4.93525 | 5.47659 | 4.67187 | 5.72882 | 5.32205 | 7.17536 | 7.07842 | 5.84164 | 6.3282 |
| 15807 | 7.73847 | 1.4738 | 1.30092 | 3.09152 | 1.28326 | 2.10509 | 2.65814 | 1.98767 | 2.19396 | 1.73857 | 2.78258 | 2.17235 | 1.93527 | 8.13991 | 10.0658 | 2.91967 | 7.55116 |
| 10680 | 6.51456 | 5.86692 | 6.73076 | 5.99195 | 6.88619 | 5.77974 | 5.8521 | 5.95985 | 6.16739 | 5.62018 | 6.15432 | 6.06002 | 6.07855 | 5.94057 | 6.1314 | 6.47044 | 7.72373 |

| strain | WT | | | | | | | | | | |
| --- | --- | --- | --- | --- | --- | --- | --- | --- | --- | --- | --- |
| condition | G\_SD | | GX\_SD | | | X\_SD | | A\_SD | | C\_SD | |
| proteinId | exp | stat | exp | trans | stat | exp | stat | exp | stat | exp | stat |
| 10469 | 0 | 0.18528 | 0.408356 | 0.92967 | 1.29037 | 0.197909 | 0.394911 | 0.963536 | 0.189081 | 1.07037 | 1.31263 |
| 10680 | 2.29488 | 3.0324 | 4.46173 | 2.23695 | 3.26574 | 1.16163 | 2.15861 | 1.92934 | 1.72291 | 3.86401 | 3.73772 |

|  | Glucose | Xylose | Arabinose | Acetate | Coumarate | Ferulate | YNB Oleic Acid | YNB Ricinoleic Acid | YNB Glucose | YNB Gluc DOC | YPD |
| --- | --- | --- | --- | --- | --- | --- | --- | --- | --- | --- | --- |
| proteinId |  |  |  |  |  |  |  |  |  |  |  |
| 10469 | 0.0479654 | 0.248788 | 0.236235 | 0.0289028 | 0.231316 | 0.138566 | 0.848711 | 0.978928 | 0.566191 | 0.343846 | -0.150579 |
| 15807 | -0.179974 | -0.0305221 | 0.0277623 | -0.088636 | -0.128047 | -0.194151 | 0.112187 | 0.0646585 | 0.0041067 | -0.117739 | -0.0236412 |

In [115]:

```
for x in temp:
    if x in model.genes:
        for r in sorted(model.genes.get_by_id(x).reactions, key=lambda x: x.id):
            print(r, r.gene_reaction_rule)
    else:
        print(x, 'no reactions')
    print()
```

```
Kt3g: h_g + k_c <=> h_c + k_g 10469
NA1Htm: h_m + na1_c <=> h_c + na1_m 10469
NAt3_1g: h_g + na1_c <=> h_c + na1_g 10469

UREA2t2: 2.0 h_e + urea_e <=> 2.0 h_c + urea_c 15807
UREAt_1: na1_e + urea_e <=> na1_c + urea_c 15807
UREAtm_1: na1_c + urea_c <=> na1_m + urea_m 15807

PDX5POi: o2_c + pdx5p_c --> h2o2_c + pydx5p_c 10680 or 8901
PYAM5PO: h2o_c + o2_c + pyam5p_c --> h2o2_c + nh4_c + pydx5p_c 10680 or 8901
PYDXNO: o2_c + pydxn_c <=> h2o2_c + pydx_c 10680 or 8901
PYDXO: 2.0 h2o_c + nh4_c + 0.5 o2_c + pydx_c <=> 2.0 h2o2_c + pydam_c 10680
PYDXO_1: h2o_c + o2_c + pydam_c <=> h2o2_c + nh4_c + pydx_c 10680 or 8901
```

In [116]:

```
model.remove_reactions(['NA1Htm'],remove_orphans=True)
```

In [117]:

```
for r in sorted(model.metabolites.get_by_id('urea_c').reactions, key=lambda x: x.id):
    if len(r.compartments) > 0:
        print(r, r.gene_reaction_rule)
print()
for r in sorted(model.metabolites.get_by_id('urea_m').reactions, key=lambda x: x.id):
    if len(r.compartments) > 0:
        print(r, r.gene_reaction_rule)
```

```
AGMT: agm_c + h2o_c --> ptrc_c + urea_c 10140
ALLTAHr: alltt_c + h2o_c <=> urdglyc_c + urea_c 14724
ARGN: arg__L_c + h2o_c --> orn_c + urea_c 16181
GUDBUTNAH: 4gudbutn_c + h2o_c --> 4abut_c + urea_c 16181
UREA2t2: 2.0 h_e + urea_e <=> 2.0 h_c + urea_c 15807
UREASE: atp_c + hco3_c + urea_c <=> adp_c + allphn_c + h_c + pi_c 9326
UREAt: urea_e <=> urea_c 13100 or 15470
UREAt_1: na1_e + urea_e <=> na1_c + urea_c 15807
UREAtm: urea_c <=> urea_m 15470
UREAtm_1: na1_c + urea_c <=> na1_m + urea_m 15807

AGMTm: agm_m + h2o_m --> ptrc_m + urea_m 10140
ARGNm: arg__L_m + h2o_m --> orn_m + urea_m 16181
UREAtm: urea_c <=> urea_m 15470
UREAtm_1: na1_c + urea_c <=> na1_m + urea_m 15807
```

In [118]:

```
temp = ['15807','10140','15470','16181']
display(Annotation.loc[temp])
Show_Data(temp)
```

|  | Combined Annotations | Signal P | Sc288c Orthologs | Human Orthologs | Sc288 Best Hit | Human Blast | Essential | WolfPSort | C Terminal |
| --- | --- | --- | --- | --- | --- | --- | --- | --- | --- |
| RTO4\_ID |  |  |  |  |  |  |  |  |  |
| 15807 | K20989: DUR3; urea-proton symporter | A | DUR3 |  | DUR3 |  | Not Essential | plas 21, vacu 3, mito 2 | GKK\* |
| 10140 | K01480: speB; agmatinase | S |  | AGMAT |  | AGMAT | Not Essential | extr 23, mito 2, plas 2 | VRV\* |
| 15470 | K03441: GLP-F; aquaglyceroporin related protei... |  | YFL054C,FPS1 | AQP3,AQP9 | YFL054C | AQP3 | Not Essential | plas 17, nucl 6, mito 1, cyto 1, E.R. 1, vacu ... | WRQ\* |
| 16181 | K01476: E3.5.3.1, rocF, arg; arginase |  | CAR1 | ARG1,ARG2 | CAR1 | ARG1 | Not Essential | cyto 25.5, cyto\_nucl 14 | TLL\* |

| strain | WT | | | | | | | | | | | | | | | | |
| --- | --- | --- | --- | --- | --- | --- | --- | --- | --- | --- | --- | --- | --- | --- | --- | --- | --- |
| condition | G\_MM | C\_MM | G\_SD | | GX\_SD | | | X\_SD | | A\_SD | | C\_SD | | MM\_CN120 | | MM\_CN5 | Diversity\_Sample |
| phase | exp | exp | exp | stat | exp | trans | stat | exp | stat | exp | stat | exp | stat | exp | stat | exp | exp |
| proteinId | Set1 | Set1 | Set2 | Set2 | Set2 | Set2 | Set2 | Set2 | Set2 | Set2 | Set2 | Set2 | Set2 | Set3 | Set3 | Set3 | Set3 |
| 15807 | 7.73847 | 1.4738 | 1.30092 | 3.09152 | 1.28326 | 2.10509 | 2.65814 | 1.98767 | 2.19396 | 1.73857 | 2.78258 | 2.17235 | 1.93527 | 8.13991 | 10.0658 | 2.91967 | 7.55116 |
| 10140 | 3.04152 | 2.81075 | 3.29284 | 2.58109 | 3.25142 | 3.39642 | 3.34463 | 3.05798 | 3.34889 | 2.76161 | 3.24483 | 3.76428 | 3.25369 | 3.11453 | 2.8434 | 2.88306 | 2.80482 |
| 15470 | 2.58482 | 2.6645 | 3.34569 | 4.55699 | 3.28507 | 3.35072 | 3.38165 | 3.06554 | 3.80438 | 2.88507 | 3.38184 | 3.12887 | 3.08643 | 3.45926 | 3.81437 | 3.23958 | 3.79991 |
| 16181 | 6.14177 | 5.45377 | 5.66317 | 5.00125 | 5.80141 | 5.23873 | 5.26963 | 5.0376 | 5.89405 | 5.1486 | 5.77584 | 6.43814 | 8.39738 | 6.36809 | 5.59229 | 6.38485 | 7.08693 |

| strain | WT | | | | | | | | | | |
| --- | --- | --- | --- | --- | --- | --- | --- | --- | --- | --- | --- |
| condition | G\_SD | | GX\_SD | | | X\_SD | | A\_SD | | C\_SD | |
| proteinId | exp | stat | exp | trans | stat | exp | stat | exp | stat | exp | stat |
| 16181 | 5.42197 | 5.51838 | 6.92697 | 5.77812 | 3.67098 | 4.50582 | 3.36132 | 8.49982 | 4.99497 | 11.78 | 13.4269 |

|  | Glucose | Xylose | Arabinose | Acetate | Coumarate | Ferulate | YNB Oleic Acid | YNB Ricinoleic Acid | YNB Glucose | YNB Gluc DOC | YPD |
| --- | --- | --- | --- | --- | --- | --- | --- | --- | --- | --- | --- |
| proteinId |  |  |  |  |  |  |  |  |  |  |  |
| 15807 | -0.179974 | -0.0305221 | 0.0277623 | -0.088636 | -0.128047 | -0.194151 | 0.112187 | 0.0646585 | 0.0041067 | -0.117739 | -0.0236412 |
| 10140 | 0.0870947 | -0.0379351 | 0.121567 | 0.232117 | 0.32541 | -0.1721 | -0.266461 | -0.274505 | 0.39974 | 0.0193737 | 0.0688812 |
| 15470 | 0.00318651 | 0.0512761 | -0.167947 | 0.0916783 | 0.0498898 | -0.185868 | 0.158407 | -0.399259 | 0.128998 | -0.140828 | -0.115934 |

In [119]:

```
for x in temp:
    if x in model.genes:
        for r in sorted(model.genes.get_by_id(x).reactions, key=lambda x: x.id):
            print(r, r.gene_reaction_rule)
    else:
        print(x, 'no reactions')
    print()
```

```
UREA2t2: 2.0 h_e + urea_e <=> 2.0 h_c + urea_c 15807
UREAt_1: na1_e + urea_e <=> na1_c + urea_c 15807
UREAtm_1: na1_c + urea_c <=> na1_m + urea_m 15807

AGMT: agm_c + h2o_c --> ptrc_c + urea_c 10140
AGMTm: agm_m + h2o_m --> ptrc_m + urea_m 10140

GLYCt: glyc_c <=> glyc_e 13100 or 15470
L_LACtcm: lac__L_c --> lac__L_m 15470
UREAt: urea_e <=> urea_c 13100 or 15470
UREAtm: urea_c <=> urea_m 15470

ARGN: arg__L_c + h2o_c --> orn_c + urea_c 16181
ARGNm: arg__L_m + h2o_m --> orn_m + urea_m 16181
GUDBUTNAH: 4gudbutn_c + h2o_c --> 4abut_c + urea_c 16181
```

In [120]:

```
model.remove_reactions(['UREAtm_1','UREAt_1','ARGNm','AGMT','L_LACtcm','LCADm','LCADi_Dm'],remove_orphans=True)
```

In [121]:

```
for r in sorted(model.metabolites.get_by_id('pydx_c').reactions, key=lambda x: x.id):
    print(r, r.gene_reaction_rule)
print()
for r in sorted(model.metabolites.get_by_id('pydam_c').reactions, key=lambda x: x.id):
    print(r, r.gene_reaction_rule)
```

```
PYDXDH_1: h2o_c + o2_c + pydx_c --> 4pyrdx_c + h2o2_c + h_c 15962
PYDXK: atp_c + pydx_c --> adp_c + h_c + pydx5p_c 14545
PYDXNO: o2_c + pydxn_c <=> h2o2_c + pydx_c 10680 or 8901
PYDXO: 2.0 h2o_c + nh4_c + 0.5 o2_c + pydx_c <=> 2.0 h2o2_c + pydam_c 10680
PYDXOR: h_c + nadph_c + pydx_c <=> nadp_c + pydxn_c 11550 or 14369
PYDXO_1: h2o_c + o2_c + pydam_c <=> h2o2_c + nh4_c + pydx_c 10680 or 8901
PYDXPP: h2o_c + pydx5p_c --> pi_c + pydx_c 13044 or 14545

HYPOE: h2o_c + pyam5p_c --> pi_c + pydam_c 13044
PYDAMK: atp_c + pydam_c --> adp_c + h_c + pyam5p_c 14545
PYDXO: 2.0 h2o_c + nh4_c + 0.5 o2_c + pydx_c <=> 2.0 h2o2_c + pydam_c 10680
PYDXO_1: h2o_c + o2_c + pydam_c <=> h2o2_c + nh4_c + pydx_c 10680 or 8901
```

In [122]:

```
temp = ['13044','14545','11550','14369','10680','15962','8901']
display(Annotation.loc[temp])
Show_Data(temp)
```

|  | Combined Annotations | Signal P | Sc288c Orthologs | Human Orthologs | Sc288 Best Hit | Human Blast | Essential | WolfPSort | C Terminal |
| --- | --- | --- | --- | --- | --- | --- | --- | --- | --- |
| RTO4\_ID |  |  |  |  |  |  |  |  |  |
| 13044 | K01101: E3.1.3.41; 4-nitrophenyl phosphatase |  | PHO13 | PGP,PDXP | PHO13 | PDXP | Not Essential | cyto 17.5, cyto\_nucl 12.5, nucl 4.5, pero 3 | LAQ\* |
| 14545 | K00868: pdxK, pdxY; pyridoxine kinase |  | BUD16,BUD17 | PDXK | BUD16 | PDXK | Not Essential | mito 10.5, cyto\_mito 10.333, cyto 9, extr 6, c... | SGV\* |
| 11550 | KOG1575: Voltage-gated shaker-like K+ channel,... | A |  |  | YPR127W | AKR7L | Not Essential | mito 8, cyto 7.5, cyto\_nucl 7.5, nucl 6.5, pero 4 | MDE\* |
| 14369 | KOG1575: Voltage-gated shaker-like K+ channel,... |  |  |  | YPR127W | KCNAB | Not Essential | cyto 23, cysk 4 | LDG\* |
| 10680 | K00275: pdxH, PNPO; pyridoxamine 5'-phosphate ... |  | PDX3 | PNPO | PDX3 | PNPO | Not Essential | cyto\_mito 10.166, cyto\_nucl 9.833, mito 9.5, c... | LSP\* |
| 15962 | K00106: XDH; xanthine dehydrogenase/oxidase |  |  | AOX1,XDH |  | XDHe | Not Essential | cyto 21.5, cyto\_nucl 12.5, nucl 2.5 | VCI\* |
| 8901 | K17759: AIBP, nnrE; NAD(P)H-hydrate epimerase |  | YNL200C | NAXE | YNL200C | NAXE | Not Essential | cyto 12.5, cyto\_nucl 9.5, extr 5, mito 4, nucl... | RKE\* |

| strain | WT | | | | | | | | | | | | | | | | |
| --- | --- | --- | --- | --- | --- | --- | --- | --- | --- | --- | --- | --- | --- | --- | --- | --- | --- |
| condition | G\_MM | C\_MM | G\_SD | | GX\_SD | | | X\_SD | | A\_SD | | C\_SD | | MM\_CN120 | | MM\_CN5 | Diversity\_Sample |
| phase | exp | exp | exp | stat | exp | trans | stat | exp | stat | exp | stat | exp | stat | exp | stat | exp | exp |
| proteinId | Set1 | Set1 | Set2 | Set2 | Set2 | Set2 | Set2 | Set2 | Set2 | Set2 | Set2 | Set2 | Set2 | Set3 | Set3 | Set3 | Set3 |
| 13044 | 7.07028 | 6.74996 | 7.16219 | 5.8811 | 7.19872 | 6.57109 | 6.45218 | 6.99004 | 6.03626 | 6.86862 | 6.18725 | 6.06425 | 5.94515 | 5.35161 | 5.26977 | 5.85498 | 5.54996 |
| 14545 | 5.86759 | 6.23356 | 5.99367 | 5.25618 | 6.2668 | 5.77917 | 5.7945 | 5.57254 | 5.78656 | 5.42948 | 5.3285 | 6.70805 | 8.09687 | 5.41984 | 5.71687 | 6.85116 | 6.09824 |
| 11550 | 1.28419 | 1.36425 | 1.0368 | 2.51252 | 1.39885 | 1.61963 | 1.57779 | 1.53525 | 2.73793 | 1.21592 | 1.52424 | 1.81606 | 1.74576 | 1.69065 | 2.07745 | 0.831968 | 4.94163 |
| 14369 | 6.37369 | 6.41949 | 7.13277 | 7.56726 | 7.36317 | 6.74208 | 6.59726 | 5.47481 | 6.02139 | 6.44692 | 6.0917 | 5.13371 | 4.9802 | 5.3732 | 5.56443 | 6.22943 | 6.06545 |
| 10680 | 6.51456 | 5.86692 | 6.73076 | 5.99195 | 6.88619 | 5.77974 | 5.8521 | 5.95985 | 6.16739 | 5.62018 | 6.15432 | 6.06002 | 6.07855 | 5.94057 | 6.1314 | 6.47044 | 7.72373 |
| 15962 | 5.75299 | 4.71703 | 5.84471 | 4.8374 | 5.5762 | 5.16905 | 5.0558 | 4.53551 | 5.66232 | 4.11391 | 4.84404 | 6.21845 | 6.05768 | 7.79894 | 6.08581 | 4.84549 | 6.2222 |
| 8901 | 8.45942 | 6.9654 | 8.38144 | 9.44002 | 8.07997 | 9.11718 | 9.14409 | 7.85926 | 9.10514 | 8.21596 | 8.99261 | 6.73511 | 7.64702 | 8.9549 | 9.81424 | 8.17586 | 8.1624 |

| strain | WT | | | | | | | | | | |
| --- | --- | --- | --- | --- | --- | --- | --- | --- | --- | --- | --- |
| condition | G\_SD | | GX\_SD | | | X\_SD | | A\_SD | | C\_SD | |
| proteinId | exp | stat | exp | trans | stat | exp | stat | exp | stat | exp | stat |
| 13044 | 4.20559 | 1.80248 | 3.87481 | 2.79768 | 2.34766 | 2.33804 | 2.98044 | 2.13068 | 1.74214 | 1.72051 | 1.29777 |
| 14545 | 11.7002 | 8.87805 | 10.3838 | 9.09559 | 8.81301 | 5.7039 | 6.1408 | 8.09437 | 7.08964 | 7.69543 | 12.1855 |
| 11550 | 0 | 0 | 0 | 0 | 0 | 7.83032 | 5.33473 | 0.966186 | 0.379285 | 0 | 0 |
| 14369 | 2.89003 | 6.11035 | 5.28807 | 6.7312 | 9.13093 | 12.3517 | 11.4727 | 7.33416 | 5.15821 | 5.98597 | 3.44867 |
| 10680 | 2.29488 | 3.0324 | 4.46173 | 2.23695 | 3.26574 | 1.16163 | 2.15861 | 1.92934 | 1.72291 | 3.86401 | 3.73772 |
| 15962 | 0.188629 | 3.38218 | 0 | 2.0322 | 1.07675 | 0.39124 | 0.404429 | 0.196014 | 0.385304 | 0 | 0 |
| 8901 | 0.985144 | 2.35447 | 1.83023 | 2.42777 | 2.56469 | 1.17479 | 2.36431 | 1.15917 | 1.338 | 1.71125 | 1.30725 |

|  | Glucose | Xylose | Arabinose | Acetate | Coumarate | Ferulate | YNB Oleic Acid | YNB Ricinoleic Acid | YNB Glucose | YNB Gluc DOC | YPD |
| --- | --- | --- | --- | --- | --- | --- | --- | --- | --- | --- | --- |
| proteinId |  |  |  |  |  |  |  |  |  |  |  |
| 14545 | 0.0209469 | -0.292783 | -0.0266595 | -0.0118151 | -0.199009 | 0.101022 | 0.0768783 | 0.290596 | 0.207443 | 0.17306 | -0.249223 |
| 11550 | 0.0774105 | 0.144846 | -0.018435 | 0.121283 | 0.226393 | 0.108317 | -0.0519291 | 0.166514 | 0.195984 | 0.0572959 | 0.122298 |
| 14369 | 0.323773 | -0.134946 | 0.179229 | 0.146106 | 0.0898245 | 0.388908 | 0.140926 | 0.0150337 | -0.223792 | 0.06217 | -0.00291277 |
| 15962 | 0.0348718 | -0.0178509 | 0.114898 | 0.0600965 | 0.0873949 | 0.05488 | 0.105901 | 0.153482 | 0.0853636 | 0.14613 | -0.246039 |
| 8901 | -0.00988828 | 0.196981 | 0.158386 | 0.096119 | -0.0526187 | -0.0215802 | 0.104289 | 0.000964794 | 0.377928 | 0.25981 | 0.0721251 |

In [123]:

```
for x in temp:
    if x in model.genes:
        for r in sorted(model.genes.get_by_id(x).reactions, key=lambda x: x.id):
            print(r, r.gene_reaction_rule)
    else:
        print(x, 'no reactions')
    print()
```

```
G3PT: glyc3p_c + h2o_c --> glyc_c + pi_c 13044 or 13413
HYPOE: h2o_c + pyam5p_c --> pi_c + pydam_c 13044
NTD2: h2o_c + ump_c --> pi_c + uri_c 13044 or 16648 or 9995
NTD4: cmp_c + h2o_c --> cytd_c + pi_c 13044 or 16648 or 9995
NTD7: amp_c + h2o_c --> adn_c + pi_c 13044 or 16648
NTD9: gmp_c + h2o_c --> gsn_c + pi_c 13044 or 16648
PDXPP: h2o_c + pdx5p_c --> pi_c + pydxn_c 13044 or 14545
PYDXPP: h2o_c + pydx5p_c --> pi_c + pydx_c 13044 or 14545
R5PP: h2o_c + r5p_c --> pi_c + rib__D_c 13044 or 14546 or 8576

PDXPP: h2o_c + pdx5p_c --> pi_c + pydxn_c 13044 or 14545
PYDAMK: atp_c + pydam_c --> adp_c + h_c + pyam5p_c 14545
PYDXK: atp_c + pydx_c --> adp_c + h_c + pydx5p_c 14545
PYDXNK: atp_c + pydxn_c --> adp_c + h_c + pdx5p_c 14545
PYDXPP: h2o_c + pydx5p_c --> pi_c + pydx_c 13044 or 14545

ALR2x: h_c + mthgxl_c + nadh_c --> acetol_c + nad_c 11550 or 14369
PYDXOR: h_c + nadph_c + pydx_c <=> nadp_c + pydxn_c 11550 or 14369

ALR2x: h_c + mthgxl_c + nadh_c --> acetol_c + nad_c 11550 or 14369
PYDXOR: h_c + nadph_c + pydx_c <=> nadp_c + pydxn_c 11550 or 14369

PDX5POi: o2_c + pdx5p_c --> h2o2_c + pydx5p_c 10680 or 8901
PYAM5PO: h2o_c + o2_c + pyam5p_c --> h2o2_c + nh4_c + pydx5p_c 10680 or 8901
PYDXNO: o2_c + pydxn_c <=> h2o2_c + pydx_c 10680 or 8901
PYDXO: 2.0 h2o_c + nh4_c + 0.5 o2_c + pydx_c <=> 2.0 h2o2_c + pydam_c 10680
PYDXO_1: h2o_c + o2_c + pydam_c <=> h2o2_c + nh4_c + pydx_c 10680 or 8901

5HOXINDACTO2OX: 5hoxindact_c + h2o_c + o2_c --> 5hoxindoa_c + h2o2_c + h_c 15962
HXAND: h2o_c + hxan_c + nad_c --> h_c + nadh_c + xan_c 15962
PYDXDH_1: h2o_c + o2_c + pydx_c --> 4pyrdx_c + h2o2_c + h_c 15962
XAND: h2o_c + nad_c + xan_c --> h_c + nadh_c + urate_c 15962
XANDp: h2o_x + nad_x + xan_x --> h_x + nadh_x + urate_x 15962
XAO2x: h2o_x + hxan_x + o2_x --> h2o2_x + xan_x 15962
XAOx: h2o_x + o2_x + xan_x --> h2o2_x + urate_x 15962

NADHXE: nadhx__S_c <=> nadhx__R_c 8901
NADPHXE: nadphx__R_c <=> nadphx__S_c 8901
PDX5POi: o2_c + pdx5p_c --> h2o2_c + pydx5p_c 10680 or 8901
PYAM5PO: h2o_c + o2_c + pyam5p_c --> h2o2_c + nh4_c + pydx5p_c 10680 or 8901
PYDXNO: o2_c + pydxn_c <=> h2o2_c + pydx_c 10680 or 8901
PYDXO_1: h2o_c + o2_c + pydam_c <=> h2o2_c + nh4_c + pydx_c 10680 or 8901
```

In [124]:

```
temp = ['13413','16648','12939','9995','11513','14546','8576']
display(Annotation.loc[temp])
Show_Data(temp)
```

|  | Combined Annotations | Signal P | Sc288c Orthologs | Human Orthologs | Sc288 Best Hit | Human Blast | Essential | WolfPSort | C Terminal |
| --- | --- | --- | --- | --- | --- | --- | --- | --- | --- |
| RTO4\_ID |  |  |  |  |  |  |  |  |  |
| 13413 | K01111: E3.1.3.68; 2-deoxyglucose-6-phosphatase |  | GPP2,GPP1,DOG2,DOG1 |  | DOG1 |  | Not Essential | cysk 17, cyto 10 | GTA\* |
| 16648 | KOG2157: Predicted tubulin-tyrosine ligase |  |  |  | PBY1 |  | Not Essential | mito 17, cyto 5, nucl 4 | WKI\* |
| 12939 | KOG4419: 5' nucleotidase |  | YHR202W |  | YHR202W |  | Not Essential | cyto 12.5, cyto\_nucl 12, nucl 10.5, pero 3 | EWN\* |
| 9995 | K18551: SDT1; pyrimidine and pyridine-specific... |  | SDT1,PHM8 |  | SDT1 |  | Not Essential | mito 17, nucl 5, pero 3 | GSS\* |
| 11513 | HMMPfam:haloacid dehalogenase-like hydrolase:P... |  |  |  |  |  | Not Essential | mito 10, cyto 7.5, cyto\_nucl 6.5, pero 5, nucl... | AYP\* |
| 14546 | K17623: HDHD1; pseudouridine 5'-phosphatase |  | YKL033W-A | PUDP | YKL033W-A | PUDP | Not Essential | cyto 10.5, cysk 9, cyto\_nucl 8, nucl 4.5 | YDQ\* |
| 8576 | K19270: yfbT, yniC; sugar-phosphatase |  |  |  | GPP2 |  | Not Essential | cyto 18, cysk 8 | FVV\* |

| strain | WT | | | | | | | | | | | | | | | | |
| --- | --- | --- | --- | --- | --- | --- | --- | --- | --- | --- | --- | --- | --- | --- | --- | --- | --- |
| condition | G\_MM | C\_MM | G\_SD | | GX\_SD | | | X\_SD | | A\_SD | | C\_SD | | MM\_CN120 | | MM\_CN5 | Diversity\_Sample |
| phase | exp | exp | exp | stat | exp | trans | stat | exp | stat | exp | stat | exp | stat | exp | stat | exp | exp |
| proteinId | Set1 | Set1 | Set2 | Set2 | Set2 | Set2 | Set2 | Set2 | Set2 | Set2 | Set2 | Set2 | Set2 | Set3 | Set3 | Set3 | Set3 |
| 13413 | 5.60139 | 6.34458 | 5.56352 | 5.20157 | 5.68008 | 5.59498 | 5.59586 | 5.83845 | 5.32538 | 5.79563 | 5.33654 | 5.97306 | 5.86648 | 5.23733 | 5.21087 | 5.93192 | 5.5741 |
| 16648 | 5.43202 | 5.96502 | 5.93189 | 5.63877 | 5.94219 | 5.47454 | 5.58875 | 5.9459 | 5.89091 | 5.92467 | 6.08698 | 5.75969 | 5.76749 | 4.69399 | 4.72659 | 5.70285 | 5.82742 |
| 12939 | 7.03722 | 6.61095 | 7.26156 | 7.13007 | 7.41427 | 6.78849 | 6.95048 | 6.53681 | 6.09522 | 6.3334 | 6.63799 | 7.48741 | 8.18115 | 6.30036 | 5.97519 | 7.32133 | 6.49743 |
| 9995 | 5.12907 | 5.69332 | 7.19278 | 6.15372 | 7.27451 | 6.1769 | 6.09996 | 6.39948 | 5.95622 | 5.65428 | 5.91294 | 6.30091 | 6.50027 | 4.67632 | 4.09637 | 6.0714 | 4.78932 |
| 11513 | 7.63976 | 6.34911 | 8.82403 | 8.57657 | 8.90305 | 8.55555 | 8.46384 | 7.2824 | 8.63429 | 6.87232 | 7.27209 | 8.07761 | 9.22487 | 8.65123 | 8.62297 | 9.0862 | 8.26419 |
| 14546 | 6.70306 | 6.64705 | 6.55577 | 6.15421 | 6.48538 | 6.55323 | 6.32456 | 6.58575 | 5.78236 | 6.22821 | 6.08386 | 6.23016 | 5.42504 | 5.40667 | 5.47056 | 5.79605 | 5.61121 |
| 8576 | 1.0794 | 0.991857 | 0.705599 | 2.30449 | 0.641345 | 0.640812 | 1.05624 | 0.535925 | 1.06389 | 0.7932 | 1.83137 | 0.86341 | 1.07793 | 0.529077 | 0.652997 | 0.461709 | 0.63206 |

| strain | WT | | | | | | | | | | |
| --- | --- | --- | --- | --- | --- | --- | --- | --- | --- | --- | --- |
| condition | G\_SD | | GX\_SD | | | X\_SD | | A\_SD | | C\_SD | |
| proteinId | exp | stat | exp | trans | stat | exp | stat | exp | stat | exp | stat |
| 13413 | 3.98763 | 1.28818 | 4.88072 | 0.737724 | 1.48285 | 3.73392 | 1.79096 | 1.93779 | 2.09957 | 4.26527 | 3.7106 |
| 16648 | 5.57663 | 3.27776 | 6.50998 | 4.07744 | 3.46827 | 6.43377 | 4.14229 | 5.02047 | 4.59626 | 3.87069 | 4.55697 |
| 12939 | 0.783453 | 1.43588 | 0.197656 | 2.41466 | 1.11165 | 1.3651 | 1.74897 | 1.93552 | 2.10219 | 0.219601 | 1.95192 |
| 9995 | 1.34068 | 0.94019 | 2.02488 | 1.8649 | 1.24834 | 0.984473 | 0.983449 | 0.967668 | 0.959383 | 1.05505 | 0.422666 |
| 11513 | 16.1014 | 20.8312 | 16.2718 | 17.1194 | 17.8821 | 7.0392 | 12.9913 | 5.60657 | 9.95543 | 20.0688 | 19.362 |
| 14546 | 5.7393 | 6.66996 | 5.30115 | 7.26182 | 7.70842 | 8.0393 | 8.65673 | 6.96264 | 7.85284 | 5.34428 | 7.41173 |

|  | Glucose | Xylose | Arabinose | Acetate | Coumarate | Ferulate | YNB Oleic Acid | YNB Ricinoleic Acid | YNB Glucose | YNB Gluc DOC | YPD |
| --- | --- | --- | --- | --- | --- | --- | --- | --- | --- | --- | --- |
| proteinId |  |  |  |  |  |  |  |  |  |  |  |
| 13413 | 0.0653619 | -0.121519 | 0.17951 | 0.0909367 | 0.399522 | 0.263557 | -0.530393 | 0.667075 | 0.651914 | 0.610799 | 0.728724 |
| 12939 | -0.0629916 | 0.0602598 | 0.0552619 | 0.0636762 | 0.383564 | -0.0264626 | -0.0932594 | 0.18259 | 0.46209 | 0.847028 | 0.508303 |
| 9995 | 0.368148 | 0.35758 | 0.0592439 | 0.409551 | 0.167506 | 0.120755 | 0.814853 | -0.430807 | -1.35726 | -0.190163 | 1.44696 |
| 11513 | 0.0450204 | 0.0302811 | -0.04327 | 0.0507433 | -0.535173 | 0.0529926 | -0.0692324 | -0.128559 | -0.175321 | -0.272272 | -0.0418747 |
| 14546 | 0.441895 | 0.052392 | 0.302713 | 0.220549 | -0.147649 | 0.167133 | -0.101398 | -0.225625 | -0.193782 | -0.238061 | 0.105055 |
| 8576 | 0.0928334 | 0.0403074 | 0.342139 | 0.0272539 | 1.10922 | 0.219086 | -0.299253 | 0.0333091 | 0.136365 | -0.0567877 | 0.190766 |

In [125]:

```
model.reactions.get_by_id('G3PT').gene_reaction_rule = '13413'
model.reactions.get_by_id('PDXPP').gene_reaction_rule = '13044'
model.reactions.get_by_id('PYDXPP').gene_reaction_rule = '13044'
model.reactions.get_by_id('R5PP').gene_reaction_rule = '14546 or 8576'
# 16648 is 5'-nucleotidase SurE
model.reactions.get_by_id('NTD2').gene_reaction_rule = '16648 or 9995'
model.reactions.get_by_id('NTD4').gene_reaction_rule = '16648 or 9995'
model.reactions.get_by_id('NTD7').gene_reaction_rule = '16648'
model.reactions.get_by_id('NTD9').gene_reaction_rule = '16648'
# 8901 is NAXE, remove it from pyridoxine reactions
model.reactions.get_by_id('PDX5POi').gene_reaction_rule = '10680'
model.reactions.get_by_id('PYAM5PO').gene_reaction_rule = '10680'
model.reactions.get_by_id('PYDXNO').gene_reaction_rule = '10680'
model.reactions.get_by_id('PYDXO_1').gene_reaction_rule = '10680'
# 11550 or 14369 YPR127W pyridoxine 4-dehydrogenase, remove ALR2x (E. coli ydjG)
# 15962 is XDH, but not AOX1, remove AOX1 reactions
model.remove_reactions(['ALR2x','PYDXO','5HOXINDACTO2OX','PYDXDH_1'],remove_orphans=True)
```

In [126]:

```
# second half
# https://www.sciencedirect.com/science/article/pii/S0005272806001630#fig3
# https://www.sciencedirect.com/science/article/pii/S0098299712000519#f0005
# https://www.sciencedirect.com/science/article/pii/S016748891630060X#f0015
temp = ['16799','11740','9011','13510','10514','16746','10635','12605','15874','8889']
display(Annotation.loc[temp])
Show_Data(temp)
```

|  | Combined Annotations | Signal P | Sc288c Orthologs | Human Orthologs | Sc288 Best Hit | Human Blast | Essential | WolfPSort | C Terminal |
| --- | --- | --- | --- | --- | --- | --- | --- | --- | --- |
| RTO4\_ID |  |  |  |  |  |  |  |  |  |
| 16799 | K15105: SLC25A12\_13, AGC; solute carrier famil... | S | AGC1 | SLC25A12,SLC25A13 | AGC1 | SLC25 | Not Essential | mito 22, cyto 3 | SQK\* |
| 11740 | K15103: UCP2\_3, SLC25A8\_9; solute carrier fami... |  |  | UCP2 | OAC1 | SLC25 | Not Essential | mito 15, cysk 6, cyto 4.5, cyto\_nucl 3 | PPV\* |
| 9011 | K15117: SLC25A34\_35, OAC1; solute carrier fami... | S | OAC1 | SLC25A35 | OAC1 | SLC25 | Not Essential | extr 14, mito 11 | ARD\* |
| 13510 | K13577: SLC25A10, DIC; solute carrier family 2... |  | DIC1 | SLC25A11,RP13-1032I1.10 | DIC1 | SLC25 | Not Essential | mito 15, extr 7, cyto 3 | GTP\* |
| 10514 | K15100: SLC25A1, CTP; solute carrier family 25... | S | CTP1 | SLC25A1 | CTP1 | SLC25A | Not Essential | extr 13, mito 5, E.R. 3, vacu 3, cyto\_mito 3 | RAV\* |
| 16746 | KOG0756: Mitochondrial tricarboxylate/dicarbox... |  | YHM2 |  | YHM2 |  | Not Essential | cyto 13, mito 10, cysk 2 | NKK\* |
| 10635 | K15110: SLC25A21, ODC; solute carrier family 2... |  | ODC2,ODC1 | SLC25A21 | ODC2 | SLC25 | Not Essential | mito 13, cyto 10, extr 3 | PYI\* |
| 12605 | K15100: SLC25A1, CTP; solute carrier family 25... | S | SFC1 |  | SFC1 | SLC25A | Not Essential | mito 11, cyto 9.5, cyto\_nucl 5.5, plas 3 | YSE\* |
| 15874 | K15102: SLC25A3, PHC, PIC; solute carrier fami... |  | MIR1,PIC2 | SLC25A3 | MIR1 | SLC25 | Not Essential | mito 12, extr 7, cyto 5.5, cyto\_nucl 3.5 | HKD\* |
| 8889 | K15102: SLC25A3, PHC, PIC; solute carrier fami... | S | MIR1,PIC2 | SLC25A3 | PIC2 | SLC25 | Essential | mito 14, extr 10, cyto 3 | TKK\* |

| strain | WT | | | | | | | | | | | | | | | | |
| --- | --- | --- | --- | --- | --- | --- | --- | --- | --- | --- | --- | --- | --- | --- | --- | --- | --- |
| condition | G\_MM | C\_MM | G\_SD | | GX\_SD | | | X\_SD | | A\_SD | | C\_SD | | MM\_CN120 | | MM\_CN5 | Diversity\_Sample |
| phase | exp | exp | exp | stat | exp | trans | stat | exp | stat | exp | stat | exp | stat | exp | stat | exp | exp |
| proteinId | Set1 | Set1 | Set2 | Set2 | Set2 | Set2 | Set2 | Set2 | Set2 | Set2 | Set2 | Set2 | Set2 | Set3 | Set3 | Set3 | Set3 |
| 16799 | 4.32711 | 5.83054 | 5.98353 | 5.46332 | 6.29856 | 5.55378 | 5.55193 | 6.03847 | 5.99353 | 5.29935 | 5.31889 | 6.47172 | 7.02717 | 5.41403 | 5.04732 | 7.50394 | 6.80801 |
| 11740 | 4.47 | 5.36422 | 4.04 | 5.02776 | 3.93205 | 4.40857 | 4.35313 | 3.67924 | 5.11429 | 4.17105 | 5.17872 | 5.43859 | 4.42604 | 3.57264 | 3.15624 | 4.36255 | 3.6936 |
| 9011 | 5.89309 | 6.55275 | 6.61983 | 4.95738 | 6.65087 | 6.41585 | 6.26478 | 6.28991 | 5.60528 | 5.76342 | 5.41461 | 5.93105 | 6.01452 | 4.38165 | 3.85428 | 4.00567 | 3.83548 |
| 13510 | 3.67785 | 5.79102 | 4.45268 | 4.79481 | 4.53865 | 5.12837 | 5.06374 | 4.59067 | 4.68711 | 4.30165 | 4.25627 | 5.78462 | 5.45423 | 4.13705 | 4.19705 | 4.23321 | 4.25478 |
| 10514 | 6.64861 | 6.36247 | 5.70844 | 6.11042 | 5.80885 | 5.78159 | 6.03447 | 5.97857 | 6.31136 | 5.74928 | 6.16677 | 6.01711 | 5.93248 | 5.65323 | 6.06213 | 5.85188 | 5.94402 |
| 16746 | 8.32844 | 7.7739 | 6.43769 | 5.79445 | 6.46432 | 6.49526 | 6.43446 | 7.0482 | 5.99569 | 6.58726 | 5.82043 | 6.58361 | 6.1044 | 9.01635 | 8.176 | 8.3526 | 7.7701 |
| 10635 | 7.33112 | 7.61288 | 7.75953 | 6.232 | 7.91259 | 7.00056 | 7.07415 | 7.612 | 6.83015 | 6.70199 | 6.34543 | 6.97135 | 6.59061 | 8.44963 | 7.55154 | 8.43238 | 7.96764 |
| 12605 | 3.68142 | 7.62714 | 6.93159 | 5.79548 | 7.109 | 6.20355 | 6.25958 | 5.07552 | 7.14139 | 5.11675 | 6.20272 | 7.1788 | 6.93089 | 4.27684 | 4.24007 | 5.1688 | 5.35612 |
| 15874 | 5.54334 | 4.37285 | 7.55907 | 6.05993 | 7.46388 | 7.0809 | 6.98891 | 6.53448 | 6.37383 | 5.792 | 5.54409 | 6.01274 | 5.50865 | 6.06785 | 6.19675 | 4.88 | 5.91761 |
| 8889 | 7.50261 | 8.3532 | 8.71937 | 8.13574 | 8.82621 | 8.66146 | 8.42327 | 8.63025 | 7.90258 | 8.22008 | 7.35762 | 8.69909 | 8.89227 | 9.35379 | 9.28452 | 9.74086 | 10.2743 |

| strain | WT | | | | | | | | | | |
| --- | --- | --- | --- | --- | --- | --- | --- | --- | --- | --- | --- |
| condition | G\_SD | | GX\_SD | | | X\_SD | | A\_SD | | C\_SD | |
| proteinId | exp | stat | exp | trans | stat | exp | stat | exp | stat | exp | stat |
| 16799 | 19.8097 | 18.5619 | 19.73 | 16.5552 | 17.1418 | 21.1478 | 21.2154 | 20.6725 | 17.3772 | 16.6663 | 21.743 |
| 9011 | 7.25412 | 6.77329 | 5.48653 | 5.20587 | 5.45571 | 6.26271 | 5.68882 | 5.22074 | 3.63032 | 3.827 | 3.9244 |
| 10514 | 6.13985 | 6.66771 | 6.71306 | 5.58373 | 4.20338 | 4.89588 | 5.27854 | 5.79215 | 6.1183 | 3.87384 | 5.66964 |
| 16746 | 10.3876 | 7.97233 | 9.98947 | 8.95814 | 9.48008 | 10.1577 | 10.4443 | 11.593 | 10.3239 | 14.2932 | 12.1912 |
| 10635 | 17.7285 | 17.3639 | 12.6183 | 14.3394 | 12.9216 | 11.952 | 10.997 | 10.433 | 7.44828 | 14.9941 | 14.5726 |
| 12605 | 7.94394 | 7.96008 | 6.70555 | 6.69664 | 6.41054 | 6.06328 | 6.21227 | 2.31934 | 4.38725 | 16.6563 | 18.1005 |
| 15874 | 11.5429 | 13.7323 | 12.7904 | 16.9395 | 14.0497 | 9.80303 | 9.11259 | 5.61108 | 6.48984 | 8.76984 | 8.91861 |
| 8889 | 40.6881 | 40.4801 | 38.6526 | 38.7215 | 36.1525 | 34.7209 | 40.3551 | 33.42 | 34.8855 | 44.7328 | 49.8519 |

|  | Glucose | Xylose | Arabinose | Acetate | Coumarate | Ferulate | YNB Oleic Acid | YNB Ricinoleic Acid | YNB Glucose | YNB Gluc DOC | YPD |
| --- | --- | --- | --- | --- | --- | --- | --- | --- | --- | --- | --- |
| proteinId |  |  |  |  |  |  |  |  |  |  |  |
| 16799 | 0.00860844 | 0.284126 | -0.110846 | -0.472156 | -0.0178013 | -0.848331 | -1.27882 | -1.48965 | -0.207239 | -0.0405575 | 0.0403966 |
| 11740 | 0.109553 | 0.00205556 | -0.00502673 | -0.149136 | 0.237068 | 0.0193826 | 0.313053 | 0.0534518 | 0.095281 | 0.428816 | 0.101248 |
| 9011 | 0.0241941 | -0.272725 | 0.161747 | -0.269852 | 0.0798061 | -0.031333 | -0.0244879 | -0.492747 | 0.287088 | 0.0815357 | -0.107048 |
| 13510 | 0.206135 | 0.686782 | 0.453603 | -0.0292148 | -0.421161 | -0.352184 | 0.219954 | -0.116016 | -0.17613 | 0.0633609 | 0.114048 |
| 10514 | 0.278631 | -0.163472 | -0.438358 | -0.0852117 | 0.465255 | -0.211769 | 0.0577349 | -1.659 | -0.743333 | -0.169441 | 0.0921063 |
| 10635 | 0.205165 | 0.0964621 | 0.203563 | 0.261393 | -3.43862 | -4.08137 | -0.480008 | -0.819799 | 0.253622 | 0.413434 | -0.0938174 |
| 12605 | -0.250531 | -1.12124 | -1.27614 | -1.68184 | -1.69155 | -1.56883 | 0.318931 | 0.287611 | 1.11429 | 0.16432 | 2.2361 |
| 15874 | -0.268098 | -0.0197262 | 0.0222415 | -0.262453 | 0.268151 | -0.511158 | 0.130008 | 0.406326 | 0.236206 | -0.0925991 | 0.264876 |

In [127]:

```
for x in temp:
    if x in model.genes:
        for r in sorted(model.genes.get_by_id(x).reactions, key=lambda x: x.id):
            print(r, r.gene_reaction_rule)
    else:
        print(x, 'no reactions')
    print()
```

```
3SALAASPm: 3sala_m + asp__L_c <=> 3sala_c + asp__L_m 16799
ASPGLU2m: asp__L_m + glu__L_c --> asp__L_c + glu__L_m 16799
ASPGLUm: asp__L_m + glu__L_c + h_c <=> asp__L_c + glu__L_m + h_m 16799
GLUt7m: glu__L_c --> glu__L_m 16799

AKGCITtm: akg_c + cit_m <=> akg_m + cit_c 11740 or 13510
AKGICITtm: akg_c + icit_m <=> akg_m + icit_c 11740 or 13510
AKGMALtm: akg_m + mal__L_c <=> akg_c + mal__L_m 11740 or 13510
CITtam: cit_c + mal__L_m <=> cit_m + mal__L_c 10514 or 11740 or 13510
CITtcm: cit_c + icit_m <=> cit_m + icit_c 10514 or 11740 or 13510
Htm: h_c --> h_m 11740
MALICITtm: icit_m + mal__L_c <=> icit_c + mal__L_m 11740 or 13510
MALOAAtm: mal__L_m + oaa_c <=> mal__L_c + oaa_m 11740 or 13510
MALtm: mal__L_c + pi_m <=> mal__L_m + pi_c 11740 or 13510
OAAAKGtm: akg_m + oaa_c <=> akg_c + oaa_m 11740 or 13510
OAACITtm: cit_m + oaa_c <=> cit_c + oaa_m 11740 or 13510
OAAICITtm: icit_m + oaa_c <=> icit_c + oaa_m 11740 or 13510
SUCCtm: pi_m + succ_c --> pi_c + succ_m 11740 or 13510
SUCFUMtm: fum_m + succ_c --> fum_c + succ_m 11740 or 12605 or 13510 or (11740 and 12605) or (12605 and 13510)

OAAt2m: h_c + oaa_c <=> h_m + oaa_m 9011

AKGCITtm: akg_c + cit_m <=> akg_m + cit_c 11740 or 13510
AKGICITtm: akg_c + icit_m <=> akg_m + icit_c 11740 or 13510
AKGMALtm: akg_m + mal__L_c <=> akg_c + mal__L_m 11740 or 13510
CITtam: cit_c + mal__L_m <=> cit_m + mal__L_c 10514 or 11740 or 13510
CITtcm: cit_c + icit_m <=> cit_m + icit_c 10514 or 11740 or 13510
FUMSO3tm: fum_c + so3_m <=> fum_m + so3_c 13510
FUMSO4tm: fum_c + so4_m <=> fum_m + so4_c 13510
FUMTSULtm: fum_c + tsul_m <=> fum_m + tsul_c 13510
FUMtm: fum_c + pi_m <=> fum_m + pi_c 13510
MALICITtm: icit_m + mal__L_c <=> icit_c + mal__L_m 11740 or 13510
MALOAAtm: mal__L_m + oaa_c <=> mal__L_c + oaa_m 11740 or 13510
MALSO3tm: mal__L_c + so3_m <=> mal__L_m + so3_c 13510
MALSO4tm: mal__L_c + so4_m <=> mal__L_m + so4_c 13510
MALTSULtm: mal__L_c + tsul_m <=> mal__L_m + tsul_c 13510
MALtm: mal__L_c + pi_m <=> mal__L_m + pi_c 11740 or 13510
OAAAKGtm: akg_m + oaa_c <=> akg_c + oaa_m 11740 or 13510
OAACITtm: cit_m + oaa_c <=> cit_c + oaa_m 11740 or 13510
OAAICITtm: icit_m + oaa_c <=> icit_c + oaa_m 11740 or 13510
SUCCtm: pi_m + succ_c --> pi_c + succ_m 11740 or 13510
SUCFUMtm: fum_m + succ_c --> fum_c + succ_m 11740 or 12605 or 13510 or (11740 and 12605) or (12605 and 13510)

CITtam: cit_c + mal__L_m <=> cit_m + mal__L_c 10514 or 11740 or 13510
CITtbm: cit_c + pep_m <=> cit_m + pep_c 10514
CITtcm: cit_c + icit_m <=> cit_m + icit_c 10514 or 11740 or 13510

16746 no reactions

2AMADPTm: L2aadp_c + akg_m <=> L2aadp_m + akg_c 10635
2OXOADPTm: 2oxoadp_c + akg_m <=> 2oxoadp_m + akg_c 10635
OXO2Ctm: akg_m + oxag_c <=> akg_c + oxag_m 10635

SUCFUMtm: fum_m + succ_c --> fum_c + succ_m 11740 or 12605 or 13510 or (11740 and 12605) or (12605 and 13510)

PIt2m: h_c + pi_c <=> h_m + pi_m 15874 or 8889

PIt2m: h_c + pi_c <=> h_m + pi_m 15874 or 8889
```

In [128]:

```
# 16799 AGC1, also acts as glutamate uniporter, ASPGLU2m incorrect
# https://onlinelibrary.wiley.com/doi/full/10.1046/j.1365-2958.2003.03742.x
# ASPGLUm asp__L_m + glu__L_c + h_c -> asp__L_c + glu__L_m + h_m correct
model.reactions.get_by_id('ASPGLUm').lower_bound = 0.0
model.remove_reactions(['ASPGLU2m'], remove_orphans=True)
# 11740 UCP2, asp/oaa/mal export in exchange with h + pi remove 11740 from other reactions
# https://www.pnas.org/content/111/3/960
# Htm is by UCP1, but no UCP1 is found in Rhodo
r = model.reactions.get_by_id('OAAt2m').copy()
r.id = 'UCP2ASPtm'
r.name = 'Uncoupling protein 2 (aspartate), mitochondrial'
r.gene_reaction_rule = '11740'
r.lower_bound = 0.0
model.add_reactions([r])
r.add_metabolites({'oaa_c': 1.0, 'oaa_m': -1.0, 'pi_c': -1.0, 'pi_m': 1.0,
                   'asp__L_m': -1.0, 'asp__L_c': 1.0})
r = model.reactions.get_by_id('OAAt2m').copy()
r.id = 'UCP2MALtm'
r.name = 'Uncoupling protein 2 (malate), mitochondrial'
r.gene_reaction_rule = '11740'
r.lower_bound = 0.0
model.add_reactions([r])
r.add_metabolites({'oaa_c': 1.0, 'oaa_m': -1.0, 'pi_c': -1.0, 'pi_m': 1.0,
                   'mal__L_m': -1.0, 'mal__L_c': 1.0})
r = model.reactions.get_by_id('OAAt2m').copy()
r.id = 'UCP2OAAtm'
r.name = 'Uncoupling protein 2 (oxaloacetate), mitochondrial'
r.gene_reaction_rule = '11740'
r.lower_bound = 0.0
model.add_reactions([r])
r.add_metabolites({'oaa_c': 2.0, 'oaa_m': -2.0, 'pi_c': -1.0, 'pi_m': 1.0})
model.remove_reactions(['Htm','OAAt2m'], remove_orphans=True)
# 9011 OAC1 transports oaa, so4, thiosulfate, 3c3hmp_m (a-isopropylmalate)
# http://www.jbc.org/content/274/32/22184.full.pdf
# http://www.jbc.org/content/283/42/28445.full
# add 3c3hmp_m export / oaa import
r = model.reactions.get_by_id('OAAAKGtm').copy()
r.id = 'OAAIPMtm'
r.name = 'Dicarboxylate/tricarboxylate carrier (oaa:3c3hmp), mitochondrial'
r.gene_reaction_rule = '9011'
r.lower_bound = 0.0
model.add_reactions([r])
r.add_metabolites({'akg_m': 1.0, 'akg_c': -1.0, '3c3hmp_m': -1.0, '3c3hmp_c': 1.0})
model.remove_reactions(['OAAAKGtm','OAACITtm','OAAICITtm'], remove_orphans=True)
# 13510 DIC1 imports malate and succinate in exchange of pi, so4, thiosulfate
model.reactions.get_by_id('MALtm').gene_reaction_rule = '13510'
model.reactions.get_by_id('SUCCtm').gene_reaction_rule = '13510'
model.reactions.get_by_id('MALtm').lower_bound = 0.0
model.reactions.get_by_id('SUCCtm').lower_bound = 0.0
model.remove_reactions(['MALOAAtm','MALSO3tm','MALSO4tm','MALTSULtm'], remove_orphans=True)
# 10514 CTP1 citrate exchange with icit / mal
model.reactions.get_by_id('CITtam').gene_reaction_rule = '10514'
model.reactions.get_by_id('CITtcm').gene_reaction_rule = '10514'
model.remove_reactions(['CITtbm','MALICITtm'], remove_orphans=True)
# 16746 YHM2 exports citrate and imports akg into mito
model.reactions.get_by_id('AKGCITtm').gene_reaction_rule = '16746'
model.reactions.get_by_id('AKGCITtm').lower_bound = 0.0
model.remove_reactions(['AKGICITtm'], remove_orphans=True)
# 10635 ODC1/ODC2 exports akg/2oxoadp_c in exchange with mal
model.reactions.get_by_id('AKGMALtm').gene_reaction_rule = '10635'
model.reactions.get_by_id('AKGMALtm').lower_bound = 0.0
model.remove_reactions(['2AMADPTm','OXO2Ctm'], remove_orphans=True)
# 12605 SFC1 imports succ and exports fum
model.reactions.get_by_id('SUCFUMtm').gene_reaction_rule = '12605'
model.reactions.get_by_id('SUCFUMtm').lower_bound = 0.0
model.remove_reactions(['FUMSO3tm','FUMSO4tm','FUMTSULtm','FUMtm'], remove_orphans=True)
# 15874 MIR1 or 8889 PIC2 imports pi
model.reactions.get_by_id('PIt2m').lower_bound = 0.0
```

In [129]:

```
temp = list(Annotation.index[Annotation['Combined Annotations'].str.contains('mitochondrial') &
                            Annotation['Combined Annotations'].str.contains('carrier')]) + \
       list(Annotation.index[Annotation['Combined Annotations'].str.contains('SLC25A')])
temp = set(temp) - set(['16799','11740','9011','13510','10514','16746','10635','12605','15874','8889'])
temp = sorted(list(temp), key=lambda x: Annotation.loc[x, 'Combined Annotations'])
display(Annotation.loc[temp])
Show_Data(temp)
```

|  | Combined Annotations | Signal P | Sc288c Orthologs | Human Orthologs | Sc288 Best Hit | Human Blast | Essential | WolfPSort | C Terminal |
| --- | --- | --- | --- | --- | --- | --- | --- | --- | --- |
| RTO4\_ID |  |  |  |  |  |  |  |  |  |
| 12704 | K05863: SLC25A4S, ANT; solute carrier family 2... |  | PET9,AAC3 | SLC25A4,SLC25A5,SLC25A6,SLC25A31 | PET9 | SLC25A | Essential | mito 17.5, cyto\_mito 11.166, extr 5, cyto 3.5 | GSG\* |
| 16095 | K13354: SLC25A17, PMP34; solute carrier family... |  |  | SLC25A17 | FLX1 | SLC25 | Not Essential | mito 11, extr 7, cyto 5, pero 4 | AKA\* |
| 9695 | K14684: SLC25A23S; solute carrier family 25 (m... |  | SAL1 | SLC25A23,SLC25A25,SLC25A24 | SAL1 | SLC25 | Not Essential | mito 10, cyto 9, cyto\_nucl 9, nucl 7 | EES\* |
| 16440 | K14684: SLC25A23S; solute carrier family 25 (m... |  | YPR011C |  | YPR011C | SLC25 | Not Essential | mito 12, cyto 12, cyto\_mito 12 | SEA\* |
| 8916 | K15084: SLC25A16, GDA, LEU5; solute carrier fa... |  | LEU5 | SLC25A16,SLC25A42 | LEU5 | SLC25 | Essential | mito 13, nucl 8, cyto 4 | EGR\* |
| 12518 | K15101: SLC25A2\_15, ORNT; solute carrier famil... |  | YMC2 |  | YMC2 | SLC25 | Not Essential | mito 10, cyto 6, mito\_nucl 6, extr 4, cyto\_nucl 4 | TSL\* |
| 9331 | K15109: SLC25A20\_29, CACT, CACL, CRC1; solute ... |  | CRC1 | SLC25A20 | CRC1 | SLC25A | Not Essential | mito 13.5, cyto\_mito 12.5, cyto 10.5 | ALF\* |
| 10149 | K15109: SLC25A20\_29, CACT, CACL, CRC1; solute ... |  |  |  | YMC2 | SLC25 | Not Essential | mito 11, plas 10, nucl 1, cyto 1, extr 1, cyto... | TSD\* |
| 12032 | K15111: SLC25A26; solute carrier family 25 (mi... |  | PET8 | SLC25A26 | PET8 | SLC25 | Not Essential | mito 10, extr 8, cyto 7 | ERD\* |
| 12446 | K15113: SLC25A28\_37, MFRN; solute carrier fami... |  | MRS3,MRS4 | SLC25A37,SLC25A28 | MRS4 | SLC25 | Essential | mito 10, cyto 5.5, extr 5, cysk 5, cyto\_nucl 3.5 | VRD\* |
| 13319 | K15114: ORT1; mitochondrial ornithine carrier ... |  | ORT1 |  | ORT1 | SLC25 | Not Essential | plas 22, E.R. 2, nucl 1, mito 1, cyto 1, cyto\_... | FYM\* |
| 14159 | K15115: SLC25A32, MFT; solute carrier family 2... |  | FLX1 | SLC25A32 | FLX1 | SLC25 | Essential | mito 12, cyto 11, nucl 2 | QVS\* |
| 10961 | K15115: SLC25A32, MFT; solute carrier family 2... |  | YEA6,YIA6 |  | YIA6 | SLC25 | Not Essential | mito 20, cyto 5 | RTS\* |
| 12387 | K15116: SLC25A33\_36, RIM2; solute carrier fami... |  | RIM2 | SLC25A36,SLC25A33 | RIM2 | SLC25 | Essential | mito 10, cyto 7.5, cyto\_nucl 7, nucl 5.5, pero 3 | RRD\* |
| 8434 | K15118: SLC25A38; solute carrier family 25, me... |  | YDL119C | SLC25A38 | YDL119C | SLC25 | Not Essential | mito 12, cyto 8.5, cyto\_nucl 6.5, nucl 3.5 | GGG\* |
| 11375 | K15119: SLC25A39\_40; solute carrier family 25,... |  | MTM1 | SLC25A40,SLC25A39 | MTM1 | SLC25 | Essential | cyto 17.5, cyto\_nucl 10.5, mito 5, nucl 2.5 | RVE\* |
| 16145 | KOG0769: Predicted mitochondrial carrier protein | S | ANT1 |  | ANT1 | SLC25 | Not Essential | mito 12, cyto 4.5, cyto\_nucl 3.5, E.R. 3, plas... | TSI\* |

| strain | WT | | | | | | | | | | | | | | | | |
| --- | --- | --- | --- | --- | --- | --- | --- | --- | --- | --- | --- | --- | --- | --- | --- | --- | --- |
| condition | G\_MM | C\_MM | G\_SD | | GX\_SD | | | X\_SD | | A\_SD | | C\_SD | | MM\_CN120 | | MM\_CN5 | Diversity\_Sample |
| phase | exp | exp | exp | stat | exp | trans | stat | exp | stat | exp | stat | exp | stat | exp | stat | exp | exp |
| proteinId | Set1 | Set1 | Set2 | Set2 | Set2 | Set2 | Set2 | Set2 | Set2 | Set2 | Set2 | Set2 | Set2 | Set3 | Set3 | Set3 | Set3 |
| 12704 | 10.7125 | 11.6295 | 11.2519 | 10.1147 | 11.409 | 11.2499 | 11.2737 | 10.9024 | 10.3916 | 10.4446 | 9.40092 | 11.2293 | 11.7268 | 12.345 | 12.2833 | 12.3893 | 13.3966 |
| 16095 | 5.33936 | 7.96058 | 6.20852 | 5.12572 | 6.17072 | 6.18045 | 5.70656 | 6.01834 | 5.54299 | 6.07046 | 5.84448 | 4.83812 | 4.92364 | 6.0347 | 6.1465 | 6.05857 | 6.14867 |
| 9695 | 4.94286 | 4.74986 | 3.08825 | 4.29393 | 3.02266 | 3.83447 | 3.90367 | 3.46026 | 4.13573 | 3.95468 | 4.35132 | 4.87629 | 4.76023 | 4.78328 | 4.92127 | 3.37891 | 5.28685 |
| 16440 | 7.61625 | 6.09961 | 6.94055 | 5.90159 | 7.29428 | 6.626 | 6.66132 | 6.22295 | 7.19372 | 6.93112 | 6.40088 | 6.80255 | 7.16465 | 6.73702 | 6.17301 | 7.09392 | 5.73373 |
| 8916 | 3.5033 | 4.86855 | 4.98601 | 5.45963 | 4.98192 | 4.79806 | 4.85235 | 4.25779 | 5.6862 | 4.28898 | 5.54346 | 5.13718 | 5.60496 | 3.298 | 3.93719 | 3.86015 | 3.70576 |
| 12518 | 6.75333 | 6.81284 | 7.46053 | 5.37109 | 7.81633 | 6.0421 | 5.9418 | 6.66481 | 5.45865 | 6.0147 | 5.42868 | 6.64352 | 6.86775 | 7.15111 | 6.35676 | 8.47584 | 6.26055 |
| 9331 | 5.75034 | 6.90719 | 6.47204 | 5.74082 | 6.55278 | 5.96035 | 6.05061 | 6.26286 | 6.44384 | 5.92166 | 6.10074 | 6.2095 | 5.9384 | 5.71808 | 5.53064 | 6.08302 | 5.74333 |
| 10149 | 4.39904 | 4.09667 | 5.06018 | 4.97188 | 5.16128 | 4.82506 | 4.96388 | 4.68437 | 5.28909 | 4.66677 | 5.26263 | 4.70503 | 3.82369 | 3.34746 | 3.44983 | 3.0779 | 3.20093 |
| 12032 | 5.75086 | 5.93131 | 5.47091 | 4.67222 | 5.52475 | 5.36429 | 5.38112 | 5.53098 | 5.09252 | 5.38708 | 5.26085 | 5.48794 | 5.2832 | 4.47949 | 4.55223 | 4.82195 | 5.28425 |
| 12446 | 3.65148 | 4.82901 | 4.50322 | 4.56572 | 4.53259 | 5.2448 | 5.27283 | 4.66011 | 5.59724 | 4.43915 | 4.85287 | 5.05288 | 5.11763 | 4.7798 | 5.20094 | 4.82951 | 5.62769 |
| 13319 | 6.06425 | 5.7694 | 5.87581 | 4.77748 | 5.95673 | 5.45114 | 5.22988 | 5.87009 | 5.51806 | 5.71512 | 5.24338 | 5.52098 | 5.39669 | 6.75526 | 6.33132 | 6.94085 | 6.57151 |
| 14159 | 4.6547 | 5.05577 | 5.2271 | 5.07696 | 5.32858 | 5.30261 | 5.58462 | 5.02017 | 5.62356 | 4.91863 | 5.21269 | 5.26316 | 5.24279 | 3.29398 | 3.73866 | 3.75775 | 3.79617 |
| 10961 | 6.55314 | 6.11476 | 6.50459 | 5.79087 | 6.48898 | 6.07591 | 6.02261 | 6.06118 | 6.00402 | 5.86337 | 5.78581 | 5.77455 | 6.37323 | 7.17515 | 7.24646 | 7.05213 | 7.27844 |
| 12387 | 3.23925 | 4.47759 | 4.11104 | 4.18912 | 4.41452 | 4.6234 | 4.70986 | 4.13891 | 4.97613 | 4.13348 | 4.53655 | 5.35313 | 6.03004 | 3.7855 | 4.57054 | 4.25143 | 5.15456 |
| 8434 | 4.54769 | 4.52163 | 8.38163 | 6.68413 | 8.30534 | 7.65852 | 7.15381 | 7.99768 | 8.06818 | 8.79968 | 8.90933 | 5.03474 | 5.55103 | 4.37797 | 4.73641 | 8.80235 | 4.64067 |
| 11375 | 3.30806 | 3.87392 | 4.68162 | 4.52479 | 5.04376 | 4.61241 | 4.88849 | 4.18832 | 5.15736 | 4.20245 | 4.67369 | 4.80212 | 5.52892 | 3.34447 | 3.8682 | 3.52549 | 4.00403 |
| 16145 | 3.60299 | 7.16959 | 5.40699 | 3.98282 | 5.45869 | 5.05104 | 4.78904 | 5.0965 | 4.85611 | 4.7079 | 4.94947 | 5.31016 | 3.92763 | 4.63476 | 5.25381 | 5.65239 | 5.11449 |

| strain | WT | | | | | | | | | | |
| --- | --- | --- | --- | --- | --- | --- | --- | --- | --- | --- | --- |
| condition | G\_SD | | GX\_SD | | | X\_SD | | A\_SD | | C\_SD | |
| proteinId | exp | stat | exp | trans | stat | exp | stat | exp | stat | exp | stat |
| 12704 | 64.7026 | 51.3062 | 59.3743 | 51.1235 | 52.6648 | 58.1976 | 59.2851 | 46.5501 | 46.719 | 59.2481 | 63.8147 |
| 16095 | 6.33625 | 6.18406 | 5.69798 | 7.80465 | 6.91427 | 5.07397 | 5.51288 | 5.99929 | 6.32416 | 9.41661 | 7.40801 |
| 9695 | 0 | 0 | 0.197656 | 0 | 0 | 0 | 0 | 0 | 0 | 0 | 0 |
| 16440 | 2.29825 | 0.333333 | 2.23672 | 1.30027 | 0.902739 | 2.14865 | 1.17189 | 2.3131 | 0.952426 | 0.649732 | 2.39012 |
| 8916 | 1.53936 | 1.29074 | 0.407932 | 1.29731 | 1.09619 | 1.17084 | 1.18543 | 1.15917 | 0.568366 | 0.642408 | 0.874071 |
| 12518 | 12.5571 | 10.4042 | 11.5842 | 10.2516 | 8.4123 | 10.2033 | 9.44531 | 11.0183 | 10.9075 | 7.05108 | 9.13304 |
| 9331 | 6.34831 | 6.32004 | 6.29991 | 7.46502 | 7.4786 | 8.03925 | 6.46805 | 5.7924 | 6.71263 | 7.27284 | 8.50065 |
| 10149 | 0.954128 | 0.351946 | 0.822239 | 1.67041 | 0.564493 | 1.17177 | 1.18291 | 0 | 0.391347 | 0.22091 | 0.44231 |
| 12032 | 2.88224 | 1.94404 | 2.03112 | 1.67305 | 1.44962 | 2.53794 | 1.78202 | 2.51069 | 1.71834 | 1.50225 | 1.52787 |
| 12446 | 0 | 0 | 0 | 0 | 0 | 0 | 0 | 0.193992 | 0 | 0 | 0 |
| 13319 | 0.76173 | 0.166667 | 1.22078 | 0.927166 | 0.177427 | 1.16914 | 0.387142 | 0.963153 | 0.770632 | 0.212277 | 0.211526 |
| 14159 | 0 | 0.195188 | 0 | 0 | 0 | 0 | 0 | 0 | 0 | 0 | 0 |
| 10961 | 2.33116 | 2.33119 | 2.02831 | 2.97633 | 4.03019 | 0.96427 | 1.94871 | 0.770172 | 1.52766 | 0.212277 | 1.10252 |
| 12387 | 0.378826 | 0.354512 | 0.409593 | 0.185549 | 0.531137 | 0 | 0.394141 | 0.388996 | 0.379285 | 0.211647 | 0.431761 |
| 8434 | 1.54834 | 0.559723 | 1.43106 | 1.8657 | 1.28256 | 1.75995 | 1.96671 | 0.971738 | 1.53325 | 0 | 0 |
| 11375 | 0.956584 | 0 | 0.612678 | 0 | 0 | 0.195962 | 0 | 0 | 0 | 0 | 0 |
| 16145 | 4.98782 | 2.7037 | 4.27454 | 3.3455 | 5.4855 | 3.51677 | 2.73553 | 3.67269 | 2.47438 | 10.4471 | 8.48171 |

|  | Glucose | Xylose | Arabinose | Acetate | Coumarate | Ferulate | YNB Oleic Acid | YNB Ricinoleic Acid | YNB Glucose | YNB Gluc DOC | YPD |
| --- | --- | --- | --- | --- | --- | --- | --- | --- | --- | --- | --- |
| proteinId |  |  |  |  |  |  |  |  |  |  |  |
| 16095 | -0.0218641 | -0.177959 | 0.247816 | 0.167411 | -0.322285 | -0.0863019 | -0.350025 | 0.0493235 | 0.345264 | 0.262751 | -0.385954 |
| 9695 | 0.0480373 | 0.164832 | 0.123385 | -0.136937 | 0.0797862 | -0.114876 | -0.0114183 | -0.668377 | -0.425499 | 0.0532996 | 0.309092 |
| 16440 | -0.0368947 | 0.0914269 | -0.305477 | -0.0597754 | -0.0287667 | -0.128169 | -0.563395 | 0.000584502 | 0.559049 | 0.77942 | 0.603735 |
| 12518 | 0.112526 | 0.0372373 | -0.100235 | 0.0203073 | 0.136688 | 0.0815494 | -0.292448 | -0.26549 | 0.0219979 | 0.091007 | 0.19193 |
| 9331 | 0.317304 | 0.660965 | -0.231468 | -1.77645 | 0.262022 | -1.83089 | -3.79725 | -6.14964 | -0.943653 | -0.572548 | -1.19392 |
| 10149 | -0.075474 | -0.4456 | -0.241651 | 0.0656419 | -0.293397 | -0.0232707 | -0.142486 | 0.752866 | -0.199261 | -0.496042 | -0.194102 |
| 12032 | -0.956871 | -0.659087 | -0.293917 | -1.14194 | -0.341075 | -0.533584 | 0.866135 | -2.15565 | -2.81679 | 1.70697 | -0.147228 |
| 13319 | -0.424986 | -0.449847 | -0.398549 | -0.266453 | -0.490897 | 0.0684878 | 0.363013 | -0.485849 | -1.56767 | -0.465167 | -0.150008 |
| 8434 | -0.288681 | -0.0976237 | -0.263692 | -0.498388 | -3.67827 | -0.708144 | -0.206495 | 0.00227462 | -0.0963591 | -0.166823 | 0.35102 |

In [130]:

```
for x in temp:
    if x in model.genes:
        for r in sorted(model.genes.get_by_id(x).reactions, key=lambda x: x.id):
            print(r, r.gene_reaction_rule)
    else:
        print(x, 'no reactions')
    print()
```

```
ATPtm: adp_c + atp_m --> adp_m + atp_c 12704

16095 no reactions

9695 no reactions

16440 no reactions

COAtim: coa_c --> coa_m 8916

12518 no reactions

ACRNtm: acrn_c --> acrn_m 9331
ARACHCRNt: arachcrn_c --> arachcrn_m 9331
C160CRNt: pmtcrn_c --> pmtcrn_m 9331
C161CRN2t: hdd2crn_c --> hdd2crn_m 9331
C161CRNt: hdcecrn_c --> hdcecrn_m 9331
C181CRNt: odecrn_c --> odecrn_m 9331
CLPNDCRNt: clpndcrn_c --> clpndcrn_m 9331
CRNtim: crn_m --> crn_c 9331
DCSPTN1CRNt: dcsptn1crn_c --> dcsptn1crn_m 9331
ELAIDCRNt: elaidcrn_c --> elaidcrn_m 9331
HEXCCRNt: hexccrn_c --> hexccrn_m 9331
HPDCACRNt: hpdcacrn_c --> hpdcacrn_m 9331
LNLNCGCRNt: lnlncgcrn_c --> lnlncgcrn_m 9331
NRVNCCRNt: nrvnccrn_c --> nrvnccrn_m 9331
PCRNtm: pcrn_c <=> pcrn_m 9331
PTDCACRNt: ptdcacrn_c --> ptdcacrn_m 9331
TETTET6CRNt: tettet6crn_c --> tettet6crn_m 9331
TTDCRNt: ttdcrn_c --> ttdcrn_m 9331

ACRNtp: acrn_x --> acrn_c 10149
PCRNtc: pcrn_x --> pcrn_c 10149

AMETt2m: ahcys_m + amet_c <=> ahcys_c + amet_m 12032
AMETtm: amet_c <=> amet_m 12032

12446 no reactions

ORNt3m: h_c + orn_m <=> h_m + orn_c 13319

FADFMNtm: fad_c + fmn_m --> fad_m + fmn_c 14159

10961 no reactions

CMPtm: cmp_c <-- cmp_m 12387
CTPtm: cmp_m + ctp_c + 2.0 h_c --> cmp_c + ctp_m + 2.0 h_m 12387
UTPtm: 2.0 h_c + ump_m + utp_c --> 2.0 h_m + ump_c + utp_m 12387

8434 no reactions

11375 no reactions

ATP2tp_H: amp_x + atp_c + h_x --> amp_c + atp_x + h_c 16145
```

In [131]:

```
# 12704 ANT, ok
# 16095 FAD, no rxn
# 9695 ADP/ATP transporter or ATP-Mg/phosphate carriers, no rxn
# 16440 YPR011C APS and PAPS (thought in exchange for pi or so4), no rxn
# 8916 CoA, ok
# 12518 SLC25A2_15, ORNT ornithine carrier or YMC2?, unclear, no rxn
# 13319 ORT1 exports ornithine in exchange of proton, can import arg or lys in exchange with orn
model.reactions.get_by_id('ORNt3m').lower_bound = 0.0
r = model.reactions.get_by_id('ORNt3m').copy()
r.id = 'ORNARGtm'
r.lower_bound = 0.0
model.add_reactions([r])
r.add_metabolites({'h_c': 1.0, 'h_m': -1.0, 'arg__L_c': -1.0, 'arg__L_m': 1.0})
r = model.reactions.get_by_id('ORNt3m').copy()
r.id = 'ORNLYStm'
r.lower_bound = 0.0
model.add_reactions([r])
r.add_metabolites({'h_c': 1.0, 'h_m': -1.0, 'lys__L_c': -1.0, 'lys__L_m': 1.0})
# 10149 basic amino acids with a preference for arg and lys, less ornithine and histidine
r = model.reactions.get_by_id('GLUt7m').copy()
r.id = 'ARGt7m'
r.lower_bound = 0.0
model.add_reactions([r])
r.add_metabolites({'glu__L_c': 1.0, 'glu__L_m': -1.0, 'arg__L_c': -1.0, 'arg__L_m': 1.0})
r = model.reactions.get_by_id('GLUt7m').copy()
r.id = 'LYSt7m'
r.lower_bound = 0.0
model.add_reactions([r])
r.add_metabolites({'glu__L_c': 1.0, 'glu__L_m': -1.0, 'lys__L_c': -1.0, 'lys__L_m': 1.0})
# 9331 carnitine, ok
# 10232 PET8 (SAM5) mito amet import by uniport or exchange with ahcys, ok
# 12446 MRS3/MRS4 mito iron transport, no rxn
r = hsa.reactions.get_by_id('FE2tm').copy()
r.gene_reaction_rule = '12446'
model.add_reactions([r])
# 14159 FLX1 FAD, ok
# 10961 YIA6 mito NAD import, no rxn
# 12387 RIM2 pyrimidine nucleotide, proton coupled, ok
# RIM2 also co-transports fe2 in MRS3 and MRS4 mutants
# http://www.biochemj.org/content/455/1/57.long
r1 = model.reactions.get_by_id('CTPtm').copy()
r1.id = 'CTPtm2'
r2 = model.reactions.get_by_id('UTPtm').copy()
r2.id = 'UTPtm2'
model.add_reactions([r1,r2])
model.reactions.get_by_id('CTPtm2').add_metabolites({'h_c': 2.0, 'h_m': -2.0, 'fe2_c': -1.0, 'fe2_m': 1.0})
model.reactions.get_by_id('UTPtm2').add_metabolites({'h_c': 2.0, 'h_m': -2.0, 'fe2_c': -1.0, 'fe2_m': 1.0})
model.remove_reactions(['CMPtm'], remove_orphans=True)
# 8434 HEM25 mito glycine
# https://journals.plos.org/plosgenetics/article?id=10.1371/journal.pgen.1005783
# mito glycine needed for heme biosynthesis
# http://www.jbc.org/content/291/38/19746/F10.expansion.html
r = hsa.reactions.get_by_id('GLYtm').copy()
r.gene_reaction_rule = '8434'
model.add_reactions([r])
# 11375 MTM1 high affinity pyridoxal 5'-phosphate transporter
r = hsa.reactions.get_by_id('PYDX5Ptm').copy()
r.gene_reaction_rule = '11375'
model.add_reactions([r])
# 16145 ANT1 perox ATP transporter is electroneutral, should bring h from cyto to pero
model.reactions.get_by_id('ATP2tp_H').add_metabolites({'h_c': -3.0, 'h_x': 3.0})
```

siroheme biosynthesis  
ALASm: gly\_m + h\_m + succoa\_m --> 5aop\_m + co2\_m + coa\_m 8677  
Add 5AOPtm: 5aop\_c <=> 5aop\_m  
PPBNGS: 2.0 5aop\_c --> 2.0 h2o\_c + h\_c + ppbng\_c 13394  
HMBS: h2o\_c + 4.0 ppbng\_c --> hmbil\_c + 4.0 nh4\_c 14718  
UPP3S: hmbil\_c --> h2o\_c + uppg3\_c 10515  
UPP3MT: 2.0 amet\_c + uppg3\_c --> 2.0 ahcys\_c + dscl\_c + h\_c 11050  
SHCHD2: dscl\_c + nad\_c --> h\_c + nadh\_c + scl\_c 11050 or 13569  
SHCHD: dscl\_c + nadp\_c --> h\_c + nadph\_c + scl\_c 13569  
SHCHF: fe2\_c + scl\_c --> 3.0 h\_c + sheme\_c 11050 or 13569

protoheme biosynthesis  
UPPDC1: 4.0 h\_c + uppg3\_c --> 4.0 co2\_c + cpppg3\_c 14179  
CPPPGO: cpppg3\_c + 2.0 h\_c + o2\_c --> 2.0 co2\_c + 2.0 h2o\_c + pppg9\_c 14197  
Add pppg9 mito transport, PPPG9tm  
PPPGOm: 3.0 o2\_m + 2.0 pppg9\_m --> 6.0 h2o\_m + 2.0 ppp9\_m 11867  
Add FCLTm: fe2\_m + ppp9\_m --> 2.0 h\_m + pheme\_m 16275  
16275 is two genes in one gene model (ATPase + HEM15) -> need to be separared  
HEMEOSm: frdp\_m + h2o\_m + pheme\_m --> hemeO\_m + ppi\_m 11103  
HEMEASm: 2.0 h\_m + hemeO\_m + 2.0 nadph\_m + 2.0 o2\_m --> 3.0 h2o\_m + hemeA\_m + 2.0 nadp\_m 10228 and 15585 and 16775

In [132]:

```
temp = ['8677','13394','14718','10515','11050','13569','14197','11867','16275','11103','10228','15585','16775']
display(Annotation.loc[temp])
Show_Data(temp)
```

|  | Combined Annotations | Signal P | Sc288c Orthologs | Human Orthologs | Sc288 Best Hit | Human Blast | Essential | WolfPSort | C Terminal |
| --- | --- | --- | --- | --- | --- | --- | --- | --- | --- |
| RTO4\_ID |  |  |  |  |  |  |  |  |  |
| 8677 | K00643: E2.3.1.37, ALAS; 5-aminolevulinate syn... |  | HEM1 | ALAS1,ALAS2,GCAT | HEM1 | ALAS2 | Essential | mito 20, extr 4, cyto 3 | ASA\* |
| 13394 | K01698: hemB, ALAD; porphobilinogen synthase |  | HEM2 | ALAD | HEM2 | ALAD | Essential | mito 12, cyto 7.5, cyto\_nucl 5.5, cysk 3, nucl... | DKQ\* |
| 14718 | K01749: hemC, HMBS; hydroxymethylbilane synthase |  | HEM3 | HMBS | HEM3 | HMBS | Essential | mito 18, cyto 6, cyto\_nucl 5.5 | VGW\* |
| 10515 | K01719: hemD, UROS; uroporphyrinogen-III synthase |  | HEM4 | UROS | HEM4 | UROS | Not Essential | mito 13, mito\_nucl 10.666, cyto\_mito 10.166, c... | QDE\* |
| 11050 | K00589: MET1; uroporphyrin-III C-methyltransfe... | S | MET1 |  | MET1 |  | Not Essential | cyto 10, mito 8, cyto\_nucl 8 | SAE\* |
| 13569 | K02304: MET8; precorrin-2 dehydrogenase / siro... |  | MET8 |  | MET8 |  | Not Essential | mito 23, cyto 4 | RRQ\* |
| 14197 | K00228: CPOX, hemF; coproporphyrinogen III oxi... | S | HEM13 | CPOX | HEM13 | CPOX | Not Essential | plas 7, mito 4, extr 4, nucl 3, E.R. 3, golg 3... | EWA\* |
| 11867 | K00231: PPOX, hemY; oxygen-dependent protoporp... | A | HEM14 | PPOX |  | PPOX | Essential | mito 22, cyto 3 | WEL\* |
| 16275 | K14951: ATP13A3\_4\_5; cation-transporting ATPas... |  | YPK9 | ATP13A2,ATP13A3,ATP13A4,ATP13A5 | YPK9 | ATP13 | Not Essential | plas 23, E.R. 2 | VSA\* |
| 11103 | K02257: COX10; protoheme IX farnesyltransferase | S | COX10 | COX10 | COX10 | COX10 | Not Essential | extr 9, plas 8, mito 7, E.R. 2 | EEV\* |
| 10228 | K02259: COX15; cytochrome c oxidase assembly p... | S | COX15 | COX15 | COX15 | COX15 | Essential | plas 14, mito 5, E.R. 5, extr 2 | IKV\* |
| 15585 | KOG3309: Ferredoxin |  | YAH1 | FDX1,FDX2 | YAH1 | FDX2 | Essential | cyto 23.5, cyto\_nucl 13.5 | THH\* |
| 16775 | K18914: FDXR; adrenodoxin-NADP+ reductase | A | ARH1 | FDXR | ARH1 | FDXR | Essential | mito 25 | VVG\* |

| strain | WT | | | | | | | | | | | | | | | | |
| --- | --- | --- | --- | --- | --- | --- | --- | --- | --- | --- | --- | --- | --- | --- | --- | --- | --- |
| condition | G\_MM | C\_MM | G\_SD | | GX\_SD | | | X\_SD | | A\_SD | | C\_SD | | MM\_CN120 | | MM\_CN5 | Diversity\_Sample |
| phase | exp | exp | exp | stat | exp | trans | stat | exp | stat | exp | stat | exp | stat | exp | stat | exp | exp |
| proteinId | Set1 | Set1 | Set2 | Set2 | Set2 | Set2 | Set2 | Set2 | Set2 | Set2 | Set2 | Set2 | Set2 | Set3 | Set3 | Set3 | Set3 |
| 8677 | 6.19253 | 5.62007 | 5.4911 | 4.68678 | 5.56086 | 3.87534 | 3.92284 | 5.7039 | 4.95523 | 5.3661 | 6.1565 | 5.08851 | 5.68171 | 6.01187 | 5.83864 | 6.51086 | 8.08013 |
| 13394 | 6.63886 | 6.40198 | 6.45606 | 6.87833 | 6.57076 | 5.73341 | 5.94384 | 5.94745 | 6.11532 | 5.91275 | 6.16191 | 6.09141 | 6.39621 | 5.71158 | 5.23281 | 6.39802 | 5.64993 |
| 14718 | 5.12418 | 5.30331 | 2.95383 | 4.15309 | 2.72221 | 4.28127 | 4.61209 | 3.11366 | 4.70528 | 3.19097 | 4.75242 | 4.00156 | 4.30669 | 3.70987 | 4.17871 | 3.33792 | 5.95977 |
| 10515 | 4.07595 | 4.19441 | 3.9687 | 4.03049 | 3.91985 | 4.30145 | 4.33985 | 4.12753 | 4.61348 | 3.79327 | 4.02273 | 4.01548 | 3.88153 | 4.21019 | 4.51894 | 3.55365 | 3.64623 |
| 11050 | 5.24113 | 4.48976 | 3.85 | 3.81267 | 3.89557 | 4.27402 | 4.18064 | 5.24587 | 3.93054 | 4.98775 | 3.97424 | 4.413 | 5.37101 | 5.939 | 5.80081 | 6.30657 | 5.83284 |
| 13569 | 5.92072 | 5.18753 | 5.17981 | 5.64671 | 5.19086 | 5.23369 | 5.36895 | 5.1783 | 5.27235 | 5.14318 | 5.40206 | 5.44419 | 5.26743 | 5.85275 | 5.78219 | 5.17617 | 5.19349 |
| 14197 | 8.33545 | 6.15877 | 7.00668 | 4.82947 | 6.95233 | 6.08713 | 6.63784 | 5.57958 | 6.27164 | 5.1462 | 5.18397 | 5.55444 | 5.23247 | 7.4205 | 8.13893 | 7.11221 | 9.55883 |
| 11867 | 5.47528 | 5.3365 | 5.51865 | 6.09489 | 5.46117 | 6.15947 | 6.01748 | 5.49858 | 5.54088 | 5.63036 | 5.67075 | 5.80503 | 5.64635 | 5.50415 | 5.70199 | 5.26357 | 5.8748 |
| 16275 | 5.51378 | 5.10443 | 4.94361 | 5.59005 | 4.91577 | 5.24466 | 5.35342 | 5.13736 | 5.34531 | 5.32426 | 5.44862 | 5.64176 | 5.4582 | 6.15898 | 6.28985 | 5.56273 | 5.8316 |
| 11103 | 4.67073 | 4.79263 | 3.052 | 3.86189 | 3.10344 | 2.82273 | 3.17948 | 2.94723 | 4.41928 | 3.85039 | 4.4562 | 4.55405 | 4.92764 | 4.52608 | 4.84307 | 4.02491 | 6.66459 |
| 10228 | 4.95232 | 5.21334 | 4.61829 | 3.28518 | 4.70431 | 3.59049 | 3.84634 | 3.23143 | 4.39559 | 3.59004 | 4.3971 | 5.49574 | 5.53657 | 3.96633 | 4.12913 | 4.11142 | 6.73363 |
| 15585 | 4.88266 | 5.56536 | 4.41696 | 4.25515 | 4.43203 | 4.59169 | 4.43999 | 4.69098 | 4.53372 | 4.34912 | 4.47085 | 4.95726 | 5.16606 | 4.53764 | 4.79042 | 5.59086 | 5.66233 |
| 16775 | 4.76107 | 4.37865 | 6.10345 | 5.14153 | 6.17496 | 5.42258 | 5.4362 | 5.27159 | 5.3704 | 4.95784 | 4.89851 | 5.75728 | 6.52048 | 6.98671 | 6.39988 | 7.59049 | 6.69027 |

| strain | WT | | | | | | | | | | |
| --- | --- | --- | --- | --- | --- | --- | --- | --- | --- | --- | --- |
| condition | G\_SD | | GX\_SD | | | X\_SD | | A\_SD | | C\_SD | |
| proteinId | exp | stat | exp | trans | stat | exp | stat | exp | stat | exp | stat |
| 8677 | 5.72937 | 1.10558 | 8.34993 | 0.748421 | 1.10854 | 1.35594 | 3.73996 | 2.1151 | 11.4857 | 3.17989 | 5.67703 |
| 13394 | 5.80784 | 7.06885 | 6.91978 | 7.2647 | 6.39548 | 7.64196 | 6.88692 | 6.96267 | 7.44862 | 12.6435 | 11.7642 |
| 14718 | 0 | 0 | 0 | 0 | 0 | 0 | 0 | 0 | 0 | 0 | 0.221651 |
| 10515 | 0 | 0.179255 | 0 | 0 | 0 | 0 | 0.387142 | 0 | 0 | 0 | 0 |
| 11050 | 1.53991 | 0.166667 | 1.83921 | 0 | 0 | 0.395869 | 0.994775 | 2.31982 | 0.952823 | 0 | 0.438555 |
| 13569 | 0.384095 | 0.179255 | 0 | 0.183791 | 0.54405 | 0 | 0 | 0 | 0.189081 | 0 | 0.221651 |
| 14197 | 6.73468 | 7.90525 | 4.86376 | 7.81501 | 9.07842 | 1.96088 | 4.69585 | 1.15964 | 2.48634 | 4.68192 | 4.09835 |
| 11867 | 0.201691 | 0.575655 | 0 | 1.66665 | 0.545334 | 0.200926 | 0.592559 | 0.388524 | 0 | 0.430131 | 0.438555 |
| 16275 | 1.92565 | 2.70801 | 2.23629 | 3.73022 | 3.08729 | 3.52661 | 4.11403 | 4.25329 | 4.21123 | 3.19692 | 2.63371 |
| 10228 | 0.190197 | 0.169232 | 0 | 0 | 0 | 0 | 0 | 0 | 0 | 0 | 0.220621 |
| 15585 | 0.566264 | 0 | 0.81119 | 0 | 0 | 0 | 0 | 0 | 0 | 0.420867 | 0.867663 |
| 16775 | 4.9841 | 5.22145 | 5.4862 | 6.33257 | 5.84208 | 4.31752 | 5.33264 | 4.24448 | 3.81961 | 4.90598 | 8.5044 |

|  | Glucose | Xylose | Arabinose | Acetate | Coumarate | Ferulate | YNB Oleic Acid | YNB Ricinoleic Acid | YNB Glucose | YNB Gluc DOC | YPD |
| --- | --- | --- | --- | --- | --- | --- | --- | --- | --- | --- | --- |
| proteinId |  |  |  |  |  |  |  |  |  |  |  |
| 11050 | -0.248795 | 0.364452 | 0.238288 | 0.242943 | 0.425463 | -1.26448 | -0.264639 | -1.31736 | -2.83255 | -0.0264744 | -0.0540932 |
| 13569 | -0.599414 | -0.697888 | -0.446222 | -0.131288 | 0.0469392 | -1.82967 | -0.719508 | -1.34086 | -3.11283 | 0.959913 | -0.789865 |
| 14197 | 0.156461 | -0.0582697 | 0.147453 | -0.0716227 | -0.190346 | -0.0878437 | -0.0200181 | -0.202957 | 0.359937 | 0.2702 | -0.0314271 |
| 16275 | -0.269023 | -0.762556 | -1.38232 | -0.0913242 | -0.0296838 | 0.0389366 | -0.116315 | -0.526475 | -0.689562 | -0.642096 | -0.0125306 |

In [133]:

```
for x in temp:
    if x in model.genes:
        for r in sorted(model.genes.get_by_id(x).reactions, key=lambda x: x.id):
            print(r, r.gene_reaction_rule)
    else:
        print(x, 'no reactions')
    print()
```

```
ALASm: gly_m + h_m + succoa_m --> 5aop_m + co2_m + coa_m 8677
GLYAT: accoa_c + gly_c <=> 2aobut_c + coa_c 8677
GLYATm: accoa_m + gly_m <=> 2aobut_m + coa_m 8677

PPBNGS: 2.0 5aop_c --> 2.0 h2o_c + h_c + ppbng_c 13394

HMBS: h2o_c + 4.0 ppbng_c --> hmbil_c + 4.0 nh4_c 14718

UPP3S: hmbil_c --> h2o_c + uppg3_c 10515

SHCHD2: dscl_c + nad_c --> h_c + nadh_c + scl_c 11050 or 13569
SHCHF: fe2_c + scl_c --> 3.0 h_c + sheme_c 11050 or 13569
UPP3MT: 2.0 amet_c + uppg3_c --> 2.0 ahcys_c + dscl_c + h_c 11050

SHCHD: dscl_c + nadp_c --> h_c + nadph_c + scl_c 13569
SHCHD2: dscl_c + nad_c --> h_c + nadh_c + scl_c 11050 or 13569
SHCHF: fe2_c + scl_c --> 3.0 h_c + sheme_c 11050 or 13569

CPPPGO: cpppg3_c + 2.0 h_c + o2_c --> 2.0 co2_c + 2.0 h2o_c + pppg9_c 14197

PPPGOm: 3.0 o2_m + 2.0 pppg9_m --> 6.0 h2o_m + 2.0 ppp9_m 11867

16275 no reactions

HEMEOSm: frdp_m + h2o_m + pheme_m --> hemeO_m + ppi_m 11103

HEMEASm: 2.0 h_m + hemeO_m + 2.0 nadph_m + 2.0 o2_m --> 3.0 h2o_m + hemeA_m + 2.0 nadp_m 10228 and 15585 and 16775

HEMEASm: 2.0 h_m + hemeO_m + 2.0 nadph_m + 2.0 o2_m --> 3.0 h2o_m + hemeA_m + 2.0 nadp_m 10228 and 15585 and 16775

HEMEASm: 2.0 h_m + hemeO_m + 2.0 nadph_m + 2.0 o2_m --> 3.0 h2o_m + hemeA_m + 2.0 nadp_m 10228 and 15585 and 16775
```

In [134]:

```
for r in sorted(model.genes.get_by_id('12731').reactions, key=lambda x: x.id):
    print(r, r.gene_reaction_rule)
print()
for r in sorted(model.metabolites.get_by_id('co2_r').reactions, key=lambda x: x.id):
    print(r, r.gene_reaction_rule)
```

```
AMAOTrm: 8aonn_m + amet_m <=> amob_m + dann_m 12731
DBTSm: atp_m + co2_m + dann_m <=> adp_m + dtbt_m + 3.0 h_m + pi_m 12731

C3STDH1Pr: 4mzym_int1_r + nadp_r --> 4mzym_int2_r + co2_r + h_r + nadph_r 13724 or 8835
C3STDH1r: 4mzym_int1_r + nad_r --> 4mzym_int2_r + co2_r + h_r + nadh_r 13724 or 8835
C3STDH2er: nad_r + zym_int1_r --> co2_r + h_r + nadh_r + zym_int2_r 13724 or 8835
CO2ter: co2_c <=> co2_r 
FAS240COAer: 9.0 h_r + 3.0 malcoa_r + 6.0 nadph_r + stcoa_r --> 3.0 co2_r + 3.0 coa_r + 3.0 h2o_r + 6.0 nadp_r + ttccoa_r (10677 and 11343 and 16241 and 16695) or (10677 and 16241 and 16655 and 16695)
FAS260COAer: 3.0 h_r + malcoa_r + 2.0 nadph_r + ttccoa_r --> co2_r + coa_r + h2o_r + hexccoa_r + 2.0 nadp_r 10677 and 11343 and 16241 and 16695
PSERDer_RT: h_r + 0.01 ps_RT_r --> co2_r + 0.01 pe_RT_r 14554
SERPTer: h_r + pmtcoa_r + ser__L_r --> 3dsphgn_r + co2_r + coa_r 10303 and 9394 and 9425
```

In [135]:

```
# 8677
r = sce.reactions.get_by_id('5AOPtm').copy()
model.add_reactions([r])
# 11050 is methyltransferase, UPP3MT is correct, remove it from SHCHD2
# 13569 MET8 this step is NADH, remove SHCHD
# SHCHF is by MET8 not MET1
model.reactions.get_by_id('SHCHD2').gene_reaction_rule = '13569'
model.reactions.get_by_id('SHCHF').gene_reaction_rule = '13569'
model.remove_reactions(['SHCHD'], remove_orphans=True)
r = sce.reactions.get_by_id('PPPG9tm').copy()
model.add_reactions([r])
r = sce.reactions.get_by_id('FCLTm').copy()
r.gene_reaction_rule = '16275'
model.add_reactions([r])
```

In [136]:

```
with model:
    sol = model.optimize()
    print(sol.objective_value)
    for k, v in sol.shadow_prices.items():
        if v and k in [m.id for m in model.reactions.BIOMASS_RT.metabolites]:
            print(k, v)
```

```
0.13657749916638628
adp_c -0.4595342375266898
atp_c -0.4708528148056722
amp_c -0.4482156602477074
cmp_c -0.05885660185070918
glu__L_c -0.028673729106759027
gmp_c -0.4746256738986702
ser__L_c -0.15996922554295429
cys__L_c -0.13733207098498712
ala__L_c -0.09658519278064931
ump_c -0.04753802457172679
gln__L_c -0.039992306385741416
dgmp_c -0.46330709661968755
pe_RT_r -264.0141153222685
his__L_c -0.25806356196080005
dtmp_c -0.18487009555671297
trp__L_c -0.2278806892168488
met__L_c -0.126013493706006
pro__L_c -0.01735515182777614
phe__L_c -0.02263715455796597
tyr__L_c -0.033955731836948666
asp__L_c -1.3322676295501878e-15
ile__L_c -0.012073149097582753
dcmp_c -0.03621944729274151
mannan_r -0.011318577278982389
damp_c -0.43689708296872476
arg__L_c -0.10488548278524032
asn__L_c -0.02263715455796611
glycogen_c -0.022637154557962183
13BDglcn_c -0.022637154557962183
thr__L_c -4.440892098500626e-16
lys__L_c -0.02716458546956224
val__L_c -0.015846008190576882
ergst_r 1.967168731087132
tre_c -0.03395573183694457
ribflv_c -0.02867372910675081
zymst_r 2.263715455796472
pa_RT_r -246.13076322147654
triglyc_RT_r -369.1961448322148
pc_RT_r -318.79602935254405
ptd1ino_RT_r -249.52633640517075
ps_RT_r -264.3914012315679
```

In [137]:

```
model.summary()
```

Out[137]:

|  | IN\_FLUXES | | OUT\_FLUXES | | OBJECTIVES | |
| --- | --- | --- | --- | --- | --- | --- |
|  | ID | FLUX | ID | FLUX | ID | FLUX |
| 0 | o2\_e | 2.000000 | h2o\_e | 4.353031 | BIOMASS\_RT | 0.136577 |
| 1 | glc\_\_D\_e | 1.438040 | co2\_e | 2.352635 | NaN | NaN |
| 2 | nh4\_e | 1.200481 | h\_e | 0.918754 | NaN | NaN |
| 3 | NaN | NaN | gly\_e | 0.124272 | NaN | NaN |
| 4 | NaN | NaN | leu\_\_L\_e | 0.086346 | NaN | NaN |
| 5 | NaN | NaN | spmd\_e | 0.075171 | NaN | NaN |

In [138]:

```
print(len(model.genes))
print(len(model.reactions))
print(len(model.metabolites))
model
```

```
1122
2115
1976
```

Out[138]:

|  |  |
| --- | --- |
| **Name** | R. toruloides |
| **Memory address** | 0x010227fdbe0 |
| **Number of metabolites** | 1976 |
| **Number of reactions** | 2115 |
| **Number of groups** | 0 |
| **Objective expression** | 1.0\*BIOMASS\_RT - 1.0\*BIOMASS\_RT\_reverse\_2b3e0 |
| **Compartments** | c, x, m, e, r, v, n, g, d |

In [139]:

```
for x in sorted(model.genes, key=lambda x: x.id):
    if not x.reactions:
        print(x)
print()
for x in sorted(model.metabolites, key=lambda x: x.id):
    if not x.reactions:
        print(x)
```

```
16454
2542_AT1

adp_x
```

In [140]:

```
cobra.manipulation.remove_genes(model, [x for x in model.genes if not x.reactions])
model.remove_metabolites([x for x in model.metabolites if not x.reactions])
```

In [141]:

```
print(len(model.genes))
print(len(model.reactions))
print(len(model.metabolites))
print(len(set([m.id.rsplit('_',1)[0] for m in model.metabolites])))
print(len(model.compartments))
model
```

```
1120
2115
1975
1236
9
```

Out[141]:

|  |  |
| --- | --- |
| **Name** | R. toruloides |
| **Memory address** | 0x010227fdbe0 |
| **Number of metabolites** | 1975 |
| **Number of reactions** | 2115 |
| **Number of groups** | 0 |
| **Objective expression** | 1.0\*BIOMASS\_RT - 1.0\*BIOMASS\_RT\_reverse\_2b3e0 |
| **Compartments** | c, x, m, e, r, v, n, g, d |

In [142]:

```
cobra.io.save_json_model(model, "IFO0880_GPR_2a.json")
```

In [143]:

```
model_old = cobra.io.load_json_model("../1.Manual_curation/IFO0880_GPR_1f.json")
model_new = cobra.io.load_json_model("IFO0880_GPR_2a.json")
```

In [144]:

```
print('Removed reactions\n')
for r in sorted(model_old.reactions, key=lambda x: x.id):
    if r not in model_new.reactions:
        print(r)
```

```
Removed reactions

2AMADPTm: L2aadp_c + akg_m <=> L2aadp_m + akg_c
2DHPFALDL: 2dhp_c <=> 3mob_c + fald_c
4ABUTtmi: 4abut_c + h_c --> 4abut_m + h_m
5HOXINDACTO2OX: 5hoxindact_c + h2o_c + o2_c --> 5hoxindoa_c + h2o2_c + h_c
AASAD2: L2aadp_c + atp_c + h_c + nadh_c --> L2aadp6sa_c + amp_c + nad_c + ppi_c
AASAD3: L2aadp6sa_c + h2o_c + nad_c --> L2aadp_c + 2.0 h_c + nadh_c
ACCOAtm: accoa_c + coa_m <=> accoa_m + coa_c
ACCOAtx: accoa_c + coa_x <=> accoa_x + coa_c
AGMT: agm_c + h2o_c --> ptrc_c + urea_c
AKGICITtm: akg_c + icit_m <=> akg_m + icit_c
ALR2x: h_c + mthgxl_c + nadh_c --> acetol_c + nad_c
ARGNm: arg__L_m + h2o_m --> orn_m + urea_m
ASNtx: asn__L_c + h_c <=> asn__L_x + h_x
ASPGLU2m: asp__L_m + glu__L_c --> asp__L_c + glu__L_m
ASPt2m: asp__L_c + h_c <=> asp__L_m + h_m
ASPtx: asp__L_x + h_x <=> asp__L_c + h_c
BALAVECSEC: 3.0 ala_B_c + atp_c + h2o_c --> adp_c + 3.0 ala_B_e + h_c + pi_c
CITtbm: cit_c + pep_m <=> cit_m + pep_c
CMPtm: cmp_c <-- cmp_m
CRNCARtm: acrn_c + crn_m --> acrn_m + crn_c
CSNATirm: accoa_m + crn_m <=> acrn_m + coa_m
CSNATr: accoa_c + crn_c <=> acrn_c + coa_c
CYSATm: glu__L_m + mercppyr_m --> akg_m + cys__L_m
DHAD1: 23dhmb_c --> 3mob_c + h2o_c
DHAD2: 23dhmp_c --> 3mop_c + h2o_c
DLYSOXGAT: akg_c + lys__D_c <=> 6a2ohxnt_c + glu__D_c
DLYSPYRAT: lys__D_c + pyr_c <=> 6a2ohxnt_c + ala__D_c
DPRm: 2dhp_m + h_m + nadph_m --> nadp_m + pant__R_m
EHGLAT: akg_c + e4hglu_c --> 4h2oglt_c + glu__L_c
EHGLATp: akg_x + e4hglu_x --> 4h2oglt_x + glu__L_x
FA160COAabcp_1: atp_c + h2o_c + pmtcoa_c --> adp_c + h_c + pi_c + pmtcoa_x
FUMSO3tm: fum_c + so3_m <=> fum_m + so3_c
FUMSO4tm: fum_c + so4_m <=> fum_m + so4_c
FUMTSULtm: fum_c + tsul_m <=> fum_m + tsul_c
FUMtm: fum_c + pi_m <=> fum_m + pi_c
GABAVESSEC: 3.0 4abut_c + atp_c + h2o_c --> 3.0 4abut_e + adp_c + h_c + pi_c
GLUt2m: glu__L_c + h_c <=> glu__L_m + h_m
GLYVESSEC: atp_c + 3.0 gly_c + h2o_c --> adp_c + 3.0 gly_e + h_c + pi_c
HCITSn: accoa_n + akg_n + h2o_n --> coa_n + h_n + hcit_n
Htm: h_c --> h_m
KARA1: 23dhmb_c + nadp_c <=> alac__S_c + h_c + nadph_c
KARA2: 2ahbut_c + h_c + nadph_c <=> 23dhmp_c + nadp_c
LCADi_Dm: h2o_m + lald__D_m + nad_m --> 2.0 h_m + lac__D_m + nadh_m
LCADm: h2o_m + lald__L_m + nad_m --> 2.0 h_m + lac__L_m + nadh_m
LEUTAi: 4mop_c + glu__L_c --> akg_c + leu__L_c
L_LACtcm: lac__L_c --> lac__L_m
MALDDH: mal__D_c + nad_c --> co2_c + nadh_c + pyr_c
MALICITtm: icit_m + mal__L_c <=> icit_c + mal__L_m
MALOAAtm: mal__L_m + oaa_c <=> mal__L_c + oaa_m
MALSO3tm: mal__L_c + so3_m <=> mal__L_m + so3_c
MALSO4tm: mal__L_c + so4_m <=> mal__L_m + so4_c
MALTSULtm: mal__L_c + tsul_m <=> mal__L_m + tsul_c
MTMOHT: 3mob_m + h2o_m + mlthf_m --> 2dhp_m + thf_m
NA1Htm: h_m + na1_c <=> h_c + na1_m
NADKm: atp_m + nad_m --> adp_m + h_m + nadp_m
OAAAKGtm: akg_m + oaa_c <=> akg_c + oaa_m
OAACITtm: cit_m + oaa_c <=> cit_c + oaa_m
OAAICITtm: icit_m + oaa_c <=> icit_c + oaa_m
OAAt2m: h_c + oaa_c <=> h_m + oaa_m
OXO2Ctm: akg_m + oxag_c <=> akg_c + oxag_m
PABB: chor_c + nh4_c --> 4adcho_c + h2o_c
PHETA1m: akg_m + phe__L_m <=> glu__L_m + phpyr_m
PNORn: nadp_n + pant__R_n <=> 2dhp_n + h_n + nadph_n
PYDXDH_1: h2o_c + o2_c + pydx_c --> 4pyrdx_c + h2o2_c + h_c
PYDXO: 2.0 h2o_c + nh4_c + 0.5 o2_c + pydx_c <=> 2.0 h2o2_c + pydam_c
RTOTAL2CRNCPT1: Rtotal2coa_c + crn_c --> Rtotal2crn_c + coa_c
RTOTAL2CRNCPT2: Rtotal2crn_m + coa_m --> Rtotal2coa_m + crn_m
RTOTAL2CRNt: Rtotal2crn_c --> Rtotal2crn_m
RTOTAL3CRNCPT1: Rtotal3coa_c + crn_c --> Rtotal3crn_c + coa_c
RTOTAL3CRNCPT2: Rtotal3crn_m + coa_m --> Rtotal3coa_m + crn_m
RTOTAL3CRNt: Rtotal3crn_c --> Rtotal3crn_m
RTOTALCRNCPT1: Rtotalcoa_c + crn_c --> Rtotalcrn_c + coa_c
RTOTALCRNCPT2: Rtotalcrn_m + coa_m --> Rtotalcoa_m + crn_m
RTOTALCRNt: Rtotalcrn_c --> Rtotalcrn_m
SER_AL: ser__L_c <=> h_c + nh3_c + pyr_c
SHCHD: dscl_c + nadp_c --> h_c + nadph_c + scl_c
TYRTAi: 34hpp_c + glu__L_c --> akg_c + tyr__L_c
TYRTAim: 34hpp_m + glu__L_m --> akg_m + tyr__L_m
UREAt_1: na1_e + urea_e <=> na1_c + urea_c
UREAtm_1: na1_c + urea_c <=> na1_m + urea_m
VALTAim: akg_m + val__L_m --> 3mob_m + glu__L_m
```

In [145]:

```
print('Updated reactions\n')
for r in sorted(model_old.reactions, key=lambda x: x.id):
    if r in model_new.reactions:
        r2 = model_new.reactions.get_by_id(r.id)
        if (r.name == r2.name and r.reaction == r2.reaction and r.gene_reaction_rule == r2.gene_reaction_rule and
            r.lower_bound == r2.lower_bound and r.upper_bound == r2.upper_bound):
            pass
        else:
            print('Old', r, r.gene_reaction_rule)
            print('New', r2, r2.gene_reaction_rule)
            print()
```

```
Updated reactions

Old 3HKYNAKGAT: akg_c + hLkynr_c --> 42A3HP24DB_c + glu__L_c 12407 or 14908 or 15839 or 8540
New 3HKYNAKGAT: akg_c + hLkynr_c --> 42A3HP24DB_c + glu__L_c 14908 or 15839 or 8540

Old 3SALACBOXL: 3sala_c + h_c --> co2_c + hyptaur_c 10722 or 9434 or 9435
New 3SALACBOXL: 3sala_c + h_c --> co2_c + hyptaur_c 10722 or 9434 or 9435

Old 5AOPt2: 5aop_e + h_e --> 5aop_c + h_c 11269 or 13423
New 5AOPt2: 5aop_e + h_e --> 5aop_c + h_c 11269

Old 5HOXINDACTOX: 5hoxindact_c + h2o_c + nad_c --> 5hoxindoa_c + 2.0 h_c + nadh_c 12042 or 13426 or 15814 or 16323
New 5HOXINDACTOX: 5hoxindact_c + h2o_c + nad_c --> 5hoxindoa_c + 2.0 h_c + nadh_c 12042 or 13426 or 16323

Old AASAD1: L2aadp_c + atp_c + h_c + nadph_c --> L2aadp6sa_c + amp_c + nadp_c + ppi_c 9495 or (10220 and 9495)
New AASAD1: L2aadp_c + atp_c + h_c + nadph_c --> L2aadp6sa_c + amp_c + nadp_c + ppi_c 10220 and 9495

Old AATA: 2oxoadp_c + glu__L_c <=> L2aadp_c + akg_c 12407 or 14908 or 15839
New AATA: 2oxoadp_c + glu__L_c <=> L2aadp_c + akg_c 14908 or 15839

Old ABUTt2r: 4abut_e + h_e <=> 4abut_c + h_c 11269 or 12743 or 13423
New ABUTt2r: 4abut_e + h_e <=> 4abut_c + h_c 11269

Old ADCS: chor_c + gln__L_c --> 4adcho_c + glu__L_c 13669 or (13669 and 16564)
New ADCS: chor_c + gln__L_c --> 4adcho_c + glu__L_c 13669

Old AKGCITtm: akg_c + cit_m <=> akg_m + cit_c 11740 or 13510
New AKGCITtm: akg_c + cit_m --> akg_m + cit_c 16746

Old AKGMALtm: akg_m + mal__L_c <=> akg_c + mal__L_m 11740 or 13510
New AKGMALtm: akg_m + mal__L_c --> akg_c + mal__L_m 10635

Old ALAt2r: ala__L_e + h_e <=> ala__L_c + h_c 12743 or 14229 or 15074 or 8962 or 9319 or 9322 or 9962
New ALAt2r: ala__L_e + h_e <=> ala__L_c + h_c 14229 or 15074 or 8962 or 9319 or 9322 or 9962

Old ALDD20x: h2o_c + id3acald_c + nad_c --> 2.0 h_c + ind3ac_c + nadh_c 12042 or 13426 or 15814 or 16323
New ALDD20x: h2o_c + id3acald_c + nad_c --> 2.0 h_c + ind3ac_c + nadh_c 12042 or 13426 or 16323

Old ALDD2x: acald_c + h2o_c + nad_c --> ac_c + 2.0 h_c + nadh_c 12042 or 13426 or 15814 or 16323
New ALDD2x: acald_c + h2o_c + nad_c --> ac_c + 2.0 h_c + nadh_c 12042 or 13426 or 16323

Old AMY1e: 8.0 h2o_e + strch1_e --> 8.0 glc__D_e + strch2_e 11625 and 9135
New AMY1e: 8.0 h2o_e + strch1_e --> 8.0 glc__D_e + strch2_e 9135

Old AMY2e: glygn2_e + 8.0 h2o_e --> 8.0 glc__D_e + glygn4_e 11625 and 9135
New AMY2e: glygn2_e + 8.0 h2o_e --> 8.0 glc__D_e + glygn4_e 9135

Old ANS: chor_c + gln__L_c --> anth_c + glu__L_c + h_c + pyr_c 15109 or 16564 or (15109 and 16564) or (15109 and 9900)
New ANS: chor_c + gln__L_c --> anth_c + glu__L_c + h_c + pyr_c 15109 and 16564

Old ARGt2r: arg__L_e + h_e <=> arg__L_c + h_c 14229 or 15074 or 8962 or 9319 or 9322 or (14229 and 8962) or (14229 and 9319) or (14229 and 9322) or (15074 and 8962) or (15074 and 9319) or (15074 and 9322)
New ARGt2r: arg__L_e + h_e <=> arg__L_c + h_c 14229 or 15074

Old ASNt2r: asn__L_e + h_e <=> asn__L_c + h_c 14229 or 15074 or 8962 or 9319 or 9322 or 9962 or (14229 and 8962) or (14229 and 9319) or (14229 and 9322) or (14229 and 9962) or (15074 and 8962) or (15074 and 9319) or (15074 and 9322) or (15074 and 9962)
New ASNt2r: asn__L_e + h_e <=> asn__L_c + h_c 14229 or 15074 or 8962 or 9319 or 9322 or 9962

Old ASPGLUm: asp__L_m + glu__L_c + h_c <=> asp__L_c + glu__L_m + h_m 16799
New ASPGLUm: asp__L_m + glu__L_c + h_c --> asp__L_c + glu__L_m + h_m 16799

Old ASPTA: akg_c + asp__L_c <=> glu__L_c + oaa_c 14281 or 8936
New ASPTA: akg_c + asp__L_c <=> glu__L_c + oaa_c 8936

Old ATP2tp_H: amp_x + atp_c + h_x --> amp_c + atp_x + h_c 16145
New ATP2tp_H: amp_x + atp_c + 2.0 h_c --> amp_c + atp_x + 2.0 h_x 16145

Old BAMPPALDOX: bamppald_c + h2o_c + nad_c --> ala_B_c + 2.0 h_c + nadh_c 12042 or 13426 or 15814 or 16323
New BAMPPALDOX: bamppald_c + h2o_c + nad_c --> ala_B_c + 2.0 h_c + nadh_c 12042 or 13426 or 16323

Old C3STKR1er: 4mzym_int2_r + h_r + nadph_r --> 4mzym_r + nadp_r ERG27
New C3STKR1er: 4mzym_int2_r + h_r + nadph_r --> 4mzym_r + nadp_r ERG27

Old C3STKR2er: h_r + nadph_r + zym_int2_r --> nadp_r + zymst_r ERG27
New C3STKR2er: h_r + nadph_r + zym_int2_r --> nadp_r + zymst_r ERG27

Old CDPDAGterm_RT: cdpdag_RT_r <=> cdpdag_RT_m 
New CDPDAGterm_RT: cdpdag_RT_r <=> cdpdag_RT_m 

Old CITtam: cit_c + mal__L_m <=> cit_m + mal__L_c 10514 or 11740 or 13510
New CITtam: cit_c + mal__L_m <=> cit_m + mal__L_c 10514

Old CITtcm: cit_c + icit_m <=> cit_m + icit_c 10514 or 11740 or 13510
New CITtcm: cit_c + icit_m <=> cit_m + icit_c 10514

Old CSNAT2x: crn_x + dmnoncoa_x <=> coa_x + dmnoncrn_x 14245
New CSNAT2x: crn_x + dmnoncoa_x <=> coa_x + dmnoncrn_x 15783

Old CYSTA: akg_c + cys__L_c --> glu__L_c + mercppyr_c 14281 or 8936
New CYSTA: akg_c + cys__L_c <=> glu__L_c + mercppyr_c 8936

Old CYSTSERex: cysi__L_e + ser__L_c --> cysi__L_c + ser__L_e 11625 and 9135
New CYSTSERex: cysi__L_e + ser__L_c --> cysi__L_c + ser__L_e 11625

Old DALAt2r: ala__D_e + h_e <=> ala__D_c + h_c 12743
New DALAt2r: ala__D_e + h_e <=> ala__D_c + h_c 14229 or 15074

Old DBTSm: atp_m + co2_m + dann_m <=> adp_m + dtbt_m + 3.0 h_m + pi_m 12731
New DBTSm: atp_m + co2_m + dann_m <=> adp_m + dtbt_m + 3.0 h_m + pi_m 12731

Old DESAT1829Z12Zer: h_r + nadh_r + o2_r + odecoa_r --> 2.0 h2o_r + nad_r + ocdycacoa_r (12471 and 16097 and 8845) or (13206 and 16097 and 8845)
New DESAT1829Z12Zer: h_r + nadh_r + o2_r + odecoa_r --> 2.0 h2o_r + nad_r + ocdycacoa_r (12471 and 16097 and 8845) or (13206 and 16097 and 8845)

Old DNTPPA: ahdt_c + h2o_c --> dhpmp_c + h_c + ppi_c 15385
New DNTPPA: ahdt_c + h2o_c --> dhpmp_c + h_c + ppi_c 15385

Old DOLPMMer: dolmanp_r --> dolp_r + h_r + mannan_r 12121 or 13840 or 8768 or (13840 and 8768)
New DOLPMMer: dolmanp_r --> dolp_r + h_r + mannan_r 12121 or 13840 or 8768

Old DPR: 2dhp_c + h_c + nadph_c --> nadp_c + pant__R_c 14277 or 16522 or 9176
New DPR: 2dhp_c + h_c + nadph_c --> nadp_c + pant__R_c 14277 or 16522

Old FA140COAabcp: atp_x + h2o_x + tdcoa_c --> adp_x + h_x + pi_x + tdcoa_x 13167 or 9637
New FA140COAabcp: atp_c + 2.0 h2o_c + tdcoa_c --> adp_c + coa_c + 2.0 h_c + pi_c + ttdca_x 13167 and 9637

Old FA141COAabcp: atp_x + h2o_x + tdecoa_c --> adp_x + h_x + pi_x + tdecoa_x 13167 or 9637
New FA141COAabcp: atp_c + 2.0 h2o_c + tdecoa_c --> adp_c + coa_c + 2.0 h_c + pi_c + ttdcea_x 13167 and 9637

Old FA160COAabcp: atp_x + h2o_x + pmtcoa_c --> adp_x + h_x + pi_x + pmtcoa_x 13167 or 9637
New FA160COAabcp: atp_c + 2.0 h2o_c + pmtcoa_c --> adp_c + coa_c + 2.0 h_c + hdca_x + pi_c 13167 and 9637

Old FA161COAabcp: atp_x + h2o_x + hdcoa_c --> adp_x + h_x + hdcoa_x + pi_x 13167 or 9637
New FA161COAabcp: atp_c + 2.0 h2o_c + hdcoa_c --> adp_c + coa_c + 2.0 h_c + hdcea_x + pi_c 13167 and 9637

Old FA180COAabcp: atp_x + h2o_x + stcoa_c --> adp_x + h_x + pi_x + stcoa_x 13167 or 9637
New FA180COAabcp: atp_c + 2.0 h2o_c + stcoa_c --> adp_c + coa_c + 2.0 h_c + ocdca_x + pi_c 13167 and 9637

Old FA181COAabcp: atp_x + h2o_x + odecoa_c --> adp_x + h_x + odecoa_x + pi_x 13167 or 9637
New FA181COAabcp: atp_c + 2.0 h2o_c + odecoa_c --> adp_c + coa_c + 2.0 h_c + ocdcea_x + pi_c 13167 and 9637

Old FA182COAabcp: atp_x + h2o_x + ocdycacoa_c --> adp_x + h_x + ocdycacoa_x + pi_x 13167 or 9637
New FA182COAabcp: atp_c + 2.0 h2o_c + ocdycacoa_c --> adp_c + coa_c + 2.0 h_c + ocdcya_x + pi_c 13167 and 9637

Old FACOAL100p: atp_x + coa_x + dca_x --> amp_x + dcacoa_x + ppi_x 12555 or 15900
New FACOAL100p: atp_x + coa_x + dca_x --> amp_x + dcacoa_x + ppi_x 15900

Old FACOAL120p: atp_x + coa_x + ddca_x --> amp_x + ddcacoa_x + ppi_x 12555 or 15900
New FACOAL120p: atp_x + coa_x + ddca_x --> amp_x + ddcacoa_x + ppi_x 12555

Old FACOAL140p: atp_x + coa_x + ttdca_x --> amp_x + ppi_x + tdcoa_x 12555 or 15900
New FACOAL140p: atp_x + coa_x + ttdca_x --> amp_x + ppi_x + tdcoa_x 12555

Old FACOAL141p: atp_x + coa_x + ttdcea_x --> amp_x + ppi_x + tdecoa_x 12555 or 15900
New FACOAL141p: atp_x + coa_x + ttdcea_x --> amp_x + ppi_x + tdecoa_x 12555

Old FACOAL160p: atp_x + coa_x + hdca_x --> amp_x + pmtcoa_x + ppi_x 12555 or 15900
New FACOAL160p: atp_x + coa_x + hdca_x --> amp_x + pmtcoa_x + ppi_x 12555

Old FACOAL161p: atp_x + coa_x + hdcea_x --> amp_x + hdcoa_x + ppi_x 12555 or 15900
New FACOAL161p: atp_x + coa_x + hdcea_x --> amp_x + hdcoa_x + ppi_x 12555

Old FACOAL240p: atp_x + coa_x + ttc_x --> amp_x + ppi_x + ttccoa_x 9912
New FACOAL240p: atp_x + coa_x + ttc_x --> amp_x + ppi_x + ttccoa_x 12555 or 9912

Old FACOAL260p: atp_x + coa_x + hexc_x --> amp_x + hexccoa_x + ppi_x 9912
New FACOAL260p: atp_x + coa_x + hexc_x --> amp_x + hexccoa_x + ppi_x 12555 or 9912

Old FACOAL80p: atp_x + coa_x + octa_x --> amp_x + occoa_x + ppi_x 12555 or 15900
New FACOAL80p: atp_x + coa_x + octa_x --> amp_x + occoa_x + ppi_x 15900

Old FAS240COAer: 9.0 h_r + 3.0 malcoa_r + 6.0 nadph_r + stcoa_r --> 3.0 co2_r + 3.0 coa_r + 3.0 h2o_r + 6.0 nadp_r + ttccoa_r (10677 and 11343 and 16241 and 16695) or (10677 and 16241 and 16655 and 16695)
New FAS240COAer: 9.0 h_r + 3.0 malcoa_r + 6.0 nadph_r + stcoa_r --> 3.0 co2_r + 3.0 coa_r + 3.0 h2o_r + 6.0 nadp_r + ttccoa_r (10677 and 11343 and 16241 and 16695) or (10677 and 16241 and 16655 and 16695)

Old FAS260COAer: 3.0 h_r + malcoa_r + 2.0 nadph_r + ttccoa_r --> co2_r + coa_r + h2o_r + hexccoa_r + 2.0 nadp_r 10677 and 11343 and 16241 and 16695
New FAS260COAer: 3.0 h_r + malcoa_r + 2.0 nadph_r + ttccoa_r --> co2_r + coa_r + h2o_r + hexccoa_r + 2.0 nadp_r 10677 and 11343 and 16241 and 16695

Old FKYNH: Lfmkynr_c + h2o_c --> Lkynr_c + for_c + h_c 8540
New FKYNH: Lfmkynr_c + h2o_c --> Lkynr_c + for_c + h_c 14788

Old FRDPtm: frdp_c <=> frdp_m 
New FRDPtm: frdp_c <=> frdp_m 

Old FTHFCLm: 5fthf_m + atp_m --> adp_m + methf_m + pi_m 12562
New FTHFCLm: 5fthf_m + atp_m --> adp_m + methf_m + pi_m 12562

Old G3PT: glyc3p_c + h2o_c --> glyc_c + pi_c 13044 or 13413
New G3PT: glyc3p_c + h2o_c --> glyc_c + pi_c 13413

Old G5SADrm: glu5sa_m <=> 1pyr5c_m + h2o_m + h_m 
New G5SADrm: glu5sa_m <=> 1pyr5c_m + h2o_m + h_m 

Old G5SADs: glu5sa_c <=> 1pyr5c_c + h2o_c + h_c 
New G5SADs: glu5sa_c <=> 1pyr5c_c + h2o_c + h_c 

Old GCALDD: gcald_c + h2o_c + nad_c --> glyclt_c + 2.0 h_c + nadh_c 12042 or 13426 or 15814 or 16323
New GCALDD: gcald_c + h2o_c + nad_c --> glyclt_c + 2.0 h_c + nadh_c 12042 or 13426 or 16323

Old GLNt2r: gln__L_e + h_e <=> gln__L_c + h_c 14229 or 15074 or 8962 or 9319 or 9322 or 9962 or (14229 and 8962) or (14229 and 9319) or (14229 and 9322) or (14229 and 9962) or (15074 and 8962) or (15074 and 9319) or (15074 and 9322) or (15074 and 9962)
New GLNt2r: gln__L_e + h_e <=> gln__L_c + h_c 14229 or 15074 or 8962 or 9319 or 9322 or 9962

Old GLYCt: glyc_c <=> glyc_e 15470
New GLYCt: glyc_c <=> glyc_e 13100 or 15470

Old GLYCtm: glyc_c <=> glyc_m 
New GLYCtm: glyc_c <=> glyc_m 

Old GLYOp: gly_x + h2o_x + o2_x --> glx_x + h2o2_x + nh4_x 15994
New GLYOp: gly_x + h2o_x + o2_x --> glx_x + h2o2_x + nh4_x 15994

Old GLYt2r: gly_e + h_e <=> gly_c + h_c 12743 or 14229 or 15074 or 8962 or 9319 or 9322 or 9962
New GLYt2r: gly_e + h_e <=> gly_c + h_c 14229 or 15074 or 8962 or 9319 or 9322 or 9962

Old H2Otm: h2o_c <=> h2o_m 13986 or 13987 or 9014 or 9015
New H2Otm: h2o_c <=> h2o_m 9014

Old H2Otp: h2o_c <=> h2o_x 13986
New H2Otp: h2o_c <=> h2o_x 

Old HACONTam: hcit_m <=> h2o_m + hacon_C_m 14600
New HACONTam: hcit_m <=> h2o_m + hacon_C_m 14600

Old HICITDm: hicit_m + nad_m <=> h_m + nadh_m + oxag_m 10428
New HICITDm: hicit_m + nad_m <=> 2oxoadp_m + co2_m + nadh_m 10428

Old ILETA: akg_c + ile__L_c <=> 3mop_c + glu__L_c 14014 or 14610 or 14853
New ILETA: akg_c + ile__L_c <=> 3mop_c + glu__L_c 14014

Old ILETAm: akg_m + ile__L_m <=> 3mop_m + glu__L_m 14014 or 14610 or 14853
New ILETAm: akg_m + ile__L_m <=> 3mop_m + glu__L_m 14610 or 14853

Old ILETRS: atp_c + ile__L_c + trnaile_c --> amp_c + iletrna_c + ppi_c 13446 or 15556
New ILETRS: atp_c + ile__L_c + trnaile_c --> amp_c + iletrna_c + ppi_c 13446

Old ILETRSm: atp_m + ile__L_m + trnaile_m --> amp_m + iletrna_m + ppi_m 13446 or 15556
New ILETRSm: atp_m + ile__L_m + trnaile_m --> amp_m + iletrna_m + ppi_m 15556

Old IMACTD: h2o_c + im4act_c + nad_c --> 2.0 h_c + im4ac_c + nadh_c 12042 or 13426 or 15814 or 16323
New IMACTD: h2o_c + im4act_c + nad_c --> 2.0 h_c + im4ac_c + nadh_c 12042 or 13426 or 16323

Old IPMD: 3c2hmp_c + nad_c --> 3c4mop_c + h_c + nadh_c 10428 or 13894
New IPMD: 3c2hmp_c + nad_c --> 3c4mop_c + h_c + nadh_c 13894

Old IPPS: 3mob_c + accoa_c + h2o_c --> 3c3hmp_c + coa_c + h_c 14856 or 15488
New IPPS: 3mob_c + accoa_c + h2o_c --> 3c3hmp_c + coa_c + h_c 15488

Old KYNAKGAT: Lkynr_c + akg_c --> 4aphdob_c + glu__L_c 12407 or 14908 or 15839 or 8540
New KYNAKGAT: Lkynr_c + akg_c --> 4aphdob_c + glu__L_c 14908 or 15839 or 8540

Old LCADi: h2o_c + lald__L_c + nad_c --> 2.0 h_c + lac__L_c + nadh_c 12042 or 13426 or 15814 or 16323
New LCADi: h2o_c + lald__L_c + nad_c --> 2.0 h_c + lac__L_c + nadh_c 12042 or 13426 or 16323

Old LCADi_D: h2o_c + lald__D_c + nad_c --> 2.0 h_c + lac__D_c + nadh_c 12042 or 13426 or 15814 or 16323
New LCADi_D: h2o_c + lald__D_c + nad_c --> 2.0 h_c + lac__D_c + nadh_c 12042 or 13426 or 16323

Old LEUTA: akg_c + leu__L_c <=> 4mop_c + glu__L_c 14014 or 14610 or 14853
New LEUTA: akg_c + leu__L_c <=> 4mop_c + glu__L_c 14014

Old LEUTAm: akg_m + leu__L_m <=> 4mop_m + glu__L_m 14014 or 14610 or 14853
New LEUTAm: akg_m + leu__L_m <=> 4mop_m + glu__L_m 14610 or 14853

Old LYSt2r: h_e + lys__L_e <=> h_c + lys__L_c 14229 or 15074 or 8962 or 9319 or 9322 or (14229 and 8962) or (14229 and 9319) or (14229 and 9322) or (15074 and 8962) or (15074 and 9319) or (15074 and 9322)
New LYSt2r: h_e + lys__L_e <=> h_c + lys__L_c 14229 or 15074

Old MALtm: mal__L_c + pi_m <=> mal__L_m + pi_c 11740 or 13510
New MALtm: mal__L_c + pi_m --> mal__L_m + pi_c 13510

Old NABTNO: h2o_c + n4abutn_c + nad_c --> 4aabutn_c + 2.0 h_c + nadh_c 12042 or 13426 or 15814 or 16323
New NABTNO: h2o_c + n4abutn_c + nad_c --> 4aabutn_c + 2.0 h_c + nadh_c 12042 or 13426 or 16323

Old NDPK10m: atp_m + didp_m <=> adp_m + ditp_m 8943
New NDPK10m: atp_m + didp_m <=> adp_m + ditp_m 8943

Old NDPK1m: atp_m + gdp_m <=> adp_m + gtp_m 8943
New NDPK1m: atp_m + gdp_m <=> adp_m + gtp_m 8943

Old NDPK2m: atp_m + udp_m <=> adp_m + utp_m 8943
New NDPK2m: atp_m + udp_m <=> adp_m + utp_m 8943

Old NDPK3m: atp_m + cdp_m <=> adp_m + ctp_m 8943
New NDPK3m: atp_m + cdp_m <=> adp_m + ctp_m 8943

Old NDPK4m: atp_m + dtdp_m <=> adp_m + dttp_m 8943
New NDPK4m: atp_m + dtdp_m <=> adp_m + dttp_m 8943

Old NDPK5m: atp_m + dgdp_m <=> adp_m + dgtp_m 8943
New NDPK5m: atp_m + dgdp_m <=> adp_m + dgtp_m 8943

Old NDPK6m: atp_m + dudp_m <=> adp_m + dutp_m 8943
New NDPK6m: atp_m + dudp_m <=> adp_m + dutp_m 8943

Old NDPK7m: atp_m + dcdp_m <=> adp_m + dctp_m 8943
New NDPK7m: atp_m + dcdp_m <=> adp_m + dctp_m 8943

Old NDPK8m: atp_m + dadp_m <=> adp_m + datp_m 8943
New NDPK8m: atp_m + dadp_m <=> adp_m + datp_m 8943

Old NDPK9m: atp_m + idp_m <=> adp_m + itp_m 8943
New NDPK9m: atp_m + idp_m <=> adp_m + itp_m 8943

Old NTD2: h2o_c + ump_c --> pi_c + uri_c 13044 or 16648 or 9995
New NTD2: h2o_c + ump_c --> pi_c + uri_c 16648 or 9995

Old NTD4: cmp_c + h2o_c --> cytd_c + pi_c 13044 or 16648 or 9995
New NTD4: cmp_c + h2o_c --> cytd_c + pi_c 16648 or 9995

Old NTD7: amp_c + h2o_c --> adn_c + pi_c 13044 or 16648
New NTD7: amp_c + h2o_c --> adn_c + pi_c 16648

Old NTD9: gmp_c + h2o_c --> gsn_c + pi_c 13044 or 16648
New NTD9: gmp_c + h2o_c --> gsn_c + pi_c 16648

Old OBDHm: 2obut_m + coa_m + nad_m --> co2_m + nadh_m + ppcoa_m (10040 and 11183 and 12566 and 15436) or (10040 and 11188 and 12566 and 15436)
New OBDHm: 2obut_m + coa_m + nad_m --> co2_m + nadh_m + ppcoa_m (10040 and 11183 and 12566 and 15436) or (10040 and 11188 and 12566 and 15436)

Old OMCDC: 3c4mop_c + h_c --> 4mop_c + co2_c 14014 or 14610 or 14853
New OMCDC: 3c4mop_c + h_c --> 4mop_c + co2_c 

Old OMCDCm: 3c4mop_m + h_m --> 4mop_m + co2_m 14014 or 14610 or 14853
New OMCDCm: 3c4mop_m + h_m --> 4mop_m + co2_m 

Old ORNt2r: h_e + orn_e <=> h_c + orn_c 14229 or 15074 or 8962 or 9319 or 9322 or (14229 and 8962) or (14229 and 9319) or (14229 and 9322) or (15074 and 8962) or (15074 and 9319) or (15074 and 9322)
New ORNt2r: h_e + orn_e <=> h_c + orn_c 14229 or 15074

Old ORNt3m: h_c + orn_m <=> h_m + orn_c 13319
New ORNt3m: h_c + orn_m --> h_m + orn_c 13319

Old PAterm_RT: pa_RT_r <=> pa_RT_m 
New PAterm_RT: pa_RT_r <=> pa_RT_m 

Old PDX5POi: o2_c + pdx5p_c --> h2o2_c + pydx5p_c 10680 or 8901
New PDX5POi: o2_c + pdx5p_c --> h2o2_c + pydx5p_c 10680

Old PDXPP: h2o_c + pdx5p_c --> pi_c + pydxn_c 13044 or 14545
New PDXPP: h2o_c + pdx5p_c --> pi_c + pydxn_c 13044

Old PEterm_RT: pe_RT_r <=> pe_RT_m 
New PEterm_RT: pe_RT_r <=> pe_RT_m 

Old PGPPAm_RT: h2o_m + 0.01 pgp_RT_m --> 0.01 pg_RT_m + pi_m 10900
New PGPPAm_RT: h2o_m + 0.01 pgp_RT_m --> 0.01 pg_RT_m + pi_m 10900

Old PHCHGSm: 1p3h5c_m + h2o_m + h_m <=> 4hglusa_m 
New PHCHGSm: 1p3h5c_m + h2o_m + h_m <=> 4hglusa_m 

Old PHETA1: akg_c + phe__L_c <=> glu__L_c + phpyr_c 12407 or 13230 or 14281 or 14610 or 14853 or 14908 or 15839 or 8936
New PHETA1: akg_c + phe__L_c <=> glu__L_c + phpyr_c 14908 or 15839

Old PI45BP5Pn_RT: h2o_n + 0.01 ptd145bp_RT_n --> pi_n + 0.01 ptd4ino_RT_n 13609 or 15545
New PI45BP5Pn_RT: h2o_n + 0.01 ptd145bp_RT_n --> pi_n + 0.01 ptd4ino_RT_n 13609 or 15545

Old PIN3Pn_RT: h2o_n + 0.01 ptd3ino_RT_n --> pi_n + 0.01 ptd1ino_RT_n 14880
New PIN3Pn_RT: h2o_n + 0.01 ptd3ino_RT_n --> pi_n + 0.01 ptd1ino_RT_n 14880

Old PIt2m: h_c + pi_c <=> h_m + pi_m 15874 or 8889
New PIt2m: h_c + pi_c --> h_m + pi_m 15874 or 8889

Old PIt9: 2.0 na1_e + pi_e <=> 2.0 na1_c + pi_c 11686
New PIt9: 2.0 na1_e + pi_e <=> 2.0 na1_c + pi_c 11686

Old PItx: pi_c <=> pi_x 
New PItx: pi_c <=> pi_x 

Old PMDPHT: 5aprbu_c + h2o_c --> 4r5au_c + pi_c 11513
New PMDPHT: 5aprbu_c + h2o_c --> 4r5au_c + pi_c 11513

Old PRODt2r: h_e + pro__D_e <=> h_c + pro__D_c 12743
New PRODt2r: h_e + pro__D_e <=> h_c + pro__D_c 14229 or 15074

Old PROt2r: h_e + pro__L_e <=> h_c + pro__L_c 12743 or 14229 or 15074
New PROt2r: h_e + pro__L_e <=> h_c + pro__L_c 14229 or 15074

Old PSterm_RT: ps_RT_r <=> ps_RT_m 
New PSterm_RT: ps_RT_r <=> ps_RT_m 

Old PTRCt3i: h_c + ptrc_e --> h_e + ptrc_c 11017 or 13128 or (11017 and 16454) or (13128 and 16454)
New PTRCt3i: h_c + ptrc_e --> h_e + ptrc_c 11017 or 11649 or 13128 or 14774

Old PTRCtex2: ptrc_c --> ptrc_e 13423 or 15991 or 16258
New PTRCtex2: ptrc_c --> ptrc_e 13423

Old PYAM5PO: h2o_c + o2_c + pyam5p_c --> h2o2_c + nh4_c + pydx5p_c 10680 or 8901
New PYAM5PO: h2o_c + o2_c + pyam5p_c --> h2o2_c + nh4_c + pydx5p_c 10680

Old PYDXNO: o2_c + pydxn_c <=> h2o2_c + pydx_c 10680 or 8901
New PYDXNO: o2_c + pydxn_c <=> h2o2_c + pydx_c 10680

Old PYDXO_1: h2o_c + o2_c + pydam_c <=> h2o2_c + nh4_c + pydx_c 10680 or 8901
New PYDXO_1: h2o_c + o2_c + pydam_c <=> h2o2_c + nh4_c + pydx_c 10680

Old PYDXPP: h2o_c + pydx5p_c --> pi_c + pydx_c 13044 or 14545
New PYDXPP: h2o_c + pydx5p_c --> pi_c + pydx_c 13044

Old PYLALDOX: h2o_c + nad_c + pylald_c --> 2.0 h_c + nadh_c + peracd_c 12042 or 13426 or 15814 or 16323
New PYLALDOX: h2o_c + nad_c + pylald_c --> 2.0 h_c + nadh_c + peracd_c 12042 or 13426 or 16323

Old R5PP: h2o_c + r5p_c --> pi_c + rib__D_c 13044 or 14546 or 8576
New R5PP: h2o_c + r5p_c --> pi_c + rib__D_c 14546 or 8576

Old SACCD1: L2aadp6sa_c + glu__L_c + h_c + nadph_c <=> h2o_c + nadp_c + saccrp__L_c 10577 or 16833
New SACCD1: L2aadp6sa_c + glu__L_c + h_c + nadph_c <=> h2o_c + nadp_c + saccrp__L_c 16833

Old SACCD3m: akg_m + h_m + lys__L_m + nadph_m --> h2o_m + nadp_m + saccrp__L_m 10577 or 16833
New SACCD3m: akg_m + h_m + lys__L_m + nadph_m --> h2o_m + nadp_m + saccrp__L_m 10577

Old SACCD4m: h2o_m + nadp_m + saccrp__L_m --> L2aadp6sa_m + glu__L_m + h_m + nadph_m 10577 or 16833
New SACCD4m: h2o_m + nadp_m + saccrp__L_m --> L2aadp6sa_m + glu__L_m + h_m + nadph_m 10577

Old SERD_L: ser__L_c --> nh4_c + pyr_c 11849 or 11909 or 9216
New SERD_L: ser__L_c --> nh4_c + pyr_c 11849 or 9216

Old SERLYSNaex: lys__L_c + na1_e + ser__L_e --> lys__L_e + na1_c + ser__L_c 11625 and 9135
New SERLYSNaex: lys__L_c + na1_e + ser__L_e --> lys__L_e + na1_c + ser__L_c 11625

Old SHCHD2: dscl_c + nad_c --> h_c + nadh_c + scl_c 11050 or 13569
New SHCHD2: dscl_c + nad_c --> h_c + nadh_c + scl_c 13569

Old SHCHF: fe2_c + scl_c --> 3.0 h_c + sheme_c 11050 or 13569
New SHCHF: fe2_c + scl_c --> 3.0 h_c + sheme_c 13569

Old SPMDt3i: h_c + spmd_e --> h_e + spmd_c 11017 or 13128 or (11017 and 16454) or (13128 and 16454)
New SPMDt3i: h_c + spmd_e --> h_e + spmd_c 11017 or 11649 or 13128 or 14774

Old SPMDtex2: spmd_c --> spmd_e 13423 or 15991 or 16258
New SPMDtex2: spmd_c --> spmd_e 13423

Old SPMS: ametam_c + ptrc_c --> 5mta_c + h_c + spmd_c 10577 or 16833
New SPMS: ametam_c + ptrc_c --> 5mta_c + h_c + spmd_c 16833

Old SPRMS: ametam_c + spmd_c --> 5mta_c + h_c + sprm_c 10577 or 16833
New SPRMS: ametam_c + spmd_c --> 5mta_c + h_c + sprm_c 16833

Old SPRMt2i: h_c + sprm_e --> h_e + sprm_c 11017 or 11649 or 13128 or 14774 or (11017 and 11649 and 16454) or (11017 and 14774 and 16454) or (11649 and 13128 and 16454) or (13128 and 14774 and 16454)
New SPRMt2i: h_c + sprm_e --> h_e + sprm_c 11017 or 11649 or 13128 or 14774

Old SUCCtm: pi_m + succ_c --> pi_c + succ_m 11740 or 13510
New SUCCtm: pi_m + succ_c --> pi_c + succ_m 13510

Old SUCFUMtm: fum_m + succ_c --> fum_c + succ_m 11740 or 12605 or 13510 or (11740 and 12605) or (12605 and 13510)
New SUCFUMtm: fum_m + succ_c --> fum_c + succ_m 12605

Old THRD_L: thr__L_c --> 2obut_c + nh4_c 11909 or 9216
New THRD_L: thr__L_c --> 2obut_c + nh4_c 9216

Old TYROXDAc: h2o_c + o2_c + tym_c --> 4hoxpacd_c + h2o2_c + nh4_c 10308 or 13959
New TYROXDAc: h2o_c + o2_c + tym_c --> 4hoxpacd_c + h2o2_c + nh4_c 10308 or 13959

Old TYRTA: akg_c + tyr__L_c <=> 34hpp_c + glu__L_c 12407 or 13230 or 14281 or 14610 or 14853 or 14908 or 15839 or 8936
New TYRTA: akg_c + tyr__L_c <=> 34hpp_c + glu__L_c 14908 or 15839 or 16065

Old UNK3: 2kmb_c + glu__L_c --> akg_c + met__L_c 12407 or 14281 or 14908 or 15839 or 8936
New UNK3: 2kmb_c + glu__L_c --> akg_c + met__L_c 8540 or 14014

Old VALTA: akg_c + val__L_c <=> 3mob_c + glu__L_c 14014 or 14610 or 14853
New VALTA: akg_c + val__L_c <=> 3mob_c + glu__L_c 14014
```

In [146]:

```
print('Added reactions\n')
for r in sorted(model_new.reactions, key=lambda x: x.id):
    if r not in model_old.reactions:
        print(r)
```

```
Added reactions

2AMACHYD: 2amac_c + h2o_c --> nh4_c + pyr_c
2OBUTt: 2obut_e + h_e <=> 2obut_c + h_c
2OBUTtm: 2obut_c <=> 2obut_m
34HPPOR: 34hpp_c + o2_c --> co2_c + hgentis_c
34HPPYRDC: 34hpp_c + h_c --> 4hoxpacd_c + co2_c
3MOBtm: 3mob_c <=> 3mob_m
3MOPtm: 3mop_c <=> 3mop_m
3SALAOX: 2.0 3sala_c + 2.0 h_c + o2_c --> 2.0 Lcyst_c
3SPYRSP: 3snpyr_c + h2o_c --> h_c + pyr_c + so3_c
3SPYRSPm: 3snpyr_m + h2o_m --> h_m + pyr_m + so3_m
4MOPtm: 4mop_c <=> 4mop_m
5AOPtm: 5aop_c <=> 5aop_m
AASAD3m: L2aadp6sa_m + h2o_m + nad_m --> L2aadp_m + 2.0 h_m + nadh_m
ACALDtm: acald_m <=> acald_c
ACRNtp: acrn_x --> acrn_c
ADPter: adp_r <=> adp_c
AHCYStr: ahcys_c <=> ahcys_r
AM6SAD: am6sa_c + h2o_c + nad_c --> amuco_c + 2.0 h_c + nadh_c
AMCOXO: amuco_c + h2o_c + h_c + nadph_c --> 2oxoadp_c + nadp_c + nh4_c
AMETr: amet_c <=> amet_r
AMPter: amp_c <=> amp_r
ARGt7m: arg__L_c --> arg__L_m
ATPM: atp_c + h2o_c --> adp_c + h_c + pi_c
ATPter: atp_c <=> atp_r
BIOMASS_RT: 1.1348 13BDglcn_c + 0.4588 ala__L_c + 0.046 amp_c + 0.1607 arg__L_c + 0.1017 asn__L_c + 0.2975 asp__L_c + 59.276 atp_c + 0.0447 cmp_c + 0.0066 cys__L_c + 0.0036 damp_c + 0.0024 dcmp_c + 0.0024 dgmp_c + 0.0036 dtmp_c + 0.0007 ergst_r + 0.1054 gln__L_c + 0.3018 glu__L_c + 0.2904 gly_c + 0.5185 glycogen_c + 0.046 gmp_c + 59.276 h2o_c + 0.0663 his__L_c + 0.1927 ile__L_c + 0.2964 leu__L_c + 0.2862 lys__L_c + 0.8079 mannan_r + 0.0507 met__L_c + 6e-06 pa_RT_r + 6e-05 pc_RT_r + 4.5e-05 pe_RT_r + 0.1339 phe__L_c + 0.1647 pro__L_c + 1.7e-05 ps_RT_r + 5.3e-05 ptd1ino_RT_r + 0.00099 ribflv_c + 0.1854 ser__L_c + 0.02 so4_c + 0.1914 thr__L_c + 0.0234 tre_c + 6.6e-05 triglyc_RT_r + 0.0284 trp__L_c + 0.102 tyr__L_c + 0.0599 ump_c + 0.2646 val__L_c + 0.0015 zymst_r --> 59.276 adp_c + 58.70001 h_c + 59.305 pi_c
C30CPT1: crn_c + ppcoa_c <=> coa_c + pcrn_c
CDPCHOLter: cdpchol_c <=> cdpchol_r
CDPter: cdp_r <=> cdp_c
CMPter: cmp_c <=> cmp_r
CO2t: co2_e <=> co2_c
CO2ter: co2_c <=> co2_r
CO2tg: co2_c <=> co2_g
CO2tm: co2_c <=> co2_m
CO2tn: co2_n <=> co2_c
CO2tp: co2_c <=> co2_x
CO2tv: co2_c <=> co2_v
COAtr: coa_c <=> coa_r
CTPter: ctp_c <=> ctp_r
CTPtm2: cmp_m + ctp_c + fe2_c --> cmp_c + ctp_m + fe2_m
DCACOAtr: dcacoa_c <=> dcacoa_r
DDCACOAtr: ddcacoa_c <=> ddcacoa_r
DDPGAm: 4h2oglt_m <=> glx_m + pyr_m
DHAPter: dhap_c <=> dhap_r
DM_aacald_c: aacald_c --> 
DOLPter: dolp_r --> dolp_c
EX_13BDglcn_e: 13BDglcn_e --> 
EX_2obut_e: 2obut_e --> 
EX_35cgmp_e: 35cgmp_e --> 
EX_4abut_e: 4abut_e --> 
EX_5adtststeroneglc_e: 5adtststeroneglc_e --> 
EX_5aop_e: 5aop_e --> 
EX_5flura_e: 5flura_e --> 
EX_6mpur_e: 6mpur_e --> 
EX_ac_e: ac_e --> 
EX_acgam_e: acgam_e --> 
EX_ach_e: ach_e --> 
EX_ade_e: ade_e --> 
EX_adn_e: adn_e --> 
EX_ahandrostanglc_e: ahandrostanglc_e --> 
EX_ala_B_e: ala_B_e --> 
EX_ala__D_e: ala__D_e --> 
EX_ala__L_e: ala__L_e --> 
EX_alltn_e: alltn_e --> 
EX_alltt_e: alltt_e --> 
EX_amet_e: amet_e --> 
EX_andrstrnglc_e: andrstrnglc_e --> 
EX_arach_e: arach_e --> 
EX_arg__L_e: arg__L_e --> 
EX_asn__L_e: asn__L_e --> 
EX_asp__L_e: asp__L_e --> 
EX_bildglcur_e: bildglcur_e --> 
EX_bilglcur_e: bilglcur_e --> 
EX_ca2_e: ca2_e --> 
EX_camp_e: camp_e --> 
EX_cgly_e: cgly_e --> 
EX_chol_e: chol_e --> 
EX_cholate_e: cholate_e --> 
EX_chtn_e: chtn_e --> 
EX_cl_e: cl_e --> 
EX_co2_e: co2_e --> 
EX_csn_e: csn_e --> 
EX_cu_e: cu_e --> 
EX_cys__L_e: cys__L_e --> 
EX_cysi__L_e: cysi__L_e --> 
EX_cytd_e: cytd_e --> 
EX_dad_2_e: dad_2_e --> 
EX_dca_e: dca_e --> 
EX_ddca_e: ddca_e --> 
EX_dhdascb_e: dhdascb_e --> 
EX_din_e: din_e --> 
EX_epist_e: epist_e --> 
EX_ergst_e: ergst_e --> 
EX_estradiolglc_e: estradiolglc_e --> 
EX_estriolglc_e: estriolglc_e --> 
EX_estroneglc_e: estroneglc_e --> 
EX_fe2_e: fe2_e --> 
EX_fe3_e: fe3_e --> 
EX_fecost_e: fecost_e --> 
EX_fmn_e: fmn_e --> 
EX_for_e: for_e --> 
EX_fru_e: fru_e --> 
EX_g3pc_e: g3pc_e --> 
EX_g3pe_e: g3pe_e --> 
EX_g3pi_e: g3pi_e --> 
EX_gal_e: gal_e --> 
EX_galur_e: galur_e --> 
EX_gam_e: gam_e --> 
EX_gchola_e: gchola_e --> 
EX_glc__D_e: glc__D_e <=> 
EX_gln__L_e: gln__L_e --> 
EX_glu__L_e: glu__L_e --> 
EX_gluala_e: gluala_e --> 
EX_gly_e: gly_e --> 
EX_glyc_e: glyc_e --> 
EX_glygn2_e: glygn2_e --> 
EX_glygn4_e: glygn4_e --> 
EX_gthrd_e: gthrd_e --> 
EX_gua_e: gua_e --> 
EX_h2o_e: h2o_e <=> 
EX_h_e: h_e <=> 
EX_hco3_e: hco3_e --> 
EX_hdca_e: hdca_e --> 
EX_hdcea_e: hdcea_e --> 
EX_his__L_e: his__L_e --> 
EX_ile__L_e: ile__L_e --> 
EX_ind3eth_e: ind3eth_e --> 
EX_inost_e: inost_e --> 
EX_ins_e: ins_e --> 
EX_k_e: k_e --> 
EX_lac__D_e: lac__D_e --> 
EX_lac__L_e: lac__L_e --> 
EX_lanost_e: lanost_e --> 
EX_leu__L_e: leu__L_e --> 
EX_lys__L_e: lys__L_e --> 
EX_malt_e: malt_e --> 
EX_man_e: man_e --> 
EX_met__L_e: met__L_e --> 
EX_na1_e: na1_e --> 
EX_nac_e: nac_e --> 
EX_nh4_e: nh4_e <=> 
EX_no2_e: no2_e --> 
EX_no3_e: no3_e --> 
EX_nrvnc_e: nrvnc_e --> 
EX_o2_e: o2_e <=> 
EX_ocdca_e: ocdca_e --> 
EX_ocdcea_e: ocdcea_e --> 
EX_ocdcya_e: ocdcya_e --> 
EX_oh1_e: oh1_e --> 
EX_orn_e: orn_e --> 
EX_oxa_e: oxa_e --> 
EX_pail1819Z160_e: pail1819Z160_e --> 
EX_pc_RT_e: pc_RT_e --> 
EX_pe1801819Z_e: pe1801819Z_e --> 
EX_pe1801829Z12Z_e: pe1801829Z12Z_e --> 
EX_pe1819Z1819Z_e: pe1819Z1819Z_e --> 
EX_pe1819Z1829Z12Z_e: pe1819Z1829Z12Z_e --> 
EX_pe_RT_e: pe_RT_e --> 
EX_pectin_e: pectin_e --> 
EX_pepd_e: pepd_e --> 
EX_pg1819Z160_e: pg1819Z160_e --> 
EX_pgp1819Z160_e: pgp1819Z160_e --> 
EX_phe__L_e: phe__L_e --> 
EX_pi_e: pi_e <=> 
EX_pnto__R_e: pnto__R_e --> 
EX_pro__D_e: pro__D_e --> 
EX_pro__L_e: pro__L_e --> 
EX_prostge1_e: prostge1_e --> 
EX_prostge2_e: prostge2_e --> 
EX_ptd1ino_RT_e: ptd1ino_RT_e --> 
EX_ptrc_e: ptrc_e --> 
EX_pyr_e: pyr_e --> 
EX_ribflv_e: ribflv_e --> 
EX_ser__L_e: ser__L_e --> 
EX_so3_e: so3_e --> 
EX_so4_e: so4_e <=> 
EX_spmd_e: spmd_e --> 
EX_sprm_e: sprm_e --> 
EX_strch1_e: strch1_e --> 
EX_strch2_e: strch2_e --> 
EX_sucr_e: sucr_e --> 
EX_tchola_e: tchola_e --> 
EX_tega_e: tega_e --> 
EX_tgua_e: tgua_e --> 
EX_thm_e: thm_e --> 
EX_thmmp_e: thmmp_e --> 
EX_thmpp_e: thmpp_e --> 
EX_thr__L_e: thr__L_e --> 
EX_trp__L_e: trp__L_e --> 
EX_tststeroneglc_e: tststeroneglc_e --> 
EX_ttc_e: ttc_e --> 
EX_ttdca_e: ttdca_e --> 
EX_tyr__L_e: tyr__L_e --> 
EX_ura_e: ura_e --> 
EX_urate_e: urate_e --> 
EX_urea_e: urea_e --> 
EX_uri_e: uri_e --> 
EX_val__L_e: val__L_e --> 
EX_xyl__D_e: xyl__D_e --> 
EX_zymst_e: zymst_e --> 
FA183COAabcp: atp_c + 2.0 h2o_c + lnlncgcoa_c --> adp_c + coa_c + 2.0 h_c + lnlncg_x + pi_c
FACOAL180p: atp_x + coa_x + ocdca_x --> amp_x + ppi_x + stcoa_x
FACOAL181p: atp_x + coa_x + ocdcea_x --> amp_x + odecoa_x + ppi_x
FACOAL182p: atp_x + coa_x + ocdcya_x --> amp_x + ocdycacoa_x + ppi_x
FACOAL1831p: atp_x + coa_x + lnlncg_x --> amp_x + lnlncgcoa_x + ppi_x
FCLTm: fe2_m + ppp9_m --> 2.0 h_m + pheme_m
FE2tm: fe2_c + h_c --> fe2_m + h_m
FORtr: for_c <=> for_r
GLYC3Pter: glyc3p_c <=> glyc3p_r
GLYtm: gly_c <=> gly_m
H2Oter: h2o_c <=> h2o_r
H2Otg: h2o_c <=> h2o_g
H2Otn: h2o_n <=> h2o_c
H2Otv: h2o_c <=> h2o_v
HCITSm: accoa_m + akg_m + h2o_m --> coa_m + h_m + hcit_m
HDCOAtr: hdcoa_c <=> hdcoa_r
HEXCCOAtr: hexccoa_c <=> hexccoa_r
HMGCOAtr: hmgcoa_c <=> hmgcoa_r
Htr: h_c <=> h_r
ILEtmi: ile__L_m <=> ile__L_c
IND3ETHt: ind3eth_c <=> ind3eth_e
INOSTter: inost_c --> inost_r
KYNATESYN: 4aphdob_c --> h2o_c + kynate_c
LEUt5m: leu__L_c <=> leu__L_m
LNLNCGCOAtr: lnlncgcoa_c <=> lnlncgcoa_r
LYSt7m: lys__L_c --> lys__L_m
MACACI: 4mlacac_c --> 4fumacac_c
MALCOAtr: malcoa_c <=> malcoa_r
NADPHtru: nadph_c --> nadph_r
NADPtru: nadp_r --> nadp_c
NH4tm: nh4_c <=> nh4_m
O2t: o2_e <=> o2_c
O2ter: o2_c <=> o2_r
O2tm: o2_c <=> o2_m
O2tn: o2_c <=> o2_n
O2tp: o2_c <=> o2_x
OAAIPMtm: 3c3hmp_m + oaa_c --> 3c3hmp_c + oaa_m
OCDYCACOAtr: ocdycacoa_c <=> ocdycacoa_r
ODECOAtr: odecoa_c <=> odecoa_r
ORNARGtm: arg__L_c + orn_m --> arg__L_m + orn_c
ORNLYStm: lys__L_c + orn_m --> lys__L_m + orn_c
PCRNtc: pcrn_x --> pcrn_c
PCRNtm: pcrn_c <=> pcrn_m
PIter: pi_r <=> pi_c
PMTCOAtr: pmtcoa_c <=> pmtcoa_r
PPItr: ppi_c <=> ppi_r
PPPG9tm: pppg9_c <=> pppg9_m
PYDX5Ptm: pydx5p_c <=> pydx5p_m
PYRt2m: h_c + pyr_c <=> h_m + pyr_m
QUILSYN: cmusa_c --> h2o_c + h_c + quln_c
SERter: ser__L_c <=> ser__L_r
STCOAtr: stcoa_c <=> stcoa_r
TDCOAtr: tdcoa_c <=> tdcoa_r
THRtm: thr__L_c <=> thr__L_m
TTCCOAtr: ttccoa_c <=> ttccoa_r
UCP2ASPtm: asp__L_m + h_c + pi_c --> asp__L_c + h_m + pi_m
UCP2MALtm: h_c + mal__L_m + pi_c --> h_m + mal__L_c + pi_m
UCP2OAAtm: h_c + oaa_m + pi_c --> h_m + oaa_c + pi_m
UREAt: urea_e <=> urea_c
UTPtm2: fe2_c + ump_m + utp_c --> fe2_m + ump_c + utp_m
VALTAm: akg_m + val__L_m <=> 3mob_m + glu__L_m
VALt5m: val__L_c <=> val__L_m
r0647: 42A3HP24DB_c <=> C02470_c + h2o_c
```
