# Supplementary material for: Multi-Omics Driven Metabolic Network Reconstruction and Analysis of Lignocellulosic Carbon Utilization in Rhodosporidium toruloides
Source: Front Bioeng Biotechnol. 2021 Jan 8;8:612832. doi: 10.3389/fbioe.2020.612832 (PMC7873862; doi:10.3389/fbioe.2020.612832)
Supplement: Supplementary File 4 — Multi-omics dataset for R. toruloides IFO0880. [file Data_Sheet_1.zip › Supplementary File S1/2.Metabolic_modeling/Refinement_2c_Update_Biomass_Composition.html]

Refinement\_2c\_Update\_Biomass\_Composition


In [1]:

```
%matplotlib inline
from matplotlib import pyplot as plt
from matplotlib import colors
import csv
import numpy as np
import pandas as pd
import cobra
```

In [2]:

```
Annotation = pd.read_excel('../../Data/R_toruloides_Data_for_Reconstruction.xlsx',
                          sheet_name='Annotation', index_col=0)
Annotation.index = Annotation.index.map(str)
Annotation = Annotation.fillna('')
Transcriptomics = pd.read_excel('../../Data/R_toruloides_Data_for_Reconstruction.xlsx',
                          sheet_name='Transcriptomics', header=[0,1,2,3], index_col=0)
Transcriptomics.index = Transcriptomics.index.map(str)
Proteomics = pd.read_excel('../../Data/R_toruloides_Data_for_Reconstruction.xlsx',
                          sheet_name='Proteomics', header=[0,1,2], index_col=0)
Proteomics.index = Proteomics.index.map(str)
Fitness = pd.read_excel('../../Data/R_toruloides_Data_for_Reconstruction.xlsx',
                          sheet_name='Fitness', index_col=0)
Fitness.index = Fitness.index.map(str)
```

In [3]:

```
model = cobra.io.load_json_model("IFO0880_GPR_2b.json")
```

In [4]:

```
eco = cobra.io.load_json_model('../../Data/BiGG_Models/iML1515.json')
sce = cobra.io.load_json_model('../../Data/BiGG_Models/iMM904.json')
hsa2 = cobra.io.load_json_model('../../Data/BiGG_Models/Recon3D.json')
```

In [5]:

```
with model:
    sol = model.optimize()
    print(sol)
```

```
<Solution 0.137 at 0x101c041048>
```

In [6]:

```
for m in model.reactions.get_by_id('BIOMASS_RT').reactants:
    print(m.id, model.reactions.get_by_id('BIOMASS_RT').get_coefficient(m.id))
```

```
13BDglcn_c -1.1348
5mthf_c -1e-06
ala__L_c -0.4588
amp_c -0.046
arg__L_c -0.1607
asn__L_c -0.1017
asp__L_c -0.2975
atp_c -59.276
btn_m -1e-06
camp_c -1e-06
cmp_c -0.0447
coa_c -1e-06
cys__L_c -0.0066
damp_c -0.0036
dcmp_c -0.0024
dgmp_c -0.0024
dtmp_c -0.0036
ergst_r -0.0007
fad_c -1e-06
gln__L_c -0.1054
glu__L_c -0.3018
gly_c -0.2904
glycogen_c -0.5185
gmp_c -0.046
gthrd_c -1e-06
h2o_c -59.276
hemeA_m -1e-06
his__L_c -0.0663
ile__L_c -0.1927
leu__L_c -0.2964
lipopb_m -1e-06
lys__L_c -0.2862
mannan_r -0.8079
met__L_c -0.0507
mlthf_c -1e-06
nad_c -1e-06
nadp_c -1e-06
pa_RT_r -6e-06
pc_RT_r -6e-05
pe_RT_r -4.5e-05
phe__L_c -0.1339
pro__L_c -0.1647
ps_RT_r -1.7e-05
ptd1ino_RT_r -5.3e-05
ptrc_c -1e-06
pydx5p_c -1e-06
q9_m -1e-06
ribflv_c -0.00099
ser__L_c -0.1854
so4_c -0.02
spmd_c -1e-06
thf_c -1e-06
thmpp_c -1e-06
thr__L_c -0.1914
tre_c -0.0234
triglyc_RT_r -6.6e-05
trp__L_c -0.0284
tyr__L_c -0.102
ump_c -0.0599
val__L_c -0.2646
zymst_r -0.0015
```

In [7]:

```
model.medium
```

Out[7]:

```
{'EX_h_e': 1000.0,
 'EX_h2o_e': 1000.0,
 'EX_nh4_e': 1000.0,
 'EX_o2_e': 2.0,
 'EX_pi_e': 1000.0,
 'EX_so4_e': 1000.0,
 'EX_glc__D_e': 10.0,
 'EX_fe2_e': 1000.0,
 'EX_fe3_e': 1000.0}
```

In [8]:

```
import BOFdat
BOFdat.__version__
```

```
/Users/joonhoonkim/anaconda3/lib/python3.6/site-packages/deap/tools/_hypervolume/pyhv.py:33 ImportWarning: Falling back to the python version of hypervolume module. Expect this to be very slow.
```

Out[8]:

```
'0.1.6'
```

In [9]:

```
# Set parameters based on dry weight composition
# DNA and RNA measurement was too low possibly due to the loss during purification
#dna_weight_fraction = 0.00190
#rna_weight_fraction = 0.00712
# Use S. cerevisiae numbers for now
dna_weight_fraction = 0.004
rna_weight_fraction = 0.063
# Protein measurement was okay
protein_weight_fraction = 0.396
```

In [10]:

```
from Bio import SeqIO, SeqUtils
```

In [11]:

```
genome = '../../Data/Rhoto_IFO0880_4/Rhoto_IFO0880_4_AssemblyScaffolds_and_Mito.fasta'
proteome = '../../Data/Rhoto_IFO0880_4/Rhoto_IFO0880_4_GeneCatalog_proteins_20170509.aa.modified.fasta'
genbank = '../../Data/Rfam/IFO0880_4_JGI_Rfam.gb'
```

In [12]:

```
for seq_record in SeqIO.parse(genome, "fasta"):
    print(seq_record.id, len(seq_record), SeqUtils.GC(seq_record.seq))
```

```
scaffold_1 2216085 61.98877750627796
scaffold_2 1973005 62.01068927853705
scaffold_3 1857541 61.91927930527509
scaffold_4 641547 61.727979399794556
scaffold_5 1666720 61.91675866372276
scaffold_6 1546801 62.13126316830672
scaffold_7 1390799 61.99644952290014
scaffold_8 1362475 61.90953962458027
scaffold_9 1352042 61.99016006899194
scaffold_10 938190 61.820313582536585
scaffold_11 938748 62.14308845398339
scaffold_12 870210 61.88632628905666
scaffold_13 784890 61.827517231714
scaffold_14 779220 61.76599676599677
scaffold_15 702308 61.732601650557875
scaffold_16 478525 61.88119742960138
scaffold_17 587612 61.96367671184387
scaffold_18 352531 61.35318596095095
scaffold_19 18910 49.68799576943416
scaffold_20 28147 59.70796177212492
scaffold_21 17271 59.730183544670254
scaffold_22 20396 49.3184938223181
scaffold_23 14857 49.17547284108501
scaffold_24 22249 59.44536833116095
scaffold_25 13957 59.86959948412983
scaffold_26 30062 49.35466702148892
scaffold_27 20541 49.3452120149944
scaffold_28 17317 49.47161748570769
scaffold_29 15828 59.988627748294164
scaffold_30 25061 49.43138741470811
scaffold_1_mito 110369 45.364187407696
```

In [13]:

```
total_bases = 0
ratio_genome = {'A': 0, 'T': 0, 'C': 0, 'G': 0}
for seq_record in SeqIO.parse(genome, "fasta"):
    total_bases = total_bases + len(seq_record)*2
    ratio_genome['A'] = ratio_genome['A'] + len(seq_record)*(1-SeqUtils.GC(seq_record.seq)/100)
    ratio_genome['T'] = ratio_genome['T'] + len(seq_record)*(1-SeqUtils.GC(seq_record.seq)/100)
    ratio_genome['C'] = ratio_genome['C'] + len(seq_record)*(SeqUtils.GC(seq_record.seq)/100)
    ratio_genome['G'] = ratio_genome['G'] + len(seq_record)*(SeqUtils.GC(seq_record.seq)/100)
for k, v in ratio_genome.items():
    ratio_genome[k] = v/total_bases
print(total_bases)
print(ratio_genome)
```

```
41588428
{'A': 0.191265392382708, 'T': 0.191265392382708, 'C': 0.308734607617292, 'G': 0.308734607617292}
```

In [14]:

```
dna_coefficients = BOFdat.core.dna._convert_to_coefficient(model, ratio_genome, 1000, dna_weight_fraction)
dna_coefficients.update({model.metabolites.get_by_id('ppi_c'): -sum(dna_coefficients.values())})
dna_coefficients
```

Out[14]:

```
{<Metabolite datp_c at 0x101d25b668>: -0.0024505605312780763,
 <Metabolite dttp_c at 0x101d29f550>: -0.0025234129702600924,
 <Metabolite dctp_c at 0x101d268438>: -0.004285393042825096,
 <Metabolite dgtp_c at 0x101d2956d8>: -0.003762784741852124,
 <Metabolite ppi_c at 0x101d25b0f0>: 0.013022151286215387}
```

In [15]:

```
rRNA_seq, rRNA_locus, rRNA_number = [], [], []
tRNA_seq, tRNA_locus, tRNA_number = [], [], []
mRNA_seq, mRNA_locus, mRNA_fraction = [], [], []
genome_record = SeqIO.parse(genbank, 'genbank')
for record in genome_record:
    for element in record.features:
        if element.type == 'mRNA':
            rna_seq = BOFdat.core.rna._get_RNA_sequence(element.location, element.strand, record)
            mRNA_seq.append(rna_seq)
            mRNA_locus.append(element.qualifiers['GeneID'][0])
            mRNA_fraction.append(BOFdat.core.rna._get_fraction(rna_seq))

        if element.type == 'tRNA':
            rna_seq = BOFdat.core.rna._get_RNA_sequence(element.location, element.strand, record)
            tRNA_seq.append(rna_seq)
            tRNA_locus.append(element.qualifiers['GeneID'][0])
            tRNA_number.append(BOFdat.core.rna._get_number(rna_seq))

        if element.type == 'rRNA':
            rna_seq = BOFdat.core.rna._get_RNA_sequence(element.location, element.strand, record)
            rRNA_seq.append(rna_seq)
            rRNA_locus.append(element.qualifiers['GeneID'][0])
            rRNA_number.append(BOFdat.core.rna._get_number(rna_seq))
tRNA_df = BOFdat.core.rna._make_number_df(tRNA_number, tRNA_locus, tRNA_seq)
rRNA_df = BOFdat.core.rna._make_number_df(rRNA_number, rRNA_locus, rRNA_seq)
mRNA_df = BOFdat.core.rna._make_number_df(mRNA_fraction, mRNA_locus, mRNA_seq)
rRNA_dict = BOFdat.core.rna._get_total_fractions(rRNA_df)
tRNA_dict = BOFdat.core.rna._get_total_fractions(tRNA_df)
all_locus = [i for i in rRNA_df['locus']] + [i for i in tRNA_df['locus']] + [i for i in mRNA_df['locus']]

temp = pd.DataFrame({'identifiers': Transcriptomics.index, 'abundances': Transcriptomics.mean(axis=1).values})
mean_abundance = pd.merge(mRNA_df, temp, left_on='locus', right_on='identifiers')
A_norm, U_norm, G_norm, C_norm = [], [], [], []
for i, row in mean_abundance.iterrows():
    # 1- Multiply fraction by abundance for each gene
    A_mean = row['A'] * row['abundances']
    U_mean = row['U'] * row['abundances']
    G_mean = row['G'] * row['abundances']
    C_mean = row['C'] * row['abundances']
    A_norm.append(A_mean)
    U_norm.append(U_mean)
    G_norm.append(G_mean)
    C_norm.append(C_mean)

mRNA_dict = {'A': 0, 'U': 0, 'C': 0, 'G': 0}
mRNA_dict['A'] = sum(A_norm) / sum(mean_abundance['abundances'])
mRNA_dict['U'] = sum(U_norm) / sum(mean_abundance['abundances'])
mRNA_dict['G'] = sum(G_norm) / sum(mean_abundance['abundances'])
mRNA_dict['C'] = sum(C_norm) / sum(mean_abundance['abundances'])
```

In [16]:

```
mRNA_weight_fraction = 0.05
tRNA_weight_fraction = 0.05
rRNA_weight_fraction = 0.9
RNA_coefficients = BOFdat.core.rna._total_coefficients(mRNA_dict, tRNA_dict, rRNA_dict,
                                                       mRNA_weight_fraction, tRNA_weight_fraction, rRNA_weight_fraction)
rna_coefficients = BOFdat.core.rna._convert_to_mmolgDW(RNA_coefficients, model, rna_weight_fraction, 1000)
rna_coefficients.update({model.metabolites.get_by_id('ppi_c'): -sum(rna_coefficients.values())})
```

In [17]:

```
rna_coefficients
```

Out[17]:

```
{<Metabolite atp_c at 0x101d243e48>: -0.04924654167536125,
 <Metabolite utp_c at 0x101d272ba8>: -0.051129186449930364,
 <Metabolite ctp_c at 0x101d268e80>: -0.04488125347893081,
 <Metabolite gtp_c at 0x101d251fd0>: -0.0510847002145461,
 <Metabolite ppi_c at 0x101d25b0f0>: 0.1963416818187685}
```

In [18]:

```
seq_dict = {}
for seq_record in SeqIO.parse(proteome, "fasta"):
    seq_dict[seq_record.id] = seq_record.seq

AMINO_ACIDS = ['A', 'C', 'D', 'E', 'F', 'G', 'H', 'I', 'K', 'L', 'M', 'N', 'P', 'Q', 'R', 'S', 'T', 'V', 'W','Y']
list_of_dict = []
for k, v in seq_dict.items():
    list_of_occurences = []
    for letter in AMINO_ACIDS:
        protein_sequence = v
        occurence_of_letter = protein_sequence.count(letter)
        list_of_occurences.append(occurence_of_letter)
    dict_of_occurences = dict(zip(AMINO_ACIDS, list_of_occurences))
    dict_per_prot = {k: dict_of_occurences}
    list_of_dict.append(dict_per_prot)
```

In [19]:

```
normalized_dict = {'A': 0., 'C': 0., 'D': 0., 'E': 0., 'F': 0., 'G': 0., 'H': 0., 'I': 0.,
                   'K': 0., 'L': 0., 'M': 0., 'N': 0., 'P': 0., 'Q': 0., 'R': 0., 'S': 0., 'T': 0., 'V': 0.,
                   'W': 0., 'Y': 0.}

proteomics = Proteomics.mean(axis=1)
for d in list_of_dict:
    coeff = proteomics.get(str(list(d.keys())[0]))
    try:
        coeff_number = float(coeff)
    except:
        coeff_number = 0.

    amino_acids = list(d.values())
    for letter in AMINO_ACIDS:
        value = float(amino_acids[0].get(letter))
        normalized_value = value * coeff_number
        new_value = normalized_dict.get(letter) + normalized_value
        normalized_dict[letter] = new_value

norm_sum = BOFdat.core.protein._get_norm_sum(normalized_dict)
ratio_dict = BOFdat.core.protein._get_ratio(normalized_dict, norm_sum, protein_weight_fraction, 1000)
protein_coefficients = BOFdat.core.protein._convert_to_coefficient(ratio_dict, model, 1000)
protein_coefficients.update({model.metabolites.get_by_id('h2o_c'): -sum(protein_coefficients.values())})
```

In [20]:

```
protein_coefficients
```

Out[20]:

```
{<Metabolite ala__L_c at 0x101d25b8d0>: -0.5775740293335541,
 <Metabolite cys__L_c at 0x101d25b5f8>: -0.03426905166485587,
 <Metabolite asp__L_c at 0x101d2952e8>: -0.19729583127170278,
 <Metabolite glu__L_c at 0x101d251710>: -0.2086730002106206,
 <Metabolite phe__L_c at 0x101d289e48>: -0.09516445920036641,
 <Metabolite gly_c at 0x101d25b208>: -0.5132201286597424,
 <Metabolite his__L_c at 0x101d272860>: -0.06116738888766259,
 <Metabolite ile__L_c at 0x101d2959b0>: -0.15856899634936897,
 <Metabolite lys__L_c at 0x101d2b6748>: -0.1723344750644607,
 <Metabolite leu__L_c at 0x101d272be0>: -0.3202054914900182,
 <Metabolite met__L_c at 0x101d27e198>: -0.057050114892004275,
 <Metabolite asn__L_c at 0x101d29fb00>: -0.10797028011920144,
 <Metabolite pro__L_c at 0x101d289668>: -0.2358971378552613,
 <Metabolite gln__L_c at 0x101d25b978>: -0.11789842203179653,
 <Metabolite arg__L_c at 0x101d29f710>: -0.14667489305150036,
 <Metabolite ser__L_c at 0x101d25b278>: -0.3368703830003513,
 <Metabolite thr__L_c at 0x101d2b6518>: -0.21813036450552883,
 <Metabolite val__L_c at 0x101d2b6c18>: -0.2751676918511302,
 <Metabolite trp__L_c at 0x101d272fd0>: -0.02331876504887462,
 <Metabolite tyr__L_c at 0x101d289f98>: -0.062363441744024195,
 <Metabolite h2o_c at 0x101d251198>: 3.919814346232026}
```

#### Estimate cellular lipid composition and lipid body composition from FAME data¶

In [8]:

```
FAME = pd.read_excel('../../Data/FAME_data_M9_YPD_SD.xlsx',
                     sheet_name='Summary', header=0, index_col=0)
FAME = FAME.iloc[:24,]
```

In [9]:

```
FAME
```

Out[9]:

|  | Media | Hours | Glucose (g/L by HPLC) | Glycerol (g/L by HPLC) | Ammonia (g/L by sigma ammonia assay) | Dryweight (mg) | Total FA | C16:0 FA | C16:1 FA | C17:0 FA | C18:0 FA | C18:1 FA | C18:2 FA | C18:3 FA | C20:0 FA | C22:0 FA | C24:0 FA |
| --- | --- | --- | --- | --- | --- | --- | --- | --- | --- | --- | --- | --- | --- | --- | --- | --- | --- |
| Sample Id |  |  |  |  |  |  |  |  |  |  |  |  |  |  |  |  |  |
| S1 | M9 | 24.0 | 1.060 | 0.000 | 0.16 | 13.5 | 0.082695 | 0.009754 | 0.000260 | 0.000136 | 0.004604 | 0.018342 | 0.031280 | 0.016215 | 0.001244 | 0.000337 | 0.000523 |
| S2 | M9 | 24.0 | 0.860 | 0.000 | 0.44 | 13.6 | 0.080732 | 0.009687 | 0.000249 | 0.001741 | 0.004252 | 0.017163 | 0.029542 | 0.015575 | 0.001876 | 0.000199 | 0.000448 |
| S3 | M9 | 24.0 | 0.820 | 0.000 | 0.57 | 13.5 | 0.081300 | 0.009755 | 0.000240 | 0.001459 | 0.004409 | 0.018193 | 0.029842 | 0.015568 | 0.001188 | 0.000178 | 0.000467 |
| S4 | M9 | 48.0 | 0.029 | 0.013 | 0.00 | 41.5 | 0.189149 | 0.024810 | 0.000887 | 0.001275 | 0.014004 | 0.070544 | 0.063840 | 0.009912 | 0.001154 | 0.000475 | 0.002249 |
| S5 | M9 | 48.0 | 0.025 | 0.011 | 0.00 | 43.0 | 0.120061 | 0.011063 | 0.000269 | 0.001229 | 0.009384 | 0.040175 | 0.045921 | 0.008263 | 0.001501 | 0.000353 | 0.001902 |
| S6 | M9 | 48.0 | 0.017 | 0.012 | 0.00 | 44.0 | 0.129923 | 0.014633 | 0.000698 | 0.000972 | 0.010344 | 0.045289 | 0.046409 | 0.007929 | 0.001350 | 0.000361 | 0.001938 |
| S7 | M9 | 72.0 | 0.000 | 0.000 | 0.00 | 44.3 | 0.112920 | 0.011042 | 0.000344 | 0.001192 | 0.007493 | 0.035627 | 0.047383 | 0.007605 | 0.000710 | 0.000240 | 0.001284 |
| S8 | M9 | 72.0 | 0.000 | 0.000 | 0.00 | 43.1 | 0.105411 | 0.009884 | 0.000229 | 0.001085 | 0.006845 | 0.035149 | 0.042158 | 0.006908 | 0.000956 | 0.000222 | 0.001974 |
| S9 | M9 | 72.0 | 0.000 | 0.000 | 0.00 | 44.6 | 0.111721 | 0.010118 | 0.000219 | 0.001095 | 0.007143 | 0.037482 | 0.044463 | 0.007605 | 0.001047 | 0.000285 | 0.002265 |
| S10 | M9 | 140.0 | 0.000 | 0.000 | 0.00 | 41.0 | 0.106181 | 0.009136 | 0.000178 | 0.001135 | 0.004922 | 0.033232 | 0.044985 | 0.006854 | 0.001334 | 0.000433 | 0.003972 |
| S11 | M9 | 140.0 | 0.000 | 0.000 | 0.00 | 41.4 | 0.110584 | 0.009649 | 0.000181 | 0.001439 | 0.004824 | 0.033644 | 0.049466 | 0.007225 | 0.001490 | 0.000303 | 0.002364 |
| S12 | M9 | 140.0 | 0.000 | 0.000 | 0.00 | 39.1 | 0.097691 | 0.008885 | 0.000165 | 0.001248 | 0.004789 | 0.031660 | 0.041994 | 0.006161 | 0.001113 | 0.000257 | 0.001417 |
| S13 | YNB CN120 | 96.0 | NaN | NaN | NaN | 69.8 | 0.626900 | 0.125903 | 0.002836 | 0.001511 | 0.071332 | 0.302896 | 0.099157 | 0.012176 | 0.003205 | 0.002338 | 0.005545 |
| S14 | YNB CN120 | 96.0 | NaN | NaN | NaN | 61.9 | 0.562239 | 0.105403 | 0.002586 | 0.001512 | 0.068760 | 0.258425 | 0.103187 | 0.010918 | 0.002983 | 0.002162 | 0.006303 |
| S15 | YNB CN120 | 96.0 | NaN | NaN | NaN | 64.5 | 0.576212 | 0.110539 | 0.002397 | 0.001379 | 0.071093 | 0.268044 | 0.099213 | 0.011687 | 0.003116 | 0.002217 | 0.006528 |
| S16 | YPD | 24.0 | NaN | NaN | NaN | 38.1 | 0.067117 | 0.010083 | 0.000428 | 0.001292 | 0.004396 | 0.033812 | 0.009936 | 0.003828 | 0.001355 | 0.000313 | 0.001675 |
| S17 | YPD | 24.0 | NaN | NaN | NaN | 37.7 | 0.065388 | 0.009300 | 0.000370 | 0.000993 | 0.003845 | 0.034151 | 0.010379 | 0.003974 | 0.001085 | 0.000294 | 0.000996 |
| S18 | YPD | 24.0 | NaN | NaN | NaN | 38.0 | 0.064597 | 0.009263 | 0.000319 | 0.001298 | 0.003821 | 0.033893 | 0.010297 | 0.003937 | 0.001172 | 0.000234 | 0.000363 |
| S19 | SD | 24.0 | NaN | NaN | NaN | 18.2 | 0.117117 | 0.016753 | 0.000152 | 0.001343 | 0.018349 | 0.050623 | 0.018826 | 0.006481 | 0.001656 | 0.000612 | 0.002324 |
| S20 | SD | 24.0 | NaN | NaN | NaN | 17.7 | 0.121068 | 0.016991 | 0.000201 | 0.001384 | 0.018351 | 0.051305 | 0.019422 | 0.006600 | 0.001638 | 0.000683 | 0.004494 |
| S21 | SD | 24.0 | NaN | NaN | NaN | 16.6 | 0.119699 | 0.016970 | 0.000187 | 0.001430 | 0.019610 | 0.050303 | 0.019980 | 0.006566 | 0.001607 | 0.000660 | 0.002386 |
| S22 | SD | 96.0 | NaN | NaN | NaN | 13.9 | 0.159894 | 0.020726 | 0.001201 | 0.001613 | 0.015207 | 0.074249 | 0.035524 | 0.006538 | 0.001609 | 0.000672 | 0.002554 |
| S23 | SD | 96.0 | NaN | NaN | NaN | 13.2 | 0.149350 | 0.019200 | 0.001157 | 0.002043 | 0.013966 | 0.068419 | 0.033698 | 0.005865 | 0.002098 | 0.000575 | 0.002329 |
| S24 | SD | 96.0 | NaN | NaN | NaN | 11.6 | 0.157526 | 0.020989 | 0.001186 | 0.002177 | 0.017115 | 0.069872 | 0.034092 | 0.006096 | 0.002380 | 0.000960 | 0.002659 |

In [10]:

```
Data = FAME
Data.index = pd.MultiIndex.from_frame(Data[['Media','Hours']])
Data = Data.drop(['Media','Hours'], axis=1)
Data['Total FA (mg)'] = Data['Dryweight (mg)']*Data['Total FA']
Data['Total FA (%)'] = 100*Data['Total FA']
Data_mean = Data.groupby(level=['Media','Hours']).mean()
Data_error = Data.groupby(level=['Media','Hours']).std()
```

In [11]:

```
fig, ax = plt.subplots(figsize=(6,4))
Data_mean.plot.bar(y=['Dryweight (mg)','Total FA (mg)'], yerr=Data_error, capsize=4, ax=ax)
ax.set_ylabel("Weight (mg)")
ax.legend(['Cell dry weight','Total Fatty Acid'])
plt.tight_layout()
plt.show()
```

In [12]:

```
mmap = {'M9': 'o', 'YNB CN120': 'x', 'YPD': '^', 'SD': 'v'}
markers = FAME['Media'].apply(lambda x: mmap[x]).values
markers
```

Out[12]:

```
array(['o', 'o', 'o', 'o', 'o', 'o', 'o', 'o', 'o', 'o', 'o', 'o', 'x',
       'x', 'x', '^', '^', '^', 'v', 'v', 'v', 'v', 'v', 'v'],
      dtype=object)
```

In [28]:

```
fig, ax = plt.subplots(figsize=(6,4))
for x in mmap:
    FAME.loc[FAME['Media'] == x].plot(x='Total FA', y='Dryweight (mg)', style=mmap[x]+'k', ax=ax, label=x, )
ax.set_xlabel("Total FA (%)")
ax.set_ylabel("Dryweight (mg)")
ax.set_xlim([0,0.7])
ax.set_ylim([0,80])
plt.legend()
plt.tight_layout()
plt.savefig('temp1.svg')
plt.show()
```

In [29]:

```
fig, (ax1,ax2) = plt.subplots(1, 2, figsize=(12,4))

for x in mmap:
    FAME.loc[FAME['Media'] == x].plot(x='Total FA', y=range(7,17), style=mmap[x], ax=ax1, legend=False)
ax1.set_xlabel("Total FA (%)")
ax1.set_ylabel("Individual FA (%)")
ax1.set_xlim([0.05,0.65])
ax1.legend(FAME.columns[7:17])

for x in mmap:
    FAME.loc[FAME['Media'] == x].plot(x='Total FA', y=range(7,17), style=mmap[x], ax=ax2, legend=False)
ax2.set_xlabel("Total FA (%)")
ax2.set_ylabel("Individual FA (%)")
#ax2.legend(FAME.columns[7:17])
ax2.set_xlim([0.05,0.2])
ax2.set_ylim([-0.01,0.1])

plt.tight_layout()
plt.savefig('temp2.svg')
plt.show()
```

In [17]:

```
from lmfit import minimize, Parameters, fit_report
```

In [18]:

```
def piecewise_linear(x, x0, k0, k1):
    return np.piecewise(x, [x < x0], [lambda x: k0, lambda x: k1*(x-x0) + k0])

def residual(p, x, y):
    p = p.valuesdict()
    result = []
    for i in range(int((len(p)-1)/2)):
        model = p['k1_'+str(i)]*(x-p['x0']) + p['k0_'+str(i)]
        model[np.where(x < p['x0'])] = p['k0_'+str(i)]
        result.append(model)
    return np.transpose(result) - y
```

In [30]:

```
# Fitting a single FA
x = FAME['Total FA'].values
y = FAME.iloc[:,7:8].values

params = Parameters()
params.add('x0', value=0.05, min=0.0)
if len(y.shape) > 1:
    k = y.shape[1]
else:
    k = 1
for i in range(k):
    params.add('k0_'+str(i), value=0.01, min=0.0)
for i in range(k):
    params.add('k1_'+str(i), value=0.2, min=0.0)

out = minimize(residual, params, args=(x, y))
print(out.success)
print(out.message)
print(fit_report(out))
p = list(out.params.valuesdict().values())

fig, ax = plt.subplots(figsize=(3,2))
ax.plot(x, y, "o")
xd = np.arange(0.00, 0.65, 0.02)
ax.plot(xd, piecewise_linear(xd, *p), linestyle='--', color='k')
ax.axvline(x=p[0], color='r', linestyle=':')
ax.axhline(y=p[1], color='b', linestyle=':')
ax.set_xlim([0.05,0.25])
ax.set_ylim([-0.01,0.06])
plt.tight_layout()
plt.savefig('temp3.svg')
plt.show()
```

```
True
Fit succeeded.
[[Fit Statistics]]
    # fitting method   = leastsq
    # function evals   = 20
    # data points      = 24
    # variables        = 3
    chi-square         = 9.1045e-05
    reduced chi-square = 4.3355e-06
    Akaike info crit   = -293.573149
    Bayesian info crit = -290.038987
[[Variables]]
    x0:    0.10325103 +/- 0.00457250 (4.43%) (init = 0.05)
    k0_0:  0.00953235 +/- 7.8699e-04 (8.26%) (init = 0.01)
    k1_0:  0.21516970 +/- 0.00285675 (1.33%) (init = 0.2)
[[Correlations]] (unreported correlations are < 0.100)
    C(x0, k0_0) =  0.800
    C(x0, k1_0) =  0.311
```

In [23]:

```
# Fitting multiple FA
x = FAME['Total FA'].values
y = FAME.iloc[:,7:17].values

params = Parameters()
params.add('x0', value=0.05, min=0.0)
if len(y.shape) > 1:
    k = y.shape[1]
else:
    k = 1
for i in range(k):
    params.add('k0_'+str(i), value=0.01, min=0.0)
for i in range(k):
    params.add('k1_'+str(i), value=0.2, min=0.0)
    
out = minimize(residual, params, args=(x, y))
print(out.success)
print(out.message)
print(fit_report(out))
```

```
True
Fit succeeded.
[[Fit Statistics]]
    # fitting method   = leastsq
    # function evals   = 344
    # data points      = 240
    # variables        = 21
    chi-square         = 0.00533726
    reduced chi-square = 2.4371e-05
    Akaike info crit   = -2529.28373
    Bayesian info crit = -2456.19032
[[Variables]]
    x0:    0.08130021 +/- 0.00453960 (5.58%) (init = 0.05)
    k0_0:  0.00643252 +/- 0.00135346 (21.04%) (init = 0.01)
    k0_1:  2.6997e-04 +/- 0.00116387 (431.11%) (init = 0.01)
    k0_2:  0.00126421 +/- 0.00116374 (92.05%) (init = 0.01)
    k0_3:  0.00520376 +/- 0.00123754 (23.78%) (init = 0.01)
    k0_4:  0.02567388 +/- 0.00199905 (7.79%) (init = 0.01)
    k0_5:  0.02890577 +/- 0.00125520 (4.34%) (init = 0.01)
    k0_6:  0.00771212 +/- 0.00116396 (15.09%) (init = 0.01)
    k0_7:  0.00128371 +/- 0.00116380 (90.66%) (init = 0.01)
    k0_8:  2.8178e-04 +/- 0.00116380 (413.01%) (init = 0.01)
    k0_9:  0.00162187 +/- 0.00116411 (71.78%) (init = 0.01)
    k1_0:  0.21080342 +/- 0.00635766 (3.02%) (init = 0.2)
    k1_1:  0.00472914 +/- 0.00631657 (133.57%) (init = 0.2)
    k1_2:  8.4347e-04 +/- 0.00631654 (748.87%) (init = 0.2)
    k1_3:  0.12841223 +/- 0.00633184 (4.93%) (init = 0.2)
    k1_4:  0.49580495 +/- 0.00654074 (1.32%) (init = 0.2)
    k1_5:  0.14348322 +/- 0.00633563 (4.42%) (init = 0.2)
    k1_6:  0.00696206 +/- 0.00631659 (90.73%) (init = 0.2)
    k1_7:  0.00365243 +/- 0.00631656 (172.94%) (init = 0.2)
    k1_8:  0.00390444 +/- 0.00631657 (161.78%) (init = 0.2)
    k1_9:  0.00905210 +/- 0.00631663 (69.78%) (init = 0.2)
[[Correlations]] (unreported correlations are < 0.100)
    C(x0, k0_4)   =  0.813
    C(x0, k0_0)   =  0.511
    C(k0_2, k1_2) = -0.500
    C(k0_7, k1_7) = -0.500
    C(k0_8, k1_8) = -0.500
    C(k0_1, k1_1) = -0.500
    C(k0_6, k1_6) = -0.500
    C(k0_9, k1_9) = -0.500
    C(k0_3, k1_3) = -0.446
    C(k0_5, k1_5) = -0.433
    C(k0_0, k0_4) =  0.415
    C(x0, k0_5)   =  0.375
    C(k0_0, k1_0) = -0.369
    C(x0, k0_3)   =  0.340
    C(k0_4, k0_5) =  0.305
    C(k0_3, k0_4) =  0.277
    C(x0, k1_4)   =  0.260
    C(k0_0, k0_5) =  0.191
    C(k0_0, k0_3) =  0.174
    C(k0_0, k1_4) =  0.133
    C(k0_3, k0_5) =  0.127
    C(x0, k1_0)   =  0.114
```

In [24]:

```
FAME_fit = pd.DataFrame(index=FAME.columns[7:17], columns=['min','cell','lipid_body'])
FAME_fit['min'] = list(out.params.valuesdict().values())[0]
FAME_fit['cell'] = list(out.params.valuesdict().values())[1:1+FAME_fit.shape[0]]
FAME_fit['lipid_body'] = list(out.params.valuesdict().values())[1+FAME_fit.shape[0]:1+2*FAME_fit.shape[0]]
FAME_fit
```

Out[24]:

|  | min | cell | lipid\_body |
| --- | --- | --- | --- |
| C16:0 FA | 0.0813 | 0.006433 | 0.210803 |
| C16:1 FA | 0.0813 | 0.000270 | 0.004729 |
| C17:0 FA | 0.0813 | 0.001264 | 0.000843 |
| C18:0 FA | 0.0813 | 0.005204 | 0.128412 |
| C18:1 FA | 0.0813 | 0.025674 | 0.495805 |
| C18:2 FA | 0.0813 | 0.028906 | 0.143483 |
| C18:3 FA | 0.0813 | 0.007712 | 0.006962 |
| C20:0 FA | 0.0813 | 0.001284 | 0.003652 |
| C22:0 FA | 0.0813 | 0.000282 | 0.003904 |
| C24:0 FA | 0.0813 | 0.001622 | 0.009052 |

In [32]:

```
fig, (ax1,ax2) = plt.subplots(1, 2, figsize=(12,4))
for x in mmap:
    FAME.loc[FAME['Media'] == x].plot(x='Total FA', y=range(7,17), style=mmap[x], ax=ax1, legend=False)

for i in range(10):
    xd = np.arange(0.05, 0.7, 0.05)
    p = FAME_fit.loc[FAME.columns[7:17][i]]
    ax1.plot(xd, piecewise_linear(xd, *p), linestyle='--')
    #ax1.axhline(y=p[1], linestyle=':')

#ax1.axvline(x=p[0], linestyle='--', color='k')

ax1.set_xlabel("Total FA (%)")
ax1.set_ylabel("Individual FA (%)")
#ax1.legend(FAME.columns[7:17], loc='center left', bbox_to_anchor=(1, 0.5))
ax1.set_xlim([0.05,0.65])

for x in mmap:
    FAME.loc[FAME['Media'] == x].plot(x='Total FA', y=range(7,17), style=mmap[x], ax=ax2, legend=False)

for i in range(10):
    xd = np.arange(0.05, 0.2, 0.015)
    p = FAME_fit.loc[FAME.columns[7:17][i]]
    ax2.plot(xd, piecewise_linear(xd, *p), linestyle='--')
    #ax.axhline(y=p[1], linestyle=':')

ax2.axvline(x=p[0], linestyle='--', color='k')

ax2.set_xlabel("Total FA (%)")
ax2.set_ylabel("Individual FA (%)")
ax2.legend(FAME.columns[7:17], loc='center left', bbox_to_anchor=(1, 0.5))
ax2.set_xlim([0.05,0.2])
ax2.set_ylim([-0.01,0.1])

plt.tight_layout()
plt.savefig('temp4.svg')
plt.show()
```

In [34]:

```
fig, ax = plt.subplots(figsize=(6,4))
for x in mmap:
    FAME.loc[FAME['Media'] == x].plot(x='Total FA', y=range(7,17), style=mmap[x], ax=ax, legend=False)

for i in range(10):
    xd = np.arange(0.05, 0.7, 0.05)
    p = FAME_fit.loc[FAME.columns[7:17][i]]
    ax.plot(xd, piecewise_linear(xd, *p), linestyle='--')
    #ax.axhline(y=p[1], linestyle=':')

ax.axvline(x=p[0], linestyle='--', color='k')

ax.set_xlabel("Total Fatty Acid (%)")
ax.set_ylabel("Individual Fatty Acid (%)")
ax.legend([x.replace(' FA','') for x in FAME.columns[7:17]], loc='center left', bbox_to_anchor=(1, 0.5))
ax.set_xlim([0.05,0.65])
ax.set_ylim([-0.02,0.32])

axins = ax.inset_axes([0.35, 0.35, 0.3, 0.6])

for x in mmap:
    FAME.loc[FAME['Media'] == x].plot(x='Total FA', y=range(7,17), style=mmap[x], ax=axins, legend=False)

for i in range(10):
    xd = np.arange(0.05, 0.15, 0.01)
    p = FAME_fit.loc[FAME.columns[7:17][i]]
    axins.plot(xd, piecewise_linear(xd, *p), linestyle='--')
    #axins.axhline(y=p[1], linestyle=':')

axins.axvline(x=p[0], linestyle='--', color='k')
axins.set_xlim([0.06,0.12])
axins.set_ylim([-0.004,0.064])

axins.set_xlabel('')
axins.set_xticklabels('')
axins.set_yticklabels('')

ax.indicate_inset_zoom(axins)

plt.tight_layout()
plt.show()
```

In [35]:

```
fig, axes = plt.subplots(2,5, figsize=(15,6))
for i in range(10):
    ax = axes[i//5,i % 5]
    for x in mmap:
        FAME.loc[FAME['Media'] == x].plot(x='Total FA', y=range(7,17)[i], style=mmap[x], ax=ax, legend=False)
    ax.set_xlabel("Total FA (%)")
    ax.set_ylabel(FAME.columns[7:17][i])

    xd = np.arange(0, .65, 0.1)
    p = FAME_fit.loc[FAME.columns[7:17][i]]
    ax.plot(xd, piecewise_linear(xd, *p))
    ax.axvline(x=p[0], color='k', linestyle='--')
    ax.axhline(y=p[1], color='r', linestyle='--')
    ax.set_ylim([-0.01,0.31])
plt.tight_layout()
plt.show()
```

In [36]:

```
fig, axes = plt.subplots(2,5, figsize=(15,6))
for i in range(10):
    ax = axes[i//5,i % 5]
    for x in mmap:
        FAME.loc[FAME['Media'] == x].plot(x='Total FA', y=range(7,17)[i], style=mmap[x], ax=ax, legend=False)
    ax.set_xlabel("Total FA (%)")
    ax.set_ylabel(FAME.columns[7:17][i])

    xd = np.arange(0, .21, 0.01)
    p = FAME_fit.loc[FAME.columns[7:17][i]]
    ax.plot(xd, piecewise_linear(xd, *p))
    ax.axvline(x=p[0], color='k', linestyle='--')
    ax.axhline(y=p[1], color='r', linestyle='--')
    ax.set_xlim([0.02,0.2])
    ax.set_ylim([-0.01,0.11])
plt.tight_layout()
plt.show()
```

In [37]:

```
# cell lipid composition
print(FAME_fit['cell'].sum())
FAME_fit['cell']/FAME_fit['cell'].sum()*100
```

```
0.07864958990370075
```

Out[37]:

```
C16:0 FA     8.178712
C16:1 FA     0.343259
C17:0 FA     1.607389
C18:0 FA     6.616382
C18:1 FA    32.643378
C18:2 FA    36.752599
C18:3 FA     9.805668
C20:0 FA     1.632184
C22:0 FA     0.358276
C24:0 FA     2.062151
Name: cell, dtype: float64
```

In [38]:

```
# lipid body composition
print(FAME_fit['lipid_body'].sum())
FAME_fit['lipid_body']/FAME_fit['lipid_body'].sum()*100
```

```
1.0076474663325126
```

Out[38]:

```
C16:0 FA    20.920354
C16:1 FA     0.469324
C17:0 FA     0.083707
C18:0 FA    12.743766
C18:1 FA    49.204207
C18:2 FA    14.239426
C18:3 FA     0.690922
C20:0 FA     0.362471
C22:0 FA     0.387481
C24:0 FA     0.898340
Name: lipid_body, dtype: float64
```

In [39]:

```
fig, axes = plt.subplots(1, 2, figsize=(8,4))

Data = FAME_fit['cell']/FAME_fit['cell'].sum()
Data.plot.pie(title='Cell Lipid', ax=axes[0])
axes[0].set_ylabel('')

Data = FAME_fit['lipid_body']/FAME_fit['lipid_body'].sum()
Data.plot.pie(title='Lipid body', ax=axes[1])
axes[1].set_ylabel('')

#plt.tight_layout()
plt.show()
```

#### Creat new fatty acid synthesis reactions¶

In [40]:

```
model.reactions.get_by_id('FAS80COA_L').reaction
```

Out[40]:

```
'accoa_c + 9.0 h_c + 3.0 malcoa_c + 6.0 nadph_c --> 3.0 co2_c + 3.0 coa_c + 3.0 h2o_c + 6.0 nadp_c + occoa_c'
```

In [41]:

```
# Add C17:0 fatty acid synthesis lumped reaction
r = model.reactions.get_by_id('FAS80COA_L').copy()
r.id = 'FAS170COA_L'
r.name = 'Fatty acyl CoA synthase n C170CoA lumped reaction'
model.add_reactions([r])
r.add_metabolites({'accoa_c': 1.0, 'ppcoa_c': -1.0, 'h_c': -12.0, 'malcoa_c': -4.0, 'nadph_c': -8.0,
                   'occoa_c': -1.0, 'hpdcacoa_c': 1.0, 'co2_c': 4.0, 'coa_c': 4.0, 'h2o_c': 4.0, 'nadp_c': 8.0})
r
```

Out[41]:

|  |  |
| --- | --- |
| **Reaction identifier** | FAS170COA\_L |
| **Name** | Fatty acyl CoA synthase n C170CoA lumped reaction |
| **Memory address** | 0x01a2aec8400 |
| **Stoichiometry** | 21.0 h\_c + 7.0 malcoa\_c + 14.0 nadph\_c + ppcoa\_c --> 7.0 co2\_c + 7.0 coa\_c + 7.0 h2o\_c + hpdcacoa\_c + 14.0 nadp\_c  21.0 H+ + 7.0 Malonyl CoA C24H33N7O19P3S + 14.0 Nicotinamide adenine dinucleotide phosphate - reduced + Propanoyl-CoA --> 7.0 CO2 CO2 + 7.0 Coenzyme A + 7.0 H2O H2O + Heptadecanoyl coa + 14.0 Nicot... |
| **GPR** | 8670 and 8777 |
| **Lower bound** | 0.0 |
| **Upper bound** | 1000.0 |

In [42]:

```
model.reactions.get_by_id('PMTCOAtr').reaction
```

Out[42]:

```
'pmtcoa_c <=> pmtcoa_r'
```

In [43]:

```
m = model.metabolites.get_by_id('hpdcacoa_c').copy()
m.id = 'hpdcacoa_r'
m.compartment = 'r'
model.add_metabolites([m])

r = model.reactions.get_by_id('PMTCOAtr').copy()
r.id = 'HPDCACOAtr'
r.name = 'Heptadecanoyl-CoA transport, endoplasmic reticulum'
model.add_reactions([r])
r.add_metabolites({'pmtcoa_c': 1.0, 'pmtcoa_r': -1.0, 'hpdcacoa_c': -1.0, 'hpdcacoa_r': 1.0})
r
```

Out[43]:

|  |  |
| --- | --- |
| **Reaction identifier** | HPDCACOAtr |
| **Name** | Heptadecanoyl-CoA transport, endoplasmic reticulum |
| **Memory address** | 0x01a2c4cd908 |
| **Stoichiometry** | hpdcacoa\_c <=> hpdcacoa\_r  Heptadecanoyl coa <=> Heptadecanoyl coa |
| **GPR** | 10946 |
| **Lower bound** | -1000.0 |
| **Upper bound** | 1000.0 |

In [44]:

```
model.reactions.get_by_id('FAS260COAer')
```

Out[44]:

|  |  |
| --- | --- |
| **Reaction identifier** | FAS260COAer |
| **Name** | Fatty acid synthase n C260 |
| **Memory address** | 0x010204426a0 |
| **Stoichiometry** | 3.0 h\_r + malcoa\_r + 2.0 nadph\_r + ttccoa\_r --> co2\_r + coa\_r + h2o\_r + hexccoa\_r + 2.0 nadp\_r  3.0 H+ + Malonyl CoA C24H33N7O19P3S + 2.0 NADPH + Tetracosanoyl CoA n C240CoA C45H78N7O17P3S --> CO2 + Coenzyme A + H2O + Hexacosanoyl CoA n C260CoA C47H82N7O17P3S + 2.0 NADP+ |
| **GPR** | 10677 and 11343 and 16241 and 16695 |
| **Lower bound** | 0.0 |
| **Upper bound** | 1000.0 |

In [45]:

```
# Add C20:0 fatty acid synthesis reaction from C18:0
m = model.metabolites.get_by_id('arachcoa_c').copy()
m.id = 'arachcoa_r'
m.compartment = 'r'
model.add_metabolites([m])
r = model.reactions.get_by_id('FAS260COAer').copy()
r.id = 'FAS200COAer'
r.name = 'Fatty acyl CoA synthase n C200CoA'
r.gene_reaction_rule = '(10677 and 11343 and 16241 and 16695) or (10677 and 16241 and 16655 and 16695)'
model.add_reactions([r])
r.add_metabolites({'ttccoa_r': 1.0, 'stcoa_r': -1.0, 'hexccoa_r': -1.0, 'arachcoa_r': 1.0})

m = hsa2.metabolites.get_by_id('docoscoa_r').copy()
model.add_metabolites([m])
r = model.reactions.get_by_id('FAS260COAer').copy()
r.id = 'FAS220COAer'
r.name = 'Fatty acyl CoA synthase n C220CoA'
r.gene_reaction_rule = '(10677 and 11343 and 16241 and 16695) or (10677 and 16241 and 16655 and 16695)'
model.add_reactions([r])
r.add_metabolites({'ttccoa_r': 1.0, 'arachcoa_r': -1.0, 'hexccoa_r': -1.0, 'docoscoa_r': 1.0})
```

In [46]:

```
model.reactions.get_by_id('FAS240COAer')
```

Out[46]:

|  |  |
| --- | --- |
| **Reaction identifier** | FAS240COAer |
| **Name** | Fatty acid synthase n C240 lumped reaction |
| **Memory address** | 0x010204427b8 |
| **Stoichiometry** | 9.0 h\_r + 3.0 malcoa\_r + 6.0 nadph\_r + stcoa\_r --> 3.0 co2\_r + 3.0 coa\_r + 3.0 h2o\_r + 6.0 nadp\_r + ttccoa\_r  9.0 H+ + 3.0 Malonyl CoA C24H33N7O19P3S + 6.0 NADPH + Stearoyl-CoA --> 3.0 CO2 + 3.0 Coenzyme A + 3.0 H2O + 6.0 NADP+ + Tetracosanoyl CoA n C240CoA C45H78N7O17P3S |
| **GPR** | (10677 and 11343 and 16241 and 16695) or (10677 and 16241 and 16655 and 16695) |
| **Lower bound** | 0.0 |
| **Upper bound** | 1000.0 |

In [47]:

```
#change FAS240 lumped to FAS240 from 22 to 24
hsa2.metabolites.get_by_id('docoscoa_r')
r = model.reactions.get_by_id('FAS240COAer')
r.name = 'Fatty acyl CoA synthase n C240CoA'
r.add_metabolites({'stcoa_r': 1.0, 'docoscoa_r': -1.0, 'h_r': 6.0, 'malcoa_r': 2.0, 'nadph_r': 4.0,
                   'co2_r': -2.0, 'coa_r': -2.0, 'h2o_r': -2.0, 'nadp_r': -4.0})
r
```

Out[47]:

|  |  |
| --- | --- |
| **Reaction identifier** | FAS240COAer |
| **Name** | Fatty acyl CoA synthase n C240CoA |
| **Memory address** | 0x010204427b8 |
| **Stoichiometry** | docoscoa\_r + 3.0 h\_r + malcoa\_r + 2.0 nadph\_r --> co2\_r + coa\_r + h2o\_r + 2.0 nadp\_r + ttccoa\_r  Docosanoyl Coenzyme A + 3.0 H+ + Malonyl CoA C24H33N7O19P3S + 2.0 NADPH --> CO2 + Coenzyme A + H2O + 2.0 NADP+ + Tetracosanoyl CoA n C240CoA C45H78N7O17P3S |
| **GPR** | (10677 and 11343 and 16241 and 16695) or (10677 and 16241 and 16655 and 16695) |
| **Lower bound** | 0.0 |
| **Upper bound** | 1000.0 |

In [48]:

```
#Add beta oxidation for C20 C22 FAs
model.reactions.get_by_id('FAOXC240200x')
```

Out[48]:

|  |  |
| --- | --- |
| **Reaction identifier** | FAOXC240200x |
| **Name** | Beta oxidation of long chain fatty acid |
| **Memory address** | 0x0102002b908 |
| **Stoichiometry** | 2.0 coa\_x + 2.0 h2o\_x + 2.0 nad\_x + 2.0 o2\_x + ttccoa\_x --> 2.0 accoa\_x + arachcoa\_x + 2.0 h2o2\_x + 2.0 h\_x + 2.0 nadh\_x  2.0 Coenzyme A + 2.0 H2O H2O + 2.0 NAD+ + 2.0 O2 O2 + Tetracosanoyl CoA n C240CoA C45H78N7O17P3S --> 2.0 Acetyl-CoA + Arachidyl coenzyme A + 2.0 Hydrogen peroxide + 2.0 H+ + 2.0 NADH |
| **GPR** | (12742 and 11362 and 13813) or (12752 and 11362 and 13813) or (9700 and 11362 and 13813) |
| **Lower bound** | 0.0 |
| **Upper bound** | 1000.0 |

In [49]:

```
# Replace lumped FAOXC240200x with FAOXC24C22x and FAOXC22C20x
r1 = hsa2.reactions.get_by_id('FAOXC24C22x').copy()
r1.gene_reaction_rule = '(12742 and 11362 and 13813) or (12752 and 11362 and 13813) or (9700 and 11362 and 13813)'
r2 = hsa2.reactions.get_by_id('FAOXC22C20x').copy()
r2.gene_reaction_rule = '(12742 and 11362 and 13813) or (12752 and 11362 and 13813) or (9700 and 11362 and 13813)'
model.add_reactions([r1,r2])
model.remove_reactions(['FAOXC240200x'], remove_orphans=True)

# Add transport reactions for C20 and C22
m = model.metabolites.get_by_id('arach_c').copy()
m.id = 'arach_x'
m.compartment = 'x'
model.add_metabolites([m])
r = model.reactions.get_by_id('FA240tp').copy()
r.id = 'FA200tp'
model.add_reactions([r])
r.add_metabolites({'ttc_c': 1.0, 'arach_c': -1.0, 'ttc_x': -1.0, 'arach_x': 1.0})
r = model.reactions.get_by_id('FACOAL240p').copy()
r.id = 'FACOAL200p'
r.name = 'Fatty acid CoA ligase n C200 peroxisomal'
model.add_reactions([r])
r.add_metabolites({'ttc_x': 1.0, 'arach_x': -1.0, 'ttccoa_x': -1.0, 'arachcoa_x': 1.0})

m1 = hsa2.metabolites.get_by_id('docosac_c').copy()
m2 = hsa2.metabolites.get_by_id('docosac_c').copy()
m2.id = 'docosac_x'
m2.compartment = 'x'
model.add_metabolites([m1,m2])
r = model.reactions.get_by_id('FA240tp').copy()
r.id = 'FA220tp'
model.add_reactions([r])
r.add_metabolites({'ttc_c': 1.0, 'docosac_c': -1.0, 'ttc_x': -1.0, 'docosac_x': 1.0})
r = model.reactions.get_by_id('FACOAL240p').copy()
r.id = 'FACOAL220p'
r.name = 'Fatty acid CoA ligase n C220 peroxisomal'
model.add_reactions([r])
r.add_metabolites({'ttc_x': 1.0, 'docosac_x': -1.0, 'ttccoa_x': -1.0, 'docoscoa_x': 1.0})

# Remove C20 transport to mitochondria
model.remove_reactions(['ARACHCPT1','ARACHCRNt','ARACHCPT2'], remove_orphans=True)
```

#### Replace the S. cer FA composition with the estimated cell lipid FA composition¶

In [50]:

```
FAME_map = {'C16:0 FA': 'pmtcoa', 'C16:1 FA': 'hdcoa', 'C17:0 FA': 'hpdcacoa', 'C18:0 FA': 'stcoa',
            'C18:1 FA': 'odecoa', 'C18:2 FA': 'ocdycacoa', 'C18:3 FA': 'lnlncgcoa', 'C20:0 FA': 'arachcoa',
            'C22:0 FA': 'docoscoa', 'C24:0 FA': 'ttccoa'}
FAcoa_FA_map = {'pmtcoa': 'hdca', 'hdcoa': 'hdcea', 'hpdcacoa': 'hpdca', 'stcoa': 'ocdca', 'odecoa': 'ocdcea',
                'ocdycacoa': 'ocdcya', 'lnlncgcoa': 'lnlncg', 'arachcoa': 'arach', 'docoscoa': 'docosac',
                'ttccoa': 'ttc'}
```

In [51]:

```
# convert Acyl-CoA formula weight to Methyl ester formula weight
# Add methyl group (+H) and subtract coa
FAME_mw = pd.Series(index=FAME_fit.index)
m = cobra.Metabolite('CH3OH')
m.formula = 'CH3OH'
temp = m.formula_weight - model.metabolites.get_by_id('coa_c').formula_weight
for k, v in FAME_map.items():
    FAME_mw[k] = model.metabolites.get_by_id(v+"_r").formula_weight + temp
FAME_mw
```

Out[51]:

```
C16:0 FA    270.45066
C16:1 FA    268.43478
C17:0 FA    284.47724
C18:0 FA    298.50382
C18:1 FA    296.48794
C18:2 FA    294.47206
C18:3 FA    292.45618
C20:0 FA    326.55698
C22:0 FA    354.61014
C24:0 FA    382.66330
dtype: float64
```

In [52]:

```
# cell lipid FAME weight fraction
FAME_cell_wf = FAME_fit['cell']/FAME_fit['cell'].sum()
FAME_cell_wf
```

Out[52]:

```
C16:0 FA    0.081787
C16:1 FA    0.003433
C17:0 FA    0.016074
C18:0 FA    0.066164
C18:1 FA    0.326434
C18:2 FA    0.367526
C18:3 FA    0.098057
C20:0 FA    0.016322
C22:0 FA    0.003583
C24:0 FA    0.020622
Name: cell, dtype: float64
```

In [53]:

```
# cell lipid FAME molar fraction
# drop fatty acids with fraction <= 1e-2
FAME_cell_mf = pd.Series()
for k, v in FAME_map.items():
    FAME_cell_mf[v] = FAME_cell_wf[k]/FAME_mw[k]
FAME_cell_mf = FAME_cell_mf/FAME_cell_mf.sum()
FAME_cell_mf = FAME_cell_mf.round(2)
print(FAME_cell_mf.sum())
FAME_cell_mf
```

```
1.0
```

Out[53]:

```
pmtcoa       0.09
hdcoa        0.00
hpdcacoa     0.02
stcoa        0.07
odecoa       0.32
ocdycacoa    0.37
lnlncgcoa    0.10
arachcoa     0.01
docoscoa     0.00
ttccoa       0.02
dtype: float64
```

In [54]:

```
# lipid body FAME weight fraction
FAME_lipid_wf = FAME_fit['lipid_body']/FAME_fit['lipid_body'].sum()
FAME_lipid_wf
```

Out[54]:

```
C16:0 FA    0.209204
C16:1 FA    0.004693
C17:0 FA    0.000837
C18:0 FA    0.127438
C18:1 FA    0.492042
C18:2 FA    0.142394
C18:3 FA    0.006909
C20:0 FA    0.003625
C22:0 FA    0.003875
C24:0 FA    0.008983
Name: lipid_body, dtype: float64
```

In [55]:

```
# lipid body FAME molar fraction
# drop fatty acids with fraction <= 1e-2
FAME_lipid_mf = pd.Series()
for k, v in FAME_map.items():
    FAME_lipid_mf[v] = FAME_lipid_wf[k]/FAME_mw[k]
FAME_lipid_mf = FAME_lipid_mf/FAME_lipid_mf.sum()
FAME_lipid_mf = FAME_lipid_mf.round(2)
print(FAME_lipid_mf.sum())
FAME_lipid_mf
```

```
1.0
```

Out[55]:

```
pmtcoa       0.23
hdcoa        0.01
hpdcacoa     0.00
stcoa        0.12
odecoa       0.48
ocdycacoa    0.14
lnlncgcoa    0.01
arachcoa     0.00
docoscoa     0.00
ttccoa       0.01
dtype: float64
```

In [56]:

```
# Calculate cell lipid average formula for acyl part scaled by 100
# Parse and sum individual acyl-CoA and subtract coa
import re
FA_cell_formula = dict()
for k, v in FAME_map.items():
    temp = re.findall(r'([A-Z][a-z]*)(\d*)', model.metabolites.get_by_id(v+"_r").formula)
    for x in temp:
        y = int(x[1]) if x[1] else 1
        FA_cell_formula[x[0]] = FA_cell_formula.setdefault(x[0], 0) + y*int(FAME_cell_mf[v]*100)
    print(k, temp)
temp = re.findall(r'([A-Z][a-z]*)(\d*)', model.metabolites.get_by_id('coa_c').formula)
for x in temp:
    y = int(x[1]) if x[1] else 1
    FA_cell_formula[x[0]] = FA_cell_formula.setdefault(x[0], 0) - y*100
for x in [k for k, v in FA_cell_formula.items() if not v]:
    FA_cell_formula.pop(x)
FA_cell_formula
```

```
C16:0 FA [('C', '37'), ('H', '62'), ('N', '7'), ('O', '17'), ('P', '3'), ('S', '')]
C16:1 FA [('C', '37'), ('H', '60'), ('N', '7'), ('O', '17'), ('P', '3'), ('S', '')]
C17:0 FA [('C', '38'), ('H', '64'), ('N', '7'), ('O', '17'), ('P', '3'), ('S', '')]
C18:0 FA [('C', '39'), ('H', '66'), ('N', '7'), ('O', '17'), ('P', '3'), ('S', '')]
C18:1 FA [('C', '39'), ('H', '64'), ('N', '7'), ('O', '17'), ('P', '3'), ('S', '')]
C18:2 FA [('C', '39'), ('H', '62'), ('N', '7'), ('O', '17'), ('P', '3'), ('S', '')]
C18:3 FA [('C', '39'), ('H', '60'), ('N', '7'), ('O', '17'), ('P', '3'), ('S', '')]
C20:0 FA [('C', '41'), ('H', '70'), ('N', '7'), ('O', '17'), ('P', '3'), ('S', '')]
C22:0 FA [('C', '43'), ('H', '74'), ('N', '7'), ('O', '17'), ('P', '3'), ('S', '')]
C24:0 FA [('C', '45'), ('H', '78'), ('N', '7'), ('O', '17'), ('P', '3'), ('S', '')]
```

Out[56]:

```
{'C': 1794, 'H': 3116, 'O': 100}
```

In [57]:

```
# Calculate average formula for 1ag3p_RT_r and compare to old one
temp = dict()
for x in re.findall(r'([A-Z][a-z]*)(\d*)', model.metabolites.get_by_id('glyc3p_r').formula):
    y = int(x[1]) if x[1] else 1
    temp[x[0]] = temp.setdefault(x[0], 0) + y*100
for k, v in FA_cell_formula.items():
    temp[k] = temp[k] + v
print(temp)
temp_old = dict()
for x in re.findall(r'([A-Z][a-z]*)(\d*)', model.metabolites.get_by_id('1ag3p_RT_r').formula):
    y = int(x[1]) if x[1] else 1
    temp_old[x[0]] = temp_old.setdefault(x[0], 0) + y
print(temp_old)
{k: temp[k] - temp_old[k] for k in temp}
```

```
{'C': 2094, 'H': 3816, 'O': 700, 'P': 100}
{'C': 1920, 'H': 3622, 'O': 700, 'P': 100}
```

Out[57]:

```
{'C': 174, 'H': 194, 'O': 0, 'P': 0}
```

On average, C 174+ and H 194+ for cell lipid

In [58]:

```
# update cell lipid formula
for r in ['1ag3p_RT_r','1agly3p_RT_r','1agpc_RT_r']:
    temp = dict()
    for x in re.findall(r'([A-Z][a-z]*)(\d*)', model.metabolites.get_by_id(r).formula):
        y = int(x[1]) if x[1] else 1
        temp[x[0]] = temp.setdefault(x[0], 0) + y
    temp['C'] = temp['C'] + 174
    temp['H'] = temp['H'] + 194
    model.metabolites.get_by_id(r).formula = ''.join(k+str(int(v)) for k, v in temp.items())
    
for r in ['pa_RT_r','pc_RT_r','pe_RT_r','ps_RT_r','ptd1ino_RT_r','ptd3ino_RT_r','ptd135bp_RT_r',
          'ptd4ino_RT_r','ptd145bp_RT_r','ptdmeeta_RT_r','ptd2meeta_RT_r','12dgr_RT_r','cdpdag_RT_r','dagpy_RT_r',
          'pa_RT_m','pe_RT_m','ps_RT_m','pg_RT_m','pgp_RT_m','cdpdag_RT_m',
          'pc_RT_e','pe_RT_e','ptd1ino_RT_e']:
    temp = dict()
    for x in re.findall(r'([A-Z][a-z]*)(\d*)', model.metabolites.get_by_id(r).formula):
        y = int(x[1]) if x[1] else 1
        temp[x[0]] = temp.setdefault(x[0], 0) + y
    temp['C'] = temp['C'] + 174*2
    temp['H'] = temp['H'] + 194*2
    model.metabolites.get_by_id(r).formula = ''.join(k+str(int(v)) for k, v in temp.items())
    
for r in ['triglyc_RT_r']:
    temp = dict()
    for x in re.findall(r'([A-Z][a-z]*)(\d*)', model.metabolites.get_by_id(r).formula):
        y = int(x[1]) if x[1] else 1
        temp[x[0]] = temp.setdefault(x[0], 0) + y
    temp['C'] = temp['C'] + 174*3
    temp['H'] = temp['H'] + 194*3
    model.metabolites.get_by_id(r).formula = ''.join(k+str(int(v)) for k, v in temp.items())
    
for r in ['clpn_RT_m']:
    temp = dict()
    for x in re.findall(r'([A-Z][a-z]*)(\d*)', model.metabolites.get_by_id(r).formula):
        y = int(x[1]) if x[1] else 1
        temp[x[0]] = temp.setdefault(x[0], 0) + y
    temp['C'] = temp['C'] + 174*4
    temp['H'] = temp['H'] + 194*4
    model.metabolites.get_by_id(r).formula = ''.join(k+str(int(v)) for k, v in temp.items())
```

In [59]:

```
# Calculate lipid body average formula for acyl part scaled by 100
# Parse and sum individual acyl-CoA and subtract coa
FA_lipid_formula = dict()
for k, v in FAME_map.items():
    temp = re.findall(r'([A-Z][a-z]*)(\d*)', model.metabolites.get_by_id(v+"_r").formula)
    for x in temp:
        y = int(x[1]) if x[1] else 1
        FA_lipid_formula[x[0]] = FA_lipid_formula.setdefault(x[0], 0) + y*int(FAME_lipid_mf[v]*100)
    print(k, temp)
temp = re.findall(r'([A-Z][a-z]*)(\d*)', model.metabolites.get_by_id('coa_c').formula)
for x in temp:
    y = int(x[1]) if x[1] else 1
    FA_lipid_formula[x[0]] = FA_lipid_formula.setdefault(x[0], 0) - y*100
for x in [k for k, v in FA_lipid_formula.items() if not v]:
    FA_lipid_formula.pop(x)
FA_lipid_formula
```

```
C16:0 FA [('C', '37'), ('H', '62'), ('N', '7'), ('O', '17'), ('P', '3'), ('S', '')]
C16:1 FA [('C', '37'), ('H', '60'), ('N', '7'), ('O', '17'), ('P', '3'), ('S', '')]
C17:0 FA [('C', '38'), ('H', '64'), ('N', '7'), ('O', '17'), ('P', '3'), ('S', '')]
C18:0 FA [('C', '39'), ('H', '66'), ('N', '7'), ('O', '17'), ('P', '3'), ('S', '')]
C18:1 FA [('C', '39'), ('H', '64'), ('N', '7'), ('O', '17'), ('P', '3'), ('S', '')]
C18:2 FA [('C', '39'), ('H', '62'), ('N', '7'), ('O', '17'), ('P', '3'), ('S', '')]
C18:3 FA [('C', '39'), ('H', '60'), ('N', '7'), ('O', '17'), ('P', '3'), ('S', '')]
C20:0 FA [('C', '41'), ('H', '70'), ('N', '7'), ('O', '17'), ('P', '3'), ('S', '')]
C22:0 FA [('C', '43'), ('H', '74'), ('N', '7'), ('O', '17'), ('P', '3'), ('S', '')]
C24:0 FA [('C', '45'), ('H', '78'), ('N', '7'), ('O', '17'), ('P', '3'), ('S', '')]
```

Out[59]:

```
{'C': 1758, 'H': 3156, 'O': 100}
```

In [60]:

```
# Calculate average formula for 1ag3p_RT_r and compare to old one
temp = dict()
for x in re.findall(r'([A-Z][a-z]*)(\d*)', model.metabolites.get_by_id('glyc3p_r').formula):
    y = int(x[1]) if x[1] else 1
    temp[x[0]] = temp.setdefault(x[0], 0) + y*100
for k, v in FA_lipid_formula.items():
    temp[k] = temp[k] + v
print(temp)
temp_old = dict()
for x in re.findall(r'([A-Z][a-z]*)(\d*)', model.metabolites.get_by_id('1ag3p_RT_r').formula):
    y = int(x[1]) if x[1] else 1
    temp_old[x[0]] = temp_old.setdefault(x[0], 0) + y
print(temp_old)
{k: temp[k] - temp_old[k] for k in temp}
```

```
{'C': 2058, 'H': 3856, 'O': 700, 'P': 100}
{'C': 2094, 'H': 3816, 'O': 700, 'P': 100}
```

Out[60]:

```
{'C': -36, 'H': 40, 'O': 0, 'P': 0}
```

In [61]:

```
# update cell lipid formula
for r in ['mag_RT_d']:
    temp = dict()
    for x in re.findall(r'([A-Z][a-z]*)(\d*)', model.metabolites.get_by_id(r).formula):
        y = int(x[1]) if x[1] else 1
        temp[x[0]] = temp.setdefault(x[0], 0) + y
    temp['C'] = temp['C'] + 138
    temp['H'] = temp['H'] + 234
    model.metabolites.get_by_id(r).formula = ''.join(k+str(int(v)) for k, v in temp.items())
    
for r in ['12dgr_RT_d']:
    temp = dict()
    for x in re.findall(r'([A-Z][a-z]*)(\d*)', model.metabolites.get_by_id(r).formula):
        y = int(x[1]) if x[1] else 1
        temp[x[0]] = temp.setdefault(x[0], 0) + y
    temp['C'] = temp['C'] + 138*2
    temp['H'] = temp['H'] + 234*2
    model.metabolites.get_by_id(r).formula = ''.join(k+str(int(v)) for k, v in temp.items())
    
for r in ['triglyc_RT_d']:
    temp = dict()
    for x in re.findall(r'([A-Z][a-z]*)(\d*)', model.metabolites.get_by_id(r).formula):
        y = int(x[1]) if x[1] else 1
        temp[x[0]] = temp.setdefault(x[0], 0) + y
    temp['C'] = temp['C'] + 138*3
    temp['H'] = temp['H'] + 234*3
    model.metabolites.get_by_id(r).formula = ''.join(k+str(int(v)) for k, v in temp.items())
```

In [62]:

```
# Difference between cell lipid and lipid body composition
for k, v in (FAME_lipid_mf - FAME_cell_mf).items():
    print(k, round(v*100))
```

```
pmtcoa 14
hdcoa 1
hpdcacoa -2
stcoa 5
odecoa 16
ocdycacoa -23
lnlncgcoa -9
arachcoa -1
docoscoa 0
ttccoa -1
```

In [63]:

```
# Simulate the accumulation of composition-specific TAG in lipid droplet by acyl-CoA exchange
r = model.reactions.get_by_id('TAGtrd')
for k, v in (FAME_lipid_mf - FAME_cell_mf).items():
    r.add_metabolites({k+'_r': -3*round(v*100)})
# Scale by 100
for m, v in model.reactions.get_by_id('TAGtrd').metabolites.items():
    model.reactions.get_by_id('TAGtrd').add_metabolites({m.id: -v})
    model.reactions.get_by_id('TAGtrd').add_metabolites({m.id: round(v/100,2)})
# Add sink reaction for lipid accumulation and mobilization
r = cobra.Reaction('SK_triglyc_RT_d')
r.name = 'Demand needed to allow triglyceride to accumulate in lipid droplet'
model.add_reactions([r])
r.add_metabolites({'triglyc_RT_d': -1.0})
```

In [64]:

```
# Old FA molar fraction for reactions incorporating acyl-coa
FAcoa_mf_old = {'dcacoa_r': 0.02, 'ddcacoa_r': 0.06, 'hdcoa_r': 0.17, 'ocdycacoa_r': 0.09, 'odecoa_r': 0.24,
                'pmtcoa_r': 0.27, 'stcoa_r': 0.05, 'tdcoa_r': 0.1}
sum(FAcoa_mf_old.values())
```

Out[64]:

```
1.0
```

In [65]:

```
for r in sorted(model.metabolites.get_by_id('pmtcoa_r').reactions, key=lambda x: x.id):
    if r.get_coefficient('pmtcoa_r') == -0.27:
        print(r, r.gene_reaction_rule)
```

```
AGATer_RT: 0.01 1ag3p_RT_r + 0.02 dcacoa_r + 0.06 ddcacoa_r + 0.17 hdcoa_r + 0.09 ocdycacoa_r + 0.24 odecoa_r + 0.27 pmtcoa_r + 0.05 stcoa_r + 0.1 tdcoa_r --> coa_r + 0.01 pa_RT_r 10427 or 16030 or 16779 or 9746
GAT1er_RT: 0.02 dcacoa_r + 0.06 ddcacoa_r + glyc3p_r + 0.17 hdcoa_r + 0.09 ocdycacoa_r + 0.24 odecoa_r + 0.27 pmtcoa_r + 0.05 stcoa_r + 0.1 tdcoa_r --> 0.01 1ag3p_RT_r + coa_r 15435
GAT2er_RT: 0.02 dcacoa_r + 0.06 ddcacoa_r + dhap_r + 0.17 hdcoa_r + 0.09 ocdycacoa_r + 0.24 odecoa_r + 0.27 pmtcoa_r + 0.05 stcoa_r + 0.1 tdcoa_r --> 0.01 1agly3p_RT_r + coa_r 13369 or 15435
LPCATer_RT: 0.01 1agpc_RT_r + 0.02 dcacoa_r + 0.06 ddcacoa_r + 0.17 hdcoa_r + 0.09 ocdycacoa_r + 0.24 odecoa_r + 0.27 pmtcoa_r + 0.05 stcoa_r + 0.1 tdcoa_r --> coa_r + 0.01 pc_RT_r 16030
TRIGSer_RT: 0.01 12dgr_RT_r + 0.02 dcacoa_r + 0.06 ddcacoa_r + 0.17 hdcoa_r + 0.09 ocdycacoa_r + 0.24 odecoa_r + 0.27 pmtcoa_r + 0.05 stcoa_r + 0.1 tdcoa_r --> coa_r + 0.01 triglyc_RT_r 16460
```

In [66]:

```
for r in sorted(model.metabolites.get_by_id('pmtcoa_r').reactions, key=lambda x: x.id):
    if r.get_coefficient('pmtcoa_r') == -0.27:
        for k, v in FAcoa_mf_old.items():
            r.add_metabolites({k: v})
        for k, v in FAME_cell_mf.items():
            r.add_metabolites({k+'_r': -v})
```

In [67]:

```
# Old FA molar fraction for reactions releasing free fatty acid
FA_mf_old = {'dca_c': 0.02, 'ddca_c': 0.06, 'hdcea_c': 0.17, 'ocdcya_c': 0.09, 'ocdcea_c': 0.24, 'hdca_c': 0.27,
             'ocdca_c': 0.05, 'ttdca_c': 0.1}
sum(FA_mf_old.values())
```

Out[67]:

```
1.0
```

In [68]:

```
for r in sorted(model.metabolites.get_by_id('hdca_c').reactions, key=lambda x: x.id):
    if r.get_coefficient('hdca_c') == 0.27:
        print(r, r.gene_reaction_rule)
```

```
DAGL_RT: 0.01 12dgr_RT_d + h2o_c --> 0.02 dca_c + 0.06 ddca_c + h_c + 0.27 hdca_c + 0.17 hdcea_c + 0.01 mag_RT_d + 0.05 ocdca_c + 0.24 ocdcea_c + 0.09 ocdcya_c + 0.1 ttdca_c 11739 or 15065
MAGL_RT: h2o_c + 0.01 mag_RT_d --> 0.02 dca_c + 0.06 ddca_c + glyc_c + h_c + 0.27 hdca_c + 0.17 hdcea_c + 0.05 ocdca_c + 0.24 ocdcea_c + 0.09 ocdcya_c + 0.1 ttdca_c 14158 or 15065 or 9728
PLBPC_RT: h2o_c + 0.005 pc_RT_r --> 0.02 dca_c + 0.06 ddca_c + 0.5 g3pc_c + h_c + 0.27 hdca_c + 0.17 hdcea_c + 0.05 ocdca_c + 0.24 ocdcea_c + 0.09 ocdcya_c + 0.1 ttdca_c 14309
TAGL_RT: h2o_c + 0.01 triglyc_RT_d --> 0.01 12dgr_RT_d + 0.02 dca_c + 0.06 ddca_c + h_c + 0.27 hdca_c + 0.17 hdcea_c + 0.05 ocdca_c + 0.24 ocdcea_c + 0.09 ocdcya_c + 0.1 ttdca_c 10393 or 14317 or 14617 or 15065 or 9746
```

In [69]:

```
# phospholipid lipase
for x in ['PLBPC_RT']:
    r = model.reactions.get_by_id(x)
    for k, v in FA_mf_old.items():
        r.add_metabolites({k: -v})
    for k, v in FAME_cell_mf.items():
        r.add_metabolites({FAcoa_FA_map[k]+'_c': v})
```

In [70]:

```
# TAG/DAG/MAG lipase
for x in ['DAGL_RT','MAGL_RT','TAGL_RT']:
    r = model.reactions.get_by_id(x)
    for k, v in FA_mf_old.items():
        r.add_metabolites({k: -v})
    for k, v in FAME_lipid_mf.items():
        r.add_metabolites({FAcoa_FA_map[k]+'_c': v})
```

In [71]:

```
for r in sorted(model.metabolites.get_by_id('hdca_e').reactions, key=lambda x: x.id):
    if r.get_coefficient('hdca_e') == 0.27:
        print(r, r.gene_reaction_rule)
```

```
PLBP1Ie_RT: h2o_e + 0.005 ptd1ino_RT_e --> 0.02 dca_e + 0.06 ddca_e + 0.5 g3pi_e + h_e + 0.27 hdca_e + 0.17 hdcea_e + 0.05 ocdca_e + 0.24 ocdcea_e + 0.09 ocdcya_e + 0.1 ttdca_e 12385
PLBPCe_RT: h2o_e + 0.005 pc_RT_e --> 0.02 dca_e + 0.06 ddca_e + 0.5 g3pc_e + h_e + 0.27 hdca_e + 0.17 hdcea_e + 0.05 ocdca_e + 0.24 ocdcea_e + 0.09 ocdcya_e + 0.1 ttdca_e 12385
PLBPEe_RT: h2o_e + 0.005 pe_RT_e --> 0.02 dca_e + 0.06 ddca_e + 0.5 g3pe_e + h_e + 0.27 hdca_e + 0.17 hdcea_e + 0.05 ocdca_e + 0.24 ocdcea_e + 0.09 ocdcya_e + 0.1 ttdca_e 12385
```

In [72]:

```
# Fatty acid transport reactions
# 9912 is SLC27A1_4, but not SLC27A2 or SLC27A5
# Add fatty acid transport by diffusion and remove fatty acid/sodium symporters

for x in ['HDCAt','HDCEAt','OCDCAt','OCDCEAt','OCDCYAt']:
    r = sce.reactions.get_by_id(x).copy()
    model.add_reactions([r])
for x in ['LNLNCGt','HPDCAt','ARACHt','DOCOSACTDe']:
    r = hsa2.reactions.get_by_id(x).copy()
    r.gene_reaction_rule = ''
    r.lower_bound = 0.0
    model.add_reactions([r])

model.reactions.get_by_id('DOCOSACTDe').id = 'DOCOSACt'
model.reactions.get_by_id('DOCOSACt').name = 'Fatty acid transport via diffusion'
r = model.reactions.get_by_id('DOCOSACt').copy()
r.id = 'TTCt'
model.add_reactions([r])
r.add_metabolites({'docosac_e': 1.0, 'docosac_c': -1.0, 'ttc_e': -1.0, 'ttc_c': 1.0})

model.remove_reactions(['FATP1t','FATP2t','FATP3t','FATP4t','FATP8t','FATP9t'], remove_orphans=True)
```

In [73]:

```
for r in sorted(model.metabolites.get_by_id('hdca_e').reactions, key=lambda x: x.id):
    if r.get_coefficient('hdca_e') == 0.27:
        for k, v in FA_mf_old.items():
            r.add_metabolites({k.replace('_c','_e'): -v})
        for k, v in FAME_cell_mf.items():
            r.add_metabolites({FAcoa_FA_map[k]+'_e': v})
```

In [74]:

```
# The sterol ester reactions look wrong - it is taking 1 sterol and 100 acyl-coa to make 1 sterol ester
model.reactions.get_by_id('EPISTATer_RT').add_metabolites({'epist_r': -0.99})
model.reactions.get_by_id('ERGSTATer_RT').add_metabolites({'ergst_r': -0.99})
model.reactions.get_by_id('FECOSTATer_RT').add_metabolites({'fecost_r': -0.99})
model.reactions.get_by_id('LANOSTATer_RT').add_metabolites({'lanost_r': -0.99})
model.reactions.get_by_id('ZYMSTATer_RT').add_metabolites({'zymst_r': -0.99})
model.reactions.get_by_id('EPISTESTH_RT').add_metabolites({'epist_c': 0.99})
model.reactions.get_by_id('ERGSTESTH_RT').add_metabolites({'ergst_c': 0.99})
model.reactions.get_by_id('FECOSTESTH_RT').add_metabolites({'fecost_c': 0.99})
model.reactions.get_by_id('LANOSTESTH_RT').add_metabolites({'lanost_c': 0.99})
model.reactions.get_by_id('ZYMSTESTH_RT').add_metabolites({'zymst_c': 0.99})
# Add sterol transport from cyto to E.R.
for x in ['epist','ergst','fecost','lanost','zymst']:
    r = model.reactions.get_by_id(x.upper()+'t').copy()
    r.id = r.id+'r'
    r.name = r.name.replace('reversible','endoplasmic reticulum')
    r.gene_reaction_rule = ''
    model.add_reactions([r])
    r.add_metabolites({x+'_e': 1.0, x+'_c': -2.0, x+'_r': 1.0})
```

In [75]:

```
# Calculate average formula for sterol ester and compare to old one
for m1, m2 in [('epist_r','epistest_RT_r'),('ergst_r','ergstest_RT_r'),('fecost_r','fecostest_RT_r'),
               ('lanost_r','lanostest_RT_r'),('zymst_r','zymstest_RT_r')]:
    temp = dict()
    for x in re.findall(r'([A-Z][a-z]*)(\d*)', model.metabolites.get_by_id(m1).formula):
        y = int(x[1]) if x[1] else 1
        temp[x[0]] = temp.setdefault(x[0], 0) + y*100
    for k, v in FA_lipid_formula.items():
        temp[k] = temp[k] + v
    temp_old = dict()
    for x in re.findall(r'([A-Z][a-z]*)(\d*)', model.metabolites.get_by_id(m2).formula):
        y = int(x[1]) if x[1] else 1
        temp_old[x[0]] = temp_old.setdefault(x[0], 0) + y
    print(m2, {k: temp[k] - temp_old[k] for k in temp})
```

```
epistest_RT_r {'C': 2863, 'H': 4761, 'O': 99}
ergstest_RT_r {'C': 2863, 'H': 4563, 'O': 99}
fecostest_RT_r {'C': 2863, 'H': 4761, 'O': 99}
lanostest_RT_r {'C': 3061, 'H': 5157, 'O': 99}
zymstest_RT_r {'C': 2764, 'H': 4563, 'O': 99}
```

In [76]:

```
for r in ['epistest_RT_r','fecostest_RT_r','epistest_RT_d','fecostest_RT_d']:
    temp = dict()
    for x in re.findall(r'([A-Z][a-z]*)(\d*)', model.metabolites.get_by_id(r).formula):
        y = int(x[1]) if x[1] else 1
        temp[x[0]] = temp.setdefault(x[0], 0) + y
    temp['C'] = temp['C'] + 2863
    temp['H'] = temp['H'] + 4761
    temp['O'] = temp['O'] + 99
    model.metabolites.get_by_id(r).formula = ''.join(k+str(int(v)) for k, v in temp.items())

for r in ['ergstest_RT_r','ergstest_RT_d']:
    temp = dict()
    for x in re.findall(r'([A-Z][a-z]*)(\d*)', model.metabolites.get_by_id(r).formula):
        y = int(x[1]) if x[1] else 1
        temp[x[0]] = temp.setdefault(x[0], 0) + y
    temp['C'] = temp['C'] + 2863
    temp['H'] = temp['H'] + 4563
    temp['O'] = temp['O'] + 99
    model.metabolites.get_by_id(r).formula = ''.join(k+str(int(v)) for k, v in temp.items())

for r in ['lanostest_RT_r','lanostest_RT_d']:
    temp = dict()
    for x in re.findall(r'([A-Z][a-z]*)(\d*)', model.metabolites.get_by_id(r).formula):
        y = int(x[1]) if x[1] else 1
        temp[x[0]] = temp.setdefault(x[0], 0) + y
    temp['C'] = temp['C'] + 3061
    temp['H'] = temp['H'] + 5157
    temp['O'] = temp['O'] + 99
    model.metabolites.get_by_id(r).formula = ''.join(k+str(int(v)) for k, v in temp.items())

for r in ['zymstest_RT_r','zymstest_RT_d']:
    temp = dict()
    for x in re.findall(r'([A-Z][a-z]*)(\d*)', model.metabolites.get_by_id(r).formula):
        y = int(x[1]) if x[1] else 1
        temp[x[0]] = temp.setdefault(x[0], 0) + y
    temp['C'] = temp['C'] + 2764
    temp['H'] = temp['H'] + 4563
    temp['O'] = temp['O'] + 99
    model.metabolites.get_by_id(r).formula = ''.join(k+str(int(v)) for k, v in temp.items())
```

In [77]:

```
# Old FA molar fraction for sterol ester reactions incorporating acyl-coa
# This appeared in iMM904, but not clear where the numbers are taken from
FA_mf_old = {'hdcoa_r': 0.655, 'hexccoa_r': 0.01, 'odecoa_r': 0.27, 'pmtcoa_r': 0.02, 'stcoa_r': 0.03,
             'tdcoa_r': 0.015}
sum(FA_mf_old.values())
```

Out[77]:

```
1.0
```

In [78]:

```
for r in sorted(model.metabolites.get_by_id('pmtcoa_r').reactions, key=lambda x: x.id):
    if r.get_coefficient('pmtcoa_r') == -0.02:
        print(r, r.gene_reaction_rule)
```

```
EPISTATer_RT: epist_r + 0.655 hdcoa_r + 0.01 hexccoa_r + 0.27 odecoa_r + 0.02 pmtcoa_r + 0.03 stcoa_r + 0.015 tdcoa_r --> coa_r + 0.01 epistest_RT_r 11799
ERGSTATer_RT: ergst_r + 0.655 hdcoa_r + 0.01 hexccoa_r + 0.27 odecoa_r + 0.02 pmtcoa_r + 0.03 stcoa_r + 0.015 tdcoa_r --> coa_r + 0.01 ergstest_RT_r 11799
FECOSTATer_RT: fecost_r + 0.655 hdcoa_r + 0.01 hexccoa_r + 0.27 odecoa_r + 0.02 pmtcoa_r + 0.03 stcoa_r + 0.015 tdcoa_r --> coa_r + 0.01 fecostest_RT_r 11799
LANOSTATer_RT: 0.655 hdcoa_r + 0.01 hexccoa_r + lanost_r + 0.27 odecoa_r + 0.02 pmtcoa_r + 0.03 stcoa_r + 0.015 tdcoa_r --> coa_r + 0.01 lanostest_RT_r 11799
ZYMSTATer_RT: 0.655 hdcoa_r + 0.01 hexccoa_r + 0.27 odecoa_r + 0.02 pmtcoa_r + 0.03 stcoa_r + 0.015 tdcoa_r + zymst_r --> coa_r + 0.01 zymstest_RT_r 11799
```

In [79]:

```
for r in sorted(model.metabolites.get_by_id('pmtcoa_r').reactions, key=lambda x: x.id):
    if r.get_coefficient('pmtcoa_r') == -0.02:
        for k, v in FA_mf_old.items():
            r.add_metabolites({k: v})
        for k, v in FAME_lipid_mf.items():
            r.add_metabolites({k+'_r': -v})
```

In [80]:

```
# Old FA molar fraction for sterol ester reactions releasing free fatty acid
# This appeared in iMM904, but not clear where the numbers are taken from
FA_mf_old = {'hdca_c': 0.02, 'hdcea_c': 0.655, 'hexc_c': 0.01, 'ocdca_c': 0.03, 'ocdcea_c': 0.27, 'ttdca_c': 0.015}
sum(FA_mf_old.values())
```

Out[80]:

```
1.0
```

In [81]:

```
for r in sorted(model.metabolites.get_by_id('hdca_c').reactions, key=lambda x: x.id):
    if r.get_coefficient('hdca_c') == 0.02:
        print(r, r.gene_reaction_rule)
```

```
EPISTESTH_RT: 0.01 epistest_RT_d + h2o_c --> epist_c + h_c + 0.02 hdca_c + 0.655 hdcea_c + 0.01 hexc_c + 0.03 ocdca_c + 0.27 ocdcea_c + 0.015 ttdca_c 14247 or 14617
ERGSTESTH_RT: 0.01 ergstest_RT_d + h2o_c --> ergst_c + h_c + 0.02 hdca_c + 0.655 hdcea_c + 0.01 hexc_c + 0.03 ocdca_c + 0.27 ocdcea_c + 0.015 ttdca_c 14247 or 14617
FECOSTESTH_RT: 0.01 fecostest_RT_d + h2o_c --> fecost_c + h_c + 0.02 hdca_c + 0.655 hdcea_c + 0.01 hexc_c + 0.03 ocdca_c + 0.27 ocdcea_c + 0.015 ttdca_c 14247 or 14617
LANOSTESTH_RT: h2o_c + 0.01 lanostest_RT_d --> h_c + 0.02 hdca_c + 0.655 hdcea_c + 0.01 hexc_c + lanost_c + 0.03 ocdca_c + 0.27 ocdcea_c + 0.015 ttdca_c 14247 or 14617
ZYMSTESTH_RT: h2o_c + 0.01 zymstest_RT_d --> h_c + 0.02 hdca_c + 0.655 hdcea_c + 0.01 hexc_c + 0.03 ocdca_c + 0.27 ocdcea_c + 0.015 ttdca_c + zymst_c 14247 or 14617
```

In [82]:

```
for r in sorted(model.metabolites.get_by_id('hdca_c').reactions, key=lambda x: x.id):
    if r.get_coefficient('hdca_c') == 0.02:
        for k, v in FA_mf_old.items():
            r.add_metabolites({k: -v})
        for k, v in FAME_lipid_mf.items():
            r.add_metabolites({FAcoa_FA_map[k]+'_c': v})
```

In [83]:

```
# Add sink reaction for lipid accumulation and mobilization
for x in ['epistest_RT_d','ergstest_RT_d','fecostest_RT_d','lanostest_RT_d','zymstest_RT_d']:
    r = cobra.Reaction('SK_'+x)
    r.name = 'Demand needed to allow ' + model.metabolites.get_by_id(x).name.split('  ')[0] + \
             ' to accumulate in lipid droplet'
    model.add_reactions([r])
    r.add_metabolites({x: -1.0})
```

In [84]:

```
for r in sorted(model.reactions, key=lambda x: x.id):
    if not r.boundary and sum(abs(x) for x in r.check_mass_balance().values()) > 1e-12:
        print(r, r.gene_reaction_rule, r.check_mass_balance())
```

```
BIOMASS_RT: 1.1348 13BDglcn_c + 1e-06 5mthf_c + 0.4588 ala__L_c + 0.046 amp_c + 0.1607 arg__L_c + 0.1017 asn__L_c + 0.2975 asp__L_c + 59.276 atp_c + 1e-06 btn_m + 1e-06 camp_c + 0.0447 cmp_c + 1e-06 coa_c + 0.0066 cys__L_c + 0.0036 damp_c + 0.0024 dcmp_c + 0.0024 dgmp_c + 0.0036 dtmp_c + 0.0007 ergst_r + 1e-06 fad_c + 0.1054 gln__L_c + 0.3018 glu__L_c + 0.2904 gly_c + 0.5185 glycogen_c + 0.046 gmp_c + 1e-06 gthrd_c + 59.276 h2o_c + 1e-06 hemeA_m + 0.0663 his__L_c + 0.1927 ile__L_c + 0.2964 leu__L_c + 1e-06 lipopb_m + 0.2862 lys__L_c + 0.8079 mannan_r + 0.0507 met__L_c + 1e-06 mlthf_c + 1e-06 nad_c + 1e-06 nadp_c + 6e-06 pa_RT_r + 6e-05 pc_RT_r + 4.5e-05 pe_RT_r + 0.1339 phe__L_c + 0.1647 pro__L_c + 1.7e-05 ps_RT_r + 5.3e-05 ptd1ino_RT_r + 1e-06 ptrc_c + 1e-06 pydx5p_c + 1e-06 q9_m + 0.00099 ribflv_c + 0.1854 ser__L_c + 0.02 so4_c + 1e-06 spmd_c + 1e-06 thf_c + 1e-06 thmpp_c + 0.1914 thr__L_c + 0.0234 tre_c + 6.6e-05 triglyc_RT_r + 0.0284 trp__L_c + 0.102 tyr__L_c + 0.0599 ump_c + 0.2646 val__L_c + 0.0015 zymst_r --> 59.276 adp_c + 58.70001 h_c + 59.305 pi_c  {'charge': -0.01617199999997561, 'C': -36.03979100000002, 'H': -65.6821809999999, 'O': -23.676359000000236, 'N': -5.596535000000022, 'P': -0.19771400000000136, 'S': -0.077306, 'Fe': -1e-06}
```

In [85]:

```
lipid_weight_fraction = 0.0813
```

In [86]:

```
# Numbers from Rh. glutinis in "Lipid Composition of 30 Speciesof Yeast"
# https://link.springer.com/article/10.1007/BF02532989
# Assume equal composition between ergst and zymst - no data
# Sphingolipid - include psphings_r as a precursor to ceramides for the unidentified 3.8% 
lipidomics = {
    'pa_RT_r': 0.413*2.8/100,
    'pc_RT_r': 0.413*40.2/100,
    'pe_RT_r': 0.413*28.7/100,
    'ps_RT_r': 0.413*4.7/100,
    'ptd1ino_RT_r': 0.413*11.9/100,
    'clpn_RT_m': 0.413*7.6/100,
    'psphings_r': 0.413*3.8/100,
    'triglyc_RT_r': 0.587*79.7/100,
    'ergst_r': 0.587*6.7/100*0.5,
    'zymst_r': 0.587*6.7/100*0.5,
    'ergstest_RT_r': 0.587*4.5/100*0.5,
    'zymstest_RT_d': 0.587*4.5/100*0.5
}
#lipidomics = pd.DataFrame(lipidomics.items(), columns=['identifiers','abundances'])
print(lipidomics)
print(sum(lipidomics.values()))
```

```
{'pa_RT_r': 0.011563999999999998, 'pc_RT_r': 0.16602599999999998, 'pe_RT_r': 0.118531, 'ps_RT_r': 0.019411, 'ptd1ino_RT_r': 0.049146999999999996, 'clpn_RT_m': 0.031388, 'psphings_r': 0.015694, 'triglyc_RT_r': 0.46783899999999995, 'ergst_r': 0.0196645, 'zymst_r': 0.0196645, 'ergstest_RT_r': 0.013207499999999999, 'zymstest_RT_d': 0.013207499999999999}
0.945344
```

In [87]:

```
lipid_coefficients = {}
for k, v in lipidomics.items():
    m = model.metabolites.get_by_id(k)
    lipid_coefficients[m] = -lipid_weight_fraction*v/m.formula_weight*1000
lipid_coefficients
```

Out[87]:

```
{<Metabolite pa_RT_r at 0x101fe99cc0>: -1.351153236901054e-05,
 <Metabolite pc_RT_r at 0x101fe99ef0>: -0.00017239181747866222,
 <Metabolite pe_RT_r at 0x101d2721d0>: -0.00013006590032135413,
 <Metabolite ps_RT_r at 0x101fea42e8>: -2.013156086582689e-05,
 <Metabolite ptd1ino_RT_r at 0x101fea41d0>: -4.6517100627855964e-05,
 <Metabolite clpn_RT_m at 0x101fbd9ba8>: -1.7602448653658758e-05,
 <Metabolite psphings_r at 0x101d2b6e80>: -0.004005845248780984,
 <Metabolite triglyc_RT_r at 0x101fe99d68>: -0.0004318611298933685,
 <Metabolite ergst_r at 0x101d2b6cc0>: -0.004030582276956849,
 <Metabolite zymst_r at 0x101fe999e8>: -0.004156441285546507,
 <Metabolite ergstest_RT_r at 0x101fe99dd8>: -1.6378266962889334e-05,
 <Metabolite zymstest_RT_d at 0x101fea4128>: -1.6683916045061826e-05}
```

In [88]:

```
# Numbers are from S. cer model
# 1,6 beta glucan and chitin numbers are taken from
# "A study of the yeast cell wall composition and structure in response to growth conditions and mode of cultivation"
# https://onlinelibrary.wiley.com/doi/full/10.1046/j.1472-765X.2003.01394.x
# 1,6 beta glucan - 15.625% of total glucan 
# Chitin - 2% of the cell wall dry mass - for simplicity (13.1+18.4)*0.2
carb_weight_fraction = {
    'glycogen_c': 8.4/100,
    'tre_c': 0.8/100,
    'mannan_r': 13.1/100,
    '13BDglcn_c': 18.4*0.84375/100,
    '16BDglcn_c': 18.4*0.15625/100,
    'chitin_c': 0.63/100,
}
print(sum(carb_weight_fraction.values()))
```

```
0.4133
```

In [89]:

```
carb_coefficients = {}
for k, v in carb_weight_fraction.items():
    m = model.metabolites.get_by_id(k)
    carb_coefficients[m] = -v/m.formula_weight*1000
carb_coefficients
```

Out[89]:

```
{<Metabolite glycogen_c at 0x101d2aa978>: -0.5180688858928609,
 <Metabolite tre_c at 0x101d2c22b0>: -0.02337155205335445,
 <Metabolite mannan_r at 0x101d29f4e0>: -0.8079407625233902,
 <Metabolite 13BDglcn_c at 0x101d2b62e8>: -0.9575023158912697,
 <Metabolite 16BDglcn_c at 0x101d29fc18>: -0.17731524368356846,
 <Metabolite chitin_c at 0x101d25b2e8>: -0.031005078336545068}
```

In [90]:

```
# Remove ATP maintenance part
for x in ['atp_c','adp_c','h_c','pi_c']:
    m = model.metabolites.get_by_id(x)
    v = model.reactions.get_by_id('BIOMASS_RT').get_coefficient(m)
    model.reactions.get_by_id('BIOMASS_RT').add_metabolites({m: -v})
```

In [91]:

```
# Remove nmp and dnmp from S. cer and replace with ntp and dntp
for x in ['amp_c','ump_c','gmp_c','cmp_c','damp_c','dtmp_c','dgmp_c','dcmp_c']:
    m = model.metabolites.get_by_id(x)
    v = model.reactions.get_by_id('BIOMASS_RT').get_coefficient(m)
    model.reactions.get_by_id('BIOMASS_RT').add_metabolites({m: -v})
```

In [92]:

```
# Existing dna and rna coefficient
for m, v in model.reactions.get_by_id('BIOMASS_RT').metabolites.items():
    if m in list({**dna_coefficients,**rna_coefficients}):
        print(m.id, v)
```

In [93]:

```
# Update dna and rna coefficient
for k, v in dna_coefficients.items():
    model.reactions.get_by_id('BIOMASS_RT').add_metabolites({k: round(v,6)})
for k, v in rna_coefficients.items():
    model.reactions.get_by_id('BIOMASS_RT').add_metabolites({k: round(v,6)})
```

In [94]:

```
# Remove existing protein, lipid, carbohydrate coefficient
for m, v in model.reactions.get_by_id('BIOMASS_RT').metabolites.items():
    if m in list({**protein_coefficients,**lipid_coefficients,**carb_coefficients}):
        model.reactions.get_by_id('BIOMASS_RT').add_metabolites({m: -v})
```

In [95]:

```
# Replace amino acids with aminoacyl-tRNA in protein coefficient
for k, v in protein_coefficients.copy().items():
    if not k.id == 'h2o_c':
        protein_coefficients.update({model.metabolites.get_by_id(k.id[0:3]+'trna_c'): v})
        protein_coefficients.pop(k)
```

In [96]:

```
# Add source reaction for tRNAs
for k, v in protein_coefficients.items():
    if not k.id == 'h2o_c':
        r = model.reactions.get_by_id('SRC_8aonn_m').copy()
        r.id = 'SRC_trna'+k.id[0:3]+'_c'
        r.name = model.metabolites.get_by_id('trna'+k.id[0:3]+'_c').name+' demand'
        model.add_reactions([r])
        r.add_metabolites({'8aonn_m': -1.0, 'trna'+k.id[0:3]+'_c': 1.0})
```

In [97]:

```
# Update protein coefficient
for k, v in protein_coefficients.items():
    model.reactions.get_by_id('BIOMASS_RT').add_metabolites({k: round(v,6)})
```

In [98]:

```
# Update lipid and carbohydrate coefficient
for k, v in lipid_coefficients.items():
    model.reactions.get_by_id('BIOMASS_RT').add_metabolites({k: round(v,6)})
for k, v in carb_coefficients.items():
    model.reactions.get_by_id('BIOMASS_RT').add_metabolites({k: round(v,6)})
```

In [99]:

```
for m, v in model.reactions.get_by_id('BIOMASS_RT').metabolites.items():
    if m in list({**dna_coefficients,**rna_coefficients,**protein_coefficients,**lipid_coefficients,
                      **carb_coefficients}):
        print(m.id, v)
print()
for m, v in model.reactions.get_by_id('BIOMASS_RT').metabolites.items():
    if m not in list({**dna_coefficients,**rna_coefficients,**protein_coefficients,**lipid_coefficients,
                      **carb_coefficients}):
        if not v == -1e-6:
            print(m.id, v)
print()
for m, v in model.reactions.get_by_id('BIOMASS_RT').metabolites.items():
    if m not in list({**dna_coefficients,**rna_coefficients,**protein_coefficients,**lipid_coefficients,
                      **carb_coefficients}):
        if v == -1e-6:
            print(m.id, v)
```

```
datp_c -0.002451
dttp_c -0.002523
dctp_c -0.004285
dgtp_c -0.003763
ppi_c 0.209364
atp_c -0.049247
utp_c -0.051129
ctp_c -0.044881
gtp_c -0.051085
h2o_c 3.919814
alatrna_c -0.577574
cystrna_c -0.034269
asptrna_c -0.197296
glutrna_c -0.208673
phetrna_c -0.095164
glytrna_c -0.51322
histrna_c -0.061167
iletrna_c -0.158569
lystrna_c -0.172334
leutrna_c -0.320205
mettrna_c -0.05705
asntrna_c -0.10797
protrna_c -0.235897
glntrna_c -0.117898
argtrna_c -0.146675
sertrna_c -0.33687
thrtrna_c -0.21813
valtrna_c -0.275168
trptrna_c -0.023319
tyrtrna_c -0.062363
pa_RT_r -1.4e-05
pc_RT_r -0.000172
pe_RT_r -0.00013
ps_RT_r -2e-05
ptd1ino_RT_r -4.7e-05
clpn_RT_m -1.8e-05
psphings_r -0.004006
triglyc_RT_r -0.000432
ergst_r -0.004031
zymst_r -0.004156
ergstest_RT_r -1.6e-05
zymstest_RT_d -1.7e-05
glycogen_c -0.518069
tre_c -0.023372
mannan_r -0.807941
13BDglcn_c -0.957502
16BDglcn_c -0.177315
chitin_c -0.031005

ribflv_c -0.00099
so4_c -0.02

5mthf_c -1e-06
btn_m -1e-06
camp_c -1e-06
coa_c -1e-06
fad_c -1e-06
gthrd_c -1e-06
hemeA_m -1e-06
lipopb_m -1e-06
mlthf_c -1e-06
nad_c -1e-06
nadp_c -1e-06
ptrc_c -1e-06
pydx5p_c -1e-06
q9_m -1e-06
spmd_c -1e-06
thf_c -1e-06
thmpp_c -1e-06
```

In [100]:

```
temp = model.reactions.get_by_id('BIOMASS_RT').check_mass_balance()
x = temp['C']
for k, v in temp.items():
    temp[k] = v/x
temp
```

Out[100]:

```
{'charge': 0.08862290756031035,
 'C': 1.0,
 'H': 1.5307670345966224,
 'N': 0.14287442070464182,
 'O': 0.437419043074669,
 'S': 0.0027601874968775104,
 'P': 0.008907180218982725,
 'Fe': 2.479395910062888e-08,
 'R': 0.09986463738029007}
```

In [101]:

```
# Remove so4_c, sulfur content seems to be enough
# C H1.769 N0.146O0.631 P0.009 S0.00162 M0.019 for S. cer
# https://onlinelibrary.wiley.com/doi/pdf/10.1002/bit.10054
# P content seems low, possible due to low DNA and RNA composition
model.reactions.get_by_id('BIOMASS_RT').add_metabolites({'so4_c': 0.02})
```

In [102]:

```
temp = model.reactions.get_by_id('BIOMASS_RT').check_mass_balance()
x = temp['C']
for k, v in temp.items():
    temp[k] = v/x
temp
```

Out[102]:

```
{'charge': 0.08961466592433551,
 'C': 1.0,
 'H': 1.5307670345966224,
 'N': 0.14287442070464182,
 'O': 0.43543552634661864,
 'S': 0.0022643083148649323,
 'P': 0.008907180218982725,
 'Fe': 2.479395910062888e-08,
 'R': 0.09986463738029007}
```

In [103]:

```
# S. cerevisiae https://onlinelibrary.wiley.com/doi/pdf/10.1002/bit.10054
# On average, the elements K, Mg, and Na made up 2.29, 0.15, and 0.06%, respectively, of the biomass.
# Ca, Fe, Mn, Cu, and Zn accounted for a total of 0.02%
# Add mg2, mn2, cu2, zn2 exchange and transport reactions from E. coli model
# Add K transporter from S. cer model
for x in ['mg2','mn2','cu2','zn2']:
    if not x+'_c' in model.metabolites:
        m = eco.metabolites.get_by_id(x+'_c').copy()
        model.add_metabolites([m])
    for y in eco.metabolites.get_by_id(x+'_e').reactions:
        r = y.copy()
        r.gene_reaction_rule = ''
        r.name = r.name.replace(' (extracellular to periplasm)','')
        model.add_reactions([r])
        if r.id.endswith('tex'):
            r.id = r.id.replace('tex','t')
            r.add_metabolites({x+'_p': -1.0, x+'_c': 1.0})
        print(r, r.name, r.gene_reaction_rule)
    model.remove_metabolites([model.metabolites.get_by_id(x+'_p')])

model.reactions.get_by_id('EX_k_e').lower_bound = -1000.0
r = sce.reactions.get_by_id('Kt2r').copy()
r.lower_bound = -1000.0
r.upper_bound = 1000.0
model.add_reactions([r])
model.reactions.get_by_id('EX_ca2_e').lower_bound = -1000.0
r = hsa2.reactions.get_by_id('CA2t').copy()
model.add_reactions([r])
model.reactions.get_by_id('EX_na1_e').lower_bound = -1000.0
```

```
EX_mg2_e: mg2_e <=>  Mg exchange 
MG2t: mg2_e <=> mg2_c Magnesium (Mg+2) transport via diffusion 
EX_mn2_e: mn2_e <=>  Mn2+ exchange 
MNt: mn2_e <=> mn2_c Manganese (Mn+2) transport via diffusion 
CU2t: cu2_e <=> cu2_c Copper (Cu+2) transport via diffusion 
EX_cu2_e: cu2_e <=>  Cu2+ exchange 
Zn2t: zn2_e <=> zn2_c Zinc (Zn+2) transport via diffusion 
EX_zn2_e: zn2_e <=>  Zinc exchange
```

In [104]:

```
# Assume equal composition for Ca2+, Fe2+, Fe3+, Mn2+, Cu2+, and Zn2+
metal_weight_fraction = {
    'k_c': 2.29/100,
    'mg2_c': 0.15/100,
    'na1_c': 0.06/100,
    'ca2_c': 0.02/100/6,
    'fe2_c': 0.02/100/6,
    'fe3_c': 0.02/100/6,
    'mn2_c': 0.02/100/6,
    'cu2_c': 0.02/100/6,
    'zn2_c': 0.02/100/6    
}
print(sum(metal_weight_fraction.values()))
```

```
0.0252
```

In [105]:

```
metal_coefficients = {}
for k, v in metal_weight_fraction.items():
    m = model.metabolites.get_by_id(k)
    metal_coefficients[m] = -v/m.formula_weight*1000
metal_coefficients
```

Out[105]:

```
{<Metabolite k_c at 0x101fbfbda0>: -0.5857032147177754,
 <Metabolite mg2_c at 0x1022b3d5f8>: -0.06171569635877392,
 <Metabolite na1_c at 0x101d2896a0>: -0.026098564709433805,
 <Metabolite ca2_c at 0x101fcbd860>: -0.0008317114959162965,
 <Metabolite fe2_c at 0x101d272208>: -0.0005968902020473335,
 <Metabolite fe3_c at 0x101fcbd0f0>: -0.0005968902020473335,
 <Metabolite mn2_c at 0x101c3a1e48>: -0.0006067440315059848,
 <Metabolite cu2_c at 0x101fc10630>: -0.0005245543910448074,
 <Metabolite zn2_c at 0x1022b35898>: -0.0005096138655740545}
```

In [106]:

```
for k, v in metal_coefficients.items():
    model.reactions.get_by_id('BIOMASS_RT').add_metabolites({k: round(v,6)})
```

In [107]:

```
fig, axes = plt.subplots(1,3, figsize=(12,4))

Data = pd.Series()
Data['DNA'] = dna_weight_fraction
Data['RNA'] = rna_weight_fraction
Data['Protein'] = protein_weight_fraction
Data['Lipid'] = lipid_weight_fraction
Data['Glycogen'] = 0.084
Data['Trehalose'] = 0.008
Data['Mannan'] = 0.131
Data['Glucan'] = 0.184
Data['Chitin'] = 0.0063
Data['Metal'] = 0.0252
#Data = Data/Data.sum()
print(Data.sum())
Data.plot.pie(title='Cell', ax=axes[0])
axes[0].set_ylabel('')

Data = FAME_fit['cell']/FAME_fit['cell'].sum()
Data.plot.pie(title='Cell Lipid', ax=axes[1])
axes[1].set_ylabel('')

#Data = FAME_fit['lipid_body'][FAME_fit['lipid_body'] > 0.01]
Data = FAME_fit['lipid_body']/FAME_fit['lipid_body'].sum()
Data.plot.pie(title='Lipid body', ax=axes[2])
axes[2].set_ylabel('')

plt.tight_layout()
plt.show()
```

```
0.9828
```

In [108]:

```
# The rest of the components and their mass fraction %
temp = 0
for m, v in model.reactions.get_by_id('BIOMASS_RT').metabolites.items():
    if m not in list({**dna_coefficients,**rna_coefficients,**protein_coefficients,**lipid_coefficients,
                      **carb_coefficients,**metal_coefficients}):
        print(m.id, v)
        temp = temp - m.formula_weight*v/1000
print()
print(temp)
```

```
5mthf_c -1e-06
btn_m -1e-06
camp_c -1e-06
coa_c -1e-06
fad_c -1e-06
gthrd_c -1e-06
hemeA_m -1e-06
lipopb_m -1e-06
mlthf_c -1e-06
nad_c -1e-06
nadp_c -1e-06
ptrc_c -1e-06
pydx5p_c -1e-06
q9_m -1e-06
ribflv_c -0.00099
spmd_c -1e-06
thf_c -1e-06
thmpp_c -1e-06

0.000380526416594
```

In [109]:

```
# Assume equal composition of cofactors and 1% total weight fraction
cofactors = ['camp_c','coa_c','fad_c','gthrd_c','hemeA_m','nad_c','nadp_c','q9_m','thf_c','thmpp_c',
             '5mthf_c','btn_m','lipopb_m','mlthf_c','ptrc_c','pydx5p_c','spmd_c']
cofactor_coefficients = dict()
for k in cofactors:
    m = model.metabolites.get_by_id(k)
    cofactor_coefficients[m] = -0.01/len(cofactors)/m.formula_weight*1000
cofactor_coefficients
```

Out[109]:

```
{<Metabolite camp_c at 0x101d29f5c0>: -0.0017923183332175356,
 <Metabolite coa_c at 0x101d25b358>: -0.0007704433183498177,
 <Metabolite fad_c at 0x101d2725c0>: -0.0007507464867136107,
 <Metabolite gthrd_c at 0x101d2683c8>: -0.001920357335176815,
 <Metabolite hemeA_m at 0x101fbf0e48>: -0.0006913735255247936,
 <Metabolite nad_c at 0x101d251e80>: -0.0008880133665945797,
 <Metabolite nadp_c at 0x101d243f60>: -0.0007945033013050617,
 <Metabolite q9_m at 0x101fe8d588>: -0.0007397078805998098,
 <Metabolite thf_c at 0x101d2724a8>: -0.0013266071203848922,
 <Metabolite thmpp_c at 0x101d2729b0>: -0.0013929633931000502,
 <Metabolite 5mthf_c at 0x101d2b67b8>: -0.0012831018430703267,
 <Metabolite btn_m at 0x101fcc85f8>: -0.0024177096847574943,
 <Metabolite lipopb_m at 0x101fe99b00>: -0.0031071245763754124,
 <Metabolite mlthf_c at 0x101d27ed30>: -0.0012916210881569782,
 <Metabolite ptrc_c at 0x101d2899e8>: -0.0065238163135490175,
 <Metabolite pydx5p_c at 0x101fbd9a90>: -0.0023997264252362017,
 <Metabolite spmd_c at 0x101d2727f0>: -0.0039673336727889825}
```

In [110]:

```
for k, v in cofactor_coefficients.items():
    v_old = model.reactions.get_by_id('BIOMASS_RT').get_coefficient(k)
    model.reactions.get_by_id('BIOMASS_RT').add_metabolites({k: -v_old})
    model.reactions.get_by_id('BIOMASS_RT').add_metabolites({k: round(v,6)})
```

In [111]:

```
temp = 0
for k, v in model.reactions.get_by_id('BIOMASS_RT').metabolites.items():
    if 'trna' in k.id:
        if 'gly' in k.id:
            temp = temp - model.metabolites.get_by_id(k.id[0:3]+'_c').formula_weight*v/1000
        else:
            temp = temp - model.metabolites.get_by_id(k.id[0:3]+'__L_c').formula_weight*v/1000
    else:
        temp = temp - k.formula_weight*v/1000
temp
```

Out[111]:

```
0.9888242707321281
```

In [112]:

```
sol = model.optimize()
print(sol.objective_value)
```

```
0.20995881730197788
```

Estimate ATP maintenance cost  
"Kinetics of continuous cultivation of the oleaginous yeast Rhodosporidium toruloides"  
https://www.ncbi.nlm.nih.gov/pubmed/23965273

In [113]:

```
model.reactions.get_by_id('ATPM').lower_bound = 0.0
model.reactions.get_by_id('ATPM').upper_bound = 1000.0
```

In [114]:

```
# maintenance cost 5.7 mg glucose / g cell mass / h
5.7/model.metabolites.glc__D_c.formula_weight
```

Out[114]:

```
0.0316392670613915
```

In [115]:

```
with model:
    model.reactions.get_by_id('EX_o2_e').lower_bound = -1000.0
    model.reactions.get_by_id('EX_glc__D_e').lower_bound = -0.0316392670613915
    model.objective = 'ATPM'
    sol = model.optimize()
    print(sol.objective_value)
    display(model.summary())
NGAM = round(sol.objective_value,2)
```

```
1.2233849930404712
```

|  | IN\_FLUXES | | OUT\_FLUXES | | OBJECTIVES | |
| --- | --- | --- | --- | --- | --- | --- |
|  | ID | FLUX | ID | FLUX | ID | FLUX |
| 0 | o2\_e | 0.189836 | co2\_e | 0.189836 | ATPM | 1.223385 |
| 1 | glc\_\_D\_e | 0.031639 | h2o\_e | 0.189836 | NaN | NaN |

In [116]:

```
# maximum cell mass yield on glucose 0.42 g cell / g glucose
# convert the number to g cell / mmol glucose
0.42*model.metabolites.glc__D_c.formula_weight/1000
```

Out[116]:

```
0.0756654696
```

In [117]:

```
# Glucose uptake range from "Kinetics of continuous cultivation of the oleaginous yeast Rhodosporidium toruloides"
print(0.05/model.metabolites.glc__D_c.formula_weight*1000)
print(0.445/model.metabolites.glc__D_c.formula_weight*1000)
```

```
0.27753743036308337
2.470083130231442
```

In [118]:

```
temp = np.arange(0.2, 2.5, 0.2)
GAM = []
for x in temp:
    with model:
        model.reactions.get_by_id('EX_o2_e').lower_bound = -1000.0
        model.reactions.get_by_id('EX_glc__D_e').lower_bound = -x
        model.reactions.get_by_id('BIOMASS_RT').lower_bound = 0.0756654696*x
        model.objective = 'ATPM'
        sol = model.optimize()
        GAM.append(sol.objective_value/0.0756654696/x)
plt.scatter(temp,GAM)
print(np.mean(GAM), np.std(GAM))
```

```
110.63239368442747 2.6828031603324685e-10
```

In [119]:

```
temp = np.arange(0.2, 2.5, 0.2)
y = []
GAM = round(np.mean(GAM), 6)
for x in temp:
    with model:
        model.reactions.get_by_id('EX_o2_e').lower_bound = -1000.0
        model.reactions.get_by_id('EX_glc__D_e').lower_bound = -x
        model.reactions.get_by_id('BIOMASS_RT').add_metabolites({'atp_c': -GAM, 'h2o_c': -GAM,
                                                                 'adp_c': GAM, 'h_c': GAM, 'pi_c': GAM})
        sol = model.optimize()
        y.append(sol.objective_value)
fit = np.polyfit(temp,y,1)
print(fit)
plt.plot(temp, y, 'o', temp, np.poly1d(fit)(temp), '--k')
```

```
[7.56654696e-02 4.72728327e-16]
```

Out[119]:

```
[<matplotlib.lines.Line2D at 0x1a2b1df9b0>,
 <matplotlib.lines.Line2D at 0x1a2b1dfb38>]
```

In [120]:

```
# Set non-growth associated ATP maintenance
model.reactions.get_by_id('ATPM').lower_bound = NGAM
model.reactions.get_by_id('ATPM').upper_bound = 1000.0
```

In [121]:

```
# Set growth associated ATP maintenance
model.reactions.get_by_id('BIOMASS_RT').add_metabolites({'atp_c': -GAM, 'h2o_c': -GAM,
                                                         'adp_c': GAM, 'h_c': GAM, 'pi_c': GAM})
```

In [122]:

```
model.reactions.get_by_id('EX_o2_e').lower_bound = -1000.0
model.reactions.get_by_id('EX_glc__D_e').lower_bound = -1.0
sol = model.optimize()
print(sol.objective_value)
```

```
0.07327809353115868
```

In [123]:

```
print(len(model.genes))
print(len(model.reactions))
print(len(model.metabolites))
model
```

```
1120
2168
1965
```

Out[123]:

|  |  |
| --- | --- |
| **Name** | R. toruloides |
| **Memory address** | 0x0101d2270f0 |
| **Number of metabolites** | 1965 |
| **Number of reactions** | 2168 |
| **Number of groups** | 0 |
| **Objective expression** | 1.0\*BIOMASS\_RT - 1.0\*BIOMASS\_RT\_reverse\_2b3e0 |
| **Compartments** | c, x, m, e, r, v, n, g, d |

In [124]:

```
for x in sorted(model.genes, key=lambda x: x.id):
    if not x.reactions:
        print(x)
print()
for x in sorted(model.metabolites, key=lambda x: x.id):
    if not x.reactions:
        print(x)
```

```

```

In [125]:

```
cobra.manipulation.remove_genes(model, [x for x in model.genes if not x.reactions])
model.remove_metabolites([x for x in model.metabolites if not x.reactions])
```

In [126]:

```
print(len(model.genes))
print(len(model.reactions))
print(len(model.metabolites))
print(len(set([m.id.rsplit('_',1)[0] for m in model.metabolites])))
print(len(model.compartments))
model
```

```
1120
2168
1965
1231
9
```

Out[126]:

|  |  |
| --- | --- |
| **Name** | R. toruloides |
| **Memory address** | 0x0101d2270f0 |
| **Number of metabolites** | 1965 |
| **Number of reactions** | 2168 |
| **Number of groups** | 0 |
| **Objective expression** | 1.0\*BIOMASS\_RT - 1.0\*BIOMASS\_RT\_reverse\_2b3e0 |
| **Compartments** | c, x, m, e, r, v, n, g, d |

In [127]:

```
cobra.io.save_json_model(model, "IFO0880_GPR_2c.json")
```

In [128]:

```
model_old = cobra.io.load_json_model("IFO0880_GPR_2b.json")
model_new = cobra.io.load_json_model("IFO0880_GPR_2c.json")
```

In [129]:

```
print('Removed reactions\n')
for r in sorted(model_old.reactions, key=lambda x: x.id):
    if r not in model_new.reactions:
        print(r)
```

```
Removed reactions

ARACHCPT1: arachcoa_c + crn_c --> arachcrn_c + coa_c
ARACHCPT2: arachcrn_m + coa_m --> arachcoa_m + crn_m
ARACHCRNt: arachcrn_c --> arachcrn_m
FAOXC240200x: 2.0 coa_x + 2.0 h2o_x + 2.0 nad_x + 2.0 o2_x + ttccoa_x --> 2.0 accoa_x + arachcoa_x + 2.0 h2o2_x + 2.0 h_x + 2.0 nadh_x
FATP1t: hdca_c + na1_c <=> hdca_e + na1_e
FATP2t: na1_c + ocdcea_c <=> na1_e + ocdcea_e
FATP3t: na1_c + ocdca_c <=> na1_e + ocdca_e
FATP4t: arach_c + na1_c <=> arach_e + na1_e
FATP8t: na1_c + ttc_c <=> na1_e + ttc_e
FATP9t: na1_c + nrvnc_c <=> na1_e + nrvnc_e
```

In [130]:

```
print('Updated reactions\n')
for r in sorted(model_old.reactions, key=lambda x: x.id):
    if r in model_new.reactions:
        r2 = model_new.reactions.get_by_id(r.id)
        if (r.name == r2.name and r.reaction == r2.reaction and r.gene_reaction_rule == r2.gene_reaction_rule and
            r.lower_bound == r2.lower_bound and r.upper_bound == r2.upper_bound):
            pass
        else:
            print('Old', r, r.gene_reaction_rule)
            print('New', r2, r2.gene_reaction_rule)
            print()
```

```
Updated reactions

Old AGATer_RT: 0.01 1ag3p_RT_r + 0.02 dcacoa_r + 0.06 ddcacoa_r + 0.17 hdcoa_r + 0.09 ocdycacoa_r + 0.24 odecoa_r + 0.27 pmtcoa_r + 0.05 stcoa_r + 0.1 tdcoa_r --> coa_r + 0.01 pa_RT_r 10427 or 16030 or 16779 or 9746
New AGATer_RT: 0.01 1ag3p_RT_r + 0.01 arachcoa_r + 0.02 hpdcacoa_r + 0.1 lnlncgcoa_r + 0.37 ocdycacoa_r + 0.32 odecoa_r + 0.09 pmtcoa_r + 0.07 stcoa_r + 0.02 ttccoa_r --> coa_r + 0.01 pa_RT_r 10427 or 16030 or 16779 or 9746

Old ATPM: atp_c + h2o_c --> adp_c + h_c + pi_c 
New ATPM: atp_c + h2o_c --> adp_c + h_c + pi_c 

Old BIOMASS_RT: 1.1348 13BDglcn_c + 1e-06 5mthf_c + 0.4588 ala__L_c + 0.046 amp_c + 0.1607 arg__L_c + 0.1017 asn__L_c + 0.2975 asp__L_c + 59.276 atp_c + 1e-06 btn_m + 1e-06 camp_c + 0.0447 cmp_c + 1e-06 coa_c + 0.0066 cys__L_c + 0.0036 damp_c + 0.0024 dcmp_c + 0.0024 dgmp_c + 0.0036 dtmp_c + 0.0007 ergst_r + 1e-06 fad_c + 0.1054 gln__L_c + 0.3018 glu__L_c + 0.2904 gly_c + 0.5185 glycogen_c + 0.046 gmp_c + 1e-06 gthrd_c + 59.276 h2o_c + 1e-06 hemeA_m + 0.0663 his__L_c + 0.1927 ile__L_c + 0.2964 leu__L_c + 1e-06 lipopb_m + 0.2862 lys__L_c + 0.8079 mannan_r + 0.0507 met__L_c + 1e-06 mlthf_c + 1e-06 nad_c + 1e-06 nadp_c + 6e-06 pa_RT_r + 6e-05 pc_RT_r + 4.5e-05 pe_RT_r + 0.1339 phe__L_c + 0.1647 pro__L_c + 1.7e-05 ps_RT_r + 5.3e-05 ptd1ino_RT_r + 1e-06 ptrc_c + 1e-06 pydx5p_c + 1e-06 q9_m + 0.00099 ribflv_c + 0.1854 ser__L_c + 0.02 so4_c + 1e-06 spmd_c + 1e-06 thf_c + 1e-06 thmpp_c + 0.1914 thr__L_c + 0.0234 tre_c + 6.6e-05 triglyc_RT_r + 0.0284 trp__L_c + 0.102 tyr__L_c + 0.0599 ump_c + 0.2646 val__L_c + 0.0015 zymst_r --> 59.276 adp_c + 58.70001 h_c + 59.305 pi_c 
New BIOMASS_RT: 0.957502 13BDglcn_c + 0.177315 16BDglcn_c + 0.001283 5mthf_c + 0.577574 alatrna_c + 0.146675 argtrna_c + 0.10797 asntrna_c + 0.197296 asptrna_c + 110.681641 atp_c + 0.002418 btn_m + 0.000832 ca2_c + 0.001792 camp_c + 0.031005 chitin_c + 1.8e-05 clpn_RT_m + 0.00077 coa_c + 0.044881 ctp_c + 0.000525 cu2_c + 0.034269 cystrna_c + 0.002451 datp_c + 0.004285 dctp_c + 0.003763 dgtp_c + 0.002523 dttp_c + 0.004031 ergst_r + 1.6e-05 ergstest_RT_r + 0.000751 fad_c + 0.000597 fe2_c + 0.000597 fe3_c + 0.117898 glntrna_c + 0.208673 glutrna_c + 0.518069 glycogen_c + 0.51322 glytrna_c + 0.00192 gthrd_c + 0.051085 gtp_c + 106.71258 h2o_c + 0.000691 hemeA_m + 0.061167 histrna_c + 0.158569 iletrna_c + 0.585703 k_c + 0.320205 leutrna_c + 0.003107 lipopb_m + 0.172334 lystrna_c + 0.807941 mannan_r + 0.05705 mettrna_c + 0.061716 mg2_c + 0.001292 mlthf_c + 0.000607 mn2_c + 0.026099 na1_c + 0.000888 nad_c + 0.000795 nadp_c + 1.4e-05 pa_RT_r + 0.000172 pc_RT_r + 0.00013 pe_RT_r + 0.095164 phetrna_c + 0.235897 protrna_c + 2e-05 ps_RT_r + 0.004006 psphings_r + 4.7e-05 ptd1ino_RT_r + 0.006524 ptrc_c + 0.0024 pydx5p_c + 0.00074 q9_m + 0.00099 ribflv_c + 0.33687 sertrna_c + 0.003967 spmd_c + 0.001327 thf_c + 0.001393 thmpp_c + 0.21813 thrtrna_c + 0.023372 tre_c + 0.000432 triglyc_RT_r + 0.023319 trptrna_c + 0.062363 tyrtrna_c + 0.051129 utp_c + 0.275168 valtrna_c + 0.00051 zn2_c + 0.004156 zymst_r + 1.7e-05 zymstest_RT_d --> 110.632394 adp_c + 110.632394 h_c + 110.632394 pi_c + 0.209364 ppi_c 

Old DAGL_RT: 0.01 12dgr_RT_d + h2o_c --> 0.02 dca_c + 0.06 ddca_c + h_c + 0.27 hdca_c + 0.17 hdcea_c + 0.01 mag_RT_d + 0.05 ocdca_c + 0.24 ocdcea_c + 0.09 ocdcya_c + 0.1 ttdca_c 11739 or 15065
New DAGL_RT: 0.01 12dgr_RT_d + h2o_c --> h_c + 0.23 hdca_c + 0.01 hdcea_c + 0.01 lnlncg_c + 0.01 mag_RT_d + 0.12 ocdca_c + 0.48 ocdcea_c + 0.14 ocdcya_c + 0.01 ttc_c 11739 or 15065

Old EPISTATer_RT: 0.01 epist_r + 0.655 hdcoa_r + 0.01 hexccoa_r + 0.27 odecoa_r + 0.02 pmtcoa_r + 0.03 stcoa_r + 0.015 tdcoa_r --> coa_r + 0.01 epistest_RT_r 11799
New EPISTATer_RT: epist_r + 0.01 hdcoa_r + 0.01 lnlncgcoa_r + 0.14 ocdycacoa_r + 0.48 odecoa_r + 0.23 pmtcoa_r + 0.12 stcoa_r + 0.01 ttccoa_r --> coa_r + 0.01 epistest_RT_r 11799

Old EPISTESTH_RT: 0.01 epistest_RT_d + h2o_c --> 0.01 epist_c + h_c + 0.02 hdca_c + 0.655 hdcea_c + 0.01 hexc_c + 0.03 ocdca_c + 0.27 ocdcea_c + 0.015 ttdca_c 14247 or 14617
New EPISTESTH_RT: 0.01 epistest_RT_d + h2o_c --> epist_c + h_c + 0.23 hdca_c + 0.01 hdcea_c + 0.01 lnlncg_c + 0.12 ocdca_c + 0.48 ocdcea_c + 0.14 ocdcya_c + 0.01 ttc_c 14247 or 14617

Old ERGSTATer_RT: 0.01 ergst_r + 0.655 hdcoa_r + 0.01 hexccoa_r + 0.27 odecoa_r + 0.02 pmtcoa_r + 0.03 stcoa_r + 0.015 tdcoa_r --> coa_r + 0.01 ergstest_RT_r 11799
New ERGSTATer_RT: ergst_r + 0.01 hdcoa_r + 0.01 lnlncgcoa_r + 0.14 ocdycacoa_r + 0.48 odecoa_r + 0.23 pmtcoa_r + 0.12 stcoa_r + 0.01 ttccoa_r --> coa_r + 0.01 ergstest_RT_r 11799

Old ERGSTESTH_RT: 0.01 ergstest_RT_d + h2o_c --> 0.01 ergst_c + h_c + 0.02 hdca_c + 0.655 hdcea_c + 0.01 hexc_c + 0.03 ocdca_c + 0.27 ocdcea_c + 0.015 ttdca_c 14247 or 14617
New ERGSTESTH_RT: 0.01 ergstest_RT_d + h2o_c --> ergst_c + h_c + 0.23 hdca_c + 0.01 hdcea_c + 0.01 lnlncg_c + 0.12 ocdca_c + 0.48 ocdcea_c + 0.14 ocdcya_c + 0.01 ttc_c 14247 or 14617

Old EX_ca2_e: ca2_e -->  
New EX_ca2_e: ca2_e <=>  

Old EX_glc__D_e: glc__D_e <=>  
New EX_glc__D_e: glc__D_e <=>  

Old EX_k_e: k_e -->  
New EX_k_e: k_e <=>  

Old EX_na1_e: na1_e -->  
New EX_na1_e: na1_e <=>  

Old EX_o2_e: o2_e <=>  
New EX_o2_e: o2_e <=>  

Old FAS240COAer: 9.0 h_r + 3.0 malcoa_r + 6.0 nadph_r + stcoa_r --> 3.0 co2_r + 3.0 coa_r + 3.0 h2o_r + 6.0 nadp_r + ttccoa_r (10677 and 11343 and 16241 and 16695) or (10677 and 16241 and 16655 and 16695)
New FAS240COAer: docoscoa_r + 3.0 h_r + malcoa_r + 2.0 nadph_r --> co2_r + coa_r + h2o_r + 2.0 nadp_r + ttccoa_r (10677 and 11343 and 16241 and 16695) or (10677 and 16241 and 16655 and 16695)

Old FECOSTATer_RT: 0.01 fecost_r + 0.655 hdcoa_r + 0.01 hexccoa_r + 0.27 odecoa_r + 0.02 pmtcoa_r + 0.03 stcoa_r + 0.015 tdcoa_r --> coa_r + 0.01 fecostest_RT_r 11799
New FECOSTATer_RT: fecost_r + 0.01 hdcoa_r + 0.01 lnlncgcoa_r + 0.14 ocdycacoa_r + 0.48 odecoa_r + 0.23 pmtcoa_r + 0.12 stcoa_r + 0.01 ttccoa_r --> coa_r + 0.01 fecostest_RT_r 11799

Old FECOSTESTH_RT: 0.01 fecostest_RT_d + h2o_c --> 0.01 fecost_c + h_c + 0.02 hdca_c + 0.655 hdcea_c + 0.01 hexc_c + 0.03 ocdca_c + 0.27 ocdcea_c + 0.015 ttdca_c 14247 or 14617
New FECOSTESTH_RT: 0.01 fecostest_RT_d + h2o_c --> fecost_c + h_c + 0.23 hdca_c + 0.01 hdcea_c + 0.01 lnlncg_c + 0.12 ocdca_c + 0.48 ocdcea_c + 0.14 ocdcya_c + 0.01 ttc_c 14247 or 14617

Old GAT1er_RT: 0.02 dcacoa_r + 0.06 ddcacoa_r + glyc3p_r + 0.17 hdcoa_r + 0.09 ocdycacoa_r + 0.24 odecoa_r + 0.27 pmtcoa_r + 0.05 stcoa_r + 0.1 tdcoa_r --> 0.01 1ag3p_RT_r + coa_r 15435
New GAT1er_RT: 0.01 arachcoa_r + glyc3p_r + 0.02 hpdcacoa_r + 0.1 lnlncgcoa_r + 0.37 ocdycacoa_r + 0.32 odecoa_r + 0.09 pmtcoa_r + 0.07 stcoa_r + 0.02 ttccoa_r --> 0.01 1ag3p_RT_r + coa_r 15435

Old GAT2er_RT: 0.02 dcacoa_r + 0.06 ddcacoa_r + dhap_r + 0.17 hdcoa_r + 0.09 ocdycacoa_r + 0.24 odecoa_r + 0.27 pmtcoa_r + 0.05 stcoa_r + 0.1 tdcoa_r --> 0.01 1agly3p_RT_r + coa_r 13369 or 15435
New GAT2er_RT: 0.01 arachcoa_r + dhap_r + 0.02 hpdcacoa_r + 0.1 lnlncgcoa_r + 0.37 ocdycacoa_r + 0.32 odecoa_r + 0.09 pmtcoa_r + 0.07 stcoa_r + 0.02 ttccoa_r --> 0.01 1agly3p_RT_r + coa_r 13369 or 15435

Old LANOSTATer_RT: 0.655 hdcoa_r + 0.01 hexccoa_r + 0.01 lanost_r + 0.27 odecoa_r + 0.02 pmtcoa_r + 0.03 stcoa_r + 0.015 tdcoa_r --> coa_r + 0.01 lanostest_RT_r 11799
New LANOSTATer_RT: 0.01 hdcoa_r + lanost_r + 0.01 lnlncgcoa_r + 0.14 ocdycacoa_r + 0.48 odecoa_r + 0.23 pmtcoa_r + 0.12 stcoa_r + 0.01 ttccoa_r --> coa_r + 0.01 lanostest_RT_r 11799

Old LANOSTESTH_RT: h2o_c + 0.01 lanostest_RT_d --> h_c + 0.02 hdca_c + 0.655 hdcea_c + 0.01 hexc_c + 0.01 lanost_c + 0.03 ocdca_c + 0.27 ocdcea_c + 0.015 ttdca_c 14247 or 14617
New LANOSTESTH_RT: h2o_c + 0.01 lanostest_RT_d --> h_c + 0.23 hdca_c + 0.01 hdcea_c + lanost_c + 0.01 lnlncg_c + 0.12 ocdca_c + 0.48 ocdcea_c + 0.14 ocdcya_c + 0.01 ttc_c 14247 or 14617

Old LPCATer_RT: 0.01 1agpc_RT_r + 0.02 dcacoa_r + 0.06 ddcacoa_r + 0.17 hdcoa_r + 0.09 ocdycacoa_r + 0.24 odecoa_r + 0.27 pmtcoa_r + 0.05 stcoa_r + 0.1 tdcoa_r --> coa_r + 0.01 pc_RT_r 16030
New LPCATer_RT: 0.01 1agpc_RT_r + 0.01 arachcoa_r + 0.02 hpdcacoa_r + 0.1 lnlncgcoa_r + 0.37 ocdycacoa_r + 0.32 odecoa_r + 0.09 pmtcoa_r + 0.07 stcoa_r + 0.02 ttccoa_r --> coa_r + 0.01 pc_RT_r 16030

Old MAGL_RT: h2o_c + 0.01 mag_RT_d --> 0.02 dca_c + 0.06 ddca_c + glyc_c + h_c + 0.27 hdca_c + 0.17 hdcea_c + 0.05 ocdca_c + 0.24 ocdcea_c + 0.09 ocdcya_c + 0.1 ttdca_c 14158 or 15065 or 9728
New MAGL_RT: h2o_c + 0.01 mag_RT_d --> glyc_c + h_c + 0.23 hdca_c + 0.01 hdcea_c + 0.01 lnlncg_c + 0.12 ocdca_c + 0.48 ocdcea_c + 0.14 ocdcya_c + 0.01 ttc_c 14158 or 15065 or 9728

Old PLBP1Ie_RT: h2o_e + 0.005 ptd1ino_RT_e --> 0.02 dca_e + 0.06 ddca_e + 0.5 g3pi_e + h_e + 0.27 hdca_e + 0.17 hdcea_e + 0.05 ocdca_e + 0.24 ocdcea_e + 0.09 ocdcya_e + 0.1 ttdca_e 12385
New PLBP1Ie_RT: h2o_e + 0.005 ptd1ino_RT_e --> 0.01 arach_e + 0.5 g3pi_e + h_e + 0.09 hdca_e + 0.02 hpdca_e + 0.1 lnlncg_e + 0.07 ocdca_e + 0.32 ocdcea_e + 0.37 ocdcya_e + 0.02 ttc_e 12385

Old PLBPC_RT: h2o_c + 0.005 pc_RT_r --> 0.02 dca_c + 0.06 ddca_c + 0.5 g3pc_c + h_c + 0.27 hdca_c + 0.17 hdcea_c + 0.05 ocdca_c + 0.24 ocdcea_c + 0.09 ocdcya_c + 0.1 ttdca_c 14309
New PLBPC_RT: h2o_c + 0.005 pc_RT_r --> 0.01 arach_c + 0.5 g3pc_c + h_c + 0.09 hdca_c + 0.02 hpdca_c + 0.1 lnlncg_c + 0.07 ocdca_c + 0.32 ocdcea_c + 0.37 ocdcya_c + 0.02 ttc_c 14309

Old PLBPCe_RT: h2o_e + 0.005 pc_RT_e --> 0.02 dca_e + 0.06 ddca_e + 0.5 g3pc_e + h_e + 0.27 hdca_e + 0.17 hdcea_e + 0.05 ocdca_e + 0.24 ocdcea_e + 0.09 ocdcya_e + 0.1 ttdca_e 12385
New PLBPCe_RT: h2o_e + 0.005 pc_RT_e --> 0.01 arach_e + 0.5 g3pc_e + h_e + 0.09 hdca_e + 0.02 hpdca_e + 0.1 lnlncg_e + 0.07 ocdca_e + 0.32 ocdcea_e + 0.37 ocdcya_e + 0.02 ttc_e 12385

Old PLBPEe_RT: h2o_e + 0.005 pe_RT_e --> 0.02 dca_e + 0.06 ddca_e + 0.5 g3pe_e + h_e + 0.27 hdca_e + 0.17 hdcea_e + 0.05 ocdca_e + 0.24 ocdcea_e + 0.09 ocdcya_e + 0.1 ttdca_e 12385
New PLBPEe_RT: h2o_e + 0.005 pe_RT_e --> 0.01 arach_e + 0.5 g3pe_e + h_e + 0.09 hdca_e + 0.02 hpdca_e + 0.1 lnlncg_e + 0.07 ocdca_e + 0.32 ocdcea_e + 0.37 ocdcya_e + 0.02 ttc_e 12385

Old TAGL_RT: h2o_c + 0.01 triglyc_RT_d --> 0.01 12dgr_RT_d + 0.02 dca_c + 0.06 ddca_c + h_c + 0.27 hdca_c + 0.17 hdcea_c + 0.05 ocdca_c + 0.24 ocdcea_c + 0.09 ocdcya_c + 0.1 ttdca_c 10393 or 14317 or 14617 or 15065 or 9746
New TAGL_RT: h2o_c + 0.01 triglyc_RT_d --> 0.01 12dgr_RT_d + h_c + 0.23 hdca_c + 0.01 hdcea_c + 0.01 lnlncg_c + 0.12 ocdca_c + 0.48 ocdcea_c + 0.14 ocdcya_c + 0.01 ttc_c 10393 or 14317 or 14617 or 15065 or 9746

Old TAGtrd: triglyc_RT_r --> triglyc_RT_d 
New TAGtrd: 0.03 hdcoa_r + 0.48 odecoa_r + 0.42 pmtcoa_r + 0.15 stcoa_r + 0.01 triglyc_RT_r --> 0.03 arachcoa_r + 0.06 hpdcacoa_r + 0.27 lnlncgcoa_r + 0.69 ocdycacoa_r + 0.01 triglyc_RT_d + 0.03 ttccoa_r 

Old TRIGSer_RT: 0.01 12dgr_RT_r + 0.02 dcacoa_r + 0.06 ddcacoa_r + 0.17 hdcoa_r + 0.09 ocdycacoa_r + 0.24 odecoa_r + 0.27 pmtcoa_r + 0.05 stcoa_r + 0.1 tdcoa_r --> coa_r + 0.01 triglyc_RT_r 16460
New TRIGSer_RT: 0.01 12dgr_RT_r + 0.01 arachcoa_r + 0.02 hpdcacoa_r + 0.1 lnlncgcoa_r + 0.37 ocdycacoa_r + 0.32 odecoa_r + 0.09 pmtcoa_r + 0.07 stcoa_r + 0.02 ttccoa_r --> coa_r + 0.01 triglyc_RT_r 16460

Old ZYMSTATer_RT: 0.655 hdcoa_r + 0.01 hexccoa_r + 0.27 odecoa_r + 0.02 pmtcoa_r + 0.03 stcoa_r + 0.015 tdcoa_r + 0.01 zymst_r --> coa_r + 0.01 zymstest_RT_r 11799
New ZYMSTATer_RT: 0.01 hdcoa_r + 0.01 lnlncgcoa_r + 0.14 ocdycacoa_r + 0.48 odecoa_r + 0.23 pmtcoa_r + 0.12 stcoa_r + 0.01 ttccoa_r + zymst_r --> coa_r + 0.01 zymstest_RT_r 11799

Old ZYMSTESTH_RT: h2o_c + 0.01 zymstest_RT_d --> h_c + 0.02 hdca_c + 0.655 hdcea_c + 0.01 hexc_c + 0.03 ocdca_c + 0.27 ocdcea_c + 0.015 ttdca_c + 0.01 zymst_c 14247 or 14617
New ZYMSTESTH_RT: h2o_c + 0.01 zymstest_RT_d --> h_c + 0.23 hdca_c + 0.01 hdcea_c + 0.01 lnlncg_c + 0.12 ocdca_c + 0.48 ocdcea_c + 0.14 ocdcya_c + 0.01 ttc_c + zymst_c 14247 or 14617
```

In [131]:

```
print('Added reactions\n')
for r in sorted(model_new.reactions, key=lambda x: x.id):
    if r not in model_old.reactions:
        print(r)
```

```
Added reactions

ARACHt: arach_e --> arach_c
CA2t: ca2_e <=> ca2_c
CU2t: cu2_e <=> cu2_c
DOCOSACt: docosac_e --> docosac_c
EPISTtr: epist_c <=> epist_r
ERGSTtr: ergst_c <=> ergst_r
EX_cu2_e: cu2_e <=> 
EX_mg2_e: mg2_e <=> 
EX_mn2_e: mn2_e <=> 
EX_zn2_e: zn2_e <=> 
FA200tp: arach_c --> arach_x
FA220tp: docosac_c --> docosac_x
FACOAL200p: arach_x + atp_x + coa_x --> amp_x + arachcoa_x + ppi_x
FACOAL220p: atp_x + coa_x + docosac_x --> amp_x + docoscoa_x + ppi_x
FAOXC22C20x: coa_x + docoscoa_x + h2o_x + nad_x + o2_x --> accoa_x + arachcoa_x + h2o2_x + h_x + nadh_x
FAOXC24C22x: coa_x + h2o_x + nad_x + o2_x + ttccoa_x --> accoa_x + docoscoa_x + h2o2_x + h_x + nadh_x
FAS170COA_L: 21.0 h_c + 7.0 malcoa_c + 14.0 nadph_c + ppcoa_c --> 7.0 co2_c + 7.0 coa_c + 7.0 h2o_c + hpdcacoa_c + 14.0 nadp_c
FAS200COAer: 3.0 h_r + malcoa_r + 2.0 nadph_r + stcoa_r --> arachcoa_r + co2_r + coa_r + h2o_r + 2.0 nadp_r
FAS220COAer: arachcoa_r + 3.0 h_r + malcoa_r + 2.0 nadph_r --> co2_r + coa_r + docoscoa_r + h2o_r + 2.0 nadp_r
FECOSTtr: fecost_c <=> fecost_r
HDCAt: hdca_e --> hdca_c
HDCEAt: hdcea_e --> hdcea_c
HPDCACOAtr: hpdcacoa_c <=> hpdcacoa_r
HPDCAt: hpdca_e --> hpdca_c
Kt2r: h_e + k_e <=> h_c + k_c
LANOSTtr: lanost_c <=> lanost_r
LNLNCGt: lnlncg_e --> lnlncg_c
MG2t: mg2_e <=> mg2_c
MNt: mn2_e <=> mn2_c
OCDCAt: ocdca_e --> ocdca_c
OCDCEAt: ocdcea_e --> ocdcea_c
OCDCYAt: ocdcya_e --> ocdcya_c
SK_epistest_RT_d: epistest_RT_d --> 
SK_ergstest_RT_d: ergstest_RT_d --> 
SK_fecostest_RT_d: fecostest_RT_d --> 
SK_lanostest_RT_d: lanostest_RT_d --> 
SK_triglyc_RT_d: triglyc_RT_d --> 
SK_zymstest_RT_d: zymstest_RT_d --> 
SRC_trnaala_c:  --> trnaala_c
SRC_trnaarg_c:  --> trnaarg_c
SRC_trnaasn_c:  --> trnaasn_c
SRC_trnaasp_c:  --> trnaasp_c
SRC_trnacys_c:  --> trnacys_c
SRC_trnagln_c:  --> trnagln_c
SRC_trnaglu_c:  --> trnaglu_c
SRC_trnagly_c:  --> trnagly_c
SRC_trnahis_c:  --> trnahis_c
SRC_trnaile_c:  --> trnaile_c
SRC_trnaleu_c:  --> trnaleu_c
SRC_trnalys_c:  --> trnalys_c
SRC_trnamet_c:  --> trnamet_c
SRC_trnaphe_c:  --> trnaphe_c
SRC_trnapro_c:  --> trnapro_c
SRC_trnaser_c:  --> trnaser_c
SRC_trnathr_c:  --> trnathr_c
SRC_trnatrp_c:  --> trnatrp_c
SRC_trnatyr_c:  --> trnatyr_c
SRC_trnaval_c:  --> trnaval_c
TTCt: ttc_e --> ttc_c
ZYMSTtr: zymst_c <=> zymst_r
Zn2t: zn2_e <=> zn2_c
```
