# Supplementary material for: Multi-Omics Driven Metabolic Network Reconstruction and Analysis of Lignocellulosic Carbon Utilization in Rhodosporidium toruloides
Source: Front Bioeng Biotechnol. 2021 Jan 8;8:612832. doi: 10.3389/fbioe.2020.612832 (PMC7873862; doi:10.3389/fbioe.2020.612832)
Supplement: Supplementary File 4 — Multi-omics dataset for R. toruloides IFO0880. [file Data_Sheet_1.zip › Supplementary File S1/0.Annotation_and_draft_reconstruction/Draft_Reconstruction_from_OrthoMCL.html]

Draft\_Reconstruction\_from\_OrthoMCL


In [1]:

```
%matplotlib inline
from matplotlib import pyplot as plt
import csv
import numpy as np
import pandas as pd
import json
import urllib
import cobra
```

In [2]:

```
cobra.__version__
```

Out[2]:

```
'0.13.4'
```

In [3]:

```
json.load(urllib.request.urlopen('http://bigg.ucsd.edu/api/v2/database_version'))
```

Out[3]:

```
{'bigg_models_version': '1.3.0',
 'api_version': 'v2',
 'last_updated': '2018-02-24 08:16:33.924054'}
```

In [6]:

```
with open("../../Data/OrthoMCL/groups.txt",'r') as f:
    reader = csv.reader(f,delimiter=':')
    line = next(reader)
    print(line)
```

```
['OG1000', ' cre|CRv4_Au5_s10_g545_t1 cre|CRv4_Au5_s10_g546_t1 cre|CRv4_Au5_s10_g547_t1 cre|CRv4_Au5_s10_g549_t1 cre|CRv4_Au5_s10_g549_t2 cre|CRv4_Au5_s10_g552_t1 cre|CRv4_Au5_s11_g2472_t1 cre|CRv4_Au5_s11_g2473_t1 cre|CRv4_Au5_s11_g2474_t1 cre|CRv4_Au5_s12_g3847_t1 cre|CRv4_Au5_s12_g3848_t1 cre|CRv4_Au5_s12_g3850_t1 cre|CRv4_Au5_s12_g3851_t1 cre|CRv4_Au5_s12_g3852_t1 cre|CRv4_Au5_s12_g3853_t1 cre|CRv4_Au5_s12_g3854_t1 cre|CRv4_Au5_s12_g3855_t1 cre|CRv4_Au5_s12_g3856_t1 cre|CRv4_Au5_s12_g3857_t1 cre|CRv4_Au5_s12_g4295_t1 cre|CRv4_Au5_s12_g4296_t1 cre|CRv4_Au5_s12_g4297_t1 cre|CRv4_Au5_s13_g4914_t1 cre|CRv4_Au5_s13_g5092_t1 cre|CRv4_Au5_s13_g5096_t1 cre|CRv4_Au5_s13_g5097_t1 cre|CRv4_Au5_s13_g5098_t1 cre|CRv4_Au5_s13_g5099_t1 cre|CRv4_Au5_s13_g5100_t1 cre|CRv4_Au5_s13_g5102_t1 cre|CRv4_Au5_s13_g5103_t1 cre|CRv4_Au5_s13_g5104_t1 cre|CRv4_Au5_s14_g5506_t1 cre|CRv4_Au5_s14_g5506_t2 cre|CRv4_Au5_s14_g5507_t1 cre|CRv4_Au5_s14_g5509_t1 cre|CRv4_Au5_s14_g5510_t1 cre|CRv4_Au5_s14_g5511_t1 cre|CRv4_Au5_s16_g6392_t1 cre|CRv4_Au5_s16_g6393_t1 cre|CRv4_Au5_s16_g6394_t1 cre|CRv4_Au5_s16_g6395_t1 cre|CRv4_Au5_s16_g6396_t1 cre|CRv4_Au5_s16_g6397_t1 cre|CRv4_Au5_s17_g7902_t1 cre|CRv4_Au5_s17_g7904_t1 cre|CRv4_Au5_s17_g7905_t1 cre|CRv4_Au5_s17_g7906_t1 cre|CRv4_Au5_s17_g7907_t1 cre|CRv4_Au5_s17_g7908_t1 cre|CRv4_Au5_s17_g7909_t1 cre|CRv4_Au5_s17_g7910_t1 cre|CRv4_Au5_s1_g1098_t1 cre|CRv4_Au5_s1_g1099_t1 cre|CRv4_Au5_s1_g1100_t1 cre|CRv4_Au5_s1_g1101_t1 cre|CRv4_Au5_s1_g1103_t1 cre|CRv4_Au5_s1_g2216_t1 cre|CRv4_Au5_s20_g8269_t1 cre|CRv4_Au5_s20_g8269_t2 cre|CRv4_Au5_s21_g9780_t1 cre|CRv4_Au5_s25_g9964_t1 cre|CRv4_Au5_s25_g9965_t1 cre|CRv4_Au5_s25_g9966_t1 cre|CRv4_Au5_s2_g8659_t1 cre|CRv4_Au5_s2_g8660_t1 cre|CRv4_Au5_s2_g8661_t1 cre|CRv4_Au5_s2_g8664_t1 cre|CRv4_Au5_s2_g8665_t1 cre|CRv4_Au5_s2_g8666_t1 cre|CRv4_Au5_s2_g9536_t1 cre|CRv4_Au5_s2_g9537_t1 cre|CRv4_Au5_s2_g9539_t1 cre|CRv4_Au5_s2_g9540_t1 cre|CRv4_Au5_s2_g9541_t1 cre|CRv4_Au5_s2_g9543_t1 cre|CRv4_Au5_s2_g9544_t1 cre|CRv4_Au5_s2_g9545_t1 cre|CRv4_Au5_s2_g9546_t1 cre|CRv4_Au5_s30_g10111_t1 cre|CRv4_Au5_s30_g10112_t1 cre|CRv4_Au5_s30_g10113_t1 cre|CRv4_Au5_s30_g10114_t1 cre|CRv4_Au5_s30_g10115_t1 cre|CRv4_Au5_s30_g10117_t1 cre|CRv4_Au5_s30_g10118_t1 cre|CRv4_Au5_s30_g10119_t1 cre|CRv4_Au5_s37_g11472_t1 cre|CRv4_Au5_s37_g11477_t1 cre|CRv4_Au5_s37_g11478_t1 cre|CRv4_Au5_s3_g11106_t1 cre|CRv4_Au5_s3_g11106_t2 cre|CRv4_Au5_s3_g11302_t1 cre|CRv4_Au5_s3_g11303_t1 cre|CRv4_Au5_s3_g11306_t1 cre|CRv4_Au5_s3_g11307_t1 cre|CRv4_Au5_s3_g11308_t1 cre|CRv4_Au5_s3_g11310_t1 cre|CRv4_Au5_s3_g11311_t1 cre|CRv4_Au5_s3_g11314_t1 cre|CRv4_Au5_s3_g11315_t1 cre|CRv4_Au5_s40_g11500_t1 cre|CRv4_Au5_s40_g11501_t1 cre|CRv4_Au5_s40_g11502_t1 cre|CRv4_Au5_s40_g11503_t1 cre|CRv4_Au5_s47_g11927_t1 cre|CRv4_Au5_s47_g11928_t1 cre|CRv4_Au5_s47_g11929_t1 cre|CRv4_Au5_s4_g11601_t1 cre|CRv4_Au5_s51_g12317_t1 cre|CRv4_Au5_s51_g12318_t1 cre|CRv4_Au5_s51_g12319_t1 cre|CRv4_Au5_s51_g12320_t1 cre|CRv4_Au5_s51_g12321_t1 cre|CRv4_Au5_s51_g12322_t1 cre|CRv4_Au5_s51_g12323_t1 cre|CRv4_Au5_s51_g12324_t1 cre|CRv4_Au5_s51_g12325_t1 cre|CRv4_Au5_s52_g12326_t1 cre|CRv4_Au5_s52_g12327_t1 cre|CRv4_Au5_s52_g12328_t1 cre|CRv4_Au5_s52_g12329_t1 cre|CRv4_Au5_s52_g12330_t1 cre|CRv4_Au5_s53_g12331_t1 cre|CRv4_Au5_s53_g12332_t1 cre|CRv4_Au5_s54_g12335_t1 cre|CRv4_Au5_s54_g12336_t1 cre|CRv4_Au5_s55_g12337_t1 cre|CRv4_Au5_s55_g12338_t1 cre|CRv4_Au5_s55_g12340_t1 cre|CRv4_Au5_s5_g12041_t1 cre|CRv4_Au5_s5_g12041_t2 cre|CRv4_Au5_s5_g12043_t1 cre|CRv4_Au5_s5_g12044_t1 cre|CRv4_Au5_s5_g12044_t2 cre|CRv4_Au5_s5_g12045_t1 cre|CRv4_Au5_s5_g12046_t1 cre|CRv4_Au5_s5_g12048_t1 cre|CRv4_Au5_s5_g12049_t1 cre|CRv4_Au5_s63_g13646_t1 cre|CRv4_Au5_s63_g13647_t1 cre|CRv4_Au5_s6_g12956_t1 cre|CRv4_Au5_s6_g12958_t1 cre|CRv4_Au5_s6_g12959_t1 cre|CRv4_Au5_s6_g12961_t1 cre|CRv4_Au5_s7_g14102_t1 cre|CRv4_Au5_s7_g14103_t1 cre|CRv4_Au5_s7_g14109_t1 cre|CRv4_Au5_s7_g14110_t1 cre|CRv4_Au5_s7_g14111_t1 cre|CRv4_Au5_s7_g14112_t1 cre|CRv4_Au5_s7_g14113_t1 cre|CRv4_Au5_s7_g14114_t1 cre|CRv4_Au5_s85_g15194_t1 cre|CRv4_Au5_s85_g15195_t1 cre|CRv4_Au5_s8_g14929_t1 cre|CRv4_Au5_s8_g14930_t1 cre|CRv4_Au5_s8_g14931_t1 cre|CRv4_Au5_s14_g5508_t1 cre|CRv4_Au5_s1_g1102_t1 cre|CRv4_Au5_s12_g3849_t1 cre|CRv4_Au5_s3_g11111_t1 cre|CRv4_Au5_s13_g5164_t1']
```

In [4]:

```
groups = dict()
with open("../../Data/OrthoMCL/groups.txt",'r') as f:
    for line in csv.reader(f,delimiter=':'):
        x = line[0]
        y = [z.strip() for z in line[1].split(' ') if z and not 'rton' in z]
        if any('rtoi|' in z for z in y) and len(y) > 1:
            groups[x] = y
```

In [5]:

```
orthologs = dict()
for v in groups.values():
    x = [z for z in v if 'rtoi|' in z]
    y = {z for z in v if 'rtoi|' not in z}
    y = {z.replace('-','_') if 'sce|' in z else z for z in y}
    for z in x:
        orthologs[z] = y
print(len(orthologs))
```

```
4316
```

In [6]:

```
# OrthoMCL groups from the eLife paper 
with open("../../Data/OrthoMCL/groups_Jeff.txt",'r') as f:
    reader = csv.reader(f,delimiter='\t')
    line = next(reader)
    x = line[0]
    temp = [line[i] for i in [3,4,5,6,13,25,26]]
    print(x, temp)
    line = next(reader)
    line = next(reader)
    line = next(reader)
    x = line[0]
    temp = [line[i] for i in [3,4,5,6,13,25,26]]
    y = sum([z.split(',') for z in temp if z and not 'rton' in z],[])
    print(x, temp, y)
```

```
Orthogroup ['Anid', 'Lsta', 'Hsap', 'Mmus', 'RTO4', 'Ylip', 'Scer']
RTO3OG16703 ['AN6208', 'Lsta_2494,Lsta_5958', '', '', '', '', ''] ['AN6208', 'Lsta_2494', 'Lsta_5958']
```

In [8]:

```
groups_Jeff = dict()
with open("../../Data/OrthoMCL/groups_Jeff.txt",'r') as f:
    reader = csv.reader(f,delimiter='\t')
    line = next(reader)
    for line in reader:
        x = line[0]
        temp = [line[i] for i in [3,4,5,6,13,25,26]]
        temp[0] = temp[0].replace('AN','ani|AN')
        temp[1] = temp[1].replace('Lsta_','lst|')
        temp[2] = temp[2].replace('Hsap_','hsa|')
        temp[3] = temp[3].replace('Mmus_','mmu|')
        temp[4] = temp[4].replace('RTO4_','rtoi|')
        temp[5] = temp[5].replace('YALI','yli|YALI')
        temp[6] = temp[6].replace('Y','sce|Y').replace('-','_')
        y = sum([z.split(',') for z in temp if z],[])
        if any('rtoi|' in z for z in y) and len(y) > 1:
            groups_Jeff[x] = y
```

In [11]:

```
orthologs_Jeff = dict()
for v in groups_Jeff.values():
    x = [z for z in v if 'rtoi|' in z]
    y = {z for z in v if 'rtoi|' not in z}
    for z in x:
        orthologs_Jeff[z] = y
print(len(orthologs_Jeff))
```

```
4560
```

In [12]:

```
orthologs_Jeff['rtoi|9326']
```

Out[12]:

```
{'ani|AN0887',
 'lst|2765',
 'lst|4946',
 'sce|YBR208C',
 'yli|YALI0E07271g',
 'yli|YALI0E35156g'}
```

In [17]:

```
sce = cobra.io.load_json_model(urllib.request.urlopen('http://bigg.ucsd.edu/static/models/iMM904.json'))
eco = cobra.io.load_json_model(urllib.request.urlopen('http://bigg.ucsd.edu/static/models/iML1515.json'))
ppu = cobra.io.load_json_model(urllib.request.urlopen('http://bigg.ucsd.edu/static/models/iJN746.json'))
cre = cobra.io.load_json_model(urllib.request.urlopen('http://bigg.ucsd.edu/static/models/iRC1080.json'))
hsa = cobra.io.load_json_model(urllib.request.urlopen('http://bigg.ucsd.edu/static/models/RECON1.json'))
mmu = cobra.io.load_json_model(urllib.request.urlopen('http://bigg.ucsd.edu/static/models/iMM1415.json'))
```

In [10]:

```
hsa_dict = {x.name:x.id for x in hsa.genes if x.name}
mmu_dict = {x.name:x.id for x in mmu.genes if x.name}
```

In [11]:

```
yli = cobra.io.load_json_model('../../Data/Other_models/iYL_2.0.json')
```

In [12]:

```
Gene_to_reaction = dict()
for k, v in orthologs.items():
    for z in v:
        for m in ['sce','eco','ppu','cre','yli']:
            if m in z:
                if z.replace(m+'|','') in eval(m).genes:
                    for x in eval(m).genes.get_by_id(z.replace(m+'|','')).reactions:
                        Gene_to_reaction.setdefault(k, dict()).setdefault(x.id, set()).add(z)
print(len(Gene_to_reaction))
```

```
1020
```

In [21]:

```
Gene_to_reaction_Jeff = dict()
for k, v in orthologs_Jeff.items():
    for z in v:
        for m in ['hsa','mmu']:
            if m in z:
                if (z.replace(m+'|','') in eval(m+'_dict')) and (eval(m+'_dict')[z.replace(m+'|','')] in eval(m).genes):
                    for x in eval(m).genes.get_by_id(eval(m+'_dict')[z.replace(m+'|','')]).reactions:
                        Gene_to_reaction_Jeff.setdefault(k, dict()).setdefault(x.id, set()).add(z)
        for m in ['sce','yli']:
            if m in z:
                if z.replace(m+'|','') in eval(m).genes:
                    for x in eval(m).genes.get_by_id(z.replace(m+'|','')).reactions:
                        Gene_to_reaction_Jeff.setdefault(k, dict()).setdefault(x.id, set()).add(z)
print(len(Gene_to_reaction_Jeff))
```

```
934
```

In [14]:

```
temp1 = set([y for y in Gene_to_reaction.keys()])
temp2 = set([y for y in Gene_to_reaction_Jeff.keys()])
print(len(temp1))
print(len(temp2))
print(len(temp1.union(temp2)))
print(len(temp1.intersection(temp2)))
print(len(temp1.difference(temp2)))
print(len(temp2.difference(temp1)))
```

```
1020
934
1137
817
203
117
```

In [15]:

```
temp1 = set([y for z in Gene_to_reaction.values() for y in z])
temp2 = set([y for z in Gene_to_reaction_Jeff.values() for y in z])
print(len(temp1))
print(len(temp2))
print(len(temp1.union(temp2)))
print(len(temp1.intersection(temp2)))
print(len(temp1.difference(temp2)))
print(len(temp2.difference(temp1)))
```

```
2995
2366
3804
1557
1438
809
```

In [16]:

```
Model_rev = dict()
for m in ['sce','eco','ppu','yli','cre','hsa','mmu']:
    for x in eval(m).reactions:
        if x.lower_bound < 0 and x.upper_bound > 0:
            temp = 'rev'
        elif x.lower_bound == 0 and x.upper_bound > 0:
            temp = 'for'
        elif x.lower_bound < 0 and x.upper_bound == 0:
            temp = 'back'
        else:
            print(m, x.id, x.lower_bound, x.upper_bound)
            temp = 'for'
        Model_rev.setdefault(x.id, dict())[m] = temp
```

```
sce ATPM 1.0 1.0
eco FHL 0.0 0.0
eco ATPM 6.86 1000.0
ppu BIOMASS_KT_TEMP 1.0 1.4
cre PRISM_solar_litho 646.06656 646.06656
cre PRISM_solar_exo 417.5912 417.5912
cre PRISM_incandescent_60W 15.94176 15.94176
cre PRISM_fluorescent_warm_18W 8.09577 8.09577
cre PRISM_fluorescent_cool_215W 44.63332 44.63332
cre PRISM_metal_halide 17.5256 17.5256
cre PRISM_high_pressure_sodium 36.15037 36.15037
cre PRISM_growth_room 58.46722 58.46722
cre PRISM_white_LED 4.59151 4.59151
cre PRISM_red_LED_array_653nm 96.62811 96.62811
cre PRISM_red_LED_674nm 3.653 3.653
cre PRISM_design_growth 51.8841087821545 51.8841087821545
cre EX_hco3_e 0.0 0.0
cre EX_rib__D_e 0.0 0.0
cre EX_no2_e 0.0 0.0
cre EX_his__L_e 0.0 0.0
cre EX_urea_e 0.0 0.0
cre EX_ad_e 0.0 0.0
cre EX_gua_e 0.0 0.0
cre EX_ade_e 0.0 0.0
cre EX_alltt_e 0.0 0.0
cre EX_alltn_e 0.0 0.0
cre EX_hxan_e 0.0 0.0
cre EX_orn_e 0.0 0.0
cre EX_gln__L_e 0.0 0.0
cre EX_urate_e 0.0 0.0
cre EX_leu__L_e 0.0 0.0
cre EX_arg__L_e 0.0 0.0
cre EX_so3_e 0.0 0.0
cre EX_s_e 0.0 0.0
cre EX_slnt_e 0.0 0.0
cre EX_lac__D_e 0.0 0.0
cre EX_6mpur_e 0.0 0.0
cre EX_tgua_e 0.0 0.0
cre EX_tega_e 0.0 0.0
cre EX_5flura_e 0.0 0.0
cre EX_cital_e 0.0 0.0
cre EX_lido_e 0.0 0.0
cre ATPM 0.183 0.183
mmu DM_13_cis_oretn_n 0.0 0.0
mmu DM_13_cis_retn_n 0.0 0.0
mmu EX_retpalm_SPACE_deleted_SPACE_10_09_2005_SPACE_SPACE_06_COLON_18_COLON_49_SPACE_PM_e 0.0 0.0
mmu SK_citr__L_c 0.0 0.0
mmu CYOOm3 0.0 0.0
mmu L_LACtcm 0.0 0.0
mmu L_LACtm 0.0 0.0
mmu PIt2m 0.0 0.0
mmu THD1m 0.0 0.0
```

In [17]:

```
Reaction_to_gene = dict()
Reaction_rev = dict()
Reaction_comp = dict()
for k, v in orthologs.items():
    for z in v:
        for m in ['sce','eco','ppu','yli','cre']:
            if m in z:
                if z.replace(m+'|','') in eval(m).genes:
                    for x in eval(m).genes.get_by_id(z.replace(m+'|','')).reactions:
                        Reaction_to_gene.setdefault(x.id, dict()).setdefault(z, set()).add(k)
                        if x.lower_bound < 0 and x.upper_bound > 0:
                            temp = 'rev'
                        elif x.lower_bound >= 0 and x.upper_bound > 0:
                            temp = 'for'
                        elif x.lower_bound < 0 and x.upper_bound == 0:
                            temp = 'back'
                        else:
                            print(m, x.id, x.lower_bound, x.upper_bound)
                            temp = 'for'
                        Reaction_rev.setdefault(x.id, dict())[m] = temp
                        Reaction_comp.setdefault(x.id, dict())[m] = x.compartments
print(len(Reaction_to_gene))
print(Reaction_to_gene['PGI'])
print(Reaction_rev['PGI'])
print(Reaction_comp['PGI'])
```

```
eco FHL 0.0 0.0
2995
{'sce|YBR196C': {'rtoi|9589'}, 'eco|b4025': {'rtoi|9589'}, 'ppu|PP_1808': {'rtoi|9589'}}
{'sce': 'rev', 'eco': 'rev', 'ppu': 'rev'}
{'sce': {'c'}, 'eco': {'c'}, 'ppu': {'c'}}
```

In [18]:

```
print(sce.compartments)
print(eco.compartments)
print(ppu.compartments)
print(yli.compartments)
print(cre.compartments)
print(hsa.compartments)
print(mmu.compartments)
```

```
{'c': 'cytosol', 'm': 'mitochondria', 'e': 'extracellular space', 'x': 'peroxisome/glyoxysome', 'r': 'endoplasmic reticulum', 'v': 'vacuole', 'n': 'nucleus', 'g': 'golgi apparatus'}
{'c': 'cytosol', 'e': 'extracellular space', 'p': 'periplasm'}
{'e': 'extracellular space', 'c': 'cytosol', 'p': 'periplasm'}
{'c': '', 'm': '', 'e': '', 'r': '', 'x': ''}
{'c': 'cytosol', 'h': 'chloroplast', 'm': 'mitochondria', 'x': 'peroxisome/glyoxysome', 'f': 'flagellum', 'e': 'extracellular space', 'n': 'nucleus', 'g': 'golgi apparatus', 's': 'eyespot', 'u': 'thylakoid'}
{'c': 'cytosol', 'e': 'extracellular space', 'l': 'lysosome', 'm': 'mitochondria', 'r': 'endoplasmic reticulum', 'n': 'nucleus', 'x': 'peroxisome/glyoxysome', 'g': 'golgi apparatus'}
{'c': 'cytosol', 'e': 'extracellular space', 'g': 'golgi apparatus', 'l': 'lysosome', 'm': 'mitochondria', 'n': 'nucleus', 'r': 'endoplasmic reticulum', 'x': 'peroxisome/glyoxysome'}
```

In [19]:

```
print(set(sum([list(x) for v in Reaction_comp.values() for x in v.values()],[])))
```

```
{'c', 'e', 'm', 'u', 'f', 'n', 'h', 'g', 'p', 'x', 'r', 's', 'v'}
```

In [20]:

```
for k, v in Reaction_comp.items():
    temp = sum([list(x) for x in v.values()],[])
    if any(x in temp for x in ['p','f','h','s','u']):
        print(k, v)
```

```
CHLDA1tu {'cre': {'u', 'h'}}
CHLDA2tu {'cre': {'u', 'h'}}
CHYA2 {'cre': {'u'}}
ZHY {'cre': {'u'}}
COLIPAabcpp {'eco': {'c', 'p'}}
PA140abcpp {'eco': {'c', 'p'}}
PA141abcpp {'eco': {'c', 'p'}}
PE181abcpp {'eco': {'c', 'p'}, 'ppu': {'c', 'p'}}
PA161abcpp {'eco': {'c', 'p'}}
PE141abcpp {'eco': {'c', 'p'}}
PG120abcpp {'eco': {'c', 'p'}}
PE140abcpp {'eco': {'c', 'p'}}
PG161abcpp {'eco': {'c', 'p'}}
PA180abcpp {'eco': {'c', 'p'}}
PGP120abcpp {'eco': {'c', 'p'}, 'ppu': {'c', 'p'}}
PGP161abcpp {'eco': {'c', 'p'}, 'ppu': {'c', 'p'}}
PE180abcpp {'eco': {'c', 'p'}, 'ppu': {'c', 'p'}}
PE160abcpp {'eco': {'c', 'p'}, 'ppu': {'c', 'p'}}
PE120abcpp {'eco': {'c', 'p'}, 'ppu': {'c', 'p'}}
PE161abcpp {'eco': {'c', 'p'}, 'ppu': {'c', 'p'}}
PG160abcpp {'eco': {'c', 'p'}}
PGP181abcpp {'eco': {'c', 'p'}, 'ppu': {'c', 'p'}}
PGP140abcpp {'eco': {'c', 'p'}}
PA160abcpp {'eco': {'c', 'p'}}
PA120abcpp {'eco': {'c', 'p'}}
PG141abcpp {'eco': {'c', 'p'}}
PA181abcpp {'eco': {'c', 'p'}}
PG140abcpp {'eco': {'c', 'p'}}
LIPAabcpp {'eco': {'c', 'p'}}
PG180abcpp {'eco': {'c', 'p'}}
PG181abcpp {'eco': {'c', 'p'}}
LIPACabcpp {'eco': {'c', 'p'}}
PGP141abcpp {'eco': {'c', 'p'}}
K2L4Aabcpp {'eco': {'c', 'p'}}
PGP160abcpp {'eco': {'c', 'p'}, 'ppu': {'c', 'p'}}
PGP180abcpp {'eco': {'c', 'p'}, 'ppu': {'c', 'p'}}
NH4tpp {'eco': {'c', 'p'}, 'ppu': {'c', 'p'}}
GALth {'cre': {'c', 'h'}}
XYLt2pp {'eco': {'c', 'p'}}
PItf {'cre': {'c', 'f'}}
PINA1th {'cre': {'c', 'h'}}
APOR {'cre': {'h'}}
CYSS_trdrd {'cre': {'h'}}
TDSRh {'cre': {'h'}}
AHAL {'cre': {'h'}}
FACOAL141t2pp {'eco': {'c', 'p'}}
FACOAL181t2pp {'eco': {'c', 'p'}}
FACOAL60t2pp {'eco': {'c', 'p'}, 'ppu': {'c', 'p'}}
FACOAL120t2pp {'eco': {'c', 'p'}, 'ppu': {'c', 'p'}}
FACOAL100t2pp {'eco': {'c', 'p'}, 'ppu': {'c', 'p'}}
FACOAL80t2pp {'eco': {'c', 'p'}, 'ppu': {'c', 'p'}}
FACOAL161t2pp {'eco': {'c', 'p'}}
FACOAL160t2pp {'eco': {'c', 'p'}, 'ppu': {'c', 'p'}}
FACOAL180t2pp {'eco': {'c', 'p'}}
FACOAL140t2pp {'eco': {'c', 'p'}, 'ppu': {'c', 'p'}}
ALLTNt2rpp {'eco': {'c', 'p'}}
LYSt2pp {'eco': {'c', 'p'}}
NI2uabcpp {'eco': {'c', 'p'}}
MG2uabcpp {'eco': {'c', 'p'}}
ACNAMt2pp {'eco': {'c', 'p'}}
MDHh {'cre': {'h'}}
MDHf {'cre': {'f'}}
NA1th {'cre': {'c', 'h'}}
LACZpp {'eco': {'p'}}
4ABUTthi {'cre': {'c', 'h'}}
XANt2pp {'eco': {'c', 'p'}}
GUAt2pp {'eco': {'c', 'p'}}
ADEt2rpp {'eco': {'c', 'p'}}
CHOLSabc {'ppu': {'c', 'p'}}
ACALDh {'cre': {'h'}}
BNORh {'cre': {'h'}}
ARBt2rpp {'eco': {'c', 'p'}}
GLCt2pp {'eco': {'c', 'p'}}
GALt2pp {'eco': {'c', 'p'}}
PTRCthr {'cre': {'c', 'h'}}
CPS {'cre': {'h'}}
G3PD1 {'cre': {'h'}}
G3PD2_1 {'cre': {'h'}}
ACt2rpp {'eco': {'c', 'p'}}
SUCCt2_2pp {'eco': {'c', 'p'}}
TKT2h {'cre': {'h'}}
TKT1h {'cre': {'h'}}
ENOf {'cre': {'f'}}
3OAR160_1 {'cre': {'h'}}
3OAR100_1 {'cre': {'h'}}
3OAR120_1 {'cre': {'h'}}
3OAR60_1 {'cre': {'h'}}
3OAR180_1 {'cre': {'h'}}
3OAR140_1 {'cre': {'h'}}
3OAR40_1 {'cre': {'h'}}
3OAR80_1 {'cre': {'h'}}
3OAR181_1 {'cre': {'h'}}
HDHh {'cre': {'h'}}
PRACHh {'cre': {'h'}}
PRADPh {'cre': {'h'}}
CYSS_1 {'cre': {'h'}}
CHOLS_ex {'ppu': {'e', 'p'}}
THRA_1 {'cre': {'h'}}
CYANSTpp {'eco': {'p'}}
ACSERLh {'cre': {'h'}}
ACSERSULLh {'cre': {'h'}}
SULR_ferr {'cre': {'h'}}
NO2R {'cre': {'h'}}
Kt2pp {'eco': {'c', 'p'}}
G6PIh {'cre': {'h'}}
PGIBh {'cre': {'h'}}
PGIAh {'cre': {'h'}}
PGLYDH {'cre': {'h'}}
ASNNpp {'eco': {'p'}}
GLUNpp {'eco': {'p'}}
RPDPK {'cre': {'h'}}
PGLh {'cre': {'h'}}
CD2t3pp {'eco': {'c', 'p'}}
COBALT2t3pp {'eco': {'c', 'p'}}
ZN2t3pp {'eco': {'c', 'p'}}
NI2t3pp {'eco': {'c', 'p'}}
MN2t3pp {'eco': {'c', 'p'}}
ATNS {'cre': {'h'}}
ATNS_nh4 {'cre': {'h'}}
IGPS_1 {'cre': {'h'}}
DHQS_1 {'cre': {'h'}}
PSCIT {'cre': {'h'}}
STARCH300S {'cre': {'h'}}
UPPDC2_1 {'cre': {'h'}}
UPPDC1_1 {'cre': {'h'}}
PGDHh {'cre': {'h'}}
STARCH300DEGRA {'cre': {'h'}}
STARCH300DEGRB {'cre': {'h'}}
STARCH300DEGR2A {'cre': {'h'}}
STARCH300DEGR2B {'cre': {'h'}}
NA1Hth {'cre': {'c', 'h'}}
PPMh {'cre': {'h'}}
PGMTh {'cre': {'h'}}
PEPPIth {'cre': {'c', 'h'}}
INOSTt4pp {'eco': {'c', 'p'}}
CLt3_2pp {'eco': {'c', 'p'}}
TAh {'cre': {'h'}}
OCTh {'cre': {'h'}}
PPATDh {'cre': {'h'}}
PDHam1hi {'cre': {'h'}}
ACAS_2ahbut {'cre': {'h'}}
APLh {'cre': {'h'}}
PDHam2hi {'cre': {'h'}}
GTPDH {'cre': {'h'}}
R5PFL {'cre': {'h'}}
HSDH {'cre': {'h'}}
ASPK_1 {'cre': {'h'}}
MDDCP5pp {'eco': {'p'}}
MDDCP1pp {'eco': {'p'}}
MDDCP4pp {'eco': {'p'}}
MDDCP3pp {'eco': {'p'}}
MDDCP2pp {'eco': {'p'}}
GAPDHh {'cre': {'h'}}
PGPS18111Z160h {'cre': {'h'}}
PGPS1819Z160h {'cre': {'h'}}
IMGPSh {'cre': {'h'}}
PRICIh {'cre': {'h'}}
PRAICh {'cre': {'h'}}
SHSL2h {'cre': {'h'}}
G6PB_pi_th {'cre': {'c', 'h'}}
G3P_pi_thr {'cre': {'c', 'h'}}
3PG_pi_thr {'cre': {'c', 'h'}}
G1P_pi_th {'cre': {'c', 'h'}}
G6PA_pi_th {'cre': {'c', 'h'}}
DHAP_pi_thr {'cre': {'c', 'h'}}
2AGPG140tipp {'eco': {'c', 'p'}}
2AGPG120tipp {'eco': {'c', 'p'}}
2AGPG160tipp {'eco': {'c', 'p'}}
2AGPE140tipp {'eco': {'c', 'p'}}
2AGPA141tipp {'eco': {'c', 'p'}}
2AGPE160tipp {'eco': {'c', 'p'}}
2AGPE120tipp {'eco': {'c', 'p'}}
2AGPG141tipp {'eco': {'c', 'p'}}
2AGPE180tipp {'eco': {'c', 'p'}}
2AGPA140tipp {'eco': {'c', 'p'}}
2AGPG161tipp {'eco': {'c', 'p'}}
2AGPE141tipp {'eco': {'c', 'p'}}
2AGPA120tipp {'eco': {'c', 'p'}}
2AGPE161tipp {'eco': {'c', 'p'}}
2AGPE181tipp {'eco': {'c', 'p'}}
2AGPG180tipp {'eco': {'c', 'p'}}
2AGPA160tipp {'eco': {'c', 'p'}}
2AGPA161tipp {'eco': {'c', 'p'}}
2AGPA180tipp {'eco': {'c', 'p'}}
2AGPG181tipp {'eco': {'c', 'p'}}
2AGPA181tipp {'eco': {'c', 'p'}}
GLUS_ferr {'cre': {'h'}}
GLUS_nadph {'cre': {'h'}}
GLUS {'cre': {'h'}}
VALTLh {'cre': {'h'}}
ORDCh {'cre': {'h'}}
PEAMNOpp {'eco': {'p'}}
TYROXDApp {'eco': {'p'}}
42A12BOOXpp {'eco': {'p'}}
PAO {'cre': {'h'}}
HSK_1 {'cre': {'h'}}
CYSAT {'cre': {'h'}}
AATC {'cre': {'h'}}
POATh {'cre': {'h'}}
AATG {'cre': {'h'}}
ASPATh {'cre': {'h'}}
TYRTAh {'cre': {'h'}}
GDR_nadp_h {'cre': {'h'}}
GDRh {'cre': {'h'}}
TREHpp {'eco': {'p'}}
PPBNGD {'cre': {'h'}}
THRS_1 {'cre': {'h'}}
TAL {'cre': {'h'}}
THRAL {'cre': {'h'}}
FE2t2pp {'eco': {'c', 'p'}}
MNt2pp {'eco': {'c', 'p'}}
G3PL {'cre': {'h'}}
TRPS2h {'cre': {'h'}}
SERH {'cre': {'h'}}
PYKf {'cre': {'f'}}
IPPSh {'cre': {'h'}}
MALSh {'cre': {'h'}}
Ftpp {'eco': {'c', 'p'}}
RPEh {'cre': {'h'}}
AACPS4_1 {'cre': {'h'}}
FA100ACPHi_1 {'cre': {'h'}}
ACOATA_1 {'cre': {'h'}}
AACPS5_1 {'cre': {'h'}}
FA120ACPHi_1 {'cre': {'h'}}
ACP1819ZD9DS {'cre': {'h'}}
3HAD60_1 {'cre': {'h'}}
EAR180x_1 {'cre': {'h'}}
FA140ACPHi_1 {'cre': {'h'}}
ACP1619ZD9DS {'cre': {'h'}}
EAR100x_1 {'cre': {'h'}}
EAR100y_1 {'cre': {'h'}}
EAR120x_1 {'cre': {'h'}}
FA160ACPHi_1 {'cre': {'h'}}
AACPS6_1 {'cre': {'h'}}
EAR120y_1 {'cre': {'h'}}
EAR181x_1 {'cre': {'h'}}
EAR140x_1 {'cre': {'h'}}
EAR181y_1 {'cre': {'h'}}
EAR140y_1 {'cre': {'h'}}
EAR40x_1 {'cre': {'h'}}
G3PAT180h {'cre': {'h'}}
EAR160x_1 {'cre': {'h'}}
EAR160y_1 {'cre': {'h'}}
FA161ACPHi_1 {'cre': {'h'}}
FA180ACPHi {'cre': {'h'}}
FA1819ZACPH {'cre': {'h'}}
EAR40y_1 {'cre': {'h'}}
FA181ACPHi {'cre': {'h'}}
FA80ACPHi_1 {'cre': {'h'}}
G3PAT1819Zh {'cre': {'h'}}
G3PAT181h {'cre': {'h'}}
AGPAT1601819Zh {'cre': {'h'}}
3OAS100_1 {'cre': {'h'}}
3OAS120_1 {'cre': {'h'}}
AGPAT160h {'cre': {'h'}}
AGPAT1801819Zh {'cre': {'h'}}
3OAS140_1 {'cre': {'h'}}
AGPAT18111Z160h {'cre': {'h'}}
AGPAT18111Z1819Zh {'cre': {'h'}}
EAR60x_1 {'cre': {'h'}}
3OAS160_1 {'cre': {'h'}}
KAS14_1 {'cre': {'h'}}
AGPAT1819Z160h {'cre': {'h'}}
3HAD120_1 {'cre': {'h'}}
EAR60y_1 {'cre': {'h'}}
AGPAT1819Z1619Zh {'cre': {'h'}}
3OAS180_1 {'cre': {'h'}}
MCOATA_1 {'cre': {'h'}}
AGPAT1819Z18111Zh {'cre': {'h'}}
EAR80x_1 {'cre': {'h'}}
3OAS181_1 {'cre': {'h'}}
AGPAT1819Z1819Zh {'cre': {'h'}}
3HAD100_1 {'cre': {'h'}}
3HAD140_1 {'cre': {'h'}}
AACP1819ZS {'cre': {'h'}}
3HAD160_1 {'cre': {'h'}}
EAR80y_1 {'cre': {'h'}}
3OAS60_1 {'cre': {'h'}}
3HAD180_1 {'cre': {'h'}}
AACPS1_1 {'cre': {'h'}}
3OAS80_1 {'cre': {'h'}}
G3PAT160h {'cre': {'h'}}
3HAD181_1 {'cre': {'h'}}
3HAD40_1 {'cre': {'h'}}
AACPS3_1 {'cre': {'h'}}
ACPS1h {'cre': {'h'}}
3HAD80_1 {'cre': {'h'}}
ATPSh {'cre': {'u', 'h'}}
ATPS4rpp {'eco': {'c', 'p'}, 'ppu': {'c', 'p'}}
NA1ATPaseh {'cre': {'c', 'h'}}
CU1abcpp {'eco': {'c', 'p'}}
PPBNGS_1 {'cre': {'h'}}
BCRPTXANH {'cre': {'u'}}
CHYA1 {'cre': {'u'}}
BCAROH {'cre': {'u'}}
UPP3MT_1 {'cre': {'h'}}
PAPA1819Z18111Zh {'cre': {'h'}}
PAPA160h {'cre': {'h'}}
PAPA1819Z160h {'cre': {'h'}}
PAPA18111Z160h {'cre': {'h'}}
PAPA18111Z1819Zh {'cre': {'h'}}
PAPA1819Z1619Zh {'cre': {'h'}}
PAPA1819Z1819Zh {'cre': {'h'}}
PAPA1801819Zh {'cre': {'h'}}
PAPA1601819Zh {'cre': {'h'}}
RIBFS {'cre': {'h'}}
SSNOh {'cre': {'h'}}
PGLYCPh {'cre': {'h'}}
NAt3pp {'eco': {'c', 'p'}}
CA2t3pp {'eco': {'c', 'p'}}
Kt3pp {'eco': {'c', 'p'}}
MDHC_nadp_hr {'cre': {'h'}}
DGDGD7DS1829Z12Z1617Z {'cre': {'h'}}
MGDGD7DS1829Z12Z1617Z {'cre': {'h'}}
MGDGD7DS1819Z1617Z {'cre': {'h'}}
DGDGD7DS1819Z1617Z {'cre': {'h'}}
FAH1819Z {'cre': {'h'}}
FAH120 {'cre': {'h'}}
FAH1829Z12Z {'cre': {'h'}}
FAH140 {'cre': {'h'}}
FAH160 {'cre': {'h'}}
GLYALDtpp {'eco': {'c', 'p'}, 'ppu': {'c', 'p'}}
GLYCtpp {'eco': {'c', 'p'}, 'ppu': {'c', 'p'}}
UREAtpp {'eco': {'c', 'p'}}
GLYtpp {'eco': {'c', 'p'}}
ANTPPT {'cre': {'h'}}
KARI_23dhmp {'cre': {'h'}}
KARI_3hmoa {'cre': {'h'}}
KARI {'cre': {'h'}}
KARI_23dhmb {'cre': {'h'}}
DMORh {'cre': {'h'}}
BFBPh {'cre': {'h'}}
GTHAMPORh {'cre': {'h'}}
FGFTh {'cre': {'h'}}
FPGFTh {'cre': {'h'}}
DHPAH {'cre': {'h'}}
G3PO {'cre': {'h'}}
ACCOAhi {'cre': {'h'}}
BTNC {'cre': {'h'}}
ACCOAC_1 {'cre': {'h'}}
ADSSh {'cre': {'h'}}
PRAISh {'cre': {'h'}}
TCYG {'cre': {'h'}}
TCYD {'cre': {'h'}}
TPIh {'cre': {'h'}}
CYTP450Rh {'cre': {'h'}}
CHRS {'cre': {'h'}}
AOOAh {'cre': {'h'}}
PGKh {'cre': {'h'}}
CPPPGO_1 {'cre': {'h'}}
DLDLBT {'cre': {'h'}}
IGPDh {'cre': {'h'}}
ASADH {'cre': {'h'}}
PPRGLh {'cre': {'h'}}
RPIh {'cre': {'h'}}
PSAT {'cre': {'h'}}
PRFGSh {'cre': {'h'}}
SQDGS160 {'cre': {'h'}}
SQDGS18111Z160 {'cre': {'h'}}
SQDGS1819Z160 {'cre': {'h'}}
CDPDAGS18111Z160h {'cre': {'h'}}
CDPDAGS1819Z160h {'cre': {'h'}}
PGMf {'cre': {'f'}}
ASPth {'cre': {'c', 'h'}}
GLUth {'cre': {'c', 'h'}}
GLUKBh {'cre': {'h'}}
AM6PTh {'cre': {'h'}}
ABFPTh {'cre': {'h'}}
GLUKAh {'cre': {'h'}}
GLYCth {'cre': {'c', 'h'}}
NADH16pp {'eco': {'c', 'p'}, 'ppu': {'c', 'p'}}
NADH18pp {'eco': {'c', 'p'}}
NADH17pp {'eco': {'c', 'p'}}
3_4DHBZt2 {'ppu': {'e', 'p'}}
CYO1_KT {'ppu': {'c', 'p'}}
AASPh {'cre': {'h'}}
GTHRDHpp {'eco': {'p'}}
ALCDH_nadp_hi {'cre': {'h'}}
IDIh {'cre': {'h'}}
ACKrh {'cre': {'h'}}
APPTh {'cre': {'h'}}
2DHGLCNkt_tpp {'ppu': {'c', 'p'}}
IDPh {'cre': {'h'}}
DNGALh {'cre': {'h'}}
GLYPT {'cre': {'h'}}
TRETINOLPMTACT {'cre': {'s'}}
CRETINOLPMTACT {'cre': {'s'}}
RETINOLACACT {'cre': {'s'}}
CBL {'cre': {'h'}}
CTINBL {'cre': {'h'}}
SELCYSTLh {'cre': {'h'}}
CYSTBL {'cre': {'h'}}
HEMELh {'cre': {'h'}}
GTHS_1 {'cre': {'h'}}
DPHS {'cre': {'h'}}
PSIIblue {'cre': {'u'}}
PSIIred {'cre': {'u'}}
UDPGth {'cre': {'c', 'h'}}
UDPGALth {'cre': {'c', 'h'}}
ATDAMh {'cre': {'h'}}
ATAMh {'cre': {'h'}}
CHRM {'cre': {'h'}}
FPPSh {'cre': {'h'}}
GALM2pp {'eco': {'p'}}
AMETt2h {'cre': {'c', 'h'}}
PSCLYSt2pp {'eco': {'c', 'p'}}
FRULYSt2pp {'eco': {'c', 'p'}}
ACCOAth {'cre': {'c', 'h'}}
PPPGO_1 {'cre': {'h'}}
G2PPpp {'eco': {'p'}}
NTD2pp {'eco': {'p'}}
G1PPpp {'eco': {'p'}}
ACP1p {'eco': {'p'}}
3NTD7pp {'eco': {'p'}}
3NTD9pp {'eco': {'p'}}
3NTD2pp {'eco': {'p'}}
NTD7pp {'eco': {'p'}}
PPTHpp {'eco': {'p'}}
PSP_Lpp {'eco': {'p'}}
NTD4pp {'eco': {'p'}}
PTHRpp {'eco': {'p'}}
R5PPpp {'eco': {'p'}}
NTD9pp {'eco': {'p'}}
3NTD4pp {'eco': {'p'}}
PAPSPAPthr {'cre': {'c', 'h'}}
NO2th {'cre': {'c', 'h'}}
ASCBOR {'cre': {'u'}}
MDDEP4pp {'eco': {'p'}}
MDDEP2pp {'eco': {'p'}}
MDDEP1pp {'eco': {'p'}}
MDDEP3pp {'eco': {'p'}}
H2Othu {'cre': {'u', 'h'}}
H2Otf {'cre': {'c', 'f'}}
H2Oth {'cre': {'c', 'h'}}
ZAXANOR {'cre': {'u'}}
ANXANOR {'cre': {'u'}}
TFENFEOR {'cre': {'h'}}
FUCtpp {'eco': {'c', 'p'}}
```

In [21]:

```
# remove reactions in irrelevant compartments now?
Reaction_subset = dict()
for k, v in Reaction_comp.items():
    temp = sum([list(x) for x in v.values()],[])
    if not any(x in temp for x in ['p','f','h','s','u']):
        Reaction_subset.setdefault(k,str())
# keep them now and remove or change compartment later
    else:
        Reaction_subset.setdefault(k,str())        
print(len(Reaction_subset))
```

```
2995
```

In [22]:

```
for k, v in sorted(Reaction_rev.items()):
    if k in Reaction_subset: 
        if len(set(v.values())) > 1:
            print(k, v)
```

```
ABTA {'ppu': 'rev', 'eco': 'for', 'yli': 'rev', 'sce': 'for'}
ACACT1r {'sce': 'for', 'eco': 'rev', 'yli': 'for', 'cre': 'for'}
ACACT2r {'ppu': 'back', 'eco': 'rev'}
ACACT3r {'ppu': 'back', 'eco': 'rev'}
ACACT4m {'yli': 'back', 'cre': 'for'}
ACACT4p {'sce': 'for', 'cre': 'rev'}
ACACT4r {'ppu': 'back', 'eco': 'rev'}
ACACT5p {'sce': 'for', 'cre': 'rev'}
ACACT5r {'ppu': 'back', 'eco': 'rev'}
ACACT6m {'yli': 'back', 'cre': 'for'}
ACACT6p {'sce': 'for', 'cre': 'rev'}
ACACT6r {'ppu': 'back', 'eco': 'rev'}
ACACT7m {'yli': 'back', 'cre': 'for'}
ACACT7p {'sce': 'for', 'cre': 'rev'}
ACACT7r {'ppu': 'back', 'eco': 'rev'}
ACALD {'eco': 'rev', 'cre': 'for'}
ACCOAC {'ppu': 'rev', 'eco': 'for', 'sce': 'rev', 'yli': 'rev'}
ACGAM6PS {'cre': 'for', 'sce': 'rev', 'yli': 'for'}
ACOAHim {'yli': 'back', 'sce': 'for'}
ACOATA {'eco': 'rev', 'sce': 'rev', 'yli': 'for'}
ACOTAim {'yli': 'for', 'sce': 'for', 'cre': 'rev'}
ACS {'cre': 'for', 'sce': 'for', 'ppu': 'rev', 'yli': 'for', 'eco': 'for'}
ADK1 {'yli': 'rev', 'eco': 'rev', 'cre': 'for', 'ppu': 'rev', 'sce': 'rev'}
ADK1m {'cre': 'for', 'sce': 'rev'}
ADNK1 {'eco': 'for', 'yli': 'rev', 'sce': 'for', 'cre': 'for'}
ADPT {'yli': 'for', 'sce': 'for', 'ppu': 'for', 'eco': 'for', 'cre': 'rev'}
AGPR {'eco': 'rev', 'cre': 'back'}
AHCi {'cre': 'rev', 'sce': 'for', 'yli': 'for'}
AHSERL2 {'yli': 'for', 'ppu': 'rev', 'sce': 'for'}
AKGDam {'cre': 'for', 'sce': 'rev'}
ALCD19y {'sce': 'for', 'cre': 'rev'}
ALLTN {'sce': 'rev', 'eco': 'for'}
AMPTASECG {'yli': 'rev', 'eco': 'for'}
ANPRT {'eco': 'for', 'sce': 'for', 'ppu': 'for', 'yli': 'rev'}
AOXSr2 {'eco': 'for', 'yli': 'rev'}
ARGSS {'ppu': 'for', 'eco': 'for', 'sce': 'rev'}
ASPK {'eco': 'rev', 'yli': 'for', 'sce': 'for', 'ppu': 'for'}
ATHRDHr {'yli': 'for', 'eco': 'rev', 'sce': 'rev'}
ATPPRT {'yli': 'for', 'ppu': 'rev', 'eco': 'for', 'sce': 'for'}
C14STR {'cre': 'rev', 'sce': 'for'}
CHORM {'eco': 'for', 'ppu': 'for', 'yli': 'rev', 'sce': 'for'}
CYSTA {'yli': 'rev', 'eco': 'for'}
CYSTGL {'yli': 'rev', 'sce': 'for', 'ppu': 'rev'}
CYTK1 {'cre': 'for', 'yli': 'rev'}
CYTK2 {'cre': 'for', 'yli': 'rev'}
DADK {'yli': 'rev', 'eco': 'rev', 'cre': 'for', 'ppu': 'rev'}
DGK1 {'eco': 'rev', 'ppu': 'rev', 'cre': 'for', 'sce': 'rev', 'yli': 'rev'}
DHORDfum {'yli': 'rev', 'sce': 'for'}
DHORDi {'ppu': 'rev', 'sce': 'for'}
DHQTi {'sce': 'for', 'yli': 'rev', 'ppu': 'rev'}
DPRm {'sce': 'for', 'cre': 'rev', 'yli': 'for'}
DTMPK {'sce': 'rev', 'cre': 'for', 'yli': 'rev', 'eco': 'rev'}
DURIPP {'yli': 'for', 'sce': 'rev', 'eco': 'rev'}
ECOAH1 {'ppu': 'rev', 'yli': 'back', 'cre': 'rev'}
ECOAH1m {'yli': 'back', 'cre': 'rev'}
FACOAL160 {'sce': 'rev', 'cre': 'for', 'yli': 'rev'}
FALDH2 {'eco': 'rev', 'yli': 'for'}
G6PDH2r {'ppu': 'for', 'sce': 'for', 'eco': 'rev'}
GALKr {'yli': 'for', 'eco': 'rev', 'cre': 'for', 'sce': 'for'}
GALUi {'yli': 'rev', 'cre': 'for', 'sce': 'rev'}
GARFT {'eco': 'rev', 'ppu': 'rev', 'sce': 'for', 'cre': 'rev', 'yli': 'for'}
GCCbim {'sce': 'for', 'yli': 'rev'}
GCCcm {'sce': 'rev', 'cre': 'for', 'yli': 'rev'}
GK1 {'eco': 'rev', 'ppu': 'rev', 'cre': 'for', 'sce': 'rev', 'yli': 'rev'}
GLUDy {'sce': 'back', 'ppu': 'rev', 'eco': 'rev', 'yli': 'back'}
GLYCLm {'sce': 'for', 'cre': 'back', 'yli': 'back'}
GTHOr {'sce': 'for', 'cre': 'for', 'ppu': 'for', 'eco': 'rev'}
GTHPi {'sce': 'rev', 'cre': 'for', 'eco': 'for', 'ppu': 'rev', 'yli': 'for'}
GTHPm {'yli': 'for', 'sce': 'rev'}
HPYRRy {'eco': 'for', 'cre': 'rev'}
HSDxi {'ppu': 'rev', 'sce': 'for', 'yli': 'rev'}
HSDy {'eco': 'rev', 'sce': 'back', 'yli': 'rev'}
HSERTA {'sce': 'rev', 'yli': 'for', 'ppu': 'rev'}
HSTPT {'sce': 'for', 'cre': 'rev', 'ppu': 'rev', 'eco': 'for', 'yli': 'rev'}
ICDHyr {'yli': 'for', 'cre': 'rev', 'sce': 'for', 'eco': 'rev', 'ppu': 'rev'}
IGPS {'yli': 'rev', 'ppu': 'for', 'sce': 'for', 'eco': 'for'}
IPDDI {'eco': 'rev', 'sce': 'rev', 'cre': 'for', 'yli': 'rev'}
IPMD {'ppu': 'rev', 'eco': 'for', 'sce': 'for', 'cre': 'for'}
KARA1im {'cre': 'for', 'sce': 'for', 'yli': 'rev'}
KARA2im {'sce': 'for', 'yli': 'rev'}
LNS14DM {'sce': 'for', 'cre': 'rev'}
MDH {'ppu': 'rev', 'yli': 'for', 'eco': 'rev'}
MHPGLUT {'ppu': 'rev', 'sce': 'for', 'yli': 'for'}
MMTSAO {'ppu': 'for', 'yli': 'rev'}
MTHFD {'sce': 'rev', 'yli': 'for', 'eco': 'rev', 'ppu': 'rev'}
NDPK1 {'yli': 'rev', 'eco': 'rev', 'sce': 'rev', 'cre': 'for', 'ppu': 'rev'}
NDPK2 {'yli': 'rev', 'eco': 'rev', 'sce': 'rev', 'cre': 'for', 'ppu': 'rev'}
NDPK3 {'yli': 'rev', 'eco': 'rev', 'sce': 'rev', 'cre': 'for', 'ppu': 'rev'}
NDPK4 {'yli': 'rev', 'eco': 'rev', 'sce': 'rev', 'cre': 'for', 'ppu': 'rev'}
NDPK5 {'yli': 'rev', 'eco': 'rev', 'sce': 'rev', 'cre': 'for', 'ppu': 'rev'}
NDPK6 {'yli': 'rev', 'eco': 'rev', 'sce': 'rev', 'cre': 'for', 'ppu': 'rev'}
NDPK7 {'yli': 'rev', 'eco': 'rev', 'sce': 'rev', 'cre': 'for', 'ppu': 'rev'}
NDPK8 {'yli': 'rev', 'eco': 'rev', 'sce': 'rev', 'cre': 'for', 'ppu': 'rev'}
NDPK9 {'yli': 'rev', 'sce': 'rev', 'cre': 'for'}
NNAM {'yli': 'for', 'cre': 'for', 'sce': 'rev', 'eco': 'for'}
NNATr {'sce': 'for', 'cre': 'rev'}
NTP10 {'cre': 'back', 'yli': 'for'}
OCBT {'eco': 'rev', 'ppu': 'rev', 'sce': 'for'}
OCOAT1 {'ppu': 'for', 'yli': 'rev'}
ORNTACim {'yli': 'rev', 'sce': 'for'}
PC {'ppu': 'for', 'cre': 'rev', 'yli': 'for', 'sce': 'for'}
PDX5POi {'sce': 'rev', 'ppu': 'for', 'yli': 'for', 'eco': 'for', 'cre': 'for'}
PGCD {'ppu': 'rev', 'sce': 'for', 'eco': 'for', 'yli': 'for'}
PPGPPDP {'cre': 'rev', 'ppu': 'for', 'eco': 'for'}
PPNDH {'sce': 'for', 'eco': 'for', 'ppu': 'for', 'yli': 'rev', 'cre': 'for'}
PRAGSr {'eco': 'rev', 'sce': 'rev', 'yli': 'for', 'ppu': 'for'}
PRASCSi {'ppu': 'rev', 'sce': 'rev', 'cre': 'for', 'eco': 'for'}
PRPPS {'sce': 'rev', 'eco': 'rev', 'cre': 'for', 'ppu': 'rev'}
PSCVT {'sce': 'for', 'ppu': 'rev', 'eco': 'rev', 'yli': 'rev'}
PSERT {'yli': 'rev', 'eco': 'for', 'ppu': 'rev', 'sce': 'for'}
PYDXNO {'sce': 'rev', 'yli': 'for', 'cre': 'for'}
PYDXO_1 {'yli': 'rev', 'cre': 'for'}
RBK {'sce': 'for', 'eco': 'for', 'cre': 'for', 'ppu': 'rev'}
SERD_L {'yli': 'for', 'ppu': 'rev', 'eco': 'for', 'sce': 'for'}
SFGTHi {'eco': 'for', 'sce': 'rev', 'yli': 'for'}
SHSL2r {'yli': 'for', 'ppu': 'rev'}
SMO {'cre': 'rev', 'yli': 'for'}
SSALy {'yli': 'for', 'sce': 'for', 'ppu': 'rev', 'eco': 'for'}
SUCD1m {'sce': 'rev', 'yli': 'rev', 'cre': 'for'}
SUCFUMtm {'sce': 'for', 'yli': 'for', 'cre': 'rev'}
SULR {'sce': 'rev', 'ppu': 'for', 'eco': 'for', 'yli': 'for'}
THRA {'eco': 'for', 'ppu': 'rev', 'sce': 'for', 'yli': 'rev'}
THRA2 {'eco': 'for', 'ppu': 'rev', 'sce': 'for', 'yli': 'rev'}
TMDS {'sce': 'for', 'yli': 'for', 'ppu': 'for', 'eco': 'for', 'cre': 'rev'}
TRPS1 {'yli': 'for', 'sce': 'for', 'eco': 'for', 'ppu': 'rev'}
TRPTA {'sce': 'rev', 'yli': 'for'}
TYRTAi {'sce': 'for', 'cre': 'rev'}
UDPGD {'eco': 'for', 'cre': 'for', 'yli': 'rev'}
UGLYCH {'yli': 'rev', 'sce': 'rev', 'eco': 'for'}
UMPK {'cre': 'for', 'sce': 'rev', 'yli': 'rev'}
UPPRT {'cre': 'rev', 'eco': 'for', 'ppu': 'for', 'yli': 'for', 'sce': 'for'}
URIDK2r {'cre': 'for', 'yli': 'rev', 'sce': 'rev'}
VALTAim {'sce': 'for', 'cre': 'rev', 'yli': 'rev'}
XPPT {'sce': 'for', 'cre': 'rev'}
XYLTD_D {'sce': 'for', 'yli': 'rev'}
```

In [23]:

```
for k, v in Reaction_rev.items():
    if k in Reaction_subset:
        if len(set(v.values())) > 1:
            if 'eco' in v:
                Reaction_subset[k] = ('eco',v['eco'])
            elif 'sce' in v:
                Reaction_subset[k] = ('sce',v['sce'])
            elif 'ppu' in v:
                Reaction_subset[k] = ('ppu',v['ppu'])
            else:
                print(k, v)
                Reaction_subset[k] = ('yli',v['yli'])
        else:
            if 'eco' in v:
                Reaction_subset[k] = ('eco',v['eco'])
            elif 'sce' in v:
                Reaction_subset[k] = ('sce',v['sce'])
            elif 'ppu' in v:
                Reaction_subset[k] = ('ppu',v['ppu'])
            elif 'cre' in v:
                Reaction_subset[k] = ('cre',v['cre'])
            elif 'yli' in v:
                Reaction_subset[k] = ('yli',v['yli'])
            else:
                print(k, v)
```

```
ACACT4m {'yli': 'back', 'cre': 'for'}
ACACT6m {'yli': 'back', 'cre': 'for'}
ACACT7m {'yli': 'back', 'cre': 'for'}
ECOAH1m {'yli': 'back', 'cre': 'rev'}
NTP10 {'cre': 'back', 'yli': 'for'}
PYDXO_1 {'yli': 'rev', 'cre': 'for'}
SMO {'cre': 'rev', 'yli': 'for'}
CYTK1 {'cre': 'for', 'yli': 'rev'}
CYTK2 {'cre': 'for', 'yli': 'rev'}
```

In [24]:

```
for k, v in Reaction_subset.items():
    if k in eco.reactions:
        if not Model_rev[k]['eco'] == v[1]:
            print(k, v, 'eco', Model_rev[k]['eco'])
    elif k in sce.reactions:
        if not Model_rev[k]['sce'] == v[1]:
            print(k, v, 'sce', Model_rev[k]['sce'])
```

```
TYRTAim ('cre', 'rev') sce for
SHK3Dr ('sce', 'for') eco rev
PRMICI ('sce', 'for') eco rev
FA141ACPHi ('sce', 'rev') eco for
FA120ACPHi ('sce', 'rev') eco for
FA160ACPHi ('sce', 'rev') eco for
FA140ACPHi ('sce', 'rev') eco for
FA161ACPHi ('sce', 'rev') eco for
DHFR ('cre', 'for') eco rev
ASAD ('sce', 'back') eco rev
NNATr ('sce', 'for') eco rev
CYSDS ('yli', 'rev') eco for
MTRI ('cre', 'for') sce rev
ACOAD1f ('ppu', 'rev') eco for
PTPATi ('yli', 'rev') eco for
PTPATim ('yli', 'rev') sce for
HXPRT ('cre', 'rev') eco for
GALUi ('sce', 'rev') eco for
UAGDP ('cre', 'rev') eco for
LALDO2x ('cre', 'rev') eco for
AP4AH ('sce', 'rev') eco for
```

In [25]:

```
for k, v in Reaction_subset.items():
    if k in eco.reactions:
        if not Model_rev[k]['eco'] == v[1]:
            Reaction_subset[k] = ('eco', Model_rev[k]['eco'])
    elif k in sce.reactions:
        if not Model_rev[k]['sce'] == v[1]:
            Reaction_subset[k] = ('sce', Model_rev[k]['sce'])
```

In [26]:

```
Reaction_to_gene_Jeff = dict()
Reaction_rev_Jeff = dict()
Reaction_comp_Jeff = dict()

for k, v in orthologs_Jeff.items():
    for z in v:
        for m in ['hsa','mmu']:
            if m in z:
                if (z.replace(m+'|','') in eval(m+'_dict')) and (eval(m+'_dict')[z.replace(m+'|','')] in eval(m).genes):
                    for x in eval(m).genes.get_by_id(eval(m+'_dict')[z.replace(m+'|','')]).reactions:
                        Reaction_to_gene_Jeff.setdefault(x.id, dict()).setdefault(z, set()).add(k)
                        if x.lower_bound < 0 and x.upper_bound > 0:
                            temp = 'rev'
                        elif x.lower_bound >= 0 and x.upper_bound > 0:
                            temp = 'for'
                        elif x.lower_bound < 0 and x.upper_bound == 0:
                            temp = 'back'
                        else:
                            print(m, x.id, x.lower_bound, x.upper_bound)
                            temp = 'for'
                        Reaction_rev_Jeff.setdefault(x.id, dict())[m] = temp
                        Reaction_comp_Jeff.setdefault(x.id, dict())[m] = x.compartments
        for m in ['sce','yli']:
            if m in z:
                if z.replace(m+'|','') in eval(m).genes:
                    for x in eval(m).genes.get_by_id(z.replace(m+'|','')).reactions:
                        Reaction_to_gene_Jeff.setdefault(x.id, dict()).setdefault(z, set()).add(k)
                        if x.lower_bound < 0 and x.upper_bound > 0:
                            temp = 'rev'
                        elif x.lower_bound >= 0 and x.upper_bound > 0:
                            temp = 'for'
                        elif x.lower_bound < 0 and x.upper_bound == 0:
                            temp = 'back'
                        else:
                            print(m, x.id, x.lower_bound, x.upper_bound)
                            temp = 'for'
                        Reaction_rev_Jeff.setdefault(x.id, dict())[m] = temp
                        Reaction_comp_Jeff.setdefault(x.id, dict())[m] = x.compartments
                        
print(len(Reaction_to_gene_Jeff))
print(Reaction_to_gene_Jeff['PGI'])
print(Reaction_rev_Jeff['PGI'])
print(Reaction_comp_Jeff['PGI'])
```

```
mmu PIt2m 0.0 0.0
mmu PIt2m 0.0 0.0
mmu L_LACtcm 0.0 0.0
2366
{'sce|YBR196C': {'rtoi|9589'}, 'mmu|Gpi1': {'rtoi|9589'}, 'hsa|GPI': {'rtoi|9589'}}
{'sce': 'rev', 'mmu': 'rev', 'hsa': 'rev'}
{'sce': {'c'}, 'mmu': {'c'}, 'hsa': {'c'}}
```

In [27]:

```
print(set(sum([list(x) for v in Reaction_comp_Jeff.values() for x in v.values()],[])))
```

```
{'c', 'e', 'm', 'n', 'l', 'g', 'x', 'r', 'v'}
```

In [28]:

```
for k, v in Reaction_comp_Jeff.items():
    temp = sum([list(x) for x in v.values()],[])
    if any(x in temp for x in ['l']):
        print(k, v)
```

```
ATPasel {'hsa': {'c', 'l'}, 'mmu': {'c', 'l'}}
B_MANNASEly {'hsa': {'l'}, 'mmu': {'l'}}
DALAt2rL {'mmu': {'c', 'l'}, 'hsa': {'c', 'l'}}
PROt2rL {'mmu': {'c', 'l'}, 'hsa': {'c', 'l'}}
PRODt2rL {'mmu': {'c', 'l'}, 'hsa': {'c', 'l'}}
ABUTt2rL {'mmu': {'c', 'l'}, 'hsa': {'c', 'l'}}
ALAt2rL {'mmu': {'c', 'l'}, 'hsa': {'c', 'l'}}
GLYt2rL {'mmu': {'c', 'l'}, 'hsa': {'c', 'l'}}
S4TASE1ly {'hsa': {'l'}, 'mmu': {'l'}}
S4TASE2ly {'hsa': {'l'}, 'mmu': {'l'}}
S4TASE3ly {'hsa': {'l'}, 'mmu': {'l'}}
S4TASE4ly {'hsa': {'l'}, 'mmu': {'l'}}
S4TASE5ly {'hsa': {'l'}, 'mmu': {'l'}}
NACHEXA6ly {'hsa': {'l'}, 'mmu': {'l'}}
NACHEX10ly {'hsa': {'l'}, 'mmu': {'l'}}
NACHEX4ly {'hsa': {'l'}, 'mmu': {'l'}}
NACHEXA11ly {'hsa': {'l'}, 'mmu': {'l'}}
NACHEX19ly {'hsa': {'l'}, 'mmu': {'l'}}
NACHEXA7ly {'hsa': {'l'}, 'mmu': {'l'}}
NACHEXA17ly {'hsa': {'l'}, 'mmu': {'l'}}
NACHEX11ly {'hsa': {'l'}, 'mmu': {'l'}}
NACHEX5ly {'hsa': {'l'}, 'mmu': {'l'}}
NACHEX1ly {'hsa': {'l'}, 'mmu': {'l'}}
NACHEXA8ly {'hsa': {'l'}, 'mmu': {'l'}}
NACHEXA18ly {'hsa': {'l'}, 'mmu': {'l'}}
NACHEX6ly {'hsa': {'l'}, 'mmu': {'l'}}
NACHEX20ly {'hsa': {'l'}, 'mmu': {'l'}}
NACHEXA9ly {'hsa': {'l'}, 'mmu': {'l'}}
NACHEXA19ly {'hsa': {'l'}, 'mmu': {'l'}}
NACHEX7ly {'hsa': {'l'}, 'mmu': {'l'}}
NACHEX21ly {'hsa': {'l'}, 'mmu': {'l'}}
NACHEXA1ly {'hsa': {'l'}, 'mmu': {'l'}}
NACHEX8ly {'hsa': {'l'}, 'mmu': {'l'}}
NACHEX22ly {'hsa': {'l'}, 'mmu': {'l'}}
NACHEXA20ly {'hsa': {'l'}, 'mmu': {'l'}}
NACHEX9ly {'hsa': {'l'}, 'mmu': {'l'}}
NACHEX23ly {'hsa': {'l'}, 'mmu': {'l'}}
NACHEXA21ly {'hsa': {'l'}, 'mmu': {'l'}}
NACHEXA10ly {'hsa': {'l'}, 'mmu': {'l'}}
NACHEX24ly {'hsa': {'l'}, 'mmu': {'l'}}
NACHEXA16ly {'hsa': {'l'}, 'mmu': {'l'}}
NACHEXA22ly {'hsa': {'l'}, 'mmu': {'l'}}
NACHEXA12ly {'hsa': {'l'}, 'mmu': {'l'}}
NACHEX25ly {'hsa': {'l'}, 'mmu': {'l'}}
NACHEX12ly {'hsa': {'l'}, 'mmu': {'l'}}
NACHEX13ly {'hsa': {'l'}, 'mmu': {'l'}}
NACHEXA2ly {'hsa': {'l'}, 'mmu': {'l'}}
NACHEXA13ly {'hsa': {'l'}, 'mmu': {'l'}}
NACHEX26ly {'hsa': {'l'}, 'mmu': {'l'}}
NACHEX14ly {'hsa': {'l'}, 'mmu': {'l'}}
NACHEX15ly {'hsa': {'l'}, 'mmu': {'l'}}
NACHEXA3ly {'hsa': {'l'}, 'mmu': {'l'}}
NACHEX16ly {'hsa': {'l'}, 'mmu': {'l'}}
NACHEXA14ly {'hsa': {'l'}, 'mmu': {'l'}}
NACHEX27ly {'hsa': {'l'}, 'mmu': {'l'}}
AHEXASE2ly {'hsa': {'l'}, 'mmu': {'l'}}
NACHEXA4ly {'hsa': {'l'}, 'mmu': {'l'}}
NACHEX17ly {'hsa': {'l'}, 'mmu': {'l'}}
NACHEXA15ly {'hsa': {'l'}, 'mmu': {'l'}}
NACHEX2ly {'hsa': {'l'}, 'mmu': {'l'}}
AHEXASEly {'hsa': {'l'}, 'mmu': {'l'}}
NACHEXA5ly {'hsa': {'l'}, 'mmu': {'l'}}
NACHEX18ly {'hsa': {'l'}, 'mmu': {'l'}}
NACHEX3ly {'hsa': {'l'}, 'mmu': {'l'}}
CYTDtl {'mmu': {'c', 'l'}, 'hsa': {'c', 'l'}}
HXANtl {'mmu': {'c', 'l'}, 'hsa': {'c', 'l'}}
ADEtl {'mmu': {'c', 'l'}, 'hsa': {'c', 'l'}}
GSNtl {'mmu': {'c', 'l'}, 'hsa': {'c', 'l'}}
INStl {'mmu': {'c', 'l'}, 'hsa': {'c', 'l'}}
THYMDtl {'mmu': {'c', 'l'}, 'hsa': {'c', 'l'}}
Uritl {'mmu': {'c', 'l'}, 'hsa': {'c', 'l'}}
ADNtl {'mmu': {'c', 'l'}, 'hsa': {'c', 'l'}}
```

In [29]:

```
Reaction_subset_Jeff = dict()
for k, v in Reaction_comp_Jeff.items():
    temp = sum([list(x) for x in v.values()],[])
    if not any(x in temp for x in ['l']):
        Reaction_subset_Jeff.setdefault(k,str())
    else:
        Reaction_subset_Jeff.setdefault(k,str())
print(len(Reaction_subset_Jeff))
```

```
2366
```

In [31]:

```
for k, v in Reaction_rev_Jeff.items():
    if k in Reaction_subset_Jeff:
        if len(set(v.values())) > 1:
            print(k, v)
```

```
XYLTD_D {'yli': 'rev', 'sce': 'for'}
GCCbim {'hsa': 'for', 'mmu': 'for', 'sce': 'for', 'yli': 'rev'}
GLYCLm {'sce': 'for', 'yli': 'back'}
PYAM5PO {'sce': 'for', 'mmu': 'rev', 'hsa': 'rev', 'yli': 'for'}
PYDXNO {'sce': 'rev', 'yli': 'for'}
PDX5POi {'sce': 'rev', 'mmu': 'rev', 'hsa': 'rev', 'yli': 'for'}
GTHPi {'sce': 'rev', 'hsa': 'rev', 'yli': 'for', 'mmu': 'rev'}
GTHPm {'hsa': 'rev', 'yli': 'for', 'mmu': 'rev', 'sce': 'rev'}
SULR {'yli': 'for', 'sce': 'rev'}
G3PD1ir {'sce': 'for', 'yli': 'for', 'hsa': 'rev', 'mmu': 'rev'}
ABTA {'yli': 'rev', 'sce': 'for'}
VALTAim {'sce': 'for', 'yli': 'rev'}
FACOAL180 {'hsa': 'for', 'mmu': 'for', 'sce': 'rev', 'yli': 'rev'}
FACOAL181 {'hsa': 'for', 'mmu': 'for', 'sce': 'rev'}
FACOAL160 {'hsa': 'for', 'mmu': 'for', 'sce': 'rev', 'yli': 'rev'}
TRPTA {'sce': 'rev', 'yli': 'for'}
TYRTAim {'yli': 'rev', 'hsa': 'rev', 'mmu': 'rev', 'sce': 'for'}
ATHRDHr {'yli': 'for', 'sce': 'rev'}
ACCOAC {'yli': 'rev', 'mmu': 'for', 'sce': 'rev', 'hsa': 'for'}
ACCOACrm {'yli': 'rev', 'sce': 'rev', 'mmu': 'for', 'hsa': 'for'}
G6PDH2r {'hsa': 'rev', 'sce': 'for', 'mmu': 'rev'}
DHQTi {'yli': 'rev', 'sce': 'for'}
PSCVT {'yli': 'rev', 'sce': 'for'}
34DHPLACOX_NADP {'hsa': 'rev', 'mmu': 'rev', 'yli': 'for'}
HSERTA {'sce': 'rev', 'yli': 'for'}
SFGTHi {'hsa': 'rev', 'sce': 'rev', 'yli': 'for', 'mmu': 'rev'}
GLCt1 {'hsa': 'rev', 'sce': 'for', 'yli': 'for'}
RDH1a {'hsa': 'rev', 'mmu': 'for'}
RDH3a {'hsa': 'rev', 'mmu': 'for'}
RDH2a {'hsa': 'rev', 'mmu': 'for'}
MTHFD {'sce': 'rev', 'mmu': 'rev', 'yli': 'for', 'hsa': 'rev'}
FTHFLi {'sce': 'for', 'mmu': 'for', 'yli': 'for', 'hsa': 'rev'}
FTHFLmi {'hsa': 'rev', 'sce': 'for', 'mmu': 'for'}
AHCi {'mmu': 'rev', 'yli': 'for', 'sce': 'for', 'hsa': 'rev'}
ARGSS {'sce': 'rev', 'hsa': 'for', 'mmu': 'for'}
PSERT {'yli': 'rev', 'hsa': 'for', 'sce': 'for', 'mmu': 'for'}
ADSL1r {'yli': 'rev', 'sce': 'rev', 'mmu': 'for', 'hsa': 'for'}
ADSL2r {'sce': 'rev', 'mmu': 'for', 'hsa': 'for'}
DURIPP {'yli': 'for', 'mmu': 'rev', 'hsa': 'rev', 'sce': 'rev'}
NP1 {'yli': 'rev', 'mmu': 'for', 'hsa': 'for'}
INSTt2 {'yli': 'for', 'mmu': 'rev', 'hsa': 'rev', 'sce': 'for'}
THRA {'sce': 'for', 'yli': 'rev'}
THRA2 {'sce': 'for', 'yli': 'rev'}
PIt2m {'hsa': 'rev', 'sce': 'rev', 'yli': 'rev', 'mmu': 'for'}
ECOAH1m {'hsa': 'rev', 'yli': 'back', 'mmu': 'rev'}
UDPGD {'hsa': 'for', 'mmu': 'for', 'yli': 'rev'}
ACOAHim {'yli': 'back', 'sce': 'for'}
ALCD2x {'yli': 'rev', 'hsa': 'for'}
34DHOXPEGOX {'yli': 'back', 'hsa': 'rev'}
HEX10 {'hsa': 'for', 'yli': 'rev', 'mmu': 'for'}
ACGAM6PS {'mmu': 'for', 'sce': 'rev', 'hsa': 'for', 'yli': 'for'}
BILGLCURte {'mmu': 'for', 'hsa': 'rev'}
BILDGLCURte {'mmu': 'for', 'hsa': 'rev'}
GARFT {'sce': 'for', 'yli': 'for', 'mmu': 'rev', 'hsa': 'rev'}
PPNDH {'sce': 'for', 'yli': 'rev'}
CHORM {'yli': 'rev', 'sce': 'for'}
KARA1im {'sce': 'for', 'yli': 'rev'}
KARA2im {'sce': 'for', 'yli': 'rev'}
NNAM {'sce': 'rev', 'yli': 'for'}
PEAMNO {'yli': 'rev', 'mmu': 'for', 'hsa': 'for'}
NMNAT {'hsa': 'rev', 'sce': 'for', 'mmu': 'rev'}
ADNK1 {'yli': 'rev', 'sce': 'for', 'mmu': 'for', 'hsa': 'for'}
SUCCtm {'mmu': 'rev', 'sce': 'for', 'yli': 'for'}
FACOAL140 {'sce': 'rev', 'mmu': 'for'}
FACOAL80 {'mmu': 'for', 'hsa': 'for', 'yli': 'rev'}
ICDHym {'yli': 'for', 'hsa': 'rev', 'sce': 'for', 'mmu': 'rev'}
HSDy {'sce': 'back', 'yli': 'rev'}
HSDxi {'sce': 'for', 'yli': 'rev'}
COAtim {'mmu': 'rev', 'yli': 'for', 'sce': 'for', 'hsa': 'rev'}
HMGCOAS {'sce': 'rev', 'hsa': 'back', 'mmu': 'back'}
HMGCOASm {'sce': 'rev', 'mmu': 'back', 'hsa': 'back'}
PRAGSr {'mmu': 'rev', 'hsa': 'rev', 'yli': 'for', 'sce': 'rev'}
ACOATA {'sce': 'rev', 'yli': 'for'}
IGPS {'sce': 'for', 'yli': 'rev'}
ANPRT {'sce': 'for', 'yli': 'rev'}
ORNTACim {'sce': 'for', 'yli': 'rev'}
CYSTGL {'yli': 'rev', 'hsa': 'for', 'mmu': 'for', 'sce': 'for'}
ASPCT {'mmu': 'rev', 'sce': 'for', 'yli': 'for', 'hsa': 'rev'}
HSTPT {'sce': 'for', 'yli': 'rev'}
ACACT1m {'hsa': 'rev', 'yli': 'for', 'sce': 'for', 'mmu': 'rev'}
```

In [32]:

```
for k, v in Reaction_rev_Jeff.items():
    if k in Reaction_subset_Jeff:
        temp = sum([list(x) for x in v.values()],[])
        if not (all(temp) or not any(temp)):
            if 'sce' in v:
                Reaction_subset_Jeff[k] = ('sce',v['sce'])
            elif 'hsa' in v:
                Reaction_subset_Jeff[k] = ('hsa',v['hsa'])
            else:
                print(k, v)
        else:
            if 'sce' in v:
                Reaction_subset_Jeff[k] = ('sce',v['sce'])
            elif 'hsa' in v:
                Reaction_subset_Jeff[k] = ('hsa',v['hsa'])
            elif 'mmu' in v:
                Reaction_subset_Jeff[k] = ('mmu',v['mmu'])
            elif 'yli' in v:
                Reaction_subset_Jeff[k] = ('yli',v['yli'])
            else:
                print(k, v)
```

In [33]:

```
for k, v in Reaction_subset_Jeff.items():
    if k in eco.reactions:
        if not Model_rev[k]['eco'] == v[1]:
            print(k, v, 'eco', Model_rev[k]['eco'])
    elif k in sce.reactions:
        if not Model_rev[k]['sce'] == v[1]:
            print(k, v, 'sce', Model_rev[k]['sce'])
```

```
GTHOr ('sce', 'for') eco rev
PTPATi ('hsa', 'rev') eco for
PTPATim ('yli', 'rev') sce for
PDX5POi ('sce', 'rev') eco for
GTHPi ('sce', 'rev') eco for
SULR ('sce', 'rev') eco for
OCBT ('sce', 'for') eco rev
AATA ('hsa', 'back') sce rev
AMPTASECG ('yli', 'rev') eco for
ACCOAC ('sce', 'rev') eco for
G6PDH2r ('sce', 'for') eco rev
CYSDS ('yli', 'rev') eco for
SHK3Dr ('sce', 'for') eco rev
PSCVT ('sce', 'for') eco rev
ALDD19xr ('sce', 'for') eco rev
PRASCSi ('sce', 'rev') eco for
SFGTHi ('sce', 'rev') eco for
MDH ('yli', 'for') eco rev
CYSTA ('hsa', 'rev') eco for
ARGSS ('sce', 'rev') eco for
ECOAH1 ('yli', 'back') eco rev
AP4AH ('sce', 'rev') eco for
ALCD2x ('hsa', 'for') eco rev
PRMICI ('sce', 'for') eco rev
ALLTN ('sce', 'rev') eco for
ASPK ('sce', 'for') eco rev
MTRI ('yli', 'for') sce rev
GALUi ('sce', 'rev') eco for
GARFT ('sce', 'for') eco rev
LCARS ('hsa', 'back') eco rev
AOXSr2 ('yli', 'rev') eco for
DHORDfum ('yli', 'rev') eco for
NNAM ('sce', 'rev') eco for
GALKr ('sce', 'for') eco rev
NNATr ('sce', 'for') eco rev
SPRMS ('yli', 'rev') sce for
ICDHyr ('sce', 'for') eco rev
HSDy ('sce', 'back') eco rev
FA141ACPHi ('sce', 'rev') eco for
FA120ACPHi ('sce', 'rev') eco for
FA160ACPHi ('sce', 'rev') eco for
FA140ACPHi ('sce', 'rev') eco for
FA161ACPHi ('sce', 'rev') eco for
ASAD ('sce', 'back') eco rev
FALDH2 ('yli', 'for') eco rev
GLUDy ('sce', 'back') eco rev
ACACT1r ('sce', 'for') eco rev
```

In [34]:

```
for k, v in Reaction_subset_Jeff.items():
    if k in eco.reactions:
        if not Model_rev[k]['eco'] == v[1]:
            Reaction_subset_Jeff[k] = ('eco', Model_rev[k]['eco'])
    elif k in sce.reactions:
        if not Model_rev[k]['sce'] == v[1]:
            Reaction_subset_Jeff[k] = ('sce', Model_rev[k]['sce'])
```

In [35]:

```
for k in set(Reaction_subset).intersection(Reaction_subset_Jeff):
    if not Reaction_subset[k][1] == Reaction_subset_Jeff[k][1]:
        print(k, Reaction_subset[k], Reaction_subset_Jeff[k])
```

```
NNATn ('cre', 'rev') ('hsa', 'for')
ECOAH9m ('cre', 'for') ('hsa', 'rev')
PPCOAOm ('cre', 'rev') ('hsa', 'for')
FACOAL1832 ('cre', 'for') ('hsa', 'rev')
PEAMNO ('yli', 'rev') ('hsa', 'for')
HEX10 ('yli', 'rev') ('hsa', 'for')
OCOAT1 ('ppu', 'for') ('yli', 'rev')
ALCD2y ('cre', 'back') ('hsa', 'for')
ECOAH12m ('cre', 'for') ('hsa', 'rev')
NP1 ('yli', 'rev') ('hsa', 'for')
GDHm ('cre', 'for') ('hsa', 'rev')
MMTSAO ('ppu', 'for') ('yli', 'rev')
LALDO ('cre', 'for') ('hsa', 'rev')
ACACT10m ('cre', 'for') ('hsa', 'rev')
FACOAL1821 ('cre', 'for') ('hsa', 'rev')
FACOAL80 ('yli', 'rev') ('hsa', 'for')
SHSL2r ('ppu', 'rev') ('yli', 'for')
ECOAH1m ('yli', 'back') ('hsa', 'rev')
34DHOXPEGOX ('yli', 'back') ('hsa', 'rev')
MAOX ('cre', 'rev') ('hsa', 'for')
PRPNCOAHYDm ('cre', 'rev') ('hsa', 'for')
FACOAL1831 ('cre', 'for') ('hsa', 'rev')
```

In [36]:

```
Reaction_subset_union = dict()
for k in set(Reaction_subset).intersection(Reaction_subset_Jeff):
    if not Reaction_subset[k][1] == Reaction_subset_Jeff[k][1]:
        if 'ppu' in Reaction_subset[k]:
            Reaction_subset_union[k] = Reaction_subset[k]
        elif 'hsa' in Reaction_subset_Jeff[k]:
            Reaction_subset_union[k] = Reaction_subset_Jeff[k]
        else:
            print(k, Reaction_subset[k], Reaction_subset_Jeff[k])
    else:
        Reaction_subset_union[k] = Reaction_subset[k]
for k in set(Reaction_subset).difference(Reaction_subset_Jeff):
    Reaction_subset_union[k] = Reaction_subset[k]
for k in set(Reaction_subset_Jeff).difference(Reaction_subset):
    Reaction_subset_union[k] = Reaction_subset_Jeff[k]
print(len(Reaction_subset_union))
```

```
3804
```

In [42]:

```
Reaction_to_gene['PGI']
```

Out[42]:

```
{'eco|b4025': {'rtoi|9589'},
 'ppu|PP_1808': {'rtoi|9589'},
 'sce|YBR196C': {'rtoi|9589'}}
```

In [37]:

```
import sympy
from sympy.logic.boolalg import to_dnf
from sympy.parsing.sympy_parser import parse_expr
Reaction_rule = dict()
for k, v in Reaction_to_gene.items():
    if k in Reaction_subset:
        for m in ['sce','eco','ppu','cre','yli']:
            if any(k2.startswith(m) for k2 in v):
                gpr = eval(m).reactions.get_by_id(k).gene_reaction_rule
                gpr = parse_expr(gpr.replace(' or ',' | ').replace(' and ',' & '))
                gpr = to_dnf(gpr)
                for k2, v2 in v.items():
                    if k2.startswith(m):
                        if len(v2) > 1:
                            temp = '('+' | '.join(x.replace('rtoi|','rtoi_') for x in v2)+')'
                        else:
                            temp = next(iter(v2)).replace('rtoi|','rtoi_')
                        temp = parse_expr(temp)
                        gpr = gpr.subs(k2.replace(m+'|',''), temp)
                gpr = to_dnf(gpr)
                gpr2 = gpr
                if isinstance(gpr, sympy.Or):
                    for x in gpr.args:
                        if isinstance(x, sympy.Symbol) and (not str(x).startswith('rtoi_')):
                            if gpr.count(x) > 1:
                                print(k, v)
                            gpr2 = to_dnf(gpr2.subs(x,0))
#                gpr2 = str(gpr2).replace(' | ',' or ').replace(' & ',' and ').replace('rtoi_','')
                Reaction_rule.setdefault(k, dict())[m] = gpr2
```

In [43]:

```
Reaction_rule['PGI']
```

Out[43]:

```
{'eco': rtoi_9589, 'ppu': rtoi_9589, 'sce': rtoi_9589}
```

In [38]:

```
print(len(Reaction_rule))
Reaction_rule_unique = dict()
for k, v in Reaction_rule.items():
    if len(set(v2 for v2 in v.values())) == 1:
        Reaction_rule_unique[k] = list(v.values())[0]
print(len(Reaction_rule_unique))
```

```
2995
2705
```

In [44]:

```
Reaction_to_gene_Jeff['PGI']
```

Out[44]:

```
{'hsa|GPI': {'rtoi|9589'},
 'mmu|Gpi1': {'rtoi|9589'},
 'sce|YBR196C': {'rtoi|9589'}}
```

In [39]:

```
hsa_dict_rev = {g.id: g.name for g in hsa.genes if g.name}
mmu_dict_rev = {g.id: g.name for g in mmu.genes if g.name}
```

In [40]:

```
import re
Reaction_rule_Jeff = dict()
for k, v in Reaction_to_gene_Jeff.items():
    if k in Reaction_subset_Jeff:
        for m in ['hsa','mmu']:
            if any(k2.startswith(m) for k2 in v):
                gpr = eval(m).reactions.get_by_id(k).gene_reaction_rule
                for x in gpr.split():
                    temp = x.strip('()')
                    if not any(y in temp for y in ['or', 'and']):
                        gpr = re.sub(r'\b'+temp+r'\b', m+'_'+temp, gpr)
                gpr = parse_expr(gpr.replace(' or ',' | ').replace(' and ',' & '))
                for x in gpr.free_symbols:
                    if str(x).replace(m+'_','') in eval(m+'_dict_rev'):
                        gpr = gpr.subs(x, eval(m+'_dict_rev')[str(x).replace(m+'_','')])
                gpr = to_dnf(gpr)
                for k2, v2 in v.items():
                    if k2.startswith(m):
                        if len(v2) > 1:
                            temp = '('+' | '.join(x.replace('rtoi|','rtoi_') for x in v2)+')'
                        else:
                            temp = next(iter(v2)).replace('rtoi|','rtoi_')
                        temp = parse_expr(temp)
                        gpr = gpr.subs(k2.replace(m+'|',''), temp)
                gpr = to_dnf(gpr)
                gpr2 = gpr
                if isinstance(gpr, sympy.Or):
                    for x in gpr.args:
                        if isinstance(x, sympy.Symbol) and (not str(x).startswith('rtoi_')):
                            gpr2 = to_dnf(gpr2.subs(x,0))
                            if gpr.count(x) > 1:
                                print(k, x, gpr, gpr2)                            
#                gpr2 = str(gpr2).replace(' | ',' or ').replace(' & ',' and ').replace('rtoi_','')
                Reaction_rule_Jeff.setdefault(k, dict())[m] = gpr2
        for m in ['sce','yli']:
            if any(k2.startswith(m) for k2 in v):
                gpr = eval(m).reactions.get_by_id(k).gene_reaction_rule
                gpr = parse_expr(gpr.replace(' or ',' | ').replace(' and ',' & '))
                gpr = to_dnf(gpr)
                for k2, v2 in v.items():
                    if k2.startswith(m):
                        if len(v2) > 1:
                            temp = '('+' | '.join(x.replace('rtoi|','rtoi_') for x in v2)+')'
                        else:
                            temp = next(iter(v2)).replace('rtoi|','rtoi_')
                        temp = parse_expr(temp)
                        gpr = gpr.subs(k2.replace(m+'|',''), temp)
                gpr = to_dnf(gpr)
                gpr2 = gpr
                if isinstance(gpr, sympy.Or):
                    for x in gpr.args:
                        if isinstance(x, sympy.Symbol) and (not str(x).startswith('rtoi_')):
                            gpr2 = to_dnf(gpr2.subs(x,0))
                            if gpr.count(x) > 1:
                                print(k, v, gpr, gpr2)
#                gpr2 = str(gpr2).replace(' | ',' or ').replace(' & ',' and ').replace('rtoi_','')
                Reaction_rule_Jeff.setdefault(k, dict())[m] = gpr2
```

```
ALCD2x ADH1A ADH1A | ADH1B | ADH1C | ADH4 | ADH6 | ADH7 | rtoi_11665 | rtoi_13657 | rtoi_14108 | rtoi_14109 | (ADH1A & ADH1B) | (ADH1A & ADH1C) | (ADH1B & ADH1C) ADH1B | ADH1C | ADH4 | ADH6 | ADH7 | rtoi_11665 | rtoi_13657 | rtoi_14108 | rtoi_14109 | (ADH1B & ADH1C)
ALCD2x ADH1B ADH1A | ADH1B | ADH1C | ADH4 | ADH6 | ADH7 | rtoi_11665 | rtoi_13657 | rtoi_14108 | rtoi_14109 | (ADH1A & ADH1B) | (ADH1A & ADH1C) | (ADH1B & ADH1C) ADH1C | ADH4 | ADH6 | ADH7 | rtoi_11665 | rtoi_13657 | rtoi_14108 | rtoi_14109
ALCD2x ADH1C ADH1A | ADH1B | ADH1C | ADH4 | ADH6 | ADH7 | rtoi_11665 | rtoi_13657 | rtoi_14108 | rtoi_14109 | (ADH1A & ADH1B) | (ADH1A & ADH1C) | (ADH1B & ADH1C) ADH4 | ADH6 | ADH7 | rtoi_11665 | rtoi_13657 | rtoi_14108 | rtoi_14109
ALCD21_D ADH1A ADH1A | ADH1B | ADH1C | ADH4 | ADH6 | ADH7 | rtoi_11665 | rtoi_13657 | rtoi_14108 | rtoi_14109 | (ADH1A & ADH1B) | (ADH1A & ADH1C) | (ADH1B & ADH1C) ADH1B | ADH1C | ADH4 | ADH6 | ADH7 | rtoi_11665 | rtoi_13657 | rtoi_14108 | rtoi_14109 | (ADH1B & ADH1C)
ALCD21_D ADH1B ADH1A | ADH1B | ADH1C | ADH4 | ADH6 | ADH7 | rtoi_11665 | rtoi_13657 | rtoi_14108 | rtoi_14109 | (ADH1A & ADH1B) | (ADH1A & ADH1C) | (ADH1B & ADH1C) ADH1C | ADH4 | ADH6 | ADH7 | rtoi_11665 | rtoi_13657 | rtoi_14108 | rtoi_14109
ALCD21_D ADH1C ADH1A | ADH1B | ADH1C | ADH4 | ADH6 | ADH7 | rtoi_11665 | rtoi_13657 | rtoi_14108 | rtoi_14109 | (ADH1A & ADH1B) | (ADH1A & ADH1C) | (ADH1B & ADH1C) ADH4 | ADH6 | ADH7 | rtoi_11665 | rtoi_13657 | rtoi_14108 | rtoi_14109
ALCD22_L ADH1A ADH1A | ADH1B | ADH1C | ADH4 | ADH6 | ADH7 | rtoi_11665 | rtoi_13657 | rtoi_14108 | rtoi_14109 | (ADH1A & ADH1B) | (ADH1A & ADH1C) | (ADH1B & ADH1C) ADH1B | ADH1C | ADH4 | ADH6 | ADH7 | rtoi_11665 | rtoi_13657 | rtoi_14108 | rtoi_14109 | (ADH1B & ADH1C)
ALCD22_L ADH1B ADH1A | ADH1B | ADH1C | ADH4 | ADH6 | ADH7 | rtoi_11665 | rtoi_13657 | rtoi_14108 | rtoi_14109 | (ADH1A & ADH1B) | (ADH1A & ADH1C) | (ADH1B & ADH1C) ADH1C | ADH4 | ADH6 | ADH7 | rtoi_11665 | rtoi_13657 | rtoi_14108 | rtoi_14109
ALCD22_L ADH1C ADH1A | ADH1B | ADH1C | ADH4 | ADH6 | ADH7 | rtoi_11665 | rtoi_13657 | rtoi_14108 | rtoi_14109 | (ADH1A & ADH1B) | (ADH1A & ADH1C) | (ADH1B & ADH1C) ADH4 | ADH6 | ADH7 | rtoi_11665 | rtoi_13657 | rtoi_14108 | rtoi_14109
LCARS ADH1A ADH1A | ADH1B | ADH1C | ADH4 | ADH6 | ADH7 | rtoi_11665 | rtoi_13657 | rtoi_14108 | rtoi_14109 | (ADH1A & ADH1B) | (ADH1A & ADH1C) | (ADH1B & ADH1C) ADH1B | ADH1C | ADH4 | ADH6 | ADH7 | rtoi_11665 | rtoi_13657 | rtoi_14108 | rtoi_14109 | (ADH1B & ADH1C)
LCARS ADH1B ADH1A | ADH1B | ADH1C | ADH4 | ADH6 | ADH7 | rtoi_11665 | rtoi_13657 | rtoi_14108 | rtoi_14109 | (ADH1A & ADH1B) | (ADH1A & ADH1C) | (ADH1B & ADH1C) ADH1C | ADH4 | ADH6 | ADH7 | rtoi_11665 | rtoi_13657 | rtoi_14108 | rtoi_14109
LCARS ADH1C ADH1A | ADH1B | ADH1C | ADH4 | ADH6 | ADH7 | rtoi_11665 | rtoi_13657 | rtoi_14108 | rtoi_14109 | (ADH1A & ADH1B) | (ADH1A & ADH1C) | (ADH1B & ADH1C) ADH4 | ADH6 | ADH7 | rtoi_11665 | rtoi_13657 | rtoi_14108 | rtoi_14109
ALCD1 ADH1A ADH1A | ADH1B | ADH1C | ADH4 | ADH6 | ADH7 | rtoi_11665 | rtoi_13657 | rtoi_14108 | rtoi_14109 | (ADH1A & ADH1B) | (ADH1A & ADH1C) | (ADH1B & ADH1C) ADH1B | ADH1C | ADH4 | ADH6 | ADH7 | rtoi_11665 | rtoi_13657 | rtoi_14108 | rtoi_14109 | (ADH1B & ADH1C)
ALCD1 ADH1B ADH1A | ADH1B | ADH1C | ADH4 | ADH6 | ADH7 | rtoi_11665 | rtoi_13657 | rtoi_14108 | rtoi_14109 | (ADH1A & ADH1B) | (ADH1A & ADH1C) | (ADH1B & ADH1C) ADH1C | ADH4 | ADH6 | ADH7 | rtoi_11665 | rtoi_13657 | rtoi_14108 | rtoi_14109
ALCD1 ADH1C ADH1A | ADH1B | ADH1C | ADH4 | ADH6 | ADH7 | rtoi_11665 | rtoi_13657 | rtoi_14108 | rtoi_14109 | (ADH1A & ADH1B) | (ADH1A & ADH1C) | (ADH1B & ADH1C) ADH4 | ADH6 | ADH7 | rtoi_11665 | rtoi_13657 | rtoi_14108 | rtoi_14109
ALCD22_D ADH1A ADH1A | ADH1B | ADH1C | ADH4 | ADH6 | ADH7 | rtoi_11665 | rtoi_13657 | rtoi_14108 | rtoi_14109 | (ADH1A & ADH1B) | (ADH1A & ADH1C) | (ADH1B & ADH1C) ADH1B | ADH1C | ADH4 | ADH6 | ADH7 | rtoi_11665 | rtoi_13657 | rtoi_14108 | rtoi_14109 | (ADH1B & ADH1C)
ALCD22_D ADH1B ADH1A | ADH1B | ADH1C | ADH4 | ADH6 | ADH7 | rtoi_11665 | rtoi_13657 | rtoi_14108 | rtoi_14109 | (ADH1A & ADH1B) | (ADH1A & ADH1C) | (ADH1B & ADH1C) ADH1C | ADH4 | ADH6 | ADH7 | rtoi_11665 | rtoi_13657 | rtoi_14108 | rtoi_14109
ALCD22_D ADH1C ADH1A | ADH1B | ADH1C | ADH4 | ADH6 | ADH7 | rtoi_11665 | rtoi_13657 | rtoi_14108 | rtoi_14109 | (ADH1A & ADH1B) | (ADH1A & ADH1C) | (ADH1B & ADH1C) ADH4 | ADH6 | ADH7 | rtoi_11665 | rtoi_13657 | rtoi_14108 | rtoi_14109
HPYRR2x LDHA LDHA | LDHAL6A | LDHAL6B | LDHB | LDHC | rtoi_13328 | (LDHA & LDHB) LDHAL6A | LDHAL6B | LDHB | LDHC | rtoi_13328
HPYRR2x LDHB LDHA | LDHAL6A | LDHAL6B | LDHB | LDHC | rtoi_13328 | (LDHA & LDHB) LDHC | rtoi_13328
HPYRR2x Ldha Ldha | Ldhal6b | Ldhb | Ldhc | mmu_160287 | rtoi_13328 | (Ldha & Ldhb) Ldhal6b | Ldhb | Ldhc | mmu_160287 | rtoi_13328
HPYRR2x Ldhb Ldha | Ldhal6b | Ldhb | Ldhc | mmu_160287 | rtoi_13328 | (Ldha & Ldhb) Ldhc | mmu_160287 | rtoi_13328
GLXO1 LDHA LDHA | LDHAL6A | LDHAL6B | LDHB | LDHC | UEVLD | rtoi_13328 | (LDHA & LDHB) LDHAL6A | LDHAL6B | LDHB | LDHC | UEVLD | rtoi_13328
GLXO1 LDHB LDHA | LDHAL6A | LDHAL6B | LDHB | LDHC | UEVLD | rtoi_13328 | (LDHA & LDHB) LDHC | UEVLD | rtoi_13328
```

In [45]:

```
Reaction_rule_Jeff['PGI']
```

Out[45]:

```
{'hsa': rtoi_9589, 'mmu': rtoi_9589, 'sce': rtoi_9589}
```

In [41]:

```
print(len(Reaction_rule_Jeff))
Reaction_rule_Jeff_unique = dict()
for k, v in Reaction_rule_Jeff.items():
    if len(set(v2 for v2 in v.values())) == 1:
        Reaction_rule_Jeff_unique[k] = list(v.values())[0]
print(len(Reaction_rule_Jeff_unique))
```

```
2366
2172
```

In [46]:

```
print(len(Reaction_rule))
print(len(Reaction_rule_Jeff))
print(len(set(Reaction_rule.keys()).union(Reaction_rule_Jeff)))
print(len(set(Reaction_rule.keys()).intersection(Reaction_rule_Jeff)))
print(len(set(Reaction_rule_unique.keys()).intersection(Reaction_rule_Jeff)))
print(len(set(Reaction_rule.keys()).intersection(Reaction_rule_Jeff_unique)))
```

```
2995
2366
3804
1557
1308
1414
```

In [47]:

```
print(len(Reaction_rule_unique))
print(len(Reaction_rule_Jeff_unique))
print(len(set(Reaction_rule_unique.keys()).intersection(Reaction_rule_Jeff_unique)))
Reaction_rule_final = dict()
for x in set(Reaction_rule_unique.keys()).intersection(Reaction_rule_Jeff_unique):
    if Reaction_rule_unique[x] == Reaction_rule_Jeff_unique[x]:
        Reaction_rule_final[x] = Reaction_rule_unique[x]
print(len(Reaction_rule_final))
```

```
2705
2172
1273
1038
```

In [48]:

```
for x in set(Reaction_rule_unique.keys()).intersection(Reaction_rule_Jeff_unique):
    if not Reaction_rule_unique[x] == Reaction_rule_Jeff_unique[x]:
        temp1 = Reaction_rule_unique[x]
        temp2 = Reaction_rule_Jeff_unique[x]
        if set(temp1.free_symbols).difference(temp2.free_symbols) and set(temp2.free_symbols).difference(temp1.free_symbols):
            if not all(str(y).startswith('rtoi_') for y in temp1.free_symbols) and \
               not all(str(y).startswith('rtoi_') for y in temp2.free_symbols):
                print(x)
                print('\t',temp1)
                print('\t',temp2)
```

```
CYOOm
	 Q0045 & Q0250 & Q0275 & YGL191W & YLR395C & YMR256C & rtoi_10685 & rtoi_11317 & rtoi_11769 & rtoi_13628 & rtoi_8802 & rtoi_9165
	 Q0045 & Q0250 & Q0275 & YLR395C & YMR256C & rtoi_10685 & rtoi_11317 & rtoi_11769 & rtoi_13007 & rtoi_13628 & rtoi_8802 & rtoi_9165
CYOR_u6m
	 Q0105 & YBL045C & YFR033C & rtoi_11618 & rtoi_12966 & rtoi_13608 & rtoi_13614 & rtoi_15231 & rtoi_15681 & rtoi_8802 & rtoi_9198
	 Q0105 & YBL045C & YHR001W_A & rtoi_11618 & rtoi_12966 & rtoi_13614 & rtoi_15231 & rtoi_15681 & rtoi_15758 & rtoi_8802 & rtoi_9198
yli_R0430
	 YALI0D14850g & YALIfMp01 & YALIfMp02 & YALIfMp16 & YALIfMp19 & YALIfMp20 & YALIfMp28 & YALIfMp29 & rtoi_10010 & rtoi_10318 & rtoi_11151 & rtoi_11702 & rtoi_12497 & rtoi_12543 & rtoi_13017 & rtoi_13214 & rtoi_13766 & rtoi_14078 & rtoi_14418 & rtoi_14900 & rtoi_15179 & rtoi_15864 & rtoi_15947 & rtoi_15973 & rtoi_8699 & rtoi_8909 & rtoi_8972 & rtoi_9348
	 YALIfMp01 & YALIfMp02 & YALIfMp16 & YALIfMp19 & YALIfMp20 & YALIfMp28 & YALIfMp29 & rtoi_10010 & rtoi_10318 & rtoi_11151 & rtoi_11702 & rtoi_12497 & rtoi_12543 & rtoi_13017 & rtoi_13214 & rtoi_13766 & rtoi_14078 & rtoi_14261 & rtoi_14418 & rtoi_14900 & rtoi_15179 & rtoi_15864 & rtoi_15947 & rtoi_15973 & rtoi_8699 & rtoi_8909 & rtoi_8972 & rtoi_9348
yli_R0432
	 YALI0D14850g & YALIfMp01 & YALIfMp02 & YALIfMp16 & YALIfMp19 & YALIfMp20 & YALIfMp28 & YALIfMp29 & rtoi_10010 & rtoi_10318 & rtoi_11151 & rtoi_11702 & rtoi_12497 & rtoi_12543 & rtoi_13017 & rtoi_13214 & rtoi_13766 & rtoi_14078 & rtoi_14418 & rtoi_14900 & rtoi_15179 & rtoi_15864 & rtoi_15947 & rtoi_15973 & rtoi_8699 & rtoi_8909 & rtoi_8972 & rtoi_9348
	 YALIfMp01 & YALIfMp02 & YALIfMp16 & YALIfMp19 & YALIfMp20 & YALIfMp28 & YALIfMp29 & rtoi_10010 & rtoi_10318 & rtoi_11151 & rtoi_11702 & rtoi_12497 & rtoi_12543 & rtoi_13017 & rtoi_13214 & rtoi_13766 & rtoi_14078 & rtoi_14261 & rtoi_14418 & rtoi_14900 & rtoi_15179 & rtoi_15864 & rtoi_15947 & rtoi_15973 & rtoi_8699 & rtoi_8909 & rtoi_8972 & rtoi_9348
```

In [49]:

```
Reaction_rule_final['CYOOm'] = parse_expr('Q0045 & Q0250 & Q0275 & YLR395C & YMR256C & rtoi_10685 & rtoi_11317 &\
                                           rtoi_11769 & rtoi_13007 & rtoi_13628 & rtoi_8802 & rtoi_9165')
Reaction_rule_final['CYOR_u6m'] = parse_expr('Q0105 & YBL045C & rtoi_11618 & rtoi_12966 & rtoi_13608 & rtoi_13614 &\
                                              rtoi_15231 & rtoi_15681 & rtoi_15758 & rtoi_8802 & rtoi_9198')
Reaction_rule_final['yli_R0430'] = parse_expr('YALIfMp01 & YALIfMp02 & YALIfMp16 & YALIfMp19 & YALIfMp20 & YALIfMp28 &\
                                               YALIfMp29 & rtoi_10010 & rtoi_10318 & rtoi_11151 & rtoi_11702 &\
                                               rtoi_12497 & rtoi_12543 & rtoi_13017 & rtoi_13214 & rtoi_13766 &\
                                               rtoi_14078 & rtoi_14261 & rtoi_14418 & rtoi_14900 & rtoi_15179 &\
                                               rtoi_15864 & rtoi_15947 & rtoi_15973 & rtoi_8699 & rtoi_8909 &\
                                               rtoi_8972 & rtoi_9348')
Reaction_rule_final['yli_R0432'] = parse_expr('YALIfMp01 & YALIfMp02 & YALIfMp16 & YALIfMp19 & YALIfMp20 & YALIfMp28 &\
                                               YALIfMp29 & rtoi_10010 & rtoi_10318 & rtoi_11151 & rtoi_11702 &\
                                               rtoi_12497 & rtoi_12543 & rtoi_13017 & rtoi_13214 & rtoi_13766 &\
                                               rtoi_14078 & rtoi_14261 & rtoi_14418 & rtoi_14900 & rtoi_15179 &\
                                               rtoi_15864 & rtoi_15947 & rtoi_15973 & rtoi_8699 & rtoi_8909 &\
                                               rtoi_8972 & rtoi_9348')
```

In [50]:

```
for x in set(Reaction_rule_unique.keys()).intersection(Reaction_rule_Jeff_unique):
    if not Reaction_rule_unique[x] == Reaction_rule_Jeff_unique[x]:
        temp1 = Reaction_rule_unique[x]
        temp2 = Reaction_rule_Jeff_unique[x]
        if set(temp1.free_symbols).difference(temp2.free_symbols) and set(temp2.free_symbols).difference(temp1.free_symbols):
            if not all(str(y).startswith('rtoi_') for y in temp1.free_symbols) and \
               all(str(y).startswith('rtoi_') for y in temp2.free_symbols):
                print(x)
                print('\t',temp1)
                print('\t',temp2)
```

```
GLYGS
	 (YJL137C & rtoi_9596) | (YKR058W & rtoi_9596)
	 rtoi_14002 & rtoi_9596
THIORDXm
	 YCR083W & rtoi_10200
	 rtoi_10200 & rtoi_15339
GLCS2
	 (YJL137C & rtoi_9596) | (YKR058W & rtoi_9596)
	 rtoi_14002 & rtoi_9596
```

In [51]:

```
for x in set(Reaction_rule_unique.keys()).intersection(Reaction_rule_Jeff_unique):
    if not Reaction_rule_unique[x] == Reaction_rule_Jeff_unique[x]:
        temp1 = Reaction_rule_unique[x]
        temp2 = Reaction_rule_Jeff_unique[x]
        if set(temp1.free_symbols).difference(temp2.free_symbols) and set(temp2.free_symbols).difference(temp1.free_symbols):
            if not all(str(y).startswith('rtoi_') for y in temp1.free_symbols) and \
               all(str(y).startswith('rtoi_') for y in temp2.free_symbols):
                Reaction_rule_final[x] = temp2
```

In [52]:

```
for x in set(Reaction_rule_unique.keys()).intersection(Reaction_rule_Jeff_unique):
    if not Reaction_rule_unique[x] == Reaction_rule_Jeff_unique[x]:
        temp1 = Reaction_rule_unique[x]
        temp2 = Reaction_rule_Jeff_unique[x]
        if set(temp1.free_symbols).difference(temp2.free_symbols) and set(temp2.free_symbols).difference(temp1.free_symbols):
            if all(str(y).startswith('rtoi_') for y in temp1.free_symbols) and \
               not all(str(y).startswith('rtoi_') for y in temp2.free_symbols):
                print(x)
                print('\t',temp1)
                print('\t',temp2)
```

```
NFTYROX
	 (rtoi_13286 & rtoi_13361) | (rtoi_13286 & rtoi_8979) | (rtoi_13286 & rtoi_9188)
	 YDR402C & rtoi_13286
```

In [53]:

```
for x in set(Reaction_rule_unique.keys()).intersection(Reaction_rule_Jeff_unique):
    if not Reaction_rule_unique[x] == Reaction_rule_Jeff_unique[x]:
        temp1 = Reaction_rule_unique[x]
        temp2 = Reaction_rule_Jeff_unique[x]
        if set(temp1.free_symbols).difference(temp2.free_symbols) and set(temp2.free_symbols).difference(temp1.free_symbols):
            if all(str(y).startswith('rtoi_') for y in temp1.free_symbols) and \
               not all(str(y).startswith('rtoi_') for y in temp2.free_symbols):
                Reaction_rule_final[x] = temp1
```

In [54]:

```
for x in set(Reaction_rule_unique.keys()).intersection(Reaction_rule_Jeff_unique):
    if not Reaction_rule_unique[x] == Reaction_rule_Jeff_unique[x]:
        temp1 = Reaction_rule_unique[x]
        temp2 = Reaction_rule_Jeff_unique[x]
        if set(temp1.free_symbols).difference(temp2.free_symbols) and set(temp2.free_symbols).difference(temp1.free_symbols):
            if all(str(y).startswith('rtoi_') for y in temp1.free_symbols) and \
               all(str(y).startswith('rtoi_') for y in temp2.free_symbols):
                print(x)
                print('\t',temp1)
                print('\t',temp2)
```

```
ALKP
	 rtoi_14546
	 rtoi_13409
G3PT
	 rtoi_13044
	 rtoi_13413
PPDOy
	 rtoi_10029 | rtoi_13554 | rtoi_13562 | rtoi_13947
	 rtoi_11882 | rtoi_12784 | rtoi_16543 | rtoi_9774
DNADDP
	 rtoi_15385
	 rtoi_14638
ECOAH1x
	 rtoi_11362
	 rtoi_11907
LALDO2x
	 rtoi_9515
	 rtoi_16543
NACt
	 rtoi_13555 | rtoi_9534 | rtoi_9938
	 rtoi_12221 | rtoi_13555
GLUt2m
	 rtoi_8766
	 rtoi_16799
HACD9m
	 rtoi_11362
	 rtoi_16284
ACOAD10m
	 rtoi_10012 | rtoi_12570
	 rtoi_12570 | rtoi_14070
ACACT10m
	 rtoi_13813
	 rtoi_8678
MAOX
	 rtoi_10308
	 rtoi_13959
LYSt6
	 rtoi_10317 | rtoi_12690 | rtoi_9277 | rtoi_9291
	 rtoi_10317 | rtoi_12219 | rtoi_12690
CATp
	 rtoi_15305
	 rtoi_9354
yli_R1420
	 rtoi_14950
	 rtoi_16607
LCARS
	 rtoi_12307 | rtoi_9101
	 rtoi_11665 | rtoi_13657 | rtoi_14108 | rtoi_14109
ALR2
	 rtoi_11882 | rtoi_15285
	 rtoi_11882 | rtoi_12784 | rtoi_16543 | rtoi_9774
HISt6
	 rtoi_10317 | rtoi_12690 | rtoi_9277 | rtoi_9291
	 rtoi_10317 | rtoi_12219 | rtoi_12690
```

In [55]:

```
for x in set(Reaction_rule_unique.keys()).intersection(Reaction_rule_Jeff_unique):
    if not Reaction_rule_unique[x] == Reaction_rule_Jeff_unique[x]:
        temp1 = Reaction_rule_unique[x]
        temp2 = Reaction_rule_Jeff_unique[x]
        if set(temp1.free_symbols).difference(temp2.free_symbols) and set(temp2.free_symbols).difference(temp1.free_symbols):
            if all(str(y).startswith('rtoi_') for y in temp1.free_symbols) and \
               all(str(y).startswith('rtoi_') for y in temp2.free_symbols):
                Reaction_rule_final[x] = sympy.Or(temp1,temp2)
```

In [56]:

```
for x in sorted(set(Reaction_rule_unique.keys()).intersection(Reaction_rule_Jeff_unique)):
    if not Reaction_rule_unique[x] == Reaction_rule_Jeff_unique[x]:
        temp1 = Reaction_rule_unique[x]
        temp2 = Reaction_rule_Jeff_unique[x]
        if set(temp1.free_symbols).difference(temp2.free_symbols) and not set(temp2.free_symbols).difference(temp1.free_symbols):
            print(x)
            print('\t',temp1)
            print('\t',temp2)
```

```
ACITL
	 CRv4_Au5_s5_g12182_t1 & rtoi_9726
	 rtoi_9726
ACOAD8m
	 rtoi_10012 | rtoi_12570
	 rtoi_10012
ACOAD9m
	 rtoi_10012 | rtoi_12570
	 rtoi_12570
AKGMALtm
	 rtoi_11740 | rtoi_13510
	 rtoi_13510
ALLTNti
	 rtoi_10921 | rtoi_11871 | rtoi_12902
	 rtoi_12902
ARAB14LO
	 rtoi_10490 | rtoi_13733
	 rtoi_10490
CITL
	 b0614 & b0615 & b0617 & rtoi_9822
	 rtoi_9822
EPISTESTH_SCe
	 rtoi_14247 | rtoi_14617
	 rtoi_14247
ERGSTESTH_SCe
	 rtoi_14247 | rtoi_14617
	 rtoi_14247
FACOAL100p
	 rtoi_11167 | rtoi_12538 | rtoi_12555 | rtoi_15748
	 rtoi_12538 | rtoi_12555
FACOAL120p
	 rtoi_11167 | rtoi_12538 | rtoi_12555 | rtoi_15748
	 rtoi_12538 | rtoi_12555
FACOAL140
	 rtoi_11167 | rtoi_12538 | rtoi_12555 | rtoi_15748
	 rtoi_11167 | rtoi_15748
FACOAL140p
	 rtoi_11167 | rtoi_12538 | rtoi_12555 | rtoi_15748
	 rtoi_12538 | rtoi_12555
FACOAL141
	 rtoi_11167 | rtoi_12538 | rtoi_12555 | rtoi_15748
	 rtoi_11167 | rtoi_15748
FACOAL141p
	 rtoi_11167 | rtoi_12538 | rtoi_12555 | rtoi_15748
	 rtoi_12538 | rtoi_12555
FACOAL160p
	 rtoi_11167 | rtoi_12538 | rtoi_12555 | rtoi_15748
	 rtoi_12538 | rtoi_12555
FACOAL161p
	 rtoi_11167 | rtoi_12538 | rtoi_12555 | rtoi_15748
	 rtoi_12538 | rtoi_12555
FACOAL182
	 rtoi_11167 | rtoi_12538 | rtoi_12555 | rtoi_15748
	 rtoi_11167 | rtoi_15748
FACOAL1821
	 rtoi_11167 | rtoi_12538 | rtoi_12555 | rtoi_15748
	 rtoi_12538 | rtoi_12555
FACOAL1831
	 rtoi_11167 | rtoi_12538 | rtoi_12555 | rtoi_15748
	 rtoi_12538 | rtoi_12555
FACOAL1832
	 rtoi_11167 | rtoi_12538 | rtoi_12555 | rtoi_15748
	 rtoi_12538 | rtoi_12555
FACOAL80
	 rtoi_11167 | rtoi_12538 | rtoi_12555 | rtoi_15748
	 rtoi_11167 | rtoi_15748
FACOAL80p
	 rtoi_11167 | rtoi_12538 | rtoi_12555 | rtoi_15748
	 rtoi_12538 | rtoi_12555
FACOAL_160_m
	 rtoi_11167 | rtoi_12538 | rtoi_12555 | rtoi_15748
	 rtoi_11167 | rtoi_15748
FAS100
	 (rtoi_12826 & rtoi_8639 & rtoi_8670 & rtoi_8777) | (rtoi_14471 & rtoi_8639 & rtoi_8670 & rtoi_8777)
	 (rtoi_12826 & rtoi_8639 & rtoi_8670) | (rtoi_12826 & rtoi_8639 & rtoi_8777)
FAS100COA
	 (rtoi_12826 & rtoi_8639 & rtoi_8670 & rtoi_8777) | (rtoi_14471 & rtoi_8639 & rtoi_8670 & rtoi_8777)
	 (rtoi_12826 & rtoi_8639 & rtoi_8670) | (rtoi_12826 & rtoi_8639 & rtoi_8777)
FAS120
	 (rtoi_12826 & rtoi_8639 & rtoi_8670 & rtoi_8777) | (rtoi_14471 & rtoi_8639 & rtoi_8670 & rtoi_8777)
	 (rtoi_12826 & rtoi_8639 & rtoi_8670) | (rtoi_12826 & rtoi_8639 & rtoi_8777)
FAS120COA
	 (rtoi_12826 & rtoi_8639 & rtoi_8670 & rtoi_8777) | (rtoi_14471 & rtoi_8639 & rtoi_8670 & rtoi_8777)
	 (rtoi_12826 & rtoi_8639 & rtoi_8670) | (rtoi_12826 & rtoi_8639 & rtoi_8777)
FAS140
	 (rtoi_12826 & rtoi_8639 & rtoi_8670 & rtoi_8777) | (rtoi_14471 & rtoi_8639 & rtoi_8670 & rtoi_8777)
	 (rtoi_12826 & rtoi_8639 & rtoi_8670) | (rtoi_12826 & rtoi_8639 & rtoi_8777)
FAS160
	 (rtoi_12826 & rtoi_8639 & rtoi_8670 & rtoi_8777) | (rtoi_14471 & rtoi_8639 & rtoi_8670 & rtoi_8777)
	 (rtoi_12826 & rtoi_8639 & rtoi_8670) | (rtoi_12826 & rtoi_8639 & rtoi_8777)
FECOSTESTH_SCe
	 rtoi_14247 | rtoi_14617
	 rtoi_14247
GLYCLTDy
	 rtoi_11036 | rtoi_12051
	 rtoi_11036
GUAD
	 rtoi_9050 | rtoi_9708
	 rtoi_9050
LALDO
	 rtoi_12286 | rtoi_14108 | rtoi_14109
	 rtoi_14108 | rtoi_14109
LANOSTESTH_SCe
	 rtoi_14247 | rtoi_14617
	 rtoi_14247
MALtm
	 rtoi_11740 | rtoi_13510
	 rtoi_13510
MBCOAi
	 rtoi_10012 | rtoi_12570
	 rtoi_10012
NADH2_u6m
	 rtoi_14261 | rtoi_9809
	 rtoi_14261
PEPAT
	 rtoi_13208 | rtoi_9438
	 rtoi_9438
PI45BP5P_SC
	 rtoi_15340 | rtoi_15545
	 rtoi_15340
PRDX
	 rtoi_15305 | rtoi_9354
	 rtoi_9354
PSERDg_SC
	 rtoi_10504 | rtoi_11446
	 rtoi_11446
PSERDv_SC
	 rtoi_10504 | rtoi_11446
	 rtoi_11446
RNDR1n
	 (YGR180C & rtoi_11290 & rtoi_15339) | (YGR180C & rtoi_11290 & rtoi_16019)
	 YGR180C & rtoi_11290 & rtoi_15339
RNDR2n
	 (YGR180C & rtoi_11290 & rtoi_15339) | (YGR180C & rtoi_11290 & rtoi_16019)
	 YGR180C & rtoi_11290 & rtoi_15339
RNDR3n
	 (YGR180C & rtoi_11290 & rtoi_15339) | (YGR180C & rtoi_11290 & rtoi_16019)
	 YGR180C & rtoi_11290 & rtoi_15339
RNDR4n
	 (YGR180C & rtoi_11290 & rtoi_15339) | (YGR180C & rtoi_11290 & rtoi_16019)
	 YGR180C & rtoi_11290 & rtoi_15339
SUCCtm
	 rtoi_11740 | rtoi_13510
	 rtoi_13510
THIORDXni
	 (rtoi_15037 & rtoi_15339) | (rtoi_15037 & rtoi_16019)
	 rtoi_15037 & rtoi_15339
THIORDXp
	 (rtoi_13262 & rtoi_15339) | (rtoi_13262 & rtoi_16019)
	 rtoi_13262 & rtoi_15339
THMt2
	 rtoi_10921 | rtoi_11871 | rtoi_12902
	 rtoi_12902
URAt2
	 rtoi_10921 | rtoi_11871 | rtoi_12902
	 rtoi_12902
ZYMSTESTH_SCe
	 rtoi_14247 | rtoi_14617
	 rtoi_14247
yli_R0141
	 rtoi_11167 | rtoi_12538 | rtoi_12555 | rtoi_15748
	 rtoi_11167 | rtoi_15748
yli_R0142
	 rtoi_11167 | rtoi_12538 | rtoi_12555 | rtoi_15748
	 rtoi_11167 | rtoi_15748
yli_R0143
	 rtoi_11167 | rtoi_12538 | rtoi_12555 | rtoi_15748
	 rtoi_11167 | rtoi_15748
yli_R0144
	 rtoi_11167 | rtoi_12538 | rtoi_12555 | rtoi_15748
	 rtoi_11167 | rtoi_15748
yli_R0146
	 rtoi_11167 | rtoi_12538 | rtoi_12555 | rtoi_15748
	 rtoi_11167 | rtoi_15748
yli_R0148
	 rtoi_11167 | rtoi_12538 | rtoi_12555 | rtoi_15748
	 rtoi_11167 | rtoi_15748
yli_R0149
	 rtoi_11167 | rtoi_12538 | rtoi_12555 | rtoi_15748
	 rtoi_11167 | rtoi_15748
yli_R0150
	 rtoi_11167 | rtoi_12538 | rtoi_12555 | rtoi_15748
	 rtoi_11167 | rtoi_15748
yli_R0151
	 rtoi_11167 | rtoi_12538 | rtoi_12555 | rtoi_15748
	 rtoi_11167 | rtoi_15748
yli_R0233
	 rtoi_11167 | rtoi_12538 | rtoi_12555 | rtoi_15748
	 rtoi_11167 | rtoi_15748
yli_R0234
	 rtoi_11167 | rtoi_12538 | rtoi_12555 | rtoi_15748
	 rtoi_11167 | rtoi_15748
yli_R0235
	 rtoi_11167 | rtoi_12538 | rtoi_12555 | rtoi_15748
	 rtoi_11167 | rtoi_15748
yli_R0236
	 rtoi_11167 | rtoi_12538 | rtoi_12555 | rtoi_15748
	 rtoi_11167 | rtoi_15748
yli_R0238
	 rtoi_11167 | rtoi_12538 | rtoi_12555 | rtoi_15748
	 rtoi_11167 | rtoi_15748
yli_R0239
	 rtoi_11167 | rtoi_12538 | rtoi_12555 | rtoi_15748
	 rtoi_11167 | rtoi_15748
yli_R0240
	 rtoi_11167 | rtoi_12538 | rtoi_12555 | rtoi_15748
	 rtoi_11167 | rtoi_15748
yli_R0241
	 rtoi_11167 | rtoi_12538 | rtoi_12555 | rtoi_15748
	 rtoi_11167 | rtoi_15748
yli_R0242
	 rtoi_11167 | rtoi_12538 | rtoi_12555 | rtoi_15748
	 rtoi_11167 | rtoi_15748
yli_R0243
	 rtoi_11167 | rtoi_12538 | rtoi_12555 | rtoi_15748
	 rtoi_11167 | rtoi_15748
yli_R0244
	 rtoi_11167 | rtoi_12538 | rtoi_12555 | rtoi_15748
	 rtoi_11167 | rtoi_15748
yli_R0338
	 rtoi_10504 | rtoi_11446 | rtoi_14554
	 rtoi_11446 | rtoi_14554
yli_R0816
	 rtoi_15305 | rtoi_9354
	 rtoi_9354
yli_R1114
	 rtoi_10921 | rtoi_11871 | rtoi_12902
	 rtoi_12902
yli_R1411
	 rtoi_10504 | rtoi_11446 | rtoi_14554
	 rtoi_11446 | rtoi_14554
yli_R1435
	 rtoi_8678 | rtoi_8885
	 rtoi_8678
```

In [57]:

```
for x in set(Reaction_rule_unique.keys()).intersection(Reaction_rule_Jeff_unique):
    if not Reaction_rule_unique[x] == Reaction_rule_Jeff_unique[x]:
        temp1 = Reaction_rule_unique[x]
        temp2 = Reaction_rule_Jeff_unique[x]
        if set(temp1.free_symbols).difference(temp2.free_symbols) and not set(temp2.free_symbols).difference(temp1.free_symbols):
            Reaction_rule_final[x] = temp1
```

In [58]:

```
for x in sorted(set(Reaction_rule_unique.keys()).intersection(Reaction_rule_Jeff_unique)):
    if not Reaction_rule_unique[x] == Reaction_rule_Jeff_unique[x]:
        temp1 = Reaction_rule_unique[x]
        temp2 = Reaction_rule_Jeff_unique[x]
        if not set(temp1.free_symbols).difference(temp2.free_symbols) and set(temp2.free_symbols).difference(temp1.free_symbols):
            print(x)
            print('\t',temp1)
            print('\t',temp2)
```

```
ACACT4m
	 rtoi_13813
	 rtoi_13813 | rtoi_9065
ACACT4p
	 rtoi_13813
	 rtoi_13813 | rtoi_9065
ACACT5p
	 rtoi_13813
	 rtoi_13813 | rtoi_9065
ACACT6m
	 rtoi_13813
	 rtoi_13813 | rtoi_9065
ACACT6p
	 rtoi_13813
	 rtoi_13813 | rtoi_9065
ACACT7m
	 rtoi_13813
	 rtoi_13813 | rtoi_9065
ACACT7p
	 rtoi_13813
	 rtoi_13813 | rtoi_9065
ACACT8p
	 rtoi_13813
	 rtoi_13813 | rtoi_9065
ACACT9p
	 rtoi_13813
	 rtoi_13813 | rtoi_9065
ACHLE1
	 rtoi_15240
	 rtoi_15133 | rtoi_15240
ACHLE2
	 rtoi_15240
	 rtoi_15133 | rtoi_15240
ACHLE3
	 rtoi_15240
	 rtoi_15133 | rtoi_15240
ACOAO4p
	 rtoi_12742 | rtoi_9700
	 rtoi_12742 | rtoi_12752 | rtoi_9700
ACOAO5p
	 rtoi_12742 | rtoi_9700
	 rtoi_12742 | rtoi_12752 | rtoi_9700
ACOAO6p
	 rtoi_12742 | rtoi_9700
	 rtoi_12742 | rtoi_12752 | rtoi_9700
ACOAO7p
	 rtoi_12742 | rtoi_9700
	 rtoi_12742 | rtoi_12752 | rtoi_9700
ACOAO8p
	 rtoi_12742 | rtoi_9700
	 rtoi_12742 | rtoi_12752 | rtoi_9700
ACOAO9p
	 rtoi_12742 | rtoi_9700
	 rtoi_12742 | rtoi_12752 | rtoi_9700
ACS2
	 rtoi_14597
	 rtoi_14597 | rtoi_15276
ALCD22yi
	 rtoi_10029 | rtoi_11882 | rtoi_13554 | rtoi_13562 | rtoi_13947
	 rtoi_10029 | rtoi_11882 | rtoi_12784 | rtoi_13554 | rtoi_13562 | rtoi_13947 | rtoi_9774
ALCD2y
	 rtoi_12784 | rtoi_9774
	 rtoi_11882 | rtoi_12784 | rtoi_9774
ALDD2x
	 rtoi_12042 | rtoi_13426
	 rtoi_12042 | rtoi_13426 | rtoi_15814 | rtoi_16323
ALLTTti
	 rtoi_15069 | rtoi_15070
	 rtoi_10379 | rtoi_15069 | rtoi_15070
AMID
	 rtoi_10276 | rtoi_10277 | rtoi_12640
	 rtoi_10276 | rtoi_10277 | rtoi_12540 | rtoi_12553 | rtoi_12640 | rtoi_12842
C3STDH1
	 rtoi_8835
	 rtoi_13724 | rtoi_8835
C3STDH2
	 rtoi_8835
	 rtoi_13724 | rtoi_8835
FAO141p_even
	 (rtoi_10293 & rtoi_11362 & rtoi_12742 & rtoi_13228 & rtoi_13813) | (rtoi_10293 & rtoi_11362 & rtoi_13228 & rtoi_13813 & rtoi_9700)
	 (rtoi_10293 & rtoi_11362 & rtoi_12742 & rtoi_13228 & rtoi_13813) | (rtoi_10293 & rtoi_11362 & rtoi_12742 & rtoi_13228 & rtoi_9065) | (rtoi_10293 & rtoi_11362 & rtoi_12752 & rtoi_13228 & rtoi_13813) | (rtoi_10293 & rtoi_11362 & rtoi_12752 & rtoi_13228 & rtoi_9065) | (rtoi_10293 & rtoi_11362 & rtoi_13228 & rtoi_13813 & rtoi_9700) | (rtoi_10293 & rtoi_11362 & rtoi_13228 & rtoi_9065 & rtoi_9700)
FAO141p_odd
	 (rtoi_10293 & rtoi_11362 & rtoi_12742 & rtoi_13813) | (rtoi_10293 & rtoi_11362 & rtoi_13813 & rtoi_9700)
	 (rtoi_10293 & rtoi_11362 & rtoi_12742 & rtoi_13813) | (rtoi_10293 & rtoi_11362 & rtoi_12742 & rtoi_9065) | (rtoi_10293 & rtoi_11362 & rtoi_12752 & rtoi_13813) | (rtoi_10293 & rtoi_11362 & rtoi_12752 & rtoi_9065) | (rtoi_10293 & rtoi_11362 & rtoi_13813 & rtoi_9700) | (rtoi_10293 & rtoi_11362 & rtoi_9065 & rtoi_9700)
FAO161p_even
	 (rtoi_10293 & rtoi_11362 & rtoi_12742 & rtoi_13228 & rtoi_13813) | (rtoi_10293 & rtoi_11362 & rtoi_13228 & rtoi_13813 & rtoi_9700)
	 (rtoi_10293 & rtoi_11362 & rtoi_12742 & rtoi_13228 & rtoi_13813) | (rtoi_10293 & rtoi_11362 & rtoi_12742 & rtoi_13228 & rtoi_9065) | (rtoi_10293 & rtoi_11362 & rtoi_12752 & rtoi_13228 & rtoi_13813) | (rtoi_10293 & rtoi_11362 & rtoi_12752 & rtoi_13228 & rtoi_9065) | (rtoi_10293 & rtoi_11362 & rtoi_13228 & rtoi_13813 & rtoi_9700) | (rtoi_10293 & rtoi_11362 & rtoi_13228 & rtoi_9065 & rtoi_9700)
FAO161p_odd
	 (rtoi_10293 & rtoi_11362 & rtoi_12742 & rtoi_13813) | (rtoi_10293 & rtoi_11362 & rtoi_13813 & rtoi_9700)
	 (rtoi_10293 & rtoi_11362 & rtoi_12742 & rtoi_13813) | (rtoi_10293 & rtoi_11362 & rtoi_12742 & rtoi_9065) | (rtoi_10293 & rtoi_11362 & rtoi_12752 & rtoi_13813) | (rtoi_10293 & rtoi_11362 & rtoi_12752 & rtoi_9065) | (rtoi_10293 & rtoi_11362 & rtoi_13813 & rtoi_9700) | (rtoi_10293 & rtoi_11362 & rtoi_9065 & rtoi_9700)
FAO181p_even
	 (rtoi_10293 & rtoi_11362 & rtoi_12742 & rtoi_13228 & rtoi_13813) | (rtoi_10293 & rtoi_11362 & rtoi_13228 & rtoi_13813 & rtoi_9700)
	 (rtoi_10293 & rtoi_11362 & rtoi_12742 & rtoi_13228 & rtoi_13813) | (rtoi_10293 & rtoi_11362 & rtoi_12742 & rtoi_13228 & rtoi_9065) | (rtoi_10293 & rtoi_11362 & rtoi_12752 & rtoi_13228 & rtoi_13813) | (rtoi_10293 & rtoi_11362 & rtoi_12752 & rtoi_13228 & rtoi_9065) | (rtoi_10293 & rtoi_11362 & rtoi_13228 & rtoi_13813 & rtoi_9700) | (rtoi_10293 & rtoi_11362 & rtoi_13228 & rtoi_9065 & rtoi_9700)
FAO181p_odd
	 (rtoi_10293 & rtoi_11362 & rtoi_12742 & rtoi_13813) | (rtoi_10293 & rtoi_11362 & rtoi_13813 & rtoi_9700)
	 (rtoi_10293 & rtoi_11362 & rtoi_12742 & rtoi_13813) | (rtoi_10293 & rtoi_11362 & rtoi_12742 & rtoi_9065) | (rtoi_10293 & rtoi_11362 & rtoi_12752 & rtoi_13813) | (rtoi_10293 & rtoi_11362 & rtoi_12752 & rtoi_9065) | (rtoi_10293 & rtoi_11362 & rtoi_13813 & rtoi_9700) | (rtoi_10293 & rtoi_11362 & rtoi_9065 & rtoi_9700)
FAO182p_eveneven
	 (rtoi_10293 & rtoi_11362 & rtoi_12742 & rtoi_13228 & rtoi_13813) | (rtoi_10293 & rtoi_11362 & rtoi_13228 & rtoi_13813 & rtoi_9700)
	 (rtoi_10293 & rtoi_11362 & rtoi_12742 & rtoi_13228 & rtoi_13813) | (rtoi_10293 & rtoi_11362 & rtoi_12742 & rtoi_13228 & rtoi_9065) | (rtoi_10293 & rtoi_11362 & rtoi_12752 & rtoi_13228 & rtoi_13813) | (rtoi_10293 & rtoi_11362 & rtoi_12752 & rtoi_13228 & rtoi_9065) | (rtoi_10293 & rtoi_11362 & rtoi_13228 & rtoi_13813 & rtoi_9700) | (rtoi_10293 & rtoi_11362 & rtoi_13228 & rtoi_9065 & rtoi_9700)
FAO182p_evenodd
	 (rtoi_10293 & rtoi_11362 & rtoi_12742 & rtoi_13228 & rtoi_13813) | (rtoi_10293 & rtoi_11362 & rtoi_13228 & rtoi_13813 & rtoi_9700)
	 (rtoi_10293 & rtoi_11362 & rtoi_12742 & rtoi_13228 & rtoi_13813) | (rtoi_10293 & rtoi_11362 & rtoi_12742 & rtoi_13228 & rtoi_9065) | (rtoi_10293 & rtoi_11362 & rtoi_12752 & rtoi_13228 & rtoi_13813) | (rtoi_10293 & rtoi_11362 & rtoi_12752 & rtoi_13228 & rtoi_9065) | (rtoi_10293 & rtoi_11362 & rtoi_13228 & rtoi_13813 & rtoi_9700) | (rtoi_10293 & rtoi_11362 & rtoi_13228 & rtoi_9065 & rtoi_9700)
FAO182p_oddodd
	 (rtoi_10293 & rtoi_11362 & rtoi_12742 & rtoi_13813) | (rtoi_10293 & rtoi_11362 & rtoi_13813 & rtoi_9700)
	 (rtoi_10293 & rtoi_11362 & rtoi_12742 & rtoi_13813) | (rtoi_10293 & rtoi_11362 & rtoi_12742 & rtoi_9065) | (rtoi_10293 & rtoi_11362 & rtoi_12752 & rtoi_13813) | (rtoi_10293 & rtoi_11362 & rtoi_12752 & rtoi_9065) | (rtoi_10293 & rtoi_11362 & rtoi_13813 & rtoi_9700) | (rtoi_10293 & rtoi_11362 & rtoi_9065 & rtoi_9700)
FAO240p
	 (rtoi_11362 & rtoi_12742 & rtoi_13813) | (rtoi_11362 & rtoi_13813 & rtoi_9700)
	 (rtoi_11362 & rtoi_12742 & rtoi_13813) | (rtoi_11362 & rtoi_12742 & rtoi_9065) | (rtoi_11362 & rtoi_12752 & rtoi_13813) | (rtoi_11362 & rtoi_12752 & rtoi_9065) | (rtoi_11362 & rtoi_13813 & rtoi_9700) | (rtoi_11362 & rtoi_9065 & rtoi_9700)
FAO80p
	 (rtoi_11362 & rtoi_12742 & rtoi_13813) | (rtoi_11362 & rtoi_13813 & rtoi_9700)
	 (rtoi_11362 & rtoi_12742 & rtoi_13813) | (rtoi_11362 & rtoi_12742 & rtoi_9065) | (rtoi_11362 & rtoi_12752 & rtoi_13813) | (rtoi_11362 & rtoi_12752 & rtoi_9065) | (rtoi_11362 & rtoi_13813 & rtoi_9700) | (rtoi_11362 & rtoi_9065 & rtoi_9700)
FRUt2
	 rtoi_11893
	 rtoi_11075 | rtoi_11893
G3PD1ir
	 rtoi_12154
	 rtoi_12154 | rtoi_14576
G3PD1irm
	 rtoi_12154
	 rtoi_12154 | rtoi_14576
GCC2cm
	 rtoi_10040
	 rtoi_10040 & rtoi_10205 & rtoi_12898 & rtoi_15184
GLUK
	 rtoi_10264
	 rtoi_10264 | rtoi_11982
GLYCDy
	 rtoi_11882
	 rtoi_11882 | rtoi_12784 | rtoi_9774
GLYO1
	 rtoi_15994
	 rtoi_15449 | rtoi_15994
H2Otm
	 rtoi_13986
	 rtoi_13986 | rtoi_13987 | rtoi_9014 | rtoi_9015
HBCO_nadp
	 rtoi_15180
	 rtoi_11203 | rtoi_15180
HEX7
	 rtoi_11982
	 rtoi_10264 | rtoi_11982
ILETA
	 rtoi_14610 | rtoi_14853
	 rtoi_14014 | rtoi_14610 | rtoi_14853
ILETAm
	 rtoi_14610 | rtoi_14853
	 rtoi_14014 | rtoi_14610 | rtoi_14853
IMACTD
	 rtoi_12042 | rtoi_13426
	 rtoi_12042 | rtoi_13426 | rtoi_15814 | rtoi_16323
KAT180_m
	 rtoi_13813
	 rtoi_13813 | rtoi_9065
LALDO3
	 rtoi_10497 | rtoi_11160 | rtoi_12151
	 rtoi_10497 | rtoi_11160 | rtoi_12151 | rtoi_12883
LEUTA
	 rtoi_14610 | rtoi_14853
	 rtoi_14014 | rtoi_14610 | rtoi_14853
LEUTAm
	 rtoi_14610 | rtoi_14853
	 rtoi_14014 | rtoi_14610 | rtoi_14853
MACCOAT
	 rtoi_13813
	 rtoi_13813 | rtoi_9065
MANt2
	 rtoi_11893
	 rtoi_11075 | rtoi_11893
ME1m
	 rtoi_13917
	 rtoi_12761 | rtoi_13917
ME2
	 rtoi_12761
	 rtoi_12761 | rtoi_13917
ME2m
	 rtoi_13917
	 rtoi_12761 | rtoi_13917
MI1PP
	 rtoi_16369
	 rtoi_13708 | rtoi_16369
MI3PP
	 rtoi_16369
	 rtoi_13708 | rtoi_16369
MI4PP
	 rtoi_16369
	 rtoi_13708 | rtoi_16369
OIVD3m
	 rtoi_11183 | rtoi_11188 | rtoi_12566 | rtoi_15436
	 (rtoi_10040 & rtoi_11183 & rtoi_12086 & rtoi_15436) | (rtoi_10040 & rtoi_11183 & rtoi_12566 & rtoi_15436) | (rtoi_10040 & rtoi_11188 & rtoi_12086 & rtoi_15436) | (rtoi_10040 & rtoi_11188 & rtoi_12566 & rtoi_15436)
OMCDC
	 rtoi_14610 | rtoi_14853
	 rtoi_14014 | rtoi_14610 | rtoi_14853
OMCDCm
	 rtoi_14610 | rtoi_14853
	 rtoi_14014 | rtoi_14610 | rtoi_14853
PIt5m
	 rtoi_8889
	 rtoi_15874 | rtoi_8889
PPCOAOm
	 rtoi_10408
	 rtoi_10408 | rtoi_12570 | rtoi_14070
SACCD1
	 rtoi_16833
	 rtoi_10577 | rtoi_16833
SACCD4m
	 rtoi_16833
	 rtoi_10577 | rtoi_16833
SELMETAT
	 rtoi_13771
	 rtoi_13771 | (rtoi_11793 & rtoi_13771)
TRPTA
	 rtoi_14908 | rtoi_15839
	 rtoi_12407 | rtoi_14908 | rtoi_15839
VALTA
	 rtoi_14610 | rtoi_14853
	 rtoi_14014 | rtoi_14610 | rtoi_14853
VALTAim
	 rtoi_14610 | rtoi_14853
	 rtoi_14014 | rtoi_14610 | rtoi_14853
yli_R0191
	 rtoi_12742 | rtoi_9700
	 rtoi_12742 | rtoi_12752 | rtoi_9700
yli_R0194
	 rtoi_13813
	 rtoi_13813 | rtoi_9065
yli_R0195
	 (rtoi_12742 & rtoi_13813 & rtoi_14805) | (rtoi_13813 & rtoi_14805 & rtoi_9700)
	 (rtoi_12742 & rtoi_13813 & rtoi_14805) | (rtoi_12742 & rtoi_14805 & rtoi_9065) | (rtoi_12752 & rtoi_13813 & rtoi_14805) | (rtoi_12752 & rtoi_14805 & rtoi_9065) | (rtoi_13813 & rtoi_14805 & rtoi_9700) | (rtoi_14805 & rtoi_9065 & rtoi_9700)
yli_R0196
	 rtoi_12742 | rtoi_9700
	 rtoi_12742 | rtoi_12752 | rtoi_9700
yli_R0200
	 rtoi_12742 | rtoi_9700
	 rtoi_12742 | rtoi_12752 | rtoi_9700
yli_R0204
	 rtoi_12742 | rtoi_9700
	 rtoi_12742 | rtoi_12752 | rtoi_9700
yli_R0208
	 rtoi_12742 | rtoi_9700
	 rtoi_12742 | rtoi_12752 | rtoi_9700
yli_R0211
	 rtoi_13813
	 rtoi_13813 | rtoi_9065
yli_R0212
	 rtoi_12742 | rtoi_9700
	 rtoi_12742 | rtoi_12752 | rtoi_9700
yli_R0216
	 (rtoi_12742 & rtoi_13813 & rtoi_14805) | (rtoi_13813 & rtoi_14805 & rtoi_9700)
	 (rtoi_12742 & rtoi_13813 & rtoi_14805) | (rtoi_12742 & rtoi_14805 & rtoi_9065) | (rtoi_12752 & rtoi_13813 & rtoi_14805) | (rtoi_12752 & rtoi_14805 & rtoi_9065) | (rtoi_13813 & rtoi_14805 & rtoi_9700) | (rtoi_14805 & rtoi_9065 & rtoi_9700)
yli_R0217
	 (rtoi_12742 & rtoi_13813 & rtoi_14805) | (rtoi_13813 & rtoi_14805 & rtoi_9700)
	 (rtoi_12742 & rtoi_13813 & rtoi_14805) | (rtoi_12742 & rtoi_14805 & rtoi_9065) | (rtoi_12752 & rtoi_13813 & rtoi_14805) | (rtoi_12752 & rtoi_14805 & rtoi_9065) | (rtoi_13813 & rtoi_14805 & rtoi_9700) | (rtoi_14805 & rtoi_9065 & rtoi_9700)
yli_R0218
	 (rtoi_12742 & rtoi_13813 & rtoi_14805) | (rtoi_13813 & rtoi_14805 & rtoi_9700)
	 (rtoi_12742 & rtoi_13813 & rtoi_14805) | (rtoi_12742 & rtoi_14805 & rtoi_9065) | (rtoi_12752 & rtoi_13813 & rtoi_14805) | (rtoi_12752 & rtoi_14805 & rtoi_9065) | (rtoi_13813 & rtoi_14805 & rtoi_9700) | (rtoi_14805 & rtoi_9065 & rtoi_9700)
yli_R0219
	 (rtoi_12742 & rtoi_13813 & rtoi_14805) | (rtoi_13813 & rtoi_14805 & rtoi_9700)
	 (rtoi_12742 & rtoi_13813 & rtoi_14805) | (rtoi_12742 & rtoi_14805 & rtoi_9065) | (rtoi_12752 & rtoi_13813 & rtoi_14805) | (rtoi_12752 & rtoi_14805 & rtoi_9065) | (rtoi_13813 & rtoi_14805 & rtoi_9700) | (rtoi_14805 & rtoi_9065 & rtoi_9700)
yli_R0220
	 (rtoi_12742 & rtoi_13813 & rtoi_14805) | (rtoi_13813 & rtoi_14805 & rtoi_9700)
	 (rtoi_12742 & rtoi_13813 & rtoi_14805) | (rtoi_12742 & rtoi_14805 & rtoi_9065) | (rtoi_12752 & rtoi_13813 & rtoi_14805) | (rtoi_12752 & rtoi_14805 & rtoi_9065) | (rtoi_13813 & rtoi_14805 & rtoi_9700) | (rtoi_14805 & rtoi_9065 & rtoi_9700)
yli_R0221
	 (rtoi_12742 & rtoi_13813 & rtoi_14805) | (rtoi_13813 & rtoi_14805 & rtoi_9700)
	 (rtoi_12742 & rtoi_13813 & rtoi_14805) | (rtoi_12742 & rtoi_14805 & rtoi_9065) | (rtoi_12752 & rtoi_13813 & rtoi_14805) | (rtoi_12752 & rtoi_14805 & rtoi_9065) | (rtoi_13813 & rtoi_14805 & rtoi_9700) | (rtoi_14805 & rtoi_9065 & rtoi_9700)
yli_R0222
	 (rtoi_12742 & rtoi_13813 & rtoi_14805) | (rtoi_13813 & rtoi_14805 & rtoi_9700)
	 (rtoi_12742 & rtoi_13813 & rtoi_14805) | (rtoi_12742 & rtoi_14805 & rtoi_9065) | (rtoi_12752 & rtoi_13813 & rtoi_14805) | (rtoi_12752 & rtoi_14805 & rtoi_9065) | (rtoi_13813 & rtoi_14805 & rtoi_9700) | (rtoi_14805 & rtoi_9065 & rtoi_9700)
yli_R0223
	 (rtoi_12742 & rtoi_13813 & rtoi_14805) | (rtoi_13813 & rtoi_14805 & rtoi_9700)
	 (rtoi_12742 & rtoi_13813 & rtoi_14805) | (rtoi_12742 & rtoi_14805 & rtoi_9065) | (rtoi_12752 & rtoi_13813 & rtoi_14805) | (rtoi_12752 & rtoi_14805 & rtoi_9065) | (rtoi_13813 & rtoi_14805 & rtoi_9700) | (rtoi_14805 & rtoi_9065 & rtoi_9700)
yli_R0224
	 (rtoi_12742 & rtoi_13813 & rtoi_14805) | (rtoi_13813 & rtoi_14805 & rtoi_9700)
	 (rtoi_12742 & rtoi_13813 & rtoi_14805) | (rtoi_12742 & rtoi_14805 & rtoi_9065) | (rtoi_12752 & rtoi_13813 & rtoi_14805) | (rtoi_12752 & rtoi_14805 & rtoi_9065) | (rtoi_13813 & rtoi_14805 & rtoi_9700) | (rtoi_14805 & rtoi_9065 & rtoi_9700)
yli_R0225
	 (rtoi_12742 & rtoi_13813 & rtoi_14805) | (rtoi_13813 & rtoi_14805 & rtoi_9700)
	 (rtoi_12742 & rtoi_13813 & rtoi_14805) | (rtoi_12742 & rtoi_14805 & rtoi_9065) | (rtoi_12752 & rtoi_13813 & rtoi_14805) | (rtoi_12752 & rtoi_14805 & rtoi_9065) | (rtoi_13813 & rtoi_14805 & rtoi_9700) | (rtoi_14805 & rtoi_9065 & rtoi_9700)
yli_R0742
	 rtoi_8835
	 rtoi_13724 | rtoi_8835
yli_R0937
	 rtoi_12566 & rtoi_15436
	 (rtoi_12086 & rtoi_15436) | (rtoi_12566 & rtoi_15436)
yli_R0938
	 rtoi_12566 & rtoi_15436
	 (rtoi_12086 & rtoi_15436) | (rtoi_12566 & rtoi_15436)
yli_R0939
	 rtoi_12566 & rtoi_15436
	 (rtoi_12086 & rtoi_15436) | (rtoi_12566 & rtoi_15436)
yli_R1133
	 rtoi_11893
	 rtoi_11075 | rtoi_11893
yli_R1397
	 rtoi_12154
	 rtoi_12154 | rtoi_14576
yli_R1442
	 rtoi_8835
	 rtoi_13724 | rtoi_8835
yli_R1587
	 rtoi_12566 & rtoi_15436
	 (rtoi_12086 & rtoi_15436) | (rtoi_12566 & rtoi_15436)
yli_R1590
	 rtoi_12566 & rtoi_15436
	 (rtoi_12086 & rtoi_15436) | (rtoi_12566 & rtoi_15436)
yli_R1591
	 rtoi_12566 & rtoi_15436
	 (rtoi_12086 & rtoi_15436) | (rtoi_12566 & rtoi_15436)
yli_R1592
	 rtoi_12566 & rtoi_15436
	 (rtoi_12086 & rtoi_15436) | (rtoi_12566 & rtoi_15436)
```

In [62]:

```
Reaction_to_gene['FAO141p_even']
```

Out[62]:

```
{'sce|YGL205W': {'rtoi|12742', 'rtoi|9700'},
 'sce|YIL160C': {'rtoi|13813'},
 'sce|YKR009C': {'rtoi|11362'},
 'sce|YLR284C': {'rtoi|10293'},
 'sce|YNL202W': {'rtoi|13228'},
 'sce|YOR180C': {'rtoi|10293'}}
```

In [63]:

```
Reaction_to_gene_Jeff['FAO141p_even']
```

Out[63]:

```
{'sce|YGL205W': {'rtoi|12742', 'rtoi|12752', 'rtoi|9700'},
 'sce|YIL160C': {'rtoi|13813', 'rtoi|9065'},
 'sce|YKR009C': {'rtoi|11362'},
 'sce|YLR284C': {'rtoi|10293'},
 'sce|YNL202W': {'rtoi|13228'},
 'sce|YOR180C': {'rtoi|10293'}}
```

In [59]:

```
for x in set(Reaction_rule_unique.keys()).intersection(Reaction_rule_Jeff_unique):
    if not Reaction_rule_unique[x] == Reaction_rule_Jeff_unique[x]:
        temp1 = Reaction_rule_unique[x]
        temp2 = Reaction_rule_Jeff_unique[x]
        if not set(temp1.free_symbols).difference(temp2.free_symbols) and set(temp2.free_symbols).difference(temp1.free_symbols):
            Reaction_rule_final[x] = temp2
```

In [60]:

```
for x in sorted(set(Reaction_rule_unique.keys()).intersection(Reaction_rule_Jeff_unique)):
    if not Reaction_rule_unique[x] == Reaction_rule_Jeff_unique[x]:
        temp1 = Reaction_rule_unique[x]
        temp2 = Reaction_rule_Jeff_unique[x]
        if not set(temp1.free_symbols).difference(temp2.free_symbols) and not set(temp2.free_symbols).difference(temp1.free_symbols):
            print(x)
            print('\t',temp1)
            print('\t',temp2)
```

```
ATPS3g
	 rtoi_11025 & rtoi_11052 & rtoi_11064 & rtoi_11117 & rtoi_11403 & rtoi_14971 & rtoi_15309 & rtoi_15351 & rtoi_15963 & rtoi_16397 & rtoi_9210 & rtoi_9622 & rtoi_9748
	 (rtoi_11025 & rtoi_11052 & rtoi_11064 & rtoi_11117 & rtoi_11403 & rtoi_14971 & rtoi_15309 & rtoi_15351 & rtoi_15963 & rtoi_16397 & rtoi_9210 & rtoi_9622) | (rtoi_11052 & rtoi_11064 & rtoi_11117 & rtoi_11403 & rtoi_14971 & rtoi_15309 & rtoi_15351 & rtoi_15963 & rtoi_16397 & rtoi_9210 & rtoi_9622 & rtoi_9748)
ATPS3v
	 rtoi_11025 & rtoi_11052 & rtoi_11064 & rtoi_11117 & rtoi_11403 & rtoi_14971 & rtoi_15309 & rtoi_15351 & rtoi_15963 & rtoi_16397 & rtoi_9210 & rtoi_9622 & rtoi_9748
	 (rtoi_11025 & rtoi_11052 & rtoi_11064 & rtoi_11117 & rtoi_11403 & rtoi_14971 & rtoi_15309 & rtoi_15351 & rtoi_15963 & rtoi_16397 & rtoi_9210 & rtoi_9622) | (rtoi_11052 & rtoi_11064 & rtoi_11117 & rtoi_11403 & rtoi_14971 & rtoi_15309 & rtoi_15351 & rtoi_15963 & rtoi_16397 & rtoi_9210 & rtoi_9622 & rtoi_9748)
FA120ACPHi
	 rtoi_8670 & rtoi_8777
	 rtoi_8670 | rtoi_8777
FA140ACPHi
	 rtoi_8670 & rtoi_8777
	 rtoi_8670 | rtoi_8777
FA141ACPHi
	 rtoi_8670 & rtoi_8777
	 rtoi_8670 | rtoi_8777
FA160ACPHi
	 rtoi_8670 & rtoi_8777
	 rtoi_8670 | rtoi_8777
FA161ACPHi
	 rtoi_8670 & rtoi_8777
	 rtoi_8670 | rtoi_8777
FA180ACPH
	 rtoi_8670 & rtoi_8777
	 rtoi_8670 | rtoi_8777
FA181ACPH
	 rtoi_8670 & rtoi_8777
	 rtoi_8670 | rtoi_8777
FA182ACPH
	 rtoi_8670 & rtoi_8777
	 rtoi_8670 | rtoi_8777
yli_R0163
	 YALI0F30679g & rtoi_8670 & rtoi_8777
	 (YALI0F30679g & rtoi_8670) | (YALI0F30679g & rtoi_8777)
yli_R0164
	 YALI0F30679g & rtoi_8670 & rtoi_8777
	 (YALI0F30679g & rtoi_8670) | (YALI0F30679g & rtoi_8777)
yli_R0165
	 YALI0F30679g & rtoi_8670 & rtoi_8777
	 (YALI0F30679g & rtoi_8670) | (YALI0F30679g & rtoi_8777)
yli_R0166
	 YALI0F30679g & rtoi_8670 & rtoi_8777
	 (YALI0F30679g & rtoi_8670) | (YALI0F30679g & rtoi_8777)
yli_R0167
	 YALI0F30679g & rtoi_8670 & rtoi_8777
	 (YALI0F30679g & rtoi_8670) | (YALI0F30679g & rtoi_8777)
yli_R0168
	 YALI0F30679g & rtoi_8670 & rtoi_8777
	 (YALI0F30679g & rtoi_8670) | (YALI0F30679g & rtoi_8777)
yli_R0169
	 YALI0F30679g & rtoi_8670 & rtoi_8777
	 (YALI0F30679g & rtoi_8670) | (YALI0F30679g & rtoi_8777)
yli_R0170
	 YALI0F30679g & rtoi_8670 & rtoi_8777
	 (YALI0F30679g & rtoi_8670) | (YALI0F30679g & rtoi_8777)
yli_R0171
	 YALI0F30679g & rtoi_8670 & rtoi_8777
	 (YALI0F30679g & rtoi_8670) | (YALI0F30679g & rtoi_8777)
yli_R0172
	 YALI0F30679g & rtoi_8670 & rtoi_8777
	 (YALI0F30679g & rtoi_8670) | (YALI0F30679g & rtoi_8777)
yli_R0173
	 rtoi_8670 & rtoi_8777
	 rtoi_8670 | rtoi_8777
yli_R0174
	 rtoi_8670 & rtoi_8777
	 rtoi_8670 | rtoi_8777
yli_R0175
	 rtoi_8670 & rtoi_8777
	 rtoi_8670 | rtoi_8777
yli_R0176
	 rtoi_8670 & rtoi_8777
	 rtoi_8670 | rtoi_8777
yli_R0177
	 rtoi_8670 & rtoi_8777
	 rtoi_8670 | rtoi_8777
yli_R0178
	 rtoi_8670 & rtoi_8777
	 rtoi_8670 | rtoi_8777
yli_R0179
	 rtoi_8670 & rtoi_8777
	 rtoi_8670 | rtoi_8777
yli_R0180
	 rtoi_8670 & rtoi_8777
	 rtoi_8670 | rtoi_8777
```

In [64]:

```
# ATPS3g and ATPS3v - 11025 & 9748 vs 11025 | 9748
# FAS - 8670 & 8777 vs 8670 & 8777
# choose subunit based on the published models
for x in sorted(set(Reaction_rule_unique.keys()).intersection(Reaction_rule_Jeff_unique)):
    if not Reaction_rule_unique[x] == Reaction_rule_Jeff_unique[x]:
        temp1 = Reaction_rule_unique[x]
        temp2 = Reaction_rule_Jeff_unique[x]
        if not set(temp1.free_symbols).difference(temp2.free_symbols) and not set(temp2.free_symbols).difference(temp1.free_symbols):
            Reaction_rule_final[x] = temp1
```

In [65]:

```
for x in sorted(set(Reaction_rule.keys()).intersection(Reaction_rule_Jeff_unique)):
    if x not in Reaction_rule_final:
        print(x)
        print('\t',Reaction_rule[x])
        print('\t',Reaction_rule_Jeff_unique[x])
```

```
2DOXG6PP
	 {'sce': rtoi_13413, 'eco': rtoi_8460 | rtoi_8576}
	 rtoi_13413
ABTA
	 {'sce': rtoi_15905, 'eco': rtoi_10937, 'ppu': rtoi_10937, 'yli': rtoi_15905}
	 rtoi_15905
ABUTt2r
	 {'sce': rtoi_11269 | rtoi_13423, 'yli': (YALI0B09537g & rtoi_11269) | (YALI0B09537g & rtoi_13423)}
	 rtoi_12743
ACACT1r
	 {'sce': rtoi_8678 | rtoi_8885, 'eco': rtoi_13813 | rtoi_8678 | rtoi_8885, 'cre': rtoi_8678 | rtoi_8885, 'yli': rtoi_8678 | rtoi_8885}
	 rtoi_8678
ACCOAC
	 {'sce': rtoi_8639, 'eco': b0185 & b2316 & b3255 & rtoi_12867, 'ppu': PP_0559 & PP_1607 & PP_1996 & rtoi_12867, 'yli': rtoi_8639}
	 rtoi_8639
ACOATA
	 {'sce': rtoi_8670 & rtoi_8777, 'eco': b1091 & rtoi_14261, 'yli': rtoi_8670 & rtoi_8777}
	 rtoi_8670 | rtoi_8777
ACP1e
	 {'sce': rtoi_10885, 'yli': rtoi_13856 | rtoi_13935}
	 rtoi_13856 | rtoi_13935
ADCS
	 {'sce': rtoi_13669, 'eco': rtoi_13669 & rtoi_16564, 'cre': rtoi_13669 & rtoi_16564, 'yli': rtoi_13669}
	 rtoi_13669
ADNK1
	 {'sce': rtoi_8385, 'eco': rtoi_15496, 'cre': rtoi_8385, 'yli': rtoi_8385}
	 rtoi_8385
ADPT
	 {'sce': rtoi_16193, 'eco': rtoi_16193, 'ppu': rtoi_16193, 'cre': rtoi_12445 | rtoi_16193, 'yli': rtoi_16193}
	 rtoi_16193
AKGDam
	 {'sce': (rtoi_10007 & rtoi_10040 & rtoi_12116) | (rtoi_10040 & rtoi_12116 & rtoi_9274), 'cre': rtoi_10007 | rtoi_9274}
	 (rtoi_10007 & rtoi_10040 & rtoi_12116) | (rtoi_10040 & rtoi_12116 & rtoi_9274)
ALCD19y
	 {'sce': rtoi_11882, 'cre': rtoi_12784 | rtoi_9774}
	 rtoi_11882 | rtoi_12784 | rtoi_9774
ALCD2ir
	 {'sce': rtoi_11665 | rtoi_15438, 'ppu': rtoi_15438, 'cre': rtoi_11665}
	 rtoi_11665 | rtoi_15438
ALCD2irm
	 {'sce': rtoi_15438, 'cre': rtoi_11665}
	 rtoi_15438
AMPTASECG
	 {'eco': rtoi_12096, 'yli': rtoi_10210}
	 rtoi_10210
ARGt2r
	 {'sce': rtoi_14229 | rtoi_15074 | rtoi_8962 | rtoi_9319 | rtoi_9322, 'yli': (rtoi_14229 & rtoi_8962) | (rtoi_14229 & rtoi_9319) | (rtoi_14229 & rtoi_9322) | (rtoi_15074 & rtoi_8962) | (rtoi_15074 & rtoi_9319) | (rtoi_15074 & rtoi_9322)}
	 rtoi_14229 | rtoi_15074
ASNN
	 {'sce': rtoi_10043 | rtoi_13627, 'eco': rtoi_8853, 'ppu': rtoi_8853}
	 rtoi_10043 | rtoi_13627
ASPK
	 {'sce': rtoi_14662, 'eco': rtoi_12080 | rtoi_14662 | rtoi_16738, 'ppu': rtoi_14662, 'yli': rtoi_14662}
	 rtoi_14662
ATPS3m
	 {'sce': (Q0080 & Q0085 & Q0130 & YPL271W & rtoi_10674 & rtoi_10675 & rtoi_11958 & rtoi_11967 & rtoi_13424 & rtoi_13759 & rtoi_14786 & rtoi_14912 & rtoi_15589 & rtoi_16359 & rtoi_9940) | (Q0080 & Q0085 & Q0130 & YOL077W_A & YPL271W & rtoi_10674 & rtoi_10675 & rtoi_11958 & rtoi_11967 & rtoi_13424 & rtoi_13759 & rtoi_14786 & rtoi_14912 & rtoi_15589 & rtoi_15880 & rtoi_16359 & rtoi_9080 & rtoi_9940), 'cre': (CRv4_Au5_s10_g33_t1 & CRv4_Au5_s10_g65_t1 & CRv4_Au5_s17_g7684_t1 & CRv4_Au5_s1_g1345_t1 & CRv4_Au5_s1_g1960_t1 & rtoi_11958 & rtoi_11967 & rtoi_13424 & rtoi_15287 & rtoi_15589) | (CRv4_Au5_s10_g33_t1 & CRv4_Au5_s10_g65_t1 & CRv4_Au5_s17_g7685_t1 & CRv4_Au5_s1_g1345_t1 & CRv4_Au5_s1_g1960_t1 & rtoi_11958 & rtoi_11967 & rtoi_13424 & rtoi_15287 & rtoi_15589) | (CRv4_Au5_s10_g65_t1 & CRv4_Au5_s17_g7684_t1 & CRv4_Au5_s1_g1345_t1 & CRv4_Au5_s1_g1960_t1 & rtoi_11958 & rtoi_11967 & rtoi_13424 & rtoi_15287 & rtoi_15589 & rtoi_16359) | (CRv4_Au5_s10_g65_t1 & CRv4_Au5_s17_g7685_t1 & CRv4_Au5_s1_g1345_t1 & CRv4_Au5_s1_g1960_t1 & rtoi_11958 & rtoi_11967 & rtoi_13424 & rtoi_15287 & rtoi_15589 & rtoi_16359)}
	 (Q0080 & Q0085 & Q0130 & YDL181W & YPL271W & rtoi_10674 & rtoi_11958 & rtoi_11967 & rtoi_13424 & rtoi_13759 & rtoi_14786 & rtoi_14912 & rtoi_15589 & rtoi_16359 & rtoi_9940) | (Q0080 & Q0085 & Q0130 & YDL181W & YOL077W_A & YPL271W & YPR020W & rtoi_10674 & rtoi_11958 & rtoi_11967 & rtoi_13424 & rtoi_13759 & rtoi_14786 & rtoi_14912 & rtoi_15589 & rtoi_15880 & rtoi_16359 & rtoi_9940)
CAT
	 {'sce': rtoi_9354, 'eco': rtoi_9354, 'ppu': rtoi_15305 | rtoi_9354}
	 rtoi_9354
CCP2m
	 {'sce': rtoi_13161 & rtoi_8802, 'cre': (rtoi_13161 & rtoi_8802) | (rtoi_13161 & rtoi_9198)}
	 (rtoi_10811 & rtoi_8802) | (rtoi_13161 & rtoi_8802)
CDPPH
	 {'cre': rtoi_9805, 'yli': rtoi_8970}
	 rtoi_8970
CITtam
	 {'sce': rtoi_10514, 'cre': rtoi_11740 | rtoi_13510, 'yli': rtoi_10514}
	 rtoi_10514
CITtcm
	 {'sce': rtoi_10514, 'cre': rtoi_11740 | rtoi_13510, 'yli': rtoi_10514}
	 rtoi_10514
CS
	 {'eco': rtoi_8548, 'ppu': rtoi_8548, 'yli': rtoi_11331}
	 rtoi_11331
CSND
	 {'sce': rtoi_14559, 'cre': rtoi_14031, 'yli': rtoi_14559}
	 rtoi_14559
CSp
	 {'sce': rtoi_11331, 'cre': rtoi_8548}
	 rtoi_11331
CYTDK1
	 {'eco': rtoi_8633, 'cre': rtoi_8633 | rtoi_9093, 'yli': rtoi_8633}
	 rtoi_8633
CYTDK2
	 {'sce': rtoi_8633, 'eco': rtoi_8633, 'cre': rtoi_8633 | rtoi_9093, 'yli': rtoi_8633}
	 rtoi_8633
DADK
	 {'eco': rtoi_15496, 'ppu': rtoi_15496, 'cre': rtoi_12300 | rtoi_15496, 'yli': rtoi_15129 | rtoi_15496}
	 rtoi_15129 | rtoi_15496
DATCY
	 {'cre': rtoi_8633 | rtoi_9093, 'yli': rtoi_8633}
	 rtoi_8633
DATUP
	 {'cre': rtoi_8633 | rtoi_9093, 'yli': rtoi_8633}
	 rtoi_8633
DCTCP
	 {'cre': rtoi_8633 | rtoi_9093, 'yli': rtoi_8633}
	 rtoi_8633
DCTUP
	 {'cre': rtoi_8633 | rtoi_9093, 'yli': rtoi_8633}
	 rtoi_8633
DGTCY
	 {'cre': rtoi_8633 | rtoi_9093, 'yli': rtoi_8633}
	 rtoi_8633
DGTUP
	 {'cre': rtoi_8633 | rtoi_9093, 'yli': rtoi_8633}
	 rtoi_8633
DHFS
	 {'eco': rtoi_10460, 'ppu': rtoi_10460, 'yli': rtoi_10460 | rtoi_14803}
	 rtoi_10460 | rtoi_14803
DHORDfum
	 {'sce': rtoi_14866, 'yli': rtoi_8814}
	 rtoi_8814
DHQTi
	 {'sce': rtoi_14252, 'ppu': rtoi_10572, 'yli': rtoi_14252}
	 rtoi_14252
DNMPPA
	 {'sce': rtoi_14615, 'cre': rtoi_15385}
	 rtoi_14615 | rtoi_14875
DPR
	 {'sce': rtoi_14277, 'eco': rtoi_14277 | rtoi_9176, 'ppu': rtoi_14277 | rtoi_16522, 'yli': rtoi_14277}
	 rtoi_14277
DTPH
	 {'cre': rtoi_9805, 'yli': rtoi_8970}
	 rtoi_8970
DTTGY
	 {'cre': rtoi_8633 | rtoi_9093, 'yli': rtoi_8633}
	 rtoi_8633
DTTUP
	 {'cre': rtoi_8633 | rtoi_9093, 'yli': rtoi_8633}
	 rtoi_8633
DURIPP
	 {'sce': rtoi_16276, 'eco': rtoi_14521, 'yli': rtoi_16276}
	 rtoi_16276
DUTCP
	 {'cre': rtoi_8633 | rtoi_9093, 'yli': rtoi_8633}
	 rtoi_8633
DUTPDP
	 {'sce': rtoi_8399, 'eco': rtoi_8399, 'ppu': rtoi_8399, 'cre': rtoi_13900 | rtoi_8399, 'yli': rtoi_8399}
	 rtoi_8399
DUTUP
	 {'cre': rtoi_8633 | rtoi_9093, 'yli': rtoi_8633}
	 rtoi_8633
ECOAH1
	 {'ppu': rtoi_14805, 'cre': rtoi_16128, 'yli': rtoi_14805}
	 rtoi_14805
ECOAH4m
	 {'cre': rtoi_11907, 'yli': rtoi_14805}
	 rtoi_14805
ECOAH5m
	 {'cre': rtoi_11907, 'yli': rtoi_14805}
	 rtoi_14805
ECOAH6m
	 {'cre': rtoi_11907, 'yli': rtoi_14805}
	 rtoi_14805
ECOAH7m
	 {'cre': rtoi_11907, 'yli': rtoi_14805}
	 rtoi_14805
FBP
	 {'sce': rtoi_15423, 'eco': rtoi_11513 | rtoi_15423, 'ppu': rtoi_15423}
	 rtoi_15423
FDH
	 {'sce': rtoi_11952, 'ppu': (PP_0490 & PP_0491 & PP_0492) | (PP_2183 & PP_2184 & PP_2186 & rtoi_13272), 'yli': rtoi_11952}
	 rtoi_11952
GLUDy
	 {'sce': rtoi_12248, 'eco': rtoi_12248 | (b3213 & rtoi_15713), 'ppu': rtoi_12248, 'yli': rtoi_12248}
	 rtoi_12248
GLUTRS
	 {'sce': rtoi_10753, 'eco': rtoi_14565, 'ppu': rtoi_14565, 'cre': rtoi_10753 | rtoi_14565}
	 rtoi_10753
GLYCTO1
	 {'ppu': PP_3746 & PP_3747 & rtoi_13328, 'yli': rtoi_14950}
	 rtoi_16607
GTPCII
	 {'sce': rtoi_12551, 'ppu': rtoi_12551 | rtoi_16666}
	 rtoi_12551
H2Ot
	 {'sce': rtoi_13987 | rtoi_9015, 'cre': rtoi_13986}
	 rtoi_13986 | rtoi_13987 | rtoi_9014 | rtoi_9015
HEX4
	 {'sce': rtoi_11982, 'cre': rtoi_10264, 'yli': rtoi_10264 | rtoi_11982}
	 rtoi_10264 | rtoi_11982
HISDr
	 {'ppu': rtoi_16462, 'cre': rtoi_15434}
	 rtoi_16462
HISTP
	 {'sce': rtoi_10206, 'eco': rtoi_8707, 'yli': rtoi_10206}
	 rtoi_10206
HPHL
	 {'cre': rtoi_16128, 'yli': rtoi_14805}
	 rtoi_14805
HPYRRy
	 {'eco': rtoi_11036 | rtoi_12051, 'cre': rtoi_9515}
	 rtoi_11036
HSDy
	 {'sce': rtoi_12080, 'eco': rtoi_12080 | rtoi_16738, 'yli': rtoi_12080}
	 rtoi_12080
ICDHyr
	 {'sce': rtoi_11129, 'eco': rtoi_11681, 'ppu': rtoi_11681, 'cre': rtoi_11129, 'yli': rtoi_11129}
	 rtoi_11129
IDPA
	 {'cre': rtoi_9805, 'yli': rtoi_8970}
	 rtoi_8970
IPMD
	 {'sce': rtoi_13894, 'eco': rtoi_10428 | rtoi_13894, 'ppu': rtoi_13894, 'cre': rtoi_13894}
	 rtoi_13894
IPPMIa
	 {'sce': rtoi_14914, 'eco': b0071 & rtoi_14914, 'ppu': PP_1986 & rtoi_14914, 'cre': CRv4_Au5_s6_g12448_t1 & rtoi_14914, 'yli': rtoi_14914}
	 rtoi_14914
IPPMIb
	 {'sce': rtoi_14914, 'eco': b0071 & rtoi_14914, 'ppu': PP_1986 & rtoi_14914, 'cre': CRv4_Au5_s6_g12448_t1 & rtoi_14914, 'yli': rtoi_14914}
	 rtoi_14914
IPPS
	 {'sce': rtoi_15488, 'eco': rtoi_14856, 'ppu': rtoi_15488, 'cre': rtoi_14856, 'yli': rtoi_15488}
	 rtoi_15488
ITCY
	 {'cre': rtoi_8633 | rtoi_9093, 'yli': rtoi_8633}
	 rtoi_8633
LNS14DM
	 {'sce': rtoi_12843 & rtoi_13286, 'cre': rtoi_12843}
	 rtoi_12843 & rtoi_13286
LYSt2r
	 {'sce': rtoi_14229 | rtoi_15074 | rtoi_8962 | rtoi_9319 | rtoi_9322, 'yli': (rtoi_14229 & rtoi_8962) | (rtoi_14229 & rtoi_9319) | (rtoi_14229 & rtoi_9322) | (rtoi_15074 & rtoi_8962) | (rtoi_15074 & rtoi_9319) | (rtoi_15074 & rtoi_9322)}
	 rtoi_14229 | rtoi_15074
MCOATA
	 {'sce': rtoi_8670 & rtoi_8777, 'eco': rtoi_14261 & rtoi_16542, 'ppu': rtoi_16542, 'yli': rtoi_8670 & rtoi_8777}
	 rtoi_8670 | rtoi_8777
METSOXR1
	 {'eco': (b3551 & rtoi_15339) | (b3551 & rtoi_16019) | (rtoi_15339 & rtoi_15902) | (rtoi_15902 & rtoi_16019), 'yli': rtoi_15469}
	 rtoi_15469
METTRS
	 {'sce': rtoi_13140, 'ppu': rtoi_13140, 'cre': rtoi_13140 | rtoi_15518}
	 rtoi_13140
MI145P6Kn
	 {'sce': rtoi_13260, 'cre': rtoi_9763}
	 rtoi_13260
MTAP
	 {'sce': rtoi_8372, 'cre': rtoi_14521, 'yli': rtoi_8372}
	 rtoi_8372
MTHFR2
	 {'eco': rtoi_9244, 'yli': rtoi_15483 | rtoi_9244}
	 rtoi_15483 | rtoi_9244
MTRI
	 {'cre': rtoi_13385 | rtoi_15595, 'yli': rtoi_13385}
	 rtoi_13385
NDP1
	 {'sce': rtoi_8970, 'cre': rtoi_9805, 'yli': rtoi_8970}
	 rtoi_8970
NDP3
	 {'sce': rtoi_8970, 'cre': rtoi_9805, 'yli': rtoi_8970}
	 rtoi_8970
NDP7
	 {'sce': rtoi_8970, 'cre': rtoi_9805, 'yli': rtoi_8970}
	 rtoi_8970
NTD2
	 {'sce': rtoi_9995, 'eco': rtoi_13044 | rtoi_16648, 'ppu': rtoi_16648}
	 rtoi_9995
NTD4
	 {'sce': rtoi_9995, 'eco': rtoi_13044 | rtoi_16648, 'ppu': rtoi_16648}
	 rtoi_9995
NTP1
	 {'cre': rtoi_9805, 'yli': rtoi_10674 & rtoi_11025 & rtoi_11052 & rtoi_11064 & rtoi_11117 & rtoi_11403 & rtoi_11958 & rtoi_11967 & rtoi_13424 & rtoi_13759 & rtoi_14786 & rtoi_14912 & rtoi_14971 & rtoi_15309 & rtoi_15351 & rtoi_15589 & rtoi_15963 & rtoi_16359 & rtoi_16397 & rtoi_9080 & rtoi_9210 & rtoi_9622 & rtoi_9748 & rtoi_9940}
	 (YALI0B21527g & rtoi_10674 & rtoi_11025 & rtoi_11052 & rtoi_11064 & rtoi_11117 & rtoi_11403 & rtoi_11958 & rtoi_11967 & rtoi_13424 & rtoi_13759 & rtoi_14786 & rtoi_14912 & rtoi_14971 & rtoi_15309 & rtoi_15351 & rtoi_15589 & rtoi_15963 & rtoi_16359 & rtoi_16397 & rtoi_9210 & rtoi_9622 & rtoi_9940) | (YALI0B21527g & rtoi_10674 & rtoi_11052 & rtoi_11064 & rtoi_11117 & rtoi_11403 & rtoi_11958 & rtoi_11967 & rtoi_13424 & rtoi_13759 & rtoi_14786 & rtoi_14912 & rtoi_14971 & rtoi_15309 & rtoi_15351 & rtoi_15589 & rtoi_15963 & rtoi_16359 & rtoi_16397 & rtoi_9210 & rtoi_9622 & rtoi_9748 & rtoi_9940)
NTP10
	 {'cre': rtoi_9805, 'yli': rtoi_8970}
	 rtoi_8970
NTP3
	 {'eco': rtoi_10886 | rtoi_16025, 'cre': rtoi_9805, 'yli': rtoi_8970}
	 rtoi_8970
NTP5
	 {'cre': rtoi_9805, 'yli': rtoi_8970}
	 rtoi_8970
NTP7
	 {'cre': rtoi_9805, 'yli': rtoi_8970}
	 rtoi_8970
NTP9
	 {'cre': rtoi_9805, 'yli': rtoi_8970}
	 rtoi_8970
ORNt2r
	 {'sce': rtoi_14229 | rtoi_15074 | rtoi_8962 | rtoi_9319 | rtoi_9322, 'yli': (rtoi_14229 & rtoi_8962) | (rtoi_14229 & rtoi_9319) | (rtoi_14229 & rtoi_9322) | (rtoi_15074 & rtoi_8962) | (rtoi_15074 & rtoi_9319) | (rtoi_15074 & rtoi_9322)}
	 rtoi_14229 | rtoi_15074
PDE1
	 {'sce': rtoi_11787 | rtoi_13690, 'cre': rtoi_11787, 'yli': rtoi_11787 | rtoi_13690}
	 rtoi_11787 | rtoi_13690
PDH
	 {'eco': b0114 & b0115 & rtoi_10040, 'ppu': PP_0338 & PP_0339 & rtoi_10040, 'yli': rtoi_13948}
	 rtoi_13948
PDX5POi
	 {'sce': rtoi_10680, 'eco': rtoi_10680, 'ppu': rtoi_10680, 'cre': rtoi_8901, 'yli': rtoi_10680}
	 rtoi_10680
PDXPP
	 {'ppu': rtoi_14545, 'cre': rtoi_13044}
	 rtoi_13044
PGL
	 {'sce': rtoi_14499, 'eco': rtoi_14132, 'ppu': rtoi_14499, 'yli': rtoi_14499}
	 rtoi_14499
PHETRS
	 {'sce': rtoi_12470 & rtoi_13249, 'ppu': PP_2470 & rtoi_12470}
	 rtoi_12470 & rtoi_13249
PIt2m
	 {'sce': rtoi_15874, 'cre': rtoi_11409 | rtoi_11410 | rtoi_11686}
	 rtoi_15874 | rtoi_8889
PPA
	 {'sce': rtoi_15879, 'eco': rtoi_16648, 'yli': rtoi_15879}
	 rtoi_15879
PPM
	 {'sce': rtoi_9959, 'cre': rtoi_13711 | rtoi_9959}
	 rtoi_9959
PPNCL2
	 {'eco': rtoi_8536, 'cre': rtoi_8878, 'yli': rtoi_8878}
	 rtoi_8878
PRMICI
	 {'sce': rtoi_11610, 'cre': rtoi_11610 | rtoi_9084, 'yli': rtoi_11610}
	 rtoi_11610
PUNP1
	 {'sce': rtoi_16276, 'eco': rtoi_14521, 'yli': rtoi_16276}
	 rtoi_16276
PUNP2
	 {'sce': rtoi_16276, 'eco': rtoi_14521, 'yli': rtoi_16276}
	 rtoi_16276
PUNP3
	 {'sce': rtoi_16276, 'eco': rtoi_14521 | rtoi_16276, 'yli': rtoi_16276}
	 rtoi_16276
PUNP4
	 {'sce': rtoi_16276, 'eco': rtoi_14521 | rtoi_16276, 'yli': rtoi_16276}
	 rtoi_16276
PUNP5
	 {'sce': rtoi_16276, 'eco': rtoi_14521 | rtoi_16276, 'yli': rtoi_16276}
	 rtoi_16276
PUNP6
	 {'sce': rtoi_16276, 'eco': rtoi_14521 | rtoi_16276, 'yli': rtoi_16276}
	 rtoi_16276
PYAM5PO
	 {'sce': rtoi_10680, 'eco': rtoi_10680, 'cre': rtoi_8901, 'yli': rtoi_10680}
	 rtoi_10680
PYDXNO
	 {'sce': rtoi_10680, 'cre': rtoi_8901, 'yli': rtoi_10680}
	 rtoi_10680
PYDXO_1
	 {'cre': rtoi_8901, 'yli': rtoi_10680}
	 rtoi_10680
PYDXPP
	 {'eco': rtoi_13044, 'ppu': rtoi_14545, 'cre': rtoi_13044}
	 rtoi_13044
RNTR1
	 {'sce': rtoi_15339 | rtoi_16019, 'cre': (CRv4_Au5_s27_g10030_t1 & CRv4_Au5_s9_g15314_t1) | (CRv4_Au5_s27_g10030_t1 & rtoi_15339) | (CRv4_Au5_s27_g10030_t1 & rtoi_16019)}
	 rtoi_15339
RNTR2
	 {'sce': rtoi_15339 | rtoi_16019, 'cre': (CRv4_Au5_s27_g10030_t1 & CRv4_Au5_s9_g15314_t1) | (CRv4_Au5_s27_g10030_t1 & rtoi_15339) | (CRv4_Au5_s27_g10030_t1 & rtoi_16019)}
	 rtoi_15339
RNTR3
	 {'sce': rtoi_15339 | rtoi_16019, 'cre': (CRv4_Au5_s27_g10030_t1 & CRv4_Au5_s9_g15314_t1) | (CRv4_Au5_s27_g10030_t1 & rtoi_15339) | (CRv4_Au5_s27_g10030_t1 & rtoi_16019)}
	 rtoi_15339
RNTR4
	 {'sce': rtoi_15339 | rtoi_16019, 'cre': (CRv4_Au5_s27_g10030_t1 & CRv4_Au5_s9_g15314_t1) | (CRv4_Au5_s27_g10030_t1 & rtoi_15339) | (CRv4_Au5_s27_g10030_t1 & rtoi_16019)}
	 rtoi_15339
SERTRS
	 {'sce': rtoi_10246 | rtoi_14225, 'ppu': rtoi_10246, 'cre': rtoi_10246 | rtoi_14225}
	 rtoi_10246 | rtoi_14225
SHCHD2
	 {'eco': rtoi_11050, 'ppu': rtoi_11050, 'yli': rtoi_13569}
	 rtoi_13569
SHCHF
	 {'sce': rtoi_13569, 'eco': rtoi_11050, 'ppu': rtoi_11050, 'yli': rtoi_13569}
	 rtoi_13569
SHKK
	 {'sce': rtoi_14252, 'eco': rtoi_13706, 'yli': rtoi_14252}
	 rtoi_14252
SHSL1
	 {'eco': rtoi_9499, 'ppu': rtoi_9499, 'yli': rtoi_11463 | rtoi_16725 | rtoi_16742}
	 rtoi_11463 | rtoi_16725 | rtoi_16742
SHSL2r
	 {'ppu': rtoi_9499, 'yli': rtoi_11463 | rtoi_16725 | rtoi_16742}
	 rtoi_11463 | rtoi_16725 | rtoi_16742
TAUDO
	 {'sce': rtoi_10226 | rtoi_8618, 'eco': rtoi_13175 | rtoi_14698, 'ppu': rtoi_13175 | rtoi_14698}
	 rtoi_10226 | rtoi_8618
THFAT
	 {'eco': rtoi_9667, 'yli': rtoi_12898}
	 rtoi_12898
THIORDXi
	 {'sce': (rtoi_12715 & rtoi_15339) | (rtoi_12715 & rtoi_16019), 'eco': rtoi_8579 | (rtoi_15037 & rtoi_15339) | (rtoi_15037 & rtoi_16019)}
	 YDR453C & rtoi_15339
THRA
	 {'sce': rtoi_16182 | rtoi_9222, 'eco': rtoi_16182 | rtoi_9222 | rtoi_9667, 'ppu': rtoi_16182 | rtoi_9222, 'yli': rtoi_16182 | rtoi_9222}
	 rtoi_16182 | rtoi_9222
THRA2
	 {'sce': rtoi_16182 | rtoi_9222, 'eco': rtoi_16182 | rtoi_9222 | rtoi_9667, 'ppu': rtoi_16182 | rtoi_9222, 'yli': rtoi_16182 | rtoi_9222}
	 rtoi_16182 | rtoi_9222
TRE6PS
	 {'sce': (YML100W & rtoi_11389 & rtoi_12034) | (YMR261C & rtoi_11389 & rtoi_12034), 'eco': rtoi_11389}
	 (YML100W & rtoi_11389 & rtoi_12034) | (YMR261C & rtoi_11389 & rtoi_12034)
TRPS1
	 {'sce': rtoi_9262, 'eco': b1260 & rtoi_9262, 'ppu': PP_0082 & rtoi_9262, 'yli': rtoi_9262}
	 rtoi_9262
TYRTAi
	 {'sce': rtoi_14908 | rtoi_15839 | rtoi_8936, 'cre': rtoi_13230 | rtoi_14281}
	 rtoi_12407 | rtoi_14908 | rtoi_15839 | rtoi_8936
TYRTRS
	 {'sce': rtoi_16641, 'ppu': rtoi_13137, 'cre': rtoi_13137 | rtoi_16641}
	 rtoi_16641
UNK3
	 {'ppu': rtoi_14281 | rtoi_8936, 'yli': rtoi_14908 | rtoi_15839}
	 rtoi_12407 | rtoi_14908 | rtoi_15839
URIK1
	 {'sce': rtoi_8633, 'eco': rtoi_8633, 'cre': rtoi_8633 | rtoi_9093, 'yli': rtoi_8633}
	 rtoi_8633
URIK2
	 {'sce': rtoi_8633, 'eco': rtoi_8633, 'cre': rtoi_8633 | rtoi_9093, 'yli': rtoi_8633}
	 rtoi_8633
URIK3
	 {'cre': rtoi_8633 | rtoi_9093, 'yli': rtoi_8633}
	 rtoi_8633
UTCY
	 {'cre': rtoi_8633 | rtoi_9093, 'yli': rtoi_8633}
	 rtoi_8633
UTUP
	 {'cre': rtoi_8633 | rtoi_9093, 'yli': rtoi_8633}
	 rtoi_8633
YUMPS
	 {'sce': rtoi_14436 | rtoi_16088 | rtoi_16423, 'eco': rtoi_16646}
	 rtoi_14436 | rtoi_16088 | rtoi_16423
```

In [66]:

```
for x in set(Reaction_rule.keys()).intersection(Reaction_rule_Jeff_unique):
    if x not in Reaction_rule_final:
        temp1 = sympy.Or(*[v for v in Reaction_rule[x].values()])
        temp2 = Reaction_rule_Jeff_unique[x]
        Reaction_rule_final[x] = sympy.Or(temp1,temp2)
```

In [73]:

```
print('ACOATA', Reaction_rule_final['ACOATA'])
print('MCOATA', Reaction_rule_final['MCOATA'])
```

```
ACOATA rtoi_8670 | rtoi_8777 | (b1091 & rtoi_14261) | (rtoi_8670 & rtoi_8777)
MCOATA rtoi_16542 | rtoi_8670 | rtoi_8777 | (rtoi_14261 & rtoi_16542) | (rtoi_8670 & rtoi_8777)
```

In [74]:

```
Reaction_rule_final['ACOATA'] = parse_expr('(b1091 & rtoi_14261) | (rtoi_8670 & rtoi_8777)')
Reaction_rule_final['MCOATA'] = parse_expr('rtoi_16542 | (rtoi_14261 & rtoi_16542) | (rtoi_8670 & rtoi_8777)')
```

In [67]:

```
for x in set(Reaction_rule_unique.keys()).intersection(Reaction_rule_Jeff):
    if x not in Reaction_rule_final:
        print(x)
        print('\t',Reaction_rule_unique[x])
        print('\t',Reaction_rule_Jeff[x])
```

```
GUAPRT
	 rtoi_16193
	 {'sce': rtoi_12445, 'yli': rtoi_16193}
FACOAL161
	 rtoi_11167 | rtoi_12538 | rtoi_12555 | rtoi_15748
	 {'hsa': rtoi_12538 | rtoi_12555, 'mmu': rtoi_12538 | rtoi_12555, 'sce': rtoi_11167 | rtoi_15748}
PEAMNO
	 rtoi_10308
	 {'hsa': rtoi_13959, 'mmu': rtoi_13959, 'yli': rtoi_10308}
MCOATAm
	 YER061C & YHR067W & YKL055C & YOR221C & rtoi_11837 & rtoi_14261
	 {'hsa': rtoi_16542, 'mmu': rtoi_16542, 'sce': YER061C & YHR067W & YKL055C & YOR221C & rtoi_11837 & rtoi_14261}
ALDD19xr
	 rtoi_12042 | rtoi_13426
	 {'hsa': rtoi_12042 | rtoi_13426 | rtoi_16323, 'mmu': rtoi_12042 | rtoi_13426 | rtoi_16323, 'sce': rtoi_12042 | rtoi_13426, 'yli': rtoi_12042 | rtoi_13426}
ACACT1m
	 rtoi_8678 | rtoi_8885
	 {'hsa': rtoi_8678 | (HADHA & HADHB), 'mmu': rtoi_8678 | (Hadha & Hadhb), 'sce': rtoi_8678, 'yli': rtoi_8678}
ALDD22x
	 rtoi_12042 | rtoi_13426 | rtoi_16323
	 {'sce': rtoi_12042 | rtoi_13426, 'yli': rtoi_12042 | rtoi_13426 | rtoi_16323}
42A12BOOX
	 rtoi_10308
	 {'hsa': rtoi_13959, 'mmu': rtoi_13959, 'yli': rtoi_10308}
ECOAH12m
	 rtoi_11907
	 {'hsa': rtoi_14805 | (HADHA & HADHB), 'mmu': rtoi_14805 | (Hadha & Hadhb)}
PMANM
	 rtoi_9241
	 {'hsa': rtoi_13711 | rtoi_9241, 'mmu': rtoi_13711 | rtoi_9241, 'sce': rtoi_9241, 'yli': rtoi_9241}
DPCOAK
	 rtoi_11114
	 {'hsa': rtoi_14849, 'mmu': rtoi_14849, 'yli': rtoi_11114}
MDHm
	 rtoi_10614 | rtoi_11178
	 {'hsa': rtoi_11178, 'mmu': rtoi_11178, 'sce': rtoi_11178, 'yli': rtoi_10614 | rtoi_11178}
SPMDtex2
	 rtoi_15991 | rtoi_16258
	 {'sce': rtoi_13423, 'yli': rtoi_15991 | rtoi_16258}
34DHOXPEGOX
	 rtoi_14108 | rtoi_14109 | rtoi_15438
	 {'hsa': rtoi_11665 | rtoi_14108 | rtoi_14109 | (ADH1A & ADH1B) | (ADH1A & ADH1C) | (ADH1B & ADH1C), 'yli': rtoi_14108 | rtoi_14109 | rtoi_15438}
GARFT
	 rtoi_13595
	 {'hsa': rtoi_14259, 'mmu': rtoi_14259, 'sce': rtoi_13595, 'yli': rtoi_13595}
ALDD20x
	 rtoi_12042 | rtoi_13426 | rtoi_16323
	 {'hsa': rtoi_12042 | rtoi_13426 | rtoi_15814 | rtoi_16323, 'mmu': rtoi_12042 | rtoi_13426 | rtoi_15814 | rtoi_16323, 'yli': rtoi_12042 | rtoi_13426 | rtoi_16323}
FACOAL181
	 rtoi_11167 | rtoi_12538 | rtoi_12555 | rtoi_15748
	 {'hsa': rtoi_11167 | rtoi_12538 | rtoi_12555 | rtoi_15748, 'mmu': rtoi_11167 | rtoi_12538 | rtoi_12555 | rtoi_15748, 'sce': rtoi_11167 | rtoi_15748}
ECOAH9m
	 rtoi_11907
	 {'hsa': rtoi_14805 | (HADHA & HADHB), 'mmu': rtoi_14805 | (Hadha & Hadhb)}
PFK26
	 rtoi_15195 | rtoi_15744
	 {'hsa': rtoi_15195, 'mmu': rtoi_15195, 'yli': rtoi_15195 | rtoi_15744}
NDPK10
	 rtoi_15679
	 {'hsa': rtoi_15679 | rtoi_8943, 'mmu': rtoi_15679 | rtoi_8943, 'yli': rtoi_15679}
PTRCtex2
	 rtoi_15991 | rtoi_16258
	 {'sce': rtoi_13423, 'yli': rtoi_15991 | rtoi_16258}
ACGSm
	 rtoi_16208 | rtoi_16267
	 {'hsa': rtoi_9377, 'mmu': rtoi_9377, 'sce': rtoi_16208 | rtoi_16267, 'yli': rtoi_16208 | rtoi_16267}
ACS
	 rtoi_14597
	 {'hsa': rtoi_14597 | rtoi_15276, 'mmu': rtoi_14597 | rtoi_15276, 'sce': rtoi_14597, 'yli': rtoi_14597}
FACOAL160
	 rtoi_11167 | rtoi_12538 | rtoi_12555 | rtoi_15748
	 {'hsa': rtoi_11167 | rtoi_12538 | rtoi_12555 | rtoi_15748, 'mmu': rtoi_11167 | rtoi_12538 | rtoi_12555 | rtoi_15748, 'sce': rtoi_11167 | rtoi_15748, 'yli': rtoi_11167 | rtoi_15748}
PRPNCOAHYDm
	 rtoi_11907
	 {'hsa': rtoi_14805 | rtoi_16128 | (HADHA & HADHB), 'mmu': rtoi_14805 | rtoi_16128 | (Hadha & Hadhb)}
PDHm
	 rtoi_10040 & rtoi_13630 & rtoi_13722 & rtoi_13948 & rtoi_14126
	 {'hsa': PDHX & rtoi_10040 & rtoi_13630 & rtoi_13948 & rtoi_14126, 'mmu': Pdhx & rtoi_10040 & rtoi_13630 & rtoi_13948 & rtoi_14126, 'sce': rtoi_10040 & rtoi_13630 & rtoi_13722 & rtoi_13948 & rtoi_14126}
NH4t
	 rtoi_12926 | rtoi_13637
	 {'sce': rtoi_12926 | rtoi_13637, 'yli': rtoi_12926 & rtoi_13637}
MALT
	 rtoi_9135
	 {'hsa': rtoi_10170, 'mmu': rtoi_10170, 'sce': rtoi_9135}
GLUCYS
	 rtoi_12007 | rtoi_12022
	 {'hsa': (GCLM & rtoi_12007) | (GCLM & rtoi_12022), 'mmu': (Gclm & rtoi_12007) | (Gclm & rtoi_12022), 'sce': rtoi_12007 | rtoi_12022, 'yli': rtoi_12007 | rtoi_12022}
SUCOAS1m
	 rtoi_15967 & rtoi_16144
	 {'hsa': SUCLG2 & rtoi_16144, 'mmu': Suclg2 & rtoi_16144, 'yli': rtoi_15967 & rtoi_16144}
ALDD20xm
	 rtoi_12042 | rtoi_13426
	 {'hsa': rtoi_12042 | rtoi_13426 | rtoi_8569, 'mmu': rtoi_12042 | rtoi_13426 | rtoi_8569, 'sce': rtoi_12042 | rtoi_13426}
TRDRm
	 YCR083W & rtoi_9688
	 {'hsa': rtoi_15482, 'mmu': rtoi_15482, 'sce': rtoi_15339 & rtoi_9688}
AGPRim
	 rtoi_9377
	 {'hsa': rtoi_16273, 'mmu': rtoi_16273, 'sce': rtoi_9377, 'yli': rtoi_9377}
FACOAL180
	 rtoi_11167 | rtoi_12538 | rtoi_12555 | rtoi_15748
	 {'hsa': rtoi_11167 | rtoi_12538 | rtoi_12555 | rtoi_15748, 'mmu': rtoi_11167 | rtoi_12538 | rtoi_12555 | rtoi_15748, 'sce': rtoi_11167 | rtoi_15748, 'yli': rtoi_11167 | rtoi_15748}
METAT
	 rtoi_13771
	 {'hsa': rtoi_13771 | (rtoi_11793 & rtoi_13771), 'mmu': rtoi_13771 | (rtoi_11793 & rtoi_13771), 'sce': rtoi_13771, 'yli': rtoi_13771}
```

In [68]:

```
for x in set(Reaction_rule_unique.keys()).intersection(Reaction_rule_Jeff):
    if x not in Reaction_rule_final:
        temp1 = Reaction_rule_unique[x]
        temp2 = sympy.Or(*[v for v in Reaction_rule_Jeff[x].values()])
        Reaction_rule_final[x] = sympy.Or(temp1,temp2)
```

In [72]:

```
for x in sorted(set(Reaction_rule.keys()).intersection(Reaction_rule_Jeff)):
    if x not in Reaction_rule_final:
        print(x)
        print('\t',Reaction_rule[x])
        print('\t',Reaction_rule_Jeff[x])
```

```
3DH5HPBMTm
	 {'sce': YLR201C & rtoi_10213 & rtoi_10633 & rtoi_11465 & rtoi_16037 & rtoi_16197 & rtoi_9681, 'yli': rtoi_11465}
	 {'sce': YLR201C & rtoi_10213 & rtoi_10633 & rtoi_11465 & rtoi_16037 & rtoi_16197 & rtoi_9681, 'yli': rtoi_11465}
AASAD1
	 {'sce': rtoi_10220 & rtoi_9495, 'yli': rtoi_9495}
	 {'sce': YGL154C & rtoi_9495, 'yli': rtoi_9495}
AASAD2
	 {'sce': rtoi_10220 & rtoi_9495, 'yli': rtoi_9495}
	 {'sce': YGL154C & rtoi_9495, 'yli': rtoi_9495}
ADEt2
	 {'sce': rtoi_14558 | rtoi_15012, 'yli': rtoi_14558 & rtoi_15012}
	 {'sce': rtoi_14461 | rtoi_14558 | rtoi_15012, 'yli': (YALI0D07392g & rtoi_14461) | (YALI0D07392g & rtoi_14558) | (YALI0D07392g & rtoi_15012)}
ADK1
	 {'sce': rtoi_15496, 'eco': rtoi_15496, 'ppu': rtoi_15496, 'cre': rtoi_12300 | rtoi_15496, 'yli': rtoi_15496}
	 {'hsa': rtoi_13190, 'mmu': rtoi_13190, 'sce': rtoi_15496, 'yli': rtoi_15496}
ADK1m
	 {'sce': rtoi_15129, 'cre': rtoi_12300}
	 {'hsa': rtoi_15129 | rtoi_15496, 'sce': rtoi_15129}
ALAt2r
	 {'sce': rtoi_14229 | rtoi_15074 | rtoi_8962 | rtoi_9319 | rtoi_9322, 'yli': (YALI0B09537g & rtoi_14229 & rtoi_8962) | (YALI0B09537g & rtoi_14229 & rtoi_9319) | (YALI0B09537g & rtoi_14229 & rtoi_9322) | (YALI0B09537g & rtoi_15074 & rtoi_8962) | (YALI0B09537g & rtoi_15074 & rtoi_9319) | (YALI0B09537g & rtoi_15074 & rtoi_9322)}
	 {'hsa': rtoi_12743, 'mmu': rtoi_12743, 'sce': rtoi_14229 | rtoi_15074 | rtoi_8962 | rtoi_9319 | rtoi_9322 | rtoi_9962, 'yli': (YALI0B09537g & rtoi_14229 & rtoi_8962) | (YALI0B09537g & rtoi_14229 & rtoi_9319) | (YALI0B09537g & rtoi_14229 & rtoi_9322) | (YALI0B09537g & rtoi_14229 & rtoi_9962) | (YALI0B09537g & rtoi_15074 & rtoi_8962) | (YALI0B09537g & rtoi_15074 & rtoi_9319) | (YALI0B09537g & rtoi_15074 & rtoi_9322) | (YALI0B09537g & rtoi_15074 & rtoi_9962)}
ALCD2x
	 {'eco': rtoi_11665 | rtoi_15438, 'yli': rtoi_14108 | rtoi_14109 | rtoi_15438}
	 {'hsa': rtoi_11665 | rtoi_13657 | rtoi_14108 | rtoi_14109, 'yli': rtoi_14108 | rtoi_14109 | rtoi_15438}
ALDD2xm
	 {'sce': rtoi_12042 | rtoi_13426, 'cre': rtoi_12042 | rtoi_13426, 'yli': rtoi_12042 | rtoi_13426 | rtoi_16323}
	 {'hsa': rtoi_12042 | rtoi_13426, 'mmu': rtoi_12042 | rtoi_13426, 'sce': rtoi_12042 | rtoi_13426, 'yli': rtoi_12042 | rtoi_13426 | rtoi_16323}
ALDD2y
	 {'sce': rtoi_12042 | rtoi_13426, 'eco': rtoi_11650 | rtoi_14700 | rtoi_15575 | rtoi_8666}
	 {'hsa': rtoi_12042 | rtoi_13426 | rtoi_16323, 'mmu': rtoi_12042 | rtoi_13426 | rtoi_16323, 'sce': rtoi_12042 | rtoi_13426}
AMID2
	 {'sce': rtoi_10276 | rtoi_10277, 'yli': rtoi_12161 | rtoi_13791}
	 {'sce': rtoi_10276 | rtoi_10277 | rtoi_12540 | rtoi_12553 | rtoi_12842, 'yli': rtoi_12161 | rtoi_13791}
AMID3
	 {'sce': rtoi_10276 | rtoi_10277, 'yli': rtoi_12161 | rtoi_13791}
	 {'sce': rtoi_10276 | rtoi_10277 | rtoi_12540 | rtoi_12553 | rtoi_12842, 'yli': rtoi_12161 | rtoi_13791}
ANS
	 {'sce': rtoi_15109 & rtoi_16564, 'eco': rtoi_15109 & rtoi_9900, 'ppu': rtoi_15109 & rtoi_16564, 'yli': rtoi_15109 | rtoi_16564}
	 {'sce': rtoi_15109 & rtoi_16564, 'yli': rtoi_15109 | rtoi_16564}
ASNS1
	 {'sce': rtoi_14762 | rtoi_15137, 'eco': rtoi_15137, 'ppu': rtoi_15137, 'cre': rtoi_14762 | rtoi_15137, 'yli': rtoi_15137}
	 {'hsa': rtoi_15137, 'mmu': rtoi_15137, 'sce': rtoi_14762 | rtoi_15137, 'yli': rtoi_15137}
ASNt2r
	 {'sce': rtoi_14229 | rtoi_15074 | rtoi_8962 | rtoi_9319 | rtoi_9322, 'yli': (rtoi_14229 & rtoi_8962) | (rtoi_14229 & rtoi_9319) | (rtoi_14229 & rtoi_9322) | (rtoi_15074 & rtoi_8962) | (rtoi_15074 & rtoi_9319) | (rtoi_15074 & rtoi_9322)}
	 {'sce': rtoi_14229 | rtoi_15074 | rtoi_8962 | rtoi_9319 | rtoi_9322 | rtoi_9962, 'yli': (rtoi_14229 & rtoi_8962) | (rtoi_14229 & rtoi_9319) | (rtoi_14229 & rtoi_9322) | (rtoi_14229 & rtoi_9962) | (rtoi_15074 & rtoi_8962) | (rtoi_15074 & rtoi_9319) | (rtoi_15074 & rtoi_9322) | (rtoi_15074 & rtoi_9962)}
ASPTA
	 {'sce': rtoi_8936, 'eco': rtoi_14281 | rtoi_8936, 'cre': rtoi_14281, 'yli': rtoi_14281 | rtoi_8936}
	 {'hsa': rtoi_8936, 'mmu': rtoi_8936, 'sce': rtoi_8936, 'yli': rtoi_14281 | rtoi_8936}
ASPt2r
	 {'sce': rtoi_14229 | rtoi_15074 | rtoi_8962 | rtoi_9319 | rtoi_9322, 'yli': (YALI0E20713g & rtoi_14229 & rtoi_8962) | (YALI0E20713g & rtoi_14229 & rtoi_9319) | (YALI0E20713g & rtoi_14229 & rtoi_9322) | (YALI0E20713g & rtoi_15074 & rtoi_8962) | (YALI0E20713g & rtoi_15074 & rtoi_9319) | (YALI0E20713g & rtoi_15074 & rtoi_9322)}
	 {'sce': rtoi_14229 | rtoi_15074 | rtoi_8962 | rtoi_9319 | rtoi_9322 | rtoi_9962, 'yli': (YALI0E20713g & rtoi_14229 & rtoi_8962) | (YALI0E20713g & rtoi_14229 & rtoi_9319) | (YALI0E20713g & rtoi_14229 & rtoi_9322) | (YALI0E20713g & rtoi_14229 & rtoi_9962) | (YALI0E20713g & rtoi_15074 & rtoi_8962) | (YALI0E20713g & rtoi_15074 & rtoi_9319) | (YALI0E20713g & rtoi_15074 & rtoi_9322) | (YALI0E20713g & rtoi_15074 & rtoi_9962)}
ATPS
	 {'sce': (YCR024C_A & YEL017C_A & rtoi_13617) | (YCR024C_A & YEL017C_A & rtoi_15584), 'cre': rtoi_12428 | rtoi_13617 | rtoi_15156 | rtoi_15584, 'yli': rtoi_13617 | rtoi_15584}
	 {'sce': (YCR024C_A & YEL017C_A & rtoi_13617) | (YCR024C_A & YEL017C_A & rtoi_15584), 'yli': rtoi_13617 | rtoi_15584}
CBPS
	 {'sce': rtoi_12302 | rtoi_16681 | (rtoi_12302 & rtoi_13297) | (rtoi_13297 & rtoi_16681), 'eco': (rtoi_12302 & rtoi_13297) | (rtoi_13297 & rtoi_16681), 'ppu': (rtoi_12302 & rtoi_13297) | (rtoi_13297 & rtoi_16681), 'cre': (rtoi_12302 & rtoi_13297) | (rtoi_13297 & rtoi_16681), 'yli': rtoi_12302 | rtoi_16681 | (rtoi_12302 & rtoi_13297) | (rtoi_13297 & rtoi_16681)}
	 {'hsa': rtoi_12302 | rtoi_16681, 'mmu': rtoi_12302 | rtoi_16681, 'sce': rtoi_12302 | rtoi_16681 | (rtoi_12302 & rtoi_13297) | (rtoi_13297 & rtoi_16681), 'yli': rtoi_12302 | rtoi_16681 | (rtoi_12302 & rtoi_13297) | (rtoi_13297 & rtoi_16681)}
CHORM
	 {'sce': rtoi_9704, 'eco': rtoi_14195 | rtoi_16225, 'ppu': rtoi_14195, 'yli': rtoi_14195 | rtoi_9704}
	 {'sce': rtoi_9704, 'yli': rtoi_14195 | rtoi_9704}
CSNATr
	 {'sce': rtoi_13580, 'yli': rtoi_13580 | rtoi_14245}
	 {'sce': rtoi_13580, 'yli': rtoi_13580 | rtoi_14245}
CSNt2
	 {'sce': rtoi_14558 | rtoi_15012, 'yli': rtoi_14558 & rtoi_15012}
	 {'sce': rtoi_14461 | rtoi_14558 | rtoi_15012, 'yli': (YALI0D07392g & rtoi_14461) | (YALI0D07392g & rtoi_14558) | (YALI0D07392g & rtoi_15012)}
CYSS
	 {'sce': rtoi_12031 | rtoi_13106, 'eco': rtoi_12031 | rtoi_13106 | rtoi_15712, 'ppu': rtoi_12031 | rtoi_13106 | rtoi_15712, 'yli': rtoi_12031 | rtoi_13106}
	 {'sce': rtoi_13106, 'yli': rtoi_12031 | rtoi_13106}
CYSTA
	 {'eco': rtoi_14281, 'yli': rtoi_14281 | rtoi_8936}
	 {'hsa': rtoi_8936, 'mmu': rtoi_8936, 'yli': rtoi_14281 | rtoi_8936}
DDPA
	 {'sce': rtoi_10602 | rtoi_12155, 'eco': rtoi_10602 | rtoi_12155, 'ppu': rtoi_10602 | rtoi_12155, 'yli': rtoi_10602 | rtoi_12155 | rtoi_15534}
	 {'sce': rtoi_10602 | rtoi_12155, 'yli': rtoi_10602 | rtoi_12155 | rtoi_15534}
DHORTS
	 {'sce': rtoi_12291 | rtoi_12302 | rtoi_16681, 'eco': rtoi_12291, 'ppu': rtoi_12291, 'yli': rtoi_12291}
	 {'hsa': rtoi_12302 | rtoi_16681, 'mmu': rtoi_12302 | rtoi_16681, 'sce': rtoi_12291 | rtoi_12302 | rtoi_16681, 'yli': rtoi_12291}
DMATT
	 {'sce': rtoi_12944, 'yli': rtoi_12944 | rtoi_16503}
	 {'hsa': rtoi_16503, 'mmu': rtoi_16503, 'sce': rtoi_12944, 'yli': rtoi_12944 | rtoi_16503}
DPRm
	 {'sce': rtoi_9176, 'cre': rtoi_14277, 'yli': rtoi_14277}
	 {'sce': rtoi_9176, 'yli': rtoi_14277}
ECOAH1m
	 {'cre': rtoi_11907, 'yli': rtoi_14805}
	 {'hsa': rtoi_14805 | rtoi_16128 | (HADHA & HADHB), 'mmu': rtoi_14805 | rtoi_16128 | (Hadha & Hadhb), 'yli': rtoi_14805}
FAS140COA
	 {'sce': (rtoi_12826 & rtoi_8639 & rtoi_8670 & rtoi_8777) | (rtoi_14471 & rtoi_8639 & rtoi_8670 & rtoi_8777), 'yli': rtoi_10677 & rtoi_16241 & rtoi_16695}
	 {'sce': (rtoi_12826 & rtoi_8639 & rtoi_8670) | (rtoi_12826 & rtoi_8639 & rtoi_8777), 'yli': rtoi_10677 & rtoi_16241 & rtoi_16695}
FAS160COA
	 {'sce': (rtoi_12826 & rtoi_8639 & rtoi_8670 & rtoi_8777) | (rtoi_14471 & rtoi_8639 & rtoi_8670 & rtoi_8777), 'yli': rtoi_10677 & rtoi_16241 & rtoi_16695}
	 {'sce': (rtoi_12826 & rtoi_8639 & rtoi_8670) | (rtoi_12826 & rtoi_8639 & rtoi_8777), 'yli': rtoi_10677 & rtoi_16241 & rtoi_16695}
FAS180
	 {'sce': (rtoi_12826 & rtoi_8639 & rtoi_8670 & rtoi_8777) | (rtoi_14471 & rtoi_8639 & rtoi_8670 & rtoi_8777), 'yli': rtoi_10677 & rtoi_16241 & rtoi_16695}
	 {'sce': (rtoi_12826 & rtoi_8639 & rtoi_8670) | (rtoi_12826 & rtoi_8639 & rtoi_8777), 'yli': rtoi_10677 & rtoi_16241 & rtoi_16695}
FAS180COA
	 {'sce': (rtoi_12826 & rtoi_8639 & rtoi_8670 & rtoi_8777) | (rtoi_14471 & rtoi_8639 & rtoi_8670 & rtoi_8777), 'yli': rtoi_10677 & rtoi_16241 & rtoi_16695}
	 {'sce': (rtoi_12826 & rtoi_8639 & rtoi_8670) | (rtoi_12826 & rtoi_8639 & rtoi_8777), 'yli': rtoi_10677 & rtoi_16241 & rtoi_16695}
FAS240_L
	 {'sce': rtoi_16241 & rtoi_16655, 'yli': rtoi_10677 & rtoi_16241 & rtoi_16695}
	 {'sce': rtoi_16241 & rtoi_16655, 'yli': rtoi_10677 & rtoi_16241 & rtoi_16695}
FAS80COA_L
	 {'sce': (rtoi_12826 & rtoi_8639 & rtoi_8670 & rtoi_8777) | (rtoi_14471 & rtoi_8639 & rtoi_8670 & rtoi_8777), 'yli': rtoi_10677 & rtoi_16241 & rtoi_16695}
	 {'sce': (rtoi_12826 & rtoi_8639 & rtoi_8670) | (rtoi_12826 & rtoi_8639 & rtoi_8777), 'yli': rtoi_10677 & rtoi_16241 & rtoi_16695}
FAS80_L
	 {'sce': (rtoi_12826 & rtoi_8639 & rtoi_8670 & rtoi_8777) | (rtoi_14471 & rtoi_8639 & rtoi_8670 & rtoi_8777), 'yli': rtoi_10677 & rtoi_16241 & rtoi_16695}
	 {'sce': (rtoi_12826 & rtoi_8639 & rtoi_8670) | (rtoi_12826 & rtoi_8639 & rtoi_8777), 'yli': rtoi_10677 & rtoi_16241 & rtoi_16695}
FBP26
	 {'sce': rtoi_15195, 'yli': rtoi_15195 | rtoi_15744}
	 {'hsa': rtoi_15195 | rtoi_15423, 'mmu': rtoi_15195 | rtoi_15423, 'sce': rtoi_15195, 'yli': rtoi_15195 | rtoi_15744}
FRDcm
	 {'sce': rtoi_11420, 'yli': rtoi_16281}
	 {'sce': rtoi_11420, 'yli': rtoi_16281}
GALt2
	 {'sce': rtoi_10704 | rtoi_11893, 'yli': rtoi_10704 & rtoi_11893}
	 {'sce': rtoi_10704 | rtoi_11075 | rtoi_11893, 'yli': (rtoi_10704 & rtoi_11075) | (rtoi_10704 & rtoi_11893)}
GCCam
	 {'sce': rtoi_10040 & rtoi_10205 & rtoi_12898 & rtoi_15184, 'yli': rtoi_10205}
	 {'hsa': rtoi_10040 & rtoi_10205 & rtoi_12898 & rtoi_15184, 'mmu': rtoi_10040 & rtoi_10205 & rtoi_12898 & rtoi_15184, 'sce': rtoi_10040 & rtoi_10205 & rtoi_12898 & rtoi_15184, 'yli': rtoi_10205}
GCCbim
	 {'sce': rtoi_10040 & rtoi_10205 & rtoi_12898 & rtoi_15184, 'yli': rtoi_12898}
	 {'hsa': rtoi_10040 & rtoi_10205 & rtoi_12898 & rtoi_15184, 'mmu': rtoi_10040 & rtoi_10205 & rtoi_12898 & rtoi_15184, 'sce': rtoi_10040 & rtoi_10205 & rtoi_12898 & rtoi_15184, 'yli': rtoi_12898}
GCCcm
	 {'sce': rtoi_10040 & rtoi_10205 & rtoi_12898 & rtoi_15184, 'cre': rtoi_10040, 'yli': rtoi_10040}
	 {'hsa': rtoi_10040 & rtoi_10205 & rtoi_12898 & rtoi_15184, 'mmu': rtoi_10040 & rtoi_10205 & rtoi_12898 & rtoi_15184, 'sce': rtoi_10040 & rtoi_10205 & rtoi_12898 & rtoi_15184, 'yli': rtoi_10040}
GLCt1
	 {'sce': rtoi_10704 | rtoi_11893, 'yli': rtoi_11893}
	 {'hsa': rtoi_10452 | rtoi_15762 | rtoi_9102 | rtoi_9841, 'sce': rtoi_10704 | rtoi_11075 | rtoi_11893, 'yli': rtoi_11075 | rtoi_11893}
GLNS
	 {'sce': rtoi_11844, 'cre': rtoi_11844 | rtoi_8959, 'yli': rtoi_11844 | rtoi_8959}
	 {'hsa': rtoi_11844 | rtoi_8959, 'mmu': rtoi_11844 | rtoi_8959, 'sce': rtoi_11844, 'yli': rtoi_11844 | rtoi_8959}
GLNt2r
	 {'sce': rtoi_14229 | rtoi_15074 | rtoi_8962 | rtoi_9319 | rtoi_9322, 'yli': (rtoi_14229 & rtoi_8962) | (rtoi_14229 & rtoi_9319) | (rtoi_14229 & rtoi_9322) | (rtoi_15074 & rtoi_8962) | (rtoi_15074 & rtoi_9319) | (rtoi_15074 & rtoi_9322)}
	 {'sce': rtoi_14229 | rtoi_15074 | rtoi_8962 | rtoi_9319 | rtoi_9322 | rtoi_9962, 'yli': (rtoi_14229 & rtoi_8962) | (rtoi_14229 & rtoi_9319) | (rtoi_14229 & rtoi_9322) | (rtoi_14229 & rtoi_9962) | (rtoi_15074 & rtoi_8962) | (rtoi_15074 & rtoi_9319) | (rtoi_15074 & rtoi_9322) | (rtoi_15074 & rtoi_9962)}
GLUt2r
	 {'sce': rtoi_10704 | rtoi_14229 | rtoi_15074 | rtoi_8962 | rtoi_9319 | rtoi_9322, 'yli': (YALI0E20713g & rtoi_10704 & rtoi_14229 & rtoi_8962) | (YALI0E20713g & rtoi_10704 & rtoi_14229 & rtoi_9319) | (YALI0E20713g & rtoi_10704 & rtoi_14229 & rtoi_9322) | (YALI0E20713g & rtoi_10704 & rtoi_15074 & rtoi_8962) | (YALI0E20713g & rtoi_10704 & rtoi_15074 & rtoi_9319) | (YALI0E20713g & rtoi_10704 & rtoi_15074 & rtoi_9322)}
	 {'sce': rtoi_10704 | rtoi_14229 | rtoi_15074 | rtoi_8962 | rtoi_9319 | rtoi_9322 | rtoi_9962, 'yli': (YALI0E20713g & rtoi_10704 & rtoi_14229 & rtoi_8962) | (YALI0E20713g & rtoi_10704 & rtoi_14229 & rtoi_9319) | (YALI0E20713g & rtoi_10704 & rtoi_14229 & rtoi_9322) | (YALI0E20713g & rtoi_10704 & rtoi_14229 & rtoi_9962) | (YALI0E20713g & rtoi_10704 & rtoi_15074 & rtoi_8962) | (YALI0E20713g & rtoi_10704 & rtoi_15074 & rtoi_9319) | (YALI0E20713g & rtoi_10704 & rtoi_15074 & rtoi_9322) | (YALI0E20713g & rtoi_10704 & rtoi_15074 & rtoi_9962)}
GLYCLm
	 {'sce': rtoi_10040 & rtoi_10205 & rtoi_12898 & rtoi_15184, 'cre': (CRv4_Au5_s12_g4121_t1 & rtoi_14894) | (rtoi_12898 & rtoi_14894), 'yli': rtoi_12898}
	 {'sce': rtoi_10040 & rtoi_10205 & rtoi_12898 & rtoi_15184, 'yli': rtoi_12898}
GLYt2r
	 {'sce': rtoi_14229 | rtoi_15074 | rtoi_8962 | rtoi_9319 | rtoi_9322, 'yli': (YALI0B09537g & rtoi_14229 & rtoi_8962) | (YALI0B09537g & rtoi_14229 & rtoi_9319) | (YALI0B09537g & rtoi_14229 & rtoi_9322) | (YALI0B09537g & rtoi_15074 & rtoi_8962) | (YALI0B09537g & rtoi_15074 & rtoi_9319) | (YALI0B09537g & rtoi_15074 & rtoi_9322)}
	 {'hsa': rtoi_12743, 'mmu': rtoi_12743, 'sce': rtoi_14229 | rtoi_15074 | rtoi_8962 | rtoi_9319 | rtoi_9322 | rtoi_9962, 'yli': (YALI0B09537g & rtoi_14229 & rtoi_8962) | (YALI0B09537g & rtoi_14229 & rtoi_9319) | (YALI0B09537g & rtoi_14229 & rtoi_9322) | (YALI0B09537g & rtoi_14229 & rtoi_9962) | (YALI0B09537g & rtoi_15074 & rtoi_8962) | (YALI0B09537g & rtoi_15074 & rtoi_9319) | (YALI0B09537g & rtoi_15074 & rtoi_9322) | (YALI0B09537g & rtoi_15074 & rtoi_9962)}
GRTT
	 {'sce': rtoi_12944, 'cre': rtoi_12944, 'yli': rtoi_12944 | rtoi_16503}
	 {'hsa': rtoi_16503, 'mmu': rtoi_16503, 'sce': rtoi_12944, 'yli': rtoi_12944 | rtoi_16503}
GTHOm
	 {'sce': rtoi_15482 & rtoi_9250, 'cre': rtoi_15482}
	 {'hsa': rtoi_15482, 'mmu': rtoi_15482, 'sce': rtoi_15482 & rtoi_9250}
GTHOr
	 {'sce': (rtoi_15038 & rtoi_15482) | (rtoi_15482 & rtoi_16549) | (rtoi_15482 & rtoi_8790), 'eco': rtoi_15482, 'ppu': rtoi_15482, 'cre': rtoi_15482}
	 {'hsa': rtoi_15482, 'mmu': rtoi_15482, 'sce': (rtoi_15038 & rtoi_15482) | (rtoi_15482 & rtoi_8790)}
GTHPi
	 {'sce': rtoi_15038 | rtoi_16549 | rtoi_8579, 'eco': rtoi_8579, 'ppu': rtoi_8579, 'cre': rtoi_8579, 'yli': rtoi_8579}
	 {'hsa': rtoi_12715 | rtoi_8579, 'mmu': rtoi_12715 | rtoi_8579, 'sce': rtoi_15038 | rtoi_8579, 'yli': rtoi_8579}
GTHPm
	 {'sce': rtoi_9250, 'yli': rtoi_8579}
	 {'hsa': rtoi_8579, 'mmu': rtoi_12715 | rtoi_8579, 'sce': rtoi_9250, 'yli': rtoi_8579}
GUAt2r
	 {'sce': rtoi_14558 | rtoi_15012, 'yli': rtoi_14558 & rtoi_15012}
	 {'sce': rtoi_14461 | rtoi_14558 | rtoi_15012, 'yli': (YALI0D07392g & rtoi_14461) | (YALI0D07392g & rtoi_14558) | (YALI0D07392g & rtoi_15012)}
HSERTA
	 {'sce': rtoi_12513, 'ppu': PP_5098 & rtoi_15248, 'yli': rtoi_12513 | rtoi_15248}
	 {'sce': rtoi_12513, 'yli': rtoi_12513 | rtoi_15248}
ICDHxm
	 {'sce': rtoi_11681 & rtoi_11682, 'yli': rtoi_11681 | rtoi_11682}
	 {'hsa': rtoi_11681 & rtoi_11682, 'mmu': rtoi_11681 & rtoi_11682, 'sce': rtoi_11681 & rtoi_11682, 'yli': rtoi_11681 | rtoi_11682}
ILETRS
	 {'sce': rtoi_13446, 'ppu': rtoi_15556, 'cre': rtoi_13446, 'yli': rtoi_13446 | rtoi_15556}
	 {'sce': rtoi_13446, 'yli': rtoi_13446 | rtoi_15556}
ILETRSm
	 {'sce': rtoi_15556, 'yli': rtoi_13446 | rtoi_15556}
	 {'sce': rtoi_15556, 'yli': rtoi_13446 | rtoi_15556}
MAN1PT
	 {'sce': rtoi_14956, 'cre': rtoi_14956, 'yli': rtoi_10964 | rtoi_14956}
	 {'sce': rtoi_14956, 'yli': rtoi_10964 | rtoi_14956}
METB1
	 {'sce': rtoi_11463, 'yli': rtoi_11463 | rtoi_16725 | rtoi_16742}
	 {'sce': rtoi_11463, 'yli': rtoi_11463 | rtoi_16725 | rtoi_16742}
METt2r
	 {'sce': rtoi_14229 | rtoi_15074, 'yli': (YALI0F25795g & rtoi_14229) | (YALI0F25795g & rtoi_15074)}
	 {'sce': rtoi_14229 | rtoi_15074, 'yli': (YALI0F25795g & rtoi_14229) | (YALI0F25795g & rtoi_15074)}
MTHFR3
	 {'sce': rtoi_15483 | rtoi_9244, 'ppu': rtoi_9244, 'yli': rtoi_15483 | rtoi_9244}
	 {'hsa': rtoi_9244, 'mmu': rtoi_9244, 'sce': rtoi_15483 | rtoi_9244, 'yli': rtoi_15483 | rtoi_9244}
NDPK1
	 {'sce': rtoi_15679, 'eco': rtoi_15496 | rtoi_15679, 'ppu': rtoi_15679, 'cre': rtoi_15679 | rtoi_8943, 'yli': rtoi_15679}
	 {'hsa': rtoi_15679 | rtoi_8943, 'mmu': rtoi_15679 | rtoi_8943, 'sce': rtoi_15679, 'yli': rtoi_15679}
NDPK2
	 {'sce': rtoi_15679, 'eco': rtoi_15496 | rtoi_15679, 'ppu': rtoi_15679, 'cre': rtoi_15679 | rtoi_8943, 'yli': rtoi_15679}
	 {'hsa': rtoi_15679 | rtoi_8943, 'mmu': rtoi_15679 | rtoi_8943, 'sce': rtoi_15679, 'yli': rtoi_15679}
NDPK3
	 {'sce': rtoi_15679, 'eco': rtoi_15496 | rtoi_15679, 'ppu': rtoi_15679, 'cre': rtoi_15679 | rtoi_8943, 'yli': rtoi_15679}
	 {'hsa': rtoi_15679 | rtoi_8943, 'mmu': rtoi_15679 | rtoi_8943, 'sce': rtoi_15679, 'yli': rtoi_15679}
NDPK4
	 {'sce': rtoi_15679, 'eco': rtoi_15496 | rtoi_15679, 'ppu': rtoi_15679, 'cre': rtoi_15679 | rtoi_8943, 'yli': rtoi_15679}
	 {'hsa': rtoi_15679 | rtoi_8943, 'mmu': rtoi_15679 | rtoi_8943, 'sce': rtoi_15679, 'yli': rtoi_15679}
NDPK5
	 {'sce': rtoi_15679, 'eco': rtoi_15496 | rtoi_15679, 'ppu': rtoi_15679, 'cre': rtoi_15679 | rtoi_8943, 'yli': rtoi_15679}
	 {'hsa': rtoi_15679 | rtoi_8943, 'mmu': rtoi_15679 | rtoi_8943, 'sce': rtoi_15679, 'yli': rtoi_15679}
NDPK6
	 {'sce': rtoi_15679, 'eco': rtoi_15496 | rtoi_15679, 'ppu': rtoi_15679, 'cre': rtoi_15679 | rtoi_8943, 'yli': rtoi_15679}
	 {'hsa': rtoi_15679 | rtoi_8943, 'mmu': rtoi_15679 | rtoi_8943, 'sce': rtoi_15679, 'yli': rtoi_15679}
NDPK7
	 {'sce': rtoi_15679, 'eco': rtoi_15496 | rtoi_15679, 'ppu': rtoi_15679, 'cre': rtoi_15679 | rtoi_8943, 'yli': rtoi_15679}
	 {'hsa': rtoi_15679 | rtoi_8943, 'mmu': rtoi_15679 | rtoi_8943, 'sce': rtoi_15679, 'yli': rtoi_15679}
NDPK8
	 {'sce': rtoi_15679, 'eco': rtoi_15496 | rtoi_15679, 'ppu': rtoi_15679, 'cre': rtoi_15679 | rtoi_8943, 'yli': rtoi_15679}
	 {'hsa': rtoi_15679 | rtoi_8943, 'mmu': rtoi_15679 | rtoi_8943, 'sce': rtoi_15679, 'yli': rtoi_15679}
NDPK9
	 {'sce': rtoi_15679, 'cre': rtoi_15679 | rtoi_8943, 'yli': rtoi_15679}
	 {'hsa': rtoi_15679 | rtoi_8943, 'mmu': rtoi_15679 | rtoi_8943, 'sce': rtoi_15679, 'yli': rtoi_15679}
ORPT
	 {'sce': rtoi_10716, 'eco': rtoi_10716, 'ppu': rtoi_10716, 'cre': rtoi_12118, 'yli': rtoi_10716}
	 {'hsa': rtoi_12118, 'mmu': rtoi_12118, 'sce': rtoi_10716, 'yli': rtoi_10716}
PAPSR
	 {'sce': (rtoi_11741 & rtoi_15339) | (rtoi_11741 & rtoi_16019), 'eco': (rtoi_11741 & rtoi_15339) | (rtoi_11741 & rtoi_16019), 'yli': rtoi_11741}
	 {'sce': rtoi_11741 & rtoi_15339, 'yli': rtoi_11741}
PDE4
	 {'sce': rtoi_11787, 'yli': rtoi_11787 | rtoi_13690}
	 {'sce': rtoi_11787, 'yli': rtoi_11787 | rtoi_13690}
PGM
	 {'sce': rtoi_9910, 'eco': rtoi_9910, 'cre': rtoi_11229 | rtoi_12393 | rtoi_9910, 'yli': rtoi_12637 | rtoi_15425 | rtoi_9910}
	 {'hsa': rtoi_9910, 'mmu': rtoi_9910, 'sce': rtoi_9910, 'yli': rtoi_12637 | rtoi_15425 | rtoi_9910}
PGMT
	 {'sce': rtoi_9959, 'eco': rtoi_14546 | rtoi_8460 | rtoi_9959}
	 {'hsa': rtoi_13711 | rtoi_9959, 'mmu': rtoi_13711 | rtoi_9959, 'sce': rtoi_9959}
PHETA1
	 {'sce': rtoi_14908 | rtoi_15839, 'eco': rtoi_14281 | rtoi_14610 | rtoi_14853 | rtoi_8936, 'ppu': rtoi_14281 | rtoi_8936, 'cre': rtoi_13230 | rtoi_14281, 'yli': rtoi_13230 | rtoi_14281 | rtoi_14908 | rtoi_15839 | rtoi_8936}
	 {'hsa': rtoi_8936, 'mmu': rtoi_8936, 'sce': rtoi_12407 | rtoi_14908 | rtoi_15839, 'yli': rtoi_12407 | rtoi_13230 | rtoi_14281 | rtoi_14908 | rtoi_15839 | rtoi_8936}
PHETA1m
	 {'cre': rtoi_13230 | rtoi_14281, 'yli': rtoi_14908 | rtoi_15839}
	 {'hsa': rtoi_14281 | rtoi_16065, 'mmu': rtoi_14281 | rtoi_16065, 'yli': rtoi_12407 | rtoi_14908 | rtoi_15839}
PIt2r
	 {'sce': rtoi_10316 | rtoi_11418 | rtoi_11534 | rtoi_11686, 'cre': rtoi_11409 | rtoi_11410 | rtoi_11686, 'yli': (rtoi_10316 & rtoi_11418 & rtoi_11686) | (rtoi_10316 & rtoi_11534 & rtoi_11686)}
	 {'sce': rtoi_10316 | rtoi_11418 | rtoi_11534 | rtoi_11686, 'yli': (rtoi_10316 & rtoi_11418 & rtoi_11686) | (rtoi_10316 & rtoi_11534 & rtoi_11686)}
PRO1xm
	 {'sce': rtoi_14602, 'yli': rtoi_13432}
	 {'hsa': rtoi_14602, 'mmu': rtoi_14602, 'yli': rtoi_13432}
PROt2r
	 {'sce': rtoi_14229 | rtoi_15074, 'yli': (YALI0B09537g & rtoi_14229) | (YALI0B09537g & rtoi_15074)}
	 {'hsa': rtoi_12743, 'mmu': rtoi_12743, 'sce': rtoi_14229 | rtoi_15074, 'yli': (YALI0B09537g & rtoi_14229) | (YALI0B09537g & rtoi_15074)}
PTRCt3i
	 {'sce': rtoi_11017 | rtoi_13128, 'yli': (rtoi_11017 & rtoi_16454) | (rtoi_13128 & rtoi_16454)}
	 {'sce': rtoi_11017, 'yli': rtoi_11017 & rtoi_16454}
RNDR1
	 {'sce': (rtoi_11172 & rtoi_11290 & rtoi_15339) | (rtoi_11172 & rtoi_11290 & rtoi_16019) | (rtoi_11290 & rtoi_14237 & rtoi_15339) | (rtoi_11290 & rtoi_14237 & rtoi_16019), 'eco': (rtoi_11172 & rtoi_11290 & rtoi_15339) | (rtoi_11172 & rtoi_11290 & rtoi_16019) | (rtoi_11290 & rtoi_14237 & rtoi_15339) | (rtoi_11290 & rtoi_14237 & rtoi_16019), 'ppu': (rtoi_11172 & rtoi_11290) | (rtoi_11290 & rtoi_14237), 'cre': (CRv4_Au5_s9_g15314_t1 & rtoi_11172 & rtoi_11290) | (CRv4_Au5_s9_g15314_t1 & rtoi_11290 & rtoi_14237) | (rtoi_11172 & rtoi_11290 & rtoi_15339) | (rtoi_11172 & rtoi_11290 & rtoi_16019) | (rtoi_11290 & rtoi_14237 & rtoi_15339) | (rtoi_11290 & rtoi_14237 & rtoi_16019), 'yli': (rtoi_11172 & rtoi_11290) | (rtoi_11290 & rtoi_14237)}
	 {'hsa': (rtoi_11172 & rtoi_11290) | (rtoi_11290 & rtoi_14237), 'mmu': (rtoi_11172 & rtoi_11290) | (rtoi_11290 & rtoi_14237), 'sce': (rtoi_11172 & rtoi_11290 & rtoi_15339) | (rtoi_11290 & rtoi_14237 & rtoi_15339), 'yli': (rtoi_11172 & rtoi_11290) | (rtoi_11290 & rtoi_14237)}
RNDR2
	 {'sce': (rtoi_11172 & rtoi_11290 & rtoi_15339) | (rtoi_11172 & rtoi_11290 & rtoi_16019) | (rtoi_11290 & rtoi_14237 & rtoi_15339) | (rtoi_11290 & rtoi_14237 & rtoi_16019), 'eco': (rtoi_11172 & rtoi_11290 & rtoi_15339) | (rtoi_11172 & rtoi_11290 & rtoi_16019) | (rtoi_11290 & rtoi_14237 & rtoi_15339) | (rtoi_11290 & rtoi_14237 & rtoi_16019), 'ppu': (rtoi_11172 & rtoi_11290) | (rtoi_11290 & rtoi_14237), 'cre': (CRv4_Au5_s9_g15314_t1 & rtoi_11172 & rtoi_11290) | (CRv4_Au5_s9_g15314_t1 & rtoi_11290 & rtoi_14237) | (rtoi_11172 & rtoi_11290 & rtoi_15339) | (rtoi_11172 & rtoi_11290 & rtoi_16019) | (rtoi_11290 & rtoi_14237 & rtoi_15339) | (rtoi_11290 & rtoi_14237 & rtoi_16019), 'yli': (rtoi_11172 & rtoi_11290) | (rtoi_11290 & rtoi_14237)}
	 {'hsa': (rtoi_11172 & rtoi_11290) | (rtoi_11290 & rtoi_14237), 'mmu': (rtoi_11172 & rtoi_11290) | (rtoi_11290 & rtoi_14237), 'sce': (rtoi_11172 & rtoi_11290 & rtoi_15339) | (rtoi_11290 & rtoi_14237 & rtoi_15339), 'yli': (rtoi_11172 & rtoi_11290) | (rtoi_11290 & rtoi_14237)}
RNDR3
	 {'sce': (rtoi_11172 & rtoi_11290 & rtoi_15339) | (rtoi_11172 & rtoi_11290 & rtoi_16019) | (rtoi_11290 & rtoi_14237 & rtoi_15339) | (rtoi_11290 & rtoi_14237 & rtoi_16019), 'eco': (rtoi_11172 & rtoi_11290 & rtoi_15339) | (rtoi_11172 & rtoi_11290 & rtoi_16019) | (rtoi_11290 & rtoi_14237 & rtoi_15339) | (rtoi_11290 & rtoi_14237 & rtoi_16019), 'ppu': (rtoi_11172 & rtoi_11290) | (rtoi_11290 & rtoi_14237), 'cre': (CRv4_Au5_s9_g15314_t1 & rtoi_11172 & rtoi_11290) | (CRv4_Au5_s9_g15314_t1 & rtoi_11290 & rtoi_14237) | (rtoi_11172 & rtoi_11290 & rtoi_15339) | (rtoi_11172 & rtoi_11290 & rtoi_16019) | (rtoi_11290 & rtoi_14237 & rtoi_15339) | (rtoi_11290 & rtoi_14237 & rtoi_16019), 'yli': (rtoi_11172 & rtoi_11290) | (rtoi_11290 & rtoi_14237)}
	 {'hsa': (rtoi_11172 & rtoi_11290) | (rtoi_11290 & rtoi_14237), 'mmu': (rtoi_11172 & rtoi_11290) | (rtoi_11290 & rtoi_14237), 'sce': (rtoi_11172 & rtoi_11290 & rtoi_15339) | (rtoi_11290 & rtoi_14237 & rtoi_15339), 'yli': (rtoi_11172 & rtoi_11290) | (rtoi_11290 & rtoi_14237)}
RNDR4
	 {'sce': (rtoi_11172 & rtoi_11290 & rtoi_15339) | (rtoi_11172 & rtoi_11290 & rtoi_16019) | (rtoi_11290 & rtoi_14237 & rtoi_15339) | (rtoi_11290 & rtoi_14237 & rtoi_16019), 'eco': (rtoi_11172 & rtoi_11290 & rtoi_15339) | (rtoi_11172 & rtoi_11290 & rtoi_16019) | (rtoi_11290 & rtoi_14237 & rtoi_15339) | (rtoi_11290 & rtoi_14237 & rtoi_16019), 'ppu': (rtoi_11172 & rtoi_11290) | (rtoi_11290 & rtoi_14237), 'cre': (CRv4_Au5_s9_g15314_t1 & rtoi_11172 & rtoi_11290) | (CRv4_Au5_s9_g15314_t1 & rtoi_11290 & rtoi_14237) | (rtoi_11172 & rtoi_11290 & rtoi_15339) | (rtoi_11172 & rtoi_11290 & rtoi_16019) | (rtoi_11290 & rtoi_14237 & rtoi_15339) | (rtoi_11290 & rtoi_14237 & rtoi_16019), 'yli': (rtoi_11172 & rtoi_11290) | (rtoi_11290 & rtoi_14237)}
	 {'hsa': (rtoi_11172 & rtoi_11290) | (rtoi_11290 & rtoi_14237), 'mmu': (rtoi_11172 & rtoi_11290) | (rtoi_11290 & rtoi_14237), 'sce': (rtoi_11172 & rtoi_11290 & rtoi_15339) | (rtoi_11290 & rtoi_14237 & rtoi_15339), 'yli': (rtoi_11172 & rtoi_11290) | (rtoi_11290 & rtoi_14237)}
SADT
	 {'sce': rtoi_13443, 'ppu': PP_1303 & rtoi_14868, 'cre': rtoi_13443, 'yli': rtoi_13443}
	 {'hsa': rtoi_8709, 'mmu': rtoi_8709, 'sce': rtoi_13443, 'yli': rtoi_13443}
SERD_L
	 {'sce': rtoi_9216, 'eco': rtoi_11849, 'ppu': rtoi_11849, 'yli': rtoi_11909 | rtoi_9216}
	 {'sce': rtoi_9216, 'yli': rtoi_11909 | rtoi_9216}
SERPT
	 {'sce': YBR058C_A & rtoi_10303 & rtoi_9425, 'cre': rtoi_10303 & rtoi_9425, 'yli': rtoi_10303 | rtoi_9425}
	 {'hsa': rtoi_10303 & rtoi_9425, 'mmu': rtoi_10303 & rtoi_9425, 'sce': YBR058C_A & rtoi_10303 & rtoi_9425, 'yli': rtoi_10303 | rtoi_9425}
SERt2r
	 {'sce': rtoi_14229 | rtoi_15074 | rtoi_8962 | rtoi_9319 | rtoi_9322, 'yli': (YALI0E20713g & rtoi_14229 & rtoi_8962) | (YALI0E20713g & rtoi_14229 & rtoi_9319) | (YALI0E20713g & rtoi_14229 & rtoi_9322) | (YALI0E20713g & rtoi_15074 & rtoi_8962) | (YALI0E20713g & rtoi_15074 & rtoi_9319) | (YALI0E20713g & rtoi_15074 & rtoi_9322)}
	 {'sce': rtoi_14229 | rtoi_15074 | rtoi_8962 | rtoi_9319 | rtoi_9322 | rtoi_9962, 'yli': (YALI0E20713g & rtoi_14229 & rtoi_8962) | (YALI0E20713g & rtoi_14229 & rtoi_9319) | (YALI0E20713g & rtoi_14229 & rtoi_9322) | (YALI0E20713g & rtoi_14229 & rtoi_9962) | (YALI0E20713g & rtoi_15074 & rtoi_8962) | (YALI0E20713g & rtoi_15074 & rtoi_9319) | (YALI0E20713g & rtoi_15074 & rtoi_9322) | (YALI0E20713g & rtoi_15074 & rtoi_9962)}
SHSL4r
	 {'sce': rtoi_11463, 'ppu': rtoi_9499, 'yli': rtoi_11463 | rtoi_16725 | rtoi_16742}
	 {'sce': rtoi_11463, 'yli': rtoi_11463 | rtoi_16725 | rtoi_16742}
SO4ti
	 {'sce': rtoi_14119 | rtoi_15736 | rtoi_16682, 'yli': (rtoi_14119 & rtoi_16682) | (rtoi_15736 & rtoi_16682)}
	 {'sce': rtoi_14119 | rtoi_15736 | rtoi_16682, 'yli': (rtoi_14119 & rtoi_16682) | (rtoi_15736 & rtoi_16682)}
SPMDt3i
	 {'sce': rtoi_11017 | rtoi_13128, 'yli': (rtoi_11017 & rtoi_16454) | (rtoi_13128 & rtoi_16454)}
	 {'sce': rtoi_11017, 'yli': rtoi_11017 & rtoi_16454}
SPRMt2i
	 {'sce': rtoi_11017 | rtoi_11649 | rtoi_13128 | rtoi_14774, 'yli': (rtoi_11017 & rtoi_11649 & rtoi_16454) | (rtoi_11017 & rtoi_14774 & rtoi_16454) | (rtoi_11649 & rtoi_13128 & rtoi_16454) | (rtoi_13128 & rtoi_14774 & rtoi_16454)}
	 {'sce': rtoi_11017 | rtoi_14774, 'yli': rtoi_11017 & rtoi_14774 & rtoi_16454}
SQLEr
	 {'sce': rtoi_13286 & rtoi_13729, 'yli': rtoi_13729}
	 {'hsa': rtoi_13729, 'mmu': rtoi_13729, 'sce': rtoi_13286 & rtoi_13729, 'yli': rtoi_13729}
SUCD1m
	 {'sce': rtoi_11629 & rtoi_11802 & rtoi_11977 & rtoi_16281, 'cre': rtoi_11629, 'yli': rtoi_11629 & rtoi_11802 & rtoi_11977 & rtoi_16281}
	 {'hsa': SDHD & rtoi_11629 & rtoi_11977 & rtoi_16281, 'mmu': Sdhd & rtoi_11629 & rtoi_11977 & rtoi_16281, 'sce': rtoi_11629 & rtoi_11802 & rtoi_11977 & rtoi_16281, 'yli': rtoi_11629 & rtoi_11802 & rtoi_11977 & rtoi_16281}
SUCFUMtm
	 {'sce': rtoi_11740 | rtoi_12605 | rtoi_13510, 'cre': rtoi_12605, 'yli': (rtoi_11740 & rtoi_12605) | (rtoi_12605 & rtoi_13510)}
	 {'sce': rtoi_12605 | rtoi_13510, 'yli': rtoi_12605 & rtoi_13510}
SULR
	 {'sce': rtoi_10374 | rtoi_8744, 'eco': rtoi_13286 & rtoi_8744, 'ppu': PP_0860 & rtoi_8744, 'yli': rtoi_10374 & rtoi_8744}
	 {'sce': rtoi_10374 | rtoi_8744, 'yli': rtoi_10374 & rtoi_8744}
THFATm
	 {'sce': rtoi_10205 & rtoi_12898 & rtoi_13630 & rtoi_13948 & rtoi_15184, 'yli': rtoi_12898}
	 {'sce': rtoi_10205 & rtoi_12898 & rtoi_13630 & rtoi_13948 & rtoi_15184, 'yli': rtoi_12898}
THRD_L
	 {'sce': rtoi_9216, 'eco': rtoi_11909, 'ppu': rtoi_11909, 'yli': rtoi_11909}
	 {'hsa': rtoi_9216, 'mmu': rtoi_9216, 'sce': rtoi_9216, 'yli': rtoi_11909}
TRDR
	 {'sce': rtoi_15339 | rtoi_16019 | rtoi_9688 | (rtoi_15339 & rtoi_9688) | (rtoi_16019 & rtoi_9688), 'eco': (rtoi_15339 & rtoi_9688) | (rtoi_16019 & rtoi_9688), 'ppu': rtoi_15339 | rtoi_16019 | rtoi_9688, 'cre': (CRv4_Au5_s2_g8777_t1 & CRv4_Au5_s9_g15314_t1) | (CRv4_Au5_s2_g8777_t1 & rtoi_15339) | (CRv4_Au5_s2_g8777_t1 & rtoi_16019) | (CRv4_Au5_s8_g14830_t1 & CRv4_Au5_s9_g15314_t1) | (CRv4_Au5_s8_g14830_t1 & rtoi_15339) | (CRv4_Au5_s8_g14830_t1 & rtoi_16019) | (CRv4_Au5_s9_g15314_t1 & rtoi_9688) | (rtoi_15339 & rtoi_9688) | (rtoi_16019 & rtoi_9688), 'yli': rtoi_9688}
	 {'hsa': rtoi_15482, 'mmu': rtoi_15482, 'sce': rtoi_15339 | rtoi_9688 | (rtoi_15339 & rtoi_9688), 'yli': rtoi_9688}
TRE6PP
	 {'sce': (YML100W & rtoi_11389 & rtoi_12034) | (YMR261C & rtoi_11389 & rtoi_12034), 'yli': rtoi_12034}
	 {'sce': (YML100W & rtoi_11389 & rtoi_12034) | (YMR261C & rtoi_11389 & rtoi_12034), 'yli': rtoi_12034}
TRPTRS
	 {'sce': rtoi_14709, 'cre': rtoi_14709 | rtoi_15533, 'yli': rtoi_14709 | rtoi_15533}
	 {'sce': rtoi_14709, 'yli': rtoi_14709 | rtoi_15533}
TYRTA
	 {'eco': rtoi_14281 | rtoi_14610 | rtoi_14853 | rtoi_8936, 'ppu': rtoi_14281 | rtoi_8936, 'cre': rtoi_14281, 'yli': rtoi_13230 | rtoi_14281 | rtoi_14908 | rtoi_15839 | rtoi_8936}
	 {'hsa': rtoi_8936, 'mmu': rtoi_8936, 'yli': rtoi_12407 | rtoi_13230 | rtoi_14281 | rtoi_14908 | rtoi_15839 | rtoi_8936}
TYRTAim
	 {'cre': rtoi_13230 | rtoi_14281, 'yli': rtoi_14908 | rtoi_15839}
	 {'hsa': rtoi_14281 | rtoi_16065, 'mmu': rtoi_14281 | rtoi_16065, 'sce': rtoi_14281, 'yli': rtoi_12407 | rtoi_14908 | rtoi_15839}
UPPRT
	 {'sce': rtoi_14241, 'eco': rtoi_15095, 'ppu': rtoi_15095, 'cre': rtoi_8633, 'yli': rtoi_14241 | rtoi_15095}
	 {'sce': rtoi_14241, 'yli': rtoi_14241 | rtoi_15095}
URIDK2r
	 {'sce': rtoi_13190, 'cre': rtoi_15252, 'yli': rtoi_15252}
	 {'sce': rtoi_13190, 'yli': rtoi_15252}
URIt2
	 {'sce': rtoi_10921 | rtoi_11871 | rtoi_12902 | rtoi_13969, 'yli': (rtoi_10921 & rtoi_13969) | (rtoi_11871 & rtoi_13969) | (rtoi_12902 & rtoi_13969)}
	 {'sce': rtoi_12902 | rtoi_13969, 'yli': rtoi_12902 & rtoi_13969}
```

In [75]:

```
for x in set(Reaction_rule.keys()).intersection(Reaction_rule_Jeff):
    if x not in Reaction_rule_final:
        temp1 = sympy.Or(*[v for v in Reaction_rule[x].values()])
        temp2 = sympy.Or(*[v for v in Reaction_rule_Jeff[x].values()])
        Reaction_rule_final[x] = sympy.Or(temp1,temp2)
```

In [78]:

```
temp = ['FAS140COA','FAS160COA','FAS180','FAS180COA','FAS80COA_L','FAS80_L']
for x in temp:
    print(x)
    print(Reaction_rule_final[x])
```

```
FAS140COA
(rtoi_10677 & rtoi_16241 & rtoi_16695) | (rtoi_12826 & rtoi_8639 & rtoi_8670) | (rtoi_12826 & rtoi_8639 & rtoi_8777) | (rtoi_12826 & rtoi_8639 & rtoi_8670 & rtoi_8777) | (rtoi_14471 & rtoi_8639 & rtoi_8670 & rtoi_8777)
FAS160COA
(rtoi_10677 & rtoi_16241 & rtoi_16695) | (rtoi_12826 & rtoi_8639 & rtoi_8670) | (rtoi_12826 & rtoi_8639 & rtoi_8777) | (rtoi_12826 & rtoi_8639 & rtoi_8670 & rtoi_8777) | (rtoi_14471 & rtoi_8639 & rtoi_8670 & rtoi_8777)
FAS180
(rtoi_10677 & rtoi_16241 & rtoi_16695) | (rtoi_12826 & rtoi_8639 & rtoi_8670) | (rtoi_12826 & rtoi_8639 & rtoi_8777) | (rtoi_12826 & rtoi_8639 & rtoi_8670 & rtoi_8777) | (rtoi_14471 & rtoi_8639 & rtoi_8670 & rtoi_8777)
FAS180COA
(rtoi_10677 & rtoi_16241 & rtoi_16695) | (rtoi_12826 & rtoi_8639 & rtoi_8670) | (rtoi_12826 & rtoi_8639 & rtoi_8777) | (rtoi_12826 & rtoi_8639 & rtoi_8670 & rtoi_8777) | (rtoi_14471 & rtoi_8639 & rtoi_8670 & rtoi_8777)
FAS80COA_L
(rtoi_10677 & rtoi_16241 & rtoi_16695) | (rtoi_12826 & rtoi_8639 & rtoi_8670) | (rtoi_12826 & rtoi_8639 & rtoi_8777) | (rtoi_12826 & rtoi_8639 & rtoi_8670 & rtoi_8777) | (rtoi_14471 & rtoi_8639 & rtoi_8670 & rtoi_8777)
FAS80_L
(rtoi_10677 & rtoi_16241 & rtoi_16695) | (rtoi_12826 & rtoi_8639 & rtoi_8670) | (rtoi_12826 & rtoi_8639 & rtoi_8777) | (rtoi_12826 & rtoi_8639 & rtoi_8670 & rtoi_8777) | (rtoi_14471 & rtoi_8639 & rtoi_8670 & rtoi_8777)
```

In [80]:

```
temp = ['FAS140COA','FAS160COA','FAS180','FAS180COA','FAS80COA_L','FAS80_L']
for x in temp:
    Reaction_rule_final[x] = parse_expr('(rtoi_10677 & rtoi_16241 & rtoi_16695) |\
                                         (rtoi_12826 & rtoi_8639 & rtoi_8670 & rtoi_8777) |\
                                         (rtoi_14471 & rtoi_8639 & rtoi_8670 & rtoi_8777)')
```

In [86]:

```
for x in set(Reaction_rule.keys()).difference(Reaction_rule_Jeff):
    if x not in Reaction_rule_final:
        temp1 = sympy.Or(*[v for v in Reaction_rule[x].values()])
        Reaction_rule_final[x] = temp1
for x in set(Reaction_rule_Jeff.keys()).difference(Reaction_rule):
    if x not in Reaction_rule_final:
        temp2 = sympy.Or(*[v for v in Reaction_rule_Jeff[x].values()])
        Reaction_rule_final[x] = temp2
```

In [87]:

```
print(len(Reaction_subset_union))
print(len(Reaction_rule_final))
```

```
3804
3804
```

In [88]:

```
print(Reaction_subset_union['PGI'])
print(Reaction_rule_final['PGI'])
```

```
('eco', 'rev')
rtoi_9589
```

In [89]:

```
IFO0880_model = cobra.Model('R. toruloides')
```

In [91]:

```
for k, v in Reaction_subset_union.items():
    temp = eval(v[0]).reactions.get_by_id(k).copy()
    if v[1] == 'rev':
        temp.lower_bound = -1000.0
        temp.upper_bound = 1000.0
    if v[1] == 'for':
        temp.lower_bound = 0.0
        temp.upper_bound = 1000.0
    if v[1] == 'back':
        temp.lower_bound = -1000.0
        temp.upper_bound = 0.0        
    temp.gene_reaction_rule = str(Reaction_rule_final[k]).replace('&','and').replace('|','or').replace('rtoi_','')
    IFO0880_model.add_reaction(temp)
```

In [92]:

```
IFO0880_model
```

Out[92]:

|  |  |
| --- | --- |
| **Name** | R. toruloides |
| **Memory address** | 0x018203cbeb8 |
| **Number of metabolites** | 3589 |
| **Number of reactions** | 3804 |
| **Objective expression** | 0 |
| **Compartments** | c, x, m, e, r, v, n, g, u, p, h, s, f, l |

In [115]:

```
print(len(Gene_to_reaction))
print(len(Gene_to_reaction_Jeff))
print(len(set(Gene_to_reaction).union(Gene_to_reaction_Jeff)))
```

```
1020
934
1137
```

In [116]:

```
print(len(IFO0880_model.genes))
print(len([x for x in IFO0880_model.genes if not x.id[0].isalpha()]))
```

```
1596
1137
```

In [117]:

```
cobra.io.save_json_model(IFO0880_model, "IFO0880_GPR.json")
```
