# Supplementary material for: Multi-Omics Driven Metabolic Network Reconstruction and Analysis of Lignocellulosic Carbon Utilization in Rhodosporidium toruloides
Source: Front Bioeng Biotechnol. 2021 Jan 8;8:612832. doi: 10.3389/fbioe.2020.612832 (PMC7873862; doi:10.3389/fbioe.2020.612832)
Supplement: Supplementary File 4 — Multi-omics dataset for R. toruloides IFO0880. [file Data_Sheet_1.zip › Supplementary File S1/0.Annotation_and_draft_reconstruction/OrthoMCL_Run.html]

OrthoMCL\_Run


Install mysql from conda  
run ./scripts/mysql\_install\_db --no-defaults from /users/joonhoonkim/bin/anaconda/pkgs/mysql-5.5.24-0  
the root is already running a mysqld instance using port 3306  
./bin/mysqld\_safe --no-defaults --port 3308 & will run mysqld using port 3308, but use ~/.my.cnf file

[mysqld]  
port=3308  
basedir=/users/joonhoonkim/bin/anaconda/pkgs/mysql-5.5.24-0  
datadir=/users/joonhoonkim/bin/anaconda/pkgs/mysql-5.5.24-0/data  
plugin-dir=/users/joonhoonkim/bin/anaconda/pkgs/mysql-5.5.24-0/lib/plugin  
log-error=/users/joonhoonkim/bin/anaconda/pkgs/mysql-5.5.24-0/data/mysql.err  
pid-file=/users/joonhoonkim/bin/anaconda/pkgs/mysql-5.5.24-0/mysql.pid

[client]  
port=3308

conda perl-dbd-mysql does not work  
conda remove perl-dbd-mysql and cpan DBD::mysql

rehash and run the server with .my.cnf and local\_infile=1  
mysqld\_safe --defaults-file=~/.my.cnf --local\_infile=1 &

connect to server  
mysql --defaults-file=~/.my.cnf

check users and set password for root  
SELECT User, Host, Password FROM mysql.user;  
SET PASSWORD FOR 'root'@'localhost' = PASSWORD('password');

create a user, a database, and assign  
CREATE USER 'joonhoonkim'@'localhost' IDENTIFIED BY 'password';  
CREATE DATABASE orthomcl;  
GRANT ALL PRIVILEGES ON orthomcl.\* TO 'joonhoonkim'@'localhost';

connect as a user and check database  
mysql --defaults-file=~/.my.cnf -u joonhoonkim -p  
SHOW DATABASES;

To shutdown  
mysqladmin --defaults-file=~/.my.cnf -u root -p shutdown

In [3]:

```
%%bash
cd ../../Data/OrthoMCL
cp ~/bin/anaconda/pkgs/orthomcl-2.0.9-pl5.22.0_0/share/orthomcl/orthomcl.config.template .
cat orthomcl.config.template
```

```
# this config assumes a mysql database named 'orthomcl'.  adjust according
# to your situation.
dbVendor=mysql 
dbConnectString=dbi:mysql:orthomcl:3307
dbLogin=
dbPassword=
similarSequencesTable=SimilarSequences
orthologTable=Ortholog
inParalogTable=InParalog
coOrthologTable=CoOrtholog
interTaxonMatchView=InterTaxonMatch
percentMatchCutoff=50
evalueExponentCutoff=-5
oracleIndexTblSpc=NONE
```

In [21]:

```
%%bash
cd ../../Data/OrthoMCL
cat orthomcl.config.template | \
    sed 's/3307/localhost:3308;mysql_local_infile=1/;
         s/dbLogin=/dbLogin=joonhoonkim/;s/dbPassword=/dbPassword=password/' \
    > orthomcl.config
cat orthomcl.config
```

```
# this config assumes a mysql database named 'orthomcl'.  adjust according
# to your situation.
dbVendor=mysql 
dbConnectString=dbi:mysql:orthomcl:localhost:3308;mysql_local_infile=1
dbLogin=joonhoonkim
dbPassword=password
similarSequencesTable=SimilarSequences
orthologTable=Ortholog
inParalogTable=InParalog
coOrthologTable=CoOrtholog
interTaxonMatchView=InterTaxonMatch
percentMatchCutoff=50
evalueExponentCutoff=-5
oracleIndexTblSpc=NONE
```

In [24]:

```
%%bash
cd ../../Data/OrthoMCL
orthomclInstallSchema orthomcl.config
```

S. cerevisiae from SGD  
R. toruloides IFO0880 and NP11, L. starkeyi, Y. lipolytica from JGI  
C. reinhardtii from http://augustus.gobics.de/predictions/chlamydomonas/ version 5  
E. coli from NCBI gb and http://rocaplab.ocean.washington.edu/tools/genbank\_to\_fasta (locus\_tag for E. coli)  
P. putida KT2440 + pDK1

In [1]:

```
%%bash
cd ../../Data/OrthoMCL/inputFasta
head -n 1 orf_trans.fasta
head -n 1 Rhoto_IFO0880_4_GeneCatalog_proteins_20170509.aa.fasta
head -n 1 Rhoto1_GeneCatalog_proteins_20141208.aa.fasta
head -n 1 Lipst1_1_GeneCatalog_proteins_20110609.aa.fasta
head -n 1 Yarli1_GeneCatalog_proteins_20130122.aa.fasta
head -n 1 augustus.u5.aa.fasta
head -n 1 NC_000913.aa.fasta
head -n 1 KT2440_pDK1.aa.fasta
```

```
>YAL001C TFC3 SGDID:S000000001, Chr I from 151006-147594,151166-151097, Genome Release 64-2-1, reverse complement, Verified ORF, "Subunit of RNA polymerase III transcription initiation factor complex; part of the TauB domain of TFIIIC that binds DNA at the BoxB promoter sites of tRNA and similar genes; cooperates with Tfc6p in DNA binding; largest of six subunits of the RNA polymerase III transcription initiation factor complex (TFIIIC)"
>jgi|Rhoto_IFO0880_4|10000|CE150238_100509_mRNA
>jgi|Rhoto1|1|RHTO_00001
>jgi|Lipst1_1|66866|fgenesh1_kg.1_#_1_#_Locus9988v2rpkm0.52
>jgi|Yarli1|64471|YALI0A00110g
>chromosome10;u5g1.t1
>b0001 | thrL | thr operon leader peptide
>PP_0001 parB chromosome-partitioning protein
```

In [2]:

```
%%bash
#format C. reinhardtii as CRv4_Au5_s2_g8330_t1
cd ../../Data/OrthoMCL/inputFasta
head augustus.u5.aa.fasta | grep '>'
head augustus.u5.aa.fasta | sed 's/Xscaf//;s/chromosome/CRv4_Au5_s/;s/;u5/_/;s/\.t/_t/' | grep '>'
cat augustus.u5.aa.fasta | sed 's/Xscaf//;s/chromosome/CRv4_Au5_s/;s/;u5/_/;s/\.t/_t/' \
    > augustus.u5.modified.aa.fasta
```

```
>chromosome10;u5g1.t1
>chromosome10;u5g2.t1
>CRv4_Au5_s10_g1_t1
>CRv4_Au5_s10_g2_t1
```

In [3]:

```
%%bash
cd ../../Data/OrthoMCL/compliantFasta
orthomclAdjustFasta sce ../inputFasta/orf_trans.fasta 1
orthomclAdjustFasta rtoi ../inputFasta/Rhoto_IFO0880_4_GeneCatalog_proteins_20170509.aa.fasta 3
orthomclAdjustFasta rton ../inputFasta/Rhoto1_GeneCatalog_proteins_20141208.aa.fasta 4
orthomclAdjustFasta lst ../inputFasta/Lipst1_1_GeneCatalog_proteins_20110609.aa.fasta 3
orthomclAdjustFasta yli ../inputFasta/Yarli1_GeneCatalog_proteins_20130122.aa.fasta 4
orthomclAdjustFasta cre ../inputFasta/augustus.u5.modified.aa.fasta 1
orthomclAdjustFasta eco ../inputFasta/NC_000913.aa.fasta 1
orthomclAdjustFasta ppu ../inputFasta/KT2440_pDK1.aa.fasta 1
head -n 1 sce.fasta
head -n 1 rtoi.fasta
head -n 1 rton.fasta
head -n 1 lst.fasta
head -n 1 yli.fasta
head -n 1 cre.fasta
head -n 1 eco.fasta
head -n 1 ppu.fasta
```

```
>sce|YAL001C
>rtoi|10000
>rton|RHTO_00001
>lst|66866
>yli|YALI0A00110g
>cre|CRv4_Au5_s10_g1_t1
>eco|b0001
>ppu|PP_0001
```

In [4]:

```
%%bash
cd ../../Data/OrthoMCL
orthomclFilterFasta compliantFasta 10 20
```

```
processing file lst.fasta
processing file ppu.fasta
processing file yli.fasta
processing file cre.fasta
processing file rtoi.fasta
processing file sce.fasta
processing file rton.fasta
processing file eco.fasta
```

In [11]:

```
%%bash
cd ../../Data/OrthoMCL
grep '>' goodProteins.fasta | wc -l
makeblastdb -in goodProteins.fasta -dbtype prot -out orthomcl_blast
```

```
64068


Building a new DB, current time: 01/31/2018 20:55:10
New DB name:   /mnt/nfsbioi.jbei/nfs/nfsbioi1/joonhoonkim/Orthomcl/orthomcl_blast
New DB title:  goodProteins.fasta
Sequence type: Protein
Deleted existing Protein BLAST database named /mnt/nfsbioi.jbei/nfs/nfsbioi1/joonhoonkim/Orthomcl/orthomcl_blast
Keep MBits: T
Maximum file size: 1000000000B
Adding sequences from FASTA; added 64068 sequences in 2.75626 seconds.
```

In [6]:

```
%%bash
cd ../../Data/OrthoMCL
pyfasta split -n 12 goodProteins.fasta
```

```
creating new files:
goodProteins.00.fasta
goodProteins.01.fasta
goodProteins.02.fasta
goodProteins.03.fasta
goodProteins.04.fasta
goodProteins.05.fasta
goodProteins.06.fasta
goodProteins.07.fasta
goodProteins.08.fasta
goodProteins.09.fasta
goodProteins.10.fasta
goodProteins.11.fasta
```

In [12]:

```
%%bash
cd ../../Data/OrthoMCL
ls goodProteins.??.fasta | parallel "blastall -p blastp -F 'm S' -v 100000 -b 100000 -e 1e-5 -d orthomcl_blast \
                                              -i {} -o {.}.tab -m 8 -z 64068"
```

```
Selenocysteine (U) at position 197 replaced by X
Selenocysteine (U) at position 196 replaced by X
Selenocysteine (U) at position 196 replaced by X
Selenocysteine (U) at position 140 replaced by X
Selenocysteine (U) at position 140 replaced by X
Selenocysteine (U) at position 197 replaced by X
Selenocysteine (U) at position 196 replaced by X
Selenocysteine (U) at position 196 replaced by X
Selenocysteine (U) at position 140 replaced by X
Selenocysteine (U) at position 197 replaced by X
Selenocysteine (U) at position 196 replaced by X
Selenocysteine (U) at position 196 replaced by X
Selenocysteine (U) at position 140 replaced by X
Selenocysteine (U) at position 197 replaced by X
Selenocysteine (U) at position 196 replaced by X
Selenocysteine (U) at position 196 replaced by X
Selenocysteine (U) at position 140 replaced by X
Selenocysteine (U) at position 197 replaced by X
Selenocysteine (U) at position 196 replaced by X
Selenocysteine (U) at position 196 replaced by X
Selenocysteine (U) at position 140 replaced by X
Selenocysteine (U) at position 140 replaced by X
Selenocysteine (U) at position 140 replaced by X
Selenocysteine (U) at position 197 replaced by X
Selenocysteine (U) at position 196 replaced by X
Selenocysteine (U) at position 196 replaced by X
Selenocysteine (U) at position 140 replaced by X
Selenocysteine (U) at position 140 replaced by X
Selenocysteine (U) at position 197 replaced by X
Selenocysteine (U) at position 196 replaced by X
Selenocysteine (U) at position 196 replaced by X
Selenocysteine (U) at position 140 replaced by X
Selenocysteine (U) at position 197 replaced by X
Selenocysteine (U) at position 196 replaced by X
Selenocysteine (U) at position 196 replaced by X
Selenocysteine (U) at position 140 replaced by X
Selenocysteine (U) at position 140 replaced by X
Selenocysteine (U) at position 197 replaced by X
Selenocysteine (U) at position 196 replaced by X
Selenocysteine (U) at position 140 replaced by X
Selenocysteine (U) at position 197 replaced by X
Selenocysteine (U) at position 196 replaced by X
Selenocysteine (U) at position 196 replaced by X
Selenocysteine (U) at position 140 replaced by X
Selenocysteine (U) at position 197 replaced by X
Selenocysteine (U) at position 196 replaced by X
Selenocysteine (U) at position 140 replaced by X
Selenocysteine (U) at position 140 replaced by X
Selenocysteine (U) at position 140 replaced by X
Selenocysteine (U) at position 197 replaced by X
Selenocysteine (U) at position 196 replaced by X
Selenocysteine (U) at position 196 replaced by X
Selenocysteine (U) at position 140 replaced by X
Selenocysteine (U) at position 197 replaced by X
Selenocysteine (U) at position 196 replaced by X
Selenocysteine (U) at position 196 replaced by X
Selenocysteine (U) at position 140 replaced by X
Selenocysteine (U) at position 140 replaced by X
Selenocysteine (U) at position 197 replaced by X
Selenocysteine (U) at position 196 replaced by X
Selenocysteine (U) at position 196 replaced by X
Selenocysteine (U) at position 140 replaced by X
Selenocysteine (U) at position 197 replaced by X
Selenocysteine (U) at position 196 replaced by X
Selenocysteine (U) at position 196 replaced by X
Selenocysteine (U) at position 140 replaced by X
Selenocysteine (U) at position 197 replaced by X
Selenocysteine (U) at position 196 replaced by X
Selenocysteine (U) at position 196 replaced by X
Selenocysteine (U) at position 140 replaced by X
Selenocysteine (U) at position 197 replaced by X
Selenocysteine (U) at position 196 replaced by X
Selenocysteine (U) at position 196 replaced by X
Selenocysteine (U) at position 140 replaced by X
Selenocysteine (U) at position 197 replaced by X
Selenocysteine (U) at position 196 replaced by X
Selenocysteine (U) at position 196 replaced by X
Selenocysteine (U) at position 140 replaced by X
```

In [13]:

```
%%bash
cd ../../Data/OrthoMCL
cat goodProteins.??.tab > blastall.tab
```

In [14]:

```
%%bash
cd ../../Data/OrthoMCL
orthomclBlastParser blastall.tab compliantFasta > similarSequences.txt
```

```
acquiring genes from lst.fasta
acquiring genes from ppu.fasta
acquiring genes from yli.fasta
acquiring genes from cre.fasta
acquiring genes from rtoi.fasta
acquiring genes from sce.fasta
acquiring genes from rton.fasta
acquiring genes from eco.fasta
```

In [25]:

```
%%bash
cd ../../Data/OrthoMCL
orthomclLoadBlast orthomcl.config similarSequences.txt
```

In [26]:

```
%%bash
cd ../../Data/OrthoMCL
orthomclPairs orthomcl.config orthomcl_pairs.log cleanup=no
```

In [28]:

```
%%bash
cd ../../Data/OrthoMCL
orthomclDumpPairsFiles orthomcl.config
```

In [29]:

```
%%bash
cd ../../Data/OrthoMCL
mcl mclInput --abc -I 1.5 -o mclOutput
```

```
.....[mcl] new tab created
[mcl] pid 1235
 ite -------------------  chaos  time hom(avg,lo,hi) m-ie m-ex i-ex fmv
  1  ...................  19.92  0.13 0.99/0.04/3.56 1.50 1.48 1.48   0
  2  ...................  29.36  0.23 0.92/0.25/2.77 1.61 1.15 1.70   1
  3  ...................  26.92  0.25 0.88/0.20/2.33 1.50 0.95 1.62   2
  4  ...................  17.70  0.22 0.85/0.18/2.54 1.39 0.91 1.47   1
  5  ...................  19.67  0.18 0.83/0.12/2.24 1.25 0.89 1.30   1
  6  ...................  14.76  0.14 0.82/0.07/1.96 1.15 0.89 1.16   0
  7  ...................  24.98  0.12 0.83/0.10/1.33 1.08 0.88 1.02   0
  8  ...................  24.56  0.10 0.84/0.24/1.37 1.05 0.88 0.90   0
  9  ...................   5.86  0.09 0.86/0.12/1.15 1.03 0.87 0.78   0
 10  ...................   9.53  0.08 0.88/0.23/1.28 1.02 0.86 0.68   0
 11  ...................   6.39  0.06 0.90/0.36/1.31 1.01 0.85 0.58   0
 12  ...................   3.81  0.05 0.92/0.33/1.21 1.01 0.85 0.49   0
 13  ...................   2.66  0.04 0.93/0.32/1.01 1.01 0.85 0.42   0
 14  ...................   2.93  0.04 0.95/0.35/1.07 1.00 0.88 0.37   0
 15  ...................   2.37  0.03 0.96/0.42/1.16 1.00 0.90 0.33   0
 16  ...................   1.73  0.03 0.97/0.52/1.00 1.00 0.92 0.30   0
 17  ...................   2.42  0.03 0.98/0.47/1.03 1.00 0.93 0.28   0
 18  ...................   2.98  0.03 0.98/0.45/1.19 1.00 0.95 0.27   0
 19  ...................   3.09  0.02 0.99/0.49/1.00 1.00 0.95 0.25   0
 20  ...................   2.10  0.03 0.99/0.56/1.00 1.00 0.95 0.24   0
 21  ...................   1.56  0.02 0.99/0.57/1.00 1.00 0.97 0.24   0
 22  ...................   0.81  0.02 0.99/0.55/1.00 1.00 0.97 0.23   0
 23  ...................   0.89  0.02 1.00/0.58/1.00 1.00 0.99 0.23   0
 24  ...................   0.73  0.02 1.00/0.67/1.00 1.00 0.99 0.22   0
 25  ...................   0.73  0.02 1.00/0.60/1.00 1.00 0.99 0.22   0
 26  ...................   0.90  0.02 1.00/0.56/1.00 1.00 0.99 0.22   0
 27  ...................   0.83  0.02 1.00/0.63/1.00 1.00 1.00 0.22   0
 28  ...................   0.50  0.02 1.00/0.63/1.00 1.00 1.00 0.22   0
 29  ...................   0.38  0.02 1.00/0.74/1.00 1.00 1.00 0.22   0
 30  ...................   0.25  0.02 1.00/0.76/1.00 1.00 1.00 0.22   0
 31  ...................   0.25  0.02 1.00/0.76/1.00 1.00 1.00 0.22   0
 32  ...................   0.25  0.02 1.00/0.77/1.00 1.00 1.00 0.22   0
 33  ...................   0.24  0.02 1.00/0.76/1.00 1.00 1.00 0.22   0
 34  ...................   0.17  0.02 1.00/0.83/1.00 1.00 1.00 0.22   0
 35  ...................   0.21  0.02 1.00/0.83/1.00 1.00 1.00 0.22   0
 36  ...................   0.25  0.02 1.00/0.77/1.00 1.00 1.00 0.22   0
 37  ...................   0.24  0.02 1.00/0.77/1.00 1.00 1.00 0.21   0
 38  ...................   0.25  0.02 1.00/0.77/1.00 1.00 1.00 0.21   0
 39  ...................   0.24  0.02 1.00/0.77/1.00 1.00 1.00 0.21   0
 40  ...................   0.17  0.02 1.00/0.83/1.00 1.00 1.00 0.21   0
 41  ...................   0.07  0.02 1.00/0.93/1.00 1.00 1.00 0.21   0
 42  ...................   0.02  0.02 1.00/0.98/1.00 1.00 1.00 0.21   0
 43  ...................   0.00  0.02 1.00/1.00/1.00 1.00 1.00 0.21   0
 44  ...................   0.00  0.02 1.00/1.00/1.00 1.00 1.00 0.21   0
[mcl] jury pruning marks: <99,99,99>, out of 100
[mcl] jury pruning synopsis: <99.0 or perfect> (cf -scheme, -do log)
[mcl] output is in mclOutput
[mcl] 12240 clusters found
[mcl] output is in mclOutput

Please cite:
    Stijn van Dongen, Graph Clustering by Flow Simulation.  PhD thesis,
    University of Utrecht, May 2000.
       (  http://www.library.uu.nl/digiarchief/dip/diss/1895620/full.pdf
       or  http://micans.org/mcl/lit/svdthesis.pdf.gz)
OR
    Stijn van Dongen, A cluster algorithm for graphs. Technical
    Report INS-R0010, National Research Institute for Mathematics
    and Computer Science in the Netherlands, Amsterdam, May 2000.
       (  http://www.cwi.nl/ftp/CWIreports/INS/INS-R0010.ps.Z
       or  http://micans.org/mcl/lit/INS-R0010.ps.Z)
```

In [30]:

```
%%bash
cd ../../Data/OrthoMCL
orthomclMclToGroups OG 1000 < mclOutput > groups.txt
```

In [31]:

```
%%bash
cd ../../Data/OrthoMCL
tail groups.txt
```

```
OG13230: sce|YOR242C yli|YALI0D06710g
OG13231: sce|YOR367W yli|YALI0E32241g
OG13232: sce|YPL034W yli|YALI0C09394g
OG13233: sce|YPL118W yli|YALI0B14993g
OG13234: sce|YPL126W yli|YALI0B04862g
OG13235: sce|YPL188W yli|YALI0E17963g
OG13236: sce|YPL215W yli|YALI0C18227g
OG13237: sce|YPR124W yli|YALI0C20295g
OG13238: sce|YPR143W yli|YALI0C12881g
OG13239: sce|YPR179C yli|YALI0E28204g
```

In [ ]:

```

```
