# Supplementary material for: Multi-Omics Driven Metabolic Network Reconstruction and Analysis of Lignocellulosic Carbon Utilization in Rhodosporidium toruloides
Source: Front Bioeng Biotechnol. 2021 Jan 8;8:612832. doi: 10.3389/fbioe.2020.612832 (PMC7873862; doi:10.3389/fbioe.2020.612832)
Supplement: Supplementary File 4 — Multi-omics dataset for R. toruloides IFO0880. [file Data_Sheet_1.zip › Supplementary File S1/0.Annotation_and_draft_reconstruction/Annotation_WoLFPSort_PTS1_PTS2_FIMO.html]

Annotation\_WoLFPSort\_PTS1\_PTS2\_FIMO


In [1]:

```
import csv
```

In [2]:

```
wolfpsort = dict()

with open("../../Data/WoLFPSort/WoLFPSort_result.txt",'r') as f:
    reader = csv.reader(f)
    line = next(reader)
    for line in reader:
        x, y = line[0].split(' ',1)
#        y = [z.strip() for z in line[1].split(',')]
        y = [y] + [z.strip() for z in line[1:]]
        wolfpsort[x] = {z.split(' ')[0]: float(z.split(' ')[1]) for z in y}

wolfpsort['10000']
```

Out[2]:

```
{'cyto_nucl': 13.5, 'nucl': 12.5, 'cyto': 11.5}
```

In [3]:

```
len(wolfpsort)
```

Out[3]:

```
8490
```

In [4]:

```
compartment = set()
for x in wolfpsort.values():
    for y in x.keys():
        if y not in compartment:
            compartment.add(y)
compartment
```

Out[4]:

```
{'E.R.',
 'cysk',
 'cyto',
 'cyto_mito',
 'cyto_nucl',
 'cyto_pero',
 'extr',
 'golg',
 'mito',
 'mito_nucl',
 'nucl',
 'pero',
 'plas',
 'vacu'}
```

In [5]:

```
pero_list_all = set()
for k, v in wolfpsort.items():
    if 'pero' in v.keys():
        pero_list_all.add(k)
print(len(pero_list_all))
```

```
1751
```

In [6]:

```
import numpy as np
import matplotlib.pyplot as plt
```

In [7]:

```
plt.hist([wolfpsort[x]['pero'] for x in pero_list_all], bins=12)
```

Out[7]:

```
(array([270., 636., 362., 227., 112.,  68.,  47.,  14.,   3.,   6.,   5.,
          1.]),
 array([ 1.,  2.,  3.,  4.,  5.,  6.,  7.,  8.,  9., 10., 11., 12., 13.]),
 <BarContainer object of 12 artists>)
```

In [8]:

```
pero_list = set()
for k, v in wolfpsort.items():
    if 'pero' in v.keys() and v['pero'] >= 6:
        pero_list.add(k)
    elif 'cyto_pero' in v.keys() and v['cyto_pero'] >= 6:
        pero_list.add(k)
print(len(pero_list))
```

```
224
```

In [9]:

```
pero_top_list = set()
for k, v in wolfpsort.items():
    if any(x in max(v.items(), key=lambda x: x[1]) for x in ['pero','cyto_pero']):
        pero_top_list.add(k)
print(len(pero_top_list))
```

```
19
```

In [10]:

```
for x in pero_top_list:
    print(x, wolfpsort[x])
```

```
14977 {'pero': 10.5, 'mito': 9.0, 'cyto_pero': 7.833, 'cyto_nucl': 4.333, 'cyto': 4.0}
14189 {'pero': 8.0, 'cyto': 7.5, 'cyto_nucl': 6.0, 'nucl': 3.5, 'mito': 3.0, 'extr': 3.0}
14081 {'pero': 9.0, 'nucl': 7.0, 'cyto_pero': 7.0, 'mito': 5.0, 'cyto': 5.0, 'cyto_mito': 5.0}
11389 {'pero': 11.0, 'cyto': 9.5, 'cyto_nucl': 8.0, 'nucl': 3.5}
14222 {'pero': 10.0, 'cyto': 7.0, 'nucl': 5.0, 'mito': 4.0}
16607 {'pero': 9.0, 'cysk': 7.0, 'cyto': 6.0, 'nucl': 5.0}
8690 {'pero': 8.0, 'cyto': 5.5, 'cyto_nucl': 5.5, 'mito': 5.0, 'nucl': 4.5, 'extr': 4.0}
15116 {'pero': 10.0, 'cyto': 8.5, 'cyto_nucl': 6.5, 'nucl': 3.5, 'mito': 3.0}
11263 {'pero': 10.0, 'cyto': 9.5, 'cyto_nucl': 7.0, 'nucl': 3.5, 'extr': 2.0}
14932 {'pero': 13.0, 'cyto_nucl': 6.0, 'nucl': 5.0, 'cyto': 5.0, 'mito': 2.0, 'extr': 2.0}
13590 {'pero': 10.0, 'nucl': 8.5, 'cyto_nucl': 8.0, 'cyto': 6.5}
9457 {'cyto_pero': 12.0, 'cyto': 11.5, 'pero': 11.5, 'nucl': 3.0}
13135 {'pero': 8.0, 'nucl': 7.5, 'cyto_nucl': 7.5, 'cyto': 6.5, 'extr': 3.0}
15829 {'pero': 7.5, 'cyto_pero': 6.5, 'cysk': 6.0, 'mito': 5.0, 'cyto': 4.5, 'nucl': 3.0}
15247 {'pero': 8.0, 'nucl': 7.5, 'cyto_nucl': 7.0, 'cyto': 5.5, 'mito': 3.0}
14506 {'pero': 9.0, 'cysk': 6.0, 'cyto': 5.0, 'mito': 4.0, 'cyto_nucl': 4.0}
13626 {'pero': 11.5, 'cyto_pero': 11.0, 'cyto': 9.5, 'mito': 4.0}
16147 {'pero': 8.0, 'cyto': 7.0, 'mito': 6.0, 'cyto_nucl': 6.0}
13779 {'pero': 6.0, 'cyto': 5.5, 'cyto_nucl': 5.0, 'cysk': 5.0, 'mito': 4.0, 'nucl': 3.5}
```

In [11]:

```
from Bio import SeqIO
```

In [12]:

```
record_dict = SeqIO.index("../../Data/Rhoto_IFO0880_4/Rhoto_IFO0880_4_GeneCatalog_proteins_20170509.aa.modified.fasta", "fasta")
print(record_dict["8690"])
print(record_dict["8690"][0:10])
```

```
ID: 8690
Name: 8690
Description: 8690 gm1.300_g_mRNA
Number of features: 0
Seq('MAPAQHHPFYVQEKAAFEQDPANAVLLALGKDNEGLAGLARDVIRDAPDPTPPR...RC*')
ID: 8690
Name: 8690
Description: 8690 gm1.300_g_mRNA
Number of features: 0
Seq('MAPAQHHPFY')
```

In [13]:

```
with open("../../Data/WoLFPSort/all_last_3.txt", "w") as output_handle:
    writer = csv.writer(output_handle)
    for x in record_dict.keys():
        writer.writerow([x, record_dict[x][-4:].seq])
```

In [14]:

```
for x in pero_top_list:
    print(x, record_dict[x][1:50].seq, record_dict[x][-4:].seq)
```

```
14977 TSFAHKSRDHGDTSKVEGRPTKIDRPSWFGSSLNPAVAISADPMEGSEF GKL*
14189 HTQDPTWDARNVLQHAGDVRHTYKSSIGALFRLSRSWSAAAAPYRFKAI TGG*
14081 VAGAEGSQGKAWATLLTKTSYLQGALVLADSLARHRSKYPLVVFATQEL DLN*
11389 ADNDSTGATRLARQRSTSSEIHQSRLSSSGQLIVVANRLPVTITADKDA GKA*
14222 DYQNRVGSKFGGGGVARADETAIDRRERLRKLALETIDLAKDPYILRNH AQK*
16607 SSDADKRPLHHSEVAKHADKDSLWVIVNGNAYDLTEFAPEHPGGMGILL ASL*
8690 APAQHHPFYVQEKAAFEQDPANAVLLALGKDNEGLAGLARDVIRDAPDP ARC*
15116 HVDHTETTPLRARLQSTTELPVDESVQAFLDKLANRIYSLESEVGRLQA GRL*
11263 SLGSCCVSGFKHEGTPSGKTQDINGVSTYISLPKGDYDKTKALLFLTDI AHL*
14932 PLLHGGSPRDPEGQSDHFAVNQKVAVDGPRGQTNAAAAMQRILADAVQQ NGR*
13590 VDSGPSGAGAAAQDALTPPAQPAKPSLLLRRGKRSFLIALPSSYADALA AKV*
9457 QAAKMSPQHNALPQGVQIHGKVEGRHQEILTPAALQFLAVLHRNFNPTR AKL*
13135 KNQQVCPPSPRPLLDSTFPDFPSSSASQIIFINDWYHDTTDTIVNSLLS ECS*
15829 VLAPPTDPSHPNDHSLIKHNNDPLHPAFLIPELCRGFYKLGWVTGTGGG TKQ*
15247 NGGKSVSNGSGGLDAMQYEGEEGFVPLWEGSNLDRREFVRLALQAFQDM AIA*
14506 SAPHALVEWVDGLLRQFGPASSQLKSWIALEDKPTKIADMMWWYYAIHP RSF*
13626 NKDDLPPRSTPTYALYQREIFLDGATKGKLPEFNTDPDEMENVAKETLS AKL*
16147 NYIQLEKLGEGTYATVHKGRSRITNELVALKEIHLDAEEGTPSTAIREI AYQ*
13779 ASLSSLIASVVDNDQGGFGPSASHLPPQFKDIPYAPFSKNDKLGRIVDW DGL*
```

In [15]:

```
temp = ["8678","8885","13813","9065","15228","10012","11989","14070","12570","12564","12742","12752","9700","12086","10408","12989","16253","14805","11362","11907","16515","11203","15180","16284","13606","11362","9469",
        "10293","13228","11338"]
for x in temp:
    print(x, record_dict[x][0:50].seq, record_dict[x][-4:].seq)
#    print(record_dict[x][-4:].seq)
```

```
8678 MLSSTASTAARRLAHIRMSSTTAQNGLREVFIVGAVRPDSFSRATRCPRA QRL*
8885 MSAVYIASAKRTAFGAFGGALKSYTASQLGGFAAKAALAELPEGTKVDSV ERV*
13813 MSSTISAAKERLSGLVSHLTPRGKAALTQQNDDDVVIVSAVRTPITRAKK AEN*
9065 MSGLISSTIDSIRGTGKSKLLKKSPDDVVITMAVRTPLQKARKGGFKDMS VAE*
15228 MRPSVPARAIADVLKKRPDDVCIVTTLRTPIGKFRGGLKDMHAEELLSHV VRD*
10012 MTHYVPRLLRTTTLAARPALAASARTLPTRPAVASSRPVSTTASSAGLGL GAL*
11989 MSLRLPLRQLAAKRVPPQALAARFASSEATAPAELAKTSSFAPYQWEDPL AFE*
14070 MLARTARSAARSLAHAHRAPQAARCFSSYIESENPVSLYSLTEEERMLKD EYS*
12570 MLSRLALRTSRTARLSTRSTRAYASEASEYTGGPTFELSEEQVGIRDLAR DYA*
12564 MASSATSYLTHLVEGLQGQYGTVGLYAAVQRLFVGFGFVGGPGSCVIDAP GVY*
12742 MSYQPPSSVPVEIPDGFKPLGTSGSRLLEIERSKASFSPADLEKYIYGDA SKL*
12752 MAPQSTLDIQAARQRASFDPDVLHRVLLAGSKDPDMRKRVAQLISASPAF AKL*
9700 MPTPLADTPTPPTPMETIAKERLNPPFDLRRMTYAMGDGEKEVKLREKHM AEE*
12086 MAQKASTFTKEQVASHTSEKDGVWIIVEGGVYDVTKFLDDHPGGKKILLN AKL*
10408 MDNSHPVVPYIGQAAWAFVQPHFSQYGKDTLAKLIAFVEGECIPAEKLFH SKL*
12989 MTFGSLVPFAEPAHCRDLPSPYYNESHDRLRKAAREWAEEHIMDVAYEWE AKL*
16253 MAARTFSLEQISEHNKEGDLWIVVNGDVYDLSRFTDIHPGGAGVLLASDI AVL*
14805 MLPALRATSMRTALPRFPRQSVVRWQSSAAAGPGEYTSILVSQPAPGVSQ TNS*
11362 MAEQLRFDKKTVVITGAGGGLGRAYSLFFASRGANVVVNDLSRENAQKVV LAQ*
11907 MSNYESKLIKCTLPSEGVLQVTLARPPVNAYNSELSAELQRHFEQASSDP AKL*
16515 MTTPQHPPVGPHLVVSYPAPKVLQLRLNRPEALNAMTDGLEADLCKMLDW ARL*
11203 MLLTRTLRTLPRSQTYNAIRTLSTGAPDIQRLTVFGGGLMGAGIAQVAAQ YSS*
15180 ADGIDKVAVIGAGQMGIGISYVAAKTAGVDVLISDRSAQQITKGLQFVDT YTAR
16284 MDVAQKVWIVTGGASGLGAAVVAALVTRGGFVAVLDLNEEAGSQAEKQYG AKM*
13606 MTSQPLTALVTGGASGIGLGIVRTLLASTSPAYNILIVDRSSASFPSLLS ASF*
11362 MAEQLRFDKKTVVITGAGGGLGRAYSLFFASRGANVVVNDLSRENAQKVV LAQ*
9469 MPAHNLDKAVGHESKPLPVAWNQRDLLLYAVGIGAKKDELAFTYEDDPNW SKL*
10293 MAPADAILVEIQAPFAIITLNQPQKKNALDSTLYKRLSSILQDIDRKPEV HKI*
13228 MAQQTASVFRPDLMKGKVAFVTGGGSGICYGITKQLMAHGANAAIFGRRK SKL*
11338 MSLAFEPVELPNGVVLKNRICKAAMEENLADIDHFLAPSHELIELYRAWG KEE*
```

In [16]:

```
with open("../../Data/WoLFPSort/pero_first_50.fasta", "w") as output_handle:
    for x in pero_list:
        SeqIO.write(record_dict[x][0:50], output_handle, "fasta")
```

In [17]:

```
with open("../../Data/WoLFPSort/pero_last_10.fasta", "w") as output_handle:
    for x in pero_list:
        SeqIO.write(record_dict[x][-10:], output_handle, "fasta")
```

In [188]:

```
%%bash
cd ../../Data/WoLFPSort/
meme pero_first_50.fasta -mod zoops -maxw 10 -nmotifs 3 -o pero_first_50_meme_zoops_10w_3
```

```
Writing results to output directory 'pero_first_50_meme_zoops_10w_3'.
Initializing the motif probability tables for 2 to 224 sites...
nsites = 224
Done initializing.
SEEDS: highwater mark: seq 223 pos 50

seqs=   224, min=  50, max=   50, total=    11200

motif=1
SEED WIDTHS: 8 10
em: w=  10, psites= 224, iter=  10 Warning: Can not convert EPS file to PNG as no install of Image Magick or Ghostscript is usable.

motif=2
SEED WIDTHS: 8 10
em: w=  10, psites= 224, iter=  10 Warning: Can not convert EPS file to PNG as no install of Image Magick or Ghostscript is usable.

motif=3
SEED WIDTHS: 8 10
em: w=  10, psites= 224, iter=  10 Warning: Can not convert EPS file to PNG as no install of Image Magick or Ghostscript is usable.
```

In [189]:

```
%%bash
open ../../Data/WoLFPSort/pero_first_50_meme_zoops_10w_3/meme.html
```

In [191]:

```
%%bash
cd ../../Data/WoLFPSort/
meme pero_last_10.fasta -mod zoops -maxw 10 -nmotifs 3 -o pero_last_10_meme_zoops_10w_3
```

```
Writing results to output directory 'pero_last_10_meme_zoops_10w_3'.
Initializing the motif probability tables for 2 to 224 sites...
nsites = 224
Done initializing.
SEEDS: highwater mark: seq 223 pos 10

seqs=   224, min=  10, max=   10, total=     2240

motif=1
SEED WIDTHS: 8 10
em: w=  10, psites= 224, iter=   0 Warning: Can not convert EPS file to PNG as no install of Image Magick or Ghostscript is usable.

motif=2
SEED WIDTHS: 8 10
em: w=  10, psites= 224, iter=   0 Warning: Can not convert EPS file to PNG as no install of Image Magick or Ghostscript is usable.

motif=3
SEED WIDTHS: 8 10
em: w=  10, psites= 224, iter=   0 Warning: Can not convert EPS file to PNG as no install of Image Magick or Ghostscript is usable.
```

In [192]:

```
%%bash
open ../../Data/WoLFPSort/pero_last_10_meme_zoops_10w_3/meme.html
```

PTS1 and PTS2 sequences from http://216.92.14.62/Target\_signal.php

In [230]:

```
%%bash
cd ../../Data/WoLFPSort/
meme PTS1_sequence.fasta -mod oops -maxw 8 -nmotifs 1 -oc PTS1_meme
```

```
The output directory 'PTS1_meme' already exists.
Its contents will be overwritten.
Initializing the motif probability tables for 2 to 98 sites...
nsites = 98
Done initializing.
SEEDS: highwater mark: seq 97 pos 8

seqs=    98, min=   8, max=    8, total=      784

motif=1
SEED WIDTHS: 8
em: w=   8, psites=  98, iter=   0 Warning: Can not convert EPS file to PNG as no install of Image Magick or Ghostscript is usable.
```

In [235]:

```
%%bash
open ../../Data/WoLFPSort/PTS1_meme/meme.html
```

In [155]:

```
%%bash
cd ../../Data/WoLFPSort/
meme PTS2_sequence.fasta -mod oops -maxw 9 -nmotifs 1 -oc PTS2_meme
```

```
The output directory 'PTS2_meme' already exists.
Its contents will be overwritten.
Initializing the motif probability tables for 2 to 9 sites...
nsites = 9
Done initializing.
SEEDS: highwater mark: seq 8 pos 9

seqs=     9, min=   9, max=    9, total=       81

motif=1
SEED WIDTHS: 8 9
em: w=   9, psites=   9, iter=   0 Warning: Can not convert EPS file to PNG as no install of Image Magick or Ghostscript is usable.
```

In [211]:

```
%%bash
open ../../Data/WoLFPSort/PTS2_meme/meme.html
```

In [19]:

```
with open("../../Data/WoLFPSort/all_first_50.fasta", "w") as output_handle:
    for x in wolfpsort.keys():
        SeqIO.write(record_dict[x][0:50], output_handle, "fasta")
```

In [20]:

```
with open("../../Data/WoLFPSort/all_last_9.fasta", "w") as output_handle:
    for x in wolfpsort.keys():
        SeqIO.write(record_dict[x][-9:], output_handle, "fasta")
```

In [233]:

```
%%bash
cd ../../Data/WoLFPSort/
fimo --oc fimo_PTS1_all_last_9 --thresh 1e-3 PTS1_meme/meme.txt all_last_9.fasta
```

```
Using motif 1 of width 8.
Computing q-values.
Estimating pi_0 from a uniformly sampled set of 8611 p-values.
Estimating pi_0.
Estimated pi_0=0.963048
```

In [234]:

```
%%bash
open ../../Data/WoLFPSort/fimo_PTS1_all_last_9/fimo.html
```

In [236]:

```
%%bash
cd ../../Data/WoLFPSort/
fimo --oc fimo_PTS2_all_first_50 PTS2_meme/meme.txt all_first_50.fasta
```

```
Using motif 1 of width 9.
Computing q-values.
Estimating pi_0 from a uniformly sampled set of 10000 p-values.
Estimating pi_0.
Estimated pi_0=0.969494
```

In [237]:

```
%%bash
open ../../Data/WoLFPSort/fimo_PTS2_all_first_50/fimo.html
```

In [ ]:

```

```
