# Supplementary material for: Multi-Omics Driven Metabolic Network Reconstruction and Analysis of Lignocellulosic Carbon Utilization in Rhodosporidium toruloides
Source: Front Bioeng Biotechnol. 2021 Jan 8;8:612832. doi: 10.3389/fbioe.2020.612832 (PMC7873862; doi:10.3389/fbioe.2020.612832)
Supplement: Supplementary File 4 — Multi-omics dataset for R. toruloides IFO0880. [file Data_Sheet_1.zip › Supplementary File S1/0.Annotation_and_draft_reconstruction/Annotation_Transporters_TCDB.html]

Annotation\_Transporters\_TCDB


In [ ]:

```
%%bash
export HMMTOP_ARCH=~/Documents/hmmtop_2.1/hmmtop.arch
export HMMTOP_PSV=~/Documents/hmmtop_2.1/hmmtop.psv
export PATH=$PATH:~/Documents/GitHub/BioVx/scripts/
gblast3.py -i ../../Data/Rhoto_IFO0880_4/Rhoto_IFO0880_4_GeneCatalog_proteins_20170509.aa.modified.fasta \
           -o ../../Data/TCDB
```

In [1]:

```
%%bash
head ../../Data/TCDB/results.tsv
```

```
#Query_id	Hit_xid	Hit_tcid	Hit_desc	Match_length	e-value	%_identity	Query_Length	Hit_Length	Query_Coverage	Hit_Coverage	Query_n_TMS	Hit_n_TMS	TM_Overlap_Score	Family_Abrv	Predicted_Substrate	row_number
13972	Q14028	1.A.1.5.3	gnl|BL_ORD_ID|9872 gnl|TC-DB|Q14028|1.A.1.5.3 Cyclic-nucleotide-gated cation channel 4 (CNG channel 4) (CNG-4) (CNG4) (Cyclic nucleotide-gated cation channel modulatory subunit) - Homo sapiens (Human).	576	3.01319e-11	23.0	729	1251	72.7	41.0	0	0	None	VIC	cations	1
14619	Q9UL51	1.A.1.5.11	gnl|BL_ORD_ID|15304 gnl|TC-DB|Q9UL51|1.A.1.5.11 Potassium/sodium hyperpolarization-activated cyclic nucleotide-gated channel 2 OS=Homo sapiens GN=HCN2 PE=1 SV=3	196	4.4374e-07	27.0	351	889	48.4	20.1	0	0	None	VIC	cations	2
15817	Q9UL51	1.A.1.5.11	gnl|BL_ORD_ID|15304 gnl|TC-DB|Q9UL51|1.A.1.5.11 Potassium/sodium hyperpolarization-activated cyclic nucleotide-gated channel 2 OS=Homo sapiens GN=HCN2 PE=1 SV=3	155	9.84162e-06	24.0	309	889	47.2	16.3	0	0	None	VIC	cations	3
13686	Q9Y3Q4	1.A.1.5.11	gnl|BL_ORD_ID|15595 gnl|TC-DB|Q9Y3Q4|1.A.1.5.11 Potassium/sodium hyperpolarization-activated cyclic nucleotide-gated channel 4 OS=Homo sapiens GN=HCN4 PE=1 SV=1	178	2.59846e-05	29.0	368	1203	46.7	13.9	0	0	None	VIC	cations	4
12675	Q9UL51	1.A.1.5.11	gnl|BL_ORD_ID|15304 gnl|TC-DB|Q9UL51|1.A.1.5.11 Potassium/sodium hyperpolarization-activated cyclic nucleotide-gated channel 2 OS=Homo sapiens GN=HCN2 PE=1 SV=3	85	0.000153194	40.0	209	889	40.2	9.0	0	0	None	VIC	cations	5
13149	Q9Y3Q4	1.A.1.5.11	gnl|BL_ORD_ID|15595 gnl|TC-DB|Q9Y3Q4|1.A.1.5.11 Potassium/sodium hyperpolarization-activated cyclic nucleotide-gated channel 4 OS=Homo sapiens GN=HCN4 PE=1 SV=1	163	0.000373194	27.0	187	1203	81.3	11.6	0	0	None	VIC	cations	6
9630	Q9Y3Q4	1.A.1.5.11	gnl|BL_ORD_ID|15595 gnl|TC-DB|Q9Y3Q4|1.A.1.5.11 Potassium/sodium hyperpolarization-activated cyclic nucleotide-gated channel 4 OS=Homo sapiens GN=HCN4 PE=1 SV=1	180	0.000445072	29.0	326	1203	53.7	14.1	0	0	None	VIC	cations	7
15047	P40310	1.A.1.7.1	gnl|BL_ORD_ID|7941 gnl|TC-DB|P40310|1.A.1.7.1 OUTWARD-RECTIFIER POTASSIUM CHANNEL TOK1 (TWO-DOMAIN OUTWARD RECTIFIER K+ CHANNEL YORK) - Saccharomyces cerevisiae (Baker's yeast).	466	1.66721e-24	22.0	720	691	59.3	59.9	0	0	None	VIC	K+	8
14173	O55017	1.A.1.11.9	gnl|BL_ORD_ID|5591 gnl|TC-DB|O55017|1.A.1.11.9 Voltage-dependent N-type calcium channel subunit alpha-1B - Mus musculus (Mouse).	115	0.000876854	30.0	226	2327	47.8	4.5	0	0	None	VIC	Ca2+	9
```

In [2]:

```
%%bash
open ../../Data/TCDB/results.html
```

In [ ]:

```

```
