# Supplementary material for: Multi-Omics Driven Metabolic Network Reconstruction and Analysis of Lignocellulosic Carbon Utilization in Rhodosporidium toruloides
Source: Front Bioeng Biotechnol. 2021 Jan 8;8:612832. doi: 10.3389/fbioe.2020.612832 (PMC7873862; doi:10.3389/fbioe.2020.612832)
Supplement: Supplementary File 4 — Multi-omics dataset for R. toruloides IFO0880. [file Data_Sheet_1.zip › Supplementary File S1/0.Annotation_and_draft_reconstruction/Annotation_ncRNA_and_mitochondria.html]

Annotation\_ncRNA\_and\_mitochondria


### Non-coding RNA annotation¶

install infernal and easel from bioconda

In [9]:

```
%%bash
cd ../../Data/Rfam
wget ftp://ftp.ebi.ac.uk/pub/databases/Rfam/CURRENT/Rfam.cm.gz
gunzip Rfam.cm.gz
wget ftp://ftp.ebi.ac.uk/pub/databases/Rfam/CURRENT/Rfam.clanin
```

```
--2019-05-06 10:21:06--  ftp://ftp.ebi.ac.uk/pub/databases/Rfam/CURRENT/Rfam.cm.gz
           => ‘Rfam.cm.gz’
Resolving ftp.ebi.ac.uk (ftp.ebi.ac.uk)... 193.62.192.4
Connecting to ftp.ebi.ac.uk (ftp.ebi.ac.uk)|193.62.192.4|:21... connected.
Logging in as anonymous ... Logged in!
==> SYST ... done.    ==> PWD ... done.
==> TYPE I ... done.  ==> CWD (1) /pub/databases/Rfam/CURRENT ... done.
==> SIZE Rfam.cm.gz ... 35670981
==> PASV ... done.    ==> RETR Rfam.cm.gz ... done.
Length: 35670981 (34M) (unauthoritative)

     0K .......... .......... .......... .......... ..........  0%  168K 3m28s
    50K .......... .......... .......... .......... ..........  0%  358K 2m32s
   100K .......... .......... .......... .......... ..........  0%  134M 1m41s
   150K .......... .......... .......... .......... ..........  0%  358K 1m40s
   200K .......... .......... .......... .......... ..........  0%  192M 80s
   250K .......... .......... .......... .......... ..........  0%  360K 83s
   300K .......... .......... .......... .......... ..........  1% 91.1M 71s
   350K .......... .......... .......... .......... ..........  1%  112M 62s
   400K .......... .......... .......... .......... ..........  1%  360K 66s
   450K .......... .......... .......... .......... ..........  1%  101M 59s
   500K .......... .......... .......... .......... ..........  1%  179K 71s
   550K .......... .......... .......... .......... ..........  1%  184M 65s
   600K .......... .......... .......... .......... ..........  1%  235M 60s
   650K .......... .......... .......... .......... ..........  2%  199M 55s
   700K .......... .......... .......... .......... ..........  2%  130M 52s
   750K .......... .......... .......... .......... ..........  2%  361K 54s
   800K .......... .......... .......... .......... ..........  2%  360K 57s
   850K .......... .......... .......... .......... ..........  2% 71.6M 53s
   900K .......... .......... .......... .......... ..........  2%  361K 55s
   950K .......... .......... .......... .......... ..........  2% 72.8M 53s
  1000K .......... .......... .......... .......... ..........  3%  360K 55s
  1050K .......... .......... .......... .......... ..........  3% 50.8M 52s
  1100K .......... .......... .......... .......... ..........  3%  361K 54s
  1150K .......... .......... .......... .......... ..........  3% 64.8M 51s
  1200K .......... .......... .......... .......... ..........  3%  361K 53s
  1250K .......... .......... .......... .......... ..........  3% 47.7M 51s
  1300K .......... .......... .......... .......... ..........  3%  361K 52s
  1350K .......... .......... .......... .......... ..........  4% 40.0M 50s
  1400K .......... .......... .......... .......... ..........  4% 98.1M 49s
  1450K .......... .......... .......... .......... ..........  4%  361K 50s
  1500K .......... .......... .......... .......... ..........  4% 80.2M 48s
  1550K .......... .......... .......... .......... ..........  4%  360K 50s
  1600K .......... .......... .......... .......... ..........  4% 75.8M 48s
  1650K .......... .......... .......... .......... ..........  4%  362K 49s
  1700K .......... .......... .......... .......... ..........  5% 25.6M 48s
  1750K .......... .......... .......... .......... ..........  5%  364K 49s
  1800K .......... .......... .......... .......... ..........  5% 22.4M 48s
  1850K .......... .......... .......... .......... ..........  5%  363K 49s
  1900K .......... .......... .......... .......... ..........  5% 26.4M 47s
  1950K .......... .......... .......... .......... ..........  5%  364K 48s
  2000K .......... .......... .......... .......... ..........  5% 29.8M 47s
  2050K .......... .......... .......... .......... ..........  6%  364K 48s
  2100K .......... .......... .......... .......... ..........  6% 27.4M 47s
  2150K .......... .......... .......... .......... ..........  6% 51.9M 46s
  2200K .......... .......... .......... .......... ..........  6%  365K 47s
  2250K .......... .......... .......... .......... ..........  6% 21.7M 46s
  2300K .......... .......... .......... .......... ..........  6%  365K 47s
  2350K .......... .......... .......... .......... ..........  6% 14.8M 46s
  2400K .......... .......... .......... .......... ..........  7%  367K 46s
  2450K .......... .......... .......... .......... ..........  7% 17.2M 45s
  2500K .......... .......... .......... .......... ..........  7%  367K 46s
  2550K .......... .......... .......... .......... ..........  7% 24.6M 45s
  2600K .......... .......... .......... .......... ..........  7% 21.7M 44s
  2650K .......... .......... .......... .......... ..........  7%  364K 45s
  2700K .......... .......... .......... .......... ..........  7% 21.9M 44s
  2750K .......... .......... .......... .......... ..........  8%  368K 45s
  2800K .......... .......... .......... .......... ..........  8% 16.0M 44s
  2850K .......... .......... .......... .......... ..........  8%  367K 45s
  2900K .......... .......... .......... .......... ..........  8% 17.4M 44s
  2950K .......... .......... .......... .......... ..........  8%  365K 45s
  3000K .......... .......... .......... .......... ..........  8% 20.6M 44s
  3050K .......... .......... .......... .......... ..........  8%  365K 44s
  3100K .......... .......... .......... .......... ..........  9% 35.7M 44s
  3150K .......... .......... .......... .......... ..........  9% 27.1M 43s
  3200K .......... .......... .......... .......... ..........  9%  364K 44s
  3250K .......... .......... .......... .......... ..........  9% 23.0M 43s
  3300K .......... .......... .......... .......... ..........  9%  365K 43s
  3350K .......... .......... .......... .......... ..........  9% 19.6M 43s
  3400K .......... .......... .......... .......... ..........  9%  365K 43s
  3450K .......... .......... .......... .......... .......... 10% 19.4M 43s
  3500K .......... .......... .......... .......... .......... 10%  365K 43s
  3550K .......... .......... .......... .......... .......... 10% 19.7M 43s
  3600K .......... .......... .......... .......... .......... 10%  365K 43s
  3650K .......... .......... .......... .......... .......... 10% 59.6M 42s
  3700K .......... .......... .......... .......... .......... 10%  353K 43s
  3750K .......... .......... .......... .......... .......... 10%  363K 43s
  3800K .......... .......... .......... .......... .......... 11%  208M 43s
  3850K .......... .......... .......... .......... .......... 11% 28.1M 42s
  3900K .......... .......... .......... .......... .......... 11%  364K 43s
  3950K .......... .......... .......... .......... .......... 11%  361K 43s
  4000K .......... .......... .......... .......... .......... 11% 20.0M 43s
  4050K .......... .......... .......... .......... .......... 11%  180K 44s
  4100K .......... .......... .......... .......... .......... 11%  204M 43s
  4150K .......... .......... .......... .......... .......... 12% 26.3M 43s
  4200K .......... .......... .......... .......... .......... 12%  179K 44s
  4250K .......... .......... .......... .......... .......... 12%  123M 44s
  4300K .......... .......... .......... .......... .......... 12%  363K 44s
  4350K .......... .......... .......... .......... .......... 12%  359K 45s
  4400K .......... .......... .......... .......... .......... 12%  359K 45s
  4450K .......... .......... .......... .......... .......... 12%  358K 45s
  4500K .......... .......... .......... .......... .......... 13%  359K 46s
  4550K .......... .......... .......... .......... .......... 13%  359K 46s
  4600K .......... .......... .......... .......... .......... 13%  359K 46s
  4650K .......... .......... .......... .......... .......... 13%  361K 47s
  4700K .......... .......... .......... .......... .......... 13%  358K 47s
  4750K .......... .......... .......... .......... .......... 13%  578K 47s
  4800K .......... .......... .......... .......... .......... 13%  901K 47s
  4850K .......... .......... .......... .......... .......... 14%  358K 47s
  4900K .......... .......... .......... .......... .......... 14%  359K 47s
  4950K .......... .......... .......... .......... .......... 14%  359K 48s
  5000K .......... .......... .......... .......... .......... 14%  359K 48s
  5050K .......... .......... .......... .......... .......... 14%  363K 48s
  5100K .......... .......... .......... .......... .......... 14%  359K 48s
  5150K .......... .......... .......... .......... .......... 14%  359K 49s
  5200K .......... .......... .......... .......... .......... 15%  360K 49s
  5250K .......... .......... .......... .......... .......... 15%  579K 49s
  5300K .......... .......... .......... .......... .......... 15%  890K 49s
  5350K .......... .......... .......... .......... .......... 15%  359K 49s
  5400K .......... .......... .......... .......... .......... 15%  363K 49s
  5450K .......... .......... .......... .......... .......... 15%  362K 49s
  5500K .......... .......... .......... .......... .......... 15%  358K 49s
  5550K .......... .......... .......... .......... .......... 16%  579K 49s
  5600K .......... .......... .......... .......... .......... 16%  891K 49s
  5650K .......... .......... .......... .......... .......... 16%  361K 49s
  5700K .......... .......... .......... .......... .......... 16%  360K 49s
  5750K .......... .......... .......... .......... .......... 16%  353K 50s
  5800K .......... .......... .......... .......... .......... 16%  370K 50s
  5850K .......... .......... .......... .......... .......... 16%  354K 50s
  5900K .......... .......... .......... .......... .......... 17%  358K 50s
  5950K .......... .......... .......... .......... .......... 17%  364K 50s
  6000K .......... .......... .......... .......... .......... 17%  385K 50s
  6050K .......... .......... .......... .......... .......... 17%  517K 50s
  6100K .......... .......... .......... .......... .......... 17%  359K 51s
  6150K .......... .......... .......... .......... .......... 17%  900K 50s
  6200K .......... .......... .......... .......... .......... 17%  360K 50s
  6250K .......... .......... .......... .......... .......... 18%  360K 51s
  6300K .......... .......... .......... .......... .......... 18%  360K 51s
  6350K .......... .......... .......... .......... .......... 18%  580K 51s
  6400K .......... .......... .......... .......... .......... 18%  359K 51s
  6450K .......... .......... .......... .......... .......... 18%  918K 51s
  6500K .......... .......... .......... .......... .......... 18%  358K 51s
  6550K .......... .......... .......... .......... .......... 18%  360K 51s
  6600K .......... .......... .......... .......... .......... 19%  581K 51s
  6650K .......... .......... .......... .......... .......... 19%  516K 51s
  6700K .......... .......... .......... .......... .......... 19%  514K 51s
  6750K .......... .......... .......... .......... .......... 19%  359K 51s
  6800K .......... .......... .......... .......... .......... 19%  397K 51s
  6850K .......... .......... .......... .......... .......... 19%  510K 51s
  6900K .......... .......... .......... .......... .......... 19%  520K 51s
  6950K .......... .......... .......... .......... .......... 20%  510K 51s
  7000K .......... .......... .......... .......... .......... 20%  359K 51s
  7050K .......... .......... .......... .......... .......... 20%  590K 51s
  7100K .......... .......... .......... .......... .......... 20%  360K 51s
  7150K .......... .......... .......... .......... .......... 20%  897K 50s
  7200K .......... .......... .......... .......... .......... 20%  360K 51s
  7250K .......... .......... .......... .......... .......... 20%  360K 51s
  7300K .......... .......... .......... .......... .......... 21%  591K 50s
  7350K .......... .......... .......... .......... .......... 21%  513K 50s
  7400K .......... .......... .......... .......... .......... 21%  515K 50s
  7450K .......... .......... .......... .......... .......... 21%  359K 50s
  7500K .......... .......... .......... .......... .......... 21%  587K 50s
  7550K .......... .......... .......... .......... .......... 21%  359K 50s
  7600K .......... .......... .......... .......... .......... 21%  918K 50s
  7650K .......... .......... .......... .......... .......... 22%  359K 50s
  7700K .......... .......... .......... .......... .......... 22%  359K 50s
  7750K .......... .......... .......... .......... .......... 22%  369K 50s
  7800K .......... .......... .......... .......... .......... 22%  350K 50s
  7850K .......... .......... .......... .......... .......... 22%  368K 50s
  7900K .......... .......... .......... .......... .......... 22%  387K 50s
  7950K .......... .......... .......... .......... .......... 22%  501K 50s
  8000K .......... .......... .......... .......... .......... 23%  514K 50s
  8050K .......... .......... .......... .......... .......... 23%  520K 50s
  8100K .......... .......... .......... .......... .......... 23%  359K 50s
  8150K .......... .......... .......... .......... .......... 23%  179K 51s
  8200K .......... .......... .......... .......... .......... 23% 1.11M 51s
  8250K .......... .......... .......... .......... .......... 23%  524K 50s
  8300K .......... .......... .......... .......... .......... 23%  273K 51s
  8350K .......... .......... .......... .......... .......... 24%  357K 51s
  8400K .......... .......... .......... .......... .......... 24%  359K 51s
  8450K .......... .......... .......... .......... .......... 24%  278K 51s
  8500K .......... .......... .......... .......... .......... 24%  504K 51s
  8550K .......... .......... .......... .......... .......... 24%  359K 51s
  8600K .......... .......... .......... .......... .......... 24%  358K 51s
  8650K .......... .......... .......... .......... .......... 24%  358K 51s
  8700K .......... .......... .......... .......... .......... 25%  365K 51s
  8750K .......... .......... .......... .......... .......... 25%  359K 51s
  8800K .......... .......... .......... .......... .......... 25%  364K 51s
  8850K .......... .......... .......... .......... .......... 25%  510K 51s
  8900K .......... .......... .......... .......... .......... 25%  359K 51s
  8950K .......... .......... .......... .......... .......... 25%  358K 51s
  9000K .......... .......... .......... .......... .......... 25%  367K 51s
  9050K .......... .......... .......... .......... .......... 26%  382K 51s
  9100K .......... .......... .......... .......... .......... 26%  359K 51s
  9150K .......... .......... .......... .......... .......... 26%  358K 51s
  9200K .......... .......... .......... .......... .......... 26%  504K 51s
  9250K .......... .......... .......... .......... .......... 26%  358K 51s
  9300K .......... .......... .......... .......... .......... 26%  359K 51s
  9350K .......... .......... .......... .......... .......... 26%  963K 51s
  9400K .......... .......... .......... .......... .......... 27%  359K 51s
  9450K .......... .......... .......... .......... .......... 27%  383K 51s
  9500K .......... .......... .......... .......... .......... 27%  363K 51s
  9550K .......... .......... .......... .......... .......... 27%  509K 51s
  9600K .......... .......... .......... .......... .......... 27%  359K 51s
  9650K .......... .......... .......... .......... .......... 27%  946K 50s
  9700K .......... .......... .......... .......... .......... 27%  371K 50s
  9750K .......... .......... .......... .......... .......... 28%  217K 51s
  9800K .......... .......... .......... .......... .......... 28%  246M 50s
  9850K .......... .......... .......... .......... .......... 28%  276K 50s
  9900K .......... .......... .......... .......... .......... 28%  358K 50s
  9950K .......... .......... .......... .......... .......... 28%  359K 50s
 10000K .......... .......... .......... .......... .......... 28%  358K 50s
 10050K .......... .......... .......... .......... .......... 28%  520K 50s
 10100K .......... .......... .......... .......... .......... 29%  360K 50s
 10150K .......... .......... .......... .......... .......... 29%  360K 50s
 10200K .......... .......... .......... .......... .......... 29%  521K 50s
 10250K .......... .......... .......... .......... .......... 29%  507K 50s
 10300K .......... .......... .......... .......... .......... 29%  388K 50s
 10350K .......... .......... .......... .......... .......... 29%  360K 50s
 10400K .......... .......... .......... .......... .......... 29%  523K 50s
 10450K .......... .......... .......... .......... .......... 30%  517K 50s
 10500K .......... .......... .......... .......... .......... 30%  514K 49s
 10550K .......... .......... .......... .......... .......... 30%  386K 49s
 10600K .......... .......... .......... .......... .......... 30%  527K 49s
 10650K .......... .......... .......... .......... .......... 30%  359K 49s
 10700K .......... .......... .......... .......... .......... 30%  518K 49s
 10750K .......... .......... .......... .......... .......... 31%  567K 49s
 10800K .......... .......... .......... .......... .......... 31%  359K 49s
 10850K .......... .......... .......... .......... .......... 31%  531K 49s
 10900K .......... .......... .......... .......... .......... 31%  212K 49s
 10950K .......... .......... .......... .......... .......... 31%  264M 49s
 11000K .......... .......... .......... .......... .......... 31%  356K 49s
 11050K .......... .......... .......... .......... .......... 31%  359K 49s
 11100K .......... .......... .......... .......... .......... 32%  358K 49s
 11150K .......... .......... .......... .......... .......... 32%  363K 49s
 11200K .......... .......... .......... .......... .......... 32%  359K 49s
 11250K .......... .......... .......... .......... .......... 32%  507K 49s
 11300K .......... .......... .......... .......... .......... 32%  392K 48s
 11350K .......... .......... .......... .......... .......... 32%  359K 48s
 11400K .......... .......... .......... .......... .......... 32%  524K 48s
 11450K .......... .......... .......... .......... .......... 33%  359K 48s
 11500K .......... .......... .......... .......... .......... 33%  503K 48s
 11550K .......... .......... .......... .......... .......... 33%  522K 48s
 11600K .......... .......... .......... .......... .......... 33%  389K 48s
 11650K .......... .......... .......... .......... .......... 33%  528K 48s
 11700K .......... .......... .......... .......... .......... 33%  360K 48s
 11750K .......... .......... .......... .......... .......... 33%  505K 48s
 11800K .......... .......... .......... .......... .......... 34%  518K 48s
 11850K .......... .......... .......... .......... .......... 34%  391K 48s
 11900K .......... .......... .......... .......... .......... 34%  526K 47s
 11950K .......... .......... .......... .......... .......... 34%  492K 47s
 12000K .......... .......... .......... .......... .......... 34%  362K 47s
 12050K .......... .......... .......... .......... .......... 34%  588K 47s
 12100K .......... .......... .......... .......... .......... 34%  373K 47s
 12150K .......... .......... .......... .......... .......... 35%  501K 47s
 12200K .......... .......... .......... .......... .......... 35%  499K 47s
 12250K .......... .......... .......... .......... .......... 35%  531K 47s
 12300K .......... .......... .......... .......... .......... 35%  385K 47s
 12350K .......... .......... .......... .......... .......... 35%  529K 47s
 12400K .......... .......... .......... .......... .......... 35%  358K 47s
 12450K .......... .......... .......... .......... .......... 35%  492K 46s
 12500K .......... .......... .......... .......... .......... 36%  540K 46s
 12550K .......... .......... .......... .......... .......... 36%  389K 46s
 12600K .......... .......... .......... .......... .......... 36%  522K 46s
 12650K .......... .......... .......... .......... .......... 36%  494K 46s
 12700K .......... .......... .......... .......... .......... 36%  372K 46s
 12750K .......... .......... .......... .......... .......... 36%  575K 46s
 12800K .......... .......... .......... .......... .......... 36%  524K 46s
 12850K .......... .......... .......... .......... .......... 37%  494K 46s
 12900K .......... .......... .......... .......... .......... 37%  369K 46s
 12950K .......... .......... .......... .......... .......... 37%  580K 45s
 13000K .......... .......... .......... .......... .......... 37%  523K 45s
 13050K .......... .......... .......... .......... .......... 37%  494K 45s
 13100K .......... .......... .......... .......... .......... 37%  533K 45s
 13150K .......... .......... .......... .......... .......... 37%  404K 45s
 13200K .......... .......... .......... .......... .......... 38%  501K 45s
 13250K .......... .......... .......... .......... .......... 38%  273K 45s
 13300K .......... .......... .......... .......... .......... 38% 4.12M 45s
 13350K .......... .......... .......... .......... .......... 38%  271K 45s
 13400K .......... .......... .......... .......... .......... 38%  280K 45s
 13450K .......... .......... .......... .......... .......... 38%  257K 45s
 13500K .......... .......... .......... .......... .......... 38%  272K 45s
 13550K .......... .......... .......... .......... .......... 39%  213K 45s
 13600K .......... .......... .......... .......... .......... 39%  279K 45s
 13650K .......... .......... .......... .......... .......... 39%  221K 45s
 13700K .......... .......... .......... .......... .......... 39%  255K 45s
 13750K .......... .......... .......... .......... .......... 39%  213K 45s
 13800K .......... .......... .......... .......... .......... 39%  273K 45s
 13850K .......... .......... .......... .......... .......... 39%  213K 45s
 13900K .......... .......... .......... .......... .......... 40%  277K 45s
 13950K .......... .......... .......... .......... .......... 40%  223K 45s
 14000K .......... .......... .......... .......... .......... 40%  257K 45s
 14050K .......... .......... .......... .......... .......... 40%  272K 45s
 14100K .......... .......... .......... .......... .......... 40%  259K 45s
 14150K .......... .......... .......... .......... .......... 40%  277K 45s
 14200K .......... .......... .......... .......... .......... 40%  358K 45s
 14250K .......... .......... .......... .......... .......... 41%  269K 45s
 14300K .......... .......... .......... .......... .......... 41%  357K 45s
 14350K .......... .......... .......... .......... .......... 41%  283K 45s
 14400K .......... .......... .......... .......... .......... 41%  327K 45s
 14450K .......... .......... .......... .......... .......... 41%  278K 45s
 14500K .......... .......... .......... .......... .......... 41%  179K 45s
 14550K .......... .......... .......... .......... .......... 41%  588K 45s
 14600K .......... .......... .......... .......... .......... 42%  258K 45s
 14650K .......... .......... .......... .......... .......... 42%  264K 45s
 14700K .......... .......... .......... .......... .......... 42%  286K 45s
 14750K .......... .......... .......... .......... .......... 42%  323K 45s
 14800K .......... .......... .......... .......... .......... 42%  226K 45s
 14850K .......... .......... .......... .......... .......... 42%  358K 45s
 14900K .......... .......... .......... .......... .......... 42%  346K 45s
 14950K .......... .......... .......... .......... .......... 43%  353K 45s
 15000K .......... .......... .......... .......... .......... 43%  287K 45s
 15050K .......... .......... .......... .......... .......... 43%  357K 45s
 15100K .......... .......... .......... .......... .......... 43%  358K 45s
 15150K .......... .......... .......... .......... .......... 43%  358K 45s
 15200K .......... .......... .......... .......... .......... 43%  179K 45s
 15250K .......... .......... .......... .......... .......... 43%  491K 44s
 15300K .......... .......... .......... .......... .......... 44%  283K 44s
 15350K .......... .......... .......... .......... .......... 44%  260K 44s
 15400K .......... .......... .......... .......... .......... 44%  265K 44s
 15450K .......... .......... .......... .......... .......... 44%  352K 44s
 15500K .......... .......... .......... .......... .......... 44%  288K 44s
 15550K .......... .......... .......... .......... .......... 44%  358K 44s
 15600K .......... .......... .......... .......... .......... 44%  259K 44s
 15650K .......... .......... .......... .......... .......... 45%  270K 44s
 15700K .......... .......... .......... .......... .......... 45%  359K 44s
 15750K .......... .......... .......... .......... .......... 45%  359K 44s
 15800K .......... .......... .......... .......... .......... 45%  346K 44s
 15850K .......... .......... .......... .......... .......... 45%  357K 44s
 15900K .......... .......... .......... .......... .......... 45%  359K 44s
 15950K .......... .......... .......... .......... .......... 45%  359K 44s
 16000K .......... .......... .......... .......... .......... 46%  286K 44s
 16050K .......... .......... .......... .......... .......... 46%  358K 44s
 16100K .......... .......... .......... .......... .......... 46%  359K 43s
 16150K .......... .......... .......... .......... .......... 46%  328K 43s
 16200K .......... .......... .......... .......... .......... 46%  280K 43s
 16250K .......... .......... .......... .......... .......... 46%  359K 43s
 16300K .......... .......... .......... .......... .......... 46%  358K 43s
 16350K .......... .......... .......... .......... .......... 47%  357K 43s
 16400K .......... .......... .......... .......... .......... 47%  358K 43s
 16450K .......... .......... .......... .......... .......... 47%  358K 43s
 16500K .......... .......... .......... .......... .......... 47%  358K 43s
 16550K .......... .......... .......... .......... .......... 47%  503K 43s
 16600K .......... .......... .......... .......... .......... 47%  358K 43s
 16650K .......... .......... .......... .......... .......... 47%  398K 42s
 16700K .......... .......... .......... .......... .......... 48%  359K 42s
 16750K .......... .......... .......... .......... .......... 48%  358K 42s
 16800K .......... .......... .......... .......... .......... 48%  480K 42s
 16850K .......... .......... .......... .......... .......... 48%  357K 42s
 16900K .......... .......... .......... .......... .......... 48%  366K 42s
 16950K .......... .......... .......... .......... .......... 48%  546K 42s
 17000K .......... .......... .......... .......... .......... 48%  360K 42s
 17050K .......... .......... .......... .......... .......... 49%  579K 42s
 17100K .......... .......... .......... .......... .......... 49%  360K 41s
 17150K .......... .......... .......... .......... .......... 49%  475K 41s
 17200K .......... .......... .......... .......... .......... 49%  361K 41s
 17250K .......... .......... .......... .......... .......... 49%  564K 41s
 17300K .......... .......... .......... .......... .......... 49%  494K 41s
 17350K .......... .......... .......... .......... .......... 49%  402K 41s
 17400K .......... .......... .......... .......... .......... 50%  480K 41s
 17450K .......... .......... .......... .......... .......... 50%  556K 41s
 17500K .......... .......... .......... .......... .......... 50%  360K 40s
 17550K .......... .......... .......... .......... .......... 50%  585K 40s
 17600K .......... .......... .......... .......... .......... 50%  478K 40s
 17650K .......... .......... .......... .......... .......... 50%  563K 40s
 17700K .......... .......... .......... .......... .......... 50%  586K 40s
 17750K .......... .......... .......... .......... .......... 51%  477K 40s
 17800K .......... .......... .......... .......... .......... 51%  564K 40s
 17850K .......... .......... .......... .......... .......... 51%  584K 39s
 17900K .......... .......... .......... .......... .......... 51%  927K 39s
 17950K .......... .......... .......... .......... .......... 51%  584K 39s
 18000K .......... .......... .......... .......... .......... 51%  479K 39s
 18050K .......... .......... .......... .......... .......... 51%  993K 39s
 18100K .......... .......... .......... .......... .......... 52%  555K 39s
 18150K .......... .......... .......... .......... .......... 52%  570K 39s
 18200K .......... .......... .......... .......... .......... 52%  969K 38s
 18250K .......... .......... .......... .......... .......... 52%  570K 38s
 18300K .......... .......... .......... .......... .......... 52%  974K 38s
 18350K .......... .......... .......... .......... .......... 52% 1008K 38s
 18400K .......... .......... .......... .......... .......... 52%  558K 38s
 18450K .......... .......... .......... .......... .......... 53% 1.41M 38s
 18500K .......... .......... .......... .......... .......... 53%  917K 37s
 18550K .......... .......... .......... .......... .......... 53%  585K 37s
 18600K .......... .......... .......... .......... .......... 53%  932K 37s
 18650K .......... .......... .......... .......... .......... 53%  585K 37s
 18700K .......... .......... .......... .......... .......... 53% 3.26M 37s
 18750K .......... .......... .......... .......... .......... 53% 1.25M 37s
 18800K .......... .......... .......... .......... .......... 54%  580K 36s
 18850K .......... .......... .......... .......... .......... 54%  938K 36s
 18900K .......... .......... .......... .......... .......... 54%  930K 36s
 18950K .......... .......... .......... .......... .......... 54% 1.51M 36s
 19000K .......... .......... .......... .......... .......... 54%  936K 36s
 19050K .......... .......... .......... .......... .......... 54%  589K 36s
 19100K .......... .......... .......... .......... .......... 54% 2.99M 35s
 19150K .......... .......... .......... .......... .......... 55% 1.28M 35s
 19200K .......... .......... .......... .......... .......... 55%  585K 35s
 19250K .......... .......... .......... .......... .......... 55% 3.13M 35s
 19300K .......... .......... .......... .......... .......... 55% 1.28M 35s
 19350K .......... .......... .......... .......... .......... 55%  909K 35s
 19400K .......... .......... .......... .......... .......... 55% 1.52M 34s
 19450K .......... .......... .......... .......... .......... 55%  959K 34s
 19500K .......... .......... .......... .......... .......... 56%  906K 34s
 19550K .......... .......... .......... .......... .......... 56% 1.53M 34s
 19600K .......... .......... .......... .......... .......... 56%  965K 34s
 19650K .......... .......... .......... .......... .......... 56% 52.5M 34s
 19700K .......... .......... .......... .......... .......... 56%  578K 33s
 19750K .......... .......... .......... .......... .......... 56% 3.26M 33s
 19800K .......... .......... .......... .......... .......... 56% 1.30M 33s
 19850K .......... .......... .......... .......... .......... 57%  903K 33s
 19900K .......... .......... .......... .......... .......... 57% 1.55M 33s
 19950K .......... .......... .......... .......... .......... 57% 3.22M 33s
 20000K .......... .......... .......... .......... .......... 57% 1.29M 32s
 20050K .......... .......... .......... .......... .......... 57%  899K 32s
 20100K .......... .......... .......... .......... .......... 57% 1.56M 32s
 20150K .......... .......... .......... .......... .......... 57%  967K 32s
 20200K .......... .......... .......... .......... .......... 58% 65.2M 32s
 20250K .......... .......... .......... .......... .......... 58%  889K 32s
 20300K .......... .......... .......... .......... .......... 58% 1.60M 31s
 20350K .......... .......... .......... .......... .......... 58% 3.33M 31s
 20400K .......... .......... .......... .......... .......... 58% 1.29M 31s
 20450K .......... .......... .......... .......... .......... 58%  889K 31s
 20500K .......... .......... .......... .......... .......... 58% 1.61M 31s
 20550K .......... .......... .......... .......... .......... 59% 3.34M 31s
 20600K .......... .......... .......... .......... .......... 59% 1.28M 31s
 20650K .......... .......... .......... .......... .......... 59%  105M 30s
 20700K .......... .......... .......... .......... .......... 59%  583K 30s
 20750K .......... .......... .......... .......... .......... 59% 61.7M 30s
 20800K .......... .......... .......... .......... .......... 59%  966K 30s
 20850K .......... .......... .......... .......... .......... 59% 46.8M 30s
 20900K .......... .......... .......... .......... .......... 60%  896K 30s
 20950K .......... .......... .......... .......... .......... 60% 1.62M 29s
 21000K .......... .......... .......... .......... .......... 60% 77.9M 29s
 21050K .......... .......... .......... .......... .......... 60%  951K 29s
 21100K .......... .......... .......... .......... .......... 60% 47.2M 29s
 21150K .......... .......... .......... .......... .......... 60%  908K 29s
 21200K .......... .......... .......... .......... .......... 61% 1.59M 29s
 21250K .......... .......... .......... .......... .......... 61% 86.1M 28s
 21300K .......... .......... .......... .......... .......... 61%  949K 28s
 21350K .......... .......... .......... .......... .......... 61% 76.1M 28s
 21400K .......... .......... .......... .......... .......... 61%  909K 28s
 21450K .......... .......... .......... .......... .......... 61% 1.59M 28s
 21500K .......... .......... .......... .......... .......... 61% 82.4M 28s
 21550K .......... .......... .......... .......... .......... 62%  948K 28s
 21600K .......... .......... .......... .......... .......... 62% 83.3M 27s
 21650K .......... .......... .......... .......... .......... 62% 1.20M 27s
 21700K .......... .......... .......... .......... .......... 62% 1.10M 27s
 21750K .......... .......... .......... .......... .......... 62%  102M 27s
 21800K .......... .......... .......... .......... .......... 62% 3.62M 27s
 21850K .......... .......... .......... .......... .......... 62% 1.22M 27s
 21900K .......... .......... .......... .......... .......... 63% 88.2M 26s
 21950K .......... .......... .......... .......... .......... 63%  921K 26s
 22000K .......... .......... .......... .......... .......... 63% 1.59M 26s
 22050K .......... .......... .......... .......... .......... 63%  101M 26s
 22100K .......... .......... .......... .......... .......... 63% 3.61M 26s
 22150K .......... .......... .......... .......... .......... 63% 1.21M 26s
 22200K .......... .......... .......... .......... .......... 63% 86.6M 26s
 22250K .......... .......... .......... .......... .......... 64%  596K 25s
 22300K .......... .......... .......... .......... .......... 64% 80.6M 25s
 22350K .......... .......... .......... .......... .......... 64% 81.4M 25s
 22400K .......... .......... .......... .......... .......... 64% 3.60M 25s
 22450K .......... .......... .......... .......... .......... 64% 1.22M 25s
 22500K .......... .......... .......... .......... .......... 64%  107M 25s
 22550K .......... .......... .......... .......... .......... 64%  781K 25s
 22600K .......... .......... .......... .......... .......... 65% 2.38M 24s
 22650K .......... .......... .......... .......... .......... 65% 78.0M 24s
 22700K .......... .......... .......... .......... .......... 65% 3.66M 24s
 22750K .......... .......... .......... .......... .......... 65% 1.21M 24s
 22800K .......... .......... .......... .......... .......... 65% 81.1M 24s
 22850K .......... .......... .......... .......... .......... 65% 1.22M 24s
 22900K .......... .......... .......... .......... .......... 65% 1.11M 24s
 22950K .......... .......... .......... .......... .......... 66% 65.8M 23s
 23000K .......... .......... .......... .......... .......... 66% 93.6M 23s
 23050K .......... .......... .......... .......... .......... 66%  942K 23s
 23100K .......... .......... .......... .......... .......... 66% 56.0M 23s
 23150K .......... .......... .......... .......... .......... 66%  113M 23s
 23200K .......... .......... .......... .......... .......... 66%  788K 23s
 23250K .......... .......... .......... .......... .......... 66% 2.39M 23s
 23300K .......... .......... .......... .......... .......... 67% 82.1M 23s
 23350K .......... .......... .......... .......... .......... 67% 77.5M 22s
 23400K .......... .......... .......... .......... .......... 67%  944K 22s
 23450K .......... .......... .......... .......... .......... 67% 70.8M 22s
 23500K .......... .......... .......... .......... .......... 67%  109M 22s
 23550K .......... .......... .......... .......... .......... 67%  787K 22s
 23600K .......... .......... .......... .......... .......... 67% 2.39M 22s
 23650K .......... .......... .......... .......... .......... 68%  103M 22s
 23700K .......... .......... .......... .......... .......... 68%  106M 21s
 23750K .......... .......... .......... .......... .......... 68% 3.55M 21s
 23800K .......... .......... .......... .......... .......... 68% 1.22M 21s
 23850K .......... .......... .......... .......... .......... 68% 79.6M 21s
 23900K .......... .......... .......... .......... .......... 68% 1.24M 21s
 23950K .......... .......... .......... .......... .......... 68% 1.09M 21s
 24000K .......... .......... .......... .......... .......... 69%  164M 21s
 24050K .......... .......... .......... .......... .......... 69%  105M 21s
 24100K .......... .......... .......... .......... .......... 69% 3.58M 20s
 24150K .......... .......... .......... .......... .......... 69% 1.22M 20s
 24200K .......... .......... .......... .......... .......... 69% 87.7M 20s
 24250K .......... .......... .......... .......... .......... 69% 63.9M 20s
 24300K .......... .......... .......... .......... .......... 69%  789K 20s
 24350K .......... .......... .......... .......... .......... 70% 2.32M 20s
 24400K .......... .......... .......... .......... .......... 70%  136M 20s
 24450K .......... .......... .......... .......... .......... 70%  113M 19s
 24500K .......... .......... .......... .......... .......... 70% 3.74M 19s
 24550K .......... .......... .......... .......... .......... 70% 1.23M 19s
 24600K .......... .......... .......... .......... .......... 70%  105M 19s
 24650K .......... .......... .......... .......... .......... 70% 66.2M 19s
 24700K .......... .......... .......... .......... .......... 71%  788K 19s
 24750K .......... .......... .......... .......... .......... 71% 2.32M 19s
 24800K .......... .......... .......... .......... .......... 71%  107M 19s
 24850K .......... .......... .......... .......... .......... 71%  114M 18s
 24900K .......... .......... .......... .......... .......... 71% 3.70M 18s
 24950K .......... .......... .......... .......... .......... 71% 1.24M 18s
 25000K .......... .......... .......... .......... .......... 71% 78.1M 18s
 25050K .......... .......... .......... .......... .......... 72% 90.6M 18s
 25100K .......... .......... .......... .......... .......... 72% 1.25M 18s
 25150K .......... .......... .......... .......... .......... 72% 1.07M 18s
 25200K .......... .......... .......... .......... .......... 72%  132M 18s
 25250K .......... .......... .......... .......... .......... 72%  112M 18s
 25300K .......... .......... .......... .......... .......... 72% 3.78M 17s
 25350K .......... .......... .......... .......... .......... 72% 1.23M 17s
 25400K .......... .......... .......... .......... .......... 73%  104M 17s
 25450K .......... .......... .......... .......... .......... 73%  106M 17s
 25500K .......... .......... .......... .......... .......... 73% 1.24M 17s
 25550K .......... .......... .......... .......... .......... 73% 1.07M 17s
 25600K .......... .......... .......... .......... .......... 73%  107M 17s
 25650K .......... .......... .......... .......... .......... 73%  102M 17s
 25700K .......... .......... .......... .......... .......... 73% 3.70M 16s
 25750K .......... .......... .......... .......... .......... 74% 1.24M 16s
 25800K .......... .......... .......... .......... .......... 74% 74.5M 16s
 25850K .......... .......... .......... .......... .......... 74%  123M 16s
 25900K .......... .......... .......... .......... .......... 74% 1.21M 16s
 25950K .......... .......... .......... .......... .......... 74% 1.10M 16s
 26000K .......... .......... .......... .......... .......... 74% 89.1M 16s
 26050K .......... .......... .......... .......... .......... 74%  131M 16s
 26100K .......... .......... .......... .......... .......... 75% 3.76M 16s
 26150K .......... .......... .......... .......... .......... 75% 1.23M 15s
 26200K .......... .......... .......... .......... .......... 75% 71.6M 15s
 26250K .......... .......... .......... .......... .......... 75% 81.6M 15s
 26300K .......... .......... .......... .......... .......... 75% 1.22M 15s
 26350K .......... .......... .......... .......... .......... 75% 1.10M 15s
 26400K .......... .......... .......... .......... .......... 75%  125M 15s
 26450K .......... .......... .......... .......... .......... 76% 86.4M 15s
 26500K .......... .......... .......... .......... .......... 76%  120M 15s
 26550K .......... .......... .......... .......... .......... 76%  931K 15s
 26600K .......... .......... .......... .......... .......... 76% 50.2M 14s
 26650K .......... .......... .......... .......... .......... 76% 69.1M 14s
 26700K .......... .......... .......... .......... .......... 76% 1.29M 14s
 26750K .......... .......... .......... .......... .......... 76% 1.08M 14s
 26800K .......... .......... .......... .......... .......... 77% 27.2M 14s
 26850K .......... .......... .......... .......... .......... 77% 60.0M 14s
 26900K .......... .......... .......... .......... .......... 77% 66.7M 14s
 26950K .......... .......... .......... .......... .......... 77%  989K 14s
 27000K .......... .......... .......... .......... .......... 77% 19.5M 14s
 27050K .......... .......... .......... .......... .......... 77% 71.3M 13s
 27100K .......... .......... .......... .......... .......... 77% 1.33M 13s
 27150K .......... .......... .......... .......... .......... 78% 1.07M 13s
 27200K .......... .......... .......... .......... .......... 78% 38.0M 13s
 27250K .......... .......... .......... .......... .......... 78% 60.0M 13s
 27300K .......... .......... .......... .......... .......... 78% 65.3M 13s
 27350K .......... .......... .......... .......... .......... 78% 3.89M 13s
 27400K .......... .......... .......... .......... .......... 78% 1.19M 13s
 27450K .......... .......... .......... .......... .......... 78% 67.4M 13s
 27500K .......... .......... .......... .......... .......... 79% 68.6M 12s
 27550K .......... .......... .......... .......... .......... 79%  812K 12s
 27600K .......... .......... .......... .......... .......... 79% 2.26M 12s
 27650K .......... .......... .......... .......... .......... 79% 66.8M 12s
 27700K .......... .......... .......... .......... .......... 79% 72.0M 12s
 27750K .......... .......... .......... .......... .......... 79% 4.04M 12s
 27800K .......... .......... .......... .......... .......... 79% 1.21M 12s
 27850K .......... .......... .......... .......... .......... 80% 35.5M 12s
 27900K .......... .......... .......... .......... .......... 80% 68.1M 12s
 27950K .......... .......... .......... .......... .......... 80%  816K 12s
 28000K .......... .......... .......... .......... .......... 80% 2.20M 11s
 28050K .......... .......... .......... .......... .......... 80%  101M 11s
 28100K .......... .......... .......... .......... .......... 80% 86.1M 11s
 28150K .......... .......... .......... .......... .......... 80% 4.21M 11s
 28200K .......... .......... .......... .......... .......... 81% 1.21M 11s
 28250K .......... .......... .......... .......... .......... 81% 50.7M 11s
 28300K .......... .......... .......... .......... .......... 81% 60.1M 11s
 28350K .......... .......... .......... .......... .......... 81% 1.29M 11s
 28400K .......... .......... .......... .......... .......... 81% 1.05M 11s
 28450K .......... .......... .......... .......... .......... 81% 66.1M 11s
 28500K .......... .......... .......... .......... .......... 81% 65.0M 10s
 28550K .......... .......... .......... .......... .......... 82% 4.07M 10s
 28600K .......... .......... .......... .......... .......... 82% 1.24M 10s
 28650K .......... .......... .......... .......... .......... 82% 42.9M 10s
 28700K .......... .......... .......... .......... .......... 82% 69.3M 10s
 28750K .......... .......... .......... .......... .......... 82% 1.29M 10s
 28800K .......... .......... .......... .......... .......... 82% 1.05M 10s
 28850K .......... .......... .......... .......... .......... 82% 52.0M 10s
 28900K .......... .......... .......... .......... .......... 83% 44.8M 10s
 28950K .......... .......... .......... .......... .......... 83%  118M 10s
 29000K .......... .......... .......... .......... .......... 83% 4.20M 10s
 29050K .......... .......... .......... .......... .......... 83% 1.23M 9s
 29100K .......... .......... .......... .......... .......... 83% 32.4M 9s
 29150K .......... .......... .......... .......... .......... 83% 1.29M 9s
 29200K .......... .......... .......... .......... .......... 83% 1.96M 9s
 29250K .......... .......... .......... .......... .......... 84% 2.20M 9s
 29300K .......... .......... .......... .......... .......... 84% 36.2M 9s
 29350K .......... .......... .......... .......... .......... 84% 75.4M 9s
 29400K .......... .......... .......... .......... .......... 84% 4.39M 9s
 29450K .......... .......... .......... .......... .......... 84% 1.23M 9s
 29500K .......... .......... .......... .......... .......... 84% 30.0M 9s
 29550K .......... .......... .......... .......... .......... 84% 92.4M 8s
 29600K .......... .......... .......... .......... .......... 85%  804K 8s
 29650K .......... .......... .......... .......... .......... 85% 2.22M 8s
 29700K .......... .......... .......... .......... .......... 85% 27.7M 8s
 29750K .......... .......... .......... .......... .......... 85% 87.4M 8s
 29800K .......... .......... .......... .......... .......... 85% 4.40M 8s
 29850K .......... .......... .......... .......... .......... 85% 1.23M 8s
 29900K .......... .......... .......... .......... .......... 85% 36.2M 8s
 29950K .......... .......... .......... .......... .......... 86% 71.8M 8s
 30000K .......... .......... .......... .......... .......... 86%  810K 8s
 30050K .......... .......... .......... .......... .......... 86% 2.15M 8s
 30100K .......... .......... .......... .......... .......... 86% 24.2M 7s
 30150K .......... .......... .......... .......... .......... 86% 92.5M 7s
 30200K .......... .......... .......... .......... .......... 86% 4.57M 7s
 30250K .......... .......... .......... .......... .......... 86% 1.24M 7s
 30300K .......... .......... .......... .......... .......... 87% 51.9M 7s
 30350K .......... .......... .......... .......... .......... 87% 50.0M 7s
 30400K .......... .......... .......... .......... .......... 87% 1.29M 7s
 30450K .......... .......... .......... .......... .......... 87% 1.05M 7s
 30500K .......... .......... .......... .......... .......... 87% 67.0M 7s
 30550K .......... .......... .......... .......... .......... 87% 21.2M 7s
 30600K .......... .......... .......... .......... .......... 87%  110M 7s
 30650K .......... .......... .......... .......... .......... 88% 1013K 7s
 30700K .......... .......... .......... .......... .......... 88% 74.8M 6s
 30750K .......... .......... .......... .......... .......... 88% 38.8M 6s
 30800K .......... .......... .......... .......... .......... 88% 1.27M 6s
 30850K .......... .......... .......... .......... .......... 88% 1.98M 6s
 30900K .......... .......... .......... .......... .......... 88% 2.23M 6s
 30950K .......... .......... .......... .......... .......... 88% 15.4M 6s
 31000K .......... .......... .......... .......... .......... 89% 72.3M 6s
 31050K .......... .......... .......... .......... .......... 89% 5.26M 6s
 31100K .......... .......... .......... .......... .......... 89% 1.22M 6s
 31150K .......... .......... .......... .......... .......... 89% 66.7M 6s
 31200K .......... .......... .......... .......... .......... 89% 67.5M 6s
 31250K .......... .......... .......... .......... .......... 89%  797K 5s
 31300K .......... .......... .......... .......... .......... 89% 2.20M 5s
 31350K .......... .......... .......... .......... .......... 90% 17.8M 5s
 31400K .......... .......... .......... .......... .......... 90% 72.7M 5s
 31450K .......... .......... .......... .......... .......... 90% 5.15M 5s
 31500K .......... .......... .......... .......... .......... 90% 1.23M 5s
 31550K .......... .......... .......... .......... .......... 90% 75.5M 5s
 31600K .......... .......... .......... .......... .......... 90% 57.5M 5s
 31650K .......... .......... .......... .......... .......... 91% 1.26M 5s
 31700K .......... .......... .......... .......... .......... 91% 1.06M 5s
 31750K .......... .......... .......... .......... .......... 91%  129M 5s
 31800K .......... .......... .......... .......... .......... 91% 16.6M 5s
 31850K .......... .......... .......... .......... .......... 91% 5.18M 4s
 31900K .......... .......... .......... .......... .......... 91% 1.22M 4s
 31950K .......... .......... .......... .......... .......... 91%  101M 4s
 32000K .......... .......... .......... .......... .......... 92% 56.4M 4s
 32050K .......... .......... .......... .......... .......... 92% 1.24M 4s
 32100K .......... .......... .......... .......... .......... 92% 1.96M 4s
 32150K .......... .......... .......... .......... .......... 92% 2.22M 4s
 32200K .......... .......... .......... .......... .......... 92% 16.6M 4s
 32250K .......... .......... .......... .......... .......... 92%  107M 4s
 32300K .......... .......... .......... .......... .......... 92% 1.00M 4s
 32350K .......... .......... .......... .......... .......... 93%  109M 4s
 32400K .......... .......... .......... .......... .......... 93%  119M 4s
 32450K .......... .......... .......... .......... .......... 93%  107M 4s
 32500K .......... .......... .......... .......... .......... 93%  777K 3s
 32550K .......... .......... .......... .......... .......... 93% 2.30M 3s
 32600K .......... .......... .......... .......... .......... 93% 14.4M 3s
 32650K .......... .......... .......... .......... .......... 93%  108M 3s
 32700K .......... .......... .......... .......... .......... 94% 5.24M 3s
 32750K .......... .......... .......... .......... .......... 94% 1.21M 3s
 32800K .......... .......... .......... .......... .......... 94%  114M 3s
 32850K .......... .......... .......... .......... .......... 94%  109M 3s
 32900K .......... .......... .......... .......... .......... 94%  785K 3s
 32950K .......... .......... .......... .......... .......... 94% 2.25M 3s
 33000K .......... .......... .......... .......... .......... 94%  117M 3s
 33050K .......... .......... .......... .......... .......... 95% 14.5M 3s
 33100K .......... .......... .......... .......... .......... 95% 5.19M 2s
 33150K .......... .......... .......... .......... .......... 95% 1.23M 2s
 33200K .......... .......... .......... .......... .......... 95%  109M 2s
 33250K .......... .......... .......... .......... .......... 95%  107M 2s
 33300K .......... .......... .......... .......... .......... 95% 1.25M 2s
 33350K .......... .......... .......... .......... .......... 95% 1.91M 2s
 33400K .......... .......... .......... .......... .......... 96% 2.20M 2s
 33450K .......... .......... .......... .......... .......... 96% 15.2M 2s
 33500K .......... .......... .......... .......... .......... 96% 79.4M 2s
 33550K .......... .......... .......... .......... .......... 96% 5.29M 2s
 33600K .......... .......... .......... .......... .......... 96% 1.25M 2s
 33650K .......... .......... .......... .......... .......... 96%  137M 2s
 33700K .......... .......... .......... .......... .......... 96% 68.9M 2s
 33750K .......... .......... .......... .......... .......... 97%  775K 2s
 33800K .......... .......... .......... .......... .......... 97% 2.25M 1s
 33850K .......... .......... .......... .......... .......... 97% 16.2M 1s
 33900K .......... .......... .......... .......... .......... 97% 69.6M 1s
 33950K .......... .......... .......... .......... .......... 97% 5.19M 1s
 34000K .......... .......... .......... .......... .......... 97% 1.23M 1s
 34050K .......... .......... .......... .......... .......... 97%  135M 1s
 34100K .......... .......... .......... .......... .......... 98%  162M 1s
 34150K .......... .......... .......... .......... .......... 98% 1.27M 1s
 34200K .......... .......... .......... .......... .......... 98% 1.89M 1s
 34250K .......... .......... .......... .......... .......... 98% 2.11M 1s
 34300K .......... .......... .......... .......... .......... 98% 23.5M 1s
 34350K .......... .......... .......... .......... .......... 98%  121M 1s
 34400K .......... .......... .......... .......... .......... 98% 1.01M 1s
 34450K .......... .......... .......... .......... .......... 99% 39.8M 0s
 34500K .......... .......... .......... .......... .......... 99%  105M 0s
 34550K .......... .......... .......... .......... .......... 99% 1.29M 0s
 34600K .......... .......... .......... .......... .......... 99% 1.88M 0s
 34650K .......... .......... .......... .......... .......... 99% 2.12M 0s
 34700K .......... .......... .......... .......... .......... 99% 27.1M 0s
 34750K .......... .......... .......... .......... .......... 99%  108M 0s
 34800K .......... .......... .......... ....                 100%  109M=50s

2019-05-06 10:21:57 (701 KB/s) - ‘Rfam.cm.gz’ saved [35670981]

--2019-05-06 10:22:00--  ftp://ftp.ebi.ac.uk/pub/databases/Rfam/CURRENT/Rfam.clanin
           => ‘Rfam.clanin’
Resolving ftp.ebi.ac.uk (ftp.ebi.ac.uk)... 193.62.192.4
Connecting to ftp.ebi.ac.uk (ftp.ebi.ac.uk)|193.62.192.4|:21... connected.
Logging in as anonymous ... Logged in!
==> SYST ... done.    ==> PWD ... done.
==> TYPE I ... done.  ==> CWD (1) /pub/databases/Rfam/CURRENT ... done.
==> SIZE Rfam.clanin ... 4034
==> PASV ... done.    ==> RETR Rfam.clanin ... done.
Length: 4034 (3.9K) (unauthoritative)

     0K ...                                                   100%  342K=0.01s

2019-05-06 10:22:01 (342 KB/s) - ‘Rfam.clanin’ saved [4034]
```

In [11]:

```
%%bash
cd ../../Data/Rfam
cmpress Rfam.cm
```

```
Working...    done.
Pressed and indexed 3016 CMs and p7 HMM filters (3016 names and 3016 accessions).
Covariance models and p7 filters pressed into binary file:  Rfam.cm.i1m
SSI index for binary covariance model file:                 Rfam.cm.i1i
Optimized p7 filter profiles (MSV part)  pressed into:      Rfam.cm.i1f
Optimized p7 filter profiles (remainder) pressed into:      Rfam.cm.i1p
```

In [19]:

```
%%bash
cd ../../Data/Rfam
cat ../../Data/Rhoto_IFO0880_4/Rhoto_IFO0880_4_AssemblyScaffolds.fasta \
    ../../Data/Rhoto_IFO0880_4/Rhoto_IFO0880_4_MitoAssemblyScaffolds.fasta \
    > Rhoto_IFO0880_4_AssemblyScaffolds_and_Mito.fasta
```

In [20]:

```
%%bash
cd ../../Data/Rfam
esl-seqstat Rhoto_IFO0880_4_AssemblyScaffolds_and_Mito.fasta
```

```
Format:              FASTA
Alphabet type:       DNA
Number of sequences: 31
Total # residues:    20794214
Smallest:            13957
Largest:             2216085
Average length:      670781.1
```

In [21]:

```
20794214*2/1000000
```

Out[21]:

```
41.588428
```

In [22]:

```
%%bash
cd ../../Data/Rfam
cmscan --cpu 24 -Z 41.588428 --cut_ga --rfam --nohmmonly --tblout IFO0880_4.tblout --fmt 2 --clanin Rfam.clanin Rfam.cm \
    Rhoto_IFO0880_4_AssemblyScaffolds_and_Mito.fasta > IFO0880_4.cmscan
```

In [23]:

```
%%bash
cd ../../Data/Rfam
head IFO0880_4.tblout
```

```
#idx target name          accession query name           accession clan name mdl mdl from   mdl to seq from   seq to strand trunc pass   gc  bias  score   E-value inc olp anyidx afrct1 afrct2 winidx wfrct1 wfrct2 description of target
#--- -------------------- --------- -------------------- --------- --------- --- -------- -------- -------- -------- ------ ----- ---- ---- ----- ------ --------- --- --- ------ ------ ------ ------ ------ ------ ---------------------
1    tRNA                 RF00005   scaffold_1           -         CL00001    cm        1       71   553894   553822      -    no    1 0.60   0.0   69.2   2.2e-14  !   *       -      -      -      -      -      - tRNA
2    tRNA                 RF00005   scaffold_1           -         CL00001    cm        1       71   566953   567025      +    no    1 0.60   0.0   69.2   2.2e-14  !   *       -      -      -      -      -      - tRNA
3    tRNA                 RF00005   scaffold_1           -         CL00001    cm        1       71   601576   601648      +    no    1 0.60   0.0   69.2   2.2e-14  !   *       -      -      -      -      -      - tRNA
4    tRNA                 RF00005   scaffold_1           -         CL00001    cm        1       71   606803   606875      +    no    1 0.60   0.0   69.2   2.2e-14  !   *       -      -      -      -      -      - tRNA
5    tRNA                 RF00005   scaffold_1           -         CL00001    cm        1       71   716467   716396      -    no    1 0.51   0.0   58.2   2.6e-11  !   *       -      -      -      -      -      - tRNA
6    tRNA                 RF00005   scaffold_1           -         CL00001    cm        1       71   736752   736681      -    no    1 0.51   0.0   58.2   2.6e-11  !   *       -      -      -      -      -      - tRNA
7    tRNA                 RF00005   scaffold_1           -         CL00001    cm        1       71  1928091  1928162      +    no    1 0.51   0.0   58.2   2.6e-11  !   *       -      -      -      -      -      - tRNA
8    tRNA                 RF00005   scaffold_1           -         CL00001    cm        1       71  1937324  1937395      +    no    1 0.51   0.0   58.2   2.6e-11  !   *       -      -      -      -      -      - tRNA
```

In [24]:

```
%%bash
cd ../../Data/Rfam
grep -v " = " IFO0880_4.tblout > IFO0880_4.deoverlapped.tblout
```

In [25]:

```
%%bash
cd ../../Data/Rfam
head IFO0880_4.deoverlapped.tblout
```

```
#idx target name          accession query name           accession clan name mdl mdl from   mdl to seq from   seq to strand trunc pass   gc  bias  score   E-value inc olp anyidx afrct1 afrct2 winidx wfrct1 wfrct2 description of target
#--- -------------------- --------- -------------------- --------- --------- --- -------- -------- -------- -------- ------ ----- ---- ---- ----- ------ --------- --- --- ------ ------ ------ ------ ------ ------ ---------------------
1    tRNA                 RF00005   scaffold_1           -         CL00001    cm        1       71   553894   553822      -    no    1 0.60   0.0   69.2   2.2e-14  !   *       -      -      -      -      -      - tRNA
2    tRNA                 RF00005   scaffold_1           -         CL00001    cm        1       71   566953   567025      +    no    1 0.60   0.0   69.2   2.2e-14  !   *       -      -      -      -      -      - tRNA
3    tRNA                 RF00005   scaffold_1           -         CL00001    cm        1       71   601576   601648      +    no    1 0.60   0.0   69.2   2.2e-14  !   *       -      -      -      -      -      - tRNA
4    tRNA                 RF00005   scaffold_1           -         CL00001    cm        1       71   606803   606875      +    no    1 0.60   0.0   69.2   2.2e-14  !   *       -      -      -      -      -      - tRNA
5    tRNA                 RF00005   scaffold_1           -         CL00001    cm        1       71   716467   716396      -    no    1 0.51   0.0   58.2   2.6e-11  !   *       -      -      -      -      -      - tRNA
6    tRNA                 RF00005   scaffold_1           -         CL00001    cm        1       71   736752   736681      -    no    1 0.51   0.0   58.2   2.6e-11  !   *       -      -      -      -      -      - tRNA
7    tRNA                 RF00005   scaffold_1           -         CL00001    cm        1       71  1928091  1928162      +    no    1 0.51   0.0   58.2   2.6e-11  !   *       -      -      -      -      -      - tRNA
8    tRNA                 RF00005   scaffold_1           -         CL00001    cm        1       71  1937324  1937395      +    no    1 0.51   0.0   58.2   2.6e-11  !   *       -      -      -      -      -      - tRNA
```

In [26]:

```
%%bash
cd ../../Data/Rfam
wc -l *.tblout
```

```
   201 IFO0880_4.deoverlapped.tblout
   330 IFO0880_4.tblout
   531 total
```

In [112]:

```
# Edit it in excel to make a gff
# Only keep tRNA and rRNA for BOFdat and use 'GeneID=tRNA_x' format
```

In [27]:

```
%%bash
cd ../../Data/Rfam/
head IFO0880_4_Rfam.gff3
```

```
scaffold_1	Infernal	tRNA	553822	553894	.	-	.	GeneID=tRNA_1
scaffold_1	Infernal	tRNA	566953	567025	.	+	.	GeneID=tRNA_2
scaffold_1	Infernal	tRNA	601576	601648	.	+	.	GeneID=tRNA_3
scaffold_1	Infernal	tRNA	606803	606875	.	+	.	GeneID=tRNA_4
scaffold_1	Infernal	tRNA	716396	716467	.	-	.	GeneID=tRNA_5
scaffold_1	Infernal	tRNA	736681	736752	.	-	.	GeneID=tRNA_6
scaffold_1	Infernal	tRNA	1149316	1149413	.	+	.	GeneID=tRNA_7
scaffold_1	Infernal	tRNA	1412446	1412545	.	-	.	GeneID=tRNA_8
scaffold_1	Infernal	tRNA	1513931	1514030	.	+	.	GeneID=tRNA_9
scaffold_1	Infernal	tRNA	1928091	1928162	.	+	.	GeneID=tRNA_10
```

In [28]:

```
%%bash
cd ../../Data/Rhoto_IFO0880_4/
head Rhoto_IFO0880_4_GeneCatalog_20170509.gff3
```

```
##gff-version 3
##sequence-region scaffold_1 1 2216085
scaffold_1	prediction	gene	1571	2453	0	-	.	ID=gene_1;Name=jgi.p|Rhoto_IFO0880_4|8369;portal_id=Rhoto_IFO0880_4;proteinId=8369;transcriptId=8497
scaffold_1	prediction	mRNA	1571	2453	.	-	.	ID=mRNA_1;Name=jgi.p|Rhoto_IFO0880_4|8369;Parent=gene_1;proteinId=8369;track=FrozenGeneCatalog;transcriptId=8497
scaffold_1	prediction	exon	2283	2453	.	-	.	ID=exon_1_1;Parent=mRNA_1
scaffold_1	prediction	CDS	2283	2453	.	-	0	ID=CDS_1;Parent=mRNA_1
scaffold_1	prediction	exon	1953	2165	.	-	.	ID=exon_1_2;Parent=mRNA_1
scaffold_1	prediction	CDS	1953	2165	.	-	0	ID=CDS_1;Parent=mRNA_1
scaffold_1	prediction	exon	1770	1895	.	-	.	ID=exon_1_3;Parent=mRNA_1
scaffold_1	prediction	CDS	1770	1895	.	-	0	ID=CDS_1;Parent=mRNA_1
```

In [130]:

```
%%bash
cd ../../Data/Rhoto_IFO0880_4/
grep $'\tmRNA' Rhoto_IFO0880_4_GeneCatalog_20170509.gff3 | wc -l
grep $'\tmRNA' Rhoto_IFO0880_4_GeneCatalog_20170509.gff3 | head
```

```
8490
scaffold_1	prediction	mRNA	1571	2453	.	-	.	ID=mRNA_1;Name=jgi.p|Rhoto_IFO0880_4|8369;Parent=gene_1;proteinId=8369;track=FrozenGeneCatalog;transcriptId=8497
scaffold_1	prediction	mRNA	3233	7809	.	-	.	ID=mRNA_2;Name=jgi.p|Rhoto_IFO0880_4|8370;Parent=gene_2;proteinId=8370;track=FrozenGeneCatalog;transcriptId=8498
scaffold_1	prediction	mRNA	8155	10023	.	+	.	ID=mRNA_3;Name=jgi.p|Rhoto_IFO0880_4|8371;Parent=gene_3;proteinId=8371;track=FrozenGeneCatalog;transcriptId=8499
scaffold_1	prediction	mRNA	10143	11954	.	+	.	ID=mRNA_4;Name=jgi.p|Rhoto_IFO0880_4|8372;Parent=gene_4;proteinId=8372;track=FrozenGeneCatalog;transcriptId=8500
scaffold_1	prediction	mRNA	12020	13736	.	+	.	ID=mRNA_5;Name=jgi.p|Rhoto_IFO0880_4|8373;Parent=gene_5;proteinId=8373;track=FrozenGeneCatalog;transcriptId=8501
scaffold_1	prediction	mRNA	13861	19221	.	-	.	ID=mRNA_6;Name=jgi.p|Rhoto_IFO0880_4|8374;Parent=gene_6;proteinId=8374;track=FrozenGeneCatalog;transcriptId=8502
scaffold_1	prediction	mRNA	20070	23052	.	+	.	ID=mRNA_7;Name=jgi.p|Rhoto_IFO0880_4|8375;Parent=gene_7;proteinId=8375;track=FrozenGeneCatalog;transcriptId=8503
scaffold_1	prediction	mRNA	23178	26734	.	-	.	ID=mRNA_8;Name=jgi.p|Rhoto_IFO0880_4|8376;Parent=gene_8;proteinId=8376;track=FrozenGeneCatalog;transcriptId=8504
scaffold_1	prediction	mRNA	28687	29599	.	-	.	ID=mRNA_9;Name=jgi.p|Rhoto_IFO0880_4|8377;Parent=gene_9;proteinId=8377;track=FrozenGeneCatalog;transcriptId=8505
scaffold_1	prediction	mRNA	29720	30804	.	+	.	ID=mRNA_10;Name=jgi.p|Rhoto_IFO0880_4|8378;Parent=gene_10;proteinId=8378;track=FrozenGeneCatalog;transcriptId=8506
```

In [134]:

```
%%bash
cd ../../Data/Rhoto_IFO0880_4/
grep $'\tmRNA' Rhoto_IFO0880_4_GeneCatalog_20170509.gff3 | sed 's/proteinId/GeneID/' | head
grep $'\tmRNA' Rhoto_IFO0880_4_GeneCatalog_20170509.gff3 | sed 's/proteinId/GeneID/' > IFO0880_4_JGI.gff3
mv IFO0880_4_JGI.gff3 ../Rfam/
```

```
scaffold_1	prediction	mRNA	1571	2453	.	-	.	ID=mRNA_1;Name=jgi.p|Rhoto_IFO0880_4|8369;Parent=gene_1;GeneID=8369;track=FrozenGeneCatalog;transcriptId=8497
scaffold_1	prediction	mRNA	3233	7809	.	-	.	ID=mRNA_2;Name=jgi.p|Rhoto_IFO0880_4|8370;Parent=gene_2;GeneID=8370;track=FrozenGeneCatalog;transcriptId=8498
scaffold_1	prediction	mRNA	8155	10023	.	+	.	ID=mRNA_3;Name=jgi.p|Rhoto_IFO0880_4|8371;Parent=gene_3;GeneID=8371;track=FrozenGeneCatalog;transcriptId=8499
scaffold_1	prediction	mRNA	10143	11954	.	+	.	ID=mRNA_4;Name=jgi.p|Rhoto_IFO0880_4|8372;Parent=gene_4;GeneID=8372;track=FrozenGeneCatalog;transcriptId=8500
scaffold_1	prediction	mRNA	12020	13736	.	+	.	ID=mRNA_5;Name=jgi.p|Rhoto_IFO0880_4|8373;Parent=gene_5;GeneID=8373;track=FrozenGeneCatalog;transcriptId=8501
scaffold_1	prediction	mRNA	13861	19221	.	-	.	ID=mRNA_6;Name=jgi.p|Rhoto_IFO0880_4|8374;Parent=gene_6;GeneID=8374;track=FrozenGeneCatalog;transcriptId=8502
scaffold_1	prediction	mRNA	20070	23052	.	+	.	ID=mRNA_7;Name=jgi.p|Rhoto_IFO0880_4|8375;Parent=gene_7;GeneID=8375;track=FrozenGeneCatalog;transcriptId=8503
scaffold_1	prediction	mRNA	23178	26734	.	-	.	ID=mRNA_8;Name=jgi.p|Rhoto_IFO0880_4|8376;Parent=gene_8;GeneID=8376;track=FrozenGeneCatalog;transcriptId=8504
scaffold_1	prediction	mRNA	28687	29599	.	-	.	ID=mRNA_9;Name=jgi.p|Rhoto_IFO0880_4|8377;Parent=gene_9;GeneID=8377;track=FrozenGeneCatalog;transcriptId=8505
scaffold_1	prediction	mRNA	29720	30804	.	+	.	ID=mRNA_10;Name=jgi.p|Rhoto_IFO0880_4|8378;Parent=gene_10;GeneID=8378;track=FrozenGeneCatalog;transcriptId=8506
```

In [31]:

```
%%bash
cd ../../Data/Rfam/
cat IFO0880_4_JGI.gff3 IFO0880_4_Rfam.gff3 > IFO0880_4_JGI_Rfam.gff3
```

In [32]:

```
from Bio import SeqIO
from Bio.Alphabet import generic_dna
from BCBio import GFF

gff_file = '../../Data/Rfam/IFO0880_4_JGI_Rfam.gff3'
fasta_file = '../../Data/Rfam/Rhoto_IFO0880_4_AssemblyScaffolds_and_Mito.fasta'
out_file = '../../Data/Rfam/IFO0880_4_JGI_Rfam.gb'
fasta_input = SeqIO.to_dict(SeqIO.parse(fasta_file, "fasta", generic_dna))
gff_iter = GFF.parse(gff_file, fasta_input)
SeqIO.write(gff_iter, out_file, "genbank")
```

Out[32]:

```
31
```

In [33]:

```
%%bash
cd ../../Data/Rfam/
head IFO0880_4_JGI_Rfam.gb
```

```
LOCUS       scaffold_1           2216085 bp    DNA              UNK 01-JAN-1980
DEFINITION  scaffold_1.
ACCESSION   scaffold_1
VERSION     scaffold_1
KEYWORDS    .
SOURCE      .
  ORGANISM  .
            .
FEATURES             Location/Qualifiers
     tRNA            complement(553822..553894)
```

IFO0880\_4\_JGI\_Rfam.gb is used in BOFdat to estimate RNA composition

### Mitochondrial genome annotation¶

download mitochondria protein sequences from http://megasun.bch.umontreal.ca/People/lang/FMGP/proteins.html  
Rhoto\_IFO0880\_3 has mitochondrial genes and annotation included in the genome assembly

In [66]:

```
%%bash
cd ../../Data/Mito/
head Mito_Proteins.fa
```

```
>cob Malawimonas jakobiformis
MVGMYPVPTTISYYWSFGSLAGLCLVIQIVTGILLAMHYTPNINLAFISVEHIMRDVNYG
WLLRYIHANGASMFFIVLYLHMFRGLYYGSYLYPRQMVWSIGVIIFLATMATAFMGYVLP
WGQMSFWGATVITNFFSAFPIVGESIVHWLWGGFSVDNATLNRFFSLHYFLPFIIAALSG
LHLAILHIPKANNPMGIRTLNDYLQFHPYFTVKDIFSLVIFIVIFAIFVFFYPNILGHAD
NYIQANPLVTPAHIVPEWYFLPFYAILRAIPDKLGGVLVMFAAIVILLFIPLLNISNVRS
SWFRPLFRPFFWLLVVDSLILAWIGGNTAEAPYVQIGQIATFFYFFYFLFLIPILAKIES
LINKY*

>cob Reclinomonas americana
```

In [69]:

```
%%bash
cd ../../Data/Mito/
reformat.sh in=Mito_Proteins.fa out=Mito_Proteins_Fixed.fa overwrite=true addunderscore ignorejunk
```

```
java -ea -Xmx200m -cp /users/joonhoonkim/anaconda3/opt/bbmap-38.22-0/current/ jgi.ReformatReads in=Mito_Proteins.fa out=Mito_Proteins_Fixed.fa overwrite=true addunderscore ignorejunk
Executing jgi.ReformatReads [in=Mito_Proteins.fa, out=Mito_Proteins_Fixed.fa, overwrite=true, addunderscore, ignorejunk]

Input is being processed as unpaired
Input:                  	227 reads          	73320 bases
Output:                 	227 reads (100.00%) 	73320 bases (100.00%)

Time:                         	0.233 seconds.
Reads Processed:         227 	0.97k reads/sec
Bases Processed:       73320 	0.31m bases/sec
```

In [71]:

```
%%bash
cd ../../Data/Mito/
cat Mito_Proteins_Fixed.fa
```

```
>cob_Malawimonas_jakobiformis
MVGMYPVPTTISYYWSFGSLAGLCLVIQIVTGILLAMHYTPNINLAFISVEHIMRDVNYGWLLRYIHANG
ASMFFIVLYLHMFRGLYYGSYLYPRQMVWSIGVIIFLATMATAFMGYVLPWGQMSFWGATVITNFFSAFP
IVGESIVHWLWGGFSVDNATLNRFFSLHYFLPFIIAALSGLHLAILHIPKANNPMGIRTLNDYLQFHPYF
TVKDIFSLVIFIVIFAIFVFFYPNILGHADNYIQANPLVTPAHIVPEWYFLPFYAILRAIPDKLGGVLVM
FAAIVILLFIPLLNISNVRSSWFRPLFRPFFWLLVVDSLILAWIGGNTAEAPYVQIGQIATFFYFFYFLF
LIPILAKIESLINKY*
>cob_Reclinomonas_americana_
MRLLKRPLIRELNSFIVDYPTPSNLSYWWNFGFIAAFCLVVQIATGIFLAMHYTPHVDLAFISVEHIMRD
VNYGWLMRYIHANGASMFFIAVYIHMFRGLYYGSYASPREFLWIIGVIILLIMILTAFMGYVLPWGQMSF
WGATVITNLASAVPYIGEHIVYWLWGGFSVDNATLNRFYSLHYLFPFIIAGLVGLHLIVLHEDGSNNPLG
IDSKVDKIPFYPYYYVKDLFGIVVFGLFFSVFVFFYPNLLGHPDNYIEANPLVTPPHIVPEWYFLPFYAI
LRSIPNKLLGVIAMLVSILILIALPFLNTSEIRSSQFRPIHRKLFWLFFVDCVILGWIGGNVPETPYLEI
GQIATVFYFAYFLVFIPLLGKLESYLINLDTNQYQKHLVK*
>cob_Rickettsia_prowazekii
MNKEIIHKKS NGIIEWIDYR LPIFSFLKHF SYYQTPKNLN YLWNLGSIAGIALVIQIITG VILAM
HYTPH VDHAFESVER IMRNVNYGWL LRYTHAVGASMFFAAIYLHI ARGLYYGSYK TPRELLWHIG
 IIIFLIMMAT AFMGYVLPWGQMSYWGATVI TNLFSAIPLV GEPIVIWLWG GFSVDNPTLN RFFA
LHYLFPFIIVVLVILH LVALHQHGSN NPKGIDVKST KDTIPFHPYY TVKDFVGFGVYFIIFAYFIF
 YAPNYLGHPD NYIPANPLVT PAHIVPEWYF LPFYAILRAVPSKLGGVFLM FGSIVVLFLL PWLD
TSKIRS GNYRPIYRIA FWIFMADCLFLGYLGSKPVS EPYITISRFA VCYYFCHFLL VLPLIGKYE
K PLPLPKVL*
>cob_Jakoba_libera_mt_cob
MRLFKLPYLRNINDFIIDYPTPSNLSYWWNFGFLAAVCLVIQLLTGIFLAMHYTPHVDLAFSSLEHIMRD
VNYGWLIRYAHANGASMFFIVVYVHIFRGLYYGSYHSPREFLWIIGVIIVFLMMATAFLGYVLPWGQMSF
WGATVITNFATAIPFVGESIVQWLWGGFSVDNATLNRFFSLHYLLPFAILGLVLLHLIVLHEDGSNNPLG
IHSKVDKVPFYPYYYFKDLFGIICFGLFFSFILFYYPNLLGHPDNYIEADPMVTPTHIVPEWYFLPFYAI
LRSIPDKLGGVIAMVLAIVILAFLPLLNTSQVRSSQFRPLHRKFFWLLLIDCFILGWIGGQVPETPYVEI
GRAATLFYFSYFLVIVPALGKLEQILMRLDTTKSFKEIWTQV*
>cob_Schizosaccharomyces_pombe_
MKILKSNPFLALANNYMIDAPEPSNISYFWNFGSLLACVLVIQIVIGILLACFYIPNMDLAFLSVERIVR
DVNYGFLLRAFHANGASFFFIFLYLHIGRGLYYGSYKYPRTMTWNIGVIIFLLTIITAFLGYCLPANQMS
FWGATVITNLLSAVPFIGDDLVHLLWGGFSVSNPTLNRFFSLHYLMPFVIAALSVMHLIALHTNGSSNPL
GVTANMDRIPMNPYYLIKDLITIFIFLIGINYMAFYNPYGFMEPDCALPADPLKTPMSIVPEWYLLPFYA
ILRAIPNFQLGVIAMLLSILVLLLLPLLDFSAIRGNSFNPFGKFFFWTFVADFVILAWIGGSHPENVFIT
IGAIATIFYFSYFFILIPVYTILGNTLIDLNLSSIKR*
>cob_Aspergillus_nidulans_(Emericella)_
MRILKSHPLLKIVNSYIIDSPQPANLSYLWNFGSLLALCLGIQIVTGVTLAMHYTPSVSEAFNSVEHIMR
DVNNGWLVRYLHSNTASAFFFLVYLHIGRGLYYGSYKTPRTLTWAIGTVILIVMMATAFLGYVLPYGQMS
LWGATVITNLMSAIPWIGQDIVEFIWGGFSVNNATLNRFFALHFLLPFVLAALALMHLIAMHDTVGSGNP
LGISANYDRLPFAPYFIFKDLITIFIFFIVLSIFVFFMPNALGDSENYVMANPMQTPPAIVPEWYLLPFY
AILRSIPNKLLGVIAMFAAILALMVMPITDLSKLRGVQFRPLSKVVFYIFVANFLILMQIGAKHVETPFI
EFGQISTIIYFAYFFVIVPVVSLIENTLVELGTKKNF*
>cob_Prototheca_wickerhamii_
MKRLSIIKQPILSILNDHIVDYPSPSNINYFWSLGSIAGICLVVQIATGIFLAMHYTPHIDLAFMSVEHI
MRDVEGGWLLRYMHANGASMFFIVVYIHMFRGLYYGSYTSPRELLWIVGVAILLLMIITAFIGYVLPWGQ
MSFWGATVITSLASAIPVVGNSIVTWLWGGFSVDNATLNRFFSLHYLLPFVLVGLSVVHLAALHQYGSNN
PLGTSSAVDKLALYPYFYVKDLVGWVGFAIFFSVFVYFFPNLLGHPDNYIPANPMSTPAHIVPEWYFLWV
YAILRSIPNKLAGVGAIALVFISLFSLPFLNTSPIRSNNFKPIHRKFFWLILADCFLLSWIGQQPVEDPY
IIIGQLASVFFFFYFLVVVPLTGKLEHYLIKYKS*
>cob_Marchantia_polymorpha_
MARRLSILKQPMFSTFNNHLMDYPTPSNMSYWWGFGSLAGLCLVIQMLTGVFLAMHYTPHVDLAFLSVEH
IMRDVKGGWLLRYMHANGASMFFIVVYLHFFRGLYYGSYASPRELVWCLGVVILLLMIVTAFMGYVLPWG
QMSFWGATVITSLASAMPVVGDTMVTWLWGGFSVDNATLNRFFSLHYLLPFMMAGASILHLAALHQYGSN
NPLGINSSVDKMAFYPYIYVKDLVGWVAFAIFFSIFVFYAPNVLGHPDNYMPANPMSTPAHMVPEWYFLP
VYAILRSMPNKLGGVAAMGLVFVSLLALPFINTSYVRSSSFRPIHQKFFWLLVADCLLLGWIGCQPVEAP
YVTIGQIASVGFFFYFAMTPILGKCEARLIKNSNACEARSVLASFLTSIGLLWW*
>cob_Porphyra_purpurea
MRLIKKPLFNIVNNHFIDYPTPINIHYAwNFGFLSAMCLIVQIITGIFLAMHYTPHVDLAFISVEHIMRD
VNFGwLLRYTHANGASMFFIVVYIHIFRGLYYGSYTSPRQFVwVIGVIILLLMIITAFIGYVLPwGQMSL
wGATVITNLVSAVPLVGDSIVAwLwGGFSVDNATLNRFFSLHYLLPFVIAAASLIHLAALHQEGSSNPLG
IDASGDKIPMYPYFIVKDLLGIVSFIIFFSFFVYFSPNLLGHPDNYIEANPMVTPAHIVPEWYFLPFYAI
LRSIPHKLGGVICMIFAIAILAFLPwIHSTEIRSSRFRPIYRVLYwSMVACCLILGwIGGMPVEDPYIII
GQIASVYYFTYFLIILPTLGSVEKFLLSYIVN*
>cob_Chondrus_crispus
MRFIKRPLISIVNDHLIDYPTPINIHYAWNFGFLSSICLIVQILTGIFLAMHYTPHVDLAFASVEHIMRD
VNYGWLLRYIHTNGASMFFIVVYIHIFRGLYFGSYIKPRHWVWVIGVLILLLMILTAFIGYVLPWGQMSL
WGATVITNLVSAVPFIGDSIVTWLWGGFSVDNATLNRFFSLHYLMPFVIAAVSLVHLAILHQDGSGNPLG
IDSNVDKVSMFPYFIVKDFLGMVIFIIFFSIFVYFSPNVLGHPDNYIEANPMVTPAHIVPEWYFLPFYAI
LRSIPHKLGGVTAMISAIAILAFLPWIHSTEIRSSRFRPLYRLFYWVMISCCLILGWIGGMPVENPYVII
GQIASIYYFIYFIILLPVLGRIEKFLLEFKI*
>cob_Phytophthora_infestans
MRWNKKSLFAVINNHLIDYPTPINLNYFFGFGSLAGIMLVVQILTGIFLAMHYTPHIDLAFNSVEHIMRD
VNNGWLIRYTHANGASFFFIVVYIHIFRGLYYGSYITPREAIWCSGVIIFILMMATAFMGYVLPWGQMSF
WGATVITNLFSAIPLIGKDIVDWLWGGFAVDNPTLNRFFSLHFTLPFIIVGAVLVHLILLHEVGSNNPLG
ITLKTENIPFYPYFYTKDLFGLMVLFLVFFIFVFYYPNTLGHPDNYIEANPMKTPLHIVPEWYFLPFYAI
LRSIPNKIGGVVAMFGSLIILLTIPFTNSSEIRSTAFRPIFKVCYWLLVIAFLILGWIGQCPVEYPYTEI
GIISMIYYFFFFIIIIPFLGKFEAYLVRYSINK*
>cob_Ochromonas_danica
MLKNFRWNRDYLLSFIDSHIIDYPTPINLNYFWSFGSTAGICLVIQILTGIFLAMHYCAEIDLAFESVEH
IMTDVNNGWLIRYIHANGASMFFIVVYCHIFRGLYYGSYIAPRGRLWATGVIIFLLMMATAFMGYVLPWG
QMSFWGATVITNLFSAIPYVGGSIVEWLWGGFSVGNATLNRFFSLHYFLPFIIAAFTIIHLSLLHKDGSN
NPLGINSNVDYIPFYPYFYVKDLFSFFIFAFIFSFFVFFYPNVLGHSDNYIPANPMVTPAHIVPEWYFLP
FYAILRSIPDKLGGVVAMVGAILVLLLLPVINTSFIRSAKFRPLYAFSYWFFVSDFILLGWIGQKPVESP
FIEVGMGATFFYFIFLLVLVPLIGILETFLLNNNDGLIEIEENAVTDFYFGQDPLDEEDTIDYDGRKFVE
KHSKLWDVEDPFEIEDYSKLTKVIFYL*
>cob_Cafeteria_roenbergensis
MQSIMLRLRQNSLFNVYVNHAEWYPSPANLNYFWGFGSIALGVLVSQIVSGVLLAMHYNANINLAFNDVE
HIMRDVVGGDGLRYLHANGASMFFISVYVHIGRGLFYGSYFYPRITLWYSGVILFFLMMAAGFLGYVLPW
GQMSFWGATVITNFFSAIPVVGGAIVEWLWGGFSVDQPTLNRFFSLHFLIPFLLAGVAIIHIALLHEHGS
NNPLGINENIKKIPFLTYYGIKDLFGIMLFIVFFVYFLVNCPNLLGHADNYIEALPLVTPAHIVPEWYFL
PFYAILRTIPDKLGGVIAMVVAILILLSLPFLDTSKIRSGLFKPLHFINFILFICVTLCLGWLGQSVVEF
PFSNLSEVVTILYFFYFISHFRLTTQYVK*
>cob_Schizophyllum_commune_
MRLLKSNSLLRLVNSYLVDSPQPGNLSYMWNFGSLLAVCLVIQILTGCFLAMHYTAHVDLAFNSVEHIMR
DVNNGWLIRYTHANVASFFFIFVYCHIARGLYYGSFKEPRTLTWSIGVIILILMMAIAFLGYVLPFGQMS
LWGATVITNLMSAIPVYGQDIVELIwGGFSVSNATLNRFFSLHYILPFLLAALAVAHMIALHVHGSGNPN
GLNAGDDRFPMHPYFIFKDLVTIFAFLLVLSIFVCFYPNALGHSDNYIPANPMVTPASIVPEWYLLPFYA
ILRSIPNKLLGVIAMFGSLLILLVLPLTDLSRVRGNQFRPLMKLSFWFFVVDFIILMWIGAEHPASPYLE
VGQIATAFYFAWFVFIVPAVGLFENTMADINKL*
>cob_Rhizopus_stolonifer_
MKLLKSHPFLSLANSYVIDSPQPSNLNYAWNFGSLLALCLGIQIVTGVTLAMHYTPNIDLAFISVEHIMR
DVNYGWMIRYLHANTASFFFLFVYLHIGRGLYYGSYKSPRALPWSIGVIILILMMATAFLGYVLPWGQMS
LWGATVITNLLSAIPWIGKDLVEFIWGGFSVDNATLNRFFSLHYLLPFILAALAVMHLLALHEHGSSNPL
GITANADRLYMHPYYTFKDLVTIFLFFLVLALFLFYAPNKLGHPDNYIPANPMQTPASIVPEWYLLPFYA
ILRSIPDKLGGVIAMFGSLLILLAMPLLDLSRVRGSAFRPLMKFFFWLLVVDFLILLWCGSQHVEEPFIT
LGQFATTFYFSWFLIIVPVVSVIENTLIDLATENKS*
>cob_Allomyces_macrogynus
MRFLKSHPVLSLANSFLIDSPLPSNITYLWNFGSLLGLCLVIQIVTGVTLAMHYAPSTSLAFVSVEHIMR
DVFYGWLIRYAHANGASFFFICVYIHMARGLYFNSYTKPRVLLWSVGVVIYILMMATAFLGYVLPWGQMS
FWGATVITNLLSAIPYIGTALVEFVWGGFSVDNATLNRFFSLHYLLPFILAALVVVHLIALHEHGSNNPL
GISSKVDRLPFHPYFTYKDLVGFFVFFLIFFGFVFYQPNLMGHPDNYIPANPLVTPVSIVPEWYFLPFYA
ILRAIPSKLGGVIGMFGALLILLALPWLETSKVRGAGFRPVMKFFFWLFVVNFFLLMYCGGQHAEEPFIT
LSRICTAYYFMYFLVIIPVIGHLENLLHRCRD*
>cob_Spizellomyces_punctatus
MKLTKRNPIlVlVNDFVIDSPLPTNlTYFWNFGSLLGVNlVILIISGLTLAMHYTPNTLLAFSSVEHIMR
DVNNGWLLRYIHANGASFFFIWVYLHIGRNLYYGSYRSPRGLLWSIGVVIFILMMATAFIGYVLPWGQMS
FWGATVITNLLSAIPWIGTDFVLFVWGGFSVDNATLNRFFSLHYLLPFILAALVVMHLLALHQDGSNNPE
GISSSSDRLRFHPYFTSKDlVGFVWMAILLSIFVFFAPYYLGHPDNSIPANPLVTPHSIVPEWYFLPFYA
ILRAIPSKLGGVIAMFGALLILFPLALIHTNNVRSNRYRPLLNLLFWIFVFNFFVLLWVGAKPIAQPYIL
IGQISTIIYFLYFVILMILGCIMTYIYTKSNCFFIYFFLYFGCSIL*
>cob_C.elegans
MKINNRLLNFVNGMLVTLPSRKTLTLRWNFGSMLGMVLIFQILTGTFLAFYYTPDRLMAFSTVQYIMYEV
NFGWVFRIFHFNGARLFFIFLYLHIFKGLFFMRYRLKKVWMSGLTIYLLVMMEAFMGYVLVWAQMRFWAA
VVITRTLRVIPIWGPTIVTWIWRGFGVTGATLKFFFVLHFLLPWAITVIVLGHLIFLHRTGRTSRLYCHG
DYDKVCFRPEYLGKDAYNIVIWLLFIVLRLIYPFNLGDAEMFIEADPMMRPVHIVPEWYFLFAYAILRAI
PNKVLGVIALLMRIVTFYFFALVNNYTSCLTKLNKFLVFMFIISSTILRWTGQCTVEDPFTILRPLFSFI
YFGLAYLMLFIFMRSKLLFK*
>cob_Drosophila_melanogaster
MNKPLRNSHPLFKIANNALVDLPAPINISSWWNFGSLLGLCLIIQILTGLFLAMHYTADINLAFYSVNHI
CRDVNYGWLLRTLHANGASFFFICIYLHVGRGIYYGSYKFTPTWLIGVIILFLVMGTAFMGYVLPWGQMS
FWVATVITNLLYAIPYLGMDLVQWLWGGFAVDNATLTRFFTFHFILPFIVLAMTMIHLLFLHQTGSNNPI
GLNSNIDKIPFHPYFTFKDIVGFIVMIFILISLVLISPNLLGDPDNFIPATPLVTPAHIQPEWYFLFAYA
ILRSIPNKLGGVIALVLSIAILMILPFYNLSKFRGIQFYPINQVMFWSMLVTVILLTWIGARPVEEPYVL
IGQILTVVYFLYYLVNPLITKWWDNLLN*
>cob_human
MTPMRKINPLMKLINRSFIDLPTPSNISAWWNFGSLLGTCLILQITTGLFLAMHYSPDASTAFSSIAHIT
RDVNYGWIIRYLHANGASMFFICLFLHIGRGLYYGSFLYSETWNIGIILLLATMATAFMGYVLPWGQMSF
WGATVITNLLSAIPYIGADLVQWIWGGYSVDSPTLTRFFTFHFILPFIIAALAALHLLFLHETGSNNPLG
ITSHSDKITFHPYYTIKDALGLLLFLLSLMTLTLFSPDLLGDPDNYTLANPLNTPPHIKPEWYFLFAYTI
LRSVPNKLGGVLALLLSILILAMIPILHMSKQQSMMFRPLSQSLYWLLAADLLILTWIGGQPVSYPFTII
GQVASVLYFTTILILMPTISLIENKMLKWA*
>cob_Xenopus_laevis
MAPNIRKSHPLIKIINNSFIDLPTPSNISSLWNFGSLLGVCLIAQIITGLFLAMHYTADTSMAFSSVAHI
CFDVNYGLLIRNLHANGLSFFFICIYLHIGRGLYYGSFLYKETWNIGVILLFLVMATAFVGYVLPWGQMS
FWGATVITNLLSAKPYIGNVLVQWSLGGFSVDNATLTRFFAFHFLLPFIIAGASILHLLFLHETGSTNPT
GLNSDPDKVPFHPYFSYKDLLGFLIMLTALTLLAMFSPNLLGDPDNFTPANPLITPPHIKPEWYFLFAYA
ILRSMNKLGGVLALVLSILILALMPLLHTSKQRSLMFRPFTQIMFWALVADTLILTWIGGQPVEDPYTMI
GQLASVIYFSIFIIMFPLMGWVENKLLNW*
>cob_sea_urchin_(Paracentrotus_lividus)
MLGPLRKEHPIFRILNSTFVDLPLPSNLSIWWNFGSLLGLCLITQILTGLFLAMHYTADISLAFSSASHI
CRDVNYGWLLRNVHANGASLFFICMYCHIGRGLYYGGSNKIETWNVGVILFLVTVLTAFVGYVLVWGRMS
FWAATVIANLVTAVPCVGTTIVQWLWGGFSVDNATLTRFFAFHFLFPFIIAALAIIDLVFLHNSGANNPV
GLNSNYDKAPFHIYYTTKDTVGFIALIAALFVLALLFPCALNDPENFIPANPLSHPPHIQPEWYFLFAYA
ILRSIPNKLGGVIALVAAILVLFLMPLLNTSKNESNSFRPLSQATFWILVATFFVLTWIGSQPVEQPFVL
IGQIASLLYFSLFIFGFPLVSSLENKMIFS*
>cox1_Rickettsia_prowazekii_
MNSLTNIEDL NCDNQHIPNG FRRWIFSTNH KDIGIMYIIF AIFAGVVGGLFSLLFRLELA MPGGT
FLNHN FQLYNVLITV HAIIMVFFMI MPALFSGFGNYFVPLLIGAP DMAFPRLNNI SFWLLIPAFL
 LLISSTFIDG GPGTGWTLYPPLSNLNGHTG AAVDVAIFSL HLTGLSSILG SINLIVTIFN MRTP
GMGLFKMPLFVWSILV TAFLIILAMP VLSGAITMLL TDRNFGTTFF KPDGGGDPLLFQHLFWFFGH
 PEVYIVILPG FGIVSQVIST FSRKPIFGYQ GMVGAMVIIGFVGFIVWAHH MFTVGLSYNA LIYF
TAGTMI IAVPTGIKIF SWIATMWGGSITFPTPMLFA IGFIILFTIG GVTGIILSNS ALDRVLHDT
Y YVVAHFHYTMSLGALFTAFA GFYYWFGKIS GKQYPEILGK IHFWITFIGV NLTFFPQHFLGLAG
MPRRIP DYPEAFAGWN MVSSIGAGIS IFAAFYFVFI VFYTLKYGKNCTANPWGDGA DTLEWKLNS
P PPFHTFETPP HIVE*
>cox1_Reclinomonas_americana
MANSFVKRWFFSTNHKDIGALYIMFGTFAGITATTISVVMRLELGLPGNQILQGNHQLYNVLITAHGLLM
LFMVVMPILLGGFGNFFVPLLIGAPDMAFPRLNNISFWLLPPALLLLFFSALVEVGAGTGWTAYPPLSGI
QSHSGASVDLAIFSLHLSGASSVLASINFITTIFNMRAPGMTMHRMPLFVWSILVTSFLLVFALPVLAGG
ITMLLTDRNFNTTFFDPAGGGDPVLFQHLFWFFGHPEVYILVIPGFGVVSHVISAFSRRPIFGYLGMVYA
MSSIGVLGFIVWAHHMYTVGMDVDTRAYFTAATMVIAIPTGIKIFSWLATMWGGSIELKAPMLFAVGFVF
LFTFGGLTGVVLSNSGLDIALHDTYYVVAHFHYVLSMGAMFAIYAAFYYWFGKITGYQYPEKLAQVQFWT
TFIGVNLTFFPMHFLGLSGMPRRIPDYPDAFSGWNAVSSYGSLVTTFSIILWFYIVYRTLTDGVKCGNDP
WGLAVGEPGKEHFATLEWTLTSPPLSHTFEEVPYIKETIKK*
>cox1_Jakoba_libera_mt_cox1_;_530_aa
MTGLTRWLYSTNHKDIGVMYIVTGAFSGILAAMISVVMRMELGLPGNQILEGNHQLYNVLITAHGLLMLF
WVLMPVLIGGFGNFFVPLLIGAPDMAFPRLNNISYWLLPASLLLLFFSAMVETGAGTGWTAYPPLSGIQS
HSGASVDLAIFSLHVSGTSSILASINFITTMLNMRAPGMNMHKMPLFCWAVFLSSFLLLLSLPVFAGGIT
MLLTDRNFNTTFFDPAGGGDPVLFQHLFWFFGHPEVYVLILPGFGIVSHIVSTFSKKPVFGHLGMVYAMC
SIGVLGSIVWAHHMYTVGLDVDTRAYFSAATMVIGVPTGIKVFSWLATLWGGSIELKTPMLYALGFIFLF
TIGGVTGIVLSNAAIDVAMHDTYYVVGHFHYVLSLGAVFSMFAAFYYWIGKMTGYQYPETLGKLQFWLMF
FGVNLTFTPMHFMGLAGMPRRIPDYPDAFAGWNLISTYGSYLSSISVLLWLYIVYRTLTDGVKCSNNPWA
WNDTSTNQTNYFTLEWTLSSPPQVHTFEEVPYVRETITKH*
>cox1_Malawimonas_jakobiformis
MTWLQRWVFSTNHKDIGTLYLIFGIFSGVIGTVFSIIIRLELAFPGNQILNGNHQLYNVIITAHGLLMVF
FSVTPALIGGFGNWLVPILIGAPDMASPRSNNISSWLLPPSLLLLLSSSLIEVGAGTGWTVYPPLSSIQS
HSGPSVDLAIFSLHLSGAGSILGAVNFITTIFNMRAPGLTMNRLPLFVWALLITAFLILLSLPVFAGAIT
MLLTDRNFNTTFYDPAGGGDPVLYQHLFWFLGHPEVHILIVPGFGIISHVIVKYSSKKSIFGYLGMVYAM
VSIGILGSIAWAHHMYTVGMDIDTRAYFTAATMIIAVPTGIKIFSWLATMWRGILVYTTPMLFAIGFIFL
FTMGGLTGVVLSNAALDVALHDTYYVVGHFHYVPSMGAVFAMFSGFYHWIYKMSGYKYPEFIGKIHFWLT
FIGVNVTFFPMHFLGLAGMPRRIPDYPDAFYLWNSVASFGSQITFVATLIFFYGVYVAFKSKIKH*
>cox1_Cafeteria_roenbergensis
MANSTSMSFSSSFRSFVMRwLFSSNHKDIGILYLIFGAFAAVIAVALSLLIRMELAAPGDQIFQGNYQLY
NVAITEHGLIMLFFVVFPILGGGFGNYFVPLMLGAPDMAFPRLNNISFwLLPRLIAFLLLSSLVESGAGT
GwTVYPPLSSIQGHSGPSVDLAIFSLHVSGASSVIGSINFIVTIFNMRAPGMFMHKIPLFAwAVLITAFL
LVISLPVVAGAITMLLTDRNLILLLDPAGGEDPNLYQHLFwFFGHPEVYILVLPAFGIISQILSRNARKP
IFGYLGMVYAIISIGILGFLVwAHHMYTVGLDVDTRAYFTAATMIIGVPTGIKIFSwIATIwGGSLDLNK
TSSLwALGFLILFSLGGITGIALANAGLDIAFHDTYYVVGHFHYVLSMGAVFGIFGGFYFwFEKIFGLSY
PEIFGKIHFwLTFLGVNITFFPMHFLGLAGMPRRIPDYPDAYAGwNFIASVGSYISIIAFLLFFVQTYLV
FFSKPEDQAFGIEEAKKNASVEKILF*
>cox1_Ochromonas_danica
MFNIHEILSKLKISETFDRLKTNENFSIIKLSEKIDTLTIDENLSKLSSRWLFSTNHKDIGVLYLIFGAF
SGVLGTMMSMLIRMELSQPGSLILNGNYQLYNVLVTGHAFIMVFFMVMPVLMGGFGNFFLPILIGAPDMA
FPRLNNISFWLLPPSLLLLLSSALVEAGAGTGWTVYPPLAGIQAHSGPSIDLAIFSLHLTGISSILGSIN
FITTTFNMRAPGLHMHRIPLFVWSMLITSFLLIISLPVFGGSITMLLTDRNFNTSFYDPAGGGDPILYQH
LFWFFGHPEVYILILPAFGIISQIIATYAKKPVFGYLGMVYAMMSIGFLGFIVWAHHMFTVGLDVDTRAY
FTAATMIIAVPTGIKIFSWIATLWGGSIKFETPMLFAIGFLFLFTIGGLTGILLSNSGINMMVHDTYYVV
GHFHYVLSMGAVFGVFAGWYYWIEKITGLKYPELLGEIHFWIFFVGVNLTFFPMHFLGLSGMPRRIPDYP
DAYAGWNSVSSFGSYVSLLSILFFFYVVYVTLTEKNNK*
>cox1_Phytophthora_infestans
MNFQNINKWSTRWLFSTNHKDIGTLYLIFSAFAGVVGTTFSLLIRMELAQPGNQIFMGNHQLYNVVVTAH
AFIMVFFLVMPALIGGFGNWFIPLMIGAPDMAFPRMNNISFWLLPPSLLLLVSSAIVESGAGTGWTVYPP
LSSVQAHSGPSVDLAIFSLHLSGISSLLGAINFISTIYNMRAPGLSFHRLPLFVWSILITAFLLLLTLPV
LAGAITMLLTDRNLNTSFYDPSGGGDPVLYQHLFWFFGHPEVYVLILPAFGIISQVSASFAKKNVFGYLG
MVYAMLSIGLLGSIVWAHHMFTVGLDVDTRAYFSAATMIIAVPTGIKIFSWLATLWGGSLKFETPLLFVL
GFILLFVMGGVTGVAMSNSGLDIALHDTYYIVGHFHYVLSMGAVFGIFTGFYFWIGKISGRRYPEILGQI
HFWLFFIGVNVTFFPMHFLGLAGMPRRIPDFPDAMSGWNAVSSFGSYISFFSALFFFYIVYVTLVHGKKI
EN*                                                            
>cox1_Prototheca_wickerhamii_
MVTRWLYSTNHKDIGTMYLIFGAFSGVLGTVFSLLIRMELAQPGNQILNGNHQLYNVIITAHAFLMIFFM
LMPALMGGFGNWFLPILIGAPDMAFPRLNNISFWLLPPSLLLLVSSALVEVGAGTGWTVYPPLASIASHS
GGSVDLAIFSLHLAGVSSILGAINFICTVFNMRAPGMSMHRLPLFVWAVFITAWLLLLCLPVLAGGITML
LTDRNFNTSFFDPAGGGDPILYQHLFWFFGHPEVYILIIPGFGIISHVIATFSKKPIFGYLGMVYAMCSI
GILGFIVWAHHMYVVGLDIDTRAYFTAATMIIAVPTGIKIFSWVATMWGGSIELRTPMLFAVGFLFLFTV
GGLTGVVLANSGLDVAFHDTYYVVAHFHYVLSMGAVFALFSGFYYWIGKITGLQYPETLGQIHFWLMFLG
VNITFFPMHFLGLAGMPRRIPDYPDCYAGWNAVASYGSYLSITAVLFFFYVVYKTLTSNEVCPRNPWETT
PGVSPTLEWMLPSPPAFHTFEEIQV*                         
>cox1_wheat_
MTNMVRWLFSTNHKDIGTLYFIFGAIAGVMGTCFSVLIRMELARPGDQILGGNHQLYNVLITAHAFLMIF
FMVMPAMIGGFGNWFVPILIGAPDMAFPRLNNISFWLLPPSLLLLLSSALVEVGSGTGWTVYPPLSGITS
HSGGAVDLAIFSLHLSGISSILGSINFITTIFNMRGPGMTMHRLPLFVWSVLVTAFLLLLSLPVLAGAIT
MLLTDRNFNTTFFDPAGGGDPILYQHLFWFFGHPEVYILILPGFGIISHIVSTFSRKPVFGYLGMVYAMI
SIGVLGFLVWAHHMFTVGLDVDTRAYFTAATMIIAVPTGIKIFSWIATMWGGSIQYKTPMLFAVGFIFLF
TIGGLTGIVLANSGLDIALHDTYYVVAHFHYVLSMGAVFALFAGFYYWVGKIFGWTYPETLGQIHFWITF
FGVNLTFFPMHFLGLSGMPRRIPDYPDAYAGWNALSSFGSYISVVGIRRFFVVVAITSSSGKNQKCAESP
WAVEQNPTTLEWLVQSPPAFHTFGELPAVKETKS*                  
>cox1_Pichia_canadensis_(Hansenula_wingei)
MYIQRWLYSTNAKDMAMLYFMFAMFSGVMGSTMSLIIRLELAAPGNQMLHGNHQLFNVLVVGHALLMIFF
LVMPGLVGGFGNYMLPLLIGASDMSFARLNNMSFWLLPPALVCLVASTLIESWAGTGWTIYPPLSGMQAH
SSPSVDLGIFAIHLTSMSSLLGAINFMATSYNMRTNGMSYSKMPLFVWAIMITAVMLLLSLPVLTAGVTM
LLMDRNFNTSFFEVAGGGDPVLYQHLFWFFGHPEVYILIVPGFGIISHIVSTYSKKPVFGEMSMVYAMAS
IAFLGFLVWSHHMYIVGLDADTRAYFTSSTMVIAVPTGIKMFSWLATLYGGSIRLAVPMTYAIAFLFLFT
MGGLTGVALANASLDVAFHDTYYVVGHFHYVLSMGAIFSLFAGYYYWSPQILGLYFNERLAQMQFWLIFV
GANVIFMPMHFLGTQGMPRRMPDYPDAYAGWNYVSSMGSVMAIMSLALFIYIMYDQLINGLTNKMDNKSV
VYSKAPDFVESNTMFANNSIKSASIEFLLNSPPAMHSFNTPAVQS*                     
>cox1_Porphyra_purpurea
MQKSLNNWIFRWIYSTNHKDIGTLYLIFGAFSGVLGACASILIRMELAQPGNQLLLGNHQVYNVLVTEHA
FLMIFFMVMPVLIGGFGNWFVPIMIGAPDMRFPRLNNISFWLLPPSLCLLLGSAMVEVGAGTGWTLYPPL
SSIQSHSGGAVDLAIFSLHLSGASSVLGAINFITTIFNMRNPGQSMYRIPLFVWSILITAFLLLLAVPVL
AGAITMLLTDRNFNTTFFDPSGGGDPVLYQHLFWFFGHPEVYILILPGFGIVSHIVSTFSRKPVFGYIGM
IYAMLSIGILGFIVWAHHMYTVGLDVDTRAYFTAATMIIAVPTGIKIFSWVATMWEGSIFLKTPMLFAIG
FIFLFTIGGLTGIILANSGLDISLHDTYYVVAHFHYVLSMGAVFAIFAGFYYWFEKISGFQYSEILGQIH
FWGTFIGVNLTFFPMHFLGLAGMPRRIPDYPDSYAGWNTIASYGSYVALFSTLFFFYLVFNTLVTPRKVP
ARNNPWNFEDSKIGSTTLEWEISSPPAYHTFNEIPLVRETEISLKIKLIYHKKKNTLLLSG*
>cox1_Chondrus_crispus
MQSFFTQWISRWIFSTNHKDIGTLYLIFGAFSGVLGGCMSMLIRMELAQPSNHLLLGNHQIYNVLITAHA
FLMIFFMVMPVMIGGFGNWLVPIMIGSPDMAFPRLNNISFWLLPPSLCLLLMSALVEVGVGTGWTVYPPL
SSIQSHSGGAVDLAIFSLHISGASSILGAVNFISTILNMRSPGQSMYRIPLFVWSILVTAFLLLLAVPVL
AGAITMLLTDRNFNTSFFDASGGGDPILYQHLFWFFGHPEVYILILPGFGMISHIVSTFS RKPVFGYIG
MVYAMVSIGVLGFIVWAHHMYTVGLDVDTRAYFTAATMIIAVPTGIKIFSWIATIWEGSIHLK TPMLFA
IGFIFLFTIGGLTGIVLANSGLDISLHDTYYVVAHFHYVLSMGAVFAIFAGFYYWFGKITGLQYPETLGQ
IHFWSTFIGVNLTFMPMHFLGLAGMPRRIPDYPDAYAGWNLIASYGSYIALFSTLFFFYIVFVSLTSNNP
CTNFPWEFNKSKTYGVSTLEWIVTSPPAYHTFEEMPLIYETKSN*                         
  
>cox1_Podospora__
MSGGVSLWIERWMLSTNAKDIGNLYLIFALFSGLLGTAFSVLIRMELSGPSVQYIADN QLYNSIITAHA
LLMIFFMVMPALIGGFGNFLLPLLVGGPDMAFPRLNNISFWLLPPSLILLVFSACIEGGAGTGWTIYPPL
SGVQSHSGPSVDLAIFALHLSGVSSLLGAMNFITTIMNMRTPSIRLHKLALFGWAVIITAVLLLLSLPVL
AGAITMLLTDRNFNTSFFETAGGGDPILFQHLFWFFGHPEVYILIIPAFGIISTTISAYS NKSVFGYIG
MVYAMMSIGILGFIVWSHHMYTVGLDVDTRAYFTAATLIIAVPTGIKIFSWLATCYGGSIRLT PSMLFA
LGFVFMFTIGGLSGVVLANASLDIAFHDTYYVVAHFHYVLSMGAVFAMFSGWYFWIPKMLGLNYNMTLSK
VQFWILFIGVNVTFFPQHFLGLQGMPRRISDYPDAFAGWNLISSFGSIISVVAAWLFLYIVYLQLVEG E
YAGRFPWLNPQFYTDTLQALLNRSYPSLEWALSSPPKPHAFVSLPLQSNILRSLF*              
  
>cox1_Schizophyllum_commune
MRWLMSTNAKEIGTLYLIYSLFAGMIGTAFSVLIRLELSAPGVQFLQGDHQLFNVIITAHAFLMIFFMVM
PGLIGGFGNYFLPIHCGAIDMAFPRLNNISFWLLVPSLLLLLLSALVGNGAGTGWTVYPPLSGIQSHSGP
SVDLAIFSLHLSGVSSLLGAINFITTALNMRTNGMGLHKLPLFVWAIFVTAILLLLSLPVLAGGITMLLT
DRNFNTSFFDPAGGGDPILYQHLFWFFGHPEVYIIIIPGFGVISHVVSTFS GKTIFGYLGMVYAMFSIG
ILGFLVWSHHMFAVGMDVDSRAYFTAATMVIAVPTGIKIFSWLATLFGGSLRFN TPLVFAMGFLALFTI
GGLTGVMLSNASLDVAFHDTYYVVAHFHYVLSMGAVFALFAGFYFWMPKIVGKTYSEFLSNVHFWTLFVG
VNLTFFPQHFLGMSGMPRRIPDAFTGWNLVSSIGSIISVVSVIIFGYIIYDMFAYQGLVSNNPWEVPSYF
VDMELFENDEQGAGSLEWSFDTPIAEHAYNMQPVQS*                         
>cox1_Spizellomyces_punctatus
MAWINRWLLSTNAKDIGTLYLIFGGFAGLIGSALSIIIRLELSAGGKVYLMGNYDQYNVVVTAHGVVMIF
FLVMPALIGGFGNWLLPVMVGSPDMAFPRLNNISFWLLPPSLILLLLVYLVSGGMGTGWTIYPPLSDTPY
HMGPAVDLGILSLHIAGVSSLMGAINLITTTINCRAPGMSFEKLPLFVWSVFITAWLLLLSLPVLAGAIT
MLLTDRNLNTSFYDPNGGGDPLLYQHLFWFFGHPEGIYLILPGFGIISHIVSRFS IKPVFGYIGMVYAM
LSIGLLGFIVWSHHMYAVGLDVDTRAYFTAATMIIALPTGIKVFSWLATLYGGQLHFY TPFMFALAFIL
LFTFGGFTGVILANASIDLALHDTYYVVAHFHYVLSMGAIYALLAAFYYWIGKITGFQYNEKWGLIHFVV
FTIAINIVFFPMHFLGIAGMPRRIPDFADGYASWNSIMTIGSFLTVISIFIFLYIVSFTVFNPKAYTLDA
TYKARWSVI*                                                            
>cox1_Aspergillus_nidulans_(Emericella)_
LIIDLNTNNVLGKKFSTSTKKENIKQIESSSFLTFKQPTEWQERWYLSSNAKDIGTLYLMFALFSGLLGT
AFSVLIRLELSGPGVQYIADN QLYNSIITAHAIMMIFFMVMPALIGGFGNFLLPLLVGGPDMAFPRLNN
ISFWLLVPSLLLFVFSATIENGAGTGWTLYPPLSGIQSHSGPSVDLAIFGLHLSGISSMLGAMNFITTIL
NMRSPGIRLHKLALFGWAVIITAVLLLLSLPVLAGGITMVLTDRNFNTSFFEVAGGGDPILFQHLFWFFG
HPEVYILIIPGFGIISTVIAAGS GKNVFGYLGMVYAMMSIGVLGFLVWSHHMYTVGLDVDTRAYFTAAT
LIIAVPTGIKIFSWLATCYGGSLHLT PPMLFALGFVVLFTIGGLSGVVLANASLDVAFHDTYYVVAHFH
YVLSMGAVFALFSGWYLWIPKLLGLSYDQFAAKVHFWILFIGVNLTFFPQHFLGLQLMPRRISDYPDAFY
GWNLLSSIGSIISVVATWYFLTIIYKQLTEG KAVSRYPWLTPQLFSDTFQVLFTRNNSSLEWCLTSPPK
PHAFASLPLQS*                       
>cox1__Rhizopus_stolonifer_
MVRWLFSTNAKDIGTLYIIFSIFAGMIGTAFSMLIRLELAGPGIQYLHGDHQLYNVIVTAHAFVMIFFLV
MPAMIGGFGNWFVPLMIGAPDMAFPRLNNISFWLLPPSLILLVASAFVENGAGTGWTVYPPLSGIASHSG
GSVDLAIFSLHLSGISSMLGAMNFITTILNMRAPGMSFHKMPLFVWAVLITAVLLLLSLPVLAGGITMLL
TDRNFNTSFYEPAGGGDPLLYQHLFWFFGHPEVYILIIPGFGIVSHVVSTFCGKPIFGYLGMVYAMLSIG
VLGFIVWSHHMYTVGLDVDTRAYFTAATMIIAVPTGIKIFSWLATLYGGSIRFTTPMLFALGFLALFTIG
GLTGVMLANASMDVALHDTYYVVAHFHYVLSMGAVFAMFAAFYYWIGKITGKTYNELLGQIHFWSLFIGV
NLTFFPQHMLGLAGMPRRIPDYPDAYAGWNLVSSFGSIISIVASLVFLYALYDLLARQEYNLANNYWYVP
QFFSSSRAIGATSTASTLEWSLTSPPSFHTVNSLPVQS*                     
>cox1_Allomyces_macrogynus_
MFQRNTVYRWLFSTNAKDIGTLYLVFSIFAGMIGTAFSVLIRFELAGPGVQYLYGDHQLYNVIITAHAFI
MIFFLVMPAMLGGFGNYFVPIMIGAPDMAFPRLNNISFWLLPPSLILLVGSAFVEQGAGTGWTVYPPLSS
IGFHSGGSVDLAIFSLHLAGISSMLGSINFITTILNMRAPGMTMHKLPLFVWSILITAILLLLSLPVLAG
AITMLLTDRNLNTTFYDPAGGGDPVLYQHLFWFFGHPEVYIIIIPGFGIISQVISTFS RKPIFGYLGMV
YAMASIGILGFIVWSHHMYTVGLDVDTRAYFTAATMIIAVPTGIKIFSWLATLYGGNILYR TPAYFALG
FLFLFTIGGVTGVMLANASLDVALHDTYYVVAHFHYVLSMGAVFALFAGFYYWIGKITGKQYNEFWGQVH
FWTMFIGVNVTFFPMHFLGLNGMPRRIPDYPDAFTQWNVISSFGSIISIVSTIVFLYGLYLTLSQPAVSL
ANNYWHVPSFFSSTHSLYGDTTQTSSSLEWVLPSPPRFHAFNHLPVQS*                     
  
>cox1_Marchantia_polymorpha_
MNNFAQRWLFSTNHKDMGTLYLIFGAIAGVMGTCFSVLIRMELAQPGNQILGGNHQLYNVLMTAHAFLMI
FFMVMPAMMGGFGNWFVPILMGSPDMAFPRLNNISFWLLPPSLLLLLSSALVEVGCGSGWTVYPPLSGMT
SHSGGSVDLAIFSLHLSGVSSILGSINFMTTIFNMRAPGLTMHRLPLFVWSVLVTAFLLLLSLPVLAGAI
TMLLTDRNFNTTFFDPAGGGDPILYQHLFWFFGHPEVYILILPGFGIISHIVSTFS RKPVFGYLGMVYA
MISIGVLGFIVWAHHMFTVGLDVDTRAYFTAATMIMAVPTGMKIFSWIATMWGGSMQYKTPMLFAVGFMF
LFTVGGLTGMVLANSGVDIALHDTYYVVAHFHYVLSMGAVFALFAGFYYWMGKMTGLQYPETLGQIHFWI
TFFGVNLTFFPMHFLGLAGMPRRIPDYPDAYAGWNAFSSFGSYVSVVGIFCFFVVVFLTLTSENKCAPSP
WAVEQNSTTLEWMVPSPPAFHTFEELPAIKESI*                    
>cox1_Neurospora_crassa_
MSSISIWTERWFLSTNAKDIGVLYLIFALFSGLLGTAFSVLIRMELSGPGVQYIADNQLYNAIITAHAIL
MIFFMVMPALIGGFGNFLLPLLVGGPDMAFPRLNNISFWLLPPSLLLLVFSACIEGGAGTGWTIYPPLSG
VQSHSGPSVDLAIFALHLSGVSSLLGSINFITTIVNMRTPGIRLHKLALFGWAVVITAVLLLLSLPVLAG
AITMLLTDRNFNTSFFETAGGGDPILFQHLFWFFGHPEVYILIIPGFGIISTTISAYS NKSVFGYIGMV
YAMMSIGILGFIVWSHHMYTVGLDVDTRAYFTAATLIIAVPTGIKIFSWLATCYGGSIRLTPSMLFALGF
VFMFTIGGLSGVVLANASLDIAFHDTYYVVAHFHYVLSMGAVFAMFSGWYHWVPKILGLNYNMVLSKAQF
WLLFIGVNLTFFPQHFLGLQGMPRRISDYPDAFSGWNLISSFGSIVSVVASWLFLYIVYIQLVQGEYAGR
YPWSIPQFYTDSLRALLNRSYPSLEWSISSPPKPHSFASLPLQSSSFFLSFFRLSSYGEQKEISGRQN*
>cox1_Saccharomyces_cerevisiae
MVQRWLYSTNAKDIAVLYFMLAIFSGMAGTAMSLIIRLELAAPGSQYLHGNSQLFNVLVVGHAVLMIFFL
VMPALIGGFGNYLLPLMIGATDTAFPRINNIAFWVLPMGLVCLVTSTLVESGAGTGWTVYPPLSSIQAHS
GPSVDLAIFALHLTSISSLLGAINFIVTTLNMRTNGMTMHKLPLFVWSIFITAFLLLLSLPVLSAGITML
LLDRNFNTSFFEVAGGGDPILYEHLFWFFGHPEVYILIIPGFGIISHVVSTYSKKPVFGEISMVYAMASI
GLLGFLVWSHHMYIVGLDADTRAYFTSATMIIAIPTGIKIFSWLATIYGGSIRLATPMLYAIAFLFLFTM
GGLTGVALANASLDVAFHDTYYVVGHFHYVLSMGAIFSLFAGYYYWSPQILGLNYNEKLAQIQFWLIFIG
ANVIFFPMHFLGINGMPRRIPDYPDAFAGWNYVASIGSFIATLSLFLFIYILYDQLVNNKSVIYAKAPDF
VESNTIFNLNTVKSSSIEFLLT SPPAVHSFNTPAVQS*                       
>cox1_Schizosaccharomyces_pombe_
MNSWWTYVNRWIFSTNAKDIAILYLLFGLVSGIIGSVFSFIIRMELSAPGSQFLSGNGQLYNVAISAHGI
LMIFFFIIPALFGAFGNYLVPLMIGAPDVAYPRVNNFTFWLLPPALMLLLISALTEEGPGGGWTVYPPLS
SITSHSGPAIDLAILSLQLTGISSTLGSVNLIATMINMRAPGLSLYQMPLFAWAIMITSILLLLTLPVLA
GGLFMLFSDRNLNTSFYAPEGGGDPVLYQHLFWFFGHPEVYILIMPAFGVVSHIIPSLAHKPIFGKEGML
WAMLSIALLGLMVWSHHLFTVGLDVDTRAYFSAATMVIAIPTGIKIFSWLATLTGGAIQWSRVPMLYAIG
FLILFTIGGLTGVILSNSVLDIAFHDTYFVVAHFHYVLSMGALFGLCGAYYWSPKMFGLMYNETLASIQF
WILFIGVNIVFGPQHFLGLNGMPRRIPDYPEAFVGWNFVSSIGSVISILSLFLFMYVMYDQFTSN RVVK
TNPYLIPSYFDDNVIFVNEKLGVAQSIEWLLHSPVHEHAFNTLPTKSI*                     
 
>cox1_C.elegans_
INLYKKYQGGLAVWLESSNHKDIGTLYFIFGLWSGMVGTSFSLLIRLELAKPGFFLSNGQLYNSVITAHA
ILMIFFMVMPTMIGGFGNWLLPLMLGAPDMSFPRLNNLSFWLLPTSMLLILDACFVDMGCGTSWTVYPPL
S TMGHPGSSVDLAIFSLHAAGLSSILGGINFMCTTKNLRSSSISLEHMTLFVWTVFVTVFLLVLSLPVL
AGAITMLLTDRNLNTSFFDPSTGGNPLIYQHLFWFFGHPEVYILILPAFGIVSQSTLYLTGKKEVFGALG
MVYAILSIGLIGCVVWAHHMYTVGMDLDSRAYFSAATMVIAVPTGVKVFSWLATLFGMKMVFNPLLLWVL
GFIFLFTLGGLTGVVLSNSSLDIILHDTYYVVSHFHYVLSLGAVFGIFTGVTLWWSFITGYVLDKLMMSA
VFILLFIGVNLTFFPLHFAGLHGFPRKYLDYPDVYSVWNIIASYGSIISTAGLFLFIYVLLESFFSYRLV
ISDYYSNSSPEYCMSNYVFGHSYQSEIYFSTTSLKN*                 
>cox1_Xenopus_laevis__
MAITRWLFSTNHKDIGTLYLVFGAWAGLVGTALSLLIRAELSQPGTLLGDDQIYNVIVTAHAFIMIFFMV
MPIMIGGFGNWLVPLMIGAPDMAFPRMNNMSFWLLPPSFLLLLASSGVEAGAGTGWTVYPPLAGNLAHAG
ASVDLTIFSLHLAGISSILGAINFITTTINMKPPAMSQYQTPLFVWSVLITAVLLLLSLPVLAAGITMLL
TDRNLNTTFFDPAGGGDPVLYQHLFWFFGHPEVYILILPGFGMISHIVTYYSGKKEPFGYMGMVWAMMSI
GLLGFIVWAHHMFTVDLNVDTRAYFTSATMIIAIPTGVKVFSWLATMHGGTIKWDAPMLWALGFIFLFTV
GGLTGIVLANSSLDIMLHDTYYVVAHFHYVLSMGAVFAIMGGFIHWFPLFTGYTLHETWAKIHFGVMFAG
VNLTFFPQHFLGLSAMPRRYSDYPDAYTLWNTVSSIGSLISLVAVIMMMFIIWEAFAAKREVTTYELTST
MLEWLQGCPTPYHTLKTSLVQINHQ*                    
>cox1_Drosophila_melanogaster
SRQWLFSTNHKDIGTLYFIFGAWAGMVGTSLSILIRAELGHPGALIGDDQIYNVIVTAHAFIMIFFMVMP
IMIGGFGNWLVPLMLGAPDMAFPRMNNMSFWLLPPALSLLLVSSMVENGAGTGWTVYPPLSAGIAHGGAS
VDLAIFSLHLAGISSILGAVNFITTVINMRSTGISLDRMPLFVWSVVITALLLLLSLPVLAGAITMLLTD
RNLNTSFFDPAGGGDPILYQHLFWFFGHPEVYILILPGFGMISHIISQESGKKETFGSLGMIYAMLAIGL
LGFIVWAHHMFTVGMDVDTRAYFTSATMIIAVPTGIKIFSWLATLHGTQLSYSPAILWALGFVFLFTVGG
LTGVVLANSSVDIILHDTYYVVAHFHYVLSMGAVFAIMAGFIHWYPLFTGLTLNNKWLKSHFIIMFIGVN
LTFFPQHFLGLAGMPRRYSDYPDAYTTWNIVSTIGSTISLLGILFFFFIIWESLVSQRQVIYPIQLNSSI
EWYQNTPPAEHSYSELPLLTN*                      
>cox1_human
MFADRWLFSTNHKDIGTLYLLFGAWAGVLGTALSLLIRAELGQPGNLLGNDHIYNVIVTAHAFVMIFFMV
MPIMIGGFGNWLVPLMIGAPDMAFPRMNNMSFWLLPPSLLLLLASAMVEAGAGTGWTVYPPLAGNYSHPG
ASVDLTIFSLHLAGVSSILGAINFITTIINMKPPAMTQYQTPLFVWSVLITAVLLLLSLPVLAAGITMLL
TDRNLNTTFFDPAGGGDPILYQHLFWFFGHPEVYILILPGFGMISHIVTYYSGKKEPFGYMGMVWAMMSI
GFLGFIVWAHHMFTVGMDVDTRAYFTSATMIIAIPTGVKVFSWLATLHGSNMKWSAAVLWALGFIFLFTV
GGLTGIVLANSSLDIVLHDTYYVVAHFHYVLSMGAVFAIMGGFIHWFPLFSGYTLDQTYAKIHFTIMFIG
VNLTFFPQHFLGLSGMPRRYSDYPDAYTTWNILSSVGSFISLTAVMLMIFMIWEAFASKRKVLMVEEPSM
NLEWLYGCPPPYHTFEEPVYMKS*                      
>cox1_sea_urchin_(Paracentrotus_lividus)
MQLSRWLFSTNHKDIGTLYLIFGAWAGMVGTAMSVIIRAELAQPGSLLNDDQIYNVVVTAHALVMIFFMV
MPIMIGGFGNWLIPLMIGAPDMAFPRMNNMSFWLIPPSFILLLASAGVESGAGTGWTIYPPLSSNIAHAG
GSVDLAIFSLHLAGASSILASINFITTIINMRTPGMSFDRLPLFVWSVFVTAFLLLLSLPVLAGAITMLL
TDRNINTTFFDPAGGGDPILFQHLFWFFGHPEVYILILPGFGMISHVIAHYSGKREPFGYLGMVYAMIAI
GVLGFLVWAHHMFTVGMDVDTRAYFTAATMIIAVPTGIKVFSWMATLQGSNLQWETPLLWALGFVFLFTL
GGLTGIVLANSSIDVVLHDTYYVVAHFHYVLSMGAVFAIFAGFTHWFPLFCGYNLHPLWGKAHFFMMFVG
VNLTFFPQHFLGLAGMPRRYSDYPDAYTLWNTVSSIGSTISLVAMLFFIFLIWEAFASQREGVTPEFANA
SLEWQYNSFPPSHHTFDETPSTVVIVK*                  
>cox1_Rhizophlyctis_rosea_(partial_sequence)
WVVPVLIGAPDMAFPRLNNISFWLLPPSLFLFTIGMFSTEAGAGTGWTIYPPLSDTPYHLGSAVDFSILA
LHIAGVSSLMGALNIITTTINMRAPGMSFEKLPLFVWSVFITAWLLVLSLPVLAGAITMLLTDRNFNTSF
YDPNGGGDPLLFQHLFWFFGHPEVYILILPGFGIISHIVSRFAIKPVFGYIGMVYAMVSIGVLGFIVWSH
HMFAVGLDVDTRAYFTAATMIIALPTGIKVFSWLATLYGGQIHYYTPFIFSLAFILLFTFGGFTGVILAN
GSIDLALHDTYYVVGHFHYVLSMGAVYALLGGLYYWVGKITGVQYNEKLGLIHLFVFTIAVNIV*
>cox2_Schizosaccharomyces_pombe_
MLFFNSILNDAPSSWALYFQDGASPSYLGVTHLNDYLMFYLTFIFIGVIYAICKAVIEYNYNSHPIAAKY
TTHGSIVEFIWTLIPALILILVALPSFKLLYLLDEVQKPSMTVKAIGRQWFWSYELNDFVTNENEPVSFD
SYMVPEEDLEEGSLRQLEVDNRLVLPIDTRIRLILTSGDVIHSWAVPSLGIKCDCIPSRLNQVSLSIDRE
GLFYGQCSELCGVLHSSMPIVVQGVSLEDFLAWLEENS*
>cox2_Reclinomonas_americana_mt_cox2_;_260_aa
MFLVALIFIIISYGMVDSLRADYAEKWQLDFQDPATPVMEGIINLHHDLWFFLILIAVFVLWILLRTLWF
FDRSRGTVPSKVVHGTVLEIVWTIAPSFILLAIAIPSFALLYSMEESTDPAITLKAIGHQWYWSYEYSDY
TTDSESLAFDSYMLPESELKEGQLRLLEVDNRIFLPTHTHLRVLITSSDVLHSWAVPSLGVKVDACPGRL
NQASIFLKREGIFYGQCSEICGVNHGFMPIVIEAVPLKEYVSWVSSQIQI*
>cox2_Jakoba_libera_mt_cox2_;_260_aa
MLQKLLFLTISFACTLGLACADAPEQWQWDFQDAASPIMEGIVDLHHDLMFFLVVIVVFVTWLLARVVIQ
FRSSVNPVPSTTNHGTTIEIIWTIIPAVILLFIAIPTFALLYSMDEGIDPAITIKAVGHQWYWSYEYSDY
TSDTESLAFESYMVPEEDLQQGQLRLLEVDNRVVVPVDTHVRVLVTSQDVLHSWAIPSLGIKMDACPGRL
NQVSMFIKREGVYYGQCSEICGVNHAFMPIVIEAVPLDDYVTWVSSELEI*
>cox2_Malawimonas_jakobiformis_mt_cox2_;_273_aa
MLNIFLNITNTDAALPNQLGFQDPATPIFAGIIDLHNEVSFYLIVVLTTVTWVIIRILWLFSGKTSKLSS
SWFYWTPKLYNGKQPRLFLKPKKYTHNTTLEIVWTITPAIILLLIAIPSFALLYSMDELIDPAMTIKAIG
HQWYWTYEYSDYNVDGKDSLIIDSYMLPEDSLNNGQLRLLDVDNRIKVPVNTHIRLITTSTDVIHSWTIP
SFGVKIDAIPGRLNQVSMFIQREGVYYGQRSEICGVNHGFMPIVVEVVSLDEYINWINNHFNK*
>cox2_Ochromonas_danica_mt_cox2_;_265_aa
MITYINNLILIVLNNIVAYCDAAEPWQISPQDPATPVMEGMIFFHNYLMFYMILIGILVFFMLFKILVTF
GENKKSVADKFTHSSLLEIIWTILPAIVLILIAVPSFTLLYSLDELVDPALTLKIIGHQWYWSYEYSDYA
ILNNGNTLSFDSYMTPTSDLDFGHLRLLEVDNRVLLPTNTHIRILVTAADVIHSWAVPSFGIKVDGTPGR
LTQGSLFIKREGVYFGQCSEICGINHAFMPIVVRTVSVPYFTEWILYRLDHFLGE*
>cox2_Aspergillus_nidulans_(Emericella)_
MFLEILKGHILMDAPTPWGIFFQDSASPQMEGIMELHNNIMFYLAIILFTVTWMMITIIRNFVAKKSPIA
HKYMNHGTLIELIWTITPAFILILIAFPSFKLLYLMDEVMDPSLVVYAEGHQWYWSYQYPDFTNEDNEFI
EFDSYIVPESDLEEGQFRMLEVDNRVIIPELTHTAFVISADVIHSYACPSLGIKADAYPGRLNQASVYIN
GPGTFFGQCSEICGILHSSMNIAIQSVSIKDFLLWLRDQMEG*
>cox2_Prototheca_wickerhamii_
MKFLLFAYALIPFVSFSDAPEAWQIGFQDPATPIMQGLIDLHHDIQFFLIAVLVFVVWMVSRALYLFHYT
RNPLPEKIIHGTLIEIVWTITPSLILIFIAVPSFALLYSLDEVVDPAVTIKAIGHQWYWSYEYSDYSIAD
DQSIAFDSYMIPDDDLELGQYRLLEVDNRVVVPVDTHIRVIITAADVLHSWAIPSLGVKCDAVPGRLNQI
PMFIKREGVFYGQCSELCGTNHAFMPIVVEAVSLENYISWVSNKLEEL*
>cox2_Marchantia_polymorpha_
MNLMWMFPIAFCDAAEPWQLGFQDPATPMMQGMIDLHNDIFFFLIVILIFVLWMLVRALWHFHYKRNPIP
ERIVHGTTMEIIWMSIFPSIILMFIAMPSFALLYSMDEVVDPAITIKAIGHQWYWTYEYSDYNSSDEQSL
TFDSYMIPEDDLELGQLRLLEVDNRVVVPAKTHLRMIITSADVLHSWAVPSLGVKCDAVPGRLNQTSIFI
KREGVYYGQCSELCGTNHGFMPIVVEAVSLDDYVSWVSNKLD*
>cox2_Chondrus_crispus
MNNILNFYPAVITTDVAENWQIGFQDPATPIMEGIINLHYDLMFFICVISVFVSWMLGRTLWHFEQNQNK
IPSSLTHGTLIEMIWTVTPAFILLIIAVPSFSLLYAMDEIISPAITIKTLGHQWYWSYEYSDYLNEDDDS
INYDSYMIPEEDLEIGQFRLLEVDNRMVIPINTHIRVIVTAADVLHSWAVPSFRSKCDAIPGRLNQTSLF
IKREGVYYGQCSEICGINHGFMPIVVEATSLPNYVSWISNKLNE*
>cox2_Phytophthora_infestans
MTITNYINNQFTFLDMAEPWQLGFQDPATPVMEGIINFHHDLMFFLIMITVFVCWMLFRVITLFDEKKNK
IPSTVVHGATIEIIWTSIPALILLIVAVPSFALLYSMDEVIDPIITLKVIGSQWYWSYEYSDNLEFSDEP
LIFDSYMVQEDDLAIGQFRLLEVDNRVVVPTNSHIRVLITASDVLHSWAIPSLGIKLDACPGRLNQTSMF
IKREGVFYGQCSEICGVNHGFMPIVIEAVSLEDYLTWLKNKINFDFNV*
>cox2_Schizophyllum_commune_
MFNLIHNLIFIFNDAPEPWQLGFQETASPAFAGLVTLHNTIGFYLVVISVGVFWFIFSIMYYFSSVKNAF
AAKYLVHGTVLELIWTITPALVLIAIAFPSFRLLYLMDEVISPSLTIKVVGHQWYWSYEYSDFISEEGDT
VEFDSYMVPDSDLELGQFRLLDVDNAVVIPVDCHVRFIVTGADVIHSFAVPSLGIKLDACPGRLNQVSFL
AERTGVYYGQCSEICGIWHGFMPIRVDVVTSSDFLVwMNNII*
>cox2_Rhizopus_stolonifer_
MLNMLKSLNIISPVWNDAPEPWQWAFQDGASPSYEGIVELHDQIMFYLVVILFGVSWVLTSIIVNFGSNK
NQIVYKYHNHGTLIELIWTITPAFVLIAIAFPSFKLLYLMDEVIDPAMTIKALGHQWYWSYEYSDFVNED
GESIEFDSYLVPTDDLEEGQLRLLEVDNRVVVPVGTHIRFIVTGADVIHSFAVPSLGLKIDAIPGRLNQT
SVLVEREGVYYGQCSEICGVHHGFMPIAVEAVSLEKYLAWLNEQA*
>cox2_Allomyces_macrogynus
MKLLNFIYNDHPQPWQIGFQDGATTTFYGIVDLHDNIIFYLIIVIIGVTWIQTSVMLDSYGNSDKHVYKY
SHHGTLVEIIWTITPALILVAIAFPSFKLLYLMDSVVDPAVTVKAIGYQWYWGYNYGDYNEDISFDSFMV
PTDELEMGQFRLLEVDNRVVVPVDTRVRIVVTAADVIHSFAVPSLGIKIDAIPGRINQASFLIDREGVYY
GQCSELCGANHGFMPIAIEAVSLDRYVAWVDAQLS*
>cox2_Spizellomyces_punctatus
MDHVSWAIKKTRWSFLQTMICTSIGWEGKLYKMFRIYADYAEAWATGFQDPASDWMYAIIDLHDRIIFYL
LIIlAVVVWFLVSSMLNTDHMAHLHHGNALELLWTITPAVILWAIGLPSLRLLYMMDEIlDPELSVKVMG
SQWFWSYEYSDYVDNGVSIAFDSFMVSDADLELGDLRTLAVDNYLVLPINTSIRLLISSNDVIHSFALPS
LAIKADAIPGRLNSTGLIINRPSTFYGQCSELCGVLHGFHAYRCSRCFYPFLPKLY*
>cox2_C.elegans
INNFFQGYNLLFQHSLFASYMDWFHRFNCSLLLGVLVFVTLLFGYLIFGTFYFKRKKIEYQFGELLCSIF
PTIILLMQMVPSLRLLYYYGLMNLDRNLTVKVTGHQWYWRYEYRDIPGLEFDSYMKSLDQLSLGEPRLLE
VDNRCVIPCDTNIRFCITSADVIHAWALNSLSVKLDAMRGILRTFSYRFPMVGVFYGQCSEICGANHSFM
PIALEVTLLDNFKRWCFGTME*
>cox2_Drosophila_melanogaster
MSTWANLGLQDSASPLMEQLIFFHDHALLILVMITVLVGYLMFMLFFNNYVNRFLLHGQLIEMIWTILPA
IILLFIALPSLRLLYLLDEINEPSVTLKSIGHQWYWSYEYSDFNNIEFDSYMIPTNELMTDGFRLLDVDN
RVVLPMNSQIRILVTAADVIHSWTVPALGVKVDGTPGRLNQTNFFINRPGLFYGQCSEICGANHSFMPIV
IESVPVNYFIKWISSNNS*
>cox2_human
MAHAAQVGLQDATSPIMEELITFHDHALMIIFLICFLVLYALFLTLTTKLTNTNISDAQEMETVWTILPA
IILVLIALPSLRILYMTDEVNDPSLTIKSIGHQWYWTYEYTDYGGLIFNSYMLPPLFLEPGDLRLLDVDN
RVVLPIEAPIRMMITSQDVLHSWAVPTLGLKTDAIPGRLNQTTFTATRPGVYYGQCSEICGANHSFMPIV
LELIPLKIFEMGPVFTL*
>cox2_Xenopus_laevis
MAHPSQLGFQDAASPIMEELLHFHDHTLMAVFLISTLVLYIITIMMTTKLTNTNLMDAQEIEMVWTIMPA
ISLIMIALPSLRILYLMDEVNDPHLTIKAIGHQWYWSYEYTNYEDLSFDSYMIPTNDLTPGQFRLLEVDN
RMVVPMESPTRLLVTAEDVLHSWAVPSLGVKTDAIPGRLHQTSFIATRPGVFYGQCSEICGANHSFMPIV
VEAVPLTDFENWSSSMLEA*
>cox2_sea_urchin_(Paracentrotus_lividus)
MATWAQFGLQDASSPLMEELTYFHDYALIVLTLITILVFYGLVSLLLSSSTNRFFLEGQELETIWTVVPA
FILIFIALPSLQLLYLMDEVNNPFLTIKAIGHQWYWSYEYTDYNDLEFDSYMVPTSDVSLGNPRLLEVDN
RLILPMQNPIRVLVSSADVLHSWAVPSLGVKMDAVPGRLNQTTFFAARAGLFYGQCSEICGANHSFMPIL
IESVPFSNFENWVAQYIEE*
>cox3_Jakoba_libera_mt_cox3_;_268_aa
MSSSLSLKRHPFHIVDPSPWPLFTTLGVLATTMGLVLYMHSYLAGGYILTAGLITILFVMYTWFRDVSRE
GTYQGHHTMAVQKGLKLGMLLFIFSEVMFFSAFFWGYFHASLAPNLEIGSIWPPQGIHPFNAWDVPFVNT
LLLLLSGATVTWAHHSMVHGNRIQCITGLILTLVLASAFTALQGYEYLEAPFSIADGIYGSTFFMATGFH
GFHVIVGSIFLAVCLLREFKYHFTTTHHVGFESAAWYWHFVDVVWLFLFVSIYWWGGA*
>cox3_Ochromonas_danica_mt_cox3;_267_aa
MSNIKNNQKHSFHIVDPSPWPLVASFAALFLTFGTVLYMHGYNGGGFLAFFGFSMVLFVMFCWWRDVIRE
ATLEGQHTKSVQKGLKIGLLLFILSEVMFFVSFFWAFFHSSLNPSPAIGGVWPPAYITTLDPWGIPLLNT
IILLSSGASVTWAHHSIVFGSKEEAIQALICTVVLAVIFTSFQGYEYVTAPFTISDGIYGSVFYMCTGFH
GLHVIIGTIFLAVCLYRVNANHYTKEHHFGFEAAAWYWHFVDVVWLFLFVSLYWWGS*
>cox3_Malawimonas_jakobiformis_mt_cox3_;_274_aa
MKNNNKILNSMSYHPFHLVDLSPWPILGATSGFIMAIGAAMSMHSYTFGPQILLFGFISLIYTMFVWWRD
VIREATFEGHHTSIVQIGLRYGMILFIISEVLFFAAFFWAFFTASLAPTIEIGAIWPPKGINVFNPWEVP
LLNTLILVLSGVTITWSHHAILAGNRSSAILSLGLTVFLGLSFTVLQVMEYLEATFNISDGIYGSTFYMA
TGFHGAHVIIGTLFLIVCLVRLIKYHFTSKHHFGFEAAAWYWHFVDVVWLFLFISIYWWGGLTA*
>cox3_Schizosaccharomyces_pombe_
MNLSTKFQGHPYHIVSASPWPFFLSVVLFFNCLAATLYLHGYKHSSVFFGISFLGLLATMYLWFRDMSTE
ANIHGAHTKAVTKGLKIGFMLFLISETFLFASIFWAFFHSSLSPTFELGAVWPPVGIADKTIDPLEVPLL
NTVILLTSGASLTYAHYSLIARNRENALKGLYMTIALSFLFLGGQAYEYWNAPFTISDSVYGASFYFATG
LHGIHIIVGTILLLVATYNIYTYHLTNTHHNGFECGIYYWHFCDVVWLFLYLTIYIWGS*
>cox3_Aspergillus_nidulans_(Emericella)_
MIYQSKRNFQNHPFHLVSPSPWPLFTSISLFILTTATVLFMHGFEGFQYLVPVAVINVMYVMGLWFRDVI
SEGTYLGNHTNAVQKGLYLGVGLFIISEVFFFLAIFWAFFHSAISPSVELGAQWPPLGIQGINPFELPLL
NTIILLSSGVTITYAHHSLIQGNRKGALYGTVVTILLAIVFTFFQGVEYTVSSFTISDSVYGSCFYFGTG
FHGLHVIIGTAFLAVGLWRLAAYHLTDHHHLGYESGILYWHFVDVVWLFLYISVYYWGY*
>cox3_Prototheca_wickerhamii_
MRKHPFHLVDPSPWPLLASFAAFLSTTGGVMYMHAYSGGGLLLAIGQLSLFYVMYVWWRDVIREGTYQGH
HTNPVQVGLRYGMLLFILSEIMFFFAFFWAFFHSSLAPTIEIGAVWPPKGIQVLNPWEIPFLNTIILLTS
GASVTWAHHAILAGNRKQGIISLAITVLLAVIFTGFQALEYLEAPFTISDGIYGSTFYLATGFHGFHVII
GTIFIAVCLFRLVLHHFSKSHHLGFEAAAWYWHMVDVVWLFLFVCIYYWGGA*
>cox3_Marchantia_polymorpha_
MSVSQKHPFHLVDPSPWPLLGSLGALASTIGGVMYMHSFTGGGTLLCLGLGMILYTMFVWWRDVMRESTY
EGHHTFVVQLGLRYGMILFIVSEVMFFLAFFWAFFHSSLAPTVEIGAIWPPKGISVLDPWGIPFLNTLIL
LSSGAAVTWAHHAMLAGLKQQAVYALMATVFLALVFTGFQGIEYMEAPFTISDGIYGSTFFLATGFHGFH
VIMGTIFLMMCGIRQYLGHFTPKHHFGFEAAAFYWHFVDVVWLFLFVSMYWWGGN*
>cox3_Chondrus_crispus
MTTLSQISKSVQRHPFHLVDPSPWPFVASLCAFSCAIGGVMYMHAYVNGSFILSISFFCLLLVMFTWWRD
VIRESTFEGHHTGIVQQGLRFGVILFIISEILFFFAFFWAFFHSSLAPTIEIGSIWPPKGINVLNPWEIP
FLNTLILLLSGCTVTWCHHSLVSNLRNQSVLSLFLTIVLAIVFTTFQAYEYSMADFRLSDGIYGSTFYMA
TGFHGFHVLVGTISLAVCLIRLLQYQLTQQHHFGFESAAWYWHFVDVVWLFLFVSIYWWGGS*
>cox3_Phytophthora_infestans
MKNYYYINKKNLQIETTTTNDSNINKFQNDLKFLKEITQNTQNTQNHPYHLVDPSPWPFVISFGLFFLTF
GGAMYLHGYIGSNFLTLTGFLMVILTMYTWFRDIVREAVYEGQHTKQVQLGLRNGMLLFIFSELLFFISF
FWAFFHSALAPTPEIGSLWPPLGIETVNAWGIPLLNTIILLSSGATITWAHHSIVFGDRKNAILSLIITI
LLAFFFSLIQAYEYIESTFSISDSIYGTTFFLLTGFHGIHVIVGTIFIIVSTLRLINHHFTKQHHFGFEA
AAWYWHFVDVVWLFLFVAVYWWGGN*
>cox3_Schizophyllum_commune_
MTSIKFYQSFSAHLVQHSPWPILVSFSLFNLAIGTVLTMHGYSHSSTTFDLGLAVTVGSILLWTRDIVIE
GSFLGDHTKQVQEGLIIGFILFIISEVFAFISVFWAYFHSALSPAVELGSTWPPVGIIPLDTFSLPLFNT
IILLSSGAFVTYGHHAIFSGKRLDSIIGLFLTVALALIFSYFQAFEYIHAGFSMSDSVFGTVFFASTGLH
GIHVMLGTLFLFVSFLRQVNYQTTKEHNIGLETSILYWHFVDLVWLFLFLVVYFWGGA*
>cox3_Rhizopus_stolonifer_
MIEMYDRNYIYNMTTKIMNRSVQAHPFHLVEASPWPIAVSFSLLVVTLSGVMTFQGYSNGLFLLTLGFIS
LVSTMTLWFKDISREGTFQGHHTFAVQKGLSLGFVLFVVSEVFFFISIFWAFFHSALAPTVELGAHWPPA
GIETLNPWEVPLLNTVILLSSGATVTYAHHSLIQGNRAGVIYGLIATVVLATVFTGFQGFEYYNAPFTFS
DGVYGSTFYMATGFHGIHVLVGTIFLTVGLFRVLSYHLTDHHHLGFEQAILYWHFVDVVWLFLFISVYWW
GG*
>cox3_Allomyces_macrogynus
MTKKLINQFKSFQVHPYHLVEPSPWPLGASVACLILTLGGVMKFHGFAAGDIGLPLGLILVLASMLLWWR
DVIREATYQGHHTKTVKYGITLGVVLFIVSEILLFFSLFWAFFHSSLAPSVELGSTWPPVGIEPLNPFEV
PLLNTIILLTSGCTITVSHAKIISGDRGATILYLILTILLAWMFLGLQWVEYVNAPFTIADSVYGSTFFV
ATGFHGLHVMIGTIFLTVSLNRILSYHLTSGHHLGYEAAIWYWHVVDVIWLFLYVSVYYWGSNV*
>cox3_Spizellomyces_punctatus
MNFNISARQAHPYHlVDVSPWPILMSMALlSTAIGLVSWLGQFPTNLVPQLVIIVLIALQWWRDVIREAK
GGYHTTLVQRGILIGFLLFLLSEVMLFFSFFWAFFHSSLSPAVElGASWPPVGINAVNPWGIPLlGSCVL
LASGFVLTLGHHAIVLGNKDlTlVSLFFTVLLGAFFLFLQFNEYYYGEFTIADSVFGSVFYMTTGLHALH
VIVGVLFLTVCLVRVYLDSFTSEHHLAAEFAIYYWHLVDVVWLLVFLIYYYWGS*
>cox3_C.elegans
MFHNFHILRLSRYAYNLFFASAGMLRSLVMFFKFGLYELFIFTLFSVLFISFAWGKDIAMEGLRGYHNFF
VMDGFKFGVILFVFREFMFFFCIFWTFFDAALVPVHELGETWSPFGMHLVNPFGVPLLNTIILLRSGVTV
TWAHHRLLRNKRCTNSMILTCLLAAYFTGIQLMEYMEARFSIADGVFGRIFYLSTGFHGIHVLCGGLFLA
FNFLRLLKNHFNYNHHLGLEFAILYWHFVDVVWLFLFVFVYWWSY*
>cox3_Drosophila_melanogaster
MSTHSNHPFHLVDYSPWPLTGAIGAMTTVSGMVKWFHQYDISLFVLGNIITILTVYQWWRDVSREGTYQG
LHTYAVTIGLRWGMILFILSEVLFFVSFFWAFFHSSLSPAIELGASWPPMGIISFNPFQIPLLNTAILLA
SGVTVTWAHHSLMENNHSQTTQGLFFTVLLGIYFTILQAYEYIEAPFTIADSIYGSTFFMATGFHGIHVL
IGTTFLLVCLLRHLNNHFSKNHHFGFEAAAWYWHFVDVVWLFLYITIYWWGG*
>cox3_human
MTHQSHAYHMVKPSPWPLTGALSALLMTSGLAMWFHFHSMTLLMLGLLTNTLTMYQWWRDVTRESTYQGH
HTPPVQKGLRYGMILFITSEIFFFAGFFWAFYHSSLAPTPQLGGHWPPTGITPLNPLEVPLLNTSVLLAS
GVSITWAHHSLMENNRNQMIQALLITILLGLYFTLLQASEYFESPFTISDGIYGSTFFVATGFHGLHVII
GSTFLTICFIRQLMFHFTSKHHFGFEAAAWYWHFVDVVWLFLYVSIYWWGS*
>cox3_Xenopus_laevis
MAHQAHAYHMVDPSPWPLTGAVAALLLTSGLAMWFHFGSMILLTLGLITMVLTMIQWWRDVIREGTFQGH
HTPPVQKGLRYGMILFITSEVFFFIGFFWAFYNSSLAPTYELGECWPPTGITPLNPFEVPLLNTAVLLAS
GVTVTWAHHSIMHGDRKEAIQSLTLTILLGLYFTALQAMEYYEAPFTIADGVYGSTFFVATGFHGLHVII
GSLFLSVCLLRQIQYHFTSKHHFGFEAAWYWHFVDVVWLFLYVSIYWWGS*
>cox3_sea_urchin_(Paracentrotus_lividus)
MAHQHPYYLVEQSPWPLTGAISGLMMNLGLVLWFHTGNIILLFTGLLLLILTLVNWWRDIVREATFQGSH
TAIVENGLRYPMILFITSEVCFFFAFFWAFFHSSLAPAVEIGVTWPPSGITPLNPFLVPLLNTAVLLSSG
VTITWSHHSILAGNRNEAIQALFLTVVLGIYFTILQAWEYIDAPFTIADSVYGSTFFVATGFHGLHVIIG
TTFLLVCLIRLSGHHFSTHHHFGFEAAAWYWHFVDVVWLFLYVCIYWWGS*
>rps2_Jakoba_libera_mt_rps2_;_222_aa
MKTYSHAIKDQMIPSRKLRIYDTEFLNELMGHEIHLGLSKTNVHPLMIKYIQGYRNEISVYRMELLIESF
RIFTNLVKQFSKDDCILFITKKREISNSIKKLAIDHHQYYMLGKWIPGLLTNFATYSKDILNLYTKKQKN
RKRVLTNTLGISSMSKLPSLIIIIGLEGNSTAIREAHKLNIPIVGFTSSREDPKKVTYCIPSNLSSSKSM
HFYLKLLQLSFK*
>rps2_Ochromonas_danica_mt_rps2_;_231_aa
MLKTKRRKSLKLTDFLKNLILSDLLVSKDLIDLNNYSEKVINPDYALALIKQFLLVTSCYIKKHPSTVPI
LLVPSKEEKSYVDFCLSKIGLSHNIRVFTSLKRLKALKGGKIIFVLDNGIIPNWNTFLKKCLLNEDFFVF
RVNNYLPFKNNFGIYTLFGQFDSLKKLLFFLLLLKRAFTLSGIPLSTTQYFYKKLFKRKINKKNKGISQS
NNYKNINYANTVSKIKRKKEI*
>rps2_Thraustochytrium_aureus_mt_rps2_;_134_aa
MIVNFKVIQEVFKIIRILSFFNKKNLPIWFVSDNPHYKLFYFLSSKRSNHFFFMNGWKNGCLTNSKQDPA
MVVFFDANLVGFHECSIKNIPSVSFNSFFSSPVTYQISFNLSNIKKSWVIYQILTHLCSLFINI*
>rps2_Rhodomonas_salina_mt_rps2_;_207_aa
MKSKILLNNLFDKKQKKDQFIEHHFVKLSALSFNFKNSSFILGKKNKVFFYKADKLVLFLNEILSFYLNF
FKSYPKSLLVVDKRLMFLVSKEASLRSFQGVFFGKYSGGMFNNNIEKYKEYKTLFSKVDMFLFLSIKDPI
FSLKELSLLKKPLIVFIEETIKLENYLFYKILFNNNSYFFNYFVLKLLSDCLIKIHMYNYVESKIVH*
>rps2_Jakoba_libera_mt_rps2_;_222_aa
MKTYSHAIKDQMIPSRKLRIYDTEFLNELMGHEIHLGLSKTNVHPLMIKYIQGYRNEISVYRMELLIESF
RIFTNLVKQFSKDDCILFITKKREISNSIKKLAIDHHQYYMLGKWIPGLLTNFATYSKDILNLYTKKQKN
RKRVLTNTLGISSMSKLPSLIIIIGLEGNSTAIREAHKLNIPIVGFTSSREDPKKVTYCIPSNLSSSKSM
HFYLKLLQLSFK*
>rps2_Ochromonas_danica_mt_rps2_;_231_aa
MLKTKRRKSLKLTDFLKNLILSDLLVSKDLIDLNNYSEKVINPDYALALIKQFLLVTSCYIKKHPSTVPI
LLVPSKEEKSYVDFCLSKIGLSHNIRVFTSLKRLKALKGGKIIFVLDNGIIPNWNTFLKKCLLNEDFFVF
RVNNYLPFKNNFGIYTLFGQFDSLKKLLFFLLLLKRAFTLSGIPLSTTQYFYKKLFKRKINKKNKGISQS
NNYKNINYANTVSKIKRKKEI*
>rps2_Thraustochytrium_aureus_mt_rps2_;_134_aa
MIVNFKVIQEVFKIIRILSFFNKKNLPIWFVSDNPHYKLFYFLSSKRSNHFFFMNGWKNGCLTNSKQDPA
MVVFFDANLVGFHECSIKNIPSVSFNSFFSSPVTYQISFNLSNIKKSWVIYQILTHLCSLFINI*
>rps2_Rhodomonas_salina_mt_rps2_;_207_aa
MKSKILLNNLFDKKQKKDQFIEHHFVKLSALSFNFKNSSFILGKKNKVFFYKADKLVLFLNEILSFYLNF
FKSYPKSLLVVDKRLMFLVSKEASLRSFQGVFFGKYSGGMFNNNIEKYKEYKTLFSKVDMFLFLSIKDPI
FSLKELSLLKKPLIVFIEETIKLENYLFYKILFNNNSYFFNYFVLKLLSDCLIKIHMYNYVESKIVH*
>Capsaspora_owczsarsaki_atp6_;_258_aa_on_
MITALLASPVEQFQVIPYFGLVTLGNVDFSYSNVVwVLLLLLLTLANISLLLGKRSNISESKVVQNNwQR
MITKVGTFVNDMSQDTIGSEGRRYVNIILGLFVFVLLLNILGMIPYSFAITSQIVVTLwISwGVwLGSLT
IGFINYGINFFSMFMPSGAPLALAPFLILIELLSYVARGISLGVRLAANITSGHILLTIIATFIFKMLNI
GGIFIPLAFLTFALLFVLTILEMAIGAIQAYVLGLLVTIYLNDSVHLH*
>Capsaspora_owczsarsaki_atp9_;_74_aa_on_
MLEAAKLIGAGLATIGVAGSGVGIGTVFGALVGATARNPSLKQQLFGYTILGFAVVEAIALFALMMAFLI
LFAF*
>Capsaspora_owczsarsaki_cob_;_381_aa_on_
MRLLKSHPILSFVNGLVVDLPAASNLSYLwNFGSLLGVCLVAQLMTGITLAMHYTPHVDLAFASVEHIMR
DVNYGwLIRYLHANGASMFFIMVYIHIGRGLYYGSYTKPRIILwSVGVIILILMMATAFLGYVLPwGQMS
FwGATVITNLFSAIPYIGQDIVLwLwGGFSVDNATLNRFFSLHYLLPFVLAALVFVHLIALHEHGSNNPL
GYNTDPDKVTFHPYFTVKDALGVLLFFIFFSGFVYFAPNVLGHSDNYIPANPLVTPPHIVPEwYFLFAYA
ILRSIPDKLGGVLALFGSLLVLLVLPFVHTAKIRVLAFRPIAKKLYwFFVVDFLILTwIGGKPVEDPYVI
VGQIATVFYFFYLIIGTPVLGwIENKLLQLN*
>Capsaspora_owczsarsaki_cox1_;_565_aa_on_
MISNFATRwLFSTNHKDIGTLYMLFGAFSGMIGTALSIIIRMELAFPGNQILNGDHQTYNVVVTAHAVLM
IFFMVMPILIGGFGNwMVPLMIGGVDMAFPRLNNISFwLLPPSLILILASSFVESGVGTGwTLYPPLSSI
IAHSGGAVDLGIFSLHIAGISSMLGAMNFICTIFNMRSPGMTLHRMPLFVwAVLITAFLLLLSLPVLAGA
LTMLITDRNFNTTFFDPAGGGDPILYQHLFwFFGHPEVYILIIPGFGIVSQIVAHGARKPVFGTIGMIYA
MLSIGVLGFIVwAHHMFTVGMDVDTRAYFTGATMVIAIPTGIKIFSwIATLYGGVITYNTAMLYTIGFIV
LFTLGGITGIVLSNASLDIALHDTYYVVGHFHYVLSLGAVFAIFAGFYYwFPKMTGYLYNETISTIQFwL
MFIGVNLTFLPQHFLGLAGAPRRYPDMPDAYAGwNLISSIGSMISLVGAIVFIYIVIDAFVKKVPAFVED
MEHVEFVQPIALTMFELESAwLKDDSNKQLEIIHYIHNMALAKATEDSHLEwKHLSPPTNHTYLDLPFVY
QAIAK*
>Capsaspora_owczsarsaki_cox2_;_277_aa_on_
MISSRPNKEVLNEKAHSALNYSCSLGLSISLLIGLTLQDQATTwQFGFQDSASFLHEEVVYLHNHIMQYA
SLIIGLSGVLLVTALTRSNGGISHRFMVHGTTLEIIwTIAPAIILILIGVPSFKLLYLQDEIVDAALTIK
AIGRQwYwSYEYSDYAENADSITFDSYMTPTADLNEGELRLLEVDNRLTLPIDTNTRFVTTASDVIHSFT
VPSLGMKVDAVPGRLNTTSTQIDRPGTFYGQCSEICGYGHGFMPIVIDAVNVQDYTTKIVSQLQELE*
>Capsaspora_owczsarsaki_cox3_;_264_aa_on_
MTKTYHTYHLVDASPwPFLGSSAALSLTAGAVMYFHGYILGELALISGLLLVIFTMIVwwRDIIREGTYQ
GHHTEVVQQGLRYGVILFIVSEVMFFFSFFwAFFHSSLAPTIEIGSVwPPVGIEALNPwTVPLLNTALLL
SSGATVTwAHHAIVAGQRREAIMGLTLTVSLGIIFTALQGLEYYEAPFTFADSVYGSTFYLATGFHGLHV
IIGTVFLAVCLVRLTHHHFTKQHHFGFEAAAwYwHFVDVVwLFLFVTIYYwGGN*
>Capsaspora_owczsarsaki_nad1_;_331_aa_on_
MSILILIIKILLVLVPVLISMAFLTLIERKVMAAMQIRLGPNVVGIQGVLQPFADALKLFVKETIIPNHS
NIAIFTIAPILSLSLALISwAVIPFGQGLVLSDINLGIMYIFAVSSLSVYTVLCSGwASNSKYAFIGALR
STAQMISYEVSIGLIIITVILCVGSLNITDIVEAQRTIwFIIPLFPAFLMFYVSALAETNRSPFDLPEGE
SELVSGYNVEYSSMTFALFFLAEYIHIILMSVMTSLLFLGGwIAPIDIYPLNLVPSIFwLSIKVVAIIYL
FIwVRASFPRYRYDQLMTLLwKSYLPLSLGLVVLVSSLLLALDALPVDAIL*
>Capsaspora_owczsarsaki_nad2_;_543_aa_on_
MNFITGTDLLILSPIIwMTVAIVLTCAIAGYTTTPISAISSLDNITIPNKMENFNISISKIIIHFVFIMS
MLITYFQYQQLETGATTIYCNNLLQTNAFIILIQTLVYITAYLVMVVYIDEISKTTYRwLKLNTYDFLTI
IFTNLVGLwIVIGSNDLITLYLGIELQTFGAFILVALRKTSEYSIESALKYFVLGAVSSAILLLGIGLVY
VSLGTTNYTNLASLIQHINDEQTQFTLQVAYVFIMVSLLFKLGAAPFHNwVPDVYQGSTTIVTTYFVSVP
KIGLISALIILLVHNIAVISNIwYQTLIICSILSMIVGTAGAVNQYNIKRLLAYSAISHTGYLIIGVLTN
SLDGVISIVIYLILYIIMSLITwPLLLNLAQKQSLNSSLSTGDTLESSIIIDNNEYHKTTQYELKGLGRS
NPVFAIIFAITLLSIAGIPPLAGFYTKwLLFSAAVDSGYSIIALIGIVTSVIGAIYYLRLIHFMYFRTPD
SKFNELSDIVGGLIPMKLSTSLIISIGLLILLIFIFIPQILIDLVTIAVLSVL*
>Capsaspora_owczsarsaki_nad3_;_117_aa_on_
MLDYSPILAVILISVIVSSILVVLSYILALQQADTEKISSYECGFNPYSDARQKFEVQFFLVGILFIIFD
LEISFLFPwTVTLSIISSFGYwTMMLFLFILTVGLYYEwKKGGLDwQ*
>Capsaspora_owczsarsaki_nad4_;_477_aa_on_
MNSFELLVVTLVLGITGVMTLKNEFQAVLFGLISTIVTFVEMIIMwSQFDSSIVGYQwETKwNLPLNwVF
GIDGLSIwLIMLTGLLIPICILSNwDSIKIMRREFVVAYILLEILLILVFSVLDILLFYVFFETILIPMF
YIIGIwGSREQKVQAAYYFFFYTLLGSVLMLIGIMwIYVTYGTTDLRLIETTNIDINVQNwLFIAFFASL
AVKVPMFPFHLwLPQAHVEAPLSGSILLAGVLLKLGGYGFIRYVLAILPEASVYFSPLVMTMSAVAVIYA
SLTTIRQIDMKRLIAYSSVAHMSIVTIGIFSGSAQGISGSIILMLAHGFVSSALFIIVTILYDRHHSRIF
KYYRGVTITMPIYSTIFFFFTLANIAAPLTANFIGEFLVLAGSLSYSFLITFLAATGMVLSAAYSLYFYN
RVAFGTMSLYLINSTENRDVTRREFwVLVPLIIPTIYFGVYPELVLNTLQTYVSSLI*
>Capsaspora_owczsarsaki_nad4L_;_99_aa_on_
MFSEYILLSVIIFLLGVLGIILNRKNIIVLLMSIELILLAINLNFIVLSVMSDDLIGQVGAILVLTVAAA
ESAIGLAILVAYYRIRGTIAVLFINLMKG*
>Capsaspora_owczsarsaki_nad5_;_662_aa_on_
MYVCRLVFDNSVYFNTPKYwILwHMYLLLVFIPMMCSMITLLFGRNIGIQGATKLTTISISSVALISLYL
GITQSQPVHINLLTWFTNDTLNVNwGLQFDSITYVMLVVVTAISALVHLYSTEYMGTDPHLPRFMSYLSL
FTFFMLILVTADNIVQLFIGwEGVGTCSYLLINYwYTRIQANKSAIKAMVVNRVGDMALSLAMFCLIYMF
NSIEFSTIFANIATVNNTIIILGLEINLISVTGILIFIGAVGKSAQLTLHTwLPDAMEGPTPVSALIHAA
TMVTAGVYLIIRCSPLYEHSNISLTLITIVGALTAFFAATVGLLQNDIKRVIAYSTCSQLGYMVFAAGLS
SFSVSLFHLFNHAFFKALLFLSAGSVIHALADEQDFRKIGGLISKVPLTYSLVLIGSLSLAGFPYLTGFY
SKDIILELAYGTYTIPSTFAYwLGTISAFCTAYYSMRLIYFTFLSAPLTSKEQFNNAGESGIAMTIPLII
LGFASIFIGFVCKDYFVGLGSQAFNQSIFISPQNNAVYEAEFLPAwIKNIPVVFSLIGAALGVYLLSAQG
SVLYNwKISPLGNAIYKFLNSKwYFDVIYNKFIVKPALNIGHDITYKIIDRGVLEYFGPNGLYSLVVKLS
QMVSTTQIGQIYNYTFTMIIGLSVLLLPTIIN*
>Capsaspora_owczsarsaki_nad6_;_198_aa_on_
MISNLIFSLFAITMIISASMVIITINPIHAVLYLISVFFNATGLLILLGFEFLGLILLIVYVGAIAILFL
FVVMMLNIKLVELNQNLTRYLPIGLLIGIALLIELIIALQLPLNNVTLSSNTNGLIEwMSQVISTPSTGI
NTLGNVLYTHYYQwFLISGLILLVAMVGAIVLTLNQRIGIKRQNLYSQLAVSADLNKF*
>Capsaspora_owczsarsaki_orf157_;_157_aa_on_
MSGNLQVLIRYYSSIIQVVFRHYSGIYQVLFRYYSSIIQVVFRHYSGIYQVwFKYYSGSIQALFRNLPGI
IQVLFRYYSGIIQVFTRCFPGILYLwLTLLwSIIQVVFRHYSGIYQVLFRYYLGIIQVNSWEITGILYLw
LTLLCLVSLLLNQVYCT*
>Capsaspora_owczsarsaki_orf165_;_165_aa_on_
MIQLPIAKIwCKLPVLKHVANYQLLKHVANCQLLKHDANCLLLKHDANYLLLKHDANYQLLKHDAIYQLP
VIYARSINFKARQVTYQNYPLDLwAIYPMSTCEVKQNPPWKIHHGKSTMENLPWKIHYRKSTMENPPWKI
HYGKPTMENPLWKIHYGKSTMENPL*
>Capsaspora_owczsarsaki_orf190_;_190_aa_on_
MILCYLHGISCYwYIwIYVTYMGFHIRYEWYFIFAMNGISYLLIYIwIYVTYMGFYIANIYMNLCNLHGI
SCYwLIIHGISYNEYIwIYVTYMGFYIANIYDFMLLTWNFMLLINNTWNFIFANIYDVLHGISYSLIYMM
LLTWNFILLIYEFMLLIWNFILLIYMMLLTWNFILAIYMNLYYLYGISYC*
>Capsaspora_owczsarsaki_orf209_;_186_aa_on_
; Warning ! Unusual start codon TTC for orf209 FVMNGISHLLIHMNLCNLHGFSY
SLwMVFHIRYEWYFIFAMKGISYLLwMAFYICYEWYFIFTNIYDFMLLTWNFMLLVHMILCNLHGILYSL
IYMILCYLHGISCYwLIIHGISYSLIYMILCYLHGISCYwLIIHGISYSLIYMILCYLHGISCYwYIwIY
VIYIGFHICYEWYFIFTNIYEFM*
>Capsaspora_owczsarsaki_orf223_;_223_aa_on_
MRYINLNKGKIRVIKKIKLNKLSHwKLRKFwNLVKIVKNNLLLTKLIKNAICNKFNLPTLNKKKLINNKI
IKGKLTQSLQGLLNKQKKTRQPKLFKNKLKFLRLKKTIKLVRAYKRLGGLYNKRQDLFFTLNQFSIKSRR
FFKKKISKIGVKQKKLGYNLRSFSASwKLISKIMKPLKSKMFNKINNTFIIHSFNQLKFHETKALKKAQI
PHSKNNSSSSLwD*
>Capsaspora_owczsarsaki_orf233_;_251_aa_on_'nu6687iac329_mt'
; Warning ! 2 stop codons found for orf233 MKQLIVKGLDFITITRKNIQKPRLKKS
SNPSLNSIKIIQRFLSKKSYNRNRFSQFNKYRKFITLKKIKKIYNLLKIKYINNVIQVPKLIKLRHIIKK
FHYLTQTLQIHNSQIKTIQSwPLMSTETSTKFMSNKERRRwIIRHIRGKRVLLDISKKRAISLwKTKKRP
KKwGFKILKYKSLKSYKSRKwFRSLKSKYINQPLISKSIIQRNVSSTLNQSNKTSKKIKLKNIVYI*LIL
ILLIIDLPN*STLQAT
>Capsaspora_owczsarsaki_orf270_;_270_aa_on_
MLYVLYTwNCLVwCEIVHEIALYDVRWYMKLPYMMwDGTwNCHIwCDMVKILHVLCIILYEMVKIwHVLY
EKGIILHVICIILCDMVKILHVLYEKSKILHVYEKGKILQVLCIILCDMVKILHVLCNILYEMVKILHVL
YEKGKNLHVLYEKGKNLHVLYEKGKILHVICIILCDMVKILYVLYEKSKILHVYEKGKILHVYEKGKILH
IYEKGKILHVYEKGKILHVYEKGKILHVICIILCEMVKFLHVLYEKGKILHALCTILwRW*
>Capsaspora_owczsarsaki_orf280_;_280_aa_on_'nu6687iac329_mt'
MIKQGIEINILAPALIKIQDIESSVSLHQSNAKQAIQESKKKQISPVKLVTNTTKHRFKKIRVTNIEPKN
VISSwTLKPRKKKSLSIKNRQGFSFwIEKSKTPLSLYKKRLLKILKKTLKNKSLIVTKKFKNNKFYKANS
KRNFNKKFGYNANYKNANYKKSGYNSGFHNKSDFNNKSEFNSKDYKKNYKSGFNSGFNKSKFNYKDNIAD
KNYSFNNKSNFNYKDNTTDKNYSFNKSKFNTNDNIGGQNFSNTKSNFNKNSRYNNYGYNKPSYNHTTYKK
*
>Capsaspora_owczsarsaki_orf517_;_517_aa_on_
MKKDSENKDIITSNTEINTDNKGNVTNNQKIVTEKKEKFTNNQGDVTNNQKIVTENKEKFTKAIKNRKHK
YDNITDLKYYIAGLFEGDGCVIMPNRTIKPKSGKIYPEFKITGNSKDIPLFDFLQVKFGHGNVVKRQGEN
TVDwTINNIEGLKKIIRwLNGKLYTPKLHVwNLFIDLINKQEKTKFKKKSLCTMSLKKNAwFCGFVDADG
ALQIRRDKGTYNGCMYLTQRRVDQYNNSYKPVMELIKNTFGFSLREITSKKLIPIDENHNETTIMNNTTN
LSANHTETIYIEDDTNIDNTTNLNATANNELNINSTGIAILKIYKGYLVASENRIANAQLIEYLDVYPFL
SYRYLDYLLwKEAIYIKEPGTKwTKEMQDKAAKLKSQMNSQRKVFTwDHLPKLSKEEMAALEVLHIKRAK
LEVNRNIRAELKAQKLENQIKLDNITLHGETEEDPVSGINTHKVNPVGDFKNTGDKVNPEGELRNTGDKV
NSEGELRNTGEGELRNTGDGENPGQTK*
>Capsaspora_owczsarsaki_rpl2_;_483_aa_on_
MTKFRFLTwGKQRINGRNNQGKITIRHRGSGHKRLYRHVSSIVTPGIwEIKEIHRDPNRTSFISLVSCKQ
LFEDGKLKPVTNSMLKYKLTAHGNKVGDLLVEFQTLEQIAIYAPLISQEISLIGSTVPIRSIPTGTKIFN
VGSKISEHGKYFKAAGSFGTLAKVDGLLKDFPTNKKDNQKLIAMKKLISAQETLQSNNQLNFANDIFTKS
KILMDKKIGNLVSIINRSSIPNIKNNITKVAPVKISSETYMNTNVLSLISQIKTNVNNSLIGKIQRKKVL
NMIPVTPIIKEDLYGLKKTLITSNDSVKTLKAKTITPIANTIHKNLIGLNETLKPSTYNSNITSSNwSKV
YAYLTMNENISTFEENVTYYSYVKLKHLGTTwRVRVKRHQLEKRTRTLRINSLALATIGQASNLNHKHEI
LGSAGASRHRGIRPHVKGEAMNVVDHPNGGRTRGGKQSRTPwGKIQRGKITARKSSARwIKLI*
>Capsaspora_owczsarsaki_rpl5_;_245_aa_on_
MMNYNKIIQRDIKSKLPHTNIYDPYPTGSKTQILFSGSSNTAAKYLKGIRMRNVTLYKEYGVNSQIVKAN
KSEALFNMRASFPIGVKGYVHKKARLGVIHRLQILMCIRTDQTVPLKKHKGNINYKSEwKVNQGTPVIIK
NVLSKHRIQKSKVIDSTFMLGYRNISSYKPHMAYVwNKSSRFVGQRQLGMNVQIKLLRPLKSREYLGwSL
NPSQLAQNNVKLVNITNSLwFLSMNHIPTLKTYSA*
>Capsaspora_owczsarsaki_rpl6_;_276_aa_on_
MKYKIFLEIQGVLSITNNDGINQLCSIYRLGKIETTILIPASISIFMLSEGATSTYVLVSTLEESSIVDK
LVTKLNNLDKTFSVKIKVTGTALKIEKINKEEIKEEQIKIGSHRYELKPLGIDEKELTNKwKISKISKHA
QIPTFLNKAKSNLwwQLLQIEQGIKDPVLQIAAKKEIKGTFAKVVTDQIQTNLQKNMIKENFVTFKSGLN
EISLCLISDQVQVTSQSGYHELISKNKDLVKKIGNDFVKLKPREPYKGKGIGFLKAPFKLKQRKKK*
>Capsaspora_owczsarsaki_rpl14_;_127_aa_on_
MQLKSKCLVADNSGVIQVECIQVKNKKNVANIGDIIKIAVKSVLPTSKIKKGAVKNGLVIKSKIGTKFKD
GSRIQFGDYEVILVERGIKGKGwVPVSNRSSGLVPYwITSTQLKTLFNwPFPKEIIK*
>Capsaspora_owczsarsaki_rpl16_;_184_aa_on_
MDYRQIKPRKTKYPNTQKSQKGRVSKYLIKSGASLLQEGIIGLRALEPGRLSAKELGSVIVYLRRKLKKV
CKITLRTFPDIGITKKPAESRLGGGKGALEFwGARCRKNQIIFELTPTGQEwSLKQTSNRDNVFIENAIS
SRMEKALIKASTKLRIKTVVVYKLPAVITTQISLTHNLSSSYEI*
>Capsaspora_owczsarsaki_rps4_;_240_aa_on_
MRIQITKKNQHIKDMKVQLLSLWNKYKRGTKQSRYDTIRISKIRKIKHDYGNLKETHTIKQACLNRNPEK
LVSTLNKRLDAILVHCNwSISIIEARNwIKLGYILVNNKIIKYGGYMPNIMDLITVKTTDSKGETIAESI
RLRNGISKHwSwLKKLRFTKKPAISKLSFLKSRKKYLGFKHKNDFFKKSGKSLNSIKSNKSLKSVKLANI
VLKKKIAMESANNAVKSLKPANIALKKKIT*
>Capsaspora_owczsarsaki_rps12_;_142_aa_on_'nu6687iac329_mt'
MVTLNQLVRKPRIRKNRKVKSAALKLLSYRNRVSTLKHHSKPFVKGICTKILIMKPKKPNSAQRKLAKVL
LKTGKTVFVSIPGIGHNLNDHATVLVRGGPVPDLPSVQYKMIRGKYDLLGVKNRRTSPSKYGVKTSTLKT
TK*
>Capsaspora_owczsarsaki_rps13_;_96_aa_on_
MKKLKLTLSKIDSTRKIKELVSNTNTKSELKLANLLQITDQLVTNILQNKVTTAILIRRSKKNTGTwHSw
RLSNGYPVHGQRTHSNAVIAKKKNKI*
>Capsaspora_owczsarsaki_rps14_;_100_aa_on_
MFKLRHKTLKDQKRREQFKEVEMTRNLYNAIIHSSIVPSIVKVYAQEKLYNLPVDSAFVRIRNRCIMTSR
PRGVIGQYNINRIKFRELVLQGKISGVKLR*
>Capsaspora_owczsarsaki_rps19_;_72_aa_on_
MKQDKKELNLDMYTRMRSAVIIPEwLNKKIGVYNGKTYIPVTIKLSMIGHKLGEYSwTTKPAIFVTKKGA
KK*
>Capsaspora_owczsarsaki_yejR_;_848_aa_on_
MYFVIMSLISIITIwKVIHKNMDLDLVERLSSITQTIEYAISGIwGNHDGSLILLLLMLIFYQIKGKNES
NMHVLSTTFLLSNNEHSNNEHSNNEHSNNEHSNNEHSNNEHSNNEHSNNEHSNNEHSNNEHFNNKHSNNK
HSNNKHSNNKHSNNEHVSKTLVTPKFVINAPNKARKGLRIIRKILKTTPLQILITIFLIFSILVAFGLKQ
STFVELKQSIFIQDETILKEIIQVLNPNLEDFFLLIHPPILYFGYLSILAIIPWITDKKALKIKLLLGMI
wLTTGISLGSwwAYHELGwGSYwYwDAVENASLwVwFFNVLFIHILLLFKYSNFNILKFQINPSwLSRSS
wLSRSSwLSRSSwLSRSSwLSRSAIFNTSLILIGGVCVTLLAIYLVRSGSLESVHSFVKDVVKSTVLGVL
IIIYLFYLILLNFLVQLNVYNIPITHDELNTNNINSIIPIFNISHTFINSKLKTHIADLNQLRVRPNKLD
MSLKwAIVTQFVILVGLILMSLPGGLPAISTwIKwAIINILIIMNSLMFKFYDKNITwDTFIIFILRLIG
IINIIYIFVTIITQPLLIIEPLMKILKILIPILGDLIQFILKVSGFTMLNHIPTYLGIDMVKTPwMISKL
IMSQISSLSILPDLSSISSMVSIPLISwILPFIKELNYYLTIENGLIFSSLLMLLTLITTLGNRNTSINK
DNVFIHIIIMFIFLCLLIMNKQTEITQVLKIGESIQLRDITLTLQNLDYIIGNNHTSIIGSLVIENYDTS
VTQVFSEKKYYFSNNQHIFKTTIMSNCFTDVFIGISNGSINTGwLVKVKIIPGVLYLwLTLLCLVSLLLN
QVYYTQQH*
>Capsaspora_owczsarsaki_yejU_;_223_aa_on_
MIILGTLSFTLICYSLEDFQQDIYwKIIFIHVPLASLSLMLYVCKVACSLLYLITSRATLAMIARNLAKI
ALTLQILCIITGSLwGKASwGTYwEFDSRLTSMLIITIITGCYVIINQTIKDETTSIKKDRILAIISIIG
SINIPIIKFSVDwwASLHQKSSINIFNVGTTKMDISIYLPLMMVLFVFVLwIIHMFSIYMMIEQSERKLQ
IIQKQIPVLPTSS*
>cox1_mt_Rhizopus_oryzae_;_528_aa
MVRWLFSTNAKDIGTLYIIFSIFAGMIGTAFSMLIRLELAGPGIQYLHGDHQLYNVIVTAHAFVMIFFLV
MPAMIGGFGNWFVPLMIGAPDMAFPRLNNISFWLLPPSLILLVASAFVENGAGTGWTVYPPLSGIASHSG
GSVDLAIFSLHLSGISSMLGAMNFITTILNMRAPGMSFHKMPLFVWAVLITAVLLLLSLPVLAGGITMLL
TDRNFNTSFYEPAGGGDPLLYQHLFWFFGHPEVYILIIPGFGIVSHVVSTFCGKPIFGYLGMVYAMLSIG
VLGFIVWSHHMYTVGLDVDTRAYFTAATMIIAVPTGIKIFSWLATLYGGSIRFTTPMLFALGFLALFTIG
GLTGVMLANASMDVALHDTYYVVAHFHYVLSMGAVFAMFAAFYYWIGKITGKTYNELLGQIHFWSLFIGV
NLTFFPQHMLGLAGMPRRIPDYPDAYAGWNLVSSFGSIISIVASLVFLYALYDLLARQEYNLANNYWYVP
QFFSSSRAIGATSTASTLEWSLTSPPSFHTVNSLPVQS*
>nad2_mt_Rhizopus_oryzae_;_523_aa
MVLFGVLTMILAIALFSLRIPAIYFNRITIILLLFSALLSYNSLYMNNIGSGVGVFGGLFQVTTITQSID
VFIYLLGALVLLLSEKANRANTLSFNKLNSTLVNKSKGLSVLAEYPLIALFSVLGMSSLISSSDLISMFL
SIELQSFAVYILATIYRESESATSAGLKYFLLGSLSSALILLGSSLLYSFTGLTSFEGLYMLCSTTETNT
AIEISVLLIMVGLLFKVSAAPFHNWAPDVYDGVPTVVTTWLTTMPKIAFLVFILEFQGFTQLANWSSWTN
LLLISSLLSLLIGTIGGLAQYRIKRLLTYSTISHVGFLLLALAINNEESVESFLFYLIQYSLTNINVFFI
LVAFGYLLGSKGLSIYSPIQLINQLKGQFKVNPLLGLSLAICLFSMAGIPPLVGFFGKQMVLYAATHNGN
FFLAFVAILVSVVSAAYYLRIIKVIHFDPVPAPSALSLIKTTEISSDLNVTETNNSLEGSSEELSTSSSL
VIATITLLLIFFIINPTPLLNSVHLITLNLFYW*
>nad3_mt_Rhizopus_oryzae_;_124_aa
MSTLTFLVLFLPILTLVLLVVNGLLAVNKPYSEKVSPYECGFTPLGDARQKFSIQFYLVAILFIVFDLEV
LFLFPFAVSLYEISTMGFWVVILFLVVLTIGFVYEFGKGALKFTKDPSTSKINL*
>nad5_mt_Rhizopus_oryzae_;_655_aa
MYLAILTLPLLSATVAGFLGRKIGKTGSHLITCSSLVLTALLALVAFYEVGLCGSPVSIKLMSWIDSEFL
LVSWGFIYDSLTVSMLLPVLIVSALVHIYSTNYMSEDPHNQRFFAYLSMFTFFMLMLVTGDNYLVMFIGW
EGVGISSYLLINFWFTRLQANKAAIKALVMNRVGDWGFSIGLWAIFWTFGNLDFTTVFSLAPFINEELIT
IISICLLVAAMGKSAQIGLHTWLPDAMEGPTPVSALIHAATMVTAGVYLLLRSSPILEFGSTALILITWV
GALTAFFAATTGLLQNDLKRVIAYSTCSQLGLLFLVCGLSQYNVALFHLVNHAWFKALLFLSAGSVIHAM
NDEQDLRKFGGLSRLLPFTYSMMVIGSLSLMALPFLTGFYSKDLIIELAYGHYSFSGNLVYWLASVAAVF
TAMYSIRSLYLTFLGYPNGPKINYNNIHEAPLIMAIPLVVLAVFSIFFGYVTKDLFVGMGTDFYNNALFI
HPNHSILVDTEFGLPMSMKFLPLIGSLLGTFGVLAIYWIFDELPNKFISTKLGRGIYRFFNQKYYFDNIY
NNLLLNKFLNFGYTTNKILDRGAIELVGPYGLVNVFKSASNKVSGLDSGFIPTYAMYIFNGLILFITLIF
FIGDPRLFVLLLWAVFLLPNNTTQK*
>nad4L_mt_Rhizopus_oryzae_;_88_aa
MNLSMVLFLIGILGFILNRKNIILMLISIEIMLLAVTLLIILSSFSFDDILGQTYGIYIIAIAGAESAIG
LGILVAYYRLRGSIAIKA*
>atp8_mt_Rhizopus_oryzae_;_48_aa
MPQLVPFYFLNQVSFAFLLLMVLLYVVSKFILPNFKLVQSSRMFLASK*
>nad4_mt_Rhizopus_oryzae_;_475_aa
MILSLLVTPILGVFGVILSGENSFLQKKVALASSLLTFVLSLVLWAGFDSNYNGFQFVSSFPTVTTQEVV
GVDGISLFFVLLTTFIIPICILASWESVKTGIKYFLIAFLVLETLLIAVFVVLDLLLFYICFESVLIPMF
LIIGIWGARERKIHAAYQFFLYTLLGSLFMLLAILVIYFEVGSTDYQVLAVADISETRQKILWLGFFLSF
AVKVPMIPFHIWLPEAHVEANLAGSIILAGILLKLAGYGFLRYSIAILPDASVFFTPLVYSLAVISIIYS
SFTTLRQVDLKKIIAYSSVGHMNITLLGLFSNTIQGIEGSLILMLAHGVVSPALFICVVILYDRYHTRLL
KYYRGLTQHMPVWSIMFFLFTLGNIAVPLSANFVGEFMTLTGAFQQNPVLTVLGGLGMILSAAYGIWLYN
RTAFGAQSKYLVPMGDINRREFMTLLPLLFLMFVMGLFPNLFLEPMHLAVSNLLI*
>atp6_mt_Rhizopus_oryzae_;_259_aa
MSTLATTFLVNNPLEQFEINDFVFILAPIFGFTKLSLTNIGFYLIMVVVITISMNVLSLNNGYVIPSRWT
IHSESVYGSILNLVREQIGPKNEVYVPFIFALFNFILISNLVGLVPYSFTTTSHLVLTISLATAILIGVT
IIGFQRHGLGFFAFFIPAGTPFALVFLLVAIELISYLARAVSLGVRLGANMIAGHSLLKIISTFTWKMVV
AGPVLLLVSLLPLLFLTALAGLEFGIGILQAYVFTILTCSYLKDAIELH*
>cox2_mt_Rhizopus_oryzae_;_255_aa
MLNMLKSLNIISPVWNDAPEPWQWAFQDGASPSYEGIVELHDQIMFYLVVILFGVSWVLTSIIVNFGSNK
NQIVYKYHNHGTLIELIWTITPAFVLIAIAFPSFKLLYLMDEVIDPAMTIKALGHQWYWSYEYSDFVNED
GESIEFDSYLVPTDDLEEGQLRLLEVDNRVVVPVGTHIRFIVTGADVIHSFAVPSLGLKIDAIPGRLNQT
SVLVEREGVYYGQCSEICGVHHGFMPIAVEAVSLEKYLAWLNEQA*
>cob_mt_Rhizopus_oryzae_;_386_aa
MKLLKSHPFLSLANSYVIDSPQPSNLNYAWNFGSLLALCLGIQIVTGVTLAMHYTPNIDLAFISVEHIMR
DVNYGWMIRYLHANTASFFFLFVYLHIGRGLYYGSYKSPRALPWSIGVIILILMMATAFLGYVLPWGQMS
LWGATVITNLLSAIPWIGKDLVEFIWGGFSVDNATLNRFFSLHYLLPFILAALAVMHLLALHEHGSSNPL
GITANADRLYMHPYYTFKDLVTIFLFFLVLALFLFYAPNKLGHPDNYIPANPMQTPASIVPEWYLLPFYA
ILRSIPDKLGGVIAMFGSLLILLAMPLLDLSRVRGSAFRPLMKFFFWLLVVDFLILLWCGSQHVEEPFIT
LGQFATTFYFSWFLIIVPVVSVIENTLIDLATENKS*
>cox3_mt_Rhizopus_oryzae_;_282_aa
MIEMYDRNYIYNMTTKIMNRSVQAHPFHLVEASPWPIAVSFSLLVVTLSGVMTFQGYSNGLFLLTLGFIS
LVSTMTLWFKDISREGTFQGHHTFAVQKGLSLGFVLFVVSEVFFFISIFWAFFHSALAPTVELGAHWPPA
GIETLNPWEVPLLNTVILLSSGATVTYAHHSLIQGNRAGVIYGLIATVVLATVFTGFQGFEYYNAPFTFS
DGVYGSTFYMATGFHGIHVLVGTIFLTVGLFRVLSYHLTDHHHLGFEQAILYWHFVDVVWLFLFISVYWW
GG*
>atp9_mt_Rhizopus_oryzae_;_74_aa
MVAAAKILGAGLATIGLAGAGVGVGLVFAALINSTSRNPSLRPQLFSYTILGFALTEAIGLFALMMAFLL
LYAA*
>nad1_mt_Rhizopus_oryzae_;_325_aa
MLLSFIEVLIVIVPLLLSVAFVTIAERKAMGSMQRRLGPNRVGYYGLLQPFADALKLFVKESVIPAHSNK
ALFLFAPIISLITSLLAWGVIPFGSGLTLADLSLGIFYLLAVGSLGIYGVVLAGWSANSKYAFLGGLRST
AQMISYEVVMGLVILTVIILTGSLNLTNIVQNQISVWFIIPLLPMGLMFLITIVAETNRAPFDLPEAESE
LVAGFFTEHSSVPFVMFFLAEYASIILMSTLYSILFLGGYLLPYFGEVNILFNIFSGLSLGIKTSLIVFI
YIWLRASFPRLRYDQLMSFCWTGMLPIVLGFIILVPCIGVAFEFV*
>nad6_mt_Rhizopus_oryzae_;_214_aa
MNAILLDLLVFGSVLSGILVITSRNPVVSVLFLISVFINVACYLILLGINFIGLAYLIVYVGAIAILFLF
VIMMLNIKLVELQDSSEDYSNPYPLAFVLGTLFVSGLSLTNKNIWGVHSEQSLFGSSNGSGIKLDLSGLF
DWVNLLSFKSNTLETLTINHSNWDNVFVSMDQINSIGQVLYTSHALFLIVASMILLLAMVGPIILCLNPT
KRVS*
>Smittium_culisetae_mt_orf327_;_327_aa
MKEIQLFNLNPNWVTGFTQADGCFNITFTKKKPNQIRLRARFIISQHIKDELLILSIKNFFNCGIIIKNK
KEIQYVVNSINDLINIIIPHFDKYPLKYGKYTSYLIFKNIIEKMKNKQHLTQKGLIDIINLTYIMNPLGK
RKINKKELFEFLKIKDFSISDENNPYTDFSLSNKFLYQNEIDINFIGGLIQGDGCFNISFRKDLKIQAQL
FIAQDMYSIELLSEIKKFFNCGNIIDKKVKMSIIEIKNINNLYNKILPLFNENLFFLDKLKQFIIFKQIV
NLLYNKQHLTKDNKLKIVDLAYNMNNNGIKRKLTKEQFIQIIINKYK*
>Smittium_culisetae_mt_atp8_;_50_aa
MPHLIPFTFFHQISFALFLISTLVYLFSVYIFPYFILRDISRLIILYPYK*
>Smittium_culisetae_mt_nad2_;_479_aa
MLTISIILFIIILSFKILKPVYINRISILISIYCSIILLTIIFNTNVFYNYGISIYNSLGYISINQIIGQ
LIVWFTITLVLLLGESGKTRKNILRSSEYTLIILLSILGMNFLLISNDLVTMSLAIELISFPLYISASLY
KNDEIYEGLGFNKNNLSGIYAGLKYLLIGSLGSVFILLGSVFIYGDTGLTNITNIIELLNIFNNKLEIYK
IGSTLIIVGLLLKLGCAPFHNWAPDVYDGVPTIVTTWISIVPKIPFLIFLSLHPLFIYPLLSNLTLFSSF
CSLIIGSIMGLLIFRIKRLLAYSSIVHLGFILLSISCIYYINTFTTIFYIVVYVITSINIFFILNSINLS
KNIELLSELKGFVYNNKLLSFSLIISFFSLAGIPPFLGFYSKYFILLDSFNLEYYFITFISLLCSVISCV
YYLRIIKIINFDDNIYMNYKSIHLNETISIVISFFTFLTLTFVLNGNLIFNLFNTPFII*
>Smittium_culisetae_mt_nad5_;_649_aa
MYTILIIIPLISAIISGLLGRYIGQKGSGIISSICIGITAILAIISFYEIGITGSPLAFSYINWIDLELL
EINWGLYLDSLTVSMAILVTLISTCVHIFSIGYMEGDPHKQRFFSYLSLFTFFMLLLISADNLLFLFIGW
EGIGICSFLLINFWYTRLEANKSAMLALIMNRVGDWSISIGIYIIFYIFGSISFTTIFSVAHNIEENTNI
TLFVIPLFIGAMAKSAQLGLNTWLPRAMEGPTPVSALLHAATMVTAGVYLLIRISPVLQYSNITLTLIIL
IGSISALLAALMALTQNDIKKIIAYSTMSQLAYCFIACGICQYDIAIFHIVNHGFFKALLFLSAGAILHS
IYDEQNIYKIGGFINIDPLIYIAIFSGSLSLMAFPFLTGFYSKDFIIAASLGTYIYIGSFAYWFGTITAF
LTSIYSIKLICLVYLIKPYGPKKHYILESNHSIYITIPIIFLTIFSIFFGYFFKDLFIGFGTPFWGNSLS
ISYSLNLLEAEFSNSNFYSLLPLCLSLIALFFTIFYYFYNSEFLAIRSTIPKLNYFSKFKYIFSNGYYLD
AIYASFIIKPYFYFSNLTNKVLDQGVFEFLGPTGFIKIFKNINISISYYDTGIIPHYGVYIILSLIIFFF
LLFIKPLIILFFVLTLLFI*
>Smittium_culisetae_mt_cob_;_396_aa
MKLLKSNPVLTLFNDYIVDSPAPSNISYLWNFGSLLGICFIIQIITGVFLAMHYTPNIDLAFLSIEHIMR
DVNNGYIIRYMHANGAGFFFICVYIHIAKGIYYGSYRKPRVFLWTIGVIIFFLMIIIGFLGYVLPFGQMS
LWGATVITNLCSAIPWIGTDLVNFIWGNYSVSNATINRFFSLHYLLPFVLAALVILHLIGLHQNGSNNPL
GISSNVDKIPFHPYFSYKDYVGFFSFFLIFFYFVFFTPNLLVEPDNYIPGNPLVTPTAIVPEFYLLPFYT
ILRSIPNKLLGVIGMVAAILIFLILPFVDISNVRSTQFKPLFKLFFYIFVVNFLILGWIGANHAELPFTI
IGQYSSIFYFSYFLFIIPLLGIIENTFSSIITKNNTPLFYFLNHKL*
>Smittium_culisetae_mt_orf224_;_224_aa
MKFICYFNSKIPILAFILPNFKAKNRIGPHNEDIISLLIGSLLGNCNGERLANGGVRFIFKQSIIHKKYL
FWLFKFLNDRGFTNNNLPKIYKNILNNKIYEYYKFNTYSYSNLLWLYKLFYSNKKKRIPLNINEYLTPLA
LAIWIMDVGYWKDNNVIIATNSFTKDEINLLILTLKKKWDLNCSIHSFTKSKQHQLYIKVNSIFLLRSIV
LPYFHKSMYYKLGL*
>Smittium_culisetae_mt_orf230_;_230_aa
; Warning ! Unusual start codon GAA for orf230 ENLPQMAENLYKFSGEYMIYSLQ
FIIPSIRRVGPHNIDLLSILIGSLLGDSYLERHGKGSRFCFQQEHTNSSYLLWLHNELSLRGYCNQKIPE
IKSRIGKKNTIRYVIRFKTWTYTSFNYFQDNFYKNNKKIIPFEIIEQYLTPLALTIWIMDDGCRSGKGLK
ISTNSFTYKEVEFLANILKKKYCLTISIHKTGSIDQYCLYIHKKCISELFKIVSPWLHPSMKYKFIL*
>Smittium_culisetae_mt_rps3_;_231_aa
MGKRHSTSLKLRHIGNWNNISYNNNNINKEKIIKFILNSYFIEKAIISEPIFIYKLNEIKIIIYYWSSFN
LLLDKDINKILLYINNILNIKVNINIIKLKKPYLSSKILSEYISINIKKYNIKKIAKKIIKQIKIEKKLL
NPLINFNYNNIKYYYINKKLYSSISGIKLVFKGRLSKRRTAERKKILTFNKGFLNKNSLFTLIDYKSPYN
FKFINGSIGIKTILNNTIHFI*
>Smittium_culisetae_mt_orf171_;_171_aa
MNKYLFMVFIILGLIQLLLIMIINIIRFIYIIYILFFTNETEVRNSPLNKAATFISQTILCFKGICGVGI
GIGSIGGTLMFADEIIKSSGRSPFFMPLVGQNMDKIFTNLGYHKMDNIEQLSREIHNLNDQILNSPLSAE
VKNKIIINNSNILSEIKNSKILSEIKNIDKK*
>Smittium_culisetae_mt_atp6_;_248_aa
MLYNPLEQFTVNKIISLYTVYYSMSLTNSSLYFIIAAIISFFIFKYSANIPYVSLINKNNYSILTESLYK
TILKMVKEQIGDKYTIYMPLIFSLFIIILVSNLVGLIPYGFSPTALFALPLGLSVTIIISVTVIGFVKYH
LKYFSVLLPSGTPLGLVPLLLVVELLSYIARAFSLGIRLAANITSGHILLNIISGFLFKTSGIALLFVII
PFTLFIALTGLELIVAILQAYVWSILTCIYIKDSLILH*
>Smittium_culisetae_mt_atp9_;_73_aa
MLASAKLIAAGLAVLSLAGTSIGIGNVFSSLLNSYSRNPSLRGQLFTYSILGFALVEAMGLFALMMSFLF
LYG*
>Smittium_culisetae_mt_cox2_;_254_aa
MNIFSFYIINNDAPEPWQICYQDSATKIMSGIDKLTGEIFYYETLLLIIVGWVLISAIIKYTKTELSYKY
FNHGTLIEILWTCSPAFILIAISFPSFKLLYLMDSIIDSQITIKVLGHQWYWSYEYSDYLDNSGDSISFD
SIMIPTDDLEPGQFRLLEVDNRIVLPIHTHIRFICTSSDVIHSFAVPSLGLKIDALPGRLNGISTYVERE
GTFYGQCSELCGVYHFGMPIVIEAVRIEKYLEWLNIHLDNTPSS*
>Smittium_culisetae_mt_nad4_;_484_aa
MYTLLLLLIPLITILLILPINNIVIIKKLALFSSIIELFIVLLIWILFDYNNPEYINTLMISNIRLGIDS
ISLIFIILTVLIMPIAILAGFNIKKKIKTFMILVLLTELIFMLIFTSLDILLFYITFESSLIPMFFLIGL
YGSDSQGMFKGKINKEKTRIYASYKFFLMTFAGSLLMLLSIIKIYTYYGTTDYLLISTFNIDNNLQIILW
LGFFISFAIKTPLLPVHQWLPLAHTEAPLTGSIILAALMLKLATYGFIKYSLILFPYASTYFLPLIMTLC
IISLIYSSFSTLRQIDMKKIIAYSSIGHMAIIIMALFSNNYQGITGALFLSFAHGLTSPALFICVGILYN
IFHTRIILYFKGLSIYMPLLSSLTFIFILANMSTPLTSNFIGEFISFISIFNYHFPFTAIIFTISIILVP
VYSIYFYIKNFFGSYSIYLKSYQDINILDFNLLITLLGFILFTGIFPNSLLNILELPVSMIIIH*
>Smittium_culisetae_mt_nad6_;_170_aa
MIITPFINILIFIFGLLIFLFINPIYSVLSLISVFILTGILFIFYGLTFIGLSYFIIYVGAISILFIFAI
MMLETDFPSTIKYLDLKKDIPLITGILCILIYYYLDLSYINNNKNIISGINNIYFYSSNIKLIGYCLYTT
HCLWLILISFILLLAMIGPIILCLKMNKNY*
>Smittium_culisetae_mt_orf170_;_170_aa
MKNVKLVRDLTPVILGSFGIVASIYNNERQIQTQKEIAKMKIENALLNNVENKTQINSALEDFYNFIEIF
SQTQMIFLYNILFILVLSSSLICLYFHFKLKSFLDYEPKWKILAFLFKYYKNYLYKFNVGFYSLIIFYSI
IILLVNNIYFLIRGIHFKYFSNRLRNLLQR*
>Smittium_culisetae_mt_nad3_;_113_aa
MNSLIIFIILTPCILSLVLIINLLLPINKPDFSKLSPYECGMDIIGSAREKFNILFYLVAILFLIFDIEV
IFLFPFATILYDISVYGYWIVFFFLIILTLGFIFELSKDILNF*
>Smittium_culisetae_mt_nad4L_;_102_aa
MLIGFFLIFIGLYGFIFNRNNYLLIVISIEIILLGTAYILSIFSINNDDIIGLLFILYIIAIAAAEVALA
LSILVTMSRLRGSIHIKTSNINTLKYNPKIIV*
>Smittium_culisetae_mt_nad1_;_323_aa
MLLSLIEILLLIFPVLFAIAFLTLTERKVMGSMQRRVGPNKVGIFGILQPIADGIKLLLKETIIPYQSNK
LLLILAPLLIFTINLIGWAPIPFSKGYQISDMELGIIYILAISSIGVVGIILAGWSGNSKYSLMGCLRTT
AQLVSYELLLGLTILTVILIINNFNINNIILFQQSIYFFIPLLPLFIIIFISAIAETNRPPFDLVESESE
LVSGAFTEYSSFSFALIFLGEYGAMITMSTLITILFLGGYLQPFFINSYFDSISLGIKICFLLFCFIWVR
STLPRVKFNQLISLCWKSLFPLVLGLFIFVSSLVLIFNLTYII*
>Smittium_culisetae_mt_cox1_;_535_aa
MINRWLLSTNAKDIGTLYLIFALFSGLLGTLLSLVIRLELMGPGIQILQGNHQFFNVVVTAHAFLMVFFF
IMPALIGGFGNYFIPVMIGAVDMAFPRLNNISFWLLPPSLLFLLASAFIENGPGTGWTVYPPLAGIQSHS
GGSVDIAIFSLHLAGISSMLGAINIITTVINMRSPGLSWHKIPLFVWAVFVTSFLLLLALPVLAGAITML
LSDRNFNSSFFDPAGGGDPILYQHLFWFFGHPEVYIIILPAFGVISQVISTFSNKPVFGYLGMVYAILSI
GILGFIVWAHHLYTVGLDTDTRAYFSAASMIIGLPTGIKIFSWLSTMYGGYIRLNTPMLFSIGFLILFTI
GGFTGIILANATLDTSLHDTYFVVAHFHYVLSMGAVFGLFAGYYYWSPKIIGRSFNDLLGKVHFWVLFIG
VNLTFFPQHFLGLAGMPRRIPDYADAYTGWNVISSYGSLISVFSTIIFLYILYKQLTNIENPISSDYWYY
PQFFNSSININTHTISSYGLEWTIASPTPLHYLYELPKVTNNINN*
>Smittium_culisetae_mt_orf355_;_355_aa
; Warning ! Unusual start codon GTA for orf355 VFIIAPALNLAICWKFLIFIGQS
ARNLINLDLLEIFRDYTPQFIYCNELIIIKNLLNKNSNQDKRYYIHKENKDIKEFNENFSYYLAGLIEGD
GTIIVPKTERSNKGRINYPSIQIVFNSKDLPLALIIQKNLGLGSISKTKGVNAYRLTINNYEGLIKLTKL
LNGKFKTVKIYYFNELIKFLNNRFSNFNILPMELNQTPFYSNSWLSGFIDADGHFNVSLNKNSVSCRFEL
VQAIEDKRGNDKKDIMIKLAEYLNKQLKIISKSYCNSKDQYNINITNLESNLIISNYLLKYPLFSSKFLN
FKDYYKVLILIKNKEHKSLIAKEQIYSIKNQMNNKRTIFTWDHLQNFYNLYK*
>Smittium_culisetae_mt_orf271_;_271_aa
; Warning ! Unusual start codon TAT for orf271 YNNLFLFKSEFQKIKKSNAPDDK
FLYWLIGFTEGDGCFSINHRKELSFILIQGIDNIELLKTIQTTLNMGNLIKQGPRVYRLIIQKREDLRLL
ILLFNGNLILPSRKVQFNLFLLNFNSKSLKKKDYTIIPYLLSNNLPSLNNTWLLGFTEAEGCFTISLLNN
SKAFRTRYILSQKGDINLPILSYLILLFKGGKVEGHSKKDNYSYIISGLDNVQFIYPYFDKYVFMGIKGL
SYLAFKQLNERIKNGEHLNLEKRKELVILSHNINRKSK*
>Smittium_culisetae_mt_orf237_;_237_aa
; Warning ! Unusual start codon GTA for orf237 VGTSETIRLLSFNNNSNKFNEWL
AGLIDGDGCFLLSKKGYASLEITMDIRDEHCLNMIKNRYEGSIKLRSNVNALRYRLHHKLGLLKIINDVN
GLIRNPVRMIQLNKICNHYNINFLFPSKLTYNNGWLVGFFDADGSITINKTNLQLAISISQKTTQILEPL
IELYGGYIYIDRSKNESFKWYITDKKSILNLIEYFKKYPSRSGKKNRLHLIPKFYELKDLKAYKASEDTL
LNRS*
>Smittium_culisetae_mt_orf279_;_279_aa
; Warning ! Unusual start codon AAA for orf279 KKIINKKYYNKKNLSTISDHVPI
HKKPLNDNDFGYYLAGLIEGDGSINKINPYISICFYELDTPLAYYIKKKIGYGTILKIKNKRAINYHLRH
KDGIIRLIDLINGKLRTNKIIDWNTYIIHKINEKYDKTYFKYNIDNSNLKNNYWLAGFTDSDGSFQIKTI
KRNNKKNNYEIRLSYQIDQKTDHILNQIKELFSGYLGFRSKLNTYYFQTVSFSSAYKVIKYLDNYHLLSS
KYLNYLKWRKTYLYIQKREHLTLKGIKKIIKIKKTMNSYSNDKFEL*
>Smittium_culisetae_mt_orf360_;_360_aa
; Warning ! Unusual start codon CAA for orf360 QNGSPFMRINGNKFHYMLKRSLN
IWFKFYKIFNLKKSKYLINQQEINRIYKLKIIFSNYAWLNIILFDLLALMENLLLNFKVYLKIGQTLNSS
ETTRETTLLILNNNNFINNNLFLNNQSNKKKKIYKDLEWLIGFTEGDGSFIKSKNRLYFVLTQKEIKILY
YIKSLLGFGKVYIYNGIGRYVVTKKEHIDILKKLFFNNLVLNKCKERFKIWNNEQIENNSIESSLLTLNN
GWLSGFIDAEGCFNVNITKNDKYITGYRVRLRFMIDQKDESEVLKKIQILLGNGRVSKRSKTTTERLTLD
TFISFPKLIDYLEKYPLKTKKRITFFKWRKIYLLCKEKKHLTIKGFNKIKKFKKLKI*
>Smittium_culisetae_mt_orf307_;_307_aa
; Warning ! Unusual start codon ACG for orf307 TFSCVLKIFKSYKIELYAGNLYF
NYLSPLGLFIKPFGKILNKEQFAGNQLINVEDIKNISDHMNKHSYPESDNDFGYYLAGLIEGDGYFGKLA
LEIIFNEKDISLAYFIKKKIGYGNIYKIKNKKAYKYYLSHKEGLKKVLDLTNGKFITNNKIDQLIKYSYN
TKFSLNIKPPASFSLLKNYWLAGFSDADSSFCITIAKSSTHKLKYSVRLEFKIKQKDPTILKKIFKEFGG
NLNIFNTENIYCYNSTNFKVAKNIINYFDNYHLNSSKYREYFRWRKAYRIIQRKEHLTLTGLIKILKLKK
NLRS*
>Smittium_culisetae_mt_orf276_;_276_aa
; Warning ! Unusual start codon AAA for orf276 KIIIFNTILPNIIKNRSINKNSI
LLPNKDYLEKFLVGLLDGDGCITVFHDKSNNYLRIKILIALLNNELNLYMFNLLKKKLEMGRISIERKDK
YVIYYIESKKDIYKTLKILDKYPLLTSRKICQLEYFKKFLENRNIDNYLNINKANIKYFKQEDIINKFNK
NFLLPDYFNGWLSGFIEAEGNFKLILYKTGGIKSRQFQIGQNNDKYLLLAIKTYLNSFHKIYIDKDSNPL
HYRISIGGEIANNNLLKHFKENPLLGNKLLSYNIWYNSFKVKN*
>Smittium_culisetae_mt_orf372_;_372_aa
; Warning ! Unusual start codon AAT for orf372 NKLAPIIIKILFNTRKSLQIYIY
DFYNFIYKKDNYSVFLLYILLICRSRFKNIIFKLFTLIKNYLYAQLVEVKNNSSILHIEKNIYKASQRLN
TKNLQWFIGFTDGDGCFSVYKEKKYQNNWRHEFSIGLQIKDIRLLYKIKNLLGCGTVKKYNNVAVFRIKK
LKHLVYQIIPLFDKFPLLTEKKRIVYLNFRNTLLNKFINSKIATNEDINFVKNLLNNNDINILYNTPIEK
FLNNIDSNYFDNWLVGFTEAEGSFYFIKNKNLKDNISQIPLKAEFRLSQNNNVILLTKIKEKLKLTRNVN
LQTNSSNHYYIVASSIDTIQNTIYFYTNPSIVKFKGIKYLKFILWLKGIKNINRYKNIKIPNNYGGDSS*
>Smittium_culisetae_mt_orf283_;_282_aa
; Warning ! 2 stop codons found for orf283 ; Warning ! Unusual start c
odon ATA for orf283 IYQIMYNLIENNLEVSQIVYSIDISINDMLSITPIYYYGPYIYPKFFKKP
IRIYSPKLDRNLIGVENRKRIIIYQWINLINGKIYIGSSMNGSTRLLSY*TTSVLKKNLPIYNNLKKYGH
NNFTLAILEDLGFTGSVNKSYLLNREQFYLNILFTEYSSLKLNCSPTAGSTFGFKHKDSFKSNRTGELNP
MFGKTFSTQFINMQIRNKVGKNNPQFGKKKSSETIAKLIKLVYVYDFKNKKFIGSYSTIDCIKKFKIGSD
TLKKYIKNGLPYKNLLFSRIKLY*
>Smittium_culisetae_mt_cox3_;_260_aa
MQLYPNHIVETSPWPIVTSISLANTLLSIVLILQGFNSLSLKISIISLILSMSLWWKDVIIESTYQGHHT
TIVMSGIKIGFLLFILSEIFVFLSVFWAQLNAALVPEIELGGLWPPLGIEAVNPFGIPLLNTLLLLSSGA
TVTWAHYSMIKNTNKKETIISLFLTILYATIFTLLQLFEYYYAPFSFVDGVYGCTFYAGTGLHALHVLVG
NTFLIVALYRIIKNHFTQTHHLGFELAIIYWHFVDIVWLLLFIIFYYWAS*
>Mortierella_verticillata_mt_atp9_;_73_aa
MLASAKIIGAGLATIGLAGAGVGIGTVFAALVNSTARNPSIKAQLFSYTILGFALTEAIGLFALMMAFLL
LYS*
>Mortierella_verticillata_mt_atp8_;_47_aa
MPQLLPFYFVNMISFSFLLFVVILYVLSTYILPTYPLLFSVRMALTK*
>Mortierella_verticillata_mt_nad3_;_199_aa
MNSFLFYFILVPVIVIALLVLNLFFAQSKPNEEKLTTFECGFSPVEQARQKFSIHFYLVGILFLVFDLEV
LLLFPAAVSMYSIGQSGFWILVFFLVVLTIGFVYEYASGALNYANKEKDSELPPINYLTLIKDKPLFKDI
NYKHNARNYSSASSKNEFKDFWLNIKPISKwYNLDKNSYFTTYINKYNSQNGIYIYRFI*
>Mortierella_verticillata_mt_nad2_;_525_aa
MLLLGIITLVMTIPLASTYLSTILIHRIAFLILLFSSLLAYNTLYVVPLSTGVGLLGGLFQTTILSQSLE
IFILIIGSIILLIAPSGEKINKELSSLNKENNFDSNKTTMSTWNYWDNLSSNVKEVLPTTKNKEGLKDYP
LIILFTTLGMSSLISSSDLITMFLAIELQSFALYILATMNRNSESAVAAGLKYFLLGGLSSCFILLGSTL
IYSYTGLTNFESLNILQSVIGQSFENNSYNYLAIGCIILTIGLLFKIGAAPLHSWAPDVYDGVPTMVTTW
LTIMPKLSILVFFIMNNFLIEPNLLMISALLSLLLGSITGLAQYKIKRLLAYSTISHVGLILLALGNESI
NGLESTIFYIVQYSITSLNIFLILILLGYTLSVNKTVSIYSPIQFISQLQGQFYQNPTLSLALAISLFSM
GGIPPLVGFFGKYFVINSALMNGNYFLSLVIVITSVISTVYYLKIIKTLFLPVANDSTSTSENNNLSPIL
AFIVAILTLFIIFFMIYPTPILNSIHLIALNTYYF*
>Mortierella_verticillata_mt_orf295_;_295_aa
MNKNNSNLTWFITGLTEAEGCFNINIYKTKAGKKTAKLRFSIAVMENDLELLKLVKDCFNCGTISESRTN
GMRYFTVSKISDINNIIIPHFQSYLLRGTKLLDFEDWVLAANIIITKSHLTEEGIEKLQLLFDGMNRKRN
KLENFLPDHCNKNSSLFIPINGNYISGFIAGDGSINIHPFSLTFDSLKFCSIFLSITQHKNNLFLMNEIK
DFFNVNNKLKIQSNNSVQLLIENKEFFRSTLIPFFNKYPLHGIKLINLNKIIKILELINKYGINRSNSYT
SDIRKEIINIWFAET*
>Mortierella_verticillata_mt_orf203_;_203_aa
MSMNKFKIIVPTIGVVASSAAAYEVGRQQSKTAMEVSIQESKTAIEIATIQANSNIEIAKIGAKSQIDQL
TVKADLSKNLVKAEANQSNVINNVESVNSPLEIGELKDLILNFDFSDLPFEILVGICIFTGTFASLWCIF
FLVIYVTVYKLDLPLENYYSGYMLKLINFVKPFSDGFIGIYLTLLVCAQMTLFILSLRLLSGY*
>Mortierella_verticillata_mt_cox1_;_534_aa
MVRWLFSTNAKDIGTLYLIFALFSGMIGTAFSMLIRLELAGPGIQYLQGDHQLYNVIVTAHAFIMIFFLV
MPALIGGFGNFLVPVMIGAVDMAFPRLNNISFWLLPPALILLLASSFVENGAGTGWTVYPPLASLQSHSG
GSVDLAIFSLHLAGISSMLGASNFITTIINMRAPGLTMHKLPLFAWAVLITAVLLLLSLPVLAGAITMLL
TDRNFNTSFYEPAGGGDPLLYQHLFWFFGHPEVYILIIPGFGIISHVISTFCGKPIFGYMGMVYAMLSIG
VLGFIVWSHHMYSVGLDVDTRAYFTAATMIIAVPTGIKIFSWLATLAGGSIRFTTPMLFALGFIFLFTIG
GVTGIVLANASIDVALHDTYYVVAHFHYVLSLGAVYALFAAYYYWVPAKILGKPYNELLGQIHFWSLSIG
VNLTFMPQHFLGLAGMPRRIPDYPDSFEGWNLVSSFGSIISIVATVLFIYIIYDTLAQPEDQLANDPWAV
PAFFTSPRGAGITKTATTLEWAITSPPAFHAFEGQLPVQSVNSP*
>Mortierella_verticillata_mt_orf296_;_296_aa
; Warning ! Unusual start codon AAAKENIFNEITNLLVIPIIGLSNSTNSFNFKNFYSMY
TKLYPNKSLPSNNFLEWFIGFTEGDGSFTITKRGELQFVISQNSLDVQVLYYIKEQLGFGTVIQQSKSDK
THRYIVQDIKNISLICSLFNGNMVFLTRNTRFLIFLAAFNDKALRSKIDIIEPISTTIIPTLNDYWLSGF
TDAEGCFSLSLLSNSSGYRLRFLLCQKWEANKPILDHICSLFGVGNVFKHSSPNNWEYIINGVKNCNSIL
PYFDKYVLFSKKKESYKLWKELRLQLIKGDHLNTNTRIKMKELANEINNVK*
>Mortierella_verticillata_mt_orf309_;_309_aa
MLLLLKNKILVITIPLASTYLSTILIRFAFLIFLSGALLSEILGGLFQTTLLTLDKSILNLDLFLSMSLL
PIVLYNSPRVLYINAKTDYKEILAENEGKSGIYVFINKINGNQYVGSAADLGDKKSGRLNRYYRPSYLTN
KKLGASKIRRAILKYDYHNFMIGILEYCSIDQLTEREQFYISVLAPKYNILTAAKSSLGYKHSYDSLKKM
SQPRPNFSPSKDHKEAIRLANANKILTLETKSKISTTLSHPIYVYTPNLSFLVKYPAITIAKLELKISPT
TIKKYCISGEIYKNKYRFSYIPLSDNKNQ*
>Mortierella_verticillata_mt_orf206_;_206_aa
; Warning ! Unusual start codon TTALFNQISQASKRRSFGSGINNTRSLEITSQEVLSNV
KPSLLDGPLTGPNDLNKYGLISRFHNSSFLDDSVNGFNNYQRIPVRTRPGGPGAIEENIKKNDLSFNLNI
NPVNNYLTPPAFSSLSYEEDKKNNKKRKRTLRKEGSCSIGVNTENTIINEAEGISNKFSANLSNVLFFFL
ILTTFIwIIIIWIIKKIHTKQDCRNNYFANK*
>Mortierella_verticillata_mt_atp6_;_256_aa
MSTLQIIVNNPLEQFEIKEFFYFGGPLIGMKFSLTNIGLYLIIVTFLILAMSYLSLNNNKVVPSRWALVQ
ESLFGSILNMINSTIGTKGQAYLPFLYTIFLYVLLSNLIGLVPYSFTPSSHFVFGIGFSVAILIGVTILG
FQTHALKFFGLFVPAGTPLGLVPVLVMIETISYLARALSLGLRLTANMIGGHVLLKIFSTFTWKAVIGGP
VLALISIIPFTFIIAFTGLELAIAFIQAYVFTMLTASYLNDAINLH*
>Mortierella_verticillata_mt_orf261_;_261_aa
MKFFNLPSINYNVTQLWRYSLRSFSLNASLWEFNKLSSTSVDPIIIYSNADIDKLKIFTDNKGKAGIYQW
KHLESGKIYIGSAIDLTKRLKDYYSFYHLDRNKYMYICNALRHYGYSAFSLSIIEYINIQNLSKNETKKL
ILKREQYYINLFNPEYNLLKVAGSLLGYQHSEETIKKISGSNNHFFGKAHTLETKVKMSIAKGTTIYVYS
SDGITLMNTFTSAREAAKFFNVEHGSILRNAKNGKLFQDKWILSTSIQSKL*
>Mortierella_verticillata_mt_cox3_;_265_aa
MNKTIQSHPFHLVEPSPWPLATSFALLTTTLSGVMYFNNYANGGLLLALGLISTVFSMSLWFRDIIIEST
FQGSHTSKVQQGLTLGFILFVISEVFFFISLFWAFFHSALSPTVELGCVWPPAGIEVMNPWEVPLLNTVL
LLSSGATVTLAHHGLIQGNRKFTIYGLFATIALAILFTGFQGFEYINAPFTIADSVYGSTFYFTTGFHGL
HVLIGTIFIAVGLYRVINYHFTDFHHVGLEAAIIYWHVVDIVWLLVFIFFYYWGS*
>Mortierella_verticillata_mt_nad4_;_495_aa
MLTALFFIPLIGALSLLFVNKNESLMKRITLGSTIINFVISLVIWGKFDNNTHEYQFVQEWAEISFCHFN
VGIDGISLFFVLLTTFLFPIIILTSWDHKNNLKSYLLNFLILESLLIATFIVLDLLLFYIFFESILIPLF
IIIINFGDLLTKEKAGFLLFLYTLFGSLFMLLSIITILILTGTTDFQILSTIDFGTDVQKLLFVGFLIAF
IVKTPLIPGHIWLGFAHVAAPIGGSVLLAGVILKLATYLALRVMIPFLPDATLYFTPLIYTLAVISIIYA
SLTCLRLIDVKAIIAYSSVSHMGVVVLGLFSNNLQGIEGAVILGLAHGVISPLLFIIVGDILYTRSHTRI
IKYYRGIATSMPILSLIFFFATLANIATPFSGNFIGEFMSFAGAFQQNPIMAILGATGIFLGTGYSIWLF
NRISFGTASRYMSTMPDISRREFFVLLPLIIVSFILGIYPNVVLDSLHLGISSLLINSPQELLLSLSNLY
FIIIw*
>Mortierella_verticillata_mt_cox2_2_;_22_aa
MPIVIEAVSLEKYMAWLNEQLS*
>Mortierella_verticillata_mt_orf342_;_342_aa
MLLLGIITLVITIPLASTYLSTILIHRIAFLILLFSASASGIDILGGLFQTSIFQTGLLLLPFSLNNKFS
LNSQVRFFSSNNTPKKKSKFQRFAFGLKKGIYLELLPDNVLIFHNSVIVRIFRVIGGISFILWISKLYLK
SNISLVLILPFVFIHLIYITIISFIKIKYLIYLWKNGKLEVRNSPIDKFATFGFRLAACAKGVCVYGAGT
GTTIALGLGIDELLAHSGRPAIFKPIFGGTLDSFLTKVGVENPNREIVNIKNEIDLLTYKIKSLNNLQEN
LKEINSIETTTVEDKILLSEVKNALNEEILSQRSELEKTIEKKIQESSIIKEIYKDGSPFKK*
>Mortierella_verticillata_mt_cox2_;_253_aa
MLSKIFNLPTVFLDVAEPWQLSFQDGASPSFDGIVDLHDQIMFYLVIIVIGVSWMLGTIILKFNTKTNPI
VYKYFTHGTVIELIWTVTPALVLVAIAFPSFRLLYLMDEVIDPAITVKAVGSQWFWSYEYSDYISDSGEP
IAFDSFMVPEADLEEGQFRLLEVDNRVVLPVDTHVRFILTGSDVIHSFAIPALGLKLDAIPGRLNQTSTF
INREGLFYGQCSEICGVNHGFMPIVIEAVSLEKYMAWLNEQLS*
>Mortierella_verticillata_mt_orf122_;_122_aa
MTNLNKLNYTVTLNTTFKATFNSFVAKSAFLGQTCFFSTEKNNEDNKLDIQFKTWLISNKPAKVYLNSAK
SKNLIYNKNCSKSGVYLWYNNINKKYYVGSSINLKHRLYYYISPTYLKKINS*
>Mortierella_verticillata_mt_nad1_;_321_aa
MLISLLEALIVIGPLLGSIAYVTIAERKVMGSMQRRIGPNKVGYYGLLQAFADALKLLVKELVLPSNTDK
ILFVLAPMISLVCGLLVWTLIPYGKGLVIADFSLGIVTILAISSVAAYGIILAGWASNNKYSFIGSLRAT
AQLISYEVVFGLIILLVIFFVGSFNLIKIIEAQKAVWYIVPLFPIALMFLIAILAESNRAPFDLPESESE
LTAGFMTEYSAFPFVFFFLAEYGAIVFLSTLSSILFLGGYLIPGVESNGLMTGLSLGFKTSILLFILIWI
RASFPRLRYDQLMAAIWCNMLPVVIAFSILIPCIIKAFEIY*
>Mortierella_verticillata_mt_cob_;_386_aa
; Warning ! Unusual start codon TTGLKLLKTHPFLSLVNSYVIDSPSPSNISYLWNYGSL
LGVVLVIQIATGVTLAMHYVPHVDLAFISVEHIMRDVNYGWMIRYFHANGAAFFFIFIYIHMAKGLYYGS
YKAPRVMLWSIGVIIFLLLIITGFLGYVLPYGQMSFWGATVITNLMSAIPWIGNDFVEFLWGGFSVGNPT
LNRFFSLHYLLPFILAALVIMHLIALHEHGSNNPLGITSNVDRIRFHPYYTFKDLVGLFVFFFLFAYFVF
FNPNFFGESDNYIPANPLVTPISIVPEFYLLPFYAILRSIPHKLLGVIGMVAAILILLATPFLDTSRIRS
MQFRPIMKFFFWLFVANFLILGWIGANHPESPLVEIGQVSTVFYFSYFLVILPVVGIIENTLFDLGTKTT
K*
>Mortierella_verticillata_mt_nad5_;_664_aa
MYLSIILIPLVGSIFAGLFGRKLGAQGSQFITTFGLIVTCLLSFTAFYEVGLNQSSVSVDLFSWIDSEYF
LVKWGFTFDSLTVSMLIPIVFISSLVHMYSIGYMGADPHTQRFFSYLSAFTFCMLLLVCGDNLLVLFVGW
EGVGVCSYLLISFWFTRIAATKSAIQAIVTNKVGDWGMSIALFAIIFVFGNLDFSNLFSLAPYINTDILT
FICLCILIGVMAKSAQLGLHAWLPTAMEGPTPVSALIHSATMVTAGIFLMIRTSPLLEYSSTALIVITCL
GSLSAFVFASIGLVQNDLKRIIAYSTASQLGYLGAVCGLSQYNIAFYHIINHAFFKALLFLAAGSVIHAM
ADEQDVRRMGGLAKLLPFTFVVMFIAALSNIAFPFLSGFYSKEIIILSGYGQYTFQGSFAYWLTTLAAFF
TSIYTTRLLYLTFFSIPNGTRNNYENTHEAPFFMAIPLGILAVLSVIFGYLSKDLFIGMGSDFFGNAVFI
HPNNIILIDAEFAIPLFIKLLPSILTICGFIGTLLFYEFVPNILVEIKLSKFGRTLYTFLNQKYFFDLVY
SRIVALILNLGYLTHKTIDRGTLELVGPTGLTNLFSNTSKTIASADSGYIPNLALYIIMSVITLTASILY
LDDARLLLVFISSLFLISSRHFTIKTNSPHFPNP*
>Mortierella_verticillata_mt_nad4L_;_87_aa
MNLSITLFLIGILGFILNRKNIILLIISIELMLLAVTFMILISSFAFDDIMGELYAIYIIAIAGAESAIG
LGIVIAFYRLRGSIRIK*
>Mortierella_verticillata_mt_orf259_;_259_aa
MININLGTKKYIEILMKNTIILFNKNLPYQPVIIYNNADTERLQILKDNKGKSGLYLWTHRETGKRYVGS
AVDLSKRLKNYYSPLKLKRADNYIARALLFHTHSAFSLTIIEFISIKDLSLEKARELILEREQVNINLLK
PEYNILKVAGSLLGYKHTQESLTKISLSNLGKTHSVETVNKIKIALRGEKNPMSKNVLVYSFDLETKDMV
LYKSFNTCIEAAKYFDCSTRNLSRYLDKNKLYKKQWILLSSLVKDNSKE*
>Mortierella_verticillata_mt_rps3_;_283_aa
MGSKHPLSLRLKTHNKWGETSYYFNSKSELHNYWGVDKIALNVIKHYFQFALISKPKFNKDSNKIVISFY
YFLNLPVSKRFNPYGQSKRIQSSLQGDVTANNKNLLPLEAKGYSKLILKLSRFYGKPVELRPVRIHYPYL
NSYILAQYIAINIRLGNFNKLMRTLFKKAKLVRNNNINTTNFRDLIKYSSLINRPQFLSGLKIQISGRLS
QRKAASRTRIVRKSIGTLRLSSSTSLIDASKFSFKGKNGAATVKVWLSSCTVNPSVNKNTLINTPPLHST
LII*
>Mortierella_verticillata_mt_nad6_;_200_aa
MNNILLDLLTFTSVLSAILVITARSPVISVLFLIAVFINIAGYLILLGVNFIALSYIMVYIGAIAILFLF
VIMMLNVRLTELHEIGSDYSKNFPLAIVVAASLSYIILSNSLTKSIDQIYLITALFDKLNFLNSGINTTT
DNYINIGYNTIYNNIFVNYEQIQAIGNIMYSSYGLYLILCSFVLLLAMLGPIIITLDKKM*
>Saitoella_complicata_mt_atp6_;_251_aa_on_
MFIFSPLDQFEINSVFQLLLPFNINISLTNIGIYFIFVLVLAVSFNLFVTNRNFVIANQWSIIRESLYAT
IHNLVLNQIGGAKGQIYFPFIFSIFTLVLFSNFLGMIPYSFAVTSHLIFTLSLSLSILIGVTILGFNLHG
LKFFSFFVPSGTPLALVPLLVLIELISYIAKGLSLGIRLGANVMSGHMLVKIIGGFIYNMMTSGIIMFLF
GLVPLSILIGVMNLELAIAAIQSYVFTILTSSYIKDSIDLH*
>Saitoella_complicata_mt_atp8_;_48_aa_on_
MPQLLPFHFMNQVVFGLSTLVALIYLFSKFILPLFLRLFLARTMISSL*
>Saitoella_complicata_mt_atp9_;_73_aa_on_
MLQAAKYIGAGLATIGLAGAGLGIGLVFGQLIAGTSRNPSLRPQIFSQAILGFALVEATGLFCLMVAFLI
LFG*
>Saitoella_complicata_mt_cob_;_549_aa_on_
MKLLKIHPLFNLFNSYLNDSPQPLNISYLWNFGSLLGVCLIIQIITGITLAMHYTPSIDLAFISVEHIMR
DVNYGWMIRYIHANVASFFFLFVYAHIGRNIYYGSYRTPRVLVFSIGVIIFLLMIITAFLGYVLPYGQMS
LWGATVITNLMSAIPWIGFDIVEFLWGGYSVGNPTLNRFFSLHFTLPFVLLALVVVHLIALHENGSSNPN
GISGNLDRAPFHPYFSYKDAVTFCLFFLVLAYFVFFAPNVLGHSDNYIPANPLSTPSSIVPEWYLLPFYA
ILRSIPNKLLGVIAMIAAILILLALPLVDTSRLRGLVFRPIGKLCYWIFIANFFILMYLGSQHVEDPYVI
LGQISTLLYFAYFLFFVPVSGIIENTLMDLALDNKKRSSSLTLSQRISYPQRPTIFIIPIIFKREFSGSA
VSPFLXRSLYSDGDYDDKTGDKIYLRXCKSLLIVERYNSLXYALFVLKLKDWEIKTYIDKPLVYKHKRFG
DVYIVSKGVNTFKDSIRDIFYRSYLSYLLNNKVSPWFLLYLFFITIFYLSLFLELYTPI*
>Saitoella_complicata_mt_cox1_;_530_aa_on_
MHGIKRWLYSTNAKDIGVLYLIFSIFSGMIGTAFSVLIRMELTSSGNQFLNGDHQLYNVIVSAHAVAMIF
FMIMPGLVGAFGNYFIPIMIGAVDMAFPRLNNVSFWLLVPSLILLLSSAFLESGAGTGWTIYPPLSSIQS
HSGGAVDLLIFSLHLSGISSMLGAINFISTVLNMRAPGMSLHKMPLFVWAILVTAVLLLLSLPILAGGLT
MLLTDRNFNTSFFDPNGGGDPLLYQHVFWLFGHPEVYILIIPGFGIVSHIMSTFSAKPVFGKIGMIYAIL
SIAVLGFIVWLHHMFQVGVDVDTRAYFTAATMIIAVPTSIKIFSWLATIFGGSIRFTVPMMFALGFILLF
TIGGLTGVVLANSALDVAFHDSYYVVAHFHYVLSMGAVFSLFAAYYYWSPKILGLSFSEYLGHLNFWLLF
IGVNVTFFPQHFLGLNGMPRRIPDFPDAFNGWNYISSMGSLISVVATIVFLYTIYDQLLNGKRVSANSWF
IPQLFTCSSTYSVSDYAFSLEWSIDSPSHSHAFNIIPITN*
>Saitoella_complicata_mt_cox2_;_251_aa_on_
MLNIFLSYISLDAPNPWALYFQDSASPSQEGIIELHDHIMFYLVIIIIGVSYMLLSIIRVYNQSSSQLVH
KYANHGTLIELIWTISPAIILVFIAFPSFKLLYLMDEVIDPDLTVKVLGHQWYWSYEYSDYLTESGAPIE
FDSYLVPEDDLEEGQLRMLEVDSRLILPVNKHIRFIISANDVIHDFAVPSLGLKVDAIPGRLNQASTKIL
RTGVFYGQCSELCGVLHSAMPIVIQAVNFENYLEWLSEQVS*
>Saitoella_complicata_mt_cox3_;_269_aa_on_
MNNITRSNFANNPFHLGGSSPWPLIISFSLFILTTSTVMFFQNYTYGSDFLMIGLFCVVSSMAFWFRDII
SEGSYQGHHTAAVQKGLTLGVALFIISEICFFLSIFWAFFHSALAPTIEIGGQWPPLGITAINPFELPLL
NTILLLSSGTSVTYSHHSLIKGNRTATLVGLIITIILAAIFTFFQYIEYVTAPFTISDGAFGSCFYFATG
FHGIHVIIGTIFLAVGLWRIRNYQLTNHHHLGFESAILYWHFVDIVWLFLFISIYYWGS*
>Saitoella_complicata_mt_nad1_;_335_aa_on_
MDFLFNIIDVFIVLVPILLGVAFITLLERKAMGSMQRRLGPNIVGYFGLLQPIADALKLIVNESILPFSS
NAVLFYIGPAVTLICALLGWAVIPFGYGLTTCDLDIGVIYSIAISSLGVYGILIAGWSSNSKYSFIGTIR
SSAQLISYELILSSAIILVIFLNGSFNYSEIISNQKVIYN
>Taphrina_deformans_mt_atp6_;_256_aa_
MTYTHIQSPLEQFEINSLMGINAPLFGYAQLSLTNIGLYFILALIVVVGFNVIANNNNQIIANKYSISQE
SLYTTILNMVQAQIASPKGQLYFPFIYCLFMLVLAGNFIGMVPYSFAITSHLIFTLSVSMTILIAVTIIG
FQEHGLKFFSYFVPAGTPLALVPLLVLIELISYIAKGLSLGIRLGANIMAGHMLTKIIGGFIYQIMAASP
LMFVIGIIPLVLLVAITGLELAIAFIQAYVFAILTCSYIKDSLDLH*
>Taphrina_deformans_mt_atp8_;_48_aa_
MPQLVPFYFVNQVTFGFAVLLVMLYLFSKYILPKYLELFLTRTFITKL*
>Taphrina_deformans_mt_atp9_;_74_aa_
MLAAAKIIGSGLATIGLAGAGVGIGLVFGNLITATSRNPALRGQLFSYAILGFALAEATGLFCLMMAFLL
LFGV*
>Taphrina_deformans_mt_cob_;_395_aa_
MKLLKTHPLLSLLNSYLIDSPQPVNISYLWNFGSLLGVCLVIQIITGITLAMHYTPSIDAAFISVEHIMR
DVNYGWLIRYLHANTASFFFLFVYAHMARGLYYGSYRSPRVLVWSIGVVIFLVMIITAFLGYVLPYGQMS
LWGATVITNLMSAIPWIGQDIVEFIWGGFSVSNATLNRFFSLHYLLPFVLAALIIMHMIALHESGSNNPV
GVTGNLDRLAMHPYFIFKDLVTIVAFFLVLSLFVFYAPNVLGHSDNYIPANPMQTPASIVPEWYLLPFYA
ILRSIPNKLLGVLAMIAAILVLFLMPIVDLSRLRGNAFKPFSKIAFAVFAVNFLLLMWLGSQHVESPYVE
IGQICTAYYFAHFLIIIPAVSVFENTLMDIALAENSNLKEGSLAA*
>Taphrina_deformans_mt_cox1_;_530_aa_
MSRWLFSTNAKDIGMLYLMFGLFSGMIGTAFSVLIRLELASPGAQYLQGDNQLYNVIVTAHAFLMIFFMV
MPAMVGGFGNWLVPLMIGAVDMAFPRLNNISFWLLPPSLILLLSSSLLEGGAGTGWTVYPPLSSIQSHSG
GAVDLAIFSLHLAGISSMLGAMNFITTILNMRAPGMTLHKMPLFVWAILVTAVLLLLSLPVLAGGITMLL
TDRNFNTSFYEPAGGGDPLLYQHLFWFFGHPEVYILIIPGFGMVSHTISAFSGKPIFGYLGMVYAMMSIG
VLGFVVWSHHMYSVGLDVDTRAYFTAATMIIAVPTGIKIFSWLATIYGGSIRFTTPMLFALGFVFLFTVG
GLTGVVLANASIDVAFHDTYYVVAHFHYVLSMGAVFALFAGWYYWSPKIFGLSYNELAGQIHFWILFIGV
NVTFLPLHFLGLMPRRIPDYPDAFAGWNLVASYGSIMSVVAVAVFIYVIYDQLANGKAVSNNPWRIPAFF
ESTAEFNLNANIASSLEWSLQSPPAHHAYNMLPAETVQTN*
>Taphrina_deformans_mt_cox2_;_259_aa_
MFLNLIINKGLGKLFSPILTDAPTPWGLYFQDGASPSFEGIVELHDQIMFYLVVILFGVAWIMGSAMKTF
SSNQTKIVHKYSNHGTLIELIWTISPALVLVAIAFPSFKLLYLMDDVLDPAMTVKAVGHQWYWSYEYSDF
VNDDGESIEFDSYMIPGEDLVDGQLRQLEVDSRVIVPVNTHIRFIVTGADVIHDFAVPALGLKIDATPGR
LNQASVLIQREGVFYGQCSEICGVLHSAMPIAIEAVSVEKYLAWLDSQA*
>Taphrina_deformans_mt_cox3_;_269_aa_
MTNLVRAQFQAHPFHLVEPSPWPLVTSFALLTLTVSGVMTMHGYAFGGYLAAIGLFSVIGSMIFWFRDII
AEGTYQGSHTFAVQKGLTIGVALFIVSEVFFFLSIFWAFFHSSLAPTVELGGHWPAKGIEAINAFELPLL
NTVLLLSSGATVTYAHHSLIQGNRKGTINGLIFTIILALIFTSLQGFEYYTAPFTISDGAFGSTFYFATG
FHGLHVIIGTIFIATMFWRILAYQLTDHHHVGFESSILYWHFVDVVWLFLYCAVYWWGS*
>Taphrina_deformans_mt_nad1_;_354_aa_
MFVLFSLFEVLIVLLPILLSVAYVTVAERKAMGSMQRRLGPNKVGYLGTLQAFADALKLIVKETIIPAHA
NSVLFFLGPIITLIFALLGWAVIPFGPGLAISDYNLGLLYSIAISSLGVYGLLIAGWAANSKYAFVGSLR
SAAQLVSYELVLSSAMLIVLFITGSLNMTTIIEYQQAVWFIIPLFPVFIIFFIGALAETNRAPFDLPEAE
SELVAGFMTEHSAVIFVFFFLAEYSNIILISTLTAILFLGGYLLPSISLGSLTSWLALDSFSFINLIGFT
SMPLVSGAINGLVIGFKATAVVFLFIWVRASFPRIRYDQLLQVCWMVLLPLVFAIIILVPCIIYSFDLLP
FNLI*
>Taphrina_deformans_mt_nad2_;_587_aa_
MLISGLFILLFISAISAKRINGIIINRAAALILLYSFILSIYTLNVSIIGSGIGVFGGLYQVTVLSKVIE
GFIYLLGAMILSNTMFVSSKVTSNGQKSNNKATEASIPTIRSEYSIIALFTVVGGILLITSNDLVSMYLS
IELQSFGLYVLATIYRDSQAATSAGLKYFLLGGLSSGFILLGSSIIYAYLGVTNLESIYLLMSQEASVGL
SNLTEKSIELSSFSFCAEQSIRSPFGADLGLLIMTVGFLFKVASAPFHNWAPDVYDGVPTKVTTWLAIMA
KISILTFLLDLVIGSGSLYLLEPSLSLVNLMLIASFLSLLVGTIVGLAQSRIKRLLAYSTISHVGFLLLA
IAINTEDSIESFIFYLIQYSLTSANAFFVLIAFGSLSLSLSKKTNSAEANGFFSYQEEDHNAEREYSPIQ
FIAQLKGQFNALEPSPLLALSMAIILFSMAGIPPLVGFFAKQAVLLSAVKNGFIFLALVGIITSVISAAY
YLRVIKVMVFDSSLEKTNDKVELNEVASNKKLVNESDKLLLLNASTGYPAHINGNTQLSNSLSFSISILT
VLTLFFVLKPTLILNSAHIMALSLFYL*
>Taphrina_deformans_mt_nad3_;_135_aa_
MATINTLSVLFILVPILGALLLVLNLLLAVHNPDNQKIQSFECGFSAFSQTRSRFSVAFYLVGILFLLFD
LEIILIFPYAVSAYNNEAYGLWMVVIFILILTLGFVFEFGKGALKISTVESDSNSASSSKNDYII*
>Taphrina_deformans_mt_nad4_;_493_aa_
MLTILLLIPLLGILSMLLVQPVSVESNTQKAESSARLYKDVALGFSIVNFFVSLVLWANFDRNTAESQFV
AEYNELSFCHFTLGVDGISLFFILLTCLLFPIIFLADYKAIKTNVRTYQIIMLLLETLLIAVFTVKDILL
FYIFFESILPPLFLLIGLFGSSKKVRASFHLFLYTLFGSLAMLLAFLTMYYLTGSTDIEILTSTNLSFDL
QKILWLAIFFSFMIKFPLVPFHLWLPLAHSEAPLGGSILLAGVVLKLALYGSLRILIPLLPEATVYFTPL
VYTIGVITIIYASLTTLRQIDMKVIIAYSSIGHVAIILMGAFSNNIQGIEGSILLGIAHGFISPALFIIT
GGCLYERYHTRVLSYYRGLSTQMPLFSVIFMLATLGNIAVPLSSNFVGEWLCLAGAFERSPIMAALAASG
IFLSAAYSIWFYNRVCGGAWSPYLAVTTDLSRREFYVLLPLIFFAFGLGIFPNVILDSIHYSVSTVLYQT
ITP*
>Taphrina_deformans_mt_nad4L_;_88_aa_
MNLSLILFLIGILGFVLNKKNIILMLMSIEIMLLAVTFMILVSSFSFDDILGQTYAIYIIVIAGAESAIG
LGILVAYYRLRGTIAIQS*
>Taphrina_deformans_mt_nad5_;_660_aa_
MYLAIITLPLLSAISAGLLGRKLGVTGAQLITSGSVIITCFLALIAFYEVGLTISPVSIKLFSWIDSESL
TIDWGFNFDALTVSMLIPVLIVSALVHVYSIGYMSEDPHQQRFFSYLSMFTFFMLILVTGDNYLVMFIGW
EGVGISSYLLVSFWFSRVQANKSAISALLFNRVGDMFLTIGLFALIFALGNIDYAIVFSIAPYLNENTVT
IIGICFLIGAMAKSAQIGLHVWLPQAMEGPTPVSALIHAATMVTAGVYLLMRSSPLLEYSPTVLLLALWI
GAITTLFAGTIGLFQNDLKKVIAYSTCSQLGMLFIAVGLSQYNVALFHLVNHAFFKALLFLGAGSIIHAM
ADEQDMRRLGGLVKLLPFSYAMMLIGSLSLAAMPFLTGFYSKDLIIELAFGQFEFSGQVVYWLAVISAIF
TMTYSIRLLFLTFLSAPNGAKINYEHSHEGPLTMAIPLIILAIMSIIFGYYARDFFVGLGSSSLAHSLFV
HPNHIISVEAEFAVPTIFKLLPVVASVLVGLTVLAIYQLAPSLLVSITKTKLGASIYAFFNQRYWLELIV
NKFIVLKGLSLGYVFNKQLDRGAIELIGPHGVVTGLSSVSGLISKLDTGIITNYALYILLGLISFISLVF
FAPLDPQGESIKAAQYSQLFILYIFALTAF*
>Taphrina_deformans_mt_nad6_;_239_aa_
MLISESLTSGFSPLGLDILSFGAILSGILVITARNPVISVLFLIALFVNIAGYLILLGISFIGLAYITVY
VGAIAILFLFVIFLLDIKLAELHQDNNKNRSEAPLGAIIGVAFLYPLYSIIPSNITEMKSFSYYVFNWLN
SLITGTTPSLKFLSITSSEPSFTGLDNSNLESVAKLEVESVFTNLWDGNFAAFSQISSIGNVMYTGYLLW
FFVASLILLLAMVAAISLTFKPAAVSNSH*
>Taphrina_deformans_mt_orf191_;_191_aa_
MLGDGYCNVRTGEGTRICLRQGAIHKEYLFYLYAFFLERGYCSKLEPIQYTRKLNHNGLTKTHYGYEFNT
YTFRSLNWIHKLFYKNGKKIVPLNIEYLITPLTLAVWISDDGGWANGGVRIATNEFTLTEVTLLANILRS
KFALDVTIQKISIENKYSIYIKKNSVSKLKEIIFPYLNSSRYYKLGLKPKV*
>Taphrina_deformans_mt_orf221_;_221_aa_
QGKLYNKTFYLILLLNFTKLIIFSFLIYIICNNDNFSSLFVTPLSISFKLRKRKNFSGFPYGPHIKPILL
KISTDSTPIRFYDNLNYNRNLIGIENRKRSIIYQWTNLITGKIYVGSSQTGSARLLTYWSPSHLKRKTPI
YLNLNYYGIHNFCLAILEDLGPAYSISSSRLSRAQSRRRQEEIFKKEQLYLNILFNKYRSAVLNLAPQAG
VLTGFKYKNKL*
>Taphrina_deformans_mt_orf241_;_241_aa_
KFCLKWPKLKSTKLNKIRSLSVITNSISLARVQSVKRIGPHNIDLLSVLIGSLLGDGHMEKDGNGSRFSF
YQSKNHGEYLLWLHKRLFELGYCKKELPLIQTRLDSKGELYYFYRFRTFTYSSFNWIHSAFYVNGRKVLP
SFIKEYLSAEALAIWIMDDGTLHKNRGLRFCTHNFILTECKFMQEILKEKFDLDTTLHKISGTVPLQYNI
YVQKASMDKLKKIVKPFIHETMLYKIGLSSL*
>Taphrina_deformans_mt_orf257_;_257_aa_
ELSCLKWLNINNLDSLSLCEIISPFLCPLIKKSNEKSSEVKNELTKLRSYQRIGPHNIDILSILIGSLLG
DGHMEKRGQGLGVRVKFEQSSKNVEYLMWFHSYLSTRGYCRLEKPEMKKRIKKDGLVIYHYLVNSYTFTS
LNWLHDMFYLHNVQTNKLKKTIPLNLSEFLTPLALAIWFMDDGSKLGQGVKIATNFFSKEEVELLSLIIK
KKFNIETSVHIAGKGKGFTLYIPKKSKPLFAEIVKPYMLSSMTYKLN*
>Taphrina_deformans_mt_orf267_;_267_aa_
KETNLADYFSNGLQAALISQNNKTVDFLKKFWVGLMDGDGSIQVNHWRKQSLQYRLVIKLSPLKSNIDML
TLISQEIGGSVKLVRNKKQELDAVLWVVNNKNNIIEICKIFDLYPPLTTRMTLQLKFLKYCLLNPDVNLY
LENRNKKFESQKELILLQQGQKNSIRAVNYFDCWLSGFIEAEGCFSLRQSNNHSFSIGQKNDKFLIDMIK
NYFNLSNNIRTIKYDFYLLEVYKKESLVKIVNHCLSPNNPLLGAKAKSLALFLTKFK*
>Taphrina_deformans_mt_orf305_;_305_aa_
KNINLSLIPCLFNYNLFIQNYEKLICTDEKERLNLNPFKASNKSWALNYPIDNEFLDWFLGFSEGDGSFI
RAKRGDISFVITQDTRDIQILNLIQNVLGFGKVIKQSNLTSRYVVQDKKGLYLLALLFNGNIITTQKRES
FKLFLSALNVHISKGRITYKPIVFKDNLCQVSLQNGWLCGFSDAESCFGVSFSSVSSNNYKIYYDIAPKG
DCNIILFDLLAKLFGVGKTYKHSQEQCWYYRVTGLANTEILFNYFDKFPLRTKKLKSYVLWKDLHCRIKN
KQHLNLQLRPGLIILAKTVNNQWAI*
>Taphrina_deformans_mt_orf327_;_327_aa_
KNIVELLNCTICTTIISNRLPTIGIISEKALTKGNKRRLESQKQEYMSIPSSFLAFLVGLIDGDGYIQIT
RTTKGFIAIKLVISLHLSDISTLKYLESILKLGKVTEYKDLKSPSCKLIINRTDLQEVLFPLFLHHGIFF
LTDTRQAQYNLAMFILNNNIKLYNEIAIFKVASLNGSRQENYDLFASVNDRQEKELSNLNQILTNPLDYV
NLPFFTNWIVGFTLAEGSFFIKSNNDGCFSLKQRIHLNLFEAFKLIFDTNRKIGTEKDLYNGFSVSSKSD
IQKVINFFSFSGYHPLIGSKGISYFKWLNALHKTDRYKNLKFPDVNF*
>Taphrina_deformans_mt_orf356_;_356_aa_
DSFILPALNLAICWELLFYIIRQSAGNLIDLNQLGILRDSMPEFIFYISCVIISPNNQSDKRFLDLNDLK
FNFTNNFAYYLTGLIEGDGSIIVPNKLRSEKGRLNYPSIQLVFHLKDLPLAMLIQKELGHGSLSRKKGVN
AYILTINNLIGLLLIVKLINGKMRTPKIVSLNKLIYWFNSKYKDLSLAEKEQNNLNKELLNNNSWLSGFI
EADGHFALRTTQIGKYPRVECKLEISQRKKDHNNLDNYSFLNDIASFLLCEVKSIRMSSKTPEYKLRTTS
LKGNLILENYLVNYPLFGSKRLDSIDWLKALNIFKEKNHLKDKKINVEILNLKSNMNDNRSLFVWDHLHE
FYKLDK*
```

In [94]:

```
%%bash
cd ../../Data/Rhoto_IFO0880_3/
head Rhoto_IFO0880_3_all_proteins_20160906.aa.fasta
```

```
>jgi|Rhoto_IFO0880_3|921252|estExt_Genewise1.C_1_t10001
MNRLFGSSSSKPKPSLADAIASTDLRVDSIEVKIRKLDAELTKYRDQLKKMRDGPGKNAVQQRALRVLKQ
KKLYESQIAQLQQQSFNMEQASMTTENLRNTMATVDAMKTANKEMKKQYGKIDIDEIEAMHDDMADLLDS
ANDVQEAMSRTYGVPEEVDEADLEAELEALGNEFEEEEGIPSYLQADATSELPDFVDEAPQKEEAPKERL
GEGVV*
>jgi|Rhoto_IFO0880_3|921253|estExt_Genewise1.C_1_t10002
MNRLFGSSSSKPKPSLADAIASTDLRVDSIEVKIRKLDAELTKYRDQLKKMRDGPGKNAVQQRALRVLKQ
KKLYESQIAQLQQQSFNMEQASMTTENLRNTMATVDAMKTANKEMKKQYGKIDIDEIEAMHDDMADLLDS
ANDVQEAMSRTYGVPEEVDEADLEAELEALGNEFEEEEGIPSYLQADATSELPDFVDEAPQKEEAPKERL
GEGVV*
```

In [95]:

```
%%bash
cd ../../Data/Rhoto_IFO0880_3/
cat Rhoto_IFO0880_3_all_proteins_20160906.aa.fasta | sed 's/jgi|Rhoto_IFO0880_3|//g' | sed 's/|/ /g' \
    > Rhoto_IFO0880_3_all_proteins_20160906.aa.modified.fasta
samtools faidx Rhoto_IFO0880_3_all_proteins_20160906.aa.modified.fasta
makeblastdb -in Rhoto_IFO0880_3_all_proteins_20160906.aa.modified.fasta -parse_seqids -dbtype prot -out Rhoto_IFO0880_3_All
```

```
Building a new DB, current time: 07/26/2019 14:19:34
New DB name:   /mnt/san-nfs/nfs/users/joonhoonkim/Data/R_toruloides/Rhoto_IFO0880_3/Rhoto_IFO0880_3_All
New DB title:  Rhoto_IFO0880_3_all_proteins_20160906.aa.modified.fasta
Sequence type: Protein
Keep MBits: T
Maximum file size: 1000000000B
Adding sequences from FASTA; added 164153 sequences in 7.53851 seconds.
```

In [97]:

```
%%bash
cd ../../Data/Mito/
blastp -query Mito_Proteins_Fixed.fa -db ../../Data/Rhoto_IFO0880_3/Rhoto_IFO0880_3_All \
    -outfmt "6 std qcovs" -num_threads 24 > Mito_Proteins_to_IFO0880_3.txt
```

```
FASTA-Reader: Ignoring invalid residues at position(s): On line 1060: 17-19
Warning: [blastp] lcl|Query_171 Smittium_culisetae_mt_orf283_;_282_aa: Warning: One or more O characters replaced by X for alignment score calculations at positions 0, 2, 8, 10
```

In [98]:

```
%%bash
cd ../../Data/Mito/
head Mito_Proteins_to_IFO0880_3.txt
```

```
cob_Malawimonas_jakobiformis	979592	54.417	283	112	6	37	308	1	277	4.21e-89	271	74
cob_Malawimonas_jakobiformis	979594	53.333	285	116	6	37	308	1	281	3.98e-88	269	74
cob_Malawimonas_jakobiformis	950128	46.784	342	120	5	22	360	11	293	9.75e-88	268	93
cob_Malawimonas_jakobiformis	946001	46.784	342	120	5	22	360	11	293	9.75e-88	268	93
cob_Malawimonas_jakobiformis	900628	57.031	128	55	0	181	308	1	128	1.03e-45	154	35
cob_Malawimonas_jakobiformis	900627	70.000	80	24	0	37	116	1	80	1.73e-33	121	22
cob_Reclinomonas_americana_	979594	53.684	285	115	6	51	322	1	281	8.18e-91	276	70
cob_Reclinomonas_americana_	979592	54.064	283	113	6	51	322	1	277	1.23e-90	276	70
cob_Reclinomonas_americana_	950128	45.892	353	129	5	34	383	9	302	1.87e-90	276	90
cob_Reclinomonas_americana_	946001	45.892	353	129	5	34	383	9	302	1.87e-90	276	90
```

In [41]:

```
import csv
import numpy as np
import matplotlib.pyplot as plt
```

In [101]:

```
# Parse blastp results
blast = "../../Data/Mito/Mito_Proteins_to_IFO0880_3.txt"
gene_list = set()
match_list = {}

min_iden = 50
min_eval = 1e-10

with open(blast,'r') as f:
    for line in csv.reader(f,delimiter='\t'):
        query = line[0]
        subject = line[1:]
        gene_list.add(query)
        if (float(subject[1]) > min_iden) and (float(subject[9]) < min_eval):
            if query in match_list:
                match_list[query].append(subject)
            else:
                match_list[query] = [subject]

print(len(gene_list),len(match_list))
```

```
201 143
```

In [102]:

```
gene_list
```

Out[102]:

```
{'Capsaspora_owczsarsaki_atp6_;_258_aa_on_',
 'Capsaspora_owczsarsaki_atp9_;_74_aa_on_',
 'Capsaspora_owczsarsaki_cob_;_381_aa_on_',
 'Capsaspora_owczsarsaki_cox1_;_565_aa_on_',
 'Capsaspora_owczsarsaki_cox2_;_277_aa_on_',
 'Capsaspora_owczsarsaki_cox3_;_264_aa_on_',
 'Capsaspora_owczsarsaki_nad1_;_331_aa_on_',
 'Capsaspora_owczsarsaki_nad2_;_543_aa_on_',
 'Capsaspora_owczsarsaki_nad3_;_117_aa_on_',
 'Capsaspora_owczsarsaki_nad4L_;_99_aa_on_',
 'Capsaspora_owczsarsaki_nad4_;_477_aa_on_',
 'Capsaspora_owczsarsaki_nad5_;_662_aa_on_',
 'Capsaspora_owczsarsaki_nad6_;_198_aa_on_',
 'Capsaspora_owczsarsaki_orf157_;_157_aa_on_',
 'Capsaspora_owczsarsaki_orf165_;_165_aa_on_',
 'Capsaspora_owczsarsaki_orf190_;_190_aa_on_',
 "Capsaspora_owczsarsaki_orf233_;_251_aa_on_'nu6687iac329_mt'",
 "Capsaspora_owczsarsaki_orf280_;_280_aa_on_'nu6687iac329_mt'",
 'Capsaspora_owczsarsaki_rpl14_;_127_aa_on_',
 'Capsaspora_owczsarsaki_rpl16_;_184_aa_on_',
 'Capsaspora_owczsarsaki_rpl2_;_483_aa_on_',
 "Capsaspora_owczsarsaki_rps12_;_142_aa_on_'nu6687iac329_mt'",
 'Capsaspora_owczsarsaki_rps13_;_96_aa_on_',
 'Capsaspora_owczsarsaki_rps14_;_100_aa_on_',
 'Capsaspora_owczsarsaki_rps19_;_72_aa_on_',
 'Mortierella_verticillata_mt_atp6_;_256_aa',
 'Mortierella_verticillata_mt_atp8_;_47_aa',
 'Mortierella_verticillata_mt_atp9_;_73_aa',
 'Mortierella_verticillata_mt_cob_;_386_aa',
 'Mortierella_verticillata_mt_cox1_;_534_aa',
 'Mortierella_verticillata_mt_cox2_2_;_22_aa',
 'Mortierella_verticillata_mt_cox2_;_253_aa',
 'Mortierella_verticillata_mt_cox3_;_265_aa',
 'Mortierella_verticillata_mt_nad1_;_321_aa',
 'Mortierella_verticillata_mt_nad2_;_525_aa',
 'Mortierella_verticillata_mt_nad3_;_199_aa',
 'Mortierella_verticillata_mt_nad4L_;_87_aa',
 'Mortierella_verticillata_mt_nad4_;_495_aa',
 'Mortierella_verticillata_mt_nad5_;_664_aa',
 'Mortierella_verticillata_mt_nad6_;_200_aa',
 'Mortierella_verticillata_mt_orf122_;_122_aa',
 'Mortierella_verticillata_mt_orf206_;_206_aa',
 'Mortierella_verticillata_mt_orf259_;_259_aa',
 'Mortierella_verticillata_mt_orf261_;_261_aa',
 'Mortierella_verticillata_mt_orf309_;_309_aa',
 'Mortierella_verticillata_mt_rps3_;_283_aa',
 'Saitoella_complicata_mt_atp6_;_251_aa_on_',
 'Saitoella_complicata_mt_atp8_;_48_aa_on_',
 'Saitoella_complicata_mt_atp9_;_73_aa_on_',
 'Saitoella_complicata_mt_cob_;_549_aa_on_',
 'Saitoella_complicata_mt_cox1_;_530_aa_on_',
 'Saitoella_complicata_mt_cox2_;_251_aa_on_',
 'Saitoella_complicata_mt_cox3_;_269_aa_on_',
 'Saitoella_complicata_mt_nad1_;_335_aa_on_',
 'Smittium_culisetae_mt_atp6_;_248_aa',
 'Smittium_culisetae_mt_atp8_;_50_aa',
 'Smittium_culisetae_mt_atp9_;_73_aa',
 'Smittium_culisetae_mt_cob_;_396_aa',
 'Smittium_culisetae_mt_cox1_;_535_aa',
 'Smittium_culisetae_mt_cox2_;_254_aa',
 'Smittium_culisetae_mt_cox3_;_260_aa',
 'Smittium_culisetae_mt_nad1_;_323_aa',
 'Smittium_culisetae_mt_nad2_;_479_aa',
 'Smittium_culisetae_mt_nad3_;_113_aa',
 'Smittium_culisetae_mt_nad4L_;_102_aa',
 'Smittium_culisetae_mt_nad4_;_484_aa',
 'Smittium_culisetae_mt_nad5_;_649_aa',
 'Smittium_culisetae_mt_nad6_;_170_aa',
 'Smittium_culisetae_mt_orf170_;_170_aa',
 'Smittium_culisetae_mt_orf171_;_171_aa',
 'Smittium_culisetae_mt_orf224_;_224_aa',
 'Smittium_culisetae_mt_orf230_;_230_aa',
 'Smittium_culisetae_mt_orf237_;_237_aa',
 'Smittium_culisetae_mt_orf276_;_276_aa',
 'Smittium_culisetae_mt_orf283_;_282_aa',
 'Smittium_culisetae_mt_orf307_;_307_aa',
 'Smittium_culisetae_mt_orf360_;_360_aa',
 'Smittium_culisetae_mt_orf372_;_372_aa',
 'Taphrina_deformans_mt_atp6_;_256_aa_',
 'Taphrina_deformans_mt_atp8_;_48_aa_',
 'Taphrina_deformans_mt_atp9_;_74_aa_',
 'Taphrina_deformans_mt_cob_;_395_aa_',
 'Taphrina_deformans_mt_cox1_;_530_aa_',
 'Taphrina_deformans_mt_cox2_;_259_aa_',
 'Taphrina_deformans_mt_cox3_;_269_aa_',
 'Taphrina_deformans_mt_nad1_;_354_aa_',
 'Taphrina_deformans_mt_nad2_;_587_aa_',
 'Taphrina_deformans_mt_nad3_;_135_aa_',
 'Taphrina_deformans_mt_nad4L_;_88_aa_',
 'Taphrina_deformans_mt_nad4_;_493_aa_',
 'Taphrina_deformans_mt_nad5_;_660_aa_',
 'Taphrina_deformans_mt_nad6_;_239_aa_',
 'Taphrina_deformans_mt_orf191_;_191_aa_',
 'Taphrina_deformans_mt_orf221_;_221_aa_',
 'Taphrina_deformans_mt_orf257_;_257_aa_',
 'Taphrina_deformans_mt_orf267_;_267_aa_',
 'Taphrina_deformans_mt_orf305_;_305_aa_',
 'Taphrina_deformans_mt_orf327_;_327_aa_',
 'atp6_mt_Rhizopus_oryzae_;_259_aa',
 'atp8_mt_Rhizopus_oryzae_;_48_aa',
 'atp9_mt_Rhizopus_oryzae_;_74_aa',
 'cob_Allomyces_macrogynus',
 'cob_Aspergillus_nidulans_(Emericella)_',
 'cob_C.elegans',
 'cob_Cafeteria_roenbergensis',
 'cob_Chondrus_crispus',
 'cob_Drosophila_melanogaster',
 'cob_Jakoba_libera_mt_cob',
 'cob_Malawimonas_jakobiformis',
 'cob_Marchantia_polymorpha_',
 'cob_Ochromonas_danica',
 'cob_Phytophthora_infestans',
 'cob_Porphyra_purpurea',
 'cob_Prototheca_wickerhamii_',
 'cob_Reclinomonas_americana_',
 'cob_Rhizopus_stolonifer_',
 'cob_Rickettsia_prowazekii',
 'cob_Schizophyllum_commune_',
 'cob_Schizosaccharomyces_pombe_',
 'cob_Spizellomyces_punctatus',
 'cob_Xenopus_laevis',
 'cob_human',
 'cob_mt_Rhizopus_oryzae_;_386_aa',
 'cob_sea_urchin_(Paracentrotus_lividus)',
 'cox1_Allomyces_macrogynus_',
 'cox1_Aspergillus_nidulans_(Emericella)_',
 'cox1_C.elegans_',
 'cox1_Cafeteria_roenbergensis',
 'cox1_Chondrus_crispus',
 'cox1_Drosophila_melanogaster',
 'cox1_Jakoba_libera_mt_cox1_;_530_aa',
 'cox1_Malawimonas_jakobiformis',
 'cox1_Marchantia_polymorpha_',
 'cox1_Neurospora_crassa_',
 'cox1_Ochromonas_danica',
 'cox1_Phytophthora_infestans',
 'cox1_Pichia_canadensis_(Hansenula_wingei)',
 'cox1_Podospora__',
 'cox1_Porphyra_purpurea',
 'cox1_Prototheca_wickerhamii_',
 'cox1_Reclinomonas_americana',
 'cox1_Rhizophlyctis_rosea_(partial_sequence)',
 'cox1_Rickettsia_prowazekii_',
 'cox1_Saccharomyces_cerevisiae',
 'cox1_Schizophyllum_commune',
 'cox1_Schizosaccharomyces_pombe_',
 'cox1_Spizellomyces_punctatus',
 'cox1_Xenopus_laevis__',
 'cox1__Rhizopus_stolonifer_',
 'cox1_human',
 'cox1_mt_Rhizopus_oryzae_;_528_aa',
 'cox1_sea_urchin_(Paracentrotus_lividus)',
 'cox1_wheat_',
 'cox2_Allomyces_macrogynus',
 'cox2_Aspergillus_nidulans_(Emericella)_',
 'cox2_C.elegans',
 'cox2_Chondrus_crispus',
 'cox2_Drosophila_melanogaster',
 'cox2_Jakoba_libera_mt_cox2_;_260_aa',
 'cox2_Malawimonas_jakobiformis_mt_cox2_;_273_aa',
 'cox2_Marchantia_polymorpha_',
 'cox2_Ochromonas_danica_mt_cox2_;_265_aa',
 'cox2_Phytophthora_infestans',
 'cox2_Prototheca_wickerhamii_',
 'cox2_Reclinomonas_americana_mt_cox2_;_260_aa',
 'cox2_Rhizopus_stolonifer_',
 'cox2_Schizophyllum_commune_',
 'cox2_Schizosaccharomyces_pombe_',
 'cox2_Spizellomyces_punctatus',
 'cox2_Xenopus_laevis',
 'cox2_human',
 'cox2_mt_Rhizopus_oryzae_;_255_aa',
 'cox2_sea_urchin_(Paracentrotus_lividus)',
 'cox3_Allomyces_macrogynus',
 'cox3_Aspergillus_nidulans_(Emericella)_',
 'cox3_C.elegans',
 'cox3_Chondrus_crispus',
 'cox3_Drosophila_melanogaster',
 'cox3_Jakoba_libera_mt_cox3_;_268_aa',
 'cox3_Malawimonas_jakobiformis_mt_cox3_;_274_aa',
 'cox3_Marchantia_polymorpha_',
 'cox3_Ochromonas_danica_mt_cox3;_267_aa',
 'cox3_Phytophthora_infestans',
 'cox3_Prototheca_wickerhamii_',
 'cox3_Rhizopus_stolonifer_',
 'cox3_Schizophyllum_commune_',
 'cox3_Schizosaccharomyces_pombe_',
 'cox3_Spizellomyces_punctatus',
 'cox3_Xenopus_laevis',
 'cox3_human',
 'cox3_mt_Rhizopus_oryzae_;_282_aa',
 'cox3_sea_urchin_(Paracentrotus_lividus)',
 'nad1_mt_Rhizopus_oryzae_;_325_aa',
 'nad2_mt_Rhizopus_oryzae_;_523_aa',
 'nad3_mt_Rhizopus_oryzae_;_124_aa',
 'nad4L_mt_Rhizopus_oryzae_;_88_aa',
 'nad4_mt_Rhizopus_oryzae_;_475_aa',
 'nad5_mt_Rhizopus_oryzae_;_655_aa',
 'nad6_mt_Rhizopus_oryzae_;_214_aa',
 'rps2_Jakoba_libera_mt_rps2_;_222_aa',
 'rps2_Thraustochytrium_aureus_mt_rps2_;_134_aa'}
```

In [121]:

```
Mito_proteinId = dict()
for k, v in match_list.items():
    #if not any(x in v[0][0] for x in Mito_proteinId.values()):
    temp = k.split('_')
    x = [y for y in temp if any(z in y for z in ['cob','cox','atp','nad'])]
    temp = Mito_proteinId.setdefault(x[0], dict())
    temp[v[0][0]] = temp.setdefault(v[0][0],float()) + float(v[0][1])
    Mito_proteinId[x[0]] = temp
    print(x[0], v[0][0:2])
```

```
cob ['979592', '54.417']
cob ['979594', '53.684']
cob ['979592', '52.297']
cob ['979592', '56.184']
cob ['979592', '51.246']
cob ['950128', '50.386']
cob ['979592', '52.448']
cob ['979592', '56.228']
cob ['979592', '54.064']
cob ['979592', '52.650']
cob ['979592', '53.710']
cob ['979592', '55.516']
cob ['900628', '54.762']
cob ['950128', '54.155']
cob ['950128', '57.990']
cob ['950128', '52.895']
cob ['979592', '59.364']
cob ['900628', '53.968']
cob ['900628', '50.781']
cob ['900627', '63.750']
cob ['900627', '57.500']
cox1 ['900659', '64.655']
cox1 ['950130', '52.727']
cox1 ['950130', '52.000']
cox1 ['950130', '56.998']
cox1 ['950130', '52.174']
cox1 ['950130', '55.624']
cox1 ['950130', '57.631']
cox1 ['950130', '55.985']
cox1 ['950130', '57.169']
cox1 ['900659', '66.667']
cox1 ['950130', '54.495']
cox1 ['950130', '53.775']
cox1 ['950130', '59.450']
cox1 ['950130', '60.886']
cox1 ['950130', '52.459']
cox1 ['950130', '59.817']
cox1 ['950130', '62.637']
cox1 ['950130', '61.792']
cox1 ['950130', '55.904']
cox1 ['950130', '59.083']
cox1 ['950130', '51.743']
cox1 ['950130', '50.549']
cox1 ['900659', '56.034']
cox1 ['950130', '51.193']
cox1 ['900659', '69.231']
cox1 ['900659', '71.795']
cox1 ['950130', '51.927']
cox1 ['950130', '52.676']
cox2 ['950130', '51.282']
cox2 ['950130', '50.403']
cox2 ['950130', '57.806']
cox2 ['950130', '50.781']
cox2 ['950130', '50.211']
cox2 ['950130', '53.306']
cox2 ['950130', '55.738']
cox2 ['950130', '55.508']
cox2 ['950130', '55.556']
cox2 ['950130', '52.991']
cox2 ['950130', '61.250']
cox2 ['950130', '70.293']
cox2 ['950130', '56.904']
cox2 ['518258', '52.041']
cox2 ['518258', '51.546']
cox2 ['518258', '58.163']
cox2 ['518258', '59.184']
cox2 ['950130', '50.673']
cox2 ['518258', '58.163']
cox3 ['946002', '51.240']
cox3 ['946002', '53.719']
cox3 ['889639', '57.143']
cox3 ['889639', '54.930']
cox3 ['946002', '60.729']
cox3 ['946002', '54.545']
cox3 ['946002', '51.240']
cox3 ['946002', '50.413']
cox3 ['946002', '50.413']
cox3 ['900631', '51.852']
cox3 ['946002', '62.698']
cox3 ['946002', '55.600']
cox3 ['900631', '52.439']
cox3 ['889639', '52.857']
cox3 ['889639', '53.571']
cox3 ['946002', '50.826']
cox3 ['946002', '54.545']
cox3 ['900631', '66.667']
atp9 ['900663', '63.014']
cob ['979592', '57.597']
cox1 ['950130', '52.166']
cox2 ['518258', '60.204']
cox3 ['946002', '56.250']
nad1 ['900641', '54.152']
nad3 ['863320', '52.874']
nad4 ['900607', '53.378']
nad4L ['518739', '55.422']
nad5 ['900668', '63.366']
cox1 ['950130', '62.637']
nad3 ['879368', '56.989']
nad5 ['900668', '66.667']
nad4L ['945993', '72.093']
nad4 ['900607', '58.108']
atp6 ['900623', '51.984']
cox2 ['950130', '70.293']
cob ['950128', '57.990']
cox3 ['946002', '62.698']
atp9 ['900663', '73.973']
nad1 ['900641', '60.498']
nad5 ['900668', '56.863']
cob ['979594', '52.982']
atp9 ['900663', '63.889']
cox2 ['950130', '54.430']
nad4 ['900607', '55.405']
nad1 ['900641', '52.143']
cox1 ['950130', '53.565']
cox3 ['889639', '50.694']
atp9 ['900663', '75.676']
atp8 ['900650', '55.319']
cox1 ['950130', '60.219']
atp6 ['945998', '51.634']
cox3 ['946002', '61.134']
nad4 ['900607', '53.378']
cox2 ['950130', '61.506']
nad1 ['900641', '55.596']
cob ['950128', '51.420']
nad5 ['900668', '60.396']
nad4L ['861805', '63.953']
atp9 ['900663', '74.324']
cob ['950128', '57.910']
cox1 ['950130', '57.982']
cox2 ['950130', '58.159']
cox3 ['946002', '60.159']
nad1 ['950129', '58.861']
atp8 ['900650', '55.102']
atp9 ['900663', '81.944']
cob ['950128', '54.124']
cox1 ['950130', '61.284']
cox2 ['950130', '66.390']
cox3 ['946002', '67.729']
nad1 ['900641', '57.756']
nad3 ['946000', '50.515']
nad4 ['900607', '58.784']
nad4L ['866713', '66.279']
nad5 ['945994', '53.959']
```

In [126]:

```
Mito_proteinId
```

Out[126]:

```
{'cob': {'979592': 655.721,
  '979594': 106.666,
  '950128': 436.87,
  '900628': 159.511,
  '900627': 121.25},
 'cox1': {'900659': 328.382, '950130': 1626.5469999999998},
 'cox2': {'950130': 1083.48, '518258': 339.301},
 'cox3': {'946002': 903.938, '889639': 269.195, '900631': 170.958},
 'atp9': {'900663': 432.82000000000005},
 'nad1': {'900641': 280.145, '950129': 58.861},
 'nad3': {'863320': 52.874, '879368': 56.989, '946000': 50.515},
 'nad4': {'900607': 279.053},
 'nad4L': {'518739': 55.422,
  '945993': 72.093,
  '861805': 63.953,
  '866713': 66.279},
 'nad5': {'900668': 247.29200000000003, '945994': 53.959},
 'atp6': {'900623': 51.984, '945998': 51.634},
 'atp8': {'900650': 110.42099999999999}}
```

In [179]:

```
targets = set(sum([list(x.keys()) for x in Mito_proteinId.values()],[]))
targets
```

Out[179]:

```
{'518258',
 '518739',
 '861805',
 '863320',
 '866713',
 '879368',
 '889639',
 '900607',
 '900623',
 '900627',
 '900628',
 '900631',
 '900641',
 '900650',
 '900659',
 '900663',
 '900668',
 '945993',
 '945994',
 '945998',
 '946000',
 '946002',
 '950128',
 '950129',
 '950130',
 '979592',
 '979594'}
```

In [136]:

```
%%bash
cd ../../Data/Rhoto_IFO0880_3/
head Rhoto_IFO0880_3_all_proteinId_transcriptId_map_20160906.txt
```

```
proteinId	transcriptId
6	157
7	158
14	165
26	177
37	188
45	196
51	202
57	208
69	220
```

In [180]:

```
# Parse blastp results
temp = "../../Data/Rhoto_IFO0880_3/Rhoto_IFO0880_3_all_proteinId_transcriptId_map_20160906.txt"
mapping = {}

with open(temp,'r') as f:
    for line in csv.reader(f,delimiter='\t'):
        query = line[0]
        subject = line[1]
        mapping[query] = subject
```

In [152]:

```
','.join([mapping[x] for x in targets])
```

Out[152]:

```
'900810,900782,900778,866864,950280,900774,900801,518890,950279,946153,979743,863471,900779,950281,946144,861956,518409,946151,879519,900758,946149,900814,900819,946145,889790,979745,900792'
```

In [145]:

```
%%bash
cd ../../Data/Rhoto_IFO0880_3/
head Rhoto_IFO0880_3_all_transcripts_20160906.nt.fasta
```

```
>jgi|Rhoto_IFO0880_3|921403|estExt_Genewise1.C_1_t10001
CACCTCTGACTGCGGTTCGAAGGTCTCTTACTAAGGTCTACAGCGTTCCTGCAGTACCAATTTCGTGCTG
TGCGGGCTCTGCGAAGCTGTAGATCGCGCCGACGTCGCCCTCGAGTTTCCACTCTCCCTTCTGCTTCTGC
CCATCTCGCTCACCTCGCTCTTCTTCGTCGCAACCGCTGCATATAGAGCTTGGACGACTCAGCGGCTGCC
TGCTACCCCTCGCACGCCAATCACTTACCTTCACGCCGGTCTCTTCCTGCCCCACCTCGACTCGGACTTC
TCGCACGCCTCGCAATCCGCTCGCACGCGTTCTCTGACAGACCAGAATCCGTCGGAGAGTATCTGAAGAT
CGACCGGCTTCCCAGTCTTACTGCCACGCACTCTCGCCACCCTCTCGCTTCGACCTTCGCTTGCCGGCCC
GCCATGAACCGCCTCTTCGGCTCGTCGTCCTCAAAACCGAAACCCTCTCTCGCCGACGCAATCGCTTCCA
CCGACCTCCGCGTCGACTCGATCGAGGTCAAGATCCGCAAGCTCGATGCAGAGCTCACCAAGTACCGCGA
CCAGCTGAAAAAGATGCGGGACGGGCCAGGGAAGAACGCCGTCCAGCAGCGCGCCCTCCGCGTCCTCAAG
```

In [146]:

```
%%bash
cd ../../Data/Rhoto_IFO0880_3/
cat Rhoto_IFO0880_3_all_transcripts_20160906.nt.fasta | sed 's/jgi|Rhoto_IFO0880_3|//g' | sed 's/|/ /g' \
    > Rhoto_IFO0880_3_all_transcripts_20160906.nt.modified.fasta
samtools faidx Rhoto_IFO0880_3_all_transcripts_20160906.nt.modified.fasta
makeblastdb -in Rhoto_IFO0880_3_all_transcripts_20160906.nt.modified.fasta -parse_seqids -dbtype nucl -out Rhoto_IFO0880_3_All_transcripts
```

```
Building a new DB, current time: 07/26/2019 14:54:59
New DB name:   /mnt/san-nfs/nfs/users/joonhoonkim/Data/R_toruloides/Rhoto_IFO0880_3/Rhoto_IFO0880_3_All_transcripts
New DB title:  Rhoto_IFO0880_3_all_transcripts_20160906.nt.modified.fasta
Sequence type: Nucleotide
Keep MBits: T
Maximum file size: 1000000000B
Adding sequences from FASTA; added 164153 sequences in 11.0689 seconds.
```

In [160]:

```
%%bash
cd ../../Data/Rhoto_IFO0880_3/
blastdbcmd -db Rhoto_IFO0880_3_All_transcripts -entry 900810,900782,900778,866864,950280,900774,900801,518890,950279,946153,979743,863471,900779,950281,946144,861956,518409,946151,879519,900758,946149,900814,900819,946145,889790,979745,900792 \
    > ../Mito/Mito_Transcripts.fa
```

In [161]:

```
%%bash
cd ../../Data/Mito
head Mito_Transcripts.fa
```

```
>900810 fgenesh1_kg.4_#_70_#_TRINITY_DN3144_c3_g8_i15
GGTTTTGGCAACTACTTAGTGCCAGTTCAAATTGGGGCGCCTGATATGGCCTTCCCACGTCTTAATAACATTAGCTTTTG
GCTACTGCCGCCGTCACTAGTGCTGCTGCTTGCAAGTGCACTAGTCGAACAGGGTGCAGGTACCGGCTGGACTATTTACC
CACCCCTGTCGGGCATTCAAAGCCATTCAGGTGGGTCGGTAGACTTAGCAATTTTTAGCCTGCACCTGTCGGGCATTAGT
AGTATGCTAGGTGCAATGAACTTCATTACTACAATTTTGAACATGCGTGCCCCTGGCATGTCAATGCACAAGCTGCCGCT
TTTTTGCTGGGCAATTTTTATTACAGCAATTTTGCTGCTGCTGTCATTGCCAGTACTAGCGAGGAGCCATTACAATGTTA
CTAACGGACCGTAACTTCAACACGTCCTTTTACGACCCTGCAGGCGGGGGTGACCCAATTTTATTCCAACACCTGTTCTG
GTTTTTTGGTCACCCTGAAGTCTACATTTTAATCATTCCTGCCTTTGGCATTGTGTCGCATGTAGTTGCAACCTTTGCTG
GTAAGCCCGTGTTTGGGTACCTAGGTATGGTCTATGCAATGTTCAGCATTGGCATTTTAGGCTTTATTGTATGTTAGCAC
CACATGTTTGCCGTGGGGCTAGACGTTGACACACGAGCCTACTTCACAGCAGCCACAATGATTATTGCTCGTCCCACAGG
```

In [172]:

```
%%bash
cd ../../Data/Mito
blastn -query Mito_Transcripts.fa -subject ../Rhoto_IFO0880_4/Rhoto_IFO0880_4_MitoAssemblyScaffolds.fasta \
    > Mito_Transcripts_to_IFO0880_4.txt
```

In [188]:

```
[(x, mapping[x]) for x in targets]
for k, v in Mito_proteinId.items():
    print(k)
    for k2, v2 in sorted(v.items(), key=lambda kv: kv[1], reverse=True):
        print(k2, mapping[k2], v2)
```

```
cob
979592 979743 655.721
950128 950279 436.87
900628 900779 159.511
900627 900778 121.25
979594 979745 106.666
cox1
950130 950281 1626.5469999999998
900659 900810 328.382
cox2
950130 950281 1083.48
518258 518409 339.301
cox3
946002 946153 903.938
889639 889790 269.195
900631 900782 170.958
atp9
900663 900814 432.82000000000005
nad1
900641 900792 280.145
950129 950280 58.861
nad3
879368 879519 56.989
863320 863471 52.874
946000 946151 50.515
nad4
900607 900758 279.053
nad4L
945993 946144 72.093
866713 866864 66.279
861805 861956 63.953
518739 518890 55.422
nad5
900668 900819 247.29200000000003
945994 946145 53.959
atp6
900623 900774 51.984
945998 946149 51.634
atp8
900650 900801 110.42099999999999
```

In [173]:

```
%%bash
cd ../../Data/Mito
cat Mito_Transcripts_to_IFO0880_4.txt
```

```
BLASTN 2.6.0+


Reference: Zheng Zhang, Scott Schwartz, Lukas Wagner, and Webb
Miller (2000), "A greedy algorithm for aligning DNA sequences", J
Comput Biol 2000; 7(1-2):203-14.


Database: User specified sequence set (Input:
/users/joonhoonkim/Data/R_toruloides/Rhoto_IFO0880_4/Rhoto_IFO0880_4
_MitoAssemblyScaffolds.fasta).
           1 sequences; 110,369 total letters


Query= 900810 fgenesh1_kg.4_#_70_#_TRINITY_DN3144_c3_g8_i15

Length=1983
                                                                      Score     E
Sequences producing significant alignments:                          (Bits)  Value

  scaffold_1_mito                                                     2135    0.0  


> scaffold_1_mito
Length=110369

 Score = 2135 bits (1156),  Expect = 0.0
 Identities = 1156/1156 (100%), Gaps = 0/1156 (0%)
 Strand=Plus/Minus

Query  828    TAACAGGTGTCGTACTTGCAAATGCTAGTCTTGACGTTGCAATTCACGATAGTACCGTGT  887
              ||||||||||||||||||||||||||||||||||||||||||||||||||||||||||||
Sbjct  95072  TAACAGGTGTCGTACTTGCAAATGCTAGTCTTGACGTTGCAATTCACGATAGTACCGTGT  95013

Query  888    CATTACACAACCTTAGCATGGGTGCAGTCAGCAGCTTTGCTGCAATAGGCTTGCGGCCCC  947
              ||||||||||||||||||||||||||||||||||||||||||||||||||||||||||||
Sbjct  95012  CATTACACAACCTTAGCATGGGTGCAGTCAGCAGCTTTGCTGCAATAGGCTTGCGGCCCC  94953

Query  948    CAATGCAATTAACTAGCAGTCAACTAGCTGCATTCACAGTAGGCCTAATCGACGGGGCCG  1007
              ||||||||||||||||||||||||||||||||||||||||||||||||||||||||||||
Sbjct  94952  CAATGCAATTAACTAGCAGTCAACTAGCTGCATTCACAGTAGGCCTAATCGACGGGGCCG  94893

Query  1008   GTTCATTGCAAGTAAACCACTGCAAAGGCAAAAATTTGCAGTACCGCCTTGTAATTAAGC  1067
              ||||||||||||||||||||||||||||||||||||||||||||||||||||||||||||
Sbjct  94892  GTTCATTGCAAGTAAACCACTGCAAAGGCAAAAATTTGCAGTACCGCCTTGTAATTAAGC  94833

Query  1068   TTAAGTACACAGCAGCAAACCATGCAATGTTGCTTAAAATTGCAGCTGTGTACCTAGGCA  1127
              ||||||||||||||||||||||||||||||||||||||||||||||||||||||||||||
Sbjct  94832  TTAAGTACACAGCAGCAAACCATGCAATGTTGCTTAAAATTGCAGCTGTGTACCTAGGCA  94773

Query  1128   GCGTACACGTGCATGGGCCTAGCAATGACCCAAAATTCGTAATGTGGGTCATTAATGACC  1187
              ||||||||||||||||||||||||||||||||||||||||||||||||||||||||||||
Sbjct  94772  GCGTACACGTGCATGGGCCTAGCAATGACCCAAAATTCGTAATGTGGGTCATTAATGACC  94713

Query  1188   GGGCTGTACTTGAAGCCCACATTTTGCCGCTGTTTGCAGCATGCCCACCACTTACAACAC  1247
              ||||||||||||||||||||||||||||||||||||||||||||||||||||||||||||
Sbjct  94712  GGGCTGTACTTGAAGCCCACATTTTGCCGCTGTTTGCAGCATGCCCACCACTTACAACAC  94653

Query  1248   GTGTGACGTTGCAACTAGCCTTCATGCTTAAAGCCATGGGCGGGCTTACGTTAAAGGACT  1307
              ||||||||||||||||||||||||||||||||||||||||||||||||||||||||||||
Sbjct  94652  GTGTGACGTTGCAACTAGCCTTCATGCTTAAAGCCATGGGCGGGCTTACGTTAAAGGACT  94593

Query  1308   ACTTTGCAACACGTGCCTTAAAATTTAATGCACGTGCCAGCATTGCACCGCTTAGCGCTT  1367
              ||||||||||||||||||||||||||||||||||||||||||||||||||||||||||||
Sbjct  94592  ACTTTGCAACACGTGCCTTAAAATTTAATGCACGTGCCAGCATTGCACCGCTTAGCGCTT  94533

Query  1368   TGCCACCGTACTTTAATGCATGACTTGCAGGCTTCATTGAAGCCAAAGGCTCGTTTGCTA  1427
              ||||||||||||||||||||||||||||||||||||||||||||||||||||||||||||
Sbjct  94532  TGCCACCGTACTTTAATGCATGACTTGCAGGCTTCATTGAAGCCAAAGGCTCGTTTGCTA  94473

Query  1428   AGCGTGCAGGCTTGCTAGGCTTTAGTTTTAGCATTGGGCAGGCCCACGACGAGTACCTGC  1487
              ||||||||||||||||||||||||||||||||||||||||||||||||||||||||||||
Sbjct  94472  AGCGTGCAGGCTTGCTAGGCTTTAGTTTTAGCATTGGGCAGGCCCACGACGAGTACCTGC  94413

Query  1488   TAGTGGCCATTTTGCAGCTGTTTAAGCTACAGCACTTAAAGGTGCAGTCTAGCATGCTTA  1547
              ||||||||||||||||||||||||||||||||||||||||||||||||||||||||||||
Sbjct  94412  TAGTGGCCATTTTGCAGCTGTTTAAGCTACAGCACTTAAAGGTGCAGTCTAGCATGCTTA  94353

Query  1548   AAAGCGGCAAGCCATTTTACTTTATTGAAGTCGGCAATTTAGCAGGCGTGACGTCTGTAG  1607
              ||||||||||||||||||||||||||||||||||||||||||||||||||||||||||||
Sbjct  94352  AAAGCGGCAAGCCATTTTACTTTATTGAAGTCGGCAATTTAGCAGGCGTGACGTCTGTAG  94293

Query  1608   TTGAACACCTGCTAGTAGTAGGCCTGCAAGGTGAAAATTACGTGCAGCTAGCACACCGCA  1667
              ||||||||||||||||||||||||||||||||||||||||||||||||||||||||||||
Sbjct  94292  TTGAACACCTGCTAGTAGTAGGCCTGCAAGGTGAAAATTACGTGCAGCTAGCACACCGCA  94233

Query  1668   TTAAGCAGTCAACGCGACTGCAGCCATTGCTAGGCAAATTTTGACCGGTGTAATGTGTCG  1727
              ||||||||||||||||||||||||||||||||||||||||||||||||||||||||||||
Sbjct  94232  TTAAGCAGTCAACGCGACTGCAGCCATTGCTAGGCAAATTTTGACCGGTGTAATGTGTCG  94173

Query  1728   TGCTGTCATACCACTGTGAAGGCTTAGCAAGCCTTCGGTCTGGGTCTAAAAGGCTGCTAG  1787
              ||||||||||||||||||||||||||||||||||||||||||||||||||||||||||||
Sbjct  94172  TGCTGTCATACCACTGTGAAGGCTTAGCAAGCCTTCGGTCTGGGTCTAAAAGGCTGCTAG  94113

Query  1788   GCTATTGGCACCAAAAGCAAGTTGCAATGTGAACACATGCAAACTACTGCCAATGCCAGC  1847
              ||||||||||||||||||||||||||||||||||||||||||||||||||||||||||||
Sbjct  94112  GCTATTGGCACCAAAAGCAAGTTGCAATGTGAACACATGCAAACTACTGCCAATGCCAGC  94053

Query  1848   AGGCGTAATTTTGAGCTTTGCATGCAAATTACACAAGCTTACTACTGCCAATGTTAGCAG  1907
              ||||||||||||||||||||||||||||||||||||||||||||||||||||||||||||
Sbjct  94052  AGGCGTAATTTTGAGCTTTGCATGCAAATTACACAAGCTTACTACTGCCAATGTTAGCAG  93993

Query  1908   GCGTAATTTTGAGCTTTGCATGCAAATTACACATGCTTACTACTGCCAATGCTAGCAGGC  1967
              ||||||||||||||||||||||||||||||||||||||||||||||||||||||||||||
Sbjct  93992  GCGTAATTTTGAGCTTTGCATGCAAATTACACATGCTTACTACTGCCAATGCTAGCAGGC  93933

Query  1968   GTAATTTTGAACTTTG  1983
              ||||||||||||||||
Sbjct  93932  GTAATTTTGAACTTTG  93917


 Score = 370 bits (200),  Expect = 7e-104
 Identities = 200/200 (100%), Gaps = 0/200 (0%)
 Strand=Plus/Minus

Query  253     GCAATGAACTTCATTACTACAATTTTGAACATGCGTGCCCCTGGCATGTCAATGCACAAG  312
               ||||||||||||||||||||||||||||||||||||||||||||||||||||||||||||
Sbjct  101768  GCAATGAACTTCATTACTACAATTTTGAACATGCGTGCCCCTGGCATGTCAATGCACAAG  101709

Query  313     CTGCCGCTTTTTTGCTGGGCAATTTTTATTACAGCAATTTTGCTGCTGCTGTCATTGCCA  372
               ||||||||||||||||||||||||||||||||||||||||||||||||||||||||||||
Sbjct  101708  CTGCCGCTTTTTTGCTGGGCAATTTTTATTACAGCAATTTTGCTGCTGCTGTCATTGCCA  101649

Query  373     GTACTAGCGAGGAGCCATTACAATGTTACTAACGGACCGTAACTTCAACACGTCCTTTTA  432
               ||||||||||||||||||||||||||||||||||||||||||||||||||||||||||||
Sbjct  101648  GTACTAGCGAGGAGCCATTACAATGTTACTAACGGACCGTAACTTCAACACGTCCTTTTA  101589

Query  433     CGACCCTGCAGGCGGGGGTG  452
               ||||||||||||||||||||
Sbjct  101588  CGACCCTGCAGGCGGGGGTG  101569


 Score = 244 bits (132),  Expect = 4e-66
 Identities = 132/132 (100%), Gaps = 0/132 (0%)
 Strand=Plus/Minus

Query  502    CTACATTTTAATCATTCCTGCCTTTGGCATTGTGTCGCATGTAGTTGCAACCTTTGCTGG  561
              ||||||||||||||||||||||||||||||||||||||||||||||||||||||||||||
Sbjct  96859  CTACATTTTAATCATTCCTGCCTTTGGCATTGTGTCGCATGTAGTTGCAACCTTTGCTGG  96800

Query  562    TAAGCCCGTGTTTGGGTACCTAGGTATGGTCTATGCAATGTTCAGCATTGGCATTTTAGG  621
              ||||||||||||||||||||||||||||||||||||||||||||||||||||||||||||
Sbjct  96799  TAAGCCCGTGTTTGGGTACCTAGGTATGGTCTATGCAATGTTCAGCATTGGCATTTTAGG  96740

Query  622    CTTTATTGTATG  633
              ||||||||||||
Sbjct  96739  CTTTATTGTATG  96728


 Score = 222 bits (120),  Expect = 2e-59
 Identities = 122/123 (99%), Gaps = 0/123 (0%)
 Strand=Plus/Minus

Query  710    CGTCCCACAGGCATTAAAATTTTTTCATGACTAGCCACTTGCTACGGCGGTGTATTGCGG  769
              ||||||||||||||||||||||||||||||||||||||||||||||||||||||||||||
Sbjct  95565  CGTCCCACAGGCATTAAAATTTTTTCATGACTAGCCACTTGCTACGGCGGTGTATTGCGG  95506

Query  770    TACACAGCACCTATGGTGTTTGCATTGGGCTTCATTGCCCTGTTCACGGTAGGTGGGTTA  829
              ||||||||||||||||||||||||||||||||||||||||||||||||||||||||||| 
Sbjct  95505  TACACAGCACCTATGGTGTTTGCATTGGGCTTCATTGCCCTGTTCACGGTAGGTGGGTTC  95446

Query  830    ACA  832
              |||
Sbjct  95445  ACA  95443


 Score = 220 bits (119),  Expect = 7e-59
 Identities = 149/164 (91%), Gaps = 0/164 (0%)
 Strand=Plus/Minus

Query  1820   ACACATGCAAACTACTGCCAATGCCAGCAGGCGTAATTTTGAGCTTTGCATGCAAATTAC  1879
              ||||| ||  |||||||||||||  |||||||||||||||||||||||||||||||||||
Sbjct  94022  ACACAAGCTTACTACTGCCAATGTTAGCAGGCGTAATTTTGAGCTTTGCATGCAAATTAC  93963

Query  1880   ACAAGCTTACTACTGCCAATGTTAGCAGGCGTAATTTTGAGCTTTGCATGCAAATTACAC  1939
              ||| ||||||||||||||||| |||||||||||||||||| |||||||||||||||| ||
Sbjct  93962  ACATGCTTACTACTGCCAATGCTAGCAGGCGTAATTTTGAACTTTGCATGCAAATTAAAC  93903

Query  1940   ATGCTTACTACTGCCAATGCTAGCAGGCGTAATTTTGAACTTTG  1983
              ||||    |||||||||||||||||||||||||||| | |||||
Sbjct  93902  ATGCAATTTACTGCCAATGCTAGCAGGCGTAATTTTAAGCTTTG  93859


 Score = 206 bits (111),  Expect = 2e-54
 Identities = 111/111 (100%), Gaps = 0/111 (0%)
 Strand=Plus/Minus

Query  142     ACCGGCTGGACTATTTACCCACCCCTGTCGGGCATTCAAAGCCATTCAGGTGGGTCGGTA  201
               ||||||||||||||||||||||||||||||||||||||||||||||||||||||||||||
Sbjct  102766  ACCGGCTGGACTATTTACCCACCCCTGTCGGGCATTCAAAGCCATTCAGGTGGGTCGGTA  102707

Query  202     GACTTAGCAATTTTTAGCCTGCACCTGTCGGGCATTAGTAGTATGCTAGGT  252
               |||||||||||||||||||||||||||||||||||||||||||||||||||
Sbjct  102706  GACTTAGCAATTTTTAGCCTGCACCTGTCGGGCATTAGTAGTATGCTAGGT  102656


 Score = 178 bits (96),  Expect = 4e-46
 Identities = 98/99 (99%), Gaps = 0/99 (0%)
 Strand=Plus/Minus

Query  46      ATGGCCTTCCCACGTCTTAATAACATTAGCTTTTGGCTACTGCCGCCGTCACTAGTGCTG  105
               ||||||||||||||||||||||||||||||||||||||||||||||||||||||||||||
Sbjct  103064  ATGGCCTTCCCACGTCTTAATAACATTAGCTTTTGGCTACTGCCGCCGTCACTAGTGCTG  103005

Query  106     CTGCTTGCAAGTGCACTAGTCGAACAGGGTGCAGGTACC  144
               |||||||||||||||||||||||||||||||||||| ||
Sbjct  103004  CTGCTTGCAAGTGCACTAGTCGAACAGGGTGCAGGTGCC  102966


 Score = 152 bits (82),  Expect = 3e-38
 Identities = 98/106 (92%), Gaps = 0/106 (0%)
 Strand=Plus/Minus

Query  1878   ACACAAGCTTACTACTGCCAATGTTAGCAGGCGTAATTTTGAGCTTTGCATGCAAATTAC  1937
              ||||| ||  |||||||||||||  |||||||||||||||||||||||||||||||||||
Sbjct  94080  ACACATGCAAACTACTGCCAATGCCAGCAGGCGTAATTTTGAGCTTTGCATGCAAATTAC  94021

Query  1938   ACATGCTTACTACTGCCAATGCTAGCAGGCGTAATTTTGAACTTTG  1983
              ||| ||||||||||||||||| |||||||||||||||||| |||||
Sbjct  94020  ACAAGCTTACTACTGCCAATGTTAGCAGGCGTAATTTTGAGCTTTG  93975


 Score = 143 bits (77),  Expect = 2e-35
 Identities = 77/77 (100%), Gaps = 0/77 (0%)
 Strand=Plus/Minus

Query  633    GTTAGCACCACATGTTTGCCGTGGGGCTAGACGTTGACACACGAGCCTACTTCACAGCAG  692
              ||||||||||||||||||||||||||||||||||||||||||||||||||||||||||||
Sbjct  96127  GTTAGCACCACATGTTTGCCGTGGGGCTAGACGTTGACACACGAGCCTACTTCACAGCAG  96068

Query  693    CCACAATGATTATTGCT  709
              |||||||||||||||||
Sbjct  96067  CCACAATGATTATTGCT  96051


 Score = 84.2 bits (45),  Expect = 1e-17
 Identities = 45/45 (100%), Gaps = 0/45 (0%)
 Strand=Plus/Minus

Query  1       GGTTTTGGCAACTACTTAGTGCCAGTTCAAATTGGGGCGCCTGAT  45
               |||||||||||||||||||||||||||||||||||||||||||||
Sbjct  103758  GGTTTTGGCAACTACTTAGTGCCAGTTCAAATTGGGGCGCCTGAT  103714


 Score = 52.8 bits (28),  Expect = 3e-08
 Identities = 28/28 (100%), Gaps = 0/28 (0%)
 Strand=Plus/Minus

Query  452     GACCCAATTTTATTCCAACACCTGTTCT  479
               ||||||||||||||||||||||||||||
Sbjct  100718  GACCCAATTTTATTCCAACACCTGTTCT  100691


Lambda      K        H
    1.33    0.621     1.12 

Gapped
Lambda      K        H
    1.28    0.460    0.850 

Effective search space used: 216727400


Query= 900782 fgenesh1_kg.4_#_42_#_TRINITY_DN1293_c0_g1_i1

Length=1565
                                                                      Score     E
Sequences producing significant alignments:                          (Bits)  Value

  scaffold_1_mito                                                     1934    0.0  


> scaffold_1_mito
Length=110369

 Score = 1934 bits (1047),  Expect = 0.0
 Identities = 1047/1047 (100%), Gaps = 0/1047 (0%)
 Strand=Plus/Plus

Query  1      GATTGTGCTTTACTTGAAATAAAGTGTGCTTAATTGCACCATGGGTTCGAATCCCATTTT  60
              ||||||||||||||||||||||||||||||||||||||||||||||||||||||||||||
Sbjct  64475  GATTGTGCTTTACTTGAAATAAAGTGTGCTTAATTGCACCATGGGTTCGAATCCCATTTT  64534

Query  61     GCCCGCAGCCCCCAGTCACGTACTGGCTCGTACAGTAGTGCGGGGTACAGCATTGCACTA  120
              ||||||||||||||||||||||||||||||||||||||||||||||||||||||||||||
Sbjct  64535  GCCCGCAGCCCCCAGTCACGTACTGGCTCGTACAGTAGTGCGGGGTACAGCATTGCACTA  64594

Query  121    GCTGCAATTGCTAATGACTGGCTACAGTCTTGTGACTAGGTCACAGCAACGTACTGCTTA  180
              ||||||||||||||||||||||||||||||||||||||||||||||||||||||||||||
Sbjct  64595  GCTGCAATTGCTAATGACTGGCTACAGTCTTGTGACTAGGTCACAGCAACGTACTGCTTA  64654

Query  181    GGCTTGTGTGCTTACACGACTTGCAACACACATACTGTTGCATTAATTTGCGAATTGTGC  240
              ||||||||||||||||||||||||||||||||||||||||||||||||||||||||||||
Sbjct  64655  GGCTTGTGTGCTTACACGACTTGCAACACACATACTGTTGCATTAATTTGCGAATTGTGC  64714

Query  241    TTGCATTGAACCACATAGTTAGACGCCAAGTGTAGCAAATTGTAAAAATTTTGGGCCCCA  300
              ||||||||||||||||||||||||||||||||||||||||||||||||||||||||||||
Sbjct  64715  TTGCATTGAACCACATAGTTAGACGCCAAGTGTAGCAAATTGTAAAAATTTTGGGCCCCA  64774

Query  301    AGGCCGCAATTTTGCAACATTTTGTTGTTGCATTGAACCGTCTAATTACAGGCTTAAGGT  360
              ||||||||||||||||||||||||||||||||||||||||||||||||||||||||||||
Sbjct  64775  AGGCCGCAATTTTGCAACATTTTGTTGTTGCATTGAACCGTCTAATTACAGGCTTAAGGT  64834

Query  361    GGCAAATTGCTAGTAATACCAACTTTACTGTTGCTACTAGTACCGCCTAATTAAGCAGTG  420
              ||||||||||||||||||||||||||||||||||||||||||||||||||||||||||||
Sbjct  64835  GGCAAATTGCTAGTAATACCAACTTTACTGTTGCTACTAGTACCGCCTAATTAAGCAGTG  64894

Query  421    GACTGTGGCAAGCTGTTGCACGGGGCCCCCGAAGGGGGCCCATTTGCACACTTTGCTGTA  480
              ||||||||||||||||||||||||||||||||||||||||||||||||||||||||||||
Sbjct  64895  GACTGTGGCAAGCTGTTGCACGGGGCCCCCGAAGGGGGCCCATTTGCACACTTTGCTGTA  64954

Query  481    GTATTGAACCGCCTAGTTAGACGCTTAATGCAGCTATTAGTAAAAATGCATGCCAAACTG  540
              ||||||||||||||||||||||||||||||||||||||||||||||||||||||||||||
Sbjct  64955  GTATTGAACCGCCTAGTTAGACGCTTAATGCAGCTATTAGTAAAAATGCATGCCAAACTG  65014

Query  541    TTGCTGTTTGTACCATGTAATTAGGCAGTTGCCTGTTACAATTTGCTACAATTTAGTCCA  600
              ||||||||||||||||||||||||||||||||||||||||||||||||||||||||||||
Sbjct  65015  TTGCTGTTTGTACCATGTAATTAGGCAGTTGCCTGTTACAATTTGCTACAATTTAGTCCA  65074

Query  601    TTTTGCATTTTGCTGTGGCATTGAACCGTCTAATTAAGGCAAATGAAGGCAAATTAGCAA  660
              ||||||||||||||||||||||||||||||||||||||||||||||||||||||||||||
Sbjct  65075  TTTTGCATTTTGCTGTGGCATTGAACCGTCTAATTAAGGCAAATGAAGGCAAATTAGCAA  65134

Query  661    CTAATGCAATTTTACTGCCCACAGGGGCTGGCAGTGTCTAACTTAAGCCGGTGTAAAAAT  720
              ||||||||||||||||||||||||||||||||||||||||||||||||||||||||||||
Sbjct  65135  CTAATGCAATTTTACTGCCCACAGGGGCTGGCAGTGTCTAACTTAAGCCGGTGTAAAAAT  65194

Query  721    GCTTCAAAATGTAACACGTGCACAATTCCAAGCACACCCGTACCACTTGGTGACGCCGTC  780
              ||||||||||||||||||||||||||||||||||||||||||||||||||||||||||||
Sbjct  65195  GCTTCAAAATGTAACACGTGCACAATTCCAAGCACACCCGTACCACTTGGTGACGCCGTC  65254

Query  781    ACCATGGCCTTTGCTGACGTCCTTTTCGTTACTAATTTTGACTGTGGCTGCTGCTATGTA  840
              ||||||||||||||||||||||||||||||||||||||||||||||||||||||||||||
Sbjct  65255  ACCATGGCCTTTGCTGACGTCCTTTTCGTTACTAATTTTGACTGTGGCTGCTGCTATGTA  65314

Query  841    CTTTAATGGCTATGCAAATGGCGGCATGCTAGTCAGCATTGGCTTCATTACAGTCGTTGC  900
              ||||||||||||||||||||||||||||||||||||||||||||||||||||||||||||
Sbjct  65315  CTTTAATGGCTATGCAAATGGCGGCATGCTAGTCAGCATTGGCTTCATTACAGTCGTTGC  65374

Query  901    GTCAATGGCATTATGGTTTCGTGACGTAATTGCAGAAGGTGCATTGCTAGGCAACCACAC  960
              ||||||||||||||||||||||||||||||||||||||||||||||||||||||||||||
Sbjct  65375  GTCAATGGCATTATGGTTTCGTGACGTAATTGCAGAAGGTGCATTGCTAGGCAACCACAC  65434

Query  961    ATTTGCGGTGCAAAAAGGACTTAACCTAGGCGTAGCCTTATTTATTGTAAGTGAGGTGtt  1020
              ||||||||||||||||||||||||||||||||||||||||||||||||||||||||||||
Sbjct  65435  ATTTGCGGTGCAAAAAGGACTTAACCTAGGCGTAGCCTTATTTATTGTAAGTGAGGTGTT  65494

Query  1021   ttttttcatttcaattttttGAGCTTA  1047
              |||||||||||||||||||||||||||
Sbjct  65495  TTTTTTCATTTCAATTTTTTGAGCTTA  65521


 Score = 791 bits (428),  Expect = 0.0
 Identities = 428/428 (100%), Gaps = 0/428 (0%)
 Strand=Plus/Plus

Query  1047   ACTTCCATTCAGCCTTAGCGCCTACAGTCGAGTTAGGCAATGCATGGCCGCCTGCTGGGG  1106
              ||||||||||||||||||||||||||||||||||||||||||||||||||||||||||||
Sbjct  66731  ACTTCCATTCAGCCTTAGCGCCTACAGTCGAGTTAGGCAATGCATGGCCGCCTGCTGGGG  66790

Query  1107   TCCAAGCATTAGACCCTTACGAAGTGCCACTACTAAACACAGTAATTTTGCTAGGGTCAG  1166
              ||||||||||||||||||||||||||||||||||||||||||||||||||||||||||||
Sbjct  66791  TCCAAGCATTAGACCCTTACGAAGTGCCACTACTAAACACAGTAATTTTGCTAGGGTCAG  66850

Query  1167   GCGCTAGCGTTACGTACGCACACCACTCATTAATTCAAGGTAGCCGTGCAGGCACAATTG  1226
              ||||||||||||||||||||||||||||||||||||||||||||||||||||||||||||
Sbjct  66851  GCGCTAGCGTTACGTACGCACACCACTCATTAATTCAAGGTAGCCGTGCAGGCACAATTG  66910

Query  1227   CAGGCCTAATTGTGACCATTGCACTAGCTGCTGTGTTCACAATGCTGCAGGGTCTTGAGT  1286
              ||||||||||||||||||||||||||||||||||||||||||||||||||||||||||||
Sbjct  66911  CAGGCCTAATTGTGACCATTGCACTAGCTGCTGTGTTCACAATGCTGCAGGGTCTTGAGT  66970

Query  1287   ACCATGAAGCTAGCTTTACAATTGCAGACGGTGCTTACGGGTCTACATTTTACTTTGCGA  1346
              ||||||||||||||||||||||||||||||||||||||||||||||||||||||||||||
Sbjct  66971  ACCATGAAGCTAGCTTTACAATTGCAGACGGTGCTTACGGGTCTACATTTTACTTTGCGA  67030

Query  1347   CTGGCTTCCATGGCCTGCACGTCATTATTGGGACCCTGTTCATTGCCGTGGCATTTGTGC  1406
              ||||||||||||||||||||||||||||||||||||||||||||||||||||||||||||
Sbjct  67031  CTGGCTTCCATGGCCTGCACGTCATTATTGGGACCCTGTTCATTGCCGTGGCATTTGTGC  67090

Query  1407   GACTACTAAGCTACCAGCTTACTGACCACCACCACTTAGGCTTTGAAGCCGCCATTTTGT  1466
              ||||||||||||||||||||||||||||||||||||||||||||||||||||||||||||
Sbjct  67091  GACTACTAAGCTACCAGCTTACTGACCACCACCACTTAGGCTTTGAAGCCGCCATTTTGT  67150

Query  1467   ACTGGCAC  1474
              ||||||||
Sbjct  67151  ACTGGCAC  67158


 Score = 172 bits (93),  Expect = 2e-44
 Identities = 93/93 (100%), Gaps = 0/93 (0%)
 Strand=Plus/Plus

Query  1473   ACTTTGTTGACGTAGTGTGGCTGTTCTTGTACGTGTCGGTGTACTGGTGAGGTGCATAAA  1532
              ||||||||||||||||||||||||||||||||||||||||||||||||||||||||||||
Sbjct  67667  ACTTTGTTGACGTAGTGTGGCTGTTCTTGTACGTGTCGGTGTACTGGTGAGGTGCATAAA  67726

Query  1533   CACAAACCCCTTGCTTGCAACGTAGCTTGCAAA  1565
              |||||||||||||||||||||||||||||||||
Sbjct  67727  CACAAACCCCTTGCTTGCAACGTAGCTTGCAAA  67759


Lambda      K        H
    1.33    0.621     1.12 

Gapped
Lambda      K        H
    1.28    0.460    0.850 

Effective search space used: 170601100


Query= 900778 fgenesh1_kg.4_#_38_#_TRINITY_DN3144_c3_g6_i1

Length=755
                                                                      Score     E
Sequences producing significant alignments:                          (Bits)  Value

  scaffold_1_mito                                                     784     0.0  


> scaffold_1_mito
Length=110369

 Score = 784 bits (424),  Expect = 0.0
 Identities = 424/424 (100%), Gaps = 0/424 (0%)
 Strand=Plus/Plus

Query  1      CACATGAACAGCATTGGCTGTTTTGCtttttttttAATGCCTGCAGGGCATTaaaaaaaa  60
              ||||||||||||||||||||||||||||||||||||||||||||||||||||||||||||
Sbjct  55183  CACATGAACAGCATTGGCTGTTTTGCTTTTTTTTTAATGCCTGCAGGGCATTAAAAAAAA  55242

Query  61     aaCTGCTGCAATTAGCTTGTGGCCGCGCTTTTAAAAATTGAAGACATTACACAGTACACA  120
              ||||||||||||||||||||||||||||||||||||||||||||||||||||||||||||
Sbjct  55243  AACTGCTGCAATTAGCTTGTGGCCGCGCTTTTAAAAATTGAAGACATTACACAGTACACA  55302

Query  121    ATTGGCACGCGTGTGGGTTTGGCACACTGTGCAGTCATTGCACACAGCATTGCAAGCTAG  180
              ||||||||||||||||||||||||||||||||||||||||||||||||||||||||||||
Sbjct  55303  ATTGGCACGCGTGTGGGTTTGGCACACTGTGCAGTCATTGCACACAGCATTGCAAGCTAG  55362

Query  181    TAGCAGCCTACTAGTAAGCACTTGCTGGCTACATTTTAATTAATGCGACTGCTTAAAACA  240
              ||||||||||||||||||||||||||||||||||||||||||||||||||||||||||||
Sbjct  55363  TAGCAGCCTACTAGTAAGCACTTGCTGGCTACATTTTAATTAATGCGACTGCTTAAAACA  55422

Query  241    CACCCCATTTTAGGTTTAGTAAATAGCTACATGGTCGACAGCCCACAACCTGCTAGCATT  300
              ||||||||||||||||||||||||||||||||||||||||||||||||||||||||||||
Sbjct  55423  CACCCCATTTTAGGTTTAGTAAATAGCTACATGGTCGACAGCCCACAACCTGCTAGCATT  55482

Query  301    ACGTACATGTGAAATTTTGGGTCGCTGCTAGGCCTGTGCTTAGTCATTCAAATTGTGACT  360
              ||||||||||||||||||||||||||||||||||||||||||||||||||||||||||||
Sbjct  55483  ACGTACATGTGAAATTTTGGGTCGCTGCTAGGCCTGTGCTTAGTCATTCAAATTGTGACT  55542

Query  361    GGCATTACGCTAGCAATGCACTACACACCGTCTATTGACTTGGCCTTTGCAAGTGTTGAG  420
              ||||||||||||||||||||||||||||||||||||||||||||||||||||||||||||
Sbjct  55543  GGCATTACGCTAGCAATGCACTACACACCGTCTATTGACTTGGCCTTTGCAAGTGTTGAG  55602

Query  421    CATA  424
              ||||
Sbjct  55603  CATA  55606


 Score = 614 bits (332),  Expect = 1e-177
 Identities = 332/332 (100%), Gaps = 0/332 (0%)
 Strand=Plus/Plus

Query  424    ATTATGCGTGACGTGCATTACGGCTGGCTAATTCGGTACTTGCATGCTAACGTGGCGTCG  483
              ||||||||||||||||||||||||||||||||||||||||||||||||||||||||||||
Sbjct  56265  ATTATGCGTGACGTGCATTACGGCTGGCTAATTCGGTACTTGCATGCTAACGTGGCGTCG  56324

Query  484    TTCTTTTTTATTTGCGTGTACTTACACATTGGTCGTGGCCTTTACTATGGGTCGTACCGG  543
              ||||||||||||||||||||||||||||||||||||||||||||||||||||||||||||
Sbjct  56325  TTCTTTTTTATTTGCGTGTACTTACACATTGGTCGTGGCCTTTACTATGGGTCGTACCGG  56384

Query  544    TCACCACGTACGCTAGTGTGGGCCATTGGGGTCGTAATTTTAGTACTTATGATAGCAACA  603
              ||||||||||||||||||||||||||||||||||||||||||||||||||||||||||||
Sbjct  56385  TCACCACGTACGCTAGTGTGGGCCATTGGGGTCGTAATTTTAGTACTTATGATAGCAACA  56444

Query  604    GCCTTTTTAGGTGACTACATTGCCTAAAATGTAATTTACGTGCTGCACAAGCCCGTGTGC  663
              ||||||||||||||||||||||||||||||||||||||||||||||||||||||||||||
Sbjct  56445  GCCTTTTTAGGTGACTACATTGCCTAAAATGTAATTTACGTGCTGCACAAGCCCGTGTGC  56504

Query  664    AATTGACTCGCGTGTGCTTGCATGAGCTTGCATTAGTAAATTTGCATGCTGCTTAAGCCC  723
              ||||||||||||||||||||||||||||||||||||||||||||||||||||||||||||
Sbjct  56505  AATTGACTCGCGTGTGCTTGCATGAGCTTGCATTAGTAAATTTGCATGCTGCTTAAGCCC  56564

Query  724    GTGTGCAATTGACTAGCGTGTGCTTGCATGGG  755
              ||||||||||||||||||||||||||||||||
Sbjct  56565  GTGTGCAATTGACTAGCGTGTGCTTGCATGGG  56596


Lambda      K        H
    1.33    0.621     1.12 

Gapped
Lambda      K        H
    1.28    0.460    0.850 

Effective search space used: 81328687


Query= 866864 gw1.4.175.1

Length=261
                                                                      Score     E
Sequences producing significant alignments:                          (Bits)  Value

  scaffold_1_mito                                                     483     1e-138


> scaffold_1_mito
Length=110369

 Score = 483 bits (261),  Expect = 1e-138
 Identities = 261/261 (100%), Gaps = 0/261 (0%)
 Strand=Plus/Plus

Query  1      AAAATGGCGCTTTCAATTGTACTGTTCGTACTAGCAGTGCTAGGCTTTGTACTAAACCGC  60
              ||||||||||||||||||||||||||||||||||||||||||||||||||||||||||||
Sbjct  11721  AAAATGGCGCTTTCAATTGTACTGTTCGTACTAGCAGTGCTAGGCTTTGTACTAAACCGC  11780

Query  61     AAAAACCTAATTTTAATGCTAATTAGCATTGAAGTTATGTTGCTTGCTGTAACGCTGCTA  120
              ||||||||||||||||||||||||||||||||||||||||||||||||||||||||||||
Sbjct  11781  AAAAACCTAATTTTAATGCTAATTAGCATTGAAGTTATGTTGCTTGCTGTAACGCTGCTA  11840

Query  121    GTCATTGTGACGTCTTATGAGTTTAATGACGTAATTGGTCAAACTTACGCTGTGTTCATT  180
              ||||||||||||||||||||||||||||||||||||||||||||||||||||||||||||
Sbjct  11841  GTCATTGTGACGTCTTATGAGTTTAATGACGTAATTGGTCAAACTTACGCTGTGTTCATT  11900

Query  181    ATTGCAATTGCAGGTGCCGAGTCAGCAATTGGACTTGGCATTTTAGTAGCCTTTTACCGT  240
              ||||||||||||||||||||||||||||||||||||||||||||||||||||||||||||
Sbjct  11901  ATTGCAATTGCAGGTGCCGAGTCAGCAATTGGACTTGGCATTTTAGTAGCCTTTTACCGT  11960

Query  241    ACACGTGGGTCAATTACACTT  261
              |||||||||||||||||||||
Sbjct  11961  ACACGTGGGTCAATTACACTT  11981


Lambda      K        H
    1.33    0.621     1.12 

Gapped
Lambda      K        H
    1.28    0.460    0.850 

Effective search space used: 26925888


Query= 950280 estExt_fgenesh1_pm.C_40011

Length=843
                                                                      Score     E
Sequences producing significant alignments:                          (Bits)  Value

  scaffold_1_mito                                                     1116    0.0  


> scaffold_1_mito
Length=110369

 Score = 1116 bits (604),  Expect = 0.0
 Identities = 604/604 (100%), Gaps = 0/604 (0%)
 Strand=Plus/Minus

Query  1      ATGCTAGCTGTAATTTTTAGTTTACTTGAAGTTTTAATTGTGTTAGTGCCTGTACTACTG  60
              ||||||||||||||||||||||||||||||||||||||||||||||||||||||||||||
Sbjct  80517  ATGCTAGCTGTAATTTTTAGTTTACTTGAAGTTTTAATTGTGTTAGTGCCTGTACTACTG  80458

Query  61     TCAGTTGCGTTCATGACCATTATTGAACGTAAGGTGCTTGCAAGCATGCAGCGACGTGTG  120
              ||||||||||||||||||||||||||||||||||||||||||||||||||||||||||||
Sbjct  80457  TCAGTTGCGTTCATGACCATTATTGAACGTAAGGTGCTTGCAAGCATGCAGCGACGTGTG  80398

Query  121    GGCCCTAACACCGTGGGCTACTTTGGTGTGCTGCAACCTTTTGCTGACGCCTTAAAATTA  180
              ||||||||||||||||||||||||||||||||||||||||||||||||||||||||||||
Sbjct  80397  GGCCCTAACACCGTGGGCTACTTTGGTGTGCTGCAACCTTTTGCTGACGCCTTAAAATTA  80338

Query  181    GTCGTTAAAGAGACCGTAGTCCCACAACATGCAACACGCAGCCTGttttttttGGCCCCT  240
              ||||||||||||||||||||||||||||||||||||||||||||||||||||||||||||
Sbjct  80337  GTCGTTAAAGAGACCGTAGTCCCACAACATGCAACACGCAGCCTGTTTTTTTTGGCCCCT  80278

Query  241    GTAATTAGCCTAGTGTTTAGCTTGCTAGGGTGGGCAGTGGTCCCATTTGCAAGCGGCCTG  300
              ||||||||||||||||||||||||||||||||||||||||||||||||||||||||||||
Sbjct  80277  GTAATTAGCCTAGTGTTTAGCTTGCTAGGGTGGGCAGTGGTCCCATTTGCAAGCGGCCTG  80218

Query  301    GCATTAAGCGACTTTAGCTTAGGCGTGCTGTACTCACTTGCAATTAGTAGCATTGGGGTT  360
              ||||||||||||||||||||||||||||||||||||||||||||||||||||||||||||
Sbjct  80217  GCATTAAGCGACTTTAGCTTAGGCGTGCTGTACTCACTTGCAATTAGTAGCATTGGGGTT  80158

Query  361    TACGGTGTACTTTTTGCAGGCTGGTCTGCAAACAGCAAGTACGCATTTTTAGGGTCGTTG  420
              ||||||||||||||||||||||||||||||||||||||||||||||||||||||||||||
Sbjct  80157  TACGGTGTACTTTTTGCAGGCTGGTCTGCAAACAGCAAGTACGCATTTTTAGGGTCGTTG  80098

Query  421    CGTAGCACAGCCCAAATGATTAGTTACGAGCTAATTTTAAGCACTGCAGTACTTGCAGTA  480
              ||||||||||||||||||||||||||||||||||||||||||||||||||||||||||||
Sbjct  80097  CGTAGCACAGCCCAAATGATTAGTTACGAGCTAATTTTAAGCACTGCAGTACTTGCAGTA  80038

Query  481    ATTTTGCTTGCAGGTACGTTTAGTTACACTGCAATTATTGAACGGCAGCAAGCAATTTAC  540
              ||||||||||||||||||||||||||||||||||||||||||||||||||||||||||||
Sbjct  80037  ATTTTGCTTGCAGGTACGTTTAGTTACACTGCAATTATTGAACGGCAGCAAGCAATTTAC  79978

Query  541    TATGCTGTGCCATTACTGCCGCTGTTCATTGTGTTTTTTATTGCAGTACTTGCTGAGACT  600
              ||||||||||||||||||||||||||||||||||||||||||||||||||||||||||||
Sbjct  79977  TATGCTGTGCCATTACTGCCGCTGTTCATTGTGTTTTTTATTGCAGTACTTGCTGAGACT  79918

Query  601    AACC  604
              ||||
Sbjct  79917  AACC  79914


 Score = 368 bits (199),  Expect = 1e-103
 Identities = 199/199 (100%), Gaps = 0/199 (0%)
 Strand=Plus/Minus

Query  645    CATTGTGTTAATGTCGGCATTGACTTCAAttttttttttAGGTGGCTATGTAATGCCACA  704
              ||||||||||||||||||||||||||||||||||||||||||||||||||||||||||||
Sbjct  79108  CATTGTGTTAATGTCGGCATTGACTTCAATTTTTTTTTTAGGTGGCTATGTAATGCCACA  79049

Query  705    GTGCATTGTAAACAACACCTTCATTAATGTGCAGGCTGTGGTACTAGCTTTAAAGACTTG  764
              ||||||||||||||||||||||||||||||||||||||||||||||||||||||||||||
Sbjct  79048  GTGCATTGTAAACAACACCTTCATTAATGTGCAGGCTGTGGTACTAGCTTTAAAGACTTG  78989

Query  765    CCTGTTTTGCTTTGTGTTTGTGTGGTTTCGTGCAACGTTGCCACGACTGCGTTATGACCA  824
              ||||||||||||||||||||||||||||||||||||||||||||||||||||||||||||
Sbjct  78988  CCTGTTTTGCTTTGTGTTTGTGTGGTTTCGTGCAACGTTGCCACGACTGCGTTATGACCA  78929

Query  825    GCTTATGCAATTTTGTTGA  843
              |||||||||||||||||||
Sbjct  78928  GCTTATGCAATTTTGTTGA  78910


 Score = 75.0 bits (40),  Expect = 2e-15
 Identities = 40/40 (100%), Gaps = 0/40 (0%)
 Strand=Plus/Minus

Query  605    TCGCAGGCTACTTTACTGAGCATTCAGGCATGATTTTTGT  644
              ||||||||||||||||||||||||||||||||||||||||
Sbjct  79175  TCGCAGGCTACTTTACTGAGCATTCAGGCATGATTTTTGT  79136


Lambda      K        H
    1.33    0.621     1.12 

Gapped
Lambda      K        H
    1.28    0.460    0.850 

Effective search space used: 91039575


Query= 900774 fgenesh1_kg.4_#_34_#_TRINITY_DN3935_c0_g1_i1

Length=1011
                                                                      Score     E
Sequences producing significant alignments:                          (Bits)  Value

  scaffold_1_mito                                                     1868    0.0  


> scaffold_1_mito
Length=110369

 Score = 1868 bits (1011),  Expect = 0.0
 Identities = 1011/1011 (100%), Gaps = 0/1011 (0%)
 Strand=Plus/Plus

Query  1      GTCCAACACGTAGTGTTGGACCTGGGACGAAACGGCCGTAATTTTACACCGGCTTAAGTT  60
              ||||||||||||||||||||||||||||||||||||||||||||||||||||||||||||
Sbjct  44419  GTCCAACACGTAGTGTTGGACCTGGGACGAAACGGCCGTAATTTTACACCGGCTTAAGTT  44478

Query  61     AGGCAGTAAGCAGTATGCCTTGCAGTTTTAAATTTGTAACCCATGTAGCTGCTAATTTGG  120
              ||||||||||||||||||||||||||||||||||||||||||||||||||||||||||||
Sbjct  44479  AGGCAGTAAGCAGTATGCCTTGCAGTTTTAAATTTGTAACCCATGTAGCTGCTAATTTGG  44538

Query  121    CTATTAGTGACTGGCGTCATTAGTCTTAAATTGCACGGCCCCCGGGTACACACACACTAA  180
              ||||||||||||||||||||||||||||||||||||||||||||||||||||||||||||
Sbjct  44539  CTATTAGTGACTGGCGTCATTAGTCTTAAATTGCACGGCCCCCGGGTACACACACACTAA  44598

Query  181    GACTGCAACATTAGTAGCACGTGCCTAACTTCACATGGTCCTTTTTACGTCACCACTTGA  240
              ||||||||||||||||||||||||||||||||||||||||||||||||||||||||||||
Sbjct  44599  GACTGCAACATTAGTAGCACGTGCCTAACTTCACATGGTCCTTTTTACGTCACCACTTGA  44658

Query  241    ACAATTTGAAGTCACGTCTTTTTTAAACGTTAACCTTACGCTATTAGGGCTTAATTTTGC  300
              ||||||||||||||||||||||||||||||||||||||||||||||||||||||||||||
Sbjct  44659  ACAATTTGAAGTCACGTCTTTTTTAAACGTTAACCTTACGCTATTAGGGCTTAATTTTGC  44718

Query  301    ATTGACTAACCTAGGCCTTTACGCCTTGCTAGTCTTAATGCTAGCGCTTAGTGTGCATGT  360
              ||||||||||||||||||||||||||||||||||||||||||||||||||||||||||||
Sbjct  44719  ATTGACTAACCTAGGCCTTTACGCCTTGCTAGTCTTAATGCTAGCGCTTAGTGTGCATGT  44778

Query  361    GCTTGCAGCAAACGACCGACGCTTGGTGCCTAGCCGCTGGTCTGTAGGCCTTGAGTCAGC  420
              ||||||||||||||||||||||||||||||||||||||||||||||||||||||||||||
Sbjct  44779  GCTTGCAGCAAACGACCGACGCTTGGTGCCTAGCCGCTGGTCTGTAGGCCTTGAGTCAGC  44838

Query  421    TTACGCGTCATTGCACGGCATGGTAAAGGAACAAATTGGCAGCAGCAACGAACGCTACCT  480
              ||||||||||||||||||||||||||||||||||||||||||||||||||||||||||||
Sbjct  44839  TTACGCGTCATTGCACGGCATGGTAAAGGAACAAATTGGCAGCAGCAACGAACGCTACCT  44898

Query  481    ACCGTTCATTTACGGGCTGTTCATGTTCGTAATTTTGGCAAATTTGACCGGCAACGTACC  540
              ||||||||||||||||||||||||||||||||||||||||||||||||||||||||||||
Sbjct  44899  ACCGTTCATTTACGGGCTGTTCATGTTCGTAATTTTGGCAAATTTGACCGGCAACGTACC  44958

Query  541    GTACTCATTTACAATTAGTACAAGTGCTGTGCTGTCAATTGGCTTAAGTTTTGCAAtttt  600
              ||||||||||||||||||||||||||||||||||||||||||||||||||||||||||||
Sbjct  44959  GTACTCATTTACAATTAGTACAAGTGCTGTGCTGTCAATTGGCTTAAGTTTTGCAATTTT  45018

Query  601    ttttGGTGTAACAGTCCTAGGACTACACAAGCATGGGGTACAtttttttgcattttttGT  660
              ||||||||||||||||||||||||||||||||||||||||||||||||||||||||||||
Sbjct  45019  TTTTGGTGTAACAGTCCTAGGACTACACAAGCATGGGGTACATTTTTTTGCATTTTTTGT  45078

Query  661    GCCTGCAGGTACACCGGTAGCTATGGTGCCCTTGCTAGTACTAATTGAGCTAATTTCGTA  720
              ||||||||||||||||||||||||||||||||||||||||||||||||||||||||||||
Sbjct  45079  GCCTGCAGGTACACCGGTAGCTATGGTGCCCTTGCTAGTACTAATTGAGCTAATTTCGTA  45138

Query  721    CCTTGCACGTGCCTTTAGCTTAGGCGTACGGCTTTTTGCTAACATGGTGGCAGGTCACAC  780
              ||||||||||||||||||||||||||||||||||||||||||||||||||||||||||||
Sbjct  45139  CCTTGCACGTGCCTTTAGCTTAGGCGTACGGCTTTTTGCTAACATGGTGGCAGGTCACAC  45198

Query  781    ACTACTAAAAATTTTAAGTGGCATGCTGTGGCCAATTTTAACGTCAGGTGTCGTTATGTT  840
              ||||||||||||||||||||||||||||||||||||||||||||||||||||||||||||
Sbjct  45199  ACTACTAAAAATTTTAAGTGGCATGCTGTGGCCAATTTTAACGTCAGGTGTCGTTATGTT  45258

Query  841    TGTGGTAGCCTTAGTGCCAATGGCAAtttttttGGCCCTAGTAGGCCTTGAAATTGCAGT  900
              ||||||||||||||||||||||||||||||||||||||||||||||||||||||||||||
Sbjct  45259  TGTGGTAGCCTTAGTGCCAATGGCAATTTTTTTGGCCCTAGTAGGCCTTGAAATTGCAGT  45318

Query  901    AAGCGTCATTCAAGCCTACGTCTTTGTAGTGCTTACATGTGTTTACTTGCGCGACGCAAT  960
              ||||||||||||||||||||||||||||||||||||||||||||||||||||||||||||
Sbjct  45319  AAGCGTCATTCAAGCCTACGTCTTTGTAGTGCTTACATGTGTTTACTTGCGCGACGCAAT  45378

Query  961    TGACTTGCACTAACCCCTTAAACCCCATTAAAATTTTACTATGACACAAAA  1011
              |||||||||||||||||||||||||||||||||||||||||||||||||||
Sbjct  45379  TGACTTGCACTAACCCCTTAAACCCCATTAAAATTTTACTATGACACAAAA  45429


 Score = 80.5 bits (43),  Expect = 6e-17
 Identities = 83/99 (84%), Gaps = 15/99 (15%)
 Strand=Plus/Plus

Query  1      GTCCAACACGTAGTGTTGGACCTGGGACGAAACGGCC------------GTAATTTTACA  48
              |||||||||||||||||||||||||||||||||||||            |||||||||||
Sbjct  44224  GTCCAACACGTAGTGTTGGACCTGGGACGAAACGGCCATGTGCATTGCAGTAATTTTACA  44283

Query  49     CCGGCTTAAGTTAGGCA-GTAAGCAGTATGCCTTGCAGT  86
              || |||||||||||||| |||||||||  ||||||||||
Sbjct  44284  CCTGCTTAAGTTAGGCAAGTAAGCAGT--GCCTTGCAGT  44320


 Score = 62.1 bits (33),  Expect = 2e-11
 Identities = 44/49 (90%), Gaps = 1/49 (2%)
 Strand=Plus/Plus

Query  35     GCCGTAATTTTACACCGGCTTAAGTTAGGC-AGTAAGCAGTATGCCTTG  82
              || ||||||||||||||||||||||||||| |||  ||||| |||||||
Sbjct  44340  GCAGTAATTTTACACCGGCTTAAGTTAGGCAAGTTTGCAGTGTGCCTTG  44388


Lambda      K        H
    1.33    0.621     1.12 

Gapped
Lambda      K        H
    1.28    0.460    0.850 

Effective search space used: 109578543


Query= 900801 fgenesh1_kg.4_#_61_#_TRINITY_DN3144_c3_g8_i12

Length=1973
                                                                      Score     E
Sequences producing significant alignments:                          (Bits)  Value

  scaffold_1_mito                                                     2316    0.0  


> scaffold_1_mito
Length=110369

 Score = 2316 bits (1254),  Expect = 0.0
 Identities = 1254/1254 (100%), Gaps = 0/1254 (0%)
 Strand=Plus/Minus

Query  720    CACGACGTATTCCTGACTACCCTGACGCCTTTGCAGGCTGGAACCTAGTGTCGAGCTTCG  779
              ||||||||||||||||||||||||||||||||||||||||||||||||||||||||||||
Sbjct  91248  CACGACGTATTCCTGACTACCCTGACGCCTTTGCAGGCTGGAACCTAGTGTCGAGCTTCG  91189

Query  780    GCTCACTAGTGTCGGTAGTAGCTACAGCAGTCTTCCTGTACGTTGTGTGGGACCAGTTTG  839
              ||||||||||||||||||||||||||||||||||||||||||||||||||||||||||||
Sbjct  91188  GCTCACTAGTGTCGGTAGTAGCTACAGCAGTCTTCCTGTACGTTGTGTGGGACCAGTTTG  91129

Query  840    TGCACGGTAAAGCCGTGGTACACAACCCATGGGCGGTGCCCCAGTACTTCACAAGCGACA  899
              ||||||||||||||||||||||||||||||||||||||||||||||||||||||||||||
Sbjct  91128  TGCACGGTAAAGCCGTGGTACACAACCCATGGGCGGTGCCCCAGTACTTCACAAGCGACA  91069

Query  900    GCCTGTTTGCAGTAGCACCACAGACCGCTAGCTCATTAGAATGGACGCTAGCAAGTCCTG  959
              ||||||||||||||||||||||||||||||||||||||||||||||||||||||||||||
Sbjct  91068  GCCTGTTTGCAGTAGCACCACAGACCGCTAGCTCATTAGAATGGACGCTAGCAAGTCCTG  91009

Query  960    TGCCATTCCATTCTTACAACACGCTGCCAGTACAGTCGTAGTGTGTGCGTGTTTGACTAG  1019
              ||||||||||||||||||||||||||||||||||||||||||||||||||||||||||||
Sbjct  91008  TGCCATTCCATTCTTACAACACGCTGCCAGTACAGTCGTAGTGTGTGCGTGTTTGACTAG  90949

Query  1020   ACTTAAGCCGGCGTGTAATTTGCATTGCAAGCAAGCCTAGTCGCAAACCCCCTGTACACC  1079
              ||||||||||||||||||||||||||||||||||||||||||||||||||||||||||||
Sbjct  90948  ACTTAAGCCGGCGTGTAATTTGCATTGCAAGCAAGCCTAGTCGCAAACCCCCTGTACACC  90889

Query  1080   AAGTCTTAGGTGCTTGCTAGGCAGTGACATAACAGCCTACGAGCCTTTGCGTGTTGCTTA  1139
              ||||||||||||||||||||||||||||||||||||||||||||||||||||||||||||
Sbjct  90888  AAGTCTTAGGTGCTTGCTAGGCAGTGACATAACAGCCTACGAGCCTTTGCGTGTTGCTTA  90829

Query  1140   CATAGCCCAAGTGCTTGCTTGGTCGTTCATAACAGCCTACGAGCCTTTGCATGACGTTAC  1199
              ||||||||||||||||||||||||||||||||||||||||||||||||||||||||||||
Sbjct  90828  CATAGCCCAAGTGCTTGCTTGGTCGTTCATAACAGCCTACGAGCCTTTGCATGACGTTAC  90769

Query  1200   TTGGGCCAAAATTAAAACACGGCCCAAGTACTTACTAAGCCGTTCATAACAGCCTGCGAG  1259
              ||||||||||||||||||||||||||||||||||||||||||||||||||||||||||||
Sbjct  90768  TTGGGCCAAAATTAAAACACGGCCCAAGTACTTACTAAGCCGTTCATAACAGCCTGCGAG  90709

Query  1260   CCTTTGCGTGACGTTTGTGTGCAAGTGTCTGCCTAGGCGTTCATAACAGCCTACGAGCCT  1319
              ||||||||||||||||||||||||||||||||||||||||||||||||||||||||||||
Sbjct  90708  CCTTTGCGTGACGTTTGTGTGCAAGTGTCTGCCTAGGCGTTCATAACAGCCTACGAGCCT  90649

Query  1320   TTGCATGACGTTTGCATGCAAGTGCTTGCTAGACTTTCATAACAGCCTACGAGCCTTTGG  1379
              ||||||||||||||||||||||||||||||||||||||||||||||||||||||||||||
Sbjct  90648  TTGCATGACGTTTGCATGCAAGTGCTTGCTAGACTTTCATAACAGCCTACGAGCCTTTGG  90589

Query  1380   CCCCAAGGCAAGGACGTTGCATGCTAGTACTTGCAGGGCTGCACATGACAGCCTACGAGC  1439
              ||||||||||||||||||||||||||||||||||||||||||||||||||||||||||||
Sbjct  90588  CCCCAAGGCAAGGACGTTGCATGCTAGTACTTGCAGGGCTGCACATGACAGCCTACGAGC  90529

Query  1440   CTTTGCGTGACGTTTACATGCAAAGTAAGACACGCTTAATTTACAGGTGGTCAATGTAGG  1499
              ||||||||||||||||||||||||||||||||||||||||||||||||||||||||||||
Sbjct  90528  CTTTGCGTGACGTTTACATGCAAAGTAAGACACGCTTAATTTACAGGTGGTCAATGTAGG  90469

Query  1500   GCAAGCCTTTGCAAACACTTAAGTACACGGGTCACTATGACCCCGACTTGCTTAATTGCA  1559
              ||||||||||||||||||||||||||||||||||||||||||||||||||||||||||||
Sbjct  90468  GCAAGCCTTTGCAAACACTTAAGTACACGGGTCACTATGACCCCGACTTGCTTAATTGCA  90409

Query  1560   AAAGGCTTCAACTTAACACCGCCTTTTCGTCCCAGGTCGAACACTGCGTGTTCGACCTGG  1619
              ||||||||||||||||||||||||||||||||||||||||||||||||||||||||||||
Sbjct  90408  AAAGGCTTCAACTTAACACCGCCTTTTCGTCCCAGGTCGAACACTGCGTGTTCGACCTGG  90349

Query  1620   GACGAAACTGCAAGCTTAGCTTAATGCAAGTTATGCCACAATTGTTGCCATTTTACTTTG  1679
              ||||||||||||||||||||||||||||||||||||||||||||||||||||||||||||
Sbjct  90348  GACGAAACTGCAAGCTTAGCTTAATGCAAGTTATGCCACAATTGTTGCCATTTTACTTTG  90289

Query  1680   TGAACCAGCTGTCATTTGCAGTCCTAGTGTTAGGTACATTAGTCTATGTGTTTGGGACTT  1739
              ||||||||||||||||||||||||||||||||||||||||||||||||||||||||||||
Sbjct  90288  TGAACCAGCTGTCATTTGCAGTCCTAGTGTTAGGTACATTAGTCTATGTGTTTGGGACTT  90229

Query  1740   ACGTGTTGCCAGTCTTTGTGCAATTGTTCGTAACACGTATGTACGTTACTAAACTATAGC  1799
              ||||||||||||||||||||||||||||||||||||||||||||||||||||||||||||
Sbjct  90228  ACGTGTTGCCAGTCTTTGTGCAATTGTTCGTAACACGTATGTACGTTACTAAACTATAGC  90169

Query  1800   AACCCCCTTGTAGCAAGGCGTAAAAATTGCTGCATTGCAAGTCATGCCGGCCCCTGGGCA  1859
              ||||||||||||||||||||||||||||||||||||||||||||||||||||||||||||
Sbjct  90168  AACCCCCTTGTAGCAAGGCGTAAAAATTGCTGCATTGCAAGTCATGCCGGCCCCTGGGCA  90109

Query  1860   GTGCTGCATTGGCTATGTAGTCAACGTGGCAAGGCGTGTAATAGCTGCATTGCTGCCCCA  1919
              ||||||||||||||||||||||||||||||||||||||||||||||||||||||||||||
Sbjct  90108  GTGCTGCATTGGCTATGTAGTCAACGTGGCAAGGCGTGTAATAGCTGCATTGCTGCCCCA  90049

Query  1920   CCCTAGTTTGCATGCTTATGTTGCATTGACTAAGTAGTCAACGTGGCAAGGCGT  1973
              ||||||||||||||||||||||||||||||||||||||||||||||||||||||
Sbjct  90048  CCCTAGTTTGCATGCTTATGTTGCATTGACTAAGTAGTCAACGTGGCAAGGCGT  89995


 Score = 981 bits (531),  Expect = 0.0
 Identities = 536/538 (99%), Gaps = 2/538 (0%)
 Strand=Plus/Minus

Query  1      TTTCGTCCCCCCTGGCCCCTACGGGGCCTGGGGGGACGAAATGGCGTAATTTTAAGTTTT  60
              ||||||||||||||||||||||||||||||||||||||||||||||||||||||||||||
Sbjct  93817  TTTCGTCCCCCCTGGCCCCTACGGGGCCTGGGGGGACGAAATGGCGTAATTTTAAGTTTT  93758

Query  61     GCATGCAAATTACACATGCAAATTACTGCCAATGCCAGCAGTTTTCGTCCCCCCTGGCCC  120
              ||||||||||||||||||||||||||||||||||||||||||||||||||||||||||||
Sbjct  93757  GCATGCAAATTACACATGCAAATTACTGCCAATGCCAGCAGTTTTCGTCCCCCCTGGCCC  93698

Query  121    CTACGGGGCCTGGGGGGACGAAATGGCGTAATTTTAAGCTTTGCATGCAAATTTGCGACT  180
              ||||||||||||||||||||||||||||||||||||||||||||||||||||||||||||
Sbjct  93697  CTACGGGGCCTGGGGGGACGAAATGGCGTAATTTTAAGCTTTGCATGCAAATTTGCGACT  93638

Query  181    AAATTGTAGCCTAAACAAGCTCATGTAATTAGACCTAGGCTAACGGTGGAACCTCACTGC  240
              ||||||||||||||||||||||||||||||||||||||||||||||||||||||||||||
Sbjct  93637  AAATTGTAGCCTAAACAAGCTCATGTAATTAGACCTAGGCTAACGGTGGAACCTCACTGC  93578

Query  241    TTGCTGCATTGCACAGCAAAACTACACCACATACCCCAGTGAGGCAATACCGTGGGAACC  300
              ||||||||||||||||||||||||||||||||||||||||||||||||||||||||||||
Sbjct  93577  TTGCTGCATTGCACAGCAAAACTACACCACATACCCCAGTGAGGCAATACCGTGGGAACC  93518

Query  301    TATTGCTGCAAATTAAAACTTGCAATTTAGGGCGCCGTAGAGACTACACGTGGTACGATT  360
              ||||||||||||||||||||||||||||||||||||||||||||||||||||||||||||
Sbjct  93517  TATTGCTGCAAATTAAAACTTGCAATTTAGGGCGCCGTAGAGACTACACGTGGTACGATT  93458

Query  361    AGTAGTCGTCATGAAGTCCGCTTCATGGGCCGGTGCTAATTGAACATATAGTCCGAACCT  420
              ||||||||||||||||||||||||||||||||||||||||||||||||||||||||||||
Sbjct  93457  AGTAGTCGTCATGAAGTCCGCTTCATGGGCCGGTGCTAATTGAACATATAGTCCGAACCT  93398

Query  421    GGCCGCGAGGCCAGCAGCTAAGCAAGTCAGGTGCAAACTGACTTGCGGCCAGCTTAAATT  480
              ||||||||||||||||||||||||||||||||||||||||||||||||||||||||||||
Sbjct  93397  GGCCGCGAGGCCAGCAGCTAAGCAAGTCAGGTGCAAACTGACTTGCGGCCAGCTTAAATT  93338

Query  481    TGCATAGGCTGCGTTTATGCAAAGCTGGCTACATTTGACATACTATGTAGTTGCACAC  538
              |||||||||||||||||||||||||||||||||||||||||||||||||||  |||||
Sbjct  93337  TGCATAGGCTGCGTTTATGCAAAGCTGGCTACATTTGACATACTATGTAGT--CACAC  93282


 Score = 350 bits (189),  Expect = 9e-98
 Identities = 189/189 (100%), Gaps = 0/189 (0%)
 Strand=Plus/Minus

Query  532    TGCACACTTCCACTATGTGCTGTCAATGGGTGCAGTCTTTGGTATGTTTGCAGGGTACTA  591
              ||||||||||||||||||||||||||||||||||||||||||||||||||||||||||||
Sbjct  92313  TGCACACTTCCACTATGTGCTGTCAATGGGTGCAGTCTTTGGTATGTTTGCAGGGTACTA  92254

Query  592    CTTTTGAAGCCCTAAAGTAATTGGCAAGACTTACAACGAGCAATTGGCACATGTACACTT  651
              ||||||||||||||||||||||||||||||||||||||||||||||||||||||||||||
Sbjct  92253  CTTTTGAAGCCCTAAAGTAATTGGCAAGACTTACAACGAGCAATTGGCACATGTACACTT  92194

Query  652    TTGGGTCATGTTTGTGGGCGTTAATGTTACATTCTTCCCACAACACTTCTTGGGCCTAGC  711
              ||||||||||||||||||||||||||||||||||||||||||||||||||||||||||||
Sbjct  92193  TTGGGTCATGTTTGTGGGCGTTAATGTTACATTCTTCCCACAACACTTCTTGGGCCTAGC  92134

Query  712    AGGTATGAC  720
              |||||||||
Sbjct  92133  AGGTATGAC  92125


 Score = 231 bits (125),  Expect = 3e-62
 Identities = 129/131 (98%), Gaps = 0/131 (0%)
 Strand=Plus/Minus

Query  43     GGCGTAATTTTAAGTTTTGCATGCAAATTACACATGCAAATTACTGCCAATGCCAGCAGT  102
              |||||||||||||| |||||||||||||||||||||||||||||||||||||||||||||
Sbjct  93877  GGCGTAATTTTAAGCTTTGCATGCAAATTACACATGCAAATTACTGCCAATGCCAGCAGT  93818

Query  103    TTTCGTCCCCCCTGGCCCCTACGGGGCCTGGGGGGACGAAATGGCGTAATTTTAAGCTTT  162
              |||||||||||||||||||||||||||||||||||||||||||||||||||||||| |||
Sbjct  93817  TTTCGTCCCCCCTGGCCCCTACGGGGCCTGGGGGGACGAAATGGCGTAATTTTAAGTTTT  93758

Query  163    GCATGCAAATT  173
              |||||||||||
Sbjct  93757  GCATGCAAATT  93747


 Score = 126 bits (68),  Expect = 2e-30
 Identities = 70/71 (99%), Gaps = 0/71 (0%)
 Strand=Plus/Minus

Query  1      TTTCGTCCCCCCTGGCCCCTACGGGGCCTGGGGGGACGAAATGGCGTAATTTTAAGTTTT  60
              |||||||||||||||||||||||||||||||||||||||||||||||||||||||| |||
Sbjct  93715  TTTCGTCCCCCCTGGCCCCTACGGGGCCTGGGGGGACGAAATGGCGTAATTTTAAGCTTT  93656

Query  61     GCATGCAAATT  71
              |||||||||||
Sbjct  93655  GCATGCAAATT  93645


 Score = 113 bits (61),  Expect = 1e-26
 Identities = 130/162 (80%), Gaps = 9/162 (6%)
 Strand=Plus/Minus

Query  1812   GCAAGGCGTAAAAATTGCTGCATTGCAAGTCATGCCGGCCCCTGGGCAGTGCTGCATTGG  1871
              ||||||||| |||||||| ||||||||| | | |   ||| |     ||||  || || |
Sbjct  90003  GCAAGGCGTTAAAATTGCAGCATTGCAA-TTAAGTACGCCAC----AAGTGTAGCCTTTG  89949

Query  1872   CTATGTAGTCAACGTGGCAAGGCGTGTAATAGCTGCATTGCTGCCCCACCCTAGTTTGCA  1931
              ||  || | |||||| ||||||||||||||||||||||||||||||||    ||||||| 
Sbjct  89948  CTTAGTGGCCAACGTAGCAAGGCGTGTAATAGCTGCATTGCTGCCCCA----AGTTTGCT  89893

Query  1932   TGCTTATGTTGCATTGACTAAGTAGTCAACGTGGCAAGGCGT  1973
              |||||   ||||||| ||||||||||||||||||||||||||
Sbjct  89892  TGCTTGGCTTGCATTTACTAAGTAGTCAACGTGGCAAGGCGT  89851


 Score = 82.4 bits (44),  Expect = 3e-17
 Identities = 76/91 (84%), Gaps = 3/91 (3%)
 Strand=Plus/Minus

Query  1107   CATAACAGCCTACGAGCCTTTGCGTGTTGCTTACATAGCCCAAGTGCTTGCTTGGTCGTT  1166
              ||||||||||| ||||||||||||||  | ||   | |  ||||||  ||| | | ||||
Sbjct  90724  CATAACAGCCTGCGAGCCTTTGCGTGACG-TT-TGT-GTGCAAGTGTCTGCCTAGGCGTT  90668

Query  1167   CATAACAGCCTACGAGCCTTTGCATGACGTT  1197
              |||||||||||||||||||||||||||||||
Sbjct  90667  CATAACAGCCTACGAGCCTTTGCATGACGTT  90637


 Score = 82.4 bits (44),  Expect = 3e-17
 Identities = 76/91 (84%), Gaps = 3/91 (3%)
 Strand=Plus/Minus

Query  1244   CATAACAGCCTGCGAGCCTTTGCGTGACG-TT-TGT-GTGCAAGTGTCTGCCTAGGCGTT  1300
              ||||||||||| ||||||||||||||  | ||   | |  ||||||  ||| | | ||||
Sbjct  90861  CATAACAGCCTACGAGCCTTTGCGTGTTGCTTACATAGCCCAAGTGCTTGCTTGGTCGTT  90802

Query  1301   CATAACAGCCTACGAGCCTTTGCATGACGTT  1331
              |||||||||||||||||||||||||||||||
Sbjct  90801  CATAACAGCCTACGAGCCTTTGCATGACGTT  90771


 Score = 76.8 bits (41),  Expect = 2e-15
 Identities = 41/41 (100%), Gaps = 0/41 (0%)
 Strand=Plus/Plus

Query  1      TTTCGTCCCCCCTGGCCCCTACGGGGCCTGGGGGGACGAAA  41
              |||||||||||||||||||||||||||||||||||||||||
Sbjct  55845  TTTCGTCCCCCCTGGCCCCTACGGGGCCTGGGGGGACGAAA  55885


 Score = 76.8 bits (41),  Expect = 2e-15
 Identities = 41/41 (100%), Gaps = 0/41 (0%)
 Strand=Plus/Plus

Query  103    TTTCGTCCCCCCTGGCCCCTACGGGGCCTGGGGGGACGAAA  143
              |||||||||||||||||||||||||||||||||||||||||
Sbjct  55845  TTTCGTCCCCCCTGGCCCCTACGGGGCCTGGGGGGACGAAA  55885


 Score = 62.1 bits (33),  Expect = 4e-11
 Identities = 39/42 (93%), Gaps = 0/42 (0%)
 Strand=Plus/Minus

Query  1860   GTGCTGCATTGGCTATGTAGTCAACGTGGCAAGGCGTGTAAT  1901
              ||| ||||||  ||||||||||||||||||||||||||||||
Sbjct  89709  GTGTTGCATTTACTATGTAGTCAACGTGGCAAGGCGTGTAAT  89668


 Score = 54.7 bits (29),  Expect = 8e-09
 Identities = 29/29 (100%), Gaps = 0/29 (0%)
 Strand=Plus/Minus

Query  145    GGCGTAATTTTAAGCTTTGCATGCAAATT  173
              |||||||||||||||||||||||||||||
Sbjct  93877  GGCGTAATTTTAAGCTTTGCATGCAAATT  93849


Lambda      K        H
    1.33    0.621     1.12 

Gapped
Lambda      K        H
    1.28    0.460    0.850 

Effective search space used: 215623900


Query= 518890 CE518738_212

Length=1015
                                                                      Score     E
Sequences producing significant alignments:                          (Bits)  Value

  scaffold_1_mito                                                     1875    0.0  


> scaffold_1_mito
Length=110369

 Score = 1875 bits (1015),  Expect = 0.0
 Identities = 1015/1015 (100%), Gaps = 0/1015 (0%)
 Strand=Plus/Plus

Query  1      AGGCAAATTAGCTTAAGCATGTAAGTACCTAACTTTACATGAGCTTGTGTGACCTATTAA  60
              ||||||||||||||||||||||||||||||||||||||||||||||||||||||||||||
Sbjct  11099  AGGCAAATTAGCTTAAGCATGTAAGTACCTAACTTTACATGAGCTTGTGTGACCTATTAA  11158

Query  61     GGGTTAAGTTGTACTTGCTTCAAATTAGTACCATGCTAACTTCACATGAGCTAGTACGAC  120
              ||||||||||||||||||||||||||||||||||||||||||||||||||||||||||||
Sbjct  11159  GGGTTAAGTTGTACTTGCTTCAAATTAGTACCATGCTAACTTCACATGAGCTAGTACGAC  11218

Query  121    CGGTCCCTAATTAAGGCATGCAAGGCTCAAGTGTGTACCATGCCAACTTCACATGAGCTT  180
              ||||||||||||||||||||||||||||||||||||||||||||||||||||||||||||
Sbjct  11219  CGGTCCCTAATTAAGGCATGCAAGGCTCAAGTGTGTACCATGCCAACTTCACATGAGCTT  11278

Query  181    GTGTAACCCGTACCTTGTAAAAGTCGACTTACTTAAATTAGGCACCTTGCTAACTCTACT  240
              ||||||||||||||||||||||||||||||||||||||||||||||||||||||||||||
Sbjct  11279  GTGTAACCCGTACCTTGTAAAAGTCGACTTACTTAAATTAGGCACCTTGCTAACTCTACT  11338

Query  241    GCCCCAAGGGGCCGAGGTAGTGTGACCGGTCCCTAATTAAGGCACACTAGCTTAAATTAG  300
              ||||||||||||||||||||||||||||||||||||||||||||||||||||||||||||
Sbjct  11339  GCCCCAAGGGGCCGAGGTAGTGTGACCGGTCCCTAATTAAGGCACACTAGCTTAAATTAG  11398

Query  301    GCACTGTGGCAACTTCACATGAGCTTATGTGTGACAGGTGTGTGCAAAATGCAAAGTCAT  360
              ||||||||||||||||||||||||||||||||||||||||||||||||||||||||||||
Sbjct  11399  GCACTGTGGCAACTTCACATGAGCTTATGTGTGACAGGTGTGTGCAAAATGCAAAGTCAT  11458

Query  361    GCTTAGTACATGCAAGCTTTATGCTAACTTGGCATGAGCTTAGTCTACACGTTTGCGCGT  420
              ||||||||||||||||||||||||||||||||||||||||||||||||||||||||||||
Sbjct  11459  GCTTAGTACATGCAAGCTTTATGCTAACTTGGCATGAGCTTAGTCTACACGTTTGCGCGT  11518

Query  421    ACTTACATGCTAGCTTCAAGCCTGTGGCATTAGGTGTTTAGTCGTGGTCGTGTACTTAGG  480
              ||||||||||||||||||||||||||||||||||||||||||||||||||||||||||||
Sbjct  11519  ACTTACATGCTAGCTTCAAGCCTGTGGCATTAGGTGTTTAGTCGTGGTCGTGTACTTAGG  11578

Query  481    TACCCGGTTTTTTAATGACCAAAACACTGCGTGTTTTGGTCATTAAAATGTAGTAAGCGT  540
              ||||||||||||||||||||||||||||||||||||||||||||||||||||||||||||
Sbjct  11579  TACCCGGTTTTTTAATGACCAAAACACTGCGTGTTTTGGTCATTAAAATGTAGTAAGCGT  11638

Query  541    CAATGCTTGCAATGTAAAACGGTGCAATTGCATGTGCATGCACACAAAATTTGCAAACAC  600
              ||||||||||||||||||||||||||||||||||||||||||||||||||||||||||||
Sbjct  11639  CAATGCTTGCAATGTAAAACGGTGCAATTGCATGTGCATGCACACAAAATTTGCAAACAC  11698

Query  601    ACCCGCAGGTACACTGCAATTAAAAATGGCGCTTTCAATTGTACTGTTCGTACTAGCAGT  660
              ||||||||||||||||||||||||||||||||||||||||||||||||||||||||||||
Sbjct  11699  ACCCGCAGGTACACTGCAATTAAAAATGGCGCTTTCAATTGTACTGTTCGTACTAGCAGT  11758

Query  661    GCTAGGCTTTGTACTAAACCGCAAAAACCTAATTTTAATGCTAATTAGCATTGAAGTTAT  720
              ||||||||||||||||||||||||||||||||||||||||||||||||||||||||||||
Sbjct  11759  GCTAGGCTTTGTACTAAACCGCAAAAACCTAATTTTAATGCTAATTAGCATTGAAGTTAT  11818

Query  721    GTTGCTTGCTGTAACGCTGCTAGTCATTGTGACGTCTTATGAGTTTAATGACGTAATTGG  780
              ||||||||||||||||||||||||||||||||||||||||||||||||||||||||||||
Sbjct  11819  GTTGCTTGCTGTAACGCTGCTAGTCATTGTGACGTCTTATGAGTTTAATGACGTAATTGG  11878

Query  781    TCAAACTTACGCTGTGTTCATTATTGCAATTGCAGGTGCCGAGTCAGCAATTGGACTTGG  840
              ||||||||||||||||||||||||||||||||||||||||||||||||||||||||||||
Sbjct  11879  TCAAACTTACGCTGTGTTCATTATTGCAATTGCAGGTGCCGAGTCAGCAATTGGACTTGG  11938

Query  841    CATTTTAGTAGCCTTTTACCGTACACGTGGGTCAATTACACTTTTTTAAATGTACCTATT  900
              ||||||||||||||||||||||||||||||||||||||||||||||||||||||||||||
Sbjct  11939  CATTTTAGTAGCCTTTTACCGTACACGTGGGTCAATTACACTTTTTTAAATGTACCTATT  11998

Query  901    GCTAATTGCTATGCCACTAGTAGGTGCTACACTTGCAGGCCTACTAGGTCGTAAGCTTGG  960
              ||||||||||||||||||||||||||||||||||||||||||||||||||||||||||||
Sbjct  11999  GCTAATTGCTATGCCACTAGTAGGTGCTACACTTGCAGGCCTACTAGGTCGTAAGCTTGG  12058

Query  961    GACCCGGGGTGCACAAGTCGTGACTTGCATTTGCATGGCTACGACTGCCGTGCTG  1015
              |||||||||||||||||||||||||||||||||||||||||||||||||||||||
Sbjct  12059  GACCCGGGGTGCACAAGTCGTGACTTGCATTTGCATGGCTACGACTGCCGTGCTG  12113


 Score = 76.8 bits (41),  Expect = 8e-16
 Identities = 41/41 (100%), Gaps = 0/41 (0%)
 Strand=Plus/Plus

Query  488    TTTTTTAATGACCAAAACACTGCGTGTTTTGGTCATTAAAA  528
              |||||||||||||||||||||||||||||||||||||||||
Sbjct  69105  TTTTTTAATGACCAAAACACTGCGTGTTTTGGTCATTAAAA  69145


 Score = 76.8 bits (41),  Expect = 8e-16
 Identities = 41/41 (100%), Gaps = 0/41 (0%)
 Strand=Plus/Plus

Query  488    TTTTTTAATGACCAAAACACTGCGTGTTTTGGTCATTAAAA  528
              |||||||||||||||||||||||||||||||||||||||||
Sbjct  73191  TTTTTTAATGACCAAAACACTGCGTGTTTTGGTCATTAAAA  73231


Lambda      K        H
    1.33    0.621     1.12 

Gapped
Lambda      K        H
    1.28    0.460    0.850 

Effective search space used: 110019947


Query= 950279 estExt_fgenesh1_pm.C_40009

Length=1134
                                                                      Score     E
Sequences producing significant alignments:                          (Bits)  Value

  scaffold_1_mito                                                     470     4e-134


> scaffold_1_mito
Length=110369

 Score = 470 bits (254),  Expect = 4e-134
 Identities = 254/254 (100%), Gaps = 0/254 (0%)
 Strand=Plus/Plus

Query  1      CACATGAACAGCATTGGCTGTTTTGCtttttttttAATGCCTGCAGGGCATTaaaaaaaa  60
              ||||||||||||||||||||||||||||||||||||||||||||||||||||||||||||
Sbjct  55183  CACATGAACAGCATTGGCTGTTTTGCTTTTTTTTTAATGCCTGCAGGGCATTAAAAAAAA  55242

Query  61     aaCTGCTGCAATTAGCTTGTGGCCGCGCTTTTAAAAATTGAAGACATTACACAGTACACA  120
              ||||||||||||||||||||||||||||||||||||||||||||||||||||||||||||
Sbjct  55243  AACTGCTGCAATTAGCTTGTGGCCGCGCTTTTAAAAATTGAAGACATTACACAGTACACA  55302

Query  121    ATTGGCACGCGTGTGGGTTTGGCACACTGTGCAGTCATTGCACACAGCATTGCAAGCTAG  180
              ||||||||||||||||||||||||||||||||||||||||||||||||||||||||||||
Sbjct  55303  ATTGGCACGCGTGTGGGTTTGGCACACTGTGCAGTCATTGCACACAGCATTGCAAGCTAG  55362

Query  181    TAGCAGCCTACTAGTAAGCACTTGCTGGCTACATTTTAATTAATGCGACTGCTTAAAACA  240
              ||||||||||||||||||||||||||||||||||||||||||||||||||||||||||||
Sbjct  55363  TAGCAGCCTACTAGTAAGCACTTGCTGGCTACATTTTAATTAATGCGACTGCTTAAAACA  55422

Query  241    CACCCCATTTTAGG  254
              ||||||||||||||
Sbjct  55423  CACCCCATTTTAGG  55436


 Score = 363 bits (196),  Expect = 6e-102
 Identities = 196/196 (100%), Gaps = 0/196 (0%)
 Strand=Plus/Plus

Query  688    GGCATTAGTGGCAACACAGACCGACTACCATTCCACCCGTACTTTACATTTAAAGACTTG  747
              ||||||||||||||||||||||||||||||||||||||||||||||||||||||||||||
Sbjct  60654  GGCATTAGTGGCAACACAGACCGACTACCATTCCACCCGTACTTTACATTTAAAGACTTG  60713

Query  748    GTAACAGTCTTCCTGTTCATGCTAGTGCTTGCAGCAATGGTCTTTTATGCGCCTAACTAC  807
              ||||||||||||||||||||||||||||||||||||||||||||||||||||||||||||
Sbjct  60714  GTAACAGTCTTCCTGTTCATGCTAGTGCTTGCAGCAATGGTCTTTTATGCGCCTAACTAC  60773

Query  808    CTGGGCCATAGTGACAATTACATTCCTGCAAACCCCATGCAAACGCCAGCGTCAATTGTG  867
              ||||||||||||||||||||||||||||||||||||||||||||||||||||||||||||
Sbjct  60774  CTGGGCCATAGTGACAATTACATTCCTGCAAACCCCATGCAAACGCCAGCGTCAATTGTG  60833

Query  868    CCTGAATGGTCAAGTG  883
              ||||||||||||||||
Sbjct  60834  CCTGAATGGTCAAGTG  60849


 Score = 355 bits (192),  Expect = 1e-99
 Identities = 192/192 (100%), Gaps = 0/192 (0%)
 Strand=Plus/Plus

Query  335    CGATTATGCGTGACGTGCATTACGGCTGGCTAATTCGGTACTTGCATGCTAACGTGGCGT  394
              ||||||||||||||||||||||||||||||||||||||||||||||||||||||||||||
Sbjct  56263  CGATTATGCGTGACGTGCATTACGGCTGGCTAATTCGGTACTTGCATGCTAACGTGGCGT  56322

Query  395    CGTTCTTTTTTATTTGCGTGTACTTACACATTGGTCGTGGCCTTTACTATGGGTCGTACC  454
              ||||||||||||||||||||||||||||||||||||||||||||||||||||||||||||
Sbjct  56323  CGTTCTTTTTTATTTGCGTGTACTTACACATTGGTCGTGGCCTTTACTATGGGTCGTACC  56382

Query  455    GGTCACCACGTACGCTAGTGTGGGCCATTGGGGTCGTAATTTTAGTACTTATGATAGCAA  514
              ||||||||||||||||||||||||||||||||||||||||||||||||||||||||||||
Sbjct  56383  GGTCACCACGTACGCTAGTGTGGGCCATTGGGGTCGTAATTTTAGTACTTATGATAGCAA  56442

Query  515    CAGCCTTTTTAG  526
              ||||||||||||
Sbjct  56443  CAGCCTTTTTAG  56454


 Score = 342 bits (185),  Expect = 8e-96
 Identities = 185/185 (100%), Gaps = 0/185 (0%)
 Strand=Plus/Plus

Query  950    CCTTTTGGCTTTTTGCTGCAAACTTTTTTGTACTAATGTACATTGGTAGCCAGCACGTTG  1009
              ||||||||||||||||||||||||||||||||||||||||||||||||||||||||||||
Sbjct  61886  CCTTTTGGCTTTTTGCTGCAAACTTTTTTGTACTAATGTACATTGGTAGCCAGCACGTTG  61945

Query  1010   AAGAGCCGTTCGTTACAGTAGGCATGATTAGCACTAGCCTGTACTTTGGCTGGTTTTTAA  1069
              ||||||||||||||||||||||||||||||||||||||||||||||||||||||||||||
Sbjct  61946  AAGAGCCGTTCGTTACAGTAGGCATGATTAGCACTAGCCTGTACTTTGGCTGGTTTTTAA  62005

Query  1070   TTATTGTGCCTGTAATTGGACTAGTCGAAAACACGCTTTTAGACTTAGCATGTGAGTCTA  1129
              ||||||||||||||||||||||||||||||||||||||||||||||||||||||||||||
Sbjct  62006  TTATTGTGCCTGTAATTGGACTAGTCGAAAACACGCTTTTAGACTTAGCATGTGAGTCTA  62065

Query  1130   AATAA  1134
              |||||
Sbjct  62066  AATAA  62070


 Score = 294 bits (159),  Expect = 2e-81
 Identities = 159/159 (100%), Gaps = 0/159 (0%)
 Strand=Plus/Plus

Query  531    CATAAGCGACCTTGGGGGTGGCTTTAGCGTCAACAACGCAACGTTAAACCGGtttttttC  590
              ||||||||||||||||||||||||||||||||||||||||||||||||||||||||||||
Sbjct  60102  CATAAGCGACCTTGGGGGTGGCTTTAGCGTCAACAACGCAACGTTAAACCGGTTTTTTTC  60161

Query  591    ATTGCATTACTTACTGCCGTTCATTTTGGCAGCACTTGCAGCTATGCACTTGCTTGCATT  650
              ||||||||||||||||||||||||||||||||||||||||||||||||||||||||||||
Sbjct  60162  ATTGCATTACTTACTGCCGTTCATTTTGGCAGCACTTGCAGCTATGCACTTGCTTGCATT  60221

Query  651    GCATGAACACGGCAGCAGCAACCCTAACAGCCTAGGGGG  689
              |||||||||||||||||||||||||||||||||||||||
Sbjct  60222  GCATGAACACGGCAGCAGCAACCCTAACAGCCTAGGGGG  60260


 Score = 156 bits (84),  Expect = 1e-39
 Identities = 84/84 (100%), Gaps = 0/84 (0%)
 Strand=Plus/Plus

Query  251    TAGGCCTGTGCTTAGTCATTCAAATTGTGACTGGCATTACGCTAGCAATGCACTACACAC  310
              ||||||||||||||||||||||||||||||||||||||||||||||||||||||||||||
Sbjct  55511  TAGGCCTGTGCTTAGTCATTCAAATTGTGACTGGCATTACGCTAGCAATGCACTACACAC  55570

Query  311    CGTCTATTGACTTGGCCTTTGCAA  334
              ||||||||||||||||||||||||
Sbjct  55571  CGTCTATTGACTTGGCCTTTGCAA  55594


 Score = 128 bits (69),  Expect = 2e-31
 Identities = 69/69 (100%), Gaps = 0/69 (0%)
 Strand=Plus/Plus

Query  881    GTGTCGTAGCCATGTTTGCTTCATTACTAATTTTACTGGCTATGCCAATTGTAGACACGT  940
              ||||||||||||||||||||||||||||||||||||||||||||||||||||||||||||
Sbjct  61784  GTGTCGTAGCCATGTTTGCTTCATTACTAATTTTACTGGCTATGCCAATTGTAGACACGT  61843

Query  941    CACGTGTAC  949
              |||||||||
Sbjct  61844  CACGTGTAC  61852


Lambda      K        H
    1.33    0.621     1.12 

Gapped
Lambda      K        H
    1.28    0.460    0.850 

Effective search space used: 123151716


Query= 946153 fgenesh1_pm.4_#_10

Length=756
                                                                      Score     E
Sequences producing significant alignments:                          (Bits)  Value

  scaffold_1_mito                                                     845     0.0  


> scaffold_1_mito
Length=110369

 Score = 845 bits (457),  Expect = 0.0
 Identities = 457/457 (100%), Gaps = 0/457 (0%)
 Strand=Plus/Plus

Query  300    TACAGACTTCCATTCAGCCTTAGCGCCTACAGTCGAGTTAGGCAATGCATGGCCGCCTGC  359
              ||||||||||||||||||||||||||||||||||||||||||||||||||||||||||||
Sbjct  66726  TACAGACTTCCATTCAGCCTTAGCGCCTACAGTCGAGTTAGGCAATGCATGGCCGCCTGC  66785

Query  360    TGGGGTCCAAGCATTAGACCCTTACGAAGTGCCACTACTAAACACAGTAATTTTGCTAGG  419
              ||||||||||||||||||||||||||||||||||||||||||||||||||||||||||||
Sbjct  66786  TGGGGTCCAAGCATTAGACCCTTACGAAGTGCCACTACTAAACACAGTAATTTTGCTAGG  66845

Query  420    GTCAGGCGCTAGCGTTACGTACGCACACCACTCATTAATTCAAGGTAGCCGTGCAGGCAC  479
              ||||||||||||||||||||||||||||||||||||||||||||||||||||||||||||
Sbjct  66846  GTCAGGCGCTAGCGTTACGTACGCACACCACTCATTAATTCAAGGTAGCCGTGCAGGCAC  66905

Query  480    AATTGCAGGCCTAATTGTGACCATTGCACTAGCTGCTGTGTTCACAATGCTGCAGGGTCT  539
              ||||||||||||||||||||||||||||||||||||||||||||||||||||||||||||
Sbjct  66906  AATTGCAGGCCTAATTGTGACCATTGCACTAGCTGCTGTGTTCACAATGCTGCAGGGTCT  66965

Query  540    TGAGTACCATGAAGCTAGCTTTACAATTGCAGACGGTGCTTACGGGTCTACATTTTACTT  599
              ||||||||||||||||||||||||||||||||||||||||||||||||||||||||||||
Sbjct  66966  TGAGTACCATGAAGCTAGCTTTACAATTGCAGACGGTGCTTACGGGTCTACATTTTACTT  67025

Query  600    TGCGACTGGCTTCCATGGCCTGCACGTCATTATTGGGACCCTGTTCATTGCCGTGGCATT  659
              ||||||||||||||||||||||||||||||||||||||||||||||||||||||||||||
Sbjct  67026  TGCGACTGGCTTCCATGGCCTGCACGTCATTATTGGGACCCTGTTCATTGCCGTGGCATT  67085

Query  660    TGTGCGACTACTAAGCTACCAGCTTACTGACCACCACCACTTAGGCTTTGAAGCCGCCAT  719
              ||||||||||||||||||||||||||||||||||||||||||||||||||||||||||||
Sbjct  67086  TGTGCGACTACTAAGCTACCAGCTTACTGACCACCACCACTTAGGCTTTGAAGCCGCCAT  67145

Query  720    TTTGTACTGGCACAGTGCCAGCTTGCTATGCAAGTAG  756
              |||||||||||||||||||||||||||||||||||||
Sbjct  67146  TTTGTACTGGCACAGTGCCAGCTTGCTATGCAAGTAG  67182


 Score = 553 bits (299),  Expect = 2e-159
 Identities = 299/299 (100%), Gaps = 0/299 (0%)
 Strand=Plus/Plus

Query  1      ATGCTTCAAAATGTAACACGTGCACAATTCCAAGCACACCCGTACCACTTGGTGACGCCG  60
              ||||||||||||||||||||||||||||||||||||||||||||||||||||||||||||
Sbjct  65193  ATGCTTCAAAATGTAACACGTGCACAATTCCAAGCACACCCGTACCACTTGGTGACGCCG  65252

Query  61     TCACCATGGCCTTTGCTGACGTCCTTTTCGTTACTAATTTTGACTGTGGCTGCTGCTATG  120
              ||||||||||||||||||||||||||||||||||||||||||||||||||||||||||||
Sbjct  65253  TCACCATGGCCTTTGCTGACGTCCTTTTCGTTACTAATTTTGACTGTGGCTGCTGCTATG  65312

Query  121    TACTTTAATGGCTATGCAAATGGCGGCATGCTAGTCAGCATTGGCTTCATTACAGTCGTT  180
              ||||||||||||||||||||||||||||||||||||||||||||||||||||||||||||
Sbjct  65313  TACTTTAATGGCTATGCAAATGGCGGCATGCTAGTCAGCATTGGCTTCATTACAGTCGTT  65372

Query  181    GCGTCAATGGCATTATGGTTTCGTGACGTAATTGCAGAAGGTGCATTGCTAGGCAACCAC  240
              ||||||||||||||||||||||||||||||||||||||||||||||||||||||||||||
Sbjct  65373  GCGTCAATGGCATTATGGTTTCGTGACGTAATTGCAGAAGGTGCATTGCTAGGCAACCAC  65432

Query  241    ACATTTGCGGTGCAAAAAGGACTTAACCTAGGCGTAGCCTTATTTATTGTAAGTGAGGT  299
              |||||||||||||||||||||||||||||||||||||||||||||||||||||||||||
Sbjct  65433  ACATTTGCGGTGCAAAAAGGACTTAACCTAGGCGTAGCCTTATTTATTGTAAGTGAGGT  65491


Lambda      K        H
    1.33    0.621     1.12 

Gapped
Lambda      K        H
    1.28    0.460    0.850 

Effective search space used: 81439038


Query= 979743 MIX18745_214_28

Length=1380
                                                                      Score     E
Sequences producing significant alignments:                          (Bits)  Value

  scaffold_1_mito                                                     763     0.0  


> scaffold_1_mito
Length=110369

 Score = 763 bits (413),  Expect = 0.0
 Identities = 413/413 (100%), Gaps = 0/413 (0%)
 Strand=Plus/Plus

Query  1      CACATGAACAGCATTGGCTGTTTTGCtttttttttAATGCCTGCAGGGCATTaaaaaaaa  60
              ||||||||||||||||||||||||||||||||||||||||||||||||||||||||||||
Sbjct  55183  CACATGAACAGCATTGGCTGTTTTGCTTTTTTTTTAATGCCTGCAGGGCATTAAAAAAAA  55242

Query  61     aaCTGCTGCAATTAGCTTGTGGCCGCGCTTTTAAAAATTGAAGACATTACACAGTACACA  120
              ||||||||||||||||||||||||||||||||||||||||||||||||||||||||||||
Sbjct  55243  AACTGCTGCAATTAGCTTGTGGCCGCGCTTTTAAAAATTGAAGACATTACACAGTACACA  55302

Query  121    ATTGGCACGCGTGTGGGTTTGGCACACTGTGCAGTCATTGCACACAGCATTGCAAGCTAG  180
              ||||||||||||||||||||||||||||||||||||||||||||||||||||||||||||
Sbjct  55303  ATTGGCACGCGTGTGGGTTTGGCACACTGTGCAGTCATTGCACACAGCATTGCAAGCTAG  55362

Query  181    TAGCAGCCTACTAGTAAGCACTTGCTGGCTACATTTTAATTAATGCGACTGCTTAAAACA  240
              ||||||||||||||||||||||||||||||||||||||||||||||||||||||||||||
Sbjct  55363  TAGCAGCCTACTAGTAAGCACTTGCTGGCTACATTTTAATTAATGCGACTGCTTAAAACA  55422

Query  241    CACCCCATTTTAGGTTTAGTAAATAGCTACATGGTCGACAGCCCACAACCTGCTAGCATT  300
              ||||||||||||||||||||||||||||||||||||||||||||||||||||||||||||
Sbjct  55423  CACCCCATTTTAGGTTTAGTAAATAGCTACATGGTCGACAGCCCACAACCTGCTAGCATT  55482

Query  301    ACGTACATGTGAAATTTTGGGTCGCTGCTAGGCCTGTGCTTAGTCATTCAAATTGTGACT  360
              ||||||||||||||||||||||||||||||||||||||||||||||||||||||||||||
Sbjct  55483  ACGTACATGTGAAATTTTGGGTCGCTGCTAGGCCTGTGCTTAGTCATTCAAATTGTGACT  55542

Query  361    GGCATTACGCTAGCAATGCACTACACACCGTCTATTGACTTGGCCTTTGCAAG  413
              |||||||||||||||||||||||||||||||||||||||||||||||||||||
Sbjct  55543  GGCATTACGCTAGCAATGCACTACACACCGTCTATTGACTTGGCCTTTGCAAG  55595


 Score = 503 bits (272),  Expect = 4e-144
 Identities = 272/272 (100%), Gaps = 0/272 (0%)
 Strand=Plus/Plus

Query  1109   GTGTCGTAGCCATGTTTGCTTCATTACTAATTTTACTGGCTATGCCAATTGTAGACACGT  1168
              ||||||||||||||||||||||||||||||||||||||||||||||||||||||||||||
Sbjct  61784  GTGTCGTAGCCATGTTTGCTTCATTACTAATTTTACTGGCTATGCCAATTGTAGACACGT  61843

Query  1169   CACGTGTACGTGGCTCGCAATTTCGACCTTTAATGCGGTGAGCCTTTTGGCTTTTTGCTG  1228
              ||||||||||||||||||||||||||||||||||||||||||||||||||||||||||||
Sbjct  61844  CACGTGTACGTGGCTCGCAATTTCGACCTTTAATGCGGTGAGCCTTTTGGCTTTTTGCTG  61903

Query  1229   CAAACTTTTTTGTACTAATGTACATTGGTAGCCAGCACGTTGAAGAGCCGTTCGTTACAG  1288
              ||||||||||||||||||||||||||||||||||||||||||||||||||||||||||||
Sbjct  61904  CAAACTTTTTTGTACTAATGTACATTGGTAGCCAGCACGTTGAAGAGCCGTTCGTTACAG  61963

Query  1289   TAGGCATGATTAGCACTAGCCTGTACTTTGGCTGGTTTTTAATTATTGTGCCTGTAATTG  1348
              ||||||||||||||||||||||||||||||||||||||||||||||||||||||||||||
Sbjct  61964  TAGGCATGATTAGCACTAGCCTGTACTTTGGCTGGTTTTTAATTATTGTGCCTGTAATTG  62023

Query  1349   GACTAGTCGAAAACACGCTTTTAGACTTAGCA  1380
              ||||||||||||||||||||||||||||||||
Sbjct  62024  GACTAGTCGAAAACACGCTTTTAGACTTAGCA  62055


 Score = 377 bits (204),  Expect = 3e-106
 Identities = 204/204 (100%), Gaps = 0/204 (0%)
 Strand=Plus/Plus

Query  413    GCTTACGTAAAGCGATTATGCGTGACGTGCATTACGGCTGGCTAATTCGGTACTTGCATG  472
              ||||||||||||||||||||||||||||||||||||||||||||||||||||||||||||
Sbjct  56251  GCTTACGTAAAGCGATTATGCGTGACGTGCATTACGGCTGGCTAATTCGGTACTTGCATG  56310

Query  473    CTAACGTGGCGTCGTTCTTTTTTATTTGCGTGTACTTACACATTGGTCGTGGCCTTTACT  532
              ||||||||||||||||||||||||||||||||||||||||||||||||||||||||||||
Sbjct  56311  CTAACGTGGCGTCGTTCTTTTTTATTTGCGTGTACTTACACATTGGTCGTGGCCTTTACT  56370

Query  533    ATGGGTCGTACCGGTCACCACGTACGCTAGTGTGGGCCATTGGGGTCGTAATTTTAGTAC  592
              ||||||||||||||||||||||||||||||||||||||||||||||||||||||||||||
Sbjct  56371  ATGGGTCGTACCGGTCACCACGTACGCTAGTGTGGGCCATTGGGGTCGTAATTTTAGTAC  56430

Query  593    TTATGATAGCAACAGCCTTTTTAG  616
              ||||||||||||||||||||||||
Sbjct  56431  TTATGATAGCAACAGCCTTTTTAG  56454


 Score = 359 bits (194),  Expect = 1e-100
 Identities = 194/194 (100%), Gaps = 0/194 (0%)
 Strand=Plus/Plus

Query  886    GGCATTAGTGGCAACACAGACCGACTACCATTCCACCCGTACTTTACATTTAAAGACTTG  945
              ||||||||||||||||||||||||||||||||||||||||||||||||||||||||||||
Sbjct  60654  GGCATTAGTGGCAACACAGACCGACTACCATTCCACCCGTACTTTACATTTAAAGACTTG  60713

Query  946    GTAACAGTCTTCCTGTTCATGCTAGTGCTTGCAGCAATGGTCTTTTATGCGCCTAACTAC  1005
              ||||||||||||||||||||||||||||||||||||||||||||||||||||||||||||
Sbjct  60714  GTAACAGTCTTCCTGTTCATGCTAGTGCTTGCAGCAATGGTCTTTTATGCGCCTAACTAC  60773

Query  1006   CTGGGCCATAGTGACAATTACATTCCTGCAAACCCCATGCAAACGCCAGCGTCAATTGTG  1065
              ||||||||||||||||||||||||||||||||||||||||||||||||||||||||||||
Sbjct  60774  CTGGGCCATAGTGACAATTACATTCCTGCAAACCCCATGCAAACGCCAGCGTCAATTGTG  60833

Query  1066   CCTGAATGGTCAAG  1079
              ||||||||||||||
Sbjct  60834  CCTGAATGGTCAAG  60847


 Score = 300 bits (162),  Expect = 6e-83
 Identities = 162/162 (100%), Gaps = 0/162 (0%)
 Strand=Plus/Plus

Query  726    CAGCATAAGCGACCTTGGGGGTGGCTTTAGCGTCAACAACGCAACGTTAAACCGGttttt  785
              ||||||||||||||||||||||||||||||||||||||||||||||||||||||||||||
Sbjct  60099  CAGCATAAGCGACCTTGGGGGTGGCTTTAGCGTCAACAACGCAACGTTAAACCGGTTTTT  60158

Query  786    ttCATTGCATTACTTACTGCCGTTCATTTTGGCAGCACTTGCAGCTATGCACTTGCTTGC  845
              ||||||||||||||||||||||||||||||||||||||||||||||||||||||||||||
Sbjct  60159  TTCATTGCATTACTTACTGCCGTTCATTTTGGCAGCACTTGCAGCTATGCACTTGCTTGC  60218

Query  846    ATTGCATGAACACGGCAGCAGCAACCCTAACAGCCTAGGGGG  887
              ||||||||||||||||||||||||||||||||||||||||||
Sbjct  60219  ATTGCATGAACACGGCAGCAGCAACCCTAACAGCCTAGGGGG  60260


 Score = 122 bits (66),  Expect = 1e-29
 Identities = 66/66 (100%), Gaps = 0/66 (0%)
 Strand=Plus/Plus

Query  615    AGCATGCAAGCGACTGTATGTGCTGCCTTACGGTCAAATGTCATTATGGGGTGAGCGCCC  674
              ||||||||||||||||||||||||||||||||||||||||||||||||||||||||||||
Sbjct  58470  AGCATGCAAGCGACTGTATGTGCTGCCTTACGGTCAAATGTCATTATGGGGTGAGCGCCC  58529

Query  675    TAAATG  680
              ||||||
Sbjct  58530  TAAATG  58535


 Score = 91.6 bits (49),  Expect = 4e-20
 Identities = 49/49 (100%), Gaps = 0/49 (0%)
 Strand=Plus/Plus

Query  680    GCTTACTGCAGCAGTCGCTACAGACTACACCACCAGTAGGCACCTGCAG  728
              |||||||||||||||||||||||||||||||||||||||||||||||||
Sbjct  58774  GCTTACTGCAGCAGTCGCTACAGACTACACCACCAGTAGGCACCTGCAG  58822


 Score = 60.2 bits (32),  Expect = 1e-10
 Identities = 32/32 (100%), Gaps = 0/32 (0%)
 Strand=Plus/Plus

Query  1079   GCAAGCCATTGCGGTTCGTCCCACAAGGCCGT  1110
              ||||||||||||||||||||||||||||||||
Sbjct  61249  GCAAGCCATTGCGGTTCGTCCCACAAGGCCGT  61280


Lambda      K        H
    1.33    0.621     1.12 

Gapped
Lambda      K        H
    1.28    0.460    0.850 

Effective search space used: 150186350


Query= 863471 gw1.4.67.1

Length=375
                                                                      Score     E
Sequences producing significant alignments:                          (Bits)  Value

  scaffold_1_mito                                                     693     0.0  


> scaffold_1_mito
Length=110369

 Score = 693 bits (375),  Expect = 0.0
 Identities = 375/375 (100%), Gaps = 0/375 (0%)
 Strand=Plus/Plus

Query  1      ACTATGGTGGTACtttttttGCTTGTGCCAGTACTAGTGGCTGTTTTGCTGTTGCTTAAT  60
              ||||||||||||||||||||||||||||||||||||||||||||||||||||||||||||
Sbjct  49557  ACTATGGTGGTACTTTTTTTGCTTGTGCCAGTACTAGTGGCTGTTTTGCTGTTGCTTAAT  49616

Query  61     GTGCTGCTTGCAGTACACCGCCCTGACACTGAAAAAGTGTCGGCTTACGAATGCGGCTTC  120
              ||||||||||||||||||||||||||||||||||||||||||||||||||||||||||||
Sbjct  49617  GTGCTGCTTGCAGTACACCGCCCTGACACTGAAAAAGTGTCGGCTTACGAATGCGGCTTC  49676

Query  121    AATGTGCTGTCGCACCAAGTACGGGCACCCTTTGCTGTGCAGTACTACCTTGTGGGCATT  180
              ||||||||||||||||||||||||||||||||||||||||||||||||||||||||||||
Sbjct  49677  AATGTGCTGTCGCACCAAGTACGGGCACCCTTTGCTGTGCAGTACTACCTTGTGGGCATT  49736

Query  181    TTGTTCTTAATTTTTGACCTTGAAATTGCAGTGCTGTACCCATTGGCTGTAACACTGTAC  240
              ||||||||||||||||||||||||||||||||||||||||||||||||||||||||||||
Sbjct  49737  TTGTTCTTAATTTTTGACCTTGAAATTGCAGTGCTGTACCCATTGGCTGTAACACTGTAC  49796

Query  241    GAGGTAACTGCCTACGGCTTTTACGTTGCAATGCTGTTTGTGGTCATGCTTACTGTAGGC  300
              ||||||||||||||||||||||||||||||||||||||||||||||||||||||||||||
Sbjct  49797  GAGGTAACTGCCTACGGCTTTTACGTTGCAATGCTGTTTGTGGTCATGCTTACTGTAGGC  49856

Query  301    TTCGTGTACGAGTACGGCAAAGGTGCTTTAAAATTTACTGACCACCGGTCGTCAATTAAC  360
              ||||||||||||||||||||||||||||||||||||||||||||||||||||||||||||
Sbjct  49857  TTCGTGTACGAGTACGGCAAAGGTGCTTTAAAATTTACTGACCACCGGTCGTCAATTAAC  49916

Query  361    CGTGCAATGCTGACT  375
              |||||||||||||||
Sbjct  49917  CGTGCAATGCTGACT  49931


Lambda      K        H
    1.33    0.621     1.12 

Gapped
Lambda      K        H
    1.28    0.460    0.850 

Effective search space used: 39506016


Query= 900779 fgenesh1_kg.4_#_39_#_TRINITY_DN3144_c3_g6_i2

Length=1838
                                                                      Score     E
Sequences producing significant alignments:                          (Bits)  Value

  scaffold_1_mito                                                     1386    0.0  


> scaffold_1_mito
Length=110369

 Score = 1386 bits (750),  Expect = 0.0
 Identities = 750/750 (100%), Gaps = 0/750 (0%)
 Strand=Plus/Plus

Query  1089   ACTAGGTGTCGTAGCCATGTTTGCTTCATTACTAATTTTACTGGCTATGCCAATTGTAGA  1148
              ||||||||||||||||||||||||||||||||||||||||||||||||||||||||||||
Sbjct  61779  ACTAGGTGTCGTAGCCATGTTTGCTTCATTACTAATTTTACTGGCTATGCCAATTGTAGA  61838

Query  1149   CACGTCACGTGTACGTGGCTCGCAATTTCGACCTTTAATGCGGTGAGCCTTTTGGCTTTT  1208
              ||||||||||||||||||||||||||||||||||||||||||||||||||||||||||||
Sbjct  61839  CACGTCACGTGTACGTGGCTCGCAATTTCGACCTTTAATGCGGTGAGCCTTTTGGCTTTT  61898

Query  1209   TGCTGCAAACTTTTTTGTACTAATGTACATTGGTAGCCAGCACGTTGAAGAGCCGTTCGT  1268
              ||||||||||||||||||||||||||||||||||||||||||||||||||||||||||||
Sbjct  61899  TGCTGCAAACTTTTTTGTACTAATGTACATTGGTAGCCAGCACGTTGAAGAGCCGTTCGT  61958

Query  1269   TACAGTAGGCATGATTAGCACTAGCCTGTACTTTGGCTGGTTTTTAATTATTGTGCCTGT  1328
              ||||||||||||||||||||||||||||||||||||||||||||||||||||||||||||
Sbjct  61959  TACAGTAGGCATGATTAGCACTAGCCTGTACTTTGGCTGGTTTTTAATTATTGTGCCTGT  62018

Query  1329   AATTGGACTAGTCGAAAACACGCTTTTAGACTTAGCATGTGAGTCTAAATAACCCCTTTT  1388
              ||||||||||||||||||||||||||||||||||||||||||||||||||||||||||||
Sbjct  62019  AATTGGACTAGTCGAAAACACGCTTTTAGACTTAGCATGTGAGTCTAAATAACCCCTTTT  62078

Query  1389   GTGACTTTACTGCCCCACCCACAACACGAACGTGTTGTGAtttttttttAATGCACTTGT  1448
              ||||||||||||||||||||||||||||||||||||||||||||||||||||||||||||
Sbjct  62079  GTGACTTTACTGCCCCACCCACAACACGAACGTGTTGTGATTTTTTTTTAATGCACTTGT  62138

Query  1449   GTGCATTaaaaaaaaaaGGGTGGGGCCAGAGCTTTTACAATGCATGCTGTTCAAAATTTT  1508
              ||||||||||||||||||||||||||||||||||||||||||||||||||||||||||||
Sbjct  62139  GTGCATTAAAAAAAAAAGGGTGGGGCCAGAGCTTTTACAATGCATGCTGTTCAAAATTTT  62198

Query  1509   TTACAGGTCGTACCCACTACCCAACTTAAATTGGCAATAGCAGGCGGTGCAATTAACAGC  1568
              ||||||||||||||||||||||||||||||||||||||||||||||||||||||||||||
Sbjct  62199  TTACAGGTCGTACCCACTACCCAACTTAAATTGGCAATAGCAGGCGGTGCAATTAACAGC  62258

Query  1569   AAATTTGAGCCAGGGCCACAAAACGTGTACATTTACGTAGTCACGTGTCAACATTAGGCA  1628
              ||||||||||||||||||||||||||||||||||||||||||||||||||||||||||||
Sbjct  62259  AAATTTGAGCCAGGGCCACAAAACGTGTACATTTACGTAGTCACGTGTCAACATTAGGCA  62318

Query  1629   GTGTAAAATTAGCAATTAATTACAGCATGTGGCCTAATTAAGCTTACTAGCAAGTCGTCA  1688
              ||||||||||||||||||||||||||||||||||||||||||||||||||||||||||||
Sbjct  62319  GTGTAAAATTAGCAATTAATTACAGCATGTGGCCTAATTAAGCTTACTAGCAAGTCGTCA  62378

Query  1689   ACATTAAACGGTACAATTGGCCTGCAAATTTGCTAGGCCGTGTTGCTTAACATACCCACT  1748
              ||||||||||||||||||||||||||||||||||||||||||||||||||||||||||||
Sbjct  62379  ACATTAAACGGTACAATTGGCCTGCAAATTTGCTAGGCCGTGTTGCTTAACATACCCACT  62438

Query  1749   ACCCCGCTTAAATTAACAATAGCAGACGGTGTAGTTTGCAACAAATTTATGCAAGGTGTG  1808
              ||||||||||||||||||||||||||||||||||||||||||||||||||||||||||||
Sbjct  62439  ACCCCGCTTAAATTAACAATAGCAGACGGTGTAGTTTGCAACAAATTTATGCAAGGTGTG  62498

Query  1809   TACCTTGGCGTTACTGCCTTAGGGCCGGTC  1838
              ||||||||||||||||||||||||||||||
Sbjct  62499  TACCTTGGCGTTACTGCCTTAGGGCCGGTC  62528


 Score = 784 bits (424),  Expect = 0.0
 Identities = 424/424 (100%), Gaps = 0/424 (0%)
 Strand=Plus/Plus

Query  1      CACATGAACAGCATTGGCTGTTTTGCtttttttttAATGCCTGCAGGGCATTaaaaaaaa  60
              ||||||||||||||||||||||||||||||||||||||||||||||||||||||||||||
Sbjct  55183  CACATGAACAGCATTGGCTGTTTTGCTTTTTTTTTAATGCCTGCAGGGCATTAAAAAAAA  55242

Query  61     aaCTGCTGCAATTAGCTTGTGGCCGCGCTTTTAAAAATTGAAGACATTACACAGTACACA  120
              ||||||||||||||||||||||||||||||||||||||||||||||||||||||||||||
Sbjct  55243  AACTGCTGCAATTAGCTTGTGGCCGCGCTTTTAAAAATTGAAGACATTACACAGTACACA  55302

Query  121    ATTGGCACGCGTGTGGGTTTGGCACACTGTGCAGTCATTGCACACAGCATTGCAAGCTAG  180
              ||||||||||||||||||||||||||||||||||||||||||||||||||||||||||||
Sbjct  55303  ATTGGCACGCGTGTGGGTTTGGCACACTGTGCAGTCATTGCACACAGCATTGCAAGCTAG  55362

Query  181    TAGCAGCCTACTAGTAAGCACTTGCTGGCTACATTTTAATTAATGCGACTGCTTAAAACA  240
              ||||||||||||||||||||||||||||||||||||||||||||||||||||||||||||
Sbjct  55363  TAGCAGCCTACTAGTAAGCACTTGCTGGCTACATTTTAATTAATGCGACTGCTTAAAACA  55422

Query  241    CACCCCATTTTAGGTTTAGTAAATAGCTACATGGTCGACAGCCCACAACCTGCTAGCATT  300
              ||||||||||||||||||||||||||||||||||||||||||||||||||||||||||||
Sbjct  55423  CACCCCATTTTAGGTTTAGTAAATAGCTACATGGTCGACAGCCCACAACCTGCTAGCATT  55482

Query  301    ACGTACATGTGAAATTTTGGGTCGCTGCTAGGCCTGTGCTTAGTCATTCAAATTGTGACT  360
              ||||||||||||||||||||||||||||||||||||||||||||||||||||||||||||
Sbjct  55483  ACGTACATGTGAAATTTTGGGTCGCTGCTAGGCCTGTGCTTAGTCATTCAAATTGTGACT  55542

Query  361    GGCATTACGCTAGCAATGCACTACACACCGTCTATTGACTTGGCCTTTGCAAGTGTTGAG  420
              ||||||||||||||||||||||||||||||||||||||||||||||||||||||||||||
Sbjct  55543  GGCATTACGCTAGCAATGCACTACACACCGTCTATTGACTTGGCCTTTGCAAGTGTTGAG  55602

Query  421    CATA  424
              ||||
Sbjct  55603  CATA  55606


 Score = 357 bits (193),  Expect = 5e-100
 Identities = 193/193 (100%), Gaps = 0/193 (0%)
 Strand=Plus/Plus

Query  850    CTAGGCATTAGTGGCAACACAGACCGACTACCATTCCACCCGTACTTTACATTTAAAGAC  909
              ||||||||||||||||||||||||||||||||||||||||||||||||||||||||||||
Sbjct  60651  CTAGGCATTAGTGGCAACACAGACCGACTACCATTCCACCCGTACTTTACATTTAAAGAC  60710

Query  910    TTGGTAACAGTCTTCCTGTTCATGCTAGTGCTTGCAGCAATGGTCTTTTATGCGCCTAAC  969
              ||||||||||||||||||||||||||||||||||||||||||||||||||||||||||||
Sbjct  60711  TTGGTAACAGTCTTCCTGTTCATGCTAGTGCTTGCAGCAATGGTCTTTTATGCGCCTAAC  60770

Query  970    TACCTGGGCCATAGTGACAATTACATTCCTGCAAACCCCATGCAAACGCCAGCGTCAATT  1029
              ||||||||||||||||||||||||||||||||||||||||||||||||||||||||||||
Sbjct  60771  TACCTGGGCCATAGTGACAATTACATTCCTGCAAACCCCATGCAAACGCCAGCGTCAATT  60830

Query  1030   GTGCCTGAATGGT  1042
              |||||||||||||
Sbjct  60831  GTGCCTGAATGGT  60843


 Score = 355 bits (192),  Expect = 2e-99
 Identities = 192/192 (100%), Gaps = 0/192 (0%)
 Strand=Plus/Plus

Query  424    ATTATGCGTGACGTGCATTACGGCTGGCTAATTCGGTACTTGCATGCTAACGTGGCGTCG  483
              ||||||||||||||||||||||||||||||||||||||||||||||||||||||||||||
Sbjct  56265  ATTATGCGTGACGTGCATTACGGCTGGCTAATTCGGTACTTGCATGCTAACGTGGCGTCG  56324

Query  484    TTCTTTTTTATTTGCGTGTACTTACACATTGGTCGTGGCCTTTACTATGGGTCGTACCGG  543
              ||||||||||||||||||||||||||||||||||||||||||||||||||||||||||||
Sbjct  56325  TTCTTTTTTATTTGCGTGTACTTACACATTGGTCGTGGCCTTTACTATGGGTCGTACCGG  56384

Query  544    TCACCACGTACGCTAGTGTGGGCCATTGGGGTCGTAATTTTAGTACTTATGATAGCAACA  603
              ||||||||||||||||||||||||||||||||||||||||||||||||||||||||||||
Sbjct  56385  TCACCACGTACGCTAGTGTGGGCCATTGGGGTCGTAATTTTAGTACTTATGATAGCAACA  56444

Query  604    GCCTTTTTAGGT  615
              ||||||||||||
Sbjct  56445  GCCTTTTTAGGT  56456


 Score = 243 bits (131),  Expect = 1e-65
 Identities = 133/134 (99%), Gaps = 0/134 (0%)
 Strand=Plus/Plus

Query  716    TTTGGGGTGGCTTTAGCGTCAACAACGCAACGTTAAACCGGtttttttCATTGCATTACT  775
              || |||||||||||||||||||||||||||||||||||||||||||||||||||||||||
Sbjct  60113  TTGGGGGTGGCTTTAGCGTCAACAACGCAACGTTAAACCGGTTTTTTTCATTGCATTACT  60172

Query  776    TACTGCCGTTCATTTTGGCAGCACTTGCAGCTATGCACTTGCTTGCATTGCATGAACACG  835
              ||||||||||||||||||||||||||||||||||||||||||||||||||||||||||||
Sbjct  60173  TACTGCCGTTCATTTTGGCAGCACTTGCAGCTATGCACTTGCTTGCATTGCATGAACACG  60232

Query  836    GCAGCAGCAACCCT  849
              ||||||||||||||
Sbjct  60233  GCAGCAGCAACCCT  60246


 Score = 124 bits (67),  Expect = 5e-30
 Identities = 67/67 (100%), Gaps = 0/67 (0%)
 Strand=Plus/Plus

Query  652    GCCACTGTAATTACTAACCTACTGTCGGCCATTCCATGAATTGGCACTGACTTTGTTCAA  711
              ||||||||||||||||||||||||||||||||||||||||||||||||||||||||||||
Sbjct  58980  GCCACTGTAATTACTAACCTACTGTCGGCCATTCCATGAATTGGCACTGACTTTGTTCAA  59039

Query  712    TTCGTTT  718
              |||||||
Sbjct  59040  TTCGTTT  59046


 Score = 89.8 bits (48),  Expect = 2e-19
 Identities = 48/48 (100%), Gaps = 0/48 (0%)
 Strand=Plus/Plus

Query  1043   ACTTGCTACCGTTTTATGCAATTTTACGATCGATCCCTAATAAGCTAC  1090
              ||||||||||||||||||||||||||||||||||||||||||||||||
Sbjct  61520  ACTTGCTACCGTTTTATGCAATTTTACGATCGATCCCTAATAAGCTAC  61567


 Score = 69.4 bits (37),  Expect = 2e-13
 Identities = 37/37 (100%), Gaps = 0/37 (0%)
 Strand=Plus/Plus

Query  616    TATGTGCTGCCTTACGGTCAAATGTCATTATGGGGTG  652
              |||||||||||||||||||||||||||||||||||||
Sbjct  58486  TATGTGCTGCCTTACGGTCAAATGTCATTATGGGGTG  58522


Lambda      K        H
    1.33    0.621     1.12 

Gapped
Lambda      K        H
    1.28    0.460    0.850 

Effective search space used: 200726650


Query= 950281 estExt_fgenesh1_pm.C_40012

Length=3334
                                                                      Score     E
Sequences producing significant alignments:                          (Bits)  Value

  scaffold_1_mito                                                     1602    0.0  


> scaffold_1_mito
Length=110369

 Score = 1602 bits (867),  Expect = 0.0
 Identities = 867/867 (100%), Gaps = 0/867 (0%)
 Strand=Plus/Minus

Query  2468   CATGCATTTTAAGCTGTGGTGTGTACCACGCCTTCATGCCCATTGTAGTCGAAGCGGTGT  2527
              ||||||||||||||||||||||||||||||||||||||||||||||||||||||||||||
Sbjct  81573  CATGCATTTTAAGCTGTGGTGTGTACCACGCCTTCATGCCCATTGTAGTCGAAGCGGTGT  81514

Query  2528   CACTCGACAAGTACCTTATGTGACTAGACAGCCAAGTCTAGTAACCCCCCTGTACAtttt  2587
              ||||||||||||||||||||||||||||||||||||||||||||||||||||||||||||
Sbjct  81513  CACTCGACAAGTACCTTATGTGACTAGACAGCCAAGTCTAGTAACCCCCCTGTACATTTT  81454

Query  2588   ttttttAAGTAGGCAGCATTGCAATAAGTGACTGTAAATTGAACCGCCTAAAGTTGTAAC  2647
              ||||||||||||||||||||||||||||||||||||||||||||||||||||||||||||
Sbjct  81453  TTTTTTAAGTAGGCAGCATTGCAATAAGTGACTGTAAATTGAACCGCCTAAAGTTGTAAC  81394

Query  2648   AAATGGTTGCAGTACGCATTTGCTGTGCTTGGCACTGTGTTGCAAGCCTAACATAGCACG  2707
              ||||||||||||||||||||||||||||||||||||||||||||||||||||||||||||
Sbjct  81393  AAATGGTTGCAGTACGCATTTGCTGTGCTTGGCACTGTGTTGCAAGCCTAACATAGCACG  81334

Query  2708   GTGTACTAGCTTGCCTAACACACTTGCGTTGCATTTTACTAGCTTTGCAAACGTCATGAC  2767
              ||||||||||||||||||||||||||||||||||||||||||||||||||||||||||||
Sbjct  81333  GTGTACTAGCTTGCCTAACACACTTGCGTTGCATTTTACTAGCTTTGCAAACGTCATGAC  81274

Query  2768   TTTAAATGGCGTACTTTAAGCAAGTTTAGTAATGTAACATAAATTACAAGTCAATGCTGT  2827
              ||||||||||||||||||||||||||||||||||||||||||||||||||||||||||||
Sbjct  81273  TTTAAATGGCGTACTTTAAGCAAGTTTAGTAATGTAACATAAATTACAAGTCAATGCTGT  81214

Query  2828   TTAAGCTCGTGTAGTGTTAGCAAATTTAGCAGCAGCATGCATTTGCTAATTTGCTAGCCT  2887
              ||||||||||||||||||||||||||||||||||||||||||||||||||||||||||||
Sbjct  81213  TTAAGCTCGTGTAGTGTTAGCAAATTTAGCAGCAGCATGCATTTGCTAATTTGCTAGCCT  81154

Query  2888   TTGCAAACTTAAGCTCATGTTGTGTTGGCTTGCTTAGTCATTGCAATGCTTGCTTCACAA  2947
              ||||||||||||||||||||||||||||||||||||||||||||||||||||||||||||
Sbjct  81153  TTGCAAACTTAAGCTCATGTTGTGTTGGCTTGCTTAGTCATTGCAATGCTTGCTTCACAA  81094

Query  2948   GTAAAAAGTTGCAAACTTAAGCTCATGTAGTATTGGCTTGCTTAGTCATTGCAATGCTTG  3007
              ||||||||||||||||||||||||||||||||||||||||||||||||||||||||||||
Sbjct  81093  GTAAAAAGTTGCAAACTTAAGCTCATGTAGTATTGGCTTGCTTAGTCATTGCAATGCTTG  81034

Query  3008   CTTTGCTAGTAAGTCTTACATTTTTAAGCTTGCGTAGTATTGGCTTGCTTAGTAATTGCA  3067
              ||||||||||||||||||||||||||||||||||||||||||||||||||||||||||||
Sbjct  81033  CTTTGCTAGTAAGTCTTACATTTTTAAGCTTGCGTAGTATTGGCTTGCTTAGTAATTGCA  80974

Query  3068   ATGCTTGCTTCGTGAGGCAAGTCTTGCATTTTTAAGCTTGCGTAGTATTGGCTTGCTTAG  3127
              ||||||||||||||||||||||||||||||||||||||||||||||||||||||||||||
Sbjct  80973  ATGCTTGCTTCGTGAGGCAAGTCTTGCATTTTTAAGCTTGCGTAGTATTGGCTTGCTTAG  80914

Query  3128   TAATTGCAATGCTTGCTAAAAATGCAAAAGTTGCTTTTTTAAAGCTCGCGTAGTATTGAC  3187
              ||||||||||||||||||||||||||||||||||||||||||||||||||||||||||||
Sbjct  80913  TAATTGCAATGCTTGCTAAAAATGCAAAAGTTGCTTTTTTAAAGCTCGCGTAGTATTGAC  80854

Query  3188   GACTTTGGCAATTGTAAGTGCTTGCAAGTGCTTATGCTGTTAATTAAAGCTCGTGTAGCA  3247
              ||||||||||||||||||||||||||||||||||||||||||||||||||||||||||||
Sbjct  80853  GACTTTGGCAATTGTAAGTGCTTGCAAGTGCTTATGCTGTTAATTAAAGCTCGTGTAGCA  80794

Query  3248   TTGACTAGTTTGGCAATTGCCTATGCTTGCTGTAAGCAAAGTTACAATTTAAAGCTTGTG  3307
              ||||||||||||||||||||||||||||||||||||||||||||||||||||||||||||
Sbjct  80793  TTGACTAGTTTGGCAATTGCCTATGCTTGCTGTAAGCAAAGTTACAATTTAAAGCTTGTG  80734

Query  3308   TAGTATTGGCTTGCTTAGTAATTGCAA  3334
              |||||||||||||||||||||||||||
Sbjct  80733  TAGTATTGGCTTGCTTAGTAATTGCAA  80707


 Score = 680 bits (368),  Expect = 0.0
 Identities = 372/374 (99%), Gaps = 0/374 (0%)
 Strand=Plus/Minus

Query  1       ATGGCTAGCAATTTGCATAAGTGCCAATACTAGCAAACTGCAACAGCTTGACTCCAGACG  60
               ||||||||||||||||||||||||||||||||||||||||||||||||||||||||||||
Sbjct  106526  ATGGCTAGCAATTTGCATAAGTGCCAATACTAGCAAACTGCAACAGCTTGACTCCAGACG  106467

Query  61      AGCTTTAATAGGCAACTAATTGCATTACTTGCACGACTAGCAAATTGTAACGACCTAACT  120
               ||||||||||||||||||||||||||||||||||||||||||||||||||||||||||||
Sbjct  106466  AGCTTTAATAGGCAACTAATTGCATTACTTGCACGACTAGCAAATTGTAACGACCTAACT  106407

Query  121     GTAGACGAGCTTAAATAGCTGCCTAATTGCATGCCTAGCATGACTCGCTAGCGGCAAGTA  180
               ||||||||||||||||||||||||||||||||||||||||||||||||||||||||||||
Sbjct  106406  GTAGACGAGCTTAAATAGCTGCCTAATTGCATGCCTAGCATGACTCGCTAGCGGCAAGTA  106347

Query  181     CCTAACTGTACACGAGCTTAAATAGCTAACTAATTGCACTACTTGCATTGCAACTACCTA  240
               ||||||||||||||||||||||||||||||||||||||||||||||||||||||||||||
Sbjct  106346  CCTAACTGTACACGAGCTTAAATAGCTAACTAATTGCACTACTTGCATTGCAACTACCTA  106287

Query  241     ACTTGGCACGAGCTTAATAGCTAGCCTTTACAATTTTGCTTAAGTAGCCATTTGCAATTT  300
               ||||||||||||||||||||||||||||||||||||||||||||||||||||||||||||
Sbjct  106286  ACTTGGCACGAGCTTAATAGCTAGCCTTTACAATTTTGCTTAAGTAGCCATTTGCAATTT  106227

Query  301     TGCCTAACTGTACATGGTGTAATGCAGCAAGTCACTAGCATAGGCTTGCAATGTGCTTTT  360
               ||||||||||||||||||||||||||||||||||||||||||||||||||||||||||||
Sbjct  106226  TGCCTAACTGTACATGGTGTAATGCAGCAAGTCACTAGCATAGGCTTGCAATGTGCTTTT  106167

Query  361     CGTCCCAGCACAAA  374
               ||||||||  ||||
Sbjct  106166  CGTCCCAGGTCAAA  106153


 Score = 503 bits (272),  Expect = 1e-143
 Identities = 272/272 (100%), Gaps = 0/272 (0%)
 Strand=Plus/Minus

Query  2196   TGACTTCATTACAGCCGACGGCGAGTCTGTTGAATTTGACAGCTACATGGTACCCGAGTC  2255
              ||||||||||||||||||||||||||||||||||||||||||||||||||||||||||||
Sbjct  83510  TGACTTCATTACAGCCGACGGCGAGTCTGTTGAATTTGACAGCTACATGGTACCCGAGTC  83451

Query  2256   TGACTTAACACAGGGTCAGCTGCGACTGCTTGAAGTCGACAACCGGGTAGTCGTGCCTGT  2315
              ||||||||||||||||||||||||||||||||||||||||||||||||||||||||||||
Sbjct  83450  TGACTTAACACAGGGTCAGCTGCGACTGCTTGAAGTCGACAACCGGGTAGTCGTGCCTGT  83391

Query  2316   AGACACGCACATTCGCTTCATTGTGACTGGCCAAGACGTCATTCATGACTGGGCTGTGCC  2375
              ||||||||||||||||||||||||||||||||||||||||||||||||||||||||||||
Sbjct  83390  AGACACGCACATTCGCTTCATTGTGACTGGCCAAGACGTCATTCATGACTGGGCTGTGCC  83331

Query  2376   TGCCTTAGGCTTAAAAATGGACGCCGTACCAGGTCGTTTAAACCAAACGTCAATTATTGT  2435
              ||||||||||||||||||||||||||||||||||||||||||||||||||||||||||||
Sbjct  83330  TGCCTTAGGCTTAAAAATGGACGCCGTACCAGGTCGTTTAAACCAAACGTCAATTATTGT  83271

Query  2436   GCAACGACCAGGTGTCTACTATGGGCAATGTA  2467
              ||||||||||||||||||||||||||||||||
Sbjct  83270  GCAACGACCAGGTGTCTACTATGGGCAATGTA  83239


 Score = 451 bits (244),  Expect = 4e-128
 Identities = 244/244 (100%), Gaps = 0/244 (0%)
 Strand=Plus/Minus

Query  1598   GCTGGAACCTAGTGTCGAGCTTCGGCTCACTAGTGTCGGTAGTAGCTACAGCAGTCTTCC  1657
              ||||||||||||||||||||||||||||||||||||||||||||||||||||||||||||
Sbjct  91212  GCTGGAACCTAGTGTCGAGCTTCGGCTCACTAGTGTCGGTAGTAGCTACAGCAGTCTTCC  91153

Query  1658   TGTACGTTGTGTGGGACCAGTTTGTGCACGGTAAAGCCGTGGTACACAACCCATGGGCGG  1717
              ||||||||||||||||||||||||||||||||||||||||||||||||||||||||||||
Sbjct  91152  TGTACGTTGTGTGGGACCAGTTTGTGCACGGTAAAGCCGTGGTACACAACCCATGGGCGG  91093

Query  1718   TGCCCCAGTACTTCACAAGCGACAGCCTGTTTGCAGTAGCACCACAGACCGCTAGCTCAT  1777
              ||||||||||||||||||||||||||||||||||||||||||||||||||||||||||||
Sbjct  91092  TGCCCCAGTACTTCACAAGCGACAGCCTGTTTGCAGTAGCACCACAGACCGCTAGCTCAT  91033

Query  1778   TAGAATGGACGCTAGCAAGTCCTGTGCCATTCCATTCTTACAACACGCTGCCAGTACAGT  1837
              ||||||||||||||||||||||||||||||||||||||||||||||||||||||||||||
Sbjct  91032  TAGAATGGACGCTAGCAAGTCCTGTGCCATTCCATTCTTACAACACGCTGCCAGTACAGT  90973

Query  1838   CGTA  1841
              ||||
Sbjct  90972  CGTA  90969


 Score = 431 bits (233),  Expect = 5e-122
 Identities = 233/233 (100%), Gaps = 0/233 (0%)
 Strand=Plus/Minus

Query  367     AGCACAAATGCAAAAGACATTGGTACCCTGTACCTAGTCTTTGCTGTGTTTAGTGGCATG  426
               ||||||||||||||||||||||||||||||||||||||||||||||||||||||||||||
Sbjct  105995  AGCACAAATGCAAAAGACATTGGTACCCTGTACCTAGTCTTTGCTGTGTTTAGTGGCATG  105936

Query  427     CTAGGTACTGCATTTAGTGTGCTAATTCGAATGGAATTGGCAGCACCAGGTGTGCAGTAC  486
               ||||||||||||||||||||||||||||||||||||||||||||||||||||||||||||
Sbjct  105935  CTAGGTACTGCATTTAGTGTGCTAATTCGAATGGAATTGGCAGCACCAGGTGTGCAGTAC  105876

Query  487     TTGCAAGGCAACCACCAACTGTACAACGTCGTAATTACAGCGCATGCCCTATTAATGAtt  546
               ||||||||||||||||||||||||||||||||||||||||||||||||||||||||||||
Sbjct  105875  TTGCAAGGCAACCACCAACTGTACAACGTCGTAATTACAGCGCATGCCCTATTAATGATT  105816

Query  547     ttttttATGGTCAGCTACCCTGCTTTTCTATACAGTAGTAATTGCGGCATGGT  599
               |||||||||||||||||||||||||||||||||||||||||||||||||||||
Sbjct  105815  TTTTTTATGGTCAGCTACCCTGCTTTTCTATACAGTAGTAATTGCGGCATGGT  105763


 Score = 307 bits (166),  Expect = 9e-85
 Identities = 166/166 (100%), Gaps = 0/166 (0%)
 Strand=Plus/Minus

Query  1062   TCCCATGGGGTTTACAGTGCAAGACCGCTACATTTTAATCATTCCTGCCTTTGGCATTGT  1121
              ||||||||||||||||||||||||||||||||||||||||||||||||||||||||||||
Sbjct  96886  TCCCATGGGGTTTACAGTGCAAGACCGCTACATTTTAATCATTCCTGCCTTTGGCATTGT  96827

Query  1122   GTCGCATGTAGTTGCAACCTTTGCTGGTAAGCCCGTGTTTGGGTACCTAGGTATGGTCTA  1181
              ||||||||||||||||||||||||||||||||||||||||||||||||||||||||||||
Sbjct  96826  GTCGCATGTAGTTGCAACCTTTGCTGGTAAGCCCGTGTTTGGGTACCTAGGTATGGTCTA  96767

Query  1182   TGCAATGTTCAGCATTGGCATTTTAGGCTTTATTGTATGGTCTTGT  1227
              ||||||||||||||||||||||||||||||||||||||||||||||
Sbjct  96766  TGCAATGTTCAGCATTGGCATTTTAGGCTTTATTGTATGGTCTTGT  96721


 Score = 292 bits (158),  Expect = 2e-80
 Identities = 158/158 (100%), Gaps = 0/158 (0%)
 Strand=Plus/Minus

Query  2038   TTGACATTAATTGAGCTAGTGTGGACCATTACGCCTGCATTAGTGTTAATTGCAATTGCA  2097
              ||||||||||||||||||||||||||||||||||||||||||||||||||||||||||||
Sbjct  84077  TTGACATTAATTGAGCTAGTGTGGACCATTACGCCTGCATTAGTGTTAATTGCAATTGCA  84018

Query  2098   TTCCCTAGCTTTAAGCTGCTGTACTTAATGGACGAAGTCATTTCACCTACTATGACCGTC  2157
              ||||||||||||||||||||||||||||||||||||||||||||||||||||||||||||
Sbjct  84017  TTCCCTAGCTTTAAGCTGCTGTACTTAATGGACGAAGTCATTTCACCTACTATGACCGTC  83958

Query  2158   AAAGTGGCAGGTCACCAATGGTACTGGTCACAAAGCTT  2195
              ||||||||||||||||||||||||||||||||||||||
Sbjct  83957  AAAGTGGCAGGTCACCAATGGTACTGGTCACAAAGCTT  83920


 Score = 276 bits (149),  Expect = 2e-75
 Identities = 149/149 (100%), Gaps = 0/149 (0%)
 Strand=Plus/Minus

Query  891     TAAGGCAATGAACTTCATTACTACAATTTTGAACATGCGTGCCCCTGGCATGTCAATGCA  950
               ||||||||||||||||||||||||||||||||||||||||||||||||||||||||||||
Sbjct  101772  TAAGGCAATGAACTTCATTACTACAATTTTGAACATGCGTGCCCCTGGCATGTCAATGCA  101713

Query  951     CAAGCTGCCGCTTTTTTGCTGGGCAATTTTTATTACAGCAATTTTGCTGCTGCTGTCATT  1010
               ||||||||||||||||||||||||||||||||||||||||||||||||||||||||||||
Sbjct  101712  CAAGCTGCCGCTTTTTTGCTGGGCAATTTTTATTACAGCAATTTTGCTGCTGCTGTCATT  101653

Query  1011    GCCAGTACTAGCGAGGAGCCATTACAATG  1039
               |||||||||||||||||||||||||||||
Sbjct  101652  GCCAGTACTAGCGAGGAGCCATTACAATG  101624


 Score = 250 bits (135),  Expect = 2e-67
 Identities = 258/317 (81%), Gaps = 9/317 (3%)
 Strand=Plus/Minus

Query  2888   TTGCAAACTTAAGCTCATGTTGTGTTGGCTTGCTTAGTCATTGCAATGCTTGCTTCACAA  2947
              |||||||||||||||||||| || |||||||||||||||||||||||||||||||  | |
Sbjct  81085  TTGCAAACTTAAGCTCATGTAGTATTGGCTTGCTTAGTCATTGCAATGCTTGCTTTGCTA  81026

Query  2948   GTAAAAAGTTGCAAACTTAAGCTCATGTAGTATTGGCTTGCTTAGTCATTGCAATGCTTG  3007
              ||||    || ||   |||||||   |||||||||||||||||||| |||||||||||||
Sbjct  81025  GTAAGTC-TTACATTTTTAAGCTTGCGTAGTATTGGCTTGCTTAGTAATTGCAATGCTTG  80967

Query  3008   CTTTGCTAGT-AAGTCTTACATTTTTAAGCTTGCGTAGTATTGGCTTGCTTAGTAATTGC  3066
              ||| |  ||  ||||||| |||||||||||||||||||||||||||||||||||||||||
Sbjct  80966  CTTCGTGAGGCAAGTCTTGCATTTTTAAGCTTGCGTAGTATTGGCTTGCTTAGTAATTGC  80907

Query  3067   AATGCTTGCTTCGTGAGGCAAGTCTTGCATTTTTAA-GCTTGCGTAGTATTGGCTTGCTT  3125
              ||||||||||     | ||||   |||| ||||||| ||| ||||||||||| |    ||
Sbjct  80906  AATGCTTGCTAAAA-ATGCAAAAGTTGCTTTTTTAAAGCTCGCGTAGTATTGACGACTTT  80848

Query  3126   AGTAATTGCAA-TGCTTGCTAAAAATGCAAAAGTTGCTTTTTTAAAGCTCGCGTAGTATT  3184
               | ||||| || ||||||| ||   |||  | | || ||  |||||||||| |||| |||
Sbjct  80847  GGCAATTGTAAGTGCTTGC-AAG--TGCTTATGCTG-TTAATTAAAGCTCGTGTAGCATT  80792

Query  3185   GACGACTTTGGCAATTG  3201
              ||| | |||||||||||
Sbjct  80791  GACTAGTTTGGCAATTG  80775


 Score = 250 bits (135),  Expect = 2e-67
 Identities = 259/318 (81%), Gaps = 11/318 (3%)
 Strand=Plus/Minus

Query  2956   TTGCAAACTTAAGCTCATGTAGTATTGGCTTGCTTAGTCATTGCAATGCTTGCTTTGCTA  3015
              |||||||||||||||||||| || |||||||||||||||||||||||||||||||  | |
Sbjct  81153  TTGCAAACTTAAGCTCATGTTGTGTTGGCTTGCTTAGTCATTGCAATGCTTGCTTCACAA  81094

Query  3016   GTAAGTC-TTACATTTTTAAGCTTGCGTAGTATTGGCTTGCTTAGTAATTGCAATGCTTG  3074
              ||||    || ||   |||||||   |||||||||||||||||||| |||||||||||||
Sbjct  81093  GTAAAAAGTTGCAAACTTAAGCTCATGTAGTATTGGCTTGCTTAGTCATTGCAATGCTTG  81034

Query  3075   CTTCGTGAGGCAAGTCTTGCATTTTTAAGCTTGCGTAGTATTGGCTTGCTTAGTAATTGC  3134
              ||| |  ||  ||||||| |||||||||||||||||||||||||||||||||||||||||
Sbjct  81033  CTTTGCTAGT-AAGTCTTACATTTTTAAGCTTGCGTAGTATTGGCTTGCTTAGTAATTGC  80975

Query  3135   AATGCTTGCTAAAA-ATGCAAAAGTTGCTTTTTTAAAGCTCGCGTAGTATTGAC-GACTT  3192
              ||||||||||     | ||||   |||| ||||||| ||| ||||||||||| |   |||
Sbjct  80974  AATGCTTGCTTCGTGAGGCAAGTCTTGCATTTTTAA-GCTTGCGTAGTATTGGCTTGCTT  80916

Query  3193   TGGCAATTGTAAGTGCTTGC-AAG--TGCTTATGCTG-TTAATTAAAGCTCGTGTAGCAT  3248
               |  ||||| || ||||||| ||   |||  | | || ||  |||||||||| |||| ||
Sbjct  80915  AGT-AATTGCAA-TGCTTGCTAAAAATGCAAAAGTTGCTTTTTTAAAGCTCGCGTAGTAT  80858

Query  3249   TGACTAGTTTGGCAATTG  3266
              |||| | |||||||||||
Sbjct  80857  TGACGACTTTGGCAATTG  80840


 Score = 220 bits (119),  Expect = 1e-58
 Identities = 122/123 (99%), Gaps = 1/123 (1%)
 Strand=Plus/Minus

Query  1480   GTAATTGGCAAGACTTACAACGAGCAATTGGCACATGTACACTTTTGGGTCATGTTTGTG  1539
              ||||||||||||||||||||||||||||||||||||||||||||||||||||||||||||
Sbjct  92237  GTAATTGGCAAGACTTACAACGAGCAATTGGCACATGTACACTTTTGGGTCATGTTTGTG  92178

Query  1540   GGCGTTAATGTTACATTCTTCCCACAACACTTCTTGGGCCTAGCAGGTATGACTTCACGC  1599
              ||||||||||||||||||||||||||||||||||||||||||||||||||||||||||| 
Sbjct  92177  GGCGTTAATGTTACATTCTTCCCACAACACTTCTTGGGCCTAGCAGGTATGACTTCACG-  92119

Query  1600   TGG  1602
              |||
Sbjct  92118  TGG  92116


 Score = 220 bits (119),  Expect = 1e-58
 Identities = 119/119 (100%), Gaps = 0/119 (0%)
 Strand=Plus/Minus

Query  656     GCAATGTACTGATGGCCTTCCCACGTCTTAATAACATTAGCTTTTGGCTACTGCCGCCGT  715
               ||||||||||||||||||||||||||||||||||||||||||||||||||||||||||||
Sbjct  103075  GCAATGTACTGATGGCCTTCCCACGTCTTAATAACATTAGCTTTTGGCTACTGCCGCCGT  103016

Query  716     CACTAGTGCTGCTGCTTGCAAGTGCACTAGTCGAACAGGGTGCAGGTGCCTGTGCCTGT  774
               |||||||||||||||||||||||||||||||||||||||||||||||||||||||||||
Sbjct  103015  CACTAGTGCTGCTGCTTGCAAGTGCACTAGTCGAACAGGGTGCAGGTGCCTGTGCCTGT  102957


 Score = 219 bits (118),  Expect = 4e-58
 Identities = 118/118 (100%), Gaps = 0/118 (0%)
 Strand=Plus/Minus

Query  1860   CTGTGACGTCCCGTACCCATGGCAAATAGGCTTCCAAGACGGTGCAACGCCCACATTTGA  1919
              ||||||||||||||||||||||||||||||||||||||||||||||||||||||||||||
Sbjct  85076  CTGTGACGTCCCGTACCCATGGCAAATAGGCTTCCAAGACGGTGCAACGCCCACATTTGA  85017

Query  1920   AGGCATTGTTGAATTGCATGACACCAtttttttttACTTAGTAGTCATTAGCTTTTTA  1977
              ||||||||||||||||||||||||||||||||||||||||||||||||||||||||||
Sbjct  85016  AGGCATTGTTGAATTGCATGACACCATTTTTTTTTACTTAGTAGTCATTAGCTTTTTA  84959


 Score = 215 bits (116),  Expect = 6e-57
 Identities = 116/116 (100%), Gaps = 0/116 (0%)
 Strand=Plus/Minus

Query  775     ACCGGCTGGACTATTTACCCACCCCTGTCGGGCATTCAAAGCCATTCAGGTGGGTCGGTA  834
               ||||||||||||||||||||||||||||||||||||||||||||||||||||||||||||
Sbjct  102766  ACCGGCTGGACTATTTACCCACCCCTGTCGGGCATTCAAAGCCATTCAGGTGGGTCGGTA  102707

Query  835     GACTTAGCAATTTTTAGCCTGCACCTGTCGGGCATTAGTAGTATGCTAGGTCACTT  890
               ||||||||||||||||||||||||||||||||||||||||||||||||||||||||
Sbjct  102706  GACTTAGCAATTTTTAGCCTGCACCTGTCGGGCATTAGTAGTATGCTAGGTCACTT  102651


 Score = 152 bits (82),  Expect = 4e-38
 Identities = 82/82 (100%), Gaps = 0/82 (0%)
 Strand=Plus/Minus

Query  1303   GCCACTTGCTACGGCGGTGTATTGCGGTACACAGCACCTATGGTGTTTGCATTGGGCTTC  1362
              ||||||||||||||||||||||||||||||||||||||||||||||||||||||||||||
Sbjct  95532  GCCACTTGCTACGGCGGTGTATTGCGGTACACAGCACCTATGGTGTTTGCATTGGGCTTC  95473

Query  1363   ATTGCCCTGTTCACGGTAGGTG  1384
              ||||||||||||||||||||||
Sbjct  95472  ATTGCCCTGTTCACGGTAGGTG  95451


 Score = 137 bits (74),  Expect = 1e-33
 Identities = 74/74 (100%), Gaps = 0/74 (0%)
 Strand=Plus/Minus

Query  1228   CACCACATGTTTGCCGTGGGGCTAGACGTTGACACACGAGCCTACTTCACAGCAGCCACA  1287
              ||||||||||||||||||||||||||||||||||||||||||||||||||||||||||||
Sbjct  96122  CACCACATGTTTGCCGTGGGGCTAGACGTTGACACACGAGCCTACTTCACAGCAGCCACA  96063

Query  1288   ATGATTATTGCTGT  1301
              ||||||||||||||
Sbjct  96062  ATGATTATTGCTGT  96049


 Score = 111 bits (60),  Expect = 7e-26
 Identities = 60/60 (100%), Gaps = 0/60 (0%)
 Strand=Plus/Minus

Query  1978   TTTAGTAGCAGCAAAGTCGGCATTGTGCACAAGTACCATAACCATGGTCGCAACCGCCAT  2037
              ||||||||||||||||||||||||||||||||||||||||||||||||||||||||||||
Sbjct  84925  TTTAGTAGCAGCAAAGTCGGCATTGTGCACAAGTACCATAACCATGGTCGCAACCGCCAT  84866


 Score = 106 bits (57),  Expect = 3e-24
 Identities = 57/57 (100%), Gaps = 0/57 (0%)
 Strand=Plus/Minus

Query  600     TCTAAGCAAGGGTTTTGGCAACTACTTAGTGCCAGTTCAAATTGGGGCGCCTGATTG  656
               |||||||||||||||||||||||||||||||||||||||||||||||||||||||||
Sbjct  103768  TCTAAGCAAGGGTTTTGGCAACTACTTAGTGCCAGTTCAAATTGGGGCGCCTGATTG  103712


 Score = 102 bits (55),  Expect = 4e-23
 Identities = 186/247 (75%), Gaps = 17/247 (7%)
 Strand=Plus/Minus

Query  2896   TTAAGCTCATGTTGTGTTGGCTTGCTTAGTCATTGCAATGCTTGCTTCACAAGTAAAAAG  2955
              |||||||   || || |||||||||||||| |||||||||||||||  | ||   |||||
Sbjct  80942  TTAAGCTTGCGTAGTATTGGCTTGCTTAGTAATTGCAATGCTTGCT-AAAAATGCAAAAG  80884

Query  2956   TTGCAAACTTAA-GCTCATGTAGTATTGGCTTGCTTAGTC-ATTGCAA-TGCTTGC-TTT  3011
              ||||    |||| ||||  ||||||||| |   ||| | | |||| || |||||||   |
Sbjct  80883  TTGCTTTTTTAAAGCTCGCGTAGTATTGAC-GACTTTGGCAATTGTAAGTGCTTGCAAGT  80825

Query  3012   GCTAGTAAG-TCTTACATTTTTAAGCTTGCGTAGTATTGGCTTGCTTAGTAATTGC-AAT  3069
              |||  || | | ||| |  || ||||| | |||| |||| || | || | ||||||  ||
Sbjct  80824  GCT--TATGCTGTTA-A--TTAAAGCTCGTGTAGCATTGACTAGTTTGGCAATTGCCTAT  80770

Query  3070   GCTTGCTTCGTGAGGCAAGTCTTGCATTTTTAAGCTTGCGTAGTATTGGCTTGCTTAGTA  3129
              |||||| | || | ||||   || || ||| ||||||| |||||||||||||||||||||
Sbjct  80769  GCTTGC-T-GT-AAGCAAAG-TTACAATTTAAAGCTTGTGTAGTATTGGCTTGCTTAGTA  80714

Query  3130   ATTGCAA  3136
              |||||||
Sbjct  80713  ATTGCAA  80707


 Score = 102 bits (55),  Expect = 4e-23
 Identities = 186/247 (75%), Gaps = 17/247 (7%)
 Strand=Plus/Minus

Query  3099   TTAAGCTTGCGTAGTATTGGCTTGCTTAGTAATTGCAATGCTTGCT-AAAAATGCAAAAG  3157
              |||||||   || || |||||||||||||| |||||||||||||||  | ||   |||||
Sbjct  81145  TTAAGCTCATGTTGTGTTGGCTTGCTTAGTCATTGCAATGCTTGCTTCACAAGTAAAAAG  81086

Query  3158   TTGCTTTTTTAAAGCTCGCGTAGTATTGAC-GACTTTGGCAATTGTAAGTGCTTGCAAGT  3216
              ||||    |||| ||||  ||||||||| |   ||| | | |||| || |||||||   |
Sbjct  81085  TTGCAAACTTAA-GCTCATGTAGTATTGGCTTGCTTAGTC-ATTGCAA-TGCTTGC-TTT  81030

Query  3217   GCT--TATGCTGTTA-A--TTAAAGCTCGTGTAGCATTGACTAGTTTGGCAATTGCCTAT  3271
              |||  || | | ||| |  || ||||| | |||| |||| || | || | ||||||  ||
Sbjct  81029  GCTAGTAAG-TCTTACATTTTTAAGCTTGCGTAGTATTGGCTTGCTTAGTAATTGC-AAT  80972

Query  3272   GCTTGC-T-GT-AAGCAAAG-TTACAATTTAAAGCTTGTGTAGTATTGGCTTGCTTAGTA  3327
              |||||| | || | ||||   || || ||| ||||||| |||||||||||||||||||||
Sbjct  80971  GCTTGCTTCGTGAGGCAAGTCTTGCATTTTTAAGCTTGCGTAGTATTGGCTTGCTTAGTA  80912

Query  3328   ATTGCAA  3334
              |||||||
Sbjct  80911  ATTGCAA  80905


 Score = 99.0 bits (53),  Expect = 6e-22
 Identities = 53/53 (100%), Gaps = 0/53 (0%)
 Strand=Plus/Minus

Query  1380   AGGTGTCGTACTTGCAAATGCTAGTCTTGACGTTGCAATTCACGATAGTACCG  1432
              |||||||||||||||||||||||||||||||||||||||||||||||||||||
Sbjct  95068  AGGTGTCGTACTTGCAAATGCTAGTCTTGACGTTGCAATTCACGATAGTACCG  95016


 Score = 91.6 bits (49),  Expect = 1e-19
 Identities = 49/49 (100%), Gaps = 0/49 (0%)
 Strand=Plus/Minus

Query  1432   GCTGGCTGTGCACACTTCCACTATGTGCTGTCAATGGGTGCAGTCTTTG  1480
              |||||||||||||||||||||||||||||||||||||||||||||||||
Sbjct  92321  GCTGGCTGTGCACACTTCCACTATGTGCTGTCAATGGGTGCAGTCTTTG  92273


 Score = 73.1 bits (39),  Expect = 4e-14
 Identities = 83/104 (80%), Gaps = 4/104 (4%)
 Strand=Plus/Minus

Query  2966   AAGCTCATGTAGTATTGGCTTGCTTAGTCATTG-CAATGCTTGCTTTGCTAGTAAGTCTT  3024
              |||||| ||||| |||| || | || |  |||| | ||||||||  ||  || ||   ||
Sbjct  80807  AAGCTCGTGTAGCATTGACTAGTTTGGCAATTGCCTATGCTTGC--TGTAAGCAAAG-TT  80751

Query  3025   ACATTTTTAAGCTTGCGTAGTATTGGCTTGCTTAGTAATTGCAA  3068
              ||| ||| ||||||| ||||||||||||||||||||||||||||
Sbjct  80750  ACAATTTAAAGCTTGTGTAGTATTGGCTTGCTTAGTAATTGCAA  80707


 Score = 73.1 bits (39),  Expect = 4e-14
 Identities = 83/104 (80%), Gaps = 4/104 (4%)
 Strand=Plus/Minus

Query  3234   AAGCTCGTGTAGCATTGACTAGTTTGGCAATTGCCTATGCTTGC--TGTAAGCAAAG-TT  3290
              |||||| ||||| |||| || | || |  |||| | ||||||||  ||  || ||   ||
Sbjct  81075  AAGCTCATGTAGTATTGGCTTGCTTAGTCATTG-CAATGCTTGCTTTGCTAGTAAGTCTT  81017

Query  3291   ACAATTTAAAGCTTGTGTAGTATTGGCTTGCTTAGTAATTGCAA  3334
              ||| ||| ||||||| ||||||||||||||||||||||||||||
Sbjct  81016  ACATTTTTAAGCTTGCGTAGTATTGGCTTGCTTAGTAATTGCAA  80973


Lambda      K        H
    1.33    0.621     1.12 

Gapped
Lambda      K        H
    1.28    0.460    0.850 

Effective search space used: 365696586


Query= 946144 fgenesh1_pm.4_#_1

Length=312
                                                                      Score     E
Sequences producing significant alignments:                          (Bits)  Value

  scaffold_1_mito                                                     577     5e-167


> scaffold_1_mito
Length=110369

 Score = 577 bits (312),  Expect = 5e-167
 Identities = 312/312 (100%), Gaps = 0/312 (0%)
 Strand=Plus/Plus

Query  1      ATGCACACAAAATTTGCAAACACACCCGCAGGTACACTGCAATTAAAAATGGCGCTTTCA  60
              ||||||||||||||||||||||||||||||||||||||||||||||||||||||||||||
Sbjct  11676  ATGCACACAAAATTTGCAAACACACCCGCAGGTACACTGCAATTAAAAATGGCGCTTTCA  11735

Query  61     ATTGTACTGTTCGTACTAGCAGTGCTAGGCTTTGTACTAAACCGCAAAAACCTAATTTTA  120
              ||||||||||||||||||||||||||||||||||||||||||||||||||||||||||||
Sbjct  11736  ATTGTACTGTTCGTACTAGCAGTGCTAGGCTTTGTACTAAACCGCAAAAACCTAATTTTA  11795

Query  121    ATGCTAATTAGCATTGAAGTTATGTTGCTTGCTGTAACGCTGCTAGTCATTGTGACGTCT  180
              ||||||||||||||||||||||||||||||||||||||||||||||||||||||||||||
Sbjct  11796  ATGCTAATTAGCATTGAAGTTATGTTGCTTGCTGTAACGCTGCTAGTCATTGTGACGTCT  11855

Query  181    TATGAGTTTAATGACGTAATTGGTCAAACTTACGCTGTGTTCATTATTGCAATTGCAGGT  240
              ||||||||||||||||||||||||||||||||||||||||||||||||||||||||||||
Sbjct  11856  TATGAGTTTAATGACGTAATTGGTCAAACTTACGCTGTGTTCATTATTGCAATTGCAGGT  11915

Query  241    GCCGAGTCAGCAATTGGACTTGGCATTTTAGTAGCCTTTTACCGTACACGTGGGTCAATT  300
              ||||||||||||||||||||||||||||||||||||||||||||||||||||||||||||
Sbjct  11916  GCCGAGTCAGCAATTGGACTTGGCATTTTAGTAGCCTTTTACCGTACACGTGGGTCAATT  11975

Query  301    ACACTTTTTTAA  312
              ||||||||||||
Sbjct  11976  ACACTTTTTTAA  11987


Lambda      K        H
    1.33    0.621     1.12 

Gapped
Lambda      K        H
    1.28    0.460    0.850 

Effective search space used: 32553840


Query= 861956 gw1.4.29.1

Length=282
                                                                      Score     E
Sequences producing significant alignments:                          (Bits)  Value

  scaffold_1_mito                                                     521     2e-150


> scaffold_1_mito
Length=110369

 Score = 521 bits (282),  Expect = 2e-150
 Identities = 282/282 (100%), Gaps = 0/282 (0%)
 Strand=Plus/Plus

Query  1      CCCGCAGGTACACTGCAATTAAAAATGGCGCTTTCAATTGTACTGTTCGTACTAGCAGTG  60
              ||||||||||||||||||||||||||||||||||||||||||||||||||||||||||||
Sbjct  11700  CCCGCAGGTACACTGCAATTAAAAATGGCGCTTTCAATTGTACTGTTCGTACTAGCAGTG  11759

Query  61     CTAGGCTTTGTACTAAACCGCAAAAACCTAATTTTAATGCTAATTAGCATTGAAGTTATG  120
              ||||||||||||||||||||||||||||||||||||||||||||||||||||||||||||
Sbjct  11760  CTAGGCTTTGTACTAAACCGCAAAAACCTAATTTTAATGCTAATTAGCATTGAAGTTATG  11819

Query  121    TTGCTTGCTGTAACGCTGCTAGTCATTGTGACGTCTTATGAGTTTAATGACGTAATTGGT  180
              ||||||||||||||||||||||||||||||||||||||||||||||||||||||||||||
Sbjct  11820  TTGCTTGCTGTAACGCTGCTAGTCATTGTGACGTCTTATGAGTTTAATGACGTAATTGGT  11879

Query  181    CAAACTTACGCTGTGTTCATTATTGCAATTGCAGGTGCCGAGTCAGCAATTGGACTTGGC  240
              ||||||||||||||||||||||||||||||||||||||||||||||||||||||||||||
Sbjct  11880  CAAACTTACGCTGTGTTCATTATTGCAATTGCAGGTGCCGAGTCAGCAATTGGACTTGGC  11939

Query  241    ATTTTAGTAGCCTTTTACCGTACACGTGGGTCAATTACACTT  282
              ||||||||||||||||||||||||||||||||||||||||||
Sbjct  11940  ATTTTAGTAGCCTTTTACCGTACACGTGGGTCAATTACACTT  11981


Lambda      K        H
    1.33    0.621     1.12 

Gapped
Lambda      K        H
    1.28    0.460    0.850 

Effective search space used: 29243280


Query= 518409 CE518257_193

Length=727
                                                                      Score     E
Sequences producing significant alignments:                          (Bits)  Value

  scaffold_1_mito                                                     1343    0.0  


> scaffold_1_mito
Length=110369

 Score = 1343 bits (727),  Expect = 0.0
 Identities = 727/727 (100%), Gaps = 0/727 (0%)
 Strand=Plus/Minus

Query  1      ACTAGGGCCATGGGTACTGACCGTAAGGCTGTGCTGCACTAGTCATTACACAAGCTAAGG  60
              ||||||||||||||||||||||||||||||||||||||||||||||||||||||||||||
Sbjct  83731  ACTAGGGCCATGGGTACTGACCGTAAGGCTGTGCTGCACTAGTCATTACACAAGCTAAGG  83672

Query  61     CCGTAAGCATTGTAACTTATGCTAAGACCACAGTACGCTAGCCACTACACAGTCACATGC  120
              ||||||||||||||||||||||||||||||||||||||||||||||||||||||||||||
Sbjct  83671  CCGTAAGCATTGTAACTTATGCTAAGACCACAGTACGCTAGCCACTACACAGTCACATGC  83612

Query  121    TAGCGCCTGTGCATTAGTAGGCTTAAGGCCCCAGCGACTGCATTACAAGTAGCCATGCCT  180
              ||||||||||||||||||||||||||||||||||||||||||||||||||||||||||||
Sbjct  83611  TAGCGCCTGTGCATTAGTAGGCTTAAGGCCCCAGCGACTGCATTACAAGTAGCCATGCCT  83552

Query  181    ACATGCCACAGTCCGGTCGTACTTGCCGCTTACGAGTACAGTGACTTCATTACAGCCGAC  240
              ||||||||||||||||||||||||||||||||||||||||||||||||||||||||||||
Sbjct  83551  ACATGCCACAGTCCGGTCGTACTTGCCGCTTACGAGTACAGTGACTTCATTACAGCCGAC  83492

Query  241    GGCGAGTCTGTTGAATTTGACAGCTACATGGTACCCGAGTCTGACTTAACACAGGGTCAG  300
              ||||||||||||||||||||||||||||||||||||||||||||||||||||||||||||
Sbjct  83491  GGCGAGTCTGTTGAATTTGACAGCTACATGGTACCCGAGTCTGACTTAACACAGGGTCAG  83432

Query  301    CTGCGACTGCTTGAAGTCGACAACCGGGTAGTCGTGCCTGTAGACACGCACATTCGCTTC  360
              ||||||||||||||||||||||||||||||||||||||||||||||||||||||||||||
Sbjct  83431  CTGCGACTGCTTGAAGTCGACAACCGGGTAGTCGTGCCTGTAGACACGCACATTCGCTTC  83372

Query  361    ATTGTGACTGGCCAAGACGTCATTCATGACTGGGCTGTGCCTGCCTTAGGCTTAAAAATG  420
              ||||||||||||||||||||||||||||||||||||||||||||||||||||||||||||
Sbjct  83371  ATTGTGACTGGCCAAGACGTCATTCATGACTGGGCTGTGCCTGCCTTAGGCTTAAAAATG  83312

Query  421    GACGCCGTACCAGGTCGTTTAAACCAAACGTCAATTATTGTGCAACGACCAGGTGTCTAC  480
              ||||||||||||||||||||||||||||||||||||||||||||||||||||||||||||
Sbjct  83311  GACGCCGTACCAGGTCGTTTAAACCAAACGTCAATTATTGTGCAACGACCAGGTGTCTAC  83252

Query  481    TATGGGCAATGTAGTGAGATTTAACAGGTCCCCAGTCATTGACTGTGCATGAATGTAAAT  540
              ||||||||||||||||||||||||||||||||||||||||||||||||||||||||||||
Sbjct  83251  TATGGGCAATGTAGTGAGATTTAACAGGTCCCCAGTCATTGACTGTGCATGAATGTAAAT  83192

Query  541    TGCAAAAACGTTATGCAACACGCCGCGTGCCCCATGTGGGCTTACGTAAGGCCACCTACA  600
              ||||||||||||||||||||||||||||||||||||||||||||||||||||||||||||
Sbjct  83191  TGCAAAAACGTTATGCAACACGCCGCGTGCCCCATGTGGGCTTACGTAAGGCCACCTACA  83132

Query  601    TTAAATTTGCAGTCATGGGTTGCATTACACCGTGTAAAGACTAGTGAGTTGCTTGCAAAG  660
              ||||||||||||||||||||||||||||||||||||||||||||||||||||||||||||
Sbjct  83131  TTAAATTTGCAGTCATGGGTTGCATTACACCGTGTAAAGACTAGTGAGTTGCTTGCAAAG  83072

Query  661    CAGGCAGACTCATTGCGTAAAAACACGCCGCGTGCCCCATGCGTGCTTGTGTAAGGTCCA  720
              ||||||||||||||||||||||||||||||||||||||||||||||||||||||||||||
Sbjct  83071  CAGGCAGACTCATTGCGTAAAAACACGCCGCGTGCCCCATGCGTGCTTGTGTAAGGTCCA  83012

Query  721    TTACATA  727
              |||||||
Sbjct  83011  TTACATA  83005


 Score = 219 bits (118),  Expect = 9e-59
 Identities = 156/174 (90%), Gaps = 4/174 (2%)
 Strand=Plus/Minus

Query  557    AACACGCCGCGTGCCCCATGTGGGCTTACGTAAGG-CCACCTACATTAAATTTGCAGTCA  615
              |||||||||||||||||||| | ||||  |||||| |||  ||||| |||||||||||||
Sbjct  83050  AACACGCCGCGTGCCCCATGCGTGCTTGTGTAAGGTCCA-TTACATAAAATTTGCAGTCA  82992

Query  616    T-GGG-TTGCATTACACCGTGTAAAGACTAGTGAGTTGCTTGCAAAGCAGGCAGACTCAT  673
              | ||| ||||||||||||||||||||||||| |||||||||||||||||||| | |||||
Sbjct  82991  TTGGGCTTGCATTACACCGTGTAAAGACTAGCGAGTTGCTTGCAAAGCAGGCTGGCTCAT  82932

Query  674    TGCGTAAAAACACGCCGCGTGCCCCATGCGTGCTTGTGTAAGGTCCATTACATA  727
              ||| |||||||||||||||||||||||||||||||  |||| | ||||||||||
Sbjct  82931  TGCTTAAAAACACGCCGCGTGCCCCATGCGTGCTTACGTAATGCCCATTACATA  82878


Lambda      K        H
    1.33    0.621     1.12 

Gapped
Lambda      K        H
    1.28    0.460    0.850 

Effective search space used: 78238859


Query= 946151 fgenesh1_pm.4_#_8

Length=390
                                                                      Score     E
Sequences producing significant alignments:                          (Bits)  Value

  scaffold_1_mito                                                     721     0.0  


> scaffold_1_mito
Length=110369

 Score = 721 bits (390),  Expect = 0.0
 Identities = 390/390 (100%), Gaps = 0/390 (0%)
 Strand=Plus/Plus

Query  1      ATGACTATGGTGGTACtttttttGCTTGTGCCAGTACTAGTGGCTGTTTTGCTGTTGCTT  60
              ||||||||||||||||||||||||||||||||||||||||||||||||||||||||||||
Sbjct  49554  ATGACTATGGTGGTACTTTTTTTGCTTGTGCCAGTACTAGTGGCTGTTTTGCTGTTGCTT  49613

Query  61     AATGTGCTGCTTGCAGTACACCGCCCTGACACTGAAAAAGTGTCGGCTTACGAATGCGGC  120
              ||||||||||||||||||||||||||||||||||||||||||||||||||||||||||||
Sbjct  49614  AATGTGCTGCTTGCAGTACACCGCCCTGACACTGAAAAAGTGTCGGCTTACGAATGCGGC  49673

Query  121    TTCAATGTGCTGTCGCACCAAGTACGGGCACCCTTTGCTGTGCAGTACTACCTTGTGGGC  180
              ||||||||||||||||||||||||||||||||||||||||||||||||||||||||||||
Sbjct  49674  TTCAATGTGCTGTCGCACCAAGTACGGGCACCCTTTGCTGTGCAGTACTACCTTGTGGGC  49733

Query  181    ATTTTGTTCTTAATTTTTGACCTTGAAATTGCAGTGCTGTACCCATTGGCTGTAACACTG  240
              ||||||||||||||||||||||||||||||||||||||||||||||||||||||||||||
Sbjct  49734  ATTTTGTTCTTAATTTTTGACCTTGAAATTGCAGTGCTGTACCCATTGGCTGTAACACTG  49793

Query  241    TACGAGGTAACTGCCTACGGCTTTTACGTTGCAATGCTGTTTGTGGTCATGCTTACTGTA  300
              ||||||||||||||||||||||||||||||||||||||||||||||||||||||||||||
Sbjct  49794  TACGAGGTAACTGCCTACGGCTTTTACGTTGCAATGCTGTTTGTGGTCATGCTTACTGTA  49853

Query  301    GGCTTCGTGTACGAGTACGGCAAAGGTGCTTTAAAATTTACTGACCACCGGTCGTCAATT  360
              ||||||||||||||||||||||||||||||||||||||||||||||||||||||||||||
Sbjct  49854  GGCTTCGTGTACGAGTACGGCAAAGGTGCTTTAAAATTTACTGACCACCGGTCGTCAATT  49913

Query  361    AACCGTGCAATGCTGACTTCAATGCAGTAA  390
              ||||||||||||||||||||||||||||||
Sbjct  49914  AACCGTGCAATGCTGACTTCAATGCAGTAA  49943


Lambda      K        H
    1.33    0.621     1.12 

Gapped
Lambda      K        H
    1.28    0.460    0.850 

Effective search space used: 41161296


Query= 879519 e_gw1.4.461.1

Length=393
                                                                      Score     E
Sequences producing significant alignments:                          (Bits)  Value

  scaffold_1_mito                                                     726     0.0  


> scaffold_1_mito
Length=110369

 Score = 726 bits (393),  Expect = 0.0
 Identities = 393/393 (100%), Gaps = 0/393 (0%)
 Strand=Plus/Plus

Query  1      CTAATGACTATGGTGGTACtttttttGCTTGTGCCAGTACTAGTGGCTGTTTTGCTGTTG  60
              ||||||||||||||||||||||||||||||||||||||||||||||||||||||||||||
Sbjct  49551  CTAATGACTATGGTGGTACTTTTTTTGCTTGTGCCAGTACTAGTGGCTGTTTTGCTGTTG  49610

Query  61     CTTAATGTGCTGCTTGCAGTACACCGCCCTGACACTGAAAAAGTGTCGGCTTACGAATGC  120
              ||||||||||||||||||||||||||||||||||||||||||||||||||||||||||||
Sbjct  49611  CTTAATGTGCTGCTTGCAGTACACCGCCCTGACACTGAAAAAGTGTCGGCTTACGAATGC  49670

Query  121    GGCTTCAATGTGCTGTCGCACCAAGTACGGGCACCCTTTGCTGTGCAGTACTACCTTGTG  180
              ||||||||||||||||||||||||||||||||||||||||||||||||||||||||||||
Sbjct  49671  GGCTTCAATGTGCTGTCGCACCAAGTACGGGCACCCTTTGCTGTGCAGTACTACCTTGTG  49730

Query  181    GGCATTTTGTTCTTAATTTTTGACCTTGAAATTGCAGTGCTGTACCCATTGGCTGTAACA  240
              ||||||||||||||||||||||||||||||||||||||||||||||||||||||||||||
Sbjct  49731  GGCATTTTGTTCTTAATTTTTGACCTTGAAATTGCAGTGCTGTACCCATTGGCTGTAACA  49790

Query  241    CTGTACGAGGTAACTGCCTACGGCTTTTACGTTGCAATGCTGTTTGTGGTCATGCTTACT  300
              ||||||||||||||||||||||||||||||||||||||||||||||||||||||||||||
Sbjct  49791  CTGTACGAGGTAACTGCCTACGGCTTTTACGTTGCAATGCTGTTTGTGGTCATGCTTACT  49850

Query  301    GTAGGCTTCGTGTACGAGTACGGCAAAGGTGCTTTAAAATTTACTGACCACCGGTCGTCA  360
              ||||||||||||||||||||||||||||||||||||||||||||||||||||||||||||
Sbjct  49851  GTAGGCTTCGTGTACGAGTACGGCAAAGGTGCTTTAAAATTTACTGACCACCGGTCGTCA  49910

Query  361    ATTAACCGTGCAATGCTGACTTCAATGCAGTAA  393
              |||||||||||||||||||||||||||||||||
Sbjct  49911  ATTAACCGTGCAATGCTGACTTCAATGCAGTAA  49943


Lambda      K        H
    1.33    0.621     1.12 

Gapped
Lambda      K        H
    1.28    0.460    0.850 

Effective search space used: 41492352


Query= 900758 fgenesh1_kg.4_#_18_#_TRINITY_DN1225_c0_g1_i1

Length=1996
                                                                      Score     E
Sequences producing significant alignments:                          (Bits)  Value

  scaffold_1_mito                                                     3687    0.0  


> scaffold_1_mito
Length=110369

 Score = 3687 bits (1996),  Expect = 0.0
 Identities = 1996/1996 (100%), Gaps = 0/1996 (0%)
 Strand=Plus/Plus

Query  1      TGCTAGCAAATGCACTACAAGTCAATGTTGCATTACAAAACACCGTAGGCTATTGACGTG  60
              ||||||||||||||||||||||||||||||||||||||||||||||||||||||||||||
Sbjct  24600  TGCTAGCAAATGCACTACAAGTCAATGTTGCATTACAAAACACCGTAGGCTATTGACGTG  24659

Query  61     TTGCAAGCATTGCATGTACTAGCAAGCAAATGTTGCATTGCAATGTACCGTACAGTGTTG  120
              ||||||||||||||||||||||||||||||||||||||||||||||||||||||||||||
Sbjct  24660  TTGCAAGCATTGCATGTACTAGCAAGCAAATGTTGCATTGCAATGTACCGTACAGTGTTG  24719

Query  121    ACGTGTTGCAATGACTTGTAAGGCTATGACCTTGACTAGTAAGCACTGCAATGCACCGTA  180
              ||||||||||||||||||||||||||||||||||||||||||||||||||||||||||||
Sbjct  24720  ACGTGTTGCAATGACTTGTAAGGCTATGACCTTGACTAGTAAGCACTGCAATGCACCGTA  24779

Query  181    CAGTGTTGGCACGTTGCAATGACTTGCAAGCCTAGCATTTTTACTAGCAAGCTAATGTAG  240
              ||||||||||||||||||||||||||||||||||||||||||||||||||||||||||||
Sbjct  24780  CAGTGTTGGCACGTTGCAATGACTTGCAAGCCTAGCATTTTTACTAGCAAGCTAATGTAG  24839

Query  241    ttttttttAATGCACAAACGACTACGTCGTTTGTGCATTaaaaaaaaaGCACGGTGCTTA  300
              ||||||||||||||||||||||||||||||||||||||||||||||||||||||||||||
Sbjct  24840  TTTTTTTTAATGCACAAACGACTACGTCGTTTGTGCATTAAAAAAAAAGCACGGTGCTTA  24899

Query  301    GTCTGTACACTTGCAAtttttttAAAATGCTTACAACTTTAATTTTGACGCCTTTAGTAG  360
              ||||||||||||||||||||||||||||||||||||||||||||||||||||||||||||
Sbjct  24900  GTCTGTACACTTGCAATTTTTTTAAAATGCTTACAACTTTAATTTTGACGCCTTTAGTAG  24959

Query  361    GTGCACTGCAATTGACTACAATGCGTGACGACACTGCACAAGCTAAGTCACGTGTTAAGC  420
              ||||||||||||||||||||||||||||||||||||||||||||||||||||||||||||
Sbjct  24960  GTGCACTGCAATTGACTACAATGCGTGACGACACTGCACAAGCTAAGTCACGTGTTAAGC  25019

Query  421    AAGTAGCCTTACTAGCTAGTGTAGTCGCATTTGCAGTCGCCATGTTGCTGTGGCTGCAAT  480
              ||||||||||||||||||||||||||||||||||||||||||||||||||||||||||||
Sbjct  25020  AAGTAGCCTTACTAGCTAGTGTAGTCGCATTTGCAGTCGCCATGTTGCTGTGGCTGCAAT  25079

Query  481    TTGACTGTGCTAGCAACGAGCTGCAATTTACTGTTAAGTACACCACTGCACTGGCCTACT  540
              ||||||||||||||||||||||||||||||||||||||||||||||||||||||||||||
Sbjct  25080  TTGACTGTGCTAGCAACGAGCTGCAATTTACTGTTAAGTACACCACTGCACTGGCCTACT  25139

Query  541    TTAACATGCACTTGGGCATTGACGGCCTGTCGCTGTACTTTGTGCTGCTTACTGCCTTTA  600
              ||||||||||||||||||||||||||||||||||||||||||||||||||||||||||||
Sbjct  25140  TTAACATGCACTTGGGCATTGACGGCCTGTCGCTGTACTTTGTGCTGCTTACTGCCTTTA  25199

Query  601    CAATGCCAATTTGCATTTTGGCAAGCTGGGCTAATGTACAGCACAGCATTAAGTCGTACA  660
              ||||||||||||||||||||||||||||||||||||||||||||||||||||||||||||
Sbjct  25200  CAATGCCAATTTGCATTTTGGCAAGCTGGGCTAATGTACAGCACAGCATTAAGTCGTACA  25259

Query  661    TGGTGGCCTTGCTTGTGCTGCAAAGCCTTTTAACTACAGCGTTCGTTGTGCAAGACTTGC  720
              ||||||||||||||||||||||||||||||||||||||||||||||||||||||||||||
Sbjct  25260  TGGTGGCCTTGCTTGTGCTGCAAAGCCTTTTAACTACAGCGTTCGTTGTGCAAGACTTGC  25319

Query  721    TACttttttacgttttttttGAAGCCGTACTAGTGCCGCTTTTTGTGCTAGTAGGTGTGT  780
              ||||||||||||||||||||||||||||||||||||||||||||||||||||||||||||
Sbjct  25320  TACTTTTTTACGTTTTTTTTGAAGCCGTACTAGTGCCGCTTTTTGTGCTAGTAGGTGTGT  25379

Query  781    GGGGTGCAAGTGCAGACCGGGTACGGGCAGCATTTTTGCTGTTCATGTACACGCTACTAG  840
              ||||||||||||||||||||||||||||||||||||||||||||||||||||||||||||
Sbjct  25380  GGGGTGCAAGTGCAGACCGGGTACGGGCAGCATTTTTGCTGTTCATGTACACGCTACTAG  25439

Query  841    GCTCACTGTTCATGTTGCTAGCCTTCATTGTAATTGCAGCCACAACAGGTACCACCGACT  900
              ||||||||||||||||||||||||||||||||||||||||||||||||||||||||||||
Sbjct  25440  GCTCACTGTTCATGTTGCTAGCCTTCATTGTAATTGCAGCCACAACAGGTACCACCGACT  25499

Query  901    TGCAAGTCCTGACTTTAGCAGGCGTAAATTTTGCAAAGCAGCAATGACTGTGACTTGCAA  960
              ||||||||||||||||||||||||||||||||||||||||||||||||||||||||||||
Sbjct  25500  TGCAAGTCCTGACTTTAGCAGGCGTAAATTTTGCAAAGCAGCAATGACTGTGACTTGCAA  25559

Query  961    TTTTTGCAAGCTTGGCTGTAAAAACGCCGCTGCTGCCAGTACACATTTGACTAAGTCGTG  1020
              ||||||||||||||||||||||||||||||||||||||||||||||||||||||||||||
Sbjct  25560  TTTTTGCAAGCTTGGCTGTAAAAACGCCGCTGCTGCCAGTACACATTTGACTAAGTCGTG  25619

Query  1021   CACACGTGCAAGCGTCGGTGGCCGTGTCAATGGTGCTTGCAGGCTTAGTGTTGAAGCTTG  1080
              ||||||||||||||||||||||||||||||||||||||||||||||||||||||||||||
Sbjct  25620  CACACGTGCAAGCGTCGGTGGCCGTGTCAATGGTGCTTGCAGGCTTAGTGTTGAAGCTTG  25679

Query  1081   CAACCTATGCCTATGTGCGCATTTTGCTGCCCTTGCTGCCTGAAGCCAGCAGCTACTTTG  1140
              ||||||||||||||||||||||||||||||||||||||||||||||||||||||||||||
Sbjct  25680  CAACCTATGCCTATGTGCGCATTTTGCTGCCCTTGCTGCCTGAAGCCAGCAGCTACTTTG  25739

Query  1141   CACCACTAGTACAAACTGTGTGTGTAGTCACCTTAATTTACAGTTCGCTTACTACATTAC  1200
              ||||||||||||||||||||||||||||||||||||||||||||||||||||||||||||
Sbjct  25740  CACCACTAGTACAAACTGTGTGTGTAGTCACCTTAATTTACAGTTCGCTTACTACATTAC  25799

Query  1201   GGCAAACTGACTTTAAAGTACTAGTGGCCTACTCGTCAGTAGCCCACATGTCGGTAGTCG  1260
              ||||||||||||||||||||||||||||||||||||||||||||||||||||||||||||
Sbjct  25800  GGCAAACTGACTTTAAAGTACTAGTGGCCTACTCGTCAGTAGCCCACATGTCGGTAGTCG  25859

Query  1261   TAATTGGCCTGTTTAGTAACACCTTGCAAGGCATTGAAGGTGCCTTACTGCTTAGCATTG  1320
              ||||||||||||||||||||||||||||||||||||||||||||||||||||||||||||
Sbjct  25860  TAATTGGCCTGTTTAGTAACACCTTGCAAGGCATTGAAGGTGCCTTACTGCTTAGCATTG  25919

Query  1321   CACACGGTGTAGTCAGCCCAGCCTTATTTTACTGCCTAGGCGGTGTGCTGTACGACCGGT  1380
              ||||||||||||||||||||||||||||||||||||||||||||||||||||||||||||
Sbjct  25920  CACACGGTGTAGTCAGCCCAGCCTTATTTTACTGCCTAGGCGGTGTGCTGTACGACCGGT  25979

Query  1381   ACCACACACGGCAGCTACGGTACTACCGTGGCTTAGTGCAGTACATGCCGGTGTTTGCAA  1440
              ||||||||||||||||||||||||||||||||||||||||||||||||||||||||||||
Sbjct  25980  ACCACACACGGCAGCTACGGTACTACCGTGGCTTAGTGCAGTACATGCCGGTGTTTGCAA  26039

Query  1441   TGCTGCTGTTCATGTTTGTGCTAGGCAACATGTCGACGCCTTTAACGTTAAATTGAATTG  1500
              ||||||||||||||||||||||||||||||||||||||||||||||||||||||||||||
Sbjct  26040  TGCTGCTGTTCATGTTTGTGCTAGGCAACATGTCGACGCCTTTAACGTTAAATTGAATTG  26099

Query  1501   GCGAGTTGCTTGCCTTACTAGGTGCAATGCAACGCAGTGCAGTAGTCGGTGTAGCCATGA  1560
              ||||||||||||||||||||||||||||||||||||||||||||||||||||||||||||
Sbjct  26100  GCGAGTTGCTTGCCTTACTAGGTGCAATGCAACGCAGTGCAGTAGTCGGTGTAGCCATGA  26159

Query  1561   GTACTGGCATTGTGCTGTCGGCATGCTACTCAATTTGGCTGTACGCCCGCATGACTGGTG  1620
              ||||||||||||||||||||||||||||||||||||||||||||||||||||||||||||
Sbjct  26160  GTACTGGCATTGTGCTGTCGGCATGCTACTCAATTTGGCTGTACGCCCGCATGACTGGTG  26219

Query  1621   GCACATGAAGCCCTTACTTAGGCTATGCTGTTGACGTTACACGGCGTGAAGTCATGGTGT  1680
              ||||||||||||||||||||||||||||||||||||||||||||||||||||||||||||
Sbjct  26220  GCACATGAAGCCCTTACTTAGGCTATGCTGTTGACGTTACACGGCGTGAAGTCATGGTGT  26279

Query  1681   TGCTGCCTTTGCTTGCAGCAATGTTCATTTTTGGTGTGTGCCCTAACGTAATTTTGACTG  1740
              ||||||||||||||||||||||||||||||||||||||||||||||||||||||||||||
Sbjct  26280  TGCTGCCTTTGCTTGCAGCAATGTTCATTTTTGGTGTGTGCCCTAACGTAATTTTGACTG  26339

Query  1741   ACTTGCACTATAGCGTTACAGCATTGCTAAACACCTAACCCCTTACTTGCATTTTGCAAA  1800
              ||||||||||||||||||||||||||||||||||||||||||||||||||||||||||||
Sbjct  26340  ACTTGCACTATAGCGTTACAGCATTGCTAAACACCTAACCCCTTACTTGCATTTTGCAAA  26399

Query  1801   GCCTAACTTTACGTGGTTCACAAATGCTGCAAATTGCTTGCATGCTAATTTTTGCAAGGT  1860
              ||||||||||||||||||||||||||||||||||||||||||||||||||||||||||||
Sbjct  26400  GCCTAACTTTACGTGGTTCACAAATGCTGCAAATTGCTTGCATGCTAATTTTTGCAAGGT  26459

Query  1861   CTAACTTCACGTGGTTCACAAAAGCAACAAATTTGTACTAGTGGCATTTTTGCTGTGGCT  1920
              ||||||||||||||||||||||||||||||||||||||||||||||||||||||||||||
Sbjct  26460  CTAACTTCACGTGGTTCACAAAAGCAACAAATTTGTACTAGTGGCATTTTTGCTGTGGCT  26519

Query  1921   TTAAGCACGACTAGCCTAACTGCACAGGGCTCACAAATGGGTCCCATTCTTAATTTGGGC  1980
              ||||||||||||||||||||||||||||||||||||||||||||||||||||||||||||
Sbjct  26520  TTAAGCACGACTAGCCTAACTGCACAGGGCTCACAAATGGGTCCCATTCTTAATTTGGGC  26579

Query  1981   CGCCATGCAATTTGTG  1996
              ||||||||||||||||
Sbjct  26580  CGCCATGCAATTTGTG  26595


 Score = 115 bits (62),  Expect = 3e-27
 Identities = 90/103 (87%), Gaps = 3/103 (3%)
 Strand=Plus/Plus

Query  3      CTAGCAAATGCACTACAAGTCAATGTTGCATTACAAAACACCGTAGGCTATTGACGTGTT  62
              ||||||||||||||||||||||||||||||||||||| |||| |||||||||| | ||||
Sbjct  24336  CTAGCAAATGCACTACAAGTCAATGTTGCATTACAAAGCACCATAGGCTATTGGCATGTT  24395

Query  63     GCAAGCATTGCA--TGTACTAGCAAGCAAATGTTGCATTGCAA  103
              ||||| | ||||  ||||||   |||| ||||||||||| |||
Sbjct  24396  GCAAGTAATGCAAGTGTACTTA-AAGCCAATGTTGCATTACAA  24437


Lambda      K        H
    1.33    0.621     1.12 

Gapped
Lambda      K        H
    1.28    0.460    0.850 

Effective search space used: 218161950


Query= 946149 fgenesh1_pm.4_#_6

Length=519
                                                                      Score     E
Sequences producing significant alignments:                          (Bits)  Value

  scaffold_1_mito                                                     848     0.0  


> scaffold_1_mito
Length=110369

 Score = 848 bits (459),  Expect = 0.0
 Identities = 459/459 (100%), Gaps = 0/459 (0%)
 Strand=Plus/Plus

Query  1      ATGGTAAAGGAACAAATTGGCAGCAGCAACGAACGCTACCTACCGTTCATTTACGGGCTG  60
              ||||||||||||||||||||||||||||||||||||||||||||||||||||||||||||
Sbjct  44858  ATGGTAAAGGAACAAATTGGCAGCAGCAACGAACGCTACCTACCGTTCATTTACGGGCTG  44917

Query  61     TTCATGTTCGTAATTTTGGCAAATTTGACCGGCAACGTACCGTACTCATTTACAATTAGT  120
              ||||||||||||||||||||||||||||||||||||||||||||||||||||||||||||
Sbjct  44918  TTCATGTTCGTAATTTTGGCAAATTTGACCGGCAACGTACCGTACTCATTTACAATTAGT  44977

Query  121    ACAAGTGCTGTGCTGTCAATTGGCTTAAGTTTTGCAAttttttttGGTGTAACAGTCCTA  180
              ||||||||||||||||||||||||||||||||||||||||||||||||||||||||||||
Sbjct  44978  ACAAGTGCTGTGCTGTCAATTGGCTTAAGTTTTGCAATTTTTTTTGGTGTAACAGTCCTA  45037

Query  181    GGACTACACAAGCATGGGGTACAtttttttgcattttttGTGCCTGCAGGTACACCGGTA  240
              ||||||||||||||||||||||||||||||||||||||||||||||||||||||||||||
Sbjct  45038  GGACTACACAAGCATGGGGTACATTTTTTTGCATTTTTTGTGCCTGCAGGTACACCGGTA  45097

Query  241    GCTATGGTGCCCTTGCTAGTACTAATTGAGCTAATTTCGTACCTTGCACGTGCCTTTAGC  300
              ||||||||||||||||||||||||||||||||||||||||||||||||||||||||||||
Sbjct  45098  GCTATGGTGCCCTTGCTAGTACTAATTGAGCTAATTTCGTACCTTGCACGTGCCTTTAGC  45157

Query  301    TTAGGCGTACGGCTTTTTGCTAACATGGTGGCAGGTCACACACTACTAAAAATTTTAAGT  360
              ||||||||||||||||||||||||||||||||||||||||||||||||||||||||||||
Sbjct  45158  TTAGGCGTACGGCTTTTTGCTAACATGGTGGCAGGTCACACACTACTAAAAATTTTAAGT  45217

Query  361    GGCATGCTGTGGCCAATTTTAACGTCAGGTGTCGTTATGTTTGTGGTAGCCTTAGTGCCA  420
              ||||||||||||||||||||||||||||||||||||||||||||||||||||||||||||
Sbjct  45218  GGCATGCTGTGGCCAATTTTAACGTCAGGTGTCGTTATGTTTGTGGTAGCCTTAGTGCCA  45277

Query  421    ATGGCAAtttttttGGCCCTAGTAGGCCTTGAAATTGCA  459
              |||||||||||||||||||||||||||||||||||||||
Sbjct  45278  ATGGCAATTTTTTTGGCCCTAGTAGGCCTTGAAATTGCA  45316


 Score = 111 bits (60),  Expect = 1e-26
 Identities = 60/60 (100%), Gaps = 0/60 (0%)
 Strand=Plus/Plus

Query  460    TCTTTGGCCCAAATTTGCTATGCAATGCACCATGGGAAGTTGCAGTCAACAAACACGTAG  519
              ||||||||||||||||||||||||||||||||||||||||||||||||||||||||||||
Sbjct  45531  TCTTTGGCCCAAATTTGCTATGCAATGCACCATGGGAAGTTGCAGTCAACAAACACGTAG  45590


Lambda      K        H
    1.33    0.621     1.12 

Gapped
Lambda      K        H
    1.28    0.460    0.850 

Effective search space used: 55396704


Query= 900814 fgenesh1_kg.4_#_74_#_TRINITY_DN274_c1_g1_i1

Length=422
                                                                      Score     E
Sequences producing significant alignments:                          (Bits)  Value

  scaffold_1_mito                                                     780     0.0  


> scaffold_1_mito
Length=110369

 Score = 780 bits (422),  Expect = 0.0
 Identities = 422/422 (100%), Gaps = 0/422 (0%)
 Strand=Plus/Minus

Query  1       TTTATGCTAGGTCATAGGACAGGTCATAGGTGTAGTGTGGCCTGCTTTGCAGTCATGTGC  60
               ||||||||||||||||||||||||||||||||||||||||||||||||||||||||||||
Sbjct  108665  TTTATGCTAGGTCATAGGACAGGTCATAGGTGTAGTGTGGCCTGCTTTGCAGTCATGTGC  108606

Query  61      TGCAATGAACGTGGCTGCACATTAAAATTTTAAAAATGCTTCAAGCTGCTAAGTACATTG  120
               ||||||||||||||||||||||||||||||||||||||||||||||||||||||||||||
Sbjct  108605  TGCAATGAACGTGGCTGCACATTAAAATTTTAAAAATGCTTCAAGCTGCTAAGTACATTG  108546

Query  121     GTTCAGGACTTGCCACAATTGGACTTGCGGGTGCCGGTGTGGGTATTGGTGTGGTGTTTG  180
               ||||||||||||||||||||||||||||||||||||||||||||||||||||||||||||
Sbjct  108545  GTTCAGGACTTGCCACAATTGGACTTGCGGGTGCCGGTGTGGGTATTGGTGTGGTGTTTG  108486

Query  181     CTGGCCTAATTACAGGTGTGTCACGTAACCCTGCAATGCGAGCCCAATTATTTAGCTACG  240
               ||||||||||||||||||||||||||||||||||||||||||||||||||||||||||||
Sbjct  108485  CTGGCCTAATTACAGGTGTGTCACGTAACCCTGCAATGCGAGCCCAATTATTTAGCTACG  108426

Query  241     CAATTCTAGGTTTTGCCCTGTCAGAAGCTACAGGCCTTTTTGCACTTATGATGTCGTTCT  300
               ||||||||||||||||||||||||||||||||||||||||||||||||||||||||||||
Sbjct  108425  CAATTCTAGGTTTTGCCCTGTCAGAAGCTACAGGCCTTTTTGCACTTATGATGTCGTTCT  108366

Query  301     TGCTACTTTACAGCTAGTAACCCCTTGCATGATAACTCCAGGCGGCGTGTAATTGCAAGT  360
               ||||||||||||||||||||||||||||||||||||||||||||||||||||||||||||
Sbjct  108365  TGCTACTTTACAGCTAGTAACCCCTTGCATGATAACTCCAGGCGGCGTGTAATTGCAAGT  108306

Query  361     TTTGCAACTTGTGTATTTTTGTGCTGCATGTAATGTAGGCTAACTCTAGGCGGCGTGTAA  420
               ||||||||||||||||||||||||||||||||||||||||||||||||||||||||||||
Sbjct  108305  TTTGCAACTTGTGTATTTTTGTGCTGCATGTAATGTAGGCTAACTCTAGGCGGCGTGTAA  108246

Query  421     TT  422
               ||
Sbjct  108245  TT  108244


 Score = 108 bits (58),  Expect = 1e-25
 Identities = 79/89 (89%), Gaps = 1/89 (1%)
 Strand=Plus/Minus

Query  333     TAACTCCAGGCGGCGTGTAATTGCAAGTTTTGCAACTTGTGTATTTTTGTGCTGCATGTA  392
               |||||| ||||||||||||||||||||||||||||||||||  |||||||| |||| || 
Sbjct  108265  TAACTCTAGGCGGCGTGTAATTGCAAGTTTTGCAACTTGTGC-TTTTTGTGTTGCACGTT  108207

Query  393     ATGTAGGCTAACTCTAGGCGGCGTGTAAT  421
               |||||||||||||| |   ||||||||||
Sbjct  108206  ATGTAGGCTAACTCCACAAGGCGTGTAAT  108178


 Score = 89.8 bits (48),  Expect = 4e-20
 Identities = 65/73 (89%), Gaps = 1/73 (1%)
 Strand=Plus/Minus

Query  333     TAACTCCAGGCGGCGTGTAATTGCAAGTTTTGCAACTTGTGTATTTTTGTGCTGCATGTA  392
               |||||  ||| |||||||||||||||||||||||||||||| ||||| ||||||||||  
Sbjct  107449  TAACTTTAGGTGGCGTGTAATTGCAAGTTTTGCAACTTGTGCATTTT-GTGCTGCATGCT  107391

Query  393     ATGTAGGCTAACT  405
               |||||| ||||||
Sbjct  107390  ATGTAGCCTAACT  107378


Lambda      K        H
    1.33    0.621     1.12 

Gapped
Lambda      K        H
    1.28    0.460    0.850 

Effective search space used: 44692560


Query= 900819 fgenesh1_kg.4_#_79_#_TRINITY_DN3144_c3_g9_i4

Length=4085
                                                                      Score     E
Sequences producing significant alignments:                          (Bits)  Value

  scaffold_1_mito                                                     2697    0.0  


> scaffold_1_mito
Length=110369

 Score = 2697 bits (1460),  Expect = 0.0
 Identities = 1479/1488 (99%), Gaps = 2/1488 (0%)
 Strand=Plus/Plus

Query  2477   CGGTGTCGGCATTAATTCATGCGGCAACGCTAGTTACGGCAGGCGTGTACCTGCTGCTGC  2536
              ||||||||||||||||||||||||||||||||||||||||||||||||||||||||||||
Sbjct  15135  CGGTGTCGGCATTAATTCATGCGGCAACGCTAGTTACGGCAGGCGTGTACCTGCTGCTGC  15194

Query  2537   GGTCAAGCCCAATGCTTGAGTACGCACCTACTGCATTGCTTGCTGTGACTTGAGTCGGTG  2596
              ||||||||||||||||||||||||||||||||||||||||||||||||||||||||||||
Sbjct  15195  GGTCAAGCCCAATGCTTGAGTACGCACCTACTGCATTGCTTGCTGTGACTTGAGTCGGTG  15254

Query  2597   CAGTCACAGCATTTTTTGCTGCAACTACAGGCCTGCTGCAAAATGACGTCAAACGAGTCA  2656
              ||||||||||||||||||||||||||||||||||||||||||||||||||||||||||||
Sbjct  15255  CAGTCACAGCATTTTTTGCTGCAACTACAGGCCTGCTGCAAAATGACGTCAAACGAGTCA  15314

Query  2657   TTGCTTACAGTACGTGTTCACAAATGGGGTACCTGTTCATGGCCGTAGGCCTGTCGCAGT  2716
              ||||||||||||||||||||||||||||||||||||||||||||||||||||||||||||
Sbjct  15315  TTGCTTACAGTACGTGTTCACAAATGGGGTACCTGTTCATGGCCGTAGGCCTGTCGCAGT  15374

Query  2717   ATTCGGTGGCATTGTTCCATTTAGTAAACCACGCATTTTTTAAAGCTGTGCtttttttGG  2776
              ||||||||||||||||||||||||||||||||||||||||||||||||||||||||||||
Sbjct  15375  ATTCGGTGGCATTGTTCCATTTAGTAAACCACGCATTTTTTAAAGCTGTGCTTTTTTTGG  15434

Query  2777   CTGCAGGCGGTGTGCTGCATTCAATGGCCGACCAGCAAGACATGCGACGCTTAGGTGGCC  2836
              ||||||||||||||||||||||||||||||||||||||||||||||||||||||||||||
Sbjct  15435  CTGCAGGCGGTGTGCTGCATTCAATGGCCGACCAGCAAGACATGCGACGCTTAGGTGGCC  15494

Query  2837   TAGTCAATGTGCTGCCATTTACGTACGTGGTAATTTTGGCAGGCTCATTAAGCTTAATGG  2896
              ||||||||||||||||||||||||||||||||||||||||||||||||||||||||||||
Sbjct  15495  TAGTCAATGTGCTGCCATTTACGTACGTGGTAATTTTGGCAGGCTCATTAAGCTTAATGG  15554

Query  2897   CAGTGCCTTTTTTAACGGGCTTTTACAGTAAAGACCTAATTTTGGAGTCGGGCTATGCTG  2956
              ||||||||||||||||||||||||||||||||||||||||||||||||||||||||||||
Sbjct  15555  CAGTGCCTTTTTTAACGGGCTTTTACAGTAAAGACCTAATTTTGGAGTCGGGCTATGCTG  15614

Query  2957   CCTTCAGCTTAAGCGGGCATGTAGTGTACTGACTGGGTACTGTAACGGCCATGCTTACTA  3016
              ||||||||||||||||||||||||||||||||||||||||||||||||||||||||||||
Sbjct  15615  CCTTCAGCTTAAGCGGGCATGTAGTGTACTGACTGGGTACTGTAACGGCCATGCTTACTA  15674

Query  3017   GCTTTTACTCATTGCGCCTAATTAGCATGACCTTTTTAACGACGCCTAATGCAGCAAAAG  3076
              ||||||||||||||||||||||||||||||||||||||||||||||||||||||||||||
Sbjct  15675  GCTTTTACTCATTGCGCCTAATTAGCATGACCTTTTTAACGACGCCTAATGCAGCAAAAG  15734

Query  3077   TGCAGTACGAACATGCACATGAACAGGACCTGCTTACTGTGCTGCCACTACTGCTGCTGG  3136
              ||||||||||||||||||||||||||||||||||||||||||||||||||||||||||||
Sbjct  15735  TGCAGTACGAACATGCACATGAACAGGACCTGCTTACTGTGCTGCCACTACTGCTGCTGG  15794

Query  3137   CCTTGCTGTCAAttttttttGGCTACGTAGCCAAAGACGCCTTTGTAGGCATGGGCAGCG  3196
              ||||||||||||||||||||||||||||||||||||||||||||||||||||||||||||
Sbjct  15795  CCTTGCTGTCAATTTTTTTTGGCTACGTAGCCAAAGACGCCTTTGTAGGCATGGGCAGCG  15854

Query  3197   ACATGCTTGCAAGCAGCCTAGTAATGCTGCCTGGCAACGTCACATTAGTCGAAGCCGAGT  3256
              ||||||||||||||||||||||||||||||||||||||||||||||||||||||||||||
Sbjct  15855  ACATGCTTGCAAGCAGCCTAGTAATGCTGCCTGGCAACGTCACATTAGTCGAAGCCGAGT  15914

Query  3257   TTGCATTGCCTACTGCCATTAAGCTACTGCCTGCAATTGGCACAGCCTGTGCTGCAGCAC  3316
              ||||||||||||||||||||||||||||||||||||||||||||||||||||||||||||
Sbjct  15915  TTGCATTGCCTACTGCCATTAAGCTACTGCCTGCAATTGGCACAGCCTGTGCTGCAGCAC  15974

Query  3317   TTGCATTGCTGCTGTACCACCAGCAAGCTAGCCTTACTGTACTAATGACGCAGCATTGAC  3376
              ||||||||||||||||||||||||||||||||||||||||||||||||||||||||||||
Sbjct  15975  TTGCATTGCTGCTGTACCACCAGCAAGCTAGCCTTACTGTACTAATGACGCAGCATTGAC  16034

Query  3377   TGGGTCAGCACATTTACGCATTTTTAAACAGCAAGTGGTACATTGACGCAGTCTACAATA  3436
              ||||||||||||||||||||||||||||||||||||||||||||||||||||||||||||
Sbjct  16035  TGGGTCAGCACATTTACGCATTTTTAAACAGCAAGTGGTACATTGACGCAGTCTACAATA  16094

Query  3437   AGCTGCTAATTACAAAAGCCTTGCAGCTTGGCCTAGTCACTGCAAACGTACTAGACCGGG  3496
              ||||||||||||||||||||||||||||||||||||||||||||||||||||||||||||
Sbjct  16095  AGCTGCTAATTACAAAAGCCTTGCAGCTTGGCCTAGTCACTGCAAACGTACTAGACCGGG  16154

Query  3497   GTGCCATTGAACTTTTAGGCCCTGCTGGTGTTACACATAGCTTGCACAATGCTAGCGCTA  3556
              ||||||||||||||||||||||||||||||||||||||||||||||||||||||||||||
Sbjct  16155  GTGCCATTGAACTTTTAGGCCCTGCTGGTGTTACACATAGCTTGCACAATGCTAGCGCTA  16214

Query  3557   GCCTTGCAAAATTAGACACGGGTGTAGTCACACACTATGCACTGTACATGACCATTGGGC  3616
              ||||||||||||||||||||||||||||||||||||||||||||||||||||||||||||
Sbjct  16215  GCCTTGCAAAATTAGACACGGGTGTAGTCACACACTATGCACTGTACATGACCATTGGGC  16274

Query  3617   TAGCCTTGCTAGTGCTGTTGCTGTTCATGCCTGTGTTTAGTGGTGTTGCAATGCATGACA  3676
              ||||||||||||||||||||||||||||||||||||||||||||||||||||||||||||
Sbjct  16275  TAGCCTTGCTAGTGCTGTTGCTGTTCATGCCTGTGTTTAGTGGTGTTGCAATGCATGACA  16334

Query  3677   GCACGTGACTATTTTACGTGCCTGCAGCCTTAATTGCTGTGGTGTACTGTCATAGCATTA  3736
              ||||||||||||||||||||||||||||||||||||||||||||||||||||||||||||
Sbjct  16335  GCACGTGACTATTTTACGTGCCTGCAGCCTTAATTGCTGTGGTGTACTGTCATAGCATTA  16394

Query  3737   GCACAGCACAGCAAAGCTAATTAGTGCCGTGTAATTACTGGCACTTACTAGCTGCATGCA  3796
              ||||||||||||||||||||||||||||||||||||||||||||||||||||||||||||
Sbjct  16395  GCACAGCACAGCAAAGCTAATTAGTGCCGTGTAATTACTGGCACTTACTAGCTGCATGCA  16454

Query  3797   ACCTAACTGTACTGCCCTGGGGCCAGGTGTTTAAAATGCCATTTTGCAGTCAAATGCCTA  3856
              ||||||||||||||||||||||||||||||||||||||||||||||||||||||||||||
Sbjct  16455  ACCTAACTGTACTGCCCTGGGGCCAGGTGTTTAAAATGCCATTTTGCAGTCAAATGCCTA  16514

Query  3857   AGTCAGGCAAGCTACACGCAACGTAACTGTACACGGTGTAAAAGGCACTACTTTGCAGTC  3916
              |||||||||||||||||||||||||||||||||||||||||| |  || |||||||||| 
Sbjct  16515  AGTCAGGCAAGCTACACGCAACGTAACTGTACACGGTGTAAA-GT-ACGACTTTGCAGTA  16572

Query  3917   AAATGCCTAAGTCAGTCATGCAACATGCAACGTAACTGTACACGGTGT  3964
              |||||| |||| |||||| || ||||||||||||||||||||||||||
Sbjct  16573  AAATGCATAAGCCAGTCAGGCTACATGCAACGTAACTGTACACGGTGT  16620


 Score = 2255 bits (1221),  Expect = 0.0
 Identities = 1262/1281 (99%), Gaps = 6/1281 (0%)
 Strand=Plus/Plus

Query  892    CTAACTTTACATGAGCTTGTGTGACCCGTACCTA--GTAAAGTCCAACTAGCTT-TAATT  948
              ||||||||||||||||||||||||||  ||   |  || ||||   ||| ||||  ||||
Sbjct  11127  CTAACTTTACATGAGCTTGTGTGACC--TATTAAGGGTTAAGTTGTACTTGCTTCAAATT  11184

Query  949    AGGCATGGTGCTAACTTCACATGAGCTAGTACGACCGGTCCCTAATTAAGGCATGCAAGG  1008
              | | |   ||||||||||||||||||||||||||||||||||||||||||||||||||||
Sbjct  11185  A-GTACCATGCTAACTTCACATGAGCTAGTACGACCGGTCCCTAATTAAGGCATGCAAGG  11243

Query  1009   CTCAAGTGTGTACCATGCCAACTTCACATGAGCTTGTGTAACCCGTACCTTGTAAAAGTC  1068
              ||||||||||||||||||||||||||||||||||||||||||||||||||||||||||||
Sbjct  11244  CTCAAGTGTGTACCATGCCAACTTCACATGAGCTTGTGTAACCCGTACCTTGTAAAAGTC  11303

Query  1069   GACTTACTTAAATTAGGCACCTTGCTAACTCTACTGCCCCAAGGGGCCGAGGTAGTGTGA  1128
              ||||||||||||||||||||||||||||||||||||||||||||||||||||||||||||
Sbjct  11304  GACTTACTTAAATTAGGCACCTTGCTAACTCTACTGCCCCAAGGGGCCGAGGTAGTGTGA  11363

Query  1129   CCGGTCCCTAATTAAGGCACACTAGCTTAAATTAGGCACTGTGGCAACTTCACATGAGCT  1188
              ||||||||||||||||||||||||||||||||||||||||||||||||||||||||||||
Sbjct  11364  CCGGTCCCTAATTAAGGCACACTAGCTTAAATTAGGCACTGTGGCAACTTCACATGAGCT  11423

Query  1189   TATGTGTGACAGGTGTGTGCAAAATGCAAAGTCATGCTTAGTACATGCAAGCTTTATGCT  1248
              ||||||||||||||||||||||||||||||||||||||||||||||||||||||||||||
Sbjct  11424  TATGTGTGACAGGTGTGTGCAAAATGCAAAGTCATGCTTAGTACATGCAAGCTTTATGCT  11483

Query  1249   AACTTGGCATGAGCTTAGTCTACACGTTTGCGCGTACTTACATGCTAGCTTCAAGCCTGT  1308
              ||||||||||||||||||||||||||||||||||||||||||||||||||||||||||||
Sbjct  11484  AACTTGGCATGAGCTTAGTCTACACGTTTGCGCGTACTTACATGCTAGCTTCAAGCCTGT  11543

Query  1309   GGCATTAGGTGTTTAGTCGTGGTCGTGTACTTAGGTACCCGGTTTTTTAATGACCAAAAC  1368
              ||||||||||||||||||||||||||||||||||||||||||||||||||||||||||||
Sbjct  11544  GGCATTAGGTGTTTAGTCGTGGTCGTGTACTTAGGTACCCGGTTTTTTAATGACCAAAAC  11603

Query  1369   ACTGCGTGTTTTGGTCATTAAAATGTAGTAAGCGTCAATGCTTGCAATGTAAAACGGTGC  1428
              ||||||||||||||||||||||||||||||||||||||||||||||||||||||||||||
Sbjct  11604  ACTGCGTGTTTTGGTCATTAAAATGTAGTAAGCGTCAATGCTTGCAATGTAAAACGGTGC  11663

Query  1429   AATTGCATGTGCATGCACACAAAATTTGCAAACACACCCGCAGGTACACTGCAATTAAAA  1488
              ||||||||||||||||||||||||||||||||||||||||||||||||||||||||||||
Sbjct  11664  AATTGCATGTGCATGCACACAAAATTTGCAAACACACCCGCAGGTACACTGCAATTAAAA  11723

Query  1489   ATGGCGCTTTCAATTGTACTGTTCGTACTAGCAGTGCTAGGCTTTGTACTAAACCGCAAA  1548
              ||||||||||||||||||||||||||||||||||||||||||||||||||||||||||||
Sbjct  11724  ATGGCGCTTTCAATTGTACTGTTCGTACTAGCAGTGCTAGGCTTTGTACTAAACCGCAAA  11783

Query  1549   AACCTAATTTTAATGCTAATTAGCATTGAAGTTATGTTGCTTGCTGTAACGCTGCTAGTC  1608
              ||||||||||||||||||||||||||||||||||||||||||||||||||||||||||||
Sbjct  11784  AACCTAATTTTAATGCTAATTAGCATTGAAGTTATGTTGCTTGCTGTAACGCTGCTAGTC  11843

Query  1609   ATTGTGACGTCTTATGAGTTTAATGACGTAATTGGTCAAACTTACGCTGTGTTCATTATT  1668
              ||||||||||||||||||||||||||||||||||||||||||||||||||||||||||||
Sbjct  11844  ATTGTGACGTCTTATGAGTTTAATGACGTAATTGGTCAAACTTACGCTGTGTTCATTATT  11903

Query  1669   GCAATTGCAGGTGCCGAGTCAGCAATTGGACTTGGCATTTTAGTAGCCTTTTACCGTACA  1728
              ||||||||||||||||||||||||||||||||||||||||||||||||||||||||||||
Sbjct  11904  GCAATTGCAGGTGCCGAGTCAGCAATTGGACTTGGCATTTTAGTAGCCTTTTACCGTACA  11963

Query  1729   CGTGGGTCAATTACACTTTTTTAAATGTACCTATTGCTAATTGCTATGCCACTAGTAGGT  1788
              ||||||||||||||||||||||||||||||||||||||||||||||||||||||||||||
Sbjct  11964  CGTGGGTCAATTACACTTTTTTAAATGTACCTATTGCTAATTGCTATGCCACTAGTAGGT  12023

Query  1789   GCTACACTTGCAGGCCTACTAGGTCGTAAGCTTGGGACCCGGGGTGCACAAGTCGTGACT  1848
              ||||||||||||||||||||||||||||||||||||||||||||||||||||||||||||
Sbjct  12024  GCTACACTTGCAGGCCTACTAGGTCGTAAGCTTGGGACCCGGGGTGCACAAGTCGTGACT  12083

Query  1849   TGCATTTGCATGGCTACGACTGCCGTGCTGGCAGCGACTGCCTTTTACGAAGTCGCATTG  1908
              ||||||||||||||||||||||||||||||||||||||||||||||||||||||||||||
Sbjct  12084  TGCATTTGCATGGCTACGACTGCCGTGCTGGCAGCGACTGCCTTTTACGAAGTCGCATTG  12143

Query  1909   TGCAATTGCAGCGTCACAGTACAGCTGCCTAGCTGACTTGACACGGGCTTACTTGCCGTT  1968
              ||||||||||||||||||||||||||||||||||||||||||||||||||||||||||||
Sbjct  12144  TGCAATTGCAGCGTCACAGTACAGCTGCCTAGCTGACTTGACACGGGCTTACTTGCCGTT  12203

Query  1969   AATTGAAGCCTAGCGTTCGACTCATTAAGCGTGGCAATGCTGCTAGCAGTCACATTTGTG  2028
              ||||||||||||||||||||||||||||||||||||||||||||||||||||||||||||
Sbjct  12204  AATTGAAGCCTAGCGTTCGACTCATTAAGCGTGGCAATGCTGCTAGCAGTCACATTTGTG  12263

Query  2029   TCGACTATGGTGCACGTGTACTCATGCAGCTACATGGCTGCTGACCCACACCAGCAACGC  2088
              ||||||||||||||||||||||||||||||||||||||||||||||||||||||||||||
Sbjct  12264  TCGACTATGGTGCACGTGTACTCATGCAGCTACATGGCTGCTGACCCACACCAGCAACGC  12323

Query  2089   TTCATGGCCTACCTGTCTATGTTTACATTTTTTATGCTAGTCTTAATTGCAGGCAACAAC  2148
              ||||||||||||||||||||||||||||||||||||||||||||||||||||||||||||
Sbjct  12324  TTCATGGCCTACCTGTCTATGTTTACATTTTTTATGCTAGTCTTAATTGCAGGCAACAAC  12383

Query  2149   TACATTGTGCTATTTTTAGGT  2169
              |||||||||||||||||||||
Sbjct  12384  TACATTGTGCTATTTTTAGGT  12404


 Score = 1801 bits (975),  Expect = 0.0
 Identities = 1027/1052 (98%), Gaps = 3/1052 (0%)
 Strand=Plus/Plus

Query  1      TTGGCAATAGCAGCAGGCGTAAATTTTAAGTAACTGCAACTTTGCAAATTGGCAGCATGT  60
              ||||||||||||||||||||||||||||||||||||||||||||||||||||||||||||
Sbjct  10103  TTGGCAATAGCAGCAGGCGTAAATTTTAAGTAACTGCAACTTTGCAAATTGGCAGCATGT  10162

Query  61     GCATTAAGTCATGGGGTACGTGGCGTTTAAACCACACGACTGTGACTTACGTTACATGCA  120
              ||||||||||||||||||||||||||||||||||||||||||||||||||||||||||||
Sbjct  10163  GCATTAAGTCATGGGGTACGTGGCGTTTAAACCACACGACTGTGACTTACGTTACATGCA  10222

Query  121    ATGTTAATTAATTTGCATGGGGCACGTGGCGTAAATTTAGCCCAATTGCGACTTGCAACA  180
              ||||||||||||||||||||||||||||||||||||||||||||||||||||||||||||
Sbjct  10223  ATGTTAATTAATTTGCATGGGGCACGTGGCGTAAATTTAGCCCAATTGCGACTTGCAACA  10282

Query  181    CCTAAGCCACAAGGCACTAGGTCATAGCCCTTAAACCCAATTTAAGCTTACGTAGCTTTA  240
              ||||||||||||||||||||||||||||||||||||||||||||||||||||||||||||
Sbjct  10283  CCTAAGCCACAAGGCACTAGGTCATAGCCCTTAAACCCAATTTAAGCTTACGTAGCTTTA  10342

Query  241    GCACTGTACACGGTTCACAGCAATTGCACAATTAGCCCGGGTACGACCAACATGACCCGT  300
              ||||||||||||||||||||||||||||||||||||||||||||||||||||||||||||
Sbjct  10343  GCACTGTACACGGTTCACAGCAATTGCACAATTAGCCCGGGTACGACCAACATGACCCGT  10402

Query  301    AAACTACACCGTATTGCCCATTGGCTAGTCTTAATGACTTCAAGCTTGCACCAAGCCAAC  360
              ||||||||||||||||||||||||||||||||||||||||||||||||||||||||||||
Sbjct  10403  AAACTACACCGTATTGCCCATTGGCTAGTCTTAATGACTTCAAGCTTGCACCAAGCCAAC  10462

Query  361    TTCTTCCCCAAAATTAGGTACCCTAATTTTGGGGACCACATGAGCTTGCATGAAGGGTTC  420
              ||||||||||||||||||||||||||||||||||||||||||||||||||||||||||||
Sbjct  10463  TTCTTCCCCAAAATTAGGTACCCTAATTTTGGGGACCACATGAGCTTGCATGAAGGGTTC  10522

Query  421    GTCATTAAGTCAAGCTAGCTTCAAGTTTGCACGTTGCTAACTTCACATGAGCTTGCATGA  480
              ||||||||||||||||||||||||||||||||||||||||||||||||||||||||||||
Sbjct  10523  GTCATTAAGTCAAGCTAGCTTCAAGTTTGCACGTTGCTAACTTCACATGAGCTTGCATGA  10582

Query  481    ACGGTACGTCATTAAGGCAGGCTAGCTTCAAATTTGCACATGCTAACTTCACATGAGCTT  540
              ||||||||||||||||||||||||||||||||||||||||||||||||||||||||||||
Sbjct  10583  ACGGTACGTCATTAAGGCAGGCTAGCTTCAAATTTGCACATGCTAACTTCACATGAGCTT  10642

Query  541    ATGCGACCTGCTAATAGTTAAGTAATGCTTGCTTCAAAATTTGCACATGCTAACTTCACA  600
              ||||||||||||||||||||||||||||||||||||||||||||||||||||||||||||
Sbjct  10643  ATGCGACCTGCTAATAGTTAAGTAATGCTTGCTTCAAAATTTGCACATGCTAACTTCACA  10702

Query  601    TGAGCTAGTGCGACCGGTACCTAATTAAGGCATGTAAGGCACCAATTTGTACCATACTAA  660
              ||||||||||||||||||||||||||||||||||||||||||||||||||||||||||||
Sbjct  10703  TGAGCTAGTGCGACCGGTACCTAATTAAGGCATGTAAGGCACCAATTTGTACCATACTAA  10762

Query  661    CTTCTTCCCCAAAATTAGGTACCCTAATTTTGGGGACCACATGAGCTATTGTGACCGGTA  720
              ||||||||||||||||||||||||||||||||||||||||||||||||||||||||||||
Sbjct  10763  CTTCTTCCCCAAAATTAGGTACCCTAATTTTGGGGACCACATGAGCTATTGTGACCGGTA  10822

Query  721    CCTAATTAATGCCGACTTGCTTAAATTAGGCACGGTGTTAACTTTACATGAGCTAGTGTG  780
              ||||||||||||||||||||||||||||||||||||||||||||||||||||||||||||
Sbjct  10823  CCTAATTAATGCCGACTTGCTTAAATTAGGCACGGTGTTAACTTTACATGAGCTAGTGTG  10882

Query  781    ACCTGTAAATTGCAAAGCCATGCTTGCTTCAAATGAGTACCATGCTAACTTTACATAAGG  840
              ||||||||||||||||||||||||||||||||||||||||||||||||||||||||||||
Sbjct  10883  ACCTGTAAATTGCAAAGCCATGCTTGCTTCAAATGAGTACCATGCTAACTTTACATAAGG  10942

Query  841    TAGTGTGGCCGGTGCTTAATTAAGGTATGCAAGCCTCAAATTTGCAGCATGCTAACTTTA  900
              ||||||||||||||||||||||||||||||||||||||||||||||||||||||||||||
Sbjct  10943  TAGTGTGGCCGGTGCTTAATTAAGGTATGCAAGCCTCAAATTTGCAGCATGCTAACTTTA  11002

Query  901    CATGAGCTTGTGTGACCCGTACCTAGTAAAGTCCAACTAGCTTTAATTAGGCATGGTGCT  960
              ||||||||||||||||||||||||||||||||||||||||||||||||||||||||||||
Sbjct  11003  CATGAGCTTGTGTGACCCGTACCTAGTAAAGTCCAACTAGCTTTAATTAGGCATGGTGCT  11062

Query  961    AACTTCACATGAGCTAGTACGACCGGTCCCTAATTAAGGCATGCAAGGCTCAAGTGTGTA  1020
              ||||  ||||||||||||| ||| |||    | | ||||||    | ||| |||  ||||
Sbjct  11063  AACTCTACATGAGCTAGTATGACAGGTTTGCAGTAAAGGCAAATTA-GCTTAAGCATGTA  11121

Query  1021   CCATGCC-AACTTCACATGAGCTTGTGTAACC  1051
                 | || ||||| |||||||||||||| |||
Sbjct  11122  AG-TACCTAACTTTACATGAGCTTGTGTGACC  11152


 Score = 569 bits (308),  Expect = 1e-163
 Identities = 308/308 (100%), Gaps = 0/308 (0%)
 Strand=Plus/Plus

Query  2170   TGGGAAGGTATTGGGGTGTCTTCGTACTTGCTAATTAATTTTTGGTTCACACGTGTGCAA  2229
              ||||||||||||||||||||||||||||||||||||||||||||||||||||||||||||
Sbjct  13692  TGGGAAGGTATTGGGGTGTCTTCGTACTTGCTAATTAATTTTTGGTTCACACGTGTGCAA  13751

Query  2230   GCCAACAAGGCAGCCATTCAGGCAATGACCGTAAACCGGGTCGGCGACATGTTTTTAAGC  2289
              ||||||||||||||||||||||||||||||||||||||||||||||||||||||||||||
Sbjct  13752  GCCAACAAGGCAGCCATTCAGGCAATGACCGTAAACCGGGTCGGCGACATGTTTTTAAGC  13811

Query  2290   ATTGCCTTTTTTGTGCTGTTTTGAACCACAGGCAGCCTAGACTATGCAAGCGTGTTTGCA  2349
              ||||||||||||||||||||||||||||||||||||||||||||||||||||||||||||
Sbjct  13812  ATTGCCTTTTTTGTGCTGTTTTGAACCACAGGCAGCCTAGACTATGCAAGCGTGTTTGCA  13871

Query  2350   GTAGCCCCGTTCATTAATGAAGCAGCATTGACTGCAGTCTGCTTGCTGTTTTTAATGGCT  2409
              ||||||||||||||||||||||||||||||||||||||||||||||||||||||||||||
Sbjct  13872  GTAGCCCCGTTCATTAATGAAGCAGCATTGACTGCAGTCTGCTTGCTGTTTTTAATGGCT  13931

Query  2410   GCAATGGGCAAGTCGGCCCAATTAGGGCTACACACATGACTACCTTCAGCTATGGAAGGC  2469
              ||||||||||||||||||||||||||||||||||||||||||||||||||||||||||||
Sbjct  13932  GCAATGGGCAAGTCGGCCCAATTAGGGCTACACACATGACTACCTTCAGCTATGGAAGGC  13991

Query  2470   TATTAAGC  2477
              ||||||||
Sbjct  13992  TATTAAGC  13999


 Score = 344 bits (186),  Expect = 8e-96
 Identities = 198/204 (97%), Gaps = 0/204 (0%)
 Strand=Plus/Plus

Query  3839   TTTGCAGTCAAATGCCTAAGTCAGGCAAGCTACACGCAACGTAACTGTACACGGTGTAAA  3898
              |||||||| |||||| |||| ||| || |||||| |||||||||||||||||||||||||
Sbjct  16564  TTTGCAGTAAAATGCATAAGCCAGTCAGGCTACATGCAACGTAACTGTACACGGTGTAAA  16623

Query  3899   AGGCACTACTTTGCAGTCAAATGCCTAAGTCAGTCATGCAACATGCAACGTAACTGTACA  3958
              ||||||||||||||||||||||||||||||||||||||||||||||||||||||||||||
Sbjct  16624  AGGCACTACTTTGCAGTCAAATGCCTAAGTCAGTCATGCAACATGCAACGTAACTGTACA  16683

Query  3959   CGGTGTTTAAAACACCACTTGCGGCAGTAAAATGTGCCAACATGTTGCCTAACTGTACTG  4018
              ||||||||||||||||||||||||||||||||||||||||||||||||||||||||||||
Sbjct  16684  CGGTGTTTAAAACACCACTTGCGGCAGTAAAATGTGCCAACATGTTGCCTAACTGTACTG  16743

Query  4019   CCCCACCAGGTGTAAAATGCGCCA  4042
              ||||||||||||||||||||||||
Sbjct  16744  CCCCACCAGGTGTAAAATGCGCCA  16767


 Score = 148 bits (80),  Expect = 7e-37
 Identities = 218/284 (77%), Gaps = 11/284 (4%)
 Strand=Plus/Plus

Query  647    TTGTACCATACTAACTTCTTCCCCAAAATTAGGTACCCTAATTTTGGGGACCACATGAGC  706
              ||| ||||  | ||||||||||||||||||||||||||||||||||||||||||||||||
Sbjct  10448  TTGCACCAAGCCAACTTCTTCCCCAAAATTAGGTACCCTAATTTTGGGGACCACATGAGC  10507

Query  707    TATTG-TGACCGGTACCTAATTAATGCCGA-CTTGCTTAAATTAGGCACGGTGTTAACTT  764
              | |   |||  ||| | | ||||| | | | || |||| || |  ||||| || ||||||
Sbjct  10508  T-TGCATGAAGGGTTCGTCATTAA-GTCAAGCTAGCTTCAAGTTTGCACGTTGCTAACTT  10565

Query  765    TACATGAGCTAGTGTGACCTGTAAATTGCAAAGCCATGCTTGCTTCAAATGAGTACCATG  824
               ||||||||| |  ||| | |||  |    ||| || ||| |||||||||  | | ||||
Sbjct  10566  CACATGAGCTTGCATGAACGGTACGTCATTAAGGCAGGCTAGCTTCAAATTTGCA-CATG  10624

Query  825    CTAACTTTACATAAGGTAGTGTGGCCGGTGCTTA-A-TTAAGGTATGCAAGCCTCAAA-T  881
              ||||||| |||| || |  || | ||  |||| | | |||||  ||||  || ||||| |
Sbjct  10625  CTAACTTCACATGAGCTTATGCGACC--TGCTAATAGTTAAGTAATGCTTGCTTCAAAAT  10682

Query  882    TTGCAGCATGCTAACTTTACATGAGCTTGTGTGACCCGTACCTA  925
              ||||| ||||||||||| ||||||||| ||| |||| |||||||
Sbjct  10683  TTGCA-CATGCTAACTTCACATGAGCTAGTGCGACCGGTACCTA  10725


 Score = 148 bits (80),  Expect = 7e-37
 Identities = 218/284 (77%), Gaps = 11/284 (4%)
 Strand=Plus/Plus

Query  346    TTGCACCAAGCCAACTTCTTCCCCAAAATTAGGTACCCTAATTTTGGGGACCACATGAGC  405
              ||| ||||  | ||||||||||||||||||||||||||||||||||||||||||||||||
Sbjct  10749  TTGTACCATACTAACTTCTTCCCCAAAATTAGGTACCCTAATTTTGGGGACCACATGAGC  10808

Query  406    T-TGCATGAAGGGTTCGTCATTAA-GTCAAGCTAGCTTCAAGTTTGCACGTTGCTAACTT  463
              | |   |||  ||| | | ||||| | | | || |||| || |  ||||| || ||||||
Sbjct  10809  TATTG-TGACCGGTACCTAATTAATGCCGA-CTTGCTTAAATTAGGCACGGTGTTAACTT  10866

Query  464    CACATGAGCTTGCATGAACGGTACGTCATTAAGGCAGGCTAGCTTCAAATTTGCA-CATG  522
               ||||||||| |  ||| | |||  |    ||| || ||| |||||||||  | | ||||
Sbjct  10867  TACATGAGCTAGTGTGACCTGTAAATTGCAAAGCCATGCTTGCTTCAAATGAGTACCATG  10926

Query  523    CTAACTTCACATGAGCTTATGCGACC--TGCTAATAGTTAAGTAATGCTTGCTTCAAAAT  580
              ||||||| |||| || |  || | ||  |||| | | |||||  ||||  || ||||| |
Sbjct  10927  CTAACTTTACATAAGGTAGTGTGGCCGGTGCTTA-A-TTAAGGTATGCAAGCCTCAAA-T  10983

Query  581    TTGCA-CATGCTAACTTCACATGAGCTAGTGCGACCGGTACCTA  623
              ||||| ||||||||||| ||||||||| ||| |||| |||||||
Sbjct  10984  TTGCAGCATGCTAACTTTACATGAGCTTGTGTGACCCGTACCTA  11027


 Score = 143 bits (77),  Expect = 3e-35
 Identities = 129/154 (84%), Gaps = 4/154 (3%)
 Strand=Plus/Plus

Query  464    CACATGAGCTTGCATGAACGGTACGTCATTAAGGCAGGCTAGCTTCAAATTTGCAC-ATG  522
              |||||||||||||||||| ||| |||||||||| || ||||||||||| |||||||  ||
Sbjct  10499  CACATGAGCTTGCATGAAGGGTTCGTCATTAAGTCAAGCTAGCTTCAAGTTTGCACGTTG  10558

Query  523    CTAACTTCACATGAGCTTATGCGACCTGCTA-ATAGTTAAGTAATGCTTGCTTCAAAATT  581
              ||||||||||||||||||    || | | ||  |  |||||  | ||| ||||| |||||
Sbjct  10559  CTAACTTCACATGAGCTTGCATGAAC-GGTACGTCATTAAGGCAGGCTAGCTTC-AAATT  10616

Query  582    TGCACATGCTAACTTCACATGAGCTAGTGCGACC  615
              |||||||||||||||||||||||||  |||||||
Sbjct  10617  TGCACATGCTAACTTCACATGAGCTTATGCGACC  10650


 Score = 143 bits (77),  Expect = 3e-35
 Identities = 129/154 (84%), Gaps = 4/154 (3%)
 Strand=Plus/Plus

Query  397    CACATGAGCTTGCATGAAGGGTTCGTCATTAAGTCAAGCTAGCTTCAAGTTTGCACGTTG  456
              |||||||||||||||||| ||| |||||||||| || ||||||||||| |||||||  ||
Sbjct  10566  CACATGAGCTTGCATGAACGGTACGTCATTAAGGCAGGCTAGCTTCAAATTTGCAC-ATG  10624

Query  457    CTAACTTCACATGAGCTTGCATGAAC-GGTACGTCATTAAGGCAGGCTAGCTTC-AAATT  514
              ||||||||||||||||||    || | | ||  |  |||||  | ||| ||||| |||||
Sbjct  10625  CTAACTTCACATGAGCTTATGCGACCTGCTA-ATAGTTAAGTAATGCTTGCTTCAAAATT  10683

Query  515    TGCACATGCTAACTTCACATGAGCTTATGCGACC  548
              |||||||||||||||||||||||||  |||||||
Sbjct  10684  TGCACATGCTAACTTCACATGAGCTAGTGCGACC  10717


 Score = 91.6 bits (49),  Expect = 1e-19
 Identities = 87/104 (84%), Gaps = 7/104 (7%)
 Strand=Plus/Plus

Query  3798   CCTAACTGTACTGCCCTGGGGCCAGGTGTTTAAAAT--GCCATTTTGCAGTCAAATGCCT  3855
              ||||||||||||||||     |||||||  ||||||  |||| |||| ||||||||| ||
Sbjct  16731  CCTAACTGTACTGCCC---CACCAGGTG--TAAAATGCGCCACTTTGTAGTCAAATGGCT  16785

Query  3856   AAGTCAGGCAAGCTACACGCAACGTAACTGTACACGGTGTAAAA  3899
              ||||||   | || ||||||||||||||||||||||||||||||
Sbjct  16786  AAGTCAATAATGCGACACGCAACGTAACTGTACACGGTGTAAAA  16829


 Score = 82.4 bits (44),  Expect = 7e-17
 Identities = 49/51 (96%), Gaps = 1/51 (2%)
 Strand=Plus/Plus

Query  658   TAACTTCTTCCCCAAAATTAGGTACCCTAATTTTGGGGA-CCACATGAGCT  707
             ||||||||||||||||||||||||||||||||||||||  |||||||||||
Sbjct  7916  TAACTTCTTCCCCAAAATTAGGTACCCTAATTTTGGGGGGCCACATGAGCT  7966


 Score = 82.4 bits (44),  Expect = 7e-17
 Identities = 51/54 (94%), Gaps = 2/54 (4%)
 Strand=Plus/Plus

Query  358   AACTTCTTCCCCAAAATTAGGTACCCTAATTTTGGGGA-CCACATGAGCTTGCA  410
             |||||||||||||||||||||||||||||||||||||  ||||||||||| |||
Sbjct  7917  AACTTCTTCCCCAAAATTAGGTACCCTAATTTTGGGGGGCCACATGAGCT-GCA  7969


 Score = 76.8 bits (41),  Expect = 3e-15
 Identities = 41/41 (100%), Gaps = 0/41 (0%)
 Strand=Plus/Plus

Query  1351   TTTTTTAATGACCAAAACACTGCGTGTTTTGGTCATTAAAA  1391
              |||||||||||||||||||||||||||||||||||||||||
Sbjct  69105  TTTTTTAATGACCAAAACACTGCGTGTTTTGGTCATTAAAA  69145


 Score = 76.8 bits (41),  Expect = 3e-15
 Identities = 41/41 (100%), Gaps = 0/41 (0%)
 Strand=Plus/Plus

Query  1351   TTTTTTAATGACCAAAACACTGCGTGTTTTGGTCATTAAAA  1391
              |||||||||||||||||||||||||||||||||||||||||
Sbjct  73191  TTTTTTAATGACCAAAACACTGCGTGTTTTGGTCATTAAAA  73231


 Score = 69.4 bits (37),  Expect = 6e-13
 Identities = 37/37 (100%), Gaps = 0/37 (0%)
 Strand=Plus/Plus

Query  4043   TTGCAACATGTAGCCTAACTGTACTGCCCCACCCCAC  4079
              |||||||||||||||||||||||||||||||||||||
Sbjct  16863  TTGCAACATGTAGCCTAACTGTACTGCCCCACCCCAC  16899


 Score = 67.6 bits (36),  Expect = 2e-12
 Identities = 36/36 (100%), Gaps = 0/36 (0%)
 Strand=Plus/Plus

Query  363    CTTCCCCAAAATTAGGTACCCTAATTTTGGGGACCA  398
              ||||||||||||||||||||||||||||||||||||
Sbjct  59723  CTTCCCCAAAATTAGGTACCCTAATTTTGGGGACCA  59758


 Score = 67.6 bits (36),  Expect = 2e-12
 Identities = 36/36 (100%), Gaps = 0/36 (0%)
 Strand=Plus/Plus

Query  664    CTTCCCCAAAATTAGGTACCCTAATTTTGGGGACCA  699
              ||||||||||||||||||||||||||||||||||||
Sbjct  59723  CTTCCCCAAAATTAGGTACCCTAATTTTGGGGACCA  59758


 Score = 60.2 bits (32),  Expect = 3e-10
 Identities = 35/36 (97%), Gaps = 1/36 (3%)
 Strand=Plus/Minus

Query  664    CTTCCCCAAAATTAGGTACCCTAATTTTGGGGACCA  699
              ||| ||||||||||||||||||||||||||||||||
Sbjct  24174  CTT-CCCAAAATTAGGTACCCTAATTTTGGGGACCA  24140


 Score = 60.2 bits (32),  Expect = 3e-10
 Identities = 35/36 (97%), Gaps = 1/36 (3%)
 Strand=Plus/Minus

Query  363    CTTCCCCAAAATTAGGTACCCTAATTTTGGGGACCA  398
              ||| ||||||||||||||||||||||||||||||||
Sbjct  24174  CTT-CCCAAAATTAGGTACCCTAATTTTGGGGACCA  24140


Lambda      K        H
    1.33    0.621     1.12 

Gapped
Lambda      K        H
    1.28    0.460    0.850 

Effective search space used: 448568685


Query= 946145 fgenesh1_pm.4_#_2

Length=1590
                                                                      Score     E
Sequences producing significant alignments:                          (Bits)  Value

  scaffold_1_mito                                                     723     0.0  


> scaffold_1_mito
Length=110369

 Score = 723 bits (391),  Expect = 0.0
 Identities = 391/391 (100%), Gaps = 0/391 (0%)
 Strand=Plus/Plus

Query  563    TCGGTGCAGTCACAGCATTTTTTGCTGCAACTACAGGCCTGCTGCAAAATGACGTCAAAC  622
              ||||||||||||||||||||||||||||||||||||||||||||||||||||||||||||
Sbjct  15249  TCGGTGCAGTCACAGCATTTTTTGCTGCAACTACAGGCCTGCTGCAAAATGACGTCAAAC  15308

Query  623    GAGTCATTGCTTACAGTACGTGTTCACAAATGGGGTACCTGTTCATGGCCGTAGGCCTGT  682
              ||||||||||||||||||||||||||||||||||||||||||||||||||||||||||||
Sbjct  15309  GAGTCATTGCTTACAGTACGTGTTCACAAATGGGGTACCTGTTCATGGCCGTAGGCCTGT  15368

Query  683    CGCAGTATTCGGTGGCATTGTTCCATTTAGTAAACCACGCATTTTTTAAAGCTGTGCttt  742
              ||||||||||||||||||||||||||||||||||||||||||||||||||||||||||||
Sbjct  15369  CGCAGTATTCGGTGGCATTGTTCCATTTAGTAAACCACGCATTTTTTAAAGCTGTGCTTT  15428

Query  743    ttttGGCTGCAGGCGGTGTGCTGCATTCAATGGCCGACCAGCAAGACATGCGACGCTTAG  802
              ||||||||||||||||||||||||||||||||||||||||||||||||||||||||||||
Sbjct  15429  TTTTGGCTGCAGGCGGTGTGCTGCATTCAATGGCCGACCAGCAAGACATGCGACGCTTAG  15488

Query  803    GTGGCCTAGTCAATGTGCTGCCATTTACGTACGTGGTAATTTTGGCAGGCTCATTAAGCT  862
              ||||||||||||||||||||||||||||||||||||||||||||||||||||||||||||
Sbjct  15489  GTGGCCTAGTCAATGTGCTGCCATTTACGTACGTGGTAATTTTGGCAGGCTCATTAAGCT  15548

Query  863    TAATGGCAGTGCCTTTTTTAACGGGCTTTTACAGTAAAGACCTAATTTTGGAGTCGGGCT  922
              ||||||||||||||||||||||||||||||||||||||||||||||||||||||||||||
Sbjct  15549  TAATGGCAGTGCCTTTTTTAACGGGCTTTTACAGTAAAGACCTAATTTTGGAGTCGGGCT  15608

Query  923    ATGCTGCCTTCAGCTTAAGCGGGCATGTAGT  953
              |||||||||||||||||||||||||||||||
Sbjct  15609  ATGCTGCCTTCAGCTTAAGCGGGCATGTAGT  15639


 Score = 623 bits (337),  Expect = 4e-180
 Identities = 340/341 (99%), Gaps = 1/341 (0%)
 Strand=Plus/Plus

Query  950    TAGTCTTTTACTCATTGCGCCTAATTAGCATGACCTTTTTAACGACGCCTAATGCAGCAA  1009
              ||| ||||||||||||||||||||||||||||||||||||||||||||||||||||||||
Sbjct  15673  TAG-CTTTTACTCATTGCGCCTAATTAGCATGACCTTTTTAACGACGCCTAATGCAGCAA  15731

Query  1010   AAGTGCAGTACGAACATGCACATGAACAGGACCTGCTTACTGTGCTGCCACTACTGCTGC  1069
              ||||||||||||||||||||||||||||||||||||||||||||||||||||||||||||
Sbjct  15732  AAGTGCAGTACGAACATGCACATGAACAGGACCTGCTTACTGTGCTGCCACTACTGCTGC  15791

Query  1070   TGGCCTTGCTGTCAAttttttttGGCTACGTAGCCAAAGACGCCTTTGTAGGCATGGGCA  1129
              ||||||||||||||||||||||||||||||||||||||||||||||||||||||||||||
Sbjct  15792  TGGCCTTGCTGTCAATTTTTTTTGGCTACGTAGCCAAAGACGCCTTTGTAGGCATGGGCA  15851

Query  1130   GCGACATGCTTGCAAGCAGCCTAGTAATGCTGCCTGGCAACGTCACATTAGTCGAAGCCG  1189
              ||||||||||||||||||||||||||||||||||||||||||||||||||||||||||||
Sbjct  15852  GCGACATGCTTGCAAGCAGCCTAGTAATGCTGCCTGGCAACGTCACATTAGTCGAAGCCG  15911

Query  1190   AGTTTGCATTGCCTACTGCCATTAAGCTACTGCCTGCAATTGGCACAGCCTGTGCTGCAG  1249
              ||||||||||||||||||||||||||||||||||||||||||||||||||||||||||||
Sbjct  15912  AGTTTGCATTGCCTACTGCCATTAAGCTACTGCCTGCAATTGGCACAGCCTGTGCTGCAG  15971

Query  1250   CACTTGCATTGCTGCTGTACCACCAGCAAGCTAGCCTTACT  1290
              |||||||||||||||||||||||||||||||||||||||||
Sbjct  15972  CACTTGCATTGCTGCTGTACCACCAGCAAGCTAGCCTTACT  16012


 Score = 555 bits (300),  Expect = 1e-159
 Identities = 300/300 (100%), Gaps = 0/300 (0%)
 Strand=Plus/Plus

Query  1291   CACATTTACGCATTTTTAAACAGCAAGTGGTACATTGACGCAGTCTACAATAAGCTGCTA  1350
              ||||||||||||||||||||||||||||||||||||||||||||||||||||||||||||
Sbjct  16043  CACATTTACGCATTTTTAAACAGCAAGTGGTACATTGACGCAGTCTACAATAAGCTGCTA  16102

Query  1351   ATTACAAAAGCCTTGCAGCTTGGCCTAGTCACTGCAAACGTACTAGACCGGGGTGCCATT  1410
              ||||||||||||||||||||||||||||||||||||||||||||||||||||||||||||
Sbjct  16103  ATTACAAAAGCCTTGCAGCTTGGCCTAGTCACTGCAAACGTACTAGACCGGGGTGCCATT  16162

Query  1411   GAACTTTTAGGCCCTGCTGGTGTTACACATAGCTTGCACAATGCTAGCGCTAGCCTTGCA  1470
              ||||||||||||||||||||||||||||||||||||||||||||||||||||||||||||
Sbjct  16163  GAACTTTTAGGCCCTGCTGGTGTTACACATAGCTTGCACAATGCTAGCGCTAGCCTTGCA  16222

Query  1471   AAATTAGACACGGGTGTAGTCACACACTATGCACTGTACATGACCATTGGGCTAGCCTTG  1530
              ||||||||||||||||||||||||||||||||||||||||||||||||||||||||||||
Sbjct  16223  AAATTAGACACGGGTGTAGTCACACACTATGCACTGTACATGACCATTGGGCTAGCCTTG  16282

Query  1531   CTAGTGCTGTTGCTGTTCATGCCTGTGTTTAGTGGTGTTGCAATGCATGACAGCACGTGA  1590
              ||||||||||||||||||||||||||||||||||||||||||||||||||||||||||||
Sbjct  16283  CTAGTGCTGTTGCTGTTCATGCCTGTGTTTAGTGGTGTTGCAATGCATGACAGCACGTGA  16342


 Score = 357 bits (193),  Expect = 4e-100
 Identities = 193/193 (100%), Gaps = 0/193 (0%)
 Strand=Plus/Plus

Query  156    CCTAGCGTTCGACTCATTAAGCGTGGCAATGCTGCTAGCAGTCACATTTGTGTCGACTAT  215
              ||||||||||||||||||||||||||||||||||||||||||||||||||||||||||||
Sbjct  12212  CCTAGCGTTCGACTCATTAAGCGTGGCAATGCTGCTAGCAGTCACATTTGTGTCGACTAT  12271

Query  216    GGTGCACGTGTACTCATGCAGCTACATGGCTGCTGACCCACACCAGCAACGCTTCATGGC  275
              ||||||||||||||||||||||||||||||||||||||||||||||||||||||||||||
Sbjct  12272  GGTGCACGTGTACTCATGCAGCTACATGGCTGCTGACCCACACCAGCAACGCTTCATGGC  12331

Query  276    CTACCTGTCTATGTTTACATTTTTTATGCTAGTCTTAATTGCAGGCAACAACTACATTGT  335
              ||||||||||||||||||||||||||||||||||||||||||||||||||||||||||||
Sbjct  12332  CTACCTGTCTATGTTTACATTTTTTATGCTAGTCTTAATTGCAGGCAACAACTACATTGT  12391

Query  336    GCTATTTTTAGGT  348
              |||||||||||||
Sbjct  12392  GCTATTTTTAGGT  12404


 Score = 287 bits (155),  Expect = 5e-79
 Identities = 155/155 (100%), Gaps = 0/155 (0%)
 Strand=Plus/Plus

Query  1      ATGTACCTATTGCTAATTGCTATGCCACTAGTAGGTGCTACACTTGCAGGCCTACTAGGT  60
              ||||||||||||||||||||||||||||||||||||||||||||||||||||||||||||
Sbjct  11988  ATGTACCTATTGCTAATTGCTATGCCACTAGTAGGTGCTACACTTGCAGGCCTACTAGGT  12047

Query  61     CGTAAGCTTGGGACCCGGGGTGCACAAGTCGTGACTTGCATTTGCATGGCTACGACTGCC  120
              ||||||||||||||||||||||||||||||||||||||||||||||||||||||||||||
Sbjct  12048  CGTAAGCTTGGGACCCGGGGTGCACAAGTCGTGACTTGCATTTGCATGGCTACGACTGCC  12107

Query  121    GTGCTGGCAGCGACTGCCTTTTACGAAGTCGCATT  155
              |||||||||||||||||||||||||||||||||||
Sbjct  12108  GTGCTGGCAGCGACTGCCTTTTACGAAGTCGCATT  12142


 Score = 246 bits (133),  Expect = 9e-67
 Identities = 138/140 (99%), Gaps = 2/140 (1%)
 Strand=Plus/Plus

Query  345    AGGTATTGGGGTGTCTTCGTACTTGCTAATTAATTTTTGGTTCACACGTGTGCAAGCCAA  404
              ||||||||||||||||||||||||||||||||||||||||||||||||||||||||||||
Sbjct  13697  AGGTATTGGGGTGTCTTCGTACTTGCTAATTAATTTTTGGTTCACACGTGTGCAAGCCAA  13756

Query  405    CAAGGCAGCCATTCAGGCAATGACCGTAAACCGGGTCGGCGACATGTTTTTAAGCATTGC  464
              ||||||||||||||||||||||||||||||||||||||||||||||||||||||||||||
Sbjct  13757  CAAGGCAGCCATTCAGGCAATGACCGTAAACCGGGTCGGCGACATGTTTTTAAGCATTGC  13816

Query  465    CTTTTTTGTGCTCGTGTTTG  484
              |||||||||||| || ||||
Sbjct  13817  CTTTTTTGTGCT-GT-TTTG  13834


 Score = 150 bits (81),  Expect = 7e-38
 Identities = 81/81 (100%), Gaps = 0/81 (0%)
 Strand=Plus/Plus

Query  477    CGTGTTTGCAGTAGCCCCGTTCATTAATGAAGCAGCATTGACTGCAGTCTGCTTGCTGTT  536
              ||||||||||||||||||||||||||||||||||||||||||||||||||||||||||||
Sbjct  13862  CGTGTTTGCAGTAGCCCCGTTCATTAATGAAGCAGCATTGACTGCAGTCTGCTTGCTGTT  13921

Query  537    TTTAATGGCTGCAATGGGCAA  557
              |||||||||||||||||||||
Sbjct  13922  TTTAATGGCTGCAATGGGCAA  13942


Lambda      K        H
    1.33    0.621     1.12 

Gapped
Lambda      K        H
    1.28    0.460    0.850 

Effective search space used: 173359850


Query= 889790 fgenesh1_pg.4_#_8

Length=894
                                                                      Score     E
Sequences producing significant alignments:                          (Bits)  Value

  scaffold_1_mito                                                     813     0.0  


> scaffold_1_mito
Length=110369

 Score = 813 bits (440),  Expect = 0.0
 Identities = 442/443 (99%), Gaps = 0/443 (0%)
 Strand=Plus/Plus

Query  452    CAACCTTAGCGCCTACAGTCGAGTTAGGCAATGCATGGCCGCCTGCTGGGGTCCAAGCAT  511
              || |||||||||||||||||||||||||||||||||||||||||||||||||||||||||
Sbjct  66740  CAGCCTTAGCGCCTACAGTCGAGTTAGGCAATGCATGGCCGCCTGCTGGGGTCCAAGCAT  66799

Query  512    TAGACCCTTACGAAGTGCCACTACTAAACACAGTAATTTTGCTAGGGTCAGGCGCTAGCG  571
              ||||||||||||||||||||||||||||||||||||||||||||||||||||||||||||
Sbjct  66800  TAGACCCTTACGAAGTGCCACTACTAAACACAGTAATTTTGCTAGGGTCAGGCGCTAGCG  66859

Query  572    TTACGTACGCACACCACTCATTAATTCAAGGTAGCCGTGCAGGCACAATTGCAGGCCTAA  631
              ||||||||||||||||||||||||||||||||||||||||||||||||||||||||||||
Sbjct  66860  TTACGTACGCACACCACTCATTAATTCAAGGTAGCCGTGCAGGCACAATTGCAGGCCTAA  66919

Query  632    TTGTGACCATTGCACTAGCTGCTGTGTTCACAATGCTGCAGGGTCTTGAGTACCATGAAG  691
              ||||||||||||||||||||||||||||||||||||||||||||||||||||||||||||
Sbjct  66920  TTGTGACCATTGCACTAGCTGCTGTGTTCACAATGCTGCAGGGTCTTGAGTACCATGAAG  66979

Query  692    CTAGCTTTACAATTGCAGACGGTGCTTACGGGTCTACATTTTACTTTGCGACTGGCTTCC  751
              ||||||||||||||||||||||||||||||||||||||||||||||||||||||||||||
Sbjct  66980  CTAGCTTTACAATTGCAGACGGTGCTTACGGGTCTACATTTTACTTTGCGACTGGCTTCC  67039

Query  752    ATGGCCTGCACGTCATTATTGGGACCCTGTTCATTGCCGTGGCATTTGTGCGACTACTAA  811
              ||||||||||||||||||||||||||||||||||||||||||||||||||||||||||||
Sbjct  67040  ATGGCCTGCACGTCATTATTGGGACCCTGTTCATTGCCGTGGCATTTGTGCGACTACTAA  67099

Query  812    GCTACCAGCTTACTGACCACCACCACTTAGGCTTTGAAGCCGCCATTTTGTACTGGCACA  871
              ||||||||||||||||||||||||||||||||||||||||||||||||||||||||||||
Sbjct  67100  GCTACCAGCTTACTGACCACCACCACTTAGGCTTTGAAGCCGCCATTTTGTACTGGCACA  67159

Query  872    GTGCCAGCTTGCTATGCAAGTAG  894
              |||||||||||||||||||||||
Sbjct  67160  GTGCCAGCTTGCTATGCAAGTAG  67182


 Score = 316 bits (171),  Expect = 4e-88
 Identities = 171/171 (100%), Gaps = 0/171 (0%)
 Strand=Plus/Plus

Query  1      ATGTACTTTAATGGCTATGCAAATGGCGGCATGCTAGTCAGCATTGGCTTCATTACAGTC  60
              ||||||||||||||||||||||||||||||||||||||||||||||||||||||||||||
Sbjct  65310  ATGTACTTTAATGGCTATGCAAATGGCGGCATGCTAGTCAGCATTGGCTTCATTACAGTC  65369

Query  61     GTTGCGTCAATGGCATTATGGTTTCGTGACGTAATTGCAGAAGGTGCATTGCTAGGCAAC  120
              ||||||||||||||||||||||||||||||||||||||||||||||||||||||||||||
Sbjct  65370  GTTGCGTCAATGGCATTATGGTTTCGTGACGTAATTGCAGAAGGTGCATTGCTAGGCAAC  65429

Query  121    CACACATTTGCGGTGCAAAAAGGACTTAACCTAGGCGTAGCCTTATTTATT  171
              |||||||||||||||||||||||||||||||||||||||||||||||||||
Sbjct  65430  CACACATTTGCGGTGCAAAAAGGACTTAACCTAGGCGTAGCCTTATTTATT  65480


 Score = 272 bits (147),  Expect = 9e-75
 Identities = 147/147 (100%), Gaps = 0/147 (0%)
 Strand=Plus/Plus

Query  308    GCAAGCTTAAGCAAGCAAATTTAAGCTACCGTGCTGCAATTGTGCTAACTCTACATGGTG  367
              ||||||||||||||||||||||||||||||||||||||||||||||||||||||||||||
Sbjct  66372  GCAAGCTTAAGCAAGCAAATTTAAGCTACCGTGCTGCAATTGTGCTAACTCTACATGGTG  66431

Query  368    CAATGTGCAAGCTTAAGCAAGCAAGTGTGAAGCTACCATGCAGCAATTGTGCTAACTCTA  427
              ||||||||||||||||||||||||||||||||||||||||||||||||||||||||||||
Sbjct  66432  CAATGTGCAAGCTTAAGCAAGCAAGTGTGAAGCTACCATGCAGCAATTGTGCTAACTCTA  66491

Query  428    CACGGTGTAATAAGCAAGCTTTGGCAA  454
              |||||||||||||||||||||||||||
Sbjct  66492  CACGGTGTAATAAGCAAGCTTTGGCAA  66518


 Score = 254 bits (137),  Expect = 3e-69
 Identities = 137/137 (100%), Gaps = 0/137 (0%)
 Strand=Plus/Plus

Query  172    TCAACCCTATGCCTTAAAGACTGCGTACTAGTGCTAAGACTAGCAGCAACCCTAGTGCCc  231
              ||||||||||||||||||||||||||||||||||||||||||||||||||||||||||||
Sbjct  65536  TCAACCCTATGCCTTAAAGACTGCGTACTAGTGCTAAGACTAGCAGCAACCCTAGTGCCC  65595

Query  232    acacacacacacacaGTACGTGGTGTGGCCTGTAATGTGGCCAGGCGTTTAATGGCAGCT  291
              ||||||||||||||||||||||||||||||||||||||||||||||||||||||||||||
Sbjct  65596  ACACACACACACACAGTACGTGGTGTGGCCTGTAATGTGGCCAGGCGTTTAATGGCAGCT  65655

Query  292    TTGCAGCTTAAAGACTG  308
              |||||||||||||||||
Sbjct  65656  TTGCAGCTTAAAGACTG  65672


Lambda      K        H
    1.33    0.621     1.12 

Gapped
Lambda      K        H
    1.28    0.460    0.850 

Effective search space used: 96667476


Query= 979745 MIX18747_214_28

Length=1398
                                                                      Score     E
Sequences producing significant alignments:                          (Bits)  Value

  scaffold_1_mito                                                     763     0.0  


> scaffold_1_mito
Length=110369

 Score = 763 bits (413),  Expect = 0.0
 Identities = 413/413 (100%), Gaps = 0/413 (0%)
 Strand=Plus/Plus

Query  1      CACATGAACAGCATTGGCTGTTTTGCtttttttttAATGCCTGCAGGGCATTaaaaaaaa  60
              ||||||||||||||||||||||||||||||||||||||||||||||||||||||||||||
Sbjct  55183  CACATGAACAGCATTGGCTGTTTTGCTTTTTTTTTAATGCCTGCAGGGCATTAAAAAAAA  55242

Query  61     aaCTGCTGCAATTAGCTTGTGGCCGCGCTTTTAAAAATTGAAGACATTACACAGTACACA  120
              ||||||||||||||||||||||||||||||||||||||||||||||||||||||||||||
Sbjct  55243  AACTGCTGCAATTAGCTTGTGGCCGCGCTTTTAAAAATTGAAGACATTACACAGTACACA  55302

Query  121    ATTGGCACGCGTGTGGGTTTGGCACACTGTGCAGTCATTGCACACAGCATTGCAAGCTAG  180
              ||||||||||||||||||||||||||||||||||||||||||||||||||||||||||||
Sbjct  55303  ATTGGCACGCGTGTGGGTTTGGCACACTGTGCAGTCATTGCACACAGCATTGCAAGCTAG  55362

Query  181    TAGCAGCCTACTAGTAAGCACTTGCTGGCTACATTTTAATTAATGCGACTGCTTAAAACA  240
              ||||||||||||||||||||||||||||||||||||||||||||||||||||||||||||
Sbjct  55363  TAGCAGCCTACTAGTAAGCACTTGCTGGCTACATTTTAATTAATGCGACTGCTTAAAACA  55422

Query  241    CACCCCATTTTAGGTTTAGTAAATAGCTACATGGTCGACAGCCCACAACCTGCTAGCATT  300
              ||||||||||||||||||||||||||||||||||||||||||||||||||||||||||||
Sbjct  55423  CACCCCATTTTAGGTTTAGTAAATAGCTACATGGTCGACAGCCCACAACCTGCTAGCATT  55482

Query  301    ACGTACATGTGAAATTTTGGGTCGCTGCTAGGCCTGTGCTTAGTCATTCAAATTGTGACT  360
              ||||||||||||||||||||||||||||||||||||||||||||||||||||||||||||
Sbjct  55483  ACGTACATGTGAAATTTTGGGTCGCTGCTAGGCCTGTGCTTAGTCATTCAAATTGTGACT  55542

Query  361    GGCATTACGCTAGCAATGCACTACACACCGTCTATTGACTTGGCCTTTGCAAG  413
              |||||||||||||||||||||||||||||||||||||||||||||||||||||
Sbjct  55543  GGCATTACGCTAGCAATGCACTACACACCGTCTATTGACTTGGCCTTTGCAAG  55595


 Score = 520 bits (281),  Expect = 4e-149
 Identities = 286/288 (99%), Gaps = 2/288 (1%)
 Strand=Plus/Plus

Query  1113   CTTTG-CTA-GTGTCGTAGCCATGTTTGCTTCATTACTAATTTTACTGGCTATGCCAATT  1170
              ||||| ||| ||||||||||||||||||||||||||||||||||||||||||||||||||
Sbjct  61774  CTTTGACTAGGTGTCGTAGCCATGTTTGCTTCATTACTAATTTTACTGGCTATGCCAATT  61833

Query  1171   GTAGACACGTCACGTGTACGTGGCTCGCAATTTCGACCTTTAATGCGGTGAGCCTTTTGG  1230
              ||||||||||||||||||||||||||||||||||||||||||||||||||||||||||||
Sbjct  61834  GTAGACACGTCACGTGTACGTGGCTCGCAATTTCGACCTTTAATGCGGTGAGCCTTTTGG  61893

Query  1231   CTTTTTGCTGCAAACTTTTTTGTACTAATGTACATTGGTAGCCAGCACGTTGAAGAGCCG  1290
              ||||||||||||||||||||||||||||||||||||||||||||||||||||||||||||
Sbjct  61894  CTTTTTGCTGCAAACTTTTTTGTACTAATGTACATTGGTAGCCAGCACGTTGAAGAGCCG  61953

Query  1291   TTCGTTACAGTAGGCATGATTAGCACTAGCCTGTACTTTGGCTGGTTTTTAATTATTGTG  1350
              ||||||||||||||||||||||||||||||||||||||||||||||||||||||||||||
Sbjct  61954  TTCGTTACAGTAGGCATGATTAGCACTAGCCTGTACTTTGGCTGGTTTTTAATTATTGTG  62013

Query  1351   CCTGTAATTGGACTAGTCGAAAACACGCTTTTAGACTTAGCATGTGAG  1398
              ||||||||||||||||||||||||||||||||||||||||||||||||
Sbjct  62014  CCTGTAATTGGACTAGTCGAAAACACGCTTTTAGACTTAGCATGTGAG  62061


 Score = 377 bits (204),  Expect = 3e-106
 Identities = 204/204 (100%), Gaps = 0/204 (0%)
 Strand=Plus/Plus

Query  413    GCTTACGTAAAGCGATTATGCGTGACGTGCATTACGGCTGGCTAATTCGGTACTTGCATG  472
              ||||||||||||||||||||||||||||||||||||||||||||||||||||||||||||
Sbjct  56251  GCTTACGTAAAGCGATTATGCGTGACGTGCATTACGGCTGGCTAATTCGGTACTTGCATG  56310

Query  473    CTAACGTGGCGTCGTTCTTTTTTATTTGCGTGTACTTACACATTGGTCGTGGCCTTTACT  532
              ||||||||||||||||||||||||||||||||||||||||||||||||||||||||||||
Sbjct  56311  CTAACGTGGCGTCGTTCTTTTTTATTTGCGTGTACTTACACATTGGTCGTGGCCTTTACT  56370

Query  533    ATGGGTCGTACCGGTCACCACGTACGCTAGTGTGGGCCATTGGGGTCGTAATTTTAGTAC  592
              ||||||||||||||||||||||||||||||||||||||||||||||||||||||||||||
Sbjct  56371  ATGGGTCGTACCGGTCACCACGTACGCTAGTGTGGGCCATTGGGGTCGTAATTTTAGTAC  56430

Query  593    TTATGATAGCAACAGCCTTTTTAG  616
              ||||||||||||||||||||||||
Sbjct  56431  TTATGATAGCAACAGCCTTTTTAG  56454


 Score = 359 bits (194),  Expect = 1e-100
 Identities = 194/194 (100%), Gaps = 0/194 (0%)
 Strand=Plus/Plus

Query  886    GGCATTAGTGGCAACACAGACCGACTACCATTCCACCCGTACTTTACATTTAAAGACTTG  945
              ||||||||||||||||||||||||||||||||||||||||||||||||||||||||||||
Sbjct  60654  GGCATTAGTGGCAACACAGACCGACTACCATTCCACCCGTACTTTACATTTAAAGACTTG  60713

Query  946    GTAACAGTCTTCCTGTTCATGCTAGTGCTTGCAGCAATGGTCTTTTATGCGCCTAACTAC  1005
              ||||||||||||||||||||||||||||||||||||||||||||||||||||||||||||
Sbjct  60714  GTAACAGTCTTCCTGTTCATGCTAGTGCTTGCAGCAATGGTCTTTTATGCGCCTAACTAC  60773

Query  1006   CTGGGCCATAGTGACAATTACATTCCTGCAAACCCCATGCAAACGCCAGCGTCAATTGTG  1065
              ||||||||||||||||||||||||||||||||||||||||||||||||||||||||||||
Sbjct  60774  CTGGGCCATAGTGACAATTACATTCCTGCAAACCCCATGCAAACGCCAGCGTCAATTGTG  60833

Query  1066   CCTGAATGGTCAAG  1079
              ||||||||||||||
Sbjct  60834  CCTGAATGGTCAAG  60847


 Score = 300 bits (162),  Expect = 6e-83
 Identities = 162/162 (100%), Gaps = 0/162 (0%)
 Strand=Plus/Plus

Query  726    CAGCATAAGCGACCTTGGGGGTGGCTTTAGCGTCAACAACGCAACGTTAAACCGGttttt  785
              ||||||||||||||||||||||||||||||||||||||||||||||||||||||||||||
Sbjct  60099  CAGCATAAGCGACCTTGGGGGTGGCTTTAGCGTCAACAACGCAACGTTAAACCGGTTTTT  60158

Query  786    ttCATTGCATTACTTACTGCCGTTCATTTTGGCAGCACTTGCAGCTATGCACTTGCTTGC  845
              ||||||||||||||||||||||||||||||||||||||||||||||||||||||||||||
Sbjct  60159  TTCATTGCATTACTTACTGCCGTTCATTTTGGCAGCACTTGCAGCTATGCACTTGCTTGC  60218

Query  846    ATTGCATGAACACGGCAGCAGCAACCCTAACAGCCTAGGGGG  887
              ||||||||||||||||||||||||||||||||||||||||||
Sbjct  60219  ATTGCATGAACACGGCAGCAGCAACCCTAACAGCCTAGGGGG  60260


 Score = 122 bits (66),  Expect = 1e-29
 Identities = 66/66 (100%), Gaps = 0/66 (0%)
 Strand=Plus/Plus

Query  615    AGCATGCAAGCGACTGTATGTGCTGCCTTACGGTCAAATGTCATTATGGGGTGAGCGCCC  674
              ||||||||||||||||||||||||||||||||||||||||||||||||||||||||||||
Sbjct  58470  AGCATGCAAGCGACTGTATGTGCTGCCTTACGGTCAAATGTCATTATGGGGTGAGCGCCC  58529

Query  675    TAAATG  680
              ||||||
Sbjct  58530  TAAATG  58535


 Score = 91.6 bits (49),  Expect = 4e-20
 Identities = 49/49 (100%), Gaps = 0/49 (0%)
 Strand=Plus/Plus

Query  680    GCTTACTGCAGCAGTCGCTACAGACTACACCACCAGTAGGCACCTGCAG  728
              |||||||||||||||||||||||||||||||||||||||||||||||||
Sbjct  58774  GCTTACTGCAGCAGTCGCTACAGACTACACCACCAGTAGGCACCTGCAG  58822


 Score = 82.4 bits (44),  Expect = 2e-17
 Identities = 44/44 (100%), Gaps = 0/44 (0%)
 Strand=Plus/Plus

Query  1079   GCAAGCCTTTGCGCAGCTTAAAACTGCAAATGTACTTTGCTAGT  1122
              ||||||||||||||||||||||||||||||||||||||||||||
Sbjct  61362  GCAAGCCTTTGCGCAGCTTAAAACTGCAAATGTACTTTGCTAGT  61405


 Score = 58.4 bits (31),  Expect = 4e-10
 Identities = 31/31 (100%), Gaps = 0/31 (0%)
 Strand=Plus/Plus

Query  1091   GCAGCTTAAAACTGCAAATGTACTTTGCTAG  1121
              |||||||||||||||||||||||||||||||
Sbjct  61300  GCAGCTTAAAACTGCAAATGTACTTTGCTAG  61330


Lambda      K        H
    1.33    0.621     1.12 

Gapped
Lambda      K        H
    1.28    0.460    0.850 

Effective search space used: 152172650


Query= 900792 fgenesh1_kg.4_#_52_#_TRINITY_DN4084_c0_g1_i1

Length=1475
                                                                      Score     E
Sequences producing significant alignments:                          (Bits)  Value

  scaffold_1_mito                                                     1652    0.0  


> scaffold_1_mito
Length=110369

 Score = 1652 bits (894),  Expect = 0.0
 Identities = 894/894 (100%), Gaps = 0/894 (0%)
 Strand=Plus/Minus

Query  1      GCAATTGCCTATGCTTGCTGTAAGCAAAGTTACAATTTAAAGCTTGTGTAGTATTGGCTT  60
              ||||||||||||||||||||||||||||||||||||||||||||||||||||||||||||
Sbjct  80781  GCAATTGCCTATGCTTGCTGTAAGCAAAGTTACAATTTAAAGCTTGTGTAGTATTGGCTT  80722

Query  61     GCTTAGTAATTGCAAATTTGCCGTTTCGTCCCAGGGTCAAACACGTAGTGTTTGACCCTG  120
              ||||||||||||||||||||||||||||||||||||||||||||||||||||||||||||
Sbjct  80721  GCTTAGTAATTGCAAATTTGCCGTTTCGTCCCAGGGTCAAACACGTAGTGTTTGACCCTG  80662

Query  121    GGACGAAAAAACAAGGTGTTCAAATTGTAAAAATTAAGCTTGCGTAGTATTGGCAAGTTC  180
              ||||||||||||||||||||||||||||||||||||||||||||||||||||||||||||
Sbjct  80661  GGACGAAAAAACAAGGTGTTCAAATTGTAAAAATTAAGCTTGCGTAGTATTGGCAAGTTC  80602

Query  181    AGTAGTCACCCTATTGCGTGTAAGTTTTGAACCCATGGGGGTTTTTAAAGCAGCATGCTG  240
              ||||||||||||||||||||||||||||||||||||||||||||||||||||||||||||
Sbjct  80601  AGTAGTCACCCTATTGCGTGTAAGTTTTGAACCCATGGGGGTTTTTAAAGCAGCATGCTG  80542

Query  241    CCTACAGTACAACGCCATAGTGTTATGCTAGCTGTAATTTTTAGTTTACTTGAAGTTTTA  300
              ||||||||||||||||||||||||||||||||||||||||||||||||||||||||||||
Sbjct  80541  CCTACAGTACAACGCCATAGTGTTATGCTAGCTGTAATTTTTAGTTTACTTGAAGTTTTA  80482

Query  301    ATTGTGTTAGTGCCTGTACTACTGTCAGTTGCGTTCATGACCATTATTGAACGTAAGGTG  360
              ||||||||||||||||||||||||||||||||||||||||||||||||||||||||||||
Sbjct  80481  ATTGTGTTAGTGCCTGTACTACTGTCAGTTGCGTTCATGACCATTATTGAACGTAAGGTG  80422

Query  361    CTTGCAAGCATGCAGCGACGTGTGGGCCCTAACACCGTGGGCTACTTTGGTGTGCTGCAA  420
              ||||||||||||||||||||||||||||||||||||||||||||||||||||||||||||
Sbjct  80421  CTTGCAAGCATGCAGCGACGTGTGGGCCCTAACACCGTGGGCTACTTTGGTGTGCTGCAA  80362

Query  421    CCTTTTGCTGACGCCTTAAAATTAGTCGTTAAAGAGACCGTAGTCCCACAACATGCAACA  480
              ||||||||||||||||||||||||||||||||||||||||||||||||||||||||||||
Sbjct  80361  CCTTTTGCTGACGCCTTAAAATTAGTCGTTAAAGAGACCGTAGTCCCACAACATGCAACA  80302

Query  481    CGCAGCCTGttttttttGGCCCCTGTAATTAGCCTAGTGTTTAGCTTGCTAGGGTGGGCA  540
              ||||||||||||||||||||||||||||||||||||||||||||||||||||||||||||
Sbjct  80301  CGCAGCCTGTTTTTTTTGGCCCCTGTAATTAGCCTAGTGTTTAGCTTGCTAGGGTGGGCA  80242

Query  541    GTGGTCCCATTTGCAAGCGGCCTGGCATTAAGCGACTTTAGCTTAGGCGTGCTGTACTCA  600
              ||||||||||||||||||||||||||||||||||||||||||||||||||||||||||||
Sbjct  80241  GTGGTCCCATTTGCAAGCGGCCTGGCATTAAGCGACTTTAGCTTAGGCGTGCTGTACTCA  80182

Query  601    CTTGCAATTAGTAGCATTGGGGTTTACGGTGTACTTTTTGCAGGCTGGTCTGCAAACAGC  660
              ||||||||||||||||||||||||||||||||||||||||||||||||||||||||||||
Sbjct  80181  CTTGCAATTAGTAGCATTGGGGTTTACGGTGTACTTTTTGCAGGCTGGTCTGCAAACAGC  80122

Query  661    AAGTACGCATTTTTAGGGTCGTTGCGTAGCACAGCCCAAATGATTAGTTACGAGCTAATT  720
              ||||||||||||||||||||||||||||||||||||||||||||||||||||||||||||
Sbjct  80121  AAGTACGCATTTTTAGGGTCGTTGCGTAGCACAGCCCAAATGATTAGTTACGAGCTAATT  80062

Query  721    TTAAGCACTGCAGTACTTGCAGTAATTTTGCTTGCAGGTACGTTTAGTTACACTGCAATT  780
              ||||||||||||||||||||||||||||||||||||||||||||||||||||||||||||
Sbjct  80061  TTAAGCACTGCAGTACTTGCAGTAATTTTGCTTGCAGGTACGTTTAGTTACACTGCAATT  80002

Query  781    ATTGAACGGCAGCAAGCAATTTACTATGCTGTGCCATTACTGCCGCTGTTCATTGTGTTT  840
              ||||||||||||||||||||||||||||||||||||||||||||||||||||||||||||
Sbjct  80001  ATTGAACGGCAGCAAGCAATTTACTATGCTGTGCCATTACTGCCGCTGTTCATTGTGTTT  79942

Query  841    TTTATTGCAGTACTTGCTGAGACTAACCGTACGCCATTTGACTTGCCTGAAGCT  894
              ||||||||||||||||||||||||||||||||||||||||||||||||||||||
Sbjct  79941  TTTATTGCAGTACTTGCTGAGACTAACCGTACGCCATTTGACTTGCCTGAAGCT  79888


 Score = 1005 bits (544),  Expect = 0.0
 Identities = 573/586 (98%), Gaps = 5/586 (1%)
 Strand=Plus/Minus

Query  895    GAGTCTGAACTAGTCGCAGGCTACTTTACTGAGCATTCAGGCATGATTTTTGTGtttttt  954
              ||||||||||||||||||||||||||||||||||||||||||||||||||||||||||||
Sbjct  79188  GAGTCTGAACTAGTCGCAGGCTACTTTACTGAGCATTCAGGCATGATTTTTGTGTTTTTT  79129

Query  955    tttttGGCTGAATACTGTAGCATTGTGTTAATGTCGGCATTGACTTCAAttttttttttA  1014
              ||||||||||||||||||||||||||||||||||||||||||||||||||||||||||||
Sbjct  79128  TTTTTGGCTGAATACTGTAGCATTGTGTTAATGTCGGCATTGACTTCAATTTTTTTTTTA  79069

Query  1015   GGTGGCTATGTAATGCCACAGTGCATTGTAAACAACACCTTCATTAATGTGCAGGCTGTG  1074
              ||||||||||||||||||||||||||||||||||||||||||||||||||||||||||||
Sbjct  79068  GGTGGCTATGTAATGCCACAGTGCATTGTAAACAACACCTTCATTAATGTGCAGGCTGTG  79009

Query  1075   GTACTAGCTTTAAAGACTTGCCTGTTTTGCTTTGTGTTTGTGTGGTTTCGTGCAACGTTG  1134
              ||||||||||||||||||||||||||||||||||||||||||||||||||||||||||||
Sbjct  79008  GTACTAGCTTTAAAGACTTGCCTGTTTTGCTTTGTGTTTGTGTGGTTTCGTGCAACGTTG  78949

Query  1135   CCACGACTGCGTTATGACCAGCTTATGCAATTTTGTTGAATGGCTATGCTGCCTGTTGCA  1194
              ||||||||||||||||||||||||||||||||||||||||||||||||||||||||||||
Sbjct  78948  CCACGACTGCGTTATGACCAGCTTATGCAATTTTGTTGAATGGCTATGCTGCCTGTTGCA  78889

Query  1195   ATTGCCTGCTTTGTGCTAGTGCTTTGCTTGCTAGTAGCCTTTGACGTAACCCCTTGCATT  1254
              ||||||||||||||||||||||||||||||||||||||||||||||||||||||||||||
Sbjct  78888  ATTGCCTGCTTTGTGCTAGTGCTTTGCTTGCTAGTAGCCTTTGACGTAACCCCTTGCATT  78829

Query  1255   ACAAGGCCCACACCTAACGTAGCACAGTGTAATAGCTTAGCAAATTTGCAATGCTAGCAA  1314
              ||||||||||||||||||||||||||||||||||||||||||||||||||||||||||||
Sbjct  78828  ACAAGGCCCACACCTAACGTAGCACAGTGTAATAGCTTAGCAAATTTGCAATGCTAGCAA  78769

Query  1315   TTGCATTAATGCAATGCAGGTCAACTGTACACAAGCCATGAGCGTGTAATTGCAAGCAAT  1374
              ||||||||||||||||||||||||||||||||||||||||||||||||||||||||||||
Sbjct  78768  TTGCATTAATGCAATGCAGGTCAACTGTACACAAGCCATGAGCGTGTAATTGCAAGCAAT  78709

Query  1375   TTGGCTGTGGCAGCAATTTGCTAGCTTAATGCAAGTACATAACTGTACAT-----GGTGT  1429
              ||||||||||||||||||||||||||||||||||||||||||||||||       |||||
Sbjct  78708  TTGGCTGTGGCAGCAATTTGCTAGCTTAATGCAAGTACATAACTGTACTGCCCCAGGTGT  78649

Query  1430   GTTACAGCACTTAATTGGGCACTAGTAGCCTGTGGCAACTTAATGC  1475
              |||||||||| ||  ||||  | |||||||||||||||||||||||
Sbjct  78648  GTTACAGCACCTACATGGGTCCGAGTAGCCTGTGGCAACTTAATGC  78603


 Score = 143 bits (77),  Expect = 1e-35
 Identities = 77/77 (100%), Gaps = 0/77 (0%)
 Strand=Plus/Minus

Query  1399   CTTAATGCAAGTACATAACTGTACATGGTGTGTTACAGCACTTAATTGGGCACTAGTAGC  1458
              ||||||||||||||||||||||||||||||||||||||||||||||||||||||||||||
Sbjct  78610  CTTAATGCAAGTACATAACTGTACATGGTGTGTTACAGCACTTAATTGGGCACTAGTAGC  78551

Query  1459   CTGTGGCAACTTAATGC  1475
              |||||||||||||||||
Sbjct  78550  CTGTGGCAACTTAATGC  78534


 Score = 87.9 bits (47),  Expect = 6e-19
 Identities = 49/50 (98%), Gaps = 0/50 (0%)
 Strand=Plus/Minus

Query  1397   AGCTTAATGCAAGTACATAACTGTACATGGTGTGTTACAGCACTTAATTG  1446
              |||||||||||||||||||||||||||||||||||||||||||||| |||
Sbjct  78161  AGCTTAATGCAAGTACATAACTGTACATGGTGTGTTACAGCACTTACTTG  78112


 Score = 69.4 bits (37),  Expect = 2e-13
 Identities = 43/46 (93%), Gaps = 0/46 (0%)
 Strand=Plus/Minus

Query  30     TTACAATTTAAAGCTTGTGTAGTATTGGCTTGCTTAGTAATTGCAA  75
              ||||| ||| ||||||| ||||||||||||||||||||||||||||
Sbjct  81018  TTACATTTTTAAGCTTGCGTAGTATTGGCTTGCTTAGTAATTGCAA  80973


 Score = 65.8 bits (35),  Expect = 3e-12
 Identities = 64/77 (83%), Gaps = 5/77 (6%)
 Strand=Plus/Minus

Query  3      AATTGCCTATGCTTGC-T-GT-AAGCAAAG-TTACAATTTAAAGCTTGTGTAGTATTGGC  58
              ||||| | |||||||| | || | ||||   || || ||| ||||||| |||||||||||
Sbjct  80980  AATTG-CAATGCTTGCTTCGTGAGGCAAGTCTTGCATTTTTAAGCTTGCGTAGTATTGGC  80922

Query  59     TTGCTTAGTAATTGCAA  75
              |||||||||||||||||
Sbjct  80921  TTGCTTAGTAATTGCAA  80905


 Score = 60.2 bits (32),  Expect = 1e-10
 Identities = 37/39 (95%), Gaps = 2/39 (5%)
 Strand=Plus/Minus

Query  1425   GGTGTGTTACAGCACTTAATTGGGCACTAGTA-GC-CTG  1461
              |||||||||||||||||||||||||||||||| || |||
Sbjct  78439  GGTGTGTTACAGCACTTAATTGGGCACTAGTAAGCACTG  78401


Lambda      K        H
    1.33    0.621     1.12 

Gapped
Lambda      K        H
    1.28    0.460    0.850 

Effective search space used: 160669600


  Database: User specified sequence set (Input:
/users/joonhoonkim/Data/R_toruloides/Rhoto_IFO0880_4/Rhoto_IFO0880_4
_MitoAssemblyScaffolds.fasta).
    Posted date:  Unknown
  Number of letters in database: 110,369
  Number of sequences in database:  1


Matrix: blastn matrix 1 -2
Gap Penalties: Existence: 0, Extension: 2.5
```

In [176]:

```
%%bash
cd ../../Data/Mito
bp_search2gff --input Mito_Transcripts_to_IFO0880_4.txt --addid --version 3 --type hit
```

```
##gff-version 3
scaffold_1_mito	BLASTN	similarity	93917	95072	1156	-	0	ID=900810;Target=Sequence:900810 828 1983
scaffold_1_mito	BLASTN	similarity	101569	101768	200	-	0	ID=900810;Target=Sequence:900810 253 452
scaffold_1_mito	BLASTN	similarity	96728	96859	132	-	0	ID=900810;Target=Sequence:900810 502 633
scaffold_1_mito	BLASTN	similarity	95443	95565	120	-	0	ID=900810;Target=Sequence:900810 710 832
scaffold_1_mito	BLASTN	similarity	93859	94022	119	-	0	ID=900810;Target=Sequence:900810 1820 1983
scaffold_1_mito	BLASTN	similarity	102656	102766	111	-	0	ID=900810;Target=Sequence:900810 142 252
scaffold_1_mito	BLASTN	similarity	102966	103064	96	-	0	ID=900810;Target=Sequence:900810 46 144
scaffold_1_mito	BLASTN	similarity	93975	94080	82	-	0	ID=900810;Target=Sequence:900810 1878 1983
scaffold_1_mito	BLASTN	similarity	96051	96127	77	-	0	ID=900810;Target=Sequence:900810 633 709
scaffold_1_mito	BLASTN	similarity	103714	103758	45	-	0	ID=900810;Target=Sequence:900810 1 45
scaffold_1_mito	BLASTN	similarity	100691	100718	28	-	0	ID=900810;Target=Sequence:900810 452 479
scaffold_1_mito	BLASTN	similarity	64475	65521	1047	+	0	ID=900782;Target=Sequence:900782 1 1047
scaffold_1_mito	BLASTN	similarity	66731	67158	428	+	0	ID=900782;Target=Sequence:900782 1047 1474
scaffold_1_mito	BLASTN	similarity	67667	67759	93	+	0	ID=900782;Target=Sequence:900782 1473 1565
scaffold_1_mito	BLASTN	similarity	55183	55606	424	+	0	ID=900778;Target=Sequence:900778 1 424
scaffold_1_mito	BLASTN	similarity	56265	56596	332	+	0	ID=900778;Target=Sequence:900778 424 755
scaffold_1_mito	BLASTN	similarity	11721	11981	261	+	0	ID=866864;Target=Sequence:866864 1 261
scaffold_1_mito	BLASTN	similarity	79914	80517	604	-	0	ID=950280;Target=Sequence:950280 1 604
scaffold_1_mito	BLASTN	similarity	78910	79108	199	-	0	ID=950280;Target=Sequence:950280 645 843
scaffold_1_mito	BLASTN	similarity	79136	79175	40	-	0	ID=950280;Target=Sequence:950280 605 644
scaffold_1_mito	BLASTN	similarity	44419	45429	1011	+	0	ID=900774;Target=Sequence:900774 1 1011
scaffold_1_mito	BLASTN	similarity	44224	44320	43	+	0	ID=900774;Target=Sequence:900774 1 86
scaffold_1_mito	BLASTN	similarity	44340	44388	33	+	0	ID=900774;Target=Sequence:900774 35 82
scaffold_1_mito	BLASTN	similarity	89995	91248	1254	-	0	ID=900801;Target=Sequence:900801 720 1973
scaffold_1_mito	BLASTN	similarity	93282	93817	531	-	0	ID=900801;Target=Sequence:900801 1 538
scaffold_1_mito	BLASTN	similarity	92125	92313	189	-	0	ID=900801;Target=Sequence:900801 532 720
scaffold_1_mito	BLASTN	similarity	93747	93877	125	-	0	ID=900801;Target=Sequence:900801 43 173
scaffold_1_mito	BLASTN	similarity	93645	93715	68	-	0	ID=900801;Target=Sequence:900801 1 71
scaffold_1_mito	BLASTN	similarity	89851	90003	61	-	0	ID=900801;Target=Sequence:900801 1812 1973
scaffold_1_mito	BLASTN	similarity	90637	90724	44	-	0	ID=900801;Target=Sequence:900801 1107 1197
scaffold_1_mito	BLASTN	similarity	90771	90861	44	-	0	ID=900801;Target=Sequence:900801 1244 1331
scaffold_1_mito	BLASTN	similarity	55845	55885	41	+	0	ID=900801;Target=Sequence:900801 1 41
scaffold_1_mito	BLASTN	similarity	55845	55885	41	+	0	ID=900801;Target=Sequence:900801 103 143
scaffold_1_mito	BLASTN	similarity	89668	89709	33	-	0	ID=900801;Target=Sequence:900801 1860 1901
scaffold_1_mito	BLASTN	similarity	93849	93877	29	-	0	ID=900801;Target=Sequence:900801 145 173
scaffold_1_mito	BLASTN	similarity	11099	12113	1015	+	0	ID=518890;Target=Sequence:518890 1 1015
scaffold_1_mito	BLASTN	similarity	69105	69145	41	+	0	ID=518890;Target=Sequence:518890 488 528
scaffold_1_mito	BLASTN	similarity	73191	73231	41	+	0	ID=518890;Target=Sequence:518890 488 528
scaffold_1_mito	BLASTN	similarity	55183	55436	254	+	0	ID=950279;Target=Sequence:950279 1 254
scaffold_1_mito	BLASTN	similarity	60654	60849	196	+	0	ID=950279;Target=Sequence:950279 688 883
scaffold_1_mito	BLASTN	similarity	56263	56454	192	+	0	ID=950279;Target=Sequence:950279 335 526
scaffold_1_mito	BLASTN	similarity	61886	62070	185	+	0	ID=950279;Target=Sequence:950279 950 1134
scaffold_1_mito	BLASTN	similarity	60102	60260	159	+	0	ID=950279;Target=Sequence:950279 531 689
scaffold_1_mito	BLASTN	similarity	55511	55594	84	+	0	ID=950279;Target=Sequence:950279 251 334
scaffold_1_mito	BLASTN	similarity	61784	61852	69	+	0	ID=950279;Target=Sequence:950279 881 949
scaffold_1_mito	BLASTN	similarity	66726	67182	457	+	0	ID=946153;Target=Sequence:946153 300 756
scaffold_1_mito	BLASTN	similarity	65193	65491	299	+	0	ID=946153;Target=Sequence:946153 1 299
scaffold_1_mito	BLASTN	similarity	55183	55595	413	+	0	ID=979743;Target=Sequence:979743 1 413
scaffold_1_mito	BLASTN	similarity	61784	62055	272	+	0	ID=979743;Target=Sequence:979743 1109 1380
scaffold_1_mito	BLASTN	similarity	56251	56454	204	+	0	ID=979743;Target=Sequence:979743 413 616
scaffold_1_mito	BLASTN	similarity	60654	60847	194	+	0	ID=979743;Target=Sequence:979743 886 1079
scaffold_1_mito	BLASTN	similarity	60099	60260	162	+	0	ID=979743;Target=Sequence:979743 726 887
scaffold_1_mito	BLASTN	similarity	58470	58535	66	+	0	ID=979743;Target=Sequence:979743 615 680
scaffold_1_mito	BLASTN	similarity	58774	58822	49	+	0	ID=979743;Target=Sequence:979743 680 728
scaffold_1_mito	BLASTN	similarity	61249	61280	32	+	0	ID=979743;Target=Sequence:979743 1079 1110
scaffold_1_mito	BLASTN	similarity	49557	49931	375	+	0	ID=863471;Target=Sequence:863471 1 375
scaffold_1_mito	BLASTN	similarity	61779	62528	750	+	0	ID=900779;Target=Sequence:900779 1089 1838
scaffold_1_mito	BLASTN	similarity	55183	55606	424	+	0	ID=900779;Target=Sequence:900779 1 424
scaffold_1_mito	BLASTN	similarity	60651	60843	193	+	0	ID=900779;Target=Sequence:900779 850 1042
scaffold_1_mito	BLASTN	similarity	56265	56456	192	+	0	ID=900779;Target=Sequence:900779 424 615
scaffold_1_mito	BLASTN	similarity	60113	60246	131	+	0	ID=900779;Target=Sequence:900779 716 849
scaffold_1_mito	BLASTN	similarity	58980	59046	67	+	0	ID=900779;Target=Sequence:900779 652 718
scaffold_1_mito	BLASTN	similarity	61520	61567	48	+	0	ID=900779;Target=Sequence:900779 1043 1090
scaffold_1_mito	BLASTN	similarity	58486	58522	37	+	0	ID=900779;Target=Sequence:900779 616 652
scaffold_1_mito	BLASTN	similarity	80707	81573	867	-	0	ID=950281;Target=Sequence:950281 2468 3334
scaffold_1_mito	BLASTN	similarity	106153	106526	368	-	0	ID=950281;Target=Sequence:950281 1 374
scaffold_1_mito	BLASTN	similarity	83239	83510	272	-	0	ID=950281;Target=Sequence:950281 2196 2467
scaffold_1_mito	BLASTN	similarity	90969	91212	244	-	0	ID=950281;Target=Sequence:950281 1598 1841
scaffold_1_mito	BLASTN	similarity	105763	105995	233	-	0	ID=950281;Target=Sequence:950281 367 599
scaffold_1_mito	BLASTN	similarity	96721	96886	166	-	0	ID=950281;Target=Sequence:950281 1062 1227
scaffold_1_mito	BLASTN	similarity	83920	84077	158	-	0	ID=950281;Target=Sequence:950281 2038 2195
scaffold_1_mito	BLASTN	similarity	101624	101772	149	-	0	ID=950281;Target=Sequence:950281 891 1039
scaffold_1_mito	BLASTN	similarity	80775	81085	135	-	0	ID=950281;Target=Sequence:950281 2888 3201
scaffold_1_mito	BLASTN	similarity	80840	81153	135	-	0	ID=950281;Target=Sequence:950281 2956 3266
scaffold_1_mito	BLASTN	similarity	92116	92237	119	-	0	ID=950281;Target=Sequence:950281 1480 1602
scaffold_1_mito	BLASTN	similarity	102957	103075	119	-	0	ID=950281;Target=Sequence:950281 656 774
scaffold_1_mito	BLASTN	similarity	84959	85076	118	-	0	ID=950281;Target=Sequence:950281 1860 1977
scaffold_1_mito	BLASTN	similarity	102651	102766	116	-	0	ID=950281;Target=Sequence:950281 775 890
scaffold_1_mito	BLASTN	similarity	95451	95532	82	-	0	ID=950281;Target=Sequence:950281 1303 1384
scaffold_1_mito	BLASTN	similarity	96049	96122	74	-	0	ID=950281;Target=Sequence:950281 1228 1301
scaffold_1_mito	BLASTN	similarity	84866	84925	60	-	0	ID=950281;Target=Sequence:950281 1978 2037
scaffold_1_mito	BLASTN	similarity	103712	103768	57	-	0	ID=950281;Target=Sequence:950281 600 656
scaffold_1_mito	BLASTN	similarity	80707	80942	55	-	0	ID=950281;Target=Sequence:950281 2896 3136
scaffold_1_mito	BLASTN	similarity	80905	81145	55	-	0	ID=950281;Target=Sequence:950281 3099 3334
scaffold_1_mito	BLASTN	similarity	95016	95068	53	-	0	ID=950281;Target=Sequence:950281 1380 1432
scaffold_1_mito	BLASTN	similarity	92273	92321	49	-	0	ID=950281;Target=Sequence:950281 1432 1480
scaffold_1_mito	BLASTN	similarity	80707	80807	39	-	0	ID=950281;Target=Sequence:950281 2966 3068
scaffold_1_mito	BLASTN	similarity	80973	81075	39	-	0	ID=950281;Target=Sequence:950281 3234 3334
scaffold_1_mito	BLASTN	similarity	11676	11987	312	+	0	ID=946144;Target=Sequence:946144 1 312
scaffold_1_mito	BLASTN	similarity	11700	11981	282	+	0	ID=861956;Target=Sequence:861956 1 282
scaffold_1_mito	BLASTN	similarity	83005	83731	727	-	0	ID=518409;Target=Sequence:518409 1 727
scaffold_1_mito	BLASTN	similarity	82878	83050	118	-	0	ID=518409;Target=Sequence:518409 557 727
scaffold_1_mito	BLASTN	similarity	49554	49943	390	+	0	ID=946151;Target=Sequence:946151 1 390
scaffold_1_mito	BLASTN	similarity	49551	49943	393	+	0	ID=879519;Target=Sequence:879519 1 393
scaffold_1_mito	BLASTN	similarity	24600	26595	1996	+	0	ID=900758;Target=Sequence:900758 1 1996
scaffold_1_mito	BLASTN	similarity	24336	24437	62	+	0	ID=900758;Target=Sequence:900758 3 103
scaffold_1_mito	BLASTN	similarity	44858	45316	459	+	0	ID=946149;Target=Sequence:946149 1 459
scaffold_1_mito	BLASTN	similarity	45531	45590	60	+	0	ID=946149;Target=Sequence:946149 460 519
scaffold_1_mito	BLASTN	similarity	108244	108665	422	-	0	ID=900814;Target=Sequence:900814 1 422
scaffold_1_mito	BLASTN	similarity	108178	108265	58	-	0	ID=900814;Target=Sequence:900814 333 421
scaffold_1_mito	BLASTN	similarity	107378	107449	48	-	0	ID=900814;Target=Sequence:900814 333 405
scaffold_1_mito	BLASTN	similarity	15135	16620	1460	+	0	ID=900819;Target=Sequence:900819 2477 3964
scaffold_1_mito	BLASTN	similarity	11127	12404	1221	+	0	ID=900819;Target=Sequence:900819 892 2169
scaffold_1_mito	BLASTN	similarity	10103	11152	975	+	0	ID=900819;Target=Sequence:900819 1 1051
scaffold_1_mito	BLASTN	similarity	13692	13999	308	+	0	ID=900819;Target=Sequence:900819 2170 2477
scaffold_1_mito	BLASTN	similarity	16564	16767	186	+	0	ID=900819;Target=Sequence:900819 3839 4042
scaffold_1_mito	BLASTN	similarity	10448	10725	80	+	0	ID=900819;Target=Sequence:900819 647 925
scaffold_1_mito	BLASTN	similarity	10749	11027	80	+	0	ID=900819;Target=Sequence:900819 346 623
scaffold_1_mito	BLASTN	similarity	10499	10650	77	+	0	ID=900819;Target=Sequence:900819 464 615
scaffold_1_mito	BLASTN	similarity	10566	10717	77	+	0	ID=900819;Target=Sequence:900819 397 548
scaffold_1_mito	BLASTN	similarity	16731	16829	49	+	0	ID=900819;Target=Sequence:900819 3798 3899
scaffold_1_mito	BLASTN	similarity	7916	7966	44	+	0	ID=900819;Target=Sequence:900819 658 707
scaffold_1_mito	BLASTN	similarity	7917	7969	44	+	0	ID=900819;Target=Sequence:900819 358 410
scaffold_1_mito	BLASTN	similarity	69105	69145	41	+	0	ID=900819;Target=Sequence:900819 1351 1391
scaffold_1_mito	BLASTN	similarity	73191	73231	41	+	0	ID=900819;Target=Sequence:900819 1351 1391
scaffold_1_mito	BLASTN	similarity	16863	16899	37	+	0	ID=900819;Target=Sequence:900819 4043 4079
scaffold_1_mito	BLASTN	similarity	59723	59758	36	+	0	ID=900819;Target=Sequence:900819 363 398
scaffold_1_mito	BLASTN	similarity	59723	59758	36	+	0	ID=900819;Target=Sequence:900819 664 699
scaffold_1_mito	BLASTN	similarity	24140	24174	32	-	0	ID=900819;Target=Sequence:900819 664 699
scaffold_1_mito	BLASTN	similarity	24140	24174	32	-	0	ID=900819;Target=Sequence:900819 363 398
scaffold_1_mito	BLASTN	similarity	15249	15639	391	+	0	ID=946145;Target=Sequence:946145 563 953
scaffold_1_mito	BLASTN	similarity	15673	16012	337	+	0	ID=946145;Target=Sequence:946145 950 1290
scaffold_1_mito	BLASTN	similarity	16043	16342	300	+	0	ID=946145;Target=Sequence:946145 1291 1590
scaffold_1_mito	BLASTN	similarity	12212	12404	193	+	0	ID=946145;Target=Sequence:946145 156 348
scaffold_1_mito	BLASTN	similarity	11988	12142	155	+	0	ID=946145;Target=Sequence:946145 1 155
scaffold_1_mito	BLASTN	similarity	13697	13834	133	+	0	ID=946145;Target=Sequence:946145 345 484
scaffold_1_mito	BLASTN	similarity	13862	13942	81	+	0	ID=946145;Target=Sequence:946145 477 557
scaffold_1_mito	BLASTN	similarity	66740	67182	440	+	0	ID=889790;Target=Sequence:889790 452 894
scaffold_1_mito	BLASTN	similarity	65310	65480	171	+	0	ID=889790;Target=Sequence:889790 1 171
scaffold_1_mito	BLASTN	similarity	66372	66518	147	+	0	ID=889790;Target=Sequence:889790 308 454
scaffold_1_mito	BLASTN	similarity	65536	65672	137	+	0	ID=889790;Target=Sequence:889790 172 308
scaffold_1_mito	BLASTN	similarity	55183	55595	413	+	0	ID=979745;Target=Sequence:979745 1 413
scaffold_1_mito	BLASTN	similarity	61774	62061	281	+	0	ID=979745;Target=Sequence:979745 1113 1398
scaffold_1_mito	BLASTN	similarity	56251	56454	204	+	0	ID=979745;Target=Sequence:979745 413 616
scaffold_1_mito	BLASTN	similarity	60654	60847	194	+	0	ID=979745;Target=Sequence:979745 886 1079
scaffold_1_mito	BLASTN	similarity	60099	60260	162	+	0	ID=979745;Target=Sequence:979745 726 887
scaffold_1_mito	BLASTN	similarity	58470	58535	66	+	0	ID=979745;Target=Sequence:979745 615 680
scaffold_1_mito	BLASTN	similarity	58774	58822	49	+	0	ID=979745;Target=Sequence:979745 680 728
scaffold_1_mito	BLASTN	similarity	61362	61405	44	+	0	ID=979745;Target=Sequence:979745 1079 1122
scaffold_1_mito	BLASTN	similarity	61300	61330	31	+	0	ID=979745;Target=Sequence:979745 1091 1121
scaffold_1_mito	BLASTN	similarity	79888	80781	894	-	0	ID=900792;Target=Sequence:900792 1 894
scaffold_1_mito	BLASTN	similarity	78603	79188	544	-	0	ID=900792;Target=Sequence:900792 895 1475
scaffold_1_mito	BLASTN	similarity	78534	78610	77	-	0	ID=900792;Target=Sequence:900792 1399 1475
scaffold_1_mito	BLASTN	similarity	78112	78161	47	-	0	ID=900792;Target=Sequence:900792 1397 1446
scaffold_1_mito	BLASTN	similarity	80973	81018	37	-	0	ID=900792;Target=Sequence:900792 30 75
scaffold_1_mito	BLASTN	similarity	80905	80980	35	-	0	ID=900792;Target=Sequence:900792 3 75
scaffold_1_mito	BLASTN	similarity	78401	78439	32	-	0	ID=900792;Target=Sequence:900792 1425 1461
```

In [177]:

```
%%bash
cd ../../Data/Mito
bp_search2gff --input Mito_Transcripts_to_IFO0880_4.txt --addid --version 3 --type hit --match
```

```
##gff-version 3
scaffold_1_mito	BLASTN	similarity	93917	95072	1156	-	0	Parent=900810;Target=Sequence:900810 828 1983
scaffold_1_mito	BLASTN	similarity	101569	101768	200	-	0	Parent=900810;Target=Sequence:900810 253 452
scaffold_1_mito	BLASTN	similarity	96728	96859	132	-	0	Parent=900810;Target=Sequence:900810 502 633
scaffold_1_mito	BLASTN	similarity	95443	95565	120	-	0	Parent=900810;Target=Sequence:900810 710 832
scaffold_1_mito	BLASTN	similarity	93859	94022	119	-	0	Parent=900810;Target=Sequence:900810 1820 1983
scaffold_1_mito	BLASTN	similarity	102656	102766	111	-	0	Parent=900810;Target=Sequence:900810 142 252
scaffold_1_mito	BLASTN	similarity	102966	103064	96	-	0	Parent=900810;Target=Sequence:900810 46 144
scaffold_1_mito	BLASTN	similarity	93975	94080	82	-	0	Parent=900810;Target=Sequence:900810 1878 1983
scaffold_1_mito	BLASTN	similarity	96051	96127	77	-	0	Parent=900810;Target=Sequence:900810 633 709
scaffold_1_mito	BLASTN	similarity	103714	103758	45	-	0	Parent=900810;Target=Sequence:900810 1 45
scaffold_1_mito	BLASTN	similarity	100691	100718	28	-	0	Parent=900810;Target=Sequence:900810 452 479
scaffold_1_mito	BLASTN	match	93859	103758	2135	-	1	ID=900810;Target=Sequence:900810
scaffold_1_mito	BLASTN	similarity	64475	65521	1047	+	0	Parent=900782;Target=Sequence:900782 1 1047
scaffold_1_mito	BLASTN	similarity	66731	67158	428	+	0	Parent=900782;Target=Sequence:900782 1047 1474
scaffold_1_mito	BLASTN	similarity	67667	67759	93	+	0	Parent=900782;Target=Sequence:900782 1473 1565
scaffold_1_mito	BLASTN	match	64475	67759	1934	+	1	ID=900782;Target=Sequence:900782
scaffold_1_mito	BLASTN	similarity	55183	55606	424	+	0	Parent=900778;Target=Sequence:900778 1 424
scaffold_1_mito	BLASTN	similarity	56265	56596	332	+	0	Parent=900778;Target=Sequence:900778 424 755
scaffold_1_mito	BLASTN	match	55183	56596	784	+	1	ID=900778;Target=Sequence:900778
scaffold_1_mito	BLASTN	similarity	11721	11981	261	+	0	Parent=866864;Target=Sequence:866864 1 261
scaffold_1_mito	BLASTN	match	11721	11981	483	+	1	ID=866864;Target=Sequence:866864
scaffold_1_mito	BLASTN	similarity	79914	80517	604	-	0	Parent=950280;Target=Sequence:950280 1 604
scaffold_1_mito	BLASTN	similarity	78910	79108	199	-	0	Parent=950280;Target=Sequence:950280 645 843
scaffold_1_mito	BLASTN	similarity	79136	79175	40	-	0	Parent=950280;Target=Sequence:950280 605 644
scaffold_1_mito	BLASTN	match	78910	80517	1116	-	1	ID=950280;Target=Sequence:950280
scaffold_1_mito	BLASTN	similarity	44419	45429	1011	+	0	Parent=900774;Target=Sequence:900774 1 1011
scaffold_1_mito	BLASTN	similarity	44224	44320	43	+	0	Parent=900774;Target=Sequence:900774 1 86
scaffold_1_mito	BLASTN	similarity	44340	44388	33	+	0	Parent=900774;Target=Sequence:900774 35 82
scaffold_1_mito	BLASTN	match	44224	45429	1868	+	1	ID=900774;Target=Sequence:900774
scaffold_1_mito	BLASTN	similarity	89995	91248	1254	-	0	Parent=900801;Target=Sequence:900801 720 1973
scaffold_1_mito	BLASTN	similarity	93282	93817	531	-	0	Parent=900801;Target=Sequence:900801 1 538
scaffold_1_mito	BLASTN	similarity	92125	92313	189	-	0	Parent=900801;Target=Sequence:900801 532 720
scaffold_1_mito	BLASTN	similarity	93747	93877	125	-	0	Parent=900801;Target=Sequence:900801 43 173
scaffold_1_mito	BLASTN	similarity	93645	93715	68	-	0	Parent=900801;Target=Sequence:900801 1 71
scaffold_1_mito	BLASTN	similarity	89851	90003	61	-	0	Parent=900801;Target=Sequence:900801 1812 1973
scaffold_1_mito	BLASTN	similarity	90637	90724	44	-	0	Parent=900801;Target=Sequence:900801 1107 1197
scaffold_1_mito	BLASTN	similarity	90771	90861	44	-	0	Parent=900801;Target=Sequence:900801 1244 1331
scaffold_1_mito	BLASTN	similarity	55845	55885	41	+	0	Parent=900801;Target=Sequence:900801 1 41
scaffold_1_mito	BLASTN	similarity	55845	55885	41	+	0	Parent=900801;Target=Sequence:900801 103 143
scaffold_1_mito	BLASTN	similarity	89668	89709	33	-	0	Parent=900801;Target=Sequence:900801 1860 1901
scaffold_1_mito	BLASTN	similarity	93849	93877	29	-	0	Parent=900801;Target=Sequence:900801 145 173
scaffold_1_mito	BLASTN	match	55845	93877	2316	-	1	ID=900801;Target=Sequence:900801
scaffold_1_mito	BLASTN	similarity	11099	12113	1015	+	0	Parent=518890;Target=Sequence:518890 1 1015
scaffold_1_mito	BLASTN	similarity	69105	69145	41	+	0	Parent=518890;Target=Sequence:518890 488 528
scaffold_1_mito	BLASTN	similarity	73191	73231	41	+	0	Parent=518890;Target=Sequence:518890 488 528
scaffold_1_mito	BLASTN	match	11099	73231	1875	+	1	ID=518890;Target=Sequence:518890
scaffold_1_mito	BLASTN	similarity	55183	55436	254	+	0	Parent=950279;Target=Sequence:950279 1 254
scaffold_1_mito	BLASTN	similarity	60654	60849	196	+	0	Parent=950279;Target=Sequence:950279 688 883
scaffold_1_mito	BLASTN	similarity	56263	56454	192	+	0	Parent=950279;Target=Sequence:950279 335 526
scaffold_1_mito	BLASTN	similarity	61886	62070	185	+	0	Parent=950279;Target=Sequence:950279 950 1134
scaffold_1_mito	BLASTN	similarity	60102	60260	159	+	0	Parent=950279;Target=Sequence:950279 531 689
scaffold_1_mito	BLASTN	similarity	55511	55594	84	+	0	Parent=950279;Target=Sequence:950279 251 334
scaffold_1_mito	BLASTN	similarity	61784	61852	69	+	0	Parent=950279;Target=Sequence:950279 881 949
scaffold_1_mito	BLASTN	match	55183	62070	470	+	1	ID=950279;Target=Sequence:950279
scaffold_1_mito	BLASTN	similarity	66726	67182	457	+	0	Parent=946153;Target=Sequence:946153 300 756
scaffold_1_mito	BLASTN	similarity	65193	65491	299	+	0	Parent=946153;Target=Sequence:946153 1 299
scaffold_1_mito	BLASTN	match	65193	67182	845	+	1	ID=946153;Target=Sequence:946153
scaffold_1_mito	BLASTN	similarity	55183	55595	413	+	0	Parent=979743;Target=Sequence:979743 1 413
scaffold_1_mito	BLASTN	similarity	61784	62055	272	+	0	Parent=979743;Target=Sequence:979743 1109 1380
scaffold_1_mito	BLASTN	similarity	56251	56454	204	+	0	Parent=979743;Target=Sequence:979743 413 616
scaffold_1_mito	BLASTN	similarity	60654	60847	194	+	0	Parent=979743;Target=Sequence:979743 886 1079
scaffold_1_mito	BLASTN	similarity	60099	60260	162	+	0	Parent=979743;Target=Sequence:979743 726 887
scaffold_1_mito	BLASTN	similarity	58470	58535	66	+	0	Parent=979743;Target=Sequence:979743 615 680
scaffold_1_mito	BLASTN	similarity	58774	58822	49	+	0	Parent=979743;Target=Sequence:979743 680 728
scaffold_1_mito	BLASTN	similarity	61249	61280	32	+	0	Parent=979743;Target=Sequence:979743 1079 1110
scaffold_1_mito	BLASTN	match	55183	62055	763	+	1	ID=979743;Target=Sequence:979743
scaffold_1_mito	BLASTN	similarity	49557	49931	375	+	0	Parent=863471;Target=Sequence:863471 1 375
scaffold_1_mito	BLASTN	match	49557	49931	693	+	1	ID=863471;Target=Sequence:863471
scaffold_1_mito	BLASTN	similarity	61779	62528	750	+	0	Parent=900779;Target=Sequence:900779 1089 1838
scaffold_1_mito	BLASTN	similarity	55183	55606	424	+	0	Parent=900779;Target=Sequence:900779 1 424
scaffold_1_mito	BLASTN	similarity	60651	60843	193	+	0	Parent=900779;Target=Sequence:900779 850 1042
scaffold_1_mito	BLASTN	similarity	56265	56456	192	+	0	Parent=900779;Target=Sequence:900779 424 615
scaffold_1_mito	BLASTN	similarity	60113	60246	131	+	0	Parent=900779;Target=Sequence:900779 716 849
scaffold_1_mito	BLASTN	similarity	58980	59046	67	+	0	Parent=900779;Target=Sequence:900779 652 718
scaffold_1_mito	BLASTN	similarity	61520	61567	48	+	0	Parent=900779;Target=Sequence:900779 1043 1090
scaffold_1_mito	BLASTN	similarity	58486	58522	37	+	0	Parent=900779;Target=Sequence:900779 616 652
scaffold_1_mito	BLASTN	match	55183	62528	1386	+	1	ID=900779;Target=Sequence:900779
scaffold_1_mito	BLASTN	similarity	80707	81573	867	-	0	Parent=950281;Target=Sequence:950281 2468 3334
scaffold_1_mito	BLASTN	similarity	106153	106526	368	-	0	Parent=950281;Target=Sequence:950281 1 374
scaffold_1_mito	BLASTN	similarity	83239	83510	272	-	0	Parent=950281;Target=Sequence:950281 2196 2467
scaffold_1_mito	BLASTN	similarity	90969	91212	244	-	0	Parent=950281;Target=Sequence:950281 1598 1841
scaffold_1_mito	BLASTN	similarity	105763	105995	233	-	0	Parent=950281;Target=Sequence:950281 367 599
scaffold_1_mito	BLASTN	similarity	96721	96886	166	-	0	Parent=950281;Target=Sequence:950281 1062 1227
scaffold_1_mito	BLASTN	similarity	83920	84077	158	-	0	Parent=950281;Target=Sequence:950281 2038 2195
scaffold_1_mito	BLASTN	similarity	101624	101772	149	-	0	Parent=950281;Target=Sequence:950281 891 1039
scaffold_1_mito	BLASTN	similarity	80775	81085	135	-	0	Parent=950281;Target=Sequence:950281 2888 3201
scaffold_1_mito	BLASTN	similarity	80840	81153	135	-	0	Parent=950281;Target=Sequence:950281 2956 3266
scaffold_1_mito	BLASTN	similarity	92116	92237	119	-	0	Parent=950281;Target=Sequence:950281 1480 1602
scaffold_1_mito	BLASTN	similarity	102957	103075	119	-	0	Parent=950281;Target=Sequence:950281 656 774
scaffold_1_mito	BLASTN	similarity	84959	85076	118	-	0	Parent=950281;Target=Sequence:950281 1860 1977
scaffold_1_mito	BLASTN	similarity	102651	102766	116	-	0	Parent=950281;Target=Sequence:950281 775 890
scaffold_1_mito	BLASTN	similarity	95451	95532	82	-	0	Parent=950281;Target=Sequence:950281 1303 1384
scaffold_1_mito	BLASTN	similarity	96049	96122	74	-	0	Parent=950281;Target=Sequence:950281 1228 1301
scaffold_1_mito	BLASTN	similarity	84866	84925	60	-	0	Parent=950281;Target=Sequence:950281 1978 2037
scaffold_1_mito	BLASTN	similarity	103712	103768	57	-	0	Parent=950281;Target=Sequence:950281 600 656
scaffold_1_mito	BLASTN	similarity	80707	80942	55	-	0	Parent=950281;Target=Sequence:950281 2896 3136
scaffold_1_mito	BLASTN	similarity	80905	81145	55	-	0	Parent=950281;Target=Sequence:950281 3099 3334
scaffold_1_mito	BLASTN	similarity	95016	95068	53	-	0	Parent=950281;Target=Sequence:950281 1380 1432
scaffold_1_mito	BLASTN	similarity	92273	92321	49	-	0	Parent=950281;Target=Sequence:950281 1432 1480
scaffold_1_mito	BLASTN	similarity	80707	80807	39	-	0	Parent=950281;Target=Sequence:950281 2966 3068
scaffold_1_mito	BLASTN	similarity	80973	81075	39	-	0	Parent=950281;Target=Sequence:950281 3234 3334
scaffold_1_mito	BLASTN	match	80707	106526	1602	-	1	ID=950281;Target=Sequence:950281
scaffold_1_mito	BLASTN	similarity	11676	11987	312	+	0	Parent=946144;Target=Sequence:946144 1 312
scaffold_1_mito	BLASTN	match	11676	11987	577	+	1	ID=946144;Target=Sequence:946144
scaffold_1_mito	BLASTN	similarity	11700	11981	282	+	0	Parent=861956;Target=Sequence:861956 1 282
scaffold_1_mito	BLASTN	match	11700	11981	521	+	1	ID=861956;Target=Sequence:861956
scaffold_1_mito	BLASTN	similarity	83005	83731	727	-	0	Parent=518409;Target=Sequence:518409 1 727
scaffold_1_mito	BLASTN	similarity	82878	83050	118	-	0	Parent=518409;Target=Sequence:518409 557 727
scaffold_1_mito	BLASTN	match	82878	83731	1343	-	1	ID=518409;Target=Sequence:518409
scaffold_1_mito	BLASTN	similarity	49554	49943	390	+	0	Parent=946151;Target=Sequence:946151 1 390
scaffold_1_mito	BLASTN	match	49554	49943	721	+	1	ID=946151;Target=Sequence:946151
scaffold_1_mito	BLASTN	similarity	49551	49943	393	+	0	Parent=879519;Target=Sequence:879519 1 393
scaffold_1_mito	BLASTN	match	49551	49943	726	+	1	ID=879519;Target=Sequence:879519
scaffold_1_mito	BLASTN	similarity	24600	26595	1996	+	0	Parent=900758;Target=Sequence:900758 1 1996
scaffold_1_mito	BLASTN	similarity	24336	24437	62	+	0	Parent=900758;Target=Sequence:900758 3 103
scaffold_1_mito	BLASTN	match	24336	26595	3687	+	1	ID=900758;Target=Sequence:900758
scaffold_1_mito	BLASTN	similarity	44858	45316	459	+	0	Parent=946149;Target=Sequence:946149 1 459
scaffold_1_mito	BLASTN	similarity	45531	45590	60	+	0	Parent=946149;Target=Sequence:946149 460 519
scaffold_1_mito	BLASTN	match	44858	45590	848	+	1	ID=946149;Target=Sequence:946149
scaffold_1_mito	BLASTN	similarity	108244	108665	422	-	0	Parent=900814;Target=Sequence:900814 1 422
scaffold_1_mito	BLASTN	similarity	108178	108265	58	-	0	Parent=900814;Target=Sequence:900814 333 421
scaffold_1_mito	BLASTN	similarity	107378	107449	48	-	0	Parent=900814;Target=Sequence:900814 333 405
scaffold_1_mito	BLASTN	match	107378	108665	780	-	1	ID=900814;Target=Sequence:900814
scaffold_1_mito	BLASTN	similarity	15135	16620	1460	+	0	Parent=900819;Target=Sequence:900819 2477 3964
scaffold_1_mito	BLASTN	similarity	11127	12404	1221	+	0	Parent=900819;Target=Sequence:900819 892 2169
scaffold_1_mito	BLASTN	similarity	10103	11152	975	+	0	Parent=900819;Target=Sequence:900819 1 1051
scaffold_1_mito	BLASTN	similarity	13692	13999	308	+	0	Parent=900819;Target=Sequence:900819 2170 2477
scaffold_1_mito	BLASTN	similarity	16564	16767	186	+	0	Parent=900819;Target=Sequence:900819 3839 4042
scaffold_1_mito	BLASTN	similarity	10448	10725	80	+	0	Parent=900819;Target=Sequence:900819 647 925
scaffold_1_mito	BLASTN	similarity	10749	11027	80	+	0	Parent=900819;Target=Sequence:900819 346 623
scaffold_1_mito	BLASTN	similarity	10499	10650	77	+	0	Parent=900819;Target=Sequence:900819 464 615
scaffold_1_mito	BLASTN	similarity	10566	10717	77	+	0	Parent=900819;Target=Sequence:900819 397 548
scaffold_1_mito	BLASTN	similarity	16731	16829	49	+	0	Parent=900819;Target=Sequence:900819 3798 3899
scaffold_1_mito	BLASTN	similarity	7916	7966	44	+	0	Parent=900819;Target=Sequence:900819 658 707
scaffold_1_mito	BLASTN	similarity	7917	7969	44	+	0	Parent=900819;Target=Sequence:900819 358 410
scaffold_1_mito	BLASTN	similarity	69105	69145	41	+	0	Parent=900819;Target=Sequence:900819 1351 1391
scaffold_1_mito	BLASTN	similarity	73191	73231	41	+	0	Parent=900819;Target=Sequence:900819 1351 1391
scaffold_1_mito	BLASTN	similarity	16863	16899	37	+	0	Parent=900819;Target=Sequence:900819 4043 4079
scaffold_1_mito	BLASTN	similarity	59723	59758	36	+	0	Parent=900819;Target=Sequence:900819 363 398
scaffold_1_mito	BLASTN	similarity	59723	59758	36	+	0	Parent=900819;Target=Sequence:900819 664 699
scaffold_1_mito	BLASTN	similarity	24140	24174	32	-	0	Parent=900819;Target=Sequence:900819 664 699
scaffold_1_mito	BLASTN	similarity	24140	24174	32	-	0	Parent=900819;Target=Sequence:900819 363 398
scaffold_1_mito	BLASTN	match	7916	73231	2697	+	1	ID=900819;Target=Sequence:900819
scaffold_1_mito	BLASTN	similarity	15249	15639	391	+	0	Parent=946145;Target=Sequence:946145 563 953
scaffold_1_mito	BLASTN	similarity	15673	16012	337	+	0	Parent=946145;Target=Sequence:946145 950 1290
scaffold_1_mito	BLASTN	similarity	16043	16342	300	+	0	Parent=946145;Target=Sequence:946145 1291 1590
scaffold_1_mito	BLASTN	similarity	12212	12404	193	+	0	Parent=946145;Target=Sequence:946145 156 348
scaffold_1_mito	BLASTN	similarity	11988	12142	155	+	0	Parent=946145;Target=Sequence:946145 1 155
scaffold_1_mito	BLASTN	similarity	13697	13834	133	+	0	Parent=946145;Target=Sequence:946145 345 484
scaffold_1_mito	BLASTN	similarity	13862	13942	81	+	0	Parent=946145;Target=Sequence:946145 477 557
scaffold_1_mito	BLASTN	match	11988	16342	723	+	1	ID=946145;Target=Sequence:946145
scaffold_1_mito	BLASTN	similarity	66740	67182	440	+	0	Parent=889790;Target=Sequence:889790 452 894
scaffold_1_mito	BLASTN	similarity	65310	65480	171	+	0	Parent=889790;Target=Sequence:889790 1 171
scaffold_1_mito	BLASTN	similarity	66372	66518	147	+	0	Parent=889790;Target=Sequence:889790 308 454
scaffold_1_mito	BLASTN	similarity	65536	65672	137	+	0	Parent=889790;Target=Sequence:889790 172 308
scaffold_1_mito	BLASTN	match	65310	67182	813	+	1	ID=889790;Target=Sequence:889790
scaffold_1_mito	BLASTN	similarity	55183	55595	413	+	0	Parent=979745;Target=Sequence:979745 1 413
scaffold_1_mito	BLASTN	similarity	61774	62061	281	+	0	Parent=979745;Target=Sequence:979745 1113 1398
scaffold_1_mito	BLASTN	similarity	56251	56454	204	+	0	Parent=979745;Target=Sequence:979745 413 616
scaffold_1_mito	BLASTN	similarity	60654	60847	194	+	0	Parent=979745;Target=Sequence:979745 886 1079
scaffold_1_mito	BLASTN	similarity	60099	60260	162	+	0	Parent=979745;Target=Sequence:979745 726 887
scaffold_1_mito	BLASTN	similarity	58470	58535	66	+	0	Parent=979745;Target=Sequence:979745 615 680
scaffold_1_mito	BLASTN	similarity	58774	58822	49	+	0	Parent=979745;Target=Sequence:979745 680 728
scaffold_1_mito	BLASTN	similarity	61362	61405	44	+	0	Parent=979745;Target=Sequence:979745 1079 1122
scaffold_1_mito	BLASTN	similarity	61300	61330	31	+	0	Parent=979745;Target=Sequence:979745 1091 1121
scaffold_1_mito	BLASTN	match	55183	62061	763	+	1	ID=979745;Target=Sequence:979745
scaffold_1_mito	BLASTN	similarity	79888	80781	894	-	0	Parent=900792;Target=Sequence:900792 1 894
scaffold_1_mito	BLASTN	similarity	78603	79188	544	-	0	Parent=900792;Target=Sequence:900792 895 1475
scaffold_1_mito	BLASTN	similarity	78534	78610	77	-	0	Parent=900792;Target=Sequence:900792 1399 1475
scaffold_1_mito	BLASTN	similarity	78112	78161	47	-	0	Parent=900792;Target=Sequence:900792 1397 1446
scaffold_1_mito	BLASTN	similarity	80973	81018	37	-	0	Parent=900792;Target=Sequence:900792 30 75
scaffold_1_mito	BLASTN	similarity	80905	80980	35	-	0	Parent=900792;Target=Sequence:900792 3 75
scaffold_1_mito	BLASTN	similarity	78401	78439	32	-	0	Parent=900792;Target=Sequence:900792 1425 1461
scaffold_1_mito	BLASTN	match	78112	81018	1652	-	1	ID=900792;Target=Sequence:900792
```

In [178]:

```
%%bash
cd ../../Data/Mito
bp_search2gff --input Mito_Transcripts_to_IFO0880_4.txt --addid --version 3 --type hit --match \
    > Mito_Transcripts_to_IFO0880_4.gff3
```

In [191]:

```
sorted(Mito_proteinId.items())
```

Out[191]:

```
[('atp6', {'900623': 51.984, '945998': 51.634}),
 ('atp8', {'900650': 110.42099999999999}),
 ('atp9', {'900663': 432.82000000000005}),
 ('cob',
  {'979592': 655.721,
   '979594': 106.666,
   '950128': 436.87,
   '900628': 159.511,
   '900627': 121.25}),
 ('cox1', {'900659': 328.382, '950130': 1626.5469999999998}),
 ('cox2', {'950130': 1083.48, '518258': 339.301}),
 ('cox3', {'946002': 903.938, '889639': 269.195, '900631': 170.958}),
 ('nad1', {'900641': 280.145, '950129': 58.861}),
 ('nad3', {'863320': 52.874, '879368': 56.989, '946000': 50.515}),
 ('nad4', {'900607': 279.053}),
 ('nad4L',
  {'518739': 55.422, '945993': 72.093, '861805': 63.953, '866713': 66.279}),
 ('nad5', {'900668': 247.29200000000003, '945994': 53.959})]
```

atp6 RTO3\_900623 Q0085 ATPeF0A, MTATP6, ATP6; F-type H+-transporting ATPase subunit a  
atp8 RTO3\_879280 Q0080 ATPase, F0 complex, subunit 8, mitochondrial, fungal (not final gene model)  
atp9 RTO3\_900663 Q0130 ATPeF0C, ATP5G, ATP9; F-type H+-transporting ATPase subunit c

COB (Q0105, CYTB) RTO3\_979594 # initially had it as 946001, but 979594 matches splicing better

COX1, COX2 (I, II) RTO3\_946004  
COX3 (III) RTO3\_946002

RTO3\_900641 ND1; NADH-ubiquinone oxidoreductase chain 1  
RTO3\_874609 ND3; NADH-ubiquinone oxidoreductase chain 3  
RTO3\_945995 ND4; NADH-ubiquinone oxidoreductase chain 4  
RTO3\_879512 NADH dehydrogenase subunit 4L and related proteins  
RTO3\_879341 NADH dehydrogenase subunit 4L and related proteins  
RTO3\_945994 ND5; NADH-ubiquinone oxidoreductase chain 5  
RTO3\_946005 ND5; NADH-ubiquinone oxidoreductase chain 5  
RTO3\_900622 ND6; NADH-ubiquinone oxidoreductase chain 6  
RTO3\_900624 nuoN; NADH-quinone oxidoreductase subunit N

In [ ]:

```

```
