# Supplementary material for: Multi-Omics Driven Metabolic Network Reconstruction and Analysis of Lignocellulosic Carbon Utilization in Rhodosporidium toruloides
Source: Front Bioeng Biotechnol. 2021 Jan 8;8:612832. doi: 10.3389/fbioe.2020.612832 (PMC7873862; doi:10.3389/fbioe.2020.612832)
Supplement: Supplementary File 4 — Multi-omics dataset for R. toruloides IFO0880. [file Data_Sheet_1.zip › Supplementary File S1/1.Manual_curation/Refinement_1b_Duplicate_Reactions.html]

Refinement\_1b\_Duplicate\_Reactions


In [1]:

```
%matplotlib inline
from matplotlib import pyplot as plt
from matplotlib import colors
import csv
import numpy as np
import pandas as pd
import cobra
```

In [2]:

```
cobra.__version__
```

Out[2]:

```
'0.15.4'
```

In [3]:

```
Annotation = pd.read_excel('../../Data/R_toruloides_Data_for_Reconstruction.xlsx',
                          sheet_name='Annotation', index_col=0)
Annotation.index = Annotation.index.map(str)
Annotation = Annotation.fillna('')
Transcriptomics = pd.read_excel('../../Data/R_toruloides_Data_for_Reconstruction.xlsx',
                          sheet_name='Transcriptomics', header=[0,1,2,3], index_col=0)
Transcriptomics.index = Transcriptomics.index.map(str)
Proteomics = pd.read_excel('../../Data/R_toruloides_Data_for_Reconstruction.xlsx',
                          sheet_name='Proteomics', header=[0,1,2], index_col=0)
Proteomics.index = Proteomics.index.map(str)
Fitness = pd.read_excel('../../Data/R_toruloides_Data_for_Reconstruction.xlsx',
                          sheet_name='Fitness', index_col=0)
Fitness.index = Fitness.index.map(str)
```

In [4]:

```
def background_gradient(s, cmap='seismic', text_color_threshold=0.408):
    lim = max(abs(s.min().min()),abs(s.max().max()))
    rng = 2.0*lim
    norm = colors.Normalize(-lim - (rng * 0.2), lim + (rng * 0.2))
    rgbas = plt.cm.get_cmap(cmap)(norm(s.values))
    def relative_luminance(rgba):
        r, g, b = (x / 12.92 if x <= 0.03928 else ((x + 0.055) / 1.055 ** 2.4) for x in rgba[:3])
        return 0.2126 * r + 0.7152 * g + 0.0722 * b
    def css(rgba):
        dark = relative_luminance(rgba) < text_color_threshold
        text_color = '#f1f1f1' if dark else '#000000'
        return 'background-color: {b};color: {c};'.format(b=colors.rgb2hex(rgba), c=text_color)

    if s.ndim == 1:
        return [css(rgba) for rgba in rgbas]
    else:
        return pd.DataFrame([[css(rgba) for rgba in row] for row in rgbas], index=s.index, columns=s.columns)

def Show_Data(x):
    display(Transcriptomics.loc[x].style.background_gradient(cmap='Reds', low=0.2, high=0.2, axis=None))
    temp = [y for y in x if y in Proteomics.index]
    display(Proteomics.loc[temp].style.background_gradient(cmap='Reds', low=0.2, high=0.2, axis=None))
    temp = [y for y in x if y in Fitness.index]
    display(Fitness.loc[temp].style.apply(background_gradient, cmap='seismic', axis=None))
    return;
```

In [5]:

```
eco = cobra.io.load_json_model('../../Data/BiGG_Models/iML1515.json')
sce = cobra.io.load_json_model('../../Data/BiGG_Models/iMM904.json')
hsa = cobra.io.load_json_model('../../Data/BiGG_Models/RECON1.json')
hsa2 = cobra.io.load_json_model('../../Data/BiGG_Models/Recon3D.json')
ptri = cobra.io.load_json_model('../../Data/BiGG_Models/iLB1027_lipid.json')
cre = cobra.io.load_json_model('../../Data/BiGG_Models/iRC1080.json')
```

In [6]:

```
model = cobra.io.load_json_model("IFO0880_GPR_1a.json")
```

In [7]:

```
print(len(model.genes))
print(len([x for x in model.genes if not x.id[0].isalpha()]))
model
```

```
1362
1149
```

Out[7]:

|  |  |
| --- | --- |
| **Name** | R. toruloides |
| **Memory address** | 0x01027435ac8 |
| **Number of metabolites** | 3409 |
| **Number of reactions** | 3510 |
| **Number of groups** | 0 |
| **Objective expression** | 0 |
| **Compartments** | c, x, m, e, r, v, n, g, p, h, s, f, l |

### Duplicate reactions¶

In [8]:

```
duplicated = set()
for i, r in enumerate(model.reactions):
    temp = 0
    for j, r2 in enumerate(model.reactions):
        if j > i and r2.id not in duplicated:
            if r.reactants == r2.reactants and r.products == r2.products:
                if temp == 0:
                    temp = 1
                    duplicated.add(r.id)
                    print(r.id, r.reaction, r.gene_reaction_rule)
                duplicated.add(r2.id)
                print(r2.id, r2.reaction, r2.gene_reaction_rule)
    if temp == 1:
        print()
```

```
ARD dhmtp_c + o2_c --> 2kmb_c + for_c + 2.0 h_c 16330
ACDO dhmtp_c + o2_c --> 2kmb_c + for_c + h_c 16330

yli_R1488 atp_c + btn_c --> btamp_c + ppi_c 16404
yli_R1487 atp_c + btn_c --> btamp_c + ppi_c 16404
yli_R1489 atp_c + btn_c --> btamp_c + ppi_c 16404
yli_R1490 atp_c + btn_c --> btamp_c + ppi_c 16404

yli_R0002 akg_c + gln__L_c + h_c + nadph_c --> glu__L_c + nadp_c 15713
GLUSy akg_c + gln__L_c + h_c + nadph_c --> 2.0 glu__L_c + nadp_c (PP_5075 and 15713) or (b3213 and 15713)

yli_R1425 co2_x + mlthf_x + nadh_x + nh4_x --> gly_x + nad_x + thf_x 12898
yli_R1378 co2_x + mlthf_x + nadh_x + nh4_x --> gly_x + nad_x + thf_x 10040
yli_R1377 co2_x + mlthf_x + nadh_x + nh4_x --> gly_x + nad_x + thf_x 10205

yli_R1466 2ippm_m + h2o_m <=> 3c2hmp_m 14914
yli_R7859 2ippm_m + h2o_m <=> 3c2hmp_m 14914

yli_R8859 3c3hmp_m <=> 2ippm_m + h2o_m 14914
yli_R1465 3c3hmp_m <=> 2ippm_m + h2o_m 14914

CERH124_copy2 cer1_24_c + h_c + nadph_c + o2_c --> cer2_24_c + h2o_c + nadp_c 9664
CERH124_copy1 cer1_24_c + h_c + nadph_c + o2_c --> cer2_24_c + h2o_c + nadp_c 15314

FMNAT atp_c + fmn_c + h_c --> fad_c + ppi_c 11542
AFAT atp_c + fmn_c + 2.0 h_c --> fad_c + ppi_c 11542 or 9298

CERH126_copy2 cer1_26_c + h_c + nadph_c + o2_c --> cer2_26_c + h2o_c + nadp_c 9664
CERH126_copy1 cer1_26_c + h_c + nadph_c + o2_c --> cer2_26_c + h2o_c + nadp_c 15314

ALDD2x acald_c + h2o_c + nad_c --> ac_c + 2.0 h_c + nadh_c 12042 or 13426 or 15814 or 16323
ALDD2x_copy1 acald_c + h2o_c + nad_c --> ac_c + 2.0 h_c + nadh_c 12042 or 13426 or 15814

GTPCI gtp_c + h2o_c --> ahdt_c + for_c + h_c 10332
GTPCI_2 gtp_c + h2o_c --> ahdt_c + for_c + 2.0 h_c 10332

GCC2cm dhlam_m + nad_m <=> h_m + lpam_m + nadh_m 10040 and 10205 and 12898 and 15184
GCC2cm_copy2 dhlam_m + nad_m --> h_m + lpam_m + nadh_m (10007 and 10040 and 12116) or (10040 and 12116 and 9274)
GCC2cm_copy1 dhlam_m + nad_m <=> h_m + lpam_m + nadh_m 10040 and 10205 and 12898 and 15184

GTHOr gthox_c + h_c + nadph_c <=> 2.0 gthrd_c + nadp_c 15482 or (15038 and 15482) or (15482 and 16549) or (15482 and 8790)
yli_R0291 gthox_c + h_c + nadph_c --> gthrd_c + nadp_c 15482

UREASE atp_c + hco3_c + urea_c <=> adp_c + allphn_c + h_c + pi_c 9326
URCB atp_c + hco3_c + urea_c --> adp_c + allphn_c + 2.0 h_c + pi_c 9326

PTPATi atp_c + h_c + pan4p_c --> dpcoa_c + ppi_c 14849
APPAT atp_c + 2.0 h_c + pan4p_c <=> dpcoa_c + ppi_c 14849

yli_R1435 2.0 accoa_x --> aacoa_x + coa_x 8678 or 8885
ACACT1x 2.0 accoa_x <=> aacoa_x + coa_x 8678 or 8885

yli_R0034 chtn_c + h2o_c --> acgam_c 13082
CHTNASE chtn_c + 2.0 h2o_c --> 3.0 acgam_c 13082

yli_R1375 4.0 h_c + pyr_c + thmpp_c --> 2ahethmpp_c + co2_c 13630 and 13948
yli_R0357 4.0 h_c + pyr_c + thmpp_c --> 2ahethmpp_c + co2_c 15791 or (13630 and 13948)

ARGSS asp__L_c + atp_c + citr__L_c --> amp_c + argsuc_c + h_c + ppi_c 16196
ARGSS_1 asp__L_c + atp_c + citr__L_c --> amp_c + argsuc_c + 2.0 h_c + ppi_c 16196

PRAGSr atp_c + gly_c + pram_c <=> adp_c + gar_c + h_c + pi_c 14259
PPRGL atp_c + gly_c + pram_c --> adp_c + gar_c + 2.0 h_c + pi_c 14259

AIRCr air_c + co2_c <=> 5aizc_c + h_c 12132
PRAIC air_c + co2_c <=> 5aizc_c + 2.0 h_c 12132

yli_R0224 8.0 coa_m + 8.0 h2o_m + 8.0 nad_m + nadph_m + 7.0 o2_m + yli_M04625_m --> 9.0 accoa_m + 7.0 h2o2_m + 7.0 h_m + 8.0 nadh_m + nadp_m (12742 and 13813 and 14805) or (12742 and 14805 and 9065) or (12752 and 13813 and 14805) or (12752 and 14805 and 9065) or (13813 and 14805 and 9700) or (14805 and 9065 and 9700)
yli_R0223 8.0 coa_m + 8.0 h2o_m + 8.0 nad_m + 2.0 nadph_m + 8.0 o2_m + yli_M04625_m --> 9.0 accoa_m + 8.0 h2o2_m + 6.0 h_m + 8.0 nadh_m + 2.0 nadp_m (12742 and 13813 and 14805) or (12742 and 14805 and 9065) or (12752 and 13813 and 14805) or (12752 and 14805 and 9065) or (13813 and 14805 and 9700) or (14805 and 9065 and 9700)

C4STMO1 44mzym_c + 3.0 h_c + 3.0 nadph_c + 3.0 o2_c --> 4mzym_int1_c + 4.0 h2o_c + 3.0 nadp_c 16640
44MZYMMO 44mzym_c + 2.0 h_c + 3.0 nadph_c + 3.0 o2_c <=> 4mzym_int1_c + 4.0 h2o_c + 3.0 nadp_c 15314

FAO182p_evenodd 8.0 coa_x + 8.0 h2o_x + 8.0 nad_x + nadph_x + 7.0 o2_x + ocdycacoa_x --> 9.0 accoa_x + 7.0 h2o2_x + 7.0 h_x + 8.0 nadh_x + nadp_x (10293 and 11362 and 12742 and 13228 and 13813) or (10293 and 11362 and 12742 and 13228 and 9065) or (10293 and 11362 and 12752 and 13228 and 13813) or (10293 and 11362 and 12752 and 13228 and 9065) or (10293 and 11362 and 13228 and 13813 and 9700) or (10293 and 11362 and 13228 and 9065 and 9700)
FAO182p_eveneven 8.0 coa_x + 8.0 h2o_x + 8.0 nad_x + 2.0 nadph_x + 8.0 o2_x + ocdycacoa_x --> 9.0 accoa_x + 8.0 h2o2_x + 6.0 h_x + 8.0 nadh_x + 2.0 nadp_x (10293 and 11362 and 12742 and 13228 and 13813) or (10293 and 11362 and 12742 and 13228 and 9065) or (10293 and 11362 and 12752 and 13228 and 13813) or (10293 and 11362 and 12752 and 13228 and 9065) or (10293 and 11362 and 13228 and 13813 and 9700) or (10293 and 11362 and 13228 and 9065 and 9700)

yli_R1510 1ag3p_SC_r + acoa_r --> coa_r + pa_EC_r 10427 or 16030 or 9746
yli_R1523 1ag3p_SC_r + acoa_r --> coa_r + pa_EC_r 16030

NMNAT atp_c + h_c + nmn_c --> nad_c + ppi_c 10430
ANNAT atp_c + 2.0 h_c + nmn_c <=> nad_c + ppi_c 10430

yli_R0848 4.0 h_m + pyr_m + thmpp_m --> 2ahethmpp_m + co2_m (13630 and 13948) or (15685 and 9800)
PDHam1mi h_m + pyr_m + thmpp_m --> 2ahethmpp_m + co2_m (13630 and 13948) or (13948 and 15791) or (15685 and 9800)

yli_R1513 h2o_r + pa_EC_r --> dag_hs_r + pi_r 12485 or 13087
yli_R1393 h2o_r + 0.01 pa_EC_r --> 0.01 dag_hs_r + pi_r 12485

NNATr atp_c + h_c + nicrnt_c <=> dnad_c + ppi_c 10430
NNATr_copy1 atp_c + h_c + nicrnt_c --> dnad_c + ppi_c 14638
NNATr_copy2 atp_c + h_c + nicrnt_c <=> dnad_c + ppi_c 10430

yli_R1508 glyald_c + h2o_c + nad_c --> glyc__R_c + h_c + nadh_c 12042 or 13426 or 16323
GLYALDDr glyald_c + h2o_c + nad_c <=> glyc__R_c + 2.0 h_c + nadh_c 12042 or 13426

HISTD h2o_c + histd_c + 2.0 nad_c --> 3.0 h_c + his__L_c + 2.0 nadh_c 11646
HDH h2o_c + histd_c + 2.0 nad_c --> 4.0 h_c + his__L_c + 2.0 nadh_c 11646

GUAD gua_c + h2o_c + h_c --> nh4_c + xan_c 9050 or 9708
GUAD_1 gua_c + h2o_c + 2.0 h_c --> nh4_c + xan_c 9050 or 9708

PANTS ala_B_c + atp_c + pant__R_c --> amp_c + h_c + pnto__R_c + ppi_c 10475
PBAL ala_B_c + atp_c + pant__R_c --> amp_c + 2.0 h_c + pnto__R_c + ppi_c 10475

NADDP h2o_c + nad_c --> amp_c + 2.0 h_c + nmn_c 12434
NPH h2o_c + nad_c --> amp_c + 3.0 h_c + nmn_c 15385

STARCH300DEGR2A 49.0 h2o_h + 250.0 pi_h + starch300_h --> 50.0 Glc_aD_h + 250.0 g1p_h (CRv4_Au5_s11_g2607_t1 and CRv4_Au5_s3_g11038_t1 and CRv4_Au5_s6_g12805_t1 and 11993 and 14256) or (CRv4_Au5_s11_g2607_t1 and CRv4_Au5_s3_g11038_t1 and CRv4_Au5_s6_g13538_t1 and 11993 and 14256) or (CRv4_Au5_s19_g8150_t1 and CRv4_Au5_s3_g11038_t1 and CRv4_Au5_s6_g12805_t1 and 11993 and 14256) or (CRv4_Au5_s19_g8150_t1 and CRv4_Au5_s3_g11038_t1 and CRv4_Au5_s6_g13538_t1 and 11993 and 14256)
STARCH300DEGRA 74.0 h2o_h + 225.0 pi_h + starch300_h --> 75.0 Glc_aD_h + 225.0 g1p_h (CRv4_Au5_s11_g2607_t1 and CRv4_Au5_s3_g11038_t1 and CRv4_Au5_s6_g12805_t1 and 11993 and 14256) or (CRv4_Au5_s11_g2607_t1 and CRv4_Au5_s3_g11038_t1 and CRv4_Au5_s6_g13538_t1 and 11993 and 14256) or (CRv4_Au5_s19_g8150_t1 and CRv4_Au5_s3_g11038_t1 and CRv4_Au5_s6_g12805_t1 and 11993 and 14256) or (CRv4_Au5_s19_g8150_t1 and CRv4_Au5_s3_g11038_t1 and CRv4_Au5_s6_g13538_t1 and 11993 and 14256)

HDC 2.0 h_c + his__L_c --> co2_c + hista_c 10722 or 9434 or 9435
HISDC h_c + his__L_c --> co2_c + hista_c 10104

STARCH300DEGR2B 49.0 h2o_h + 250.0 pi_h + starch300_h --> 250.0 g1p_h + 50.0 glc__bD_h (CRv4_Au5_s11_g2607_t1 and CRv4_Au5_s3_g11038_t1 and CRv4_Au5_s6_g12805_t1 and 11993 and 14256) or (CRv4_Au5_s11_g2607_t1 and CRv4_Au5_s3_g11038_t1 and CRv4_Au5_s6_g13538_t1 and 11993 and 14256) or (CRv4_Au5_s19_g8150_t1 and CRv4_Au5_s3_g11038_t1 and CRv4_Au5_s6_g12805_t1 and 11993 and 14256) or (CRv4_Au5_s19_g8150_t1 and CRv4_Au5_s3_g11038_t1 and CRv4_Au5_s6_g13538_t1 and 11993 and 14256)
STARCH300DEGRB 74.0 h2o_h + 225.0 pi_h + starch300_h --> 225.0 g1p_h + 75.0 glc__bD_h (CRv4_Au5_s11_g2607_t1 and CRv4_Au5_s3_g11038_t1 and CRv4_Au5_s6_g12805_t1 and 11993 and 14256) or (CRv4_Au5_s11_g2607_t1 and CRv4_Au5_s3_g11038_t1 and CRv4_Au5_s6_g13538_t1 and 11993 and 14256) or (CRv4_Au5_s19_g8150_t1 and CRv4_Au5_s3_g11038_t1 and CRv4_Au5_s6_g12805_t1 and 11993 and 14256) or (CRv4_Au5_s19_g8150_t1 and CRv4_Au5_s3_g11038_t1 and CRv4_Au5_s6_g13538_t1 and 11993 and 14256)

ANNATn atp_n + 2.0 h_n + nmn_n <=> nad_n + ppi_n 10430
NMNATn atp_n + h_n + nmn_n --> nad_n + ppi_n 10430

CLHCOtex cl_e + 2.0 hco3_c --> cl_c + 2.0 hco3_e 14119 or 15736
CLHCO3tex2 2.0 cl_e + hco3_c --> 2.0 cl_c + hco3_e 16202

FACOAL204_copy1 arachd_c + atp_c + coa_c <=> amp_c + arachdcoa_c + ppi_c 12538 or 12555
FACOAL204_copy2 arachd_c + atp_c + coa_c --> amp_c + arachdcoa_c + ppi_c 11167 or 12538 or 12555 or 15748
```

#### ARD¶

In [9]:

```
for r in sorted(model.genes.get_by_id('16330').reactions, key=lambda x: x.id):
        print(r.id, r.reaction, r.gene_reaction_rule)
```

```
ACDO dhmtp_c + o2_c --> 2kmb_c + for_c + h_c 16330
ARD dhmtp_c + o2_c --> 2kmb_c + for_c + 2.0 h_c 16330
ARD1 dhmtp_c + o2_c --> co_c + for_c + h_c + mtpp_c 16330
DKMPPD2 dkmpp_c + 3.0 h2o_c --> 2kmb_c + for_c + 6.0 h_c + pi_c YEL038W and 16330
yli_R1495 dhmtp_c + o2_c --> co_c + for_c + mtpp_c 16330
```

In [10]:

```
# 5mta -> 5mdr1p -> 5mdru1p -> dkmpp
for r in sorted(model.metabolites.get_by_id('5mta_c').reactions, key=lambda x: x.id):
    print(r.id, r.reaction, r.gene_reaction_rule)
print()
for r in sorted(model.metabolites.get_by_id('5mdru1p_c').reactions, key=lambda x: x.id):
    print(r.id, r.reaction, r.gene_reaction_rule)
print()
for r in sorted(model.metabolites.get_by_id('dkmpp_c').reactions, key=lambda x: x.id):
    print(r.id, r.reaction, r.gene_reaction_rule)
print()
for r in sorted(model.metabolites.get_by_id('dhmtp_c').reactions, key=lambda x: x.id):
    print(r.id, r.reaction, r.gene_reaction_rule)
print()
for r in sorted(model.metabolites.get_by_id('2kmb_c').reactions, key=lambda x: x.id):
    print(r.id, r.reaction, r.gene_reaction_rule)
print()
for r in sorted(model.metabolites.get_by_id('mtpp_c').reactions, key=lambda x: x.id):
    print(r.id, r.reaction, r.gene_reaction_rule)
```

```
ACPCS amet_c --> 1acpc_c + 5mta_c + h_c 8613
ACPT16018111Z 12dgr16018111Z_c + amet_c --> 5mta_c + dghs16018111Z_c + h_c 10576
ACPT1601819Z 12dgr1601819Z_c + amet_c --> 5mta_c + dghs1601819Z_c + h_c 10576
ACPT18111Z18111Z 12dgr18111Z18111Z_c + amet_c --> 5mta_c + dghs18111Z18111Z_c + h_c 10576
ACPT18111Z1819Z 12dgr18111Z1819Z_c + amet_c --> 5mta_c + dghs18111Z1819Z_c + h_c 10576
ACPT1819Z18111Z 12dgr1819Z18111Z_c + amet_c --> 5mta_c + dghs1819Z18111Z_c + h_c 10576
ACPT1819Z1819Z 12dgr1819Z1819Z_c + amet_c --> 5mta_c + dghs1819Z1819Z_c + h_c 10576
MTAP 5mta_c + pi_c --> 5mdr1p_c + ade_c 14521 or 8372
SPMS ametam_c + ptrc_c --> 5mta_c + h_c + spmd_c 10577 or 16833
SPRMS ametam_c + spmd_c --> 5mta_c + h_c + sprm_c 10577 or 16833

MDRPD 5mdru1p_c --> dkmpp_c + h2o_c 15829
MTRI 5mdr1p_c <=> 5mdru1p_c 13385 or 15595

DKMPPD2 dkmpp_c + 3.0 h2o_c --> 2kmb_c + for_c + 6.0 h_c + pi_c YEL038W and 16330
MDRPD 5mdru1p_c --> dkmpp_c + h2o_c 15829

ACDO dhmtp_c + o2_c --> 2kmb_c + for_c + h_c 16330
ARD dhmtp_c + o2_c --> 2kmb_c + for_c + 2.0 h_c 16330
ARD1 dhmtp_c + o2_c --> co_c + for_c + h_c + mtpp_c 16330
yli_R1495 dhmtp_c + o2_c --> co_c + for_c + mtpp_c 16330

ACDO dhmtp_c + o2_c --> 2kmb_c + for_c + h_c 16330
ARD dhmtp_c + o2_c --> 2kmb_c + for_c + 2.0 h_c 16330
DKMPPD2 dkmpp_c + 3.0 h2o_c --> 2kmb_c + for_c + 6.0 h_c + pi_c YEL038W and 16330
UNK3 2kmb_c + glu__L_c --> akg_c + met__L_c 12407 or 14281 or 14908 or 15839 or 8936

ARD1 dhmtp_c + o2_c --> co_c + for_c + h_c + mtpp_c 16330
yli_R1495 dhmtp_c + o2_c --> co_c + for_c + mtpp_c 16330
```

In [11]:

```
temp = ['8372','14521','13385','15595','15829','11455','16330','12407','14281','14908','15839','8936']
display(Annotation.loc[temp])
Show_Data(temp)
```

|  | Combined Annotations | Signal P | Sc288c Orthologs | Human Orthologs | Sc288 Best Hit | Human Blast | Essential | WolfPSort | C Terminal |
| --- | --- | --- | --- | --- | --- | --- | --- | --- | --- |
| RTO4\_ID |  |  |  |  |  |  |  |  |  |
| 8372 | K00772: mtaP, MTAP; 5'-methylthioadenosine pho... |  | MEU1 | MTAP | MEU1 | MTAP | Not Essential | cysk 19, cyto 7 | GRQ\* |
| 14521 | K00757: udp, UPP; uridine phosphorylase |  |  |  |  |  | Not Essential | extr 11, mito 6, cyto 6, cyto\_mito 6 | RGE\* |
| 13385 | K08963: mtnA; methylthioribose-1-phosphate iso... |  | MRI1 | MRI1 | MRI1 | MRI1 | Not Essential | cyto 20, cysk 4, mito 2 | YDLR |
| 15595 | K03239: EIF2B1; translation initiation factor ... |  | GCN3 | EIF2B1 | GCN3 | EIF2B | Not Essential | mito 8, cyto 7, cyto\_nucl 5.5, extr 4, nucl 2,... | YAE\* |
| 15829 | K08964: mtnB; methylthioribulose-1-phosphate d... |  | MDE1 | APIP | MDE1 | APIP | Not Essential | pero 7.5, cyto\_pero 6.5, cysk 6, mito 5, cyto ... | TKQ\* |
| 11455 | HMMPfam:haloacid dehalogenase-like hydrolase:P... |  | YNL010W |  | YNL010W |  | Not Essential | cyto 19.5, cyto\_nucl 12.333, cyto\_mito 11.666,... | AKK\* |
| 16330 | K08967: mtnD, mtnZ, ADI1; 1,2-dihydroxy-3-keto... |  | ADI1 | ADI1 | ADI1 | ADI1 | Not Essential | nucl 13.5, cyto\_nucl 12.5, cyto 8.5, pero 3 | IKA\* |
| 12407 | K00825: AADAT, KAT2; kynurenine/2-aminoadipate... |  | ARO8 | AADAT | ARO8 | AADAT | Not Essential | cyto 11.5, cyto\_nucl 11.5, nucl 8.5, pero 6 | EKA\* |
| 14281 | K14455: GOT2; aspartate aminotransferase, mito... |  | AAT1 | GOT2 | AAT2 | GOT2 | Not Essential | mito 26 | NDA\* |
| 14908 | K00838: ARO8; aromatic amino acid aminotransfe... |  | ARO8 | AADAT | ARO8 | AADAT | Not Essential | cyto 7, cysk 7, mito 5, cyto\_nucl 5, pero 4, m... | WAY\* |
| 15839 | K00838: ARO8; aromatic amino acid aminotransfe... |  | ARO8 | AADAT | ARO8 | AADAT | Not Essential | cysk 9, cyto 6, nucl 4, mito 4, pero 4, mito\_n... | FKD\* |
| 8936 | K14454: GOT1; aspartate aminotransferase, cyto... |  | AAT2 | GOT1 | AAT2 | GOT2 | Not Essential | cyto 18.5, cyto\_nucl 12.5, nucl 3.5, pero 3 | GQL\* |

| strain | WT | | | | | | | | | | | | | | | | |
| --- | --- | --- | --- | --- | --- | --- | --- | --- | --- | --- | --- | --- | --- | --- | --- | --- | --- |
| condition | G\_MM | C\_MM | G\_SD | | GX\_SD | | | X\_SD | | A\_SD | | C\_SD | | MM\_CN120 | | MM\_CN5 | Diversity\_Sample |
| phase | exp | exp | exp | stat | exp | trans | stat | exp | stat | exp | stat | exp | stat | exp | stat | exp | exp |
| proteinId | Set1 | Set1 | Set2 | Set2 | Set2 | Set2 | Set2 | Set2 | Set2 | Set2 | Set2 | Set2 | Set2 | Set3 | Set3 | Set3 | Set3 |
| 8372 | 6.9668 | 8.6149 | 7.5227 | 6.94273 | 7.63791 | 5.97126 | 5.82998 | 7.05879 | 6.46796 | 7.00966 | 6.86565 | 9.7135 | 11.1034 | 7.02843 | 6.88484 | 9.35325 | 8.7758 |
| 14521 | 4.92771 | 4.63256 | 6.20515 | 4.58461 | 6.13068 | 4.95113 | 5.05278 | 4.80099 | 5.02868 | 4.52795 | 5.27059 | 3.80246 | 2.61298 | 5.31658 | 5.0291 | 5.45984 | 5.58418 |
| 13385 | 5.45841 | 5.35091 | 5.21125 | 4.10017 | 5.32312 | 4.84168 | 4.75369 | 5.41555 | 4.38092 | 5.04833 | 4.38311 | 5.18267 | 4.65664 | 6.04377 | 5.26202 | 6.08433 | 6.31766 |
| 15595 | 5.30502 | 5.67088 | 5.54515 | 5.13821 | 5.72176 | 5.15199 | 5.12278 | 5.23845 | 4.59422 | 5.04685 | 4.70691 | 5.60793 | 5.44442 | 4.64934 | 4.71259 | 5.59848 | 4.88013 |
| 15829 | 5.71022 | 5.61538 | 4.81934 | 4.86122 | 4.82549 | 5.26229 | 5.31035 | 5.13874 | 4.98628 | 4.89304 | 5.03267 | 5.18725 | 4.5538 | 4.28038 | 4.27139 | 3.72316 | 4.55088 |
| 11455 | 4.51583 | 7.1095 | 4.92978 | 4.67304 | 5.01291 | 5.01874 | 4.8773 | 5.7837 | 4.9818 | 5.48922 | 4.59463 | 5.57519 | 5.2435 | 4.48176 | 4.88905 | 5.98095 | 5.98961 |
| 16330 | 6.74299 | 6.53172 | 7.80554 | 7.25876 | 7.85778 | 6.99319 | 7.02419 | 7.81486 | 6.95768 | 7.46804 | 7.00714 | 7.71381 | 8.26394 | 5.75118 | 5.50821 | 7.22107 | 5.57589 |
| 12407 | 7.16156 | 6.96812 | 7.22568 | 5.61243 | 7.56705 | 6.22319 | 6.25552 | 6.21589 | 5.90204 | 5.8889 | 6.07415 | 7.3238 | 8.10277 | 5.28312 | 5.16308 | 7.46782 | 5.58338 |
| 14281 | 6.58722 | 7.20393 | 8.20142 | 6.09041 | 8.52252 | 7.21017 | 7.01216 | 7.85368 | 6.20268 | 7.13853 | 6.15274 | 7.58338 | 7.76082 | 9.02 | 8.16336 | 10.7998 | 10.0833 |
| 14908 | 4.96188 | 5.39382 | 6.32914 | 4.57254 | 6.87228 | 3.93282 | 4.6105 | 2.86689 | 4.55744 | 3.57167 | 4.54445 | 5.45156 | 8.85544 | 4.48318 | 3.71453 | 9.27804 | 5.94028 |
| 15839 | 8.11963 | 6.83965 | 6.43567 | 6.23959 | 6.4065 | 6.29378 | 6.4164 | 6.59727 | 6.49423 | 6.81951 | 6.69291 | 6.85344 | 6.82691 | 7.91155 | 7.40486 | 6.5373 | 6.73561 |
| 8936 | 7.86637 | 6.64116 | 7.91375 | 7.07647 | 8.09211 | 6.95363 | 6.89341 | 6.93058 | 7.53621 | 6.38309 | 7.15592 | 7.01203 | 7.52195 | 7.45909 | 6.9531 | 7.81801 | 7.00633 |

| strain | WT | | | | | | | | | | |
| --- | --- | --- | --- | --- | --- | --- | --- | --- | --- | --- | --- |
| condition | G\_SD | | GX\_SD | | | X\_SD | | A\_SD | | C\_SD | |
| proteinId | exp | stat | exp | trans | stat | exp | stat | exp | stat | exp | stat |
| 8372 | 24.675 | 18.4238 | 27.041 | 12.2971 | 14.9615 | 21.0034 | 21.6759 | 22.2163 | 18.3886 | 45.346 | 54.2423 |
| 14521 | 0.574292 | 0 | 0.812953 | 0 | 0 | 0 | 0 | 0 | 0 | 0.212277 | 0 |
| 13385 | 4.41349 | 4.51406 | 4.89274 | 3.34735 | 1.85699 | 5.26361 | 2.57821 | 3.48245 | 3.66181 | 3.63522 | 2.17222 |
| 15595 | 4.04648 | 3.75102 | 4.26627 | 2.98904 | 2.1921 | 3.17169 | 1.94469 | 2.12589 | 2.10247 | 1.91009 | 3.03033 |
| 15829 | 2.87666 | 3.17265 | 2.85973 | 2.40829 | 3.04813 | 3.36396 | 4.11224 | 4.04582 | 2.6777 | 2.35232 | 3.02807 |
| 11455 | 11.0128 | 7.94298 | 8.14194 | 7.04743 | 4.72869 | 9.02372 | 7.32346 | 10.0314 | 9.5905 | 9.60551 | 6.55712 |
| 16330 | 4.05593 | 1.97571 | 4.06276 | 1.30027 | 1.83783 | 3.53645 | 2.35373 | 2.70632 | 2.29842 | 3.41324 | 3.06325 |
| 12407 | 7.11912 | 7.07756 | 7.33971 | 5.944 | 5.30084 | 6.87491 | 5.50339 | 7.70989 | 5.56004 | 7.71286 | 9.59344 |
| 14281 | 44.8268 | 45.1951 | 51.0269 | 41.2985 | 42.3767 | 45.6448 | 45.2421 | 43.2537 | 42.8765 | 41.581 | 46.6144 |
| 14908 | 9.31562 | 0 | 11.3891 | 0 | 0 | 3.31911 | 0 | 1.54769 | 0 | 2.57326 | 8.71334 |
| 15839 | 4.43453 | 11.9185 | 6.09339 | 17.1048 | 18.0361 | 9.79935 | 13.8093 | 7.91426 | 12.8202 | 11.5438 | 12.6204 |
| 8936 | 14.1864 | 16.949 | 11.5716 | 18.0127 | 17.684 | 12.1475 | 12.4499 | 13.8959 | 12.0653 | 10.9247 | 14.1547 |

|  | Glucose | Xylose | Arabinose | Acetate | Coumarate | Ferulate | YNB Oleic Acid | YNB Ricinoleic Acid | YNB Glucose | YNB Gluc DOC | YPD |
| --- | --- | --- | --- | --- | --- | --- | --- | --- | --- | --- | --- |
| proteinId |  |  |  |  |  |  |  |  |  |  |  |
| 8372 | -0.268657 | -0.234283 | 0.00572781 | 0.494423 | -0.527027 | -0.116889 | -0.46519 | -1.91569 | 0.170461 | -0.556624 | -0.682021 |
| 14521 | -0.00706592 | 0.25772 | 0.022661 | 0.24152 | 0.653536 | 0.454526 | 0.31852 | -0.151502 | -0.000959473 | 0.252956 | 0.0155481 |
| 13385 | -0.183169 | 0.131527 | -0.0895433 | 0.189403 | 0.740175 | -0.27821 | 0.517699 | -0.922633 | -0.283394 | 0.265367 | 1.42844 |
| 15595 | 0.248649 | 0.278266 | -0.180005 | 0.217383 | -0.814564 | -0.133952 | -0.679194 | -0.553147 | 0.591779 | 0.279251 | 0.00149673 |
| 15829 | -0.0422372 | -0.103604 | -0.219452 | -0.227858 | 1.56949 | -0.186447 | 0.945233 | 0.946233 | 0.0554741 | 0.471732 | -0.282393 |
| 11455 | 0.387876 | 0.457422 | 0.271382 | 0.32656 | 0.17532 | 0.0348011 | 0.0796446 | 0.0216117 | 0.206991 | 0.228272 | -0.227832 |
| 16330 | 0.120232 | 0.474802 | -0.0189183 | -0.0584658 | 0.317082 | 0.258953 | 0.116167 | -0.135116 | 0.167846 | 0.258171 | 0.412204 |
| 12407 | -0.120884 | 0.0459414 | -0.0417789 | -0.263358 | -0.162155 | -0.400014 | 0.275532 | 0.206367 | 0.0858757 | 0.335526 | 1.09564 |
| 14281 | -0.134443 | 0.0025888 | -0.314789 | -0.228214 | -0.0206698 | -0.461234 | -0.785788 | -2.1105 | -0.415618 | -0.136043 | -0.195287 |
| 14908 | 0.0451271 | -0.280956 | 0.220759 | -0.0377905 | 0.21851 | -0.259388 | -0.292657 | 0.206026 | 0.339453 | 0.398586 | -0.0659921 |
| 15839 | -0.0847761 | -0.12423 | 0.269542 | 0.0647239 | 0.154571 | 0.146919 | -0.346466 | -0.214054 | -0.177142 | -0.229588 | -0.0156292 |
| 8936 | -0.354295 | -0.277708 | -0.923495 | -1.45071 | -4.24826 | -4.247 | -0.440484 | -0.917334 | -0.37578 | 0.0703331 | -0.272326 |

S-methyl-5'-thioadenosine degradation  
MTAP 5mta\_c + pi\_c --> 5mdr1p\_c + ade\_c 14521 or 8372  
8372 cysk MEU1  
14521 extr uridine phosphorylase (also catalyze deoxy) -> not MTAP

MTRI 5mdr1p\_c <=> 5mdru1p\_c 13385 or 15595  
13385 cyto mtnA/MRI1  
15595 cito GCN3 translation initiation factor eIF-2B subunit alpha EIF2B1 -> not MTRI

MDRPD 5mdru1p\_c --> dkmpp\_c + h2o\_c 15829  
15829 cyto\_pero mtnB/MDE1

DKMPPD2 dkmpp\_c + 3.0 h2o\_c --> 2kmb\_c + for\_c + 6.0 h\_c + pi\_c YEL038W and 16330  
ACRS, 2OH3K5MPPISO, ACDO lumped, incorrect stoichiometry

DKMPPD3\_1 - dkmpp + h2o -> dhmtp + pi + h by mtnC (ACRS, 2OH3K5MPPISO lumped)  
ACRS - dkmpp -> hkmpp + h by mtnW/mtnC  
2OH3K5MPPISO - hkmpp + h2o -> dhmtp + pi by mtnX/mtnC  
11455 cyto mtnX blast to 11455

ACDO dhmtp\_c + o2\_c --> 2kmb\_c + for\_c + h\_c 16330, fe2+ requiring  
ARD dhmtp\_c + o2\_c --> 2kmb\_c + for\_c + 2.0 h\_c 16330, incorrect stoichiometry  
ARD1 dhmtp\_c + o2\_c --> co\_c + for\_c + h\_c + mtpp\_c 16330, ni2+ requiring  
16330 cyto\_nucl mtnD/ADI1 is fe2+ requiring and produce 2kmb and for -> keep ACDO

UNK3 2kmb\_c + glu**L\_c --> akg\_c + met**L\_c 12407 or 14281 or 14908 or 15839 or 8936  
UNK3 reaction is different in the pathway page, it is two step.  
2kmb + gln\_L -> 2ogm + met\_L by mtnE  
2ogm + h2o -> akg + nh4 by mtnU  
The enzyme page 2.6.1.57 is consistent with UNK3, but 2.6.1.57 is not this reaction.  
Blast of known enzymes results in mixed results  
KEGG says this reaction is possible by 2.6.1.5 or 2.6.1.57  
12407 cyto\_nucl ARO8 tryptophan aminotransferase 2.6.1.27  
14281 mito AAT1/AAT2 aspartate aminotransferase, mitochondrial 2.6.1.1  
14908 cyto ARO8 aromatic amino acid aminotransferase I / 2-aminoadipate transaminase 2.6.1.57/2.6.1.39/2.6.1.27/2.6.1.5  
15839 cysk ARO8 aromatic amino acid aminotransferase I / 2-aminoadipate transaminase 2.6.1.57/2.6.1.39/2.6.1.27/2.6.1.5  
8936 cyto AAT2 aspartate aminotransferase, cytoplasmic 2.6.1.1  
Keep genes for now

Change MTAP genes to '8372'  
Change MTRI genes to '13385'  
Add ACRS and 2OH3K5MPPISO, change genes '11455'  
Remove DKMPPD2, ARD, ARD1, yli\_R1495

In [12]:

```
model.reactions.get_by_id('MTAP').gene_reaction_rule = '8372'
model.reactions.get_by_id('MTRI').gene_reaction_rule = '13385'
r1 = cre.reactions.get_by_id('ACRS').copy()
r1.gene_reaction_rule = '11455'
r2 = cre.reactions.get_by_id('2OH3K5MPPISO').copy()
r2.gene_reaction_rule = '11455'
model.add_reactions([r1,r2])
model.remove_reactions(['DKMPPD2','ARD','ARD1','yli_R1495'],remove_orphans=True)
```

In [13]:

```
for r in sorted(model.genes.get_by_id('8372').reactions, key=lambda x: x.id):
    print(r.id, r.reaction, r.gene_reaction_rule)
print()
for r in sorted(model.genes.get_by_id('13385').reactions, key=lambda x: x.id):
    print(r.id, r.reaction, r.gene_reaction_rule)
print()
for r in sorted(model.genes.get_by_id('15829').reactions, key=lambda x: x.id):
    print(r.id, r.reaction, r.gene_reaction_rule)
print()
for r in sorted(model.genes.get_by_id('11455').reactions, key=lambda x: x.id):
    print(r.id, r.reaction, r.gene_reaction_rule)
print()
for r in sorted(model.genes.get_by_id('16330').reactions, key=lambda x: x.id):
    print(r.id, r.reaction, r.gene_reaction_rule)
```

```
MTAP 5mta_c + pi_c --> 5mdr1p_c + ade_c 8372

MTRI 5mdr1p_c <=> 5mdru1p_c 13385
MTRK 5mtr_c + atp_c --> 5mdr1p_c + adp_c + h_c 13385

MDRPD 5mdru1p_c --> dkmpp_c + h2o_c 15829

2OH3K5MPPISO h2o_c + hkmpp_c --> dhmtp_c + pi_c 11455
ACRS dkmpp_c --> h_c + hkmpp_c 11455

ACDO dhmtp_c + o2_c --> 2kmb_c + for_c + h_c 16330
```

In [14]:

```
for r in sorted(model.metabolites.get_by_id('5mtr_c').reactions, key=lambda x: x.id):
    print(r.id, r.reaction, r.gene_reaction_rule)
print()
for r in sorted(model.metabolites.get_by_id('5mdr1p_c').reactions, key=lambda x: x.id):
    print(r.id, r.reaction, r.gene_reaction_rule)
```

```
MTRK 5mtr_c + atp_c --> 5mdr1p_c + adp_c + h_c 13385

MTAP 5mta_c + pi_c --> 5mdr1p_c + ade_c 8372
MTRI 5mdr1p_c <=> 5mdru1p_c 13385
MTRK 5mtr_c + atp_c --> 5mdr1p_c + adp_c + h_c 13385
```

MTRK is catalyzed by mtnK/MTK1  
Blast of uniprot reviewed mtnK seqs results in no hit

In [15]:

```
model.reactions.get_by_id('MTRK').remove_from_model(remove_orphans=True)
```

#### btn¶

In [16]:

```
for r in sorted(model.genes.get_by_id('16404').reactions, key=lambda x: x.id):
    print(r.id, r.reaction, r.gene_reaction_rule)
```

```
BACCL atp_c + btn_c + h_c --> btamp_c + ppi_c 16404
BACCLm atp_m + btn_m + h_m --> btamp_m + ppi_m 16404
BTNPL apoC_Lys_c + btamp_c --> amp_c + apoC_Lys_btn_c + h_c 16404
BTNPLm apoC_Lys_m + btamp_m --> amp_m + apoC_Lys_btn_m + h_m 16404
yli_R1487 atp_c + btn_c --> btamp_c + ppi_c 16404
yli_R1488 atp_c + btn_c --> btamp_c + ppi_c 16404
yli_R1489 atp_c + btn_c --> btamp_c + ppi_c 16404
yli_R1490 atp_c + btn_c --> btamp_c + ppi_c 16404
```

In [17]:

```
for r in sorted(model.metabolites.get_by_id('btn_c').reactions, key=lambda x: x.id):
    print(r.id, r.reaction, r.gene_reaction_rule)
print()
for r in sorted(model.metabolites.get_by_id('btamp_c').reactions, key=lambda x: x.id):
    print(r.id, r.reaction, r.gene_reaction_rule)
print()
for r in sorted(model.metabolites.get_by_id('apoC_Lys_c').reactions, key=lambda x: x.id):
    print(r.id, r.reaction, r.gene_reaction_rule)
print()
for r in sorted(model.metabolites.get_by_id('apoC_Lys_btn_c').reactions, key=lambda x: x.id):
    print(r.id, r.reaction, r.gene_reaction_rule)
```

```
BACCL atp_c + btn_c + h_c --> btamp_c + ppi_c 16404
BTS5 2fe2s_c + amet_c + dtbt_c --> 2fe1s_c + btn_c + dad_5_c + h_c + met__L_c 15908
BTSr dtbt_c + s_c <=> btn_c + 2.0 h_c 15908
yli_R1487 atp_c + btn_c --> btamp_c + ppi_c 16404
yli_R1488 atp_c + btn_c --> btamp_c + ppi_c 16404
yli_R1489 atp_c + btn_c --> btamp_c + ppi_c 16404
yli_R1490 atp_c + btn_c --> btamp_c + ppi_c 16404

BACCL atp_c + btn_c + h_c --> btamp_c + ppi_c 16404
BTNPL apoC_Lys_c + btamp_c --> amp_c + apoC_Lys_btn_c + h_c 16404
yli_R1487 atp_c + btn_c --> btamp_c + ppi_c 16404
yli_R1488 atp_c + btn_c --> btamp_c + ppi_c 16404
yli_R1489 atp_c + btn_c --> btamp_c + ppi_c 16404
yli_R1490 atp_c + btn_c --> btamp_c + ppi_c 16404

BTNPL apoC_Lys_c + btamp_c --> amp_c + apoC_Lys_btn_c + h_c 16404

BTNPL apoC_Lys_c + btamp_c --> amp_c + apoC_Lys_btn_c + h_c 16404
```

In [18]:

```
for r in sorted(model.metabolites.get_by_id('btn_m').reactions, key=lambda x: x.id):
    print(r.id, r.reaction, r.gene_reaction_rule)
print()
for r in sorted(model.metabolites.get_by_id('btamp_m').reactions, key=lambda x: x.id):
    print(r.id, r.reaction, r.gene_reaction_rule)
print()
for r in sorted(model.metabolites.get_by_id('apoC_Lys_m').reactions, key=lambda x: x.id):
    print(r.id, r.reaction, r.gene_reaction_rule)
print()
for r in sorted(model.metabolites.get_by_id('apoC_Lys_btn_m').reactions, key=lambda x: x.id):
    print(r.id, r.reaction, r.gene_reaction_rule)
```

```
BACCLm atp_m + btn_m + h_m --> btamp_m + ppi_m 16404

BACCLm atp_m + btn_m + h_m --> btamp_m + ppi_m 16404
BTNPLm apoC_Lys_m + btamp_m --> amp_m + apoC_Lys_btn_m + h_m 16404

BTNPLm apoC_Lys_m + btamp_m --> amp_m + apoC_Lys_btn_m + h_m 16404

BTNPLm apoC_Lys_m + btamp_m --> amp_m + apoC_Lys_btn_m + h_m 16404
```

In [19]:

```
temp = ['12736','12731','15908','16404']
display(Annotation.loc[temp])
Show_Data(temp)
```

|  | Combined Annotations | Signal P | Sc288c Orthologs | Human Orthologs | Sc288 Best Hit | Human Blast | Essential | WolfPSort | C Terminal |
| --- | --- | --- | --- | --- | --- | --- | --- | --- | --- |
| RTO4\_ID |  |  |  |  |  |  |  |  |  |
| 12736 | K00652: bioF; 8-amino-7-oxononanoate synthase |  |  |  | LCB2 | GCAT | Essential | mito 22, cyto 3 | AKL\* |
| 12731 | K19562: BIO3-BIO1; bifunctional dethiobiotin s... |  |  |  | BIO3 |  | Essential | mito 11, extr 10, cyto 3, pero 2 | GAS\* |
| 15908 | K01012: bioB; biotin synthase |  | BIO2 |  | BIO2 |  | Essential | mito 26.5, cyto\_mito 14 | VAA\* |
| 16404 | K01942: HLCS; biotin--protein ligase |  | BPL1 | HLCS | BPL1 | HLCS | Essential | extr 12, cyto 6, cyto\_nucl 5.5, mito 4, nucl 3 | KSG\* |

| strain | WT | | | | | | | | | | | | | | | | |
| --- | --- | --- | --- | --- | --- | --- | --- | --- | --- | --- | --- | --- | --- | --- | --- | --- | --- |
| condition | G\_MM | C\_MM | G\_SD | | GX\_SD | | | X\_SD | | A\_SD | | C\_SD | | MM\_CN120 | | MM\_CN5 | Diversity\_Sample |
| phase | exp | exp | exp | stat | exp | trans | stat | exp | stat | exp | stat | exp | stat | exp | stat | exp | exp |
| proteinId | Set1 | Set1 | Set2 | Set2 | Set2 | Set2 | Set2 | Set2 | Set2 | Set2 | Set2 | Set2 | Set2 | Set3 | Set3 | Set3 | Set3 |
| 12736 | 6.18417 | 4.82841 | 6.18295 | 4.4444 | 6.23411 | 5.46759 | 5.23184 | 5.77831 | 4.92094 | 4.9809 | 4.63317 | 4.74662 | 4.93048 | 6.46169 | 5.98598 | 6.47904 | 5.51455 |
| 12731 | 6.02185 | 4.60555 | 6.45171 | 5.35008 | 6.39028 | 6.2194 | 5.81525 | 6.0943 | 4.79923 | 4.92084 | 4.63383 | 4.19327 | 3.7021 | 7.16018 | 6.81399 | 7.2256 | 6.71275 |
| 15908 | 7.52668 | 7.0794 | 6.1455 | 4.45929 | 6.24934 | 4.98317 | 4.78694 | 6.18894 | 4.23801 | 5.67966 | 4.58943 | 5.42106 | 6.46046 | 7.44985 | 6.84422 | 7.47498 | 6.98186 |
| 16404 | 5.14898 | 5.19209 | 5.68806 | 4.45038 | 5.78277 | 5.12319 | 4.79587 | 5.48776 | 4.40562 | 5.33835 | 4.57964 | 4.22056 | 4.62725 | 5.50854 | 5.4644 | 6.08156 | 6.255 |

| strain | WT | | | | | | | | | | |
| --- | --- | --- | --- | --- | --- | --- | --- | --- | --- | --- | --- |
| condition | G\_SD | | GX\_SD | | | X\_SD | | A\_SD | | C\_SD | |
| proteinId | exp | stat | exp | trans | stat | exp | stat | exp | stat | exp | stat |
| 12736 | 1.2967 | 0.524211 | 1.23017 | 0 | 0 | 0 | 0 | 0 | 0 | 0 | 0 |
| 12731 | 10.3494 | 10.6314 | 10.7826 | 13.9404 | 14.2423 | 9.78049 | 10.1996 | 5.98241 | 7.46515 | 8.755 | 7.20604 |
| 15908 | 4.95587 | 0.18528 | 3.66997 | 0.187227 | 0 | 0.795632 | 0 | 0 | 0 | 1.91908 | 0.666647 |
| 16404 | 0.579561 | 0 | 1.42376 | 0.185549 | 0.173732 | 0.200926 | 0.189187 | 0.579683 | 0.19525 | 0 | 0 |

|  | Glucose | Xylose | Arabinose | Acetate | Coumarate | Ferulate | YNB Oleic Acid | YNB Ricinoleic Acid | YNB Glucose | YNB Gluc DOC | YPD |
| --- | --- | --- | --- | --- | --- | --- | --- | --- | --- | --- | --- |
| proteinId |  |  |  |  |  |  |  |  |  |  |  |
| 16404 | 0.224466 | -0.772008 | -0.234875 | -0.5554 | -0.0770478 | 0.701184 | 0.14778 | -0.208173 | -1.19924 | -0.980346 | 0.058461 |

In [20]:

```
for x in temp:
    if x in model.genes:
        for r in sorted(model.genes.get_by_id(x).reactions, key=lambda x: x.id):
            print(r, r.gene_reaction_rule)
    else:
        print(x, 'no reactions')
    print()
```

```
AOXSr2: ala__L_c + pimACP_c --> 8aonn_c + ACP_c + co2_c 12736

AMAOTr: 8aonn_c + amet_c <=> amob_c + dann_c 12731

BTS5: 2fe2s_c + amet_c + dtbt_c --> 2fe1s_c + btn_c + dad_5_c + h_c + met__L_c 15908
BTSr: dtbt_c + s_c <=> btn_c + 2.0 h_c 15908

BACCL: atp_c + btn_c + h_c --> btamp_c + ppi_c 16404
BACCLm: atp_m + btn_m + h_m --> btamp_m + ppi_m 16404
BTNPL: apoC_Lys_c + btamp_c --> amp_c + apoC_Lys_btn_c + h_c 16404
BTNPLm: apoC_Lys_m + btamp_m --> amp_m + apoC_Lys_btn_m + h_m 16404
yli_R1487: atp_c + btn_c --> btamp_c + ppi_c 16404
yli_R1488: atp_c + btn_c --> btamp_c + ppi_c 16404
yli_R1489: atp_c + btn_c --> btamp_c + ppi_c 16404
yli_R1490: atp_c + btn_c --> btamp_c + ppi_c 16404
```

Biotin  
16404 extr 12 cyto 6 cyto\_nucl 5.5 mito 4 BPL1 biotin---protein ligase  
required for acetyl-CoA carboxylase (ACC1, cyto), should it be part of that reaction?  
BACCL has correct stoichiomtery

bioF is peroxisomal http://www.jbc.org/content/286/35/30455  
pimACP or pimcoa works as a substrate for 8aonn synthesis  
It is not clear how pimelate or pimcoa is synthesized  
pimeloyl-CoA biosynthesis in peroxisome? beta-oxidation? http://www.jbc.org/content/286/49/42133  
Check genes near 12731-12736, maybe a cluster  
In E. coli, it is fatty acid synthesis https://www.nature.com/articles/nchembio.420  
In B. subtilis, it is from long chain acyl-acp by C450 enzyme https://www.ncbi.nlm.nih.gov/pubmed/11368323  
The rest of the biotin biosynthesis is mitochondrial  
15908 mito BIO2 biotin synthase -> check 2Fe-2S ferredoxin metabolites  
BTS5 has correct stoichiomtery

Remove BTSr, yli\_R1487, yli\_R1488, yli\_R1489, yli\_R1490, BACCLm, BTNPLm

In [21]:

```
m = model.metabolites.get_by_id('pimACP_c')
m.id = 'pimcoa_c'
m.name = 'Pimeloyl-CoA'
m.formula = 'C28H41N7O19P3S'
m.charge = -5

r = model.reactions.get_by_id('AOXSr2').copy()
r.id = 'AOXSp'
model.add_reactions([r])
r.add_metabolites({'ACP_c': -1.0, 'coa_c': 1.0, 'h_c': -1.0})
for m in r.metabolites:
    if not m.id.replace('_c','_x') in model.metabolites:
        m2 = m.copy()
        m2.id = m.id.replace('_c','_x')
        m2.compartment = 'x'
        model.add_metabolites([m2])
    r.add_metabolites({m.id: -r.get_coefficient(m.id), m.id.replace('_c','_x'): r.get_coefficient(m.id)})

r = sce.reactions.get_by_id('DBTS').copy()
r.gene_reaction_rule = '12731'
model.add_reactions([r])

for x in ['AMAOTr','DBTS','BTS5']:
    r = model.reactions.get_by_id(x)
    r.id = r.id + 'm'
    for m in r.metabolites:
        if not m.id.replace('_c','_m') in model.metabolites:
            m2 = m.copy()
            m2.id = m.id.replace('_c','_m')
            m2.compartment = 'm'
            model.add_metabolites([m2])
        r.add_metabolites({m.id: -r.get_coefficient(m.id), m.id.replace('_c','_m'): r.get_coefficient(m.id)})

model.remove_reactions(['AOXSr2','BTSr','yli_R1487','yli_R1488','yli_R1489','yli_R1490'],remove_orphans=True)
```

#### GLUSy¶

In [22]:

```
for r in sorted(model.genes.get_by_id('15713').reactions, key=lambda x: x.id):
    print(r.id, r.reaction, r.gene_reaction_rule)
print()
for r in sorted(model.genes.get_by_id('12248').reactions, key=lambda x: x.id):
    print(r.id, r.reaction, r.gene_reaction_rule)
print()
for r in sorted(model.genes.get_by_id('9856').reactions, key=lambda x: x.id):
    print(r.id, r.reaction, r.gene_reaction_rule)
```

```
GLUDy glu__L_c + h2o_c + nadp_c <=> akg_c + h_c + nadph_c + nh4_c 12248 or (b3213 and 15713)
GLUS akg_h + gln__L_h + h_h + nadh_h --> 2.0 glu__L_h + nad_h 15713
GLUS_ferr akg_h + 2.0 fdxrd_h + gln__L_h --> 2.0 fdxox_h + 2.0 glu__L_h + 2.0 h_h (CRv4_Au5_s16_g6229_t1 and 15713) or (CRv4_Au5_s17_g7064_t1 and 15713) or (CRv4_Au5_s3_g10824_t1 and 15713) or (CRv4_Au5_s6_g13523_t1 and 15713) or (CRv4_Au5_s7_g14133_t1 and 15713)
GLUS_nadph akg_h + gln__L_h + h_h + nadph_h --> 2.0 glu__L_h + nadp_h 15713
GLUSx akg_c + gln__L_c + h_c + nadh_c --> 2.0 glu__L_c + nad_c 15713
GLUSy akg_c + gln__L_c + h_c + nadph_c --> 2.0 glu__L_c + nadp_c (PP_5075 and 15713) or (b3213 and 15713)
yli_R0002 akg_c + gln__L_c + h_c + nadph_c --> glu__L_c + nadp_c 15713

GDHm glu__L_m + h2o_m + nad_m <=> akg_m + h_m + nadh_m + nh4_m 12248
GLUDy glu__L_c + h2o_c + nadp_c <=> akg_c + h_c + nadph_c + nh4_c 12248 or (b3213 and 15713)
GLUDym glu__L_m + h2o_m + nadp_m <=> akg_m + h_m + nadph_m + nh4_m 12248

GLUDxi glu__L_c + h2o_c + nad_c --> akg_c + h_c + nadh_c + nh4_c 9856
```

15713 cyto GLT1 glutamate synthase NAD -> GLUSx  
12248 cyto GDH1, GDH3 glutamate dehydrogenase NADP -> GLUDy  
9856 cyto GDH2 glutamate dehydrogenase NAD -> GLUDxi

Change GLUDy genes to '12248'  
Remove GLUS, GLUS\_ferr, GLUS\_nadph, GLUSy, yli\_R0002, GDHm, GLUDym

In [23]:

```
model.reactions.get_by_id('GLUDy').gene_reaction_rule = '12248'
model.remove_reactions(['GLUS','GLUS_ferr','GLUS_nadph','GLUSy','yli_R0002','GDHm','GLUDym'],remove_orphans=True)
```

#### mlthf / GCC2cm¶

In [24]:

```
for r in sorted(model.metabolites.get_by_id('mlthf_x').reactions, key=lambda x: x.id):
    print(r.id, r.reaction, r.gene_reaction_rule)
```

```
yli_R1377 co2_x + mlthf_x + nadh_x + nh4_x --> gly_x + nad_x + thf_x 10205
yli_R1378 co2_x + mlthf_x + nadh_x + nh4_x --> gly_x + nad_x + thf_x 10040
yli_R1387 gly_x + h2o_x + mlthf_x <=> ser__L_x + thf_x 9667
yli_R1425 co2_x + mlthf_x + nadh_x + nh4_x --> gly_x + nad_x + thf_x 12898
```

In [25]:

```
for r in sorted(model.genes.get_by_id('10205').reactions, key=lambda x: x.id):
    print(r.id, r.reaction, r.gene_reaction_rule)
print()
for r in sorted(model.genes.get_by_id('10040').reactions, key=lambda x: x.id):
    print(r.id, r.reaction, r.gene_reaction_rule)
print()
for r in sorted(model.genes.get_by_id('9667').reactions, key=lambda x: x.id):
    print(r.id, r.reaction, r.gene_reaction_rule)
print()
for r in sorted(model.genes.get_by_id('12898').reactions, key=lambda x: x.id):
    print(r.id, r.reaction, r.gene_reaction_rule)
```

```
GCC2am gly_m + h_m + lpam_m <=> alpam_m + co2_m 10040 and 10205 and 12898 and 15184
GCC2bim alpam_m + thf_m --> dhlam_m + mlthf_m + nh4_m 10040 and 10205 and 12898 and 15184
GCC2cm dhlam_m + nad_m <=> h_m + lpam_m + nadh_m 10040 and 10205 and 12898 and 15184
GCC2cm_copy1 dhlam_m + nad_m <=> h_m + lpam_m + nadh_m 10040 and 10205 and 12898 and 15184
GCCam gly_m + h_m + lpro_m <=> alpro_m + co2_m 10205 or (10040 and 10205 and 12898 and 15184)
GCCbim alpro_m + thf_m --> dhlpro_m + mlthf_m + nh4_m 12898 or (10040 and 10205 and 12898 and 15184)
GCCcm dhlpro_m + nad_m <=> h_m + lpro_m + nadh_m 10040 or (10040 and 10205 and 12898 and 15184)
GLYCL gly_c + nad_c + thf_c --> co2_c + mlthf_c + nadh_c + nh4_c 10040 and 10205 and 12898 and 15184
GLYCLm gly_m + nad_m + thf_m --> co2_m + mlthf_m + nadh_m + nh4_m 12898 or (CRv4_Au5_s12_g4121_t1 and 14894) or (12898 and 14894) or (10040 and 10205 and 12898 and 15184)
GLYDHD gly_m + lpro_m --> alpro_m + co2_m 10205 and 15184
THFATm h2o_m + methf_m --> 5fthf_m + h_m 12898 or (10205 and 12898 and 13630 and 13948 and 15184)
yli_R1377 co2_x + mlthf_x + nadh_x + nh4_x --> gly_x + nad_x + thf_x 10205

2OXOADOXm 2oxoadp_m + coa_m + nad_m --> co2_m + glutcoa_m + nadh_m (PDHX and 10007 and 10040 and 12116) or (PDHX and 10040 and 12116 and 9274) or (Pdhx and 10007 and 10040 and 12116) or (Pdhx and 10040 and 12116 and 9274)
AKGDH akg_c + coa_c + nad_c --> co2_c + nadh_c + succoa_c (10007 and 10040 and 12116) or (10040 and 12116 and 9274)
AKGDam akg_m + h_m + lpam_m <=> co2_m + sdhlam_m 10007 or 9274 or (10007 and 10040 and 12116) or (10040 and 12116 and 9274)
AKGDbm coa_m + sdhlam_m --> dhlam_m + succoa_m (10007 and 10040 and 12116) or (10040 and 12116 and 9274)
AKGDm akg_m + coa_m + nad_m --> co2_m + nadh_m + succoa_m (PDHX and 10007 and 10040 and 12116) or (PDHX and 10040 and 12116 and 9274) or (Pdhx and 10007 and 10040 and 12116) or (Pdhx and 10040 and 12116 and 9274)
GCC2am gly_m + h_m + lpam_m <=> alpam_m + co2_m 10040 and 10205 and 12898 and 15184
GCC2bim alpam_m + thf_m --> dhlam_m + mlthf_m + nh4_m 10040 and 10205 and 12898 and 15184
GCC2cm dhlam_m + nad_m <=> h_m + lpam_m + nadh_m 10040 and 10205 and 12898 and 15184
GCC2cm_copy1 dhlam_m + nad_m <=> h_m + lpam_m + nadh_m 10040 and 10205 and 12898 and 15184
GCC2cm_copy2 dhlam_m + nad_m --> h_m + lpam_m + nadh_m (10007 and 10040 and 12116) or (10040 and 12116 and 9274)
GCCam gly_m + h_m + lpro_m <=> alpro_m + co2_m 10205 or (10040 and 10205 and 12898 and 15184)
GCCbim alpro_m + thf_m --> dhlpro_m + mlthf_m + nh4_m 12898 or (10040 and 10205 and 12898 and 15184)
GCCcm dhlpro_m + nad_m <=> h_m + lpro_m + nadh_m 10040 or (10040 and 10205 and 12898 and 15184)
GLYCL gly_c + nad_c + thf_c --> co2_c + mlthf_c + nadh_c + nh4_c 10040 and 10205 and 12898 and 15184
GLYCLm gly_m + nad_m + thf_m --> co2_m + mlthf_m + nadh_m + nh4_m 12898 or (CRv4_Au5_s12_g4121_t1 and 14894) or (12898 and 14894) or (10040 and 10205 and 12898 and 15184)
OIVD1m 4mop_m + coa_m + nad_m --> co2_m + ivcoa_m + nadh_m (10040 and 11183 and 12086 and 15436) or (10040 and 11183 and 12566 and 15436) or (10040 and 11188 and 12086 and 15436) or (10040 and 11188 and 12566 and 15436)
OIVD2m 3mob_m + coa_m + nad_m --> co2_m + ibcoa_m + nadh_m (10040 and 11183 and 12086 and 15436) or (10040 and 11183 and 12566 and 15436) or (10040 and 11188 and 12086 and 15436) or (10040 and 11188 and 12566 and 15436)
OIVD3m 3mop_m + coa_m + nad_m --> 2mbcoa_m + co2_m + nadh_m (10040 and 11183 and 12086 and 15436) or (10040 and 11183 and 12566 and 15436) or (10040 and 11188 and 12086 and 15436) or (10040 and 11188 and 12566 and 15436)
PDH coa_c + nad_c + pyr_c --> accoa_c + co2_c + nadh_c 13948 or (PP_0338 and PP_0339 and 10040) or (b0114 and b0115 and 10040)
PDHcr dhlam_c + nad_c <=> h_c + lpam_c + nadh_c 10040
PDHm coa_m + nad_m + pyr_m --> accoa_m + co2_m + nadh_m (PDHX and 10040 and 13630 and 13948 and 14126) or (Pdhx and 10040 and 13630 and 13948 and 14126) or (10040 and 13630 and 13722 and 13948 and 14126)
yli_R0381 nad_m + yli_M03934_m --> h_m + nadh_m + yli_M03933_m 10040
yli_R1378 co2_x + mlthf_x + nadh_x + nh4_x --> gly_x + nad_x + thf_x 10040
yli_R1419 nad_c + yli_M03934_c --> h_c + nadh_c + yli_M03933_c 10040

ALATA_D2 ala__D_c + pydx5p_c --> pyam5p_c + pyr_c 16182 or 9222 or 9667
ALATA_L2 ala__L_c + pydx5p_c --> pyam5p_c + pyr_c 16182 or 9222 or 9667
GHMT2r ser__L_c + thf_c <=> gly_c + h2o_c + mlthf_c 9667
GHMT2rm ser__L_m + thf_m <=> gly_m + h2o_m + mlthf_m 9667
GHMT3 3htmelys_c + h_c --> 4tmeabut_c + gly_c 9667
GHMT3m 3htmelys_m + h_m --> 4tmeabut_m + gly_m 9667
THFAT h2o_c + methf_c --> 5fthf_c + h_c 12898 or 9667
THRA thr__L_c --> acald_c + gly_c 16182 or 9222 or 9667
THRA2 athr__L_c --> acald_c + gly_c 16182 or 9222 or 9667
yli_R1387 gly_x + h2o_x + mlthf_x <=> ser__L_x + thf_x 9667

GCC2am gly_m + h_m + lpam_m <=> alpam_m + co2_m 10040 and 10205 and 12898 and 15184
GCC2bim alpam_m + thf_m --> dhlam_m + mlthf_m + nh4_m 10040 and 10205 and 12898 and 15184
GCC2cm dhlam_m + nad_m <=> h_m + lpam_m + nadh_m 10040 and 10205 and 12898 and 15184
GCC2cm_copy1 dhlam_m + nad_m <=> h_m + lpam_m + nadh_m 10040 and 10205 and 12898 and 15184
GCCam gly_m + h_m + lpro_m <=> alpro_m + co2_m 10205 or (10040 and 10205 and 12898 and 15184)
GCCbim alpro_m + thf_m --> dhlpro_m + mlthf_m + nh4_m 12898 or (10040 and 10205 and 12898 and 15184)
GCCcm dhlpro_m + nad_m <=> h_m + lpro_m + nadh_m 10040 or (10040 and 10205 and 12898 and 15184)
GLYCL gly_c + nad_c + thf_c --> co2_c + mlthf_c + nadh_c + nh4_c 10040 and 10205 and 12898 and 15184
GLYCL_2 co2_c + mlthf_c + nadh_c + nh4_c --> gly_c + nad_c + thf_c 12898
GLYCLm gly_m + nad_m + thf_m --> co2_m + mlthf_m + nadh_m + nh4_m 12898 or (CRv4_Au5_s12_g4121_t1 and 14894) or (12898 and 14894) or (10040 and 10205 and 12898 and 15184)
MTAM 5fthf_m + 2.0 h_m --> h2o_m + methf_m (CRv4_Au5_s12_g4121_t1 and 14894) or (12898 and 14894)
MTAM_nh4 alpro_m + h_m + thf_m <=> dhlpro_m + mlthf_m + nh4_m (CRv4_Au5_s12_g4121_t1 and 14894) or (12898 and 14894)
THFAT h2o_c + methf_c --> 5fthf_c + h_c 12898 or 9667
THFATm h2o_m + methf_m --> 5fthf_m + h_m 12898 or (10205 and 12898 and 13630 and 13948 and 15184)
yli_R1425 co2_x + mlthf_x + nadh_x + nh4_x --> gly_x + nad_x + thf_x 12898
```

10040 mito 9 cyto 8 LPD1  
glycine cleavage complex  
12898 mito GCV1  
10205 mito GCV2  
15184 mito GCV3  
alpha keto-glutarate dehydrogenase  
10007 mito KGD1  
12116 mito KGD2  
oxoadipate dehydrogenase  
9724 nucl KGD1 probable 2-oxoglutarate dehydrogenase E1 component DHKTD1  
pyruvate dehydrogenase  
13630 mito PDA1  
13948 cysk PDB1  
13722 mito PDX1  
14126 cyto LAT1  
serine hydroxymethyltransferase  
9667 cyto SHM1, SHM2 (GHMT2r)  
L-threonine aldolase  
16182 cyto GLY1  
9222 mito GLY1, hydroxytrimethyllysine aldolase

14894 is IBA57 protein involved in incorporating iron-sulfur clusters into proteins

GCC2am, GCC2bim, GCC2cm (GCC2cm\_copy1, GCC2cm\_copy2) is GLYCLm  
GCCam, GCCbim, GCCcm is GLYCLm

MTAM is wrong (FTHFCLm is correct with ATP)  
Replace FTHFCL with FTHFCLm and change genes to '12562' mito fau1  
MTAM\_nh4 is GCCbim

THFAT/THFATm reaction is catalyzed by serine hydroxymethyltransferase, not gcv or pda/pdb  
It is not known that yeast SHM has THFAT activity, and S. cerevisiae biocyc does not have this reaction.  
THFATm and FTHFCLm make a futile cycle, and the role of 5fthf is not clear.

GHMT2r only cyto SHMT is present in Rhodo  
GHMT3/GHMT3m is part of L-carnitine biosynthesis -> by mito GLY1 (paperblast to hydroxytrimethyllysine aldolase)  
https://www.ncbi.nlm.nih.gov/pubmed/19289605  
S. cer cannot synthesize carnitine, is it important for lipid for its role in shuttle?  
GHMT3/GHMT3m has incorrect stoichiometry (proton), replace with HTMLA\_m from iLB1027\_lipid

ALATA\_D2, ALATA\_L2 is inactivation of the enzyme in E. coli, activity is low  
https://onlinelibrary.wiley.com/doi/full/10.1046/j.0014-2956.2001.02606.x

Change GLYCLm genes to '10040 and 10205 and 12898 and 15184'  
Remove GCC2am, GCC2bim, GCC2cm, GCC2cm\_copy1, GCC2cm\_copy2, GCCam, GCCbim, GCCcm  
Remove GLYCL, GLYCL\_2, GLYDHD, MTAM, MTAM\_nh4, THFAT, THFATm, yli\_R1377, yli\_R1378, yli\_R1425

Add FTHFCLm and change genes to '12562'  
Remove FTHFCL

Change AKGDm genes to '10007 and 10040 and 12116'  
Change 2OXOADOXm genes to '10040 and 12116 and 9274'  
Remove AKGDH, AKGDam, AKGDbm

Change PDHm genes to '10040 and 13630 and 13722 and 13948 and 14126'  
Remove PDH, PDHcr, yli\_R0381, yli\_R1419

Remove GHMT2rm, GHMT3, GHMT3m, ALATA\_D2, ALATA\_L2, yli\_R1387

Change THRA genes to '16182'  
Change THRA2 genes to '16182'  
Add HTMLA\_m from iLB1027\_lipid, and change genes to '9222'

In [26]:

```
model.reactions.get_by_id('GLYCLm').gene_reaction_rule = '10040 and 10205 and 12898 and 15184'
model.remove_reactions(['GCC2am','GCC2bim','GCC2cm','GCC2cm_copy1','GCC2cm_copy2','GCCam','GCCbim','GCCcm'],remove_orphans=True)
model.remove_reactions(['GLYCL','GLYCL_2','GLYDHD','MTAM','MTAM_nh4','THFAT','THFATm','yli_R1377','yli_R1378','yli_R1425'],remove_orphans=True)
r = sce.reactions.get_by_id('FTHFCLm').copy()
r.gene_reaction_rule = '12562'
model.add_reactions([r])
model.remove_reactions(['FTHFCL'],remove_orphans=True)
model.reactions.get_by_id('AKGDm').gene_reaction_rule = '10007 and 10040 and 12116'
model.reactions.get_by_id('2OXOADOXm').gene_reaction_rule = '10040 and 12116 and 9274'
model.remove_reactions(['AKGDH','AKGDam','AKGDbm'],remove_orphans=True)
model.reactions.get_by_id('PDHm').gene_reaction_rule = '10040 and 13630 and 13722 and 13948 and 14126'
model.remove_reactions(['PDH','PDHcr','yli_R0381','yli_R1419'],remove_orphans=True)
model.remove_reactions(['GHMT2rm','GHMT3','GHMT3m','ALATA_D2','ALATA_L2','yli_R1387'],remove_orphans=True)
model.reactions.get_by_id('THRA').gene_reaction_rule = '16182'
model.reactions.get_by_id('THRA2').gene_reaction_rule = '16182'
r = ptri.reactions.get_by_id('HTMLA_m').copy()
r.gene_reaction_rule = '9222'
model.add_reactions([r])
```

In [27]:

```
for r in sorted(model.genes.get_by_id('10040').reactions, key=lambda x: x.id):
    print(r.id, r.reaction, r.gene_reaction_rule)
print()
for r in sorted(model.genes.get_by_id('10205').reactions, key=lambda x: x.id):
    print(r.id, r.reaction, r.gene_reaction_rule)
print()
for r in sorted(model.genes.get_by_id('12898').reactions, key=lambda x: x.id):
    print(r.id, r.reaction, r.gene_reaction_rule)
print()
for r in sorted(model.genes.get_by_id('15184').reactions, key=lambda x: x.id):
    print(r.id, r.reaction, r.gene_reaction_rule)
print()
for r in sorted(model.genes.get_by_id('12562').reactions, key=lambda x: x.id):
    print(r.id, r.reaction, r.gene_reaction_rule)
print()
for r in sorted(model.genes.get_by_id('10007').reactions, key=lambda x: x.id):
    print(r.id, r.reaction, r.gene_reaction_rule)
print()
for r in sorted(model.genes.get_by_id('12116').reactions, key=lambda x: x.id):
    print(r.id, r.reaction, r.gene_reaction_rule)
print()
for r in sorted(model.genes.get_by_id('9274').reactions, key=lambda x: x.id):
    print(r.id, r.reaction, r.gene_reaction_rule)
print()
for r in sorted(model.genes.get_by_id('13630').reactions, key=lambda x: x.id):
    print(r.id, r.reaction, r.gene_reaction_rule)
print()
for r in sorted(model.genes.get_by_id('13722').reactions, key=lambda x: x.id):
    print(r.id, r.reaction, r.gene_reaction_rule)
print()
for r in sorted(model.genes.get_by_id('13948').reactions, key=lambda x: x.id):
    print(r.id, r.reaction, r.gene_reaction_rule)
print()
for r in sorted(model.genes.get_by_id('14126').reactions, key=lambda x: x.id):
    print(r.id, r.reaction, r.gene_reaction_rule)
print()
for r in sorted(model.genes.get_by_id('9667').reactions, key=lambda x: x.id):
    print(r.id, r.reaction, r.gene_reaction_rule)
print()
for r in sorted(model.genes.get_by_id('16182').reactions, key=lambda x: x.id):
    print(r.id, r.reaction, r.gene_reaction_rule)
print()
for r in sorted(model.genes.get_by_id('9222').reactions, key=lambda x: x.id):
    print(r.id, r.reaction, r.gene_reaction_rule)
```

```
2OXOADOXm 2oxoadp_m + coa_m + nad_m --> co2_m + glutcoa_m + nadh_m 10040 and 12116 and 9274
AKGDm akg_m + coa_m + nad_m --> co2_m + nadh_m + succoa_m 10007 and 10040 and 12116
GLYCLm gly_m + nad_m + thf_m --> co2_m + mlthf_m + nadh_m + nh4_m 10040 and 10205 and 12898 and 15184
OIVD1m 4mop_m + coa_m + nad_m --> co2_m + ivcoa_m + nadh_m (10040 and 11183 and 12086 and 15436) or (10040 and 11183 and 12566 and 15436) or (10040 and 11188 and 12086 and 15436) or (10040 and 11188 and 12566 and 15436)
OIVD2m 3mob_m + coa_m + nad_m --> co2_m + ibcoa_m + nadh_m (10040 and 11183 and 12086 and 15436) or (10040 and 11183 and 12566 and 15436) or (10040 and 11188 and 12086 and 15436) or (10040 and 11188 and 12566 and 15436)
OIVD3m 3mop_m + coa_m + nad_m --> 2mbcoa_m + co2_m + nadh_m (10040 and 11183 and 12086 and 15436) or (10040 and 11183 and 12566 and 15436) or (10040 and 11188 and 12086 and 15436) or (10040 and 11188 and 12566 and 15436)
PDHm coa_m + nad_m + pyr_m --> accoa_m + co2_m + nadh_m 10040 and 13630 and 13722 and 13948 and 14126

GLYCLm gly_m + nad_m + thf_m --> co2_m + mlthf_m + nadh_m + nh4_m 10040 and 10205 and 12898 and 15184

GLYCLm gly_m + nad_m + thf_m --> co2_m + mlthf_m + nadh_m + nh4_m 10040 and 10205 and 12898 and 15184

GLYCLm gly_m + nad_m + thf_m --> co2_m + mlthf_m + nadh_m + nh4_m 10040 and 10205 and 12898 and 15184

FOMETRi 5fthf_c + h_c --> h2o_c + methf_c 12562
FTHFCLm 5fthf_m + atp_m --> adp_m + methf_m + pi_m 12562

AKGDa akg_c + h_c + lpam_c <=> co2_c + sdhlam_c 10007 or 9274
AKGDm akg_m + coa_m + nad_m --> co2_m + nadh_m + succoa_m 10007 and 10040 and 12116
GLCOASYNT S_gtrdhdlp_c + coa_c --> dhlam_c + glutcoa_c 10007 or 9274
yli_R0428 2oxoadp_c + coa_c + nad_c --> co2_c + glutcoa_c + nadh_c 10007 or 9274
yli_R0788 akg_m + h_m + yli_M03933_m --> co2_m + yli_M04007_m 10007 or 9274
yli_R1575 HC01435_m + yli_M03933_m --> thmpp_m + yli_M04007_m 10007 or 9274
yli_R1576 akg_m + thmpp_m --> HC01435_m + co2_m 10007 or 9274

2OXOADOXm 2oxoadp_m + coa_m + nad_m --> co2_m + glutcoa_m + nadh_m 10040 and 12116 and 9274
AKGDHe2r coa_m + h_m + sdhlam_m <=> dhlam_m + succoa_m 12116
AKGDb coa_c + sdhlam_c <=> dhlam_c + succoa_c 12116
AKGDm akg_m + coa_m + nad_m --> co2_m + nadh_m + succoa_m 10007 and 10040 and 12116
OXOADLR 2oxoadp_c + h_c + lpam_c --> S_gtrdhdlp_c + co2_c 12116
yli_R0784 succoa_m + yli_M03934_m <=> coa_m + yli_M04007_m 12116
yli_R1532 glutcoa_m + yli_M03934_m <=> S_gtrdhdlp_m + coa_m 12116

2OXOADOXm 2oxoadp_m + coa_m + nad_m --> co2_m + glutcoa_m + nadh_m 10040 and 12116 and 9274
AKGDa akg_c + h_c + lpam_c <=> co2_c + sdhlam_c 10007 or 9274
GLCOASYNT S_gtrdhdlp_c + coa_c --> dhlam_c + glutcoa_c 10007 or 9274
yli_R0428 2oxoadp_c + coa_c + nad_c --> co2_c + glutcoa_c + nadh_c 10007 or 9274
yli_R0788 akg_m + h_m + yli_M03933_m --> co2_m + yli_M04007_m 10007 or 9274
yli_R1575 HC01435_m + yli_M03933_m --> thmpp_m + yli_M04007_m 10007 or 9274
yli_R1576 akg_m + thmpp_m --> HC01435_m + co2_m 10007 or 9274

PDHam1hi h_h + pyr_h + thmpp_h --> 2ahethmpp_h + co2_h (CRv4_Au5_s3_g11028_t1 and 13630) or (15685 and 9800)
PDHam1mi h_m + pyr_m + thmpp_m --> 2ahethmpp_m + co2_m (13630 and 13948) or (13948 and 15791) or (15685 and 9800)
PDHam2hi 2ahethmpp_h + lpam_h --> adhlam_h + thmpp_h (CRv4_Au5_s3_g11028_t1 and 13630) or (15685 and 9800)
PDHam2mi 2ahethmpp_m + lpam_m --> adhlam_m + thmpp_m (13630 and 13948) or (13948 and 15791) or (15685 and 9800)
PDHm coa_m + nad_m + pyr_m --> accoa_m + co2_m + nadh_m 10040 and 13630 and 13722 and 13948 and 14126
yli_R0357 4.0 h_c + pyr_c + thmpp_c --> 2ahethmpp_c + co2_c 15791 or (13630 and 13948)
yli_R0377 2ahethmpp_m + yli_M03933_m --> 3.0 h_c + thmpp_m + yli_M04008_m 13630 and 13948
yli_R0848 4.0 h_m + pyr_m + thmpp_m --> 2ahethmpp_m + co2_m (13630 and 13948) or (15685 and 9800)
yli_R1375 4.0 h_c + pyr_c + thmpp_c --> 2ahethmpp_c + co2_c 13630 and 13948

PDHm coa_m + nad_m + pyr_m --> accoa_m + co2_m + nadh_m 10040 and 13630 and 13722 and 13948 and 14126

PDHam1mi h_m + pyr_m + thmpp_m --> 2ahethmpp_m + co2_m (13630 and 13948) or (13948 and 15791) or (15685 and 9800)
PDHam2mi 2ahethmpp_m + lpam_m --> adhlam_m + thmpp_m (13630 and 13948) or (13948 and 15791) or (15685 and 9800)
PDHm coa_m + nad_m + pyr_m --> accoa_m + co2_m + nadh_m 10040 and 13630 and 13722 and 13948 and 14126
yli_R0357 4.0 h_c + pyr_c + thmpp_c --> 2ahethmpp_c + co2_c 15791 or (13630 and 13948)
yli_R0377 2ahethmpp_m + yli_M03933_m --> 3.0 h_c + thmpp_m + yli_M04008_m 13630 and 13948
yli_R0848 4.0 h_m + pyr_m + thmpp_m --> 2ahethmpp_m + co2_m (13630 and 13948) or (15685 and 9800)
yli_R1375 4.0 h_c + pyr_c + thmpp_c --> 2ahethmpp_c + co2_c 13630 and 13948

PDHe2r adhlam_m + coa_m <=> accoa_m + dhlam_m 14126
PDHm coa_m + nad_m + pyr_m --> accoa_m + co2_m + nadh_m 10040 and 13630 and 13722 and 13948 and 14126
yli_R0374 accoa_m + yli_M03934_m <=> coa_m + yli_M04008_m 14126

GHMT2r ser__L_c + thf_c <=> gly_c + h2o_c + mlthf_c 9667

4HTHRA 4hthr_c <=> gcald_c + gly_c 16182 or 9222
THRA thr__L_c --> acald_c + gly_c 16182
THRA2 athr__L_c --> acald_c + gly_c 16182
THRA_1 thr__L_h <=> acald_h + gly_h 16182 or 9222

4HTHRA 4hthr_c <=> gcald_c + gly_c 16182 or 9222
HTMLA_m 3htmelys_m --> 4tmeabut_m + gly_m 9222
THRA_1 thr__L_h <=> acald_h + gly_h 16182 or 9222
```

In [28]:

```
for r in sorted(model.metabolites.get_by_id('4hthr_c').reactions, key=lambda x: x.id):
        print(r.id, r.reaction, r.gene_reaction_rule)
print()
for r in sorted(model.metabolites.get_by_id('phthr_c').reactions, key=lambda x: x.id):
        print(r.id, r.reaction, r.gene_reaction_rule)
```

```
4HTHRA 4hthr_c <=> gcald_c + gly_c 16182 or 9222
4HTHRK 4hthr_c + atp_c --> adp_c + h_c + phthr_c 8651
4HTHRS h2o_c + phthr_c --> 4hthr_c + pi_c 9742

4HTHRK 4hthr_c + atp_c --> adp_c + h_c + phthr_c 8651
4HTHRS h2o_c + phthr_c --> 4hthr_c + pi_c 9742
```

In [29]:

```
for r in sorted(model.genes.get_by_id('8651').reactions, key=lambda x: x.id):
    print(r.id, r.reaction, r.gene_reaction_rule)
print()
for r in sorted(model.genes.get_by_id('9742').reactions, key=lambda x: x.id):
    print(r.id, r.reaction, r.gene_reaction_rule)
```

```
4HTHRK 4hthr_c + atp_c --> adp_c + h_c + phthr_c 8651
HSK atp_c + hom__L_c --> adp_c + h_c + phom_c 8651
HSK_1 atp_h + hom__L_h --> adp_h + h_h + phom_h 8651

4HTHRS h2o_c + phthr_c --> 4hthr_c + pi_c 9742
THRS h2o_c + phom_c --> pi_c + thr__L_c 9742
THRS_1 h2o_h + phom_h --> pi_h + thr__L_h 9742
```

Remove FOMETRi, AKGDa, GLCOASYNT, yli\_R0428, yli\_R0788, yli\_R1575, yli\_R1576  
Remove AKGDHe2r, AKGDb, OXOADLR, yli\_R0784, yli\_R1532  
Remove PDHam1hi, PDHam1mi, PDHam2hi, PDHam2mi, yli\_R0357, yli\_R0377, yli\_R0848, yli\_R1375, PDHe2r, yli\_R0374  
Remove THRA\_1, 4HTHRA, 4HTHRK, HSK\_1, 4HTHRS, THRS\_1  
4HTHRA is by E. coli itaE, not by glyA (GLY1) and not in S. cer biocyc. Similar for 4HTHRK, 4HTHRS

In [30]:

```
model.remove_reactions(['FOMETRi','AKGDa','GLCOASYNT','yli_R0428','yli_R0788','yli_R1575','yli_R1576'],remove_orphans=True)
model.remove_reactions(['AKGDHe2r','AKGDb','OXOADLR','yli_R0784','yli_R1532'],remove_orphans=True)
model.remove_reactions(['PDHam1hi','PDHam1mi','PDHam2hi','PDHam2mi','yli_R0357','yli_R0377','yli_R0848','yli_R1375','PDHe2r','yli_R0374'],remove_orphans=True)
model.remove_reactions(['THRA_1','4HTHRA','4HTHRK','HSK_1','4HTHRS','THRS_1'],remove_orphans=True)
```

In [31]:

```
# Search for reactions involiving tmlys, 3htmelys 
for r in sorted(model.genes.get_by_id('14530').reactions, key=lambda x: x.id):
    print(r.id, r.reaction, r.gene_reaction_rule)
print()
for r in sorted(model.genes.get_by_id('16853').reactions, key=lambda x: x.id):
    print(r.id, r.reaction, r.gene_reaction_rule)
print()
for r in sorted(model.genes.get_by_id('9180').reactions, key=lambda x: x.id):
    print(r.id, r.reaction, r.gene_reaction_rule)
print()
for r in sorted(model.genes.get_by_id('9222').reactions, key=lambda x: x.id):
    print(r.id, r.reaction, r.gene_reaction_rule)
```

```
LYSMTF1n amet_n + peplys_n --> Nmelys_n + ahcys_n 14530
LYSMTF2n Nmelys_n + amet_n --> Ndmelys_n + ahcys_n 14530
LYSMTF3n Ndmelys_n + amet_n --> Ntmelys_n + ahcys_n 14530

PLYSPSer Ntmelys_r + h2o_r --> pepslys_r + tmlys_r (Spcs2 and Spcs3 and 16853 and 9881) or (SEC11A and SPCS2 and 10517 and 16853 and 9881)

TMLYSOX akg_c + o2_c + tmlys_c --> 3htmelys_c + co2_c + succ_c 9180

HTMLA_m 3htmelys_m --> 4tmeabut_m + gly_m 9222
```

L-carnitine biosynthesis  
14530 mito 22, nucl 2, cyto 2, cyto\_nucl 2 K11427: DOT1L, DOT1; histone-lysine N-methyltransferase, H3 lysine-79 specific  
9180 mito TMLHE, trimethyllysine dioxygenase -> replace TMLYSOX with TMLOX\_m from iLB1027\_lipid  
9222 mito GLY1, hydroxytrimethyllysine aldolase  
13426 mito blast of 4-trimethylammoniobutyraldehyde dehydrogenase ALDH9A1 hit -> TMABDH1\_m  
15060 cyto 10, nucl 7, pero 7, extr 2 BBOX1, gamma-butyrobetaine dioxygenase -> BBHOX exist in BiGG, but assume mito and use GBBOX\_m  
Longer RNA-extended gene model is predicted to be mitochondrial  
Remove upstream reactions (by 14530) since it is not clear where tmlys\_c is coming from

TMLOX\_m akg\_m + o2\_m + tmlys\_m ⇌ co2\_m + succ\_m + 3htmelys\_m 9180  
HTMLA\_m 3htmelys\_m --> 4tmeabut\_m + gly\_m 9222  
TMABDH1\_m h2o\_m + nad\_m + 4tmeabut\_m ⇌ 2.0 h\_m + nadh\_m + gbbtn\_m 13426  
GBBOX\_m akg\_m + o2\_m + gbbtn\_m → co2\_m + crn\_m + succ\_m 15060

Add TMLOX\_m from iLB1027\_lipid and change genes to '9180'  
Add TMABDH1\_m from iLB1027\_lipid and change genes to '13426'  
Add GBBOX\_m from iLB1027\_lipid and change genes to '15060'  
Remove TMLYSOX, LYSMTF1n, LYSMTF2n, LYSMTF3n, PLYSPSer

In [32]:

```
r = ptri.reactions.get_by_id('TMLOX_m').copy()
r.gene_reaction_rule = '9180'
model.add_reactions([r])
r = ptri.reactions.get_by_id('TMABDH1_m').copy()
r.gene_reaction_rule = '13426'
model.add_reactions([r])
r = ptri.reactions.get_by_id('GBBOX_m').copy()
r.gene_reaction_rule = '15060'
model.add_reactions([r])
model.remove_reactions(['TMLYSOX','LYSMTF1n','LYSMTF2n','LYSMTF3n','PLYSPSer'],remove_orphans=True)
```

In [33]:

```
for r in sorted(model.genes.get_by_id('12566').reactions, key=lambda x: x.id):
    print(r.id, r.reaction, r.gene_reaction_rule)
print()
for r in sorted(model.genes.get_by_id('15436').reactions, key=lambda x: x.id):
    print(r.id, r.reaction, r.gene_reaction_rule)
print()
for r in sorted(model.genes.get_by_id('11183').reactions, key=lambda x: x.id):
    print(r.id, r.reaction, r.gene_reaction_rule)
print()
for r in sorted(model.genes.get_by_id('12086').reactions, key=lambda x: x.id):
    print(r.id, r.reaction, r.gene_reaction_rule)
print()
for r in sorted(model.genes.get_by_id('11188').reactions, key=lambda x: x.id):
    print(r.id, r.reaction, r.gene_reaction_rule)
```

```
MOD 3mob_m + h_m + thmpp_m --> 2mhop_m + co2_m 12566 and 15436
MOD_2mbdhl 2mhob_m + lpam_m --> 2mbdhl_m + thmpp_m 12566 and 15436
MOD_2mhop 2mhop_m + lpam_m --> 2mpdhl_m + thmpp_m 12566 and 15436
MOD_3mhtpp 3mhtpp_m + lpam_m --> 3mbdhl_m + thmpp_m 12566 and 15436
MOD_3mop 3mop_m + h_m + thmpp_m --> 2mhob_m + co2_m 12566 and 15436
MOD_4mop 4mop_m + h_m + thmpp_m --> 3mhtpp_m + co2_m 12566 and 15436
OIVD1m 4mop_m + coa_m + nad_m --> co2_m + ivcoa_m + nadh_m (10040 and 11183 and 12086 and 15436) or (10040 and 11183 and 12566 and 15436) or (10040 and 11188 and 12086 and 15436) or (10040 and 11188 and 12566 and 15436)
OIVD1r 4mop_c + coa_c + nad_c <=> co2_c + ivcoa_c + nadh_c (PP_4404 and 11183 and 12566 and 15436) or (PP_4404 and 11188 and 12566 and 15436)
OIVD2 3mob_c + coa_c + nad_c --> co2_c + ibcoa_c + nadh_c (PP_4404 and 11183 and 12566 and 15436) or (PP_4404 and 11188 and 12566 and 15436)
OIVD2m 3mob_m + coa_m + nad_m --> co2_m + ibcoa_m + nadh_m (10040 and 11183 and 12086 and 15436) or (10040 and 11183 and 12566 and 15436) or (10040 and 11188 and 12086 and 15436) or (10040 and 11188 and 12566 and 15436)
OIVD3 3mop_c + coa_c + nad_c --> 2mbcoa_c + co2_c + nadh_c (PP_4404 and 11183 and 12566 and 15436) or (PP_4404 and 11188 and 12566 and 15436)
OIVD3m 3mop_m + coa_m + nad_m --> 2mbcoa_m + co2_m + nadh_m (10040 and 11183 and 12086 and 15436) or (10040 and 11183 and 12566 and 15436) or (10040 and 11188 and 12086 and 15436) or (10040 and 11188 and 12566 and 15436)
yli_R0937 4mop_c + h_c + yli_M03933_c --> co2_c + yli_M03936_c (12086 and 15436) or (12566 and 15436)
yli_R0938 3mob_c + h_c + yli_M03933_c --> co2_c + yli_M03938_c (12086 and 15436) or (12566 and 15436)
yli_R0939 3mop_c + h_c + yli_M03933_c --> co2_c + yli_M03940_c (12086 and 15436) or (12566 and 15436)
yli_R1587 4mop_c + thmpp_c <=> 3mhtpp_c + co2_c (12086 and 15436) or (12566 and 15436)
yli_R1590 2mhop_c + yli_M03933_c --> thmpp_c + yli_M03938_c (12086 and 15436) or (12566 and 15436)
yli_R1591 3mhtpp_c + yli_M03933_c --> thmpp_c + yli_M03936_c (12086 and 15436) or (12566 and 15436)
yli_R1592 2mhob_c + yli_M03933_c --> thmpp_c + yli_M03940_c (12086 and 15436) or (12566 and 15436)

MOD 3mob_m + h_m + thmpp_m --> 2mhop_m + co2_m 12566 and 15436
MOD_2mbdhl 2mhob_m + lpam_m --> 2mbdhl_m + thmpp_m 12566 and 15436
MOD_2mhop 2mhop_m + lpam_m --> 2mpdhl_m + thmpp_m 12566 and 15436
MOD_3mhtpp 3mhtpp_m + lpam_m --> 3mbdhl_m + thmpp_m 12566 and 15436
MOD_3mop 3mop_m + h_m + thmpp_m --> 2mhob_m + co2_m 12566 and 15436
MOD_4mop 4mop_m + h_m + thmpp_m --> 3mhtpp_m + co2_m 12566 and 15436
OIVD1m 4mop_m + coa_m + nad_m --> co2_m + ivcoa_m + nadh_m (10040 and 11183 and 12086 and 15436) or (10040 and 11183 and 12566 and 15436) or (10040 and 11188 and 12086 and 15436) or (10040 and 11188 and 12566 and 15436)
OIVD1r 4mop_c + coa_c + nad_c <=> co2_c + ivcoa_c + nadh_c (PP_4404 and 11183 and 12566 and 15436) or (PP_4404 and 11188 and 12566 and 15436)
OIVD2 3mob_c + coa_c + nad_c --> co2_c + ibcoa_c + nadh_c (PP_4404 and 11183 and 12566 and 15436) or (PP_4404 and 11188 and 12566 and 15436)
OIVD2m 3mob_m + coa_m + nad_m --> co2_m + ibcoa_m + nadh_m (10040 and 11183 and 12086 and 15436) or (10040 and 11183 and 12566 and 15436) or (10040 and 11188 and 12086 and 15436) or (10040 and 11188 and 12566 and 15436)
OIVD3 3mop_c + coa_c + nad_c --> 2mbcoa_c + co2_c + nadh_c (PP_4404 and 11183 and 12566 and 15436) or (PP_4404 and 11188 and 12566 and 15436)
OIVD3m 3mop_m + coa_m + nad_m --> 2mbcoa_m + co2_m + nadh_m (10040 and 11183 and 12086 and 15436) or (10040 and 11183 and 12566 and 15436) or (10040 and 11188 and 12086 and 15436) or (10040 and 11188 and 12566 and 15436)
yli_R0937 4mop_c + h_c + yli_M03933_c --> co2_c + yli_M03936_c (12086 and 15436) or (12566 and 15436)
yli_R0938 3mob_c + h_c + yli_M03933_c --> co2_c + yli_M03938_c (12086 and 15436) or (12566 and 15436)
yli_R0939 3mop_c + h_c + yli_M03933_c --> co2_c + yli_M03940_c (12086 and 15436) or (12566 and 15436)
yli_R1587 4mop_c + thmpp_c <=> 3mhtpp_c + co2_c (12086 and 15436) or (12566 and 15436)
yli_R1590 2mhop_c + yli_M03933_c --> thmpp_c + yli_M03938_c (12086 and 15436) or (12566 and 15436)
yli_R1591 3mhtpp_c + yli_M03933_c --> thmpp_c + yli_M03936_c (12086 and 15436) or (12566 and 15436)
yli_R1592 2mhob_c + yli_M03933_c --> thmpp_c + yli_M03940_c (12086 and 15436) or (12566 and 15436)

DHRT_2mbcoa 2mbdhl_m + coa_m --> 2mbcoa_m + dhlam_m 11183 or 11188
DHRT_ibcoa 2mpdhl_m + coa_m --> dhlam_m + ibcoa_m 11183 or 11188
DHRT_ivcoa 3mbdhl_m + coa_m --> dhlam_m + ivcoa_m 11183 or 11188
OIVD1m 4mop_m + coa_m + nad_m --> co2_m + ivcoa_m + nadh_m (10040 and 11183 and 12086 and 15436) or (10040 and 11183 and 12566 and 15436) or (10040 and 11188 and 12086 and 15436) or (10040 and 11188 and 12566 and 15436)
OIVD1r 4mop_c + coa_c + nad_c <=> co2_c + ivcoa_c + nadh_c (PP_4404 and 11183 and 12566 and 15436) or (PP_4404 and 11188 and 12566 and 15436)
OIVD2 3mob_c + coa_c + nad_c --> co2_c + ibcoa_c + nadh_c (PP_4404 and 11183 and 12566 and 15436) or (PP_4404 and 11188 and 12566 and 15436)
OIVD2m 3mob_m + coa_m + nad_m --> co2_m + ibcoa_m + nadh_m (10040 and 11183 and 12086 and 15436) or (10040 and 11183 and 12566 and 15436) or (10040 and 11188 and 12086 and 15436) or (10040 and 11188 and 12566 and 15436)
OIVD3 3mop_c + coa_c + nad_c --> 2mbcoa_c + co2_c + nadh_c (PP_4404 and 11183 and 12566 and 15436) or (PP_4404 and 11188 and 12566 and 15436)
OIVD3m 3mop_m + coa_m + nad_m --> 2mbcoa_m + co2_m + nadh_m (10040 and 11183 and 12086 and 15436) or (10040 and 11183 and 12566 and 15436) or (10040 and 11188 and 12086 and 15436) or (10040 and 11188 and 12566 and 15436)
yli_R0878 coa_c + yli_M03938_c --> ibcoa_c + yli_M03934_c 11183 or 11188
yli_R0879 coa_c + yli_M03940_c --> yli_M03934_c + yli_M03941_c 11183 or 11188
yli_R0880 coa_c + yli_M03936_c --> ivcoa_c + yli_M03934_c 11183 or 11188

OIVD1m 4mop_m + coa_m + nad_m --> co2_m + ivcoa_m + nadh_m (10040 and 11183 and 12086 and 15436) or (10040 and 11183 and 12566 and 15436) or (10040 and 11188 and 12086 and 15436) or (10040 and 11188 and 12566 and 15436)
OIVD2m 3mob_m + coa_m + nad_m --> co2_m + ibcoa_m + nadh_m (10040 and 11183 and 12086 and 15436) or (10040 and 11183 and 12566 and 15436) or (10040 and 11188 and 12086 and 15436) or (10040 and 11188 and 12566 and 15436)
OIVD3m 3mop_m + coa_m + nad_m --> 2mbcoa_m + co2_m + nadh_m (10040 and 11183 and 12086 and 15436) or (10040 and 11183 and 12566 and 15436) or (10040 and 11188 and 12086 and 15436) or (10040 and 11188 and 12566 and 15436)
yli_R0937 4mop_c + h_c + yli_M03933_c --> co2_c + yli_M03936_c (12086 and 15436) or (12566 and 15436)
yli_R0938 3mob_c + h_c + yli_M03933_c --> co2_c + yli_M03938_c (12086 and 15436) or (12566 and 15436)
yli_R0939 3mop_c + h_c + yli_M03933_c --> co2_c + yli_M03940_c (12086 and 15436) or (12566 and 15436)
yli_R1587 4mop_c + thmpp_c <=> 3mhtpp_c + co2_c (12086 and 15436) or (12566 and 15436)
yli_R1590 2mhop_c + yli_M03933_c --> thmpp_c + yli_M03938_c (12086 and 15436) or (12566 and 15436)
yli_R1591 3mhtpp_c + yli_M03933_c --> thmpp_c + yli_M03936_c (12086 and 15436) or (12566 and 15436)
yli_R1592 2mhob_c + yli_M03933_c --> thmpp_c + yli_M03940_c (12086 and 15436) or (12566 and 15436)

DHRT_2mbcoa 2mbdhl_m + coa_m --> 2mbcoa_m + dhlam_m 11183 or 11188
DHRT_ibcoa 2mpdhl_m + coa_m --> dhlam_m + ibcoa_m 11183 or 11188
DHRT_ivcoa 3mbdhl_m + coa_m --> dhlam_m + ivcoa_m 11183 or 11188
OIVD1m 4mop_m + coa_m + nad_m --> co2_m + ivcoa_m + nadh_m (10040 and 11183 and 12086 and 15436) or (10040 and 11183 and 12566 and 15436) or (10040 and 11188 and 12086 and 15436) or (10040 and 11188 and 12566 and 15436)
OIVD1r 4mop_c + coa_c + nad_c <=> co2_c + ivcoa_c + nadh_c (PP_4404 and 11183 and 12566 and 15436) or (PP_4404 and 11188 and 12566 and 15436)
OIVD2 3mob_c + coa_c + nad_c --> co2_c + ibcoa_c + nadh_c (PP_4404 and 11183 and 12566 and 15436) or (PP_4404 and 11188 and 12566 and 15436)
OIVD2m 3mob_m + coa_m + nad_m --> co2_m + ibcoa_m + nadh_m (10040 and 11183 and 12086 and 15436) or (10040 and 11183 and 12566 and 15436) or (10040 and 11188 and 12086 and 15436) or (10040 and 11188 and 12566 and 15436)
OIVD3 3mop_c + coa_c + nad_c --> 2mbcoa_c + co2_c + nadh_c (PP_4404 and 11183 and 12566 and 15436) or (PP_4404 and 11188 and 12566 and 15436)
OIVD3m 3mop_m + coa_m + nad_m --> 2mbcoa_m + co2_m + nadh_m (10040 and 11183 and 12086 and 15436) or (10040 and 11183 and 12566 and 15436) or (10040 and 11188 and 12086 and 15436) or (10040 and 11188 and 12566 and 15436)
yli_R0878 coa_c + yli_M03938_c --> ibcoa_c + yli_M03934_c 11183 or 11188
yli_R0879 coa_c + yli_M03940_c --> yli_M03934_c + yli_M03941_c 11183 or 11188
yli_R0880 coa_c + yli_M03936_c --> ivcoa_c + yli_M03934_c 11183 or 11188
```

branched-chain alpha-keto acid dehydrogenase (missing in S. cer)  
12566 mito bkdA1 Branched chain alpha-keto acid dehydrogenase E1, alpha subunit  
15436 mito bkdA2 Branched chain alpha-keto acid dehydrogenase E1, beta subunit  
11183 mito bkdB Dihydrolipoamide transacylase (alpha-keto acid dehydrogenase E2 subunit)  
extra cyto subunits?  
12086 cyto ortholog of E1, Short-chain acyl-CoA dehydrogenase (butyryl) has cytochrome b5-like domain -> fatty acid oxidation  
11188 cyto Dihydrolipoamide transacylase (alpha-keto acid dehydrogenase E2 subunit) -> keep as isozyme for now

Keep mito OIVDs (OIVD1m, OIVD2m, OIVD3m), and change genes to '(10040 and 11183 and 12566 and 15436) or (10040 and 11188 and 12566 and 15436)'

BCAA additional reactions, e.g., OBDHm in S. cer is missing genes  
Add OBDHm from iMM904 and change genes to OIVD1m genes

Remove cyto OIVDs (OIVD1r, OIVD2, OIVD3), MOD, MOD\_2mbdhl, MOD\_2mhop, MOD\_3mhtpp, MOD\_3mop, MOD\_4mop  
Remove yli\_R0937, yli\_R0938, yli\_R0939, yli\_R1587, yli\_R1590, yli\_R1591, yli\_R1592  
Remove DHRT\_2mbcoa, DHRT\_ibcoa, DHRT\_ivcoa, yli\_R0878, yli\_R0879, yli\_R0880

In [34]:

```
model.reactions.get_by_id('OIVD1m').gene_reaction_rule = '(10040 and 11183 and 12566 and 15436) or (10040 and 11188 and 12566 and 15436)'
model.reactions.get_by_id('OIVD2m').gene_reaction_rule = '(10040 and 11183 and 12566 and 15436) or (10040 and 11188 and 12566 and 15436)'
model.reactions.get_by_id('OIVD3m').gene_reaction_rule = '(10040 and 11183 and 12566 and 15436) or (10040 and 11188 and 12566 and 15436)'
r = sce.reactions.get_by_id('OBDHm').copy()
r.gene_reaction_rule = '(10040 and 11183 and 12566 and 15436) or (10040 and 11188 and 12566 and 15436)'
model.add_reactions([r])
model.remove_reactions(['OIVD1r','OIVD2','OIVD3','MOD','MOD_2mbdhl','MOD_2mhop','MOD_3mhtpp','MOD_3mop','MOD_4mop'],remove_orphans=True)
model.remove_reactions(['yli_R0937','yli_R0938','yli_R0939','yli_R1587','yli_R1590','yli_R1591','yli_R1592'],remove_orphans=True)
model.remove_reactions(['DHRT_2mbcoa','DHRT_ibcoa','DHRT_ivcoa','yli_R0878','yli_R0879','yli_R0880'],remove_orphans=True)
```

In [35]:

```
for r in sorted(model.genes.get_by_id('15685').reactions, key=lambda x: x.id):
    print(r.id, r.reaction, r.gene_reaction_rule)
print()
for r in sorted(model.genes.get_by_id('9800').reactions, key=lambda x: x.id):
    print(r.id, r.reaction, r.gene_reaction_rule)
```

```
ACAS_2ahbut 2ahethmpp_h + 2obut_h --> 2ahbut_h + thmpp_h 15685 and 9800
ACHBS 2obut_c + h_c + pyr_c --> 2ahbut_c + co2_c (b3670 and 15685) or (15685 and 9800)
ACHBSm 2obut_m + h_m + pyr_m --> 2ahbut_m + co2_m 15685 and 9800
ACLS h_c + 2.0 pyr_c --> alac__S_c + co2_c (b3670 and 15685) or (15685 and 9800)
ACLSm h_m + 2.0 pyr_m --> alac__S_m + co2_m 15685 and 9800
APLh 2ahethmpp_h + pyr_h --> alac__S_h + thmpp_h 15685 and 9800
APLm 2ahethmpp_m + pyr_m --> alac__S_m + thmpp_m 15685 and 9800
PPATDh h_h + 2.0 pyr_h --> alac__S_h + co2_h 15685 and 9800
yli_R0861 2ahethmpp_m + pyr_m --> alac__S_m + 3.0 h_m + thmpp_m 15685 and 9800
yli_R0862 2ahethmpp_m + 2obut_m --> 2ahbut_m + 3.0 h_m + thmpp_m 15685 and 9800
yli_R1588 2obut_m + pyr_m --> 2ahbut_m + co2_m 15685 and 9800

ACAS_2ahbut 2ahethmpp_h + 2obut_h --> 2ahbut_h + thmpp_h 15685 and 9800
ACHBS 2obut_c + h_c + pyr_c --> 2ahbut_c + co2_c (b3670 and 15685) or (15685 and 9800)
ACHBSm 2obut_m + h_m + pyr_m --> 2ahbut_m + co2_m 15685 and 9800
ACLS h_c + 2.0 pyr_c --> alac__S_c + co2_c (b3670 and 15685) or (15685 and 9800)
ACLSm h_m + 2.0 pyr_m --> alac__S_m + co2_m 15685 and 9800
APLh 2ahethmpp_h + pyr_h --> alac__S_h + thmpp_h 15685 and 9800
APLm 2ahethmpp_m + pyr_m --> alac__S_m + thmpp_m 15685 and 9800
PPATDh h_h + 2.0 pyr_h --> alac__S_h + co2_h 15685 and 9800
yli_R0861 2ahethmpp_m + pyr_m --> alac__S_m + 3.0 h_m + thmpp_m 15685 and 9800
yli_R0862 2ahethmpp_m + 2obut_m --> 2ahbut_m + 3.0 h_m + thmpp_m 15685 and 9800
yli_R1588 2obut_m + pyr_m --> 2ahbut_m + co2_m 15685 and 9800
```

15685 mito ILV2  
9800 mito ILV6  
ILV2 and ILV6 catalyze ACHBSm and ACLSm  
Remove ACAS\_2ahbut, ACHBS, ACLS, APLh, APLm, PPATDh, yli\_R0861, yli\_R0862, yli\_R1588

In [36]:

```
model.remove_reactions(['ACAS_2ahbut','ACHBS','ACLS','APLh','APLm','PPATDh','yli_R0861','yli_R0862','yli_R1588'],remove_orphans=True)
```

In [37]:

```
for r in sorted(model.metabolites.get_by_id('2ahethmpp_m').reactions, key=lambda x: x.id):
        print(r.id, r.reaction, r.gene_reaction_rule)
print()
for r in sorted(model.metabolites.get_by_id('2ahethmpp_c').reactions, key=lambda x: x.id):
        print(r.id, r.reaction, r.gene_reaction_rule)
```

```
PDCm 2ahethmpp_m --> acald_m + thmpp_m 15791

yli_R0364 2ahethmpp_c --> acald_c + 3.0 h_c + thmpp_c 15791
```

In [38]:

```
for r in sorted(model.genes.get_by_id('15791').reactions, key=lambda x: x.id):
    print(r.id, r.reaction, r.gene_reaction_rule)
```

```
3MOBDC 3mob_c + h_c --> 2mppal_c + co2_c 15791
3MOPDC 3mop_c + h_c --> 2mbald_c + co2_c 15791
4MOPDC 4mop_c + h_c --> 3mbald_c + co2_c 15791
ACALDCD 2.0 acald_c --> actn__R_c 15791
INDPYRD h_c + indpyr_c <=> co2_c + id3acald_c 15791
PDCm 2ahethmpp_m --> acald_m + thmpp_m 15791
PPYRDC h_c + phpyr_c --> co2_c + pacald_c 15791
PYRDC h_c + pyr_c --> acald_c + co2_c 15791
PYRDC2 acald_c + h_c + pyr_c --> actn__R_c + co2_c 15791
PYRDC_1 h_m + pyr_m --> acald_m + co2_m 15791
yli_R0364 2ahethmpp_c --> acald_c + 3.0 h_c + thmpp_c 15791
```

15791 cyto PDC  
Remove PDCm, PYRDC\_1, yli\_R0364

In [39]:

```
model.remove_reactions(['PDCm','PYRDC_1','yli_R0364'],remove_orphans=True)
```

#### 2ippm¶

In [40]:

```
for r in sorted(model.genes.get_by_id('14914').reactions, key=lambda x: x.id):
    print(r.id, r.reaction, r.gene_reaction_rule)
```

```
IPPMIa 3c2hmp_c <=> 2ippm_c + h2o_c 14914 or (CRv4_Au5_s6_g12448_t1 and 14914) or (PP_1986 and 14914) or (b0071 and 14914)
IPPMIb 2ippm_c + h2o_c <=> 3c3hmp_c 14914 or (CRv4_Au5_s6_g12448_t1 and 14914) or (PP_1986 and 14914) or (b0071 and 14914)
yli_R1465 3c3hmp_m <=> 2ippm_m + h2o_m 14914
yli_R1466 2ippm_m + h2o_m <=> 3c2hmp_m 14914
yli_R7859 2ippm_m + h2o_m <=> 3c2hmp_m 14914
yli_R8859 3c3hmp_m <=> 2ippm_m + h2o_m 14914
```

In [41]:

```
# 14914 is cytosolic, and contains both large (leuC) and small (leuD) subunits
model.reactions.get_by_id('IPPMIa').gene_reaction_rule = '14914'
model.reactions.get_by_id('IPPMIb').gene_reaction_rule = '14914'
model.remove_reactions(['yli_R1465','yli_R1466','yli_R7859','yli_R8859'], remove_orphans=True)
```

#### cer1\_24¶

In [42]:

```
for r in sorted(model.genes.get_by_id('9664').reactions, key=lambda x: x.id):
    print(r.id, r.reaction, r.gene_reaction_rule)
print()
for r in sorted(model.genes.get_by_id('15314').reactions, key=lambda x: x.id):
    print(r.id, r.reaction, r.gene_reaction_rule)
```

```
CERH124_copy2 cer1_24_c + h_c + nadph_c + o2_c --> cer2_24_c + h2o_c + nadp_c 9664
CERH126_copy2 cer1_26_c + h_c + nadph_c + o2_c --> cer2_26_c + h2o_c + nadp_c 9664
CERS324 cer2_24_c + h_c + nadph_c + o2_c --> cer3_24_c + h2o_c + nadp_c 9664
CERS326 cer2_26_c + h_c + nadph_c + o2_c --> cer3_26_c + h2o_c + nadp_c 9664

44MZYMMO 44mzym_c + 2.0 h_c + 3.0 nadph_c + 3.0 o2_c <=> 4mzym_int1_c + 4.0 h2o_c + 3.0 nadp_c 15314
CERH124_copy1 cer1_24_c + h_c + nadph_c + o2_c --> cer2_24_c + h2o_c + nadp_c 15314
CERH126_copy1 cer1_26_c + h_c + nadph_c + o2_c --> cer2_26_c + h2o_c + nadp_c 15314
PSPHS h_c + nadph_c + o2_c + sphgn_c --> h2o_c + nadp_c + psphings_c 15314
yli_R0702 h_c + nadph_c + o2_c + yli_M05742_c --> h2o_c + nadp_c + yli_M05749_c 15314
yli_R0703 h_c + nadph_c + o2_c + yli_M05741_c --> h2o_c + nadp_c + yli_M05748_c 15314
yli_R0704 h_c + nadph_c + o2_c + yli_M05749_c --> h2o_c + nadp_c + yli_M05725_c 15314
yli_R0705 h_c + nadph_c + o2_c + yli_M05748_c --> h2o_c + nadp_c + yli_M05724_c 15314
yli_R1441 h_r + nadph_r + o2_r + sphgn_r --> h2o_r + nadp_r + psphings_r 15314
yli_R1571 dhcrm_cho_r + h_r + nadph_r + o2_r --> h2o_r + nadp_r + phcrm_hs_r 15314
```

Sphingolipid biosynthesis  
https://onlinelibrary.wiley.com/doi/pdf/10.1111/tra.12239  
https://www.sciencedirect.com/science/article/pii/S0022283616303746  
https://link.springer.com/content/pdf/10.1007%2F978-3-319-43676-0\_21-1.pdf  
https://www.ncbi.nlm.nih.gov/pmc/articles/PMC3683901/  
https://pdfs.semanticscholar.org/e239/05c20c98c108bd8a7613dde68d283e7cba22.pdf  
SERPT  
10303 cyto LCB1 (SPOTS complex in ER membrane) serine palmitoyltransferase  
9425 cyto LCB2 (SPOTS complex in ER membrane) serine palmitoyltransferase  
9394 mito Small subunit of serine palmitoyltransferase-like -> TSC3  
3DSPHR  
9979 extr TSC10 (lipid droplet)  
PSPHS  
15314 mito 9, plas 5, E.R. 4 SUR2 (uses cytochrome b5)  
CERS124/CERS124er  
11391 plas LAC1, LAG1 (ER) ceramide synthase  
15168 plas ceramide synthase  
can't find LIP1  
SBPP1/SBPP1er  
13412 plas LCB3, YSR3 (ER) check for isozyme  
16352 DAG/PA phosphatase LPP1?  
12927 AUR1 Phosphatidylinositol:ceramide phosphoinositol transferase  
13172 IPT1 Phosphatidylinositol:ceramide phosphoinositol transferase  
13087 DPP1 DAG/PA phosphatase  
15202 PA phosphatase 3 LPP3 in Arabidopsis  
14049 dolichyldiphosphatase  
SLCBK1/SLCBK2  
10391 cyto LCB4 (plasma, ER, golgi), LCB5 (golgi) Sphingosine kinase  
16228 cyto Sphingosine kinase  
CERH124\_copy2/CERS324  
9664 mito 9 pero 7 cyto\_mito 7 mito\_nucl 7 SCS7 (membrane, ER)  
PSPHPL  
11925 DPL1 (ER) sphinganine-1-phosphate aldolase

10303 cyto 11, cyto\_nucl 9.5, mito 7, nucl 6 K00654: SPT; serine palmitoyltransferase GKK *9425 cyto 11.5, mito 10, cyto\_nucl 9, nucl 5.5 K00654: SPT; serine palmitoyltransferase EHA*  
9394 mito 12, extr 7, mito\_nucl 7 HMMPfam:Protein of unknown function (DUF3317):PF11779 ELL *9979 extr 16, nucl 3, mito 3, mito\_nucl 3 K04708: E1.1.1.102; 3-dehydrosphinganine reductase VVG*  
15314 mito 9, plas 5, E.R. 4, extr 3, golg 3, cyto 2 K04713: SUR2; sphinganine C4-monooxygenase KTE *11391 plas 22, mito 2, cyto 1, pero 1, E.R. 1, mito\_nucl 1, cyto\_pero 1 K04709: LAG1; Acyl-CoA-dependent ceramide synthase KKR*  
15168 plas 20, mito 3, vacu 2 K04709: LAG1; Acyl-CoA-dependent ceramide synthase KER *13412 plas 26 K04717: SGPP2; sphingosine-1-phosphate phosphotase 2 SVR*  
16352 plas 14, extr 6, E.R. 3, mito 2, vacu 2 KOG3030: Lipid phosphate phosphatase and related enzymes of the PAP2 family AVV *12927 plas 22, mito 2, vacu 2 HMMPfam:PAP2 superfamily:PF01569,SMART:Acid phosphatase homologues:SM00014,SUPERFAMILY::SSF48317 RRD*  
13172 extr 10, mito 7, plas 4, cyto\_mito 4 HMMPfam:PAP2 superfamily:PF01569 SLA *13087 plas 23, mito 2 K18693: DPP1; diacylglycerol diphosphate phosphatase / phosphatidate phosphatase GYY*  
15202 plas 27 KOG3030: Lipid phosphate phosphatase and related enzymes of the PAP2 family RMV *14049 mito 13, extr 12 KOG3146: Dolichyl pyrophosphate phosphatase and related acid phosphatases GEL*  
10391 cyto 13.5, cyto\_nucl 12, nucl 5.5, pero 4, mito 3 K04718: SPHK; sphingosine kinase GFD *16228 cyto 12, nucl 8, mito 5 HMMPfam:Diacylglycerol kinase catalytic domain:PF00781,ProSiteProfiles:DAG-kinase catalytic (DAGKc) domain profile.:PS50146,SUPERFAMILY::SSF111331 EKE*  
9664 mito 9, pero 7, cyto\_mito 7, mito\_nucl 7 K19703: FA2H, SCS7; 4-hydroxysphinganine ceramide fatty acyl 2-hydroxylase AKA *11925 cyto 17.5, cyto\_nucl 9.5, mito 9 K01634: SGPL1, DPL1; sphinganine-1-phosphate aldolase LYA*

In [43]:

```
for r in sorted(model.genes.get_by_id('10303').reactions, key=lambda x: x.id):
    print(r.id, r.reaction, r.gene_reaction_rule)
print()
for r in sorted(model.genes.get_by_id('9425').reactions, key=lambda x: x.id):
    print(r.id, r.reaction, r.gene_reaction_rule)
print()
for r in sorted(model.genes.get_by_id('9979').reactions, key=lambda x: x.id):
    print(r.id, r.reaction, r.gene_reaction_rule)
print()
for r in sorted(model.genes.get_by_id('15314').reactions, key=lambda x: x.id):
    print(r.id, r.reaction, r.gene_reaction_rule)
print()
for r in sorted(model.genes.get_by_id('11391').reactions, key=lambda x: x.id):
    print(r.id, r.reaction, r.gene_reaction_rule)
print()
for r in sorted(model.genes.get_by_id('15168').reactions, key=lambda x: x.id):
    print(r.id, r.reaction, r.gene_reaction_rule)
print()
for r in sorted(model.genes.get_by_id('13412').reactions, key=lambda x: x.id):
    print(r.id, r.reaction, r.gene_reaction_rule)
print()
for r in sorted(model.genes.get_by_id('10391').reactions, key=lambda x: x.id):
    print(r.id, r.reaction, r.gene_reaction_rule)
print()
for r in sorted(model.genes.get_by_id('9664').reactions, key=lambda x: x.id):
    print(r.id, r.reaction, r.gene_reaction_rule)
print()
for r in sorted(model.genes.get_by_id('11925').reactions, key=lambda x: x.id):
    print(r.id, r.reaction, r.gene_reaction_rule)
```

```
GLYATx accoa_x + gly_x <=> 2aobut_x + coa_x + h_x 10303
SERPT h_c + pmtcoa_c + ser__L_c --> 3dsphgn_c + co2_c + coa_c 10303 or 9425 or (10303 and 9425) or (YBR058C_A and 10303 and 9425)
yli_R1438 h_r + pmtcoa_r + ser__L_r --> 3dsphgn_r + co2_r + coa_r 10303 or 9425

SERPT h_c + pmtcoa_c + ser__L_c --> 3dsphgn_c + co2_c + coa_c 10303 or 9425 or (10303 and 9425) or (YBR058C_A and 10303 and 9425)
yli_R1438 h_r + pmtcoa_r + ser__L_r --> 3dsphgn_r + co2_r + coa_r 10303 or 9425

3DSPHR 3dsphgn_c + h_c + nadph_c --> nadp_c + sphgn_c 9979
yli_R1440 3dsphgn_r + h_r + nadph_r --> nadp_r + sphgn_r 9979

44MZYMMO 44mzym_c + 2.0 h_c + 3.0 nadph_c + 3.0 o2_c <=> 4mzym_int1_c + 4.0 h2o_c + 3.0 nadp_c 15314
CERH124_copy1 cer1_24_c + h_c + nadph_c + o2_c --> cer2_24_c + h2o_c + nadp_c 15314
CERH126_copy1 cer1_26_c + h_c + nadph_c + o2_c --> cer2_26_c + h2o_c + nadp_c 15314
PSPHS h_c + nadph_c + o2_c + sphgn_c --> h2o_c + nadp_c + psphings_c 15314
yli_R0702 h_c + nadph_c + o2_c + yli_M05742_c --> h2o_c + nadp_c + yli_M05749_c 15314
yli_R0703 h_c + nadph_c + o2_c + yli_M05741_c --> h2o_c + nadp_c + yli_M05748_c 15314
yli_R0704 h_c + nadph_c + o2_c + yli_M05749_c --> h2o_c + nadp_c + yli_M05725_c 15314
yli_R0705 h_c + nadph_c + o2_c + yli_M05748_c --> h2o_c + nadp_c + yli_M05724_c 15314
yli_R1441 h_r + nadph_r + o2_r + sphgn_r --> h2o_r + nadp_r + psphings_r 15314
yli_R1571 dhcrm_cho_r + h_r + nadph_r + o2_r --> h2o_r + nadp_r + phcrm_hs_r 15314

CERS124 sphgn_c + ttccoa_c --> cer1_24_c + coa_c + h_c 11391
CERS124er sphgn_r + ttccoa_r --> cer1_24_r + coa_r + h_r 11391
CERS126 hexccoa_c + sphgn_c --> cer1_26_c + coa_c + h_c 11391
CERS126er hexccoa_r + sphgn_r --> cer1_26_r + coa_r + h_r 11391
CERS224 psphings_c + ttccoa_c --> cer2_24_c + coa_c + h_c 11391
CERS224er psphings_r + ttccoa_r --> cer2_24_r + coa_r + h_r 11391
CERS226 hexccoa_c + psphings_c --> cer2_26_c + coa_c + h_c 11391
CERS226er hexccoa_r + psphings_r --> cer2_26_r + coa_r + h_r 11391
yli_R0698 sphgn_c + yli_M04597_c --> coa_c + h_c + yli_M05742_c 11391 or 15168
yli_R0699 sphgn_c + yli_M04095_c --> coa_c + h_c + yli_M05741_c 11391 or 15168
yli_R0700 psphings_c + yli_M04095_c --> coa_c + h_c + yli_M05748_c 11391 or 15168
yli_R0701 psphings_c + yli_M04597_c --> coa_c + h_c + yli_M05749_c 11391 or 15168
yli_R1570 acoa_r + sphgn_r --> coa_r + dhcrm_cho_r 11391 or 15168

yli_R0698 sphgn_c + yli_M04597_c --> coa_c + h_c + yli_M05742_c 11391 or 15168
yli_R0699 sphgn_c + yli_M04095_c --> coa_c + h_c + yli_M05741_c 11391 or 15168
yli_R0700 psphings_c + yli_M04095_c --> coa_c + h_c + yli_M05748_c 11391 or 15168
yli_R0701 psphings_c + yli_M04597_c --> coa_c + h_c + yli_M05749_c 11391 or 15168
yli_R1570 acoa_r + sphgn_r --> coa_r + dhcrm_cho_r 11391 or 15168

SBPP1 h2o_c + sph1p_c --> pi_c + sphgn_c 13412
SBPP1er h2o_r + sph1p_r --> pi_r + sphgn_r 13412
SBPP2er h2o_r + psph1p_r --> pi_r + psphings_r 13412

SLCBK1 atp_c + sphgn_c --> adp_c + h_c + sph1p_c 10391
SLCBK2 atp_c + psphings_c --> adp_c + h_c + psph1p_c 10391
SPHK21c atp_c + sphings_c --> adp_c + h_c + sphs1p_c 10391
yli_R1439 atp_r + sphgn_r --> adp_r + h_r + sph1p_r 10391
yli_R1569 atp_r + yli_M07050_r --> adp_r + sphs1p_r 10391

CERH124_copy2 cer1_24_c + h_c + nadph_c + o2_c --> cer2_24_c + h2o_c + nadp_c 9664
CERH126_copy2 cer1_26_c + h_c + nadph_c + o2_c --> cer2_26_c + h2o_c + nadp_c 9664
CERS324 cer2_24_c + h_c + nadph_c + o2_c --> cer3_24_c + h2o_c + nadp_c 9664
CERS326 cer2_26_c + h_c + nadph_c + o2_c --> cer3_26_c + h2o_c + nadp_c 9664

PSPHPL psph1p_c --> 2hhxdal_c + ethamp_c 11925
SGPL11r sph1p_r --> ethamp_r + hxdcal_r 11925
SGPL12r h2o_r + sphs1p_r --> ethamp_r + h_r + hdca_r 11925
SGPL13 sphs1p_c --> ethamp_c + hxdceal_c 11925
SPHPL sph1p_c --> ethamp_c + hxdcal_c 11925
yli_R1567 sphs1p_r --> ethamp_r + hxdceal_r 11925
yli_R1568 sph1p_r --> ethamp_r + yli_M07049_r 11925
```

Change yli\_R1438 to SERPTer  
Change SERPTer genes to '10303 and 9394 and 9425'  
Remove GLYATx, yli\_R1438  
Change yli\_R1440 to 3DSPHRer  
Remove 3DSPHR  
Change CERH124\_copy1 to CERH124er, and change metabolites from c to r  
Change CERH126\_copy1 to CERH126er, and change metabolites from c to r  
Change yli\_R1441 to PSPHSer  
Remove PSPHS, yli\_R0702, yli\_R0703, yli\_R0704, yli\_R0705, yli\_R1571  
Change CERS124er genes to '11391 or 15168'  
Change CERS126er genes to '11391 or 15168'  
Change CERS224er genes to '11391 or 15168'  
Change CERS226er genes to '11391 or 15168'  
Remove CERS124, CERS126, CERS224, CERS226, yli\_R0698, yli\_R0699, yli\_R0700, yli\_R0701, yli\_R1570  
Remove SBPP1  
Change SLCBK1 to SLCBK1er, and change genes to '10391 or 16228'  
Change SLCBK2 to SLCBK2er, and change genes to '10391 or 16228'  
Remove SPHK21c, SGOR, yli\_R1439, yli\_R1569  
Change CERH124\_copy2 to CERS2p24, change metabolites from c to r, and change cer2\_24 to cer2p\_24  
Change CERH126\_copy2 to CERS2p26, change metabolites from c to r, and change cer2\_26 to cer2p\_26  
Change CERS324 to CERS324er, and change metabolites from c to r  
Change CERS326 to CERS326er, and change metabolites from c to r  
Change PSPHPL to PSPHPLer, and change metabolites from c to r  
Change SPHPL to SPHPLer, and change metabolites from c to r  
Remove SGPL11r, SGPL12r, SGPL13, yli\_R1567, yli\_R1568

In [44]:

```
model.reactions.get_by_id('yli_R1438').id = 'SERPTer'
model.reactions.get_by_id('SERPTer').gene_reaction_rule = '10303 and 9394 and 9425'
model.remove_reactions(['GLYATx','SERPT'], remove_orphans=True)
model.reactions.get_by_id('yli_R1440').id = '3DSPHRer'
model.remove_reactions(['3DSPHR'], remove_orphans=True)
r = model.reactions.get_by_id('CERH124_copy1').copy()
model.reactions.get_by_id('CERH124_copy1').id = 'CERH124er'
for m in r.metabolites:
    model.reactions.get_by_id('CERH124er').add_metabolites({m.id: -r.get_coefficient(m.id), m.id.replace('_c','_r'): r.get_coefficient(m.id)})
r = model.reactions.get_by_id('CERH126_copy1').copy()
model.reactions.get_by_id('CERH126_copy1').id = 'CERH126er'
for m in r.metabolites:
    model.reactions.get_by_id('CERH126er').add_metabolites({m.id: -r.get_coefficient(m.id), m.id.replace('_c','_r'): r.get_coefficient(m.id)})
model.reactions.get_by_id('yli_R1441').id = 'PSPHSer'
model.remove_reactions(['PSPHS','yli_R0702','yli_R0703','yli_R0704','yli_R0705','yli_R1571'], remove_orphans=True)
model.reactions.get_by_id('CERS124er').gene_reaction_rule = '11391 or 15168'
model.reactions.get_by_id('CERS126er').gene_reaction_rule = '11391 or 15168'
model.reactions.get_by_id('CERS224er').gene_reaction_rule = '11391 or 15168'
model.reactions.get_by_id('CERS226er').gene_reaction_rule = '11391 or 15168'
model.remove_reactions(['CERS124','CERS126','CERS224','CERS226','yli_R0698','yli_R0699','yli_R0700','yli_R0701','yli_R1570','SBPP1'], remove_orphans=True)
r = model.reactions.get_by_id('SLCBK1').copy()
model.reactions.get_by_id('SLCBK1').id = 'SLCBK1er'
model.reactions.get_by_id('SLCBK1er').gene_reaction_rule = '10391 or 16228'
for m in r.metabolites:
    model.reactions.get_by_id('SLCBK1er').add_metabolites({m.id: -r.get_coefficient(m.id), m.id.replace('_c','_r'): r.get_coefficient(m.id)})
r = model.reactions.get_by_id('SLCBK2').copy()
model.reactions.get_by_id('SLCBK2').id = 'SLCBK2er'
model.reactions.get_by_id('SLCBK2er').gene_reaction_rule = '10391 or 16228'
for m in r.metabolites:
    model.reactions.get_by_id('SLCBK2er').add_metabolites({m.id: -r.get_coefficient(m.id), m.id.replace('_c','_r'): r.get_coefficient(m.id)})
model.remove_reactions(['SPHK21c','SGOR','yli_R1439','yli_R1569'], remove_orphans=True)
m1 = model.metabolites.get_by_id('cer2_24_c').copy()
m2 = model.metabolites.get_by_id('cer2_26_c').copy()
m1.id = 'cer2p_24_r'
m1.compartment = 'r'
m2.id = 'cer2p_26_r'
m2.compartment = 'r'
m1.name = 'Ceramide 2p (Sphinganine:n-C24:0OH)'
m2.name = 'Ceramide 2p (Sphinganine:n-C26:0OH)'
model.add_metabolites([m1,m2])
r = model.reactions.get_by_id('CERH124_copy2').copy()
model.reactions.get_by_id('CERH124_copy2').id = 'CERS2p24er'
model.reactions.get_by_id('CERS2p24er').name = 'Ceramide 2p synthase  24C'
for m in r.metabolites:
    model.reactions.get_by_id('CERS2p24er').add_metabolites({m.id: -r.get_coefficient(m.id), m.id.replace('_c','_r'): r.get_coefficient(m.id)})
model.reactions.get_by_id('CERS2p24er').add_metabolites({'cer2_24_r': -1.0, 'cer2p_24_r': 1.0})
r = model.reactions.get_by_id('CERH126_copy2').copy()
model.reactions.get_by_id('CERH126_copy2').id = 'CERS2p26er'
model.reactions.get_by_id('CERS2p26er').name = 'Ceramide 2p synthase  26C'
for m in r.metabolites:
    model.reactions.get_by_id('CERS2p26er').add_metabolites({m.id: -r.get_coefficient(m.id), m.id.replace('_c','_r'): r.get_coefficient(m.id)})
model.reactions.get_by_id('CERS2p26er').add_metabolites({'cer2_26_r': -1.0, 'cer2p_26_r': 1.0})
m1 = model.metabolites.get_by_id('cer3_24_c').copy()
m2 = model.metabolites.get_by_id('cer3_26_c').copy()
m1.id = 'cer3_24_r'
m1.compartment = 'r'
m2.id = 'cer3_26_r'
m2.compartment = 'r'
model.add_metabolites([m1,m2])
r = model.reactions.get_by_id('CERS324').copy()
model.reactions.get_by_id('CERS324').id = 'CERS324er'
for m in r.metabolites:
    model.reactions.get_by_id('CERS324er').add_metabolites({m.id: -r.get_coefficient(m.id), m.id.replace('_c','_r'): r.get_coefficient(m.id)})

r = model.reactions.get_by_id('CERS326').copy()
model.reactions.get_by_id('CERS326').id = 'CERS326er'
for m in r.metabolites:
    model.reactions.get_by_id('CERS326er').add_metabolites({m.id: -r.get_coefficient(m.id), m.id.replace('_c','_r'): r.get_coefficient(m.id)})
m1 = model.metabolites.get_by_id('2hhxdal_c').copy()
m2 = model.metabolites.get_by_id('hxdcal_c').copy()
m1.id = '2hhxdal_r'
m1.compartment = 'r'
model.add_metabolites([m1])
r = model.reactions.get_by_id('PSPHPL').copy()
model.reactions.get_by_id('PSPHPL').id = 'PSPHPLer'
for m in r.metabolites:
    model.reactions.get_by_id('PSPHPLer').add_metabolites({m.id: -r.get_coefficient(m.id), m.id.replace('_c','_r'): r.get_coefficient(m.id)})
r = model.reactions.get_by_id('SPHPL').copy()
model.reactions.get_by_id('SPHPL').id = 'SPHPLer'
for m in r.metabolites:
    model.reactions.get_by_id('SPHPLer').add_metabolites({m.id: -r.get_coefficient(m.id), m.id.replace('_c','_r'): r.get_coefficient(m.id)})
model.remove_reactions(['SGPL11r','SGPL12r','SGPL13','yli_R1567','yli_R1568'], remove_orphans=True)
```

#### FMNAT¶

In [45]:

```
for r in sorted(model.genes.get_by_id('11542').reactions, key=lambda x: x.id):
    print(r.id, r.reaction, r.gene_reaction_rule)
print()
for r in sorted(model.genes.get_by_id('9298').reactions, key=lambda x: x.id):
    print(r.id, r.reaction, r.gene_reaction_rule)
```

```
AFAT atp_c + fmn_c + 2.0 h_c --> fad_c + ppi_c 11542 or 9298
FMNAT atp_c + fmn_c + h_c --> fad_c + ppi_c 11542
FMNATm atp_m + fmn_m + h_m --> fad_m + ppi_m 11542

AFAT atp_c + fmn_c + 2.0 h_c --> fad_c + ppi_c 11542 or 9298
RBFK atp_c + ribflv_c --> adp_c + fmn_c + h_c 9298
RBFKm atp_m + ribflv_m --> adp_m + fmn_m + h_m 9298
```

11542 cyto FAD1  
16092 mito FAD synthetase  
9298 mito 11, cyto\_mito 10.666 FMN1 Riboflavin kinase

FMNAT has correct stoichiometry, AFAT incorrect  
Change FMNATm genes to '16092'
Remove AFAT

In [46]:

```
model.reactions.get_by_id('FMNATm').gene_reaction_rule = '16092'
model.remove_reactions(['AFAT'], remove_orphans=True)
```

#### ALDD2x¶

In [47]:

```
model.remove_reactions(['ALDD2x_copy1','GTPCI_2'], remove_orphans=True)
```

#### GTHOr¶

In [48]:

```
for r in sorted(model.genes.get_by_id('15482').reactions, key=lambda x: x.id):
    print(r.id, r.reaction, r.gene_reaction_rule)
print()
for r in sorted(model.genes.get_by_id('15038').reactions, key=lambda x: x.id):
    print(r.id, r.reaction, r.gene_reaction_rule)
print()
for r in sorted(model.genes.get_by_id('16549').reactions, key=lambda x: x.id):
    print(r.id, r.reaction, r.gene_reaction_rule)
print()
for r in sorted(model.genes.get_by_id('8790').reactions, key=lambda x: x.id):
    print(r.id, r.reaction, r.gene_reaction_rule)
print()
for r in sorted(model.genes.get_by_id('9250').reactions, key=lambda x: x.id):
    print(r.id, r.reaction, r.gene_reaction_rule)
```

```
GDR gthox_c + h_c + nadh_c --> 2.0 gthrd_c + nad_c 15482
GDR_nadp_h gthox_h + h_h + nadph_h --> 2.0 gthrd_h + nadp_h 15482
GDRh gthox_h + h_h + nadh_h --> 2.0 gthrd_h + nad_h 15482
GDRm gthox_m + h_m + nadh_m --> 2.0 gthrd_m + nad_m 15482
GTHOm gthox_m + h_m + nadph_m --> 2.0 gthrd_m + nadp_m 15482 or (15482 and 9250)
GTHOr gthox_c + h_c + nadph_c <=> 2.0 gthrd_c + nadp_c 15482 or (15038 and 15482) or (15482 and 16549) or (15482 and 8790)
TRDR h_c + nadph_c + trdox_c --> nadp_c + trdrd_c 15339 or 15482 or 16019 or 9688 or (CRv4_Au5_s2_g8777_t1 and CRv4_Au5_s9_g15314_t1) or (CRv4_Au5_s2_g8777_t1 and 15339) or (CRv4_Au5_s2_g8777_t1 and 16019) or (CRv4_Au5_s8_g14830_t1 and CRv4_Au5_s9_g15314_t1) or (CRv4_Au5_s8_g14830_t1 and 15339) or (CRv4_Au5_s8_g14830_t1 and 16019) or (CRv4_Au5_s9_g15314_t1 and 9688) or (15339 and 9688) or (16019 and 9688)
TRDRm h_m + nadph_m + trdox_m --> nadp_m + trdrd_m 15482 or (YCR083W and 9688) or (15339 and 9688)
yli_R0291 gthox_c + h_c + nadph_c --> gthrd_c + nadp_c 15482

DASCBR dhdascb_c + h_c + nadph_c --> ascb__L_c + nadp_c 15038
GRXR grxox_c + 2.0 gthrd_c --> grxrd_c + gthox_c 15038 or 16549 or 9250
GTHOr gthox_c + h_c + nadph_c <=> 2.0 gthrd_c + nadp_c 15482 or (15038 and 15482) or (15482 and 16549) or (15482 and 8790)
GTHPi 2.0 gthrd_c + h2o2_c --> gthox_c + 2.0 h2o_c 12715 or 15038 or 16549 or 8579
PAPSR2 grxrd_c + paps_c --> grxox_c + 2.0 h_c + pap_c + so3_c (b0849 and 11741) or (b1064 and 11741) or (11741 and 15038) or (11741 and 16549) or (11741 and 9250)
RNDR1b adp_c + grxrd_c --> dadp_c + grxox_c + h2o_c (b0849 and b2675 and b2676) or (b1064 and b2675 and b2676) or (b2675 and b2676 and 15038) or (b2675 and b2676 and 16549) or (b2675 and b2676 and 9250)
RNDR2b gdp_c + grxrd_c --> dgdp_c + grxox_c + h2o_c (b0849 and b2675 and b2676) or (b1064 and b2675 and b2676) or (b2675 and b2676 and 15038) or (b2675 and b2676 and 16549) or (b2675 and b2676 and 9250)
RNDR3b cdp_c + grxrd_c --> dcdp_c + grxox_c + h2o_c (b0849 and b2675 and b2676) or (b1064 and b2675 and b2676) or (b2675 and b2676 and 15038) or (b2675 and b2676 and 16549) or (b2675 and b2676 and 9250)
RNDR4b grxrd_c + udp_c --> dudp_c + grxox_c + h2o_c (b0849 and b2675 and b2676) or (b1064 and b2675 and b2676) or (b2675 and b2676 and 15038) or (b2675 and b2676 and 16549) or (b2675 and b2676 and 9250)

GRXR grxox_c + 2.0 gthrd_c --> grxrd_c + gthox_c 15038 or 16549 or 9250
GTHOr gthox_c + h_c + nadph_c <=> 2.0 gthrd_c + nadp_c 15482 or (15038 and 15482) or (15482 and 16549) or (15482 and 8790)
GTHPi 2.0 gthrd_c + h2o2_c --> gthox_c + 2.0 h2o_c 12715 or 15038 or 16549 or 8579
PAPSR2 grxrd_c + paps_c --> grxox_c + 2.0 h_c + pap_c + so3_c (b0849 and 11741) or (b1064 and 11741) or (11741 and 15038) or (11741 and 16549) or (11741 and 9250)
RNDR1b adp_c + grxrd_c --> dadp_c + grxox_c + h2o_c (b0849 and b2675 and b2676) or (b1064 and b2675 and b2676) or (b2675 and b2676 and 15038) or (b2675 and b2676 and 16549) or (b2675 and b2676 and 9250)
RNDR2b gdp_c + grxrd_c --> dgdp_c + grxox_c + h2o_c (b0849 and b2675 and b2676) or (b1064 and b2675 and b2676) or (b2675 and b2676 and 15038) or (b2675 and b2676 and 16549) or (b2675 and b2676 and 9250)
RNDR3b cdp_c + grxrd_c --> dcdp_c + grxox_c + h2o_c (b0849 and b2675 and b2676) or (b1064 and b2675 and b2676) or (b2675 and b2676 and 15038) or (b2675 and b2676 and 16549) or (b2675 and b2676 and 9250)
RNDR4b grxrd_c + udp_c --> dudp_c + grxox_c + h2o_c (b0849 and b2675 and b2676) or (b1064 and b2675 and b2676) or (b2675 and b2676 and 15038) or (b2675 and b2676 and 16549) or (b2675 and b2676 and 9250)

GTHOr gthox_c + h_c + nadph_c <=> 2.0 gthrd_c + nadp_c 15482 or (15038 and 15482) or (15482 and 16549) or (15482 and 8790)

GRXR grxox_c + 2.0 gthrd_c --> grxrd_c + gthox_c 15038 or 16549 or 9250
GTHOm gthox_m + h_m + nadph_m --> 2.0 gthrd_m + nadp_m 15482 or (15482 and 9250)
GTHPm 2.0 gthrd_m + h2o2_m <=> gthox_m + 2.0 h2o_m 12715 or 8579 or 9250
PAPSR2 grxrd_c + paps_c --> grxox_c + 2.0 h_c + pap_c + so3_c (b0849 and 11741) or (b1064 and 11741) or (11741 and 15038) or (11741 and 16549) or (11741 and 9250)
RNDR1b adp_c + grxrd_c --> dadp_c + grxox_c + h2o_c (b0849 and b2675 and b2676) or (b1064 and b2675 and b2676) or (b2675 and b2676 and 15038) or (b2675 and b2676 and 16549) or (b2675 and b2676 and 9250)
RNDR2b gdp_c + grxrd_c --> dgdp_c + grxox_c + h2o_c (b0849 and b2675 and b2676) or (b1064 and b2675 and b2676) or (b2675 and b2676 and 15038) or (b2675 and b2676 and 16549) or (b2675 and b2676 and 9250)
RNDR3b cdp_c + grxrd_c --> dcdp_c + grxox_c + h2o_c (b0849 and b2675 and b2676) or (b1064 and b2675 and b2676) or (b2675 and b2676 and 15038) or (b2675 and b2676 and 16549) or (b2675 and b2676 and 9250)
RNDR4b grxrd_c + udp_c --> dudp_c + grxox_c + h2o_c (b0849 and b2675 and b2676) or (b1064 and b2675 and b2676) or (b2675 and b2676 and 15038) or (b2675 and b2676 and 16549) or (b2675 and b2676 and 9250)
```

In [49]:

```
for r in sorted(model.metabolites.get_by_id('dhdascb_c').reactions, key=lambda x: x.id):
    print(r.id, r.reaction, r.gene_reaction_rule)
print()
for r in sorted(model.metabolites.get_by_id('paps_c').reactions, key=lambda x: x.id):
    print(r.id, r.reaction, r.gene_reaction_rule)
```

```
DASCBR dhdascb_c + h_c + nadph_c --> ascb__L_c + nadp_c 15038
DHAAt1r dhdascb_e <=> dhdascb_c 15762

ADSK aps_c + atp_c --> adp_c + h_c + paps_c 8709
BPNT2 h2o_c + paps_c --> aps_c + pi_c 10673
PAPSPAPthr pap_c + paps_h <=> pap_h + paps_c 10998 or 11267
PAPSR paps_c + trdrd_c --> 2.0 h_c + pap_c + so3_c + trdox_c 11741 or (11741 and 15339) or (11741 and 16019)
PAPSR2 grxrd_c + paps_c --> grxox_c + 2.0 h_c + pap_c + so3_c (b0849 and 11741) or (b1064 and 11741) or (11741 and 15038) or (11741 and 16549) or (11741 and 9250)
PAPStg paps_c <=> paps_g 10998 or 11267
```

Glutathione  
15482 cyto GLR1 cytosolic and mitochondrial glutathione oxidoreductase  
15038 nucl 10.5, cyto\_nucl 9.333, mito 8.5, cyto\_mito 8.333 GRX1 glutaredoxin  
8790 cyto\_nucl 13.333, cyto 12.5, nucl 11, mito\_nucl 6.999 GRX3,GRX4 glutaredoxin  
16549 mito (no sigP) GRX2 glutaredoxin  
9250 mito GRX5 glutaredoxin  
13734 mito (anchor, 416 aa) glutaredoxin  
8579 cyto 17, cyto\_nucl 12.5 GPX2,HYR1

12715 cyto 15.5, cyto\_mito 9.5 TSA1,TSA2 -> THIORDXi

Change GTHOr genes to '15482'  
Change GRXR genes to '15038 or 8790'  
Change GTHPi genes to '8579'  
Change GTHOm genes to '15482'  
Add GRXRm, and change genes to '13734 or 16549 or 9250'  
Remove GDR, GDR\_nadp\_h, GDRh, GDRm, yli\_R0291, GTHPm

Replace DASCBR (nadph) with DHAOX\_c (gthrd) from iLB1027\_lipid  
PAPSR, 11741 cyto 13.5, cyto\_nucl 12.333 MET16 uses thioredoxin

Thioredoxin reductase  
9688 mito 25.5, cyto\_mito 14 (no sigP) TRR1,TRR2 -> cyto\_mito  
9687 has sigP, but it seems truncated (~20% coverage), downstream of 9688, long intron could make another isoform  
Thioredoxins  
15339 mito 11, cyto\_mito 9.166, pero 7, cyto\_nucl 5.166, cyto 5 (no sigP) TRX1,TRX2 -> pero  
16019 mito 25 thioredoxin by orthomcl (TRX3?) -> mito  
10848 cyto 17.5, cyto\_nucl 12.5 thioredoxin -> cyto\_nucl  
12730 cyto 11.5, cyto\_nucl 8.5 thioredoxin -> cyto\_nucl  
12737 cyto 16, cyto\_nucl 12 thioredoxin -> cyto\_nucl

Change PAPSR genes to '(10848 and 11741) or (11741 and 12730) or (11741 and 12737) or (11741 and 15339)'  
Remove PAPSPAPthr, PAPSR2

In S. cer, cyto TRDR trx1, trx2, trr1 / mito TRDRm trx3, trr2
Change TRDR genes to '(10848 and 9688) or (12730 and 9688) or (12737 and 9688)'  
Change TRDRm genes to '16019 and 9688'  
RNDRs use thioredoxins  
Remove RNDR1b, RNDR2b, RNDR3b, RNDR3b

In [50]:

```
model.reactions.get_by_id('GTHOr').gene_reaction_rule = '15482'
model.reactions.get_by_id('GRXR').gene_reaction_rule = '15038 or 8790'
model.reactions.get_by_id('GTHPi').gene_reaction_rule = '8579'
model.reactions.get_by_id('GTHOm').gene_reaction_rule = '15482'
m1 = model.metabolites.get_by_id('grxox_c').copy()
m2 = model.metabolites.get_by_id('grxrd_c').copy()
m1.id = 'grxox_m'
m1.compartment = 'm'
m2.id = 'grxrd_m'
m2.compartment = 'm'
model.add_metabolites([m1,m2])
r = model.reactions.get_by_id('GRXR').copy()
r.id = 'GRXRm'
r.gene_reaction_rule = '13734 or 16549 or 9250'
model.add_reactions([r])
for m in model.reactions.get_by_id('GRXR').metabolites:
    model.reactions.get_by_id('GRXRm').add_metabolites({m.id: -r.get_coefficient(m.id), m.id.replace('_c','_m'): r.get_coefficient(m.id)})
model.remove_reactions(['GDR','GDR_nadp_h','GDRh','GDRm','yli_R0291','GTHPm'], remove_orphans=True)
model.add_reactions([ptri.reactions.get_by_id('DHAOX_c').copy()])
model.reactions.get_by_id('DHAOX_c').gene_reaction_rule = '15038'
model.reactions.get_by_id('DASCBR').remove_from_model(remove_orphans=True)
model.reactions.get_by_id('PAPSR').gene_reaction_rule = '(10848 and 11741) or (11741 and 12730) or (11741 and 12737) or (11741 and 15339)'
model.reactions.get_by_id('PAPSPAPthr').remove_from_model(remove_orphans=True)
model.reactions.get_by_id('PAPSR2').remove_from_model(remove_orphans=True)
model.reactions.get_by_id('TRDR').gene_reaction_rule = '(10848 and 9688) or (12730 and 9688) or (12737 and 9688)'
model.reactions.get_by_id('TRDRm').gene_reaction_rule = '16019 and 9688'
model.remove_reactions(['RNDR1b','RNDR2b','RNDR3b','RNDR4b'], remove_orphans=True)
```

In [51]:

```
for r in sorted(model.genes.get_by_id('9688').reactions, key=lambda x: x.id):
    print(r.id, r.reaction, r.gene_reaction_rule)
print()
for r in sorted(model.genes.get_by_id('15339').reactions, key=lambda x: x.id):
    print(r.id, r.reaction, r.gene_reaction_rule)
```

```
TDSRh h_h + nadph_h + trdox_h --> nadp_h + trdrd_h (CRv4_Au5_s23_g9860_t1 and CRv4_Au5_s2_g8777_t1) or (CRv4_Au5_s23_g9860_t1 and CRv4_Au5_s8_g14830_t1) or (CRv4_Au5_s23_g9860_t1 and 9688) or (CRv4_Au5_s2_g8777_t1 and CRv4_Au5_s5_g12205_t1) or (CRv4_Au5_s2_g8777_t1 and 15339) or (CRv4_Au5_s2_g8777_t1 and 16019) or (CRv4_Au5_s5_g12205_t1 and CRv4_Au5_s8_g14830_t1) or (CRv4_Au5_s5_g12205_t1 and 9688) or (CRv4_Au5_s8_g14830_t1 and 15339) or (CRv4_Au5_s8_g14830_t1 and 16019) or (15339 and 9688) or (16019 and 9688)
TRDR h_c + nadph_c + trdox_c --> nadp_c + trdrd_c (10848 and 9688) or (12730 and 9688) or (12737 and 9688)
TRDRm h_m + nadph_m + trdox_m --> nadp_m + trdrd_m 16019 and 9688

AHAL achms_h + trdrd_h + tsul_h --> ac_h + h_h + hcys__L_h + so3_h + trdox_h (CRv4_Au5_s23_g9860_t1 and CRv4_Au5_s60_g12367_t1) or (CRv4_Au5_s5_g12205_t1 and CRv4_Au5_s60_g12367_t1) or (CRv4_Au5_s5_g12205_t2 and CRv4_Au5_s60_g12367_t1) or (CRv4_Au5_s60_g12367_t1 and 15339) or (CRv4_Au5_s60_g12367_t1 and 16019)
CYSS_trdrd acser_h + trdrd_h + tsul_h --> ac_h + cys__L_h + h_h + so3_h + trdox_h (CRv4_Au5_s23_g9860_t1 and 12031) or (CRv4_Au5_s23_g9860_t1 and 13106) or (CRv4_Au5_s23_g9860_t1 and 15712) or (CRv4_Au5_s5_g12205_t1 and 12031) or (CRv4_Au5_s5_g12205_t1 and 13106) or (CRv4_Au5_s5_g12205_t1 and 15712) or (CRv4_Au5_s5_g12205_t2 and 12031) or (CRv4_Au5_s5_g12205_t2 and 13106) or (CRv4_Au5_s5_g12205_t2 and 15712) or (12031 and 15339) or (12031 and 16019) or (13106 and 15339) or (13106 and 16019) or (15339 and 15712) or (15712 and 16019)
DSBDR dsbdox_c + trdrd_c --> dsbdrd_c + trdox_c (b4136 and 15339) or (b4136 and 16019)
METSOXR1 metsox_S__L_c + trdrd_c --> h2o_c + met__L_c + trdox_c 15469 or (b3551 and 15339) or (b3551 and 16019) or (15339 and 15902) or (15902 and 16019)
METSOXR2 metsox_R__L_c + trdrd_c --> h2o_c + met__L_c + trdox_c (15339 and 15469) or (15339 and 9153) or (15469 and 16019) or (16019 and 9153)
PAPSR paps_c + trdrd_c --> 2.0 h_c + pap_c + so3_c + trdox_c (10848 and 11741) or (11741 and 12730) or (11741 and 12737) or (11741 and 15339)
RNDR1 adp_c + trdrd_c --> dadp_c + h2o_c + trdox_c (11172 and 11290) or (11290 and 14237) or (CRv4_Au5_s9_g15314_t1 and 11172 and 11290) or (CRv4_Au5_s9_g15314_t1 and 11290 and 14237) or (11172 and 11290 and 15339) or (11172 and 11290 and 16019) or (11290 and 14237 and 15339) or (11290 and 14237 and 16019)
RNDR1n adp_n + trdrd_n --> dadp_n + h2o_n + trdox_n (YGR180C and 11290 and 15339) or (YGR180C and 11290 and 16019)
RNDR2 gdp_c + trdrd_c --> dgdp_c + h2o_c + trdox_c (11172 and 11290) or (11290 and 14237) or (CRv4_Au5_s9_g15314_t1 and 11172 and 11290) or (CRv4_Au5_s9_g15314_t1 and 11290 and 14237) or (11172 and 11290 and 15339) or (11172 and 11290 and 16019) or (11290 and 14237 and 15339) or (11290 and 14237 and 16019)
RNDR2n gdp_n + trdrd_n --> dgdp_n + h2o_n + trdox_n (YGR180C and 11290 and 15339) or (YGR180C and 11290 and 16019)
RNDR3 cdp_c + trdrd_c --> dcdp_c + h2o_c + trdox_c (11172 and 11290) or (11290 and 14237) or (CRv4_Au5_s9_g15314_t1 and 11172 and 11290) or (CRv4_Au5_s9_g15314_t1 and 11290 and 14237) or (11172 and 11290 and 15339) or (11172 and 11290 and 16019) or (11290 and 14237 and 15339) or (11290 and 14237 and 16019)
RNDR3n cdp_n + trdrd_n --> dcdp_n + h2o_n + trdox_n (YGR180C and 11290 and 15339) or (YGR180C and 11290 and 16019)
RNDR4 trdrd_c + udp_c --> dudp_c + h2o_c + trdox_c (11172 and 11290) or (11290 and 14237) or (CRv4_Au5_s9_g15314_t1 and 11172 and 11290) or (CRv4_Au5_s9_g15314_t1 and 11290 and 14237) or (11172 and 11290 and 15339) or (11172 and 11290 and 16019) or (11290 and 14237 and 15339) or (11290 and 14237 and 16019)
RNDR4n trdrd_n + udp_n --> dudp_n + h2o_n + trdox_n (YGR180C and 11290 and 15339) or (YGR180C and 11290 and 16019)
RNTR1 atp_c + trdrd_c --> datp_c + h2o_c + trdox_c 15339 or 16019 or (CRv4_Au5_s27_g10030_t1 and CRv4_Au5_s9_g15314_t1) or (CRv4_Au5_s27_g10030_t1 and 15339) or (CRv4_Au5_s27_g10030_t1 and 16019)
RNTR2 gtp_c + trdrd_c --> dgtp_c + h2o_c + trdox_c 15339 or 16019 or (CRv4_Au5_s27_g10030_t1 and CRv4_Au5_s9_g15314_t1) or (CRv4_Au5_s27_g10030_t1 and 15339) or (CRv4_Au5_s27_g10030_t1 and 16019)
RNTR3 ctp_c + trdrd_c --> dctp_c + h2o_c + trdox_c 15339 or 16019 or (CRv4_Au5_s27_g10030_t1 and CRv4_Au5_s9_g15314_t1) or (CRv4_Au5_s27_g10030_t1 and 15339) or (CRv4_Au5_s27_g10030_t1 and 16019)
RNTR4 trdrd_c + utp_c --> dutp_c + h2o_c + trdox_c 15339 or 16019 or (CRv4_Au5_s27_g10030_t1 and CRv4_Au5_s9_g15314_t1) or (CRv4_Au5_s27_g10030_t1 and 15339) or (CRv4_Au5_s27_g10030_t1 and 16019)
TDSRh h_h + nadph_h + trdox_h --> nadp_h + trdrd_h (CRv4_Au5_s23_g9860_t1 and CRv4_Au5_s2_g8777_t1) or (CRv4_Au5_s23_g9860_t1 and CRv4_Au5_s8_g14830_t1) or (CRv4_Au5_s23_g9860_t1 and 9688) or (CRv4_Au5_s2_g8777_t1 and CRv4_Au5_s5_g12205_t1) or (CRv4_Au5_s2_g8777_t1 and 15339) or (CRv4_Au5_s2_g8777_t1 and 16019) or (CRv4_Au5_s5_g12205_t1 and CRv4_Au5_s8_g14830_t1) or (CRv4_Au5_s5_g12205_t1 and 9688) or (CRv4_Au5_s8_g14830_t1 and 15339) or (CRv4_Au5_s8_g14830_t1 and 16019) or (15339 and 9688) or (16019 and 9688)
THIORDXi h2o2_c + trdrd_c --> 2.0 h2o_c + trdox_c 8579 or (YDR453C and 15339) or (12715 and 15339) or (12715 and 16019) or (15037 and 15339) or (15037 and 16019)
THIORDXm h2o2_m + trdrd_m <=> 2.0 h2o_m + trdox_m 10200 and 15339
THIORDXni h2o2_n + trdrd_n --> 2.0 h2o_n + trdox_n (15037 and 15339) or (15037 and 16019)
THIORDXp h2o2_x + trdrd_x <=> 2.0 h2o_x + trdox_x (13262 and 15339) or (13262 and 16019)
```

Thioredoxin  
Remove TDSRh, CYSS\_trdrd, AHAL -> check cysteine biosynthesis  
Remove DSBDR (E. coli specific)  
15902 mito 8, cysk 6, cyto\_mito 6 MXR1 (cyto) methionine-S-sulfoxide reductase (peptide methionine, non-peptide)  
15469 cyto\_mito (no sigP) YKL069W (cyto) methionine-R-sulfoxide reductase (non-peptide methionine)  
9153 mito MXR2 (mito) methionine-R-sulfoxide reductase (peptide methionine)  
Change METSOXR1 genes to '(10848 and 15902) or (12730 and 15902) or (12737 and 15902) or (15339 and 15902)'  
Change METSOXR2 genes to '(10848 and 15469) or (12730 and 15469) or (12737 and 15469) or (15339 and 15469)'

RNDR needs small heterodimer and large homodimer  
11290 cyto 14.5, cyto\_mito 12.5 RNR1,RNR3 ribonucleotide reductase, alpha subunit (large)  
14237 cyto 13.5, cyto\_nucl 10.5 RNR2 ribonucleotide reductase, beta subunit (small)  
11172 cyto\_nucl RNR2 ribonucleotide reductase, beta subunit (small)  
Change RNDRx genes to '11172 and 11290 and 14237 and trdrd\_cyto (10848 or 12730 or 12737 or 15339)'  
'(10848 and 11172 and 11290 and 14237) or (11172 and 11290 and 12730 and 14237) or (11172 and 11290 and 12737 and 14237) or (11172 and 11290 and 14237 and 15339)'  
Change RNDRnx genes to '11172 and 11290 and 14237 and trdrd\_nucl (10848 or 12730 or 12737)'  
'(10848 and 11172 and 11290 and 14237) or (11172 and 11290 and 12730 and 14237) or (11172 and 11290 and 12737 and 14237)'  
Remove RNTRx (not present in S. cer and no genes in Rhodo)

Thioredoxin peroxidase  
beta-oxidation http://www.jbc.org/content/274/6/3402.long  
12715 cyto 15.5, cyto\_mito 9.5 TSA1,TSA2 (cyto) -> THIORDXi  
15037 nucl 12.5, cyto\_nucl 11 DOT5 (nucl) nuclear thiol peroxidase -> THIORDXni  
13262 cyto 20.5, cyto\_nucl 12.5, pero 4 AHP1 (pero) -> THIORDXp  
ahp1-trx2 https://www.ncbi.nlm.nih.gov/pmc/articles/PMC3366830/  
10200 nucl 11, cyto 8, pero 5, cyto\_mito 5 PRX1 (mito) -> THIORDXm  
Change THIORDXi genes to '(10848 and 12715) or (12715 and 12730) or (12715 and 12737)'  
Change THIORDXni genes to '(10848 and 15037) or (12730 and 15037) or (12737 and 15037)'  
Change THIORDXp genes to '13262 and 15339'
Change THIORDXm genes to '10200 and 16019'

In [52]:

```
model.remove_reactions(['TDSRh','CYSS_trdrd','AHAL','DSBDR'], remove_orphans=True)
model.reactions.get_by_id('METSOXR1').gene_reaction_rule = '(10848 and 15902) or (12730 and 15902) or (12737 and 15902) or (15339 and 15902)'
model.reactions.get_by_id('METSOXR2').gene_reaction_rule = '(10848 and 15469) or (12730 and 15469) or (12737 and 15469) or (15339 and 15469)'
model.reactions.get_by_id('RNDR1').gene_reaction_rule = '(10848 and 11172 and 11290 and 14237) or (11172 and 11290 and 12730 and 14237) or (11172 and 11290 and 12737 and 14237) or (11172 and 11290 and 14237 and 15339)'
model.reactions.get_by_id('RNDR2').gene_reaction_rule = '(10848 and 11172 and 11290 and 14237) or (11172 and 11290 and 12730 and 14237) or (11172 and 11290 and 12737 and 14237) or (11172 and 11290 and 14237 and 15339)'
model.reactions.get_by_id('RNDR3').gene_reaction_rule = '(10848 and 11172 and 11290 and 14237) or (11172 and 11290 and 12730 and 14237) or (11172 and 11290 and 12737 and 14237) or (11172 and 11290 and 14237 and 15339)'
model.reactions.get_by_id('RNDR4').gene_reaction_rule = '(10848 and 11172 and 11290 and 14237) or (11172 and 11290 and 12730 and 14237) or (11172 and 11290 and 12737 and 14237) or (11172 and 11290 and 14237 and 15339)'
model.reactions.get_by_id('RNDR1n').gene_reaction_rule = '(10848 and 11172 and 11290 and 14237) or (11172 and 11290 and 12730 and 14237) or (11172 and 11290 and 12737 and 14237)'
model.reactions.get_by_id('RNDR2n').gene_reaction_rule = '(10848 and 11172 and 11290 and 14237) or (11172 and 11290 and 12730 and 14237) or (11172 and 11290 and 12737 and 14237)'
model.reactions.get_by_id('RNDR3n').gene_reaction_rule = '(10848 and 11172 and 11290 and 14237) or (11172 and 11290 and 12730 and 14237) or (11172 and 11290 and 12737 and 14237)'
model.reactions.get_by_id('RNDR4n').gene_reaction_rule = '(10848 and 11172 and 11290 and 14237) or (11172 and 11290 and 12730 and 14237) or (11172 and 11290 and 12737 and 14237)'
model.remove_reactions(['RNTR1','RNTR2','RNTR3','RNTR4'], remove_orphans=True)
model.reactions.get_by_id('THIORDXi').gene_reaction_rule = '(10848 and 12715) or (12715 and 12730) or (12715 and 12737)'
model.reactions.get_by_id('THIORDXni').gene_reaction_rule = '(10848 and 15037) or (12730 and 15037) or (12737 and 15037)'
model.reactions.get_by_id('THIORDXp').gene_reaction_rule = '13262 and 15339'
model.reactions.get_by_id('THIORDXm').gene_reaction_rule = '10200 and 16019'
```

In [53]:

```
for r in sorted(model.genes.get_by_id('15679').reactions, key=lambda x: x.id):
    print(r.id, r.reaction, r.gene_reaction_rule)
print()
for r in sorted(model.genes.get_by_id('15496').reactions, key=lambda x: x.id):
    print(r.id, r.reaction, r.gene_reaction_rule)
print()
for r in sorted(model.genes.get_by_id('8943').reactions, key=lambda x: x.id):
    print(r.id, r.reaction, r.gene_reaction_rule)
```

```
ATDGDm atp_m + dgdp_m + h_m --> adp_m + dgtp_m 15679
ATGDm atp_m + gdp_m + h_m --> adp_m + gtp_m 15679
NDPK1 atp_c + gdp_c <=> adp_c + gtp_c 15496 or 15679 or 8943
NDPK10 atp_c + didp_c <=> adp_c + ditp_c 15679 or 8943
NDPK10n atp_n + didp_n <=> adp_n + ditp_n 15679
NDPK1n atp_n + gdp_n <=> adp_n + gtp_n 15679
NDPK2 atp_c + udp_c <=> adp_c + utp_c 15496 or 15679 or 8943
NDPK2m atp_m + udp_m --> adp_m + utp_m 15679
NDPK2n atp_n + udp_n <=> adp_n + utp_n 15679
NDPK3 atp_c + cdp_c <=> adp_c + ctp_c 15496 or 15679 or 8943
NDPK3m atp_m + cdp_m --> adp_m + ctp_m 15679
NDPK3n atp_n + cdp_n <=> adp_n + ctp_n 15679
NDPK4 atp_c + dtdp_c <=> adp_c + dttp_c 15496 or 15679 or 8943
NDPK4m atp_m + dtdp_m --> adp_m + dttp_m 15679
NDPK4n atp_n + dtdp_n <=> adp_n + dttp_n 15679
NDPK5 atp_c + dgdp_c <=> adp_c + dgtp_c 15496 or 15679 or 8943
NDPK5n atp_n + dgdp_n <=> adp_n + dgtp_n 15679
NDPK6 atp_c + dudp_c <=> adp_c + dutp_c 15496 or 15679 or 8943
NDPK6m atp_m + dudp_m --> adp_m + dutp_m 15679
NDPK6n atp_n + dudp_n <=> adp_n + dutp_n 15679
NDPK7 atp_c + dcdp_c <=> adp_c + dctp_c 15496 or 15679 or 8943
NDPK7m atp_m + dcdp_m --> adp_m + dctp_m 15679
NDPK7n atp_n + dcdp_n <=> adp_n + dctp_n 15679
NDPK8 atp_c + dadp_c <=> adp_c + datp_c 15496 or 15679 or 8943
NDPK8m atp_m + dadp_m --> adp_m + datp_m 15679
NDPK8n atp_n + dadp_n <=> adp_n + datp_n 15679
NDPK9 atp_c + idp_c <=> adp_c + itp_c 15679 or 8943
NDPK9m atp_m + idp_m --> adp_m + itp_m 15679
NDPK9n atp_n + idp_n <=> adp_n + itp_n 15679

ADK1 amp_c + atp_c <=> 2.0 adp_c 12300 or 13190 or 15496
ADK1m amp_m + atp_m <=> 2.0 adp_m 12300 or 15129 or 15496
ADK3 amp_c + gtp_c <=> adp_c + gdp_c 15496
ADK4 amp_c + itp_c <=> adp_c + idp_c 15496
ADNK1 adn_c + atp_c --> adp_c + amp_c + h_c 15496 or 8385
DADK atp_c + damp_c <=> adp_c + dadp_c 12300 or 15129 or 15496
NDPK1 atp_c + gdp_c <=> adp_c + gtp_c 15496 or 15679 or 8943
NDPK2 atp_c + udp_c <=> adp_c + utp_c 15496 or 15679 or 8943
NDPK3 atp_c + cdp_c <=> adp_c + ctp_c 15496 or 15679 or 8943
NDPK4 atp_c + dtdp_c <=> adp_c + dttp_c 15496 or 15679 or 8943
NDPK5 atp_c + dgdp_c <=> adp_c + dgtp_c 15496 or 15679 or 8943
NDPK6 atp_c + dudp_c <=> adp_c + dutp_c 15496 or 15679 or 8943
NDPK7 atp_c + dcdp_c <=> adp_c + dctp_c 15496 or 15679 or 8943
NDPK8 atp_c + dadp_c <=> adp_c + datp_c 15496 or 15679 or 8943

NDPK1 atp_c + gdp_c <=> adp_c + gtp_c 15496 or 15679 or 8943
NDPK10 atp_c + didp_c <=> adp_c + ditp_c 15679 or 8943
NDPK2 atp_c + udp_c <=> adp_c + utp_c 15496 or 15679 or 8943
NDPK3 atp_c + cdp_c <=> adp_c + ctp_c 15496 or 15679 or 8943
NDPK4 atp_c + dtdp_c <=> adp_c + dttp_c 15496 or 15679 or 8943
NDPK5 atp_c + dgdp_c <=> adp_c + dgtp_c 15496 or 15679 or 8943
NDPK6 atp_c + dudp_c <=> adp_c + dutp_c 15496 or 15679 or 8943
NDPK7 atp_c + dcdp_c <=> adp_c + dctp_c 15496 or 15679 or 8943
NDPK8 atp_c + dadp_c <=> adp_c + datp_c 15496 or 15679 or 8943
NDPK9 atp_c + idp_c <=> adp_c + itp_c 15679 or 8943
```

15679 cyto YNK1 (cyto, mito intermembrane space) nucleoside diphosphate kinase  
8943 mito nucleoside diphosphate kinase

Remove NPDKxm, NDPKxn  
Add NPDKxm (reversible) from Recon1  
Change NDPKx genes to '15679'  
Change NDPKxm genes to '8943'

In [54]:

```
model.remove_reactions([r.id for r in model.reactions if r.id.startswith('NDPK') and r.id.endswith('n')], remove_orphans=True)
model.remove_reactions([r.id for r in model.reactions if r.id.startswith('NDPK') and r.id.endswith('m')], remove_orphans=True)
model.add_reactions([r.copy() for r in hsa.reactions if r.id.startswith('NDPK') and r.id.endswith('m')])
for r in model.reactions:
    if r.id.startswith('NDPK') and r.id.endswith('m'):
        r.gene_reaction_rule = '8943'
    elif r.id.startswith('NDPK'):
        r.gene_reaction_rule = '15679'
```

In [55]:

```
for r in sorted(model.genes.get_by_id('15496').reactions, key=lambda x: x.id):
    print(r.id, r.reaction, r.gene_reaction_rule)
print()
for r in sorted(model.genes.get_by_id('15129').reactions, key=lambda x: x.id):
    print(r.id, r.reaction, r.gene_reaction_rule)
print()
for r in sorted(model.genes.get_by_id('12300').reactions, key=lambda x: x.id):
    print(r.id, r.reaction, r.gene_reaction_rule)
print()
for r in sorted(model.genes.get_by_id('8385').reactions, key=lambda x: x.id):
    print(r.id, r.reaction, r.gene_reaction_rule)
```

```
ADK1 amp_c + atp_c <=> 2.0 adp_c 12300 or 13190 or 15496
ADK1m amp_m + atp_m <=> 2.0 adp_m 12300 or 15129 or 15496
ADK3 amp_c + gtp_c <=> adp_c + gdp_c 15496
ADK4 amp_c + itp_c <=> adp_c + idp_c 15496
ADNK1 adn_c + atp_c --> adp_c + amp_c + h_c 15496 or 8385
DADK atp_c + damp_c <=> adp_c + dadp_c 12300 or 15129 or 15496

ADK1m amp_m + atp_m <=> 2.0 adp_m 12300 or 15129 or 15496
ADK3m amp_m + gtp_m <=> adp_m + gdp_m 15129
ADK4m amp_m + itp_m <=> adp_m + idp_m 15129
DADK atp_c + damp_c <=> adp_c + dadp_c 12300 or 15129 or 15496

ADK1 amp_c + atp_c <=> 2.0 adp_c 12300 or 13190 or 15496
ADK1m amp_m + atp_m <=> 2.0 adp_m 12300 or 15129 or 15496
ATAMh amp_h + atp_h --> 2.0 adp_h 12300
ATDAMh atp_h + damp_h --> adp_h + dadp_h 12300
ATDAMm atp_m + damp_m --> adp_m + dadp_m 12300
DADK atp_c + damp_c <=> adp_c + dadp_c 12300 or 15129 or 15496

ADNK1 adn_c + atp_c --> adp_c + amp_c + h_c 15496 or 8385
ADNK1m adn_m + atp_m --> adp_m + amp_m + h_m 8385
```

15496 cyto ADK1 adenylate kinase (also mito intermembrane space)
15129 mito ADK2 adenylate kinase  
12300 mito FAP7 Essential NTPase required for small ribosome subunit synthesis  
8385 extr (sigP) ADO1 adenosine kinase (cyto and nucl?)

S. cer ADK1 catalyze ADK1 and DADK, and ADK2 catalyze and ADK3m and ADK4m  
http://www.jbc.org/content/280/19/18604.full  
Change ADK1 genes to '15496'  
Change DADK genes to '15496'  
Change ADK3m genes to '15129'  
Remove ADK1m, ADK3, ADK4, ATAMh, ATDAMh, ATDAMm

Change ADNK1 genes to '8385'
Remove ADNK1m

In [56]:

```
model.reactions.get_by_id('ADK1').gene_reaction_rule = '15496'
model.reactions.get_by_id('DADK').gene_reaction_rule = '15496'
model.reactions.get_by_id('ADK3m').gene_reaction_rule = '15129'
model.reactions.get_by_id('ADK4m').gene_reaction_rule = '15129'
model.remove_reactions(['ADK1m','ADK3','ADK4','ATAMh','ATDAMh','ATDAMm'], remove_orphans=True)
model.reactions.get_by_id('ADNK1').gene_reaction_rule = '8385'
model.remove_reactions(['ADNK1m'], remove_orphans=True)
```

In [57]:

```
for r in sorted(model.genes.get_by_id('15252').reactions, key=lambda x: x.id):
    print(r.id, r.reaction, r.gene_reaction_rule)
print()
for r in sorted(model.genes.get_by_id('13190').reactions, key=lambda x: x.id):
    print(r.id, r.reaction, r.gene_reaction_rule)
```

```
DTMPK atp_c + dtmp_c <=> adp_c + dtdp_c 15252
NDP8 dudp_c + h2o_c --> dump_c + h_c + pi_c 15252
URIDK2r atp_c + dump_c <=> adp_c + dudp_c 13190 or 15252

CPK1 cmp_c + ctp_c <=> 2.0 cdp_c 13190
CYTK1 atp_c + cmp_c <=> adp_c + cdp_c 13190
CYTK10 cmp_c + dgtp_c <=> cdp_c + dgdp_c 13190
CYTK10n cmp_n + dgtp_n <=> cdp_n + dgdp_n 13190
CYTK11 dcmp_c + dgtp_c <=> dcdp_c + dgdp_c 13190
CYTK11n dcmp_n + dgtp_n <=> dcdp_n + dgdp_n 13190
CYTK12 dcmp_c + dctp_c <=> 2.0 dcdp_c 13190
CYTK12n dcmp_n + dctp_n <=> 2.0 dcdp_n 13190
CYTK13 datp_c + dcmp_c <=> dadp_c + dcdp_c 13190
CYTK13n datp_n + dcmp_n <=> dadp_n + dcdp_n 13190
CYTK14 dcmp_c + utp_c <=> dcdp_c + udp_c 13190
CYTK14n dcmp_n + utp_n <=> dcdp_n + udp_n 13190
CYTK1n atp_n + cmp_n <=> adp_n + cdp_n 13190
CYTK2 atp_c + dcmp_c <=> adp_c + dcdp_c 13190
CYTK2_1 ctp_c + dcmp_c <=> cdp_c + dcdp_c 13190
CYTK2n atp_n + dcmp_n <=> adp_n + dcdp_n 13190
CYTK3n ctp_n + dcmp_n <=> cdp_n + dcdp_n 13190
CYTK4n dcmp_n + gtp_n <=> dcdp_n + gdp_n 13190
CYTK5n cmp_n + gtp_n <=> cdp_n + gdp_n 13190
CYTK6n cmp_n + ctp_n <=> 2.0 cdp_n 13190
CYTK7 cmp_c + utp_c <=> cdp_c + udp_c 13190
CYTK7n cmp_n + utp_n <=> cdp_n + udp_n 13190
CYTK8 cmp_c + datp_c <=> cdp_c + dadp_c 13190
CYTK8n cmp_n + datp_n <=> cdp_n + dadp_n 13190
CYTK9 cmp_c + dctp_c <=> cdp_c + dcdp_c 13190
CYTK9n cmp_n + dctp_n <=> cdp_n + dcdp_n 13190
UMPK atp_c + ump_c <=> adp_c + udp_c 13190
UMPK2 ctp_c + ump_c <=> cdp_c + udp_c 13190
UMPK2n ctp_n + ump_n <=> cdp_n + udp_n 13190
UMPK3 ump_c + utp_c <=> 2.0 udp_c 13190
UMPK3n ump_n + utp_n <=> 2.0 udp_n 13190
UMPK4 gtp_c + ump_c <=> gdp_c + udp_c 13190
UMPK4n gtp_n + ump_n <=> gdp_n + udp_n 13190
UMPK5 datp_c + ump_c <=> dadp_c + udp_c 13190
UMPK5n datp_n + ump_n <=> dadp_n + udp_n 13190
UMPK6 dctp_c + ump_c <=> dcdp_c + udp_c 13190
UMPK6n dctp_n + ump_n <=> dcdp_n + udp_n 13190
UMPK7 dgtp_c + ump_c <=> dgdp_c + udp_c 13190
UMPK7n dgtp_n + ump_n <=> dgdp_n + udp_n 13190
UMPKn atp_n + ump_n <=> adp_n + udp_n 13190
URIDK2r atp_c + dump_c <=> adp_c + dudp_c 13190 or 15252
URIDK2rn atp_n + dump_n <=> adp_n + dudp_n 13190
```

15252 mito (no sigP) CDC8 thymidylate kinase (nucl and cyto) -> DTMPK  
13190 cysk 10, cyto 8, mito 7 (no sigP) URA6 Uridylate kinase/adenylate kinase (cyto predominantly and nucl) / KEGG UMP-CMP kinase  
URA6 activity controversy -> keep these reactions for now

Change URIDK2r genes to '13190'

In [58]:

```
model.reactions.get_by_id('URIDK2r').gene_reaction_rule = '13190'
```

In [59]:

```
for r in sorted(model.metabolites.get_by_id('hom__L_c').reactions, key=lambda x: x.id):
    print(r.id, r.reaction, r.gene_reaction_rule)
print()
for r in sorted(model.metabolites.get_by_id('achms_c').reactions, key=lambda x: x.id):
    print(r.id, r.reaction, r.gene_reaction_rule)
print()
for r in sorted(model.metabolites.get_by_id('hcys__L_c').reactions, key=lambda x: x.id):
    print(r.id, r.reaction, r.gene_reaction_rule)
print()
for r in sorted(model.metabolites.get_by_id('cyst__L_c').reactions, key=lambda x: x.id):
    print(r.id, r.reaction, r.gene_reaction_rule)
print()
for r in sorted(model.genes.get_by_id('16618').reactions, key=lambda x: x.id):
    print(r.id, r.reaction, r.gene_reaction_rule)
```

```
HSDxi aspsa_c + h_c + nadh_c --> hom__L_c + nad_c 12080
HSDy hom__L_c + nadp_c <=> aspsa_c + h_c + nadph_c 12080 or 16738
HSERTA accoa_c + hom__L_c <=> achms_c + coa_c 12513 or 15248 or (PP_5098 and 15248)
HSK atp_c + hom__L_c --> adp_c + h_c + phom_c 8651

AHSERL achms_c + ch4s_c --> ac_c + h_c + met__L_c 16618
AHSERL2 achms_c + h2s_c --> ac_c + h_c + hcys__L_c 16618
HSERTA accoa_c + hom__L_c <=> achms_c + coa_c 12513 or 15248 or (PP_5098 and 15248)
METB1 achms_c + cys__L_c --> ac_c + cyst__L_c + h_c 11463 or 16725 or 16742
yli_R0094 achms_c + h_c + trdrd_c + tsul_c --> ac_c + hcys__L_c + so3_c + trdox_c 11463 or 16725 or 16742

AHCi ahcys_c + h2o_c --> adn_c + hcys__L_c 12912
AHSERL2 achms_c + h2s_c --> ac_c + h_c + hcys__L_c 16618
CYSTL cyst__L_c + h2o_c --> hcys__L_c + nh4_c + pyr_c 8759
CYSTS hcys__L_c + ser__L_c --> cyst__L_c + h2o_c 15712
HCYSMT amet_c + hcys__L_c --> ahcys_c + h_c + met__L_c 15759
HCYSMT2 hcys__L_c + mmet_c --> h_c + 2.0 met__L_c 15759
METS 5mthf_c + hcys__L_c --> h_c + met__L_c + thf_c 9825
MHPGLUT hcys__L_c + mhpglu_c --> hpglu_c + met__L_c 9825
SHSL2r h2s_c + suchms_c <=> h_c + hcys__L_c + succ_c 11463 or 16725 or 16742 or 9499
yli_R0094 achms_c + h_c + trdrd_c + tsul_c --> ac_c + hcys__L_c + so3_c + trdox_c 11463 or 16725 or 16742

CYSTGL cyst__L_c + h2o_c --> 2obut_c + cys__L_c + nh4_c 9499
CYSTL cyst__L_c + h2o_c --> hcys__L_c + nh4_c + pyr_c 8759
CYSTS hcys__L_c + ser__L_c --> cyst__L_c + h2o_c 15712
METB1 achms_c + cys__L_c --> ac_c + cyst__L_c + h_c 11463 or 16725 or 16742
SHSL1 cys__L_c + suchms_c --> cyst__L_c + h_c + succ_c 11463 or 16725 or 16742 or 9499

AHSERL achms_c + ch4s_c --> ac_c + h_c + met__L_c 16618
AHSERL2 achms_c + h2s_c --> ac_c + h_c + hcys__L_c 16618
AHSERL4 acser_c + trdrd_c + tsul_c --> ac_c + cys__L_c + h_c + so3_c + trdox_c 12031 or 13106 or 16618
```

Cysteine biosynthesis

12080 extr HOM6 (cyto) homoserine dehydrogenase  
16738 cyto homoserine dehydrogenase  
8651 mito THR1 homoserine kinase

12513 cyto\_nucl MET2 L-homoserine O-acetyltransferase  
15248 nucl MET2 L-homoserine O-acetyltransferase  
16618 cyto MET17 O-acetylhomoserine sulfhydrylase  
15712 cyto CYS4 cystathionine beta-synthase  
9499 mito CYS3 cystathionine gamma-lyase  
8759 mito (no sigP) STR3 (pero) cystathionine beta-lyase (also involved in 3-mercaptohexanol)  
11463 mito (no sigP) STR2 (cyto),YLL058W,YML082W (cyto) cystathionine gamma-synthase
16725 cyto YHR112C cystathionine beta-lyase/gamma-synthase, unknown function in S. cer  
16742 plas YHR112C cystathionine beta-lyase/gamma-synthase, unknown function in S. cer

serine O-acetyltransferase is missing in Rhodo, 9734 is maltose O-acetyltransferase  
12031 mito (no sigP) MCY1 cysteine synthase A (O-acetyl-L-serine sulfhydrylase)  
13106 cyto\_mito (anchor) MCY1 cysteine synthase A (O-acetyl-L-serine sulfhydrylase)

12912 cyto SAH1 S-adenosyl-L-homocysteine hydrolase  
15759 extr MHT1,SAM4,YMR321C S-methylmethionine-homocysteine methyltransferase  
9825 mito (non-sigP) MET6 cobalamin-independent methionine synthase  
12876 cyto cobalamin-independent methionine synthase  
12920 cyto cobalamin-independent methionine synthase

Change HSDxi genes to '12080 or 16738'  
Change HSERTA genes to '12513 or 15248'  
AHSERL, AHSERL2 is correct  
Remove AHSERL4, METB1, yli\_R0094, SHSL2r (P. putida specific)  
Change METS genes to '12876 or 12920 or 9825'  
MHPGLUT is a specific 5mthf (mhpglu\_c) blocked rxn  
Remove MHPGLUT  
Change CYSTL genes to '16725 or 16742 or 8759'  
Remove CYSTLp  
CHange SHSL1 genes to '11463'

In [60]:

```
model.reactions.get_by_id('HSDxi').gene_reaction_rule = '12080 or 16738'
model.reactions.get_by_id('HSERTA').gene_reaction_rule = '12513 or 15248'
model.remove_reactions(['AHSERL4','METB1','yli_R0094','SHSL2r'], remove_orphans=True)
model.reactions.get_by_id('METS').gene_reaction_rule = '12876 or 12920 or 9825'
model.remove_reactions(['MHPGLUT'], remove_orphans=True)
model.reactions.get_by_id('CYSTL').gene_reaction_rule = '16725 or 16742 or 8759'
model.remove_reactions(['CYSTLp'], remove_orphans=True)
model.reactions.get_by_id('SHSL1').gene_reaction_rule = '11463'
```

In [61]:

```
temp = ['12080','14662','16738','8651','12513','15248','16618','15712','9499','8759','11463','16725','16742',
        '9734','12031','13106','12912','15759','9825','12876','12920']
display(Annotation.loc[temp])
Show_Data(temp)
```

|  | Combined Annotations | Signal P | Sc288c Orthologs | Human Orthologs | Sc288 Best Hit | Human Blast | Essential | WolfPSort | C Terminal |
| --- | --- | --- | --- | --- | --- | --- | --- | --- | --- |
| RTO4\_ID |  |  |  |  |  |  |  |  |  |
| 12080 | K00003: E1.1.1.3; homoserine dehydrogenase | S | HOM6 |  | HOM6 |  | Essential | extr 10, cyto 7.5, mito 7, cyto\_nucl 4.5 | VHL\* |
| 14662 | K00928: lysC; aspartate kinase |  | HOM3 |  | HOM3 |  | Essential | cyto 13, mito 12 | VFS\* |
| 16738 | K12525: metL; bifunctional aspartokinase / hom... |  |  |  | HOM6 |  | Not Essential | cyto 13, cyto\_mito 8.833, extr 8, cyto\_nucl 7.... | VGL\* |
| 8651 | K00872: thrB1; homoserine kinase |  | THR1 |  | THR1 |  | Essential | mito 14, cyto 7.5, cyto\_nucl 4.5, extr 2, pero 2 | EHK\* |
| 12513 | K00641: metX; homoserine O-acetyltransferase |  | MET2 |  | MET2 |  | Not Essential | cyto\_nucl 12, nucl 11.5, cyto 11.5, pero 2 | TRW\* |
| 15248 | K00641: metX; homoserine O-acetyltransferase |  |  |  | MET2 |  | Not Essential | nucl 20, cyto 4, mito 2 | ADP\* |
| 16618 | K17069: MET17; O-acetylhomoserine/O-acetylseri... |  | MET17 |  | MET17 | CTH | Not Essential | cyto 19, cyto\_nucl 12.5, pero 3 | TAL\* |
| 15712 | K01697: E4.2.1.22, CBS; cystathionine beta-syn... |  | CYS4 | CBS,CBSL | CYS4 | CBSL | Not Essential | cyto 20.5, cyto\_mito 13, mito 4.5 | GDA\* |
| 9499 | K01758: CTH; cystathionine gamma-lyase |  | CYS3 | CTH | CYS3 | CTH | Not Essential | mito 21, cyto 6 | VKA\* |
| 8759 | K01760: metC; cystathionine beta-lyase |  | STR3 |  | STR3 | CTH | Not Essential | mito 13, cyto 9.5, cyto\_nucl 8.5, nucl 4.5 | PADL |
| 11463 | K01739: metB; cystathionine gamma-synthase |  | STR2,YML082W,YLL058W |  | STR2 |  | Not Essential | mito 10, nucl 5, cyto 4, extr 4, plas 2 | TVE\* |
| 16725 | K01739: metB; cystathionine gamma-synthase |  | YHR112C |  | YHR112C | CTH | Not Essential | cyto 13, cyto\_nucl 9.5, nucl 4, plas 3, extr 2... | TLQ\* |
| 16742 | KOG0053: Cystathionine beta-lyases/cystathioni... |  | YHR112C |  | YHR112C | CTH | Not Essential | plas 9, nucl 5.5, cyto\_nucl 5.5, cyto 4.5, mit... | FLS\* |
| 9734 | K00661: maa; maltose O-acetyltransferase |  | YJL218W |  | YJL218W |  | Not Essential | cyto 14, cyto\_nucl 9.5, mito 4, nucl 3, extr 3 | AKK\* |
| 12031 | K01738: cysK; cysteine synthase A |  |  |  | YGR012W | CBSL | Not Essential | mito 23.5, cyto\_mito 13.5 | VLP\* |
| 13106 | K01738: cysK; cysteine synthase A | A | YGR012W |  | YGR012W | CBSL | Not Essential | mito 12.5, cyto\_mito 12, cyto 10.5, extr 2 | GQS\* |
| 12912 | K01251: E3.3.1.1, ahcY; adenosylhomocysteinase |  | SAH1 | AHCY | SAH1 | AHCY | Essential | cyto 22, cysk 5 | YRY\* |
| 15759 | K00547: mmuM, BHMT2; homocysteine S-methyltran... |  | MHT1,SAM4 |  |  |  | Not Essential | extr 16, cyto 7.5, cyto\_nucl 5, mito 2 | AKE\* |
| 9825 | K00549: metE; 5-methyltetrahydropteroyltriglut... |  | MET6 |  | MET6 |  | Not Essential | mito 20, nucl 3, plas 3 | EYA\* |
| 12876 | KOG2263: Methionine synthase II (cobalamin-ind... |  |  |  |  |  | Not Essential | cyto 16.5, cyto\_nucl 12, nucl 4.5, mito 3 | SGA\* |
| 12920 | KOG2263: Methionine synthase II (cobalamin-ind... |  |  |  |  |  | Not Essential | cyto 20.5, cyto\_nucl 14, nucl 4.5 | SDA\* |

| strain | WT | | | | | | | | | | | | | | | | |
| --- | --- | --- | --- | --- | --- | --- | --- | --- | --- | --- | --- | --- | --- | --- | --- | --- | --- |
| condition | G\_MM | C\_MM | G\_SD | | GX\_SD | | | X\_SD | | A\_SD | | C\_SD | | MM\_CN120 | | MM\_CN5 | Diversity\_Sample |
| phase | exp | exp | exp | stat | exp | trans | stat | exp | stat | exp | stat | exp | stat | exp | stat | exp | exp |
| proteinId | Set1 | Set1 | Set2 | Set2 | Set2 | Set2 | Set2 | Set2 | Set2 | Set2 | Set2 | Set2 | Set2 | Set3 | Set3 | Set3 | Set3 |
| 12080 | 5.37711 | 6.33989 | 5.88653 | 5.6913 | 5.97397 | 6.65735 | 6.47209 | 6.98792 | 5.8263 | 6.22241 | 5.82229 | 6.10347 | 6.45378 | 7.03104 | 6.98462 | 7.71813 | 7.49153 |
| 14662 | 5.87502 | 7.09812 | 6.53007 | 3.66946 | 6.86932 | 5.38637 | 5.66047 | 6.0607 | 4.16865 | 4.93445 | 3.56787 | 7.76938 | 8.23471 | 4.90925 | 5.04472 | 7.5581 | 6.23579 |
| 16738 | 3.28859 | 3.11251 | 2.45657 | 2.92321 | 2.67339 | 2.53217 | 2.73548 | 2.92483 | 2.20724 | 2.7738 | 2.5712 | 2.63698 | 2.13162 | 3.55115 | 3.85247 | 4.5266 | 3.45568 |
| 8651 | 8.18701 | 7.80988 | 8.58186 | 6.84117 | 8.82886 | 8.01419 | 7.84369 | 7.6978 | 6.75902 | 6.88132 | 6.83704 | 7.52587 | 8.06456 | 8.5044 | 8.09553 | 9.53158 | 8.31281 |
| 12513 | 5.50435 | 5.78477 | 5.73772 | 4.29915 | 6.16302 | 4.85313 | 5.02116 | 4.93071 | 5.17655 | 4.82062 | 4.8534 | 6.23245 | 7.56468 | 4.04676 | 4.26599 | 6.44857 | 4.86474 |
| 15248 | 4.77579 | 5.09828 | 5.16052 | 4.65071 | 5.25529 | 5.05503 | 4.55137 | 6.37016 | 4.61116 | 6.15557 | 4.92458 | 4.89249 | 5.73376 | 4.79598 | 4.61193 | 7.82305 | 5.67427 |
| 16618 | 3.66863 | 6.39746 | 2.79199 | 2.04492 | 2.42701 | 2.64934 | 2.15409 | 5.73185 | 2.14058 | 5.64536 | 2.68149 | 2.39947 | 2.04014 | 1.96034 | 2.34301 | 6.24548 | 4.59359 |
| 15712 | 5.6776 | 6.08222 | 5.63322 | 6.23119 | 5.20726 | 5.38816 | 5.79508 | 4.47276 | 5.98925 | 4.94022 | 6.61947 | 5.7921 | 3.85307 | 5.60701 | 5.19008 | 5.23074 | 4.40741 |
| 9499 | 5.90147 | 5.7589 | 7.08215 | 8.0137 | 7.06648 | 7.01341 | 7.59232 | 6.16686 | 7.74408 | 5.91491 | 7.62068 | 5.91259 | 6.0471 | 6.83041 | 6.56295 | 6.6224 | 5.98304 |
| 8759 | 6.07945 | 6.48278 | 6.43719 | 5.73961 | 6.53685 | 6.26359 | 5.99018 | 6.97311 | 5.33091 | 6.44274 | 5.62708 | 7.04163 | 7.54766 | 6.23801 | 6.3152 | 8.39031 | 7.22902 |
| 11463 | 6.26796 | 6.80364 | 6.82844 | 5.05871 | 7.16494 | 6.19096 | 6.24618 | 5.84525 | 6.2683 | 5.42335 | 5.51979 | 7.62914 | 8.16505 | 6.37033 | 6.36179 | 7.74938 | 6.71233 |
| 16725 | 7.6487 | 7.90998 | 6.50228 | 5.41198 | 6.91568 | 4.7491 | 5.71719 | 4.94148 | 6.10528 | 4.55164 | 6.15479 | 9.09453 | 10.1214 | 5.42176 | 4.64949 | 7.4025 | 6.98818 |
| 16742 | 4.69493 | 4.92812 | 4.33268 | 4.13424 | 4.42182 | 4.4894 | 4.43189 | 4.96499 | 4.8482 | 4.81596 | 4.28086 | 4.71773 | 4.83134 | 5.05017 | 5.03618 | 5.72767 | 5.38089 |
| 9734 | 6.68877 | 6.72088 | 6.30316 | 6.65029 | 6.35076 | 5.69218 | 5.37726 | 7.42113 | 5.53744 | 6.00093 | 4.68887 | 7.48333 | 6.80222 | 6.71915 | 6.27535 | 7.11265 | 6.5848 |
| 12031 | 6.54744 | 7.01991 | 6.8446 | 5.78562 | 6.90134 | 6.36052 | 6.17766 | 7.53894 | 5.99978 | 6.80221 | 5.90004 | 6.67107 | 6.82057 | 5.18359 | 4.83158 | 6.87458 | 6.17331 |
| 13106 | 5.12136 | 4.78057 | 5.23997 | 5.14658 | 5.17287 | 5.40534 | 5.39306 | 5.35166 | 5.24983 | 5.16388 | 5.14096 | 5.47319 | 5.14301 | 4.63839 | 4.78748 | 4.34427 | 4.71479 |
| 12912 | 5.16791 | 8.05987 | 6.33628 | 4.70387 | 6.52555 | 5.73959 | 5.68239 | 7.86419 | 4.6314 | 6.80592 | 4.1811 | 6.3894 | 6.13098 | 6.68766 | 6.59696 | 9.35737 | 10.0375 |
| 15759 | 4.34019 | 4.92188 | 3.78193 | 3.943 | 3.91627 | 3.95133 | 4.10319 | 4.08098 | 4.63768 | 4.2025 | 4.45422 | 5.78753 | 5.28312 | 4.25929 | 4.67933 | 4.69738 | 5.21708 |
| 9825 | 8.08125 | 8.57941 | 9.15364 | 6.38367 | 9.61999 | 7.26769 | 7.08625 | 9.08874 | 7.16258 | 8.63623 | 6.82618 | 8.33517 | 9.27305 | 9.48394 | 8.75169 | 13.3867 | 11.868 |
| 12876 | 5.31563 | 4.34853 | 5.50322 | 5.15707 | 5.46341 | 5.72393 | 5.4655 | 5.48696 | 4.97269 | 5.15462 | 4.95104 | 4.29206 | 3.3658 | 5.47926 | 5.26055 | 5.36543 | 5.17199 |
| 12920 | 2.51917 | 6.69393 | 2.97962 | 5.52922 | 3.13258 | 3.93896 | 4.18614 | 4.14075 | 4.60901 | 3.71207 | 5.01365 | 6.46747 | 5.37976 | 3.23846 | 4.05295 | 4.04091 | 6.53819 |

| strain | WT | | | | | | | | | | |
| --- | --- | --- | --- | --- | --- | --- | --- | --- | --- | --- | --- |
| condition | G\_SD | | GX\_SD | | | X\_SD | | A\_SD | | C\_SD | |
| proteinId | exp | stat | exp | trans | stat | exp | stat | exp | stat | exp | stat |
| 12080 | 10.6044 | 12.6567 | 12.205 | 9.8583 | 12.4625 | 14.8322 | 13.5979 | 10.2408 | 10.3284 | 9.58668 | 8.46391 |
| 14662 | 9.78159 | 6.29342 | 10.0098 | 4.83991 | 3.6552 | 4.08951 | 5.29466 | 6.18133 | 5.57223 | 6.81094 | 17.4591 |
| 16738 | 0 | 0 | 0 | 0.185549 | 0 | 0 | 0 | 0 | 0 | 0 | 0 |
| 8651 | 10.3111 | 8.67011 | 9.15026 | 12.6485 | 10.7663 | 7.63738 | 4.8965 | 7.35493 | 3.07457 | 8.58997 | 6.71573 |
| 12513 | 2.48229 | 2.15279 | 1.63361 | 0.928468 | 0.732118 | 0.592166 | 0.592866 | 1.54524 | 0.580428 | 0.430131 | 2.58841 |
| 15248 | 1.73011 | 0.888746 | 1.62685 | 2.04615 | 1.11011 | 2.75537 | 2.71511 | 2.32256 | 2.66282 | 0.219601 | 0 |
| 16618 | 1.72428 | 0.188312 | 2.24005 | 0 | 0 | 4.68175 | 5.30851 | 13.9005 | 10.483 | 0 | 0 |
| 15712 | 3.6986 | 2.69554 | 3.04448 | 2.7841 | 3.09086 | 4.50527 | 4.35295 | 4.83214 | 4.02227 | 11.5709 | 9.36007 |
| 9499 | 9.97024 | 0.5368 | 12.8252 | 0.189313 | 0.707701 | 5.49381 | 0 | 0.19251 | 0 | 9.15044 | 3.04898 |
| 8759 | 5.17508 | 4.2963 | 5.10278 | 3.5426 | 2.76525 | 4.51727 | 4.34802 | 5.40713 | 2.85807 | 2.97116 | 3.92858 |
| 11463 | 13.0551 | 7.77488 | 13.2244 | 6.12373 | 6.1623 | 8.62003 | 4.70329 | 7.91998 | 4.58854 | 5.97118 | 10.0237 |
| 16725 | 5.74913 | 1.80418 | 6.10236 | 2.03207 | 1.27617 | 3.7115 | 2.76114 | 2.70418 | 0.384727 | 8.77578 | 19.5346 |
| 16742 | 2.10215 | 0.543291 | 3.25818 | 0.189769 | 0 | 1.35131 | 0.566061 | 0.966186 | 0.189478 | 0.853425 | 1.53534 |
| 9734 | 3.26005 | 4.97547 | 4.88114 | 6.15589 | 6.04647 | 8.03626 | 7.27435 | 7.91965 | 8.05353 | 9.18415 | 10.8972 |
| 12031 | 16.5999 | 14.047 | 19.1026 | 13.5921 | 12.9868 | 16.8116 | 16.5235 | 17.7712 | 18.7866 | 9.00422 | 11.1239 |
| 13106 | 0.391889 | 0.521646 | 0.604673 | 0.940764 | 1.06511 | 0.782146 | 0.79857 | 0 | 0 | 0.439202 | 1.53201 |
| 12912 | 25.5789 | 19.8823 | 28.4605 | 14.9095 | 14.926 | 26.0359 | 26.5885 | 26.2701 | 23.3587 | 24.9849 | 22.0152 |
| 15759 | 1.5508 | 0.738127 | 1.01437 | 0.185549 | 0.35399 | 0.586657 | 0.401158 | 0 | 0.769635 | 3.21216 | 3.4937 |
| 9825 | 87.1739 | 44.8769 | 91.2944 | 32.9405 | 35.0678 | 69.1417 | 30.9277 | 53.5098 | 18.9466 | 76.4782 | 89.7412 |
| 12876 | 5.52738 | 8.13522 | 5.08813 | 10.2232 | 10.4317 | 10.9109 | 9.50073 | 7.72533 | 7.84721 | 13.4494 | 10.0418 |
| 12920 | 7.42042 | 6.85077 | 7.92501 | 5.21191 | 6.76608 | 11.9306 | 10.8448 | 7.71747 | 7.09638 | 31.449 | 25.2632 |

|  | Glucose | Xylose | Arabinose | Acetate | Coumarate | Ferulate | YNB Oleic Acid | YNB Ricinoleic Acid | YNB Glucose | YNB Gluc DOC | YPD |
| --- | --- | --- | --- | --- | --- | --- | --- | --- | --- | --- | --- |
| proteinId |  |  |  |  |  |  |  |  |  |  |  |
| 16738 | 0.558274 | 0.307275 | 0.626728 | 0.565536 | 0.507002 | 0.365426 | 0.298877 | -0.137587 | 0.672879 | 0.366337 | -0.169222 |
| 12513 | -0.835478 | -1.0497 | -1.03052 | 0.326171 | -0.144332 | 0.0229116 | -3.87828 | -4.15135 | -5.17453 | -0.357745 | -0.323942 |
| 15248 | 0.0989456 | -0.365113 | 0.00543596 | 0.136312 | -0.432678 | -1.73711 | -0.382425 | -1.90233 | -2.30563 | 0.256682 | 0.310651 |
| 16618 | 0.0419636 | 0.0304368 | -0.143477 | -0.158729 | -0.0705999 | -0.052245 | 0.209139 | 0.397923 | 0.195869 | 0.231633 | 0.0845513 |
| 15712 | -0.231243 | -0.613382 | -0.146129 | -0.19365 | 0.19931 | -0.126857 | 0.648164 | -0.66015 | -0.333046 | -0.497314 | -0.0583468 |
| 9499 | -0.0362174 | -0.108912 | -0.420679 | -0.254394 | 0.149364 | 0.116627 | -0.365395 | -0.485208 | -0.0270825 | 0.108624 | 0.206243 |
| 11463 | -0.167618 | -0.0282166 | -0.0612006 | -0.182825 | 0.106678 | -0.0196322 | 0.0520783 | -0.172054 | 0.0643711 | -0.14793 | 0.0628106 |
| 16725 | -0.0611906 | -0.56125 | 0.0652367 | -0.119765 | -0.245465 | -0.155754 | -0.148042 | -0.632691 | 0.447575 | 0.495966 | 0.353243 |
| 16742 | -0.143095 | 0.0413233 | -0.0158122 | 0.116166 | 0.384373 | 0.0921014 | -0.0265362 | -0.240916 | -0.377589 | -0.582753 | -0.481596 |
| 9734 | -0.0852503 | 0.117004 | -0.251187 | -0.0296711 | -0.257072 | -0.09008 | -0.0715038 | 0.295928 | -0.183571 | -0.0811435 | 0.0786046 |
| 12031 | -0.0683232 | -0.52423 | -0.224546 | -0.229108 | -0.478246 | -2.47907 | -0.466508 | -2.59065 | -2.88539 | -0.605957 | -0.479297 |
| 13106 | -0.01865 | 0.063463 | -0.0134732 | 0.0484738 | -0.960612 | -0.16424 | 0.0888878 | -0.36252 | -0.495907 | -0.112539 | -0.342573 |
| 15759 | -0.670316 | -0.477989 | -0.387906 | -0.470753 | -0.401093 | -0.171006 | -0.0941091 | 0.184942 | -0.0276698 | 0.162868 | -1.37311 |
| 9825 | -1.14217 | -1.54222 | -1.4924 | -0.733303 | -2.35847 | -0.242351 | -2.57016 | -2.20835 | -2.29425 | -0.665664 | -0.66676 |
| 12876 | 0.0526246 | 0.112894 | 0.00776545 | -0.205377 | -0.0576661 | -0.137189 | 0.152013 | 0.13217 | 0.52675 | 0.356051 | -0.0977661 |
| 12920 | 0.128847 | 0.210065 | 0.15477 | 0.0910073 | 0.588857 | -0.41173 | 0.220943 | 0.0828859 | -0.625153 | -0.267929 | 0.120482 |

In [62]:

```
for x in temp:
    if x in model.genes:
        for r in sorted(model.genes.get_by_id(x).reactions, key=lambda x: x.id):
            print(r, r.gene_reaction_rule)
    else:
        print(x, 'no reactions')
    print()
```

```
ASPK: asp__L_c + atp_c <=> 4pasp_c + adp_c 12080 or 14662 or 16738
ASPK_1: asp__L_h + atp_h --> 4pasp_h + adp_h 12080 or 14662
HSDH: aspsa_h + h_h + nadph_h --> hom__L_h + nadp_h 12080
HSDxi: aspsa_c + h_c + nadh_c --> hom__L_c + nad_c 12080 or 16738
HSDy: hom__L_c + nadp_c <=> aspsa_c + h_c + nadph_c 12080 or 16738

ASPK: asp__L_c + atp_c <=> 4pasp_c + adp_c 12080 or 14662 or 16738
ASPK_1: asp__L_h + atp_h --> 4pasp_h + adp_h 12080 or 14662

ASPK: asp__L_c + atp_c <=> 4pasp_c + adp_c 12080 or 14662 or 16738
HSDxi: aspsa_c + h_c + nadh_c --> hom__L_c + nad_c 12080 or 16738
HSDy: hom__L_c + nadp_c <=> aspsa_c + h_c + nadph_c 12080 or 16738

HSK: atp_c + hom__L_c --> adp_c + h_c + phom_c 8651

HSERTA: accoa_c + hom__L_c <=> achms_c + coa_c 12513 or 15248

HSERTA: accoa_c + hom__L_c <=> achms_c + coa_c 12513 or 15248

AHSERL: achms_c + ch4s_c --> ac_c + h_c + met__L_c 16618
AHSERL2: achms_c + h2s_c --> ac_c + h_c + hcys__L_c 16618

ACSERL: acser_c + seln_c <=> ac_c + 2.0 h_c + selcys_c 12031 or 13106 or 15712
ACSERLh: acser_h + seln_h <=> ac_h + 2.0 h_h + selcys_h 15712
ACSERLm: acser_m + seln_m <=> ac_m + 2.0 h_m + selcys_m 15712
ACSERSULL: acser_c + tsul_c --> ac_c + h_c + scys__L_c 12031 or 13106 or 15712
ACSERSULLh: acser_h + tsul_h --> ac_h + h_h + scys__L_h 15712
ACSERSULLm: acser_m + tsul_m --> ac_m + h_m + scys__L_m 15712
CYS: h2s_c + ser__L_c --> cys__L_c + h2o_c 15712
CYSS: acser_c + h2s_c --> ac_c + cys__L_c + h_c 12031 or 13106 or 15712
CYSS_1: acser_h + h2s_h --> ac_h + cys__L_h 12031 or 13106 or 15712
CYSTS: hcys__L_c + ser__L_c --> cyst__L_c + h2o_c 15712
SELCYSTS: selhcys_c + ser__L_c --> h2o_c + selcyst_c 15712

CYSDS: cys__L_c + h2o_c --> h2s_c + nh4_c + pyr_c 8759 or 9499
CYSTGL: cyst__L_c + h2o_c --> 2obut_c + cys__L_c + nh4_c 9499
SELCYSTGL: h2o_c + selcyst_c --> 2obut_c + nh4_c + selcys_c 9499
SHSL4r: h2o_c + suchms_c <=> 2obut_c + h_c + nh4_c + succ_c 11463 or 16725 or 16742 or 9499

CBL: cyst__L_h + h2o_h --> hcys__L_h + nh4_h + pyr_h 8759
CTINBL: cysi__L_h + h2o_h --> nh4_h + pyr_h + thcys_h 8759
CYSDS: cys__L_c + h2o_c --> h2s_c + nh4_c + pyr_c 8759 or 9499
CYSTBL: h2s_h + h_h + nh4_h + pyr_h --> cys__L_h + h2o_h 8759
CYSTL: cyst__L_c + h2o_c --> hcys__L_c + nh4_c + pyr_c 16725 or 16742 or 8759
SELCYSTL: h2o_c + selcyst_c --> h_c + nh4_c + pyr_c + selhcys_c 8759
SELCYSTLh: h2o_h + selcyst_h --> h_h + nh4_h + pyr_h + selhcys_h 8759

SHSL1: cys__L_c + suchms_c --> cyst__L_c + h_c + succ_c 11463
SHSL4r: h2o_c + suchms_c <=> 2obut_c + h_c + nh4_c + succ_c 11463 or 16725 or 16742 or 9499

CYSTL: cyst__L_c + h2o_c --> hcys__L_c + nh4_c + pyr_c 16725 or 16742 or 8759
SHSL4r: h2o_c + suchms_c <=> 2obut_c + h_c + nh4_c + succ_c 11463 or 16725 or 16742 or 9499

CYSTL: cyst__L_c + h2o_c --> hcys__L_c + nh4_c + pyr_c 16725 or 16742 or 8759
SHSL4r: h2o_c + suchms_c <=> 2obut_c + h_c + nh4_c + succ_c 11463 or 16725 or 16742 or 9499

GLCATr: accoa_c + glc__D_c <=> acglc__D_c + coa_c 9734
MALTATr: accoa_c + malt_c <=> acmalt_c + coa_c 9734

ACSERL: acser_c + seln_c <=> ac_c + 2.0 h_c + selcys_c 12031 or 13106 or 15712
ACSERSULL: acser_c + tsul_c --> ac_c + h_c + scys__L_c 12031 or 13106 or 15712
CHOLS_ex: chols_e <=> chols_p 12031 or 13106
CYSS: acser_c + h2s_c --> ac_c + cys__L_c + h_c 12031 or 13106 or 15712
CYSS_1: acser_h + h2s_h --> ac_h + cys__L_h 12031 or 13106 or 15712
SLCYSS: acser_c + tsul_c --> ac_c + scys__L_c 12031 or 13106

ACSERL: acser_c + seln_c <=> ac_c + 2.0 h_c + selcys_c 12031 or 13106 or 15712
ACSERSULL: acser_c + tsul_c --> ac_c + h_c + scys__L_c 12031 or 13106 or 15712
CHOLS_ex: chols_e <=> chols_p 12031 or 13106
CYSS: acser_c + h2s_c --> ac_c + cys__L_c + h_c 12031 or 13106 or 15712
CYSS_1: acser_h + h2s_h --> ac_h + cys__L_h 12031 or 13106 or 15712
SLCYSS: acser_c + tsul_c --> ac_c + scys__L_c 12031 or 13106

ADSHm: ahcys_m + h2o_m <=> adn_m + hcys__L_m 12912
AHCi: ahcys_c + h2o_c --> adn_c + hcys__L_c 12912
SEAHCYSHYD: h2o_c + seahcys_c --> adn_c + selhcys_c 12912
SEAHCYSHYD_1: h2o_c + seahcys_c <=> adn_c + h_c + selhcys_c 12912

HCYSMT: amet_c + hcys__L_c --> ahcys_c + h_c + met__L_c 15759
HCYSMT2: hcys__L_c + mmet_c --> h_c + 2.0 met__L_c 15759

METS: 5mthf_c + hcys__L_c --> h_c + met__L_c + thf_c 12876 or 12920 or 9825
MS: h_m + hcys__L_m + mhpglu_m --> hpglu_m + met__L_m 9825

METS: 5mthf_c + hcys__L_c --> h_c + met__L_c + thf_c 12876 or 12920 or 9825

METS: 5mthf_c + hcys__L_c --> h_c + met__L_c + thf_c 12876 or 12920 or 9825
```

In [63]:

```
# 14662 aspartate kinase
model.reactions.get_by_id('ASPK').gene_reaction_rule = '14662'
model.remove_reactions(['ASPK_1','HSDH'], remove_orphans=True)
# 15712 CYS4 cystathionine beta-synthase (4.2.1.22)
# L-Serine + L-Homocysteine <=> L-Cystathionine + H2O (CYSTS, correct)
# 12031 and 13106 cysteine synthase A / O-acetyl-L-serine sulfhydrylase (2.5.1.47)
# O-Acetyl-L-serine + Hydrogen sulfide <=> L-Cysteine + Acetate (CYSS)
model.reactions.get_by_id('CYSS').gene_reaction_rule = '12031 or 13106'
# ACSERL/SLCYSS is by selenocysteine synthase, and ACSERSULL by cysteine synthase B only
# CHOLS_ex and CYSS_1 wrong compartment, CYS wrong, SELCYSTS not known
model.remove_reactions(['ACSERL','ACSERLh','ACSERLm','SLCYSS','ACSERSULL','ACSERSULLh','ACSERSULLm',
                        'CHOLS_ex','CYSS_1','CYS','SELCYSTS'], remove_orphans=True)
# 9499 CYS3 cystathionine gamma-lyase -> CYSTGL
model.reactions.get_by_id('CYSTGL').gene_reaction_rule = '9499'
# 8759 STR3 cystathionine beta-lyase -> CYSTL
model.reactions.get_by_id('CYSTL').gene_reaction_rule = '8759'
# 11463 STR2 cystathionine gamma-synthase -> SHSL1
# SHSLr4 is sum of CYSTL and CYSTGL
model.remove_reactions(['SELCYSTGL','SHSL4r','CBL','CTINBL','CYSTBL','SELCYSTL','SELCYSTLh'], remove_orphans=True)
# 16725 and 16742 best hit is A. fum cysteine-S-conjugate beta-lyase -> CYSDS
model.reactions.get_by_id('CYSDS').gene_reaction_rule = '16725 or 16742'
# Remove the rest of seleno-reactions
# MS incorrect proton, mhpglu is correct in biocyc but MS is the only reaction using this
model.remove_reactions(['SEAHCYSHYD','SEAHCYSHYD_1','MS'], remove_orphans=True)
```

In [64]:

```
for r in sorted(model.metabolites.get_by_id('cys__L_c').reactions, key=lambda x: x.id):
    print(r, r.gene_reaction_rule)
```

```
AMPTASECG: cgly_c + h2o_c --> cys__L_c + gly_c 10210 or 12096
CYSDS: cys__L_c + h2o_c --> h2s_c + nh4_c + pyr_c 16725 or 16742
CYSS: acser_c + h2s_c --> ac_c + cys__L_c + h_c 12031 or 13106
CYSTA: akg_c + cys__L_c --> glu__L_c + mercppyr_c 14281 or 8936
CYSTGL: cyst__L_c + h2o_c --> 2obut_c + cys__L_c + nh4_c 9499
CYSTRS: atp_c + cys__L_c + trnacys_c --> amp_c + cystrna_c + ppi_c 9855
CYSt2r: cys__L_e + h_e <=> cys__L_c + h_c 14229 or 15074
GLUCYS: atp_c + cys__L_c + glu__L_c --> adp_c + glucys_c + h_c + pi_c 12007 or 12022 or (GCLM and 12007) or (GCLM and 12022) or (Gclm and 12007) or (Gclm and 12022)
ICYSDS: cys__L_c + iscs_c --> ala__L_c + iscssh_c 13740
PPNCL: 4ppan_c + ctp_c + cys__L_c --> 4ppcys_c + cdp_c + h_c + pi_c 8536
PPNCL2: 4ppan_c + ctp_c + cys__L_c --> 4ppcys_c + cmp_c + h_c + ppi_c 8536 or 8878
PPNCL3: 4ppan_c + atp_c + cys__L_c --> 4ppcys_c + amp_c + h_c + ppi_c 8878
SHSL1: cys__L_c + suchms_c --> cyst__L_c + h_c + succ_c 11463
```

In [65]:

```
# 12007 ~100% coverage, but 12002 only ~50% coverage
model.reactions.get_by_id('GLUCYS').gene_reaction_rule = '12007'
```

#### UREASE¶

In [66]:

```
for r in sorted(model.genes.get_by_id('9326').reactions, key=lambda x: x.id):
    print(r.id, r.reaction, r.gene_reaction_rule)
```

```
ALPHNH allphn_c + h2o_c + 3.0 h_c --> 2.0 co2_c + 2.0 nh4_c 9326
ALPHm allphn_m + h2o_m + 3.0 h_m --> 2.0 co2_m + 2.0 nh4_m 9326
URCB atp_c + hco3_c + urea_c --> adp_c + allphn_c + 2.0 h_c + pi_c 9326
URCBm atp_m + hco3_m + urea_m --> adp_m + allphn_m + 2.0 h_m + pi_m 9326
UREASE atp_c + hco3_c + urea_c <=> adp_c + allphn_c + h_c + pi_c 9326
```

9326 cyto 13.5, cyto\_mito 13 (no sigP) DUR1,2 urea amidolyase  
UREASE is correct, URCB is incorrect
Remove ALPHm, URCB, URCBm

In [67]:

```
model.remove_reactions(['ALPHm','URCB','URCBm'], remove_orphans=True)
```

#### PTPATi¶

In [68]:

```
for r in sorted(model.genes.get_by_id('14849').reactions, key=lambda x: x.id):
    print(r.id, r.reaction, r.gene_reaction_rule)
print()
for r in sorted(model.genes.get_by_id('11114').reactions, key=lambda x: x.id):
    print(r.id, r.reaction, r.gene_reaction_rule)
```

```
APPAT atp_c + 2.0 h_c + pan4p_c <=> dpcoa_c + ppi_c 14849
DPCOAK atp_c + dpcoa_c --> adp_c + coa_c + h_c 11114 or 14849
PTPATi atp_c + h_c + pan4p_c --> dpcoa_c + ppi_c 14849
PTPATim atp_m + h_m + pan4p_m --> dpcoa_m + ppi_m 14849

DPCOAK atp_c + dpcoa_c --> adp_c + coa_c + h_c 11114 or 14849
DPCOAKm atp_m + dpcoa_m --> adp_m + coa_m + h_m 11114
```

14849 mito 13, cyto 10 (sigP) CAB4 pantetheine-phosphate adenylyltransferase  
11114 cyto (no sigP) CAB5 dephospho-CoA kinase  
PTPATi is correct, APPAT is incorrect, mito reactions are orphans  
Change DPCOAK genes to '11114'  
Remove APPAT, PTPATim, DPCOAKm

In [69]:

```
model.reactions.get_by_id('DPCOAK').gene_reaction_rule = '11114'
model.remove_reactions(['APPAT','PTPATim','DPCOAKm'], remove_orphans=True)
```

#### ACACT1x¶

In [70]:

```
for r in sorted(model.genes.get_by_id('8678').reactions, key=lambda x: x.id):
    print(r.id, r.reaction, r.gene_reaction_rule)
print()
for r in sorted(model.genes.get_by_id('8885').reactions, key=lambda x: x.id):
    print(r.id, r.reaction, r.gene_reaction_rule)
```

```
ACACT10m 2maacoa_m + coa_m <=> accoa_m + ppcoa_m 13813 or 8678
ACACT1m 2.0 accoa_m --> aacoa_m + coa_m 8678 or 8885 or (HADHA and HADHB) or (Hadha and Hadhb)
ACACT1r 2.0 accoa_c <=> aacoa_c + coa_c 13813 or 8678 or 8885
ACACT1x 2.0 accoa_x <=> aacoa_x + coa_x 8678 or 8885
ACACT2 3ohcoa_x + coa_x <=> accoa_x + btcoa_x 13813 or 8678 or 8885
ACACT2r accoa_c + btcoa_c <=> 3ohcoa_c + coa_c 13813 or 8678 or 8885
KAT1 aacoa_c + coa_c --> 2.0 accoa_c 13813 or 8678 or 8885
yli_R1435 2.0 accoa_x --> aacoa_x + coa_x 8678 or 8885

ACACT1m 2.0 accoa_m --> aacoa_m + coa_m 8678 or 8885 or (HADHA and HADHB) or (Hadha and Hadhb)
ACACT1r 2.0 accoa_c <=> aacoa_c + coa_c 13813 or 8678 or 8885
ACACT1x 2.0 accoa_x <=> aacoa_x + coa_x 8678 or 8885
ACACT2 3ohcoa_x + coa_x <=> accoa_x + btcoa_x 13813 or 8678 or 8885
ACACT2r accoa_c + btcoa_c <=> 3ohcoa_c + coa_c 13813 or 8678 or 8885
KAT1 aacoa_c + coa_c --> 2.0 accoa_c 13813 or 8678 or 8885
yli_R1435 2.0 accoa_x --> aacoa_x + coa_x 8678 or 8885
```

In [71]:

```
# Fix simple things now and curate fatty acid metabolism later
model.reactions.get_by_id('ACACT1m').gene_reaction_rule = '8678 or 8885'
model.remove_reactions(['KAT1','yli_R1435'], remove_orphans=True)
```

In [72]:

```
#### Rest of duplicated
```

In [73]:

```
duplicated = set()
for i, r in enumerate(model.reactions):
    temp = 0
    for j, r2 in enumerate(model.reactions):
        if j > i and r2.id not in duplicated:
            if r.reactants == r2.reactants and r.products == r2.products:
                if temp == 0:
                    temp = 1
                    duplicated.add(r.id)
                    print(r.id, r.reaction, r.gene_reaction_rule)
                duplicated.add(r2.id)
                print(r2.id, r2.reaction, r2.gene_reaction_rule)
    if temp == 1:
        print()
```

```
yli_R0034 chtn_c + h2o_c --> acgam_c 13082
CHTNASE chtn_c + 2.0 h2o_c --> 3.0 acgam_c 13082

ARGSS asp__L_c + atp_c + citr__L_c --> amp_c + argsuc_c + h_c + ppi_c 16196
ARGSS_1 asp__L_c + atp_c + citr__L_c --> amp_c + argsuc_c + 2.0 h_c + ppi_c 16196

PRAGSr atp_c + gly_c + pram_c <=> adp_c + gar_c + h_c + pi_c 14259
PPRGL atp_c + gly_c + pram_c --> adp_c + gar_c + 2.0 h_c + pi_c 14259

AIRCr air_c + co2_c <=> 5aizc_c + h_c 12132
PRAIC air_c + co2_c <=> 5aizc_c + 2.0 h_c 12132

yli_R0224 8.0 coa_m + 8.0 h2o_m + 8.0 nad_m + nadph_m + 7.0 o2_m + yli_M04625_m --> 9.0 accoa_m + 7.0 h2o2_m + 7.0 h_m + 8.0 nadh_m + nadp_m (12742 and 13813 and 14805) or (12742 and 14805 and 9065) or (12752 and 13813 and 14805) or (12752 and 14805 and 9065) or (13813 and 14805 and 9700) or (14805 and 9065 and 9700)
yli_R0223 8.0 coa_m + 8.0 h2o_m + 8.0 nad_m + 2.0 nadph_m + 8.0 o2_m + yli_M04625_m --> 9.0 accoa_m + 8.0 h2o2_m + 6.0 h_m + 8.0 nadh_m + 2.0 nadp_m (12742 and 13813 and 14805) or (12742 and 14805 and 9065) or (12752 and 13813 and 14805) or (12752 and 14805 and 9065) or (13813 and 14805 and 9700) or (14805 and 9065 and 9700)

C4STMO1 44mzym_c + 3.0 h_c + 3.0 nadph_c + 3.0 o2_c --> 4mzym_int1_c + 4.0 h2o_c + 3.0 nadp_c 16640
44MZYMMO 44mzym_c + 2.0 h_c + 3.0 nadph_c + 3.0 o2_c <=> 4mzym_int1_c + 4.0 h2o_c + 3.0 nadp_c 15314

FAO182p_evenodd 8.0 coa_x + 8.0 h2o_x + 8.0 nad_x + nadph_x + 7.0 o2_x + ocdycacoa_x --> 9.0 accoa_x + 7.0 h2o2_x + 7.0 h_x + 8.0 nadh_x + nadp_x (10293 and 11362 and 12742 and 13228 and 13813) or (10293 and 11362 and 12742 and 13228 and 9065) or (10293 and 11362 and 12752 and 13228 and 13813) or (10293 and 11362 and 12752 and 13228 and 9065) or (10293 and 11362 and 13228 and 13813 and 9700) or (10293 and 11362 and 13228 and 9065 and 9700)
FAO182p_eveneven 8.0 coa_x + 8.0 h2o_x + 8.0 nad_x + 2.0 nadph_x + 8.0 o2_x + ocdycacoa_x --> 9.0 accoa_x + 8.0 h2o2_x + 6.0 h_x + 8.0 nadh_x + 2.0 nadp_x (10293 and 11362 and 12742 and 13228 and 13813) or (10293 and 11362 and 12742 and 13228 and 9065) or (10293 and 11362 and 12752 and 13228 and 13813) or (10293 and 11362 and 12752 and 13228 and 9065) or (10293 and 11362 and 13228 and 13813 and 9700) or (10293 and 11362 and 13228 and 9065 and 9700)

yli_R1510 1ag3p_SC_r + acoa_r --> coa_r + pa_EC_r 10427 or 16030 or 9746
yli_R1523 1ag3p_SC_r + acoa_r --> coa_r + pa_EC_r 16030

NMNAT atp_c + h_c + nmn_c --> nad_c + ppi_c 10430
ANNAT atp_c + 2.0 h_c + nmn_c <=> nad_c + ppi_c 10430

yli_R1513 h2o_r + pa_EC_r --> dag_hs_r + pi_r 12485 or 13087
yli_R1393 h2o_r + 0.01 pa_EC_r --> 0.01 dag_hs_r + pi_r 12485

NNATr atp_c + h_c + nicrnt_c <=> dnad_c + ppi_c 10430
NNATr_copy1 atp_c + h_c + nicrnt_c --> dnad_c + ppi_c 14638
NNATr_copy2 atp_c + h_c + nicrnt_c <=> dnad_c + ppi_c 10430

yli_R1508 glyald_c + h2o_c + nad_c --> glyc__R_c + h_c + nadh_c 12042 or 13426 or 16323
GLYALDDr glyald_c + h2o_c + nad_c <=> glyc__R_c + 2.0 h_c + nadh_c 12042 or 13426

HISTD h2o_c + histd_c + 2.0 nad_c --> 3.0 h_c + his__L_c + 2.0 nadh_c 11646
HDH h2o_c + histd_c + 2.0 nad_c --> 4.0 h_c + his__L_c + 2.0 nadh_c 11646

GUAD gua_c + h2o_c + h_c --> nh4_c + xan_c 9050 or 9708
GUAD_1 gua_c + h2o_c + 2.0 h_c --> nh4_c + xan_c 9050 or 9708

PANTS ala_B_c + atp_c + pant__R_c --> amp_c + h_c + pnto__R_c + ppi_c 10475
PBAL ala_B_c + atp_c + pant__R_c --> amp_c + 2.0 h_c + pnto__R_c + ppi_c 10475

NADDP h2o_c + nad_c --> amp_c + 2.0 h_c + nmn_c 12434
NPH h2o_c + nad_c --> amp_c + 3.0 h_c + nmn_c 15385

STARCH300DEGR2A 49.0 h2o_h + 250.0 pi_h + starch300_h --> 50.0 Glc_aD_h + 250.0 g1p_h (CRv4_Au5_s11_g2607_t1 and CRv4_Au5_s3_g11038_t1 and CRv4_Au5_s6_g12805_t1 and 11993 and 14256) or (CRv4_Au5_s11_g2607_t1 and CRv4_Au5_s3_g11038_t1 and CRv4_Au5_s6_g13538_t1 and 11993 and 14256) or (CRv4_Au5_s19_g8150_t1 and CRv4_Au5_s3_g11038_t1 and CRv4_Au5_s6_g12805_t1 and 11993 and 14256) or (CRv4_Au5_s19_g8150_t1 and CRv4_Au5_s3_g11038_t1 and CRv4_Au5_s6_g13538_t1 and 11993 and 14256)
STARCH300DEGRA 74.0 h2o_h + 225.0 pi_h + starch300_h --> 75.0 Glc_aD_h + 225.0 g1p_h (CRv4_Au5_s11_g2607_t1 and CRv4_Au5_s3_g11038_t1 and CRv4_Au5_s6_g12805_t1 and 11993 and 14256) or (CRv4_Au5_s11_g2607_t1 and CRv4_Au5_s3_g11038_t1 and CRv4_Au5_s6_g13538_t1 and 11993 and 14256) or (CRv4_Au5_s19_g8150_t1 and CRv4_Au5_s3_g11038_t1 and CRv4_Au5_s6_g12805_t1 and 11993 and 14256) or (CRv4_Au5_s19_g8150_t1 and CRv4_Au5_s3_g11038_t1 and CRv4_Au5_s6_g13538_t1 and 11993 and 14256)

HDC 2.0 h_c + his__L_c --> co2_c + hista_c 10722 or 9434 or 9435
HISDC h_c + his__L_c --> co2_c + hista_c 10104

STARCH300DEGR2B 49.0 h2o_h + 250.0 pi_h + starch300_h --> 250.0 g1p_h + 50.0 glc__bD_h (CRv4_Au5_s11_g2607_t1 and CRv4_Au5_s3_g11038_t1 and CRv4_Au5_s6_g12805_t1 and 11993 and 14256) or (CRv4_Au5_s11_g2607_t1 and CRv4_Au5_s3_g11038_t1 and CRv4_Au5_s6_g13538_t1 and 11993 and 14256) or (CRv4_Au5_s19_g8150_t1 and CRv4_Au5_s3_g11038_t1 and CRv4_Au5_s6_g12805_t1 and 11993 and 14256) or (CRv4_Au5_s19_g8150_t1 and CRv4_Au5_s3_g11038_t1 and CRv4_Au5_s6_g13538_t1 and 11993 and 14256)
STARCH300DEGRB 74.0 h2o_h + 225.0 pi_h + starch300_h --> 225.0 g1p_h + 75.0 glc__bD_h (CRv4_Au5_s11_g2607_t1 and CRv4_Au5_s3_g11038_t1 and CRv4_Au5_s6_g12805_t1 and 11993 and 14256) or (CRv4_Au5_s11_g2607_t1 and CRv4_Au5_s3_g11038_t1 and CRv4_Au5_s6_g13538_t1 and 11993 and 14256) or (CRv4_Au5_s19_g8150_t1 and CRv4_Au5_s3_g11038_t1 and CRv4_Au5_s6_g12805_t1 and 11993 and 14256) or (CRv4_Au5_s19_g8150_t1 and CRv4_Au5_s3_g11038_t1 and CRv4_Au5_s6_g13538_t1 and 11993 and 14256)

ANNATn atp_n + 2.0 h_n + nmn_n <=> nad_n + ppi_n 10430
NMNATn atp_n + h_n + nmn_n --> nad_n + ppi_n 10430

CLHCOtex cl_e + 2.0 hco3_c --> cl_c + 2.0 hco3_e 14119 or 15736
CLHCO3tex2 2.0 cl_e + hco3_c --> 2.0 cl_c + hco3_e 16202

FACOAL204_copy1 arachd_c + atp_c + coa_c <=> amp_c + arachdcoa_c + ppi_c 12538 or 12555
FACOAL204_copy2 arachd_c + atp_c + coa_c --> amp_c + arachdcoa_c + ppi_c 11167 or 12538 or 12555 or 15748
```

In [74]:

```
for x in ['13082','16196','14259','12132','10430','14638','16323','11646','9050','9708',
          '10475','12434','15385','11993','14256','10722','9434','9435','10104','14119','15736','16202']:
    for r in sorted(model.genes.get_by_id(x).reactions, key=lambda x: x.id):
        print(r.id, r.reaction, r.gene_reaction_rule)
    print()
```

```
CHTNASE chtn_c + 2.0 h2o_c --> 3.0 acgam_c 13082
CHTNASEe chtn_e + 2.0 h2o_e --> 3.0 acgam_e 13082
yli_R0034 chtn_c + h2o_c --> acgam_c 13082

ARGSS asp__L_c + atp_c + citr__L_c --> amp_c + argsuc_c + h_c + ppi_c 16196
ARGSS_1 asp__L_c + atp_c + citr__L_c --> amp_c + argsuc_c + 2.0 h_c + ppi_c 16196

FGFTh fgam_h + 3.0 h_h + thf_h --> gar_h + h2o_h + methf_h 13595 or 14259
FPGFTh 10fthf_h + gar_h <=> fgam_h + h_h + thf_h 13595 or 14259
GARFT 10fthf_c + gar_c <=> fgam_c + h_c + thf_c 13595 or 14259
PPRGL atp_c + gly_c + pram_c --> adp_c + gar_c + 2.0 h_c + pi_c 14259
PPRGLh atp_h + gly_h + pram_h --> adp_h + gar_h + h_h + pi_h 14259
PRAGSr atp_c + gly_c + pram_c <=> adp_c + gar_c + h_c + pi_c 14259
PRAIS atp_c + fpram_c --> adp_c + air_c + 2.0 h_c + pi_c 14259

AIRC2 air_c + atp_c + hco3_c --> 5caiz_c + adp_c + h_c + pi_c 12132
AIRC3 5aizc_c <=> 5caiz_c 12132
AIRCr air_c + co2_c <=> 5aizc_c + h_c 12132
PRAIC air_c + co2_c <=> 5aizc_c + 2.0 h_c 12132
PRAICh air_h + co2_h <=> 5aizc_h + h_h 12132

ANNAT atp_c + 2.0 h_c + nmn_c <=> nad_c + ppi_c 10430
ANNATn atp_n + 2.0 h_n + nmn_n <=> nad_n + ppi_n 10430
NMNAT atp_c + h_c + nmn_c --> nad_c + ppi_c 10430
NMNATm atp_m + h_m + nmn_m --> nad_m + ppi_m 10430
NMNATn atp_n + h_n + nmn_n --> nad_n + ppi_n 10430
NNATm atp_m + h_m + nicrnt_m --> dnad_m + ppi_m 10430
NNATn atp_n + h_n + nicrnt_n --> dnad_n + ppi_n 10430
NNATr atp_c + h_c + nicrnt_c <=> dnad_c + ppi_c 10430
NNATr_copy2 atp_c + h_c + nicrnt_c <=> dnad_c + ppi_c 10430

DNADDP dnad_c + h2o_c --> amp_c + 2.0 h_c + nicrnt_c 14638 or 15385
FADDP fad_c + h2o_c --> amp_c + fmn_c + 2.0 h_c 14638
NNATr_copy1 atp_c + h_c + nicrnt_c --> dnad_c + ppi_c 14638
UDPGP h2o_c + udpg_c --> g1p_c + 2.0 h_c + ump_c 14638

34DHALDD 34dhpac_c + h2o_c + nad_c --> 34dhpha_c + 2.0 h_c + nadh_c 12042 or 13426 or 16323
34DHPLACOX_NADP 34dhpac_c + h2o_c + nadp_c <=> 34dhpha_c + 2.0 h_c + nadph_c 12042 or 13426 or 16323
3M4HDXPAC 3mox4hpac_c + h2o_c + nad_c <=> 2.0 h_c + homoval_c + nadh_c 12042 or 13426 or 16323
3MOX4HOXPGALDOX 3m4hpga_c + h2o_c + nad_c --> 3mox4hoxm_c + 2.0 h_c + nadh_c 12042 or 13426 or 16323
3MOX4HOXPGALDOX_NADP 3m4hpga_c + h2o_c + nadp_c <=> 3mox4hoxm_c + 2.0 h_c + nadph_c 12042 or 13426 or 16323
4HOXPACDOX_NADP 4hoxpacd_c + h2o_c + nadp_c <=> 4hphac_c + 2.0 h_c + nadph_c 12042 or 13426 or 16323
5HOXINDACTOX 5hoxindact_c + h2o_c + nad_c --> 5hoxindoa_c + 2.0 h_c + nadh_c 12042 or 13426 or 15814 or 16323
ABUTD 4abutn_c + h2o_c + nad_c --> 4abut_c + 2.0 h_c + nadh_c 12042 or 13426 or 16323
ALDD19x_P h2o_c + nadp_c + pacald_c --> 2.0 h_c + nadph_c + pac_c 12042 or 13426 or 16323
ALDD19xr h2o_c + nad_c + pacald_c <=> 2.0 h_c + nadh_c + pac_c 12042 or 13426 or 16323
ALDD20x h2o_c + id3acald_c + nad_c --> 2.0 h_c + ind3ac_c + nadh_c 12042 or 13426 or 15814 or 16323
ALDD21 h2o_c + nad_c + pristanal_c --> 2.0 h_c + nadh_c + prist_c 16323
ALDD2x acald_c + h2o_c + nad_c --> ac_c + 2.0 h_c + nadh_c 12042 or 13426 or 15814 or 16323
ALDD2xm acald_m + h2o_m + nad_m --> ac_m + 2.0 h_m + nadh_m 12042 or 13426 or 16323
ALDD2y acald_c + h2o_c + nadp_c --> ac_c + 2.0 h_c + nadph_c 11650 or 12042 or 13426 or 14700 or 16323 or 8666
BAMPPALDOX bamppald_c + h2o_c + nad_c --> ala_B_c + 2.0 h_c + nadh_c 12042 or 13426 or 15814 or 16323
COALDDH conialdh_c + h2o_c + nad_c --> fer_c + 2.0 h_c + nadh_c 16323
GCALDD gcald_c + h2o_c + nad_c --> glyclt_c + 2.0 h_c + nadh_c 12042 or 13426 or 15814 or 16323
GLACO glac_c + 2.0 h2o_c + nad_c --> glcr_c + 3.0 h_c + nadh_c 12042 or 13426 or 16323
IMACTD h2o_c + im4act_c + nad_c --> 2.0 h_c + im4ac_c + nadh_c 12042 or 13426 or 15814 or 16323
LCADi h2o_c + lald__L_c + nad_c --> 2.0 h_c + lac__L_c + nadh_c 12042 or 13426 or 15814 or 16323
LCADi_D h2o_c + lald__D_c + nad_c --> 2.0 h_c + lac__D_c + nadh_c 12042 or 13426 or 15814 or 16323
MACOXO 3mldz_c + h2o_c + nad_c --> 3mlda_c + 2.0 h_c + nadh_c 12042 or 13426 or 16323
NABTNO h2o_c + n4abutn_c + nad_c --> 4aabutn_c + 2.0 h_c + nadh_c 12042 or 13426 or 15814 or 16323
PYLALDOX h2o_c + nad_c + pylald_c --> 2.0 h_c + nadh_c + peracd_c 12042 or 13426 or 15814 or 16323
yli_R0303 glyald_c + h_c + nadh_c <=> glyc_c + h2o_c + nad_c 12042 or 13426 or 16323
yli_R1508 glyald_c + h2o_c + nad_c --> glyc__R_c + h_c + nadh_c 12042 or 13426 or 16323

HDH h2o_c + histd_c + 2.0 nad_c --> 4.0 h_c + his__L_c + 2.0 nadh_c 11646
HDHh h2o_h + histd_h + 2.0 nad_h --> 3.0 h_h + his__L_h + 2.0 nadh_h 11646
HISTD h2o_c + histd_c + 2.0 nad_c --> 3.0 h_c + his__L_c + 2.0 nadh_c 11646
PRACHh h2o_h + prbamp_h --> prfp_h 11646
PRADPh h2o_h + prbatp_h --> 4.0 h_h + ppi_h + prbamp_h 11646
PRAMPC h2o_c + prbamp_c --> prfp_c 11646
PRATPP h2o_c + prbatp_c --> h_c + ppi_c + prbamp_c 11646

GUAD gua_c + h2o_c + h_c --> nh4_c + xan_c 9050 or 9708
GUAD_1 gua_c + h2o_c + 2.0 h_c --> nh4_c + xan_c 9050 or 9708

GUAD gua_c + h2o_c + h_c --> nh4_c + xan_c 9050 or 9708
GUAD_1 gua_c + h2o_c + 2.0 h_c --> nh4_c + xan_c 9050 or 9708

PANTS ala_B_c + atp_c + pant__R_c --> amp_c + h_c + pnto__R_c + ppi_c 10475
PBAL ala_B_c + atp_c + pant__R_c --> amp_c + 2.0 h_c + pnto__R_c + ppi_c 10475
PBALm ala_B_m + atp_m + pant__R_m --> amp_m + 3.0 h_m + pnto__R_m + ppi_m 10475

NADDP h2o_c + nad_c --> amp_c + 2.0 h_c + nmn_c 12434
NADDPp h2o_x + nad_x --> amp_x + 2.0 h_x + nmn_x 12434
yli_R1538 h2o_c + nad_c --> amp_c + nmn_c 12434
yli_R1539 dnad_c + h2o_c --> amp_c + nicrnt_c 12434

DNADDP dnad_c + h2o_c --> amp_c + 2.0 h_c + nicrnt_c 14638 or 15385
DNMPPA dhpmp_c + h2o_c --> dhnpt_c + pi_c 14615 or 14875 or 15385
DNTPPA_1 ahdt_c + h2o_c --> dhpmp_c + ppi_c 15385
NPH h2o_c + nad_c --> amp_c + 3.0 h_c + nmn_c 15385

AAMYL 14glucan_c --> malthx_c 11993
STARCH300DEGR2A 49.0 h2o_h + 250.0 pi_h + starch300_h --> 50.0 Glc_aD_h + 250.0 g1p_h (CRv4_Au5_s11_g2607_t1 and CRv4_Au5_s3_g11038_t1 and CRv4_Au5_s6_g12805_t1 and 11993 and 14256) or (CRv4_Au5_s11_g2607_t1 and CRv4_Au5_s3_g11038_t1 and CRv4_Au5_s6_g13538_t1 and 11993 and 14256) or (CRv4_Au5_s19_g8150_t1 and CRv4_Au5_s3_g11038_t1 and CRv4_Au5_s6_g12805_t1 and 11993 and 14256) or (CRv4_Au5_s19_g8150_t1 and CRv4_Au5_s3_g11038_t1 and CRv4_Au5_s6_g13538_t1 and 11993 and 14256)
STARCH300DEGR2B 49.0 h2o_h + 250.0 pi_h + starch300_h --> 250.0 g1p_h + 50.0 glc__bD_h (CRv4_Au5_s11_g2607_t1 and CRv4_Au5_s3_g11038_t1 and CRv4_Au5_s6_g12805_t1 and 11993 and 14256) or (CRv4_Au5_s11_g2607_t1 and CRv4_Au5_s3_g11038_t1 and CRv4_Au5_s6_g13538_t1 and 11993 and 14256) or (CRv4_Au5_s19_g8150_t1 and CRv4_Au5_s3_g11038_t1 and CRv4_Au5_s6_g12805_t1 and 11993 and 14256) or (CRv4_Au5_s19_g8150_t1 and CRv4_Au5_s3_g11038_t1 and CRv4_Au5_s6_g13538_t1 and 11993 and 14256)
STARCH300DEGRA 74.0 h2o_h + 225.0 pi_h + starch300_h --> 75.0 Glc_aD_h + 225.0 g1p_h (CRv4_Au5_s11_g2607_t1 and CRv4_Au5_s3_g11038_t1 and CRv4_Au5_s6_g12805_t1 and 11993 and 14256) or (CRv4_Au5_s11_g2607_t1 and CRv4_Au5_s3_g11038_t1 and CRv4_Au5_s6_g13538_t1 and 11993 and 14256) or (CRv4_Au5_s19_g8150_t1 and CRv4_Au5_s3_g11038_t1 and CRv4_Au5_s6_g12805_t1 and 11993 and 14256) or (CRv4_Au5_s19_g8150_t1 and CRv4_Au5_s3_g11038_t1 and CRv4_Au5_s6_g13538_t1 and 11993 and 14256)
STARCH300DEGRB 74.0 h2o_h + 225.0 pi_h + starch300_h --> 225.0 g1p_h + 75.0 glc__bD_h (CRv4_Au5_s11_g2607_t1 and CRv4_Au5_s3_g11038_t1 and CRv4_Au5_s6_g12805_t1 and 11993 and 14256) or (CRv4_Au5_s11_g2607_t1 and CRv4_Au5_s3_g11038_t1 and CRv4_Au5_s6_g13538_t1 and 11993 and 14256) or (CRv4_Au5_s19_g8150_t1 and CRv4_Au5_s3_g11038_t1 and CRv4_Au5_s6_g12805_t1 and 11993 and 14256) or (CRv4_Au5_s19_g8150_t1 and CRv4_Au5_s3_g11038_t1 and CRv4_Au5_s6_g13538_t1 and 11993 and 14256)

GLCP glycogen_c + pi_c --> g1p_c 14256
GLCP2 bglycogen_c + pi_c --> g1p_c 14256
GLPASE1 glygn2_c + 3.0 pi_c --> dxtrn_c + 3.0 g1p_c 14256
GLPASE2 glygn3_c + 7.0 h2o_c --> Tyr_ggn_c + 7.0 glc__D_c 14256
MLTP1 maltpt_c + pi_c <=> g1p_c + maltttr_c 14256
MLTP2 malthx_c + pi_c <=> g1p_c + maltpt_c 14256
MLTP3 malthp_c + pi_c <=> g1p_c + malthx_c 14256
STARCH300DEGR2A 49.0 h2o_h + 250.0 pi_h + starch300_h --> 50.0 Glc_aD_h + 250.0 g1p_h (CRv4_Au5_s11_g2607_t1 and CRv4_Au5_s3_g11038_t1 and CRv4_Au5_s6_g12805_t1 and 11993 and 14256) or (CRv4_Au5_s11_g2607_t1 and CRv4_Au5_s3_g11038_t1 and CRv4_Au5_s6_g13538_t1 and 11993 and 14256) or (CRv4_Au5_s19_g8150_t1 and CRv4_Au5_s3_g11038_t1 and CRv4_Au5_s6_g12805_t1 and 11993 and 14256) or (CRv4_Au5_s19_g8150_t1 and CRv4_Au5_s3_g11038_t1 and CRv4_Au5_s6_g13538_t1 and 11993 and 14256)
STARCH300DEGR2B 49.0 h2o_h + 250.0 pi_h + starch300_h --> 250.0 g1p_h + 50.0 glc__bD_h (CRv4_Au5_s11_g2607_t1 and CRv4_Au5_s3_g11038_t1 and CRv4_Au5_s6_g12805_t1 and 11993 and 14256) or (CRv4_Au5_s11_g2607_t1 and CRv4_Au5_s3_g11038_t1 and CRv4_Au5_s6_g13538_t1 and 11993 and 14256) or (CRv4_Au5_s19_g8150_t1 and CRv4_Au5_s3_g11038_t1 and CRv4_Au5_s6_g12805_t1 and 11993 and 14256) or (CRv4_Au5_s19_g8150_t1 and CRv4_Au5_s3_g11038_t1 and CRv4_Au5_s6_g13538_t1 and 11993 and 14256)
STARCH300DEGRA 74.0 h2o_h + 225.0 pi_h + starch300_h --> 75.0 Glc_aD_h + 225.0 g1p_h (CRv4_Au5_s11_g2607_t1 and CRv4_Au5_s3_g11038_t1 and CRv4_Au5_s6_g12805_t1 and 11993 and 14256) or (CRv4_Au5_s11_g2607_t1 and CRv4_Au5_s3_g11038_t1 and CRv4_Au5_s6_g13538_t1 and 11993 and 14256) or (CRv4_Au5_s19_g8150_t1 and CRv4_Au5_s3_g11038_t1 and CRv4_Au5_s6_g12805_t1 and 11993 and 14256) or (CRv4_Au5_s19_g8150_t1 and CRv4_Au5_s3_g11038_t1 and CRv4_Au5_s6_g13538_t1 and 11993 and 14256)
STARCH300DEGRB 74.0 h2o_h + 225.0 pi_h + starch300_h --> 225.0 g1p_h + 75.0 glc__bD_h (CRv4_Au5_s11_g2607_t1 and CRv4_Au5_s3_g11038_t1 and CRv4_Au5_s6_g12805_t1 and 11993 and 14256) or (CRv4_Au5_s11_g2607_t1 and CRv4_Au5_s3_g11038_t1 and CRv4_Au5_s6_g13538_t1 and 11993 and 14256) or (CRv4_Au5_s19_g8150_t1 and CRv4_Au5_s3_g11038_t1 and CRv4_Au5_s6_g12805_t1 and 11993 and 14256) or (CRv4_Au5_s19_g8150_t1 and CRv4_Au5_s3_g11038_t1 and CRv4_Au5_s6_g13538_t1 and 11993 and 14256)
yli_R0732 pi_c + starch_c --> g1p_c 14256

ASP1DC asp__L_c + h_c --> ala_B_c + co2_c 10722 or 9434 or 9435
GLUDC glu__L_c + h_c --> 4abut_c + co2_c 10722 or 9434 or 9435
HDC 2.0 h_c + his__L_c --> co2_c + hista_c 10722 or 9434 or 9435
LCYSTCBOXL Lcyst_c + h_c --> co2_c + taur_c 10722 or 9434 or 9435
SERDC h_c + ser__L_c --> co2_c + etha_c 10722 or 9434 or 9435
yli_R0777 3sala_c + h_c --> co2_c + yli_M00487_c 10722 or 9434 or 9435

ASP1DC asp__L_c + h_c --> ala_B_c + co2_c 10722 or 9434 or 9435
GLUDC glu__L_c + h_c --> 4abut_c + co2_c 10722 or 9434 or 9435
HDC 2.0 h_c + his__L_c --> co2_c + hista_c 10722 or 9434 or 9435
LCYSTCBOXL Lcyst_c + h_c --> co2_c + taur_c 10722 or 9434 or 9435
SERDC h_c + ser__L_c --> co2_c + etha_c 10722 or 9434 or 9435
yli_R0777 3sala_c + h_c --> co2_c + yli_M00487_c 10722 or 9434 or 9435

ASP1DC asp__L_c + h_c --> ala_B_c + co2_c 10722 or 9434 or 9435
GLUDC glu__L_c + h_c --> 4abut_c + co2_c 10722 or 9434 or 9435
HDC 2.0 h_c + his__L_c --> co2_c + hista_c 10722 or 9434 or 9435
LCYSTCBOXL Lcyst_c + h_c --> co2_c + taur_c 10722 or 9434 or 9435
SERDC h_c + ser__L_c --> co2_c + etha_c 10722 or 9434 or 9435
yli_R0777 3sala_c + h_c --> co2_c + yli_M00487_c 10722 or 9434 or 9435

3HLYTCL 34dhphe_c + h_c --> co2_c + dopa_c 10104
3HXKYNDCL hLkynr_c + h_c --> 3hxkynam_c + co2_c 10104
5HLTDL 5htrp_c + h_c --> co2_c + srtn_c 10104
5HXKYNDCL 5hxkyn_c + h_c --> 5hxkynam_c + co2_c 10104
HISDC h_c + his__L_c --> co2_c + hista_c 10104
LTDCL h_c + trp__L_c --> co2_c + trypta_c 10104
PHYCBOXL h_c + phe__L_c --> co2_c + peamn_c 10104
TYRCBOX h_c + tyr__L_c --> co2_c + tym_c 10104

CHOLSabc atp_c + chols_p + h2o_c --> adp_c + chols_c + h_c + pi_c (PP_0076 and 14119) or (PP_0076 and 15736) or (PP_0868 and PP_0869 and PP_0870) or (PP_0868 and PP_0870 and PP_0871)
CLHCOtex cl_e + 2.0 hco3_c --> cl_c + 2.0 hco3_e 14119 or 15736
OXAHCOtex 2.0 hco3_c + oxa_e --> 2.0 hco3_e + oxa_c 14119 or 15736
SO4HCOtex 2.0 hco3_c + so4_e --> 2.0 hco3_e + so4_c 14119 or 15736
SO4t2 h_e + so4_e <=> h_c + so4_c 14119 or 15736
SO4ti so4_e --> so4_c 14119 or 15736 or 16682 or (14119 and 16682) or (15736 and 16682)

CHOLSabc atp_c + chols_p + h2o_c --> adp_c + chols_c + h_c + pi_c (PP_0076 and 14119) or (PP_0076 and 15736) or (PP_0868 and PP_0869 and PP_0870) or (PP_0868 and PP_0870 and PP_0871)
CLHCOtex cl_e + 2.0 hco3_c --> cl_c + 2.0 hco3_e 14119 or 15736
OXAHCOtex 2.0 hco3_c + oxa_e --> 2.0 hco3_e + oxa_c 14119 or 15736
SO4HCOtex 2.0 hco3_c + so4_e --> 2.0 hco3_e + so4_c 14119 or 15736
SO4t2 h_e + so4_e <=> h_c + so4_c 14119 or 15736
SO4ti so4_e --> so4_c 14119 or 15736 or 16682 or (14119 and 16682) or (15736 and 16682)

CLFORtex2 2.0 cl_e + for_c --> 2.0 cl_c + for_e 16202
CLHCO3tex2 2.0 cl_e + hco3_c --> 2.0 cl_c + hco3_e 16202
CLOHtex2 2.0 cl_e + oh1_c --> 2.0 cl_c + oh1_e 16202
CLOXAtex2 2.0 cl_e + oxa_c --> 2.0 cl_c + oxa_e 16202
SO4CLtex2 cl_c + 2.0 so4_e --> cl_e + 2.0 so4_c 16202
SO4OXAtex2 oxa_c + 2.0 so4_e --> oxa_e + 2.0 so4_c 16202
```

13082 extr 23, golg 2 K01183: E3.2.1.14; chitinase GMQ\*  
ARGSS\_1 incorrect stoich  
PRAGSr, PRAIS correct (air\_c -2 in BiGG, but -1 in Metacyc)  
AIRCr correct (AIRC2+AIRC3 bacteria, PRAIC with air\_c -1)

NMA1,NMA2 10430 cyto 13.5, cyto\_mito 10.5, nucl 7, mito 6.5 K06210: NMNAT; nicotinamide mononucleotide adenylyltransferase SAS *NPP1,NPP2 14638 cyto 8.5, extr 7, mito 6, cyto\_nucl 5, plas 2 KOG2645: Type I phosphodiesterase/nucleotide pyrophosphatase EGV*  
15385 cyto\_mito 7, mito 6.5, cyto 6.5, pero 5, nucl 4, extr 2, cysk 2 K03574: mutT, NUDT15, MTH2; 8-oxo-dGTP diphosphatase LVL\*

NMNAT/NMNATm/NMNATn, NNATr/NNATm/NNATn correct  
Change NNATr to NNAT, and make it irreversible (deltaG -54 kcal/mol in MetaCyc)

GLYALDDr is not clear -> remove for now

12434 mito 13.5, cyto\_mito 10.833, cyto 7, cyto\_nucl 5.333, pero 3 K03426: E3.6.1.22, NUDT12, nudC; NAD+ diphosphatase SKM\*

14119 plas 22, E.R. 4 K14708: SLC26A11; solute carrier family 26 (sodium-independent sulfate anion transporter), member 11 TKA *15736 plas 25 K14708: SLC26A11; solute carrier family 26 (sodium-independent sulfate anion transporter), member 11 DDW*  
16682 plas 22, E.R. 3 K03321: TC.SULP; sulfate permease, SulP family ASL\*

In [75]:

```
temp = ['yli_R0034','ARGSS_1','FGFTh','FPGFTh','PPRGL','PPRGLh','AIRC2','AIRC3','PRAIC','PRAICh','ANNAT','ANNATn',
        'NNATr_copy1','NNATr_copy2','GLYALDDr','yli_R0303','yli_R1508','HDH','HDHh','PRACHh','PRADPh','GUAD_1','PBAL',
        'PBALm','NADDP','yli_R1538','yli_R1539','NPH','DNTPPA_1','STARCH300DEGR2A','STARCH300DEGR2B','STARCH300DEGRA',
        'STARCH300DEGRB','HDC','yli_R0777','CHOLSabc']
model.remove_reactions(temp, remove_orphans=True)

model.reactions.get_by_id('NNATr').id = 'NNAT'
model.reactions.get_by_id('NNAT').lower_bound = 0.0

r = sce.reactions.get_by_id('DNTPPA').copy()
r.gene_reaction_rule = '15385'
model.add_reactions([r])
r = hsa.reactions.get_by_id('3SALACBOXL').copy() # why is this not picked up? check
r.gene_reaction_rule = '10722 or 9434 or 9435'
model.add_reactions([r])

model.reactions.get_by_id('SO4ti').gene_reaction_rule = '14119 or 15736 or 16682'
```

#### Fix fatty acid and sterol in the next step¶

In [76]:

```
print(len(model.genes))
print(len(model.reactions))
print(len(model.metabolites))
model
```

```
1332
3264
3290
```

Out[76]:

|  |  |
| --- | --- |
| **Name** | R. toruloides |
| **Memory address** | 0x01027435ac8 |
| **Number of metabolites** | 3290 |
| **Number of reactions** | 3264 |
| **Number of groups** | 0 |
| **Objective expression** | 0 |
| **Compartments** | c, x, m, e, r, v, n, g, p, h, s, f, l |

In [77]:

```
for x in sorted(model.genes, key=lambda x: x.id):
    if not x.reactions:
        print(x)
print()
for x in sorted(model.metabolites, key=lambda x: x.id):
    if not x.reactions:
        print(x)
```

```
12022
15595
4833_AT1
9153
CRv4_Au5_s6_g12448_t1
GCLM
Gclm
PDHX
PHATRDRAFT_34976
PHATRDRAFT_36641
PHATRDRAFT_37658
PHATRDRAFT_46880
PP_1986
PP_5098
Pdhx
YCR083W
YDR453C
YGR180C
b0071
b3551

2hhxdal_c
amob_c
dann_c
hxdcal_c
psph1p_c
psphings_c
sph1p_c
```

In [78]:

```
cobra.manipulation.remove_genes(model, [x for x in model.genes if not x.reactions])
model.remove_metabolites([x for x in model.metabolites if not x.reactions])
```

In [79]:

```
print(len(model.genes))
print(len(model.reactions))
print(len(model.metabolites))
model
```

```
1312
3264
3283
```

Out[79]:

|  |  |
| --- | --- |
| **Name** | R. toruloides |
| **Memory address** | 0x01027435ac8 |
| **Number of metabolites** | 3283 |
| **Number of reactions** | 3264 |
| **Number of groups** | 0 |
| **Objective expression** | 0 |
| **Compartments** | c, x, m, e, r, v, n, g, p, h, s, f, l |

In [80]:

```
for x in sorted(model.genes, key=lambda x: x.id):
    if not x.reactions:
        print(x)
print()
for x in sorted(model.metabolites, key=lambda x: x.id):
    if not x.reactions:
        print(x)
```

```

```

In [81]:

```
cobra.io.save_json_model(model, "IFO0880_GPR_1b.json")
```

In [82]:

```
model_old = cobra.io.load_json_model("IFO0880_GPR_1a.json")
model_new = cobra.io.load_json_model("IFO0880_GPR_1b.json")
```

In [83]:

```
print('Removed reactions\n')
for r in sorted(model_old.reactions, key=lambda x: x.id):
    if r not in model_new.reactions:
        print(r)
```

```
Removed reactions

3DSPHR: 3dsphgn_c + h_c + nadph_c --> nadp_c + sphgn_c
4HTHRA: 4hthr_c <=> gcald_c + gly_c
4HTHRK: 4hthr_c + atp_c --> adp_c + h_c + phthr_c
4HTHRS: h2o_c + phthr_c --> 4hthr_c + pi_c
ACAS_2ahbut: 2ahethmpp_h + 2obut_h --> 2ahbut_h + thmpp_h
ACHBS: 2obut_c + h_c + pyr_c --> 2ahbut_c + co2_c
ACLS: h_c + 2.0 pyr_c --> alac__S_c + co2_c
ACSERL: acser_c + seln_c <=> ac_c + 2.0 h_c + selcys_c
ACSERLh: acser_h + seln_h <=> ac_h + 2.0 h_h + selcys_h
ACSERLm: acser_m + seln_m <=> ac_m + 2.0 h_m + selcys_m
ACSERSULL: acser_c + tsul_c --> ac_c + h_c + scys__L_c
ACSERSULLh: acser_h + tsul_h --> ac_h + h_h + scys__L_h
ACSERSULLm: acser_m + tsul_m --> ac_m + h_m + scys__L_m
ADK1m: amp_m + atp_m <=> 2.0 adp_m
ADK3: amp_c + gtp_c <=> adp_c + gdp_c
ADK4: amp_c + itp_c <=> adp_c + idp_c
ADNK1m: adn_m + atp_m --> adp_m + amp_m + h_m
AFAT: atp_c + fmn_c + 2.0 h_c --> fad_c + ppi_c
AHAL: achms_h + trdrd_h + tsul_h --> ac_h + h_h + hcys__L_h + so3_h + trdox_h
AHSERL4: acser_c + trdrd_c + tsul_c --> ac_c + cys__L_c + h_c + so3_c + trdox_c
AIRC2: air_c + atp_c + hco3_c --> 5caiz_c + adp_c + h_c + pi_c
AIRC3: 5aizc_c <=> 5caiz_c
AKGDH: akg_c + coa_c + nad_c --> co2_c + nadh_c + succoa_c
AKGDHe2r: coa_m + h_m + sdhlam_m <=> dhlam_m + succoa_m
AKGDa: akg_c + h_c + lpam_c <=> co2_c + sdhlam_c
AKGDam: akg_m + h_m + lpam_m <=> co2_m + sdhlam_m
AKGDb: coa_c + sdhlam_c <=> dhlam_c + succoa_c
AKGDbm: coa_m + sdhlam_m --> dhlam_m + succoa_m
ALATA_D2: ala__D_c + pydx5p_c --> pyam5p_c + pyr_c
ALATA_L2: ala__L_c + pydx5p_c --> pyam5p_c + pyr_c
ALDD2x_copy1: acald_c + h2o_c + nad_c --> ac_c + 2.0 h_c + nadh_c
ALPHm: allphn_m + h2o_m + 3.0 h_m --> 2.0 co2_m + 2.0 nh4_m
AMAOTr: 8aonn_c + amet_c <=> amob_c + dann_c
ANNAT: atp_c + 2.0 h_c + nmn_c <=> nad_c + ppi_c
ANNATn: atp_n + 2.0 h_n + nmn_n <=> nad_n + ppi_n
AOXSr2: ala__L_c + pimACP_c --> 8aonn_c + ACP_c + co2_c
APLh: 2ahethmpp_h + pyr_h --> alac__S_h + thmpp_h
APLm: 2ahethmpp_m + pyr_m --> alac__S_m + thmpp_m
APPAT: atp_c + 2.0 h_c + pan4p_c <=> dpcoa_c + ppi_c
ARD: dhmtp_c + o2_c --> 2kmb_c + for_c + 2.0 h_c
ARD1: dhmtp_c + o2_c --> co_c + for_c + h_c + mtpp_c
ARGSS_1: asp__L_c + atp_c + citr__L_c --> amp_c + argsuc_c + 2.0 h_c + ppi_c
ASPK_1: asp__L_h + atp_h --> 4pasp_h + adp_h
ATAMh: amp_h + atp_h --> 2.0 adp_h
ATDAMh: atp_h + damp_h --> adp_h + dadp_h
ATDAMm: atp_m + damp_m --> adp_m + dadp_m
BTS5: 2fe2s_c + amet_c + dtbt_c --> 2fe1s_c + btn_c + dad_5_c + h_c + met__L_c
BTSr: dtbt_c + s_c <=> btn_c + 2.0 h_c
CBL: cyst__L_h + h2o_h --> hcys__L_h + nh4_h + pyr_h
CERH124_copy1: cer1_24_c + h_c + nadph_c + o2_c --> cer2_24_c + h2o_c + nadp_c
CERH124_copy2: cer1_24_c + h_c + nadph_c + o2_c --> cer2_24_c + h2o_c + nadp_c
CERH126_copy1: cer1_26_c + h_c + nadph_c + o2_c --> cer2_26_c + h2o_c + nadp_c
CERH126_copy2: cer1_26_c + h_c + nadph_c + o2_c --> cer2_26_c + h2o_c + nadp_c
CERS124: sphgn_c + ttccoa_c --> cer1_24_c + coa_c + h_c
CERS126: hexccoa_c + sphgn_c --> cer1_26_c + coa_c + h_c
CERS224: psphings_c + ttccoa_c --> cer2_24_c + coa_c + h_c
CERS226: hexccoa_c + psphings_c --> cer2_26_c + coa_c + h_c
CERS324: cer2_24_c + h_c + nadph_c + o2_c --> cer3_24_c + h2o_c + nadp_c
CERS326: cer2_26_c + h_c + nadph_c + o2_c --> cer3_26_c + h2o_c + nadp_c
CHOLS_ex: chols_e <=> chols_p
CHOLSabc: atp_c + chols_p + h2o_c --> adp_c + chols_c + h_c + pi_c
CTINBL: cysi__L_h + h2o_h --> nh4_h + pyr_h + thcys_h
CYS: h2s_c + ser__L_c --> cys__L_c + h2o_c
CYSS_1: acser_h + h2s_h --> ac_h + cys__L_h
CYSS_trdrd: acser_h + trdrd_h + tsul_h --> ac_h + cys__L_h + h_h + so3_h + trdox_h
CYSTBL: h2s_h + h_h + nh4_h + pyr_h --> cys__L_h + h2o_h
CYSTLp: cyst__L_x + h2o_x --> hcys__L_x + nh4_x + pyr_x
DASCBR: dhdascb_c + h_c + nadph_c --> ascb__L_c + nadp_c
DHRT_2mbcoa: 2mbdhl_m + coa_m --> 2mbcoa_m + dhlam_m
DHRT_ibcoa: 2mpdhl_m + coa_m --> dhlam_m + ibcoa_m
DHRT_ivcoa: 3mbdhl_m + coa_m --> dhlam_m + ivcoa_m
DKMPPD2: dkmpp_c + 3.0 h2o_c --> 2kmb_c + for_c + 6.0 h_c + pi_c
DNTPPA_1: ahdt_c + h2o_c --> dhpmp_c + ppi_c
DPCOAKm: atp_m + dpcoa_m --> adp_m + coa_m + h_m
DSBDR: dsbdox_c + trdrd_c --> dsbdrd_c + trdox_c
FGFTh: fgam_h + 3.0 h_h + thf_h --> gar_h + h2o_h + methf_h
FOMETRi: 5fthf_c + h_c --> h2o_c + methf_c
FPGFTh: 10fthf_h + gar_h <=> fgam_h + h_h + thf_h
FTHFCL: 5fthf_c + atp_c --> adp_c + methf_c + pi_c
GCC2am: gly_m + h_m + lpam_m <=> alpam_m + co2_m
GCC2bim: alpam_m + thf_m --> dhlam_m + mlthf_m + nh4_m
GCC2cm: dhlam_m + nad_m <=> h_m + lpam_m + nadh_m
GCC2cm_copy1: dhlam_m + nad_m <=> h_m + lpam_m + nadh_m
GCC2cm_copy2: dhlam_m + nad_m --> h_m + lpam_m + nadh_m
GCCam: gly_m + h_m + lpro_m <=> alpro_m + co2_m
GCCbim: alpro_m + thf_m --> dhlpro_m + mlthf_m + nh4_m
GCCcm: dhlpro_m + nad_m <=> h_m + lpro_m + nadh_m
GDHm: glu__L_m + h2o_m + nad_m <=> akg_m + h_m + nadh_m + nh4_m
GDR: gthox_c + h_c + nadh_c --> 2.0 gthrd_c + nad_c
GDR_nadp_h: gthox_h + h_h + nadph_h --> 2.0 gthrd_h + nadp_h
GDRh: gthox_h + h_h + nadh_h --> 2.0 gthrd_h + nad_h
GDRm: gthox_m + h_m + nadh_m --> 2.0 gthrd_m + nad_m
GHMT2rm: ser__L_m + thf_m <=> gly_m + h2o_m + mlthf_m
GHMT3: 3htmelys_c + h_c --> 4tmeabut_c + gly_c
GHMT3m: 3htmelys_m + h_m --> 4tmeabut_m + gly_m
GLCOASYNT: S_gtrdhdlp_c + coa_c --> dhlam_c + glutcoa_c
GLUDym: glu__L_m + h2o_m + nadp_m <=> akg_m + h_m + nadph_m + nh4_m
GLUS: akg_h + gln__L_h + h_h + nadh_h --> 2.0 glu__L_h + nad_h
GLUS_ferr: akg_h + 2.0 fdxrd_h + gln__L_h --> 2.0 fdxox_h + 2.0 glu__L_h + 2.0 h_h
GLUS_nadph: akg_h + gln__L_h + h_h + nadph_h --> 2.0 glu__L_h + nadp_h
GLUSy: akg_c + gln__L_c + h_c + nadph_c --> 2.0 glu__L_c + nadp_c
GLYALDDr: glyald_c + h2o_c + nad_c <=> glyc__R_c + 2.0 h_c + nadh_c
GLYATx: accoa_x + gly_x <=> 2aobut_x + coa_x + h_x
GLYCL: gly_c + nad_c + thf_c --> co2_c + mlthf_c + nadh_c + nh4_c
GLYCL_2: co2_c + mlthf_c + nadh_c + nh4_c --> gly_c + nad_c + thf_c
GLYDHD: gly_m + lpro_m --> alpro_m + co2_m
GTHPm: 2.0 gthrd_m + h2o2_m <=> gthox_m + 2.0 h2o_m
GTPCI_2: gtp_c + h2o_c --> ahdt_c + for_c + 2.0 h_c
GUAD_1: gua_c + h2o_c + 2.0 h_c --> nh4_c + xan_c
HDC: 2.0 h_c + his__L_c --> co2_c + hista_c
HDH: h2o_c + histd_c + 2.0 nad_c --> 4.0 h_c + his__L_c + 2.0 nadh_c
HDHh: h2o_h + histd_h + 2.0 nad_h --> 3.0 h_h + his__L_h + 2.0 nadh_h
HSDH: aspsa_h + h_h + nadph_h --> hom__L_h + nadp_h
HSK_1: atp_h + hom__L_h --> adp_h + h_h + phom_h
KAT1: aacoa_c + coa_c --> 2.0 accoa_c
LYSMTF1n: amet_n + peplys_n --> Nmelys_n + ahcys_n
LYSMTF2n: Nmelys_n + amet_n --> Ndmelys_n + ahcys_n
LYSMTF3n: Ndmelys_n + amet_n --> Ntmelys_n + ahcys_n
METB1: achms_c + cys__L_c --> ac_c + cyst__L_c + h_c
MHPGLUT: hcys__L_c + mhpglu_c --> hpglu_c + met__L_c
MOD: 3mob_m + h_m + thmpp_m --> 2mhop_m + co2_m
MOD_2mbdhl: 2mhob_m + lpam_m --> 2mbdhl_m + thmpp_m
MOD_2mhop: 2mhop_m + lpam_m --> 2mpdhl_m + thmpp_m
MOD_3mhtpp: 3mhtpp_m + lpam_m --> 3mbdhl_m + thmpp_m
MOD_3mop: 3mop_m + h_m + thmpp_m --> 2mhob_m + co2_m
MOD_4mop: 4mop_m + h_m + thmpp_m --> 3mhtpp_m + co2_m
MS: h_m + hcys__L_m + mhpglu_m --> hpglu_m + met__L_m
MTAM: 5fthf_m + 2.0 h_m --> h2o_m + methf_m
MTAM_nh4: alpro_m + h_m + thf_m <=> dhlpro_m + mlthf_m + nh4_m
MTRK: 5mtr_c + atp_c --> 5mdr1p_c + adp_c + h_c
NADDP: h2o_c + nad_c --> amp_c + 2.0 h_c + nmn_c
NDPK10n: atp_n + didp_n <=> adp_n + ditp_n
NDPK1n: atp_n + gdp_n <=> adp_n + gtp_n
NDPK2n: atp_n + udp_n <=> adp_n + utp_n
NDPK3n: atp_n + cdp_n <=> adp_n + ctp_n
NDPK4n: atp_n + dtdp_n <=> adp_n + dttp_n
NDPK5n: atp_n + dgdp_n <=> adp_n + dgtp_n
NDPK6n: atp_n + dudp_n <=> adp_n + dutp_n
NDPK7n: atp_n + dcdp_n <=> adp_n + dctp_n
NDPK8n: atp_n + dadp_n <=> adp_n + datp_n
NDPK9n: atp_n + idp_n <=> adp_n + itp_n
NNATr: atp_c + h_c + nicrnt_c <=> dnad_c + ppi_c
NNATr_copy1: atp_c + h_c + nicrnt_c --> dnad_c + ppi_c
NNATr_copy2: atp_c + h_c + nicrnt_c <=> dnad_c + ppi_c
NPH: h2o_c + nad_c --> amp_c + 3.0 h_c + nmn_c
OIVD1r: 4mop_c + coa_c + nad_c <=> co2_c + ivcoa_c + nadh_c
OIVD2: 3mob_c + coa_c + nad_c --> co2_c + ibcoa_c + nadh_c
OIVD3: 3mop_c + coa_c + nad_c --> 2mbcoa_c + co2_c + nadh_c
OXOADLR: 2oxoadp_c + h_c + lpam_c --> S_gtrdhdlp_c + co2_c
PAPSPAPthr: pap_c + paps_h <=> pap_h + paps_c
PAPSR2: grxrd_c + paps_c --> grxox_c + 2.0 h_c + pap_c + so3_c
PBAL: ala_B_c + atp_c + pant__R_c --> amp_c + 2.0 h_c + pnto__R_c + ppi_c
PBALm: ala_B_m + atp_m + pant__R_m --> amp_m + 3.0 h_m + pnto__R_m + ppi_m
PDCm: 2ahethmpp_m --> acald_m + thmpp_m
PDH: coa_c + nad_c + pyr_c --> accoa_c + co2_c + nadh_c
PDHam1hi: h_h + pyr_h + thmpp_h --> 2ahethmpp_h + co2_h
PDHam1mi: h_m + pyr_m + thmpp_m --> 2ahethmpp_m + co2_m
PDHam2hi: 2ahethmpp_h + lpam_h --> adhlam_h + thmpp_h
PDHam2mi: 2ahethmpp_m + lpam_m --> adhlam_m + thmpp_m
PDHcr: dhlam_c + nad_c <=> h_c + lpam_c + nadh_c
PDHe2r: adhlam_m + coa_m <=> accoa_m + dhlam_m
PLYSPSer: Ntmelys_r + h2o_r --> pepslys_r + tmlys_r
PPATDh: h_h + 2.0 pyr_h --> alac__S_h + co2_h
PPRGL: atp_c + gly_c + pram_c --> adp_c + gar_c + 2.0 h_c + pi_c
PPRGLh: atp_h + gly_h + pram_h --> adp_h + gar_h + h_h + pi_h
PRACHh: h2o_h + prbamp_h --> prfp_h
PRADPh: h2o_h + prbatp_h --> 4.0 h_h + ppi_h + prbamp_h
PRAIC: air_c + co2_c <=> 5aizc_c + 2.0 h_c
PRAICh: air_h + co2_h <=> 5aizc_h + h_h
PSPHPL: psph1p_c --> 2hhxdal_c + ethamp_c
PSPHS: h_c + nadph_c + o2_c + sphgn_c --> h2o_c + nadp_c + psphings_c
PTPATim: atp_m + h_m + pan4p_m --> dpcoa_m + ppi_m
PYRDC_1: h_m + pyr_m --> acald_m + co2_m
RNDR1b: adp_c + grxrd_c --> dadp_c + grxox_c + h2o_c
RNDR2b: gdp_c + grxrd_c --> dgdp_c + grxox_c + h2o_c
RNDR3b: cdp_c + grxrd_c --> dcdp_c + grxox_c + h2o_c
RNDR4b: grxrd_c + udp_c --> dudp_c + grxox_c + h2o_c
RNTR1: atp_c + trdrd_c --> datp_c + h2o_c + trdox_c
RNTR2: gtp_c + trdrd_c --> dgtp_c + h2o_c + trdox_c
RNTR3: ctp_c + trdrd_c --> dctp_c + h2o_c + trdox_c
RNTR4: trdrd_c + utp_c --> dutp_c + h2o_c + trdox_c
SBPP1: h2o_c + sph1p_c --> pi_c + sphgn_c
SEAHCYSHYD: h2o_c + seahcys_c --> adn_c + selhcys_c
SEAHCYSHYD_1: h2o_c + seahcys_c <=> adn_c + h_c + selhcys_c
SELCYSTGL: h2o_c + selcyst_c --> 2obut_c + nh4_c + selcys_c
SELCYSTL: h2o_c + selcyst_c --> h_c + nh4_c + pyr_c + selhcys_c
SELCYSTLh: h2o_h + selcyst_h --> h_h + nh4_h + pyr_h + selhcys_h
SELCYSTS: selhcys_c + ser__L_c --> h2o_c + selcyst_c
SERPT: h_c + pmtcoa_c + ser__L_c --> 3dsphgn_c + co2_c + coa_c
SGOR: fad_c + sphgn_c <=> fadh2_c + sphings_c
SGPL11r: sph1p_r --> ethamp_r + hxdcal_r
SGPL12r: h2o_r + sphs1p_r --> ethamp_r + h_r + hdca_r
SGPL13: sphs1p_c --> ethamp_c + hxdceal_c
SHSL2r: h2s_c + suchms_c <=> h_c + hcys__L_c + succ_c
SHSL4r: h2o_c + suchms_c <=> 2obut_c + h_c + nh4_c + succ_c
SLCBK1: atp_c + sphgn_c --> adp_c + h_c + sph1p_c
SLCBK2: atp_c + psphings_c --> adp_c + h_c + psph1p_c
SLCYSS: acser_c + tsul_c --> ac_c + scys__L_c
SPHK21c: atp_c + sphings_c --> adp_c + h_c + sphs1p_c
SPHPL: sph1p_c --> ethamp_c + hxdcal_c
STARCH300DEGR2A: 49.0 h2o_h + 250.0 pi_h + starch300_h --> 50.0 Glc_aD_h + 250.0 g1p_h
STARCH300DEGR2B: 49.0 h2o_h + 250.0 pi_h + starch300_h --> 250.0 g1p_h + 50.0 glc__bD_h
STARCH300DEGRA: 74.0 h2o_h + 225.0 pi_h + starch300_h --> 75.0 Glc_aD_h + 225.0 g1p_h
STARCH300DEGRB: 74.0 h2o_h + 225.0 pi_h + starch300_h --> 225.0 g1p_h + 75.0 glc__bD_h
TDSRh: h_h + nadph_h + trdox_h --> nadp_h + trdrd_h
THFAT: h2o_c + methf_c --> 5fthf_c + h_c
THFATm: h2o_m + methf_m --> 5fthf_m + h_m
THRA_1: thr__L_h <=> acald_h + gly_h
THRS_1: h2o_h + phom_h --> pi_h + thr__L_h
TMLYSOX: akg_c + o2_c + tmlys_c --> 3htmelys_c + co2_c + succ_c
URCB: atp_c + hco3_c + urea_c --> adp_c + allphn_c + 2.0 h_c + pi_c
URCBm: atp_m + hco3_m + urea_m --> adp_m + allphn_m + 2.0 h_m + pi_m
yli_R0002: akg_c + gln__L_c + h_c + nadph_c --> glu__L_c + nadp_c
yli_R0034: chtn_c + h2o_c --> acgam_c
yli_R0094: achms_c + h_c + trdrd_c + tsul_c --> ac_c + hcys__L_c + so3_c + trdox_c
yli_R0291: gthox_c + h_c + nadph_c --> gthrd_c + nadp_c
yli_R0303: glyald_c + h_c + nadh_c <=> glyc_c + h2o_c + nad_c
yli_R0357: 4.0 h_c + pyr_c + thmpp_c --> 2ahethmpp_c + co2_c
yli_R0364: 2ahethmpp_c --> acald_c + 3.0 h_c + thmpp_c
yli_R0374: accoa_m + yli_M03934_m <=> coa_m + yli_M04008_m
yli_R0377: 2ahethmpp_m + yli_M03933_m --> 3.0 h_c + thmpp_m + yli_M04008_m
yli_R0381: nad_m + yli_M03934_m --> h_m + nadh_m + yli_M03933_m
yli_R0428: 2oxoadp_c + coa_c + nad_c --> co2_c + glutcoa_c + nadh_c
yli_R0698: sphgn_c + yli_M04597_c --> coa_c + h_c + yli_M05742_c
yli_R0699: sphgn_c + yli_M04095_c --> coa_c + h_c + yli_M05741_c
yli_R0700: psphings_c + yli_M04095_c --> coa_c + h_c + yli_M05748_c
yli_R0701: psphings_c + yli_M04597_c --> coa_c + h_c + yli_M05749_c
yli_R0702: h_c + nadph_c + o2_c + yli_M05742_c --> h2o_c + nadp_c + yli_M05749_c
yli_R0703: h_c + nadph_c + o2_c + yli_M05741_c --> h2o_c + nadp_c + yli_M05748_c
yli_R0704: h_c + nadph_c + o2_c + yli_M05749_c --> h2o_c + nadp_c + yli_M05725_c
yli_R0705: h_c + nadph_c + o2_c + yli_M05748_c --> h2o_c + nadp_c + yli_M05724_c
yli_R0777: 3sala_c + h_c --> co2_c + yli_M00487_c
yli_R0784: succoa_m + yli_M03934_m <=> coa_m + yli_M04007_m
yli_R0788: akg_m + h_m + yli_M03933_m --> co2_m + yli_M04007_m
yli_R0848: 4.0 h_m + pyr_m + thmpp_m --> 2ahethmpp_m + co2_m
yli_R0861: 2ahethmpp_m + pyr_m --> alac__S_m + 3.0 h_m + thmpp_m
yli_R0862: 2ahethmpp_m + 2obut_m --> 2ahbut_m + 3.0 h_m + thmpp_m
yli_R0878: coa_c + yli_M03938_c --> ibcoa_c + yli_M03934_c
yli_R0879: coa_c + yli_M03940_c --> yli_M03934_c + yli_M03941_c
yli_R0880: coa_c + yli_M03936_c --> ivcoa_c + yli_M03934_c
yli_R0937: 4mop_c + h_c + yli_M03933_c --> co2_c + yli_M03936_c
yli_R0938: 3mob_c + h_c + yli_M03933_c --> co2_c + yli_M03938_c
yli_R0939: 3mop_c + h_c + yli_M03933_c --> co2_c + yli_M03940_c
yli_R1375: 4.0 h_c + pyr_c + thmpp_c --> 2ahethmpp_c + co2_c
yli_R1377: co2_x + mlthf_x + nadh_x + nh4_x --> gly_x + nad_x + thf_x
yli_R1378: co2_x + mlthf_x + nadh_x + nh4_x --> gly_x + nad_x + thf_x
yli_R1387: gly_x + h2o_x + mlthf_x <=> ser__L_x + thf_x
yli_R1419: nad_c + yli_M03934_c --> h_c + nadh_c + yli_M03933_c
yli_R1425: co2_x + mlthf_x + nadh_x + nh4_x --> gly_x + nad_x + thf_x
yli_R1435: 2.0 accoa_x --> aacoa_x + coa_x
yli_R1438: h_r + pmtcoa_r + ser__L_r --> 3dsphgn_r + co2_r + coa_r
yli_R1439: atp_r + sphgn_r --> adp_r + h_r + sph1p_r
yli_R1440: 3dsphgn_r + h_r + nadph_r --> nadp_r + sphgn_r
yli_R1441: h_r + nadph_r + o2_r + sphgn_r --> h2o_r + nadp_r + psphings_r
yli_R1465: 3c3hmp_m <=> 2ippm_m + h2o_m
yli_R1466: 2ippm_m + h2o_m <=> 3c2hmp_m
yli_R1487: atp_c + btn_c --> btamp_c + ppi_c
yli_R1488: atp_c + btn_c --> btamp_c + ppi_c
yli_R1489: atp_c + btn_c --> btamp_c + ppi_c
yli_R1490: atp_c + btn_c --> btamp_c + ppi_c
yli_R1495: dhmtp_c + o2_c --> co_c + for_c + mtpp_c
yli_R1508: glyald_c + h2o_c + nad_c --> glyc__R_c + h_c + nadh_c
yli_R1532: glutcoa_m + yli_M03934_m <=> S_gtrdhdlp_m + coa_m
yli_R1538: h2o_c + nad_c --> amp_c + nmn_c
yli_R1539: dnad_c + h2o_c --> amp_c + nicrnt_c
yli_R1567: sphs1p_r --> ethamp_r + hxdceal_r
yli_R1568: sph1p_r --> ethamp_r + yli_M07049_r
yli_R1569: atp_r + yli_M07050_r --> adp_r + sphs1p_r
yli_R1570: acoa_r + sphgn_r --> coa_r + dhcrm_cho_r
yli_R1571: dhcrm_cho_r + h_r + nadph_r + o2_r --> h2o_r + nadp_r + phcrm_hs_r
yli_R1575: HC01435_m + yli_M03933_m --> thmpp_m + yli_M04007_m
yli_R1576: akg_m + thmpp_m --> HC01435_m + co2_m
yli_R1587: 4mop_c + thmpp_c <=> 3mhtpp_c + co2_c
yli_R1588: 2obut_m + pyr_m --> 2ahbut_m + co2_m
yli_R1590: 2mhop_c + yli_M03933_c --> thmpp_c + yli_M03938_c
yli_R1591: 3mhtpp_c + yli_M03933_c --> thmpp_c + yli_M03936_c
yli_R1592: 2mhob_c + yli_M03933_c --> thmpp_c + yli_M03940_c
yli_R7859: 2ippm_m + h2o_m <=> 3c2hmp_m
yli_R8859: 3c3hmp_m <=> 2ippm_m + h2o_m
```

In [84]:

```
print('Updated reactions\n')
for r in sorted(model_old.reactions, key=lambda x: x.id):
    if r in model_new.reactions:
        r2 = model_new.reactions.get_by_id(r.id)
        if (r.name == r2.name and r.reaction == r2.reaction and r.gene_reaction_rule == r2.gene_reaction_rule and
            r.lower_bound == r2.lower_bound and r.upper_bound == r2.upper_bound):
            pass
        else:
            print('Old', r, r.gene_reaction_rule)
            print('New', r2, r2.gene_reaction_rule)
            print()
```

```
Updated reactions

Old 2OXOADOXm: 2oxoadp_m + coa_m + nad_m --> co2_m + glutcoa_m + nadh_m (PDHX and 10007 and 10040 and 12116) or (PDHX and 10040 and 12116 and 9274) or (Pdhx and 10007 and 10040 and 12116) or (Pdhx and 10040 and 12116 and 9274)
New 2OXOADOXm: 2oxoadp_m + coa_m + nad_m --> co2_m + glutcoa_m + nadh_m 10040 and 12116 and 9274

Old ACACT1m: 2.0 accoa_m --> aacoa_m + coa_m 8678 or 8885 or (HADHA and HADHB) or (Hadha and Hadhb)
New ACACT1m: 2.0 accoa_m --> aacoa_m + coa_m 8678 or 8885

Old ADK1: amp_c + atp_c <=> 2.0 adp_c 12300 or 13190 or 15496
New ADK1: amp_c + atp_c <=> 2.0 adp_c 15496

Old ADNK1: adn_c + atp_c --> adp_c + amp_c + h_c 15496 or 8385
New ADNK1: adn_c + atp_c --> adp_c + amp_c + h_c 8385

Old AKGDm: akg_m + coa_m + nad_m --> co2_m + nadh_m + succoa_m (PDHX and 10007 and 10040 and 12116) or (PDHX and 10040 and 12116 and 9274) or (Pdhx and 10007 and 10040 and 12116) or (Pdhx and 10040 and 12116 and 9274)
New AKGDm: akg_m + coa_m + nad_m --> co2_m + nadh_m + succoa_m 10007 and 10040 and 12116

Old ASPK: asp__L_c + atp_c <=> 4pasp_c + adp_c 12080 or 14662 or 16738
New ASPK: asp__L_c + atp_c <=> 4pasp_c + adp_c 14662

Old CERS124er: sphgn_r + ttccoa_r --> cer1_24_r + coa_r + h_r 11391
New CERS124er: sphgn_r + ttccoa_r --> cer1_24_r + coa_r + h_r 11391 or 15168

Old CERS126er: hexccoa_r + sphgn_r --> cer1_26_r + coa_r + h_r 11391
New CERS126er: hexccoa_r + sphgn_r --> cer1_26_r + coa_r + h_r 11391 or 15168

Old CERS224er: psphings_r + ttccoa_r --> cer2_24_r + coa_r + h_r 11391
New CERS224er: psphings_r + ttccoa_r --> cer2_24_r + coa_r + h_r 11391 or 15168

Old CERS226er: hexccoa_r + psphings_r --> cer2_26_r + coa_r + h_r 11391
New CERS226er: hexccoa_r + psphings_r --> cer2_26_r + coa_r + h_r 11391 or 15168

Old CYSDS: cys__L_c + h2o_c --> h2s_c + nh4_c + pyr_c 8759 or 9499
New CYSDS: cys__L_c + h2o_c --> h2s_c + nh4_c + pyr_c 16725 or 16742

Old CYSS: acser_c + h2s_c --> ac_c + cys__L_c + h_c 12031 or 13106 or 15712
New CYSS: acser_c + h2s_c --> ac_c + cys__L_c + h_c 12031 or 13106

Old DADK: atp_c + damp_c <=> adp_c + dadp_c 12300 or 15129 or 15496
New DADK: atp_c + damp_c <=> adp_c + dadp_c 15496

Old DPCOAK: atp_c + dpcoa_c --> adp_c + coa_c + h_c 11114 or 14849
New DPCOAK: atp_c + dpcoa_c --> adp_c + coa_c + h_c 11114

Old FMNATm: atp_m + fmn_m + h_m --> fad_m + ppi_m 11542
New FMNATm: atp_m + fmn_m + h_m --> fad_m + ppi_m 16092

Old GLUCYS: atp_c + cys__L_c + glu__L_c --> adp_c + glucys_c + h_c + pi_c 12007 or 12022 or (GCLM and 12007) or (GCLM and 12022) or (Gclm and 12007) or (Gclm and 12022)
New GLUCYS: atp_c + cys__L_c + glu__L_c --> adp_c + glucys_c + h_c + pi_c 12007

Old GLUDy: glu__L_c + h2o_c + nadp_c <=> akg_c + h_c + nadph_c + nh4_c 12248 or (b3213 and 15713)
New GLUDy: glu__L_c + h2o_c + nadp_c <=> akg_c + h_c + nadph_c + nh4_c 12248

Old GLYCLm: gly_m + nad_m + thf_m --> co2_m + mlthf_m + nadh_m + nh4_m 12898 or (CRv4_Au5_s12_g4121_t1 and 14894) or (12898 and 14894) or (10040 and 10205 and 12898 and 15184)
New GLYCLm: gly_m + nad_m + thf_m --> co2_m + mlthf_m + nadh_m + nh4_m 10040 and 10205 and 12898 and 15184

Old GRXR: grxox_c + 2.0 gthrd_c --> grxrd_c + gthox_c 15038 or 16549 or 9250
New GRXR: grxox_c + 2.0 gthrd_c --> grxrd_c + gthox_c 15038 or 8790

Old GTHOm: gthox_m + h_m + nadph_m --> 2.0 gthrd_m + nadp_m 15482 or (15482 and 9250)
New GTHOm: gthox_m + h_m + nadph_m --> 2.0 gthrd_m + nadp_m 15482

Old GTHOr: gthox_c + h_c + nadph_c <=> 2.0 gthrd_c + nadp_c 15482 or (15038 and 15482) or (15482 and 16549) or (15482 and 8790)
New GTHOr: gthox_c + h_c + nadph_c <=> 2.0 gthrd_c + nadp_c 15482

Old GTHPi: 2.0 gthrd_c + h2o2_c --> gthox_c + 2.0 h2o_c 12715 or 15038 or 16549 or 8579
New GTHPi: 2.0 gthrd_c + h2o2_c --> gthox_c + 2.0 h2o_c 8579

Old HSDxi: aspsa_c + h_c + nadh_c --> hom__L_c + nad_c 12080
New HSDxi: aspsa_c + h_c + nadh_c --> hom__L_c + nad_c 12080 or 16738

Old HSERTA: accoa_c + hom__L_c <=> achms_c + coa_c 12513 or 15248 or (PP_5098 and 15248)
New HSERTA: accoa_c + hom__L_c <=> achms_c + coa_c 12513 or 15248

Old IPPMIa: 3c2hmp_c <=> 2ippm_c + h2o_c 14914 or (CRv4_Au5_s6_g12448_t1 and 14914) or (PP_1986 and 14914) or (b0071 and 14914)
New IPPMIa: 3c2hmp_c <=> 2ippm_c + h2o_c 14914

Old IPPMIb: 2ippm_c + h2o_c <=> 3c3hmp_c 14914 or (CRv4_Au5_s6_g12448_t1 and 14914) or (PP_1986 and 14914) or (b0071 and 14914)
New IPPMIb: 2ippm_c + h2o_c <=> 3c3hmp_c 14914

Old METS: 5mthf_c + hcys__L_c --> h_c + met__L_c + thf_c 9825
New METS: 5mthf_c + hcys__L_c --> h_c + met__L_c + thf_c 12876 or 12920 or 9825

Old METSOXR1: metsox_S__L_c + trdrd_c --> h2o_c + met__L_c + trdox_c 15469 or (b3551 and 15339) or (b3551 and 16019) or (15339 and 15902) or (15902 and 16019)
New METSOXR1: metsox_S__L_c + trdrd_c --> h2o_c + met__L_c + trdox_c (10848 and 15902) or (12730 and 15902) or (12737 and 15902) or (15339 and 15902)

Old METSOXR2: metsox_R__L_c + trdrd_c --> h2o_c + met__L_c + trdox_c (15339 and 15469) or (15339 and 9153) or (15469 and 16019) or (16019 and 9153)
New METSOXR2: metsox_R__L_c + trdrd_c --> h2o_c + met__L_c + trdox_c (10848 and 15469) or (12730 and 15469) or (12737 and 15469) or (15339 and 15469)

Old MTAP: 5mta_c + pi_c --> 5mdr1p_c + ade_c 14521 or 8372
New MTAP: 5mta_c + pi_c --> 5mdr1p_c + ade_c 8372

Old MTRI: 5mdr1p_c <=> 5mdru1p_c 13385 or 15595
New MTRI: 5mdr1p_c <=> 5mdru1p_c 13385

Old NDPK1: atp_c + gdp_c <=> adp_c + gtp_c 15496 or 15679 or 8943
New NDPK1: atp_c + gdp_c <=> adp_c + gtp_c 15679

Old NDPK10: atp_c + didp_c <=> adp_c + ditp_c 15679 or 8943
New NDPK10: atp_c + didp_c <=> adp_c + ditp_c 15679

Old NDPK2: atp_c + udp_c <=> adp_c + utp_c 15496 or 15679 or 8943
New NDPK2: atp_c + udp_c <=> adp_c + utp_c 15679

Old NDPK2m: atp_m + udp_m --> adp_m + utp_m 15679
New NDPK2m: atp_m + udp_m <=> adp_m + utp_m 8943

Old NDPK3: atp_c + cdp_c <=> adp_c + ctp_c 15496 or 15679 or 8943
New NDPK3: atp_c + cdp_c <=> adp_c + ctp_c 15679

Old NDPK3m: atp_m + cdp_m --> adp_m + ctp_m 15679
New NDPK3m: atp_m + cdp_m <=> adp_m + ctp_m 8943

Old NDPK4: atp_c + dtdp_c <=> adp_c + dttp_c 15496 or 15679 or 8943
New NDPK4: atp_c + dtdp_c <=> adp_c + dttp_c 15679

Old NDPK4m: atp_m + dtdp_m --> adp_m + dttp_m 15679
New NDPK4m: atp_m + dtdp_m <=> adp_m + dttp_m 8943

Old NDPK5: atp_c + dgdp_c <=> adp_c + dgtp_c 15496 or 15679 or 8943
New NDPK5: atp_c + dgdp_c <=> adp_c + dgtp_c 15679

Old NDPK6: atp_c + dudp_c <=> adp_c + dutp_c 15496 or 15679 or 8943
New NDPK6: atp_c + dudp_c <=> adp_c + dutp_c 15679

Old NDPK6m: atp_m + dudp_m --> adp_m + dutp_m 15679
New NDPK6m: atp_m + dudp_m <=> adp_m + dutp_m 8943

Old NDPK7: atp_c + dcdp_c <=> adp_c + dctp_c 15496 or 15679 or 8943
New NDPK7: atp_c + dcdp_c <=> adp_c + dctp_c 15679

Old NDPK7m: atp_m + dcdp_m --> adp_m + dctp_m 15679
New NDPK7m: atp_m + dcdp_m <=> adp_m + dctp_m 8943

Old NDPK8: atp_c + dadp_c <=> adp_c + datp_c 15496 or 15679 or 8943
New NDPK8: atp_c + dadp_c <=> adp_c + datp_c 15679

Old NDPK8m: atp_m + dadp_m --> adp_m + datp_m 15679
New NDPK8m: atp_m + dadp_m <=> adp_m + datp_m 8943

Old NDPK9: atp_c + idp_c <=> adp_c + itp_c 15679 or 8943
New NDPK9: atp_c + idp_c <=> adp_c + itp_c 15679

Old NDPK9m: atp_m + idp_m --> adp_m + itp_m 15679
New NDPK9m: atp_m + idp_m <=> adp_m + itp_m 8943

Old OIVD1m: 4mop_m + coa_m + nad_m --> co2_m + ivcoa_m + nadh_m (10040 and 11183 and 12086 and 15436) or (10040 and 11183 and 12566 and 15436) or (10040 and 11188 and 12086 and 15436) or (10040 and 11188 and 12566 and 15436)
New OIVD1m: 4mop_m + coa_m + nad_m --> co2_m + ivcoa_m + nadh_m (10040 and 11183 and 12566 and 15436) or (10040 and 11188 and 12566 and 15436)

Old OIVD2m: 3mob_m + coa_m + nad_m --> co2_m + ibcoa_m + nadh_m (10040 and 11183 and 12086 and 15436) or (10040 and 11183 and 12566 and 15436) or (10040 and 11188 and 12086 and 15436) or (10040 and 11188 and 12566 and 15436)
New OIVD2m: 3mob_m + coa_m + nad_m --> co2_m + ibcoa_m + nadh_m (10040 and 11183 and 12566 and 15436) or (10040 and 11188 and 12566 and 15436)

Old OIVD3m: 3mop_m + coa_m + nad_m --> 2mbcoa_m + co2_m + nadh_m (10040 and 11183 and 12086 and 15436) or (10040 and 11183 and 12566 and 15436) or (10040 and 11188 and 12086 and 15436) or (10040 and 11188 and 12566 and 15436)
New OIVD3m: 3mop_m + coa_m + nad_m --> 2mbcoa_m + co2_m + nadh_m (10040 and 11183 and 12566 and 15436) or (10040 and 11188 and 12566 and 15436)

Old PAPSR: paps_c + trdrd_c --> 2.0 h_c + pap_c + so3_c + trdox_c 11741 or (11741 and 15339) or (11741 and 16019)
New PAPSR: paps_c + trdrd_c --> 2.0 h_c + pap_c + so3_c + trdox_c (10848 and 11741) or (11741 and 12730) or (11741 and 12737) or (11741 and 15339)

Old PDHm: coa_m + nad_m + pyr_m --> accoa_m + co2_m + nadh_m (PDHX and 10040 and 13630 and 13948 and 14126) or (Pdhx and 10040 and 13630 and 13948 and 14126) or (10040 and 13630 and 13722 and 13948 and 14126)
New PDHm: coa_m + nad_m + pyr_m --> accoa_m + co2_m + nadh_m 10040 and 13630 and 13722 and 13948 and 14126

Old RNDR1: adp_c + trdrd_c --> dadp_c + h2o_c + trdox_c (11172 and 11290) or (11290 and 14237) or (CRv4_Au5_s9_g15314_t1 and 11172 and 11290) or (CRv4_Au5_s9_g15314_t1 and 11290 and 14237) or (11172 and 11290 and 15339) or (11172 and 11290 and 16019) or (11290 and 14237 and 15339) or (11290 and 14237 and 16019)
New RNDR1: adp_c + trdrd_c --> dadp_c + h2o_c + trdox_c (10848 and 11172 and 11290 and 14237) or (11172 and 11290 and 12730 and 14237) or (11172 and 11290 and 12737 and 14237) or (11172 and 11290 and 14237 and 15339)

Old RNDR1n: adp_n + trdrd_n --> dadp_n + h2o_n + trdox_n (YGR180C and 11290 and 15339) or (YGR180C and 11290 and 16019)
New RNDR1n: adp_n + trdrd_n --> dadp_n + h2o_n + trdox_n (10848 and 11172 and 11290 and 14237) or (11172 and 11290 and 12730 and 14237) or (11172 and 11290 and 12737 and 14237)

Old RNDR2: gdp_c + trdrd_c --> dgdp_c + h2o_c + trdox_c (11172 and 11290) or (11290 and 14237) or (CRv4_Au5_s9_g15314_t1 and 11172 and 11290) or (CRv4_Au5_s9_g15314_t1 and 11290 and 14237) or (11172 and 11290 and 15339) or (11172 and 11290 and 16019) or (11290 and 14237 and 15339) or (11290 and 14237 and 16019)
New RNDR2: gdp_c + trdrd_c --> dgdp_c + h2o_c + trdox_c (10848 and 11172 and 11290 and 14237) or (11172 and 11290 and 12730 and 14237) or (11172 and 11290 and 12737 and 14237) or (11172 and 11290 and 14237 and 15339)

Old RNDR2n: gdp_n + trdrd_n --> dgdp_n + h2o_n + trdox_n (YGR180C and 11290 and 15339) or (YGR180C and 11290 and 16019)
New RNDR2n: gdp_n + trdrd_n --> dgdp_n + h2o_n + trdox_n (10848 and 11172 and 11290 and 14237) or (11172 and 11290 and 12730 and 14237) or (11172 and 11290 and 12737 and 14237)

Old RNDR3: cdp_c + trdrd_c --> dcdp_c + h2o_c + trdox_c (11172 and 11290) or (11290 and 14237) or (CRv4_Au5_s9_g15314_t1 and 11172 and 11290) or (CRv4_Au5_s9_g15314_t1 and 11290 and 14237) or (11172 and 11290 and 15339) or (11172 and 11290 and 16019) or (11290 and 14237 and 15339) or (11290 and 14237 and 16019)
New RNDR3: cdp_c + trdrd_c --> dcdp_c + h2o_c + trdox_c (10848 and 11172 and 11290 and 14237) or (11172 and 11290 and 12730 and 14237) or (11172 and 11290 and 12737 and 14237) or (11172 and 11290 and 14237 and 15339)

Old RNDR3n: cdp_n + trdrd_n --> dcdp_n + h2o_n + trdox_n (YGR180C and 11290 and 15339) or (YGR180C and 11290 and 16019)
New RNDR3n: cdp_n + trdrd_n --> dcdp_n + h2o_n + trdox_n (10848 and 11172 and 11290 and 14237) or (11172 and 11290 and 12730 and 14237) or (11172 and 11290 and 12737 and 14237)

Old RNDR4: trdrd_c + udp_c --> dudp_c + h2o_c + trdox_c (11172 and 11290) or (11290 and 14237) or (CRv4_Au5_s9_g15314_t1 and 11172 and 11290) or (CRv4_Au5_s9_g15314_t1 and 11290 and 14237) or (11172 and 11290 and 15339) or (11172 and 11290 and 16019) or (11290 and 14237 and 15339) or (11290 and 14237 and 16019)
New RNDR4: trdrd_c + udp_c --> dudp_c + h2o_c + trdox_c (10848 and 11172 and 11290 and 14237) or (11172 and 11290 and 12730 and 14237) or (11172 and 11290 and 12737 and 14237) or (11172 and 11290 and 14237 and 15339)

Old RNDR4n: trdrd_n + udp_n --> dudp_n + h2o_n + trdox_n (YGR180C and 11290 and 15339) or (YGR180C and 11290 and 16019)
New RNDR4n: trdrd_n + udp_n --> dudp_n + h2o_n + trdox_n (10848 and 11172 and 11290 and 14237) or (11172 and 11290 and 12730 and 14237) or (11172 and 11290 and 12737 and 14237)

Old SHSL1: cys__L_c + suchms_c --> cyst__L_c + h_c + succ_c 11463 or 16725 or 16742 or 9499
New SHSL1: cys__L_c + suchms_c --> cyst__L_c + h_c + succ_c 11463

Old SO4ti: so4_e --> so4_c 14119 or 15736 or 16682 or (14119 and 16682) or (15736 and 16682)
New SO4ti: so4_e --> so4_c 14119 or 15736 or 16682

Old THIORDXi: h2o2_c + trdrd_c --> 2.0 h2o_c + trdox_c 8579 or (YDR453C and 15339) or (12715 and 15339) or (12715 and 16019) or (15037 and 15339) or (15037 and 16019)
New THIORDXi: h2o2_c + trdrd_c --> 2.0 h2o_c + trdox_c (10848 and 12715) or (12715 and 12730) or (12715 and 12737)

Old THIORDXm: h2o2_m + trdrd_m <=> 2.0 h2o_m + trdox_m 10200 and 15339
New THIORDXm: h2o2_m + trdrd_m <=> 2.0 h2o_m + trdox_m 10200 and 16019

Old THIORDXni: h2o2_n + trdrd_n --> 2.0 h2o_n + trdox_n (15037 and 15339) or (15037 and 16019)
New THIORDXni: h2o2_n + trdrd_n --> 2.0 h2o_n + trdox_n (10848 and 15037) or (12730 and 15037) or (12737 and 15037)

Old THIORDXp: h2o2_x + trdrd_x <=> 2.0 h2o_x + trdox_x (13262 and 15339) or (13262 and 16019)
New THIORDXp: h2o2_x + trdrd_x <=> 2.0 h2o_x + trdox_x 13262 and 15339

Old THRA: thr__L_c --> acald_c + gly_c 16182 or 9222 or 9667
New THRA: thr__L_c --> acald_c + gly_c 16182

Old THRA2: athr__L_c --> acald_c + gly_c 16182 or 9222 or 9667
New THRA2: athr__L_c --> acald_c + gly_c 16182

Old TRDR: h_c + nadph_c + trdox_c --> nadp_c + trdrd_c 15339 or 15482 or 16019 or 9688 or (CRv4_Au5_s2_g8777_t1 and CRv4_Au5_s9_g15314_t1) or (CRv4_Au5_s2_g8777_t1 and 15339) or (CRv4_Au5_s2_g8777_t1 and 16019) or (CRv4_Au5_s8_g14830_t1 and CRv4_Au5_s9_g15314_t1) or (CRv4_Au5_s8_g14830_t1 and 15339) or (CRv4_Au5_s8_g14830_t1 and 16019) or (CRv4_Au5_s9_g15314_t1 and 9688) or (15339 and 9688) or (16019 and 9688)
New TRDR: h_c + nadph_c + trdox_c --> nadp_c + trdrd_c (10848 and 9688) or (12730 and 9688) or (12737 and 9688)

Old TRDRm: h_m + nadph_m + trdox_m --> nadp_m + trdrd_m 15482 or (YCR083W and 9688) or (15339 and 9688)
New TRDRm: h_m + nadph_m + trdox_m --> nadp_m + trdrd_m 16019 and 9688

Old URIDK2r: atp_c + dump_c <=> adp_c + dudp_c 13190 or 15252
New URIDK2r: atp_c + dump_c <=> adp_c + dudp_c 13190
```

In [85]:

```
print('Added reactions\n')
for r in sorted(model_new.reactions, key=lambda x: x.id):
    if r not in model_old.reactions:
        print(r)
```

```
Added reactions

2OH3K5MPPISO: h2o_c + hkmpp_c --> dhmtp_c + pi_c
3DSPHRer: 3dsphgn_r + h_r + nadph_r --> nadp_r + sphgn_r
3SALACBOXL: 3sala_c + h_c --> co2_c + hyptaur_c
ACRS: dkmpp_c --> h_c + hkmpp_c
AMAOTrm: 8aonn_m + amet_m <=> amob_m + dann_m
AOXSp: ala__L_x + h_x + pimcoa_x --> 8aonn_x + co2_x + coa_x
BTS5m: 2fe2s_m + amet_m + dtbt_m --> 2fe1s_m + btn_m + dad_5_m + h_m + met__L_m
CERH124er: cer1_24_r + h_r + nadph_r + o2_r --> cer2_24_r + h2o_r + nadp_r
CERH126er: cer1_26_r + h_r + nadph_r + o2_r --> cer2_26_r + h2o_r + nadp_r
CERS2p24er: cer1_24_r + h_r + nadph_r + o2_r --> cer2p_24_r + h2o_r + nadp_r
CERS2p26er: cer1_26_r + h_r + nadph_r + o2_r --> cer2p_26_r + h2o_r + nadp_r
CERS324er: cer2_24_r + h_r + nadph_r + o2_r --> cer3_24_r + h2o_r + nadp_r
CERS326er: cer2_26_r + h_r + nadph_r + o2_r --> cer3_26_r + h2o_r + nadp_r
DBTSm: atp_m + co2_m + dann_m <=> adp_m + dtbt_m + 3.0 h_m + pi_m
DHAOX_c: dhdascb_c + 2.0 gthrd_c --> ascb__L_c + gthox_c + h_c
DNTPPA: ahdt_c + h2o_c --> dhpmp_c + h_c + ppi_c
FTHFCLm: 5fthf_m + atp_m --> adp_m + methf_m + pi_m
GBBOX_m: akg_m + gbbtn_m + o2_m --> co2_m + crn_m + succ_m
GRXRm: grxox_m + 2.0 gthrd_m --> grxrd_m + gthox_m
HTMLA_m: 3htmelys_m --> 4tmeabut_m + gly_m
NDPK10m: atp_m + didp_m <=> adp_m + ditp_m
NDPK1m: atp_m + gdp_m <=> adp_m + gtp_m
NDPK5m: atp_m + dgdp_m <=> adp_m + dgtp_m
NNAT: atp_c + h_c + nicrnt_c --> dnad_c + ppi_c
OBDHm: 2obut_m + coa_m + nad_m --> co2_m + nadh_m + ppcoa_m
PSPHPLer: psph1p_r --> 2hhxdal_r + ethamp_r
PSPHSer: h_r + nadph_r + o2_r + sphgn_r --> h2o_r + nadp_r + psphings_r
SERPTer: h_r + pmtcoa_r + ser__L_r --> 3dsphgn_r + co2_r + coa_r
SLCBK1er: atp_r + sphgn_r --> adp_r + h_r + sph1p_r
SLCBK2er: atp_r + psphings_r --> adp_r + h_r + psph1p_r
SPHPLer: sph1p_r --> ethamp_r + hxdcal_r
TMABDH1_m: 4tmeabut_m + h2o_m + nad_m --> gbbtn_m + 2.0 h_m + nadh_m
TMLOX_m: akg_m + o2_m + tmlys_m --> 3htmelys_m + co2_m + succ_m
```
