# Supplementary material for: Multi-Omics Driven Metabolic Network Reconstruction and Analysis of Lignocellulosic Carbon Utilization in Rhodosporidium toruloides
Source: Front Bioeng Biotechnol. 2021 Jan 8;8:612832. doi: 10.3389/fbioe.2020.612832 (PMC7873862; doi:10.3389/fbioe.2020.612832)
Supplement: Supplementary File 4 — Multi-omics dataset for R. toruloides IFO0880. [file Data_Sheet_1.zip › Supplementary File S1/1.Manual_curation/Refinement_1d_Central_Metabolism.html]

Refinement\_1d\_Central\_Metabolism


In [1]:

```
%matplotlib inline
from matplotlib import pyplot as plt
from matplotlib import colors
import csv
import numpy as np
import pandas as pd
import cobra
```

In [2]:

```
cobra.__version__
```

Out[2]:

```
'0.17.1'
```

In [3]:

```
Annotation = pd.read_excel('../../Data/R_toruloides_Data_for_Reconstruction.xlsx',
                          sheet_name='Annotation', index_col=0)
Annotation.index = Annotation.index.map(str)
Annotation = Annotation.fillna('')
Transcriptomics = pd.read_excel('../../Data/R_toruloides_Data_for_Reconstruction.xlsx',
                          sheet_name='Transcriptomics', header=[0,1,2,3], index_col=0)
Transcriptomics.index = Transcriptomics.index.map(str)
Proteomics = pd.read_excel('../../Data/R_toruloides_Data_for_Reconstruction.xlsx',
                          sheet_name='Proteomics', header=[0,1,2], index_col=0)
Proteomics.index = Proteomics.index.map(str)
Fitness = pd.read_excel('../../Data/R_toruloides_Data_for_Reconstruction.xlsx',
                          sheet_name='Fitness', index_col=0)
Fitness.index = Fitness.index.map(str)
```

In [4]:

```
def background_gradient(s, cmap='seismic', text_color_threshold=0.408):
    lim = max(abs(s.min().min()),abs(s.max().max()))
    rng = 2.0*lim
    norm = colors.Normalize(-lim - (rng * 0.2), lim + (rng * 0.2))
    rgbas = plt.cm.get_cmap(cmap)(norm(s.values))
    def relative_luminance(rgba):
        r, g, b = (x / 12.92 if x <= 0.03928 else ((x + 0.055) / 1.055 ** 2.4) for x in rgba[:3])
        return 0.2126 * r + 0.7152 * g + 0.0722 * b
    def css(rgba):
        dark = relative_luminance(rgba) < text_color_threshold
        text_color = '#f1f1f1' if dark else '#000000'
        return 'background-color: {b};color: {c};'.format(b=colors.rgb2hex(rgba), c=text_color)

    if s.ndim == 1:
        return [css(rgba) for rgba in rgbas]
    else:
        return pd.DataFrame([[css(rgba) for rgba in row] for row in rgbas], index=s.index, columns=s.columns)

def Show_Data(x):
    display(Transcriptomics.loc[x].style.background_gradient(cmap='Reds', low=0.2, high=0.2, axis=None))
    temp = [y for y in x if y in Proteomics.index]
    display(Proteomics.loc[temp].style.background_gradient(cmap='Reds', low=0.2, high=0.2, axis=None))
    temp = [y for y in x if y in Fitness.index]
    display(Fitness.loc[temp].style.apply(background_gradient, cmap='seismic', axis=None))
    return;
```

In [5]:

```
eco = cobra.io.load_json_model('../../Data/BiGG_Models/iML1515.json')
sce = cobra.io.load_json_model('../../Data/BiGG_Models/iMM904.json')
hsa = cobra.io.load_json_model('../../Data/BiGG_Models/RECON1.json')
hsa2 = cobra.io.load_json_model('../../Data/BiGG_Models/Recon3D.json')
```

In [6]:

```
model = cobra.io.load_json_model("IFO0880_GPR_1c.json")
```

In [7]:

```
print(len(model.genes))
print(len([x for x in model.genes if not x.id[0].isalpha()]))
model
```

```
1231
1154
```

Out[7]:

|  |  |
| --- | --- |
| **Name** | R. toruloides |
| **Memory address** | 0x0102d579748 |
| **Number of metabolites** | 2585 |
| **Number of reactions** | 2402 |
| **Number of groups** | 0 |
| **Objective expression** | 0 |
| **Compartments** | c, x, m, e, r, v, n, g, p, h, f, l, d |

Glycolysis  
Glucose transport  
STL1 10704 plas 20, E.R. 3, mito 2, vacu 2 AQV *HXT2 11075 plas 21, E.R. 4 KOG0254: Predicted transporter (major facilitator superfamily) EDV*  
GAL2 11893 plas 25 K08139: HXT; MFS transporter, SP family, sugar:H+ symporter EVH *Hexokinase (D-glucose:ATP)  
GLK1 10264 cyto 10, cyto\_mito 8.333, cyto\_nucl 6.833, mito 5.5, pero 4, cysk 3, nucl 2.5 K00844: HK; hexokinase VTK*  
HXK2 11982 mito 20, cyto 4, pero 2 K00844: HK; hexokinase AHV *Glucose-6-phosphate isomerase  
PGI1 9589 cyto 14, pero 10, vacu 2 K01810: GPI, pgi; glucose-6-phosphate isomerase SRA*  
Phosphofructokinase  
PFK1 8859 cyto 21, pero 3, mito 2 K00850: pfkA, PFK; 6-phosphofructokinase 1 QTS *PFK2 8863 nucl 11, cyto\_nucl 10.5, cyto 8, pero 4, mito 3 K00850: pfkA, PFK; 6-phosphofructokinase 1 QTS*  
PFK3 8867 cyto 16, mito 8, cyto\_nucl 8 QTS *Fructose-bisphosphatase  
FBP1 15423 cyto 17.5, cyto\_nucl 15.5, nucl 6.5 KDQ*  
Fructose-bisphosphate aldolase  
FBA1 15420 cyto 15.5, cyto\_mito 11.666, cyto\_nucl 10.833, mito 6.5, nucl 4 GTL *Triose-phosphate isomerase  
TPI1 9300 mito 17, cyto 6, pero 4 K01803: TPI, tpiA; triosephosphate isomerase (TIM) AKL*  
Glyceraldehyde-3-phosphate dehydrogenase  
TDH1 10613 cyto 27 K00134: GAPDH, gapA; glyceraldehyde 3-phosphate dehydrogenase GKL *Phosphoglycerate kinase  
PGK1 15721 cyto 13.5, mito 12, cyto\_nucl 7.5 K00927: PGK, pgk; phosphoglycerate kinase EKQ*  
Phosphoglycerate mutase  
GPM1 9910 mito 16, cyto 8, nucl 2 K01834: PGAM, gpmA; 2,3-bisphosphoglycerate-dependent phosphoglycerate mutase ESK *GPM2 12393 mito 15.5, cyto\_mito 11.666, cyto\_nucl 6.833, cyto 6.5, nucl 5 K15634: gpmB; probable phosphoglycerate mutase GAQ*  
GPM3 11229 nucl 19.5, cyto\_nucl 13, cyto 5.5 KOG4754: Predicted phosphoglycerate mutase RTY *GPM4? 15425 mito 19, nucl 5, cyto 3 K15634: gpmB; probable phosphoglycerate mutase AQI*  
Enolase  
ENO1 9037 mito 19.5, cyto\_mito 14, cyto 7.5 K01689: ENO, eno; enolase SRK\*

In [8]:

```
temp = ['10704','11075','11893','10264','11982','9589','8859','8863','8867','11513','15423','15195','15744',
        '15420','9300','10613','15721','9910','11229','12393','12637','15425','9037']
Annotation.loc[temp]
```

Out[8]:

|  | Combined Annotations | Signal P | Sc288c Orthologs | Human Orthologs | Sc288 Best Hit | Human Blast | Essential | WolfPSort | C Terminal |
| --- | --- | --- | --- | --- | --- | --- | --- | --- | --- |
| RTO4\_ID |  |  |  |  |  |  |  |  |  |
| 10704 | KOG0254: Predicted transporter (major facilita... | S | STL1 |  | STL1 | SLC2A | Not Essential | plas 20, E.R. 3, mito 2, vacu 2 | AQV\* |
| 11075 | KOG0254: Predicted transporter (major facilita... |  | YDL245C,YDR342C,YDR343C,HXT3,YEL069C,HXT10,HXT... |  | GAL2 | SLC2A | Not Essential | plas 21, E.R. 4 | EDV\* |
| 11893 | K08139: HXT; MFS transporter, SP family, sugar... | S | YDL245C,YDR342C,YDR343C,HXT3,YEL069C,HXT10,HXT... |  | HXT7 | SLC2A | Not Essential | plas 25 | EVH\* |
| 10264 | K00844: HK; hexokinase |  | GLK1,EMI2,HXK1,HXK2 | HK1,HK2,HK3,HKDC1 | HXK2 | HK1 | Not Essential | cyto 10, cyto\_mito 8.333, cyto\_nucl 6.833, mit... | VTK\* |
| 11982 | K00844: HK; hexokinase |  | GLK1,EMI2,HXK1,HXK2 | HK1,HK2,HK3,HKDC1 | HXK2 | HK2e | Not Essential | mito 20, cyto 4, pero 2 | AHV\* |
| 9589 | K01810: GPI, pgi; glucose-6-phosphate isomerase |  | PGI1 | GPI | PGI1 | GPI | Essential | cyto 14, pero 10, vacu 2 | SRA\* |
| 8859 | K00850: pfkA, PFK; 6-phosphofructokinase 1 |  | PFK1,PFK2 | PFKL,PFKM,PFKP | PFK2 | PFKL | Unclear (ambiguous TDNA mapping) | cyto 21, pero 3, mito 2 | QTS\* |
| 8863 | K00850: pfkA, PFK; 6-phosphofructokinase 1 |  | PFK1,PFK2 | PFKL,PFKM,PFKP | PFK2 | PFKL | Unclear (ambiguous TDNA mapping) | nucl 11, cyto\_nucl 10.5, cyto 8, pero 4, mito 3 | QTS\* |
| 8867 | K00850: pfkA, PFK; 6-phosphofructokinase 1 |  | PFK1,PFK2 | PFKL,PFKM,PFKP | PFK2 | PFKL | Unclear (ambiguous TDNA mapping) | cyto 16, mito 8, cyto\_nucl 8 | QTS\* |
| 11513 | HMMPfam:haloacid dehalogenase-like hydrolase:P... |  |  |  |  |  | Not Essential | mito 10, cyto 7.5, cyto\_nucl 6.5, pero 5, nucl... | AYP\* |
| 15423 | K03841: FBP, fbp; fructose-1,6-bisphosphatase I |  | FBP1 | FBP1,FBP2 | FBP1 | FBP2 | Not Essential | cyto 17.5, cyto\_nucl 15.5, nucl 6.5 | KDQ\* |
| 15195 | K19029: PFKFB2; 6-phosphofructo-2-kinase / fru... |  | FBP26 | PFKFB1,PFKFB2,PFKFB3,PFKFB4 | FBP26 | PFKFB | Not Essential | mito 22, cyto\_nucl 3.5, nucl 3 | YDA\* |
| 15744 | K19030: PFKFB4; 6-phosphofructo-2-kinase / fru... | S | YLR345W |  | YLR345W | PFKFB | Not Essential | cyto 8, nucl 7, mito 5, pero 4, plas 1, extr 1... | FWQ\* |
| 15420 | K01624: FBA, fbaA; fructose-bisphosphate aldol... |  | FBA1 |  | FBA1 |  | Essential | cyto 15.5, cyto\_mito 11.666, cyto\_nucl 10.833,... | GTL\* |
| 9300 | K01803: TPI, tpiA; triosephosphate isomerase (... |  | TPI1 | TPI1 | TPI1 | TPI1 | Essential | mito 17, cyto 6, pero 4 | AKL\* |
| 10613 | K00134: GAPDH, gapA; glyceraldehyde 3-phosphat... |  | TDH3,TDH1,TDH2 | GAPDH | TDH1 | GAPDH | Essential | cyto 27 | GKL\* |
| 15721 | K00927: PGK, pgk; phosphoglycerate kinase |  | PGK1 | PGK1,PGK2 | PGK1 | PGK1 | Essential | cyto 13.5, mito 12, cyto\_nucl 7.5 | EKQ\* |
| 9910 | K01834: PGAM, gpmA; 2,3-bisphosphoglycerate-de... |  | GPM1 | PGAM2,PGAM1 | GPM1 | PGAM1 | Essential | mito 16, cyto 8, nucl 2 | ESK\* |
| 11229 | KOG4754: Predicted phosphoglycerate mutase |  |  |  |  |  | Not Essential | nucl 19.5, cyto\_nucl 13, cyto 5.5 | RTY\* |
| 12393 | K15634: gpmB; probable phosphoglycerate mutase |  |  |  | GPM1 | PGAM2 | Not Essential | mito 15.5, cyto\_mito 11.666, cyto\_nucl 6.833, ... | GAQ\* |
| 12637 | K15634: gpmB; probable phosphoglycerate mutase |  | SHB17 |  | SHB17 |  | Not Essential | mito 16.5, mito\_nucl 12, nucl 6.5, cyto 4 | LPN\* |
| 15425 | K15634: gpmB; probable phosphoglycerate mutase |  | YOR283W |  | YOR283W | TIGAR | Not Essential | mito 19, nucl 5, cyto 3 | AQI\* |
| 9037 | K01689: ENO, eno; enolase |  | ENO1,ENO2,ERR3,YOR393W,YPL281C | ENO1,ENO2,ENO3 | ENO1 | ENO2 | Not Essential | mito 19.5, cyto\_mito 14, cyto 7.5 | SRK\* |

In [9]:

```
for x in temp:
    if x in model.genes:
        for r in sorted(model.genes.get_by_id(x).reactions, key=lambda x: x.id):
            print(r.id, r.reaction, r.gene_reaction_rule)
    else:
        print(x, 'no reactions')
    print()
```

```
GALt2 gal_e + h_e --> gal_c + h_c 10704 or 11075 or 11893 or (10704 and 11075) or (10704 and 11893)
GLCt1 glc__D_e --> glc__D_c 10452 or 10704 or 11075 or 11893 or 15762 or 9102 or 9841
GLUt2r glu__L_e + h_e <=> glu__L_c + h_c 10704 or 14229 or 15074 or 8962 or 9319 or 9322 or 9962 or (YALI0E20713g and 10704 and 14229 and 8962) or (YALI0E20713g and 10704 and 14229 and 9319) or (YALI0E20713g and 10704 and 14229 and 9322) or (YALI0E20713g and 10704 and 14229 and 9962) or (YALI0E20713g and 10704 and 15074 and 8962) or (YALI0E20713g and 10704 and 15074 and 9319) or (YALI0E20713g and 10704 and 15074 and 9322) or (YALI0E20713g and 10704 and 15074 and 9962)

FRUt2 fru_e + h_e --> fru_c + h_c 11075 or 11893
GALt2 gal_e + h_e --> gal_c + h_c 10704 or 11075 or 11893 or (10704 and 11075) or (10704 and 11893)
GLCt1 glc__D_e --> glc__D_c 10452 or 10704 or 11075 or 11893 or 15762 or 9102 or 9841
MANt2 h_e + man_e --> h_c + man_c 11075 or 11893

FRUt2 fru_e + h_e --> fru_c + h_c 11075 or 11893
GALt2 gal_e + h_e --> gal_c + h_c 10704 or 11075 or 11893 or (10704 and 11075) or (10704 and 11893)
GALth gal_c + h_c <=> gal_h + h_h 11893
GLCt1 glc__D_e --> glc__D_c 10452 or 10704 or 11075 or 11893 or 15762 or 9102 or 9841
MANt2 h_e + man_e --> h_c + man_c 11075 or 11893
XYLt2pp h_p + xyl__D_p --> h_c + xyl__D_c 11893

ABFPTh atp_h + fru_B_h --> adp_h + f6p_B_h + h_h 10264
AM6PTh atp_h + man_h --> adp_h + h_h + man6p_h 10264
GLUKAh Glc_aD_h + atp_h --> adp_h + g6p_A_h + h_h 10264
GLUKBh atp_h + glc__bD_h --> adp_h + g6p_B_h + h_h 10264
HEX1 atp_c + glc__D_c --> adp_c + g6p_c + h_c 10264 or 11982
HEX10 atp_c + gam_c --> adp_c + gam6p_c + h_c 10264 or 11982
HEX4 atp_c + man_c --> adp_c + h_c + man6p_c 10264 or 11982
HEX7 atp_c + fru_c --> adp_c + f6p_c + h_c 10264 or 11982

HEX1 atp_c + glc__D_c --> adp_c + g6p_c + h_c 10264 or 11982
HEX10 atp_c + gam_c --> adp_c + gam6p_c + h_c 10264 or 11982
HEX4 atp_c + man_c --> adp_c + h_c + man6p_c 10264 or 11982
HEX7 atp_c + fru_c --> adp_c + f6p_c + h_c 10264 or 11982

G6PIh g6p_A_h <=> g6p_B_h 9589
PGI g6p_c <=> f6p_c 9589
PGIAh g6p_A_h <=> f6p_B_h 9589
PGIBh g6p_B_h <=> f6p_B_h 9589

PFK atp_c + f6p_c --> adp_c + fdp_c + h_c 8859 or 8863 or 8867
PFK_2 atp_c + tag6p__D_c --> adp_c + h_c + tagdp__D_c 8859 or 8863 or 8867
PFK_3 atp_c + s7p_c --> adp_c + h_c + s17bp_c 8859 or 8863 or 8867

PFK atp_c + f6p_c --> adp_c + fdp_c + h_c 8859 or 8863 or 8867
PFK_2 atp_c + tag6p__D_c --> adp_c + h_c + tagdp__D_c 8859 or 8863 or 8867
PFK_3 atp_c + s7p_c --> adp_c + h_c + s17bp_c 8859 or 8863 or 8867

PFK atp_c + f6p_c --> adp_c + fdp_c + h_c 8859 or 8863 or 8867
PFK_2 atp_c + tag6p__D_c --> adp_c + h_c + tagdp__D_c 8859 or 8863 or 8867
PFK_3 atp_c + s7p_c --> adp_c + h_c + s17bp_c 8859 or 8863 or 8867

F1PP f1p_c + h2o_c --> fru_c + pi_c 11513 or 14546 or 8576
F6PP f6p_c + h2o_c --> fru_c + pi_c 11513 or 14546 or 8576
FBP fdp_c + h2o_c --> f6p_c + pi_c 11513 or 15423
G1PP g1p_c + h2o_c --> glc__D_c + pi_c 11513 or 8576
MN6PP h2o_c + man6p_c --> man_c + pi_c 11513 or 8460
R5PP h2o_c + r5p_c --> pi_c + rib__D_c 11513 or 13044 or 14546 or 8576

BFBPh fdp_B_h + h2o_h --> f6p_B_h + pi_h 15423
FBP fdp_c + h2o_c --> f6p_c + pi_c 11513 or 15423
FBP26 f26bp_c + h2o_c --> f6p_c + pi_c 15195 or 15423 or 15744

FBP26 f26bp_c + h2o_c --> f6p_c + pi_c 15195 or 15423 or 15744
PFK26 atp_c + f6p_c --> adp_c + f26bp_c + h_c 15195 or 15744
RZ5PP 5prdmbz_c + h2o_c --> pi_c + rdmbzi_c 12393 or 15195

FBP26 f26bp_c + h2o_c --> f6p_c + pi_c 15195 or 15423 or 15744
PFK26 atp_c + f6p_c --> adp_c + f26bp_c + h_c 15195 or 15744

FBA fdp_c <=> dhap_c + g3p_c 15420
FBA2 f1p_c <=> dhap_c + glyald_c 15420
FBA3 s17bp_c <=> dhap_c + e4p_c 15420

TPI dhap_c <=> g3p_c 9300
TPIh dhap_h <=> g3p_h 9300

E4PD e4p_c + h2o_c + nad_c <=> 4per_c + 2.0 h_c + nadh_c 10613
GAPD g3p_c + nad_c + pi_c <=> 13dpg_c + h_c + nadh_c 10613
NADHHR h2o_c + nadh_c --> nadhx__R_c 10613
NADHHS h2o_c + nadh_c --> nadhx__S_c 10613
NADPHHR h2o_c + nadph_c --> nadphx__R_c 10613
NADPHHS h2o_c + nadph_c --> nadphx__S_c 10613

PGK 3pg_c + atp_c <=> 13dpg_c + adp_c 15721
PGKh 13dpg_h + adp_h <=> 3pg_h + atp_h 15721

DPGM 13dpg_c <=> 23dpg_c + h_c 9910
DPGase 23dpg_c + h2o_c --> 3pg_c + pi_c 9910
PGM 2pg_c <=> 3pg_c 11229 or 12393 or 12637 or 15425 or 9910
PGMf 3pg_f <=> 2pg_f 11229 or 12393 or 9910
PGMm 3pg_m <=> 2pg_m 11229 or 12393 or 9910

PGM 2pg_c <=> 3pg_c 11229 or 12393 or 12637 or 15425 or 9910
PGMf 3pg_f <=> 2pg_f 11229 or 12393 or 9910
PGMm 3pg_m <=> 2pg_m 11229 or 12393 or 9910

PGM 2pg_c <=> 3pg_c 11229 or 12393 or 12637 or 15425 or 9910
PGMf 3pg_f <=> 2pg_f 11229 or 12393 or 9910
PGMm 3pg_m <=> 2pg_m 11229 or 12393 or 9910
RZ5PP 5prdmbz_c + h2o_c --> pi_c + rdmbzi_c 12393 or 15195

PGM 2pg_c <=> 3pg_c 11229 or 12393 or 12637 or 15425 or 9910

PGM 2pg_c <=> 3pg_c 11229 or 12393 or 12637 or 15425 or 9910

ENO 2pg_c <=> h2o_c + pep_c 9037
ENOf 2pg_f <=> h2o_f + pep_f 9037
ENOm 2pg_m <=> h2o_m + pep_m 9037
```

In [10]:

```
# 10704 STL1 glycerol proton symporter of plasma membrane
# 10704 and 10705 gene models wrong, they are one gene
# GLYCt2 has 12792 GUP1/GUP2, which is O-acyltransferase involved in remodeling of the GPI anchor
model.reactions.get_by_id('GLYCt2').gene_reaction_rule = '10704 and 10705'
model.reactions.get_by_id('GALt2').gene_reaction_rule = '11075 or 11893'
model.reactions.get_by_id('GLCt1').gene_reaction_rule = '10452 or 11075 or 11893 or 15762 or 9102 or 9841'
model.reactions.get_by_id('GLUt2r').gene_reaction_rule = '14229 or 15074 or 8962 or 9319 or 9322 or 9962'
model.remove_reactions(['GALth','XYLt2pp','ABFPTh','AM6PTh','GLUKAh','GLUKBh'], remove_orphans=True)
```

In [11]:

```
# 11513 is putative phosphatase - ycsE in B. subtilis, 5-amino-6-(5-phospho-D-ribitylamino)uracil phosphatase
# Blast of known E. coli and B. subtilis genes hit to 11513
# PMDPHT is needed for riboflavin biosynthesis
r = sce.reactions.get_by_id('PMDPHT').copy()
model.add_reactions([r])
model.reactions.get_by_id('PMDPHT').gene_reaction_rule = '11513'
# Remove 11513 from other reactions
model.reactions.get_by_id('F1PP').gene_reaction_rule = '14546 or 8576'
model.reactions.get_by_id('F6PP').gene_reaction_rule = '14546 or 8576'
model.reactions.get_by_id('FBP').gene_reaction_rule = '15423'
model.reactions.get_by_id('G1PP').gene_reaction_rule = '8576'
model.reactions.get_by_id('MN6PP').gene_reaction_rule = '8460'
model.reactions.get_by_id('R5PP').gene_reaction_rule = '13044 or 14546 or 8576'
# 15195 or 15744 are Fructose-6-phosphate 2-kinase/fructose-2,6-biphosphatase -> FBP26 and PFK26
model.reactions.get_by_id('FBP26').gene_reaction_rule = '15195 or 15744'
model.remove_reactions(['BFBPh','RZ5PP','TPIh','PGKh'], remove_orphans=True)
# 9910 PGAM1, 12393 PGAM2, 11229 predicted phosphoglycerate mutase, 15425 broad substrate specificity phosphatase
# 12637 is sedoheptulose bisphosphatase
r = model.reactions.get_by_id('FBP26').copy()
r.id = 'SBP'
r.name = 'Sedoheptulose-bisphosphatase'
r.subsystem = 'Calvin cycle/Pentose phosphate pathway'
r.gene_reaction_rule = '12637'
model.add_reactions([r])
r.add_metabolites({'f26bp_c': 1.0, 'f6p_c': -1.0, 's17bp_c': -1.0, 's7p_c': 1.0})
# Remove 12637 from PGM
model.reactions.get_by_id('PGM').gene_reaction_rule = '11229 or 12393 or 15425 or 9910'
model.remove_reactions(['PGMf','PGMm','ENOf','ENOm'], remove_orphans=True)
```

Pentose phosphate pathway  
Glucose 6-phosphate dehydrogenase  
ZWF1 10000 K00036: G6PD, zwf; glucose-6-phosphate 1-dehydrogenase cyto\_nucl 13.5, nucl 12.5, cyto 11.5 SVL *6-phosphogluconolactonase  
SOL1 14499 K01057: PGLS, pgl, devB; 6-phosphogluconolactonase cyto\_nucl 10.833, cyto 10.5, nucl 7, cyto\_mito 6.832, pero 5, extr 2 SKL*  
SOL2 14132 K07404: pgl; 6-phosphogluconolactonase cyto 13, cyto\_mito 10.833, cyto\_nucl 9.333, mito 7.5, nucl 4.5 WLP *Phosphogluconate dehydrogenase  
GND1 15167 K00033: PGD, gnd, gntZ; 6-phosphogluconate dehydrogenase cyto 21, cysk 4 YNV*  
GND2 10592 KOG2653: 6-phosphogluconate dehydrogenase
Ribulose 5-phosphate 3-epimerase  
RPE1 16264 K01783: rpe, RPE; ribulose-phosphate 3-epimerase cyto 19, extr 4, nucl 2, mito 2, mito\_nucl 2 EGL *Ribose-5-phosphate isomerase  
RKI1 13288 K01807: rpiA; ribose 5-phosphate isomerase A mito 21, cyto 6 QRV*  
Transketolase  
TKL1 15631 K00615: E2.2.1.1, tktA, tktB; transketolase cyto 20, cyto\_nucl 12, pero 4 IHA *Transaldolase  
TAL1 10173 K00616: E2.2.1.2, talA, talB; transaldolase cyto 20, cyto\_nucl 13, nucl 4 KLQ*  
Pyruvate kinase
CDC19 13002 K00873: PK, pyk; pyruvate kinase cyto 15.5, cyto\_nucl 9.833, cyto\_pero 9.166, cysk 6, nucl 2, mito 2 PVE *Pyruvate decarboxylase  
PDC1 15791 K01568: PDC, pdc; pyruvate decarboxylase cyto 21.5, cyto\_mito 11.5, nucl 3 NAA*  
Aldehyde dehydrogenase  
ALDH 12042 K00128: ALDH; aldehyde dehydrogenase (NAD+) cyto 18.5, cyto\_nucl 10, mito 4, pero 4 NPL *Acetyl-CoA synthetase  
ACS2 14597 K01895: ACSS, acs; acetyl-CoA synthetase cyto 13, plas 5, mito 3, pero 2, mito\_nucl 2 SSE*  
ACS1 15276 K01907: AACS, acsA; acetoacetyl-CoA synthetase plas 9, pero 6, mito 5, cyto 5, cyto\_mito 5 SKL *Butanediol dehydrogenase  
BDH1 13641 K00004: BDH, butB; (R,R)-butanediol dehydrogenase / meso-butanediol dehydrogenase / diacetyl reductase plas 11, cyto 8.5, cyto\_nucl 5, mito 3, E.R. 2 QPF*  
BDH2 9634 K00004: BDH, butB; (R,R)-butanediol dehydrogenase / meso-butanediol dehydrogenase / diacetyl reductase cyto 16, mito 7, nucl 2 EEK *Pyruvate carboxylase  
PYC2 16630 K01958: PC, pyc; pyruvate carboxylase mito 19, cyto 7 VHS*  
Phosphoenolpyruvate carboxykinase  
PCK1 10841 K01610: E4.1.1.49, pckA; phosphoenolpyruvate carboxykinase (ATP) mito 17.5, cyto\_mito 12, cyto 5.5, pero 3 PKI\*

In [12]:

```
temp = ['10000','14499','14132','15167','10592','16264','13288','15631','10173','13002','15791','12042','14597',
        '12272','15276','13641','9634','16630','10841']
display(Annotation.loc[temp])
Show_Data(temp)
```

|  | Combined Annotations | Signal P | Sc288c Orthologs | Human Orthologs | Sc288 Best Hit | Human Blast | Essential | WolfPSort | C Terminal |
| --- | --- | --- | --- | --- | --- | --- | --- | --- | --- |
| RTO4\_ID |  |  |  |  |  |  |  |  |  |
| 10000 | K00036: G6PD, zwf; glucose-6-phosphate 1-dehyd... |  | ZWF1 | G6PD | ZWF1 | G6PD | Essential | cyto\_nucl 13.5, nucl 12.5, cyto 11.5 | SVL\* |
| 14499 | K01057: PGLS, pgl, devB; 6-phosphogluconolacto... |  | SOL1,SOL3,SOL2 | PGLS | SOL1 | PGLS | Essential | cyto\_nucl 10.833, cyto 10.5, nucl 7, cyto\_mito... | SKL\* |
| 14132 | K07404: pgl; 6-phosphogluconolactonase |  |  |  |  |  | Not Essential | cyto 13, cyto\_mito 10.833, cyto\_nucl 9.333, mi... | WLP\* |
| 15167 | K00033: PGD, gnd, gntZ; 6-phosphogluconate deh... |  | GND2,GND1 | PGD | GND1 | PGD | Essential | cyto 21, cysk 4 | YNV\* |
| 10592 | KOG2653: 6-phosphogluconate dehydrogenase |  |  |  | GND2 | PGD | Not Essential | cyto 19.5, cyto\_mito 12, mito 3.5, nucl 2, pero 2 | VKR\* |
| 16264 | K01783: rpe, RPE; ribulose-phosphate 3-epimerase |  | RPE1 | RPE,RPEL1 | RPE1 | RPE | Essential | cyto 19, extr 4, nucl 2, mito 2, mito\_nucl 2 | EGL\* |
| 13288 | K01807: rpiA; ribose 5-phosphate isomerase A |  | RKI1 | RPIA | RKI1 | RPIA | Essential | mito 21, cyto 6 | QRV\* |
| 15631 | K00615: E2.2.1.1, tktA, tktB; transketolase |  | TKL2,TKL1 | TKT,TKTL1,TKTL2 | TKL1 | TKT | Essential | cyto 20, cyto\_nucl 12, pero 4 | IHA\* |
| 10173 | K00616: E2.2.1.2, talA, talB; transaldolase |  | TAL1,NQM1 | TALDO1 | TAL1 | TALDO | Essential | cyto 20, cyto\_nucl 13, nucl 4 | KLQ\* |
| 13002 | K00873: PK, pyk; pyruvate kinase |  | CDC19,PYK2 | PKLR,PKM | CDC19 | PKM | Essential | cyto 15.5, cyto\_nucl 9.833, cyto\_pero 9.166, c... | PVE\* |
| 15791 | K01568: PDC, pdc; pyruvate decarboxylase |  | THI3,PDC6,PDC1,PDC5 |  | PDC1 |  | Not Essential | cyto 21.5, cyto\_mito 11.5, nucl 3 | NAA\* |
| 12042 | K00128: ALDH; aldehyde dehydrogenase (NAD+) |  | ALD5,ALD4,ALD6,ALD3,ALD2 | ALDH1A1,ALDH1A2,ALDH1A3,ALDH1B1,ALDH2 | ALD5 | ALDH2 | Not Essential | cyto 18.5, cyto\_nucl 10, mito 4, pero 4 | NPL\* |
| 14597 | K01895: ACSS, acs; acetyl-CoA synthetase |  | ACS1,ACS2 | ACSS1,ACSS2 | ACS2 | ACSS1 | Not Essential | cyto 13, plas 5, mito 3, pero 2, mito\_nucl 2 | SSE\* |
| 12272 | KOG1176: Acyl-CoA synthetase |  |  |  |  |  | Not Essential | mito 27 | MSQ\* |
| 15276 | K01907: AACS, acsA; acetoacetyl-CoA synthetase |  |  | AACS | ACS1 | AACS | Not Essential | plas 9, pero 6, mito 5, cyto 5, cyto\_mito 5 | SKL\* |
| 13641 | K00004: BDH, butB; (R,R)-butanediol dehydrogen... |  | YAL061W,BDH1 |  | BDH1 | SORD | Not Essential | plas 11, cyto 8.5, cyto\_nucl 5, mito 3, E.R. 2 | QPF\* |
| 9634 | K00004: BDH, butB; (R,R)-butanediol dehydrogen... |  | YAL061W,BDH1 |  | BDH2 | SORD | Not Essential | cyto 16, mito 7, nucl 2 | EEK\* |
| 16630 | K01958: PC, pyc; pyruvate carboxylase |  | PYC2,PYC1 | PC | PYC2 | PCe | Not Essential | mito 19, cyto 7 | VHS\* |
| 10841 | K01610: E4.1.1.49, pckA; phosphoenolpyruvate c... | S | PCK1 |  | PCK1 |  | Not Essential | mito 17.5, cyto\_mito 12, cyto 5.5, pero 3 | PKI\* |

| strain | WT | | | | | | | | | | | | | | | | |
| --- | --- | --- | --- | --- | --- | --- | --- | --- | --- | --- | --- | --- | --- | --- | --- | --- | --- |
| condition | G\_MM | C\_MM | G\_SD | | GX\_SD | | | X\_SD | | A\_SD | | C\_SD | | MM\_CN120 | | MM\_CN5 | Diversity\_Sample |
| phase | exp | exp | exp | stat | exp | trans | stat | exp | stat | exp | stat | exp | stat | exp | stat | exp | exp |
| proteinId | Set1 | Set1 | Set2 | Set2 | Set2 | Set2 | Set2 | Set2 | Set2 | Set2 | Set2 | Set2 | Set2 | Set3 | Set3 | Set3 | Set3 |
| 10000 | 8.17341 | 7.56828 | 8.48119 | 7.56243 | 8.57013 | 7.38039 | 7.34324 | 7.98479 | 6.85949 | 7.57151 | 6.58238 | 7.50307 | 7.59802 | 8.97582 | 8.61849 | 9.52322 | 8.74003 |
| 14499 | 7.9603 | 6.93284 | 8.27572 | 7.71507 | 8.36168 | 7.68692 | 7.90567 | 7.92291 | 7.92277 | 7.53002 | 7.48456 | 7.16792 | 7.33507 | 8.50151 | 8.56664 | 8.66587 | 8.78677 |
| 14132 | 4.37585 | 4.13659 | 5.96959 | 5.81081 | 6.34321 | 5.49882 | 5.67866 | 5.64995 | 6.36196 | 5.33224 | 5.41727 | 5.46633 | 5.8739 | 5.02813 | 5.23587 | 6.20566 | 4.64237 |
| 15167 | 8.54247 | 8.15633 | 9.37082 | 9.17898 | 9.44735 | 8.88378 | 8.84713 | 9.14596 | 8.52569 | 8.57528 | 7.93606 | 8.88545 | 8.9537 | 10.1147 | 9.83233 | 10.3693 | 10.2505 |
| 10592 | 4.20948 | 4.68681 | 3.92077 | 5.04418 | 3.91406 | 4.07911 | 4.22296 | 4.19904 | 4.2393 | 4.03972 | 4.91128 | 4.18668 | 4.25664 | 2.04735 | 2.18052 | 2.49167 | 2.18094 |
| 16264 | 7.37279 | 7.10592 | 6.82398 | 6.94046 | 6.8181 | 6.5394 | 6.61615 | 6.85964 | 6.40193 | 6.90669 | 6.76079 | 6.81824 | 6.74303 | 5.7131 | 5.83298 | 6.05124 | 5.8063 |
| 13288 | 5.84168 | 5.7298 | 6.15228 | 5.54092 | 6.27327 | 5.68548 | 5.80491 | 5.70565 | 5.89688 | 5.35867 | 5.77555 | 6.62452 | 7.03709 | 5.4092 | 5.44378 | 5.93017 | 5.33237 |
| 15631 | 9.23851 | 8.30643 | 9.22744 | 8.18399 | 9.28233 | 8.49606 | 8.28703 | 9.09759 | 7.87844 | 8.63321 | 7.54606 | 8.43199 | 7.91792 | 10.526 | 10.0093 | 10.4202 | 10.2477 |
| 10173 | 9.29313 | 8.78775 | 9.10703 | 8.59375 | 9.25514 | 8.69499 | 8.66493 | 9.21152 | 8.11539 | 8.82828 | 8.12043 | 8.44188 | 8.1976 | 9.38936 | 9.29014 | 10.3864 | 9.90979 |
| 13002 | 8.12051 | 6.22005 | 8.34341 | 4.24559 | 8.44416 | 6.95567 | 6.64084 | 8.42667 | 4.96456 | 7.19982 | 4.35827 | 5.6981 | 5.3494 | 9.12098 | 8.44723 | 9.26396 | 8.46837 |
| 15791 | 7.84603 | 5.59 | 8.00875 | 7.69016 | 7.99933 | 7.37604 | 7.53144 | 7.37936 | 7.66072 | 7.3849 | 7.52336 | 6.39243 | 7.14277 | 7.57892 | 7.20347 | 7.69795 | 7.22035 |
| 12042 | 8.00849 | 8.563 | 8.41698 | 9.34145 | 8.34137 | 8.32176 | 8.06734 | 8.44974 | 8.36047 | 8.84866 | 8.59447 | 7.17248 | 5.67475 | 7.81326 | 7.51984 | 8.29774 | 8.31731 |
| 14597 | 8.42238 | 7.97378 | 8.51818 | 7.99019 | 8.64056 | 7.40482 | 7.62933 | 7.37675 | 8.46789 | 7.83928 | 8.54106 | 7.90993 | 8.02345 | 8.92605 | 8.10393 | 9.91014 | 9.38791 |
| 12272 | 7.16396 | 6.06768 | 9.30378 | 7.71896 | 9.27654 | 9.15473 | 8.82481 | 8.04888 | 7.04121 | 7.27369 | 6.71586 | 5.31259 | 3.33195 | 7.049 | 7.87296 | 6.5763 | 7.19854 |
| 15276 | 5.29782 | 7.5276 | 6.02897 | 6.3398 | 5.8819 | 5.96372 | 5.9225 | 5.9897 | 5.36727 | 6.33017 | 5.89141 | 6.06136 | 5.30571 | 4.91543 | 4.54293 | 6.28014 | 5.10407 |
| 13641 | 5.66714 | 5.91845 | 2.17964 | 7.20987 | 1.49877 | 3.75845 | 3.49875 | 5.1321 | 4.19655 | 5.78775 | 4.32498 | 3.77193 | 0.576553 | 4.1106 | 4.07305 | 1.27722 | 3.97769 |
| 9634 | 4.19811 | 5.04256 | 2.35237 | 4.31974 | 2.16794 | 3.22784 | 3.34341 | 2.77526 | 4.79765 | 2.94779 | 5.13518 | 5.28646 | 4.57313 | 2.71676 | 2.60882 | 1.96379 | 2.9692 |
| 16630 | 8.6001 | 6.01767 | 9.14438 | 6.22731 | 9.34469 | 7.83324 | 7.58559 | 8.4822 | 5.89563 | 6.96413 | 5.59145 | 5.69896 | 4.66498 | 10.5956 | 10.2485 | 10.3326 | 9.33882 |
| 10841 | 6.39312 | 8.92655 | 8.20573 | 8.25224 | 8.27021 | 7.93778 | 8.05292 | 6.99663 | 8.90226 | 6.97456 | 8.14806 | 9.01701 | 10.0196 | 7.65739 | 7.30454 | 8.41918 | 8.83815 |

| strain | WT | | | | | | | | | | |
| --- | --- | --- | --- | --- | --- | --- | --- | --- | --- | --- | --- |
| condition | G\_SD | | GX\_SD | | | X\_SD | | A\_SD | | C\_SD | |
| proteinId | exp | stat | exp | trans | stat | exp | stat | exp | stat | exp | stat |
| 10000 | 28.4853 | 24.4134 | 34.9922 | 26.0213 | 27.5068 | 31.7194 | 33.2809 | 29.9574 | 29.4618 | 32.0917 | 27.3956 |
| 14499 | 19.9578 | 19.5681 | 19.9227 | 14.5189 | 19.1815 | 15.6529 | 17.0948 | 14.8836 | 17.0135 | 19.2347 | 22.248 |
| 14132 | 3.44906 | 5.80737 | 5.28024 | 6.85546 | 4.70556 | 5.8747 | 6.69982 | 6.37179 | 6.32646 | 2.57659 | 5.42357 |
| 15167 | 77.8848 | 72.1529 | 76.4837 | 67.7072 | 67.2912 | 75.9026 | 73.8327 | 71.4907 | 75.615 | 74.1825 | 73.5808 |
| 10592 | 1.92401 | 0.726389 | 2.02978 | 2.04319 | 1.46878 | 1.56145 | 0.765517 | 0.776352 | 0.574656 | 1.71584 | 1.09615 |
| 16264 | 6.53448 | 6.46553 | 5.50632 | 6.50618 | 7.66784 | 6.62665 | 7.50541 | 7.71201 | 7.47854 | 9.60996 | 8.28117 |
| 13288 | 8.59267 | 6.40288 | 9.15912 | 5.19659 | 5.79748 | 5.49508 | 6.90597 | 6.38257 | 7.07151 | 5.12131 | 10.433 |
| 15631 | 57.461 | 56.5774 | 60.6412 | 54.8836 | 53.7254 | 62.8295 | 57.6377 | 55.4563 | 62.2754 | 79.5313 | 66.4131 |
| 10173 | 37.8979 | 35.4896 | 38.2662 | 27.1971 | 28.8483 | 39.9558 | 44.538 | 44.8023 | 46.8749 | 36.7126 | 34.4136 |
| 13002 | 50.1794 | 43.8725 | 45.3775 | 37.0336 | 34.3579 | 41.2554 | 40.7139 | 37.6311 | 39.8143 | 24.982 | 23.0943 |
| 15791 | 18.6789 | 22.6393 | 20.5475 | 21.5711 | 21.3974 | 18.4088 | 20.9127 | 17.6003 | 20.8646 | 9.62964 | 10.8778 |
| 12042 | 33.9947 | 44.5614 | 32.5616 | 41.5121 | 40.2993 | 56.5808 | 63.4798 | 61.9973 | 72.5595 | 57.7357 | 40.7488 |
| 14597 | 33.4132 | 33.1333 | 28.0397 | 32.0427 | 29.6961 | 27.1912 | 30.3839 | 29.3511 | 35.2144 | 32.6888 | 37.6604 |
| 12272 | 9.63519 | 8.57386 | 8.57174 | 10.4172 | 9.6036 | 10.1791 | 7.89223 | 2.51425 | 3.24356 | 3.19408 | 1.5411 |
| 15276 | 11.0591 | 15.3254 | 10.9798 | 19.3402 | 20.029 | 18.4148 | 19.0534 | 17.1797 | 17.9891 | 29.9096 | 20.8835 |
| 13641 | 0 | 0.367567 | 0 | 0 | 0 | 0 | 0 | 1.35521 | 1.14348 | 1.48433 | 0.211526 |
| 9634 | 0 | 0 | 0 | 0 | 0 | 0 | 0 | 0 | 0 | 0.418441 | 0.435902 |
| 16630 | 88.9877 | 79.2711 | 88.286 | 80.7258 | 73.9948 | 68.8684 | 69.7168 | 63.9439 | 57.8313 | 53.5691 | 45.6917 |
| 10841 | 36.3205 | 38.1821 | 31.5244 | 35.9228 | 39.1203 | 37.7643 | 39.8016 | 27.424 | 32.3648 | 67.0475 | 72.3901 |

|  | Glucose | Xylose | Arabinose | Acetate | Coumarate | Ferulate | YNB Oleic Acid | YNB Ricinoleic Acid | YNB Glucose | YNB Gluc DOC | YPD |
| --- | --- | --- | --- | --- | --- | --- | --- | --- | --- | --- | --- |
| proteinId |  |  |  |  |  |  |  |  |  |  |  |
| 14132 | 0.30626 | 0.114532 | 0.30298 | 0.319481 | -0.0189789 | 0.226079 | -0.0707322 | -0.0179278 | -0.0110356 | -0.159417 | 0.144169 |
| 15167 | -0.0792831 | -0.0317026 | -0.686468 | 0.322317 | -0.237042 | 0.241671 | 0.0290566 | -0.879584 | 1.9236 | 1.7504 | 1.4516 |
| 10592 | -0.196362 | -0.134398 | -0.106302 | -0.138755 | 0.467126 | -0.0350862 | -0.114681 | -0.465819 | 0.159834 | 0.152704 | 0.0162792 |
| 15791 | -0.154956 | -0.0421572 | -0.0907286 | -0.0167367 | -0.120817 | -0.129008 | -0.170373 | -0.0587459 | -0.473236 | -0.697073 | -0.423493 |
| 12042 | 0.170666 | -0.143043 | 0.000953096 | -0.0230376 | -0.521499 | 0.0146534 | 0.144875 | -0.295284 | 0.392859 | 0.454577 | -0.242457 |
| 14597 | 0.134527 | 0.0997527 | 0.107444 | -1.05713 | 0.0951078 | -0.0881028 | 0.151616 | 0.954851 | 0.0158946 | -0.116256 | 0.000665712 |
| 12272 | -0.197686 | -0.196943 | -0.00208274 | 0.0746683 | 0.025135 | 0.0984501 | -0.335589 | -0.358104 | -0.0593179 | -0.0656978 | 0.0740662 |
| 15276 | -0.192767 | 0.221437 | 0.0764749 | -0.176646 | 0.00381209 | -0.117964 | -0.0335189 | -0.260812 | -0.126874 | -0.0575812 | -0.262471 |
| 13641 | -0.300208 | -0.0385665 | -0.282213 | -0.148702 | -0.265214 | -0.257009 | 0.0145788 | 0.0578672 | -0.315068 | -0.0920345 | -0.261348 |
| 9634 | -0.3042 | -0.786183 | -0.152149 | -0.255707 | -0.311738 | 0.488645 | -1.12467 | -0.704742 | -0.0783104 | -1.02173 | 0.285965 |
| 16630 | -1.81658 | -1.35136 | -2.0206 | 0.241922 | 0.338143 | 0.00905369 | -0.1375 | 0.217258 | -3.24805 | -0.0672098 | -0.368913 |
| 10841 | -0.0793112 | 0.0278154 | -0.00240243 | -0.996561 | -1.36306 | -0.798607 | -0.763344 | -0.977337 | -0.136229 | -0.287091 | -0.3009 |

In [13]:

```
for x in temp:
    if x in model.genes:
        for r in sorted(model.genes.get_by_id(x).reactions, key=lambda x: x.id):
            print(r.id, r.reaction, r.gene_reaction_rule)
    else:
        print(x, 'no reactions')
    print()
```

```
G6PDH2r g6p_c + nadp_c <=> 6pgl_c + h_c + nadph_c 10000

PGL 6pgl_c + h2o_c --> 6pgc_c + h_c 14132 or 14499
PGLh 6pgl_h + h2o_h --> 6pgc_h + h_h 14499

PGL 6pgl_c + h2o_c --> 6pgc_c + h_c 14132 or 14499

GND 6pgc_c + nadp_c --> co2_c + nadph_c + ru5p__D_c 15167
PGDHh 6pgc_h + nadp_h --> co2_h + nadph_h + ru5p__D_h 15167
yli_R1379 6pgc_c + nad_c --> co2_c + nadh_c + ru5p__D_c 15167

10592 no reactions

RPE ru5p__D_c <=> xu5p__D_c 16264
RPEh ru5p__D_h <=> xu5p__D_h 16264

RPI r5p_c <=> ru5p__D_c 13288
RPIh r5p_h <=> ru5p__D_h 13288
yli_R0482 yli_M00116_c <=> ru5p__D_c 13288

TKT1 r5p_c + xu5p__D_c <=> g3p_c + s7p_c 15631
TKT1h r5p_h + xu5p__D_h <=> g3p_h + s7p_h 15631
TKT2 e4p_c + xu5p__D_c <=> f6p_c + g3p_c 15631
TKT2h e4p_h + xu5p__D_h <=> f6p_B_h + g3p_h 15631
yli_R0485 g3p_c + s7p_c <=> xu5p__D_c + yli_M00116_c 15631

TALA g3p_c + s7p_c <=> e4p_c + f6p_c 10173
TAh g3p_h + s7p_h <=> e4p_h + f6p_B_h 10173

AGPOP dgdp_c + h_c + pep_c --> dgtp_c + pyr_c 13002
AGPOPm dgdp_m + 2.0 h_m + pep_m --> dgtp_m + pyr_m 13002
DAPOP dadp_m + h_m + pep_m --> datp_m + pyr_m 13002
GTPOPm gdp_m + 2.0 h_m + pep_m --> gtp_m + pyr_m 13002
PYK adp_c + h_c + pep_c --> atp_c + pyr_c 13002
PYK3 gdp_c + h_c + pep_c --> gtp_c + pyr_c 13002
PYKf adp_f + h_f + pep_f --> atp_f + pyr_f 13002
PYKm adp_m + h_m + pep_m --> atp_m + pyr_m 13002
yli_R1553 dgtp_c + pyr_c <=> dgdp_c + pep_c 13002
yli_R1554 gtp_c + pyr_c <=> gdp_c + pep_c 13002
yli_R1557 datp_c + pyr_c <=> dadp_c + pep_c 13002

3MOBDC 3mob_c + h_c --> 2mppal_c + co2_c 15791
3MOPDC 3mop_c + h_c --> 2mbald_c + co2_c 15791
4MOPDC 4mop_c + h_c --> 3mbald_c + co2_c 15791
ACALDCD 2.0 acald_c --> actn__R_c 15791
INDPYRD h_c + indpyr_c <=> co2_c + id3acald_c 15791
PPYRDC h_c + phpyr_c --> co2_c + pacald_c 15791
PYRDC h_c + pyr_c --> acald_c + co2_c 15791
PYRDC2 acald_c + h_c + pyr_c --> actn__R_c + co2_c 15791

34DHALDD 34dhpac_c + h2o_c + nad_c --> 34dhpha_c + 2.0 h_c + nadh_c 12042 or 13426 or 16323
34DHPLACOX_NADP 34dhpac_c + h2o_c + nadp_c <=> 34dhpha_c + 2.0 h_c + nadph_c 12042 or 13426 or 16323
3HPADHi 3hppnl_c + h2o_c + nad_c --> 3hpp_c + 2.0 h_c + nadh_c 12042 or 13426
3M4HDXPAC 3mox4hpac_c + h2o_c + nad_c <=> 2.0 h_c + homoval_c + nadh_c 12042 or 13426 or 16323
3MOX4HOXPGALDOX 3m4hpga_c + h2o_c + nad_c --> 3mox4hoxm_c + 2.0 h_c + nadh_c 12042 or 13426 or 16323
3MOX4HOXPGALDOX_NADP 3m4hpga_c + h2o_c + nadp_c <=> 3mox4hoxm_c + 2.0 h_c + nadph_c 12042 or 13426 or 16323
4HOXPACDOX_NADP 4hoxpacd_c + h2o_c + nadp_c <=> 4hphac_c + 2.0 h_c + nadph_c 12042 or 13426 or 16323
5HOXINDACTOX 5hoxindact_c + h2o_c + nad_c --> 5hoxindoa_c + 2.0 h_c + nadh_c 12042 or 13426 or 15814 or 16323
5HOXINDACTOXm 5hoxindact_m + h2o_m + nad_m --> 5hoxindoa_m + 2.0 h_m + nadh_m 12042 or 13426
ABOR 4abutn_m + h2o_m + nadp_m --> 4abut_m + 2.0 h_m + nadph_m 12042 or 13426
ABUTD 4abutn_c + h2o_c + nad_c --> 4abut_c + 2.0 h_c + nadh_c 12042 or 13426 or 16323
ABUTDm 4abutn_m + h2o_m + nad_m --> 4abut_m + 2.0 h_m + nadh_m 12042 or 13426
ALDD19x_P h2o_c + nadp_c + pacald_c --> 2.0 h_c + nadph_c + pac_c 12042 or 13426 or 16323
ALDD19xr h2o_c + nad_c + pacald_c <=> 2.0 h_c + nadh_c + pac_c 12042 or 13426 or 16323
ALDD20x h2o_c + id3acald_c + nad_c --> 2.0 h_c + ind3ac_c + nadh_c 12042 or 13426 or 15814 or 16323
ALDD20xm h2o_m + id3acald_m + nad_m --> 2.0 h_m + ind3ac_m + nadh_m 12042 or 13426
ALDD20y h2o_c + id3acald_c + nadp_c --> 2.0 h_c + ind3ac_c + nadph_c 12042 or 13426
ALDD20ym h2o_m + id3acald_m + nadp_m --> 2.0 h_m + ind3ac_m + nadph_m 12042 or 13426
ALDD2x acald_c + h2o_c + nad_c --> ac_c + 2.0 h_c + nadh_c 12042 or 13426 or 15814 or 16323
ALDD2xm acald_m + h2o_m + nad_m --> ac_m + 2.0 h_m + nadh_m 12042 or 13426 or 16323
ALDD2y acald_c + h2o_c + nadp_c --> ac_c + 2.0 h_c + nadph_c 11650 or 12042 or 13426 or 14700 or 16323 or 8666
ALDD2ym acald_m + h2o_m + nadp_m --> ac_m + 2.0 h_m + nadph_m 12042 or 13426
BAMPPALDOX bamppald_c + h2o_c + nad_c --> ala_B_c + 2.0 h_c + nadh_c 12042 or 13426 or 15814 or 16323
BAMPPALDOXm bamppald_m + h2o_m + nad_m --> ala_B_m + 2.0 h_m + nadh_m 12042 or 13426
FTHFDH 10fthf_c + h2o_c + nadp_c --> co2_c + h_c + nadph_c + thf_c 12042 or 13426
GCALDD gcald_c + h2o_c + nad_c --> glyclt_c + 2.0 h_c + nadh_c 12042 or 13426 or 15814 or 16323
GCALDDm gcald_m + h2o_m + nad_m --> glyclt_m + 2.0 h_m + nadh_m 12042 or 13426
GDBTALDH gdbtal_c + h2o_c + nad_c --> 4gudbutn_c + 2.0 h_c + nadh_c 12042 or 13426
GGGABADr ggbutal_c + h2o_c + nadp_c <=> gg4abut_c + 2.0 h_c + nadph_c 12042 or 13426
GLACO glac_c + 2.0 h2o_c + nad_c --> glcr_c + 3.0 h_c + nadh_c 12042 or 13426 or 16323
GLACOm glac_m + 2.0 h2o_m + nad_m --> glcr_m + 3.0 h_m + nadh_m 12042 or 13426
IMACTD h2o_c + im4act_c + nad_c --> 2.0 h_c + im4ac_c + nadh_c 12042 or 13426 or 15814 or 16323
IMACTD_m h2o_m + im4act_m + nad_m --> 2.0 h_m + im4ac_m + nadh_m 12042 or 13426
LCADi h2o_c + lald__L_c + nad_c --> 2.0 h_c + lac__L_c + nadh_c 12042 or 13426 or 15814 or 16323
LCADi_D h2o_c + lald__D_c + nad_c --> 2.0 h_c + lac__D_c + nadh_c 12042 or 13426 or 15814 or 16323
LCADi_Dm h2o_m + lald__D_m + nad_m --> 2.0 h_m + lac__D_m + nadh_m 12042 or 13426
LCADm h2o_m + lald__L_m + nad_m --> 2.0 h_m + lac__L_m + nadh_m 12042 or 13426
MACOXO 3mldz_c + h2o_c + nad_c --> 3mlda_c + 2.0 h_c + nadh_c 12042 or 13426 or 16323
NABTNO h2o_c + n4abutn_c + nad_c --> 4aabutn_c + 2.0 h_c + nadh_c 12042 or 13426 or 15814 or 16323
NABTNOm h2o_m + n4abutn_m + nad_m --> 4aabutn_m + 2.0 h_m + nadh_m 12042 or 13426
PYLALDOX h2o_c + nad_c + pylald_c --> 2.0 h_c + nadh_c + peracd_c 12042 or 13426 or 15814 or 16323
PYLALDOXm h2o_m + nad_m + pylald_m --> 2.0 h_m + nadh_m + peracd_m 12042 or 13426

ACS ac_c + atp_c + coa_c --> accoa_c + amp_c + ppi_c 14597
ACS2 atp_c + coa_c + ppa_c --> amp_c + ppcoa_c + ppi_c 14597
ACSm ac_m + atp_m + coa_m --> accoa_m + amp_m + ppi_m 12272 or 14597

ACSm ac_m + atp_m + coa_m --> accoa_m + amp_m + ppi_m 12272 or 14597
FACOAL40im atp_m + but_m + coa_m --> amp_m + btcoa_m + ppi_m 12272

AACOATx acac_x + atp_x + coa_x <=> aacoa_x + amp_x + ppi_x 15276

BTDD_RR btd_RR_c + nad_c <=> actn__R_c + h_c + nadh_c 13641 or 9634

BTDD_RR btd_RR_c + nad_c <=> actn__R_c + h_c + nadh_c 13641 or 9634

OAADC h_c + oaa_c --> co2_c + pyr_c 16630
PC atp_c + hco3_c + pyr_c --> adp_c + h_c + oaa_c + pi_c 16630
PCm atp_m + hco3_m + pyr_m --> adp_m + h_m + oaa_m + pi_m 16630

PPCK atp_c + oaa_c --> adp_c + co2_c + pep_c 10841
PPCKm atp_m + oaa_m --> adp_m + co2_m + pep_m 10841
```

Dual localization of oxidative PP pathway in cyto and pero  
https://academic.oup.com/femsyr/article/12/1/61/642733  
PYK1 in yeast has no entries in brenda for other cofactors than ADP  
PYRDC2 is lumped reaction of PYRDC and ACALDCD  
OAADC is a sub-reaction of malate dehydrogenase

In [14]:

```
r = model.reactions.get_by_id('G6PDH2r').copy()
r.id = 'G6PDH2rp'
model.add_reactions([r])
for m in r.metabolites:
    if not m.id.replace('_c','_x') in model.metabolites:
        m2 = m.copy()
        m2.id = m.id.replace('_c','_x')
        m2.compartment = 'x'
        model.add_metabolites([m2])
    r.add_metabolites({m.id: -r.get_coefficient(m.id), m.id.replace('_c','_x'): r.get_coefficient(m.id)})

model.reactions.get_by_id('PGL').gene_reaction_rule = '14132'
r = model.reactions.get_by_id('PGL').copy()
r.id = 'PGLp'
r.gene_reaction_rule = '14499'
model.add_reactions([r])
for m in r.metabolites:
    if not m.id.replace('_c','_x') in model.metabolites:
        m2 = m.copy()
        m2.id = m.id.replace('_c','_x')
        m2.compartment = 'x'
        model.add_metabolites([m2])
    r.add_metabolites({m.id: -r.get_coefficient(m.id), m.id.replace('_c','_x'): r.get_coefficient(m.id)})

r = model.reactions.get_by_id('GND').copy()
r.id = 'GNDp'
r.gene_reaction_rule = '10592'
model.add_reactions([r])
for m in r.metabolites:
    if not m.id.replace('_c','_x') in model.metabolites:
        m2 = m.copy()
        m2.id = m.id.replace('_c','_x')
        m2.compartment = 'x'
        model.add_metabolites([m2])
    r.add_metabolites({m.id: -r.get_coefficient(m.id), m.id.replace('_c','_x'): r.get_coefficient(m.id)})
remove = ['PGLh','PGDHh','yli_R1379','RPEh','RPIh','yli_R0482','TKT1h','TKT2h','yli_R0485','TAh','AGPOP','AGPOPm',
          'DAPOP','GTPOPm','PYK3','PYKf','PYKm','yli_R1553','yli_R1554','yli_R1557','PYRDC2','PCm','PPCKm']
model.remove_reactions(remove, remove_orphans=True)
```

In [15]:

```
temp = ['12761','13917','11178','10614']
display(Annotation.loc[temp])
Show_Data(temp)
```

|  | Combined Annotations | Signal P | Sc288c Orthologs | Human Orthologs | Sc288 Best Hit | Human Blast | Essential | WolfPSort | C Terminal |
| --- | --- | --- | --- | --- | --- | --- | --- | --- | --- |
| RTO4\_ID |  |  |  |  |  |  |  |  |  |
| 12761 | K00029: E1.1.1.40, maeB; malate dehydrogenase ... |  | MAE1 | ME1,ME2,ME3 | MAE1 | ME1e | Not Essential | mito 8, cyto 7.5, cyto\_nucl 7.5, nucl 6.5, pero 4 | QAQ\* |
| 13917 | K00027: ME2, sfcA, maeA; malate dehydrogenase ... | S | MAE1 | ME1,ME2,ME3 | MAE1 | ME1e | Not Essential | mito 18, cyto 6.5, cyto\_nucl 4.5 | DRH\* |
| 11178 | K00026: MDH2; malate dehydrogenase |  | MDH1 | MDH2 | MDH1 | MDH2 | Essential | mito 27 | MSA\* |
| 10614 | K00026: MDH2; malate dehydrogenase |  |  |  | MDH1 | MDH2 | Not Essential | cyto 14, mito 7, cyto\_nucl 7 | PKL\* |

| strain | WT | | | | | | | | | | | | | | | | |
| --- | --- | --- | --- | --- | --- | --- | --- | --- | --- | --- | --- | --- | --- | --- | --- | --- | --- |
| condition | G\_MM | C\_MM | G\_SD | | GX\_SD | | | X\_SD | | A\_SD | | C\_SD | | MM\_CN120 | | MM\_CN5 | Diversity\_Sample |
| phase | exp | exp | exp | stat | exp | trans | stat | exp | stat | exp | stat | exp | stat | exp | stat | exp | exp |
| proteinId | Set1 | Set1 | Set2 | Set2 | Set2 | Set2 | Set2 | Set2 | Set2 | Set2 | Set2 | Set2 | Set2 | Set3 | Set3 | Set3 | Set3 |
| 12761 | 6.06035 | 6.86758 | 5.35077 | 5.80532 | 5.46901 | 5.38484 | 5.91764 | 6.30228 | 5.18916 | 5.49928 | 5.19408 | 7.77164 | 8.61656 | 6.34657 | 6.52166 | 8.27188 | 8.08078 |
| 13917 | 4.3637 | 2.51542 | 7.17473 | 3.73426 | 7.29327 | 5.23853 | 4.87626 | 4.89474 | 3.91726 | 3.07649 | 3.2265 | 2.66923 | 2.22177 | 6.99097 | 6.26771 | 7.7727 | 7.10322 |
| 11178 | 7.03543 | 7.97856 | 9.46982 | 7.786 | 9.46998 | 9.20134 | 8.82189 | 9.58209 | 7.68509 | 8.40458 | 7.05307 | 8.35846 | 8.32769 | 9.3256 | 9.41544 | 10.5412 | 10.6966 |
| 10614 | 8.64545 | 8.32781 | 9.21071 | 8.6178 | 9.23594 | 8.92871 | 8.94549 | 8.35765 | 8.83237 | 8.15525 | 8.11422 | 9.39967 | 8.94239 | 9.51798 | 8.67715 | 9.19906 | 9.41826 |

| strain | WT | | | | | | | | | | |
| --- | --- | --- | --- | --- | --- | --- | --- | --- | --- | --- | --- |
| condition | G\_SD | | GX\_SD | | | X\_SD | | A\_SD | | C\_SD | |
| proteinId | exp | stat | exp | trans | stat | exp | stat | exp | stat | exp | stat |
| 12761 | 7.87783 | 12.551 | 8.34815 | 7.4678 | 7.31806 | 11.7468 | 11.4396 | 16.6158 | 16.4617 | 22.1843 | 34.1449 |
| 13917 | 16.6451 | 13.958 | 16.2782 | 14.5306 | 10.5669 | 1.56501 | 1.37134 | 0 | 0 | 0 | 0 |
| 11178 | 68.0062 | 59.6944 | 67.3574 | 60.436 | 61.5583 | 64.9688 | 66.1383 | 56.6021 | 57.2142 | 57.7355 | 57.9989 |
| 10614 | 34.2711 | 35.2867 | 34.5652 | 31.2482 | 35.1281 | 31.5759 | 35.2867 | 30.1388 | 31.7645 | 56.1891 | 60.5102 |

|  | Glucose | Xylose | Arabinose | Acetate | Coumarate | Ferulate | YNB Oleic Acid | YNB Ricinoleic Acid | YNB Glucose | YNB Gluc DOC | YPD |
| --- | --- | --- | --- | --- | --- | --- | --- | --- | --- | --- | --- |
| proteinId |  |  |  |  |  |  |  |  |  |  |  |
| 12761 | 0.231734 | 0.270871 | -0.00401769 | -0.00800572 | 0.236081 | 0.218122 | -0.520763 | 0.299513 | -0.0948034 | -0.0522589 | 0.186543 |
| 13917 | 0.0368201 | -0.149196 | -0.0875023 | -0.192422 | 0.121169 | -0.145843 | -0.220337 | 0.176785 | 0.280122 | 0.474473 | -0.0862589 |
| 10614 | -0.489826 | 0.863084 | -0.36301 | -0.671649 | -0.929687 | 0.0981584 | -0.627571 | -0.185759 | -1.13085 | -0.441528 | -0.184541 |

In [16]:

```
for x in temp:
    for r in sorted(model.genes.get_by_id(x).reactions, key=lambda x: x.id):
        print(r.id, r.reaction, r.gene_reaction_rule)
    print()
```

```
MDHC_nadp_hr mal__L_h + nadp_h <=> co2_h + nadph_h + pyr_h 12761
ME1 mal__L_c + nad_c --> co2_c + nadh_c + pyr_c 12761 or 13917
ME1m mal__L_m + nad_m --> co2_m + nadh_m + pyr_m 12761 or 13917
ME2 mal__L_c + nadp_c --> co2_c + nadph_c + pyr_c 12761 or 13917
ME2m mal__L_m + nadp_m --> co2_m + nadph_m + pyr_m 12761 or 13917

ME1 mal__L_c + nad_c --> co2_c + nadh_c + pyr_c 12761 or 13917
ME1m mal__L_m + nad_m --> co2_m + nadh_m + pyr_m 12761 or 13917
ME2 mal__L_c + nadp_c --> co2_c + nadph_c + pyr_c 12761 or 13917
ME2m mal__L_m + nadp_m --> co2_m + nadph_m + pyr_m 12761 or 13917

MDH mal__L_c + nad_c <=> h_c + nadh_c + oaa_c 10614 or 11178
MDHf h_f + nadh_f + oaa_f <=> mal__L_f + nad_f 10614 or 11178
MDHh h_h + nadh_h + oaa_h <=> mal__L_h + nad_h 10614 or 11178
MDHm mal__L_m + nad_m <=> h_m + nadh_m + oaa_m 10614 or 11178
MDHp mal__L_x + nad_x <=> h_x + nadh_x + oaa_x 10614 or 11178
SLDxm nad_m + sl__L_m <=> 3spyr_m + h_m + nadh_m 11178
yli_R1494 nad_c + yli_M07013_c --> h_c + nadh_c + yli_M02657_c 10614 or 11178

MDH mal__L_c + nad_c <=> h_c + nadh_c + oaa_c 10614 or 11178
MDHf h_f + nadh_f + oaa_f <=> mal__L_f + nad_f 10614 or 11178
MDHh h_h + nadh_h + oaa_h <=> mal__L_h + nad_h 10614 or 11178
MDHm mal__L_m + nad_m <=> h_m + nadh_m + oaa_m 10614 or 11178
MDHp mal__L_x + nad_x <=> h_x + nadh_x + oaa_x 10614 or 11178
yli_R1494 nad_c + yli_M07013_c --> h_c + nadh_c + yli_M02657_c 10614 or 11178
```

In [17]:

```
# 12671 ME1/maeB 1.1.1.40 cyto NADP, and 13917 ME2/maeA 1.1.1.38 mito NAD
model.reactions.get_by_id('OAADC').gene_reaction_rule = '12761'
r = model.reactions.get_by_id('OAADC').copy()
r.id = 'OAADCm'
r.name = 'Oxaloacetate decarboxylase mitochondrial'
r.gene_reaction_rule = '13917'
model.add_reactions([r])
for m in r.metabolites:
    if not m.id.replace('_c','_m') in model.metabolites:
        m2 = m.copy()
        m2.id = m.id.replace('_c','_m')
        m2.compartment = 'm'
        model.add_metabolites([m2])
    r.add_metabolites({m.id: -r.get_coefficient(m.id), m.id.replace('_c','_m'): r.get_coefficient(m.id)})
model.reactions.get_by_id('ME2').gene_reaction_rule = '12761'
model.reactions.get_by_id('ME1m').gene_reaction_rule = '13917'
model.remove_reactions(['MDHC_nadp_hr','ME1','ME2m'], remove_orphans=True)
# 11178 MDH1 yeast / MDH2 human 1.1.1.37 mito, 10614 weak sigP and PKL* -> MDH3 yeast?
model.reactions.get_by_id('MDHm').gene_reaction_rule = '11178'
model.reactions.get_by_id('MDHp').gene_reaction_rule = '10614'
model.remove_reactions(['MDH','MDHf','MDHh','SLDxm','yli_R1494'], remove_orphans=True)
```

Pyruvate metabolism and the TCA cycle  
Pyruvate transport in via proton symport  
JEN1 10184 K08178: JEN; MFS transporter, SHS family, lactate transporter plas 25 SAY *Pyruvate dehydrogenase mitochondrial  
LPD1 10040 K00382: DLD, lpd, pdhD; dihydrolipoamide dehydrogenase mito 9, cyto 8, extr 7, pero 2 INA*  
PDA1 13630 K00161: PDHA, pdhA; pyruvate dehydrogenase E1 component alpha subunit mito 25.5, cyto\_mito 13.5 STV *PDX1 13722 KOG0557: Dihydrolipoamide acetyltransferase mito 23.5, cyto\_mito 12.833, cyto\_nucl 1.833 LGL*  
PDB1 13948 K00162: PDHB, pdhB; pyruvate dehydrogenase E1 component beta subunit cysk 14, cyto 7, cyto\_nucl 5.5, mito 4 YRV *LAT1 14126 K00627: DLAT, aceF, pdhC; pyruvate dehydrogenase E2 component (dihydrolipoamide acetyltransferase) cyto 24.5, cyto\_nucl 13.333 FML*  
Citrate synthase  
CIT1 11331 K01647: CS, gltA; citrate synthase mito 26.5, cyto\_mito 14 LVK *CIT2 8548 K01647: CS, gltA; citrate synthase cyto 12, cyto\_nucl 8, mito 5, pero 4, cysk 4 SKL*  
Citrate transport mitochondrial
CTP1 10514 K15100: SLC25A1, CTP; solute carrier family 25 (mitochondrial citrate transporter), member 1 extr 13, mito 5, E.R. 3, vacu 3, cyto\_mito 3 RAV *Aconitate hydratase
ACO1 11624 K01681: ACO, acnA; aconitate hydratase mito 18.5, cyto\_mito 13, cyto 6.5 SKQ*  
ACO2 14600 K17450: ACO2; homoaconitase mito 20, cyto 4.5, cyto\_nucl 3.5 GQA\*

In [18]:

```
temp = ['10184','10040','13630','13722','13948','14126','11331','8548','10514','11624','14600']
display(Annotation.loc[temp])
Show_Data(temp)
```

|  | Combined Annotations | Signal P | Sc288c Orthologs | Human Orthologs | Sc288 Best Hit | Human Blast | Essential | WolfPSort | C Terminal |
| --- | --- | --- | --- | --- | --- | --- | --- | --- | --- |
| RTO4\_ID |  |  |  |  |  |  |  |  |  |
| 10184 | K08178: JEN; MFS transporter, SHS family, lact... |  | JEN1 |  | JEN1 |  | Not Essential | plas 25 | SAY\* |
| 10040 | K00382: DLD, lpd, pdhD; dihydrolipoamide dehyd... | S | LPD1 | DLD | LPD1 | DLD | Essential | mito 9, cyto 8, extr 7, pero 2 | INA\* |
| 13630 | K00161: PDHA, pdhA; pyruvate dehydrogenase E1 ... |  | PDA1 | PDHA1,PDHA2 | PDA1 | PDHA2 | Essential | mito 25.5, cyto\_mito 13.5 | STV\* |
| 13722 | KOG0557: Dihydrolipoamide acetyltransferase |  | PDX1 |  | PDX1 | PDHX | Not Essential | mito 23.5, cyto\_mito 12.833, cyto\_nucl 1.833 | LGL\* |
| 13948 | K00162: PDHB, pdhB; pyruvate dehydrogenase E1 ... |  | PDB1 | PDHB | PDB1 | PDHB | Essential | cysk 14, cyto 7, cyto\_nucl 5.5, mito 4 | YRV\* |
| 14126 | K00627: DLAT, aceF, pdhC; pyruvate dehydrogena... |  | LAT1 | DLAT | LAT1 | DLAT | Essential | cyto 24.5, cyto\_nucl 13.333 | FML\* |
| 11331 | K01647: CS, gltA; citrate synthase |  | CIT2,CIT1,CIT3 | CS | CIT1 | CSe | Essential | mito 26.5, cyto\_mito 14 | LVK\* |
| 8548 | K01647: CS, gltA; citrate synthase |  |  | HDDC3 | CIT1 |  | Not Essential | cyto 12, cyto\_nucl 8, mito 5, pero 4, cysk 4 | SKL\* |
| 10514 | K15100: SLC25A1, CTP; solute carrier family 25... | S | CTP1 | SLC25A1 | CTP1 | SLC25A | Not Essential | extr 13, mito 5, E.R. 3, vacu 3, cyto\_mito 3 | RAV\* |
| 11624 | K01681: ACO, acnA; aconitate hydratase |  | ACO2,ACO1 | ACO1,IREB2,ACO2 | ACO1 | ACO2 | Not Essential | mito 18.5, cyto\_mito 13, cyto 6.5 | SKQ\* |
| 14600 | K17450: ACO2; homoaconitase |  | ACO2,ACO1 | ACO1,IREB2,ACO2 | ACO2 | ACO2 | Not Essential | mito 20, cyto 4.5, cyto\_nucl 3.5 | GQA\* |

| strain | WT | | | | | | | | | | | | | | | | |
| --- | --- | --- | --- | --- | --- | --- | --- | --- | --- | --- | --- | --- | --- | --- | --- | --- | --- |
| condition | G\_MM | C\_MM | G\_SD | | GX\_SD | | | X\_SD | | A\_SD | | C\_SD | | MM\_CN120 | | MM\_CN5 | Diversity\_Sample |
| phase | exp | exp | exp | stat | exp | trans | stat | exp | stat | exp | stat | exp | stat | exp | stat | exp | exp |
| proteinId | Set1 | Set1 | Set2 | Set2 | Set2 | Set2 | Set2 | Set2 | Set2 | Set2 | Set2 | Set2 | Set2 | Set3 | Set3 | Set3 | Set3 |
| 10184 | 4.58216 | 4.15146 | 2.59409 | 5.99922 | 2.47565 | 4.45012 | 4.2663 | 5.70524 | 6.06956 | 6.88161 | 6.66917 | 0.679086 | 1.31305 | 2.07764 | 1.21593 | 3.74866 | 3.21065 |
| 10040 | 8.00662 | 7.31735 | 8.13214 | 6.97186 | 8.20048 | 7.36158 | 7.42582 | 7.56306 | 7.36598 | 7.19991 | 6.96873 | 7.35137 | 7.14045 | 8.72929 | 8.27061 | 9.01736 | 8.6493 |
| 13630 | 8.48359 | 7.58054 | 7.96555 | 6.02301 | 8.09102 | 7.39264 | 7.10697 | 7.98586 | 6.30924 | 7.38822 | 6.04112 | 7.15937 | 6.84481 | 9.46081 | 9.0204 | 9.6851 | 9.29698 |
| 13722 | 7.42057 | 5.828 | 6.9185 | 5.29614 | 6.92697 | 6.19558 | 6.1106 | 6.71591 | 5.59054 | 6.07582 | 5.34279 | 5.44552 | 4.79128 | 7.44355 | 6.7497 | 7.42908 | 6.56676 |
| 13948 | 8.3982 | 7.54127 | 7.74636 | 5.53748 | 7.79889 | 7.08864 | 6.69894 | 7.69181 | 5.81407 | 7.12451 | 5.59537 | 6.25868 | 5.94264 | 10.3788 | 9.73252 | 10.7617 | 10.1126 |
| 14126 | 8.10052 | 7.03471 | 7.88739 | 5.76332 | 8.02155 | 6.80631 | 6.54223 | 7.8752 | 5.90326 | 7.26807 | 5.83753 | 6.75984 | 6.80684 | 9.53979 | 8.53346 | 9.854 | 9.09821 |
| 11331 | 7.9659 | 8.38591 | 9.88165 | 7.7434 | 9.92018 | 8.31748 | 8.07868 | 8.98807 | 7.84055 | 7.75622 | 7.02527 | 7.95241 | 7.48739 | 10.6424 | 10.0994 | 11.5202 | 11.4734 |
| 8548 | 7.52186 | 7.85554 | 6.55094 | 7.08984 | 6.42217 | 6.61907 | 6.86413 | 6.10908 | 5.8416 | 6.10943 | 5.82702 | 7.40186 | 6.86364 | 6.82012 | 6.15303 | 7.00354 | 6.78515 |
| 10514 | 6.64861 | 6.36247 | 5.70844 | 6.11042 | 5.80885 | 5.78159 | 6.03447 | 5.97857 | 6.31136 | 5.74928 | 6.16677 | 6.01711 | 5.93248 | 5.65323 | 6.06213 | 5.85188 | 5.94402 |
| 11624 | 7.51552 | 7.94222 | 5.95564 | 6.36926 | 5.94043 | 6.18795 | 5.92054 | 5.51375 | 6.11534 | 5.6773 | 6.09101 | 6.31763 | 6.20146 | 7.48602 | 8.08796 | 7.88672 | 9.83162 |
| 14600 | 4.23002 | 6.82861 | 3.04574 | 3.75571 | 2.88192 | 4.01126 | 4.14943 | 3.80416 | 3.69202 | 3.80241 | 4.05072 | 4.9755 | 4.72552 | 4.21007 | 4.34772 | 4.50387 | 6.4354 |

| strain | WT | | | | | | | | | | |
| --- | --- | --- | --- | --- | --- | --- | --- | --- | --- | --- | --- |
| condition | G\_SD | | GX\_SD | | | X\_SD | | A\_SD | | C\_SD | |
| proteinId | exp | stat | exp | trans | stat | exp | stat | exp | stat | exp | stat |
| 10184 | 0 | 0.562755 | 0 | 0.93093 | 0.180258 | 0.974881 | 1.18215 | 1.15917 | 1.14892 | 0 | 0 |
| 10040 | 47.0337 | 54.5942 | 39.0915 | 48.1654 | 53.3645 | 35.4178 | 41.1307 | 33.4162 | 36.9135 | 42.3765 | 44.1734 |
| 13630 | 31.1424 | 29.3179 | 31.7646 | 28.6595 | 28.5254 | 28.364 | 35.5131 | 29.1694 | 27.9411 | 23.9754 | 25.0472 |
| 13722 | 9.01341 | 7.65947 | 9.58594 | 7.26433 | 8.40491 | 5.47845 | 5.88606 | 7.73304 | 7.84819 | 5.77765 | 6.12104 |
| 13948 | 24.1495 | 22.2025 | 26.4382 | 19.525 | 20.7251 | 21.7231 | 19.8865 | 17.5688 | 15.9145 | 18.5711 | 16.1251 |
| 14126 | 26.1505 | 23.8033 | 23.4011 | 19.1179 | 19.7413 | 19.5951 | 19.8326 | 18.1422 | 18.192 | 20.882 | 15.4575 |
| 11331 | 73.6604 | 63.3635 | 65.7316 | 58.2509 | 62.2853 | 48.7414 | 48.8113 | 39.796 | 39.8205 | 67.4978 | 63.1003 |
| 8548 | 15.4971 | 17.9775 | 17.9043 | 17.8454 | 19.741 | 18.837 | 15.1446 | 13.712 | 13.4056 | 38.3064 | 37.4182 |
| 10514 | 6.13985 | 6.66771 | 6.71306 | 5.58373 | 4.20338 | 4.89588 | 5.27854 | 5.79215 | 6.1183 | 3.87384 | 5.66964 |
| 11624 | 34.2951 | 27.3851 | 32.3467 | 23.8186 | 25.0857 | 27.593 | 31.6434 | 34.5659 | 32.318 | 31.4151 | 30.6923 |
| 14600 | 0.382292 | 0 | 0.41045 | 0 | 0 | 0 | 0 | 0.776735 | 0 | 0 | 0 |

|  | Glucose | Xylose | Arabinose | Acetate | Coumarate | Ferulate | YNB Oleic Acid | YNB Ricinoleic Acid | YNB Glucose | YNB Gluc DOC | YPD |
| --- | --- | --- | --- | --- | --- | --- | --- | --- | --- | --- | --- |
| proteinId |  |  |  |  |  |  |  |  |  |  |  |
| 10184 | -0.105762 | -0.0555069 | -0.0970765 | 0.0146873 | -0.41921 | -0.14372 | -0.30588 | 0.0848488 | 0.408314 | 0.34009 | -0.371832 |
| 13722 | 0.0649694 | 1.51508 | -1.82634 | 0.487225 | 1.01451 | -0.669915 | 0.322796 | -0.905681 | 0.170222 | -0.301801 | 0.367548 |
| 8548 | -0.0322813 | -0.0315513 | 0.033696 | 0.0394766 | -0.870274 | 0.10789 | -0.0464371 | -0.475277 | -0.285286 | -0.285364 | -0.198662 |
| 10514 | 0.278631 | -0.163472 | -0.438358 | -0.0852117 | 0.465255 | -0.211769 | 0.0577349 | -1.659 | -0.743333 | -0.169441 | 0.0921063 |
| 11624 | 0.00931414 | -0.0227372 | 0.0866395 | 0.0830134 | 0.105858 | 0.262747 | 0.224679 | 0.527917 | 0.0699586 | -0.0471849 | 0.0128419 |
| 14600 | -0.10018 | 0.289792 | -0.141329 | 0.0684176 | -0.00584732 | -0.323588 | -0.218815 | -1.45316 | -1.32814 | -0.239199 | 0.545846 |

In [19]:

```
for x in temp:
    for r in sorted(model.genes.get_by_id(x).reactions, key=lambda x: x.id):
        print(r.id, r.reaction, r.gene_reaction_rule)
    print()
```

```
ACNAMt2pp acnam_p + h_p --> acnam_c + h_c 10184
D_LACt2 h_e + lac__D_e <=> h_c + lac__D_c 10184
L_LACt2r h_e + lac__L_e <=> h_c + lac__L_c 10184
PYRt2 h_e + pyr_e --> h_c + pyr_c 10184

2OXOADOXm 2oxoadp_m + coa_m + nad_m --> co2_m + glutcoa_m + nadh_m 10040 and 12116 and 9274
AKGDm akg_m + coa_m + nad_m --> co2_m + nadh_m + succoa_m 10007 and 10040 and 12116
GLYCLm gly_m + nad_m + thf_m --> co2_m + mlthf_m + nadh_m + nh4_m 10040 and 10205 and 12898 and 15184
OBDHm 2obut_m + coa_m + nad_m --> co2_m + nadh_m + ppcoa_m (10040 and 11183 and 12566 and 15436) or (10040 and 11188 and 12566 and 15436)
OIVD1m 4mop_m + coa_m + nad_m --> co2_m + ivcoa_m + nadh_m (10040 and 11183 and 12566 and 15436) or (10040 and 11188 and 12566 and 15436)
OIVD2m 3mob_m + coa_m + nad_m --> co2_m + ibcoa_m + nadh_m (10040 and 11183 and 12566 and 15436) or (10040 and 11188 and 12566 and 15436)
OIVD3m 3mop_m + coa_m + nad_m --> 2mbcoa_m + co2_m + nadh_m (10040 and 11183 and 12566 and 15436) or (10040 and 11188 and 12566 and 15436)
PDHm coa_m + nad_m + pyr_m --> accoa_m + co2_m + nadh_m 10040 and 13630 and 13722 and 13948 and 14126

PDHm coa_m + nad_m + pyr_m --> accoa_m + co2_m + nadh_m 10040 and 13630 and 13722 and 13948 and 14126

PDHm coa_m + nad_m + pyr_m --> accoa_m + co2_m + nadh_m 10040 and 13630 and 13722 and 13948 and 14126

PDHm coa_m + nad_m + pyr_m --> accoa_m + co2_m + nadh_m 10040 and 13630 and 13722 and 13948 and 14126

PDHm coa_m + nad_m + pyr_m --> accoa_m + co2_m + nadh_m 10040 and 13630 and 13722 and 13948 and 14126

CS accoa_c + h2o_c + oaa_c --> cit_c + coa_c + h_c 11331 or 8548
CSm accoa_m + h2o_m + oaa_m --> cit_m + coa_m + h_m 11331
CSp accoa_x + h2o_x + oaa_x --> cit_x + coa_x + h_x 11331 or 8548
MCITSm h2o_m + oaa_m + ppcoa_m --> 2mcit_m + coa_m + h_m 11331

CS accoa_c + h2o_c + oaa_c --> cit_c + coa_c + h_c 11331 or 8548
CSp accoa_x + h2o_x + oaa_x --> cit_x + coa_x + h_x 11331 or 8548

CITtam cit_c + mal__L_m <=> cit_m + mal__L_c 10514 or 11740 or 13510
CITtbm cit_c + pep_m <=> cit_m + pep_c 10514
CITtcm cit_c + icit_m <=> cit_m + icit_c 10514 or 11740 or 13510

ACN_a_m acon_C_m + h2o_m <=> cit_m 11624 or 14600
ACN_b_m acon_C_m + h2o_m <=> icit_m 11624 or 14600
ACONT cit_c <=> icit_c 11624 or 14600
ACONTa cit_c <=> acon_C_c + h2o_c 11624 or 14600
ACONTb acon_C_c + h2o_c <=> icit_c 11624 or 14600
ACONTm cit_m <=> icit_m 11624 or 14600
MICITDr 2mcacn_c + h2o_c <=> micit_c 11624 or 14600

ACN_a_m acon_C_m + h2o_m <=> cit_m 11624 or 14600
ACN_b_m acon_C_m + h2o_m <=> icit_m 11624 or 14600
ACONT cit_c <=> icit_c 11624 or 14600
ACONTa cit_c <=> acon_C_c + h2o_c 11624 or 14600
ACONTb acon_C_c + h2o_c <=> icit_c 11624 or 14600
ACONTm cit_m <=> icit_m 11624 or 14600
MICITDr 2mcacn_c + h2o_c <=> micit_c 11624 or 14600
```

In [20]:

```
temp = ['11328','12642','16016']
display(Annotation.loc[temp])
```

|  | Combined Annotations | Signal P | Sc288c Orthologs | Human Orthologs | Sc288 Best Hit | Human Blast | Essential | WolfPSort | C Terminal |
| --- | --- | --- | --- | --- | --- | --- | --- | --- | --- |
| RTO4\_ID |  |  |  |  |  |  |  |  |  |
| 11328 | KOG2617: Citrate synthase | S |  |  | CIT3 | CSe | Not Essential | extr 10, mito 7, cyto 3.5, cyto\_nucl 3, pero 3... | GLPI |
| 12642 | K01720: prpD; 2-methylcitrate dehydratase | S | PDH1 |  | PDH1 |  | Not Essential | mito 26 | HKP\* |
| 16016 | K01705: LYS4; homoaconitate hydratase |  | LYS4 |  | LYS4 | ACO2 | Not Essential | mito 23, cyto 4 | ISA\* |

In [21]:

```
model.remove_reactions(['ACNAMt2pp'], remove_orphans=True)
# 11331 CIT1 mito, 8548 CIT2 pero, 11328 CIT3 mito 2-methylcitrate
model.reactions.get_by_id('CSm').gene_reaction_rule = '11331'
model.reactions.get_by_id('CSp').gene_reaction_rule = '8548'
model.reactions.get_by_id('MCITSm').gene_reaction_rule = '11328'
model.remove_reactions(['CS'], remove_orphans=True)
# 11624 ACO1 mito citrate
model.reactions.get_by_id('ACN_a_m').id = 'ACONTam'
model.reactions.get_by_id('ACN_b_m').id = 'ACONTbm'
model.reactions.get_by_id('ACONTam').gene_reaction_rule = '11624'
model.reactions.get_by_id('ACONTbm').gene_reaction_rule = '11624'
model.remove_reactions(['ACONT','ACONTa','ACONTb','ACONTm'], remove_orphans=True)
# 14600 ACO2 mito homocitrate (first half)
# 16016 LYS4 homoaconitate hydratase (second half)
# Change b124tc to hacon_C
r = sce.reactions.get_by_id('MCITDm').copy()
r.id = 'HACONTam'
r.name = 'Homocitrate hydrolase mitochondrial'
r.gene_reaction_rule = '14600'
model.add_reactions([r])
model.reactions.get_by_id('HACNHm').id = 'HACONTbm'
model.metabolites.get_by_id('b124tc_m').id = 'hacon_C_m'
model.metabolites.get_by_id('hacon_C_m').name = 'Cis-Homoaconitate'
model.remove_reactions(['yli_R0417'], remove_orphans=True)
# 12642 PDH1 mito 2-methylcitrate dehydratase
# ACONT3m is correct, remove MCITD and MICITDr
model.remove_reactions(['MCITD','MICITDr'], remove_orphans=True)
```

Rest of the TCA cycle  
Isocitrate dehydrogenase  
IDH2 11681 K00030: IDH3; isocitrate dehydrogenase (NAD+) mito 26 KKL *IDH1 11682 K00030: IDH3; isocitrate dehydrogenase (NAD+) mito 24.5, cyto\_mito 13.5 GLQ*  
IDP1 11129 K00031: IDH1, IDH2, icd; isocitrate dehydrogenase cyto 13, pero 8, nucl 4 PKL *Oxoglutarate dehydrogenase mitochondrial  
KGD1 10007 K00164: OGDH, sucA; 2-oxoglutarate dehydrogenase E1 component mito 25 GDF*  
KGD2 12116 K00658: DLST, sucB; 2-oxoglutarate dehydrogenase E2 component (dihydrolipoamide succinyltransferase) mito 23, cyto\_mito 14.5 LYN *Glycine cleavage complex mitochondrial
GCV1 12898 K00605: gcvT, AMT; aminomethyltransferase mito 23, cyto 4 RGE*  
GCV2 10205 K00281: GLDC, gcvP; glycine dehydrogenase mito 21, cyto 2, plas 1, extr 1, cyto\_nucl 1, pero 1, E.R. 1 VDA *GCV3 15184 K02437: gcvH, GCSH; glycine cleavage system H protein mito 27 EAS*  
Succinate CoA ligase mitochondrial  
LSC2 15967 K01900: LSC2; succinyl-CoA synthetase beta subunit mito 18.5, cyto\_mito 12.5, cyto 5.5 AAQ *LSC1 16144 K01899: LSC1; succinyl-CoA synthetase alpha subunit mito 24, pero 2 GLA*  
Succinate dehydrogenase mitochondrial  
SDH1 16281 K00234: SDHA, SDH1; succinate dehydrogenase (ubiquinone) flavoprotein subunit mito 27 VRG *SDH2 11977 K00235: SDHB, SDH2; succinate dehydrogenase (ubiquinone) iron-sulfur subunit nucl 9, mito 7, cyto 7, cyto\_mito 7 ASA*  
SHH3 11629 K00236: SDHC, SDH3; succinate dehydrogenase (ubiquinone) cytochrome b560 subunit mito 22.5, cyto\_mito 13.5, cyto 3.5 LAM *SHH4 11802 K00237: SDHD, SDH4; succinate dehydrogenase (ubiquinone) membrane anchor subunit mito 10, extr 8, cyto\_mito 8 KQE*  
Succinate fumarate transport mitochondrial  
SFC1 12605 K15100: SLC25A1, CTP; solute carrier family 25 (mitochondrial citrate transporter), member 1 mito 11, cyto 9.5, cyto\_nucl 5.5, plas 3 YSE *UCP2 11740 K15103: UCP2\_3, SLC25A8\_9; solute carrier family 25 (mitochondrial uncoupling protein), member 8/9 mito 15, cysk 6, cyto 4.5, cyto\_nucl 3 PPV*  
DIC1 13510 K13577: SLC25A10, DIC; solute carrier family 25 (mitochondrial dicarboxylate transporter), member 10 mito 15, extr 7, cyto 3 GTP *Fumarate reductase  
FRD1 11420 KOG2404: Fumarate reductase, flavoprotein subunit cyto 16.5, cyto\_nucl 9, extr 8 YKQ*  
Fumarase  
FUM1 14001 K01679: E4.2.1.2B, fumC; fumarate hydratase, class II cyto 17, cyto\_nucl 14, nucl 7 SDA *Oxaloacetate transport mitochondrial  
OAC1 9011 K15117: SLC25A34\_35, OAC1; solute carrier family 25, member 34/35 extr 14, mito 11 ARD*  
Isocitrate lyase  
ICL1 14022 K01637: E4.1.3.1, aceA; isocitrate lyase cyto\_nucl 9.5, cyto 8.5, nucl 7.5, pero 7, mito 2 QFK *ICL2 14162 K01637: E4.1.3.1, aceA; isocitrate lyase mito 26 HTF*  
Malate synthase
MLS1 9457 K01638: aceB, glcB; malate synthase cyto\_pero 12, cyto 11.5, pero 11.5, nucl 3 AKL *Phosphate transporter mitochondrial
MIR1 15874 K15102: SLC25A3, PHC, PIC; solute carrier family 25 (mitochondrial phosphate transporter), member 3 mito 12, extr 7, cyto 5.5, cyto\_nucl 3.5 HKD*  
ATP-Citrate lyase  
ACLY 9726 K01648: ACLY; ATP citrate (pro-S)-lyase cyto 13.5, mito 8, cyto\_nucl 7.5, extr 2, pero 2 QRQ\*

In [22]:

```
temp = ['11681','11682','11129','10007','12116','10040','12898','10205','15184','15967','16144','16281',
        '11977','11629','11802','12605','11740','13510','11420','11536','14001','9011','14022','14162','9457',
        '15874','9726']
display(Annotation.loc[temp])
Show_Data(temp)
```

|  | Combined Annotations | Signal P | Sc288c Orthologs | Human Orthologs | Sc288 Best Hit | Human Blast | Essential | WolfPSort | C Terminal |
| --- | --- | --- | --- | --- | --- | --- | --- | --- | --- |
| RTO4\_ID |  |  |  |  |  |  |  |  |  |
| 11681 | K00030: IDH3; isocitrate dehydrogenase (NAD+) |  | IDH2 | IDH3A | IDH2 | IDH3A | Essential | mito 26 | KKL\* |
| 11682 | K00030: IDH3; isocitrate dehydrogenase (NAD+) |  | IDH1 | IDH3B,IDH3G | IDH1 | IDH3G | Essential | mito 24.5, cyto\_mito 13.5 | GLQ\* |
| 11129 | K00031: IDH1, IDH2, icd; isocitrate dehydrogenase |  | IDP1,IDP2,IDP3 | IDH1,IDH2 | IDP1 | IDH2 | Not Essential | cyto 13, pero 8, nucl 4 | PKL\* |
| 10007 | K00164: OGDH, sucA; 2-oxoglutarate dehydrogena... | S | KGD1 | DHTKD1,OGDH,OGDHL | KGD1 | OGDH | Essential | mito 25 | GDF\* |
| 12116 | K00658: DLST, sucB; 2-oxoglutarate dehydrogena... |  | KGD2 | DLST | KGD2 | DLST | Essential | mito 23, cyto\_mito 14.5 | LYN\* |
| 10040 | K00382: DLD, lpd, pdhD; dihydrolipoamide dehyd... | S | LPD1 | DLD | LPD1 | DLD | Essential | mito 9, cyto 8, extr 7, pero 2 | INA\* |
| 12898 | K00605: gcvT, AMT; aminomethyltransferase |  | GCV1 | AMT,RP11-949J7.8 | GCV1 | AMT | Not Essential | mito 23, cyto 4 | RGE\* |
| 10205 | K00281: GLDC, gcvP; glycine dehydrogenase |  | GCV2 | GLDC | GCV2 | GLDC | Not Essential | mito 21, cyto 2, plas 1, extr 1, cyto\_nucl 1, ... | VDA\* |
| 15184 | K02437: gcvH, GCSH; glycine cleavage system H ... |  | GCV3 | GCSH | GCV3 | GCSH | Essential | mito 27 | EAS\* |
| 15967 | K01900: LSC2; succinyl-CoA synthetase beta sub... |  | LSC2 | SUCLA2 | LSC2 | SUCLA | Essential | mito 18.5, cyto\_mito 12.5, cyto 5.5 | AAQ\* |
| 16144 | K01899: LSC1; succinyl-CoA synthetase alpha su... |  | LSC1 | SUCLG1 | LSC1 | SUCLG | Essential | mito 24, pero 2 | GLA\* |
| 16281 | K00234: SDHA, SDH1; succinate dehydrogenase (u... |  | YJL045W,SDH1 | SDHA | SDH1 | SDHA | Not Essential | mito 27 | VRG\* |
| 11977 | K00235: SDHB, SDH2; succinate dehydrogenase (u... |  | SDH2 | SDHB | SDH2 | SDHB | Essential | nucl 9, mito 7, cyto 7, cyto\_mito 7 | ASA\* |
| 11629 | K00236: SDHC, SDH3; succinate dehydrogenase (u... | A | YMR118C,SDH3 | SDHC | SHH3 |  | Essential | mito 22.5, cyto\_mito 13.5, cyto 3.5 | LAM\* |
| 11802 | K00237: SDHD, SDH4; succinate dehydrogenase (u... | S | SDH4,SHH4,TIM18 |  | SHH4 |  | Not Essential | mito 10, extr 8, cyto\_mito 8 | KQE\* |
| 12605 | K15100: SLC25A1, CTP; solute carrier family 25... | S | SFC1 |  | SFC1 | SLC25A | Not Essential | mito 11, cyto 9.5, cyto\_nucl 5.5, plas 3 | YSE\* |
| 11740 | K15103: UCP2\_3, SLC25A8\_9; solute carrier fami... |  |  | UCP2 | OAC1 | SLC25 | Not Essential | mito 15, cysk 6, cyto 4.5, cyto\_nucl 3 | PPV\* |
| 13510 | K13577: SLC25A10, DIC; solute carrier family 2... |  | DIC1 | SLC25A11,RP13-1032I1.10 | DIC1 | SLC25 | Not Essential | mito 15, extr 7, cyto 3 | GTP\* |
| 11420 | KOG2404: Fumarate reductase, flavoprotein subunit | S | FRD1,OSM1 |  | FRD1 | CYB5B | Not Essential | cyto 16.5, cyto\_nucl 9, extr 8 | YKQ\* |
| 11536 | KOG2404: Fumarate reductase, flavoprotein subunit |  |  |  | FRD1 |  | Essential | nucl 17.5, cyto\_nucl 12.5, cyto 6.5 | YKQ\* |
| 14001 | K01679: E4.2.1.2B, fumC; fumarate hydratase, c... |  | FUM1 | FH | FUM1 | FHes | Essential | cyto 17, cyto\_nucl 14, nucl 7 | SDA\* |
| 9011 | K15117: SLC25A34\_35, OAC1; solute carrier fami... | S | OAC1 | SLC25A35 | OAC1 | SLC25 | Not Essential | extr 14, mito 11 | ARD\* |
| 14022 | K01637: E4.1.3.1, aceA; isocitrate lyase |  | ICL1,ICL2 |  | ICL1 |  | Not Essential | cyto\_nucl 9.5, cyto 8.5, nucl 7.5, pero 7, mito 2 | QFK\* |
| 14162 | K01637: E4.1.3.1, aceA; isocitrate lyase | S | ICL1,ICL2 |  | ICL2 |  | Not Essential | mito 26 | HTF\* |
| 9457 | K01638: aceB, glcB; malate synthase |  | DAL7,MLS1 |  | MLS1 |  | Not Essential | cyto\_pero 12, cyto 11.5, pero 11.5, nucl 3 | AKL\* |
| 15874 | K15102: SLC25A3, PHC, PIC; solute carrier fami... |  | MIR1,PIC2 | SLC25A3 | MIR1 | SLC25 | Not Essential | mito 12, extr 7, cyto 5.5, cyto\_nucl 3.5 | HKD\* |
| 9726 | K01648: ACLY; ATP citrate (pro-S)-lyase |  |  | ACLY | LSC1 | ACLY | Not Essential | cyto 13.5, mito 8, cyto\_nucl 7.5, extr 2, pero 2 | QRQ\* |

| strain | WT | | | | | | | | | | | | | | | | |
| --- | --- | --- | --- | --- | --- | --- | --- | --- | --- | --- | --- | --- | --- | --- | --- | --- | --- |
| condition | G\_MM | C\_MM | G\_SD | | GX\_SD | | | X\_SD | | A\_SD | | C\_SD | | MM\_CN120 | | MM\_CN5 | Diversity\_Sample |
| phase | exp | exp | exp | stat | exp | trans | stat | exp | stat | exp | stat | exp | stat | exp | stat | exp | exp |
| proteinId | Set1 | Set1 | Set2 | Set2 | Set2 | Set2 | Set2 | Set2 | Set2 | Set2 | Set2 | Set2 | Set2 | Set3 | Set3 | Set3 | Set3 |
| 11681 | 7.45459 | 6.84887 | 8.53566 | 6.97744 | 8.5233 | 7.58163 | 7.50924 | 7.89875 | 6.9442 | 7.09675 | 6.32633 | 6.87558 | 6.46939 | 8.46814 | 8.46421 | 8.99975 | 8.87032 |
| 11682 | 8.26657 | 7.69142 | 9.00712 | 8.35665 | 9.06935 | 8.25899 | 8.2924 | 8.37928 | 8.24521 | 7.77321 | 7.77306 | 7.26903 | 7.09893 | 8.35991 | 8.51722 | 8.6074 | 8.4353 |
| 11129 | 7.16101 | 9.69917 | 9.09782 | 9.1897 | 9.10341 | 8.25755 | 8.17244 | 9.13677 | 8.14724 | 8.15581 | 7.36988 | 9.45626 | 9.137 | 8.17518 | 7.65132 | 9.33382 | 9.10174 |
| 10007 | 6.13987 | 7.10137 | 8.36676 | 7.55675 | 8.42709 | 7.92907 | 7.79415 | 7.36553 | 7.67486 | 6.97414 | 6.88326 | 7.72291 | 7.57654 | 7.53589 | 7.29696 | 8.09195 | 8.12393 |
| 12116 | 5.86941 | 6.89481 | 7.97752 | 7.34651 | 8.02189 | 7.50709 | 7.27856 | 7.15223 | 7.34773 | 6.7324 | 6.88036 | 7.7731 | 7.80868 | 7.42645 | 7.10966 | 7.60385 | 7.67867 |
| 10040 | 8.00662 | 7.31735 | 8.13214 | 6.97186 | 8.20048 | 7.36158 | 7.42582 | 7.56306 | 7.36598 | 7.19991 | 6.96873 | 7.35137 | 7.14045 | 8.72929 | 8.27061 | 9.01736 | 8.6493 |
| 12898 | 5.30779 | 6.16177 | 7.47357 | 6.71084 | 7.64377 | 6.0455 | 6.11362 | 6.04373 | 6.20104 | 5.73529 | 6.24391 | 6.82944 | 6.50432 | 7.56733 | 5.70301 | 8.5194 | 6.16256 |
| 10205 | 4.83123 | 5.54924 | 7.97399 | 5.98862 | 8.12491 | 6.41649 | 6.46535 | 6.74541 | 5.83306 | 5.84004 | 5.77441 | 7.26908 | 7.56145 | 9.29324 | 6.68953 | 9.87107 | 7.22708 |
| 15184 | 9.04257 | 8.85836 | 8.81924 | 8.06792 | 8.79698 | 8.14148 | 8.03457 | 8.47687 | 8.03811 | 8.35723 | 8.05271 | 8.24789 | 8.0256 | 8.24429 | 7.81031 | 8.50946 | 7.61067 |
| 15967 | 7.37575 | 7.91135 | 7.95544 | 7.67736 | 8.02084 | 7.39503 | 7.24731 | 8.02792 | 7.42953 | 7.65532 | 7.25906 | 7.60014 | 7.07549 | 7.52794 | 7.2357 | 8.22197 | 7.88226 |
| 16144 | 6.61282 | 7.5429 | 7.67383 | 6.96173 | 7.78493 | 7.19572 | 7.0448 | 7.21593 | 7.01728 | 6.83247 | 6.68711 | 7.66425 | 7.32798 | 8.49413 | 8.10215 | 9.28111 | 9.01928 |
| 16281 | 7.58134 | 8.45851 | 6.40678 | 6.77233 | 6.51478 | 6.1033 | 6.35029 | 5.44833 | 7.282 | 6.03135 | 7.08228 | 7.52031 | 7.84622 | 7.61919 | 7.80471 | 6.73201 | 9.53915 |
| 11977 | 7.22236 | 8.61599 | 5.0314 | 6.94706 | 5.05521 | 4.29576 | 5.48362 | 3.32552 | 6.60665 | 3.57239 | 6.84809 | 6.61717 | 6.73366 | 4.65907 | 5.6235 | 3.77661 | 7.44867 |
| 11629 | 8.43373 | 9.10772 | 6.80434 | 6.7177 | 6.91899 | 6.15402 | 6.34597 | 5.9431 | 6.78599 | 6.41761 | 7.16141 | 7.44507 | 7.8897 | 7.4823 | 7.78889 | 7.66595 | 9.63313 |
| 11802 | 7.1725 | 8.09016 | 5.85127 | 6.36987 | 5.89663 | 5.477 | 5.61949 | 4.87642 | 6.53998 | 5.49395 | 6.9056 | 6.95091 | 7.11065 | 4.18029 | 4.94741 | 3.72961 | 6.10689 |
| 12605 | 3.68142 | 7.62714 | 6.93159 | 5.79548 | 7.109 | 6.20355 | 6.25958 | 5.07552 | 7.14139 | 5.11675 | 6.20272 | 7.1788 | 6.93089 | 4.27684 | 4.24007 | 5.1688 | 5.35612 |
| 11740 | 4.47 | 5.36422 | 4.04 | 5.02776 | 3.93205 | 4.40857 | 4.35313 | 3.67924 | 5.11429 | 4.17105 | 5.17872 | 5.43859 | 4.42604 | 3.57264 | 3.15624 | 4.36255 | 3.6936 |
| 13510 | 3.67785 | 5.79102 | 4.45268 | 4.79481 | 4.53865 | 5.12837 | 5.06374 | 4.59067 | 4.68711 | 4.30165 | 4.25627 | 5.78462 | 5.45423 | 4.13705 | 4.19705 | 4.23321 | 4.25478 |
| 11420 | 7.51955 | 8.42336 | 6.17733 | 8.13326 | 5.86578 | 7.09933 | 7.38715 | 6.27839 | 7.26226 | 7.73716 | 7.94284 | 7.48497 | 7.83102 | 6.55528 | 8.53299 | 6.58298 | 7.75061 |
| 11536 | 3.01496 | 3.87201 | 3.74397 | 5.56463 | 3.37396 | 4.44283 | 4.75823 | 3.80302 | 4.75689 | 5.19004 | 5.4241 | 4.97552 | 5.32097 | 4.57482 | 6.35728 | 4.17283 | 5.21604 |
| 14001 | 6.96801 | 7.8509 | 9.11379 | 6.90613 | 9.17873 | 8.06654 | 7.87683 | 7.5725 | 7.90521 | 7.14557 | 7.09781 | 8.10116 | 7.7614 | 9.35207 | 8.56516 | 10.0237 | 10.1293 |
| 9011 | 5.89309 | 6.55275 | 6.61983 | 4.95738 | 6.65087 | 6.41585 | 6.26478 | 6.28991 | 5.60528 | 5.76342 | 5.41461 | 5.93105 | 6.01452 | 4.38165 | 3.85428 | 4.00567 | 3.83548 |
| 14022 | 4.6865 | 7.31059 | 8.98622 | 7.36314 | 9.16563 | 7.82857 | 8.00893 | 7.72855 | 8.21391 | 6.12419 | 7.44567 | 7.05071 | 8.07862 | 6.22935 | 7.5451 | 10.1141 | 6.86669 |
| 14162 | 5.97913 | 5.90127 | 8.37789 | 5.48236 | 8.34099 | 6.41778 | 6.10498 | 7.34865 | 6.09534 | 5.70719 | 5.82756 | 5.91579 | 6.65088 | 6.07938 | 5.94597 | 7.06487 | 6.11391 |
| 9457 | 6.68318 | 7.22895 | 8.58027 | 7.16444 | 8.71042 | 7.64187 | 7.67559 | 7.55327 | 7.80155 | 6.6003 | 7.15658 | 6.86652 | 6.30312 | 8.15008 | 8.4776 | 8.68898 | 7.68689 |
| 15874 | 5.54334 | 4.37285 | 7.55907 | 6.05993 | 7.46388 | 7.0809 | 6.98891 | 6.53448 | 6.37383 | 5.792 | 5.54409 | 6.01274 | 5.50865 | 6.06785 | 6.19675 | 4.88 | 5.91761 |
| 9726 | 10.038 | 8.23152 | 7.43503 | 3.98289 | 7.55493 | 7.25406 | 6.61106 | 8.41288 | 4.1543 | 7.72571 | 3.22215 | 6.12272 | 5.16932 | 11.0778 | 10.3324 | 10.2421 | 9.5927 |

| strain | WT | | | | | | | | | | |
| --- | --- | --- | --- | --- | --- | --- | --- | --- | --- | --- | --- |
| condition | G\_SD | | GX\_SD | | | X\_SD | | A\_SD | | C\_SD | |
| proteinId | exp | stat | exp | trans | stat | exp | stat | exp | stat | exp | stat |
| 11681 | 27.3224 | 29.563 | 26.4545 | 27.169 | 28.016 | 23.2651 | 22.825 | 18.9208 | 22.6259 | 20.0628 | 20.9346 |
| 11682 | 26.8825 | 26.8794 | 30.5095 | 28.2578 | 26.4306 | 26.406 | 25.9484 | 23.7577 | 23.949 | 21.775 | 21.334 |
| 11129 | 50.0393 | 47.3779 | 46.9643 | 37.7576 | 36.8082 | 43.7864 | 48.1441 | 41.5317 | 40.1972 | 59.3245 | 57.0295 |
| 10007 | 54.3251 | 59.3669 | 55.947 | 58.6255 | 62.5259 | 46.3245 | 51.4574 | 44.2218 | 50.5898 | 57.6952 | 60.1059 |
| 12116 | 16.272 | 18.4584 | 16.0752 | 20.1197 | 22.9965 | 15.6424 | 18.5041 | 14.8723 | 17.6016 | 23.4829 | 22.4095 |
| 10040 | 47.0337 | 54.5942 | 39.0915 | 48.1654 | 53.3645 | 35.4178 | 41.1307 | 33.4162 | 36.9135 | 42.3765 | 44.1734 |
| 12898 | 16.9291 | 17.1668 | 13.4308 | 12.083 | 13.1344 | 8.18911 | 11.8329 | 9.289 | 8.25817 | 12.1783 | 18.3095 |
| 10205 | 51.3167 | 57.9704 | 48.0144 | 48.9303 | 53.9603 | 33.6046 | 36.974 | 26.4449 | 31.7185 | 35.7099 | 46.8955 |
| 15184 | 2.30911 | 1.80601 | 1.81837 | 2.22304 | 3.40839 | 3.53917 | 4.73889 | 2.12517 | 4.02187 | 5.97787 | 4.98359 |
| 15967 | 27.3133 | 27.7524 | 30.4987 | 28.2636 | 31.3009 | 28.9617 | 29.0322 | 28.7885 | 27.3592 | 32.9221 | 33.2916 |
| 16144 | 19.126 | 23.5643 | 19.5279 | 24.391 | 21.9345 | 21.728 | 21.0388 | 21.8481 | 21.7698 | 21.4086 | 21.3652 |
| 16281 | 16.6006 | 14.0312 | 15.2742 | 10.2248 | 11.6842 | 14.083 | 13.9523 | 16.4172 | 14.9306 | 26.3156 | 27.8606 |
| 11977 | 5.75637 | 4.13767 | 5.89084 | 2.04695 | 1.99736 | 4.70008 | 6.3283 | 6.17849 | 7.48059 | 6.62294 | 7.19917 |
| 11629 | 3.67161 | 2.16569 | 4.47279 | 2.41429 | 3.11952 | 3.14482 | 3.16557 | 4.82394 | 3.45265 | 4.48951 | 4.12003 |
| 11802 | 1.72171 | 0.3835 | 2.44866 | 1.11096 | 0.7422 | 0.593778 | 1.19944 | 1.544 | 1.53482 | 2.12457 | 2.6054 |
| 12605 | 7.94394 | 7.96008 | 6.70555 | 6.69664 | 6.41054 | 6.06328 | 6.21227 | 2.31934 | 4.38725 | 16.6563 | 18.1005 |
| 11420 | 8.79832 | 31.3079 | 11.37 | 25.8866 | 36.4802 | 26.8003 | 38.463 | 36.1123 | 50.7262 | 38.0531 | 42.2965 |
| 11536 | 2.49989 | 9.21834 | 3.6683 | 5.77266 | 11.4831 | 8.78577 | 15.1321 | 13.9214 | 20.8712 | 13.6488 | 14.133 |
| 14001 | 20.7005 | 22.5044 | 19.9585 | 21.9462 | 20.9492 | 20.9423 | 21.5847 | 14.8815 | 15.5226 | 28.3881 | 30.074 |
| 9011 | 7.25412 | 6.77329 | 5.48653 | 5.20587 | 5.45571 | 6.26271 | 5.68882 | 5.22074 | 3.63032 | 3.827 | 3.9244 |
| 14022 | 28.9649 | 36.0553 | 32.5962 | 34.2525 | 32.1963 | 21.6915 | 34.5075 | 14.3133 | 21.2862 | 39.8994 | 40.7649 |
| 14162 | 35.1016 | 33.9706 | 35.9799 | 34.259 | 35.7012 | 31.1206 | 37.6102 | 22.9865 | 23.5662 | 33.1761 | 27.9325 |
| 9457 | 35.4255 | 32.7945 | 39.2524 | 39.9616 | 42.0678 | 28.1449 | 35.6202 | 25.7065 | 23.3625 | 47.2338 | 42.4634 |
| 15874 | 11.5429 | 13.7323 | 12.7904 | 16.9395 | 14.0497 | 9.80303 | 9.11259 | 5.61108 | 6.48984 | 8.76984 | 8.91861 |
| 9726 | 84.5812 | 85.4518 | 81.5967 | 84.8369 | 79.9097 | 100.57 | 93.1329 | 101.606 | 96.6858 | 94.1006 | 68.1666 |

|  | Glucose | Xylose | Arabinose | Acetate | Coumarate | Ferulate | YNB Oleic Acid | YNB Ricinoleic Acid | YNB Glucose | YNB Gluc DOC | YPD |
| --- | --- | --- | --- | --- | --- | --- | --- | --- | --- | --- | --- |
| proteinId |  |  |  |  |  |  |  |  |  |  |  |
| 11681 | 0.281262 | 0.952127 | 0.251201 | 0.352229 | -0.926581 | 1.00517 | -0.018426 | -1.53492 | -0.654143 | -1.5936 | -1.7233 |
| 11129 | -0.393612 | 0.510054 | -0.255185 | -0.254103 | 0.183252 | -0.0410789 | 0.33642 | 0.792335 | -1.65387 | -0.460691 | -0.0902951 |
| 12898 | -0.542249 | -1.88155 | -1.44918 | -0.407967 | -1.06514 | -0.406853 | -0.495496 | -1.37202 | 0.206331 | -0.653699 | 0.171222 |
| 10205 | -0.104693 | -0.602127 | -0.515849 | -0.120529 | -0.305359 | 0.0575178 | -0.24409 | -1.08858 | -0.136329 | 0.151202 | 0.119006 |
| 12605 | -0.250531 | -1.12124 | -1.27614 | -1.68184 | -1.69155 | -1.56883 | 0.318931 | 0.287611 | 1.11429 | 0.16432 | 2.2361 |
| 11740 | 0.109553 | 0.00205556 | -0.00502673 | -0.149136 | 0.237068 | 0.0193826 | 0.313053 | 0.0534518 | 0.095281 | 0.428816 | 0.101248 |
| 13510 | 0.206135 | 0.686782 | 0.453603 | -0.0292148 | -0.421161 | -0.352184 | 0.219954 | -0.116016 | -0.17613 | 0.0633609 | 0.114048 |
| 11420 | -0.0270622 | -0.0934326 | -0.0197034 | -0.0572847 | 0.13398 | 0.0496476 | -0.160719 | -0.236253 | -0.248188 | -0.122242 | -0.166463 |
| 9011 | 0.0241941 | -0.272725 | 0.161747 | -0.269852 | 0.0798061 | -0.031333 | -0.0244879 | -0.492747 | 0.287088 | 0.0815357 | -0.107048 |
| 14022 | -0.0684361 | -0.0406226 | 0.00566276 | -3.53877 | -0.672274 | -0.646294 | -3.49352 | -4.31542 | -0.143782 | -0.190331 | 0.0376189 |
| 14162 | -0.351181 | 0.486327 | -2.48228 | -3.32582 | -2.68378 | -3.7193 | -0.577084 | -0.579107 | 0.265499 | 0.289154 | -0.0659644 |
| 9457 | -0.0285278 | 0.126065 | -0.0124983 | -1.86103 | -0.467399 | -0.545453 | -2.92614 | -2.60194 | -0.143079 | -0.0561765 | -0.23821 |
| 15874 | -0.268098 | -0.0197262 | 0.0222415 | -0.262453 | 0.268151 | -0.511158 | 0.130008 | 0.406326 | 0.236206 | -0.0925991 | 0.264876 |

In [23]:

```
for x in temp:
    for r in sorted(model.genes.get_by_id(x).reactions, key=lambda x: x.id):
        print(r.id, r.reaction, r.gene_reaction_rule)
    print()
```

```
ICDHxm icit_m + nad_m --> akg_m + co2_m + nadh_m 11681 or 11682 or (11681 and 11682)
ICDHyr icit_c + nadp_c <=> akg_c + co2_c + nadph_c 11129 or 11681

ICDHxm icit_m + nad_m --> akg_m + co2_m + nadh_m 11681 or 11682 or (11681 and 11682)

ICDHym icit_m + nadp_m --> akg_m + co2_m + nadph_m 11129
ICDHyp icit_x + nadp_x --> akg_x + co2_x + nadph_x 11129
ICDHyr icit_c + nadp_c <=> akg_c + co2_c + nadph_c 11129 or 11681

AKGDm akg_m + coa_m + nad_m --> co2_m + nadh_m + succoa_m 10007 and 10040 and 12116

2OXOADOXm 2oxoadp_m + coa_m + nad_m --> co2_m + glutcoa_m + nadh_m 10040 and 12116 and 9274
AKGDm akg_m + coa_m + nad_m --> co2_m + nadh_m + succoa_m 10007 and 10040 and 12116

2OXOADOXm 2oxoadp_m + coa_m + nad_m --> co2_m + glutcoa_m + nadh_m 10040 and 12116 and 9274
AKGDm akg_m + coa_m + nad_m --> co2_m + nadh_m + succoa_m 10007 and 10040 and 12116
GLYCLm gly_m + nad_m + thf_m --> co2_m + mlthf_m + nadh_m + nh4_m 10040 and 10205 and 12898 and 15184
OBDHm 2obut_m + coa_m + nad_m --> co2_m + nadh_m + ppcoa_m (10040 and 11183 and 12566 and 15436) or (10040 and 11188 and 12566 and 15436)
OIVD1m 4mop_m + coa_m + nad_m --> co2_m + ivcoa_m + nadh_m (10040 and 11183 and 12566 and 15436) or (10040 and 11188 and 12566 and 15436)
OIVD2m 3mob_m + coa_m + nad_m --> co2_m + ibcoa_m + nadh_m (10040 and 11183 and 12566 and 15436) or (10040 and 11188 and 12566 and 15436)
OIVD3m 3mop_m + coa_m + nad_m --> 2mbcoa_m + co2_m + nadh_m (10040 and 11183 and 12566 and 15436) or (10040 and 11188 and 12566 and 15436)
PDHm coa_m + nad_m + pyr_m --> accoa_m + co2_m + nadh_m 10040 and 13630 and 13722 and 13948 and 14126

GLYCLm gly_m + nad_m + thf_m --> co2_m + mlthf_m + nadh_m + nh4_m 10040 and 10205 and 12898 and 15184

GLYCLm gly_m + nad_m + thf_m --> co2_m + mlthf_m + nadh_m + nh4_m 10040 and 10205 and 12898 and 15184

GLYCLm gly_m + nad_m + thf_m --> co2_m + mlthf_m + nadh_m + nh4_m 10040 and 10205 and 12898 and 15184

SUCOASm atp_m + coa_m + succ_m <=> adp_m + pi_m + succoa_m 15967 and 16144

SUCOASm atp_m + coa_m + succ_m <=> adp_m + pi_m + succoa_m 15967 and 16144

SUCD2_u9m q9_m + succ_m --> fum_m + q9h2_m 10953 and 11205 and 11629 and 11802 and 11977 and 12093 and 16281 and 8486

SUCD2_u9m q9_m + succ_m --> fum_m + q9h2_m 10953 and 11205 and 11629 and 11802 and 11977 and 12093 and 16281 and 8486

SUCD2_u9m q9_m + succ_m --> fum_m + q9h2_m 10953 and 11205 and 11629 and 11802 and 11977 and 12093 and 16281 and 8486

SUCD2_u9m q9_m + succ_m --> fum_m + q9h2_m 10953 and 11205 and 11629 and 11802 and 11977 and 12093 and 16281 and 8486

SUCFUMtm fum_m + succ_c --> fum_c + succ_m 11740 or 12605 or 13510 or (11740 and 12605) or (12605 and 13510)

AKGCITtm akg_c + cit_m <=> akg_m + cit_c 11740 or 13510
AKGICITtm akg_c + icit_m <=> akg_m + icit_c 11740 or 13510
AKGMALtm akg_m + mal__L_c <=> akg_c + mal__L_m 11740 or 13510
CITtam cit_c + mal__L_m <=> cit_m + mal__L_c 10514 or 11740 or 13510
CITtcm cit_c + icit_m <=> cit_m + icit_c 10514 or 11740 or 13510
Htm h_c --> h_m 11740
MALICITtm icit_m + mal__L_c <=> icit_c + mal__L_m 11740 or 13510
MALOAAtm mal__L_m + oaa_c <=> mal__L_c + oaa_m 11740 or 13510
MALtm mal__L_c + pi_m <=> mal__L_m + pi_c 11740 or 13510
OAAAKGtm akg_m + oaa_c <=> akg_c + oaa_m 11740 or 13510
OAACITtm cit_m + oaa_c <=> cit_c + oaa_m 11740 or 13510
OAAICITtm icit_m + oaa_c <=> icit_c + oaa_m 11740 or 13510
SUCCtm pi_m + succ_c --> pi_c + succ_m 11740 or 13510
SUCFUMtm fum_m + succ_c --> fum_c + succ_m 11740 or 12605 or 13510 or (11740 and 12605) or (12605 and 13510)

AKGCITtm akg_c + cit_m <=> akg_m + cit_c 11740 or 13510
AKGICITtm akg_c + icit_m <=> akg_m + icit_c 11740 or 13510
AKGMALtm akg_m + mal__L_c <=> akg_c + mal__L_m 11740 or 13510
CITtam cit_c + mal__L_m <=> cit_m + mal__L_c 10514 or 11740 or 13510
CITtcm cit_c + icit_m <=> cit_m + icit_c 10514 or 11740 or 13510
FUMSO3tm fum_c + so3_m <=> fum_m + so3_c 13510
FUMSO4tm fum_c + so4_m <=> fum_m + so4_c 13510
FUMTSULtm fum_c + tsul_m <=> fum_m + tsul_c 13510
FUMtm fum_c + pi_m <=> fum_m + pi_c 13510
MALICITtm icit_m + mal__L_c <=> icit_c + mal__L_m 11740 or 13510
MALOAAtm mal__L_m + oaa_c <=> mal__L_c + oaa_m 11740 or 13510
MALSO3tm mal__L_c + so3_m <=> mal__L_m + so3_c 13510
MALSO4tm mal__L_c + so4_m <=> mal__L_m + so4_c 13510
MALTSULtm mal__L_c + tsul_m <=> mal__L_m + tsul_c 13510
MALtm mal__L_c + pi_m <=> mal__L_m + pi_c 11740 or 13510
OAAAKGtm akg_m + oaa_c <=> akg_c + oaa_m 11740 or 13510
OAACITtm cit_m + oaa_c <=> cit_c + oaa_m 11740 or 13510
OAAICITtm icit_m + oaa_c <=> icit_c + oaa_m 11740 or 13510
SUCCtm pi_m + succ_c --> pi_c + succ_m 11740 or 13510
SUCFUMtm fum_m + succ_c --> fum_c + succ_m 11740 or 12605 or 13510 or (11740 and 12605) or (12605 and 13510)

FRDcm fadh2_m + fum_c --> fad_m + succ_c 11420

FRDm fadh2_m + fum_m --> fad_m + succ_m 11536

FUM fum_c + h2o_c <=> mal__L_c 14001
FUMm fum_m + h2o_m <=> mal__L_m 14001

OAAt2m h_c + oaa_c <=> h_m + oaa_m 9011

ICLp icit_x --> glx_x + succ_x 14022

MCITL2m micit_m --> pyr_m + succ_m 14162

MALSp accoa_x + glx_x + h2o_x --> coa_x + h_x + mal__L_x 9457

PIt2m h_c + pi_c <=> h_m + pi_m 15874 or 8889

ACITL atp_c + cit_c + coa_c --> accoa_c + adp_c + oaa_c + pi_c 9726
```

Isocitrate dehydrogenase http://emboj.embopress.org/content/17/3/677  
11681 IDH2 mito nadh alpha subunit  
11682 IDH1 mito nadh gamma subunit  
11129 IDP1/IDP2/IDP3 cyto 13 pero 8 nadph  
A. nidulans three isoforms http://www.jbc.org/content/276/40/37722  
y. lipolytica one isoform https://link.springer.com/article/10.1007%2Fs12010-013-0373-1  
Gene structure shows three isoforms possible  
RNA-Seq shows the slicing of short N-terminal (no MTS) and long C-terminal (PTS1)  
Change ICDHxm genes to '11681 and 11682', ICDHyp genes to '11129'  
Remove ICDHyr, ICDHym

Fumarate reductase  
11420 has sigP and cytochrome b5-like domain. In S. cer, mito Osm1 localizes to mitochondrial intermembrane space  
https://www.ncbi.nlm.nih.gov/pmc/articles/PMC5638582/pdf/2773.pdf  
11536 has cytochrome b5-like domain but FRD domain is ~50% coverage, located at the end of scaffold 16, essential  
These two look like the same sequence incorrectly assembled  
S. cer FRD1 and OSM1 do not have C-terminal cytochrome b5-like domain. Osm1 facilitates the transfer of electrons from Erv1 to fumarate, osm1 and erv1 are synthetic lethal in anaerobic conditions in S. cer  
16224 K17783: ERV1, GFER, ALR; mitochondrial FAD-linked sulfhydryl oxidase  
http://www.ebi.ac.uk/interpro/entry/IPR010960/proteins-matched?ida=392051

In [24]:

```
model.reactions.get_by_id('ICDHxm').gene_reaction_rule = '11681 and 11682'
model.reactions.get_by_id('ICDHyp').gene_reaction_rule = '11129'
model.remove_reactions(['ICDHyr','ICDHym'], remove_orphans=True)
model.reactions.get_by_id('FRDcm').gene_reaction_rule = '11420 or 11536'
model.remove_reactions(['FRDm','FUM'], remove_orphans=True)
```

In [25]:

```
print(len(model.genes))
print(len(model.reactions))
print(len(model.metabolites))
model
```

```
1234
2351
2551
```

Out[25]:

|  |  |
| --- | --- |
| **Name** | R. toruloides |
| **Memory address** | 0x0102d579748 |
| **Number of metabolites** | 2551 |
| **Number of reactions** | 2351 |
| **Number of groups** | 0 |
| **Objective expression** | 0 |
| **Compartments** | c, x, m, e, r, v, n, g, p, h, f, l, d |

In [26]:

```
for x in sorted(model.genes, key=lambda x: x.id):
    if not x.reactions:
        print(x)
print()
for x in sorted(model.metabolites, key=lambda x: x.id):
    if not x.reactions:
        print(x)
```

```

```

In [27]:

```
cobra.manipulation.remove_genes(model, [x for x in model.genes if not x.reactions])
model.remove_metabolites([x for x in model.metabolites if not x.reactions])
```

In [28]:

```
print(len(model.genes))
print(len(model.reactions))
print(len(model.metabolites))
model
```

```
1234
2351
2551
```

Out[28]:

|  |  |
| --- | --- |
| **Name** | R. toruloides |
| **Memory address** | 0x0102d579748 |
| **Number of metabolites** | 2551 |
| **Number of reactions** | 2351 |
| **Number of groups** | 0 |
| **Objective expression** | 0 |
| **Compartments** | c, x, m, e, r, v, n, g, p, h, f, l, d |

In [29]:

```
for x in sorted(model.genes, key=lambda x: x.id):
    if not x.reactions:
        print(x)
print()
for x in sorted(model.metabolites, key=lambda x: x.id):
    if not x.reactions:
        print(x)
```

```

```

In [30]:

```
cobra.io.save_json_model(model, "IFO0880_GPR_1d.json")
```

In [31]:

```
model_old = cobra.io.load_json_model("IFO0880_GPR_1c.json")
model_new = cobra.io.load_json_model("IFO0880_GPR_1d.json")
```

In [32]:

```
print('Removed reactions\n')
for r in sorted(model_old.reactions, key=lambda x: x.id):
    if r not in model_new.reactions:
        print(r)
```

```
Removed reactions

ABFPTh: atp_h + fru_B_h --> adp_h + f6p_B_h + h_h
ACNAMt2pp: acnam_p + h_p --> acnam_c + h_c
ACN_a_m: acon_C_m + h2o_m <=> cit_m
ACN_b_m: acon_C_m + h2o_m <=> icit_m
ACONT: cit_c <=> icit_c
ACONTa: cit_c <=> acon_C_c + h2o_c
ACONTb: acon_C_c + h2o_c <=> icit_c
ACONTm: cit_m <=> icit_m
AGPOP: dgdp_c + h_c + pep_c --> dgtp_c + pyr_c
AGPOPm: dgdp_m + 2.0 h_m + pep_m --> dgtp_m + pyr_m
AM6PTh: atp_h + man_h --> adp_h + h_h + man6p_h
BFBPh: fdp_B_h + h2o_h --> f6p_B_h + pi_h
CS: accoa_c + h2o_c + oaa_c --> cit_c + coa_c + h_c
DAPOP: dadp_m + h_m + pep_m --> datp_m + pyr_m
ENOf: 2pg_f <=> h2o_f + pep_f
ENOm: 2pg_m <=> h2o_m + pep_m
FRDm: fadh2_m + fum_m --> fad_m + succ_m
FUM: fum_c + h2o_c <=> mal__L_c
GALth: gal_c + h_c <=> gal_h + h_h
GLUKAh: Glc_aD_h + atp_h --> adp_h + g6p_A_h + h_h
GLUKBh: atp_h + glc__bD_h --> adp_h + g6p_B_h + h_h
GTPOPm: gdp_m + 2.0 h_m + pep_m --> gtp_m + pyr_m
HACNHm: b124tc_m + h2o_m <=> hicit_m
ICDHym: icit_m + nadp_m --> akg_m + co2_m + nadph_m
ICDHyr: icit_c + nadp_c <=> akg_c + co2_c + nadph_c
MCITD: 2mcit_c --> 2mcacn_c + h2o_c
MDH: mal__L_c + nad_c <=> h_c + nadh_c + oaa_c
MDHC_nadp_hr: mal__L_h + nadp_h <=> co2_h + nadph_h + pyr_h
MDHf: h_f + nadh_f + oaa_f <=> mal__L_f + nad_f
MDHh: h_h + nadh_h + oaa_h <=> mal__L_h + nad_h
ME1: mal__L_c + nad_c --> co2_c + nadh_c + pyr_c
ME2m: mal__L_m + nadp_m --> co2_m + nadph_m + pyr_m
MICITDr: 2mcacn_c + h2o_c <=> micit_c
PCm: atp_m + hco3_m + pyr_m --> adp_m + h_m + oaa_m + pi_m
PGDHh: 6pgc_h + nadp_h --> co2_h + nadph_h + ru5p__D_h
PGKh: 13dpg_h + adp_h <=> 3pg_h + atp_h
PGLh: 6pgl_h + h2o_h --> 6pgc_h + h_h
PGMf: 3pg_f <=> 2pg_f
PGMm: 3pg_m <=> 2pg_m
PPCKm: atp_m + oaa_m --> adp_m + co2_m + pep_m
PYK3: gdp_c + h_c + pep_c --> gtp_c + pyr_c
PYKf: adp_f + h_f + pep_f --> atp_f + pyr_f
PYKm: adp_m + h_m + pep_m --> atp_m + pyr_m
PYRDC2: acald_c + h_c + pyr_c --> actn__R_c + co2_c
RPEh: ru5p__D_h <=> xu5p__D_h
RPIh: r5p_h <=> ru5p__D_h
RZ5PP: 5prdmbz_c + h2o_c --> pi_c + rdmbzi_c
SLDxm: nad_m + sl__L_m <=> 3spyr_m + h_m + nadh_m
TAh: g3p_h + s7p_h <=> e4p_h + f6p_B_h
TKT1h: r5p_h + xu5p__D_h <=> g3p_h + s7p_h
TKT2h: e4p_h + xu5p__D_h <=> f6p_B_h + g3p_h
TPIh: dhap_h <=> g3p_h
XYLt2pp: h_p + xyl__D_p --> h_c + xyl__D_c
yli_R0417: yli_M01059_m <=> hicit_m
yli_R0482: yli_M00116_c <=> ru5p__D_c
yli_R0485: g3p_c + s7p_c <=> xu5p__D_c + yli_M00116_c
yli_R1379: 6pgc_c + nad_c --> co2_c + nadh_c + ru5p__D_c
yli_R1494: nad_c + yli_M07013_c --> h_c + nadh_c + yli_M02657_c
yli_R1553: dgtp_c + pyr_c <=> dgdp_c + pep_c
yli_R1554: gtp_c + pyr_c <=> gdp_c + pep_c
yli_R1557: datp_c + pyr_c <=> dadp_c + pep_c
```

In [33]:

```
print('Updated reactions\n')
for r in sorted(model_old.reactions, key=lambda x: x.id):
    if r in model_new.reactions:
        r2 = model_new.reactions.get_by_id(r.id)
        if (r.name == r2.name and r.reaction == r2.reaction and r.gene_reaction_rule == r2.gene_reaction_rule and
            r.lower_bound == r2.lower_bound and r.upper_bound == r2.upper_bound):
            pass
        else:
            print('Old', r, r.gene_reaction_rule)
            print('New', r2, r2.gene_reaction_rule)
            print()
```

```
Updated reactions

Old CSp: accoa_x + h2o_x + oaa_x --> cit_x + coa_x + h_x 11331 or 8548
New CSp: accoa_x + h2o_x + oaa_x --> cit_x + coa_x + h_x 8548

Old F1PP: f1p_c + h2o_c --> fru_c + pi_c 11513 or 14546 or 8576
New F1PP: f1p_c + h2o_c --> fru_c + pi_c 14546 or 8576

Old F6PP: f6p_c + h2o_c --> fru_c + pi_c 11513 or 14546 or 8576
New F6PP: f6p_c + h2o_c --> fru_c + pi_c 14546 or 8576

Old FBP: fdp_c + h2o_c --> f6p_c + pi_c 11513 or 15423
New FBP: fdp_c + h2o_c --> f6p_c + pi_c 15423

Old FBP26: f26bp_c + h2o_c --> f6p_c + pi_c 15195 or 15423 or 15744
New FBP26: f26bp_c + h2o_c --> f6p_c + pi_c 15195 or 15744

Old FRDcm: fadh2_m + fum_c --> fad_m + succ_c 11420
New FRDcm: fadh2_m + fum_c --> fad_m + succ_c 11420 or 11536

Old G1PP: g1p_c + h2o_c --> glc__D_c + pi_c 11513 or 8576
New G1PP: g1p_c + h2o_c --> glc__D_c + pi_c 8576

Old GALt2: gal_e + h_e --> gal_c + h_c 10704 or 11075 or 11893 or (10704 and 11075) or (10704 and 11893)
New GALt2: gal_e + h_e --> gal_c + h_c 11075 or 11893

Old GLCt1: glc__D_e --> glc__D_c 10452 or 10704 or 11075 or 11893 or 15762 or 9102 or 9841
New GLCt1: glc__D_e --> glc__D_c 10452 or 11075 or 11893 or 15762 or 9102 or 9841

Old GLUt2r: glu__L_e + h_e <=> glu__L_c + h_c 10704 or 14229 or 15074 or 8962 or 9319 or 9322 or 9962 or (YALI0E20713g and 10704 and 14229 and 8962) or (YALI0E20713g and 10704 and 14229 and 9319) or (YALI0E20713g and 10704 and 14229 and 9322) or (YALI0E20713g and 10704 and 14229 and 9962) or (YALI0E20713g and 10704 and 15074 and 8962) or (YALI0E20713g and 10704 and 15074 and 9319) or (YALI0E20713g and 10704 and 15074 and 9322) or (YALI0E20713g and 10704 and 15074 and 9962)
New GLUt2r: glu__L_e + h_e <=> glu__L_c + h_c 14229 or 15074 or 8962 or 9319 or 9322 or 9962

Old GLYCt2: glyc_e + h_e --> glyc_c + h_c 12792
New GLYCt2: glyc_e + h_e --> glyc_c + h_c 10704 and 10705

Old ICDHxm: icit_m + nad_m --> akg_m + co2_m + nadh_m 11681 or 11682 or (11681 and 11682)
New ICDHxm: icit_m + nad_m --> akg_m + co2_m + nadh_m 11681 and 11682

Old MCITSm: h2o_m + oaa_m + ppcoa_m --> 2mcit_m + coa_m + h_m 11331
New MCITSm: h2o_m + oaa_m + ppcoa_m --> 2mcit_m + coa_m + h_m 11328

Old MDHm: mal__L_m + nad_m <=> h_m + nadh_m + oaa_m 10614 or 11178
New MDHm: mal__L_m + nad_m <=> h_m + nadh_m + oaa_m 11178

Old MDHp: mal__L_x + nad_x <=> h_x + nadh_x + oaa_x 10614 or 11178
New MDHp: mal__L_x + nad_x <=> h_x + nadh_x + oaa_x 10614

Old ME1m: mal__L_m + nad_m --> co2_m + nadh_m + pyr_m 12761 or 13917
New ME1m: mal__L_m + nad_m --> co2_m + nadh_m + pyr_m 13917

Old ME2: mal__L_c + nadp_c --> co2_c + nadph_c + pyr_c 12761 or 13917
New ME2: mal__L_c + nadp_c --> co2_c + nadph_c + pyr_c 12761

Old MN6PP: h2o_c + man6p_c --> man_c + pi_c 11513 or 8460
New MN6PP: h2o_c + man6p_c --> man_c + pi_c 8460

Old OAADC: h_c + oaa_c --> co2_c + pyr_c 16630
New OAADC: h_c + oaa_c --> co2_c + pyr_c 12761

Old PGL: 6pgl_c + h2o_c --> 6pgc_c + h_c 14132 or 14499
New PGL: 6pgl_c + h2o_c --> 6pgc_c + h_c 14132

Old PGM: 2pg_c <=> 3pg_c 11229 or 12393 or 12637 or 15425 or 9910
New PGM: 2pg_c <=> 3pg_c 11229 or 12393 or 15425 or 9910

Old R5PP: h2o_c + r5p_c --> pi_c + rib__D_c 11513 or 13044 or 14546 or 8576
New R5PP: h2o_c + r5p_c --> pi_c + rib__D_c 13044 or 14546 or 8576
```

In [34]:

```
print('Added reactions\n')
for r in sorted(model_new.reactions, key=lambda x: x.id):
    if r not in model_old.reactions:
        print(r)
```

```
Added reactions

ACONTam: acon_C_m + h2o_m <=> cit_m
ACONTbm: acon_C_m + h2o_m <=> icit_m
G6PDH2rp: g6p_x + nadp_x <=> 6pgl_x + h_x + nadph_x
GNDp: 6pgc_x + nadp_x --> co2_x + nadph_x + ru5p__D_x
HACONTam: hcit_m <=> h2o_m + hacon_C_m
HACONTbm: h2o_m + hacon_C_m <=> hicit_m
OAADCm: h_m + oaa_m --> co2_m + pyr_m
PGLp: 6pgl_x + h2o_x --> 6pgc_x + h_x
PMDPHT: 5aprbu_c + h2o_c --> 4r5au_c + pi_c
SBP: h2o_c + s17bp_c --> pi_c + s7p_c
```
