# Supplementary material for: Multi-Omics Driven Metabolic Network Reconstruction and Analysis of Lignocellulosic Carbon Utilization in Rhodosporidium toruloides
Source: Front Bioeng Biotechnol. 2021 Jan 8;8:612832. doi: 10.3389/fbioe.2020.612832 (PMC7873862; doi:10.3389/fbioe.2020.612832)
Supplement: Supplementary File 4 — Multi-omics dataset for R. toruloides IFO0880. [file Data_Sheet_1.zip › Supplementary File S1/1.Manual_curation/Refinement_1a_Duplicate_Metabolites.html]

Refinement\_1a\_Duplicate\_Metabolites


In [1]:

```
%matplotlib inline
from matplotlib import pyplot as plt
from matplotlib import colors
import csv
import numpy as np
import pandas as pd
import json
import urllib
import cobra
```

In [2]:

```
cobra.__version__
```

Out[2]:

```
'0.15.4'
```

In [3]:

```
Annotation = pd.read_excel('../../Data/R_toruloides_Data_for_Reconstruction.xlsx',
                          sheet_name='Annotation', index_col=0)
Annotation.index = Annotation.index.map(str)
Annotation = Annotation.fillna('')
Transcriptomics = pd.read_excel('../../Data/R_toruloides_Data_for_Reconstruction.xlsx',
                          sheet_name='Transcriptomics', header=[0,1,2,3], index_col=0)
Transcriptomics.index = Transcriptomics.index.map(str)
Proteomics = pd.read_excel('../../Data/R_toruloides_Data_for_Reconstruction.xlsx',
                          sheet_name='Proteomics', header=[0,1,2], index_col=0)
Proteomics.index = Proteomics.index.map(str)
Fitness = pd.read_excel('../../Data/R_toruloides_Data_for_Reconstruction.xlsx',
                          sheet_name='Fitness', index_col=0)
Fitness.index = Fitness.index.map(str)
```

In [4]:

```
def background_gradient(s, cmap='seismic', text_color_threshold=0.408):
    lim = max(abs(s.min().min()),abs(s.max().max()))
    rng = 2.0*lim
    norm = colors.Normalize(-lim - (rng * 0.2), lim + (rng * 0.2))
    rgbas = plt.cm.get_cmap(cmap)(norm(s.values))
    def relative_luminance(rgba):
        r, g, b = (x / 12.92 if x <= 0.03928 else ((x + 0.055) / 1.055 ** 2.4) for x in rgba[:3])
        return 0.2126 * r + 0.7152 * g + 0.0722 * b
    def css(rgba):
        dark = relative_luminance(rgba) < text_color_threshold
        text_color = '#f1f1f1' if dark else '#000000'
        return 'background-color: {b};color: {c};'.format(b=colors.rgb2hex(rgba), c=text_color)

    if s.ndim == 1:
        return [css(rgba) for rgba in rgbas]
    else:
        return pd.DataFrame([[css(rgba) for rgba in row] for row in rgbas], index=s.index, columns=s.columns)

def Show_Data(x):
    display(Transcriptomics.loc[x].style.background_gradient(cmap='Reds', low=0.2, high=0.2, axis=None))
    temp = [y for y in x if y in Proteomics.index]
    display(Proteomics.loc[temp].style.background_gradient(cmap='Reds', low=0.2, high=0.2, axis=None))
    temp = [y for y in x if y in Fitness.index]
    display(Fitness.loc[temp].style.apply(background_gradient, cmap='seismic', axis=None))
    return;
```

In [5]:

```
temp = ['10212','15218','11208','12042','13426','8678','14070','8975']
#temp = Annotation.index[Annotation['Combined Annotations'].str.contains('aspartate')]
#temp = Annotation.index[Annotation['Sc288c Orthologs'].str.contains('GPD1', na=False)]
display(Annotation.loc[temp])
Show_Data(temp)
```

|  | Combined Annotations | Signal P | Sc288c Orthologs | Human Orthologs | Sc288 Best Hit | Human Blast | Essential | WolfPSort | C Terminal |
| --- | --- | --- | --- | --- | --- | --- | --- | --- | --- |
| RTO4\_ID |  |  |  |  |  |  |  |  |  |
| 10212 | K16066: ydfG; 3-hydroxy acid dehydrogenase / m... | S | YMR226C | DHRS11 | YMR226C | DHRS11 | Not Essential | mito 12, cyto 8, extr 5, cyto\_pero 5 | GGK\* |
| 15218 | K05605: HIBCH; 3-hydroxyisobutyryl-CoA hydrolase |  | EHD3 | HIBCH | EHD3 | HIBCH | Not Essential | mito 22.5, cyto\_mito 14, cyto 4.5 | WVS\* |
| 11208 | KOG4178: Soluble epoxide hydrolase |  |  |  |  |  | Not Essential | cysk 20, cyto 5 | EAK\* |
| 12042 | K00128: ALDH; aldehyde dehydrogenase (NAD+) |  | ALD5,ALD4,ALD6,ALD3,ALD2 | ALDH1A1,ALDH1A2,ALDH1A3,ALDH1B1,ALDH2 | ALD5 | ALDH2 | Not Essential | cyto 18.5, cyto\_nucl 10, mito 4, pero 4 | NPL\* |
| 13426 | K07249: E1.2.1.36; retinal dehydrogenase |  | ALD5,ALD4,ALD6,ALD3,ALD2 | ALDH1A1,ALDH1A2,ALDH1A3,ALDH1B1,ALDH2 | ALD5 | ALDH1 | Not Essential | mito 25.5, cyto\_mito 14 | WPL\* |
| 8678 | K00626: E2.3.1.9, atoB; acetyl-CoA C-acetyltra... |  | ERG10 | ACAT1 | ERG10 | ACAT1 | Not Essential | mito 23.5, cyto\_mito 14 | QRL\* |
| 14070 | K09478: ACADSB; short/branched chain acyl-CoA ... |  |  | ACADSB |  | ACADS | Not Essential | mito 26, cyto\_mito 14.5 | EYS\* |
| 8975 | K00140: mmsA, iolA, ALDH6A1; malonate-semialde... |  |  | ALDH6A1 | UGA2 | ALDH6 | Not Essential | mito 27 | THH\* |

| strain | WT | | | | | | | | | | | | | | | | |
| --- | --- | --- | --- | --- | --- | --- | --- | --- | --- | --- | --- | --- | --- | --- | --- | --- | --- |
| condition | G\_MM | C\_MM | G\_SD | | GX\_SD | | | X\_SD | | A\_SD | | C\_SD | | MM\_CN120 | | MM\_CN5 | Diversity\_Sample |
| phase | exp | exp | exp | stat | exp | trans | stat | exp | stat | exp | stat | exp | stat | exp | stat | exp | exp |
| proteinId | Set1 | Set1 | Set2 | Set2 | Set2 | Set2 | Set2 | Set2 | Set2 | Set2 | Set2 | Set2 | Set2 | Set3 | Set3 | Set3 | Set3 |
| 10212 | 6.53836 | 6.95944 | 6.24124 | 5.45969 | 6.30796 | 5.39226 | 4.99547 | 6.86667 | 5.50641 | 6.68701 | 5.75112 | 5.57276 | 4.60922 | 3.85041 | 2.89144 | 6.3788 | 5.01429 |
| 15218 | 6.86467 | 7.09307 | 6.60272 | 6.70549 | 6.55215 | 6.34078 | 6.39006 | 6.66868 | 6.49998 | 6.38468 | 6.70848 | 7.22747 | 7.16794 | 5.92126 | 5.82553 | 6.53206 | 6.51419 |
| 11208 | 6.29253 | 5.23641 | 4.78325 | 4.38211 | 4.58861 | 5.07727 | 4.86509 | 5.45579 | 4.59222 | 5.66995 | 4.74112 | 4.80782 | 4.00174 | 6.37036 | 6.3473 | 5.44117 | 5.76806 |
| 12042 | 8.00849 | 8.563 | 8.41698 | 9.34145 | 8.34137 | 8.32176 | 8.06734 | 8.44974 | 8.36047 | 8.84866 | 8.59447 | 7.17248 | 5.67475 | 7.81326 | 7.51984 | 8.29774 | 8.31731 |
| 13426 | 7.30781 | 4.89844 | 3.84807 | 2.79984 | 3.78882 | 3.98344 | 3.73207 | 4.58197 | 3.36082 | 4.5625 | 3.35098 | 4.25235 | 3.1633 | 6.93547 | 6.81825 | 3.29168 | 5.38811 |
| 8678 | 7.02165 | 8.49604 | 6.56773 | 5.30595 | 6.67599 | 5.76971 | 5.63167 | 7.10664 | 5.26798 | 5.7248 | 5.42382 | 5.88536 | 5.87647 | 7.0048 | 6.36022 | 8.25583 | 7.88214 |
| 14070 | 5.47087 | 6.86451 | 8.73838 | 7.09927 | 8.64357 | 7.57784 | 7.86415 | 7.23047 | 7.00805 | 5.82687 | 7.45331 | 5.41054 | 5.40283 | 5.58343 | 6.95021 | 6.80154 | 5.95799 |
| 8975 | 5.66327 | 6.02765 | 6.70623 | 7.30337 | 6.7434 | 6.22721 | 6.22823 | 6.24526 | 6.03253 | 5.57225 | 6.31843 | 5.45415 | 5.95381 | 6.50537 | 5.94092 | 7.64182 | 6.56961 |

| strain | WT | | | | | | | | | | |
| --- | --- | --- | --- | --- | --- | --- | --- | --- | --- | --- | --- |
| condition | G\_SD | | GX\_SD | | | X\_SD | | A\_SD | | C\_SD | |
| proteinId | exp | stat | exp | trans | stat | exp | stat | exp | stat | exp | stat |
| 10212 | 5.54566 | 5.59178 | 4.68671 | 4.64674 | 5.31618 | 8.80939 | 9.46517 | 9.6673 | 9.19188 | 6.83544 | 3.68413 |
| 15218 | 21.1773 | 18.7988 | 21.94 | 18.4431 | 20.2026 | 20.9192 | 22.5031 | 20.6773 | 20.4994 | 26.9393 | 28.0827 |
| 11208 | 0.579959 | 0 | 0.203508 | 0 | 0.180258 | 0 | 0 | 0.769789 | 0.196097 | 0.429907 | 0.433177 |
| 12042 | 33.9947 | 44.5614 | 32.5616 | 41.5121 | 40.2993 | 56.5808 | 63.4798 | 61.9973 | 72.5595 | 57.7357 | 40.7488 |
| 13426 | 0.757094 | 0.533653 | 1.41886 | 0.929251 | 0.72686 | 2.14514 | 0.960201 | 3.46858 | 3.64334 | 1.06907 | 0.864976 |
| 8678 | 24.2091 | 19.2125 | 22.5986 | 15.4605 | 14.3787 | 24.8578 | 22.1975 | 26.0698 | 22.4098 | 28.6911 | 21.4138 |
| 14070 | 34.4501 | 30.6889 | 29.0831 | 31.7846 | 35.7825 | 24.6024 | 29.0654 | 25.091 | 18.1594 | 28.1585 | 21.7638 |
| 8975 | 15.1685 | 20.4773 | 14.4448 | 16.0147 | 15.9748 | 17.0593 | 17.9072 | 17.7641 | 18.2123 | 22.8784 | 18.4812 |

|  | Glucose | Xylose | Arabinose | Acetate | Coumarate | Ferulate | YNB Oleic Acid | YNB Ricinoleic Acid | YNB Glucose | YNB Gluc DOC | YPD |
| --- | --- | --- | --- | --- | --- | --- | --- | --- | --- | --- | --- |
| proteinId |  |  |  |  |  |  |  |  |  |  |  |
| 10212 | 0.537244 | 0.955703 | 0.339564 | -0.0161788 | 0.965219 | -0.128446 | 0.259048 | 1.42188 | 0.331166 | -0.0426479 | 0.592278 |
| 15218 | 0.0745291 | -0.0219716 | -0.274042 | -1.22526 | -0.919181 | -0.37866 | -0.549135 | 0.000358951 | -0.184537 | -0.260827 | 0.21517 |
| 11208 | -0.147252 | -0.119349 | -0.215916 | -0.214711 | -0.2992 | 0.191603 | 0.300566 | 0.424416 | 0.037048 | -0.199725 | 0.142372 |
| 12042 | 0.170666 | -0.143043 | 0.000953096 | -0.0230376 | -0.521499 | 0.0146534 | 0.144875 | -0.295284 | 0.392859 | 0.454577 | -0.242457 |
| 13426 | -0.226122 | -0.181863 | -0.169891 | -0.0897023 | -0.0133507 | -0.00809092 | -1.1502 | -2.84044 | -0.85113 | -0.458514 | -0.0243377 |
| 14070 | -0.215941 | -0.275626 | -0.128586 | -0.914107 | -1.70534 | -0.685301 | -1.95523 | -2.096 | -0.0821845 | -0.69886 | -0.125942 |
| 8975 | -0.183461 | 0.337248 | -0.0360008 | -0.0852432 | 0.164365 | 0.133413 | -0.0728905 | -0.353268 | -0.148485 | -0.0951318 | 0.168732 |

In [6]:

```
json.load(urllib.request.urlopen('http://bigg.ucsd.edu/api/v2/database_version'))
```

Out[6]:

```
{'last_updated': '2018-02-24 08:16:33.924054',
 'bigg_models_version': '1.3.0',
 'api_version': 'v2'}
```

In [7]:

```
eco = cobra.io.load_json_model(urllib.request.urlopen('http://bigg.ucsd.edu/static/models/iML1515.json'))
sce = cobra.io.load_json_model(urllib.request.urlopen('http://bigg.ucsd.edu/static/models/iMM904.json'))
hsa = cobra.io.load_json_model(urllib.request.urlopen('http://bigg.ucsd.edu/static/models/RECON1.json'))
hsa2 = cobra.io.load_json_model(urllib.request.urlopen('http://bigg.ucsd.edu/static/models/Recon3D.json'))
ppu = cobra.io.load_json_model(urllib.request.urlopen('http://bigg.ucsd.edu/static/models/iJN746.json'))
ptri = cobra.io.load_json_model(urllib.request.urlopen('http://bigg.ucsd.edu/static/models/iLB1027_lipid.json'))
cre = cobra.io.load_json_model(urllib.request.urlopen('http://bigg.ucsd.edu/static/models/iRC1080.json'))
```

In [8]:

```
cobra.io.save_json_model(eco, "../../Data/BiGG_Models/iML1515.json")
cobra.io.save_json_model(sce, "../../Data/BiGG_Models/iMM904.json")
cobra.io.save_json_model(hsa, "../../Data/BiGG_Models/RECON1.json")
cobra.io.save_json_model(hsa2, "../../Data/BiGG_Models/Recon3D.json")
cobra.io.save_json_model(ppu, "../../Data/BiGG_Models/iJN746.json")
cobra.io.save_json_model(ptri, "../../Data/BiGG_Models/iLB1027_lipid.json")
cobra.io.save_json_model(cre, "../../Data/BiGG_Models/iRC1080.json")
```

In [9]:

```
model = cobra.io.load_json_model("../0.Annotation_and_Draft_reconstruction/IFO0880_GPR.json")
```

In [10]:

```
print(len(model.genes))
print(len([x for x in model.genes if not x.id[0].isalpha()]))
model
```

```
1596
1137
```

Out[10]:

|  |  |
| --- | --- |
| **Name** | R. toruloides |
| **Memory address** | 0x0102e8aaa20 |
| **Number of metabolites** | 3589 |
| **Number of reactions** | 3804 |
| **Number of groups** | 0 |
| **Objective expression** | 0 |
| **Compartments** | c, x, m, e, r, v, n, g, u, p, h, s, f, l |

### Duplicate metabolites¶

In [11]:

```
# Duplicate metabolite names
mets = sorted([m.id for m in model.metabolites])
mets_uniq = set()
for x in mets:
    if x.lower() not in mets_uniq:
        mets_uniq.add(x.lower())
    else:
        print(x)
```

```
ficytc_m
focytc_m
```

In [12]:

```
print('ficytC')
for r in sorted(model.metabolites.get_by_id('ficytC_m').reactions, key=lambda x: x.id):
    print(r.id, r.reaction)
print()
print('focytC')
for r in sorted(model.metabolites.get_by_id('focytC_m').reactions, key=lambda x: x.id):
    print(r.id, r.reaction)
```

```
ficytC
CYOOm3 4.0 focytC_m + 7.92 h_m + o2_m --> 4.0 ficytC_m + 1.96 h2o_m + 4.0 h_c + 0.02 o2s_m
CYOR_u10m 2.0 ficytC_m + 2.0 h_m + q10h2_m --> 2.0 focytC_m + 4.0 h_c + q10_m
SULFOX 2.0 ficytC_m + h2o_c + so3_c --> 2.0 focytC_m + 2.0 h_c + so4_c

focytC
CYOOm3 4.0 focytC_m + 7.92 h_m + o2_m --> 4.0 ficytC_m + 1.96 h2o_m + 4.0 h_c + 0.02 o2s_m
CYOR_u10m 2.0 ficytC_m + 2.0 h_m + q10h2_m --> 2.0 focytC_m + 4.0 h_c + q10_m
SULFOX 2.0 ficytC_m + h2o_c + so3_c --> 2.0 focytC_m + 2.0 h_c + so4_c
yli_R0431 2.0 ficytc_m + 1.5 h_m + yli_M04633_m --> 2.0 focytC_m + 1.5 h_c + q6_m
yli_R0667 2.0 ficytc_m + lac__L_m --> 2.0 focytC_m + pyr_m
yli_R0668 2.0 ficytc_m + lac__D_m --> 2.0 focytC_m + pyr_m
```

In [13]:

```
for r in model.reactions:
    for x in ['ficytC_m','focytC_m']:
        if x in [m.id for m in r.metabolites]:
            r.add_metabolites({x: -r.get_coefficient(x), x.replace('C','c'): r.get_coefficient(x)})
```

In [14]:

```
for x in model.metabolites:
    if not x.reactions:
        print(x)
```

```
focytC_m
ficytC_m
```

In [15]:

```
for x in model.metabolites:
    if not x.reactions:
        x.remove_from_model()
```

In [16]:

```
print('ficytc')
for r in sorted(model.metabolites.get_by_id('ficytc_m').reactions, key=lambda x: x.id):
    print(r.id + '   \t', r.reaction)
print()
for r in sorted(model.metabolites.get_by_id('ficytc_m').reactions, key=lambda x: x.id):
    print(r.id + '   \t', r.gene_reaction_rule)
print()
print('focytc')
for r in model.metabolites.get_by_id('focytc_m').reactions:
    if r not in model.metabolites.get_by_id('ficytc_m').reactions:
        print(r.id + '  ', '\t', r.reaction, r.gene_reaction_rule)
```

```
ficytc
CCP2m   	 2.0 focytc_m + h2o2_m --> 2.0 ficytc_m + 2.0 h2o_m
CYOO6m   	 4.0 focytc_m + 8.0 h_m + o2_m --> 4.0 ficytc_m + 2.0 h2o_m + 4.0 h_c
CYOOm   	 4.0 focytc_m + 6.0 h_m + o2_m --> 4.0 ficytc_m + 2.0 h2o_m + 6.0 h_c
CYOOm3   	 4.0 focytc_m + 7.92 h_m + o2_m --> 4.0 ficytc_m + 1.96 h2o_m + 4.0 h_c + 0.02 o2s_m
CYOR_q8_m   	 2.0 ficytc_m + 2.0 h_m + q8h2_m --> 2.0 focytc_m + 4.0 h_c + q8_m
CYOR_u10m   	 2.0 ficytc_m + 2.0 h_m + q10h2_m --> 2.0 focytc_m + 4.0 h_c + q10_m
CYOR_u6m   	 2.0 ficytc_m + 1.5 h_m + q6h2_m --> 2.0 focytc_m + 1.5 h_c + q6_m
D_LACDHm   	 2.0 ficytc_m + lac__D_m --> 2.0 focytc_m + 2.0 h_m + pyr_m
D_LACDcm   	 2.0 ficytc_m + lac__D_c --> 2.0 focytc_m + pyr_c
D_LACDm   	 2.0 ficytc_m + lac__D_m --> 2.0 focytc_m + pyr_m
LLDH_ferr_m   	 2.0 ficytc_m + lac__L_m --> 2.0 focytc_m + 2.0 h_m + pyr_m
L_LACD2cm   	 2.0 ficytc_m + lac__L_c --> 2.0 focytc_m + pyr_c
SULFOX   	 2.0 ficytc_m + h2o_c + so3_c --> 2.0 focytc_m + 2.0 h_c + so4_c
yli_R0431   	 2.0 ficytc_m + 1.5 h_m + yli_M04633_m --> 2.0 focytc_m + 1.5 h_c + q6_m
yli_R0667   	 2.0 ficytc_m + lac__L_m --> 2.0 focytc_m + pyr_m
yli_R0668   	 2.0 ficytc_m + lac__D_m --> 2.0 focytc_m + pyr_m

CCP2m   	 (10811 and 8802) or (13161 and 8802) or (13161 and 9198)
CYOO6m   	 (CRv4_Au5_s1_g1958_t1 and CRv4_Au5_s3_g10243_t1 and CRv4_Au5_s4_g11710_t1 and 11317 and 8802) or (CRv4_Au5_s1_g1958_t1 and CRv4_Au5_s3_g10243_t1 and CRv4_Au5_s4_g11710_t1 and 11317 and 9198)
CYOOm   	 Q0045 and Q0250 and Q0275 and YLR395C and YMR256C and 10685 and 11317 and 11769 and 13007 and 13628 and 8802 and 9165
CYOOm3   	 COX1 and COX2 and COX3 and COX4I1 and COX4I2 and COX6A1 and COX6A2 and COX6C and COX7A1 and COX7A2 and COX7A2L and COX7B and COX7B2 and COX7C and COX8A and COX8C and 10685 and 11317 and 9165
CYOR_q8_m   	 (13614 and 15681 and 8802) or (13614 and 15681 and 9198)
CYOR_u10m   	 (CYTB and UQCR11 and UQCRH and UQCRQ and 11618 and 13614 and 15231 and 15681 and 9198 and 9705) or (CYTB and Uqcr11 and Uqcrq and mmu_100042918 and 11618 and 13614 and 15231 and 15681 and 9198 and 9705)
CYOR_u6m   	 Q0105 and YBL045C and 11618 and 12966 and 13608 and 13614 and 15231 and 15681 and 15758 and 8802 and 9198
D_LACDHm   	 (13328 and 8802) or (13328 and 9198) or (16643 and 8802) or (16643 and 9198)
D_LACDcm   	 (13328 and 8802) or (16643 and 8802)
D_LACDm   	 16643 and 8802
LLDH_ferr_m   	 (CRv4_Au5_s9_g15537_t1 and 8802) or (CRv4_Au5_s9_g15537_t1 and 9198)
L_LACD2cm   	 16607 and 8802
SULFOX   	 16758
yli_R0431   	 13614
yli_R0667   	 16607
yli_R0668   	 13328

focytc
```

### Lactate dehydrogenase and related reactions¶

In [17]:

```
# D-lactate reactions
for r in sorted(model.metabolites.get_by_id('lac__D_m').reactions, key=lambda x: x.id):
        print(r.id, r.reaction, r.gene_reaction_rule)
print()
for r in sorted(model.metabolites.get_by_id('lac__D_c').reactions, key=lambda x: x.id):
        print(r.id, r.reaction, r.gene_reaction_rule)
```

```
D_LACDHm 2.0 ficytc_m + lac__D_m --> 2.0 focytc_m + 2.0 h_m + pyr_m (13328 and 8802) or (13328 and 9198) or (16643 and 8802) or (16643 and 9198)
D_LACDm 2.0 ficytc_m + lac__D_m --> 2.0 focytc_m + pyr_m 16643 and 8802
GLYOXm h2o_m + lgt__S_m --> gthrd_m + h_m + lac__D_m 13041
LCADi_Dm h2o_m + lald__D_m + nad_m --> 2.0 h_m + lac__D_m + nadh_m 12042 or 13426 or 16273 or 8569 or 8692 or 8975
yli_R0668 2.0 ficytc_m + lac__D_m --> 2.0 focytc_m + pyr_m 13328

D_LACDcm 2.0 ficytc_m + lac__D_c --> 2.0 focytc_m + pyr_c (13328 and 8802) or (16643 and 8802)
D_LACt2 h_e + lac__D_e <=> h_c + lac__D_c 10184
GLYOX h2o_c + lgt__S_c --> gthrd_c + h_c + lac__D_c 13041
GLYOX3 h2o_c + mthgxl_c --> h_c + lac__D_c 12381
LCADi_D h2o_c + lald__D_c + nad_c --> 2.0 h_c + lac__D_c + nadh_c 12042 or 13426 or 15814 or 16323
LDH_D lac__D_c + nad_c <=> h_c + nadh_c + pyr_c 13328
```

In [18]:

```
temp = ['13328','16643','13239','8802','9198']
display(Annotation.loc[temp])
Show_Data(temp)
```

|  | Combined Annotations | Signal P | Sc288c Orthologs | Human Orthologs | Sc288 Best Hit | Human Blast | Essential | WolfPSort | C Terminal |
| --- | --- | --- | --- | --- | --- | --- | --- | --- | --- |
| RTO4\_ID |  |  |  |  |  |  |  |  |  |
| 13328 | K00102: dld, LDHD; D-lactate dehydrogenase (cy... | A | DLD1 | LDHD | DLD1 | LDHD | Not Essential | mito 24, cyto 3 | ASH\* |
| 16643 | K00102: dld, LDHD; D-lactate dehydrogenase (cy... | S | DLD2,DLD3 | D2HGDH | DLD2 | D2HGD | Not Essential | mito 24.5, cyto\_mito 14 | AEQ\* |
| 13239 | K03777: dld; D-lactate dehydrogenase | S |  |  | DLD1 | LDHD | Not Essential | mito 19, cyto 6 | WDA\* |
| 8802 | K08738: CYC; cytochrome c |  | CYC7,CYC1 | CYCS | CYC7 | CYCS | Not Essential | mito 19, cyto 6 | STA\* |
| 9198 | K00413: CYC1, CYT1, petC; ubiquinol-cytochrome... | S | CYT1 | CYC1 | CYT1 | CYC1 | Not Essential | mito 27 | PAK\* |

| strain | WT | | | | | | | | | | | | | | | | |
| --- | --- | --- | --- | --- | --- | --- | --- | --- | --- | --- | --- | --- | --- | --- | --- | --- | --- |
| condition | G\_MM | C\_MM | G\_SD | | GX\_SD | | | X\_SD | | A\_SD | | C\_SD | | MM\_CN120 | | MM\_CN5 | Diversity\_Sample |
| phase | exp | exp | exp | stat | exp | trans | stat | exp | stat | exp | stat | exp | stat | exp | stat | exp | exp |
| proteinId | Set1 | Set1 | Set2 | Set2 | Set2 | Set2 | Set2 | Set2 | Set2 | Set2 | Set2 | Set2 | Set2 | Set3 | Set3 | Set3 | Set3 |
| 13328 | 5.71937 | 5.82878 | 7.47435 | 4.88877 | 7.78181 | 6.76757 | 6.48163 | 6.33395 | 4.73111 | 6.23592 | 3.81796 | 4.93261 | 4.30363 | 5.09755 | 4.80473 | 5.58755 | 5.60639 |
| 16643 | 6.66421 | 6.56722 | 6.72194 | 6.15557 | 6.81848 | 6.97251 | 6.80884 | 7.07628 | 5.78036 | 6.0989 | 5.75058 | 5.89099 | 5.77695 | 6.20075 | 5.73998 | 6.51135 | 6.39697 |
| 13239 | 4.8232 | 5.6639 | 5.01459 | 5.72345 | 4.9999 | 5.49857 | 5.16634 | 5.86086 | 4.47739 | 5.51275 | 5.14905 | 5.6161 | 6.52771 | 3.44985 | 3.04082 | 4.19596 | 4.16836 |
| 8802 | 8.67558 | 10.1071 | 5.36904 | 6.82226 | 5.56482 | 6.09659 | 6.99801 | 4.52932 | 7.13324 | 5.94802 | 7.83162 | 7.07509 | 6.46397 | 4.75847 | 5.6766 | 4.60598 | 9.80594 |
| 9198 | 7.54537 | 8.14958 | 5.67577 | 6.43 | 5.97028 | 4.89267 | 5.61202 | 5.49101 | 6.75841 | 6.52274 | 7.32225 | 7.45546 | 7.36181 | 5.6883 | 6.20024 | 6.69287 | 8.33871 |

| strain | WT | | | | | | | | | | |
| --- | --- | --- | --- | --- | --- | --- | --- | --- | --- | --- | --- |
| condition | G\_SD | | GX\_SD | | | X\_SD | | A\_SD | | C\_SD | |
| proteinId | exp | stat | exp | trans | stat | exp | stat | exp | stat | exp | stat |
| 13328 | 13.6298 | 14.295 | 16.6749 | 16.9246 | 14.9358 | 16.0815 | 9.82871 | 9.86624 | 10.339 | 3.18519 | 1.96207 |
| 16643 | 14.0562 | 16.7944 | 12.4059 | 12.9843 | 16.7837 | 12.5553 | 14.3733 | 11.7959 | 9.94229 | 8.134 | 10.8617 |
| 13239 | 0.385663 | 0.5 | 0 | 0 | 0.357685 | 0.981841 | 0.205723 | 0.190305 | 0.574781 | 0.421498 | 0.217934 |
| 8802 | 10.7513 | 7.03085 | 11.0145 | 3.51378 | 4.58806 | 6.88666 | 6.1489 | 7.53591 | 4.02746 | 11.5408 | 8.92371 |
| 9198 | 18.8769 | 15.308 | 22.1688 | 12.6493 | 12.7867 | 12.316 | 11.7816 | 16.0313 | 15.6705 | 18.8639 | 21.5202 |

|  | Glucose | Xylose | Arabinose | Acetate | Coumarate | Ferulate | YNB Oleic Acid | YNB Ricinoleic Acid | YNB Glucose | YNB Gluc DOC | YPD |
| --- | --- | --- | --- | --- | --- | --- | --- | --- | --- | --- | --- |
| proteinId |  |  |  |  |  |  |  |  |  |  |  |
| 13328 | 0.180118 | -0.0871126 | 0.209748 | 0.130933 | 0.182657 | 0.137433 | 0.132868 | 0.101425 | 0.308586 | 0.285462 | 0.0422158 |
| 16643 | -1.06968 | 0.253369 | -0.281544 | -0.493532 | 1.18377 | -0.50392 | 0.285326 | -1.2611 | -0.879881 | -0.055865 | 0.0582271 |
| 13239 | 0.210698 | 0.0956797 | 0.126082 | 0.160421 | 0.192898 | 0.226086 | -0.0240317 | 0.230848 | -0.0563568 | -0.00250703 | 0.182905 |

In [19]:

```
for x in temp:
    if x in model.genes:
        for r in sorted(model.genes.get_by_id(x).reactions, key=lambda x: x.id):
            print(r.id, r.reaction, r.gene_reaction_rule)
    else:
        print(x, 'no reactions')
    print()
```

```
D_LACDHm 2.0 ficytc_m + lac__D_m --> 2.0 focytc_m + 2.0 h_m + pyr_m (13328 and 8802) or (13328 and 9198) or (16643 and 8802) or (16643 and 9198)
D_LACDcm 2.0 ficytc_m + lac__D_c --> 2.0 focytc_m + pyr_c (13328 and 8802) or (16643 and 8802)
GLXO1 glx_c + h2o_c + nad_c --> 2.0 h_c + nadh_c + oxa_c 13328
GLYCTO1 glyclt_c + o2_c --> glx_c + h2o2_c 14950 or 16607 or (PP_3746 and PP_3747 and 13328)
GLYCTO2 glyclt_c + q8_c --> glx_c + q8h2_c b4467 and b4468 and 13328
GLYCTO3 glyclt_c + mqn8_c --> glx_c + mql8_c b4467 and b4468 and 13328
GLYCTO4 2dmmq8_c + glyclt_c --> 2dmmql8_c + glx_c b4467 and b4468 and 13328
HPYRR2x h_c + hpyr_c + nadh_c --> glyc__S_c + nad_c 13328
LDH_D lac__D_c + nad_c <=> h_c + nadh_c + pyr_c 13328
yli_R0668 2.0 ficytc_m + lac__D_m --> 2.0 focytc_m + pyr_m 13328

D_LACDHm 2.0 ficytc_m + lac__D_m --> 2.0 focytc_m + 2.0 h_m + pyr_m (13328 and 8802) or (13328 and 9198) or (16643 and 8802) or (16643 and 9198)
D_LACDcm 2.0 ficytc_m + lac__D_c --> 2.0 focytc_m + pyr_c (13328 and 8802) or (16643 and 8802)
D_LACDm 2.0 ficytc_m + lac__D_m --> 2.0 focytc_m + pyr_m 16643 and 8802

13239 no reactions

CCP2m 2.0 focytc_m + h2o2_m --> 2.0 ficytc_m + 2.0 h2o_m (10811 and 8802) or (13161 and 8802) or (13161 and 9198)
CYOO6m 4.0 focytc_m + 8.0 h_m + o2_m --> 4.0 ficytc_m + 2.0 h2o_m + 4.0 h_c (CRv4_Au5_s1_g1958_t1 and CRv4_Au5_s3_g10243_t1 and CRv4_Au5_s4_g11710_t1 and 11317 and 8802) or (CRv4_Au5_s1_g1958_t1 and CRv4_Au5_s3_g10243_t1 and CRv4_Au5_s4_g11710_t1 and 11317 and 9198)
CYOOm 4.0 focytc_m + 6.0 h_m + o2_m --> 4.0 ficytc_m + 2.0 h2o_m + 6.0 h_c Q0045 and Q0250 and Q0275 and YLR395C and YMR256C and 10685 and 11317 and 11769 and 13007 and 13628 and 8802 and 9165
CYOR_q8_m 2.0 ficytc_m + 2.0 h_m + q8h2_m --> 2.0 focytc_m + 4.0 h_c + q8_m (13614 and 15681 and 8802) or (13614 and 15681 and 9198)
CYOR_u6m 2.0 ficytc_m + 1.5 h_m + q6h2_m --> 2.0 focytc_m + 1.5 h_c + q6_m Q0105 and YBL045C and 11618 and 12966 and 13608 and 13614 and 15231 and 15681 and 15758 and 8802 and 9198
D_LACDHm 2.0 ficytc_m + lac__D_m --> 2.0 focytc_m + 2.0 h_m + pyr_m (13328 and 8802) or (13328 and 9198) or (16643 and 8802) or (16643 and 9198)
D_LACDcm 2.0 ficytc_m + lac__D_c --> 2.0 focytc_m + pyr_c (13328 and 8802) or (16643 and 8802)
D_LACDm 2.0 ficytc_m + lac__D_m --> 2.0 focytc_m + pyr_m 16643 and 8802
LLDH_ferr_m 2.0 ficytc_m + lac__L_m --> 2.0 focytc_m + 2.0 h_m + pyr_m (CRv4_Au5_s9_g15537_t1 and 8802) or (CRv4_Au5_s9_g15537_t1 and 9198)
L_LACD2cm 2.0 ficytc_m + lac__L_c --> 2.0 focytc_m + pyr_c 16607 and 8802

CCP2m 2.0 focytc_m + h2o2_m --> 2.0 ficytc_m + 2.0 h2o_m (10811 and 8802) or (13161 and 8802) or (13161 and 9198)
CYO1_KT 2.0 ficytc_c + 2.0 h_c + q8h2_c --> 2.0 focytc_c + 2.0 h_p + q8_c PP_1318 and 13614 and 9198
CYOO6m 4.0 focytc_m + 8.0 h_m + o2_m --> 4.0 ficytc_m + 2.0 h2o_m + 4.0 h_c (CRv4_Au5_s1_g1958_t1 and CRv4_Au5_s3_g10243_t1 and CRv4_Au5_s4_g11710_t1 and 11317 and 8802) or (CRv4_Au5_s1_g1958_t1 and CRv4_Au5_s3_g10243_t1 and CRv4_Au5_s4_g11710_t1 and 11317 and 9198)
CYOR_q8_m 2.0 ficytc_m + 2.0 h_m + q8h2_m --> 2.0 focytc_m + 4.0 h_c + q8_m (13614 and 15681 and 8802) or (13614 and 15681 and 9198)
CYOR_u10m 2.0 ficytc_m + 2.0 h_m + q10h2_m --> 2.0 focytc_m + 4.0 h_c + q10_m (CYTB and UQCR11 and UQCRH and UQCRQ and 11618 and 13614 and 15231 and 15681 and 9198 and 9705) or (CYTB and Uqcr11 and Uqcrq and mmu_100042918 and 11618 and 13614 and 15231 and 15681 and 9198 and 9705)
CYOR_u6m 2.0 ficytc_m + 1.5 h_m + q6h2_m --> 2.0 focytc_m + 1.5 h_c + q6_m Q0105 and YBL045C and 11618 and 12966 and 13608 and 13614 and 15231 and 15681 and 15758 and 8802 and 9198
D_LACDHm 2.0 ficytc_m + lac__D_m --> 2.0 focytc_m + 2.0 h_m + pyr_m (13328 and 8802) or (13328 and 9198) or (16643 and 8802) or (16643 and 9198)
LLDH_ferr_m 2.0 ficytc_m + lac__L_m --> 2.0 focytc_m + 2.0 h_m + pyr_m (CRv4_Au5_s9_g15537_t1 and 8802) or (CRv4_Au5_s9_g15537_t1 and 9198)
```

D-lactate dehydrogenase (cytochrome)  
13328 (mito, DLD1, anchor)  
13239 (mito, no reactions, secreted), no rxns  
16643 (mito, DLD2/DLD3, secreted) -> (R)-2-hydroxyglutarate---pyruvate transhydrogenase [EC:1.1.99.40]

13328 is dld1, anchored, D-lac\_c + ficyt\_m -> pyr\_c  
13239 is dld1, secreted, assign it to the same reaction as 13328

8802 (mito, CYC1/CYC7) is cytochrome c  
9198 (mito, CYT1) is cytochrome c1

Add two protons as product to D\_LACDcm  
Change D\_LACDcm genes to '(13239 and 8802) or (13328 and 8802)'  
Remove D\_LACDHm, yli\_R0668, LDH\_D

GLXO1 is incorrect, it should be either of the following  
glyoxylate + H2O + O2 <==> oxalate + H2O2  
glyoxylate + CoA + NADP+ <==> oxalyl-CoA + NADPH + H+  
No enzyme exist for HPYRR2x (S)-glycerate producing

In [20]:

```
model.reactions.get_by_id('D_LACDcm').add_metabolites({'h_c': 2.0})
model.reactions.get_by_id('D_LACDcm').gene_reaction_rule = '(13239 and 8802) or (13328 and 8802)'
model.remove_reactions(['D_LACDHm','yli_R0668','LDH_D','D_LACDm'],remove_orphans=True)
model.remove_reactions(['GLXO1','HPYRR2x'],remove_orphans=True)
```

In [21]:

```
# 2-hydroxyglutarate
for r in sorted(model.metabolites.get_by_id('r2hglut_c').reactions, key=lambda x: x.id):
        print(r.id, r.reaction, r.gene_reaction_rule)
print()
for r in sorted(model.metabolites.get_by_id('S2hglut_c').reactions, key=lambda x: x.id):
        print(r.id, r.reaction, r.gene_reaction_rule)
```

```
ARHGDx nad_c + r2hglut_c <=> akg_c + h_c + nadh_c 9085

AHGDx S2hglut_c + nad_c <=> akg_c + h_c + nadh_c 9085
SHGO S2hglut_c + o2_c --> akg_c + h2o2_c 10251
```

In [22]:

```
temp = ['16643','9085','10251']
display(Annotation.loc[temp])
Show_Data(temp)
```

|  | Combined Annotations | Signal P | Sc288c Orthologs | Human Orthologs | Sc288 Best Hit | Human Blast | Essential | WolfPSort | C Terminal |
| --- | --- | --- | --- | --- | --- | --- | --- | --- | --- |
| RTO4\_ID |  |  |  |  |  |  |  |  |  |
| 16643 | K00102: dld, LDHD; D-lactate dehydrogenase (cy... | S | DLD2,DLD3 | D2HGDH | DLD2 | D2HGD | Not Essential | mito 24.5, cyto\_mito 14 | AEQ\* |
| 9085 | K00058: serA, PHGDH; D-3-phosphoglycerate dehy... |  | SER3,SER33 | PHGDH | SER33 | PHGDH | Not Essential | extr 12, mito 10, pero 2, nucl 1, cyto 1, cyto... | MLF\* |
| 10251 | KOG2665: Predicted FAD-dependent oxidoreductase | A |  | L2HGDH |  | L2HGD | Not Essential | extr 10, mito 6, cyto 4, E.R. 4, pero 2 | SWA\* |

| strain | WT | | | | | | | | | | | | | | | | |
| --- | --- | --- | --- | --- | --- | --- | --- | --- | --- | --- | --- | --- | --- | --- | --- | --- | --- |
| condition | G\_MM | C\_MM | G\_SD | | GX\_SD | | | X\_SD | | A\_SD | | C\_SD | | MM\_CN120 | | MM\_CN5 | Diversity\_Sample |
| phase | exp | exp | exp | stat | exp | trans | stat | exp | stat | exp | stat | exp | stat | exp | stat | exp | exp |
| proteinId | Set1 | Set1 | Set2 | Set2 | Set2 | Set2 | Set2 | Set2 | Set2 | Set2 | Set2 | Set2 | Set2 | Set3 | Set3 | Set3 | Set3 |
| 16643 | 6.66421 | 6.56722 | 6.72194 | 6.15557 | 6.81848 | 6.97251 | 6.80884 | 7.07628 | 5.78036 | 6.0989 | 5.75058 | 5.89099 | 5.77695 | 6.20075 | 5.73998 | 6.51135 | 6.39697 |
| 9085 | 5.51865 | 6.79119 | 6.42727 | 4.30297 | 6.80539 | 5.31543 | 5.37244 | 6.29024 | 4.93145 | 5.78997 | 4.86602 | 6.70617 | 7.41309 | 6.22817 | 6.30417 | 8.68456 | 7.8997 |
| 10251 | 7.71869 | 6.58336 | 6.59926 | 7.49333 | 6.63223 | 6.63498 | 7.6772 | 6.33673 | 6.51955 | 6.45842 | 6.32927 | 7.34894 | 7.98136 | 5.62066 | 5.44957 | 5.3021 | 4.78868 |

| strain | WT | | | | | | | | | | |
| --- | --- | --- | --- | --- | --- | --- | --- | --- | --- | --- | --- |
| condition | G\_SD | | GX\_SD | | | X\_SD | | A\_SD | | C\_SD | |
| proteinId | exp | stat | exp | trans | stat | exp | stat | exp | stat | exp | stat |
| 16643 | 14.0562 | 16.7944 | 12.4059 | 12.9843 | 16.7837 | 12.5553 | 14.3733 | 11.7959 | 9.94229 | 8.134 | 10.8617 |
| 9085 | 29.7241 | 20.5488 | 29.6855 | 15.2734 | 13.5295 | 18.3694 | 17.9218 | 23.9533 | 19.7199 | 17.6986 | 22.2046 |
| 10251 | 1.53151 | 2.52285 | 2.64528 | 1.6748 | 1.83158 | 1.15969 | 0.992987 | 1.73645 | 1.15509 | 3.20749 | 3.28149 |

|  | Glucose | Xylose | Arabinose | Acetate | Coumarate | Ferulate | YNB Oleic Acid | YNB Ricinoleic Acid | YNB Glucose | YNB Gluc DOC | YPD |
| --- | --- | --- | --- | --- | --- | --- | --- | --- | --- | --- | --- |
| proteinId |  |  |  |  |  |  |  |  |  |  |  |
| 16643 | -1.06968 | 0.253369 | -0.281544 | -0.493532 | 1.18377 | -0.50392 | 0.285326 | -1.2611 | -0.879881 | -0.055865 | 0.0582271 |
| 10251 | 0.112779 | 0.123063 | -0.019406 | 0.206727 | 0.105546 | 0.0701885 | -0.0265131 | 0.175547 | 0.0826319 | -0.39978 | -0.143696 |

In [23]:

```
for x in temp:
    if x in model.genes:
        for r in sorted(model.genes.get_by_id(x).reactions, key=lambda x: x.id):
            print(r.id, r.reaction, r.gene_reaction_rule)
    else:
        print(x, 'no reactions')
    print()
```

```
16643 no reactions

AHGDx S2hglut_c + nad_c <=> akg_c + h_c + nadh_c 9085
ARHGDx nad_c + r2hglut_c <=> akg_c + h_c + nadh_c 9085
PGCD 3pg_c + nad_c --> 3php_c + h_c + nadh_c 9085
PGLYDH 3pg_h + nad_h --> 3php_h + h_h + nadh_h 9085

SHGO S2hglut_c + o2_c --> akg_c + h2o2_c 10251
```

16643 (mito, DLD2/DLD3, secreted) -> mito version of ARHGDx  
https://www.ncbi.nlm.nih.gov/pubmed/19586914  
r2hglut -> pyr using fad\_m  
r2hglut\_m + fad\_m <-> akg\_m + fadh2\_m  
Saccharomyces cerevisiae Forms D-2-Hydroxyglutarate and Couples Its Degradation to D-Lactate Formation via a Cytosolic Transhydrogenase. https://www.ncbi.nlm.nih.gov/pubmed/26774271  
DLD2 converts D-2HG to akg, DLD3 coupled to DLD1 makes transhydrogenase  
(R)-2-hydroxyglutarate---pyruvate transhydrogenase [EC:1.1.99.40]  
(R)-2-hydroxyglutarate + pyruvate = 2-oxoglutarate + (R)-lactate  
make ARHGDm with fad as a cofactor, and set genes to 16643

9085 is cyto D-isomer-specific 2-hydroxy acid dehydrogenase -> PGCD, ARHGDx

10251 is mito L-2-hydroxyglutarate dehydrogenase, mitochondrial; EC 1.1.99.2, FAD dependent  
(S)-2-Hydroxyglutarate + FAD <=> 2-Oxoglutarate + FADH2
Change AHGDx to AHGDm, make it mito, and change nad to fad

Remove PGLYDH, SHGO

In mammals, d-2HG is formed as a degradation product of aminolevulinate (39) and it was also shown to be formed within the mitochondria from 2-ketoglutarate through a hydroxyacid-oxoacid-transhydrogenase in a reaction in which γ-hydroxybutyrate is converted to succinic semialdehyde (40). However, no ortholog of this enzyme has been found in plants.

The production of d-2HG could be the result of the condensation of propionyl-CoA and glyoxylate through the action of a 2-hydroxyglutarate synthase (EC 2.3.3.11) in peroxisomes. The existence of this activity was previously reported in E. coli and Aspergillus glaucus grown on propionate (47, 48). Propionyl-CoA is produced in the final step of odd-chain fatty acids, Val and Leu catabolism through β-oxidation. Propionyl-CoA is also a final product of metabolism of the methyl branched acids, phytanic acid, derived from the degradation of phytol, the hydrophobic tail of chlorophyll (49).

In [24]:

```
m1 = model.metabolites.get_by_id('r2hglut_c').copy()
m1.id = 'r2hglut_m'
m1.compartment = 'm'
m2 = model.metabolites.get_by_id('S2hglut_c').copy()
m2.id = 's2hglut_m'
m2.compartment = 'm'
model.add_metabolites([m1,m2])
r = model.reactions.get_by_id('ARHGDx').copy()
r.id = 'ARHGDxm'
model.add_reactions([r])
model.reactions.get_by_id('ARHGDxm').add_metabolites({'r2hglut_c': 1.0, 'r2hglut_m': -1.0, 'akg_c': -1.0, 'akg_m': 1.0,
                                                      'nad_c': 1.0, 'fad_m': -1.0, 'nadh_c': -1.0, 'fadh2_m': 1.0, 'h_c': -1.0})
model.reactions.get_by_id('ARHGDxm').gene_reaction_rule = '16643'
model.reactions.get_by_id('AHGDx').id = 'AHGDxm'
model.reactions.get_by_id('AHGDxm').add_metabolites({'S2hglut_c': 1.0, 's2hglut_m': -1.0, 'akg_c': -1.0, 'akg_m': 1.0,
                                                     'nad_c': 1.0, 'fad_m': -1.0, 'nadh_c': -1.0, 'fadh2_m': 1.0, 'h_c': -1.0})
model.reactions.get_by_id('AHGDxm').gene_reaction_rule = '10251'
model.remove_reactions(['PGLYDH','SHGO'],remove_orphans=True)
```

In [25]:

```
temp = ['11665']
display(Annotation.loc[temp])
Show_Data(temp)
```

|  | Combined Annotations | Signal P | Sc288c Orthologs | Human Orthologs | Sc288 Best Hit | Human Blast | Essential | WolfPSort | C Terminal |
| --- | --- | --- | --- | --- | --- | --- | --- | --- | --- |
| RTO4\_ID |  |  |  |  |  |  |  |  |  |
| 11665 | K11173: ADHFE1; hydroxyacid-oxoacid transhydro... | S | ADH4 | ADHFE1 | ADH4 | ADHFE | Not Essential | mito 27 | MEF\* |

| strain | WT | | | | | | | | | | | | | | | | |
| --- | --- | --- | --- | --- | --- | --- | --- | --- | --- | --- | --- | --- | --- | --- | --- | --- | --- |
| condition | G\_MM | C\_MM | G\_SD | | GX\_SD | | | X\_SD | | A\_SD | | C\_SD | | MM\_CN120 | | MM\_CN5 | Diversity\_Sample |
| phase | exp | exp | exp | stat | exp | trans | stat | exp | stat | exp | stat | exp | stat | exp | stat | exp | exp |
| proteinId | Set1 | Set1 | Set2 | Set2 | Set2 | Set2 | Set2 | Set2 | Set2 | Set2 | Set2 | Set2 | Set2 | Set3 | Set3 | Set3 | Set3 |
| 11665 | 4.22641 | 5.55435 | 4.75735 | 7.1575 | 4.79726 | 5.12656 | 5.30819 | 3.88146 | 5.38366 | 4.03613 | 5.58852 | 5.77764 | 5.29814 | 5.56967 | 5.26707 | 6.37168 | 6.10535 |

| strain | WT | | | | | | | | | | |
| --- | --- | --- | --- | --- | --- | --- | --- | --- | --- | --- | --- |
| condition | G\_SD | | GX\_SD | | | X\_SD | | A\_SD | | C\_SD | |
| proteinId | exp | stat | exp | trans | stat | exp | stat | exp | stat | exp | stat |
| 11665 | 3.07433 | 10.4278 | 3.05938 | 9.12547 | 9.99706 | 6.25788 | 6.66646 | 5.40882 | 6.32383 | 10.6608 | 12.2356 |

|  | Glucose | Xylose | Arabinose | Acetate | Coumarate | Ferulate | YNB Oleic Acid | YNB Ricinoleic Acid | YNB Glucose | YNB Gluc DOC | YPD |
| --- | --- | --- | --- | --- | --- | --- | --- | --- | --- | --- | --- |
| proteinId |  |  |  |  |  |  |  |  |  |  |  |
| 11665 | -0.166964 | -0.0391266 | -0.0862561 | -0.116515 | -0.184212 | -0.437342 | -0.0459631 | 0.103025 | 0.112559 | -0.0250673 | 0.240574 |

In [26]:

```
for r in sorted(model.genes.get_by_id('11665').reactions, key=lambda x: x.id):
    print(r.id, r.reaction, r.gene_reaction_rule)
```

```
34DHOXPEGOX 34dhmald_c + h_c + nadh_c <=> 34dhoxpeg_c + nad_c 11665 or 14108 or 14109 or 15438 or (ADH1A and ADH1B) or (ADH1A and ADH1C) or (ADH1B and ADH1C)
ACALD acald_c + coa_c + nad_c <=> accoa_c + h_c + nadh_c 11665
ACALDh acald_h + coa_h + nad_h --> accoa_h + h_h + nadh_h 11665
ACDHmi accoa_m + 2.0 h_m + 2.0 nadh_m --> coa_m + etoh_m + 2.0 nad_m 11665
ADHEr accoa_c + 2.0 h_c + 2.0 nadh_c --> coa_c + etoh_c + 2.0 nad_c 11665
ALCD1 meoh_c + nad_c <=> fald_c + h_c + nadh_c 11665 or 13657 or 14108 or 14109
ALCD19 glyald_c + h_c + nadh_c <=> glyc_c + nad_c 11665 or 12784 or 15438 or 9774
ALCD21_D 12ppd__R_c + nad_c --> h_c + lald__D_c + nadh_c 11665 or 13657 or 14108 or 14109
ALCD22_D lald__D_c + nad_c --> h_c + mthgxl_c + nadh_c 11665 or 13657 or 14108 or 14109
ALCD22_L lald__L_c + nad_c --> h_c + mthgxl_c + nadh_c 11665 or 13657 or 14108 or 14109
ALCD22xi 2mbald_c + h_c + nadh_c --> 2mbtoh_c + nad_c 11665 or 14108 or 14109 or 15438
ALCD23xi 2mppal_c + h_c + nadh_c --> ibutoh_c + nad_c 11665 or 14108 or 14109 or 15438
ALCD24xi 3mbald_c + h_c + nadh_c --> iamoh_c + nad_c 11665 or 14108 or 14109 or 15438
ALCD25xi h_c + nadh_c + pacald_c --> 2phetoh_c + nad_c 11665 or 14108 or 14109 or 15438
ALCD26xi h_c + id3acald_c + nadh_c --> ind3eth_c + nad_c 11665 or 14108 or 14109 or 15438
ALCD2ir acald_c + h_c + nadh_c --> etoh_c + nad_c 11665 or 15438
ALCD2irm acald_m + h_m + nadh_m --> etoh_m + nad_m 11665 or 15438
ALCD2x etoh_c + nad_c <=> acald_c + h_c + nadh_c 11665 or 13657 or 14108 or 14109 or 15438
BNORh btal_h + coa_h + nad_h --> btcoa_h + h_h + nadh_h 11665
BTCOARx btcoa_c + h_c + nadh_c <-- btal_c + coa_c + nad_c 11665
LCARS h_c + lald__L_c + nadh_c <=> 12ppd__S_c + nad_c 11665 or 12307 or 13657 or 14108 or 14109 or 9101
THRD nad_c + thr__L_c --> 2aobut_c + h_c + nadh_c 11665 or 16452
```

11665 is mito hydroxyacid-oxoacid transhydrogenase  
(S)-3-hydroxybutanoate + 2-oxoglutarate = acetoacetate + (R)-2-hydroxyglutarate  
2-oxoglutarate + 4-hydroxybutanoate → succinate semialdehyde + (R)-2-hydroxyglutarate  
4-Hydroxybutanoate and (R)-2-hydroxyglutarate can also act as donors; 4-oxobutanoate can also act as acceptor

In [27]:

```
# Make two reactions for 11665
m1 = model.metabolites.get_by_id('bhb_m').copy()
m1.id = 's3hb_m'
m1.compartment = 'm'
m1.name = '(S)-3-Hydroxybutanoate'
m1.formula = 'C4H7O3'
m1.charge = -1
m2 = model.metabolites.get_by_id('ghb_c').copy()
m2.id = 'ghb_m'
m2.compartment = 'm'
model.add_metabolites([m1,m2])
r = model.reactions.get_by_id('ARHGDxm').copy()
r.id = 'S3HBTRHDm'
model.add_reactions([r])
model.reactions.get_by_id('S3HBTRHDm').add_metabolites({'r2hglut_m': 2.0, 'fad_m': 1.0, 'akg_m': -2.0, 'fadh2_m': -1.0,
                                                        's3hb_m': -1.0, 'acac_m': 1.0})
model.reactions.get_by_id('S3HBTRHDm').gene_reaction_rule = '11665'
r = model.reactions.get_by_id('S3HBTRHDm').copy()
r.id = 'GHBTRHDm'
model.add_reactions([r])
model.reactions.get_by_id('GHBTRHDm').add_metabolites({'s3hb_m': 1.0, 'acac_m': -1.0, 'ghb_m': -1.0, 'sucsal_m': 1.0})
model.reactions.get_by_id('GHBTRHDm').gene_reaction_rule = '11665'
```

In [28]:

```
#temp = Annotation.index[Annotation['Combined Annotations'].str.contains('alcohol dehydrogenase')]
#temp = Annotation.index[Annotation['Sc288c Orthologs'].str.startswith('ADH')]
temp = ['14108','14109','15438','13657','12784','9774','11882','12307','9101','16452']
display(Annotation.loc[temp])
Show_Data(temp)
```

|  | Combined Annotations | Signal P | Sc288c Orthologs | Human Orthologs | Sc288 Best Hit | Human Blast | Essential | WolfPSort | C Terminal |
| --- | --- | --- | --- | --- | --- | --- | --- | --- | --- |
| RTO4\_ID |  |  |  |  |  |  |  |  |  |
| 14108 | K00121: frmA, ADH5, adhC; S-(hydroxymethyl)glu... |  | SFA1 | ADH5 | SFA1 | ADH5 | Unclear (ambiguous TDNA mapping) | cyto 19, extr 6 | MWA\* |
| 14109 | K00121: frmA, ADH5, adhC; S-(hydroxymethyl)glu... |  | SFA1 | ADH5 | SFA1 | ADH5 | Unclear (ambiguous TDNA mapping) | cyto 19, extr 6 | MWA\* |
| 15438 | K13953: adhP; alcohol dehydrogenase, propanol-... |  | ADH5,ADH3,ADH2,ADH1 |  | ADH3 | ZADH2 | Not Essential | cyto 25.5, cyto\_nucl 13.5 | KNF\* |
| 13657 | K00344: qor, CRYZ; NADPH2:quinone reductase |  | ZTA1 | ZADH2,CRYZ | ZTA1 | CRYZ | Not Essential | cyto 15.5, cyto\_nucl 9, mito 3, pero 3, extr 2 | KVA\* |
| 12784 | K00002: AKR1A1, adh; alcohol dehydrogenase (NA... |  | YPR1,GCY1 | AKR1A1 | YPR1 | AKR1A | Not Essential | mito 13.5, cyto\_mito 12, cyto 9.5, pero 4 | AKL\* |
| 9774 | K00002: AKR1A1, adh; alcohol dehydrogenase (NA... |  | YPR1,GCY1 | AKR1A1 | YPR1 | AKR1A | Not Essential | cyto 17.5, cyto\_mito 13.666, cyto\_nucl 10.333,... | KIK\* |
| 11882 | K18097: GCY1; glycerol 2-dehydrogenase (NADP+) |  | YPR1,GCY1 | AKR1A1 | YPR1 | AKR1A | Not Essential | cyto 24.5, cyto\_nucl 13.5 | SHW\* |
| 12307 | K13954: yiaY; alcohol dehydrogenase |  |  |  | ADH4 |  | Not Essential | cyto 13.5, mito 8, cyto\_nucl 7.5, extr 2, pero 2 | RPE\* |
| 9101 | K13954: yiaY; alcohol dehydrogenase |  |  |  | ADH4 |  | Not Essential | extr 11, mito 8, cyto 6 | RPS\* |
| 16452 | K05351: E1.1.1.9; D-xylulose reductase |  | YDL246C,SOR1,XYL2 | SORD | SOR1 | SORD | Not Essential | cyto 16, cyto\_nucl 10, mito 4, pero 3, nucl 2 | DKF\* |

| strain | WT | | | | | | | | | | | | | | | | |
| --- | --- | --- | --- | --- | --- | --- | --- | --- | --- | --- | --- | --- | --- | --- | --- | --- | --- |
| condition | G\_MM | C\_MM | G\_SD | | GX\_SD | | | X\_SD | | A\_SD | | C\_SD | | MM\_CN120 | | MM\_CN5 | Diversity\_Sample |
| phase | exp | exp | exp | stat | exp | trans | stat | exp | stat | exp | stat | exp | stat | exp | stat | exp | exp |
| proteinId | Set1 | Set1 | Set2 | Set2 | Set2 | Set2 | Set2 | Set2 | Set2 | Set2 | Set2 | Set2 | Set2 | Set3 | Set3 | Set3 | Set3 |
| 14108 | 7.23326 | 7.01358 | 5.64402 | 6.69668 | 5.53004 | 5.99657 | 6.08719 | 5.36359 | 6.35115 | 5.52325 | 6.40565 | 6.79692 | 6.55453 | 6.97915 | 7.14294 | 5.17651 | 7.49575 |
| 14109 | 8.1968 | 7.85284 | 6.09397 | 7.21435 | 5.98603 | 6.42928 | 6.60693 | 5.78854 | 6.82888 | 5.9483 | 6.79863 | 7.20147 | 6.86586 | 7.25272 | 7.30374 | 5.27859 | 7.86155 |
| 15438 | 10.1609 | 8.53977 | 5.43727 | 5.78674 | 5.14966 | 5.82878 | 5.74105 | 5.70476 | 6.17528 | 5.33619 | 5.18161 | 5.6251 | 3.78025 | 8.62195 | 9.06836 | 5.22118 | 8.47463 |
| 13657 | 7.23952 | 7.83455 | 7.34693 | 6.07437 | 7.36528 | 6.73633 | 6.6343 | 7.11205 | 6.31758 | 6.32286 | 5.97201 | 6.63067 | 5.73863 | 6.89159 | 6.5466 | 7.06777 | 6.5451 |
| 12784 | 2.08671 | 1.96656 | 1.05974 | 1.28618 | 1.01927 | 0.689282 | 0.833234 | 1.3947 | 1.87113 | 2.53738 | 1.88953 | 1.63086 | 3.2399 | 0.662714 | 0.423146 | 2.00301 | 1.06163 |
| 9774 | 9.27193 | 8.0739 | 9.06855 | 8.59521 | 9.81437 | 9.92808 | 9.23804 | 9.63717 | 7.83349 | 10.5623 | 7.02255 | 5.69079 | 5.81959 | 10.0636 | 9.3345 | 10.8455 | 10.3765 |
| 11882 | 4.07818 | 5.71861 | 5.83615 | 5.62544 | 5.77337 | 5.29629 | 5.15703 | 7.02755 | 5.00344 | 4.97816 | 5.2813 | 4.36109 | 4.34722 | 6.19133 | 6.37203 | 7.21044 | 7.40048 |
| 12307 | 5.09528 | 6.17958 | 3.99001 | 4.89936 | 4.08211 | 4.38468 | 3.94334 | 4.45024 | 3.96891 | 4.51818 | 4.26915 | 5.63684 | 5.56806 | 5.67756 | 5.39759 | 6.24192 | 5.54614 |
| 9101 | 1.97852 | 6.14363 | 1.87446 | 2.05433 | 1.60698 | 1.70345 | 1.85845 | 2.07739 | 2.67502 | 2.71788 | 3.11233 | 4.03246 | 2.69707 | 5.52818 | 4.27444 | 4.90806 | 4.22376 |
| 16452 | 4.493 | 5.78462 | 3.48315 | 3.87701 | 6.36506 | 8.41286 | 8.08551 | 5.87633 | 2.0546 | 8.57031 | 1.59692 | 4.77431 | 4.80377 | 4.40777 | 4.12949 | 3.02469 | 7.50315 |

| strain | WT | | | | | | | | | | |
| --- | --- | --- | --- | --- | --- | --- | --- | --- | --- | --- | --- |
| condition | G\_SD | | GX\_SD | | | X\_SD | | A\_SD | | C\_SD | |
| proteinId | exp | stat | exp | trans | stat | exp | stat | exp | stat | exp | stat |
| 14108 | 18.0857 | 18.8911 | 18.7317 | 20.2976 | 20.2797 | 27.8013 | 24.3021 | 25.503 | 21.4289 | 38.0378 | 35.2758 |
| 15438 | 1.92087 | 3.42103 | 2.02435 | 4.84423 | 5.98016 | 5.47116 | 7.05095 | 7.14776 | 7.4443 | 7.46885 | 9.36764 |
| 13657 | 12.9764 | 15.1758 | 13.4118 | 11.9115 | 16.4293 | 15.278 | 13.745 | 11.7937 | 13.9831 | 18.366 | 13.2745 |
| 12784 | 0.384095 | 0 | 0.204746 | 0 | 0 | 0 | 0 | 0 | 0.189081 | 0 | 0.21114 |
| 9774 | 24.0361 | 20.651 | 27.2528 | 17.3246 | 17.6781 | 36.9396 | 32.9039 | 40.9485 | 37.8727 | 18.5732 | 14.8411 |
| 11882 | 7.28788 | 9.64582 | 6.91339 | 7.61119 | 9.64307 | 14.6905 | 14.9097 | 6.37514 | 6.3363 | 10.6828 | 9.16872 |
| 12307 | 2.30228 | 1.75716 | 2.64057 | 3.16653 | 2.95559 | 1.35696 | 2.35404 | 1.15431 | 3.63657 | 4.47629 | 4.13224 |
| 16452 | 0 | 0.335899 | 4.65906 | 14.7083 | 18.0417 | 44.5046 | 34.693 | 37.8708 | 31.5359 | 2.77938 | 2.84436 |

|  | Glucose | Xylose | Arabinose | Acetate | Coumarate | Ferulate | YNB Oleic Acid | YNB Ricinoleic Acid | YNB Glucose | YNB Gluc DOC | YPD |
| --- | --- | --- | --- | --- | --- | --- | --- | --- | --- | --- | --- |
| proteinId |  |  |  |  |  |  |  |  |  |  |  |
| 14108 | -0.211659 | -0.531723 | -0.079527 | 0.0229318 | -0.277901 | -0.0256706 | 0.156921 | 0.219306 | -0.220427 | -0.0578182 | 0.207023 |
| 15438 | 0.0161124 | -0.202524 | -0.327434 | -0.164034 | -0.309855 | 0.00473032 | -0.0374177 | -0.0303498 | 0.195573 | 0.369537 | -0.187797 |
| 13657 | -0.0681022 | -0.149991 | -0.121465 | -0.14913 | 0.237315 | 0.0848525 | -0.0812734 | -0.20384 | 0.423301 | 0.387808 | 0.151714 |
| 12784 | -0.0694909 | -0.243761 | 0.0933115 | 0.0897106 | 0.117505 | -0.125559 | 0.0784752 | -0.493313 | -0.483602 | -0.36137 | -0.0103173 |
| 9774 | 0.0235694 | -0.71521 | -3.89519 | -0.087274 | -0.0710531 | -0.0880198 | -0.00217939 | -0.26233 | 0.0739166 | -0.149324 | -0.00933948 |
| 11882 | 0.155027 | 0.00241144 | 0.151468 | -0.00673348 | 0.60387 | -0.0309193 | 0.675184 | 0.843 | 0.320601 | 0.27912 | 0.086355 |
| 12307 | -0.103262 | -0.203923 | -0.151666 | -0.029787 | -0.0801901 | 0.219843 | -0.283153 | -0.353235 | -0.36946 | 0.122514 | 0.0606745 |
| 9101 | 0.0714984 | -0.178956 | -0.0614084 | -0.0684494 | -0.327682 | -0.168501 | 0.1433 | 0.10887 | -0.0535352 | -0.194092 | -0.201912 |
| 16452 | -0.30032 | -0.278447 | -0.209842 | 0.129556 | -0.122194 | 0.300891 | -0.304882 | -0.426796 | -0.41936 | -0.138067 | -0.295863 |

In [29]:

```
temp = ['14108','14109','15438','13657','12784','9774','12307','9101','16452']
for x in temp:
    print(x)
    for r in model.genes.get_by_id(x).reactions:
        if not '11665' in [g.id for g in r.genes]:
            print(r.id, r.reaction, r.gene_reaction_rule)
```

```
14108
LALDO gthrd_c + lald__D_c + nad_c <=> h_c + lgt__S_c + nadh_c 12286 or 14108 or 14109
FALDH fald_c + gthrd_c + nad_c <=> Sfglutth_c + h_c + nadh_c 14108 or 14109
ALCD2x_copy2 etoh_c + nad_c <=> acald_c + h_c + nadh_c 14108 or 14109
FALDH2 hmgth_c + nad_c <=> Sfglutth_c + h_c + nadh_c 14108 or 14109
14109
LALDO gthrd_c + lald__D_c + nad_c <=> h_c + lgt__S_c + nadh_c 12286 or 14108 or 14109
FALDH fald_c + gthrd_c + nad_c <=> Sfglutth_c + h_c + nadh_c 14108 or 14109
ALCD2x_copy2 etoh_c + nad_c <=> acald_c + h_c + nadh_c 14108 or 14109
FALDH2 hmgth_c + nad_c <=> Sfglutth_c + h_c + nadh_c 14108 or 14109
15438
ALCD23xim 2mppal_m + h_m + nadh_m --> ibutoh_m + nad_m 15438
ALCD2x_copy1 etoh_c + nad_c --> acald_c + h_c + nadh_c 15438
ALCD24xim 3mbald_m + h_m + nadh_m --> iamoh_m + nad_m 15438
ALCD26xim h_m + id3acald_m + nadh_m --> ind3eth_m + nad_m 15438
ALCD22xim 2mbald_m + h_m + nadh_m --> 2mbtoh_m + nad_m 15438
ALCD25xim h_m + nadh_m + pacald_m --> 2phetoh_m + nad_m 15438
13657
NADPHQR2 h_c + nadph_c + q8_c --> nadp_c + q8h2_c 13657
12784
ALCDH_nadp_hi acald_h + h_h + nadph_h --> etoh_h + nadp_h 12784 or 9774
ALCD22yi 2mbald_c + h_c + nadph_c --> 2mbtoh_c + nadp_c 10029 or 11882 or 12784 or 13554 or 13562 or 13947 or 9774
LALDO2 h_c + mthgxl_c + nadph_c --> lald__D_c + nadp_c 11882 or 12784 or 16543 or 9774
ARABR arab__L_c + h_c + nadph_c --> abt_c + nadp_c 11882 or 12784 or 16543 or 9774
GLYCDy glyc_c + nadp_c --> dha_c + h_c + nadph_c 11882 or 12784 or 9774
ALCD19y glyald_c + h_c + nadph_c --> glyc_c + nadp_c 11882 or 12784 or 9774
PPDOy h_c + lald__D_c + nadph_c --> 12ppd__R_c + nadp_c 10029 or 11882 or 12784 or 13554 or 13562 or 13947 or 16543 or 9774
ALR2 h_c + mthgxl_c + nadph_c --> acetol_c + nadp_c 11882 or 12784 or 15285 or 16543 or 9774
GALOR gal_c + h_c + nadph_c <=> galt_c + nadp_c 11882 or 12784 or 9774
ALR3 acetol_c + h_c + nadph_c --> 12ppd__S_c + nadp_c 11882 or 12784 or 16543 or 9774
SBTR glc__D_c + h_c + nadph_c --> nadp_c + sbt__D_c 11882 or 12784 or 16543 or 9774
ALCD2y etoh_c + nadp_c --> acald_c + h_c + nadph_c 11882 or 12784 or 9774
9774
ALCDH_nadp_hi acald_h + h_h + nadph_h --> etoh_h + nadp_h 12784 or 9774
ALCD22yi 2mbald_c + h_c + nadph_c --> 2mbtoh_c + nadp_c 10029 or 11882 or 12784 or 13554 or 13562 or 13947 or 9774
LALDO2 h_c + mthgxl_c + nadph_c --> lald__D_c + nadp_c 11882 or 12784 or 16543 or 9774
ARABR arab__L_c + h_c + nadph_c --> abt_c + nadp_c 11882 or 12784 or 16543 or 9774
GLYCDy glyc_c + nadp_c --> dha_c + h_c + nadph_c 11882 or 12784 or 9774
ALCD19y glyald_c + h_c + nadph_c --> glyc_c + nadp_c 11882 or 12784 or 9774
PPDOy h_c + lald__D_c + nadph_c --> 12ppd__R_c + nadp_c 10029 or 11882 or 12784 or 13554 or 13562 or 13947 or 16543 or 9774
ALR2 h_c + mthgxl_c + nadph_c --> acetol_c + nadp_c 11882 or 12784 or 15285 or 16543 or 9774
GALOR gal_c + h_c + nadph_c <=> galt_c + nadp_c 11882 or 12784 or 9774
ALR3 acetol_c + h_c + nadph_c --> 12ppd__S_c + nadp_c 11882 or 12784 or 16543 or 9774
SBTR glc__D_c + h_c + nadph_c --> nadp_c + sbt__D_c 11882 or 12784 or 16543 or 9774
ALCD2y etoh_c + nadp_c --> acald_c + h_c + nadph_c 11882 or 12784 or 9774
12307
9101
16452
SBTD_D2 nad_c + sbt__D_c --> fru_c + h_c + nadh_c 16452
yli_R0260 nad_c + sbt__D_c <=> fru_B_c + h_c + nadh_c 16452
XYLTD_D nad_c + xylt_c --> h_c + nadh_c + xylu__D_c 16452
SBTD_L nad_c + sbt__L_c --> h_c + nadh_c + srb__L_c 16452
```

In [30]:

```
# remove norepinephrine and epinephrine degradation reactions
model.remove_reactions(['34DHOXPEGOX','34DHXMANDACOX','34DHXMANDACOX_NADP','yli_R1583','yli_R1584','yli_R1585'],remove_orphans=True)
# Remove acald/btal dehydrogenase - no genes
model.remove_reactions(['ACALD','ACALDh','ACDHmi','ADHEr','BNORh','BTCOARx'],remove_orphans=True)
# 14108 14109 cyto ADH5 S-(hydroxymethyl)glutathione dehydrogenase / alcohol dehydrogenase [EC:1.1.1.284 1.1.1.1]
# Add FALGTHLs from E. coli model (spontaneous), keep FALDH2, SFGTHi, remove ALCD1, FALDH
# Remove ALCD21_D, ALCD22_D, ALCD22_L - wrong direction in yeast, Remove LALDO - lumped rxn 
r = eco.reactions.get_by_id('FALGTHLs').copy()
r.gene_reaction_rule = ''
model.add_reactions([r])
model.remove_reactions(['ALCD1','FALDH','ALCD21_D','ALCD22_D','ALCD22_L','LALDO'],remove_orphans=True)
# 15438 cyto alcohol dehydrogenase, but mito ADH3 ortholog, splicing does not match gene model
# Keep cyto reactions and remove the same mito reactions
model.remove_reactions(['ALCD23xim','ALCD24xim','ALCD22xim','ALCD26xim','ALCD25xim','ALCD2irm'],remove_orphans=True)
# Keep ALCD2x with '15438' and remove ALCD2x_copy1, ALCD2x_copy2, ALCD2ir
model.remove_reactions(['ALCD2x_copy1','ALCD2x_copy2','ALCD2ir'],remove_orphans=True)
# 9774 is cyto L-glyceraldehyde reductase (NADP+), but fitness defect in arabinose, why?
# 11882 is cyto glycerol 2-dehydrogenase (NADP+)
# 12784 is (likely perox) aldehyde reductase / aldo-keto reductase / Aldose reductase
model.remove_reactions(['ALCDH_nadp_hi'],remove_orphans=True)
# 12307 and 9101 are maleylacetate reductase
# Remove LCARS - no gene for L-lactaldehyde reductase or L-1,2-propanediol oxidoreductase
# 16452 is D-xylulose reductase / Sorbitol dehydrogenase
# Remove THRD - no gene for L-threonine dehydrogenase
model.remove_reactions(['LCARS','THRD','yli_R0260'],remove_orphans=True)
model.reactions.get_by_id('ALCD19').gene_reaction_rule = '15438'
model.reactions.get_by_id('ALCD22xi').gene_reaction_rule = '14108 or 14109 or 15438'
model.reactions.get_by_id('ALCD23xi').gene_reaction_rule = '14108 or 14109 or 15438'
model.reactions.get_by_id('ALCD24xi').gene_reaction_rule = '14108 or 14109 or 15438'
model.reactions.get_by_id('ALCD25xi').gene_reaction_rule = '14108 or 14109 or 15438'
model.reactions.get_by_id('ALCD26xi').gene_reaction_rule = '14108 or 14109 or 15438'
model.reactions.get_by_id('ALCD2x').gene_reaction_rule = '15438'
```

In [31]:

```
for r in sorted(model.metabolites.get_by_id('bhb_c').reactions, key=lambda x: x.id):
        print(r.id, r.reaction, r.gene_reaction_rule)
print()
for r in sorted(model.metabolites.get_by_id('bhb_m').reactions, key=lambda x: x.id):
        print(r.id, r.reaction, r.gene_reaction_rule)
print()
for r in sorted(model.metabolites.get_by_id('ghb_c').reactions, key=lambda x: x.id):
        print(r.id, r.reaction, r.gene_reaction_rule)
print()
for r in sorted(model.metabolites.get_by_id('ghb_m').reactions, key=lambda x: x.id):
        print(r.id, r.reaction, r.gene_reaction_rule)
print()
for r in sorted(model.metabolites.get_by_id('sucsal_c').reactions, key=lambda x: x.id):
        print(r.id, r.reaction, r.gene_reaction_rule)
print()
for r in sorted(model.metabolites.get_by_id('sucsal_m').reactions, key=lambda x: x.id):
        print(r.id, r.reaction, r.gene_reaction_rule)
```

```
BDH bhb_c + nad_c <=> acac_c + h_c + nadh_c 12795

BDHm bhb_m + nad_m <=> acac_m + h_m + nadh_m 15575

GHBDHx h_c + nadh_c + sucsal_c <=> ghb_c + nad_c 13694

GHBTRHDm akg_m + ghb_m <=> r2hglut_m + sucsal_m 11665

ABTA 4abut_c + akg_c --> glu__L_c + sucsal_c 10937 or 15905
GHBDHx h_c + nadh_c + sucsal_c <=> ghb_c + nad_c 13694
SSALy h2o_c + nadp_c + sucsal_c --> 2.0 h_c + nadph_c + succ_c 8692
yli_R1491 h2o_c + nad_c + sucsal_c --> h_c + nadh_c + succ_c 8692

ABTArm 4abut_m + akg_m <=> glu__L_m + sucsal_m 15905
GHBTRHDm akg_m + ghb_m <=> r2hglut_m + sucsal_m 11665
SSALxm h2o_m + nad_m + sucsal_m --> 2.0 h_m + nadh_m + succ_m 8692
```

In [32]:

```
temp = ['12795','15575','13694','10937','15905','8692']
display(Annotation.loc[temp])
Show_Data(temp)
```

|  | Combined Annotations | Signal P | Sc288c Orthologs | Human Orthologs | Sc288 Best Hit | Human Blast | Essential | WolfPSort | C Terminal |
| --- | --- | --- | --- | --- | --- | --- | --- | --- | --- |
| RTO4\_ID |  |  |  |  |  |  |  |  |  |
| 12795 | K11539: CBR4; carbonyl reductase 4 |  |  |  | YMR226C | CBR4 | Not Essential | cyto 16, mito 9, cyto\_nucl 9 | WTA\* |
| 15575 | KOG1209: 1-Acyl dihydroxyacetone phosphate red... | A | AYR1 | HSD17B1,HSD17B2 | YMR226C | DHRS7 | Not Essential | plas 10, mito 8, cyto 5, E.R. 3 | KRR\* |
| 13694 | K00020: mmsB, HIBADH; 3-hydroxyisobutyrate deh... | S |  | HIBADH |  | HIBADH | Not Essential | mito 17, extr 5, E.R. 2, nucl 1, cyto 1, cyto\_... | KGE\* |
| 10937 | K07250: gabT; 4-aminobutyrate aminotransferase... |  |  | AGXT2 | ARG8 | AGXT2 | Not Essential | cyto 15, cyto\_mito 12.333, cyto\_nucl 8.833, mi... | HEG\* |
| 15905 | K13524: ABAT; 4-aminobutyrate aminotransferase... |  | UGA1 | ABAT | UGA1 | ABAT | Not Essential | mito 26 | NMS\* |
| 8692 | K00135: gabD; succinate-semialdehyde dehydroge... |  | UGA2 | ALDH5A1 | UGA2 | ALDH5 | Not Essential | cyto 17, mito 7, pero 2 | GGL\* |

| strain | WT | | | | | | | | | | | | | | | | |
| --- | --- | --- | --- | --- | --- | --- | --- | --- | --- | --- | --- | --- | --- | --- | --- | --- | --- |
| condition | G\_MM | C\_MM | G\_SD | | GX\_SD | | | X\_SD | | A\_SD | | C\_SD | | MM\_CN120 | | MM\_CN5 | Diversity\_Sample |
| phase | exp | exp | exp | stat | exp | trans | stat | exp | stat | exp | stat | exp | stat | exp | stat | exp | exp |
| proteinId | Set1 | Set1 | Set2 | Set2 | Set2 | Set2 | Set2 | Set2 | Set2 | Set2 | Set2 | Set2 | Set2 | Set3 | Set3 | Set3 | Set3 |
| 12795 | 6.00923 | 5.77737 | 5.10942 | 5.36956 | 5.02784 | 4.82022 | 4.7727 | 4.88363 | 5.32896 | 4.88673 | 5.57821 | 4.71098 | 4.05599 | 4.4627 | 4.48829 | 4.14736 | 3.90909 |
| 15575 | 5.00789 | 5.55921 | 3.78109 | 4.40763 | 3.72864 | 4.0156 | 4.09444 | 3.81637 | 4.88951 | 4.45461 | 5.14918 | 3.33321 | 3.7216 | 4.60306 | 4.95769 | 4.57903 | 4.36877 |
| 13694 | 7.4348 | 7.06562 | 7.90504 | 7.326 | 7.75159 | 6.91036 | 6.96889 | 6.71789 | 7.18266 | 6.48413 | 7.58691 | 6.10716 | 5.24905 | 6.34472 | 5.75207 | 6.70962 | 6.51354 |
| 10937 | 5.34598 | 5.55945 | 6.51015 | 6.67274 | 7.00851 | 6.72084 | 6.82701 | 6.38013 | 6.7731 | 6.07713 | 6.8177 | 7.30439 | 7.56095 | 4.77018 | 5.34697 | 7.52402 | 5.67411 |
| 15905 | 7.00614 | 6.60151 | 5.42188 | 4.85478 | 5.9331 | 3.2202 | 3.52389 | 4.11749 | 4.27075 | 4.56288 | 5.03435 | 5.76963 | 5.55895 | 4.79904 | 4.96018 | 6.42678 | 6.38085 |
| 8692 | 6.79493 | 6.35249 | 5.65812 | 7.05874 | 5.69294 | 6.19946 | 6.32583 | 6.28829 | 6.54245 | 6.38575 | 6.7305 | 6.79402 | 6.70036 | 7.14562 | 6.62425 | 7.3439 | 6.40971 |

| strain | WT | | | | | | | | | | |
| --- | --- | --- | --- | --- | --- | --- | --- | --- | --- | --- | --- |
| condition | G\_SD | | GX\_SD | | | X\_SD | | A\_SD | | C\_SD | |
| proteinId | exp | stat | exp | trans | stat | exp | stat | exp | stat | exp | stat |
| 12795 | 1.73231 | 0.90553 | 1.82803 | 0.366379 | 0 | 0.985019 | 0.957682 | 1.15917 | 1.33813 | 0.212277 | 0 |
| 15575 | 1.16385 | 1.79086 | 0.810334 | 2.23727 | 2.94794 | 2.54748 | 2.55275 | 2.13327 | 1.71052 | 2.57083 | 2.63149 |
| 13694 | 14.8316 | 15.2448 | 14.2045 | 13.9738 | 11.6537 | 15.109 | 15.5383 | 13.9109 | 10.9221 | 18.8048 | 13.7153 |
| 10937 | 16.5246 | 15.5808 | 17.6871 | 18.2392 | 18.7639 | 18.3695 | 17.2973 | 20.1003 | 19.5082 | 14.4206 | 20.8634 |
| 15905 | 11.4696 | 8.46692 | 11.182 | 4.46295 | 4.38324 | 7.67606 | 9.04404 | 17.194 | 13.3959 | 11.9937 | 10.2171 |
| 8692 | 3.6361 | 8.51211 | 6.91092 | 8.74287 | 9.78357 | 9.78847 | 11.236 | 11.7871 | 15.8764 | 10.7064 | 13.5355 |

|  | Glucose | Xylose | Arabinose | Acetate | Coumarate | Ferulate | YNB Oleic Acid | YNB Ricinoleic Acid | YNB Glucose | YNB Gluc DOC | YPD |
| --- | --- | --- | --- | --- | --- | --- | --- | --- | --- | --- | --- |
| proteinId |  |  |  |  |  |  |  |  |  |  |  |
| 12795 | 0.0673547 | 0.953909 | 0.137586 | 0.306703 | -0.0694808 | 0.291715 | -0.227028 | -0.390367 | -0.260799 | -0.155835 | -0.199948 |
| 15575 | -0.151587 | -0.275145 | -0.0921302 | -0.128402 | 0.0368643 | -0.320583 | -0.153631 | 0.0916747 | 0.171425 | 0.151195 | 0.226969 |
| 13694 | -0.121242 | 0.0898307 | 0.047509 | 0.00938531 | -0.317171 | 0.252759 | -0.124098 | -0.448979 | -0.11424 | -0.0883009 | -0.0388408 |
| 10937 | 0.125487 | 0.362162 | 0.242396 | 0.177756 | 0.0130856 | 0.391006 | -0.254579 | 0.281721 | -0.244334 | -0.480414 | -0.324646 |
| 15905 | -0.0550694 | 0.0820966 | 0.104398 | 0.0213273 | -0.363552 | -0.282914 | -0.248534 | 0.46351 | -0.303281 | -0.307461 | -0.0522098 |
| 8692 | 0.206583 | 0.109573 | 0.11735 | -0.20828 | -0.679619 | -0.0919705 | -0.441194 | -0.863605 | -0.219306 | -0.14764 | 0.364484 |

In [33]:

```
for r in sorted(model.genes.get_by_id('12795').reactions, key=lambda x: x.id):
    print(r.id, r.reaction, r.gene_reaction_rule)
print()
for r in sorted(model.genes.get_by_id('15575').reactions, key=lambda x: x.id):
    print(r.id, r.reaction, r.gene_reaction_rule)
print()
for r in sorted(model.genes.get_by_id('13694').reactions, key=lambda x: x.id):
    print(r.id, r.reaction, r.gene_reaction_rule)
print()
for r in sorted(model.genes.get_by_id('10937').reactions, key=lambda x: x.id):
    print(r.id, r.reaction, r.gene_reaction_rule)
print()
for r in sorted(model.genes.get_by_id('15905').reactions, key=lambda x: x.id):
    print(r.id, r.reaction, r.gene_reaction_rule)
print()
for r in sorted(model.genes.get_by_id('8692').reactions, key=lambda x: x.id):
    print(r.id, r.reaction, r.gene_reaction_rule)
```

```
BDH bhb_c + nad_c <=> acac_c + h_c + nadh_c 12795

ADHAPR_SC 0.01 1agly3p_SC_c + h_c + nadph_c --> 0.01 1ag3p_SC_c + nadp_c 15575
ALDD2y acald_c + h2o_c + nadp_c --> ac_c + 2.0 h_c + nadph_c 11650 or 12042 or 13426 or 14700 or 15575 or 16323 or 8666
BDHm bhb_m + nad_m <=> acac_m + h_m + nadh_m 15575
HBNOx bhb_x + nad_x <=> acac_x + h_x + nadh_x 15575
HSD17B1 estrone_c + h_c + nadph_c <=> estradiol_c + nadp_c 15575
HSD17B2r andrstndn_r + h_r + nadh_r --> nad_r + tststerone_r 15575
HSD17B8r estrone_r + h_r + nadh_r --> estradiol_r + nad_r 15575
RDH1a nadp_c + retinol_c <=> h_c + nadph_c + retinal_c 10008 or 11621 or 12858 or 13775 or 13867 or 15575 or 15911
RDH2a nadp_c + retinol_9_cis_c <=> h_c + nadph_c + retinal_cis_9_c 10008 or 11621 or 12858 or 13775 or 13867 or 15575 or 15911

3SLAR h_c + nadh_c + sla_c --> dhps_c + nad_c 13694
GHBDHx h_c + nadh_c + sucsal_c <=> ghb_c + nad_c 13694
HIBDkt 3hmp_c + nad_c --> h_c + mmtsa_c + nadh_c 13694
HIBDm 3hmp_m + nad_m <=> 2mop_m + h_m + nadh_m 13694
TRSARr 2h3oppan_c + h_c + nadh_c <=> glyc__R_c + nad_c 13694
yli_R0867 3hib_c + nad_c --> h_c + mmtsa_c + nadh_c 13694

3PG_pi_thr 3pg_c + pi_h <=> 3pg_h + pi_c 10888 or 10937
ABTA 4abut_c + akg_c --> glu__L_c + sucsal_c 10937 or 15905
ACOTA acorn_c + akg_c <=> acg5sa_c + glu__L_c 10937 or 14878
AGTim ala__L_m + glx_m --> gly_m + pyr_m 10937
APTNAT 5aptn_c + akg_c <=> glu__L_c + oxptn_c 10937
DHAP_pi_thr dhap_c + pi_h <=> dhap_h + pi_c 10888 or 10937
G1P_pi_th g1p_c + pi_h <=> g1p_h + pi_c 10888 or 10937
G3P_pi_thr g3p_c + pi_h <=> g3p_h + pi_c 10888 or 10937
G6PA_pi_th g6p_A_c + pi_h <=> g6p_A_h + pi_c 10888 or 10937
G6PB_pi_th g6p_B_c + pi_h <=> g6p_B_h + pi_c 10888 or 10937
SDPTA akg_c + sl26da_c <=> glu__L_c + sl2a6o_c 10937 or 14878 or 15404

3AIBTm 2mop_m + glu__L_m <=> 3aib_m + akg_m 15905
ABTA 4abut_c + akg_c --> glu__L_c + sucsal_c 10937 or 15905
ABTArm 4abut_m + akg_m <=> glu__L_m + sucsal_m 15905
APAT2rm akg_m + ala_B_m <=> glu__L_m + msa_m 15905
yli_R0593 akg_c + ala_B_c <=> glu__L_c + msa_c 15905
yli_R1594 akg_c + yli_M07070_c <=> glu__L_c + mmtsa_c 15905

GCALDDm gcald_m + h2o_m + nad_m --> glyclt_m + 2.0 h_m + nadh_m 12042 or 13426 or 16273 or 8569 or 8692 or 8975
LCADi_Dm h2o_m + lald__D_m + nad_m --> 2.0 h_m + lac__D_m + nadh_m 12042 or 13426 or 16273 or 8569 or 8692 or 8975
LCADm h2o_m + lald__L_m + nad_m --> 2.0 h_m + lac__L_m + nadh_m 12042 or 13426 or 16273 or 8569 or 8692 or 8975
OXPTNDH h2o_c + nad_c + oxptn_c <=> glutar_c + 2.0 h_c + nadh_c 8692
SSALxm h2o_m + nad_m + sucsal_m --> 2.0 h_m + nadh_m + succ_m 8692
SSALy h2o_c + nadp_c + sucsal_c --> 2.0 h_c + nadph_c + succ_c 8692
SSNOh h2o_h + nad_h + sucsal_h --> 2.0 h_h + nadh_h + succ_h 8692
yli_R1491 h2o_c + nad_c + sucsal_c --> h_c + nadh_c + succ_c 8692
```

In [34]:

```
for r in sorted(model.metabolites.get_by_id('2h3oppan_c').reactions, key=lambda x: x.id):
        print(r.id, r.reaction, r.gene_reaction_rule)
print()
for r in sorted(model.genes.get_by_id('16266').reactions, key=lambda x: x.id):
        print(r.id, r.reaction, r.gene_reaction_rule)
print()
for r in sorted(model.genes.get_by_id('14878').reactions, key=lambda x: x.id):
    print(r.id, r.reaction, r.gene_reaction_rule)
print()
for r in sorted(model.genes.get_by_id('15404').reactions, key=lambda x: x.id):
    print(r.id, r.reaction, r.gene_reaction_rule)
print()
for r in sorted(model.genes.get_by_id('8975').reactions, key=lambda x: x.id):
    print(r.id, r.reaction, r.gene_reaction_rule)
print()
for r in sorted(model.genes.get_by_id('8819').reactions, key=lambda x: x.id):
    print(r.id, r.reaction, r.gene_reaction_rule)
print()
for r in sorted(model.genes.get_by_id('9635').reactions, key=lambda x: x.id):
    print(r.id, r.reaction, r.gene_reaction_rule)
```

```
GLCRAL 5dh4dglc_c --> 2h3oppan_c + pyr_c 16266
TRSARr 2h3oppan_c + h_c + nadh_c <=> glyc__R_c + nad_c 13694

GLCRAL 5dh4dglc_c --> 2h3oppan_c + pyr_c 16266
LKDRA lkdr_c <=> lald__L_c + pyr_c 16266

ACOTA acorn_c + akg_c <=> acg5sa_c + glu__L_c 10937 or 14878
ACOTAim acg5sa_m + glu__L_m --> acorn_m + akg_m 14878
SDPTA akg_c + sl26da_c <=> glu__L_c + sl2a6o_c 10937 or 14878 or 15404
SOTA akg_c + sucorn_c --> glu__L_c + sucgsa_c 14878

OHPBAT glu__L_c + ohpb_c <=> akg_c + phthr_c 15404
PSAT 3php_h + glu__L_h --> akg_h + pser__L_h 15404
PSERT 3php_c + glu__L_c --> akg_c + pser__L_c 15404
SDPTA akg_c + sl26da_c <=> glu__L_c + sl2a6o_c 10937 or 14878 or 15404

GCALDDm gcald_m + h2o_m + nad_m --> glyclt_m + 2.0 h_m + nadh_m 12042 or 13426 or 16273 or 8569 or 8692 or 8975
LCADi_Dm h2o_m + lald__D_m + nad_m --> 2.0 h_m + lac__D_m + nadh_m 12042 or 13426 or 16273 or 8569 or 8692 or 8975
LCADm h2o_m + lald__L_m + nad_m --> 2.0 h_m + lac__L_m + nadh_m 12042 or 13426 or 16273 or 8569 or 8692 or 8975
MMSAD1m 2mop_m + coa_m + nad_m --> co2_m + nadh_m + ppcoa_m 8975
MMSAD3 coa_c + msa_c + nad_c --> accoa_c + co2_c + nadh_c 8975
MMSAD3m coa_m + msa_m + nad_m --> accoa_m + co2_m + nadh_m 8975
MMSDH coa_m + mmtsa_m + nad_m --> co2_m + nadh_m + ppcoa_m 8975
MMTSAO coa_c + mmtsa_c + nad_c --> co2_c + nadh_c + ppcoa_c 8975
yli_R0598 coa_c + msa_c + nadp_c <=> h_c + malcoa_c + nadph_c 8975

AGTi ala__L_c + glx_c --> gly_c + pyr_c 8819
AGTix ala__L_x + glx_x --> gly_x + pyr_x 8819
GLYTA_1 glu__L_m + glx_m --> akg_m + gly_m 8819 or 9635
SGAT glx_m + ser__L_m <=> gly_m + hpyr_m 8819
SPT_syn glx_c + ser__L_c <=> gly_c + hpyr_c 8819
SPTc pyr_c + ser__L_c <=> ala__L_c + hpyr_c 8819
SPTix pyr_x + ser__L_x --> ala__L_x + hpyr_x 8819
yli_R1388 glx_x + ser__L_x <=> gly_x + hpyr_x 8819

ALATA_L akg_c + ala__L_c <=> glu__L_c + pyr_c 9635
ALATA_Lm akg_m + ala__L_m <=> glu__L_m + pyr_m 9635
GLYTA_1 glu__L_m + glx_m --> akg_m + gly_m 8819 or 9635
```

In [35]:

```
# 12795 is cyto carbonyl reductase? mixed annotation
# MycoCosm has Mitochondrial/plastidial beta-ketoacyl-ACP reductase / 3-hydroxybutyrate dehydrogenase
# Remove BDH and keep BDHm and change gene to 12795
# 15575 is 1-Acyl dihydroxyacetone phosphate reductase
# Remove 15575 from other reactions
model.reactions.get_by_id('BDHm').gene_reaction_rule = '12795'
model.reactions.get_by_id('ALDD2y').gene_reaction_rule = '11650 or 12042 or 13426 or 14700 or 16323 or 8666'
model.remove_reactions(['BDH','HBNOx','HSD17B1','HSD17B2r','HSD17B8r','RDH1a','RDH2a','RDH3a'], remove_orphans=True)
# 13694 is mito 3-hydroxyisobutyrate dehydrogenase
# remove 3SLAR, GHBDHx, HIBDkt, yli_R0867 - no genes or duplicates
# 16266 is 2-keto-3-deoxy-L-rhamnonate aldolase / 4-hydroxy-2-oxoheptanedioate aldolase -> LKDRA
# remove GLCRAL + TRSARr
model.remove_reactions(['3SLAR','GHBDHx','HIBDkt','GLCRAL','TRSARr','yli_R0867'], remove_orphans=True)
# 10937 is cyto 4-aminobutyrate aminotransferase / (S)-3-amino-2-methylpropionate transaminase
# 15905 is mito 4-aminobutyrate aminotransferase / (S)-3-amino-2-methylpropionate transaminase, also b-alanine
# Change ABTA genes to '10937'
# Remove APTNAT - no annotation for this reaction 
# Remove 3PG_pi_thr, DHAP_pi_thr, G1P_pi_th, G3P_pi_thr, G6PA_pi_th, G6PB_pi_th, PEPPIth
# This removes 10888 - probably cyto to golgi nucleotide sugar translocator, add reactions for new compartment?
# Also removes 16411 SLC35E1
# Remove yli_R0593 (cyto), yli_R1594 (cyto, sameas 3AIBTm)  
model.reactions.get_by_id('ABTA').gene_reaction_rule = '10937'
model.remove_reactions(['APTNAT'], remove_orphans=True)
model.remove_reactions(['3PG_pi_thr','DHAP_pi_thr','G1P_pi_th','G3P_pi_thr','G6PA_pi_th','G6PB_pi_th','PEPPIth'], remove_orphans=True)
model.remove_reactions(['yli_R0593','yli_R1594'], remove_orphans=True)
# 14878 is mito acetylornithine aminotransferase
# 15404 is cyto phosphoserine aminotransferase
# Keep ACOTAim and remove ACOTA
# Change SDPTA to '15404' (10937 not annotated with this function, 14878 is mito)
# Remove SOTA (cyto, orphan), OHPBAT (bacterial, orphan), PSAT
model.reactions.get_by_id('SDPTA').gene_reaction_rule = '15404'
model.remove_reactions(['ACOTA','SOTA','OHPBAT','PSAT'], remove_orphans=True)
# 8692 is cyto NADP succinate-semialdehyde dehydrogenase
# Remove OXPTNDH, SSALxm, SSNOh, yli_R1491
model.remove_reactions(['OXPTNDH','SSALxm','SSNOh','yli_R1491'], remove_orphans=True)
# 8975 is mito malonate-semialdehyde dehydrogenase (acetylating) / methylmalonate-semialdehyde dehydrogenase
# Remove 8975 and 8692 from GCALDDm, LCADi_Dm, LCADm
# Keep MMSAD1m and MMSAD3m, remove cyto and duplicates (mmtsa is same as 3mop)
model.reactions.get_by_id('GCALDDm').gene_reaction_rule = '12042 or 13426 or 16273 or 8569'
model.reactions.get_by_id('LCADi_Dm').gene_reaction_rule = '12042 or 13426 or 16273 or 8569'
model.reactions.get_by_id('LCADm').gene_reaction_rule = '12042 or 13426 or 16273 or 8569'
model.remove_reactions(['MMSAD3','MMSDH','MMTSAO','yli_R0598'], remove_orphans=True)
# 8819 cyto AGX1 Alanine-glyoxylate aminotransferase AGT1 -> AGTi, SPT_syn, SPTc
# Yeast AGX1 is cyto/mito, human AGXT is perox, paperblast higher %id to AGX1
# Change AGTim gene to 8819
model.reactions.get_by_id('AGTim').gene_reaction_rule = '8819'
# GLYTA_1 not major reaction catalyzed by 8819, no genes for glycine transaminase
# 9635 mito (sigP) ALT1,ALT2 alanine transaminase -> ALATA_Lm genes are correct '9635'
# Remove AGTix, GLYTA_1, SGAT, SPT_syn, SPTc, SPTix, yli_R1388, ALATA_L
model.remove_reactions(['AGTix','GLYTA_1','SGAT','SPT_syn','SPTc','SPTix','yli_R1388','ALATA_L'],remove_orphans=True)
```

In [36]:

```
# 3hmp and 3hib are the same (S)-3-hydroxy-2-methylpropanoate / (S)-3-hydroxyisobutyrate
for r in sorted(model.metabolites.get_by_id('3hmp_m').reactions, key=lambda x: x.id):
        print(r.id, r.reaction, r.gene_reaction_rule)
print()
for r in sorted(model.metabolites.get_by_id('3hib_m').reactions, key=lambda x: x.id):
        print(r.id, r.reaction, r.gene_reaction_rule)
```

```
HIBDm 3hmp_m + nad_m <=> 2mop_m + h_m + nadh_m 13694

HIBH 3hibutcoa_m + h2o_m --> 3hib_m + coa_m + h_m 15218
HMNO 3hib_m + nad_m <=> h_m + mmtsa_m + nadh_m 11362
```

In [37]:

```
temp = ['11362','15218']
display(Annotation.loc[temp])
Show_Data(temp)
```

|  | Combined Annotations | Signal P | Sc288c Orthologs | Human Orthologs | Sc288 Best Hit | Human Blast | Essential | WolfPSort | C Terminal |
| --- | --- | --- | --- | --- | --- | --- | --- | --- | --- |
| RTO4\_ID |  |  |  |  |  |  |  |  |  |
| 11362 | K14729: FOX2; multifunctional beta-oxidation p... |  | FOX2 | HSD17B4 | FOX2 | HSD17 | Not Essential | cyto 15, mito 6, pero 4, mito\_nucl 4 | LAQ\* |
| 15218 | K05605: HIBCH; 3-hydroxyisobutyryl-CoA hydrolase |  | EHD3 | HIBCH | EHD3 | HIBCH | Not Essential | mito 22.5, cyto\_mito 14, cyto 4.5 | WVS\* |

| strain | WT | | | | | | | | | | | | | | | | |
| --- | --- | --- | --- | --- | --- | --- | --- | --- | --- | --- | --- | --- | --- | --- | --- | --- | --- |
| condition | G\_MM | C\_MM | G\_SD | | GX\_SD | | | X\_SD | | A\_SD | | C\_SD | | MM\_CN120 | | MM\_CN5 | Diversity\_Sample |
| phase | exp | exp | exp | stat | exp | trans | stat | exp | stat | exp | stat | exp | stat | exp | stat | exp | exp |
| proteinId | Set1 | Set1 | Set2 | Set2 | Set2 | Set2 | Set2 | Set2 | Set2 | Set2 | Set2 | Set2 | Set2 | Set3 | Set3 | Set3 | Set3 |
| 11362 | 6.4714 | 7.8679 | 6.21284 | 7.41652 | 6.15013 | 6.7725 | 7.06199 | 6.33216 | 6.88691 | 6.87267 | 7.25799 | 7.02773 | 6.87898 | 6.72127 | 7.06759 | 5.96352 | 6.44637 |
| 15218 | 6.86467 | 7.09307 | 6.60272 | 6.70549 | 6.55215 | 6.34078 | 6.39006 | 6.66868 | 6.49998 | 6.38468 | 6.70848 | 7.22747 | 7.16794 | 5.92126 | 5.82553 | 6.53206 | 6.51419 |

| strain | WT | | | | | | | | | | |
| --- | --- | --- | --- | --- | --- | --- | --- | --- | --- | --- | --- |
| condition | G\_SD | | GX\_SD | | | X\_SD | | A\_SD | | C\_SD | |
| proteinId | exp | stat | exp | trans | stat | exp | stat | exp | stat | exp | stat |
| 11362 | 20.4831 | 39.7562 | 20.574 | 30.8562 | 31.4551 | 28.1334 | 34.0703 | 34.5879 | 34.4629 | 67.7196 | 51.5122 |
| 15218 | 21.1773 | 18.7988 | 21.94 | 18.4431 | 20.2026 | 20.9192 | 22.5031 | 20.6773 | 20.4994 | 26.9393 | 28.0827 |

|  | Glucose | Xylose | Arabinose | Acetate | Coumarate | Ferulate | YNB Oleic Acid | YNB Ricinoleic Acid | YNB Glucose | YNB Gluc DOC | YPD |
| --- | --- | --- | --- | --- | --- | --- | --- | --- | --- | --- | --- |
| proteinId |  |  |  |  |  |  |  |  |  |  |  |
| 11362 | 0.0751211 | 0.0521463 | 0.00810646 | -0.0907637 | -0.150565 | 0.0238548 | -1.49407 | 0.0551923 | 0.104864 | -0.187183 | -0.0642188 |
| 15218 | 0.0745291 | -0.0219716 | -0.274042 | -1.22526 | -0.919181 | -0.37866 | -0.549135 | 0.000358951 | -0.184537 | -0.260827 | 0.21517 |

In [38]:

```
#Replace HIBH with 3HBCOAHLm from recon3D, keep HIBDm and remove HMNO
r = hsa2.reactions.get_by_id('3HBCOAHLm').copy()
r.gene_reaction_rule = '15218'
model.add_reactions([r])
model.remove_reactions(['HIBH','HMNO'],remove_orphans=True)
```

In [39]:

```
# mmtsa and 2mop are the same S-methylmalonate semialdehyde / (S)-2-methyl-3-oxopropanoate
for r in sorted(model.metabolites.get_by_id('mmtsa_c').reactions, key=lambda x: x.id):
        print(r.id, r.reaction, r.gene_reaction_rule)
print()
for r in sorted(model.metabolites.get_by_id('mmtsa_m').reactions, key=lambda x: x.id):
        print(r.id, r.reaction, r.gene_reaction_rule)
print()
for r in sorted(model.metabolites.get_by_id('2mop_m').reactions, key=lambda x: x.id):
        print(r.id, r.reaction, r.gene_reaction_rule)
```

```
yli_R0869 h2o_c + mmtsa_c + nad_c --> mmal_c + nadh_c 12042 or 13426 or 16323

ALDH h2o_m + mmtsa_m + nad_m --> 2.0 h_m + mm_m + nadh_m 12042 or 13426

3AIBTm 2mop_m + glu__L_m <=> 3aib_m + akg_m 15905
HIBDm 3hmp_m + nad_m <=> 2mop_m + h_m + nadh_m 13694
MMSAD1m 2mop_m + coa_m + nad_m --> co2_m + nadh_m + ppcoa_m 8975
```

In [40]:

```
temp = ['12042','13426','16323']
display(Annotation.loc[temp])
Show_Data(temp)
```

|  | Combined Annotations | Signal P | Sc288c Orthologs | Human Orthologs | Sc288 Best Hit | Human Blast | Essential | WolfPSort | C Terminal |
| --- | --- | --- | --- | --- | --- | --- | --- | --- | --- |
| RTO4\_ID |  |  |  |  |  |  |  |  |  |
| 12042 | K00128: ALDH; aldehyde dehydrogenase (NAD+) |  | ALD5,ALD4,ALD6,ALD3,ALD2 | ALDH1A1,ALDH1A2,ALDH1A3,ALDH1B1,ALDH2 | ALD5 | ALDH2 | Not Essential | cyto 18.5, cyto\_nucl 10, mito 4, pero 4 | NPL\* |
| 13426 | K07249: E1.2.1.36; retinal dehydrogenase |  | ALD5,ALD4,ALD6,ALD3,ALD2 | ALDH1A1,ALDH1A2,ALDH1A3,ALDH1B1,ALDH2 | ALD5 | ALDH1 | Not Essential | mito 25.5, cyto\_mito 14 | WPL\* |
| 16323 | K00128: ALDH; aldehyde dehydrogenase (NAD+) |  | HFD1 | ALDH3A1,ALDH3A2,ALDH3B1,ALDH3B2 | HFD1 | ALDH3 | Not Essential | cyto 10.5, plas 7, cyto\_nucl 7, mito 3, nucl 2... | GQA\* |

| strain | WT | | | | | | | | | | | | | | | | |
| --- | --- | --- | --- | --- | --- | --- | --- | --- | --- | --- | --- | --- | --- | --- | --- | --- | --- |
| condition | G\_MM | C\_MM | G\_SD | | GX\_SD | | | X\_SD | | A\_SD | | C\_SD | | MM\_CN120 | | MM\_CN5 | Diversity\_Sample |
| phase | exp | exp | exp | stat | exp | trans | stat | exp | stat | exp | stat | exp | stat | exp | stat | exp | exp |
| proteinId | Set1 | Set1 | Set2 | Set2 | Set2 | Set2 | Set2 | Set2 | Set2 | Set2 | Set2 | Set2 | Set2 | Set3 | Set3 | Set3 | Set3 |
| 12042 | 8.00849 | 8.563 | 8.41698 | 9.34145 | 8.34137 | 8.32176 | 8.06734 | 8.44974 | 8.36047 | 8.84866 | 8.59447 | 7.17248 | 5.67475 | 7.81326 | 7.51984 | 8.29774 | 8.31731 |
| 13426 | 7.30781 | 4.89844 | 3.84807 | 2.79984 | 3.78882 | 3.98344 | 3.73207 | 4.58197 | 3.36082 | 4.5625 | 3.35098 | 4.25235 | 3.1633 | 6.93547 | 6.81825 | 3.29168 | 5.38811 |
| 16323 | 5.54194 | 5.5053 | 6.37226 | 5.68644 | 6.28507 | 6.1392 | 5.86895 | 6.06104 | 5.82576 | 5.50985 | 6.17279 | 4.76433 | 4.74944 | 5.59107 | 6.40434 | 5.99953 | 6.38502 |

| strain | WT | | | | | | | | | | |
| --- | --- | --- | --- | --- | --- | --- | --- | --- | --- | --- | --- |
| condition | G\_SD | | GX\_SD | | | X\_SD | | A\_SD | | C\_SD | |
| proteinId | exp | stat | exp | trans | stat | exp | stat | exp | stat | exp | stat |
| 12042 | 33.9947 | 44.5614 | 32.5616 | 41.5121 | 40.2993 | 56.5808 | 63.4798 | 61.9973 | 72.5595 | 57.7357 | 40.7488 |
| 13426 | 0.757094 | 0.533653 | 1.41886 | 0.929251 | 0.72686 | 2.14514 | 0.960201 | 3.46858 | 3.64334 | 1.06907 | 0.864976 |
| 16323 | 10.9902 | 12.5683 | 13.6351 | 14.1342 | 18.2252 | 11.3209 | 13.9247 | 9.27817 | 10.1675 | 6.60796 | 3.93398 |

|  | Glucose | Xylose | Arabinose | Acetate | Coumarate | Ferulate | YNB Oleic Acid | YNB Ricinoleic Acid | YNB Glucose | YNB Gluc DOC | YPD |
| --- | --- | --- | --- | --- | --- | --- | --- | --- | --- | --- | --- |
| proteinId |  |  |  |  |  |  |  |  |  |  |  |
| 12042 | 0.170666 | -0.143043 | 0.000953096 | -0.0230376 | -0.521499 | 0.0146534 | 0.144875 | -0.295284 | 0.392859 | 0.454577 | -0.242457 |
| 13426 | -0.226122 | -0.181863 | -0.169891 | -0.0897023 | -0.0133507 | -0.00809092 | -1.1502 | -2.84044 | -0.85113 | -0.458514 | -0.0243377 |
| 16323 | 0.000210473 | -0.0174318 | 0.0140589 | -0.110443 | 0.00440814 | 0.0892768 | 0.155043 | 0.103889 | -0.177274 | -0.243731 | -0.140368 |

In [41]:

```
# Remove yli_R0869, ALDH - wrong reactions involving methylmalonate
model.remove_reactions(['yli_R0869','ALDH'],remove_orphans=True)
```

In [42]:

```
# L-lactate reactions
for r in sorted(model.metabolites.get_by_id('lac__L_m').reactions, key=lambda x: x.id):
        print(r.id, r.reaction, r.gene_reaction_rule)
print()
for r in sorted(model.metabolites.get_by_id('lac__L_c').reactions, key=lambda x: x.id):
        print(r.id, r.reaction, r.gene_reaction_rule)
```

```
LCADm h2o_m + lald__L_m + nad_m --> 2.0 h_m + lac__L_m + nadh_m 12042 or 13426 or 16273 or 8569
LLDH_ferr_m 2.0 ficytc_m + lac__L_m --> 2.0 focytc_m + 2.0 h_m + pyr_m (CRv4_Au5_s9_g15537_t1 and 8802) or (CRv4_Au5_s9_g15537_t1 and 9198)
L_LACtcm lac__L_c --> lac__L_m 15470
yli_R0667 2.0 ficytc_m + lac__L_m --> 2.0 focytc_m + pyr_m 16607

LCADi h2o_c + lald__L_c + nad_c --> 2.0 h_c + lac__L_c + nadh_c 12042 or 13426 or 15814 or 16323
L_LACD2 lac__L_c + q8_c --> pyr_c + q8h2_c 14950 or (b0306 and b0307 and b0308)
L_LACD2cm 2.0 ficytc_m + lac__L_c --> 2.0 focytc_m + pyr_c 16607 and 8802
L_LACD3 lac__L_c + mqn8_c --> mql8_c + pyr_c 14950 or (b0306 and b0307 and b0308)
L_LACt2r h_e + lac__L_e <=> h_c + lac__L_c 10184
L_LACtcm lac__L_c --> lac__L_m 15470
```

In [43]:

```
#temp = Annotation.index[Annotation['Combined Annotations'].str.contains('L-lactate')]
temp = ['9113','16607','14950','14997','13626']
display(Annotation.loc[temp])
Show_Data(temp)
```

|  | Combined Annotations | Signal P | Sc288c Orthologs | Human Orthologs | Sc288 Best Hit | Human Blast | Essential | WolfPSort | C Terminal |
| --- | --- | --- | --- | --- | --- | --- | --- | --- | --- |
| RTO4\_ID |  |  |  |  |  |  |  |  |  |
| 9113 | K00101: E1.1.2.3, lldD; L-lactate dehydrogenas... | S |  |  | CYB2 | HAO1 | Not Essential | mito 23, cyto 4 | VPV\* |
| 16607 | K00101: E1.1.2.3, lldD; L-lactate dehydrogenas... |  | CYB2 | HAO1 | CYB2 | HAO1 | Not Essential | pero 9, cysk 7, cyto 6, nucl 5 | ASL\* |
| 14950 | K11517: HAO; (S)-2-hydroxy-acid oxidase |  |  |  | CYB2 | HAO1 | Not Essential | cyto 11, cyto\_nucl 7.5, pero 7, cysk 5, nucl 2... | AKL\* |
| 14997 | KOG0538: Glycolate oxidase |  |  |  | CYB2 | HAO2 | Not Essential | cyto 15.5, cyto\_nucl 9.5, mito 8 | ARL\* |
| 13626 | KOG0538: Glycolate oxidase |  |  |  | CYB2 | HAO2 | Not Essential | pero 11.5, cyto\_pero 11, cyto 9.5, mito 4 | AKL\* |

| strain | WT | | | | | | | | | | | | | | | | |
| --- | --- | --- | --- | --- | --- | --- | --- | --- | --- | --- | --- | --- | --- | --- | --- | --- | --- |
| condition | G\_MM | C\_MM | G\_SD | | GX\_SD | | | X\_SD | | A\_SD | | C\_SD | | MM\_CN120 | | MM\_CN5 | Diversity\_Sample |
| phase | exp | exp | exp | stat | exp | trans | stat | exp | stat | exp | stat | exp | stat | exp | stat | exp | exp |
| proteinId | Set1 | Set1 | Set2 | Set2 | Set2 | Set2 | Set2 | Set2 | Set2 | Set2 | Set2 | Set2 | Set2 | Set3 | Set3 | Set3 | Set3 |
| 9113 | 3.9869 | 6.98984 | 3.19077 | 5.93817 | 3.00268 | 3.66599 | 3.76099 | 3.97558 | 4.08613 | 4.32791 | 3.89147 | 5.87759 | 5.05788 | 5.64509 | 4.10136 | 3.4434 | 5.01179 |
| 16607 | 5.05797 | 5.46352 | 4.24956 | 5.20096 | 4.33762 | 3.83773 | 3.62912 | 4.77443 | 4.82202 | 5.21026 | 5.39558 | 5.09955 | 5.34864 | 4.80888 | 4.67216 | 6.27967 | 5.58178 |
| 14950 | 4.395 | 5.06233 | 4.86469 | 5.61348 | 4.78182 | 5.35029 | 5.01644 | 5.25824 | 5.05175 | 5.09182 | 5.23989 | 4.80372 | 4.43783 | 4.01235 | 4.07732 | 4.63339 | 4.62603 |
| 14997 | 4.43374 | 5.61554 | 4.67024 | 4.24974 | 5.08419 | 3.88591 | 3.93588 | 4.00926 | 4.5154 | 3.97919 | 4.53537 | 5.66186 | 7.9514 | 2.6772 | 2.87154 | 6.22678 | 4.75189 |
| 13626 | 2.73177 | 4.96101 | 1.8473 | 2.80243 | 1.60752 | 1.32151 | 1.91091 | 2.16777 | 2.58412 | 1.34847 | 2.28479 | 2.89502 | 3.31131 | 1.74527 | 1.279 | 3.42562 | 1.31132 |

| strain | WT | | | | | | | | | | |
| --- | --- | --- | --- | --- | --- | --- | --- | --- | --- | --- | --- |
| condition | G\_SD | | GX\_SD | | | X\_SD | | A\_SD | | C\_SD | |
| proteinId | exp | stat | exp | trans | stat | exp | stat | exp | stat | exp | stat |
| 9113 | 0 | 0 | 0 | 0 | 0.180258 | 0 | 0.205723 | 0.190305 | 0 | 2.12744 | 0.877787 |
| 16607 | 1.74751 | 1.28611 | 1.21693 | 1.4811 | 1.09591 | 3.91086 | 4.33224 | 5.21808 | 3.45253 | 5.7682 | 4.80133 |
| 14950 | 0.560996 | 0.712057 | 0.408254 | 0.185549 | 1.30543 | 0.971319 | 1.59031 | 0.388524 | 1.35079 | 1.05073 | 0.851741 |
| 14997 | 0.382292 | 0 | 0.204746 | 0 | 0 | 0 | 0 | 0 | 0 | 0.862688 | 1.74952 |

|  | Glucose | Xylose | Arabinose | Acetate | Coumarate | Ferulate | YNB Oleic Acid | YNB Ricinoleic Acid | YNB Glucose | YNB Gluc DOC | YPD |
| --- | --- | --- | --- | --- | --- | --- | --- | --- | --- | --- | --- |
| proteinId |  |  |  |  |  |  |  |  |  |  |  |
| 9113 | 0.256547 | 0.0431719 | 0.164607 | 0.30401 | 0.13412 | -0.00780829 | 0.0339626 | 0.364485 | 0.351905 | -0.166731 | 0.153316 |
| 16607 | -0.177708 | 0.134844 | 0.0122926 | 0.0435882 | -0.549301 | 0.0942183 | 0.119487 | 0.248538 | -0.338643 | -0.0214949 | -0.593175 |
| 14950 | 0.200317 | 0.0789993 | 0.400746 | 0.296287 | 0.281775 | 0.0427567 | 0.115909 | 0.208815 | 0.829543 | 0.771701 | -0.128595 |
| 14997 | 0.173702 | -0.171627 | 0.0411792 | 0.0914154 | 0.0993831 | 0.113042 | 0.0507829 | 0.383122 | 0.0352678 | 0.179534 | -0.445492 |
| 13626 | 0.128917 | -0.198102 | 0.195125 | 0.261057 | 0.387961 | 0.26444 | -0.251658 | -0.00121394 | 0.135075 | -0.31572 | -0.357786 |

In [44]:

```
for x in temp:
    if x in model.genes:
        for r in sorted(model.genes.get_by_id(x).reactions, key=lambda x: x.id):
            print(r.id, r.reaction, r.gene_reaction_rule)
    else:
        print(x, 'no reactions')
    print()
```

```
9113 no reactions

GLXO2p glx_x + h2o_x + o2_x --> h2o2_x + h_x + oxa_x 16607
GLYCTO1 glyclt_c + o2_c --> glx_c + h2o2_c 14950 or 16607 or (PP_3746 and PP_3747 and 13328)
GLYCTO1p glyclt_x + o2_x --> glx_x + h2o2_x 16607
L_LACD2cm 2.0 ficytc_m + lac__L_c --> 2.0 focytc_m + pyr_c 16607 and 8802
yli_R0667 2.0 ficytc_m + lac__L_m --> 2.0 focytc_m + pyr_m 16607
yli_R1420 glyclt_x + o2_x --> glx_x + h2o2_x 14950 or 16607

GLYCTO1 glyclt_c + o2_c --> glx_c + h2o2_c 14950 or 16607 or (PP_3746 and PP_3747 and 13328)
L_LACD2 lac__L_c + q8_c --> pyr_c + q8h2_c 14950 or (b0306 and b0307 and b0308)
L_LACD3 lac__L_c + mqn8_c --> mql8_c + pyr_c 14950 or (b0306 and b0307 and b0308)
yli_R1420 glyclt_x + o2_x --> glx_x + h2o2_x 14950 or 16607

14997 no reactions

13626 no reactions
```

L-lactate dehydrogenase (cytochrome) 1.1.2.3 / (S)-2-hydroxy-acid oxidase 1.1.3.15  
9113 (mito, sigpep probability: 0.504, anchor probability: 0.483), no rxn  
16607 (pero, CYB2/HAO1, multiloc2 and cello say cyto)  
14950 (cyto/pero)  
Glycolate oxidase
14997 (cyto? pero?, missing b5-like domain), no rxn  
13626 (pero/cyto\_pero, missing b5-like domain), no rxn

9113 is not an ortholog of CYB2, but it is mito  
16607 is the best ortholog to CYB2, but it is not mito  
9113, 16607, and 14950 have cytochrome b5-like domain, but glycolate oxidase does not have one  
14997 and 13626 are missing cytochrome b5-like domain, likely glycolate oxidase

Set 9113 as L-lactate dehydrogenase since it is mito and has b5-like domain  
Set 14950 and 16607 as peroxisomal glycolate oxidase  
HAO2 14997, 13626 (no b5-like domain) most active with 2-hydroxypalmitate, fatty acid oxidation?

Add L\_LACDcm from Recon3D and change ficytC/focytC to ficytc/focytc  
Change L\_LACDcm genes to '9113 and 8802'  
Remove LLDH\_ferr\_m, yli\_R0667, L\_LACD2cm, L\_LACD2, L\_LACD3

In [45]:

```
r = hsa2.reactions.get_by_id('L_LACDcm').copy()
r.gene_reaction_rule = '9113 and 8802'
model.add_reactions([r])
for x in ['ficytC_m','focytC_m']:
    r.add_metabolites({x: -model.reactions.get_by_id('L_LACDcm').get_coefficient(x),
                       x.replace('C','c'): model.reactions.get_by_id('L_LACDcm').get_coefficient(x)})
model.remove_reactions(['LLDH_ferr_m','yli_R0667','L_LACD2','L_LACD2cm','L_LACD3'],remove_orphans=True)
```

In [46]:

```
for r in sorted(model.metabolites.get_by_id('glyclt_c').reactions, key=lambda x: x.id):
        print(r.id, r.reaction, r.gene_reaction_rule)
print()
for r in sorted(model.metabolites.get_by_id('glyclt_m').reactions, key=lambda x: x.id):
        print(r.id, r.reaction, r.gene_reaction_rule)
print()
for r in sorted(model.metabolites.get_by_id('glyclt_x').reactions, key=lambda x: x.id):
        print(r.id, r.reaction, r.gene_reaction_rule)
```

```
GCALDD gcald_c + h2o_c + nad_c --> glyclt_c + 2.0 h_c + nadh_c 12042 or 13426 or 15814 or 16323
GLYCLTDx glx_c + h_c + nadh_c --> glyclt_c + nad_c 11036 or 12051
GLYCLTDy glx_c + h_c + nadph_c --> glyclt_c + nadp_c 11036 or 12051
GLYCTO1 glyclt_c + o2_c --> glx_c + h2o2_c 14950 or 16607 or (PP_3746 and PP_3747 and 13328)
GLYCTO2 glyclt_c + q8_c --> glx_c + q8h2_c b4467 and b4468 and 13328
GLYCTO3 glyclt_c + mqn8_c --> glx_c + mql8_c b4467 and b4468 and 13328
GLYCTO4 2dmmq8_c + glyclt_c --> 2dmmql8_c + glx_c b4467 and b4468 and 13328

GCALDDm gcald_m + h2o_m + nad_m --> glyclt_m + 2.0 h_m + nadh_m 12042 or 13426 or 16273 or 8569
GCLDH fdxox_m + glyclt_m --> fdxrd_m + glx_m CRv4_Au5_s6_g13173_t1 and 15585

GLYCTO1p glyclt_x + o2_x --> glx_x + h2o2_x 16607
PGLYCPx 2pglyc_x + h2o_x --> glyclt_x + pi_x 13044
yli_R1420 glyclt_x + o2_x --> glx_x + h2o2_x 14950 or 16607
```

In [47]:

```
temp = ['15585','13044']
display(Annotation.loc[temp])
Show_Data(temp)
```

|  | Combined Annotations | Signal P | Sc288c Orthologs | Human Orthologs | Sc288 Best Hit | Human Blast | Essential | WolfPSort | C Terminal |
| --- | --- | --- | --- | --- | --- | --- | --- | --- | --- |
| RTO4\_ID |  |  |  |  |  |  |  |  |  |
| 15585 | KOG3309: Ferredoxin |  | YAH1 | FDX1,FDX2 | YAH1 | FDX2 | Essential | cyto 23.5, cyto\_nucl 13.5 | THH\* |
| 13044 | K01101: E3.1.3.41; 4-nitrophenyl phosphatase |  | PHO13 | PGP,PDXP | PHO13 | PDXP | Not Essential | cyto 17.5, cyto\_nucl 12.5, nucl 4.5, pero 3 | LAQ\* |

| strain | WT | | | | | | | | | | | | | | | | |
| --- | --- | --- | --- | --- | --- | --- | --- | --- | --- | --- | --- | --- | --- | --- | --- | --- | --- |
| condition | G\_MM | C\_MM | G\_SD | | GX\_SD | | | X\_SD | | A\_SD | | C\_SD | | MM\_CN120 | | MM\_CN5 | Diversity\_Sample |
| phase | exp | exp | exp | stat | exp | trans | stat | exp | stat | exp | stat | exp | stat | exp | stat | exp | exp |
| proteinId | Set1 | Set1 | Set2 | Set2 | Set2 | Set2 | Set2 | Set2 | Set2 | Set2 | Set2 | Set2 | Set2 | Set3 | Set3 | Set3 | Set3 |
| 15585 | 4.88266 | 5.56536 | 4.41696 | 4.25515 | 4.43203 | 4.59169 | 4.43999 | 4.69098 | 4.53372 | 4.34912 | 4.47085 | 4.95726 | 5.16606 | 4.53764 | 4.79042 | 5.59086 | 5.66233 |
| 13044 | 7.07028 | 6.74996 | 7.16219 | 5.8811 | 7.19872 | 6.57109 | 6.45218 | 6.99004 | 6.03626 | 6.86862 | 6.18725 | 6.06425 | 5.94515 | 5.35161 | 5.26977 | 5.85498 | 5.54996 |

| strain | WT | | | | | | | | | | |
| --- | --- | --- | --- | --- | --- | --- | --- | --- | --- | --- | --- |
| condition | G\_SD | | GX\_SD | | | X\_SD | | A\_SD | | C\_SD | |
| proteinId | exp | stat | exp | trans | stat | exp | stat | exp | stat | exp | stat |
| 15585 | 0.566264 | 0 | 0.81119 | 0 | 0 | 0 | 0 | 0 | 0 | 0.420867 | 0.867663 |
| 13044 | 4.20559 | 1.80248 | 3.87481 | 2.79768 | 2.34766 | 2.33804 | 2.98044 | 2.13068 | 1.74214 | 1.72051 | 1.29777 |

|  | Glucose | Xylose | Arabinose | Acetate | Coumarate | Ferulate | YNB Oleic Acid | YNB Ricinoleic Acid | YNB Glucose | YNB Gluc DOC | YPD |
| --- | --- | --- | --- | --- | --- | --- | --- | --- | --- | --- | --- |
| proteinId |  |  |  |  |  |  |  |  |  |  |  |

In [48]:

```
for x in temp:
    if x in model.genes:
        for r in sorted(model.genes.get_by_id(x).reactions, key=lambda x: x.id):
            print(r.id, r.reaction, r.gene_reaction_rule)
    else:
        print(x, 'no reactions')
    print()
```

```
GCLDH fdxox_m + glyclt_m --> fdxrd_m + glx_m CRv4_Au5_s6_g13173_t1 and 15585
HEMEOMOm hemeO_m + nadh_m + o2_m --> h2o_m + hemeA_m + nad_m 10228 and 15585 and 16775
HYDAm 2.0 fdxrd_m <=> 2.0 fdxox_m + h2_m + 2.0 h_m (CRv4_Au5_s3_g11137_t1 and 15585) or (CRv4_Au5_s9_g15404_t1 and 15585)
SELTORm 6.0 fdxrd_m + slnt_m --> 6.0 fdxox_m + 3.0 h2o_m + 6.0 h_m + seln_m 15585 and 8744

G3PT glyc3p_c + h2o_c --> glyc_c + pi_c 13044 or 13413
HYPOE h2o_c + pyam5p_c --> pi_c + pydam_c 13044
NTD2 h2o_c + ump_c --> pi_c + uri_c 13044 or 16648 or 9995
NTD4 cmp_c + h2o_c --> cytd_c + pi_c 13044 or 16648 or 9995
NTD7 amp_c + h2o_c --> adn_c + pi_c 13044 or 16648
NTD9 gmp_c + h2o_c --> gsn_c + pi_c 13044 or 16648
PDXPP h2o_c + pdx5p_c --> pi_c + pydxn_c 13044 or 14545
PGLYCPh 2pglyc_h + h2o_h --> glyclt_h + pi_h 13044
PGLYCPx 2pglyc_x + h2o_x --> glyclt_x + pi_x 13044
PYDXPP h2o_c + pydx5p_c --> pi_c + pydx_c 13044 or 14545
R5PP h2o_c + r5p_c --> pi_c + rib__D_c 11513 or 13044 or 14546 or 8576
```

HAO2 does not have activity on glycolate and glyoxylate  
Change GLYCTO1p genes to '14950 or 16607'  
No specific enzyme for glyoxylate oxidase in Rhodo  
In literature, this is side reaction of glycolate oxidase or lactate dehydrogenase  
Change GLXO2p genes to '14950 or 16607'  
Remove Remove GLYCTO1, GLYCTO2, GLYCTO3, GLYCTO4, yli\_R1420

No mitochondrial glycolate oxidase in Rhodo and 15585 is mito ferredoxin YAH1  
Remove GCLDH, also remove HYDAm (no gene)  
Remove PGLYCPx (blocked, 13044 4-nitrophenyl phosphatase), PGLYCPh

In [49]:

```
model.reactions.get_by_id('GLYCTO1p').gene_reaction_rule = '14950 or 16607'
model.reactions.get_by_id('GLXO2p').gene_reaction_rule = '14950 or 16607'
model.remove_reactions(['GLYCTO1','GLYCTO2','GLYCTO3','GLYCTO4','yli_R1420'],remove_orphans=True)
model.remove_reactions(['GCLDH','HYDAm','PGLYCPx','PGLYCPh'],remove_orphans=True)
```

In [50]:

```
for r in sorted(model.metabolites.get_by_id('glx_c').reactions, key=lambda x: x.id):
        print(r.id, r.reaction, r.gene_reaction_rule)
print()
for r in sorted(model.metabolites.get_by_id('glx_m').reactions, key=lambda x: x.id):
        print(r.id, r.reaction, r.gene_reaction_rule)
print()
for r in sorted(model.metabolites.get_by_id('glx_x').reactions, key=lambda x: x.id):
        print(r.id, r.reaction, r.gene_reaction_rule)
```

```
AGTi ala__L_c + glx_c --> gly_c + pyr_c 8819
GLYCLTDx glx_c + h_c + nadh_c --> glyclt_c + nad_c 11036 or 12051
GLYCLTDy glx_c + h_c + nadph_c --> glyclt_c + nadp_c 11036 or 12051
GLYO1 gly_c + h2o_c + o2_c --> glx_c + h2o2_c + nh4_c 15449 or 15994
ICL icit_c --> glx_c + succ_c 14022 or 14162
MALS accoa_c + glx_c + h2o_c --> coa_c + h_c + mal__L_c 9457
UGLYCH h2o_c + 2.0 h_c + urdglyc_c --> co2_c + glx_c + 2.0 nh4_c 14724

AGTim ala__L_m + glx_m --> gly_m + pyr_m 8819
ICL_1 icit_m --> glx_m + succ_m 14022 or 14162
MALSm accoa_m + glx_m + h2o_m --> coa_m + h_m + mal__L_m 9457
yli_R0672 accoa_m + glx_m + h2o_m --> coa_m + h_c + mal__L_m 9457

GLXO2p glx_x + h2o_x + o2_x --> h2o2_x + h_x + oxa_x 14950 or 16607
GLYCTO1p glyclt_x + o2_x --> glx_x + h2o2_x 14950 or 16607
MALSp accoa_x + glx_x + h2o_x --> coa_x + h_x + mal__L_x 9457
yli_R1434 accoa_x + glx_x + h2o_x --> coa_x + h_c + mal__L_x 9457
yli_R1480 icit_x --> glx_x + succ_x 14022 or 14162
```

In [51]:

```
for r in sorted(model.metabolites.get_by_id('hpyr_c').reactions, key=lambda x: x.id):
        print(r.id, r.reaction, r.gene_reaction_rule)
print()
for r in sorted(model.metabolites.get_by_id('hpyr_m').reactions, key=lambda x: x.id):
        print(r.id, r.reaction, r.gene_reaction_rule)
```

```
HPYRP 3php_c + h2o_c --> hpyr_c + pi_c 13350
HPYRRx h_c + hpyr_c + nadh_c --> glyc__R_c + nad_c 11036 or 12051
HPYRRy h_c + hpyr_c + nadph_c --> glyc__R_c + nadp_c 11036 or 12051 or 9515

GLYCDH_1 h_m + hpyr_m + nadh_m --> glyc__R_m + nad_m 11036 or 12051
```

In [52]:

```
temp = ['11036','12051','9515','9520','13784']
display(Annotation.loc[temp])
Show_Data(temp)
```

|  | Combined Annotations | Signal P | Sc288c Orthologs | Human Orthologs | Sc288 Best Hit | Human Blast | Essential | WolfPSort | C Terminal |
| --- | --- | --- | --- | --- | --- | --- | --- | --- | --- |
| RTO4\_ID |  |  |  |  |  |  |  |  |  |
| 11036 | K00049: GRHPR; glyoxylate/hydroxypyruvate redu... |  |  | GRHPR | GOR1 | GRHPR | Not Essential | mito 16, cyto 6, cyto\_nucl 6, nucl 4 | VKL\* |
| 12051 | KOG0069: Glyoxylate/hydroxypyruvate reductase ... | S | GOR1 |  | GOR1 | CTBP1 | Not Essential | mito 23, pero 3 | GHF\* |
| 9515 | KOG0069: Glyoxylate/hydroxypyruvate reductase ... |  |  |  |  | GRHPR | Not Essential | cyto 12.5, extr 8, cyto\_nucl 7, mito 4 | MGQ\* |
| 9520 | KOG0069: Glyoxylate/hydroxypyruvate reductase ... |  |  |  |  | GRHPR | Not Essential | cysk 12, cyto 7, cyto\_nucl 5.5, nucl 2, mito 2... | EDD\* |
| 13784 | K04496: CTBP; C-terminal binding protein |  |  |  | GOR1 | CTBP1 | Not Essential | mito 10, cyto 9, nucl 3, cysk 3 | KAQ\* |

| strain | WT | | | | | | | | | | | | | | | | |
| --- | --- | --- | --- | --- | --- | --- | --- | --- | --- | --- | --- | --- | --- | --- | --- | --- | --- |
| condition | G\_MM | C\_MM | G\_SD | | GX\_SD | | | X\_SD | | A\_SD | | C\_SD | | MM\_CN120 | | MM\_CN5 | Diversity\_Sample |
| phase | exp | exp | exp | stat | exp | trans | stat | exp | stat | exp | stat | exp | stat | exp | stat | exp | exp |
| proteinId | Set1 | Set1 | Set2 | Set2 | Set2 | Set2 | Set2 | Set2 | Set2 | Set2 | Set2 | Set2 | Set2 | Set3 | Set3 | Set3 | Set3 |
| 11036 | 5.11456 | 5.78736 | 5.15205 | 4.84847 | 5.30596 | 5.38477 | 5.27825 | 6.37514 | 4.84187 | 5.12534 | 5.75428 | 4.69817 | 5.58944 | 5.84831 | 5.04025 | 5.87783 | 5.70169 |
| 12051 | 5.49005 | 4.90948 | 5.86562 | 5.91837 | 5.84087 | 5.63879 | 5.52379 | 5.56949 | 5.18737 | 5.33109 | 5.54119 | 5.05274 | 4.46329 | 4.63929 | 4.72545 | 4.90033 | 4.5544 |
| 9515 | 2.71916 | 3.5578 | 5.18056 | 5.24105 | 5.06491 | 5.09825 | 5.10857 | 4.85814 | 3.51104 | 2.73644 | 4.11322 | 2.47156 | 2.76983 | 4.12845 | 1.32602 | 4.12971 | 2.37129 |
| 9520 | 6.66177 | 5.95628 | 7.03704 | 6.5122 | 7.07629 | 6.76808 | 6.74117 | 6.70268 | 6.10968 | 6.32404 | 5.99824 | 5.77727 | 5.29713 | 6.64209 | 6.89673 | 7.35984 | 6.75127 |
| 13784 | 2.47173 | 4.0591 | 2.05595 | 4.18474 | 2.02782 | 2.37046 | 2.9283 | 2.70685 | 3.01487 | 3.49305 | 3.27197 | 2.69645 | 1.75522 | 1.82313 | 1.6273 | 2.51972 | 1.84215 |

| strain | WT | | | | | | | | | | |
| --- | --- | --- | --- | --- | --- | --- | --- | --- | --- | --- | --- |
| condition | G\_SD | | GX\_SD | | | X\_SD | | A\_SD | | C\_SD | |
| proteinId | exp | stat | exp | trans | stat | exp | stat | exp | stat | exp | stat |
| 11036 | 6.17929 | 9.83562 | 4.27324 | 8.55531 | 9.15595 | 9.97659 | 12.2792 | 7.33418 | 8.9816 | 7.48053 | 8.04947 |
| 12051 | 2.69133 | 5.59065 | 3.66411 | 3.52904 | 4.00252 | 7.64768 | 7.49411 | 5.79818 | 6.31793 | 2.99219 | 5.23037 |
| 9515 | 0 | 1.40122 | 0 | 0.548411 | 1.46906 | 2.343 | 1.37028 | 0 | 0 | 0 | 0 |
| 9520 | 8.06354 | 10.8323 | 11.5747 | 11.5268 | 13.2741 | 12.5392 | 11.8586 | 10.6319 | 10.1327 | 7.03946 | 6.72713 |

|  | Glucose | Xylose | Arabinose | Acetate | Coumarate | Ferulate | YNB Oleic Acid | YNB Ricinoleic Acid | YNB Glucose | YNB Gluc DOC | YPD |
| --- | --- | --- | --- | --- | --- | --- | --- | --- | --- | --- | --- |
| proteinId |  |  |  |  |  |  |  |  |  |  |  |
| 11036 | 0.024601 | -0.161983 | 0.0796525 | -0.0395243 | -0.0943849 | -0.0338847 | -0.251425 | 0.458271 | 0.301021 | 0.0204884 | 0.214889 |
| 12051 | 0.0973852 | -0.0878814 | 0.0927382 | 0.0871653 | -0.0747326 | 0.184323 | -0.00356084 | -0.179646 | -0.0359196 | 0.0345598 | -0.0304602 |
| 9515 | -0.26238 | -0.479496 | -0.0976874 | -0.228915 | 0.176125 | -0.103218 | 0.189812 | -0.217378 | 0.230198 | 0.0290391 | -0.364222 |
| 9520 | 0.200351 | -0.0295315 | 0.140982 | 0.104999 | -0.0996001 | -0.219486 | -0.0664831 | 0.509412 | -0.135098 | 0.0938895 | 0.035346 |
| 13784 | 0.109426 | -0.256949 | -0.218291 | 0.0569437 | -0.196125 | 0.143119 | 0.158861 | 0.06832 | 0.114423 | 0.00930607 | -0.199088 |

In [53]:

```
for x in temp:
    if x in model.genes:
        for r in sorted(model.genes.get_by_id(x).reactions, key=lambda x: x.id):
            print(r.id, r.reaction, r.gene_reaction_rule)
    else:
        print(x, 'no reactions')
    print()
```

```
2DGULRGx 2dhguln_c + h_c + nadh_c --> glcn_c + nad_c 11036 or 12051
2DGULRGy 2dhguln_c + h_c + nadph_c --> glcn_c + nadp_c 11036 or 12051
2DGULRx 2dhguln_c + h_c + nadh_c --> idon__L_c + nad_c 11036 or 12051
2DGULRy 2dhguln_c + h_c + nadph_c --> idon__L_c + nadp_c 11036 or 12051
DKGLCNR2x 25dkglcn_c + h_c + nadh_c --> 5dglcn_c + nad_c 11036 or 12051
DKGLCNR2y 25dkglcn_c + h_c + nadph_c --> 5dglcn_c + nadp_c 11036 or 12051
GLYCDH_1 h_m + hpyr_m + nadh_m --> glyc__R_m + nad_m 11036 or 12051
GLYCLTDx glx_c + h_c + nadh_c --> glyclt_c + nad_c 11036 or 12051
GLYCLTDy glx_c + h_c + nadph_c --> glyclt_c + nadp_c 11036 or 12051
HPYRRx h_c + hpyr_c + nadh_c --> glyc__R_c + nad_c 11036 or 12051
HPYRRy h_c + hpyr_c + nadph_c --> glyc__R_c + nadp_c 11036 or 12051 or 9515
LALDD lald__D_c + nadp_c <=> h_c + mthgxl_c + nadph_c 11036
PGLCNDH 6p2dhglcn_c + nadph_c <=> 6pgc_c + 2.0 h_c + nadp_c 11036 or 12051

2DGULRGx 2dhguln_c + h_c + nadh_c --> glcn_c + nad_c 11036 or 12051
2DGULRGy 2dhguln_c + h_c + nadph_c --> glcn_c + nadp_c 11036 or 12051
2DGULRx 2dhguln_c + h_c + nadh_c --> idon__L_c + nad_c 11036 or 12051
2DGULRy 2dhguln_c + h_c + nadph_c --> idon__L_c + nadp_c 11036 or 12051
DKGLCNR2x 25dkglcn_c + h_c + nadh_c --> 5dglcn_c + nad_c 11036 or 12051
DKGLCNR2y 25dkglcn_c + h_c + nadph_c --> 5dglcn_c + nadp_c 11036 or 12051
GLYCDH_1 h_m + hpyr_m + nadh_m --> glyc__R_m + nad_m 11036 or 12051
GLYCLTDx glx_c + h_c + nadh_c --> glyclt_c + nad_c 11036 or 12051
GLYCLTDy glx_c + h_c + nadph_c --> glyclt_c + nadp_c 11036 or 12051
HPYRRx h_c + hpyr_c + nadh_c --> glyc__R_c + nad_c 11036 or 12051
HPYRRy h_c + hpyr_c + nadph_c --> glyc__R_c + nadp_c 11036 or 12051 or 9515
PGLCNDH 6p2dhglcn_c + nadph_c <=> 6pgc_c + 2.0 h_c + nadp_c 11036 or 12051

HPYRRy h_c + hpyr_c + nadph_c --> glyc__R_c + nadp_c 11036 or 12051 or 9515
LALDO2x h_c + mthgxl_c + nadh_c --> lald__D_c + nad_c 16543 or 9515

9520 no reactions

13784 no reactions
```

glyoxylate/hydroxypyruvate reductase (NADP)  
1.1.1.79  
Glycolate + NADP+ <=> Glyoxylate + NADPH + H+  
D-Glycerate + NADP+ <=> Hydroxypyruvate + NADPH + H+  
1.1.1.81 (D-Glycerate is (R)-glycerate)  
D-Glycerate + NAD+ <=> Hydroxypyruvate + NADH + H+  
D-Glycerate + NADP+ <=> Hydroxypyruvate + NADPH + H+  
1.1.1.215 (E. coli multifunctional, but not others)
D-Gluconic acid + NADP+ <=> 2-Keto-D-gluconic acid + NADPH + H+  
5-Dehydro-D-gluconate + NADP+ <=> 2,5-Didehydro-D-gluconate + NADPH + H+  
L-Idonate + NADP+ <=> 2-Dehydro-L-idonate + NADPH + H+  
11036 cyto/pero GRHPR  
9515 cyto (also glyoxylate/hydroxypyruvate/2-ketogluconate reductase)  
9520 cysk (also glyoxylate/hydroxypyruvate/2-ketogluconate reductase)

glyoxylate reductase (NAD)  
1.1.1.26  
Glycolate + NAD+ <=> Glyoxylate + NADH + H+  
12051 mito (sigP) GOR1  
13784 mito 10, cyto 9 (no sigP) - paperblast to R-mandelate dehydrogenase in Rhodotorula graminis  
11952 cyto FDH1 formate dehydrogenase (search hit by glyoxylate reductase)

Change GLYCLTDy genes to '11036 or 9515 or 9520'
Change HPYRRx, HPYRRy genes to '11036 or 9515 or 9520'  
Make GLYCLTDyp and set genes to '11036'
Change GLYCLTDx to GLYCLTDxm and change genes to '12051'
Remove 2DGULRGx, 2DGULRGy, 2DGULRx, 2DGULRy, DKGLCNR2x, DKGLCNR2y, GLYCDH\_1, LALDD, PGLCNDH, LALDO2x

In [54]:

```
model.reactions.get_by_id('GLYCLTDy').name = 'Glyoxylate reductase (NADP)'
model.reactions.get_by_id('GLYCLTDy').gene_reaction_rule = '11036 or 9515 or 9520'
model.reactions.get_by_id('HPYRRx').name = 'Hydroxypyruvate reductase (NAD)'
model.reactions.get_by_id('HPYRRx').gene_reaction_rule = '11036 or 9515 or 9520'
model.reactions.get_by_id('HPYRRy').name = 'Hydroxypyruvate reductase (NADP)'
model.reactions.get_by_id('HPYRRy').gene_reaction_rule = '11036 or 9515 or 9520'

r = model.reactions.get_by_id('GLYCLTDy').copy()
r.id = 'GLYCLTDyp'
r.name = 'Glyoxylate reductase (NADP), peroxisomal'
r.gene_reaction_rule = '11036'
model.add_reactions([r])
for m in r.metabolites:
    if not m.id.replace('_c','_x') in model.metabolites:
        m2 = m.copy()
        m2.id = m.id.replace('_c','_x')
        m2.compartment = 'x'
        model.add_metabolites([m2])
    r.add_metabolites({m.id: -r.get_coefficient(m.id), m.id.replace('_c','_x'): r.get_coefficient(m.id)})

r = model.reactions.get_by_id('GLYCLTDx')
r.id = 'GLYCLTDxm'
r.name = 'Glyoxylate reductase (NAD), mitochondrial'
r.gene_reaction_rule = '12051'
for m in r.metabolites:
    if not m.id.replace('_c','_m') in model.metabolites:
        m2 = m.copy()
        m2.id = m.id.replace('_c','_m')
        m2.compartment = 'm'
        model.add_metabolites([m2])
    r.add_metabolites({m.id: -r.get_coefficient(m.id), m.id.replace('_c','_m'): r.get_coefficient(m.id)})

Remove = ['2DGULRGx','2DGULRGy','2DGULRx','2DGULRy','DKGLCNR2x','DKGLCNR2y','GLYCDH_1','LALDD','PGLCNDH','LALDO2x']
model.remove_reactions(Remove,remove_orphans=True)
```

In [55]:

```
temp = ['16543','11882','12784','15285','9774']
display(Annotation.loc[temp])
Show_Data(temp)
```

|  | Combined Annotations | Signal P | Sc288c Orthologs | Human Orthologs | Sc288 Best Hit | Human Blast | Essential | WolfPSort | C Terminal |
| --- | --- | --- | --- | --- | --- | --- | --- | --- | --- |
| RTO4\_ID |  |  |  |  |  |  |  |  |  |
| 16543 | K15303: AKR7; aflatoxin B1 aldehyde reductase |  |  | AKR7A2,AKR7A3,AKR7L | AAD4 | AKR7A | Not Essential | cyto 19.5, cyto\_mito 12.166, cyto\_nucl 11.333,... | IKI\* |
| 11882 | K18097: GCY1; glycerol 2-dehydrogenase (NADP+) |  | YPR1,GCY1 | AKR1A1 | YPR1 | AKR1A | Not Essential | cyto 24.5, cyto\_nucl 13.5 | SHW\* |
| 12784 | K00002: AKR1A1, adh; alcohol dehydrogenase (NA... |  | YPR1,GCY1 | AKR1A1 | YPR1 | AKR1A | Not Essential | mito 13.5, cyto\_mito 12, cyto 9.5, pero 4 | AKL\* |
| 15285 | K04883: KCNAB2; potassium voltage-gated channe... |  |  | KCNAB1,KCNAB2,KCNAB3 | YPL088W | KCNAB | Not Essential | cyto 12, cyto\_nucl 9.5, pero 7, nucl 5 | GRA\* |
| 9774 | K00002: AKR1A1, adh; alcohol dehydrogenase (NA... |  | YPR1,GCY1 | AKR1A1 | YPR1 | AKR1A | Not Essential | cyto 17.5, cyto\_mito 13.666, cyto\_nucl 10.333,... | KIK\* |

| strain | WT | | | | | | | | | | | | | | | | |
| --- | --- | --- | --- | --- | --- | --- | --- | --- | --- | --- | --- | --- | --- | --- | --- | --- | --- |
| condition | G\_MM | C\_MM | G\_SD | | GX\_SD | | | X\_SD | | A\_SD | | C\_SD | | MM\_CN120 | | MM\_CN5 | Diversity\_Sample |
| phase | exp | exp | exp | stat | exp | trans | stat | exp | stat | exp | stat | exp | stat | exp | stat | exp | exp |
| proteinId | Set1 | Set1 | Set2 | Set2 | Set2 | Set2 | Set2 | Set2 | Set2 | Set2 | Set2 | Set2 | Set2 | Set3 | Set3 | Set3 | Set3 |
| 16543 | 5.20225 | 5.78907 | 4.49264 | 3.31023 | 4.63261 | 4.31056 | 3.8116 | 4.17967 | 2.89776 | 4.48752 | 2.2772 | 3.85104 | 2.36305 | 3.68526 | 3.35679 | 4.29924 | 4.62616 |
| 11882 | 4.07818 | 5.71861 | 5.83615 | 5.62544 | 5.77337 | 5.29629 | 5.15703 | 7.02755 | 5.00344 | 4.97816 | 5.2813 | 4.36109 | 4.34722 | 6.19133 | 6.37203 | 7.21044 | 7.40048 |
| 12784 | 2.08671 | 1.96656 | 1.05974 | 1.28618 | 1.01927 | 0.689282 | 0.833234 | 1.3947 | 1.87113 | 2.53738 | 1.88953 | 1.63086 | 3.2399 | 0.662714 | 0.423146 | 2.00301 | 1.06163 |
| 15285 | 8.92263 | 9.07417 | 9.76112 | 9.11167 | 9.7707 | 9.05986 | 9.1026 | 10.2928 | 8.98926 | 10.618 | 10.3852 | 7.59314 | 6.02817 | 8.5145 | 7.8427 | 10.2757 | 8.89486 |
| 9774 | 9.27193 | 8.0739 | 9.06855 | 8.59521 | 9.81437 | 9.92808 | 9.23804 | 9.63717 | 7.83349 | 10.5623 | 7.02255 | 5.69079 | 5.81959 | 10.0636 | 9.3345 | 10.8455 | 10.3765 |

| strain | WT | | | | | | | | | | |
| --- | --- | --- | --- | --- | --- | --- | --- | --- | --- | --- | --- |
| condition | G\_SD | | GX\_SD | | | X\_SD | | A\_SD | | C\_SD | |
| proteinId | exp | stat | exp | trans | stat | exp | stat | exp | stat | exp | stat |
| 16543 | 3.29547 | 1.75926 | 2.45532 | 0.925835 | 0.540356 | 7.26453 | 6.69903 | 4.24471 | 4.01328 | 4.04274 | 1.52518 |
| 11882 | 7.28788 | 9.64582 | 6.91339 | 7.61119 | 9.64307 | 14.6905 | 14.9097 | 6.37514 | 6.3363 | 10.6828 | 9.16872 |
| 12784 | 0.384095 | 0 | 0.204746 | 0 | 0 | 0 | 0 | 0 | 0.189081 | 0 | 0.21114 |
| 15285 | 24.0568 | 25.4138 | 25.4561 | 25.3228 | 28.0987 | 33.4373 | 33.4815 | 34.5599 | 37.3536 | 22.6963 | 20.2845 |
| 9774 | 24.0361 | 20.651 | 27.2528 | 17.3246 | 17.6781 | 36.9396 | 32.9039 | 40.9485 | 37.8727 | 18.5732 | 14.8411 |

|  | Glucose | Xylose | Arabinose | Acetate | Coumarate | Ferulate | YNB Oleic Acid | YNB Ricinoleic Acid | YNB Glucose | YNB Gluc DOC | YPD |
| --- | --- | --- | --- | --- | --- | --- | --- | --- | --- | --- | --- |
| proteinId |  |  |  |  |  |  |  |  |  |  |  |
| 11882 | 0.155027 | 0.00241144 | 0.151468 | -0.00673348 | 0.60387 | -0.0309193 | 0.675184 | 0.843 | 0.320601 | 0.27912 | 0.086355 |
| 12784 | -0.0694909 | -0.243761 | 0.0933115 | 0.0897106 | 0.117505 | -0.125559 | 0.0784752 | -0.493313 | -0.483602 | -0.36137 | -0.0103173 |
| 15285 | 0.39531 | 0.569787 | 0.226131 | 0.077798 | -0.720378 | 0.357177 | 0.959645 | 0.247658 | 1.22572 | 1.04478 | 0.365732 |
| 9774 | 0.0235694 | -0.71521 | -3.89519 | -0.087274 | -0.0710531 | -0.0880198 | -0.00217939 | -0.26233 | 0.0739166 | -0.149324 | -0.00933948 |

In [56]:

```
for x in temp:
    if x in model.genes:
        for r in sorted(model.genes.get_by_id(x).reactions, key=lambda x: x.id):
            print(r.id, r.reaction, r.gene_reaction_rule)
    else:
        print(x, 'no reactions')
    print()
```

```
ALR2 h_c + mthgxl_c + nadph_c --> acetol_c + nadp_c 11882 or 12784 or 15285 or 16543 or 9774
ALR3 acetol_c + h_c + nadph_c --> 12ppd__S_c + nadp_c 11882 or 12784 or 16543 or 9774
ARABR arab__L_c + h_c + nadph_c --> abt_c + nadp_c 11882 or 12784 or 16543 or 9774
LALDO2 h_c + mthgxl_c + nadph_c --> lald__D_c + nadp_c 11882 or 12784 or 16543 or 9774
LCARR h_c + lald__D_c + nadh_c --> 12ppd__R_c + nad_c 16543
PPDOy h_c + lald__D_c + nadph_c --> 12ppd__R_c + nadp_c 10029 or 11882 or 12784 or 13554 or 13562 or 13947 or 16543 or 9774
SBTR glc__D_c + h_c + nadph_c --> nadp_c + sbt__D_c 11882 or 12784 or 16543 or 9774

ALCD19y glyald_c + h_c + nadph_c --> glyc_c + nadp_c 11882 or 12784 or 9774
ALCD22yi 2mbald_c + h_c + nadph_c --> 2mbtoh_c + nadp_c 10029 or 11882 or 12784 or 13554 or 13562 or 13947 or 9774
ALCD2y etoh_c + nadp_c --> acald_c + h_c + nadph_c 11882 or 12784 or 9774
ALR2 h_c + mthgxl_c + nadph_c --> acetol_c + nadp_c 11882 or 12784 or 15285 or 16543 or 9774
ALR3 acetol_c + h_c + nadph_c --> 12ppd__S_c + nadp_c 11882 or 12784 or 16543 or 9774
ARABR arab__L_c + h_c + nadph_c --> abt_c + nadp_c 11882 or 12784 or 16543 or 9774
DKGLCNR1 25dkglcn_c + h_c + nadph_c --> 2dhguln_c + nadp_c 11882
GALOR gal_c + h_c + nadph_c <=> galt_c + nadp_c 11882 or 12784 or 9774
GLYCDy glyc_c + nadp_c --> dha_c + h_c + nadph_c 11882 or 12784 or 9774
LALDO2 h_c + mthgxl_c + nadph_c --> lald__D_c + nadp_c 11882 or 12784 or 16543 or 9774
PPDOy h_c + lald__D_c + nadph_c --> 12ppd__R_c + nadp_c 10029 or 11882 or 12784 or 13554 or 13562 or 13947 or 16543 or 9774
SBTR glc__D_c + h_c + nadph_c --> nadp_c + sbt__D_c 11882 or 12784 or 16543 or 9774

ALCD19y glyald_c + h_c + nadph_c --> glyc_c + nadp_c 11882 or 12784 or 9774
ALCD22yi 2mbald_c + h_c + nadph_c --> 2mbtoh_c + nadp_c 10029 or 11882 or 12784 or 13554 or 13562 or 13947 or 9774
ALCD2y etoh_c + nadp_c --> acald_c + h_c + nadph_c 11882 or 12784 or 9774
ALR2 h_c + mthgxl_c + nadph_c --> acetol_c + nadp_c 11882 or 12784 or 15285 or 16543 or 9774
ALR3 acetol_c + h_c + nadph_c --> 12ppd__S_c + nadp_c 11882 or 12784 or 16543 or 9774
ARABR arab__L_c + h_c + nadph_c --> abt_c + nadp_c 11882 or 12784 or 16543 or 9774
GALOR gal_c + h_c + nadph_c <=> galt_c + nadp_c 11882 or 12784 or 9774
GLYCDy glyc_c + nadp_c --> dha_c + h_c + nadph_c 11882 or 12784 or 9774
LALDO2 h_c + mthgxl_c + nadph_c --> lald__D_c + nadp_c 11882 or 12784 or 16543 or 9774
PPDOy h_c + lald__D_c + nadph_c --> 12ppd__R_c + nadp_c 10029 or 11882 or 12784 or 13554 or 13562 or 13947 or 16543 or 9774
SBTR glc__D_c + h_c + nadph_c --> nadp_c + sbt__D_c 11882 or 12784 or 16543 or 9774

ALR2 h_c + mthgxl_c + nadph_c --> acetol_c + nadp_c 11882 or 12784 or 15285 or 16543 or 9774

ALCD19y glyald_c + h_c + nadph_c --> glyc_c + nadp_c 11882 or 12784 or 9774
ALCD22yi 2mbald_c + h_c + nadph_c --> 2mbtoh_c + nadp_c 10029 or 11882 or 12784 or 13554 or 13562 or 13947 or 9774
ALCD2y etoh_c + nadp_c --> acald_c + h_c + nadph_c 11882 or 12784 or 9774
ALR2 h_c + mthgxl_c + nadph_c --> acetol_c + nadp_c 11882 or 12784 or 15285 or 16543 or 9774
ALR3 acetol_c + h_c + nadph_c --> 12ppd__S_c + nadp_c 11882 or 12784 or 16543 or 9774
ARABR arab__L_c + h_c + nadph_c --> abt_c + nadp_c 11882 or 12784 or 16543 or 9774
GALOR gal_c + h_c + nadph_c <=> galt_c + nadp_c 11882 or 12784 or 9774
GLYCDy glyc_c + nadp_c --> dha_c + h_c + nadph_c 11882 or 12784 or 9774
LALDO2 h_c + mthgxl_c + nadph_c --> lald__D_c + nadp_c 11882 or 12784 or 16543 or 9774
PPDOy h_c + lald__D_c + nadph_c --> 12ppd__R_c + nadp_c 10029 or 11882 or 12784 or 13554 or 13562 or 13947 or 16543 or 9774
SBTR glc__D_c + h_c + nadph_c --> nadp_c + sbt__D_c 11882 or 12784 or 16543 or 9774
```

In [57]:

```
model.reactions.get_by_id('ALR2').gene_reaction_rule = '11882 or 12784 or 9774'
model.reactions.get_by_id('ALR3').gene_reaction_rule = '11882 or 12784 or 9774'
model.reactions.get_by_id('ARABR').gene_reaction_rule = '11882 or 12784 or 9774'
model.reactions.get_by_id('LALDO2').gene_reaction_rule = '11882 or 12784 or 9774'
model.reactions.get_by_id('SBTR').gene_reaction_rule = '11882 or 12784 or 9774'
model.reactions.get_by_id('PPDOy').gene_reaction_rule = '10029 or 11882 or 12784 or 13554 or 13562 or 13947 or 9774'
model.remove_reactions(['LCARR'],remove_orphans=True)
```

In [58]:

```
temp = ['11952','13272','15007','15008']
display(Annotation.loc[temp])
Show_Data(temp)
```

|  | Combined Annotations | Signal P | Sc288c Orthologs | Human Orthologs | Sc288 Best Hit | Human Blast | Essential | WolfPSort | C Terminal |
| --- | --- | --- | --- | --- | --- | --- | --- | --- | --- |
| RTO4\_ID |  |  |  |  |  |  |  |  |  |
| 11952 | K00122: FDH; formate dehydrogenase |  | YOR388C | CTBP1,CTBP2 | FDH1 | PHGDH | Not Essential | cyto 11.5, extr 7, cyto\_nucl 6.5, mito 4, E.R. 3 | RTK\* |
| 13272 | K00367: narB; ferredoxin-nitrate reductase |  |  |  |  |  | Not Essential | cyto 11.5, cyto\_nucl 9, pero 7, nucl 5.5 | VAC\* |
| 15007 | K00362: nirB; nitrite reductase (NADH) large s... |  |  |  |  |  | Not Essential | cyto 24, cysk 2 | LEW\* |
| 15008 | K10534: NR; nitrate reductase (NAD(P)H) | S |  |  | PGA3 | CYB5R | Not Essential | mito 21, cyto 5 | VIF\* |

| strain | WT | | | | | | | | | | | | | | | | |
| --- | --- | --- | --- | --- | --- | --- | --- | --- | --- | --- | --- | --- | --- | --- | --- | --- | --- |
| condition | G\_MM | C\_MM | G\_SD | | GX\_SD | | | X\_SD | | A\_SD | | C\_SD | | MM\_CN120 | | MM\_CN5 | Diversity\_Sample |
| phase | exp | exp | exp | stat | exp | trans | stat | exp | stat | exp | stat | exp | stat | exp | stat | exp | exp |
| proteinId | Set1 | Set1 | Set2 | Set2 | Set2 | Set2 | Set2 | Set2 | Set2 | Set2 | Set2 | Set2 | Set2 | Set3 | Set3 | Set3 | Set3 |
| 11952 | 4.57567 | 7.27387 | 4.76383 | 8.53668 | 4.95554 | 7.00437 | 6.68261 | 5.18364 | 7.26494 | 7.31759 | 6.81568 | 1.47344 | 0.120661 | 3.45384 | 4.76656 | 3.95292 | 9.06122 |
| 13272 | 3.02443 | 2.50911 | 1.78546 | 2.44612 | 2.01004 | 1.04589 | 1.61621 | 0.55722 | 3.1837 | 0.789172 | 3.34266 | 0.937457 | 1.7181 | 0.726956 | 0.58165 | 1.77959 | 2.02696 |
| 15007 | 5.50837 | 2.9135 | 1.56512 | 1.40343 | 1.44328 | 1.98531 | 2.17451 | 0.966222 | 1.83827 | 0.973603 | 1.1798 | 2.11016 | 0.829466 | 3.8543 | 4.98892 | 1.30082 | 3.16838 |
| 15008 | 5.97049 | 3.88484 | 3.29302 | 3.89837 | 3.15466 | 4.28548 | 4.21181 | 3.58056 | 4.21685 | 3.56895 | 3.94672 | 4.20845 | 3.07188 | 5.36662 | 6.24016 | 3.0239 | 3.79289 |

| strain | WT | | | | | | | | | | |
| --- | --- | --- | --- | --- | --- | --- | --- | --- | --- | --- | --- |
| condition | G\_SD | | GX\_SD | | | X\_SD | | A\_SD | | C\_SD | |
| proteinId | exp | stat | exp | trans | stat | exp | stat | exp | stat | exp | stat |
| 11952 | 0.390932 | 14.9396 | 1.01051 | 17.0997 | 21.3445 | 26.6118 | 30.8528 | 24.7112 | 36.5614 | 3.43348 | 10.0021 |
| 15007 | 0 | 0 | 0 | 0 | 0.192891 | 0 | 0 | 0 | 0 | 0 | 0 |

|  | Glucose | Xylose | Arabinose | Acetate | Coumarate | Ferulate | YNB Oleic Acid | YNB Ricinoleic Acid | YNB Glucose | YNB Gluc DOC | YPD |
| --- | --- | --- | --- | --- | --- | --- | --- | --- | --- | --- | --- |
| proteinId |  |  |  |  |  |  |  |  |  |  |  |
| 11952 | -0.356403 | 0.0154854 | -0.188449 | -0.106291 | -0.000393056 | -0.34403 | 0.161914 | 0.25669 | -0.0745119 | 0.0596839 | 0.297454 |
| 13272 | -0.225078 | -0.0580958 | -0.17741 | -0.165378 | -0.114317 | -0.0735069 | -0.381231 | -0.444803 | -0.238885 | -0.277819 | -0.100003 |
| 15007 | 0.0642667 | -0.0678666 | 0.0192604 | 0.0280947 | 0.189085 | 0.0945154 | 0.0482013 | 0.0973294 | 0.17198 | 0.424331 | -0.0749723 |
| 15008 | -0.109736 | -0.129731 | -0.0835689 | -0.050578 | -0.0556491 | -0.0262034 | 0.0517597 | 0.281359 | 0.13525 | 0.164921 | -0.0497346 |

In [59]:

```
for x in temp:
    if x in model.genes:
        for r in sorted(model.genes.get_by_id(x).reactions, key=lambda x: x.id):
            print(r.id, r.reaction, r.gene_reaction_rule)
    else:
        print(x, 'no reactions')
    print()
```

```
FDH for_c + nad_c --> co2_c + nadh_c 11952 or (PP_0490 and PP_0491 and PP_0492) or (PP_2183 and PP_2184 and PP_2186 and 13272)

FDH for_c + nad_c --> co2_c + nadh_c 11952 or (PP_0490 and PP_0491 and PP_0492) or (PP_2183 and PP_2184 and PP_2186 and 13272)
FHL for_c + h_c --> co2_c + h2_c (b2719 and b2720 and b2721 and b2722 and b2723 and b2724 and 13272) or (b2481 and b2482 and b2483 and b2484 and b2485 and b2486 and b2487 and b2488 and b2489 and b2490 and 13272)

NTRIR2x 5.0 h_c + 3.0 nadh_c + no2_c --> 2.0 h2o_c + 3.0 nad_c + nh4_c (PP_1706 and 15007) or (b3366 and 15007)

NITR h_c + nadh_c + no3_c --> h2o_c + nad_c + no2_c 15008
```

In [60]:

```
for r in sorted(model.metabolites.get_by_id('no3_c').reactions, key=lambda x: x.id):
        print(r.id, r.reaction, r.gene_reaction_rule)
print()
for r in sorted(model.metabolites.get_by_id('no2_c').reactions, key=lambda x: x.id):
        print(r.id, r.reaction, r.gene_reaction_rule)
```

```
NITR h_c + nadh_c + no3_c --> h2o_c + nad_c + no2_c 15008
NO3t no3_e <=> no3_c 15006

NARK no2_c <=> no2_e 15006
NITR h_c + nadh_c + no3_c --> h2o_c + nad_c + no2_c 15008
NO2th no2_c <=> no2_h 15006
NTRIR2x 5.0 h_c + 3.0 nadh_c + no2_c --> 2.0 h2o_c + 3.0 nad_c + nh4_c (PP_1706 and 15007) or (b3366 and 15007)
```

11952 cyto FDH1 formate dehydrogenase (search hit by glyoxylate reductase)  
13272 cyto nitrate reductase narB (bacterial)  
15007 cyto nitrite reductase  
15008 mito (sigP) nitrate reductase

Change FDH genes to '11952'  
Change NITR genes to '13272 or 15008'
Change NTRIR2x genes to '15007'  
Remove FHL, NO2th

In [61]:

```
model.reactions.get_by_id('FDH').gene_reaction_rule = '11952'
model.reactions.get_by_id('NITR').gene_reaction_rule = '13272 or 15008'
model.reactions.get_by_id('NTRIR2x').gene_reaction_rule = '15007'
model.remove_reactions(['FHL','NO2th'],remove_orphans=True)
```

In [62]:

```
# GLYO1 gly_c + h2o_c + o2_c --> glx_c + h2o2_c + nh4_c 15449 or 15994
temp = ['15449','15994','11876']
display(Annotation.loc[temp])
Show_Data(temp)
```

|  | Combined Annotations | Signal P | Sc288c Orthologs | Human Orthologs | Sc288 Best Hit | Human Blast | Essential | WolfPSort | C Terminal |
| --- | --- | --- | --- | --- | --- | --- | --- | --- | --- |
| RTO4\_ID |  |  |  |  |  |  |  |  |  |
| 15449 | K00272: DDO; D-aspartate oxidase | S |  | DDO |  | DDOe | Not Essential | extr 14, cyto 6, mito 2, pero 2, E.R. 2 | SKL\* |
| 15994 | K00273: DAO, aao; D-amino-acid oxidase | S |  | DDO |  | DAOe | Not Essential | extr 21, E.R. 3, mito 2 | SKL\* |
| 11876 | K00273: DAO, aao; D-amino-acid oxidase |  |  |  |  | DDOe | Not Essential | mito 14, cyto 9, pero 3 | AKL\* |

| strain | WT | | | | | | | | | | | | | | | | |
| --- | --- | --- | --- | --- | --- | --- | --- | --- | --- | --- | --- | --- | --- | --- | --- | --- | --- |
| condition | G\_MM | C\_MM | G\_SD | | GX\_SD | | | X\_SD | | A\_SD | | C\_SD | | MM\_CN120 | | MM\_CN5 | Diversity\_Sample |
| phase | exp | exp | exp | stat | exp | trans | stat | exp | stat | exp | stat | exp | stat | exp | stat | exp | exp |
| proteinId | Set1 | Set1 | Set2 | Set2 | Set2 | Set2 | Set2 | Set2 | Set2 | Set2 | Set2 | Set2 | Set2 | Set3 | Set3 | Set3 | Set3 |
| 15449 | 6.1225 | 5.78668 | 6.44346 | 3.85059 | 6.94475 | 4.41766 | 4.57665 | 4.45656 | 3.93578 | 4.1826 | 4.2266 | 6.63182 | 7.66142 | 4.96215 | 4.95433 | 7.7273 | 5.76154 |
| 15994 | 6.51978 | 5.49754 | 5.70631 | 5.34674 | 5.72016 | 5.08648 | 5.10305 | 5.18769 | 4.98045 | 5.1336 | 5.29073 | 4.29946 | 3.8442 | 4.71473 | 3.94407 | 5.29857 | 4.41342 |
| 11876 | 3.65606 | 4.35629 | 3.76853 | 4.65057 | 3.69181 | 4.1704 | 4.15454 | 4.08782 | 3.90054 | 4.03363 | 4.34147 | 4.26817 | 4.03997 | 4.97051 | 5.31194 | 4.39063 | 4.56138 |

| strain | WT | | | | | | | | | | |
| --- | --- | --- | --- | --- | --- | --- | --- | --- | --- | --- | --- |
| condition | G\_SD | | GX\_SD | | | X\_SD | | A\_SD | | C\_SD | |
| proteinId | exp | stat | exp | trans | stat | exp | stat | exp | stat | exp | stat |
| 15449 | 0.378267 | 0 | 0.817423 | 0 | 0 | 0 | 0 | 0 | 0 | 1.71453 | 3.47682 |
| 15994 | 3.87171 | 2.68302 | 4.26751 | 2.2325 | 2.90691 | 4.68459 | 2.35837 | 2.70351 | 3.25813 | 2.13755 | 2.17732 |
| 11876 | 0 | 0 | 0 | 0 | 0 | 0 | 0.189187 | 0 | 0 | 0 | 0 |

|  | Glucose | Xylose | Arabinose | Acetate | Coumarate | Ferulate | YNB Oleic Acid | YNB Ricinoleic Acid | YNB Glucose | YNB Gluc DOC | YPD |
| --- | --- | --- | --- | --- | --- | --- | --- | --- | --- | --- | --- |
| proteinId |  |  |  |  |  |  |  |  |  |  |  |
| 15449 | 0.031875 | -0.210235 | 0.0691616 | 0.19753 | -0.106863 | -0.0201389 | 0.410968 | -0.267938 | 0.265497 | -0.037821 | -0.223983 |
| 15994 | 0.0735229 | 0.171963 | 0.39364 | 0.289509 | 0.389464 | 0.615552 | -0.264389 | 0.0114502 | -0.0151002 | 0.0532391 | -0.0110871 |
| 11876 | -0.122031 | -0.0199141 | -0.141354 | -0.100667 | -0.0500189 | -0.200556 | -0.198248 | 0.158059 | -0.120557 | -0.0800513 | 0.399097 |

In [63]:

```
for x in temp:
    if x in model.genes:
        for r in sorted(model.genes.get_by_id(x).reactions, key=lambda x: x.id):
            print(r.id, r.reaction, r.gene_reaction_rule)
    else:
        print(x, 'no reactions')
    print()
```

```
DASPO1p asp__D_x + h2o_x + o2_x --> h2o2_x + nh4_x + oaa_x 15449 or 15994
GLYO1 gly_c + h2o_c + o2_c --> glx_c + h2o2_c + nh4_c 15449 or 15994

DASPO1p asp__D_x + h2o_x + o2_x --> h2o2_x + nh4_x + oaa_x 15449 or 15994
GLYO1 gly_c + h2o_c + o2_c --> glx_c + h2o2_c + nh4_c 15449 or 15994

11876 no reactions
```

D-aspartate oxidase 1.4.3.1, acts on acidic AAs D-asp, D-glu  
11876, 15449  
D-amino-acid oxidase 1.4.3.3, acts on neutral and basic AAs, KEGG says 1.4.3.3 also acts on glycine  
15994

Change DASPO1p genes to '11876 or 15449'  
Add GLYOp from Recon Change GLYOp genes to '15994'  
Remove GLYO1

In [64]:

```
model.reactions.get_by_id('DASPO1p').gene_reaction_rule = '11876 or 15449 or 15994'
r = hsa.reactions.get_by_id('GLYOp').copy()
r.gene_reaction_rule = '15994'
model.add_reactions([r])
model.remove_reactions(['GLYO1'],remove_orphans=True)
```

In [65]:

```
for r in sorted(model.metabolites.get_by_id('mthgxl_c').reactions, key=lambda x: x.id):
        print(r.id, r.reaction, r.gene_reaction_rule)
print()
for r in sorted(model.metabolites.get_by_id('mthgxl_x').reactions, key=lambda x: x.id):
        print(r.id, r.reaction, r.gene_reaction_rule)
print()
for r in sorted(model.metabolites.get_by_id('lgt__S_c').reactions, key=lambda x: x.id):
        print(r.id, r.reaction, r.gene_reaction_rule)
print()
for r in sorted(model.metabolites.get_by_id('lgt__S_m').reactions, key=lambda x: x.id):
        print(r.id, r.reaction, r.gene_reaction_rule)
```

```
AACTOOR aact_c + h2o_c + o2_c --> h2o2_c + mthgxl_c + nh4_c 13959
ACTLMO acetol_c + h_c + nadph_c + o2_c --> 2.0 h2o_c + mthgxl_c + nadp_c 11057 or 12565 or 13289
ALR2 h_c + mthgxl_c + nadph_c --> acetol_c + nadp_c 11882 or 12784 or 9774
ALR2x h_c + mthgxl_c + nadh_c --> acetol_c + nad_c 11550 or 14369
GLYOX3 h2o_c + mthgxl_c --> h_c + lac__D_c 12381
GLYOX_1 lgt__S_c <=> gthrd_c + mthgxl_c 13265
LALDO2 h_c + mthgxl_c + nadph_c --> lald__D_c + nadp_c 11882 or 12784 or 9774
LALDO3 h_c + mthgxl_c + nadph_c --> lald__L_c + nadp_c 10497 or 11160 or 12151 or 12883
LGTHL gthrd_c + mthgxl_c --> lgt__S_c 13265

AMO aact_x + h2o_x + h_x + o2_x --> h2o2_x + mthgxl_x + nh4_x 15939

GLYOX h2o_c + lgt__S_c --> gthrd_c + h_c + lac__D_c 13041
GLYOX_1 lgt__S_c <=> gthrd_c + mthgxl_c 13265
LGTHL gthrd_c + mthgxl_c --> lgt__S_c 13265

GLYOXm h2o_m + lgt__S_m --> gthrd_m + h_m + lac__D_m 13041
```

In [66]:

```
temp = ['12381','13265','13041','16520','13959','10308','15939']
display(Annotation.loc[temp])
Show_Data(temp)
```

|  | Combined Annotations | Signal P | Sc288c Orthologs | Human Orthologs | Sc288 Best Hit | Human Blast | Essential | WolfPSort | C Terminal |
| --- | --- | --- | --- | --- | --- | --- | --- | --- | --- |
| RTO4\_ID |  |  |  |  |  |  |  |  |  |
| 12381 | HMMPfam:DJ-1/PfpI family:PF01965,HMMPfam:N-ter... |  | HSP31 |  | HSP31 |  | Not Essential | cyto 21, pero 4 | LGN\* |
| 13265 | K01759: GLO1, gloA; lactoylglutathione lyase |  | GLO1 | GLO1 | GLO1 | GLO1 | Not Essential | nucl 12, cyto 11, mito 3 | KGM\* |
| 13041 | K01069: E3.1.2.6, gloB; hydroxyacylglutathione... |  | GLO2,GLO4 | HAGH | GLO2 | HAGH | Not Essential | cyto 18, cyto\_nucl 12, mito 5 | NKG\* |
| 16520 | KOG2944: Glyoxalase |  |  |  | GLO1 | GLO1 | Not Essential | cyto 14, nucl 4, pero 3, cysk 3, mito 1, extr ... | EKH\* |
| 13959 | K11182: AOC1, ABP1; diamine oxidase | S |  | AOC2,AOC3 |  | AOC1 | Not Essential | extr 23, E.R. 2 | SIV\* |
| 10308 | K00276: AOC3, AOC2, tynA; primary-amine oxidase |  |  |  |  | AOC2 | Not Essential | mito 13.5, cyto\_mito 11.5, cyto 8.5, pero 2, c... | CGK\* |
| 15939 | K11450: KDM1A, AOF2, LSD1; lysine-specific his... | S | FMS1 |  | FMS1 | KDM1B | Not Essential | cyto 19, mito 4, extr 4 | ALL\* |

| strain | WT | | | | | | | | | | | | | | | | |
| --- | --- | --- | --- | --- | --- | --- | --- | --- | --- | --- | --- | --- | --- | --- | --- | --- | --- |
| condition | G\_MM | C\_MM | G\_SD | | GX\_SD | | | X\_SD | | A\_SD | | C\_SD | | MM\_CN120 | | MM\_CN5 | Diversity\_Sample |
| phase | exp | exp | exp | stat | exp | trans | stat | exp | stat | exp | stat | exp | stat | exp | stat | exp | exp |
| proteinId | Set1 | Set1 | Set2 | Set2 | Set2 | Set2 | Set2 | Set2 | Set2 | Set2 | Set2 | Set2 | Set2 | Set3 | Set3 | Set3 | Set3 |
| 12381 | 9.32121 | 8.57203 | 9.66446 | 9.52849 | 9.51541 | 8.30505 | 8.59746 | 8.32684 | 8.80683 | 7.66164 | 7.30112 | 7.992 | 7.44688 | 9.64137 | 9.563 | 8.16632 | 9.81655 |
| 13265 | 7.2015 | 7.80303 | 7.09486 | 7.61599 | 7.16645 | 7.13492 | 7.023 | 7.27315 | 6.55851 | 7.26113 | 6.68156 | 7.31305 | 6.87529 | 6.16306 | 6.5693 | 5.59852 | 7.11009 |
| 13041 | 6.81725 | 7.10231 | 5.84298 | 7.47232 | 5.78491 | 6.44631 | 6.57302 | 5.93472 | 6.49338 | 6.21389 | 6.57323 | 6.5248 | 6.13513 | 5.15084 | 5.80909 | 4.2897 | 4.95039 |
| 16520 | 3.37914 | 4.2603 | 3.2019 | 4.06907 | 2.82205 | 3.17436 | 3.20266 | 3.20677 | 3.76993 | 3.8244 | 4.15439 | 3.90875 | 3.10343 | 2.90832 | 2.79097 | 2.43094 | 2.74808 |
| 13959 | 8.41303 | 2.52398 | 4.38379 | 2.42129 | 4.66271 | 1.80071 | 2.42655 | 1.4511 | 2.03383 | 1.25304 | 2.43998 | 2.38103 | 4.094 | 10.8349 | 9.92979 | 7.10287 | 9.10938 |
| 10308 | 2.86399 | 3.45578 | 3.22767 | 5.58837 | 3.10407 | 2.05203 | 3.18883 | 2.77963 | 3.63453 | 2.51632 | 3.88029 | 4.31793 | 3.63946 | 2.21286 | 1.83573 | 2.9379 | 2.57152 |
| 15939 | 4.57229 | 5.15652 | 4.6989 | 4.82628 | 4.76811 | 4.43851 | 4.35223 | 4.73077 | 4.46523 | 4.80015 | 4.74923 | 5.19912 | 4.56138 | 3.46246 | 3.32524 | 3.74167 | 3.76899 |

| strain | WT | | | | | | | | | | |
| --- | --- | --- | --- | --- | --- | --- | --- | --- | --- | --- | --- |
| condition | G\_SD | | GX\_SD | | | X\_SD | | A\_SD | | C\_SD | |
| proteinId | exp | stat | exp | trans | stat | exp | stat | exp | stat | exp | stat |
| 12381 | 8.65192 | 14.9124 | 10.3776 | 10.7883 | 13.4941 | 10.9831 | 13.1609 | 7.35886 | 8.60536 | 17.5303 | 13.0501 |
| 13265 | 3.06843 | 5.41866 | 3.86187 | 5.22416 | 5.477 | 6.07926 | 6.46058 | 4.64607 | 4.97384 | 5.988 | 6.97931 |
| 13041 | 0.572723 | 2.72977 | 0.204847 | 2.02798 | 4.33751 | 5.3144 | 4.11252 | 2.51631 | 1.34417 | 8.10748 | 8.71218 |
| 15939 | 1.13435 | 0.924143 | 0.616433 | 1.30072 | 1.27049 | 2.13974 | 2.78393 | 1.92747 | 2.68362 | 3.19072 | 3.49228 |

|  | Glucose | Xylose | Arabinose | Acetate | Coumarate | Ferulate | YNB Oleic Acid | YNB Ricinoleic Acid | YNB Glucose | YNB Gluc DOC | YPD |
| --- | --- | --- | --- | --- | --- | --- | --- | --- | --- | --- | --- |
| proteinId |  |  |  |  |  |  |  |  |  |  |  |
| 12381 | -0.410772 | -0.68355 | -0.322993 | -0.329437 | -1.46935 | -0.177134 | 0.238126 | 1.17331 | -0.0468796 | 0.580134 | 1.78305 |
| 13265 | -0.557694 | -0.0572244 | -0.640371 | -0.998739 | -0.935246 | -0.270976 | 0.0683897 | -0.91845 | 0.301362 | 0.358795 | -0.368673 |
| 13041 | -0.465208 | 0.0557896 | -0.0945668 | -0.785122 | -0.28307 | -0.417524 | 1.07073 | 0.171952 | 0.531161 | 0.250705 | -0.0515562 |
| 16520 | -0.0579346 | -0.38443 | 0.0950234 | -0.0807645 | -0.386138 | -0.236659 | 0.242956 | -0.177448 | 0.0819643 | 0.118931 | 0.085333 |
| 13959 | 0.0611431 | -0.102722 | -0.0778613 | -0.209695 | -0.169321 | -0.209236 | 0.133836 | 0.259305 | 0.034285 | 0.0318075 | 0.199766 |
| 10308 | -0.015976 | -0.0984928 | 0.0423567 | 0.10555 | 0.0916015 | -0.11559 | -0.00433155 | 0.293239 | 0.0480233 | 0.108416 | -0.186236 |
| 15939 | -0.904433 | -0.501351 | -1.22538 | -0.906687 | -1.9922 | -0.59751 | 1.06478 | -0.696643 | 0.98022 | 1.29529 | 0.49661 |

In [67]:

```
for r in sorted(model.genes.get_by_id('12381').reactions, key=lambda x: x.id):
    print(r.id, r.reaction, r.gene_reaction_rule)
print()
for r in sorted(model.genes.get_by_id('13265').reactions, key=lambda x: x.id):
    print(r.id, r.reaction, r.gene_reaction_rule)
print()
for r in sorted(model.genes.get_by_id('13041').reactions, key=lambda x: x.id):
    print(r.id, r.reaction, r.gene_reaction_rule)
print()
for r in sorted(model.genes.get_by_id('13959').reactions, key=lambda x: x.id):
    print(r.id, r.reaction, r.gene_reaction_rule)
print()
for r in sorted(model.genes.get_by_id('10308').reactions, key=lambda x: x.id):
    print(r.id, r.reaction, r.gene_reaction_rule)
print()
for r in sorted(model.genes.get_by_id('15939').reactions, key=lambda x: x.id):
    print(r.id, r.reaction, r.gene_reaction_rule)
```

```
GLYOX3 h2o_c + mthgxl_c --> h_c + lac__D_c 12381

GLYOX_1 lgt__S_c <=> gthrd_c + mthgxl_c 13265
LGTHL gthrd_c + mthgxl_c --> lgt__S_c 13265

GLYOX h2o_c + lgt__S_c --> gthrd_c + h_c + lac__D_c 13041
GLYOXm h2o_m + lgt__S_m --> gthrd_m + h_m + lac__D_m 13041

13DAMPPOX 13dampp_c + h2o_c + o2_c --> bamppald_c + h2o2_c + nh4_c 13959
42A12BOOX dopa_c + h2o_c + o2_c --> 34dhpac_c + h2o2_c + nh4_c 10308 or 13959
AACTOOR aact_c + h2o_c + o2_c --> h2o2_c + mthgxl_c + nh4_c 13959
AO h2o_c + hista_c + o2_c --> h2o2_c + h_c + im4act_c + nh4_c 13959
HISTASE h2o_c + hista_c + o2_c --> h2o2_c + im4act_c + nh4_c 13959
MAOX h2o_c + mma_c + o2_c --> fald_c + h2o2_c + nh4_c 10308 or 13959
MHISOR h2o_c + mhista_c + o2_c --> 3mldz_c + h2o2_c + nh4_c 13959
NMPTRCOX nmptrc_c + o2_c --> 1mpyr_c + h2o2_c + nh4_c 13959
PEAMNO h2o_c + o2_c + peamn_c --> h2o2_c + nh4_c + pacald_c 10308 or 13959
PTOR h2o_m + o2_m + ptrc_m <=> 4abutn_m + h2o2_m + nh4_m 13959
PTRCOX1 h2o_c + o2_c + ptrc_c --> 4abutn_c + h2o2_c + nh4_c 13959
TRYPTAOX h2o_c + o2_c + trypta_c --> h2o2_c + id3acald_c + nh4_c 13959

42A12BOOX dopa_c + h2o_c + o2_c --> 34dhpac_c + h2o2_c + nh4_c 10308 or 13959
42A12BOOXpp dopa_p + h2o_p + o2_p --> 34dhpac_p + h2o2_p + nh4_p 10308
MAOX h2o_c + mma_c + o2_c --> fald_c + h2o2_c + nh4_c 10308 or 13959
PAO 13dampp_h + h2o_h + o2_h --> bamppald_h + h2o2_h + nh4_h 10308
PEAMNO h2o_c + o2_c + peamn_c --> h2o2_c + nh4_c + pacald_c 10308 or 13959
PEAMNOpp h2o_p + o2_p + peamn_p --> h2o2_p + nh4_p + pacald_p 10308
TYROXDApp h2o_p + o2_p + tym_p --> 4hoxpacd_p + h2o2_p + nh4_p 10308
yli_R0078 h2o_c + o2_c + yli_M00858_c --> aproa_c + h2o2_c + nh4_c 10308

AMO aact_x + h2o_x + h_x + o2_x --> h2o2_x + mthgxl_x + nh4_x 15939
CITALOR cital_c + h2o_c + o2_c --> citalald_c + dma_c + h2o2_c 15939
DDMCITALOR ddmcital_c + h2o_c + o2_c --> citalald_c + h2o2_c + nh4_c 15939
DMCITALOR dmcital_c + h2o_c + o2_c --> citalald_c + h2o2_c + mma_c 15939
POLYAO N1aspmd_c + h2o_c + o2_c --> aproa_c + aprut_c + h2o2_c 15939
POLYAO2 N1sprm_c + h2o_c + o2_c --> N1aspmd_c + aproa_c + h2o2_c 15939
POLYAO3 h2o_c + o2_c + sprm_c --> aproa_c + h2o2_c + spmd_c 15939
```

In [68]:

```
for r in sorted(model.metabolites.get_by_id('bamppald_c').reactions, key=lambda x: x.id):
        print(r.id, r.reaction, r.gene_reaction_rule)
print()
for r in sorted(model.metabolites.get_by_id('bamppald_m').reactions, key=lambda x: x.id):
        print(r.id, r.reaction, r.gene_reaction_rule)
print()
for r in sorted(model.metabolites.get_by_id('bamppald_h').reactions, key=lambda x: x.id):
        print(r.id, r.reaction, r.gene_reaction_rule)
print()
for r in sorted(model.metabolites.get_by_id('aproa_c').reactions, key=lambda x: x.id):
        print(r.id, r.reaction, r.gene_reaction_rule)
```

```
13DAMPPOX 13dampp_c + h2o_c + o2_c --> bamppald_c + h2o2_c + nh4_c 13959
BAMPPALDOX bamppald_c + h2o_c + nad_c --> ala_B_c + 2.0 h_c + nadh_c 12042 or 13426 or 15814 or 16323

BAMPPALDOXm bamppald_m + h2o_m + nad_m --> ala_B_m + 2.0 h_m + nadh_m 12042 or 13426 or 8569

APOR bamppald_h + h2o_h + nad_h --> ala_B_h + 2.0 h_h + nadh_h 12042 or 13426
PAO 13dampp_h + h2o_h + o2_h --> bamppald_h + h2o2_h + nh4_h 10308

ALDD22x aproa_c + h2o_c + nad_c --> ala_B_c + 2.0 h_c + nadh_c 12042 or 13426 or 16323
POLYAO N1aspmd_c + h2o_c + o2_c --> aproa_c + aprut_c + h2o2_c 15939
POLYAO2 N1sprm_c + h2o_c + o2_c --> N1aspmd_c + aproa_c + h2o2_c 15939
POLYAO3 h2o_c + o2_c + sprm_c --> aproa_c + h2o2_c + spmd_c 15939
yli_R0078 h2o_c + o2_c + yli_M00858_c --> aproa_c + h2o2_c + nh4_c 10308
```

GLYOX3 h2o\_c + mthgxl\_c --> h\_c + lac\_\_D\_c 12381  
GLYOX3 is lumped reaction and 12381 is heat shock protein

LGTHL gthrd\_c + mthgxl\_c --> lgt**S\_c 13265  
GLYOX h2o\_c + lgt**S\_c --> gthrd\_c + h\_c + lac\_\_D\_c 13041

GLYOX\_1 lgt**S\_c <=> gthrd\_c + mthgxl\_c 13265  
GLYOXm h2o\_m + lgt**S\_m --> gthrd\_m + h\_m + lac\_\_D\_m 13041

Glyoxalase / glyoxylase  
13265 GLO1 nucl12 cyto 11 [c] : gthrd + mthgxl --> lgt-S (glyoxalase I)  
13041 GLO2, GLO4 cyto [c] : h2o + lgt-S --> gthrd + h + lac-D (glyoxalase II)  
16520 GLO1 cyto glyoxalase I, no rxn

Change LGTHL genes to '13265 or 16520'  
Remove GLYOX3, GLYOX\_1, GLYOXm  
Remove AMO (same as AACTOOR, but in pero with extra proton)  
Remove AO (same as HISTASE, but with extra proton)  
Remove PTOR (same as PTRCOX1, but in mito)  
Remove 42A12BOOXpp, PAO, PEAMNOpp (wrong comp)  
Replace TYROXDApp with TYROXDAc from Recon1  
bamppald is same as aproa, replace aproa\_c with bamppald\_c  
Remove APOR, ALDD22x (same as BAMPPALDOX), yli\_R0078 (same as 13DAMPPOX, 13959 is correct)  
Remove citalopram reactions - irrelevant and no direct annotation

In [69]:

```
model.reactions.get_by_id('LGTHL').gene_reaction_rule = '13265 or 16520'
model.remove_reactions(['GLYOX3','GLYOX_1','GLYOXm'],remove_orphans=True)
model.remove_reactions(['AMO','AO','PTOR','42A12BOOXpp','PAO','PEAMNOpp','TYROXDApp'],remove_orphans=True)
r = hsa.reactions.get_by_id('TYROXDAc').copy()
r.gene_reaction_rule = '10308 or 13959'
model.add_reactions([r])
model.remove_reactions(['APOR','ALDD22x','yli_R0078'],remove_orphans=True)
model.reactions.get_by_id('POLYAO').add_metabolites({'aproa_c': -1.0, 'bamppald_c': 1.0})
model.reactions.get_by_id('POLYAO2').add_metabolites({'aproa_c': -1.0, 'bamppald_c': 1.0})
model.reactions.get_by_id('POLYAO3').add_metabolites({'aproa_c': -1.0, 'bamppald_c': 1.0})
model.remove_reactions(['CITALOR','DDMCITALOR','DMCITALOR','CITALALDOR'],remove_orphans=True)
```

In [70]:

```
# glyoxylate shunt
for r in sorted(model.genes.get_by_id('14022').reactions, key=lambda x: x.id):
    print(r.id, r.reaction, r.gene_reaction_rule)
print()
for r in sorted(model.genes.get_by_id('14162').reactions, key=lambda x: x.id):
    print(r.id, r.reaction, r.gene_reaction_rule)
print()
for r in sorted(model.genes.get_by_id('9457').reactions, key=lambda x: x.id):
    print(r.id, r.reaction, r.gene_reaction_rule)
```

```
ICL icit_c --> glx_c + succ_c 14022 or 14162
ICL_1 icit_m --> glx_m + succ_m 14022 or 14162
MCITL2m micit_m --> pyr_m + succ_m 14022 or 14162
yli_R1480 icit_x --> glx_x + succ_x 14022 or 14162

ICL icit_c --> glx_c + succ_c 14022 or 14162
ICL_1 icit_m --> glx_m + succ_m 14022 or 14162
MCITL2m micit_m --> pyr_m + succ_m 14022 or 14162
yli_R1480 icit_x --> glx_x + succ_x 14022 or 14162

MALS accoa_c + glx_c + h2o_c --> coa_c + h_c + mal__L_c 9457
MALSh accoa_h + glx_h + h2o_h --> coa_h + h_h + mal__L_h 9457
MALSm accoa_m + glx_m + h2o_m --> coa_m + h_m + mal__L_m 9457
MALSp accoa_x + glx_x + h2o_x --> coa_x + h_x + mal__L_x 9457
yli_R0672 accoa_m + glx_m + h2o_m --> coa_m + h_c + mal__L_m 9457
yli_R1434 accoa_x + glx_x + h2o_x --> coa_x + h_c + mal__L_x 9457
```

In [71]:

```
temp = ['14022','14162','9457','11129']
display(Annotation.loc[temp])
Show_Data(temp)
```

|  | Combined Annotations | Signal P | Sc288c Orthologs | Human Orthologs | Sc288 Best Hit | Human Blast | Essential | WolfPSort | C Terminal |
| --- | --- | --- | --- | --- | --- | --- | --- | --- | --- |
| RTO4\_ID |  |  |  |  |  |  |  |  |  |
| 14022 | K01637: E4.1.3.1, aceA; isocitrate lyase |  | ICL1,ICL2 |  | ICL1 |  | Not Essential | cyto\_nucl 9.5, cyto 8.5, nucl 7.5, pero 7, mito 2 | QFK\* |
| 14162 | K01637: E4.1.3.1, aceA; isocitrate lyase | S | ICL1,ICL2 |  | ICL2 |  | Not Essential | mito 26 | HTF\* |
| 9457 | K01638: aceB, glcB; malate synthase |  | DAL7,MLS1 |  | MLS1 |  | Not Essential | cyto\_pero 12, cyto 11.5, pero 11.5, nucl 3 | AKL\* |
| 11129 | K00031: IDH1, IDH2, icd; isocitrate dehydrogenase |  | IDP1,IDP2,IDP3 | IDH1,IDH2 | IDP1 | IDH2 | Not Essential | cyto 13, pero 8, nucl 4 | PKL\* |

| strain | WT | | | | | | | | | | | | | | | | |
| --- | --- | --- | --- | --- | --- | --- | --- | --- | --- | --- | --- | --- | --- | --- | --- | --- | --- |
| condition | G\_MM | C\_MM | G\_SD | | GX\_SD | | | X\_SD | | A\_SD | | C\_SD | | MM\_CN120 | | MM\_CN5 | Diversity\_Sample |
| phase | exp | exp | exp | stat | exp | trans | stat | exp | stat | exp | stat | exp | stat | exp | stat | exp | exp |
| proteinId | Set1 | Set1 | Set2 | Set2 | Set2 | Set2 | Set2 | Set2 | Set2 | Set2 | Set2 | Set2 | Set2 | Set3 | Set3 | Set3 | Set3 |
| 14022 | 4.6865 | 7.31059 | 8.98622 | 7.36314 | 9.16563 | 7.82857 | 8.00893 | 7.72855 | 8.21391 | 6.12419 | 7.44567 | 7.05071 | 8.07862 | 6.22935 | 7.5451 | 10.1141 | 6.86669 |
| 14162 | 5.97913 | 5.90127 | 8.37789 | 5.48236 | 8.34099 | 6.41778 | 6.10498 | 7.34865 | 6.09534 | 5.70719 | 5.82756 | 5.91579 | 6.65088 | 6.07938 | 5.94597 | 7.06487 | 6.11391 |
| 9457 | 6.68318 | 7.22895 | 8.58027 | 7.16444 | 8.71042 | 7.64187 | 7.67559 | 7.55327 | 7.80155 | 6.6003 | 7.15658 | 6.86652 | 6.30312 | 8.15008 | 8.4776 | 8.68898 | 7.68689 |
| 11129 | 7.16101 | 9.69917 | 9.09782 | 9.1897 | 9.10341 | 8.25755 | 8.17244 | 9.13677 | 8.14724 | 8.15581 | 7.36988 | 9.45626 | 9.137 | 8.17518 | 7.65132 | 9.33382 | 9.10174 |

| strain | WT | | | | | | | | | | |
| --- | --- | --- | --- | --- | --- | --- | --- | --- | --- | --- | --- |
| condition | G\_SD | | GX\_SD | | | X\_SD | | A\_SD | | C\_SD | |
| proteinId | exp | stat | exp | trans | stat | exp | stat | exp | stat | exp | stat |
| 14022 | 28.9649 | 36.0553 | 32.5962 | 34.2525 | 32.1963 | 21.6915 | 34.5075 | 14.3133 | 21.2862 | 39.8994 | 40.7649 |
| 14162 | 35.1016 | 33.9706 | 35.9799 | 34.259 | 35.7012 | 31.1206 | 37.6102 | 22.9865 | 23.5662 | 33.1761 | 27.9325 |
| 9457 | 35.4255 | 32.7945 | 39.2524 | 39.9616 | 42.0678 | 28.1449 | 35.6202 | 25.7065 | 23.3625 | 47.2338 | 42.4634 |
| 11129 | 50.0393 | 47.3779 | 46.9643 | 37.7576 | 36.8082 | 43.7864 | 48.1441 | 41.5317 | 40.1972 | 59.3245 | 57.0295 |

|  | Glucose | Xylose | Arabinose | Acetate | Coumarate | Ferulate | YNB Oleic Acid | YNB Ricinoleic Acid | YNB Glucose | YNB Gluc DOC | YPD |
| --- | --- | --- | --- | --- | --- | --- | --- | --- | --- | --- | --- |
| proteinId |  |  |  |  |  |  |  |  |  |  |  |
| 14022 | -0.0684361 | -0.0406226 | 0.00566276 | -3.53877 | -0.672274 | -0.646294 | -3.49352 | -4.31542 | -0.143782 | -0.190331 | 0.0376189 |
| 14162 | -0.351181 | 0.486327 | -2.48228 | -3.32582 | -2.68378 | -3.7193 | -0.577084 | -0.579107 | 0.265499 | 0.289154 | -0.0659644 |
| 9457 | -0.0285278 | 0.126065 | -0.0124983 | -1.86103 | -0.467399 | -0.545453 | -2.92614 | -2.60194 | -0.143079 | -0.0561765 | -0.23821 |
| 11129 | -0.393612 | 0.510054 | -0.255185 | -0.254103 | 0.183252 | -0.0410789 | 0.33642 | 0.792335 | -1.65387 | -0.460691 | -0.0902951 |

Isocitrate lyase  
Check sequence alignment to ICL and MICL  
https://www.ncbi.nlm.nih.gov/pubmed/16151139
14022 pero ICL1 cytosolic ICL in S. cer  
14162 mito ICL2 mitochondrial MCITL in S. cer

ICL1 cyto in S. cer, but pero in other, blast matches are annotated as pero in uniprot  
Fatty acid beta-oxidation and glyoxylate cycle  
https://www.ncbi.nlm.nih.gov/pmc/articles/PMC1167536/  
Add peroxisomal citrate, isocitrate, and succinate transport reactions

Malate synthase  
9457 cyto\_pero DAL7, MLS1

Change ICL genes to '14022'  
Change MCITL2m genes to '14162'  
Remove ICL\_1, yli\_R1480, MALSh, MALSm, yli\_R0672, yli\_R1434

In [72]:

```
r = model.reactions.get_by_id('ICL')
r.id = 'ICLp'
r.gene_reaction_rule = '14022'
for m in r.metabolites:
    if not m.id.replace('_c','_x') in model.metabolites:
        m2 = m.copy()
        m2.id = m.id.replace('_c','_x')
        m2.compartment = 'x'
        model.add_metabolites([m2])
    r.add_metabolites({m.id: -r.get_coefficient(m.id), m.id.replace('_c','_x'): r.get_coefficient(m.id)})
r = hsa2.reactions.get_by_id('SUCCtp').copy()
model.add_reactions([r])
r = hsa2.reactions.get_by_id('SUCCtp').copy()
r.id = 'CITtp'
r.name = 'Citrate transporter, peroxisome'
model.add_reactions([r])
r.add_metabolites({'succ_c': 1.0, 'succ_x': -1.0, 'cit_c': -1.0, 'cit_x': 1.0})
r = hsa2.reactions.get_by_id('SUCCtp').copy()
r.id = 'ICITtp'
r.name = 'Isocitrate transporter, peroxisome'
model.add_reactions([r])
r.add_metabolites({'succ_c': 1.0, 'succ_x': -1.0, 'icit_c': -1.0, 'icit_x': 1.0})

model.reactions.get_by_id('MCITL2m').gene_reaction_rule = '14162'
model.remove_reactions(['ICL_1','yli_R1480','MALS','MALSh','MALSm','yli_R0672','yli_R1434'],remove_orphans=True)
```

In [73]:

```
print('ficytc')
for r in sorted(model.metabolites.get_by_id('ficytc_m').reactions, key=lambda x: x.id):
    print(r.id + '   \t', r.reaction)
print()
for r in sorted(model.metabolites.get_by_id('ficytc_m').reactions, key=lambda x: x.id):
    print(r.id + '   \t', r.gene_reaction_rule)
print()
print('focytc')
for r in model.metabolites.get_by_id('focytc_m').reactions:
    if r not in model.metabolites.get_by_id('ficytc_m').reactions:
        print(r.id + '  ', '\t', r.reaction, r.gene_reaction_rule)
```

```
ficytc
CCP2m   	 2.0 focytc_m + h2o2_m --> 2.0 ficytc_m + 2.0 h2o_m
CYOO6m   	 4.0 focytc_m + 8.0 h_m + o2_m --> 4.0 ficytc_m + 2.0 h2o_m + 4.0 h_c
CYOOm   	 4.0 focytc_m + 6.0 h_m + o2_m --> 4.0 ficytc_m + 2.0 h2o_m + 6.0 h_c
CYOOm3   	 4.0 focytc_m + 7.92 h_m + o2_m --> 4.0 ficytc_m + 1.96 h2o_m + 4.0 h_c + 0.02 o2s_m
CYOR_q8_m   	 2.0 ficytc_m + 2.0 h_m + q8h2_m --> 2.0 focytc_m + 4.0 h_c + q8_m
CYOR_u10m   	 2.0 ficytc_m + 2.0 h_m + q10h2_m --> 2.0 focytc_m + 4.0 h_c + q10_m
CYOR_u6m   	 2.0 ficytc_m + 1.5 h_m + q6h2_m --> 2.0 focytc_m + 1.5 h_c + q6_m
D_LACDcm   	 2.0 ficytc_m + lac__D_c --> 2.0 focytc_m + 2.0 h_c + pyr_c
L_LACDcm   	 2.0 ficytc_m + lac__L_c --> 2.0 focytc_m + 2.0 h_c + pyr_c
SULFOX   	 2.0 ficytc_m + h2o_c + so3_c --> 2.0 focytc_m + 2.0 h_c + so4_c
yli_R0431   	 2.0 ficytc_m + 1.5 h_m + yli_M04633_m --> 2.0 focytc_m + 1.5 h_c + q6_m

CCP2m   	 (10811 and 8802) or (13161 and 8802) or (13161 and 9198)
CYOO6m   	 (CRv4_Au5_s1_g1958_t1 and CRv4_Au5_s3_g10243_t1 and CRv4_Au5_s4_g11710_t1 and 11317 and 8802) or (CRv4_Au5_s1_g1958_t1 and CRv4_Au5_s3_g10243_t1 and CRv4_Au5_s4_g11710_t1 and 11317 and 9198)
CYOOm   	 Q0045 and Q0250 and Q0275 and YLR395C and YMR256C and 10685 and 11317 and 11769 and 13007 and 13628 and 8802 and 9165
CYOOm3   	 COX1 and COX2 and COX3 and COX4I1 and COX4I2 and COX6A1 and COX6A2 and COX6C and COX7A1 and COX7A2 and COX7A2L and COX7B and COX7B2 and COX7C and COX8A and COX8C and 10685 and 11317 and 9165
CYOR_q8_m   	 (13614 and 15681 and 8802) or (13614 and 15681 and 9198)
CYOR_u10m   	 (CYTB and UQCR11 and UQCRH and UQCRQ and 11618 and 13614 and 15231 and 15681 and 9198 and 9705) or (CYTB and Uqcr11 and Uqcrq and mmu_100042918 and 11618 and 13614 and 15231 and 15681 and 9198 and 9705)
CYOR_u6m   	 Q0105 and YBL045C and 11618 and 12966 and 13608 and 13614 and 15231 and 15681 and 15758 and 8802 and 9198
D_LACDcm   	 (13239 and 8802) or (13328 and 8802)
L_LACDcm   	 9113 and 8802
SULFOX   	 16758
yli_R0431   	 13614

focytc
```

In [74]:

```
print('ficytc')
for r in sorted(model.metabolites.get_by_id('ficytc_c').reactions, key=lambda x: x.id):
    print(r.id + '   \t', r.reaction)
print()
for r in sorted(model.metabolites.get_by_id('ficytc_c').reactions, key=lambda x: x.id):
    print(r.id + '   \t', r.gene_reaction_rule)
print()
print('focytc')
for r in model.metabolites.get_by_id('focytc_c').reactions:
    if r not in model.metabolites.get_by_id('ficytc_c').reactions:
        print(r.id + '  ', '\t', r.reaction, r.gene_reaction_rule)
```

```
ficytc
CYO1_KT   	 2.0 ficytc_c + 2.0 h_c + q8h2_c --> 2.0 focytc_c + 2.0 h_p + q8_c
CYO1b   	 2.0 focytc_c + 0.5 o2_c --> 2.0 ficytc_c + h2o_c

CYO1_KT   	 PP_1318 and 13614 and 9198
CYO1b   	 (PP_0103 and PP_0104 and PP_0106 and 8721) or (PP_4250 and PP_4251 and PP_4252 and PP_4253) or (PP_4255 and PP_4256 and PP_4257 and PP_4258)

focytc
```

#### Cytochrome C and related reactions¶

In [75]:

```
for r in sorted(model.genes.get_by_id('10811').reactions, key=lambda x: x.id):
    print(r.id, r.reaction, r.gene_reaction_rule)
print()
for r in sorted(model.genes.get_by_id('13161').reactions, key=lambda x: x.id):
    print(r.id, r.reaction, r.gene_reaction_rule)
```

```
CCP2m 2.0 focytc_m + h2o2_m --> 2.0 ficytc_m + 2.0 h2o_m (10811 and 8802) or (13161 and 8802) or (13161 and 9198)

CCP2m 2.0 focytc_m + h2o2_m --> 2.0 ficytc_m + 2.0 h2o_m (10811 and 8802) or (13161 and 8802) or (13161 and 9198)
```

In [76]:

```
print(ptri.reactions.get_by_id('CCP2_m'))
print()
for r in sorted(model.metabolites.get_by_id('ficytc_c').reactions, key=lambda x: x.id):
        print(r.id, r.reaction, r.gene_reaction_rule)
```

```
CCP2_m: 2.0 focytc_m + h2o2_m + 2.0 h_m --> 2.0 ficytc_m + 2.0 h2o_m

CYO1_KT 2.0 ficytc_c + 2.0 h_c + q8h2_c --> 2.0 focytc_c + 2.0 h_p + q8_c PP_1318 and 13614 and 9198
CYO1b 2.0 focytc_c + 0.5 o2_c --> 2.0 ficytc_c + h2o_c (PP_0103 and PP_0104 and PP_0106 and 8721) or (PP_4250 and PP_4251 and PP_4252 and PP_4253) or (PP_4255 and PP_4256 and PP_4257 and PP_4258)
```

10811 cyto 11, mito 8, cyto\_nucl 8, pero 5 HMMPfam:Peroxidase:PF00141,PRINTS:Haem peroxidase superfamily signature:PR00458,PRINTS:Plant ascorbate peroxidase signature:PR00459,ProSitePatterns:Peroxidases proximal heme-ligand signature.:PS00435,ProSitePatterns:Peroxidases active site signature.:PS00436,ProSiteProfiles:Plant heme peroxidase family profile.:PS50873,SUPERFAMILY::SSF48113 SKM*13161 mito 21, extr 6 HMMPfam:Peroxidase:PF00141,PRINTS:Haem peroxidase superfamily signature:PR00458,PRINTS:Plant ascorbate peroxidase signature:PR00459,ProSitePatterns:Peroxidases proximal heme-ligand signature.:PS00435,ProSitePatterns:Peroxidases active site signature.:PS00436,ProSiteProfiles:Plant heme peroxidase family profile.:PS50873,SUPERFAMILY::SSF48113 ASA*

10811 is cytochrome c peroxidase without sigpep  
13161 is mito cytochrome c peroxidase

CCP2\_m from iLB1027\_lipid has 2 protons consistent with metacyc  
Replace CCP2m with CCP2\_m  
Change CCP2\_m genes to '(10811 and 8802) or (13161 and 8802)'

Remove cytosolic CYO1\_KT and CYO1b from P. putida  
8721 is COX11 cytochrome c oxidase assembly protein subunit 11

In [77]:

```
r = ptri.reactions.get_by_id('CCP2_m').copy()
r.gene_reaction_rule = '(10811 and 8802) or (13161 and 8802)'
model.add_reactions([r])
model.remove_reactions(['CCP2m','CYO1_KT','CYO1b'],remove_orphans=True)
```

In [78]:

```
for r in sorted(model.reactions, key=lambda x: x.id):
    if r.id.startswith("CYOO"):
        print(r.id, r.reaction, r.gene_reaction_rule)
```

```
CYOO6m 4.0 focytc_m + 8.0 h_m + o2_m --> 4.0 ficytc_m + 2.0 h2o_m + 4.0 h_c (CRv4_Au5_s1_g1958_t1 and CRv4_Au5_s3_g10243_t1 and CRv4_Au5_s4_g11710_t1 and 11317 and 8802) or (CRv4_Au5_s1_g1958_t1 and CRv4_Au5_s3_g10243_t1 and CRv4_Au5_s4_g11710_t1 and 11317 and 9198)
CYOOm 4.0 focytc_m + 6.0 h_m + o2_m --> 4.0 ficytc_m + 2.0 h2o_m + 6.0 h_c Q0045 and Q0250 and Q0275 and YLR395C and YMR256C and 10685 and 11317 and 11769 and 13007 and 13628 and 8802 and 9165
CYOOm3 4.0 focytc_m + 7.92 h_m + o2_m --> 4.0 ficytc_m + 1.96 h2o_m + 4.0 h_c + 0.02 o2s_m COX1 and COX2 and COX3 and COX4I1 and COX4I2 and COX6A1 and COX6A2 and COX6C and COX7A1 and COX7A2 and COX7A2L and COX7B and COX7B2 and COX7C and COX8A and COX8C and 10685 and 11317 and 9165
```

CYOOm, CYOOm3, CYOO6m are cytochrome c oxidase  
CYOOm yeast genes are  
Q0045 and Q0250 and Q0275 and YDL067C and YGL187C and YGL191W and YHR051W and YLR038C and YLR395C and YMR256C and (YNL052W or YIL111W) and (YEL039C or YJR048W)  
COX1 and COX2 and COX3 and COX4 and (COX5A or COX5B) and COX6 and COX7 and COX8 and COX9 and COX12 and COX13 and (CYC1 or CYC7)

COX1, COX2, COX3 genes are missing in RTO4, but they can be found in RTO3 all gene models

COX1, COX2 (I, II) RTO3\_946004  
COX3 (III) RTO3\_946002  
COX4 (Vb) 9165  
COX5A (IV)  
COX5B (IV) 13628  
COX6 (Va) 10685  
COX7 (VIIa) 12506  
COX8 (VIIc) 11352 13275  
COX9 (VIIa) 11769  
COX12 (VIb) 11317 12210  
COX13 (VIa) 13007

CYC1/CYC7 8802

COX10 (Heme A farnesyltransferase) 11103  
COX11 (assembly protein CtaG) 8721  
COX14 (assembly) 15954  
COX15 (assembly, Heme A) 10228  
COX16 (assembly) 13680  
COX17 (assembly protein / Cu2+ chaperone) 10086  
COX18 (membrane insert / assembly protein 18) 9694  
COX19 (assembly) 15263  
COX23 (assembly) 12162

SCO1 (assembly, cu) 14064  
PET191 (assembly) 14895  
COA1 (assembly) 14176  
COA3 (assembly) 15286  
SURF1 (assembly) 14230  
CMC1 (biogenesis) 12873  
LRPPRC (assembly) 12949

CYOO6m has correct stoichiometry  
Change CYOO6m genes to 'RTO3\_946004 and RTO3\_946002 and 9165 and 13628 and 10685 and 12506 and 11352 and 13275 and 11769 and 11317 and 12210 and 13007 and 8802'  
Remove CYOOm, CYOOm3

In [79]:

```
model.reactions.get_by_id('CYOO6m').gene_reaction_rule = 'RTO3_946004 and RTO3_946002 and 9165 and 13628 and 10685 and 12506 and 11352 and 13275 and 11769 and 11317 and 12210 and 13007 and 8802'
model.remove_reactions(['CYOOm','CYOOm3'],remove_orphans=True)
```

In [80]:

```
for r in sorted(model.genes.get_by_id('11103').reactions, key=lambda x: x.id):
    print(r.id, r.reaction, r.gene_reaction_rule)
print()
for r in sorted(model.genes.get_by_id('10228').reactions, key=lambda x: x.id):
    print(r.id, r.reaction, r.gene_reaction_rule)
```

```
HEMEOS frdp_c + h2o_c + pheme_c --> hemeO_c + ppi_c 11103
HEMEOSm frdp_m + h2o_m + pheme_m --> hemeO_m + ppi_m 11103
HEMEOSm_1 frdp_m + h2o_m + pheme_m --> h_m + hemeO_m + ppi_m 11103

HEMEASm h2o_m + hemeO_m --> 5.0 h_m + hemeA_m 10228
HEMEOMOm hemeO_m + nadh_m + o2_m --> h2o_m + hemeA_m + nad_m 10228 and 15585 and 16775
```

In [81]:

```
for r in sorted(model.metabolites.get_by_id('pheme_c').reactions, key=lambda x: x.id):
        print(r.id, r.reaction, r.gene_reaction_rule)
print()
for r in sorted(model.metabolites.get_by_id('pheme_m').reactions, key=lambda x: x.id):
        print(r.id, r.reaction, r.gene_reaction_rule)
print()
for r in sorted(model.metabolites.get_by_id('hemeO_c').reactions, key=lambda x: x.id):
        print(r.id, r.reaction, r.gene_reaction_rule)
print()
for r in sorted(model.metabolites.get_by_id('hemeO_m').reactions, key=lambda x: x.id):
        print(r.id, r.reaction, r.gene_reaction_rule)
```

```
HEMEOS frdp_c + h2o_c + pheme_c --> hemeO_c + ppi_c 11103

HEMELm apocytc_m + pheme_m <=> cytc_m 9830
HEMEOSm frdp_m + h2o_m + pheme_m --> hemeO_m + ppi_m 11103
HEMEOSm_1 frdp_m + h2o_m + pheme_m --> h_m + hemeO_m + ppi_m 11103

HEMEOS frdp_c + h2o_c + pheme_c --> hemeO_c + ppi_c 11103

HEMEASm h2o_m + hemeO_m --> 5.0 h_m + hemeA_m 10228
HEMEOMOm hemeO_m + nadh_m + o2_m --> h2o_m + hemeA_m + nad_m 10228 and 15585 and 16775
HEMEOSm frdp_m + h2o_m + pheme_m --> hemeO_m + ppi_m 11103
HEMEOSm_1 frdp_m + h2o_m + pheme_m --> h_m + hemeO_m + ppi_m 11103
```

In [82]:

```
for r in sorted(model.metabolites.get_by_id('apocytc_m').reactions, key=lambda x: x.id):
        print(r.id, r.reaction, r.gene_reaction_rule)
print()
for r in sorted(model.metabolites.get_by_id('cytc_m').reactions, key=lambda x: x.id):
        print(r.id, r.reaction, r.gene_reaction_rule)
print()
for r in sorted(model.genes.get_by_id('9830').reactions, key=lambda x: x.id):
    print(r.id, r.reaction, r.gene_reaction_rule)
```

```
HEMELm apocytc_m + pheme_m <=> cytc_m 9830

HEMELm apocytc_m + pheme_m <=> cytc_m 9830

HEMELh apocytc_h + pheme_h <=> cytc_h 9830
HEMELm apocytc_m + pheme_m <=> cytc_m 9830
```

HEMEOSm has correct stoichiometry  
HEMEOSm frdp\_m + h2o\_m + pheme\_m --> hemeO\_m + ppi\_m 11103 (COX10)

HEMEOMOm incorrect stoichiometry  
HEMEOMOm hemeO\_m + nadh\_m + o2\_m --> h2o\_m + hemeA\_m + nad\_m 10228 and 15585 and 16775  
HEMEASM\_m from iLB1027\_lipid has nadp/nadph, but backward  
Update reaction?  
https://febs.onlinelibrary.wiley.com/doi/full/10.1016/S0014-5793%2801%2902249-9  
HEMEOMOm hemeO\_m + 2.0 nadph\_m + 2.0 o2\_m + 2.0 h\_m --> 3.0 h2o\_m + hemeA\_m + 2.0 nadp\_m  
10228 and 15585 and 16775 (COX15 and YAH1 and ARH1)

HEMEL is for heme c, not protoheme (or heme b)

Remove HEMEOS, HEMEOSm\_1, HEMELh, HEMEASm, HEMEOMOm  
Update HEMEOMOm and change name to HEMEASm  
Add FRDPtm from sce  
Update HEMELm to replace pheme\_m with hemeC\_m

In [83]:

```
model.remove_reactions(['HEMEOS','HEMEOSm_1','HEMELh','HEMEASm'], remove_orphans=True)
model.reactions.get_by_id('HEMEOMOm').id = 'HEMEASm'
model.reactions.get_by_id('HEMEASm').add_metabolites({'nadh_m': 1.0, 'nad_m': -1.0,
                                                      'nadph_m': -2.0, 'nadp_m': 2.0, 'h_m': -2.0,
                                                      'o2_m': -1.0, 'h2o_m': 2.0})
model.add_reactions([sce.reactions.get_by_id('FRDPtm').copy()])
m = model.metabolites.get_by_id('pheme_m').copy()
m.id = 'hemeC_m'
m.formula = 'C34H34FeN4O4S2'
model.add_metabolites([m])
model.reactions.get_by_id('HEMELm').add_metabolites({'pheme_m': 1.0, 'hemeC_m': -1.0})
```

NADH - ubiquinone - cytochrome c - oxygen electron transfer chain  
10069 mito 20, cyto 5 K03522: fixB, etfA; electron transfer flavoprotein alpha subunit  
9886 cyto 19, cyto\_mito 14.5, mito 8 K03521: fixA, etfB; electron transfer flavoprotein beta subunit  
10218 mito 27 K00311: ETFDH; electron-transferring-flavoprotein dehydrogenase

In [84]:

```
for r in sorted(model.genes.get_by_id('10069').reactions, key=lambda x: x.id):
    print(r.id, r.reaction, r.gene_reaction_rule)
print()
for r in sorted(model.genes.get_by_id('9886').reactions, key=lambda x: x.id):
    print(r.id, r.reaction, r.gene_reaction_rule)
print()
for r in sorted(model.genes.get_by_id('10218').reactions, key=lambda x: x.id):
    print(r.id, r.reaction, r.gene_reaction_rule)
```

```
ETF etfox_m + fadh2_m --> etfrd_m + fad_m 10069 and 9886

ETF etfox_m + fadh2_m --> etfrd_m + fad_m 10069 and 9886

ETFQO etfrd_m + q10_m --> etfox_m + q10h2_m 10218
ETFQO_1 etfrd_m + q8_m --> etfox_m + q8h2_m 10218
```

R. toruloides has Q9 quinone  
https://www.jstage.jst.go.jp/article/jgam1955/35/5/35\_5\_377/\_pdf
Add q9 reactions from iLB1027\_lipid and remove other quinone reactions

In [85]:

```
temp = Annotation.index[Annotation['Combined Annotations'].str.contains('COQ')]
temp = list(temp) + ['10633','12467']
display(Annotation.loc[temp])
Show_Data(temp)
```

|  | Combined Annotations | Signal P | Sc288c Orthologs | Human Orthologs | Sc288 Best Hit | Human Blast | Essential | WolfPSort | C Terminal |
| --- | --- | --- | --- | --- | --- | --- | --- | --- | --- |
| RTO4\_ID |  |  |  |  |  |  |  |  |  |
| 8559 | K18588: COQ10; coenzyme Q-binding protein COQ10 |  | COQ10 | COQ10B,COQ10A |  | COQ10 | Essential | mito 23, nucl 2 | AQR\* |
| 8873 | HMMPfam:ubiE/COQ5 methyltransferase family:PF0... |  |  |  |  |  | Not Essential | mito 25 | KAE\* |
| 8900 | K05355: hexPS, COQ1; hexaprenyl-diphosphate sy... | S | COQ1 | PDSS1 | COQ1 | PDSS1 | Not Essential | mito 26 | RKK\* |
| 9681 | K18586: COQ4; ubiquinone biosynthesis protein ... | A | COQ4 | COQ4 | COQ4 | COQ4 | Essential | mito 25.5, cyto\_mito 14 | HAV\* |
| 10213 | K06127: COQ5; 2-methoxy-6-polyprenyl-1,4-benzo... |  | COQ5 | COQ5 | COQ5 | COQ5 | Essential | cyto 13, cyto\_nucl 10.833, cyto\_mito 8.999, nu... | VKV\* |
| 11465 | K00591: COQ3; polyprenyldihydroxybenzoate meth... |  | COQ3 | COQ3 | COQ3 | COQ3 | Not Essential | mito 24, cyto 2 | RVQ\* |
| 15237 | K06125: COQ2; 4-hydroxybenzoate polyprenyltran... | S | COQ2 | COQ2,CLPB | COQ2 | COQ2 | Essential | extr 12, mito 7, cyto 7, cyto\_mito 7 | GMM\* |
| 16037 | K06134: COQ7; ubiquinone biosynthesis monooxyg... | S | CAT5 | COQ7 | CAT5 | COQ7 | Essential | mito 24.5, cyto\_mito 14 | EKI\* |
| 16197 | K06126: COQ6; ubiquinone biosynthesis monooxyg... | A | COQ6 | COQ6 | COQ6 | COQ6 | Not Essential | mito 15, nucl 3, cyto 3, cyto\_nucl 3, plas 2, ... | KGR\* |
| 10633 | K08869: ADCK, ABC1; aarF domain-containing kinase | S | COQ8 | COQ8A,COQ8B | COQ8 | COQ8A | Essential | nucl 9, cysk 5, mito 4, cyto 4, pero 4, cyto\_m... | CAL\* |
| 12467 | HMMPfam:Phenolic acid decarboxylase (PAD):PF05... |  |  |  |  |  | Not Essential | nucl 10, cyto 7, mito 3, extr 2, pero 2, golg ... | RQC\* |

| strain | WT | | | | | | | | | | | | | | | | |
| --- | --- | --- | --- | --- | --- | --- | --- | --- | --- | --- | --- | --- | --- | --- | --- | --- | --- |
| condition | G\_MM | C\_MM | G\_SD | | GX\_SD | | | X\_SD | | A\_SD | | C\_SD | | MM\_CN120 | | MM\_CN5 | Diversity\_Sample |
| phase | exp | exp | exp | stat | exp | trans | stat | exp | stat | exp | stat | exp | stat | exp | stat | exp | exp |
| proteinId | Set1 | Set1 | Set2 | Set2 | Set2 | Set2 | Set2 | Set2 | Set2 | Set2 | Set2 | Set2 | Set2 | Set3 | Set3 | Set3 | Set3 |
| 8559 | 4.98231 | 4.8054 | 4.92269 | 4.89372 | 5.15268 | 4.84301 | 4.79234 | 4.92001 | 5.31358 | 4.82196 | 4.99107 | 4.52811 | 4.8137 | 4.35084 | 4.37049 | 4.82846 | 4.9565 |
| 8873 | 8.20309 | 6.61612 | 11.655 | 11.5917 | 11.6346 | 11.6821 | 11.5272 | 11.9982 | 11.7929 | 12.2408 | 12.483 | 8.51614 | 6.50514 | 9.12112 | 8.10491 | 11.3005 | 7.23669 |
| 8900 | 3.76882 | 4.79292 | 4.71375 | 4.50448 | 4.73272 | 5.11952 | 5.1903 | 4.8356 | 5.21722 | 4.8738 | 5.05437 | 5.0194 | 4.57674 | 4.85067 | 5.35525 | 5.7989 | 6.43808 |
| 9681 | 5.42333 | 5.01663 | 5.57472 | 6.07486 | 5.56132 | 5.53112 | 5.56131 | 5.32794 | 5.30248 | 5.44333 | 5.63275 | 5.25601 | 4.84897 | 5.02399 | 5.39393 | 5.03042 | 5.16545 |
| 10213 | 5.88172 | 6.19762 | 5.20243 | 5.41788 | 5.40004 | 5.09596 | 5.36683 | 5.22643 | 5.87529 | 5.45692 | 6.33501 | 6.07895 | 6.37616 | 5.18369 | 5.5507 | 5.6635 | 6.47709 |
| 11465 | 7.4794 | 7.13858 | 7.40843 | 7.5156 | 7.3893 | 7.71744 | 7.72724 | 7.54219 | 7.44602 | 7.54623 | 7.62899 | 7.31122 | 7.00981 | 6.00139 | 6.31205 | 6.18779 | 6.3065 |
| 15237 | 4.63293 | 4.97849 | 4.49714 | 5.13291 | 4.58174 | 5.20347 | 5.32735 | 4.91819 | 5.0203 | 4.73988 | 4.75243 | 5.14378 | 5.12482 | 4.67861 | 5.62419 | 4.82184 | 4.65626 |
| 16037 | 5.32662 | 5.09173 | 5.35107 | 5.81367 | 5.46109 | 5.72672 | 5.90303 | 5.27901 | 5.70869 | 5.24768 | 5.74401 | 5.25462 | 5.64777 | 4.99727 | 5.29083 | 4.97936 | 4.76005 |
| 16197 | 5.06906 | 4.90822 | 5.61278 | 5.98002 | 5.66651 | 5.49685 | 5.57607 | 5.55264 | 5.5659 | 5.50356 | 5.70092 | 6.0029 | 6.18666 | 4.70664 | 4.93049 | 5.00847 | 4.81022 |
| 10633 | 5.52412 | 5.26398 | 5.93796 | 5.71076 | 5.91134 | 5.99457 | 5.90323 | 5.64884 | 5.85435 | 5.35007 | 5.63426 | 5.34526 | 5.11936 | 6.10842 | 5.82006 | 5.90191 | 5.61811 |
| 12467 | 5.95492 | 7.48868 | 2.77403 | 3.08432 | 2.61809 | 2.57179 | 2.79028 | 2.00026 | 3.04079 | 2.13197 | 2.02207 | 3.97072 | 4.20645 | 3.60515 | 2.29811 | 4.70542 | 2.88573 |

| strain | WT | | | | | | | | | | |
| --- | --- | --- | --- | --- | --- | --- | --- | --- | --- | --- | --- |
| condition | G\_SD | | GX\_SD | | | X\_SD | | A\_SD | | C\_SD | |
| proteinId | exp | stat | exp | trans | stat | exp | stat | exp | stat | exp | stat |
| 8559 | 0.77039 | 0.937198 | 1.82846 | 0.736522 | 0.371882 | 1.18492 | 0.604768 | 0.770643 | 0.378889 | 0.20922 | 0.422666 |
| 8873 | 30.3949 | 46.7521 | 29.5257 | 35.2803 | 54.6975 | 36.3751 | 47.7358 | 33.0017 | 52.4487 | 11.7702 | 15.4302 |
| 8900 | 5.55566 | 4.39154 | 4.46196 | 3.14647 | 3.63901 | 2.9543 | 2.97448 | 2.32553 | 0.959689 | 3.41671 | 3.04707 |
| 9681 | 0.583984 | 0.524211 | 0.808994 | 1.29818 | 1.27589 | 1.16712 | 0.393103 | 0 | 0 | 0.208996 | 0.647395 |
| 10213 | 1.36956 | 0.897803 | 3.04748 | 0.18083 | 0.173732 | 0.794704 | 0.978258 | 0.388524 | 0.189478 | 0.850774 | 1.95832 |
| 11465 | 3.24589 | 4.30586 | 2.03122 | 2.41637 | 2.57762 | 1.56943 | 2.3576 | 0.969307 | 1.14228 | 1.49165 | 1.74411 |
| 16037 | 0.766387 | 1.46782 | 0.40074 | 0.555692 | 0.173732 | 0.197909 | 1.18915 | 0.190305 | 0.758174 | 0.424555 | 1.31507 |
| 16197 | 5.56249 | 6.13957 | 2.83468 | 5.37893 | 4.57359 | 5.49113 | 5.88006 | 3.67567 | 4.38268 | 4.49395 | 7.86677 |
| 10633 | 0.783778 | 2.9032 | 2.64975 | 1.49046 | 2.01965 | 1.36773 | 3.55973 | 1.16176 | 0.569093 | 0.842365 | 0.42228 |
| 12467 | 0 | 0 | 0.206941 | 0 | 0 | 0 | 0 | 0 | 0 | 0 | 0 |

|  | Glucose | Xylose | Arabinose | Acetate | Coumarate | Ferulate | YNB Oleic Acid | YNB Ricinoleic Acid | YNB Glucose | YNB Gluc DOC | YPD |
| --- | --- | --- | --- | --- | --- | --- | --- | --- | --- | --- | --- |
| proteinId |  |  |  |  |  |  |  |  |  |  |  |
| 8873 | 0.0589421 | 0.254546 | 0.113626 | -0.19223 | -0.212035 | -0.297574 | 0.715953 | 0.157643 | 0.00942156 | -0.0939315 | -0.0249116 |
| 11465 | -0.0314687 | 0.0888688 | 0.0271293 | 0.123384 | -0.116852 | 0.317395 | -0.211165 | -0.183164 | -0.370499 | -0.0712369 | -0.266982 |
| 16197 | -0.670635 | -0.291277 | 0.598587 | 0.109946 | -0.215684 | 0.775421 | -0.9498 | -1.78661 | -1.14682 | -1.09647 | 0.494682 |
| 12467 | 0.451049 | 0.124327 | -0.0207068 | 0.217107 | -0.0819564 | 0.361946 | 0.0230384 | -0.707581 | -0.166716 | -0.0388523 | 0.679939 |

In [86]:

```
for x in temp:
    if x in model.genes:
        for r in sorted(model.genes.get_by_id(x).reactions, key=lambda x: x.id):
            print(r.id, r.reaction, r.gene_reaction_rule)
    else:
        print(x, 'no reactions')
    print()
```

```
8559 no reactions

8873 no reactions

OCTDPS frdp_c + 5.0 ipdp_c --> octdp_c + 5.0 ppi_c 8900
PPTTm ipdp_m + pendp_m --> hexdp_m + ppi_m 8900
yli_R0769 frdp_c + 3.0 ipdp_c --> hexdp_c + 3.0 ppi_c 8900
yli_R0770 ggdp_c + 2.0 ipdp_c --> hexdp_c + 2.0 ppi_c 8900

2HMHMBQMTm 2hpmhmbq_m + amet_m --> ahcys_m + h_m + q6_m YLR201C and 10213 and 10633 and 11465 and 16037 and 16197 and 9681
2HP6MPMOm 2hp6mp_m + o2_m --> 2hp6mbq_m + h2o_m YLR201C and 10213 and 10633 and 11465 and 16037 and 16197 and 9681
2HPMBQMTm 2hp6mbq_m + amet_m --> 2hpmmbq_m + ahcys_m + h_m YLR201C and 10213 and 10633 and 11465 and 16037 and 16197 and 9681
2HPMMBQMOm 2hpmmbq_m + 0.5 o2_m --> 2hpmhmbq_m YLR201C and 10213 and 10633 and 11465 and 16037 and 16197 and 9681
3DH5HPBMTm 3dh5hpb_m + amet_m --> 3hph5mb_m + ahcys_m + h_m 11465 or (YLR201C and 10213 and 10633 and 11465 and 16037 and 16197 and 9681)

2HMHMBQMTm 2hpmhmbq_m + amet_m --> ahcys_m + h_m + q6_m YLR201C and 10213 and 10633 and 11465 and 16037 and 16197 and 9681
2HP6MPMOm 2hp6mp_m + o2_m --> 2hp6mbq_m + h2o_m YLR201C and 10213 and 10633 and 11465 and 16037 and 16197 and 9681
2HPMBQMTm 2hp6mbq_m + amet_m --> 2hpmmbq_m + ahcys_m + h_m YLR201C and 10213 and 10633 and 11465 and 16037 and 16197 and 9681
2HPMMBQMOm 2hpmmbq_m + 0.5 o2_m --> 2hpmhmbq_m YLR201C and 10213 and 10633 and 11465 and 16037 and 16197 and 9681
3DH5HPBMTm 3dh5hpb_m + amet_m --> 3hph5mb_m + ahcys_m + h_m 11465 or (YLR201C and 10213 and 10633 and 11465 and 16037 and 16197 and 9681)
AMMQLT8 2dmmql8_c + amet_c --> ahcys_c + h_c + mql8_c 10213
OMBZLM 2ombzl_c + amet_c --> 2ommbl_c + ahcys_c + h_c 10213
yli_R0945 amet_m + yli_M02815_m --> ahcys_m + h_m + yli_M02816_m 10213

2HMHMBQMTm 2hpmhmbq_m + amet_m --> ahcys_m + h_m + q6_m YLR201C and 10213 and 10633 and 11465 and 16037 and 16197 and 9681
2HP6MPMOm 2hp6mp_m + o2_m --> 2hp6mbq_m + h2o_m YLR201C and 10213 and 10633 and 11465 and 16037 and 16197 and 9681
2HPMBQMTm 2hp6mbq_m + amet_m --> 2hpmmbq_m + ahcys_m + h_m YLR201C and 10213 and 10633 and 11465 and 16037 and 16197 and 9681
2HPMMBQMOm 2hpmmbq_m + 0.5 o2_m --> 2hpmhmbq_m YLR201C and 10213 and 10633 and 11465 and 16037 and 16197 and 9681
3DH5HPBMTm 3dh5hpb_m + amet_m --> 3hph5mb_m + ahcys_m + h_m 11465 or (YLR201C and 10213 and 10633 and 11465 and 16037 and 16197 and 9681)
COQ3m 2dpmhobq_m + amet_m --> ahcys_m + h_m + q10_m 11465
DHDPBMTm 3dpdhb_m + amet_m --> 3dpdhb_me_m + ahcys_m + h_m 11465
DMQMT 2omhmbl_c + amet_c --> ahcys_c + h_c + q8h2_c 11465
OHPHM 2ohph_c + amet_c --> 2omph_c + ahcys_c + h_c 11465
yli_R0947 amet_m + yli_M02817_m --> ahcys_m + h_m + q6_m 11465

HBZOPT 4hbz_c + octdp_c --> 3ophb_c + ppi_c 15237
HBZOPT10m 4hbz_m + decdp_m --> 3dphb_m + ppi_m 15237
HBZOPT6m 4hbz_m + hexdp_m --> 3ophb_5_m + ppi_m 15237

2HMHMBQMTm 2hpmhmbq_m + amet_m --> ahcys_m + h_m + q6_m YLR201C and 10213 and 10633 and 11465 and 16037 and 16197 and 9681
2HP6MPMOm 2hp6mp_m + o2_m --> 2hp6mbq_m + h2o_m YLR201C and 10213 and 10633 and 11465 and 16037 and 16197 and 9681
2HPMBQMTm 2hp6mbq_m + amet_m --> 2hpmmbq_m + ahcys_m + h_m YLR201C and 10213 and 10633 and 11465 and 16037 and 16197 and 9681
2HPMMBQMOm 2hpmmbq_m + 0.5 o2_m --> 2hpmhmbq_m YLR201C and 10213 and 10633 and 11465 and 16037 and 16197 and 9681
3DH5HPBMTm 3dh5hpb_m + amet_m --> 3hph5mb_m + ahcys_m + h_m 11465 or (YLR201C and 10213 and 10633 and 11465 and 16037 and 16197 and 9681)
COQ7m 2dp6mobq_me_m + h_m + nadph_m + o2_m --> 2dpmhobq_m + h2o_m + nadp_m 16037
yli_R0946 h_m + nadph_m + o2_m + yli_M02816_m --> h2o_m + nadp_m + yli_M02817_m 16037

2HMHMBQMTm 2hpmhmbq_m + amet_m --> ahcys_m + h_m + q6_m YLR201C and 10213 and 10633 and 11465 and 16037 and 16197 and 9681
2HP6MPMOm 2hp6mp_m + o2_m --> 2hp6mbq_m + h2o_m YLR201C and 10213 and 10633 and 11465 and 16037 and 16197 and 9681
2HPMBQMTm 2hp6mbq_m + amet_m --> 2hpmmbq_m + ahcys_m + h_m YLR201C and 10213 and 10633 and 11465 and 16037 and 16197 and 9681
2HPMMBQMOm 2hpmmbq_m + 0.5 o2_m --> 2hpmhmbq_m YLR201C and 10213 and 10633 and 11465 and 16037 and 16197 and 9681
3DH5HPBMTm 3dh5hpb_m + amet_m --> 3hph5mb_m + ahcys_m + h_m 11465 or (YLR201C and 10213 and 10633 and 11465 and 16037 and 16197 and 9681)
BCAROH caro_u + h_u + nadph_u + o2_u <=> bcryptox_u + h2o_u + nadp_u 16197
BCRPTXANH bcryptox_u + h_u + nadph_u + o2_u <=> h2o_u + nadp_u + zeax_u 16197
CHYA1 acaro_u + h_u + nadph_u + o2_u --> h2o_u + nadp_u + zxan_u 16197
COQ6m 2dp6mep_m + o2_m --> 2dp6mobq_m + h2o_m 16197
OMMBLHXy 2ommbl_c + h_c + nadph_c + o2_c --> 2omhmbl_c + h2o_c + nadp_c 16197
OPHHXy 2oph_c + h_c + nadph_c + o2_c --> 2ohph_c + h2o_c + nadp_c 16197
yli_R0944 2hp6mp_m + o2_m --> h2o_m + yli_M02815_m 16197

2HMHMBQMTm 2hpmhmbq_m + amet_m --> ahcys_m + h_m + q6_m YLR201C and 10213 and 10633 and 11465 and 16037 and 16197 and 9681
2HP6MPMOm 2hp6mp_m + o2_m --> 2hp6mbq_m + h2o_m YLR201C and 10213 and 10633 and 11465 and 16037 and 16197 and 9681
2HPMBQMTm 2hp6mbq_m + amet_m --> 2hpmmbq_m + ahcys_m + h_m YLR201C and 10213 and 10633 and 11465 and 16037 and 16197 and 9681
2HPMMBQMOm 2hpmmbq_m + 0.5 o2_m --> 2hpmhmbq_m YLR201C and 10213 and 10633 and 11465 and 16037 and 16197 and 9681
3DH5HPBMTm 3dh5hpb_m + amet_m --> 3hph5mb_m + ahcys_m + h_m 11465 or (YLR201C and 10213 and 10633 and 11465 and 16037 and 16197 and 9681)

12467 no reactions
```

Gene for decarboxylation step is unknown  
12467 Phenolic acid decarboxylase (PAD) nucl 10, cyto 7, mito 3, extr 2, pero 2, golg 1, cysk 1, vacu 1  
10633 COQ8

Add q9 reactions (iLB1027\_lipid)  
NPDPS\_m (COQ1, 7 ipp + gpp -> npp + 8 ppi), HBZNPT\_m (COQ2), 3NPHBH2\_m (COQ6), DHNPBMT\_m (COQ3), NPHMBDC\_m (4.1.1.-), COQ6\_m, COQ5\_m,COQ7\_m,COQ3\_m

Remove q6 reactions  
PENDPtm,HEXDPtm,PPTTm,HBZOPT6m,3OPHB5Hm,3DH5HPBMTm,3HPH5MBDCm,2HP6MPMOm,2HPMBQMTm,2HPMMBQMOm,2HMHMBQMTm  
Remove q8 reactions  
OCTDPS,HBZOPT,OPHBDC,OPHHX3,OPHHXy,OHPHM,OMPHHXy,OMBZLM,OMMBLHXy,DMQMT  
Remove q10 reactions  
DPPS,DECDPtm,HBZOPT10m,3DPHBH1,3DPHBH2,DHDPBMTm,DPHMBDCm,COQ6m,COQ5m,COQ7m,COQ3m

Add q9 reaction ETFQO\_m from iLB1027\_lipid and remove q8 and q10 reactions ETFQO, ETFQO\_1  
ETFQO\_m: fadh2\_m + q9\_m --> fad\_m + q9h2\_m -> ETFQOm: etfrd\_m + q9\_m --> etfox\_m + q9h2\_m

In [87]:

```
for x in ['IPDPtm','GRDPt_c','NPDPS_m','HBZNPT_m','3NPHBH2_m','DHNPBMT_m','NPHMBDC_m','COQ6_m','COQ5_m','COQ7_m','COQ3_m']:
    r = ptri.reactions.get_by_id(x).copy()
    model.add_reactions([r])
model.reactions.get_by_id('GRDPt_c').id = 'GRDPtm'
model.reactions.get_by_id('GRDPtm').name = 'Geranyl diphosphate transport mitochondrial'
model.reactions.get_by_id('NPDPS_m').gene_reaction_rule = '8900' # COQ1
model.reactions.get_by_id('HBZNPT_m').gene_reaction_rule = '15237' # COQ2
model.reactions.get_by_id('3NPHBH2_m').gene_reaction_rule = '16197' # COQ6
model.reactions.get_by_id('DHNPBMT_m').gene_reaction_rule = '11465' # COQ3
model.reactions.get_by_id('NPHMBDC_m').gene_reaction_rule = '' # Unknown decarboxylase, maybe 12467
model.reactions.get_by_id('COQ6_m').gene_reaction_rule = '16197'
model.reactions.get_by_id('COQ5_m').gene_reaction_rule = '10213'
model.reactions.get_by_id('COQ7_m').gene_reaction_rule = '16037'
model.reactions.get_by_id('COQ3_m').gene_reaction_rule = '11465'
for x in ['PENDPtm','HEXDPtm','PPTTm','HBZOPT6m','3OPHB5Hm','3DH5HPBMTm','3HPH5MBDCm','2HP6MPMOm','2HPMBQMTm','2HPMMBQMOm','2HMHMBQMTm']:
    if x in model.reactions:
        model.remove_reactions([x], remove_orphans=True)
for x in ['OCTDPS','HBZOPT','OPHBDC','OPHHX3','OPHHXy','OHPHM','OMPHHXy','OMBZLM','OMMBLHXy','DMQMT']:
    if x in model.reactions:
        model.remove_reactions([x], remove_orphans=True)
for x in ['DPPS','DECDPtm','HBZOPT10m','3DPHBH1','3DPHBH2','DHDPBMTm','DPHMBDCm','COQ6m','COQ5m','COQ7m','COQ3m']:
    if x in model.reactions:
        model.remove_reactions([x], remove_orphans=True)
model.add_reactions([ptri.reactions.get_by_id('ETFQO_m').copy()])
model.reactions.get_by_id('ETFQO_m').add_metabolites({'fadh2_m': 1.0, 'fad_m': -1.0, 'etfrd_m': -1.0, 'etfox_m': 1.0})
model.reactions.get_by_id('ETFQO_m').gene_reaction_rule = '10218'
model.remove_reactions(['ETFQO','ETFQO_1'], remove_orphans=True)
```

In [88]:

```
for r in sorted(model.genes.get_by_id('8900').reactions, key=lambda x: x.id):
    print(r.id, r.reaction, r.gene_reaction_rule)
print()
for r in sorted(model.genes.get_by_id('15237').reactions, key=lambda x: x.id):
    print(r.id, r.reaction, r.gene_reaction_rule)
print()
for r in sorted(model.genes.get_by_id('16197').reactions, key=lambda x: x.id):
    print(r.id, r.reaction, r.gene_reaction_rule)
print()
for r in sorted(model.genes.get_by_id('11465').reactions, key=lambda x: x.id):
    print(r.id, r.reaction, r.gene_reaction_rule)
print()
for r in sorted(model.genes.get_by_id('10213').reactions, key=lambda x: x.id):
    print(r.id, r.reaction, r.gene_reaction_rule)
print()
for r in sorted(model.genes.get_by_id('16037').reactions, key=lambda x: x.id):
    print(r.id, r.reaction, r.gene_reaction_rule)
```

```
NPDPS_m grdp_m + 7.0 ipdp_m --> npdp_m + 7.0 ppi_m 8900
yli_R0769 frdp_c + 3.0 ipdp_c --> hexdp_c + 3.0 ppi_c 8900
yli_R0770 ggdp_c + 2.0 ipdp_c --> hexdp_c + 2.0 ppi_c 8900

HBZNPT_m 4hbz_m + npdp_m --> 3nphb_m + ppi_m 15237

3NPHBH2_m 3nphb_m + h_m + nadph_m + o2_m --> 3npdhb_m + h2o_m + nadp_m 16197
BCAROH caro_u + h_u + nadph_u + o2_u <=> bcryptox_u + h2o_u + nadp_u 16197
BCRPTXANH bcryptox_u + h_u + nadph_u + o2_u <=> h2o_u + nadp_u + zeax_u 16197
CHYA1 acaro_u + h_u + nadph_u + o2_u --> h2o_u + nadp_u + zxan_u 16197
COQ6_m 2np6mep_m + o2_m --> 2np6mobq_m + h2o_m 16197
yli_R0944 2hp6mp_m + o2_m --> h2o_m + yli_M02815_m 16197

COQ3_m 2npmhmobq_m + amet_m --> ahcys_m + h_m + q9_m 11465
DHNPBMT_m 3npdhb_m + amet_m --> ahcys_m + h_m + me3dhnpdh_m 11465
yli_R0947 amet_m + yli_M02817_m --> ahcys_m + h_m + q6_m 11465

AMMQLT8 2dmmql8_c + amet_c --> ahcys_c + h_c + mql8_c 10213
COQ5_m 2np6mobq_m + amet_m --> ahcys_m + h_m + me2np6mobq_m 10213
yli_R0945 amet_m + yli_M02815_m --> ahcys_m + h_m + yli_M02816_m 10213

COQ7_m h_m + me2np6mobq_m + nadph_m + o2_m --> 2npmhmobq_m + h2o_m + nadp_m 16037
yli_R0946 h_m + nadph_m + o2_m + yli_M02816_m --> h2o_m + nadp_m + yli_M02817_m 16037
```

In [89]:

```
model.remove_reactions(['yli_R0769','yli_R0770','yli_R0944','yli_R0947','AMMQLT8','yli_R0945','yli_R0946'], remove_orphans=True)
```

In [90]:

```
for r in model.reactions:
    if 'u' in r.compartments:
        print(r.id, r.reaction, r.gene_reaction_rule)
```

```
ZHY h_u + nadph_u + o2_u + zxan_u --> h2o_u + lut_u + nadp_u 13361 or 8979 or 9188
ATPSh adp_h + 4.0 h_u + pi_h --> atp_h + h2o_u + 3.0 h_h CRv4_Au5_s11_g2708_t1 and 11958 and 13424
ANXANOR anxan_u + h_u + nadph_u + o2_u --> h2o_u + nadp_u + vioxan_u 11963
ZAXANOR h_u + nadph_u + o2_u + zeax_u --> anxan_u + h2o_u + nadp_u 11963
CHLDA2tu atp_h + chlld_h + h2o_h --> adp_h + chlld_u + h_h + pi_h 11266 or 13264 or 14111 or 14463 or 14844 or 8693 or 9145
CHYA1 acaro_u + h_u + nadph_u + o2_u --> h2o_u + nadp_u + zxan_u 16197
H2Othu h2o_u <=> h2o_h 13986
BCRPTXANH bcryptox_u + h_u + nadph_u + o2_u <=> h2o_u + nadp_u + zeax_u 16197
PSIIred 2.0 h2o_u + 4.0 photon673_u + 2.0 pq_u --> o2D_u + 2.0 pqh2_u CRv4_Au5_s10_g452_t1 and CRv4_Au5_s10_g683_t1 and CRv4_Au5_s11_g2578_t1 and CRv4_Au5_s12_g3255_t1 and CRv4_Au5_s12_g4032_t1 and CRv4_Au5_s12_g4080_t1 and CRv4_Au5_s16_g6524_t1 and CRv4_Au5_s16_g6629_t1 and CRv4_Au5_s17_g7448_t1 and CRv4_Au5_s1_g1303_t1 and CRv4_Au5_s1_g1306_t1 and CRv4_Au5_s2_g8458_t1 and CRv4_Au5_s2_g9294_t1 and CRv4_Au5_s2_g9451_t1 and CRv4_Au5_s3_g11119_t1 and CRv4_Au5_s5_g12218_t1 and CRv4_Au5_s6_g12617_t1 and CRv4_Au5_s6_g12872_t1 and CRv4_Au5_s6_g13079_t1 and CRv4_Au5_s7_g14002_t1 and CRv4_Au5_s7_g14003_t1 and CRv4_Au5_s8_g14723_t1 and CRv4_Au5_s8_g14912_t1 and CRv4_Au5_s9_g15785_t1 and 13812
PSIIblue 2.0 h2o_u + 4.0 photon438_u + 2.0 pq_u --> o2D_u + 2.0 pqh2_u CRv4_Au5_s10_g452_t1 and CRv4_Au5_s10_g683_t1 and CRv4_Au5_s11_g2578_t1 and CRv4_Au5_s12_g3255_t1 and CRv4_Au5_s12_g4032_t1 and CRv4_Au5_s12_g4080_t1 and CRv4_Au5_s16_g6524_t1 and CRv4_Au5_s16_g6629_t1 and CRv4_Au5_s17_g7448_t1 and CRv4_Au5_s1_g1303_t1 and CRv4_Au5_s1_g1306_t1 and CRv4_Au5_s2_g8458_t1 and CRv4_Au5_s2_g9294_t1 and CRv4_Au5_s2_g9451_t1 and CRv4_Au5_s3_g11119_t1 and CRv4_Au5_s5_g12218_t1 and CRv4_Au5_s6_g12617_t1 and CRv4_Au5_s6_g12872_t1 and CRv4_Au5_s6_g13079_t1 and CRv4_Au5_s7_g14002_t1 and CRv4_Au5_s7_g14003_t1 and CRv4_Au5_s8_g14723_t1 and CRv4_Au5_s8_g14912_t1 and CRv4_Au5_s9_g15785_t1 and 13812
CHYA2 acaro_u + h_u + nadph_u + o2_u --> crpxan_u + h2o_u + nadp_u 13361 or 8979 or 9188
BCAROH caro_u + h_u + nadph_u + o2_u <=> bcryptox_u + h2o_u + nadp_u 16197
ASCBOR 2.0 ascb__L_u + o2_u --> 2.0 dhdascb_u + 2.0 h2o_u 13857
CHLDA1tu atp_h + chlld_u + h2o_h --> adp_h + chlld_h + h_h + pi_h 11266 or 13264 or 14111 or 14463 or 14844 or 8693 or 9145
```

13361 or 8979 or 9188 are cytochrome p450s, but assigned to NFTYROX (homology to DIT2)  
13812 is Eukaryotic cytochrome b561, no other rxns  
13857 annotated as L-ascorbate oxidase, similar to iron transport multicopper oxidase, laccase I/II, assigned to FE3t

In [91]:

```
for r in model.reactions:
    if 'u' in r.compartments:
        model.remove_reactions([r], remove_orphans=True)
```

In [92]:

```
for r in sorted(model.metabolites.get_by_id('q6_m').reactions, key=lambda x: x.id):
    print(r.id, r.reaction, r.gene_reaction_rule)
print()
for r in sorted(model.metabolites.get_by_id('q8_m').reactions, key=lambda x: x.id):
    print(r.id, r.reaction, r.gene_reaction_rule)
print()
for r in sorted(model.metabolites.get_by_id('q10_m').reactions, key=lambda x: x.id):
    print(r.id, r.reaction, r.gene_reaction_rule)
print()
for r in sorted(model.metabolites.get_by_id('q8_c').reactions, key=lambda x: x.id):
    print(r.id, r.reaction, r.gene_reaction_rule)
print()
for r in sorted(model.metabolites.get_by_id('q9_m').reactions, key=lambda x: x.id):
    print(r.id, r.reaction, r.gene_reaction_rule)
```

```
CYOR_u6m 2.0 ficytc_m + 1.5 h_m + q6h2_m --> 2.0 focytc_m + 1.5 h_c + q6_m Q0105 and YBL045C and 11618 and 12966 and 13608 and 13614 and 15231 and 15681 and 15758 and 8802 and 9198
DHORD4i dhor__S_c + q6_m --> orot_c + q6h2_m 14866
DXHPScm h2o_c + q6_m + spmd_c --> 13dampp_c + 4abutn_c + q6h2_m 10198
NADH2_u6cm h_c + nadh_c + q6_m --> nad_c + q6h2_m 8972
NADH2_u6m h_m + nadh_m + q6_m --> nad_m + q6h2_m 14261 or 9809
SUCD2_u6m q6_m + succ_m <=> fum_m + q6h2_m 11629 and 11802 and 11977 and 16281
SUCD3_u6m fadh2_m + q6_m <=> fad_m + q6h2_m 11629 and 11802 and 11977 and 16281
yli_R0430 h_m + nadh_m + q6_m --> nad_m + yli_M04633_m YALIfMp01 and YALIfMp02 and YALIfMp16 and YALIfMp19 and YALIfMp20 and YALIfMp28 and YALIfMp29 and 10010 and 10318 and 11151 and 11702 and 12497 and 12543 and 13017 and 13214 and 13766 and 14078 and 14261 and 14418 and 14900 and 15179 and 15864 and 15947 and 15973 and 8699 and 8909 and 8972 and 9348
yli_R0431 2.0 ficytc_m + 1.5 h_m + yli_M04633_m --> 2.0 focytc_m + 1.5 h_c + q6_m 13614
yli_R0432 h_c + nadh_c + q6_m --> nad_c + yli_M04633_m YALIfMp01 and YALIfMp02 and YALIfMp16 and YALIfMp19 and YALIfMp20 and YALIfMp28 and YALIfMp29 and 10010 and 10318 and 11151 and 11702 and 12497 and 12543 and 13017 and 13214 and 13766 and 14078 and 14261 and 14418 and 14900 and 15179 and 15864 and 15947 and 15973 and 8699 and 8909 and 8972 and 9348

CYOR_q8_m 2.0 ficytc_m + 2.0 h_m + q8h2_m --> 2.0 focytc_m + 4.0 h_c + q8_m (13614 and 15681 and 8802) or (13614 and 15681 and 9198)
NADHOR h_m + nadh_m + q8_m --> nad_m + q8h2_m 13766 or 8972 or (CRv4_Au5_s2_g9603_t1 and CRv4_Au5_s6_g12740_t1 and CRv4_Au5_s6_g13274_t1 and CRv4_Au5_s7_g14116_t1 and CRv4_Au5_s8_g15041_t1 and 10010 and 10541 and 11151 and 11702 and 12497 and 13017 and 13214 and 14078 and 14418 and 14900 and 15179 and 15973 and 15998 and 8699) or (CRv4_Au5_s2_g9603_t1 and CRv4_Au5_s6_g12740_t1 and CRv4_Au5_s7_g14116_t1 and CRv4_Au5_s8_g15041_t1 and 10010 and 10541 and 11151 and 11702 and 12497 and 13017 and 13214 and 14078 and 14418 and 14828 and 14900 and 15179 and 15973 and 15998 and 8699) or (CRv4_Au5_s2_g9603_t1 and CRv4_Au5_s6_g12740_t1 and CRv4_Au5_s7_g14116_t1 and CRv4_Au5_s8_g15041_t1 and 10010 and 10541 and 11151 and 11702 and 12497 and 13017 and 13214 and 14078 and 14418 and 14831 and 14900 and 15179 and 15973 and 15998 and 8699)
SUCDH_q8_m q8_m + succ_m <=> fum_m + q8h2_m 11977 and 16281

CYOR_u10m 2.0 ficytc_m + 2.0 h_m + q10h2_m --> 2.0 focytc_m + 4.0 h_c + q10_m (CYTB and UQCR11 and UQCRH and UQCRQ and 11618 and 13614 and 15231 and 15681 and 9198 and 9705) or (CYTB and Uqcr11 and Uqcrq and mmu_100042918 and 11618 and 13614 and 15231 and 15681 and 9198 and 9705)
DHORD9 dhor__S_c + q10_m --> orot_c + q10h2_m 8814
NADH2_u10m 5.0 h_m + nadh_m + q10_m --> 4.0 h_c + nad_m + q10h2_m (ND1 and ND2 and ND3 and ND4 and ND4L and ND5 and ND6 and NDUFA1 and NDUFA10 and NDUFA11 and NDUFA3 and NDUFA4 and NDUFA7 and NDUFB1 and NDUFB10 and NDUFB2 and NDUFB3 and NDUFB4 and NDUFB5 and NDUFB6 and NDUFB7 and NDUFB8 and NDUFC1 and NDUFC2 and NDUFS5 and NDUFV3 and TUSC3 and 10010 and 10318 and 10541 and 11702 and 12497 and 12543 and 13017 and 13214 and 14261 and 14418 and 14900 and 15179 and 15864 and 15947 and 15973 and 15998 and 8699 and 9348) or (ND1 and ND2 and ND3 and ND4 and ND4L and ND5 and Ndufa1 and Ndufa10 and Ndufa11 and Ndufa13 and Ndufa3 and Ndufa4 and Ndufa7 and Ndufb10 and Ndufb2 and Ndufb3 and Ndufb5 and Ndufb6 and Ndufb7 and Ndufb8 and Ndufc1 and Ndufc2 and Ndufs5 and Ndufv3 and Tusc3 and mmu_100043472 and mmu_4541 and mmu_4707 and mmu_4710 and 10010 and 10318 and 10541 and 11702 and 12497 and 12543 and 13017 and 13214 and 14418 and 14900 and 15179 and 15864 and 15947 and 15973 and 8699 and 9348)

DHORD2 dhor__S_c + q8_c --> orot_c + q8h2_c 8814
G3PD5 glyc3p_c + q8_c --> dhap_c + q8h2_c 11039 or (b2241 and b2242 and b2243)
NADH16pp 4.0 h_c + nadh_c + q8_c --> 3.0 h_p + nad_c + q8h2_c (PP_4119 and PP_4125 and PP_4127 and PP_4128 and PP_4129 and PP_4130 and PP_4131 and 13017 and 13214 and 14418 and 15864 and 15973 and 8699) or (b2276 and b2277 and b2278 and b2279 and b2280 and b2282 and b2288 and 13017 and 13214 and 14418 and 15864 and 15973 and 8699)
NADH5 h_c + nadh_c + q8_c --> nad_c + q8h2_c 14898 or 9809
NADPHQR2 h_c + nadph_c + q8_c --> nadp_c + q8h2_c 13657
PROD3 pro__L_c + q8_c --> 1pyr5c_c + h_c + q8h2_c 8569
SUCDi q8_c + succ_c --> fum_c + q8h2_c (PP_4192 and PP_4193 and 11977 and 16281) or (b0721 and b0722 and 11977 and 16281)

COQ3_m 2npmhmobq_m + amet_m --> ahcys_m + h_m + q9_m 11465
ETFQO_m etfrd_m + q9_m --> etfox_m + q9h2_m 10218
```

In [93]:

```
for r in sorted(model.reactions, key=lambda x: x.id):
    if r.id.startswith("CYOR"):
        print(r.id, r.reaction, r.gene_reaction_rule)
```

```
CYOR_q8_m 2.0 ficytc_m + 2.0 h_m + q8h2_m --> 2.0 focytc_m + 4.0 h_c + q8_m (13614 and 15681 and 8802) or (13614 and 15681 and 9198)
CYOR_u10m 2.0 ficytc_m + 2.0 h_m + q10h2_m --> 2.0 focytc_m + 4.0 h_c + q10_m (CYTB and UQCR11 and UQCRH and UQCRQ and 11618 and 13614 and 15231 and 15681 and 9198 and 9705) or (CYTB and Uqcr11 and Uqcrq and mmu_100042918 and 11618 and 13614 and 15231 and 15681 and 9198 and 9705)
CYOR_u6m 2.0 ficytc_m + 1.5 h_m + q6h2_m --> 2.0 focytc_m + 1.5 h_c + q6_m Q0105 and YBL045C and 11618 and 12966 and 13608 and 13614 and 15231 and 15681 and 15758 and 8802 and 9198
```

Cytochrome c reductase  
COB (Q0105, CYTB) RTO3\_979594 # initially had it as 946001, but 979594 matches splicing better  
COR1 (YBL045C) 9705 blast to MAS1 homolog in Rhodo  
CYT1 9198  
QCR2 11618  
QCR6 15758  
QCR7 15681  
QCR8 12966  
QCR9 15231  
QCR10 13608  
RIP1 13614  
CYC1/CYC7 8802

CBP3 (assembly) 12140

CYOR\_u6m is yeast ubiquinol 6 cytochome c reductase, incorect stoichiometry, Rhodo is q9  
CYOR\_u6m 2.0 ficytc\_m + 1.5 h\_m + q6h2\_m --> 2.0 focytc\_m + 1.5 h\_c + q6\_m  
Update reaction to  
CYOR\_u9m 2.0 ficytc\_m + 2.0 h\_m + q9h2\_m --> 2.0 focytc\_m + 4.0 h\_c + q9\_m  
Change genes to 'RTO3\_979594 and 9705 and 9198 and 11618 and 15758 and 15681 and 12966 and 15231 and 13608 and 13614 and 8802'  
Remove CYOR\_q8\_m, CYOR\_u10m, yli\_R0431

In [94]:

```
model.reactions.get_by_id('CYOR_u6m').id = 'CYOR_u9m'
model.reactions.get_by_id('CYOR_u9m').gene_reaction_rule = 'RTO3_979594 and 9705 and 9198 and 11618 and 15758 and 15681 and 12966 and 15231 and 13608 and 13614 and 8802'
model.reactions.get_by_id('CYOR_u9m').add_metabolites({'h_m': -0.5, 'h_c': 2.5, 'q6h2_m': 1.0, 'q6_m': -1.0, 'q9h2_m': -1.0, 'q9_m': 1.0})
model.remove_reactions(['CYOR_q8_m','CYOR_u10m','yli_R0431'],remove_orphans=True)
```

In [95]:

```
for r in sorted(model.metabolites.get_by_id('dhor__S_c').reactions, key=lambda x: x.id):
    print(r.id, r.reaction, r.gene_reaction_rule)
print()
for r in sorted(model.metabolites.get_by_id('dhor__S_m').reactions, key=lambda x: x.id):
    print(r.id, r.reaction, r.gene_reaction_rule)
```

```
DHORD2 dhor__S_c + q8_c --> orot_c + q8h2_c 8814
DHORD4i dhor__S_c + q6_m --> orot_c + q6h2_m 14866
DHORD5 dhor__S_c + mqn8_c --> mql8_c + orot_c 8814
DHORD9 dhor__S_c + q10_m --> orot_c + q10h2_m 8814
DHORDfum dhor__S_c + fum_c --> orot_c + succ_c 14866 or 8814
DHORDi dhor__S_c + o2_c --> h2o2_c + orot_c 14866 or 8814
DHORTS dhor__S_c + h2o_c <=> cbasp_c + h_c 12291 or 12302 or 16681
yli_R1559 dhor__S_c + yli_M07040_c <=> hqn_c + orot_c 8814

DHR cbasp_m <=> dhor__S_m + h2o_m + h_m 12291
DHROm dhor__S_m + h_m + o2_m <=> h2o2_m + orot_m CRv4_Au5_s6_g13154_t1 and 8814
```

In [96]:

```
for r in sorted(model.metabolites.get_by_id('orot_c').reactions, key=lambda x: x.id):
    print(r.id, r.reaction, r.gene_reaction_rule)
print()
for r in sorted(model.metabolites.get_by_id('orot5p_c').reactions, key=lambda x: x.id):
    print(r.id, r.reaction, r.gene_reaction_rule)
print()
for r in sorted(model.metabolites.get_by_id('orot_m').reactions, key=lambda x: x.id):
    print(r.id, r.reaction, r.gene_reaction_rule)
```

```
DHORD2 dhor__S_c + q8_c --> orot_c + q8h2_c 8814
DHORD4i dhor__S_c + q6_m --> orot_c + q6h2_m 14866
DHORD5 dhor__S_c + mqn8_c --> mql8_c + orot_c 8814
DHORD9 dhor__S_c + q10_m --> orot_c + q10h2_m 8814
DHORDfum dhor__S_c + fum_c --> orot_c + succ_c 14866 or 8814
DHORDi dhor__S_c + o2_c --> h2o2_c + orot_c 14866 or 8814
ORPT orot5p_c + ppi_c <=> orot_c + prpp_c 10716 or 12118
yli_R1559 dhor__S_c + yli_M07040_c <=> hqn_c + orot_c 8814

OMPDC h_c + orot5p_c --> co2_c + ump_c 12118
ORPT orot5p_c + ppi_c <=> orot_c + prpp_c 10716 or 12118

DHROm dhor__S_m + h_m + o2_m <=> h2o2_m + orot_m CRv4_Au5_s6_g13154_t1 and 8814
```

In [97]:

```
for r in sorted(model.genes.get_by_id('16681').reactions, key=lambda x: x.id):
    print(r.id, r.reaction, r.gene_reaction_rule)
print()
for r in sorted(model.genes.get_by_id('12302').reactions, key=lambda x: x.id):
    print(r.id, r.reaction, r.gene_reaction_rule)
print()
for r in sorted(model.genes.get_by_id('13297').reactions, key=lambda x: x.id):
    print(r.id, r.reaction, r.gene_reaction_rule)
print()
for r in sorted(model.genes.get_by_id('12291').reactions, key=lambda x: x.id):
    print(r.id, r.reaction, r.gene_reaction_rule)
print()
for r in sorted(model.genes.get_by_id('14866').reactions, key=lambda x: x.id):
    print(r.id, r.reaction, r.gene_reaction_rule)
print()
for r in sorted(model.genes.get_by_id('8814').reactions, key=lambda x: x.id):
    print(r.id, r.reaction, r.gene_reaction_rule)
print()
for r in sorted(model.genes.get_by_id('10716').reactions, key=lambda x: x.id):
    print(r.id, r.reaction, r.gene_reaction_rule)
print()
for r in sorted(model.genes.get_by_id('12118').reactions, key=lambda x: x.id):
    print(r.id, r.reaction, r.gene_reaction_rule)
print()
```

```
ASPCT asp__L_c + cbp_c --> cbasp_c + h_c + pi_c 12302 or 16681
CBPS 2.0 atp_c + gln__L_c + h2o_c + hco3_c --> 2.0 adp_c + cbp_c + glu__L_c + 2.0 h_c + pi_c 12302 or 16681 or (12302 and 13297) or (13297 and 16681)
CBPSam 2.0 atp_m + hco3_m + nh4_m --> 2.0 adp_m + cbp_m + 2.0 h_m + pi_m 12302 or 16681
CPS 2.0 atp_h + co2_h + h2o_h + nh4_h --> 2.0 adp_h + cbp_h + 3.0 h_h + pi_h 12302 or 16681
DHORTS dhor__S_c + h2o_c <=> cbasp_c + h_c 12291 or 12302 or 16681
HCGALm 2.0 atp_m + gln__L_m + h2o_m + hco3_m --> 2.0 adp_m + cbp_m + glu__L_m + 2.0 h_m + pi_m (12302 and 13297) or (13297 and 16681)

ASPCT asp__L_c + cbp_c --> cbasp_c + h_c + pi_c 12302 or 16681
CBPS 2.0 atp_c + gln__L_c + h2o_c + hco3_c --> 2.0 adp_c + cbp_c + glu__L_c + 2.0 h_c + pi_c 12302 or 16681 or (12302 and 13297) or (13297 and 16681)
CBPSam 2.0 atp_m + hco3_m + nh4_m --> 2.0 adp_m + cbp_m + 2.0 h_m + pi_m 12302 or 16681
CPS 2.0 atp_h + co2_h + h2o_h + nh4_h --> 2.0 adp_h + cbp_h + 3.0 h_h + pi_h 12302 or 16681
DHORTS dhor__S_c + h2o_c <=> cbasp_c + h_c 12291 or 12302 or 16681
HCGALm 2.0 atp_m + gln__L_m + h2o_m + hco3_m --> 2.0 adp_m + cbp_m + glu__L_m + 2.0 h_m + pi_m (12302 and 13297) or (13297 and 16681)

CBPS 2.0 atp_c + gln__L_c + h2o_c + hco3_c --> 2.0 adp_c + cbp_c + glu__L_c + 2.0 h_c + pi_c 12302 or 16681 or (12302 and 13297) or (13297 and 16681)
HCGALm 2.0 atp_m + gln__L_m + h2o_m + hco3_m --> 2.0 adp_m + cbp_m + glu__L_m + 2.0 h_m + pi_m (12302 and 13297) or (13297 and 16681)

DHORTS dhor__S_c + h2o_c <=> cbasp_c + h_c 12291 or 12302 or 16681
DHR cbasp_m <=> dhor__S_m + h2o_m + h_m 12291

DHORD4i dhor__S_c + q6_m --> orot_c + q6h2_m 14866
DHORDfum dhor__S_c + fum_c --> orot_c + succ_c 14866 or 8814
DHORDi dhor__S_c + o2_c --> h2o2_c + orot_c 14866 or 8814

DHORD2 dhor__S_c + q8_c --> orot_c + q8h2_c 8814
DHORD5 dhor__S_c + mqn8_c --> mql8_c + orot_c 8814
DHORD9 dhor__S_c + q10_m --> orot_c + q10h2_m 8814
DHORDfum dhor__S_c + fum_c --> orot_c + succ_c 14866 or 8814
DHORDi dhor__S_c + o2_c --> h2o2_c + orot_c 14866 or 8814
DHROm dhor__S_m + h_m + o2_m <=> h2o2_m + orot_m CRv4_Au5_s6_g13154_t1 and 8814
yli_R1559 dhor__S_c + yli_M07040_c <=> hqn_c + orot_c 8814

ORPT orot5p_c + ppi_c <=> orot_c + prpp_c 10716 or 12118

5FLURAPRT 5flura_c + prpp_c --> 5flurimp_c + h_c + ppi_c 12118
OMPDC h_c + orot5p_c --> co2_c + ump_c 12118
ORPT orot5p_c + ppi_c <=> orot_c + prpp_c 10716 or 12118
```

UMP biosynthesis  
16681 cyto 14.5, cyto\_mito 9.166, cyto\_nucl 8.833, plas 4, E.R. 3, mito 2.5 (no sigP) URA2 K11541: URA2; carbamoyl-phosphate synthase 6.3.5.5 / aspartate carbamoyltransferase 2.1.3.2 -> CBPS / ASPCT  
12302 mito 21, cyto 4 (no sigP) CPA2 K01955: carB, CPA2; carbamoyl-phosphate synthase large subunit 6.3.5.5 -> CBPS  
13297 mito 15, extr 10 (sigP) CPA1 K01956: carA, CPA1; carbamoyl-phosphate synthase small subunit -> CBPS  
12291 cyto 14.5, cyto\_nucl 10.5, mito 8, nucl 3.5 (no sigP) URA4 K01465: URA4, pyrC; dihydroorotase 3.5.2.3 -> DHORTS, DHR incorrect stoic

14866 mito 20, nucl 4, cyto 2 (sigP) URA1 K00226: pyrD; dihydroorotate dehydrogenase (fumarate) -> DHORDfum, this is known as cytosolic in literature, but sigP is there  
8814 mito 22, cyto 3 (sigP) K00254: DHODH, pyrD; dihydroorotate dehydrogenase (quinone) -> DHORD4i  
10716 cyto 21, cyto\_mito 12.833, cyto\_nucl 11.833, mito 3.5 (no sigP) URA10,URA5 K00762: pyrE; orotate phosphoribosyltransferase -> ORPT  
12118 mito 22, cyto 5 (no sigP) URA3 K13421: UMPS; uridine monophosphate synthetase -> OMPDC

Change CBPS genes to '16681'  
Change HCGALm to make CBPSm, change genes to '12302 and 13297'  
Remove CBPSam, CPS  
Change ASPCT genes to '16681'  
Change DHORTS genes to '12291'  
Remove DHR  
DHORD localization  
http://www.plantcell.org/content/24/4/1549  
https://beyondthedish.wordpress.com/2017/01/03/hitting-acute-myeloid-leukemia-where-it-hurts/  
Change DHORD4i to DHORD\_u9m, and replace q6 with q9\_m, change genes to '8814'  
Change DHORDfum genes to '14866'  
Remove DHORDi, DHORD2, DHORD5, DHORD9, DHROm, yli\_R1559
Change ORPT genes to '10716'  
Change OMPDC genes to '12118'  
Remove 5FLURAPRT

In [98]:

```
model.reactions.get_by_id('CBPS').gene_reaction_rule = '16681'
model.reactions.get_by_id('HCGALm').id = 'CBPSm'
model.reactions.get_by_id('CBPSm').gene_reaction_rule = '12302 and 13297'
model.remove_reactions(['CBPSam','CPS'], remove_orphans=True)
model.reactions.get_by_id('ASPCT').gene_reaction_rule = '16681'
model.reactions.get_by_id('DHORTS').gene_reaction_rule = '12291'
model.remove_reactions(['DHR'], remove_orphans=True)
model.reactions.get_by_id('DHORD4i').id = 'DHORD_u9m'
model.reactions.get_by_id('DHORD_u9m').add_metabolites({'q6_m': 1.0, 'q6h2_m': -1.0, 'q9_m': -1.0, 'q9h2_m': 1.0})
model.reactions.get_by_id('DHORD_u9m').gene_reaction_rule = '8814'
model.reactions.get_by_id('DHORDfum').gene_reaction_rule = '14866'
model.remove_reactions(['DHORDi','DHORD2','DHORD5','DHORD9','DHROm','yli_R1559'], remove_orphans=True)
model.reactions.get_by_id('ORPT').gene_reaction_rule = '10716'
model.reactions.get_by_id('OMPDC').gene_reaction_rule = '12118'
model.remove_reactions(['5FLURAPRT'], remove_orphans=True)
```

In [99]:

```
for r in sorted(model.reactions, key=lambda x: x.id):
    if r.id.startswith('NADH'):
        print(r.id, r.reaction, r.gene_reaction_rule)
print()
for r in sorted(model.reactions, key=lambda x: x.id):
    if r.id.startswith('NADPH'):
        print(r.id, r.reaction, r.gene_reaction_rule)
print()
for x in ['yli_R0430','yli_R0432']:
    r = model.reactions.get_by_id(x)
    print(r.id, r.reaction, r.gene_reaction_rule)
```

```
NADH10 h_c + mqn8_c + nadh_c --> mql8_c + nad_c 14898 or 9809
NADH16pp 4.0 h_c + nadh_c + q8_c --> 3.0 h_p + nad_c + q8h2_c (PP_4119 and PP_4125 and PP_4127 and PP_4128 and PP_4129 and PP_4130 and PP_4131 and 13017 and 13214 and 14418 and 15864 and 15973 and 8699) or (b2276 and b2277 and b2278 and b2279 and b2280 and b2282 and b2288 and 13017 and 13214 and 14418 and 15864 and 15973 and 8699)
NADH17pp 4.0 h_c + mqn8_c + nadh_c --> 3.0 h_p + mql8_c + nad_c b2276 and b2277 and b2278 and b2279 and b2280 and b2282 and b2288 and 13017 and 13214 and 14418 and 15864 and 15973 and 8699
NADH18pp 2dmmq8_c + 4.0 h_c + nadh_c --> 2dmmql8_c + 3.0 h_p + nad_c b2276 and b2277 and b2278 and b2279 and b2280 and b2282 and b2288 and 13017 and 13214 and 14418 and 15864 and 15973 and 8699
NADH2_u10m 5.0 h_m + nadh_m + q10_m --> 4.0 h_c + nad_m + q10h2_m (ND1 and ND2 and ND3 and ND4 and ND4L and ND5 and ND6 and NDUFA1 and NDUFA10 and NDUFA11 and NDUFA3 and NDUFA4 and NDUFA7 and NDUFB1 and NDUFB10 and NDUFB2 and NDUFB3 and NDUFB4 and NDUFB5 and NDUFB6 and NDUFB7 and NDUFB8 and NDUFC1 and NDUFC2 and NDUFS5 and NDUFV3 and TUSC3 and 10010 and 10318 and 10541 and 11702 and 12497 and 12543 and 13017 and 13214 and 14261 and 14418 and 14900 and 15179 and 15864 and 15947 and 15973 and 15998 and 8699 and 9348) or (ND1 and ND2 and ND3 and ND4 and ND4L and ND5 and Ndufa1 and Ndufa10 and Ndufa11 and Ndufa13 and Ndufa3 and Ndufa4 and Ndufa7 and Ndufb10 and Ndufb2 and Ndufb3 and Ndufb5 and Ndufb6 and Ndufb7 and Ndufb8 and Ndufc1 and Ndufc2 and Ndufs5 and Ndufv3 and Tusc3 and mmu_100043472 and mmu_4541 and mmu_4707 and mmu_4710 and 10010 and 10318 and 10541 and 11702 and 12497 and 12543 and 13017 and 13214 and 14418 and 14900 and 15179 and 15864 and 15947 and 15973 and 8699 and 9348)
NADH2_u6cm h_c + nadh_c + q6_m --> nad_c + q6h2_m 8972
NADH2_u6m h_m + nadh_m + q6_m --> nad_m + q6h2_m 14261 or 9809
NADH5 h_c + nadh_c + q8_c --> nad_c + q8h2_c 14898 or 9809
NADH9 2dmmq8_c + h_c + nadh_c --> 2dmmql8_c + nad_c 14898 or 9809
NADHHR h2o_c + nadh_c --> nadhx__R_c 10613
NADHHS h2o_c + nadh_c --> nadhx__S_c 10613
NADHOR h_m + nadh_m + q8_m --> nad_m + q8h2_m 13766 or 8972 or (CRv4_Au5_s2_g9603_t1 and CRv4_Au5_s6_g12740_t1 and CRv4_Au5_s6_g13274_t1 and CRv4_Au5_s7_g14116_t1 and CRv4_Au5_s8_g15041_t1 and 10010 and 10541 and 11151 and 11702 and 12497 and 13017 and 13214 and 14078 and 14418 and 14900 and 15179 and 15973 and 15998 and 8699) or (CRv4_Au5_s2_g9603_t1 and CRv4_Au5_s6_g12740_t1 and CRv4_Au5_s7_g14116_t1 and CRv4_Au5_s8_g15041_t1 and 10010 and 10541 and 11151 and 11702 and 12497 and 13017 and 13214 and 14078 and 14418 and 14828 and 14900 and 15179 and 15973 and 15998 and 8699) or (CRv4_Au5_s2_g9603_t1 and CRv4_Au5_s6_g12740_t1 and CRv4_Au5_s7_g14116_t1 and CRv4_Au5_s8_g15041_t1 and 10010 and 10541 and 11151 and 11702 and 12497 and 13017 and 13214 and 14078 and 14418 and 14831 and 14900 and 15179 and 15973 and 15998 and 8699)
NADHPO h2o2_c + h_c + nadh_c --> 2.0 h2o_c + nad_c b0606 and 12715
NADHXD adp_c + nadhx__S_c --> amp_c + h_c + nadh_c + pi_c 16345
NADHXE nadhx__S_c <=> nadhx__R_c 16345

NADPHHR h2o_c + nadph_c --> nadphx__R_c 10613
NADPHHS h2o_c + nadph_c --> nadphx__S_c 10613
NADPHQR2 h_c + nadph_c + q8_c --> nadp_c + q8h2_c 13657
NADPHXD adp_c + nadphx__S_c --> amp_c + h_c + nadph_c + pi_c 16345
NADPHXE nadphx__R_c <=> nadphx__S_c 16345

yli_R0430 h_m + nadh_m + q6_m --> nad_m + yli_M04633_m YALIfMp01 and YALIfMp02 and YALIfMp16 and YALIfMp19 and YALIfMp20 and YALIfMp28 and YALIfMp29 and 10010 and 10318 and 11151 and 11702 and 12497 and 12543 and 13017 and 13214 and 13766 and 14078 and 14261 and 14418 and 14900 and 15179 and 15864 and 15947 and 15973 and 8699 and 8909 and 8972 and 9348
yli_R0432 h_c + nadh_c + q6_m --> nad_c + yli_M04633_m YALIfMp01 and YALIfMp02 and YALIfMp16 and YALIfMp19 and YALIfMp20 and YALIfMp28 and YALIfMp29 and 10010 and 10318 and 11151 and 11702 and 12497 and 12543 and 13017 and 13214 and 13766 and 14078 and 14261 and 14418 and 14900 and 15179 and 15864 and 15947 and 15973 and 8699 and 8909 and 8972 and 9348
```

NADH:ubiquinone reductase  
8972 NDE1/NDE2 cytosolic NADH, ndh1; NADH:ubiquinone reductase (non-electrogenic) -> NADH2\_u6cm  
13766 ndh1; NADH:ubiquinone reductase (non-electrogenic), blast to External alternative NAD(P)H-ubiquinone oxidoreductase B1, mitochondrial; EC 1.6.5.9; External alternative NADH dehydrogenase NDB1; NADH:ubiquinone reductase (non-electrogenic) NDB1  
14261 ACP1 / NADH-ubiquinone oxidoreductase 9.6 kDa subunit, it is acp1 in yeast, but NDUFAB1 in human  
9809 NDI1? blast to Internal alternative NAD(P)H-ubiquinone oxidoreductase A2, mitochondrial; EC 1.6.5.9; Internal alternative NADH dehydrogenase NDA2; NADH:ubiquinone reductase (non-electrogenic) NDA2  
Change NADH2\_u6cm to NADH2\_u9cm, replace q6 with q9, change genes to '8972 or 13766'  
Change NADH2\_u6m to NADH2\_u9m, replace q6 with q9, change genes to '9809'  
14898 wrbA; NAD(P)H dehydrogenase (quinone) -> blast 1,4-benzoquinone reductase (NADPH) -> no reaction in BiGG  
15859 qorB; NAD(P)H dehydrogenase (quinone) -> low cov hit (half and half) to two different functions

Find all subunits for NADH dehydrogenase in v4 and v3 (mito)  
14261 Acyl carrier protein/NADH-ubiquinone oxidoreductase, NDUFAB1/SDAP subunit  
13656 Complex I intermediate-associated protein 30 (CIA30):PF08547,SUPERFAMILY::SSF49785  
8817 ESSS subunit of NADH:ubiquinone oxidoreductase (complex I):PF10183  
11151 NADH dehydrogenase (ubiquinone) Fe-S protein 5  
8530 NADH dehydrogenase [ubiquinone] 1 alpha subcomplex subunit  
14717 NADH dehydrogenase [ubiquinone] 1 beta subcomplex  
10017 NADH-dehydrogenase (ubiquinone)  
13411 NADH-ubiquinone oxidoreductase 21 kDa subunit  
8909 NADH-ubiquinone oxidoreductase ASHI subunit (CI-ASHI or NDUFB8):PF05821  
13268 NADH-ubiquinone oxidoreductase B12 subunit family:PF08122  
8446 NADH-ubiquinone oxidoreductase B15 subunit (NDUFB4):PF07225  
12482 NADH-ubiquinone reductase complex 1 MLRQ subunit:PF06522  
9144 NADH:ubiquinone oxidoreductase kD subunit  
11857 NADH:ubiquinone oxidoreductase, 17.2kDa subunit  
14900 NDUFA12; NADH dehydrogenase (ubiquinone) 1 alpha subcomplex subunit 12  
15998 NDUFA13; NADH dehydrogenase (ubiquinone) 1 alpha subcomplex subunit 13  
9348 NDUFA2; NADH dehydrogenase (ubiquinone) 1 alpha subcomplex subunit 2  
12497 NDUFA5; NADH dehydrogenase (ubiquinone) 1 alpha subcomplex subunit 5  
10541 NDUFA6; NADH dehydrogenase (ubiquinone) 1 alpha subcomplex subunit 6  
15947 NDUFA8; NADH dehydrogenase (ubiquinone) 1 alpha subcomplex subunit 8  
15179 NDUFA9; NADH dehydrogenase (ubiquinone) 1 alpha subcomplex subunit 9  
11411 NDUFAF1, CIA30; NADH dehydrogenase [ubiquinone] 1 alpha subcomplex assembly factor 1  
13925 NDUFAF3; NADH dehydrogenase [ubiquinone] 1 alpha subcomplex assembly factor 3  
12653 NDUFAF5; NADH dehydrogenase [ubiquinone] 1 alpha subcomplex assembly factor 5  
10607 NDUFAF6; NADH dehydrogenase [ubiquinone] 1 alpha subcomplex assembly factor 6  
8444 NDUFAF7; NADH dehydrogenase [ubiquinone] 1 alpha subcomplex assembly factor 7  
14078 NDUFB7; NADH dehydrogenase (ubiquinone) 1 beta subcomplex subunit 7  
10318 NDUFB9; NADH dehydrogenase (ubiquinone) 1 beta subcomplex subunit 9  
13017 NDUFS1; NADH dehydrogenase (ubiquinone) Fe-S protein 1  
15864 NDUFS2; NADH dehydrogenase (ubiquinone) Fe-S protein 2  
11702 NDUFS3; NADH dehydrogenase (ubiquinone) Fe-S protein 3  
10010 NDUFS4; NADH dehydrogenase (ubiquinone) Fe-S protein 4  
12543 NDUFS6; NADH dehydrogenase (ubiquinone) Fe-S protein 6  
8699 NDUFS7; NADH dehydrogenase (ubiquinone) Fe-S protein 7  
15973 NDUFS8; NADH dehydrogenase (ubiquinone) Fe-S protein 8  
14418 NDUFV1; NADH dehydrogenase (ubiquinone) flavoprotein 1  
13214 NDUFV2; NADH dehydrogenase (ubiquinone) flavoprotein 2  
RTO3\_879512 NADH dehydrogenase subunit 4L and related proteins  
RTO3\_879341 NADH dehydrogenase subunit 4L and related proteins  
RTO3\_900641 ND1; NADH-ubiquinone oxidoreductase chain 1  
RTO3\_874609 ND3; NADH-ubiquinone oxidoreductase chain 3  
RTO3\_945995 ND4; NADH-ubiquinone oxidoreductase chain 4  
RTO3\_945994 ND5; NADH-ubiquinone oxidoreductase chain 5  
RTO3\_946005 ND5; NADH-ubiquinone oxidoreductase chain 5  
RTO3\_900622 ND6; NADH-ubiquinone oxidoreductase chain 6  
RTO3\_900624 nuoN; NADH-quinone oxidoreductase subunit N  
Change NADH2\_u10m to NADH2\_u9m, replace q10 with q9, change genes to  
'10010 and 10017 and 10318 and 10541 and 10607 and 11151 and 11411 and 11702 and 11857 and 12482 and 12497 and 12543 and 12653 and 13017 and 13214 and 13268 and 13411 and 13656 and 13925 and 14078 and 14261 and 14418 and 14717 and 14900 and 15179 and 15864 and 15947 and 15973 and 15998 and 8444 and 8446 and 8530 and 8699 and 8817 and 8909 and 9144 and 9348 and RTO3\_874609 and RTO3\_879341 and RTO3\_879512 and RTO3\_900622 and RTO3\_900624 and RTO3\_900641 and RTO3\_945994 and RTO3\_945995 and RTO3\_946005'  
Remove NADH10, NADH16pp, NADH17pp, NADH18pp, NADH5, NADH9, NADHOR, yli\_R0430, yli\_R0432

10613 cyto GAPDH (reactions only in E. coli model, but NADH reactions also in metacyc by human GAPDH)  
Remove GAPDHh, GAPDHm  
16345 mito 21, cyto 5.5, cyto\_nucl 3.5 (no sigP) CARKD ATP-dependent NAD(P)H-hydrate dehydratase, NADPHXD is with ADP (bacteria), eukaryotic CARKD is with ATP  
Change NADHXD to NADHXD2, NADPHXD to NADPHXD2, replace adp/amp with atp/adp  
8901 cyto 12.5, cyto\_nucl 9.5, extr 5, mito 4, nucl 3.5 NAD(P)H-hydrate epimerase  
Change NADHXE, NADPHXE genes to 8901  
13657 cyto 15.5, cyto\_nucl 9, mito 3, pero 3, extr 2 (weak sigP) qor, CRYZ; NADPH2:quinone reductase, NADPHQR2 is full reduction whereas 1.6.5.5 is one-electron reduction (semiquinone), may be non-enzymatically reduced to hydroquinone  
Remove NADPHQR2, since orthoquinones, such as 1,2-naphthoquinone or 9,10-phenanthrenequinone, are the best substrates  
12715 is Alkyl hydroperoxide reductase, thiol specific antioxidant and related enzymes, no NADH peroxidase gene in Rhodo  
Remove NADHPO

In [100]:

```
model.reactions.get_by_id('NADH2_u6cm').id = 'NADH2_u9cm'
model.reactions.get_by_id('NADH2_u9cm').add_metabolites({'q6_m': 1.0, 'q6h2_m': -1.0, 'q9_m': -1.0, 'q9h2_m': 1.0})
model.reactions.get_by_id('NADH2_u9cm').gene_reaction_rule = '8972 or 13766'
model.reactions.get_by_id('NADH2_u6m').id = 'NADH2_u9m1'
model.reactions.get_by_id('NADH2_u9m1').add_metabolites({'q6_m': 1.0, 'q6h2_m': -1.0, 'q9_m': -1.0, 'q9h2_m': 1.0})
model.reactions.get_by_id('NADH2_u9m1').gene_reaction_rule = '9809'
model.reactions.get_by_id('NADH2_u10m').id = 'NADH2_u9m2'
model.reactions.get_by_id('NADH2_u9m2').add_metabolites({'q10_m': 1.0, 'q10h2_m': -1.0, 'q9_m': -1.0, 'q9h2_m': 1.0})
model.reactions.get_by_id('NADH2_u9m2').gene_reaction_rule = '10010 and 10017 and 10318 and 10541 and 10607 and 11151 and 11411 and 11702 and 11857 and 12482 and 12497 and 12543 and 12653 and 13017 and 13214 and 13268 and 13411 and 13656 and 13925 and 14078 and 14261 and 14418 and 14717 and 14900 and 15179 and 15864 and 15947 and 15973 and 15998 and 8444 and 8446 and 8530 and 8699 and 8817 and 8909 and 9144 and 9348 and RTO3_874609 and RTO3_879341 and RTO3_879512 and RTO3_900622 and RTO3_900624 and RTO3_900641 and RTO3_945994 and RTO3_945995 and RTO3_946005'
model.remove_reactions(['NADH10','NADH16pp','NADH17pp','NADH18pp','NADH5','NADH9','NADHOR','yli_R0430','yli_R0432'], remove_orphans=True)
model.remove_reactions(['GAPDHh','GAPDHm'], remove_orphans=True)
model.reactions.get_by_id('NADHXD').id = 'NADHXD2'
model.reactions.get_by_id('NADHXD2').add_metabolites({'adp_c': 2.0, 'amp_c': -1.0, 'atp_c': -1.0})
model.reactions.get_by_id('NADPHXD').id = 'NADPHXD2'
model.reactions.get_by_id('NADPHXD2').add_metabolites({'adp_c': 2.0, 'amp_c': -1.0, 'atp_c': -1.0})
model.reactions.get_by_id('NADHXE').gene_reaction_rule = '8901'
model.reactions.get_by_id('NADPHXE').gene_reaction_rule = '8901'
model.remove_reactions(['NADPHQR2'], remove_orphans=True)
model.remove_reactions(['NADHPO'], remove_orphans=True)
```

In [101]:

```
for r in sorted(model.reactions, key=lambda x: x.id):
    if r.id.startswith('NADH'):
        print(r.id, r.reaction, r.gene_reaction_rule)
print()
for r in sorted(model.reactions, key=lambda x: x.id):
    if r.id.startswith('NADPH'):
        print(r.id, r.reaction, r.gene_reaction_rule)
```

```
NADH2_u9cm h_c + nadh_c + q9_m --> nad_c + q9h2_m 8972 or 13766
NADH2_u9m1 h_m + nadh_m + q9_m --> nad_m + q9h2_m 9809
NADH2_u9m2 5.0 h_m + nadh_m + q9_m --> 4.0 h_c + nad_m + q9h2_m 10010 and 10017 and 10318 and 10541 and 10607 and 11151 and 11411 and 11702 and 11857 and 12482 and 12497 and 12543 and 12653 and 13017 and 13214 and 13268 and 13411 and 13656 and 13925 and 14078 and 14261 and 14418 and 14717 and 14900 and 15179 and 15864 and 15947 and 15973 and 15998 and 8444 and 8446 and 8530 and 8699 and 8817 and 8909 and 9144 and 9348 and RTO3_874609 and RTO3_879341 and RTO3_879512 and RTO3_900622 and RTO3_900624 and RTO3_900641 and RTO3_945994 and RTO3_945995 and RTO3_946005
NADHHR h2o_c + nadh_c --> nadhx__R_c 10613
NADHHS h2o_c + nadh_c --> nadhx__S_c 10613
NADHXD2 atp_c + nadhx__S_c --> adp_c + h_c + nadh_c + pi_c 16345
NADHXE nadhx__S_c <=> nadhx__R_c 8901

NADPHHR h2o_c + nadph_c --> nadphx__R_c 10613
NADPHHS h2o_c + nadph_c --> nadphx__S_c 10613
NADPHXD2 atp_c + nadphx__S_c --> adp_c + h_c + nadph_c + pi_c 16345
NADPHXE nadphx__R_c <=> nadphx__S_c 8901
```

In [102]:

```
for r in sorted(model.genes.get_by_id('11629').reactions, key=lambda x: x.id):
    print(r.id, r.reaction, r.gene_reaction_rule)
print()
for r in sorted(model.genes.get_by_id('11802').reactions, key=lambda x: x.id):
    print(r.id, r.reaction, r.gene_reaction_rule)
print()
for r in sorted(model.genes.get_by_id('11977').reactions, key=lambda x: x.id):
    print(r.id, r.reaction, r.gene_reaction_rule)
print()
for r in sorted(model.genes.get_by_id('16281').reactions, key=lambda x: x.id):
    print(r.id, r.reaction, r.gene_reaction_rule)
```

```
SUCD1m fad_m + succ_m <=> fadh2_m + fum_m 11629 or (SDHD and 11629 and 11977 and 16281) or (Sdhd and 11629 and 11977 and 16281) or (11629 and 11802 and 11977 and 16281)
SUCD2_u6m q6_m + succ_m <=> fum_m + q6h2_m 11629 and 11802 and 11977 and 16281
SUCD3_u6m fadh2_m + q6_m <=> fad_m + q6h2_m 11629 and 11802 and 11977 and 16281

SUCD1m fad_m + succ_m <=> fadh2_m + fum_m 11629 or (SDHD and 11629 and 11977 and 16281) or (Sdhd and 11629 and 11977 and 16281) or (11629 and 11802 and 11977 and 16281)
SUCD2_u6m q6_m + succ_m <=> fum_m + q6h2_m 11629 and 11802 and 11977 and 16281
SUCD3_u6m fadh2_m + q6_m <=> fad_m + q6h2_m 11629 and 11802 and 11977 and 16281

SUCD1 fad_c + succ_c <=> fadh2_c + fum_c PP_4192 and PP_4193 and 11977 and 16281
SUCD1m fad_m + succ_m <=> fadh2_m + fum_m 11629 or (SDHD and 11629 and 11977 and 16281) or (Sdhd and 11629 and 11977 and 16281) or (11629 and 11802 and 11977 and 16281)
SUCD2_u6m q6_m + succ_m <=> fum_m + q6h2_m 11629 and 11802 and 11977 and 16281
SUCD3_u6m fadh2_m + q6_m <=> fad_m + q6h2_m 11629 and 11802 and 11977 and 16281
SUCDH_q8_m q8_m + succ_m <=> fum_m + q8h2_m 11977 and 16281
SUCDi q8_c + succ_c --> fum_c + q8h2_c (PP_4192 and PP_4193 and 11977 and 16281) or (b0721 and b0722 and 11977 and 16281)

FRDcm fadh2_m + fum_c --> fad_m + succ_c 11420 or 16281
SUCD1 fad_c + succ_c <=> fadh2_c + fum_c PP_4192 and PP_4193 and 11977 and 16281
SUCD1m fad_m + succ_m <=> fadh2_m + fum_m 11629 or (SDHD and 11629 and 11977 and 16281) or (Sdhd and 11629 and 11977 and 16281) or (11629 and 11802 and 11977 and 16281)
SUCD2_u6m q6_m + succ_m <=> fum_m + q6h2_m 11629 and 11802 and 11977 and 16281
SUCD3_u6m fadh2_m + q6_m <=> fad_m + q6h2_m 11629 and 11802 and 11977 and 16281
SUCDH_q8_m q8_m + succ_m <=> fum_m + q8h2_m 11977 and 16281
SUCDi q8_c + succ_c --> fum_c + q8h2_c (PP_4192 and PP_4193 and 11977 and 16281) or (b0721 and b0722 and 11977 and 16281)
```

16281 mito 27 SDH1; succinate dehydrogenase (ubiquinone) flavoprotein subunit  
11977 nucl 9, mito 7, cyto 7, cyto\_mito 7 SDH2; succinate dehydrogenase (ubiquinone) iron-sulfur subunit  
11629 mito 22.5, cyto\_mito 13.5, cyto 3.5 SDH3; succinate dehydrogenase (ubiquinone) cytochrome b560 subunit  
11802 mito 10, extr 8, cyto\_mito 8 SDH4; succinate dehydrogenase (ubiquinone) membrane anchor subunit  
10953 mito 26 SDH6 succinate dehydrogenase assembly factor 1  
12093 mito 16, nucl 7.5, cyto\_nucl 5, cyto 1.5 SDH5 succinate dehydrogenase assembly factor 2  
11205 mito 23.5, cyto\_mito 13.5 SDH7 succinate dehydrogenase assembly factor 3  
8486 mito 18, nucl 7 SDH8 succinate dehydrogenase assembly factor 4  
Change SUCD2\_u6m to SUCD2\_u9m, replace q6 with q9, make it irreversible, change genes to '10953 and 11205 and 11629 and 11802 and 11977 and 12093 and 16281 and 8486'  
Remove 'SUCD1m','SUCD3\_u6m','SUCD1','SUCDH\_q8\_m','SUCDi'

11420 cyto 16.5, cyto\_nucl 9, extr 8 (sigP) FRD1,OSM1 KOG2404: Fumarate reductase, flavoprotein subunit  
11536 nucl 17.5, cyto\_nucl 12.5, cyto 6.5 (weak sigP) KOG2404: Fumarate reductase, flavoprotein subunit (shorter, missing cytochrome b5 domains, half coverage, very at the end of scaffold 16)  
osm1 localizes to mitochondrial intermembrane space  
https://www.ncbi.nlm.nih.gov/pubmed/28814504  
Change FRDcm genes to 11420  
Change FRDm genes to 11536

In [103]:

```
model.reactions.get_by_id('SUCD2_u6m').id = 'SUCD2_u9m'
model.reactions.get_by_id('SUCD2_u9m').add_metabolites({'q6_m': 1.0, 'q6h2_m': -1.0, 'q9_m': -1.0, 'q9h2_m': 1.0})
model.reactions.get_by_id('SUCD2_u9m').lower_bound = 0.0
model.reactions.get_by_id('SUCD2_u9m').gene_reaction_rule = '10953 and 11205 and 11629 and 11802 and 11977 and 12093 and 16281 and 8486'
model.remove_reactions(['SUCD1m','SUCD3_u6m','SUCD1','SUCDH_q8_m','SUCDi'], remove_orphans=True)
model.reactions.get_by_id('FRDcm').gene_reaction_rule = '11420'
model.reactions.get_by_id('FRDm').gene_reaction_rule = '11536'
```

In [104]:

```
for r in sorted(model.metabolites.get_by_id('q6_m').reactions, key=lambda x: x.id):
    print(r.id, r.reaction, r.gene_reaction_rule)
print()
for r in sorted(model.metabolites.get_by_id('q8_c').reactions, key=lambda x: x.id):
    print(r.id, r.reaction, r.gene_reaction_rule)
print()
for r in sorted(model.metabolites.get_by_id('q10_c').reactions, key=lambda x: x.id):
    print(r.id, r.reaction, r.gene_reaction_rule)
print()
for r in sorted(model.metabolites.get_by_id('q10_m').reactions, key=lambda x: x.id):
    print(r.id, r.reaction, r.gene_reaction_rule)
print()
for r in sorted(model.metabolites.get_by_id('q9_m').reactions, key=lambda x: x.id):
    print(r.id, r.reaction, r.gene_reaction_rule)
```

```
DXHPScm h2o_c + q6_m + spmd_c --> 13dampp_c + 4abutn_c + q6h2_m 10198

G3PD5 glyc3p_c + q8_c --> dhap_c + q8h2_c 11039 or (b2241 and b2242 and b2243)
PROD3 pro__L_c + q8_c --> 1pyr5c_c + h_c + q8h2_c 8569

TRDR2 h_c + nadph_c + q10_c --> nadp_c + q10h2_c 15482
TRDR3 h_c + nadh_c + q10_c --> nad_c + q10h2_c 15482


COQ3_m 2npmhmobq_m + amet_m --> ahcys_m + h_m + q9_m 11465
CYOR_u9m 2.0 ficytc_m + 2.0 h_m + q9h2_m --> 2.0 focytc_m + 4.0 h_c + q9_m RTO3_979594 and 9705 and 9198 and 11618 and 15758 and 15681 and 12966 and 15231 and 13608 and 13614 and 8802
DHORD_u9m dhor__S_c + q9_m --> orot_c + q9h2_m 8814
ETFQO_m etfrd_m + q9_m --> etfox_m + q9h2_m 10218
NADH2_u9cm h_c + nadh_c + q9_m --> nad_c + q9h2_m 8972 or 13766
NADH2_u9m1 h_m + nadh_m + q9_m --> nad_m + q9h2_m 9809
NADH2_u9m2 5.0 h_m + nadh_m + q9_m --> 4.0 h_c + nad_m + q9h2_m 10010 and 10017 and 10318 and 10541 and 10607 and 11151 and 11411 and 11702 and 11857 and 12482 and 12497 and 12543 and 12653 and 13017 and 13214 and 13268 and 13411 and 13656 and 13925 and 14078 and 14261 and 14418 and 14717 and 14900 and 15179 and 15864 and 15947 and 15973 and 15998 and 8444 and 8446 and 8530 and 8699 and 8817 and 8909 and 9144 and 9348 and RTO3_874609 and RTO3_879341 and RTO3_879512 and RTO3_900622 and RTO3_900624 and RTO3_900641 and RTO3_945994 and RTO3_945995 and RTO3_946005
SUCD2_u9m q9_m + succ_m --> fum_m + q9h2_m 10953 and 11205 and 11629 and 11802 and 11977 and 12093 and 16281 and 8486
```

In [105]:

```
for r in sorted(model.genes.get_by_id('10198').reactions, key=lambda x: x.id):
    print(r.id, r.reaction, r.gene_reaction_rule)
print()
for r in sorted(model.genes.get_by_id('11039').reactions, key=lambda x: x.id):
    print(r.id, r.reaction, r.gene_reaction_rule)
print()
for r in sorted(model.genes.get_by_id('14576').reactions, key=lambda x: x.id):
    print(r.id, r.reaction, r.gene_reaction_rule)
```

```
DXHPScm h2o_c + q6_m + spmd_c --> 13dampp_c + 4abutn_c + q6h2_m 10198
yli_R0888 h2o_c + o2_c + spmd_c --> 4abutn_c + h2o2_c + yli_M00858_c 10198

G3PD fad_c + glyc3p_c --> dhap_c + fadh2_c 11039
G3PD2m fad_m + glyc3p_c --> dhap_c + fadh2_m 11039
G3PD5 glyc3p_c + q8_c --> dhap_c + q8h2_c 11039 or (b2241 and b2242 and b2243)
G3PDm fad_m + glyc3p_m --> dhap_m + fadh2_m 11039
G3PO dhap_h + h2o2_h <=> glyc3p_h + o2_h 11039
yli_R1398 fad_r + glyc3p_r --> dhap_r + fadh2_r 11039

G3PD1ir dhap_c + h_c + nadh_c --> glyc3p_c + nad_c 12154 or 14576
G3PD1irm dhap_m + h_m + nadh_m --> glyc3p_m + nad_m 12154 or 14576
yli_R1397 dhap_r + h_r + nadh_r --> glyc3p_r + nad_r 12154 or 14576
```

10198 cysk 11, cyto 9, cyto\_pero 7.333, cyto\_nucl 6.333, pero 4.5 (no sigP) DYS1 K00809: DHPS, dys; deoxyhypusine synthase  
deoxyhypusine synthase is modification of eukaryotic translation initiation factor 5A (eIF-5A), and reactions are incorrect  
Remove DXHPScm,yli\_R0888

11039 mito 22, cyto\_mito 14, cyto 4 (anchor) GUT2 K00111: glpA, glpD; glycerol-3-phosphate dehydrogenase  
GUT2 uses quinone  
Change G3PD5 to G3PD\_u9cm, replace fad\_c with q9\_m  
Remove G3PD2m,G3PD5,G3PDm,G3PO,yli\_R1398

12154 cyto 23, pero 4 (weak sigP) K00006: GPD1; glycerol-3-phosphate dehydrogenase (NAD+)  
14576 cyto 17.5, cyto\_nucl 14, nucl 5.5 (no sigP) K00006: GPD1; glycerol-3-phosphate dehydrogenase (NAD+)  
14575 mito 23.5, cyto\_mito 13.5 (sigP) KOG2711: Glycerol-3-phosphate dehydrogenase/dihydroxyacetone 3-phosphate reductase, partial sequence (~20% coverage)  
Remove G3PD1,G3PD1irm,G3PD2,G3PD2\_1,yli\_R1397

In [106]:

```
model.remove_reactions(['DXHPScm','yli_R0888'], remove_orphans=True)
model.reactions.get_by_id('G3PD').id = 'G3PD_u9cm'
model.reactions.get_by_id('G3PD_u9cm').add_metabolites({'fad_c': 1.0, 'fadh2_c': -1.0, 'q9_m': -1.0, 'q9h2_m': 1.0})
model.remove_reactions(['G3PD2m','G3PD5','G3PDm','G3PO','yli_R1398'], remove_orphans=True)
model.remove_reactions(['G3PD1','G3PD1irm','G3PD2','G3PD2_1','yli_R1397'], remove_orphans=True)
```

In [107]:

```
for r in sorted(model.genes.get_by_id('14602').reactions, key=lambda x: x.id):
    print(r.id, r.reaction, r.gene_reaction_rule)
print()
for r in sorted(model.genes.get_by_id('8569').reactions, key=lambda x: x.id):
    if len(r.genes) < 2:
        print(r.id, r.reaction)
```

```
PRO1xm nad_m + pro__L_m --> 1pyr5c_m + 2.0 h_m + nadh_m 13432 or 14602
PROD2m fad_m + pro__L_m --> 1pyr5c_m + fadh2_m + h_m 14602

4HGLSDm 4hglusa_m + h2o_m + nad_m <=> e4hglu_m + 2.0 h_m + nadh_m
HPROxm 4hpro_LT_m + nad_m --> 1p3h5c_m + 2.0 h_m + nadh_m
HPROym 4hpro_LT_m + nadp_m --> 1p3h5c_m + 2.0 h_m + nadph_m
P5CD 1pyr5c_c + 2.0 h2o_c + nad_c --> glu__L_c + h_c + nadh_c
P5CDm 1pyr5c_m + 2.0 h2o_m + nad_m --> glu__L_m + h_m + nadh_m
PHCDm 1p3h5c_m + 2.0 h2o_m + nad_m --> e4hglu_m + h_m + nadh_m
PROD2 fad_c + pro__L_c --> 1pyr5c_c + fadh2_c + h_c
PROD3 pro__L_c + q8_c --> 1pyr5c_c + h_c + q8h2_c
PYR5CDm glu5sa_m + h2o_m + nadp_m --> glu__L_m + 2.0 h_m + nadph_m
r0074 glu5sa_m + h2o_m + nad_m --> glu__L_m + 2.0 h_m + nadh_m
yli_R0011 1pyr5c_c + 2.0 h2o_c + nadp_c --> glu__L_c + h_c + nadph_c
```

In [108]:

```
for r in sorted(model.genes.get_by_id('8569').reactions, key=lambda x: x.id):
    if len(r.genes) > 1:
        print("model.reactions.get_by_id('", r.id, "').gene_reaction_rule = '", r.gene_reaction_rule.replace(' or 8569','').replace(' or 16273',''), "'", sep='')
```

```
model.reactions.get_by_id('5HOXINDACTOXm').gene_reaction_rule = '12042 or 13426'
model.reactions.get_by_id('ALDD20xm').gene_reaction_rule = '12042 or 13426'
model.reactions.get_by_id('BAMPPALDOXm').gene_reaction_rule = '12042 or 13426'
model.reactions.get_by_id('GCALDDm').gene_reaction_rule = '12042 or 13426'
model.reactions.get_by_id('GLACOm').gene_reaction_rule = '12042 or 13426'
model.reactions.get_by_id('IMACTD_m').gene_reaction_rule = '12042 or 13426'
model.reactions.get_by_id('LCADi_Dm').gene_reaction_rule = '12042 or 13426'
model.reactions.get_by_id('LCADm').gene_reaction_rule = '12042 or 13426'
model.reactions.get_by_id('NABTNOm').gene_reaction_rule = '12042 or 13426'
model.reactions.get_by_id('PYLALDOXm').gene_reaction_rule = '12042 or 13426'
```

In [109]:

```
for r in sorted(model.genes.get_by_id('15188').reactions, key=lambda x: x.id):
    print(r.id, r.reaction, r.gene_reaction_rule)
print()
for r in sorted(model.genes.get_by_id('16273').reactions, key=lambda x: x.id):
    print(r.id, r.reaction, r.gene_reaction_rule)
print()
for r in sorted(model.genes.get_by_id('13432').reactions, key=lambda x: x.id):
    print(r.id, r.reaction, r.gene_reaction_rule)
```

```
GLU5K atp_c + glu__L_c --> adp_c + glu5p_c 15188

AGPRim acg5p_m + h_m + nadph_m --> acg5sa_m + nadp_m + pi_m 16273 or 9377
G5DHx glu5p_x + h_x + nadph_x --> glu5sa_x + nadp_x + pi_x 16273
G5SD glu5p_c + h_c + nadph_c --> glu5sa_c + nadp_c + pi_c 16273
G5SD2 glu5p_c + h_c + nadh_c --> glu5sa_c + nad_c + pi_c 16273
G5SDym glu5p_m + h_m + nadph_m --> glu5sa_m + nadp_m + pi_m 16273
GCALDDm gcald_m + h2o_m + nad_m --> glyclt_m + 2.0 h_m + nadh_m 12042 or 13426 or 16273 or 8569
GLU5Km atp_m + glu__L_m --> adp_m + glu5p_m 16273
LCADi_Dm h2o_m + lald__D_m + nad_m --> 2.0 h_m + lac__D_m + nadh_m 12042 or 13426 or 16273 or 8569
LCADm h2o_m + lald__L_m + nad_m --> 2.0 h_m + lac__L_m + nadh_m 12042 or 13426 or 16273 or 8569

HPROa 1p3h5c_c + 2.0 h_c + nadh_c --> 4hpro_LT_c + nad_c 13432
HPROb 1p3h5c_c + 2.0 h_c + nadph_c --> 4hpro_LT_c + nadp_c 13432
P5CR 1pyr5c_c + 2.0 h_c + nadph_c --> nadp_c + pro__L_c 13432
P5CRm 1pyr5c_m + 2.0 h_m + nadph_m --> nadp_m + pro__L_m 13432
P5CRx 1pyr5c_c + 2.0 h_c + nadh_c --> nad_c + pro__L_c 13432
P5CRxm 1pyr5c_m + 2.0 h_m + nadh_m --> nad_m + pro__L_m 13432
PRO1x nad_c + pro__L_c --> 1pyr5c_c + 2.0 h_c + nadh_c 13432
PRO1xm nad_m + pro__L_m --> 1pyr5c_m + 2.0 h_m + nadh_m 13432 or 14602
```

8569 mito 12, cyto 10.5, cyto\_nucl 6.5, pero 2 (no sigP) PUT2 K00294: E1.2.1.88; 1-pyrroline-5-carboxylate dehydrogenase  
14602 mito 14, extr 5, nucl 2, cyto 2, cyto\_nucl 2 PUT1 K00318: PRODH; proline dehydrogenase  
15188 cyto 15, cyto\_nucl 9.5, mito 8, nucl 2, extr 2 PRO1 K00931: proB; glutamate 5-kinase  
16273 cyto 13, cyto\_nucl 11, mito 8, nucl 5 PRO2 K00147: proA; glutamate-5-semialdehyde dehydrogenase  
13432 plas 10, cyto 4, extr 4, E.R. 4, vacu 4 PRO3 K00286: proC; pyrroline-5-carboxylate reductase  
12042 cyto 18.5, cyto\_nucl 10, mito 4, pero 4 ALD4,ALD5,ALD6 K00128: ALDH; aldehyde dehydrogenase (NAD+)  
13426 mito 25.5, cyto\_mito 14 ALD2,ALD3 K07249: E1.2.1.36; retinal dehydrogenase

PUT2 4HGLSDm,HPROxm,HPROym,PHCDm,PYR5CDm in iMM904 -> HPROxm, HPROym are by PUT1 (as PRO1xm), not PUT2  
PUT1 uses quinone in metacyc  
Change PROD2m to PROD\_u9m, genes to '14602', change reactions from  
fad\_m + pro**L\_m --> 1pyr5c\_m + fadh2\_m + h\_m to  
q9\_m + pro**L\_m --> 1pyr5c\_m + q9h2\_m + h\_m
Change HPROxm to HPRO\_u9m, genes to '14602', change reactions from  
4hpro\_LT\_m + nad\_m --> 1p3h5c\_m + 2.0 h\_m + nadh\_m to  
4hpro\_LT\_m + q9\_m --> 1p3h5c\_m + h\_m + q9h2\_m  
Remove PRO1x,PRO1xm,PROD2,PROD3,HPROym

PUT2 uses NAD in metacyc/KEGG (first step is spontaneous)  
r0074, 4HGLSDm is correct  
Add G5SADs,G5SADrm,PHCHGSm from iMM904  
Change r0074 to G5SADm  
Change 4HGLSDm to irreversible  
Remove PYR5CDm (NADP), P5CD,yli\_R0011 (overall cyto), P5CDm, PHCDm (overall mito)

PRO2 uses NADP, G5SD correct  
Remove G5DHx, G5SD2, G5SDym, GLU5Km  
Remove 16273 from AGPRim genes -> Change AGPRim '9377'

PRO3 uses NAD/NADP, P5CR/P5CRx and HPROa/HPROb correct  
Remove P5CRm,P5CRxm

Remove 16273 and 8569 from aldehyde reactions

15482 cyto GLR1 cytosolic and mitochondrial glutathione oxidoreductase  
Remove TRDR2,TRDR3

In [110]:

```
model.reactions.get_by_id('PROD2m').id = 'PROD_u9m'
model.reactions.get_by_id('PROD_u9m').add_metabolites({'fad_m': 1.0, 'fadh2_m': -1.0, 'q9_m': -1.0, 'q9h2_m': 1.0})
model.reactions.get_by_id('PROD_u9m').gene_reaction_rule = '14602'
model.reactions.get_by_id('HPROxm').id = 'HPRO_u9m'
model.reactions.get_by_id('HPRO_u9m').add_metabolites({'nad_m': 1.0, 'nadh_m': -1.0, 'h_m': -1.0, 'q9_m': -1.0, 'q9h2_m': 1.0})
model.reactions.get_by_id('HPRO_u9m').gene_reaction_rule = '14602'
model.remove_reactions(['PRO1x','PRO1xm','PROD2','PROD3','HPROym'], remove_orphans=True)
r = sce.reactions.get_by_id('G5SADs').copy()
model.add_reactions([r])
r = sce.reactions.get_by_id('G5SADrm').copy()
model.add_reactions([r])
r = sce.reactions.get_by_id('PHCHGSm').copy()
model.add_reactions([r])
model.reactions.get_by_id('r0074').id = 'G5SADm'
model.reactions.get_by_id('4HGLSDm').lower_bound = 0.0
model.remove_reactions(['PYR5CDm','P5CD','yli_R0011','P5CDm','PHCDm'], remove_orphans=True)
model.remove_reactions(['G5DHx','G5SD2','G5SDym','GLU5Km'], remove_orphans=True)
model.reactions.get_by_id('AGPRim').gene_reaction_rule = '9377'
model.remove_reactions(['P5CRm','P5CRxm'], remove_orphans=True)
model.reactions.get_by_id('5HOXINDACTOXm').gene_reaction_rule = '12042 or 13426'
model.reactions.get_by_id('ALDD20xm').gene_reaction_rule = '12042 or 13426'
model.reactions.get_by_id('BAMPPALDOXm').gene_reaction_rule = '12042 or 13426'
model.reactions.get_by_id('GCALDDm').gene_reaction_rule = '12042 or 13426'
model.reactions.get_by_id('GLACOm').gene_reaction_rule = '12042 or 13426'
model.reactions.get_by_id('IMACTD_m').gene_reaction_rule = '12042 or 13426'
model.reactions.get_by_id('LCADi_Dm').gene_reaction_rule = '12042 or 13426'
model.reactions.get_by_id('LCADm').gene_reaction_rule = '12042 or 13426'
model.reactions.get_by_id('NABTNOm').gene_reaction_rule = '12042 or 13426'
model.reactions.get_by_id('PYLALDOXm').gene_reaction_rule = '12042 or 13426'
model.remove_reactions(['TRDR2','TRDR3'], remove_orphans=True)
```

In [111]:

```
for r in sorted(model.reactions, key=lambda x: x.id):
    if r.id.startswith("ATP"):
        print(r.id, r.reaction, r.gene_reaction_rule)
```

```
ATP2tp_H amp_x + atp_c + h_x --> amp_c + atp_x + h_c 16145
ATPATF1 adp_c + atp_c + h_c --> ap4a_c + pi_c 16552
ATPATF2 adp_c + gtp_c + h_c --> ap4g_c + pi_c 16552
ATPATF3 gdp_c + gtp_c + h_c --> gp4g_c + pi_c 16552
ATPH1 atp_c + 2.0 h2o_c --> amp_c + 2.0 h_c + 2.0 pi_c 8970
ATPH1e atp_e + 2.0 h2o_e --> amp_e + 2.0 h_e + 2.0 pi_e 8970 or 9805
ATPH2e adp_e + h2o_e --> amp_e + h_e + pi_e 8970 or 9805
ATPPHm atp_m + h2o_m --> adp_m + h_m + pi_m 9805
ATPPRT atp_c + prpp_c --> ppi_c + prbatp_c 9888
ATPS atp_c + h2o_c --> adp_c + h_e + pi_c 12428 or 13617 or 15156 or 15584 or (YCR024C_A and YEL017C_A and 13617) or (YCR024C_A and YEL017C_A and 15584)
ATPS3g adp_g + 3.0 h_c + pi_g --> atp_g + h2o_g + 2.0 h_g 11025 and 11052 and 11064 and 11117 and 11403 and 14971 and 15309 and 15351 and 15963 and 16397 and 9210 and 9622 and 9748
ATPS3m adp_m + 3.0 h_c + pi_m --> atp_m + h2o_m + 2.0 h_m (CRv4_Au5_s10_g33_t1 and CRv4_Au5_s10_g65_t1 and CRv4_Au5_s17_g7684_t1 and CRv4_Au5_s1_g1345_t1 and CRv4_Au5_s1_g1960_t1 and 11958 and 11967 and 13424 and 15287 and 15589) or (CRv4_Au5_s10_g33_t1 and CRv4_Au5_s10_g65_t1 and CRv4_Au5_s17_g7685_t1 and CRv4_Au5_s1_g1345_t1 and CRv4_Au5_s1_g1960_t1 and 11958 and 11967 and 13424 and 15287 and 15589) or (CRv4_Au5_s10_g65_t1 and CRv4_Au5_s17_g7684_t1 and CRv4_Au5_s1_g1345_t1 and CRv4_Au5_s1_g1960_t1 and 11958 and 11967 and 13424 and 15287 and 15589 and 16359) or (CRv4_Au5_s10_g65_t1 and CRv4_Au5_s17_g7685_t1 and CRv4_Au5_s1_g1345_t1 and CRv4_Au5_s1_g1960_t1 and 11958 and 11967 and 13424 and 15287 and 15589 and 16359) or (Q0080 and Q0085 and Q0130 and YDL181W and YPL271W and 10674 and 11958 and 11967 and 13424 and 13759 and 14786 and 14912 and 15589 and 16359 and 9940) or (Q0080 and Q0085 and Q0130 and YPL271W and 10674 and 10675 and 11958 and 11967 and 13424 and 13759 and 14786 and 14912 and 15589 and 16359 and 9940) or (Q0080 and Q0085 and Q0130 and YDL181W and YOL077W_A and YPL271W and YPR020W and 10674 and 11958 and 11967 and 13424 and 13759 and 14786 and 14912 and 15589 and 15880 and 16359 and 9940) or (Q0080 and Q0085 and Q0130 and YOL077W_A and YPL271W and 10674 and 10675 and 11958 and 11967 and 13424 and 13759 and 14786 and 14912 and 15589 and 15880 and 16359 and 9080 and 9940)
ATPS3v adp_v + 3.0 h_c + pi_v --> atp_v + h2o_v + 2.0 h_v 11025 and 11052 and 11064 and 11117 and 11403 and 14971 and 15309 and 15351 and 15963 and 16397 and 9210 and 9622 and 9748
ATPS4m adp_m + 4.0 h_c + pi_m --> atp_m + h2o_m + 3.0 h_m (ATP5E and ATP5F1 and ATP5G1 and ATP5H and ATP5I and ATP5J and ATP5J2 and ATP5L and 11749 and 11958 and 11967 and 15589 and 16359) or (ATP5E and ATP5F1 and ATP5G2 and ATP5H and ATP5I and ATP5J and ATP5J2 and ATP5L and 11749 and 11958 and 11967 and 15589 and 16359) or (ATP5E and ATP5F1 and ATP5G3 and ATP5H and ATP5I and ATP5J and ATP5J2 and ATP5L and 11749 and 11958 and 11967 and 15589 and 16359) or (Atp5f1 and Atp5g1 and Atp5j and Atp5j2 and Atp5k and mmu_10476 and mmu_514 and mmu_627998 and 11749 and 11958 and 11967 and 15589 and 16359) or (Atp5f1 and Atp5g3 and Atp5j and Atp5j2 and Atp5k and mmu_10476 and mmu_514 and mmu_627998 and 11749 and 11958 and 11967 and 15589 and 16359) or (Atp5f1 and Atp5j and Atp5j2 and Atp5k and mmu_100039108 and mmu_10476 and mmu_514 and mmu_627998 and 11749 and 11958 and 11967 and 15589 and 16359)
ATPS4rpp adp_c + 4.0 h_p + pi_c <=> atp_c + h2o_c + 3.0 h_c (PP_5417 and PP_5418 and PP_5419 and 11958 and 11967 and 13424 and 15589 and 16359) or (b3736 and b3737 and b3738 and 11958 and 11967 and 13424 and 15589 and 16359) or (b3736 and b3737 and b3738 and b3739 and 11958 and 11967 and 13424 and 15589 and 16359)
ATP_3h_tm adp_m + atp_c + 3.0 h_m --> adp_c + atp_m + 3.0 h_c 12704
ATPasel atp_c + h2o_c + 3.0 h_c --> adp_c + 4.0 h_l + pi_c (ATP6V0E1 and ATP6V1C2 and ATP6V1E2 and ATP6V1G2 and 11025 and 11052 and 11064 and 11117 and 11403 and 14971 and 15309 and 15351 and 15963) or (ATP6V0E1 and ATP6V1C2 and ATP6V1E2 and ATP6V1G2 and 11052 and 11064 and 11117 and 11403 and 14971 and 15309 and 15351 and 15963 and 9748) or (ATP6V0E1 and ATP6V1C2 and ATP6V1E2 and ATP6V1G3 and 11025 and 11052 and 11064 and 11117 and 11403 and 14971 and 15309 and 15351 and 15963) or (ATP6V0E1 and ATP6V1C2 and ATP6V1E2 and ATP6V1G3 and 11052 and 11064 and 11117 and 11403 and 14971 and 15309 and 15351 and 15963 and 9748) or (ATP6V0E1 and ATP6V1C2 and ATP6V1E2 and 11025 and 11052 and 11064 and 11117 and 11403 and 14971 and 15309 and 15351 and 15963 and 16397) or (ATP6V0E1 and ATP6V1C2 and ATP6V1E2 and 11052 and 11064 and 11117 and 11403 and 14971 and 15309 and 15351 and 15963 and 16397 and 9748) or (ATP6V0E1 and ATP6V1C2 and ATP6V1G2 and 11025 and 11052 and 11064 and 11117 and 11403 and 14971 and 15309 and 15351 and 15963 and 9622) or (ATP6V0E1 and ATP6V1C2 and ATP6V1G2 and 11052 and 11064 and 11117 and 11403 and 14971 and 15309 and 15351 and 15963 and 9622 and 9748) or (ATP6V0E1 and ATP6V1C2 and ATP6V1G3 and 11025 and 11052 and 11064 and 11117 and 11403 and 14971 and 15309 and 15351 and 15963 and 9622) or (ATP6V0E1 and ATP6V1C2 and ATP6V1G3 and 11052 and 11064 and 11117 and 11403 and 14971 and 15309 and 15351 and 15963 and 9622 and 9748) or (ATP6V0E1 and ATP6V1C2 and 11025 and 11052 and 11064 and 11117 and 11403 and 14971 and 15309 and 15351 and 15963 and 16397 and 9622) or (ATP6V0E1 and ATP6V1C2 and 11052 and 11064 and 11117 and 11403 and 14971 and 15309 and 15351 and 15963 and 16397 and 9622 and 9748) or (ATP6V0E1 and ATP6V1E2 and ATP6V1G2 and 11025 and 11052 and 11064 and 11117 and 11403 and 14971 and 15309 and 15351 and 15963 and 9210) or (ATP6V0E1 and ATP6V1E2 and ATP6V1G2 and 11052 and 11064 and 11117 and 11403 and 14971 and 15309 and 15351 and 15963 and 9210 and 9748) or (ATP6V0E1 and ATP6V1E2 and ATP6V1G3 and 11025 and 11052 and 11064 and 11117 and 11403 and 14971 and 15309 and 15351 and 15963 and 9210) or (ATP6V0E1 and ATP6V1E2 and ATP6V1G3 and 11052 and 11064 and 11117 and 11403 and 14971 and 15309 and 15351 and 15963 and 9210 and 9748) or (ATP6V0E1 and ATP6V1E2 and 11025 and 11052 and 11064 and 11117 and 11403 and 14971 and 15309 and 15351 and 15963 and 16397 and 9210) or (ATP6V0E1 and ATP6V1E2 and 11052 and 11064 and 11117 and 11403 and 14971 and 15309 and 15351 and 15963 and 16397 and 9210 and 9748) or (ATP6V0E1 and ATP6V1G2 and 11025 and 11052 and 11064 and 11117 and 11403 and 14971 and 15309 and 15351 and 15963 and 9210 and 9622) or (ATP6V0E1 and ATP6V1G2 and 11052 and 11064 and 11117 and 11403 and 14971 and 15309 and 15351 and 15963 and 9210 and 9622 and 9748) or (ATP6V0E1 and ATP6V1G3 and 11025 and 11052 and 11064 and 11117 and 11403 and 14971 and 15309 and 15351 and 15963 and 9210 and 9622) or (ATP6V0E1 and ATP6V1G3 and 11052 and 11064 and 11117 and 11403 and 14971 and 15309 and 15351 and 15963 and 9210 and 9622 and 9748) or (ATP6V0E1 and 11025 and 11052 and 11064 and 11117 and 11403 and 14971 and 15309 and 15351 and 15963 and 16397 and 9210 and 9622) or (ATP6V0E1 and 11052 and 11064 and 11117 and 11403 and 14971 and 15309 and 15351 and 15963 and 16397 and 9210 and 9622 and 9748) or (Atp6v0e and Atp6v1c2 and Atp6v1e2 and Atp6v1g2 and 11025 and 11052 and 11064 and 11117 and 11403 and 14971 and 15309 and 15351 and 15963) or (Atp6v0e and Atp6v1c2 and Atp6v1e2 and Atp6v1g2 and 11052 and 11064 and 11117 and 11403 and 14971 and 15309 and 15351 and 15963 and 9748) or (Atp6v0e and Atp6v1c2 and Atp6v1e2 and Atp6v1g3 and 11025 and 11052 and 11064 and 11117 and 11403 and 14971 and 15309 and 15351 and 15963) or (Atp6v0e and Atp6v1c2 and Atp6v1e2 and Atp6v1g3 and 11052 and 11064 and 11117 and 11403 and 14971 and 15309 and 15351 and 15963 and 9748) or (Atp6v0e and Atp6v1c2 and Atp6v1e2 and 11025 and 11052 and 11064 and 11117 and 11403 and 14971 and 15309 and 15351 and 15963 and 16397) or (Atp6v0e and Atp6v1c2 and Atp6v1e2 and 11052 and 11064 and 11117 and 11403 and 14971 and 15309 and 15351 and 15963 and 16397 and 9748) or (Atp6v0e and Atp6v1c2 and Atp6v1g2 and 11025 and 11052 and 11064 and 11117 and 11403 and 14971 and 15309 and 15351 and 15963 and 9622) or (Atp6v0e and Atp6v1c2 and Atp6v1g2 and 11052 and 11064 and 11117 and 11403 and 14971 and 15309 and 15351 and 15963 and 9622 and 9748) or (Atp6v0e and Atp6v1c2 and Atp6v1g3 and 11025 and 11052 and 11064 and 11117 and 11403 and 14971 and 15309 and 15351 and 15963 and 9622) or (Atp6v0e and Atp6v1c2 and Atp6v1g3 and 11052 and 11064 and 11117 and 11403 and 14971 and 15309 and 15351 and 15963 and 9622 and 9748) or (Atp6v0e and Atp6v1c2 and 11025 and 11052 and 11064 and 11117 and 11403 and 14971 and 15309 and 15351 and 15963 and 16397 and 9622) or (Atp6v0e and Atp6v1c2 and 11052 and 11064 and 11117 and 11403 and 14971 and 15309 and 15351 and 15963 and 16397 and 9622 and 9748) or (Atp6v0e and Atp6v1e2 and Atp6v1g2 and 11025 and 11052 and 11064 and 11117 and 11403 and 14971 and 15309 and 15351 and 15963 and 9210) or (Atp6v0e and Atp6v1e2 and Atp6v1g2 and 11052 and 11064 and 11117 and 11403 and 14971 and 15309 and 15351 and 15963 and 9210 and 9748) or (Atp6v0e and Atp6v1e2 and Atp6v1g3 and 11025 and 11052 and 11064 and 11117 and 11403 and 14971 and 15309 and 15351 and 15963 and 9210) or (Atp6v0e and Atp6v1e2 and Atp6v1g3 and 11052 and 11064 and 11117 and 11403 and 14971 and 15309 and 15351 and 15963 and 9210 and 9748) or (Atp6v0e and Atp6v1e2 and 11025 and 11052 and 11064 and 11117 and 11403 and 14971 and 15309 and 15351 and 15963 and 16397 and 9210) or (Atp6v0e and Atp6v1e2 and 11052 and 11064 and 11117 and 11403 and 14971 and 15309 and 15351 and 15963 and 16397 and 9210 and 9748) or (Atp6v0e and Atp6v1g2 and 11025 and 11052 and 11064 and 11117 and 11403 and 14971 and 15309 and 15351 and 15963 and 9210 and 9622) or (Atp6v0e and Atp6v1g2 and 11052 and 11064 and 11117 and 11403 and 14971 and 15309 and 15351 and 15963 and 9210 and 9622 and 9748) or (Atp6v0e and Atp6v1g3 and 11025 and 11052 and 11064 and 11117 and 11403 and 14971 and 15309 and 15351 and 15963 and 9210 and 9622) or (Atp6v0e and Atp6v1g3 and 11052 and 11064 and 11117 and 11403 and 14971 and 15309 and 15351 and 15963 and 9210 and 9622 and 9748) or (Atp6v0e and 11025 and 11052 and 11064 and 11117 and 11403 and 14971 and 15309 and 15351 and 15963 and 16397 and 9210 and 9622) or (Atp6v0e and 11052 and 11064 and 11117 and 11403 and 14971 and 15309 and 15351 and 15963 and 16397 and 9210 and 9622 and 9748)
ATPtm adp_c + atp_m --> adp_m + atp_c 12704
ATPtm_H adp_c + atp_m + h_c --> adp_m + atp_c + h_m 12704
ATPtp_H adp_x + atp_c + h_x --> adp_c + atp_x + h_c 16145
```

In [112]:

```
for r in sorted(model.genes.get_by_id('16145').reactions, key=lambda x: x.id):
    print(r.id, r.reaction, r.gene_reaction_rule)
print()
for r in sorted(model.genes.get_by_id('12704').reactions, key=lambda x: x.id):
    print(r.id, r.reaction, r.gene_reaction_rule)
```

```
ATP2tp_H amp_x + atp_c + h_x --> amp_c + atp_x + h_c 16145
ATPtp_H adp_x + atp_c + h_x --> adp_c + atp_x + h_c 16145

ATP_3h_tm adp_m + atp_c + 3.0 h_m --> adp_c + atp_m + 3.0 h_c 12704
ATPtm adp_c + atp_m --> adp_m + atp_c 12704
ATPtm_H adp_c + atp_m + h_c --> adp_m + atp_c + h_m 12704
```

ADP/ATP translocase  
mitochondria adp/atp no proton, peroxisome amp/atp pump proton  
https://link.springer.com/article/10.1007/s00018-010-0612-3  
http://www.biochemj.org/content/381/3/581

16145 ANT1 mito 12, cyto 4.5, cyto\_nucl 3.5, E.R. 3, plas 2, extr 2, nucl 1.5 KOG0769: Predicted mitochondrial carrier protein, ANT1 is Peroxisomal adenine nucleotide transporter  
12704 PET9 mito 17.5, cyto\_mito 11.166, extr 5, cyto 3.5 K05863: SLC25A4S, ANT; solute carrier family 25 (mitochondrial adenine nucleotide translocator), member 4/5/6/31

ATP2tp\_H amp\_x + atp\_c + h\_x --> amp\_c + atp\_x + h\_c 16145  
ATPtm adp\_c + atp\_m --> adp\_m + atp\_c 12704

Remove ATP\_3h\_tm,ATPtm\_H,ATPtp\_H

In [113]:

```
model.remove_reactions(['ATP_3h_tm','ATPtm_H','ATPtp_H'], remove_orphans=True)
```

In [114]:

```
# Phosphate transport
temp = ['8889','11409','11410','11686','15874','10316','11418','11534']
display(Annotation.loc[temp])
Show_Data(temp)
```

|  | Combined Annotations | Signal P | Sc288c Orthologs | Human Orthologs | Sc288 Best Hit | Human Blast | Essential | WolfPSort | C Terminal |
| --- | --- | --- | --- | --- | --- | --- | --- | --- | --- |
| RTO4\_ID |  |  |  |  |  |  |  |  |  |
| 8889 | K15102: SLC25A3, PHC, PIC; solute carrier fami... | S | MIR1,PIC2 | SLC25A3 | PIC2 | SLC25 | Essential | mito 14, extr 10, cyto 3 | TKK\* |
| 11409 | KOG0252: Inorganic phosphate transporter |  |  |  | PHO84 |  | Not Essential | plas 26 | GGI\* |
| 11410 | KOG0252: Inorganic phosphate transporter |  |  |  | PHO84 |  | Not Essential | plas 26 | GQQ\* |
| 11686 | K14640: SLC20A, PIT; solute carrier family 20 ... | S | PHO89 | SLC20A1,SLC20A2 | PHO89 | SLC20 | Not Essential | plas 21, vacu 4 | PSQ\* |
| 15874 | K15102: SLC25A3, PHC, PIC; solute carrier fami... |  | MIR1,PIC2 | SLC25A3 | MIR1 | SLC25 | Not Essential | mito 12, extr 7, cyto 5.5, cyto\_nucl 3.5 | HKD\* |
| 10316 | K08176: PHO84; MFS transporter, PHS family, in... |  | PHO84 |  | PHO84 | SV2B | Not Essential | plas 27 | ARV\* |
| 11418 | K14430: PHO87\_91; phosphate transporter |  | PHO87,PHO90,PHO91 |  | PHO91 |  | Unclear (ambiguous TDNA mapping) | plas 22, mito 2, cyto 2, cyto\_mito 2 | IGL\* |
| 11534 | K14430: PHO87\_91; phosphate transporter |  | PHO87,PHO90,PHO91 |  | PHO91 |  | Unclear (ambiguous TDNA mapping) | plas 22, mito 2, cyto 2, cyto\_mito 2 | IGL\* |

| strain | WT | | | | | | | | | | | | | | | | |
| --- | --- | --- | --- | --- | --- | --- | --- | --- | --- | --- | --- | --- | --- | --- | --- | --- | --- |
| condition | G\_MM | C\_MM | G\_SD | | GX\_SD | | | X\_SD | | A\_SD | | C\_SD | | MM\_CN120 | | MM\_CN5 | Diversity\_Sample |
| phase | exp | exp | exp | stat | exp | trans | stat | exp | stat | exp | stat | exp | stat | exp | stat | exp | exp |
| proteinId | Set1 | Set1 | Set2 | Set2 | Set2 | Set2 | Set2 | Set2 | Set2 | Set2 | Set2 | Set2 | Set2 | Set3 | Set3 | Set3 | Set3 |
| 8889 | 7.50261 | 8.3532 | 8.71937 | 8.13574 | 8.82621 | 8.66146 | 8.42327 | 8.63025 | 7.90258 | 8.22008 | 7.35762 | 8.69909 | 8.89227 | 9.35379 | 9.28452 | 9.74086 | 10.2743 |
| 11409 | 6.96951 | 6.87533 | 7.03642 | 5.36386 | 7.23756 | 5.20705 | 6.67118 | 7.17724 | 7.73882 | 7.45786 | 6.49465 | 7.40853 | 7.09777 | 7.89794 | 6.34863 | 7.11465 | 8.26566 |
| 11410 | 0.772833 | 1.45613 | 0.992991 | 2.90554 | 0.916697 | 1.36278 | 1.98927 | 0.722476 | 1.41644 | 0.708742 | 2.02642 | 4.97262 | 4.55759 | 1.26139 | 1.78483 | 0.966718 | 2.51643 |
| 11686 | 1.84789 | 4.28676 | 1.97867 | 3.4195 | 1.83397 | 2.53965 | 2.50218 | 2.01009 | 2.4845 | 1.65211 | 3.04948 | 7.45399 | 7.49075 | 2.27837 | 2.42843 | 1.39887 | 5.69554 |
| 15874 | 5.54334 | 4.37285 | 7.55907 | 6.05993 | 7.46388 | 7.0809 | 6.98891 | 6.53448 | 6.37383 | 5.792 | 5.54409 | 6.01274 | 5.50865 | 6.06785 | 6.19675 | 4.88 | 5.91761 |
| 10316 | 1.63247 | 2.34385 | 2.68823 | 2.84986 | 2.86063 | 1.25294 | 1.52562 | 2.51572 | 2.9827 | 3.91034 | 2.67479 | 3.23968 | 4.54752 | 3.02417 | 1.99194 | 1.90409 | 6.52659 |
| 11418 | 5.6266 | 5.40598 | 5.8364 | 7.37594 | 5.76185 | 6.039 | 6.42426 | 5.81269 | 6.39796 | 5.71996 | 6.32532 | 7.18477 | 6.97118 | 6.8158 | 6.81807 | 6.40947 | 6.7254 |
| 11534 | 5.61984 | 5.35956 | 5.83909 | 7.37349 | 5.77158 | 6.01678 | 6.41316 | 5.83861 | 6.40244 | 5.69987 | 6.32131 | 7.18764 | 6.99396 | 6.83218 | 6.81035 | 6.42476 | 6.67932 |

| strain | WT | | | | | | | | | | |
| --- | --- | --- | --- | --- | --- | --- | --- | --- | --- | --- | --- |
| condition | G\_SD | | GX\_SD | | | X\_SD | | A\_SD | | C\_SD | |
| proteinId | exp | stat | exp | trans | stat | exp | stat | exp | stat | exp | stat |
| 8889 | 40.6881 | 40.4801 | 38.6526 | 38.7215 | 36.1525 | 34.7209 | 40.3551 | 33.42 | 34.8855 | 44.7328 | 49.8519 |
| 11409 | 0.568465 | 0 | 1.21944 | 0 | 0 | 0.400832 | 0.195435 | 0.190305 | 0 | 0 | 0 |
| 15874 | 11.5429 | 13.7323 | 12.7904 | 16.9395 | 14.0497 | 9.80303 | 9.11259 | 5.61108 | 6.48984 | 8.76984 | 8.91861 |
| 11418 | 4.61235 | 6.55342 | 5.50993 | 5.39511 | 6.89455 | 4.68336 | 5.73437 | 4.83149 | 7.08929 | 9.8026 | 9.58593 |

|  | Glucose | Xylose | Arabinose | Acetate | Coumarate | Ferulate | YNB Oleic Acid | YNB Ricinoleic Acid | YNB Glucose | YNB Gluc DOC | YPD |
| --- | --- | --- | --- | --- | --- | --- | --- | --- | --- | --- | --- |
| proteinId |  |  |  |  |  |  |  |  |  |  |  |
| 11409 | -0.446797 | -0.436451 | -0.19646 | -0.0972827 | -0.181678 | -0.165113 | -0.246158 | -0.322861 | 0.223899 | -0.0773217 | 0.129112 |
| 11410 | -0.0747803 | -0.140179 | -0.273866 | -0.148383 | 0.174274 | 0.162573 | 0.134582 | -0.31194 | -0.242375 | 0.046449 | -0.253539 |
| 11686 | -0.278682 | -0.192103 | -0.226724 | 0.0377009 | -0.464625 | -0.210029 | -0.131382 | -0.707703 | 0.219386 | 0.162949 | 0.0907714 |
| 15874 | -0.268098 | -0.0197262 | 0.0222415 | -0.262453 | 0.268151 | -0.511158 | 0.130008 | 0.406326 | 0.236206 | -0.0925991 | 0.264876 |
| 10316 | 0.00147071 | 0.192773 | -0.0409058 | 0.00544164 | 0.190309 | 0.22018 | 0.14609 | 0.00987175 | 0.232658 | -0.02026 | 0.0898483 |

MIR1/PIC2 mito pi H+ symport, 8889 15874
PHO84 high affinity pi transporter, 10316 11409 11410  
PHO89 Plasma membrane Na+/Pi cotransporter, 11686  
PHO87/PHO90 low affinity pi transporter  
PHO91 low affinity vacuolar phosphate transporter

In [115]:

```
for r in sorted(model.genes.get_by_id('8889').reactions, key=lambda x: x.id):
    print(r.id, r.reaction, r.gene_reaction_rule)
print()
for r in sorted(model.genes.get_by_id('11409').reactions, key=lambda x: x.id):
    print(r.id, r.reaction, r.gene_reaction_rule)
print()
for r in sorted(model.genes.get_by_id('11410').reactions, key=lambda x: x.id):
    print(r.id, r.reaction, r.gene_reaction_rule)
print()
for r in sorted(model.genes.get_by_id('11686').reactions, key=lambda x: x.id):
    print(r.id, r.reaction, r.gene_reaction_rule)
print()
for r in sorted(model.genes.get_by_id('15874').reactions, key=lambda x: x.id):
    print(r.id, r.reaction, r.gene_reaction_rule)
print()
for r in sorted(model.genes.get_by_id('10316').reactions, key=lambda x: x.id):
    print(r.id, r.reaction, r.gene_reaction_rule)
print()
for r in sorted(model.genes.get_by_id('11418').reactions, key=lambda x: x.id):
    print(r.id, r.reaction, r.gene_reaction_rule)
print()
for r in sorted(model.genes.get_by_id('11534').reactions, key=lambda x: x.id):
    print(r.id, r.reaction, r.gene_reaction_rule)
```

```
PIcm pi_c <=> pi_m 8889
PIt2m h_c + pi_c <=> h_m + pi_m 11409 or 11410 or 11686 or 15874 or 8889
PIt5m oh1_m + pi_c <=> oh1_c + pi_m 15874 or 8889
PItn pi_n <=> pi_c 8889

PIt2m h_c + pi_c <=> h_m + pi_m 11409 or 11410 or 11686 or 15874 or 8889
PIt2p h_c + pi_c <=> h_x + pi_x 11409 or 11410 or 11686
PIt2r h_e + pi_e <=> h_c + pi_c 10316 or 11409 or 11410 or 11418 or 11534 or 11686 or (10316 and 11418 and 11686) or (10316 and 11534 and 11686)
PItf h_c + pi_c <=> h_f + pi_f 11409 or 11410 or 11686

PIt2m h_c + pi_c <=> h_m + pi_m 11409 or 11410 or 11686 or 15874 or 8889
PIt2p h_c + pi_c <=> h_x + pi_x 11409 or 11410 or 11686
PIt2r h_e + pi_e <=> h_c + pi_c 10316 or 11409 or 11410 or 11418 or 11534 or 11686 or (10316 and 11418 and 11686) or (10316 and 11534 and 11686)
PItf h_c + pi_c <=> h_f + pi_f 11409 or 11410 or 11686

PINA1th na1_h + pi_h --> na1_c + pi_c 11686
PINA1tm na1_c + pi_c <=> na1_m + pi_m 11686
PIt2m h_c + pi_c <=> h_m + pi_m 11409 or 11410 or 11686 or 15874 or 8889
PIt2p h_c + pi_c <=> h_x + pi_x 11409 or 11410 or 11686
PIt2r h_e + pi_e <=> h_c + pi_c 10316 or 11409 or 11410 or 11418 or 11534 or 11686 or (10316 and 11418 and 11686) or (10316 and 11534 and 11686)
PIt8 1.5 na1_e + pi_e <=> 1.5 na1_c + pi_c 11686
PItf h_c + pi_c <=> h_f + pi_f 11409 or 11410 or 11686

PIt2m h_c + pi_c <=> h_m + pi_m 11409 or 11410 or 11686 or 15874 or 8889
PIt5m oh1_m + pi_c <=> oh1_c + pi_m 15874 or 8889

PIt2r h_e + pi_e <=> h_c + pi_c 10316 or 11409 or 11410 or 11418 or 11534 or 11686 or (10316 and 11418 and 11686) or (10316 and 11534 and 11686)

PIt2r h_e + pi_e <=> h_c + pi_c 10316 or 11409 or 11410 or 11418 or 11534 or 11686 or (10316 and 11418 and 11686) or (10316 and 11534 and 11686)

PIt2r h_e + pi_e <=> h_c + pi_c 10316 or 11409 or 11410 or 11418 or 11534 or 11686 or (10316 and 11418 and 11686) or (10316 and 11534 and 11686)
```

Remove PIcm, PIt5m, PINA1tm - PIt2m is correct  
Change PIt2m genes to '15874 or 8889'  
Remove PIt2p, add PItx from hsa1 (no gene)  
Change PItn genes to ''  
Change PIt2r genes to '10316 or 11409 or 11410 or 11418 or 11534'  
Replace PIt8 with PIt9 (2 Na+), change genes to '11686'
Remove PItf, PINA1th

In [116]:

```
model.reactions.get_by_id('PIt2m').gene_reaction_rule = '15874 or 8889'
r = hsa.reactions.get_by_id('PItx').copy()
model.add_reactions([r])
model.reactions.get_by_id('PItn').gene_reaction_rule = ''
model.reactions.get_by_id('PIt2r').gene_reaction_rule = '10316 or 11409 or 11410 or 11418 or 11534'
r = hsa.reactions.get_by_id('PIt9').copy()
r.gene_reaction_rule = '11686'
model.add_reactions([r])
model.remove_reactions(['PIcm','PIt5m','PINA1tm','PIt2p','PIt8','PItf','PINA1th'], remove_orphans=True)
```

In [117]:

```
for r in sorted(model.genes.get_by_id('16552').reactions, key=lambda x: x.id):
    print(r.id, r.reaction, r.gene_reaction_rule)
print()
for r in sorted(model.genes.get_by_id('8970').reactions, key=lambda x: x.id):
    print(r.id, r.reaction, r.gene_reaction_rule)
print()
for r in sorted(model.genes.get_by_id('9805').reactions, key=lambda x: x.id):
    print(r.id, r.reaction, r.gene_reaction_rule)
```

```
ADATT adp_n + atp_n + h_n --> ap4a_n + pi_n 16552
APATT aps_n + atp_n --> ap4a_n + so4_n 16552
ATPATF1 adp_c + atp_c + h_c --> ap4a_c + pi_c 16552
ATPATF2 adp_c + gtp_c + h_c --> ap4g_c + pi_c 16552
ATPATF3 gdp_c + gtp_c + h_c --> gp4g_c + pi_c 16552
SLFAT adp_c + h_c + so4_c <=> aps_c + pi_c 16552
yli_R1555 aps_c + atp_c --> ap4a_c + so4_c 16552
yli_R1556 adp_c + atp_c --> ap4a_c + pi_c 16552

ATPH1 atp_c + 2.0 h2o_c --> amp_c + 2.0 h_c + 2.0 pi_c 8970
ATPH1e atp_e + 2.0 h2o_e --> amp_e + 2.0 h_e + 2.0 pi_e 8970 or 9805
ATPH2e adp_e + h2o_e --> amp_e + h_e + pi_e 8970 or 9805
CDPPH cdp_c + h2o_c --> cmp_c + h_c + pi_c 8970 or 9805
DTPH dtdp_c + h2o_c --> dtmp_c + h_c + pi_c 8970 or 9805
GTPH1 gtp_c + 2.0 h2o_c --> gmp_c + 2.0 h_c + 2.0 pi_c 8970
IDPA h2o_c + idp_c --> h_c + imp_c + pi_c 8970 or 9805
NDP1 adp_c + h2o_c --> amp_c + h_c + pi_c 8970 or 9805
NDP3 gdp_c + h2o_c --> gmp_c + h_c + pi_c 8970 or 9805
NDP7 h2o_c + udp_c --> h_c + pi_c + ump_c 8970 or 9805
NDP7g h2o_g + udp_g --> h_g + pi_g + ump_g 8970
NTP10 h2o_c + itp_c --> h_c + idp_c + pi_c 8970 or 9805
NTP3 gtp_c + h2o_c --> gdp_c + h_c + pi_c 10886 or 16025 or 8970 or 9805
NTP5 ctp_c + h2o_c --> cdp_c + h_c + pi_c 8970 or 9805
NTP7 h2o_c + utp_c --> h_c + pi_c + udp_c 8970 or 9805
NTP9 dttp_c + h2o_c --> dtdp_c + h_c + pi_c 8970 or 9805
UTPH1 2.0 h2o_c + utp_c --> 2.0 h_c + 2.0 pi_c + ump_c 8970

ATPH1e atp_e + 2.0 h2o_e --> amp_e + 2.0 h_e + 2.0 pi_e 8970 or 9805
ATPH2e adp_e + h2o_e --> amp_e + h_e + pi_e 8970 or 9805
ATPPHm atp_m + h2o_m --> adp_m + h_m + pi_m 9805
CDPPH cdp_c + h2o_c --> cmp_c + h_c + pi_c 8970 or 9805
DTPH dtdp_c + h2o_c --> dtmp_c + h_c + pi_c 8970 or 9805
IDPA h2o_c + idp_c --> h_c + imp_c + pi_c 8970 or 9805
NDP1 adp_c + h2o_c --> amp_c + h_c + pi_c 8970 or 9805
NDP3 gdp_c + h2o_c --> gmp_c + h_c + pi_c 8970 or 9805
NDP3g gdp_g + h2o_g --> gmp_g + h_g + pi_g 9805
NDP7 h2o_c + udp_c --> h_c + pi_c + ump_c 8970 or 9805
NTP1 atp_c + h2o_c --> adp_c + h_c + pi_c 9805 or (YALI0B21527g and 10674 and 11025 and 11052 and 11064 and 11117 and 11403 and 11958 and 11967 and 13424 and 13759 and 14786 and 14912 and 14971 and 15309 and 15351 and 15589 and 15963 and 16359 and 16397 and 9210 and 9622 and 9940) or (YALI0B21527g and 10674 and 11052 and 11064 and 11117 and 11403 and 11958 and 11967 and 13424 and 13759 and 14786 and 14912 and 14971 and 15309 and 15351 and 15589 and 15963 and 16359 and 16397 and 9210 and 9622 and 9748 and 9940) or (10674 and 11025 and 11052 and 11064 and 11117 and 11403 and 11958 and 11967 and 13424 and 13759 and 14786 and 14912 and 14971 and 15309 and 15351 and 15589 and 15963 and 16359 and 16397 and 9080 and 9210 and 9622 and 9748 and 9940)
NTP10 h2o_c + itp_c --> h_c + idp_c + pi_c 8970 or 9805
NTP3 gtp_c + h2o_c --> gdp_c + h_c + pi_c 10886 or 16025 or 8970 or 9805
NTP5 ctp_c + h2o_c --> cdp_c + h_c + pi_c 8970 or 9805
NTP7 h2o_c + utp_c --> h_c + pi_c + udp_c 8970 or 9805
NTP9 dttp_c + h2o_c --> dtdp_c + h_c + pi_c 8970 or 9805
```

In [118]:

```
for r in sorted(model.genes.get_by_id('10886').reactions, key=lambda x: x.id):
    print(r.id, r.reaction, r.gene_reaction_rule)
print()
for r in sorted(model.genes.get_by_id('16025').reactions, key=lambda x: x.id):
    print(r.id, r.reaction, r.gene_reaction_rule)
```

```
NTP3 gtp_c + h2o_c --> gdp_c + h_c + pi_c 10886 or 16025 or 8970 or 9805

NTP3 gtp_c + h2o_c --> gdp_c + h_c + pi_c 10886 or 16025 or 8970 or 9805
```

16552 APA1,APA2 cyto 18.5, cyto\_nucl 11.5, mito 4, nucl 3.5 K00988: APA1\_2; ATP adenylyltransferase  
8970 YND1 mito 8, plas 5, nucl 4, extr 4, cyto 3, pero 2 K14642: YND1; golgi apyrase  
9805 GDA1 cyto 9.5, cysk 9, cyto\_pero 7.166, cyto\_nucl 6.833, pero 3.5, mito 3 K01526: E3.6.1.42; guanosine-diphosphatase  
10886 cyto 15, cyto\_mito 12.665, cyto\_nucl 11.333, mito 8.5 K03189: ureG; urease accessory protein  
16025 cyto\_nucl 14, nucl 11.5, cyto 11.5 KOG2743: Cobalamin synthesis protein

remove ADATT,APATT 16552 is cyto and these are only in iRC1080
remove yli\_R1555,yli\_R1556 incorrect reactions

NDP7g h2o\_g + udp\_g --> h\_g + pi\_g + ump\_g 8970
NDP3g gdp\_g + h2o\_g --> gmp\_g + h\_g + pi\_g 9805

Remove ATPH1,CDPPH,DTPH,GTPH1,IDPA,UTPH1,NDP1,NDP3,NDP7,NTP1,NTP3,NTP5,NTP7,NTP9,NTP10,ATPPHm

In [119]:

```
model.remove_reactions(['ADATT','APATT','yli_R1555','yli_R1556'], remove_orphans=True)
model.remove_reactions(['ATPH1','ATPH1e','ATPH2e','CDPPH','DTPH','GTPH1','IDPA','UTPH1','NDP1','NDP3','NDP7',
                                'NTP1','NTP3','NTP5','NTP7','NTP9','NTP10','ATPPHm'], remove_orphans=True)
```

ATP synthase  
http://emboj.embopress.org/content/embojnl/33/15/1617/F1.large.jpg  
Yeast ATP synthase 2.9 +/- 0.2 H+ translocated per ATP  
ATPS3m translocates 3 cyto H+ to 3 mito H+  
https://www.ncbi.nlm.nih.gov/pmc/articles/PMC3396544/

atp1 16359 mito 24, cyto 3 K02132: ATPeF1A, ATP5A1, ATP1; F-type H+-transporting ATPase subunit alpha  
atp2 15589 mito 26 K02133: ATPeF1B, ATP5B, ATP2; F-type H+-transporting ATPase subunit beta  
atp3 11958 mito 24, cyto\_mito 13.833, mito\_nucl 12.833 K02136: ATPeF1G, ATP5C1, ATP3; F-type H+-transporting ATPase subunit gamma  
atp15 13842 mito 26 HMMPfam:Mitochondrial ATP synthase epsilon chain:PF04627,SUPERFAMILY::SSF48690  
atp16 11967 mito 24, cyto\_nucl 2, nucl 1.5, cyto 1.5 K02134: ATPeF1D, ATP5D, ATP16; F-type H+-transporting ATPase subunit delta (also epsilon)

atp6 RTO3\_900623 Q0085 ATPeF0A, MTATP6, ATP6; F-type H+-transporting ATPase subunit a  
atp8 RTO3\_879280 Q0080 ATPase, F0 complex, subunit 8, mitochondrial, fungal (not final gene model)  
atp9 RTO3\_900663 Q0130 ATPeF0C, ATP5G, ATP9; F-type H+-transporting ATPase subunit c

atp4 14786 mito 17, cyto 5, cyto\_nucl 5, nucl 3 K02127: ATPeF0B, ATP5F1, ATP4; F-type H+-transporting ATPase subunit b  
atp5 13424 mito 22, cyto\_nucl 3, nucl 2.5, cyto 2.5 K02137: ATPeF0O, ATP5O, ATP5; F-type H+-transporting ATPase subunit O  
atp7/d 14912 mito 15.5, cyto\_mito 11.333, cyto 6, cyto\_nucl 5.333, nucl 3.5 K02138: ATPeF0D, ATP5H, ATP7; F-type H+-transporting ATPase subunit d  
atp14/h 10674 mito 25 HMMPfam:ATP synthase complex subunit h:PF10775

atp17/f 9940 mito 21, cyto\_mito 12.5, extr 3 K02139: ATPeFF, ATP17; F-type H+-transporting ATPase subunit f  
atp18/i/j 13759 mito 11, extr 9, nucl 3, cyto 3, cyto\_nucl 3 HMMPfam:ATP synthase j chain:PF04911  
atp19 9619 cyto 23.5, cyto\_nucl 12.5 HMMPfam:Protein of unknown function (DUF2611):PF11022
atp20/g 9080 mito 25 KOG4103: Mitochondrial F1F0-ATP synthase, subunit g/ATP20  
atp21/e 15880 mito 13.5, cyto\_mito 10.833, cyto 7, cyto\_nucl 6.333, nucl 4.5 HMMPfam:ATP synthase E chain:PF05680

atp10 13053 K18192: ATP10; mitochondrial ATPase complex subunit ATP10 Inner membrane protein required for assembly of the F0 sector of ATP synthase  
atp11 13252 K07555: ATPeAF1, ATPAF1, ATP11; ATP synthase mitochondrial F1 complex assembly factor 1  
atp12 15287 mito 24, cyto 1, extr 1, pero 1, cyto\_pero 1 K07556: ATPeAF2, ATPAF2, ATP12; ATP synthase mitochondrial F1 complex assembly factor 2

10675 nucl 19, cyto\_nucl 14.5, cyto 6 HMMPfam:Mitochondrial ATPase inhibitor, IATP:PF04568,SUPERFAMILY::SSF64602

Change ATPS3m 'RTO3\_879280 and RTO3\_900623 and RTO3\_900663 and 9080 and 9619 and 9940 and 10674 and 11958 and 11967 and 13053 and 13252 and 13424 and 13759 and 13842 and 14786 and 14912 and 15287 and 15589 and 15880 and 16359'
Remove ATPS4m,ATPS4rpp,ATPasel

In [120]:

```
model.reactions.get_by_id('ATPS3m').gene_reaction_rule = 'RTO3_879280 and RTO3_900623 and RTO3_900663 and 9080 and 9619 and 9940 and 10674 and 11958 and 11967 and 13053 and 13252 and 13424 and 13759 and 13842 and 14786 and 14912 and 15287 and 15589 and 15880 and 16359'
model.remove_reactions(['ATPS4m','ATPS4rpp','ATPasel'], remove_orphans=True)
```

Plasma membrane H+-ATPase

ATPS atp\_c + h2o\_c --> adp\_c + h\_e + pi\_c 12428 or 13617 or 15156 or 15584 or (YCR024C\_A and YEL017C\_A and 13617) or (YCR024C\_A and YEL017C\_A and 15584)
iMM904 PMP1 and PMP2 and (PMA1 or PMA2)  
PMP1 and PMP2 are Regulatory subunit for the plasma membrane H(+)-ATPase Pma1p  
13617 PMA1, PMA2 plas 24, E.R. 1, golg 1, vacu 1 K01535: PMA1, PMA2; H+-transporting ATPase
15584 PMA1, PMA2 plas 21, nucl 2, cyto 2, cyto\_nucl 2 K01535: PMA1, PMA2; H+-transporting ATPase

Change ATPS genes to '13617 or 15584'

Vacuolar H+-ATPase  
http://mmbr.asm.org/content/70/1/177.long  
V-ATPase translocates 2 H+ into vacuole per ATP
https://www.ncbi.nlm.nih.gov/pmc/articles/PMC1218484/

VMA1 15351 cyto\_mito 8.166, mito 8, cyto 8, cyto\_nucl 6.833, cyto\_pero 5.333 K02145: ATPeV1A, ATP6A; V-type H+-transporting ATPase subunit A  
VMA2 11064 cysk 10, cyto 9.5, cyto\_nucl 9, nucl 5.5 K02147: ATPeV1B, ATP6B; V-type H+-transporting ATPase subunit B  
VMA5 9210 nucl 14, cyto\_nucl 11.333, cyto 6.5, cyto\_mito 6.166, mito 4.5 K02148: ATPeV1C, ATP6C; V-type H+-transporting ATPase subunit C  
VMA8 14971 mito 20.5, cyto\_mito 13, cyto 4.5 K02149: ATPeV1D, ATP6M; V-type H+-transporting ATPase subunit D  
VMA4 9622 nucl 15, cyto 11 K02150: ATPeV1E, ATP6E; V-type H+-transporting ATPase subunit E  
VMA7 15963 cyto\_nucl 16.5, cyto 16, nucl 7 K02151: ATPeV1F, ATP6S14; V-type H+-transporting ATPase subunit F  
VMA10 16397 nucl 18, cyto\_nucl 14.5, cyto 7 K02152: ATPeV1G, ATP6G; V-type H+-transporting ATPase subunit G  
VMA13 11052 mito 9, cyto\_nucl 8.5, cyto 7.5, nucl 6.5, pero 4 K02144: ATPeV1H; V-type H+-transporting ATPase subunit H

STV1,VPH1 (VPH1 is best hit) 15309 plas 17, mito 4, E.R. 3, vacu 2, cyto\_mito 2, mito\_nucl 2 K02154: ATPeV0A, ATP6N; V-type H+-transporting ATPase subunit a  
VMA16 11403 plas 13, mito 8, vacu 4, cyto\_mito 4, mito\_nucl 4 K03661: ATPeV0B, ATP6F; V-type H+-transporting ATPase 21kDa proteolipid subunit  
VMA3 9748 plas 12, mito 7, vacu 4, E.R. 3 K02155: ATPeV0C, ATP6L; V-type H+-transporting ATPase 16kDa proteolipid subunit  
VMA11 11025 plas 14, mito 5, vacu 4, E.R. 3 K02155: ATPeV0C, ATP6L; V-type H+-transporting ATPase 16kDa proteolipid subunit  
VMA6 11117 cyto\_nucl 10.333, cyto 9.5, nucl 9, cyto\_mito 6.666, pero 4, mito 2.5 K02146: ATPeV0D, ATP6D; V-type H+-transporting ATPase subunit d  
VMA9 10802 extr 12, plas 9, vacu 4 HMMPfam:ATP synthase subunit H:PF05493

Change ATPS3v adp\_v + 3.0 h\_c + pi\_v --> atp\_v + h2o\_v + 2.0 h\_v to  
ATPS2v atp\_c + h2o\_c + h\_c --> adp\_c + 2.0 h\_v + pi\_c  
Change ATPS2v genes to '10802 and 11025 and 11052 and 11064 and 11117 and 11403 and 14971 and 15309 and 15351 and 15963 and 16397 and 9210 and 9622 and 9748'

Golgi H+-ATPase  
ATPS3g adp\_g + 3.0 h\_c + pi\_g --> atp\_g + h2o\_g + 2.0 h\_g 11025 and 11052 and 11064 and 11117 and 11403 and 14971 and 15309 and 15351 and 15963 and 16397 and 9210 and 9622 and 9748  
(these are same as vaculoar H+-ATPase genes)  
S. cerevisiae STV1 is responsible for golgi localization, and need W83KY sequence which is missing in 15309 (VPH1)  
http://www.jbc.org/content/287/23/19487.full  
Remove ATPS3g

Other transporting/translocating ATPases

NA+/K+  
13049 plas 27 K01539: ATP1A; sodium/potassium-transporting ATPase subunit alpha  
12846 ENA1,ENA2,ENA5 plas 25 K01537: E3.6.3.7; Na+-exporting ATPase

NaKt needs ATP1A1 and ATP1B1, and ATP1B1 is an auxiliary non-catalytic beta subunit  
Sodium/potassium-transporting ATPase 3.6.3.9 is known to exist in animals, and H+-ATPase is known to exist in plants and fungi  
Blast of 13049 hit to NA+exporting ATPase 3.6.3.7 ACU1 in Ustilago maydis, which transports NA+ and K+  
http://ec.asm.org/content/3/2/359.full  
Sodium efflux ATPase also pumps potassium, probably electroneutral  
https://www.sciencedirect.com/science/article/pii/S0005273610002439

Change NaKt atp\_c + h2o\_c + 2.0 k\_e + 3.0 na1\_c --> adp\_c + h\_c + 2.0 k\_c + 3.0 na1\_e + pi\_c, (ATP1B1 and 13049) or (ATP1B2 and 13049) or (ATP1B3 and 13049) or (ATP1B4 and 13049) to  
Na1ATPase atp\_c + h2o\_c + h\_e + na1\_c --> adp\_c + 2.0 h\_c + na1\_e + pi\_c  
Change HKt atp\_c + h2o\_c + k\_e --> adp\_c + h\_e + k\_c + pi\_c (ATP4B and 13049) or (Atp4b and 13049) to  
KATPase atp\_c + h2o\_c + h\_e + k\_c --> adp\_c + 2.0 h\_c + k\_e + pi\_c  
Remove NA1ATPaseh,NA1ATPasem

Ca2+  
8631 PMC1 plas 19, E.R. 4, cyto 2.5, cyto\_nucl 2 K01537: E3.6.3.8; Ca2+-transporting ATPase  
12428 PMR1 plas 22, E.R. 2, nucl 1, cyto 1, cyto\_nucl 1, golg 1 K01537: E3.6.3.8; Ca2+-transporting ATPase  
15156 PMR1 plas 9, cyto 7, E.R. 7, mito 2 K05853: ATP2A; Ca2+ transporting ATPase, sarcoplasmic/endoplasmic reticulum
15140 mito 17, cyto 4.5, cyto\_nucl 4, nucl 2.5 KOG0202: Ca2+ transporting ATPase sarcoplasmic/endoplasmic reticulum (15140 + 15141 looks like one gene)  
15141 nucl 10, mito 8, cyto 5, pero 4 HMMPfam:Cation transporter/ATPase, N-terminus:PF00690,SUPERFAMILY::SSF81665

S. cerevisiae contains two Ca2+ ATPases: Pmc1p and Pmr1p. Pmr1p is the principal Ca2+ ATPase that is expressed under normal growth conditions and Pmc1p is induced in the absence of PMR1 or with calcium overload (2).
PMR1 encodes the major Golgi membrane P-type ATPase ion pump responsible for transporting calcium [Ca(2+)] and manganese [Mn(2+)] ions into the Golgi apparatus. Ca(2+) is required to sustain protein sorting while Mn(2+) is required for protein glycosylation. In addition, Pmr1p provides a major route for cellular detoxification of Mn(2+). Excess levels of cytosolic Mn(2+) are transported into the Golgi and then exit the cell via secretory pathway vesicles.  
PMC1 encodes a Ca2+ ATPase that transports calcium from the cytoplasm into the vacuole and participates in calcium homeostasis.

PMCA (plasma membrane Ca2+-ATPase) pump 1 Ca2+ into, 1 H+ out of extracellular (another H+ by ATP hydrolysis)  
SERCA (sarco(endo)plasmic reticulum Ca2+-ATPase) pump 2 Ca2+ into, 2 H+ out of ER (another H+ by ATP hydrolysis)  
https://www.sciencedirect.com/science/article/pii/S0968000408000431  
https://link.springer.com/article/10.1007/s002490050132

Change CAATPS atp\_c + 2.0 ca2\_c + h2o\_c --> adp\_c + 2.0 ca2\_e + h\_e + pi\_c, 8631 to  
PMCA atp\_c + ca2\_c + h2o\_c + h\_e --> adp\_c + ca2\_e + 2.0 h\_c + pi\_c, genes to '8631 or 12428'  
SERCA atp\_c + 2.0 ca2\_c + h2o\_c + 2.0 h\_r --> adp\_c + 2.0 ca2\_r + 3.0 h\_c + pi\_c, genes to '(15140 and 15141) or 15156'

15342 plas 22, E.R. 4 CCC2 K17686: copA, ATP7; Cu+-exporting ATPase  
Change CU1abcpp atp\_c + cu\_c + h2o\_c --> adp\_c + cu\_p + h\_c + pi\_c 15342 to  
Cu1ATPase atp\_c + cu\_c + h2o\_c --> adp\_c + cu\_e + h\_c + pi\_c 15342 (gene is correct)

14615 nucl 10.5, cyto\_nucl 10.5, cyto 9.5, cysk 5 K01551: arsA, ASNA1; arsenite-transporting ATPase, GET3 in S. cer, Guanine nucleotide exchange factor for Gpa1p  
8483 SPF1 plas 12, E.R. 7, mito 4, vacu 2, cyto\_mito 2, mito\_nucl 2 K14950: ATP13A1; cation-transporting ATPase 13A1 (manganese transport into ER)  
16275 YPK9 plas 23, E.R. 2 K14951: ATP13A3\_4\_5; cation-transporting ATPase 13A3/4/5 (vacuolar)

9428 DNF1 plas 21, nucl 2, mito 2, mito\_nucl 2 K01530: E3.6.3.1; phospholipid-translocating ATPase  
10754 NEO1 plas 16, mito 7, E.R. 2 K01530: E3.6.3.1; phospholipid-translocating ATPase  
12680 DRS2 plas 26 K14802: DRS2, ATP8A; phospholipid-transporting ATPase  
15426 DNF2 plas 15, mito 8, nucl 1, cyto 1, cyto\_nucl 1, pero 1, E.R. 1, cyto\_pero 1 KOG0206: P-type ATPase (DNF2 flippase)

In [121]:

```
model.reactions.get_by_id('ATPS').gene_reaction_rule = '13617 or 15584'
model.reactions.get_by_id('ATPS3v').id = 'ATPS2v'
model.reactions.get_by_id('ATPS2v').name = 'Vacuolar H+-ATPase'
model.reactions.get_by_id('ATPS2v').add_metabolites({'atp_v': -1.0, 'atp_c': -1.0, 'adp_v': 1.0, 'adp_c': 1.0, 'pi_v': 1.0, 'pi_c': 1.0, 'h2o_v': -1.0, 'h2o_c': -1.0, 'h_c': 2.0})
model.reactions.get_by_id('ATPS2v').gene_reaction_rule = '10802 and 11025 and 11052 and 11064 and 11117 and 11403 and 14971 and 15309 and 15351 and 15963 and 16397 and 9210 and 9622 and 9748'
model.remove_reactions(['ATPS3g'], remove_orphans=True)
model.reactions.get_by_id('NaKt').id = 'Na1ATPase'
model.reactions.get_by_id('Na1ATPase').name = 'Na+-exporting ATPase'
model.reactions.get_by_id('Na1ATPase').add_metabolites({'k_e': 2.0, 'na1_c': 2.0, 'k_c': -2.0, 'na1_e': -2.0, 'h_e': -1.0, 'h_c': 1.0})
model.reactions.get_by_id('Na1ATPase').gene_reaction_rule = '12846 or 13049'
model.reactions.get_by_id('HKt').id = 'KATPase'
model.reactions.get_by_id('KATPase').name = 'Na+-exporting ATPase (K+)'
model.reactions.get_by_id('KATPase').add_metabolites({'k_e': 2.0, 'k_c': -2.0, 'h_e': -2.0, 'h_c': 2.0})
model.reactions.get_by_id('KATPase').gene_reaction_rule = '12846 or 13049'
model.remove_reactions(['NA1ATPaseh','NA1ATPasem'], remove_orphans=True)
r = model.reactions.get_by_id('CAATPS').copy()
r.id = 'PMCA'
r.name = 'Plasma membrane Ca2+-ATPase'
model.add_reactions([r])
model.reactions.get_by_id('PMCA').add_metabolites({'ca2_c': 1.0, 'ca2_e': -1.0, 'h_e': -2.0, 'h_c': 2.0})
model.reactions.get_by_id('PMCA').gene_reaction_rule = '8631 or 12428'
m = model.metabolites.get_by_id('ca2_c').copy()
m.id = 'ca2_r'
m.compartment = 'r'
model.add_metabolites([m])
model.reactions.get_by_id('CAATPS').id = 'SERCA'
model.reactions.get_by_id('SERCA').name = 'Sarcoplasmic/endoplasmic reticulum Ca2+-ATPase'
model.reactions.get_by_id('SERCA').add_metabolites({'ca2_e': -2.0, 'ca2_r': 2.0, 'h_e': -1.0, 'h_r': -2.0, 'h_c': 3.0})
model.reactions.get_by_id('SERCA').gene_reaction_rule = '(15140 and 15141) or 15156'
m = model.metabolites.get_by_id('cu_p').copy()
m.id = 'cu_e'
m.compartment = 'e'
model.add_metabolites([m])
model.reactions.get_by_id('CU1abcpp').id = 'Cu1ATPase'
model.reactions.get_by_id('Cu1ATPase').name = 'Cu+-exporting ATPase'
model.reactions.get_by_id('Cu1ATPase').add_metabolites({'cu_p': -1.0, 'cu_e': 1.0})
```

In [122]:

```
temp = ['ATPS3m','ATPS2v','Na1ATPase','KATPase','PMCA','SERCA','Cu1ATPase']
for x in temp:
    r = model.reactions.get_by_id(x)
    for g in r.genes:
        if set(r.id for r in g.reactions).difference(temp):
            print(g.id, set(r.id for r in g.reactions), set((r.id, r.gene_reaction_rule) for r in g.reactions).difference(temp))
```

```
12428 {'MG2uabcpp', 'NI2uabcpp', 'PMCA'} {('MG2uabcpp', '12428 or 15156'), ('PMCA', '8631 or 12428'), ('NI2uabcpp', '12428 or 15156 or (b3476 and b3477 and b3478 and b3479 and b3480)')}
15156 {'MG2uabcpp', 'SERCA', 'NI2uabcpp'} {('MG2uabcpp', '12428 or 15156'), ('SERCA', '(15140 and 15141) or 15156'), ('NI2uabcpp', '12428 or 15156 or (b3476 and b3477 and b3478 and b3479 and b3480)')}
```

In [123]:

```
model.remove_reactions(['MG2uabcpp','NI2uabcpp'], remove_orphans=True)
```

In [124]:

```
for r in sorted(model.metabolites.get_by_id('glc__D_e').reactions, key=lambda x: x.id):
    print(r.id, r.reaction, r.gene_reaction_rule)
```

```
13BGHe 13BDglcn_e + h2o_e --> glc__D_e 13553
AMY1e 8.0 h2o_e + strch1_e --> 8.0 glc__D_e + strch2_e 11625 and 9135
AMY2e glygn2_e + 8.0 h2o_e --> 8.0 glc__D_e + glygn4_e 11625 and 9135
GLCt1 glc__D_e --> glc__D_c 10452 or 10704 or 11075 or 11893 or 15762 or 9102 or 9841
SUCRe h2o_e + sucr_e --> fru_e + glc__D_e 14826
```

In [125]:

```
for r in sorted(model.metabolites.get_by_id('Glc_aD_c').reactions, key=lambda x: x.id):
    print(r.id, r.reaction, r.gene_reaction_rule)
print()
for r in sorted(model.metabolites.get_by_id('glc__bD_c').reactions, key=lambda x: x.id):
    print(r.id, r.reaction, r.gene_reaction_rule)
print()
for r in sorted(model.metabolites.get_by_id('glc__D_c').reactions, key=lambda x: x.id):
    print(r.id, r.reaction, r.gene_reaction_rule)
```

```
A1E Glc_aD_c <=> glc__bD_c 14480
AATHA h2o_c + tre_c --> 2.0 Glc_aD_c 8580
GLUKA Glc_aD_c + atp_c --> adp_c + g6p_A_c + h_c 10264
MG1A asnglcnacglcnacman_man_manman_manman_manmanmanglcglcglc_c + h2o_c --> Glc_aD_c + asnglcnacglcnacman_man_manman_manman_manmanmanglcglc_c 9981
MG2A asnglcnacglcnacman_man_manman_manman_manmanmanglcglc_c + h2o_c --> Glc_aD_c + asnglcnacglcnacman_man_manman_manman_manmanmanglc_c 10170
MG3A asnglcnacglcnacman_man_manman_manman_manmanmanglc_c + h2o_c --> Glc_aD_c + asnglcnacglcnacman_man_manman_manman_manmanman_c 10170

A1E Glc_aD_c <=> glc__bD_c 14480
AATHB h2o_c + tre_c --> 2.0 glc__bD_c 8580
GLUK_syn atp_c + glc__bD_c --> adp_c + g6p_B_c + h_c 10264
MG1B asnglcnacglcnacman_man_manman_manman_manmanmanglcglcglc_c + h2o_c --> asnglcnacglcnacman_man_manman_manman_manmanmanglcglc_c + glc__bD_c 9981
MG2B asnglcnacglcnacman_man_manman_manman_manmanmanglcglc_c + h2o_c --> asnglcnacglcnacman_man_manman_manman_manmanmanglc_c + glc__bD_c 10170
MG3B asnglcnacglcnacman_man_manman_manman_manmanmanglc_c + h2o_c --> asnglcnacglcnacman_man_manman_manman_manmanman_c + glc__bD_c 10170

BGLA cellb_c + h2o_c --> 2.0 glc__D_c 13486 or 9639
DGGH 6dg_c + h2o_c --> gal_c + glc__D_c 9135
G1PP g1p_c + h2o_c --> glc__D_c + pi_c 11513 or 8576
G6PP g6p_c + h2o_c --> glc__D_c + pi_c 8576
GLCATr accoa_c + glc__D_c <=> acglc__D_c + coa_c 9734
GLCGSD glycogen_c + h2o_c --> glc__D_c 12639
GLCt1 glc__D_e --> glc__D_c 10452 or 10704 or 11075 or 11893 or 15762 or 9102 or 9841
GLCt2pp glc__D_p + h_p --> glc__D_c + h_c 13042 or 8608
GLCtg glc__D_c <=> glc__D_g 15762
GLDBRAN dxtrn_c + h2o_c --> glc__D_c + glygn3_c 12639
GLPASE2 glygn3_c + 7.0 h2o_c --> Tyr_ggn_c + 7.0 glc__D_c 14256
GLUK atp_c + glc__D_c --> adp_c + g6p_B_c + h_c 10264 or 11982
HEX1 atp_c + glc__D_c --> adp_c + g6p_c + h_c 10264 or 11982
MALT h2o_c + malt_c --> 2.0 glc__D_c 10170 or 9135
MLTG1 h2o_c + malttr_c --> glc__D_c + malt_c 10170
SBTR glc__D_c + h_c + nadph_c --> nadp_c + sbt__D_c 11882 or 12784 or 9774
TRE6PH h2o_c + tre6p_c --> g6p_c + glc__D_c 9135
TREH h2o_c + tre_c --> 2.0 glc__D_c 8580
XYHDL h2o_c + iprimv_c --> glc__D_c + xyl__D_c 10170
yli_R0371 atp_c + glc__D_c --> adp_c + g6p_A_c + h_c 10264 or 11982
yli_R0728 h2o_c + starch_c --> glc__D_c 14743
yli_R0735 h2o_c + yli_M00682_c --> glc__D_c 13486 or 9639
yli_R0736 h2o_c + yli_M00844_c --> glc__D_c 13553
yli_R1573 malt_c --> amylose_c + glc__D_c 12639
yli_R1574 glycogen_c + h2o_c --> amylose_c + glc__D_c 12639
```

In [126]:

```
for r in sorted(model.metabolites.get_by_id('glc__bD_c').reactions, key=lambda x: x.id):
    r.add_metabolites({'glc__bD_c': -r.get_coefficient('glc__bD_c'),
                       'glc__D_c': r.get_coefficient('glc__bD_c')})

# 13042 and 8608 are ITR1 and ITR2 myo-inositol transporters
model.remove_reactions(['GLCt2pp'], remove_orphans=True)
# remove redundant reactions
remove = ['AATHB','GLUKA','GLUK_syn','GLUK','yli_R0371','yli_R0728','yli_R0735','yli_R0736','yli_R1573','yli_R1574']
model.remove_reactions(remove, remove_orphans=True)
```

In [127]:

```
for r in sorted(model.metabolites.get_by_id('g6p_A_c').reactions, key=lambda x: x.id):
    print(r.id, r.reaction, r.gene_reaction_rule)
print()
for r in sorted(model.metabolites.get_by_id('g6p_B_c').reactions, key=lambda x: x.id):
    print(r.id, r.reaction, r.gene_reaction_rule)
print()
for r in sorted(model.metabolites.get_by_id('g6p_c').reactions, key=lambda x: x.id):
    print(r.id, r.reaction, r.gene_reaction_rule)
```

```
BFFS h2o_c + suc6p_c <=> fru_B_c + g6p_A_c 14826
G6PADH g6p_A_c + nadp_c --> 6pgl_c + h_c + nadph_c 10000
G6PI_1 g6p_A_c <=> g6p_B_c 9589
MI3PSA g6p_A_c --> mi3p__D_c 10136
PGCM g1p_c <=> g6p_A_c 13711 or 9959
PGIA g6p_A_c <=> f6p_B_c 9589
UG6PGT g6p_A_c + udpg_c --> h_c + tre6p_c + udp_c 11389 or 12034
yli_R0376 g6p_A_c <=> f6p_c 9589

G6PBDH g6p_B_c + nadp_c --> 6pgl_c + h_c + nadph_c 10000
G6PI g6p_c <=> g6p_B_c 9589
G6PI2 g6p_B_c <=> f6p_B_c 9589
G6PI3 g6p_B_c <=> f6p_c 9589
G6PI_1 g6p_A_c <=> g6p_B_c 9589
MI3PSB g6p_B_c --> mi3p__D_c 10136

G6PDH2r g6p_c + nadp_c <=> 6pgl_c + h_c + nadph_c 10000
G6PI g6p_c <=> g6p_B_c 9589
G6PP g6p_c + h2o_c --> glc__D_c + pi_c 8576
HEX1 atp_c + glc__D_c --> adp_c + g6p_c + h_c 10264 or 11982
MI1PS g6p_c --> mi1p__D_c 10136
PGI g6p_c <=> f6p_c 9589
PGMT g1p_c <=> g6p_c 13711 or 14546 or 8460 or 9959
TRE6PH h2o_c + tre6p_c --> g6p_c + glc__D_c 9135
TRE6PS g6p_c + udpg_c --> h_c + tre6p_c + udp_c 11389 or (YML100W and 11389 and 12034) or (YMR261C and 11389 and 12034)
```

In [128]:

```
model.reactions.get_by_id('BFFS').add_metabolites({'fru_B_c': -1.0, 'fru_c': 1.0,
                                                   'g6p_A_c': -1.0, 'g6p_c': 1.0})
r = hsa2.reactions.get_by_id('MI3PS').copy()
r.gene_reaction_rule = '10136'
model.add_reactions([r])
model.reactions.get_by_id('TRE6PS').gene_reaction_rule = '11389 or 12034'
remove = ['G6PADH','G6PBDH','G6PI_1','G6PI','G6PI2','G6PI3','MI3PSA','MI3PSB','MI1PS','PGCM','PGIA','UG6PGT','yli_R0376']
model.remove_reactions(remove, remove_orphans=True)
```

In [129]:

```
for r in sorted(model.metabolites.get_by_id('fru_B_c').reactions, key=lambda x: x.id):
    print(r.id, r.reaction, r.gene_reaction_rule)
print()
for r in sorted(model.metabolites.get_by_id('fru_c').reactions, key=lambda x: x.id):
    print(r.id, r.reaction, r.gene_reaction_rule)
```

```
ABFPT atp_c + fru_B_c --> adp_c + f6p_B_c + h_c 10264
yli_R0268 atp_c + fru_B_c --> adp_c + f6p_c + h_c 10264 or 11982
yli_R1133 fru_B_e + h_e --> fru_B_c + h_c 11075 or 11893

BFFS h2o_c + suc6p_c <=> fru_c + g6p_c 14826
F1PP f1p_c + h2o_c --> fru_c + pi_c 11513 or 14546 or 8576
F6PP f6p_c + h2o_c --> fru_c + pi_c 11513 or 14546 or 8576
FRUt1r fru_e <=> fru_c 15762
FRUt2 fru_e + h_e --> fru_c + h_c 11075 or 11893
HEX7 atp_c + fru_c --> adp_c + f6p_c + h_c 10264 or 11982
SBTD_D2 nad_c + sbt__D_c --> fru_c + h_c + nadh_c 16452
```

In [130]:

```
for r in sorted(model.metabolites.get_by_id('f6p_B_c').reactions, key=lambda x: x.id):
    print(r.id, r.reaction, r.gene_reaction_rule)
print()
for r in sorted(model.metabolites.get_by_id('f6p_c').reactions, key=lambda x: x.id):
    print(r.id, r.reaction, r.gene_reaction_rule)
```

```
ABFPT atp_c + fru_B_c --> adp_c + f6p_B_c + h_c 10264
FBPPH f26bp_c + h2o_c --> f6p_B_c + pi_c 15195
G6PDA_1 gam6p_c + h2o_c <=> f6p_B_c + nh4_c 14499
GF6PTA_1 f6p_B_c + gln__L_c --> gam6p_c + glu__L_c 12099
MPAKI man6p_c <=> f6p_B_c 8607
PFK26_1 atp_c + f6p_B_c --> adp_c + f26bp_c + h_c 15195

F6PP f6p_c + h2o_c --> fru_c + pi_c 11513 or 14546 or 8576
FBP fdp_c + h2o_c --> f6p_c + pi_c 11513 or 15423
FBP26 f26bp_c + h2o_c --> f6p_c + pi_c 15195 or 15423 or 15744
GF6PTA f6p_c + gln__L_c --> gam6p_c + glu__L_c 12099
HEX7 atp_c + fru_c --> adp_c + f6p_c + h_c 10264 or 11982
MAN6PI man6p_c <=> f6p_c 8607
PFK atp_c + f6p_c --> adp_c + fdp_c + h_c 8859 or 8863 or 8867
PFK26 atp_c + f6p_c --> adp_c + f26bp_c + h_c 15195 or 15744
PGI g6p_c <=> f6p_c 9589
TALA g3p_c + s7p_c <=> e4p_c + f6p_c 10173
TKT2 e4p_c + xu5p__D_c <=> f6p_c + g3p_c 15631
yli_R0268 atp_c + fru_B_c --> adp_c + f6p_c + h_c 10264 or 11982
yli_R0379 atp_c + f6p_c --> adp_c + fdp_B_c + h_c 8859 or 8863 or 8867
yli_R0380 fdp_B_c + h2o_c --> f6p_c + pi_c 15423
```

In [131]:

```
# Remove G6PDA_1, 14499 is PGL
remove = ['ABFPT','yli_R0268','yli_R1133','FBPPH','G6PDA_1','GF6PTA_1','MPAKI','PFK26_1']
model.remove_reactions(remove, remove_orphans=True)
```

In [132]:

```
for r in sorted(model.metabolites.get_by_id('fdp_B_c').reactions, key=lambda x: x.id):
    print(r.id, r.reaction, r.gene_reaction_rule)
print()
for r in sorted(model.metabolites.get_by_id('fdp_c').reactions, key=lambda x: x.id):
    print(r.id, r.reaction, r.gene_reaction_rule)
```

```
yli_R0368 fdp_B_c <=> dhap_c + g3p_c 15420
yli_R0379 atp_c + f6p_c --> adp_c + fdp_B_c + h_c 8859 or 8863 or 8867
yli_R0380 fdp_B_c + h2o_c --> f6p_c + pi_c 15423

FBA fdp_c <=> dhap_c + g3p_c 15420
FBP fdp_c + h2o_c --> f6p_c + pi_c 11513 or 15423
PFK atp_c + f6p_c --> adp_c + fdp_c + h_c 8859 or 8863 or 8867
```

In [133]:

```
remove = ['yli_R0368','yli_R0379','yli_R0380']
model.remove_reactions(remove, remove_orphans=True)
```

In [134]:

```
# Duplicate reaction names
rxns = sorted([r.id for r in model.reactions])
rxns_uniq = set()
for x in rxns:
    if x.lower() not in rxns_uniq:
        rxns_uniq.add(x.lower())
    else:
        print(x)
```

```
IGPDh
```

In [135]:

```
model.reactions.get_by_id('IGPDh')
```

Out[135]:

|  |  |
| --- | --- |
| **Reaction identifier** | IGPDh |
| **Name** | Imidazoleglycerol-phosphate dehydratase, chloroplast |
| **Memory address** | 0x0102f59cdd8 |
| **Stoichiometry** | eig3p\_h --> h2o\_h + imacp\_h  D-erythro-1-(Imidazol-4-yl)glycerol 3-phosphate --> H2O H2O + 3-(Imidazol-4-yl)-2-oxopropyl phosphate |
| **GPR** | 8707 |
| **Lower bound** | 0.0 |
| **Upper bound** | 1000.0 |

In [136]:

```
for r in sorted(model.genes.get_by_id('8707').reactions, key=lambda x: x.id):
    print(r.id, r.reaction, r.gene_reaction_rule)
```

```
HISTP h2o_c + hisp_c --> histd_c + pi_c 10206 or 8707
IGPDH eig3p_c --> h2o_c + imacp_c 8707
IGPDh eig3p_h --> h2o_h + imacp_h 8707
```

In [137]:

```
for r in sorted(model.genes.get_by_id('10206').reactions, key=lambda x: x.id):
    print(r.id, r.reaction, r.gene_reaction_rule)
```

```
HISTP h2o_c + hisp_c --> histd_c + pi_c 10206 or 8707
```

8707 cyto imidazoleglycerol-phosphate dehydratase  
10206 cyto histidinol-phosphatase  
Change HISTP gene to '10206'  
Remove IGPDHh

In [138]:

```
model.reactions.get_by_id('HISTP').gene_reaction_rule = '10206'
model.remove_reactions(['IGPDh'], remove_orphans=True)
```

In [139]:

```
print(len(model.genes))
print(len(model.reactions))
print(len(model.metabolites))
model
```

```
1459
3510
3416
```

Out[139]:

|  |  |
| --- | --- |
| **Name** | R. toruloides |
| **Memory address** | 0x0102e8aaa20 |
| **Number of metabolites** | 3416 |
| **Number of reactions** | 3510 |
| **Number of groups** | 0 |
| **Objective expression** | 0 |
| **Compartments** | c, x, m, e, r, v, n, g, p, h, s, f, l |

In [140]:

```
for x in sorted(model.genes, key=lambda x: x.id):
    if not x.reactions:
        print(x)
print()
for x in sorted(model.metabolites, key=lambda x: x.id):
    if not x.reactions:
        print(x)
```

```
10675
15285
ATP1B1
ATP1B2
ATP1B3
ATP1B4
ATP4B
Atp4b
CRv4_Au5_s10_g65_t1
CRv4_Au5_s17_g7684_t1
CRv4_Au5_s17_g7685_t1
CRv4_Au5_s1_g1345_t1
CRv4_Au5_s1_g1958_t1
CRv4_Au5_s1_g1960_t1
CRv4_Au5_s3_g10243_t1
CRv4_Au5_s4_g11710_t1
ND1
ND2
ND3
ND4
ND4L
ND5
ND6
NDUFA1
NDUFA10
NDUFA11
NDUFA3
NDUFA4
NDUFA7
NDUFB1
NDUFB10
NDUFB2
NDUFB3
NDUFB4
NDUFB5
NDUFB6
NDUFB7
NDUFB8
NDUFC1
NDUFC2
NDUFS5
NDUFV3
Ndufa1
Ndufa10
Ndufa11
Ndufa13
Ndufa3
Ndufa4
Ndufa7
Ndufb10
Ndufb2
Ndufb3
Ndufb5
Ndufb6
Ndufb7
Ndufb8
Ndufc1
Ndufc2
Ndufs5
Ndufv3
PHATRDRAFT_12269
PHATRDRAFT_12373
PHATRDRAFT_16283
PHATRDRAFT_16615
PHATRDRAFT_17860
PHATRDRAFT_35064
PHATRDRAFT_38985
PHATRDRAFT_42245
PHATRDRAFT_47199
PHATRDRAFT_48932
PHATRDRAFT_9984
PP_0490
PP_0491
PP_0492
PP_1706
PP_2183
PP_2184
PP_2186
Phatr3_EG00791
Q0080
Q0085
Q0105
Q0130
TUSC3
Tusc3
YBL045C
YCR024C_A
YDL181W
YEL017C_A
YOL077W_A
YPL271W
YPR020W
b3366
mmu_100043472
mmu_4541
mmu_4707
mmu_4710

aproa_c
cu_p
ficytC_m
focytC_m
glc__bD_c
q10_m
q10h2_m
```

In [141]:

```
cobra.manipulation.remove_genes(model, [x for x in model.genes if not x.reactions])
model.remove_metabolites([x for x in model.metabolites if not x.reactions])
```

In [142]:

```
for x in sorted(model.genes, key=lambda x: x.id):
    if not x.reactions:
        print(x)
print()
for x in sorted(model.metabolites, key=lambda x: x.id):
    if not x.reactions:
        print(x)
```

```

```

In [143]:

```
print(len(model.genes))
print(len(model.reactions))
print(len(model.metabolites))
print(len(set([m.id.rsplit('_',1)[0] for m in model.metabolites])))
print(len(model.compartments))
model
```

```
1362
3510
3409
1960
13
```

Out[143]:

|  |  |
| --- | --- |
| **Name** | R. toruloides |
| **Memory address** | 0x0102e8aaa20 |
| **Number of metabolites** | 3409 |
| **Number of reactions** | 3510 |
| **Number of groups** | 0 |
| **Objective expression** | 0 |
| **Compartments** | c, x, m, e, r, v, n, g, p, h, s, f, l |

In [144]:

```
cobra.io.save_json_model(model, "IFO0880_GPR_1a.json")
```

In [145]:

```
model_old = cobra.io.load_json_model("../0.Annotation_and_Draft_reconstruction/IFO0880_GPR.json")
model_new = cobra.io.load_json_model("IFO0880_GPR_1a.json")
```

In [146]:

```
print('Removed reactions\n')
for r in sorted(model_old.reactions, key=lambda x: x.id):
    if r not in model_new.reactions:
        print(r)
```

```
Removed reactions

2DGULRGx: 2dhguln_c + h_c + nadh_c --> glcn_c + nad_c
2DGULRGy: 2dhguln_c + h_c + nadph_c --> glcn_c + nadp_c
2DGULRx: 2dhguln_c + h_c + nadh_c --> idon__L_c + nad_c
2DGULRy: 2dhguln_c + h_c + nadph_c --> idon__L_c + nadp_c
2HMHMBQMTm: 2hpmhmbq_m + amet_m --> ahcys_m + h_m + q6_m
2HP6MPMOm: 2hp6mp_m + o2_m --> 2hp6mbq_m + h2o_m
2HPMBQMTm: 2hp6mbq_m + amet_m --> 2hpmmbq_m + ahcys_m + h_m
2HPMMBQMOm: 2hpmmbq_m + 0.5 o2_m --> 2hpmhmbq_m
34DHOXPEGOX: 34dhmald_c + h_c + nadh_c <=> 34dhoxpeg_c + nad_c
34DHXMANDACOX: 34dhmald_c + h2o_c + nad_c --> 34dhoxmand_c + 2.0 h_c + nadh_c
34DHXMANDACOX_NADP: 34dhmald_c + h2o_c + nadp_c <=> 34dhoxmand_c + 2.0 h_c + nadph_c
3DH5HPBMTm: 3dh5hpb_m + amet_m --> 3hph5mb_m + ahcys_m + h_m
3PG_pi_thr: 3pg_c + pi_h <=> 3pg_h + pi_c
3SLAR: h_c + nadh_c + sla_c --> dhps_c + nad_c
42A12BOOXpp: dopa_p + h2o_p + o2_p --> 34dhpac_p + h2o2_p + nh4_p
5FLURAPRT: 5flura_c + prpp_c --> 5flurimp_c + h_c + ppi_c
AATHB: h2o_c + tre_c --> 2.0 glc__bD_c
ABFPT: atp_c + fru_B_c --> adp_c + f6p_B_c + h_c
ACALD: acald_c + coa_c + nad_c <=> accoa_c + h_c + nadh_c
ACALDh: acald_h + coa_h + nad_h --> accoa_h + h_h + nadh_h
ACDHmi: accoa_m + 2.0 h_m + 2.0 nadh_m --> coa_m + etoh_m + 2.0 nad_m
ACOTA: acorn_c + akg_c <=> acg5sa_c + glu__L_c
ADATT: adp_n + atp_n + h_n --> ap4a_n + pi_n
ADHEr: accoa_c + 2.0 h_c + 2.0 nadh_c --> coa_c + etoh_c + 2.0 nad_c
AGTix: ala__L_x + glx_x --> gly_x + pyr_x
AHGDx: S2hglut_c + nad_c <=> akg_c + h_c + nadh_c
ALATA_L: akg_c + ala__L_c <=> glu__L_c + pyr_c
ALCD1: meoh_c + nad_c <=> fald_c + h_c + nadh_c
ALCD21_D: 12ppd__R_c + nad_c --> h_c + lald__D_c + nadh_c
ALCD22_D: lald__D_c + nad_c --> h_c + mthgxl_c + nadh_c
ALCD22_L: lald__L_c + nad_c --> h_c + mthgxl_c + nadh_c
ALCD22xim: 2mbald_m + h_m + nadh_m --> 2mbtoh_m + nad_m
ALCD23xim: 2mppal_m + h_m + nadh_m --> ibutoh_m + nad_m
ALCD24xim: 3mbald_m + h_m + nadh_m --> iamoh_m + nad_m
ALCD25xim: h_m + nadh_m + pacald_m --> 2phetoh_m + nad_m
ALCD26xim: h_m + id3acald_m + nadh_m --> ind3eth_m + nad_m
ALCD2ir: acald_c + h_c + nadh_c --> etoh_c + nad_c
ALCD2irm: acald_m + h_m + nadh_m --> etoh_m + nad_m
ALCD2x_copy1: etoh_c + nad_c --> acald_c + h_c + nadh_c
ALCD2x_copy2: etoh_c + nad_c <=> acald_c + h_c + nadh_c
ALCDH_nadp_hi: acald_h + h_h + nadph_h --> etoh_h + nadp_h
ALDD22x: aproa_c + h2o_c + nad_c --> ala_B_c + 2.0 h_c + nadh_c
ALDH: h2o_m + mmtsa_m + nad_m --> 2.0 h_m + mm_m + nadh_m
AMMQLT8: 2dmmql8_c + amet_c --> ahcys_c + h_c + mql8_c
AMO: aact_x + h2o_x + h_x + o2_x --> h2o2_x + mthgxl_x + nh4_x
ANXANOR: anxan_u + h_u + nadph_u + o2_u --> h2o_u + nadp_u + vioxan_u
AO: h2o_c + hista_c + o2_c --> h2o2_c + h_c + im4act_c + nh4_c
APATT: aps_n + atp_n --> ap4a_n + so4_n
APOR: bamppald_h + h2o_h + nad_h --> ala_B_h + 2.0 h_h + nadh_h
APTNAT: 5aptn_c + akg_c <=> glu__L_c + oxptn_c
ASCBOR: 2.0 ascb__L_u + o2_u --> 2.0 dhdascb_u + 2.0 h2o_u
ATPH1: atp_c + 2.0 h2o_c --> amp_c + 2.0 h_c + 2.0 pi_c
ATPH1e: atp_e + 2.0 h2o_e --> amp_e + 2.0 h_e + 2.0 pi_e
ATPH2e: adp_e + h2o_e --> amp_e + h_e + pi_e
ATPPHm: atp_m + h2o_m --> adp_m + h_m + pi_m
ATPS3g: adp_g + 3.0 h_c + pi_g --> atp_g + h2o_g + 2.0 h_g
ATPS3v: adp_v + 3.0 h_c + pi_v --> atp_v + h2o_v + 2.0 h_v
ATPS4m: adp_m + 4.0 h_c + pi_m --> atp_m + h2o_m + 3.0 h_m
ATPS4rpp: adp_c + 4.0 h_p + pi_c <=> atp_c + h2o_c + 3.0 h_c
ATPSh: adp_h + 4.0 h_u + pi_h --> atp_h + h2o_u + 3.0 h_h
ATP_3h_tm: adp_m + atp_c + 3.0 h_m --> adp_c + atp_m + 3.0 h_c
ATPasel: atp_c + h2o_c + 3.0 h_c --> adp_c + 4.0 h_l + pi_c
ATPtm_H: adp_c + atp_m + h_c --> adp_m + atp_c + h_m
ATPtp_H: adp_x + atp_c + h_x --> adp_c + atp_x + h_c
BCAROH: caro_u + h_u + nadph_u + o2_u <=> bcryptox_u + h2o_u + nadp_u
BCRPTXANH: bcryptox_u + h_u + nadph_u + o2_u <=> h2o_u + nadp_u + zeax_u
BDH: bhb_c + nad_c <=> acac_c + h_c + nadh_c
BNORh: btal_h + coa_h + nad_h --> btcoa_h + h_h + nadh_h
BTCOARx: btcoa_c + h_c + nadh_c <-- btal_c + coa_c + nad_c
CAATPS: atp_c + 2.0 ca2_c + h2o_c --> adp_c + 2.0 ca2_e + h_e + pi_c
CBPSam: 2.0 atp_m + hco3_m + nh4_m --> 2.0 adp_m + cbp_m + 2.0 h_m + pi_m
CCP2m: 2.0 focytc_m + h2o2_m --> 2.0 ficytc_m + 2.0 h2o_m
CDPPH: cdp_c + h2o_c --> cmp_c + h_c + pi_c
CHLDA1tu: atp_h + chlld_u + h2o_h --> adp_h + chlld_h + h_h + pi_h
CHLDA2tu: atp_h + chlld_h + h2o_h --> adp_h + chlld_u + h_h + pi_h
CHYA1: acaro_u + h_u + nadph_u + o2_u --> h2o_u + nadp_u + zxan_u
CHYA2: acaro_u + h_u + nadph_u + o2_u --> crpxan_u + h2o_u + nadp_u
CITALALDOR: citalald_c + h2o_c + o2_c --> citalppa_c + h2o2_c + h_c
CITALOR: cital_c + h2o_c + o2_c --> citalald_c + dma_c + h2o2_c
COQ3m: 2dpmhobq_m + amet_m --> ahcys_m + h_m + q10_m
COQ6m: 2dp6mep_m + o2_m --> 2dp6mobq_m + h2o_m
COQ7m: 2dp6mobq_me_m + h_m + nadph_m + o2_m --> 2dpmhobq_m + h2o_m + nadp_m
CPS: 2.0 atp_h + co2_h + h2o_h + nh4_h --> 2.0 adp_h + cbp_h + 3.0 h_h + pi_h
CU1abcpp: atp_c + cu_c + h2o_c --> adp_c + cu_p + h_c + pi_c
CYO1_KT: 2.0 ficytc_c + 2.0 h_c + q8h2_c --> 2.0 focytc_c + 2.0 h_p + q8_c
CYO1b: 2.0 focytc_c + 0.5 o2_c --> 2.0 ficytc_c + h2o_c
CYOOm: 4.0 focytc_m + 6.0 h_m + o2_m --> 4.0 ficytc_m + 2.0 h2o_m + 6.0 h_c
CYOOm3: 4.0 focytC_m + 7.92 h_m + o2_m --> 4.0 ficytC_m + 1.96 h2o_m + 4.0 h_c + 0.02 o2s_m
CYOR_q8_m: 2.0 ficytc_m + 2.0 h_m + q8h2_m --> 2.0 focytc_m + 4.0 h_c + q8_m
CYOR_u10m: 2.0 ficytC_m + 2.0 h_m + q10h2_m --> 2.0 focytC_m + 4.0 h_c + q10_m
CYOR_u6m: 2.0 ficytc_m + 1.5 h_m + q6h2_m --> 2.0 focytc_m + 1.5 h_c + q6_m
DDMCITALOR: ddmcital_c + h2o_c + o2_c --> citalald_c + h2o2_c + nh4_c
DHAP_pi_thr: dhap_c + pi_h <=> dhap_h + pi_c
DHDPBMTm: 3dpdhb_m + amet_m --> 3dpdhb_me_m + ahcys_m + h_m
DHORD2: dhor__S_c + q8_c --> orot_c + q8h2_c
DHORD4i: dhor__S_c + q6_m --> orot_c + q6h2_m
DHORD5: dhor__S_c + mqn8_c --> mql8_c + orot_c
DHORD9: dhor__S_c + q10_m --> orot_c + q10h2_m
DHORDi: dhor__S_c + o2_c --> h2o2_c + orot_c
DHR: cbasp_m <=> dhor__S_m + h2o_m + h_m
DHROm: dhor__S_m + h_m + o2_m <=> h2o2_m + orot_m
DKGLCNR2x: 25dkglcn_c + h_c + nadh_c --> 5dglcn_c + nad_c
DKGLCNR2y: 25dkglcn_c + h_c + nadph_c --> 5dglcn_c + nadp_c
DMCITALOR: dmcital_c + h2o_c + o2_c --> citalald_c + h2o2_c + mma_c
DMQMT: 2omhmbl_c + amet_c --> ahcys_c + h_c + q8h2_c
DTPH: dtdp_c + h2o_c --> dtmp_c + h_c + pi_c
DXHPScm: h2o_c + q6_m + spmd_c --> 13dampp_c + 4abutn_c + q6h2_m
D_LACDHm: 2.0 ficytc_m + lac__D_m --> 2.0 focytc_m + 2.0 h_m + pyr_m
D_LACDm: 2.0 ficytc_m + lac__D_m --> 2.0 focytc_m + pyr_m
ETFQO: etfrd_m + q10_m --> etfox_m + q10h2_m
ETFQO_1: etfrd_m + q8_m --> etfox_m + q8h2_m
FALDH: fald_c + gthrd_c + nad_c <=> Sfglutth_c + h_c + nadh_c
FBPPH: f26bp_c + h2o_c --> f6p_B_c + pi_c
FHL: for_c + h_c --> co2_c + h2_c
G1P_pi_th: g1p_c + pi_h <=> g1p_h + pi_c
G3PD: fad_c + glyc3p_c --> dhap_c + fadh2_c
G3PD1: dhap_h + h_h + nadh_h <=> glyc3p_h + nad_h
G3PD1irm: dhap_m + h_m + nadh_m --> glyc3p_m + nad_m
G3PD2: glyc3p_c + nadp_c <=> dhap_c + h_c + nadph_c
G3PD2_1: dhap_h + h_h + nadph_h <=> glyc3p_h + nadp_h
G3PD2m: fad_m + glyc3p_c --> dhap_c + fadh2_m
G3PD5: glyc3p_c + q8_c --> dhap_c + q8h2_c
G3PDm: fad_m + glyc3p_m --> dhap_m + fadh2_m
G3PO: dhap_h + h2o2_h <=> glyc3p_h + o2_h
G3P_pi_thr: g3p_c + pi_h <=> g3p_h + pi_c
G5DHx: glu5p_x + h_x + nadph_x --> glu5sa_x + nadp_x + pi_x
G5SD2: glu5p_c + h_c + nadh_c --> glu5sa_c + nad_c + pi_c
G5SDym: glu5p_m + h_m + nadph_m --> glu5sa_m + nadp_m + pi_m
G6PADH: g6p_A_c + nadp_c --> 6pgl_c + h_c + nadph_c
G6PA_pi_th: g6p_A_c + pi_h <=> g6p_A_h + pi_c
G6PBDH: g6p_B_c + nadp_c --> 6pgl_c + h_c + nadph_c
G6PB_pi_th: g6p_B_c + pi_h <=> g6p_B_h + pi_c
G6PDA_1: gam6p_c + h2o_c <=> f6p_B_c + nh4_c
G6PI: g6p_c <=> g6p_B_c
G6PI2: g6p_B_c <=> f6p_B_c
G6PI3: g6p_B_c <=> f6p_c
G6PI_1: g6p_A_c <=> g6p_B_c
GAPDHh: g3p_h + nad_h + pi_h <=> 13dpg_h + h_h + nadh_h
GAPDHm: g3p_m + nad_m + pi_m <=> 13dpg_m + h_m + nadh_m
GCLDH: fdxox_m + glyclt_m --> fdxrd_m + glx_m
GF6PTA_1: f6p_B_c + gln__L_c --> gam6p_c + glu__L_c
GHBDHx: h_c + nadh_c + sucsal_c <=> ghb_c + nad_c
GLCRAL: 5dh4dglc_c --> 2h3oppan_c + pyr_c
GLCt2pp: glc__D_p + h_p --> glc__D_c + h_c
GLU5Km: atp_m + glu__L_m --> adp_m + glu5p_m
GLUK: atp_c + glc__D_c --> adp_c + g6p_B_c + h_c
GLUKA: Glc_aD_c + atp_c --> adp_c + g6p_A_c + h_c
GLUK_syn: atp_c + glc__bD_c --> adp_c + g6p_B_c + h_c
GLXO1: glx_c + h2o_c + nad_c --> 2.0 h_c + nadh_c + oxa_c
GLYCDH_1: h_m + hpyr_m + nadh_m --> glyc__R_m + nad_m
GLYCLTDx: glx_c + h_c + nadh_c --> glyclt_c + nad_c
GLYCTO1: glyclt_c + o2_c --> glx_c + h2o2_c
GLYCTO2: glyclt_c + q8_c --> glx_c + q8h2_c
GLYCTO3: glyclt_c + mqn8_c --> glx_c + mql8_c
GLYCTO4: 2dmmq8_c + glyclt_c --> 2dmmql8_c + glx_c
GLYO1: gly_c + h2o_c + o2_c --> glx_c + h2o2_c + nh4_c
GLYOX3: h2o_c + mthgxl_c --> h_c + lac__D_c
GLYOX_1: lgt__S_c <=> gthrd_c + mthgxl_c
GLYOXm: h2o_m + lgt__S_m --> gthrd_m + h_m + lac__D_m
GLYTA_1: glu__L_m + glx_m --> akg_m + gly_m
GTPH1: gtp_c + 2.0 h2o_c --> gmp_c + 2.0 h_c + 2.0 pi_c
H2Othu: h2o_u <=> h2o_h
HBNOx: bhb_x + nad_x <=> acac_x + h_x + nadh_x
HBZOPT: 4hbz_c + octdp_c --> 3ophb_c + ppi_c
HBZOPT10m: 4hbz_m + decdp_m --> 3dphb_m + ppi_m
HBZOPT6m: 4hbz_m + hexdp_m --> 3ophb_5_m + ppi_m
HCGALm: 2.0 atp_m + gln__L_m + h2o_m + hco3_m --> 2.0 adp_m + cbp_m + glu__L_m + 2.0 h_m + pi_m
HEMELh: apocytc_h + pheme_h <=> cytc_h
HEMEOMOm: hemeO_m + nadh_m + o2_m --> h2o_m + hemeA_m + nad_m
HEMEOS: frdp_c + h2o_c + pheme_c --> hemeO_c + ppi_c
HEMEOSm_1: frdp_m + h2o_m + pheme_m --> h_m + hemeO_m + ppi_m
HIBDkt: 3hmp_c + nad_c --> h_c + mmtsa_c + nadh_c
HIBH: 3hibutcoa_m + h2o_m --> 3hib_m + coa_m + h_m
HKt: atp_c + h2o_c + k_e --> adp_c + h_e + k_c + pi_c
HMNO: 3hib_m + nad_m <=> h_m + mmtsa_m + nadh_m
HPROxm: 4hpro_LT_m + nad_m --> 1p3h5c_m + 2.0 h_m + nadh_m
HPROym: 4hpro_LT_m + nadp_m --> 1p3h5c_m + 2.0 h_m + nadph_m
HPYRR2x: h_c + hpyr_c + nadh_c --> glyc__S_c + nad_c
HSD17B1: estrone_c + h_c + nadph_c <=> estradiol_c + nadp_c
HSD17B2r: andrstndn_r + h_r + nadh_r --> nad_r + tststerone_r
HSD17B8r: estrone_r + h_r + nadh_r --> estradiol_r + nad_r
HYDAm: 2.0 fdxrd_m <=> 2.0 fdxox_m + h2_m + 2.0 h_m
ICL: icit_c --> glx_c + succ_c
ICL_1: icit_m --> glx_m + succ_m
IDPA: h2o_c + idp_c --> h_c + imp_c + pi_c
IGPDh: eig3p_h --> h2o_h + imacp_h
LALDD: lald__D_c + nadp_c <=> h_c + mthgxl_c + nadph_c
LALDO: gthrd_c + lald__D_c + nad_c <=> h_c + lgt__S_c + nadh_c
LALDO2x: h_c + mthgxl_c + nadh_c --> lald__D_c + nad_c
LCARR: h_c + lald__D_c + nadh_c --> 12ppd__R_c + nad_c
LCARS: h_c + lald__L_c + nadh_c <=> 12ppd__S_c + nad_c
LDH_D: lac__D_c + nad_c <=> h_c + nadh_c + pyr_c
LLDH_ferr_m: 2.0 ficytc_m + lac__L_m --> 2.0 focytc_m + 2.0 h_m + pyr_m
L_LACD2: lac__L_c + q8_c --> pyr_c + q8h2_c
L_LACD2cm: 2.0 ficytc_m + lac__L_c --> 2.0 focytc_m + pyr_c
L_LACD3: lac__L_c + mqn8_c --> mql8_c + pyr_c
MALS: accoa_c + glx_c + h2o_c --> coa_c + h_c + mal__L_c
MALSh: accoa_h + glx_h + h2o_h --> coa_h + h_h + mal__L_h
MALSm: accoa_m + glx_m + h2o_m --> coa_m + h_m + mal__L_m
MG2uabcpp: atp_c + h2o_c + mg2_p --> adp_c + h_c + mg2_c + pi_c
MI1PS: g6p_c --> mi1p__D_c
MI3PSA: g6p_A_c --> mi3p__D_c
MI3PSB: g6p_B_c --> mi3p__D_c
MMSAD3: coa_c + msa_c + nad_c --> accoa_c + co2_c + nadh_c
MMSDH: coa_m + mmtsa_m + nad_m --> co2_m + nadh_m + ppcoa_m
MMTSAO: coa_c + mmtsa_c + nad_c --> co2_c + nadh_c + ppcoa_c
MPAKI: man6p_c <=> f6p_B_c
NA1ATPaseh: atp_h + h2o_h + na1_h --> adp_h + h_h + na1_c + pi_h
NA1ATPasem: atp_m + h2o_m + na1_m --> adp_m + h_m + na1_c + pi_m
NADH10: h_c + mqn8_c + nadh_c --> mql8_c + nad_c
NADH16pp: 4.0 h_c + nadh_c + q8_c --> 3.0 h_p + nad_c + q8h2_c
NADH17pp: 4.0 h_c + mqn8_c + nadh_c --> 3.0 h_p + mql8_c + nad_c
NADH18pp: 2dmmq8_c + 4.0 h_c + nadh_c --> 2dmmql8_c + 3.0 h_p + nad_c
NADH2_u10m: 5.0 h_m + nadh_m + q10_m --> 4.0 h_c + nad_m + q10h2_m
NADH2_u6cm: h_c + nadh_c + q6_m --> nad_c + q6h2_m
NADH2_u6m: h_m + nadh_m + q6_m --> nad_m + q6h2_m
NADH5: h_c + nadh_c + q8_c --> nad_c + q8h2_c
NADH9: 2dmmq8_c + h_c + nadh_c --> 2dmmql8_c + nad_c
NADHOR: h_m + nadh_m + q8_m --> nad_m + q8h2_m
NADHPO: h2o2_c + h_c + nadh_c --> 2.0 h2o_c + nad_c
NADHXD: adp_c + nadhx__S_c --> amp_c + h_c + nadh_c + pi_c
NADPHQR2: h_c + nadph_c + q8_c --> nadp_c + q8h2_c
NADPHXD: adp_c + nadphx__S_c --> amp_c + h_c + nadph_c + pi_c
NDP1: adp_c + h2o_c --> amp_c + h_c + pi_c
NDP3: gdp_c + h2o_c --> gmp_c + h_c + pi_c
NDP7: h2o_c + udp_c --> h_c + pi_c + ump_c
NI2uabcpp: atp_c + h2o_c + ni2_p --> adp_c + h_c + ni2_c + pi_c
NO2th: no2_c <=> no2_h
NTP1: atp_c + h2o_c --> adp_c + h_c + pi_c
NTP10: h2o_c + itp_c --> h_c + idp_c + pi_c
NTP3: gtp_c + h2o_c --> gdp_c + h_c + pi_c
NTP5: ctp_c + h2o_c --> cdp_c + h_c + pi_c
NTP7: h2o_c + utp_c --> h_c + pi_c + udp_c
NTP9: dttp_c + h2o_c --> dtdp_c + h_c + pi_c
NaKt: atp_c + h2o_c + 2.0 k_e + 3.0 na1_c --> adp_c + h_c + 2.0 k_c + 3.0 na1_e + pi_c
OCTDPS: frdp_c + 5.0 ipdp_c --> octdp_c + 5.0 ppi_c
OHPBAT: glu__L_c + ohpb_c <=> akg_c + phthr_c
OHPHM: 2ohph_c + amet_c --> 2omph_c + ahcys_c + h_c
OMBZLM: 2ombzl_c + amet_c --> 2ommbl_c + ahcys_c + h_c
OMMBLHXy: 2ommbl_c + h_c + nadph_c + o2_c --> 2omhmbl_c + h2o_c + nadp_c
OPHHXy: 2oph_c + h_c + nadph_c + o2_c --> 2ohph_c + h2o_c + nadp_c
OXPTNDH: h2o_c + nad_c + oxptn_c <=> glutar_c + 2.0 h_c + nadh_c
P5CD: 1pyr5c_c + 2.0 h2o_c + nad_c --> glu__L_c + h_c + nadh_c
P5CDm: 1pyr5c_m + 2.0 h2o_m + nad_m --> glu__L_m + h_m + nadh_m
P5CRm: 1pyr5c_m + 2.0 h_m + nadph_m --> nadp_m + pro__L_m
P5CRxm: 1pyr5c_m + 2.0 h_m + nadh_m --> nad_m + pro__L_m
PAO: 13dampp_h + h2o_h + o2_h --> bamppald_h + h2o2_h + nh4_h
PEAMNOpp: h2o_p + o2_p + peamn_p --> h2o2_p + nh4_p + pacald_p
PEPPIth: pep_c + pi_h --> pep_h + pi_c
PFK26_1: atp_c + f6p_B_c --> adp_c + f26bp_c + h_c
PGCM: g1p_c <=> g6p_A_c
PGIA: g6p_A_c <=> f6p_B_c
PGLCNDH: 6p2dhglcn_c + nadph_c <=> 6pgc_c + 2.0 h_c + nadp_c
PGLYCPh: 2pglyc_h + h2o_h --> glyclt_h + pi_h
PGLYCPx: 2pglyc_x + h2o_x --> glyclt_x + pi_x
PGLYDH: 3pg_h + nad_h --> 3php_h + h_h + nadh_h
PHCDm: 1p3h5c_m + 2.0 h2o_m + nad_m --> e4hglu_m + h_m + nadh_m
PINA1th: na1_h + pi_h --> na1_c + pi_c
PINA1tm: na1_c + pi_c <=> na1_m + pi_m
PIcm: pi_c <=> pi_m
PIt2p: h_c + pi_c <=> h_x + pi_x
PIt5m: oh1_m + pi_c <=> oh1_c + pi_m
PIt8: 1.5 na1_e + pi_e <=> 1.5 na1_c + pi_c
PItf: h_c + pi_c <=> h_f + pi_f
PPTTm: ipdp_m + pendp_m --> hexdp_m + ppi_m
PRO1x: nad_c + pro__L_c --> 1pyr5c_c + 2.0 h_c + nadh_c
PRO1xm: nad_m + pro__L_m --> 1pyr5c_m + 2.0 h_m + nadh_m
PROD2: fad_c + pro__L_c --> 1pyr5c_c + fadh2_c + h_c
PROD2m: fad_m + pro__L_m --> 1pyr5c_m + fadh2_m + h_m
PROD3: pro__L_c + q8_c --> 1pyr5c_c + h_c + q8h2_c
PSAT: 3php_h + glu__L_h --> akg_h + pser__L_h
PSIIblue: 2.0 h2o_u + 4.0 photon438_u + 2.0 pq_u --> o2D_u + 2.0 pqh2_u
PSIIred: 2.0 h2o_u + 4.0 photon673_u + 2.0 pq_u --> o2D_u + 2.0 pqh2_u
PTOR: h2o_m + o2_m + ptrc_m <=> 4abutn_m + h2o2_m + nh4_m
PYR5CDm: glu5sa_m + h2o_m + nadp_m --> glu__L_m + 2.0 h_m + nadph_m
RDH1a: nadp_c + retinol_c <=> h_c + nadph_c + retinal_c
RDH2a: nadp_c + retinol_9_cis_c <=> h_c + nadph_c + retinal_cis_9_c
RDH3a: nadp_c + retinol_cis_11_c <=> h_c + nadph_c + retinal_11_cis_c
SGAT: glx_m + ser__L_m <=> gly_m + hpyr_m
SHGO: S2hglut_c + o2_c --> akg_c + h2o2_c
SOTA: akg_c + sucorn_c --> glu__L_c + sucgsa_c
SPT_syn: glx_c + ser__L_c <=> gly_c + hpyr_c
SPTc: pyr_c + ser__L_c <=> ala__L_c + hpyr_c
SPTix: pyr_x + ser__L_x --> ala__L_x + hpyr_x
SSALxm: h2o_m + nad_m + sucsal_m --> 2.0 h_m + nadh_m + succ_m
SSNOh: h2o_h + nad_h + sucsal_h --> 2.0 h_h + nadh_h + succ_h
SUCD1: fad_c + succ_c <=> fadh2_c + fum_c
SUCD1m: fad_m + succ_m <=> fadh2_m + fum_m
SUCD2_u6m: q6_m + succ_m <=> fum_m + q6h2_m
SUCD3_u6m: fadh2_m + q6_m <=> fad_m + q6h2_m
SUCDH_q8_m: q8_m + succ_m <=> fum_m + q8h2_m
SUCDi: q8_c + succ_c --> fum_c + q8h2_c
THRD: nad_c + thr__L_c --> 2aobut_c + h_c + nadh_c
TRDR2: h_c + nadph_c + q10_c --> nadp_c + q10h2_c
TRDR3: h_c + nadh_c + q10_c --> nad_c + q10h2_c
TRSARr: 2h3oppan_c + h_c + nadh_c <=> glyc__R_c + nad_c
TYROXDApp: h2o_p + o2_p + tym_p --> 4hoxpacd_p + h2o2_p + nh4_p
UG6PGT: g6p_A_c + udpg_c --> h_c + tre6p_c + udp_c
UTPH1: 2.0 h2o_c + utp_c --> 2.0 h_c + 2.0 pi_c + ump_c
ZAXANOR: h_u + nadph_u + o2_u + zeax_u --> anxan_u + h2o_u + nadp_u
ZHY: h_u + nadph_u + o2_u + zxan_u --> h2o_u + lut_u + nadp_u
r0074: glu5sa_m + h2o_m + nad_m --> glu__L_m + 2.0 h_m + nadh_m
yli_R0011: 1pyr5c_c + 2.0 h2o_c + nadp_c --> glu__L_c + h_c + nadph_c
yli_R0078: h2o_c + o2_c + yli_M00858_c --> aproa_c + h2o2_c + nh4_c
yli_R0260: nad_c + sbt__D_c <=> fru_B_c + h_c + nadh_c
yli_R0268: atp_c + fru_B_c --> adp_c + f6p_c + h_c
yli_R0368: fdp_B_c <=> dhap_c + g3p_c
yli_R0371: atp_c + glc__D_c --> adp_c + g6p_A_c + h_c
yli_R0376: g6p_A_c <=> f6p_c
yli_R0379: atp_c + f6p_c --> adp_c + fdp_B_c + h_c
yli_R0380: fdp_B_c + h2o_c --> f6p_c + pi_c
yli_R0430: h_m + nadh_m + q6_m --> nad_m + yli_M04633_m
yli_R0431: 2.0 ficytc_m + 1.5 h_m + yli_M04633_m --> 2.0 focytC_m + 1.5 h_c + q6_m
yli_R0432: h_c + nadh_c + q6_m --> nad_c + yli_M04633_m
yli_R0593: akg_c + ala_B_c <=> glu__L_c + msa_c
yli_R0598: coa_c + msa_c + nadp_c <=> h_c + malcoa_c + nadph_c
yli_R0667: 2.0 ficytc_m + lac__L_m --> 2.0 focytC_m + pyr_m
yli_R0668: 2.0 ficytc_m + lac__D_m --> 2.0 focytC_m + pyr_m
yli_R0672: accoa_m + glx_m + h2o_m --> coa_m + h_c + mal__L_m
yli_R0728: h2o_c + starch_c --> glc__D_c
yli_R0735: h2o_c + yli_M00682_c --> glc__D_c
yli_R0736: h2o_c + yli_M00844_c --> glc__D_c
yli_R0769: frdp_c + 3.0 ipdp_c --> hexdp_c + 3.0 ppi_c
yli_R0770: ggdp_c + 2.0 ipdp_c --> hexdp_c + 2.0 ppi_c
yli_R0867: 3hib_c + nad_c --> h_c + mmtsa_c + nadh_c
yli_R0869: h2o_c + mmtsa_c + nad_c --> mmal_c + nadh_c
yli_R0888: h2o_c + o2_c + spmd_c --> 4abutn_c + h2o2_c + yli_M00858_c
yli_R0944: 2hp6mp_m + o2_m --> h2o_m + yli_M02815_m
yli_R0945: amet_m + yli_M02815_m --> ahcys_m + h_m + yli_M02816_m
yli_R0946: h_m + nadph_m + o2_m + yli_M02816_m --> h2o_m + nadp_m + yli_M02817_m
yli_R0947: amet_m + yli_M02817_m --> ahcys_m + h_m + q6_m
yli_R1133: fru_B_e + h_e --> fru_B_c + h_c
yli_R1388: glx_x + ser__L_x <=> gly_x + hpyr_x
yli_R1397: dhap_r + h_r + nadh_r --> glyc3p_r + nad_r
yli_R1398: fad_r + glyc3p_r --> dhap_r + fadh2_r
yli_R1420: glyclt_x + o2_x --> glx_x + h2o2_x
yli_R1434: accoa_x + glx_x + h2o_x --> coa_x + h_c + mal__L_x
yli_R1480: icit_x --> glx_x + succ_x
yli_R1491: h2o_c + nad_c + sucsal_c --> h_c + nadh_c + succ_c
yli_R1555: aps_c + atp_c --> ap4a_c + so4_c
yli_R1556: adp_c + atp_c --> ap4a_c + pi_c
yli_R1559: dhor__S_c + yli_M07040_c <=> hqn_c + orot_c
yli_R1573: malt_c --> amylose_c + glc__D_c
yli_R1574: glycogen_c + h2o_c --> amylose_c + glc__D_c
yli_R1583: 34dhoxpeg_c + amet_c --> ahcys_c + yli_M07061_c
yli_R1584: amet_c + yli_M07062_c --> ahcys_c + yli_M07063_c
yli_R1585: 34dhmald_c + h2o_c + nad_c --> h_c + nadh_c + yli_M07062_c
yli_R1594: akg_c + yli_M07070_c <=> glu__L_c + mmtsa_c
```

In [147]:

```
print('Updated reactions\n')
for r in sorted(model_old.reactions, key=lambda x: x.id):
    if r in model_new.reactions:
        r2 = model_new.reactions.get_by_id(r.id)
        if (r.name == r2.name and r.reaction == r2.reaction and r.gene_reaction_rule == r2.gene_reaction_rule and
            r.lower_bound == r2.lower_bound and r.upper_bound == r2.upper_bound):
            pass
        else:
            print('Old', r, r.gene_reaction_rule)
            print('New', r2, r2.gene_reaction_rule)
            print()
```

```
Updated reactions

Old 4HGLSDm: 4hglusa_m + h2o_m + nad_m <=> e4hglu_m + 2.0 h_m + nadh_m 8569
New 4HGLSDm: 4hglusa_m + h2o_m + nad_m --> e4hglu_m + 2.0 h_m + nadh_m 8569

Old 5HOXINDACTOXm: 5hoxindact_m + h2o_m + nad_m --> 5hoxindoa_m + 2.0 h_m + nadh_m 12042 or 13426 or 8569
New 5HOXINDACTOXm: 5hoxindact_m + h2o_m + nad_m --> 5hoxindoa_m + 2.0 h_m + nadh_m 12042 or 13426

Old A1E: Glc_aD_c <=> glc__bD_c 14480
New A1E: Glc_aD_c <=> glc__D_c 14480

Old ABTA: 4abut_c + akg_c --> glu__L_c + sucsal_c 10937 or 15905
New ABTA: 4abut_c + akg_c --> glu__L_c + sucsal_c 10937

Old AGPRim: acg5p_m + h_m + nadph_m --> acg5sa_m + nadp_m + pi_m 16273 or 9377
New AGPRim: acg5p_m + h_m + nadph_m --> acg5sa_m + nadp_m + pi_m 9377

Old AGTim: ala__L_m + glx_m --> gly_m + pyr_m 10937
New AGTim: ala__L_m + glx_m --> gly_m + pyr_m 8819

Old ALCD19: glyald_c + h_c + nadh_c <=> glyc_c + nad_c 11665 or 12784 or 15438 or 9774
New ALCD19: glyald_c + h_c + nadh_c <=> glyc_c + nad_c 15438

Old ALCD22xi: 2mbald_c + h_c + nadh_c --> 2mbtoh_c + nad_c 11665 or 14108 or 14109 or 15438
New ALCD22xi: 2mbald_c + h_c + nadh_c --> 2mbtoh_c + nad_c 14108 or 14109 or 15438

Old ALCD23xi: 2mppal_c + h_c + nadh_c --> ibutoh_c + nad_c 11665 or 14108 or 14109 or 15438
New ALCD23xi: 2mppal_c + h_c + nadh_c --> ibutoh_c + nad_c 14108 or 14109 or 15438

Old ALCD24xi: 3mbald_c + h_c + nadh_c --> iamoh_c + nad_c 11665 or 14108 or 14109 or 15438
New ALCD24xi: 3mbald_c + h_c + nadh_c --> iamoh_c + nad_c 14108 or 14109 or 15438

Old ALCD25xi: h_c + nadh_c + pacald_c --> 2phetoh_c + nad_c 11665 or 14108 or 14109 or 15438
New ALCD25xi: h_c + nadh_c + pacald_c --> 2phetoh_c + nad_c 14108 or 14109 or 15438

Old ALCD26xi: h_c + id3acald_c + nadh_c --> ind3eth_c + nad_c 11665 or 14108 or 14109 or 15438
New ALCD26xi: h_c + id3acald_c + nadh_c --> ind3eth_c + nad_c 14108 or 14109 or 15438

Old ALCD2x: etoh_c + nad_c <=> acald_c + h_c + nadh_c 11665 or 13657 or 14108 or 14109 or 15438
New ALCD2x: etoh_c + nad_c <=> acald_c + h_c + nadh_c 15438

Old ALDD20xm: h2o_m + id3acald_m + nad_m --> 2.0 h_m + ind3ac_m + nadh_m 12042 or 13426 or 8569
New ALDD20xm: h2o_m + id3acald_m + nad_m --> 2.0 h_m + ind3ac_m + nadh_m 12042 or 13426

Old ALDD2y: acald_c + h2o_c + nadp_c --> ac_c + 2.0 h_c + nadph_c 11650 or 12042 or 13426 or 14700 or 15575 or 16323 or 8666
New ALDD2y: acald_c + h2o_c + nadp_c --> ac_c + 2.0 h_c + nadph_c 11650 or 12042 or 13426 or 14700 or 16323 or 8666

Old ALR2: h_c + mthgxl_c + nadph_c --> acetol_c + nadp_c 11882 or 12784 or 15285 or 16543 or 9774
New ALR2: h_c + mthgxl_c + nadph_c --> acetol_c + nadp_c 11882 or 12784 or 9774

Old ALR3: acetol_c + h_c + nadph_c --> 12ppd__S_c + nadp_c 11882 or 12784 or 16543 or 9774
New ALR3: acetol_c + h_c + nadph_c --> 12ppd__S_c + nadp_c 11882 or 12784 or 9774

Old ARABR: arab__L_c + h_c + nadph_c --> abt_c + nadp_c 11882 or 12784 or 16543 or 9774
New ARABR: arab__L_c + h_c + nadph_c --> abt_c + nadp_c 11882 or 12784 or 9774

Old ASPCT: asp__L_c + cbp_c --> cbasp_c + h_c + pi_c 12302 or 16681
New ASPCT: asp__L_c + cbp_c --> cbasp_c + h_c + pi_c 16681

Old ATPS: atp_c + h2o_c --> adp_c + h_e + pi_c 12428 or 13617 or 15156 or 15584 or (YCR024C_A and YEL017C_A and 13617) or (YCR024C_A and YEL017C_A and 15584)
New ATPS: atp_c + h2o_c --> adp_c + h_e + pi_c 13617 or 15584

Old ATPS3m: adp_m + 3.0 h_c + pi_m --> atp_m + h2o_m + 2.0 h_m (CRv4_Au5_s10_g33_t1 and CRv4_Au5_s10_g65_t1 and CRv4_Au5_s17_g7684_t1 and CRv4_Au5_s1_g1345_t1 and CRv4_Au5_s1_g1960_t1 and 11958 and 11967 and 13424 and 15287 and 15589) or (CRv4_Au5_s10_g33_t1 and CRv4_Au5_s10_g65_t1 and CRv4_Au5_s17_g7685_t1 and CRv4_Au5_s1_g1345_t1 and CRv4_Au5_s1_g1960_t1 and 11958 and 11967 and 13424 and 15287 and 15589) or (CRv4_Au5_s10_g65_t1 and CRv4_Au5_s17_g7684_t1 and CRv4_Au5_s1_g1345_t1 and CRv4_Au5_s1_g1960_t1 and 11958 and 11967 and 13424 and 15287 and 15589 and 16359) or (CRv4_Au5_s10_g65_t1 and CRv4_Au5_s17_g7685_t1 and CRv4_Au5_s1_g1345_t1 and CRv4_Au5_s1_g1960_t1 and 11958 and 11967 and 13424 and 15287 and 15589 and 16359) or (Q0080 and Q0085 and Q0130 and YDL181W and YPL271W and 10674 and 11958 and 11967 and 13424 and 13759 and 14786 and 14912 and 15589 and 16359 and 9940) or (Q0080 and Q0085 and Q0130 and YPL271W and 10674 and 10675 and 11958 and 11967 and 13424 and 13759 and 14786 and 14912 and 15589 and 16359 and 9940) or (Q0080 and Q0085 and Q0130 and YDL181W and YOL077W_A and YPL271W and YPR020W and 10674 and 11958 and 11967 and 13424 and 13759 and 14786 and 14912 and 15589 and 15880 and 16359 and 9940) or (Q0080 and Q0085 and Q0130 and YOL077W_A and YPL271W and 10674 and 10675 and 11958 and 11967 and 13424 and 13759 and 14786 and 14912 and 15589 and 15880 and 16359 and 9080 and 9940)
New ATPS3m: adp_m + 3.0 h_c + pi_m --> atp_m + h2o_m + 2.0 h_m RTO3_879280 and RTO3_900623 and RTO3_900663 and 9080 and 9619 and 9940 and 10674 and 11958 and 11967 and 13053 and 13252 and 13424 and 13759 and 13842 and 14786 and 14912 and 15287 and 15589 and 15880 and 16359

Old BAMPPALDOXm: bamppald_m + h2o_m + nad_m --> ala_B_m + 2.0 h_m + nadh_m 12042 or 13426 or 8569
New BAMPPALDOXm: bamppald_m + h2o_m + nad_m --> ala_B_m + 2.0 h_m + nadh_m 12042 or 13426

Old BDHm: bhb_m + nad_m <=> acac_m + h_m + nadh_m 15575
New BDHm: bhb_m + nad_m <=> acac_m + h_m + nadh_m 12795

Old BFFS: h2o_c + suc6p_c <=> fru_B_c + g6p_A_c 14826
New BFFS: h2o_c + suc6p_c <=> fru_c + g6p_c 14826

Old CBPS: 2.0 atp_c + gln__L_c + h2o_c + hco3_c --> 2.0 adp_c + cbp_c + glu__L_c + 2.0 h_c + pi_c 12302 or 16681 or (12302 and 13297) or (13297 and 16681)
New CBPS: 2.0 atp_c + gln__L_c + h2o_c + hco3_c --> 2.0 adp_c + cbp_c + glu__L_c + 2.0 h_c + pi_c 16681

Old CYOO6m: 4.0 focytc_m + 8.0 h_m + o2_m --> 4.0 ficytc_m + 2.0 h2o_m + 4.0 h_c (CRv4_Au5_s1_g1958_t1 and CRv4_Au5_s3_g10243_t1 and CRv4_Au5_s4_g11710_t1 and 11317 and 8802) or (CRv4_Au5_s1_g1958_t1 and CRv4_Au5_s3_g10243_t1 and CRv4_Au5_s4_g11710_t1 and 11317 and 9198)
New CYOO6m: 4.0 focytc_m + 8.0 h_m + o2_m --> 4.0 ficytc_m + 2.0 h2o_m + 4.0 h_c RTO3_946004 and RTO3_946002 and 9165 and 13628 and 10685 and 12506 and 11352 and 13275 and 11769 and 11317 and 12210 and 13007 and 8802

Old DASPO1p: asp__D_x + h2o_x + o2_x --> h2o2_x + nh4_x + oaa_x 15449 or 15994
New DASPO1p: asp__D_x + h2o_x + o2_x --> h2o2_x + nh4_x + oaa_x 11876 or 15449 or 15994

Old DHORDfum: dhor__S_c + fum_c --> orot_c + succ_c 14866 or 8814
New DHORDfum: dhor__S_c + fum_c --> orot_c + succ_c 14866

Old DHORTS: dhor__S_c + h2o_c <=> cbasp_c + h_c 12291 or 12302 or 16681
New DHORTS: dhor__S_c + h2o_c <=> cbasp_c + h_c 12291

Old D_LACDcm: 2.0 ficytc_m + lac__D_c --> 2.0 focytc_m + pyr_c (13328 and 8802) or (16643 and 8802)
New D_LACDcm: 2.0 ficytc_m + lac__D_c --> 2.0 focytc_m + 2.0 h_c + pyr_c (13239 and 8802) or (13328 and 8802)

Old FDH: for_c + nad_c --> co2_c + nadh_c 11952 or (PP_0490 and PP_0491 and PP_0492) or (PP_2183 and PP_2184 and PP_2186 and 13272)
New FDH: for_c + nad_c --> co2_c + nadh_c 11952

Old FRDcm: fadh2_m + fum_c --> fad_m + succ_c 11420 or 16281
New FRDcm: fadh2_m + fum_c --> fad_m + succ_c 11420

Old FRDm: fadh2_m + fum_m --> fad_m + succ_m 11420
New FRDm: fadh2_m + fum_m --> fad_m + succ_m 11536

Old GCALDDm: gcald_m + h2o_m + nad_m --> glyclt_m + 2.0 h_m + nadh_m 12042 or 13426 or 16273 or 8569 or 8692 or 8975
New GCALDDm: gcald_m + h2o_m + nad_m --> glyclt_m + 2.0 h_m + nadh_m 12042 or 13426

Old GLACOm: glac_m + 2.0 h2o_m + nad_m --> glcr_m + 3.0 h_m + nadh_m 12042 or 13426 or 8569
New GLACOm: glac_m + 2.0 h2o_m + nad_m --> glcr_m + 3.0 h_m + nadh_m 12042 or 13426

Old GLXO2p: glx_x + h2o_x + o2_x --> h2o2_x + h_x + oxa_x 16607
New GLXO2p: glx_x + h2o_x + o2_x --> h2o2_x + h_x + oxa_x 14950 or 16607

Old GLYCLTDy: glx_c + h_c + nadph_c --> glyclt_c + nadp_c 11036 or 12051
New GLYCLTDy: glx_c + h_c + nadph_c --> glyclt_c + nadp_c 11036 or 9515 or 9520

Old GLYCTO1p: glyclt_x + o2_x --> glx_x + h2o2_x 16607
New GLYCTO1p: glyclt_x + o2_x --> glx_x + h2o2_x 14950 or 16607

Old HEMEASm: h2o_m + hemeO_m --> 5.0 h_m + hemeA_m 10228
New HEMEASm: 2.0 h_m + hemeO_m + 2.0 nadph_m + 2.0 o2_m --> 3.0 h2o_m + hemeA_m + 2.0 nadp_m 10228 and 15585 and 16775

Old HEMELm: apocytc_m + pheme_m <=> cytc_m 9830
New HEMELm: apocytc_m + hemeC_m <=> cytc_m 9830

Old HISTP: h2o_c + hisp_c --> histd_c + pi_c 10206 or 8707
New HISTP: h2o_c + hisp_c --> histd_c + pi_c 10206

Old HPYRRx: h_c + hpyr_c + nadh_c --> glyc__R_c + nad_c 11036 or 12051
New HPYRRx: h_c + hpyr_c + nadh_c --> glyc__R_c + nad_c 11036 or 9515 or 9520

Old HPYRRy: h_c + hpyr_c + nadph_c --> glyc__R_c + nadp_c 11036 or 12051 or 9515
New HPYRRy: h_c + hpyr_c + nadph_c --> glyc__R_c + nadp_c 11036 or 9515 or 9520

Old IMACTD_m: h2o_m + im4act_m + nad_m --> 2.0 h_m + im4ac_m + nadh_m 12042 or 13426 or 8569
New IMACTD_m: h2o_m + im4act_m + nad_m --> 2.0 h_m + im4ac_m + nadh_m 12042 or 13426

Old LALDO2: h_c + mthgxl_c + nadph_c --> lald__D_c + nadp_c 11882 or 12784 or 16543 or 9774
New LALDO2: h_c + mthgxl_c + nadph_c --> lald__D_c + nadp_c 11882 or 12784 or 9774

Old LCADi_Dm: h2o_m + lald__D_m + nad_m --> 2.0 h_m + lac__D_m + nadh_m 12042 or 13426 or 16273 or 8569 or 8692 or 8975
New LCADi_Dm: h2o_m + lald__D_m + nad_m --> 2.0 h_m + lac__D_m + nadh_m 12042 or 13426

Old LCADm: h2o_m + lald__L_m + nad_m --> 2.0 h_m + lac__L_m + nadh_m 12042 or 13426 or 16273 or 8569 or 8692 or 8975
New LCADm: h2o_m + lald__L_m + nad_m --> 2.0 h_m + lac__L_m + nadh_m 12042 or 13426

Old LGTHL: gthrd_c + mthgxl_c --> lgt__S_c 13265
New LGTHL: gthrd_c + mthgxl_c --> lgt__S_c 13265 or 16520

Old MCITL2m: micit_m --> pyr_m + succ_m 14022 or 14162
New MCITL2m: micit_m --> pyr_m + succ_m 14162

Old MG1B: asnglcnacglcnacman_man_manman_manman_manmanmanglcglcglc_c + h2o_c --> asnglcnacglcnacman_man_manman_manman_manmanmanglcglc_c + glc__bD_c 9981
New MG1B: asnglcnacglcnacman_man_manman_manman_manmanmanglcglcglc_c + h2o_c --> asnglcnacglcnacman_man_manman_manman_manmanmanglcglc_c + glc__D_c 9981

Old MG2B: asnglcnacglcnacman_man_manman_manman_manmanmanglcglc_c + h2o_c --> asnglcnacglcnacman_man_manman_manman_manmanmanglc_c + glc__bD_c 10170
New MG2B: asnglcnacglcnacman_man_manman_manman_manmanmanglcglc_c + h2o_c --> asnglcnacglcnacman_man_manman_manman_manmanmanglc_c + glc__D_c 10170

Old MG3B: asnglcnacglcnacman_man_manman_manman_manmanmanglc_c + h2o_c --> asnglcnacglcnacman_man_manman_manman_manmanman_c + glc__bD_c 10170
New MG3B: asnglcnacglcnacman_man_manman_manman_manmanmanglc_c + h2o_c --> asnglcnacglcnacman_man_manman_manman_manmanman_c + glc__D_c 10170

Old NABTNOm: h2o_m + n4abutn_m + nad_m --> 4aabutn_m + 2.0 h_m + nadh_m 12042 or 13426 or 8569
New NABTNOm: h2o_m + n4abutn_m + nad_m --> 4aabutn_m + 2.0 h_m + nadh_m 12042 or 13426

Old NADHXE: nadhx__S_c <=> nadhx__R_c 16345
New NADHXE: nadhx__S_c <=> nadhx__R_c 8901

Old NADPHXE: nadphx__R_c <=> nadphx__S_c 16345
New NADPHXE: nadphx__R_c <=> nadphx__S_c 8901

Old NITR: h_c + nadh_c + no3_c --> h2o_c + nad_c + no2_c 15008
New NITR: h_c + nadh_c + no3_c --> h2o_c + nad_c + no2_c 13272 or 15008

Old NTRIR2x: 5.0 h_c + 3.0 nadh_c + no2_c --> 2.0 h2o_c + 3.0 nad_c + nh4_c (PP_1706 and 15007) or (b3366 and 15007)
New NTRIR2x: 5.0 h_c + 3.0 nadh_c + no2_c --> 2.0 h2o_c + 3.0 nad_c + nh4_c 15007

Old ORPT: orot5p_c + ppi_c <=> orot_c + prpp_c 10716 or 12118
New ORPT: orot5p_c + ppi_c <=> orot_c + prpp_c 10716

Old PIt2m: h_c + pi_c <=> h_m + pi_m 11409 or 11410 or 11686 or 15874 or 8889
New PIt2m: h_c + pi_c <=> h_m + pi_m 15874 or 8889

Old PIt2r: h_e + pi_e <=> h_c + pi_c 10316 or 11409 or 11410 or 11418 or 11534 or 11686 or (10316 and 11418 and 11686) or (10316 and 11534 and 11686)
New PIt2r: h_e + pi_e <=> h_c + pi_c 10316 or 11409 or 11410 or 11418 or 11534

Old PItn: pi_n <=> pi_c 8889
New PItn: pi_n <=> pi_c 

Old POLYAO: N1aspmd_c + h2o_c + o2_c --> aproa_c + aprut_c + h2o2_c 15939
New POLYAO: N1aspmd_c + h2o_c + o2_c --> aprut_c + bamppald_c + h2o2_c 15939

Old POLYAO2: N1sprm_c + h2o_c + o2_c --> N1aspmd_c + aproa_c + h2o2_c 15939
New POLYAO2: N1sprm_c + h2o_c + o2_c --> N1aspmd_c + bamppald_c + h2o2_c 15939

Old POLYAO3: h2o_c + o2_c + sprm_c --> aproa_c + h2o2_c + spmd_c 15939
New POLYAO3: h2o_c + o2_c + sprm_c --> bamppald_c + h2o2_c + spmd_c 15939

Old PPDOy: h_c + lald__D_c + nadph_c --> 12ppd__R_c + nadp_c 10029 or 11882 or 12784 or 13554 or 13562 or 13947 or 16543 or 9774
New PPDOy: h_c + lald__D_c + nadph_c --> 12ppd__R_c + nadp_c 10029 or 11882 or 12784 or 13554 or 13562 or 13947 or 9774

Old PYLALDOXm: h2o_m + nad_m + pylald_m --> 2.0 h_m + nadh_m + peracd_m 12042 or 13426 or 8569
New PYLALDOXm: h2o_m + nad_m + pylald_m --> 2.0 h_m + nadh_m + peracd_m 12042 or 13426

Old SBTR: glc__D_c + h_c + nadph_c --> nadp_c + sbt__D_c 11882 or 12784 or 16543 or 9774
New SBTR: glc__D_c + h_c + nadph_c --> nadp_c + sbt__D_c 11882 or 12784 or 9774

Old SDPTA: akg_c + sl26da_c <=> glu__L_c + sl2a6o_c 10937 or 14878 or 15404
New SDPTA: akg_c + sl26da_c <=> glu__L_c + sl2a6o_c 15404

Old SULFOX: 2.0 ficytC_m + h2o_c + so3_c --> 2.0 focytC_m + 2.0 h_c + so4_c 16758
New SULFOX: 2.0 ficytc_m + h2o_c + so3_c --> 2.0 focytc_m + 2.0 h_c + so4_c 16758

Old TRE6PS: g6p_c + udpg_c --> h_c + tre6p_c + udp_c 11389 or (YML100W and 11389 and 12034) or (YMR261C and 11389 and 12034)
New TRE6PS: g6p_c + udpg_c --> h_c + tre6p_c + udp_c 11389 or 12034
```

In [148]:

```
print('Added reactions\n')
for r in sorted(model_new.reactions, key=lambda x: x.id):
    if r not in model_old.reactions:
        print(r)
```

```
Added reactions

3HBCOAHLm: 3hibutcoa_m + h2o_m --> 3hmp_m + coa_m + h_m
3NPHBH2_m: 3nphb_m + h_m + nadph_m + o2_m --> 3npdhb_m + h2o_m + nadp_m
AHGDxm: fad_m + s2hglut_m <=> akg_m + fadh2_m
ARHGDxm: fad_m + r2hglut_m <=> akg_m + fadh2_m
ATPS2v: atp_c + h2o_c + h_c --> adp_c + 2.0 h_v + pi_c
CBPSm: 2.0 atp_m + gln__L_m + h2o_m + hco3_m --> 2.0 adp_m + cbp_m + glu__L_m + 2.0 h_m + pi_m
CCP2_m: 2.0 focytc_m + h2o2_m + 2.0 h_m --> 2.0 ficytc_m + 2.0 h2o_m
CITtp: cit_c <=> cit_x
COQ3_m: 2npmhmobq_m + amet_m --> ahcys_m + h_m + q9_m
COQ5_m: 2np6mobq_m + amet_m --> ahcys_m + h_m + me2np6mobq_m
COQ6_m: 2np6mep_m + o2_m --> 2np6mobq_m + h2o_m
COQ7_m: h_m + me2np6mobq_m + nadph_m + o2_m --> 2npmhmobq_m + h2o_m + nadp_m
CYOR_u9m: 2.0 ficytc_m + 2.0 h_m + q9h2_m --> 2.0 focytc_m + 4.0 h_c + q9_m
Cu1ATPase: atp_c + cu_c + h2o_c --> adp_c + cu_e + h_c + pi_c
DHNPBMT_m: 3npdhb_m + amet_m --> ahcys_m + h_m + me3dhnpdh_m
DHORD_u9m: dhor__S_c + q9_m --> orot_c + q9h2_m
ETFQO_m: etfrd_m + q9_m --> etfox_m + q9h2_m
FALGTHLs: fald_c + gthrd_c <=> hmgth_c
FRDPtm: frdp_c <=> frdp_m
G3PD_u9cm: glyc3p_c + q9_m --> dhap_c + q9h2_m
G5SADm: glu5sa_m + h2o_m + nad_m --> glu__L_m + 2.0 h_m + nadh_m
G5SADrm: glu5sa_m <=> 1pyr5c_m + h2o_m + h_m
G5SADs: glu5sa_c <=> 1pyr5c_c + h2o_c + h_c
GHBTRHDm: akg_m + ghb_m <=> r2hglut_m + sucsal_m
GLYCLTDxm: glx_m + h_m + nadh_m --> glyclt_m + nad_m
GLYCLTDyp: glx_x + h_x + nadph_x --> glyclt_x + nadp_x
GLYOp: gly_x + h2o_x + o2_x --> glx_x + h2o2_x + nh4_x
GRDPtm: grdp_c <=> grdp_m
HBZNPT_m: 4hbz_m + npdp_m --> 3nphb_m + ppi_m
HPRO_u9m: 4hpro_LT_m + q9_m --> 1p3h5c_m + h_m + q9h2_m
ICITtp: icit_c <=> icit_x
ICLp: icit_x --> glx_x + succ_x
IPDPtm: ipdp_c <=> ipdp_m
KATPase: atp_c + h2o_c + h_e + k_c --> adp_c + 2.0 h_c + k_e + pi_c
L_LACDcm: 2.0 ficytc_m + lac__L_c --> 2.0 focytc_m + 2.0 h_c + pyr_c
MI3PS: g6p_c --> mi3p__D_c
NADH2_u9cm: h_c + nadh_c + q9_m --> nad_c + q9h2_m
NADH2_u9m1: h_m + nadh_m + q9_m --> nad_m + q9h2_m
NADH2_u9m2: 5.0 h_m + nadh_m + q9_m --> 4.0 h_c + nad_m + q9h2_m
NADHXD2: atp_c + nadhx__S_c --> adp_c + h_c + nadh_c + pi_c
NADPHXD2: atp_c + nadphx__S_c --> adp_c + h_c + nadph_c + pi_c
NPDPS_m: grdp_m + 7.0 ipdp_m --> npdp_m + 7.0 ppi_m
NPHMBDC_m: h_m + me3dhnpdh_m --> 2np6mep_m + co2_m
Na1ATPase: atp_c + h2o_c + h_e + na1_c --> adp_c + 2.0 h_c + na1_e + pi_c
PHCHGSm: 1p3h5c_m + h2o_m + h_m <=> 4hglusa_m
PIt9: 2.0 na1_e + pi_e <=> 2.0 na1_c + pi_c
PItx: pi_c <=> pi_x
PMCA: atp_c + ca2_c + h2o_c + h_e --> adp_c + ca2_e + 2.0 h_c + pi_c
PROD_u9m: pro__L_m + q9_m --> 1pyr5c_m + h_m + q9h2_m
S3HBTRHDm: akg_m + s3hb_m <=> acac_m + r2hglut_m
SERCA: atp_c + 2.0 ca2_c + h2o_c + 2.0 h_r --> adp_c + 2.0 ca2_r + 3.0 h_c + pi_c
SUCCtp: succ_c <=> succ_x
SUCD2_u9m: q9_m + succ_m --> fum_m + q9h2_m
TYROXDAc: h2o_c + o2_c + tym_c --> 4hoxpacd_c + h2o2_c + nh4_c
```

In [ ]:

```

```
