# Supplementary material for: Multi-Omics Driven Metabolic Network Reconstruction and Analysis of Lignocellulosic Carbon Utilization in Rhodosporidium toruloides
Source: Front Bioeng Biotechnol. 2021 Jan 8;8:612832. doi: 10.3389/fbioe.2020.612832 (PMC7873862; doi:10.3389/fbioe.2020.612832)
Supplement: Supplementary File 4 — Multi-omics dataset for R. toruloides IFO0880. [file Data_Sheet_1.zip › Supplementary File S1/1.Manual_curation/Refinement_1e_Compartments.html]

Refinement\_1e\_Compartments


In [1]:

```
%matplotlib inline
from matplotlib import pyplot as plt
from matplotlib import colors
import csv
import numpy as np
import pandas as pd
import cobra
```

In [2]:

```
cobra.__version__
```

Out[2]:

```
'0.17.1'
```

In [3]:

```
Annotation = pd.read_excel('../../Data/R_toruloides_Data_for_Reconstruction.xlsx',
                          sheet_name='Annotation', index_col=0)
Annotation.index = Annotation.index.map(str)
Annotation = Annotation.fillna('')
```

In [4]:

```
model = cobra.io.load_json_model("IFO0880_GPR_1d.json")
```

In [5]:

```
print(len(model.genes))
print(len(model.reactions))
print(len(model.metabolites))
model
```

```
1234
2351
2551
```

Out[5]:

|  |  |
| --- | --- |
| **Name** | R. toruloides |
| **Memory address** | 0x010296144e0 |
| **Number of metabolites** | 2551 |
| **Number of reactions** | 2351 |
| **Number of groups** | 0 |
| **Objective expression** | 0 |
| **Compartments** | c, x, m, e, r, v, n, g, p, h, f, l, d |

### Compartments¶

In [6]:

```
for x in model.compartments:
    print(x, len([m for m in model.metabolites if m.compartment == x]))
```

```
c 1089
x 140
m 431
e 157
r 204
v 23
n 69
g 66
p 116
h 146
f 1
l 101
d 8
```

In [7]:

```
temp = list()
for r in sorted(model.reactions, key=lambda x: x.id):
    if 'p' in r.compartments:
        print(r.id, r.reaction, r.gene_reaction_rule)
        for g in r.genes:
            if g.id in Annotation.index and g.id not in temp:
                temp.append(g.id)
            for r2 in g.reactions:
                if r2 is not r and 'p' not in r2.compartments:
                    print(' ',r2.id, r2.reaction, r2.gene_reaction_rule)
        print()
```

```
2DHGLCNkt_tpp 2dhglcn_p + h_p --> 2dhglcn_c + h_c 16161

3NTD2pp 3ump_p + h2o_p --> pi_p + uri_p 13409
  ALKP dhap_c + h2o_c --> dha_c + pi_c 13409 or 14546
  AKP1 ahdt_c + 3.0 h2o_c --> dhnpt_c + 2.0 h_c + 3.0 pi_c 13409

3NTD4pp 3cmp_p + h2o_p --> cytd_p + pi_p 13409
  ALKP dhap_c + h2o_c --> dha_c + pi_c 13409 or 14546
  AKP1 ahdt_c + 3.0 h2o_c --> dhnpt_c + 2.0 h_c + 3.0 pi_c 13409

3NTD7pp 3amp_p + h2o_p --> adn_p + pi_p 13409
  ALKP dhap_c + h2o_c --> dha_c + pi_c 13409 or 14546
  AKP1 ahdt_c + 3.0 h2o_c --> dhnpt_c + 2.0 h_c + 3.0 pi_c 13409

3NTD9pp 3gmp_p + h2o_p --> gsn_p + pi_p 13409
  ALKP dhap_c + h2o_c --> dha_c + pi_c 13409 or 14546
  AKP1 ahdt_c + 3.0 h2o_c --> dhnpt_c + 2.0 h_c + 3.0 pi_c 13409

3_4DHBZt2 34dhbz_e <=> 34dhbz_p 15873

ACP1p fmn_p + h2o_p --> pi_p + ribflv_p 13409
  ALKP dhap_c + h2o_c --> dha_c + pi_c 13409 or 14546
  AKP1 ahdt_c + 3.0 h2o_c --> dhnpt_c + 2.0 h_c + 3.0 pi_c 13409

ACt2rpp ac_p + h_p <=> ac_c + h_c 10658 or 13824
  ACtr ac_e <=> ac_c 10658 or 13824
  ACtr ac_e <=> ac_c 10658 or 13824

ADEt2rpp ade_p + h_p <=> ade_c + h_c 10529
  5FLURAt 5flura_e <=> 5flura_c 10529
  TEGAt tega_e <=> tega_c 10529
  TGUAt tgua_e <=> tgua_c 10529
  6MPURt 6mpur_e <=> 6mpur_c 10529
  URATEtm h_c + urate_c <=> h_m + urate_m 10529
  URATEt_1 h_e + urate_e <=> h_c + urate_c 10529

ALLTNt2rpp alltn_p + h_p <=> alltn_c + h_c 12909
  ALLTNtm alltn_c + h_c <=> alltn_m + 2.0 h_m 12909

ARBt2rpp arab__L_p + h_p <=> arab__L_c + h_c 13042 or 8608
  INSTt2 h_e + inost_e --> h_c + inost_c 13042 or 8608
  INSTt2 h_e + inost_e --> h_c + inost_c 13042 or 8608

ASNNpp asn__L_p + h2o_p --> asp__L_p + nh4_p 10043 or 13627
  ASNN asn__L_c + h2o_c --> asp__L_c + nh4_c 10043 or 13627 or 8853
  GLUN gln__L_c + h2o_c --> glu__L_c + nh4_c 10043 or 13627
  ASNNe asn__L_e + h2o_e --> asp__L_e + nh4_e 10043 or 13627
  ASNN asn__L_c + h2o_c --> asp__L_c + nh4_c 10043 or 13627 or 8853
  GLUN gln__L_c + h2o_c --> glu__L_c + nh4_c 10043 or 13627
  ASNNe asn__L_e + h2o_e --> asp__L_e + nh4_e 10043 or 13627

CA2t3pp ca2_c + h_p --> ca2_p + h_c 15553

CD2t3pp cd2_c + h_p --> cd2_p + h_c 11397

CLt3_2pp 2.0 cl_p + h_c --> 2.0 cl_c + h_p 14836

COBALT2t3pp cobalt2_c + h_p --> cobalt2_p + h_c 11397

COLIPAabcpp atp_c + colipa_c + h2o_c --> adp_c + colipa_p + h_c + pi_c 11777 or 9854
  PCt atp_c + h2o_c + pchol_hs_c <=> adp_c + h_c + pchol_hs_e + pi_c 11777
  GCHOLAt3 atp_c + gchola_c + h2o_c --> adp_c + gchola_e + h_c + pi_c 11266 or 11777 or 13264 or 14111 or 14463 or 14844 or 8693 or 9145
  TCHOLAt3 atp_c + h2o_c + tchola_c --> adp_c + h_c + pi_c + tchola_e 11266 or 11777 or 13264 or 14111 or 14463 or 14844 or 8693 or 9145
  CHOLATEt3 atp_c + cholate_c + h2o_c --> adp_c + cholate_e + h_c + pi_c 11266 or 11777 or 13264 or 14111 or 14463 or 14844 or 8693 or 9145

CYANSTpp cyan_p + tsul_p --> h_p + so3_p + tcynt_p 15100

FE2t2pp fe2_p + h_p --> fe2_c + h_c 15928 or 15934

FRULYSt2pp frulys_p + h_p --> frulys_c + h_c 11625
  AMY2e glygn2_e + 8.0 h2o_e --> 8.0 glc__D_e + glygn4_e 11625 and 9135
  METLEUex leu__L_c + met__L_e --> leu__L_e + met__L_c (SLC3A2 and 11625) or (Slc3a2 and 11625)
  CYSTSERex cysi__L_e + ser__L_c --> cysi__L_c + ser__L_e 11625 and 9135
  SERLYSNaex lys__L_c + na1_e + ser__L_e --> lys__L_e + na1_c + ser__L_c 11625 and 9135
  ARGLYSex arg__L_e + lys__L_c --> arg__L_c + lys__L_e (SLC3A2 and 11625) or (Slc3a2 and 11625)
  AMY1e 8.0 h2o_e + strch1_e --> 8.0 glc__D_e + strch2_e 11625 and 9135

FUCtpp fuc__L_p + h_p <=> fuc__L_c + h_c 10339

Ftpp f_c --> f_p 9978

G1PPpp g1p_p + h2o_p --> glc__D_p + pi_p 13409
  ALKP dhap_c + h2o_c --> dha_c + pi_c 13409 or 14546
  AKP1 ahdt_c + 3.0 h2o_c --> dhnpt_c + 2.0 h_c + 3.0 pi_c 13409

G2PPpp glyc2p_p + h2o_p --> glyc_p + pi_p 13409
  ALKP dhap_c + h2o_c --> dha_c + pi_c 13409 or 14546
  AKP1 ahdt_c + 3.0 h2o_c --> dhnpt_c + 2.0 h_c + 3.0 pi_c 13409

GALM2pp gal_bD_p --> gal_p 14480
  A1E Glc_aD_c <=> glc__D_c 14480

GALt2pp gal_p + h_p --> gal_c + h_c 13042 or 8608
  INSTt2 h_e + inost_e --> h_c + inost_c 13042 or 8608
  INSTt2 h_e + inost_e --> h_c + inost_c 13042 or 8608

GLUNpp gln__L_p + h2o_p --> glu__L_p + nh4_p 10043 or 13627
  ASNN asn__L_c + h2o_c --> asp__L_c + nh4_c 10043 or 13627 or 8853
  GLUN gln__L_c + h2o_c --> glu__L_c + nh4_c 10043 or 13627
  ASNNe asn__L_e + h2o_e --> asp__L_e + nh4_e 10043 or 13627
  ASNN asn__L_c + h2o_c --> asp__L_c + nh4_c 10043 or 13627 or 8853
  GLUN gln__L_c + h2o_c --> glu__L_c + nh4_c 10043 or 13627
  ASNNe asn__L_e + h2o_e --> asp__L_e + nh4_e 10043 or 13627

GLYALDtpp glyald_p <=> glyald_c 15470
  UREAtm urea_c <=> urea_m 15470
  L_LACtcm lac__L_c --> lac__L_m 15470
  GLYCt glyc_c <=> glyc_e 15470

GLYCtpp glyc_c <=> glyc_p 15470
  UREAtm urea_c <=> urea_m 15470
  L_LACtcm lac__L_c --> lac__L_m 15470
  GLYCt glyc_c <=> glyc_e 15470

GLYtpp gly_c <=> gly_p 15470
  UREAtm urea_c <=> urea_m 15470
  L_LACtcm lac__L_c --> lac__L_m 15470
  GLYCt glyc_c <=> glyc_e 15470

GTHRDHpp gthrd_p + h2o_p --> cgly_p + glu__L_p 9838
  GTHRDH_syn gthrd_c + h2o_c --> cgly_c + glu__L_c 10380 or 9838
  GTMLTe ala__L_c + gthrd_c --> cgly_e + gluala_e 9838
  GTMLT ala__L_c + gthrd_c --> cgly_c + gluala_c 9838

GUAt2pp gua_p + h_p --> gua_c + h_c 10529
  5FLURAt 5flura_e <=> 5flura_c 10529
  TEGAt tega_e <=> tega_c 10529
  TGUAt tgua_e <=> tgua_c 10529
  6MPURt 6mpur_e <=> 6mpur_c 10529
  URATEtm h_c + urate_c <=> h_m + urate_m 10529
  URATEt_1 h_e + urate_e <=> h_c + urate_c 10529

INOSTt4pp inost_p + na1_p --> inost_c + na1_c 15807
  UREA2t2 2.0 h_e + urea_e <=> 2.0 h_c + urea_c 15807
  UREAt_1 na1_e + urea_e <=> na1_c + urea_c 15807
  UREAtm_1 na1_c + urea_c <=> na1_m + urea_m 15807

K2L4Aabcpp atp_c + h2o_c + kdo2lipid4_c --> adp_c + h_c + kdo2lipid4_p + pi_c 11777 or 9854
  PCt atp_c + h2o_c + pchol_hs_c <=> adp_c + h_c + pchol_hs_e + pi_c 11777
  GCHOLAt3 atp_c + gchola_c + h2o_c --> adp_c + gchola_e + h_c + pi_c 11266 or 11777 or 13264 or 14111 or 14463 or 14844 or 8693 or 9145
  TCHOLAt3 atp_c + h2o_c + tchola_c --> adp_c + h_c + pi_c + tchola_e 11266 or 11777 or 13264 or 14111 or 14463 or 14844 or 8693 or 9145
  CHOLATEt3 atp_c + cholate_c + h2o_c --> adp_c + cholate_e + h_c + pi_c 11266 or 11777 or 13264 or 14111 or 14463 or 14844 or 8693 or 9145

Kt2pp h_p + k_p --> h_c + k_c 9810 or (b1291 and b1363 and b3290) or (b1291 and b3290 and b3849)

Kt3pp h_p + k_c --> h_c + k_p 15553

LACZpp h2o_p + lcts_p --> gal_p + glc__D_p 16716 or 16717 or 8528

LIPACabcpp atp_c + h2o_c + lipa_cold_c --> adp_c + h_c + lipa_cold_p + pi_c 11777 or 9854
  PCt atp_c + h2o_c + pchol_hs_c <=> adp_c + h_c + pchol_hs_e + pi_c 11777
  GCHOLAt3 atp_c + gchola_c + h2o_c --> adp_c + gchola_e + h_c + pi_c 11266 or 11777 or 13264 or 14111 or 14463 or 14844 or 8693 or 9145
  TCHOLAt3 atp_c + h2o_c + tchola_c --> adp_c + h_c + pi_c + tchola_e 11266 or 11777 or 13264 or 14111 or 14463 or 14844 or 8693 or 9145
  CHOLATEt3 atp_c + cholate_c + h2o_c --> adp_c + cholate_e + h_c + pi_c 11266 or 11777 or 13264 or 14111 or 14463 or 14844 or 8693 or 9145

LIPAabcpp atp_c + h2o_c + lipa_c --> adp_c + h_c + lipa_p + pi_c 11777 or 9854
  PCt atp_c + h2o_c + pchol_hs_c <=> adp_c + h_c + pchol_hs_e + pi_c 11777
  GCHOLAt3 atp_c + gchola_c + h2o_c --> adp_c + gchola_e + h_c + pi_c 11266 or 11777 or 13264 or 14111 or 14463 or 14844 or 8693 or 9145
  TCHOLAt3 atp_c + h2o_c + tchola_c --> adp_c + h_c + pi_c + tchola_e 11266 or 11777 or 13264 or 14111 or 14463 or 14844 or 8693 or 9145
  CHOLATEt3 atp_c + cholate_c + h2o_c --> adp_c + cholate_e + h_c + pi_c 11266 or 11777 or 13264 or 14111 or 14463 or 14844 or 8693 or 9145

LYSt2pp h_p + lys__L_p --> h_c + lys__L_c 8962 or 9319 or 9322
  GLNt2r gln__L_e + h_e <=> gln__L_c + h_c 14229 or 15074 or 8962 or 9319 or 9322 or 9962 or (14229 and 8962) or (14229 and 9319) or (14229 and 9322) or (14229 and 9962) or (15074 and 8962) or (15074 and 9319) or (15074 and 9322) or (15074 and 9962)
  ALAt2r ala__L_e + h_e <=> ala__L_c + h_c 12743 or 14229 or 15074 or 8962 or 9319 or 9322 or 9962 or (YALI0B09537g and 14229 and 8962) or (YALI0B09537g and 14229 and 9319) or (YALI0B09537g and 14229 and 9322) or (YALI0B09537g and 14229 and 9962) or (YALI0B09537g and 15074 and 8962) or (YALI0B09537g and 15074 and 9319) or (YALI0B09537g and 15074 and 9322) or (YALI0B09537g and 15074 and 9962)
  ARGt2r arg__L_e + h_e <=> arg__L_c + h_c 14229 or 15074 or 8962 or 9319 or 9322 or (14229 and 8962) or (14229 and 9319) or (14229 and 9322) or (15074 and 8962) or (15074 and 9319) or (15074 and 9322)
  ASPt2r asp__L_e + h_e <=> asp__L_c + h_c 14229 or 15074 or 8962 or 9319 or 9322 or 9962 or (YALI0E20713g and 14229 and 8962) or (YALI0E20713g and 14229 and 9319) or (YALI0E20713g and 14229 and 9322) or (YALI0E20713g and 14229 and 9962) or (YALI0E20713g and 15074 and 8962) or (YALI0E20713g and 15074 and 9319) or (YALI0E20713g and 15074 and 9322) or (YALI0E20713g and 15074 and 9962)
  GLYt2r gly_e + h_e <=> gly_c + h_c 12743 or 14229 or 15074 or 8962 or 9319 or 9322 or 9962 or (YALI0B09537g and 14229 and 8962) or (YALI0B09537g and 14229 and 9319) or (YALI0B09537g and 14229 and 9322) or (YALI0B09537g and 14229 and 9962) or (YALI0B09537g and 15074 and 8962) or (YALI0B09537g and 15074 and 9319) or (YALI0B09537g and 15074 and 9322) or (YALI0B09537g and 15074 and 9962)
  ASNt2r asn__L_e + h_e <=> asn__L_c + h_c 14229 or 15074 or 8962 or 9319 or 9322 or 9962 or (14229 and 8962) or (14229 and 9319) or (14229 and 9322) or (14229 and 9962) or (15074 and 8962) or (15074 and 9319) or (15074 and 9322) or (15074 and 9962)
  SERt2r h_e + ser__L_e <=> h_c + ser__L_c 14229 or 15074 or 8962 or 9319 or 9322 or 9962 or (YALI0E20713g and 14229 and 8962) or (YALI0E20713g and 14229 and 9319) or (YALI0E20713g and 14229 and 9322) or (YALI0E20713g and 14229 and 9962) or (YALI0E20713g and 15074 and 8962) or (YALI0E20713g and 15074 and 9319) or (YALI0E20713g and 15074 and 9322) or (YALI0E20713g and 15074 and 9962)
  GLUt2r glu__L_e + h_e <=> glu__L_c + h_c 14229 or 15074 or 8962 or 9319 or 9322 or 9962
  ORNt2r h_e + orn_e <=> h_c + orn_c 14229 or 15074 or 8962 or 9319 or 9322 or (14229 and 8962) or (14229 and 9319) or (14229 and 9322) or (15074 and 8962) or (15074 and 9319) or (15074 and 9322)
  LYSt2r h_e + lys__L_e <=> h_c + lys__L_c 14229 or 15074 or 8962 or 9319 or 9322 or (14229 and 8962) or (14229 and 9319) or (14229 and 9322) or (15074 and 8962) or (15074 and 9319) or (15074 and 9322)
  GLNt2r gln__L_e + h_e <=> gln__L_c + h_c 14229 or 15074 or 8962 or 9319 or 9322 or 9962 or (14229 and 8962) or (14229 and 9319) or (14229 and 9322) or (14229 and 9962) or (15074 and 8962) or (15074 and 9319) or (15074 and 9322) or (15074 and 9962)
  ALAt2r ala__L_e + h_e <=> ala__L_c + h_c 12743 or 14229 or 15074 or 8962 or 9319 or 9322 or 9962 or (YALI0B09537g and 14229 and 8962) or (YALI0B09537g and 14229 and 9319) or (YALI0B09537g and 14229 and 9322) or (YALI0B09537g and 14229 and 9962) or (YALI0B09537g and 15074 and 8962) or (YALI0B09537g and 15074 and 9319) or (YALI0B09537g and 15074 and 9322) or (YALI0B09537g and 15074 and 9962)
  ARGt2r arg__L_e + h_e <=> arg__L_c + h_c 14229 or 15074 or 8962 or 9319 or 9322 or (14229 and 8962) or (14229 and 9319) or (14229 and 9322) or (15074 and 8962) or (15074 and 9319) or (15074 and 9322)
  ASPt2r asp__L_e + h_e <=> asp__L_c + h_c 14229 or 15074 or 8962 or 9319 or 9322 or 9962 or (YALI0E20713g and 14229 and 8962) or (YALI0E20713g and 14229 and 9319) or (YALI0E20713g and 14229 and 9322) or (YALI0E20713g and 14229 and 9962) or (YALI0E20713g and 15074 and 8962) or (YALI0E20713g and 15074 and 9319) or (YALI0E20713g and 15074 and 9322) or (YALI0E20713g and 15074 and 9962)
  GLYt2r gly_e + h_e <=> gly_c + h_c 12743 or 14229 or 15074 or 8962 or 9319 or 9322 or 9962 or (YALI0B09537g and 14229 and 8962) or (YALI0B09537g and 14229 and 9319) or (YALI0B09537g and 14229 and 9322) or (YALI0B09537g and 14229 and 9962) or (YALI0B09537g and 15074 and 8962) or (YALI0B09537g and 15074 and 9319) or (YALI0B09537g and 15074 and 9322) or (YALI0B09537g and 15074 and 9962)
  ASNt2r asn__L_e + h_e <=> asn__L_c + h_c 14229 or 15074 or 8962 or 9319 or 9322 or 9962 or (14229 and 8962) or (14229 and 9319) or (14229 and 9322) or (14229 and 9962) or (15074 and 8962) or (15074 and 9319) or (15074 and 9322) or (15074 and 9962)
  SERt2r h_e + ser__L_e <=> h_c + ser__L_c 14229 or 15074 or 8962 or 9319 or 9322 or 9962 or (YALI0E20713g and 14229 and 8962) or (YALI0E20713g and 14229 and 9319) or (YALI0E20713g and 14229 and 9322) or (YALI0E20713g and 14229 and 9962) or (YALI0E20713g and 15074 and 8962) or (YALI0E20713g and 15074 and 9319) or (YALI0E20713g and 15074 and 9322) or (YALI0E20713g and 15074 and 9962)
  GLUt2r glu__L_e + h_e <=> glu__L_c + h_c 14229 or 15074 or 8962 or 9319 or 9322 or 9962
  ORNt2r h_e + orn_e <=> h_c + orn_c 14229 or 15074 or 8962 or 9319 or 9322 or (14229 and 8962) or (14229 and 9319) or (14229 and 9322) or (15074 and 8962) or (15074 and 9319) or (15074 and 9322)
  LYSt2r h_e + lys__L_e <=> h_c + lys__L_c 14229 or 15074 or 8962 or 9319 or 9322 or (14229 and 8962) or (14229 and 9319) or (14229 and 9322) or (15074 and 8962) or (15074 and 9319) or (15074 and 9322)
  GLNt2r gln__L_e + h_e <=> gln__L_c + h_c 14229 or 15074 or 8962 or 9319 or 9322 or 9962 or (14229 and 8962) or (14229 and 9319) or (14229 and 9322) or (14229 and 9962) or (15074 and 8962) or (15074 and 9319) or (15074 and 9322) or (15074 and 9962)
  ALAt2r ala__L_e + h_e <=> ala__L_c + h_c 12743 or 14229 or 15074 or 8962 or 9319 or 9322 or 9962 or (YALI0B09537g and 14229 and 8962) or (YALI0B09537g and 14229 and 9319) or (YALI0B09537g and 14229 and 9322) or (YALI0B09537g and 14229 and 9962) or (YALI0B09537g and 15074 and 8962) or (YALI0B09537g and 15074 and 9319) or (YALI0B09537g and 15074 and 9322) or (YALI0B09537g and 15074 and 9962)
  ARGt2r arg__L_e + h_e <=> arg__L_c + h_c 14229 or 15074 or 8962 or 9319 or 9322 or (14229 and 8962) or (14229 and 9319) or (14229 and 9322) or (15074 and 8962) or (15074 and 9319) or (15074 and 9322)
  ASPt2r asp__L_e + h_e <=> asp__L_c + h_c 14229 or 15074 or 8962 or 9319 or 9322 or 9962 or (YALI0E20713g and 14229 and 8962) or (YALI0E20713g and 14229 and 9319) or (YALI0E20713g and 14229 and 9322) or (YALI0E20713g and 14229 and 9962) or (YALI0E20713g and 15074 and 8962) or (YALI0E20713g and 15074 and 9319) or (YALI0E20713g and 15074 and 9322) or (YALI0E20713g and 15074 and 9962)
  GLYt2r gly_e + h_e <=> gly_c + h_c 12743 or 14229 or 15074 or 8962 or 9319 or 9322 or 9962 or (YALI0B09537g and 14229 and 8962) or (YALI0B09537g and 14229 and 9319) or (YALI0B09537g and 14229 and 9322) or (YALI0B09537g and 14229 and 9962) or (YALI0B09537g and 15074 and 8962) or (YALI0B09537g and 15074 and 9319) or (YALI0B09537g and 15074 and 9322) or (YALI0B09537g and 15074 and 9962)
  ASNt2r asn__L_e + h_e <=> asn__L_c + h_c 14229 or 15074 or 8962 or 9319 or 9322 or 9962 or (14229 and 8962) or (14229 and 9319) or (14229 and 9322) or (14229 and 9962) or (15074 and 8962) or (15074 and 9319) or (15074 and 9322) or (15074 and 9962)
  SERt2r h_e + ser__L_e <=> h_c + ser__L_c 14229 or 15074 or 8962 or 9319 or 9322 or 9962 or (YALI0E20713g and 14229 and 8962) or (YALI0E20713g and 14229 and 9319) or (YALI0E20713g and 14229 and 9322) or (YALI0E20713g and 14229 and 9962) or (YALI0E20713g and 15074 and 8962) or (YALI0E20713g and 15074 and 9319) or (YALI0E20713g and 15074 and 9322) or (YALI0E20713g and 15074 and 9962)
  GLUt2r glu__L_e + h_e <=> glu__L_c + h_c 14229 or 15074 or 8962 or 9319 or 9322 or 9962
  ORNt2r h_e + orn_e <=> h_c + orn_c 14229 or 15074 or 8962 or 9319 or 9322 or (14229 and 8962) or (14229 and 9319) or (14229 and 9322) or (15074 and 8962) or (15074 and 9319) or (15074 and 9322)
  LYSt2r h_e + lys__L_e <=> h_c + lys__L_c 14229 or 15074 or 8962 or 9319 or 9322 or (14229 and 8962) or (14229 and 9319) or (14229 and 9322) or (15074 and 8962) or (15074 and 9319) or (15074 and 9322)

MDDCP1pp h2o_p + murein5px4p_p --> ala__D_p + murein4px4p_p 12994 or 13295 or 15126 or 15127

MDDCP2pp h2o_p + murein5px4px4p_p --> ala__D_p + murein4px4px4p_p 12994 or 13295 or 15126 or 15127

MDDCP3pp h2o_p + murein5p5p_p --> ala__D_p + murein5p4p_p 12994 or 13295 or 15126 or 15127

MDDCP4pp h2o_p + murein5p4p_p --> ala__D_p + murein4p4p_p 12994 or 13295 or 15126 or 15127

MDDCP5pp h2o_p + murein5p3p_p --> ala__D_p + murein4p3p_p 12994 or 13295 or 15126 or 15127

MDDEP1pp h2o_p + murein4px4p_p --> murein4p4p_p 13295

MDDEP2pp h2o_p + murein3px4p_p --> murein4p3p_p 13295

MDDEP3pp h2o_p + murein5px4p_p --> murein5p4p_p 13295

MDDEP4pp h2o_p + murein4px4px4p_p --> murein4px4p4p_p 13295

MN2t3pp h_p + mn2_c --> h_c + mn2_p 11397

MNt2pp h_p + mn2_p --> h_c + mn2_c 15928 or 15934

NAt3pp h_p + na1_c --> h_c + na1_p 15553

NH4tpp nh4_p <=> nh4_c 12926 or 13637
  NH4t nh4_e <=> nh4_c 12926 or 13637 or (12926 and 13637)
  NH4tp nh4_c <=> nh4_x 12926 or 13637
  NH4t nh4_e <=> nh4_c 12926 or 13637 or (12926 and 13637)
  NH4tp nh4_c <=> nh4_x 12926 or 13637

NI2t3pp h_p + ni2_c --> h_c + ni2_p 11397

NTD2pp h2o_p + ump_p --> pi_p + uri_p 13409
  ALKP dhap_c + h2o_c --> dha_c + pi_c 13409 or 14546
  AKP1 ahdt_c + 3.0 h2o_c --> dhnpt_c + 2.0 h_c + 3.0 pi_c 13409

NTD4pp cmp_p + h2o_p --> cytd_p + pi_p 13409
  ALKP dhap_c + h2o_c --> dha_c + pi_c 13409 or 14546
  AKP1 ahdt_c + 3.0 h2o_c --> dhnpt_c + 2.0 h_c + 3.0 pi_c 13409

NTD7pp amp_p + h2o_p --> adn_p + pi_p 13409
  ALKP dhap_c + h2o_c --> dha_c + pi_c 13409 or 14546
  AKP1 ahdt_c + 3.0 h2o_c --> dhnpt_c + 2.0 h_c + 3.0 pi_c 13409

NTD9pp gmp_p + h2o_p --> gsn_p + pi_p 13409
  ALKP dhap_c + h2o_c --> dha_c + pi_c 13409 or 14546
  AKP1 ahdt_c + 3.0 h2o_c --> dhnpt_c + 2.0 h_c + 3.0 pi_c 13409

PA120abcpp atp_c + h2o_c + pa120_c --> adp_c + h_c + pa120_p + pi_c 11777 or 9854
  PCt atp_c + h2o_c + pchol_hs_c <=> adp_c + h_c + pchol_hs_e + pi_c 11777
  GCHOLAt3 atp_c + gchola_c + h2o_c --> adp_c + gchola_e + h_c + pi_c 11266 or 11777 or 13264 or 14111 or 14463 or 14844 or 8693 or 9145
  TCHOLAt3 atp_c + h2o_c + tchola_c --> adp_c + h_c + pi_c + tchola_e 11266 or 11777 or 13264 or 14111 or 14463 or 14844 or 8693 or 9145
  CHOLATEt3 atp_c + cholate_c + h2o_c --> adp_c + cholate_e + h_c + pi_c 11266 or 11777 or 13264 or 14111 or 14463 or 14844 or 8693 or 9145

PA140abcpp atp_c + h2o_c + pa140_c --> adp_c + h_c + pa140_p + pi_c 11777 or 9854
  PCt atp_c + h2o_c + pchol_hs_c <=> adp_c + h_c + pchol_hs_e + pi_c 11777
  GCHOLAt3 atp_c + gchola_c + h2o_c --> adp_c + gchola_e + h_c + pi_c 11266 or 11777 or 13264 or 14111 or 14463 or 14844 or 8693 or 9145
  TCHOLAt3 atp_c + h2o_c + tchola_c --> adp_c + h_c + pi_c + tchola_e 11266 or 11777 or 13264 or 14111 or 14463 or 14844 or 8693 or 9145
  CHOLATEt3 atp_c + cholate_c + h2o_c --> adp_c + cholate_e + h_c + pi_c 11266 or 11777 or 13264 or 14111 or 14463 or 14844 or 8693 or 9145

PA141abcpp atp_c + h2o_c + pa141_c --> adp_c + h_c + pa141_p + pi_c 11777 or 9854
  PCt atp_c + h2o_c + pchol_hs_c <=> adp_c + h_c + pchol_hs_e + pi_c 11777
  GCHOLAt3 atp_c + gchola_c + h2o_c --> adp_c + gchola_e + h_c + pi_c 11266 or 11777 or 13264 or 14111 or 14463 or 14844 or 8693 or 9145
  TCHOLAt3 atp_c + h2o_c + tchola_c --> adp_c + h_c + pi_c + tchola_e 11266 or 11777 or 13264 or 14111 or 14463 or 14844 or 8693 or 9145
  CHOLATEt3 atp_c + cholate_c + h2o_c --> adp_c + cholate_e + h_c + pi_c 11266 or 11777 or 13264 or 14111 or 14463 or 14844 or 8693 or 9145

PA160abcpp atp_c + h2o_c + pa160_c --> adp_c + h_c + pa160_p + pi_c 11777 or 9854
  PCt atp_c + h2o_c + pchol_hs_c <=> adp_c + h_c + pchol_hs_e + pi_c 11777
  GCHOLAt3 atp_c + gchola_c + h2o_c --> adp_c + gchola_e + h_c + pi_c 11266 or 11777 or 13264 or 14111 or 14463 or 14844 or 8693 or 9145
  TCHOLAt3 atp_c + h2o_c + tchola_c --> adp_c + h_c + pi_c + tchola_e 11266 or 11777 or 13264 or 14111 or 14463 or 14844 or 8693 or 9145
  CHOLATEt3 atp_c + cholate_c + h2o_c --> adp_c + cholate_e + h_c + pi_c 11266 or 11777 or 13264 or 14111 or 14463 or 14844 or 8693 or 9145

PA161abcpp atp_c + h2o_c + pa161_c --> adp_c + h_c + pa161_p + pi_c 11777 or 9854
  PCt atp_c + h2o_c + pchol_hs_c <=> adp_c + h_c + pchol_hs_e + pi_c 11777
  GCHOLAt3 atp_c + gchola_c + h2o_c --> adp_c + gchola_e + h_c + pi_c 11266 or 11777 or 13264 or 14111 or 14463 or 14844 or 8693 or 9145
  TCHOLAt3 atp_c + h2o_c + tchola_c --> adp_c + h_c + pi_c + tchola_e 11266 or 11777 or 13264 or 14111 or 14463 or 14844 or 8693 or 9145
  CHOLATEt3 atp_c + cholate_c + h2o_c --> adp_c + cholate_e + h_c + pi_c 11266 or 11777 or 13264 or 14111 or 14463 or 14844 or 8693 or 9145

PA180abcpp atp_c + h2o_c + pa180_c --> adp_c + h_c + pa180_p + pi_c 11777 or 9854
  PCt atp_c + h2o_c + pchol_hs_c <=> adp_c + h_c + pchol_hs_e + pi_c 11777
  GCHOLAt3 atp_c + gchola_c + h2o_c --> adp_c + gchola_e + h_c + pi_c 11266 or 11777 or 13264 or 14111 or 14463 or 14844 or 8693 or 9145
  TCHOLAt3 atp_c + h2o_c + tchola_c --> adp_c + h_c + pi_c + tchola_e 11266 or 11777 or 13264 or 14111 or 14463 or 14844 or 8693 or 9145
  CHOLATEt3 atp_c + cholate_c + h2o_c --> adp_c + cholate_e + h_c + pi_c 11266 or 11777 or 13264 or 14111 or 14463 or 14844 or 8693 or 9145

PA181abcpp atp_c + h2o_c + pa181_c --> adp_c + h_c + pa181_p + pi_c 11777 or 9854
  PCt atp_c + h2o_c + pchol_hs_c <=> adp_c + h_c + pchol_hs_e + pi_c 11777
  GCHOLAt3 atp_c + gchola_c + h2o_c --> adp_c + gchola_e + h_c + pi_c 11266 or 11777 or 13264 or 14111 or 14463 or 14844 or 8693 or 9145
  TCHOLAt3 atp_c + h2o_c + tchola_c --> adp_c + h_c + pi_c + tchola_e 11266 or 11777 or 13264 or 14111 or 14463 or 14844 or 8693 or 9145
  CHOLATEt3 atp_c + cholate_c + h2o_c --> adp_c + cholate_e + h_c + pi_c 11266 or 11777 or 13264 or 14111 or 14463 or 14844 or 8693 or 9145

PE120abcpp atp_c + h2o_c + pe120_c --> adp_c + h_c + pe120_p + pi_c 11777 or 9854
  PCt atp_c + h2o_c + pchol_hs_c <=> adp_c + h_c + pchol_hs_e + pi_c 11777
  GCHOLAt3 atp_c + gchola_c + h2o_c --> adp_c + gchola_e + h_c + pi_c 11266 or 11777 or 13264 or 14111 or 14463 or 14844 or 8693 or 9145
  TCHOLAt3 atp_c + h2o_c + tchola_c --> adp_c + h_c + pi_c + tchola_e 11266 or 11777 or 13264 or 14111 or 14463 or 14844 or 8693 or 9145
  CHOLATEt3 atp_c + cholate_c + h2o_c --> adp_c + cholate_e + h_c + pi_c 11266 or 11777 or 13264 or 14111 or 14463 or 14844 or 8693 or 9145

PE140abcpp atp_c + h2o_c + pe140_c --> adp_c + h_c + pe140_p + pi_c 11777 or 9854
  PCt atp_c + h2o_c + pchol_hs_c <=> adp_c + h_c + pchol_hs_e + pi_c 11777
  GCHOLAt3 atp_c + gchola_c + h2o_c --> adp_c + gchola_e + h_c + pi_c 11266 or 11777 or 13264 or 14111 or 14463 or 14844 or 8693 or 9145
  TCHOLAt3 atp_c + h2o_c + tchola_c --> adp_c + h_c + pi_c + tchola_e 11266 or 11777 or 13264 or 14111 or 14463 or 14844 or 8693 or 9145
  CHOLATEt3 atp_c + cholate_c + h2o_c --> adp_c + cholate_e + h_c + pi_c 11266 or 11777 or 13264 or 14111 or 14463 or 14844 or 8693 or 9145

PE141abcpp atp_c + h2o_c + pe141_c --> adp_c + h_c + pe141_p + pi_c 11777 or 9854
  PCt atp_c + h2o_c + pchol_hs_c <=> adp_c + h_c + pchol_hs_e + pi_c 11777
  GCHOLAt3 atp_c + gchola_c + h2o_c --> adp_c + gchola_e + h_c + pi_c 11266 or 11777 or 13264 or 14111 or 14463 or 14844 or 8693 or 9145
  TCHOLAt3 atp_c + h2o_c + tchola_c --> adp_c + h_c + pi_c + tchola_e 11266 or 11777 or 13264 or 14111 or 14463 or 14844 or 8693 or 9145
  CHOLATEt3 atp_c + cholate_c + h2o_c --> adp_c + cholate_e + h_c + pi_c 11266 or 11777 or 13264 or 14111 or 14463 or 14844 or 8693 or 9145

PE160abcpp atp_c + h2o_c + pe160_c --> adp_c + h_c + pe160_p + pi_c 11777 or 9854
  PCt atp_c + h2o_c + pchol_hs_c <=> adp_c + h_c + pchol_hs_e + pi_c 11777
  GCHOLAt3 atp_c + gchola_c + h2o_c --> adp_c + gchola_e + h_c + pi_c 11266 or 11777 or 13264 or 14111 or 14463 or 14844 or 8693 or 9145
  TCHOLAt3 atp_c + h2o_c + tchola_c --> adp_c + h_c + pi_c + tchola_e 11266 or 11777 or 13264 or 14111 or 14463 or 14844 or 8693 or 9145
  CHOLATEt3 atp_c + cholate_c + h2o_c --> adp_c + cholate_e + h_c + pi_c 11266 or 11777 or 13264 or 14111 or 14463 or 14844 or 8693 or 9145

PE161abcpp atp_c + h2o_c + pe161_c --> adp_c + h_c + pe161_p + pi_c 11777 or 9854
  PCt atp_c + h2o_c + pchol_hs_c <=> adp_c + h_c + pchol_hs_e + pi_c 11777
  GCHOLAt3 atp_c + gchola_c + h2o_c --> adp_c + gchola_e + h_c + pi_c 11266 or 11777 or 13264 or 14111 or 14463 or 14844 or 8693 or 9145
  TCHOLAt3 atp_c + h2o_c + tchola_c --> adp_c + h_c + pi_c + tchola_e 11266 or 11777 or 13264 or 14111 or 14463 or 14844 or 8693 or 9145
  CHOLATEt3 atp_c + cholate_c + h2o_c --> adp_c + cholate_e + h_c + pi_c 11266 or 11777 or 13264 or 14111 or 14463 or 14844 or 8693 or 9145

PE180abcpp atp_c + h2o_c + pe180_c --> adp_c + h_c + pe180_p + pi_c 11777 or 9854
  PCt atp_c + h2o_c + pchol_hs_c <=> adp_c + h_c + pchol_hs_e + pi_c 11777
  GCHOLAt3 atp_c + gchola_c + h2o_c --> adp_c + gchola_e + h_c + pi_c 11266 or 11777 or 13264 or 14111 or 14463 or 14844 or 8693 or 9145
  TCHOLAt3 atp_c + h2o_c + tchola_c --> adp_c + h_c + pi_c + tchola_e 11266 or 11777 or 13264 or 14111 or 14463 or 14844 or 8693 or 9145
  CHOLATEt3 atp_c + cholate_c + h2o_c --> adp_c + cholate_e + h_c + pi_c 11266 or 11777 or 13264 or 14111 or 14463 or 14844 or 8693 or 9145

PE181abcpp atp_c + h2o_c + pe181_c --> adp_c + h_c + pe181_p + pi_c 11777 or 9854
  PCt atp_c + h2o_c + pchol_hs_c <=> adp_c + h_c + pchol_hs_e + pi_c 11777
  GCHOLAt3 atp_c + gchola_c + h2o_c --> adp_c + gchola_e + h_c + pi_c 11266 or 11777 or 13264 or 14111 or 14463 or 14844 or 8693 or 9145
  TCHOLAt3 atp_c + h2o_c + tchola_c --> adp_c + h_c + pi_c + tchola_e 11266 or 11777 or 13264 or 14111 or 14463 or 14844 or 8693 or 9145
  CHOLATEt3 atp_c + cholate_c + h2o_c --> adp_c + cholate_e + h_c + pi_c 11266 or 11777 or 13264 or 14111 or 14463 or 14844 or 8693 or 9145

PG120abcpp atp_c + h2o_c + pg120_c --> adp_c + h_c + pg120_p + pi_c 11777 or 9854
  PCt atp_c + h2o_c + pchol_hs_c <=> adp_c + h_c + pchol_hs_e + pi_c 11777
  GCHOLAt3 atp_c + gchola_c + h2o_c --> adp_c + gchola_e + h_c + pi_c 11266 or 11777 or 13264 or 14111 or 14463 or 14844 or 8693 or 9145
  TCHOLAt3 atp_c + h2o_c + tchola_c --> adp_c + h_c + pi_c + tchola_e 11266 or 11777 or 13264 or 14111 or 14463 or 14844 or 8693 or 9145
  CHOLATEt3 atp_c + cholate_c + h2o_c --> adp_c + cholate_e + h_c + pi_c 11266 or 11777 or 13264 or 14111 or 14463 or 14844 or 8693 or 9145

PG140abcpp atp_c + h2o_c + pg140_c --> adp_c + h_c + pg140_p + pi_c 11777 or 9854
  PCt atp_c + h2o_c + pchol_hs_c <=> adp_c + h_c + pchol_hs_e + pi_c 11777
  GCHOLAt3 atp_c + gchola_c + h2o_c --> adp_c + gchola_e + h_c + pi_c 11266 or 11777 or 13264 or 14111 or 14463 or 14844 or 8693 or 9145
  TCHOLAt3 atp_c + h2o_c + tchola_c --> adp_c + h_c + pi_c + tchola_e 11266 or 11777 or 13264 or 14111 or 14463 or 14844 or 8693 or 9145
  CHOLATEt3 atp_c + cholate_c + h2o_c --> adp_c + cholate_e + h_c + pi_c 11266 or 11777 or 13264 or 14111 or 14463 or 14844 or 8693 or 9145

PG141abcpp atp_c + h2o_c + pg141_c --> adp_c + h_c + pg141_p + pi_c 11777 or 9854
  PCt atp_c + h2o_c + pchol_hs_c <=> adp_c + h_c + pchol_hs_e + pi_c 11777
  GCHOLAt3 atp_c + gchola_c + h2o_c --> adp_c + gchola_e + h_c + pi_c 11266 or 11777 or 13264 or 14111 or 14463 or 14844 or 8693 or 9145
  TCHOLAt3 atp_c + h2o_c + tchola_c --> adp_c + h_c + pi_c + tchola_e 11266 or 11777 or 13264 or 14111 or 14463 or 14844 or 8693 or 9145
  CHOLATEt3 atp_c + cholate_c + h2o_c --> adp_c + cholate_e + h_c + pi_c 11266 or 11777 or 13264 or 14111 or 14463 or 14844 or 8693 or 9145

PG160abcpp atp_c + h2o_c + pg160_c --> adp_c + h_c + pg160_p + pi_c 11777 or 9854
  PCt atp_c + h2o_c + pchol_hs_c <=> adp_c + h_c + pchol_hs_e + pi_c 11777
  GCHOLAt3 atp_c + gchola_c + h2o_c --> adp_c + gchola_e + h_c + pi_c 11266 or 11777 or 13264 or 14111 or 14463 or 14844 or 8693 or 9145
  TCHOLAt3 atp_c + h2o_c + tchola_c --> adp_c + h_c + pi_c + tchola_e 11266 or 11777 or 13264 or 14111 or 14463 or 14844 or 8693 or 9145
  CHOLATEt3 atp_c + cholate_c + h2o_c --> adp_c + cholate_e + h_c + pi_c 11266 or 11777 or 13264 or 14111 or 14463 or 14844 or 8693 or 9145

PG161abcpp atp_c + h2o_c + pg161_c --> adp_c + h_c + pg161_p + pi_c 11777 or 9854
  PCt atp_c + h2o_c + pchol_hs_c <=> adp_c + h_c + pchol_hs_e + pi_c 11777
  GCHOLAt3 atp_c + gchola_c + h2o_c --> adp_c + gchola_e + h_c + pi_c 11266 or 11777 or 13264 or 14111 or 14463 or 14844 or 8693 or 9145
  TCHOLAt3 atp_c + h2o_c + tchola_c --> adp_c + h_c + pi_c + tchola_e 11266 or 11777 or 13264 or 14111 or 14463 or 14844 or 8693 or 9145
  CHOLATEt3 atp_c + cholate_c + h2o_c --> adp_c + cholate_e + h_c + pi_c 11266 or 11777 or 13264 or 14111 or 14463 or 14844 or 8693 or 9145

PG180abcpp atp_c + h2o_c + pg180_c --> adp_c + h_c + pg180_p + pi_c 11777 or 9854
  PCt atp_c + h2o_c + pchol_hs_c <=> adp_c + h_c + pchol_hs_e + pi_c 11777
  GCHOLAt3 atp_c + gchola_c + h2o_c --> adp_c + gchola_e + h_c + pi_c 11266 or 11777 or 13264 or 14111 or 14463 or 14844 or 8693 or 9145
  TCHOLAt3 atp_c + h2o_c + tchola_c --> adp_c + h_c + pi_c + tchola_e 11266 or 11777 or 13264 or 14111 or 14463 or 14844 or 8693 or 9145
  CHOLATEt3 atp_c + cholate_c + h2o_c --> adp_c + cholate_e + h_c + pi_c 11266 or 11777 or 13264 or 14111 or 14463 or 14844 or 8693 or 9145

PG181abcpp atp_c + h2o_c + pg181_c --> adp_c + h_c + pg181_p + pi_c 11777 or 9854
  PCt atp_c + h2o_c + pchol_hs_c <=> adp_c + h_c + pchol_hs_e + pi_c 11777
  GCHOLAt3 atp_c + gchola_c + h2o_c --> adp_c + gchola_e + h_c + pi_c 11266 or 11777 or 13264 or 14111 or 14463 or 14844 or 8693 or 9145
  TCHOLAt3 atp_c + h2o_c + tchola_c --> adp_c + h_c + pi_c + tchola_e 11266 or 11777 or 13264 or 14111 or 14463 or 14844 or 8693 or 9145
  CHOLATEt3 atp_c + cholate_c + h2o_c --> adp_c + cholate_e + h_c + pi_c 11266 or 11777 or 13264 or 14111 or 14463 or 14844 or 8693 or 9145

PGP120abcpp atp_c + h2o_c + pgp120_c --> adp_c + h_c + pgp120_p + pi_c 11777 or 9854
  PCt atp_c + h2o_c + pchol_hs_c <=> adp_c + h_c + pchol_hs_e + pi_c 11777
  GCHOLAt3 atp_c + gchola_c + h2o_c --> adp_c + gchola_e + h_c + pi_c 11266 or 11777 or 13264 or 14111 or 14463 or 14844 or 8693 or 9145
  TCHOLAt3 atp_c + h2o_c + tchola_c --> adp_c + h_c + pi_c + tchola_e 11266 or 11777 or 13264 or 14111 or 14463 or 14844 or 8693 or 9145
  CHOLATEt3 atp_c + cholate_c + h2o_c --> adp_c + cholate_e + h_c + pi_c 11266 or 11777 or 13264 or 14111 or 14463 or 14844 or 8693 or 9145

PGP140abcpp atp_c + h2o_c + pgp140_c --> adp_c + h_c + pgp140_p + pi_c 11777 or 9854
  PCt atp_c + h2o_c + pchol_hs_c <=> adp_c + h_c + pchol_hs_e + pi_c 11777
  GCHOLAt3 atp_c + gchola_c + h2o_c --> adp_c + gchola_e + h_c + pi_c 11266 or 11777 or 13264 or 14111 or 14463 or 14844 or 8693 or 9145
  TCHOLAt3 atp_c + h2o_c + tchola_c --> adp_c + h_c + pi_c + tchola_e 11266 or 11777 or 13264 or 14111 or 14463 or 14844 or 8693 or 9145
  CHOLATEt3 atp_c + cholate_c + h2o_c --> adp_c + cholate_e + h_c + pi_c 11266 or 11777 or 13264 or 14111 or 14463 or 14844 or 8693 or 9145

PGP141abcpp atp_c + h2o_c + pgp141_c --> adp_c + h_c + pgp141_p + pi_c 11777 or 9854
  PCt atp_c + h2o_c + pchol_hs_c <=> adp_c + h_c + pchol_hs_e + pi_c 11777
  GCHOLAt3 atp_c + gchola_c + h2o_c --> adp_c + gchola_e + h_c + pi_c 11266 or 11777 or 13264 or 14111 or 14463 or 14844 or 8693 or 9145
  TCHOLAt3 atp_c + h2o_c + tchola_c --> adp_c + h_c + pi_c + tchola_e 11266 or 11777 or 13264 or 14111 or 14463 or 14844 or 8693 or 9145
  CHOLATEt3 atp_c + cholate_c + h2o_c --> adp_c + cholate_e + h_c + pi_c 11266 or 11777 or 13264 or 14111 or 14463 or 14844 or 8693 or 9145

PGP160abcpp atp_c + h2o_c + pgp160_c --> adp_c + h_c + pgp160_p + pi_c 11777 or 9854
  PCt atp_c + h2o_c + pchol_hs_c <=> adp_c + h_c + pchol_hs_e + pi_c 11777
  GCHOLAt3 atp_c + gchola_c + h2o_c --> adp_c + gchola_e + h_c + pi_c 11266 or 11777 or 13264 or 14111 or 14463 or 14844 or 8693 or 9145
  TCHOLAt3 atp_c + h2o_c + tchola_c --> adp_c + h_c + pi_c + tchola_e 11266 or 11777 or 13264 or 14111 or 14463 or 14844 or 8693 or 9145
  CHOLATEt3 atp_c + cholate_c + h2o_c --> adp_c + cholate_e + h_c + pi_c 11266 or 11777 or 13264 or 14111 or 14463 or 14844 or 8693 or 9145

PGP161abcpp atp_c + h2o_c + pgp161_c --> adp_c + h_c + pgp161_p + pi_c 11777 or 9854
  PCt atp_c + h2o_c + pchol_hs_c <=> adp_c + h_c + pchol_hs_e + pi_c 11777
  GCHOLAt3 atp_c + gchola_c + h2o_c --> adp_c + gchola_e + h_c + pi_c 11266 or 11777 or 13264 or 14111 or 14463 or 14844 or 8693 or 9145
  TCHOLAt3 atp_c + h2o_c + tchola_c --> adp_c + h_c + pi_c + tchola_e 11266 or 11777 or 13264 or 14111 or 14463 or 14844 or 8693 or 9145
  CHOLATEt3 atp_c + cholate_c + h2o_c --> adp_c + cholate_e + h_c + pi_c 11266 or 11777 or 13264 or 14111 or 14463 or 14844 or 8693 or 9145

PGP180abcpp atp_c + h2o_c + pgp180_c --> adp_c + h_c + pgp180_p + pi_c 11777 or 9854
  PCt atp_c + h2o_c + pchol_hs_c <=> adp_c + h_c + pchol_hs_e + pi_c 11777
  GCHOLAt3 atp_c + gchola_c + h2o_c --> adp_c + gchola_e + h_c + pi_c 11266 or 11777 or 13264 or 14111 or 14463 or 14844 or 8693 or 9145
  TCHOLAt3 atp_c + h2o_c + tchola_c --> adp_c + h_c + pi_c + tchola_e 11266 or 11777 or 13264 or 14111 or 14463 or 14844 or 8693 or 9145
  CHOLATEt3 atp_c + cholate_c + h2o_c --> adp_c + cholate_e + h_c + pi_c 11266 or 11777 or 13264 or 14111 or 14463 or 14844 or 8693 or 9145

PGP181abcpp atp_c + h2o_c + pgp181_c --> adp_c + h_c + pgp181_p + pi_c 11777 or 9854
  PCt atp_c + h2o_c + pchol_hs_c <=> adp_c + h_c + pchol_hs_e + pi_c 11777
  GCHOLAt3 atp_c + gchola_c + h2o_c --> adp_c + gchola_e + h_c + pi_c 11266 or 11777 or 13264 or 14111 or 14463 or 14844 or 8693 or 9145
  TCHOLAt3 atp_c + h2o_c + tchola_c --> adp_c + h_c + pi_c + tchola_e 11266 or 11777 or 13264 or 14111 or 14463 or 14844 or 8693 or 9145
  CHOLATEt3 atp_c + cholate_c + h2o_c --> adp_c + cholate_e + h_c + pi_c 11266 or 11777 or 13264 or 14111 or 14463 or 14844 or 8693 or 9145

PPTHpp h2o_p + ppt_p --> h2_p + pi_p 13409
  ALKP dhap_c + h2o_c --> dha_c + pi_c 13409 or 14546
  AKP1 ahdt_c + 3.0 h2o_c --> dhnpt_c + 2.0 h_c + 3.0 pi_c 13409

PSCLYSt2pp h_p + psclys_p --> h_c + psclys_c 11625
  AMY2e glygn2_e + 8.0 h2o_e --> 8.0 glc__D_e + glygn4_e 11625 and 9135
  METLEUex leu__L_c + met__L_e --> leu__L_e + met__L_c (SLC3A2 and 11625) or (Slc3a2 and 11625)
  CYSTSERex cysi__L_e + ser__L_c --> cysi__L_c + ser__L_e 11625 and 9135
  SERLYSNaex lys__L_c + na1_e + ser__L_e --> lys__L_e + na1_c + ser__L_c 11625 and 9135
  ARGLYSex arg__L_e + lys__L_c --> arg__L_c + lys__L_e (SLC3A2 and 11625) or (Slc3a2 and 11625)
  AMY1e 8.0 h2o_e + strch1_e --> 8.0 glc__D_e + strch2_e 11625 and 9135

PSP_Lpp h2o_p + pser__L_p --> pi_p + ser__L_p 13409
  ALKP dhap_c + h2o_c --> dha_c + pi_c 13409 or 14546
  AKP1 ahdt_c + 3.0 h2o_c --> dhnpt_c + 2.0 h_c + 3.0 pi_c 13409

PTHRpp h2o_p + thrp_p --> pi_p + thr__L_p 13409
  ALKP dhap_c + h2o_c --> dha_c + pi_c 13409 or 14546
  AKP1 ahdt_c + 3.0 h2o_c --> dhnpt_c + 2.0 h_c + 3.0 pi_c 13409

R5PPpp h2o_p + r5p_p --> pi_p + rib__D_p 13409
  ALKP dhap_c + h2o_c --> dha_c + pi_c 13409 or 14546
  AKP1 ahdt_c + 3.0 h2o_c --> dhnpt_c + 2.0 h_c + 3.0 pi_c 13409

SUCCt2_2pp 2.0 h_p + succ_p --> 2.0 h_c + succ_c 10658 or 13824
  ACtr ac_e <=> ac_c 10658 or 13824
  ACtr ac_e <=> ac_c 10658 or 13824

TREHpp h2o_p + tre_p --> 2.0 glc__D_p 8580
  TREH h2o_c + tre_c --> 2.0 glc__D_c 8580
  AATHA h2o_c + tre_c --> 2.0 Glc_aD_c 8580

UREAtpp urea_p <=> urea_c 15470
  UREAtm urea_c <=> urea_m 15470
  L_LACtcm lac__L_c --> lac__L_m 15470
  GLYCt glyc_c <=> glyc_e 15470

XANt2pp h_p + xan_p --> h_c + xan_c 10529
  5FLURAt 5flura_e <=> 5flura_c 10529
  TEGAt tega_e <=> tega_c 10529
  TGUAt tgua_e <=> tgua_c 10529
  6MPURt 6mpur_e <=> 6mpur_c 10529
  URATEtm h_c + urate_c <=> h_m + urate_m 10529
  URATEt_1 h_e + urate_e <=> h_c + urate_c 10529

ZN2t3pp h_p + zn2_c --> h_c + zn2_p 11397
```

In [8]:

```
Annotation.loc[temp]
```

Out[8]:

|  | Combined Annotations | Signal P | Sc288c Orthologs | Human Orthologs | Sc288 Best Hit | Human Blast | Essential | WolfPSort | C Terminal |
| --- | --- | --- | --- | --- | --- | --- | --- | --- | --- |
| RTO4\_ID |  |  |  |  |  |  |  |  |  |
| 16161 | KOG2533: Permease of the major facilitator sup... |  |  |  | TNA1 |  | Not Essential | plas 27 | YLL\* |
| 13409 | K01077: E3.1.3.1, phoA, phoB; alkaline phospha... |  | PHO8 | ALPI,ALPL,ALPP,ALPPL2 | PHO8 | ALPL | Not Essential | plas 14, nucl 4.5, cyto\_nucl 4.5, cyto 3.5, mi... | GDF\* |
| 15873 | KOG3292: Predicted membrane protein | A | MPO1 |  |  |  | Not Essential | plas 17, E.R. 4, extr 3, vacu 3 | KKA\* |
| 10658 | HMMPfam:GPR1/FUN34/yaaH family:PF01184 | A | ADY2,ATO2 |  | ATO2 |  | Not Essential | plas 25 | GDLS |
| 13824 | K07034: K07034; uncharacterized protein |  | ADY2,ATO2 |  | ADY2 |  | Not Essential | plas 25 | KKD\* |
| 10529 | K03458: TC.NCS2; nucleobase:cation symporter-2... | A |  |  |  |  | Not Essential | plas 26 | DRK\* |
| 12909 | K03457: TC.NCS1; nucleobase:cation symporter-1... | A |  |  | FUR4 |  | Not Essential | plas 25 | AEV\* |
| 13042 | K08150: SLC2A13, ITR; MFS transporter, SP fami... | A | ITR1,ITR2 | SLC2A13 | ITR2 | SLC2A | Not Essential | plas 27 | ATV\* |
| 8608 | KOG0254: Predicted transporter (major facilita... | S | ITR1,ITR2 | SLC2A13 | ITR1 | SLC2A | Not Essential | plas 20, extr 2, E.R. 2, vacu 2 | HPD\* |
| 10043 | K01424: E3.5.1.1, ansA, ansB; L-asparaginase | S | YLR155C,ASP3-2,ASP3-3,ASP3-4,ASP1 |  | ASP3-4 |  | Not Essential | extr 26 | SYY\* |
| 13627 | K01424: E3.5.1.1, ansA, ansB; L-asparaginase | S | YLR155C,ASP3-2,ASP3-3,ASP3-4,ASP1 |  | ASP3-4 |  | Not Essential | extr 24, cyto 2 | GSS\* |
| 15553 | K07300: chaA, CAX; Ca2+:H+ antiporter | S | VCX1 |  | VCX1 |  | Not Essential | plas 13, extr 6, vacu 3, mito 2, E.R. 2 | FTG\* |
| 11397 | K14688: SLC30A1, ZNT1; solute carrier family 3... | S | ZRC1,COT1 | SLC30A1,SLC30A10 | ZRC1 | SLC30A | Not Essential | extr 21, plas 4 | RKR\* |
| 14836 | K05012: CLCN3\_4\_5; chloride channel 3/4/5 |  | GEF1 | CLCN3,CLCN4,CLCN5,CLCN7 | GEF1 | CLCN5 | Not Essential | plas 22, E.R. 3 | ARR\* |
| 11777 | K05658: ABCB1, CD243; ATP-binding cassette, su... | S | STE6 | ABCB1,ABCB11,ABCB4,ABCB5 | STE6 | ABCB1 | Not Essential | plas 11, E.R. 7, extr 5, mito 3 | KAH\* |
| 9854 | K11085: msbA; ATP-binding cassette, subfamily ... |  |  |  | STE6 | ABCB1 | Not Essential | plas 20, mito 3, E.R. 3 | ETS\* |
| 15100 | KOG1530: Rhodanese-related sulfurtransferase | S | RDL1,RDL2 | TSTD1,TSTD3 | RDL2 | TSTD1 | Essential | mito 20, cyto 4, extr 2, cyto\_pero 2 | GDD\* |
| 15928 | K12346: SMF; metal iron transporter |  | SMF1,SMF2,SMF3 | SLC11A1,SLC11A2 | SMF1 | SLC11 | Unclear (ambiguous TDNA mapping) | plas 22, nucl 2, E.R. 2 | QGA\* |
| 15934 | K12347: SLC11A, NRAMP; natural resistance-asso... |  | SMF1,SMF2,SMF3 | SLC11A1,SLC11A2 | SMF2 | SLC11 | Unclear (ambiguous TDNA mapping) | plas 20, E.R. 3, nucl 2 | CSR\* |
| 11625 | K13869: SLC7A11; solute carrier family 7 (L-ty... |  |  | SLC7A6,SLC7A7,SLC7A9,SLC7A5 | MUP1 | SLC7A | Not Essential | plas 27 | EMT\* |
| 10339 | K02429: fucP; MFS transporter, FHS family, L-f... | S |  |  |  |  | Not Essential | plas 15, E.R. 7, mito 4 | SRV\* |
| 9978 | HMMPfam:CrcB-like protein:PF02537 |  | FEX1,FEX2 |  | FEX1 |  | Not Essential | plas 26 | GAA\* |
| 14480 | K01785: galM, GALM; aldose 1-epimerase | A |  | GALM | GAL10 | GALM | Not Essential | mito 11, extr 4, nucl 3, golg 3, plas 2, pero ... | QLD\* |
| 15470 | K03441: GLP-F; aquaglyceroporin related protei... |  | YFL054C,FPS1 | AQP3,AQP9 | YFL054C | AQP3 | Not Essential | plas 17, nucl 6, mito 1, cyto 1, E.R. 1, vacu ... | WRQ\* |
| 9838 | K18592: GGT1\_5, CD224; gamma-glutamyltranspept... |  | ECM38 | GGT1,GGT2 | ECM38 | GGT1 | Not Essential | mito 15, cyto 4.5, cyto\_nucl 3.5, extr 3, pero 2 | ILE\* |
| 15807 | K20989: DUR3; urea-proton symporter | A | DUR3 |  | DUR3 |  | Not Essential | plas 21, vacu 3, mito 2 | GKK\* |
| 9810 | K03549: kup; KUP system potassium uptake protein |  |  |  |  |  | Not Essential | plas 26 | ASL\* |
| 16716 | K05349: bglX; beta-glucosidase | S |  |  |  |  | Not Essential | extr 26 | FSY\* |
| 8528 | K05349: bglX; beta-glucosidase |  |  |  |  |  | Not Essential | cyto 16.5, cyto\_nucl 9, mito 6, extr 3 | FVY\* |
| 16717 | K05349: bglX; beta-glucosidase | S |  |  |  |  | Not Essential | extr 24, cyto 2 | FSY\* |
| 9322 | K16261: YAT; yeast amino acid transporter | A | DIP5 |  | DIP5 |  | Not Essential | plas 27 | WLM\* |
| 9319 | K16261: YAT; yeast amino acid transporter | A | DIP5 |  | DIP5 |  | Not Essential | plas 21, mito 2, vacu 2 | WLM\* |
| 8962 | K16261: YAT; yeast amino acid transporter |  | DIP5 |  | DIP5 |  | Not Essential | plas 27 | AIM\* |
| 13295 | HMMPfam:Beta-lactamase:PF00144,SUPERFAMILY::SS... | S |  |  |  |  | Not Essential | mito 9, cyto 8.5, cyto\_nucl 6, pero 4, nucl 2.5 | AKV\* |
| 12994 | HMMPfam:Beta-lactamase:PF00144,SUPERFAMILY::SS... |  |  |  |  |  | Not Essential | cyto 13, pero 5, cysk 5, nucl 3 | RAE\* |
| 15126 | HMMPfam:Beta-lactamase:PF00144,SUPERFAMILY::SS... |  |  |  |  |  | Not Essential | cyto 11, mito 5, E.R. 5, pero 2, nucl 1, plas ... | GLA\* |
| 15127 | HMMPfam:Beta-lactamase:PF00144,SUPERFAMILY::SS... |  |  |  |  |  | Not Essential | cyto 12, extr 5, E.R. 4, mito 2, pero 2, golg 2 | LKE\* |
| 13637 | K03320: amt, AMT, MEP; ammonium transporter, A... | S | MEP1,MEP3 |  | MEP2 |  | Not Essential | plas 19, E.R. 4, mito 3 | FRG\* |
| 12926 | K03320: amt, AMT, MEP; ammonium transporter, A... |  | MEP2 |  | MEP2 |  | Not Essential | plas 25 | AAV\* |
| 8580 | K01194: TREH, treA, treF; alpha,alpha-trehalase |  | NTH2,NTH1 |  | NTH1 | TREH | Not Essential | cyto 10, cyto\_nucl 9.833, nucl 8.5, cyto\_mito ... | HQQ\* |

In [9]:

```
temp = list()
for r in sorted(model.reactions, key=lambda x: x.id):
    if 'h' in r.compartments:
        print(r.id, r.reaction, r.gene_reaction_rule)
        for g in r.genes:
            if g.id in Annotation.index and g.id not in temp:
                temp.append(g.id)
            for r2 in g.reactions:
                if r2 is not r and 'h' not in r2.compartments:
                    print(' ',r2.id, r2.reaction, r2.gene_reaction_rule)
        print()
```

```
4ABUTthi 4abut_c + h_c --> 4abut_h + h_h 11269 or 13423
  4ABUTtmi 4abut_c + h_c --> 4abut_m + h_m 11269 or 13423
  5AOPt2 5aop_e + h_e --> 5aop_c + h_c 11269 or 13423
  SPMDtex2 spmd_c --> spmd_e 13423 or 15991 or 16258
  ABUTt2r 4abut_e + h_e <=> 4abut_c + h_c 11269 or 12743 or 13423 or (YALI0B09537g and 11269) or (YALI0B09537g and 13423)
  PTRCtex2 ptrc_c --> ptrc_e 13423 or 15991 or 16258
  5AOPt2 5aop_e + h_e --> 5aop_c + h_c 11269 or 13423
  4ABUTtmi 4abut_c + h_c --> 4abut_m + h_m 11269 or 13423
  ABUTt2r 4abut_e + h_e <=> 4abut_c + h_c 11269 or 12743 or 13423 or (YALI0B09537g and 11269) or (YALI0B09537g and 13423)

AASPh aps_h + atp_h --> adp_h + h_h + paps_h 8709
  SELADT atp_c + h_c + sel_c --> adsel_c + ppi_c 8709
  SADT atp_c + h_c + so4_c --> aps_c + ppi_c 13443 or 8709 or (PP_1303 and 14868)
  AASPm aps_m + atp_m --> adp_m + h_m + paps_m 8709
  ADSK aps_c + atp_c --> adp_c + h_c + paps_c 8709
  ADSELK adsel_c + atp_c --> 3padsel_c + adp_c + h_c 8709

AATC Lcyst_h + akg_h <=> 3spyr_h + glu__L_h 14281
  PHETA1 akg_c + phe__L_c <=> glu__L_c + phpyr_c 12407 or 13230 or 14281 or 14610 or 14853 or 14908 or 15839 or 8936
  ASPTAm akg_m + asp__L_m <=> glu__L_m + oaa_m 14281
  yli_R0097 3sala_c + akg_c + h_c --> glu__L_c + yli_M02657_c 14281 or 8936
  ASPTA akg_c + asp__L_c <=> glu__L_c + oaa_c 14281 or 8936
  yli_R1493 Lcyst_c + akg_c <=> glu__L_c + yli_M02657_c 14281 or 8936
  CYSTAm akg_m + cys__L_m <=> glu__L_m + mercppyr_m 14281
  UNK3 2kmb_c + glu__L_c --> akg_c + met__L_c 12407 or 14281 or 14908 or 15839 or 8936
  CYSATm glu__L_m + mercppyr_m --> akg_m + cys__L_m 14281
  TYRTAi 34hpp_c + glu__L_c --> akg_c + tyr__L_c 12407 or 13230 or 14281 or 14908 or 15839 or 8936
  CYSTA akg_c + cys__L_c --> glu__L_c + mercppyr_c 14281 or 8936
  EHGLAT2m e4hglu_m + oaa_m --> 4h2oglt_m + asp__L_m 14281
  PHETA1m akg_m + phe__L_m <=> glu__L_m + phpyr_m 12407 or 13230 or 14281 or 14908 or 15839 or 16065
  EHGLATm akg_m + e4hglu_m --> 4h2oglt_m + glu__L_m 14281
  TYRTAim 34hpp_m + glu__L_m --> akg_m + tyr__L_m 12407 or 13230 or 14281 or 14908 or 15839 or 16065
  LCYSTATm Lcyst_m + akg_m <=> 3spyr_m + glu__L_m 14281
  TYRTA akg_c + tyr__L_c <=> 34hpp_c + glu__L_c 12407 or 13230 or 14281 or 14610 or 14853 or 14908 or 15839 or 8936
  3SALATAim 3sala_m + akg_m + h_m --> 3snpyr_m + glu__L_m 14281
  AATGm 3sala_m + akg_m --> 3snpyr_m + glu__L_m 14281

AATG 3sala_h + akg_h --> 3snpyr_h + glu__L_h 14281
  PHETA1 akg_c + phe__L_c <=> glu__L_c + phpyr_c 12407 or 13230 or 14281 or 14610 or 14853 or 14908 or 15839 or 8936
  ASPTAm akg_m + asp__L_m <=> glu__L_m + oaa_m 14281
  yli_R0097 3sala_c + akg_c + h_c --> glu__L_c + yli_M02657_c 14281 or 8936
  ASPTA akg_c + asp__L_c <=> glu__L_c + oaa_c 14281 or 8936
  yli_R1493 Lcyst_c + akg_c <=> glu__L_c + yli_M02657_c 14281 or 8936
  CYSTAm akg_m + cys__L_m <=> glu__L_m + mercppyr_m 14281
  UNK3 2kmb_c + glu__L_c --> akg_c + met__L_c 12407 or 14281 or 14908 or 15839 or 8936
  CYSATm glu__L_m + mercppyr_m --> akg_m + cys__L_m 14281
  TYRTAi 34hpp_c + glu__L_c --> akg_c + tyr__L_c 12407 or 13230 or 14281 or 14908 or 15839 or 8936
  CYSTA akg_c + cys__L_c --> glu__L_c + mercppyr_c 14281 or 8936
  EHGLAT2m e4hglu_m + oaa_m --> 4h2oglt_m + asp__L_m 14281
  PHETA1m akg_m + phe__L_m <=> glu__L_m + phpyr_m 12407 or 13230 or 14281 or 14908 or 15839 or 16065
  EHGLATm akg_m + e4hglu_m --> 4h2oglt_m + glu__L_m 14281
  TYRTAim 34hpp_m + glu__L_m --> akg_m + tyr__L_m 12407 or 13230 or 14281 or 14908 or 15839 or 16065
  LCYSTATm Lcyst_m + akg_m <=> 3spyr_m + glu__L_m 14281
  TYRTA akg_c + tyr__L_c <=> 34hpp_c + glu__L_c 12407 or 13230 or 14281 or 14610 or 14853 or 14908 or 15839 or 8936
  3SALATAim 3sala_m + akg_m + h_m --> 3snpyr_m + glu__L_m 14281
  AATGm 3sala_m + akg_m --> 3snpyr_m + glu__L_m 14281

ACCOAth accoa_c + coa_h <=> accoa_h + coa_c 10946
  GAO1 accoa_c + gd3_hs_c --> coa_c + oagd3_hs_c 10946
  GAO2 accoa_c + gt3_hs_c --> coa_c + oagt3_hs_c 10946
  ACCOAtm accoa_c + coa_m <=> accoa_m + coa_c 10946
  ACCOAtx accoa_c + coa_x <=> accoa_x + coa_c 10946
  ACCOAgt accoa_c <=> accoa_g 10946
  GAO2g accoa_g + gt3_hs_g --> coa_g + oagt3_hs_g 10946
  GAO1g accoa_g + gd3_hs_g --> coa_g + oagd3_hs_g 10946
  ACCOAtr accoa_c <=> accoa_r 10946

ACKrh ac_h + atp_h <=> actp_h + adp_h 13383
  APPTm atp_m + ppa_m <=> adp_m + ppap_m 13383
  ACKr ac_c + atp_c <=> actp_c + adp_c 13383
  ACKrm ac_m + atp_m <=> actp_m + adp_m 13383

ADSSh asp__L_h + gtp_h + imp_h --> dcamp_h + gdp_h + 3.0 h_h + pi_h 12170
  ADSS asp__L_c + gtp_c + imp_c --> dcamp_c + gdp_c + 2.0 h_c + pi_c 12170

AMETt2h ahcys_c + amet_h <=> ahcys_h + amet_c 12032
  AMETt2m ahcys_m + amet_c <=> ahcys_c + amet_m 12032
  AMETtm amet_c <=> amet_m 12032

ANTPPT anth_h + prpp_h --> h_h + ppi_h + pran_h 9900
  ANPRT anth_c + prpp_c --> ppi_c + pran_c 9900
  ANS chor_c + gln__L_c --> anth_c + glu__L_c + h_c + pyr_c 15109 or 16564 or (15109 and 16564) or (15109 and 9900)

AOOAh akg_h + hisp_h <=> glu__L_h + imacp_h 13230
  TYRTAim 34hpp_m + glu__L_m --> akg_m + tyr__L_m 12407 or 13230 or 14281 or 14908 or 15839 or 16065
  PHETA1m akg_m + phe__L_m <=> glu__L_m + phpyr_m 12407 or 13230 or 14281 or 14908 or 15839 or 16065
  PHETA1 akg_c + phe__L_c <=> glu__L_c + phpyr_c 12407 or 13230 or 14281 or 14610 or 14853 or 14908 or 15839 or 8936
  TYRTA akg_c + tyr__L_c <=> 34hpp_c + glu__L_c 12407 or 13230 or 14281 or 14610 or 14853 or 14908 or 15839 or 8936
  TYRTAi 34hpp_c + glu__L_c --> akg_c + tyr__L_c 12407 or 13230 or 14281 or 14908 or 15839 or 8936
  HSTPT glu__L_c + imacp_c --> akg_c + hisp_c 13230

APPTh atp_h + ppa_h <=> adp_h + ppap_h 13383
  APPTm atp_m + ppa_m <=> adp_m + ppap_m 13383
  ACKr ac_c + atp_c <=> actp_c + adp_c 13383
  ACKrm ac_m + atp_m <=> actp_m + adp_m 13383

ASADH 4pasp_h + h_h + nadph_h --> aspsa_h + nadp_h + pi_h 10330
  ASAD aspsa_c + nadp_c + pi_c <=> 4pasp_c + h_c + nadph_c 10330

ASPATh akg_h + asp__L_h <=> glu__L_h + oaa_h 14281
  PHETA1 akg_c + phe__L_c <=> glu__L_c + phpyr_c 12407 or 13230 or 14281 or 14610 or 14853 or 14908 or 15839 or 8936
  ASPTAm akg_m + asp__L_m <=> glu__L_m + oaa_m 14281
  yli_R0097 3sala_c + akg_c + h_c --> glu__L_c + yli_M02657_c 14281 or 8936
  ASPTA akg_c + asp__L_c <=> glu__L_c + oaa_c 14281 or 8936
  yli_R1493 Lcyst_c + akg_c <=> glu__L_c + yli_M02657_c 14281 or 8936
  CYSTAm akg_m + cys__L_m <=> glu__L_m + mercppyr_m 14281
  UNK3 2kmb_c + glu__L_c --> akg_c + met__L_c 12407 or 14281 or 14908 or 15839 or 8936
  CYSATm glu__L_m + mercppyr_m --> akg_m + cys__L_m 14281
  TYRTAi 34hpp_c + glu__L_c --> akg_c + tyr__L_c 12407 or 13230 or 14281 or 14908 or 15839 or 8936
  CYSTA akg_c + cys__L_c --> glu__L_c + mercppyr_c 14281 or 8936
  EHGLAT2m e4hglu_m + oaa_m --> 4h2oglt_m + asp__L_m 14281
  PHETA1m akg_m + phe__L_m <=> glu__L_m + phpyr_m 12407 or 13230 or 14281 or 14908 or 15839 or 16065
  EHGLATm akg_m + e4hglu_m --> 4h2oglt_m + glu__L_m 14281
  TYRTAim 34hpp_m + glu__L_m --> akg_m + tyr__L_m 12407 or 13230 or 14281 or 14908 or 15839 or 16065
  LCYSTATm Lcyst_m + akg_m <=> 3spyr_m + glu__L_m 14281
  TYRTA akg_c + tyr__L_c <=> 34hpp_c + glu__L_c 12407 or 13230 or 14281 or 14610 or 14853 or 14908 or 15839 or 8936
  3SALATAim 3sala_m + akg_m + h_m --> 3snpyr_m + glu__L_m 14281
  AATGm 3sala_m + akg_m --> 3snpyr_m + glu__L_m 14281

ASPth asp__L_c + h_c <=> asp__L_h + h_h 8766
  ASPt7 asp__L_v + h_v --> asp__L_c + h_c 8766
  ASPt2m asp__L_c + h_c <=> asp__L_m + h_m 8766
  GLUt7 glu__L_v + h_v --> glu__L_c + h_c 8766
  GLUt2m glu__L_c + h_c <=> glu__L_m + h_m 16799 or 8766
  ASPtx asp__L_x + h_x <=> asp__L_c + h_c 8766

ATNS chor_h + gln__L_h --> anth_h + glu__L_h + h_h + pyr_h 16564
  IGPS 2cpr5p_c + h_c --> 3ig3p_c + co2_c + h2o_c 16564
  PRAIi pran_c --> 2cpr5p_c 16564
  ANS chor_c + gln__L_c --> anth_c + glu__L_c + h_c + pyr_c 15109 or 16564 or (15109 and 16564) or (15109 and 9900)
  ADCS chor_c + gln__L_c --> 4adcho_c + glu__L_c 13669 or (13669 and 16564)

ATNS_nh4 chor_h + nh4_h --> anth_h + h2o_h + h_h + pyr_h 16564
  IGPS 2cpr5p_c + h_c --> 3ig3p_c + co2_c + h2o_c 16564
  PRAIi pran_c --> 2cpr5p_c 16564
  ANS chor_c + gln__L_c --> anth_c + glu__L_c + h_c + pyr_c 15109 or 16564 or (15109 and 16564) or (15109 and 9900)
  ADCS chor_c + gln__L_c --> 4adcho_c + glu__L_c 13669 or (13669 and 16564)

CHRM chor_h <=> pphn_h 9704
  CHORM chor_c --> pphn_c 14195 or 16225 or 9704

CHRS 3psme_h --> chor_h + pi_h 14037
  CHORS 3psme_c --> chor_c + pi_c 14037

CPPPGO_1 cpppg3_h + 2.0 h_h + 2.0 o2_h --> 2.0 co2_h + 2.0 h2o2_h + pppg9_h 14197
  CPPPGO cpppg3_c + 2.0 h_c + o2_c --> 2.0 co2_c + 2.0 h2o_c + pppg9_c 14197

CYSAT glu__L_h + mercppyr_h --> akg_h + cys__L_h 14281
  PHETA1 akg_c + phe__L_c <=> glu__L_c + phpyr_c 12407 or 13230 or 14281 or 14610 or 14853 or 14908 or 15839 or 8936
  ASPTAm akg_m + asp__L_m <=> glu__L_m + oaa_m 14281
  yli_R0097 3sala_c + akg_c + h_c --> glu__L_c + yli_M02657_c 14281 or 8936
  ASPTA akg_c + asp__L_c <=> glu__L_c + oaa_c 14281 or 8936
  yli_R1493 Lcyst_c + akg_c <=> glu__L_c + yli_M02657_c 14281 or 8936
  CYSTAm akg_m + cys__L_m <=> glu__L_m + mercppyr_m 14281
  UNK3 2kmb_c + glu__L_c --> akg_c + met__L_c 12407 or 14281 or 14908 or 15839 or 8936
  CYSATm glu__L_m + mercppyr_m --> akg_m + cys__L_m 14281
  TYRTAi 34hpp_c + glu__L_c --> akg_c + tyr__L_c 12407 or 13230 or 14281 or 14908 or 15839 or 8936
  CYSTA akg_c + cys__L_c --> glu__L_c + mercppyr_c 14281 or 8936
  EHGLAT2m e4hglu_m + oaa_m --> 4h2oglt_m + asp__L_m 14281
  PHETA1m akg_m + phe__L_m <=> glu__L_m + phpyr_m 12407 or 13230 or 14281 or 14908 or 15839 or 16065
  EHGLATm akg_m + e4hglu_m --> 4h2oglt_m + glu__L_m 14281
  TYRTAim 34hpp_m + glu__L_m --> akg_m + tyr__L_m 12407 or 13230 or 14281 or 14908 or 15839 or 16065
  LCYSTATm Lcyst_m + akg_m <=> 3spyr_m + glu__L_m 14281
  TYRTA akg_c + tyr__L_c <=> 34hpp_c + glu__L_c 12407 or 13230 or 14281 or 14610 or 14853 or 14908 or 15839 or 8936
  3SALATAim 3sala_m + akg_m + h_m --> 3snpyr_m + glu__L_m 14281
  AATGm 3sala_m + akg_m --> 3snpyr_m + glu__L_m 14281

DHPAH 25dhpp_h + h2o_h --> 5apru_h + nh4_h 16837
  APRAUR 5apru_c + h_c + nadph_c --> 5aprbu_c + nadp_c 16837
  yli_R0683 25drapp_c + 3.0 h_c + nadh_c --> nad_c + yli_M04300_c 16837
  DHPPDA2 25drapp_c + h2o_c + h_c --> 5apru_c + nh4_c 16837
  DHPPDA 25dhpp_c + h2o_c + h_c --> 5apru_c + nh4_c 16837
  DROPPRy 25dhpp_c + h_c + nadph_c --> 25dthpp_c + nadp_c 16837
  yli_R0684 25drapp_c + 3.0 h_c + nadph_c --> nadp_c + yli_M04300_c 16837

DHQS_1 2dda7p_h --> 3dhq_h + pi_h 14252
  SHK3Dr 3dhsk_c + h_c + nadph_c <=> nadp_c + skm_c 14252
  PPND nad_c + pphn_c --> 34hpp_c + co2_c + nadh_c 14252 or 16225
  DHQS 2dda7p_c --> 3dhq_c + pi_c 14252
  PSCVT pep_c + skm5p_c <=> 3psme_c + pi_c 14252
  DHQTi 3dhq_c --> 3dhsk_c + h2o_c 10572 or 14252
  SHKK atp_c + skm_c --> adp_c + h_c + skm5p_c 13706 or 14252

DLDLBT 4r5au_h + db4p_h --> dmlz_h + 2.0 h2o_h + h_h + pi_h 9278
  RBFSa 4r5au_c + db4p_c --> dmlz_c + 2.0 h2o_c + pi_c 9278

DMORh alac__S_h + h_h + nadph_h --> 23dhmb_h + nadp_h 9176
  KARA2 2ahbut_c + h_c + nadph_c <=> 23dhmp_c + nadp_c 9176
  KARA1 23dhmb_c + nadp_c <=> alac__S_c + h_c + nadph_c 9176
  DPR 2dhp_c + h_c + nadph_c --> nadp_c + pant__R_c 14277 or 16522 or 9176
  KARA1im alac__S_m + h_m + nadph_m --> 23dhmb_m + nadp_m 9176
  DPRm 2dhp_m + h_m + nadph_m --> nadp_m + pant__R_m 14277 or 9176
  yli_R1589 alac__S_m <=> 3hmoa_m 9176
  KARA2im 2ahbut_m + h_m + nadph_m --> 23dhmp_m + nadp_m 9176

DNGALh atp_h + dnad_h + gln__L_h + h2o_h --> amp_h + glu__L_h + 2.0 h_h + nad_h + ppi_h 9283
  NADS2 atp_c + dnad_c + gln__L_c + h2o_c --> amp_c + glu__L_c + h_c + nad_c + ppi_c 9283
  NADS1n atp_n + dnad_n + nh4_n --> amp_n + h_n + nad_n + ppi_n 9283
  NADS1 atp_c + dnad_c + nh4_c --> amp_c + h_c + nad_c + ppi_c 9283

DPHS e4p_h + h2o_h + pep_h --> 2dda7p_h + pi_h 15534
  DDPA e4p_c + h2o_c + pep_c --> 2dda7p_c + pi_c 10602 or 12155 or 15534

G3PL 3ig3p_h --> g3p_h + indole_h CRv4_Au5_s12_g3638_t1 and 9262
  TRPS3 3ig3p_c --> g3p_c + indole_c b1260 and 9262
  TRPS1 3ig3p_c + ser__L_c --> g3p_c + h2o_c + trp__L_c 9262 or (PP_0082 and 9262) or (b1260 and 9262)
  TRPS2 indole_c + ser__L_c --> h2o_c + trp__L_c 9262 or (b1260 and 9262)

G6PIh g6p_A_h <=> g6p_B_h 9589
  PGI g6p_c <=> f6p_c 9589

GLUth glu__L_h + h_h --> glu__L_c + h_c 8766
  ASPt7 asp__L_v + h_v --> asp__L_c + h_c 8766
  ASPt2m asp__L_c + h_c <=> asp__L_m + h_m 8766
  GLUt7 glu__L_v + h_v --> glu__L_c + h_c 8766
  GLUt2m glu__L_c + h_c <=> glu__L_m + h_m 16799 or 8766
  ASPtx asp__L_x + h_x <=> asp__L_c + h_c 8766

GLYCth glyc_c + h_c <=> glyc_h + h_h 12792

GLYPT atp_h + glyc__R_h --> 3pg_h + adp_h + h_h 10038
  GLYCK atp_c + glyc__R_c --> 3pg_c + adp_c + h_c 10038
  yli_R1507 atp_c + glyc__R_c --> 3pg_c + adp_c 10038

GTHAMPORh amp_h + gthox_h + h_h + so3_h <=> aps_h + 2.0 gthrd_h 11741
  GTHAMPORm amp_m + gthox_m + h_m + so3_m <=> aps_m + 2.0 gthrd_m 11741
  PAPSR paps_c + trdrd_c --> 2.0 h_c + pap_c + so3_c + trdox_c (10848 and 11741) or (11741 and 12730) or (11741 and 12737) or (11741 and 15339)
  APSR aps_c + trdrd_c --> amp_c + 2.0 h_c + so3_c + trdox_c 11741
  GTHAMPOR amp_c + gthox_c + h_c + so3_c <=> aps_c + 2.0 gthrd_c 11741

GTHS_1 atp_h + glucys_h + gly_h --> adp_h + gthrd_h + 2.0 h_h + pi_h 9929
  GTHS atp_c + glucys_c + gly_c --> adp_c + gthrd_c + h_c + pi_c 9929

GTPDH gtp_h + 3.0 h2o_h --> 25dhpp_h + for_h + 2.0 h_h + ppi_h 12551 or 16666
  GTPCII gtp_c + 3.0 h2o_c --> 25dhpp_c + for_c + 2.0 h_c + ppi_c 12551 or 16666
  GTPCII2 gtp_c + 3.0 h2o_c --> 25drapp_c + for_c + 2.0 h_c + ppi_c 12551
  GTPCII gtp_c + 3.0 h2o_c --> 25dhpp_c + for_c + 2.0 h_c + ppi_c 12551 or 16666
  DB4PS ru5p__D_c --> db4p_c + for_c + h_c 16666

H2Oth h2o_c <=> h2o_h 13986
  H2Ot h2o_e <=> h2o_c 13986 or 13987 or 9014 or 9015
  H2Otp h2o_c <=> h2o_x 13986
  H2Otf h2o_c <=> h2o_f 13986
  H2Otm h2o_c <=> h2o_m 13986 or 13987 or 9014 or 9015

IDPh h2o_h + ppi_h --> 2.0 pi_h 15879
  PPA h2o_c + ppi_c --> h_c + 2.0 pi_c 15879 or 16648
  IDPm h2o_m + ppi_m --> 2.0 pi_m 15879
  PPAm h2o_m + ppi_m --> h_m + 2.0 pi_m 15879

IGPS_1 2cpr5p_h + h_h --> 3ig3p_h + co2_h + h2o_h 16564
  IGPS 2cpr5p_c + h_c --> 3ig3p_c + co2_c + h2o_c 16564
  PRAIi pran_c --> 2cpr5p_c 16564
  ANS chor_c + gln__L_c --> anth_c + glu__L_c + h_c + pyr_c 15109 or 16564 or (15109 and 16564) or (15109 and 9900)
  ADCS chor_c + gln__L_c --> 4adcho_c + glu__L_c 13669 or (13669 and 16564)

IMGPSh gln__L_h + prlp_h --> aicar_h + eig3p_h + glu__L_h + h_h 9084
  IG3PS gln__L_c + prlp_c --> aicar_c + eig3p_c + glu__L_c + h_c 9084
  PRMICI prfp_c <=> prlp_c 11610 or 9084

IPPSh 3mob_h + accoa_h + h2o_h --> 3c3hmp_h + coa_h + h_h 14856
  IPPS 3mob_c + accoa_c + h2o_c --> 3c3hmp_c + coa_c + h_c 14856 or 15488
  yli_R0419 accoa_m + akg_m + h2o_m --> coa_m + h_m + yli_M01059_m 14856
  HCITSn accoa_n + akg_n + h2o_n --> coa_n + h_n + hcit_n 14856

KARI 2ahbut_h <=> 3hmop_h 9176
  KARA2 2ahbut_c + h_c + nadph_c <=> 23dhmp_c + nadp_c 9176
  KARA1 23dhmb_c + nadp_c <=> alac__S_c + h_c + nadph_c 9176
  DPR 2dhp_c + h_c + nadph_c --> nadp_c + pant__R_c 14277 or 16522 or 9176
  KARA1im alac__S_m + h_m + nadph_m --> 23dhmb_m + nadp_m 9176
  DPRm 2dhp_m + h_m + nadph_m --> nadp_m + pant__R_m 14277 or 9176
  yli_R1589 alac__S_m <=> 3hmoa_m 9176
  KARA2im 2ahbut_m + h_m + nadph_m --> 23dhmp_m + nadp_m 9176

KARI_23dhmb 3hmoa_h + h_h + nadph_h --> 23dhmb_h + nadp_h 9176
  KARA2 2ahbut_c + h_c + nadph_c <=> 23dhmp_c + nadp_c 9176
  KARA1 23dhmb_c + nadp_c <=> alac__S_c + h_c + nadph_c 9176
  DPR 2dhp_c + h_c + nadph_c --> nadp_c + pant__R_c 14277 or 16522 or 9176
  KARA1im alac__S_m + h_m + nadph_m --> 23dhmb_m + nadp_m 9176
  DPRm 2dhp_m + h_m + nadph_m --> nadp_m + pant__R_m 14277 or 9176
  yli_R1589 alac__S_m <=> 3hmoa_m 9176
  KARA2im 2ahbut_m + h_m + nadph_m --> 23dhmp_m + nadp_m 9176

KARI_23dhmp 3hmop_h + h_h + nadph_h --> 23dhmp_h + nadp_h 9176
  KARA2 2ahbut_c + h_c + nadph_c <=> 23dhmp_c + nadp_c 9176
  KARA1 23dhmb_c + nadp_c <=> alac__S_c + h_c + nadph_c 9176
  DPR 2dhp_c + h_c + nadph_c --> nadp_c + pant__R_c 14277 or 16522 or 9176
  KARA1im alac__S_m + h_m + nadph_m --> 23dhmb_m + nadp_m 9176
  DPRm 2dhp_m + h_m + nadph_m --> nadp_m + pant__R_m 14277 or 9176
  yli_R1589 alac__S_m <=> 3hmoa_m 9176
  KARA2im 2ahbut_m + h_m + nadph_m --> 23dhmp_m + nadp_m 9176

KARI_3hmoa alac__S_h <=> 3hmoa_h 9176
  KARA2 2ahbut_c + h_c + nadph_c <=> 23dhmp_c + nadp_c 9176
  KARA1 23dhmb_c + nadp_c <=> alac__S_c + h_c + nadph_c 9176
  DPR 2dhp_c + h_c + nadph_c --> nadp_c + pant__R_c 14277 or 16522 or 9176
  KARA1im alac__S_m + h_m + nadph_m --> 23dhmb_m + nadp_m 9176
  DPRm 2dhp_m + h_m + nadph_m --> nadp_m + pant__R_m 14277 or 9176
  yli_R1589 alac__S_m <=> 3hmoa_m 9176
  KARA2im 2ahbut_m + h_m + nadph_m --> 23dhmp_m + nadp_m 9176

NA1Hth h_h + na1_c <=> h_c + na1_h 10469
  NA1Htm h_m + na1_c <=> h_c + na1_m 10469
  Kt3g h_g + k_c <=> h_c + k_g 10469
  NAt3_1g h_g + na1_c <=> h_c + na1_g 10469

NA1th na1_c <=> na1_h 15860
  NA1tm na1_c <=> na1_m 15860

OCTh cbp_h + orn_h <=> citr__L_h + pi_h 13302
  OCTm cbp_m + orn_m <=> citr__L_m + pi_m 13302
  OCT cbp_c + orn_c <=> citr__L_c + pi_c 13302
  OCBT cbp_c + orn_c <=> citr__L_c + h_c + pi_c 13302
  OCBTm cbp_m + orn_m --> citr__L_m + h_m + pi_m 13302

ORDCh h_h + orn_h --> co2_h + ptrc_h 12635
  ORNDC h_c + orn_c --> co2_c + ptrc_c 12635

PGIAh g6p_A_h <=> f6p_B_h 9589
  PGI g6p_c <=> f6p_c 9589

PGIBh g6p_B_h <=> f6p_B_h 9589
  PGI g6p_c <=> f6p_c 9589

PGMTh g1p_h <=> g6p_A_h 9959
  PPM r1p_c <=> r5p_c 13711 or 9959
  PGMT g1p_c <=> g6p_c 13711 or 14546 or 8460 or 9959
  yli_R0552 r1p_c <=> yli_M00116_c 13711 or 9959

POATh glu__L_h + phpyr_h <=> akg_h + phe__L_h 13230 or 14281
  PHETA1 akg_c + phe__L_c <=> glu__L_c + phpyr_c 12407 or 13230 or 14281 or 14610 or 14853 or 14908 or 15839 or 8936
  ASPTAm akg_m + asp__L_m <=> glu__L_m + oaa_m 14281
  yli_R0097 3sala_c + akg_c + h_c --> glu__L_c + yli_M02657_c 14281 or 8936
  ASPTA akg_c + asp__L_c <=> glu__L_c + oaa_c 14281 or 8936
  yli_R1493 Lcyst_c + akg_c <=> glu__L_c + yli_M02657_c 14281 or 8936
  CYSTAm akg_m + cys__L_m <=> glu__L_m + mercppyr_m 14281
  UNK3 2kmb_c + glu__L_c --> akg_c + met__L_c 12407 or 14281 or 14908 or 15839 or 8936
  CYSATm glu__L_m + mercppyr_m --> akg_m + cys__L_m 14281
  TYRTAi 34hpp_c + glu__L_c --> akg_c + tyr__L_c 12407 or 13230 or 14281 or 14908 or 15839 or 8936
  CYSTA akg_c + cys__L_c --> glu__L_c + mercppyr_c 14281 or 8936
  EHGLAT2m e4hglu_m + oaa_m --> 4h2oglt_m + asp__L_m 14281
  PHETA1m akg_m + phe__L_m <=> glu__L_m + phpyr_m 12407 or 13230 or 14281 or 14908 or 15839 or 16065
  EHGLATm akg_m + e4hglu_m --> 4h2oglt_m + glu__L_m 14281
  TYRTAim 34hpp_m + glu__L_m --> akg_m + tyr__L_m 12407 or 13230 or 14281 or 14908 or 15839 or 16065
  LCYSTATm Lcyst_m + akg_m <=> 3spyr_m + glu__L_m 14281
  TYRTA akg_c + tyr__L_c <=> 34hpp_c + glu__L_c 12407 or 13230 or 14281 or 14610 or 14853 or 14908 or 15839 or 8936
  3SALATAim 3sala_m + akg_m + h_m --> 3snpyr_m + glu__L_m 14281
  AATGm 3sala_m + akg_m --> 3snpyr_m + glu__L_m 14281
  TYRTAim 34hpp_m + glu__L_m --> akg_m + tyr__L_m 12407 or 13230 or 14281 or 14908 or 15839 or 16065
  PHETA1m akg_m + phe__L_m <=> glu__L_m + phpyr_m 12407 or 13230 or 14281 or 14908 or 15839 or 16065
  PHETA1 akg_c + phe__L_c <=> glu__L_c + phpyr_c 12407 or 13230 or 14281 or 14610 or 14853 or 14908 or 15839 or 8936
  TYRTA akg_c + tyr__L_c <=> 34hpp_c + glu__L_c 12407 or 13230 or 14281 or 14610 or 14853 or 14908 or 15839 or 8936
  TYRTAi 34hpp_c + glu__L_c --> akg_c + tyr__L_c 12407 or 13230 or 14281 or 14908 or 15839 or 8936
  HSTPT glu__L_c + imacp_c --> akg_c + hisp_c 13230

PPBNGD h2o_h + 4.0 ppbng_h <=> hmbil_h + 4.0 nh4_h 14718
  HMBS h2o_c + 4.0 ppbng_c --> hmbil_c + 4.0 nh4_c 14718

PPBNGS_1 2.0 5aop_h + h_h --> 2.0 h2o_h + ppbng_h 13394
  PPBNGS 2.0 5aop_c --> 2.0 h2o_c + h_c + ppbng_c 13394

PPMh r1p_h <=> r5p_h 9959
  PPM r1p_c <=> r5p_c 13711 or 9959
  PGMT g1p_c <=> g6p_c 13711 or 14546 or 8460 or 9959
  yli_R0552 r1p_c <=> yli_M00116_c 13711 or 9959

PPPGO_1 3.0 o2_h + pppg9_h --> 3.0 h2o2_h + ppp9_h 11867
  PPPGOm 3.0 o2_m + 2.0 pppg9_m --> 6.0 h2o_m + 2.0 ppp9_m 11867
  yli_R0586 3.0 o2_m + pppg9_m --> 3.0 h2o2_m + ppp9_m 11867

PRAISh 5aizc_h + asp__L_h + atp_h --> 25aics_h + adp_h + h_h + pi_h 9220
  PRASCSi 5aizc_c + asp__L_c + atp_c --> 25aics_c + adp_c + h_c + pi_c 9220
  yli_R0553 5aizc_c + asp__L_c + atp_c <=> adp_c + h_c + pi_c + yli_M02454_c 9220

PRFGSh atp_h + fgam_h + gln__L_h + h2o_h --> adp_h + fpram_h + glu__L_h + h_h + pi_h 15033
  PRFGS atp_c + fgam_c + gln__L_c + h2o_c --> adp_c + fpram_c + glu__L_c + h_c + pi_c 15033

PRICIh prfp_h --> prlp_h 11610 or 9084
  IG3PS gln__L_c + prlp_c --> aicar_c + eig3p_c + glu__L_c + h_c 9084
  PRMICI prfp_c <=> prlp_c 11610 or 9084
  PRMICI prfp_c <=> prlp_c 11610 or 9084

PSCIT pep_h + skm5p_h <=> 3psme_h + pi_h 14252
  SHK3Dr 3dhsk_c + h_c + nadph_c <=> nadp_c + skm_c 14252
  PPND nad_c + pphn_c --> 34hpp_c + co2_c + nadh_c 14252 or 16225
  DHQS 2dda7p_c --> 3dhq_c + pi_c 14252
  PSCVT pep_c + skm5p_c <=> 3psme_c + pi_c 14252
  DHQTi 3dhq_c --> 3dhsk_c + h2o_c 10572 or 14252
  SHKK atp_c + skm_c --> adp_c + h_c + skm5p_c 13706 or 14252

PTRCthr h_h + ptrc_c <=> h_c + ptrc_h 11649 or 14774
  SPMDtmr h_m + spmd_c <=> h_c + spmd_m 11649 or 14774
  SPRMt2i h_c + sprm_e --> h_e + sprm_c 11017 or 11649 or 13128 or 14774 or (11017 and 11649 and 16454) or (11017 and 14774 and 16454) or (11649 and 13128 and 16454) or (13128 and 14774 and 16454)
  SPMDtmr h_m + spmd_c <=> h_c + spmd_m 11649 or 14774
  SPRMt2i h_c + sprm_e --> h_e + sprm_c 11017 or 11649 or 13128 or 14774 or (11017 and 11649 and 16454) or (11017 and 14774 and 16454) or (11649 and 13128 and 16454) or (13128 and 14774 and 16454)

R5PFL r5p_h --> db4p_h + for_h + h_h 16666
  GTPCII gtp_c + 3.0 h2o_c --> 25dhpp_c + for_c + 2.0 h_c + ppi_c 12551 or 16666
  DB4PS ru5p__D_c --> db4p_c + for_c + h_c 16666

RIBFS 2.0 dmlz_h + h_h --> 4r5au_h + ribflv_h 12495
  RBFSb 2.0 dmlz_c --> 4r5au_c + ribflv_c 12495

RPDPK atp_h + r5p_h <=> amp_h + h_h + prpp_h 11145 or 16592
  PRPPS atp_c + r5p_c <=> amp_c + h_c + prpp_c 11145 or 16592
  yli_R0521 atp_c + yli_M00116_c <=> amp_c + h_c + prpp_c 11145 or 16592
  PRPPS atp_c + r5p_c <=> amp_c + h_c + prpp_c 11145 or 16592
  yli_R0521 atp_c + yli_M00116_c <=> amp_c + h_c + prpp_c 11145 or 16592

SERH 3ig3p_h + ser__L_h --> g3p_h + h2o_h + trp__L_h CRv4_Au5_s12_g3638_t1 and 9262
  TRPS3 3ig3p_c --> g3p_c + indole_c b1260 and 9262
  TRPS1 3ig3p_c + ser__L_c --> g3p_c + h2o_c + trp__L_c 9262 or (PP_0082 and 9262) or (b1260 and 9262)
  TRPS2 indole_c + ser__L_c --> h2o_c + trp__L_c 9262 or (b1260 and 9262)

SHSL2h h2s_h + suchms_h --> hcys__L_h + succ_h 12649 or 14061
  FT frdp_c + ipdp_c --> ppi_c + ttc_ggdp_c 12649

SQDGS160 12dgr160_h + udpsq_h --> h_h + sqdg160_h + udp_h 9212

SQDGS1819Z160 12dgr1819Z160_h + udpsq_h --> h_h + sqdg1819Z160_h + udp_h 9212

STARCH300S 300.0 adpglc_h + h2o_h --> 300.0 adp_h + 300.0 h_h + starch300_h (CRv4_Au5_s12_g3500_t1 and 12594) or (CRv4_Au5_s16_g6335_t1 and 12594) or (CRv4_Au5_s16_g6375_t1 and 12594) or (CRv4_Au5_s3_g10852_t1 and 12594) or (CRv4_Au5_s6_g13040_t1 and 12594)
  GBEZ 14glun_c --> glycogen_c + h2o_c 12594
  GLBRAN glygn1_c --> glygn2_c 12594
  yli_R0731 amylose_c --> h2o_c + starch_c 12594
  GLBRAN2 glycogen_c --> bglycogen_c 12594

TAL thr__L_h --> 2obut_h + nh4_h 11909
  SERD_L ser__L_c --> nh4_c + pyr_c 11849 or 11909 or 9216
  THRD_Lm thr__L_m --> 2obut_m + nh4_m 11909
  THRD_L thr__L_c --> 2obut_c + nh4_c 11909 or 9216
  SER_AL ser__L_c <=> h_c + nh3_c + pyr_c 11909

TCYD 2m6phol_h --> dtocophe_h 13847 or 16138

TCYG 23dmphol_h --> gtocophe_h 13847 or 16138

TFENFEOR h_h + nadh_h + tfenfe3_h <=> nad_h + tfenfe2_h 8656

THRAL ser__L_h <=> nh4_h + pyr_h 11909
  SERD_L ser__L_c --> nh4_c + pyr_c 11849 or 11909 or 9216
  THRD_Lm thr__L_m --> 2obut_m + nh4_m 11909
  THRD_L thr__L_c --> 2obut_c + nh4_c 11909 or 9216
  SER_AL ser__L_c <=> h_c + nh3_c + pyr_c 11909

TRPS2h indole_h + ser__L_h --> h2o_h + trp__L_h CRv4_Au5_s12_g3638_t1 and 9262
  TRPS3 3ig3p_c --> g3p_c + indole_c b1260 and 9262
  TRPS1 3ig3p_c + ser__L_c --> g3p_c + h2o_c + trp__L_c 9262 or (PP_0082 and 9262) or (b1260 and 9262)
  TRPS2 indole_c + ser__L_c --> h2o_c + trp__L_c 9262 or (b1260 and 9262)

TYRTAh 34hpp_h + glu__L_h <=> akg_h + tyr__L_h 13230 or 14281
  PHETA1 akg_c + phe__L_c <=> glu__L_c + phpyr_c 12407 or 13230 or 14281 or 14610 or 14853 or 14908 or 15839 or 8936
  ASPTAm akg_m + asp__L_m <=> glu__L_m + oaa_m 14281
  yli_R0097 3sala_c + akg_c + h_c --> glu__L_c + yli_M02657_c 14281 or 8936
  ASPTA akg_c + asp__L_c <=> glu__L_c + oaa_c 14281 or 8936
  yli_R1493 Lcyst_c + akg_c <=> glu__L_c + yli_M02657_c 14281 or 8936
  CYSTAm akg_m + cys__L_m <=> glu__L_m + mercppyr_m 14281
  UNK3 2kmb_c + glu__L_c --> akg_c + met__L_c 12407 or 14281 or 14908 or 15839 or 8936
  CYSATm glu__L_m + mercppyr_m --> akg_m + cys__L_m 14281
  TYRTAi 34hpp_c + glu__L_c --> akg_c + tyr__L_c 12407 or 13230 or 14281 or 14908 or 15839 or 8936
  CYSTA akg_c + cys__L_c --> glu__L_c + mercppyr_c 14281 or 8936
  EHGLAT2m e4hglu_m + oaa_m --> 4h2oglt_m + asp__L_m 14281
  PHETA1m akg_m + phe__L_m <=> glu__L_m + phpyr_m 12407 or 13230 or 14281 or 14908 or 15839 or 16065
  EHGLATm akg_m + e4hglu_m --> 4h2oglt_m + glu__L_m 14281
  TYRTAim 34hpp_m + glu__L_m --> akg_m + tyr__L_m 12407 or 13230 or 14281 or 14908 or 15839 or 16065
  LCYSTATm Lcyst_m + akg_m <=> 3spyr_m + glu__L_m 14281
  TYRTA akg_c + tyr__L_c <=> 34hpp_c + glu__L_c 12407 or 13230 or 14281 or 14610 or 14853 or 14908 or 15839 or 8936
  3SALATAim 3sala_m + akg_m + h_m --> 3snpyr_m + glu__L_m 14281
  AATGm 3sala_m + akg_m --> 3snpyr_m + glu__L_m 14281
  TYRTAim 34hpp_m + glu__L_m --> akg_m + tyr__L_m 12407 or 13230 or 14281 or 14908 or 15839 or 16065
  PHETA1m akg_m + phe__L_m <=> glu__L_m + phpyr_m 12407 or 13230 or 14281 or 14908 or 15839 or 16065
  PHETA1 akg_c + phe__L_c <=> glu__L_c + phpyr_c 12407 or 13230 or 14281 or 14610 or 14853 or 14908 or 15839 or 8936
  TYRTA akg_c + tyr__L_c <=> 34hpp_c + glu__L_c 12407 or 13230 or 14281 or 14610 or 14853 or 14908 or 15839 or 8936
  TYRTAi 34hpp_c + glu__L_c --> akg_c + tyr__L_c 12407 or 13230 or 14281 or 14908 or 15839 or 8936
  HSTPT glu__L_c + imacp_c --> akg_c + hisp_c 13230

UDPGALth udpgal_c + ump_h <=> udpgal_h + ump_c 15942
  UDPGALt2g udpgal_c --> udpgal_g 15942

UDPGth udp_h + udpg_c <=> udp_c + udpg_h 15942
  UDPGALt2g udpgal_c --> udpgal_g 15942

UPP3MT_1 2.0 amet_h + uppg3_h --> 2.0 ahcys_h + dscl_h + 2.0 h_h 11050
  UPP3MT 2.0 amet_c + uppg3_c --> 2.0 ahcys_c + dscl_c + h_c 11050
  SHCHF fe2_c + scl_c --> 3.0 h_c + sheme_c 11050 or 13569
  SHCHD2 dscl_c + nad_c --> h_c + nadh_c + scl_c 11050 or 13569

UPPDC1_1 4.0 h_h + uppg3_h --> 4.0 co2_h + cpppg3_h 14179
  UPPDC1 4.0 h_c + uppg3_c --> 4.0 co2_c + cpppg3_c 14179
  UPPDC2 4.0 h_c + uppg1_c --> 4.0 co2_c + cpppg1_c 14179

UPPDC2_1 4.0 h_h + uppg1_h --> 4.0 co2_h + cpppg1_h 14179
  UPPDC1 4.0 h_c + uppg3_c --> 4.0 co2_c + cpppg3_c 14179
  UPPDC2 4.0 h_c + uppg1_c --> 4.0 co2_c + cpppg1_c 14179

VALTLh atp_h + trnaval_h + val__L_h --> amp_h + ppi_h + valtrna_h 16528
  VALTRS atp_c + trnaval_c + val__L_c --> amp_c + ppi_c + valtrna_c 16528
  VALTRSm atp_m + trnaval_m + val__L_m --> amp_m + ppi_m + valtrna_m 16528
```

In [10]:

```
Annotation.loc[temp]
```

Out[10]:

|  | Combined Annotations | Signal P | Sc288c Orthologs | Human Orthologs | Sc288 Best Hit | Human Blast | Essential | WolfPSort | C Terminal |
| --- | --- | --- | --- | --- | --- | --- | --- | --- | --- |
| RTO4\_ID |  |  |  |  |  |  |  |  |  |
| 13423 | KOG1289: Amino acid transporters |  | TPO5 |  | HNM1 |  | Not Essential | plas 27 | HHR\* |
| 11269 | KOG1289: Amino acid transporters |  |  |  | UGA4 |  | Not Essential | plas 27 | EVK\* |
| 8709 | K00860: cysC; adenylylsulfate kinase |  | MET14 | PAPSS1,PAPSS2 | MET14 | PAPSS1 | Not Essential | cyto 15, cyto\_mito 11.999, cyto\_nucl 10.833, m... | NFI\* |
| 14281 | K14455: GOT2; aspartate aminotransferase, mito... |  | AAT1 | GOT2 | AAT2 | GOT2 | Not Essential | mito 26 | NDA\* |
| 10946 | KOG3574: Acetyl-CoA transporter |  | YBR220C | SLC33A1 | YBR220C | SLC33 | Not Essential | plas 26 | NGR\* |
| ... | ... | ... | ... | ... | ... | ... | ... | ... | ... |
| 8656 | K00521: E1.16.1.7; ferric-chelate reductase | A |  |  | FRE5 |  | Not Essential | plas 23, E.R. 2 | FGW\* |
| 15942 | K15275: SLC35B1; solute carrier family 35 (UDP... | A | HUT1 | SLC35B1 | HUT1 | SLC35 | Essential | plas 17, E.R. 4, golg 3, mito 2 | KEA\* |
| 11050 | K00589: MET1; uroporphyrin-III C-methyltransfe... | S | MET1 |  | MET1 |  | Not Essential | cyto 10, mito 8, cyto\_nucl 8 | SAE\* |
| 14179 | K01599: hemE, UROD; uroporphyrinogen decarboxy... |  | HEM12 | UROD | HEM12 | UROD | Essential | cyto 8.5, pero 8, cyto\_nucl 6.5, mito 5, nucl 3.5 | AYA\* |
| 16528 | K01873: VARS, valS; valyl-tRNA synthetase | S | VAS1 | VARS,VARS2 | VAS1 | VARS | Essential | mito 15.5, cyto\_mito 11.833, cyto 7, cyto\_nucl... | LRV\* |

62 rows × 9 columns

In [11]:

```
temp = list()
for r in sorted(model.reactions, key=lambda x: x.id):
    if 'f' in r.compartments:
        print(r.id, r.reaction, r.gene_reaction_rule)
        for g in r.genes:
            if g.id in Annotation.index and g.id not in temp:
                temp.append(g.id)
            for r2 in g.reactions:
                if r2 is not r and 'f' not in r2.compartments:
                    print(' ',r2.id, r2.reaction, r2.gene_reaction_rule)
        print()
```

```
H2Otf h2o_c <=> h2o_f 13986
  H2Oth h2o_c <=> h2o_h 13986
  H2Ot h2o_e <=> h2o_c 13986 or 13987 or 9014 or 9015
  H2Otp h2o_c <=> h2o_x 13986
  H2Otm h2o_c <=> h2o_m 13986 or 13987 or 9014 or 9015
```

In [12]:

```
Annotation.loc[temp]
```

Out[12]:

|  | Combined Annotations | Signal P | Sc288c Orthologs | Human Orthologs | Sc288 Best Hit | Human Blast | Essential | WolfPSort | C Terminal |
| --- | --- | --- | --- | --- | --- | --- | --- | --- | --- |
| RTO4\_ID |  |  |  |  |  |  |  |  |  |
| 13986 | K09866: AQP4; aquaporin-4 |  | AQY1 | AQP4,AQP1,AQP8 | AQY1 | AQP4 | Not Essential | plas 23, E.R. 3 | SPV\* |

In [13]:

```
temp = list()
for r in sorted(model.reactions, key=lambda x: x.id):
    if 'l' in r.compartments:
        print(r.id, r.reaction, r.gene_reaction_rule)
        for g in r.genes:
            if g.id in Annotation.index and g.id not in temp:
                temp.append(g.id)
            for r2 in g.reactions:
                if r2 is not r and 'l' not in r2.compartments:
                    print(' ',r2.id, r2.reaction, r2.gene_reaction_rule)
        print()
```

```
ABUTt2rL 4abut_l + h_l <=> 4abut_c + h_c 12743
  ILEt7 h_v + ile__L_v --> h_c + ile__L_c 12743
  ASNt7 asn__L_v + h_v --> asn__L_c + h_c 12743
  ALAt2r ala__L_e + h_e <=> ala__L_c + h_c 12743 or 14229 or 15074 or 8962 or 9319 or 9322 or 9962 or (YALI0B09537g and 14229 and 8962) or (YALI0B09537g and 14229 and 9319) or (YALI0B09537g and 14229 and 9322) or (YALI0B09537g and 14229 and 9962) or (YALI0B09537g and 15074 and 8962) or (YALI0B09537g and 15074 and 9319) or (YALI0B09537g and 15074 and 9322) or (YALI0B09537g and 15074 and 9962)
  LEUt7 h_v + leu__L_v --> h_c + leu__L_c 12743
  GLYt2r gly_e + h_e <=> gly_c + h_c 12743 or 14229 or 15074 or 8962 or 9319 or 9322 or 9962 or (YALI0B09537g and 14229 and 8962) or (YALI0B09537g and 14229 and 9319) or (YALI0B09537g and 14229 and 9322) or (YALI0B09537g and 14229 and 9962) or (YALI0B09537g and 15074 and 8962) or (YALI0B09537g and 15074 and 9319) or (YALI0B09537g and 15074 and 9322) or (YALI0B09537g and 15074 and 9962)
  PRODt2r h_e + pro__D_e <=> h_c + pro__D_c 12743
  TYRt7 h_v + tyr__L_v --> h_c + tyr__L_c 12743
  GLNt7 gln__L_v + h_v --> gln__L_c + h_c 12743
  DALAt2r ala__D_e + h_e <=> ala__D_c + h_c 12743
  ABUTt2r 4abut_e + h_e <=> 4abut_c + h_c 11269 or 12743 or 13423 or (YALI0B09537g and 11269) or (YALI0B09537g and 13423)
  PROt2r h_e + pro__L_e <=> h_c + pro__L_c 12743 or 14229 or 15074 or (YALI0B09537g and 14229) or (YALI0B09537g and 15074)

ADEtl ade_l <=> ade_c 13969
  ADNt2 adn_e + h_e --> adn_c + h_c 13969
  URIt2 h_e + uri_e --> h_c + uri_c 10921 or 11871 or 12902 or 13969 or (10921 and 13969) or (11871 and 13969) or (12902 and 13969)
  CYTDt2 cytd_e + h_e --> cytd_c + h_c 13969
  ADNtm adn_c <=> adn_m 13969

ADNtl adn_l <=> adn_c 13969
  ADNt2 adn_e + h_e --> adn_c + h_c 13969
  URIt2 h_e + uri_e --> h_c + uri_c 10921 or 11871 or 12902 or 13969 or (10921 and 13969) or (11871 and 13969) or (12902 and 13969)
  CYTDt2 cytd_e + h_e --> cytd_c + h_c 13969
  ADNtm adn_c <=> adn_m 13969

AHEXASE2ly 3.0 h2o_l + n2m2nmn_l --> 3.0 acgam_l + m2mn_l 12375

AHEXASEly 2.0 h2o_l + n2m2mn_l --> 2.0 acgam_l + m2mn_l 12375

ALAt2rL ala__L_l + h_l <=> ala__L_c + h_c 12743
  ILEt7 h_v + ile__L_v --> h_c + ile__L_c 12743
  ASNt7 asn__L_v + h_v --> asn__L_c + h_c 12743
  ALAt2r ala__L_e + h_e <=> ala__L_c + h_c 12743 or 14229 or 15074 or 8962 or 9319 or 9322 or 9962 or (YALI0B09537g and 14229 and 8962) or (YALI0B09537g and 14229 and 9319) or (YALI0B09537g and 14229 and 9322) or (YALI0B09537g and 14229 and 9962) or (YALI0B09537g and 15074 and 8962) or (YALI0B09537g and 15074 and 9319) or (YALI0B09537g and 15074 and 9322) or (YALI0B09537g and 15074 and 9962)
  LEUt7 h_v + leu__L_v --> h_c + leu__L_c 12743
  GLYt2r gly_e + h_e <=> gly_c + h_c 12743 or 14229 or 15074 or 8962 or 9319 or 9322 or 9962 or (YALI0B09537g and 14229 and 8962) or (YALI0B09537g and 14229 and 9319) or (YALI0B09537g and 14229 and 9322) or (YALI0B09537g and 14229 and 9962) or (YALI0B09537g and 15074 and 8962) or (YALI0B09537g and 15074 and 9319) or (YALI0B09537g and 15074 and 9322) or (YALI0B09537g and 15074 and 9962)
  PRODt2r h_e + pro__D_e <=> h_c + pro__D_c 12743
  TYRt7 h_v + tyr__L_v --> h_c + tyr__L_c 12743
  GLNt7 gln__L_v + h_v --> gln__L_c + h_c 12743
  DALAt2r ala__D_e + h_e <=> ala__D_c + h_c 12743
  ABUTt2r 4abut_e + h_e <=> 4abut_c + h_c 11269 or 12743 or 13423 or (YALI0B09537g and 11269) or (YALI0B09537g and 13423)
  PROt2r h_e + pro__L_e <=> h_c + pro__L_c 12743 or 14229 or 15074 or (YALI0B09537g and 14229) or (YALI0B09537g and 15074)

B_MANNASEly h2o_l + mn_l --> acgam_l + man_l 15421

CYTDtl cytd_l <=> cytd_c 13969
  ADNt2 adn_e + h_e --> adn_c + h_c 13969
  URIt2 h_e + uri_e --> h_c + uri_c 10921 or 11871 or 12902 or 13969 or (10921 and 13969) or (11871 and 13969) or (12902 and 13969)
  CYTDt2 cytd_e + h_e --> cytd_c + h_c 13969
  ADNtm adn_c <=> adn_m 13969

DALAt2rL ala__D_l + h_l <=> ala__D_c + h_c 12743
  ILEt7 h_v + ile__L_v --> h_c + ile__L_c 12743
  ASNt7 asn__L_v + h_v --> asn__L_c + h_c 12743
  ALAt2r ala__L_e + h_e <=> ala__L_c + h_c 12743 or 14229 or 15074 or 8962 or 9319 or 9322 or 9962 or (YALI0B09537g and 14229 and 8962) or (YALI0B09537g and 14229 and 9319) or (YALI0B09537g and 14229 and 9322) or (YALI0B09537g and 14229 and 9962) or (YALI0B09537g and 15074 and 8962) or (YALI0B09537g and 15074 and 9319) or (YALI0B09537g and 15074 and 9322) or (YALI0B09537g and 15074 and 9962)
  LEUt7 h_v + leu__L_v --> h_c + leu__L_c 12743
  GLYt2r gly_e + h_e <=> gly_c + h_c 12743 or 14229 or 15074 or 8962 or 9319 or 9322 or 9962 or (YALI0B09537g and 14229 and 8962) or (YALI0B09537g and 14229 and 9319) or (YALI0B09537g and 14229 and 9322) or (YALI0B09537g and 14229 and 9962) or (YALI0B09537g and 15074 and 8962) or (YALI0B09537g and 15074 and 9319) or (YALI0B09537g and 15074 and 9322) or (YALI0B09537g and 15074 and 9962)
  PRODt2r h_e + pro__D_e <=> h_c + pro__D_c 12743
  TYRt7 h_v + tyr__L_v --> h_c + tyr__L_c 12743
  GLNt7 gln__L_v + h_v --> gln__L_c + h_c 12743
  DALAt2r ala__D_e + h_e <=> ala__D_c + h_c 12743
  ABUTt2r 4abut_e + h_e <=> 4abut_c + h_c 11269 or 12743 or 13423 or (YALI0B09537g and 11269) or (YALI0B09537g and 13423)
  PROt2r h_e + pro__L_e <=> h_c + pro__L_c 12743 or 14229 or 15074 or (YALI0B09537g and 14229) or (YALI0B09537g and 15074)

GLYt2rL gly_l + h_l <=> gly_c + h_c 12743
  ILEt7 h_v + ile__L_v --> h_c + ile__L_c 12743
  ASNt7 asn__L_v + h_v --> asn__L_c + h_c 12743
  ALAt2r ala__L_e + h_e <=> ala__L_c + h_c 12743 or 14229 or 15074 or 8962 or 9319 or 9322 or 9962 or (YALI0B09537g and 14229 and 8962) or (YALI0B09537g and 14229 and 9319) or (YALI0B09537g and 14229 and 9322) or (YALI0B09537g and 14229 and 9962) or (YALI0B09537g and 15074 and 8962) or (YALI0B09537g and 15074 and 9319) or (YALI0B09537g and 15074 and 9322) or (YALI0B09537g and 15074 and 9962)
  LEUt7 h_v + leu__L_v --> h_c + leu__L_c 12743
  GLYt2r gly_e + h_e <=> gly_c + h_c 12743 or 14229 or 15074 or 8962 or 9319 or 9322 or 9962 or (YALI0B09537g and 14229 and 8962) or (YALI0B09537g and 14229 and 9319) or (YALI0B09537g and 14229 and 9322) or (YALI0B09537g and 14229 and 9962) or (YALI0B09537g and 15074 and 8962) or (YALI0B09537g and 15074 and 9319) or (YALI0B09537g and 15074 and 9322) or (YALI0B09537g and 15074 and 9962)
  PRODt2r h_e + pro__D_e <=> h_c + pro__D_c 12743
  TYRt7 h_v + tyr__L_v --> h_c + tyr__L_c 12743
  GLNt7 gln__L_v + h_v --> gln__L_c + h_c 12743
  DALAt2r ala__D_e + h_e <=> ala__D_c + h_c 12743
  ABUTt2r 4abut_e + h_e <=> 4abut_c + h_c 11269 or 12743 or 13423 or (YALI0B09537g and 11269) or (YALI0B09537g and 13423)
  PROt2r h_e + pro__L_e <=> h_c + pro__L_c 12743 or 14229 or 15074 or (YALI0B09537g and 14229) or (YALI0B09537g and 15074)

GSNtl gsn_l <=> gsn_c 13969
  ADNt2 adn_e + h_e --> adn_c + h_c 13969
  URIt2 h_e + uri_e --> h_c + uri_c 10921 or 11871 or 12902 or 13969 or (10921 and 13969) or (11871 and 13969) or (12902 and 13969)
  CYTDt2 cytd_e + h_e --> cytd_c + h_c 13969
  ADNtm adn_c <=> adn_m 13969

HXANtl hxan_l <=> hxan_c 13969
  ADNt2 adn_e + h_e --> adn_c + h_c 13969
  URIt2 h_e + uri_e --> h_c + uri_c 10921 or 11871 or 12902 or 13969 or (10921 and 13969) or (11871 and 13969) or (12902 and 13969)
  CYTDt2 cytd_e + h_e --> cytd_c + h_c 13969
  ADNtm adn_c <=> adn_m 13969

INStl ins_l <=> ins_c 13969
  ADNt2 adn_e + h_e --> adn_c + h_c 13969
  URIt2 h_e + uri_e --> h_c + uri_c 10921 or 11871 or 12902 or 13969 or (10921 and 13969) or (11871 and 13969) or (12902 and 13969)
  CYTDt2 cytd_e + h_e --> cytd_c + h_c 13969
  ADNtm adn_c <=> adn_m 13969

NACHEX10ly 2.0 h2o_l + ksi_deg7_l --> 2.0 acgam_l + ksi_deg8_l 12375

NACHEX11ly h2o_l + ksi_deg10_l --> acgam_l + ksi_deg11_l 12375

NACHEX12ly h2o_l + ksi_deg13_l --> acgam_l + ksi_deg14_l 12375

NACHEX13ly h2o_l + ksi_deg16_l --> acgam_l + ksi_deg17_l 12375

NACHEX14ly h2o_l + ksi_deg19_l --> acgam_l + ksi_deg20_l 12375

NACHEX15ly h2o_l + ksi_deg22_l --> acgam_l + ksi_deg23_l 12375

NACHEX16ly h2o_l + ksi_deg25_l --> acgam_l + ksi_deg26_l 12375

NACHEX17ly h2o_l + ksi_deg28_l --> acgam_l + ksi_deg29_l 12375

NACHEX18ly h2o_l + ksi_deg31_l --> acgam_l + ksi_deg32_l 12375

NACHEX19ly h2o_l + ksi_deg34_l --> acgam_l + ksi_deg35_l 12375

NACHEX1ly cs_a_deg1_l + h2o_l --> acgal_l + cs_a_deg2_l 12375

NACHEX20ly h2o_l + ksi_deg37_l --> acgam_l + ksi_deg38_l 12375

NACHEX21ly h2o_l + ksi_deg39_l --> acgam_l + ksi_deg40_l 12375

NACHEX22ly h2o_l + ksi_deg41_l --> acgam_l + m2mn_l 12375

NACHEX23ly h2o_l + ksii_core2_deg4_l --> acgam_l + ksii_core2_deg5_l 12375

NACHEX24ly h2o_l + ksii_core2_deg7_l --> acgam_l + ksii_core2_deg8_l 12375

NACHEX25ly h2o_l + ksii_core2_deg9_l --> acgam_l + f1a_l 12375

NACHEX26ly 2.0 h2o_l + ksii_core4_deg4_l --> 2.0 acgam_l + ksii_core2_deg5_l 12375

NACHEX27ly h2o_l + ha_deg1_l --> acgam_l + ha_pre1_l 12375

NACHEX2ly cs_a_deg4_l + h2o_l --> acgal_l + cs_a_deg5_l 12375

NACHEX3ly cs_b_deg1_l + h2o_l --> acgal_l + cs_b_deg2_l 12375

NACHEX4ly cs_c_deg1_l + h2o_l --> acgal_l + cs_c_deg2_l 12375

NACHEX5ly cs_c_deg4_l + h2o_l --> acgal_l + cs_c_deg5_l 12375

NACHEX6ly cs_d_deg1_l + h2o_l --> acgal_l + cs_d_deg2_l 12375

NACHEX7ly cs_d_deg5_l + h2o_l --> acgal_l + cs_d_deg6_l 12375

NACHEX8ly cs_e_deg2_l + h2o_l --> acgal_l + cs_e_deg3_l 12375

NACHEX9ly cs_e_deg6_l + h2o_l --> acgal_l + cs_e_deg7_l 12375

NACHEXA10ly 2.0 h2o_l + ksi_deg9_l --> acgam_l + h_l + ksi_deg11_l + so4_l 12375

NACHEXA11ly 2.0 h2o_l + ksi_deg12_l --> acgam_l + h_l + ksi_deg14_l + so4_l 12375

NACHEXA12ly 2.0 h2o_l + ksi_deg15_l --> acgam_l + h_l + ksi_deg17_l + so4_l 12375

NACHEXA13ly 2.0 h2o_l + ksi_deg18_l --> acgam_l + h_l + ksi_deg20_l + so4_l 12375

NACHEXA14ly 2.0 h2o_l + ksi_deg21_l --> acgam_l + h_l + ksi_deg23_l + so4_l 12375

NACHEXA15ly 2.0 h2o_l + ksi_deg24_l --> acgam_l + h_l + ksi_deg26_l + so4_l 12375

NACHEXA16ly 2.0 h2o_l + ksi_deg27_l --> acgam_l + h_l + ksi_deg29_l + so4_l 12375

NACHEXA17ly 2.0 h2o_l + ksi_deg30_l --> acgam_l + h_l + ksi_deg32_l + so4_l 12375

NACHEXA18ly 2.0 h2o_l + ksi_deg33_l --> acgam_l + h_l + ksi_deg35_l + so4_l 12375

NACHEXA19ly 2.0 h2o_l + ksi_deg36_l --> acgam_l + h_l + ksi_deg38_l + so4_l 12375

NACHEXA1ly cs_a_l + 2.0 h2o_l --> acgal_l + cs_a_deg2_l + h_l + so4_l 12375

NACHEXA20ly 2.0 h2o_l + ksii_core2_deg3_l --> acgam_l + h_l + ksii_core2_deg5_l + so4_l 12375

NACHEXA21ly 2.0 h2o_l + ksii_core2_deg6_l --> acgam_l + h_l + ksii_core2_deg8_l + so4_l 12375

NACHEXA22ly 3.0 h2o_l + ksii_core4_deg3_l --> 2.0 acgam_l + h_l + ksii_core2_deg5_l + so4_l 12375

NACHEXA2ly cs_b_l + 2.0 h2o_l --> acgal_l + cs_b_deg2_l + h_l + so4_l 12375

NACHEXA3ly cs_c_l + 2.0 h2o_l --> acgal_l + cs_c_deg2_l + h_l + so4_l 12375

NACHEXA4ly cs_c_deg3_l + 2.0 h2o_l --> acgal_l + cs_c_deg5_l + h_l + so4_l 12375

NACHEXA5ly cs_d_l + 2.0 h2o_l --> acgal_l + cs_d_deg2_l + h_l + so4_l 12375

NACHEXA6ly cs_d_deg4_l + 2.0 h2o_l --> acgal_l + cs_d_deg6_l + h_l + so4_l 12375

NACHEXA7ly cs_e_l + 3.0 h2o_l --> acgal_l + cs_e_deg3_l + 2.0 h_l + 2.0 so4_l 12375

NACHEXA8ly cs_e_deg4_l + 3.0 h2o_l --> acgal_l + cs_e_deg7_l + 2.0 h_l + 2.0 so4_l 12375

NACHEXA9ly 3.0 h2o_l + ksi_deg6_l --> 2.0 acgam_l + h_l + ksi_deg8_l + so4_l 12375

PRODt2rL h_l + pro__D_l <=> h_c + pro__D_c 12743
  ILEt7 h_v + ile__L_v --> h_c + ile__L_c 12743
  ASNt7 asn__L_v + h_v --> asn__L_c + h_c 12743
  ALAt2r ala__L_e + h_e <=> ala__L_c + h_c 12743 or 14229 or 15074 or 8962 or 9319 or 9322 or 9962 or (YALI0B09537g and 14229 and 8962) or (YALI0B09537g and 14229 and 9319) or (YALI0B09537g and 14229 and 9322) or (YALI0B09537g and 14229 and 9962) or (YALI0B09537g and 15074 and 8962) or (YALI0B09537g and 15074 and 9319) or (YALI0B09537g and 15074 and 9322) or (YALI0B09537g and 15074 and 9962)
  LEUt7 h_v + leu__L_v --> h_c + leu__L_c 12743
  GLYt2r gly_e + h_e <=> gly_c + h_c 12743 or 14229 or 15074 or 8962 or 9319 or 9322 or 9962 or (YALI0B09537g and 14229 and 8962) or (YALI0B09537g and 14229 and 9319) or (YALI0B09537g and 14229 and 9322) or (YALI0B09537g and 14229 and 9962) or (YALI0B09537g and 15074 and 8962) or (YALI0B09537g and 15074 and 9319) or (YALI0B09537g and 15074 and 9322) or (YALI0B09537g and 15074 and 9962)
  PRODt2r h_e + pro__D_e <=> h_c + pro__D_c 12743
  TYRt7 h_v + tyr__L_v --> h_c + tyr__L_c 12743
  GLNt7 gln__L_v + h_v --> gln__L_c + h_c 12743
  DALAt2r ala__D_e + h_e <=> ala__D_c + h_c 12743
  ABUTt2r 4abut_e + h_e <=> 4abut_c + h_c 11269 or 12743 or 13423 or (YALI0B09537g and 11269) or (YALI0B09537g and 13423)
  PROt2r h_e + pro__L_e <=> h_c + pro__L_c 12743 or 14229 or 15074 or (YALI0B09537g and 14229) or (YALI0B09537g and 15074)

PROt2rL h_l + pro__L_l <=> h_c + pro__L_c 12743
  ILEt7 h_v + ile__L_v --> h_c + ile__L_c 12743
  ASNt7 asn__L_v + h_v --> asn__L_c + h_c 12743
  ALAt2r ala__L_e + h_e <=> ala__L_c + h_c 12743 or 14229 or 15074 or 8962 or 9319 or 9322 or 9962 or (YALI0B09537g and 14229 and 8962) or (YALI0B09537g and 14229 and 9319) or (YALI0B09537g and 14229 and 9322) or (YALI0B09537g and 14229 and 9962) or (YALI0B09537g and 15074 and 8962) or (YALI0B09537g and 15074 and 9319) or (YALI0B09537g and 15074 and 9322) or (YALI0B09537g and 15074 and 9962)
  LEUt7 h_v + leu__L_v --> h_c + leu__L_c 12743
  GLYt2r gly_e + h_e <=> gly_c + h_c 12743 or 14229 or 15074 or 8962 or 9319 or 9322 or 9962 or (YALI0B09537g and 14229 and 8962) or (YALI0B09537g and 14229 and 9319) or (YALI0B09537g and 14229 and 9322) or (YALI0B09537g and 14229 and 9962) or (YALI0B09537g and 15074 and 8962) or (YALI0B09537g and 15074 and 9319) or (YALI0B09537g and 15074 and 9322) or (YALI0B09537g and 15074 and 9962)
  PRODt2r h_e + pro__D_e <=> h_c + pro__D_c 12743
  TYRt7 h_v + tyr__L_v --> h_c + tyr__L_c 12743
  GLNt7 gln__L_v + h_v --> gln__L_c + h_c 12743
  DALAt2r ala__D_e + h_e <=> ala__D_c + h_c 12743
  ABUTt2r 4abut_e + h_e <=> 4abut_c + h_c 11269 or 12743 or 13423 or (YALI0B09537g and 11269) or (YALI0B09537g and 13423)
  PROt2r h_e + pro__L_e <=> h_c + pro__L_c 12743 or 14229 or 15074 or (YALI0B09537g and 14229) or (YALI0B09537g and 15074)

S4TASE1ly cs_a_l + h2o_l <=> cs_a_deg1_l + h_l + so4_l 15267 or 15283

S4TASE2ly cs_a_deg3_l + h2o_l <=> cs_a_deg4_l + h_l + so4_l 15267 or 15283

S4TASE3ly cs_b_l + h2o_l <=> cs_b_deg1_l + h_l + so4_l 15267 or 15283

S4TASE4ly cs_e_l + h2o_l <=> cs_e_deg1_l + h_l + so4_l 15267 or 15283

S4TASE5ly cs_e_deg4_l + h2o_l <=> cs_e_deg5_l + h_l + so4_l 15267 or 15283

THYMDtl thymd_l <=> thymd_c 13969
  ADNt2 adn_e + h_e --> adn_c + h_c 13969
  URIt2 h_e + uri_e --> h_c + uri_c 10921 or 11871 or 12902 or 13969 or (10921 and 13969) or (11871 and 13969) or (12902 and 13969)
  CYTDt2 cytd_e + h_e --> cytd_c + h_c 13969
  ADNtm adn_c <=> adn_m 13969

Uritl uri_l <=> uri_c 13969
  ADNt2 adn_e + h_e --> adn_c + h_c 13969
  URIt2 h_e + uri_e --> h_c + uri_c 10921 or 11871 or 12902 or 13969 or (10921 and 13969) or (11871 and 13969) or (12902 and 13969)
  CYTDt2 cytd_e + h_e --> cytd_c + h_c 13969
  ADNtm adn_c <=> adn_m 13969
```

In [14]:

```
Annotation.loc[temp]
```

Out[14]:

|  | Combined Annotations | Signal P | Sc288c Orthologs | Human Orthologs | Sc288 Best Hit | Human Blast | Essential | WolfPSort | C Terminal |
| --- | --- | --- | --- | --- | --- | --- | --- | --- | --- |
| RTO4\_ID |  |  |  |  |  |  |  |  |  |
| 12743 | K14209: SLC36A, PAT; solute carrier family 36 ... |  | AVT3,AVT4 | SLC36A1,SLC36A2,SLC36A3,SLC36A4 | AVT3 | SLC36 | Not Essential | plas 13, E.R. 6, extr 5, vacu 2 | PRA\* |
| 13969 | K15014: SLC29A1\_2\_3, ENT1\_2\_3; solute carrier ... |  | FUN26 | SLC29A3 |  | SLC29 | Not Essential | plas 17, E.R. 8 | PFV\* |
| 12375 | K12373: HEXA\_B; hexosaminidase | S |  | HEXA,HEXB |  | HEXB | Not Essential | mito 13, extr 10, cyto 4 | VEL\* |
| 15421 | K01192: E3.2.1.25, MANBA, manB; beta-mannosidase |  |  | MANBA |  | MANBA | Not Essential | mito 14.5, cyto\_mito 10.833, cyto 6, cyto\_nucl... | LGD\* |
| 15283 | K01130: E3.1.6.1, aslA; arylsulfatase |  |  | ARSB,ARSI,ARSJ |  | ARSJ | Not Essential | mito 7.5, cyto\_mito 7.5, cyto 6.5, cysk 4, nuc... | TEQ\* |
| 15267 | K01130: E3.1.6.1, aslA; arylsulfatase |  |  | ARSB,ARSI,ARSJ |  | ARSJ | Not Essential | cyto 7.5, cyto\_mito 6.5, nucl 5, mito 4.5, cys... | TEQ\* |

In [15]:

```
model.remove_reactions([r for r in model.reactions if 'p' in r.compartments], remove_orphans=True)
model.remove_reactions([r for r in model.reactions if 'h' in r.compartments], remove_orphans=True)
model.remove_reactions([r for r in model.reactions if 's' in r.compartments], remove_orphans=True)
model.remove_reactions([r for r in model.reactions if 'f' in r.compartments], remove_orphans=True)
model.remove_reactions([r for r in model.reactions if 'l' in r.compartments], remove_orphans=True)
```

In [16]:

```
print(len(model.genes))
print(len(model.reactions))
print(len(model.metabolites))
model
```

```
1195
2102
2144
```

Out[16]:

|  |  |
| --- | --- |
| **Name** | R. toruloides |
| **Memory address** | 0x010296144e0 |
| **Number of metabolites** | 2144 |
| **Number of reactions** | 2102 |
| **Number of groups** | 0 |
| **Objective expression** | 0 |
| **Compartments** | c, x, m, e, r, v, n, g, d |

In [17]:

```
PTS1 = pd.read_csv('../../Data/WoLFPSort/fimo_PTS1_all_last_9/fimo.txt', sep='\t')
PTS1
```

Out[17]:

|  | #pattern name | sequence name | start | stop | strand | score | p-value | q-value | matched sequence |
| --- | --- | --- | --- | --- | --- | --- | --- | --- | --- |
| 0 | 1 | 13926 | 1 | 8 | + | 12.95950 | 0.000004 | 0.0336 | IKKDKAKL |
| 1 | 1 | 13437 | 1 | 8 | + | 12.56760 | 0.000008 | 0.0336 | VKKQKSKL |
| 2 | 1 | 15900 | 1 | 8 | + | 11.98650 | 0.000019 | 0.0448 | VKQQKAKL |
| 3 | 1 | 14802 | 1 | 8 | + | 11.72970 | 0.000027 | 0.0448 | EDMTKSKL |
| 4 | 1 | 15992 | 1 | 8 | + | 11.71620 | 0.000027 | 0.0448 | KGFAKAKL |
| 5 | 1 | 15449 | 1 | 8 | + | 11.36490 | 0.000042 | 0.0520 | ALSGKSKL |
| 6 | 1 | 15684 | 1 | 8 | + | 11.32430 | 0.000044 | 0.0520 | SSPRKAKL |
| 7 | 1 | 12555 | 1 | 8 | + | 11.10810 | 0.000056 | 0.0552 | EGKARAKL |
| 8 | 1 | 11130 | 1 | 8 | + | 11.05410 | 0.000060 | 0.0552 | KRKRPSRL |
| 9 | 1 | 16481 | 1 | 8 | + | 10.93240 | 0.000069 | 0.0569 | ESSGKAKL |
| 10 | 1 | 10991 | 1 | 8 | + | 10.71620 | 0.000086 | 0.0651 | AQTVKAKL |
| 11 | 1 | 9457 | 1 | 8 | + | 10.56760 | 0.000100 | 0.0694 | APSQPAKL |
| 12 | 1 | 16635 | 1 | 8 | + | 10.37840 | 0.000121 | 0.0726 | EEKPRAKL |
| 13 | 1 | 16515 | 1 | 8 | + | 10.36490 | 0.000123 | 0.0726 | SQPRKARL |
| 14 | 1 | 8782 | 1 | 8 | + | 10.18920 | 0.000145 | 0.0773 | LKNAQSKL |
| 15 | 1 | 11907 | 1 | 8 | + | 10.06760 | 0.000162 | 0.0773 | PLPGPAKL |
| 16 | 1 | 14245 | 1 | 8 | + | 10.05410 | 0.000164 | 0.0773 | PSADKAKL |
| 17 | 1 | 8888 | 1 | 8 | + | 10.02700 | 0.000168 | 0.0773 | LPTATARL |
| 18 | 1 | 10570 | 1 | 8 | + | 9.79730 | 0.000205 | 0.0829 | TREPKAKL |
| 19 | 1 | 12399 | 1 | 8 | + | 9.72973 | 0.000217 | 0.0829 | PEAASSKL |
| 20 | 1 | 13802 | 1 | 8 | + | 9.70270 | 0.000222 | 0.0829 | TRFAIAKL |
| 21 | 1 | 14934 | 1 | 8 | + | 9.67568 | 0.000227 | 0.0829 | PRQKRARL |
| 22 | 1 | 11263 | 1 | 8 | + | 9.66216 | 0.000230 | 0.0829 | VNFIKAHL |
| 23 | 1 | 8548 | 1 | 8 | + | 9.36486 | 0.000292 | 0.0980 | FLARQSKL |
| 24 | 1 | 14567 | 1 | 8 | + | 9.28378 | 0.000311 | 0.0980 | AASSRAKL |
| 25 | 1 | 9300 | 1 | 8 | + | 9.27027 | 0.000314 | 0.0980 | AYKSKAKL |
| 26 | 1 | 11237 | 1 | 8 | + | 9.22973 | 0.000324 | 0.0980 | WETVKAKL |
| 27 | 1 | 10727 | 2 | 9 | + | 9.20270 | 0.000331 | 0.0980 | QLRPKKKL |
| 28 | 1 | 14040 | 1 | 8 | + | 9.10811 | 0.000355 | 0.1020 | ALQLESKL |
| 29 | 1 | 15693 | 1 | 8 | + | 8.95946 | 0.000396 | 0.1090 | AEKQKAKL |
| 30 | 1 | 16101 | 1 | 8 | + | 8.89189 | 0.000416 | 0.1110 | ESKGLSKL |
| 31 | 1 | 12265 | 1 | 8 | + | 8.78378 | 0.000449 | 0.1140 | KVKRESKL |
| 32 | 1 | 13626 | 1 | 8 | + | 8.77027 | 0.000453 | 0.1140 | EATLPAKL |
| 33 | 1 | 8416 | 1 | 8 | + | 8.54054 | 0.000530 | 0.1260 | GDTPFSRL |
| 34 | 1 | 13228 | 1 | 8 | + | 8.51351 | 0.000539 | 0.1260 | GEAPTSKL |
| 35 | 1 | 15276 | 1 | 8 | + | 8.45946 | 0.000559 | 0.1260 | DVPEKSKL |
| 36 | 1 | 10360 | 1 | 8 | + | 8.40541 | 0.000579 | 0.1260 | WSLRPARL |
| 37 | 1 | 12820 | 1 | 8 | + | 8.37838 | 0.000589 | 0.1260 | AKDEPSKL |
| 38 | 1 | 15540 | 1 | 8 | + | 8.35135 | 0.000599 | 0.1260 | SDLFPAKL |
| 39 | 1 | 9163 | 1 | 8 | + | 8.32432 | 0.000610 | 0.1260 | VSGGRSKL |
| 40 | 1 | 13803 | 1 | 8 | + | 8.10811 | 0.000698 | 0.1390 | FSIRKPRL |
| 41 | 1 | 14553 | 1 | 8 | + | 8.08108 | 0.000710 | 0.1390 | PTFARAKL |
| 42 | 1 | 14499 | 1 | 8 | + | 8.02703 | 0.000734 | 0.1390 | KFEDSSKL |
| 43 | 1 | 15966 | 1 | 8 | + | 8.01351 | 0.000740 | 0.1390 | ETPLKAKL |
| 44 | 1 | 11833 | 1 | 8 | + | 7.93243 | 0.000777 | 0.1400 | ASNQPAKL |
| 45 | 1 | 12784 | 1 | 8 | + | 7.93243 | 0.000777 | 0.1400 | KDVPDAKL |
| 46 | 1 | 10338 | 1 | 8 | + | 7.85135 | 0.000815 | 0.1440 | ADQEFAKL |
| 47 | 1 | 14950 | 1 | 8 | + | 7.79730 | 0.000842 | 0.1440 | PVRALAKL |
| 48 | 1 | 14262 | 1 | 8 | + | 7.77027 | 0.000855 | 0.1440 | ESRPPPHL |
| 49 | 1 | 15783 | 1 | 8 | + | 7.70270 | 0.000889 | 0.1440 | VEEAKAKL |
| 50 | 1 | 12434 | 1 | 8 | + | 7.68919 | 0.000896 | 0.1440 | VPVSVSKM |
| 51 | 1 | 12742 | 1 | 8 | + | 7.67568 | 0.000903 | 0.1440 | GVRAWSKL |

In [18]:

```
for x in PTS1['sequence name']:
    if str(x) in model.genes:
        print(x)
        for r in model.genes.get_by_id(str(x)).reactions:
            print(r.id, r.reaction, r.gene_reaction_rule)
        print()
```

```
15900
FACOAL161p atp_x + coa_x + hdcea_x --> amp_x + hdcoa_x + ppi_x 12555 or 15900
FACOAL140p atp_x + coa_x + ttdca_x --> amp_x + ppi_x + tdcoa_x 12555 or 15900
FACOAL100p atp_x + coa_x + dca_x --> amp_x + dcacoa_x + ppi_x 12555 or 15900
FACOAL141p atp_x + coa_x + ttdcea_x --> amp_x + ppi_x + tdecoa_x 12555 or 15900
FACOAL80p atp_x + coa_x + octa_x --> amp_x + occoa_x + ppi_x 12555 or 15900
FACOAL160p atp_x + coa_x + hdca_x --> amp_x + pmtcoa_x + ppi_x 12555 or 15900
FACOAL120p atp_x + coa_x + ddca_x --> amp_x + ddcacoa_x + ppi_x 12555 or 15900

14802
4CMCOAS T4hcinnm_c + atp_c + coa_c --> amp_c + coucoa_c + ppi_c 11833 or 13700 or 14802 or 16129

15449
DASPO1p asp__D_x + h2o_x + o2_x --> h2o2_x + nh4_x + oaa_x 11876 or 15449 or 15994

12555
FACOAL161p atp_x + coa_x + hdcea_x --> amp_x + hdcoa_x + ppi_x 12555 or 15900
FACOAL140p atp_x + coa_x + ttdca_x --> amp_x + ppi_x + tdcoa_x 12555 or 15900
FACOAL100p atp_x + coa_x + dca_x --> amp_x + dcacoa_x + ppi_x 12555 or 15900
FACOAL141p atp_x + coa_x + ttdcea_x --> amp_x + ppi_x + tdecoa_x 12555 or 15900
FACOAL80p atp_x + coa_x + octa_x --> amp_x + occoa_x + ppi_x 12555 or 15900
FACOAL160p atp_x + coa_x + hdca_x --> amp_x + pmtcoa_x + ppi_x 12555 or 15900
FACOAL120p atp_x + coa_x + ddca_x --> amp_x + ddcacoa_x + ppi_x 12555 or 15900

9457
MALSp accoa_x + glx_x + h2o_x --> coa_x + h_x + mal__L_x 9457

16635
COUCOAFm T4hcinnm_m + atp_m + coa_m --> amp_m + coucoa_m + ppi_m 16635

11907
FAO181p_even 8.0 coa_x + 8.0 h2o_x + 8.0 nad_x + nadph_x + 8.0 o2_x + odecoa_x --> 9.0 accoa_x + 8.0 h2o2_x + 7.0 h_x + 8.0 nadh_x + nadp_x (10293 and 11362 and 11907 and 12742 and 13228 and 13813) or (10293 and 11362 and 11907 and 12752 and 13228 and 13813) or (10293 and 11362 and 11907 and 13228 and 13813 and 9700)
FAO183p_even 8.0 coa_x + 8.0 h2o_x + lnlncgcoa_x + 8.0 nad_x + 3.0 nadph_x + 8.0 o2_x --> 9.0 accoa_x + 8.0 h2o2_x + 5.0 h_x + 8.0 nadh_x + 3.0 nadp_x (10293 and 11362 and 11907 and 12742 and 13228 and 13813) or (10293 and 11362 and 11907 and 12752 and 13228 and 13813) or (10293 and 11362 and 11907 and 13228 and 13813 and 9700)
FAO161p_even 7.0 coa_x + 7.0 h2o_x + hdcoa_x + 7.0 nad_x + nadph_x + 7.0 o2_x --> 8.0 accoa_x + 7.0 h2o2_x + 6.0 h_x + 7.0 nadh_x + nadp_x (10293 and 11362 and 11907 and 12742 and 13228 and 13813) or (10293 and 11362 and 11907 and 12752 and 13228 and 13813) or (10293 and 11362 and 11907 and 13228 and 13813 and 9700)
FAO141p_even 6.0 coa_x + 6.0 h2o_x + 6.0 nad_x + nadph_x + 6.0 o2_x + tdecoa_x --> 7.0 accoa_x + 6.0 h2o2_x + 5.0 h_x + 6.0 nadh_x + nadp_x (10293 and 11362 and 11907 and 12742 and 13228 and 13813) or (10293 and 11362 and 11907 and 12752 and 13228 and 13813) or (10293 and 11362 and 11907 and 13228 and 13813 and 9700)
FAO182p_even 8.0 coa_x + 8.0 h2o_x + 8.0 nad_x + 2.0 nadph_x + 8.0 o2_x + ocdycacoa_x --> 9.0 accoa_x + 8.0 h2o2_x + 6.0 h_x + 8.0 nadh_x + 2.0 nadp_x (10293 and 11362 and 11907 and 12742 and 13228 and 13813) or (10293 and 11362 and 11907 and 12752 and 13228 and 13813) or (10293 and 11362 and 11907 and 13228 and 13813 and 9700)

14245
CSNATr accoa_c + crn_c <=> acrn_c + coa_c 13580 or 14245
CSNAT2m coa_m + pcrn_m <=> crn_m + ppcoa_m 14245
CSNAT2x crn_x + dmnoncoa_x <=> coa_x + dmnoncrn_x 14245
CSNATp accoa_x + crn_x <=> acrn_x + coa_x 14245
CSNATer accoa_r + crn_r <=> acrn_r + coa_r 14245
CSNAT3x crn_x + ppcoa_x <=> coa_x + pcrn_x 14245
CSNATm acrn_m + coa_m <=> accoa_m + crn_m 14245
CSNATirm accoa_m + crn_m <=> acrn_m + coa_m 13580 or 14245

10570
yli_R0495 h2o_c + o2_c + urate_c --> 5hiu_c + h2o2_c 10570
URO h2o_m + o2_m + urate_m --> 5hiu_m + h2o2_m + h_m 10570

8548
CSp accoa_x + h2o_x + oaa_x --> cit_x + coa_x + h_x 8548

9300
TPI dhap_c <=> g3p_c 9300

15693
HMGLm hmgcoa_m --> acac_m + accoa_m 15693
HMGLx hmgcoa_x --> acac_x + accoa_x 15693

13228
FAO181p_even 8.0 coa_x + 8.0 h2o_x + 8.0 nad_x + nadph_x + 8.0 o2_x + odecoa_x --> 9.0 accoa_x + 8.0 h2o2_x + 7.0 h_x + 8.0 nadh_x + nadp_x (10293 and 11362 and 11907 and 12742 and 13228 and 13813) or (10293 and 11362 and 11907 and 12752 and 13228 and 13813) or (10293 and 11362 and 11907 and 13228 and 13813 and 9700)
FAO183p_even 8.0 coa_x + 8.0 h2o_x + lnlncgcoa_x + 8.0 nad_x + 3.0 nadph_x + 8.0 o2_x --> 9.0 accoa_x + 8.0 h2o2_x + 5.0 h_x + 8.0 nadh_x + 3.0 nadp_x (10293 and 11362 and 11907 and 12742 and 13228 and 13813) or (10293 and 11362 and 11907 and 12752 and 13228 and 13813) or (10293 and 11362 and 11907 and 13228 and 13813 and 9700)
FAO161p_even 7.0 coa_x + 7.0 h2o_x + hdcoa_x + 7.0 nad_x + nadph_x + 7.0 o2_x --> 8.0 accoa_x + 7.0 h2o2_x + 6.0 h_x + 7.0 nadh_x + nadp_x (10293 and 11362 and 11907 and 12742 and 13228 and 13813) or (10293 and 11362 and 11907 and 12752 and 13228 and 13813) or (10293 and 11362 and 11907 and 13228 and 13813 and 9700)
FAO141p_even 6.0 coa_x + 6.0 h2o_x + 6.0 nad_x + nadph_x + 6.0 o2_x + tdecoa_x --> 7.0 accoa_x + 6.0 h2o2_x + 5.0 h_x + 6.0 nadh_x + nadp_x (10293 and 11362 and 11907 and 12742 and 13228 and 13813) or (10293 and 11362 and 11907 and 12752 and 13228 and 13813) or (10293 and 11362 and 11907 and 13228 and 13813 and 9700)
FAO182p_even 8.0 coa_x + 8.0 h2o_x + 8.0 nad_x + 2.0 nadph_x + 8.0 o2_x + ocdycacoa_x --> 9.0 accoa_x + 8.0 h2o2_x + 6.0 h_x + 8.0 nadh_x + 2.0 nadp_x (10293 and 11362 and 11907 and 12742 and 13228 and 13813) or (10293 and 11362 and 11907 and 12752 and 13228 and 13813) or (10293 and 11362 and 11907 and 13228 and 13813 and 9700)
FAO182p_odd 8.0 coa_x + 8.0 h2o_x + 8.0 nad_x + nadph_x + 7.0 o2_x + ocdycacoa_x --> 9.0 accoa_x + 7.0 h2o2_x + 7.0 h_x + 8.0 nadh_x + nadp_x (10293 and 11362 and 12742 and 13228 and 13813) or (10293 and 11362 and 12752 and 13228 and 13813) or (10293 and 11362 and 13228 and 13813 and 9700)
FAO183p_odd 8.0 coa_x + 8.0 h2o_x + lnlncgcoa_x + 8.0 nad_x + 2.0 nadph_x + 7.0 o2_x --> 9.0 accoa_x + 7.0 h2o2_x + 6.0 h_x + 8.0 nadh_x + 2.0 nadp_x (10293 and 11362 and 12742 and 13228 and 13813) or (10293 and 11362 and 12752 and 13228 and 13813) or (10293 and 11362 and 13228 and 13813 and 9700)

15276
AACOATx acac_x + atp_x + coa_x <=> aacoa_x + amp_x + ppi_x 15276

14499
PGLp 6pgl_x + h2o_x --> 6pgc_x + h_x 14499

11833
4CMCOAS T4hcinnm_c + atp_c + coa_c --> amp_c + coucoa_c + ppi_c 11833 or 13700 or 14802 or 16129

12784
ALR2 h_c + mthgxl_c + nadph_c --> acetol_c + nadp_c 11882 or 12784 or 9774
SBTR glc__D_c + h_c + nadph_c --> nadp_c + sbt__D_c 11882 or 12784 or 9774
ALCD2y etoh_c + nadp_c --> acald_c + h_c + nadph_c 11882 or 12784 or 9774
ALCD22yi 2mbald_c + h_c + nadph_c --> 2mbtoh_c + nadp_c 10029 or 11882 or 12784 or 13554 or 13562 or 13947 or 9774
ARABR arab__L_c + h_c + nadph_c --> abt_c + nadp_c 11882 or 12784 or 9774
GALOR gal_c + h_c + nadph_c <=> galt_c + nadp_c 11882 or 12784 or 9774
GLYCDy glyc_c + nadp_c --> dha_c + h_c + nadph_c 11882 or 12784 or 9774
ALR3 acetol_c + h_c + nadph_c --> 12ppd__S_c + nadp_c 11882 or 12784 or 9774
LALDO2 h_c + mthgxl_c + nadph_c --> lald__D_c + nadp_c 11882 or 12784 or 9774
ALCD19y glyald_c + h_c + nadph_c --> glyc_c + nadp_c 11882 or 12784 or 9774
PPDOy h_c + lald__D_c + nadph_c --> 12ppd__R_c + nadp_c 10029 or 11882 or 12784 or 13554 or 13562 or 13947 or 9774

14950
GLXO2p glx_x + h2o_x + o2_x --> h2o2_x + h_x + oxa_x 14950 or 16607
GLYCTO1p glyclt_x + o2_x --> glx_x + h2o2_x 14950 or 16607

12434
NADDPp h2o_x + nad_x --> amp_x + 2.0 h_x + nmn_x 12434

12742
FAOXC241181x 3.0 coa_x + 3.0 h2o_x + 3.0 nad_x + nrvnccoa_x + 3.0 o2_x --> 3.0 accoa_x + 3.0 h2o2_x + 3.0 h_x + 3.0 nadh_x + odecoa_x (12742 and 11362 and 13813) or (12752 and 11362 and 13813) or (9700 and 11362 and 13813)
ACOX22x dhcholoylcoa_x + fadh2_x + 0.5 o2_x --> fad_x + thcholoylcoa_x 12742 or 12752 or 9700
FAO161p_odd 7.0 coa_x + 7.0 h2o_x + hdcoa_x + 7.0 nad_x + 6.0 o2_x --> 8.0 accoa_x + 6.0 h2o2_x + 7.0 h_x + 7.0 nadh_x (10293 and 11362 and 12742 and 13813) or (10293 and 11362 and 12752 and 13813) or (10293 and 11362 and 13813 and 9700)
FAO182p_even 8.0 coa_x + 8.0 h2o_x + 8.0 nad_x + 2.0 nadph_x + 8.0 o2_x + ocdycacoa_x --> 9.0 accoa_x + 8.0 h2o2_x + 6.0 h_x + 8.0 nadh_x + 2.0 nadp_x (10293 and 11362 and 11907 and 12742 and 13228 and 13813) or (10293 and 11362 and 11907 and 12752 and 13228 and 13813) or (10293 and 11362 and 11907 and 13228 and 13813 and 9700)
FAO182p_odd 8.0 coa_x + 8.0 h2o_x + 8.0 nad_x + nadph_x + 7.0 o2_x + ocdycacoa_x --> 9.0 accoa_x + 7.0 h2o2_x + 7.0 h_x + 8.0 nadh_x + nadp_x (10293 and 11362 and 12742 and 13228 and 13813) or (10293 and 11362 and 12752 and 13228 and 13813) or (10293 and 11362 and 13228 and 13813 and 9700)
FAOXC2442246x coa_x + h2o_x + nad_x + o2_x + tettet6coa_x --> accoa_x + adrncoa_x + h2o2_x + h_x + nadh_x (12742 and 11362 and 13813) or (12752 and 11362 and 13813) or (9700 and 11362 and 13813)
FAOXC2452256x coa_x + h2o_x + nad_x + o2_x + tetpent6coa_x --> accoa_x + dcsptn1coa_x + h2o2_x + h_x + nadh_x (12742 and 11362 and 13813) or (12752 and 11362 and 13813) or (9700 and 11362 and 13813)
FAOXC200180x arachcoa_x + coa_x + h2o_x + nad_x + o2_x --> accoa_x + h2o2_x + h_x + nadh_x + stcoa_x (12742 and 11362 and 13813) or (12752 and 11362 and 13813) or (9700 and 11362 and 13813)
FAO181p_even 8.0 coa_x + 8.0 h2o_x + 8.0 nad_x + nadph_x + 8.0 o2_x + odecoa_x --> 9.0 accoa_x + 8.0 h2o2_x + 7.0 h_x + 8.0 nadh_x + nadp_x (10293 and 11362 and 11907 and 12742 and 13228 and 13813) or (10293 and 11362 and 11907 and 12752 and 13228 and 13813) or (10293 and 11362 and 11907 and 13228 and 13813 and 9700)
FAO80p 3.0 coa_x + 3.0 h2o_x + 3.0 nad_x + 3.0 o2_x + occoa_x --> 4.0 accoa_x + 3.0 h2o2_x + 3.0 h_x + 3.0 nadh_x (11362 and 12742 and 13813) or (11362 and 12742 and 9065) or (11362 and 12752 and 13813) or (11362 and 12752 and 9065) or (11362 and 13813 and 9700) or (11362 and 9065 and 9700)
ACOAO7p o2_x + pmtcoa_x --> h2o2_x + hdd2coa_x 12742 or 12752 or 9700
FAOXC2452253x coa_x + h2o_x + nad_x + o2_x + tetpent3coa_x --> accoa_x + clpndcoa_x + h2o2_x + h_x + nadh_x (12742 and 11362 and 13813) or (12752 and 11362 and 13813) or (9700 and 11362 and 13813)
FAO183p_even 8.0 coa_x + 8.0 h2o_x + lnlncgcoa_x + 8.0 nad_x + 3.0 nadph_x + 8.0 o2_x --> 9.0 accoa_x + 8.0 h2o2_x + 5.0 h_x + 8.0 nadh_x + 3.0 nadp_x (10293 and 11362 and 11907 and 12742 and 13228 and 13813) or (10293 and 11362 and 11907 and 12752 and 13228 and 13813) or (10293 and 11362 and 11907 and 13228 and 13813 and 9700)
FAO161p_even 7.0 coa_x + 7.0 h2o_x + hdcoa_x + 7.0 nad_x + nadph_x + 7.0 o2_x --> 8.0 accoa_x + 7.0 h2o2_x + 6.0 h_x + 7.0 nadh_x + nadp_x (10293 and 11362 and 11907 and 12742 and 13228 and 13813) or (10293 and 11362 and 11907 and 12752 and 13228 and 13813) or (10293 and 11362 and 11907 and 13228 and 13813 and 9700)
FAO141p_even 6.0 coa_x + 6.0 h2o_x + 6.0 nad_x + nadph_x + 6.0 o2_x + tdecoa_x --> 7.0 accoa_x + 6.0 h2o2_x + 5.0 h_x + 6.0 nadh_x + nadp_x (10293 and 11362 and 11907 and 12742 and 13228 and 13813) or (10293 and 11362 and 11907 and 12752 and 13228 and 13813) or (10293 and 11362 and 11907 and 13228 and 13813 and 9700)
ACOAO9p hexccoa_x + o2_x --> h2o2_x + hxc2coa_x 12742 or 12752 or 9700
ACOAO5p ddcacoa_x + o2_x --> dd2coa_x + h2o2_x 12742 or 12752 or 9700
FAOXC240200x 2.0 coa_x + 2.0 h2o_x + 2.0 nad_x + 2.0 o2_x + ttccoa_x --> 2.0 accoa_x + arachcoa_x + 2.0 h2o2_x + 2.0 h_x + 2.0 nadh_x (12742 and 11362 and 13813) or (12752 and 11362 and 13813) or (9700 and 11362 and 13813)
ACOAO4p dcacoa_x + o2_x --> dc2coa_x + h2o2_x 12742 or 12752 or 9700
ACOAO6p o2_x + tdcoa_x --> h2o2_x + td2coa_x 12742 or 12752 or 9700
FAO181p_odd 8.0 coa_x + 8.0 h2o_x + 8.0 nad_x + 7.0 o2_x + odecoa_x --> 9.0 accoa_x + 7.0 h2o2_x + 8.0 h_x + 8.0 nadh_x (10293 and 11362 and 12742 and 13813) or (10293 and 11362 and 12752 and 13813) or (10293 and 11362 and 13813 and 9700)
FAO141p_odd 6.0 coa_x + 6.0 h2o_x + 6.0 nad_x + 5.0 o2_x + tdecoa_x --> 7.0 accoa_x + 5.0 h2o2_x + 6.0 h_x + 6.0 nadh_x (10293 and 11362 and 12742 and 13813) or (10293 and 11362 and 12752 and 13813) or (10293 and 11362 and 13813 and 9700)
ACOX2x cholcoas_x + fadh2_x + o2_x --> cholcoads_x + fad_x + 2.0 h2o_x 12742 or 12752 or 9700
ACOAO8p o2_x + stcoa_x --> h2o2_x + od2coa_x 12742 or 12752 or 9700
FAO183p_odd 8.0 coa_x + 8.0 h2o_x + lnlncgcoa_x + 8.0 nad_x + 2.0 nadph_x + 7.0 o2_x --> 9.0 accoa_x + 7.0 h2o2_x + 6.0 h_x + 8.0 nadh_x + 2.0 nadp_x (10293 and 11362 and 12742 and 13228 and 13813) or (10293 and 11362 and 12752 and 13228 and 13813) or (10293 and 11362 and 13228 and 13813 and 9700)
```

In [19]:

```
PTS2 = pd.read_csv('../../Data/WoLFPSort/fimo_PTS2_all_first_50/fimo.txt', sep='\t')
PTS2
```

Out[19]:

|  | #pattern name | sequence name | start | stop | strand | score | p-value | q-value | matched sequence |
| --- | --- | --- | --- | --- | --- | --- | --- | --- | --- |
| 0 | 1 | 10551 | 6 | 14 | + | 9.6250 | 0.000001 | 0.166 | RLQQVQGQL |
| 1 | 1 | 16092 | 8 | 16 | + | 8.8375 | 0.000002 | 0.166 | RLRSVAAHL |
| 2 | 1 | 9211 | 4 | 12 | + | 8.7125 | 0.000002 | 0.166 | RSQVVLRLL |
| 3 | 1 | 12390 | 5 | 13 | + | 8.3625 | 0.000002 | 0.166 | RLQPLLRPL |
| 4 | 1 | 9739 | 7 | 15 | + | 8.2750 | 0.000002 | 0.166 | AHQQVLDHL |
| ... | ... | ... | ... | ... | ... | ... | ... | ... | ... |
| 66 | 1 | 11426 | 13 | 21 | + | -2.7375 | 0.000093 | 0.480 | ALQVLLRRP |
| 67 | 1 | 14402 | 28 | 36 | + | -2.8250 | 0.000097 | 0.480 | RKQAVKDLL |
| 68 | 1 | 11183 | 39 | 47 | + | -2.8250 | 0.000097 | 0.480 | RIQQLNRYL |
| 69 | 1 | 10699 | 35 | 43 | + | -2.8500 | 0.000097 | 0.480 | RLRILLFAA |
| 70 | 1 | 12414 | 36 | 44 | + | -2.9125 | 0.000100 | 0.487 | LDQSLLGEL |

71 rows × 9 columns

In [20]:

```
for x in PTS2['sequence name']:
    if str(x) in model.genes:
        print(x)
        for r in model.genes.get_by_id(str(x)).reactions:
            print(r.id, r.reaction, r.gene_reaction_rule)
        print()
```

```
16092
FMNATm atp_m + fmn_m + h_m --> fad_m + ppi_m 16092

9596
GGNG Tyr_ggn_c + 8.0 udpg_c --> ggn_c + 8.0 h_c + 8.0 udp_c (mmu_8908 and 9596) or (14002 and 9596)
GLGNS1 ggn_c + 3.0 udpg_c --> glygn1_c + 3.0 h_c + 3.0 udp_c (mmu_8908 and 9596) or (14002 and 9596)
GLCS2 udpg_c --> glycogen_c + h_c + udp_c 14002 and 9596
yli_R0726 h2o_c + udpg_c --> amylose_c + h_c + udp_c 9596
GLYGS h2o_c + udpg_c --> 14glun_c + h_c + udp_c 14002 and 9596

12272
ACSm ac_m + atp_m + coa_m --> accoa_m + amp_m + ppi_m 12272 or 14597
FACOAL40im atp_m + but_m + coa_m --> amp_m + btcoa_m + ppi_m 12272

16648
NTD2 h2o_c + ump_c --> pi_c + uri_c 13044 or 16648 or 9995
GDTP gdptp_c + h2o_c <=> 2.0 h_c + pi_c + ppgpp_c 16648
NTD7 amp_c + h2o_c --> adn_c + pi_c 13044 or 16648
NTD10 h2o_c + xmp_c --> pi_c + xtsn_c 16648
NTD12 dimp_c + h2o_c --> din_c + pi_c 16648
NTD4 cmp_c + h2o_c --> cytd_c + pi_c 13044 or 16648 or 9995
NTD1 dump_c + h2o_c --> duri_c + pi_c 16648
NTD11 h2o_c + imp_c --> ins_c + pi_c 16648
NTD3 dcmp_c + h2o_c --> dcyt_c + pi_c 16648
NTD6 damp_c + h2o_c --> dad_2_c + pi_c 16648
PPA h2o_c + ppi_c --> h_c + 2.0 pi_c 15879 or 16648
NTD9 gmp_c + h2o_c --> gsn_c + pi_c 13044 or 16648
NTD8 dgmp_c + h2o_c --> dgsn_c + pi_c 16648
PPA2 h2o_c + pppi_c --> h_c + pi_c + ppi_c 16648
NTD5 dtmp_c + h2o_c --> pi_c + thymd_c 16648

13252
ATPS3m adp_m + 3.0 h_c + pi_m --> atp_m + h2o_m + 2.0 h_m RTO3_879280 and RTO3_900623 and RTO3_900663 and 9080 and 9619 and 9940 and 10674 and 11958 and 11967 and 13053 and 13252 and 13424 and 13759 and 13842 and 14786 and 14912 and 15287 and 15589 and 15880 and 16359

10558
RHACOAR100 3odcoa_c + h_c + nadph_c <=> R_3hdcoa_c + nadp_c 10558
3OAR160 3opalmACP_c + h_c + nadph_c <=> 3hpalmACP_c + nadp_c 10558
3OAR40 actACP_c + h_c + nadph_c <=> 3haACP_c + nadp_c 10558
3OAR180 3ooctdACP_c + h_c + nadph_c <=> 3hoctaACP_c + nadp_c 10558
3OAR100 3odecACP_c + h_c + nadph_c <=> 3hdecACP_c + nadp_c 10558
3OAR161 3ocpalm9eACP_c + h_c + nadph_c --> 3hcpalm9eACP_c + nadp_c 10558
3OAR141 3ocmrs7eACP_c + h_c + nadph_c --> 3hcmrs7eACP_c + nadp_c 10558
3OACOAR 3ohodcoa_c + h_c + nadph_c --> 3hodcoa_c + nadp_c 10558
3OAR120 3oddecACP_c + h_c + nadph_c <=> 3hddecACP_c + nadp_c 10558
3OAR80 3ooctACP_c + h_c + nadph_c <=> 3hoctACP_c + nadp_c 10558
3OAR121 3ocddec5eACP_c + h_c + nadph_c --> 3hcddec5eACP_c + nadp_c 10558
3OAR140 3omrsACP_c + h_c + nadph_c <=> 3hmrsACP_c + nadp_c 10558
RHACOAR140 3otdcoa_c + h_c + nadph_c <=> R_3hmrscoa_c + nadp_c 10558
RHACOAR120 3oddcoa_c + h_c + nadph_c <=> R_3hddcoa_c + nadp_c 10558
OGMEACPR h_c + nadph_c + ogmeACP_c --> hgmeACP_c + nadp_c 10558
3OAR60 3ohexACP_c + h_c + nadph_c <=> 3hhexACP_c + nadp_c 10558
OPMEACPR h_c + nadph_c + opmeACP_c --> hpmeACP_c + nadp_c 10558
RHACOAR60 3ohcoa_c + h_c + nadph_c <=> R_3hhcoa_c + nadp_c 10558
3OAR181 3ocvac11eACP_c + h_c + nadph_c --> 3hcvac11eACP_c + nadp_c 10558
RHACOAR80 3oocoa_c + h_c + nadph_c <=> R_3hocoa_c + nadp_c 10558

12393
PGM 2pg_c <=> 3pg_c 11229 or 12393 or 15425 or 9910

11183
OIVD3m 3mop_m + coa_m + nad_m --> 2mbcoa_m + co2_m + nadh_m (10040 and 11183 and 12566 and 15436) or (10040 and 11188 and 12566 and 15436)
OBDHm 2obut_m + coa_m + nad_m --> co2_m + nadh_m + ppcoa_m (10040 and 11183 and 12566 and 15436) or (10040 and 11188 and 12566 and 15436)
OIVD2m 3mob_m + coa_m + nad_m --> co2_m + ibcoa_m + nadh_m (10040 and 11183 and 12566 and 15436) or (10040 and 11188 and 12566 and 15436)
OIVD1m 4mop_m + coa_m + nad_m --> co2_m + ivcoa_m + nadh_m (10040 and 11183 and 12566 and 15436) or (10040 and 11188 and 12566 and 15436)
```

### Genes¶

In [21]:

```
for g in sorted(model.genes, key=lambda x: x.id):
    if not g.id.isnumeric():
        print(g.id)
```

```
COPII
DPM3
Dpm3
ERG27
P4HA1
P4HA2
P4ha1
P4ha2
PAFAH1B2
PAFAH1B3
PIGH
PIGX
PP_0082
PP_0932
PP_1303
PP_2470
PP_2843
PP_2844
Pafah1b2
Pafah1b3
Pigh
Pigx
RPN2
RTO3_874609
RTO3_879280
RTO3_879341
RTO3_879512
RTO3_900622
RTO3_900623
RTO3_900624
RTO3_900641
RTO3_900663
RTO3_945994
RTO3_945995
RTO3_946002
RTO3_946004
RTO3_946005
RTO3_979594
Rpn2
SLC3A2
Slc3a2
YALI0A18656g
YALI0B09537g
YALI0D07392g
YALI0E20713g
YALI0F07260g
YALI0F25795g
YGL154C
YML100W
YMR261C
b0614
b0615
b0617
b0783
b0784
b1198
b1199
b1260
b1338
b2415
b2416
b2538
b2539
b2540
b2752
b2868
mmu_8908
```

In [22]:

```
excluded = ['COPII','ERG27','RTO3_874609','RTO3_879280','RTO3_879341','RTO3_879512','RTO3_900622','RTO3_900623',
            'RTO3_900624','RTO3_900641','RTO3_900663','RTO3_945994','RTO3_945995','RTO3_946002','RTO3_946004',
            'RTO3_946005','RTO3_979594']

for g in model.genes:
    if (not g.id.isnumeric()) and (not g.id in excluded):
        print(g.id)
        for r in g.reactions:
            print(' ', r, r.gene_reaction_rule)
```

```
YALI0F07260g
  yli_R0712: gdpmann_c + 0.01 yli_M04599_c --> gdp_c + h_c + 0.01 yli_M04615_c YALI0F07260g and 12953
  yli_R0716: gdpmann_c + 0.01 yli_M04603_c --> gdp_c + h_c + 0.01 yli_M04619_c YALI0F07260g and 12953
  yli_R0713: gdpmann_c + 0.01 yli_M04600_c --> gdp_c + h_c + 0.01 yli_M04616_c YALI0F07260g and 12953
  yli_R0715: gdpmann_c + 0.01 yli_M04602_c --> gdp_c + h_c + 0.01 yli_M04618_c YALI0F07260g and 12953
  yli_R0714: gdpmann_c + 0.01 yli_M04601_c --> gdp_c + h_c + 0.01 yli_M04617_c YALI0F07260g and 12953
  yli_R0717: gdpmann_c + 0.01 yli_M04604_c --> gdp_c + h_c + 0.01 yli_M04620_c YALI0F07260g and 12953
PP_2470
  PHETRS: atp_c + phe__L_c + trnaphe_c --> amp_c + phetrna_c + ppi_c (PP_2470 and 12470) or (12470 and 13249)
YALI0A18656g
  yli_R1477: gdpmann_r + yli_M00109_r --> dolmanp_r + gdp_r YALI0A18656g and 13484 and 9386
  yli_R0935: gdpmann_c + yli_M00109_c --> dolmanp_c + gdp_c YALI0A18656g and 13484 and 9386
YALI0F25795g
  METt2r: h_e + met__L_e <=> h_c + met__L_c 14229 or 15074 or (YALI0F25795g and 14229) or (YALI0F25795g and 15074)
b0615
  CITL: cit_c --> ac_c + oaa_c b0614 and b0615 and b0617 and 9822
b0617
  CITL: cit_c --> ac_c + oaa_c b0614 and b0615 and b0617 and 9822
b0614
  CITL: cit_c --> ac_c + oaa_c b0614 and b0615 and b0617 and 9822
YGL154C
  AASAD1: L2aadp_c + atp_c + h_c + nadph_c --> L2aadp6sa_c + amp_c + nadp_c + ppi_c 9495 or (YGL154C and 9495) or (10220 and 9495)
  AASAD2: L2aadp_c + atp_c + h_c + nadh_c --> L2aadp6sa_c + amp_c + nad_c + ppi_c 9495 or (YGL154C and 9495) or (10220 and 9495)
YALI0D07392g
  ADEt2: ade_e + h_e --> ade_c + h_c 14461 or 14558 or 15012 or (YALI0D07392g and 14461) or (YALI0D07392g and 14558) or (YALI0D07392g and 15012) or (14558 and 15012)
  GUAt2r: gua_e + h_e <=> gua_c + h_c 14461 or 14558 or 15012 or (YALI0D07392g and 14461) or (YALI0D07392g and 14558) or (YALI0D07392g and 15012) or (14558 and 15012)
  CSNt2: csn_e + h_e --> csn_c + h_c 14461 or 14558 or 15012 or (YALI0D07392g and 14461) or (YALI0D07392g and 14558) or (YALI0D07392g and 15012) or (14558 and 15012)
YALI0E20713g
  ASPt2r: asp__L_e + h_e <=> asp__L_c + h_c 14229 or 15074 or 8962 or 9319 or 9322 or 9962 or (YALI0E20713g and 14229 and 8962) or (YALI0E20713g and 14229 and 9319) or (YALI0E20713g and 14229 and 9322) or (YALI0E20713g and 14229 and 9962) or (YALI0E20713g and 15074 and 8962) or (YALI0E20713g and 15074 and 9319) or (YALI0E20713g and 15074 and 9322) or (YALI0E20713g and 15074 and 9962)
  SERt2r: h_e + ser__L_e <=> h_c + ser__L_c 14229 or 15074 or 8962 or 9319 or 9322 or 9962 or (YALI0E20713g and 14229 and 8962) or (YALI0E20713g and 14229 and 9319) or (YALI0E20713g and 14229 and 9322) or (YALI0E20713g and 14229 and 9962) or (YALI0E20713g and 15074 and 8962) or (YALI0E20713g and 15074 and 9319) or (YALI0E20713g and 15074 and 9322) or (YALI0E20713g and 15074 and 9962)
YALI0B09537g
  GLYt2r: gly_e + h_e <=> gly_c + h_c 12743 or 14229 or 15074 or 8962 or 9319 or 9322 or 9962 or (YALI0B09537g and 14229 and 8962) or (YALI0B09537g and 14229 and 9319) or (YALI0B09537g and 14229 and 9322) or (YALI0B09537g and 14229 and 9962) or (YALI0B09537g and 15074 and 8962) or (YALI0B09537g and 15074 and 9319) or (YALI0B09537g and 15074 and 9322) or (YALI0B09537g and 15074 and 9962)
  PROt2r: h_e + pro__L_e <=> h_c + pro__L_c 12743 or 14229 or 15074 or (YALI0B09537g and 14229) or (YALI0B09537g and 15074)
  ALAt2r: ala__L_e + h_e <=> ala__L_c + h_c 12743 or 14229 or 15074 or 8962 or 9319 or 9322 or 9962 or (YALI0B09537g and 14229 and 8962) or (YALI0B09537g and 14229 and 9319) or (YALI0B09537g and 14229 and 9322) or (YALI0B09537g and 14229 and 9962) or (YALI0B09537g and 15074 and 8962) or (YALI0B09537g and 15074 and 9319) or (YALI0B09537g and 15074 and 9322) or (YALI0B09537g and 15074 and 9962)
  ABUTt2r: 4abut_e + h_e <=> 4abut_c + h_c 11269 or 12743 or 13423 or (YALI0B09537g and 11269) or (YALI0B09537g and 13423)
YMR261C
  TRE6PP: h2o_c + tre6p_c --> pi_c + tre_c 12034 or (YML100W and 11389 and 12034) or (YMR261C and 11389 and 12034)
YML100W
  TRE6PP: h2o_c + tre6p_c --> pi_c + tre_c 12034 or (YML100W and 11389 and 12034) or (YMR261C and 11389 and 12034)
PP_1303
  SADT: atp_c + h_c + so4_c --> aps_c + ppi_c 13443 or 8709 or (PP_1303 and 14868)
b1260
  TRPS1: 3ig3p_c + ser__L_c --> g3p_c + h2o_c + trp__L_c 9262 or (PP_0082 and 9262) or (b1260 and 9262)
  TRPS2: indole_c + ser__L_c --> h2o_c + trp__L_c 9262 or (b1260 and 9262)
  TRPS3: 3ig3p_c --> g3p_c + indole_c b1260 and 9262
PP_0082
  TRPS1: 3ig3p_c + ser__L_c --> g3p_c + h2o_c + trp__L_c 9262 or (PP_0082 and 9262) or (b1260 and 9262)
PP_2844
  UREA: h2o_c + 2.0 h_c + urea_c --> co2_c + 2.0 nh4_c PP_2843 and PP_2844 and 11207
PP_2843
  UREA: h2o_c + 2.0 h_c + urea_c --> co2_c + 2.0 nh4_c PP_2843 and PP_2844 and 11207
b2752
  SADT2: atp_c + gtp_c + h2o_c + so4_c --> aps_c + gdp_c + pi_c + ppi_c b2752 and 14868
b2415
  DHAPT: dha_c + pep_c --> dhap_c + pyr_c b1198 and b1199 and b2415 and b2416 and 12269
b1198
  DHAPT: dha_c + pep_c --> dhap_c + pyr_c b1198 and b1199 and b2415 and b2416 and 12269
b1199
  DHAPT: dha_c + pep_c --> dhap_c + pyr_c b1198 and b1199 and b2415 and b2416 and 12269
b2416
  DHAPT: dha_c + pep_c --> dhap_c + pyr_c b1198 and b1199 and b2415 and b2416 and 12269
PP_0932
  GLNTRAT: atp_c + gln__L_c + glutrna_gln_c + h2o_c --> adp_c + glntrna_c + glu__L_c + h_c + pi_c PP_0932 and 11436 and 12640
b0783
  CPMPS: gtp_c + h2o_c --> cpmp_c + ppi_c b0783 and 15692
b2868
  HXAND: h2o_c + hxan_c + nad_c --> h_c + nadh_c + xan_c 15962 or (b2868 and 15962)
  XAND: h2o_c + nad_c + xan_c --> h_c + nadh_c + urate_c 15962 or (b2868 and 15962)
b0784
  MPTS: cpmp_c + cu2_c + 2.0 moadcosh_c --> 5.0 h_c + 2.0 moadcoo_c + mpt_c b0784 and 12718
b2540
  CINNDO: cinnm_c + h_c + nadh_c + o2_c --> cenchddd_c + nad_c b2538 and b2539 and b2540 and 10147
  PPPNDO: h_c + nadh_c + o2_c + pppn_c --> cechddd_c + nad_c b2538 and b2539 and b2540 and 10147
b2539
  CINNDO: cinnm_c + h_c + nadh_c + o2_c --> cenchddd_c + nad_c b2538 and b2539 and b2540 and 10147
  PPPNDO: h_c + nadh_c + o2_c + pppn_c --> cechddd_c + nad_c b2538 and b2539 and b2540 and 10147
b2538
  CINNDO: cinnm_c + h_c + nadh_c + o2_c --> cenchddd_c + nad_c b2538 and b2539 and b2540 and 10147
  PPPNDO: h_c + nadh_c + o2_c + pppn_c --> cechddd_c + nad_c b2538 and b2539 and b2540 and 10147
b1338
  4ABZGLUH: 4abzglu_c + h2o_c <=> 4abz_c + glu__L_c (b1338 and 15067) or (b1338 and 15072)
SLC3A2
  METLEUex: leu__L_c + met__L_e --> leu__L_e + met__L_c (SLC3A2 and 11625) or (Slc3a2 and 11625)
  ARGLYSex: arg__L_e + lys__L_c --> arg__L_c + lys__L_e (SLC3A2 and 11625) or (Slc3a2 and 11625)
Slc3a2
  METLEUex: leu__L_c + met__L_e --> leu__L_e + met__L_c (SLC3A2 and 11625) or (Slc3a2 and 11625)
  ARGLYSex: arg__L_e + lys__L_c --> arg__L_c + lys__L_e (SLC3A2 and 11625) or (Slc3a2 and 11625)
mmu_8908
  GGNG: Tyr_ggn_c + 8.0 udpg_c --> ggn_c + 8.0 h_c + 8.0 udp_c (mmu_8908 and 9596) or (14002 and 9596)
  GLGNS1: ggn_c + 3.0 udpg_c --> glygn1_c + 3.0 h_c + 3.0 udp_c (mmu_8908 and 9596) or (14002 and 9596)
P4HA1
  PROAKGOX1r: akg_r + o2_r + pro__L_r --> 4hpro_LT_r + co2_r + succ_r (P4HA1 and 10499) or (P4HA2 and 10499) or (P4ha1 and 10499) or (P4ha2 and 10499)
P4ha1
  PROAKGOX1r: akg_r + o2_r + pro__L_r --> 4hpro_LT_r + co2_r + succ_r (P4HA1 and 10499) or (P4HA2 and 10499) or (P4ha1 and 10499) or (P4ha2 and 10499)
P4HA2
  PROAKGOX1r: akg_r + o2_r + pro__L_r --> 4hpro_LT_r + co2_r + succ_r (P4HA1 and 10499) or (P4HA2 and 10499) or (P4ha1 and 10499) or (P4ha2 and 10499)
P4ha2
  PROAKGOX1r: akg_r + o2_r + pro__L_r --> 4hpro_LT_r + co2_r + succ_r (P4HA1 and 10499) or (P4HA2 and 10499) or (P4ha1 and 10499) or (P4ha2 and 10499)
RPN2
  DOLASNT_Ler: Asn_X_Ser_Thr_r + 0.1 g3m8mpdol__L_r --> 0.1 doldp__L_r + g3m8masn_r + h_r (RPN2 and 10629 and 12815 and 14867) or (Rpn2 and 10629 and 12815 and 14867)
  DOLASNT_Uer: Asn_X_Ser_Thr_r + 0.1 g3m8mpdol_U_r --> 0.1 doldp_U_r + g3m8masn_r + h_r (RPN2 and 10629 and 12815 and 14867) or (Rpn2 and 10629 and 12815 and 14867)
Rpn2
  DOLASNT_Ler: Asn_X_Ser_Thr_r + 0.1 g3m8mpdol__L_r --> 0.1 doldp__L_r + g3m8masn_r + h_r (RPN2 and 10629 and 12815 and 14867) or (Rpn2 and 10629 and 12815 and 14867)
  DOLASNT_Uer: Asn_X_Ser_Thr_r + 0.1 g3m8mpdol_U_r --> 0.1 doldp_U_r + g3m8masn_r + h_r (RPN2 and 10629 and 12815 and 14867) or (Rpn2 and 10629 and 12815 and 14867)
PIGH
  PIACGT: pail_hs_c + uacgam_c --> acgpail_hs_c + h_c + udp_c (PIGH and 11826 and 11850 and 13739 and 15144 and 9386) or (PIGH and 11826 and 11850 and 13739 and 15153 and 9386) or (Pigh and 11826 and 11850 and 13739 and 15144 and 9386) or (Pigh and 11826 and 11850 and 13739 and 15153 and 9386)
Pigh
  PIACGT: pail_hs_c + uacgam_c --> acgpail_hs_c + h_c + udp_c (PIGH and 11826 and 11850 and 13739 and 15144 and 9386) or (PIGH and 11826 and 11850 and 13739 and 15153 and 9386) or (Pigh and 11826 and 11850 and 13739 and 15144 and 9386) or (Pigh and 11826 and 11850 and 13739 and 15153 and 9386)
PIGX
  GPIMTer_U: 0.1 dolmanp_U_r + gacpail_hs_r --> 0.1 dolp_U_r + h_r + mgacpail_hs_r (PIGX and 9905) or (Pigx and 9905)
  GPIMTer_L: 0.1 dolmanp__L_r + gacpail_hs_r --> 0.1 dolp__L_r + h_r + mgacpail_hs_r (PIGX and 9905) or (Pigx and 9905)
Pigx
  GPIMTer_U: 0.1 dolmanp_U_r + gacpail_hs_r --> 0.1 dolp_U_r + h_r + mgacpail_hs_r (PIGX and 9905) or (Pigx and 9905)
  GPIMTer_L: 0.1 dolmanp__L_r + gacpail_hs_r --> 0.1 dolp__L_r + h_r + mgacpail_hs_r (PIGX and 9905) or (Pigx and 9905)
Dpm3
  DOLPMT_L: 0.1 dolp__L_c + gdpmann_c --> 0.1 dolmanp__L_c + gdp_c (DPM3 and 13484 and 9386) or (Dpm3 and 13484 and 9386)
  DOLPMT_U: 0.1 dolp_U_c + gdpmann_c --> 0.1 dolmanp_U_c + gdp_c (DPM3 and 13484 and 9386) or (Dpm3 and 13484 and 9386)
DPM3
  DOLPMT_L: 0.1 dolp__L_c + gdpmann_c --> 0.1 dolmanp__L_c + gdp_c (DPM3 and 13484 and 9386) or (Dpm3 and 13484 and 9386)
  DOLPMT_U: 0.1 dolp_U_c + gdpmann_c --> 0.1 dolmanp_U_c + gdp_c (DPM3 and 13484 and 9386) or (Dpm3 and 13484 and 9386)
Pafah1b2
  PAFH: h2o_c + paf_hs_c --> ac_c + ak2lgchol_hs_c + h_c 12283 or (PAFAH1B2 and PAFAH1B3) or (Pafah1b2 and Pafah1b3) or (PAFAH1B2 and PAFAH1B3 and 15227) or (Pafah1b2 and Pafah1b3 and 15227)
PAFAH1B3
  PAFH: h2o_c + paf_hs_c --> ac_c + ak2lgchol_hs_c + h_c 12283 or (PAFAH1B2 and PAFAH1B3) or (Pafah1b2 and Pafah1b3) or (PAFAH1B2 and PAFAH1B3 and 15227) or (Pafah1b2 and Pafah1b3 and 15227)
PAFAH1B2
  PAFH: h2o_c + paf_hs_c --> ac_c + ak2lgchol_hs_c + h_c 12283 or (PAFAH1B2 and PAFAH1B3) or (Pafah1b2 and Pafah1b3) or (PAFAH1B2 and PAFAH1B3 and 15227) or (Pafah1b2 and Pafah1b3 and 15227)
Pafah1b3
  PAFH: h2o_c + paf_hs_c --> ac_c + ak2lgchol_hs_c + h_c 12283 or (PAFAH1B2 and PAFAH1B3) or (Pafah1b2 and Pafah1b3) or (PAFAH1B2 and PAFAH1B3 and 15227) or (Pafah1b2 and Pafah1b3 and 15227)
```

In [23]:

```
temp = model.copy()
excluded = ['COPII','ERG27','RTO3_874609','RTO3_879280','RTO3_879341','RTO3_879512','RTO3_900622','RTO3_900623',
            'RTO3_900624','RTO3_900641','RTO3_900663','RTO3_945994','RTO3_945995','RTO3_946002','RTO3_946004',
            'RTO3_946005','RTO3_979594']

cobra.manipulation.remove_genes(temp, [g for g in model.genes 
                                       if (not g.id.isnumeric()) and (not g.id in excluded)])
temp
```

Out[23]:

|  |  |
| --- | --- |
| **Name** | R. toruloides |
| **Memory address** | 0x010296ff780 |
| **Number of metabolites** | 2144 |
| **Number of reactions** | 2073 |
| **Number of groups** | 0 |
| **Objective expression** | 0 |
| **Compartments** | c, x, m, e, r, v, n, g, d |

In [24]:

```
for r in sorted(model.reactions, key=lambda x: x.id):
    if not r in temp.reactions:
        print(r.id, r.reaction, r.gene_reaction_rule)
```

```
4ABZGLUH 4abzglu_c + h2o_c <=> 4abz_c + glu__L_c (b1338 and 15067) or (b1338 and 15072)
ARGLYSex arg__L_e + lys__L_c --> arg__L_c + lys__L_e (SLC3A2 and 11625) or (Slc3a2 and 11625)
CINNDO cinnm_c + h_c + nadh_c + o2_c --> cenchddd_c + nad_c b2538 and b2539 and b2540 and 10147
CITL cit_c --> ac_c + oaa_c b0614 and b0615 and b0617 and 9822
CPMPS gtp_c + h2o_c --> cpmp_c + ppi_c b0783 and 15692
DHAPT dha_c + pep_c --> dhap_c + pyr_c b1198 and b1199 and b2415 and b2416 and 12269
DOLASNT_Ler Asn_X_Ser_Thr_r + 0.1 g3m8mpdol__L_r --> 0.1 doldp__L_r + g3m8masn_r + h_r (RPN2 and 10629 and 12815 and 14867) or (Rpn2 and 10629 and 12815 and 14867)
DOLASNT_Uer Asn_X_Ser_Thr_r + 0.1 g3m8mpdol_U_r --> 0.1 doldp_U_r + g3m8masn_r + h_r (RPN2 and 10629 and 12815 and 14867) or (Rpn2 and 10629 and 12815 and 14867)
DOLPMT_L 0.1 dolp__L_c + gdpmann_c --> 0.1 dolmanp__L_c + gdp_c (DPM3 and 13484 and 9386) or (Dpm3 and 13484 and 9386)
DOLPMT_U 0.1 dolp_U_c + gdpmann_c --> 0.1 dolmanp_U_c + gdp_c (DPM3 and 13484 and 9386) or (Dpm3 and 13484 and 9386)
GLNTRAT atp_c + gln__L_c + glutrna_gln_c + h2o_c --> adp_c + glntrna_c + glu__L_c + h_c + pi_c PP_0932 and 11436 and 12640
GPIMTer_L 0.1 dolmanp__L_r + gacpail_hs_r --> 0.1 dolp__L_r + h_r + mgacpail_hs_r (PIGX and 9905) or (Pigx and 9905)
GPIMTer_U 0.1 dolmanp_U_r + gacpail_hs_r --> 0.1 dolp_U_r + h_r + mgacpail_hs_r (PIGX and 9905) or (Pigx and 9905)
METLEUex leu__L_c + met__L_e --> leu__L_e + met__L_c (SLC3A2 and 11625) or (Slc3a2 and 11625)
MPTS cpmp_c + cu2_c + 2.0 moadcosh_c --> 5.0 h_c + 2.0 moadcoo_c + mpt_c b0784 and 12718
PIACGT pail_hs_c + uacgam_c --> acgpail_hs_c + h_c + udp_c (PIGH and 11826 and 11850 and 13739 and 15144 and 9386) or (PIGH and 11826 and 11850 and 13739 and 15153 and 9386) or (Pigh and 11826 and 11850 and 13739 and 15144 and 9386) or (Pigh and 11826 and 11850 and 13739 and 15153 and 9386)
PPPNDO h_c + nadh_c + o2_c + pppn_c --> cechddd_c + nad_c b2538 and b2539 and b2540 and 10147
PROAKGOX1r akg_r + o2_r + pro__L_r --> 4hpro_LT_r + co2_r + succ_r (P4HA1 and 10499) or (P4HA2 and 10499) or (P4ha1 and 10499) or (P4ha2 and 10499)
SADT2 atp_c + gtp_c + h2o_c + so4_c --> aps_c + gdp_c + pi_c + ppi_c b2752 and 14868
TRPS3 3ig3p_c --> g3p_c + indole_c b1260 and 9262
UREA h2o_c + 2.0 h_c + urea_c --> co2_c + 2.0 nh4_c PP_2843 and PP_2844 and 11207
yli_R0712 gdpmann_c + 0.01 yli_M04599_c --> gdp_c + h_c + 0.01 yli_M04615_c YALI0F07260g and 12953
yli_R0713 gdpmann_c + 0.01 yli_M04600_c --> gdp_c + h_c + 0.01 yli_M04616_c YALI0F07260g and 12953
yli_R0714 gdpmann_c + 0.01 yli_M04601_c --> gdp_c + h_c + 0.01 yli_M04617_c YALI0F07260g and 12953
yli_R0715 gdpmann_c + 0.01 yli_M04602_c --> gdp_c + h_c + 0.01 yli_M04618_c YALI0F07260g and 12953
yli_R0716 gdpmann_c + 0.01 yli_M04603_c --> gdp_c + h_c + 0.01 yli_M04619_c YALI0F07260g and 12953
yli_R0717 gdpmann_c + 0.01 yli_M04604_c --> gdp_c + h_c + 0.01 yli_M04620_c YALI0F07260g and 12953
yli_R0935 gdpmann_c + yli_M00109_c --> dolmanp_c + gdp_c YALI0A18656g and 13484 and 9386
yli_R1477 gdpmann_r + yli_M00109_r --> dolmanp_r + gdp_r YALI0A18656g and 13484 and 9386
```

In [25]:

```
excluded = ['COPII','ERG27','RTO3_874609','RTO3_879280','RTO3_879341','RTO3_879512','RTO3_900622','RTO3_900623',
            'RTO3_900624','RTO3_900641','RTO3_900663','RTO3_945994','RTO3_945995','RTO3_946002','RTO3_946004',
            'RTO3_946005','RTO3_979594']

cobra.manipulation.remove_genes(model, [g for g in model.genes 
                                        if (not g.id.isnumeric()) and (not g.id in excluded)])
```

### Metabolites¶

In [26]:

```
for m in sorted(model.metabolites, key=lambda x: x.id):
    if '_hs' in m.id:
        print(m.id)
        for r in sorted(m.reactions, key=lambda x: x.id):
            print(r.id, r.reaction, r.gene_reaction_rule)
            for g in r.genes:
                for r2 in g.reactions:
                    if r2 is not r:
                        print(' ',r2.id, r2.reaction, r2.gene_reaction_rule)
        print()
```

```
acgpail_hs_c
ACGPID acgpail_hs_c + h2o_c --> ac_c + gpail_hs_c 9887

ak2lgchol_hs_c
PAFH h2o_c + paf_hs_c --> ac_c + ak2lgchol_hs_c + h_c 12283
  PAFHe h2o_e + paf_hs_e --> ac_e + ak2lgchol_hs_e + h_e 12283

ak2lgchol_hs_e
PAFHe h2o_e + paf_hs_e --> ac_e + ak2lgchol_hs_e + h_e 12283
  PAFH h2o_c + paf_hs_c --> ac_c + ak2lgchol_hs_c + h_c 12283

cs_hs_linkage_g
GLCATg l2xser_g + udpglcur_g --> cs_hs_linkage_g + h_g + udp_g 10942

dag_hs_c
yli_R0350 100.0 h2o_c + pail_cho_c --> dag_hs_c + 100.0 h_c + 100.0 mi1p__D_c 11755

dag_hs_r
H2ETer mgacpail_hs_r + pe_hs_r --> dag_hs_r + emgacpail_hs_r 16789
  H7_ETer emem2gacpail_hs_r + pe_hs_r --> dag_hs_r + gpi_hs_r 16789
  H3ETer m2gacpail_hs_r + pe_hs_r --> dag_hs_r + memgacpail_hs_r 16789
  H4ETer m3gacpail_hs_r + pe_hs_r --> dag_hs_r + m2emgacpail_hs_r 16789
  H6_ETer em3gacpail_hs_r + pe_hs_r --> dag_hs_r + em2emgacpail_hs_r 16789
H3ETer m2gacpail_hs_r + pe_hs_r --> dag_hs_r + memgacpail_hs_r 16789
  H7_ETer emem2gacpail_hs_r + pe_hs_r --> dag_hs_r + gpi_hs_r 16789
  H2ETer mgacpail_hs_r + pe_hs_r --> dag_hs_r + emgacpail_hs_r 16789
  H4ETer m3gacpail_hs_r + pe_hs_r --> dag_hs_r + m2emgacpail_hs_r 16789
  H6_ETer em3gacpail_hs_r + pe_hs_r --> dag_hs_r + em2emgacpail_hs_r 16789
H4ET3er m3gacpail_hs_r + pe_hs_r --> dag_hs_r + em3gacpail_hs_r 12437 and 8931
  M4BET2er mem2emgacpail_hs_r + pe_hs_r --> dag_hs_r + m_em_3gacpail_hs_r 14092 and 8931
  H6_ET2er em3gacpail_hs_r + pe_hs_r --> dag_hs_r + emem2gacpail_hs_r 14092 and 8931
  M4CET3er m3emgacpail_hs_r + pe_hs_r --> dag_hs_r + mem2emgacpail_hs_r 12437 and 8931
  H7ET2er em2emgacpail_hs_r + pe_hs_r --> dag_hs_r + gpi_hs_r 14092 and 8931
  H6ET3er m2emgacpail_hs_r + pe_hs_r --> dag_hs_r + em2emgacpail_hs_r 12437 and 8931
  M4CET3er m3emgacpail_hs_r + pe_hs_r --> dag_hs_r + mem2emgacpail_hs_r 12437 and 8931
  H6ET3er m2emgacpail_hs_r + pe_hs_r --> dag_hs_r + em2emgacpail_hs_r 12437 and 8931
H4ETer m3gacpail_hs_r + pe_hs_r --> dag_hs_r + m2emgacpail_hs_r 16789
  H7_ETer emem2gacpail_hs_r + pe_hs_r --> dag_hs_r + gpi_hs_r 16789
  H2ETer mgacpail_hs_r + pe_hs_r --> dag_hs_r + emgacpail_hs_r 16789
  H3ETer m2gacpail_hs_r + pe_hs_r --> dag_hs_r + memgacpail_hs_r 16789
  H6_ETer em3gacpail_hs_r + pe_hs_r --> dag_hs_r + em2emgacpail_hs_r 16789
H6ET3er m2emgacpail_hs_r + pe_hs_r --> dag_hs_r + em2emgacpail_hs_r 12437 and 8931
  H4ET3er m3gacpail_hs_r + pe_hs_r --> dag_hs_r + em3gacpail_hs_r 12437 and 8931
  M4BET2er mem2emgacpail_hs_r + pe_hs_r --> dag_hs_r + m_em_3gacpail_hs_r 14092 and 8931
  H6_ET2er em3gacpail_hs_r + pe_hs_r --> dag_hs_r + emem2gacpail_hs_r 14092 and 8931
  M4CET3er m3emgacpail_hs_r + pe_hs_r --> dag_hs_r + mem2emgacpail_hs_r 12437 and 8931
  H7ET2er em2emgacpail_hs_r + pe_hs_r --> dag_hs_r + gpi_hs_r 14092 and 8931
  H4ET3er m3gacpail_hs_r + pe_hs_r --> dag_hs_r + em3gacpail_hs_r 12437 and 8931
  M4CET3er m3emgacpail_hs_r + pe_hs_r --> dag_hs_r + mem2emgacpail_hs_r 12437 and 8931
H6_ET2er em3gacpail_hs_r + pe_hs_r --> dag_hs_r + emem2gacpail_hs_r 14092 and 8931
  H7ET2er em2emgacpail_hs_r + pe_hs_r --> dag_hs_r + gpi_hs_r 14092 and 8931
  M4BET2er mem2emgacpail_hs_r + pe_hs_r --> dag_hs_r + m_em_3gacpail_hs_r 14092 and 8931
  H4ET3er m3gacpail_hs_r + pe_hs_r --> dag_hs_r + em3gacpail_hs_r 12437 and 8931
  M4BET2er mem2emgacpail_hs_r + pe_hs_r --> dag_hs_r + m_em_3gacpail_hs_r 14092 and 8931
  M4CET3er m3emgacpail_hs_r + pe_hs_r --> dag_hs_r + mem2emgacpail_hs_r 12437 and 8931
  H7ET2er em2emgacpail_hs_r + pe_hs_r --> dag_hs_r + gpi_hs_r 14092 and 8931
  H6ET3er m2emgacpail_hs_r + pe_hs_r --> dag_hs_r + em2emgacpail_hs_r 12437 and 8931
H6_ETer em3gacpail_hs_r + pe_hs_r --> dag_hs_r + em2emgacpail_hs_r 16789
  H7_ETer emem2gacpail_hs_r + pe_hs_r --> dag_hs_r + gpi_hs_r 16789
  H2ETer mgacpail_hs_r + pe_hs_r --> dag_hs_r + emgacpail_hs_r 16789
  H3ETer m2gacpail_hs_r + pe_hs_r --> dag_hs_r + memgacpail_hs_r 16789
  H4ETer m3gacpail_hs_r + pe_hs_r --> dag_hs_r + m2emgacpail_hs_r 16789
H7ET2er em2emgacpail_hs_r + pe_hs_r --> dag_hs_r + gpi_hs_r 14092 and 8931
  H6_ET2er em3gacpail_hs_r + pe_hs_r --> dag_hs_r + emem2gacpail_hs_r 14092 and 8931
  M4BET2er mem2emgacpail_hs_r + pe_hs_r --> dag_hs_r + m_em_3gacpail_hs_r 14092 and 8931
  H4ET3er m3gacpail_hs_r + pe_hs_r --> dag_hs_r + em3gacpail_hs_r 12437 and 8931
  M4BET2er mem2emgacpail_hs_r + pe_hs_r --> dag_hs_r + m_em_3gacpail_hs_r 14092 and 8931
  H6_ET2er em3gacpail_hs_r + pe_hs_r --> dag_hs_r + emem2gacpail_hs_r 14092 and 8931
  M4CET3er m3emgacpail_hs_r + pe_hs_r --> dag_hs_r + mem2emgacpail_hs_r 12437 and 8931
  H6ET3er m2emgacpail_hs_r + pe_hs_r --> dag_hs_r + em2emgacpail_hs_r 12437 and 8931
H7_ETer emem2gacpail_hs_r + pe_hs_r --> dag_hs_r + gpi_hs_r 16789
  H2ETer mgacpail_hs_r + pe_hs_r --> dag_hs_r + emgacpail_hs_r 16789
  H3ETer m2gacpail_hs_r + pe_hs_r --> dag_hs_r + memgacpail_hs_r 16789
  H4ETer m3gacpail_hs_r + pe_hs_r --> dag_hs_r + m2emgacpail_hs_r 16789
  H6_ETer em3gacpail_hs_r + pe_hs_r --> dag_hs_r + em2emgacpail_hs_r 16789
M4BET2er mem2emgacpail_hs_r + pe_hs_r --> dag_hs_r + m_em_3gacpail_hs_r 14092 and 8931
  H6_ET2er em3gacpail_hs_r + pe_hs_r --> dag_hs_r + emem2gacpail_hs_r 14092 and 8931
  H7ET2er em2emgacpail_hs_r + pe_hs_r --> dag_hs_r + gpi_hs_r 14092 and 8931
  H4ET3er m3gacpail_hs_r + pe_hs_r --> dag_hs_r + em3gacpail_hs_r 12437 and 8931
  H6_ET2er em3gacpail_hs_r + pe_hs_r --> dag_hs_r + emem2gacpail_hs_r 14092 and 8931
  M4CET3er m3emgacpail_hs_r + pe_hs_r --> dag_hs_r + mem2emgacpail_hs_r 12437 and 8931
  H7ET2er em2emgacpail_hs_r + pe_hs_r --> dag_hs_r + gpi_hs_r 14092 and 8931
  H6ET3er m2emgacpail_hs_r + pe_hs_r --> dag_hs_r + em2emgacpail_hs_r 12437 and 8931
M4CET3er m3emgacpail_hs_r + pe_hs_r --> dag_hs_r + mem2emgacpail_hs_r 12437 and 8931
  H4ET3er m3gacpail_hs_r + pe_hs_r --> dag_hs_r + em3gacpail_hs_r 12437 and 8931
  M4BET2er mem2emgacpail_hs_r + pe_hs_r --> dag_hs_r + m_em_3gacpail_hs_r 14092 and 8931
  H6_ET2er em3gacpail_hs_r + pe_hs_r --> dag_hs_r + emem2gacpail_hs_r 14092 and 8931
  H7ET2er em2emgacpail_hs_r + pe_hs_r --> dag_hs_r + gpi_hs_r 14092 and 8931
  H6ET3er m2emgacpail_hs_r + pe_hs_r --> dag_hs_r + em2emgacpail_hs_r 12437 and 8931
  H4ET3er m3gacpail_hs_r + pe_hs_r --> dag_hs_r + em3gacpail_hs_r 12437 and 8931
  H6ET3er m2emgacpail_hs_r + pe_hs_r --> dag_hs_r + em2emgacpail_hs_r 12437 and 8931

em2emgacpail_hs_r
H6ET3er m2emgacpail_hs_r + pe_hs_r --> dag_hs_r + em2emgacpail_hs_r 12437 and 8931
  H4ET3er m3gacpail_hs_r + pe_hs_r --> dag_hs_r + em3gacpail_hs_r 12437 and 8931
  M4BET2er mem2emgacpail_hs_r + pe_hs_r --> dag_hs_r + m_em_3gacpail_hs_r 14092 and 8931
  H6_ET2er em3gacpail_hs_r + pe_hs_r --> dag_hs_r + emem2gacpail_hs_r 14092 and 8931
  M4CET3er m3emgacpail_hs_r + pe_hs_r --> dag_hs_r + mem2emgacpail_hs_r 12437 and 8931
  H7ET2er em2emgacpail_hs_r + pe_hs_r --> dag_hs_r + gpi_hs_r 14092 and 8931
  H4ET3er m3gacpail_hs_r + pe_hs_r --> dag_hs_r + em3gacpail_hs_r 12437 and 8931
  M4CET3er m3emgacpail_hs_r + pe_hs_r --> dag_hs_r + mem2emgacpail_hs_r 12437 and 8931
H6_ETer em3gacpail_hs_r + pe_hs_r --> dag_hs_r + em2emgacpail_hs_r 16789
  H7_ETer emem2gacpail_hs_r + pe_hs_r --> dag_hs_r + gpi_hs_r 16789
  H2ETer mgacpail_hs_r + pe_hs_r --> dag_hs_r + emgacpail_hs_r 16789
  H3ETer m2gacpail_hs_r + pe_hs_r --> dag_hs_r + memgacpail_hs_r 16789
  H4ETer m3gacpail_hs_r + pe_hs_r --> dag_hs_r + m2emgacpail_hs_r 16789
H7ET2er em2emgacpail_hs_r + pe_hs_r --> dag_hs_r + gpi_hs_r 14092 and 8931
  H6_ET2er em3gacpail_hs_r + pe_hs_r --> dag_hs_r + emem2gacpail_hs_r 14092 and 8931
  M4BET2er mem2emgacpail_hs_r + pe_hs_r --> dag_hs_r + m_em_3gacpail_hs_r 14092 and 8931
  H4ET3er m3gacpail_hs_r + pe_hs_r --> dag_hs_r + em3gacpail_hs_r 12437 and 8931
  M4BET2er mem2emgacpail_hs_r + pe_hs_r --> dag_hs_r + m_em_3gacpail_hs_r 14092 and 8931
  H6_ET2er em3gacpail_hs_r + pe_hs_r --> dag_hs_r + emem2gacpail_hs_r 14092 and 8931
  M4CET3er m3emgacpail_hs_r + pe_hs_r --> dag_hs_r + mem2emgacpail_hs_r 12437 and 8931
  H6ET3er m2emgacpail_hs_r + pe_hs_r --> dag_hs_r + em2emgacpail_hs_r 12437 and 8931
H7MTer_L 0.1 dolmanp__L_r + em2emgacpail_hs_r --> 0.1 dolp__L_r + h_r + mem2emgacpail_hs_r 8605
  H7MTer_U 0.1 dolmanp_U_r + em2emgacpail_hs_r --> 0.1 dolp_U_r + h_r + mem2emgacpail_hs_r 8605
  H8MTer_U 0.1 dolmanp_U_r + gpi_hs_r --> 0.1 dolp_U_r + h_r + m_em_3gacpail_hs_r 8605
  H8MTer_L 0.1 dolmanp__L_r + gpi_hs_r --> 0.1 dolp__L_r + h_r + m_em_3gacpail_hs_r 8605
  H6MTer_L 0.1 dolmanp__L_r + m2emgacpail_hs_r --> 0.1 dolp__L_r + h_r + m3emgacpail_hs_r 8605
  H6MTer_U 0.1 dolmanp_U_r + m2emgacpail_hs_r --> 0.1 dolp_U_r + h_r + m3emgacpail_hs_r 8605
H7MTer_U 0.1 dolmanp_U_r + em2emgacpail_hs_r --> 0.1 dolp_U_r + h_r + mem2emgacpail_hs_r 8605
  H8MTer_U 0.1 dolmanp_U_r + gpi_hs_r --> 0.1 dolp_U_r + h_r + m_em_3gacpail_hs_r 8605
  H8MTer_L 0.1 dolmanp__L_r + gpi_hs_r --> 0.1 dolp__L_r + h_r + m_em_3gacpail_hs_r 8605
  H6MTer_L 0.1 dolmanp__L_r + m2emgacpail_hs_r --> 0.1 dolp__L_r + h_r + m3emgacpail_hs_r 8605
  H7MTer_L 0.1 dolmanp__L_r + em2emgacpail_hs_r --> 0.1 dolp__L_r + h_r + mem2emgacpail_hs_r 8605
  H6MTer_U 0.1 dolmanp_U_r + m2emgacpail_hs_r --> 0.1 dolp_U_r + h_r + m3emgacpail_hs_r 8605
H7_TAer em2emgacpail_hs_r + pre_prot_r --> em2emgacpail_prot_hs_r + gpi_sig_r 10664 and 11490 and 12838 and 12988 and 13065
  M4BTAer mem2emgacpail_hs_r + pre_prot_r --> gpi_sig_r + mem2emgacpail_prot_hs_r 10664 and 11490 and 12838 and 12988 and 13065
  H8TAer gpi_hs_r + pre_prot_r --> gpi_prot_hs_r + gpi_sig_r 10664 and 11490 and 12838 and 12988 and 13065
  M4ATAer m_em_3gacpail_hs_r + pre_prot_r --> gpi_sig_r + m_em_3gacpail_prot_hs_r 10664 and 11490 and 12988 and 13065
  M4BTAer mem2emgacpail_hs_r + pre_prot_r --> gpi_sig_r + mem2emgacpail_prot_hs_r 10664 and 11490 and 12838 and 12988 and 13065
  H8TAer gpi_hs_r + pre_prot_r --> gpi_prot_hs_r + gpi_sig_r 10664 and 11490 and 12838 and 12988 and 13065
  M4BTAer mem2emgacpail_hs_r + pre_prot_r --> gpi_sig_r + mem2emgacpail_prot_hs_r 10664 and 11490 and 12838 and 12988 and 13065
  H8TAer gpi_hs_r + pre_prot_r --> gpi_prot_hs_r + gpi_sig_r 10664 and 11490 and 12838 and 12988 and 13065
  M4ATAer m_em_3gacpail_hs_r + pre_prot_r --> gpi_sig_r + m_em_3gacpail_prot_hs_r 10664 and 11490 and 12988 and 13065
  M4BTAer mem2emgacpail_hs_r + pre_prot_r --> gpi_sig_r + mem2emgacpail_prot_hs_r 10664 and 11490 and 12838 and 12988 and 13065
  H8TAer gpi_hs_r + pre_prot_r --> gpi_prot_hs_r + gpi_sig_r 10664 and 11490 and 12838 and 12988 and 13065
  M4ATAer m_em_3gacpail_hs_r + pre_prot_r --> gpi_sig_r + m_em_3gacpail_prot_hs_r 10664 and 11490 and 12988 and 13065
  M4BTAer mem2emgacpail_hs_r + pre_prot_r --> gpi_sig_r + mem2emgacpail_prot_hs_r 10664 and 11490 and 12838 and 12988 and 13065
  H8TAer gpi_hs_r + pre_prot_r --> gpi_prot_hs_r + gpi_sig_r 10664 and 11490 and 12838 and 12988 and 13065
  M4ATAer m_em_3gacpail_hs_r + pre_prot_r --> gpi_sig_r + m_em_3gacpail_prot_hs_r 10664 and 11490 and 12988 and 13065

em2emgacpail_prot_hs_r
H7_TAer em2emgacpail_hs_r + pre_prot_r --> em2emgacpail_prot_hs_r + gpi_sig_r 10664 and 11490 and 12838 and 12988 and 13065
  M4BTAer mem2emgacpail_hs_r + pre_prot_r --> gpi_sig_r + mem2emgacpail_prot_hs_r 10664 and 11490 and 12838 and 12988 and 13065
  H8TAer gpi_hs_r + pre_prot_r --> gpi_prot_hs_r + gpi_sig_r 10664 and 11490 and 12838 and 12988 and 13065
  M4ATAer m_em_3gacpail_hs_r + pre_prot_r --> gpi_sig_r + m_em_3gacpail_prot_hs_r 10664 and 11490 and 12988 and 13065
  M4BTAer mem2emgacpail_hs_r + pre_prot_r --> gpi_sig_r + mem2emgacpail_prot_hs_r 10664 and 11490 and 12838 and 12988 and 13065
  H8TAer gpi_hs_r + pre_prot_r --> gpi_prot_hs_r + gpi_sig_r 10664 and 11490 and 12838 and 12988 and 13065
  M4BTAer mem2emgacpail_hs_r + pre_prot_r --> gpi_sig_r + mem2emgacpail_prot_hs_r 10664 and 11490 and 12838 and 12988 and 13065
  H8TAer gpi_hs_r + pre_prot_r --> gpi_prot_hs_r + gpi_sig_r 10664 and 11490 and 12838 and 12988 and 13065
  M4ATAer m_em_3gacpail_hs_r + pre_prot_r --> gpi_sig_r + m_em_3gacpail_prot_hs_r 10664 and 11490 and 12988 and 13065
  M4BTAer mem2emgacpail_hs_r + pre_prot_r --> gpi_sig_r + mem2emgacpail_prot_hs_r 10664 and 11490 and 12838 and 12988 and 13065
  H8TAer gpi_hs_r + pre_prot_r --> gpi_prot_hs_r + gpi_sig_r 10664 and 11490 and 12838 and 12988 and 13065
  M4ATAer m_em_3gacpail_hs_r + pre_prot_r --> gpi_sig_r + m_em_3gacpail_prot_hs_r 10664 and 11490 and 12988 and 13065
  M4BTAer mem2emgacpail_hs_r + pre_prot_r --> gpi_sig_r + mem2emgacpail_prot_hs_r 10664 and 11490 and 12838 and 12988 and 13065
  H8TAer gpi_hs_r + pre_prot_r --> gpi_prot_hs_r + gpi_sig_r 10664 and 11490 and 12838 and 12988 and 13065
  M4ATAer m_em_3gacpail_hs_r + pre_prot_r --> gpi_sig_r + m_em_3gacpail_prot_hs_r 10664 and 11490 and 12988 and 13065

em3gacpail_hs_r
H4ET3er m3gacpail_hs_r + pe_hs_r --> dag_hs_r + em3gacpail_hs_r 12437 and 8931
  M4BET2er mem2emgacpail_hs_r + pe_hs_r --> dag_hs_r + m_em_3gacpail_hs_r 14092 and 8931
  H6_ET2er em3gacpail_hs_r + pe_hs_r --> dag_hs_r + emem2gacpail_hs_r 14092 and 8931
  M4CET3er m3emgacpail_hs_r + pe_hs_r --> dag_hs_r + mem2emgacpail_hs_r 12437 and 8931
  H7ET2er em2emgacpail_hs_r + pe_hs_r --> dag_hs_r + gpi_hs_r 14092 and 8931
  H6ET3er m2emgacpail_hs_r + pe_hs_r --> dag_hs_r + em2emgacpail_hs_r 12437 and 8931
  M4CET3er m3emgacpail_hs_r + pe_hs_r --> dag_hs_r + mem2emgacpail_hs_r 12437 and 8931
  H6ET3er m2emgacpail_hs_r + pe_hs_r --> dag_hs_r + em2emgacpail_hs_r 12437 and 8931
H6_ET2er em3gacpail_hs_r + pe_hs_r --> dag_hs_r + emem2gacpail_hs_r 14092 and 8931
  H7ET2er em2emgacpail_hs_r + pe_hs_r --> dag_hs_r + gpi_hs_r 14092 and 8931
  M4BET2er mem2emgacpail_hs_r + pe_hs_r --> dag_hs_r + m_em_3gacpail_hs_r 14092 and 8931
  H4ET3er m3gacpail_hs_r + pe_hs_r --> dag_hs_r + em3gacpail_hs_r 12437 and 8931
  M4BET2er mem2emgacpail_hs_r + pe_hs_r --> dag_hs_r + m_em_3gacpail_hs_r 14092 and 8931
  M4CET3er m3emgacpail_hs_r + pe_hs_r --> dag_hs_r + mem2emgacpail_hs_r 12437 and 8931
  H7ET2er em2emgacpail_hs_r + pe_hs_r --> dag_hs_r + gpi_hs_r 14092 and 8931
  H6ET3er m2emgacpail_hs_r + pe_hs_r --> dag_hs_r + em2emgacpail_hs_r 12437 and 8931
H6_ETer em3gacpail_hs_r + pe_hs_r --> dag_hs_r + em2emgacpail_hs_r 16789
  H7_ETer emem2gacpail_hs_r + pe_hs_r --> dag_hs_r + gpi_hs_r 16789
  H2ETer mgacpail_hs_r + pe_hs_r --> dag_hs_r + emgacpail_hs_r 16789
  H3ETer m2gacpail_hs_r + pe_hs_r --> dag_hs_r + memgacpail_hs_r 16789
  H4ETer m3gacpail_hs_r + pe_hs_r --> dag_hs_r + m2emgacpail_hs_r 16789

emem2gacpail_hs_r
H6_ET2er em3gacpail_hs_r + pe_hs_r --> dag_hs_r + emem2gacpail_hs_r 14092 and 8931
  H7ET2er em2emgacpail_hs_r + pe_hs_r --> dag_hs_r + gpi_hs_r 14092 and 8931
  M4BET2er mem2emgacpail_hs_r + pe_hs_r --> dag_hs_r + m_em_3gacpail_hs_r 14092 and 8931
  H4ET3er m3gacpail_hs_r + pe_hs_r --> dag_hs_r + em3gacpail_hs_r 12437 and 8931
  M4BET2er mem2emgacpail_hs_r + pe_hs_r --> dag_hs_r + m_em_3gacpail_hs_r 14092 and 8931
  M4CET3er m3emgacpail_hs_r + pe_hs_r --> dag_hs_r + mem2emgacpail_hs_r 12437 and 8931
  H7ET2er em2emgacpail_hs_r + pe_hs_r --> dag_hs_r + gpi_hs_r 14092 and 8931
  H6ET3er m2emgacpail_hs_r + pe_hs_r --> dag_hs_r + em2emgacpail_hs_r 12437 and 8931
H7_ETer emem2gacpail_hs_r + pe_hs_r --> dag_hs_r + gpi_hs_r 16789
  H2ETer mgacpail_hs_r + pe_hs_r --> dag_hs_r + emgacpail_hs_r 16789
  H3ETer m2gacpail_hs_r + pe_hs_r --> dag_hs_r + memgacpail_hs_r 16789
  H4ETer m3gacpail_hs_r + pe_hs_r --> dag_hs_r + m2emgacpail_hs_r 16789
  H6_ETer em3gacpail_hs_r + pe_hs_r --> dag_hs_r + em2emgacpail_hs_r 16789

emgacpail_hs_r
H2ETer mgacpail_hs_r + pe_hs_r --> dag_hs_r + emgacpail_hs_r 16789
  H7_ETer emem2gacpail_hs_r + pe_hs_r --> dag_hs_r + gpi_hs_r 16789
  H3ETer m2gacpail_hs_r + pe_hs_r --> dag_hs_r + memgacpail_hs_r 16789
  H4ETer m3gacpail_hs_r + pe_hs_r --> dag_hs_r + m2emgacpail_hs_r 16789
  H6_ETer em3gacpail_hs_r + pe_hs_r --> dag_hs_r + em2emgacpail_hs_r 16789
H5MTer_L 0.1 dolmanp__L_r + emgacpail_hs_r --> 0.1 dolp__L_r + h_r + memgacpail_hs_r 11445
  H2MTer_U 0.1 dolmanp_U_r + mgacpail_hs_r --> 0.1 dolp_U_r + h_r + m2gacpail_hs_r 11445
  H5MTer_U 0.1 dolmanp_U_r + emgacpail_hs_r --> 0.1 dolp_U_r + h_r + memgacpail_hs_r 11445
  H2MTer_L 0.1 dolmanp__L_r + mgacpail_hs_r --> 0.1 dolp__L_r + h_r + m2gacpail_hs_r 11445
H5MTer_U 0.1 dolmanp_U_r + emgacpail_hs_r --> 0.1 dolp_U_r + h_r + memgacpail_hs_r 11445
  H2MTer_U 0.1 dolmanp_U_r + mgacpail_hs_r --> 0.1 dolp_U_r + h_r + m2gacpail_hs_r 11445
  H5MTer_L 0.1 dolmanp__L_r + emgacpail_hs_r --> 0.1 dolp__L_r + h_r + memgacpail_hs_r 11445
  H2MTer_L 0.1 dolmanp__L_r + mgacpail_hs_r --> 0.1 dolp__L_r + h_r + m2gacpail_hs_r 11445

gacpail_hs_c
GPIAT gpail_hs_c + pmtcoa_c --> coa_c + gacpail_hs_c 11701

gacpail_hs_r

gd3_hs_c
GAO1 accoa_c + gd3_hs_c --> coa_c + oagd3_hs_c 10946
  GAO2 accoa_c + gt3_hs_c --> coa_c + oagt3_hs_c 10946
  ACCOAtm accoa_c + coa_m <=> accoa_m + coa_c 10946
  ACCOAtx accoa_c + coa_x <=> accoa_x + coa_c 10946
  ACCOAgt accoa_c <=> accoa_g 10946
  GAO2g accoa_g + gt3_hs_g --> coa_g + oagt3_hs_g 10946
  GAO1g accoa_g + gd3_hs_g --> coa_g + oagd3_hs_g 10946
  ACCOAtr accoa_c <=> accoa_r 10946

gd3_hs_g
GAO1g accoa_g + gd3_hs_g --> coa_g + oagd3_hs_g 10946
  GAO1 accoa_c + gd3_hs_c --> coa_c + oagd3_hs_c 10946
  GAO2 accoa_c + gt3_hs_c --> coa_c + oagt3_hs_c 10946
  ACCOAtm accoa_c + coa_m <=> accoa_m + coa_c 10946
  ACCOAtx accoa_c + coa_x <=> accoa_x + coa_c 10946
  ACCOAgt accoa_c <=> accoa_g 10946
  GAO2g accoa_g + gt3_hs_g --> coa_g + oagt3_hs_g 10946
  ACCOAtr accoa_c <=> accoa_r 10946

gpail_hs_c
ACGPID acgpail_hs_c + h2o_c --> ac_c + gpail_hs_c 9887
GPIAT gpail_hs_c + pmtcoa_c --> coa_c + gacpail_hs_c 11701

gpi_hs_r
H7ET2er em2emgacpail_hs_r + pe_hs_r --> dag_hs_r + gpi_hs_r 14092 and 8931
  H6_ET2er em3gacpail_hs_r + pe_hs_r --> dag_hs_r + emem2gacpail_hs_r 14092 and 8931
  M4BET2er mem2emgacpail_hs_r + pe_hs_r --> dag_hs_r + m_em_3gacpail_hs_r 14092 and 8931
  H4ET3er m3gacpail_hs_r + pe_hs_r --> dag_hs_r + em3gacpail_hs_r 12437 and 8931
  M4BET2er mem2emgacpail_hs_r + pe_hs_r --> dag_hs_r + m_em_3gacpail_hs_r 14092 and 8931
  H6_ET2er em3gacpail_hs_r + pe_hs_r --> dag_hs_r + emem2gacpail_hs_r 14092 and 8931
  M4CET3er m3emgacpail_hs_r + pe_hs_r --> dag_hs_r + mem2emgacpail_hs_r 12437 and 8931
  H6ET3er m2emgacpail_hs_r + pe_hs_r --> dag_hs_r + em2emgacpail_hs_r 12437 and 8931
H7_ETer emem2gacpail_hs_r + pe_hs_r --> dag_hs_r + gpi_hs_r 16789
  H2ETer mgacpail_hs_r + pe_hs_r --> dag_hs_r + emgacpail_hs_r 16789
  H3ETer m2gacpail_hs_r + pe_hs_r --> dag_hs_r + memgacpail_hs_r 16789
  H4ETer m3gacpail_hs_r + pe_hs_r --> dag_hs_r + m2emgacpail_hs_r 16789
  H6_ETer em3gacpail_hs_r + pe_hs_r --> dag_hs_r + em2emgacpail_hs_r 16789
H8MTer_L 0.1 dolmanp__L_r + gpi_hs_r --> 0.1 dolp__L_r + h_r + m_em_3gacpail_hs_r 8605
  H7MTer_U 0.1 dolmanp_U_r + em2emgacpail_hs_r --> 0.1 dolp_U_r + h_r + mem2emgacpail_hs_r 8605
  H8MTer_U 0.1 dolmanp_U_r + gpi_hs_r --> 0.1 dolp_U_r + h_r + m_em_3gacpail_hs_r 8605
  H6MTer_L 0.1 dolmanp__L_r + m2emgacpail_hs_r --> 0.1 dolp__L_r + h_r + m3emgacpail_hs_r 8605
  H7MTer_L 0.1 dolmanp__L_r + em2emgacpail_hs_r --> 0.1 dolp__L_r + h_r + mem2emgacpail_hs_r 8605
  H6MTer_U 0.1 dolmanp_U_r + m2emgacpail_hs_r --> 0.1 dolp_U_r + h_r + m3emgacpail_hs_r 8605
H8MTer_U 0.1 dolmanp_U_r + gpi_hs_r --> 0.1 dolp_U_r + h_r + m_em_3gacpail_hs_r 8605
  H7MTer_U 0.1 dolmanp_U_r + em2emgacpail_hs_r --> 0.1 dolp_U_r + h_r + mem2emgacpail_hs_r 8605
  H8MTer_L 0.1 dolmanp__L_r + gpi_hs_r --> 0.1 dolp__L_r + h_r + m_em_3gacpail_hs_r 8605
  H6MTer_L 0.1 dolmanp__L_r + m2emgacpail_hs_r --> 0.1 dolp__L_r + h_r + m3emgacpail_hs_r 8605
  H7MTer_L 0.1 dolmanp__L_r + em2emgacpail_hs_r --> 0.1 dolp__L_r + h_r + mem2emgacpail_hs_r 8605
  H6MTer_U 0.1 dolmanp_U_r + m2emgacpail_hs_r --> 0.1 dolp_U_r + h_r + m3emgacpail_hs_r 8605
H8TAer gpi_hs_r + pre_prot_r --> gpi_prot_hs_r + gpi_sig_r 10664 and 11490 and 12838 and 12988 and 13065
  M4BTAer mem2emgacpail_hs_r + pre_prot_r --> gpi_sig_r + mem2emgacpail_prot_hs_r 10664 and 11490 and 12838 and 12988 and 13065
  H7_TAer em2emgacpail_hs_r + pre_prot_r --> em2emgacpail_prot_hs_r + gpi_sig_r 10664 and 11490 and 12838 and 12988 and 13065
  M4ATAer m_em_3gacpail_hs_r + pre_prot_r --> gpi_sig_r + m_em_3gacpail_prot_hs_r 10664 and 11490 and 12988 and 13065
  M4BTAer mem2emgacpail_hs_r + pre_prot_r --> gpi_sig_r + mem2emgacpail_prot_hs_r 10664 and 11490 and 12838 and 12988 and 13065
  H7_TAer em2emgacpail_hs_r + pre_prot_r --> em2emgacpail_prot_hs_r + gpi_sig_r 10664 and 11490 and 12838 and 12988 and 13065
  M4BTAer mem2emgacpail_hs_r + pre_prot_r --> gpi_sig_r + mem2emgacpail_prot_hs_r 10664 and 11490 and 12838 and 12988 and 13065
  H7_TAer em2emgacpail_hs_r + pre_prot_r --> em2emgacpail_prot_hs_r + gpi_sig_r 10664 and 11490 and 12838 and 12988 and 13065
  M4ATAer m_em_3gacpail_hs_r + pre_prot_r --> gpi_sig_r + m_em_3gacpail_prot_hs_r 10664 and 11490 and 12988 and 13065
  M4BTAer mem2emgacpail_hs_r + pre_prot_r --> gpi_sig_r + mem2emgacpail_prot_hs_r 10664 and 11490 and 12838 and 12988 and 13065
  H7_TAer em2emgacpail_hs_r + pre_prot_r --> em2emgacpail_prot_hs_r + gpi_sig_r 10664 and 11490 and 12838 and 12988 and 13065
  M4ATAer m_em_3gacpail_hs_r + pre_prot_r --> gpi_sig_r + m_em_3gacpail_prot_hs_r 10664 and 11490 and 12988 and 13065
  M4BTAer mem2emgacpail_hs_r + pre_prot_r --> gpi_sig_r + mem2emgacpail_prot_hs_r 10664 and 11490 and 12838 and 12988 and 13065
  H7_TAer em2emgacpail_hs_r + pre_prot_r --> em2emgacpail_prot_hs_r + gpi_sig_r 10664 and 11490 and 12838 and 12988 and 13065
  M4ATAer m_em_3gacpail_hs_r + pre_prot_r --> gpi_sig_r + m_em_3gacpail_prot_hs_r 10664 and 11490 and 12988 and 13065

gpi_prot_hs_r
H8TAer gpi_hs_r + pre_prot_r --> gpi_prot_hs_r + gpi_sig_r 10664 and 11490 and 12838 and 12988 and 13065
  M4BTAer mem2emgacpail_hs_r + pre_prot_r --> gpi_sig_r + mem2emgacpail_prot_hs_r 10664 and 11490 and 12838 and 12988 and 13065
  H7_TAer em2emgacpail_hs_r + pre_prot_r --> em2emgacpail_prot_hs_r + gpi_sig_r 10664 and 11490 and 12838 and 12988 and 13065
  M4ATAer m_em_3gacpail_hs_r + pre_prot_r --> gpi_sig_r + m_em_3gacpail_prot_hs_r 10664 and 11490 and 12988 and 13065
  M4BTAer mem2emgacpail_hs_r + pre_prot_r --> gpi_sig_r + mem2emgacpail_prot_hs_r 10664 and 11490 and 12838 and 12988 and 13065
  H7_TAer em2emgacpail_hs_r + pre_prot_r --> em2emgacpail_prot_hs_r + gpi_sig_r 10664 and 11490 and 12838 and 12988 and 13065
  M4BTAer mem2emgacpail_hs_r + pre_prot_r --> gpi_sig_r + mem2emgacpail_prot_hs_r 10664 and 11490 and 12838 and 12988 and 13065
  H7_TAer em2emgacpail_hs_r + pre_prot_r --> em2emgacpail_prot_hs_r + gpi_sig_r 10664 and 11490 and 12838 and 12988 and 13065
  M4ATAer m_em_3gacpail_hs_r + pre_prot_r --> gpi_sig_r + m_em_3gacpail_prot_hs_r 10664 and 11490 and 12988 and 13065
  M4BTAer mem2emgacpail_hs_r + pre_prot_r --> gpi_sig_r + mem2emgacpail_prot_hs_r 10664 and 11490 and 12838 and 12988 and 13065
  H7_TAer em2emgacpail_hs_r + pre_prot_r --> em2emgacpail_prot_hs_r + gpi_sig_r 10664 and 11490 and 12838 and 12988 and 13065
  M4ATAer m_em_3gacpail_hs_r + pre_prot_r --> gpi_sig_r + m_em_3gacpail_prot_hs_r 10664 and 11490 and 12988 and 13065
  M4BTAer mem2emgacpail_hs_r + pre_prot_r --> gpi_sig_r + mem2emgacpail_prot_hs_r 10664 and 11490 and 12838 and 12988 and 13065
  H7_TAer em2emgacpail_hs_r + pre_prot_r --> em2emgacpail_prot_hs_r + gpi_sig_r 10664 and 11490 and 12838 and 12988 and 13065
  M4ATAer m_em_3gacpail_hs_r + pre_prot_r --> gpi_sig_r + m_em_3gacpail_prot_hs_r 10664 and 11490 and 12988 and 13065

gt3_hs_c
GAO2 accoa_c + gt3_hs_c --> coa_c + oagt3_hs_c 10946
  GAO1 accoa_c + gd3_hs_c --> coa_c + oagd3_hs_c 10946
  ACCOAtm accoa_c + coa_m <=> accoa_m + coa_c 10946
  ACCOAtx accoa_c + coa_x <=> accoa_x + coa_c 10946
  ACCOAgt accoa_c <=> accoa_g 10946
  GAO2g accoa_g + gt3_hs_g --> coa_g + oagt3_hs_g 10946
  GAO1g accoa_g + gd3_hs_g --> coa_g + oagd3_hs_g 10946
  ACCOAtr accoa_c <=> accoa_r 10946

gt3_hs_g
GAO2g accoa_g + gt3_hs_g --> coa_g + oagt3_hs_g 10946
  GAO1 accoa_c + gd3_hs_c --> coa_c + oagd3_hs_c 10946
  GAO2 accoa_c + gt3_hs_c --> coa_c + oagt3_hs_c 10946
  ACCOAtm accoa_c + coa_m <=> accoa_m + coa_c 10946
  ACCOAtx accoa_c + coa_x <=> accoa_x + coa_c 10946
  ACCOAgt accoa_c <=> accoa_g 10946
  GAO1g accoa_g + gd3_hs_g --> coa_g + oagd3_hs_g 10946
  ACCOAtr accoa_c <=> accoa_r 10946

m2emgacpail_hs_r
BMTer_L 0.1 dolmanp__L_r + memgacpail_hs_r --> 0.1 dolp__L_r + h_r + m2emgacpail_hs_r 12961
  BMTer_U 0.1 dolmanp_U_r + memgacpail_hs_r --> 0.1 dolp_U_r + h_r + m2emgacpail_hs_r 12961
  H3MTer_U 0.1 dolmanp_U_r + m2gacpail_hs_r --> 0.1 dolp_U_r + h_r + m3gacpail_hs_r 12961
  H3MTer_L 0.1 dolmanp__L_r + m2gacpail_hs_r --> 0.1 dolp__L_r + h_r + m3gacpail_hs_r 12961
BMTer_U 0.1 dolmanp_U_r + memgacpail_hs_r --> 0.1 dolp_U_r + h_r + m2emgacpail_hs_r 12961
  BMTer_L 0.1 dolmanp__L_r + memgacpail_hs_r --> 0.1 dolp__L_r + h_r + m2emgacpail_hs_r 12961
  H3MTer_U 0.1 dolmanp_U_r + m2gacpail_hs_r --> 0.1 dolp_U_r + h_r + m3gacpail_hs_r 12961
  H3MTer_L 0.1 dolmanp__L_r + m2gacpail_hs_r --> 0.1 dolp__L_r + h_r + m3gacpail_hs_r 12961
H4ETer m3gacpail_hs_r + pe_hs_r --> dag_hs_r + m2emgacpail_hs_r 16789
  H7_ETer emem2gacpail_hs_r + pe_hs_r --> dag_hs_r + gpi_hs_r 16789
  H2ETer mgacpail_hs_r + pe_hs_r --> dag_hs_r + emgacpail_hs_r 16789
  H3ETer m2gacpail_hs_r + pe_hs_r --> dag_hs_r + memgacpail_hs_r 16789
  H6_ETer em3gacpail_hs_r + pe_hs_r --> dag_hs_r + em2emgacpail_hs_r 16789
H6ET3er m2emgacpail_hs_r + pe_hs_r --> dag_hs_r + em2emgacpail_hs_r 12437 and 8931
  H4ET3er m3gacpail_hs_r + pe_hs_r --> dag_hs_r + em3gacpail_hs_r 12437 and 8931
  M4BET2er mem2emgacpail_hs_r + pe_hs_r --> dag_hs_r + m_em_3gacpail_hs_r 14092 and 8931
  H6_ET2er em3gacpail_hs_r + pe_hs_r --> dag_hs_r + emem2gacpail_hs_r 14092 and 8931
  M4CET3er m3emgacpail_hs_r + pe_hs_r --> dag_hs_r + mem2emgacpail_hs_r 12437 and 8931
  H7ET2er em2emgacpail_hs_r + pe_hs_r --> dag_hs_r + gpi_hs_r 14092 and 8931
  H4ET3er m3gacpail_hs_r + pe_hs_r --> dag_hs_r + em3gacpail_hs_r 12437 and 8931
  M4CET3er m3emgacpail_hs_r + pe_hs_r --> dag_hs_r + mem2emgacpail_hs_r 12437 and 8931
H6MTer_L 0.1 dolmanp__L_r + m2emgacpail_hs_r --> 0.1 dolp__L_r + h_r + m3emgacpail_hs_r 8605
  H7MTer_U 0.1 dolmanp_U_r + em2emgacpail_hs_r --> 0.1 dolp_U_r + h_r + mem2emgacpail_hs_r 8605
  H8MTer_U 0.1 dolmanp_U_r + gpi_hs_r --> 0.1 dolp_U_r + h_r + m_em_3gacpail_hs_r 8605
  H8MTer_L 0.1 dolmanp__L_r + gpi_hs_r --> 0.1 dolp__L_r + h_r + m_em_3gacpail_hs_r 8605
  H7MTer_L 0.1 dolmanp__L_r + em2emgacpail_hs_r --> 0.1 dolp__L_r + h_r + mem2emgacpail_hs_r 8605
  H6MTer_U 0.1 dolmanp_U_r + m2emgacpail_hs_r --> 0.1 dolp_U_r + h_r + m3emgacpail_hs_r 8605
H6MTer_U 0.1 dolmanp_U_r + m2emgacpail_hs_r --> 0.1 dolp_U_r + h_r + m3emgacpail_hs_r 8605
  H7MTer_U 0.1 dolmanp_U_r + em2emgacpail_hs_r --> 0.1 dolp_U_r + h_r + mem2emgacpail_hs_r 8605
  H8MTer_U 0.1 dolmanp_U_r + gpi_hs_r --> 0.1 dolp_U_r + h_r + m_em_3gacpail_hs_r 8605
  H8MTer_L 0.1 dolmanp__L_r + gpi_hs_r --> 0.1 dolp__L_r + h_r + m_em_3gacpail_hs_r 8605
  H6MTer_L 0.1 dolmanp__L_r + m2emgacpail_hs_r --> 0.1 dolp__L_r + h_r + m3emgacpail_hs_r 8605
  H7MTer_L 0.1 dolmanp__L_r + em2emgacpail_hs_r --> 0.1 dolp__L_r + h_r + mem2emgacpail_hs_r 8605

m2gacpail_hs_r
H2MTer_L 0.1 dolmanp__L_r + mgacpail_hs_r --> 0.1 dolp__L_r + h_r + m2gacpail_hs_r 11445
  H2MTer_U 0.1 dolmanp_U_r + mgacpail_hs_r --> 0.1 dolp_U_r + h_r + m2gacpail_hs_r 11445
  H5MTer_U 0.1 dolmanp_U_r + emgacpail_hs_r --> 0.1 dolp_U_r + h_r + memgacpail_hs_r 11445
  H5MTer_L 0.1 dolmanp__L_r + emgacpail_hs_r --> 0.1 dolp__L_r + h_r + memgacpail_hs_r 11445
H2MTer_U 0.1 dolmanp_U_r + mgacpail_hs_r --> 0.1 dolp_U_r + h_r + m2gacpail_hs_r 11445
  H5MTer_U 0.1 dolmanp_U_r + emgacpail_hs_r --> 0.1 dolp_U_r + h_r + memgacpail_hs_r 11445
  H5MTer_L 0.1 dolmanp__L_r + emgacpail_hs_r --> 0.1 dolp__L_r + h_r + memgacpail_hs_r 11445
  H2MTer_L 0.1 dolmanp__L_r + mgacpail_hs_r --> 0.1 dolp__L_r + h_r + m2gacpail_hs_r 11445
H3ETer m2gacpail_hs_r + pe_hs_r --> dag_hs_r + memgacpail_hs_r 16789
  H7_ETer emem2gacpail_hs_r + pe_hs_r --> dag_hs_r + gpi_hs_r 16789
  H2ETer mgacpail_hs_r + pe_hs_r --> dag_hs_r + emgacpail_hs_r 16789
  H4ETer m3gacpail_hs_r + pe_hs_r --> dag_hs_r + m2emgacpail_hs_r 16789
  H6_ETer em3gacpail_hs_r + pe_hs_r --> dag_hs_r + em2emgacpail_hs_r 16789
H3MTer_L 0.1 dolmanp__L_r + m2gacpail_hs_r --> 0.1 dolp__L_r + h_r + m3gacpail_hs_r 12961
  BMTer_L 0.1 dolmanp__L_r + memgacpail_hs_r --> 0.1 dolp__L_r + h_r + m2emgacpail_hs_r 12961
  BMTer_U 0.1 dolmanp_U_r + memgacpail_hs_r --> 0.1 dolp_U_r + h_r + m2emgacpail_hs_r 12961
  H3MTer_U 0.1 dolmanp_U_r + m2gacpail_hs_r --> 0.1 dolp_U_r + h_r + m3gacpail_hs_r 12961
H3MTer_U 0.1 dolmanp_U_r + m2gacpail_hs_r --> 0.1 dolp_U_r + h_r + m3gacpail_hs_r 12961
  BMTer_L 0.1 dolmanp__L_r + memgacpail_hs_r --> 0.1 dolp__L_r + h_r + m2emgacpail_hs_r 12961
  BMTer_U 0.1 dolmanp_U_r + memgacpail_hs_r --> 0.1 dolp_U_r + h_r + m2emgacpail_hs_r 12961
  H3MTer_L 0.1 dolmanp__L_r + m2gacpail_hs_r --> 0.1 dolp__L_r + h_r + m3gacpail_hs_r 12961

m3emgacpail_hs_r
H6MTer_L 0.1 dolmanp__L_r + m2emgacpail_hs_r --> 0.1 dolp__L_r + h_r + m3emgacpail_hs_r 8605
  H7MTer_U 0.1 dolmanp_U_r + em2emgacpail_hs_r --> 0.1 dolp_U_r + h_r + mem2emgacpail_hs_r 8605
  H8MTer_U 0.1 dolmanp_U_r + gpi_hs_r --> 0.1 dolp_U_r + h_r + m_em_3gacpail_hs_r 8605
  H8MTer_L 0.1 dolmanp__L_r + gpi_hs_r --> 0.1 dolp__L_r + h_r + m_em_3gacpail_hs_r 8605
  H7MTer_L 0.1 dolmanp__L_r + em2emgacpail_hs_r --> 0.1 dolp__L_r + h_r + mem2emgacpail_hs_r 8605
  H6MTer_U 0.1 dolmanp_U_r + m2emgacpail_hs_r --> 0.1 dolp_U_r + h_r + m3emgacpail_hs_r 8605
H6MTer_U 0.1 dolmanp_U_r + m2emgacpail_hs_r --> 0.1 dolp_U_r + h_r + m3emgacpail_hs_r 8605
  H7MTer_U 0.1 dolmanp_U_r + em2emgacpail_hs_r --> 0.1 dolp_U_r + h_r + mem2emgacpail_hs_r 8605
  H8MTer_U 0.1 dolmanp_U_r + gpi_hs_r --> 0.1 dolp_U_r + h_r + m_em_3gacpail_hs_r 8605
  H8MTer_L 0.1 dolmanp__L_r + gpi_hs_r --> 0.1 dolp__L_r + h_r + m_em_3gacpail_hs_r 8605
  H6MTer_L 0.1 dolmanp__L_r + m2emgacpail_hs_r --> 0.1 dolp__L_r + h_r + m3emgacpail_hs_r 8605
  H7MTer_L 0.1 dolmanp__L_r + em2emgacpail_hs_r --> 0.1 dolp__L_r + h_r + mem2emgacpail_hs_r 8605
M4CET3er m3emgacpail_hs_r + pe_hs_r --> dag_hs_r + mem2emgacpail_hs_r 12437 and 8931
  H4ET3er m3gacpail_hs_r + pe_hs_r --> dag_hs_r + em3gacpail_hs_r 12437 and 8931
  M4BET2er mem2emgacpail_hs_r + pe_hs_r --> dag_hs_r + m_em_3gacpail_hs_r 14092 and 8931
  H6_ET2er em3gacpail_hs_r + pe_hs_r --> dag_hs_r + emem2gacpail_hs_r 14092 and 8931
  H7ET2er em2emgacpail_hs_r + pe_hs_r --> dag_hs_r + gpi_hs_r 14092 and 8931
  H6ET3er m2emgacpail_hs_r + pe_hs_r --> dag_hs_r + em2emgacpail_hs_r 12437 and 8931
  H4ET3er m3gacpail_hs_r + pe_hs_r --> dag_hs_r + em3gacpail_hs_r 12437 and 8931
  H6ET3er m2emgacpail_hs_r + pe_hs_r --> dag_hs_r + em2emgacpail_hs_r 12437 and 8931

m3gacpail_hs_r
H3MTer_L 0.1 dolmanp__L_r + m2gacpail_hs_r --> 0.1 dolp__L_r + h_r + m3gacpail_hs_r 12961
  BMTer_L 0.1 dolmanp__L_r + memgacpail_hs_r --> 0.1 dolp__L_r + h_r + m2emgacpail_hs_r 12961
  BMTer_U 0.1 dolmanp_U_r + memgacpail_hs_r --> 0.1 dolp_U_r + h_r + m2emgacpail_hs_r 12961
  H3MTer_U 0.1 dolmanp_U_r + m2gacpail_hs_r --> 0.1 dolp_U_r + h_r + m3gacpail_hs_r 12961
H3MTer_U 0.1 dolmanp_U_r + m2gacpail_hs_r --> 0.1 dolp_U_r + h_r + m3gacpail_hs_r 12961
  BMTer_L 0.1 dolmanp__L_r + memgacpail_hs_r --> 0.1 dolp__L_r + h_r + m2emgacpail_hs_r 12961
  BMTer_U 0.1 dolmanp_U_r + memgacpail_hs_r --> 0.1 dolp_U_r + h_r + m2emgacpail_hs_r 12961
  H3MTer_L 0.1 dolmanp__L_r + m2gacpail_hs_r --> 0.1 dolp__L_r + h_r + m3gacpail_hs_r 12961
H4ET3er m3gacpail_hs_r + pe_hs_r --> dag_hs_r + em3gacpail_hs_r 12437 and 8931
  M4BET2er mem2emgacpail_hs_r + pe_hs_r --> dag_hs_r + m_em_3gacpail_hs_r 14092 and 8931
  H6_ET2er em3gacpail_hs_r + pe_hs_r --> dag_hs_r + emem2gacpail_hs_r 14092 and 8931
  M4CET3er m3emgacpail_hs_r + pe_hs_r --> dag_hs_r + mem2emgacpail_hs_r 12437 and 8931
  H7ET2er em2emgacpail_hs_r + pe_hs_r --> dag_hs_r + gpi_hs_r 14092 and 8931
  H6ET3er m2emgacpail_hs_r + pe_hs_r --> dag_hs_r + em2emgacpail_hs_r 12437 and 8931
  M4CET3er m3emgacpail_hs_r + pe_hs_r --> dag_hs_r + mem2emgacpail_hs_r 12437 and 8931
  H6ET3er m2emgacpail_hs_r + pe_hs_r --> dag_hs_r + em2emgacpail_hs_r 12437 and 8931
H4ETer m3gacpail_hs_r + pe_hs_r --> dag_hs_r + m2emgacpail_hs_r 16789
  H7_ETer emem2gacpail_hs_r + pe_hs_r --> dag_hs_r + gpi_hs_r 16789
  H2ETer mgacpail_hs_r + pe_hs_r --> dag_hs_r + emgacpail_hs_r 16789
  H3ETer m2gacpail_hs_r + pe_hs_r --> dag_hs_r + memgacpail_hs_r 16789
  H6_ETer em3gacpail_hs_r + pe_hs_r --> dag_hs_r + em2emgacpail_hs_r 16789

m_em_3gacpail_hs_r
H8MTer_L 0.1 dolmanp__L_r + gpi_hs_r --> 0.1 dolp__L_r + h_r + m_em_3gacpail_hs_r 8605
  H7MTer_U 0.1 dolmanp_U_r + em2emgacpail_hs_r --> 0.1 dolp_U_r + h_r + mem2emgacpail_hs_r 8605
  H8MTer_U 0.1 dolmanp_U_r + gpi_hs_r --> 0.1 dolp_U_r + h_r + m_em_3gacpail_hs_r 8605
  H6MTer_L 0.1 dolmanp__L_r + m2emgacpail_hs_r --> 0.1 dolp__L_r + h_r + m3emgacpail_hs_r 8605
  H7MTer_L 0.1 dolmanp__L_r + em2emgacpail_hs_r --> 0.1 dolp__L_r + h_r + mem2emgacpail_hs_r 8605
  H6MTer_U 0.1 dolmanp_U_r + m2emgacpail_hs_r --> 0.1 dolp_U_r + h_r + m3emgacpail_hs_r 8605
H8MTer_U 0.1 dolmanp_U_r + gpi_hs_r --> 0.1 dolp_U_r + h_r + m_em_3gacpail_hs_r 8605
  H7MTer_U 0.1 dolmanp_U_r + em2emgacpail_hs_r --> 0.1 dolp_U_r + h_r + mem2emgacpail_hs_r 8605
  H8MTer_L 0.1 dolmanp__L_r + gpi_hs_r --> 0.1 dolp__L_r + h_r + m_em_3gacpail_hs_r 8605
  H6MTer_L 0.1 dolmanp__L_r + m2emgacpail_hs_r --> 0.1 dolp__L_r + h_r + m3emgacpail_hs_r 8605
  H7MTer_L 0.1 dolmanp__L_r + em2emgacpail_hs_r --> 0.1 dolp__L_r + h_r + mem2emgacpail_hs_r 8605
  H6MTer_U 0.1 dolmanp_U_r + m2emgacpail_hs_r --> 0.1 dolp_U_r + h_r + m3emgacpail_hs_r 8605
M4ATAer m_em_3gacpail_hs_r + pre_prot_r --> gpi_sig_r + m_em_3gacpail_prot_hs_r 10664 and 11490 and 12988 and 13065
  M4BTAer mem2emgacpail_hs_r + pre_prot_r --> gpi_sig_r + mem2emgacpail_prot_hs_r 10664 and 11490 and 12838 and 12988 and 13065
  H7_TAer em2emgacpail_hs_r + pre_prot_r --> em2emgacpail_prot_hs_r + gpi_sig_r 10664 and 11490 and 12838 and 12988 and 13065
  H8TAer gpi_hs_r + pre_prot_r --> gpi_prot_hs_r + gpi_sig_r 10664 and 11490 and 12838 and 12988 and 13065
  M4BTAer mem2emgacpail_hs_r + pre_prot_r --> gpi_sig_r + mem2emgacpail_prot_hs_r 10664 and 11490 and 12838 and 12988 and 13065
  H7_TAer em2emgacpail_hs_r + pre_prot_r --> em2emgacpail_prot_hs_r + gpi_sig_r 10664 and 11490 and 12838 and 12988 and 13065
  H8TAer gpi_hs_r + pre_prot_r --> gpi_prot_hs_r + gpi_sig_r 10664 and 11490 and 12838 and 12988 and 13065
  M4BTAer mem2emgacpail_hs_r + pre_prot_r --> gpi_sig_r + mem2emgacpail_prot_hs_r 10664 and 11490 and 12838 and 12988 and 13065
  H7_TAer em2emgacpail_hs_r + pre_prot_r --> em2emgacpail_prot_hs_r + gpi_sig_r 10664 and 11490 and 12838 and 12988 and 13065
  H8TAer gpi_hs_r + pre_prot_r --> gpi_prot_hs_r + gpi_sig_r 10664 and 11490 and 12838 and 12988 and 13065
  M4BTAer mem2emgacpail_hs_r + pre_prot_r --> gpi_sig_r + mem2emgacpail_prot_hs_r 10664 and 11490 and 12838 and 12988 and 13065
  H7_TAer em2emgacpail_hs_r + pre_prot_r --> em2emgacpail_prot_hs_r + gpi_sig_r 10664 and 11490 and 12838 and 12988 and 13065
  H8TAer gpi_hs_r + pre_prot_r --> gpi_prot_hs_r + gpi_sig_r 10664 and 11490 and 12838 and 12988 and 13065
M4BET2er mem2emgacpail_hs_r + pe_hs_r --> dag_hs_r + m_em_3gacpail_hs_r 14092 and 8931
  H6_ET2er em3gacpail_hs_r + pe_hs_r --> dag_hs_r + emem2gacpail_hs_r 14092 and 8931
  H7ET2er em2emgacpail_hs_r + pe_hs_r --> dag_hs_r + gpi_hs_r 14092 and 8931
  H4ET3er m3gacpail_hs_r + pe_hs_r --> dag_hs_r + em3gacpail_hs_r 12437 and 8931
  H6_ET2er em3gacpail_hs_r + pe_hs_r --> dag_hs_r + emem2gacpail_hs_r 14092 and 8931
  M4CET3er m3emgacpail_hs_r + pe_hs_r --> dag_hs_r + mem2emgacpail_hs_r 12437 and 8931
  H7ET2er em2emgacpail_hs_r + pe_hs_r --> dag_hs_r + gpi_hs_r 14092 and 8931
  H6ET3er m2emgacpail_hs_r + pe_hs_r --> dag_hs_r + em2emgacpail_hs_r 12437 and 8931

m_em_3gacpail_prot_hs_r
M4ATAer m_em_3gacpail_hs_r + pre_prot_r --> gpi_sig_r + m_em_3gacpail_prot_hs_r 10664 and 11490 and 12988 and 13065
  M4BTAer mem2emgacpail_hs_r + pre_prot_r --> gpi_sig_r + mem2emgacpail_prot_hs_r 10664 and 11490 and 12838 and 12988 and 13065
  H7_TAer em2emgacpail_hs_r + pre_prot_r --> em2emgacpail_prot_hs_r + gpi_sig_r 10664 and 11490 and 12838 and 12988 and 13065
  H8TAer gpi_hs_r + pre_prot_r --> gpi_prot_hs_r + gpi_sig_r 10664 and 11490 and 12838 and 12988 and 13065
  M4BTAer mem2emgacpail_hs_r + pre_prot_r --> gpi_sig_r + mem2emgacpail_prot_hs_r 10664 and 11490 and 12838 and 12988 and 13065
  H7_TAer em2emgacpail_hs_r + pre_prot_r --> em2emgacpail_prot_hs_r + gpi_sig_r 10664 and 11490 and 12838 and 12988 and 13065
  H8TAer gpi_hs_r + pre_prot_r --> gpi_prot_hs_r + gpi_sig_r 10664 and 11490 and 12838 and 12988 and 13065
  M4BTAer mem2emgacpail_hs_r + pre_prot_r --> gpi_sig_r + mem2emgacpail_prot_hs_r 10664 and 11490 and 12838 and 12988 and 13065
  H7_TAer em2emgacpail_hs_r + pre_prot_r --> em2emgacpail_prot_hs_r + gpi_sig_r 10664 and 11490 and 12838 and 12988 and 13065
  H8TAer gpi_hs_r + pre_prot_r --> gpi_prot_hs_r + gpi_sig_r 10664 and 11490 and 12838 and 12988 and 13065
  M4BTAer mem2emgacpail_hs_r + pre_prot_r --> gpi_sig_r + mem2emgacpail_prot_hs_r 10664 and 11490 and 12838 and 12988 and 13065
  H7_TAer em2emgacpail_hs_r + pre_prot_r --> em2emgacpail_prot_hs_r + gpi_sig_r 10664 and 11490 and 12838 and 12988 and 13065
  H8TAer gpi_hs_r + pre_prot_r --> gpi_prot_hs_r + gpi_sig_r 10664 and 11490 and 12838 and 12988 and 13065

mag_hs_c
LPS3 h2o_c + mag_hs_c --> Rtotal2_c + glyc_c + h_c 14158 or 9728
  MAGAH160 h2o_c + mag160_c --> glyc_c + h_c + hdca_c 9728
  MAGAH180 h2o_c + mag180_c --> glyc_c + h_c + ocdca_c 9728
  MAGAH1819Z h2o_c + mag1819Z_c --> glyc_c + h_c + ocdcea_c 9728
  MAGL_RT h2o_c + 0.01 mag_RT_d --> 0.02 dca_c + 0.06 ddca_c + glyc_c + h_c + 0.27 hdca_c + 0.17 hdcea_c + 0.05 ocdca_c + 0.24 ocdcea_c + 0.09 ocdcya_c + 0.1 ttdca_c 14158 or 15065 or 9728
  MAGL_RT h2o_c + 0.01 mag_RT_d --> 0.02 dca_c + 0.06 ddca_c + glyc_c + h_c + 0.27 hdca_c + 0.17 hdcea_c + 0.05 ocdca_c + 0.24 ocdcea_c + 0.09 ocdcya_c + 0.1 ttdca_c 14158 or 15065 or 9728

mem2emgacpail_hs_r
H7MTer_L 0.1 dolmanp__L_r + em2emgacpail_hs_r --> 0.1 dolp__L_r + h_r + mem2emgacpail_hs_r 8605
  H7MTer_U 0.1 dolmanp_U_r + em2emgacpail_hs_r --> 0.1 dolp_U_r + h_r + mem2emgacpail_hs_r 8605
  H8MTer_U 0.1 dolmanp_U_r + gpi_hs_r --> 0.1 dolp_U_r + h_r + m_em_3gacpail_hs_r 8605
  H8MTer_L 0.1 dolmanp__L_r + gpi_hs_r --> 0.1 dolp__L_r + h_r + m_em_3gacpail_hs_r 8605
  H6MTer_L 0.1 dolmanp__L_r + m2emgacpail_hs_r --> 0.1 dolp__L_r + h_r + m3emgacpail_hs_r 8605
  H6MTer_U 0.1 dolmanp_U_r + m2emgacpail_hs_r --> 0.1 dolp_U_r + h_r + m3emgacpail_hs_r 8605
H7MTer_U 0.1 dolmanp_U_r + em2emgacpail_hs_r --> 0.1 dolp_U_r + h_r + mem2emgacpail_hs_r 8605
  H8MTer_U 0.1 dolmanp_U_r + gpi_hs_r --> 0.1 dolp_U_r + h_r + m_em_3gacpail_hs_r 8605
  H8MTer_L 0.1 dolmanp__L_r + gpi_hs_r --> 0.1 dolp__L_r + h_r + m_em_3gacpail_hs_r 8605
  H6MTer_L 0.1 dolmanp__L_r + m2emgacpail_hs_r --> 0.1 dolp__L_r + h_r + m3emgacpail_hs_r 8605
  H7MTer_L 0.1 dolmanp__L_r + em2emgacpail_hs_r --> 0.1 dolp__L_r + h_r + mem2emgacpail_hs_r 8605
  H6MTer_U 0.1 dolmanp_U_r + m2emgacpail_hs_r --> 0.1 dolp_U_r + h_r + m3emgacpail_hs_r 8605
M4BET2er mem2emgacpail_hs_r + pe_hs_r --> dag_hs_r + m_em_3gacpail_hs_r 14092 and 8931
  H6_ET2er em3gacpail_hs_r + pe_hs_r --> dag_hs_r + emem2gacpail_hs_r 14092 and 8931
  H7ET2er em2emgacpail_hs_r + pe_hs_r --> dag_hs_r + gpi_hs_r 14092 and 8931
  H4ET3er m3gacpail_hs_r + pe_hs_r --> dag_hs_r + em3gacpail_hs_r 12437 and 8931
  H6_ET2er em3gacpail_hs_r + pe_hs_r --> dag_hs_r + emem2gacpail_hs_r 14092 and 8931
  M4CET3er m3emgacpail_hs_r + pe_hs_r --> dag_hs_r + mem2emgacpail_hs_r 12437 and 8931
  H7ET2er em2emgacpail_hs_r + pe_hs_r --> dag_hs_r + gpi_hs_r 14092 and 8931
  H6ET3er m2emgacpail_hs_r + pe_hs_r --> dag_hs_r + em2emgacpail_hs_r 12437 and 8931
M4BTAer mem2emgacpail_hs_r + pre_prot_r --> gpi_sig_r + mem2emgacpail_prot_hs_r 10664 and 11490 and 12838 and 12988 and 13065
  H7_TAer em2emgacpail_hs_r + pre_prot_r --> em2emgacpail_prot_hs_r + gpi_sig_r 10664 and 11490 and 12838 and 12988 and 13065
  H8TAer gpi_hs_r + pre_prot_r --> gpi_prot_hs_r + gpi_sig_r 10664 and 11490 and 12838 and 12988 and 13065
  M4ATAer m_em_3gacpail_hs_r + pre_prot_r --> gpi_sig_r + m_em_3gacpail_prot_hs_r 10664 and 11490 and 12988 and 13065
  H7_TAer em2emgacpail_hs_r + pre_prot_r --> em2emgacpail_prot_hs_r + gpi_sig_r 10664 and 11490 and 12838 and 12988 and 13065
  H8TAer gpi_hs_r + pre_prot_r --> gpi_prot_hs_r + gpi_sig_r 10664 and 11490 and 12838 and 12988 and 13065
  H7_TAer em2emgacpail_hs_r + pre_prot_r --> em2emgacpail_prot_hs_r + gpi_sig_r 10664 and 11490 and 12838 and 12988 and 13065
  H8TAer gpi_hs_r + pre_prot_r --> gpi_prot_hs_r + gpi_sig_r 10664 and 11490 and 12838 and 12988 and 13065
  M4ATAer m_em_3gacpail_hs_r + pre_prot_r --> gpi_sig_r + m_em_3gacpail_prot_hs_r 10664 and 11490 and 12988 and 13065
  H7_TAer em2emgacpail_hs_r + pre_prot_r --> em2emgacpail_prot_hs_r + gpi_sig_r 10664 and 11490 and 12838 and 12988 and 13065
  H8TAer gpi_hs_r + pre_prot_r --> gpi_prot_hs_r + gpi_sig_r 10664 and 11490 and 12838 and 12988 and 13065
  M4ATAer m_em_3gacpail_hs_r + pre_prot_r --> gpi_sig_r + m_em_3gacpail_prot_hs_r 10664 and 11490 and 12988 and 13065
  H7_TAer em2emgacpail_hs_r + pre_prot_r --> em2emgacpail_prot_hs_r + gpi_sig_r 10664 and 11490 and 12838 and 12988 and 13065
  H8TAer gpi_hs_r + pre_prot_r --> gpi_prot_hs_r + gpi_sig_r 10664 and 11490 and 12838 and 12988 and 13065
  M4ATAer m_em_3gacpail_hs_r + pre_prot_r --> gpi_sig_r + m_em_3gacpail_prot_hs_r 10664 and 11490 and 12988 and 13065
M4CET3er m3emgacpail_hs_r + pe_hs_r --> dag_hs_r + mem2emgacpail_hs_r 12437 and 8931
  H4ET3er m3gacpail_hs_r + pe_hs_r --> dag_hs_r + em3gacpail_hs_r 12437 and 8931
  M4BET2er mem2emgacpail_hs_r + pe_hs_r --> dag_hs_r + m_em_3gacpail_hs_r 14092 and 8931
  H6_ET2er em3gacpail_hs_r + pe_hs_r --> dag_hs_r + emem2gacpail_hs_r 14092 and 8931
  H7ET2er em2emgacpail_hs_r + pe_hs_r --> dag_hs_r + gpi_hs_r 14092 and 8931
  H6ET3er m2emgacpail_hs_r + pe_hs_r --> dag_hs_r + em2emgacpail_hs_r 12437 and 8931
  H4ET3er m3gacpail_hs_r + pe_hs_r --> dag_hs_r + em3gacpail_hs_r 12437 and 8931
  H6ET3er m2emgacpail_hs_r + pe_hs_r --> dag_hs_r + em2emgacpail_hs_r 12437 and 8931

mem2emgacpail_prot_hs_r
M4BTAer mem2emgacpail_hs_r + pre_prot_r --> gpi_sig_r + mem2emgacpail_prot_hs_r 10664 and 11490 and 12838 and 12988 and 13065
  H7_TAer em2emgacpail_hs_r + pre_prot_r --> em2emgacpail_prot_hs_r + gpi_sig_r 10664 and 11490 and 12838 and 12988 and 13065
  H8TAer gpi_hs_r + pre_prot_r --> gpi_prot_hs_r + gpi_sig_r 10664 and 11490 and 12838 and 12988 and 13065
  M4ATAer m_em_3gacpail_hs_r + pre_prot_r --> gpi_sig_r + m_em_3gacpail_prot_hs_r 10664 and 11490 and 12988 and 13065
  H7_TAer em2emgacpail_hs_r + pre_prot_r --> em2emgacpail_prot_hs_r + gpi_sig_r 10664 and 11490 and 12838 and 12988 and 13065
  H8TAer gpi_hs_r + pre_prot_r --> gpi_prot_hs_r + gpi_sig_r 10664 and 11490 and 12838 and 12988 and 13065
  H7_TAer em2emgacpail_hs_r + pre_prot_r --> em2emgacpail_prot_hs_r + gpi_sig_r 10664 and 11490 and 12838 and 12988 and 13065
  H8TAer gpi_hs_r + pre_prot_r --> gpi_prot_hs_r + gpi_sig_r 10664 and 11490 and 12838 and 12988 and 13065
  M4ATAer m_em_3gacpail_hs_r + pre_prot_r --> gpi_sig_r + m_em_3gacpail_prot_hs_r 10664 and 11490 and 12988 and 13065
  H7_TAer em2emgacpail_hs_r + pre_prot_r --> em2emgacpail_prot_hs_r + gpi_sig_r 10664 and 11490 and 12838 and 12988 and 13065
  H8TAer gpi_hs_r + pre_prot_r --> gpi_prot_hs_r + gpi_sig_r 10664 and 11490 and 12838 and 12988 and 13065
  M4ATAer m_em_3gacpail_hs_r + pre_prot_r --> gpi_sig_r + m_em_3gacpail_prot_hs_r 10664 and 11490 and 12988 and 13065
  H7_TAer em2emgacpail_hs_r + pre_prot_r --> em2emgacpail_prot_hs_r + gpi_sig_r 10664 and 11490 and 12838 and 12988 and 13065
  H8TAer gpi_hs_r + pre_prot_r --> gpi_prot_hs_r + gpi_sig_r 10664 and 11490 and 12838 and 12988 and 13065
  M4ATAer m_em_3gacpail_hs_r + pre_prot_r --> gpi_sig_r + m_em_3gacpail_prot_hs_r 10664 and 11490 and 12988 and 13065

memgacpail_hs_r
BMTer_L 0.1 dolmanp__L_r + memgacpail_hs_r --> 0.1 dolp__L_r + h_r + m2emgacpail_hs_r 12961
  BMTer_U 0.1 dolmanp_U_r + memgacpail_hs_r --> 0.1 dolp_U_r + h_r + m2emgacpail_hs_r 12961
  H3MTer_U 0.1 dolmanp_U_r + m2gacpail_hs_r --> 0.1 dolp_U_r + h_r + m3gacpail_hs_r 12961
  H3MTer_L 0.1 dolmanp__L_r + m2gacpail_hs_r --> 0.1 dolp__L_r + h_r + m3gacpail_hs_r 12961
BMTer_U 0.1 dolmanp_U_r + memgacpail_hs_r --> 0.1 dolp_U_r + h_r + m2emgacpail_hs_r 12961
  BMTer_L 0.1 dolmanp__L_r + memgacpail_hs_r --> 0.1 dolp__L_r + h_r + m2emgacpail_hs_r 12961
  H3MTer_U 0.1 dolmanp_U_r + m2gacpail_hs_r --> 0.1 dolp_U_r + h_r + m3gacpail_hs_r 12961
  H3MTer_L 0.1 dolmanp__L_r + m2gacpail_hs_r --> 0.1 dolp__L_r + h_r + m3gacpail_hs_r 12961
H3ETer m2gacpail_hs_r + pe_hs_r --> dag_hs_r + memgacpail_hs_r 16789
  H7_ETer emem2gacpail_hs_r + pe_hs_r --> dag_hs_r + gpi_hs_r 16789
  H2ETer mgacpail_hs_r + pe_hs_r --> dag_hs_r + emgacpail_hs_r 16789
  H4ETer m3gacpail_hs_r + pe_hs_r --> dag_hs_r + m2emgacpail_hs_r 16789
  H6_ETer em3gacpail_hs_r + pe_hs_r --> dag_hs_r + em2emgacpail_hs_r 16789
H5MTer_L 0.1 dolmanp__L_r + emgacpail_hs_r --> 0.1 dolp__L_r + h_r + memgacpail_hs_r 11445
  H2MTer_U 0.1 dolmanp_U_r + mgacpail_hs_r --> 0.1 dolp_U_r + h_r + m2gacpail_hs_r 11445
  H5MTer_U 0.1 dolmanp_U_r + emgacpail_hs_r --> 0.1 dolp_U_r + h_r + memgacpail_hs_r 11445
  H2MTer_L 0.1 dolmanp__L_r + mgacpail_hs_r --> 0.1 dolp__L_r + h_r + m2gacpail_hs_r 11445
H5MTer_U 0.1 dolmanp_U_r + emgacpail_hs_r --> 0.1 dolp_U_r + h_r + memgacpail_hs_r 11445
  H2MTer_U 0.1 dolmanp_U_r + mgacpail_hs_r --> 0.1 dolp_U_r + h_r + m2gacpail_hs_r 11445
  H5MTer_L 0.1 dolmanp__L_r + emgacpail_hs_r --> 0.1 dolp__L_r + h_r + memgacpail_hs_r 11445
  H2MTer_L 0.1 dolmanp__L_r + mgacpail_hs_r --> 0.1 dolp__L_r + h_r + m2gacpail_hs_r 11445

mgacpail_hs_r
H2ETer mgacpail_hs_r + pe_hs_r --> dag_hs_r + emgacpail_hs_r 16789
  H7_ETer emem2gacpail_hs_r + pe_hs_r --> dag_hs_r + gpi_hs_r 16789
  H3ETer m2gacpail_hs_r + pe_hs_r --> dag_hs_r + memgacpail_hs_r 16789
  H4ETer m3gacpail_hs_r + pe_hs_r --> dag_hs_r + m2emgacpail_hs_r 16789
  H6_ETer em3gacpail_hs_r + pe_hs_r --> dag_hs_r + em2emgacpail_hs_r 16789
H2MTer_L 0.1 dolmanp__L_r + mgacpail_hs_r --> 0.1 dolp__L_r + h_r + m2gacpail_hs_r 11445
  H2MTer_U 0.1 dolmanp_U_r + mgacpail_hs_r --> 0.1 dolp_U_r + h_r + m2gacpail_hs_r 11445
  H5MTer_U 0.1 dolmanp_U_r + emgacpail_hs_r --> 0.1 dolp_U_r + h_r + memgacpail_hs_r 11445
  H5MTer_L 0.1 dolmanp__L_r + emgacpail_hs_r --> 0.1 dolp__L_r + h_r + memgacpail_hs_r 11445
H2MTer_U 0.1 dolmanp_U_r + mgacpail_hs_r --> 0.1 dolp_U_r + h_r + m2gacpail_hs_r 11445
  H5MTer_U 0.1 dolmanp_U_r + emgacpail_hs_r --> 0.1 dolp_U_r + h_r + memgacpail_hs_r 11445
  H5MTer_L 0.1 dolmanp__L_r + emgacpail_hs_r --> 0.1 dolp__L_r + h_r + memgacpail_hs_r 11445
  H2MTer_L 0.1 dolmanp__L_r + mgacpail_hs_r --> 0.1 dolp__L_r + h_r + m2gacpail_hs_r 11445

oagd3_hs_c
GAO1 accoa_c + gd3_hs_c --> coa_c + oagd3_hs_c 10946
  GAO2 accoa_c + gt3_hs_c --> coa_c + oagt3_hs_c 10946
  ACCOAtm accoa_c + coa_m <=> accoa_m + coa_c 10946
  ACCOAtx accoa_c + coa_x <=> accoa_x + coa_c 10946
  ACCOAgt accoa_c <=> accoa_g 10946
  GAO2g accoa_g + gt3_hs_g --> coa_g + oagt3_hs_g 10946
  GAO1g accoa_g + gd3_hs_g --> coa_g + oagd3_hs_g 10946
  ACCOAtr accoa_c <=> accoa_r 10946

oagd3_hs_g
GAO1g accoa_g + gd3_hs_g --> coa_g + oagd3_hs_g 10946
  GAO1 accoa_c + gd3_hs_c --> coa_c + oagd3_hs_c 10946
  GAO2 accoa_c + gt3_hs_c --> coa_c + oagt3_hs_c 10946
  ACCOAtm accoa_c + coa_m <=> accoa_m + coa_c 10946
  ACCOAtx accoa_c + coa_x <=> accoa_x + coa_c 10946
  ACCOAgt accoa_c <=> accoa_g 10946
  GAO2g accoa_g + gt3_hs_g --> coa_g + oagt3_hs_g 10946
  ACCOAtr accoa_c <=> accoa_r 10946

oagt3_hs_c
GAO2 accoa_c + gt3_hs_c --> coa_c + oagt3_hs_c 10946
  GAO1 accoa_c + gd3_hs_c --> coa_c + oagd3_hs_c 10946
  ACCOAtm accoa_c + coa_m <=> accoa_m + coa_c 10946
  ACCOAtx accoa_c + coa_x <=> accoa_x + coa_c 10946
  ACCOAgt accoa_c <=> accoa_g 10946
  GAO2g accoa_g + gt3_hs_g --> coa_g + oagt3_hs_g 10946
  GAO1g accoa_g + gd3_hs_g --> coa_g + oagd3_hs_g 10946
  ACCOAtr accoa_c <=> accoa_r 10946

oagt3_hs_g
GAO2g accoa_g + gt3_hs_g --> coa_g + oagt3_hs_g 10946
  GAO1 accoa_c + gd3_hs_c --> coa_c + oagd3_hs_c 10946
  GAO2 accoa_c + gt3_hs_c --> coa_c + oagt3_hs_c 10946
  ACCOAtm accoa_c + coa_m <=> accoa_m + coa_c 10946
  ACCOAtx accoa_c + coa_x <=> accoa_x + coa_c 10946
  ACCOAgt accoa_c <=> accoa_g 10946
  GAO1g accoa_g + gd3_hs_g --> coa_g + oagd3_hs_g 10946
  ACCOAtr accoa_c <=> accoa_r 10946

paf_hs_c
PAFH h2o_c + paf_hs_c --> ac_c + ak2lgchol_hs_c + h_c 12283
  PAFHe h2o_e + paf_hs_e --> ac_e + ak2lgchol_hs_e + h_e 12283

paf_hs_e
PAFHe h2o_e + paf_hs_e --> ac_e + ak2lgchol_hs_e + h_e 12283
  PAFH h2o_c + paf_hs_c --> ac_c + ak2lgchol_hs_c + h_c 12283

pail_hs_c

pchol_hs_c
PCt atp_c + h2o_c + pchol_hs_c <=> adp_c + h_c + pchol_hs_e + pi_c 11777
  CHOLATEt3 atp_c + cholate_c + h2o_c --> adp_c + cholate_e + h_c + pi_c 11266 or 11777 or 13264 or 14111 or 14463 or 14844 or 8693 or 9145
  GCHOLAt3 atp_c + gchola_c + h2o_c --> adp_c + gchola_e + h_c + pi_c 11266 or 11777 or 13264 or 14111 or 14463 or 14844 or 8693 or 9145
  TCHOLAt3 atp_c + h2o_c + tchola_c --> adp_c + h_c + pi_c + tchola_e 11266 or 11777 or 13264 or 14111 or 14463 or 14844 or 8693 or 9145

pchol_hs_e
PCt atp_c + h2o_c + pchol_hs_c <=> adp_c + h_c + pchol_hs_e + pi_c 11777
  CHOLATEt3 atp_c + cholate_c + h2o_c --> adp_c + cholate_e + h_c + pi_c 11266 or 11777 or 13264 or 14111 or 14463 or 14844 or 8693 or 9145
  GCHOLAt3 atp_c + gchola_c + h2o_c --> adp_c + gchola_e + h_c + pi_c 11266 or 11777 or 13264 or 14111 or 14463 or 14844 or 8693 or 9145
  TCHOLAt3 atp_c + h2o_c + tchola_c --> adp_c + h_c + pi_c + tchola_e 11266 or 11777 or 13264 or 14111 or 14463 or 14844 or 8693 or 9145

pe_hs_c
PEFLIP atp_c + h2o_c + pe_hs_e --> adp_c + h_c + pe_hs_c + pi_c 12680 or 15426 or 9428
  PE1801819Zt atp_c + h2o_c + pe1801819Z_c --> adp_c + h_c + pe1801819Z_e + pi_c 12680 or 15426 or 9428
  PSFLIPm atp_c + h2o_c + ps_hs_c --> adp_c + h_c + pi_c + ps_hs_m 12680 or 15426 or 9428
  PE1819Z1819Zt atp_c + h2o_c + pe1819Z1819Z_c --> adp_c + h_c + pe1819Z1819Z_e + pi_c 12680 or 15426 or 9428
  PE1801829Z12Zt atp_c + h2o_c + pe1801829Z12Z_c --> adp_c + h_c + pe1801829Z12Z_e + pi_c 12680 or 15426 or 9428
  PAIL1819Z160t atp_c + h2o_c + pail1819Z160_c --> adp_c + h_c + pail1819Z160_e + pi_c 12680 or 15426 or 9428
  PEFLIPm atp_c + h2o_c + pe_hs_c --> adp_c + h_c + pe_hs_m + pi_c 12680 or 15426 or 9428
  PGP1819Z160t atp_c + h2o_c + pgp1819Z160_c --> adp_c + h_c + pgp1819Z160_e + pi_c 12680 or 15426 or 9428
  PSFLIP atp_c + h2o_c + ps_hs_e --> adp_c + h_c + pi_c + ps_hs_c 12680 or 15426 or 9428
  PG1819Z160t atp_c + h2o_c + pg1819Z160_c --> adp_c + h_c + pg1819Z160_e + pi_c 12680 or 15426 or 9428
  PE1819Z1829Z12Zt atp_c + h2o_c + pe1819Z1829Z12Z_c --> adp_c + h_c + pe1819Z1829Z12Z_e + pi_c 12680 or 15426 or 9428
  PE1801819Zt atp_c + h2o_c + pe1801819Z_c --> adp_c + h_c + pe1801819Z_e + pi_c 12680 or 15426 or 9428
  PSFLIPm atp_c + h2o_c + ps_hs_c --> adp_c + h_c + pi_c + ps_hs_m 12680 or 15426 or 9428
  PE1819Z1819Zt atp_c + h2o_c + pe1819Z1819Z_c --> adp_c + h_c + pe1819Z1819Z_e + pi_c 12680 or 15426 or 9428
  PE1801829Z12Zt atp_c + h2o_c + pe1801829Z12Z_c --> adp_c + h_c + pe1801829Z12Z_e + pi_c 12680 or 15426 or 9428
  PAIL1819Z160t atp_c + h2o_c + pail1819Z160_c --> adp_c + h_c + pail1819Z160_e + pi_c 12680 or 15426 or 9428
  PEFLIPm atp_c + h2o_c + pe_hs_c --> adp_c + h_c + pe_hs_m + pi_c 12680 or 15426 or 9428
  PGP1819Z160t atp_c + h2o_c + pgp1819Z160_c --> adp_c + h_c + pgp1819Z160_e + pi_c 12680 or 15426 or 9428
  PSFLIP atp_c + h2o_c + ps_hs_e --> adp_c + h_c + pi_c + ps_hs_c 12680 or 15426 or 9428
  PG1819Z160t atp_c + h2o_c + pg1819Z160_c --> adp_c + h_c + pg1819Z160_e + pi_c 12680 or 15426 or 9428
  PE1819Z1829Z12Zt atp_c + h2o_c + pe1819Z1829Z12Z_c --> adp_c + h_c + pe1819Z1829Z12Z_e + pi_c 12680 or 15426 or 9428
  PE1801819Zt atp_c + h2o_c + pe1801819Z_c --> adp_c + h_c + pe1801819Z_e + pi_c 12680 or 15426 or 9428
  PSFLIPm atp_c + h2o_c + ps_hs_c --> adp_c + h_c + pi_c + ps_hs_m 12680 or 15426 or 9428
  PE1819Z1819Zt atp_c + h2o_c + pe1819Z1819Z_c --> adp_c + h_c + pe1819Z1819Z_e + pi_c 12680 or 15426 or 9428
  PE1801829Z12Zt atp_c + h2o_c + pe1801829Z12Z_c --> adp_c + h_c + pe1801829Z12Z_e + pi_c 12680 or 15426 or 9428
  PAIL1819Z160t atp_c + h2o_c + pail1819Z160_c --> adp_c + h_c + pail1819Z160_e + pi_c 12680 or 15426 or 9428
  PEFLIPm atp_c + h2o_c + pe_hs_c --> adp_c + h_c + pe_hs_m + pi_c 12680 or 15426 or 9428
  PGP1819Z160t atp_c + h2o_c + pgp1819Z160_c --> adp_c + h_c + pgp1819Z160_e + pi_c 12680 or 15426 or 9428
  PSFLIP atp_c + h2o_c + ps_hs_e --> adp_c + h_c + pi_c + ps_hs_c 12680 or 15426 or 9428
  PG1819Z160t atp_c + h2o_c + pg1819Z160_c --> adp_c + h_c + pg1819Z160_e + pi_c 12680 or 15426 or 9428
  PE1819Z1829Z12Zt atp_c + h2o_c + pe1819Z1829Z12Z_c --> adp_c + h_c + pe1819Z1829Z12Z_e + pi_c 12680 or 15426 or 9428
PEFLIPm atp_c + h2o_c + pe_hs_c --> adp_c + h_c + pe_hs_m + pi_c 12680 or 15426 or 9428
  PE1801819Zt atp_c + h2o_c + pe1801819Z_c --> adp_c + h_c + pe1801819Z_e + pi_c 12680 or 15426 or 9428
  PSFLIPm atp_c + h2o_c + ps_hs_c --> adp_c + h_c + pi_c + ps_hs_m 12680 or 15426 or 9428
  PE1819Z1819Zt atp_c + h2o_c + pe1819Z1819Z_c --> adp_c + h_c + pe1819Z1819Z_e + pi_c 12680 or 15426 or 9428
  PE1801829Z12Zt atp_c + h2o_c + pe1801829Z12Z_c --> adp_c + h_c + pe1801829Z12Z_e + pi_c 12680 or 15426 or 9428
  PAIL1819Z160t atp_c + h2o_c + pail1819Z160_c --> adp_c + h_c + pail1819Z160_e + pi_c 12680 or 15426 or 9428
  PEFLIP atp_c + h2o_c + pe_hs_e --> adp_c + h_c + pe_hs_c + pi_c 12680 or 15426 or 9428
  PGP1819Z160t atp_c + h2o_c + pgp1819Z160_c --> adp_c + h_c + pgp1819Z160_e + pi_c 12680 or 15426 or 9428
  PSFLIP atp_c + h2o_c + ps_hs_e --> adp_c + h_c + pi_c + ps_hs_c 12680 or 15426 or 9428
  PG1819Z160t atp_c + h2o_c + pg1819Z160_c --> adp_c + h_c + pg1819Z160_e + pi_c 12680 or 15426 or 9428
  PE1819Z1829Z12Zt atp_c + h2o_c + pe1819Z1829Z12Z_c --> adp_c + h_c + pe1819Z1829Z12Z_e + pi_c 12680 or 15426 or 9428
  PE1801819Zt atp_c + h2o_c + pe1801819Z_c --> adp_c + h_c + pe1801819Z_e + pi_c 12680 or 15426 or 9428
  PSFLIPm atp_c + h2o_c + ps_hs_c --> adp_c + h_c + pi_c + ps_hs_m 12680 or 15426 or 9428
  PE1819Z1819Zt atp_c + h2o_c + pe1819Z1819Z_c --> adp_c + h_c + pe1819Z1819Z_e + pi_c 12680 or 15426 or 9428
  PE1801829Z12Zt atp_c + h2o_c + pe1801829Z12Z_c --> adp_c + h_c + pe1801829Z12Z_e + pi_c 12680 or 15426 or 9428
  PAIL1819Z160t atp_c + h2o_c + pail1819Z160_c --> adp_c + h_c + pail1819Z160_e + pi_c 12680 or 15426 or 9428
  PEFLIP atp_c + h2o_c + pe_hs_e --> adp_c + h_c + pe_hs_c + pi_c 12680 or 15426 or 9428
  PGP1819Z160t atp_c + h2o_c + pgp1819Z160_c --> adp_c + h_c + pgp1819Z160_e + pi_c 12680 or 15426 or 9428
  PSFLIP atp_c + h2o_c + ps_hs_e --> adp_c + h_c + pi_c + ps_hs_c 12680 or 15426 or 9428
  PG1819Z160t atp_c + h2o_c + pg1819Z160_c --> adp_c + h_c + pg1819Z160_e + pi_c 12680 or 15426 or 9428
  PE1819Z1829Z12Zt atp_c + h2o_c + pe1819Z1829Z12Z_c --> adp_c + h_c + pe1819Z1829Z12Z_e + pi_c 12680 or 15426 or 9428
  PE1801819Zt atp_c + h2o_c + pe1801819Z_c --> adp_c + h_c + pe1801819Z_e + pi_c 12680 or 15426 or 9428
  PSFLIPm atp_c + h2o_c + ps_hs_c --> adp_c + h_c + pi_c + ps_hs_m 12680 or 15426 or 9428
  PE1819Z1819Zt atp_c + h2o_c + pe1819Z1819Z_c --> adp_c + h_c + pe1819Z1819Z_e + pi_c 12680 or 15426 or 9428
  PE1801829Z12Zt atp_c + h2o_c + pe1801829Z12Z_c --> adp_c + h_c + pe1801829Z12Z_e + pi_c 12680 or 15426 or 9428
  PAIL1819Z160t atp_c + h2o_c + pail1819Z160_c --> adp_c + h_c + pail1819Z160_e + pi_c 12680 or 15426 or 9428
  PEFLIP atp_c + h2o_c + pe_hs_e --> adp_c + h_c + pe_hs_c + pi_c 12680 or 15426 or 9428
  PGP1819Z160t atp_c + h2o_c + pgp1819Z160_c --> adp_c + h_c + pgp1819Z160_e + pi_c 12680 or 15426 or 9428
  PSFLIP atp_c + h2o_c + ps_hs_e --> adp_c + h_c + pi_c + ps_hs_c 12680 or 15426 or 9428
  PG1819Z160t atp_c + h2o_c + pg1819Z160_c --> adp_c + h_c + pg1819Z160_e + pi_c 12680 or 15426 or 9428
  PE1819Z1829Z12Zt atp_c + h2o_c + pe1819Z1829Z12Z_c --> adp_c + h_c + pe1819Z1829Z12Z_e + pi_c 12680 or 15426 or 9428

pe_hs_e
PEFLIP atp_c + h2o_c + pe_hs_e --> adp_c + h_c + pe_hs_c + pi_c 12680 or 15426 or 9428
  PE1801819Zt atp_c + h2o_c + pe1801819Z_c --> adp_c + h_c + pe1801819Z_e + pi_c 12680 or 15426 or 9428
  PSFLIPm atp_c + h2o_c + ps_hs_c --> adp_c + h_c + pi_c + ps_hs_m 12680 or 15426 or 9428
  PE1819Z1819Zt atp_c + h2o_c + pe1819Z1819Z_c --> adp_c + h_c + pe1819Z1819Z_e + pi_c 12680 or 15426 or 9428
  PE1801829Z12Zt atp_c + h2o_c + pe1801829Z12Z_c --> adp_c + h_c + pe1801829Z12Z_e + pi_c 12680 or 15426 or 9428
  PAIL1819Z160t atp_c + h2o_c + pail1819Z160_c --> adp_c + h_c + pail1819Z160_e + pi_c 12680 or 15426 or 9428
  PEFLIPm atp_c + h2o_c + pe_hs_c --> adp_c + h_c + pe_hs_m + pi_c 12680 or 15426 or 9428
  PGP1819Z160t atp_c + h2o_c + pgp1819Z160_c --> adp_c + h_c + pgp1819Z160_e + pi_c 12680 or 15426 or 9428
  PSFLIP atp_c + h2o_c + ps_hs_e --> adp_c + h_c + pi_c + ps_hs_c 12680 or 15426 or 9428
  PG1819Z160t atp_c + h2o_c + pg1819Z160_c --> adp_c + h_c + pg1819Z160_e + pi_c 12680 or 15426 or 9428
  PE1819Z1829Z12Zt atp_c + h2o_c + pe1819Z1829Z12Z_c --> adp_c + h_c + pe1819Z1829Z12Z_e + pi_c 12680 or 15426 or 9428
  PE1801819Zt atp_c + h2o_c + pe1801819Z_c --> adp_c + h_c + pe1801819Z_e + pi_c 12680 or 15426 or 9428
  PSFLIPm atp_c + h2o_c + ps_hs_c --> adp_c + h_c + pi_c + ps_hs_m 12680 or 15426 or 9428
  PE1819Z1819Zt atp_c + h2o_c + pe1819Z1819Z_c --> adp_c + h_c + pe1819Z1819Z_e + pi_c 12680 or 15426 or 9428
  PE1801829Z12Zt atp_c + h2o_c + pe1801829Z12Z_c --> adp_c + h_c + pe1801829Z12Z_e + pi_c 12680 or 15426 or 9428
  PAIL1819Z160t atp_c + h2o_c + pail1819Z160_c --> adp_c + h_c + pail1819Z160_e + pi_c 12680 or 15426 or 9428
  PEFLIPm atp_c + h2o_c + pe_hs_c --> adp_c + h_c + pe_hs_m + pi_c 12680 or 15426 or 9428
  PGP1819Z160t atp_c + h2o_c + pgp1819Z160_c --> adp_c + h_c + pgp1819Z160_e + pi_c 12680 or 15426 or 9428
  PSFLIP atp_c + h2o_c + ps_hs_e --> adp_c + h_c + pi_c + ps_hs_c 12680 or 15426 or 9428
  PG1819Z160t atp_c + h2o_c + pg1819Z160_c --> adp_c + h_c + pg1819Z160_e + pi_c 12680 or 15426 or 9428
  PE1819Z1829Z12Zt atp_c + h2o_c + pe1819Z1829Z12Z_c --> adp_c + h_c + pe1819Z1829Z12Z_e + pi_c 12680 or 15426 or 9428
  PE1801819Zt atp_c + h2o_c + pe1801819Z_c --> adp_c + h_c + pe1801819Z_e + pi_c 12680 or 15426 or 9428
  PSFLIPm atp_c + h2o_c + ps_hs_c --> adp_c + h_c + pi_c + ps_hs_m 12680 or 15426 or 9428
  PE1819Z1819Zt atp_c + h2o_c + pe1819Z1819Z_c --> adp_c + h_c + pe1819Z1819Z_e + pi_c 12680 or 15426 or 9428
  PE1801829Z12Zt atp_c + h2o_c + pe1801829Z12Z_c --> adp_c + h_c + pe1801829Z12Z_e + pi_c 12680 or 15426 or 9428
  PAIL1819Z160t atp_c + h2o_c + pail1819Z160_c --> adp_c + h_c + pail1819Z160_e + pi_c 12680 or 15426 or 9428
  PEFLIPm atp_c + h2o_c + pe_hs_c --> adp_c + h_c + pe_hs_m + pi_c 12680 or 15426 or 9428
  PGP1819Z160t atp_c + h2o_c + pgp1819Z160_c --> adp_c + h_c + pgp1819Z160_e + pi_c 12680 or 15426 or 9428
  PSFLIP atp_c + h2o_c + ps_hs_e --> adp_c + h_c + pi_c + ps_hs_c 12680 or 15426 or 9428
  PG1819Z160t atp_c + h2o_c + pg1819Z160_c --> adp_c + h_c + pg1819Z160_e + pi_c 12680 or 15426 or 9428
  PE1819Z1829Z12Zt atp_c + h2o_c + pe1819Z1829Z12Z_c --> adp_c + h_c + pe1819Z1829Z12Z_e + pi_c 12680 or 15426 or 9428

pe_hs_m
PEFLIPm atp_c + h2o_c + pe_hs_c --> adp_c + h_c + pe_hs_m + pi_c 12680 or 15426 or 9428
  PE1801819Zt atp_c + h2o_c + pe1801819Z_c --> adp_c + h_c + pe1801819Z_e + pi_c 12680 or 15426 or 9428
  PSFLIPm atp_c + h2o_c + ps_hs_c --> adp_c + h_c + pi_c + ps_hs_m 12680 or 15426 or 9428
  PE1819Z1819Zt atp_c + h2o_c + pe1819Z1819Z_c --> adp_c + h_c + pe1819Z1819Z_e + pi_c 12680 or 15426 or 9428
  PE1801829Z12Zt atp_c + h2o_c + pe1801829Z12Z_c --> adp_c + h_c + pe1801829Z12Z_e + pi_c 12680 or 15426 or 9428
  PAIL1819Z160t atp_c + h2o_c + pail1819Z160_c --> adp_c + h_c + pail1819Z160_e + pi_c 12680 or 15426 or 9428
  PEFLIP atp_c + h2o_c + pe_hs_e --> adp_c + h_c + pe_hs_c + pi_c 12680 or 15426 or 9428
  PGP1819Z160t atp_c + h2o_c + pgp1819Z160_c --> adp_c + h_c + pgp1819Z160_e + pi_c 12680 or 15426 or 9428
  PSFLIP atp_c + h2o_c + ps_hs_e --> adp_c + h_c + pi_c + ps_hs_c 12680 or 15426 or 9428
  PG1819Z160t atp_c + h2o_c + pg1819Z160_c --> adp_c + h_c + pg1819Z160_e + pi_c 12680 or 15426 or 9428
  PE1819Z1829Z12Zt atp_c + h2o_c + pe1819Z1829Z12Z_c --> adp_c + h_c + pe1819Z1829Z12Z_e + pi_c 12680 or 15426 or 9428
  PE1801819Zt atp_c + h2o_c + pe1801819Z_c --> adp_c + h_c + pe1801819Z_e + pi_c 12680 or 15426 or 9428
  PSFLIPm atp_c + h2o_c + ps_hs_c --> adp_c + h_c + pi_c + ps_hs_m 12680 or 15426 or 9428
  PE1819Z1819Zt atp_c + h2o_c + pe1819Z1819Z_c --> adp_c + h_c + pe1819Z1819Z_e + pi_c 12680 or 15426 or 9428
  PE1801829Z12Zt atp_c + h2o_c + pe1801829Z12Z_c --> adp_c + h_c + pe1801829Z12Z_e + pi_c 12680 or 15426 or 9428
  PAIL1819Z160t atp_c + h2o_c + pail1819Z160_c --> adp_c + h_c + pail1819Z160_e + pi_c 12680 or 15426 or 9428
  PEFLIP atp_c + h2o_c + pe_hs_e --> adp_c + h_c + pe_hs_c + pi_c 12680 or 15426 or 9428
  PGP1819Z160t atp_c + h2o_c + pgp1819Z160_c --> adp_c + h_c + pgp1819Z160_e + pi_c 12680 or 15426 or 9428
  PSFLIP atp_c + h2o_c + ps_hs_e --> adp_c + h_c + pi_c + ps_hs_c 12680 or 15426 or 9428
  PG1819Z160t atp_c + h2o_c + pg1819Z160_c --> adp_c + h_c + pg1819Z160_e + pi_c 12680 or 15426 or 9428
  PE1819Z1829Z12Zt atp_c + h2o_c + pe1819Z1829Z12Z_c --> adp_c + h_c + pe1819Z1829Z12Z_e + pi_c 12680 or 15426 or 9428
  PE1801819Zt atp_c + h2o_c + pe1801819Z_c --> adp_c + h_c + pe1801819Z_e + pi_c 12680 or 15426 or 9428
  PSFLIPm atp_c + h2o_c + ps_hs_c --> adp_c + h_c + pi_c + ps_hs_m 12680 or 15426 or 9428
  PE1819Z1819Zt atp_c + h2o_c + pe1819Z1819Z_c --> adp_c + h_c + pe1819Z1819Z_e + pi_c 12680 or 15426 or 9428
  PE1801829Z12Zt atp_c + h2o_c + pe1801829Z12Z_c --> adp_c + h_c + pe1801829Z12Z_e + pi_c 12680 or 15426 or 9428
  PAIL1819Z160t atp_c + h2o_c + pail1819Z160_c --> adp_c + h_c + pail1819Z160_e + pi_c 12680 or 15426 or 9428
  PEFLIP atp_c + h2o_c + pe_hs_e --> adp_c + h_c + pe_hs_c + pi_c 12680 or 15426 or 9428
  PGP1819Z160t atp_c + h2o_c + pgp1819Z160_c --> adp_c + h_c + pgp1819Z160_e + pi_c 12680 or 15426 or 9428
  PSFLIP atp_c + h2o_c + ps_hs_e --> adp_c + h_c + pi_c + ps_hs_c 12680 or 15426 or 9428
  PG1819Z160t atp_c + h2o_c + pg1819Z160_c --> adp_c + h_c + pg1819Z160_e + pi_c 12680 or 15426 or 9428
  PE1819Z1829Z12Zt atp_c + h2o_c + pe1819Z1829Z12Z_c --> adp_c + h_c + pe1819Z1829Z12Z_e + pi_c 12680 or 15426 or 9428

pe_hs_r
H2ETer mgacpail_hs_r + pe_hs_r --> dag_hs_r + emgacpail_hs_r 16789
  H7_ETer emem2gacpail_hs_r + pe_hs_r --> dag_hs_r + gpi_hs_r 16789
  H3ETer m2gacpail_hs_r + pe_hs_r --> dag_hs_r + memgacpail_hs_r 16789
  H4ETer m3gacpail_hs_r + pe_hs_r --> dag_hs_r + m2emgacpail_hs_r 16789
  H6_ETer em3gacpail_hs_r + pe_hs_r --> dag_hs_r + em2emgacpail_hs_r 16789
H3ETer m2gacpail_hs_r + pe_hs_r --> dag_hs_r + memgacpail_hs_r 16789
  H7_ETer emem2gacpail_hs_r + pe_hs_r --> dag_hs_r + gpi_hs_r 16789
  H2ETer mgacpail_hs_r + pe_hs_r --> dag_hs_r + emgacpail_hs_r 16789
  H4ETer m3gacpail_hs_r + pe_hs_r --> dag_hs_r + m2emgacpail_hs_r 16789
  H6_ETer em3gacpail_hs_r + pe_hs_r --> dag_hs_r + em2emgacpail_hs_r 16789
H4ET3er m3gacpail_hs_r + pe_hs_r --> dag_hs_r + em3gacpail_hs_r 12437 and 8931
  M4BET2er mem2emgacpail_hs_r + pe_hs_r --> dag_hs_r + m_em_3gacpail_hs_r 14092 and 8931
  H6_ET2er em3gacpail_hs_r + pe_hs_r --> dag_hs_r + emem2gacpail_hs_r 14092 and 8931
  M4CET3er m3emgacpail_hs_r + pe_hs_r --> dag_hs_r + mem2emgacpail_hs_r 12437 and 8931
  H7ET2er em2emgacpail_hs_r + pe_hs_r --> dag_hs_r + gpi_hs_r 14092 and 8931
  H6ET3er m2emgacpail_hs_r + pe_hs_r --> dag_hs_r + em2emgacpail_hs_r 12437 and 8931
  M4CET3er m3emgacpail_hs_r + pe_hs_r --> dag_hs_r + mem2emgacpail_hs_r 12437 and 8931
  H6ET3er m2emgacpail_hs_r + pe_hs_r --> dag_hs_r + em2emgacpail_hs_r 12437 and 8931
H4ETer m3gacpail_hs_r + pe_hs_r --> dag_hs_r + m2emgacpail_hs_r 16789
  H7_ETer emem2gacpail_hs_r + pe_hs_r --> dag_hs_r + gpi_hs_r 16789
  H2ETer mgacpail_hs_r + pe_hs_r --> dag_hs_r + emgacpail_hs_r 16789
  H3ETer m2gacpail_hs_r + pe_hs_r --> dag_hs_r + memgacpail_hs_r 16789
  H6_ETer em3gacpail_hs_r + pe_hs_r --> dag_hs_r + em2emgacpail_hs_r 16789
H6ET3er m2emgacpail_hs_r + pe_hs_r --> dag_hs_r + em2emgacpail_hs_r 12437 and 8931
  H4ET3er m3gacpail_hs_r + pe_hs_r --> dag_hs_r + em3gacpail_hs_r 12437 and 8931
  M4BET2er mem2emgacpail_hs_r + pe_hs_r --> dag_hs_r + m_em_3gacpail_hs_r 14092 and 8931
  H6_ET2er em3gacpail_hs_r + pe_hs_r --> dag_hs_r + emem2gacpail_hs_r 14092 and 8931
  M4CET3er m3emgacpail_hs_r + pe_hs_r --> dag_hs_r + mem2emgacpail_hs_r 12437 and 8931
  H7ET2er em2emgacpail_hs_r + pe_hs_r --> dag_hs_r + gpi_hs_r 14092 and 8931
  H4ET3er m3gacpail_hs_r + pe_hs_r --> dag_hs_r + em3gacpail_hs_r 12437 and 8931
  M4CET3er m3emgacpail_hs_r + pe_hs_r --> dag_hs_r + mem2emgacpail_hs_r 12437 and 8931
H6_ET2er em3gacpail_hs_r + pe_hs_r --> dag_hs_r + emem2gacpail_hs_r 14092 and 8931
  H7ET2er em2emgacpail_hs_r + pe_hs_r --> dag_hs_r + gpi_hs_r 14092 and 8931
  M4BET2er mem2emgacpail_hs_r + pe_hs_r --> dag_hs_r + m_em_3gacpail_hs_r 14092 and 8931
  H4ET3er m3gacpail_hs_r + pe_hs_r --> dag_hs_r + em3gacpail_hs_r 12437 and 8931
  M4BET2er mem2emgacpail_hs_r + pe_hs_r --> dag_hs_r + m_em_3gacpail_hs_r 14092 and 8931
  M4CET3er m3emgacpail_hs_r + pe_hs_r --> dag_hs_r + mem2emgacpail_hs_r 12437 and 8931
  H7ET2er em2emgacpail_hs_r + pe_hs_r --> dag_hs_r + gpi_hs_r 14092 and 8931
  H6ET3er m2emgacpail_hs_r + pe_hs_r --> dag_hs_r + em2emgacpail_hs_r 12437 and 8931
H6_ETer em3gacpail_hs_r + pe_hs_r --> dag_hs_r + em2emgacpail_hs_r 16789
  H7_ETer emem2gacpail_hs_r + pe_hs_r --> dag_hs_r + gpi_hs_r 16789
  H2ETer mgacpail_hs_r + pe_hs_r --> dag_hs_r + emgacpail_hs_r 16789
  H3ETer m2gacpail_hs_r + pe_hs_r --> dag_hs_r + memgacpail_hs_r 16789
  H4ETer m3gacpail_hs_r + pe_hs_r --> dag_hs_r + m2emgacpail_hs_r 16789
H7ET2er em2emgacpail_hs_r + pe_hs_r --> dag_hs_r + gpi_hs_r 14092 and 8931
  H6_ET2er em3gacpail_hs_r + pe_hs_r --> dag_hs_r + emem2gacpail_hs_r 14092 and 8931
  M4BET2er mem2emgacpail_hs_r + pe_hs_r --> dag_hs_r + m_em_3gacpail_hs_r 14092 and 8931
  H4ET3er m3gacpail_hs_r + pe_hs_r --> dag_hs_r + em3gacpail_hs_r 12437 and 8931
  M4BET2er mem2emgacpail_hs_r + pe_hs_r --> dag_hs_r + m_em_3gacpail_hs_r 14092 and 8931
  H6_ET2er em3gacpail_hs_r + pe_hs_r --> dag_hs_r + emem2gacpail_hs_r 14092 and 8931
  M4CET3er m3emgacpail_hs_r + pe_hs_r --> dag_hs_r + mem2emgacpail_hs_r 12437 and 8931
  H6ET3er m2emgacpail_hs_r + pe_hs_r --> dag_hs_r + em2emgacpail_hs_r 12437 and 8931
H7_ETer emem2gacpail_hs_r + pe_hs_r --> dag_hs_r + gpi_hs_r 16789
  H2ETer mgacpail_hs_r + pe_hs_r --> dag_hs_r + emgacpail_hs_r 16789
  H3ETer m2gacpail_hs_r + pe_hs_r --> dag_hs_r + memgacpail_hs_r 16789
  H4ETer m3gacpail_hs_r + pe_hs_r --> dag_hs_r + m2emgacpail_hs_r 16789
  H6_ETer em3gacpail_hs_r + pe_hs_r --> dag_hs_r + em2emgacpail_hs_r 16789
M4BET2er mem2emgacpail_hs_r + pe_hs_r --> dag_hs_r + m_em_3gacpail_hs_r 14092 and 8931
  H6_ET2er em3gacpail_hs_r + pe_hs_r --> dag_hs_r + emem2gacpail_hs_r 14092 and 8931
  H7ET2er em2emgacpail_hs_r + pe_hs_r --> dag_hs_r + gpi_hs_r 14092 and 8931
  H4ET3er m3gacpail_hs_r + pe_hs_r --> dag_hs_r + em3gacpail_hs_r 12437 and 8931
  H6_ET2er em3gacpail_hs_r + pe_hs_r --> dag_hs_r + emem2gacpail_hs_r 14092 and 8931
  M4CET3er m3emgacpail_hs_r + pe_hs_r --> dag_hs_r + mem2emgacpail_hs_r 12437 and 8931
  H7ET2er em2emgacpail_hs_r + pe_hs_r --> dag_hs_r + gpi_hs_r 14092 and 8931
  H6ET3er m2emgacpail_hs_r + pe_hs_r --> dag_hs_r + em2emgacpail_hs_r 12437 and 8931
M4CET3er m3emgacpail_hs_r + pe_hs_r --> dag_hs_r + mem2emgacpail_hs_r 12437 and 8931
  H4ET3er m3gacpail_hs_r + pe_hs_r --> dag_hs_r + em3gacpail_hs_r 12437 and 8931
  M4BET2er mem2emgacpail_hs_r + pe_hs_r --> dag_hs_r + m_em_3gacpail_hs_r 14092 and 8931
  H6_ET2er em3gacpail_hs_r + pe_hs_r --> dag_hs_r + emem2gacpail_hs_r 14092 and 8931
  H7ET2er em2emgacpail_hs_r + pe_hs_r --> dag_hs_r + gpi_hs_r 14092 and 8931
  H6ET3er m2emgacpail_hs_r + pe_hs_r --> dag_hs_r + em2emgacpail_hs_r 12437 and 8931
  H4ET3er m3gacpail_hs_r + pe_hs_r --> dag_hs_r + em3gacpail_hs_r 12437 and 8931
  H6ET3er m2emgacpail_hs_r + pe_hs_r --> dag_hs_r + em2emgacpail_hs_r 12437 and 8931

ps_hs_c
PSFLIP atp_c + h2o_c + ps_hs_e --> adp_c + h_c + pi_c + ps_hs_c 12680 or 15426 or 9428
  PE1801819Zt atp_c + h2o_c + pe1801819Z_c --> adp_c + h_c + pe1801819Z_e + pi_c 12680 or 15426 or 9428
  PSFLIPm atp_c + h2o_c + ps_hs_c --> adp_c + h_c + pi_c + ps_hs_m 12680 or 15426 or 9428
  PE1819Z1819Zt atp_c + h2o_c + pe1819Z1819Z_c --> adp_c + h_c + pe1819Z1819Z_e + pi_c 12680 or 15426 or 9428
  PE1801829Z12Zt atp_c + h2o_c + pe1801829Z12Z_c --> adp_c + h_c + pe1801829Z12Z_e + pi_c 12680 or 15426 or 9428
  PAIL1819Z160t atp_c + h2o_c + pail1819Z160_c --> adp_c + h_c + pail1819Z160_e + pi_c 12680 or 15426 or 9428
  PEFLIP atp_c + h2o_c + pe_hs_e --> adp_c + h_c + pe_hs_c + pi_c 12680 or 15426 or 9428
  PEFLIPm atp_c + h2o_c + pe_hs_c --> adp_c + h_c + pe_hs_m + pi_c 12680 or 15426 or 9428
  PGP1819Z160t atp_c + h2o_c + pgp1819Z160_c --> adp_c + h_c + pgp1819Z160_e + pi_c 12680 or 15426 or 9428
  PG1819Z160t atp_c + h2o_c + pg1819Z160_c --> adp_c + h_c + pg1819Z160_e + pi_c 12680 or 15426 or 9428
  PE1819Z1829Z12Zt atp_c + h2o_c + pe1819Z1829Z12Z_c --> adp_c + h_c + pe1819Z1829Z12Z_e + pi_c 12680 or 15426 or 9428
  PE1801819Zt atp_c + h2o_c + pe1801819Z_c --> adp_c + h_c + pe1801819Z_e + pi_c 12680 or 15426 or 9428
  PSFLIPm atp_c + h2o_c + ps_hs_c --> adp_c + h_c + pi_c + ps_hs_m 12680 or 15426 or 9428
  PE1819Z1819Zt atp_c + h2o_c + pe1819Z1819Z_c --> adp_c + h_c + pe1819Z1819Z_e + pi_c 12680 or 15426 or 9428
  PE1801829Z12Zt atp_c + h2o_c + pe1801829Z12Z_c --> adp_c + h_c + pe1801829Z12Z_e + pi_c 12680 or 15426 or 9428
  PAIL1819Z160t atp_c + h2o_c + pail1819Z160_c --> adp_c + h_c + pail1819Z160_e + pi_c 12680 or 15426 or 9428
  PEFLIP atp_c + h2o_c + pe_hs_e --> adp_c + h_c + pe_hs_c + pi_c 12680 or 15426 or 9428
  PEFLIPm atp_c + h2o_c + pe_hs_c --> adp_c + h_c + pe_hs_m + pi_c 12680 or 15426 or 9428
  PGP1819Z160t atp_c + h2o_c + pgp1819Z160_c --> adp_c + h_c + pgp1819Z160_e + pi_c 12680 or 15426 or 9428
  PG1819Z160t atp_c + h2o_c + pg1819Z160_c --> adp_c + h_c + pg1819Z160_e + pi_c 12680 or 15426 or 9428
  PE1819Z1829Z12Zt atp_c + h2o_c + pe1819Z1829Z12Z_c --> adp_c + h_c + pe1819Z1829Z12Z_e + pi_c 12680 or 15426 or 9428
  PE1801819Zt atp_c + h2o_c + pe1801819Z_c --> adp_c + h_c + pe1801819Z_e + pi_c 12680 or 15426 or 9428
  PSFLIPm atp_c + h2o_c + ps_hs_c --> adp_c + h_c + pi_c + ps_hs_m 12680 or 15426 or 9428
  PE1819Z1819Zt atp_c + h2o_c + pe1819Z1819Z_c --> adp_c + h_c + pe1819Z1819Z_e + pi_c 12680 or 15426 or 9428
  PE1801829Z12Zt atp_c + h2o_c + pe1801829Z12Z_c --> adp_c + h_c + pe1801829Z12Z_e + pi_c 12680 or 15426 or 9428
  PAIL1819Z160t atp_c + h2o_c + pail1819Z160_c --> adp_c + h_c + pail1819Z160_e + pi_c 12680 or 15426 or 9428
  PEFLIP atp_c + h2o_c + pe_hs_e --> adp_c + h_c + pe_hs_c + pi_c 12680 or 15426 or 9428
  PEFLIPm atp_c + h2o_c + pe_hs_c --> adp_c + h_c + pe_hs_m + pi_c 12680 or 15426 or 9428
  PGP1819Z160t atp_c + h2o_c + pgp1819Z160_c --> adp_c + h_c + pgp1819Z160_e + pi_c 12680 or 15426 or 9428
  PG1819Z160t atp_c + h2o_c + pg1819Z160_c --> adp_c + h_c + pg1819Z160_e + pi_c 12680 or 15426 or 9428
  PE1819Z1829Z12Zt atp_c + h2o_c + pe1819Z1829Z12Z_c --> adp_c + h_c + pe1819Z1829Z12Z_e + pi_c 12680 or 15426 or 9428
PSFLIPm atp_c + h2o_c + ps_hs_c --> adp_c + h_c + pi_c + ps_hs_m 12680 or 15426 or 9428
  PE1801819Zt atp_c + h2o_c + pe1801819Z_c --> adp_c + h_c + pe1801819Z_e + pi_c 12680 or 15426 or 9428
  PE1819Z1819Zt atp_c + h2o_c + pe1819Z1819Z_c --> adp_c + h_c + pe1819Z1819Z_e + pi_c 12680 or 15426 or 9428
  PE1801829Z12Zt atp_c + h2o_c + pe1801829Z12Z_c --> adp_c + h_c + pe1801829Z12Z_e + pi_c 12680 or 15426 or 9428
  PAIL1819Z160t atp_c + h2o_c + pail1819Z160_c --> adp_c + h_c + pail1819Z160_e + pi_c 12680 or 15426 or 9428
  PEFLIP atp_c + h2o_c + pe_hs_e --> adp_c + h_c + pe_hs_c + pi_c 12680 or 15426 or 9428
  PEFLIPm atp_c + h2o_c + pe_hs_c --> adp_c + h_c + pe_hs_m + pi_c 12680 or 15426 or 9428
  PGP1819Z160t atp_c + h2o_c + pgp1819Z160_c --> adp_c + h_c + pgp1819Z160_e + pi_c 12680 or 15426 or 9428
  PSFLIP atp_c + h2o_c + ps_hs_e --> adp_c + h_c + pi_c + ps_hs_c 12680 or 15426 or 9428
  PG1819Z160t atp_c + h2o_c + pg1819Z160_c --> adp_c + h_c + pg1819Z160_e + pi_c 12680 or 15426 or 9428
  PE1819Z1829Z12Zt atp_c + h2o_c + pe1819Z1829Z12Z_c --> adp_c + h_c + pe1819Z1829Z12Z_e + pi_c 12680 or 15426 or 9428
  PE1801819Zt atp_c + h2o_c + pe1801819Z_c --> adp_c + h_c + pe1801819Z_e + pi_c 12680 or 15426 or 9428
  PE1819Z1819Zt atp_c + h2o_c + pe1819Z1819Z_c --> adp_c + h_c + pe1819Z1819Z_e + pi_c 12680 or 15426 or 9428
  PE1801829Z12Zt atp_c + h2o_c + pe1801829Z12Z_c --> adp_c + h_c + pe1801829Z12Z_e + pi_c 12680 or 15426 or 9428
  PAIL1819Z160t atp_c + h2o_c + pail1819Z160_c --> adp_c + h_c + pail1819Z160_e + pi_c 12680 or 15426 or 9428
  PEFLIP atp_c + h2o_c + pe_hs_e --> adp_c + h_c + pe_hs_c + pi_c 12680 or 15426 or 9428
  PEFLIPm atp_c + h2o_c + pe_hs_c --> adp_c + h_c + pe_hs_m + pi_c 12680 or 15426 or 9428
  PGP1819Z160t atp_c + h2o_c + pgp1819Z160_c --> adp_c + h_c + pgp1819Z160_e + pi_c 12680 or 15426 or 9428
  PSFLIP atp_c + h2o_c + ps_hs_e --> adp_c + h_c + pi_c + ps_hs_c 12680 or 15426 or 9428
  PG1819Z160t atp_c + h2o_c + pg1819Z160_c --> adp_c + h_c + pg1819Z160_e + pi_c 12680 or 15426 or 9428
  PE1819Z1829Z12Zt atp_c + h2o_c + pe1819Z1829Z12Z_c --> adp_c + h_c + pe1819Z1829Z12Z_e + pi_c 12680 or 15426 or 9428
  PE1801819Zt atp_c + h2o_c + pe1801819Z_c --> adp_c + h_c + pe1801819Z_e + pi_c 12680 or 15426 or 9428
  PE1819Z1819Zt atp_c + h2o_c + pe1819Z1819Z_c --> adp_c + h_c + pe1819Z1819Z_e + pi_c 12680 or 15426 or 9428
  PE1801829Z12Zt atp_c + h2o_c + pe1801829Z12Z_c --> adp_c + h_c + pe1801829Z12Z_e + pi_c 12680 or 15426 or 9428
  PAIL1819Z160t atp_c + h2o_c + pail1819Z160_c --> adp_c + h_c + pail1819Z160_e + pi_c 12680 or 15426 or 9428
  PEFLIP atp_c + h2o_c + pe_hs_e --> adp_c + h_c + pe_hs_c + pi_c 12680 or 15426 or 9428
  PEFLIPm atp_c + h2o_c + pe_hs_c --> adp_c + h_c + pe_hs_m + pi_c 12680 or 15426 or 9428
  PGP1819Z160t atp_c + h2o_c + pgp1819Z160_c --> adp_c + h_c + pgp1819Z160_e + pi_c 12680 or 15426 or 9428
  PSFLIP atp_c + h2o_c + ps_hs_e --> adp_c + h_c + pi_c + ps_hs_c 12680 or 15426 or 9428
  PG1819Z160t atp_c + h2o_c + pg1819Z160_c --> adp_c + h_c + pg1819Z160_e + pi_c 12680 or 15426 or 9428
  PE1819Z1829Z12Zt atp_c + h2o_c + pe1819Z1829Z12Z_c --> adp_c + h_c + pe1819Z1829Z12Z_e + pi_c 12680 or 15426 or 9428

ps_hs_e
PSFLIP atp_c + h2o_c + ps_hs_e --> adp_c + h_c + pi_c + ps_hs_c 12680 or 15426 or 9428
  PE1801819Zt atp_c + h2o_c + pe1801819Z_c --> adp_c + h_c + pe1801819Z_e + pi_c 12680 or 15426 or 9428
  PSFLIPm atp_c + h2o_c + ps_hs_c --> adp_c + h_c + pi_c + ps_hs_m 12680 or 15426 or 9428
  PE1819Z1819Zt atp_c + h2o_c + pe1819Z1819Z_c --> adp_c + h_c + pe1819Z1819Z_e + pi_c 12680 or 15426 or 9428
  PE1801829Z12Zt atp_c + h2o_c + pe1801829Z12Z_c --> adp_c + h_c + pe1801829Z12Z_e + pi_c 12680 or 15426 or 9428
  PAIL1819Z160t atp_c + h2o_c + pail1819Z160_c --> adp_c + h_c + pail1819Z160_e + pi_c 12680 or 15426 or 9428
  PEFLIP atp_c + h2o_c + pe_hs_e --> adp_c + h_c + pe_hs_c + pi_c 12680 or 15426 or 9428
  PEFLIPm atp_c + h2o_c + pe_hs_c --> adp_c + h_c + pe_hs_m + pi_c 12680 or 15426 or 9428
  PGP1819Z160t atp_c + h2o_c + pgp1819Z160_c --> adp_c + h_c + pgp1819Z160_e + pi_c 12680 or 15426 or 9428
  PG1819Z160t atp_c + h2o_c + pg1819Z160_c --> adp_c + h_c + pg1819Z160_e + pi_c 12680 or 15426 or 9428
  PE1819Z1829Z12Zt atp_c + h2o_c + pe1819Z1829Z12Z_c --> adp_c + h_c + pe1819Z1829Z12Z_e + pi_c 12680 or 15426 or 9428
  PE1801819Zt atp_c + h2o_c + pe1801819Z_c --> adp_c + h_c + pe1801819Z_e + pi_c 12680 or 15426 or 9428
  PSFLIPm atp_c + h2o_c + ps_hs_c --> adp_c + h_c + pi_c + ps_hs_m 12680 or 15426 or 9428
  PE1819Z1819Zt atp_c + h2o_c + pe1819Z1819Z_c --> adp_c + h_c + pe1819Z1819Z_e + pi_c 12680 or 15426 or 9428
  PE1801829Z12Zt atp_c + h2o_c + pe1801829Z12Z_c --> adp_c + h_c + pe1801829Z12Z_e + pi_c 12680 or 15426 or 9428
  PAIL1819Z160t atp_c + h2o_c + pail1819Z160_c --> adp_c + h_c + pail1819Z160_e + pi_c 12680 or 15426 or 9428
  PEFLIP atp_c + h2o_c + pe_hs_e --> adp_c + h_c + pe_hs_c + pi_c 12680 or 15426 or 9428
  PEFLIPm atp_c + h2o_c + pe_hs_c --> adp_c + h_c + pe_hs_m + pi_c 12680 or 15426 or 9428
  PGP1819Z160t atp_c + h2o_c + pgp1819Z160_c --> adp_c + h_c + pgp1819Z160_e + pi_c 12680 or 15426 or 9428
  PG1819Z160t atp_c + h2o_c + pg1819Z160_c --> adp_c + h_c + pg1819Z160_e + pi_c 12680 or 15426 or 9428
  PE1819Z1829Z12Zt atp_c + h2o_c + pe1819Z1829Z12Z_c --> adp_c + h_c + pe1819Z1829Z12Z_e + pi_c 12680 or 15426 or 9428
  PE1801819Zt atp_c + h2o_c + pe1801819Z_c --> adp_c + h_c + pe1801819Z_e + pi_c 12680 or 15426 or 9428
  PSFLIPm atp_c + h2o_c + ps_hs_c --> adp_c + h_c + pi_c + ps_hs_m 12680 or 15426 or 9428
  PE1819Z1819Zt atp_c + h2o_c + pe1819Z1819Z_c --> adp_c + h_c + pe1819Z1819Z_e + pi_c 12680 or 15426 or 9428
  PE1801829Z12Zt atp_c + h2o_c + pe1801829Z12Z_c --> adp_c + h_c + pe1801829Z12Z_e + pi_c 12680 or 15426 or 9428
  PAIL1819Z160t atp_c + h2o_c + pail1819Z160_c --> adp_c + h_c + pail1819Z160_e + pi_c 12680 or 15426 or 9428
  PEFLIP atp_c + h2o_c + pe_hs_e --> adp_c + h_c + pe_hs_c + pi_c 12680 or 15426 or 9428
  PEFLIPm atp_c + h2o_c + pe_hs_c --> adp_c + h_c + pe_hs_m + pi_c 12680 or 15426 or 9428
  PGP1819Z160t atp_c + h2o_c + pgp1819Z160_c --> adp_c + h_c + pgp1819Z160_e + pi_c 12680 or 15426 or 9428
  PG1819Z160t atp_c + h2o_c + pg1819Z160_c --> adp_c + h_c + pg1819Z160_e + pi_c 12680 or 15426 or 9428
  PE1819Z1829Z12Zt atp_c + h2o_c + pe1819Z1829Z12Z_c --> adp_c + h_c + pe1819Z1829Z12Z_e + pi_c 12680 or 15426 or 9428

ps_hs_m
PSFLIPm atp_c + h2o_c + ps_hs_c --> adp_c + h_c + pi_c + ps_hs_m 12680 or 15426 or 9428
  PE1801819Zt atp_c + h2o_c + pe1801819Z_c --> adp_c + h_c + pe1801819Z_e + pi_c 12680 or 15426 or 9428
  PE1819Z1819Zt atp_c + h2o_c + pe1819Z1819Z_c --> adp_c + h_c + pe1819Z1819Z_e + pi_c 12680 or 15426 or 9428
  PE1801829Z12Zt atp_c + h2o_c + pe1801829Z12Z_c --> adp_c + h_c + pe1801829Z12Z_e + pi_c 12680 or 15426 or 9428
  PAIL1819Z160t atp_c + h2o_c + pail1819Z160_c --> adp_c + h_c + pail1819Z160_e + pi_c 12680 or 15426 or 9428
  PEFLIP atp_c + h2o_c + pe_hs_e --> adp_c + h_c + pe_hs_c + pi_c 12680 or 15426 or 9428
  PEFLIPm atp_c + h2o_c + pe_hs_c --> adp_c + h_c + pe_hs_m + pi_c 12680 or 15426 or 9428
  PGP1819Z160t atp_c + h2o_c + pgp1819Z160_c --> adp_c + h_c + pgp1819Z160_e + pi_c 12680 or 15426 or 9428
  PSFLIP atp_c + h2o_c + ps_hs_e --> adp_c + h_c + pi_c + ps_hs_c 12680 or 15426 or 9428
  PG1819Z160t atp_c + h2o_c + pg1819Z160_c --> adp_c + h_c + pg1819Z160_e + pi_c 12680 or 15426 or 9428
  PE1819Z1829Z12Zt atp_c + h2o_c + pe1819Z1829Z12Z_c --> adp_c + h_c + pe1819Z1829Z12Z_e + pi_c 12680 or 15426 or 9428
  PE1801819Zt atp_c + h2o_c + pe1801819Z_c --> adp_c + h_c + pe1801819Z_e + pi_c 12680 or 15426 or 9428
  PE1819Z1819Zt atp_c + h2o_c + pe1819Z1819Z_c --> adp_c + h_c + pe1819Z1819Z_e + pi_c 12680 or 15426 or 9428
  PE1801829Z12Zt atp_c + h2o_c + pe1801829Z12Z_c --> adp_c + h_c + pe1801829Z12Z_e + pi_c 12680 or 15426 or 9428
  PAIL1819Z160t atp_c + h2o_c + pail1819Z160_c --> adp_c + h_c + pail1819Z160_e + pi_c 12680 or 15426 or 9428
  PEFLIP atp_c + h2o_c + pe_hs_e --> adp_c + h_c + pe_hs_c + pi_c 12680 or 15426 or 9428
  PEFLIPm atp_c + h2o_c + pe_hs_c --> adp_c + h_c + pe_hs_m + pi_c 12680 or 15426 or 9428
  PGP1819Z160t atp_c + h2o_c + pgp1819Z160_c --> adp_c + h_c + pgp1819Z160_e + pi_c 12680 or 15426 or 9428
  PSFLIP atp_c + h2o_c + ps_hs_e --> adp_c + h_c + pi_c + ps_hs_c 12680 or 15426 or 9428
  PG1819Z160t atp_c + h2o_c + pg1819Z160_c --> adp_c + h_c + pg1819Z160_e + pi_c 12680 or 15426 or 9428
  PE1819Z1829Z12Zt atp_c + h2o_c + pe1819Z1829Z12Z_c --> adp_c + h_c + pe1819Z1829Z12Z_e + pi_c 12680 or 15426 or 9428
  PE1801819Zt atp_c + h2o_c + pe1801819Z_c --> adp_c + h_c + pe1801819Z_e + pi_c 12680 or 15426 or 9428
  PE1819Z1819Zt atp_c + h2o_c + pe1819Z1819Z_c --> adp_c + h_c + pe1819Z1819Z_e + pi_c 12680 or 15426 or 9428
  PE1801829Z12Zt atp_c + h2o_c + pe1801829Z12Z_c --> adp_c + h_c + pe1801829Z12Z_e + pi_c 12680 or 15426 or 9428
  PAIL1819Z160t atp_c + h2o_c + pail1819Z160_c --> adp_c + h_c + pail1819Z160_e + pi_c 12680 or 15426 or 9428
  PEFLIP atp_c + h2o_c + pe_hs_e --> adp_c + h_c + pe_hs_c + pi_c 12680 or 15426 or 9428
  PEFLIPm atp_c + h2o_c + pe_hs_c --> adp_c + h_c + pe_hs_m + pi_c 12680 or 15426 or 9428
  PGP1819Z160t atp_c + h2o_c + pgp1819Z160_c --> adp_c + h_c + pgp1819Z160_e + pi_c 12680 or 15426 or 9428
  PSFLIP atp_c + h2o_c + ps_hs_e --> adp_c + h_c + pi_c + ps_hs_c 12680 or 15426 or 9428
  PG1819Z160t atp_c + h2o_c + pg1819Z160_c --> adp_c + h_c + pg1819Z160_e + pi_c 12680 or 15426 or 9428
  PE1819Z1829Z12Zt atp_c + h2o_c + pe1819Z1829Z12Z_c --> adp_c + h_c + pe1819Z1829Z12Z_e + pi_c 12680 or 15426 or 9428
```

In [27]:

```
for m in sorted(model.metabolites, key=lambda x: x.id):
    if '_cho' in m.id:
        print(m.id)
        for r in sorted(m.reactions, key=lambda x: x.id):
            print(r.id, r.reaction, r.gene_reaction_rule)
            for g in r.genes:
                for r2 in g.reactions:
                    if r2 is not r:
                        print(' ',r2.id, r2.reaction, r2.gene_reaction_rule)
        print()
```

```
pail_cho_c
yli_R0350 100.0 h2o_c + pail_cho_c --> dag_hs_c + 100.0 h_c + 100.0 mi1p__D_c 11755
```

In [28]:

```
for m in sorted(model.metabolites, key=lambda x: x.id):
    if 'yli' in m.id:
        print(m.id)
        for r in sorted(m.reactions, key=lambda x: x.id):
            print(r.id, r.reaction, r.gene_reaction_rule)
            for g in r.genes:
                for r2 in g.reactions:
                    if r2 is not r:
                        print(' ',r2.id, r2.reaction, r2.gene_reaction_rule)
        print()
```

```
yli_M00109_c
yli_R0936 dolmanp_c --> h_c + mannan_c + yli_M00109_c 12121 or 13840 or 8768
  DOLPMMer dolmanp_r --> dolp_r + h_r + mannan_r 12121 or 13840 or 8768 or (13840 and 8768)
  yli_R1478 dolmanp_r --> h_r + mannan_r + yli_M00109_r 12121 or 13840 or 8768
  DOLPMMer dolmanp_r --> dolp_r + h_r + mannan_r 12121 or 13840 or 8768 or (13840 and 8768)
  yli_R1478 dolmanp_r --> h_r + mannan_r + yli_M00109_r 12121 or 13840 or 8768
  DOLPMMer dolmanp_r --> dolp_r + h_r + mannan_r 12121 or 13840 or 8768 or (13840 and 8768)
  yli_R1478 dolmanp_r --> h_r + mannan_r + yli_M00109_r 12121 or 13840 or 8768

yli_M00109_r
yli_R1478 dolmanp_r --> h_r + mannan_r + yli_M00109_r 12121 or 13840 or 8768
  yli_R0936 dolmanp_c --> h_c + mannan_c + yli_M00109_c 12121 or 13840 or 8768
  DOLPMMer dolmanp_r --> dolp_r + h_r + mannan_r 12121 or 13840 or 8768 or (13840 and 8768)
  yli_R0936 dolmanp_c --> h_c + mannan_c + yli_M00109_c 12121 or 13840 or 8768
  DOLPMMer dolmanp_r --> dolp_r + h_r + mannan_r 12121 or 13840 or 8768 or (13840 and 8768)
  yli_R0936 dolmanp_c --> h_c + mannan_c + yli_M00109_c 12121 or 13840 or 8768
  DOLPMMer dolmanp_r --> dolp_r + h_r + mannan_r 12121 or 13840 or 8768 or (13840 and 8768)
yli_R1533 udpg_r + yli_M00109_r <=> dolglcp_r + udp_r 11809
  UDPDOLPT_U 0.1 dolp_U_c + udpg_c --> 0.1 dolglcp_U_c + udp_c 11809
  UDPDOLPT_L 0.1 dolp__L_c + udpg_c --> 0.1 dolglcp__L_c + udp_c 11809
  UDPDOLPT dolp_c + udpg_c --> dolpglc_c + udp_c 11809
yli_R1534 uacgam_r + yli_M00109_r <=> naglc2p_r + ump_r 11202
  GLCNACPT_U 0.1 dolp_U_c + uacgam_c --> 0.1 naglc2p_U_c + ump_c 11202
  GLCNACPT_L 0.1 dolp__L_c + uacgam_c --> 0.1 naglc2p__L_c + ump_c 11202
  GLCNACPT dolp_c + 2.0 h_c + uacgam_c --> doldpglcnac_c + ump_c 11202

yli_M00116_c
yli_R0481 atp_c + rib__D_c --> adp_c + h_c + yli_M00116_c 10869
  DRBK atp_c + drib_c --> 2dr5p_c + adp_c + h_c 10869
  RBK atp_c + rib__D_c --> adp_c + h_c + r5p_c 10869
yli_R0521 atp_c + yli_M00116_c <=> amp_c + h_c + prpp_c 11145 or 16592
  PRPPS atp_c + r5p_c <=> amp_c + h_c + prpp_c 11145 or 16592
  PRPPS atp_c + r5p_c <=> amp_c + h_c + prpp_c 11145 or 16592
yli_R0540 adprib_c + h2o_c --> amp_c + 2.0 h_c + yli_M00116_c 16511
  ADPRDP adprib_c + h2o_c --> amp_c + 2.0 h_c + r5p_c 16511
  ADPGLC adpglc_c + h2o_c --> amp_c + g1p_c + 2.0 h_c 16511
  ADPMAN adpman_c + h2o_c --> amp_c + 2.0 h_c + man1p_c 16511
yli_R0552 r1p_c <=> yli_M00116_c 13711 or 9959
  PPM r1p_c <=> r5p_c 13711 or 9959
  PGMT g1p_c <=> g6p_c 13711 or 14546 or 8460 or 9959
  PPM r1p_c <=> r5p_c 13711 or 9959
  PGMT g1p_c <=> g6p_c 13711 or 14546 or 8460 or 9959
  PMANM man1p_c <=> man6p_c 13711 or 9241

yli_M00844_c
yli_R0738 udpg_c --> h_c + udp_c + yli_M00844_c 15984
  13GS udpg_c --> 13BDglcn_c + h_c + udp_c 15984

yli_M01059_m
yli_R0419 accoa_m + akg_m + h2o_m --> coa_m + h_m + yli_M01059_m 14856
  IPPS 3mob_c + accoa_c + h2o_c --> 3c3hmp_c + coa_c + h_c 14856 or 15488
  HCITSn accoa_n + akg_n + h2o_n --> coa_n + h_n + hcit_n 14856

yli_M01609_c
yli_R0535 h2o_c + yli_M01609_c <=> alltt_c + h_c 11884
  ATNAH alltn_m + h2o_m + h_m --> alltt_m 11884
  ALLTN alltn_c + h2o_c --> alltt_c + h_c 11884
yli_R1114 yli_M01609_e --> yli_M01609_c 10921 or 11871 or 12902
  ALLTNti alltn_e --> alltn_c 10921 or 11871 or 12902
  URIt2 h_e + uri_e --> h_c + uri_c 10921 or 11871 or 12902 or 13969 or (10921 and 13969) or (11871 and 13969) or (12902 and 13969)
  THMt2 h_e + thm_e --> h_c + thm_c 10921 or 11871 or 12902
  URAt2 h_e + ura_e --> h_c + ura_c 10921 or 11871 or 12902
  ALLTNti alltn_e --> alltn_c 10921 or 11871 or 12902
  URIt2 h_e + uri_e --> h_c + uri_c 10921 or 11871 or 12902 or 13969 or (10921 and 13969) or (11871 and 13969) or (12902 and 13969)
  THMt2 h_e + thm_e --> h_c + thm_c 10921 or 11871 or 12902
  URAt2 h_e + ura_e --> h_c + ura_c 10921 or 11871 or 12902
  ALLTNti alltn_e --> alltn_c 10921 or 11871 or 12902
  URIt2 h_e + uri_e --> h_c + uri_c 10921 or 11871 or 12902 or 13969 or (10921 and 13969) or (11871 and 13969) or (12902 and 13969)
  THMt2 h_e + thm_e --> h_c + thm_c 10921 or 11871 or 12902
  URAt2 h_e + ura_e --> h_c + ura_c 10921 or 11871 or 12902

yli_M01609_e
yli_R1114 yli_M01609_e --> yli_M01609_c 10921 or 11871 or 12902
  ALLTNti alltn_e --> alltn_c 10921 or 11871 or 12902
  URIt2 h_e + uri_e --> h_c + uri_c 10921 or 11871 or 12902 or 13969 or (10921 and 13969) or (11871 and 13969) or (12902 and 13969)
  THMt2 h_e + thm_e --> h_c + thm_c 10921 or 11871 or 12902
  URAt2 h_e + ura_e --> h_c + ura_c 10921 or 11871 or 12902
  ALLTNti alltn_e --> alltn_c 10921 or 11871 or 12902
  URIt2 h_e + uri_e --> h_c + uri_c 10921 or 11871 or 12902 or 13969 or (10921 and 13969) or (11871 and 13969) or (12902 and 13969)
  THMt2 h_e + thm_e --> h_c + thm_c 10921 or 11871 or 12902
  URAt2 h_e + ura_e --> h_c + ura_c 10921 or 11871 or 12902
  ALLTNti alltn_e --> alltn_c 10921 or 11871 or 12902
  URIt2 h_e + uri_e --> h_c + uri_c 10921 or 11871 or 12902 or 13969 or (10921 and 13969) or (11871 and 13969) or (12902 and 13969)
  THMt2 h_e + thm_e --> h_c + thm_c 10921 or 11871 or 12902
  URAt2 h_e + ura_e --> h_c + ura_c 10921 or 11871 or 12902

yli_M02355_c
yli_R0026 h_c + utp_c + yli_M02355_c --> ppi_c + uacgam_c 9079
  UDPACGLP acgam1p_c + h_c + utp_c <=> ppi_c + udpacgal_c 9079
  UAGDP acgam1p_c + h_c + utp_c --> ppi_c + uacgam_c 9079
yli_R0032 acgam6p_c <=> yli_M02355_c 12965
  PGAMT gam1p_c <=> gam6p_c 12965
  ACGAMPM acgam6p_c <=> acgam1p_c 12965

yli_M02454_c
yli_R0549 yli_M02454_c <=> aicar_c + fum_c 15805
  ADSL2r 25aics_c <=> aicar_c + fum_c 15805
  MUCCY_kt CCbuttc_c + h_c --> 4cml_c 15805
  ADSL1r dcamp_c <=> amp_c + fum_c 15805
yli_R0553 5aizc_c + asp__L_c + atp_c <=> adp_c + h_c + pi_c + yli_M02454_c 9220
  PRASCSi 5aizc_c + asp__L_c + atp_c --> 25aics_c + adp_c + h_c + pi_c 9220

yli_M02657_c
yli_R0097 3sala_c + akg_c + h_c --> glu__L_c + yli_M02657_c 14281 or 8936
  PHETA1 akg_c + phe__L_c <=> glu__L_c + phpyr_c 12407 or 13230 or 14281 or 14610 or 14853 or 14908 or 15839 or 8936
  ASPTAm akg_m + asp__L_m <=> glu__L_m + oaa_m 14281
  ASPTA akg_c + asp__L_c <=> glu__L_c + oaa_c 14281 or 8936
  yli_R1493 Lcyst_c + akg_c <=> glu__L_c + yli_M02657_c 14281 or 8936
  CYSTAm akg_m + cys__L_m <=> glu__L_m + mercppyr_m 14281
  UNK3 2kmb_c + glu__L_c --> akg_c + met__L_c 12407 or 14281 or 14908 or 15839 or 8936
  CYSATm glu__L_m + mercppyr_m --> akg_m + cys__L_m 14281
  TYRTAi 34hpp_c + glu__L_c --> akg_c + tyr__L_c 12407 or 13230 or 14281 or 14908 or 15839 or 8936
  CYSTA akg_c + cys__L_c --> glu__L_c + mercppyr_c 14281 or 8936
  EHGLAT2m e4hglu_m + oaa_m --> 4h2oglt_m + asp__L_m 14281
  PHETA1m akg_m + phe__L_m <=> glu__L_m + phpyr_m 12407 or 13230 or 14281 or 14908 or 15839 or 16065
  EHGLATm akg_m + e4hglu_m --> 4h2oglt_m + glu__L_m 14281
  TYRTAim 34hpp_m + glu__L_m --> akg_m + tyr__L_m 12407 or 13230 or 14281 or 14908 or 15839 or 16065
  LCYSTATm Lcyst_m + akg_m <=> 3spyr_m + glu__L_m 14281
  TYRTA akg_c + tyr__L_c <=> 34hpp_c + glu__L_c 12407 or 13230 or 14281 or 14610 or 14853 or 14908 or 15839 or 8936
  3SALATAim 3sala_m + akg_m + h_m --> 3snpyr_m + glu__L_m 14281
  AATGm 3sala_m + akg_m --> 3snpyr_m + glu__L_m 14281
  LEUTAi 4mop_c + glu__L_c --> akg_c + leu__L_c 14610 or 14853 or 8936
  EHGLATp akg_x + e4hglu_x --> 4h2oglt_x + glu__L_x 8936
  PHETA1 akg_c + phe__L_c <=> glu__L_c + phpyr_c 12407 or 13230 or 14281 or 14610 or 14853 or 14908 or 15839 or 8936
  ASPTA akg_c + asp__L_c <=> glu__L_c + oaa_c 14281 or 8936
  3SALATAi 3sala_c + akg_c + h_c --> 3snpyr_c + glu__L_c 8936
  yli_R1493 Lcyst_c + akg_c <=> glu__L_c + yli_M02657_c 14281 or 8936
  EHGLAT akg_c + e4hglu_c --> 4h2oglt_c + glu__L_c 8936
  LCYSTAT Lcyst_c + akg_c <=> 3spyr_c + glu__L_c 8936
  UNK3 2kmb_c + glu__L_c --> akg_c + met__L_c 12407 or 14281 or 14908 or 15839 or 8936
  TYRTAi 34hpp_c + glu__L_c --> akg_c + tyr__L_c 12407 or 13230 or 14281 or 14908 or 15839 or 8936
  CYSTA akg_c + cys__L_c --> glu__L_c + mercppyr_c 14281 or 8936
  TYRTAip 34hpp_x + glu__L_x --> akg_x + tyr__L_x 8936
  DLYSOXGAT akg_c + lys__D_c <=> 6a2ohxnt_c + glu__D_c 8936
  TYRTA akg_c + tyr__L_c <=> 34hpp_c + glu__L_c 12407 or 13230 or 14281 or 14610 or 14853 or 14908 or 15839 or 8936
  ASPTAp akg_x + asp__L_x <=> glu__L_x + oaa_x 8936
  DLYSPYRAT lys__D_c + pyr_c <=> 6a2ohxnt_c + ala__D_c 8936
yli_R1493 Lcyst_c + akg_c <=> glu__L_c + yli_M02657_c 14281 or 8936
  PHETA1 akg_c + phe__L_c <=> glu__L_c + phpyr_c 12407 or 13230 or 14281 or 14610 or 14853 or 14908 or 15839 or 8936
  ASPTAm akg_m + asp__L_m <=> glu__L_m + oaa_m 14281
  yli_R0097 3sala_c + akg_c + h_c --> glu__L_c + yli_M02657_c 14281 or 8936
  ASPTA akg_c + asp__L_c <=> glu__L_c + oaa_c 14281 or 8936
  CYSTAm akg_m + cys__L_m <=> glu__L_m + mercppyr_m 14281
  UNK3 2kmb_c + glu__L_c --> akg_c + met__L_c 12407 or 14281 or 14908 or 15839 or 8936
  CYSATm glu__L_m + mercppyr_m --> akg_m + cys__L_m 14281
  TYRTAi 34hpp_c + glu__L_c --> akg_c + tyr__L_c 12407 or 13230 or 14281 or 14908 or 15839 or 8936
  CYSTA akg_c + cys__L_c --> glu__L_c + mercppyr_c 14281 or 8936
  EHGLAT2m e4hglu_m + oaa_m --> 4h2oglt_m + asp__L_m 14281
  PHETA1m akg_m + phe__L_m <=> glu__L_m + phpyr_m 12407 or 13230 or 14281 or 14908 or 15839 or 16065
  EHGLATm akg_m + e4hglu_m --> 4h2oglt_m + glu__L_m 14281
  TYRTAim 34hpp_m + glu__L_m --> akg_m + tyr__L_m 12407 or 13230 or 14281 or 14908 or 15839 or 16065
  LCYSTATm Lcyst_m + akg_m <=> 3spyr_m + glu__L_m 14281
  TYRTA akg_c + tyr__L_c <=> 34hpp_c + glu__L_c 12407 or 13230 or 14281 or 14610 or 14853 or 14908 or 15839 or 8936
  3SALATAim 3sala_m + akg_m + h_m --> 3snpyr_m + glu__L_m 14281
  AATGm 3sala_m + akg_m --> 3snpyr_m + glu__L_m 14281
  LEUTAi 4mop_c + glu__L_c --> akg_c + leu__L_c 14610 or 14853 or 8936
  EHGLATp akg_x + e4hglu_x --> 4h2oglt_x + glu__L_x 8936
  PHETA1 akg_c + phe__L_c <=> glu__L_c + phpyr_c 12407 or 13230 or 14281 or 14610 or 14853 or 14908 or 15839 or 8936
  yli_R0097 3sala_c + akg_c + h_c --> glu__L_c + yli_M02657_c 14281 or 8936
  ASPTA akg_c + asp__L_c <=> glu__L_c + oaa_c 14281 or 8936
  3SALATAi 3sala_c + akg_c + h_c --> 3snpyr_c + glu__L_c 8936
  EHGLAT akg_c + e4hglu_c --> 4h2oglt_c + glu__L_c 8936
  LCYSTAT Lcyst_c + akg_c <=> 3spyr_c + glu__L_c 8936
  UNK3 2kmb_c + glu__L_c --> akg_c + met__L_c 12407 or 14281 or 14908 or 15839 or 8936
  TYRTAi 34hpp_c + glu__L_c --> akg_c + tyr__L_c 12407 or 13230 or 14281 or 14908 or 15839 or 8936
  CYSTA akg_c + cys__L_c --> glu__L_c + mercppyr_c 14281 or 8936
  TYRTAip 34hpp_x + glu__L_x --> akg_x + tyr__L_x 8936
  DLYSOXGAT akg_c + lys__D_c <=> 6a2ohxnt_c + glu__D_c 8936
  TYRTA akg_c + tyr__L_c <=> 34hpp_c + glu__L_c 12407 or 13230 or 14281 or 14610 or 14853 or 14908 or 15839 or 8936
  ASPTAp akg_x + asp__L_x <=> glu__L_x + oaa_x 8936
  DLYSPYRAT lys__D_c + pyr_c <=> 6a2ohxnt_c + ala__D_c 8936

yli_M02714_c
yli_R0816 2.0 3hanthrn_c + 4.0 o2_c --> 2.0 h2o2_c + 2.0 h_c + 2.0 o2s_c + yli_M02714_c 15305 or 9354
  CATp 2.0 h2o2_x --> 2.0 h2o_x + o2_x 15305 or 9354
  PRDX h2o2_c + meoh_c --> fald_c + 2.0 h2o_c 15305 or 9354
  CAT 2.0 h2o2_c --> 2.0 h2o_c + o2_c 15305 or 9354
  CATm 2.0 h2o2_m --> 2.0 h2o_m + o2_m 9354
  CAT2p etoh_x + h2o2_x --> acald_x + 2.0 h2o_x 9354
  PRDX h2o2_c + meoh_c --> fald_c + 2.0 h2o_c 15305 or 9354
  CAT 2.0 h2o2_c --> 2.0 h2o_c + o2_c 15305 or 9354
  CATp 2.0 h2o2_x --> 2.0 h2o_x + o2_x 15305 or 9354

yli_M03378_c
yli_R0258 atp_c + glu__L_c + thf_c <=> adp_c + h_c + pi_c + yli_M03378_c 10460 or 14803
  FPGS8m 10fthf5glu_m + atp_m + glu__L_m --> 10fthf6glu_m + adp_m + h_m + pi_m 14803
  FPGS3m 6thf_m + atp_m + glu__L_m --> 7thf_m + adp_m + h_m + pi_m 14803
  FPGS5m 5dhf_m + atp_m + glu__L_m --> 6dhf_m + adp_m + h_m + pi_m 14803
  10FTHFGLULLm 10fthf_m + atp_m + glu__L_m --> 10fthfglu__L_m + adp_m + pi_m 14803
  FPGS4 4.0 atp_c + dhf_c + 4.0 glu__L_c --> 5dhf_c + 4.0 adp_c + 4.0 h_c + 4.0 pi_c 14803
  FPGS2 5thf_c + atp_c + glu__L_c --> 6thf_c + adp_c + h_c + pi_c 14803
  FPGS3 6thf_c + atp_c + glu__L_c --> 7thf_c + adp_c + h_c + pi_c 14803
  yli_R1386 atp_m + dhpt_m + glu__L_m --> adp_m + dhf_m + h_m + pi_m 10460 or 14803
  FPGS2m 5thf_m + atp_m + glu__L_m --> 6thf_m + adp_m + h_m + pi_m 14803
  FPGS5 5dhf_c + atp_c + glu__L_c --> 6dhf_c + adp_c + h_c + pi_c 14803
  FPGS6 6dhf_c + atp_c + glu__L_c --> 7dhf_c + adp_c + h_c + pi_c 14803
  FPGS7 10fthf_c + 4.0 atp_c + 4.0 glu__L_c --> 10fthf5glu_c + 4.0 adp_c + 4.0 h_c + 4.0 pi_c 14803
  FPGS4m 4.0 atp_m + dhf_m + 4.0 glu__L_m --> 5dhf_m + 4.0 adp_m + 4.0 h_m + 4.0 pi_m 14803
  FPGS9m 10fthf6glu_m + atp_m + glu__L_m --> 10fthf7glu_m + adp_m + h_m + pi_m 14803
  yli_R1385 atp_m + glu__L_m + thf_m <=> adp_m + h_m + pi_m + yli_M03378_m 10460 or 14803
  THFGLUS atp_c + glu__L_c + thf_c <=> adp_c + h_c + pi_c + thfglu_c 10460 or 14803
  DHFS atp_c + dhpt_c + glu__L_c --> adp_c + dhf_c + h_c + pi_c 10460 or 14803
  FPGS8 10fthf5glu_c + atp_c + glu__L_c --> 10fthf6glu_c + adp_c + h_c + pi_c 14803
  FPGS7m 10fthf_m + 4.0 atp_m + 4.0 glu__L_m --> 10fthf5glu_m + 4.0 adp_m + 4.0 h_m + 4.0 pi_m 14803
  FPGS 4.0 atp_c + 4.0 glu__L_c + thf_c --> 5thf_c + 4.0 adp_c + 4.0 h_c + 4.0 pi_c 14803
  FPGS9 10fthf6glu_c + atp_c + glu__L_c --> 10fthf7glu_c + adp_c + h_c + pi_c 14803
  FPGS6m 6dhf_m + atp_m + glu__L_m --> 7dhf_m + adp_m + h_m + pi_m 14803
  FPGSm 4.0 atp_m + 4.0 glu__L_m + thf_m --> 5thf_m + 4.0 adp_m + 4.0 h_m + 4.0 pi_m 14803
  yli_R1385 atp_m + glu__L_m + thf_m <=> adp_m + h_m + pi_m + yli_M03378_m 10460 or 14803
  yli_R1386 atp_m + dhpt_m + glu__L_m --> adp_m + dhf_m + h_m + pi_m 10460 or 14803
  THFGLUS atp_c + glu__L_c + thf_c <=> adp_c + h_c + pi_c + thfglu_c 10460 or 14803
  DHFS atp_c + dhpt_c + glu__L_c --> adp_c + dhf_c + h_c + pi_c 10460 or 14803

yli_M03378_m
yli_R1385 atp_m + glu__L_m + thf_m <=> adp_m + h_m + pi_m + yli_M03378_m 10460 or 14803
  FPGS8m 10fthf5glu_m + atp_m + glu__L_m --> 10fthf6glu_m + adp_m + h_m + pi_m 14803
  FPGS3m 6thf_m + atp_m + glu__L_m --> 7thf_m + adp_m + h_m + pi_m 14803
  FPGS5m 5dhf_m + atp_m + glu__L_m --> 6dhf_m + adp_m + h_m + pi_m 14803
  10FTHFGLULLm 10fthf_m + atp_m + glu__L_m --> 10fthfglu__L_m + adp_m + pi_m 14803
  FPGS4 4.0 atp_c + dhf_c + 4.0 glu__L_c --> 5dhf_c + 4.0 adp_c + 4.0 h_c + 4.0 pi_c 14803
  FPGS2 5thf_c + atp_c + glu__L_c --> 6thf_c + adp_c + h_c + pi_c 14803
  FPGS3 6thf_c + atp_c + glu__L_c --> 7thf_c + adp_c + h_c + pi_c 14803
  yli_R1386 atp_m + dhpt_m + glu__L_m --> adp_m + dhf_m + h_m + pi_m 10460 or 14803
  FPGS2m 5thf_m + atp_m + glu__L_m --> 6thf_m + adp_m + h_m + pi_m 14803
  FPGS5 5dhf_c + atp_c + glu__L_c --> 6dhf_c + adp_c + h_c + pi_c 14803
  FPGS6 6dhf_c + atp_c + glu__L_c --> 7dhf_c + adp_c + h_c + pi_c 14803
  FPGS7 10fthf_c + 4.0 atp_c + 4.0 glu__L_c --> 10fthf5glu_c + 4.0 adp_c + 4.0 h_c + 4.0 pi_c 14803
  FPGS4m 4.0 atp_m + dhf_m + 4.0 glu__L_m --> 5dhf_m + 4.0 adp_m + 4.0 h_m + 4.0 pi_m 14803
  FPGS9m 10fthf6glu_m + atp_m + glu__L_m --> 10fthf7glu_m + adp_m + h_m + pi_m 14803
  THFGLUS atp_c + glu__L_c + thf_c <=> adp_c + h_c + pi_c + thfglu_c 10460 or 14803
  DHFS atp_c + dhpt_c + glu__L_c --> adp_c + dhf_c + h_c + pi_c 10460 or 14803
  FPGS8 10fthf5glu_c + atp_c + glu__L_c --> 10fthf6glu_c + adp_c + h_c + pi_c 14803
  FPGS7m 10fthf_m + 4.0 atp_m + 4.0 glu__L_m --> 10fthf5glu_m + 4.0 adp_m + 4.0 h_m + 4.0 pi_m 14803
  FPGS 4.0 atp_c + 4.0 glu__L_c + thf_c --> 5thf_c + 4.0 adp_c + 4.0 h_c + 4.0 pi_c 14803
  yli_R0258 atp_c + glu__L_c + thf_c <=> adp_c + h_c + pi_c + yli_M03378_c 10460 or 14803
  FPGS9 10fthf6glu_c + atp_c + glu__L_c --> 10fthf7glu_c + adp_c + h_c + pi_c 14803
  FPGS6m 6dhf_m + atp_m + glu__L_m --> 7dhf_m + adp_m + h_m + pi_m 14803
  FPGSm 4.0 atp_m + 4.0 glu__L_m + thf_m --> 5thf_m + 4.0 adp_m + 4.0 h_m + 4.0 pi_m 14803
  yli_R0258 atp_c + glu__L_c + thf_c <=> adp_c + h_c + pi_c + yli_M03378_c 10460 or 14803
  yli_R1386 atp_m + dhpt_m + glu__L_m --> adp_m + dhf_m + h_m + pi_m 10460 or 14803
  THFGLUS atp_c + glu__L_c + thf_c <=> adp_c + h_c + pi_c + thfglu_c 10460 or 14803
  DHFS atp_c + dhpt_c + glu__L_c --> adp_c + dhf_c + h_c + pi_c 10460 or 14803

yli_M04300_c
yli_R0683 25drapp_c + 3.0 h_c + nadh_c --> nad_c + yli_M04300_c 16837
  APRAUR 5apru_c + h_c + nadph_c --> 5aprbu_c + nadp_c 16837
  DHPPDA2 25drapp_c + h2o_c + h_c --> 5apru_c + nh4_c 16837
  DHPPDA 25dhpp_c + h2o_c + h_c --> 5apru_c + nh4_c 16837
  DROPPRy 25dhpp_c + h_c + nadph_c --> 25dthpp_c + nadp_c 16837
  yli_R0684 25drapp_c + 3.0 h_c + nadph_c --> nadp_c + yli_M04300_c 16837
yli_R0684 25drapp_c + 3.0 h_c + nadph_c --> nadp_c + yli_M04300_c 16837
  APRAUR 5apru_c + h_c + nadph_c --> 5aprbu_c + nadp_c 16837
  yli_R0683 25drapp_c + 3.0 h_c + nadh_c --> nad_c + yli_M04300_c 16837
  DHPPDA2 25drapp_c + h2o_c + h_c --> 5apru_c + nh4_c 16837
  DHPPDA 25dhpp_c + h2o_c + h_c --> 5apru_c + nh4_c 16837
  DROPPRy 25dhpp_c + h_c + nadph_c --> 25dthpp_c + nadp_c 16837
yli_R0685 h2o_c + yli_M04300_c --> 5aprbu_c + h_c + nh4_c 12663
  DRTPPD 25dthpp_c + h2o_c + h_c --> 5aprbu_c + nh4_c 12663

yli_M04599_c

yli_M04600_c

yli_M04601_c

yli_M04602_c

yli_M04603_c

yli_M04604_c

yli_M04615_c

yli_M04616_c

yli_M04617_c

yli_M04618_c

yli_M04619_c

yli_M04620_c

yli_M07024_c
yli_R1529 L2aadp_c + atp_c <=> ppi_c + yli_M07024_c 9495
  yli_R1531 h_m + nadph_m + yli_M07026_m --> L2aadp6sa_m + nadp_m + yli_M07025_m 9495
  AASAD2 L2aadp_c + atp_c + h_c + nadh_c --> L2aadp6sa_c + amp_c + nad_c + ppi_c 9495 or (10220 and 9495)
  yli_R1528 L2aadp_m + atp_m <=> ppi_m + yli_M07024_m 9495
  AASAD1 L2aadp_c + atp_c + h_c + nadph_c --> L2aadp6sa_c + amp_c + nadp_c + ppi_c 9495 or (10220 and 9495)
  yli_R1530 yli_M07024_m + yli_M07025_m --> amp_m + yli_M07026_m 9495

yli_M07024_m
yli_R1528 L2aadp_m + atp_m <=> ppi_m + yli_M07024_m 9495
  yli_R1531 h_m + nadph_m + yli_M07026_m --> L2aadp6sa_m + nadp_m + yli_M07025_m 9495
  yli_R1529 L2aadp_c + atp_c <=> ppi_c + yli_M07024_c 9495
  AASAD2 L2aadp_c + atp_c + h_c + nadh_c --> L2aadp6sa_c + amp_c + nad_c + ppi_c 9495 or (10220 and 9495)
  AASAD1 L2aadp_c + atp_c + h_c + nadph_c --> L2aadp6sa_c + amp_c + nadp_c + ppi_c 9495 or (10220 and 9495)
  yli_R1530 yli_M07024_m + yli_M07025_m --> amp_m + yli_M07026_m 9495
yli_R1530 yli_M07024_m + yli_M07025_m --> amp_m + yli_M07026_m 9495
  yli_R1531 h_m + nadph_m + yli_M07026_m --> L2aadp6sa_m + nadp_m + yli_M07025_m 9495
  yli_R1529 L2aadp_c + atp_c <=> ppi_c + yli_M07024_c 9495
  AASAD2 L2aadp_c + atp_c + h_c + nadh_c --> L2aadp6sa_c + amp_c + nad_c + ppi_c 9495 or (10220 and 9495)
  yli_R1528 L2aadp_m + atp_m <=> ppi_m + yli_M07024_m 9495
  AASAD1 L2aadp_c + atp_c + h_c + nadph_c --> L2aadp6sa_c + amp_c + nadp_c + ppi_c 9495 or (10220 and 9495)

yli_M07025_m
yli_R1530 yli_M07024_m + yli_M07025_m --> amp_m + yli_M07026_m 9495
  yli_R1531 h_m + nadph_m + yli_M07026_m --> L2aadp6sa_m + nadp_m + yli_M07025_m 9495
  yli_R1529 L2aadp_c + atp_c <=> ppi_c + yli_M07024_c 9495
  AASAD2 L2aadp_c + atp_c + h_c + nadh_c --> L2aadp6sa_c + amp_c + nad_c + ppi_c 9495 or (10220 and 9495)
  yli_R1528 L2aadp_m + atp_m <=> ppi_m + yli_M07024_m 9495
  AASAD1 L2aadp_c + atp_c + h_c + nadph_c --> L2aadp6sa_c + amp_c + nadp_c + ppi_c 9495 or (10220 and 9495)
yli_R1531 h_m + nadph_m + yli_M07026_m --> L2aadp6sa_m + nadp_m + yli_M07025_m 9495
  yli_R1529 L2aadp_c + atp_c <=> ppi_c + yli_M07024_c 9495
  AASAD2 L2aadp_c + atp_c + h_c + nadh_c --> L2aadp6sa_c + amp_c + nad_c + ppi_c 9495 or (10220 and 9495)
  yli_R1528 L2aadp_m + atp_m <=> ppi_m + yli_M07024_m 9495
  AASAD1 L2aadp_c + atp_c + h_c + nadph_c --> L2aadp6sa_c + amp_c + nadp_c + ppi_c 9495 or (10220 and 9495)
  yli_R1530 yli_M07024_m + yli_M07025_m --> amp_m + yli_M07026_m 9495

yli_M07026_m
yli_R1530 yli_M07024_m + yli_M07025_m --> amp_m + yli_M07026_m 9495
  yli_R1531 h_m + nadph_m + yli_M07026_m --> L2aadp6sa_m + nadp_m + yli_M07025_m 9495
  yli_R1529 L2aadp_c + atp_c <=> ppi_c + yli_M07024_c 9495
  AASAD2 L2aadp_c + atp_c + h_c + nadh_c --> L2aadp6sa_c + amp_c + nad_c + ppi_c 9495 or (10220 and 9495)
  yli_R1528 L2aadp_m + atp_m <=> ppi_m + yli_M07024_m 9495
  AASAD1 L2aadp_c + atp_c + h_c + nadph_c --> L2aadp6sa_c + amp_c + nadp_c + ppi_c 9495 or (10220 and 9495)
yli_R1531 h_m + nadph_m + yli_M07026_m --> L2aadp6sa_m + nadp_m + yli_M07025_m 9495
  yli_R1529 L2aadp_c + atp_c <=> ppi_c + yli_M07024_c 9495
  AASAD2 L2aadp_c + atp_c + h_c + nadh_c --> L2aadp6sa_c + amp_c + nad_c + ppi_c 9495 or (10220 and 9495)
  yli_R1528 L2aadp_m + atp_m <=> ppi_m + yli_M07024_m 9495
  AASAD1 L2aadp_c + atp_c + h_c + nadph_c --> L2aadp6sa_c + amp_c + nadp_c + ppi_c 9495 or (10220 and 9495)
  yli_R1530 yli_M07024_m + yli_M07025_m --> amp_m + yli_M07026_m 9495

yli_M07057_c
yli_R1579 Lfmkynr_c + h2o_c <=> ala__L_c + yli_M07057_c 8725
  yli_R1578 Lkynr_c + h2o_c <=> ala__L_c + anth_c 8725
  KYN Lkynr_c + h2o_c --> ala__L_c + anth_c + h_c 8725
  HKYNH h2o_c + hLkynr_c --> 3hanthrn_c + ala__L_c 8725
  LFORKYNHYD Lfmkynr_c + h2o_c --> ala__L_c + h_c + nformanth_c 8725

yli_M07058_c
yli_R1580 Lkynr_c + akg_c <=> glu__L_c + yli_M07058_c 8540
  KYNAKGAT Lkynr_c + akg_c --> 4aphdob_c + glu__L_c 12407 or 14908 or 15839 or 8540
  yli_R1581 akg_c + hLkynr_c <=> glu__L_c + yli_M07059_c 8540
  3HKYNAKGAT akg_c + hLkynr_c --> 42A3HP24DB_c + glu__L_c 12407 or 14908 or 15839 or 8540
  FKYNH Lfmkynr_c + h2o_c --> Lkynr_c + for_c + h_c 8540

yli_M07059_c
yli_R1581 akg_c + hLkynr_c <=> glu__L_c + yli_M07059_c 8540
  yli_R1580 Lkynr_c + akg_c <=> glu__L_c + yli_M07058_c 8540
  KYNAKGAT Lkynr_c + akg_c --> 4aphdob_c + glu__L_c 12407 or 14908 or 15839 or 8540
  3HKYNAKGAT akg_c + hLkynr_c --> 42A3HP24DB_c + glu__L_c 12407 or 14908 or 15839 or 8540
  FKYNH Lfmkynr_c + h2o_c --> Lkynr_c + for_c + h_c 8540
```

In [29]:

```
model.remove_metabolites([m for m in model.metabolites if '_hs' in m.id], destructive=True)
model.remove_metabolites([m for m in model.metabolites if '_cho' in m.id], destructive=True)
model.remove_metabolites([m for m in model.metabolites if 'yli_' in m.id], destructive=True)
```

In [30]:

```
for m in sorted(model.metabolites, key=lambda x: x.id):
    if '_SC' in m.id:
        print(m.id)
        for r in sorted(m.reactions, key=lambda x: x.id):
            print(r.id, r.reaction, r.gene_reaction_rule)
        print()
```

```
12dgr_SC_g
IPCS124g_RT cer1_24_g + 0.01 ptd1ino_SC_g --> 0.01 12dgr_SC_g + 0.01 ipc124_SC_g 12927 and 8747
IPCS126g_RT cer1_26_g + 0.01 ptd1ino_SC_g --> 0.01 12dgr_SC_g + 0.01 ipc126_SC_g 12927 and 8747
IPCS224g_RT cer2_24_g + 0.01 ptd1ino_SC_g --> 0.01 12dgr_SC_g + 0.01 ipc224_SC_g 12927 and 8747
IPCS226g_RT cer2_26_g + 0.01 ptd1ino_SC_g --> 0.01 12dgr_SC_g + 0.01 ipc226_SC_g 12927 and 8747
IPCS324g_RT cer3_24_g + 0.01 ptd1ino_SC_g --> 0.01 12dgr_SC_g + 0.01 ipc324_SC_g 12927 and 8747
IPCS326g_RT cer3_26_g + 0.01 ptd1ino_SC_g --> 0.01 12dgr_SC_g + 0.01 ipc326_SC_g 12927 and 8747
MIP2CS124g_RT 0.01 mipc124_SC_g + 0.01 ptd1ino_SC_g --> 0.01 12dgr_SC_g + 0.01 mip2c124_SC_g 13172
MIP2CS126g_RT 0.01 mipc126_SC_g + 0.01 ptd1ino_SC_g --> 0.01 12dgr_SC_g + 0.01 mip2c126_SC_g 13172
MIP2CS224g_RT 0.01 mipc224_SC_g + 0.01 ptd1ino_SC_g --> 0.01 12dgr_SC_g + 0.01 mip2c224_SC_g 13172
MIP2CS226g_RT 0.01 mipc226_SC_g + 0.01 ptd1ino_SC_g --> 0.01 12dgr_SC_g + 0.01 mip2c226_SC_g 13172
MIP2CS324g_RT 0.01 mipc324_SC_g + 0.01 ptd1ino_SC_g --> 0.01 12dgr_SC_g + 0.01 mip2c324_SC_g 13172
MIP2CS326g_RT 0.01 mipc326_SC_g + 0.01 ptd1ino_SC_g --> 0.01 12dgr_SC_g + 0.01 mip2c326_SC_g 13172

12dgr_SC_n
PI45BPPn_RT h2o_n + 0.01 ptd145bp_SC_n --> 0.01 12dgr_SC_n + h_n + mi145p_n 12855

12dgr_SC_r
DAGCPTer_RT 0.01 12dgr_SC_r + cdpchol_r --> cmp_r + h_r + 0.01 pc_SC_r 11351 or 9088
DAGKer_RT 0.01 12dgr_SC_r + ctp_r --> cdp_r + h_r + 0.01 pa_SC_r 10156
DAGPYPer_RT h2o_r + 0.01 pa_SC_r --> 0.01 12dgr_SC_r + pi_r 12485 or 13087
ETHAPTer_RT 0.01 12dgr_SC_r + cdpea_r --> cmp_r + h_r + 0.01 pe_SC_r 11351
PCDAGATer_RT 12dgr_SC_r + pc_SC_r --> 1agpc_SC_r + triglyc_SC_r 16477
TRIGSer_RT 0.01 12dgr_SC_r + 0.02 dcacoa_r + 0.06 ddcacoa_r + 0.17 hdcoa_r + 0.09 ocdycacoa_r + 0.24 odecoa_r + 0.27 pmtcoa_r + 0.05 stcoa_r + 0.1 tdcoa_r --> coa_r + 0.01 triglyc_SC_r 16460

1ag3p_SC_r
ADHAPRer_RT 0.01 1agly3p_SC_r + h_r + nadph_r --> 0.01 1ag3p_SC_r + nadp_r 15575
AGATer_RT 0.01 1ag3p_SC_r + 0.02 dcacoa_r + 0.06 ddcacoa_r + 0.17 hdcoa_r + 0.09 ocdycacoa_r + 0.24 odecoa_r + 0.27 pmtcoa_r + 0.05 stcoa_r + 0.1 tdcoa_r --> coa_r + 0.01 pa_SC_r 10427 or 16030 or 16779 or 9746
GAT1er_RT 0.02 dcacoa_r + 0.06 ddcacoa_r + glyc3p_r + 0.17 hdcoa_r + 0.09 ocdycacoa_r + 0.24 odecoa_r + 0.27 pmtcoa_r + 0.05 stcoa_r + 0.1 tdcoa_r --> 0.01 1ag3p_SC_r + coa_r 15435

1agly3p_SC_r
ADHAPRer_RT 0.01 1agly3p_SC_r + h_r + nadph_r --> 0.01 1ag3p_SC_r + nadp_r 15575
GAT2er_RT 0.02 dcacoa_r + 0.06 ddcacoa_r + dhap_r + 0.17 hdcoa_r + 0.09 ocdycacoa_r + 0.24 odecoa_r + 0.27 pmtcoa_r + 0.05 stcoa_r + 0.1 tdcoa_r --> 0.01 1agly3p_SC_r + coa_r 13369 or 15435

1agpc_SC_r
LPCATer_RT 0.01 1agpc_SC_r + 0.02 dcacoa_r + 0.06 ddcacoa_r + 0.17 hdcoa_r + 0.09 ocdycacoa_r + 0.24 odecoa_r + 0.27 pmtcoa_r + 0.05 stcoa_r + 0.1 tdcoa_r --> coa_r + 0.01 pc_SC_r 16030
PCDAGATer_RT 12dgr_SC_r + pc_SC_r --> 1agpc_SC_r + triglyc_SC_r 16477

cdpdag_SC_m
CDPDAGterm_RT cdpdag_SC_r <=> cdpdag_SC_m 
CDPDGPm_RT 0.01 cdpdag_SC_m + glyc3p_m --> cmp_m + h_m + 0.01 pgp_SC_m 12596
CLPNSm_SC 0.01 cdpdag_SC_m + 0.01 pg_SC_m --> 0.01 clpn_SC_m + cmp_m + h_m 16415
DASYNm_RT ctp_m + h_m + 0.01 pa_SC_m --> 0.01 cdpdag_SC_m + ppi_m 11307
PSERSm_RT 0.01 cdpdag_SC_m + ser__L_m --> cmp_m + h_m + 0.01 ps_SC_m 11583

cdpdag_SC_r
CDPDAGterm_RT cdpdag_SC_r <=> cdpdag_SC_m 
DASYNer_RT ctp_r + h_r + 0.01 pa_SC_r --> 0.01 cdpdag_SC_r + ppi_r 12881
PINOSer_RT 0.01 cdpdag_SC_r + inost_r --> cmp_r + h_r + 0.01 ptd1ino_SC_r 15121
PSERSer_RT 0.01 cdpdag_SC_r + ser__L_r --> cmp_r + h_r + 0.01 ps_SC_r 11583

clpn_SC_m
CLPNSm_SC 0.01 cdpdag_SC_m + 0.01 pg_SC_m --> 0.01 clpn_SC_m + cmp_m + h_m 16415

dagpy_SC_r
LPPer_RT 0.01 dagpy_SC_r + h2o_r --> h_r + 0.01 pa_SC_r + pi_r 13087

epistest_SC_r
EPISTATer_RT 0.01 epist_r + 0.655 hdcoa_r + 0.01 hexccoa_r + 0.27 odecoa_r + 0.02 pmtcoa_r + 0.03 stcoa_r + 0.015 tdcoa_r --> coa_r + 0.01 epistest_SC_r 11799
EPISTESTtrd epistest_SC_r --> epistest_RT_d 

ergstest_SC_r
ERGSTATer_RT 0.01 ergst_r + 0.655 hdcoa_r + 0.01 hexccoa_r + 0.27 odecoa_r + 0.02 pmtcoa_r + 0.03 stcoa_r + 0.015 tdcoa_r --> coa_r + 0.01 ergstest_SC_r 11799
ERGSTESTtrd ergstest_SC_r --> ergstest_RT_d 

fecostest_SC_r
FECOSTATer_RT 0.01 fecost_r + 0.655 hdcoa_r + 0.01 hexccoa_r + 0.27 odecoa_r + 0.02 pmtcoa_r + 0.03 stcoa_r + 0.015 tdcoa_r --> coa_r + 0.01 fecostest_SC_r 11799
FECOSTESTtrd fecostest_SC_r --> fecostest_RT_d 

ipc124_SC_g
IPC124PLCg_RT h2o_g + 0.01 ipc124_SC_g --> cer1_24_g + h_g + mi1p__D_g 15857
IPCS124g_RT cer1_24_g + 0.01 ptd1ino_SC_g --> 0.01 12dgr_SC_g + 0.01 ipc124_SC_g 12927 and 8747
MIPCS124g_RT gdpmann_g + 0.01 ipc124_SC_g --> gdp_g + h_g + 0.01 mipc124_SC_g 16453

ipc126_SC_g
IPC126PLCg_RT h2o_g + 0.01 ipc126_SC_g --> cer1_26_g + h_g + mi1p__D_g 15857
IPCS126g_RT cer1_26_g + 0.01 ptd1ino_SC_g --> 0.01 12dgr_SC_g + 0.01 ipc126_SC_g 12927 and 8747
MIPCS126g_RT gdpmann_g + 0.01 ipc126_SC_g --> gdp_g + h_g + 0.01 mipc126_SC_g 16453

ipc224_SC_g
IPC224PLCg_RT h2o_g + 0.01 ipc224_SC_g --> cer2_24_g + h_g + mi1p__D_g 15857
IPCS224g_RT cer2_24_g + 0.01 ptd1ino_SC_g --> 0.01 12dgr_SC_g + 0.01 ipc224_SC_g 12927 and 8747
MIPCS224g_RT gdpmann_g + 0.01 ipc224_SC_g --> gdp_g + h_g + 0.01 mipc224_SC_g 16453

ipc226_SC_g
IPC226PLCg_RT h2o_g + 0.01 ipc226_SC_g --> cer2_26_g + h_g + mi1p__D_g 15857
IPCS226g_RT cer2_26_g + 0.01 ptd1ino_SC_g --> 0.01 12dgr_SC_g + 0.01 ipc226_SC_g 12927 and 8747
MIPCS226g_RT gdpmann_g + 0.01 ipc226_SC_g --> gdp_g + h_g + 0.01 mipc226_SC_g 16453

ipc324_SC_g
IPC324PLCg_RT h2o_g + 0.01 ipc324_SC_g --> cer3_24_g + h_g + mi1p__D_g 15857
IPCS324g_RT cer3_24_g + 0.01 ptd1ino_SC_g --> 0.01 12dgr_SC_g + 0.01 ipc324_SC_g 12927 and 8747
MIPCS324g_RT gdpmann_g + 0.01 ipc324_SC_g --> gdp_g + h_g + 0.01 mipc324_SC_g 16453

ipc326_SC_g
IPC326PLCg_RT h2o_g + 0.01 ipc326_SC_g --> cer3_26_g + h_g + mi1p__D_g 15857
IPCS326g_RT cer3_26_g + 0.01 ptd1ino_SC_g --> 0.01 12dgr_SC_g + 0.01 ipc326_SC_g 12927 and 8747
MIPCS326g_RT gdpmann_g + 0.01 ipc326_SC_g --> gdp_g + h_g + 0.01 mipc326_SC_g 16453

lanostest_SC_r
LANOSTATer_RT 0.655 hdcoa_r + 0.01 hexccoa_r + 0.01 lanost_r + 0.27 odecoa_r + 0.02 pmtcoa_r + 0.03 stcoa_r + 0.015 tdcoa_r --> coa_r + 0.01 lanostest_SC_r 11799
LANOSTESTtrd lanostest_SC_r --> lanostest_RT_d 

mip2c124_SC_g
MIP2C124PLCg_RT h2o_g + 0.01 mip2c124_SC_g --> cer1_24_g + h_g + man2mi1p__D_g 15857
MIP2CS124g_RT 0.01 mipc124_SC_g + 0.01 ptd1ino_SC_g --> 0.01 12dgr_SC_g + 0.01 mip2c124_SC_g 13172

mip2c126_SC_g
MIP2C126PLCg_RT h2o_g + 0.01 mip2c126_SC_g --> cer1_26_g + h_g + man2mi1p__D_g 15857
MIP2CS126g_RT 0.01 mipc126_SC_g + 0.01 ptd1ino_SC_g --> 0.01 12dgr_SC_g + 0.01 mip2c126_SC_g 13172

mip2c224_SC_g
MIP2C224PLCg_RT h2o_g + 0.01 mip2c224_SC_g --> cer2_24_g + h_g + man2mi1p__D_g 15857
MIP2CS224g_RT 0.01 mipc224_SC_g + 0.01 ptd1ino_SC_g --> 0.01 12dgr_SC_g + 0.01 mip2c224_SC_g 13172

mip2c226_SC_g
MIP2C226PLCg_RT h2o_g + 0.01 mip2c226_SC_g --> cer2_26_g + h_g + man2mi1p__D_g 15857
MIP2CS226g_RT 0.01 mipc226_SC_g + 0.01 ptd1ino_SC_g --> 0.01 12dgr_SC_g + 0.01 mip2c226_SC_g 13172

mip2c324_SC_g
MIP2C324PLCg_RT h2o_g + 0.01 mip2c324_SC_g --> cer3_24_g + h_g + man2mi1p__D_g 15857
MIP2CS324g_RT 0.01 mipc324_SC_g + 0.01 ptd1ino_SC_g --> 0.01 12dgr_SC_g + 0.01 mip2c324_SC_g 13172

mip2c326_SC_g
MIP2C326PLCg_RT h2o_g + 0.01 mip2c326_SC_g --> cer3_26_g + h_g + man2mi1p__D_g 15857
MIP2CS326g_RT 0.01 mipc326_SC_g + 0.01 ptd1ino_SC_g --> 0.01 12dgr_SC_g + 0.01 mip2c326_SC_g 13172

mipc124_SC_g
MIP2CS124g_RT 0.01 mipc124_SC_g + 0.01 ptd1ino_SC_g --> 0.01 12dgr_SC_g + 0.01 mip2c124_SC_g 13172
MIPC124PLCg_RT h2o_g + 0.01 mipc124_SC_g --> cer1_24_g + h_g + manmi1p__D_g 15857
MIPCS124g_RT gdpmann_g + 0.01 ipc124_SC_g --> gdp_g + h_g + 0.01 mipc124_SC_g 16453

mipc126_SC_g
MIP2CS126g_RT 0.01 mipc126_SC_g + 0.01 ptd1ino_SC_g --> 0.01 12dgr_SC_g + 0.01 mip2c126_SC_g 13172
MIPC126PLCg_RT h2o_g + 0.01 mipc126_SC_g --> cer1_26_g + h_g + manmi1p__D_g 15857
MIPCS126g_RT gdpmann_g + 0.01 ipc126_SC_g --> gdp_g + h_g + 0.01 mipc126_SC_g 16453

mipc224_SC_g
MIP2CS224g_RT 0.01 mipc224_SC_g + 0.01 ptd1ino_SC_g --> 0.01 12dgr_SC_g + 0.01 mip2c224_SC_g 13172
MIPC224PLCg_RT h2o_g + 0.01 mipc224_SC_g --> cer2_24_g + h_g + manmi1p__D_g 15857
MIPCS224g_RT gdpmann_g + 0.01 ipc224_SC_g --> gdp_g + h_g + 0.01 mipc224_SC_g 16453

mipc226_SC_g
MIP2CS226g_RT 0.01 mipc226_SC_g + 0.01 ptd1ino_SC_g --> 0.01 12dgr_SC_g + 0.01 mip2c226_SC_g 13172
MIPC226PLCg_RT h2o_g + 0.01 mipc226_SC_g --> cer2_26_g + h_g + manmi1p__D_g 15857
MIPCS226g_RT gdpmann_g + 0.01 ipc226_SC_g --> gdp_g + h_g + 0.01 mipc226_SC_g 16453

mipc324_SC_g
MIP2CS324g_RT 0.01 mipc324_SC_g + 0.01 ptd1ino_SC_g --> 0.01 12dgr_SC_g + 0.01 mip2c324_SC_g 13172
MIPC324PLCg_RT h2o_g + 0.01 mipc324_SC_g --> cer3_24_g + h_g + manmi1p__D_g 15857
MIPCS324g_RT gdpmann_g + 0.01 ipc324_SC_g --> gdp_g + h_g + 0.01 mipc324_SC_g 16453

mipc326_SC_g
MIP2CS326g_RT 0.01 mipc326_SC_g + 0.01 ptd1ino_SC_g --> 0.01 12dgr_SC_g + 0.01 mip2c326_SC_g 13172
MIPC326PLCg_RT h2o_g + 0.01 mipc326_SC_g --> cer3_26_g + h_g + manmi1p__D_g 15857
MIPCS326g_RT gdpmann_g + 0.01 ipc326_SC_g --> gdp_g + h_g + 0.01 mipc326_SC_g 16453

pa_SC_m
DASYNm_RT ctp_m + h_m + 0.01 pa_SC_m --> 0.01 cdpdag_SC_m + ppi_m 11307
PAterm_RT pa_SC_r <=> pa_SC_m 

pa_SC_n
PLDn_RT h2o_n + 0.01 pc_SC_n --> chol_n + h_n + 0.01 pa_SC_n 14023

pa_SC_r
AGATer_RT 0.01 1ag3p_SC_r + 0.02 dcacoa_r + 0.06 ddcacoa_r + 0.17 hdcoa_r + 0.09 ocdycacoa_r + 0.24 odecoa_r + 0.27 pmtcoa_r + 0.05 stcoa_r + 0.1 tdcoa_r --> coa_r + 0.01 pa_SC_r 10427 or 16030 or 16779 or 9746
DAGKer_RT 0.01 12dgr_SC_r + ctp_r --> cdp_r + h_r + 0.01 pa_SC_r 10156
DAGPYPer_RT h2o_r + 0.01 pa_SC_r --> 0.01 12dgr_SC_r + pi_r 12485 or 13087
DASYNer_RT ctp_r + h_r + 0.01 pa_SC_r --> 0.01 cdpdag_SC_r + ppi_r 12881
LPPer_RT 0.01 dagpy_SC_r + h2o_r --> h_r + 0.01 pa_SC_r + pi_r 13087
PAterm_RT pa_SC_r <=> pa_SC_m 

pc_SC_e
PLBPCe_RT h2o_e + 0.005 pc_SC_e --> 0.02 dca_e + 0.06 ddca_e + 0.5 g3pc_e + h_e + 0.27 hdca_e + 0.17 hdcea_e + 0.05 ocdca_e + 0.24 ocdcea_e + 0.09 ocdcya_e + 0.1 ttdca_e 12385

pc_SC_n
PLDn_RT h2o_n + 0.01 pc_SC_n --> chol_n + h_n + 0.01 pa_SC_n 14023

pc_SC_r
DAGCPTer_RT 0.01 12dgr_SC_r + cdpchol_r --> cmp_r + h_r + 0.01 pc_SC_r 11351 or 9088
LPCATer_RT 0.01 1agpc_SC_r + 0.02 dcacoa_r + 0.06 ddcacoa_r + 0.17 hdcoa_r + 0.09 ocdycacoa_r + 0.24 odecoa_r + 0.27 pmtcoa_r + 0.05 stcoa_r + 0.1 tdcoa_r --> coa_r + 0.01 pc_SC_r 16030
PCDAGATer_RT 12dgr_SC_r + pc_SC_r --> 1agpc_SC_r + triglyc_SC_r 16477
PLBPC_RT h2o_c + 0.005 pc_SC_r --> 0.02 dca_c + 0.06 ddca_c + 0.5 g3pc_c + h_c + 0.27 hdca_c + 0.17 hdcea_c + 0.05 ocdca_c + 0.24 ocdcea_c + 0.09 ocdcya_c + 0.1 ttdca_c 14309
PMETMer_RT amet_r + 0.01 ptd2meeta_SC_r --> ahcys_r + h_r + 0.01 pc_SC_r 12748

pe_SC_e
PLBPEe_RT h2o_e + 0.005 pe_SC_e --> 0.02 dca_e + 0.06 ddca_e + 0.5 g3pe_e + h_e + 0.27 hdca_e + 0.17 hdcea_e + 0.05 ocdca_e + 0.24 ocdcea_e + 0.09 ocdcya_e + 0.1 ttdca_e 12385

pe_SC_g
PSERDg_RT h_g + 0.01 ps_SC_g --> co2_g + 0.01 pe_SC_g 10504 or 11446

pe_SC_m
PEterm_RT pe_SC_r <=> pe_SC_m 
PSERDm_SC h_m + 0.01 ps_SC_m --> co2_m + 0.01 pe_SC_m 14554

pe_SC_r
ETHAPTer_RT 0.01 12dgr_SC_r + cdpea_r --> cmp_r + h_r + 0.01 pe_SC_r 11351
PETOHMer_RT amet_r + 0.01 pe_SC_r --> ahcys_r + h_r + 0.01 ptdmeeta_SC_r 10647
PEterm_RT pe_SC_r <=> pe_SC_m 
PSERDer_RT h_r + 0.01 ps_SC_r --> co2_r + 0.01 pe_SC_r 14554

pe_SC_v
PSERDv_RT h_v + 0.01 ps_SC_v --> co2_v + 0.01 pe_SC_v 10504 or 11446

pg_SC_m
CLPNSm_SC 0.01 cdpdag_SC_m + 0.01 pg_SC_m --> 0.01 clpn_SC_m + cmp_m + h_m 16415
PGPPAm_SC h2o_m + 0.01 pgp_SC_m --> 0.01 pg_SC_m + pi_m 10900

pgp_SC_m
CDPDGPm_RT 0.01 cdpdag_SC_m + glyc3p_m --> cmp_m + h_m + 0.01 pgp_SC_m 12596
PGPPAm_SC h2o_m + 0.01 pgp_SC_m --> 0.01 pg_SC_m + pi_m 10900

ps_SC_g
PSERDg_RT h_g + 0.01 ps_SC_g --> co2_g + 0.01 pe_SC_g 10504 or 11446

ps_SC_m
PSERDm_SC h_m + 0.01 ps_SC_m --> co2_m + 0.01 pe_SC_m 14554
PSERSm_RT 0.01 cdpdag_SC_m + ser__L_m --> cmp_m + h_m + 0.01 ps_SC_m 11583
PSterm_RT ps_SC_r <=> ps_SC_m 

ps_SC_r
PSERDer_RT h_r + 0.01 ps_SC_r --> co2_r + 0.01 pe_SC_r 14554
PSERSer_RT 0.01 cdpdag_SC_r + ser__L_r --> cmp_r + h_r + 0.01 ps_SC_r 11583
PSterm_RT ps_SC_r <=> ps_SC_m 

ps_SC_v
PSERDv_RT h_v + 0.01 ps_SC_v --> co2_v + 0.01 pe_SC_v 10504 or 11446

ptd135bp_SC_n
PI3P5Kn_RT atp_n + 0.01 ptd3ino_SC_n --> adp_n + h_n + 0.01 ptd135bp_SC_n 14139

ptd135bp_SC_r
PI35BP5Per_RT h2o_r + 0.01 ptd135bp_SC_r --> pi_r + 0.01 ptd3ino_SC_r 11381 or 15340 or 16619

ptd145bp_SC_n
PI45BP5Pn_RT h2o_n + 0.01 ptd145bp_SC_n --> pi_n + 0.01 ptd4ino_SC_n 13609 or 15545
PI45BPPn_RT h2o_n + 0.01 ptd145bp_SC_n --> 0.01 12dgr_SC_n + h_n + mi145p_n 12855
PI4P5Kn_RT atp_n + 0.01 ptd4ino_SC_n --> adp_n + h_n + 0.01 ptd145bp_SC_n 11878

ptd145bp_SC_r
PI45BP5Per_RT h2o_r + 0.01 ptd145bp_SC_r --> pi_r + 0.01 ptd4ino_SC_r 15340

ptd1ino_SC_e
PLBP1Ie_RT h2o_e + 0.005 ptd1ino_SC_e --> 0.02 dca_e + 0.06 ddca_e + 0.5 g3pi_e + h_e + 0.27 hdca_e + 0.17 hdcea_e + 0.05 ocdca_e + 0.24 ocdcea_e + 0.09 ocdcya_e + 0.1 ttdca_e 12385

ptd1ino_SC_g
IPCS124g_RT cer1_24_g + 0.01 ptd1ino_SC_g --> 0.01 12dgr_SC_g + 0.01 ipc124_SC_g 12927 and 8747
IPCS126g_RT cer1_26_g + 0.01 ptd1ino_SC_g --> 0.01 12dgr_SC_g + 0.01 ipc126_SC_g 12927 and 8747
IPCS224g_RT cer2_24_g + 0.01 ptd1ino_SC_g --> 0.01 12dgr_SC_g + 0.01 ipc224_SC_g 12927 and 8747
IPCS226g_RT cer2_26_g + 0.01 ptd1ino_SC_g --> 0.01 12dgr_SC_g + 0.01 ipc226_SC_g 12927 and 8747
IPCS324g_RT cer3_24_g + 0.01 ptd1ino_SC_g --> 0.01 12dgr_SC_g + 0.01 ipc324_SC_g 12927 and 8747
IPCS326g_RT cer3_26_g + 0.01 ptd1ino_SC_g --> 0.01 12dgr_SC_g + 0.01 ipc326_SC_g 12927 and 8747
MIP2CS124g_RT 0.01 mipc124_SC_g + 0.01 ptd1ino_SC_g --> 0.01 12dgr_SC_g + 0.01 mip2c124_SC_g 13172
MIP2CS126g_RT 0.01 mipc126_SC_g + 0.01 ptd1ino_SC_g --> 0.01 12dgr_SC_g + 0.01 mip2c126_SC_g 13172
MIP2CS224g_RT 0.01 mipc224_SC_g + 0.01 ptd1ino_SC_g --> 0.01 12dgr_SC_g + 0.01 mip2c224_SC_g 13172
MIP2CS226g_RT 0.01 mipc226_SC_g + 0.01 ptd1ino_SC_g --> 0.01 12dgr_SC_g + 0.01 mip2c226_SC_g 13172
MIP2CS324g_RT 0.01 mipc324_SC_g + 0.01 ptd1ino_SC_g --> 0.01 12dgr_SC_g + 0.01 mip2c324_SC_g 13172
MIP2CS326g_RT 0.01 mipc326_SC_g + 0.01 ptd1ino_SC_g --> 0.01 12dgr_SC_g + 0.01 mip2c326_SC_g 13172

ptd1ino_SC_n
PIN3Kn_RT atp_n + 0.01 ptd1ino_SC_n --> adp_n + h_n + 0.01 ptd3ino_SC_n 8712
PIN3Pn_RT h2o_n + 0.01 ptd3ino_SC_n --> pi_n + 0.01 ptd1ino_SC_n 14880
PIN4Kn_RT atp_n + 0.01 ptd1ino_SC_n --> adp_n + h_n + 0.01 ptd4ino_SC_n 15086 or 9782

ptd1ino_SC_r
PIN3Per_RT h2o_r + 0.01 ptd3ino_SC_r --> pi_r + 0.01 ptd1ino_SC_r 11381
PIN4Ker_RT atp_r + 0.01 ptd1ino_SC_r --> adp_r + h_r + 0.01 ptd4ino_SC_r 15159
PIN4Per_RT h2o_r + 0.01 ptd4ino_SC_r --> pi_r + 0.01 ptd1ino_SC_r 11381
PINOSer_RT 0.01 cdpdag_SC_r + inost_r --> cmp_r + h_r + 0.01 ptd1ino_SC_r 15121

ptd2meeta_SC_r
MFAPSer_RT amet_r + 0.01 ptdmeeta_SC_r --> ahcys_r + h_r + 0.01 ptd2meeta_SC_r 12748
PMETMer_RT amet_r + 0.01 ptd2meeta_SC_r --> ahcys_r + h_r + 0.01 pc_SC_r 12748

ptd3ino_SC_n
PI3P5Kn_RT atp_n + 0.01 ptd3ino_SC_n --> adp_n + h_n + 0.01 ptd135bp_SC_n 14139
PIN3Kn_RT atp_n + 0.01 ptd1ino_SC_n --> adp_n + h_n + 0.01 ptd3ino_SC_n 8712
PIN3Pn_RT h2o_n + 0.01 ptd3ino_SC_n --> pi_n + 0.01 ptd1ino_SC_n 14880

ptd3ino_SC_r
PI35BP5Per_RT h2o_r + 0.01 ptd135bp_SC_r --> pi_r + 0.01 ptd3ino_SC_r 11381 or 15340 or 16619
PIN3Per_RT h2o_r + 0.01 ptd3ino_SC_r --> pi_r + 0.01 ptd1ino_SC_r 11381

ptd4ino_SC_n
PI45BP5Pn_RT h2o_n + 0.01 ptd145bp_SC_n --> pi_n + 0.01 ptd4ino_SC_n 13609 or 15545
PI4P5Kn_RT atp_n + 0.01 ptd4ino_SC_n --> adp_n + h_n + 0.01 ptd145bp_SC_n 11878
PIN4Kn_RT atp_n + 0.01 ptd1ino_SC_n --> adp_n + h_n + 0.01 ptd4ino_SC_n 15086 or 9782

ptd4ino_SC_r
PI45BP5Per_RT h2o_r + 0.01 ptd145bp_SC_r --> pi_r + 0.01 ptd4ino_SC_r 15340
PIN4Ker_RT atp_r + 0.01 ptd1ino_SC_r --> adp_r + h_r + 0.01 ptd4ino_SC_r 15159
PIN4Per_RT h2o_r + 0.01 ptd4ino_SC_r --> pi_r + 0.01 ptd1ino_SC_r 11381

ptdmeeta_SC_r
MFAPSer_RT amet_r + 0.01 ptdmeeta_SC_r --> ahcys_r + h_r + 0.01 ptd2meeta_SC_r 12748
PETOHMer_RT amet_r + 0.01 pe_SC_r --> ahcys_r + h_r + 0.01 ptdmeeta_SC_r 10647

triglyc_SC_r
PCDAGATer_RT 12dgr_SC_r + pc_SC_r --> 1agpc_SC_r + triglyc_SC_r 16477
TAGtrd triglyc_SC_r --> triglyc_RT_d 
TRIGSer_RT 0.01 12dgr_SC_r + 0.02 dcacoa_r + 0.06 ddcacoa_r + 0.17 hdcoa_r + 0.09 ocdycacoa_r + 0.24 odecoa_r + 0.27 pmtcoa_r + 0.05 stcoa_r + 0.1 tdcoa_r --> coa_r + 0.01 triglyc_SC_r 16460

zymstest_SC_r
ZYMSTATer_RT 0.655 hdcoa_r + 0.01 hexccoa_r + 0.27 odecoa_r + 0.02 pmtcoa_r + 0.03 stcoa_r + 0.015 tdcoa_r + 0.01 zymst_r --> coa_r + 0.01 zymstest_SC_r 11799
ZYMSTESTtrd zymstest_SC_r --> zymstest_RT_d
```

In [31]:

```
for m in sorted(model.metabolites, key=lambda x: x.id):
    if '_SC' in m.id:
        m.id = m.id.replace('_SC','_RT')
```

### Reactions¶

In [32]:

```
for r in sorted(model.reactions, key=lambda x: x.id):
    if '_SC' in r.id:
        print(r)
```

```
CLPNSm_SC: 0.01 cdpdag_RT_m + 0.01 pg_RT_m --> 0.01 clpn_RT_m + cmp_m + h_m
PGPPAm_SC: h2o_m + 0.01 pgp_RT_m --> 0.01 pg_RT_m + pi_m
PSERDm_SC: h_m + 0.01 ps_RT_m --> co2_m + 0.01 pe_RT_m
```

In [33]:

```
for r in sorted(model.reactions, key=lambda x: x.id):
    if '_SC' in r.id:
        r.id = r.id.replace('_SC','_RT')
```

In [34]:

```
for r in sorted(model.reactions, key=lambda x: x.id):
    if 'yli' in r.id:
        print(r.id, r.reaction, r.gene_reaction_rule)
        for g in r.genes:
            for r2 in g.reactions:
                if r2 is not r:
                    print(' ',r2.id, r2.reaction, r2.gene_reaction_rule)
        print()
```

```
yli_R0031 uacgam_c --> chtn_c + h_c + udp_c 14541 or 14960 or 15828 or 8441 or 8512 or 8908 or 9711 or 9756
  CHTNS udpacgal_c --> chitin_c + h_c + udp_c 14541 or 14960 or 15828 or 8441 or 9711 or 9756
  CHTNS udpacgal_c --> chitin_c + h_c + udp_c 14541 or 14960 or 15828 or 8441 or 9711 or 9756
  CHTNS udpacgal_c --> chitin_c + h_c + udp_c 14541 or 14960 or 15828 or 8441 or 9711 or 9756
  CHTNS udpacgal_c --> chitin_c + h_c + udp_c 14541 or 14960 or 15828 or 8441 or 9711 or 9756
  CHTNS udpacgal_c --> chitin_c + h_c + udp_c 14541 or 14960 or 15828 or 8441 or 9711 or 9756
  CHTNS udpacgal_c --> chitin_c + h_c + udp_c 14541 or 14960 or 15828 or 8441 or 9711 or 9756

yli_R0248 4abz_m + 6hmhpt_m --> dhpt_m + h2o_m 14377
  HPPKm 2ahhmp_m + atp_m --> 2ahhmd_m + amp_m + h_m 14377
  DHPSm 2ahhmp_m + 4abz_m --> dhpt_m + h2o_m 14377
  FOLD3m 2ahhmd_m + 4abz_m --> dhpt_m + ppi_m 14377
  yli_R0257 dhnpt_m --> 6hmhpt_m + gcald_m 14377
  FOLD3_1 2ahhmd_c + 4abz_c --> dhpt_c + h_c + ppi_c 14377
  DHPS 2ahhmp_c + 4abz_c --> dhpt_c + h2o_c 14377
  DHPS2 4abz_c + 6hmhptpp_c --> dhpt_c + ppi_c 14377
  yli_R0251 6hmhpt_m + atp_m --> 2ahhmd_m + amp_m + h_m 14377
  DHNPAm dhnpt_m --> 2ahhmp_m + gcald_m + h_m 14377
  FOLD3 2ahhmd_c + 4abz_c --> dhpt_c + ppi_c 14377

yli_R0251 6hmhpt_m + atp_m --> 2ahhmd_m + amp_m + h_m 14377
  yli_R0248 4abz_m + 6hmhpt_m --> dhpt_m + h2o_m 14377
  HPPKm 2ahhmp_m + atp_m --> 2ahhmd_m + amp_m + h_m 14377
  DHPSm 2ahhmp_m + 4abz_m --> dhpt_m + h2o_m 14377
  FOLD3m 2ahhmd_m + 4abz_m --> dhpt_m + ppi_m 14377
  yli_R0257 dhnpt_m --> 6hmhpt_m + gcald_m 14377
  FOLD3_1 2ahhmd_c + 4abz_c --> dhpt_c + h_c + ppi_c 14377
  DHPS 2ahhmp_c + 4abz_c --> dhpt_c + h2o_c 14377
  DHPS2 4abz_c + 6hmhptpp_c --> dhpt_c + ppi_c 14377
  DHNPAm dhnpt_m --> 2ahhmp_m + gcald_m + h_m 14377
  FOLD3 2ahhmd_c + 4abz_c --> dhpt_c + ppi_c 14377

yli_R0257 dhnpt_m --> 6hmhpt_m + gcald_m 14377
  yli_R0248 4abz_m + 6hmhpt_m --> dhpt_m + h2o_m 14377
  HPPKm 2ahhmp_m + atp_m --> 2ahhmd_m + amp_m + h_m 14377
  DHPSm 2ahhmp_m + 4abz_m --> dhpt_m + h2o_m 14377
  FOLD3m 2ahhmd_m + 4abz_m --> dhpt_m + ppi_m 14377
  FOLD3_1 2ahhmd_c + 4abz_c --> dhpt_c + h_c + ppi_c 14377
  DHPS 2ahhmp_c + 4abz_c --> dhpt_c + h2o_c 14377
  DHPS2 4abz_c + 6hmhptpp_c --> dhpt_c + ppi_c 14377
  yli_R0251 6hmhpt_m + atp_m --> 2ahhmd_m + amp_m + h_m 14377
  DHNPAm dhnpt_m --> 2ahhmp_m + gcald_m + h_m 14377
  FOLD3 2ahhmd_c + 4abz_c --> dhpt_c + ppi_c 14377

yli_R0415 hicit_m + nad_m <=> 2oxoadp_m + co2_m + nadh_m 10428
  MALDDH mal__D_c + nad_c --> co2_c + nadh_c + pyr_c 10428
  HICITDm hicit_m + nad_m <=> h_m + nadh_m + oxag_m 10428
  IPMD 3c2hmp_c + nad_c --> 3c4mop_c + h_c + nadh_c 10428 or 13894

yli_R0459 gar_c + h2o_c + methf_c --> fgam_c + 2.0 h_c + thf_c 13595
  GARFT 10fthf_c + gar_c <=> fgam_c + h_c + thf_c 13595 or 14259
  FPGFTm 10fthf_m + gar_m <=> fgam_m + h_m + thf_m 13595
  FGFTm fgam_m + 3.0 h_m + thf_m --> gar_m + h2o_m + methf_m 13595
  yli_R1541 gar_c + h2o_c + methf_c --> fgam_c + thf_c 13595

yli_R0495 h2o_c + o2_c + urate_c --> 5hiu_c + h2o2_c 10570
  URO h2o_m + o2_m + urate_m --> 5hiu_m + h2o2_m + h_m 10570

yli_R0586 3.0 o2_m + pppg9_m --> 3.0 h2o2_m + ppp9_m 11867
  PPPGOm 3.0 o2_m + 2.0 pppg9_m --> 6.0 h2o_m + 2.0 ppp9_m 11867

yli_R0592 gly_m + h_c + succoa_m --> 5aop_m + co2_m + coa_m 8677
  GLYATm accoa_m + gly_m <=> 2aobut_m + coa_m 8677
  GLYAT accoa_c + gly_c <=> 2aobut_c + coa_c 8677
  ALASm gly_m + h_m + succoa_m --> 5aop_m + co2_m + coa_m 8677

yli_R0726 h2o_c + udpg_c --> amylose_c + h_c + udp_c 9596
  GGNG Tyr_ggn_c + 8.0 udpg_c --> ggn_c + 8.0 h_c + 8.0 udp_c 14002 and 9596
  GLGNS1 ggn_c + 3.0 udpg_c --> glygn1_c + 3.0 h_c + 3.0 udp_c 14002 and 9596
  GLCS2 udpg_c --> glycogen_c + h_c + udp_c 14002 and 9596
  GLYGS h2o_c + udpg_c --> 14glun_c + h_c + udp_c 14002 and 9596

yli_R0731 amylose_c --> h2o_c + starch_c 12594
  GBEZ 14glun_c --> glycogen_c + h2o_c 12594
  GLBRAN glygn1_c --> glygn2_c 12594
  GLBRAN2 glycogen_c --> bglycogen_c 12594

yli_R0732 pi_c + starch_c --> g1p_c 14256
  GLPASE2 glygn3_c + 7.0 h2o_c --> Tyr_ggn_c + 7.0 glc__D_c 14256
  MLTP2 malthx_c + pi_c <=> g1p_c + maltpt_c 14256
  MLTP3 malthp_c + pi_c <=> g1p_c + malthx_c 14256
  MLTP1 maltpt_c + pi_c <=> g1p_c + maltttr_c 14256
  GLPASE1 glygn2_c + 3.0 pi_c --> dxtrn_c + 3.0 g1p_c 14256
  GLCP2 bglycogen_c + pi_c --> g1p_c 14256
  GLCP glycogen_c + pi_c --> g1p_c 14256

yli_R1092 akg_m <=> akg_c 10635
  OXO2Ctm akg_m + oxag_c <=> akg_c + oxag_m 10635
  2OXOADPTm 2oxoadp_c + akg_m <=> 2oxoadp_m + akg_c 10635
  2AMADPTm L2aadp_c + akg_m <=> L2aadp_m + akg_c 10635

yli_R1386 atp_m + dhpt_m + glu__L_m --> adp_m + dhf_m + h_m + pi_m 10460 or 14803
  FPGS8m 10fthf5glu_m + atp_m + glu__L_m --> 10fthf6glu_m + adp_m + h_m + pi_m 14803
  FPGS3m 6thf_m + atp_m + glu__L_m --> 7thf_m + adp_m + h_m + pi_m 14803
  FPGS5m 5dhf_m + atp_m + glu__L_m --> 6dhf_m + adp_m + h_m + pi_m 14803
  10FTHFGLULLm 10fthf_m + atp_m + glu__L_m --> 10fthfglu__L_m + adp_m + pi_m 14803
  FPGS4 4.0 atp_c + dhf_c + 4.0 glu__L_c --> 5dhf_c + 4.0 adp_c + 4.0 h_c + 4.0 pi_c 14803
  FPGS2 5thf_c + atp_c + glu__L_c --> 6thf_c + adp_c + h_c + pi_c 14803
  FPGS3 6thf_c + atp_c + glu__L_c --> 7thf_c + adp_c + h_c + pi_c 14803
  FPGS2m 5thf_m + atp_m + glu__L_m --> 6thf_m + adp_m + h_m + pi_m 14803
  FPGS5 5dhf_c + atp_c + glu__L_c --> 6dhf_c + adp_c + h_c + pi_c 14803
  FPGS6 6dhf_c + atp_c + glu__L_c --> 7dhf_c + adp_c + h_c + pi_c 14803
  FPGS7 10fthf_c + 4.0 atp_c + 4.0 glu__L_c --> 10fthf5glu_c + 4.0 adp_c + 4.0 h_c + 4.0 pi_c 14803
  FPGS4m 4.0 atp_m + dhf_m + 4.0 glu__L_m --> 5dhf_m + 4.0 adp_m + 4.0 h_m + 4.0 pi_m 14803
  FPGS9m 10fthf6glu_m + atp_m + glu__L_m --> 10fthf7glu_m + adp_m + h_m + pi_m 14803
  THFGLUS atp_c + glu__L_c + thf_c <=> adp_c + h_c + pi_c + thfglu_c 10460 or 14803
  DHFS atp_c + dhpt_c + glu__L_c --> adp_c + dhf_c + h_c + pi_c 10460 or 14803
  FPGS8 10fthf5glu_c + atp_c + glu__L_c --> 10fthf6glu_c + adp_c + h_c + pi_c 14803
  FPGS7m 10fthf_m + 4.0 atp_m + 4.0 glu__L_m --> 10fthf5glu_m + 4.0 adp_m + 4.0 h_m + 4.0 pi_m 14803
  FPGS 4.0 atp_c + 4.0 glu__L_c + thf_c --> 5thf_c + 4.0 adp_c + 4.0 h_c + 4.0 pi_c 14803
  FPGS9 10fthf6glu_c + atp_c + glu__L_c --> 10fthf7glu_c + adp_c + h_c + pi_c 14803
  FPGS6m 6dhf_m + atp_m + glu__L_m --> 7dhf_m + adp_m + h_m + pi_m 14803
  FPGSm 4.0 atp_m + 4.0 glu__L_m + thf_m --> 5thf_m + 4.0 adp_m + 4.0 h_m + 4.0 pi_m 14803
  THFGLUS atp_c + glu__L_c + thf_c <=> adp_c + h_c + pi_c + thfglu_c 10460 or 14803
  DHFS atp_c + dhpt_c + glu__L_c --> adp_c + dhf_c + h_c + pi_c 10460 or 14803

yli_R1390 atp_r + glyc_r --> adp_r + glyc3p_r + h_r 11022
  GLYK atp_c + glyc_c --> adp_c + glyc3p_c + h_c 11022
  GLYKm atp_m + glyc_m --> adp_m + glyc3p_m + h_m 11022

yli_R1399 atp_r + chol_r --> adp_r + cholp_r + h_r 15982
  CHOLK atp_c + chol_c --> adp_c + cholp_c + h_c 15982
  ETHAK atp_c + etha_c --> adp_c + ethamp_c + h_c 15982

yli_R1400 g3pc_r + h2o_r --> chol_r + glyc3p_r + h_r 11723
  GPDDA1 g3pc_c + h2o_c --> chol_c + glyc3p_c + h_c 11723
  GPDDA5 g3pi_c + h2o_c --> glyc3p_c + h_c + inost_c 11723
  GPDDA4 g3pg_c + h2o_c --> glyc3p_c + glyc_c + h_c 11723
  GPDDA2 g3pe_c + h2o_c --> etha_c + glyc3p_c + h_c 11723
  GPDDA3 g3ps_c + h2o_c --> glyc3p_c + h_c + ser__L_c 11723

yli_R1404 cholp_r + ctp_r + h_r --> cdpchol_r + ppi_r 10540
  CPCTDTX ctp_c + h_c + ntm2amep_c --> cmpntm2amep_c + ppi_c 10540
  CHLPCTD cholp_c + ctp_c + h_c --> cdpchol_c + ppi_c 10540

yli_R1421 h2o_m + nad_m + saccrp__L_m --> akg_m + h_m + lys__L_m + nadh_m 14087
  SACCD2 h2o_c + nad_c + saccrp__L_c <=> akg_c + h_c + lys__L_c + nadh_c 14087

yli_R1467 gtp_c + h2o_c --> HC01651_c 10332
  GTPCIn gtp_n + h2o_n --> ahdt_n + for_n + h_n 10332
  yli_R1470 HC01710_c --> ahdt_c + h2o_c 10332
  HPPK_1 2ahhmp_c + atp_c --> 2ahhmd_c + amp_c 10332
  yli_R1468 HC01651_c + h2o_c --> HC01652_c + for_c 10332
  GTPCI gtp_c + h2o_c --> ahdt_c + for_c + h_c 10332
  yli_R1469 HC01652_c --> HC01710_c 10332

yli_R1468 HC01651_c + h2o_c --> HC01652_c + for_c 10332
  GTPCIn gtp_n + h2o_n --> ahdt_n + for_n + h_n 10332
  yli_R1470 HC01710_c --> ahdt_c + h2o_c 10332
  HPPK_1 2ahhmp_c + atp_c --> 2ahhmd_c + amp_c 10332
  yli_R1467 gtp_c + h2o_c --> HC01651_c 10332
  GTPCI gtp_c + h2o_c --> ahdt_c + for_c + h_c 10332
  yli_R1469 HC01652_c --> HC01710_c 10332

yli_R1469 HC01652_c --> HC01710_c 10332
  GTPCIn gtp_n + h2o_n --> ahdt_n + for_n + h_n 10332
  yli_R1470 HC01710_c --> ahdt_c + h2o_c 10332
  HPPK_1 2ahhmp_c + atp_c --> 2ahhmd_c + amp_c 10332
  yli_R1468 HC01651_c + h2o_c --> HC01652_c + for_c 10332
  yli_R1467 gtp_c + h2o_c --> HC01651_c 10332
  GTPCI gtp_c + h2o_c --> ahdt_c + for_c + h_c 10332

yli_R1470 HC01710_c --> ahdt_c + h2o_c 10332
  GTPCIn gtp_n + h2o_n --> ahdt_n + for_n + h_n 10332
  HPPK_1 2ahhmp_c + atp_c --> 2ahhmd_c + amp_c 10332
  yli_R1468 HC01651_c + h2o_c --> HC01652_c + for_c 10332
  yli_R1467 gtp_c + h2o_c --> HC01651_c 10332
  GTPCI gtp_c + h2o_c --> ahdt_c + for_c + h_c 10332
  yli_R1469 HC01652_c --> HC01710_c 10332

yli_R1492 f26bp_c --> 2amac_c + h2o_c 9216
  SERD_L ser__L_c --> nh4_c + pyr_c 11849 or 11909 or 9216
  THRD_L thr__L_c --> 2obut_c + nh4_c 11909 or 9216
  SERHL ser__L_c --> 2amac_c + h2o_c 9216

yli_R1507 atp_c + glyc__R_c --> 3pg_c + adp_c 10038
  GLYCK atp_c + glyc__R_c --> 3pg_c + adp_c + h_c 10038

yli_R1535 naglc2p_r + uacgam_r --> chito2pdol_r + udp_r 14170
  GLCNACT doldpglcnac_c + uacgam_c --> doldpglcnacglcnac_c + h_c + udp_c 13348 or 14170

yli_R1536 h2o_c + rnam_c --> ncam_c + rib__D_c 12384
  yli_R1537 h2o_c + nicrns_c --> nac_c + rib__D_c 12384
  URIH h2o_c + uri_c --> rib__D_c + ura_c 12384
  INSH h2o_c + ins_c --> hxan_c + rib__D_c 12384
  ADNUC adn_c + h2o_c --> ade_c + rib__D_c 12384
  CYTDH cytd_c + h2o_c --> csn_c + rib__D_c 12384
  GNNUC gsn_c + h2o_c --> gua_c + rib__D_c 12384
  XTSNH h2o_c + xtsn_c --> rib__D_c + xan_c 12384

yli_R1537 h2o_c + nicrns_c --> nac_c + rib__D_c 12384
  URIH h2o_c + uri_c --> rib__D_c + ura_c 12384
  INSH h2o_c + ins_c --> hxan_c + rib__D_c 12384
  yli_R1536 h2o_c + rnam_c --> ncam_c + rib__D_c 12384
  ADNUC adn_c + h2o_c --> ade_c + rib__D_c 12384
  CYTDH cytd_c + h2o_c --> csn_c + rib__D_c 12384
  GNNUC gsn_c + h2o_c --> gua_c + rib__D_c 12384
  XTSNH h2o_c + xtsn_c --> rib__D_c + xan_c 12384

yli_R1540 atp_c + nad_c --> adp_c + nadp_c 10182
  NADK atp_c + nad_c --> adp_c + h_c + nadp_c 10182
  NADKm atp_m + nad_m --> adp_m + h_m + nadp_m 10182

yli_R1541 gar_c + h2o_c + methf_c --> fgam_c + thf_c 13595
  GARFT 10fthf_c + gar_c <=> fgam_c + h_c + thf_c 13595 or 14259
  FPGFTm 10fthf_m + gar_m <=> fgam_m + h_m + thf_m 13595
  FGFTm fgam_m + 3.0 h_m + thf_m --> gar_m + h2o_m + methf_m 13595
  yli_R0459 gar_c + h2o_c + methf_c --> fgam_c + 2.0 h_c + thf_c 13595

yli_R1545 abt__D_c + nad_c <=> h_c + nadh_c + rbl__D_c 8988 or 9990

yli_R1547 3hpcoa_c + h2o_c <=> 3hpp_c + coa_c 15218
  3HPCOAHYD 3hpcoa_c + h2o_c --> 3hpp_c + coa_c + h_c 15218
  3HBCOAHLm 3hibutcoa_m + h2o_m --> 3hmp_m + coa_m + h_m 15218

yli_R1550 gtp_c + h2o_c --> gmp_c + ppi_c 13900
  NTPP9 h2o_c + itp_c --> h_c + imp_c + ppi_c 13900
  DUTPDP dutp_c + h2o_c --> dump_c + h_c + ppi_c 13900 or 8399
  NTPP2 gtp_c + h2o_c --> gmp_c + h_c + ppi_c 13900
  NTPP1 dgtp_c + h2o_c --> dgmp_c + h_c + ppi_c 13900
  yli_R1552 h2o_c + itp_c --> imp_c + ppi_c 13900
  NTPP8 h2o_c + utp_c --> h_c + ppi_c + ump_c 13900 or 8601
  NTPP11 h2o_c + xtp_c --> h_c + ppi_c + xmp_c 13900
  NTPP10 ditp_c + h2o_c --> dimp_c + h_c + ppi_c 13900
  yli_R1551 dgtp_c + h2o_c --> dgmp_c + ppi_c 13900

yli_R1551 dgtp_c + h2o_c --> dgmp_c + ppi_c 13900
  NTPP9 h2o_c + itp_c --> h_c + imp_c + ppi_c 13900
  DUTPDP dutp_c + h2o_c --> dump_c + h_c + ppi_c 13900 or 8399
  NTPP2 gtp_c + h2o_c --> gmp_c + h_c + ppi_c 13900
  NTPP1 dgtp_c + h2o_c --> dgmp_c + h_c + ppi_c 13900
  yli_R1552 h2o_c + itp_c --> imp_c + ppi_c 13900
  NTPP8 h2o_c + utp_c --> h_c + ppi_c + ump_c 13900 or 8601
  NTPP11 h2o_c + xtp_c --> h_c + ppi_c + xmp_c 13900
  NTPP10 ditp_c + h2o_c --> dimp_c + h_c + ppi_c 13900
  yli_R1550 gtp_c + h2o_c --> gmp_c + ppi_c 13900

yli_R1552 h2o_c + itp_c --> imp_c + ppi_c 13900
  NTPP9 h2o_c + itp_c --> h_c + imp_c + ppi_c 13900
  DUTPDP dutp_c + h2o_c --> dump_c + h_c + ppi_c 13900 or 8399
  NTPP2 gtp_c + h2o_c --> gmp_c + h_c + ppi_c 13900
  NTPP1 dgtp_c + h2o_c --> dgmp_c + h_c + ppi_c 13900
  NTPP8 h2o_c + utp_c --> h_c + ppi_c + ump_c 13900 or 8601
  NTPP11 h2o_c + xtp_c --> h_c + ppi_c + xmp_c 13900
  NTPP10 ditp_c + h2o_c --> dimp_c + h_c + ppi_c 13900
  yli_R1551 dgtp_c + h2o_c --> dgmp_c + ppi_c 13900
  yli_R1550 gtp_c + h2o_c --> gmp_c + ppi_c 13900

yli_R1562 nh4_c + thym_c <=> 5mcsn_c + h2o_c 14559
  CSND csn_c + h2o_c + h_c --> nh4_c + ura_c 14031 or 14559

yli_R1572 udpglcur_c --> co2_c + udpxyl_c 15418
  ULA4NFT 10fthf_c + udpLa4n_c --> h_c + thf_c + udpLa4fn_c 15418
  UDPGLDC h_c + udpglcur_c --> co2_c + udpxyl_c 15418
  UDPGDC nad_c + udpglcur_c --> co2_c + nadh_c + udpLa4o_c 15418
  UDPGLDCg h_g + udpglcur_g --> co2_g + udpxyl_g 15418

yli_R1578 Lkynr_c + h2o_c <=> ala__L_c + anth_c 8725
  KYN Lkynr_c + h2o_c --> ala__L_c + anth_c + h_c 8725
  HKYNH h2o_c + hLkynr_c --> 3hanthrn_c + ala__L_c 8725
  LFORKYNHYD Lfmkynr_c + h2o_c --> ala__L_c + h_c + nformanth_c 8725

yli_R1589 alac__S_m <=> 3hmoa_m 9176
  KARA2 2ahbut_c + h_c + nadph_c <=> 23dhmp_c + nadp_c 9176
  KARA1 23dhmb_c + nadp_c <=> alac__S_c + h_c + nadph_c 9176
  DPR 2dhp_c + h_c + nadph_c --> nadp_c + pant__R_c 14277 or 16522 or 9176
  KARA1im alac__S_m + h_m + nadph_m --> 23dhmb_m + nadp_m 9176
  KARA2im 2ahbut_m + h_m + nadph_m --> 23dhmp_m + nadp_m 9176
  DPRm 2dhp_m + h_m + nadph_m --> nadp_m + pant__R_m 14277 or 9176
```

In [35]:

```
for r in sorted(model.reactions, key=lambda x: x.id):
    if 'yli' in r.id:
        count = 0
        for g in r.genes:
            for r2 in g.reactions:
                if r2 is not r and 'yli' not in r2.id:
                    count = count + 1
            if not count and g.id in Annotation.index:
                print(r.id, r.reaction, r.gene_reaction_rule)
                display(Annotation.loc[[g.id]])
```

```
yli_R1545 abt__D_c + nad_c <=> h_c + nadh_c + rbl__D_c 8988 or 9990
```

|  | Combined Annotations | Signal P | Sc288c Orthologs | Human Orthologs | Sc288 Best Hit | Human Blast | Essential | WolfPSort | C Terminal |
| --- | --- | --- | --- | --- | --- | --- | --- | --- | --- |
| RTO4\_ID |  |  |  |  |  |  |  |  |  |
| 8988 | K17742: SOU1; sorbose reductase |  |  |  |  | DHRS4 | Not Essential | cyto 17, cyto\_nucl 10, mito 8 | TLS\* |

```
yli_R1545 abt__D_c + nad_c <=> h_c + nadh_c + rbl__D_c 8988 or 9990
```

|  | Combined Annotations | Signal P | Sc288c Orthologs | Human Orthologs | Sc288 Best Hit | Human Blast | Essential | WolfPSort | C Terminal |
| --- | --- | --- | --- | --- | --- | --- | --- | --- | --- |
| RTO4\_ID |  |  |  |  |  |  |  |  |  |
| 9990 | K17738: ARD; D-arabinitol 2-dehydrogenase | S |  |  | SPS19 | CBR4 | Not Essential | mito 21, cyto 5 | TLT\* |

In [36]:

```
model.reactions.get_by_id('yli_R1545').id = 'DABT2D'
```

In [37]:

```
model.remove_reactions([r for r in model.reactions if 'yli' in r.id], remove_orphans=True)
```

In [38]:

```
for r in model.reactions:
    if r.compartments == {'e'} and len(r.metabolites) > 1:
        print(r, r.gene_reaction_rule)
```

```
PLBPCe_RT: h2o_e + 0.005 pc_RT_e --> 0.02 dca_e + 0.06 ddca_e + 0.5 g3pc_e + h_e + 0.27 hdca_e + 0.17 hdcea_e + 0.05 ocdca_e + 0.24 ocdcea_e + 0.09 ocdcya_e + 0.1 ttdca_e 12385
ACP1e: fmn_e + h2o_e --> pi_e + ribflv_e 10885 or 13856 or 13935
SUCRe: h2o_e + sucr_e --> fru_e + glc__D_e 14826
PLBP1Ie_RT: h2o_e + 0.005 ptd1ino_RT_e --> 0.02 dca_e + 0.06 ddca_e + 0.5 g3pi_e + h_e + 0.27 hdca_e + 0.17 hdcea_e + 0.05 ocdca_e + 0.24 ocdcea_e + 0.09 ocdcya_e + 0.1 ttdca_e 12385
EPGALURSe: h2o_e + pectin_e --> galur_e 10484
ASNNe: asn__L_e + h2o_e --> asp__L_e + nh4_e 10043 or 13627
13BGHe: 13BDglcn_e + h2o_e --> glc__D_e 13553
THMPe: h2o_e + thmmp_e --> pi_e + thm_e 10885
THMDPe: 2.0 h2o_e + thmpp_e --> h_e + 2.0 pi_e + thm_e 10885
ACHEe: ach_e + h2o_e --> ac_e + chol_e + h_e 9571
AMY2e: glygn2_e + 8.0 h2o_e --> 8.0 glc__D_e + glygn4_e 11625 and 9135
DADAe: dad_2_e + h2o_e + h_e --> din_e + nh4_e 16732
CHTNASEe: chtn_e + 2.0 h2o_e --> 3.0 acgam_e 13082
ADAe: adn_e + h2o_e + h_e --> ins_e + nh4_e 16732
AMY1e: 8.0 h2o_e + strch1_e --> 8.0 glc__D_e + strch2_e 11625 and 9135
PLBPEe_RT: h2o_e + 0.005 pe_RT_e --> 0.02 dca_e + 0.06 ddca_e + 0.5 g3pe_e + h_e + 0.27 hdca_e + 0.17 hdcea_e + 0.05 ocdca_e + 0.24 ocdcea_e + 0.09 ocdcya_e + 0.1 ttdca_e 12385
```

In [39]:

```
print(len(model.genes))
print(len(model.reactions))
print(len(model.metabolites))
model
```

```
1143
1959
2046
```

Out[39]:

|  |  |
| --- | --- |
| **Name** | R. toruloides |
| **Memory address** | 0x010296144e0 |
| **Number of metabolites** | 2046 |
| **Number of reactions** | 1959 |
| **Number of groups** | 0 |
| **Objective expression** | 0 |
| **Compartments** | c, x, m, e, r, v, n, g, d |

In [40]:

```
for x in sorted(model.genes, key=lambda x: x.id):
    if not x.reactions:
        print(x)
print()
for x in sorted(model.metabolites, key=lambda x: x.id):
    if not x.reactions:
        print(x)
```

```
10147
10499
10664
10942
11207
11436
11445
11490
11701
11755
11826
11850
12283
12437
12718
12838
12953
12961
12988
13065
13739
14092
14867
14868
15067
15072
15144
15153
15227
15692
16789
8605
8931
9386
9822
9887

4abzglu_c
4hpro_LT_r
Asn_X_Ser_Thr_r
Rtotal2_c
akg_r
cechddd_c
cenchddd_c
cinnm_c
coa_g
cpmp_c
doldp_U_r
doldp__L_r
dolglcp_r
dolmanp_U_c
dolmanp__L_c
gdp_r
gdpmann_r
glutrna_gln_c
gpi_sig_r
l2xser_g
mannan_c
moadcosh_c
mpt_c
pppn_c
pre_prot_r
pro__L_r
succ_r
udpg_r
ump_r
```

In [41]:

```
cobra.manipulation.remove_genes(model, [x for x in model.genes if not x.reactions])
model.remove_metabolites([x for x in model.metabolites if not x.reactions])
```

In [42]:

```
for x in sorted(model.genes, key=lambda x: x.id):
    if not x.reactions:
        print(x)
print()
for x in sorted(model.metabolites, key=lambda x: x.id):
    if not x.reactions:
        print(x)
```

```

```

In [43]:

```
print(len(model.genes))
print(len(model.reactions))
print(len(model.metabolites))
print(len(set([m.id.rsplit('_',1)[0] for m in model.metabolites])))
print(len(model.compartments))
model
```

```
1107
1959
2017
1248
9
```

Out[43]:

|  |  |
| --- | --- |
| **Name** | R. toruloides |
| **Memory address** | 0x010296144e0 |
| **Number of metabolites** | 2017 |
| **Number of reactions** | 1959 |
| **Number of groups** | 0 |
| **Objective expression** | 0 |
| **Compartments** | c, x, m, e, r, v, n, g, d |

In [44]:

```
for x in model.compartments:
    print(x, len([m for m in model.metabolites if m.compartment == x]))
```

```
c 985
x 140
m 422
e 150
r 161
v 23
n 69
g 59
d 8
```

In [45]:

```
cobra.io.save_json_model(model, "IFO0880_GPR_1e.json")
```

In [46]:

```
model_old = cobra.io.load_json_model("IFO0880_GPR_1d.json")
model_new = cobra.io.load_json_model("IFO0880_GPR_1e.json")
```

In [47]:

```
print('Removed reactions\n')
for r in sorted(model_old.reactions, key=lambda x: x.id):
    if r not in model_new.reactions:
        print(r)
```

```
Removed reactions

2DHGLCNkt_tpp: 2dhglcn_p + h_p --> 2dhglcn_c + h_c
3NTD2pp: 3ump_p + h2o_p --> pi_p + uri_p
3NTD4pp: 3cmp_p + h2o_p --> cytd_p + pi_p
3NTD7pp: 3amp_p + h2o_p --> adn_p + pi_p
3NTD9pp: 3gmp_p + h2o_p --> gsn_p + pi_p
3_4DHBZt2: 34dhbz_e <=> 34dhbz_p
4ABUTthi: 4abut_c + h_c --> 4abut_h + h_h
4ABZGLUH: 4abzglu_c + h2o_c <=> 4abz_c + glu__L_c
AASPh: aps_h + atp_h --> adp_h + h_h + paps_h
AATC: Lcyst_h + akg_h <=> 3spyr_h + glu__L_h
AATG: 3sala_h + akg_h --> 3snpyr_h + glu__L_h
ABUTt2rL: 4abut_l + h_l <=> 4abut_c + h_c
ACCOAth: accoa_c + coa_h <=> accoa_h + coa_c
ACGPID: acgpail_hs_c + h2o_c --> ac_c + gpail_hs_c
ACKrh: ac_h + atp_h <=> actp_h + adp_h
ACP1p: fmn_p + h2o_p --> pi_p + ribflv_p
ACt2rpp: ac_p + h_p <=> ac_c + h_c
ADEt2rpp: ade_p + h_p <=> ade_c + h_c
ADEtl: ade_l <=> ade_c
ADNtl: adn_l <=> adn_c
ADSSh: asp__L_h + gtp_h + imp_h --> dcamp_h + gdp_h + 3.0 h_h + pi_h
AHEXASE2ly: 3.0 h2o_l + n2m2nmn_l --> 3.0 acgam_l + m2mn_l
AHEXASEly: 2.0 h2o_l + n2m2mn_l --> 2.0 acgam_l + m2mn_l
ALAt2rL: ala__L_l + h_l <=> ala__L_c + h_c
ALLTNt2rpp: alltn_p + h_p <=> alltn_c + h_c
AMETt2h: ahcys_c + amet_h <=> ahcys_h + amet_c
ANTPPT: anth_h + prpp_h --> h_h + ppi_h + pran_h
AOOAh: akg_h + hisp_h <=> glu__L_h + imacp_h
APPTh: atp_h + ppa_h <=> adp_h + ppap_h
ARBt2rpp: arab__L_p + h_p <=> arab__L_c + h_c
ARGLYSex: arg__L_e + lys__L_c --> arg__L_c + lys__L_e
ASADH: 4pasp_h + h_h + nadph_h --> aspsa_h + nadp_h + pi_h
ASNNpp: asn__L_p + h2o_p --> asp__L_p + nh4_p
ASPATh: akg_h + asp__L_h <=> glu__L_h + oaa_h
ASPth: asp__L_c + h_c <=> asp__L_h + h_h
ATNS: chor_h + gln__L_h --> anth_h + glu__L_h + h_h + pyr_h
ATNS_nh4: chor_h + nh4_h --> anth_h + h2o_h + h_h + pyr_h
BMTer_L: 0.1 dolmanp__L_r + memgacpail_hs_r --> 0.1 dolp__L_r + h_r + m2emgacpail_hs_r
BMTer_U: 0.1 dolmanp_U_r + memgacpail_hs_r --> 0.1 dolp_U_r + h_r + m2emgacpail_hs_r
B_MANNASEly: h2o_l + mn_l --> acgam_l + man_l
CA2t3pp: ca2_c + h_p --> ca2_p + h_c
CD2t3pp: cd2_c + h_p --> cd2_p + h_c
CHRM: chor_h <=> pphn_h
CHRS: 3psme_h --> chor_h + pi_h
CINNDO: cinnm_c + h_c + nadh_c + o2_c --> cenchddd_c + nad_c
CITL: cit_c --> ac_c + oaa_c
CLPNSm_SC: 0.01 cdpdag_SC_m + 0.01 pg_SC_m --> 0.01 clpn_SC_m + cmp_m + h_m
CLt3_2pp: 2.0 cl_p + h_c --> 2.0 cl_c + h_p
COBALT2t3pp: cobalt2_c + h_p --> cobalt2_p + h_c
COLIPAabcpp: atp_c + colipa_c + h2o_c --> adp_c + colipa_p + h_c + pi_c
CPMPS: gtp_c + h2o_c --> cpmp_c + ppi_c
CPPPGO_1: cpppg3_h + 2.0 h_h + 2.0 o2_h --> 2.0 co2_h + 2.0 h2o2_h + pppg9_h
CYANSTpp: cyan_p + tsul_p --> h_p + so3_p + tcynt_p
CYSAT: glu__L_h + mercppyr_h --> akg_h + cys__L_h
CYTDtl: cytd_l <=> cytd_c
DALAt2rL: ala__D_l + h_l <=> ala__D_c + h_c
DHAPT: dha_c + pep_c --> dhap_c + pyr_c
DHPAH: 25dhpp_h + h2o_h --> 5apru_h + nh4_h
DHQS_1: 2dda7p_h --> 3dhq_h + pi_h
DLDLBT: 4r5au_h + db4p_h --> dmlz_h + 2.0 h2o_h + h_h + pi_h
DMORh: alac__S_h + h_h + nadph_h --> 23dhmb_h + nadp_h
DNGALh: atp_h + dnad_h + gln__L_h + h2o_h --> amp_h + glu__L_h + 2.0 h_h + nad_h + ppi_h
DOLASNT_Ler: Asn_X_Ser_Thr_r + 0.1 g3m8mpdol__L_r --> 0.1 doldp__L_r + g3m8masn_r + h_r
DOLASNT_Uer: Asn_X_Ser_Thr_r + 0.1 g3m8mpdol_U_r --> 0.1 doldp_U_r + g3m8masn_r + h_r
DOLPMT_L: 0.1 dolp__L_c + gdpmann_c --> 0.1 dolmanp__L_c + gdp_c
DOLPMT_U: 0.1 dolp_U_c + gdpmann_c --> 0.1 dolmanp_U_c + gdp_c
DPHS: e4p_h + h2o_h + pep_h --> 2dda7p_h + pi_h
FE2t2pp: fe2_p + h_p --> fe2_c + h_c
FRULYSt2pp: frulys_p + h_p --> frulys_c + h_c
FUCtpp: fuc__L_p + h_p <=> fuc__L_c + h_c
Ftpp: f_c --> f_p
G1PPpp: g1p_p + h2o_p --> glc__D_p + pi_p
G2PPpp: glyc2p_p + h2o_p --> glyc_p + pi_p
G3PL: 3ig3p_h --> g3p_h + indole_h
G6PIh: g6p_A_h <=> g6p_B_h
GALM2pp: gal_bD_p --> gal_p
GALt2pp: gal_p + h_p --> gal_c + h_c
GAO1: accoa_c + gd3_hs_c --> coa_c + oagd3_hs_c
GAO1g: accoa_g + gd3_hs_g --> coa_g + oagd3_hs_g
GAO2: accoa_c + gt3_hs_c --> coa_c + oagt3_hs_c
GAO2g: accoa_g + gt3_hs_g --> coa_g + oagt3_hs_g
GLCATg: l2xser_g + udpglcur_g --> cs_hs_linkage_g + h_g + udp_g
GLNTRAT: atp_c + gln__L_c + glutrna_gln_c + h2o_c --> adp_c + glntrna_c + glu__L_c + h_c + pi_c
GLUNpp: gln__L_p + h2o_p --> glu__L_p + nh4_p
GLUth: glu__L_h + h_h --> glu__L_c + h_c
GLYALDtpp: glyald_p <=> glyald_c
GLYCth: glyc_c + h_c <=> glyc_h + h_h
GLYCtpp: glyc_c <=> glyc_p
GLYPT: atp_h + glyc__R_h --> 3pg_h + adp_h + h_h
GLYt2rL: gly_l + h_l <=> gly_c + h_c
GLYtpp: gly_c <=> gly_p
GPIAT: gpail_hs_c + pmtcoa_c --> coa_c + gacpail_hs_c
GPIMTer_L: 0.1 dolmanp__L_r + gacpail_hs_r --> 0.1 dolp__L_r + h_r + mgacpail_hs_r
GPIMTer_U: 0.1 dolmanp_U_r + gacpail_hs_r --> 0.1 dolp_U_r + h_r + mgacpail_hs_r
GSNtl: gsn_l <=> gsn_c
GTHAMPORh: amp_h + gthox_h + h_h + so3_h <=> aps_h + 2.0 gthrd_h
GTHRDHpp: gthrd_p + h2o_p --> cgly_p + glu__L_p
GTHS_1: atp_h + glucys_h + gly_h --> adp_h + gthrd_h + 2.0 h_h + pi_h
GTPDH: gtp_h + 3.0 h2o_h --> 25dhpp_h + for_h + 2.0 h_h + ppi_h
GUAt2pp: gua_p + h_p --> gua_c + h_c
H2ETer: mgacpail_hs_r + pe_hs_r --> dag_hs_r + emgacpail_hs_r
H2MTer_L: 0.1 dolmanp__L_r + mgacpail_hs_r --> 0.1 dolp__L_r + h_r + m2gacpail_hs_r
H2MTer_U: 0.1 dolmanp_U_r + mgacpail_hs_r --> 0.1 dolp_U_r + h_r + m2gacpail_hs_r
H2Otf: h2o_c <=> h2o_f
H2Oth: h2o_c <=> h2o_h
H3ETer: m2gacpail_hs_r + pe_hs_r --> dag_hs_r + memgacpail_hs_r
H3MTer_L: 0.1 dolmanp__L_r + m2gacpail_hs_r --> 0.1 dolp__L_r + h_r + m3gacpail_hs_r
H3MTer_U: 0.1 dolmanp_U_r + m2gacpail_hs_r --> 0.1 dolp_U_r + h_r + m3gacpail_hs_r
H4ET3er: m3gacpail_hs_r + pe_hs_r --> dag_hs_r + em3gacpail_hs_r
H4ETer: m3gacpail_hs_r + pe_hs_r --> dag_hs_r + m2emgacpail_hs_r
H5MTer_L: 0.1 dolmanp__L_r + emgacpail_hs_r --> 0.1 dolp__L_r + h_r + memgacpail_hs_r
H5MTer_U: 0.1 dolmanp_U_r + emgacpail_hs_r --> 0.1 dolp_U_r + h_r + memgacpail_hs_r
H6ET3er: m2emgacpail_hs_r + pe_hs_r --> dag_hs_r + em2emgacpail_hs_r
H6MTer_L: 0.1 dolmanp__L_r + m2emgacpail_hs_r --> 0.1 dolp__L_r + h_r + m3emgacpail_hs_r
H6MTer_U: 0.1 dolmanp_U_r + m2emgacpail_hs_r --> 0.1 dolp_U_r + h_r + m3emgacpail_hs_r
H6_ET2er: em3gacpail_hs_r + pe_hs_r --> dag_hs_r + emem2gacpail_hs_r
H6_ETer: em3gacpail_hs_r + pe_hs_r --> dag_hs_r + em2emgacpail_hs_r
H7ET2er: em2emgacpail_hs_r + pe_hs_r --> dag_hs_r + gpi_hs_r
H7MTer_L: 0.1 dolmanp__L_r + em2emgacpail_hs_r --> 0.1 dolp__L_r + h_r + mem2emgacpail_hs_r
H7MTer_U: 0.1 dolmanp_U_r + em2emgacpail_hs_r --> 0.1 dolp_U_r + h_r + mem2emgacpail_hs_r
H7_ETer: emem2gacpail_hs_r + pe_hs_r --> dag_hs_r + gpi_hs_r
H7_TAer: em2emgacpail_hs_r + pre_prot_r --> em2emgacpail_prot_hs_r + gpi_sig_r
H8MTer_L: 0.1 dolmanp__L_r + gpi_hs_r --> 0.1 dolp__L_r + h_r + m_em_3gacpail_hs_r
H8MTer_U: 0.1 dolmanp_U_r + gpi_hs_r --> 0.1 dolp_U_r + h_r + m_em_3gacpail_hs_r
H8TAer: gpi_hs_r + pre_prot_r --> gpi_prot_hs_r + gpi_sig_r
HXANtl: hxan_l <=> hxan_c
IDPh: h2o_h + ppi_h --> 2.0 pi_h
IGPS_1: 2cpr5p_h + h_h --> 3ig3p_h + co2_h + h2o_h
IMGPSh: gln__L_h + prlp_h --> aicar_h + eig3p_h + glu__L_h + h_h
INOSTt4pp: inost_p + na1_p --> inost_c + na1_c
INStl: ins_l <=> ins_c
IPPSh: 3mob_h + accoa_h + h2o_h --> 3c3hmp_h + coa_h + h_h
K2L4Aabcpp: atp_c + h2o_c + kdo2lipid4_c --> adp_c + h_c + kdo2lipid4_p + pi_c
KARI: 2ahbut_h <=> 3hmop_h
KARI_23dhmb: 3hmoa_h + h_h + nadph_h --> 23dhmb_h + nadp_h
KARI_23dhmp: 3hmop_h + h_h + nadph_h --> 23dhmp_h + nadp_h
KARI_3hmoa: alac__S_h <=> 3hmoa_h
Kt2pp: h_p + k_p --> h_c + k_c
Kt3pp: h_p + k_c --> h_c + k_p
LACZpp: h2o_p + lcts_p --> gal_p + glc__D_p
LIPACabcpp: atp_c + h2o_c + lipa_cold_c --> adp_c + h_c + lipa_cold_p + pi_c
LIPAabcpp: atp_c + h2o_c + lipa_c --> adp_c + h_c + lipa_p + pi_c
LPS3: h2o_c + mag_hs_c --> Rtotal2_c + glyc_c + h_c
LYSt2pp: h_p + lys__L_p --> h_c + lys__L_c
M4ATAer: m_em_3gacpail_hs_r + pre_prot_r --> gpi_sig_r + m_em_3gacpail_prot_hs_r
M4BET2er: mem2emgacpail_hs_r + pe_hs_r --> dag_hs_r + m_em_3gacpail_hs_r
M4BTAer: mem2emgacpail_hs_r + pre_prot_r --> gpi_sig_r + mem2emgacpail_prot_hs_r
M4CET3er: m3emgacpail_hs_r + pe_hs_r --> dag_hs_r + mem2emgacpail_hs_r
MDDCP1pp: h2o_p + murein5px4p_p --> ala__D_p + murein4px4p_p
MDDCP2pp: h2o_p + murein5px4px4p_p --> ala__D_p + murein4px4px4p_p
MDDCP3pp: h2o_p + murein5p5p_p --> ala__D_p + murein5p4p_p
MDDCP4pp: h2o_p + murein5p4p_p --> ala__D_p + murein4p4p_p
MDDCP5pp: h2o_p + murein5p3p_p --> ala__D_p + murein4p3p_p
MDDEP1pp: h2o_p + murein4px4p_p --> murein4p4p_p
MDDEP2pp: h2o_p + murein3px4p_p --> murein4p3p_p
MDDEP3pp: h2o_p + murein5px4p_p --> murein5p4p_p
MDDEP4pp: h2o_p + murein4px4px4p_p --> murein4px4p4p_p
METLEUex: leu__L_c + met__L_e --> leu__L_e + met__L_c
MN2t3pp: h_p + mn2_c --> h_c + mn2_p
MNt2pp: h_p + mn2_p --> h_c + mn2_c
MPTS: cpmp_c + cu2_c + 2.0 moadcosh_c --> 5.0 h_c + 2.0 moadcoo_c + mpt_c
NA1Hth: h_h + na1_c <=> h_c + na1_h
NA1th: na1_c <=> na1_h
NACHEX10ly: 2.0 h2o_l + ksi_deg7_l --> 2.0 acgam_l + ksi_deg8_l
NACHEX11ly: h2o_l + ksi_deg10_l --> acgam_l + ksi_deg11_l
NACHEX12ly: h2o_l + ksi_deg13_l --> acgam_l + ksi_deg14_l
NACHEX13ly: h2o_l + ksi_deg16_l --> acgam_l + ksi_deg17_l
NACHEX14ly: h2o_l + ksi_deg19_l --> acgam_l + ksi_deg20_l
NACHEX15ly: h2o_l + ksi_deg22_l --> acgam_l + ksi_deg23_l
NACHEX16ly: h2o_l + ksi_deg25_l --> acgam_l + ksi_deg26_l
NACHEX17ly: h2o_l + ksi_deg28_l --> acgam_l + ksi_deg29_l
NACHEX18ly: h2o_l + ksi_deg31_l --> acgam_l + ksi_deg32_l
NACHEX19ly: h2o_l + ksi_deg34_l --> acgam_l + ksi_deg35_l
NACHEX1ly: cs_a_deg1_l + h2o_l --> acgal_l + cs_a_deg2_l
NACHEX20ly: h2o_l + ksi_deg37_l --> acgam_l + ksi_deg38_l
NACHEX21ly: h2o_l + ksi_deg39_l --> acgam_l + ksi_deg40_l
NACHEX22ly: h2o_l + ksi_deg41_l --> acgam_l + m2mn_l
NACHEX23ly: h2o_l + ksii_core2_deg4_l --> acgam_l + ksii_core2_deg5_l
NACHEX24ly: h2o_l + ksii_core2_deg7_l --> acgam_l + ksii_core2_deg8_l
NACHEX25ly: h2o_l + ksii_core2_deg9_l --> acgam_l + f1a_l
NACHEX26ly: 2.0 h2o_l + ksii_core4_deg4_l --> 2.0 acgam_l + ksii_core2_deg5_l
NACHEX27ly: h2o_l + ha_deg1_l --> acgam_l + ha_pre1_l
NACHEX2ly: cs_a_deg4_l + h2o_l --> acgal_l + cs_a_deg5_l
NACHEX3ly: cs_b_deg1_l + h2o_l --> acgal_l + cs_b_deg2_l
NACHEX4ly: cs_c_deg1_l + h2o_l --> acgal_l + cs_c_deg2_l
NACHEX5ly: cs_c_deg4_l + h2o_l --> acgal_l + cs_c_deg5_l
NACHEX6ly: cs_d_deg1_l + h2o_l --> acgal_l + cs_d_deg2_l
NACHEX7ly: cs_d_deg5_l + h2o_l --> acgal_l + cs_d_deg6_l
NACHEX8ly: cs_e_deg2_l + h2o_l --> acgal_l + cs_e_deg3_l
NACHEX9ly: cs_e_deg6_l + h2o_l --> acgal_l + cs_e_deg7_l
NACHEXA10ly: 2.0 h2o_l + ksi_deg9_l --> acgam_l + h_l + ksi_deg11_l + so4_l
NACHEXA11ly: 2.0 h2o_l + ksi_deg12_l --> acgam_l + h_l + ksi_deg14_l + so4_l
NACHEXA12ly: 2.0 h2o_l + ksi_deg15_l --> acgam_l + h_l + ksi_deg17_l + so4_l
NACHEXA13ly: 2.0 h2o_l + ksi_deg18_l --> acgam_l + h_l + ksi_deg20_l + so4_l
NACHEXA14ly: 2.0 h2o_l + ksi_deg21_l --> acgam_l + h_l + ksi_deg23_l + so4_l
NACHEXA15ly: 2.0 h2o_l + ksi_deg24_l --> acgam_l + h_l + ksi_deg26_l + so4_l
NACHEXA16ly: 2.0 h2o_l + ksi_deg27_l --> acgam_l + h_l + ksi_deg29_l + so4_l
NACHEXA17ly: 2.0 h2o_l + ksi_deg30_l --> acgam_l + h_l + ksi_deg32_l + so4_l
NACHEXA18ly: 2.0 h2o_l + ksi_deg33_l --> acgam_l + h_l + ksi_deg35_l + so4_l
NACHEXA19ly: 2.0 h2o_l + ksi_deg36_l --> acgam_l + h_l + ksi_deg38_l + so4_l
NACHEXA1ly: cs_a_l + 2.0 h2o_l --> acgal_l + cs_a_deg2_l + h_l + so4_l
NACHEXA20ly: 2.0 h2o_l + ksii_core2_deg3_l --> acgam_l + h_l + ksii_core2_deg5_l + so4_l
NACHEXA21ly: 2.0 h2o_l + ksii_core2_deg6_l --> acgam_l + h_l + ksii_core2_deg8_l + so4_l
NACHEXA22ly: 3.0 h2o_l + ksii_core4_deg3_l --> 2.0 acgam_l + h_l + ksii_core2_deg5_l + so4_l
NACHEXA2ly: cs_b_l + 2.0 h2o_l --> acgal_l + cs_b_deg2_l + h_l + so4_l
NACHEXA3ly: cs_c_l + 2.0 h2o_l --> acgal_l + cs_c_deg2_l + h_l + so4_l
NACHEXA4ly: cs_c_deg3_l + 2.0 h2o_l --> acgal_l + cs_c_deg5_l + h_l + so4_l
NACHEXA5ly: cs_d_l + 2.0 h2o_l --> acgal_l + cs_d_deg2_l + h_l + so4_l
NACHEXA6ly: cs_d_deg4_l + 2.0 h2o_l --> acgal_l + cs_d_deg6_l + h_l + so4_l
NACHEXA7ly: cs_e_l + 3.0 h2o_l --> acgal_l + cs_e_deg3_l + 2.0 h_l + 2.0 so4_l
NACHEXA8ly: cs_e_deg4_l + 3.0 h2o_l --> acgal_l + cs_e_deg7_l + 2.0 h_l + 2.0 so4_l
NACHEXA9ly: 3.0 h2o_l + ksi_deg6_l --> 2.0 acgam_l + h_l + ksi_deg8_l + so4_l
NAt3pp: h_p + na1_c --> h_c + na1_p
NH4tpp: nh4_p <=> nh4_c
NI2t3pp: h_p + ni2_c --> h_c + ni2_p
NTD2pp: h2o_p + ump_p --> pi_p + uri_p
NTD4pp: cmp_p + h2o_p --> cytd_p + pi_p
NTD7pp: amp_p + h2o_p --> adn_p + pi_p
NTD9pp: gmp_p + h2o_p --> gsn_p + pi_p
OCTh: cbp_h + orn_h <=> citr__L_h + pi_h
ORDCh: h_h + orn_h --> co2_h + ptrc_h
PA120abcpp: atp_c + h2o_c + pa120_c --> adp_c + h_c + pa120_p + pi_c
PA140abcpp: atp_c + h2o_c + pa140_c --> adp_c + h_c + pa140_p + pi_c
PA141abcpp: atp_c + h2o_c + pa141_c --> adp_c + h_c + pa141_p + pi_c
PA160abcpp: atp_c + h2o_c + pa160_c --> adp_c + h_c + pa160_p + pi_c
PA161abcpp: atp_c + h2o_c + pa161_c --> adp_c + h_c + pa161_p + pi_c
PA180abcpp: atp_c + h2o_c + pa180_c --> adp_c + h_c + pa180_p + pi_c
PA181abcpp: atp_c + h2o_c + pa181_c --> adp_c + h_c + pa181_p + pi_c
PAFH: h2o_c + paf_hs_c --> ac_c + ak2lgchol_hs_c + h_c
PAFHe: h2o_e + paf_hs_e --> ac_e + ak2lgchol_hs_e + h_e
PCt: atp_c + h2o_c + pchol_hs_c <=> adp_c + h_c + pchol_hs_e + pi_c
PE120abcpp: atp_c + h2o_c + pe120_c --> adp_c + h_c + pe120_p + pi_c
PE140abcpp: atp_c + h2o_c + pe140_c --> adp_c + h_c + pe140_p + pi_c
PE141abcpp: atp_c + h2o_c + pe141_c --> adp_c + h_c + pe141_p + pi_c
PE160abcpp: atp_c + h2o_c + pe160_c --> adp_c + h_c + pe160_p + pi_c
PE161abcpp: atp_c + h2o_c + pe161_c --> adp_c + h_c + pe161_p + pi_c
PE180abcpp: atp_c + h2o_c + pe180_c --> adp_c + h_c + pe180_p + pi_c
PE181abcpp: atp_c + h2o_c + pe181_c --> adp_c + h_c + pe181_p + pi_c
PEFLIP: atp_c + h2o_c + pe_hs_e --> adp_c + h_c + pe_hs_c + pi_c
PEFLIPm: atp_c + h2o_c + pe_hs_c --> adp_c + h_c + pe_hs_m + pi_c
PG120abcpp: atp_c + h2o_c + pg120_c --> adp_c + h_c + pg120_p + pi_c
PG140abcpp: atp_c + h2o_c + pg140_c --> adp_c + h_c + pg140_p + pi_c
PG141abcpp: atp_c + h2o_c + pg141_c --> adp_c + h_c + pg141_p + pi_c
PG160abcpp: atp_c + h2o_c + pg160_c --> adp_c + h_c + pg160_p + pi_c
PG161abcpp: atp_c + h2o_c + pg161_c --> adp_c + h_c + pg161_p + pi_c
PG180abcpp: atp_c + h2o_c + pg180_c --> adp_c + h_c + pg180_p + pi_c
PG181abcpp: atp_c + h2o_c + pg181_c --> adp_c + h_c + pg181_p + pi_c
PGIAh: g6p_A_h <=> f6p_B_h
PGIBh: g6p_B_h <=> f6p_B_h
PGMTh: g1p_h <=> g6p_A_h
PGP120abcpp: atp_c + h2o_c + pgp120_c --> adp_c + h_c + pgp120_p + pi_c
PGP140abcpp: atp_c + h2o_c + pgp140_c --> adp_c + h_c + pgp140_p + pi_c
PGP141abcpp: atp_c + h2o_c + pgp141_c --> adp_c + h_c + pgp141_p + pi_c
PGP160abcpp: atp_c + h2o_c + pgp160_c --> adp_c + h_c + pgp160_p + pi_c
PGP161abcpp: atp_c + h2o_c + pgp161_c --> adp_c + h_c + pgp161_p + pi_c
PGP180abcpp: atp_c + h2o_c + pgp180_c --> adp_c + h_c + pgp180_p + pi_c
PGP181abcpp: atp_c + h2o_c + pgp181_c --> adp_c + h_c + pgp181_p + pi_c
PGPPAm_SC: h2o_m + 0.01 pgp_SC_m --> 0.01 pg_SC_m + pi_m
PIACGT: pail_hs_c + uacgam_c --> acgpail_hs_c + h_c + udp_c
POATh: glu__L_h + phpyr_h <=> akg_h + phe__L_h
PPBNGD: h2o_h + 4.0 ppbng_h <=> hmbil_h + 4.0 nh4_h
PPBNGS_1: 2.0 5aop_h + h_h --> 2.0 h2o_h + ppbng_h
PPMh: r1p_h <=> r5p_h
PPPGO_1: 3.0 o2_h + pppg9_h --> 3.0 h2o2_h + ppp9_h
PPPNDO: h_c + nadh_c + o2_c + pppn_c --> cechddd_c + nad_c
PPTHpp: h2o_p + ppt_p --> h2_p + pi_p
PRAISh: 5aizc_h + asp__L_h + atp_h --> 25aics_h + adp_h + h_h + pi_h
PRFGSh: atp_h + fgam_h + gln__L_h + h2o_h --> adp_h + fpram_h + glu__L_h + h_h + pi_h
PRICIh: prfp_h --> prlp_h
PROAKGOX1r: akg_r + o2_r + pro__L_r --> 4hpro_LT_r + co2_r + succ_r
PRODt2rL: h_l + pro__D_l <=> h_c + pro__D_c
PROt2rL: h_l + pro__L_l <=> h_c + pro__L_c
PSCIT: pep_h + skm5p_h <=> 3psme_h + pi_h
PSCLYSt2pp: h_p + psclys_p --> h_c + psclys_c
PSERDm_SC: h_m + 0.01 ps_SC_m --> co2_m + 0.01 pe_SC_m
PSFLIP: atp_c + h2o_c + ps_hs_e --> adp_c + h_c + pi_c + ps_hs_c
PSFLIPm: atp_c + h2o_c + ps_hs_c --> adp_c + h_c + pi_c + ps_hs_m
PSP_Lpp: h2o_p + pser__L_p --> pi_p + ser__L_p
PTHRpp: h2o_p + thrp_p --> pi_p + thr__L_p
PTRCthr: h_h + ptrc_c <=> h_c + ptrc_h
R5PFL: r5p_h --> db4p_h + for_h + h_h
R5PPpp: h2o_p + r5p_p --> pi_p + rib__D_p
RIBFS: 2.0 dmlz_h + h_h --> 4r5au_h + ribflv_h
RPDPK: atp_h + r5p_h <=> amp_h + h_h + prpp_h
S4TASE1ly: cs_a_l + h2o_l <=> cs_a_deg1_l + h_l + so4_l
S4TASE2ly: cs_a_deg3_l + h2o_l <=> cs_a_deg4_l + h_l + so4_l
S4TASE3ly: cs_b_l + h2o_l <=> cs_b_deg1_l + h_l + so4_l
S4TASE4ly: cs_e_l + h2o_l <=> cs_e_deg1_l + h_l + so4_l
S4TASE5ly: cs_e_deg4_l + h2o_l <=> cs_e_deg5_l + h_l + so4_l
SADT2: atp_c + gtp_c + h2o_c + so4_c --> aps_c + gdp_c + pi_c + ppi_c
SERH: 3ig3p_h + ser__L_h --> g3p_h + h2o_h + trp__L_h
SHSL2h: h2s_h + suchms_h --> hcys__L_h + succ_h
SQDGS160: 12dgr160_h + udpsq_h --> h_h + sqdg160_h + udp_h
SQDGS1819Z160: 12dgr1819Z160_h + udpsq_h --> h_h + sqdg1819Z160_h + udp_h
STARCH300S: 300.0 adpglc_h + h2o_h --> 300.0 adp_h + 300.0 h_h + starch300_h
SUCCt2_2pp: 2.0 h_p + succ_p --> 2.0 h_c + succ_c
TAL: thr__L_h --> 2obut_h + nh4_h
TCYD: 2m6phol_h --> dtocophe_h
TCYG: 23dmphol_h --> gtocophe_h
TFENFEOR: h_h + nadh_h + tfenfe3_h <=> nad_h + tfenfe2_h
THRAL: ser__L_h <=> nh4_h + pyr_h
THYMDtl: thymd_l <=> thymd_c
TREHpp: h2o_p + tre_p --> 2.0 glc__D_p
TRPS2h: indole_h + ser__L_h --> h2o_h + trp__L_h
TRPS3: 3ig3p_c --> g3p_c + indole_c
TYRTAh: 34hpp_h + glu__L_h <=> akg_h + tyr__L_h
UDPGALth: udpgal_c + ump_h <=> udpgal_h + ump_c
UDPGth: udp_h + udpg_c <=> udp_c + udpg_h
UPP3MT_1: 2.0 amet_h + uppg3_h --> 2.0 ahcys_h + dscl_h + 2.0 h_h
UPPDC1_1: 4.0 h_h + uppg3_h --> 4.0 co2_h + cpppg3_h
UPPDC2_1: 4.0 h_h + uppg1_h --> 4.0 co2_h + cpppg1_h
UREA: h2o_c + 2.0 h_c + urea_c --> co2_c + 2.0 nh4_c
UREAtpp: urea_p <=> urea_c
Uritl: uri_l <=> uri_c
VALTLh: atp_h + trnaval_h + val__L_h --> amp_h + ppi_h + valtrna_h
XANt2pp: h_p + xan_p --> h_c + xan_c
ZN2t3pp: h_p + zn2_c --> h_c + zn2_p
yli_R0026: h_c + utp_c + yli_M02355_c --> ppi_c + uacgam_c
yli_R0031: uacgam_c --> chtn_c + h_c + udp_c
yli_R0032: acgam6p_c <=> yli_M02355_c
yli_R0097: 3sala_c + akg_c + h_c --> glu__L_c + yli_M02657_c
yli_R0248: 4abz_m + 6hmhpt_m --> dhpt_m + h2o_m
yli_R0251: 6hmhpt_m + atp_m --> 2ahhmd_m + amp_m + h_m
yli_R0257: dhnpt_m --> 6hmhpt_m + gcald_m
yli_R0258: atp_c + glu__L_c + thf_c <=> adp_c + h_c + pi_c + yli_M03378_c
yli_R0350: 100.0 h2o_c + pail_cho_c --> dag_hs_c + 100.0 h_c + 100.0 mi1p__D_c
yli_R0415: hicit_m + nad_m <=> 2oxoadp_m + co2_m + nadh_m
yli_R0419: accoa_m + akg_m + h2o_m --> coa_m + h_m + yli_M01059_m
yli_R0459: gar_c + h2o_c + methf_c --> fgam_c + 2.0 h_c + thf_c
yli_R0481: atp_c + rib__D_c --> adp_c + h_c + yli_M00116_c
yli_R0495: h2o_c + o2_c + urate_c --> 5hiu_c + h2o2_c
yli_R0521: atp_c + yli_M00116_c <=> amp_c + h_c + prpp_c
yli_R0535: h2o_c + yli_M01609_c <=> alltt_c + h_c
yli_R0540: adprib_c + h2o_c --> amp_c + 2.0 h_c + yli_M00116_c
yli_R0549: yli_M02454_c <=> aicar_c + fum_c
yli_R0552: r1p_c <=> yli_M00116_c
yli_R0553: 5aizc_c + asp__L_c + atp_c <=> adp_c + h_c + pi_c + yli_M02454_c
yli_R0586: 3.0 o2_m + pppg9_m --> 3.0 h2o2_m + ppp9_m
yli_R0592: gly_m + h_c + succoa_m --> 5aop_m + co2_m + coa_m
yli_R0683: 25drapp_c + 3.0 h_c + nadh_c --> nad_c + yli_M04300_c
yli_R0684: 25drapp_c + 3.0 h_c + nadph_c --> nadp_c + yli_M04300_c
yli_R0685: h2o_c + yli_M04300_c --> 5aprbu_c + h_c + nh4_c
yli_R0712: gdpmann_c + 0.01 yli_M04599_c --> gdp_c + h_c + 0.01 yli_M04615_c
yli_R0713: gdpmann_c + 0.01 yli_M04600_c --> gdp_c + h_c + 0.01 yli_M04616_c
yli_R0714: gdpmann_c + 0.01 yli_M04601_c --> gdp_c + h_c + 0.01 yli_M04617_c
yli_R0715: gdpmann_c + 0.01 yli_M04602_c --> gdp_c + h_c + 0.01 yli_M04618_c
yli_R0716: gdpmann_c + 0.01 yli_M04603_c --> gdp_c + h_c + 0.01 yli_M04619_c
yli_R0717: gdpmann_c + 0.01 yli_M04604_c --> gdp_c + h_c + 0.01 yli_M04620_c
yli_R0726: h2o_c + udpg_c --> amylose_c + h_c + udp_c
yli_R0731: amylose_c --> h2o_c + starch_c
yli_R0732: pi_c + starch_c --> g1p_c
yli_R0738: udpg_c --> h_c + udp_c + yli_M00844_c
yli_R0816: 2.0 3hanthrn_c + 4.0 o2_c --> 2.0 h2o2_c + 2.0 h_c + 2.0 o2s_c + yli_M02714_c
yli_R0935: gdpmann_c + yli_M00109_c --> dolmanp_c + gdp_c
yli_R0936: dolmanp_c --> h_c + mannan_c + yli_M00109_c
yli_R1092: akg_m <=> akg_c
yli_R1114: yli_M01609_e --> yli_M01609_c
yli_R1385: atp_m + glu__L_m + thf_m <=> adp_m + h_m + pi_m + yli_M03378_m
yli_R1386: atp_m + dhpt_m + glu__L_m --> adp_m + dhf_m + h_m + pi_m
yli_R1390: atp_r + glyc_r --> adp_r + glyc3p_r + h_r
yli_R1399: atp_r + chol_r --> adp_r + cholp_r + h_r
yli_R1400: g3pc_r + h2o_r --> chol_r + glyc3p_r + h_r
yli_R1404: cholp_r + ctp_r + h_r --> cdpchol_r + ppi_r
yli_R1421: h2o_m + nad_m + saccrp__L_m --> akg_m + h_m + lys__L_m + nadh_m
yli_R1467: gtp_c + h2o_c --> HC01651_c
yli_R1468: HC01651_c + h2o_c --> HC01652_c + for_c
yli_R1469: HC01652_c --> HC01710_c
yli_R1470: HC01710_c --> ahdt_c + h2o_c
yli_R1477: gdpmann_r + yli_M00109_r --> dolmanp_r + gdp_r
yli_R1478: dolmanp_r --> h_r + mannan_r + yli_M00109_r
yli_R1492: f26bp_c --> 2amac_c + h2o_c
yli_R1493: Lcyst_c + akg_c <=> glu__L_c + yli_M02657_c
yli_R1507: atp_c + glyc__R_c --> 3pg_c + adp_c
yli_R1528: L2aadp_m + atp_m <=> ppi_m + yli_M07024_m
yli_R1529: L2aadp_c + atp_c <=> ppi_c + yli_M07024_c
yli_R1530: yli_M07024_m + yli_M07025_m --> amp_m + yli_M07026_m
yli_R1531: h_m + nadph_m + yli_M07026_m --> L2aadp6sa_m + nadp_m + yli_M07025_m
yli_R1533: udpg_r + yli_M00109_r <=> dolglcp_r + udp_r
yli_R1534: uacgam_r + yli_M00109_r <=> naglc2p_r + ump_r
yli_R1535: naglc2p_r + uacgam_r --> chito2pdol_r + udp_r
yli_R1536: h2o_c + rnam_c --> ncam_c + rib__D_c
yli_R1537: h2o_c + nicrns_c --> nac_c + rib__D_c
yli_R1540: atp_c + nad_c --> adp_c + nadp_c
yli_R1541: gar_c + h2o_c + methf_c --> fgam_c + thf_c
yli_R1545: abt__D_c + nad_c <=> h_c + nadh_c + rbl__D_c
yli_R1547: 3hpcoa_c + h2o_c <=> 3hpp_c + coa_c
yli_R1550: gtp_c + h2o_c --> gmp_c + ppi_c
yli_R1551: dgtp_c + h2o_c --> dgmp_c + ppi_c
yli_R1552: h2o_c + itp_c --> imp_c + ppi_c
yli_R1562: nh4_c + thym_c <=> 5mcsn_c + h2o_c
yli_R1572: udpglcur_c --> co2_c + udpxyl_c
yli_R1578: Lkynr_c + h2o_c <=> ala__L_c + anth_c
yli_R1579: Lfmkynr_c + h2o_c <=> ala__L_c + yli_M07057_c
yli_R1580: Lkynr_c + akg_c <=> glu__L_c + yli_M07058_c
yli_R1581: akg_c + hLkynr_c <=> glu__L_c + yli_M07059_c
yli_R1589: alac__S_m <=> 3hmoa_m
```

In [48]:

```
print('Updated reactions\n')
for r in sorted(model_old.reactions, key=lambda x: x.id):
    if r in model_new.reactions:
        r2 = model_new.reactions.get_by_id(r.id)
        if (r.name == r2.name and r.reaction == r2.reaction and r.gene_reaction_rule == r2.gene_reaction_rule and
            r.lower_bound == r2.lower_bound and r.upper_bound == r2.upper_bound):
            pass
        else:
            print('Old', r, r.gene_reaction_rule)
            print('New', r2, r2.gene_reaction_rule)
            print()
```

```
Updated reactions

Old AASAD1: L2aadp_c + atp_c + h_c + nadph_c --> L2aadp6sa_c + amp_c + nadp_c + ppi_c 9495 or (YGL154C and 9495) or (10220 and 9495)
New AASAD1: L2aadp_c + atp_c + h_c + nadph_c --> L2aadp6sa_c + amp_c + nadp_c + ppi_c 9495 or (10220 and 9495)

Old AASAD2: L2aadp_c + atp_c + h_c + nadh_c --> L2aadp6sa_c + amp_c + nad_c + ppi_c 9495 or (YGL154C and 9495) or (10220 and 9495)
New AASAD2: L2aadp_c + atp_c + h_c + nadh_c --> L2aadp6sa_c + amp_c + nad_c + ppi_c 9495 or (10220 and 9495)

Old ABUTt2r: 4abut_e + h_e <=> 4abut_c + h_c 11269 or 12743 or 13423 or (YALI0B09537g and 11269) or (YALI0B09537g and 13423)
New ABUTt2r: 4abut_e + h_e <=> 4abut_c + h_c 11269 or 12743 or 13423

Old ADEt2: ade_e + h_e --> ade_c + h_c 14461 or 14558 or 15012 or (YALI0D07392g and 14461) or (YALI0D07392g and 14558) or (YALI0D07392g and 15012) or (14558 and 15012)
New ADEt2: ade_e + h_e --> ade_c + h_c 14461 or 14558 or 15012 or (14558 and 15012)

Old ADHAPRer_RT: 0.01 1agly3p_SC_r + h_r + nadph_r --> 0.01 1ag3p_SC_r + nadp_r 15575
New ADHAPRer_RT: 0.01 1agly3p_RT_r + h_r + nadph_r --> 0.01 1ag3p_RT_r + nadp_r 15575

Old AGATer_RT: 0.01 1ag3p_SC_r + 0.02 dcacoa_r + 0.06 ddcacoa_r + 0.17 hdcoa_r + 0.09 ocdycacoa_r + 0.24 odecoa_r + 0.27 pmtcoa_r + 0.05 stcoa_r + 0.1 tdcoa_r --> coa_r + 0.01 pa_SC_r 10427 or 16030 or 16779 or 9746
New AGATer_RT: 0.01 1ag3p_RT_r + 0.02 dcacoa_r + 0.06 ddcacoa_r + 0.17 hdcoa_r + 0.09 ocdycacoa_r + 0.24 odecoa_r + 0.27 pmtcoa_r + 0.05 stcoa_r + 0.1 tdcoa_r --> coa_r + 0.01 pa_RT_r 10427 or 16030 or 16779 or 9746

Old ALAt2r: ala__L_e + h_e <=> ala__L_c + h_c 12743 or 14229 or 15074 or 8962 or 9319 or 9322 or 9962 or (YALI0B09537g and 14229 and 8962) or (YALI0B09537g and 14229 and 9319) or (YALI0B09537g and 14229 and 9322) or (YALI0B09537g and 14229 and 9962) or (YALI0B09537g and 15074 and 8962) or (YALI0B09537g and 15074 and 9319) or (YALI0B09537g and 15074 and 9322) or (YALI0B09537g and 15074 and 9962)
New ALAt2r: ala__L_e + h_e <=> ala__L_c + h_c 12743 or 14229 or 15074 or 8962 or 9319 or 9322 or 9962

Old ASPt2r: asp__L_e + h_e <=> asp__L_c + h_c 14229 or 15074 or 8962 or 9319 or 9322 or 9962 or (YALI0E20713g and 14229 and 8962) or (YALI0E20713g and 14229 and 9319) or (YALI0E20713g and 14229 and 9322) or (YALI0E20713g and 14229 and 9962) or (YALI0E20713g and 15074 and 8962) or (YALI0E20713g and 15074 and 9319) or (YALI0E20713g and 15074 and 9322) or (YALI0E20713g and 15074 and 9962)
New ASPt2r: asp__L_e + h_e <=> asp__L_c + h_c 14229 or 15074 or 8962 or 9319 or 9322 or 9962

Old CDPDAGterm_RT: cdpdag_SC_r <=> cdpdag_SC_m 
New CDPDAGterm_RT: cdpdag_RT_r <=> cdpdag_RT_m 

Old CDPDGPm_RT: 0.01 cdpdag_SC_m + glyc3p_m --> cmp_m + h_m + 0.01 pgp_SC_m 12596
New CDPDGPm_RT: 0.01 cdpdag_RT_m + glyc3p_m --> cmp_m + h_m + 0.01 pgp_RT_m 12596

Old CSNt2: csn_e + h_e --> csn_c + h_c 14461 or 14558 or 15012 or (YALI0D07392g and 14461) or (YALI0D07392g and 14558) or (YALI0D07392g and 15012) or (14558 and 15012)
New CSNt2: csn_e + h_e --> csn_c + h_c 14461 or 14558 or 15012 or (14558 and 15012)

Old DAGCPTer_RT: 0.01 12dgr_SC_r + cdpchol_r --> cmp_r + h_r + 0.01 pc_SC_r 11351 or 9088
New DAGCPTer_RT: 0.01 12dgr_RT_r + cdpchol_r --> cmp_r + h_r + 0.01 pc_RT_r 11351 or 9088

Old DAGKer_RT: 0.01 12dgr_SC_r + ctp_r --> cdp_r + h_r + 0.01 pa_SC_r 10156
New DAGKer_RT: 0.01 12dgr_RT_r + ctp_r --> cdp_r + h_r + 0.01 pa_RT_r 10156

Old DAGPYPer_RT: h2o_r + 0.01 pa_SC_r --> 0.01 12dgr_SC_r + pi_r 12485 or 13087
New DAGPYPer_RT: h2o_r + 0.01 pa_RT_r --> 0.01 12dgr_RT_r + pi_r 12485 or 13087

Old DASYNer_RT: ctp_r + h_r + 0.01 pa_SC_r --> 0.01 cdpdag_SC_r + ppi_r 12881
New DASYNer_RT: ctp_r + h_r + 0.01 pa_RT_r --> 0.01 cdpdag_RT_r + ppi_r 12881

Old DASYNm_RT: ctp_m + h_m + 0.01 pa_SC_m --> 0.01 cdpdag_SC_m + ppi_m 11307
New DASYNm_RT: ctp_m + h_m + 0.01 pa_RT_m --> 0.01 cdpdag_RT_m + ppi_m 11307

Old EPISTATer_RT: 0.01 epist_r + 0.655 hdcoa_r + 0.01 hexccoa_r + 0.27 odecoa_r + 0.02 pmtcoa_r + 0.03 stcoa_r + 0.015 tdcoa_r --> coa_r + 0.01 epistest_SC_r 11799
New EPISTATer_RT: 0.01 epist_r + 0.655 hdcoa_r + 0.01 hexccoa_r + 0.27 odecoa_r + 0.02 pmtcoa_r + 0.03 stcoa_r + 0.015 tdcoa_r --> coa_r + 0.01 epistest_RT_r 11799

Old EPISTESTtrd: epistest_SC_r --> epistest_RT_d 
New EPISTESTtrd: epistest_RT_r --> epistest_RT_d 

Old ERGSTATer_RT: 0.01 ergst_r + 0.655 hdcoa_r + 0.01 hexccoa_r + 0.27 odecoa_r + 0.02 pmtcoa_r + 0.03 stcoa_r + 0.015 tdcoa_r --> coa_r + 0.01 ergstest_SC_r 11799
New ERGSTATer_RT: 0.01 ergst_r + 0.655 hdcoa_r + 0.01 hexccoa_r + 0.27 odecoa_r + 0.02 pmtcoa_r + 0.03 stcoa_r + 0.015 tdcoa_r --> coa_r + 0.01 ergstest_RT_r 11799

Old ERGSTESTtrd: ergstest_SC_r --> ergstest_RT_d 
New ERGSTESTtrd: ergstest_RT_r --> ergstest_RT_d 

Old ETHAPTer_RT: 0.01 12dgr_SC_r + cdpea_r --> cmp_r + h_r + 0.01 pe_SC_r 11351
New ETHAPTer_RT: 0.01 12dgr_RT_r + cdpea_r --> cmp_r + h_r + 0.01 pe_RT_r 11351

Old FECOSTATer_RT: 0.01 fecost_r + 0.655 hdcoa_r + 0.01 hexccoa_r + 0.27 odecoa_r + 0.02 pmtcoa_r + 0.03 stcoa_r + 0.015 tdcoa_r --> coa_r + 0.01 fecostest_SC_r 11799
New FECOSTATer_RT: 0.01 fecost_r + 0.655 hdcoa_r + 0.01 hexccoa_r + 0.27 odecoa_r + 0.02 pmtcoa_r + 0.03 stcoa_r + 0.015 tdcoa_r --> coa_r + 0.01 fecostest_RT_r 11799

Old FECOSTESTtrd: fecostest_SC_r --> fecostest_RT_d 
New FECOSTESTtrd: fecostest_RT_r --> fecostest_RT_d 

Old GAT1er_RT: 0.02 dcacoa_r + 0.06 ddcacoa_r + glyc3p_r + 0.17 hdcoa_r + 0.09 ocdycacoa_r + 0.24 odecoa_r + 0.27 pmtcoa_r + 0.05 stcoa_r + 0.1 tdcoa_r --> 0.01 1ag3p_SC_r + coa_r 15435
New GAT1er_RT: 0.02 dcacoa_r + 0.06 ddcacoa_r + glyc3p_r + 0.17 hdcoa_r + 0.09 ocdycacoa_r + 0.24 odecoa_r + 0.27 pmtcoa_r + 0.05 stcoa_r + 0.1 tdcoa_r --> 0.01 1ag3p_RT_r + coa_r 15435

Old GAT2er_RT: 0.02 dcacoa_r + 0.06 ddcacoa_r + dhap_r + 0.17 hdcoa_r + 0.09 ocdycacoa_r + 0.24 odecoa_r + 0.27 pmtcoa_r + 0.05 stcoa_r + 0.1 tdcoa_r --> 0.01 1agly3p_SC_r + coa_r 13369 or 15435
New GAT2er_RT: 0.02 dcacoa_r + 0.06 ddcacoa_r + dhap_r + 0.17 hdcoa_r + 0.09 ocdycacoa_r + 0.24 odecoa_r + 0.27 pmtcoa_r + 0.05 stcoa_r + 0.1 tdcoa_r --> 0.01 1agly3p_RT_r + coa_r 13369 or 15435

Old GGNG: Tyr_ggn_c + 8.0 udpg_c --> ggn_c + 8.0 h_c + 8.0 udp_c (mmu_8908 and 9596) or (14002 and 9596)
New GGNG: Tyr_ggn_c + 8.0 udpg_c --> ggn_c + 8.0 h_c + 8.0 udp_c 14002 and 9596

Old GLGNS1: ggn_c + 3.0 udpg_c --> glygn1_c + 3.0 h_c + 3.0 udp_c (mmu_8908 and 9596) or (14002 and 9596)
New GLGNS1: ggn_c + 3.0 udpg_c --> glygn1_c + 3.0 h_c + 3.0 udp_c 14002 and 9596

Old GLYt2r: gly_e + h_e <=> gly_c + h_c 12743 or 14229 or 15074 or 8962 or 9319 or 9322 or 9962 or (YALI0B09537g and 14229 and 8962) or (YALI0B09537g and 14229 and 9319) or (YALI0B09537g and 14229 and 9322) or (YALI0B09537g and 14229 and 9962) or (YALI0B09537g and 15074 and 8962) or (YALI0B09537g and 15074 and 9319) or (YALI0B09537g and 15074 and 9322) or (YALI0B09537g and 15074 and 9962)
New GLYt2r: gly_e + h_e <=> gly_c + h_c 12743 or 14229 or 15074 or 8962 or 9319 or 9322 or 9962

Old GUAt2r: gua_e + h_e <=> gua_c + h_c 14461 or 14558 or 15012 or (YALI0D07392g and 14461) or (YALI0D07392g and 14558) or (YALI0D07392g and 15012) or (14558 and 15012)
New GUAt2r: gua_e + h_e <=> gua_c + h_c 14461 or 14558 or 15012 or (14558 and 15012)

Old HXAND: h2o_c + hxan_c + nad_c --> h_c + nadh_c + xan_c 15962 or (b2868 and 15962)
New HXAND: h2o_c + hxan_c + nad_c --> h_c + nadh_c + xan_c 15962

Old IPC124PLCg_RT: h2o_g + 0.01 ipc124_SC_g --> cer1_24_g + h_g + mi1p__D_g 15857
New IPC124PLCg_RT: h2o_g + 0.01 ipc124_RT_g --> cer1_24_g + h_g + mi1p__D_g 15857

Old IPC126PLCg_RT: h2o_g + 0.01 ipc126_SC_g --> cer1_26_g + h_g + mi1p__D_g 15857
New IPC126PLCg_RT: h2o_g + 0.01 ipc126_RT_g --> cer1_26_g + h_g + mi1p__D_g 15857

Old IPC224PLCg_RT: h2o_g + 0.01 ipc224_SC_g --> cer2_24_g + h_g + mi1p__D_g 15857
New IPC224PLCg_RT: h2o_g + 0.01 ipc224_RT_g --> cer2_24_g + h_g + mi1p__D_g 15857

Old IPC226PLCg_RT: h2o_g + 0.01 ipc226_SC_g --> cer2_26_g + h_g + mi1p__D_g 15857
New IPC226PLCg_RT: h2o_g + 0.01 ipc226_RT_g --> cer2_26_g + h_g + mi1p__D_g 15857

Old IPC324PLCg_RT: h2o_g + 0.01 ipc324_SC_g --> cer3_24_g + h_g + mi1p__D_g 15857
New IPC324PLCg_RT: h2o_g + 0.01 ipc324_RT_g --> cer3_24_g + h_g + mi1p__D_g 15857

Old IPC326PLCg_RT: h2o_g + 0.01 ipc326_SC_g --> cer3_26_g + h_g + mi1p__D_g 15857
New IPC326PLCg_RT: h2o_g + 0.01 ipc326_RT_g --> cer3_26_g + h_g + mi1p__D_g 15857

Old IPCS124g_RT: cer1_24_g + 0.01 ptd1ino_SC_g --> 0.01 12dgr_SC_g + 0.01 ipc124_SC_g 12927 and 8747
New IPCS124g_RT: cer1_24_g + 0.01 ptd1ino_RT_g --> 0.01 12dgr_RT_g + 0.01 ipc124_RT_g 12927 and 8747

Old IPCS126g_RT: cer1_26_g + 0.01 ptd1ino_SC_g --> 0.01 12dgr_SC_g + 0.01 ipc126_SC_g 12927 and 8747
New IPCS126g_RT: cer1_26_g + 0.01 ptd1ino_RT_g --> 0.01 12dgr_RT_g + 0.01 ipc126_RT_g 12927 and 8747

Old IPCS224g_RT: cer2_24_g + 0.01 ptd1ino_SC_g --> 0.01 12dgr_SC_g + 0.01 ipc224_SC_g 12927 and 8747
New IPCS224g_RT: cer2_24_g + 0.01 ptd1ino_RT_g --> 0.01 12dgr_RT_g + 0.01 ipc224_RT_g 12927 and 8747

Old IPCS226g_RT: cer2_26_g + 0.01 ptd1ino_SC_g --> 0.01 12dgr_SC_g + 0.01 ipc226_SC_g 12927 and 8747
New IPCS226g_RT: cer2_26_g + 0.01 ptd1ino_RT_g --> 0.01 12dgr_RT_g + 0.01 ipc226_RT_g 12927 and 8747

Old IPCS324g_RT: cer3_24_g + 0.01 ptd1ino_SC_g --> 0.01 12dgr_SC_g + 0.01 ipc324_SC_g 12927 and 8747
New IPCS324g_RT: cer3_24_g + 0.01 ptd1ino_RT_g --> 0.01 12dgr_RT_g + 0.01 ipc324_RT_g 12927 and 8747

Old IPCS326g_RT: cer3_26_g + 0.01 ptd1ino_SC_g --> 0.01 12dgr_SC_g + 0.01 ipc326_SC_g 12927 and 8747
New IPCS326g_RT: cer3_26_g + 0.01 ptd1ino_RT_g --> 0.01 12dgr_RT_g + 0.01 ipc326_RT_g 12927 and 8747

Old LANOSTATer_RT: 0.655 hdcoa_r + 0.01 hexccoa_r + 0.01 lanost_r + 0.27 odecoa_r + 0.02 pmtcoa_r + 0.03 stcoa_r + 0.015 tdcoa_r --> coa_r + 0.01 lanostest_SC_r 11799
New LANOSTATer_RT: 0.655 hdcoa_r + 0.01 hexccoa_r + 0.01 lanost_r + 0.27 odecoa_r + 0.02 pmtcoa_r + 0.03 stcoa_r + 0.015 tdcoa_r --> coa_r + 0.01 lanostest_RT_r 11799

Old LANOSTESTtrd: lanostest_SC_r --> lanostest_RT_d 
New LANOSTESTtrd: lanostest_RT_r --> lanostest_RT_d 

Old LPCATer_RT: 0.01 1agpc_SC_r + 0.02 dcacoa_r + 0.06 ddcacoa_r + 0.17 hdcoa_r + 0.09 ocdycacoa_r + 0.24 odecoa_r + 0.27 pmtcoa_r + 0.05 stcoa_r + 0.1 tdcoa_r --> coa_r + 0.01 pc_SC_r 16030
New LPCATer_RT: 0.01 1agpc_RT_r + 0.02 dcacoa_r + 0.06 ddcacoa_r + 0.17 hdcoa_r + 0.09 ocdycacoa_r + 0.24 odecoa_r + 0.27 pmtcoa_r + 0.05 stcoa_r + 0.1 tdcoa_r --> coa_r + 0.01 pc_RT_r 16030

Old LPPer_RT: 0.01 dagpy_SC_r + h2o_r --> h_r + 0.01 pa_SC_r + pi_r 13087
New LPPer_RT: 0.01 dagpy_RT_r + h2o_r --> h_r + 0.01 pa_RT_r + pi_r 13087

Old METt2r: h_e + met__L_e <=> h_c + met__L_c 14229 or 15074 or (YALI0F25795g and 14229) or (YALI0F25795g and 15074)
New METt2r: h_e + met__L_e <=> h_c + met__L_c 14229 or 15074

Old MFAPSer_RT: amet_r + 0.01 ptdmeeta_SC_r --> ahcys_r + h_r + 0.01 ptd2meeta_SC_r 12748
New MFAPSer_RT: amet_r + 0.01 ptdmeeta_RT_r --> ahcys_r + h_r + 0.01 ptd2meeta_RT_r 12748

Old MIP2C124PLCg_RT: h2o_g + 0.01 mip2c124_SC_g --> cer1_24_g + h_g + man2mi1p__D_g 15857
New MIP2C124PLCg_RT: h2o_g + 0.01 mip2c124_RT_g --> cer1_24_g + h_g + man2mi1p__D_g 15857

Old MIP2C126PLCg_RT: h2o_g + 0.01 mip2c126_SC_g --> cer1_26_g + h_g + man2mi1p__D_g 15857
New MIP2C126PLCg_RT: h2o_g + 0.01 mip2c126_RT_g --> cer1_26_g + h_g + man2mi1p__D_g 15857

Old MIP2C224PLCg_RT: h2o_g + 0.01 mip2c224_SC_g --> cer2_24_g + h_g + man2mi1p__D_g 15857
New MIP2C224PLCg_RT: h2o_g + 0.01 mip2c224_RT_g --> cer2_24_g + h_g + man2mi1p__D_g 15857

Old MIP2C226PLCg_RT: h2o_g + 0.01 mip2c226_SC_g --> cer2_26_g + h_g + man2mi1p__D_g 15857
New MIP2C226PLCg_RT: h2o_g + 0.01 mip2c226_RT_g --> cer2_26_g + h_g + man2mi1p__D_g 15857

Old MIP2C324PLCg_RT: h2o_g + 0.01 mip2c324_SC_g --> cer3_24_g + h_g + man2mi1p__D_g 15857
New MIP2C324PLCg_RT: h2o_g + 0.01 mip2c324_RT_g --> cer3_24_g + h_g + man2mi1p__D_g 15857

Old MIP2C326PLCg_RT: h2o_g + 0.01 mip2c326_SC_g --> cer3_26_g + h_g + man2mi1p__D_g 15857
New MIP2C326PLCg_RT: h2o_g + 0.01 mip2c326_RT_g --> cer3_26_g + h_g + man2mi1p__D_g 15857

Old MIP2CS124g_RT: 0.01 mipc124_SC_g + 0.01 ptd1ino_SC_g --> 0.01 12dgr_SC_g + 0.01 mip2c124_SC_g 13172
New MIP2CS124g_RT: 0.01 mipc124_RT_g + 0.01 ptd1ino_RT_g --> 0.01 12dgr_RT_g + 0.01 mip2c124_RT_g 13172

Old MIP2CS126g_RT: 0.01 mipc126_SC_g + 0.01 ptd1ino_SC_g --> 0.01 12dgr_SC_g + 0.01 mip2c126_SC_g 13172
New MIP2CS126g_RT: 0.01 mipc126_RT_g + 0.01 ptd1ino_RT_g --> 0.01 12dgr_RT_g + 0.01 mip2c126_RT_g 13172

Old MIP2CS224g_RT: 0.01 mipc224_SC_g + 0.01 ptd1ino_SC_g --> 0.01 12dgr_SC_g + 0.01 mip2c224_SC_g 13172
New MIP2CS224g_RT: 0.01 mipc224_RT_g + 0.01 ptd1ino_RT_g --> 0.01 12dgr_RT_g + 0.01 mip2c224_RT_g 13172

Old MIP2CS226g_RT: 0.01 mipc226_SC_g + 0.01 ptd1ino_SC_g --> 0.01 12dgr_SC_g + 0.01 mip2c226_SC_g 13172
New MIP2CS226g_RT: 0.01 mipc226_RT_g + 0.01 ptd1ino_RT_g --> 0.01 12dgr_RT_g + 0.01 mip2c226_RT_g 13172

Old MIP2CS324g_RT: 0.01 mipc324_SC_g + 0.01 ptd1ino_SC_g --> 0.01 12dgr_SC_g + 0.01 mip2c324_SC_g 13172
New MIP2CS324g_RT: 0.01 mipc324_RT_g + 0.01 ptd1ino_RT_g --> 0.01 12dgr_RT_g + 0.01 mip2c324_RT_g 13172

Old MIP2CS326g_RT: 0.01 mipc326_SC_g + 0.01 ptd1ino_SC_g --> 0.01 12dgr_SC_g + 0.01 mip2c326_SC_g 13172
New MIP2CS326g_RT: 0.01 mipc326_RT_g + 0.01 ptd1ino_RT_g --> 0.01 12dgr_RT_g + 0.01 mip2c326_RT_g 13172

Old MIPC124PLCg_RT: h2o_g + 0.01 mipc124_SC_g --> cer1_24_g + h_g + manmi1p__D_g 15857
New MIPC124PLCg_RT: h2o_g + 0.01 mipc124_RT_g --> cer1_24_g + h_g + manmi1p__D_g 15857

Old MIPC126PLCg_RT: h2o_g + 0.01 mipc126_SC_g --> cer1_26_g + h_g + manmi1p__D_g 15857
New MIPC126PLCg_RT: h2o_g + 0.01 mipc126_RT_g --> cer1_26_g + h_g + manmi1p__D_g 15857

Old MIPC224PLCg_RT: h2o_g + 0.01 mipc224_SC_g --> cer2_24_g + h_g + manmi1p__D_g 15857
New MIPC224PLCg_RT: h2o_g + 0.01 mipc224_RT_g --> cer2_24_g + h_g + manmi1p__D_g 15857

Old MIPC226PLCg_RT: h2o_g + 0.01 mipc226_SC_g --> cer2_26_g + h_g + manmi1p__D_g 15857
New MIPC226PLCg_RT: h2o_g + 0.01 mipc226_RT_g --> cer2_26_g + h_g + manmi1p__D_g 15857

Old MIPC324PLCg_RT: h2o_g + 0.01 mipc324_SC_g --> cer3_24_g + h_g + manmi1p__D_g 15857
New MIPC324PLCg_RT: h2o_g + 0.01 mipc324_RT_g --> cer3_24_g + h_g + manmi1p__D_g 15857

Old MIPC326PLCg_RT: h2o_g + 0.01 mipc326_SC_g --> cer3_26_g + h_g + manmi1p__D_g 15857
New MIPC326PLCg_RT: h2o_g + 0.01 mipc326_RT_g --> cer3_26_g + h_g + manmi1p__D_g 15857

Old MIPCS124g_RT: gdpmann_g + 0.01 ipc124_SC_g --> gdp_g + h_g + 0.01 mipc124_SC_g 16453
New MIPCS124g_RT: gdpmann_g + 0.01 ipc124_RT_g --> gdp_g + h_g + 0.01 mipc124_RT_g 16453

Old MIPCS126g_RT: gdpmann_g + 0.01 ipc126_SC_g --> gdp_g + h_g + 0.01 mipc126_SC_g 16453
New MIPCS126g_RT: gdpmann_g + 0.01 ipc126_RT_g --> gdp_g + h_g + 0.01 mipc126_RT_g 16453

Old MIPCS224g_RT: gdpmann_g + 0.01 ipc224_SC_g --> gdp_g + h_g + 0.01 mipc224_SC_g 16453
New MIPCS224g_RT: gdpmann_g + 0.01 ipc224_RT_g --> gdp_g + h_g + 0.01 mipc224_RT_g 16453

Old MIPCS226g_RT: gdpmann_g + 0.01 ipc226_SC_g --> gdp_g + h_g + 0.01 mipc226_SC_g 16453
New MIPCS226g_RT: gdpmann_g + 0.01 ipc226_RT_g --> gdp_g + h_g + 0.01 mipc226_RT_g 16453

Old MIPCS324g_RT: gdpmann_g + 0.01 ipc324_SC_g --> gdp_g + h_g + 0.01 mipc324_SC_g 16453
New MIPCS324g_RT: gdpmann_g + 0.01 ipc324_RT_g --> gdp_g + h_g + 0.01 mipc324_RT_g 16453

Old MIPCS326g_RT: gdpmann_g + 0.01 ipc326_SC_g --> gdp_g + h_g + 0.01 mipc326_SC_g 16453
New MIPCS326g_RT: gdpmann_g + 0.01 ipc326_RT_g --> gdp_g + h_g + 0.01 mipc326_RT_g 16453

Old PAterm_RT: pa_SC_r <=> pa_SC_m 
New PAterm_RT: pa_RT_r <=> pa_RT_m 

Old PCDAGATer_RT: 12dgr_SC_r + pc_SC_r --> 1agpc_SC_r + triglyc_SC_r 16477
New PCDAGATer_RT: 12dgr_RT_r + pc_RT_r --> 1agpc_RT_r + triglyc_RT_r 16477

Old PETOHMer_RT: amet_r + 0.01 pe_SC_r --> ahcys_r + h_r + 0.01 ptdmeeta_SC_r 10647
New PETOHMer_RT: amet_r + 0.01 pe_RT_r --> ahcys_r + h_r + 0.01 ptdmeeta_RT_r 10647

Old PEterm_RT: pe_SC_r <=> pe_SC_m 
New PEterm_RT: pe_RT_r <=> pe_RT_m 

Old PHETRS: atp_c + phe__L_c + trnaphe_c --> amp_c + phetrna_c + ppi_c (PP_2470 and 12470) or (12470 and 13249)
New PHETRS: atp_c + phe__L_c + trnaphe_c --> amp_c + phetrna_c + ppi_c 12470 and 13249

Old PI35BP5Per_RT: h2o_r + 0.01 ptd135bp_SC_r --> pi_r + 0.01 ptd3ino_SC_r 11381 or 15340 or 16619
New PI35BP5Per_RT: h2o_r + 0.01 ptd135bp_RT_r --> pi_r + 0.01 ptd3ino_RT_r 11381 or 15340 or 16619

Old PI3P5Kn_RT: atp_n + 0.01 ptd3ino_SC_n --> adp_n + h_n + 0.01 ptd135bp_SC_n 14139
New PI3P5Kn_RT: atp_n + 0.01 ptd3ino_RT_n --> adp_n + h_n + 0.01 ptd135bp_RT_n 14139

Old PI45BP5Per_RT: h2o_r + 0.01 ptd145bp_SC_r --> pi_r + 0.01 ptd4ino_SC_r 15340
New PI45BP5Per_RT: h2o_r + 0.01 ptd145bp_RT_r --> pi_r + 0.01 ptd4ino_RT_r 15340

Old PI45BP5Pn_RT: h2o_n + 0.01 ptd145bp_SC_n --> pi_n + 0.01 ptd4ino_SC_n 13609 or 15545
New PI45BP5Pn_RT: h2o_n + 0.01 ptd145bp_RT_n --> pi_n + 0.01 ptd4ino_RT_n 13609 or 15545

Old PI45BPPn_RT: h2o_n + 0.01 ptd145bp_SC_n --> 0.01 12dgr_SC_n + h_n + mi145p_n 12855
New PI45BPPn_RT: h2o_n + 0.01 ptd145bp_RT_n --> 0.01 12dgr_RT_n + h_n + mi145p_n 12855

Old PI4P5Kn_RT: atp_n + 0.01 ptd4ino_SC_n --> adp_n + h_n + 0.01 ptd145bp_SC_n 11878
New PI4P5Kn_RT: atp_n + 0.01 ptd4ino_RT_n --> adp_n + h_n + 0.01 ptd145bp_RT_n 11878

Old PIN3Kn_RT: atp_n + 0.01 ptd1ino_SC_n --> adp_n + h_n + 0.01 ptd3ino_SC_n 8712
New PIN3Kn_RT: atp_n + 0.01 ptd1ino_RT_n --> adp_n + h_n + 0.01 ptd3ino_RT_n 8712

Old PIN3Per_RT: h2o_r + 0.01 ptd3ino_SC_r --> pi_r + 0.01 ptd1ino_SC_r 11381
New PIN3Per_RT: h2o_r + 0.01 ptd3ino_RT_r --> pi_r + 0.01 ptd1ino_RT_r 11381

Old PIN3Pn_RT: h2o_n + 0.01 ptd3ino_SC_n --> pi_n + 0.01 ptd1ino_SC_n 14880
New PIN3Pn_RT: h2o_n + 0.01 ptd3ino_RT_n --> pi_n + 0.01 ptd1ino_RT_n 14880

Old PIN4Ker_RT: atp_r + 0.01 ptd1ino_SC_r --> adp_r + h_r + 0.01 ptd4ino_SC_r 15159
New PIN4Ker_RT: atp_r + 0.01 ptd1ino_RT_r --> adp_r + h_r + 0.01 ptd4ino_RT_r 15159

Old PIN4Kn_RT: atp_n + 0.01 ptd1ino_SC_n --> adp_n + h_n + 0.01 ptd4ino_SC_n 15086 or 9782
New PIN4Kn_RT: atp_n + 0.01 ptd1ino_RT_n --> adp_n + h_n + 0.01 ptd4ino_RT_n 15086 or 9782

Old PIN4Per_RT: h2o_r + 0.01 ptd4ino_SC_r --> pi_r + 0.01 ptd1ino_SC_r 11381
New PIN4Per_RT: h2o_r + 0.01 ptd4ino_RT_r --> pi_r + 0.01 ptd1ino_RT_r 11381

Old PINOSer_RT: 0.01 cdpdag_SC_r + inost_r --> cmp_r + h_r + 0.01 ptd1ino_SC_r 15121
New PINOSer_RT: 0.01 cdpdag_RT_r + inost_r --> cmp_r + h_r + 0.01 ptd1ino_RT_r 15121

Old PLBP1Ie_RT: h2o_e + 0.005 ptd1ino_SC_e --> 0.02 dca_e + 0.06 ddca_e + 0.5 g3pi_e + h_e + 0.27 hdca_e + 0.17 hdcea_e + 0.05 ocdca_e + 0.24 ocdcea_e + 0.09 ocdcya_e + 0.1 ttdca_e 12385
New PLBP1Ie_RT: h2o_e + 0.005 ptd1ino_RT_e --> 0.02 dca_e + 0.06 ddca_e + 0.5 g3pi_e + h_e + 0.27 hdca_e + 0.17 hdcea_e + 0.05 ocdca_e + 0.24 ocdcea_e + 0.09 ocdcya_e + 0.1 ttdca_e 12385

Old PLBPC_RT: h2o_c + 0.005 pc_SC_r --> 0.02 dca_c + 0.06 ddca_c + 0.5 g3pc_c + h_c + 0.27 hdca_c + 0.17 hdcea_c + 0.05 ocdca_c + 0.24 ocdcea_c + 0.09 ocdcya_c + 0.1 ttdca_c 14309
New PLBPC_RT: h2o_c + 0.005 pc_RT_r --> 0.02 dca_c + 0.06 ddca_c + 0.5 g3pc_c + h_c + 0.27 hdca_c + 0.17 hdcea_c + 0.05 ocdca_c + 0.24 ocdcea_c + 0.09 ocdcya_c + 0.1 ttdca_c 14309

Old PLBPCe_RT: h2o_e + 0.005 pc_SC_e --> 0.02 dca_e + 0.06 ddca_e + 0.5 g3pc_e + h_e + 0.27 hdca_e + 0.17 hdcea_e + 0.05 ocdca_e + 0.24 ocdcea_e + 0.09 ocdcya_e + 0.1 ttdca_e 12385
New PLBPCe_RT: h2o_e + 0.005 pc_RT_e --> 0.02 dca_e + 0.06 ddca_e + 0.5 g3pc_e + h_e + 0.27 hdca_e + 0.17 hdcea_e + 0.05 ocdca_e + 0.24 ocdcea_e + 0.09 ocdcya_e + 0.1 ttdca_e 12385

Old PLBPEe_RT: h2o_e + 0.005 pe_SC_e --> 0.02 dca_e + 0.06 ddca_e + 0.5 g3pe_e + h_e + 0.27 hdca_e + 0.17 hdcea_e + 0.05 ocdca_e + 0.24 ocdcea_e + 0.09 ocdcya_e + 0.1 ttdca_e 12385
New PLBPEe_RT: h2o_e + 0.005 pe_RT_e --> 0.02 dca_e + 0.06 ddca_e + 0.5 g3pe_e + h_e + 0.27 hdca_e + 0.17 hdcea_e + 0.05 ocdca_e + 0.24 ocdcea_e + 0.09 ocdcya_e + 0.1 ttdca_e 12385

Old PLDn_RT: h2o_n + 0.01 pc_SC_n --> chol_n + h_n + 0.01 pa_SC_n 14023
New PLDn_RT: h2o_n + 0.01 pc_RT_n --> chol_n + h_n + 0.01 pa_RT_n 14023

Old PMETMer_RT: amet_r + 0.01 ptd2meeta_SC_r --> ahcys_r + h_r + 0.01 pc_SC_r 12748
New PMETMer_RT: amet_r + 0.01 ptd2meeta_RT_r --> ahcys_r + h_r + 0.01 pc_RT_r 12748

Old PROt2r: h_e + pro__L_e <=> h_c + pro__L_c 12743 or 14229 or 15074 or (YALI0B09537g and 14229) or (YALI0B09537g and 15074)
New PROt2r: h_e + pro__L_e <=> h_c + pro__L_c 12743 or 14229 or 15074

Old PSERDer_RT: h_r + 0.01 ps_SC_r --> co2_r + 0.01 pe_SC_r 14554
New PSERDer_RT: h_r + 0.01 ps_RT_r --> co2_r + 0.01 pe_RT_r 14554

Old PSERDg_RT: h_g + 0.01 ps_SC_g --> co2_g + 0.01 pe_SC_g 10504 or 11446
New PSERDg_RT: h_g + 0.01 ps_RT_g --> co2_g + 0.01 pe_RT_g 10504 or 11446

Old PSERDv_RT: h_v + 0.01 ps_SC_v --> co2_v + 0.01 pe_SC_v 10504 or 11446
New PSERDv_RT: h_v + 0.01 ps_RT_v --> co2_v + 0.01 pe_RT_v 10504 or 11446

Old PSERSer_RT: 0.01 cdpdag_SC_r + ser__L_r --> cmp_r + h_r + 0.01 ps_SC_r 11583
New PSERSer_RT: 0.01 cdpdag_RT_r + ser__L_r --> cmp_r + h_r + 0.01 ps_RT_r 11583

Old PSERSm_RT: 0.01 cdpdag_SC_m + ser__L_m --> cmp_m + h_m + 0.01 ps_SC_m 11583
New PSERSm_RT: 0.01 cdpdag_RT_m + ser__L_m --> cmp_m + h_m + 0.01 ps_RT_m 11583

Old PSterm_RT: ps_SC_r <=> ps_SC_m 
New PSterm_RT: ps_RT_r <=> ps_RT_m 

Old SADT: atp_c + h_c + so4_c --> aps_c + ppi_c 13443 or 8709 or (PP_1303 and 14868)
New SADT: atp_c + h_c + so4_c --> aps_c + ppi_c 13443 or 8709

Old SERt2r: h_e + ser__L_e <=> h_c + ser__L_c 14229 or 15074 or 8962 or 9319 or 9322 or 9962 or (YALI0E20713g and 14229 and 8962) or (YALI0E20713g and 14229 and 9319) or (YALI0E20713g and 14229 and 9322) or (YALI0E20713g and 14229 and 9962) or (YALI0E20713g and 15074 and 8962) or (YALI0E20713g and 15074 and 9319) or (YALI0E20713g and 15074 and 9322) or (YALI0E20713g and 15074 and 9962)
New SERt2r: h_e + ser__L_e <=> h_c + ser__L_c 14229 or 15074 or 8962 or 9319 or 9322 or 9962

Old TAGtrd: triglyc_SC_r --> triglyc_RT_d 
New TAGtrd: triglyc_RT_r --> triglyc_RT_d 

Old TRE6PP: h2o_c + tre6p_c --> pi_c + tre_c 12034 or (YML100W and 11389 and 12034) or (YMR261C and 11389 and 12034)
New TRE6PP: h2o_c + tre6p_c --> pi_c + tre_c 12034

Old TRIGSer_RT: 0.01 12dgr_SC_r + 0.02 dcacoa_r + 0.06 ddcacoa_r + 0.17 hdcoa_r + 0.09 ocdycacoa_r + 0.24 odecoa_r + 0.27 pmtcoa_r + 0.05 stcoa_r + 0.1 tdcoa_r --> coa_r + 0.01 triglyc_SC_r 16460
New TRIGSer_RT: 0.01 12dgr_RT_r + 0.02 dcacoa_r + 0.06 ddcacoa_r + 0.17 hdcoa_r + 0.09 ocdycacoa_r + 0.24 odecoa_r + 0.27 pmtcoa_r + 0.05 stcoa_r + 0.1 tdcoa_r --> coa_r + 0.01 triglyc_RT_r 16460

Old TRPS1: 3ig3p_c + ser__L_c --> g3p_c + h2o_c + trp__L_c 9262 or (PP_0082 and 9262) or (b1260 and 9262)
New TRPS1: 3ig3p_c + ser__L_c --> g3p_c + h2o_c + trp__L_c 9262

Old TRPS2: indole_c + ser__L_c --> h2o_c + trp__L_c 9262 or (b1260 and 9262)
New TRPS2: indole_c + ser__L_c --> h2o_c + trp__L_c 9262

Old XAND: h2o_c + nad_c + xan_c --> h_c + nadh_c + urate_c 15962 or (b2868 and 15962)
New XAND: h2o_c + nad_c + xan_c --> h_c + nadh_c + urate_c 15962

Old ZYMSTATer_RT: 0.655 hdcoa_r + 0.01 hexccoa_r + 0.27 odecoa_r + 0.02 pmtcoa_r + 0.03 stcoa_r + 0.015 tdcoa_r + 0.01 zymst_r --> coa_r + 0.01 zymstest_SC_r 11799
New ZYMSTATer_RT: 0.655 hdcoa_r + 0.01 hexccoa_r + 0.27 odecoa_r + 0.02 pmtcoa_r + 0.03 stcoa_r + 0.015 tdcoa_r + 0.01 zymst_r --> coa_r + 0.01 zymstest_RT_r 11799

Old ZYMSTESTtrd: zymstest_SC_r --> zymstest_RT_d 
New ZYMSTESTtrd: zymstest_RT_r --> zymstest_RT_d
```

In [49]:

```
print('Added reactions\n')
for r in sorted(model_new.reactions, key=lambda x: x.id):
    if r not in model_old.reactions:
        print(r)
```

```
Added reactions

CLPNSm_RT: 0.01 cdpdag_RT_m + 0.01 pg_RT_m --> 0.01 clpn_RT_m + cmp_m + h_m
DABT2D: abt__D_c + nad_c <=> h_c + nadh_c + rbl__D_c
PGPPAm_RT: h2o_m + 0.01 pgp_RT_m --> 0.01 pg_RT_m + pi_m
PSERDm_RT: h_m + 0.01 ps_RT_m --> co2_m + 0.01 pe_RT_m
```
