# Supplementary material for: Multi-Omics Driven Metabolic Network Reconstruction and Analysis of Lignocellulosic Carbon Utilization in Rhodosporidium toruloides
Source: Front Bioeng Biotechnol. 2021 Jan 8;8:612832. doi: 10.3389/fbioe.2020.612832 (PMC7873862; doi:10.3389/fbioe.2020.612832)
Supplement: Supplementary File 4 — Multi-omics dataset for R. toruloides IFO0880. [file Data_Sheet_1.zip › Supplementary File S1/1.Manual_curation/Refinement_1c_Fatty_Acids_and_Lipid_Metabolism.html]

Refinement\_1c\_Fatty\_Acids\_and\_Lipid\_Metabolism


In [1]:

```
%matplotlib inline
from matplotlib import pyplot as plt
from matplotlib import colors
import csv
import numpy as np
import pandas as pd
import cobra
```

In [2]:

```
cobra.__version__
```

Out[2]:

```
'0.17.1'
```

In [3]:

```
Annotation = pd.read_excel('../../Data/R_toruloides_Data_for_Reconstruction.xlsx',
                          sheet_name='Annotation', index_col=0)
Annotation.index = Annotation.index.map(str)
Annotation = Annotation.fillna('')
Transcriptomics = pd.read_excel('../../Data/R_toruloides_Data_for_Reconstruction.xlsx',
                          sheet_name='Transcriptomics', header=[0,1,2,3], index_col=0)
Transcriptomics.index = Transcriptomics.index.map(str)
Proteomics = pd.read_excel('../../Data/R_toruloides_Data_for_Reconstruction.xlsx',
                          sheet_name='Proteomics', header=[0,1,2], index_col=0)
Proteomics.index = Proteomics.index.map(str)
Fitness = pd.read_excel('../../Data/R_toruloides_Data_for_Reconstruction.xlsx',
                          sheet_name='Fitness', index_col=0)
Fitness.index = Fitness.index.map(str)
```

In [4]:

```
def background_gradient(s, cmap='seismic', text_color_threshold=0.408):
    lim = max(abs(s.min().min()),abs(s.max().max()))
    rng = 2.0*lim
    norm = colors.Normalize(-lim - (rng * 0.2), lim + (rng * 0.2))
    rgbas = plt.cm.get_cmap(cmap)(norm(s.values))
    def relative_luminance(rgba):
        r, g, b = (x / 12.92 if x <= 0.03928 else ((x + 0.055) / 1.055 ** 2.4) for x in rgba[:3])
        return 0.2126 * r + 0.7152 * g + 0.0722 * b
    def css(rgba):
        dark = relative_luminance(rgba) < text_color_threshold
        text_color = '#f1f1f1' if dark else '#000000'
        return 'background-color: {b};color: {c};'.format(b=colors.rgb2hex(rgba), c=text_color)

    if s.ndim == 1:
        return [css(rgba) for rgba in rgbas]
    else:
        return pd.DataFrame([[css(rgba) for rgba in row] for row in rgbas], index=s.index, columns=s.columns)

def Show_Data(x):
    display(Transcriptomics.loc[x].style.background_gradient(cmap='Reds', low=0.2, high=0.2, axis=None))
    temp = [y for y in x if y in Proteomics.index]
    display(Proteomics.loc[temp].style.background_gradient(cmap='Reds', low=0.2, high=0.2, axis=None))
    temp = [y for y in x if y in Fitness.index]
    display(Fitness.loc[temp].style.apply(background_gradient, cmap='seismic', axis=None))
    return;
```

In [5]:

```
eco = cobra.io.load_json_model('../../Data/BiGG_Models/iML1515.json')
sce = cobra.io.load_json_model('../../Data/BiGG_Models/iMM904.json')
hsa = cobra.io.load_json_model('../../Data/BiGG_Models/RECON1.json')
hsa2 = cobra.io.load_json_model('../../Data/BiGG_Models/Recon3D.json')
ptri = cobra.io.load_json_model('../../Data/BiGG_Models/iLB1027_lipid.json')
```

In [6]:

```
model = cobra.io.load_json_model("IFO0880_GPR_1b.json")
```

In [7]:

```
print(len(model.genes))
print(len([x for x in model.genes if not x.id[0].isalpha()]))
model
```

```
1312
1150
```

Out[7]:

|  |  |
| --- | --- |
| **Name** | R. toruloides |
| **Memory address** | 0x0102f395e80 |
| **Number of metabolites** | 3283 |
| **Number of reactions** | 3264 |
| **Number of groups** | 0 |
| **Objective expression** | 0 |
| **Compartments** | c, x, m, e, r, v, n, g, p, h, s, f, l |

### Fatty acid degradation¶

In [8]:

```
for r in sorted(model.reactions, key=lambda x: x.id):
    if r.id.startswith('ACACT') and r.id.endswith('m'):
        print(r.id, r.reaction, r.gene_reaction_rule)
print()
for r in sorted(model.reactions, key=lambda x: x.id):
    if r.id.startswith('ACACT') and r.id.endswith('r'):
        print(r.id, r.reaction, r.gene_reaction_rule)
print()
for r in sorted(model.reactions, key=lambda x: x.id):
    if r.id.startswith('ACACT') and not (r.id.endswith('m') or r.id.endswith('r')):
        print(r.id, r.reaction, r.gene_reaction_rule)
```

```
ACACT10m 2maacoa_m + coa_m <=> accoa_m + ppcoa_m 13813 or 8678
ACACT1m 2.0 accoa_m --> aacoa_m + coa_m 8678 or 8885
ACACT2m accoa_m + btcoa_m --> 3ohcoa_m + coa_m 13813
ACACT3m accoa_m + hxcoa_m --> 3oocoa_m + coa_m 13813
ACACT4m accoa_m + occoa_m <-- 3odcoa_m + coa_m 13813 or 9065
ACACT5m accoa_m + dcacoa_m --> 3oddcoa_m + coa_m 13813
ACACT6m accoa_m + ddcacoa_m <-- 3otdcoa_m + coa_m 13813 or 9065
ACACT7m accoa_m + tdcoa_m <-- 3ohdcoa_m + coa_m 13813 or 9065

ACACT1r 2.0 accoa_c <=> aacoa_c + coa_c 13813 or 8678 or 8885
ACACT2r accoa_c + btcoa_c <=> 3ohcoa_c + coa_c 13813 or 8678 or 8885
ACACT3r accoa_c + hxcoa_c <=> 3oocoa_c + coa_c 13813
ACACT4r accoa_c + occoa_c <=> 3odcoa_c + coa_c 13813
ACACT5r accoa_c + dcacoa_c <=> 3oddcoa_c + coa_c 13813
ACACT6r accoa_c + ddcacoa_c <=> 3otdcoa_c + coa_c 13813
ACACT7r accoa_c + tdcoa_c <=> 3ohdcoa_c + coa_c 13813
ACACT8r 3ohodcoa_c + coa_c <=> accoa_c + pmtcoa_c 13813

ACACT1x 2.0 accoa_x <=> aacoa_x + coa_x 8678 or 8885
ACACT2 3ohcoa_x + coa_x <=> accoa_x + btcoa_x 13813 or 8678 or 8885
ACACT3 3oocoa_x + coa_x <=> accoa_x + hxcoa_x 13813
ACACT4p 3odcoa_x + coa_x --> accoa_x + occoa_x 13813 or 9065
ACACT5p 3oddcoa_x + coa_x --> accoa_x + dcacoa_x 13813 or 9065
ACACT6p 3otdcoa_x + coa_x --> accoa_x + ddcacoa_x 13813 or 9065
ACACT7p 3ohdcoa_x + coa_x --> accoa_x + tdcoa_x 13813 or 9065
ACACT8p 3ohodcoa_x + coa_x --> accoa_x + pmtcoa_x 13813 or 9065
ACACT9p 3ohxccoa_x + coa_x --> accoa_x + ttccoa_x 13813 or 9065
```

In [9]:

```
temp = ['8678','8885','13813','9065','15228','14055','11237']
display(Annotation.loc[temp])
Show_Data(temp)
```

|  | Combined Annotations | Signal P | Sc288c Orthologs | Human Orthologs | Sc288 Best Hit | Human Blast | Essential | WolfPSort | C Terminal |
| --- | --- | --- | --- | --- | --- | --- | --- | --- | --- |
| RTO4\_ID |  |  |  |  |  |  |  |  |  |
| 8678 | K00626: E2.3.1.9, atoB; acetyl-CoA C-acetyltra... |  | ERG10 | ACAT1 | ERG10 | ACAT1 | Not Essential | mito 23.5, cyto\_mito 14 | QRL\* |
| 8885 | K07508: ACAA2; acetyl-CoA acyltransferase 2 |  |  | ACAA2 | ERG10 | ACAA2 | Not Essential | mito 12, cyto 11, pero 3 | ERV\* |
| 13813 | K07513: ACAA1; acetyl-CoA acyltransferase 1 |  | POT1 | ACAA1 | POT1 | ACAA1 | Not Essential | mito 18.5, cyto\_mito 13, cyto 6.5 | AEN\* |
| 9065 | KOG1389: 3-oxoacyl CoA thiolase |  | POT1 | ACAA1 | POT1 | ACAA1 | Not Essential | mito 13, cyto 10, nucl 2 | VAE\* |
| 15228 | K07513: ACAA1; acetyl-CoA acyltransferase 1 |  |  |  | POT1 | ACAA1 | Not Essential | cyto 14, mito 13 | VRD\* |
| 14055 | K08764: SCP2, SCPX; sterol carrier protein 2 |  |  | SCP2 |  | SCP2 | Not Essential | cyto\_mito 13.333, mito 13, cyto 11.5, cyto\_nuc... | ANL\* |
| 11237 | KOG4170: 2-enoyl-CoA hydratase/3-hydroxyacyl-C... |  |  |  |  |  | Not Essential | mito\_nucl 10.166, nucl 10, mito 10, cyto\_nucl 8 | AKL\* |

| strain | WT | | | | | | | | | | | | | | | | |
| --- | --- | --- | --- | --- | --- | --- | --- | --- | --- | --- | --- | --- | --- | --- | --- | --- | --- |
| condition | G\_MM | C\_MM | G\_SD | | GX\_SD | | | X\_SD | | A\_SD | | C\_SD | | MM\_CN120 | | MM\_CN5 | Diversity\_Sample |
| phase | exp | exp | exp | stat | exp | trans | stat | exp | stat | exp | stat | exp | stat | exp | stat | exp | exp |
| proteinId | Set1 | Set1 | Set2 | Set2 | Set2 | Set2 | Set2 | Set2 | Set2 | Set2 | Set2 | Set2 | Set2 | Set3 | Set3 | Set3 | Set3 |
| 8678 | 7.02165 | 8.49604 | 6.56773 | 5.30595 | 6.67599 | 5.76971 | 5.63167 | 7.10664 | 5.26798 | 5.7248 | 5.42382 | 5.88536 | 5.87647 | 7.0048 | 6.36022 | 8.25583 | 7.88214 |
| 8885 | 5.50456 | 6.56512 | 8.06594 | 6.66451 | 8.04905 | 7.5543 | 7.73885 | 7.49931 | 6.91044 | 5.93075 | 6.44877 | 5.70682 | 5.01682 | 5.69492 | 7.32498 | 7.22296 | 6.63295 |
| 13813 | 5.1178 | 7.3876 | 6.03318 | 5.96724 | 5.98504 | 6.21365 | 6.14483 | 6.10575 | 6.68139 | 6.45958 | 7.17877 | 4.89153 | 5.56731 | 6.09138 | 6.73907 | 6.06941 | 5.88532 |
| 9065 | 6.87944 | 10.1979 | 5.14482 | 6.8755 | 5.59546 | 5.11208 | 5.34363 | 5.54889 | 6.85322 | 6.1589 | 6.98373 | 7.89676 | 7.21514 | 6.43406 | 5.64064 | 6.48087 | 5.47448 |
| 15228 | 5.08351 | 8.82976 | 6.51084 | 6.27447 | 6.57848 | 6.36614 | 6.38556 | 5.34693 | 6.75073 | 5.03638 | 6.14225 | 8.95082 | 8.02647 | 5.47011 | 4.79342 | 5.22158 | 5.51855 |
| 14055 | 4.9469 | 5.44289 | 4.90852 | 7.01407 | 4.66565 | 5.30569 | 5.40196 | 4.76118 | 6.01986 | 5.85943 | 6.59248 | 4.97965 | 3.45072 | 5.13243 | 4.58448 | 4.16971 | 5.83072 |
| 11237 | 6.86008 | 6.54251 | 6.03446 | 6.90183 | 5.95762 | 6.07863 | 6.08366 | 5.98974 | 6.61385 | 6.50627 | 6.95316 | 6.05677 | 6.11439 | 6.16105 | 6.40974 | 6.13112 | 5.53001 |

| strain | WT | | | | | | | | | | |
| --- | --- | --- | --- | --- | --- | --- | --- | --- | --- | --- | --- |
| condition | G\_SD | | GX\_SD | | | X\_SD | | A\_SD | | C\_SD | |
| proteinId | exp | stat | exp | trans | stat | exp | stat | exp | stat | exp | stat |
| 8678 | 24.2091 | 19.2125 | 22.5986 | 15.4605 | 14.3787 | 24.8578 | 22.1975 | 26.0698 | 22.4098 | 28.6911 | 21.4138 |
| 8885 | 23.1969 | 23.6191 | 20.995 | 28.2507 | 31.2032 | 18.9939 | 22.0911 | 13.5247 | 12.6383 | 22.6354 | 20.6502 |
| 13813 | 9.63957 | 15.6393 | 7.31655 | 15.6204 | 17.2048 | 12.5533 | 15.9549 | 15.0541 | 17.6173 | 24.763 | 22.1905 |
| 9065 | 8.79404 | 15.4411 | 8.72689 | 10.2283 | 9.64574 | 8.99429 | 13.9761 | 15.269 | 19.7318 | 67.2783 | 45.319 |
| 15228 | 12.5114 | 17.7373 | 14.8422 | 18.9819 | 18.4454 | 14.6812 | 15.1221 | 11.2071 | 10.7048 | 37.8231 | 38.5613 |
| 14055 | 0.382292 | 0.901646 | 0.197656 | 1.1041 | 1.06255 | 0.796651 | 2.19945 | 2.31789 | 4.17809 | 2.5547 | 0.42946 |
| 11237 | 2.09351 | 5.81273 | 2.83688 | 3.90854 | 4.59655 | 4.70509 | 6.8702 | 5.40381 | 6.52007 | 4.72439 | 3.94011 |

|  | Glucose | Xylose | Arabinose | Acetate | Coumarate | Ferulate | YNB Oleic Acid | YNB Ricinoleic Acid | YNB Glucose | YNB Gluc DOC | YPD |
| --- | --- | --- | --- | --- | --- | --- | --- | --- | --- | --- | --- |
| proteinId |  |  |  |  |  |  |  |  |  |  |  |
| 8885 | -0.216781 | 0.0868698 | 0.107291 | -0.144912 | -0.0878781 | 0.256151 | -0.409361 | -0.996266 | -0.417173 | -0.594114 | 0.200075 |
| 13813 | 0.12641 | 0.107964 | 0.192915 | 0.252853 | 0.192276 | 0.251335 | -2.49127 | -1.6891 | -0.0652642 | -0.2766 | 0.225342 |
| 9065 | -0.350988 | -0.271906 | -0.134182 | -0.284826 | -5.6088 | -3.42636 | -0.290069 | 0.247955 | 0.258871 | 0.400966 | -0.20317 |
| 15228 | -0.223091 | -0.11819 | -0.335936 | -0.277735 | -4.99507 | -4.67996 | 0.138344 | 0.163114 | 0.296363 | 0.54175 | 0.402816 |
| 14055 | 0.39435 | 0.131153 | 0.25378 | 0.0630405 | 0.300374 | -0.158759 | -0.304284 | -0.624025 | 0.255405 | -0.263458 | -0.21954 |
| 11237 | -0.0579288 | -0.355731 | 0.0689474 | 0.0852672 | 0.215514 | -0.159126 | -0.115729 | -0.0764419 | 0.094597 | 0.206729 | -0.335975 |

8678 mito 23.5, cyto\_mito 14 (no sigP) ERG10 Acetyl-CoA acetyltransferase, KEGG acetyl-CoA C-acetyltransferase 2.3.1.9 (ACAT1) QRL *8885 mito 12, cyto 11, pero 3 (no sigP) Acetyl-CoA acetyltransferase, KEGG acetyl-CoA acyltransferase 2 2.3.1.16 (ACAA2) -> mito fatty acid beta-oxidation ERV*  
13813 mito 18.5, cyto\_mito 13, cyto 6.5 POT1 (sigP) 3-oxoacyl CoA thiolase, KEGG acetyl-CoA acyltransferase 1 2.3.1.16 (ACAA1) -> peroxisomal fatty acid beta-oxidation AEN\*

These two have significant fitness defect in coumarate or ferulate - 4HB-acetyl-CoA to 4HB-CoA / 3-oxoadipyl CoA thiolase  
9065 mito 13, cyto 10, nucl 2 POT1 (no sigP) 3-oxoacyl CoA thiolase, KEGG acetyl-CoA acyltransferase 1 2.3.1.16 (ACAA1) -> 4HB-acetyl-CoA to 4HB-CoA  
15228 cyto 14, mito 13 (no sigP) 3-oxoacyl CoA thiolase, KEGG acetyl-CoA acyltransferase 1 2.3.1.16 (ACAA1), no rxn -> blast hit to 3-oxoadipyl CoA thiolase

14055 cyto\_mito 13.333, mito 13, cyto 11.5, cyto\_nucl 7.166 Peroxisomal 3-ketoacyl-CoA-thiolase P-44/SCP2, KEGG propanoyl-CoA C-acyltransferase 2.3.1.176 (SCP2) -> check reactions  
11237 mito\_nucl 10.166, nucl 10, mito 10, cyto\_nucl 8 sterol-binding domain and related enzymes, shorter only SCP-2 sterol transfer family domain, KEGG 2.3.1.176, no rxn

ERG10 is cyto and human ACAT1 is mito, works on ACACT1 and ACACT10  
Change ACACT1r genes to '8678'  
Remove ACACT2r-8r  
Change ACACT1m genes to '8678', make it reversible  
Change ACACT2m, ACACT3m, ACACT5m irreversible in the reverse direction  
Change ACACT2m-7m genes to '8885'  
Change ACACT10m genes to '8678'  
Reverse ACACT1x  
Change ACACT1x, ACACT2, ACACT3 to ACACT1p, ACACT2p, ACACT3p  
Change ACACTxp genes to '13813'

In [10]:

```
model.reactions.get_by_id('ACACT1r').gene_reaction_rule = '8678'
model.remove_reactions(['ACACT2r','ACACT3r','ACACT4r','ACACT5r','ACACT6r','ACACT7r','ACACT8r'], remove_orphans=True)
model.reactions.get_by_id('ACACT1m').gene_reaction_rule = '8678'
model.reactions.get_by_id('ACACT1m').lower_bound = -1000.0
model.reactions.get_by_id('ACACT2m').upper_bound = 0.0
model.reactions.get_by_id('ACACT2m').lower_bound = -1000.0
model.reactions.get_by_id('ACACT3m').upper_bound = 0.0
model.reactions.get_by_id('ACACT3m').lower_bound = -1000.0
model.reactions.get_by_id('ACACT5m').upper_bound = 0.0
model.reactions.get_by_id('ACACT5m').lower_bound = -1000.0
for r in sorted(model.reactions, key=lambda x: x.id):
    if r.id.startswith('ACACT') and not r.id.startswith('ACACT1') and r.id.endswith('m'):
        r.gene_reaction_rule = '8885'
model.reactions.get_by_id('ACACT10m').gene_reaction_rule = '8678'
for m in model.reactions.get_by_id('ACACT1x').metabolites:
    model.reactions.get_by_id('ACACT1x').add_metabolites({m.id: -2*model.reactions.get_by_id('ACACT1x').get_coefficient(m.id)})
model.reactions.get_by_id('ACACT1x').id = 'ACACT1p'
model.reactions.get_by_id('ACACT2').id = 'ACACT2p'
model.reactions.get_by_id('ACACT3').id = 'ACACT3p'
model.reactions.get_by_id('ACACT1p').lower_bound = 0.0
model.reactions.get_by_id('ACACT2p').lower_bound = 0.0
model.reactions.get_by_id('ACACT3p').lower_bound = 0.0
for r in sorted(model.reactions, key=lambda x: x.id):
    if r.id.startswith('ACACT') and r.id.endswith('p'):
        r.gene_reaction_rule = '13813'
```

In [11]:

```
for r in sorted(model.genes.get_by_id('8678').reactions, key=lambda x: x.id):
    print(r.id, r.reaction, r.gene_reaction_rule)
print()
for r in sorted(model.genes.get_by_id('8885').reactions, key=lambda x: x.id):
    print(r.id, r.reaction, r.gene_reaction_rule)
print()
for r in sorted(model.genes.get_by_id('13813').reactions, key=lambda x: x.id):
    if 'FAO' not in r.id:
        print(r.id, r.reaction, r.gene_reaction_rule)
print()
for r in sorted(model.genes.get_by_id('9065').reactions, key=lambda x: x.id):
    if 'FAO' not in r.id:
        print(r.id, r.reaction, r.gene_reaction_rule)
```

```
ACACT10m 2maacoa_m + coa_m <=> accoa_m + ppcoa_m 8678
ACACT1m 2.0 accoa_m <=> aacoa_m + coa_m 8678
ACACT1r 2.0 accoa_c <=> aacoa_c + coa_c 8678

ACACT2m accoa_m + btcoa_m <-- 3ohcoa_m + coa_m 8885
ACACT3m accoa_m + hxcoa_m <-- 3oocoa_m + coa_m 8885
ACACT4m accoa_m + occoa_m <-- 3odcoa_m + coa_m 8885
ACACT5m accoa_m + dcacoa_m <-- 3oddcoa_m + coa_m 8885
ACACT6m accoa_m + ddcacoa_m <-- 3otdcoa_m + coa_m 8885
ACACT7m accoa_m + tdcoa_m <-- 3ohdcoa_m + coa_m 8885

ACACT1p aacoa_x + coa_x --> 2.0 accoa_x 13813
ACACT2p 3ohcoa_x + coa_x --> accoa_x + btcoa_x 13813
ACACT3p 3oocoa_x + coa_x --> accoa_x + hxcoa_x 13813
ACACT4p 3odcoa_x + coa_x --> accoa_x + occoa_x 13813
ACACT5p 3oddcoa_x + coa_x --> accoa_x + dcacoa_x 13813
ACACT6p 3otdcoa_x + coa_x --> accoa_x + ddcacoa_x 13813
ACACT7p 3ohdcoa_x + coa_x --> accoa_x + tdcoa_x 13813
ACACT8p 3ohodcoa_x + coa_x --> accoa_x + pmtcoa_x 13813
ACACT9p 3ohxccoa_x + coa_x --> accoa_x + ttccoa_x 13813
KAT180_m 3ohodcoa_m + coa_m --> accoa_m + pmtcoa_m 13813 or 9065
MACCOAT 2maacoa_c + coa_c --> accoa_c + ppcoa_c 13813 or 9065
PEROXx 3.0 coa_x + 3.0 h2o_x + 3.0 nad_x + 2.0 o2_x + pristcoa_x --> accoa_x + dmnoncoa_x + 2.0 h2o2_x + 3.0 h_x + 3.0 nadh_x + 2.0 ppcoa_x (ACOX3 and EHHADH and 11362 and 13813) or (ACOX3 and EHHADH and 11362 and 9065) or (Acox3 and Ehhadh and 11362 and 13813) or (Acox3 and Ehhadh and 11362 and 9065)
yli_R0194 coa_m + yli_M04585_m --> accoa_m + yli_M04095_m 13813 or 9065
yli_R0195 3.0 coa_m + 3.0 h2o_m + 3.0 nad_m + 3.0 o2_m + yli_M04095_m --> 3.0 accoa_m + 3.0 h2o2_m + 3.0 h_m + 3.0 nadh_m + stcoa_m (12742 and 13813 and 14805) or (12742 and 14805 and 9065) or (12752 and 13813 and 14805) or (12752 and 14805 and 9065) or (13813 and 14805 and 9700) or (14805 and 9065 and 9700)
yli_R0211 3oddcoa_m + coa_m --> accoa_m + dccoa_m 13813 or 9065
yli_R0216 3.0 coa_m + 3.0 h2o_m + 3.0 nad_m + 3.0 o2_m + occoa_m --> 4.0 accoa_m + 3.0 h2o2_m + 3.0 h_m + 3.0 nadh_m (12742 and 13813 and 14805) or (12742 and 14805 and 9065) or (12752 and 13813 and 14805) or (12752 and 14805 and 9065) or (13813 and 14805 and 9700) or (14805 and 9065 and 9700)
yli_R0217 6.0 coa_m + 6.0 h2o_m + 6.0 nad_m + nadph_m + 6.0 o2_m + yli_M04635_m --> 7.0 accoa_m + 6.0 h2o2_m + 5.0 h_m + 6.0 nadh_m + nadp_m (12742 and 13813 and 14805) or (12742 and 14805 and 9065) or (12752 and 13813 and 14805) or (12752 and 14805 and 9065) or (13813 and 14805 and 9700) or (14805 and 9065 and 9700)
yli_R0218 6.0 coa_m + 6.0 h2o_m + 6.0 nad_m + 5.0 o2_m + yli_M04635_m --> 7.0 accoa_m + 5.0 h2o2_m + 6.0 h_m + 6.0 nadh_m (12742 and 13813 and 14805) or (12742 and 14805 and 9065) or (12752 and 13813 and 14805) or (12752 and 14805 and 9065) or (13813 and 14805 and 9700) or (14805 and 9065 and 9700)
yli_R0219 7.0 coa_m + 7.0 h2o_m + 7.0 nad_m + nadph_m + 7.0 o2_m + yli_M04594_m --> 8.0 accoa_m + 7.0 h2o2_m + 6.0 h_m + 7.0 nadh_m + nadp_m (12742 and 13813 and 14805) or (12742 and 14805 and 9065) or (12752 and 13813 and 14805) or (12752 and 14805 and 9065) or (13813 and 14805 and 9700) or (14805 and 9065 and 9700)
yli_R0220 7.0 coa_m + 7.0 h2o_m + 7.0 nad_m + 6.0 o2_m + yli_M04594_m --> 8.0 accoa_m + 6.0 h2o2_m + 7.0 h_m + 7.0 nadh_m (12742 and 13813 and 14805) or (12742 and 14805 and 9065) or (12752 and 13813 and 14805) or (12752 and 14805 and 9065) or (13813 and 14805 and 9700) or (14805 and 9065 and 9700)
yli_R0221 8.0 coa_m + 8.0 h2o_m + 8.0 nad_m + nadph_m + 8.0 o2_m + yli_M04626_m --> 9.0 accoa_m + 8.0 h2o2_m + 7.0 h_m + 8.0 nadh_m + nadp_m (12742 and 13813 and 14805) or (12742 and 14805 and 9065) or (12752 and 13813 and 14805) or (12752 and 14805 and 9065) or (13813 and 14805 and 9700) or (14805 and 9065 and 9700)
yli_R0222 8.0 coa_m + 8.0 h2o_m + 8.0 nad_m + 7.0 o2_m + yli_M04626_m --> 9.0 accoa_m + 7.0 h2o2_m + 8.0 h_m + 8.0 nadh_m (12742 and 13813 and 14805) or (12742 and 14805 and 9065) or (12752 and 13813 and 14805) or (12752 and 14805 and 9065) or (13813 and 14805 and 9700) or (14805 and 9065 and 9700)
yli_R0223 8.0 coa_m + 8.0 h2o_m + 8.0 nad_m + 2.0 nadph_m + 8.0 o2_m + yli_M04625_m --> 9.0 accoa_m + 8.0 h2o2_m + 6.0 h_m + 8.0 nadh_m + 2.0 nadp_m (12742 and 13813 and 14805) or (12742 and 14805 and 9065) or (12752 and 13813 and 14805) or (12752 and 14805 and 9065) or (13813 and 14805 and 9700) or (14805 and 9065 and 9700)
yli_R0224 8.0 coa_m + 8.0 h2o_m + 8.0 nad_m + nadph_m + 7.0 o2_m + yli_M04625_m --> 9.0 accoa_m + 7.0 h2o2_m + 7.0 h_m + 8.0 nadh_m + nadp_m (12742 and 13813 and 14805) or (12742 and 14805 and 9065) or (12752 and 13813 and 14805) or (12752 and 14805 and 9065) or (13813 and 14805 and 9700) or (14805 and 9065 and 9700)
yli_R0225 8.0 coa_m + 8.0 h2o_m + 8.0 nad_m + 6.0 o2_m + yli_M04625_m --> 9.0 accoa_m + 6.0 h2o2_m + 8.0 h_m + 8.0 nadh_m (12742 and 13813 and 14805) or (12742 and 14805 and 9065) or (12752 and 13813 and 14805) or (12752 and 14805 and 9065) or (13813 and 14805 and 9700) or (14805 and 9065 and 9700)

KAT180_m 3ohodcoa_m + coa_m --> accoa_m + pmtcoa_m 13813 or 9065
MACCOAT 2maacoa_c + coa_c --> accoa_c + ppcoa_c 13813 or 9065
PEROXx 3.0 coa_x + 3.0 h2o_x + 3.0 nad_x + 2.0 o2_x + pristcoa_x --> accoa_x + dmnoncoa_x + 2.0 h2o2_x + 3.0 h_x + 3.0 nadh_x + 2.0 ppcoa_x (ACOX3 and EHHADH and 11362 and 13813) or (ACOX3 and EHHADH and 11362 and 9065) or (Acox3 and Ehhadh and 11362 and 13813) or (Acox3 and Ehhadh and 11362 and 9065)
yli_R0194 coa_m + yli_M04585_m --> accoa_m + yli_M04095_m 13813 or 9065
yli_R0195 3.0 coa_m + 3.0 h2o_m + 3.0 nad_m + 3.0 o2_m + yli_M04095_m --> 3.0 accoa_m + 3.0 h2o2_m + 3.0 h_m + 3.0 nadh_m + stcoa_m (12742 and 13813 and 14805) or (12742 and 14805 and 9065) or (12752 and 13813 and 14805) or (12752 and 14805 and 9065) or (13813 and 14805 and 9700) or (14805 and 9065 and 9700)
yli_R0211 3oddcoa_m + coa_m --> accoa_m + dccoa_m 13813 or 9065
yli_R0216 3.0 coa_m + 3.0 h2o_m + 3.0 nad_m + 3.0 o2_m + occoa_m --> 4.0 accoa_m + 3.0 h2o2_m + 3.0 h_m + 3.0 nadh_m (12742 and 13813 and 14805) or (12742 and 14805 and 9065) or (12752 and 13813 and 14805) or (12752 and 14805 and 9065) or (13813 and 14805 and 9700) or (14805 and 9065 and 9700)
yli_R0217 6.0 coa_m + 6.0 h2o_m + 6.0 nad_m + nadph_m + 6.0 o2_m + yli_M04635_m --> 7.0 accoa_m + 6.0 h2o2_m + 5.0 h_m + 6.0 nadh_m + nadp_m (12742 and 13813 and 14805) or (12742 and 14805 and 9065) or (12752 and 13813 and 14805) or (12752 and 14805 and 9065) or (13813 and 14805 and 9700) or (14805 and 9065 and 9700)
yli_R0218 6.0 coa_m + 6.0 h2o_m + 6.0 nad_m + 5.0 o2_m + yli_M04635_m --> 7.0 accoa_m + 5.0 h2o2_m + 6.0 h_m + 6.0 nadh_m (12742 and 13813 and 14805) or (12742 and 14805 and 9065) or (12752 and 13813 and 14805) or (12752 and 14805 and 9065) or (13813 and 14805 and 9700) or (14805 and 9065 and 9700)
yli_R0219 7.0 coa_m + 7.0 h2o_m + 7.0 nad_m + nadph_m + 7.0 o2_m + yli_M04594_m --> 8.0 accoa_m + 7.0 h2o2_m + 6.0 h_m + 7.0 nadh_m + nadp_m (12742 and 13813 and 14805) or (12742 and 14805 and 9065) or (12752 and 13813 and 14805) or (12752 and 14805 and 9065) or (13813 and 14805 and 9700) or (14805 and 9065 and 9700)
yli_R0220 7.0 coa_m + 7.0 h2o_m + 7.0 nad_m + 6.0 o2_m + yli_M04594_m --> 8.0 accoa_m + 6.0 h2o2_m + 7.0 h_m + 7.0 nadh_m (12742 and 13813 and 14805) or (12742 and 14805 and 9065) or (12752 and 13813 and 14805) or (12752 and 14805 and 9065) or (13813 and 14805 and 9700) or (14805 and 9065 and 9700)
yli_R0221 8.0 coa_m + 8.0 h2o_m + 8.0 nad_m + nadph_m + 8.0 o2_m + yli_M04626_m --> 9.0 accoa_m + 8.0 h2o2_m + 7.0 h_m + 8.0 nadh_m + nadp_m (12742 and 13813 and 14805) or (12742 and 14805 and 9065) or (12752 and 13813 and 14805) or (12752 and 14805 and 9065) or (13813 and 14805 and 9700) or (14805 and 9065 and 9700)
yli_R0222 8.0 coa_m + 8.0 h2o_m + 8.0 nad_m + 7.0 o2_m + yli_M04626_m --> 9.0 accoa_m + 7.0 h2o2_m + 8.0 h_m + 8.0 nadh_m (12742 and 13813 and 14805) or (12742 and 14805 and 9065) or (12752 and 13813 and 14805) or (12752 and 14805 and 9065) or (13813 and 14805 and 9700) or (14805 and 9065 and 9700)
yli_R0223 8.0 coa_m + 8.0 h2o_m + 8.0 nad_m + 2.0 nadph_m + 8.0 o2_m + yli_M04625_m --> 9.0 accoa_m + 8.0 h2o2_m + 6.0 h_m + 8.0 nadh_m + 2.0 nadp_m (12742 and 13813 and 14805) or (12742 and 14805 and 9065) or (12752 and 13813 and 14805) or (12752 and 14805 and 9065) or (13813 and 14805 and 9700) or (14805 and 9065 and 9700)
yli_R0224 8.0 coa_m + 8.0 h2o_m + 8.0 nad_m + nadph_m + 7.0 o2_m + yli_M04625_m --> 9.0 accoa_m + 7.0 h2o2_m + 7.0 h_m + 8.0 nadh_m + nadp_m (12742 and 13813 and 14805) or (12742 and 14805 and 9065) or (12752 and 13813 and 14805) or (12752 and 14805 and 9065) or (13813 and 14805 and 9700) or (14805 and 9065 and 9700)
yli_R0225 8.0 coa_m + 8.0 h2o_m + 8.0 nad_m + 6.0 o2_m + yli_M04625_m --> 9.0 accoa_m + 6.0 h2o2_m + 8.0 h_m + 8.0 nadh_m (12742 and 13813 and 14805) or (12742 and 14805 and 9065) or (12752 and 13813 and 14805) or (12752 and 14805 and 9065) or (13813 and 14805 and 9700) or (14805 and 9065 and 9700)
```

In [12]:

```
# MACCOAT is cyto version of ACACT10m, mitochondrial ppcoa from beta oxidation or branched aa degradation
# KAT180_m long chain beta oxidation should be in peroxisome
model.remove_reactions(['KAT180_m','MACCOAT'], remove_orphans=True)
# Other yli mito reactions redundant or use o2 like pero -> remove
# genes are '(12742 or 12752 or 9700)/POX1 and 14805/ECHS1 and (13813 or 9065)/ACAA1', they are all covered below
model.remove_reactions(['yli_R0194','yli_R0195','yli_R0211','yli_R0216','yli_R0217','yli_R0218','yli_R0219','yli_R0220','yli_R0221','yli_R0222','yli_R0223','yli_R0224','yli_R0225'], remove_orphans=True)
```

In [13]:

```
for r in sorted(model.reactions, key=lambda x: x.id):
    if r.id.startswith('ACOAO') and r.id.endswith('p'):
        print(r.id, r.reaction, r.gene_reaction_rule)
print()
for r in sorted(model.reactions, key=lambda x: x.id):
    if r.id.startswith('ACOA') and r.id.endswith('OR'):
        print(r.id, r.reaction, r.gene_reaction_rule)
print()
for r in sorted(model.reactions, key=lambda x: x.id):
    if r.id.startswith('ACOAD') and not r.id.startswith('ACOADAGAT'):
        print(r.id, r.reaction, r.gene_reaction_rule)
```

```
ACOAO4p dcacoa_x + o2_x --> dc2coa_x + h2o2_x 12742 or 12752 or 9700
ACOAO5p ddcacoa_x + o2_x --> dd2coa_x + h2o2_x 12742 or 12752 or 9700
ACOAO6p o2_x + tdcoa_x --> h2o2_x + td2coa_x 12742 or 12752 or 9700
ACOAO7p o2_x + pmtcoa_x --> h2o2_x + hdd2coa_x 12742 or 12752 or 9700
ACOAO8p o2_x + stcoa_x --> h2o2_x + od2coa_x 12742 or 12752 or 9700
ACOAO9p hexccoa_x + o2_x --> h2o2_x + hxc2coa_x 12742 or 12752 or 9700

ACOA100OR dcacoa_x + fad_x --> dc2coa_x + fadh2_x 11989 or 12564 or 12742 or 9700
ACOA120OR ddcacoa_x + fad_x --> dd2coa_x + fadh2_x 11989 or 12564 or 12742 or 9700
ACOA140OR fad_x + tdcoa_x --> fadh2_x + td2coa_x 11989 or 12564 or 12742 or 9700
ACOA160OR fad_x + pmtcoa_x --> fadh2_x + hdd2coa_x 11989 or 12564 or 12742 or 9700
ACOA40OR btcoa_x + fad_x --> b2coa_x + fadh2_x 10012 or 11989 or 12564 or 12570 or 12742 or 9700
ACOA60OR fad_x + hxcoa_x --> fadh2_x + hx2coa_x 10012 or 11989 or 12564 or 12570 or 12742 or 9700
ACOA80OR fad_x + occoa_x --> fadh2_x + oc2coa_x 11989 or 12564 or 12742 or 9700

ACOAD1 b2coa_c + h_c + nadh_c <=> btcoa_c + nad_c 10012 or 12570
ACOAD10m 2mbcoa_m + fad_m --> 2mb2coa_m + fadh2_m 10012 or 12570 or 14070
ACOAD1f btcoa_c + fad_c --> b2coa_c + fadh2_c 14070
ACOAD1fm btcoa_m + fad_m --> b2coa_m + fadh2_m 10408 or 12570 or 14070
ACOAD1fr b2coa_c + fadh2_c --> btcoa_c + fad_c 10012 or 12570
ACOAD2f fad_c + hxcoa_c <=> fadh2_c + hx2coa_c 14070
ACOAD8m fad_m + ivcoa_m --> 3mb2coa_m + fadh2_m 10012 or 12570
ACOAD9m fad_m + ibcoa_m --> 2mp2coa_m + fadh2_m 10012 or 12570
```

In [14]:

```
# Add 8963 acyl-CoA dehydrogenase, citronellyl-CoA dehydrogenase?
Annotation.loc[['8963']]
```

Out[14]:

|  | Combined Annotations | Signal P | Sc288c Orthologs | Human Orthologs | Sc288 Best Hit | Human Blast | Essential | WolfPSort | C Terminal |
| --- | --- | --- | --- | --- | --- | --- | --- | --- | --- |
| RTO4\_ID |  |  |  |  |  |  |  |  |  |
| 8963 | KOG0137: Very-long-chain acyl-CoA dehydrogenase |  |  |  | CYB5 | IVDe | Not Essential | cyto 16.5, cyto\_nucl 9, mito 6, pero 4 | AVL\* |

# mito¶

10012 mito 25.5, cyto\_mito 14 (sigP) IVD, ivd; isovaleryl-CoA dehydrogenase (FAD)  
11989 mito 26.5, cyto\_mito 14 (sigP) GCDH, gcdH; glutaryl-CoA dehydrogenase (FAD)  
14070 mito 26, cyto\_mito 14.5 (no sigP) ACADSB; short/branched chain acyl-CoA dehydrogenase (branched and C4-C6)  
12570 mito 26 (no sigP) ACADM, acd; medium-chain acyl-CoA dehydrogenase (C4-C16, most active with C6-C12)  
12564 mito 11, cyto 6.5, cyto\_nucl 5.5, extr 4, nucl 3.5 (sigP) SRD5A1; 3-oxo-5-alpha-steroid 4-dehydrogenase 1

# pero¶

12742 cyto 7, pero 6, cysk 6, nucl 5, cyto\_mito 5 (no sigP) POX1 K00232: E1.3.3.6, ACOX1, ACOX3; acyl-CoA oxidase  
12752 mito 11, cyto 8, pero 4, nucl 3 (no sigP) E1.3.3.6, ACOX1, ACOX3; acyl-CoA oxidase  
9700 cyto 9.5, cyto\_nucl 7.5, cysk 7, nucl 4.5, pero 4 (no sigP) POX1 K00232: E1.3.3.6, ACOX1, ACOX3; acyl-CoA oxidase  
12086 cyto 17, cyto\_nucl 10, pero 4, cysk 3 (no sigP) ACADS, acd; short-chain acyl-CoA dehydrogenase, mitochondrial, KEGG butyryl-CoA dehydrogenase butyryl hexanoyl (has cytochrome b5 domain), no rxn (C4-C6)  
10408 cyto 17.5, cyto\_nucl 10.5, pero 6 (no sigP) ACAD10; acyl-CoA dehydrogenase family member 10, KEGG acyl-CoA dehydrogenase (medium-chain EC, assign C4-C12)  
12989 cyto 15.5, cyto\_nucl 8.5, cysk 6, pero 3 (sigP) ACADL, long-chain acyl-CoA dehydrogenase (C8-C22, most active with C14-C16)  
16253 mito 9, cysk 8, cyto 5.5, pero 4, cyto\_nucl 3.5 (sigP) ACADVL, very-long-chain acyl-CoA dehydrogenase (C14-C24, most active with C14-C18)

# peroxisomal acyl-CoA dehydrogenases - use fadh? where is the electron going?¶

# mito¶

short/branched chain, C3-C5  
Change ACOAD8m genes to '10012'  
Change ACOAD9m, ACOAD10m genes to '14070'  
short chain, C4-C6  
Change ACOAD1fm to ACOAD1m, and change genes to '12570 or 14070'  
Remove ACOAD1, ACOAD1f, ACOAD1fr  
Change ACOAD2f to ACOAD2m, change metabolites to mito, make it irreversible  
Change ACOAD2m genes to '12570 or 14070'  
Medium chain, C6-C12  
Make ACOAD3m - ACOAD7m, and add genes '12570'

# pero¶

Change ACOA40OR to ACOAD1p
Change ACOA60OR to ACOAD2p
Change ACOA80OR to ACOAD3p
Change ACOA100OR to ACOAD4p
Change ACOA120OR to ACOAD5p
Change ACOA140OR to ACOAD6p
Change ACOA160OR to ACOAD7p
ACOA40OR - ACOA60OR '10408 or 12086'  
ACOA80OR - ACOA120OR '10408 or 12989'  
ACOA140OR - ACOA160OR '12989 or 16253'  
Add ACOAD8p, and genes '12989 or 16253'

In [15]:

```
model.reactions.get_by_id('ACOAD8m').gene_reaction_rule = '10012'
model.reactions.get_by_id('ACOAD9m').gene_reaction_rule = '14070'
model.reactions.get_by_id('ACOAD10m').gene_reaction_rule = '14070'
model.reactions.get_by_id('ACOAD1fm').id = 'ACOAD1m'
model.reactions.get_by_id('ACOAD1m').gene_reaction_rule = '12570 or 14070'
model.remove_reactions(['ACOAD1','ACOAD1f','ACOAD1fr'], remove_orphans=True)
r = model.reactions.get_by_id('ACOAD2f').copy()
model.reactions.get_by_id('ACOAD2f').id = 'ACOAD2m'
for m in r.metabolites:
    model.reactions.get_by_id('ACOAD2m').add_metabolites({m.id: -r.get_coefficient(m.id), m.id.replace('_c','_m'): r.get_coefficient(m.id)})
model.reactions.get_by_id('ACOAD2m').lower_bound = 0.0
model.reactions.get_by_id('ACOAD2m').gene_reaction_rule = '12570 or 14070'
r = model.reactions.get_by_id('ACOAD2m').copy()
r.gene_reaction_rule = '12570'
r.id = 'ACOAD3m'
r.name = 'Acyl-CoA dehydrogenase (octanoyl-CoA)'
model.add_reactions([r])
model.reactions.get_by_id('ACOAD3m').add_metabolites({'hxcoa_m': 1.0, 'hx2coa_m': -1.0, 'occoa_m': -1.0, 'oc2coa_m': 1.0})
r = model.reactions.get_by_id('ACOAD2m').copy()
r.gene_reaction_rule = '12570'
r.id = 'ACOAD4m'
r.name = 'Acyl-CoA dehydrogenase (decanoyl-CoA)'
model.add_reactions([r])
model.reactions.get_by_id('ACOAD4m').add_metabolites({'hxcoa_m': 1.0, 'hx2coa_m': -1.0, 'dcacoa_m': -1.0, 'dc2coa_m': 1.0})
r = model.reactions.get_by_id('ACOAD2m').copy()
r.gene_reaction_rule = '12570'
r.id = 'ACOAD5m'
r.name = 'Acyl-CoA dehydrogenase (dodecanoyl-CoA)'
model.add_reactions([r])
model.reactions.get_by_id('ACOAD5m').add_metabolites({'hxcoa_m': 1.0, 'hx2coa_m': -1.0, 'ddcacoa_m': -1.0, 'dd2coa_m': 1.0})
r = model.reactions.get_by_id('ACOAD2m').copy()
r.gene_reaction_rule = '12570'
r.id = 'ACOAD6m'
r.name = 'Acyl-CoA dehydrogenase (tetradecanoyl-CoA)'
model.add_reactions([r])
model.reactions.get_by_id('ACOAD6m').add_metabolites({'hxcoa_m': 1.0, 'hx2coa_m': -1.0, 'tdcoa_m': -1.0, 'td2coa_m': 1.0})
r = model.reactions.get_by_id('ACOAD2m').copy()
r.gene_reaction_rule = '12570'
r.id = 'ACOAD7m'
r.name = 'Acyl-CoA dehydrogenase (palmitoyl-CoA)'
model.add_reactions([r])
model.reactions.get_by_id('ACOAD7m').add_metabolites({'hxcoa_m': 1.0, 'hx2coa_m': -1.0, 'pmtcoa_m': -1.0, 'hdd2coa_m': 1.0})
model.reactions.get_by_id('ACOA40OR').id = 'ACOAD1p'
model.reactions.get_by_id('ACOAD1p').name = 'Acyl-CoA dehydrogenase (butanoyl-CoA)'
model.reactions.get_by_id('ACOAD1p').gene_reaction_rule = '10408 or 12086'
model.reactions.get_by_id('ACOA60OR').id = 'ACOAD2p'
model.reactions.get_by_id('ACOAD2p').name = 'Acyl-CoA dehydrogenase (hexanoyl-CoA)'
model.reactions.get_by_id('ACOAD2p').gene_reaction_rule = '10408 or 12086'
model.reactions.get_by_id('ACOA80OR').id = 'ACOAD3p'
model.reactions.get_by_id('ACOAD3p').name = 'Acyl-CoA dehydrogenase (octanoyl-CoA)'
model.reactions.get_by_id('ACOAD3p').gene_reaction_rule = '10408 or 12989'
model.reactions.get_by_id('ACOA100OR').id = 'ACOAD4p'
model.reactions.get_by_id('ACOAD4p').name = 'Acyl-CoA dehydrogenase (decanoyl-CoA)'
model.reactions.get_by_id('ACOAD4p').gene_reaction_rule = '10408 or 12989'
model.reactions.get_by_id('ACOA120OR').id = 'ACOAD5p'
model.reactions.get_by_id('ACOAD5p').name = 'Acyl-CoA dehydrogenase (dodecanoyl-CoA)'
model.reactions.get_by_id('ACOAD5p').gene_reaction_rule = '10408 or 12989'
model.reactions.get_by_id('ACOA140OR').id = 'ACOAD6p'
model.reactions.get_by_id('ACOAD6p').name = 'Acyl-CoA dehydrogenase (tetradecanoyl-CoA)'
model.reactions.get_by_id('ACOAD6p').gene_reaction_rule = '12989 or 16253'
model.reactions.get_by_id('ACOA160OR').id = 'ACOAD7p'
model.reactions.get_by_id('ACOAD7p').name = 'Acyl-CoA dehydrogenase (palmitoyl-CoA)'
model.reactions.get_by_id('ACOAD7p').gene_reaction_rule = '12989 or 16253'
r = model.reactions.get_by_id('ACOAD7p').copy()
r.id = 'ACOAD8p'
r.name = 'Acyl-CoA dehydrogenase (stearoyl-CoA)'
model.add_reactions([r])
model.reactions.get_by_id('ACOAD8p').add_metabolites({'pmtcoa_x': 1.0, 'hdd2coa_x': -1.0, 'stcoa_x': -1.0, 'od2coa_x': 1.0})
```

In [16]:

```
for r in sorted(model.genes.get_by_id('10012').reactions, key=lambda x: x.id):
    print(r.id, r.reaction, r.gene_reaction_rule)
print()
for r in sorted(model.genes.get_by_id('11989').reactions, key=lambda x: x.id):
    print(r.id, r.reaction, r.gene_reaction_rule)
print()
for r in sorted(model.genes.get_by_id('14070').reactions, key=lambda x: x.id):
    print(r.id, r.reaction, r.gene_reaction_rule)
print()
for r in sorted(model.genes.get_by_id('12570').reactions, key=lambda x: x.id):
    if not r.id.startswith('FAO'):
        print(r.id, r.reaction, r.gene_reaction_rule)
print()
for r in sorted(model.genes.get_by_id('12564').reactions, key=lambda x: x.id):
    print(r.id, r.reaction, r.gene_reaction_rule)
```

```
ACOAD8m fad_m + ivcoa_m --> 3mb2coa_m + fadh2_m 10012
MBCOAi fad_c + ivcoa_c --> 3mb2coa_c + fadh2_c 10012 or 12570

GLUTCOADHc fad_c + glutcoa_c + h_c --> b2coa_c + co2_c + fadh2_c 11989
GLUTCOADHm fad_m + glutcoa_m + h_m --> b2coa_m + co2_m + fadh2_m 11989

ACOAD10m 2mbcoa_m + fad_m --> 2mb2coa_m + fadh2_m 14070
ACOAD1m btcoa_m + fad_m --> b2coa_m + fadh2_m 12570 or 14070
ACOAD2m fad_m + hxcoa_m --> fadh2_m + hx2coa_m 12570 or 14070
ACOAD9m fad_m + ibcoa_m --> 2mp2coa_m + fadh2_m 14070
PPCOAOm fad_m + ppcoa_m --> fadh2_m + prpncoa_m 10408 or 12570 or 14070

ACOAD1m btcoa_m + fad_m --> b2coa_m + fadh2_m 12570 or 14070
ACOAD2m fad_m + hxcoa_m --> fadh2_m + hx2coa_m 12570 or 14070
ACOAD3m fad_m + occoa_m --> fadh2_m + oc2coa_m 12570
ACOAD4m dcacoa_m + fad_m --> dc2coa_m + fadh2_m 12570
ACOAD5m ddcacoa_m + fad_m --> dd2coa_m + fadh2_m 12570
ACOAD6m fad_m + tdcoa_m --> fadh2_m + td2coa_m 12570
ACOAD7m fad_m + pmtcoa_m --> fadh2_m + hdd2coa_m 12570
MBCOAi fad_c + ivcoa_c --> 3mb2coa_c + fadh2_c 10012 or 12570
PPCOAOm fad_m + ppcoa_m --> fadh2_m + prpncoa_m 10408 or 12570 or 14070

SR5AR2r andrstndn_r + h_r + nadph_r --> andrstandn_r + nadp_r 12564
SR5ARr h_r + nadph_r + tststerone_r --> 5adtststerone_r + nadp_r 12564
```

Remove MBCOAi, GLUTCOADHc (orphan), GLUTCOADHm has correct gene  
Change PPCOAOm genes to '14070'

In [17]:

```
model.remove_reactions(['MBCOAi','GLUTCOADHc'], remove_orphans=True)
model.reactions.get_by_id('PPCOAOm').gene_reaction_rule = '14070'
```

In [18]:

```
for r in sorted(model.metabolites.get_by_id('ppcoa_x').reactions, key=lambda x: x.id):
    print(r.id, r.reaction, r.gene_reaction_rule)
```

```
CSNAT3x crn_x + ppcoa_x <=> coa_x + pcrn_x 14245
PEROXx 3.0 coa_x + 3.0 h2o_x + 3.0 nad_x + 2.0 o2_x + pristcoa_x --> accoa_x + dmnoncoa_x + 2.0 h2o2_x + 3.0 h_x + 3.0 nadh_x + 2.0 ppcoa_x (ACOX3 and EHHADH and 11362 and 13813) or (ACOX3 and EHHADH and 11362 and 9065) or (Acox3 and Ehhadh and 11362 and 13813) or (Acox3 and Ehhadh and 11362 and 9065)
SCP2x coa_x + dhocholoylcoa_x --> dgcholcoa_x + ppcoa_x 14055
SCP3x coa_x + dhcholestancoa_x + o2_x --> dcholcoa_x + h2o_x + ppcoa_x 14055
SCPx cholcoaone_x + coa_x --> cholcoa_x + ppcoa_x 14055
```

In [19]:

```
for r in sorted(model.metabolites.get_by_id('ppcoa_c').reactions, key=lambda x: x.id):
    print(r.id, r.reaction, r.gene_reaction_rule)
print()
for r in sorted(model.metabolites.get_by_id('3mb2coa_c').reactions, key=lambda x: x.id):
    print(r.id, r.reaction, r.gene_reaction_rule)
print()
for r in sorted(model.metabolites.get_by_id('3mgcoa_c').reactions, key=lambda x: x.id):
    print(r.id, r.reaction, r.gene_reaction_rule)
```

```
ACCOAL atp_c + coa_c + ppa_c --> adp_c + pi_c + ppcoa_c 14597
ACS2 atp_c + coa_c + ppa_c --> amp_c + ppcoa_c + ppi_c 14597 or 15276
PPCSCT ppcoa_c + succ_c --> ppa_c + succoa_c 13797
yli_R0594 coa_c + ppad_c <=> amp_c + 2.0 h_c + ppcoa_c 14597

MCCC 3mb2coa_c + atp_c + hco3_c --> 3mgcoa_c + adp_c + h_c + pi_c 12867
yli_R1593 3hivcoa_c <=> 3mb2coa_c + h2o_c 14805

MCCC 3mb2coa_c + atp_c + hco3_c --> 3mgcoa_c + adp_c + h_c + pi_c 12867
```

In [20]:

```
for r in sorted(model.metabolites.get_by_id('ppcoa_m').reactions, key=lambda x: x.id):
    print(r.id, r.reaction, r.gene_reaction_rule)
print()
for r in sorted(model.metabolites.get_by_id('ivcoa_m').reactions, key=lambda x: x.id):
    print(r.id, r.reaction, r.gene_reaction_rule)
print()
for r in sorted(model.metabolites.get_by_id('3mb2coa_m').reactions, key=lambda x: x.id):
    print(r.id, r.reaction, r.gene_reaction_rule)
print()
for r in sorted(model.metabolites.get_by_id('3mgcoa_m').reactions, key=lambda x: x.id):
    print(r.id, r.reaction, r.gene_reaction_rule)
```

```
ACACT10m 2maacoa_m + coa_m <=> accoa_m + ppcoa_m 8678
ACCOALm atp_m + coa_m + ppa_m --> amp_m + ppcoa_m + ppi_m 14597
CSNAT2m coa_m + pcrn_m <=> crn_m + ppcoa_m 14245
FAOXC150m 6.0 coa_m + 6.0 fad_m + 6.0 h2o_m + 6.0 nad_m + ptdcacoa_m --> 6.0 accoa_m + 6.0 fadh2_m + 6.0 h_m + 6.0 nadh_m + ppcoa_m 12570
FAOXC170m 7.0 coa_m + 7.0 fad_m + 7.0 h2o_m + hpdcacoa_m + 7.0 nad_m --> 7.0 accoa_m + 7.0 fadh2_m + 7.0 h_m + 7.0 nadh_m + ppcoa_m 12570
MCITSm h2o_m + oaa_m + ppcoa_m --> 2mcit_m + coa_m + h_m 11331
MMSAD1m 2mop_m + coa_m + nad_m --> co2_m + nadh_m + ppcoa_m 8975
OBDHm 2obut_m + coa_m + nad_m --> co2_m + nadh_m + ppcoa_m (10040 and 11183 and 12566 and 15436) or (10040 and 11188 and 12566 and 15436)
PACPT coa_m + ppad_m <=> amp_m + h_m + ppcoa_m 14597
PPACOALm atp_m + coa_m + ppa_m --> amp_m + h_m + ppcoa_m + ppi_m 14597
PPCOACm atp_m + hco3_m + ppcoa_m --> adp_m + h_m + mmcoa__S_m + pi_m (PCCB and 13009) or (Pccb and 13009)
PPCOAOm fad_m + ppcoa_m --> fadh2_m + prpncoa_m 14070

ACOAD8m fad_m + ivcoa_m --> 3mb2coa_m + fadh2_m 10012
OIVD1m 4mop_m + coa_m + nad_m --> co2_m + ivcoa_m + nadh_m (10040 and 11183 and 12566 and 15436) or (10040 and 11188 and 12566 and 15436)

ACOAD8m fad_m + ivcoa_m --> 3mb2coa_m + fadh2_m 10012
ECH_3hivcoa 3mb2coa_m + h2o_m <=> 3hivcoa_m 11907
MCCCrm 3mb2coa_m + atp_m + hco3_m <=> 3mgcoa_m + adp_m + h_m + pi_m 12867 and 15694
MCTC 3mb2coa_m + atp_m + hco3_m --> 3mgcoa_m + adp_m + pi_m 12867 and 15694

MCCCrm 3mb2coa_m + atp_m + hco3_m <=> 3mgcoa_m + adp_m + h_m + pi_m 12867 and 15694
MCTC 3mb2coa_m + atp_m + hco3_m --> 3mgcoa_m + adp_m + pi_m 12867 and 15694
MGCHrm 3mgcoa_m + h2o_m <=> hmgcoa_m 16128
```

In [21]:

```
temp = ['14597','15276','8975','13797','13009']
display(Annotation.loc[temp])
Show_Data(temp)
```

|  | Combined Annotations | Signal P | Sc288c Orthologs | Human Orthologs | Sc288 Best Hit | Human Blast | Essential | WolfPSort | C Terminal |
| --- | --- | --- | --- | --- | --- | --- | --- | --- | --- |
| RTO4\_ID |  |  |  |  |  |  |  |  |  |
| 14597 | K01895: ACSS, acs; acetyl-CoA synthetase |  | ACS1,ACS2 | ACSS1,ACSS2 | ACS2 | ACSS1 | Not Essential | cyto 13, plas 5, mito 3, pero 2, mito\_nucl 2 | SSE\* |
| 15276 | K01907: AACS, acsA; acetoacetyl-CoA synthetase |  |  | AACS | ACS1 | AACS | Not Essential | plas 9, pero 6, mito 5, cyto 5, cyto\_mito 5 | SKL\* |
| 8975 | K00140: mmsA, iolA, ALDH6A1; malonate-semialde... |  |  | ALDH6A1 | UGA2 | ALDH6 | Not Essential | mito 27 | THH\* |
| 13797 | K01067: E3.1.2.1, ACH1; acetyl-CoA hydrolase |  | ACH1 |  | ACH1 |  | Not Essential | mito 23, cyto 3 | GWS\* |
| 13009 | KOG1161: Protein involved in vacuolar polyphos... |  | VTC4 | PCCA | VTC4 |  | Not Essential | plas 11, cyto 5.5, cyto\_nucl 5.5, pero 5, nucl... | QGI\* |

| strain | WT | | | | | | | | | | | | | | | | |
| --- | --- | --- | --- | --- | --- | --- | --- | --- | --- | --- | --- | --- | --- | --- | --- | --- | --- |
| condition | G\_MM | C\_MM | G\_SD | | GX\_SD | | | X\_SD | | A\_SD | | C\_SD | | MM\_CN120 | | MM\_CN5 | Diversity\_Sample |
| phase | exp | exp | exp | stat | exp | trans | stat | exp | stat | exp | stat | exp | stat | exp | stat | exp | exp |
| proteinId | Set1 | Set1 | Set2 | Set2 | Set2 | Set2 | Set2 | Set2 | Set2 | Set2 | Set2 | Set2 | Set2 | Set3 | Set3 | Set3 | Set3 |
| 14597 | 8.42238 | 7.97378 | 8.51818 | 7.99019 | 8.64056 | 7.40482 | 7.62933 | 7.37675 | 8.46789 | 7.83928 | 8.54106 | 7.90993 | 8.02345 | 8.92605 | 8.10393 | 9.91014 | 9.38791 |
| 15276 | 5.29782 | 7.5276 | 6.02897 | 6.3398 | 5.8819 | 5.96372 | 5.9225 | 5.9897 | 5.36727 | 6.33017 | 5.89141 | 6.06136 | 5.30571 | 4.91543 | 4.54293 | 6.28014 | 5.10407 |
| 8975 | 5.66327 | 6.02765 | 6.70623 | 7.30337 | 6.7434 | 6.22721 | 6.22823 | 6.24526 | 6.03253 | 5.57225 | 6.31843 | 5.45415 | 5.95381 | 6.50537 | 5.94092 | 7.64182 | 6.56961 |
| 13797 | 5.62849 | 4.81503 | 8.17125 | 5.05096 | 8.08706 | 6.64768 | 6.37872 | 7.05384 | 5.34017 | 5.19003 | 4.77626 | 5.67236 | 5.47792 | 6.75758 | 7.34726 | 8.41857 | 7.00014 |
| 13009 | 6.71566 | 6.55691 | 6.50033 | 6.24381 | 6.53976 | 6.06693 | 6.37051 | 6.43926 | 5.83359 | 6.48263 | 6.07399 | 7.25084 | 7.25259 | 5.9355 | 5.97244 | 6.3321 | 6.29758 |

| strain | WT | | | | | | | | | | |
| --- | --- | --- | --- | --- | --- | --- | --- | --- | --- | --- | --- |
| condition | G\_SD | | GX\_SD | | | X\_SD | | A\_SD | | C\_SD | |
| proteinId | exp | stat | exp | trans | stat | exp | stat | exp | stat | exp | stat |
| 14597 | 33.4132 | 33.1333 | 28.0397 | 32.0427 | 29.6961 | 27.1912 | 30.3839 | 29.3511 | 35.2144 | 32.6888 | 37.6604 |
| 15276 | 11.0591 | 15.3254 | 10.9798 | 19.3402 | 20.029 | 18.4148 | 19.0534 | 17.1797 | 17.9891 | 29.9096 | 20.8835 |
| 8975 | 15.1685 | 20.4773 | 14.4448 | 16.0147 | 15.9748 | 17.0593 | 17.9072 | 17.7641 | 18.2123 | 22.8784 | 18.4812 |
| 13797 | 38.7939 | 30.9017 | 34.0227 | 34.8301 | 33.2894 | 18.1948 | 23.4433 | 8.11648 | 10.1511 | 13.2507 | 13.2613 |
| 13009 | 3.85129 | 4.36663 | 2.84907 | 4.47436 | 6.34297 | 1.9389 | 2.95716 | 3.07608 | 4.20551 | 3.83276 | 3.49638 |

|  | Glucose | Xylose | Arabinose | Acetate | Coumarate | Ferulate | YNB Oleic Acid | YNB Ricinoleic Acid | YNB Glucose | YNB Gluc DOC | YPD |
| --- | --- | --- | --- | --- | --- | --- | --- | --- | --- | --- | --- |
| proteinId |  |  |  |  |  |  |  |  |  |  |  |
| 14597 | 0.134527 | 0.0997527 | 0.107444 | -1.05713 | 0.0951078 | -0.0881028 | 0.151616 | 0.954851 | 0.0158946 | -0.116256 | 0.000665712 |
| 15276 | -0.192767 | 0.221437 | 0.0764749 | -0.176646 | 0.00381209 | -0.117964 | -0.0335189 | -0.260812 | -0.126874 | -0.0575812 | -0.262471 |
| 8975 | -0.183461 | 0.337248 | -0.0360008 | -0.0852432 | 0.164365 | 0.133413 | -0.0728905 | -0.353268 | -0.148485 | -0.0951318 | 0.168732 |
| 13797 | -0.0172407 | -0.175817 | -0.123985 | -0.429914 | -0.168931 | -0.0772301 | -0.0119267 | 0.333993 | -0.0622429 | -0.0438979 | -0.158202 |
| 13009 | 0.0956749 | -0.138998 | -0.38607 | 0.0215119 | -0.68409 | 0.176859 | -1.08209 | -0.72106 | -1.62232 | -1.54764 | 0.721124 |

14597 cyto 13, plas 5, mito 3, pero 2, mito\_nucl 2 K01895: ACSS, acs; acetyl-CoA synthetase  
15276 plas 9, pero 6, mito 5, cyto 5, cyto\_mito 5 K01907: AACS, acsA; acetoacetyl-CoA synthetase  
8975 mito 27 K00140: mmsA, iolA, ALDH6A1; malonate-semialdehyde dehydrogenase (acetylating) / methylmalonate-semialdehyde dehydrogenase  
13797 mito 23, cyto 3 K01067: E3.1.2.1, ACH1; acetyl-CoA hydrolase  
13009 plas 11, cyto 5.5, cyto\_nucl 5.5, pero 5, nucl 4.5 KOG1161: Protein involved in vacuolar polyphosphate accumulation, contains SPX domain

12867 mito 27 K01968: E6.4.1.4A; 3-methylcrotonyl-CoA carboxylase alpha subunit  
15694 mito 27 K01969: E6.4.1.4B; 3-methylcrotonyl-CoA carboxylase beta subunit  
16128 mito 26 K05607: AUH; methylglutaconyl-CoA hydratase

14805 mito 26.5, cyto\_mito 14 K07511: ECHS1; enoyl-CoA hydratase (mitochondria), both degradation and biosynthesis  
11362 cyto 15, mito 6, pero 4, mito\_nucl 4 K14729: FOX2; multifunctional beta-oxidation protein (peroxisome)  
11907 extr 9, mito 8, cyto 5.5, cyto\_nucl 4, pero 3 K12663: ECH1; Delta(3,5)-Delta(2,4)-dienoyl-CoA isomerase (peroxisome), no correct rxns in BiGG

PPACOALm (incorrect stoic) = ACCOALm (incorrect stoic), ACCOAL (adp, cyto) = APAT + PACPT  
13009 not relevant remove PPCOACm  
ECH\_3hivcoa is incorrect, 3hivcoa can be made from 2mb2coa by ech  
yli\_R1593 is cyto version of ECH\_3hivcoa  
MCCCrm is correct, remove MCCC and MCTC
Change ACS, ACS2 genes to '14597'

In [22]:

```
model.remove_reactions(['ACCOAL','ACCOALm','PPACOALm','APAT','PACPT','PPCOACm',
                        'ECH_3hivcoa','yli_R1593','MCCC','MCTC'], remove_orphans=True)
model.reactions.get_by_id('ACS').gene_reaction_rule = '14597'
model.reactions.get_by_id('ACS2').gene_reaction_rule = '14597'
```

In [23]:

```
for r in sorted(model.genes.get_by_id('14597').reactions, key=lambda x: x.id):
    print(r.id, r.reaction, r.gene_reaction_rule)
print()
for r in sorted(model.genes.get_by_id('15276').reactions, key=lambda x: x.id):
    print(r.id, r.reaction, r.gene_reaction_rule)
print()
for r in sorted(model.genes.get_by_id('8975').reactions, key=lambda x: x.id):
    print(r.id, r.reaction, r.gene_reaction_rule)
print()
for r in sorted(model.genes.get_by_id('13797').reactions, key=lambda x: x.id):
    print(r.id, r.reaction, r.gene_reaction_rule)
```

```
ACS ac_c + atp_c + coa_c --> accoa_c + amp_c + ppi_c 14597
ACS2 atp_c + coa_c + ppa_c --> amp_c + ppcoa_c + ppi_c 14597
ACSm ac_m + atp_m + coa_m --> accoa_m + amp_m + ppi_m 14597
ACSp ac_x + atp_x + coa_x --> accoa_x + amp_x + ppi_x 14597
yli_R0594 coa_c + ppad_c <=> amp_c + 2.0 h_c + ppcoa_c 14597
yli_R0596 atp_c + 2.0 h_c + yli_M00161_c <=> ppad_c + ppi_c 14597
yli_R1565 HC01672_c + coa_c --> accoa_c + amp_c 14597
yli_R1566 ac_c + atp_c <=> HC01672_c + ppi_c 14597

AACOAT acac_c + atp_c + coa_c <=> aacoa_c + amp_c + ppi_c 15276

MMSAD1m 2mop_m + coa_m + nad_m --> co2_m + nadh_m + ppcoa_m 8975
MMSAD3m coa_m + msa_m + nad_m --> accoa_m + co2_m + nadh_m 8975

ACOAH ac_c + coa_c + h_c --> accoa_c + h2o_c 13797
ACOAHim accoa_m + h2o_m --> ac_m + coa_m + h_m 13797
PPCSCT ppcoa_c + succ_c --> ppa_c + succoa_c 13797
```

ACS is cyto, mito, nucl in S. cer remove ACSp  
HC01672 is acetyl-AMP remove yli  
13797 is mito acetyl-coa hydrolase  
13048 cyto 20.5, cyto\_mito 11, nucl 5 K01067: E3.1.2.1, ACH1; acetyl-CoA hydrolase -> ACOAH with reverse direction
Remove PPCSCT

In [24]:

```
model.remove_reactions(['ACSp','yli_R0594','yli_R0596','yli_R1565','yli_R1566'], remove_orphans=True)
model.reactions.get_by_id('ACOAH').gene_reaction_rule = '13048'
model.reactions.get_by_id('ACOAH').lower_bound = -1000.0
model.reactions.get_by_id('ACOAH').upper_bound = 0.0
model.remove_reactions(['PPCSCT'], remove_orphans=True)
```

In [25]:

```
for r in sorted(model.genes.get_by_id('12867').reactions, key=lambda x: x.id):
    print(r.id, r.reaction, r.gene_reaction_rule)
print()
for r in sorted(model.genes.get_by_id('15694').reactions, key=lambda x: x.id):
    print(r.id, r.reaction, r.gene_reaction_rule)
print()
for r in sorted(model.genes.get_by_id('16128').reactions, key=lambda x: x.id):
    print(r.id, r.reaction, r.gene_reaction_rule)
```

```
ACCOAC accoa_c + atp_c + hco3_c --> adp_c + h_c + malcoa_c + pi_c 8639 or (PP_0559 and PP_1607 and PP_1996 and 12867) or (b0185 and b2316 and b3255 and 12867)
ACCOAC_1 accoa_h + cbtnCCP_h + h_h <=> btnCCP_h + malcoa_h (CRv4_Au5_s12_g2759_t1 and CRv4_Au5_s17_g7349_t1) or (CRv4_Au5_s12_g2759_t1 and CRv4_Au5_s1_g1722_t1) or (CRv4_Au5_s12_g3449_t1 and CRv4_Au5_s17_g7349_t1) or (CRv4_Au5_s12_g3449_t1 and CRv4_Au5_s1_g1722_t1) or (CRv4_Au5_s17_g7349_t1 and 12867) or (CRv4_Au5_s1_g1722_t1 and 12867)
ACCOAhi accoa_h + atp_h + hco3_h --> adp_h + malcoa_h + pi_h (CRv4_Au5_s12_g2759_t1 and CRv4_Au5_s17_g7349_t1) or (CRv4_Au5_s12_g3449_t1 and CRv4_Au5_s17_g7349_t1) or (CRv4_Au5_s17_g7349_t1 and 12867)
BTNC atp_h + btnCCP_h + hco3_h --> adp_h + cbtnCCP_h + h_h + pi_h (CRv4_Au5_s17_g7349_t1 and 12867) or (CRv4_Au5_s1_g1722_t1 and 12867)
MCCCrm 3mb2coa_m + atp_m + hco3_m <=> 3mgcoa_m + adp_m + h_m + pi_m 12867 and 15694

MCCCrm 3mb2coa_m + atp_m + hco3_m <=> 3mgcoa_m + adp_m + h_m + pi_m 12867 and 15694

3HBCDm b2coa_m + h2o_m <=> 3hbcoa__R_m 16128
C2M26DCOAHLm c2m26dcoa_m + h2o_m <=> 3h26dm5coa_m 16128 or (HADHA and HADHB) or (Hadha and Hadhb)
ECOAH1 3hbcoa_c <=> b2coa_c + h2o_c 14805 or 16128
ECOAH1m 3hbcoa_m <=> b2coa_m + h2o_m 11907 or 14805 or 16128 or (HADHA and HADHB) or (Hadha and Hadhb)
HBCHLR 3hbcoa__R_c <=> b2coa_c + h2o_c 16128
HPHL 3hpcoa_c <=> h2o_c + prpncoa_c 14805 or 16128
MGCHrm 3mgcoa_m + h2o_m <=> hmgcoa_m 16128
PRPNCOAHYDm h2o_m + prpncoa_m --> 3hpcoa_m 11907 or 14805 or 16128 or (HADHA and HADHB) or (Hadha and Hadhb)
T2M26DCOAHLm h2o_m + t2m26dcoa_m <=> 3h26dm5coa_m 16128 or (HADHA and HADHB) or (Hadha and Hadhb)
```

Change ACCOAC genes to '8639'  
Remove ACCOAC\_1, ACCOAhi, BTNC  
Remove 3HBCDm (ECOAH1m), HBCHLR (ECOAH1)  
Remove C2M26DCOAHLm, T2M26DCOAHLm, C2M26DCOAHLx, T2M26DCOAHLx (orphans)  
Remove HPHL (only in iRC, part of autotrophic co2 fixation)  
Change PRPNCOAHYDm genes to '14805'

In [26]:

```
model.reactions.get_by_id('ACCOAC').gene_reaction_rule = '8639'
model.remove_reactions(['ACCOAC_1','ACCOAhi','BTNC','3HBCDm','HBCHLR','C2M26DCOAHLm','T2M26DCOAHLm','C2M26DCOAHLx','T2M26DCOAHLx','HPHL'], remove_orphans=True)
model.reactions.get_by_id('PRPNCOAHYDm').gene_reaction_rule = '14805'
```

In [27]:

```
for r in sorted(model.genes.get_by_id('14805').reactions, key=lambda x: x.id):
    print(r.id, r.reaction, r.gene_reaction_rule)
print()
for r in sorted(model.genes.get_by_id('11362').reactions, key=lambda x: x.id):
    if not r.id.startswith('FAO'):
        print(r.id, r.reaction, r.gene_reaction_rule)
print()
for r in sorted(model.genes.get_by_id('11907').reactions, key=lambda x: x.id):
    print(r.id, r.reaction, r.gene_reaction_rule)
```

```
DHACOAH 23dhacoa_c + h2o_c <=> 3hadpcoa_c 14805
ECOAH1 3hbcoa_c <=> b2coa_c + h2o_c 14805 or 16128
ECOAH12 2mp2coa_c + h2o_c --> 3hibutcoa_c 14805
ECOAH12m 2mp2coa_m + h2o_m <=> 3hibutcoa_m 11907 or 14805 or (HADHA and HADHB) or (Hadha and Hadhb)
ECOAH1m 3hbcoa_m <=> b2coa_m + h2o_m 11907 or 14805 or 16128 or (HADHA and HADHB) or (Hadha and Hadhb)
ECOAH2 3hhcoa_c <=> h2o_c + hx2coa_c 14805
ECOAH3 3hocoa_c <=> h2o_c + oc2coa_c 14805
ECOAH4 3hdcoa_c <=> dc2coa_c + h2o_c 14805
ECOAH4m 3hdcoa_m <=> dc2coa_m + h2o_m 11907 or 14805
ECOAH5 3hddcoa_c <=> dd2coa_c + h2o_c 14805
ECOAH5m 3hddcoa_m <=> dd2coa_m + h2o_m 11907 or 14805
ECOAH6 3htdcoa_c <=> h2o_c + td2coa_c 14805
ECOAH6m 3htdcoa_m <=> h2o_m + td2coa_m 11907 or 14805
ECOAH7 3hhdcoa_c <=> h2o_c + hdd2coa_c 14805
ECOAH7m 3hhdcoa_m <=> h2o_m + hdd2coa_m 11907 or 14805
ECOAH9ir 2mb2coa_c + h2o_c --> 3hmbcoa_c 14805
ECOAH9m 2mb2coa_m + h2o_m <=> 3hmbcoa_m 11907 or 14805 or (HADHA and HADHB) or (Hadha and Hadhb)
HADPCOADH 3hadpcoa_c --> h2_c + oxadpcoa_c 14805 and 15180
HMR_3116 h2o_m + od2coa_m <=> CE2248_m 14805
PRPNCOAHYDm h2o_m + prpncoa_m --> 3hpcoa_m 14805
yli_R0192 h2o_m + yli_M04598_m <=> yli_M06408_m 14805

C3STKR2r h_r + nadph_r + zym_int2_r --> nadp_r + zymst_r 11362
ECOAH11p h2o_x + hxc2coa_x <=> 3hxccoa_x 11362
ECOAH1x 3hbcoa_x <=> b2coa_x + h2o_x 11362 or 11907
ECOAH2_1 h2o_x + hx2coa_x <=> 3hhcoa_x 11362
ECOAH3_1 h2o_x + oc2coa_x <=> 3hocoa_x 11362
ECOAH4p 3hdcoa_x <=> dc2coa_x + h2o_x 11362
ECOAH5p 3hddcoa_x <=> dd2coa_x + h2o_x 11362
ECOAH6p 3htdcoa_x <=> h2o_x + td2coa_x 11362
ECOAH7p 3hhdcoa_x <=> h2o_x + hdd2coa_x 11362
ECOAH8p 3hodcoa_x <=> h2o_x + od2coa_x 11362
HACD10p 3hxccoa_x + nad_x <=> 3ohxccoa_x + h_x + nadh_x 11362
HACD1x aacoa_x + h_x + nadh_x <=> 3hbcoa_x + nad_x 11362
HACD2_1 3hhcoa_x + nad_x <=> 3ohcoa_x + h_x + nadh_x 11362
HACD2m 3ohcoa_m + h_m + nadh_m <=> 3hhcoa_m + nad_m 11362
HACD3_1 3hocoa_x + nad_x <=> 3oocoa_x + h_x + nadh_x 11362
HACD3m 3oocoa_m + h_m + nadh_m <=> 3hocoa_m + nad_m 11362
HACD4m 3odcoa_m + h_m + nadh_m <=> 3hdcoa_m + nad_m 11362
HACD4p 3odcoa_x + h_x + nadh_x <=> 3hdcoa_x + nad_x 11362
HACD5m 3oddcoa_m + h_m + nadh_m <=> 3hddcoa_m + nad_m 11362
HACD5p 3oddcoa_x + h_x + nadh_x <=> 3hddcoa_x + nad_x 11362
HACD6m 3otdcoa_m + h_m + nadh_m <=> 3htdcoa_m + nad_m 11362
HACD6p 3otdcoa_x + h_x + nadh_x <=> 3htdcoa_x + nad_x 11362
HACD7m 3ohdcoa_m + h_m + nadh_m <=> 3hhdcoa_m + nad_m 11362
HACD7p 3ohdcoa_x + h_x + nadh_x <=> 3hhdcoa_x + nad_x 11362
HACD8p 3ohodcoa_x + h_x + nadh_x <=> 3hodcoa_x + nad_x 11362
HACD9m 3hmbcoa_m + nad_m <=> 2maacoa_m + h_m + nadh_m 11362 or 16284
HSD17B42x h_x + nadph_x + o2_x + thcholoylcoa_x --> dhocholoylcoa_x + 2.0 h2o_x + nadp_x 11362
HSD17B4x cholcoads_x + h_x + nadph_x + o2_x --> cholcoaone_x + h2o_x + nadp_x 11362
PEROXx 3.0 coa_x + 3.0 h2o_x + 3.0 nad_x + 2.0 o2_x + pristcoa_x --> accoa_x + dmnoncoa_x + 2.0 h2o2_x + 3.0 h_x + 3.0 nadh_x + 2.0 ppcoa_x (ACOX3 and EHHADH and 11362 and 13813) or (ACOX3 and EHHADH and 11362 and 9065) or (Acox3 and Ehhadh and 11362 and 13813) or (Acox3 and Ehhadh and 11362 and 9065)

ECOAH12m 2mp2coa_m + h2o_m <=> 3hibutcoa_m 11907 or 14805 or (HADHA and HADHB) or (Hadha and Hadhb)
ECOAH1m 3hbcoa_m <=> b2coa_m + h2o_m 11907 or 14805 or 16128 or (HADHA and HADHB) or (Hadha and Hadhb)
ECOAH1x 3hbcoa_x <=> b2coa_x + h2o_x 11362 or 11907
ECOAH2m 3hhcoa_m <=> h2o_m + hx2coa_m 11907
ECOAH3m 3hocoa_m <=> h2o_m + oc2coa_m 11907
ECOAH4m 3hdcoa_m <=> dc2coa_m + h2o_m 11907 or 14805
ECOAH5m 3hddcoa_m <=> dd2coa_m + h2o_m 11907 or 14805
ECOAH6m 3htdcoa_m <=> h2o_m + td2coa_m 11907 or 14805
ECOAH7m 3hhdcoa_m <=> h2o_m + hdd2coa_m 11907 or 14805
ECOAH9m 2mb2coa_m + h2o_m <=> 3hmbcoa_m 11907 or 14805 or (HADHA and HADHB) or (Hadha and Hadhb)
```

In [28]:

```
# Remove DHACOAH (part of phenylacetate degradation), HADPCOADH (wrong) cyto rxns 
# Remove HMR_3116 (C18 fatty acid mito degradation)
# Remove yli_R0191,yli_R0192,yli_R0243 (C26 fatty acid mito degradation)
model.remove_reactions(['DHACOAH','HADPCOADH','HMR_3116','yli_R0191','yli_R0192','yli_R0243'], remove_orphans=True)
# Remove C3STKR2r, ERG27 is missing
model.remove_reactions(['C3STKR2r'], remove_orphans=True)
# PEROXx is wrong, need 3 o2
model.remove_reactions(['PEROXx'])
```

In [29]:

```
temp = Annotation.index[Annotation['Sc288c Orthologs'].str.contains('ERG')]
display(Annotation.loc[temp])
Show_Data(temp)
```

|  | Combined Annotations | Signal P | Sc288c Orthologs | Human Orthologs | Sc288 Best Hit | Human Blast | Essential | WolfPSort | C Terminal |
| --- | --- | --- | --- | --- | --- | --- | --- | --- | --- |
| RTO4\_ID |  |  |  |  |  |  |  |  |  |
| 8671 | K00222: TM7SF2, ERG24; Delta14-sterol reductase | A | ERG24 | LBR,TM7SF2 | ERG24 | LBR | Essential | plas 13, mito 9, cyto 3 | YVY\* |
| 8678 | K00626: E2.3.1.9, atoB; acetyl-CoA C-acetyltra... |  | ERG10 | ACAT1 | ERG10 | ACAT1 | Not Essential | mito 23.5, cyto\_mito 14 | QRL\* |
| 8702 | K00938: E2.7.4.2, mvaK2; phosphomevalonate kinase |  | ERG8 |  | ERG8 |  | Essential | cyto 16, cyto\_mito 11.833, cyto\_nucl 9.833, mi... | KKR\* |
| 8758 | K00869: E2.7.1.36, MVK, mvaK1; mevalonate kinase |  | ERG12 | MVK | ERG12 | MVK | Essential | cyto 13, extr 9, cyto\_nucl 8.833, cyto\_mito 7.... | VCA\* |
| 8781 | K00227: SC5DL, ERG3; Delta7-sterol 5-desaturase |  | ERG3 | SC5D | ERG3 | SC5D | Not Essential | plas 26 | ARK\* |
| 8835 | K07748: E1.1.1.170, NSDHL, ERG26; sterol-4alph... |  | ERG26 | NSDHL | ERG26 | NSDHL | Essential | cyto 13, cyto\_nucl 8.833, cyto\_mito 8.833, per... | KKA\* |
| 9240 | K01852: LSS, ERG7; lanosterol synthase |  | ERG7 | LSS | ERG7 | LSS | Essential | cysk 22, cyto 5 | EGW\* |
| 10113 | K09829: ERG2; C-8 sterol isomerase | S | ERG2 | SIGMAR1 | ERG2 | SIGMA | Essential | plas 17, E.R. 5, mito 2, extr 2 | GKI\* |
| 10981 | K00223: ERG4; Delta24(24(1))-sterol reductase |  | ERG4 |  | ERG4 | LBR | Not Essential | plas 18, E.R. 5, mito 3 | YVF\* |
| 11716 | K09831: ERG5, CYP61A; sterol 22-desaturase | A | ERG5 |  | ERG5 | CYP26C | Not Essential | plas 14, E.R. 8, vacu 3 | QDL\* |
| 12035 | KOG3455: Predicted membrane protein |  | ERG28 | C14orf1 | ERG28 | C14orf | Not Essential | mito 17, extr 6, pero 2 | VRL\* |
| 12122 | K01641: E2.3.3.10; hydroxymethylglutaryl-CoA s... |  | ERG13 | HMGCS1,HMGCS2 | ERG13 | HMGCS | Essential | nucl 11, cyto\_nucl 10, mito 9, cyto 7 | HVA\* |
| 12843 | K05917: CYP51; sterol 14-demethylase | S | ERG11 | CYP51A1 | ERG11 | CYP51 | Essential | cyto 9, E.R. 5, mito 3, extr 3, plas 2, pero 2... | KVL\* |
| 12944 | K00787: FDPS; farnesyl diphosphate synthase |  | ERG20 | FDPS | ERG20 | FDPS | Essential | cyto 16.5, cyto\_nucl 13.5, nucl 9.5 | RQK\* |
| 13060 | K00559: E2.1.1.41, SMT1, ERG6; sterol 24-C-met... |  | ERG6 |  | ERG6 |  | Essential | mito 11, cyto 8, cyto\_pero 7.333, cyto\_nucl 5.... | HSQ\* |
| 13724 | K07748: E1.1.1.170, NSDHL, ERG26; sterol-4alph... |  | ERG26 | NSDHL | ERG26 | NSDHL | Not Essential | mito 16, nucl 5, cyto 3, pero 2 | VVA\* |
| 13729 | K00511: SQLE, ERG1; squalene monooxygenase |  | ERG1 | SQLE | ERG1 | SQLE | Essential | plas 18, extr 3, E.R. 3, cyto 2 | GQM\* |
| 16591 | K00801: FDFT1; farnesyl-diphosphate farnesyltr... | S | ERG9 | FDFT1 | ERG9 | FDFT1 | Essential | mito 16.5, cyto\_mito 11.5, cyto 5.5, pero 3 | KQLP |
| 16640 | K07750: E1.14.13.72, SC4MOL, ERG25; methylster... |  | ERG25 | MSMO1 | ERG25 | MSMO1 | Essential | cyto 14, cyto\_nucl 9.333, cyto\_pero 8.833, mit... | KAQ\* |

| strain | WT | | | | | | | | | | | | | | | | |
| --- | --- | --- | --- | --- | --- | --- | --- | --- | --- | --- | --- | --- | --- | --- | --- | --- | --- |
| condition | G\_MM | C\_MM | G\_SD | | GX\_SD | | | X\_SD | | A\_SD | | C\_SD | | MM\_CN120 | | MM\_CN5 | Diversity\_Sample |
| phase | exp | exp | exp | stat | exp | trans | stat | exp | stat | exp | stat | exp | stat | exp | stat | exp | exp |
| proteinId | Set1 | Set1 | Set2 | Set2 | Set2 | Set2 | Set2 | Set2 | Set2 | Set2 | Set2 | Set2 | Set2 | Set3 | Set3 | Set3 | Set3 |
| RTO4\_ID |  |  |  |  |  |  |  |  |  |  |  |  |  |  |  |  |  |
| 8671 | 6.22688 | 5.3299 | 5.4943 | 6.2628 | 5.37695 | 6.56024 | 6.5853 | 6.84475 | 6.21731 | 6.07989 | 6.21377 | 6.07059 | 4.1731 | 6.36718 | 6.48947 | 6.32487 | 5.68874 |
| 8678 | 7.02165 | 8.49604 | 6.56773 | 5.30595 | 6.67599 | 5.76971 | 5.63167 | 7.10664 | 5.26798 | 5.7248 | 5.42382 | 5.88536 | 5.87647 | 7.0048 | 6.36022 | 8.25583 | 7.88214 |
| 8702 | 5.04259 | 6.39317 | 5.95629 | 3.72055 | 6.00004 | 6.15773 | 6.32827 | 6.54327 | 5.62512 | 6.34048 | 5.30701 | 4.31951 | 3.72451 | 6.00056 | 6.36577 | 6.27223 | 5.78955 |
| 8758 | 6.05612 | 6.51837 | 6.52645 | 5.97515 | 6.61353 | 6.36493 | 6.05844 | 7.1059 | 5.44352 | 6.61199 | 5.81527 | 7.18794 | 7.87935 | 6.92465 | 7.00717 | 9.18327 | 7.82502 |
| 8781 | 6.91069 | 7.62283 | 7.74517 | 7.92974 | 7.6617 | 7.96594 | 7.8762 | 9.08473 | 7.35312 | 8.02506 | 6.65301 | 7.95883 | 8.69648 | 8.77672 | 8.11224 | 8.81603 | 8.29644 |
| 8835 | 6.94104 | 7.02409 | 6.53469 | 5.64546 | 6.61285 | 6.38041 | 6.2487 | 6.85079 | 6.14123 | 6.69882 | 6.33309 | 6.33766 | 6.16494 | 6.77891 | 6.72212 | 7.04337 | 6.8664 |
| 9240 | 6.27628 | 7.17445 | 5.19435 | 3.89795 | 5.25968 | 4.62605 | 4.26754 | 6.63079 | 3.81925 | 4.97026 | 3.71263 | 4.94754 | 4.22375 | 4.39143 | 4.34407 | 6.49852 | 5.40605 |
| 10113 | 6.58987 | 6.82561 | 5.97173 | 5.49611 | 5.93444 | 7.16545 | 7.07916 | 8.15846 | 5.94353 | 6.09353 | 4.69977 | 7.45638 | 7.17751 | 6.79082 | 6.33323 | 7.32705 | 6.37336 |
| 10981 | 5.75248 | 5.70882 | 5.18639 | 3.6203 | 5.26081 | 5.01531 | 4.73675 | 5.76294 | 4.20981 | 5.83276 | 3.46674 | 4.88481 | 4.34642 | 6.81521 | 6.18613 | 6.79042 | 6.36206 |
| 11716 | 7.41316 | 7.23343 | 8.80868 | 8.20412 | 8.68849 | 8.83638 | 8.6301 | 9.5245 | 7.63442 | 8.26888 | 7.00839 | 8.74623 | 8.66727 | 10.1272 | 9.52401 | 9.91396 | 9.14061 |
| 12035 | 6.61905 | 6.1629 | 5.81921 | 5.51006 | 5.96139 | 5.4299 | 5.3171 | 6.11865 | 5.01889 | 6.19185 | 5.14718 | 5.55678 | 5.30881 | 6.95005 | 6.88488 | 7.07122 | 6.73123 |
| 12122 | 7.27828 | 9.03224 | 5.77881 | 4.82011 | 5.92352 | 5.9146 | 5.80187 | 7.55064 | 5.42163 | 6.2539 | 4.92862 | 5.77986 | 5.90457 | 7.43531 | 6.61498 | 9.2174 | 8.65524 |
| 12843 | 6.486 | 6.96988 | 6.1351 | 6.18602 | 6.13006 | 6.96058 | 7.04161 | 7.55664 | 6.48138 | 6.35175 | 5.47921 | 6.94232 | 6.56708 | 5.93759 | 6.03206 | 6.5059 | 6.46395 |
| 12944 | 6.43529 | 7.9721 | 6.0436 | 4.11601 | 6.0929 | 5.87684 | 5.52768 | 6.87639 | 4.78162 | 5.98477 | 4.19075 | 5.73411 | 5.15128 | 6.06274 | 5.61949 | 7.42711 | 7.11724 |
| 13060 | 7.41609 | 9.39363 | 8.28261 | 7.5897 | 8.34389 | 8.49817 | 8.44249 | 9.7372 | 8.11474 | 8.02964 | 7.51526 | 8.50089 | 7.91035 | 6.1719 | 5.88267 | 7.24977 | 6.91492 |
| 13724 | 5.77097 | 6.63121 | 5.2038 | 5.82574 | 5.04416 | 5.75528 | 5.79708 | 5.67087 | 5.94701 | 5.61872 | 6.03465 | 7.47942 | 7.78363 | 5.30706 | 5.92808 | 4.00152 | 4.54492 |
| 13729 | 6.20854 | 6.41227 | 6.16577 | 5.49102 | 6.34313 | 6.07532 | 6.19202 | 6.62304 | 6.32505 | 5.56108 | 6.40165 | 6.05029 | 6.05355 | 6.10731 | 6.04757 | 6.8401 | 7.68251 |
| 16591 | 6.14216 | 6.46437 | 5.77347 | 5.2392 | 5.8368 | 5.65713 | 5.56164 | 6.39469 | 5.52036 | 5.92511 | 5.40235 | 5.75465 | 5.11788 | 5.48298 | 5.04262 | 5.88335 | 5.29871 |
| 16640 | 7.47454 | 7.59915 | 5.19448 | 4.94612 | 5.02339 | 6.02117 | 6.92894 | 7.69658 | 6.1765 | 5.20923 | 5.24584 | 6.33509 | 6.64899 | 7.06264 | 7.00659 | 7.14331 | 8.70479 |

| strain | WT | | | | | | | | | | |
| --- | --- | --- | --- | --- | --- | --- | --- | --- | --- | --- | --- |
| condition | G\_SD | | GX\_SD | | | X\_SD | | A\_SD | | C\_SD | |
| proteinId | exp | stat | exp | trans | stat | exp | stat | exp | stat | exp | stat |
| 8671 | 0.378826 | 1.26921 | 0.605588 | 1.1169 | 0.913083 | 0.976828 | 1.18917 | 0.388524 | 1.54027 | 1.28265 | 1.51877 |
| 8678 | 24.2091 | 19.2125 | 22.5986 | 15.4605 | 14.3787 | 24.8578 | 22.1975 | 26.0698 | 22.4098 | 28.6911 | 21.4138 |
| 8702 | 5.35647 | 4.16918 | 3.86435 | 3.9005 | 4.0275 | 6.06516 | 4.30719 | 3.66721 | 4.19765 | 1.27869 | 0.42946 |
| 8758 | 8.88763 | 4.69977 | 6.9225 | 5.94877 | 4.39143 | 7.42494 | 7.05941 | 6.1854 | 5.15683 | 3.19426 | 2.83003 |
| 8781 | 1.74507 | 0.169232 | 3.26119 | 0.746335 | 0 | 1.36456 | 0 | 0.773675 | 0 | 0 | 0 |
| 8835 | 6.36706 | 5.56431 | 8.13106 | 6.32614 | 5.44875 | 6.26603 | 5.30629 | 7.72028 | 5.72505 | 7.88326 | 4.98186 |
| 9240 | 5.5153 | 6.04072 | 3.86976 | 8.38208 | 5.81651 | 5.07218 | 3.54243 | 4.43557 | 3.83626 | 5.3441 | 3.46252 |
| 10113 | 1.13939 | 2.64436 | 2.6535 | 2.60645 | 2.1921 | 2.5317 | 1.75848 | 2.12084 | 0.378889 | 3.43348 | 3.69117 |
| 10981 | 3.07613 | 1.97 | 2.64099 | 4.27489 | 1.81269 | 2.74336 | 1.96421 | 3.28394 | 1.33873 | 1.27051 | 1.06971 |
| 11716 | 8.05551 | 3.44656 | 8.53581 | 8.21657 | 6.72594 | 8.8237 | 1.17916 | 5.40311 | 1.14163 | 6.39978 | 3.47306 |
| 12035 | 0 | 0.528521 | 0.197656 | 0.751858 | 0.711395 | 0.395869 | 0.403372 | 1.15917 | 0.95367 | 0 | 0 |
| 12122 | 20.27 | 14.932 | 23.5961 | 12.0966 | 10.9676 | 19.539 | 14.988 | 15.2721 | 13.7782 | 21.5047 | 17.6543 |
| 12843 | 4.01357 | 3.19686 | 4.48063 | 4.08683 | 3.114 | 6.46135 | 3.70913 | 4.63011 | 2.68886 | 3.84886 | 3.90851 |
| 12944 | 6.92054 | 5.03038 | 9.98717 | 8.75389 | 6.74706 | 8.028 | 7.61565 | 6.95617 | 6.116 | 12.1709 | 9.5866 |
| 13060 | 11.9544 | 18.2422 | 13.8508 | 20.2755 | 18.7096 | 17.8224 | 17.4639 | 13.9154 | 14.1827 | 17.5495 | 17.8389 |
| 13724 | 0.180601 | 0.354512 | 0 | 0 | 0 | 0.190315 | 0 | 0 | 0 | 0 | 0 |
| 13729 | 1.97343 | 1.78912 | 1.62319 | 1.10754 | 1.82548 | 1.9468 | 0.593616 | 1.16285 | 0.385575 | 0.208996 | 0 |
| 16591 | 8.26785 | 5.80068 | 10.3742 | 8.38926 | 6.7848 | 7.84075 | 5.89943 | 5.79581 | 7.10046 | 9.62065 | 7.19414 |
| 16640 | 0 | 0 | 0 | 0 | 0 | 0.200926 | 0 | 0 | 0 | 0 | 0 |

|  | Glucose | Xylose | Arabinose | Acetate | Coumarate | Ferulate | YNB Oleic Acid | YNB Ricinoleic Acid | YNB Glucose | YNB Gluc DOC | YPD |
| --- | --- | --- | --- | --- | --- | --- | --- | --- | --- | --- | --- |
| proteinId |  |  |  |  |  |  |  |  |  |  |  |
| 13724 | -0.0242052 | -0.13888 | -0.054681 | 0.0237792 | -0.219687 | 0.208523 | -0.0744516 | -0.261135 | -0.0276679 | -0.136039 | -0.093421 |

In [30]:

```
temp = Annotation.index[Annotation['Combined Annotations'].str.contains('hydroxysteroid')]
display(Annotation.loc[temp])
Show_Data(temp)
```

|  | Combined Annotations | Signal P | Sc288c Orthologs | Human Orthologs | Sc288 Best Hit | Human Blast | Essential | WolfPSort | C Terminal |
| --- | --- | --- | --- | --- | --- | --- | --- | --- | --- |
| RTO4\_ID |  |  |  |  |  |  |  |  |  |
| 11599 | KOG1430: C-3 sterol dehydrogenase/3-beta-hydro... |  |  |  |  |  | Not Essential | cyto 25 | VGG\* |
| 11765 | K13370: HSD17B8; 17beta-estradiol 17-dehydroge... |  |  | BDH2 | SPS19 | DHRS4 | Not Essential | mito 19.5, cyto\_mito 12, cyto 3.5, extr 2 | TPT\* |
| 13356 | KOG1014: 17 beta-hydroxysteroid dehydrogenase ... |  |  |  |  |  | Not Essential | cyto 9.5, cyto\_nucl 6.5, mito 6, extr 6, nucl 2.5 | EKF\* |
| 14267 | K00038: E1.1.1.53; 3alpha(or 20beta)-hydroxyst... |  |  |  | SPS19 | DHRS4 | Not Essential | cyto 16.5, cyto\_nucl 9, extr 6, mito 4 | KWC\* |
| 15346 | KOG1014: 17 beta-hydroxysteroid dehydrogenase ... | S |  |  |  |  | Not Essential | mito 19, extr 5, cyto 2 | EKW\* |

| strain | WT | | | | | | | | | | | | | | | | |
| --- | --- | --- | --- | --- | --- | --- | --- | --- | --- | --- | --- | --- | --- | --- | --- | --- | --- |
| condition | G\_MM | C\_MM | G\_SD | | GX\_SD | | | X\_SD | | A\_SD | | C\_SD | | MM\_CN120 | | MM\_CN5 | Diversity\_Sample |
| phase | exp | exp | exp | stat | exp | trans | stat | exp | stat | exp | stat | exp | stat | exp | stat | exp | exp |
| proteinId | Set1 | Set1 | Set2 | Set2 | Set2 | Set2 | Set2 | Set2 | Set2 | Set2 | Set2 | Set2 | Set2 | Set3 | Set3 | Set3 | Set3 |
| RTO4\_ID |  |  |  |  |  |  |  |  |  |  |  |  |  |  |  |  |  |
| 11599 | 4.82999 | 5.08182 | 4.49364 | 4.00277 | 4.5463 | 4.30542 | 4.08564 | 4.31568 | 4.0655 | 4.09063 | 3.79212 | 4.24546 | 3.16578 | 4.08266 | 3.90386 | 4.27322 | 4.51167 |
| 11765 | 2.91192 | 4.07621 | 3.3524 | 5.31952 | 3.13082 | 3.12018 | 3.73719 | 2.95409 | 4.1501 | 3.0469 | 4.7533 | 4.54123 | 4.05936 | 2.31793 | 2.82038 | 2.99813 | 3.28174 |
| 13356 | 6.46727 | 5.57704 | 6.94148 | 5.3097 | 7.04575 | 4.15141 | 4.32071 | 4.02975 | 4.58712 | 4.14919 | 4.50569 | 4.97449 | 6.00263 | 4.18146 | 2.4398 | 6.33141 | 3.6281 |
| 14267 | 5.82984 | 5.76013 | 4.63059 | 4.66884 | 4.81662 | 3.5412 | 3.4523 | 5.6219 | 5.50923 | 5.35077 | 5.61054 | 4.89981 | 3.90712 | 3.32986 | 2.61718 | 5.45814 | 3.87456 |
| 15346 | 6.01785 | 6.45278 | 4.63753 | 5.15621 | 4.74766 | 4.19259 | 4.14313 | 4.5853 | 4.63056 | 4.96963 | 5.2584 | 5.16036 | 4.42525 | 3.23561 | 3.34519 | 3.65999 | 4.36013 |

| strain | WT | | | | | | | | | | |
| --- | --- | --- | --- | --- | --- | --- | --- | --- | --- | --- | --- |
| condition | G\_SD | | GX\_SD | | | X\_SD | | A\_SD | | C\_SD | |
| proteinId | exp | stat | exp | trans | stat | exp | stat | exp | stat | exp | stat |
| 11599 | 0.180601 | 0 | 0 | 0 | 0 | 0.200926 | 0.189187 | 0.196014 | 0 | 0.219601 | 0.217934 |
| 11765 | 0 | 0 | 0 | 0 | 0 | 0 | 0 | 0 | 0 | 0.854055 | 0.431761 |
| 13356 | 0 | 0.357545 | 0 | 0 | 0 | 0 | 0 | 0 | 0 | 0.853831 | 0 |
| 14267 | 1.72517 | 1.25716 | 2.02988 | 1.11352 | 0.722761 | 2.74281 | 2.37729 | 2.70142 | 2.86548 | 3.43113 | 3.06087 |
| 15346 | 5.20685 | 5.60335 | 4.87938 | 7.80691 | 7.66486 | 6.85773 | 7.45759 | 6.96033 | 5.93885 | 9.00023 | 7.18862 |

|  | Glucose | Xylose | Arabinose | Acetate | Coumarate | Ferulate | YNB Oleic Acid | YNB Ricinoleic Acid | YNB Glucose | YNB Gluc DOC | YPD |
| --- | --- | --- | --- | --- | --- | --- | --- | --- | --- | --- | --- |
| proteinId |  |  |  |  |  |  |  |  |  |  |  |
| 11599 | -0.149168 | 0.565516 | 0.0343418 | 0.138936 | 0.313571 | 0.0936143 | 0.0472852 | -0.335484 | -0.0458369 | -0.0802779 | -0.310492 |
| 11765 | 0.107044 | 0.135627 | 0.101293 | 0.0450894 | 0.0149791 | -0.0316738 | 0.0112884 | 0.0701067 | -0.118633 | -0.0299555 | 0.387225 |
| 13356 | 0.0353567 | 0.0712028 | -0.20468 | -0.327876 | 0.127818 | -0.0408538 | -0.806669 | -0.136497 | 0.0486512 | -0.135179 | 0.261526 |
| 14267 | 0.207207 | -0.248473 | 0.215494 | 0.063958 | -0.173733 | 0.246239 | 0.0646736 | 0.0278355 | -0.138097 | -0.164489 | 0.459204 |
| 15346 | -0.0721767 | 0.153242 | -0.0470953 | -0.000743865 | -0.246982 | -0.123396 | 0.0771705 | -0.0813489 | 0.222447 | -0.0457231 | 0.163785 |

In [31]:

```
for r in sorted(model.reactions, key=lambda x: x.id):
    if r.id.startswith('ECOAH') and r.id.endswith('m'):
        print(r.id, r.reaction, r.gene_reaction_rule)
print()
for r in sorted(model.reactions, key=lambda x: x.id):
    if r.id.startswith('ECOAH') and (r.id.endswith('p') or r.id.endswith('x') or r.id.endswith('_1')):
        print(r.id, r.reaction, r.gene_reaction_rule)
print()
for r in sorted(model.reactions, key=lambda x: x.id):
    if r.id.startswith('ECOAH') and not (r.id.endswith('m') or r.id.endswith('p') or r.id.endswith('x') or r.id.endswith('_1')):
        print(r.id, r.reaction, r.gene_reaction_rule)
```

```
ECOAH12m 2mp2coa_m + h2o_m <=> 3hibutcoa_m 11907 or 14805 or (HADHA and HADHB) or (Hadha and Hadhb)
ECOAH1m 3hbcoa_m <=> b2coa_m + h2o_m 11907 or 14805 or 16128 or (HADHA and HADHB) or (Hadha and Hadhb)
ECOAH2m 3hhcoa_m <=> h2o_m + hx2coa_m 11907
ECOAH3m 3hocoa_m <=> h2o_m + oc2coa_m 11907
ECOAH4m 3hdcoa_m <=> dc2coa_m + h2o_m 11907 or 14805
ECOAH5m 3hddcoa_m <=> dd2coa_m + h2o_m 11907 or 14805
ECOAH6m 3htdcoa_m <=> h2o_m + td2coa_m 11907 or 14805
ECOAH7m 3hhdcoa_m <=> h2o_m + hdd2coa_m 11907 or 14805
ECOAH9m 2mb2coa_m + h2o_m <=> 3hmbcoa_m 11907 or 14805 or (HADHA and HADHB) or (Hadha and Hadhb)

ECOAH11p h2o_x + hxc2coa_x <=> 3hxccoa_x 11362
ECOAH1x 3hbcoa_x <=> b2coa_x + h2o_x 11362 or 11907
ECOAH2_1 h2o_x + hx2coa_x <=> 3hhcoa_x 11362
ECOAH3_1 h2o_x + oc2coa_x <=> 3hocoa_x 11362
ECOAH4p 3hdcoa_x <=> dc2coa_x + h2o_x 11362
ECOAH5p 3hddcoa_x <=> dd2coa_x + h2o_x 11362
ECOAH6p 3htdcoa_x <=> h2o_x + td2coa_x 11362
ECOAH7p 3hhdcoa_x <=> h2o_x + hdd2coa_x 11362
ECOAH8p 3hodcoa_x <=> h2o_x + od2coa_x 11362

ECOAH1 3hbcoa_c <=> b2coa_c + h2o_c 14805 or 16128
ECOAH12 2mp2coa_c + h2o_c --> 3hibutcoa_c 14805
ECOAH2 3hhcoa_c <=> h2o_c + hx2coa_c 14805
ECOAH3 3hocoa_c <=> h2o_c + oc2coa_c 14805
ECOAH4 3hdcoa_c <=> dc2coa_c + h2o_c 14805
ECOAH5 3hddcoa_c <=> dd2coa_c + h2o_c 14805
ECOAH6 3htdcoa_c <=> h2o_c + td2coa_c 14805
ECOAH7 3hhdcoa_c <=> h2o_c + hdd2coa_c 14805
ECOAH9ir 2mb2coa_c + h2o_c --> 3hmbcoa_c 14805
```

In [32]:

```
temp = ['11907','14805','16515','11362','9469']
display(Annotation.loc[temp])
Show_Data(temp)
```

|  | Combined Annotations | Signal P | Sc288c Orthologs | Human Orthologs | Sc288 Best Hit | Human Blast | Essential | WolfPSort | C Terminal |
| --- | --- | --- | --- | --- | --- | --- | --- | --- | --- |
| RTO4\_ID |  |  |  |  |  |  |  |  |  |
| 11907 | K12663: ECH1; Delta(3,5)-Delta(2,4)-dienoyl-Co... |  |  | ECH1 |  | ECH1 | Not Essential | extr 9, mito 8, cyto 5.5, cyto\_nucl 4, pero 3 | AKL\* |
| 14805 | K07511: ECHS1; enoyl-CoA hydratase |  |  | ECHS1 | EHD3 | ECHS1 | Not Essential | mito 26.5, cyto\_mito 14 | TNS\* |
| 16515 | HMMPfam:Enoyl-CoA hydratase/isomerase family:P... |  |  |  |  | ECHS1 | Not Essential | mito 10.5, cyto\_mito 9.833, cyto 8, cyto\_nucl ... | ARL\* |
| 11362 | K14729: FOX2; multifunctional beta-oxidation p... |  | FOX2 | HSD17B4 | FOX2 | HSD17 | Not Essential | cyto 15, mito 6, pero 4, mito\_nucl 4 | LAQ\* |
| 9469 | KOG1206: Peroxisomal multifunctional beta-oxid... |  |  |  | FOX2 | HSD17 | Not Essential | cyto 25 | SKL\* |

| strain | WT | | | | | | | | | | | | | | | | |
| --- | --- | --- | --- | --- | --- | --- | --- | --- | --- | --- | --- | --- | --- | --- | --- | --- | --- |
| condition | G\_MM | C\_MM | G\_SD | | GX\_SD | | | X\_SD | | A\_SD | | C\_SD | | MM\_CN120 | | MM\_CN5 | Diversity\_Sample |
| phase | exp | exp | exp | stat | exp | trans | stat | exp | stat | exp | stat | exp | stat | exp | stat | exp | exp |
| proteinId | Set1 | Set1 | Set2 | Set2 | Set2 | Set2 | Set2 | Set2 | Set2 | Set2 | Set2 | Set2 | Set2 | Set3 | Set3 | Set3 | Set3 |
| 11907 | 4.74678 | 5.70781 | 4.46348 | 4.96393 | 4.30815 | 5.01839 | 4.92947 | 4.82019 | 5.30592 | 5.57951 | 5.3894 | 4.73704 | 4.90627 | 4.67855 | 4.75084 | 4.79321 | 4.29008 |
| 14805 | 6.66336 | 7.46868 | 7.28983 | 7.01391 | 7.23743 | 6.95771 | 6.90675 | 7.20778 | 6.45434 | 6.80526 | 6.96181 | 6.4687 | 5.2893 | 6.9611 | 6.88428 | 7.38075 | 7.07681 |
| 16515 | 4.60341 | 7.72482 | 4.50872 | 4.61116 | 4.53397 | 4.19523 | 4.02373 | 4.13397 | 4.83742 | 4.78941 | 5.23086 | 5.63921 | 4.56216 | 4.16295 | 4.14747 | 4.49176 | 4.83887 |
| 11362 | 6.4714 | 7.8679 | 6.21284 | 7.41652 | 6.15013 | 6.7725 | 7.06199 | 6.33216 | 6.88691 | 6.87267 | 7.25799 | 7.02773 | 6.87898 | 6.72127 | 7.06759 | 5.96352 | 6.44637 |
| 9469 | 5.82005 | 10.5948 | 4.68671 | 5.15186 | 4.83711 | 4.53727 | 4.40813 | 4.97297 | 5.62221 | 6.1261 | 6.42486 | 6.93686 | 6.62484 | 3.8895 | 4.31166 | 5.62877 | 4.85907 |

| strain | WT | | | | | | | | | | |
| --- | --- | --- | --- | --- | --- | --- | --- | --- | --- | --- | --- |
| condition | G\_SD | | GX\_SD | | | X\_SD | | A\_SD | | C\_SD | |
| proteinId | exp | stat | exp | trans | stat | exp | stat | exp | stat | exp | stat |
| 11907 | 0.564696 | 3.61281 | 0.615297 | 3.15118 | 4.54856 | 4.34278 | 4.34294 | 4.44065 | 4.79797 | 3.635 | 2.8388 |
| 14805 | 11.7011 | 12.7691 | 12.8252 | 14.7036 | 16.0384 | 15.837 | 14.9541 | 14.8732 | 14.9406 | 17.9733 | 16.775 |
| 16515 | 0 | 0.333333 | 0 | 0.183791 | 1.08045 | 0 | 0.403372 | 0 | 0 | 6.19528 | 3.45342 |
| 11362 | 20.4831 | 39.7562 | 20.574 | 30.8562 | 31.4551 | 28.1334 | 34.0703 | 34.5879 | 34.4629 | 67.7196 | 51.5122 |
| 9469 | 2.11428 | 1.99518 | 1.01594 | 0.735718 | 1.80731 | 2.53315 | 2.74191 | 5.02793 | 5.18811 | 26.743 | 14.3845 |

|  | Glucose | Xylose | Arabinose | Acetate | Coumarate | Ferulate | YNB Oleic Acid | YNB Ricinoleic Acid | YNB Glucose | YNB Gluc DOC | YPD |
| --- | --- | --- | --- | --- | --- | --- | --- | --- | --- | --- | --- |
| proteinId |  |  |  |  |  |  |  |  |  |  |  |
| 11907 | 0.114424 | -0.0555856 | 0.084374 | 0.0957274 | 0.0248728 | 0.0169171 | -1.15316 | 0.0380234 | -0.189194 | -0.250849 | -0.147514 |
| 14805 | 0.195211 | 0.271089 | -0.627815 | -1.43546 | -0.649913 | -0.937919 | -2.90688 | -4.21411 | 0.0902761 | -0.0765189 | 0.461105 |
| 16515 | -0.00111379 | 0.314535 | 0.231734 | 0.369911 | 0.142545 | 0.523174 | -0.0657867 | 0.443237 | -0.0131392 | -0.0949175 | 0.317089 |
| 11362 | 0.0751211 | 0.0521463 | 0.00810646 | -0.0907637 | -0.150565 | 0.0238548 | -1.49407 | 0.0551923 | 0.104864 | -0.187183 | -0.0642188 |
| 9469 | -0.132763 | 0.0961697 | -0.120022 | -0.0748623 | 0.240087 | -0.313024 | 0.0222837 | 0.0728534 | 0.0136585 | -0.0957982 | 0.0982957 |

11907 perox Delta3,5-Delta2,4-dienoyl-CoA isomerase  
14805 ECHS1 mito enoyl-coA hydratase  
16515 Enoyl-CoA hydratase/isomerase family, no rxn, (paperblast to Mevalonyl-coenzyme A hydratase sidH in pero), protein upregulated in p-coumarate, couA?  
11362 FOX2 HSD17B4 perox multifunctional beta-oxidation protein  
9469 perox multifunctional beta-oxidation protein, but only last two domains, blast to peroxisomal dehydratase / enoyl-CoA hydratase 2, no rxn, upregulated in coumarate

Change ECOAH1x to ECOAH1p  
Change ECOAH2\_1 to ECOAH2p  
Change ECOAH3\_1 to ECOAH3p

Change ECOAHxp genes to '11362'  
Change ECOAHxm genes to '14805'  
ECOAH8m is missing, add one?  
Remove ECOAHx cyto reactions

In [33]:

```
model.reactions.get_by_id('ECOAH1x').id = 'ECOAH1p'
model.reactions.get_by_id('ECOAH2_1').id = 'ECOAH2p'
model.reactions.get_by_id('ECOAH3_1').id = 'ECOAH3p'
model.reactions.get_by_id('ECOAH1p').gene_reaction_rule = '11362'
model.reactions.get_by_id('ECOAH2p').gene_reaction_rule = '11362'
model.reactions.get_by_id('ECOAH3p').gene_reaction_rule = '11362'
model.reactions.get_by_id('ECOAH4p').gene_reaction_rule = '11362'
model.reactions.get_by_id('ECOAH5p').gene_reaction_rule = '11362'
model.reactions.get_by_id('ECOAH6p').gene_reaction_rule = '11362'
model.reactions.get_by_id('ECOAH7p').gene_reaction_rule = '11362'
model.reactions.get_by_id('ECOAH8p').gene_reaction_rule = '11362'
model.reactions.get_by_id('ECOAH11p').gene_reaction_rule = '11362'
model.reactions.get_by_id('ECOAH1m').gene_reaction_rule = '14805'
model.reactions.get_by_id('ECOAH2m').gene_reaction_rule = '14805'
model.reactions.get_by_id('ECOAH3m').gene_reaction_rule = '14805'
model.reactions.get_by_id('ECOAH4m').gene_reaction_rule = '14805'
model.reactions.get_by_id('ECOAH5m').gene_reaction_rule = '14805'
model.reactions.get_by_id('ECOAH6m').gene_reaction_rule = '14805'
model.reactions.get_by_id('ECOAH7m').gene_reaction_rule = '14805'
model.reactions.get_by_id('ECOAH9m').gene_reaction_rule = '14805'
model.reactions.get_by_id('ECOAH12m').gene_reaction_rule = '14805'
model.remove_reactions(['ECOAH1','ECOAH2','ECOAH3','ECOAH4','ECOAH5','ECOAH6','ECOAH7','ECOAH9ir','ECOAH12'], remove_orphans=True)
```

In [34]:

```
for r in sorted(model.reactions, key=lambda x: x.id):
    if r.id.startswith('HACD') and r.id.endswith('m'):
        print(r.id, r.reaction, r.gene_reaction_rule)
print()
for r in sorted(model.reactions, key=lambda x: x.id):
    if r.id.startswith('HACD') and (r.id.endswith('p') or r.id.endswith('x') or r.id.endswith('_1')):
        print(r.id, r.reaction, r.gene_reaction_rule)
print()
for r in sorted(model.reactions, key=lambda x: x.id):
    if r.id.startswith('HACD') and not (r.id.endswith('m') or r.id.endswith('p') or r.id.endswith('x') or r.id.endswith('_1')):
        print(r.id, r.reaction, r.gene_reaction_rule)
```

```
HACD1m aacoa_m + h_m + nadh_m <=> 3hbcoa_m + nad_m 11203 or 15180 or 16284 or (HADHA and HADHB) or (Hadha and Hadhb)
HACD2m 3ohcoa_m + h_m + nadh_m <=> 3hhcoa_m + nad_m 11362
HACD3m 3oocoa_m + h_m + nadh_m <=> 3hocoa_m + nad_m 11362
HACD4m 3odcoa_m + h_m + nadh_m <=> 3hdcoa_m + nad_m 11362
HACD5m 3oddcoa_m + h_m + nadh_m <=> 3hddcoa_m + nad_m 11362
HACD6m 3otdcoa_m + h_m + nadh_m <=> 3htdcoa_m + nad_m 11362
HACD7m 3ohdcoa_m + h_m + nadh_m <=> 3hhdcoa_m + nad_m 11362
HACD9m 3hmbcoa_m + nad_m <=> 2maacoa_m + h_m + nadh_m 11362 or 16284

HACD10p 3hxccoa_x + nad_x <=> 3ohxccoa_x + h_x + nadh_x 11362
HACD1x aacoa_x + h_x + nadh_x <=> 3hbcoa_x + nad_x 11362
HACD2_1 3hhcoa_x + nad_x <=> 3ohcoa_x + h_x + nadh_x 11362
HACD3_1 3hocoa_x + nad_x <=> 3oocoa_x + h_x + nadh_x 11362
HACD4p 3odcoa_x + h_x + nadh_x <=> 3hdcoa_x + nad_x 11362
HACD5p 3oddcoa_x + h_x + nadh_x <=> 3hddcoa_x + nad_x 11362
HACD6p 3otdcoa_x + h_x + nadh_x <=> 3htdcoa_x + nad_x 11362
HACD7p 3ohdcoa_x + h_x + nadh_x <=> 3hhdcoa_x + nad_x 11362
HACD8p 3ohodcoa_x + h_x + nadh_x <=> 3hodcoa_x + nad_x 11362

HACD1i 3hbcoa_c + nad_c --> aacoa_c + h_c + nadh_c 16284
HACD2i 3hhcoa_c + nad_c --> 3ohcoa_c + h_c + nadh_c 16284
HACD3i 3hocoa_c + nad_c --> 3oocoa_c + h_c + nadh_c 16284
HACD4i 3hdcoa_c + nad_c --> 3odcoa_c + h_c + nadh_c 16284
HACD5i 3hddcoa_c + nad_c --> 3oddcoa_c + h_c + nadh_c 16284
HACD6i 3htdcoa_c + nad_c --> 3otdcoa_c + h_c + nadh_c 16284
HACD7i 3hhdcoa_c + nad_c --> 3ohdcoa_c + h_c + nadh_c 16284
```

In [35]:

```
temp = ['11203','15180','16284','13606','11362']
display(Annotation.loc[temp])
Show_Data(temp)
```

|  | Combined Annotations | Signal P | Sc288c Orthologs | Human Orthologs | Sc288 Best Hit | Human Blast | Essential | WolfPSort | C Terminal |
| --- | --- | --- | --- | --- | --- | --- | --- | --- | --- |
| RTO4\_ID |  |  |  |  |  |  |  |  |  |
| 11203 | K00022: HADH; 3-hydroxyacyl-CoA dehydrogenase |  |  | HADH |  | HADH | Not Essential | mito 20, cyto\_mito 13.333, cyto 4.5, cyto\_nucl... | YSS\* |
| 15180 | K00074: paaH, hbd, fadB, mmgB; 3-hydroxybutyry... |  |  | HADH |  | HADH | Not Essential | cyto 13, cyto\_nucl 8, cysk 8, mito 4 | YTAR |
| 16284 | K08683: HSD17B10; 3-hydroxyacyl-CoA dehydrogen... | S |  | HSD17B10 |  | HSD17 | Not Essential | extr 11, mito 5, plas 5, E.R. 4 | AKM\* |
| 13606 | KOG1199: Short-chain alcohol dehydrogenase/3-h... | S |  |  |  | HSD17 | Not Essential | cyto 11.5, cyto\_mito 10, mito 7.5, extr 7 | ASF\* |
| 11362 | K14729: FOX2; multifunctional beta-oxidation p... |  | FOX2 | HSD17B4 | FOX2 | HSD17 | Not Essential | cyto 15, mito 6, pero 4, mito\_nucl 4 | LAQ\* |

| strain | WT | | | | | | | | | | | | | | | | |
| --- | --- | --- | --- | --- | --- | --- | --- | --- | --- | --- | --- | --- | --- | --- | --- | --- | --- |
| condition | G\_MM | C\_MM | G\_SD | | GX\_SD | | | X\_SD | | A\_SD | | C\_SD | | MM\_CN120 | | MM\_CN5 | Diversity\_Sample |
| phase | exp | exp | exp | stat | exp | trans | stat | exp | stat | exp | stat | exp | stat | exp | stat | exp | exp |
| proteinId | Set1 | Set1 | Set2 | Set2 | Set2 | Set2 | Set2 | Set2 | Set2 | Set2 | Set2 | Set2 | Set2 | Set3 | Set3 | Set3 | Set3 |
| 11203 | 6.04144 | 6.52539 | 6.87702 | 5.38344 | 6.84931 | 6.03213 | 5.78184 | 6.32067 | 5.58855 | 5.70619 | 5.51458 | 6.04538 | 5.47375 | 5.18492 | 5.6258 | 5.69338 | 5.76141 |
| 15180 | 6.39131 | 6.93904 | 8.45481 | 7.90698 | 8.28786 | 7.85758 | 7.92101 | 7.72636 | 6.73246 | 6.58912 | 7.20451 | 5.11868 | 2.99123 | 6.78467 | 7.32609 | 6.31068 | 5.76589 |
| 16284 | 3.35477 | 5.76758 | 3.27426 | 3.20131 | 3.25902 | 3.6194 | 3.47793 | 2.82846 | 4.36417 | 4.26054 | 4.47659 | 3.27286 | 3.57433 | 1.86628 | 2.28615 | 1.63316 | 1.93157 |
| 13606 | 4.56417 | 3.98019 | 4.30784 | 4.24056 | 4.15453 | 4.49967 | 4.81746 | 4.13308 | 3.96345 | 4.57589 | 4.57166 | 2.84995 | 2.82936 | 3.00183 | 3.6982 | 2.69136 | 2.92017 |
| 11362 | 6.4714 | 7.8679 | 6.21284 | 7.41652 | 6.15013 | 6.7725 | 7.06199 | 6.33216 | 6.88691 | 6.87267 | 7.25799 | 7.02773 | 6.87898 | 6.72127 | 7.06759 | 5.96352 | 6.44637 |

| strain | WT | | | | | | | | | | |
| --- | --- | --- | --- | --- | --- | --- | --- | --- | --- | --- | --- |
| condition | G\_SD | | GX\_SD | | | X\_SD | | A\_SD | | C\_SD | |
| proteinId | exp | stat | exp | trans | stat | exp | stat | exp | stat | exp | stat |
| 11203 | 9.79647 | 11.4043 | 8.12888 | 8.92969 | 11.3169 | 9.5734 | 13.3616 | 9.65801 | 9.38026 | 9.40164 | 11.1454 |
| 15180 | 12.6329 | 11.1282 | 14.6691 | 13.9489 | 14.7616 | 14.4663 | 16.3257 | 12.9436 | 12.4499 | 11.9861 | 13.0816 |
| 16284 | 0.791247 | 1.02934 | 0.41045 | 1.29645 | 1.28101 | 0.789529 | 0.57633 | 1.54284 | 1.34541 | 4.90066 | 3.49707 |
| 11362 | 20.4831 | 39.7562 | 20.574 | 30.8562 | 31.4551 | 28.1334 | 34.0703 | 34.5879 | 34.4629 | 67.7196 | 51.5122 |

|  | Glucose | Xylose | Arabinose | Acetate | Coumarate | Ferulate | YNB Oleic Acid | YNB Ricinoleic Acid | YNB Glucose | YNB Gluc DOC | YPD |
| --- | --- | --- | --- | --- | --- | --- | --- | --- | --- | --- | --- |
| proteinId |  |  |  |  |  |  |  |  |  |  |  |
| 11203 | -0.0203069 | -0.0319005 | 0.142411 | 0.0669948 | 0.031557 | 0.0807583 | -1.34826 | -1.89792 | 0.232648 | -0.109088 | 0.193759 |
| 15180 | -0.0297025 | 0.104901 | -0.197956 | -0.248617 | -0.285895 | -0.000632661 | -0.205114 | -0.216211 | -0.184384 | 0.068575 | -0.0350937 |
| 16284 | 0.26627 | 0.405879 | 0.10098 | 0.288009 | 0.532942 | 0.271564 | 0.124402 | 0.270842 | -0.788518 | -0.311085 | -0.82854 |
| 13606 | 0.279153 | -0.0459621 | 0.327194 | 0.354471 | 0.719525 | 0.273068 | -0.217612 | 0.0319699 | 0.37306 | 0.295145 | 0.0925538 |
| 11362 | 0.0751211 | 0.0521463 | 0.00810646 | -0.0907637 | -0.150565 | 0.0238548 | -1.49407 | 0.0551923 | 0.104864 | -0.187183 | -0.0642188 |

11203 HADH 3-hydroxyacyl-CoA dehydrogenase 1.1.1.35 beta oxidation (mito, medium and short chain)  
15180 HADH paaH, hbd, fadB, mmgB; 3-hydroxybutyryl-CoA dehydrogenase 1.1.1.157 (S)-3-Hydroxybutanoyl-CoA + NADP+ <=> Acetoacetyl-CoA + NADPH + H+, also (3S)-3-Hydroxyadipyl-CoA + NAD+ <=> 3-Oxoadipyl-CoA + NADH + H+  
16284 HSD17B10 3-hydroxyacyl-CoA dehydrogenase / 3-hydroxy-2-methylbutyryl-CoA dehydrogenase 1.1.1.35 beta oxidation 1.1.1.178 (2S,3S)-3-Hydroxy-2-methylbutanoyl-CoA + NAD+ <=> 2-Methylacetoacetyl-CoA + NADH + H+  
13606 3-hydroxyacyl-CoA dehydrogenase / 3-hydroxy-2-methylbutyryl-CoA dehydrogenase 1.1.1.35 beta oxidation 1.1.1.178 (2S,3S)-3-Hydroxy-2-methylbutanoyl-CoA + NAD+ <=> 2-Methylacetoacetyl-CoA + NADH + H+, no rxn  
11362 FOX2 HSD17B4 pero multifunctional beta-oxidation protein

Change HACD1x to HACD1p  
Change HACD2\_1 to HACD2p  
Change HACD3\_1 to HACD3p  
Change HACDxp genes to '11362'  
Change HACDxm genes to '11203'  
HACD9m is for 2-methylacetoacetyl-CoA, change genes to '13606 or 16284'  
Change HBCO\_nadp genes to '15180', HADPCOADH3 is correct  
Remove HBCO\_nadp\_m

In [36]:

```
model.reactions.get_by_id('HACD1x').id = 'HACD1p'
model.reactions.get_by_id('HACD2_1').id = 'HACD2p'
model.reactions.get_by_id('HACD3_1').id = 'HACD3p'
model.reactions.get_by_id('HACD1p').gene_reaction_rule = '11362'
model.reactions.get_by_id('HACD2p').gene_reaction_rule = '11362'
model.reactions.get_by_id('HACD3p').gene_reaction_rule = '11362'
model.reactions.get_by_id('HACD4p').gene_reaction_rule = '11362'
model.reactions.get_by_id('HACD5p').gene_reaction_rule = '11362'
model.reactions.get_by_id('HACD6p').gene_reaction_rule = '11362'
model.reactions.get_by_id('HACD7p').gene_reaction_rule = '11362'
model.reactions.get_by_id('HACD8p').gene_reaction_rule = '11362'
model.reactions.get_by_id('HACD10p').gene_reaction_rule = '11362'
model.reactions.get_by_id('HACD1m').gene_reaction_rule = '11203'
model.reactions.get_by_id('HACD2m').gene_reaction_rule = '11203'
model.reactions.get_by_id('HACD3m').gene_reaction_rule = '11203'
model.reactions.get_by_id('HACD4m').gene_reaction_rule = '11203'
model.reactions.get_by_id('HACD5m').gene_reaction_rule = '11203'
model.reactions.get_by_id('HACD6m').gene_reaction_rule = '11203'
model.reactions.get_by_id('HACD7m').gene_reaction_rule = '11203'
model.reactions.get_by_id('HACD9m').gene_reaction_rule = '13606 or 16284'
model.remove_reactions(['HACD1i','HACD2i','HACD3i','HACD4i','HACD5i','HACD6i','HACD7i'], remove_orphans=True)
model.reactions.get_by_id('HBCO_nadp').gene_reaction_rule = '15180'
model.remove_reactions(['HBCO_nadp_m'], remove_orphans=True)
```

In [37]:

```
for r in sorted(model.genes.get_by_id('11203').reactions, key=lambda x: x.id):
    print(r.id, r.reaction, r.gene_reaction_rule)
print()
for r in sorted(model.genes.get_by_id('15180').reactions, key=lambda x: x.id):
    print(r.id, r.reaction, r.gene_reaction_rule)
print()
for r in sorted(model.genes.get_by_id('11362').reactions, key=lambda x: x.id):
    if not r.id.startswith('FAO'):
        print(r.id, r.reaction, r.gene_reaction_rule)
```

```
HACD1m aacoa_m + h_m + nadh_m <=> 3hbcoa_m + nad_m 11203
HACD2m 3ohcoa_m + h_m + nadh_m <=> 3hhcoa_m + nad_m 11203
HACD3m 3oocoa_m + h_m + nadh_m <=> 3hocoa_m + nad_m 11203
HACD4m 3odcoa_m + h_m + nadh_m <=> 3hdcoa_m + nad_m 11203
HACD5m 3oddcoa_m + h_m + nadh_m <=> 3hddcoa_m + nad_m 11203
HACD6m 3otdcoa_m + h_m + nadh_m <=> 3htdcoa_m + nad_m 11203
HACD7m 3ohdcoa_m + h_m + nadh_m <=> 3hhdcoa_m + nad_m 11203

HADPCOADH3 3hadpcoa_c + nad_c <=> h_c + nadh_c + oxadpcoa_c 15180
HBCO_nadp aacoa_c + h_c + nadph_c <=> 3hbcoa_c + nadp_c 15180

ECOAH11p h2o_x + hxc2coa_x <=> 3hxccoa_x 11362
ECOAH1p 3hbcoa_x <=> b2coa_x + h2o_x 11362
ECOAH2p h2o_x + hx2coa_x <=> 3hhcoa_x 11362
ECOAH3p h2o_x + oc2coa_x <=> 3hocoa_x 11362
ECOAH4p 3hdcoa_x <=> dc2coa_x + h2o_x 11362
ECOAH5p 3hddcoa_x <=> dd2coa_x + h2o_x 11362
ECOAH6p 3htdcoa_x <=> h2o_x + td2coa_x 11362
ECOAH7p 3hhdcoa_x <=> h2o_x + hdd2coa_x 11362
ECOAH8p 3hodcoa_x <=> h2o_x + od2coa_x 11362
HACD10p 3hxccoa_x + nad_x <=> 3ohxccoa_x + h_x + nadh_x 11362
HACD1p aacoa_x + h_x + nadh_x <=> 3hbcoa_x + nad_x 11362
HACD2p 3hhcoa_x + nad_x <=> 3ohcoa_x + h_x + nadh_x 11362
HACD3p 3hocoa_x + nad_x <=> 3oocoa_x + h_x + nadh_x 11362
HACD4p 3odcoa_x + h_x + nadh_x <=> 3hdcoa_x + nad_x 11362
HACD5p 3oddcoa_x + h_x + nadh_x <=> 3hddcoa_x + nad_x 11362
HACD6p 3otdcoa_x + h_x + nadh_x <=> 3htdcoa_x + nad_x 11362
HACD7p 3ohdcoa_x + h_x + nadh_x <=> 3hhdcoa_x + nad_x 11362
HACD8p 3ohodcoa_x + h_x + nadh_x <=> 3hodcoa_x + nad_x 11362
HSD17B42x h_x + nadph_x + o2_x + thcholoylcoa_x --> dhocholoylcoa_x + 2.0 h2o_x + nadp_x 11362
HSD17B4x cholcoads_x + h_x + nadph_x + o2_x --> cholcoaone_x + h2o_x + nadp_x 11362
```

mitochondrial beta oxidation genes  
ACADSB (branched and C4-C6) 14070  
ACADM (C4-C16) 12570  
ECHS1 14805  
HADH 11203  
ACAA2 8885  
FAOXCm - '12570 and 14805 and 11203 and 8885'  
FAOXCm (short) - '(12570 and 14805 and 11203 and 8885) or (14070 and 14805 and 11203 and 8885)'

peroxisomal beta oxidation genes  
POX1/ACOX1 12742, 12752, 9700  
ACADS (C4-C6) 12086  
ACAD10 (C4-C12) 10408  
ACADL (C8-C22) 12989  
ACADVL (C14-C24) 16253  
FOX2 11362  
POT1/ACAA1 13813  
FAOXCx (o2) - '(12742 and 11362 and 13813) or (12752 and 11362 and 13813) or (9700 and 11362 and 13813)'

Unsaturated genes  
13228 SPS19 cyto 10, mito 7, cyto\_nucl 7, pero 4, nucl 2, cysk 2 K13237: DECR2; peroxisomal 2,4-dienoyl-CoA reductase SKL*, uses NADPH  
10293 ECI1 mito 8, cyto\_nucl 7.5, extr 7, cyto 6, nucl 5 K13239: PECI; peroxisomal 3,2-trans-enoyl-CoA isomerase HKI*  
11907 DCI1 extr 9, mito 8, cyto 5.5, cyto\_nucl 4, pero 3 K12663: ECH1; Delta(3,5)-Delta(2,4)-dienoyl-CoA isomerase AKL*, trans cis alk-3,5-dienoyl-CoA to trans trans alk-2,4-dienoyl-CoA  
11338 cyto 18.5, cyto\_nucl 10, mito 4, cysk 4 K00219: fadH; 2,4-dienoyl-CoA reductase (NADPH2) KEE*

Unsaturated, even number trans  
regular oxidation  
First route uses one less O2 compared to saturated  
or  
POX1 trans alk-4-enoyl-CoA to trans trans alk-2,4-dienoyl-CoA, uses O2  
SPS19 trans trans alk-2,4-dienoyl-CoA to trans alk-3-enoyl-CoA, uses NADPH  
ECH1 trans alk-3-enoyl-CoA to trans alk-2-enoyl-CoA  
Second route uses one additional NADPH compared to saturated

Unsaturated, even number cis  
POX1 cis alk-4-enoyl-CoA to trans cis alk-2,4-dienoyl-CoA, uses O2  
SPS19 trans cis alk-2,4-dienoyl-CoA to trans alk-3-enoyl-CoA, uses NADPH  
ECH1 trans alk-3-enoyl-CoA to trans alk-2-enoyl-CoA  
uses one additional nadph compared to saturated

Unsaturated, odd number cis needs either  
ECI1 cis alk-3-enoyl-CoA to trans alk-2-enoyl-CoA  
First route uses one less O2 compared to saturated (FAO161p\_odd)  
or  
POX1 cis alk-5-enoyl-CoA to trans cis alk-2,5-dienoyl-CoA, uses O2  
ECI1 trans cis alk-2,5-dienoyl-CoA to trans cis alk-3,5-dienoyl-CoA  
DCI1 trans cis alk-3,5-dienoyl-CoA to trans trans alk-2,4-dienoyl-CoA  
SPS19 trans trans alk-2,4-dienoyl-CoA to trans alk-3-enoyl-CoA, uses NADPH  
ECH1 trans alk-3-dienoyl-CoA to trans alk-2-dienoyl-CoA  
Second route uses one additional nadph compared to saturated (FAO161p\_even)

Unsaturated, odd number trans needs ECH1 (trans alk-3-enoyl-CoA to trans alk-2-enoyl-CoA)  
use one less O2 compared to saturated

FAO181p\_odd (9 cis) - 10293 + FAOXCx genes, one less O2  
FAO181p\_even (9 cis) - 10293 and 11907 and 13228 + FAOXCx genes, one additional nadph  
FAO182p\_odd (9 cis, 12 cis) - 10293 and 13228 + FAOXCx genes, one less O2 and one additional nadph  
FAO182p\_even (9 cis, 12 cis) - 10293 and 11907 and 13228 + FAOXCx genes, two additional nadph  
FAO183p\_odd (6 cis, 9 cis, 12 cis) - 10293 and 13228 + FAOXCx genes, one less O2 and two additional nadph  
FAO183p\_even (6 cis, 9 cis, 12 cis) - 10293 and 11907 and 13228 + FAOXCx genes, three additional nadph

Genes are peroxisomal -> remove mito unsaturated, odd number fatty acid degradation

In [38]:

```
temp = [r.id for r in sorted(model.reactions, key=lambda x: x.id) if r.id.startswith('FAOXC')]
temp_uniq = set(x.split('m')[0] if 'm' in x else x.split('x')[0] for x in temp)
for x in sorted(temp_uniq):
    print(x if x in model.reactions else '\t', end='\t')
    print(x+'m' if x+'m' in model.reactions else '\t', end='\t')
    print(x+'x' if x+'x' in model.reactions else '')
```

```
FAOXC11			
FAOXC140			
		FAOXC150m	
FAOXC160			
		FAOXC16080m	FAOXC16080x
		FAOXC16180m	
		FAOXC161802m	
		FAOXC170m	
FAOXC180			FAOXC180x
		FAOXC1811601m	
		FAOXC1811602m	
		FAOXC1811603m	
		FAOXC18280m	
		FAOXC182806m	
		FAOXC183803m	
		FAOXC183806m	FAOXC183806x
		FAOXC18480m	FAOXC18480x
		FAOXC200180m	FAOXC200180x
		FAOXC2031836m	
FAOXC204			
		FAOXC204184m	
		FAOXC2051843m	FAOXC2051843x
		FAOXC2242046m	FAOXC2242046x
		FAOXC2251836m	FAOXC2251836x
		FAOXC2252053m	FAOXC2252053x
FAOXC226			
		FAOXC226205m	FAOXC226205x
				FAOXC240200x
				FAOXC241181x
				FAOXC2442246x
				FAOXC2452253x
				FAOXC2452256x
				FAOXC246226x
				FAOXC260240x
FAOXC80			
```

In [39]:

```
# Mitochondrial FAOXC
for r in sorted(model.reactions, key=lambda x: x.id):
    if r.id.startswith('FAOXC') and not (r.id.endswith('m') or r.id.endswith('x')):
        print(r.id, r.reaction)
#        print("\'" + r.id + "\'", end=",")
```

```
FAOXC11 coa_m + dmnoncoa_m + h2o_m + nad_m --> accoa_m + dmhptcoa_m + h_m + nadh_m
FAOXC140 6.0 coa_m + 6.0 fad_m + 6.0 h2o_m + 6.0 nad_m + tdcoa_m --> 7.0 accoa_m + 6.0 fadh2_m + 6.0 h_m + 6.0 nadh_m
FAOXC160 7.0 coa_m + 7.0 fad_m + 7.0 h2o_m + 7.0 nad_m + pmtcoa_m --> 8.0 accoa_m + 7.0 fadh2_m + 7.0 h_m + 7.0 nadh_m
FAOXC180 coa_m + fad_m + h2o_m + nad_m + stcoa_m --> accoa_m + fadh2_m + h_m + nadh_m + pmtcoa_m
FAOXC204 arachdcoa_m + 9.0 coa_m + 5.0 fad_m + 9.0 h2o_m + 9.0 nad_m --> 10.0 accoa_m + 5.0 fadh2_m + 9.0 h_m + 9.0 nadh_m
FAOXC226 c226coa_m + 10.0 coa_m + 4.0 fad_m + 10.0 h2o_m + 10.0 nad_m --> 11.0 accoa_m + 4.0 fadh2_m + 10.0 h_m + 10.0 nadh_m
FAOXC80 3.0 coa_m + 3.0 fad_m + 3.0 h2o_m + 3.0 nad_m + occoa_m --> 4.0 accoa_m + 3.0 fadh2_m + 3.0 h_m + 3.0 nadh_m
```

FAOXC11 orphan  
FAOXC80, FAOXC140, FAOXC160 redundant with ACOAD/ECOAH/HACD/ACACT
FAOXC180, FAOXC204, FAOXC226 long

In [40]:

```
model.remove_reactions(['FAOXC11','FAOXC80','FAOXC140','FAOXC160','FAOXC180','FAOXC204','FAOXC226'], remove_orphans=True)
```

In [41]:

```
for r in sorted(model.reactions, key=lambda x: x.id):
    if r.id.startswith('FAOXC') and r.id.endswith('m'):
        print(r.id, r.reaction, r.gene_reaction_rule)
```

```
FAOXC150m 6.0 coa_m + 6.0 fad_m + 6.0 h2o_m + 6.0 nad_m + ptdcacoa_m --> 6.0 accoa_m + 6.0 fadh2_m + 6.0 h_m + 6.0 nadh_m + ppcoa_m 12570
FAOXC16080m 4.0 coa_m + 4.0 fad_m + 4.0 h2o_m + 4.0 nad_m + pmtcoa_m --> 4.0 accoa_m + 4.0 fadh2_m + 4.0 h_m + 4.0 nadh_m + occoa_m 12570
FAOXC161802m 4.0 coa_m + 3.0 fad_m + 4.0 h2o_m + hdd2coa_m + 4.0 nad_m --> 4.0 accoa_m + 3.0 fadh2_m + 4.0 h_m + 4.0 nadh_m + occoa_m 12570
FAOXC16180m 4.0 coa_m + 3.0 fad_m + 4.0 h2o_m + hdcoa_m + 4.0 nad_m --> 4.0 accoa_m + 3.0 fadh2_m + 4.0 h_m + 4.0 nadh_m + occoa_m 12570
FAOXC170m 7.0 coa_m + 7.0 fad_m + 7.0 h2o_m + hpdcacoa_m + 7.0 nad_m --> 7.0 accoa_m + 7.0 fadh2_m + 7.0 h_m + 7.0 nadh_m + ppcoa_m 12570
FAOXC1811601m 5.0 coa_m + 4.0 fad_m + 5.0 h2o_m + 5.0 nad_m + od2coa_m --> 5.0 accoa_m + 4.0 fadh2_m + 5.0 h_m + 5.0 nadh_m + occoa_m 12570
FAOXC1811602m 5.0 coa_m + 4.0 fad_m + 5.0 h2o_m + 5.0 nad_m + vacccoa_m --> 5.0 accoa_m + 4.0 fadh2_m + 5.0 h_m + 5.0 nadh_m + occoa_m 12570
FAOXC1811603m 5.0 coa_m + 4.0 fad_m + 5.0 h2o_m + 5.0 nad_m + odecoa_m --> 5.0 accoa_m + 4.0 fadh2_m + 5.0 h_m + 5.0 nadh_m + occoa_m 12570
FAOXC182806m 5.0 coa_m + 3.0 fad_m + 5.0 h2o_m + lnlccoa_m + 5.0 nad_m --> 5.0 accoa_m + 3.0 fadh2_m + 5.0 h_m + 5.0 nadh_m + occoa_m 12570
FAOXC18280m 5.0 coa_m + 3.0 fad_m + 5.0 h2o_m + lneldccoa_m + 5.0 nad_m --> 5.0 accoa_m + 3.0 fadh2_m + 5.0 h_m + 5.0 nadh_m + occoa_m 12570
FAOXC183803m 5.0 coa_m + 2.0 fad_m + 5.0 h2o_m + lnlncacoa_m + 5.0 nad_m --> 5.0 accoa_m + 2.0 fadh2_m + 5.0 h_m + 5.0 nadh_m + occoa_m 12570
FAOXC183806m 5.0 coa_m + 5.0 h2o_m + lnlncgcoa_m + 5.0 nad_m + 2.0 o2_m --> 5.0 accoa_m + 2.0 h2o2_m + 5.0 h_m + 5.0 nadh_m + occoa_m 12570
FAOXC18480m 5.0 coa_m + fad_m + 5.0 h2o_m + 5.0 nad_m + strdnccoa_m --> 5.0 accoa_m + fadh2_m + 5.0 h_m + 5.0 nadh_m + occoa_m 12570
FAOXC200180m arachcoa_m + coa_m + fad_m + h2o_m + nad_m --> accoa_m + fadh2_m + h_m + nadh_m + stcoa_m 12570
FAOXC2031836m coa_m + dlnlcgcoa_m + fad_m + h2o_m + nad_m --> accoa_m + fadh2_m + h_m + lnlncgcoa_m + nadh_m 12570
FAOXC204184m coa_m + eicostetcoa_m + fad_m + h2o_m + nad_m --> accoa_m + fadh2_m + h_m + nadh_m + strdnccoa_m 12570
FAOXC2051843m coa_m + h2o_m + nad_m + tmndnccoa_m --> accoa_m + h_m + nadh_m + strdnccoa_m 12570
FAOXC2242046m adrncoa_m + coa_m + fad_m + h2o_m + nad_m --> accoa_m + arachdcoa_m + fadh2_m + h_m + nadh_m 12570
FAOXC2251836m 2.0 coa_m + dcsptn1coa_m + 2.0 h2o_m + 2.0 nad_m --> 2.0 accoa_m + 2.0 h_m + lnlncgcoa_m + 2.0 nadh_m 12570
FAOXC2252053m coa_m + dcsptn1coa_m + h2o_m + nad_m + o2_m --> accoa_m + h2o2_m + h_m + nadh_m + tmndnccoa_m 12570
FAOXC226205m c226coa_m + coa_m + h2o_m + nad_m --> accoa_m + h_m + nadh_m + tmndnccoa_m 12570
```

FAOXC16080m - redundant with ACOAD/ECOAH/HACD/ACACT  
FAOXC16180m - hdcoa\_m is from (Z)-hexadec-9-enoyl-CoA (n-C16:1, cis), odd number unsaturated  
FAOXC161802m - hdd2coa\_m is trans-2-hexadecenoyl-CoA, even number unsaturated, but redundant  
FAOXC1811601m - od2coa is Trans-octadec-2-enoyl-CoA, even number unsaturated, long  
FAOXC1811602m - vacccoa is Trans-octadec-11-enoyl-CoA, odd number unsaturated, long  
FAOXC1811603m - odecoa is oleate, (Z)-octadec-9-enoyl-CoA (n-C18:1, cis), odd number unsaturated, long  
FAOXC182806m, FAOXC18280m, FAOXC183803m, FAOXC183806m, FAOXC18480m - long  
FAOXC200180m, FAOXC2031836m, FAOXC204184m, FAOXC2051843m - long  
FAOXC2242046m, FAOXC2251836m, FAOXC2252053m, FAOXC226205m - long

Remove mito unsaturated odd number oxidation rxns, and long-chain (> C17) rxns  
Add odd number fatty acid mito oxidation from Recon3D and change genes to '12570 and 14805 and 11203 and 8885'
Change FAOXC5C3x to FAOXC5C3m, and change genes to '(12570 and 14805 and 11203 and 8885) or (14070 and 14805 and 11203 and 8885)'

In [42]:

```
model.remove_reactions(['FAOXC150m','FAOXC16080m','FAOXC161802m','FAOXC16180m','FAOXC170m','FAOXC1811601m',
                        'FAOXC1811602m','FAOXC1811603m','FAOXC182806m','FAOXC18280m','FAOXC183803m','FAOXC183806m',
                        'FAOXC18480m','FAOXC200180m','FAOXC2031836m','FAOXC204184m','FAOXC2051843m','FAOXC2242046m',
                        'FAOXC2251836m','FAOXC2252053m','FAOXC226205m'], remove_orphans=True)
for x in ['FAOXC5C3x','FAOXC7C5m','FAOXC9C7m','FAOXC11C9m','FAOXC13C11m','FAOXC15C13m','FAOXC170150m']:
    model.add_reactions([hsa2.reactions.get_by_id(x).copy()])
    model.reactions.get_by_id(x).gene_reaction_rule = '12570 and 14805 and 11203 and 8885'
model.reactions.get_by_id('FAOXC5C3x').id = 'FAOXC5C3m'
model.reactions.get_by_id('FAOXC5C3m').gene_reaction_rule = '(12570 and 14805 and 11203 and 8885) or (14070 and 14805 and 11203 and 8885)'
```

In [43]:

```
for r in sorted(model.reactions, key=lambda x: x.id):
    if r.id.startswith('FAOXC') and 'm' in r.compartments:
        print(r.id, r.reaction, r.gene_reaction_rule)
```

```
FAOXC11C9m coa_m + fad_m + h2o_m + nad_m + undcoa_m --> accoa_m + fadh2_m + h_m + nadh_m + noncoa_m 12570 and 14805 and 11203 and 8885
FAOXC13C11m coa_m + fad_m + h2o_m + nad_m + tridcoa_m --> accoa_m + fadh2_m + h_m + nadh_m + undcoa_m 12570 and 14805 and 11203 and 8885
FAOXC15C13m coa_m + fad_m + h2o_m + nad_m + ptdcacoa_m --> accoa_m + fadh2_m + h_m + nadh_m + tridcoa_m 12570 and 14805 and 11203 and 8885
FAOXC170150m coa_m + fad_m + h2o_m + hpdcacoa_m + nad_m --> accoa_m + fadh2_m + h_m + nadh_m + ptdcacoa_m 12570 and 14805 and 11203 and 8885
FAOXC5C3m coa_m + fad_m + h2o_m + nad_m + pentcoa_m --> accoa_m + fadh2_m + h_m + nadh_m + ppcoa_m (12570 and 14805 and 11203 and 8885) or (14070 and 14805 and 11203 and 8885)
FAOXC7C5m coa_m + fad_m + h2o_m + hepcoa_m + nad_m --> accoa_m + fadh2_m + h_m + nadh_m + pentcoa_m 12570 and 14805 and 11203 and 8885
FAOXC9C7m coa_m + fad_m + h2o_m + nad_m + noncoa_m --> accoa_m + fadh2_m + h_m + hepcoa_m + nadh_m 12570 and 14805 and 11203 and 8885
```

In [44]:

```
# Now peroxisomal FAOXC
for r in sorted(model.reactions, key=lambda x: x.id):
    if r.id.startswith('FAO') and not r.id.startswith('FAOXC'):
        print(r.id, r.reaction, r.gene_reaction_rule)
#        print("\'" + r.id + "\'", end=",")
```

```
FAO141p_even 6.0 coa_x + 6.0 h2o_x + 6.0 nad_x + nadph_x + 6.0 o2_x + tdecoa_x --> 7.0 accoa_x + 6.0 h2o2_x + 5.0 h_x + 6.0 nadh_x + nadp_x (10293 and 11362 and 12742 and 13228 and 13813) or (10293 and 11362 and 12742 and 13228 and 9065) or (10293 and 11362 and 12752 and 13228 and 13813) or (10293 and 11362 and 12752 and 13228 and 9065) or (10293 and 11362 and 13228 and 13813 and 9700) or (10293 and 11362 and 13228 and 9065 and 9700)
FAO141p_odd 6.0 coa_x + 6.0 h2o_x + 6.0 nad_x + 5.0 o2_x + tdecoa_x --> 7.0 accoa_x + 5.0 h2o2_x + 6.0 h_x + 6.0 nadh_x (10293 and 11362 and 12742 and 13813) or (10293 and 11362 and 12742 and 9065) or (10293 and 11362 and 12752 and 13813) or (10293 and 11362 and 12752 and 9065) or (10293 and 11362 and 13813 and 9700) or (10293 and 11362 and 9065 and 9700)
FAO161p_even 7.0 coa_x + 7.0 h2o_x + hdcoa_x + 7.0 nad_x + nadph_x + 7.0 o2_x --> 8.0 accoa_x + 7.0 h2o2_x + 6.0 h_x + 7.0 nadh_x + nadp_x (10293 and 11362 and 12742 and 13228 and 13813) or (10293 and 11362 and 12742 and 13228 and 9065) or (10293 and 11362 and 12752 and 13228 and 13813) or (10293 and 11362 and 12752 and 13228 and 9065) or (10293 and 11362 and 13228 and 13813 and 9700) or (10293 and 11362 and 13228 and 9065 and 9700)
FAO161p_odd 7.0 coa_x + 7.0 h2o_x + hdcoa_x + 7.0 nad_x + 6.0 o2_x --> 8.0 accoa_x + 6.0 h2o2_x + 7.0 h_x + 7.0 nadh_x (10293 and 11362 and 12742 and 13813) or (10293 and 11362 and 12742 and 9065) or (10293 and 11362 and 12752 and 13813) or (10293 and 11362 and 12752 and 9065) or (10293 and 11362 and 13813 and 9700) or (10293 and 11362 and 9065 and 9700)
FAO181p_even 8.0 coa_x + 8.0 h2o_x + 8.0 nad_x + nadph_x + 8.0 o2_x + odecoa_x --> 9.0 accoa_x + 8.0 h2o2_x + 7.0 h_x + 8.0 nadh_x + nadp_x (10293 and 11362 and 12742 and 13228 and 13813) or (10293 and 11362 and 12742 and 13228 and 9065) or (10293 and 11362 and 12752 and 13228 and 13813) or (10293 and 11362 and 12752 and 13228 and 9065) or (10293 and 11362 and 13228 and 13813 and 9700) or (10293 and 11362 and 13228 and 9065 and 9700)
FAO181p_odd 8.0 coa_x + 8.0 h2o_x + 8.0 nad_x + 7.0 o2_x + odecoa_x --> 9.0 accoa_x + 7.0 h2o2_x + 8.0 h_x + 8.0 nadh_x (10293 and 11362 and 12742 and 13813) or (10293 and 11362 and 12742 and 9065) or (10293 and 11362 and 12752 and 13813) or (10293 and 11362 and 12752 and 9065) or (10293 and 11362 and 13813 and 9700) or (10293 and 11362 and 9065 and 9700)
FAO182p_eveneven 8.0 coa_x + 8.0 h2o_x + 8.0 nad_x + 2.0 nadph_x + 8.0 o2_x + ocdycacoa_x --> 9.0 accoa_x + 8.0 h2o2_x + 6.0 h_x + 8.0 nadh_x + 2.0 nadp_x (10293 and 11362 and 12742 and 13228 and 13813) or (10293 and 11362 and 12742 and 13228 and 9065) or (10293 and 11362 and 12752 and 13228 and 13813) or (10293 and 11362 and 12752 and 13228 and 9065) or (10293 and 11362 and 13228 and 13813 and 9700) or (10293 and 11362 and 13228 and 9065 and 9700)
FAO182p_evenodd 8.0 coa_x + 8.0 h2o_x + 8.0 nad_x + nadph_x + 7.0 o2_x + ocdycacoa_x --> 9.0 accoa_x + 7.0 h2o2_x + 7.0 h_x + 8.0 nadh_x + nadp_x (10293 and 11362 and 12742 and 13228 and 13813) or (10293 and 11362 and 12742 and 13228 and 9065) or (10293 and 11362 and 12752 and 13228 and 13813) or (10293 and 11362 and 12752 and 13228 and 9065) or (10293 and 11362 and 13228 and 13813 and 9700) or (10293 and 11362 and 13228 and 9065 and 9700)
FAO182p_oddodd 8.0 coa_x + 8.0 h2o_x + 8.0 nad_x + 6.0 o2_x + ocdycacoa_x --> 9.0 accoa_x + 6.0 h2o2_x + 8.0 h_x + 8.0 nadh_x (10293 and 11362 and 12742 and 13813) or (10293 and 11362 and 12742 and 9065) or (10293 and 11362 and 12752 and 13813) or (10293 and 11362 and 12752 and 9065) or (10293 and 11362 and 13813 and 9700) or (10293 and 11362 and 9065 and 9700)
FAO240p 3.0 coa_x + 3.0 h2o_x + 3.0 nad_x + 3.0 o2_x + ttccoa_x --> 3.0 accoa_x + 3.0 h2o2_x + 3.0 h_x + 3.0 nadh_x + stcoa_x (11362 and 12742 and 13813) or (11362 and 12742 and 9065) or (11362 and 12752 and 13813) or (11362 and 12752 and 9065) or (11362 and 13813 and 9700) or (11362 and 9065 and 9700)
FAO80p 3.0 coa_x + 3.0 h2o_x + 3.0 nad_x + 3.0 o2_x + occoa_x --> 4.0 accoa_x + 3.0 h2o2_x + 3.0 h_x + 3.0 nadh_x (11362 and 12742 and 13813) or (11362 and 12742 and 9065) or (11362 and 12752 and 13813) or (11362 and 12752 and 9065) or (11362 and 13813 and 9700) or (11362 and 9065 and 9700)
```

14:1, 16:1, 18:1 are 9Z odd unsaturated, can use one less o2 (odd) or one additional nadph (even)  
18:2 is 9Z12Z, can be one less o2 and one additional nadph (oddeven), or two additional nadph (eveneven)  
18:3 is 6Z9Z12Z, can be one less o2 and two additional nadph (evenoddeven), or three additional nadph (eveneveneven)  
change FAO182p\_eveneven to FAO182p\_even and FAO182p\_evenodd to FAO182p\_odd, remove FAO182p\_oddodd  
add FAO183p\_even and FAO183p\_odd

In [45]:

```
model.reactions.get_by_id('FAO141p_even').gene_reaction_rule = '(10293 and 11362 and 11907 and 12742 and 13228 and 13813) or (10293 and 11362 and 11907 and 12752 and 13228 and 13813) or (10293 and 11362 and 11907 and 13228 and 13813 and 9700)'
model.reactions.get_by_id('FAO161p_even').gene_reaction_rule = '(10293 and 11362 and 11907 and 12742 and 13228 and 13813) or (10293 and 11362 and 11907 and 12752 and 13228 and 13813) or (10293 and 11362 and 11907 and 13228 and 13813 and 9700)'
model.reactions.get_by_id('FAO181p_even').gene_reaction_rule = '(10293 and 11362 and 11907 and 12742 and 13228 and 13813) or (10293 and 11362 and 11907 and 12752 and 13228 and 13813) or (10293 and 11362 and 11907 and 13228 and 13813 and 9700)'
model.reactions.get_by_id('FAO182p_eveneven').id = 'FAO182p_even'
model.reactions.get_by_id('FAO182p_even').gene_reaction_rule = '(10293 and 11362 and 11907 and 12742 and 13228 and 13813) or (10293 and 11362 and 11907 and 12752 and 13228 and 13813) or (10293 and 11362 and 11907 and 13228 and 13813 and 9700)'

model.reactions.get_by_id('FAO141p_odd').gene_reaction_rule = '(10293 and 11362 and 12742 and 13813) or (10293 and 11362 and 12752 and 13813) or (10293 and 11362 and 13813 and 9700)'
model.reactions.get_by_id('FAO161p_odd').gene_reaction_rule = '(10293 and 11362 and 12742 and 13813) or (10293 and 11362 and 12752 and 13813) or (10293 and 11362 and 13813 and 9700)'
model.reactions.get_by_id('FAO181p_odd').gene_reaction_rule = '(10293 and 11362 and 12742 and 13813) or (10293 and 11362 and 12752 and 13813) or (10293 and 11362 and 13813 and 9700)'
model.reactions.get_by_id('FAO182p_evenodd').id = 'FAO182p_odd'
model.reactions.get_by_id('FAO182p_odd').gene_reaction_rule = '(10293 and 11362 and 12742 and 13228 and 13813) or (10293 and 11362 and 12752 and 13228 and 13813) or (10293 and 11362 and 13228 and 13813 and 9700)'

model.remove_reactions(['FAO182p_oddodd'], remove_orphans=True)
r1 = model.reactions.get_by_id('FAO182p_even').copy()
r1.id = 'FAO183p_even'
r2 = model.reactions.get_by_id('FAO182p_odd').copy()
r2.id = 'FAO183p_odd'
model.add_reactions([r1,r2])
r1.add_metabolites({'ocdycacoa_x': 1.0, 'lnlncgcoa_x': -1.0, 'nadph_x': -1.0, 'nadp_x': 1.0, 'h_x': -1.0})
r2.add_metabolites({'ocdycacoa_x': 1.0, 'lnlncgcoa_x': -1.0, 'nadph_x': -1.0, 'nadp_x': 1.0, 'h_x': -1.0})
```

In [46]:

```
for r in sorted(model.reactions, key=lambda x: x.id):
    if r.id.startswith('FAOXC') and r.id.endswith('x'):
        print(r.id, r.reaction, r.gene_reaction_rule)
```

```
FAOXC16080x 4.0 coa_x + 4.0 h2o_x + 4.0 nad_x + 4.0 o2_x + pmtcoa_x --> 4.0 accoa_x + 4.0 h2o2_x + 4.0 h_x + 4.0 nadh_x + occoa_x (EHHADH and 12742 and 13813) or (EHHADH and 12742 and 9065) or (EHHADH and 12752 and 13813) or (EHHADH and 12752 and 9065) or (EHHADH and 13813 and 9700) or (EHHADH and 9065 and 9700) or (Ehhadh and 12742 and 13813) or (Ehhadh and 12742 and 9065) or (Ehhadh and 12752 and 13813) or (Ehhadh and 12752 and 9065) or (Ehhadh and 13813 and 9700) or (Ehhadh and 9065 and 9700) or (11362 and 12742 and 13813) or (11362 and 12742 and 9065) or (11362 and 12752 and 13813) or (11362 and 12752 and 9065) or (11362 and 13813 and 9700) or (11362 and 9065 and 9700)
FAOXC180x coa_x + h2o_x + nad_x + o2_x + stcoa_x --> accoa_x + h2o2_x + h_x + nadh_x + pmtcoa_x (EHHADH and 12742 and 13813) or (EHHADH and 12742 and 9065) or (EHHADH and 12752 and 13813) or (EHHADH and 12752 and 9065) or (EHHADH and 13813 and 9700) or (EHHADH and 9065 and 9700) or (Ehhadh and 12742 and 13813) or (Ehhadh and 12742 and 9065) or (Ehhadh and 12752 and 13813) or (Ehhadh and 12752 and 9065) or (Ehhadh and 13813 and 9700) or (Ehhadh and 9065 and 9700) or (11362 and 12742 and 13813) or (11362 and 12742 and 9065) or (11362 and 12752 and 13813) or (11362 and 12752 and 9065) or (11362 and 13813 and 9700) or (11362 and 9065 and 9700)
FAOXC183806x 5.0 coa_x + 5.0 h2o_x + lnlncgcoa_x + 5.0 nad_x + 2.0 o2_x --> 5.0 accoa_x + 2.0 h2o2_x + 5.0 h_x + 5.0 nadh_x + occoa_x (EHHADH and 12742 and 13813) or (EHHADH and 12742 and 9065) or (EHHADH and 12752 and 13813) or (EHHADH and 12752 and 9065) or (EHHADH and 13813 and 9700) or (EHHADH and 9065 and 9700) or (Ehhadh and 12742 and 13813) or (Ehhadh and 12742 and 9065) or (Ehhadh and 12752 and 13813) or (Ehhadh and 12752 and 9065) or (Ehhadh and 13813 and 9700) or (Ehhadh and 9065 and 9700) or (11362 and 12742 and 13813) or (11362 and 12742 and 9065) or (11362 and 12752 and 13813) or (11362 and 12752 and 9065) or (11362 and 13813 and 9700) or (11362 and 9065 and 9700)
FAOXC18480x 5.0 coa_x + 5.0 h2o_x + 5.0 nad_x + o2_x + strdnccoa_x --> 5.0 accoa_x + h2o2_x + 5.0 h_x + 5.0 nadh_x + occoa_x (EHHADH and 12742 and 13813) or (EHHADH and 12742 and 9065) or (EHHADH and 12752 and 13813) or (EHHADH and 12752 and 9065) or (EHHADH and 13813 and 9700) or (EHHADH and 9065 and 9700) or (Ehhadh and 12742 and 13813) or (Ehhadh and 12742 and 9065) or (Ehhadh and 12752 and 13813) or (Ehhadh and 12752 and 9065) or (Ehhadh and 13813 and 9700) or (Ehhadh and 9065 and 9700) or (11362 and 12742 and 13813) or (11362 and 12742 and 9065) or (11362 and 12752 and 13813) or (11362 and 12752 and 9065) or (11362 and 13813 and 9700) or (11362 and 9065 and 9700)
FAOXC200180x arachcoa_x + coa_x + h2o_x + nad_x + o2_x --> accoa_x + h2o2_x + h_x + nadh_x + stcoa_x (EHHADH and 12742 and 13813) or (EHHADH and 12742 and 9065) or (EHHADH and 12752 and 13813) or (EHHADH and 12752 and 9065) or (EHHADH and 13813 and 9700) or (EHHADH and 9065 and 9700) or (Ehhadh and 12742 and 13813) or (Ehhadh and 12742 and 9065) or (Ehhadh and 12752 and 13813) or (Ehhadh and 12752 and 9065) or (Ehhadh and 13813 and 9700) or (Ehhadh and 9065 and 9700) or (11362 and 12742 and 13813) or (11362 and 12742 and 9065) or (11362 and 12752 and 13813) or (11362 and 12752 and 9065) or (11362 and 13813 and 9700) or (11362 and 9065 and 9700)
FAOXC2051843x coa_x + h2o_x + nad_x + tmndnccoa_x --> accoa_x + h_x + nadh_x + strdnccoa_x (EHHADH and 12742 and 13813) or (EHHADH and 12742 and 9065) or (EHHADH and 12752 and 13813) or (EHHADH and 12752 and 9065) or (EHHADH and 13813 and 9700) or (EHHADH and 9065 and 9700) or (Ehhadh and 12742 and 13813) or (Ehhadh and 12742 and 9065) or (Ehhadh and 12752 and 13813) or (Ehhadh and 12752 and 9065) or (Ehhadh and 13813 and 9700) or (Ehhadh and 9065 and 9700) or (11362 and 12742 and 13813) or (11362 and 12742 and 9065) or (11362 and 12752 and 13813) or (11362 and 12752 and 9065) or (11362 and 13813 and 9700) or (11362 and 9065 and 9700)
FAOXC2242046x adrncoa_x + coa_x + h2o_x + nad_x + o2_x --> accoa_x + arachdcoa_x + h2o2_x + h_x + nadh_x (EHHADH and 12742 and 13813) or (EHHADH and 12742 and 9065) or (EHHADH and 12752 and 13813) or (EHHADH and 12752 and 9065) or (EHHADH and 13813 and 9700) or (EHHADH and 9065 and 9700) or (Ehhadh and 12742 and 13813) or (Ehhadh and 12742 and 9065) or (Ehhadh and 12752 and 13813) or (Ehhadh and 12752 and 9065) or (Ehhadh and 13813 and 9700) or (Ehhadh and 9065 and 9700) or (11362 and 12742 and 13813) or (11362 and 12742 and 9065) or (11362 and 12752 and 13813) or (11362 and 12752 and 9065) or (11362 and 13813 and 9700) or (11362 and 9065 and 9700)
FAOXC2251836x 2.0 coa_x + dcsptn1coa_x + 2.0 h2o_x + 2.0 nad_x --> 2.0 accoa_x + 2.0 h_x + lnlncgcoa_x + 2.0 nadh_x (EHHADH and 12742 and 13813) or (EHHADH and 12742 and 9065) or (EHHADH and 12752 and 13813) or (EHHADH and 12752 and 9065) or (EHHADH and 13813 and 9700) or (EHHADH and 9065 and 9700) or (Ehhadh and 12742 and 13813) or (Ehhadh and 12742 and 9065) or (Ehhadh and 12752 and 13813) or (Ehhadh and 12752 and 9065) or (Ehhadh and 13813 and 9700) or (Ehhadh and 9065 and 9700) or (11362 and 12742 and 13813) or (11362 and 12742 and 9065) or (11362 and 12752 and 13813) or (11362 and 12752 and 9065) or (11362 and 13813 and 9700) or (11362 and 9065 and 9700)
FAOXC2252053x coa_x + dcsptn1coa_x + h2o_x + nad_x + o2_x --> accoa_x + h2o2_x + h_x + nadh_x + tmndnccoa_x (EHHADH and 12742 and 13813) or (EHHADH and 12742 and 9065) or (EHHADH and 12752 and 13813) or (EHHADH and 12752 and 9065) or (EHHADH and 13813 and 9700) or (EHHADH and 9065 and 9700) or (Ehhadh and 12742 and 13813) or (Ehhadh and 12742 and 9065) or (Ehhadh and 12752 and 13813) or (Ehhadh and 12752 and 9065) or (Ehhadh and 13813 and 9700) or (Ehhadh and 9065 and 9700) or (11362 and 12742 and 13813) or (11362 and 12742 and 9065) or (11362 and 12752 and 13813) or (11362 and 12752 and 9065) or (11362 and 13813 and 9700) or (11362 and 9065 and 9700)
FAOXC226205x c226coa_x + coa_x + h2o_x + nad_x --> accoa_x + h_x + nadh_x + tmndnccoa_x (EHHADH and 12742 and 13813) or (EHHADH and 12742 and 9065) or (EHHADH and 12752 and 13813) or (EHHADH and 12752 and 9065) or (EHHADH and 13813 and 9700) or (EHHADH and 9065 and 9700) or (Ehhadh and 12742 and 13813) or (Ehhadh and 12742 and 9065) or (Ehhadh and 12752 and 13813) or (Ehhadh and 12752 and 9065) or (Ehhadh and 13813 and 9700) or (Ehhadh and 9065 and 9700) or (11362 and 12742 and 13813) or (11362 and 12742 and 9065) or (11362 and 12752 and 13813) or (11362 and 12752 and 9065) or (11362 and 13813 and 9700) or (11362 and 9065 and 9700)
FAOXC240200x 2.0 coa_x + 2.0 h2o_x + lgnccoa_x + 2.0 nad_x + 2.0 o2_x --> 2.0 accoa_x + arachcoa_x + 2.0 h2o2_x + 2.0 h_x + 2.0 nadh_x (EHHADH and 12742 and 13813) or (EHHADH and 12742 and 9065) or (EHHADH and 12752 and 13813) or (EHHADH and 12752 and 9065) or (EHHADH and 13813 and 9700) or (EHHADH and 9065 and 9700) or (Ehhadh and 12742 and 13813) or (Ehhadh and 12742 and 9065) or (Ehhadh and 12752 and 13813) or (Ehhadh and 12752 and 9065) or (Ehhadh and 13813 and 9700) or (Ehhadh and 9065 and 9700) or (11362 and 12742 and 13813) or (11362 and 12742 and 9065) or (11362 and 12752 and 13813) or (11362 and 12752 and 9065) or (11362 and 13813 and 9700) or (11362 and 9065 and 9700)
FAOXC241181x 3.0 coa_x + 3.0 h2o_x + 3.0 nad_x + nrvnccoa_x + 3.0 o2_x --> 3.0 accoa_x + 3.0 h2o2_x + 3.0 h_x + 3.0 nadh_x + odecoa_x (EHHADH and 12742 and 13813) or (EHHADH and 12742 and 9065) or (EHHADH and 12752 and 13813) or (EHHADH and 12752 and 9065) or (EHHADH and 13813 and 9700) or (EHHADH and 9065 and 9700) or (Ehhadh and 12742 and 13813) or (Ehhadh and 12742 and 9065) or (Ehhadh and 12752 and 13813) or (Ehhadh and 12752 and 9065) or (Ehhadh and 13813 and 9700) or (Ehhadh and 9065 and 9700) or (11362 and 12742 and 13813) or (11362 and 12742 and 9065) or (11362 and 12752 and 13813) or (11362 and 12752 and 9065) or (11362 and 13813 and 9700) or (11362 and 9065 and 9700)
FAOXC2442246x coa_x + h2o_x + nad_x + o2_x + tettet6coa_x --> accoa_x + adrncoa_x + h2o2_x + h_x + nadh_x (EHHADH and 12742 and 13813) or (EHHADH and 12742 and 9065) or (EHHADH and 12752 and 13813) or (EHHADH and 12752 and 9065) or (EHHADH and 13813 and 9700) or (EHHADH and 9065 and 9700) or (Ehhadh and 12742 and 13813) or (Ehhadh and 12742 and 9065) or (Ehhadh and 12752 and 13813) or (Ehhadh and 12752 and 9065) or (Ehhadh and 13813 and 9700) or (Ehhadh and 9065 and 9700) or (11362 and 12742 and 13813) or (11362 and 12742 and 9065) or (11362 and 12752 and 13813) or (11362 and 12752 and 9065) or (11362 and 13813 and 9700) or (11362 and 9065 and 9700)
FAOXC2452253x coa_x + h2o_x + nad_x + o2_x + tetpent3coa_x --> accoa_x + clpndcoa_x + h2o2_x + h_x + nadh_x (EHHADH and 12742 and 13813) or (EHHADH and 12742 and 9065) or (EHHADH and 12752 and 13813) or (EHHADH and 12752 and 9065) or (EHHADH and 13813 and 9700) or (EHHADH and 9065 and 9700) or (Ehhadh and 12742 and 13813) or (Ehhadh and 12742 and 9065) or (Ehhadh and 12752 and 13813) or (Ehhadh and 12752 and 9065) or (Ehhadh and 13813 and 9700) or (Ehhadh and 9065 and 9700) or (11362 and 12742 and 13813) or (11362 and 12742 and 9065) or (11362 and 12752 and 13813) or (11362 and 12752 and 9065) or (11362 and 13813 and 9700) or (11362 and 9065 and 9700)
FAOXC2452256x coa_x + h2o_x + nad_x + o2_x + tetpent6coa_x --> accoa_x + dcsptn1coa_x + h2o2_x + h_x + nadh_x (EHHADH and 12742 and 13813) or (EHHADH and 12742 and 9065) or (EHHADH and 12752 and 13813) or (EHHADH and 12752 and 9065) or (EHHADH and 13813 and 9700) or (EHHADH and 9065 and 9700) or (Ehhadh and 12742 and 13813) or (Ehhadh and 12742 and 9065) or (Ehhadh and 12752 and 13813) or (Ehhadh and 12752 and 9065) or (Ehhadh and 13813 and 9700) or (Ehhadh and 9065 and 9700) or (11362 and 12742 and 13813) or (11362 and 12742 and 9065) or (11362 and 12752 and 13813) or (11362 and 12752 and 9065) or (11362 and 13813 and 9700) or (11362 and 9065 and 9700)
FAOXC246226x coa_x + h2o_x + nad_x + o2_x + tethex3coa_x --> accoa_x + c226coa_x + h2o2_x + h_x + nadh_x (EHHADH and 12742 and 13813) or (EHHADH and 12742 and 9065) or (EHHADH and 12752 and 13813) or (EHHADH and 12752 and 9065) or (EHHADH and 13813 and 9700) or (EHHADH and 9065 and 9700) or (Ehhadh and 12742 and 13813) or (Ehhadh and 12742 and 9065) or (Ehhadh and 12752 and 13813) or (Ehhadh and 12752 and 9065) or (Ehhadh and 13813 and 9700) or (Ehhadh and 9065 and 9700) or (11362 and 12742 and 13813) or (11362 and 12742 and 9065) or (11362 and 12752 and 13813) or (11362 and 12752 and 9065) or (11362 and 13813 and 9700) or (11362 and 9065 and 9700)
FAOXC260240x coa_x + h2o_x + hexccoa_x + nad_x + o2_x --> accoa_x + h2o2_x + h_x + lgnccoa_x + nadh_x (EHHADH and 12742 and 13813) or (EHHADH and 12742 and 9065) or (EHHADH and 12752 and 13813) or (EHHADH and 12752 and 9065) or (EHHADH and 13813 and 9700) or (EHHADH and 9065 and 9700) or (Ehhadh and 12742 and 13813) or (Ehhadh and 12742 and 9065) or (Ehhadh and 12752 and 13813) or (Ehhadh and 12752 and 9065) or (Ehhadh and 13813 and 9700) or (Ehhadh and 9065 and 9700) or (11362 and 12742 and 13813) or (11362 and 12742 and 9065) or (11362 and 12752 and 13813) or (11362 and 12752 and 9065) or (11362 and 13813 and 9700) or (11362 and 9065 and 9700)
```

FAOXC16080x, FAOXC180x, FAO240p, FAOXC260240x are redundant  
ttccoa and lgnccoa / ttc and lgnc C24:0 are the same -> replace it with ttccoa / ttc

In [47]:

```
model.remove_reactions(['FAOXC16080x','FAOXC180x','FAO240p','FAOXC260240x'], remove_orphans=True)
```

In [48]:

```
for m in sorted(model.metabolites, key=lambda x: x.id):
    if m.id.startswith('ttc'):
        print(m.id)
for m in sorted(model.metabolites, key=lambda x: x.id):
    if m.id.startswith('lgnc'):
        print(m.id)
```

```
ttc_c
ttc_ggdp_c
ttc_m
ttc_x
ttccoa_r
ttccoa_x
lgnc_c
lgnc_e
lgnccoa_c
lgnccoa_m
lgnccoa_x
lgnccrn_c
lgnccrn_m
```

In [49]:

```
for r in sorted(model.metabolites.get_by_id('ttc_c').reactions, key=lambda x: x.id):
    print(r.id, r.reaction, r.gene_reaction_rule)
print()
for r in sorted(model.metabolites.get_by_id('ttc_m').reactions, key=lambda x: x.id):
    print(r.id, r.reaction, r.gene_reaction_rule)
print()
for r in sorted(model.metabolites.get_by_id('ttc_x').reactions, key=lambda x: x.id):
    print(r.id, r.reaction, r.gene_reaction_rule)
```

```
FA240tp ttc_c --> ttc_x 9912
FAS240_L 9.0 h_c + 3.0 malcoa_c + 6.0 nadph_c + ocdca_c --> 3.0 co2_c + 3.0 coa_c + 3.0 h2o_c + 6.0 nadp_c + ttc_c (16241 and 16655) or (10677 and 16241 and 16695)
FAS260 3.0 h_c + malcoa_c + 2.0 nadph_c + ttc_c --> co2_c + coa_c + h2o_c + hexc_c + 2.0 nadp_c 16241 and 16655
yli_R0137 3.0 h_c + malcoa_c + 2.0 nadph_c + ttc_c --> co2_c + coa_c + h2o_c + 2.0 nadp_c + yli_M05672_c 10677 and 16241 and 16695
yli_R0150 atp_c + coa_c + ttc_c <=> amp_c + ppi_c + yli_M04095_c 11167 or 12538 or 12555 or 15748

yli_R0228 h2o_m + yli_M04095_m --> coa_m + h_m + ttc_m 16048
yli_R0242 atp_m + coa_m + ttc_m <=> amp_m + ppi_m + yli_M04095_m 11167 or 12538 or 12555 or 15748

FA240tp ttc_c --> ttc_x 9912
FACOAL240p atp_x + coa_x + ttc_x <=> amp_x + ppi_x + ttccoa_x 9912
PTE12x h2o_x + ttccoa_x --> coa_x + h_x + ttc_x 16048
```

In [50]:

```
for r in sorted(model.metabolites.get_by_id('lgnc_c').reactions, key=lambda x: x.id):
    print(r.id, r.reaction, r.gene_reaction_rule)
print()
for r in sorted(model.metabolites.get_by_id('lgnc_e').reactions, key=lambda x: x.id):
    print(r.id, r.reaction, r.gene_reaction_rule)
```

```
FACOAL240 atp_c + coa_c + lgnc_c <=> amp_c + lgnccoa_c + ppi_c 12538 or 12555
FATP8t lgnc_c + na1_c <=> lgnc_e + na1_e 9912

FATP8t lgnc_c + na1_c <=> lgnc_e + na1_e 9912
```

yli\_R0137 and FAS260 are the same  
Remove yli\_R0137 and change FAS260 genes to '(16241 and 16655) or (10677 and 16241 and 16695)'

yli\_R0150 and FACOAL240 are the same (cyto)  
Remove yli\_R0150 and change FACOAL240 genes to '11167 or 12538 or 12555 or 15748'

Replace lgnc with ttc

In [51]:

```
model.remove_reactions(['yli_R0137','yli_R0150'], remove_orphans=True)
model.reactions.get_by_id('FAS260').gene_reaction_rule = '(16241 and 16655) or (10677 and 16241 and 16695)'
for r in model.metabolites.get_by_id('lgnc_c').reactions:
    r.add_metabolites({'lgnc_c': -r.get_coefficient('lgnc_c'), 'ttc_c': r.get_coefficient('lgnc_c')})
m = model.metabolites.get_by_id('ttc_c').copy()
m.id = 'ttc_e'
m.compartment = 'e'
model.add_metabolites([m])
for r in model.metabolites.get_by_id('lgnc_e').reactions:
    r.add_metabolites({'lgnc_e': -r.get_coefficient('lgnc_e'), 'ttc_e': r.get_coefficient('lgnc_e')})
```

In [52]:

```
for r in sorted(model.metabolites.get_by_id('ttccoa_r').reactions, key=lambda x: x.id):
    print(r.id, r.reaction, r.gene_reaction_rule)
print()
for r in sorted(model.metabolites.get_by_id('ttccoa_x').reactions, key=lambda x: x.id):
    print(r.id, r.reaction, r.gene_reaction_rule)
```

```
CERS124er sphgn_r + ttccoa_r --> cer1_24_r + coa_r + h_r 11391 or 15168
CERS224er psphings_r + ttccoa_r --> cer2_24_r + coa_r + h_r 11391 or 15168

ACACT9p 3ohxccoa_x + coa_x --> accoa_x + ttccoa_x 13813
FACOAL240p atp_x + coa_x + ttc_x <=> amp_x + ppi_x + ttccoa_x 9912
PTE12x h2o_x + ttccoa_x --> coa_x + h_x + ttc_x 16048
```

In [53]:

```
for r in sorted(model.metabolites.get_by_id('lgnccoa_c').reactions, key=lambda x: x.id):
    print(r.id, r.reaction, r.gene_reaction_rule)
for r in sorted(model.metabolites.get_by_id('lgnccoa_m').reactions, key=lambda x: x.id):
    print(r.id, r.reaction, r.gene_reaction_rule)
for r in sorted(model.metabolites.get_by_id('lgnccoa_x').reactions, key=lambda x: x.id):
    print(r.id, r.reaction, r.gene_reaction_rule)
print()
for r in sorted(model.metabolites.get_by_id('lgnccrn_c').reactions, key=lambda x: x.id):
    print(r.id, r.reaction, r.gene_reaction_rule)
for r in sorted(model.metabolites.get_by_id('lgnccrn_m').reactions, key=lambda x: x.id):
    print(r.id, r.reaction, r.gene_reaction_rule)
```

```
FACOAL240 atp_c + coa_c + ttc_c <=> amp_c + lgnccoa_c + ppi_c 12538 or 12555
LGNCCPT1 crn_c + lgnccoa_c --> coa_c + lgnccrn_c 9315
LGNCCPT2 coa_m + lgnccrn_m --> crn_m + lgnccoa_m 13580
FAOXC240200x 2.0 coa_x + 2.0 h2o_x + lgnccoa_x + 2.0 nad_x + 2.0 o2_x --> 2.0 accoa_x + arachcoa_x + 2.0 h2o2_x + 2.0 h_x + 2.0 nadh_x (EHHADH and 12742 and 13813) or (EHHADH and 12742 and 9065) or (EHHADH and 12752 and 13813) or (EHHADH and 12752 and 9065) or (EHHADH and 13813 and 9700) or (EHHADH and 9065 and 9700) or (Ehhadh and 12742 and 13813) or (Ehhadh and 12742 and 9065) or (Ehhadh and 12752 and 13813) or (Ehhadh and 12752 and 9065) or (Ehhadh and 13813 and 9700) or (Ehhadh and 9065 and 9700) or (11362 and 12742 and 13813) or (11362 and 12742 and 9065) or (11362 and 12752 and 13813) or (11362 and 12752 and 9065) or (11362 and 13813 and 9700) or (11362 and 9065 and 9700)

LGNCCPT1 crn_c + lgnccoa_c --> coa_c + lgnccrn_c 9315
LGNCCRNt lgnccrn_c --> lgnccrn_m 9331
LGNCCPT2 coa_m + lgnccrn_m --> crn_m + lgnccoa_m 13580
LGNCCRNt lgnccrn_c --> lgnccrn_m 9331
```

ttccoa not in mitochondria in Recon3D (C24), remove crn transport reactions  
replace lgnccoa with ttccoa

In [54]:

```
model.remove_reactions(['LGNCCPT1','LGNCCPT2','LGNCCRNt'], remove_orphans=True)
m = model.metabolites.get_by_id('ttccoa_x').copy()
m.id = 'ttccoa_c'
m.compartment = 'c'
model.add_metabolites([m])
for x in ['lgnccoa_c','lgnccoa_x']:
    for r in model.metabolites.get_by_id(x).reactions:
        r.add_metabolites({x: -r.get_coefficient(x), x.replace('lgnc','ttc'): r.get_coefficient(x)})
```

In [55]:

```
for r in sorted(model.reactions, key=lambda x: x.id):
    if r.id.startswith('FAOXC') and 'x' in r.compartments:
        print(r.id, r.reaction, r.gene_reaction_rule, sep='\n')
        print()
```

```
FAOXC183806x
5.0 coa_x + 5.0 h2o_x + lnlncgcoa_x + 5.0 nad_x + 2.0 o2_x --> 5.0 accoa_x + 2.0 h2o2_x + 5.0 h_x + 5.0 nadh_x + occoa_x
(EHHADH and 12742 and 13813) or (EHHADH and 12742 and 9065) or (EHHADH and 12752 and 13813) or (EHHADH and 12752 and 9065) or (EHHADH and 13813 and 9700) or (EHHADH and 9065 and 9700) or (Ehhadh and 12742 and 13813) or (Ehhadh and 12742 and 9065) or (Ehhadh and 12752 and 13813) or (Ehhadh and 12752 and 9065) or (Ehhadh and 13813 and 9700) or (Ehhadh and 9065 and 9700) or (11362 and 12742 and 13813) or (11362 and 12742 and 9065) or (11362 and 12752 and 13813) or (11362 and 12752 and 9065) or (11362 and 13813 and 9700) or (11362 and 9065 and 9700)

FAOXC18480x
5.0 coa_x + 5.0 h2o_x + 5.0 nad_x + o2_x + strdnccoa_x --> 5.0 accoa_x + h2o2_x + 5.0 h_x + 5.0 nadh_x + occoa_x
(EHHADH and 12742 and 13813) or (EHHADH and 12742 and 9065) or (EHHADH and 12752 and 13813) or (EHHADH and 12752 and 9065) or (EHHADH and 13813 and 9700) or (EHHADH and 9065 and 9700) or (Ehhadh and 12742 and 13813) or (Ehhadh and 12742 and 9065) or (Ehhadh and 12752 and 13813) or (Ehhadh and 12752 and 9065) or (Ehhadh and 13813 and 9700) or (Ehhadh and 9065 and 9700) or (11362 and 12742 and 13813) or (11362 and 12742 and 9065) or (11362 and 12752 and 13813) or (11362 and 12752 and 9065) or (11362 and 13813 and 9700) or (11362 and 9065 and 9700)

FAOXC200180x
arachcoa_x + coa_x + h2o_x + nad_x + o2_x --> accoa_x + h2o2_x + h_x + nadh_x + stcoa_x
(EHHADH and 12742 and 13813) or (EHHADH and 12742 and 9065) or (EHHADH and 12752 and 13813) or (EHHADH and 12752 and 9065) or (EHHADH and 13813 and 9700) or (EHHADH and 9065 and 9700) or (Ehhadh and 12742 and 13813) or (Ehhadh and 12742 and 9065) or (Ehhadh and 12752 and 13813) or (Ehhadh and 12752 and 9065) or (Ehhadh and 13813 and 9700) or (Ehhadh and 9065 and 9700) or (11362 and 12742 and 13813) or (11362 and 12742 and 9065) or (11362 and 12752 and 13813) or (11362 and 12752 and 9065) or (11362 and 13813 and 9700) or (11362 and 9065 and 9700)

FAOXC2051843x
coa_x + h2o_x + nad_x + tmndnccoa_x --> accoa_x + h_x + nadh_x + strdnccoa_x
(EHHADH and 12742 and 13813) or (EHHADH and 12742 and 9065) or (EHHADH and 12752 and 13813) or (EHHADH and 12752 and 9065) or (EHHADH and 13813 and 9700) or (EHHADH and 9065 and 9700) or (Ehhadh and 12742 and 13813) or (Ehhadh and 12742 and 9065) or (Ehhadh and 12752 and 13813) or (Ehhadh and 12752 and 9065) or (Ehhadh and 13813 and 9700) or (Ehhadh and 9065 and 9700) or (11362 and 12742 and 13813) or (11362 and 12742 and 9065) or (11362 and 12752 and 13813) or (11362 and 12752 and 9065) or (11362 and 13813 and 9700) or (11362 and 9065 and 9700)

FAOXC2242046x
adrncoa_x + coa_x + h2o_x + nad_x + o2_x --> accoa_x + arachdcoa_x + h2o2_x + h_x + nadh_x
(EHHADH and 12742 and 13813) or (EHHADH and 12742 and 9065) or (EHHADH and 12752 and 13813) or (EHHADH and 12752 and 9065) or (EHHADH and 13813 and 9700) or (EHHADH and 9065 and 9700) or (Ehhadh and 12742 and 13813) or (Ehhadh and 12742 and 9065) or (Ehhadh and 12752 and 13813) or (Ehhadh and 12752 and 9065) or (Ehhadh and 13813 and 9700) or (Ehhadh and 9065 and 9700) or (11362 and 12742 and 13813) or (11362 and 12742 and 9065) or (11362 and 12752 and 13813) or (11362 and 12752 and 9065) or (11362 and 13813 and 9700) or (11362 and 9065 and 9700)

FAOXC2251836x
2.0 coa_x + dcsptn1coa_x + 2.0 h2o_x + 2.0 nad_x --> 2.0 accoa_x + 2.0 h_x + lnlncgcoa_x + 2.0 nadh_x
(EHHADH and 12742 and 13813) or (EHHADH and 12742 and 9065) or (EHHADH and 12752 and 13813) or (EHHADH and 12752 and 9065) or (EHHADH and 13813 and 9700) or (EHHADH and 9065 and 9700) or (Ehhadh and 12742 and 13813) or (Ehhadh and 12742 and 9065) or (Ehhadh and 12752 and 13813) or (Ehhadh and 12752 and 9065) or (Ehhadh and 13813 and 9700) or (Ehhadh and 9065 and 9700) or (11362 and 12742 and 13813) or (11362 and 12742 and 9065) or (11362 and 12752 and 13813) or (11362 and 12752 and 9065) or (11362 and 13813 and 9700) or (11362 and 9065 and 9700)

FAOXC2252053x
coa_x + dcsptn1coa_x + h2o_x + nad_x + o2_x --> accoa_x + h2o2_x + h_x + nadh_x + tmndnccoa_x
(EHHADH and 12742 and 13813) or (EHHADH and 12742 and 9065) or (EHHADH and 12752 and 13813) or (EHHADH and 12752 and 9065) or (EHHADH and 13813 and 9700) or (EHHADH and 9065 and 9700) or (Ehhadh and 12742 and 13813) or (Ehhadh and 12742 and 9065) or (Ehhadh and 12752 and 13813) or (Ehhadh and 12752 and 9065) or (Ehhadh and 13813 and 9700) or (Ehhadh and 9065 and 9700) or (11362 and 12742 and 13813) or (11362 and 12742 and 9065) or (11362 and 12752 and 13813) or (11362 and 12752 and 9065) or (11362 and 13813 and 9700) or (11362 and 9065 and 9700)

FAOXC226205x
c226coa_x + coa_x + h2o_x + nad_x --> accoa_x + h_x + nadh_x + tmndnccoa_x
(EHHADH and 12742 and 13813) or (EHHADH and 12742 and 9065) or (EHHADH and 12752 and 13813) or (EHHADH and 12752 and 9065) or (EHHADH and 13813 and 9700) or (EHHADH and 9065 and 9700) or (Ehhadh and 12742 and 13813) or (Ehhadh and 12742 and 9065) or (Ehhadh and 12752 and 13813) or (Ehhadh and 12752 and 9065) or (Ehhadh and 13813 and 9700) or (Ehhadh and 9065 and 9700) or (11362 and 12742 and 13813) or (11362 and 12742 and 9065) or (11362 and 12752 and 13813) or (11362 and 12752 and 9065) or (11362 and 13813 and 9700) or (11362 and 9065 and 9700)

FAOXC240200x
2.0 coa_x + 2.0 h2o_x + 2.0 nad_x + 2.0 o2_x + ttccoa_x --> 2.0 accoa_x + arachcoa_x + 2.0 h2o2_x + 2.0 h_x + 2.0 nadh_x
(EHHADH and 12742 and 13813) or (EHHADH and 12742 and 9065) or (EHHADH and 12752 and 13813) or (EHHADH and 12752 and 9065) or (EHHADH and 13813 and 9700) or (EHHADH and 9065 and 9700) or (Ehhadh and 12742 and 13813) or (Ehhadh and 12742 and 9065) or (Ehhadh and 12752 and 13813) or (Ehhadh and 12752 and 9065) or (Ehhadh and 13813 and 9700) or (Ehhadh and 9065 and 9700) or (11362 and 12742 and 13813) or (11362 and 12742 and 9065) or (11362 and 12752 and 13813) or (11362 and 12752 and 9065) or (11362 and 13813 and 9700) or (11362 and 9065 and 9700)

FAOXC241181x
3.0 coa_x + 3.0 h2o_x + 3.0 nad_x + nrvnccoa_x + 3.0 o2_x --> 3.0 accoa_x + 3.0 h2o2_x + 3.0 h_x + 3.0 nadh_x + odecoa_x
(EHHADH and 12742 and 13813) or (EHHADH and 12742 and 9065) or (EHHADH and 12752 and 13813) or (EHHADH and 12752 and 9065) or (EHHADH and 13813 and 9700) or (EHHADH and 9065 and 9700) or (Ehhadh and 12742 and 13813) or (Ehhadh and 12742 and 9065) or (Ehhadh and 12752 and 13813) or (Ehhadh and 12752 and 9065) or (Ehhadh and 13813 and 9700) or (Ehhadh and 9065 and 9700) or (11362 and 12742 and 13813) or (11362 and 12742 and 9065) or (11362 and 12752 and 13813) or (11362 and 12752 and 9065) or (11362 and 13813 and 9700) or (11362 and 9065 and 9700)

FAOXC2442246x
coa_x + h2o_x + nad_x + o2_x + tettet6coa_x --> accoa_x + adrncoa_x + h2o2_x + h_x + nadh_x
(EHHADH and 12742 and 13813) or (EHHADH and 12742 and 9065) or (EHHADH and 12752 and 13813) or (EHHADH and 12752 and 9065) or (EHHADH and 13813 and 9700) or (EHHADH and 9065 and 9700) or (Ehhadh and 12742 and 13813) or (Ehhadh and 12742 and 9065) or (Ehhadh and 12752 and 13813) or (Ehhadh and 12752 and 9065) or (Ehhadh and 13813 and 9700) or (Ehhadh and 9065 and 9700) or (11362 and 12742 and 13813) or (11362 and 12742 and 9065) or (11362 and 12752 and 13813) or (11362 and 12752 and 9065) or (11362 and 13813 and 9700) or (11362 and 9065 and 9700)

FAOXC2452253x
coa_x + h2o_x + nad_x + o2_x + tetpent3coa_x --> accoa_x + clpndcoa_x + h2o2_x + h_x + nadh_x
(EHHADH and 12742 and 13813) or (EHHADH and 12742 and 9065) or (EHHADH and 12752 and 13813) or (EHHADH and 12752 and 9065) or (EHHADH and 13813 and 9700) or (EHHADH and 9065 and 9700) or (Ehhadh and 12742 and 13813) or (Ehhadh and 12742 and 9065) or (Ehhadh and 12752 and 13813) or (Ehhadh and 12752 and 9065) or (Ehhadh and 13813 and 9700) or (Ehhadh and 9065 and 9700) or (11362 and 12742 and 13813) or (11362 and 12742 and 9065) or (11362 and 12752 and 13813) or (11362 and 12752 and 9065) or (11362 and 13813 and 9700) or (11362 and 9065 and 9700)

FAOXC2452256x
coa_x + h2o_x + nad_x + o2_x + tetpent6coa_x --> accoa_x + dcsptn1coa_x + h2o2_x + h_x + nadh_x
(EHHADH and 12742 and 13813) or (EHHADH and 12742 and 9065) or (EHHADH and 12752 and 13813) or (EHHADH and 12752 and 9065) or (EHHADH and 13813 and 9700) or (EHHADH and 9065 and 9700) or (Ehhadh and 12742 and 13813) or (Ehhadh and 12742 and 9065) or (Ehhadh and 12752 and 13813) or (Ehhadh and 12752 and 9065) or (Ehhadh and 13813 and 9700) or (Ehhadh and 9065 and 9700) or (11362 and 12742 and 13813) or (11362 and 12742 and 9065) or (11362 and 12752 and 13813) or (11362 and 12752 and 9065) or (11362 and 13813 and 9700) or (11362 and 9065 and 9700)

FAOXC246226x
coa_x + h2o_x + nad_x + o2_x + tethex3coa_x --> accoa_x + c226coa_x + h2o2_x + h_x + nadh_x
(EHHADH and 12742 and 13813) or (EHHADH and 12742 and 9065) or (EHHADH and 12752 and 13813) or (EHHADH and 12752 and 9065) or (EHHADH and 13813 and 9700) or (EHHADH and 9065 and 9700) or (Ehhadh and 12742 and 13813) or (Ehhadh and 12742 and 9065) or (Ehhadh and 12752 and 13813) or (Ehhadh and 12752 and 9065) or (Ehhadh and 13813 and 9700) or (Ehhadh and 9065 and 9700) or (11362 and 12742 and 13813) or (11362 and 12742 and 9065) or (11362 and 12752 and 13813) or (11362 and 12752 and 9065) or (11362 and 13813 and 9700) or (11362 and 9065 and 9700)
```

These unsaturaed reactions are currently all blocked, need to add transport reactions (no gene in hsa)  
FAOXC2251836x (4Z,7Z,10Z,13Z,16Z)-docosapentaenoyl-CoA to (6Z,9Z,12Z)-octadecatrienoyl-CoA, wrong need o2  
FAOXC183806x Gamma-Linolenoyl-CoA, (6Z,9Z,12Z)-octadecatrienoyl-CoA to occoa, wrong need nadph  
FAOXC2252053x (4Z,7Z,10Z,13Z,16Z)-docosapentaenoyl-CoA to (5Z,8Z,11Z,14Z,17Z)-icosapentaenoyl-CoA, wrong need o2  
FAOXC2051843x (5Z,8Z,11Z,14Z,17Z)-icosapentaenoyl-CoA to (6Z,9Z,12Z,15Z)-octadecatetraenoyl-CoA, wrong need o2, nadph  
FAOXC18480x (6Z,9Z,12Z,15Z)-octadecatetraenoyl-CoA to occoa, wrong cannot make occoa  
FAOXC2442246x (9Z,12Z,15Z,18Z)-tetracosatetraenoyl-CoA to (7Z,10Z,13Z,16Z)-docosatetraenoyl CoA, OK normal  
FAOXC2242046x (7Z,10Z,13Z,16Z)-docosatetraenoyl-CoA to (5Z,8Z,11Z,14Z)-eicosatetraenoyl-CoA, OK normal  
FAOXC246226x (6Z,9Z,12Z,15Z,18Z,21Z)-tetracosahexaenoyl-CoA to (4Z,7Z,10Z,13Z,16Z,19Z)-docosahexaenoyl CoA, OK normal  
FAOXC226205x (4Z,7Z,10Z,13Z,16Z,19Z)-docosahexaenoyl-CoA to (5Z,8Z,11Z,14Z,17Z)-icosapentaenoyl-CoA, wrong need o2, nadph  
FAOXC241181x (15Z)-tetracosenoyl-CoA to (9Z)-octadecenoyl-CoA, OK normal  
FAOXC2452253x (9Z,12Z,15Z,18Z,21Z)-tetracosapentaenoyl-CoA to (7Z,10Z,13Z,16Z,19Z)-Docosapentaenoyl-CoA, OK normal  
FAOXC2452256x (6Z,9Z,12Z,15Z,18Z)-tetracosapentaenoyl-CoA to (4Z,7Z,10Z,13Z,16Z)-docosapentaenoyl-CoA, OK normal

In [56]:

```
model.remove_reactions(['FAOXC2251836x','FAOXC183806x','FAOXC2252053x','FAOXC2051843x','FAOXC18480x','FAOXC226205x'], remove_orphans=True)
```

In [57]:

```
# peroxisomal beta oxidation lumped reactions
model.reactions.get_by_id('FAOXC200180x').gene_reaction_rule = '(12742 and 11362 and 13813) or (12752 and 11362 and 13813) or (9700 and 11362 and 13813)'
model.reactions.get_by_id('FAOXC2242046x').gene_reaction_rule = '(12742 and 11362 and 13813) or (12752 and 11362 and 13813) or (9700 and 11362 and 13813)'
model.reactions.get_by_id('FAOXC240200x').gene_reaction_rule = '(12742 and 11362 and 13813) or (12752 and 11362 and 13813) or (9700 and 11362 and 13813)'
model.reactions.get_by_id('FAOXC241181x').gene_reaction_rule = '(12742 and 11362 and 13813) or (12752 and 11362 and 13813) or (9700 and 11362 and 13813)'
model.reactions.get_by_id('FAOXC2442246x').gene_reaction_rule = '(12742 and 11362 and 13813) or (12752 and 11362 and 13813) or (9700 and 11362 and 13813)'
model.reactions.get_by_id('FAOXC2452253x').gene_reaction_rule = '(12742 and 11362 and 13813) or (12752 and 11362 and 13813) or (9700 and 11362 and 13813)'
model.reactions.get_by_id('FAOXC2452256x').gene_reaction_rule = '(12742 and 11362 and 13813) or (12752 and 11362 and 13813) or (9700 and 11362 and 13813)'
model.reactions.get_by_id('FAOXC246226x').gene_reaction_rule = '(12742 and 11362 and 13813) or (12752 and 11362 and 13813) or (9700 and 11362 and 13813)'
```

In [58]:

```
temp = ['16048','8456','14585']
display(Annotation.loc[temp])
Show_Data(temp)
```

|  | Combined Annotations | Signal P | Sc288c Orthologs | Human Orthologs | Sc288 Best Hit | Human Blast | Essential | WolfPSort | C Terminal |
| --- | --- | --- | --- | --- | --- | --- | --- | --- | --- |
| RTO4\_ID |  |  |  |  |  |  |  |  |  |
| 16048 | K11992: ACOT8, PTE; acyl-CoA thioesterase 8 |  | TES1 | ACOT8 |  | ACOT8 | Not Essential | nucl 8.5, cyto\_nucl 7, pero 7, mito 5, cyto 4.5 | AAL\* |
| 8456 | K17361: ACOT9; acyl-coenzyme A thioesterase 9 |  |  | ACOT9 |  | ACOT9 | Not Essential | nucl 13.5, cyto\_nucl 11.5, cyto 8.5, mito 3 | QAP\* |
| 14585 | K17362: ACOT13; acyl-coenzyme A thioesterase 13 |  |  | ACOT13 |  | ACOT1 | Not Essential | cysk 9, mito 8, cyto 5.5, cyto\_nucl 5, nucl 3.5 | PSE\* |

| strain | WT | | | | | | | | | | | | | | | | |
| --- | --- | --- | --- | --- | --- | --- | --- | --- | --- | --- | --- | --- | --- | --- | --- | --- | --- |
| condition | G\_MM | C\_MM | G\_SD | | GX\_SD | | | X\_SD | | A\_SD | | C\_SD | | MM\_CN120 | | MM\_CN5 | Diversity\_Sample |
| phase | exp | exp | exp | stat | exp | trans | stat | exp | stat | exp | stat | exp | stat | exp | stat | exp | exp |
| proteinId | Set1 | Set1 | Set2 | Set2 | Set2 | Set2 | Set2 | Set2 | Set2 | Set2 | Set2 | Set2 | Set2 | Set3 | Set3 | Set3 | Set3 |
| 16048 | 4.84988 | 6.19867 | 4.63217 | 4.18146 | 4.58936 | 4.42248 | 4.28251 | 4.51311 | 4.76714 | 4.23634 | 5.04363 | 4.88331 | 4.60716 | 6.28102 | 6.33049 | 5.40791 | 5.35123 |
| 8456 | 6.43335 | 5.59125 | 6.49661 | 5.41488 | 6.73554 | 5.8529 | 5.85338 | 6.21259 | 6.03027 | 5.99749 | 5.68253 | 6.23893 | 6.98304 | 6.45522 | 5.94157 | 7.75667 | 5.57259 |
| 14585 | 6.51562 | 6.99916 | 6.165 | 5.66356 | 6.05827 | 5.69262 | 5.83295 | 6.36267 | 6.16031 | 6.6796 | 6.86733 | 6.27465 | 5.64081 | 3.79309 | 3.92494 | 4.87148 | 2.6195 |

| strain | WT | | | | | | | | | | |
| --- | --- | --- | --- | --- | --- | --- | --- | --- | --- | --- | --- |
| condition | G\_SD | | GX\_SD | | | X\_SD | | A\_SD | | C\_SD | |
| proteinId | exp | stat | exp | trans | stat | exp | stat | exp | stat | exp | stat |
| 16048 | 3.24463 | 3.7886 | 3.85281 | 6.5133 | 5.63143 | 4.10592 | 3.89505 | 3.28571 | 2.68834 | 9.42534 | 5.01209 |
| 8456 | 5.38215 | 6.42375 | 5.10016 | 4.64596 | 3.41619 | 4.32861 | 2.53948 | 4.24464 | 2.10247 | 1.06795 | 1.96108 |
| 14585 | 1.12232 | 1.26933 | 1.83732 | 0.751858 | 0.357685 | 0.956129 | 1.17187 | 1.35253 | 1.52793 | 1.71125 | 1.95556 |

|  | Glucose | Xylose | Arabinose | Acetate | Coumarate | Ferulate | YNB Oleic Acid | YNB Ricinoleic Acid | YNB Glucose | YNB Gluc DOC | YPD |
| --- | --- | --- | --- | --- | --- | --- | --- | --- | --- | --- | --- |
| proteinId |  |  |  |  |  |  |  |  |  |  |  |
| 16048 | -0.0941699 | -0.0375465 | 0.0121643 | -0.147875 | -0.0126005 | -0.0406894 | 0.172548 | 0.0362263 | -0.245912 | -0.040811 | 0.228448 |
| 8456 | -0.0934737 | -0.207925 | -0.306311 | -0.212615 | -0.296622 | -0.0315301 | 0.0612052 | 0.0553147 | -0.326957 | -0.394361 | -0.0124277 |
| 14585 | -0.518157 | 0.276727 | -0.224457 | 0.0451634 | -0.339184 | 0.298084 | -0.011245 | -0.0680009 | -0.110504 | -0.149529 | 0.172852 |

In [59]:

```
# 16048 ACOT8, PTE; acyl-CoA thioesterase 8, ends with AAL* -> pero
# 8456 ACOT9; acyl-coenzyme A thioesterase 9 -> cyto
# 14585 ACOT13; acyl-coenzyme A thioesterase 13, no rxns -> mito
for r in sorted(model.genes.get_by_id('16048').reactions, key=lambda x: x.id):
    print(r.id, r.reaction, r.gene_reaction_rule)
print()
for r in sorted(model.genes.get_by_id('8456').reactions, key=lambda x: x.id):
    print(r.id, r.reaction, r.gene_reaction_rule)
```

```
FACOAE100 dcacoa_c + h2o_c --> coa_c + dca_c + h_c 16048
FACOAE120 ddcacoa_c + h2o_c --> coa_c + ddca_c + h_c 16048
FACOAE140 h2o_c + tdcoa_c --> coa_c + h_c + ttdca_c 16048
FACOAE141 h2o_c + tdecoa_c --> coa_c + h_c + ttdcea_c 16048
FACOAE160 h2o_c + pmtcoa_c --> coa_c + h_c + hdca_c 16048 or 8456
FACOAE161 h2o_c + hdcoa_c --> coa_c + h_c + hdcea_c 16048
FACOAE180 h2o_c + stcoa_c --> coa_c + h_c + ocdca_c 16048 or 8456
FACOAE181 h2o_c + odecoa_c --> coa_c + h_c + ocdcea_c 16048
FACOAE60 h2o_c + hxcoa_c --> coa_c + h_c + hxa_c 16048
FACOAE80 h2o_c + occoa_c --> coa_c + h_c + octa_c 16048
PTE10x dcacoa_x + h2o_x --> coa_x + dca_x + h_x 16048
PTE11x ddcacoa_x + h2o_x --> coa_x + ddca_x + h_x 16048
PTE12x h2o_x + ttccoa_x --> coa_x + h_x + ttc_x 16048
PTE2x h2o_x + pmtcoa_x --> coa_x + h_x + hdca_x 16048
PTE7x h2o_x + tdcoa_x --> coa_x + h_x + ttdca_x 16048
PTE8x h2o_x + stcoa_x --> coa_x + h_x + ocdca_x 16048
PTE9x h2o_x + occoa_x --> coa_x + h_x + octa_x 16048
RE0344M h2o_m + stcoa_m --> coa_m + h_m + ocdca_m 16048
yli_R0226 dccoa_m + h2o_m --> coa_m + h_m + yli_M01250_m 16048
yli_R0227 ddcacoa_m + h2o_m --> coa_m + h_m + yli_M01723_m 16048
yli_R0228 h2o_m + yli_M04095_m --> coa_m + h_m + ttc_m 16048
yli_R0229 h2o_m + pmtcoa_m --> coa_m + h_m + hdca_m 16048
yli_R0230 h2o_m + tdcoa_m --> coa_m + h_m + yli_M03073_m 16048
yli_R0232 h2o_m + occoa_m --> coa_m + h_m + octa_m 16048

FACOAE160 h2o_c + pmtcoa_c --> coa_c + h_c + hdca_c 16048 or 8456
FACOAE180 h2o_c + stcoa_c --> coa_c + h_c + ocdca_c 16048 or 8456
FACOAE1819Z h2o_c + ocdce9coa_c --> coa_c + h_c + ocdce9a_c 8456
FACOAE1829Z12Z h2o_c + lnlccoa_c --> coa_c + h_c + lnlc_c 8456
FACOAE1836Z9Z12Z h2o_c + lnlncgcoa_c --> coa_c + h_c + lnlncg_c 8456
FACOAE1839Z12Z15Z h2o_c + lnlncacoa_c --> coa_c + h_c + lnlnca_c 8456
```

In [60]:

```
for r in sorted(model.reactions, key=lambda x: x.id):
    if r.id.startswith('FACOAE'):
        print(r.id, r.reaction, r.gene_reaction_rule)
```

```
FACOAE100 dcacoa_c + h2o_c --> coa_c + dca_c + h_c 16048
FACOAE120 ddcacoa_c + h2o_c --> coa_c + ddca_c + h_c 16048
FACOAE140 h2o_c + tdcoa_c --> coa_c + h_c + ttdca_c 16048
FACOAE141 h2o_c + tdecoa_c --> coa_c + h_c + ttdcea_c 16048
FACOAE160 h2o_c + pmtcoa_c --> coa_c + h_c + hdca_c 16048 or 8456
FACOAE161 h2o_c + hdcoa_c --> coa_c + h_c + hdcea_c 16048
FACOAE180 h2o_c + stcoa_c --> coa_c + h_c + ocdca_c 16048 or 8456
FACOAE181 h2o_c + odecoa_c --> coa_c + h_c + ocdcea_c 16048
FACOAE1819Z h2o_c + ocdce9coa_c --> coa_c + h_c + ocdce9a_c 8456
FACOAE1829Z12Z h2o_c + lnlccoa_c --> coa_c + h_c + lnlc_c 8456
FACOAE1836Z9Z12Z h2o_c + lnlncgcoa_c --> coa_c + h_c + lnlncg_c 8456
FACOAE1839Z12Z15Z h2o_c + lnlncacoa_c --> coa_c + h_c + lnlnca_c 8456
FACOAE60 h2o_c + hxcoa_c --> coa_c + h_c + hxa_c 16048
FACOAE80 h2o_c + occoa_c --> coa_c + h_c + octa_c 16048
```

In [61]:

```
for r in sorted(model.reactions, key=lambda x: x.id):
    if r.id.startswith('FACOAE'):
        r.gene_reaction_rule = '8456'
```

In [62]:

```
for r in sorted(model.reactions, key=lambda x: x.id):
    if r.id.startswith('PTE') and r.id.endswith('x'):
        print(r.id, r.reaction, r.gene_reaction_rule)
```

```
PTE10x dcacoa_x + h2o_x --> coa_x + dca_x + h_x 16048
PTE11x ddcacoa_x + h2o_x --> coa_x + ddca_x + h_x 16048
PTE12x h2o_x + ttccoa_x --> coa_x + h_x + ttc_x 16048
PTE2x h2o_x + pmtcoa_x --> coa_x + h_x + hdca_x 16048
PTE7x h2o_x + tdcoa_x --> coa_x + h_x + ttdca_x 16048
PTE8x h2o_x + stcoa_x --> coa_x + h_x + ocdca_x 16048
PTE9x h2o_x + occoa_x --> coa_x + h_x + octa_x 16048
```

In [63]:

```
for r in sorted(model.reactions, key=lambda x: x.id):
    if 'h2o_m' in [m.id for m in r.reactants] and 'coa_m' in [m.id for m in r.products] and len(r.reactants) == 2:
        print(r.id, r.reaction, r.gene_reaction_rule)
```

```
3HBCOAHLm 3hibutcoa_m + h2o_m --> 3hmp_m + coa_m + h_m 15218
ACOAHim accoa_m + h2o_m --> ac_m + coa_m + h_m 13797
RE0344M h2o_m + stcoa_m --> coa_m + h_m + ocdca_m 16048
yli_R0226 dccoa_m + h2o_m --> coa_m + h_m + yli_M01250_m 16048
yli_R0227 ddcacoa_m + h2o_m --> coa_m + h_m + yli_M01723_m 16048
yli_R0228 h2o_m + yli_M04095_m --> coa_m + h_m + ttc_m 16048
yli_R0229 h2o_m + pmtcoa_m --> coa_m + h_m + hdca_m 16048
yli_R0230 h2o_m + tdcoa_m --> coa_m + h_m + yli_M03073_m 16048
yli_R0232 h2o_m + occoa_m --> coa_m + h_m + octa_m 16048
```

In [64]:

```
# 15218 K05605: HIBCH; 3-hydroxyisobutyryl-CoA hydrolase, mito
model.reactions.get_by_id('RE0344M').id = 'PTE8m' # C18
model.reactions.get_by_id('yli_R0226').id = 'PTE10m' # C10
model.reactions.get_by_id('yli_R0227').id = 'PTE11m' # C12
model.reactions.get_by_id('yli_R0228').id = 'PTE12m' # C24
model.reactions.get_by_id('yli_R0229').id = 'PTE2m' # C16
model.reactions.get_by_id('yli_R0230').id = 'PTE7m' # C14
model.reactions.get_by_id('yli_R0232').id = 'PTE9m' # C8
```

In [65]:

```
# C18 and C24 mito reactions -> long chain, remove
for r in model.metabolites.get_by_id('stcoa_m').reactions:
    print(r.id, r.reaction)
print()
for r in model.metabolites.get_by_id('stcrn_m').reactions:
    print(r.id, r.reaction)
print()
for r in model.metabolites.get_by_id('stcrn_c').reactions:
    print(r.id, r.reaction)
print()
for r in model.metabolites.get_by_id('ocdca_m').reactions:
    print(r.id, r.reaction)
print()
for r in model.metabolites.get_by_id('yli_M04095_m').reactions:
    print(r.id, r.reaction)
print()
for r in model.metabolites.get_by_id('ttc_m').reactions:
    print(r.id, r.reaction)
```

```
PTE8m h2o_m + stcoa_m --> coa_m + h_m + ocdca_m
yli_R0196 o2_m + stcoa_m --> h2o2_m + od2coa_m
yli_R0239 atp_m + coa_m + ocdca_m <=> amp_m + ppi_m + stcoa_m
C180CPT2 coa_m + stcrn_m --> crn_m + stcoa_m

C180CRNt stcrn_c --> stcrn_m
C180CPT2 coa_m + stcrn_m --> crn_m + stcoa_m

C180CRNt stcrn_c --> stcrn_m
C180CPT1 crn_c + stcoa_c --> coa_c + stcrn_c

yli_R0239 atp_m + coa_m + ocdca_m <=> amp_m + ppi_m + stcoa_m
yli_R1473 h2o_m + 0.005 pchol_cho_m --> 0.5 g3pc_m + h_m + 0.27 hdca_m + 0.05 ocdca_m + 0.02 yli_M01250_m + 0.06 yli_M01723_m + 0.1 yli_M03073_m + 0.17 yli_M03299_m + 0.24 yli_M04622_m + 0.09 yli_M05526_m
PTE8m h2o_m + stcoa_m --> coa_m + h_m + ocdca_m

PTE12m h2o_m + yli_M04095_m --> coa_m + h_m + ttc_m
yli_R0242 atp_m + coa_m + ttc_m <=> amp_m + ppi_m + yli_M04095_m

PTE12m h2o_m + yli_M04095_m --> coa_m + h_m + ttc_m
yli_R0242 atp_m + coa_m + ttc_m <=> amp_m + ppi_m + yli_M04095_m
```

In [66]:

```
model.remove_reactions(['PTE8m','C180CPT2','yli_R0239','yli_R0196','C180CRNt','C180CPT1','yli_R1473',
                        'yli_R0242','PTE12m'], remove_orphans=True)
```

In [67]:

```
for r in sorted(model.reactions, key=lambda x: x.id):
    if r.id.startswith('PTE') and r.id.endswith('m'):
        print(r.id, r.reaction, r.gene_reaction_rule)
```

```
PTE10m dccoa_m + h2o_m --> coa_m + h_m + yli_M01250_m 16048
PTE11m ddcacoa_m + h2o_m --> coa_m + h_m + yli_M01723_m 16048
PTE2m h2o_m + pmtcoa_m --> coa_m + h_m + hdca_m 16048
PTE7m h2o_m + tdcoa_m --> coa_m + h_m + yli_M03073_m 16048
PTE9m h2o_m + occoa_m --> coa_m + h_m + octa_m 16048
```

In [68]:

```
for k, v in {'yli_M01250': 'dca', 'yli_M01723': 'ddca', 'yli_M03073': 'ttdca'}.items():
    model.metabolites.get_by_id(k+'_m').id = v+'_m'
```

In [69]:

```
for k, v in {'yli_M01250': 'dca', 'yli_M01723': 'ddca', 'yli_M03073': 'ttdca'}.items():
    for m in model.metabolites:
        if k in m.id:
            print(m.id)
            for r in sorted(m.reactions, key=lambda x: x.id):
                print(r.id, r.reaction)
            temp = v+'_'+m.id.rsplit('_',1)[1]
            print(temp)
            if temp in model.metabolites:
                for r in sorted(model.metabolites.get_by_id(temp).reactions, key=lambda x: x.id):
                    print(r.id, r.reaction)
            print()
```

```
yli_M01250_c
yli_R0127 3.0 h_c + malcoa_c + 2.0 nadph_c + octa_c --> co2_c + coa_c + h2o_c + 2.0 nadp_c + yli_M01250_c
yli_R0128 3.0 h_c + malcoa_c + 2.0 nadph_c + yli_M01250_c --> co2_c + coa_c + h2o_c + 2.0 nadp_c + yli_M01723_c
yli_R0141 atp_c + coa_c + yli_M01250_c <=> amp_c + dccoa_c + ppi_c
yli_R0312 h2o_c + 0.01 tag_cho_c --> 0.01 dag_hs_c + h_c + 0.27 hdca_c + 0.05 ocdca_c + 0.02 yli_M01250_c + 0.06 yli_M01723_c + 0.1 yli_M03073_c + 0.17 yli_M03299_c + 0.24 yli_M04622_c + 0.09 yli_M05526_c
yli_R0895 h2o_c + 0.005 pchol_cho_c --> 0.5 g3pc_c + h_c + 0.27 hdca_c + 0.05 ocdca_c + 0.02 yli_M01250_c + 0.06 yli_M01723_c + 0.1 yli_M03073_c + 0.17 yli_M03299_c + 0.24 yli_M04622_c + 0.09 yli_M05526_c
dca_c
AACPS8 ACP_c + atp_c + dca_c --> amp_c + dcaACP_c + ppi_c
FACOAE100 dcacoa_c + h2o_c --> coa_c + dca_c + h_c
FAS100 3.0 h_c + malcoa_c + 2.0 nadph_c + octa_c --> co2_c + coa_c + dca_c + h2o_c + 2.0 nadp_c
FAS120 dca_c + 3.0 h_c + malcoa_c + 2.0 nadph_c --> co2_c + coa_c + ddca_c + h2o_c + 2.0 nadp_c
PLBPC_SC h2o_c + 0.005 pc_SC_c --> 0.02 dca_c + 0.06 ddca_c + 0.5 g3pc_c + h_c + 0.27 hdca_c + 0.17 hdcea_c + 0.05 ocdca_c + 0.24 ocdcea_c + 0.09 ocdcya_c + 0.1 ttdca_c
TAGL_SC h2o_c + 0.01 triglyc_SC_c --> 0.01 12dgr_SC_c + 0.02 dca_c + 0.06 ddca_c + h_c + 0.27 hdca_c + 0.17 hdcea_c + 0.05 ocdca_c + 0.24 ocdcea_c + 0.09 ocdcya_c + 0.1 ttdca_c

yli_M01250_r
yli_R1396 h2o_r + 0.01 tag_cho_r --> 0.01 dag_hs_r + h_r + 0.27 hdca_r + 0.05 ocdca_r + 0.02 yli_M01250_r + 0.06 yli_M01723_r + 0.1 yli_M03073_r + 0.17 yli_M03299_r + 0.24 yli_M04622_r + 0.09 yli_M05526_r
dca_r

yli_M01723_c
yli_R0128 3.0 h_c + malcoa_c + 2.0 nadph_c + yli_M01250_c --> co2_c + coa_c + h2o_c + 2.0 nadp_c + yli_M01723_c
yli_R0130 3.0 h_c + malcoa_c + 2.0 nadph_c + yli_M01723_c --> co2_c + coa_c + h2o_c + 2.0 nadp_c + yli_M03073_c
yli_R0138 4.0 h_c + malcoa_c + 3.0 nadph_c + o2_c + yli_M01723_c --> co2_c + coa_c + 3.0 h2o_c + 3.0 nadp_c + yli_M04636_c
yli_R0142 atp_c + coa_c + yli_M01723_c <=> amp_c + ddcacoa_c + ppi_c
yli_R0173 h2o_c + yli_M02516_c <=> ACP_c + h_c + yli_M01723_c
yli_R0312 h2o_c + 0.01 tag_cho_c --> 0.01 dag_hs_c + h_c + 0.27 hdca_c + 0.05 ocdca_c + 0.02 yli_M01250_c + 0.06 yli_M01723_c + 0.1 yli_M03073_c + 0.17 yli_M03299_c + 0.24 yli_M04622_c + 0.09 yli_M05526_c
yli_R0895 h2o_c + 0.005 pchol_cho_c --> 0.5 g3pc_c + h_c + 0.27 hdca_c + 0.05 ocdca_c + 0.02 yli_M01250_c + 0.06 yli_M01723_c + 0.1 yli_M03073_c + 0.17 yli_M03299_c + 0.24 yli_M04622_c + 0.09 yli_M05526_c
ddca_c
AACPS7 ACP_c + atp_c + ddca_c --> amp_c + ddcaACP_c + ppi_c
FA120ACPHi ddcaACP_c + h2o_c --> ACP_c + ddca_c + h_c
FACOAE120 ddcacoa_c + h2o_c --> coa_c + ddca_c + h_c
FAS120 dca_c + 3.0 h_c + malcoa_c + 2.0 nadph_c --> co2_c + coa_c + ddca_c + h2o_c + 2.0 nadp_c
FAS140 ddca_c + 3.0 h_c + malcoa_c + 2.0 nadph_c --> co2_c + coa_c + h2o_c + 2.0 nadp_c + ttdca_c
FAS141 ddca_c + 4.0 h_c + malcoa_c + 3.0 nadph_c + o2_c --> co2_c + coa_c + 3.0 h2o_c + 3.0 nadp_c + ttdcea_c
PLBPC_SC h2o_c + 0.005 pc_SC_c --> 0.02 dca_c + 0.06 ddca_c + 0.5 g3pc_c + h_c + 0.27 hdca_c + 0.17 hdcea_c + 0.05 ocdca_c + 0.24 ocdcea_c + 0.09 ocdcya_c + 0.1 ttdca_c
TAGL_SC h2o_c + 0.01 triglyc_SC_c --> 0.01 12dgr_SC_c + 0.02 dca_c + 0.06 ddca_c + h_c + 0.27 hdca_c + 0.17 hdcea_c + 0.05 ocdca_c + 0.24 ocdcea_c + 0.09 ocdcya_c + 0.1 ttdca_c

yli_M01723_r
yli_R1396 h2o_r + 0.01 tag_cho_r --> 0.01 dag_hs_r + h_r + 0.27 hdca_r + 0.05 ocdca_r + 0.02 yli_M01250_r + 0.06 yli_M01723_r + 0.1 yli_M03073_r + 0.17 yli_M03299_r + 0.24 yli_M04622_r + 0.09 yli_M05526_r
ddca_r

yli_M03073_c
yli_R0130 3.0 h_c + malcoa_c + 2.0 nadph_c + yli_M01723_c --> co2_c + coa_c + h2o_c + 2.0 nadp_c + yli_M03073_c
yli_R0132 3.0 h_c + malcoa_c + 2.0 nadph_c + yli_M03073_c --> co2_c + coa_c + h2o_c + hdca_c + 2.0 nadp_c
yli_R0139 4.0 h_c + malcoa_c + 3.0 nadph_c + o2_c + yli_M03073_c --> co2_c + coa_c + 3.0 h2o_c + 3.0 nadp_c + yli_M03299_c
yli_R0143 atp_c + coa_c + yli_M03073_c <=> amp_c + ppi_c + tdcoa_c
yli_R0174 h2o_c + yli_M02786_c <=> ACP_c + h_c + yli_M03073_c
yli_R0312 h2o_c + 0.01 tag_cho_c --> 0.01 dag_hs_c + h_c + 0.27 hdca_c + 0.05 ocdca_c + 0.02 yli_M01250_c + 0.06 yli_M01723_c + 0.1 yli_M03073_c + 0.17 yli_M03299_c + 0.24 yli_M04622_c + 0.09 yli_M05526_c
yli_R0895 h2o_c + 0.005 pchol_cho_c --> 0.5 g3pc_c + h_c + 0.27 hdca_c + 0.05 ocdca_c + 0.02 yli_M01250_c + 0.06 yli_M01723_c + 0.1 yli_M03073_c + 0.17 yli_M03299_c + 0.24 yli_M04622_c + 0.09 yli_M05526_c
ttdca_c
AACPS1 ACP_c + atp_c + ttdca_c --> amp_c + myrsACP_c + ppi_c
EPISTESTH_SC 0.01 epistest_SC_c + h2o_c --> 0.01 epist_c + h_c + 0.02 hdca_c + 0.655 hdcea_c + 0.01 hexc_c + 0.03 ocdca_c + 0.27 ocdcea_c + 0.015 ttdca_c
ERGSTESTH_SC 0.01 ergstest_SC_c + h2o_c --> 0.01 ergst_c + h_c + 0.02 hdca_c + 0.655 hdcea_c + 0.01 hexc_c + 0.03 ocdca_c + 0.27 ocdcea_c + 0.015 ttdca_c
FA140ACPHi h2o_c + myrsACP_c --> ACP_c + h_c + ttdca_c
FACOAE140 h2o_c + tdcoa_c --> coa_c + h_c + ttdca_c
FACOAL140 atp_c + coa_c + ttdca_c <=> amp_c + ppi_c + tdcoa_c
FAS140 ddca_c + 3.0 h_c + malcoa_c + 2.0 nadph_c --> co2_c + coa_c + h2o_c + 2.0 nadp_c + ttdca_c
FAS160 3.0 h_c + malcoa_c + 2.0 nadph_c + ttdca_c --> co2_c + coa_c + h2o_c + hdca_c + 2.0 nadp_c
FAS161 4.0 h_c + malcoa_c + 3.0 nadph_c + o2_c + ttdca_c --> co2_c + coa_c + 3.0 h2o_c + hdcea_c + 3.0 nadp_c
FECOSTESTH_SC 0.01 fecostest_SC_c + h2o_c --> 0.01 fecost_c + h_c + 0.02 hdca_c + 0.655 hdcea_c + 0.01 hexc_c + 0.03 ocdca_c + 0.27 ocdcea_c + 0.015 ttdca_c
LANOSTESTH_SC h2o_c + 0.01 lanostest_SC_c --> h_c + 0.02 hdca_c + 0.655 hdcea_c + 0.01 hexc_c + 0.01 lanost_c + 0.03 ocdca_c + 0.27 ocdcea_c + 0.015 ttdca_c
PLBPC_SC h2o_c + 0.005 pc_SC_c --> 0.02 dca_c + 0.06 ddca_c + 0.5 g3pc_c + h_c + 0.27 hdca_c + 0.17 hdcea_c + 0.05 ocdca_c + 0.24 ocdcea_c + 0.09 ocdcya_c + 0.1 ttdca_c
TAGL_SC h2o_c + 0.01 triglyc_SC_c --> 0.01 12dgr_SC_c + 0.02 dca_c + 0.06 ddca_c + h_c + 0.27 hdca_c + 0.17 hdcea_c + 0.05 ocdca_c + 0.24 ocdcea_c + 0.09 ocdcya_c + 0.1 ttdca_c
ZYMSTESTH_SC h2o_c + 0.01 zymstest_SC_c --> h_c + 0.02 hdca_c + 0.655 hdcea_c + 0.01 hexc_c + 0.03 ocdca_c + 0.27 ocdcea_c + 0.015 ttdca_c + 0.01 zymst_c

yli_M03073_r
yli_R1396 h2o_r + 0.01 tag_cho_r --> 0.01 dag_hs_r + h_r + 0.27 hdca_r + 0.05 ocdca_r + 0.02 yli_M01250_r + 0.06 yli_M01723_r + 0.1 yli_M03073_r + 0.17 yli_M03299_r + 0.24 yli_M04622_r + 0.09 yli_M05526_r
ttdca_r
```

In [70]:

```
# Remove redundant yli reactions and irrelevant reactions
model.remove_reactions(['yli_R0127','yli_R0128','yli_R0312','yli_R0895','yli_R1396','yli_R0130',
                        'yli_R0138','yli_R0173','yli_R0132','yli_R0139','yli_R0143','yli_R0174'], remove_orphans=True)
# FACOAL100 and FACOAL120
model.reactions.get_by_id('yli_R0141').id = 'FACOAL100'
model.reactions.get_by_id('FACOAL100').add_metabolites({'yli_M01250_c': 1.0, 'dca_c': -1.0,
                                                        'dccoa_c': -1.0, 'dcacoa_c': 1.0})
model.reactions.get_by_id('yli_R0142').id = 'FACOAL120'
model.reactions.get_by_id('FACOAL120').add_metabolites({'yli_M01723_c': 1.0, 'ddca_c': -1.0})
```

In [71]:

```
for r in model.metabolites.get_by_id('dccoa_c').reactions:
    print(r.id, r.reaction)
print()
for r in model.metabolites.get_by_id('dccoa_m').reactions:
    print(r.id, r.reaction)
print()
for r in model.metabolites.get_by_id('dccoa_r').reactions:
    print(r.id, r.reaction)
```

```
yli_R0335 0.02 dccoa_c + 0.06 ddcacoa_c + dhap_c + 0.27 pmtcoa_c + 0.05 stcoa_c + 0.1 tdcoa_c + 0.17 yli_M04594_c + 0.09 yli_M04625_c + 0.24 yli_M04626_c --> 0.01 adhap_hs_c + coa_c
yli_R0307 0.02 dccoa_c + 0.06 ddcacoa_c + glyc3p_c + 0.27 pmtcoa_c + 0.05 stcoa_c + 0.1 tdcoa_c + 0.17 yli_M04594_c + 0.09 yli_M04625_c + 0.24 yli_M04626_c --> 0.01 1ag3p_SC_c + coa_c
yli_R0129 dccoa_c + 3.0 h_c + malcoa_c + 2.0 nadph_c --> co2_c + coa_c + ddcacoa_c + h2o_c + 2.0 nadp_c
yli_R0308 0.01 1ag3p_SC_c + 0.02 dccoa_c + 0.06 ddcacoa_c + 0.27 pmtcoa_c + 0.05 stcoa_c + 0.1 tdcoa_c + 0.17 yli_M04594_c + 0.09 yli_M04625_c + 0.24 yli_M04626_c --> coa_c + 0.01 pa_EC_c
yli_R0126 3.0 h_c + malcoa_c + 2.0 nadph_c + occoa_c --> co2_c + coa_c + dccoa_c + h2o_c + 2.0 nadp_c
yli_R0319 0.02 dccoa_c + 0.06 ddcacoa_c + 0.27 pmtcoa_c + 0.05 stcoa_c + 0.1 tdcoa_c + 0.01 yli_M02277_c + 0.17 yli_M04594_c + 0.09 yli_M04625_c + 0.24 yli_M04626_c --> coa_c + 0.01 pchol_cho_c

yli_R0233 atp_m + coa_m + dca_m <=> amp_m + dccoa_m + ppi_m
yli_R0212 dccoa_m + o2_m --> dc2coa_m + h2o2_m
PTE10m dccoa_m + h2o_m --> coa_m + dca_m + h_m

yli_R1402 0.02 dccoa_r + 0.06 ddcacoa_r + 0.27 pmtcoa_r + 0.05 stcoa_r + 0.1 tdcoa_r + 0.01 yli_M02277_r + 0.17 yli_M04594_r + 0.09 yli_M04625_r + 0.24 yli_M04626_r --> coa_r + 0.01 pchol_cho_r
yli_R1408 0.02 dccoa_r + 0.06 ddcacoa_r + dhap_r + 0.27 pmtcoa_r + 0.05 stcoa_r + 0.1 tdcoa_r + 0.17 yli_M04594_r + 0.09 yli_M04625_r + 0.24 yli_M04626_r --> 0.01 adhap_hs_r + coa_r
yli_R1392 0.01 1ag3p_SC_r + 0.02 dccoa_r + 0.06 ddcacoa_r + 0.27 pmtcoa_r + 0.05 stcoa_r + 0.1 tdcoa_r + 0.17 yli_M04594_r + 0.09 yli_M04625_r + 0.24 yli_M04626_r --> coa_r + 0.01 pa_EC_r
yli_R1391 0.02 dccoa_r + 0.06 ddcacoa_r + glyc3p_r + 0.27 pmtcoa_r + 0.05 stcoa_r + 0.1 tdcoa_r + 0.17 yli_M04594_r + 0.09 yli_M04625_r + 0.24 yli_M04626_r --> 0.01 1ag3p_SC_r + coa_r
```

In [72]:

```
for r in model.metabolites.get_by_id('dcacoa_c').reactions:
    print(r.id, r.reaction)
print()
for r in model.metabolites.get_by_id('dcacoa_m').reactions:
    print(r.id, r.reaction)
print()
for r in model.metabolites.get_by_id('dcacoa_x').reactions:
    print(r.id, r.reaction)
```

```
GAT2_SC 0.02 dcacoa_c + 0.06 ddcacoa_c + dhap_c + 0.17 hdcoa_c + 0.09 ocdycacoa_c + 0.24 odecoa_c + 0.27 pmtcoa_c + 0.05 stcoa_c + 0.1 tdcoa_c --> 0.01 1agly3p_SC_c + coa_c
LPCAT_SC 0.01 1agpc_SC_c + 0.02 dcacoa_c + 0.06 ddcacoa_c + 0.17 hdcoa_c + 0.09 ocdycacoa_c + 0.24 odecoa_c + 0.27 pmtcoa_c + 0.05 stcoa_c + 0.1 tdcoa_c --> coa_c + 0.01 pc_SC_c
FACOAE100 dcacoa_c + h2o_c --> coa_c + dca_c + h_c
FAS120COA dcacoa_c + 3.0 h_c + malcoa_c + 2.0 nadph_c --> co2_c + coa_c + ddcacoa_c + h2o_c + 2.0 nadp_c
AGAT_SC 0.01 1ag3p_SC_c + 0.02 dcacoa_c + 0.06 ddcacoa_c + 0.17 hdcoa_c + 0.09 ocdycacoa_c + 0.24 odecoa_c + 0.27 pmtcoa_c + 0.05 stcoa_c + 0.1 tdcoa_c --> coa_c + 0.01 pa_SC_c
FAS100COA 3.0 h_c + malcoa_c + 2.0 nadph_c + occoa_c --> co2_c + coa_c + dcacoa_c + h2o_c + 2.0 nadp_c
FACOAL100 atp_c + coa_c + dca_c <=> amp_c + dcacoa_c + ppi_c
TRIGS_SC 0.01 12dgr_SC_c + 0.02 dcacoa_c + 0.06 ddcacoa_c + 0.17 hdcoa_c + 0.09 ocdycacoa_c + 0.24 odecoa_c + 0.27 pmtcoa_c + 0.05 stcoa_c + 0.1 tdcoa_c --> coa_c + 0.01 triglyc_SC_c
GAT1_SC 0.02 dcacoa_c + 0.06 ddcacoa_c + glyc3p_c + 0.17 hdcoa_c + 0.09 ocdycacoa_c + 0.24 odecoa_c + 0.27 pmtcoa_c + 0.05 stcoa_c + 0.1 tdcoa_c --> 0.01 1ag3p_SC_c + coa_c
FACOAL100t2pp atp_c + coa_c + dca_p + h_p --> amp_c + dcacoa_c + h_c + ppi_c

ACOAR4m dc2coa_m + h_m + nadph_m --> dcacoa_m + nadp_m
ACACT5m accoa_m + dcacoa_m <-- 3oddcoa_m + coa_m
ACOAD4m dcacoa_m + fad_m --> dc2coa_m + fadh2_m

ACACT5p 3oddcoa_x + coa_x --> accoa_x + dcacoa_x
ACOAO4p dcacoa_x + o2_x --> dc2coa_x + h2o2_x
PTE10x dcacoa_x + h2o_x --> coa_x + dca_x + h_x
FACOAL100p atp_x + coa_x + dca_x <=> amp_x + dcacoa_x + ppi_x
ACOAD4p dcacoa_x + fad_x --> dc2coa_x + fadh2_x
```

In [73]:

```
# replace dccoa with dcacoa
model.reactions.get_by_id('PTE10m').add_metabolites({'dccoa_m': 1.0, 'dcacoa_m': -1.0})
```

In [74]:

```
for r in sorted(model.reactions, key=lambda x: x.id):
    if r.id.startswith('PTE') and r.id.endswith('m'):
        r.gene_reaction_rule = '14585'
```

Acyl-CoA synthetase  
11167 cyto 11, cyto\_nucl 8, mito 7, nucl 3, plas 3 K01897: ACSL, fadD; long-chain acyl-CoA synthetase VYP *15745 cyto\_nucl 11.333, nucl 11, cyto 7.5, cyto\_mito 6.332, extr 4, mito 3.5 KOG1256: Long-chain acyl-CoA synthetases (AMP-forming) VDS*  
15746 cyto 13, mito 5, cysk 4, mito\_nucl 4 KOG1180: Acyl-CoA synthetase EPY *15748 cyto 23, mito 3 K01897: ACSL, fadD; long-chain acyl-CoA synthetase VYP*  
15745 and 15746 look like splitted genes -> 899170 SigP cyto 15.5, cyto\_mito 9, nucl 7, pero 2, mito 1.5, cysk 1 VDS\*

12555 cyto 15.5, cyto\_nucl 11.5, pero 6, nucl 4.5 K01897: ACSL, fadD; long-chain acyl-CoA synthetase AKL *15900 cyto 13, pero 11, cyto\_nucl 8.5 K01896: ACSM; medium-chain acyl-CoA synthetase AKL*  
9912 extr 12, plas 8, pero 4, mito 1, cyto 1, E.R. 1, cyto\_mito 1 K08745: SLC27A1\_4, FATP1, FATP4; solute carrier family 27 (fatty acid transporter), member 1/4, Very long-chain acyl-CoA synthetase/fatty acid transporter
VRL\*

12538 mito 18.5, cyto\_mito 12.5, cyto 5.5 KOG1256: Long-chain acyl-CoA synthetases (AMP-forming) GEQ *12272 mito 27 KOG1176: Acyl-CoA synthetase, blast to acetate/butyrate-CoA ligase, paperblast to 3-(methylthio)propionyl-CoA ligase MSQ*

15306 extr 20, cyto 4, mito 3 SUPERFAMILY::SSF56801, blast to acetyl-CoA synthetase-like protein WLQ\*

12555 has fitness defect in oleic acid, and less defect in ricinoleic acid  
15746 has fitness defect in ricinoleic acid, but not in oleic acid

In [75]:

```
temp = ['11167','15745','15746','15748','12555','12538','15900','9912','12272','15306']
display(Annotation.loc[temp])
Show_Data(temp)
```

|  | Combined Annotations | Signal P | Sc288c Orthologs | Human Orthologs | Sc288 Best Hit | Human Blast | Essential | WolfPSort | C Terminal |
| --- | --- | --- | --- | --- | --- | --- | --- | --- | --- |
| RTO4\_ID |  |  |  |  |  |  |  |  |  |
| 11167 | K01897: ACSL, fadD; long-chain acyl-CoA synthe... |  | FAA3,FAA4,FAA1 | ACSL3,ACSL4 | FAA1 | ACSL4 | Not Essential | cyto 11, cyto\_nucl 8, mito 7, nucl 3, plas 3 | VYP\* |
| 15745 | KOG1256: Long-chain acyl-CoA synthetases (AMP-... |  |  |  |  |  | Not Essential | cyto\_nucl 11.333, nucl 11, cyto 7.5, cyto\_mito... | VDS\* |
| 15746 | KOG1180: Acyl-CoA synthetase |  |  |  | FAA1 | ACSL1 | Not Essential | cyto 13, mito 5, cysk 4, mito\_nucl 4 | EPY\* |
| 15748 | K01897: ACSL, fadD; long-chain acyl-CoA synthe... |  | FAA3,FAA4,FAA1 | ACSL3,ACSL4 | FAA1 | ACSL4 | Not Essential | cyto 23, mito 3 | VYP\* |
| 12555 | K01897: ACSL, fadD; long-chain acyl-CoA synthe... |  | FAA2 | ACSL1,ACSL5,ACSL6,CTB-127M13.1 | FAA2 | ACSL1 | Not Essential | cyto 15.5, cyto\_nucl 11.5, pero 6, nucl 4.5 | AKL\* |
| 12538 | KOG1256: Long-chain acyl-CoA synthetases (AMP-... |  | FAA2 | ACSL1,ACSL5,ACSL6,CTB-127M13.1 | FAA2 | ACSL5 | Not Essential | mito 18.5, cyto\_mito 12.5, cyto 5.5 | GEQ\* |
| 15900 | K01896: ACSM; medium-chain acyl-CoA synthetase |  |  | ACSM1,ACSM2A,ACSM2B,ACSM3,ACSM4,ACSM5 | ACS2 | ACSM3 | Not Essential | cyto 13, pero 11, cyto\_nucl 8.5 | AKL\* |
| 9912 | K08745: SLC27A1\_4, FATP1, FATP4; solute carrie... | S | FAT1 | SLC27A1,SLC27A4,SLC27A2,SLC27A5,SLC27A6 | FAT1 | SLC27 | Not Essential | extr 12, plas 8, pero 4, mito 1, cyto 1, E.R. ... | VRL\* |
| 12272 | KOG1176: Acyl-CoA synthetase |  |  |  |  |  | Not Essential | mito 27 | MSQ\* |
| 15306 | SUPERFAMILY::SSF56801 | S |  |  |  |  | Not Essential | extr 20, cyto 4, mito 3 | WLQ\* |

| strain | WT | | | | | | | | | | | | | | | | |
| --- | --- | --- | --- | --- | --- | --- | --- | --- | --- | --- | --- | --- | --- | --- | --- | --- | --- |
| condition | G\_MM | C\_MM | G\_SD | | GX\_SD | | | X\_SD | | A\_SD | | C\_SD | | MM\_CN120 | | MM\_CN5 | Diversity\_Sample |
| phase | exp | exp | exp | stat | exp | trans | stat | exp | stat | exp | stat | exp | stat | exp | stat | exp | exp |
| proteinId | Set1 | Set1 | Set2 | Set2 | Set2 | Set2 | Set2 | Set2 | Set2 | Set2 | Set2 | Set2 | Set2 | Set3 | Set3 | Set3 | Set3 |
| 11167 | 5.9621 | 7.09987 | 4.84994 | 6.05118 | 4.80079 | 5.32025 | 5.45006 | 5.13851 | 5.54572 | 6.10497 | 6.08465 | 7.84655 | 6.74645 | 5.27842 | 5.5176 | 6.01611 | 5.95804 |
| 15745 | 6.9564 | 6.06286 | 5.63402 | 3.39281 | 5.80044 | 3.44405 | 3.8522 | 5.46955 | 4.14345 | 6.26085 | 4.35092 | 5.6221 | 5.24879 | 7.506 | 6.53542 | 6.97181 | 5.85927 |
| 15746 | 6.17513 | 5.00871 | 5.35736 | 7.01893 | 5.22708 | 6.1675 | 6.49496 | 5.4928 | 6.05738 | 5.80054 | 6.30284 | 4.90705 | 4.68716 | 5.61671 | 6.11376 | 4.26197 | 4.28049 |
| 15748 | 7.66396 | 6.19238 | 6.48567 | 8.82502 | 6.43027 | 7.53242 | 8.15709 | 6.40942 | 7.66299 | 6.28743 | 8.0696 | 7.01832 | 7.07566 | 7.78243 | 7.12869 | 7.43588 | 6.18399 |
| 12555 | 6.4532 | 6.51166 | 5.43327 | 5.78233 | 5.42694 | 5.52551 | 5.26328 | 5.25738 | 6.1227 | 6.15634 | 6.45249 | 4.76408 | 5.10246 | 6.78726 | 5.6908 | 6.00758 | 6.18519 |
| 12538 | 5.1932 | 5.55322 | 4.16034 | 3.68415 | 4.67797 | 4.37239 | 4.52657 | 3.91004 | 4.36289 | 4.96869 | 3.65995 | 6.54618 | 6.53583 | 5.10212 | 4.96231 | 5.5082 | 5.45748 |
| 15900 | 4.89589 | 6.00099 | 6.46859 | 5.98835 | 6.37204 | 5.19183 | 7.41643 | 6.18244 | 4.59866 | 4.89713 | 4.91078 | 4.42504 | 3.57437 | 3.85314 | 3.9658 | 4.41517 | 3.66558 |
| 9912 | 5.93073 | 5.40845 | 5.89641 | 6.2248 | 5.87895 | 5.80068 | 5.93113 | 5.50431 | 5.91601 | 5.5566 | 6.22915 | 5.26476 | 4.97236 | 5.88904 | 5.91406 | 5.56329 | 5.5721 |
| 12272 | 7.16396 | 6.06768 | 9.30378 | 7.71896 | 9.27654 | 9.15473 | 8.82481 | 8.04888 | 7.04121 | 7.27369 | 6.71586 | 5.31259 | 3.33195 | 7.049 | 7.87296 | 6.5763 | 7.19854 |
| 15306 | 5.25329 | 5.66891 | 5.70489 | 4.7986 | 5.65928 | 5.0391 | 4.75814 | 5.63818 | 4.57786 | 5.39485 | 5.01639 | 7.11439 | 6.07255 | 5.61175 | 5.09153 | 5.61606 | 5.41798 |

| strain | WT | | | | | | | | | | |
| --- | --- | --- | --- | --- | --- | --- | --- | --- | --- | --- | --- |
| condition | G\_SD | | GX\_SD | | | X\_SD | | A\_SD | | C\_SD | |
| proteinId | exp | stat | exp | trans | stat | exp | stat | exp | stat | exp | stat |
| 11167 | 3.85307 | 9.08389 | 3.2467 | 6.1521 | 6.18454 | 6.85565 | 7.09515 | 9.27911 | 10.1393 | 24.0002 | 18.9346 |
| 15746 | 1.36121 | 2.67347 | 1.42772 | 3.34468 | 4.9162 | 1.58181 | 3.36504 | 1.93098 | 3.23872 | 4.05097 | 4.57385 |
| 15748 | 11.1351 | 19.2 | 10.3844 | 22.1379 | 22.4536 | 9.62578 | 17.1545 | 9.47102 | 17.0241 | 22.1999 | 22.6504 |
| 12555 | 5.37108 | 12.3435 | 4.25136 | 12.0863 | 10.9404 | 7.12026 | 11.2197 | 12.1786 | 13.0103 | 17.5464 | 11.1295 |
| 12538 | 0 | 0.335899 | 0 | 0.189313 | 0 | 0 | 0.393854 | 0.576179 | 1.338 | 0.439202 | 0.221651 |
| 15900 | 3.25523 | 1.45139 | 3.2441 | 2.98097 | 4.38989 | 4.30749 | 5.07897 | 2.12175 | 1.72318 | 2.54453 | 1.75055 |
| 9912 | 4.40189 | 9.06281 | 3.66274 | 8.91569 | 10.3772 | 5.86924 | 6.49892 | 5.02317 | 5.93995 | 4.96027 | 4.99924 |
| 12272 | 9.63519 | 8.57386 | 8.57174 | 10.4172 | 9.6036 | 10.1791 | 7.89223 | 2.51425 | 3.24356 | 3.19408 | 1.5411 |
| 15306 | 3.85368 | 2.72931 | 4.27094 | 4.27685 | 2.88504 | 3.90546 | 3.70142 | 2.51217 | 4.59471 | 7.49028 | 8.66921 |

|  | Glucose | Xylose | Arabinose | Acetate | Coumarate | Ferulate | YNB Oleic Acid | YNB Ricinoleic Acid | YNB Glucose | YNB Gluc DOC | YPD |
| --- | --- | --- | --- | --- | --- | --- | --- | --- | --- | --- | --- |
| proteinId |  |  |  |  |  |  |  |  |  |  |  |
| 11167 | 0.0946003 | -0.123058 | -0.258405 | -0.0485982 | -0.0949716 | 0.12414 | 0.437731 | 0.018182 | -0.147964 | -0.0276757 | -0.200199 |
| 15745 | -0.1045 | -0.0341638 | 0.065697 | -0.0468312 | 0.0131684 | -0.155592 | -0.0930866 | -0.0553649 | -0.322587 | -0.129447 | 0.125032 |
| 15746 | -0.292855 | -0.044816 | -0.192011 | -0.333648 | -0.131629 | 0.0496685 | 0.183731 | -1.1449 | 0.233525 | 0.530271 | 1.0953 |
| 15748 | -0.455473 | 0.172527 | -0.163183 | -0.136613 | -0.855098 | -0.410175 | -0.256403 | -0.113934 | -1.02932 | -0.810487 | 0.0346502 |
| 12555 | -0.311448 | -0.162485 | -0.131826 | -0.274268 | -0.854248 | -0.027038 | -1.52861 | -0.659443 | -0.300839 | -0.371389 | -0.434224 |
| 12538 | -0.0228742 | -0.00284459 | 0.0444012 | 0.0245939 | 0.0476792 | -0.0249695 | 0.0981869 | 0.240673 | 0.0573442 | 0.0435764 | 0.0668027 |
| 15900 | -0.402251 | 0.0381164 | -0.120976 | -0.323788 | -0.290967 | -0.292245 | 0.0775032 | 0.423076 | 0.367482 | -0.304034 | -0.394707 |
| 9912 | 0.0677425 | -0.270103 | -0.302936 | -0.051684 | -0.404229 | 0.0682701 | -0.0630695 | -0.272301 | -0.340334 | -0.0743227 | -0.0288945 |
| 12272 | -0.197686 | -0.196943 | -0.00208274 | 0.0746683 | 0.025135 | 0.0984501 | -0.335589 | -0.358104 | -0.0593179 | -0.0656978 | 0.0740662 |
| 15306 | -0.366886 | -0.531324 | -1.04962 | -0.303438 | -0.887738 | -0.433277 | 0.342058 | 0.387281 | 0.30455 | -0.228431 | -1.0505 |

In [76]:

```
for r in sorted(model.reactions, key=lambda x: x.id):
    if r.id.startswith('FACOAL'):
        print(r.id, r.reaction, r.gene_reaction_rule)
```

```
FACOAL100 atp_c + coa_c + dca_c <=> amp_c + dcacoa_c + ppi_c 11167 or 12538 or 12555 or 15748
FACOAL100p atp_x + coa_x + dca_x <=> amp_x + dcacoa_x + ppi_x 11167 or 12538 or 12555 or 15748
FACOAL100t2pp atp_c + coa_c + dca_p + h_p --> amp_c + dcacoa_c + h_c + ppi_c 11833 or 13700 or 14802 or 15900
FACOAL120 atp_c + coa_c + ddca_c <=> amp_c + ddcacoa_c + ppi_c 11167 or 12538 or 12555 or 15748
FACOAL120p atp_x + coa_x + ddca_x <=> amp_x + ddcacoa_x + ppi_x 11167 or 12538 or 12555 or 15748
FACOAL120t2pp atp_c + coa_c + ddca_p + h_p --> amp_c + ddcacoa_c + h_c + ppi_c 11833 or 13700 or 14802 or 15900
FACOAL140 atp_c + coa_c + ttdca_c <=> amp_c + ppi_c + tdcoa_c 11167 or 12538 or 12555 or 15748
FACOAL140p atp_x + coa_x + ttdca_x <=> amp_x + ppi_x + tdcoa_x 11167 or 12538 or 12555 or 15748
FACOAL140t2pp atp_c + coa_c + h_p + ttdca_p --> amp_c + h_c + ppi_c + tdcoa_c 11833 or 13700 or 14802 or 15900
FACOAL141 atp_c + coa_c + ttdcea_c <=> amp_c + ppi_c + tdecoa_c 11167 or 12538 or 12555 or 15748
FACOAL141p atp_x + coa_x + ttdcea_x <=> amp_x + ppi_x + tdecoa_x 11167 or 12538 or 12555 or 15748
FACOAL141t2pp atp_c + coa_c + h_p + ttdcea_p --> amp_c + h_c + ppi_c + tdecoa_c 11833 or 13700 or 14802
FACOAL150 atp_c + coa_c + ptdca_c <=> amp_c + ppi_c + ptdcacoa_c 11167 or 12538 or 12555 or 15748
FACOAL160 atp_c + coa_c + hdca_c <=> amp_c + pmtcoa_c + ppi_c 11167 or 12538 or 12555 or 15748
FACOAL160p atp_x + coa_x + hdca_x <=> amp_x + pmtcoa_x + ppi_x 11167 or 12538 or 12555 or 15748
FACOAL160t2pp atp_c + coa_c + h_p + hdca_p --> amp_c + h_c + pmtcoa_c + ppi_c 11833 or 13700 or 14802 or 15900
FACOAL161 atp_c + coa_c + hdcea_c <=> amp_c + hdcoa_c + ppi_c 11167 or 12538 or 12555 or 15748
FACOAL161p atp_x + coa_x + hdcea_x <=> amp_x + hdcoa_x + ppi_x 11167 or 12538 or 12555 or 15748
FACOAL161t2pp atp_c + coa_c + h_p + hdcea_p --> amp_c + h_c + hdcoa_c + ppi_c 11833 or 13700 or 14802
FACOAL170 atp_c + coa_c + hpdca_c <=> amp_c + hpdcacoa_c + ppi_c 11167 or 12538 or 12555 or 15748
FACOAL180 atp_c + coa_c + ocdca_c <=> amp_c + ppi_c + stcoa_c 11167 or 12538 or 12555 or 15748
FACOAL180_1 atp_c + coa_c + ocdca_c --> amp_c + ocdccoa_c + ppi_c 11167 or 12538 or 12555 or 15748
FACOAL180t2pp atp_c + coa_c + h_p + ocdca_p --> amp_c + h_c + ppi_c + stcoa_c 11833 or 13700 or 14802
FACOAL181 atp_c + coa_c + ocdcea_c <=> amp_c + odecoa_c + ppi_c 11167 or 12538 or 12555 or 15748
FACOAL18111Z atp_c + coa_c + ocdcea_c --> amp_c + ocdcecoa_c + ppi_c 11167 or 12538 or 12555 or 15748
FACOAL1812 atp_c + coa_c + vacc_c <=> amp_c + ppi_c + vacccoa_c 12538 or 12555
FACOAL1813 atp_c + coa_c + elaid_c <=> amp_c + od2coa_c + ppi_c 12538 or 12555
FACOAL181_1 atp_c + coa_c + ocdce9a_c --> amp_c + ocdce9coa_c + ppi_c 11167 or 12538 or 12555 or 15748
FACOAL181t2pp atp_c + coa_c + h_p + ocdcea_p --> amp_c + h_c + odecoa_c + ppi_c 11833 or 13700 or 14802
FACOAL182 atp_c + coa_c + ocdcya_c <=> amp_c + ocdycacoa_c + ppi_c 11167 or 12538 or 12555 or 15748
FACOAL1821 atp_c + coa_c + lnlc_c <=> amp_c + lnlccoa_c + ppi_c 11167 or 12538 or 12555 or 15748
FACOAL1822 atp_c + coa_c + lneldc_c <=> amp_c + lneldccoa_c + ppi_c 12538 or 12555
FACOAL1831 atp_c + coa_c + lnlncg_c <=> amp_c + lnlncgcoa_c + ppi_c 11167 or 12538 or 12555 or 15748
FACOAL1832 atp_c + coa_c + lnlnca_c <=> amp_c + lnlncacoa_c + ppi_c 11167 or 12538 or 12555 or 15748
FACOAL184 atp_c + coa_c + strdnc_c <=> amp_c + ppi_c + strdnccoa_c 12538 or 12555
FACOAL191 atp_c + coa_c + prist_c --> amp_c + ppi_c + pristcoa_c 9912
FACOAL200 arach_c + atp_c + coa_c <=> amp_c + arachcoa_c + ppi_c 12538 or 12555
FACOAL203 atp_c + coa_c + dlnlcg_c <=> amp_c + dlnlcgcoa_c + ppi_c 12538 or 12555
FACOAL2042 atp_c + coa_c + eicostet_c <=> amp_c + eicostetcoa_c + ppi_c 12538 or 12555
FACOAL204_copy1 arachd_c + atp_c + coa_c <=> amp_c + arachdcoa_c + ppi_c 12538 or 12555
FACOAL204_copy2 arachd_c + atp_c + coa_c --> amp_c + arachdcoa_c + ppi_c 11167 or 12538 or 12555 or 15748
FACOAL205 atp_c + coa_c + tmndnc_c <=> amp_c + ppi_c + tmndnccoa_c 12538 or 12555
FACOAL206 atp_c + coa_c + phyt_c <=> amp_c + phytcoa_c + ppi_c 9912
FACOAL224 adrn_c + atp_c + coa_c <=> adrncoa_c + amp_c + ppi_c 12538 or 12555
FACOAL2251 atp_c + coa_c + dcsptn1_c <=> amp_c + dcsptn1coa_c + ppi_c 12538 or 12555
FACOAL2252 atp_c + clpnd_c + coa_c <=> amp_c + clpndcoa_c + ppi_c 12538 or 12555
FACOAL226_copy1 atp_c + coa_c + crvnc_c <=> amp_c + c226coa_c + ppi_c 12538 or 12555
FACOAL240 atp_c + coa_c + ttc_c <=> amp_c + ppi_c + ttccoa_c 12538 or 12555
FACOAL240p atp_x + coa_x + ttc_x <=> amp_x + ppi_x + ttccoa_x 9912
FACOAL241 atp_c + coa_c + nrvnc_c <=> amp_c + nrvnccoa_c + ppi_c 12538 or 12555
FACOAL244_1 atp_c + coa_c + tettet6_c <=> amp_c + ppi_c + tettet6coa_c 12538 or 12555
FACOAL245_1 atp_c + coa_c + tetpent6_c <=> amp_c + ppi_c + tetpent6coa_c 12538 or 12555
FACOAL245_2 atp_c + coa_c + tetpent3_c <=> amp_c + ppi_c + tetpent3coa_c 12538 or 12555
FACOAL246_1 atp_c + coa_c + tethex3_c <=> amp_c + ppi_c + tethex3coa_c 12538 or 12555
FACOAL260 atp_c + coa_c + hexc_c <=> amp_c + hexccoa_c + ppi_c 12538 or 12555
FACOAL260p atp_x + coa_x + hexc_x <=> amp_x + hexccoa_x + ppi_x 9912
FACOAL40im atp_m + but_m + coa_m --> amp_m + btcoa_m + ppi_m 15900
FACOAL60t2pp atp_c + coa_c + h_p + hxa_p --> amp_c + h_c + hxcoa_c + ppi_c 11833 or 13700 or 14802 or 15900
FACOAL80 atp_c + coa_c + octa_c --> amp_c + occoa_c + ppi_c 11167 or 12538 or 12555 or 15748
FACOAL80p atp_x + coa_x + octa_x <=> amp_x + occoa_x + ppi_x 11167 or 12538 or 12555 or 15748
FACOAL80t2pp atp_c + coa_c + h_p + octa_p --> amp_c + h_c + occoa_c + ppi_c 11833 or 13700 or 14802 or 15900
FACOAL_160_m atp_m + coa_m + hdca_m <=> amp_m + pmtcoa_m + ppi_m 11167 or 12538 or 12555 or 15748
```

In [77]:

```
for r in sorted(model.reactions, key=lambda x: x.id):
    if r.id.startswith('FACOAL') and 'c' in r.compartments:
        print(r.id, r.reaction, r.gene_reaction_rule)
```

```
FACOAL100 atp_c + coa_c + dca_c <=> amp_c + dcacoa_c + ppi_c 11167 or 12538 or 12555 or 15748
FACOAL100t2pp atp_c + coa_c + dca_p + h_p --> amp_c + dcacoa_c + h_c + ppi_c 11833 or 13700 or 14802 or 15900
FACOAL120 atp_c + coa_c + ddca_c <=> amp_c + ddcacoa_c + ppi_c 11167 or 12538 or 12555 or 15748
FACOAL120t2pp atp_c + coa_c + ddca_p + h_p --> amp_c + ddcacoa_c + h_c + ppi_c 11833 or 13700 or 14802 or 15900
FACOAL140 atp_c + coa_c + ttdca_c <=> amp_c + ppi_c + tdcoa_c 11167 or 12538 or 12555 or 15748
FACOAL140t2pp atp_c + coa_c + h_p + ttdca_p --> amp_c + h_c + ppi_c + tdcoa_c 11833 or 13700 or 14802 or 15900
FACOAL141 atp_c + coa_c + ttdcea_c <=> amp_c + ppi_c + tdecoa_c 11167 or 12538 or 12555 or 15748
FACOAL141t2pp atp_c + coa_c + h_p + ttdcea_p --> amp_c + h_c + ppi_c + tdecoa_c 11833 or 13700 or 14802
FACOAL150 atp_c + coa_c + ptdca_c <=> amp_c + ppi_c + ptdcacoa_c 11167 or 12538 or 12555 or 15748
FACOAL160 atp_c + coa_c + hdca_c <=> amp_c + pmtcoa_c + ppi_c 11167 or 12538 or 12555 or 15748
FACOAL160t2pp atp_c + coa_c + h_p + hdca_p --> amp_c + h_c + pmtcoa_c + ppi_c 11833 or 13700 or 14802 or 15900
FACOAL161 atp_c + coa_c + hdcea_c <=> amp_c + hdcoa_c + ppi_c 11167 or 12538 or 12555 or 15748
FACOAL161t2pp atp_c + coa_c + h_p + hdcea_p --> amp_c + h_c + hdcoa_c + ppi_c 11833 or 13700 or 14802
FACOAL170 atp_c + coa_c + hpdca_c <=> amp_c + hpdcacoa_c + ppi_c 11167 or 12538 or 12555 or 15748
FACOAL180 atp_c + coa_c + ocdca_c <=> amp_c + ppi_c + stcoa_c 11167 or 12538 or 12555 or 15748
FACOAL180_1 atp_c + coa_c + ocdca_c --> amp_c + ocdccoa_c + ppi_c 11167 or 12538 or 12555 or 15748
FACOAL180t2pp atp_c + coa_c + h_p + ocdca_p --> amp_c + h_c + ppi_c + stcoa_c 11833 or 13700 or 14802
FACOAL181 atp_c + coa_c + ocdcea_c <=> amp_c + odecoa_c + ppi_c 11167 or 12538 or 12555 or 15748
FACOAL18111Z atp_c + coa_c + ocdcea_c --> amp_c + ocdcecoa_c + ppi_c 11167 or 12538 or 12555 or 15748
FACOAL1812 atp_c + coa_c + vacc_c <=> amp_c + ppi_c + vacccoa_c 12538 or 12555
FACOAL1813 atp_c + coa_c + elaid_c <=> amp_c + od2coa_c + ppi_c 12538 or 12555
FACOAL181_1 atp_c + coa_c + ocdce9a_c --> amp_c + ocdce9coa_c + ppi_c 11167 or 12538 or 12555 or 15748
FACOAL181t2pp atp_c + coa_c + h_p + ocdcea_p --> amp_c + h_c + odecoa_c + ppi_c 11833 or 13700 or 14802
FACOAL182 atp_c + coa_c + ocdcya_c <=> amp_c + ocdycacoa_c + ppi_c 11167 or 12538 or 12555 or 15748
FACOAL1821 atp_c + coa_c + lnlc_c <=> amp_c + lnlccoa_c + ppi_c 11167 or 12538 or 12555 or 15748
FACOAL1822 atp_c + coa_c + lneldc_c <=> amp_c + lneldccoa_c + ppi_c 12538 or 12555
FACOAL1831 atp_c + coa_c + lnlncg_c <=> amp_c + lnlncgcoa_c + ppi_c 11167 or 12538 or 12555 or 15748
FACOAL1832 atp_c + coa_c + lnlnca_c <=> amp_c + lnlncacoa_c + ppi_c 11167 or 12538 or 12555 or 15748
FACOAL184 atp_c + coa_c + strdnc_c <=> amp_c + ppi_c + strdnccoa_c 12538 or 12555
FACOAL191 atp_c + coa_c + prist_c --> amp_c + ppi_c + pristcoa_c 9912
FACOAL200 arach_c + atp_c + coa_c <=> amp_c + arachcoa_c + ppi_c 12538 or 12555
FACOAL203 atp_c + coa_c + dlnlcg_c <=> amp_c + dlnlcgcoa_c + ppi_c 12538 or 12555
FACOAL2042 atp_c + coa_c + eicostet_c <=> amp_c + eicostetcoa_c + ppi_c 12538 or 12555
FACOAL204_copy1 arachd_c + atp_c + coa_c <=> amp_c + arachdcoa_c + ppi_c 12538 or 12555
FACOAL204_copy2 arachd_c + atp_c + coa_c --> amp_c + arachdcoa_c + ppi_c 11167 or 12538 or 12555 or 15748
FACOAL205 atp_c + coa_c + tmndnc_c <=> amp_c + ppi_c + tmndnccoa_c 12538 or 12555
FACOAL206 atp_c + coa_c + phyt_c <=> amp_c + phytcoa_c + ppi_c 9912
FACOAL224 adrn_c + atp_c + coa_c <=> adrncoa_c + amp_c + ppi_c 12538 or 12555
FACOAL2251 atp_c + coa_c + dcsptn1_c <=> amp_c + dcsptn1coa_c + ppi_c 12538 or 12555
FACOAL2252 atp_c + clpnd_c + coa_c <=> amp_c + clpndcoa_c + ppi_c 12538 or 12555
FACOAL226_copy1 atp_c + coa_c + crvnc_c <=> amp_c + c226coa_c + ppi_c 12538 or 12555
FACOAL240 atp_c + coa_c + ttc_c <=> amp_c + ppi_c + ttccoa_c 12538 or 12555
FACOAL241 atp_c + coa_c + nrvnc_c <=> amp_c + nrvnccoa_c + ppi_c 12538 or 12555
FACOAL244_1 atp_c + coa_c + tettet6_c <=> amp_c + ppi_c + tettet6coa_c 12538 or 12555
FACOAL245_1 atp_c + coa_c + tetpent6_c <=> amp_c + ppi_c + tetpent6coa_c 12538 or 12555
FACOAL245_2 atp_c + coa_c + tetpent3_c <=> amp_c + ppi_c + tetpent3coa_c 12538 or 12555
FACOAL246_1 atp_c + coa_c + tethex3_c <=> amp_c + ppi_c + tethex3coa_c 12538 or 12555
FACOAL260 atp_c + coa_c + hexc_c <=> amp_c + hexccoa_c + ppi_c 12538 or 12555
FACOAL60t2pp atp_c + coa_c + h_p + hxa_p --> amp_c + h_c + hxcoa_c + ppi_c 11833 or 13700 or 14802 or 15900
FACOAL80 atp_c + coa_c + octa_c --> amp_c + occoa_c + ppi_c 11167 or 12538 or 12555 or 15748
FACOAL80t2pp atp_c + coa_c + h_p + octa_p --> amp_c + h_c + occoa_c + ppi_c 11833 or 13700 or 14802 or 15900
```

In [78]:

```
for r in sorted(model.reactions, key=lambda x: x.id):
    if r.id.startswith('FACOAL') and r.id.endswith('t2pp'):
        print(r.id, r.reaction, r.gene_reaction_rule)
```

```
FACOAL100t2pp atp_c + coa_c + dca_p + h_p --> amp_c + dcacoa_c + h_c + ppi_c 11833 or 13700 or 14802 or 15900
FACOAL120t2pp atp_c + coa_c + ddca_p + h_p --> amp_c + ddcacoa_c + h_c + ppi_c 11833 or 13700 or 14802 or 15900
FACOAL140t2pp atp_c + coa_c + h_p + ttdca_p --> amp_c + h_c + ppi_c + tdcoa_c 11833 or 13700 or 14802 or 15900
FACOAL141t2pp atp_c + coa_c + h_p + ttdcea_p --> amp_c + h_c + ppi_c + tdecoa_c 11833 or 13700 or 14802
FACOAL160t2pp atp_c + coa_c + h_p + hdca_p --> amp_c + h_c + pmtcoa_c + ppi_c 11833 or 13700 or 14802 or 15900
FACOAL161t2pp atp_c + coa_c + h_p + hdcea_p --> amp_c + h_c + hdcoa_c + ppi_c 11833 or 13700 or 14802
FACOAL180t2pp atp_c + coa_c + h_p + ocdca_p --> amp_c + h_c + ppi_c + stcoa_c 11833 or 13700 or 14802
FACOAL181t2pp atp_c + coa_c + h_p + ocdcea_p --> amp_c + h_c + odecoa_c + ppi_c 11833 or 13700 or 14802
FACOAL60t2pp atp_c + coa_c + h_p + hxa_p --> amp_c + h_c + hxcoa_c + ppi_c 11833 or 13700 or 14802 or 15900
FACOAL80t2pp atp_c + coa_c + h_p + octa_p --> amp_c + h_c + occoa_c + ppi_c 11833 or 13700 or 14802 or 15900
```

In [79]:

```
temp = [r.id for r in model.reactions if r.id.startswith('FACOAL') and r.id.endswith('t2pp')]
model.remove_reactions(temp, remove_orphans=True)
```

In [80]:

```
for r in sorted(model.reactions, key=lambda x: x.id):
    if r.id.startswith('FACOAL'):
        r.lower_bound = 0.0
```

FACOAL180\_1 ocdccoa\_c is stcoa\_c -> replace ocdccoa\_c with stcoa, and remove FACOAL180\_1  
FACOAL18111Z ocdcecoa\_c is 11Z and same as vacccoa\_c, but coming from ocdcea\_c 9Z  
-> replace ocdcecoa\_c with vacccoa\_c, and remove FACOAL18111Z,  
replace ocdcea\_c with vacc\_c in 11Z reactions  
FACOAL181\_1 ocdce9a\_c is the same as ocdcea\_c, and ocdce9coa\_c is the same as odecoa\_c  
-> replace ocdce9a\_c with ocdcea\_c and ocdce9coa\_c with odecoa\_c, and remove FACOAL181\_1, FACOAE1819Z  
COA1819ZD9DS is transferring electrons from O2 to focytb5\_c  
FACOAL204\_copy1 -> FACOAL204 and remove FACOAL204\_copy2  
FACOAL226\_copy1 -> FACOAL226

In [81]:

```
for r in model.metabolites.get_by_id('ocdccoa_c').reactions:
    print(r.id, r.reaction)
print()
for r in model.metabolites.get_by_id('ocdcecoa_c').reactions:
    print(r.id, r.reaction)
print()
for r in model.metabolites.get_by_id('vacccoa_c').reactions:
    print(r.id, r.reaction)
```

```
ACOADAGAT1819Z18111Z180 12dgr1819Z18111Z_c + ocdccoa_c --> coa_c + tag1819Z18111Z180_c
ACOADAGAT1601819Z180 12dgr1601819Z_c + ocdccoa_c --> coa_c + tag1601819Z180_c
ACOADAGAT18111Z1819Z180 12dgr18111Z1819Z_c + ocdccoa_c --> coa_c + tag18111Z1819Z180_c
FACOAL180_1 atp_c + coa_c + ocdca_c --> amp_c + ocdccoa_c + ppi_c
ACOADAGAT18111Z18111Z180 12dgr18111Z18111Z_c + ocdccoa_c --> coa_c + tag18111Z18111Z180_c
ACOADAGAT16018111Z180 12dgr16018111Z_c + ocdccoa_c --> coa_c + tag16018111Z180_c
ACOADAGAT1801819Z180 12dgr1801819Z_c + ocdccoa_c --> coa_c + tag1801819Z180_c
ACOADAGAT1819Z1819Z180 12dgr1819Z1819Z_c + ocdccoa_c --> coa_c + tag1819Z1819Z180_c

ACOADAGAT18111Z18111Z18111Z 12dgr18111Z18111Z_c + ocdcecoa_c --> coa_c + tag18111Z18111Z18111Z_c
ACOADAGAT1601819Z18111Z 12dgr1601819Z_c + ocdcecoa_c --> coa_c + tag1601819Z18111Z_c
AGPATCOA1819Z18111Z 1odec9eg3p_c + ocdcecoa_c --> coa_c + pa1819Z18111Z_c
ACOADAGAT18111Z1819Z18111Z 12dgr18111Z1819Z_c + ocdcecoa_c --> coa_c + tag18111Z1819Z18111Z_c
ACOADAGAT1801819Z18111Z 12dgr1801819Z_c + ocdcecoa_c --> coa_c + tag1801819Z18111Z_c
AGPATCOA16018111Z 1hdecg3p_c + ocdcecoa_c --> coa_c + pa16018111Z_c
ACOADAGAT16018111Z18111Z 12dgr16018111Z_c + ocdcecoa_c --> coa_c + tag16018111Z18111Z_c
ACOADAGAT1819Z18111Z18111Z 12dgr1819Z18111Z_c + ocdcecoa_c --> coa_c + tag1819Z18111Z18111Z_c
ACOADAGAT1819Z1819Z18111Z 12dgr1819Z1819Z_c + ocdcecoa_c --> coa_c + tag1819Z1819Z18111Z_c
AGPATCOA18111Z18111Z 1odec11eg3p_c + ocdcecoa_c --> coa_c + pa18111Z18111Z_c
FACOAL18111Z atp_c + coa_c + ocdcea_c --> amp_c + ocdcecoa_c + ppi_c

DESAT18_4 h_c + nadh_c + o2_c + stcoa_c --> 2.0 h2o_c + nad_c + vacccoa_c
FACOAL1812 atp_c + coa_c + vacc_c --> amp_c + ppi_c + vacccoa_c
DESAT18_7 h_c + nadh_c + o2_c + vacccoa_c --> 2.0 h2o_c + lneldccoa_c + nad_c
VACCCPT1 crn_c + vacccoa_c --> coa_c + vacccrn_c
```

In [82]:

```
for r in model.metabolites.get_by_id('ocdce9a_c').reactions:
    print(r.id, r.reaction)
print()
for r in model.metabolites.get_by_id('ocdcea_c').reactions:
    print(r.id, r.reaction)
print()
for r in model.metabolites.get_by_id('ocdce9coa_c').reactions:
    print(r.id, r.reaction)
print()
for r in model.metabolites.get_by_id('odecoa_c').reactions:
    print(r.id, r.reaction)
```

```
PLPSA21801819Z h2o_c + pe1801819Z_c --> 1agpe180_c + h_c + ocdce9a_c
FACOAE1819Z h2o_c + ocdce9coa_c --> coa_c + h_c + ocdce9a_c
MAGAH1819Z h2o_c + mag1819Z_c --> glyc_c + h_c + ocdce9a_c
FACOAL181_1 atp_c + coa_c + ocdce9a_c --> amp_c + ocdce9coa_c + ppi_c
PLPSA21819Z1819Z h2o_c + pe1819Z1819Z_c --> 1agpe1819Z_c + h_c + ocdce9a_c
PLPSA218111Z1819Z h2o_c + pe18111Z1819Z_c --> 1agpe18111Z_c + h_c + ocdce9a_c
LPLPS1AGPE1819Z 1agpe1819Z_c + h2o_c --> g3pe_c + h_c + ocdce9a_c

FA181ACPH h2o_c + octeACP_c <=> ACP_c + h_c + ocdcea_c
FATP2t na1_c + ocdcea_c <=> na1_e + ocdcea_e
FAS181 4.0 h_c + hdca_c + malcoa_c + 3.0 nadph_c + o2_c --> co2_c + coa_c + 3.0 h2o_c + 3.0 nadp_c + ocdcea_c
FECOSTESTH_SC 0.01 fecostest_SC_c + h2o_c --> 0.01 fecost_c + h_c + 0.02 hdca_c + 0.655 hdcea_c + 0.01 hexc_c + 0.03 ocdca_c + 0.27 ocdcea_c + 0.015 ttdca_c
TAGL_SC h2o_c + 0.01 triglyc_SC_c --> 0.01 12dgr_SC_c + 0.02 dca_c + 0.06 ddca_c + h_c + 0.27 hdca_c + 0.17 hdcea_c + 0.05 ocdca_c + 0.24 ocdcea_c + 0.09 ocdcya_c + 0.1 ttdca_c
AACPS5 ACP_c + atp_c + ocdcea_c --> amp_c + octeACP_c + ppi_c
ERGSTESTH_SC 0.01 ergstest_SC_c + h2o_c --> 0.01 ergst_c + h_c + 0.02 hdca_c + 0.655 hdcea_c + 0.01 hexc_c + 0.03 ocdca_c + 0.27 ocdcea_c + 0.015 ttdca_c
FACOAL18111Z atp_c + coa_c + ocdcea_c --> amp_c + ocdcecoa_c + ppi_c
MAGAH18111Z h2o_c + mag18111Z_c --> glyc_c + h_c + ocdcea_c
FACOAL181 atp_c + coa_c + ocdcea_c --> amp_c + odecoa_c + ppi_c
PLBPC_SC h2o_c + 0.005 pc_SC_c --> 0.02 dca_c + 0.06 ddca_c + 0.5 g3pc_c + h_c + 0.27 hdca_c + 0.17 hdcea_c + 0.05 ocdca_c + 0.24 ocdcea_c + 0.09 ocdcya_c + 0.1 ttdca_c
LANOSTESTH_SC h2o_c + 0.01 lanostest_SC_c --> h_c + 0.02 hdca_c + 0.655 hdcea_c + 0.01 hexc_c + 0.01 lanost_c + 0.03 ocdca_c + 0.27 ocdcea_c + 0.015 ttdca_c
LPLPS1AGPE18111Z 1agpe18111Z_c + h2o_c --> g3pe_c + h_c + ocdcea_c
ZYMSTESTH_SC h2o_c + 0.01 zymstest_SC_c --> h_c + 0.02 hdca_c + 0.655 hdcea_c + 0.01 hexc_c + 0.03 ocdca_c + 0.27 ocdcea_c + 0.015 ttdca_c + 0.01 zymst_c
FACOAE181 h2o_c + odecoa_c --> coa_c + h_c + ocdcea_c
EPISTESTH_SC 0.01 epistest_SC_c + h2o_c --> 0.01 epist_c + h_c + 0.02 hdca_c + 0.655 hdcea_c + 0.01 hexc_c + 0.03 ocdca_c + 0.27 ocdcea_c + 0.015 ttdca_c

1AGPEAT1801819Z 1agpe180_c + ocdce9coa_c --> coa_c + pe1801819Z_c
ACOADAGAT1819Z18111Z1819Z 12dgr1819Z18111Z_c + ocdce9coa_c --> coa_c + tag1819Z18111Z1819Z_c
COA1819ZD9DS 2.0 focytb5_c + 2.0 h_c + o2_c + stcoa_c --> 2.0 ficytb5_c + 2.0 h2o_c + ocdce9coa_c
ACOADAGAT1801819Z1819Z 12dgr1801819Z_c + ocdce9coa_c --> coa_c + tag1801819Z1819Z_c
FACOAL181_1 atp_c + coa_c + ocdce9a_c --> amp_c + ocdce9coa_c + ppi_c
AGPATCOA18111Z1819Z 1odec11eg3p_c + ocdce9coa_c --> coa_c + pa18111Z1819Z_c
ACOADAGAT1819Z1819Z1819Z 12dgr1819Z1819Z_c + ocdce9coa_c --> coa_c + tag1819Z1819Z1819Z_c
1AGPEAT1819Z1819Z 1agpe1819Z_c + ocdce9coa_c --> coa_c + pe1819Z1819Z_c
AGPATCOA1601819Z 1hdecg3p_c + ocdce9coa_c --> coa_c + pa1601819Z_c
AGPATCOA1819Z1819Z 1odec9eg3p_c + ocdce9coa_c --> coa_c + pa1819Z1819Z_c
FACOAE1819Z h2o_c + ocdce9coa_c --> coa_c + h_c + ocdce9a_c
ACOADAGAT1601819Z1819Z 12dgr1601819Z_c + ocdce9coa_c --> coa_c + tag1601819Z1819Z_c
1AGPEAT18111Z1819Z 1agpe18111Z_c + ocdce9coa_c --> coa_c + pe18111Z1819Z_c
AGPATCOA1801819Z 1odecg3p_c + ocdce9coa_c --> coa_c + pa1801819Z_c
ACOADAGAT16018111Z1819Z 12dgr16018111Z_c + ocdce9coa_c --> coa_c + tag16018111Z1819Z_c
ACOADAGAT18111Z1819Z1819Z 12dgr18111Z1819Z_c + ocdce9coa_c --> coa_c + tag18111Z1819Z1819Z_c
ACOADAGAT18111Z18111Z1819Z 12dgr18111Z18111Z_c + ocdce9coa_c --> coa_c + tag18111Z18111Z1819Z_c

DESAT18 h_c + nadph_c + o2_c + stcoa_c --> 2.0 h2o_c + nadp_c + odecoa_c
LANOSTAT_SC 0.655 hdcoa_c + 0.01 hexccoa_c + 0.01 lanost_c + 0.27 odecoa_c + 0.02 pmtcoa_c + 0.03 stcoa_c + 0.015 tdcoa_c --> coa_c + 0.01 lanostest_SC_c
ERGSTAT_SC 0.01 ergst_c + 0.655 hdcoa_c + 0.01 hexccoa_c + 0.27 odecoa_c + 0.02 pmtcoa_c + 0.03 stcoa_c + 0.015 tdcoa_c --> coa_c + 0.01 ergstest_SC_c
GAT2_SC 0.02 dcacoa_c + 0.06 ddcacoa_c + dhap_c + 0.17 hdcoa_c + 0.09 ocdycacoa_c + 0.24 odecoa_c + 0.27 pmtcoa_c + 0.05 stcoa_c + 0.1 tdcoa_c --> 0.01 1agly3p_SC_c + coa_c
DESAT18_6 h_c + nadh_c + o2_c + odecoa_c --> 2.0 h2o_c + lneldccoa_c + nad_c
FA181COAabcp atp_x + h2o_x + odecoa_c --> adp_x + h_x + odecoa_x + pi_x
EPISTAT_SC 0.01 epist_c + 0.655 hdcoa_c + 0.01 hexccoa_c + 0.27 odecoa_c + 0.02 pmtcoa_c + 0.03 stcoa_c + 0.015 tdcoa_c --> coa_c + 0.01 epistest_SC_c
TRIGS_SC 0.01 12dgr_SC_c + 0.02 dcacoa_c + 0.06 ddcacoa_c + 0.17 hdcoa_c + 0.09 ocdycacoa_c + 0.24 odecoa_c + 0.27 pmtcoa_c + 0.05 stcoa_c + 0.1 tdcoa_c --> coa_c + 0.01 triglyc_SC_c
ZYMSTAT_SC 0.655 hdcoa_c + 0.01 hexccoa_c + 0.27 odecoa_c + 0.02 pmtcoa_c + 0.03 stcoa_c + 0.015 tdcoa_c + 0.01 zymst_c --> coa_c + 0.01 zymstest_SC_c
FACOAL181 atp_c + coa_c + ocdcea_c --> amp_c + odecoa_c + ppi_c
LPCAT_SC 0.01 1agpc_SC_c + 0.02 dcacoa_c + 0.06 ddcacoa_c + 0.17 hdcoa_c + 0.09 ocdycacoa_c + 0.24 odecoa_c + 0.27 pmtcoa_c + 0.05 stcoa_c + 0.1 tdcoa_c --> coa_c + 0.01 pc_SC_c
C181CPT1 crn_c + odecoa_c --> coa_c + odecrn_c
AGAT_SC 0.01 1ag3p_SC_c + 0.02 dcacoa_c + 0.06 ddcacoa_c + 0.17 hdcoa_c + 0.09 ocdycacoa_c + 0.24 odecoa_c + 0.27 pmtcoa_c + 0.05 stcoa_c + 0.1 tdcoa_c --> coa_c + 0.01 pa_SC_c
FACOAE181 h2o_c + odecoa_c --> coa_c + h_c + ocdcea_c
DESAT18_3 h_c + nadh_c + o2_c + stcoa_c --> 2.0 h2o_c + nad_c + odecoa_c
GAT1_SC 0.02 dcacoa_c + 0.06 ddcacoa_c + glyc3p_c + 0.17 hdcoa_c + 0.09 ocdycacoa_c + 0.24 odecoa_c + 0.27 pmtcoa_c + 0.05 stcoa_c + 0.1 tdcoa_c --> 0.01 1ag3p_SC_c + coa_c
FECOSTAT_SC 0.01 fecost_c + 0.655 hdcoa_c + 0.01 hexccoa_c + 0.27 odecoa_c + 0.02 pmtcoa_c + 0.03 stcoa_c + 0.015 tdcoa_c --> coa_c + 0.01 fecostest_SC_c
```

In [83]:

```
for r in model.metabolites.get_by_id('ocdccoa_c').reactions:
    r.add_metabolites({'ocdccoa_c': -r.get_coefficient('ocdccoa_c'), 'stcoa_c': r.get_coefficient('ocdccoa_c')})
model.remove_reactions(['FACOAL180_1'], remove_orphans=True)
for r in model.metabolites.get_by_id('ocdcecoa_c').reactions:
    r.add_metabolites({'ocdcecoa_c': -r.get_coefficient('ocdcecoa_c'), 'vacccoa_c': r.get_coefficient('ocdcecoa_c')})
model.remove_reactions(['FACOAL18111Z'], remove_orphans=True)
for r in model.metabolites.get_by_id('ocdcea_c').reactions:
    if '18111Z' in r.id:
        r.add_metabolites({'ocdcea_c': -r.get_coefficient('ocdcea_c'), 'vacc_c': r.get_coefficient('ocdcea_c')})
for r in model.metabolites.get_by_id('ocdce9a_c').reactions:
    r.add_metabolites({'ocdce9a_c': -r.get_coefficient('ocdce9a_c'), 'ocdcea_c': r.get_coefficient('ocdce9a_c')})
for r in model.metabolites.get_by_id('ocdce9coa_c').reactions:
    r.add_metabolites({'ocdce9coa_c': -r.get_coefficient('ocdce9coa_c'), 'odecoa_c': r.get_coefficient('ocdce9coa_c')})
model.remove_reactions(['FACOAL181_1','FACOAE1819Z'], remove_orphans=True)
model.reactions.get_by_id('FACOAL204_copy1').id = 'FACOAL204'
model.reactions.get_by_id('FACOAL226_copy1').id = 'FACOAL226'
model.remove_reactions(['FACOAL204_copy2'], remove_orphans=True)
```

In [84]:

```
for r in sorted(model.reactions, key=lambda x: x.id):
    if r.id.startswith('FACOAL') and 'c' in r.compartments:
        r.gene_reaction_rule = '11167 or 15746 or 15748'
```

In [85]:

```
for r in sorted(model.reactions, key=lambda x: x.id):
    if (not r.id.startswith('FACOAL')) and 'atp_c' in [m.id for m in r.reactants] and 'coa_c' in [m.id for m in r.reactants]:
        print(r.id, r.reaction, r.gene_reaction_rule)
```

```
AACOAT acac_c + atp_c + coa_c <=> aacoa_c + amp_c + ppi_c 15276
ACITL atp_c + cit_c + coa_c --> accoa_c + adp_c + oaa_c + pi_c CRv4_Au5_s5_g12182_t1 and 9726
ACS ac_c + atp_c + coa_c --> accoa_c + amp_c + ppi_c 14597
ACS2 atp_c + coa_c + ppa_c --> amp_c + ppcoa_c + ppi_c 14597
CACOAL atp_c + ca_c + coa_c --> amp_c + cacoa_c + h_c + ppi_c 11167 or 12538 or 12555 or 15748
CRNCAL2 atp_c + coa_c + crn_c --> adp_c + crncoa_c + pi_c 11833 or 13700
CRNDCAL2 atp_c + coa_c + crn__D_c --> adp_c + crnDcoa_c + pi_c 11833 or 13700
CTBTCAL2 atp_c + coa_c + ctbt_c --> adp_c + ctbtcoa_c + pi_c 11833 or 13700
PACOAL atp_c + coa_c + pa_c --> amp_c + pacoa_c + ppi_c 11167 or 12538 or 12555 or 15748
SUCOAS atp_c + coa_c + succ_c <=> adp_c + pi_c + succoa_c 15967 and 16144
yli_R0144 atp_c + coa_c + yli_M04636_c <=> amp_c + ppi_c + yli_M04635_c 11167 or 12538 or 12555 or 15748
yli_R0146 atp_c + coa_c + yli_M03299_c <=> amp_c + ppi_c + yli_M04594_c 11167 or 12538 or 12555 or 15748
yli_R0148 atp_c + coa_c + yli_M04622_c <=> amp_c + ppi_c + yli_M04626_c 11167 or 12538 or 12555 or 15748
yli_R0149 atp_c + coa_c + yli_M05526_c <=> amp_c + ppi_c + yli_M04625_c 11167 or 12538 or 12555 or 15748
yli_R0151 atp_c + coa_c + yli_M05672_c <=> amp_c + ppi_c + yli_M04597_c 11167 or 12538 or 12555 or 15748
```

In [86]:

```
# Fix ACITL GPR and remove 5Z fatty acid reactions and redundant yli reactions
model.reactions.get_by_id('ACITL').gene_reaction_rule = '9726'
# Pinolenic acid and Coniferonic acid only in iRC model
model.remove_reactions(['PACOAL','CACOAL'], remove_orphans=True)
model.remove_reactions(['yli_R0144','yli_R0146','yli_R0148','yli_R0149','yli_R0151'], remove_orphans=True)
```

Other Acyl-CoA synthetase  
14597 cyto 13, plas 5, mito 3, pero 2, mito\_nucl 2 K01895: ACSS, acs; acetyl-CoA synthetase SSE *-> OK  
15276 plas 9, pero 6, mito 5, cyto 5, cyto\_mito 5 K01907: AACS, acsA; acetoacetyl-CoA synthetase SKL* -> OK  
12122 nucl 11, cyto\_nucl 10, mito 9, cyto 7 K01641: E2.3.3.10; hydroxymethylglutaryl-CoA synthase HVA\* -> Fix

In [87]:

```
temp = ['14597','15276','12122']
display(Annotation.loc[temp])
Show_Data(temp)
```

|  | Combined Annotations | Signal P | Sc288c Orthologs | Human Orthologs | Sc288 Best Hit | Human Blast | Essential | WolfPSort | C Terminal |
| --- | --- | --- | --- | --- | --- | --- | --- | --- | --- |
| RTO4\_ID |  |  |  |  |  |  |  |  |  |
| 14597 | K01895: ACSS, acs; acetyl-CoA synthetase |  | ACS1,ACS2 | ACSS1,ACSS2 | ACS2 | ACSS1 | Not Essential | cyto 13, plas 5, mito 3, pero 2, mito\_nucl 2 | SSE\* |
| 15276 | K01907: AACS, acsA; acetoacetyl-CoA synthetase |  |  | AACS | ACS1 | AACS | Not Essential | plas 9, pero 6, mito 5, cyto 5, cyto\_mito 5 | SKL\* |
| 12122 | K01641: E2.3.3.10; hydroxymethylglutaryl-CoA s... |  | ERG13 | HMGCS1,HMGCS2 | ERG13 | HMGCS | Essential | nucl 11, cyto\_nucl 10, mito 9, cyto 7 | HVA\* |

| strain | WT | | | | | | | | | | | | | | | | |
| --- | --- | --- | --- | --- | --- | --- | --- | --- | --- | --- | --- | --- | --- | --- | --- | --- | --- |
| condition | G\_MM | C\_MM | G\_SD | | GX\_SD | | | X\_SD | | A\_SD | | C\_SD | | MM\_CN120 | | MM\_CN5 | Diversity\_Sample |
| phase | exp | exp | exp | stat | exp | trans | stat | exp | stat | exp | stat | exp | stat | exp | stat | exp | exp |
| proteinId | Set1 | Set1 | Set2 | Set2 | Set2 | Set2 | Set2 | Set2 | Set2 | Set2 | Set2 | Set2 | Set2 | Set3 | Set3 | Set3 | Set3 |
| 14597 | 8.42238 | 7.97378 | 8.51818 | 7.99019 | 8.64056 | 7.40482 | 7.62933 | 7.37675 | 8.46789 | 7.83928 | 8.54106 | 7.90993 | 8.02345 | 8.92605 | 8.10393 | 9.91014 | 9.38791 |
| 15276 | 5.29782 | 7.5276 | 6.02897 | 6.3398 | 5.8819 | 5.96372 | 5.9225 | 5.9897 | 5.36727 | 6.33017 | 5.89141 | 6.06136 | 5.30571 | 4.91543 | 4.54293 | 6.28014 | 5.10407 |
| 12122 | 7.27828 | 9.03224 | 5.77881 | 4.82011 | 5.92352 | 5.9146 | 5.80187 | 7.55064 | 5.42163 | 6.2539 | 4.92862 | 5.77986 | 5.90457 | 7.43531 | 6.61498 | 9.2174 | 8.65524 |

| strain | WT | | | | | | | | | | |
| --- | --- | --- | --- | --- | --- | --- | --- | --- | --- | --- | --- |
| condition | G\_SD | | GX\_SD | | | X\_SD | | A\_SD | | C\_SD | |
| proteinId | exp | stat | exp | trans | stat | exp | stat | exp | stat | exp | stat |
| 14597 | 33.4132 | 33.1333 | 28.0397 | 32.0427 | 29.6961 | 27.1912 | 30.3839 | 29.3511 | 35.2144 | 32.6888 | 37.6604 |
| 15276 | 11.0591 | 15.3254 | 10.9798 | 19.3402 | 20.029 | 18.4148 | 19.0534 | 17.1797 | 17.9891 | 29.9096 | 20.8835 |
| 12122 | 20.27 | 14.932 | 23.5961 | 12.0966 | 10.9676 | 19.539 | 14.988 | 15.2721 | 13.7782 | 21.5047 | 17.6543 |

|  | Glucose | Xylose | Arabinose | Acetate | Coumarate | Ferulate | YNB Oleic Acid | YNB Ricinoleic Acid | YNB Glucose | YNB Gluc DOC | YPD |
| --- | --- | --- | --- | --- | --- | --- | --- | --- | --- | --- | --- |
| proteinId |  |  |  |  |  |  |  |  |  |  |  |
| 14597 | 0.134527 | 0.0997527 | 0.107444 | -1.05713 | 0.0951078 | -0.0881028 | 0.151616 | 0.954851 | 0.0158946 | -0.116256 | 0.000665712 |
| 15276 | -0.192767 | 0.221437 | 0.0764749 | -0.176646 | 0.00381209 | -0.117964 | -0.0335189 | -0.260812 | -0.126874 | -0.0575812 | -0.262471 |

In [88]:

```
for r in sorted(model.genes.get_by_id('14597').reactions, key=lambda x: x.id):
    print(r.id, r.reaction, r.gene_reaction_rule)
print()
for r in sorted(model.genes.get_by_id('15276').reactions, key=lambda x: x.id):
    print(r.id, r.reaction, r.gene_reaction_rule)
print()
for r in sorted(model.genes.get_by_id('12122').reactions, key=lambda x: x.id):
    print(r.id, r.reaction, r.gene_reaction_rule)
```

```
ACS ac_c + atp_c + coa_c --> accoa_c + amp_c + ppi_c 14597
ACS2 atp_c + coa_c + ppa_c --> amp_c + ppcoa_c + ppi_c 14597
ACSm ac_m + atp_m + coa_m --> accoa_m + amp_m + ppi_m 14597

AACOAT acac_c + atp_c + coa_c <=> aacoa_c + amp_c + ppi_c 15276

HMGCOAS coa_c + h_c + hmgcoa_c <=> aacoa_c + accoa_c + h2o_c 12122
HMGCOASm coa_m + h_m + hmgcoa_m <=> aacoa_m + accoa_m + h2o_m 12122
MHGS aacoa_c + accoa_c + h2o_c --> coa_c + hmgcoa_c 12122
yli_R0771 aacoa_c + accoa_c + h2o_c --> coa_c + h_c + yli_M00348_c 12122
yli_R1457 aacoa_m + accoa_m + h2o_m --> coa_m + h_m + yli_M00348_m 12122
```

In [89]:

```
for r in sorted(model.metabolites.get_by_id('hmgcoa_c').reactions, key=lambda x: x.id):
    print(r.id, r.reaction, r.gene_reaction_rule)
print()
for r in sorted(model.metabolites.get_by_id('yli_M00348_c').reactions, key=lambda x: x.id):
    print(r.id, r.reaction, r.gene_reaction_rule)
print()
for r in sorted(model.metabolites.get_by_id('hmgcoa_m').reactions, key=lambda x: x.id):
    print(r.id, r.reaction, r.gene_reaction_rule)
print()
for r in sorted(model.metabolites.get_by_id('yli_M00348_m').reactions, key=lambda x: x.id):
    print(r.id, r.reaction, r.gene_reaction_rule)
print()
for r in sorted(model.metabolites.get_by_id('hmgcoa_x').reactions, key=lambda x: x.id):
    print(r.id, r.reaction, r.gene_reaction_rule)
print()
for r in sorted(model.metabolites.get_by_id('hmgcoa_r').reactions, key=lambda x: x.id):
    print(r.id, r.reaction, r.gene_reaction_rule)
```

```
HMGCOAR coa_c + mev__R_c + 2.0 nadp_c <=> 2.0 h_c + hmgcoa_c + 2.0 nadph_c 9574
HMGCOAS coa_c + h_c + hmgcoa_c <=> aacoa_c + accoa_c + h2o_c 12122
HMGL hmgcoa_c --> acac_c + accoa_c 15693
MHGS aacoa_c + accoa_c + h2o_c --> coa_c + hmgcoa_c 12122

yli_R0765 2.0 h_c + 2.0 nadph_c + yli_M00348_c --> coa_c + mev__R_c + 2.0 nadp_c 9574
yli_R0771 aacoa_c + accoa_c + h2o_c --> coa_c + h_c + yli_M00348_c 12122
yli_R0773 yli_M00348_c --> acac_c + accoa_c 15693

HMGCOASm coa_m + h_m + hmgcoa_m <=> aacoa_m + accoa_m + h2o_m 12122
HMGL_1 hmgcoa_m --> acac_m + accoa_m + h_m 15693
HMGLm hmgcoa_m --> acac_m + accoa_m 15693
MGCHrm 3mgcoa_m + h2o_m <=> hmgcoa_m 16128

yli_R1457 aacoa_m + accoa_m + h2o_m --> coa_m + h_m + yli_M00348_m 12122
yli_R1458 yli_M00348_m --> acac_m + accoa_m 15693

HMGCOARx 2.0 h_x + hmgcoa_x + 2.0 nadph_x --> coa_x + mev__R_x + 2.0 nadp_x 9574
HMGLx hmgcoa_x --> acac_x + accoa_x 15693

HMGCOARr 2.0 h_r + hmgcoa_r + 2.0 nadph_r --> coa_r + mev__R_r + 2.0 nadp_r 9574
```

In [90]:

```
model.remove_reactions(['HMGL_1','yli_R0765','yli_R0771','yli_R0773','yli_R1457','yli_R1458'], remove_orphans=True)
```

In [91]:

```
temp = ['11833','13700','14802','16129','16635']
display(Annotation.loc[temp])
Show_Data(temp)
```

|  | Combined Annotations | Signal P | Sc288c Orthologs | Human Orthologs | Sc288 Best Hit | Human Blast | Essential | WolfPSort | C Terminal |
| --- | --- | --- | --- | --- | --- | --- | --- | --- | --- |
| RTO4\_ID |  |  |  |  |  |  |  |  |  |
| 11833 | KOG1176: Acyl-CoA synthetase |  |  |  | PCS60 | ACSF2 | Not Essential | cyto 16, cyto\_nucl 11.5, nucl 5, pero 5 | AKL\* |
| 13700 | K01904: 4CL; 4-coumarate--CoA ligase |  |  |  | PCS60 | ACSM3 | Not Essential | cyto 16.5, cyto\_nucl 12.5, nucl 5.5, mito 3 | LPS\* |
| 14802 | K01904: 4CL; 4-coumarate--CoA ligase |  |  |  | PCS60 | ACSF2 | Not Essential | cyto 17.5, cyto\_nucl 9.5, plas 5, pero 2 | SKL\* |
| 16129 | KOG1176: Acyl-CoA synthetase |  |  |  | PCS60 | ACSF2 | Not Essential | nucl 6.5, cyto\_nucl 6.5, plas 6, cyto 5.5, mit... | AKL\* |
| 16635 | K01897: ACSL, fadD; long-chain acyl-CoA synthe... |  |  |  | PCS60 | ACSF2 | Not Essential | mito 18, cyto 5.5, cyto\_nucl 3.5, pero 3 | AKL\* |

| strain | WT | | | | | | | | | | | | | | | | |
| --- | --- | --- | --- | --- | --- | --- | --- | --- | --- | --- | --- | --- | --- | --- | --- | --- | --- |
| condition | G\_MM | C\_MM | G\_SD | | GX\_SD | | | X\_SD | | A\_SD | | C\_SD | | MM\_CN120 | | MM\_CN5 | Diversity\_Sample |
| phase | exp | exp | exp | stat | exp | trans | stat | exp | stat | exp | stat | exp | stat | exp | stat | exp | exp |
| proteinId | Set1 | Set1 | Set2 | Set2 | Set2 | Set2 | Set2 | Set2 | Set2 | Set2 | Set2 | Set2 | Set2 | Set3 | Set3 | Set3 | Set3 |
| 11833 | 2.46223 | 4.20737 | 3.19576 | 4.4752 | 3.20897 | 3.7118 | 3.10995 | 3.17637 | 4.55459 | 3.82578 | 4.68766 | 3.84813 | 3.23193 | 4.23124 | 3.29641 | 4.26261 | 3.40104 |
| 13700 | 5.74721 | 5.32873 | 7.4201 | 7.10001 | 7.2645 | 6.37234 | 6.23686 | 6.78221 | 5.65711 | 6.70406 | 6.02738 | 5.3426 | 5.03044 | 5.26247 | 5.06899 | 7.32532 | 6.07408 |
| 14802 | 6.19315 | 5.98462 | 6.74698 | 5.39926 | 6.62515 | 5.87436 | 5.80604 | 5.90673 | 6.0751 | 6.20344 | 6.0472 | 5.27798 | 5.42542 | 6.35048 | 5.71086 | 5.52788 | 6.21151 |
| 16129 | 2.39236 | 4.22282 | 3.55249 | 4.7968 | 3.39643 | 3.75488 | 3.95684 | 2.9184 | 4.91399 | 3.57913 | 5.22696 | 1.34886 | 1.27195 | 3.00063 | 2.09969 | 3.78102 | 3.0124 |
| 16635 | 4.27464 | 9.22331 | 3.95243 | 5.18043 | 4.38087 | 5.23868 | 5.00857 | 4.03567 | 4.42434 | 5.10762 | 4.80736 | 7.59196 | 6.55645 | 4.19255 | 3.54929 | 4.91759 | 3.73605 |

| strain | WT | | | | | | | | | | |
| --- | --- | --- | --- | --- | --- | --- | --- | --- | --- | --- | --- |
| condition | G\_SD | | GX\_SD | | | X\_SD | | A\_SD | | C\_SD | |
| proteinId | exp | stat | exp | trans | stat | exp | stat | exp | stat | exp | stat |
| 11833 | 0 | 0.883033 | 0 | 0 | 0 | 0 | 0 | 0 | 0 | 0.22091 | 0 |
| 13700 | 10.7633 | 12.1799 | 12.5986 | 15.4698 | 15.5193 | 17.2325 | 17.7115 | 14.2936 | 13.9775 | 13.4594 | 11.3454 |
| 14802 | 11.9014 | 16.5288 | 11.6078 | 11.8906 | 13.6084 | 12.3254 | 14.6507 | 11.2001 | 11.6792 | 14.5351 | 14.1431 |
| 16129 | 0 | 0 | 0 | 0 | 0 | 0 | 0 | 0 | 0 | 0.431248 | 0 |
| 16635 | 0.738459 | 4.50162 | 2.43018 | 6.30879 | 8.59084 | 4.91411 | 4.7286 | 3.66781 | 6.73056 | 29.9041 | 22.8617 |

|  | Glucose | Xylose | Arabinose | Acetate | Coumarate | Ferulate | YNB Oleic Acid | YNB Ricinoleic Acid | YNB Glucose | YNB Gluc DOC | YPD |
| --- | --- | --- | --- | --- | --- | --- | --- | --- | --- | --- | --- |
| proteinId |  |  |  |  |  |  |  |  |  |  |  |
| 11833 | 0.218588 | -0.139988 | 0.12494 | -0.131598 | -0.0323055 | 0.0393466 | 0.176377 | 0.128813 | 0.120682 | -0.0829543 | -0.133672 |
| 13700 | -0.107884 | -0.155441 | -0.25423 | -0.0388004 | 0.149475 | -0.0544939 | -0.0665896 | -0.16933 | -0.25386 | -0.32858 | -0.324718 |
| 14802 | 0.318433 | 0.250797 | 0.241559 | 0.171794 | -0.0119312 | 0.176682 | -0.1177 | -0.0917166 | 0.131314 | -0.0334729 | -0.125359 |
| 16129 | -0.223651 | -0.0133421 | 0.159221 | -0.114595 | -0.0296944 | -0.117031 | 0.0551636 | 0.0633251 | 0.415751 | 0.445395 | -0.195331 |
| 16635 | -0.386094 | 0.0748675 | -0.24649 | -0.872329 | -1.76568 | -3.60067 | -0.198188 | -0.0374127 | 0.311272 | 0.402185 | 0.87277 |

11833 Acyl-CoA synthetase, blast to 4-coumarate--CoA ligase AKL *13700 K01904: 4CL; 4-coumarate--CoA ligase LPS*  
14802 K01904: 4CL; 4-coumarate--CoA ligase SKL*, no reaction  
16129 KOG1176: Acyl-CoA synthetase, blast to 4-coumarate--CoA ligase / succinylbenzoate--CoA ligase AKL*  
16635 K01897: ACSL, fadD; long-chain acyl-CoA synthetase, blast to 4-coumarate--CoA ligase AKL16635 has fitness defect and is upregulated in coumarate and ferulate, while 11183, 13700, 14802 do/are not

In [92]:

```
for r in sorted(model.genes.get_by_id('11833').reactions, key=lambda x: x.id):
    print(r.id, r.reaction, r.gene_reaction_rule)
```

```
CRNCAL2 atp_c + coa_c + crn_c --> adp_c + crncoa_c + pi_c 11833 or 13700
CRNDCAL2 atp_c + coa_c + crn__D_c --> adp_c + crnDcoa_c + pi_c 11833 or 13700
CTBTCAL2 atp_c + coa_c + ctbt_c --> adp_c + ctbtcoa_c + pi_c 11833 or 13700
```

In [93]:

```
print(hsa2.reactions.get_by_id('4CMCOAS'))
print(hsa2.reactions.get_by_id('COUCOAFm'))
```

```
4CMCOAS: T4hcinnm_c + atp_c + coa_c --> amp_c + coucoa_c + ppi_c
COUCOAFm: T4hcinnm_m + atp_m + coa_m --> amp_m + coucoa_m + ppi_m
```

In [94]:

```
model.remove_reactions(['CRNCAL2','CRNDCAL2','CTBTCAL2'], remove_orphans=True)
r1 = hsa2.reactions.get_by_id('4CMCOAS').copy() # 4-Coumarate-coA ligase
r1.gene_reaction_rule = '11833 or 13700 or 14802 or 16129'
r2 = hsa2.reactions.get_by_id('COUCOAFm').copy() # P coumaroyl CoA formation
r2.gene_reaction_rule = '16635'
model.add_reactions([r1,r2])
```

15900 cyto 13, pero 11, cyto\_nucl 8.5 K01896: ACSM; medium-chain acyl-CoA synthetase AKL *12555 cyto 15.5, cyto\_nucl 11.5, pero 6, nucl 4.5 K01897: ACSL, fadD; long-chain acyl-CoA synthetase AKL*  
9912 extr 12, plas 8, pero 4, mito 1, cyto 1, E.R. 1, cyto\_mito 1 K08745: SLC27A1\_4, FATP1, FATP4; solute carrier family 27 (fatty acid transporter), member 1/4, Very long-chain acyl-CoA synthetase/fatty acid transporter
VRL\*

In [95]:

```
for r in sorted(model.reactions, key=lambda x: x.id):
    if r.id.startswith('FACOAL') and 'x' in r.compartments:
        print(r.id, r.reaction, r.gene_reaction_rule)
```

```
FACOAL100p atp_x + coa_x + dca_x --> amp_x + dcacoa_x + ppi_x 11167 or 12538 or 12555 or 15748
FACOAL120p atp_x + coa_x + ddca_x --> amp_x + ddcacoa_x + ppi_x 11167 or 12538 or 12555 or 15748
FACOAL140p atp_x + coa_x + ttdca_x --> amp_x + ppi_x + tdcoa_x 11167 or 12538 or 12555 or 15748
FACOAL141p atp_x + coa_x + ttdcea_x --> amp_x + ppi_x + tdecoa_x 11167 or 12538 or 12555 or 15748
FACOAL160p atp_x + coa_x + hdca_x --> amp_x + pmtcoa_x + ppi_x 11167 or 12538 or 12555 or 15748
FACOAL161p atp_x + coa_x + hdcea_x --> amp_x + hdcoa_x + ppi_x 11167 or 12538 or 12555 or 15748
FACOAL240p atp_x + coa_x + ttc_x --> amp_x + ppi_x + ttccoa_x 9912
FACOAL260p atp_x + coa_x + hexc_x --> amp_x + hexccoa_x + ppi_x 9912
FACOAL80p atp_x + coa_x + octa_x --> amp_x + occoa_x + ppi_x 11167 or 12538 or 12555 or 15748
```

In [96]:

```
for r in sorted(model.reactions, key=lambda x: x.id):
    if r.id.startswith('FACOAL') and 'x' in r.compartments and (not r.id.startswith('FACOAL2')):
        r.gene_reaction_rule = '12555 or 15900'
```

In [97]:

```
for r in sorted(model.reactions, key=lambda x: x.id):
    if (not r.id.startswith('FACOAL')) and 'atp_x' in [m.id for m in r.reactants] and 'coa_x' in [m.id for m in r.reactants]:
        print(r.id, r.reaction, r.gene_reaction_rule)
```

```
VLCS2p atp_x + coa_x + dhcholestanate_x --> amp_x + dhcholestancoa_x + ppi_x 9912
VLCSp atp_x + coa_x + thcholstoic_x --> amp_x + cholcoar_x + ppi_x 9912
```

In [98]:

```
for r in sorted(model.genes.get_by_id('9912').reactions, key=lambda x: x.id):
    print(r.id, r.reaction, r.gene_reaction_rule)
```

```
FA240tp ttc_c --> ttc_x 9912
FA260tp hexc_c --> hexc_x 9912
FACOAL240p atp_x + coa_x + ttc_x --> amp_x + ppi_x + ttccoa_x 9912
FACOAL260p atp_x + coa_x + hexc_x --> amp_x + hexccoa_x + ppi_x 9912
FATP1t hdca_c + na1_c <=> hdca_e + na1_e 9912
FATP2t na1_c + ocdcea_c <=> na1_e + ocdcea_e 9912
FATP3t na1_c + ocdca_c <=> na1_e + ocdca_e 9912
FATP4t arach_c + na1_c <=> arach_e + na1_e 9912
FATP5t arachd_c + na1_c <=> arachd_e + na1_e 9912
FATP6t adrn_c + na1_c <=> adrn_e + na1_e 9912
FATP7t crvnc_c + na1_c <=> crvnc_e + na1_e 9912
FATP8t na1_c + ttc_c <=> na1_e + ttc_e 9912
FATP9t na1_c + nrvnc_c <=> na1_e + nrvnc_e 9912
VLCS2p atp_x + coa_x + dhcholestanate_x --> amp_x + dhcholestancoa_x + ppi_x 9912
VLCS2r atp_r + coa_r + dhcholestanate_r --> amp_r + dhcholestancoa_r + ppi_r 9912
VLCSp atp_x + coa_x + thcholstoic_x --> amp_x + cholcoar_x + ppi_x 9912
VLCSr atp_r + coa_r + thcholstoic_r --> amp_r + cholcoar_r + ppi_r 9912
```

12538 mito 18.5, cyto\_mito 12.5, cyto 5.5 KOG1256: Long-chain acyl-CoA synthetases (AMP-forming) GEQ *12272 mito 27 KOG1176: Acyl-CoA synthetase, blast to acetate/butyrate-CoA ligase, paperblast to 3-(methylthio)propionyl-CoA ligase MSQ*

12538 expression does not match the gene model and the gene model is < 50% coverage blast

In [99]:

```
for r in sorted(model.reactions, key=lambda x: x.id):
    if r.id.startswith('FACOAL') and 'm' in r.compartments:
        print(r.id, r.reaction, r.gene_reaction_rule)
```

```
FACOAL40im atp_m + but_m + coa_m --> amp_m + btcoa_m + ppi_m 15900
FACOAL_160_m atp_m + coa_m + hdca_m --> amp_m + pmtcoa_m + ppi_m 11167 or 12538 or 12555 or 15748
```

In [100]:

```
for r in sorted(model.reactions, key=lambda x: x.id):
    if (not r.id.startswith('FACOAL')) and 'atp_m' in [m.id for m in r.reactants] and 'coa_m' in [m.id for m in r.reactants]:
        print(r.id, r.reaction, r.gene_reaction_rule)
```

```
ACSm ac_m + atp_m + coa_m --> accoa_m + amp_m + ppi_m 14597
COUCOAFm T4hcinnm_m + atp_m + coa_m --> amp_m + coucoa_m + ppi_m 16635
ITCOALm atp_m + coa_m + itacon_m <=> adp_m + itaccoa_m + pi_m 15967 and 16144
MECOALm atp_m + coa_m + mescon_m <=> adp_m + mescoa_m + pi_m 15967 and 16144
SUCLm atp_m + coa_m + h_m + succ_m <=> adp_m + pi_m + succoa_m 15967 and 16144
SUCOASm atp_m + coa_m + succ_m <=> adp_m + pi_m + succoa_m 15967 and 16144
yli_R0233 atp_m + coa_m + dca_m <=> amp_m + dccoa_m + ppi_m 11167 or 12538 or 12555 or 15748
yli_R0234 atp_m + coa_m + ddca_m <=> amp_m + ddcacoa_m + ppi_m 11167 or 12538 or 12555 or 15748
yli_R0235 atp_m + coa_m + ttdca_m <=> amp_m + ppi_m + tdcoa_m 11167 or 12538 or 12555 or 15748
yli_R0236 atp_m + coa_m + yli_M04636_m <=> amp_m + ppi_m + yli_M04635_m 11167 or 12538 or 12555 or 15748
yli_R0238 atp_m + coa_m + yli_M03299_m <=> amp_m + ppi_m + yli_M04594_m 11167 or 12538 or 12555 or 15748
yli_R0240 atp_m + coa_m + yli_M04622_m <=> amp_m + ppi_m + yli_M04626_m 11167 or 12538 or 12555 or 15748
yli_R0241 atp_m + coa_m + yli_M05526_m <=> amp_m + ppi_m + yli_M04625_m 11167 or 12538 or 12555 or 15748
yli_R0244 atp_m + coa_m + octa_m <=> amp_m + occoa_m + ppi_m 11167 or 12538 or 12555 or 15748
```

In [101]:

```
model.reactions.get_by_id('ACSm').gene_reaction_rule = '12272 or 14597'
model.reactions.get_by_id('FACOAL40im').gene_reaction_rule = '12272'
```

In [102]:

```
model.remove_reactions(['FACOAL_160_m','yli_R0233','yli_R0234','yli_R0235','yli_R0236','yli_R0238',
                        'yli_R0240','yli_R0241','yli_R0244'], remove_orphans=True)
```

15967 mito 18.5, cyto\_mito 12.5, cyto 5.5 K01900: LSC2; succinyl-CoA synthetase beta subunit AAQ *16144 mito 24, pero 2 K01899: LSC1; succinyl-CoA synthetase alpha subunit GLA*

In [103]:

```
for r in sorted(model.genes.get_by_id('15967').reactions, key=lambda x: x.id):
    print(r.id, r.reaction, r.gene_reaction_rule)
print()
for r in sorted(model.genes.get_by_id('16144').reactions, key=lambda x: x.id):
    print(r.id, r.reaction, r.gene_reaction_rule)
```

```
ITCOALm atp_m + coa_m + itacon_m <=> adp_m + itaccoa_m + pi_m 15967 and 16144
MECOALm atp_m + coa_m + mescon_m <=> adp_m + mescoa_m + pi_m 15967 and 16144
SUCLm atp_m + coa_m + h_m + succ_m <=> adp_m + pi_m + succoa_m 15967 and 16144
SUCOAS atp_c + coa_c + succ_c <=> adp_c + pi_c + succoa_c 15967 and 16144
SUCOAS1m coa_m + gtp_m + succ_m <=> gdp_m + pi_m + succoa_m (SUCLG2 and 16144) or (Suclg2 and 16144) or (15967 and 16144)
SUCOASm atp_m + coa_m + succ_m <=> adp_m + pi_m + succoa_m 15967 and 16144

ITCOAL1m coa_m + gtp_m + itacon_m <=> gdp_m + itaccoa_m + pi_m (SUCLG2 and 16144) or (Suclg2 and 16144)
ITCOALm atp_m + coa_m + itacon_m <=> adp_m + itaccoa_m + pi_m 15967 and 16144
MECOALm atp_m + coa_m + mescon_m <=> adp_m + mescoa_m + pi_m 15967 and 16144
MECOAS1m coa_m + gtp_m + mescon_m <=> gdp_m + mescoa_m + pi_m (SUCLG2 and 16144) or (Suclg2 and 16144)
SUCLm atp_m + coa_m + h_m + succ_m <=> adp_m + pi_m + succoa_m 15967 and 16144
SUCOAS atp_c + coa_c + succ_c <=> adp_c + pi_c + succoa_c 15967 and 16144
SUCOAS1m coa_m + gtp_m + succ_m <=> gdp_m + pi_m + succoa_m (SUCLG2 and 16144) or (Suclg2 and 16144) or (15967 and 16144)
SUCOASm atp_m + coa_m + succ_m <=> adp_m + pi_m + succoa_m 15967 and 16144
```

In [104]:

```
# Remove irrelevant and wrong reactions
model.remove_reactions(['ITCOALm','MECOALm','SUCLm','SUCOAS','SUCOAS1m','ITCOAL1m','MECOAS1m'], remove_orphans=True)
```

In [105]:

```
for r in sorted(model.reactions, key=lambda x: x.id):
    if r.id.startswith('DESAT'):
        print(r.id, r.reaction, r.gene_reaction_rule)
```

```
DESAT16 h_c + nadph_c + o2_c + pmtcoa_c --> 2.0 h2o_c + hdcoa_c + nadp_c 9730
DESAT16_2 h_c + nadh_c + o2_c + pmtcoa_c --> 2.0 h2o_c + hdcoa_c + nad_c 9730
DESAT18 h_c + nadph_c + o2_c + stcoa_c --> 2.0 h2o_c + nadp_c + odecoa_c 9730
DESAT18_10 h_c + lnlncacoa_c + nadh_c + o2_c --> 2.0 h2o_c + nad_c + strdnccoa_c 9578
DESAT18_3 h_c + nadh_c + o2_c + stcoa_c --> 2.0 h2o_c + nad_c + odecoa_c 9730
DESAT18_4 h_c + nadh_c + o2_c + stcoa_c --> 2.0 h2o_c + nad_c + vacccoa_c 9730
DESAT18_5 h_c + nadh_c + o2_c + stcoa_c --> 2.0 h2o_c + nad_c + od2coa_c 9730
DESAT18_6 h_c + nadh_c + o2_c + odecoa_c --> 2.0 h2o_c + lneldccoa_c + nad_c 9578
DESAT18_7 h_c + nadh_c + o2_c + vacccoa_c --> 2.0 h2o_c + lneldccoa_c + nad_c 9578
DESAT18_8 h_c + nadh_c + o2_c + od2coa_c --> 2.0 h2o_c + lneldccoa_c + nad_c 9578
DESAT18_9 h_c + lnlccoa_c + nadh_c + o2_c --> 2.0 h2o_c + lnlncgcoa_c + nad_c 9578
DESAT20_1 dlnlcgcoa_c + h_c + nadh_c + o2_c --> arachdcoa_c + 2.0 h2o_c + nad_c 9578
DESAT20_2 eicostetcoa_c + h_c + nadh_c + o2_c --> 2.0 h2o_c + nad_c + tmndnccoa_c 9578
DESAT24_1 h_c + nadh_c + o2_c + tetpent3coa_c --> 2.0 h2o_c + nad_c + tethex3coa_c 9578
```

9730 mito 8, plas 8, cyto 7.5, cyto\_nucl 5, nucl 1.5 K00507: SCD, desC; stearoyl-CoA desaturase (Delta-9 desaturase), has cytochrome b5-like domain VKA *8845 plas 12, mito 9, cyto 2, pero 2, cyto\_pero 2 K10256: FAD2; omega-6 fatty acid desaturase / acyl-lipid omega-6 desaturase (Delta-12 desaturase), no cytochrome b5-like domain, introduces a cis double bond at position 12 of fatty-acyl-CoAs that contain a cis double bond at position 9, Requires cytochrome b5 as the electron donor, introduces a cis double bond in fatty acids attached to lipid molecules at a location 6 carbons away from the methyl end EGL*  
9578 plas 22, mito 3 K13076: SLD; Delta 6-fatty acid desaturase/delta-8 sphingolipid desaturase, has cytochrome b5-like domain, introduces a double bond at Delta 6 of fatty acids, introduces a trans double bond at the 8-position of sphingoid bases in sphingolipids LHH\*

Fatty acid desaturase  
9730 - saturated -> 9Z  
8845 - 9Z -> 9Z, 12Z  
9578 - 9Z,12Z -> 6Z,9Z,12Z

In [106]:

```
for r in sorted(model.genes.get_by_id('9730').reactions, key=lambda x: x.id):
    print(r.id, r.reaction, r.gene_reaction_rule)
print()
for r in sorted(model.genes.get_by_id('8845').reactions, key=lambda x: x.id):
    print(r.id, r.reaction, r.gene_reaction_rule)
print()
for r in sorted(model.genes.get_by_id('9578').reactions, key=lambda x: x.id):
    print(r.id, r.reaction, r.gene_reaction_rule)
```

```
DESAT16 h_c + nadph_c + o2_c + pmtcoa_c --> 2.0 h2o_c + hdcoa_c + nadp_c 9730
DESAT16_2 h_c + nadh_c + o2_c + pmtcoa_c --> 2.0 h2o_c + hdcoa_c + nad_c 9730
DESAT18 h_c + nadph_c + o2_c + stcoa_c --> 2.0 h2o_c + nadp_c + odecoa_c 9730
DESAT18_3 h_c + nadh_c + o2_c + stcoa_c --> 2.0 h2o_c + nad_c + odecoa_c 9730
DESAT18_4 h_c + nadh_c + o2_c + stcoa_c --> 2.0 h2o_c + nad_c + vacccoa_c 9730
DESAT18_5 h_c + nadh_c + o2_c + stcoa_c --> 2.0 h2o_c + nad_c + od2coa_c 9730
DGDGD7DS1819Z1617Z dgdg1819Z160_h + fdxox_h + 4.0 nadph_h + o2_h --> dgdg1819Z1617Z_h + fdxrd_h + 2.0 h2o_h + 4.0 nadp_h (CRv4_Au5_s13_g4871_t1 and CRv4_Au5_s16_g6229_t1) or (CRv4_Au5_s13_g4871_t1 and CRv4_Au5_s17_g7064_t1) or (CRv4_Au5_s13_g4871_t1 and CRv4_Au5_s3_g10824_t1) or (CRv4_Au5_s13_g4871_t1 and CRv4_Au5_s6_g13523_t1) or (CRv4_Au5_s13_g4871_t1 and CRv4_Au5_s7_g14133_t1) or (CRv4_Au5_s16_g6229_t1 and 9730) or (CRv4_Au5_s17_g7064_t1 and 9730) or (CRv4_Au5_s3_g10824_t1 and 9730) or (CRv4_Au5_s6_g13523_t1 and 9730) or (CRv4_Au5_s7_g14133_t1 and 9730)
DGDGD7DS1829Z12Z1617Z dgdg1829Z12Z160_h + fdxox_h + 4.0 nadph_h + o2_h --> dgdg1829Z12Z1617Z_h + fdxrd_h + 2.0 h2o_h + 4.0 nadp_h (CRv4_Au5_s13_g4871_t1 and CRv4_Au5_s16_g6229_t1) or (CRv4_Au5_s13_g4871_t1 and CRv4_Au5_s17_g7064_t1) or (CRv4_Au5_s13_g4871_t1 and CRv4_Au5_s3_g10824_t1) or (CRv4_Au5_s13_g4871_t1 and CRv4_Au5_s6_g13523_t1) or (CRv4_Au5_s13_g4871_t1 and CRv4_Au5_s7_g14133_t1) or (CRv4_Au5_s16_g6229_t1 and 9730) or (CRv4_Au5_s17_g7064_t1 and 9730) or (CRv4_Au5_s3_g10824_t1 and 9730) or (CRv4_Au5_s6_g13523_t1 and 9730) or (CRv4_Au5_s7_g14133_t1 and 9730)
FAH120 cytP450r_h + ddca_h + o2_h --> cytP450o_h + h2o_h + whddca_h CRv4_Au5_s3_g10234_t1 and 9730
FAH140 cytP450r_h + o2_h + ttdca_h --> cytP450o_h + h2o_h + whttdca_h CRv4_Au5_s3_g10234_t1 and 9730
FAH160 cytP450r_h + hdca_h + o2_h --> cytP450o_h + h2o_h + whhdca_h CRv4_Au5_s3_g10234_t1 and 9730
FAH1819Z cytP450r_h + o2_h + ocdce9a_h --> cytP450o_h + h2o_h + whodce9a_h CRv4_Au5_s3_g10234_t1 and 9730
FAH1829Z12Z cytP450r_h + lnlc_h + o2_h --> cytP450o_h + h2o_h + whlnlc_h CRv4_Au5_s3_g10234_t1 and 9730
MGDGD7DS1819Z1617Z fdxox_h + mgdg1819Z160_h + 4.0 nadph_h + o2_h --> fdxrd_h + 2.0 h2o_h + mgdg1819Z1617Z_h + 4.0 nadp_h (CRv4_Au5_s13_g4871_t1 and CRv4_Au5_s16_g6229_t1) or (CRv4_Au5_s13_g4871_t1 and CRv4_Au5_s17_g7064_t1) or (CRv4_Au5_s13_g4871_t1 and CRv4_Au5_s3_g10824_t1) or (CRv4_Au5_s13_g4871_t1 and CRv4_Au5_s6_g13523_t1) or (CRv4_Au5_s13_g4871_t1 and CRv4_Au5_s7_g14133_t1) or (CRv4_Au5_s16_g6229_t1 and 9730) or (CRv4_Au5_s17_g7064_t1 and 9730) or (CRv4_Au5_s3_g10824_t1 and 9730) or (CRv4_Au5_s6_g13523_t1 and 9730) or (CRv4_Au5_s7_g14133_t1 and 9730)
MGDGD7DS1829Z12Z1617Z fdxox_h + mgdg1829Z12Z160_h + 4.0 nadph_h + o2_h --> fdxrd_h + 2.0 h2o_h + mgdg1829Z12Z1617Z_h + 4.0 nadp_h (CRv4_Au5_s13_g4871_t1 and CRv4_Au5_s16_g6229_t1) or (CRv4_Au5_s13_g4871_t1 and CRv4_Au5_s17_g7064_t1) or (CRv4_Au5_s13_g4871_t1 and CRv4_Au5_s3_g10824_t1) or (CRv4_Au5_s13_g4871_t1 and CRv4_Au5_s6_g13523_t1) or (CRv4_Au5_s13_g4871_t1 and CRv4_Au5_s7_g14133_t1) or (CRv4_Au5_s16_g6229_t1 and 9730) or (CRv4_Au5_s17_g7064_t1 and 9730) or (CRv4_Au5_s3_g10824_t1 and 9730) or (CRv4_Au5_s6_g13523_t1 and 9730) or (CRv4_Au5_s7_g14133_t1 and 9730)
yli_R0154 h_c + nadph_c + o2_c + pmtcoa_c --> 2.0 h2o_c + nadp_c + yli_M04594_c 9730
yli_R0155 h_c + nadph_c + o2_c + stcoa_c --> 2.0 h2o_c + nadp_c + yli_M04626_c 9730

ASQDCADS1829Z12Z160 asqdca1819Z160_c + 2.0 nadh_c + o2_c --> asqdca1829Z12Z160_c + 2.0 h2o_c + 2.0 nad_c 8845
ASQDCADS1839Z12Z15Z160 asqdca1829Z12Z160_c + 2.0 nadh_c + o2_c --> asqdca1839Z12Z15Z160_c + 2.0 h2o_c + 2.0 nad_c 8845
ASQDPADS1829Z12Z160 asqdpa1819Z160_c + 2.0 nadh_c + o2_c --> asqdpa1829Z12Z160_c + 2.0 h2o_c + 2.0 nad_c 8845
ASQDPADS1839Z12Z15Z160 asqdpa1829Z12Z160_c + 2.0 nadh_c + o2_c --> asqdpa1839Z12Z15Z160_c + 2.0 h2o_c + 2.0 nad_c 8845
DGTSD5DS1601835Z9Z12Z dgts1601829Z12Z_c + 2.0 nadh_c + o2_c --> dgts1601835Z9Z12Z_c + 2.0 h2o_c + 2.0 nad_c 8845
DGTSD5DS18111Z1835Z9Z12Z dgts18111Z1829Z12Z_c + 2.0 nadh_c + o2_c --> dgts18111Z1835Z9Z12Z_c + 2.0 h2o_c + 2.0 nad_c 8845
DGTSD5DS1819Z1835Z9Z12Z dgts1819Z1829Z12Z_c + 2.0 nadh_c + o2_c --> dgts1819Z1835Z9Z12Z_c + 2.0 h2o_c + 2.0 nad_c 8845
DGTSD5DS1829Z12Z1835Z9Z12Z dgts1829Z12Z1829Z12Z_c + 2.0 nadh_c + o2_c --> dgts1829Z12Z1835Z9Z12Z_c + 2.0 h2o_c + 2.0 nad_c 8845
DGTSDS1601829Z12Z dgts1601819Z_c + 2.0 nadh_c + o2_c --> dgts1601829Z12Z_c + 2.0 h2o_c + 2.0 nad_c 8845
DGTSDS1601845Z9Z12Z15Z dgts1601835Z9Z12Z_c + 2.0 nadh_c + o2_c --> dgts1601845Z9Z12Z15Z_c + 2.0 h2o_c + 2.0 nad_c 8845
DGTSDS18111Z1829Z12Z dgts18111Z1819Z_c + 2.0 nadh_c + o2_c --> dgts18111Z1829Z12Z_c + 2.0 h2o_c + 2.0 nad_c 8845
DGTSDS18111Z1845Z9Z12Z15Z dgts18111Z1835Z9Z12Z_c + 2.0 nadh_c + o2_c --> dgts18111Z1845Z9Z12Z15Z_c + 2.0 h2o_c + 2.0 nad_c 8845
DGTSDS1819Z1829Z12Z dgts1819Z1819Z_c + 2.0 nadh_c + o2_c --> dgts1819Z1829Z12Z_c + 2.0 h2o_c + 2.0 nad_c 8845
DGTSDS1819Z1845Z9Z12Z15Z dgts1819Z1835Z9Z12Z_c + 2.0 nadh_c + o2_c --> dgts1819Z1845Z9Z12Z15Z_c + 2.0 h2o_c + 2.0 nad_c 8845
DGTSDS1829Z12Z18111Z dgts1819Z18111Z_c + 2.0 nadh_c + o2_c --> dgts1829Z12Z18111Z_c + 2.0 h2o_c + 2.0 nad_c 8845
DGTSDS1829Z12Z1819Z dgts1819Z1819Z_c + 2.0 nadh_c + o2_c --> dgts1829Z12Z1819Z_c + 2.0 h2o_c + 2.0 nad_c 8845
DGTSDS1829Z12Z1829Z12Z1 dgts1829Z12Z1819Z_c + 2.0 nadh_c + o2_c --> dgts1829Z12Z1829Z12Z_c + 2.0 h2o_c + 2.0 nad_c 8845
DGTSDS1829Z12Z1829Z12Z2 dgts1819Z1829Z12Z_c + 2.0 nadh_c + o2_c --> dgts1829Z12Z1829Z12Z_c + 2.0 h2o_c + 2.0 nad_c 8845
DGTSDS1829Z12Z1835Z9Z12Z dgts1819Z1835Z9Z12Z_c + 2.0 nadh_c + o2_c --> dgts1829Z12Z1835Z9Z12Z_c + 2.0 h2o_c + 2.0 nad_c 8845
DGTSDS1829Z12Z1845Z9Z12Z15Z1 dgts1819Z1845Z9Z12Z15Z_c + 2.0 nadh_c + o2_c --> dgts1829Z12Z1845Z9Z12Z15Z_c + 2.0 h2o_c + 2.0 nad_c 8845
DGTSDS1829Z12Z1845Z9Z12Z15Z2 dgts1829Z12Z1835Z9Z12Z_c + 2.0 nadh_c + o2_c --> dgts1829Z12Z1845Z9Z12Z15Z_c + 2.0 h2o_c + 2.0 nad_c 8845
DGTSDS1839Z12Z15Z18111Z dgts1829Z12Z18111Z_c + 2.0 nadh_c + o2_c --> dgts1839Z12Z15Z18111Z_c + 2.0 h2o_c + 2.0 nad_c 8845
DGTSDS1839Z12Z15Z1819Z dgts1829Z12Z1819Z_c + 2.0 nadh_c + o2_c --> dgts1839Z12Z15Z1819Z_c + 2.0 h2o_c + 2.0 nad_c 8845
DGTSDS1839Z12Z15Z1835Z9Z12Z dgts1829Z12Z1835Z9Z12Z_c + 2.0 nadh_c + o2_c --> dgts1839Z12Z15Z1835Z9Z12Z_c + 2.0 h2o_c + 2.0 nad_c 8845
DGTSDS1839Z12Z15Z1845Z9Z12Z15Z1 dgts1829Z12Z1845Z9Z12Z15Z_c + 2.0 nadh_c + o2_c --> dgts1839Z12Z15Z1845Z9Z12Z15Z_c + 2.0 h2o_c + 2.0 nad_c 8845
DGTSDS1839Z12Z15Z1845Z9Z12Z15Z2 dgts1839Z12Z15Z1835Z9Z12Z_c + 2.0 nadh_c + o2_c --> dgts1839Z12Z15Z1845Z9Z12Z15Z_c + 2.0 h2o_c + 2.0 nad_c 8845
PED5DS1801835Z9Z12Z 2.0 nadh_c + o2_c + pe1801829Z12Z_c --> 2.0 h2o_c + 2.0 nad_c + pe1801835Z9Z12Z_c 8845
PED5DS18111Z1835Z9Z12Z 2.0 nadh_c + o2_c + pe18111Z1829Z12Z_c --> 2.0 h2o_c + 2.0 nad_c + pe18111Z1835Z9Z12Z_c 8845
PED5DS1819Z1835Z9Z12Z 2.0 nadh_c + o2_c + pe1819Z1829Z12Z_c --> 2.0 h2o_c + 2.0 nad_c + pe1819Z1835Z9Z12Z_c 8845
PEDS1801829Z12Z 2.0 nadh_c + o2_c + pe1801819Z_c --> 2.0 h2o_c + 2.0 nad_c + pe1801829Z12Z_c 8845
PEDS1801845Z9Z12Z15Z 2.0 nadh_c + o2_c + pe1801835Z9Z12Z_c --> 2.0 h2o_c + 2.0 nad_c + pe1801845Z9Z12Z15Z_c 8845
PEDS18111Z1829Z12Z 2.0 nadh_c + o2_c + pe18111Z1819Z_c --> 2.0 h2o_c + 2.0 nad_c + pe18111Z1829Z12Z_c 8845
PEDS18111Z1845Z9Z12Z15Z 2.0 nadh_c + o2_c + pe18111Z1835Z9Z12Z_c --> 2.0 h2o_c + 2.0 nad_c + pe18111Z1845Z9Z12Z15Z_c 8845
PEDS1819Z1829Z12Z 2.0 nadh_c + o2_c + pe1819Z1819Z_c --> 2.0 h2o_c + 2.0 nad_c + pe1819Z1829Z12Z_c 8845
PEDS1819Z1845Z9Z12Z15Z 2.0 nadh_c + o2_c + pe1819Z1835Z9Z12Z_c --> 2.0 h2o_c + 2.0 nad_c + pe1819Z1845Z9Z12Z15Z_c 8845
PEDS1829Z12Z1835Z9Z12Z 2.0 nadh_c + o2_c + pe1819Z1835Z9Z12Z_c --> 2.0 h2o_c + 2.0 nad_c + pe1829Z12Z1835Z9Z12Z_c 8845

DESAT18_10 h_c + lnlncacoa_c + nadh_c + o2_c --> 2.0 h2o_c + nad_c + strdnccoa_c 9578
DESAT18_6 h_c + nadh_c + o2_c + odecoa_c --> 2.0 h2o_c + lneldccoa_c + nad_c 9578
DESAT18_7 h_c + nadh_c + o2_c + vacccoa_c --> 2.0 h2o_c + lneldccoa_c + nad_c 9578
DESAT18_8 h_c + nadh_c + o2_c + od2coa_c --> 2.0 h2o_c + lneldccoa_c + nad_c 9578
DESAT18_9 h_c + lnlccoa_c + nadh_c + o2_c --> 2.0 h2o_c + lnlncgcoa_c + nad_c 9578
DESAT20_1 dlnlcgcoa_c + h_c + nadh_c + o2_c --> arachdcoa_c + 2.0 h2o_c + nad_c 9578
DESAT20_2 eicostetcoa_c + h_c + nadh_c + o2_c --> 2.0 h2o_c + nad_c + tmndnccoa_c 9578
DESAT24_1 h_c + nadh_c + o2_c + tetpent3coa_c --> 2.0 h2o_c + nad_c + tethex3coa_c 9578
```

In [107]:

```
# Remove h compartment reactions, redundant reactions, sulfoquinovosyl-DAGs, and DAG-O4-NNN-trimetyl homoserine
# Remove delta 5 desaturase
model.remove_reactions(['DGDGD7DS1819Z1617Z','DGDGD7DS1829Z12Z1617Z','FAH120','FAH140','FAH160','FAH1819Z',
                        'FAH1829Z12Z','MGDGD7DS1819Z1617Z','MGDGD7DS1829Z12Z1617Z','yli_R0154','yli_R0155',
                        'ASQDCADS1829Z12Z160','ASQDCADS1839Z12Z15Z160','ASQDPADS1829Z12Z160','ASQDPADS1839Z12Z15Z160',
                        'DGTSD5DS1601835Z9Z12Z','DGTSD5DS18111Z1835Z9Z12Z','DGTSD5DS1819Z1835Z9Z12Z',
                        'DGTSD5DS1829Z12Z1835Z9Z12Z','DGTSDS1601829Z12Z','DGTSDS1601845Z9Z12Z15Z',
                        'DGTSDS18111Z1829Z12Z','DGTSDS18111Z1845Z9Z12Z15Z','DGTSDS1819Z1829Z12Z',
                        'DGTSDS1819Z1845Z9Z12Z15Z','DGTSDS1829Z12Z18111Z','DGTSDS1829Z12Z1819Z',
                        'DGTSDS1829Z12Z1829Z12Z1','DGTSDS1829Z12Z1829Z12Z2','DGTSDS1829Z12Z1835Z9Z12Z',
                        'DGTSDS1829Z12Z1845Z9Z12Z15Z1','DGTSDS1829Z12Z1845Z9Z12Z15Z2','DGTSDS1839Z12Z15Z18111Z',
                        'DGTSDS1839Z12Z15Z1819Z','DGTSDS1839Z12Z15Z1835Z9Z12Z','DGTSDS1839Z12Z15Z1845Z9Z12Z15Z1',
                        'DGTSDS1839Z12Z15Z1845Z9Z12Z15Z2',
                               ], remove_orphans=True)
# Remove 5Z fatty acid reactions
temp = [r.id for r in sorted(model.reactions, key=lambda x: x.id) if '5Z9Z' in r.id]
model.remove_reactions(temp, remove_orphans=True)
```

In [108]:

```
for r in sorted(model.genes.get_by_id('9730').reactions, key=lambda x: x.id):
    print(r.id, r.reaction, r.gene_reaction_rule)
print()
for r in sorted(model.genes.get_by_id('8845').reactions, key=lambda x: x.id):
    print(r.id, r.reaction, r.gene_reaction_rule)
print()
for r in sorted(model.genes.get_by_id('9578').reactions, key=lambda x: x.id):
    print(r.id, r.reaction, r.gene_reaction_rule)
```

```
DESAT16 h_c + nadph_c + o2_c + pmtcoa_c --> 2.0 h2o_c + hdcoa_c + nadp_c 9730
DESAT16_2 h_c + nadh_c + o2_c + pmtcoa_c --> 2.0 h2o_c + hdcoa_c + nad_c 9730
DESAT18 h_c + nadph_c + o2_c + stcoa_c --> 2.0 h2o_c + nadp_c + odecoa_c 9730
DESAT18_3 h_c + nadh_c + o2_c + stcoa_c --> 2.0 h2o_c + nad_c + odecoa_c 9730
DESAT18_4 h_c + nadh_c + o2_c + stcoa_c --> 2.0 h2o_c + nad_c + vacccoa_c 9730
DESAT18_5 h_c + nadh_c + o2_c + stcoa_c --> 2.0 h2o_c + nad_c + od2coa_c 9730

PEDS1801829Z12Z 2.0 nadh_c + o2_c + pe1801819Z_c --> 2.0 h2o_c + 2.0 nad_c + pe1801829Z12Z_c 8845
PEDS18111Z1829Z12Z 2.0 nadh_c + o2_c + pe18111Z1819Z_c --> 2.0 h2o_c + 2.0 nad_c + pe18111Z1829Z12Z_c 8845
PEDS1819Z1829Z12Z 2.0 nadh_c + o2_c + pe1819Z1819Z_c --> 2.0 h2o_c + 2.0 nad_c + pe1819Z1829Z12Z_c 8845

DESAT18_10 h_c + lnlncacoa_c + nadh_c + o2_c --> 2.0 h2o_c + nad_c + strdnccoa_c 9578
DESAT18_6 h_c + nadh_c + o2_c + odecoa_c --> 2.0 h2o_c + lneldccoa_c + nad_c 9578
DESAT18_7 h_c + nadh_c + o2_c + vacccoa_c --> 2.0 h2o_c + lneldccoa_c + nad_c 9578
DESAT18_8 h_c + nadh_c + o2_c + od2coa_c --> 2.0 h2o_c + lneldccoa_c + nad_c 9578
DESAT18_9 h_c + lnlccoa_c + nadh_c + o2_c --> 2.0 h2o_c + lnlncgcoa_c + nad_c 9578
DESAT20_1 dlnlcgcoa_c + h_c + nadh_c + o2_c --> arachdcoa_c + 2.0 h2o_c + nad_c 9578
DESAT20_2 eicostetcoa_c + h_c + nadh_c + o2_c --> 2.0 h2o_c + nad_c + tmndnccoa_c 9578
DESAT24_1 h_c + nadh_c + o2_c + tetpent3coa_c --> 2.0 h2o_c + nad_c + tethex3coa_c 9578
```

In [109]:

```
# Keep delta-9 NADH reactions for 9730
model.reactions.get_by_id('DESAT16_2').id = 'DESAT1619Z'
model.reactions.get_by_id('DESAT18_3').id = 'DESAT1819Z'
model.remove_reactions(['DESAT16','DESAT18','DESAT18_4','DESAT18_5'], remove_orphans=True)
# Keep omega-6/delta-12 reactions for 8845
# 8845 needs cytchrome b5 and reductase 10123 and 16097
# C18:1 9Z -> C18:2 9Z12Z are missing -> add, how about C16:1 9Z -> C16:2 9Z12Z?
# Remove 5Z unsaturated fatty acids - no gene 
# C18:2 is named ocdcya/ocdycacoa in iMM904, but lnlc/lnlccoa in Recon1/3D 
# replace ocdcya/ocdycacoa with lnlc/lnlccoa 
r = sce.reactions.get_by_id('DESAT18_2').copy()
r.id = 'DESAT1829Z12Z'
model.add_reactions([r])
model.reactions.get_by_id('DESAT1829Z12Z').add_metabolites({'nadph_c': -r.get_coefficient('nadph_c'),
                                                            'nadh_c': r.get_coefficient('nadph_c'),
                                                            'nadp_c': -r.get_coefficient('nadp_c'),
                                                            'nad_c': r.get_coefficient('nadp_c')})
model.reactions.get_by_id('DESAT1829Z12Z').gene_reaction_rule = '10123 and 16097 and 8845'
# 8845 is known to desaturate PCs, but not PEs
model.remove_reactions(['PEDS1801829Z12Z','PEDS18111Z1829Z12Z','PEDS1819Z1829Z12Z'], remove_orphans=True)
# Keep delta-6 fatty acid and delta-8 sphingolipid reactions for 9578
# lneldc_c is all trans C18:2, but reaction says 6Z,9Z -> remove lneldc reactions 
model.reactions.get_by_id('DESAT18_9').id = 'DESAT1836Z9Z12Z'
model.remove_reactions(['DESAT18_6','DESAT18_7','DESAT18_8'], remove_orphans=True)
# DESAT18_10, DESAT24_1 are 6Z okay but irrelevant, DESAT20_1, DESAT20_2 is acyl-lipid 5 desaturase for arachidonate 
model.remove_reactions(['DESAT18_10','DESAT20_1','DESAT20_2','DESAT24_1'], remove_orphans=True)
# Desaturase localized to ER, change cyto to ER
for r in sorted(model.reactions, key=lambda x: x.id):
    if r.id.startswith('DESAT'):
        r.id = r.id+'er'
        for m in r.metabolites:
            if not m.id.replace('_c','_r') in model.metabolites:
                m2 = m.copy()
                m2.id = m.id.replace('_c','_r')
                m2.compartment = 'r'
                model.add_metabolites([m2])
            r.add_metabolites({m.id: -r.get_coefficient(m.id), m.id.replace('_c','_r'): r.get_coefficient(m.id)})
```

In [110]:

```
for r in sorted(model.metabolites.get_by_id('ocdcya_c').reactions, key=lambda x: x.id):
    print(r.id, r.reaction)
print()
for r in sorted(model.metabolites.get_by_id('ocdcya_e').reactions, key=lambda x: x.id):
    print(r.id, r.reaction)
print()
for r in sorted(model.metabolites.get_by_id('lnlc_c').reactions, key=lambda x: x.id):
    print(r.id, r.reaction)
```

```
FA182ACPH h2o_c + ocdcyaACP_c <=> ACP_c + h_c + ocdcya_c
FACOAL182 atp_c + coa_c + ocdcya_c --> amp_c + ocdycacoa_c + ppi_c
PLBPC_SC h2o_c + 0.005 pc_SC_c --> 0.02 dca_c + 0.06 ddca_c + 0.5 g3pc_c + h_c + 0.27 hdca_c + 0.17 hdcea_c + 0.05 ocdca_c + 0.24 ocdcea_c + 0.09 ocdcya_c + 0.1 ttdca_c
TAGL_SC h2o_c + 0.01 triglyc_SC_c --> 0.01 12dgr_SC_c + 0.02 dca_c + 0.06 ddca_c + h_c + 0.27 hdca_c + 0.17 hdcea_c + 0.05 ocdca_c + 0.24 ocdcea_c + 0.09 ocdcya_c + 0.1 ttdca_c

PLBP1I_SCe h2o_e + 0.005 ptd1ino_SC_e --> 0.02 dca_e + 0.06 ddca_e + 0.5 g3pi_e + h_e + 0.27 hdca_e + 0.17 hdcea_e + 0.05 ocdca_e + 0.24 ocdcea_e + 0.09 ocdcya_e + 0.1 ttdca_e
PLBPC_SCe h2o_e + 0.005 pc_SC_e --> 0.02 dca_e + 0.06 ddca_e + 0.5 g3pc_e + h_e + 0.27 hdca_e + 0.17 hdcea_e + 0.05 ocdca_e + 0.24 ocdcea_e + 0.09 ocdcya_e + 0.1 ttdca_e

FACOAE1829Z12Z h2o_c + lnlccoa_c --> coa_c + h_c + lnlc_c
FACOAL1821 atp_c + coa_c + lnlc_c --> amp_c + lnlccoa_c + ppi_c
LPLPS1AGPE1829Z12Z 1agpe1829Z12Z_c + h2o_c --> g3pe_c + h_c + lnlc_c
PLPSA21801829Z12Z h2o_c + pe1801829Z12Z_c --> 1agpe180_c + h_c + lnlc_c
PLPSA218111Z1829Z12Z h2o_c + pe18111Z1829Z12Z_c --> 1agpe18111Z_c + h_c + lnlc_c
PLPSA21819Z1829Z12Z h2o_c + pe1819Z1829Z12Z_c --> 1agpe1819Z_c + h_c + lnlc_c
```

In [111]:

```
# replace lnlc_c with ocdcya_c and remove redundant reactions
for r in model.metabolites.get_by_id('lnlc_c').reactions:
    r.add_metabolites({'lnlc_c': -r.get_coefficient('lnlc_c'), 'ocdcya_c': r.get_coefficient('lnlc_c')})
model.remove_reactions(['FACOAL1821'], remove_orphans=True)
```

In [112]:

```
for r in sorted(model.metabolites.get_by_id('ocdycacoa_c').reactions, key=lambda x: x.id):
    print(r.id, r.reaction)
for r in sorted(model.metabolites.get_by_id('ocdycacoa_x').reactions, key=lambda x: x.id):
    print(r.id, r.reaction)
for r in sorted(model.metabolites.get_by_id('ocdycacoa_r').reactions, key=lambda x: x.id):
    print(r.id, r.reaction)
print()
for r in sorted(model.metabolites.get_by_id('lnlccoa_c').reactions, key=lambda x: x.id):
    print(r.id, r.reaction)
for r in sorted(model.metabolites.get_by_id('lnlccoa_m').reactions, key=lambda x: x.id):
    print(r.id, r.reaction)
for r in sorted(model.metabolites.get_by_id('lnlccoa_r').reactions, key=lambda x: x.id):
    print(r.id, r.reaction)
for r in sorted(model.metabolites.get_by_id('lnlccrn_c').reactions, key=lambda x: x.id):
    print(r.id, r.reaction)
for r in sorted(model.metabolites.get_by_id('lnlccrn_m').reactions, key=lambda x: x.id):
    print(r.id, r.reaction)
```

```
AGAT_SC 0.01 1ag3p_SC_c + 0.02 dcacoa_c + 0.06 ddcacoa_c + 0.17 hdcoa_c + 0.09 ocdycacoa_c + 0.24 odecoa_c + 0.27 pmtcoa_c + 0.05 stcoa_c + 0.1 tdcoa_c --> coa_c + 0.01 pa_SC_c
FA182COAabcp atp_x + h2o_x + ocdycacoa_c --> adp_x + h_x + ocdycacoa_x + pi_x
FACOAL182 atp_c + coa_c + ocdcya_c --> amp_c + ocdycacoa_c + ppi_c
GAT1_SC 0.02 dcacoa_c + 0.06 ddcacoa_c + glyc3p_c + 0.17 hdcoa_c + 0.09 ocdycacoa_c + 0.24 odecoa_c + 0.27 pmtcoa_c + 0.05 stcoa_c + 0.1 tdcoa_c --> 0.01 1ag3p_SC_c + coa_c
GAT2_SC 0.02 dcacoa_c + 0.06 ddcacoa_c + dhap_c + 0.17 hdcoa_c + 0.09 ocdycacoa_c + 0.24 odecoa_c + 0.27 pmtcoa_c + 0.05 stcoa_c + 0.1 tdcoa_c --> 0.01 1agly3p_SC_c + coa_c
LPCAT_SC 0.01 1agpc_SC_c + 0.02 dcacoa_c + 0.06 ddcacoa_c + 0.17 hdcoa_c + 0.09 ocdycacoa_c + 0.24 odecoa_c + 0.27 pmtcoa_c + 0.05 stcoa_c + 0.1 tdcoa_c --> coa_c + 0.01 pc_SC_c
TRIGS_SC 0.01 12dgr_SC_c + 0.02 dcacoa_c + 0.06 ddcacoa_c + 0.17 hdcoa_c + 0.09 ocdycacoa_c + 0.24 odecoa_c + 0.27 pmtcoa_c + 0.05 stcoa_c + 0.1 tdcoa_c --> coa_c + 0.01 triglyc_SC_c
FA182COAabcp atp_x + h2o_x + ocdycacoa_c --> adp_x + h_x + ocdycacoa_x + pi_x
FAO182p_even 8.0 coa_x + 8.0 h2o_x + 8.0 nad_x + 2.0 nadph_x + 8.0 o2_x + ocdycacoa_x --> 9.0 accoa_x + 8.0 h2o2_x + 6.0 h_x + 8.0 nadh_x + 2.0 nadp_x
FAO182p_odd 8.0 coa_x + 8.0 h2o_x + 8.0 nad_x + nadph_x + 7.0 o2_x + ocdycacoa_x --> 9.0 accoa_x + 7.0 h2o2_x + 7.0 h_x + 8.0 nadh_x + nadp_x
DESAT1829Z12Zer h_r + nadh_r + o2_r + odecoa_r --> 2.0 h2o_r + nad_r + ocdycacoa_r

1AGPEAT1801829Z12Z 1agpe180_c + lnlccoa_c --> coa_c + pe1801829Z12Z_c
1AGPEAT18111Z1829Z12Z 1agpe18111Z_c + lnlccoa_c --> coa_c + pe18111Z1829Z12Z_c
1AGPEAT1819Z1829Z12Z 1agpe1819Z_c + lnlccoa_c --> coa_c + pe1819Z1829Z12Z_c
FACOAE1829Z12Z h2o_c + lnlccoa_c --> coa_c + h_c + ocdcya_c
LNLCCPT1 crn_c + lnlccoa_c --> coa_c + lnlccrn_c
LNLCCPT2 coa_m + lnlccrn_m --> crn_m + lnlccoa_m
DESAT1836Z9Z12Zer h_r + lnlccoa_r + nadh_r + o2_r --> 2.0 h2o_r + lnlncgcoa_r + nad_r
LNLCCPT1 crn_c + lnlccoa_c --> coa_c + lnlccrn_c
LNLCCRNt lnlccrn_c --> lnlccrn_m
LNLCCPT2 coa_m + lnlccrn_m --> crn_m + lnlccoa_m
LNLCCRNt lnlccrn_c --> lnlccrn_m
```

In [113]:

```
# replace lnlccoa_c with ocdycacoa_c and remove mito reactions C18 long
for r in model.metabolites.get_by_id('lnlccoa_c').reactions:
    r.add_metabolites({'lnlccoa_c': -r.get_coefficient('lnlccoa_c'), 'ocdycacoa_c': r.get_coefficient('lnlccoa_c')})
for r in model.metabolites.get_by_id('lnlccoa_r').reactions:
    r.add_metabolites({'lnlccoa_r': -r.get_coefficient('lnlccoa_r'), 'ocdycacoa_r': r.get_coefficient('lnlccoa_r')})
model.remove_reactions(['LNLCCPT1','LNLCCPT2','LNLCCRNt'], remove_orphans=True)
```

In [114]:

```
# lneldc_c is all trans C18:2, but reaction says 6Z,9Z -> remove lneldc reactions 
for r in sorted(model.metabolites.get_by_id('lneldc_c').reactions, key=lambda x: x.id):
    print(r.id, r.reaction)
for r in sorted(model.metabolites.get_by_id('lneldccoa_c').reactions, key=lambda x: x.id):
    print(r.id, r.reaction)
for r in sorted(model.metabolites.get_by_id('lneldccoa_m').reactions, key=lambda x: x.id):
    print(r.id, r.reaction)
for r in sorted(model.metabolites.get_by_id('lneldccrn_c').reactions, key=lambda x: x.id):
    print(r.id, r.reaction)
for r in sorted(model.metabolites.get_by_id('lneldccrn_m').reactions, key=lambda x: x.id):
    print(r.id, r.reaction)
```

```
FACOAL1822 atp_c + coa_c + lneldc_c --> amp_c + lneldccoa_c + ppi_c
FACOAL1822 atp_c + coa_c + lneldc_c --> amp_c + lneldccoa_c + ppi_c
LNELDCCPT1 crn_c + lneldccoa_c --> coa_c + lneldccrn_c
LNELDCCPT2 coa_m + lneldccrn_m --> crn_m + lneldccoa_m
LNELDCCPT1 crn_c + lneldccoa_c --> coa_c + lneldccrn_c
LNELDCCRNt lneldccrn_c --> lneldccrn_m
LNELDCCPT2 coa_m + lneldccrn_m --> crn_m + lneldccoa_m
LNELDCCRNt lneldccrn_c --> lneldccrn_m
```

In [115]:

```
model.remove_reactions(['FACOAL1822','LNELDCCPT1','LNELDCCPT2','LNELDCCRNt'], remove_orphans=True)
```

DESAT18\_6 h\_c + nadh\_c + o2\_c + odecoa\_c --> 2.0 h2o\_c + lneldccoa\_c + nad\_c 9578  
DESAT18\_7 h\_c + nadh\_c + o2\_c + vacccoa\_c --> 2.0 h2o\_c + lneldccoa\_c + nad\_c 9578  
DESAT18\_8 h\_c + nadh\_c + o2\_c + od2coa\_c --> 2.0 h2o\_c + lneldccoa\_c + nad\_c 9578

DESAT18\_10 h\_c + lnlncacoa\_c + nadh\_c + o2\_c --> 2.0 h2o\_c + nad\_c + strdnccoa\_c 9578  
DESAT20\_1 dlnlcgcoa\_c + h\_c + nadh\_c + o2\_c --> arachdcoa\_c + 2.0 h2o\_c + nad\_c 9578  
DESAT20\_2 eicostetcoa\_c + h\_c + nadh\_c + o2\_c --> 2.0 h2o\_c + nad\_c + tmndnccoa\_c 9578  
DESAT24\_1 h\_c + nadh\_c + o2\_c + tetpent3coa\_c --> 2.0 h2o\_c + nad\_c + tethex3coa\_c 9578

In [116]:

```
for r in sorted(model.metabolites.get_by_id('vacccoa_c').reactions, key=lambda x: x.id):
    print(r.id, r.reaction)
for r in sorted(model.metabolites.get_by_id('vacccoa_m').reactions, key=lambda x: x.id):
    print(r.id, r.reaction)
for r in sorted(model.metabolites.get_by_id('vacccrn_m').reactions, key=lambda x: x.id):
    print(r.id, r.reaction)
print()
for r in sorted(model.reactions, key=lambda x: x.id):
    if '18111Z' in r.id:
        print(r.id, r.reaction)
```

```
ACOADAGAT16018111Z18111Z 12dgr16018111Z_c + vacccoa_c --> coa_c + tag16018111Z18111Z_c
ACOADAGAT1601819Z18111Z 12dgr1601819Z_c + vacccoa_c --> coa_c + tag1601819Z18111Z_c
ACOADAGAT1801819Z18111Z 12dgr1801819Z_c + vacccoa_c --> coa_c + tag1801819Z18111Z_c
ACOADAGAT18111Z18111Z18111Z 12dgr18111Z18111Z_c + vacccoa_c --> coa_c + tag18111Z18111Z18111Z_c
ACOADAGAT18111Z1819Z18111Z 12dgr18111Z1819Z_c + vacccoa_c --> coa_c + tag18111Z1819Z18111Z_c
ACOADAGAT1819Z18111Z18111Z 12dgr1819Z18111Z_c + vacccoa_c --> coa_c + tag1819Z18111Z18111Z_c
ACOADAGAT1819Z1819Z18111Z 12dgr1819Z1819Z_c + vacccoa_c --> coa_c + tag1819Z1819Z18111Z_c
AGPATCOA16018111Z 1hdecg3p_c + vacccoa_c --> coa_c + pa16018111Z_c
AGPATCOA18111Z18111Z 1odec11eg3p_c + vacccoa_c --> coa_c + pa18111Z18111Z_c
AGPATCOA1819Z18111Z 1odec9eg3p_c + vacccoa_c --> coa_c + pa1819Z18111Z_c
FACOAL1812 atp_c + coa_c + vacc_c --> amp_c + ppi_c + vacccoa_c
VACCCPT1 crn_c + vacccoa_c --> coa_c + vacccrn_c
VACCCPT2 coa_m + vacccrn_m --> crn_m + vacccoa_m
VACCCPT2 coa_m + vacccrn_m --> crn_m + vacccoa_m
VACCCRNt vacccrn_c --> vacccrn_m

1AGPEAT18111Z1819Z 1agpe18111Z_c + odecoa_c --> coa_c + pe18111Z1819Z_c
1AGPEAT18111Z1829Z12Z 1agpe18111Z_c + ocdycacoa_c --> coa_c + pe18111Z1829Z12Z_c
ACOADAGAT16018111Z160 12dgr16018111Z_c + pmtcoa_c --> coa_c + tag16018111Z160_c
ACOADAGAT16018111Z180 12dgr16018111Z_c + stcoa_c --> coa_c + tag16018111Z180_c
ACOADAGAT16018111Z18111Z 12dgr16018111Z_c + vacccoa_c --> coa_c + tag16018111Z18111Z_c
ACOADAGAT16018111Z1819Z 12dgr16018111Z_c + odecoa_c --> coa_c + tag16018111Z1819Z_c
ACOADAGAT1601819Z18111Z 12dgr1601819Z_c + vacccoa_c --> coa_c + tag1601819Z18111Z_c
ACOADAGAT1801819Z18111Z 12dgr1801819Z_c + vacccoa_c --> coa_c + tag1801819Z18111Z_c
ACOADAGAT18111Z18111Z160 12dgr18111Z18111Z_c + pmtcoa_c --> coa_c + tag18111Z18111Z160_c
ACOADAGAT18111Z18111Z180 12dgr18111Z18111Z_c + stcoa_c --> coa_c + tag18111Z18111Z180_c
ACOADAGAT18111Z18111Z18111Z 12dgr18111Z18111Z_c + vacccoa_c --> coa_c + tag18111Z18111Z18111Z_c
ACOADAGAT18111Z18111Z1819Z 12dgr18111Z18111Z_c + odecoa_c --> coa_c + tag18111Z18111Z1819Z_c
ACOADAGAT18111Z1819Z160 12dgr18111Z1819Z_c + pmtcoa_c --> coa_c + tag18111Z1819Z160_c
ACOADAGAT18111Z1819Z180 12dgr18111Z1819Z_c + stcoa_c --> coa_c + tag18111Z1819Z180_c
ACOADAGAT18111Z1819Z18111Z 12dgr18111Z1819Z_c + vacccoa_c --> coa_c + tag18111Z1819Z18111Z_c
ACOADAGAT18111Z1819Z1819Z 12dgr18111Z1819Z_c + odecoa_c --> coa_c + tag18111Z1819Z1819Z_c
ACOADAGAT1819Z18111Z160 12dgr1819Z18111Z_c + pmtcoa_c --> coa_c + tag1819Z18111Z160_c
ACOADAGAT1819Z18111Z180 12dgr1819Z18111Z_c + stcoa_c --> coa_c + tag1819Z18111Z180_c
ACOADAGAT1819Z18111Z18111Z 12dgr1819Z18111Z_c + vacccoa_c --> coa_c + tag1819Z18111Z18111Z_c
ACOADAGAT1819Z18111Z1819Z 12dgr1819Z18111Z_c + odecoa_c --> coa_c + tag1819Z18111Z1819Z_c
ACOADAGAT1819Z1819Z18111Z 12dgr1819Z1819Z_c + vacccoa_c --> coa_c + tag1819Z1819Z18111Z_c
ACPT16018111Z 12dgr16018111Z_c + amet_c --> 5mta_c + dghs16018111Z_c + h_c
ACPT18111Z18111Z 12dgr18111Z18111Z_c + amet_c --> 5mta_c + dghs18111Z18111Z_c + h_c
ACPT18111Z1819Z 12dgr18111Z1819Z_c + amet_c --> 5mta_c + dghs18111Z1819Z_c + h_c
ACPT1819Z18111Z 12dgr1819Z18111Z_c + amet_c --> 5mta_c + dghs1819Z18111Z_c + h_c
AGPAT18111Z160h 1odec11eg3p_h + palmACP_h --> ACP_h + pa18111Z160_h
AGPAT18111Z1819Zh 1odec11eg3p_h + octe9ACP_h --> ACP_h + pa18111Z1819Z_h
AGPAT1819Z18111Zh 1odec9eg3p_h + octeACP_h --> ACP_h + pa1819Z18111Z_h
AGPATCOA16018111Z 1hdecg3p_c + vacccoa_c --> coa_c + pa16018111Z_c
AGPATCOA18111Z160 1odec11eg3p_c + pmtcoa_c --> coa_c + pa18111Z160_c
AGPATCOA18111Z18111Z 1odec11eg3p_c + vacccoa_c --> coa_c + pa18111Z18111Z_c
AGPATCOA18111Z1819Z 1odec11eg3p_c + odecoa_c --> coa_c + pa18111Z1819Z_c
AGPATCOA1819Z18111Z 1odec9eg3p_c + vacccoa_c --> coa_c + pa1819Z18111Z_c
CDIPT18111Z160 cdp12dgr18111Z160_c + inost_c --> cmp_c + 2.0 h_c + pail18111Z160_c
CDPDAGS18111Z160 ctp_c + 3.0 h_c + pa18111Z160_c --> cdp12dgr18111Z160_c + ppi_c
CDPDAGS18111Z160h ctp_h + 2.0 h_h + pa18111Z160_h --> cdp12dgr18111Z160_h + ppi_h
ETHAPT18111Z1819Z 12dgr18111Z1819Z_c + cdpea_c --> cmp_c + h_c + pe18111Z1819Z_c
LPLPS1AGPE18111Z 1agpe18111Z_c + h2o_c --> g3pe_c + h_c + vacc_c
MAGAH18111Z h2o_c + mag18111Z_c --> glyc_c + h_c + vacc_c
PAIL18111Z160t atp_c + h2o_c + pail18111Z160_c --> adp_c + h_c + pail18111Z160_e + pi_c
PAPA18111Z160h h2o_h + pa18111Z160_h --> 12dgr18111Z160_h + pi_h
PAPA18111Z1819Zh h2o_h + pa18111Z1819Z_h --> 12dgr18111Z1819Z_h + pi_h
PAPA1819Z18111Zh h2o_h + pa1819Z18111Z_h --> 12dgr1819Z18111Z_h + pi_h
PE18111Z1819Zt atp_c + h2o_c + pe18111Z1819Z_c --> adp_c + h_c + pe18111Z1819Z_e + pi_c
PE18111Z1829Z12Zt atp_c + h2o_c + pe18111Z1829Z12Z_c --> adp_c + h_c + pe18111Z1829Z12Z_e + pi_c
PG18111Z160t atp_c + h2o_c + pg18111Z160_c --> adp_c + h_c + pg18111Z160_e + pi_c
PGP18111Z160t atp_c + h2o_c + pgp18111Z160_c --> adp_c + h_c + pgp18111Z160_e + pi_c
PGPS18111Z160 cdp12dgr18111Z160_c + glyc3p_c --> cmp_c + pgp18111Z160_c
PGPS18111Z160h cdp12dgr18111Z160_h + glyc3p_h --> cmp_h + pgp18111Z160_h
PLDAGAT16018111Z1601 12dgr16018111Z_c + pa18111Z160_c --> 1odec11eg3p_c + tag16018111Z160_c
PLDAGAT16018111Z1602 12dgr16018111Z_c + pa1819Z160_c --> 1odec9eg3p_c + tag16018111Z160_c
PLDAGAT16018111Z18111Z1 12dgr16018111Z_c + pa16018111Z_c --> 1hdecg3p_c + tag16018111Z18111Z_c
PLDAGAT16018111Z18111Z2 12dgr16018111Z_c + pa18111Z18111Z_c --> 1odec11eg3p_c + tag16018111Z18111Z_c
PLDAGAT16018111Z18111Z3 12dgr16018111Z_c + pa1819Z18111Z_c --> 1odec9eg3p_c + tag16018111Z18111Z_c
PLDAGAT16018111Z1819Z1 12dgr16018111Z_c + pa1601819Z_c --> 1hdecg3p_c + tag16018111Z1819Z_c
PLDAGAT16018111Z1819Z2 12dgr16018111Z_c + pa1801819Z_c --> 1odecg3p_c + tag16018111Z1819Z_c
PLDAGAT16018111Z1819Z3 12dgr16018111Z_c + pa18111Z1819Z_c --> 1odec11eg3p_c + tag16018111Z1819Z_c
PLDAGAT16018111Z1819Z4 12dgr16018111Z_c + pa1819Z1819Z_c --> 1odec9eg3p_c + tag16018111Z1819Z_c
PLDAGAT1601819Z18111Z1 12dgr1601819Z_c + pa16018111Z_c --> 1hdecg3p_c + tag1601819Z18111Z_c
PLDAGAT1601819Z18111Z2 12dgr1601819Z_c + pa18111Z18111Z_c --> 1odec11eg3p_c + tag1601819Z18111Z_c
PLDAGAT1601819Z18111Z3 12dgr1601819Z_c + pa1819Z18111Z_c --> 1odec9eg3p_c + tag1601819Z18111Z_c
PLDAGAT1801819Z18111Z1 12dgr1801819Z_c + pa16018111Z_c --> 1hdecg3p_c + tag1801819Z18111Z_c
PLDAGAT1801819Z18111Z2 12dgr1801819Z_c + pa18111Z18111Z_c --> 1odec11eg3p_c + tag1801819Z18111Z_c
PLDAGAT1801819Z18111Z3 12dgr1801819Z_c + pa1819Z18111Z_c --> 1odec9eg3p_c + tag1801819Z18111Z_c
PLDAGAT18111Z18111Z1601 12dgr18111Z18111Z_c + pa18111Z160_c --> 1odec11eg3p_c + tag18111Z18111Z160_c
PLDAGAT18111Z18111Z1602 12dgr18111Z18111Z_c + pa1819Z160_c --> 1odec9eg3p_c + tag18111Z18111Z160_c
PLDAGAT18111Z18111Z18111Z1 12dgr18111Z18111Z_c + pa16018111Z_c --> 1hdecg3p_c + tag18111Z18111Z18111Z_c
PLDAGAT18111Z18111Z18111Z2 12dgr18111Z18111Z_c + pa18111Z18111Z_c --> 1odec11eg3p_c + tag18111Z18111Z18111Z_c
PLDAGAT18111Z18111Z18111Z3 12dgr18111Z18111Z_c + pa1819Z18111Z_c --> 1odec9eg3p_c + tag18111Z18111Z18111Z_c
PLDAGAT18111Z18111Z1819Z1 12dgr18111Z18111Z_c + pa1601819Z_c --> 1hdecg3p_c + tag18111Z18111Z1819Z_c
PLDAGAT18111Z18111Z1819Z2 12dgr18111Z18111Z_c + pa1801819Z_c --> 1odecg3p_c + tag18111Z18111Z1819Z_c
PLDAGAT18111Z18111Z1819Z3 12dgr18111Z18111Z_c + pa18111Z1819Z_c --> 1odec11eg3p_c + tag18111Z18111Z1819Z_c
PLDAGAT18111Z18111Z1819Z4 12dgr18111Z18111Z_c + pa1819Z1819Z_c --> 1odec9eg3p_c + tag18111Z18111Z1819Z_c
PLDAGAT18111Z1819Z1601 12dgr18111Z1819Z_c + pa18111Z160_c --> 1odec11eg3p_c + tag18111Z1819Z160_c
PLDAGAT18111Z1819Z1602 12dgr18111Z1819Z_c + pa1819Z160_c --> 1odec9eg3p_c + tag18111Z1819Z160_c
PLDAGAT18111Z1819Z18111Z1 12dgr18111Z1819Z_c + pa16018111Z_c --> 1hdecg3p_c + tag18111Z1819Z18111Z_c
PLDAGAT18111Z1819Z18111Z2 12dgr18111Z1819Z_c + pa18111Z18111Z_c --> 1odec11eg3p_c + tag18111Z1819Z18111Z_c
PLDAGAT18111Z1819Z18111Z3 12dgr18111Z1819Z_c + pa1819Z18111Z_c --> 1odec9eg3p_c + tag18111Z1819Z18111Z_c
PLDAGAT18111Z1819Z1819Z1 12dgr18111Z1819Z_c + pa1601819Z_c --> 1hdecg3p_c + tag18111Z1819Z1819Z_c
PLDAGAT18111Z1819Z1819Z2 12dgr18111Z1819Z_c + pa1801819Z_c --> 1odecg3p_c + tag18111Z1819Z1819Z_c
PLDAGAT18111Z1819Z1819Z3 12dgr18111Z1819Z_c + pa18111Z1819Z_c --> 1odec11eg3p_c + tag18111Z1819Z1819Z_c
PLDAGAT18111Z1819Z1819Z4 12dgr18111Z1819Z_c + pa1819Z1819Z_c --> 1odec9eg3p_c + tag18111Z1819Z1819Z_c
PLDAGAT1819Z18111Z1601 12dgr1819Z18111Z_c + pa18111Z160_c --> 1odec11eg3p_c + tag1819Z18111Z160_c
PLDAGAT1819Z18111Z1602 12dgr1819Z18111Z_c + pa1819Z160_c --> 1odec9eg3p_c + tag1819Z18111Z160_c
PLDAGAT1819Z18111Z18111Z1 12dgr1819Z18111Z_c + pa16018111Z_c --> 1hdecg3p_c + tag1819Z18111Z18111Z_c
PLDAGAT1819Z18111Z18111Z2 12dgr1819Z18111Z_c + pa18111Z18111Z_c --> 1odec11eg3p_c + tag1819Z18111Z18111Z_c
PLDAGAT1819Z18111Z18111Z3 12dgr1819Z18111Z_c + pa1819Z18111Z_c --> 1odec9eg3p_c + tag1819Z18111Z18111Z_c
PLDAGAT1819Z18111Z1819Z1 12dgr1819Z18111Z_c + pa1601819Z_c --> 1hdecg3p_c + tag1819Z18111Z1819Z_c
PLDAGAT1819Z18111Z1819Z2 12dgr1819Z18111Z_c + pa1801819Z_c --> 1odecg3p_c + tag1819Z18111Z1819Z_c
PLDAGAT1819Z18111Z1819Z3 12dgr1819Z18111Z_c + pa18111Z1819Z_c --> 1odec11eg3p_c + tag1819Z18111Z1819Z_c
PLDAGAT1819Z18111Z1819Z4 12dgr1819Z18111Z_c + pa1819Z1819Z_c --> 1odec9eg3p_c + tag1819Z18111Z1819Z_c
PLDAGAT1819Z1819Z18111Z1 12dgr1819Z1819Z_c + pa16018111Z_c --> 1hdecg3p_c + tag1819Z1819Z18111Z_c
PLDAGAT1819Z1819Z18111Z2 12dgr1819Z1819Z_c + pa18111Z18111Z_c --> 1odec11eg3p_c + tag1819Z1819Z18111Z_c
PLDAGAT1819Z1819Z18111Z3 12dgr1819Z1819Z_c + pa1819Z18111Z_c --> 1odec9eg3p_c + tag1819Z1819Z18111Z_c
PLPSA218111Z1819Z h2o_c + pe18111Z1819Z_c --> 1agpe18111Z_c + h_c + ocdcea_c
PLPSA218111Z1829Z12Z h2o_c + pe18111Z1829Z12Z_c --> 1agpe18111Z_c + h_c + ocdcya_c
SQDGS18111Z160 12dgr18111Z160_h + udpsq_h --> h_h + sqdg18111Z160_h + udp_h
TM16018111Z 3.0 amet_c + dghs16018111Z_c --> 3.0 ahcys_c + dgts16018111Z_c + 3.0 h_c
TM18111Z18111Z 3.0 amet_c + dghs18111Z18111Z_c --> 3.0 ahcys_c + dgts18111Z18111Z_c + 3.0 h_c
TM18111Z1819Z 3.0 amet_c + dghs18111Z1819Z_c --> 3.0 ahcys_c + dgts18111Z1819Z_c + 3.0 h_c
TM1819Z18111Z 3.0 amet_c + dghs1819Z18111Z_c --> 3.0 ahcys_c + dgts1819Z18111Z_c + 3.0 h_c
```

In [117]:

```
# Remove vacccoa - 18:1 11Z produced by bacteria and plants - no enzyme in Rhodo
model.remove_reactions(['FACOAL1812','VACCCPT1','VACCCPT2','VACCCRNt'], remove_orphans=True)
# Remove 18111Z fatty acid reactions
temp = [r.id for r in sorted(model.reactions, key=lambda x: x.id) if '18111Z' in r.id]
model.remove_reactions(temp, remove_orphans=True)
```

In [118]:

```
for r in sorted(model.metabolites.get_by_id('lnlnca_c').reactions, key=lambda x: x.id):
    print(r.id, r.reaction)
for r in sorted(model.metabolites.get_by_id('lnlncacoa_c').reactions, key=lambda x: x.id):
    print(r.id, r.reaction)
for r in sorted(model.metabolites.get_by_id('lnlncacoa_m').reactions, key=lambda x: x.id):
    print(r.id, r.reaction)
for r in sorted(model.metabolites.get_by_id('lnlncacrn_m').reactions, key=lambda x: x.id):
    print(r.id, r.reaction)
print()
for r in sorted(model.metabolites.get_by_id('strdnc_c').reactions, key=lambda x: x.id):
    print(r.id, r.reaction)
for r in sorted(model.metabolites.get_by_id('strdnccoa_c').reactions, key=lambda x: x.id):
    print(r.id, r.reaction)
for r in sorted(model.metabolites.get_by_id('strdnccoa_m').reactions, key=lambda x: x.id):
    print(r.id, r.reaction)
for r in sorted(model.metabolites.get_by_id('strdnccrn_m').reactions, key=lambda x: x.id):
    print(r.id, r.reaction)
print()
for r in sorted(model.metabolites.get_by_id('dlnlcg_c').reactions, key=lambda x: x.id):
    print(r.id, r.reaction)
for r in sorted(model.metabolites.get_by_id('dlnlcgcoa_c').reactions, key=lambda x: x.id):
    print(r.id, r.reaction)
for r in sorted(model.metabolites.get_by_id('dlnlcgcoa_m').reactions, key=lambda x: x.id):
    print(r.id, r.reaction)
for r in sorted(model.metabolites.get_by_id('dlnlcgcrn_m').reactions, key=lambda x: x.id):
    print(r.id, r.reaction)
print()
for r in sorted(model.metabolites.get_by_id('arachd_e').reactions, key=lambda x: x.id):
    print(r.id, r.reaction)
for r in sorted(model.metabolites.get_by_id('arachd_c').reactions, key=lambda x: x.id):
    print(r.id, r.reaction)
for r in sorted(model.metabolites.get_by_id('arachd_r').reactions, key=lambda x: x.id):
    print(r.id, r.reaction)
for r in sorted(model.metabolites.get_by_id('arachdcoa_c').reactions, key=lambda x: x.id):
    print(r.id, r.reaction)
for r in sorted(model.metabolites.get_by_id('arachdcoa_m').reactions, key=lambda x: x.id):
    print(r.id, r.reaction)
for r in sorted(model.metabolites.get_by_id('arachdcoa_x').reactions, key=lambda x: x.id):
    print(r.id, r.reaction)
for r in sorted(model.metabolites.get_by_id('arachdcrn_m').reactions, key=lambda x: x.id):
    print(r.id, r.reaction)
print()
for r in sorted(model.metabolites.get_by_id('adrn_e').reactions, key=lambda x: x.id):
    print(r.id, r.reaction)
for r in sorted(model.metabolites.get_by_id('adrn_c').reactions, key=lambda x: x.id):
    print(r.id, r.reaction)
for r in sorted(model.metabolites.get_by_id('adrncoa_c').reactions, key=lambda x: x.id):
    print(r.id, r.reaction)
for r in sorted(model.metabolites.get_by_id('adrncoa_m').reactions, key=lambda x: x.id):
    print(r.id, r.reaction)
for r in sorted(model.metabolites.get_by_id('adrncrn_m').reactions, key=lambda x: x.id):
    print(r.id, r.reaction)
```

```
FACOAE1839Z12Z15Z h2o_c + lnlncacoa_c --> coa_c + h_c + lnlnca_c
FACOAL1832 atp_c + coa_c + lnlnca_c --> amp_c + lnlncacoa_c + ppi_c
FACOAE1839Z12Z15Z h2o_c + lnlncacoa_c --> coa_c + h_c + lnlnca_c
FACOAL1832 atp_c + coa_c + lnlnca_c --> amp_c + lnlncacoa_c + ppi_c
LNLNCACPT1 crn_c + lnlncacoa_c --> coa_c + lnlncacrn_c
LNLNCACPT2 coa_m + lnlncacrn_m --> crn_m + lnlncacoa_m
LNLNCACPT2 coa_m + lnlncacrn_m --> crn_m + lnlncacoa_m
LNLNCACRNt lnlncacrn_c --> lnlncacrn_m

FACOAL184 atp_c + coa_c + strdnc_c --> amp_c + ppi_c + strdnccoa_c
FACOAL184 atp_c + coa_c + strdnc_c --> amp_c + ppi_c + strdnccoa_c
STRDNCCPT1 crn_c + strdnccoa_c --> coa_c + strdnccrn_c
STRDNCCPT2 coa_m + strdnccrn_m --> crn_m + strdnccoa_m
STRDNCCPT2 coa_m + strdnccrn_m --> crn_m + strdnccoa_m
STRDNCCRNt strdnccrn_c --> strdnccrn_m

FACOAL203 atp_c + coa_c + dlnlcg_c --> amp_c + dlnlcgcoa_c + ppi_c
DLNLCGCPT1 crn_c + dlnlcgcoa_c --> coa_c + dlnlcgcrn_c
FACOAL203 atp_c + coa_c + dlnlcg_c --> amp_c + dlnlcgcoa_c + ppi_c
DLNLCGCPT2 coa_m + dlnlcgcrn_m --> crn_m + dlnlcgcoa_m
DLNLCGCPT2 coa_m + dlnlcgcrn_m --> crn_m + dlnlcgcoa_m
DLNLCGCRNt dlnlcgcrn_c --> dlnlcgcrn_m

FATP5t arachd_c + na1_c <=> arachd_e + na1_e
FACOAL204 arachd_c + atp_c + coa_c --> amp_c + arachdcoa_c + ppi_c
FATP5t arachd_c + na1_c <=> arachd_e + na1_e
P4504B1r arachd_r + h_r + nadph_r + o2_r --> 12harachd_r + h2o_r + nadp_r
C204CPT1 arachdcoa_c + crn_c --> arachdcrn_c + coa_c
FACOAL204 arachd_c + atp_c + coa_c --> amp_c + arachdcoa_c + ppi_c
C204CPT2 arachdcrn_m + coa_m --> arachdcoa_m + crn_m
FAOXC2242046x adrncoa_x + coa_x + h2o_x + nad_x + o2_x --> accoa_x + arachdcoa_x + h2o2_x + h_x + nadh_x
C204CPT2 arachdcrn_m + coa_m --> arachdcoa_m + crn_m

FATP6t adrn_c + na1_c <=> adrn_e + na1_e
FACOAL224 adrn_c + atp_c + coa_c --> adrncoa_c + amp_c + ppi_c
FATP6t adrn_c + na1_c <=> adrn_e + na1_e
ADRNCPT1 adrncoa_c + crn_c --> adrncrn_c + coa_c
FACOAL224 adrn_c + atp_c + coa_c --> adrncoa_c + amp_c + ppi_c
ADRNCPT2 adrncrn_m + coa_m --> adrncoa_m + crn_m
ADRNCPT2 adrncrn_m + coa_m --> adrncoa_m + crn_m
ADRNCRNt adrncrn_c --> adrncrn_m
```

In [119]:

```
model.remove_reactions(['FACOAE1839Z12Z15Z','FACOAL1832','LNLNCACPT1','LNLNCACPT2','LNLNCACRNt',
                        'FACOAL184','STRDNCCPT1','STRDNCCPT2','STRDNCCRNt',
                        'FACOAL203','DLNLCGCPT1','DLNLCGCPT2','DLNLCGCRNt',
                        'FATP5t','FACOAL204','P4504B1r','C204CPT1','C204CPT2',
                        'FAOXC2242046x','FATP6t','FACOAL224','ADRNCPT1','ADRNCPT2','ADRNCRNt'], remove_orphans=True)
```

In [120]:

```
for r in sorted(model.metabolites.get_by_id('eicostet_c').reactions, key=lambda x: x.id):
    print(r.id, r.reaction)
for r in sorted(model.metabolites.get_by_id('eicostetcoa_c').reactions, key=lambda x: x.id):
    print(r.id, r.reaction)
for r in sorted(model.metabolites.get_by_id('eicostetcoa_m').reactions, key=lambda x: x.id):
    print(r.id, r.reaction)
for r in sorted(model.metabolites.get_by_id('eicostetcrn_m').reactions, key=lambda x: x.id):
    print(r.id, r.reaction)
print()
for r in sorted(model.metabolites.get_by_id('tmndnc_c').reactions, key=lambda x: x.id):
    print(r.id, r.reaction)
for r in sorted(model.metabolites.get_by_id('tmndnccoa_c').reactions, key=lambda x: x.id):
    print(r.id, r.reaction)
for r in sorted(model.metabolites.get_by_id('tmndnccoa_m').reactions, key=lambda x: x.id):
    print(r.id, r.reaction)
for r in sorted(model.metabolites.get_by_id('tmndnccrn_m').reactions, key=lambda x: x.id):
    print(r.id, r.reaction)
print()
for r in sorted(model.metabolites.get_by_id('tetpent3_c').reactions, key=lambda x: x.id):
    print(r.id, r.reaction)
for r in sorted(model.metabolites.get_by_id('tetpent3coa_c').reactions, key=lambda x: x.id):
    print(r.id, r.reaction)
for r in sorted(model.metabolites.get_by_id('tetpent3coa_m').reactions, key=lambda x: x.id):
    print(r.id, r.reaction)
for r in sorted(model.metabolites.get_by_id('tetpent3crn_m').reactions, key=lambda x: x.id):
    print(r.id, r.reaction)
print()
for r in sorted(model.metabolites.get_by_id('tetpent6_c').reactions, key=lambda x: x.id):
    print(r.id, r.reaction)
for r in sorted(model.metabolites.get_by_id('tetpent6coa_c').reactions, key=lambda x: x.id):
    print(r.id, r.reaction)
for r in sorted(model.metabolites.get_by_id('tetpent6coa_m').reactions, key=lambda x: x.id):
    print(r.id, r.reaction)
for r in sorted(model.metabolites.get_by_id('tetpent6crn_m').reactions, key=lambda x: x.id):
    print(r.id, r.reaction)
print()
for r in sorted(model.metabolites.get_by_id('tethex3_c').reactions, key=lambda x: x.id):
    print(r.id, r.reaction)
for r in sorted(model.metabolites.get_by_id('tethex3coa_c').reactions, key=lambda x: x.id):
    print(r.id, r.reaction)
for r in sorted(model.metabolites.get_by_id('tethex3coa_x').reactions, key=lambda x: x.id):
    print(r.id, r.reaction)
print()
for r in sorted(model.metabolites.get_by_id('crvnc_e').reactions, key=lambda x: x.id):
    print(r.id, r.reaction)
for r in sorted(model.metabolites.get_by_id('crvnc_c').reactions, key=lambda x: x.id):
    print(r.id, r.reaction)
for r in sorted(model.metabolites.get_by_id('c226coa_c').reactions, key=lambda x: x.id):
    print(r.id, r.reaction)
for r in sorted(model.metabolites.get_by_id('c226coa_m').reactions, key=lambda x: x.id):
    print(r.id, r.reaction)
for r in sorted(model.metabolites.get_by_id('c226coa_x').reactions, key=lambda x: x.id):
    print(r.id, r.reaction)
for r in sorted(model.metabolites.get_by_id('c226crn_m').reactions, key=lambda x: x.id):
    print(r.id, r.reaction)
```

```
FACOAL2042 atp_c + coa_c + eicostet_c --> amp_c + eicostetcoa_c + ppi_c
EICOSTETCPT1 crn_c + eicostetcoa_c --> coa_c + eicostetcrn_c
FACOAL2042 atp_c + coa_c + eicostet_c --> amp_c + eicostetcoa_c + ppi_c
EICOSTETCPT2 coa_m + eicostetcrn_m --> crn_m + eicostetcoa_m
EICOSTETCPT2 coa_m + eicostetcrn_m --> crn_m + eicostetcoa_m
EICOSTETCRNt eicostetcrn_c --> eicostetcrn_m

FACOAL205 atp_c + coa_c + tmndnc_c --> amp_c + ppi_c + tmndnccoa_c
FACOAL205 atp_c + coa_c + tmndnc_c --> amp_c + ppi_c + tmndnccoa_c
TMNDNCCPT1 crn_c + tmndnccoa_c --> coa_c + tmndnccrn_c
TMNDNCCPT2 coa_m + tmndnccrn_m --> crn_m + tmndnccoa_m
TMNDNCCPT2 coa_m + tmndnccrn_m --> crn_m + tmndnccoa_m
TMNDNCCRNt tmndnccrn_c --> tmndnccrn_m

FACOAL245_2 atp_c + coa_c + tetpent3_c --> amp_c + ppi_c + tetpent3coa_c
FACOAL245_2 atp_c + coa_c + tetpent3_c --> amp_c + ppi_c + tetpent3coa_c
TETPENT3CPT1 crn_c + tetpent3coa_c --> coa_c + tetpent3crn_c
TETPENT3CPT2 coa_m + tetpent3crn_m --> crn_m + tetpent3coa_m
TETPENT3CPT2 coa_m + tetpent3crn_m --> crn_m + tetpent3coa_m
TETPENT3CRNt tetpent3crn_c --> tetpent3crn_m

FACOAL245_1 atp_c + coa_c + tetpent6_c --> amp_c + ppi_c + tetpent6coa_c
FACOAL245_1 atp_c + coa_c + tetpent6_c --> amp_c + ppi_c + tetpent6coa_c
TETPENT6CPT1 crn_c + tetpent6coa_c --> coa_c + tetpent6crn_c
TETPENT6CPT2 coa_m + tetpent6crn_m --> crn_m + tetpent6coa_m
TETPENT6CPT2 coa_m + tetpent6crn_m --> crn_m + tetpent6coa_m
TETPENT6CRNt tetpent6crn_c --> tetpent6crn_m

FACOAL246_1 atp_c + coa_c + tethex3_c --> amp_c + ppi_c + tethex3coa_c
FACOAL246_1 atp_c + coa_c + tethex3_c --> amp_c + ppi_c + tethex3coa_c
FAOXC246226x coa_x + h2o_x + nad_x + o2_x + tethex3coa_x --> accoa_x + c226coa_x + h2o2_x + h_x + nadh_x

FATP7t crvnc_c + na1_c <=> crvnc_e + na1_e
FACOAL226 atp_c + coa_c + crvnc_c --> amp_c + c226coa_c + ppi_c
FATP7t crvnc_c + na1_c <=> crvnc_e + na1_e
C226CPT1 c226coa_c + crn_c --> c226crn_c + coa_c
FACOAL226 atp_c + coa_c + crvnc_c --> amp_c + c226coa_c + ppi_c
C226CPT2 c226crn_m + coa_m --> c226coa_m + crn_m
FAOXC246226x coa_x + h2o_x + nad_x + o2_x + tethex3coa_x --> accoa_x + c226coa_x + h2o2_x + h_x + nadh_x
C226CPT2 c226crn_m + coa_m --> c226coa_m + crn_m
```

In [121]:

```
model.remove_reactions(['FACOAL2042','EICOSTETCPT1','EICOSTETCPT2','EICOSTETCRNt',
                        'FACOAL205','TMNDNCCPT1','TMNDNCCPT2','TMNDNCCRNt',
                        'FACOAL245_2','TETPENT3CPT1','TETPENT3CPT2','TETPENT3CRNt',
                        'FACOAL245_1','TETPENT6CPT1','TETPENT6CPT2','TETPENT6CRNt',
                        'FACOAL246_1','FAOXC246226x','FATP7t','FACOAL226','C226CPT1','C226CPT2'], remove_orphans=True)
```

In [122]:

```
for r in sorted(model.genes.get_by_id('9730').reactions, key=lambda x: x.id):
    print(r.id, r.reaction, r.gene_reaction_rule)
print()
for r in sorted(model.genes.get_by_id('8845').reactions, key=lambda x: x.id):
    print(r.id, r.reaction, r.gene_reaction_rule)
print()
for r in sorted(model.genes.get_by_id('9578').reactions, key=lambda x: x.id):
    print(r.id, r.reaction, r.gene_reaction_rule)
```

```
DESAT1619Zer h_r + nadh_r + o2_r + pmtcoa_r --> 2.0 h2o_r + hdcoa_r + nad_r 9730
DESAT1819Zer h_r + nadh_r + o2_r + stcoa_r --> 2.0 h2o_r + nad_r + odecoa_r 9730

DESAT1829Z12Zer h_r + nadh_r + o2_r + odecoa_r --> 2.0 h2o_r + nad_r + ocdycacoa_r 10123 and 16097 and 8845

DESAT1836Z9Z12Zer h_r + nadh_r + o2_r + ocdycacoa_r --> 2.0 h2o_r + lnlncgcoa_r + nad_r 9578
```

9629 plas 21, mito 2, E.R. 2 K04712: DEGS; sphingolipid Delta-4 desaturase, no cytochrome b5-like domain, generates a trans double bond at position 4 of sphinganine bases in sphingolipids, requires an external cytochrome b5 KDE *9578 plas 22, mito 3 K13076: SLD; Delta 6-fatty acid desaturase/delta-8 sphingolipid desaturase, has cytochrome b5-like domain, introduces a double bond at Delta 6 of fatty acids, introduces a trans double bond at the 8-position of sphingoid bases in sphingolipids LHH*  
10197 cyto 11.5, cyto\_nucl 7.5, mito 4, extr 4, E.R. 3, nucl 2.5 K20238: E2.1.1.317; sphingolipid C9-methyltransferase LVV *15324 plas 17, extr 6, vacu 2 K00720: UGCG; ceramide glucosyltransferase EPR*

Sphingolipid  
9629 dihydroceramide -> 4E-sphingosine ceramide  
9578 4E-sphingosine ceramide -> 4E,8E-sphingadienine ceramide  
10197 4E,8E-sphingadienine ceramide -> 9-methyl-4E,8E-sphingadienine ceramide  
15324 9-methyl-4E,8E-sphingadienine ceramide -> glucosylceramide
https://www.ncbi.nlm.nih.gov/pubmed/20019081

Ceramide transport from ER to golgi via vesicle (COPII) or CERT-like Nvj2 (14324)
https://www.ncbi.nlm.nih.gov/pubmed/11733544
https://www.ncbi.nlm.nih.gov/pubmed/28011845

In [123]:

```
for r in sorted(model.genes.get_by_id('9629').reactions, key=lambda x: x.id):
    print(r.id, r.reaction, r.gene_reaction_rule)
print()
for r in sorted(model.genes.get_by_id('9578').reactions, key=lambda x: x.id):
    print(r.id, r.reaction, r.gene_reaction_rule)
print()
for r in sorted(model.genes.get_by_id('10197').reactions, key=lambda x: x.id):
    print(r.id, r.reaction, r.gene_reaction_rule)
print()
for r in sorted(model.genes.get_by_id('15324').reactions, key=lambda x: x.id):
    print(r.id, r.reaction, r.gene_reaction_rule)
```

```
DHCRD1 dhcrm_hs_c + nadp_c --> crm_hs_c + h_c + nadph_c 9629
DHCRD2 dhcrm_hs_c + fad_c --> crm_hs_c + fadh2_c 9629
yli_R0706 0.01 pail_cho_c + yli_M05741_c --> 0.01 dag_hs_c + 0.01 yli_M04599_c 9629
yli_R0707 0.01 pail_cho_c + yli_M05742_c --> 0.01 dag_hs_c + 0.01 yli_M04600_c 9629
yli_R0708 0.01 pail_cho_c + yli_M05748_c --> 0.01 dag_hs_c + 0.01 yli_M04601_c 9629
yli_R0709 0.01 pail_cho_c + yli_M05749_c --> 0.01 dag_hs_c + 0.01 yli_M04602_c 9629
yli_R0710 0.01 pail_cho_c + yli_M05724_c --> 0.01 dag_hs_c + 0.01 yli_M04603_c 9629
yli_R0711 0.01 pail_cho_c + yli_M05725_c --> 0.01 dag_hs_c + 0.01 yli_M04604_c 9629

DESAT1836Z9Z12Zer h_r + nadh_r + o2_r + ocdycacoa_r --> 2.0 h2o_r + lnlncgcoa_r + nad_r 9578

CFAS160E 2.0 amet_c + pe161_c --> 2.0 ahcys_c + cpe160_c + 2.0 h_c 10197
CFAS160G 2.0 amet_c + pg161_c --> 2.0 ahcys_c + cpg160_c + 2.0 h_c 10197
CFAS180E 2.0 amet_c + pe181_c --> 2.0 ahcys_c + cpe180_c + 2.0 h_c 10197
CFAS180G 2.0 amet_c + pg181_c --> 2.0 ahcys_c + cpg180_c + 2.0 h_c 10197

UGCG crm_hs_r + udpg_r --> gluside_hs_r + h_r + udp_r 15324
```

In [124]:

```
# Remove irrelevant, human general, bacteria/plant specific reactions
# CFAS cyclopropane FA synthase are specific to bacteria/plant
model.remove_reactions(['DHCRD1','DHCRD2','yli_R0706','yli_R0707','yli_R0708','yli_R0709','yli_R0710',
                        'yli_R0711','CFAS160E','CFAS160G','CFAS180E','CFAS180G','UGCG'], remove_orphans=True)
# Need to add reactions for sphingolipid desaturases
```

In [125]:

```
for r in sorted(model.reactions, key=lambda x: x.id):
    if 'CERS' in r.id:
        print(r.id, r.reaction, r.compartments, r.subsystem, r.gene_reaction_rule)
```

```
CERS124er sphgn_r + ttccoa_r --> cer1_24_r + coa_r + h_r {'r'} S_Sphingolipid_Metabolism 11391 or 15168
CERS126er hexccoa_r + sphgn_r --> cer1_26_r + coa_r + h_r {'r'} S_Sphingolipid_Metabolism 11391 or 15168
CERS224er psphings_r + ttccoa_r --> cer2_24_r + coa_r + h_r {'r'} S_Sphingolipid_Metabolism 11391 or 15168
CERS226er hexccoa_r + psphings_r --> cer2_26_r + coa_r + h_r {'r'} S_Sphingolipid_Metabolism 11391 or 15168
CERS2p24er cer1_24_r + h_r + nadph_r + o2_r --> cer2p_24_r + h2o_r + nadp_r {'r'} S_Sphingolipid_Metabolism 9664
CERS2p26er cer1_26_r + h_r + nadph_r + o2_r --> cer2p_26_r + h2o_r + nadp_r {'r'} S_Sphingolipid_Metabolism 9664
CERS324er cer2_24_r + h_r + nadph_r + o2_r --> cer3_24_r + h2o_r + nadp_r {'r'} S_Sphingolipid_Metabolism 9664
CERS326er cer2_26_r + h_r + nadph_r + o2_r --> cer3_26_r + h2o_r + nadp_r {'r'} S_Sphingolipid_Metabolism 9664
```

In [126]:

```
for r in model.genes.get_by_id('15685').reactions:
    print(r)
model.metabolites.get_by_id('34dhbz_p')
```

```
ACLSm: h_m + 2.0 pyr_m --> alac__S_m + co2_m
ACHBSm: 2obut_m + h_m + pyr_m --> 2ahbut_m + co2_m
```

Out[126]:

|  |  |
| --- | --- |
| **Metabolite identifier** | 34dhbz\_p |
| **Name** | 3,4-Dihydroxybenzoate |
| **Memory address** | 0x0102f725978 |
| **Formula** | C7H5O4 |
| **Compartment** | p |
| **In 1 reaction(s)** | 3\_4DHBZt2 |

In [127]:

```
# check nadh/nadph since sphingolipid reactions use cytochrome b5
# may need to replace nadph with nadh in all reactions

# CERS118er sphgn_r + stcoa_r --> cer1_18_r + coa_r + h_r
# CERS2p18er cer1_18_r + h_r + nadph_r + o2_r --> cer2p_18_r + h2o_r + nadp_r
r1 = model.reactions.get_by_id('CERS124er').copy()
r2 = model.reactions.get_by_id('CERS2p24er').copy()
r1.id = 'CERS118er'
r2.id = 'CERS2p18er'
r1.name = 'Ceramide 1 synthase  18C   endoplasmic reticular'
r2.name = 'Ceramide 2p synthase  18C'
model.add_reactions([r1,r2])
m1 = model.metabolites.get_by_id('cer1_24_r').copy()
m2 = model.metabolites.get_by_id('cer2p_24_r').copy()
m1.id = 'cer1_18_r'
m2.id = 'cer2p_18_r'
m1.name = 'Ceramide 1 (Sphinganine:n-C18:0, Cer(d18:0/18:0))'
m2.name = 'Ceramide 2p (Sphinganine:n-C18:0OH, Cer(d18:0/18:0(2OH)))'
m1.formula = 'C36H73NO3'
m2.formula = 'C36H73NO4'
model.add_metabolites([m1,m2])
model.reactions.get_by_id('CERS118er').add_metabolites({'ttccoa_r': 1.0, 'stcoa_r': -1.0,
                                                        'cer1_24_r': -1.0, 'cer1_18_r': 1.0})
model.reactions.get_by_id('CERS2p18er').add_metabolites({'cer1_24_r': 1.0, 'cer1_18_r': -1.0,
                                                         'cer2p_24_r': -1.0, 'cer2p_18_r': 1.0})
# CERD418er cer2p_18_r + h_r + nadph_r + o2_r --> cer4_18_r + 2 h2o_r + nadp_r
# CERD518er cer4_18_r + h_r + nadph_r + o2_r --> cer5_18_r + 2 h2o_r + nadp_r
r1 = model.reactions.get_by_id('CERS2p18er').copy()
r2 = model.reactions.get_by_id('CERS2p18er').copy()
r1.id = 'CERD418er'
r2.id = 'CERD518er'
r1.name = 'Sphingolipid 4-desaturase  18C'
r2.name = 'Sphingolipid 8-desaturase  18C'
r1.gene_reaction_rule = '10123 and 16097 and 9629'
r2.gene_reaction_rule = '9578'
model.add_reactions([r1,r2])
m1 = model.metabolites.get_by_id('cer2p_18_r').copy()
m2 = model.metabolites.get_by_id('cer2p_18_r').copy()
m1.id = 'cer4_18_r'
m2.id = 'cer5_18_r'
m1.name = 'Ceramide 4 (Sphingosine:n-C18:0OH, Cer(d18:1(4E)/18:0(2OH)))'
m2.name = 'Ceramide 5 (Sphinga-4,8-dienine:n-C18:0OH, Cer(d18:2(4E,8E)/18:0(2OH)))'
m1.formula = 'C36H71NO4'
m2.formula = 'C36H69NO4'
model.add_metabolites([m1,m2])
model.reactions.get_by_id('CERD418er').add_metabolites({'cer1_18_r': 1.0, 'cer2p_18_r': -2.0,
                                                        'cer4_18_r': 1.0, 'h2o_r': 1.0})
model.reactions.get_by_id('CERD518er').add_metabolites({'cer1_18_r': 1.0, 'cer2p_18_r': -1.0,
                                                        'cer4_18_r': -1.0, 'cer5_18_r': 1.0, 'h2o_r': 1.0})
# CERMT618er cer5_18_r + amet_r --> cer6_18_r + ahcys_r + h_r
# CER618tg cer6_18_r --> cer6_18_g
# CERGT718g cer6_18_g + udpg_g --> glccer_18_g + h_g + udp_g
r1 = cobra.Reaction('CERMT618er')
r2 = cobra.Reaction('CER618trg')
r3 = cobra.Reaction('CERGT18g')
r1.name = 'Sphingolipid C9-methyltransferase  18C'
r2.name = 'Ceramide transport from ER to golgi'
r3.name = 'Ceramide glucosyltransferase  18C'
r1.subsystem = 'S_Sphingolipid_Metabolism'
r2.subsystem = 'S_Sphingolipid_Metabolism'
r3.subsystem = 'S_Sphingolipid_Metabolism'
r1.gene_reaction_rule = '10197'
r2.gene_reaction_rule = '14324 or COPII'
r3.gene_reaction_rule = '15324'
model.add_reactions([r1,r2,r3])
m1 = model.metabolites.get_by_id('cer5_18_r').copy()
m2 = model.metabolites.get_by_id('cer5_18_r').copy()
m3 = model.metabolites.get_by_id('cer5_18_r').copy()
m1.id = 'cer6_18_r'
m2.id = 'cer6_18_g'
m3.id = 'glccer_18_g'
m2.compartment = 'g'
m3.compartment = 'g'
m1.name = 'Ceramide 6 (9-Methyl-sphinga-4,8-dienine:n-C18:0OH, Cer(d18:2(4E,8E)(9Me)/18:0(2OH)))'
m2.name = 'Ceramide 6 (9-Methyl-sphinga-4,8-dienine:n-C18:0OH, Cer(d18:2(4E,8E)(9Me)/18:0(2OH)))'
m3.name = 'Glucosylceramide (Glucosyl 9-methyl-sphinga-4,8-dienine:n-C18:0OH, GlcCer(d18:2(4E,8E)(9Me)/18:0(2OH)))'
m1.formula = 'C37H71NO4'
m2.formula = 'C37H71NO4'
m3.formula = 'C43H81NO9'
model.add_metabolites([m1,m2,m3])
model.reactions.get_by_id('CERMT618er').add_metabolites({'cer5_18_r': -1.0, 'amet_r': -1.0,
                                                         'cer6_18_r': 1.0, 'ahcys_r': 1.0, 'h_r': 1.0})
model.reactions.get_by_id('CER618trg').add_metabolites({'cer6_18_r': -1.0, 'cer6_18_g': 1.0})
model.reactions.get_by_id('CERGT18g').add_metabolites({'cer6_18_g': -1.0, 'udpg_g': -1.0,
                                                        'glccer_18_g': 1.0, 'h_g': 1.0, 'udp_g': 1.0})
```

In [128]:

```
for r in sorted(model.reactions, key=lambda x: x.id):
    if 'CER' in r.id:
        print(r.id, r.reaction, r.compartments, r.gene_reaction_rule)
```

```
CER618trg cer6_18_r --> cer6_18_g {'g', 'r'} 14324 or COPII
CERD418er cer2p_18_r + h_r + nadph_r + o2_r --> cer4_18_r + 2.0 h2o_r + nadp_r {'r'} 10123 and 16097 and 9629
CERD518er cer4_18_r + h_r + nadph_r + o2_r --> cer5_18_r + 2.0 h2o_r + nadp_r {'r'} 9578
CERGT18g cer6_18_g + udpg_g --> glccer_18_g + h_g + udp_g {'g'} 15324
CERH124er cer1_24_r + h_r + nadph_r + o2_r --> cer2_24_r + h2o_r + nadp_r {'r'} 15314
CERH126er cer1_26_r + h_r + nadph_r + o2_r --> cer2_26_r + h2o_r + nadp_r {'r'} 15314
CERMT618er amet_r + cer5_18_r --> ahcys_r + cer6_18_r + h_r {'r'} 10197
CERS118er sphgn_r + stcoa_r --> cer1_18_r + coa_r + h_r {'r'} 11391 or 15168
CERS124er sphgn_r + ttccoa_r --> cer1_24_r + coa_r + h_r {'r'} 11391 or 15168
CERS126er hexccoa_r + sphgn_r --> cer1_26_r + coa_r + h_r {'r'} 11391 or 15168
CERS224er psphings_r + ttccoa_r --> cer2_24_r + coa_r + h_r {'r'} 11391 or 15168
CERS226er hexccoa_r + psphings_r --> cer2_26_r + coa_r + h_r {'r'} 11391 or 15168
CERS2p18er cer1_18_r + h_r + nadph_r + o2_r --> cer2p_18_r + h2o_r + nadp_r {'r'} 9664
CERS2p24er cer1_24_r + h_r + nadph_r + o2_r --> cer2p_24_r + h2o_r + nadp_r {'r'} 9664
CERS2p26er cer1_26_r + h_r + nadph_r + o2_r --> cer2p_26_r + h2o_r + nadp_r {'r'} 9664
CERS324er cer2_24_r + h_r + nadph_r + o2_r --> cer3_24_r + h2o_r + nadp_r {'r'} 9664
CERS326er cer2_26_r + h_r + nadph_r + o2_r --> cer3_26_r + h2o_r + nadp_r {'r'} 9664
```

In [129]:

```
for m in sorted(model.metabolites, key=lambda x: x.id):
    if m.id.startswith('cer'):
        print(m.id, m.name, m.formula, m.formula_weight)
print()
for r in sorted(model.reactions, key=lambda x: x.id):
    if r.id.startswith('CER'):
        print(r.id, r.name, r.gene_reaction_rule)
```

```
cer1_18_r Ceramide 1 (Sphinganine:n-C18:0, Cer(d18:0/18:0)) C36H73NO3 567.96972
cer1_24_c Ceramide 1  Sphinganinen C240  C42H85NO3 C42H85NO3 652.1292
cer1_24_r Ceramide 1  Sphinganinen C240  C42H85NO3 C42H85NO3 652.1292
cer1_26_c Ceramide 1  Sphinganinen C260  C44H89NO3 C44H89NO3 680.1823600000001
cer1_26_r Ceramide 1  Sphinganinen C260  C44H89NO3 C44H89NO3 680.1823600000001
cer2_24_c Ceramide 2  Phytosphingosinen C240  C42H85NO4 C42H85NO4 668.1286
cer2_24_r Ceramide 2  Phytosphingosinen C240  C42H85NO4 C42H85NO4 668.1286
cer2_26_c Ceramide 2  Phytosphingosinen C260  C44H89NO4 C44H89NO4 696.1817600000002
cer2_26_r Ceramide 2  Phytosphingosinen C260  C44H89NO4 C44H89NO4 696.1817600000002
cer2p_18_r Ceramide 2p (Sphinganine:n-C18:0OH, Cer(d18:0/18:0(2OH))) C36H73NO4 583.9691200000001
cer2p_24_r Ceramide 2p (Sphinganine:n-C24:0OH) C42H85NO4 668.1286
cer2p_26_r Ceramide 2p (Sphinganine:n-C26:0OH) C44H89NO4 696.1817600000002
cer3_24_c Ceramide 3  Phytosphingosinen C240OH  C42H85NO5 C42H85NO5 684.1279999999999
cer3_24_r Ceramide 3  Phytosphingosinen C240OH  C42H85NO5 C42H85NO5 684.1279999999999
cer3_26_c Ceramide 3  Phytosphingosinen C260OH  C44H89NO5 C44H89NO5 712.1811600000001
cer3_26_r Ceramide 3  Phytosphingosinen C260OH  C44H89NO5 C44H89NO5 712.1811600000001
cer4_18_r Ceramide 4 (Sphingosine:n-C18:0OH, Cer(d18:1(4E)/18:0(2OH))) C36H71NO4 581.95324
cer5_18_r Ceramide 5 (Sphinga-4,8-dienine:n-C18:0OH, Cer(d18:2(4E,8E)/18:0(2OH))) C36H69NO4 579.93736
cer6_18_g Ceramide 6 (9-Methyl-sphinga-4,8-dienine:n-C18:0OH, Cer(d18:2(4E,8E)(9Me)/18:0(2OH))) C37H71NO4 593.9639400000001
cer6_18_r Ceramide 6 (9-Methyl-sphinga-4,8-dienine:n-C18:0OH, Cer(d18:2(4E,8E)(9Me)/18:0(2OH))) C37H71NO4 593.9639400000001

CER618trg Ceramide transport from ER to golgi 14324 or COPII
CERD418er Sphingolipid 4-desaturase  18C 10123 and 16097 and 9629
CERD518er Sphingolipid 8-desaturase  18C 9578
CERGT18g Ceramide glucosyltransferase  18C 15324
CERH124er Ceramide 1 hydroxylase  24C  15314
CERH126er Ceramide 1 hydroxylase  26C  15314
CERMT618er Sphingolipid C9-methyltransferase  18C 10197
CERS118er Ceramide 1 synthase  18C   endoplasmic reticular 11391 or 15168
CERS124er Ceramide 1 synthase  24C   endoplasmic reticular 11391 or 15168
CERS126er Ceramide 1 synthase  26C   endoplasmic reticular 11391 or 15168
CERS224er Ceramide 2 synthase  24C   endoplasmic reticular 11391 or 15168
CERS226er Ceramide 2 synthase  26C   endoplasmic reticular 11391 or 15168
CERS2p18er Ceramide 2p synthase  18C 9664
CERS2p24er Ceramide 2p synthase  24C 9664
CERS2p26er Ceramide 2p synthase  26C 9664
CERS324er Ceramide 3 synthase  24C  9664
CERS326er Ceramide 3 synthase  26C  9664
```

In [130]:

```
for r in sorted(model.reactions, key=lambda x: x.id):
    if '124' in r.id:
        print(r.id, r.reaction, r.gene_reaction_rule)
```

```
CERH124er cer1_24_r + h_r + nadph_r + o2_r --> cer2_24_r + h2o_r + nadp_r 15314
CERS124er sphgn_r + ttccoa_r --> cer1_24_r + coa_r + h_r 11391 or 15168
IPC124PLC_SC h2o_c + 0.01 ipc124_SC_c --> cer1_24_c + h_c + mi1p__D_c 15857
IPCS124_SC cer1_24_c + 0.01 ptd1ino_SC_c --> 0.01 12dgr_SC_c + 0.01 ipc124_SC_c 12927
MIP2C124PLC_SC h2o_c + 0.01 mip2c124_SC_c --> cer1_24_c + h_c + man2mi1p__D_c 15857
MIP2CS124_SC 0.01 mipc124_SC_c + 0.01 ptd1ino_SC_c --> 0.01 12dgr_SC_c + 0.01 mip2c124_SC_c 13172
MIPC124PLC_SC h2o_c + 0.01 mipc124_SC_c --> cer1_24_c + h_c + manmi1p__D_c 15857
MIPCS124_SC gdpmann_c + 0.01 ipc124_SC_c --> gdp_c + h_c + 0.01 mipc124_SC_c YBR036C and 16453
```

Inositol phosphorylceramide synthase needs two subunits, Aur1 and Kei1 (12927 and 8747)
12927 plas 22, mito 2, vacu 2 Aur1, HMMPfam:PAP2 superfamily:PF01569,SMART:Acid phosphatase homologues:SM00014,SUPERFAMILY::SSF48317 RRD *8747 mito 7, extr 5, nucl 4, cyto\_mito 4, plas 3, pero 3 Kei1, HMMPfam:Inositolphosphorylceramide synthase subunit Kei1:PF08552 TPR*  
16453 extr 13, plas 12 CSH1/SUR1, HMMPfam:Glycosyltransferase sugar-binding region containing DXD motif:PF04488,SUPERFAMILY::SSF53448 ESP *13172 extr 10, mito 7, plas 4, cyto\_mito 4 Ipt1, HMMPfam:PAP2 superfamily:PF01569 SLA*  
15857 plas 14, mito 4, extr 4, cyto 3 Isc1, K12351: SMPD2; sphingomyelin phosphodiesterase 2 WTR\*

Change IPCSxxx\_SC genes to '12927 and 8747'  
Cannot find YBR036C (CSG2 regulatory subunit), change MIPCSxxx\_SC genes to '16453'  
Change compartment from cyto to golgi

Change IPCxxx\_SC / MIPCxxx\_SC to xxxg\_RT

In [131]:

```
for r in sorted(model.genes.get_by_id('12927').reactions, key=lambda x: x.id):
    print(r.id, r.reaction, r.gene_reaction_rule)
print()
for r in sorted(model.genes.get_by_id('16453').reactions, key=lambda x: x.id):
    print(r.id, r.reaction, r.gene_reaction_rule)
print()
for r in sorted(model.genes.get_by_id('13172').reactions, key=lambda x: x.id):
    print(r.id, r.reaction, r.gene_reaction_rule)
print()
for r in sorted(model.genes.get_by_id('15857').reactions, key=lambda x: x.id):
    print(r.id, r.reaction, r.gene_reaction_rule)
```

```
IPCS124_SC cer1_24_c + 0.01 ptd1ino_SC_c --> 0.01 12dgr_SC_c + 0.01 ipc124_SC_c 12927
IPCS126_SC cer1_26_c + 0.01 ptd1ino_SC_c --> 0.01 12dgr_SC_c + 0.01 ipc126_SC_c 12927
IPCS224_SC cer2_24_c + 0.01 ptd1ino_SC_c --> 0.01 12dgr_SC_c + 0.01 ipc224_SC_c 12927
IPCS226_SC cer2_26_c + 0.01 ptd1ino_SC_c --> 0.01 12dgr_SC_c + 0.01 ipc226_SC_c 12927
IPCS324_SC cer3_24_c + 0.01 ptd1ino_SC_c --> 0.01 12dgr_SC_c + 0.01 ipc324_SC_c 12927
IPCS326_SC cer3_26_c + 0.01 ptd1ino_SC_c --> 0.01 12dgr_SC_c + 0.01 ipc326_SC_c 12927
yli_R0718 0.01 pail_cho_c + 0.01 yli_M04615_c --> 0.01 dag_hs_c + 0.01 yli_M04609_c 12927
yli_R0719 0.01 pail_cho_c + 0.01 yli_M04616_c --> 0.01 dag_hs_c + 0.01 yli_M04610_c 12927
yli_R0720 0.01 pail_cho_c + 0.01 yli_M04617_c --> 0.01 dag_hs_c + 0.01 yli_M04611_c 12927
yli_R0721 0.01 pail_cho_c + 0.01 yli_M04618_c --> 0.01 dag_hs_c + 0.01 yli_M04612_c 12927
yli_R0722 0.01 pail_cho_c + 0.01 yli_M04619_c --> 0.01 dag_hs_c + 0.01 yli_M04613_c 12927
yli_R0723 0.01 pail_cho_c + 0.01 yli_M04620_c --> 0.01 dag_hs_c + 0.01 yli_M04614_c 12927

MIPCS124_SC gdpmann_c + 0.01 ipc124_SC_c --> gdp_c + h_c + 0.01 mipc124_SC_c YBR036C and 16453
MIPCS126_SC gdpmann_c + 0.01 ipc126_SC_c --> gdp_c + h_c + 0.01 mipc126_SC_c YBR036C and 16453
MIPCS224_SC gdpmann_c + 0.01 ipc224_SC_c --> gdp_c + h_c + 0.01 mipc224_SC_c YBR036C and 16453
MIPCS226_SC gdpmann_c + 0.01 ipc226_SC_c --> gdp_c + h_c + 0.01 mipc226_SC_c YBR036C and 16453
MIPCS324_SC gdpmann_c + 0.01 ipc324_SC_c --> gdp_c + h_c + 0.01 mipc324_SC_c YBR036C and 16453
MIPCS326_SC gdpmann_c + 0.01 ipc326_SC_c --> gdp_c + h_c + 0.01 mipc326_SC_c YBR036C and 16453

MIP2CS124_SC 0.01 mipc124_SC_c + 0.01 ptd1ino_SC_c --> 0.01 12dgr_SC_c + 0.01 mip2c124_SC_c 13172
MIP2CS126_SC 0.01 mipc126_SC_c + 0.01 ptd1ino_SC_c --> 0.01 12dgr_SC_c + 0.01 mip2c126_SC_c 13172
MIP2CS224_SC 0.01 mipc224_SC_c + 0.01 ptd1ino_SC_c --> 0.01 12dgr_SC_c + 0.01 mip2c224_SC_c 13172
MIP2CS226_SC 0.01 mipc226_SC_c + 0.01 ptd1ino_SC_c --> 0.01 12dgr_SC_c + 0.01 mip2c226_SC_c 13172
MIP2CS324_SC 0.01 mipc324_SC_c + 0.01 ptd1ino_SC_c --> 0.01 12dgr_SC_c + 0.01 mip2c324_SC_c 13172
MIP2CS326_SC 0.01 mipc326_SC_c + 0.01 ptd1ino_SC_c --> 0.01 12dgr_SC_c + 0.01 mip2c326_SC_c 13172

IPC124PLC_SC h2o_c + 0.01 ipc124_SC_c --> cer1_24_c + h_c + mi1p__D_c 15857
IPC126PLC_SC h2o_c + 0.01 ipc126_SC_c --> cer1_26_c + h_c + mi1p__D_c 15857
IPC224PLC_SC h2o_c + 0.01 ipc224_SC_c --> cer2_24_c + h_c + mi1p__D_c 15857
IPC226PLC_SC h2o_c + 0.01 ipc226_SC_c --> cer2_26_c + h_c + mi1p__D_c 15857
IPC324PLC_SC h2o_c + 0.01 ipc324_SC_c --> cer3_24_c + h_c + mi1p__D_c 15857
IPC326PLC_SC h2o_c + 0.01 ipc326_SC_c --> cer3_26_c + h_c + mi1p__D_c 15857
MIP2C124PLC_SC h2o_c + 0.01 mip2c124_SC_c --> cer1_24_c + h_c + man2mi1p__D_c 15857
MIP2C126PLC_SC h2o_c + 0.01 mip2c126_SC_c --> cer1_26_c + h_c + man2mi1p__D_c 15857
MIP2C224PLC_SC h2o_c + 0.01 mip2c224_SC_c --> cer2_24_c + h_c + man2mi1p__D_c 15857
MIP2C226PLC_SC h2o_c + 0.01 mip2c226_SC_c --> cer2_26_c + h_c + man2mi1p__D_c 15857
MIP2C324PLC_SC h2o_c + 0.01 mip2c324_SC_c --> cer3_24_c + h_c + man2mi1p__D_c 15857
MIP2C326PLC_SC h2o_c + 0.01 mip2c326_SC_c --> cer3_26_c + h_c + man2mi1p__D_c 15857
MIPC124PLC_SC h2o_c + 0.01 mipc124_SC_c --> cer1_24_c + h_c + manmi1p__D_c 15857
MIPC126PLC_SC h2o_c + 0.01 mipc126_SC_c --> cer1_26_c + h_c + manmi1p__D_c 15857
MIPC224PLC_SC h2o_c + 0.01 mipc224_SC_c --> cer2_24_c + h_c + manmi1p__D_c 15857
MIPC226PLC_SC h2o_c + 0.01 mipc226_SC_c --> cer2_26_c + h_c + manmi1p__D_c 15857
MIPC324PLC_SC h2o_c + 0.01 mipc324_SC_c --> cer3_24_c + h_c + manmi1p__D_c 15857
MIPC326PLC_SC h2o_c + 0.01 mipc326_SC_c --> cer3_26_c + h_c + manmi1p__D_c 15857
```

In [132]:

```
# Remove redundant yli reactions
model.remove_reactions(['yli_R0718','yli_R0719','yli_R0720','yli_R0721','yli_R0722','yli_R0723'], remove_orphans=True)
```

In [133]:

```
for g in ['12927','16453','13172','15857']:
    for r in model.genes.get_by_id(g).reactions:
        r.id = r.id.replace('_SC','g_RT')
        if g == '12927':
            r.gene_reaction_rule = '12927 and 8747'
        if g == '16453':
            r.gene_reaction_rule = '16453'
        for m in r.metabolites:
            if not m.id.replace('_c','_g') in model.metabolites:
                m2 = m.copy()
                m2.id = m.id.replace('_c','_g')
                m2.compartment = 'g'
                model.add_metabolites([m2])
            r.add_metabolites({m.id: -r.get_coefficient(m.id), m.id.replace('_c','_g'): r.get_coefficient(m.id)})
```

In [134]:

```
r = model.reactions.get_by_id('CER618trg').copy()
r.id = 'CER124trg'
model.add_reactions([r])
r.add_metabolites({'cer6_18_r': 1.0, 'cer6_18_g': -1.0, 'cer1_24_r': -1.0, 'cer1_24_g': 1.0})
r = model.reactions.get_by_id('CER618trg').copy()
r.id = 'CER126trg'
model.add_reactions([r])
r.add_metabolites({'cer6_18_r': 1.0, 'cer6_18_g': -1.0, 'cer1_26_r': -1.0, 'cer1_26_g': 1.0})
r = model.reactions.get_by_id('CER618trg').copy()
r.id = 'CER224trg'
model.add_reactions([r])
r.add_metabolites({'cer6_18_r': 1.0, 'cer6_18_g': -1.0, 'cer2_24_r': -1.0, 'cer2_24_g': 1.0})
r = model.reactions.get_by_id('CER618trg').copy()
r.id = 'CER226trg'
model.add_reactions([r])
r.add_metabolites({'cer6_18_r': 1.0, 'cer6_18_g': -1.0, 'cer2_26_r': -1.0, 'cer2_26_g': 1.0})
r = model.reactions.get_by_id('CER618trg').copy()
r.id = 'CER324trg'
model.add_reactions([r])
r.add_metabolites({'cer6_18_r': 1.0, 'cer6_18_g': -1.0, 'cer3_24_r': -1.0, 'cer3_24_g': 1.0})
r = model.reactions.get_by_id('CER618trg').copy()
r.id = 'CER326trg'
model.add_reactions([r])
r.add_metabolites({'cer6_18_r': 1.0, 'cer6_18_g': -1.0, 'cer3_26_r': -1.0, 'cer3_26_g': 1.0})
```

In [135]:

```
for r in sorted(model.genes.get_by_id('12927').reactions, key=lambda x: x.id):
    print(r.id, r.reaction, r.gene_reaction_rule)
print()
for r in sorted(model.genes.get_by_id('16453').reactions, key=lambda x: x.id):
    print(r.id, r.reaction, r.gene_reaction_rule)
print()
for r in sorted(model.genes.get_by_id('13172').reactions, key=lambda x: x.id):
    print(r.id, r.reaction, r.gene_reaction_rule)
print()
for r in sorted(model.genes.get_by_id('15857').reactions, key=lambda x: x.id):
    print(r.id, r.reaction, r.gene_reaction_rule)
```

```
IPCS124g_RT cer1_24_g + 0.01 ptd1ino_SC_g --> 0.01 12dgr_SC_g + 0.01 ipc124_SC_g 12927 and 8747
IPCS126g_RT cer1_26_g + 0.01 ptd1ino_SC_g --> 0.01 12dgr_SC_g + 0.01 ipc126_SC_g 12927 and 8747
IPCS224g_RT cer2_24_g + 0.01 ptd1ino_SC_g --> 0.01 12dgr_SC_g + 0.01 ipc224_SC_g 12927 and 8747
IPCS226g_RT cer2_26_g + 0.01 ptd1ino_SC_g --> 0.01 12dgr_SC_g + 0.01 ipc226_SC_g 12927 and 8747
IPCS324g_RT cer3_24_g + 0.01 ptd1ino_SC_g --> 0.01 12dgr_SC_g + 0.01 ipc324_SC_g 12927 and 8747
IPCS326g_RT cer3_26_g + 0.01 ptd1ino_SC_g --> 0.01 12dgr_SC_g + 0.01 ipc326_SC_g 12927 and 8747

MIPCS124g_RT gdpmann_g + 0.01 ipc124_SC_g --> gdp_g + h_g + 0.01 mipc124_SC_g 16453
MIPCS126g_RT gdpmann_g + 0.01 ipc126_SC_g --> gdp_g + h_g + 0.01 mipc126_SC_g 16453
MIPCS224g_RT gdpmann_g + 0.01 ipc224_SC_g --> gdp_g + h_g + 0.01 mipc224_SC_g 16453
MIPCS226g_RT gdpmann_g + 0.01 ipc226_SC_g --> gdp_g + h_g + 0.01 mipc226_SC_g 16453
MIPCS324g_RT gdpmann_g + 0.01 ipc324_SC_g --> gdp_g + h_g + 0.01 mipc324_SC_g 16453
MIPCS326g_RT gdpmann_g + 0.01 ipc326_SC_g --> gdp_g + h_g + 0.01 mipc326_SC_g 16453

MIP2CS124g_RT 0.01 mipc124_SC_g + 0.01 ptd1ino_SC_g --> 0.01 12dgr_SC_g + 0.01 mip2c124_SC_g 13172
MIP2CS126g_RT 0.01 mipc126_SC_g + 0.01 ptd1ino_SC_g --> 0.01 12dgr_SC_g + 0.01 mip2c126_SC_g 13172
MIP2CS224g_RT 0.01 mipc224_SC_g + 0.01 ptd1ino_SC_g --> 0.01 12dgr_SC_g + 0.01 mip2c224_SC_g 13172
MIP2CS226g_RT 0.01 mipc226_SC_g + 0.01 ptd1ino_SC_g --> 0.01 12dgr_SC_g + 0.01 mip2c226_SC_g 13172
MIP2CS324g_RT 0.01 mipc324_SC_g + 0.01 ptd1ino_SC_g --> 0.01 12dgr_SC_g + 0.01 mip2c324_SC_g 13172
MIP2CS326g_RT 0.01 mipc326_SC_g + 0.01 ptd1ino_SC_g --> 0.01 12dgr_SC_g + 0.01 mip2c326_SC_g 13172

IPC124PLCg_RT h2o_g + 0.01 ipc124_SC_g --> cer1_24_g + h_g + mi1p__D_g 15857
IPC126PLCg_RT h2o_g + 0.01 ipc126_SC_g --> cer1_26_g + h_g + mi1p__D_g 15857
IPC224PLCg_RT h2o_g + 0.01 ipc224_SC_g --> cer2_24_g + h_g + mi1p__D_g 15857
IPC226PLCg_RT h2o_g + 0.01 ipc226_SC_g --> cer2_26_g + h_g + mi1p__D_g 15857
IPC324PLCg_RT h2o_g + 0.01 ipc324_SC_g --> cer3_24_g + h_g + mi1p__D_g 15857
IPC326PLCg_RT h2o_g + 0.01 ipc326_SC_g --> cer3_26_g + h_g + mi1p__D_g 15857
MIP2C124PLCg_RT h2o_g + 0.01 mip2c124_SC_g --> cer1_24_g + h_g + man2mi1p__D_g 15857
MIP2C126PLCg_RT h2o_g + 0.01 mip2c126_SC_g --> cer1_26_g + h_g + man2mi1p__D_g 15857
MIP2C224PLCg_RT h2o_g + 0.01 mip2c224_SC_g --> cer2_24_g + h_g + man2mi1p__D_g 15857
MIP2C226PLCg_RT h2o_g + 0.01 mip2c226_SC_g --> cer2_26_g + h_g + man2mi1p__D_g 15857
MIP2C324PLCg_RT h2o_g + 0.01 mip2c324_SC_g --> cer3_24_g + h_g + man2mi1p__D_g 15857
MIP2C326PLCg_RT h2o_g + 0.01 mip2c326_SC_g --> cer3_26_g + h_g + man2mi1p__D_g 15857
MIPC124PLCg_RT h2o_g + 0.01 mipc124_SC_g --> cer1_24_g + h_g + manmi1p__D_g 15857
MIPC126PLCg_RT h2o_g + 0.01 mipc126_SC_g --> cer1_26_g + h_g + manmi1p__D_g 15857
MIPC224PLCg_RT h2o_g + 0.01 mipc224_SC_g --> cer2_24_g + h_g + manmi1p__D_g 15857
MIPC226PLCg_RT h2o_g + 0.01 mipc226_SC_g --> cer2_26_g + h_g + manmi1p__D_g 15857
MIPC324PLCg_RT h2o_g + 0.01 mipc324_SC_g --> cer3_24_g + h_g + manmi1p__D_g 15857
MIPC326PLCg_RT h2o_g + 0.01 mipc326_SC_g --> cer3_26_g + h_g + manmi1p__D_g 15857
```

cytchrome b5 reductase and cyt b5 10123 and 16097  
10123 cyto 14.5, cyto\_nucl 9.5, mito 5, nucl 3.5, extr 3 IRC21, 22% cov of CYB5R4, K00326: E1.6.2.2; cytochrome-b5 reductase VPE *13206 extr 8, cyto 7, E.R. 3, nucl 2, mito 2, plas 2, golg 2, mito\_nucl 2 CBR1, K00326: E1.6.2.2; cytochrome-b5 reductase FCF*  
12471 extr 11, mito 9, cyto 4, nucl 1, pero 1, E.R. 1 MCR1, K00326: E1.6.2.2; cytochrome-b5 reductase VKL*, no rxn  
15098 mito 24, cyto 3 MCR1, K00326: E1.6.2.2; cytochrome-b5 reductase FRF*

16097 cyto 23.5, cyto\_nucl 13 CYB5, KOG0537: Cytochrome b5 VAD  
15652 cyto 11, cyto\_nucl 10.333, cyto\_mito 9.333, nucl 8.5, mito 6.5 KOG0537: Cytochrome b5 GSL*, no rxn  
15808 mito 19, pero 4, cyto 2.5, cyto\_nucl 2.5 20% cov of LDH, KOG0537: Cytochrome b5 SKL*, no rxn

16097 wrong gene model / different isoform -> 960651, ~100% cov of cyt b5  
15652 low cov ~43% of cyt b5  
15808 wrong gene model / different isoform -> 742088, still low cov ~60% of cyt b5

For ER/golgi, (12471 or 13206) and 16097

In [136]:

```
for r in sorted(model.genes.get_by_id('10123').reactions, key=lambda x: x.id):
    print(r.id, r.reaction, r.gene_reaction_rule)
print()
for r in sorted(model.genes.get_by_id('13206').reactions, key=lambda x: x.id):
    print(r.id, r.reaction, r.gene_reaction_rule)
print()
for r in sorted(model.genes.get_by_id('15098').reactions, key=lambda x: x.id):
    print(r.id, r.reaction, r.gene_reaction_rule)
```

```
CERD418er cer2p_18_r + h_r + nadph_r + o2_r --> cer4_18_r + 2.0 h2o_r + nadp_r 10123 and 16097 and 9629
COA1819ZD9DS 2.0 focytb5_c + 2.0 h_c + o2_c + stcoa_c --> 2.0 ficytb5_c + 2.0 h2o_c + odecoa_c 10123 or 16097
CY_focytb5_c ficytb5_c <=> focytb5_c 10123 or 16097
DESAT1829Z12Zer h_r + nadh_r + o2_r + odecoa_r --> 2.0 h2o_r + nad_r + ocdycacoa_r 10123 and 16097 and 8845

C22STDSx ergtrol_c + h_c + nadh_c + o2_c --> ergtetrol_c + 2.0 h2o_c + nad_c (11716 and 13206 and 16097) or (11716 and 15098 and 16097)
LNS14DMx 2.0 h_c + lanost_c + 3.0 nadh_c + 3.0 o2_c --> 44mctr_c + for_c + 4.0 h2o_c + 3.0 nad_c (12843 and 13206 and 16097) or (12843 and 15098 and 16097)
SQLErx h_r + nadh_r + o2_r + sql_r --> Ssq23epx_r + h2o_r + nad_r (13206 and 13729 and 16097) or (13729 and 15098 and 16097)

C22STDSx ergtrol_c + h_c + nadh_c + o2_c --> ergtetrol_c + 2.0 h2o_c + nad_c (11716 and 13206 and 16097) or (11716 and 15098 and 16097)
LNS14DMx 2.0 h_c + lanost_c + 3.0 nadh_c + 3.0 o2_c --> 44mctr_c + for_c + 4.0 h2o_c + 3.0 nad_c (12843 and 13206 and 16097) or (12843 and 15098 and 16097)
SQLErx h_r + nadh_r + o2_r + sql_r --> Ssq23epx_r + h2o_r + nad_r (13206 and 13729 and 16097) or (13729 and 15098 and 16097)
```

In [137]:

```
model.reactions.get_by_id('DESAT1829Z12Zer').gene_reaction_rule = '(12471 and 16097 and 8845) or (13206 and 16097 and 8845)'
model.reactions.get_by_id('CERD418er').gene_reaction_rule = '(12471 and 16097 and 9629) or (13206 and 16097 and 9629)'
model.reactions.get_by_id('C22STDSx').gene_reaction_rule = '(11716 and 12471 and 16097) or (11716 and 13206 and 16097)'
model.reactions.get_by_id('LNS14DMx').gene_reaction_rule = '(12843 and 12471 and 16097) or (12843 and 13206 and 16097)'
model.reactions.get_by_id('SQLErx').gene_reaction_rule = '(13729 and 12471 and 16097) or (13729 and 13206 and 16097)'
```

In [138]:

```
for x in ['C22STDSx','LNS14DMx']:
    r = model.reactions.get_by_id(x)
    r.id = r.id.replace('x','rx')
    for m in r.metabolites:
        if not m.id.replace('_c','_r') in model.metabolites:
            m2 = m.copy()
            m2.id = m.id.replace('_c','_r')
            m2.compartment = 'r'
            model.add_metabolites([m2])
        r.add_metabolites({m.id: -r.get_coefficient(m.id), m.id.replace('_c','_r'): r.get_coefficient(m.id)})
```

In [139]:

```
for r in sorted(model.metabolites.get_by_id('ficytb5_c').reactions, key=lambda x: x.id):
    print(r.id, r.reaction, r.gene_reaction_rule)
```

```
COA1819ZD9DS 2.0 focytb5_c + 2.0 h_c + o2_c + stcoa_c --> 2.0 ficytb5_c + 2.0 h2o_c + odecoa_c 10123 or 16097
CY_focytb5_c ficytb5_c <=> focytb5_c 10123 or 16097
```

In [140]:

```
model.remove_reactions(['COA1819ZD9DS','CY_focytb5_c'], remove_orphans=True)
```

non-x reactions use NADPH, needs 13286 instead of cyt b5 and reductase  
13286 extr 12, E.R. 8, cyto 2, vacu 2, plas 1, cyto\_nucl 1, pero 1, golg 1, cyto\_mito 1 K00327: POR; NADPH-ferrihemoprotein reductase VWS\*

In [141]:

```
for r in sorted(model.genes.get_by_id('16591').reactions, key=lambda x: x.id):
    print(r.id, r.reaction, r.gene_reaction_rule)
print()
for r in sorted(model.genes.get_by_id('13729').reactions, key=lambda x: x.id):
    print(r.id, r.reaction, r.gene_reaction_rule)
print()
for r in sorted(model.genes.get_by_id('9240').reactions, key=lambda x: x.id):
    print(r.id, r.reaction, r.gene_reaction_rule)
print()
for r in sorted(model.genes.get_by_id('12843').reactions, key=lambda x: x.id):
    print(r.id, r.reaction, r.gene_reaction_rule)
print()
for r in sorted(model.genes.get_by_id('8671').reactions, key=lambda x: x.id):
    print(r.id, r.reaction, r.gene_reaction_rule)
print()
for r in sorted(model.genes.get_by_id('16640').reactions, key=lambda x: x.id):
    print(r.id, r.reaction, r.gene_reaction_rule)
print()
for r in sorted(model.genes.get_by_id('13724').reactions, key=lambda x: x.id):
    print(r.id, r.reaction, r.gene_reaction_rule)
print()
for r in sorted(model.genes.get_by_id('8835').reactions, key=lambda x: x.id):
    print(r.id, r.reaction, r.gene_reaction_rule)
```

```
PSPPS 2.0 frdp_c --> ppi_c + psqldp_c 16591
SQLS 2.0 frdp_c + h_c + nadph_c --> nadp_c + 2.0 ppi_c + sql_c 16591
SQLSr 2.0 frdp_r + h_r + nadph_r --> nadp_r + 2.0 ppi_r + sql_r 16591
SS h_c + nadph_c + psqldp_c --> nadp_c + ppi_c + sql_c 16591
yli_R0755 2.0 frdp_c --> nadp_c + ppi_c + psqldp_c 16591
yli_R1451 2.0 frdp_r --> nadp_r + ppi_r + psqldp_r 16591
yli_R1452 h_r + nadph_r + psqldp_r --> nadp_r + ppi_r + sql_r 16591

SMO h_c + nadph_c + o2_c + sql_c --> Ssq23epx_c + h2o_c + nadp_c 13729
SQLEr h_r + nadph_r + o2_r + sql_r --> Ssq23epx_r + h2o_r + nadp_r 13729 or (13286 and 13729)
SQLErx h_r + nadh_r + o2_r + sql_r --> Ssq23epx_r + h2o_r + nad_r (13729 and 12471 and 16097) or (13729 and 13206 and 16097)

CAS Ssq23epx_c <=> cyart_c 9240
LNSTLS Ssq23epx_c --> lanost_c 9240
LNSTLSr Ssq23epx_r --> lanost_r 9240

LNS14DM 2.0 h_c + lanost_c + 3.0 nadph_c + 3.0 o2_c --> 44mctr_c + for_c + 4.0 h2o_c + 3.0 nadp_c 12843 or (12843 and 13286)
LNS14DMr 2.0 h_r + lanost_r + 3.0 nadph_r + 3.0 o2_r --> 44mctr_r + for_r + 4.0 h2o_r + 3.0 nadp_r 12843
LNS14DMrx 2.0 h_r + lanost_r + 3.0 nadh_r + 3.0 o2_r --> 44mctr_r + for_r + 4.0 h2o_r + 3.0 nad_r (12843 and 12471 and 16097) or (12843 and 13206 and 16097)
OBFOOLOR 2.0 h_c + 3.0 nadph_c + 3.0 o2_c + obfool_c --> for_c + 4.0 h2o_c + mergtrol_c + 3.0 nadp_c 12843
yli_R0744 2.0 h_c + lanost_c + 3.0 nadph_c + 3.0 o2_c --> for_c + 4.0 h2o_c + 3.0 nadp_c + yli_M03503_c 12843
yli_R1444 2.0 h_r + lanost_r + 3.0 nadph_r + 3.0 o2_r --> for_r + 4.0 h2o_r + 3.0 nadp_r + yli_M03503_r 12843

C14STR 44mctr_c + h_c + nadph_c --> 44mzym_c + nadp_c 8671
C14STRr 44mctr_r + h_r + nadph_r --> 44mzym_r + nadp_r 8671
MERGTROLR h_c + mergtrol_c + nadph_c <=> mfecostrl_c + nadp_c 8671
yli_R0748 h_c + nadph_c + yli_M03503_c --> nadp_c + yli_M02486_c 8671
yli_R1447 h_r + nadph_r + yli_M03503_r --> nadp_r + yli_M02486_r 8671

C4STMO1 44mzym_c + 3.0 h_c + 3.0 nadph_c + 3.0 o2_c --> 4mzym_int1_c + 4.0 h2o_c + 3.0 nadp_c 16640
C4STMO1r 44mzym_r + 3.0 h_r + 3.0 nadph_r + 3.0 o2_r --> 4mzym_int1_r + 4.0 h2o_r + 3.0 nadp_r 16640
C4STMO2 4mzym_c + 3.0 h_c + 3.0 nadph_c + 3.0 o2_c --> 4.0 h2o_c + 3.0 nadp_c + zym_int1_c 16640
C4STMO2r 4mzym_int2_r + nad_r + o2_r --> co2_r + h_r + nadh_r + zym_int2_r 13724 or 16640 or 8835
yli_R0746 3.0 h_c + 3.0 nadph_c + 3.0 o2_c + yli_M02486_c --> 4.0 h2o_c + 3.0 nadp_c + yli_M03891_c 16640
yli_R1445 3.0 h_r + 3.0 nadph_r + 3.0 o2_r + yli_M02486_r --> 4.0 h2o_r + 3.0 nadp_r + yli_M03891_r 16640

C3STDH1 4mzym_int1_c + nad_c --> 4mzym_int2_c + co2_c + h_c + nadh_c 13724 or 8835
C3STDH1Pr 4mzym_int1_r + nadp_r --> 4mzym_int2_r + co2_r + h_r + nadph_r 13724 or 8835
C3STDH1r 4mzym_int1_r + nad_r --> 4mzym_int2_r + co2_r + h_r + nadh_r 13724 or 8835
C3STDH2 nad_c + zym_int1_c --> co2_c + h_c + nadh_c + zym_int2_c 13724 or 8835
C4STMO2Pr 4mzym_int2_r + nadp_r + o2_r --> co2_r + h_r + nadph_r + zym_int2_r 13724 or 8835
C4STMO2r 4mzym_int2_r + nad_r + o2_r --> co2_r + h_r + nadh_r + zym_int2_r 13724 or 16640 or 8835
yli_R0742 nadp_c + yli_M03891_c --> co2_c + h_c + nadph_c + yli_M03899_c 13724 or 8835
yli_R1442 nadp_r + yli_M03891_r --> co2_r + h_r + nadph_r + yli_M03899_r 13724 or 8835

4MZYMCODH 4mzym_int1_c + nadp_c --> 4mzym_int2_c + co2_c + nadph_c 8835
C3STDH1 4mzym_int1_c + nad_c --> 4mzym_int2_c + co2_c + h_c + nadh_c 13724 or 8835
C3STDH1Pr 4mzym_int1_r + nadp_r --> 4mzym_int2_r + co2_r + h_r + nadph_r 13724 or 8835
C3STDH1r 4mzym_int1_r + nad_r --> 4mzym_int2_r + co2_r + h_r + nadh_r 13724 or 8835
C3STDH2 nad_c + zym_int1_c --> co2_c + h_c + nadh_c + zym_int2_c 13724 or 8835
C4STMO2Pr 4mzym_int2_r + nadp_r + o2_r --> co2_r + h_r + nadph_r + zym_int2_r 13724 or 8835
C4STMO2r 4mzym_int2_r + nad_r + o2_r --> co2_r + h_r + nadh_r + zym_int2_r 13724 or 16640 or 8835
yli_R0742 nadp_c + yli_M03891_c --> co2_c + h_c + nadph_c + yli_M03899_c 13724 or 8835
yli_R1442 nadp_r + yli_M03891_r --> co2_r + h_r + nadph_r + yli_M03899_r 13724 or 8835
```

In [142]:

```
for r in sorted(model.genes.get_by_id('13060').reactions, key=lambda x: x.id):
    print(r.id, r.reaction, r.gene_reaction_rule)
print()
for r in sorted(model.genes.get_by_id('10113').reactions, key=lambda x: x.id):
    print(r.id, r.reaction, r.gene_reaction_rule)
print()
for r in sorted(model.genes.get_by_id('8781').reactions, key=lambda x: x.id):
    print(r.id, r.reaction, r.gene_reaction_rule)
print()
for r in sorted(model.genes.get_by_id('11716').reactions, key=lambda x: x.id):
    print(r.id, r.reaction, r.gene_reaction_rule)
print()
for r in sorted(model.genes.get_by_id('10981').reactions, key=lambda x: x.id):
    print(r.id, r.reaction, r.gene_reaction_rule)
print()
for r in sorted(model.genes.get_by_id('12791').reactions, key=lambda x: x.id):
    print(r.id, r.reaction, r.gene_reaction_rule)
print()
for r in sorted(model.genes.get_by_id('13559').reactions, key=lambda x: x.id):
    print(r.id, r.reaction, r.gene_reaction_rule)
```

```
SAM24MT amet_c + zymst_c --> ahcys_c + fecost_c + h_c 13060
yli_R1449 amet_r + zymst_r --> ahcys_r + fecost_r + h_r 13060

C8STI fecost_c --> epist_c 10113
yli_R1454 fecost_r --> epist_r 10113

C5STDS epist_c + h_c + nadph_c + o2_c --> ergtrol_c + 2.0 h2o_c + nadp_c 8781
LSTO1r chlstol_r + h_r + nadph_r + o2_r --> ddsmsterol_r + 2.0 h2o_r + nadp_r 8781
LSTO2r h_r + lthstrl_r + nadph_r + o2_r --> 7dhchsterol_r + 2.0 h2o_r + nadp_r 8781
LTHSTRLOR h_c + lthstrl_c + nadph_c + o2_c --> 7dhchsterol_c + 2.0 h2o_c + nadp_c 8781
yli_R0758 epist_c + nadp_c --> h_c + nadph_c + yli_M03873_c 8781
yli_R1455 epist_r + nadp_r --> h_r + nadph_r + yli_M03873_r 8781

C22STDS ergtrol_c + h_c + nadph_c + o2_c --> ergtetrol_c + 2.0 h2o_c + nadp_c 11716 and 13286
C22STDSrx ergtrol_r + h_r + nadh_r + o2_r --> ergtetrol_r + 2.0 h2o_r + nad_r (11716 and 12471 and 16097) or (11716 and 13206 and 16097)
yli_R0759 nadp_c + yli_M03873_c --> ergtetrol_c + h_c + nadph_c 11716
yli_R1456 nadp_r + yli_M03873_r --> ergtetrol_r + h_r + nadph_r 11716

C24STRer ergtetrol_r + h_r + nadph_r --> ergst_r + nadp_r 10981
ERGSTOLR_c ergtetrol_c + h_c + nadph_c --> ergst_c + nadp_c 10981

CHLSTD78I zymstnl_c --> lthstrl_c 12791 or 13559
CHLSTI_1 zymst_c --> chlstol_c 12791 or 13559

CHLSTD78I zymstnl_c --> lthstrl_c 12791 or 13559
CHLSTI_1 zymst_c --> chlstol_c 12791 or 13559
```

In [143]:

```
# Remove cyto/redundant sterol reactions
# ERG27 is missing - potential function of succinate semialdehyde dehydrogenase in ERG27-missing organisms 
# https://www.ncbi.nlm.nih.gov/pmc/articles/PMC2817430/
Remove = ['yli_R0755','PSPPS','SS','SQLS','yli_R1451','yli_R1452','SMO','CAS','LNSTLS','yli_R0744','yli_R0748',
          'yli_R0746','yli_R0742','yli_R1444','yli_R1447','yli_R1445','yli_R1442','LNS14DM','C14STR','C4STMO1',
          'C3STDH1','OBFOOLOR','MERGTROLR','44MZYMMO','4MZYMCODH','yli_R1449','yli_R1454','yli_R1455','yli_R1456',
          'yli_R0758','yli_R0759','ERGSTOLR_c','CHLSTD78I','LSTO2r','LTHSTRLOR']
model.remove_reactions(Remove, remove_orphans=True)
#C4STMO2r, C4STMO2Pr wrong -> replace with C4STMO2 and C3STDH2, and change to ER 
model.remove_reactions(['C4STMO2r','C4STMO2Pr'], remove_orphans=True)
# check nadh/nadph for reactions
model.reactions.get_by_id('SQLEr').gene_reaction_rule = '13286 and 13729'
model.reactions.get_by_id('LNS14DMr').gene_reaction_rule = '12843 and 13286'
# 12791 DnaJ homolog, 13559 C-8,7 sterol isomerase
model.reactions.get_by_id('CHLSTI_1').gene_reaction_rule = '13559'
# 8781 can convert chlstol to ddsmsterol -> keep LSTO1r
# Add ERG27 reactions from sce
r1 = sce.reactions.get_by_id('C3STKR1')
r2 = sce.reactions.get_by_id('C3STKR2')
r1.gene_reaction_rule = 'ERG27'
r2.gene_reaction_rule = 'ERG27'
model.add_reactions([r1,r2])
# Change cyto to ER
for x in ['C3STKR1','C4STMO2','C3STDH2','C3STKR2','SAM24MT','C8STI','C5STDS','C22STDS','CHLSTI_1']:
    r = model.reactions.get_by_id(x)
    r.id = r.id+'er'
    for m in r.metabolites:
        if not m.id.replace('_c','_r') in model.metabolites:
            m2 = m.copy()
            m2.id = m.id.replace('_c','_r')
            m2.compartment = 'r'
            model.add_metabolites([m2])
        r.add_metabolites({m.id: -r.get_coefficient(m.id), m.id.replace('_c','_r'): r.get_coefficient(m.id)})
```

In [144]:

```
for r in sorted(model.genes.get_by_id('13286').reactions, key=lambda x: x.id):
    print(r.id, r.reaction, r.gene_reaction_rule)
```

```
C22STDSer ergtrol_r + h_r + nadph_r + o2_r --> ergtetrol_r + 2.0 h2o_r + nadp_r 11716 and 13286
CYTP450R cytP450o_c + h_c + nadph_c <=> cytP450r_c + nadp_c 13286
CYTP450Rh cytP450o_h + h_h + nadph_h <=> cytP450r_h + nadp_h 13286
FADRx2 fad_c + h_c + nadph_c --> fadh2_c + nadp_c 13286 and 8744
FLVR h_c + nadph_c + ribflv_c --> nadp_c + rbflvrd_c 13286 and 8744
FMNRx2 fmn_c + h_c + nadph_c --> fmnh2_c + nadp_c 13286 and 8744
LNS14DMr 2.0 h_r + lanost_r + 3.0 nadph_r + 3.0 o2_r --> 44mctr_r + for_r + 4.0 h2o_r + 3.0 nadp_r 12843 and 13286
NFTYROX 2.0 Nfortyr_c + h_c + nadph_c --> Nbfortyr_c + nadp_c (13286 and 13361) or (13286 and 8979) or (13286 and 9188)
SQLEr h_r + nadph_r + o2_r + sql_r --> Ssq23epx_r + h2o_r + nadp_r 13286 and 13729
SULR 5.0 h_c + 3.0 nadph_c + so3_c --> 3.0 h2o_c + h2s_c + 3.0 nadp_c 10374 or 8744 or (PP_0860 and 8744) or (10374 and 8744) or (13286 and 8744)
```

13361 extr 7, E.R. 7, cyto 4, plas 3, golg 2, nucl 1, mito 1, pero 1, vacu 1, mito\_nucl 1 K20660: CYP709; cytochrome P450, family 709 DEE *8979 plas 8, extr 5, cyto 3, pero 3, E.R. 3, cyto\_pero 3 K00490: CYP4F; cytochrome P450, family 4, subfamily F DEV*  
9188 nucl 8, pero 5, cyto\_mito 5, mito 4.5, cyto 4.5, extr 4 K20495: CYP704B1; long-chain fatty acid omega-monooxygenase KRA *10374 cyto 16, cyto\_mito 11.333, cyto\_nucl 9.833, mito 5.5, nucl 2.5 K00380: cysJ; sulfite reductase (NADPH) flavoprotein alpha-component HTQ*  
8744 mito 9, cyto 8, nucl 4, extr 4 K00381: cysI; sulfite reductase (NADPH) hemoprotein beta-component CPQ\*

9188 CYP704B1; long-chain fatty acid omega-monooxygenase expression is up in bisabolene strains at 48h, need to add reactions for this enzyme

In [145]:

```
for r in sorted(model.genes.get_by_id('13361').reactions, key=lambda x: x.id):
    print(r.id, r.reaction, r.gene_reaction_rule)
print()
for r in sorted(model.genes.get_by_id('8979').reactions, key=lambda x: x.id):
    print(r.id, r.reaction, r.gene_reaction_rule)
print()
for r in sorted(model.genes.get_by_id('9188').reactions, key=lambda x: x.id):
    print(r.id, r.reaction, r.gene_reaction_rule)
print()
for r in sorted(model.genes.get_by_id('10374').reactions, key=lambda x: x.id):
    print(r.id, r.reaction, r.gene_reaction_rule)
print()
for r in sorted(model.genes.get_by_id('8744').reactions, key=lambda x: x.id):
    print(r.id, r.reaction, r.gene_reaction_rule)
```

```
NFTYROX 2.0 Nfortyr_c + h_c + nadph_c --> Nbfortyr_c + nadp_c (13286 and 13361) or (13286 and 8979) or (13286 and 9188)

NFTYROX 2.0 Nfortyr_c + h_c + nadph_c --> Nbfortyr_c + nadp_c (13286 and 13361) or (13286 and 8979) or (13286 and 9188)

NFTYROX 2.0 Nfortyr_c + h_c + nadph_c --> Nbfortyr_c + nadp_c (13286 and 13361) or (13286 and 8979) or (13286 and 9188)

SULR 5.0 h_c + 3.0 nadph_c + so3_c --> 3.0 h2o_c + h2s_c + 3.0 nadp_c 10374 or 8744 or (PP_0860 and 8744) or (10374 and 8744) or (13286 and 8744)

FADRx2 fad_c + h_c + nadph_c --> fadh2_c + nadp_c 13286 and 8744
FLVR h_c + nadph_c + ribflv_c --> nadp_c + rbflvrd_c 13286 and 8744
FMNRx2 fmn_c + h_c + nadph_c --> fmnh2_c + nadp_c 13286 and 8744
NO2R 6.0 fdxrd_h + no2_h --> 6.0 fdxox_h + 2.0 h2o_h + 4.0 h_h + nh4_h (CRv4_Au5_s16_g6229_t1 and 8744) or (CRv4_Au5_s17_g7064_t1 and 8744) or (CRv4_Au5_s3_g10824_t1 and 8744) or (CRv4_Au5_s6_g13523_t1 and 8744) or (CRv4_Au5_s7_g14133_t1 and 8744)
SELTORc 6.0 fdxrd_c + slnt_c --> 6.0 fdxox_c + 3.0 h2o_c + 6.0 h_c + seln_c (CRv4_Au5_s12_g2838_t1 and 8744) or (CRv4_Au5_s1_g1092_t1 and 8744) or (CRv4_Au5_s6_g13230_t1 and 8744)
SELTORm 6.0 fdxrd_m + slnt_m --> 6.0 fdxox_m + 3.0 h2o_m + 6.0 h_m + seln_m 15585 and 8744
SULR 5.0 h_c + 3.0 nadph_c + so3_c --> 3.0 h2o_c + h2s_c + 3.0 nadp_c 10374 or 8744 or (PP_0860 and 8744) or (10374 and 8744) or (13286 and 8744)
SULR_ferr 6.0 fdxrd_h + so3_h --> 6.0 fdxox_h + 3.0 h2o_h + h2s_h + 6.0 h_h (CRv4_Au5_s12_g2838_t1 and 8744) or (CRv4_Au5_s1_g1092_t1 and 8744) or (CRv4_Au5_s6_g13230_t1 and 8744)
```

In [146]:

```
# CYTP450 - NADPH reductase
model.reactions.get_by_id('SULR').gene_reaction_rule = '10374 and 8744'
# CYTP450R/h blocked, FADRx2 and FLVR by E. coli cysJ blocked
# Need to update Iron-sulfur cluster biosynthesis and riboflavin biosynthesis
# NFTYROX only present in iMM904 and involved in spore wall maturation, ortholog not found in IFO0880
model.remove_reactions(['CYTP450R','CYTP450Rh','FADRx2','FLVR','FMNRx2','NFTYROX'], remove_orphans=True)
model.remove_reactions(['NO2R','SELTORc','SELTORm','SULR_ferr'], remove_orphans=True)
```

Clean up mevalonate pathway  
ERG10 8678 mito 23.5, cyto\_mito 14 K00626: E2.3.1.9, atoB; acetyl-CoA C-acetyltransferase QRL *ERG13 12122 nucl 11, cyto\_nucl 10, mito 9, cyto 7 K01641: E2.3.3.10; hydroxymethylglutaryl-CoA synthase HVA*  
HMG2 9574 plas 16, E.R. 7, mito 3 K00021: HMGCR; hydroxymethylglutaryl-CoA reductase (NADPH) GKD *HMGL 15693 mito 27 K01640: E4.1.3.4, HMGCL, hmgL; hydroxymethylglutaryl-CoA lyase AKL*  
AACS 15276 plas 9, pero 6, mito 5, cyto 5, cyto\_mito 5 K01907: AACS, acsA; acetoacetyl-CoA synthetase SKL *ERG12 8758 cyto 13, extr 9, cyto\_nucl 8.833, cyto\_mito 7.833, nucl 3.5 K00869: E2.7.1.36, MVK, mvaK1; mevalonate kinase VCA*  
ERG8 8702 cyto 16, cyto\_mito 11.833, cyto\_nucl 9.833, mito 6.5 K00938: E2.7.4.2, mvaK2; phosphomevalonate kinase KKR *MVD1 11910 cyto 14.5, cyto\_mito 13, mito 10.5 K01597: MVD, mvaD; diphosphomevalonate decarboxylase SLK*  
IDI1 16203 cyto 11, cyto\_nucl 10.5, nucl 8, cysk 5 K01823: idi, IDI; isopentenyl-diphosphate Delta-isomerase IRM\*

# IDI2 16219 cyto 17, cyto\_nucl 13, nucl 7 KOG0142: Isopentenyl pyrophosphate:dimethylallyl pyrophosphate isomerase IRM\* (only ~50% coverage)¶

ERG20 12944 cyto 16.5, cyto\_nucl 13.5, nucl 9.5 K00787: FDPS; farnesyl diphosphate synthase RQK *BTS1 16503 cysk 9, cyto 8.5, cyto\_nucl 7.5, nucl 5.5, pero 2 K00804: GGPS1; geranylgeranyl diphosphate synthase, type III PKA*

# PSY1 8589 plas 18, extr 3, mito 2, E.R. 2 HMMPfam:Squalene/phytoene synthase:PF00494,ProSitePatterns:Squalene and phytoene synthases signature 2.:PS01045,SUPERFAMILY::SSF48576,TIGRFAM:CarR\_dom\_SF: lycopene cyclase domain:TIGR03462 QAL\*¶

# CRTI 8585 mito 7, plas 5, cyto 4, extr 4, E.R. 4, golg 3 K02292: crtO; beta-carotene ketolase (CrtO type) LRG\*¶

ERG9 16591 mito 16.5, cyto\_mito 11.5, cyto 5.5, pero 3 K00801: FDFT1; farnesyl-diphosphate farnesyltransferase KQLP  
ERG1 13729 plas 18, extr 3, E.R. 3, cyto 2 K00511: SQLE, ERG1; squalene monooxygenase GQM *ERG7 9240 cysk 22, cyto 5 K01852: LSS, ERG7; lanosterol synthase EGW*  
ERG11 12843 cyto 9, E.R. 5, mito 3, extr 3, plas 2, pero 2, vacu 2, mito\_nucl 2 K05917: CYP51; sterol 14-demethylase KVL *ERG24 8671 plas 13, mito 9, cyto 3 K00222: TM7SF2, ERG24; Delta14-sterol reductase YVY*  
ERG25 16640 cyto 14, cyto\_nucl 9.333, cyto\_pero 8.833, mito 6, nucl 3.5 K07750: E1.14.13.72, SC4MOL, ERG25; methylsterol monooxygenase KAQ *ERG26 8835 cyto 13, cyto\_nucl 8.833, cyto\_mito 8.833, pero 6, nucl 3.5, mito 3.5 K07748: E1.1.1.170, NSDHL, ERG26; sterol-4alpha-carboxylate 3-dehydrogenase (decarboxylating) KKA*  
ERG26 13724 mito 16, nucl 5, cyto 3, pero 2 K07748: E1.1.1.170, NSDHL, ERG26; sterol-4alpha-carboxylate 3-dehydrogenase (decarboxylating) VVA *ERG6 13060 mito 11, cyto 8, cyto\_pero 7.333, cyto\_nucl 5.833, pero 5.5 K00559: E2.1.1.41, SMT1, ERG6; sterol 24-C-methyltransferase HSQ*  
ERG2 10113 plas 17, E.R. 5, mito 2, extr 2 K09829: ERG2; C-8 sterol isomerase GKI *ERG3 8781 plas 26 K00227: SC5DL, ERG3; Delta7-sterol 5-desaturase ARK*  
ERG5 11716 plas 14, E.R. 8, vacu 3 K09831: ERG5, CYP61A; sterol 22-desaturase QDL *ERG4 10981 plas 18, E.R. 5, mito 3 K00223: ERG4; Delta24(24(1))-sterol reductase YVF*

In [147]:

```
for x in ['8678','12122','9574','15693','15276','8758','8702','11910','16203','12944','16503','16591','13729','9240','12843','8671','16640','8835','13724','13060','10113','8781','11716','10981']:
    for r in sorted(model.genes.get_by_id(x).reactions, key=lambda x: x.id):
        print(r.id, r.reaction, r.gene_reaction_rule)
    print()
```

```
ACACT10m 2maacoa_m + coa_m <=> accoa_m + ppcoa_m 8678
ACACT1m 2.0 accoa_m <=> aacoa_m + coa_m 8678
ACACT1r 2.0 accoa_c <=> aacoa_c + coa_c 8678

HMGCOAS coa_c + h_c + hmgcoa_c <=> aacoa_c + accoa_c + h2o_c 12122
HMGCOASm coa_m + h_m + hmgcoa_m <=> aacoa_m + accoa_m + h2o_m 12122
MHGS aacoa_c + accoa_c + h2o_c --> coa_c + hmgcoa_c 12122

HMGCOAR coa_c + mev__R_c + 2.0 nadp_c <=> 2.0 h_c + hmgcoa_c + 2.0 nadph_c 9574
HMGCOARr 2.0 h_r + hmgcoa_r + 2.0 nadph_r --> coa_r + mev__R_r + 2.0 nadp_r 9574
HMGCOARx 2.0 h_x + hmgcoa_x + 2.0 nadph_x --> coa_x + mev__R_x + 2.0 nadp_x 9574

HMGL hmgcoa_c --> acac_c + accoa_c 15693
HMGLm hmgcoa_m --> acac_m + accoa_m 15693
HMGLx hmgcoa_x --> acac_x + accoa_x 15693

AACOAT acac_c + atp_c + coa_c <=> aacoa_c + amp_c + ppi_c 15276

MEVK1 atp_c + mev__R_c --> 5pmev_c + adp_c + h_c 8758
MEVK1x atp_x + mev__R_x --> 5pmev_x + adp_x + h_x 8758
MEVK2 ctp_c + mev__R_c --> 5pmev_c + cdp_c + h_c 8758
MEVK3 gtp_c + mev__R_c --> 5pmev_c + gdp_c + h_c 8758
MEVK4 mev__R_c + utp_c --> 5pmev_c + h_c + udp_c 8758

PMEVK 5pmev_c + atp_c --> 5dpmev_c + adp_c 8702

DPMVD 5dpmev_c + atp_c --> adp_c + co2_c + ipdp_c + pi_c 11910
DPMVDx 5dpmev_x + atp_x --> adp_x + co2_x + ipdp_x + pi_x 11910

IDIh ipdp_h --> dmpp_h 16203
IPDDI ipdp_c <=> dmpp_c 16203
IPDDIx ipdp_x <=> dmpp_x 16203

DMATT dmpp_c + ipdp_c --> grdp_c + ppi_c 12944 or 16503
DMATTx dmpp_x + ipdp_x --> grdp_x + ppi_x 12944
FPPSh grdp_h + ipdp_h --> frdp_h + h_h + ppi_h 12944
GRTT grdp_c + ipdp_c --> frdp_c + ppi_c 12944 or 16503
GRTTx grdp_x + ipdp_x --> frdp_x + ppi_x 12944

DMATT dmpp_c + ipdp_c --> grdp_c + ppi_c 12944 or 16503
FRTT frdp_c + ipdp_c --> ggdp_c + ppi_c 16503
GRTT grdp_c + ipdp_c --> frdp_c + ppi_c 12944 or 16503

SQLSr 2.0 frdp_r + h_r + nadph_r --> nadp_r + 2.0 ppi_r + sql_r 16591

SQLEr h_r + nadph_r + o2_r + sql_r --> Ssq23epx_r + h2o_r + nadp_r 13286 and 13729
SQLErx h_r + nadh_r + o2_r + sql_r --> Ssq23epx_r + h2o_r + nad_r (13729 and 12471 and 16097) or (13729 and 13206 and 16097)

LNSTLSr Ssq23epx_r --> lanost_r 9240

LNS14DMr 2.0 h_r + lanost_r + 3.0 nadph_r + 3.0 o2_r --> 44mctr_r + for_r + 4.0 h2o_r + 3.0 nadp_r 12843 and 13286
LNS14DMrx 2.0 h_r + lanost_r + 3.0 nadh_r + 3.0 o2_r --> 44mctr_r + for_r + 4.0 h2o_r + 3.0 nad_r (12843 and 12471 and 16097) or (12843 and 13206 and 16097)

C14STRr 44mctr_r + h_r + nadph_r --> 44mzym_r + nadp_r 8671

C4STMO1r 44mzym_r + 3.0 h_r + 3.0 nadph_r + 3.0 o2_r --> 4mzym_int1_r + 4.0 h2o_r + 3.0 nadp_r 16640
C4STMO2er 4mzym_r + 3.0 h_r + 3.0 nadph_r + 3.0 o2_r --> 4.0 h2o_r + 3.0 nadp_r + zym_int1_r 16640

C3STDH1Pr 4mzym_int1_r + nadp_r --> 4mzym_int2_r + co2_r + h_r + nadph_r 13724 or 8835
C3STDH1r 4mzym_int1_r + nad_r --> 4mzym_int2_r + co2_r + h_r + nadh_r 13724 or 8835
C3STDH2er nad_r + zym_int1_r --> co2_r + h_r + nadh_r + zym_int2_r 13724 or 8835

C3STDH1Pr 4mzym_int1_r + nadp_r --> 4mzym_int2_r + co2_r + h_r + nadph_r 13724 or 8835
C3STDH1r 4mzym_int1_r + nad_r --> 4mzym_int2_r + co2_r + h_r + nadh_r 13724 or 8835
C3STDH2er nad_r + zym_int1_r --> co2_r + h_r + nadh_r + zym_int2_r 13724 or 8835

SAM24MTer amet_r + zymst_r --> ahcys_r + fecost_r + h_r 13060

C8STIer fecost_r --> epist_r 10113

C5STDSer epist_r + h_r + nadph_r + o2_r --> ergtrol_r + 2.0 h2o_r + nadp_r 8781
LSTO1r chlstol_r + h_r + nadph_r + o2_r --> ddsmsterol_r + 2.0 h2o_r + nadp_r 8781

C22STDSer ergtrol_r + h_r + nadph_r + o2_r --> ergtetrol_r + 2.0 h2o_r + nadp_r 11716 and 13286
C22STDSrx ergtrol_r + h_r + nadh_r + o2_r --> ergtetrol_r + 2.0 h2o_r + nad_r (11716 and 12471 and 16097) or (11716 and 13206 and 16097)

C24STRer ergtetrol_r + h_r + nadph_r --> ergst_r + nadp_r 10981
```

In [148]:

```
r = model.reactions.get_by_id('AACOAT')
r.id = 'AACOATx'
for m in r.metabolites:
    if not m.id.replace('_c','_x') in model.metabolites:
        m2 = m.copy()
        m2.id = m.id.replace('_c','_x')
        m2.compartment = 'x'
        model.add_metabolites([m2])
    r.add_metabolites({m.id: -r.get_coefficient(m.id), m.id.replace('_c','_x'): r.get_coefficient(m.id)})
model.reactions.get_by_id('DMATT').gene_reaction_rule = '12944'
model.reactions.get_by_id('GRTT').gene_reaction_rule = '12944'
r = hsa2.reactions.get_by_id('FRDPtcr').copy()
model.add_reactions([r])
model.remove_reactions(['MHGS','HMGCOARx','HMGL','OCOAT1','MEVK1x','MEVK2','MEVK3','MEVK4','DPMVDx',
                        'IDIh','IPDDIx','DMATTx','FPPSh','GRTTx','UDCPDPS','yli_R0768'], remove_orphans=True)
```

13090 mito 27 K01027: OXCT; 3-oxoacid CoA-transferase, Succinyl-CoA:3-ketoacid coenzyme A transferase 1, mitochondrial MDA *12795 cyto 16, mito 9, cyto\_nucl 9 K11539: CBR4; carbonyl reductase 4 WTA*  
15180 cyto 13, cyto\_nucl 8, cysk 8, mito 4 K00074: paaH, hbd, fadB, mmgB; 3-hydroxybutyryl-CoA dehydrogenase YTAR  
11962 cyto 7.5, mito 6, cyto\_nucl 6, cysk 6, extr 3, pero 3 K01555: FAH, fahA; fumarylacetoacetase RMA\*

In [149]:

```
for r in sorted(model.genes.get_by_id('13090').reactions, key=lambda x: x.id):
    print(r.id, r.reaction, r.gene_reaction_rule)
print()
for r in sorted(model.genes.get_by_id('12795').reactions, key=lambda x: x.id):
    print(r.id, r.reaction, r.gene_reaction_rule)
print()
for r in sorted(model.genes.get_by_id('15180').reactions, key=lambda x: x.id):
    print(r.id, r.reaction, r.gene_reaction_rule)
```

```
ACACCT acac_c + accoa_c --> aacoa_c + ac_c b2221 and 13090
BUTCT accoa_c + but_c --> ac_c + btcoa_c b2221 and 13090
HXCT accoa_c + hxa_c --> ac_c + hxcoa_c b2221 and 13090
OCOAT1m acac_m + succoa_m <=> aacoa_m + succ_m 13090

BDHm bhb_m + nad_m <=> acac_m + h_m + nadh_m 12795

HADPCOADH3 3hadpcoa_c + nad_c <=> h_c + nadh_c + oxadpcoa_c 15180
HBCO_nadp aacoa_c + h_c + nadph_c <=> 3hbcoa_c + nadp_c 15180
```

In [150]:

```
model.remove_reactions(['ACACCT','BUTCT','HXCT'], remove_orphans=True)
```

In [151]:

```
for r in sorted(model.metabolites.get_by_id('stcoa_c').reactions, key=lambda x: x.id):
    print(r.id, r.reaction, r.gene_reaction_rule)
```

```
ACOADAGAT1601819Z180 12dgr1601819Z_c + stcoa_c --> coa_c + tag1601819Z180_c 16460
ACOADAGAT1801819Z180 12dgr1801819Z_c + stcoa_c --> coa_c + tag1801819Z180_c 16460
ACOADAGAT1819Z1819Z180 12dgr1819Z1819Z_c + stcoa_c --> coa_c + tag1819Z1819Z180_c 16460
AGAT_SC 0.01 1ag3p_SC_c + 0.02 dcacoa_c + 0.06 ddcacoa_c + 0.17 hdcoa_c + 0.09 ocdycacoa_c + 0.24 odecoa_c + 0.27 pmtcoa_c + 0.05 stcoa_c + 0.1 tdcoa_c --> coa_c + 0.01 pa_SC_c 10427
ECOAR h_c + nadph_c + od2coa_c --> nadp_c + stcoa_c 16241
EPISTAT_SC 0.01 epist_c + 0.655 hdcoa_c + 0.01 hexccoa_c + 0.27 odecoa_c + 0.02 pmtcoa_c + 0.03 stcoa_c + 0.015 tdcoa_c --> coa_c + 0.01 epistest_SC_c 11799
ERGSTAT_SC 0.01 ergst_c + 0.655 hdcoa_c + 0.01 hexccoa_c + 0.27 odecoa_c + 0.02 pmtcoa_c + 0.03 stcoa_c + 0.015 tdcoa_c --> coa_c + 0.01 ergstest_SC_c 11799
FA180COAabcp atp_x + h2o_x + stcoa_c --> adp_x + h_x + pi_x + stcoa_x 13167 or 9637
FACOAE180 h2o_c + stcoa_c --> coa_c + h_c + ocdca_c 8456
FACOAL180 atp_c + coa_c + ocdca_c --> amp_c + ppi_c + stcoa_c 11167 or 15746 or 15748
FAS180COA 3.0 h_c + malcoa_c + 2.0 nadph_c + pmtcoa_c --> co2_c + coa_c + h2o_c + 2.0 nadp_c + stcoa_c (10677 and 16241 and 16695) or (12826 and 8639 and 8670 and 8777) or (14471 and 8639 and 8670 and 8777)
FECOSTAT_SC 0.01 fecost_c + 0.655 hdcoa_c + 0.01 hexccoa_c + 0.27 odecoa_c + 0.02 pmtcoa_c + 0.03 stcoa_c + 0.015 tdcoa_c --> coa_c + 0.01 fecostest_SC_c 11799
GAT1_SC 0.02 dcacoa_c + 0.06 ddcacoa_c + glyc3p_c + 0.17 hdcoa_c + 0.09 ocdycacoa_c + 0.24 odecoa_c + 0.27 pmtcoa_c + 0.05 stcoa_c + 0.1 tdcoa_c --> 0.01 1ag3p_SC_c + coa_c 15435
GAT2_SC 0.02 dcacoa_c + 0.06 ddcacoa_c + dhap_c + 0.17 hdcoa_c + 0.09 ocdycacoa_c + 0.24 odecoa_c + 0.27 pmtcoa_c + 0.05 stcoa_c + 0.1 tdcoa_c --> 0.01 1agly3p_SC_c + coa_c 15435
LANOSTAT_SC 0.655 hdcoa_c + 0.01 hexccoa_c + 0.01 lanost_c + 0.27 odecoa_c + 0.02 pmtcoa_c + 0.03 stcoa_c + 0.015 tdcoa_c --> coa_c + 0.01 lanostest_SC_c 11799
LPCAT_SC 0.01 1agpc_SC_c + 0.02 dcacoa_c + 0.06 ddcacoa_c + 0.17 hdcoa_c + 0.09 ocdycacoa_c + 0.24 odecoa_c + 0.27 pmtcoa_c + 0.05 stcoa_c + 0.1 tdcoa_c --> coa_c + 0.01 pc_SC_c 16474
TRIGS_SC 0.01 12dgr_SC_c + 0.02 dcacoa_c + 0.06 ddcacoa_c + 0.17 hdcoa_c + 0.09 ocdycacoa_c + 0.24 odecoa_c + 0.27 pmtcoa_c + 0.05 stcoa_c + 0.1 tdcoa_c --> coa_c + 0.01 triglyc_SC_c 11799 or 16460
ZYMSTAT_SC 0.655 hdcoa_c + 0.01 hexccoa_c + 0.27 odecoa_c + 0.02 pmtcoa_c + 0.03 stcoa_c + 0.015 tdcoa_c + 0.01 zymst_c --> coa_c + 0.01 zymstest_SC_c 11799
yli_R0307 0.02 dccoa_c + 0.06 ddcacoa_c + glyc3p_c + 0.27 pmtcoa_c + 0.05 stcoa_c + 0.1 tdcoa_c + 0.17 yli_M04594_c + 0.09 yli_M04625_c + 0.24 yli_M04626_c --> 0.01 1ag3p_SC_c + coa_c 15435
yli_R0308 0.01 1ag3p_SC_c + 0.02 dccoa_c + 0.06 ddcacoa_c + 0.27 pmtcoa_c + 0.05 stcoa_c + 0.1 tdcoa_c + 0.17 yli_M04594_c + 0.09 yli_M04625_c + 0.24 yli_M04626_c --> coa_c + 0.01 pa_EC_c 10427 or 16030 or 9746
yli_R0319 0.02 dccoa_c + 0.06 ddcacoa_c + 0.27 pmtcoa_c + 0.05 stcoa_c + 0.1 tdcoa_c + 0.01 yli_M02277_c + 0.17 yli_M04594_c + 0.09 yli_M04625_c + 0.24 yli_M04626_c --> coa_c + 0.01 pchol_cho_c 16030
yli_R0335 0.02 dccoa_c + 0.06 ddcacoa_c + dhap_c + 0.27 pmtcoa_c + 0.05 stcoa_c + 0.1 tdcoa_c + 0.17 yli_M04594_c + 0.09 yli_M04625_c + 0.24 yli_M04626_c --> 0.01 adhap_hs_c + coa_c 15435
```

In [152]:

```
model.remove_reactions(['yli_R0307','yli_R0308','yli_R0319','yli_R0335'], remove_orphans=True)
```

Fatty acid biosynthesis  
ACC1 8639 cyto 12.5, cyto\_nucl 10, nucl 6.5, pero 5 K11262: ACACA; acetyl-CoA carboxylase / biotin carboxylase 1 ILA *ACH1 13797 mito 23, cyto 3 K01067: E3.1.2.1, ACH1; acetyl-CoA hydrolase GWS*  
FAS1 8670 mito 22, cyto 3.5, cyto\_nucl 2.5 K00668: FAS1; fatty acid synthase subunit beta, fungi type GGL *FAS2 8777 cyto 15.5, cyto\_nucl 9.5, mito 3, nucl 2.5, E.R. 2, plas 1, extr 1, pero 1, vacu 1 K00667: FAS2; fatty acid synthase subunit alpha, fungi type AQK*

FAA1 11167 cyto 11, cyto\_nucl 8, mito 7, nucl 3, plas 3 K01897: ACSL, fadD; long-chain acyl-CoA synthetase VYP *FAA2 12555 cyto 15.5, cyto\_nucl 11.5, pero 6, nucl 4.5 K01897: ACSL, fadD; long-chain acyl-CoA synthetase AKL*  
FAA4 15748 cyto 23, mito 3 K01897: ACSL, fadD; long-chain acyl-CoA synthetase VYP\*

ELO2 16655 plas 22, E.R. 2, nucl 1, mito 1, vacu 1, mito\_nucl 1 K10245: ELO2; fatty acid elongase 2 KTN *ELO3 11343 mito 13, extr 5, plas 4, E.R. 2, nucl 1, cyto 1, cyto\_nucl 1, pero 1, cyto\_pero 1 KOG3072: Long chain fatty acid elongase KAN*, no rxn  
IFA38 16695 cyto 12, cyto\_nucl 8.5, mito 7, pero 4, nucl 3 K10251: HSD17B12, KAR, IFA38; 17beta-estradiol 17-dehydrogenase / very-long-chain 3-oxoacyl-CoA reductase KGE *PHS1 10677 mito 23, cyto 2 K10703: PHS1, PAS2; very-long-chain (3R)-3-hydroxyacyl-CoA dehydratase KSE*  
TSC13 16241 mito 20.5, cyto\_mito 12.333, cyto 3, cyto\_nucl 2.833 K10258: TER, TSC13, CER10; very-long-chain enoyl-CoA reductase FIA\*

Mitochondrial FA biosynthesis
Missing HFA1 (ACCOACrm), CEM1 (malACP + acACP -> acacACP + CO2), HTD2 (3hbACP -> b2ACP, CoA rxn can be done by ECOAH)  
MCT1 16542 mito 26 K00645: fabD; [acyl-carrier-protein] S-malonyltransferase DGQ *ETR1 11837 mito 27 K07512: MECR, NRBF1; mitochondrial trans-2-enoyl-CoA reductase KDE*  
ACP1 14261 mito 24, cyto 2 KOG1748: Acyl carrier protein/NADH-ubiquinone oxidoreductase, NDUFAB1/SDAP subunit EAH *CBR4+HSD17B8 (OAR1, acetocetyl-ACP -> 3hbACP, CoA rxn can be done by HACD)  
CBR4 12795 cyto 16, mito 9, cyto\_nucl 9 K11539: CBR4; carbonyl reductase 4 WTA*  
HSD17B8 11765 mito 19.5, cyto\_mito 12, cyto 3.5, extr 2 K13370: HSD17B8; 17beta-estradiol 17-dehydrogenase / 3alpha(17beta)-hydroxysteroid dehydrogenase (NAD+) TPT *AIM22 13899 mito 20, nucl 4, cyto 1, plas 1, pero 1, cyto\_pero 1 K03800: lplA, lplJ; lipoate---protein ligase GEM*  
LIP2 14821 mito 27 K03801: lipB; lipoyl(octanoyl) transferase LGV *LIP5 14096 mito 25 K03644: lipA; lipoyl synthase VRV*

Many 3-oxoacyl-[acyl-carrier protein] reductase genes  
incomplete? remove them for now, and check later  
https://www.ncbi.nlm.nih.gov/pubmed/19028688  
https://www.ncbi.nlm.nih.gov/pubmed/27553474

Mitochondrial fatty acid synthesis is needed for lipoic acid biosynthesis in S. cer  
S. cer lacks mitochondrial beta-oxidation, therefore cannot make C8 FA needed for lipoate  
R. toruloides can degrade FA in mitochondria, check the Mito FAS in other fungi

In [153]:

```
for x in ['8639','13797','8670','8777','11167','12555','15748','16655','16695','10677','16241',
          '16542','11837','14261','12795','13899','14821','14096',]:
    for r in sorted(model.genes.get_by_id(x).reactions, key=lambda x: x.id):
        print(r.id, r.reaction, r.gene_reaction_rule)
    print()
```

```
ACCOAC accoa_c + atp_c + hco3_c --> adp_c + h_c + malcoa_c + pi_c 8639
ACCOACrm accoa_m + atp_m + hco3_m <=> adp_m + h_m + malcoa_m + pi_m 8639
FAS100 3.0 h_c + malcoa_c + 2.0 nadph_c + octa_c --> co2_c + coa_c + dca_c + h2o_c + 2.0 nadp_c (12826 and 8639 and 8670 and 8777) or (14471 and 8639 and 8670 and 8777)
FAS100COA 3.0 h_c + malcoa_c + 2.0 nadph_c + occoa_c --> co2_c + coa_c + dcacoa_c + h2o_c + 2.0 nadp_c (12826 and 8639 and 8670 and 8777) or (14471 and 8639 and 8670 and 8777)
FAS120 dca_c + 3.0 h_c + malcoa_c + 2.0 nadph_c --> co2_c + coa_c + ddca_c + h2o_c + 2.0 nadp_c (12826 and 8639 and 8670 and 8777) or (14471 and 8639 and 8670 and 8777)
FAS120COA dcacoa_c + 3.0 h_c + malcoa_c + 2.0 nadph_c --> co2_c + coa_c + ddcacoa_c + h2o_c + 2.0 nadp_c (12826 and 8639 and 8670 and 8777) or (14471 and 8639 and 8670 and 8777)
FAS140 ddca_c + 3.0 h_c + malcoa_c + 2.0 nadph_c --> co2_c + coa_c + h2o_c + 2.0 nadp_c + ttdca_c (12826 and 8639 and 8670 and 8777) or (14471 and 8639 and 8670 and 8777)
FAS140COA ddcacoa_c + 3.0 h_c + malcoa_c + 2.0 nadph_c --> co2_c + coa_c + h2o_c + 2.0 nadp_c + tdcoa_c (10677 and 16241 and 16695) or (12826 and 8639 and 8670 and 8777) or (14471 and 8639 and 8670 and 8777)
FAS160 3.0 h_c + malcoa_c + 2.0 nadph_c + ttdca_c --> co2_c + coa_c + h2o_c + hdca_c + 2.0 nadp_c (12826 and 8639 and 8670 and 8777) or (14471 and 8639 and 8670 and 8777)
FAS160COA 3.0 h_c + malcoa_c + 2.0 nadph_c + tdcoa_c --> co2_c + coa_c + h2o_c + 2.0 nadp_c + pmtcoa_c (10677 and 16241 and 16695) or (12826 and 8639 and 8670 and 8777) or (14471 and 8639 and 8670 and 8777)
FAS180 3.0 h_c + hdca_c + malcoa_c + 2.0 nadph_c --> co2_c + coa_c + h2o_c + 2.0 nadp_c + ocdca_c (10677 and 16241 and 16695) or (12826 and 8639 and 8670 and 8777) or (14471 and 8639 and 8670 and 8777)
FAS180COA 3.0 h_c + malcoa_c + 2.0 nadph_c + pmtcoa_c --> co2_c + coa_c + h2o_c + 2.0 nadp_c + stcoa_c (10677 and 16241 and 16695) or (12826 and 8639 and 8670 and 8777) or (14471 and 8639 and 8670 and 8777)
FAS80COA_L accoa_c + 9.0 h_c + 3.0 malcoa_c + 6.0 nadph_c --> 3.0 co2_c + 3.0 coa_c + 3.0 h2o_c + 6.0 nadp_c + occoa_c (10677 and 16241 and 16695) or (12826 and 8639 and 8670 and 8777) or (14471 and 8639 and 8670 and 8777)
FAS80_L accoa_c + 8.0 h_c + 3.0 malcoa_c + 6.0 nadph_c --> 3.0 co2_c + 4.0 coa_c + 2.0 h2o_c + 6.0 nadp_c + octa_c (10677 and 16241 and 16695) or (12826 and 8639 and 8670 and 8777) or (14471 and 8639 and 8670 and 8777)

ACOAHim accoa_m + h2o_m --> ac_m + coa_m + h_m 13797

ACOATA ACP_c + accoa_c <=> acACP_c + coa_c (b1091 and 14261) or (8670 and 8777)
FA120ACPHi ddcaACP_c + h2o_c --> ACP_c + ddca_c + h_c 8670 and 8777
FA140ACPHi h2o_c + myrsACP_c --> ACP_c + h_c + ttdca_c 8670 and 8777
FA141ACPHi h2o_c + tdeACP_c --> ACP_c + h_c + ttdcea_c 8670 and 8777
FA160ACPHi h2o_c + palmACP_c --> ACP_c + h_c + hdca_c 8670 and 8777
FA161ACPHi h2o_c + hdeACP_c --> ACP_c + h_c + hdcea_c 8670 and 8777
FA180ACPH h2o_c + ocdcaACP_c <=> ACP_c + h_c + ocdca_c 8670 and 8777
FA181ACPH h2o_c + octeACP_c <=> ACP_c + h_c + ocdcea_c 8670 and 8777
FA182ACPH h2o_c + ocdcyaACP_c <=> ACP_c + h_c + ocdcya_c 8670 and 8777
FAS100 3.0 h_c + malcoa_c + 2.0 nadph_c + octa_c --> co2_c + coa_c + dca_c + h2o_c + 2.0 nadp_c (12826 and 8639 and 8670 and 8777) or (14471 and 8639 and 8670 and 8777)
FAS100COA 3.0 h_c + malcoa_c + 2.0 nadph_c + occoa_c --> co2_c + coa_c + dcacoa_c + h2o_c + 2.0 nadp_c (12826 and 8639 and 8670 and 8777) or (14471 and 8639 and 8670 and 8777)
FAS120 dca_c + 3.0 h_c + malcoa_c + 2.0 nadph_c --> co2_c + coa_c + ddca_c + h2o_c + 2.0 nadp_c (12826 and 8639 and 8670 and 8777) or (14471 and 8639 and 8670 and 8777)
FAS120COA dcacoa_c + 3.0 h_c + malcoa_c + 2.0 nadph_c --> co2_c + coa_c + ddcacoa_c + h2o_c + 2.0 nadp_c (12826 and 8639 and 8670 and 8777) or (14471 and 8639 and 8670 and 8777)
FAS140 ddca_c + 3.0 h_c + malcoa_c + 2.0 nadph_c --> co2_c + coa_c + h2o_c + 2.0 nadp_c + ttdca_c (12826 and 8639 and 8670 and 8777) or (14471 and 8639 and 8670 and 8777)
FAS140COA ddcacoa_c + 3.0 h_c + malcoa_c + 2.0 nadph_c --> co2_c + coa_c + h2o_c + 2.0 nadp_c + tdcoa_c (10677 and 16241 and 16695) or (12826 and 8639 and 8670 and 8777) or (14471 and 8639 and 8670 and 8777)
FAS160 3.0 h_c + malcoa_c + 2.0 nadph_c + ttdca_c --> co2_c + coa_c + h2o_c + hdca_c + 2.0 nadp_c (12826 and 8639 and 8670 and 8777) or (14471 and 8639 and 8670 and 8777)
FAS160COA 3.0 h_c + malcoa_c + 2.0 nadph_c + tdcoa_c --> co2_c + coa_c + h2o_c + 2.0 nadp_c + pmtcoa_c (10677 and 16241 and 16695) or (12826 and 8639 and 8670 and 8777) or (14471 and 8639 and 8670 and 8777)
FAS180 3.0 h_c + hdca_c + malcoa_c + 2.0 nadph_c --> co2_c + coa_c + h2o_c + 2.0 nadp_c + ocdca_c (10677 and 16241 and 16695) or (12826 and 8639 and 8670 and 8777) or (14471 and 8639 and 8670 and 8777)
FAS180COA 3.0 h_c + malcoa_c + 2.0 nadph_c + pmtcoa_c --> co2_c + coa_c + h2o_c + 2.0 nadp_c + stcoa_c (10677 and 16241 and 16695) or (12826 and 8639 and 8670 and 8777) or (14471 and 8639 and 8670 and 8777)
FAS80COA_L accoa_c + 9.0 h_c + 3.0 malcoa_c + 6.0 nadph_c --> 3.0 co2_c + 3.0 coa_c + 3.0 h2o_c + 6.0 nadp_c + occoa_c (10677 and 16241 and 16695) or (12826 and 8639 and 8670 and 8777) or (14471 and 8639 and 8670 and 8777)
FAS80_L accoa_c + 8.0 h_c + 3.0 malcoa_c + 6.0 nadph_c --> 3.0 co2_c + 4.0 coa_c + 2.0 h2o_c + 6.0 nadp_c + octa_c (10677 and 16241 and 16695) or (12826 and 8639 and 8670 and 8777) or (14471 and 8639 and 8670 and 8777)
MCOATA ACP_c + malcoa_c <=> coa_c + malACP_c 16542 or (14261 and 16542) or (8670 and 8777)
yli_R0163 acACP_c + 9.0 h_c + 3.0 malACP_c + 6.0 nadph_c --> 3.0 ACP_c + 3.0 co2_c + 3.0 h2o_c + 6.0 nadp_c + yli_M02777_c YALI0F30679g and 8670 and 8777
yli_R0164 3.0 h_c + malACP_c + 2.0 nadph_c + yli_M02777_c --> ACP_c + co2_c + h2o_c + 2.0 nadp_c + yli_M02780_c YALI0F30679g and 8670 and 8777
yli_R0165 3.0 h_c + malACP_c + 2.0 nadph_c + yli_M02780_c --> ACP_c + co2_c + h2o_c + 2.0 nadp_c + yli_M02516_c YALI0F30679g and 8670 and 8777
yli_R0166 3.0 h_c + malACP_c + 2.0 nadph_c + yli_M02516_c --> ACP_c + co2_c + h2o_c + 2.0 nadp_c + yli_M02786_c YALI0F30679g and 8670 and 8777
yli_R0167 4.0 h_c + malACP_c + 3.0 nadph_c + o2_c + yli_M02516_c --> ACP_c + co2_c + 3.0 h2o_c + 3.0 nadp_c + yli_M04634_c YALI0F30679g and 8670 and 8777
yli_R0168 4.0 h_c + malACP_c + 3.0 nadph_c + o2_c + yli_M02786_c --> ACP_c + co2_c + 3.0 h2o_c + 3.0 nadp_c + yli_M02318_c YALI0F30679g and 8670 and 8777
yli_R0169 3.0 h_c + malACP_c + 2.0 nadph_c + yli_M02786_c --> ACP_c + co2_c + h2o_c + 2.0 nadp_c + yli_M02789_c YALI0F30679g and 8670 and 8777
yli_R0170 5.0 h_c + malACP_c + 4.0 nadph_c + 2.0 o2_c + yli_M02789_c --> ACP_c + co2_c + 5.0 h2o_c + 4.0 nadp_c + yli_M04624_c YALI0F30679g and 8670 and 8777
yli_R0171 4.0 h_c + malACP_c + 3.0 nadph_c + o2_c + yli_M02789_c --> ACP_c + co2_c + 3.0 h2o_c + 3.0 nadp_c + yli_M01019_c YALI0F30679g and 8670 and 8777
yli_R0172 3.0 h_c + malACP_c + 2.0 nadph_c + yli_M02789_c --> ACP_c + co2_c + h2o_c + 2.0 nadp_c + yli_M02235_c YALI0F30679g and 8670 and 8777
yli_R0175 h2o_c + yli_M04634_c <=> ACP_c + h_c + yli_M04636_c 8670 and 8777
yli_R0176 h2o_c + yli_M02789_c <=> ACP_c + h_c + hdca_c 8670 and 8777
yli_R0177 h2o_c + yli_M02318_c <=> ACP_c + h_c + yli_M03299_c 8670 and 8777
yli_R0178 h2o_c + yli_M02235_c <=> ACP_c + h_c + ocdca_c 8670 and 8777
yli_R0179 h2o_c + yli_M01019_c <=> ACP_c + h_c + yli_M04622_c 8670 and 8777
yli_R0180 h2o_c + yli_M04624_c <=> ACP_c + h_c + yli_M05526_c 8670 and 8777

ACOATA ACP_c + accoa_c <=> acACP_c + coa_c (b1091 and 14261) or (8670 and 8777)
ACPS1 apoACP_c + coa_c --> ACP_c + h_c + pap_c 8777
FA120ACPHi ddcaACP_c + h2o_c --> ACP_c + ddca_c + h_c 8670 and 8777
FA140ACPHi h2o_c + myrsACP_c --> ACP_c + h_c + ttdca_c 8670 and 8777
FA141ACPHi h2o_c + tdeACP_c --> ACP_c + h_c + ttdcea_c 8670 and 8777
FA160ACPHi h2o_c + palmACP_c --> ACP_c + h_c + hdca_c 8670 and 8777
FA161ACPHi h2o_c + hdeACP_c --> ACP_c + h_c + hdcea_c 8670 and 8777
FA180ACPH h2o_c + ocdcaACP_c <=> ACP_c + h_c + ocdca_c 8670 and 8777
FA181ACPH h2o_c + octeACP_c <=> ACP_c + h_c + ocdcea_c 8670 and 8777
FA182ACPH h2o_c + ocdcyaACP_c <=> ACP_c + h_c + ocdcya_c 8670 and 8777
FAS100 3.0 h_c + malcoa_c + 2.0 nadph_c + octa_c --> co2_c + coa_c + dca_c + h2o_c + 2.0 nadp_c (12826 and 8639 and 8670 and 8777) or (14471 and 8639 and 8670 and 8777)
FAS100COA 3.0 h_c + malcoa_c + 2.0 nadph_c + occoa_c --> co2_c + coa_c + dcacoa_c + h2o_c + 2.0 nadp_c (12826 and 8639 and 8670 and 8777) or (14471 and 8639 and 8670 and 8777)
FAS120 dca_c + 3.0 h_c + malcoa_c + 2.0 nadph_c --> co2_c + coa_c + ddca_c + h2o_c + 2.0 nadp_c (12826 and 8639 and 8670 and 8777) or (14471 and 8639 and 8670 and 8777)
FAS120COA dcacoa_c + 3.0 h_c + malcoa_c + 2.0 nadph_c --> co2_c + coa_c + ddcacoa_c + h2o_c + 2.0 nadp_c (12826 and 8639 and 8670 and 8777) or (14471 and 8639 and 8670 and 8777)
FAS140 ddca_c + 3.0 h_c + malcoa_c + 2.0 nadph_c --> co2_c + coa_c + h2o_c + 2.0 nadp_c + ttdca_c (12826 and 8639 and 8670 and 8777) or (14471 and 8639 and 8670 and 8777)
FAS140COA ddcacoa_c + 3.0 h_c + malcoa_c + 2.0 nadph_c --> co2_c + coa_c + h2o_c + 2.0 nadp_c + tdcoa_c (10677 and 16241 and 16695) or (12826 and 8639 and 8670 and 8777) or (14471 and 8639 and 8670 and 8777)
FAS160 3.0 h_c + malcoa_c + 2.0 nadph_c + ttdca_c --> co2_c + coa_c + h2o_c + hdca_c + 2.0 nadp_c (12826 and 8639 and 8670 and 8777) or (14471 and 8639 and 8670 and 8777)
FAS160COA 3.0 h_c + malcoa_c + 2.0 nadph_c + tdcoa_c --> co2_c + coa_c + h2o_c + 2.0 nadp_c + pmtcoa_c (10677 and 16241 and 16695) or (12826 and 8639 and 8670 and 8777) or (14471 and 8639 and 8670 and 8777)
FAS180 3.0 h_c + hdca_c + malcoa_c + 2.0 nadph_c --> co2_c + coa_c + h2o_c + 2.0 nadp_c + ocdca_c (10677 and 16241 and 16695) or (12826 and 8639 and 8670 and 8777) or (14471 and 8639 and 8670 and 8777)
FAS180COA 3.0 h_c + malcoa_c + 2.0 nadph_c + pmtcoa_c --> co2_c + coa_c + h2o_c + 2.0 nadp_c + stcoa_c (10677 and 16241 and 16695) or (12826 and 8639 and 8670 and 8777) or (14471 and 8639 and 8670 and 8777)
FAS80COA_L accoa_c + 9.0 h_c + 3.0 malcoa_c + 6.0 nadph_c --> 3.0 co2_c + 3.0 coa_c + 3.0 h2o_c + 6.0 nadp_c + occoa_c (10677 and 16241 and 16695) or (12826 and 8639 and 8670 and 8777) or (14471 and 8639 and 8670 and 8777)
FAS80_L accoa_c + 8.0 h_c + 3.0 malcoa_c + 6.0 nadph_c --> 3.0 co2_c + 4.0 coa_c + 2.0 h2o_c + 6.0 nadp_c + octa_c (10677 and 16241 and 16695) or (12826 and 8639 and 8670 and 8777) or (14471 and 8639 and 8670 and 8777)
MCOATA ACP_c + malcoa_c <=> coa_c + malACP_c 16542 or (14261 and 16542) or (8670 and 8777)
yli_R0163 acACP_c + 9.0 h_c + 3.0 malACP_c + 6.0 nadph_c --> 3.0 ACP_c + 3.0 co2_c + 3.0 h2o_c + 6.0 nadp_c + yli_M02777_c YALI0F30679g and 8670 and 8777
yli_R0164 3.0 h_c + malACP_c + 2.0 nadph_c + yli_M02777_c --> ACP_c + co2_c + h2o_c + 2.0 nadp_c + yli_M02780_c YALI0F30679g and 8670 and 8777
yli_R0165 3.0 h_c + malACP_c + 2.0 nadph_c + yli_M02780_c --> ACP_c + co2_c + h2o_c + 2.0 nadp_c + yli_M02516_c YALI0F30679g and 8670 and 8777
yli_R0166 3.0 h_c + malACP_c + 2.0 nadph_c + yli_M02516_c --> ACP_c + co2_c + h2o_c + 2.0 nadp_c + yli_M02786_c YALI0F30679g and 8670 and 8777
yli_R0167 4.0 h_c + malACP_c + 3.0 nadph_c + o2_c + yli_M02516_c --> ACP_c + co2_c + 3.0 h2o_c + 3.0 nadp_c + yli_M04634_c YALI0F30679g and 8670 and 8777
yli_R0168 4.0 h_c + malACP_c + 3.0 nadph_c + o2_c + yli_M02786_c --> ACP_c + co2_c + 3.0 h2o_c + 3.0 nadp_c + yli_M02318_c YALI0F30679g and 8670 and 8777
yli_R0169 3.0 h_c + malACP_c + 2.0 nadph_c + yli_M02786_c --> ACP_c + co2_c + h2o_c + 2.0 nadp_c + yli_M02789_c YALI0F30679g and 8670 and 8777
yli_R0170 5.0 h_c + malACP_c + 4.0 nadph_c + 2.0 o2_c + yli_M02789_c --> ACP_c + co2_c + 5.0 h2o_c + 4.0 nadp_c + yli_M04624_c YALI0F30679g and 8670 and 8777
yli_R0171 4.0 h_c + malACP_c + 3.0 nadph_c + o2_c + yli_M02789_c --> ACP_c + co2_c + 3.0 h2o_c + 3.0 nadp_c + yli_M01019_c YALI0F30679g and 8670 and 8777
yli_R0172 3.0 h_c + malACP_c + 2.0 nadph_c + yli_M02789_c --> ACP_c + co2_c + h2o_c + 2.0 nadp_c + yli_M02235_c YALI0F30679g and 8670 and 8777
yli_R0175 h2o_c + yli_M04634_c <=> ACP_c + h_c + yli_M04636_c 8670 and 8777
yli_R0176 h2o_c + yli_M02789_c <=> ACP_c + h_c + hdca_c 8670 and 8777
yli_R0177 h2o_c + yli_M02318_c <=> ACP_c + h_c + yli_M03299_c 8670 and 8777
yli_R0178 h2o_c + yli_M02235_c <=> ACP_c + h_c + ocdca_c 8670 and 8777
yli_R0179 h2o_c + yli_M01019_c <=> ACP_c + h_c + yli_M04622_c 8670 and 8777
yli_R0180 h2o_c + yli_M04624_c <=> ACP_c + h_c + yli_M05526_c 8670 and 8777

FACOAL100 atp_c + coa_c + dca_c --> amp_c + dcacoa_c + ppi_c 11167 or 15746 or 15748
FACOAL120 atp_c + coa_c + ddca_c --> amp_c + ddcacoa_c + ppi_c 11167 or 15746 or 15748
FACOAL140 atp_c + coa_c + ttdca_c --> amp_c + ppi_c + tdcoa_c 11167 or 15746 or 15748
FACOAL141 atp_c + coa_c + ttdcea_c --> amp_c + ppi_c + tdecoa_c 11167 or 15746 or 15748
FACOAL150 atp_c + coa_c + ptdca_c --> amp_c + ppi_c + ptdcacoa_c 11167 or 15746 or 15748
FACOAL160 atp_c + coa_c + hdca_c --> amp_c + pmtcoa_c + ppi_c 11167 or 15746 or 15748
FACOAL161 atp_c + coa_c + hdcea_c --> amp_c + hdcoa_c + ppi_c 11167 or 15746 or 15748
FACOAL170 atp_c + coa_c + hpdca_c --> amp_c + hpdcacoa_c + ppi_c 11167 or 15746 or 15748
FACOAL180 atp_c + coa_c + ocdca_c --> amp_c + ppi_c + stcoa_c 11167 or 15746 or 15748
FACOAL181 atp_c + coa_c + ocdcea_c --> amp_c + odecoa_c + ppi_c 11167 or 15746 or 15748
FACOAL1813 atp_c + coa_c + elaid_c --> amp_c + od2coa_c + ppi_c 11167 or 15746 or 15748
FACOAL182 atp_c + coa_c + ocdcya_c --> amp_c + ocdycacoa_c + ppi_c 11167 or 15746 or 15748
FACOAL1831 atp_c + coa_c + lnlncg_c --> amp_c + lnlncgcoa_c + ppi_c 11167 or 15746 or 15748
FACOAL191 atp_c + coa_c + prist_c --> amp_c + ppi_c + pristcoa_c 11167 or 15746 or 15748
FACOAL200 arach_c + atp_c + coa_c --> amp_c + arachcoa_c + ppi_c 11167 or 15746 or 15748
FACOAL206 atp_c + coa_c + phyt_c --> amp_c + phytcoa_c + ppi_c 11167 or 15746 or 15748
FACOAL2251 atp_c + coa_c + dcsptn1_c --> amp_c + dcsptn1coa_c + ppi_c 11167 or 15746 or 15748
FACOAL2252 atp_c + clpnd_c + coa_c --> amp_c + clpndcoa_c + ppi_c 11167 or 15746 or 15748
FACOAL240 atp_c + coa_c + ttc_c --> amp_c + ppi_c + ttccoa_c 11167 or 15746 or 15748
FACOAL241 atp_c + coa_c + nrvnc_c --> amp_c + nrvnccoa_c + ppi_c 11167 or 15746 or 15748
FACOAL244_1 atp_c + coa_c + tettet6_c --> amp_c + ppi_c + tettet6coa_c 11167 or 15746 or 15748
FACOAL260 atp_c + coa_c + hexc_c --> amp_c + hexccoa_c + ppi_c 11167 or 15746 or 15748
FACOAL80 atp_c + coa_c + octa_c --> amp_c + occoa_c + ppi_c 11167 or 15746 or 15748

FACOAL100p atp_x + coa_x + dca_x --> amp_x + dcacoa_x + ppi_x 12555 or 15900
FACOAL120p atp_x + coa_x + ddca_x --> amp_x + ddcacoa_x + ppi_x 12555 or 15900
FACOAL140p atp_x + coa_x + ttdca_x --> amp_x + ppi_x + tdcoa_x 12555 or 15900
FACOAL141p atp_x + coa_x + ttdcea_x --> amp_x + ppi_x + tdecoa_x 12555 or 15900
FACOAL160p atp_x + coa_x + hdca_x --> amp_x + pmtcoa_x + ppi_x 12555 or 15900
FACOAL161p atp_x + coa_x + hdcea_x --> amp_x + hdcoa_x + ppi_x 12555 or 15900
FACOAL80p atp_x + coa_x + octa_x --> amp_x + occoa_x + ppi_x 12555 or 15900

FACOAL100 atp_c + coa_c + dca_c --> amp_c + dcacoa_c + ppi_c 11167 or 15746 or 15748
FACOAL120 atp_c + coa_c + ddca_c --> amp_c + ddcacoa_c + ppi_c 11167 or 15746 or 15748
FACOAL140 atp_c + coa_c + ttdca_c --> amp_c + ppi_c + tdcoa_c 11167 or 15746 or 15748
FACOAL141 atp_c + coa_c + ttdcea_c --> amp_c + ppi_c + tdecoa_c 11167 or 15746 or 15748
FACOAL150 atp_c + coa_c + ptdca_c --> amp_c + ppi_c + ptdcacoa_c 11167 or 15746 or 15748
FACOAL160 atp_c + coa_c + hdca_c --> amp_c + pmtcoa_c + ppi_c 11167 or 15746 or 15748
FACOAL161 atp_c + coa_c + hdcea_c --> amp_c + hdcoa_c + ppi_c 11167 or 15746 or 15748
FACOAL170 atp_c + coa_c + hpdca_c --> amp_c + hpdcacoa_c + ppi_c 11167 or 15746 or 15748
FACOAL180 atp_c + coa_c + ocdca_c --> amp_c + ppi_c + stcoa_c 11167 or 15746 or 15748
FACOAL181 atp_c + coa_c + ocdcea_c --> amp_c + odecoa_c + ppi_c 11167 or 15746 or 15748
FACOAL1813 atp_c + coa_c + elaid_c --> amp_c + od2coa_c + ppi_c 11167 or 15746 or 15748
FACOAL182 atp_c + coa_c + ocdcya_c --> amp_c + ocdycacoa_c + ppi_c 11167 or 15746 or 15748
FACOAL1831 atp_c + coa_c + lnlncg_c --> amp_c + lnlncgcoa_c + ppi_c 11167 or 15746 or 15748
FACOAL191 atp_c + coa_c + prist_c --> amp_c + ppi_c + pristcoa_c 11167 or 15746 or 15748
FACOAL200 arach_c + atp_c + coa_c --> amp_c + arachcoa_c + ppi_c 11167 or 15746 or 15748
FACOAL206 atp_c + coa_c + phyt_c --> amp_c + phytcoa_c + ppi_c 11167 or 15746 or 15748
FACOAL2251 atp_c + coa_c + dcsptn1_c --> amp_c + dcsptn1coa_c + ppi_c 11167 or 15746 or 15748
FACOAL2252 atp_c + clpnd_c + coa_c --> amp_c + clpndcoa_c + ppi_c 11167 or 15746 or 15748
FACOAL240 atp_c + coa_c + ttc_c --> amp_c + ppi_c + ttccoa_c 11167 or 15746 or 15748
FACOAL241 atp_c + coa_c + nrvnc_c --> amp_c + nrvnccoa_c + ppi_c 11167 or 15746 or 15748
FACOAL244_1 atp_c + coa_c + tettet6_c --> amp_c + ppi_c + tettet6coa_c 11167 or 15746 or 15748
FACOAL260 atp_c + coa_c + hexc_c --> amp_c + hexccoa_c + ppi_c 11167 or 15746 or 15748
FACOAL80 atp_c + coa_c + octa_c --> amp_c + occoa_c + ppi_c 11167 or 15746 or 15748

13GS udpg_c --> 13BDglcn_c + h_c + udp_c (YLR343W and 15984 and 16655) or (YMR215W and 15984 and 16655) or (YMR307W and 15984 and 16655) or (YOL030W and 15984 and 16655) or (YOL132W and 15984 and 16655)
FAS141 ddca_c + 4.0 h_c + malcoa_c + 3.0 nadph_c + o2_c --> co2_c + coa_c + 3.0 h2o_c + 3.0 nadp_c + ttdcea_c 16655
FAS161 4.0 h_c + malcoa_c + 3.0 nadph_c + o2_c + ttdca_c --> co2_c + coa_c + 3.0 h2o_c + hdcea_c + 3.0 nadp_c 16655
FAS181 4.0 h_c + hdca_c + malcoa_c + 3.0 nadph_c + o2_c --> co2_c + coa_c + 3.0 h2o_c + 3.0 nadp_c + ocdcea_c 16655
FAS240_L 9.0 h_c + 3.0 malcoa_c + 6.0 nadph_c + ocdca_c --> 3.0 co2_c + 3.0 coa_c + 3.0 h2o_c + 6.0 nadp_c + ttc_c (16241 and 16655) or (10677 and 16241 and 16695)
FAS260 3.0 h_c + malcoa_c + 2.0 nadph_c + ttc_c --> co2_c + coa_c + h2o_c + hexc_c + 2.0 nadp_c (16241 and 16655) or (10677 and 16241 and 16695)

FAS140COA ddcacoa_c + 3.0 h_c + malcoa_c + 2.0 nadph_c --> co2_c + coa_c + h2o_c + 2.0 nadp_c + tdcoa_c (10677 and 16241 and 16695) or (12826 and 8639 and 8670 and 8777) or (14471 and 8639 and 8670 and 8777)
FAS160COA 3.0 h_c + malcoa_c + 2.0 nadph_c + tdcoa_c --> co2_c + coa_c + h2o_c + 2.0 nadp_c + pmtcoa_c (10677 and 16241 and 16695) or (12826 and 8639 and 8670 and 8777) or (14471 and 8639 and 8670 and 8777)
FAS180 3.0 h_c + hdca_c + malcoa_c + 2.0 nadph_c --> co2_c + coa_c + h2o_c + 2.0 nadp_c + ocdca_c (10677 and 16241 and 16695) or (12826 and 8639 and 8670 and 8777) or (14471 and 8639 and 8670 and 8777)
FAS180COA 3.0 h_c + malcoa_c + 2.0 nadph_c + pmtcoa_c --> co2_c + coa_c + h2o_c + 2.0 nadp_c + stcoa_c (10677 and 16241 and 16695) or (12826 and 8639 and 8670 and 8777) or (14471 and 8639 and 8670 and 8777)
FAS240_L 9.0 h_c + 3.0 malcoa_c + 6.0 nadph_c + ocdca_c --> 3.0 co2_c + 3.0 coa_c + 3.0 h2o_c + 6.0 nadp_c + ttc_c (16241 and 16655) or (10677 and 16241 and 16695)
FAS260 3.0 h_c + malcoa_c + 2.0 nadph_c + ttc_c --> co2_c + coa_c + h2o_c + hexc_c + 2.0 nadp_c (16241 and 16655) or (10677 and 16241 and 16695)
FAS80COA_L accoa_c + 9.0 h_c + 3.0 malcoa_c + 6.0 nadph_c --> 3.0 co2_c + 3.0 coa_c + 3.0 h2o_c + 6.0 nadp_c + occoa_c (10677 and 16241 and 16695) or (12826 and 8639 and 8670 and 8777) or (14471 and 8639 and 8670 and 8777)
FAS80_L accoa_c + 8.0 h_c + 3.0 malcoa_c + 6.0 nadph_c --> 3.0 co2_c + 4.0 coa_c + 2.0 h2o_c + 6.0 nadp_c + octa_c (10677 and 16241 and 16695) or (12826 and 8639 and 8670 and 8777) or (14471 and 8639 and 8670 and 8777)
yli_R0126 3.0 h_c + malcoa_c + 2.0 nadph_c + occoa_c --> co2_c + coa_c + dccoa_c + h2o_c + 2.0 nadp_c 10677 and 16241 and 16695
yli_R0129 dccoa_c + 3.0 h_c + malcoa_c + 2.0 nadph_c --> co2_c + coa_c + ddcacoa_c + h2o_c + 2.0 nadp_c 10677 and 16241 and 16695
yli_R0140 4.0 h_c + hdca_c + malcoa_c + 3.0 nadph_c + o2_c --> co2_c + coa_c + 3.0 h2o_c + 3.0 nadp_c + yli_M04622_c 10677 and 16241 and 16695

FAS140COA ddcacoa_c + 3.0 h_c + malcoa_c + 2.0 nadph_c --> co2_c + coa_c + h2o_c + 2.0 nadp_c + tdcoa_c (10677 and 16241 and 16695) or (12826 and 8639 and 8670 and 8777) or (14471 and 8639 and 8670 and 8777)
FAS160COA 3.0 h_c + malcoa_c + 2.0 nadph_c + tdcoa_c --> co2_c + coa_c + h2o_c + 2.0 nadp_c + pmtcoa_c (10677 and 16241 and 16695) or (12826 and 8639 and 8670 and 8777) or (14471 and 8639 and 8670 and 8777)
FAS180 3.0 h_c + hdca_c + malcoa_c + 2.0 nadph_c --> co2_c + coa_c + h2o_c + 2.0 nadp_c + ocdca_c (10677 and 16241 and 16695) or (12826 and 8639 and 8670 and 8777) or (14471 and 8639 and 8670 and 8777)
FAS180COA 3.0 h_c + malcoa_c + 2.0 nadph_c + pmtcoa_c --> co2_c + coa_c + h2o_c + 2.0 nadp_c + stcoa_c (10677 and 16241 and 16695) or (12826 and 8639 and 8670 and 8777) or (14471 and 8639 and 8670 and 8777)
FAS240_L 9.0 h_c + 3.0 malcoa_c + 6.0 nadph_c + ocdca_c --> 3.0 co2_c + 3.0 coa_c + 3.0 h2o_c + 6.0 nadp_c + ttc_c (16241 and 16655) or (10677 and 16241 and 16695)
FAS260 3.0 h_c + malcoa_c + 2.0 nadph_c + ttc_c --> co2_c + coa_c + h2o_c + hexc_c + 2.0 nadp_c (16241 and 16655) or (10677 and 16241 and 16695)
FAS80COA_L accoa_c + 9.0 h_c + 3.0 malcoa_c + 6.0 nadph_c --> 3.0 co2_c + 3.0 coa_c + 3.0 h2o_c + 6.0 nadp_c + occoa_c (10677 and 16241 and 16695) or (12826 and 8639 and 8670 and 8777) or (14471 and 8639 and 8670 and 8777)
FAS80_L accoa_c + 8.0 h_c + 3.0 malcoa_c + 6.0 nadph_c --> 3.0 co2_c + 4.0 coa_c + 2.0 h2o_c + 6.0 nadp_c + octa_c (10677 and 16241 and 16695) or (12826 and 8639 and 8670 and 8777) or (14471 and 8639 and 8670 and 8777)
yli_R0126 3.0 h_c + malcoa_c + 2.0 nadph_c + occoa_c --> co2_c + coa_c + dccoa_c + h2o_c + 2.0 nadp_c 10677 and 16241 and 16695
yli_R0129 dccoa_c + 3.0 h_c + malcoa_c + 2.0 nadph_c --> co2_c + coa_c + ddcacoa_c + h2o_c + 2.0 nadp_c 10677 and 16241 and 16695
yli_R0140 4.0 h_c + hdca_c + malcoa_c + 3.0 nadph_c + o2_c --> co2_c + coa_c + 3.0 h2o_c + 3.0 nadp_c + yli_M04622_c 10677 and 16241 and 16695

ACOAR2m h_m + hx2coa_m + nadph_m --> hxcoa_m + nadp_m 16241
ACOAR3m h_m + nadph_m + oc2coa_m --> nadp_m + occoa_m 16241
ACOAR4m dc2coa_m + h_m + nadph_m --> dcacoa_m + nadp_m 16241
ACOAR5m dd2coa_m + h_m + nadph_m --> ddcacoa_m + nadp_m 16241
ACOAR6m h_m + nadph_m + td2coa_m --> nadp_m + tdcoa_m 16241
ACOAR7m h_m + hdd2coa_m + nadph_m --> nadp_m + pmtcoa_m 16241
ECOAR h_c + nadph_c + od2coa_c --> nadp_c + stcoa_c 16241
FAS140COA ddcacoa_c + 3.0 h_c + malcoa_c + 2.0 nadph_c --> co2_c + coa_c + h2o_c + 2.0 nadp_c + tdcoa_c (10677 and 16241 and 16695) or (12826 and 8639 and 8670 and 8777) or (14471 and 8639 and 8670 and 8777)
FAS160COA 3.0 h_c + malcoa_c + 2.0 nadph_c + tdcoa_c --> co2_c + coa_c + h2o_c + 2.0 nadp_c + pmtcoa_c (10677 and 16241 and 16695) or (12826 and 8639 and 8670 and 8777) or (14471 and 8639 and 8670 and 8777)
FAS180 3.0 h_c + hdca_c + malcoa_c + 2.0 nadph_c --> co2_c + coa_c + h2o_c + 2.0 nadp_c + ocdca_c (10677 and 16241 and 16695) or (12826 and 8639 and 8670 and 8777) or (14471 and 8639 and 8670 and 8777)
FAS180COA 3.0 h_c + malcoa_c + 2.0 nadph_c + pmtcoa_c --> co2_c + coa_c + h2o_c + 2.0 nadp_c + stcoa_c (10677 and 16241 and 16695) or (12826 and 8639 and 8670 and 8777) or (14471 and 8639 and 8670 and 8777)
FAS240_L 9.0 h_c + 3.0 malcoa_c + 6.0 nadph_c + ocdca_c --> 3.0 co2_c + 3.0 coa_c + 3.0 h2o_c + 6.0 nadp_c + ttc_c (16241 and 16655) or (10677 and 16241 and 16695)
FAS260 3.0 h_c + malcoa_c + 2.0 nadph_c + ttc_c --> co2_c + coa_c + h2o_c + hexc_c + 2.0 nadp_c (16241 and 16655) or (10677 and 16241 and 16695)
FAS80COA_L accoa_c + 9.0 h_c + 3.0 malcoa_c + 6.0 nadph_c --> 3.0 co2_c + 3.0 coa_c + 3.0 h2o_c + 6.0 nadp_c + occoa_c (10677 and 16241 and 16695) or (12826 and 8639 and 8670 and 8777) or (14471 and 8639 and 8670 and 8777)
FAS80_L accoa_c + 8.0 h_c + 3.0 malcoa_c + 6.0 nadph_c --> 3.0 co2_c + 4.0 coa_c + 2.0 h2o_c + 6.0 nadp_c + octa_c (10677 and 16241 and 16695) or (12826 and 8639 and 8670 and 8777) or (14471 and 8639 and 8670 and 8777)
yli_R0126 3.0 h_c + malcoa_c + 2.0 nadph_c + occoa_c --> co2_c + coa_c + dccoa_c + h2o_c + 2.0 nadp_c 10677 and 16241 and 16695
yli_R0129 dccoa_c + 3.0 h_c + malcoa_c + 2.0 nadph_c --> co2_c + coa_c + ddcacoa_c + h2o_c + 2.0 nadp_c 10677 and 16241 and 16695
yli_R0140 4.0 h_c + hdca_c + malcoa_c + 3.0 nadph_c + o2_c --> co2_c + coa_c + 3.0 h2o_c + 3.0 nadp_c + yli_M04622_c 10677 and 16241 and 16695

MCOATA ACP_c + malcoa_c <=> coa_c + malACP_c 16542 or (14261 and 16542) or (8670 and 8777)
MCOATA_1 ACP_h + malcoa_h <=> coa_h + malACP_h (CRv4_Au5_s16_g6129_t1 and 14261) or (CRv4_Au5_s17_g7486_t2 and 14261) or (CRv4_Au5_s2_g8568_t1 and 14261) or (14261 and 16542)
MCOATAm ACP_m + malcoa_m <=> coa_m + malACP_m 16542 or (YER061C and YHR067W and YKL055C and YOR221C and 11837 and 14261)

ACOATAm ACP_m + accoa_m <=> acACP_m + coa_m YER061C and YHR067W and YKL055C and YOR221C and 11837 and 14261
EAR100y_1 h_h + nadph_h + tdec2eACP_h --> dcaACP_h + nadp_h (CRv4_Au5_s16_g6129_t1 and 14261) or (CRv4_Au5_s17_g7486_t2 and 14261) or (11837 and 14261)
EAR120y_1 h_h + nadph_h + tddec2eACP_h --> ddcaACP_h + nadp_h (CRv4_Au5_s16_g6129_t1 and 14261) or (CRv4_Au5_s17_g7486_t2 and 14261) or (11837 and 14261)
EAR140y_1 h_h + nadph_h + tmrs2eACP_h --> myrsACP_h + nadp_h (CRv4_Au5_s16_g6129_t1 and 14261) or (CRv4_Au5_s17_g7486_t2 and 14261) or (11837 and 14261)
EAR160y_1 h_h + nadph_h + tpalm2eACP_h --> nadp_h + palmACP_h (CRv4_Au5_s16_g6129_t1 and 14261) or (CRv4_Au5_s17_g7486_t2 and 14261) or (11837 and 14261)
EAR181y_1 h_h + nadph_h + t3c11vaceACP_h --> nadp_h + octeACP_h (CRv4_Au5_s16_g6129_t1 and 14261) or (CRv4_Au5_s17_g7486_t2 and 14261) or (11837 and 14261)
EAR40y_1 but2eACP_h + h_h + nadph_h --> butACP_h + nadp_h 11837 and 14261
EAR60y_1 h_h + nadph_h + thex2eACP_h --> hexACP_h + nadp_h (CRv4_Au5_s16_g6129_t1 and 14261) or (CRv4_Au5_s17_g7486_t2 and 14261) or (11837 and 14261)
EAR80y_1 h_h + nadph_h + toct2eACP_h --> nadp_h + ocACP_h (CRv4_Au5_s16_g6129_t1 and 14261) or (CRv4_Au5_s17_g7486_t2 and 14261) or (11837 and 14261)
FAS100ACPm 3.0 h_m + malACP_m + 2.0 nadph_m + ocACP_m --> ACP_m + co2_m + dcaACP_m + h2o_m + 2.0 nadp_m YER061C and YHR067W and YKL055C and YOR221C and 11837 and 14261
FAS120ACPm dcaACP_m + 3.0 h_m + malACP_m + 2.0 nadph_m --> ACP_m + co2_m + ddcaACP_m + h2o_m + 2.0 nadp_m YER061C and YHR067W and YKL055C and YOR221C and 11837 and 14261
FAS140ACPm ddcaACP_m + 3.0 h_m + malACP_m + 2.0 nadph_m --> ACP_m + co2_m + h2o_m + myrsACP_m + 2.0 nadp_m YER061C and YHR067W and YKL055C and YOR221C and 11837 and 14261
FAS141ACPm ddcaACP_m + 4.0 h_m + malACP_m + 3.0 nadph_m + o2_m --> ACP_m + co2_m + 3.0 h2o_m + 3.0 nadp_m + tdeACP_m YER061C and YHR067W and YKL055C and YOR221C and 11837 and 14261
FAS160ACPm 3.0 h_m + malACP_m + myrsACP_m + 2.0 nadph_m --> ACP_m + co2_m + h2o_m + 2.0 nadp_m + palmACP_m YER061C and YHR067W and YKL055C and YOR221C and 11837 and 14261
FAS161ACPm 4.0 h_m + malACP_m + myrsACP_m + 3.0 nadph_m + o2_m --> ACP_m + co2_m + 3.0 h2o_m + hdeACP_m + 3.0 nadp_m YER061C and YHR067W and YKL055C and YOR221C and 11837 and 14261
FAS180ACPm 3.0 h_m + malACP_m + 2.0 nadph_m + palmACP_m --> ACP_m + co2_m + h2o_m + 2.0 nadp_m + ocdcaACP_m YER061C and YHR067W and YKL055C and YOR221C and 11837 and 14261
FAS181ACPm 4.0 h_m + malACP_m + 3.0 nadph_m + o2_m + palmACP_m --> ACP_m + co2_m + 3.0 h2o_m + 3.0 nadp_m + octeACP_m YER061C and YHR067W and YKL055C and YOR221C and 11837 and 14261
FAS182ACPm 5.0 h_m + malACP_m + 4.0 nadph_m + 2.0 o2_m + palmACP_m --> ACP_m + co2_m + 5.0 h2o_m + 4.0 nadp_m + ocdcyaACP_m YER061C and YHR067W and YKL055C and YOR221C and 11837 and 14261
FAS80ACPm_L acACP_m + 9.0 h_m + 3.0 malACP_m + 6.0 nadph_m --> 3.0 ACP_m + 3.0 co2_m + 3.0 h2o_m + 6.0 nadp_m + ocACP_m YER061C and YHR067W and YKL055C and YOR221C and 11837 and 14261
MCOATAm ACP_m + malcoa_m <=> coa_m + malACP_m 16542 or (YER061C and YHR067W and YKL055C and YOR221C and 11837 and 14261)

3HAD100_1 3hdecACP_h <=> h2o_h + tdec2eACP_h (CRv4_Au5_s16_g6129_t1 and 14261) or (CRv4_Au5_s17_g7486_t2 and 14261) or (CRv4_Au5_s3_g11300_t1 and 14261)
3HAD120_1 3hddecACP_h <=> h2o_h + tddec2eACP_h (CRv4_Au5_s16_g6129_t1 and 14261) or (CRv4_Au5_s17_g7486_t2 and 14261) or (CRv4_Au5_s3_g11300_t1 and 14261)
3HAD140_1 3hmrsACP_h <=> h2o_h + tmrs2eACP_h (CRv4_Au5_s16_g6129_t1 and 14261) or (CRv4_Au5_s17_g7486_t2 and 14261)
3HAD160_1 3hpalmACP_h <=> h2o_h + tpalm2eACP_h (CRv4_Au5_s16_g6129_t1 and 14261) or (CRv4_Au5_s17_g7486_t2 and 14261)
3HAD180_1 3hoctaACP_h --> h2o_h + toctd2eACP_h (CRv4_Au5_s16_g6129_t1 and 14261) or (CRv4_Au5_s17_g7486_t2 and 14261)
3HAD181_1 3hcvac11eACP_h --> h2o_h + t3c11vaceACP_h (CRv4_Au5_s16_g6129_t1 and 14261) or (CRv4_Au5_s17_g7486_t2 and 14261)
3HAD40_1 3hbutACP_h <=> but2eACP_h + h2o_h (CRv4_Au5_s16_g6129_t1 and 14261) or (CRv4_Au5_s17_g7486_t2 and 14261)
3HAD60_1 3hhexACP_h <=> h2o_h + thex2eACP_h (CRv4_Au5_s16_g6129_t1 and 14261) or (CRv4_Au5_s17_g7486_t2 and 14261)
3HAD80_1 3hoctACP_h <=> h2o_h + toct2eACP_h (CRv4_Au5_s16_g6129_t1 and 14261) or (CRv4_Au5_s17_g7486_t2 and 14261) or (CRv4_Au5_s3_g11300_t1 and 14261)
3OAR100_1 3odecACP_h + h_h + nadph_h <=> 3hdecACP_h + nadp_h (CRv4_Au5_s16_g6129_t1 and 14261) or (CRv4_Au5_s17_g7486_t2 and 14261) or (10558 and 14261)
3OAR120_1 3oddecACP_h + h_h + nadph_h <=> 3hddecACP_h + nadp_h (CRv4_Au5_s16_g6129_t1 and 14261) or (CRv4_Au5_s17_g7486_t2 and 14261) or (10558 and 14261)
3OAR140_1 3omrsACP_h + h_h + nadph_h <=> 3hmrsACP_h + nadp_h (CRv4_Au5_s16_g6129_t1 and 14261) or (CRv4_Au5_s17_g7486_t2 and 14261) or (10558 and 14261)
3OAR160_1 3opalmACP_h + h_h + nadph_h <=> 3hpalmACP_h + nadp_h (CRv4_Au5_s16_g6129_t1 and 14261) or (CRv4_Au5_s17_g7486_t2 and 14261) or (10558 and 14261)
3OAR180_1 3ooctdACP_h + h_h + nadph_h --> 3hoctaACP_h + nadp_h (CRv4_Au5_s16_g6129_t1 and 14261) or (CRv4_Au5_s17_g7486_t2 and 14261) or (10558 and 14261)
3OAR181_1 3ocvac11eACP_h + nadph_h --> 3hcvac11eACP_h + nadp_h (CRv4_Au5_s16_g6129_t1 and 14261) or (CRv4_Au5_s17_g7486_t2 and 14261) or (10558 and 14261)
3OAR40_1 actACP_h + h_h + nadph_h <=> 3hbutACP_h + nadp_h (CRv4_Au5_s16_g6129_t1 and 14261) or (CRv4_Au5_s17_g7486_t2 and 14261) or (10558 and 14261)
3OAR60_1 3ohexACP_h + h_h + nadph_h <=> 3hhexACP_h + nadp_h (CRv4_Au5_s16_g6129_t1 and 14261) or (CRv4_Au5_s17_g7486_t2 and 14261) or (10558 and 14261)
3OAR80_1 3ooctACP_h + h_h + nadph_h <=> 3hoctACP_h + nadp_h (CRv4_Au5_s16_g6129_t1 and 14261) or (CRv4_Au5_s17_g7486_t2 and 14261) or (10558 and 14261)
3OAS100_1 malACP_h + ocACP_h --> 3odecACP_h + ACP_h + co2_h (CRv4_Au5_s10_g406_t1 and 14261) or (CRv4_Au5_s16_g6129_t1 and 14261) or (CRv4_Au5_s17_g7486_t2 and 14261) or (CRv4_Au5_s22_g9840_t1 and 14261) or (CRv4_Au5_s4_g11616_t1 and 14261) or (CRv4_Au5_s7_g14144_t1 and 14261)
3OAS120_1 dcaACP_h + malACP_h --> 3oddecACP_h + ACP_h + co2_h (CRv4_Au5_s10_g406_t1 and 14261) or (CRv4_Au5_s16_g6129_t1 and 14261) or (CRv4_Au5_s17_g7486_t2 and 14261) or (CRv4_Au5_s22_g9840_t1 and 14261) or (CRv4_Au5_s4_g11616_t1 and 14261) or (CRv4_Au5_s7_g14144_t1 and 14261)
3OAS140_1 ddcaACP_h + malACP_h --> 3omrsACP_h + ACP_h + co2_h (CRv4_Au5_s10_g406_t1 and 14261) or (CRv4_Au5_s16_g6129_t1 and 14261) or (CRv4_Au5_s17_g7486_t2 and 14261) or (CRv4_Au5_s22_g9840_t1 and 14261) or (CRv4_Au5_s4_g11616_t1 and 14261) or (CRv4_Au5_s7_g14144_t1 and 14261)
3OAS160_1 malACP_h + myrsACP_h --> 3opalmACP_h + ACP_h + co2_h (CRv4_Au5_s10_g406_t1 and 14261) or (CRv4_Au5_s16_g6129_t1 and 14261) or (CRv4_Au5_s17_g7486_t2 and 14261) or (CRv4_Au5_s22_g9840_t1 and 14261) or (CRv4_Au5_s4_g11616_t1 and 14261) or (CRv4_Au5_s7_g14144_t1 and 14261)
3OAS180_1 malACP_h + palmACP_h --> 3ooctdACP_h + ACP_h + co2_h (CRv4_Au5_s10_g406_t1 and 14261) or (CRv4_Au5_s16_g6129_t1 and 14261) or (CRv4_Au5_s17_g7486_t2 and 14261) or (CRv4_Au5_s22_g9840_t1 and 14261) or (CRv4_Au5_s7_g14144_t1 and 14261)
3OAS181_1 h_h + hdeACP_h + malACP_h --> 3ocvac11eACP_h + ACP_h + co2_h (CRv4_Au5_s10_g406_t1 and 14261) or (CRv4_Au5_s16_g6129_t1 and 14261) or (CRv4_Au5_s17_g7486_t2 and 14261) or (CRv4_Au5_s22_g9840_t1 and 14261) or (CRv4_Au5_s7_g14144_t1 and 14261)
3OAS60_1 butACP_h + malACP_h --> 3ohexACP_h + ACP_h + co2_h (CRv4_Au5_s10_g406_t1 and 14261) or (CRv4_Au5_s16_g6129_t1 and 14261) or (CRv4_Au5_s17_g7486_t2 and 14261) or (CRv4_Au5_s22_g9840_t1 and 14261) or (CRv4_Au5_s4_g11616_t1 and 14261) or (CRv4_Au5_s7_g14144_t1 and 14261)
3OAS80_1 hexACP_h + malACP_h --> 3ooctACP_h + ACP_h + co2_h (CRv4_Au5_s10_g406_t1 and 14261) or (CRv4_Au5_s16_g6129_t1 and 14261) or (CRv4_Au5_s17_g7486_t2 and 14261) or (CRv4_Au5_s22_g9840_t1 and 14261) or (CRv4_Au5_s4_g11616_t1 and 14261) or (CRv4_Au5_s7_g14144_t1 and 14261)
AACP1819ZS ACP_h + atp_h + ocdce9a_h --> amp_h + h_h + octe9ACP_h + ppi_h CRv4_Au5_s6_g13391_t1 and 14261
AACPS1 ACP_c + atp_c + ttdca_c --> amp_c + myrsACP_c + ppi_c b2836 and 14261
AACPS1_1 ACP_h + atp_h + ttdca_h --> amp_h + h_h + myrsACP_h + ppi_h CRv4_Au5_s6_g13391_t1 and 14261
AACPS2 ACP_c + atp_c + ttdcea_c --> amp_c + ppi_c + tdeACP_c b2836 and 14261
AACPS3 ACP_c + atp_c + hdca_c --> amp_c + palmACP_c + ppi_c b2836 and 14261
AACPS3_1 ACP_h + atp_h + hdca_h --> amp_h + h_h + palmACP_h + ppi_h CRv4_Au5_s6_g13391_t1 and 14261
AACPS4 ACP_c + atp_c + hdcea_c --> amp_c + hdeACP_c + ppi_c b2836 and 14261
AACPS4_1 ACP_h + atp_h + hdcea_h --> amp_h + h_h + hdeACP_h + ppi_h CRv4_Au5_s6_g13391_t1 and 14261
AACPS5 ACP_c + atp_c + ocdcea_c --> amp_c + octeACP_c + ppi_c b2836 and 14261
AACPS5_1 ACP_h + atp_h + ocdcea_h --> amp_h + h_h + octeACP_h + ppi_h CRv4_Au5_s6_g13391_t1 and 14261
AACPS6 ACP_c + atp_c + ocdca_c --> amp_c + ocdcaACP_c + ppi_c b2836 and 14261
AACPS6_1 ACP_h + atp_h + ocdca_h --> amp_h + h_h + ocdcaACP_h + ppi_h CRv4_Au5_s6_g13391_t1 and 14261
AACPS7 ACP_c + atp_c + ddca_c --> amp_c + ddcaACP_c + ppi_c b2836 and 14261
AACPS8 ACP_c + atp_c + dca_c --> amp_c + dcaACP_c + ppi_c b2836 and 14261
AACPS9 ACP_c + atp_c + octa_c --> amp_c + ocACP_c + ppi_c b2836 and 14261
ACOATA ACP_c + accoa_c <=> acACP_c + coa_c (b1091 and 14261) or (8670 and 8777)
ACOATA_1 ACP_h + accoa_h --> acACP_h + coa_h (CRv4_Au5_s16_g6129_t1 and 14261) or (CRv4_Au5_s17_g7486_t2 and 14261) or (CRv4_Au5_s4_g11616_t1 and 14261)
ACOATAm ACP_m + accoa_m <=> acACP_m + coa_m YER061C and YHR067W and YKL055C and YOR221C and 11837 and 14261
ACP1619ZD9DS fdxox_h + 4.0 nadph_h + o2_h + palmACP_h --> fdxrd_h + 2.0 h2o_h + hdeACP_h + 4.0 nadp_h (CRv4_Au5_s16_g6229_t1 and CRv4_Au5_s17_g7079_t1 and 14261) or (CRv4_Au5_s17_g7064_t1 and CRv4_Au5_s17_g7079_t1 and 14261) or (CRv4_Au5_s17_g7079_t1 and CRv4_Au5_s3_g10824_t1 and 14261) or (CRv4_Au5_s17_g7079_t1 and CRv4_Au5_s6_g13523_t1 and 14261) or (CRv4_Au5_s17_g7079_t1 and CRv4_Au5_s7_g14133_t1 and 14261)
ACP1819ZD9DS fdxox_h + 4.0 nadph_h + o2_h + ocdcaACP_h --> fdxrd_h + 2.0 h2o_h + 4.0 nadp_h + octe9ACP_h (CRv4_Au5_s16_g6229_t1 and CRv4_Au5_s17_g7079_t1 and 14261) or (CRv4_Au5_s17_g7064_t1 and CRv4_Au5_s17_g7079_t1 and 14261) or (CRv4_Au5_s17_g7079_t1 and CRv4_Au5_s3_g10824_t1 and 14261) or (CRv4_Au5_s17_g7079_t1 and CRv4_Au5_s6_g13523_t1 and 14261) or (CRv4_Au5_s17_g7079_t1 and CRv4_Au5_s7_g14133_t1 and 14261)
ACPPAT120 ddcaACP_c + h_c + pi_c --> ACP_c + ddcap_c b1090 and 14261
ACPPAT140 h_c + myrsACP_c + pi_c --> ACP_c + ttdcap_c b1090 and 14261
ACPPAT141 h_c + pi_c + tdeACP_c --> ACP_c + ttdceap_c b1090 and 14261
ACPPAT160 h_c + palmACP_c + pi_c --> ACP_c + hdcap_c b1090 and 14261
ACPPAT161 h_c + hdeACP_c + pi_c --> ACP_c + hdceap_c b1090 and 14261
ACPPAT180 h_c + ocdcaACP_c + pi_c --> ACP_c + ocdcap_c b1090 and 14261
ACPPAT181 h_c + octeACP_c + pi_c --> ACP_c + ocdceap_c b1090 and 14261
ACPS1_1 apoACP_c + coa_c <=> ACP_c + pap_c CRv4_Au5_s1_g1339_t1 and 14261
ACPS1h apoACP_h + coa_h <=> ACP_h + pap_h CRv4_Au5_s1_g1339_t1 and 14261
AGPAT1601819Zh 1hdecg3p_h + octe9ACP_h --> ACP_h + pa1601819Z_h 10427 and 14261
AGPAT160h 1hdecg3p_h + palmACP_h --> ACP_h + pa160_h 10427 and 14261
AGPAT1801819Zh 1odecg3p_h + octe9ACP_h --> ACP_h + pa1801819Z_h 10427 and 14261
AGPAT1819Z160h 1odec9eg3p_h + palmACP_h --> ACP_h + pa1819Z160_h 10427 and 14261
AGPAT1819Z1619Zh 1odec9eg3p_h + hdeACP_h --> ACP_h + pa1819Z1619Z_h 10427 and 14261
AGPAT1819Z1819Zh 1odec9eg3p_h + octe9ACP_h --> ACP_h + pa1819Z1819Z_h 10427 and 14261
EAR100x_1 h_h + nadh_h + tdec2eACP_h --> dcaACP_h + nad_h (CRv4_Au5_s16_g6129_t1 and 14261) or (CRv4_Au5_s17_g7486_t2 and 14261) or (CRv4_Au5_s6_g13296_t1 and 14261)
EAR100y_1 h_h + nadph_h + tdec2eACP_h --> dcaACP_h + nadp_h (CRv4_Au5_s16_g6129_t1 and 14261) or (CRv4_Au5_s17_g7486_t2 and 14261) or (11837 and 14261)
EAR120x_1 h_h + nadh_h + tddec2eACP_h --> ddcaACP_h + nad_h (CRv4_Au5_s16_g6129_t1 and 14261) or (CRv4_Au5_s17_g7486_t2 and 14261) or (CRv4_Au5_s6_g13296_t1 and 14261)
EAR120y_1 h_h + nadph_h + tddec2eACP_h --> ddcaACP_h + nadp_h (CRv4_Au5_s16_g6129_t1 and 14261) or (CRv4_Au5_s17_g7486_t2 and 14261) or (11837 and 14261)
EAR140x_1 h_h + nadh_h + tmrs2eACP_h --> myrsACP_h + nad_h (CRv4_Au5_s16_g6129_t1 and 14261) or (CRv4_Au5_s17_g7486_t2 and 14261) or (CRv4_Au5_s6_g13296_t1 and 14261)
EAR140y_1 h_h + nadph_h + tmrs2eACP_h --> myrsACP_h + nadp_h (CRv4_Au5_s16_g6129_t1 and 14261) or (CRv4_Au5_s17_g7486_t2 and 14261) or (11837 and 14261)
EAR160x_1 h_h + nadh_h + tpalm2eACP_h --> nad_h + palmACP_h (CRv4_Au5_s16_g6129_t1 and 14261) or (CRv4_Au5_s17_g7486_t2 and 14261) or (CRv4_Au5_s6_g13296_t1 and 14261)
EAR160y_1 h_h + nadph_h + tpalm2eACP_h --> nadp_h + palmACP_h (CRv4_Au5_s16_g6129_t1 and 14261) or (CRv4_Au5_s17_g7486_t2 and 14261) or (11837 and 14261)
EAR180x_1 h_h + nadh_h + toctd2eACP_h --> nad_h + ocdcaACP_h (CRv4_Au5_s16_g6129_t1 and 14261) or (CRv4_Au5_s17_g7486_t2 and 14261) or (CRv4_Au5_s6_g13296_t1 and 14261)
EAR181x_1 h_h + nadh_h + t3c11vaceACP_h --> nad_h + octeACP_h (CRv4_Au5_s16_g6129_t1 and 14261) or (CRv4_Au5_s17_g7486_t2 and 14261) or (CRv4_Au5_s6_g13296_t1 and 14261)
EAR181y_1 h_h + nadph_h + t3c11vaceACP_h --> nadp_h + octeACP_h (CRv4_Au5_s16_g6129_t1 and 14261) or (CRv4_Au5_s17_g7486_t2 and 14261) or (11837 and 14261)
EAR40x_1 but2eACP_h + h_h + nadh_h --> butACP_h + nad_h (CRv4_Au5_s16_g6129_t1 and 14261) or (CRv4_Au5_s17_g7486_t2 and 14261) or (CRv4_Au5_s6_g13296_t1 and 14261)
EAR40y_1 but2eACP_h + h_h + nadph_h --> butACP_h + nadp_h 11837 and 14261
EAR60x_1 h_h + nadh_h + thex2eACP_h --> hexACP_h + nad_h (CRv4_Au5_s16_g6129_t1 and 14261) or (CRv4_Au5_s17_g7486_t2 and 14261) or (CRv4_Au5_s6_g13296_t1 and 14261)
EAR60y_1 h_h + nadph_h + thex2eACP_h --> hexACP_h + nadp_h (CRv4_Au5_s16_g6129_t1 and 14261) or (CRv4_Au5_s17_g7486_t2 and 14261) or (11837 and 14261)
EAR80x_1 h_h + nadh_h + toct2eACP_h --> nad_h + ocACP_h (CRv4_Au5_s16_g6129_t1 and 14261) or (CRv4_Au5_s17_g7486_t2 and 14261) or (CRv4_Au5_s6_g13296_t1 and 14261)
EAR80y_1 h_h + nadph_h + toct2eACP_h --> nadp_h + ocACP_h (CRv4_Au5_s16_g6129_t1 and 14261) or (CRv4_Au5_s17_g7486_t2 and 14261) or (11837 and 14261)
FA100ACPHi_1 dcaACP_h + h2o_h --> ACP_h + dca_h + h_h (CRv4_Au5_s6_g12531_t1 and 14261) or (CRv4_Au5_s9_g15253_t1 and 14261)
FA120ACPHi_1 ddcaACP_h + h2o_h --> ACP_h + ddca_h + h_h (CRv4_Au5_s6_g12531_t1 and 14261) or (CRv4_Au5_s9_g15253_t1 and 14261)
FA140ACPHi_1 h2o_h + myrsACP_h --> ACP_h + h_h + ttdca_h (CRv4_Au5_s6_g12531_t1 and 14261) or (CRv4_Au5_s9_g15253_t1 and 14261)
FA160ACPHi_1 h2o_h + palmACP_h --> ACP_h + h_h + hdca_h (CRv4_Au5_s16_g6129_t1 and 14261) or (CRv4_Au5_s17_g7486_t2 and 14261) or (CRv4_Au5_s6_g12531_t1 and 14261) or (CRv4_Au5_s9_g15253_t1 and 14261)
FA161ACPHi_1 h2o_h + hdeACP_h --> ACP_h + h_h + hdcea_h (CRv4_Au5_s6_g12531_t1 and 14261) or (CRv4_Au5_s9_g15253_t1 and 14261)
FA180ACPHi h2o_h + ocdcaACP_h --> ACP_h + h_h + ocdca_h (CRv4_Au5_s16_g6129_t1 and 14261) or (CRv4_Au5_s17_g7486_t2 and 14261) or (CRv4_Au5_s6_g12531_t1 and 14261) or (CRv4_Au5_s9_g15253_t1 and 14261)
FA1819ZACPH h2o_h + octe9ACP_h --> ACP_h + h_h + ocdce9a_h (CRv4_Au5_s6_g12531_t1 and 14261) or (CRv4_Au5_s9_g15253_t1 and 14261)
FA181ACPHi h2o_h + octeACP_h --> ACP_h + h_h + ocdcea_h (CRv4_Au5_s6_g12531_t1 and 14261) or (CRv4_Au5_s9_g15253_t1 and 14261)
FA80ACPHi_1 h2o_h + ocACP_h --> ACP_h + h_h + octa_h (CRv4_Au5_s6_g12531_t1 and 14261) or (CRv4_Au5_s9_g15253_t1 and 14261)
FAS100ACPm 3.0 h_m + malACP_m + 2.0 nadph_m + ocACP_m --> ACP_m + co2_m + dcaACP_m + h2o_m + 2.0 nadp_m YER061C and YHR067W and YKL055C and YOR221C and 11837 and 14261
FAS120ACPm dcaACP_m + 3.0 h_m + malACP_m + 2.0 nadph_m --> ACP_m + co2_m + ddcaACP_m + h2o_m + 2.0 nadp_m YER061C and YHR067W and YKL055C and YOR221C and 11837 and 14261
FAS140ACPm ddcaACP_m + 3.0 h_m + malACP_m + 2.0 nadph_m --> ACP_m + co2_m + h2o_m + myrsACP_m + 2.0 nadp_m YER061C and YHR067W and YKL055C and YOR221C and 11837 and 14261
FAS141ACPm ddcaACP_m + 4.0 h_m + malACP_m + 3.0 nadph_m + o2_m --> ACP_m + co2_m + 3.0 h2o_m + 3.0 nadp_m + tdeACP_m YER061C and YHR067W and YKL055C and YOR221C and 11837 and 14261
FAS160ACPm 3.0 h_m + malACP_m + myrsACP_m + 2.0 nadph_m --> ACP_m + co2_m + h2o_m + 2.0 nadp_m + palmACP_m YER061C and YHR067W and YKL055C and YOR221C and 11837 and 14261
FAS161ACPm 4.0 h_m + malACP_m + myrsACP_m + 3.0 nadph_m + o2_m --> ACP_m + co2_m + 3.0 h2o_m + hdeACP_m + 3.0 nadp_m YER061C and YHR067W and YKL055C and YOR221C and 11837 and 14261
FAS180ACPm 3.0 h_m + malACP_m + 2.0 nadph_m + palmACP_m --> ACP_m + co2_m + h2o_m + 2.0 nadp_m + ocdcaACP_m YER061C and YHR067W and YKL055C and YOR221C and 11837 and 14261
FAS181ACPm 4.0 h_m + malACP_m + 3.0 nadph_m + o2_m + palmACP_m --> ACP_m + co2_m + 3.0 h2o_m + 3.0 nadp_m + octeACP_m YER061C and YHR067W and YKL055C and YOR221C and 11837 and 14261
FAS182ACPm 5.0 h_m + malACP_m + 4.0 nadph_m + 2.0 o2_m + palmACP_m --> ACP_m + co2_m + 5.0 h2o_m + 4.0 nadp_m + ocdcyaACP_m YER061C and YHR067W and YKL055C and YOR221C and 11837 and 14261
FAS80ACPm_L acACP_m + 9.0 h_m + 3.0 malACP_m + 6.0 nadph_m --> 3.0 ACP_m + 3.0 co2_m + 3.0 h2o_m + 6.0 nadp_m + ocACP_m YER061C and YHR067W and YKL055C and YOR221C and 11837 and 14261
G3PAT120 ddcaACP_c + glyc3p_c --> 1ddecg3p_c + ACP_c 13369 or (13369 and 14261)
G3PAT140 glyc3p_c + myrsACP_c --> 1tdecg3p_c + ACP_c 13369 and 14261
G3PAT141 glyc3p_c + tdeACP_c --> 1tdec7eg3p_c + ACP_c 13369 and 14261
G3PAT160 glyc3p_c + palmACP_c --> 1hdecg3p_c + ACP_c 13369 or (13369 and 14261)
G3PAT160h glyc3p_h + palmACP_h --> 1hdecg3p_h + ACP_h CRv4_Au5_s2_g9657_t1 and 14261
G3PAT161 glyc3p_c + hdeACP_c --> 1hdec9eg3p_c + ACP_c 13369 or (13369 and 14261)
G3PAT180 glyc3p_c + ocdcaACP_c --> 1odecg3p_c + ACP_c 13369 or (13369 and 14261)
G3PAT180h glyc3p_h + ocdcaACP_h --> 1odecg3p_h + ACP_h CRv4_Au5_s2_g9657_t1 and 14261
G3PAT181 glyc3p_c + octeACP_c --> 1odec11eg3p_c + ACP_c 13369 or (13369 and 14261)
G3PAT1819Zh glyc3p_h + octe9ACP_h --> 1odec9eg3p_h + ACP_h CRv4_Au5_s2_g9657_t1 and 14261
G3PAT181h glyc3p_h + octeACP_h --> 1odec11eg3p_h + ACP_h CRv4_Au5_s2_g9657_t1 and 14261
KAS14_1 acACP_h + malACP_h --> ACP_h + actACP_h + co2_h (CRv4_Au5_s10_g406_t1 and 14261) or (CRv4_Au5_s16_g6129_t1 and 14261) or (CRv4_Au5_s17_g7486_t2 and 14261) or (CRv4_Au5_s22_g9840_t1 and 14261) or (CRv4_Au5_s4_g11616_t1 and 14261) or (CRv4_Au5_s7_g14144_t1 and 14261)
MCOATA ACP_c + malcoa_c <=> coa_c + malACP_c 16542 or (14261 and 16542) or (8670 and 8777)
MCOATA_1 ACP_h + malcoa_h <=> coa_h + malACP_h (CRv4_Au5_s16_g6129_t1 and 14261) or (CRv4_Au5_s17_g7486_t2 and 14261) or (CRv4_Au5_s2_g8568_t1 and 14261) or (14261 and 16542)
MCOATAm ACP_m + malcoa_m <=> coa_m + malACP_m 16542 or (YER061C and YHR067W and YKL055C and YOR221C and 11837 and 14261)
NADH2_u9m2 5.0 h_m + nadh_m + q9_m --> 4.0 h_c + nad_m + q9h2_m 10010 and 10017 and 10318 and 10541 and 10607 and 11151 and 11411 and 11702 and 11857 and 12482 and 12497 and 12543 and 12653 and 13017 and 13214 and 13268 and 13411 and 13656 and 13925 and 14078 and 14261 and 14418 and 14717 and 14900 and 15179 and 15864 and 15947 and 15973 and 15998 and 8444 and 8446 and 8530 and 8699 and 8817 and 8909 and 9144 and 9348 and RTO3_874609 and RTO3_879341 and RTO3_879512 and RTO3_900622 and RTO3_900624 and RTO3_900641 and RTO3_945994 and RTO3_945995 and RTO3_946005
UAGAAT 3hmrsACP_c + uacgam_c <=> ACP_c + u3aga_c b0181 and 14261

BDHm bhb_m + nad_m <=> acac_m + h_m + nadh_m 12795

LIPAMPL lipoamp_c --> amp_c + lipopb_c 13899
LIPATPT atp_c + lipoate_c --> lipoamp_c + ppi_c 13899
OCTNLL atp_c + h_c + octa_c --> amp_c + octapb_c + ppi_c 13899

LIPOCT h_c + ocACP_c --> ACP_c + octapb_c 14821

LIPOS 4fe4s_c + 2.0 amet_c + h_c + nad_c + octapb_c --> 2fe2s_c + 2.0 dad_5_c + 2.0 fe2_c + lipopb_c + 2.0 met__L_c + nadh_c 14096
```

In [154]:

```
temp = ['13899','14821','14096']
display(Annotation.loc[temp])
Show_Data(temp)
```

|  | Combined Annotations | Signal P | Sc288c Orthologs | Human Orthologs | Sc288 Best Hit | Human Blast | Essential | WolfPSort | C Terminal |
| --- | --- | --- | --- | --- | --- | --- | --- | --- | --- |
| RTO4\_ID |  |  |  |  |  |  |  |  |  |
| 13899 | K03800: lplA, lplJ; lipoate---protein ligase |  | AIM22 | LIPT1 | AIM22 | LIPT1 | Essential | mito 20, nucl 4, cyto 1, plas 1, pero 1, cyto\_... | GEM\* |
| 14821 | K03801: lipB; lipoyl(octanoyl) transferase | S | LIP2 | LIPT2 |  |  | Not Essential | mito 27 | LGV\* |
| 14096 | K03644: lipA; lipoyl synthase | S | LIP5 | LIAS | LIP5 | LIAS | Essential | mito 25 | VRV\* |

| strain | WT | | | | | | | | | | | | | | | | |
| --- | --- | --- | --- | --- | --- | --- | --- | --- | --- | --- | --- | --- | --- | --- | --- | --- | --- |
| condition | G\_MM | C\_MM | G\_SD | | GX\_SD | | | X\_SD | | A\_SD | | C\_SD | | MM\_CN120 | | MM\_CN5 | Diversity\_Sample |
| phase | exp | exp | exp | stat | exp | trans | stat | exp | stat | exp | stat | exp | stat | exp | stat | exp | exp |
| proteinId | Set1 | Set1 | Set2 | Set2 | Set2 | Set2 | Set2 | Set2 | Set2 | Set2 | Set2 | Set2 | Set2 | Set3 | Set3 | Set3 | Set3 |
| 13899 | 6.13776 | 5.38147 | 5.93976 | 7.0956 | 5.87833 | 6.48813 | 6.6631 | 6.19008 | 6.10676 | 6.23258 | 6.50523 | 5.72275 | 4.91908 | 4.86625 | 4.95779 | 4.72677 | 3.95335 |
| 14821 | 6.53356 | 5.33866 | 5.50701 | 5.67997 | 5.33663 | 5.61933 | 5.85488 | 5.79781 | 5.85629 | 5.78479 | 5.48136 | 5.74651 | 5.91636 | 5.27182 | 4.83702 | 5.43191 | 4.60573 |
| 14096 | 7.10419 | 6.51245 | 8.56407 | 5.85355 | 8.60978 | 6.5451 | 6.14237 | 8.11409 | 7.52566 | 8.44285 | 7.8142 | 6.40188 | 6.80827 | 6.00415 | 5.03101 | 7.72315 | 5.25478 |

| strain | WT | | | | | | | | | | |
| --- | --- | --- | --- | --- | --- | --- | --- | --- | --- | --- | --- |
| condition | G\_SD | | GX\_SD | | | X\_SD | | A\_SD | | C\_SD | |
| proteinId | exp | stat | exp | trans | stat | exp | stat | exp | stat | exp | stat |
| 13899 | 0.587987 | 1.08393 | 1.22078 | 1.30235 | 1.09591 | 1.17479 | 1.18215 | 0.775498 | 1.14892 | 0.208996 | 0.42946 |
| 14821 | 1.70565 | 2.36609 | 1.22036 | 1.30203 | 2.90107 | 3.70917 | 4.51993 | 2.70495 | 3.6437 | 3.41936 | 5.40703 |
| 14096 | 3.65981 | 1.06144 | 4.67462 | 0.379082 | 0.177427 | 0.783547 | 0.57633 | 0.196014 | 0 | 3.21014 | 3.25467 |

|  | Glucose | Xylose | Arabinose | Acetate | Coumarate | Ferulate | YNB Oleic Acid | YNB Ricinoleic Acid | YNB Glucose | YNB Gluc DOC | YPD |
| --- | --- | --- | --- | --- | --- | --- | --- | --- | --- | --- | --- |
| proteinId |  |  |  |  |  |  |  |  |  |  |  |

In [155]:

```
temp = ['12826','14471','8639','14261','16542','11837']
display(Annotation.loc[temp])
Show_Data(temp)
```

|  | Combined Annotations | Signal P | Sc288c Orthologs | Human Orthologs | Sc288 Best Hit | Human Blast | Essential | WolfPSort | C Terminal |
| --- | --- | --- | --- | --- | --- | --- | --- | --- | --- |
| RTO4\_ID |  |  |  |  |  |  |  |  |  |
| 12826 | K08762: DBI, ACBP; diazepam-binding inhibitor ... |  | ACB1 | ACBD7,DBI | ACB1 | DBI | Essential | cyto 11, mito 8, cyto\_nucl 8 | LAA\* |
| 14471 | K08762: DBI, ACBP; diazepam-binding inhibitor ... |  |  |  | ACB1 | DBI | Not Essential | mito 16, nucl 8, cyto 1, pero 1, vacu 1, cyto\_... | VRR\* |
| 8639 | K11262: ACACA; acetyl-CoA carboxylase / biotin... |  | HFA1,ACC1 | ACACA,ACACB | ACC1 | ACACB | Essential | cyto 12.5, cyto\_nucl 10, nucl 6.5, pero 5 | ILA\* |
| 14261 | KOG1748: Acyl carrier protein/NADH-ubiquinone ... | S | ACP1 | NDUFAB1 |  |  | Essential | mito 24, cyto 2 | EAH\* |
| 16542 | K00645: fabD; [acyl-carrier-protein] S-malonyl... |  |  | MCAT |  |  | Not Essential | mito 26 | DGQ\* |
| 11837 | K07512: MECR, NRBF1; mitochondrial trans-2-eno... | S | ETR1 | MECR | ETR1 | MECR | Not Essential | mito 27 | KDE\* |

| strain | WT | | | | | | | | | | | | | | | | |
| --- | --- | --- | --- | --- | --- | --- | --- | --- | --- | --- | --- | --- | --- | --- | --- | --- | --- |
| condition | G\_MM | C\_MM | G\_SD | | GX\_SD | | | X\_SD | | A\_SD | | C\_SD | | MM\_CN120 | | MM\_CN5 | Diversity\_Sample |
| phase | exp | exp | exp | stat | exp | trans | stat | exp | stat | exp | stat | exp | stat | exp | stat | exp | exp |
| proteinId | Set1 | Set1 | Set2 | Set2 | Set2 | Set2 | Set2 | Set2 | Set2 | Set2 | Set2 | Set2 | Set2 | Set3 | Set3 | Set3 | Set3 |
| 12826 | 9.7204 | 8.38765 | 8.06527 | 7.21671 | 8.1067 | 7.76682 | 7.53594 | 8.00913 | 6.91493 | 7.79403 | 6.95957 | 7.27194 | 6.76142 | 10.0211 | 9.6537 | 9.74544 | 9.03866 |
| 14471 | 5.38584 | 4.71576 | 4.89959 | 4.73157 | 4.97295 | 4.88314 | 4.99331 | 5.04261 | 4.74117 | 5.1103 | 4.89383 | 4.64276 | 4.98066 | 3.58689 | 3.33266 | 3.27839 | 3.036 |
| 8639 | 8.30349 | 6.25226 | 6.43919 | 3.88921 | 6.55602 | 5.93308 | 5.09146 | 6.6301 | 3.79039 | 6.29497 | 2.94944 | 5.90405 | 5.28307 | 10.2872 | 9.31204 | 9.43556 | 8.59167 |
| 14261 | 6.45017 | 6.83438 | 6.62746 | 6.54923 | 6.74052 | 6.61555 | 6.25684 | 6.98528 | 6.28791 | 6.69514 | 6.37707 | 6.6481 | 6.49898 | 6.05858 | 6.20728 | 6.67069 | 6.10672 |
| 16542 | 3.76843 | 6.03584 | 6.54621 | 6.38456 | 6.57713 | 6.74757 | 6.53774 | 5.5785 | 7.52331 | 6.37397 | 6.70748 | 7.04746 | 7.71185 | 4.94897 | 5.56324 | 5.87501 | 5.33605 |
| 11837 | 5.81663 | 6.65673 | 7.65714 | 5.54687 | 7.60434 | 6.78631 | 6.48507 | 6.80744 | 5.56124 | 5.44452 | 5.18646 | 8.16712 | 8.12259 | 3.6351 | 4.30887 | 4.10351 | 3.74949 |

| strain | WT | | | | | | | | | | |
| --- | --- | --- | --- | --- | --- | --- | --- | --- | --- | --- | --- |
| condition | G\_SD | | GX\_SD | | | X\_SD | | A\_SD | | C\_SD | |
| proteinId | exp | stat | exp | trans | stat | exp | stat | exp | stat | exp | stat |
| 12826 | 10.6303 | 9.01705 | 11.7985 | 7.44737 | 7.90346 | 8.40092 | 7.44324 | 7.34512 | 7.26534 | 16.4545 | 11.3345 |
| 14471 | 0.399358 | 0.166667 | 0 | 0.379082 | 0.549309 | 0.976878 | 0.387893 | 0 | 0.574986 | 0 | 0 |
| 8639 | 96.9235 | 99.2644 | 94.2202 | 102.31 | 89.9616 | 105.428 | 87.8344 | 99.502 | 97.8051 | 141.544 | 109.253 |
| 14261 | 2.29118 | 2.13504 | 2.64631 | 2.0454 | 1.44835 | 3.52321 | 3.1483 | 2.12405 | 3.0623 | 2.57326 | 2.61411 |
| 16542 | 1.54741 | 3.74275 | 1.02313 | 2.98607 | 3.8552 | 0.791638 | 2.18464 | 0.772465 | 0.966333 | 4.70709 | 8.96087 |
| 11837 | 10.3929 | 9.18375 | 8.76423 | 12.4579 | 13.339 | 5.46971 | 8.0934 | 2.30837 | 2.6771 | 10.0432 | 10.676 |

|  | Glucose | Xylose | Arabinose | Acetate | Coumarate | Ferulate | YNB Oleic Acid | YNB Ricinoleic Acid | YNB Glucose | YNB Gluc DOC | YPD |
| --- | --- | --- | --- | --- | --- | --- | --- | --- | --- | --- | --- |
| proteinId |  |  |  |  |  |  |  |  |  |  |  |
| 14471 | 0.0630059 | 0.0458949 | -0.162117 | -0.0445085 | -0.107805 | -0.252623 | -0.220519 | -1.11705 | -0.439887 | -0.362255 | 0.227652 |
| 16542 | -0.161755 | -0.168402 | -0.475257 | 0.334468 | -0.474717 | -0.326335 | -0.154747 | -1.28835 | -0.424862 | -0.0347438 | -0.659424 |
| 11837 | -0.00746914 | -0.0836084 | -0.0138207 | -0.0768996 | -0.241361 | 0.0511913 | -0.0700867 | 0.287896 | -0.04288 | -0.327269 | 0.112745 |

In [156]:

```
# Remove FAS reactions with free fatty acid as product
for r in sorted(model.reactions, key=lambda x: x.id):
    if r.id.startswith('FAS') and not ('COA' in r.id or 'ACP' in r.id):
        print(r.id, r.reaction, r.gene_reaction_rule)
        model.remove_reactions([r])
```

```
FAS100 3.0 h_c + malcoa_c + 2.0 nadph_c + octa_c --> co2_c + coa_c + dca_c + h2o_c + 2.0 nadp_c (12826 and 8639 and 8670 and 8777) or (14471 and 8639 and 8670 and 8777)
FAS120 dca_c + 3.0 h_c + malcoa_c + 2.0 nadph_c --> co2_c + coa_c + ddca_c + h2o_c + 2.0 nadp_c (12826 and 8639 and 8670 and 8777) or (14471 and 8639 and 8670 and 8777)
FAS140 ddca_c + 3.0 h_c + malcoa_c + 2.0 nadph_c --> co2_c + coa_c + h2o_c + 2.0 nadp_c + ttdca_c (12826 and 8639 and 8670 and 8777) or (14471 and 8639 and 8670 and 8777)
FAS141 ddca_c + 4.0 h_c + malcoa_c + 3.0 nadph_c + o2_c --> co2_c + coa_c + 3.0 h2o_c + 3.0 nadp_c + ttdcea_c 16655
FAS160 3.0 h_c + malcoa_c + 2.0 nadph_c + ttdca_c --> co2_c + coa_c + h2o_c + hdca_c + 2.0 nadp_c (12826 and 8639 and 8670 and 8777) or (14471 and 8639 and 8670 and 8777)
FAS161 4.0 h_c + malcoa_c + 3.0 nadph_c + o2_c + ttdca_c --> co2_c + coa_c + 3.0 h2o_c + hdcea_c + 3.0 nadp_c 16655
FAS180 3.0 h_c + hdca_c + malcoa_c + 2.0 nadph_c --> co2_c + coa_c + h2o_c + 2.0 nadp_c + ocdca_c (10677 and 16241 and 16695) or (12826 and 8639 and 8670 and 8777) or (14471 and 8639 and 8670 and 8777)
FAS181 4.0 h_c + hdca_c + malcoa_c + 3.0 nadph_c + o2_c --> co2_c + coa_c + 3.0 h2o_c + 3.0 nadp_c + ocdcea_c 16655
FAS240_L 9.0 h_c + 3.0 malcoa_c + 6.0 nadph_c + ocdca_c --> 3.0 co2_c + 3.0 coa_c + 3.0 h2o_c + 6.0 nadp_c + ttc_c (16241 and 16655) or (10677 and 16241 and 16695)
FAS260 3.0 h_c + malcoa_c + 2.0 nadph_c + ttc_c --> co2_c + coa_c + h2o_c + hexc_c + 2.0 nadp_c (16241 and 16655) or (10677 and 16241 and 16695)
FAS80_L accoa_c + 8.0 h_c + 3.0 malcoa_c + 6.0 nadph_c --> 3.0 co2_c + 4.0 coa_c + 2.0 h2o_c + 6.0 nadp_c + octa_c (10677 and 16241 and 16695) or (12826 and 8639 and 8670 and 8777) or (14471 and 8639 and 8670 and 8777)
```

In [157]:

```
# Remove cyto ACP reactions
model.remove_reactions(['ACOATA','FA120ACPHi','FA140ACPHi','FA141ACPHi','FA160ACPHi','FA161ACPHi','FA180ACPH',
                        'FA181ACPH','FA182ACPH','MCOATA','yli_R0163','yli_R0164','yli_R0165','yli_R0166',
                        'yli_R0167','yli_R0168','yli_R0169','yli_R0170','yli_R0171','yli_R0172','yli_R0175',
                        'yli_R0176','yli_R0177','yli_R0178','yli_R0179','yli_R0180'], remove_orphans=True)
# Remove mito FA ACP synthesis
model.remove_reactions(['ACCOACrm','FAS100ACPm','FAS120ACPm','FAS140ACPm','FAS141ACPm','FAS160ACPm','FAS161ACPm',
                        'FAS180ACPm','FAS181ACPm','FAS182ACPm','FAS80ACPm_L'], remove_orphans=True)
model.remove_reactions(['ECOAR','yli_R0126','yli_R0129','yli_R0140','MCOATA_1','ACOATAm','ACPS1_1'], remove_orphans=True)
for r in sorted(model.genes.get_by_id('14261').reactions, key=lambda x: x.id):
    if 'h' in r.compartments:
        model.remove_reactions([r])
    if 'b' in r.gene_reaction_rule:
        model.remove_reactions([r])
```

In [158]:

```
for r in sorted(model.reactions, key=lambda x: x.id):
    if r.id.startswith('FAS') and 'COA' in r.id:
        r.gene_reaction_rule = '8670 and 8777'
        
r = sce.reactions.get_by_id('FAS240_L').copy()
r.id = 'FAS240COAer'
r.gene_reaction_rule = '(10677 and 11343 and 16241 and 16695) or (10677 and 16241 and 16655 and 16695)'
model.add_reactions([r])
r.add_metabolites({'ocdca_c': 1.0, 'stcoa_c': -1.0, 'ttc_c': -1.0, 'ttccoa_c': 1.0})
for m in r.metabolites:
    if not m.id.replace('_c','_r') in model.metabolites:
        m2 = m.copy()
        m2.id = m.id.replace('_c','_r')
        m2.compartment = 'r'
        model.add_metabolites([m2])
    r.add_metabolites({m.id: -r.get_coefficient(m.id), m.id.replace('_c','_r'): r.get_coefficient(m.id)})
    
r = sce.reactions.get_by_id('FAS260').copy()
r.id = 'FAS260COAer'
r.gene_reaction_rule = '10677 and 11343 and 16241 and 16695'
model.add_reactions([r])
r.add_metabolites({'ttc_c': 1.0, 'ttccoa_c': -1.0, 'hexc_c': -1.0, 'hexccoa_c': 1.0})
for m in r.metabolites:
    if not m.id.replace('_c','_r') in model.metabolites:
        m2 = m.copy()
        m2.id = m.id.replace('_c','_r')
        m2.compartment = 'r'
        model.add_metabolites([m2])
    r.add_metabolites({m.id: -r.get_coefficient(m.id), m.id.replace('_c','_r'): r.get_coefficient(m.id)})
    
for r in sorted(model.reactions, key=lambda x: x.id):
    if r.id.startswith('ACOAR'):
        r.gene_reaction_rule = '11837'
        
model.reactions.get_by_id('13GS').gene_reaction_rule = '15984'
model.reactions.get_by_id('MCOATAm').gene_reaction_rule = '14261 and 16542'

for x in ['LIPAMPL','LIPATPT','OCTNLL','LIPOCT','LIPOS']:
    r = model.reactions.get_by_id(x)
    r.id = r.id + 'm'
    for m in r.metabolites:
        if not m.id.replace('_c','_m') in model.metabolites:
            m2 = m.copy()
            m2.id = m.id.replace('_c','_m')
            m2.compartment = 'm'
            model.add_metabolites([m2])
        r.add_metabolites({m.id: -r.get_coefficient(m.id), m.id.replace('_c','_m'): r.get_coefficient(m.id)})
```

TAG biosynthesis (E.R.)
GPD1 12154 cyto 23, pero 4 K00006: GPD1; glycerol-3-phosphate dehydrogenase (NAD+) AHI *GPD2 14576 cyto 17.5, cyto\_nucl 14, nucl 5.5 K00006: GPD1; glycerol-3-phosphate dehydrogenase (NAD+) EGI*  
SCT1 15435 plas 8, cyto 7.5, cyto\_nucl 6.5, nucl 4.5, mito 4 K13507: GAT; glycerol-3-phosphate O-acyltransferase / dihydroxyacetone phosphate acyltransferase AQE *GNPAT 13369 cyto 12.5, cyto\_nucl 10.5, nucl 7.5, mito 5 K00649: GNPAT; glyceronephosphate O-acyltransferase AAL*  
AYR1 15575 plas 10, mito 8, cyto 5, E.R. 3 KOG1209: 1-Acyl dihydroxyacetone phosphate reductase and related dehydrogenases KRR *SLC1 10427 mito 18, E.R. 4, extr 2, plas 1, golg 1, vacu 1 K13509: AGPAT1\_2; lysophosphatidate acyltransferase PSE*  
ALE1 16030 plas 18, E.R. 4, vacu 3 K13519: LPT1, ALE1; lysophospholipid acyltransferase KEL *16030 is the blast hit of known lysoPC AT -> LPCAT\_SC, TAZ1 is mitochondrial  
LOA1 16779 extr 11, mito 8, cyto 5, plas 2 KOG2898: Predicted phosphate acyltransferase, contains PlsC domain KGR*  
TGL4,TGL5 9746 plas 22, nucl 2, mito 1, cyto 1, vacu 1, cyto\_mito 1 KOG2214: Predicted esterase of the alpha-beta hydrolase superfamily PWH *16779, 9746 oleoyl-CoA specific  
DPP1 13087 plas 23, mito 2 K18693: DPP1; diacylglycerol diphosphate phosphatase / phosphatidate phosphatase GYY*  
PAH1 12485 nucl 12.5, cyto\_nucl 10.5, cyto 7.5, mito 5 K15728: LPIN; phosphatidate phosphatase LPIN PYL *DGK1 10156 mito 10, plas 8, cyto 3, pero 2, E.R. 2 K16368: DGK1; diacylglycerol kinase (CTP) ILG*  
DGA1 16460 plas 12, mito 5, extr 3, E.R. 3, mito\_nucl 3 K14457: MOGAT2, MGAT2; 2-acylglycerol O-acyltransferase 2 IIA *ARE1,ARE2 11799 plas 24, E.R. 2 K11155: DGAT1; diacylglycerol O-acyltransferase 1 LRY*  
LRO1 16477 cyto 9.5, cyto\_nucl 9.5, nucl 8.5, pero 5, mito 3 K00679: E2.3.1.158; phospholipid:diacylglycerol acyltransferase WEG *TAZ1 16474 mito 13, cyto\_nucl 5, nucl 4.5, cyto 4.5, plas 2, pero 2 K13511: TAZ; monolysocardiolipin acyltransferase TGW*  
TGL2 14317 mito 12, cyto 7.5, cyto\_nucl 5, pero 3, extr 2 K01046: E3.1.1.3; triacylglycerol lipase EGF *10393 best hit TGL2  
TGL4,TGL5 9746 plas 22, nucl 2, mito 1, cyto 1, vacu 1, cyto\_mito 1 KOG2214: Predicted esterase of the alpha-beta hydrolase superfamily PWH*  
TGL1 14617 plas 8, mito 6, nucl 4.5, cyto\_nucl 4.5, cyto 3.5, pero 3 K01052: LIPA; lysosomal acid lipase/cholesteryl ester hydrolase DRV *YEH1,YEH2 14247 plas 12, mito 5, golg 3, cyto\_mito 3, nucl 2, KOG2624: Triglyceride lipase-cholesterol esterase EGY*
NTE1 14309 extr 8, plas 7, cyto 6, nucl 2, mito 2, mito\_nucl 2 K14676: NTE, NRE; lysophospholipid hydrolase RLT *PLB1,PLB2,PLB3 12385 extr 27 K13333: PLB; lysophospholipase LLL*

LIPE 15065 extr 10, plas 5, mito 4, cyto 2, E.R. 2, golg 2 K07188: LIPE, HSL; hormone-sensitive lipase  
ATG15 9000 nucl 16.5, cyto\_nucl 12.5, cyto 7.5 K17900: ATG15, AUT5; lipase ATG15 TYV\*

In [159]:

```
temp = ['14317','9746','14617','14247','14309','12385','9000']
display(Annotation.loc[temp])
Show_Data(temp)
```

|  | Combined Annotations | Signal P | Sc288c Orthologs | Human Orthologs | Sc288 Best Hit | Human Blast | Essential | WolfPSort | C Terminal |
| --- | --- | --- | --- | --- | --- | --- | --- | --- | --- |
| RTO4\_ID |  |  |  |  |  |  |  |  |  |
| 14317 | K01046: E3.1.1.3; triacylglycerol lipase |  | TGL2 |  | TGL2 |  | Not Essential | mito 12, cyto 7.5, cyto\_nucl 5, pero 3, extr 2 | EGF\* |
| 9746 | KOG2214: Predicted esterase of the alpha-beta ... |  | TGL4,TGL5 |  | TGL5 |  | Not Essential | plas 22, nucl 2, mito 1, cyto 1, vacu 1, cyto\_... | PWH\* |
| 14617 | K01052: LIPA; lysosomal acid lipase/cholestery... |  | TGL1 | LIPF,LIPK,LIPA,LIPJ,LIPM | YEH2 | LIPK | Not Essential | plas 8, mito 6, nucl 4.5, cyto\_nucl 4.5, cyto ... | DRV\* |
| 14247 | KOG2624: Triglyceride lipase-cholesterol esterase |  | YEH1,YEH2 |  | YEH2 | LIPA | Not Essential | plas 12, mito 5, golg 3, cyto\_mito 3, nucl 2, ... | EGY\* |
| 14309 | K14676: NTE, NRE; lysophospholipid hydrolase | A | NTE1 | PNPLA6,PNPLA7 | NTE1 | PNPLA | Not Essential | extr 8, plas 7, cyto 6, nucl 2, mito 2, mito\_n... | RLT\* |
| 12385 | K13333: PLB; lysophospholipase | S | PLB2,PLB1,PLB3 |  | PLB1 |  | Not Essential | extr 27 | LLL\* |
| 9000 | K17900: ATG15, AUT5; lipase ATG15 |  | ATG15 |  | ATG15 |  | Not Essential | nucl 16.5, cyto\_nucl 12.5, cyto 7.5 | TYV\* |

| strain | WT | | | | | | | | | | | | | | | | |
| --- | --- | --- | --- | --- | --- | --- | --- | --- | --- | --- | --- | --- | --- | --- | --- | --- | --- |
| condition | G\_MM | C\_MM | G\_SD | | GX\_SD | | | X\_SD | | A\_SD | | C\_SD | | MM\_CN120 | | MM\_CN5 | Diversity\_Sample |
| phase | exp | exp | exp | stat | exp | trans | stat | exp | stat | exp | stat | exp | stat | exp | stat | exp | exp |
| proteinId | Set1 | Set1 | Set2 | Set2 | Set2 | Set2 | Set2 | Set2 | Set2 | Set2 | Set2 | Set2 | Set2 | Set3 | Set3 | Set3 | Set3 |
| 14317 | 5.53716 | 4.71851 | 5.40352 | 6.56022 | 5.16009 | 5.51135 | 5.91985 | 5.09931 | 6.05995 | 5.25388 | 5.95932 | 6.00256 | 6.49049 | 4.15021 | 4.78742 | 3.69045 | 3.53569 |
| 9746 | 5.93373 | 4.6165 | 7.08407 | 6.25703 | 6.95978 | 5.57002 | 5.58595 | 5.69823 | 5.11517 | 5.65796 | 4.99609 | 5.14196 | 5.66 | 5.98327 | 5.936 | 5.29933 | 5.08927 |
| 14617 | 3.41627 | 4.67516 | 3.72431 | 5.46232 | 3.61852 | 3.78678 | 3.30436 | 4.19751 | 4.52198 | 5.18761 | 4.91892 | 3.15926 | 4.03184 | 3.39192 | 3.26358 | 4.64783 | 4.29629 |
| 14247 | 5.55026 | 4.70183 | 5.01385 | 5.51859 | 5.02875 | 4.59472 | 5.10083 | 5.2668 | 5.2138 | 5.34583 | 4.97352 | 5.34094 | 6.37942 | 5.82305 | 5.88148 | 6.25107 | 5.08601 |
| 14309 | 4.33033 | 4.17914 | 5.34365 | 5.59446 | 5.29318 | 4.72329 | 4.9651 | 4.5146 | 5.01812 | 4.42968 | 4.40949 | 4.46505 | 4.64398 | 4.80295 | 4.67312 | 4.81812 | 5.00013 |
| 12385 | 6.13491 | 5.44431 | 7.0996 | 7.03359 | 7.13414 | 6.43367 | 6.76806 | 6.20608 | 6.86488 | 6.13468 | 6.86102 | 5.49135 | 4.40896 | 7.74736 | 6.90343 | 7.22961 | 7.11422 |
| 9000 | 6.54297 | 5.19522 | 6.4752 | 6.77044 | 6.48789 | 6.22495 | 6.05873 | 6.04516 | 6.44972 | 6.25869 | 6.38835 | 6.23907 | 6.98871 | 6.93196 | 6.82833 | 6.08579 | 5.7604 |

| strain | WT | | | | | | | | | | |
| --- | --- | --- | --- | --- | --- | --- | --- | --- | --- | --- | --- |
| condition | G\_SD | | GX\_SD | | | X\_SD | | A\_SD | | C\_SD | |
| proteinId | exp | stat | exp | trans | stat | exp | stat | exp | stat | exp | stat |
| 14317 | 0.190197 | 0.169232 | 0 | 0.742919 | 1.64152 | 0 | 0.583347 | 0.581889 | 0.568492 | 0 | 0 |
| 9746 | 1.33485 | 3.59683 | 1.01208 | 3.55199 | 4.92287 | 3.11276 | 2.37027 | 2.31982 | 2.87839 | 1.28265 | 2.61856 |
| 14617 | 0 | 0 | 0 | 0 | 0 | 0 | 0 | 0 | 0.189081 | 0 | 0 |
| 14247 | 0.763931 | 0.879575 | 0.816186 | 0.547209 | 0.173732 | 0.199906 | 0 | 0.773293 | 0 | 0 | 0.211526 |
| 14309 | 5.36818 | 8.08828 | 6.10811 | 7.61419 | 6.76024 | 4.5048 | 3.94628 | 3.48229 | 4.21122 | 3.61861 | 3.27973 |
| 12385 | 2.54615 | 0.354979 | 3.86344 | 0 | 0.192891 | 2.352 | 1.98563 | 2.32294 | 0.953549 | 1.91602 | 1.74315 |
| 9000 | 0 | 0.188312 | 0 | 1.12542 | 0.192891 | 0 | 0.188437 | 0.193364 | 0 | 0 | 0 |

|  | Glucose | Xylose | Arabinose | Acetate | Coumarate | Ferulate | YNB Oleic Acid | YNB Ricinoleic Acid | YNB Glucose | YNB Gluc DOC | YPD |
| --- | --- | --- | --- | --- | --- | --- | --- | --- | --- | --- | --- |
| proteinId |  |  |  |  |  |  |  |  |  |  |  |
| 9746 | -0.0223147 | -0.0041398 | -0.068294 | 0.0642932 | 0.319812 | -0.0303963 | 0.178785 | 0.0256476 | 0.146721 | 0.192792 | 0.0446445 |
| 14617 | -0.0419116 | -0.0971542 | -0.0660963 | 0.0316563 | 0.0744906 | 0.202458 | -0.111763 | -0.368591 | -0.188215 | -0.313815 | -0.0952425 |
| 14247 | -0.0246227 | -0.175606 | 0.00642496 | -0.0811898 | -0.822125 | -0.476904 | -0.140187 | 0.0623536 | -0.188237 | -0.0181106 | -0.327808 |
| 14309 | -0.0421803 | -0.1025 | -0.0699972 | -0.446404 | 0.313427 | -0.223928 | 0.170773 | -0.526365 | -0.067268 | -0.221199 | -0.447931 |
| 12385 | 0.168375 | 0.221694 | 0.0416109 | 0.0721808 | -0.572405 | 0.258112 | -0.0124852 | 0.0609392 | -0.0540949 | 0.00780372 | 0.358722 |
| 9000 | -0.169705 | -0.259195 | -0.196552 | -0.197213 | -0.273888 | -0.414895 | -0.470405 | -0.990165 | 0.145391 | 0.110963 | 0.400523 |

In [160]:

```
for x in ['12154','14576','15435','13369','15575','10427','16030','9746','13087','12485','10156','16460','11799',
          '16477','16474','14317','14617','14247','14309','12385']:
    for r in sorted(model.genes.get_by_id(x).reactions, key=lambda x: x.id):
        print(r.id, r.reaction, r.gene_reaction_rule)
    print()
```

```
G3PD1ir dhap_c + h_c + nadh_c --> glyc3p_c + nad_c 12154 or 14576

G3PD1ir dhap_c + h_c + nadh_c --> glyc3p_c + nad_c 12154 or 14576

GAT1_SC 0.02 dcacoa_c + 0.06 ddcacoa_c + glyc3p_c + 0.17 hdcoa_c + 0.09 ocdycacoa_c + 0.24 odecoa_c + 0.27 pmtcoa_c + 0.05 stcoa_c + 0.1 tdcoa_c --> 0.01 1ag3p_SC_c + coa_c 15435
GAT2_SC 0.02 dcacoa_c + 0.06 ddcacoa_c + dhap_c + 0.17 hdcoa_c + 0.09 ocdycacoa_c + 0.24 odecoa_c + 0.27 pmtcoa_c + 0.05 stcoa_c + 0.1 tdcoa_c --> 0.01 1agly3p_SC_c + coa_c 15435
yli_R1391 0.02 dccoa_r + 0.06 ddcacoa_r + glyc3p_r + 0.27 pmtcoa_r + 0.05 stcoa_r + 0.1 tdcoa_r + 0.17 yli_M04594_r + 0.09 yli_M04625_r + 0.24 yli_M04626_r --> 0.01 1ag3p_SC_r + coa_r 15435
yli_R1408 0.02 dccoa_r + 0.06 ddcacoa_r + dhap_r + 0.27 pmtcoa_r + 0.05 stcoa_r + 0.1 tdcoa_r + 0.17 yli_M04594_r + 0.09 yli_M04625_r + 0.24 yli_M04626_r --> 0.01 adhap_hs_r + coa_r 15435
yli_R1509 acoa_r + glyc3p_r --> 1ag3p_SC_r + coa_r 15435

DHAPA Rtotalcoa_c + dhap_c --> adhap_hs_c + coa_c 13369
DHAPAx Rtotalcoa_x + dhap_x --> adhap_hs_x + coa_x 13369
G3PAT120 ddcaACP_c + glyc3p_c --> 1ddecg3p_c + ACP_c 13369 or (13369 and 14261)
G3PAT140 glyc3p_c + myrsACP_c --> 1tdecg3p_c + ACP_c 13369 and 14261
G3PAT141 glyc3p_c + tdeACP_c --> 1tdec7eg3p_c + ACP_c 13369 and 14261
G3PAT160 glyc3p_c + palmACP_c --> 1hdecg3p_c + ACP_c 13369 or (13369 and 14261)
G3PAT161 glyc3p_c + hdeACP_c --> 1hdec9eg3p_c + ACP_c 13369 or (13369 and 14261)
G3PAT180 glyc3p_c + ocdcaACP_c --> 1odecg3p_c + ACP_c 13369 or (13369 and 14261)
G3PAT181 glyc3p_c + octeACP_c --> 1odec11eg3p_c + ACP_c 13369 or (13369 and 14261)
GPAM_hs Rtotalcoa_c + glyc3p_c --> alpa_hs_c + coa_c 13369
GPAMm_hs Rtotalcoa_m + glyc3p_m --> alpa_hs_m + coa_m 13369

ADHAPR_SC 0.01 1agly3p_SC_c + h_c + nadph_c --> 0.01 1ag3p_SC_c + nadp_c 15575

AGAT_SC 0.01 1ag3p_SC_c + 0.02 dcacoa_c + 0.06 ddcacoa_c + 0.17 hdcoa_c + 0.09 ocdycacoa_c + 0.24 odecoa_c + 0.27 pmtcoa_c + 0.05 stcoa_c + 0.1 tdcoa_c --> coa_c + 0.01 pa_SC_c 10427
AGPAT1 Rtotal2coa_c + alpa_hs_c --> coa_c + pa_hs_c 10427
AGPAT120 1ddecg3p_c + ddcaACP_c --> ACP_c + pa120_c 10427
AGPAT140 1tdecg3p_c + myrsACP_c --> ACP_c + pa140_c 10427
AGPAT141 1tdec7eg3p_c + tdeACP_c --> ACP_c + pa141_c 10427
AGPAT160 1hdecg3p_c + palmACP_c --> ACP_c + pa160_c 10427
AGPAT161 1hdec9eg3p_c + hdeACP_c --> ACP_c + pa161_c 10427
AGPAT180 1odecg3p_c + ocdcaACP_c --> ACP_c + pa180_c 10427
AGPAT181 1odec11eg3p_c + octeACP_c --> ACP_c + pa181_c 10427
AGPATCOA1601819Z 1hdecg3p_c + odecoa_c --> coa_c + pa1601819Z_c 10427
AGPATCOA1801819Z 1odecg3p_c + odecoa_c --> coa_c + pa1801819Z_c 10427
AGPATCOA1819Z160 1odec9eg3p_c + pmtcoa_c --> coa_c + pa1819Z160_c 10427
AGPATCOA1819Z1819Z 1odec9eg3p_c + odecoa_c --> coa_c + pa1819Z1819Z_c 10427
yli_R1392 0.01 1ag3p_SC_r + 0.02 dccoa_r + 0.06 ddcacoa_r + 0.27 pmtcoa_r + 0.05 stcoa_r + 0.1 tdcoa_r + 0.17 yli_M04594_r + 0.09 yli_M04625_r + 0.24 yli_M04626_r --> coa_r + 0.01 pa_EC_r 10427 or 16030 or 9746
yli_R1510 1ag3p_SC_r + acoa_r --> coa_r + pa_EC_r 10427 or 16030 or 9746

1AGPEAT1801819Z 1agpe180_c + odecoa_c --> coa_c + pe1801819Z_c 16030 or 16474
1AGPEAT1801829Z12Z 1agpe180_c + ocdycacoa_c --> coa_c + pe1801829Z12Z_c 16030 or 16474
1AGPEAT1819Z1819Z 1agpe1819Z_c + odecoa_c --> coa_c + pe1819Z1819Z_c 16030 or 16474
1AGPEAT1819Z1829Z12Z 1agpe1819Z_c + ocdycacoa_c --> coa_c + pe1819Z1829Z12Z_c 16030 or 16474
yli_R1392 0.01 1ag3p_SC_r + 0.02 dccoa_r + 0.06 ddcacoa_r + 0.27 pmtcoa_r + 0.05 stcoa_r + 0.1 tdcoa_r + 0.17 yli_M04594_r + 0.09 yli_M04625_r + 0.24 yli_M04626_r --> coa_r + 0.01 pa_EC_r 10427 or 16030 or 9746
yli_R1402 0.02 dccoa_r + 0.06 ddcacoa_r + 0.27 pmtcoa_r + 0.05 stcoa_r + 0.1 tdcoa_r + 0.01 yli_M02277_r + 0.17 yli_M04594_r + 0.09 yli_M04625_r + 0.24 yli_M04626_r --> coa_r + 0.01 pchol_cho_r 16030
yli_R1510 1ag3p_SC_r + acoa_r --> coa_r + pa_EC_r 10427 or 16030 or 9746
yli_R1517 acoa_r + yli_M02277_r --> coa_r + pchol_cho_r 16030
yli_R1518 acoa_r + yli_M07019_r --> coa_r + pe_SC_r 16030
yli_R1523 1ag3p_SC_r + acoa_r --> coa_r + pa_EC_r 16030

yli_R1392 0.01 1ag3p_SC_r + 0.02 dccoa_r + 0.06 ddcacoa_r + 0.27 pmtcoa_r + 0.05 stcoa_r + 0.1 tdcoa_r + 0.17 yli_M04594_r + 0.09 yli_M04625_r + 0.24 yli_M04626_r --> coa_r + 0.01 pa_EC_r 10427 or 16030 or 9746
yli_R1510 1ag3p_SC_r + acoa_r --> coa_r + pa_EC_r 10427 or 16030 or 9746
yli_R1511 h2o_r + tag_cho_r --> dag_hs_r + hdca_r 14317 or 9000 or 9746
yli_R1512 dag_hs_r + h2o_r --> M07018_r + hdca_r 14317 or 9000 or 9746
yli_R1515 h2o_r + pchol_cho_r --> hdca_r + yli_M02277_r 9746
yli_R1516 h2o_r + pe_SC_r --> hdca_r + yli_M07019_r 9746

DAGPYP_SC h2o_c + 0.01 pa_SC_c --> 0.01 12dgr_SC_c + pi_c 13087
LPP_SC 0.01 dagpy_SC_c + h2o_c --> h_c + 0.01 pa_SC_c + pi_c 13087
PAPA1601819Zh h2o_h + pa1601819Z_h --> 12dgr1601819Z_h + pi_h 13087
PAPA160h h2o_h + pa160_h --> 12dgr160_h + pi_h 13087
PAPA1801819Zh h2o_h + pa1801819Z_h --> 12dgr1801819Z_h + pi_h 13087
PAPA1819Z160h h2o_h + pa1819Z160_h --> 12dgr1819Z160_h + pi_h 13087
PAPA1819Z1619Zh h2o_h + pa1819Z1619Z_h --> 12dgr1819Z1619Z_h + pi_h 13087
PAPA1819Z1819Zh h2o_h + pa1819Z1819Z_h --> 12dgr1819Z1819Z_h + pi_h 13087
yli_R1513 h2o_r + pa_EC_r --> dag_hs_r + pi_r 12485 or 13087

yli_R0309 h2o_c + 0.01 pa_EC_c --> 0.01 dag_hs_c + pi_c 12485
yli_R1393 h2o_r + 0.01 pa_EC_r --> 0.01 dag_hs_r + pi_r 12485
yli_R1513 h2o_r + pa_EC_r --> dag_hs_r + pi_r 12485 or 13087

yli_R1522 ctp_r + dag_hs_r --> cdp_r + pa_EC_r 10156

ACOADAGAT1601819Z160 12dgr1601819Z_c + pmtcoa_c --> coa_c + tag1601819Z160_c 16460
ACOADAGAT1601819Z180 12dgr1601819Z_c + stcoa_c --> coa_c + tag1601819Z180_c 16460
ACOADAGAT1601819Z1819Z 12dgr1601819Z_c + odecoa_c --> coa_c + tag1601819Z1819Z_c 16460
ACOADAGAT1801819Z160 12dgr1801819Z_c + pmtcoa_c --> coa_c + tag1801819Z160_c 16460
ACOADAGAT1801819Z180 12dgr1801819Z_c + stcoa_c --> coa_c + tag1801819Z180_c 16460
ACOADAGAT1801819Z1819Z 12dgr1801819Z_c + odecoa_c --> coa_c + tag1801819Z1819Z_c 16460
ACOADAGAT1819Z1819Z160 12dgr1819Z1819Z_c + pmtcoa_c --> coa_c + tag1819Z1819Z160_c 16460
ACOADAGAT1819Z1819Z180 12dgr1819Z1819Z_c + stcoa_c --> coa_c + tag1819Z1819Z180_c 16460
ACOADAGAT1819Z1819Z1819Z 12dgr1819Z1819Z_c + odecoa_c --> coa_c + tag1819Z1819Z1819Z_c 16460
CRETINOLPMTACT pmtcoa_s + retinol_cis_11_s --> coa_s + retpalm_11_cis_s 16460
DGAT Rtotal3coa_c + dag_hs_c --> coa_c + tag_hs_c 16460
MOGAT Rtotalcoa_c + mag_hs_c --> coa_c + dag_hs_c 16460
RETINOLACACT accoa_s + retinol_s --> coa_s + retac_s 16460
TRETINOLPMTACT pmtcoa_s + retinol_s --> coa_s + retpalm_s 16460
TRIGS_SC 0.01 12dgr_SC_c + 0.02 dcacoa_c + 0.06 ddcacoa_c + 0.17 hdcoa_c + 0.09 ocdycacoa_c + 0.24 odecoa_c + 0.27 pmtcoa_c + 0.05 stcoa_c + 0.1 tdcoa_c --> coa_c + 0.01 triglyc_SC_c 11799 or 16460

EPISTAT_SC 0.01 epist_c + 0.655 hdcoa_c + 0.01 hexccoa_c + 0.27 odecoa_c + 0.02 pmtcoa_c + 0.03 stcoa_c + 0.015 tdcoa_c --> coa_c + 0.01 epistest_SC_c 11799
ERGSTAT_SC 0.01 ergst_c + 0.655 hdcoa_c + 0.01 hexccoa_c + 0.27 odecoa_c + 0.02 pmtcoa_c + 0.03 stcoa_c + 0.015 tdcoa_c --> coa_c + 0.01 ergstest_SC_c 11799
FECOSTAT_SC 0.01 fecost_c + 0.655 hdcoa_c + 0.01 hexccoa_c + 0.27 odecoa_c + 0.02 pmtcoa_c + 0.03 stcoa_c + 0.015 tdcoa_c --> coa_c + 0.01 fecostest_SC_c 11799
LANOSTAT_SC 0.655 hdcoa_c + 0.01 hexccoa_c + 0.01 lanost_c + 0.27 odecoa_c + 0.02 pmtcoa_c + 0.03 stcoa_c + 0.015 tdcoa_c --> coa_c + 0.01 lanostest_SC_c 11799
TRIGS_SC 0.01 12dgr_SC_c + 0.02 dcacoa_c + 0.06 ddcacoa_c + 0.17 hdcoa_c + 0.09 ocdycacoa_c + 0.24 odecoa_c + 0.27 pmtcoa_c + 0.05 stcoa_c + 0.1 tdcoa_c --> coa_c + 0.01 triglyc_SC_c 11799 or 16460
ZYMSTAT_SC 0.655 hdcoa_c + 0.01 hexccoa_c + 0.27 odecoa_c + 0.02 pmtcoa_c + 0.03 stcoa_c + 0.015 tdcoa_c + 0.01 zymst_c --> coa_c + 0.01 zymstest_SC_c 11799

PCDAGAT 12dgr_SC_c + pc_SC_c --> 1agpc_SC_c + triglyc_SC_c 16477
PLDAGAT1601819Z1601 12dgr1601819Z_c + pa18111Z160_c --> 1odec11eg3p_c + tag1601819Z160_c 16477
PLDAGAT1601819Z1602 12dgr1601819Z_c + pa1819Z160_c --> 1odec9eg3p_c + tag1601819Z160_c 16477
PLDAGAT1601819Z1819Z1 12dgr1601819Z_c + pa1601819Z_c --> 1hdecg3p_c + tag1601819Z1819Z_c 16477
PLDAGAT1601819Z1819Z2 12dgr1601819Z_c + pa1801819Z_c --> 1odecg3p_c + tag1601819Z1819Z_c 16477
PLDAGAT1601819Z1819Z3 12dgr1601819Z_c + pa18111Z1819Z_c --> 1odec11eg3p_c + tag1601819Z1819Z_c 16477
PLDAGAT1601819Z1819Z4 12dgr1601819Z_c + pa1819Z1819Z_c --> 1odec9eg3p_c + tag1601819Z1819Z_c 16477
PLDAGAT1801819Z1601 12dgr1801819Z_c + pa18111Z160_c --> 1odec11eg3p_c + tag1801819Z160_c 16477
PLDAGAT1801819Z1602 12dgr1801819Z_c + pa1819Z160_c --> 1odec9eg3p_c + tag1801819Z160_c 16477
PLDAGAT1801819Z1819Z1 12dgr1801819Z_c + pa1601819Z_c --> 1hdecg3p_c + tag1801819Z1819Z_c 16477
PLDAGAT1801819Z1819Z2 12dgr1801819Z_c + pa1801819Z_c --> 1odecg3p_c + tag1801819Z1819Z_c 16477
PLDAGAT1801819Z1819Z3 12dgr1801819Z_c + pa18111Z1819Z_c --> 1odec11eg3p_c + tag1801819Z1819Z_c 16477
PLDAGAT1801819Z1819Z4 12dgr1801819Z_c + pa1819Z1819Z_c --> 1odec9eg3p_c + tag1801819Z1819Z_c 16477
PLDAGAT1819Z1819Z1601 12dgr1819Z1819Z_c + pa18111Z160_c --> 1odec11eg3p_c + tag1819Z1819Z160_c 16477
PLDAGAT1819Z1819Z1602 12dgr1819Z1819Z_c + pa1819Z160_c --> 1odec9eg3p_c + tag1819Z1819Z160_c 16477
PLDAGAT1819Z1819Z1819Z1 12dgr1819Z1819Z_c + pa1601819Z_c --> 1hdecg3p_c + tag1819Z1819Z1819Z_c 16477
PLDAGAT1819Z1819Z1819Z2 12dgr1819Z1819Z_c + pa1801819Z_c --> 1odecg3p_c + tag1819Z1819Z1819Z_c 16477
PLDAGAT1819Z1819Z1819Z3 12dgr1819Z1819Z_c + pa18111Z1819Z_c --> 1odec11eg3p_c + tag1819Z1819Z1819Z_c 16477
PLDAGAT1819Z1819Z1819Z4 12dgr1819Z1819Z_c + pa1819Z1819Z_c --> 1odec9eg3p_c + tag1819Z1819Z1819Z_c 16477
yli_R0311 dag_hs_c + pchol_cho_c --> tag_cho_c + yli_M02277_c 16477
yli_R1395 dag_hs_r + pchol_cho_r --> tag_cho_r + yli_M02277_r 16477

1AGPEAT1801819Z 1agpe180_c + odecoa_c --> coa_c + pe1801819Z_c 16030 or 16474
1AGPEAT1801829Z12Z 1agpe180_c + ocdycacoa_c --> coa_c + pe1801829Z12Z_c 16030 or 16474
1AGPEAT1819Z1819Z 1agpe1819Z_c + odecoa_c --> coa_c + pe1819Z1819Z_c 16030 or 16474
1AGPEAT1819Z1829Z12Z 1agpe1819Z_c + ocdycacoa_c --> coa_c + pe1819Z1829Z12Z_c 16030 or 16474
2AGPA120tipp 2ddecg3p_p --> 2ddecg3p_c 16474
2AGPA140tipp 2tdecg3p_p --> 2tdecg3p_c 16474
2AGPA141tipp 2tdec7eg3p_p --> 2tdec7eg3p_c 16474
2AGPA160tipp 2hdecg3p_p --> 2hdecg3p_c 16474
2AGPA161tipp 2hdec9eg3p_p --> 2hdec9eg3p_c 16474
2AGPA180tipp 2odecg3p_p --> 2odecg3p_c 16474
2AGPA181tipp 2odec11eg3p_p --> 2odec11eg3p_c 16474
2AGPE120tipp 2agpe120_p --> 2agpe120_c 16474
2AGPE140tipp 2agpe140_p --> 2agpe140_c 16474
2AGPE141tipp 2agpe141_p --> 2agpe141_c 16474
2AGPE160tipp 2agpe160_p --> 2agpe160_c 16474
2AGPE161tipp 2agpe161_p --> 2agpe161_c 16474
2AGPE180tipp 2agpe180_p --> 2agpe180_c 16474
2AGPE181tipp 2agpe181_p --> 2agpe181_c 16474
2AGPG120tipp 2agpg120_p --> 2agpg120_c 16474
2AGPG140tipp 2agpg140_p --> 2agpg140_c 16474
2AGPG141tipp 2agpg141_p --> 2agpg141_c 16474
2AGPG160tipp 2agpg160_p --> 2agpg160_c 16474
2AGPG161tipp 2agpg161_p --> 2agpg161_c 16474
2AGPG180tipp 2agpg180_p --> 2agpg180_c 16474
2AGPG181tipp 2agpg181_p --> 2agpg181_c 16474
LPCAT_SC 0.01 1agpc_SC_c + 0.02 dcacoa_c + 0.06 ddcacoa_c + 0.17 hdcoa_c + 0.09 ocdycacoa_c + 0.24 odecoa_c + 0.27 pmtcoa_c + 0.05 stcoa_c + 0.1 tdcoa_c --> coa_c + 0.01 pc_SC_c 16474

TAGL_SC h2o_c + 0.01 triglyc_SC_c --> 0.01 12dgr_SC_c + 0.02 dca_c + 0.06 ddca_c + h_c + 0.27 hdca_c + 0.17 hdcea_c + 0.05 ocdca_c + 0.24 ocdcea_c + 0.09 ocdcya_c + 0.1 ttdca_c 14317
yli_R1511 h2o_r + tag_cho_r --> dag_hs_r + hdca_r 14317 or 9000 or 9746
yli_R1512 dag_hs_r + h2o_r --> M07018_r + hdca_r 14317 or 9000 or 9746

EPISTESTH_SC 0.01 epistest_SC_c + h2o_c --> 0.01 epist_c + h_c + 0.02 hdca_c + 0.655 hdcea_c + 0.01 hexc_c + 0.03 ocdca_c + 0.27 ocdcea_c + 0.015 ttdca_c 14247 or 14617
EPISTESTH_SCe 0.01 epistest_SC_e + h2o_e --> 0.01 epist_e + h_e + 0.02 hdca_e + 0.655 hdcea_e + 0.01 hexc_e + 0.03 ocdca_e + 0.27 ocdcea_e + 0.015 ttdca_e 14247 or 14617
ERGSTESTH_SC 0.01 ergstest_SC_c + h2o_c --> 0.01 ergst_c + h_c + 0.02 hdca_c + 0.655 hdcea_c + 0.01 hexc_c + 0.03 ocdca_c + 0.27 ocdcea_c + 0.015 ttdca_c 14247 or 14617
ERGSTESTH_SCe 0.01 ergstest_SC_e + h2o_e --> 0.01 ergst_e + h_e + 0.02 hdca_e + 0.655 hdcea_e + 0.01 hexc_e + 0.03 ocdca_e + 0.27 ocdcea_e + 0.015 ttdca_e 14247 or 14617
FECOSTESTH_SC 0.01 fecostest_SC_c + h2o_c --> 0.01 fecost_c + h_c + 0.02 hdca_c + 0.655 hdcea_c + 0.01 hexc_c + 0.03 ocdca_c + 0.27 ocdcea_c + 0.015 ttdca_c 14247 or 14617
FECOSTESTH_SCe 0.01 fecostest_SC_e + h2o_e --> 0.01 fecost_e + h_e + 0.02 hdca_e + 0.655 hdcea_e + 0.01 hexc_e + 0.03 ocdca_e + 0.27 ocdcea_e + 0.015 ttdca_e 14247 or 14617
LANOSTESTH_SC h2o_c + 0.01 lanostest_SC_c --> h_c + 0.02 hdca_c + 0.655 hdcea_c + 0.01 hexc_c + 0.01 lanost_c + 0.03 ocdca_c + 0.27 ocdcea_c + 0.015 ttdca_c 14247 or 14617
LANOSTESTH_SCe h2o_e + 0.01 lanostest_SC_e --> h_e + 0.02 hdca_e + 0.655 hdcea_e + 0.01 hexc_e + 0.01 lanost_e + 0.03 ocdca_e + 0.27 ocdcea_e + 0.015 ttdca_e 14247 or 14617
LPSe h2o_e + tag_hs_e --> Rtotal3_e + dag_hs_e + h_e 14617
ZYMSTESTH_SC h2o_c + 0.01 zymstest_SC_c --> h_c + 0.02 hdca_c + 0.655 hdcea_c + 0.01 hexc_c + 0.03 ocdca_c + 0.27 ocdcea_c + 0.015 ttdca_c + 0.01 zymst_c 14247 or 14617
ZYMSTESTH_SCe h2o_e + 0.01 zymstest_SC_e --> h_e + 0.02 hdca_e + 0.655 hdcea_e + 0.01 hexc_e + 0.03 ocdca_e + 0.27 ocdcea_e + 0.015 ttdca_e + 0.01 zymst_e 14247 or 14617

EPISTESTH_SC 0.01 epistest_SC_c + h2o_c --> 0.01 epist_c + h_c + 0.02 hdca_c + 0.655 hdcea_c + 0.01 hexc_c + 0.03 ocdca_c + 0.27 ocdcea_c + 0.015 ttdca_c 14247 or 14617
[truncated: 231,415 more chars]
