# Supplementary material for: Multi-Omics Driven Metabolic Network Reconstruction and Analysis of Lignocellulosic Carbon Utilization in Rhodosporidium toruloides
Source: Front Bioeng Biotechnol. 2021 Jan 8;8:612832. doi: 10.3389/fbioe.2020.612832 (PMC7873862; doi:10.3389/fbioe.2020.612832)
Supplement: Supplementary File 4 — Multi-omics dataset for R. toruloides IFO0880. [file Data_Sheet_1.zip › Supplementary File S1/1.Manual_curation/Refinement_1f_Mass_and_Charge_Balance.html]

Refinement\_1f\_Mass\_and\_Charge\_Balance


In [1]:

```
%matplotlib inline
from matplotlib import pyplot as plt
import numpy as np
import pandas as pd
import json
import urllib
import cobra
```

In [2]:

```
json.load(urllib.request.urlopen('http://bigg.ucsd.edu/api/v2/database_version'))
```

Out[2]:

```
{'api_version': 'v2',
 'last_updated': '2019-10-31 10:05:54.157598',
 'bigg_models_version': '1.6.0'}
```

In [3]:

```
model = cobra.io.load_json_model("IFO0880_GPR_1e.json")
```

In [4]:

```
temp = set(m.id for m in model.metabolites if not m.formula)
temp
```

Out[4]:

```
{'2mb2coa_m',
 '2mbcoa_m',
 '3hdcoa_m',
 '3hddcoa_m',
 '3hhcoa_m',
 '3hhcoa_x',
 '3hhdcoa_m',
 '3hocoa_m',
 '3hocoa_x',
 '3htdcoa_m',
 '3mb2coa_m',
 '3mgcoa_m',
 '3ohcoa_m',
 '3ohcoa_x',
 '3oocoa_m',
 '3oocoa_x',
 '3sala_m',
 '3snpyr_m',
 '3spyr_m',
 '5hiu_m',
 '5mthf_m',
 'Lcyst_m',
 'Ncbmpts_m',
 'actp_m',
 'alatrna_m',
 'apocytc_m',
 'apppa_n',
 'asptrna_m',
 'bhb_m',
 'cytc_m',
 'dcacoa_m',
 'dd2coa_m',
 'dmnoncrn_x',
 'etfox_m',
 'etfrd_m',
 'fe2_c',
 'fe2_e',
 'fe2_m',
 'ficytc_m',
 'focytc_m',
 'galctn__D_n',
 'hemeA_m',
 'hemeO_m',
 'hx2coa_m',
 'hx2coa_x',
 'hxcoa_m',
 'hxcoa_x',
 'ibcoa_m',
 'ivcoa_m',
 'lystrna_m',
 'mercppyr_m',
 'msa_m',
 'na1_c',
 'na1_e',
 'oc2coa_m',
 'oc2coa_x',
 'pcrn_m',
 'pcrn_x',
 'pheme_m',
 'ppap_m',
 'sertrna_sec_m',
 'sucsal_m',
 'td2coa_m',
 'trnaala_m',
 'trnaasp_m',
 'trnalys_m',
 'trnasecys_m'}
```

In [5]:

```
for x in sorted(temp):
    for m in model.metabolites:
        if m.id.rsplit('_',1)[0] == x.rsplit('_',1)[0] and m.formula:
            print(x, model.metabolites.get_by_id(x).charge, m.id, m.formula, m.charge)
            break
```

```
3hdcoa_m None 3hdcoa_x C31H50N7O18P3S -4
3hddcoa_m None 3hddcoa_x C33H54N7O18P3S -4
3hhdcoa_m None 3hhdcoa_x C37H62N7O18P3S -4
3htdcoa_m None 3htdcoa_x C35H58N7O18P3S -4
3ohcoa_m None 3ohcoa_c C27H40N7O18P3S -4
3ohcoa_x None 3ohcoa_c C27H40N7O18P3S -4
3oocoa_m None 3oocoa_c C29H44N7O18P3S -4
3oocoa_x None 3oocoa_c C29H44N7O18P3S -4
3sala_m None 3sala_c C3H5NO4S -2
3snpyr_m None 3snpyr_c C3H2O5S -2
3spyr_m None 3spyr_c C3H2O6S -2
5mthf_m None 5mthf_c C20H24N7O6 -1
Lcyst_m None Lcyst_c C3H6NO5S -1
actp_m None actp_c C2H3O5P -2
alatrna_m None alatrna_c C3H6NOR 1
asptrna_m None asptrna_c C4H5NO3R 0
dcacoa_m None dcacoa_x C31H50N7O17P3S -4
dd2coa_m None dd2coa_x C33H52N7O17P3S -4
hxcoa_m None hxcoa_c C27H42N7O17P3S None
hxcoa_x None hxcoa_c C27H42N7O17P3S None
lystrna_m None lystrna_c C6H14N2OR 2
mercppyr_m None mercppyr_c C3H3O3S -1
msa_m None msa_c C3H3O3 -1
na1_c 1 na1_m Na None
na1_e 1 na1_m Na None
sucsal_m None sucsal_c C4H5O3 -1
td2coa_m None td2coa_x C35H56N7O17P3S -4
trnaala_m None trnaala_c R 0
trnaasp_m None trnaasp_c R 0
trnalys_m None trnalys_c R 0
```

In [6]:

```
model.metabolites.get_by_id('hxcoa_c').charge = -4
model.metabolites.get_by_id('na1_m').charge = 1
for x in temp:
    for m in model.metabolites:
        if m.id.rsplit('_',1)[0] == x.rsplit('_',1)[0] and m.formula:
            model.metabolites.get_by_id(x).formula = m.formula
            model.metabolites.get_by_id(x).charge = m.charge
            break
```

In [7]:

```
temp = set(m.id for m in model.metabolites if not m.formula)
temp
```

Out[7]:

```
{'2mb2coa_m',
 '2mbcoa_m',
 '3hhcoa_m',
 '3hhcoa_x',
 '3hocoa_m',
 '3hocoa_x',
 '3mb2coa_m',
 '3mgcoa_m',
 '5hiu_m',
 'Ncbmpts_m',
 'apocytc_m',
 'apppa_n',
 'bhb_m',
 'cytc_m',
 'dmnoncrn_x',
 'etfox_m',
 'etfrd_m',
 'fe2_c',
 'fe2_e',
 'fe2_m',
 'ficytc_m',
 'focytc_m',
 'galctn__D_n',
 'hemeA_m',
 'hemeO_m',
 'hx2coa_m',
 'hx2coa_x',
 'ibcoa_m',
 'ivcoa_m',
 'oc2coa_m',
 'oc2coa_x',
 'pcrn_m',
 'pcrn_x',
 'pheme_m',
 'ppap_m',
 'sertrna_sec_m',
 'trnasecys_m'}
```

In [8]:

```
bigg_met = dict()
for x in set(y.rsplit('_',1)[0] for y in temp):
    bigg_met[x] = json.load(urllib.request.urlopen('http://bigg.ucsd.edu/api/v2/universal/metabolites/'+x))
```

In [9]:

```
bigg_met['2mb2coa']
```

Out[9]:

```
{'database_links': {'InChI Key': [{'link': 'https://identifiers.org/inchikey/PMWATMXOQQZNBX-DKBZLLMOSA-J',
    'id': 'PMWATMXOQQZNBX-DKBZLLMOSA-J'}],
  'Reactome Compound': [{'link': 'http://identifiers.org/reactome/R-ALL-70799',
    'id': '70799'}],
  'SEED Compound': [{'link': 'http://identifiers.org/seed.compound/cpd02125',
    'id': 'cpd02125'},
   {'link': 'http://identifiers.org/seed.compound/cpd29594',
    'id': 'cpd29594'}],
  'CHEBI': [{'link': 'http://identifiers.org/chebi/CHEBI:10949',
    'id': 'CHEBI:10949'},
   {'link': 'http://identifiers.org/chebi/CHEBI:11614', 'id': 'CHEBI:11614'},
   {'link': 'http://identifiers.org/chebi/CHEBI:11619', 'id': 'CHEBI:11619'},
   {'link': 'http://identifiers.org/chebi/CHEBI:1199', 'id': 'CHEBI:1199'},
   {'link': 'http://identifiers.org/chebi/CHEBI:1204', 'id': 'CHEBI:1204'},
   {'link': 'http://identifiers.org/chebi/CHEBI:15478', 'id': 'CHEBI:15478'},
   {'link': 'http://identifiers.org/chebi/CHEBI:19691', 'id': 'CHEBI:19691'},
   {'link': 'http://identifiers.org/chebi/CHEBI:19697', 'id': 'CHEBI:19697'},
   {'link': 'http://identifiers.org/chebi/CHEBI:57260', 'id': 'CHEBI:57260'},
   {'link': 'http://identifiers.org/chebi/CHEBI:57337', 'id': 'CHEBI:57337'}],
  'Human Metabolome Database': [{'link': 'http://identifiers.org/hmdb/HMDB00993',
    'id': 'HMDB00993'},
   {'link': 'http://identifiers.org/hmdb/HMDB02054', 'id': 'HMDB02054'},
   {'link': 'http://identifiers.org/hmdb/HMDB02371', 'id': 'HMDB02371'},
   {'link': 'http://identifiers.org/hmdb/HMDB06871', 'id': 'HMDB06871'},
   {'link': 'http://identifiers.org/hmdb/HMDB62753', 'id': 'HMDB62753'}],
  'LipidMaps': [{'link': 'http://identifiers.org/lipidmaps/LMFA07050191',
    'id': 'LMFA07050191'},
   {'link': 'http://identifiers.org/lipidmaps/LMFA07050374',
    'id': 'LMFA07050374'}],
  'MetaNetX (MNX) Chemical': [{'link': 'http://identifiers.org/metanetx.chemical/MNXM609',
    'id': 'MNXM609'}],
  'KEGG Compound': [{'link': 'http://identifiers.org/kegg.compound/C03345',
    'id': 'C03345'}],
  'BioCyc': [{'link': 'http://identifiers.org/biocyc/META:CPD-1083',
    'id': 'META:CPD-1083'}]},
 'bigg_id': '2mb2coa',
 'formulae': ['C26H38N7O17P3S'],
 'old_identifiers': ['2mb2coa'],
 'charges': [-4],
 'name': 'Trans-2-Methylbut-2-enoyl-CoA',
 'compartments_in_models': [{'bigg_id': 'c',
   'organism': 'Pseudomonas putida KT2440',
   'model_bigg_id': 'iJN746'},
  {'bigg_id': 'c',
   'organism': 'Bacillus subtilis subsp. subtilis str. 168',
   'model_bigg_id': 'iYO844'},
  {'bigg_id': 'c',
   'organism': 'Cricetulus griseus',
   'model_bigg_id': 'iCHOv1'},
  {'bigg_id': 'c', 'organism': 'Homo sapiens', 'model_bigg_id': 'Recon3D'},
  {'bigg_id': 'c',
   'organism': 'Mycobacterium tuberculosis H37Rv',
   'model_bigg_id': 'iEK1008'},
  {'bigg_id': 'c',
   'organism': 'Cricetulus griseus',
   'model_bigg_id': 'iCHOv1_DG44'},
  {'bigg_id': 'c',
   'organism': 'Staphylococcus aureus subsp. aureus USA300_TCH1516',
   'model_bigg_id': 'iYS854'},
  {'bigg_id': 'c',
   'organism': 'Acinetobacter baumannii AYE',
   'model_bigg_id': 'iCN718'},
  {'bigg_id': 'c',
   'organism': 'Plasmodium falciparum 3D7',
   'model_bigg_id': 'iAM_Pf480'},
  {'bigg_id': 'c',
   'organism': 'Plasmodium vivax Sal-1',
   'model_bigg_id': 'iAM_Pv461'},
  {'bigg_id': 'c',
   'organism': 'Plasmodium berghei',
   'model_bigg_id': 'iAM_Pb448'},
  {'bigg_id': 'c',
   'organism': 'Plasmodium cynomolgi strain B',
   'model_bigg_id': 'iAM_Pc455'},
  {'bigg_id': 'c',
   'organism': 'Plasmodium knowlesi strain H',
   'model_bigg_id': 'iAM_Pk459'},
  {'bigg_id': 'c',
   'organism': 'Clostridioides difficile 630',
   'model_bigg_id': 'iCN900'},
  {'bigg_id': 'c',
   'organism': 'Pseudomonas putida KT2440',
   'model_bigg_id': 'iJN1463'},
  {'bigg_id': 'm', 'organism': 'Mus musculus', 'model_bigg_id': 'iMM1415'},
  {'bigg_id': 'm',
   'organism': 'Chlamydomonas reinhardtii',
   'model_bigg_id': 'iRC1080'},
  {'bigg_id': 'm', 'organism': 'Homo sapiens', 'model_bigg_id': 'RECON1'},
  {'bigg_id': 'm', 'organism': 'Homo sapiens', 'model_bigg_id': 'iAT_PLT_636'},
  {'bigg_id': 'm',
   'organism': 'Cricetulus griseus',
   'model_bigg_id': 'iCHOv1'},
  {'bigg_id': 'm',
   'organism': 'Phaeodactylum tricornutum CCAP 1055/1',
   'model_bigg_id': 'iLB1027_lipid'},
  {'bigg_id': 'm', 'organism': 'Homo sapiens', 'model_bigg_id': 'Recon3D'},
  {'bigg_id': 'm',
   'organism': 'Cricetulus griseus',
   'model_bigg_id': 'iCHOv1_DG44'}]}
```

In [10]:

```
for k, v in sorted(bigg_met.items()):
    if len(v['formulae']) == 1:
        print(k, v['formulae'], v['charges'])
        for x in temp:
            if x.rsplit('_',1)[0] == k:
                print('\t', x, model.metabolites.get_by_id(x).formula, model.metabolites.get_by_id(x).charge)
```

```
2mb2coa ['C26H38N7O17P3S'] [-4]
	 2mb2coa_m None None
2mbcoa ['C26H40N7O17P3S'] [0, -4]
	 2mbcoa_m None None
3hhcoa ['C27H42N7O18P3S'] [-4]
	 3hhcoa_m None None
	 3hhcoa_x None None
3hocoa ['C29H46N7O18P3S'] [-4]
	 3hocoa_x None None
	 3hocoa_m None None
3mb2coa ['C26H38N7O17P3S'] [-4]
	 3mb2coa_m None None
3mgcoa ['C27H37N7O19P3S'] [-5]
	 3mgcoa_m None None
5hiu ['C5H3N4O4'] [-1]
	 5hiu_m None None
apppa ['C20H24N10O16P3'] [-3]
	 apppa_n None None
bhb ['C4H7O3'] [0, -1]
	 bhb_m None None
etfox ['R'] [0]
	 etfox_m None None
fe2 ['Fe'] [2]
	 fe2_e None 2
	 fe2_m None 2
	 fe2_c None 2
galctn__D ['C6H11O7'] [-1]
	 galctn__D_n None None
hx2coa ['C27H40N7O17P3S'] [-4]
	 hx2coa_x None None
	 hx2coa_m None None
ibcoa ['C25H38N7O17P3S'] [0, -4]
	 ibcoa_m None None
ivcoa ['C26H40N7O17P3S'] [0, -4]
	 ivcoa_m None None
oc2coa ['C29H44N7O17P3S'] [-4]
	 oc2coa_x None None
	 oc2coa_m None None
pcrn ['C10H19NO4'] [0]
	 pcrn_m None None
	 pcrn_x None None
ppap ['C3H5O5P'] [-2]
	 ppap_m None None
```

In [11]:

```
for k, v in sorted(bigg_met.items()):
    if len(v['formulae']) == 1:
        for x in temp:
            if x.rsplit('_',1)[0] == k:
                model.metabolites.get_by_id(x).formula = v['formulae'][0]
                model.metabolites.get_by_id(x).charge = v['charges'][-1]
```

In [12]:

```
for k, v in sorted(bigg_met.items()):
    if len(v['formulae']) > 1:
        print(k, v['formulae'], v['charges'])
        for x in temp:
            if x.rsplit('_',1)[0] == k:
                print(x, model.metabolites.get_by_id(x).formula, model.metabolites.get_by_id(x).charge)
```

```
Ncbmpts ['C5H14N3O', 'C5H13N3O'] [0, 1]
Ncbmpts_m None None
dmnoncrn ['C18H37NO4', 'C18H35NO4'] [0]
dmnoncrn_x None None
etfrd ['H2R', 'RH2'] [0]
etfrd_m None None
ficytc ['C34H32FeN4O4R4', 'C42H52FeN8O6S2'] [1, -1]
ficytc_m None 1
focytc ['C42H53FeN8O6S2', 'C34H32FeN4O4R4', 'C34H32FeN4O4'] [1, 2, -2]
focytc_m None 1
hemeA ['C49FeH54N4O6', 'C49H54FeN4O6', 'C49H55FeN4O6'] [-6, -3, -2]
hemeA_m None -3
hemeO ['C49FeH56N4O5', 'C49H56FeN4O5'] [0, -2]
hemeO_m None -2
pheme ['C34H32FeN4O4', 'C34FeH30N4O4', 'C34H30FeN4O4'] [0, -2, -1]
pheme_m None -2
sertrna_sec ['C3H6NO2R', 'C3H7NO2R'] [1]
sertrna_sec_m None None
trnasecys ['R', 'HR'] [0]
trnasecys_m None None
```

In [13]:

```
model.metabolites.get_by_id('Ncbmpts_m').formula = 'C5H14N3O'
model.metabolites.get_by_id('Ncbmpts_m').charge = 1
model.metabolites.get_by_id('dmnoncrn_x').formula = 'C18H35NO4'
model.metabolites.get_by_id('dmnoncrn_x').charge = 0
model.metabolites.get_by_id('dmnoncoa_x').formula = 'C32H52N7O17P3S'
model.metabolites.get_by_id('dmnoncoa_x').charge = -4
model.metabolites.get_by_id('dmnoncoa_c').formula = 'C32H52N7O17P3S'
model.metabolites.get_by_id('dmnoncoa_c').charge = -4
model.metabolites.get_by_id('etfrd_m').formula = 'RH2'
model.metabolites.get_by_id('etfrd_m').charge = 0
model.metabolites.get_by_id('ficytc_m').formula = 'C42H54FeN8O6S2' # Fe3+
model.metabolites.get_by_id('ficytc_m').charge = 3
model.metabolites.get_by_id('focytc_m').formula = 'C42H54FeN8O6S2' # Fe2+
model.metabolites.get_by_id('focytc_m').charge = 2
model.metabolites.get_by_id('hemeA_m').formula = 'C49H54FeN4O6'
model.metabolites.get_by_id('hemeA_m').charge = -2
model.metabolites.get_by_id('hemeO_m').formula = 'C49H56FeN4O5'
model.metabolites.get_by_id('hemeO_m').charge = -2
model.metabolites.get_by_id('pheme_m').formula = 'C34H30FeN4O4'
model.metabolites.get_by_id('pheme_m').charge = -2
model.metabolites.get_by_id('apocytc_m').formula = 'R'
model.metabolites.get_by_id('apocytc_m').charge = 0
model.metabolites.get_by_id('cytc_m').formula = 'C34H34FeN4O4S2R'
model.metabolites.get_by_id('cytc_m').charge = -2
model.metabolites.get_by_id('sertrna_sec_m').formula = 'C3H6NO2R'
model.metabolites.get_by_id('sertrna_sec_m').charge = 1
model.metabolites.get_by_id('trnasecys_m').formula = 'R'
model.metabolites.get_by_id('trnasecys_m').charge = 0
```

In [14]:

```
temp = set(m.id for m in model.metabolites if not m.formula)
temp
```

Out[14]:

```
set()
```

In [15]:

```
temp = set(m.id.rsplit('_',1)[0] for m in model.metabolites)
for x in sorted(temp):
    if len(set(m.formula for m in model.metabolites if m.id.rsplit('_',1)[0] == x)) > 1:
        print([(m.id, m.formula, m.charge) for m in model.metabolites if m.id.rsplit('_',1)[0] == x])
```

```
[('2ahhmd_m', 'C7H8N5O8P2', '-3'), ('2ahhmd_c', 'C7H9N5O8P2', None)]
[('35cgmp_c', 'C10H11N5O7P', -1), ('35cgmp_n', 'C10H10N5O7P', None), ('35cgmp_e', 'C10H11N5O7P', -1)]
[('alltn_c', 'C4H6N4O3', 0), ('alltn_e', 'C4H6N4O3', 0), ('alltn_m', 'C4H5N4O3', None)]
[('btamp_c', 'C20H28N7O9PS', '0'), ('btamp_m', 'C20H27N7O9PS', -1)]
[('btn_c', 'C10H16N2O3S', '0'), ('btn_m', 'C10H15N2O3S', -1)]
[('chtn_c', 'C8H13NO5', '0'), ('chtn_e', 'C24H41N3O16', 0)]
[('fgam_c', 'C8H13N2O9P', -2), ('fgam_m', 'C8H12N2O9P', None)]
[('for_c', 'CH1O2', '-1'), ('for_r', 'CH1O2', '-1'), ('for_m', 'CH1O2', -1), ('for_x', 'CHO2', None), ('for_e', 'CH1O2', -1), ('for_n', 'CH1O2', -1)]
[('gar_c', 'C7H14N2O8P', -1), ('gar_m', 'C7H13N2O8P', None)]
[('methf_m', 'C20H20N7O6', -1), ('methf_c', 'C20H20N7O6', -1), ('methf_x', 'C20H21N7O6', None)]
[('nmn_x', 'C11H14N2O8P', -1), ('nmn_c', 'C11H14N2O8P', -1), ('nmn_m', 'C11H14N2O8P', -1), ('nmn_n', 'C11H13N2O8P', None)]
[('pi_m', 'HO4P', '-2'), ('pi_c', 'HO4P', -2), ('pi_v', 'HO4P', -2), ('pi_x', 'HO4P', -2), ('pi_e', 'HO4P', -2), ('pi_g', 'HO4P', -2), ('pi_r', 'HO4P', '-2'), ('pi_n', 'HPO4', None)]
[('so3_c', 'O3S', -2), ('so3_e', 'O3S', -2), ('so3_m', 'HSO3', None)]
[('so4_c', 'O4S', '-2'), ('so4_e', 'O4S', -2), ('so4_m', 'SO4', None)]
[('trnagly_c', 'R', 0), ('trnagly_m', 'C10H17O10PR2', None)]
[('trnathr_c', 'R', 0), ('trnathr_m', 'C10H17O10PR2', None)]
[('tsul_c', 'O3S2', '-2'), ('tsul_m', 'HS2O3', None)]
[('udpxyl_c', 'C14H22N2O16P2', '0'), ('udpxyl_g', 'C14H20N2O16P2', -2)]
[('urea_c', 'CH4N2O', 0), ('urea_e', 'CH4N2O', 0), ('urea_m', 'CH5N2O', None)]
```

In [16]:

```
model.metabolites.get_by_id('2ahhmd_c').formula = 'C7H8N5O8P2'
model.metabolites.get_by_id('2ahhmd_c').charge = -3
model.metabolites.get_by_id('35cgmp_n').formula = 'C10H11N5O7P'
model.metabolites.get_by_id('35cgmp_n').charge = -1
model.metabolites.get_by_id('acon_C_m').formula = 'C6H3O6'
model.metabolites.get_by_id('acon_C_m').charge = -3
model.metabolites.get_by_id('alltn_m').formula = 'C4H6N4O3'
model.metabolites.get_by_id('alltn_m').charge = 0
model.metabolites.get_by_id('btamp_c').formula = 'C20H27N7O9PS'
model.metabolites.get_by_id('btamp_c').charge = -1
model.metabolites.get_by_id('btn_c').formula = 'C10H15N2O3S'
model.metabolites.get_by_id('btn_c').charge = -1
model.metabolites.get_by_id('chtn_c').formula = 'C24H41N3O16'
model.metabolites.get_by_id('chtn_c').charge = 0
model.metabolites.get_by_id('fgam_m').formula = 'C8H13N2O9P'
model.metabolites.get_by_id('fgam_m').charge = -2
model.metabolites.get_by_id('for_x').formula = 'CH1O2'
model.metabolites.get_by_id('for_x').charge = -1
model.metabolites.get_by_id('gar_m').formula = 'C7H14N2O8P'
model.metabolites.get_by_id('gar_m').charge = -1
model.metabolites.get_by_id('glyc__R_c').formula = 'C3H5O4'
model.metabolites.get_by_id('glyc__R_c').charge = -1
model.metabolites.get_by_id('methf_x').formula = 'C20H20N7O6'
model.metabolites.get_by_id('methf_x').charge = -1
model.metabolites.get_by_id('nmn_n').formula = 'C11H14N2O8P'
model.metabolites.get_by_id('nmn_n').charge = -1
model.metabolites.get_by_id('pi_n').formula = 'HO4P'
model.metabolites.get_by_id('pi_n').charge = -2
model.metabolites.get_by_id('so3_m').formula = 'O3S'
model.metabolites.get_by_id('so3_m').charge = -2
model.metabolites.get_by_id('so4_m').formula = 'O4S'
model.metabolites.get_by_id('so4_m').charge = -2
model.metabolites.get_by_id('trnagly_m').formula = 'R'
model.metabolites.get_by_id('trnagly_m').charge = 0
model.metabolites.get_by_id('trnathr_m').formula = 'R'
model.metabolites.get_by_id('trnathr_m').charge = 0
model.metabolites.get_by_id('thrtrna_m').formula = 'C4H8NO2R'
model.metabolites.get_by_id('thrtrna_m').charge = 1
model.metabolites.get_by_id('tsul_m').formula = 'O3S2'
model.metabolites.get_by_id('tsul_m').charge = -2
model.metabolites.get_by_id('udpxyl_c').formula = 'C14H20N2O16P2'
model.metabolites.get_by_id('udpxyl_c').charge = -2
model.metabolites.get_by_id('udparab_c').formula = 'C14H20N2O16P2'
model.metabolites.get_by_id('udparab_c').charge = -2
model.metabolites.get_by_id('urea_m').formula = 'CH4N2O'
model.metabolites.get_by_id('urea_m').charge = 0
```

In [17]:

```
for m in model.metabolites:
    if m.charge and not isinstance(m.charge,int):
        print(m.id, m.charge)
```

```
2kmb_c -1
dhmtp_c 0
for_c -1
o2_c 0
akg_c -2
glu__L_c -1
adp_m -3
atp_m -4
glu__L_m -1
pi_m -2
thf_m -2
ACP_c -1
ppi_c -3
L2aadp_c -1
3odcoa_m -4
occoa_m -4
glx_c -1
gly_c 0
hpyr_c -1
ser__L_c 0
acser_c 0
cys__L_c 0
tsul_c -2
h2o_r 0
h_r 1
nadp_r -3
nadph_r -4
o2_r 0
gln__L_c 0
co2_x 0
gly_x 0
nad_x -1
nadh_x -2
nh4_x 1
tdcoa_c -4
25drapp_c -2
h2o2_m 0
o2_m 0
od2coa_m -4
cgly_c 0
gthrd_c -1
ppi_r -3
sql_r 0
co2_r 0
pe_RT_r 0
Lfmkynr_c 0
adp_r -3
atp_r -4
sph1p_r -1
34dhpac_c 0
dopa_c 1
f6p_c -2
gdpmann_c -2
thmpp_c -2
glc__D_c 0
ddcacoa_m -4
ddca_m -1
cellb_c 0
amet_e 1
acald_c 0
akg_m -2
ala_B_c 0
msa_c -1
hdca_c -1
ahcys_m 0
amet_m 1
idp_c -3
itp_c -4
3c3hmp_m -2
dhf_m -2
dhpt_m -1
didp_c -3
ditp_c -4
cmp_m -2
dhnpt_m 0
gcald_m 0
glx_x -1
mal__L_x -2
L2aadp6sa_m 0
saccrp__L_m -1
chol_c 1
1ag3p_RT_r -200
ddcacoa_r -4
pmtcoa_r -4
stcoa_r -4
tdcoa_r -4
dolmanp_r -1
uacgam_c -2
o2s_c -1
glyc3p_r -2
dcmp_c -2
ahcys_r 0
amet_r 1
4abut_c 0
cdpea_r -1
cmp_r -2
Lcyst_c -1
acrn_m 0
crn_m 0
dhap_r -2
ppcoa_c -4
3sala_c -2
octa_m -1
sbt__D_c 0
L2aadp_m -1
acac_m -1
glx_m -1
3otdcoa_m -4
cdp_r -3
ctp_r -4
cbp_m -2
citr__L_m 0
2ahhmd_m -3
pi_r -2
r1p_c -2
id3acald_c 0
ind3ac_c -1
aacoa_x -4
hdd2coa_m -4
pmtcoa_m -4
dc2coa_m -4
acgam_c 0
ergtetrol_r 0
dca_m -1
ppp9_m -2
pppg9_m -2
malt_c 0
psphings_r 1
2amac_c 0
f26bp_c -4
nad_r -1
nadh_r -2
2oxoadp_c -2
suc6p_c -2
ethamp_r -1
tre6p_c -2
3dsphgn_r 1
ser__L_r 0
lac__D_m -1
leutrna_m 1
trnaleu_m 0
dhor__S_c -1
orot_c -1
2ahbut_m -1
so4_c -2
cdpchol_r -1
thm_e 1
rnam_c 1
msa_m -1
for_r -1
lanost_r 0
lys__L_m 1
frdp_r -3
inost_r 0
5oxpro_c -1
dolmanp_c -1
tdcoa_m -4
ttdca_m -1
succ_x -2
meoh_c 0
lac__L_m -1
hdca_m -1
etha_c 1
glutcoa_m -5
3ohdcoa_m -4
urate_c 0
Lcyst_m -1
2oxoadp_m -2
3oddcoa_m -4
34dhpha_c -1
homoval_c -1
3sala_m -2
abt__D_c 0
8aonn_x 0
8aonn_m 0
amob_m 0
dann_m 0
chol_n 1
```

In [18]:

```
for m in model.metabolites:
    if isinstance(m.charge,str):
        m.charge = int(m.charge)
```

In [19]:

```
temp = set(m.id for m in model.metabolites if m.charge is None)
print(len(temp))
temp
```

```
212
```

Out[19]:

```
{'10fthf_x',
 '10fthfglu__L_m',
 '12dgr1601819Z_c',
 '12dgr1819Z1819Z_c',
 '1acpc_c',
 '1btol_c',
 '1pyr5c_m',
 '2ahhmp_c',
 '2dhp_n',
 '34dhbald_c',
 '34dhbz_c',
 '3hbcoa_c',
 '3hbcoa_x',
 '3hodcoa_c',
 '3hpcoa_c',
 '3hpp_c',
 '3odcoa_c',
 '3oddcoa_c',
 '3ohodcoa_c',
 '3oxoadp_c',
 '4cml_c',
 '4fumacac_c',
 '4hbald_c',
 '4mlacac_c',
 '4pyrdx_c',
 '56dh5flura_c',
 '56dura_c',
 '5flura_c',
 '5flura_e',
 '5odhf2a_c',
 '6a2ohxnt_c',
 '6mpur_c',
 '6mpur_e',
 '6tgsnmp_c',
 '6tins5mp_c',
 '6txan5mp_c',
 'Asn_X_Ser_Thr_c',
 'C04051_c',
 'CCbuttc_c',
 'Glc_aD_c',
 'R_3hdcoa_c',
 'R_3hddcoa_c',
 'R_3hhcoa_c',
 'R_3hmrscoa_c',
 'R_3hocoa_c',
 'aacoa_c',
 'acac_c',
 'acac_x',
 'acglu_c',
 'ad_c',
 'aflbala_c',
 'aflburppa_c',
 'ala_B_m',
 'ala__D_c',
 'ala__L_c',
 'ala__L_x',
 'alltt_m',
 'ans_c',
 'apoACP_c',
 'aps_c',
 'aps_m',
 'arso4_c',
 'asn__L_x',
 'asnglcnacglcnacman_man_man_c',
 'asnglcnacglcnacman_man_man_man_man_c',
 'asnglcnacglcnacman_man_manman_man_manmanman_c',
 'asnglcnacglcnacman_man_manman_manman_manmanman_c',
 'asnglcnacglcnacman_man_manman_manman_manmanmanglc_c',
 'asnglcnacglcnacman_man_manman_manman_manmanmanglcglc_c',
 'asnglcnacglcnacman_man_manman_manman_manmanmanglcglcglc_c',
 'asp__L_m',
 'b2coa_x',
 'btal_c',
 'btcoa_m',
 'btcoa_x',
 'cala_c',
 'camp_n',
 'catechol_c',
 'ccmuac_c',
 'chols_c',
 'conialdh_c',
 'cpppg1_c',
 'cys__L_m',
 'dadp_c',
 'dadp_m',
 'datp_m',
 'dgdp_m',
 'dghs1601819Z_c',
 'dghs1819Z1819Z_c',
 'dgtp_c',
 'dgtp_m',
 'dgts1601819Z_c',
 'dgts1819Z1819Z_c',
 'dna5mtc_c',
 'dna_c',
 'dnad_c',
 'doldp_c',
 'doldpglcnac_c',
 'doldpglcnacglcnac_c',
 'doldpglcnacglcnacman_c',
 'doldpglcnacglcnacman_man_man_c',
 'doldpglcnacglcnacman_man_man_manman_manmanman_c',
 'doldpglcnacglcnacman_man_manman_c',
 'doldpglcnacglcnacman_man_manman_manman_manmanman_c',
 'doldpglcnacglcnacman_man_manman_manman_manmanmanglcglcglc_c',
 'doldpglcnacglcnacman_man_manmanman_c',
 'doldpglcnacglcnacman_manman_manmanman_c',
 'doldpglcnacglcnacman_manmanman_manmanman_c',
 'doldpglcnacglcnacmanman_c',
 'dolpglc_c',
 'dttp_c',
 'dutp_m',
 'fad_c',
 'fad_m',
 'fad_x',
 'fadh2_m',
 'fadh2_x',
 'fe3_c',
 'fe3_e',
 'fer_c',
 'fmettrna_c',
 'fol_c',
 'frmd_c',
 'frmd_x',
 'g3pe_c',
 'g3pe_e',
 'gal_n',
 'gdbtal_c',
 'gln__L_m',
 'glu__D_c',
 'glyclt_m',
 'glyclt_x',
 'glytrna_m',
 'gmp_m',
 'gmp_n',
 'h2co3_c',
 'hcys__L_m',
 'hdd2coa_c',
 'hgentis_c',
 'hista_c',
 'hkmpp_c',
 'im4ac_c',
 'im4act_c',
 'imp_m',
 'lys__D_c',
 'mag160_c',
 'mag180_c',
 'mag1819Z_c',
 'mi134p_n',
 'mi14p_c',
 'mi14p_n',
 'mi3p__D_c',
 'mi4p__D_c',
 'nad_m',
 'nadh_m',
 'nadp_n',
 'nadph_n',
 'nh3_c',
 'no2_c',
 'no2_e',
 'no3_c',
 'no3_e',
 'o2_n',
 'od2coa_c',
 'oxadpcoa_c',
 'pail1819Z160_c',
 'pail1819Z160_e',
 'pant__R_n',
 'paps_m',
 'pdx5p_c',
 'pe1801819Z_c',
 'pe1801819Z_e',
 'pe1801829Z12Z_c',
 'pe1801829Z12Z_e',
 'pe1819Z1819Z_c',
 'pe1819Z1819Z_e',
 'pe1819Z1829Z12Z_c',
 'pe1819Z1829Z12Z_e',
 'pg1819Z160_c',
 'pg1819Z160_e',
 'pgp1819Z160_c',
 'pgp1819Z160_e',
 'phenol_c',
 'phpyr_m',
 'ppa_c',
 'ppa_m',
 'ppgpp_c',
 'pro__L_m',
 'ptcys_c',
 'ptrc_m',
 'ptth_c',
 'pyam5p_c',
 'pydam_c',
 'pydxn_c',
 'seasmet_c',
 'selmeth_c',
 'spmd_m',
 'succ_m',
 'tega_c',
 'tega_e',
 'tgua_c',
 'tgua_e',
 'ttc_ggdp_c',
 'udpgalur_c',
 'ump_c',
 'uppg1_c',
 'urate_e',
 'urate_m',
 'urcan_c',
 'vanln_c',
 'vanlt_c',
 'xmp_m'}
```

In [20]:

```
for x in sorted(temp):
    for m in model.metabolites:
        if m.id.rsplit('_',1)[0] == x.rsplit('_',1)[0] and m.charge is not None:
            print(x, model.metabolites.get_by_id(x).formula, m.id, m.formula, m.charge)
            break
```

```
10fthf_x C20H21N7O7 10fthf_m C20H21N7O7 -2
1pyr5c_m C5H6NO2 1pyr5c_c C5H6NO2 -1
2ahhmp_c C7H9N5O2 2ahhmp_m C7H9N5O2 0
2dhp_n C6H9O4 2dhp_c C6H9O4 -1
3hbcoa_c C25H38N7O18P3S 3hbcoa_m C25H38N7O18P3S -4
3hbcoa_x C25H38N7O18P3S 3hbcoa_m C25H38N7O18P3S -4
3hodcoa_c C39H66N7O18P3S 3hodcoa_x C39H66N7O18P3S -4
3hpcoa_c C24H36N7O18P3S 3hpcoa_m C24H36N7O18P3S -4
3odcoa_c C31H48N7O18P3S 3odcoa_m C31H48N7O18P3S -4
3oddcoa_c C33H52N7O18P3S 3oddcoa_x C33H52N7O18P3S -4
3ohodcoa_c C39H64N7O18P3S 3ohodcoa_x C39H64N7O18P3S -4
aacoa_c C25H36N7O18P3S aacoa_m C25H36N7O18P3S -4
acac_c C4H5O3 acac_m C4H5O3 -1
acac_x C4H5O3 acac_m C4H5O3 -1
acglu_c C7H9NO5 acglu_m C7H9NO5 -2
ala_B_m C3H7NO2 ala_B_c C3H7NO2 0
ala__D_c C3H7NO2 ala__D_e C3H7NO2 0
ala__L_c C3H7NO2 ala__L_e C3H7NO2 0
ala__L_x C3H7NO2 ala__L_e C3H7NO2 0
alltt_m C4H7N4O4 alltt_c C4H7N4O4 -1
asn__L_x C4H8N2O3 asn__L_c C4H8N2O3 0
asp__L_m C4H6NO4 asp__L_c C4H6NO4 -1
b2coa_x C25H36N7O17P3S b2coa_m C25H36N7O17P3S -4
camp_n C10H11N5O6P camp_c C10H11N5O6P -1
cys__L_m C3H7NO2S cys__L_c C3H7NO2S 0
dadp_c C10H12N5O9P2 dadp_n C10H12N5O9P2 -3
dadp_m C10H12N5O9P2 dadp_n C10H12N5O9P2 -3
datp_m C10H12N5O12P3 datp_c C10H12N5O12P3 -4
dgdp_m C10H12N5O10P2 dgdp_c C10H12N5O10P2 -3
dgtp_c C10H12N5O13P3 dgtp_n C10H12N5O13P3 -4
dgtp_m C10H12N5O13P3 dgtp_n C10H12N5O13P3 -4
dnad_c C21H24N6O15P2 dnad_n C21H24N6O15P2 -2
dttp_c C10H13N2O14P3 dttp_m C10H13N2O14P3 -4
dutp_m C9H11N2O14P3 dutp_c C9H11N2O14P3 -4
fmettrna_c C6H9NO2SR fmettrna_m C6H9NO2SR 0
gal_n C6H12O6 gal_c C6H12O6 0
gln__L_m C5H10N2O3 gln__L_c C5H10N2O3 0
glyclt_m C2H3O3 glyclt_c C2H3O3 -1
glyclt_x C2H3O3 glyclt_c C2H3O3 -1
glytrna_m C2H4NOR glytrna_c C2H4NOR 1
gmp_m C10H12N5O8P gmp_c C10H12N5O8P -2
gmp_n C10H12N5O8P gmp_c C10H12N5O8P -2
hcys__L_m C4H9NO2S hcys__L_c C4H9NO2S 0
hdd2coa_c C37H60N7O17P3S hdd2coa_x C37H60N7O17P3S -4
im4ac_c C5H5N2O2 im4ac_m C5H5N2O2 -1
im4act_c C5H6N2O im4act_m C5H6N2O 0
imp_m C10H11N4O8P imp_c C10H11N4O8P -2
nad_m C21H26N7O14P2 nad_c C21H26N7O14P2 -1
nadh_m C21H27N7O14P2 nadh_c C21H27N7O14P2 -2
nadp_n C21H25N7O17P3 nadp_c C21H25N7O17P3 -3
nadph_n C21H26N7O17P3 nadph_c C21H26N7O17P3 -4
o2_n O2 o2_c O2 0
od2coa_c C39H64N7O17P3S od2coa_m C39H64N7O17P3S -4
pant__R_n C6H11O4 pant__R_c C6H11O4 -1
paps_m C10H11N5O13P2S paps_c C10H11N5O13P2S -4
phpyr_m C9H7O3 phpyr_c C9H7O3 -1
pro__L_m C5H9NO2 pro__L_c C5H9NO2 0
ptrc_m C4H14N2 ptrc_c C4H14N2 2
spmd_m C7H22N3 spmd_c C7H22N3 3
succ_m C4H4O4 succ_c C4H4O4 -2
ump_c C9H11N2O9P ump_n C9H11N2O9P -2
urate_e C5H4N4O3 urate_c C5H4N4O3 0
urate_m C5H4N4O3 urate_c C5H4N4O3 0
xmp_m C10H11N4O9P xmp_c C10H11N4O9P -2
```

In [21]:

```
for x in temp:
    for m in model.metabolites:
        if m.id.rsplit('_',1)[0] == x.rsplit('_',1)[0] and m.charge is not None:
            model.metabolites.get_by_id(x).charge = m.charge
            break
```

In [22]:

```
temp = set(m.id for m in model.metabolites if m.charge is None)
print(len(temp))
temp
```

```
148
```

Out[22]:

```
{'10fthfglu__L_m',
 '12dgr1601819Z_c',
 '12dgr1819Z1819Z_c',
 '1acpc_c',
 '1btol_c',
 '34dhbald_c',
 '34dhbz_c',
 '3hpp_c',
 '3oxoadp_c',
 '4cml_c',
 '4fumacac_c',
 '4hbald_c',
 '4mlacac_c',
 '4pyrdx_c',
 '56dh5flura_c',
 '56dura_c',
 '5flura_c',
 '5flura_e',
 '5odhf2a_c',
 '6a2ohxnt_c',
 '6mpur_c',
 '6mpur_e',
 '6tgsnmp_c',
 '6tins5mp_c',
 '6txan5mp_c',
 'Asn_X_Ser_Thr_c',
 'C04051_c',
 'CCbuttc_c',
 'Glc_aD_c',
 'R_3hdcoa_c',
 'R_3hddcoa_c',
 'R_3hhcoa_c',
 'R_3hmrscoa_c',
 'R_3hocoa_c',
 'ad_c',
 'aflbala_c',
 'aflburppa_c',
 'ans_c',
 'apoACP_c',
 'aps_c',
 'aps_m',
 'arso4_c',
 'asnglcnacglcnacman_man_man_c',
 'asnglcnacglcnacman_man_man_man_man_c',
 'asnglcnacglcnacman_man_manman_man_manmanman_c',
 'asnglcnacglcnacman_man_manman_manman_manmanman_c',
 'asnglcnacglcnacman_man_manman_manman_manmanmanglc_c',
 'asnglcnacglcnacman_man_manman_manman_manmanmanglcglc_c',
 'asnglcnacglcnacman_man_manman_manman_manmanmanglcglcglc_c',
 'btal_c',
 'btcoa_m',
 'btcoa_x',
 'cala_c',
 'catechol_c',
 'ccmuac_c',
 'chols_c',
 'conialdh_c',
 'cpppg1_c',
 'dghs1601819Z_c',
 'dghs1819Z1819Z_c',
 'dgts1601819Z_c',
 'dgts1819Z1819Z_c',
 'dna5mtc_c',
 'dna_c',
 'doldp_c',
 'doldpglcnac_c',
 'doldpglcnacglcnac_c',
 'doldpglcnacglcnacman_c',
 'doldpglcnacglcnacman_man_man_c',
 'doldpglcnacglcnacman_man_man_manman_manmanman_c',
 'doldpglcnacglcnacman_man_manman_c',
 'doldpglcnacglcnacman_man_manman_manman_manmanman_c',
 'doldpglcnacglcnacman_man_manman_manman_manmanmanglcglcglc_c',
 'doldpglcnacglcnacman_man_manmanman_c',
 'doldpglcnacglcnacman_manman_manmanman_c',
 'doldpglcnacglcnacman_manmanman_manmanman_c',
 'doldpglcnacglcnacmanman_c',
 'dolpglc_c',
 'fad_c',
 'fad_m',
 'fad_x',
 'fadh2_m',
 'fadh2_x',
 'fe3_c',
 'fe3_e',
 'fer_c',
 'fol_c',
 'frmd_c',
 'frmd_x',
 'g3pe_c',
 'g3pe_e',
 'gdbtal_c',
 'glu__D_c',
 'h2co3_c',
 'hgentis_c',
 'hista_c',
 'hkmpp_c',
 'lys__D_c',
 'mag160_c',
 'mag180_c',
 'mag1819Z_c',
 'mi134p_n',
 'mi14p_c',
 'mi14p_n',
 'mi3p__D_c',
 'mi4p__D_c',
 'nh3_c',
 'no2_c',
 'no2_e',
 'no3_c',
 'no3_e',
 'oxadpcoa_c',
 'pail1819Z160_c',
 'pail1819Z160_e',
 'pdx5p_c',
 'pe1801819Z_c',
 'pe1801819Z_e',
 'pe1801829Z12Z_c',
 'pe1801829Z12Z_e',
 'pe1819Z1819Z_c',
 'pe1819Z1819Z_e',
 'pe1819Z1829Z12Z_c',
 'pe1819Z1829Z12Z_e',
 'pg1819Z160_c',
 'pg1819Z160_e',
 'pgp1819Z160_c',
 'pgp1819Z160_e',
 'phenol_c',
 'ppa_c',
 'ppa_m',
 'ppgpp_c',
 'ptcys_c',
 'ptth_c',
 'pyam5p_c',
 'pydam_c',
 'pydxn_c',
 'seasmet_c',
 'selmeth_c',
 'tega_c',
 'tega_e',
 'tgua_c',
 'tgua_e',
 'ttc_ggdp_c',
 'udpgalur_c',
 'uppg1_c',
 'urcan_c',
 'vanln_c',
 'vanlt_c'}
```

In [23]:

```
bigg_met = dict()
for x in set(y.rsplit('_',1)[0] for y in temp):
    bigg_met[x] = json.load(urllib.request.urlopen('http://bigg.ucsd.edu/api/v2/universal/metabolites/'+x))
```

In [24]:

```
for k, v in sorted(bigg_met.items()):
    if len(v['charges']) == 1:
        print(k, v['formulae'], v['charges'])
        for x in temp:
            if x.rsplit('_',1)[0] == k:
                print('\t', x, model.metabolites.get_by_id(x).formula, model.metabolites.get_by_id(x).charge)
```

```
12dgr1601819Z ['C37H70O5'] [0]
	 12dgr1601819Z_c C37H70O5 None
1acpc ['C4H7NO2'] [0]
	 1acpc_c C4H7NO2 None
34dhbald ['C7H6O3'] [0]
	 34dhbald_c C7H6O3 None
34dhbz ['C7H5O4'] [-1]
	 34dhbz_c C7H5O4 None
3hpp ['C3H5O3'] [-1]
	 3hpp_c C3H5O3 None
3oxoadp ['C6H6O5'] [-2]
	 3oxoadp_c C6H6O5 None
4fumacac ['C8H6O6'] [-2]
	 4fumacac_c C8H6O6 None
4hbald ['C7H6O2'] [0]
	 4hbald_c C7H6O2 None
4mlacac ['C8H6O6'] [-2]
	 4mlacac_c C8H6O6 None
4pyrdx ['C8H8NO4', 'C8H9NO4'] [0]
	 4pyrdx_c C8H8NO4 None
56dura ['C4H6N2O2'] [0]
	 56dura_c C4H6N2O2 None
5odhf2a ['C6H5O4'] [-1]
	 5odhf2a_c C6H5O4 None
6a2ohxnt ['C6H11NO3'] [0]
	 6a2ohxnt_c C6H11NO3 None
Asn_X_Ser_Thr ['XH', 'C4H6N2O2X', 'C5H7N3O3R2'] [0]
	 Asn_X_Ser_Thr_c C5H7N3O3R2 None
C04051 ['C4H6N4O'] [0]
	 C04051_c C4H6N4O None
CCbuttc ['C7H3O6'] [-3]
	 CCbuttc_c C7H3O6 None
R_3hdcoa ['C31H50N7O18P3S'] [-4]
	 R_3hdcoa_c C31H50N7O18P3S None
R_3hddcoa ['C33H54N7O18P3S'] [-4]
	 R_3hddcoa_c C33H54N7O18P3S None
R_3hhcoa ['C27H42N7O18P3S'] [-4]
	 R_3hhcoa_c C27H42N7O18P3S None
R_3hmrscoa ['C35H58N7O18P3S'] [-4]
	 R_3hmrscoa_c C35H58N7O18P3S None
R_3hocoa ['C29H46N7O18P3S'] [-4]
	 R_3hocoa_c C29H46N7O18P3S None
ad ['C2H6NO', 'C2H5NO'] [0]
	 ad_c C2H6NO None
apoACP ['HSR', 'RHO', 'C373H583N94O136S2'] [0]
	 apoACP_c HSR None
aps ['C10H12N5O10PS'] [-2]
	 aps_c C10H12N5O10PS None
	 aps_m C10H12N5O10PS None
btal ['C4H8O'] [0]
	 btal_c C4H8O None
btcoa ['C25H38N7O17P3S'] [-4]
	 btcoa_m C25H38N7O17P3S None
	 btcoa_x C25H38N7O17P3S None
cala ['C4H7N2O3', 'C4H8N2O3', 'C4H6N2O2'] [-1]
	 cala_c C4H8N2O3 None
catechol ['C6H6O2'] [0]
	 catechol_c C6H6O2 None
ccmuac ['C6H4O4'] [-2]
	 ccmuac_c C6H4O4 None
chols ['C5H13NO4S'] [0]
	 chols_c C5H13NO4S None
conialdh ['C10H10O3'] [0]
	 conialdh_c C10H10O3 None
cpppg1 ['C36H40N4O8'] [-4]
	 cpppg1_c C36H40N4O8 None
dna ['C10H17O8PR2', 'RH'] [0]
	 dna_c C10H17O8PR2 None
dna5mtc ['C11H19O8PR2', 'CH3R'] [0]
	 dna5mtc_c C11H19O8PR2 None
doldp ['C95H155O7P2', 'C55H91O7P2'] [-3]
	 doldp_c C55H91O7P2 None
dolpglc ['C61H102O9P'] [-1]
	 dolpglc_c C61H102O9P None
fadh2 ['C27H33N9O15P2'] [-2]
	 fadh2_x C27H33N9O15P2 None
	 fadh2_m C27H33N9O15P2 None
fe3 ['Fe'] [3]
	 fe3_c Fe None
	 fe3_e Fe None
fer ['C10H9O4'] [-1]
	 fer_c C10H9O4 None
frmd ['CH3NO', 'CH4NO'] [0]
	 frmd_c CH4NO None
	 frmd_x CH4NO None
g3pe ['C5H14NO6P'] [0]
	 g3pe_e C5H14NO6P None
	 g3pe_c C5H14NO6P None
gdbtal ['C5H12N3O'] [1]
	 gdbtal_c C5H12N3O None
glu__D ['C5H8NO4'] [-1]
	 glu__D_c C5H8NO4 None
h2co3 ['H2CO3', 'CH2O3'] [0]
	 h2co3_c H2CO3 None
hista ['C5H11N3', 'C5H10N3'] [1]
	 hista_c C5H11N3 None
hkmpp ['C6H9O6PS', 'C6H8O6PS'] [-2]
	 hkmpp_c C6H8O6PS None
lys__D ['C6H15N2O2'] [1]
	 lys__D_c C6H15N2O2 None
mag160 ['C19H38O4'] [0]
	 mag160_c C19H38O4 None
mag180 ['C21H42O4'] [0]
	 mag180_c C21H42O4 None
mag1819Z ['C21H40O4'] [0]
	 mag1819Z_c C21H40O4 None
mi134p ['C6H9O15P3'] [-6]
	 mi134p_n C6H9O15P3 None
mi14p ['C6H10O12P2'] [-4]
	 mi14p_n C6H10O12P2 None
	 mi14p_c C6H10O12P2 None
nh3 ['H3N'] [0]
	 nh3_c H3N None
no3 ['NO3'] [-1]
	 no3_e NO3 None
	 no3_c NO3 None
oxadpcoa ['C27H37N7O20P3S'] [-5]
	 oxadpcoa_c C27H37N7O20P3S None
pdx5p ['C8H10NO6P'] [-2]
	 pdx5p_c C8H10NO6P None
pe1801819Z ['C41H80NO8P'] [0]
	 pe1801819Z_c C41H80NO8P None
	 pe1801819Z_e C41H80NO8P None
pg1819Z160 ['C40H77O10P', 'C40H76O10P'] [-1]
	 pg1819Z160_e C40H77O10P None
	 pg1819Z160_c C40H77O10P None
pgp1819Z160 ['C40H78O13P2', 'C40H75O13P2'] [-3]
	 pgp1819Z160_c C40H78O13P2 None
	 pgp1819Z160_e C40H78O13P2 None
ppa ['C3H5O2'] [-1]
	 ppa_c C3H5O2 None
	 ppa_m C3H5O2 None
ptth ['C11H21N2O4S', 'C11H22N2O4S'] [0]
	 ptth_c C11H21N2O4S None
pyam5p ['C8H12N2O5P'] [-1]
	 pyam5p_c C8H12N2O5P None
pydam ['C8H13N2O2'] [1]
	 pydam_c C8H13N2O2 None
pydxn ['C8H11NO3'] [0]
	 pydxn_c C8H11NO3 None
seasmet ['C15H23N6O5Se'] [1]
	 seasmet_c C15H23N6O5Se None
selmeth ['C5H11NO2Se'] [0]
	 selmeth_c C5H11NO2Se None
ttc_ggdp ['C20H33O7P2'] [-3]
	 ttc_ggdp_c C20H33O7P2 None
udpgalur ['C15H19N2O18P2', 'C15H22N2O18P2'] [0]
	 udpgalur_c C15H19N2O18P2 None
uppg1 ['C40H36N4O16'] [-8]
	 uppg1_c C40H36N4O16 None
urcan ['C6H5N2O2'] [-1]
	 urcan_c C6H5N2O2 None
vanln ['C8H8O3'] [0]
	 vanln_c C8H8O3 None
vanlt ['C8H7O4'] [-1]
	 vanlt_c C8H7O4 None
```

In [25]:

```
for k, v in sorted(bigg_met.items()):
    if len(v['charges']) == 1:
        for x in temp:
            if x.rsplit('_',1)[0] == k:
                model.metabolites.get_by_id(x).charge = v['charges'][0]
```

In [26]:

```
for k, v in sorted(bigg_met.items()):
    if len(v['charges']) > 1:
        print(k, v['formulae'], v['charges'])
        for x in temp:
            if x.rsplit('_',1)[0] == k:
                print(x, model.metabolites.get_by_id(x).formula, model.metabolites.get_by_id(x).charge)
```

```
4cml ['C7H6O6', 'C7H4O6'] [0, -2]
4cml_c C7H4O6 None
fad ['C27H31N9O15P2'] [-3, -2]
fad_m C27H31N9O15P2 None
fad_c C27H31N9O15P2 None
fad_x C27H31N9O15P2 None
fol ['C19H17N7O6', 'C19H18N7O6'] [-1, -2]
fol_c C19H17N7O6 None
hgentis ['C8H7O4'] [0, -1]
hgentis_c C8H7O4 None
mi3p__D ['C6H11O9P'] [0, -2, -1]
mi3p__D_c C6H11O9P None
mi4p__D ['C6H11O9P'] [0, -2, -1]
mi4p__D_c C6H11O9P None
no2 ['NO2'] [0, -1]
no2_e NO2 None
no2_c NO2 None
ppgpp ['C10H11N5O17P4', 'C10H12N5O17P4'] [-6, -5]
ppgpp_c C10H11N5O17P4 None
```

In [27]:

```
model.metabolites.get_by_id('4cml_c').charge = -2
model.metabolites.get_by_id('fad_m').charge = -2
model.metabolites.get_by_id('fad_x').charge = -2
model.metabolites.get_by_id('fad_c').charge = -2
model.metabolites.get_by_id('fol_c').charge = -2
model.metabolites.get_by_id('hgentis_c').charge = -1
model.metabolites.get_by_id('mi3p__D_c').charge = -2
model.metabolites.get_by_id('mi4p__D_c').charge = -2
model.metabolites.get_by_id('no2_e').charge = -1
model.metabolites.get_by_id('no2_c').charge = -1
model.metabolites.get_by_id('ppgpp_c').charge = -6
```

In [28]:

```
for r in sorted(model.reactions, key=lambda x: x.id):
    if r.check_mass_balance() and not r.boundary:
        print(r, r.check_mass_balance())
```

```
10FTHFGLULLm: 10fthf_m + atp_m + glu__L_m --> 10fthfglu__L_m + adp_m + pi_m {'charge': 2.0}
2OH3K5MPPISO: h2o_c + hkmpp_c --> dhmtp_c + pi_c {'H': 1.0}
56DH5FLURAAMH: 56dh5flura_c + h2o_c + h_c --> aflburppa_c {'charge': -1.0}
6MPURPRT: 6mpur_c + prpp_c --> 6tins5mp_c + ppi_c {'charge': 2.0}
AATGm: 3sala_m + akg_m --> 3snpyr_m + glu__L_m {'charge': 1.0, 'H': 1.0}
ACDO: dhmtp_c + o2_c --> 2kmb_c + for_c + h_c {'charge': -1.0, 'H': -1.0}
ACPS1: apoACP_c + coa_c --> ACP_c + h_c + pap_c {'O': 1.0, 'S': -1.0}
ACRS: dkmpp_c --> h_c + hkmpp_c {'charge': 1.0}
AGATer_RT: 0.01 1ag3p_RT_r + 0.02 dcacoa_r + 0.06 ddcacoa_r + 0.17 hdcoa_r + 0.09 ocdycacoa_r + 0.24 odecoa_r + 0.27 pmtcoa_r + 0.05 stcoa_r + 0.1 tdcoa_r --> coa_r + 0.01 pa_RT_r {'charge': 1.6653345369377348e-16, 'C': -2.6645352591003757e-15, 'H': -7.105427357601002e-15, 'O': -4.440892098500626e-16, 'P': -1.1102230246251565e-16, 'N': 1.1102230246251565e-16, 'S': -9.71445146547012e-17}
ALLTNtm: alltn_c + h_c <=> alltn_m + 2.0 h_m {'charge': 1.0, 'H': 1.0}
AMAOTrm: 8aonn_m + amet_m <=> amob_m + dann_m {'charge': -1.0}
AMID_1: ad_c + h2o_c --> ac_c + h_c + nh4_c {'charge': 1.0}
APCPT: atp_c + ptcys_c --> 4ppcys_c + adp_c + h_c {'charge': -1.0, 'H': 1.0}
APNPT: atp_c + ptth_c --> adp_c + h_c + pan4p_c {'H': 1.0}
ASNTRS: asn__L_c + atp_c + trnaasn_c --> amp_c + asntrna_c + ppi_c {'H': -1.0}
ASNTRSm: asn__L_m + atp_m + trnaasn_m --> amp_m + asntrna_m + ppi_m {'H': -1.0}
ASNtx: asn__L_c + h_c <=> asn__L_x + 2.0 h_x {'charge': 1.0, 'H': 1.0}
ATDGDm: atp_m + dgdp_m + h_m --> adp_m + dgtp_m {'charge': -1.0, 'H': -1.0}
ATGDm: atp_m + gdp_m + h_m --> adp_m + gtp_m {'charge': -1.0, 'H': -1.0}
ATNAH: alltn_m + h2o_m + h_m --> alltt_m {'charge': -2.0, 'H': -2.0}
BUPN: cala_c + h2o_c + h_c --> ala_B_c + co2_c + nh4_c {'charge': 1.0}
CMPA: Ncbmpts_m + h2o_m + h_m --> co2_m + nh4_m + ptrc_m {'charge': 1.0, 'H': 1.0}
DAGL_RT: 0.01 12dgr_RT_d + h2o_c --> 0.02 dca_c + 0.06 ddca_c + h_c + 0.27 hdca_c + 0.17 hdcea_c + 0.01 mag_RT_d + 0.05 ocdca_c + 0.24 ocdcea_c + 0.09 ocdcya_c + 0.1 ttdca_c {'C': 2.220446049250313e-15, 'H': -3.552713678800501e-15}
DBTSm: atp_m + co2_m + dann_m <=> adp_m + dtbt_m + 3.0 h_m + pi_m {'charge': 1.0, 'H': 1.0}
DHAOX_c: dhdascb_c + 2.0 gthrd_c --> ascb__L_c + gthox_c + h_c {'charge': 1.0, 'H': 1.0}
DHNPAm: dhnpt_m --> 2ahhmp_m + gcald_m + h_m {'charge': 1.0, 'H': 1.0}
DHPD: 56dura_c + h2o_c <=> cala_c {'charge': -1.0}
DHPM1: 56dura_c + h2o_c --> cala_c + h_c {'H': 1.0}
EPISTATer_RT: 0.01 epist_r + 0.655 hdcoa_r + 0.01 hexccoa_r + 0.27 odecoa_r + 0.02 pmtcoa_r + 0.03 stcoa_r + 0.015 tdcoa_r --> coa_r + 0.01 epistest_RT_r {'charge': 2.220446049250313e-16, 'C': 5.551115123125783e-16, 'H': -1.099120794378905e-14, 'N': 6.938893903907228e-17, 'O': 3.3306690738754696e-16, 'P': -1.5265566588595902e-16, 'S': -5.551115123125783e-17}
EPISTESTH_RT: 0.01 epistest_RT_d + h2o_c --> 0.01 epist_c + h_c + 0.02 hdca_c + 0.655 hdcea_c + 0.01 hexc_c + 0.03 ocdca_c + 0.27 ocdcea_c + 0.015 ttdca_c {'charge': -9.71445146547012e-17, 'C': 2.6922908347160046e-15, 'H': 4.163336342344337e-15, 'O': 1.942890293094024e-16}
ERGSTATer_RT: 0.01 ergst_r + 0.655 hdcoa_r + 0.01 hexccoa_r + 0.27 odecoa_r + 0.02 pmtcoa_r + 0.03 stcoa_r + 0.015 tdcoa_r --> coa_r + 0.01 ergstest_RT_r {'charge': 2.220446049250313e-16, 'C': 5.551115123125783e-16, 'H': -1.099120794378905e-14, 'N': 6.938893903907228e-17, 'O': 3.3306690738754696e-16, 'P': -1.5265566588595902e-16, 'S': -5.551115123125783e-17}
ERGSTESTH_RT: 0.01 ergstest_RT_d + h2o_c --> 0.01 ergst_c + h_c + 0.02 hdca_c + 0.655 hdcea_c + 0.01 hexc_c + 0.03 ocdca_c + 0.27 ocdcea_c + 0.015 ttdca_c {'charge': -9.71445146547012e-17, 'C': 2.6922908347160046e-15, 'H': 4.163336342344337e-15, 'O': 1.942890293094024e-16}
FECOSTATer_RT: 0.01 fecost_r + 0.655 hdcoa_r + 0.01 hexccoa_r + 0.27 odecoa_r + 0.02 pmtcoa_r + 0.03 stcoa_r + 0.015 tdcoa_r --> coa_r + 0.01 fecostest_RT_r {'charge': 2.220446049250313e-16, 'C': 5.551115123125783e-16, 'H': -1.099120794378905e-14, 'N': 6.938893903907228e-17, 'O': 3.3306690738754696e-16, 'P': -1.5265566588595902e-16, 'S': -5.551115123125783e-17}
FECOSTESTH_RT: 0.01 fecostest_RT_d + h2o_c --> 0.01 fecost_c + h_c + 0.02 hdca_c + 0.655 hdcea_c + 0.01 hexc_c + 0.03 ocdca_c + 0.27 ocdcea_c + 0.015 ttdca_c {'charge': -9.71445146547012e-17, 'C': 2.6922908347160046e-15, 'H': 4.163336342344337e-15, 'O': 1.942890293094024e-16}
FGFTm: fgam_m + 3.0 h_m + thf_m --> gar_m + h2o_m + methf_m {'charge': -1.0, 'H': -1.0}
FOLD3_1: 2ahhmd_c + 4abz_c --> dhpt_c + h_c + ppi_c {'charge': 1.0, 'H': 1.0}
FORA: frmd_c + h2o_c --> for_c + h_c + nh4_c {'charge': 1.0}
FORAMDx: frmd_x + h2o_x --> for_x + nh4_x {'H': -1.0}
GAT1er_RT: 0.02 dcacoa_r + 0.06 ddcacoa_r + glyc3p_r + 0.17 hdcoa_r + 0.09 ocdycacoa_r + 0.24 odecoa_r + 0.27 pmtcoa_r + 0.05 stcoa_r + 0.1 tdcoa_r --> 0.01 1ag3p_RT_r + coa_r {'charge': 3.885780586188048e-16, 'C': 1.1546319456101628e-14, 'H': -7.105427357601002e-15, 'O': 4.440892098500626e-16, 'P': -1.1102230246251565e-16, 'N': 1.1102230246251565e-16, 'S': -9.71445146547012e-17}
GAT2er_RT: 0.02 dcacoa_r + 0.06 ddcacoa_r + dhap_r + 0.17 hdcoa_r + 0.09 ocdycacoa_r + 0.24 odecoa_r + 0.27 pmtcoa_r + 0.05 stcoa_r + 0.1 tdcoa_r --> 0.01 1agly3p_RT_r + coa_r {'charge': 3.885780586188048e-16, 'C': 1.1546319456101628e-14, 'H': -7.105427357601002e-15, 'O': 4.440892098500626e-16, 'P': -1.1102230246251565e-16, 'N': 1.1102230246251565e-16, 'S': -9.71445146547012e-17}
GCPN: 35cgmp_c + h2o_c --> gmp_c {'charge': -1.0, 'H': -1.0}
GDTP: gdptp_c + h2o_c <=> 2.0 h_c + pi_c + ppgpp_c {'charge': 1.0, 'H': 1.0}
GLCNACPT: dolp_c + 2.0 h_c + uacgam_c --> doldpglcnac_c + ump_c {'C': 40.0, 'H': 64.0}
GLPT: glp_c + tdcoa_c --> coa_c + tglp_c {'H': -1.0}
GMPS_glu_m: atp_m + gln__L_m + h2o_m + xmp_m --> amp_m + glu__L_m + gmp_m + 4.0 h_m + ppi_m {'charge': 2.0, 'H': 2.0}
GMPSm: atp_m + nh4_m + xmp_m --> amp_m + gmp_m + 4.0 h_m + ppi_m {'charge': 2.0, 'H': 2.0}
GPAR: gua_c + h_c + prpp_c <=> gmp_c + ppi_c {'charge': -1.0, 'H': -1.0}
GTHAMPOR: amp_c + gthox_c + h_c + so3_c <=> aps_c + 2.0 gthrd_c {'charge': 1.0, 'H': 1.0}
GTHAMPORm: amp_m + gthox_m + h_m + so3_m <=> aps_m + 2.0 gthrd_m {'charge': 1.0, 'H': 1.0}
GTPDPK_1: atp_c + gtp_c <=> amp_c + gdptp_c {'charge': -1.0, 'H': -1.0}
HISDC: h_c + his__L_c --> co2_c + hista_c {'H': 1.0}
HISTASE: h2o_c + hista_c + o2_c --> h2o2_c + im4act_c + nh4_c {'H': -1.0}
HPPK_1: 2ahhmp_c + atp_c --> 2ahhmd_c + amp_c {'charge': -1.0, 'H': -1.0}
IDPm: h2o_m + ppi_m --> 2.0 pi_m {'charge': -1.0, 'H': -1.0}
LANOSTATer_RT: 0.655 hdcoa_r + 0.01 hexccoa_r + 0.01 lanost_r + 0.27 odecoa_r + 0.02 pmtcoa_r + 0.03 stcoa_r + 0.015 tdcoa_r --> coa_r + 0.01 lanostest_RT_r {'charge': 2.220446049250313e-16, 'C': -1.2212453270876722e-15, 'H': -3.885780586188048e-15, 'N': 6.938893903907228e-17, 'O': 3.3306690738754696e-16, 'P': -1.5265566588595902e-16, 'S': -5.551115123125783e-17}
LANOSTESTH_RT: h2o_c + 0.01 lanostest_RT_d --> h_c + 0.02 hdca_c + 0.655 hdcea_c + 0.01 hexc_c + 0.01 lanost_c + 0.03 ocdca_c + 0.27 ocdcea_c + 0.015 ttdca_c {'charge': -9.71445146547012e-17, 'H': 2.3869795029440866e-15, 'O': 1.942890293094024e-16, 'C': 2.6922908347160046e-15}
LPCATer_RT: 0.01 1agpc_RT_r + 0.02 dcacoa_r + 0.06 ddcacoa_r + 0.17 hdcoa_r + 0.09 ocdycacoa_r + 0.24 odecoa_r + 0.27 pmtcoa_r + 0.05 stcoa_r + 0.1 tdcoa_r --> coa_r + 0.01 pc_RT_r {'charge': 3.885780586188048e-16, 'C': -2.6645352591003757e-15, 'N': 1.1102230246251565e-16, 'O': -4.440892098500626e-16, 'P': -1.1102230246251565e-16, 'S': -9.71445146547012e-17}
MAGL_RT: h2o_c + 0.01 mag_RT_d --> 0.02 dca_c + 0.06 ddca_c + glyc_c + h_c + 0.27 hdca_c + 0.17 hdcea_c + 0.05 ocdca_c + 0.24 ocdcea_c + 0.09 ocdcya_c + 0.1 ttdca_c {'C': 2.220446049250313e-15, 'H': 3.552713678800501e-15}
MATm: atp_m + h2o_m + met__L_m --> amet_m + h_m + pi_m + ppi_m {'charge': 1.0, 'H': 1.0}
MIP2CS124g_RT: 0.01 mipc124_RT_g + 0.01 ptd1ino_RT_g --> 0.01 12dgr_RT_g + 0.01 mip2c124_RT_g {'C': 7.105427357601002e-15}
MIP2CS126g_RT: 0.01 mipc126_RT_g + 0.01 ptd1ino_RT_g --> 0.01 12dgr_RT_g + 0.01 mip2c126_RT_g {'C': 7.105427357601002e-15}
MIP2CS224g_RT: 0.01 mipc224_RT_g + 0.01 ptd1ino_RT_g --> 0.01 12dgr_RT_g + 0.01 mip2c224_RT_g {'C': 7.105427357601002e-15}
MIP2CS226g_RT: 0.01 mipc226_RT_g + 0.01 ptd1ino_RT_g --> 0.01 12dgr_RT_g + 0.01 mip2c226_RT_g {'C': 7.105427357601002e-15}
MIP2CS324g_RT: 0.01 mipc324_RT_g + 0.01 ptd1ino_RT_g --> 0.01 12dgr_RT_g + 0.01 mip2c324_RT_g {'C': 7.105427357601002e-15}
MIP2CS326g_RT: 0.01 mipc326_RT_g + 0.01 ptd1ino_RT_g --> 0.01 12dgr_RT_g + 0.01 mip2c326_RT_g {'C': 7.105427357601002e-15}
MTHFO: h_m + mlthf_m + nadh_m --> 5mthf_m + nad_m {'charge': 1.0, 'H': 1.0}
MTHFO_nadp: h_m + mlthf_m + nadph_m --> 5mthf_m + nadp_m {'charge': 1.0, 'H': 1.0}
OCT: cbp_c + orn_c <=> citr__L_c + pi_c {'charge': -1.0, 'H': -1.0}
OCTm: cbp_m + orn_m <=> citr__L_m + pi_m {'charge': -1.0, 'H': -1.0}
PLBP1Ie_RT: h2o_e + 0.005 ptd1ino_RT_e --> 0.02 dca_e + 0.06 ddca_e + 0.5 g3pi_e + h_e + 0.27 hdca_e + 0.17 hdcea_e + 0.05 ocdca_e + 0.24 ocdcea_e + 0.09 ocdcya_e + 0.1 ttdca_e {'charge': -2.7755575615628914e-17, 'C': 1.5543122344752192e-15, 'H': 4.440892098500626e-15, 'O': -1.0547118733938987e-15}
PLBPC_RT: h2o_c + 0.005 pc_RT_r --> 0.02 dca_c + 0.06 ddca_c + 0.5 g3pc_c + h_c + 0.27 hdca_c + 0.17 hdcea_c + 0.05 ocdca_c + 0.24 ocdcea_c + 0.09 ocdcya_c + 0.1 ttdca_c {'C': 1.5543122344752192e-15, 'H': 4.440892098500626e-15, 'O': 2.7755575615628914e-16}
PLBPCe_RT: h2o_e + 0.005 pc_RT_e --> 0.02 dca_e + 0.06 ddca_e + 0.5 g3pc_e + h_e + 0.27 hdca_e + 0.17 hdcea_e + 0.05 ocdca_e + 0.24 ocdcea_e + 0.09 ocdcya_e + 0.1 ttdca_e {'C': 1.5543122344752192e-15, 'H': 4.440892098500626e-15, 'O': 2.7755575615628914e-16}
PLBPEe_RT: h2o_e + 0.005 pe_RT_e --> 0.02 dca_e + 0.06 ddca_e + 0.5 g3pe_e + h_e + 0.27 hdca_e + 0.17 hdcea_e + 0.05 ocdca_e + 0.24 ocdcea_e + 0.09 ocdcya_e + 0.1 ttdca_e {'C': 1.5543122344752192e-15, 'H': 4.440892098500626e-15, 'O': 2.7755575615628914e-16}
PYDXDH: h2o_c + o2_c + pydx_c --> 4pyrdx_c + h2o2_c {'H': -1.0}
PYDXDH_1: h2o_c + o2_c + pydx_c --> 4pyrdx_c + h2o2_c + h_c {'charge': 1.0}
SULO: h2o_c + o2_c + so3_c --> h2o2_c + h_c + so4_c {'charge': 1.0, 'H': 1.0}
SULOm: h2o_m + o2_m + so3_m --> h2o2_m + h_m + so4_m {'charge': 1.0, 'H': 1.0}
TAGL_RT: h2o_c + 0.01 triglyc_RT_d --> 0.01 12dgr_RT_d + 0.02 dca_c + 0.06 ddca_c + h_c + 0.27 hdca_c + 0.17 hdcea_c + 0.05 ocdca_c + 0.24 ocdcea_c + 0.09 ocdcya_c + 0.1 ttdca_c {'C': -5.551115123125783e-15, 'H': 1.1546319456101628e-14, 'O': -1.0547118733938987e-15}
TGUAPRT: prpp_c + tgua_c --> 6tgsnmp_c + ppi_c {'charge': 2.0}
TRIGSer_RT: 0.01 12dgr_RT_r + 0.02 dcacoa_r + 0.06 ddcacoa_r + 0.17 hdcoa_r + 0.09 ocdycacoa_r + 0.24 odecoa_r + 0.27 pmtcoa_r + 0.05 stcoa_r + 0.1 tdcoa_r --> coa_r + 0.01 triglyc_RT_r {'charge': 3.885780586188048e-16, 'O': -8.881784197001252e-16, 'N': 1.1102230246251565e-16, 'P': -1.1102230246251565e-16, 'S': -9.71445146547012e-17}
UDPDOLPT: dolp_c + udpg_c --> dolpglc_c + udp_c {'C': 40.0, 'H': 64.0}
UDPGALOR: h2o_c + 2.0 nad_c + udpgal_c --> 3.0 h_c + 2.0 nadh_c + udpgalur_c {'charge': 3.0}
ZYMSTATer_RT: 0.655 hdcoa_r + 0.01 hexccoa_r + 0.27 odecoa_r + 0.02 pmtcoa_r + 0.03 stcoa_r + 0.015 tdcoa_r + 0.01 zymst_r --> coa_r + 0.01 zymstest_RT_r {'charge': 2.220446049250313e-16, 'H': -7.105427357601002e-15, 'N': 6.938893903907228e-17, 'O': 4.440892098500626e-16, 'P': -1.5265566588595902e-16, 'S': -5.551115123125783e-17}
ZYMSTESTH_RT: h2o_c + 0.01 zymstest_RT_d --> h_c + 0.02 hdca_c + 0.655 hdcea_c + 0.01 hexc_c + 0.03 ocdca_c + 0.27 ocdcea_c + 0.015 ttdca_c + 0.01 zymst_c {'charge': -9.71445146547012e-17, 'H': 7.105427357601002e-15, 'O': 2.220446049250313e-16}
```

In [29]:

```
for r in sorted(model.reactions, key=lambda x: x.id):
    if r.check_mass_balance() and not r.boundary:
        if sum(abs(x) for x in r.check_mass_balance().values()) > 1e-6:
            print(r, r.check_mass_balance())
            print('\t', [(m.id, m.formula, m.charge) for m in r.reactants])
            print('\t', [(m.id, m.formula, m.charge) for m in r.products])
```

```
10FTHFGLULLm: 10fthf_m + atp_m + glu__L_m --> 10fthfglu__L_m + adp_m + pi_m {'charge': 2.0}
	 [('10fthf_m', 'C20H21N7O7', -2), ('atp_m', 'C10H12N5O13P3', -4), ('glu__L_m', 'C5H8NO4', -1)]
	 [('10fthfglu__L_m', 'C25H28N8O10', None), ('adp_m', 'C10H12N5O10P2', -3), ('pi_m', 'HO4P', -2)]
2OH3K5MPPISO: h2o_c + hkmpp_c --> dhmtp_c + pi_c {'H': 1.0}
	 [('h2o_c', 'H2O', 0), ('hkmpp_c', 'C6H8O6PS', -2)]
	 [('dhmtp_c', 'C6H10O3S', 0), ('pi_c', 'HO4P', -2)]
56DH5FLURAAMH: 56dh5flura_c + h2o_c + h_c --> aflburppa_c {'charge': -1.0}
	 [('56dh5flura_c', 'C4H4FN2O2', None), ('h2o_c', 'H2O', 0), ('h_c', 'H', 1)]
	 [('aflburppa_c', 'C4H7FN2O3', None)]
6MPURPRT: 6mpur_c + prpp_c --> 6tins5mp_c + ppi_c {'charge': 2.0}
	 [('6mpur_c', 'C5H4N4S', None), ('prpp_c', 'C5H8O14P3', -5)]
	 [('6tins5mp_c', 'C10H11N4O7PS', None), ('ppi_c', 'HO7P2', -3)]
AATGm: 3sala_m + akg_m --> 3snpyr_m + glu__L_m {'charge': 1.0, 'H': 1.0}
	 [('3sala_m', 'C3H5NO4S', -2), ('akg_m', 'C5H4O5', -2)]
	 [('3snpyr_m', 'C3H2O5S', -2), ('glu__L_m', 'C5H8NO4', -1)]
ACDO: dhmtp_c + o2_c --> 2kmb_c + for_c + h_c {'charge': -1.0, 'H': -1.0}
	 [('dhmtp_c', 'C6H10O3S', 0), ('o2_c', 'O2', 0)]
	 [('2kmb_c', 'C5H7O3S', -1), ('for_c', 'CH1O2', -1), ('h_c', 'H', 1)]
ACPS1: apoACP_c + coa_c --> ACP_c + h_c + pap_c {'O': 1.0, 'S': -1.0}
	 [('apoACP_c', 'HSR', 0), ('coa_c', 'C21H32N7O16P3S', -4)]
	 [('ACP_c', 'C11H21N2O7PRS', -1), ('h_c', 'H', 1), ('pap_c', 'C10H11N5O10P2', -4)]
ACRS: dkmpp_c --> h_c + hkmpp_c {'charge': 1.0}
	 [('dkmpp_c', 'C6H9O6PS', -2)]
	 [('h_c', 'H', 1), ('hkmpp_c', 'C6H8O6PS', -2)]
ALLTNtm: alltn_c + h_c <=> alltn_m + 2.0 h_m {'charge': 1.0, 'H': 1.0}
	 [('alltn_c', 'C4H6N4O3', 0), ('h_c', 'H', 1)]
	 [('alltn_m', 'C4H6N4O3', 0), ('h_m', 'H', 1)]
AMAOTrm: 8aonn_m + amet_m <=> amob_m + dann_m {'charge': -1.0}
	 [('8aonn_m', 'C9H17NO3', 0), ('amet_m', 'C15H23N6O5S', 1)]
	 [('amob_m', 'C15H20N5O6S', 0), ('dann_m', 'C9H20N2O2', 0)]
AMID_1: ad_c + h2o_c --> ac_c + h_c + nh4_c {'charge': 1.0}
	 [('ad_c', 'C2H6NO', 0), ('h2o_c', 'H2O', 0)]
	 [('ac_c', 'C2H3O2', -1), ('h_c', 'H', 1), ('nh4_c', 'H4N', 1)]
APCPT: atp_c + ptcys_c --> 4ppcys_c + adp_c + h_c {'charge': -1.0, 'H': 1.0}
	 [('atp_c', 'C10H12N5O13P3', -4), ('ptcys_c', 'C12H20N2O6S', None)]
	 [('4ppcys_c', 'C12H20N2O9PS', -3), ('adp_c', 'C10H12N5O10P2', -3), ('h_c', 'H', 1)]
APNPT: atp_c + ptth_c --> adp_c + h_c + pan4p_c {'H': 1.0}
	 [('atp_c', 'C10H12N5O13P3', -4), ('ptth_c', 'C11H21N2O4S', 0)]
	 [('adp_c', 'C10H12N5O10P2', -3), ('h_c', 'H', 1), ('pan4p_c', 'C11H21N2O7PS', -2)]
ASNTRS: asn__L_c + atp_c + trnaasn_c --> amp_c + asntrna_c + ppi_c {'H': -1.0}
	 [('asn__L_c', 'C4H8N2O3', 0), ('atp_c', 'C10H12N5O13P3', -4), ('trnaasn_c', 'C10H17O10PR2', 0)]
	 [('amp_c', 'C10H12N5O7P', -2), ('asntrna_c', 'C14H23N2O12PR2', 1), ('ppi_c', 'HO7P2', -3)]
ASNTRSm: asn__L_m + atp_m + trnaasn_m --> amp_m + asntrna_m + ppi_m {'H': -1.0}
	 [('asn__L_m', 'C4H8N2O3', 0), ('atp_m', 'C10H12N5O13P3', -4), ('trnaasn_m', 'C10H17O10PR2', 0)]
	 [('amp_m', 'C10H12N5O7P', -2), ('asntrna_m', 'C14H23N2O12PR2', 1), ('ppi_m', 'HO7P2', -3)]
ASNtx: asn__L_c + h_c <=> asn__L_x + 2.0 h_x {'charge': 1.0, 'H': 1.0}
	 [('asn__L_c', 'C4H8N2O3', 0), ('h_c', 'H', 1)]
	 [('asn__L_x', 'C4H8N2O3', 0), ('h_x', 'H', 1)]
ATDGDm: atp_m + dgdp_m + h_m --> adp_m + dgtp_m {'charge': -1.0, 'H': -1.0}
	 [('atp_m', 'C10H12N5O13P3', -4), ('dgdp_m', 'C10H12N5O10P2', -3), ('h_m', 'H', 1)]
	 [('adp_m', 'C10H12N5O10P2', -3), ('dgtp_m', 'C10H12N5O13P3', -4)]
ATGDm: atp_m + gdp_m + h_m --> adp_m + gtp_m {'charge': -1.0, 'H': -1.0}
	 [('atp_m', 'C10H12N5O13P3', -4), ('gdp_m', 'C10H12N5O11P2', -3), ('h_m', 'H', 1)]
	 [('adp_m', 'C10H12N5O10P2', -3), ('gtp_m', 'C10H12N5O14P3', -4)]
ATNAH: alltn_m + h2o_m + h_m --> alltt_m {'charge': -2.0, 'H': -2.0}
	 [('alltn_m', 'C4H6N4O3', 0), ('h2o_m', 'H2O', 0), ('h_m', 'H', 1)]
	 [('alltt_m', 'C4H7N4O4', -1)]
BUPN: cala_c + h2o_c + h_c --> ala_B_c + co2_c + nh4_c {'charge': 1.0}
	 [('cala_c', 'C4H8N2O3', -1), ('h2o_c', 'H2O', 0), ('h_c', 'H', 1)]
	 [('ala_B_c', 'C3H7NO2', 0), ('co2_c', 'CO2', 0), ('nh4_c', 'H4N', 1)]
CMPA: Ncbmpts_m + h2o_m + h_m --> co2_m + nh4_m + ptrc_m {'charge': 1.0, 'H': 1.0}
	 [('Ncbmpts_m', 'C5H14N3O', 1), ('h2o_m', 'H2O', 0), ('h_m', 'H', 1)]
	 [('co2_m', 'CO2', 0), ('nh4_m', 'H4N', 1), ('ptrc_m', 'C4H14N2', 2)]
DBTSm: atp_m + co2_m + dann_m <=> adp_m + dtbt_m + 3.0 h_m + pi_m {'charge': 1.0, 'H': 1.0}
	 [('atp_m', 'C10H12N5O13P3', -4), ('co2_m', 'CO2', 0), ('dann_m', 'C9H20N2O2', 0)]
	 [('adp_m', 'C10H12N5O10P2', -3), ('dtbt_m', 'C10H17N2O3', -1), ('h_m', 'H', 1), ('pi_m', 'HO4P', -2)]
DHAOX_c: dhdascb_c + 2.0 gthrd_c --> ascb__L_c + gthox_c + h_c {'charge': 1.0, 'H': 1.0}
	 [('dhdascb_c', 'C6H6O6', 0), ('gthrd_c', 'C10H16N3O6S', -1)]
	 [('ascb__L_c', 'C6H8O6', 0), ('gthox_c', 'C20H30N6O12S2', -2), ('h_c', 'H', 1)]
DHNPAm: dhnpt_m --> 2ahhmp_m + gcald_m + h_m {'charge': 1.0, 'H': 1.0}
	 [('dhnpt_m', 'C9H13N5O4', 0)]
	 [('2ahhmp_m', 'C7H9N5O2', 0), ('gcald_m', 'C2H4O2', 0), ('h_m', 'H', 1)]
DHPD: 56dura_c + h2o_c <=> cala_c {'charge': -1.0}
	 [('56dura_c', 'C4H6N2O2', 0), ('h2o_c', 'H2O', 0)]
	 [('cala_c', 'C4H8N2O3', -1)]
DHPM1: 56dura_c + h2o_c --> cala_c + h_c {'H': 1.0}
	 [('56dura_c', 'C4H6N2O2', 0), ('h2o_c', 'H2O', 0)]
	 [('cala_c', 'C4H8N2O3', -1), ('h_c', 'H', 1)]
FGFTm: fgam_m + 3.0 h_m + thf_m --> gar_m + h2o_m + methf_m {'charge': -1.0, 'H': -1.0}
	 [('fgam_m', 'C8H13N2O9P', -2), ('h_m', 'H', 1), ('thf_m', 'C19H21N7O6', -2)]
	 [('gar_m', 'C7H14N2O8P', -1), ('h2o_m', 'H2O', 0), ('methf_m', 'C20H20N7O6', -1)]
FOLD3_1: 2ahhmd_c + 4abz_c --> dhpt_c + h_c + ppi_c {'charge': 1.0, 'H': 1.0}
	 [('2ahhmd_c', 'C7H8N5O8P2', -3), ('4abz_c', 'C7H6NO2', -1)]
	 [('dhpt_c', 'C14H13N6O3', -1), ('h_c', 'H', 1), ('ppi_c', 'HO7P2', -3)]
FORA: frmd_c + h2o_c --> for_c + h_c + nh4_c {'charge': 1.0}
	 [('frmd_c', 'CH4NO', 0), ('h2o_c', 'H2O', 0)]
	 [('for_c', 'CH1O2', -1), ('h_c', 'H', 1), ('nh4_c', 'H4N', 1)]
FORAMDx: frmd_x + h2o_x --> for_x + nh4_x {'H': -1.0}
	 [('frmd_x', 'CH4NO', 0), ('h2o_x', 'H2O', 0)]
	 [('for_x', 'CH1O2', -1), ('nh4_x', 'H4N', 1)]
GCPN: 35cgmp_c + h2o_c --> gmp_c {'charge': -1.0, 'H': -1.0}
	 [('35cgmp_c', 'C10H11N5O7P', -1), ('h2o_c', 'H2O', 0)]
	 [('gmp_c', 'C10H12N5O8P', -2)]
GDTP: gdptp_c + h2o_c <=> 2.0 h_c + pi_c + ppgpp_c {'charge': 1.0, 'H': 1.0}
	 [('gdptp_c', 'C10H11N5O20P5', -7), ('h2o_c', 'H2O', 0)]
	 [('h_c', 'H', 1), ('pi_c', 'HO4P', -2), ('ppgpp_c', 'C10H11N5O17P4', -6)]
GLCNACPT: dolp_c + 2.0 h_c + uacgam_c --> doldpglcnac_c + ump_c {'C': 40.0, 'H': 64.0}
	 [('dolp_c', 'C15H27O4P', -2), ('h_c', 'H', 1), ('uacgam_c', 'C17H25N3O17P2', -2)]
	 [('doldpglcnac_c', 'C63H107NO12P2', None), ('ump_c', 'C9H11N2O9P', -2)]
GLPT: glp_c + tdcoa_c --> coa_c + tglp_c {'H': -1.0}
	 [('glp_c', 'C4H7N2O3R', 0), ('tdcoa_c', 'C35H58N7O17P3S', -4)]
	 [('coa_c', 'C21H32N7O16P3S', -4), ('tglp_c', 'C18H32N2O4R', 0)]
GMPS_glu_m: atp_m + gln__L_m + h2o_m + xmp_m --> amp_m + glu__L_m + gmp_m + 4.0 h_m + ppi_m {'charge': 2.0, 'H': 2.0}
	 [('atp_m', 'C10H12N5O13P3', -4), ('gln__L_m', 'C5H10N2O3', 0), ('h2o_m', 'H2O', 0), ('xmp_m', 'C10H11N4O9P', -2)]
	 [('amp_m', 'C10H12N5O7P', -2), ('glu__L_m', 'C5H8NO4', -1), ('gmp_m', 'C10H12N5O8P', -2), ('h_m', 'H', 1), ('ppi_m', 'HO7P2', -3)]
GMPSm: atp_m + nh4_m + xmp_m --> amp_m + gmp_m + 4.0 h_m + ppi_m {'charge': 2.0, 'H': 2.0}
	 [('atp_m', 'C10H12N5O13P3', -4), ('nh4_m', 'H4N', 1), ('xmp_m', 'C10H11N4O9P', -2)]
	 [('amp_m', 'C10H12N5O7P', -2), ('gmp_m', 'C10H12N5O8P', -2), ('h_m', 'H', 1), ('ppi_m', 'HO7P2', -3)]
GPAR: gua_c + h_c + prpp_c <=> gmp_c + ppi_c {'charge': -1.0, 'H': -1.0}
	 [('gua_c', 'C5H5N5O', 0), ('h_c', 'H', 1), ('prpp_c', 'C5H8O14P3', -5)]
	 [('gmp_c', 'C10H12N5O8P', -2), ('ppi_c', 'HO7P2', -3)]
GTHAMPOR: amp_c + gthox_c + h_c + so3_c <=> aps_c + 2.0 gthrd_c {'charge': 1.0, 'H': 1.0}
	 [('amp_c', 'C10H12N5O7P', -2), ('gthox_c', 'C20H30N6O12S2', -2), ('h_c', 'H', 1), ('so3_c', 'O3S', -2)]
	 [('aps_c', 'C10H12N5O10PS', -2), ('gthrd_c', 'C10H16N3O6S', -1)]
GTHAMPORm: amp_m + gthox_m + h_m + so3_m <=> aps_m + 2.0 gthrd_m {'charge': 1.0, 'H': 1.0}
	 [('amp_m', 'C10H12N5O7P', -2), ('gthox_m', 'C20H30N6O12S2', -2), ('h_m', 'H', 1), ('so3_m', 'O3S', -2)]
	 [('aps_m', 'C10H12N5O10PS', -2), ('gthrd_m', 'C10H16N3O6S', -1)]
GTPDPK_1: atp_c + gtp_c <=> amp_c + gdptp_c {'charge': -1.0, 'H': -1.0}
	 [('atp_c', 'C10H12N5O13P3', -4), ('gtp_c', 'C10H12N5O14P3', -4)]
	 [('amp_c', 'C10H12N5O7P', -2), ('gdptp_c', 'C10H11N5O20P5', -7)]
HISDC: h_c + his__L_c --> co2_c + hista_c {'H': 1.0}
	 [('h_c', 'H', 1), ('his__L_c', 'C6H9N3O2', 0)]
	 [('co2_c', 'CO2', 0), ('hista_c', 'C5H11N3', 1)]
HISTASE: h2o_c + hista_c + o2_c --> h2o2_c + im4act_c + nh4_c {'H': -1.0}
	 [('h2o_c', 'H2O', 0), ('hista_c', 'C5H11N3', 1), ('o2_c', 'O2', 0)]
	 [('h2o2_c', 'H2O2', 0), ('im4act_c', 'C5H6N2O', 0), ('nh4_c', 'H4N', 1)]
HPPK_1: 2ahhmp_c + atp_c --> 2ahhmd_c + amp_c {'charge': -1.0, 'H': -1.0}
	 [('2ahhmp_c', 'C7H9N5O2', 0), ('atp_c', 'C10H12N5O13P3', -4)]
	 [('2ahhmd_c', 'C7H8N5O8P2', -3), ('amp_c', 'C10H12N5O7P', -2)]
IDPm: h2o_m + ppi_m --> 2.0 pi_m {'charge': -1.0, 'H': -1.0}
	 [('h2o_m', 'H2O', 0), ('ppi_m', 'HO7P2', -3)]
	 [('pi_m', 'HO4P', -2)]
MATm: atp_m + h2o_m + met__L_m --> amet_m + h_m + pi_m + ppi_m {'charge': 1.0, 'H': 1.0}
	 [('atp_m', 'C10H12N5O13P3', -4), ('h2o_m', 'H2O', 0), ('met__L_m', 'C5H11NO2S', 0)]
	 [('amet_m', 'C15H23N6O5S', 1), ('h_m', 'H', 1), ('pi_m', 'HO4P', -2), ('ppi_m', 'HO7P2', -3)]
MTHFO: h_m + mlthf_m + nadh_m --> 5mthf_m + nad_m {'charge': 1.0, 'H': 1.0}
	 [('h_m', 'H', 1), ('mlthf_m', 'C20H21N7O6', -2), ('nadh_m', 'C21H27N7O14P2', -2)]
	 [('5mthf_m', 'C20H24N7O6', -1), ('nad_m', 'C21H26N7O14P2', -1)]
MTHFO_nadp: h_m + mlthf_m + nadph_m --> 5mthf_m + nadp_m {'charge': 1.0, 'H': 1.0}
	 [('h_m', 'H', 1), ('mlthf_m', 'C20H21N7O6', -2), ('nadph_m', 'C21H26N7O17P3', -4)]
	 [('5mthf_m', 'C20H24N7O6', -1), ('nadp_m', 'C21H25N7O17P3', -3)]
OCT: cbp_c + orn_c <=> citr__L_c + pi_c {'charge': -1.0, 'H': -1.0}
	 [('cbp_c', 'CH2NO5P', -2), ('orn_c', 'C5H13N2O2', 1)]
	 [('citr__L_c', 'C6H13N3O3', 0), ('pi_c', 'HO4P', -2)]
OCTm: cbp_m + orn_m <=> citr__L_m + pi_m {'charge': -1.0, 'H': -1.0}
	 [('cbp_m', 'CH2NO5P', -2), ('orn_m', 'C5H13N2O2', 1)]
	 [('citr__L_m', 'C6H13N3O3', 0), ('pi_m', 'HO4P', -2)]
PYDXDH: h2o_c + o2_c + pydx_c --> 4pyrdx_c + h2o2_c {'H': -1.0}
	 [('h2o_c', 'H2O', 0), ('o2_c', 'O2', 0), ('pydx_c', 'C8H9NO3', 0)]
	 [('4pyrdx_c', 'C8H8NO4', 0), ('h2o2_c', 'H2O2', 0)]
PYDXDH_1: h2o_c + o2_c + pydx_c --> 4pyrdx_c + h2o2_c + h_c {'charge': 1.0}
	 [('h2o_c', 'H2O', 0), ('o2_c', 'O2', 0), ('pydx_c', 'C8H9NO3', 0)]
	 [('4pyrdx_c', 'C8H8NO4', 0), ('h2o2_c', 'H2O2', 0), ('h_c', 'H', 1)]
SULO: h2o_c + o2_c + so3_c --> h2o2_c + h_c + so4_c {'charge': 1.0, 'H': 1.0}
	 [('h2o_c', 'H2O', 0), ('o2_c', 'O2', 0), ('so3_c', 'O3S', -2)]
	 [('h2o2_c', 'H2O2', 0), ('h_c', 'H', 1), ('so4_c', 'O4S', -2)]
SULOm: h2o_m + o2_m + so3_m --> h2o2_m + h_m + so4_m {'charge': 1.0, 'H': 1.0}
	 [('h2o_m', 'H2O', 0), ('o2_m', 'O2', 0), ('so3_m', 'O3S', -2)]
	 [('h2o2_m', 'H2O2', 0), ('h_m', 'H', 1), ('so4_m', 'O4S', -2)]
TGUAPRT: prpp_c + tgua_c --> 6tgsnmp_c + ppi_c {'charge': 2.0}
	 [('prpp_c', 'C5H8O14P3', -5), ('tgua_c', 'C5H5N5S', None)]
	 [('6tgsnmp_c', 'C10H12N5O7PS', None), ('ppi_c', 'HO7P2', -3)]
UDPDOLPT: dolp_c + udpg_c --> dolpglc_c + udp_c {'C': 40.0, 'H': 64.0}
	 [('dolp_c', 'C15H27O4P', -2), ('udpg_c', 'C15H22N2O17P2', -2)]
	 [('dolpglc_c', 'C61H102O9P', -1), ('udp_c', 'C9H11N2O12P2', -3)]
UDPGALOR: h2o_c + 2.0 nad_c + udpgal_c --> 3.0 h_c + 2.0 nadh_c + udpgalur_c {'charge': 3.0}
	 [('h2o_c', 'H2O', 0), ('nad_c', 'C21H26N7O14P2', -1), ('udpgal_c', 'C15H22N2O17P2', -2)]
	 [('h_c', 'H', 1), ('nadh_c', 'C21H27N7O14P2', -2), ('udpgalur_c', 'C15H19N2O18P2', 0)]
```

In [30]:

```
# Remove ALLTNtm and ATNAH - ALLTN is correct
model.remove_reactions(['ALLTNtm','ATNAH'], remove_orphans=True)
# 3SALATAim is correct, remove AATGm (wrong)
model.remove_reactions(['AATGm'], remove_orphans=True)
# ATDGDm and ATGDm by 15679 incorrect - correct cyto reactions exist
model.remove_reactions(['ATDGDm','ATGDm'], remove_orphans=True)
# GDTP and GTPDPK_1 incorrect, GDPDPK and GDPTPDP correct
model.remove_reactions(['GDTP','GTPDPK_1'], remove_orphans=True)
# DHPD incorrect, DHPM1 correct
model.remove_reactions(['DHPD'], remove_orphans=True)
# FGFTm incorrect, GARFT by 13595 but not 14259
model.reactions.get_by_id('GARFT').gene_reaction_rule = '13595'
model.remove_reactions(['FGFTm'], remove_orphans=True)
# FOLD3_1 incorrect, FOLD3 correct
model.remove_reactions(['FOLD3_1'], remove_orphans=True)
# GCPN incorrect, PDE4 correct
model.remove_reactions(['GCPN'], remove_orphans=True)
# GMPS2, GMPS, GUAPRT correct
model.remove_reactions(['GMPS_glu_m','GMPSm','GPAR'], remove_orphans=True)
# GTHAMPOR and GTHAMPORm incorrect, APSR correct
# AASPm incorrect, ADSK correct
model.remove_reactions(['GTHAMPOR','GTHAMPORm','AASPm'], remove_orphans=True)
# HPPK_1 incorrect reaction and gene
model.remove_reactions(['HPPK_1'], remove_orphans=True)
# IDPm incorrect, PPAm correct
model.remove_reactions(['IDPm'], remove_orphans=True) 
# MATm incorrect, METAT correct
# Remove SELMETAT (removed in Recon3D)
model.remove_reactions(['MATm','SELMETAT'], remove_orphans=True)
# OCBT, OCBTm correct
model.remove_reactions(['OCT','OCTm'], remove_orphans=True)
# PYDXDH incorrect, PYDXDH_1 correct
model.remove_reactions(['PYDXDH'], remove_orphans=True)
# 16758 uses o2 not cytochrome c, remove SULFOX
model.remove_reactions(['SULFOX'], remove_orphans=True)
```

In [31]:

```
model.metabolites.get_by_id('10fthfglu__L_m').charge = -2
model.metabolites.get_by_id('hkmpp_c').charge = -3
model.metabolites.get_by_id('dhmtp_c').formula = 'C6H9O3S'
model.metabolites.get_by_id('dhmtp_c').charge = -1
model.metabolites.get_by_id('34dhbz_c').charge = -1
model.metabolites.get_by_id('56dh5flura_c').formula = 'C4H5FN2O2'
model.metabolites.get_by_id('56dh5flura_c').charge = 0
model.metabolites.get_by_id('aflburppa_c').formula = 'C4H7FN2O3'
model.metabolites.get_by_id('aflburppa_c').charge = 0
model.reactions.get_by_id('56DH5FLURAAMH').add_metabolites({'h_c': 1.0})
model.metabolites.get_by_id('6mpur_c').charge = 0
model.metabolites.get_by_id('6tins5mp_c').charge = -2
model.metabolites.get_by_id('6txan5mp_c').charge = -2
model.metabolites.get_by_id('6tgsnmp_c').charge = -2
model.metabolites.get_by_id('apoACP_c').formula = 'ROH'
model.metabolites.get_by_id('ACP_c').formula = 'C11H21N2O7PRS'
model.metabolites.get_by_id('amob_m').formula = 'C15H19N5O6S'
model.metabolites.get_by_id('amob_m').charge = 0
model.metabolites.get_by_id('dann_m').formula = 'C9H21N2O2'
model.metabolites.get_by_id('dann_m').charge = 1
model.metabolites.get_by_id('ad_c').formula = 'C2H5NO'
model.metabolites.get_by_id('ad_c').charge = 0
model.reactions.get_by_id('AMID_1').add_metabolites({'h_c': -1.0})
model.metabolites.get_by_id('hista_c').formula = 'C5H10N3'
model.metabolites.get_by_id('ptcys_c').formula = 'C12H21N2O6S'
model.metabolites.get_by_id('ptcys_c').charge = -1
model.metabolites.get_by_id('ptth_c').formula = 'C11H22N2O4S'
model.metabolites.get_by_id('asntrna_c').formula = 'C14H24N2O12PR2'
model.metabolites.get_by_id('asntrna_m').formula = 'C14H24N2O12PR2'
model.reactions.get_by_id('ASNtx').add_metabolites({'h_x': -1.0})
```

In [32]:

```
model.metabolites.get_by_id('bhb_m').charge = -1
model.metabolites.get_by_id('cala_c').formula = 'C4H7N2O3'
model.reactions.get_by_id('BUPN').add_metabolites({'h_c': -1.0})
model.metabolites.get_by_id('catechol_c').charge = 0
model.metabolites.get_by_id('ccmuac_c').charge = -2
model.reactions.get_by_id('CMPA').add_metabolites({'h_m': -1.0})
model.metabolites.get_by_id('conialdh_c').charge = 0
model.metabolites.get_by_id('fer_c').charge = -1
model.metabolites.get_by_id('ascb__L_c').formula = 'C6H7O6'
model.metabolites.get_by_id('ascb__L_c').charge = -1
model.reactions.get_by_id('DHNPAm').add_metabolites({'h_m': -1.0})
model.metabolites.get_by_id('lys__D_c').charge = 1
model.metabolites.get_by_id('frmd_c').formula = 'CH3NO'
model.reactions.get_by_id('FORA').add_metabolites({'h_c': -1.0})
model.metabolites.get_by_id('frmd_x').formula = 'CH3NO'
model.metabolites.get_by_id('gdbtal_c').charge = 1
model.metabolites.get_by_id('dolp_c').formula = 'C15H27O4P'
model.metabolites.get_by_id('dolpglc_c').formula = 'C21H38O9P'
model.metabolites.get_by_id('doldpglcnac_c').formula = 'C23H43NO12P2'
model.metabolites.get_by_id('doldpglcnacglcnac_c').formula = 'C31H56N2O17P2'
model.metabolites.get_by_id('doldpglcnacglcnacman_c').formula = 'C37H66N2O22P2'
model.metabolites.get_by_id('doldpglcnacglcnacmanman_c').formula = 'C43H76N2O27P2'
model.metabolites.get_by_id('doldpglcnacglcnacman_man_man_c').formula = 'C49H86N2O32P2'
model.metabolites.get_by_id('doldpglcnacglcnacman_man_manman_c').formula = 'C55H96N2O37P2'
model.metabolites.get_by_id('doldpglcnacglcnacman_man_manmanman_c').formula = 'C61H106N2O42P2'
model.reactions.get_by_id('GLPT').add_metabolites({'h_c': 1.0})
model.metabolites.get_by_id('glp_c').charge = 1
```

In [33]:

```
model.reactions.get_by_id('MTHFO').id = 'MTHFR2m'
model.reactions.get_by_id('MTHFR2m').add_metabolites({'h_m': -1.0})
model.reactions.get_by_id('MTHFO_nadp').id = 'MTHFR3m'
model.reactions.get_by_id('MTHFR3m').add_metabolites({'h_m': -1.0})
model.metabolites.get_by_id('CCbuttc_c').charge = -3
model.metabolites.get_by_id('3oxoadp_c').charge = -2
model.metabolites.get_by_id('4pyrdx_c').charge = -1
model.metabolites.get_by_id('R_3hdcoa_c').charge = -4
model.metabolites.get_by_id('R_3hddcoa_c').charge = -4
model.metabolites.get_by_id('R_3hmrscoa_c').charge = -4
model.metabolites.get_by_id('R_3hhcoa_c').charge = -4
model.metabolites.get_by_id('R_3hocoa_c').charge = -4
model.reactions.get_by_id('SULO').add_metabolites({'h_c': -1.0})
model.reactions.get_by_id('SULOm').add_metabolites({'h_m': -1.0})
model.metabolites.get_by_id('udpgalur_c').charge = -3
model.metabolites.get_by_id('vanln_c').charge = 0
model.metabolites.get_by_id('vanlt_c').charge = -1
```

In [34]:

```
for r in sorted(model.reactions, key=lambda x: x.id):
    if r.check_mass_balance() and not r.boundary:
        print(r, r.check_mass_balance())
```

```
AGATer_RT: 0.01 1ag3p_RT_r + 0.02 dcacoa_r + 0.06 ddcacoa_r + 0.17 hdcoa_r + 0.09 ocdycacoa_r + 0.24 odecoa_r + 0.27 pmtcoa_r + 0.05 stcoa_r + 0.1 tdcoa_r --> coa_r + 0.01 pa_RT_r {'charge': 1.6653345369377348e-16, 'C': -2.6645352591003757e-15, 'H': -7.105427357601002e-15, 'O': -4.440892098500626e-16, 'P': -1.1102230246251565e-16, 'N': 1.1102230246251565e-16, 'S': -9.71445146547012e-17}
DAGL_RT: 0.01 12dgr_RT_d + h2o_c --> 0.02 dca_c + 0.06 ddca_c + h_c + 0.27 hdca_c + 0.17 hdcea_c + 0.01 mag_RT_d + 0.05 ocdca_c + 0.24 ocdcea_c + 0.09 ocdcya_c + 0.1 ttdca_c {'C': 2.220446049250313e-15, 'H': -3.552713678800501e-15}
EPISTATer_RT: 0.01 epist_r + 0.655 hdcoa_r + 0.01 hexccoa_r + 0.27 odecoa_r + 0.02 pmtcoa_r + 0.03 stcoa_r + 0.015 tdcoa_r --> coa_r + 0.01 epistest_RT_r {'charge': 2.220446049250313e-16, 'C': 5.551115123125783e-16, 'H': -1.099120794378905e-14, 'N': 6.938893903907228e-17, 'O': 3.3306690738754696e-16, 'P': -1.5265566588595902e-16, 'S': -5.551115123125783e-17}
EPISTESTH_RT: 0.01 epistest_RT_d + h2o_c --> 0.01 epist_c + h_c + 0.02 hdca_c + 0.655 hdcea_c + 0.01 hexc_c + 0.03 ocdca_c + 0.27 ocdcea_c + 0.015 ttdca_c {'charge': -9.71445146547012e-17, 'C': 2.6922908347160046e-15, 'H': 4.163336342344337e-15, 'O': 1.942890293094024e-16}
ERGSTATer_RT: 0.01 ergst_r + 0.655 hdcoa_r + 0.01 hexccoa_r + 0.27 odecoa_r + 0.02 pmtcoa_r + 0.03 stcoa_r + 0.015 tdcoa_r --> coa_r + 0.01 ergstest_RT_r {'charge': 2.220446049250313e-16, 'C': 5.551115123125783e-16, 'H': -1.099120794378905e-14, 'N': 6.938893903907228e-17, 'O': 3.3306690738754696e-16, 'P': -1.5265566588595902e-16, 'S': -5.551115123125783e-17}
ERGSTESTH_RT: 0.01 ergstest_RT_d + h2o_c --> 0.01 ergst_c + h_c + 0.02 hdca_c + 0.655 hdcea_c + 0.01 hexc_c + 0.03 ocdca_c + 0.27 ocdcea_c + 0.015 ttdca_c {'charge': -9.71445146547012e-17, 'C': 2.6922908347160046e-15, 'H': 4.163336342344337e-15, 'O': 1.942890293094024e-16}
FECOSTATer_RT: 0.01 fecost_r + 0.655 hdcoa_r + 0.01 hexccoa_r + 0.27 odecoa_r + 0.02 pmtcoa_r + 0.03 stcoa_r + 0.015 tdcoa_r --> coa_r + 0.01 fecostest_RT_r {'charge': 2.220446049250313e-16, 'C': 5.551115123125783e-16, 'H': -1.099120794378905e-14, 'N': 6.938893903907228e-17, 'O': 3.3306690738754696e-16, 'P': -1.5265566588595902e-16, 'S': -5.551115123125783e-17}
FECOSTESTH_RT: 0.01 fecostest_RT_d + h2o_c --> 0.01 fecost_c + h_c + 0.02 hdca_c + 0.655 hdcea_c + 0.01 hexc_c + 0.03 ocdca_c + 0.27 ocdcea_c + 0.015 ttdca_c {'charge': -9.71445146547012e-17, 'C': 2.6922908347160046e-15, 'H': 4.163336342344337e-15, 'O': 1.942890293094024e-16}
GAT1er_RT: 0.02 dcacoa_r + 0.06 ddcacoa_r + glyc3p_r + 0.17 hdcoa_r + 0.09 ocdycacoa_r + 0.24 odecoa_r + 0.27 pmtcoa_r + 0.05 stcoa_r + 0.1 tdcoa_r --> 0.01 1ag3p_RT_r + coa_r {'charge': 3.885780586188048e-16, 'C': 1.1546319456101628e-14, 'H': -7.105427357601002e-15, 'O': 4.440892098500626e-16, 'P': -1.1102230246251565e-16, 'N': 1.1102230246251565e-16, 'S': -9.71445146547012e-17}
GAT2er_RT: 0.02 dcacoa_r + 0.06 ddcacoa_r + dhap_r + 0.17 hdcoa_r + 0.09 ocdycacoa_r + 0.24 odecoa_r + 0.27 pmtcoa_r + 0.05 stcoa_r + 0.1 tdcoa_r --> 0.01 1agly3p_RT_r + coa_r {'charge': 3.885780586188048e-16, 'C': 1.1546319456101628e-14, 'H': -7.105427357601002e-15, 'O': 4.440892098500626e-16, 'P': -1.1102230246251565e-16, 'N': 1.1102230246251565e-16, 'S': -9.71445146547012e-17}
LANOSTATer_RT: 0.655 hdcoa_r + 0.01 hexccoa_r + 0.01 lanost_r + 0.27 odecoa_r + 0.02 pmtcoa_r + 0.03 stcoa_r + 0.015 tdcoa_r --> coa_r + 0.01 lanostest_RT_r {'charge': 2.220446049250313e-16, 'C': -1.2212453270876722e-15, 'H': -3.885780586188048e-15, 'N': 6.938893903907228e-17, 'O': 3.3306690738754696e-16, 'P': -1.5265566588595902e-16, 'S': -5.551115123125783e-17}
LANOSTESTH_RT: h2o_c + 0.01 lanostest_RT_d --> h_c + 0.02 hdca_c + 0.655 hdcea_c + 0.01 hexc_c + 0.01 lanost_c + 0.03 ocdca_c + 0.27 ocdcea_c + 0.015 ttdca_c {'charge': -9.71445146547012e-17, 'H': 2.3869795029440866e-15, 'O': 1.942890293094024e-16, 'C': 2.6922908347160046e-15}
LPCATer_RT: 0.01 1agpc_RT_r + 0.02 dcacoa_r + 0.06 ddcacoa_r + 0.17 hdcoa_r + 0.09 ocdycacoa_r + 0.24 odecoa_r + 0.27 pmtcoa_r + 0.05 stcoa_r + 0.1 tdcoa_r --> coa_r + 0.01 pc_RT_r {'charge': 3.885780586188048e-16, 'C': -2.6645352591003757e-15, 'N': 1.1102230246251565e-16, 'O': -4.440892098500626e-16, 'P': -1.1102230246251565e-16, 'S': -9.71445146547012e-17}
MAGL_RT: h2o_c + 0.01 mag_RT_d --> 0.02 dca_c + 0.06 ddca_c + glyc_c + h_c + 0.27 hdca_c + 0.17 hdcea_c + 0.05 ocdca_c + 0.24 ocdcea_c + 0.09 ocdcya_c + 0.1 ttdca_c {'C': 2.220446049250313e-15, 'H': 3.552713678800501e-15}
MIP2CS124g_RT: 0.01 mipc124_RT_g + 0.01 ptd1ino_RT_g --> 0.01 12dgr_RT_g + 0.01 mip2c124_RT_g {'C': 7.105427357601002e-15}
MIP2CS126g_RT: 0.01 mipc126_RT_g + 0.01 ptd1ino_RT_g --> 0.01 12dgr_RT_g + 0.01 mip2c126_RT_g {'C': 7.105427357601002e-15}
MIP2CS224g_RT: 0.01 mipc224_RT_g + 0.01 ptd1ino_RT_g --> 0.01 12dgr_RT_g + 0.01 mip2c224_RT_g {'C': 7.105427357601002e-15}
MIP2CS226g_RT: 0.01 mipc226_RT_g + 0.01 ptd1ino_RT_g --> 0.01 12dgr_RT_g + 0.01 mip2c226_RT_g {'C': 7.105427357601002e-15}
MIP2CS324g_RT: 0.01 mipc324_RT_g + 0.01 ptd1ino_RT_g --> 0.01 12dgr_RT_g + 0.01 mip2c324_RT_g {'C': 7.105427357601002e-15}
MIP2CS326g_RT: 0.01 mipc326_RT_g + 0.01 ptd1ino_RT_g --> 0.01 12dgr_RT_g + 0.01 mip2c326_RT_g {'C': 7.105427357601002e-15}
PLBP1Ie_RT: h2o_e + 0.005 ptd1ino_RT_e --> 0.02 dca_e + 0.06 ddca_e + 0.5 g3pi_e + h_e + 0.27 hdca_e + 0.17 hdcea_e + 0.05 ocdca_e + 0.24 ocdcea_e + 0.09 ocdcya_e + 0.1 ttdca_e {'charge': -2.7755575615628914e-17, 'C': 1.5543122344752192e-15, 'H': 4.440892098500626e-15, 'O': -1.0547118733938987e-15}
PLBPC_RT: h2o_c + 0.005 pc_RT_r --> 0.02 dca_c + 0.06 ddca_c + 0.5 g3pc_c + h_c + 0.27 hdca_c + 0.17 hdcea_c + 0.05 ocdca_c + 0.24 ocdcea_c + 0.09 ocdcya_c + 0.1 ttdca_c {'C': 1.5543122344752192e-15, 'H': 4.440892098500626e-15, 'O': 2.7755575615628914e-16}
PLBPCe_RT: h2o_e + 0.005 pc_RT_e --> 0.02 dca_e + 0.06 ddca_e + 0.5 g3pc_e + h_e + 0.27 hdca_e + 0.17 hdcea_e + 0.05 ocdca_e + 0.24 ocdcea_e + 0.09 ocdcya_e + 0.1 ttdca_e {'C': 1.5543122344752192e-15, 'H': 4.440892098500626e-15, 'O': 2.7755575615628914e-16}
PLBPEe_RT: h2o_e + 0.005 pe_RT_e --> 0.02 dca_e + 0.06 ddca_e + 0.5 g3pe_e + h_e + 0.27 hdca_e + 0.17 hdcea_e + 0.05 ocdca_e + 0.24 ocdcea_e + 0.09 ocdcya_e + 0.1 ttdca_e {'C': 1.5543122344752192e-15, 'H': 4.440892098500626e-15, 'O': 2.7755575615628914e-16}
TAGL_RT: h2o_c + 0.01 triglyc_RT_d --> 0.01 12dgr_RT_d + 0.02 dca_c + 0.06 ddca_c + h_c + 0.27 hdca_c + 0.17 hdcea_c + 0.05 ocdca_c + 0.24 ocdcea_c + 0.09 ocdcya_c + 0.1 ttdca_c {'C': -5.551115123125783e-15, 'H': 1.1546319456101628e-14, 'O': -1.0547118733938987e-15}
TRIGSer_RT: 0.01 12dgr_RT_r + 0.02 dcacoa_r + 0.06 ddcacoa_r + 0.17 hdcoa_r + 0.09 ocdycacoa_r + 0.24 odecoa_r + 0.27 pmtcoa_r + 0.05 stcoa_r + 0.1 tdcoa_r --> coa_r + 0.01 triglyc_RT_r {'charge': 3.885780586188048e-16, 'O': -8.881784197001252e-16, 'N': 1.1102230246251565e-16, 'P': -1.1102230246251565e-16, 'S': -9.71445146547012e-17}
ZYMSTATer_RT: 0.655 hdcoa_r + 0.01 hexccoa_r + 0.27 odecoa_r + 0.02 pmtcoa_r + 0.03 stcoa_r + 0.015 tdcoa_r + 0.01 zymst_r --> coa_r + 0.01 zymstest_RT_r {'charge': 2.220446049250313e-16, 'H': -7.105427357601002e-15, 'N': 6.938893903907228e-17, 'O': 4.440892098500626e-16, 'P': -1.5265566588595902e-16, 'S': -5.551115123125783e-17}
ZYMSTESTH_RT: h2o_c + 0.01 zymstest_RT_d --> h_c + 0.02 hdca_c + 0.655 hdcea_c + 0.01 hexc_c + 0.03 ocdca_c + 0.27 ocdcea_c + 0.015 ttdca_c + 0.01 zymst_c {'charge': -9.71445146547012e-17, 'H': 7.105427357601002e-15, 'O': 2.220446049250313e-16}
```

In [35]:

```
for r in sorted(model.reactions, key=lambda x: x.id):
    if r.check_mass_balance() and not r.boundary:
        if sum(abs(x) for x in r.check_mass_balance().values()) > 1e-6:
            print(r, r.check_mass_balance())
```

In [36]:

```
for r in sorted(model.reactions, key=lambda x: x.id):
    if r.check_mass_balance() and not r.boundary:
        print(r.id, sum(abs(x) for x in r.check_mass_balance().values()))
```

```
AGATer_RT 1.0699774399824946e-14
DAGL_RT 5.773159728050814e-15
EPISTATer_RT 1.2378986724570495e-14
EPISTESTH_RT 7.147060721024445e-15
ERGSTATer_RT 1.2378986724570495e-14
ERGSTESTH_RT 7.147060721024445e-15
FECOSTATer_RT 1.2378986724570495e-14
FECOSTESTH_RT 7.147060721024445e-15
GAT1er_RT 1.980360320175123e-14
GAT2er_RT 1.980360320175123e-14
LANOSTATer_RT 5.9396931817445875e-15
LANOSTESTH_RT 5.370703881624195e-15
LPCATer_RT 3.816391647148976e-15
MAGL_RT 5.773159728050814e-15
MIP2CS124g_RT 7.105427357601002e-15
MIP2CS126g_RT 7.105427357601002e-15
MIP2CS224g_RT 7.105427357601002e-15
MIP2CS226g_RT 7.105427357601002e-15
MIP2CS324g_RT 7.105427357601002e-15
MIP2CS326g_RT 7.105427357601002e-15
PLBP1Ie_RT 7.077671781985373e-15
PLBPC_RT 6.2727600891321345e-15
PLBPCe_RT 6.2727600891321345e-15
PLBPEe_RT 6.2727600891321345e-15
TAGL_RT 1.815214645262131e-14
TRIGSer_RT 1.5959455978986625e-15
ZYMSTATer_RT 8.049116928532385e-15
ZYMSTESTH_RT 7.424616477180734e-15
```

In [37]:

```
for m in model.metabolites:
    if m.charge is None:
        print(m.id, m.formula, m.charge)
```

```
asnglcnacglcnacman_man_manman_man_manmanman_c C69H113N5O53R2 None
asnglcnacglcnacman_man_manman_manman_manmanman_c C75H123N5O58R2 None
pe1801829Z12Z_c C41H78NO8P None
Glc_aD_c C6H12O6 None
12dgr1819Z1819Z_c C39H72O5 None
tgua_c C5H5N5S None
tgua_e C5H5N5S None
doldpglcnacglcnacman_manman_manmanman_c C107H180N2O47P2 None
doldpglcnacglcnacman_manmanman_manmanman_c C113H190N2O52P2 None
doldpglcnac_c C23H43NO12P2 None
doldpglcnacglcnac_c C31H56N2O17P2 None
dghs1819Z1819Z_c C43H79NO7 None
1btol_c C4H10O None
pe1819Z1829Z12Z_c C41H76NO8P None
ans_c C10H16N4O3 None
asnglcnacglcnacman_man_manman_manman_manmanmanglc_c C81H133N5O63R2 None
pe1819Z1819Z_c C41H78NO8P None
pe1819Z1819Z_e C41H78NO8P None
pail1819Z160_c C43H81O13P None
pail1819Z160_e C43H81O13P None
dghs1601819Z_c C41H77NO7 None
dgts1601819Z_c C44H83NO7 None
doldpglcnacglcnacman_c C37H66N2O22P2 None
asnglcnacglcnacman_man_man_man_man_c C51H83N5O38R2 None
5flura_c C4H3FN2O2 None
5flura_e C4H3FN2O2 None
asnglcnacglcnacman_man_man_c C39H63N5O28R2 None
asnglcnacglcnacman_man_manman_manman_manmanmanglcglc_c C87H143N5O68R2 None
asnglcnacglcnacman_man_manman_manman_manmanmanglcglcglc_c C93H153N5O73R2 None
dgts1819Z1819Z_c C46H85NO7 None
6mpur_e C5H4N4S None
doldpglcnacglcnacman_man_manman_manman_manmanmanglcglcglc_c C143H240N2O77P2 None
pe1801829Z12Z_e C41H78NO8P None
doldpglcnacglcnacman_man_man_manman_manmanman_c C119H200N2O57P2 None
doldpglcnacglcnacman_man_manman_manman_manmanman_c C125H210N2O62P2 None
arso4_c C6H6O4S None
phenol_c C6H6O None
doldpglcnacglcnacman_man_man_c C49H86N2O32P2 None
doldpglcnacglcnacmanman_c C43H76N2O27P2 None
aflbala_c C3H6FNO2 None
pe1819Z1829Z12Z_e C41H76NO8P None
tega_c C8H8FN2O3 None
tega_e C8H8FN2O3 None
doldpglcnacglcnacman_man_manman_c C55H96N2O37P2 None
doldpglcnacglcnacman_man_manmanman_c C61H106N2O42P2 None
```

In [38]:

```
for m in model.metabolites:
    if m.charge is None:
        m.charge = 0
```

In [39]:

```
print(len(model.genes))
print(len(model.reactions))
print(len(model.metabolites))
model
```

```
1106
1934
2010
```

Out[39]:

|  |  |
| --- | --- |
| **Name** | R. toruloides |
| **Memory address** | 0x010266687f0 |
| **Number of metabolites** | 2010 |
| **Number of reactions** | 1934 |
| **Number of groups** | 0 |
| **Objective expression** | 0 |
| **Compartments** | c, x, m, e, r, v, n, g, d |

In [40]:

```
for x in sorted(model.genes, key=lambda x: x.id):
    if not x.reactions:
        print(x)
print()
for x in sorted(model.metabolites, key=lambda x: x.id):
    if not x.reactions:
        print(x)
```

```

```

In [41]:

```
cobra.manipulation.remove_genes(model, [x for x in model.genes if not x.reactions])
model.remove_metabolites([x for x in model.metabolites if not x.reactions])
```

In [42]:

```
print(len(model.genes))
print(len(model.reactions))
print(len(model.metabolites))
print(len(set([m.id.rsplit('_',1)[0] for m in model.metabolites])))
print(len(model.compartments))
model
```

```
1106
1934
2010
1246
9
```

Out[42]:

|  |  |
| --- | --- |
| **Name** | R. toruloides |
| **Memory address** | 0x010266687f0 |
| **Number of metabolites** | 2010 |
| **Number of reactions** | 1934 |
| **Number of groups** | 0 |
| **Objective expression** | 0 |
| **Compartments** | c, x, m, e, r, v, n, g, d |

In [43]:

```
cobra.io.save_json_model(model, "IFO0880_GPR_1f.json")
```

In [44]:

```
model_old = cobra.io.load_json_model("IFO0880_GPR_1e.json")
model_new = cobra.io.load_json_model("IFO0880_GPR_1f.json")
```

In [45]:

```
print('Removed reactions\n')
for r in sorted(model_old.reactions, key=lambda x: x.id):
    if r not in model_new.reactions:
        print(r)
```

```
Removed reactions

AASPm: aps_m + atp_m --> adp_m + h_m + paps_m
AATGm: 3sala_m + akg_m --> 3snpyr_m + glu__L_m
ALLTNtm: alltn_c + h_c <=> alltn_m + 2.0 h_m
ATDGDm: atp_m + dgdp_m + h_m --> adp_m + dgtp_m
ATGDm: atp_m + gdp_m + h_m --> adp_m + gtp_m
ATNAH: alltn_m + h2o_m + h_m --> alltt_m
DHPD: 56dura_c + h2o_c <=> cala_c
FGFTm: fgam_m + 3.0 h_m + thf_m --> gar_m + h2o_m + methf_m
FOLD3_1: 2ahhmd_c + 4abz_c --> dhpt_c + h_c + ppi_c
GCPN: 35cgmp_c + h2o_c --> gmp_c
GDTP: gdptp_c + h2o_c <=> 2.0 h_c + pi_c + ppgpp_c
GMPS_glu_m: atp_m + gln__L_m + h2o_m + xmp_m --> amp_m + glu__L_m + gmp_m + 4.0 h_m + ppi_m
GMPSm: atp_m + nh4_m + xmp_m --> amp_m + gmp_m + 4.0 h_m + ppi_m
GPAR: gua_c + h_c + prpp_c <=> gmp_c + ppi_c
GTHAMPOR: amp_c + gthox_c + h_c + so3_c <=> aps_c + 2.0 gthrd_c
GTHAMPORm: amp_m + gthox_m + h_m + so3_m <=> aps_m + 2.0 gthrd_m
GTPDPK_1: atp_c + gtp_c <=> amp_c + gdptp_c
HPPK_1: 2ahhmp_c + atp_c --> 2ahhmd_c + amp_c
IDPm: h2o_m + ppi_m --> 2.0 pi_m
MATm: atp_m + h2o_m + met__L_m --> amet_m + h_m + pi_m + ppi_m
MTHFO: h_m + mlthf_m + nadh_m --> 5mthf_m + nad_m
MTHFO_nadp: h_m + mlthf_m + nadph_m --> 5mthf_m + nadp_m
OCT: cbp_c + orn_c <=> citr__L_c + pi_c
OCTm: cbp_m + orn_m <=> citr__L_m + pi_m
PYDXDH: h2o_c + o2_c + pydx_c --> 4pyrdx_c + h2o2_c
SELMETAT: atp_c + h2o_c + selmeth_c --> pi_c + ppi_c + seasmet_c
SULFOX: 2.0 ficytc_m + h2o_c + so3_c --> 2.0 focytc_m + 2.0 h_c + so4_c
```

In [46]:

```
print('Updated reactions\n')
for r in sorted(model_old.reactions, key=lambda x: x.id):
    if r in model_new.reactions:
        r2 = model_new.reactions.get_by_id(r.id)
        if (r.name == r2.name and r.reaction == r2.reaction and r.gene_reaction_rule == r2.gene_reaction_rule and
            r.lower_bound == r2.lower_bound and r.upper_bound == r2.upper_bound):
            pass
        else:
            print('Old', r, r.gene_reaction_rule)
            print('New', r2, r2.gene_reaction_rule)
            print()
```

```
Updated reactions

Old 56DH5FLURAAMH: 56dh5flura_c + h2o_c + h_c --> aflburppa_c 11877
New 56DH5FLURAAMH: 56dh5flura_c + h2o_c --> aflburppa_c 11877

Old AMID_1: ad_c + h2o_c --> ac_c + h_c + nh4_c 13791
New AMID_1: ad_c + h2o_c --> ac_c + nh4_c 13791

Old ASNtx: asn__L_c + h_c <=> asn__L_x + 2.0 h_x 16187
New ASNtx: asn__L_c + h_c <=> asn__L_x + h_x 16187

Old BUPN: cala_c + h2o_c + h_c --> ala_B_c + co2_c + nh4_c 14839
New BUPN: cala_c + h2o_c + 2.0 h_c --> ala_B_c + co2_c + nh4_c 14839

Old CMPA: Ncbmpts_m + h2o_m + h_m --> co2_m + nh4_m + ptrc_m 14839
New CMPA: Ncbmpts_m + h2o_m + 2.0 h_m --> co2_m + nh4_m + ptrc_m 14839

Old DHNPAm: dhnpt_m --> 2ahhmp_m + gcald_m + h_m 14377
New DHNPAm: dhnpt_m --> 2ahhmp_m + gcald_m 14377

Old FORA: frmd_c + h2o_c --> for_c + h_c + nh4_c 15806
New FORA: frmd_c + h2o_c --> for_c + nh4_c 15806

Old GARFT: 10fthf_c + gar_c <=> fgam_c + h_c + thf_c 13595 or 14259
New GARFT: 10fthf_c + gar_c <=> fgam_c + h_c + thf_c 13595

Old GLPT: glp_c + tdcoa_c --> coa_c + tglp_c 15158
New GLPT: glp_c + tdcoa_c --> coa_c + h_c + tglp_c 15158

Old SULO: h2o_c + o2_c + so3_c --> h2o2_c + h_c + so4_c 16758
New SULO: h2o_c + o2_c + so3_c --> h2o2_c + so4_c 16758

Old SULOm: h2o_m + o2_m + so3_m --> h2o2_m + h_m + so4_m 16758
New SULOm: h2o_m + o2_m + so3_m --> h2o2_m + so4_m 16758
```

In [47]:

```
print('Added reactions\n')
for r in sorted(model_new.reactions, key=lambda x: x.id):
    if r not in model_old.reactions:
        print(r)
```

```
Added reactions

MTHFR2m: 2.0 h_m + mlthf_m + nadh_m --> 5mthf_m + nad_m
MTHFR3m: 2.0 h_m + mlthf_m + nadph_m --> 5mthf_m + nadp_m
```
